# Novel robust biomarkers for human bladder cancer based on activation of intracellular signaling pathways

**DOI:** 10.18632/oncotarget.2493

**Published:** 2014-09-16

**Authors:** Ksenia Lezhnina, Olga Kovalchuk, Alexander A. Zhavoronkov, Mikhail B. Korzinkin, Anastasia A. Zabolotneva, Peter V. Shegay, Dmitry G. Sokov, Nurshat M. Gaifullin, Igor G. Rusakov, Alexander M. Aliper, Sergey A. Roumiantsev, Boris Y. Alekseev, Nikolay M. Borisov, Anton A. Buzdin

**Affiliations:** ^1^ Pathway Pharmaceuticals, Wan Chai, Hong Kong, Hong Kong SAR; ^2^ Laboratory of Bioinformatics, D. Rogachyov Federal Research Center of Pediatric Hematology, Oncology and Immunology, Moscow, Russia; ^3^ Department of Biological Sciences, University of Lethbridge, 4401 University Drive, Lethbridge, AB, T1K 3M4; ^4^ Canada Cancer and Aging Research Laboratories, Lethbridge, AB, Canada; ^5^ Insilico Medicine, Inc, ETC, Johns Hopkins University, Baltimore, MD; ^6^ Faculty of Biological and Medical Physics, Moscow Institute of Physics and Technology; ^7^ Group for Genomic Regulation of Cell Signaling Systems, Shemyakn-Ovchinnikov Institute of Bioorganic Chemistry, Moscow, Russia; ^8^ P.A. Herzen Moscow Oncological Research Institute, Moscow, Russia; ^9^ Moscow 1st Oncological Hospital, Moscow, Russia; ^10^ Lomonosov Moscow State University, Faculty of Fundamental Medicine, Moscow, Russia; ^11^ Russian medical postgraduate academy, Moscow, Russia; ^12^ Laboratory of Systems Biology, A.I. Burnasyan Federal Medical Biophysical Center, Moscow, Russia

**Keywords:** Bladder cancer, Intracellular signaling pathway activation, Gene expression, Transcriptome profiling, Molecular markers, AUC

## Abstract

We recently proposed a new bioinformatic algorithm called OncoFinder for quantifying the activation of intracellular signaling pathways. It was proved advantageous for minimizing errors of high-throughput gene expression analyses and showed strong potential for identifying new biomarkers. Here, for the first time, we applied OncoFinder for normal and cancerous tissues of the human bladder to identify biomarkers of bladder cancer. Using Illumina HT12v4 microarrays, we profiled gene expression in 17 cancer and seven non-cancerous bladder tissue samples. These experiments were done in two independent laboratories located in Russia and Canada. We calculated pathway activation strength values for the investigated transcriptomes and identified signaling pathways that were regulated differently in bladder cancer (BC) tissues compared with normal controls. We found, for both experimental datasets, 44 signaling pathways that serve as excellent new biomarkers of BC, supported by high area under the curve (AUC) values. We conclude that the OncoFinder approach is highly efficient in finding new biomarkers for cancer. These markers are mathematical functions involving multiple gene products, which distinguishes them from “traditional” expression biomarkers that only assess concentrations of single genes.

## INTRODUCTION

Bladder cancer (BC) is the second most frequent urological cancer and the ninth most common of all cancers. Approximately 356,000 new BC cases are reported annually worldwide [1], with the majority observed in males. BC incidence varies greatly among different geographic regions (ranging between 1.8–27.1 per 100,000 males and 0.5–4.1 per 100,000 females), with the highest incidences in countries where the dominant population is Caucasoid [2]. BC accounts for 3.1% and 1.8% of the overall cancer mortality in males and females, respectively.

Early diagnosis is a prerequisite for successful BC treatment. In advanced stages, the effectiveness of BC treatment is dramatically decreased and is associated with poor quality of life. In contrast, early diagnosis of BC can significantly prolong lifespan as well as quality of life. Existing methods of clinical BC diagnostics are, in general, not efficient for detecting BC in its early stages; as a result, there is an urgent need and opportunity to develop novel diagnostic tools that would efficiently detect early-stage BC [3-5]. Moreover, associating marker expression with successful medical treatment may provide clues to a more efficient, patient-oriented cancer treatment therapy [6-9].

Recently, we developed a new bioinformatic technique called OncoFinder [10-11]. Based on large-scale transcriptomic data, this novel approach enables quantitative measurement of intracellular signaling pathway (ISP) activation in many cell/tissue physiological and pathological conditions, including cancer. OncoFinder operates similarly to another recently published approach termed Pathifier [12], which also quantitatively analyzes the extent of signaling pathway activation basing on gene expression data. However, the Pathifier algorithm utilizes different mathematical formulae for calculation of pathway activation scores and does not take into account specific roles (stimulatory, inhibitory, ambivalent, unknown, etc) of individual gene products forming a pathway, which may produce a biased output. In OncoFinder, we use a manually curated database of molecular signaling pathways that includes the functional roles present in a pathway [10-11].

Signaling pathways regulate all major cellular events in health and disease [13-17]. OncoFinder calculates a quantitative measurement of the signaling pathway activation termed “pathway activation strength” (PAS) for the ISPs under investigation. PAS measures the cumulative value of perturbations in a signaling pathway and may serve as a distinct indicator of pathological changes in the intracellular signaling machinery at the cellular, tissue, or organ level. In previous studies we confirmed the robustness of this approach and its applicability to analyzing intracellular signaling [10,18]. The PAS calculation algorithm dramatically diminished the discrepancies between the microarray and deep sequencing data obtained using various experimental platforms [19]. The PAS value itself may serve as a new type of biomarker that can distinguish between the ISP activation profiles in different cancer types [20]. However, no such work has been published so far using the PAS approach to identify new cancer biomarkers or establish signaling pathways relevant to cancer progression.

In this study, we aimed to evaluate whether PAS values serve as efficient markers for human BC or not. To this end, we generated gene expression profiles for non-cancerous and cancerous bladder tissues using the Illumina HT12v4 microarray platform (Illumina, USA) in a research laboratory in Moscow, Russia, and in duplicate in another laboratory in Lethbridge, Canada. According to universal criteria, we compared the two gene expression datasets and found 36 new PAS biomarkers for BC with high area-under-the-curve (AUC) scores >0.75. We also report on a new method of applying the OncoFinder algorithm for finding new cancer-specific biomarkers.

## RESULTS AND DISCUSSION

### Building intracellular signaling pathway activation profiles

We investigated gene expression profiles generated from 17 clinical BC tissue samples and seven non-cancerous bladder tissue samples using Illumina human HT 12 v4 bead arrays. The bladder cancer patients were treated at the P.A. Herzen Oncology Institute (Moscow). The non- cancerous tissue samples were taken from healthy donors killed in road accidents. To minimize the batch effect error, eight cancer and four non-cancerous samples were analyzed Dr. Kovalchuk's laboratory in Lethbridge (Canada), and nine cancer and three normal bladder tissue samples were analyzed in Dr. Buzdin's laboratory in Moscow (Russia). The hybridization signals were quantile normalized according to [21]. The normalized gene expression data from Lethbridge and Moscow are shown in Supplemental Files 1 and 2, respecrively. The data were next processed using the OncoFinder algorithm to establish pathway activation strength (PAS) profiles. The formula for PAS calculation accounts for gene expression data and for information on the protein interactions in a pathway, namely, individual protein activator or repressor roles in a pathway [10]; for pathway *p*, $PAS_{p}=\sum_{n}ARR_{p}\cdot lg(CNR_{n})$. The relative role of a gene product in signal transduction is reflected by a discrete flag *activator/repressor role* (*ARR*), which equals 1 for an activator gene product, –1 for a repressor, and shows intermediate values −0,5; 0,5 and 0 for the gene products that have repressor, activator, or unknown roles, respectively. The *CNR_n_* value (*case-to-normal ratio)* is the ratio of the expression level of a gene *n* in the sample under investigation to the average expression level in the reference sampling. The positive value of PAS indicates abnormal activation of a signaling pathway, and the negative value - its repression.

We analyzed activations of 271 intracellular signaling pathways. For PAS calculations, the Moscow cancer samples were normalized to the averaged Moscow normal samples, and Lethbridge cancer samples were normalized to the averaged Lethbridge normal samples. This type of analysis resulted in a cloud of PAS values for *cancer* samples denoted as PAS(Mos_BC/Mos_norm) (Supplemental file 3) and PAS(Leth_BC/Leth_norm) (Supplemental file 4). For calculating the cloud of PAS values for *non-cancerous* samples – PAS(Leth_norm/Mos_norm) (Supplemental file 5) and PAS(Mos_norm/Leth_norm) (Supplemental file 6) - we used reciprocal normalization of the Moscow normal samples to the averaged Lethbridge normal samples, and vice versa.

### Assessment of the biomarker potential of PAS

We next investigated if the uncovered signaling pathway activation strength values may serve as the biomarkers of bladder cancer. To this end, we calculated area under the curve (AUC) values. The AUC value is the universal test of biomarker robustness. It positively correlates with biomarker quality and depends on the sensitivity and specificity of a biomarker. AUC may vary within an interval from 0.5 to 1. The AUC threshold for discriminating good and poor biomarkers is typically 0.7 or 0.75, with higher AUC scores considered to be good-quality biomarkers and vice-versa [22]. We calculated AUC scores for the comparison of (i) PAS(Mos_BC/Mos_­norm) vs PAS(Leth_norm/Mos_norm) and (ii) PAS(Leth_BC/Leth_norm) vs PAS(Mos_norm/Leth_norm) (Supplemental Files 7 and 8 for (i) and (ii), respectively). We next compared the results for both obtained AUC datasets. We found that many PAS scores showed strong AUC values simultaneously in both comparisons. Overall, there were 102 pathways with AUC > 0.75 for comparison (i) (Supplemental Files 9) and 113 pathways with AUC > 0.75 for comparison (ii) (Supplemental Files 10). The distributions of the PAS values for the differential pathways among cancer vs non-cancer samples is shown in Supplemental files 11 and 12 for the comparisons (i) and (ii), respectively.

**Figure 1:**
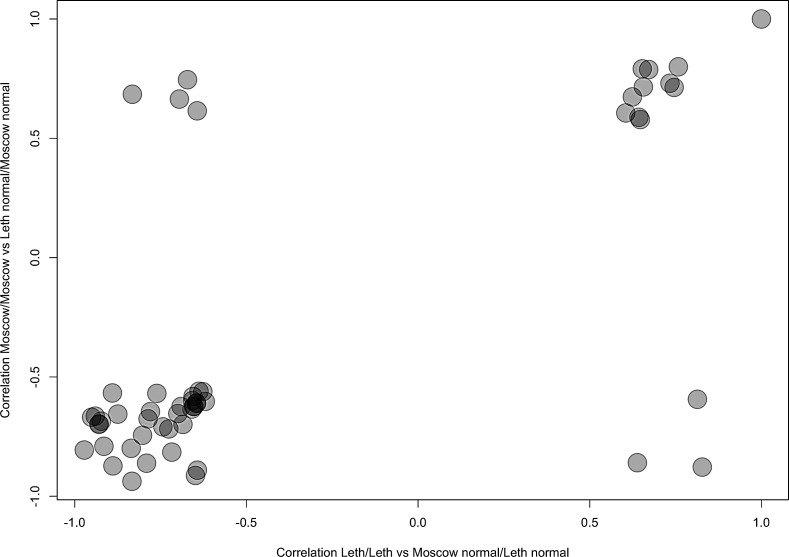
Representation of the Correlation coefficients for pathway activation states depending on the normal/cancerous nature of BC tissue specimens, calculated for the PAS values of the different intracellular signaling pathways for the Comparisons (i) and (ii) Negative correlation coefficients mean downregulation of a pathway in BC, positive – upregulation. Correlation Moscow/Moscow means correlation coefficients calculated for the Comparison (i); correlation Leth/Leth means correlation coefficients calculated for the Comparison (ii).

Of these, 44 top AUC-scoring pathways overlapped, which suggests they may serve as efficient BC biomarkers (Table 1). The use of two independent gene expression profiling procedures (in Moscow and in Lethbridge) strengthens the significance of these results. These good-quality common markers (AUC > 0.75) showed congruent activation patterns in both comparisons (i) and (ii), as supported by similar correlation coefficients between pathway activation strengths and cancerous/non-cancerous state of the bladder tissue (Fig. 1). This means that in both comparisons the 44 different pathways showed similar characteristics of up/downregulation in cancer. Among the 44 overlapping PAS biomarkers, 10 (23%) were upregulated and 34 (77%) were downregulated in BC (Table 1). Eight differential PAS biomarkers (18%) represented independent regulatory networks, whereas the rest, thirty-six (82%), were terminal branches of larger molecular signaling pathways (Table 1).

**Table 1:** AUC scores of the differential BC-specific pathways for the comparisons (i) -AUC_L and (ii) – AUC_M

Status in BC	Pathway	AUC_L	AUC_M
Upregulated	AHR_Pathway	0,88	0,92
Upregulated	AHR_Pathway_C_Myc_Expression	1,00	0,97
Upregulated	AHR_Pathway_Cath_Repression	1,00	0,97
Downregulated	AKT_Pathway_Cell_Cycle	0,96	0,97
Downregulated	Androgen_receptor_Pathway	1,00	0,94
Downregulated	Androgen_receptor_Pathway_AR_Degradation	1,00	0,92
Downregulated	ATM_Pathway_Apoptosis	1,00	1,00
Downregulated	cAMP_Pathway_Endothelial_Cell_Regulation	1,00	0,92
Downregulated	cAMP_Pathway_Glycolysis	0,88	1,00
Downregulated	CREB_Pathway	1,00	0,97
Downregulated	CREB_Pathway_Gene_Expression	0,92	1,00
Upregulated	Glucocorticoid_Receptor_Pathway_Cell_cycle_arrest	0,88	0,86
Downregulated	GSK3_Pathway_Degradation	1,00	1,00
Downregulated	HIF1Alpha_Pathway_Gene_expression	0,96	0,94
Downregulated	HIF1Alpha_Pathway_NOS_pathway	0,96	0,97
Downregulated	HIF1Alpha_Pathway_Pyruvate	0,96	0,97
Downregulated	IGF1R_Signaling_Pathway	1,00	1,00
Downregulated	IGF1R_Signaling_Pathway_Cell_survival	1,00	0,97
Downregulated	ILK_Pathway_Apoptosis	0,88	0,92
Downregulated	ILK_Pathway_Cell_adhesion_cell_motility opsonization	0,88	0,86
Downregulated	ILK_Pathway_Cell_cycle_proliferation	0,88	0,86
Downregulated	ILK_Pathway_Cell_migration.retraction	0,88	0,89
Downregulated	ILK_Pathway_Cell_motility	0,92	0,92
Downregulated	ILK_Pathway_Cytoskeletal_reorganiation	0,88	0,92
Downregulated	ILK_Pathway_G2_phase_arrest	0,88	0,86
Downregulated	ILK_Pathway_Regulation_of intermediate_filaments	0,88	0,86
Downregulated	ILK_Pathway_Regulation_of_junction assembly_of_desmosomes	0,88	0,86
Downregulated	ILK_Pathway_Wound_healing	0,88	0,86
Downregulated	Integrin_SIgnaling_Pathway Focal_adhesion_and_stress_fibers	1,00	1,00
Downregulated	IP3_Pathway	1,00	0,89
Downregulated	IP3_Pathway_Gene_expression	1,00	1,00
Upregulated	JAK_mStat_Pathway_JAK_degradation	1,00	1,00
Downregulated	mTOR_Pathway_Translation_on	0,92	0,83
Upregulated	mTOR_Pathway_VEGF_pathway	0,96	0,89
Upregulated	p53_Signaling_m_Pathway_p53_Degradation	1,00	1,00
Downregulated	PAK_Pathway_Myosin_Activation	1,00	1,00
Upregulated	RNA_Polymerase_II_Complex_Pathway	1,00	0,89
Downregulated	SMAD_Pathway	1,00	0,97
Downregulated	TGF_beta_Pathway_Epithelial_mesehchymal transdifferentiation	0,96	0,97
Downregulated	TGF_beta_Pathway_Post_transcriptional G1_arrest	1,00	1,00
Upregulated	Transition_and_termination_of DNA_replication_effect	1,00	1,00
Downregulated	VEGF_Pathway	1,00	0,92
Downregulated	VEGF_Pathway_Actin_Reorganization	1,00	0,92
Upregulated	Wnt_Pathway_Ctnn.B_Degradation	1,00	1,00

### Biological effect of the differential pathways

The up/downregulation of the 44 differential pathways seen in the BC samples could lead to contradictory effects on the survival and proliferation of cancer cells (Table 2). Information in the literature indicates that seven (16%) of the changes in the affected pathways promote cancer cell growth, survival, and proliferation while 12 (27%) of the changes exert negative effects on cancer cells (references shown on Table 2). The rest of the pathways play contradictory roles on cancer cells, which prevents us from unambiguous labeling them as “positive” or “negative” regulators of BC progression (Table 2).

**Table 2:** Functional statuses of the marker differential pathways in BC and their overall effects on proliferation of cancer cells

Pathway	Status in BC	Effect on Proliferation	References
AHR_Pathway	Upregulated	Controversial	[39]
AHR_Pathway_C_Myc_Expression	Upregulated	Positive	[40-41]
AHR_Pathway_Cath_D_Repression	Upregulated	Controversial	[42-43]
AKT_Pathway_Cell_Cycle	Downregulated	Negative	[44-45]
Androgen_receptor_Pathway	Downregulated	Controversial	[46-47]
Androgen_receptor_Pathway_AR_Degradation	Downregulated	Positive	[47-46]
ATM_Pathway_Apoptosis	Downregulated	Positive	[48-49]
cAMP_Pathway_Endothelial_Cell_Regulation	Downregulated	Controversial	[50]
cAMP_Pathway_Glycolysis	Downregulated	Negative	[51]
CREB_Pathway	Downregulated	Controversial	[52-53]
CREB_Pathway_Gene_Expression	Downregulated	Controversial	[52-53]
Glucocorticoid_Receptor_Pathway_Cell_cycle_arrest	Upregulated	Negative	[54]
GSK3_Pathway_ Degradation of Ctnn_B.	Downregulated	Controversial	[55-57]
HIF1Alpha_Pathway_Gene_expression	Downregulated	Controversial	[58]
HIF1Alpha_Pathway_NOS_pathway	Downregulated	Controversial	[58]
HIF1Alpha_Pathway_Pyruvate	Downregulated	Controversial	[58]
IGF1R_Signaling_Pathway	Downregulated	Negative	[59-60]
IGF1R_Signaling_Pathway_Cell_survival	Downregulated	Negative	[59-60]
ILK_Pathway_Regulation of Apoptosis	Downregulated	Negative	[61]
ILK_Pathway_Cell_adhesion_cell_motility opsonization	Downregulated	Controversial	[62]
ILK_Pathway_Cell_cycle_proliferation	Downregulated	Negative	[61]
ILK_Pathway_Cell_migration.retraction	Downregulated	Controversial	[63]
ILK_Pathway_Cell_motility	Downregulated	Controversial	[62]
ILK_Pathway_Cytoskeletal_reorganiation	Downregulated	Controversial	[64]
ILK_Pathway_G2_phase_arrest regulation	Downregulated	Negative	[65]
ILK_Pathway_Regulation_of_intermediate_filaments	Downregulated	Controversial	[64]
ILK_Pathway_Regulation_of_junction_assembly of_desmosomes	Downregulated	Controversial	[64]
ILK_Pathway_Wound_healing	Downregulated	Controversial	[64]
Integrin_SIgnaling_Pathway_Focal_adhesion_and_ stress_fibers	Downregulated	Controversial	[64]
IP3_Pathway	Downregulated	Controversial	[66-68]
IP3_Pathway_Gene_expression	Downregulated	Controversial	[66-68]
JAK_mStat_Pathway_JAK_degradation	Upregulated	Controversial	[69-72]
mTOR_Pathway_Translation_on	Downregulated	Negative	[73-74]
mTOR_Pathway_VEGF_pathway activation	Upregulated	Positive	[73-74]
p53_Signaling_m_Pathway_p53_Degradation	Upregulated	Positive	[75-76]
PAK_Pathway_Myosin_Activation	Downregulated	Controversial	[77]
RNA_Polymerase_II_Complex_Pathway	Upregulated	Controversial	[78]
SMAD_Pathway	Downregulated	Positive	[79-80]
TGF_beta_Pathway_Epithelial_mesehchymal_ transdifferentiation	Downregulated	Negative	[81]
TGF_beta_Pathway_Post_transcriptional_ G1_arrest	Downregulated	Positive	[81]
Transition_and_termination_of_DNA_replication_ effect	Upregulated	Negative	[82-83]
VEGF_Pathway	Downregulated	Negative	[84]
VEGF_Pathway_Actin_Reorganization	Downregulated	Controversial	[84]
Wnt_Pathway_Ctnn.B_Degradation	Upregulated	Controversial	[57-58, 85]

However, overall we observed a clearly enhanced proportion of negative BC regulatory changes among the marker pathways. This finding may be explained by the following general factors in cancer biology. Initial and further steps of cancer malignization activate internal sensory protective systems of the affected cells, which leads to upregulation of the anticancer signaling [23-26]. Normally, these anticancer changes in intracellular signaling should result in decreased proliferation and motility, growth and cell cycle arrest, intense proapoptotic signaling and pause in biosynthesis. However, cancer cells surmount these barriers using a wide variety of strategies, e.g., by blocking cell death via induction of inhibitor of apoptosis (IAP) proteins like Survivin, and by ubiquitination-mediated targeted proteolysis of key tumor suppressor proteins [27-30]. These effects act in concert with the variety of molecular mechanisms that simultaneously promote cancer cell growth and proliferation, invasion, and vascularization of cancer tissues. Importantly, the activated cancer-promoting mechanisms are very diverse among the individual cancer cases and may vary greatly among samples, even in the same cancer type [31-33]. This illustrates the well-known observation that individual cancers are largely unique and that personalized approaches are needed to increase efficiency of therapy and diagnostics [34-35]. Anticancer protective regulatory mechanisms have evolved for hundreds of millions of years and represent a conserved set of regulatory pathway activation features [36-37]. Cancer-promoting mechanisms are, in contrast, significantly more variable within individual cancer cases as they reflect unique deleterious changes of the intracellular regulatory network [31-33].

Good-quality PAS biomarkers of BC are signaling pathway activation features that clearly distinguish cancer vs non-cancer bladder tissues. According to the above model, PAS biomarkers must be enriched in protective (more conserved) changes in intracellular signaling, which is in good agreement with our observations for bladder cancer. However, this model remains to be further verified for other cancer types.

The functional role for most of the BC-specific features of intracellular signaling remains unknown (Table 2). However, the above theory predicts that many are protective cancer suppressor mechanisms.

## CONCLUSION

In this study, for the first time we performed large-scale quantitative and qualitative profiling of the intracellular signaling pathways, which distinguish normal and cancerous tissues. This analysis was carried out for human bladder tissues, but the same algorithm can be employed to investigate any tissue type or any pathology of interest. To determine pathology-specific signaling pathways, the following approach is suggested. First, one should determine gene expression levels in a pool of pathological samples and in a pool of matching normal samples. The results obtained for the normal pool are then divided into at least two subsets, of which one is used as the “normal” reference for the PAS calculation, and another is used as the “false-pathological” sampling that is formally treated as “pathological” during the application of OncoFinder algorithm. The, the clouds of PAS values are determined for the true pathological samples and for the false-pathological samples, which are compared to each other, followed by calculation of AUC scores, which support pathology-specific PAS biomarkers. We used this approach to identify forty-four new BC-specific PAS biomarkers, all of which showed significant AUC values. This pool of biomarkers was enriched in anticancer protective regulatory features, which is consistent with the concept that they are more conserved compared to the highly variable tumorigenic molecular mechanisms.

## METHODS

### Planning the experimental procedures

Microarray analyses were preformed on tumor samples from 17 BC patients treated at the P.A. Herzen Moscow Oncological Research Institute. Of these samples, nine were examined at the Institute of Bioorganic Chemistry (IBC; Moscow, Russia) and eight at the University of Lethbridge (UL; Alberta, Canada). All tumor samples were examined using Illumina microarrays (series Illumina **Human HT-12 v4**). On each Illumina microchip (both at IBC and at UL), we investigated tumor samples and the control samples from the intact bladder: three at IBC and four at UL.

### Tissue collection and RNA isolation

This study was approved by the local ethical committee at Shemyakin-Ovchinnikov IBC. Tissue samples from malignant tumors were obtained from patients who had undergone surgery for BC at the P.A. Hertzen Moscow Clinical Oncology Institute between 2009 and 2013. All patients provided written informed consent to participate in this study. The consent procedure was approved by the ethical committee of the P.A. Hertzen Moscow Clinical Oncology Institute. Tissue samples from non-cancer controls were collected from autopsies at the Department of Pathology at the Faculty of Medicine, Moscow State University. Both the tumors and normal tissues were evaluated by a pathologist to confirm the diagnosis and estimate the tumor cell numbers. All tumor samples used in this study contained at least 80% tumor cells. Tissue samples were immediately stabilized in RNAlater (Qiagen, Germany) and then stored at −80°C. Seventeen samples from tumors and seven from normal bladder tissues were analyzed. The mean age of the cancer patients at the time of surgical tumor resection was 64.6 years, with a median age of 64 years (range 48­–77 years). Six patients had stage T1, two had stage T2, six had stage T3, and three had stage T4 BC. Grades of disease were G1 in one patient, G2 in three, and G3 in 13. Nine of 17 patients had recurrent tumor growth (Supplemental file 13). The mean age of the healthy tissue donors was 42.11 years, with a median of 45 years (range 20–71 years). Tissue samples were stabilized in RNAlater and then stored at −80°C. Frozen tissue was homogenized in TRIzol Reagent (Life Technologies, Inc., CA, USA). RNA was isolated following the manufacturer's protocol. Purified RNA was dissolved in RNase-free water and stored at −80°C.

### Gene expression microarray experiments

A total of 24 tissue samples—17 cancer and seven normal bladder mucosa specimens—were selected for microarray analysis. Total RNA was extracted using TRIzol Reagent and then reverse-transcribed to cDNA and cRNA using the Ambion TotalPrep cRNA Amplification Kit (Invitrogen, USA). The cRNA concentration was quantified and adjusted to 150 ng/ml using an ND-1000 Spectrophotometer (NanoDrop Technologies, USA). A total 750 ng of each RNA library was hybridized onto the bead arrays.

Gene expression experiments were performed by Genoanalytica (Moscow, Russia) and the O. Kovalchuk Laboratory (Lethbridge, Canada) using the Illumina HumanHT-12v4 Expression BeadChip (Illumina, Inc.). This gene expression platform contains more than 25,000 annotated genes and more than 48,000 probes derived from the National Center for Biotechnology Information RefSeq (build 36.2, release 22) and the UniGene (build 199) databases.

### Source datasets

The signaling pathways knowledge base developed by SABiosciences (http://www.sabiosciences.com/pathwaycentral.php) was used to determine structures of intracellular pathways, which were used for OncoFinder as described previously [10,18].

### Functional annotation of gene expression data

We applied OncoFinder's original algorithm [10] for functional annotation of the primary expression data and for calculating PAS scores. The microarray gene expression data were quantile normalized according to [21]. Our approach to the transcriptome-wide gene expression analysis entailed processing of these scores using a scheme that considered the overall impact of each gene product in the signaling pathway but ignored its position in the pathway graph. The formula used to calculate the PAS for a given sample and a given pathway *p* is as follows:

$$PAS_{p}=\sum_{n}ARR_{p}\cdot BTIF_{n}\cdot lg(CNR_{n})$$

Here the case-to-normal ratio, *CNR_n_*, is the ratio of expression levels for a gene *n* in the sample under investigation to the same average value of the control group of samples. The Boolean flag of *BTIF* (beyond tolerance interval flag) equals zero when the *CNR* value has simultaneously passed the two criteria that demark the significantly perturbed expression level from essentially normal. The first criterion is the expression level for the sample that lies within the tolerance interval, where p>0.05. The second criterion is the discrete value of the activator/repressor role that equals the following fixed values: −1, when the gene/protein *n* is a repressor of pathway excitation; 1, if the gene/protein *n* is an activator of pathway excitation; 0, when the gene/protein *n* can be both an activator and a repressor of the pathway; and 0.5 and −0.5, respectively, if the gene/protein *n* is instead an activator or repressor of the signaling pathway *p*, respectively. Results for the 271 pathways were obtained for each sample (listed in Supplemental Dataset 2). Statistical tests were determined using the R software package. The AUC values were calculated according to [38].

## SUPPLEMENTARY, TABLE AND FIGURE


























