# Supplementary material for: Novel robust biomarkers for human bladder cancer based on activation of intracellular signaling pathways
Source: Oncotarget. 2014 Sep 16;5(19):9022–32. doi: 10.18632/oncotarget.2493 (PMC4253415; doi:10.18632/oncotarget.2493)
Supplement: Supplementary file 13 [file oncotarget-05-9022-s013.pdf]

Supplementary file 13. Patient information and characteristics of clinical material.

BC sample ID	Gender (F/M)	Age, years	Tumor TNM classification and grade, G	Primary or recurrent tumor growth
2	M	60	T1N0M0, G3	Recurrence
3	F	77	T2N0M0, G2	Primary
4	M	62	T4aNxM0, G3	Recurrence
5	M	58	T4aN1M0, G3	Primary
6	M	57	T3aN0M0, G2	Recurrence
7	M	59	T1NxM0, G3	Recurrence
8	M	48	T4aN2M0, G3	Recurrence
54	M	71	T3aN1M0, G3	Primary
55	M	55	T1N0M0, G3	Primary
56	F	66	T3bN0M0, G3	Primary
57	M	65	T3bNxM0, G3	Primary
58	M	75	T1NxM0, G3	Recurrence
59	M	72	T2bN0M0, G3	Primary
60	M	71	T1NxM0, G1	Primary
61	M	63	T3bN0M0, G2	Recurrence
62	M	60	T3bN0M0, G3	Recurrence
63	F	74	T1N0M0, G3	Recurrence
