# Supplementary material for: Novel robust biomarkers for human bladder cancer based on activation of intracellular signaling pathways
Source: Oncotarget. 2014 Sep 16;5(19):9022–32. doi: 10.18632/oncotarget.2493 (PMC4253415; doi:10.18632/oncotarget.2493)
Supplement: Supplementary file 1 [file oncotarget-05-9022-s001.pdf]

SYMBOL	Normal_pool_A.AVG_Signal	Normal_pool_B.AVG_Signal	Normal_13.AVG_Signal	Normal_43.AVG_Signal	Tumour_55.AVG_Signal	Tumour_56.AVG_Signal	Tumour_60.AVG_Signal	Tumour_64.AVG_Signal	Tumour_66.AVG_Signal	Tumour_68.AVG_Signal	Tumour_69.AVG_Signal	Tumour_72.AVG_Signal
7A5	148.497497694754	126.103561089901	148.13657736444	149.091925032548	145.530904040477	146.143941384453	164.659092629962	154.193086881801	133.802898895457	146.944628763232	130.390722272955	155.883386742315
A1BG	142.77817647952	162.484777758676	160.27511502306	135.934976183762	146.171094379173	141.779801021888	162.586012723045	138.824082857747	140.163936299053	139.609751976657	145.625205055601	145.442914818456
A1CF	140.775447163954	146.311966562883	131.972669163481	142.902838068479	143.569894807386	136.244995503799	138.017249996128	134.060214479265	143.785769607842	132.164132609317	137.27298184582	133.150660840801
A26C3	141.308299074832	140.510889880872	141.609037401299	143.447768653042	136.754116864655	143.13304460184	138.22823004719	135.771423315597	140.622130978368	138.82269002406	139.159510392614	132.996622574111
A2BP1	128.604270382206	134.527165454543	132.741859397511	126.482660154852	130.249430984172	126.180714796187	126.566165822427	129.455347273068	130.114464233633	126.531502877517	130.564962937297	130.874283538818
A2LD1	186.156006020985	170.941404165185	143.772376623996	170.590817818414	303.724140156439	173.790848780011	172.521737547052	162.085813676373	204.770742504612	166.606522485953	176.340265963032	212.5119818331
A2M	7699.37963536892	7992.84111657183	3515.36256599555	5361.3600315322	565.609400092543	1810.68433539154	605.583144451322	2698.5455305009	2723.55722258794	505.404271675438	619.75986112089	744.523059417351
A2ML1	103.09089100768	122.460829835477	108.404733392914	101.316813921703	121.067907968175	113.222264855561	122.996616773523	170.376620730856	206.35895739543	1880.35608492553	158.002906093899	109.599589420575
A3GALT2	129.487458179949	134.758752741831	142.677386111317	150.198869833614	135.127041919869	143.772376623996	152.207601283271	138.737332306848	141.703410265546	150.756776447358	124.772219140795	135.99579763634
A4GALT	366.408565514304	336.212224495877	249.339449789861	371.00603789759	515.327420453564	140.664303738113	545.566596665916	162.082166316002	340.193863653875	277.406445570925	357.972320648217	130.574019477669
A4GNT	156.71199811686	136.504438803108	151.627496656808	141.047709861055	165.737618983611	147.038700869156	145.82249684619	166.341564850299	149.962143642332	134.201392031199	169.220974415101	155.069048442532
AAA1	137.06192448185	134.224781719944	135.105147647223	133.682715540066	133.906867716171	132.741859397511	136.77666893627	134.452938571435	128.318545300038	126.728375196449	136.436762791912	139.870306695324
AAAS	196.16056297158	200.506615977654	207.718619151226	212.312154721246	252.514569129392	148.424970450231	175.442400672352	179.060189557496	180.986305037222	167.764352429391	220.578790067967	175.857952663383
AACS	162.327039624723	157.155105022743	149.999838961906	152.654938328424	231.314944217099	234.661864595717	195.16856673253	178.928873342925	209.487240895674	327.324711245289	189.840366340622	219.441959146815
AACSL	179.389797867379	161.577683729655	155.07594431078	162.06940121135	149.832456450186	162.259941098076	166.040918339519	147.54950981909	158.14434287956	185.661496988658	166.835922279372	169.612203020686

AADAC 152.818046945164 143.607579034109 154.011708696954 142.976123206776
174.717158790668 237.171424222756 149.622695843398 198.863347487219
147.218415065692 143.338628928675 145.892876412762 133.268239762624
AADACL1 232.276667029184 230.91747428831 246.989526028884
233.464856303761 309.798246248863 293.986292438714 283.191303666907
352.22815095475 439.727046291381 257.5097052677 315.863232876382
332.798130757632
AADACL2 157.010145291837 144.259643132933 155.290648078877
148.729753117861 137.549175595441 135.536810684197 140.916876460413
142.261639670072 154.466614452651 151.09974848374 146.129805607031
149.615585907229
AADACL3 119.057157701097 129.340996658802 140.020294940544
173.408784502027 132.906201428703 136.482609639417 133.667852983262
148.388093331273 126.247378598596 132.087183382523 115.80656409185
133.404671629495
AADACL4 140.330400168187 120.686906898624 138.947760022173
124.134916773123 119.049388179929 120.428171428188 120.201521341263
114.826501559828 129.121598974852 123.926293128246 139.293775885106
120.77691423934
AADAT 190.809584998617 189.307823427362 242.037132958656 185.154876233931
155.53992663898 300.358738353443 262.349315496653 294.653534388707
202.622747952763 144.727773039491 187.893420010787 271.989060206794
AAGAB 431.413030534188 427.637083424453 326.055727207966 368.879825610754
434.792010353541 503.757797957538 508.127470400078 400.23671843715
419.4017188518 273.61337724143 525.287367366192 603.906237227931
AAK1 186.268881719287 172.583615020042 185.071775840607 163.885645883066
236.292053621233 247.236004734567 202.524668687686 206.999289979742
189.0608798771 619.995008098949 212.959246855759 205.341121539273
AAMP 480.101253056873 470.578335285993 483.174262289559 524.725307261999
600.162650426961 400.98290237416 565.059609779245 415.113340335205
563.490027149224 429.268202514543 528.767644181119 490.464012303239
AANAT 110.024815508703 110.536316697732 104.780306305981 118.21084414402
114.960460615926 105.925737068775 122.089332300031 115.80656409185
106.393497890486 114.378420394004 120.497796762933 104.154182930018
AARS 3019.57419253979 2758.01526530661 3083.98169220838 3219.27851139047
2648.41012401723 1382.12750708801 2794.89048827025 1637.66306286151
2715.55326746591 3033.50962589321 1727.7144253077 1336.75600604068
AARS2 283.163770658619 289.21739941764 263.47760086159 328.33387481068
290.867822301134 242.221324619171 408.758021864761 287.703569453502
416.933146258428 280.127735578097 281.44159425243 299.935166870211
AARSD1 561.062594540005 619.635734567779 589.409235971915
779.623566217277 325.642275889649 461.229315272354 384.566987097657
620.760947818536 461.413469623811 365.140886302762 301.369680426563
369.275635424092
AASDH 252.222481637238 230.053292316517 269.853718252574 222.156471143857
221.18757533703 231.769926214655 299.513950278952 273.353254857377
265.712482648121 225.4575611466 224.981439707657 198.925776755081
AASDHPPT 284.715092831843 265.110264596425 309.283897272661
250.885949055834 254.517135747445 319.685597963267 412.648926259488
326.055727207966 300.602422224047 409.592855687405 364.331860640225
419.690542859168
AASS 214.295260811046 184.223623297663 227.092319765009 191.38608906576
158.74180418577 164.389447533163 230.015981563049 141.181931072161
171.130215858803 187.069074575776 164.332073056902 158.826907233022

AATF 401.267425721838 435.037306125887 468.496972957503 548.014025722956
651.43207839949 517.42417250172 685.354305381323 818.761934233826
596.3549500121 413.398641688413 616.014975366684 442.334886941795
AATK 167.164675439456 162.10411532202 162.819608252826 170.035994634371
154.227433553131 156.123528587917 170.202597821861 170.636731478608
154.935741493562 159.154102271517 166.863589702453 166.788336808924
ABAT 126.256716138724 128.533900744619 134.640783050966 130.735223763219
160.892163220381 172.783430414523 180.867208892996 131.308384127079
152.952479729682 128.289680999565 252.593574250965 183.26554404668
ABCA1 2135.04382065446 1742.84803427086 2267.0818302379 1619.67820476414
1179.42590997099 378.037190077449 1169.43742964112 826.314416168552
1736.03342040646 412.129826478453 920.083835296898 365.441289363381
ABCA10 168.783725505491 163.074919710259 173.951605889009
159.0794075765 131.532660547328 145.654559623732 150.132420238581
162.620593985693 161.481227197715 135.982797108848 163.102043862117
212.591357254196
ABCA11 156.916784141484 154.746081871718 155.268650584431
154.598520288855 133.138744413911 153.328585916625 154.524588318927
164.905467905433 159.320692108734 150.203281104753 164.991522994763
175.469375624274
ABCA12 114.084543168359 125.025064237439 116.5272207025
115.002176041974 116.514299377365 114.790717658188 115.491262364277
114.334059060506 121.144174389418 170.233084517898 131.114995025516
112.666812944192
ABCA13 120.170269310738 115.649568330705 107.221547998974
105.712302595131 138.680447175582 115.041324824728 112.456644814178
144.642717750351 118.578205738502 220.098071755353 138.116568200293
111.10737497653
ABCA2 174.866036336378 177.862368731138 153.271518348258 191.029458520006
188.062732621031 127.04217189075 142.643027992419 135.507247740621
131.302225305148 141.470472968171 182.969563278473 147.369111678068
ABCA3 263.448958595597 301.591430919567 327.010503005631 313.412131722189
237.641190133427 156.702300398105 163.904137395349 179.432235153344
190.031339775209 157.895460674072 156.128116786072 163.141010815086
ABCA4 140.113334247294 141.331397551086 151.026905311756 143.576177625265
155.214638707046 137.510586346207 132.195666100325 132.829401072883
153.276938815536 146.21362402936 154.594339065082 131.468537312778
ABCA5 131.22421475185 130.2867846895 127.924195488323 119.9652990395
115.725323816619 134.053685488055 140.222121251146 126.599418127637
124.025104622631 132.344296085487 147.132625797946 131.965184111199
ABCA6 328.680312364877 289.037033338975 330.405026390912 231.955750250711
144.387375194186 166.582931148846 273.709160508205 252.280435525505
166.842970309527 152.933230159216 442.334886941795 168.582192754233
ABCA7 155.011931456736 165.143417235314 144.468333012278 148.038291195414
179.96623248843 153.398587738216 146.559502785264 162.61434899634
160.499355902773 180.392337212428 164.022021048626 146.897528834303
ABCA8 473.60579090007 367.825328832492 351.998551165704 320.936905584424
140.820807603291 202.887417145119 158.308558656144 174.698724064671
161.700258550244 140.485102148597 150.339554736141 155.997210529742
ABCA9 230.399207047948 193.124599671798 224.62335524729 203.563098303937
138.633019006784 164.690739760426 156.433603187722 162.033909093358
158.499787920522 152.939759795631 198.783034238774 228.524222740306

ABCB1 166.173429365582 159.603586227897 127.842177770276 134.928967716193
133.223310036542 140.965498009244 129.532122459128 143.290642777702
137.936605651724 133.944538335319 124.513891912986 130.033927910392
ABCB10 292.524552445564 236.164692035094 344.575967665039
248.690121805116 273.353254857377 429.096075419528 329.82030842286
354.705553237406 282.561381978659 303.380479700334 258.998065427002
342.935878735854
ABCB11 147.526701500887 151.159145294127 157.089650890944
175.055634340766 149.173121214851 161.281195929918 141.418737063013
178.966169998125 160.667815093292 161.922462797736 147.178598908199
158.7912155022
ABCB4 165.763459146818 153.585849286113 159.39197109516 152.987939791009
132.407736899761 118.039780360151 151.09670688533 123.042372128864
143.458937009197 142.558109812638 112.75167046116 126.595668758744
ABCB5 98.1433670546536 105.900294919664 109.242622591321 110.553759711075
106.066897223031 102.024765473404 110.678680817471 101.713609383766
107.520000793184 101.761615925097 107.884870799936 105.976284291356
ABCB6 473.057488824363 478.479988875208 602.371843434907 618.437282203498
424.025588636796 271.615621030842 507.95331185029 256.687384001447
241.039766283972 330.457534783492 402.366797969833 323.788197265954
ABCB7 453.875259610173 496.328898534802 368.879825610754 708.268962600305
325.011269531407 303.638516587721 394.65807885612 238.978845152474
300.094509257518 511.670320118232 346.118765910093 336.279209908669
ABCB8 133.079767347356 124.347680638586 140.951031275943 131.575356402693
163.373180928181 142.984243805285 149.563405421366 148.012353232918
163.239195865198 149.091925032548 131.679566454851 135.155955441604
ABCB9 201.893722965557 222.526610842047 180.231462528702 296.75326294587
417.731815310276 272.351487980415 263.888891822857 251.787648609546
230.399207047948 214.132445321197 188.20675818131 245.317436234586
ABCC1 163.558869203898 155.507030276749 150.409919207059 150.105503384881
154.792156982219 168.443386067427 160.822287037366 157.963634535239
158.160604786093 152.411545237204 155.878493935166 155.041173289759
ABCC10 172.319604146201 178.819486867379 168.156002330441
188.891032176388 260.489744360233 184.041062308263 171.795878750365
153.748035612441 153.551359970644 163.340435089911 190.5930909277
201.931760074628
ABCC11 136.041605158822 134.619470709414 135.271290872348
133.505261458701 133.879079395215 136.891612156271 134.688504981603
141.259186690966 136.332399354517 139.734784859495 127.023543953724
131.827899207029
ABCC12 113.980716384814 124.302581985661 115.613348109569
115.30786313391 125.231476412101 116.678969343492 114.841964705981
119.060186838239 114.504409331018 114.056147008769 123.351112976814
114.44610607807
ABCC13 132.971844952199 139.435000959919 156.417000271727
153.033582154882 128.578494940165 140.012295249756 133.397734409167
131.80257432775 140.857793157932 141.169908353507 140.125575976708
136.614620534169
ABCC2 148.564655847496 128.259909378964 126.806537241253 129.985797334276
112.534864678892 131.715579653796 128.52473663996 128.630613404354
108.483284007316 131.954310449801 137.826458479398 118.134614195753
ABCC3 213.468420853218 229.881164774875 226.35854874278 165.960176666275
731.284098301795 285.980446338971 573.074116810032 1294.18114559128
959.126054626181 226.919545367682 719.369561410372 1235.75946952752

ABCC4 315.863232876382 299.390443444504 300.358738353443 271.791126011275
222.605195861218 813.454826901794 665.106631364961 270.201322204641
325.859917318516 203.089661928125 724.777166238211 697.97116981445
ABCC5 337.487980551006 357.140791982825 334.709049890042 320.390351199433
274.01377986185 508.449904285771 521.215655189324 715.43295523922
423.152420192284 302.637859140061 1114.10687039922 324.25775261173
ABCC6 136.218750083407 140.421945574721 139.675292022085 135.485867373592
140.051904905695 145.768857553634 135.675570204709 131.326536951329
138.266328639467 138.081268025435 141.408545519654 137.652358744527
ABCC6P1 149.483826392464 128.26423663004 147.371905597494
137.354782768068 143.891725249061 141.86370253437 159.012011848781
146.112388101621 138.858138379065 141.712693059287 163.331424612891
142.598106777707
ABCC6P2 144.316475814221 140.946008063839 161.225608630791
140.115154069602 148.000706956532 164.736294372006 142.802965008073
155.333294650676 145.937733795336 148.224587582189 162.893396525252
161.8627732644
ABCC8 125.593595066736 138.393565473857 111.276543583667 126.553693803748
112.14768481333 107.580425848009 111.837468699281 105.62620788923
110.234403581151 122.251178920649 111.204065131669 107.365390754133
ABCC9 153.118492567137 163.579282884905 165.942761337382 145.095023408224
136.263658288289 149.127963327999 138.664401460739 143.784022884904
162.026757124868 148.158062155247 147.338615809214 144.585120811896
ABCD1 209.596792486876 247.806371601734 155.150904036206 336.70893120135
371.941204080246 118.740346254406 141.78867917709 139.989830921287
165.504532455205 136.254966566424 168.111124820968 128.683044953833
ABCD2 132.649409216699 121.049927973123 131.215202712283 128.669887479835
122.642077625035 122.989642938474 123.96665768708 132.767474034305
123.13812550459 124.679444852938 110.925532078514 130.782296492278
ABCD3 228.675099004411 243.501083338279 241.251507026982 204.057019122032
494.538861626895 979.019125719101 472.793332506369 474.14051635531
888.04996477314 242.096692715029 453.875259610173 329.604568691377
ABCD4 131.984467471242 138.263361518905 134.182902752156 136.77260462185
135.673080643346 137.52653395793 128.700516033555 133.63022526522
136.121873179928 135.705904966172 141.917318809044 134.904123162692
ABCE1 335.239364345057 277.129985959184 300.985037429772 229.017256104437
317.12120265631 936.221322010828 465.029298672645 602.703887178113
654.881990232975 547.516635695872 441.578261054602 609.309339417625
ABCF1 925.009157785129 1060.24125879951 968.548243081111 1201.82026045778
1013.60354455825 943.677679525947 972.468110850466 922.877217016704
1033.03719406038 865.435843446989 1046.13353853089 1000.91520225219
ABCF2 172.077522197357 174.255919030841 172.319604146201 195.019706886336
196.16056297158 220.047563627558 195.618369827396 218.839681873515
181.771654391653 233.71520737817 168.841313096346 205.821101661022
ABCF3 152.323927317575 165.219393390909 134.801507994667 184.694741621812
251.472556189141 164.236417852165 181.578228610334 152.577131215027
173.816408146725 165.131463797217 207.453490385508 155.247452081206
ABCG1 178.377461551319 182.929326147069 174.903798946103 168.753660955229
260.356834114611 157.13859713415 367.567681162661 179.451637129672
242.695286989672 285.172001915966 395.133831206474 197.427788552936
ABCG2 130.208619015193 126.841836614341 105.096927149239 106.923686318112
114.209249333221 132.576755070703 113.427848757112 107.32960937124
122.905225081965 111.867464669155 138.149037534929 119.161972571516

ABCG4 151.325068155715 155.883386742315 159.43317827517 162.57736365619
151.196713920345 176.516540713888 150.544779197947 171.350620456505
159.563866056798 164.87433908877 162.439052954676 167.912321820083
ABCG5 142.493119780028 160.234704406302 142.329137800558 139.258468432364
135.544048708523 137.429832103492 133.930997975106 154.904424749528
148.435419356331 148.807422049327 133.272540012108 147.264690436042
ABCG8 154.594339065082 175.128935809466 141.094578556895 159.724824188037
182.02250582556 183.493455947795 161.094103878416 150.354401669138
161.870389635266 148.654099017805 148.42845062376 152.933230159216
ABHD1 159.26101905957 141.606501859513 130.018928906449 137.959775678716
147.617354114584 135.691862612951 147.932875151331 145.97512573326
148.446949168794 130.551216910933 147.314687795555 140.204023682096
ABHD10 364.766154056243 444.374521095495 359.256193328366
391.500696444149 232.918330930051 396.611050163857 258.036064876416
235.372592051464 304.479999301061 195.384273821324 265.536429548886
241.527811817146
ABHD11 142.771962099818 141.226901872221 146.058477355527
150.431052959951 171.86363382331 150.82596027483 259.146673652222
248.761038207278 159.257221624668 148.611352905643 172.175584707792
246.049729366987
ABHD12 263.47760086159 283.874600697211 253.463871742303
258.109876262342 673.824770556885 1055.22233969473 498.05778289794
425.229044880287 1043.14253624883 272.968792195442 744.808064061459
451.380376296813
ABHD12B 149.267019532847 141.484730939562 147.716353308549
150.841261270382 145.481279504679 141.306418675404 147.393178410954
152.142909854773 141.972403120632 141.987635042691 137.832597613423
140.000880942015
ABHD13 145.142331981651 147.261153294747 148.993620368781
148.825790628891 144.472969298036 163.413365060063 142.114181233875
148.391885541349 141.33919297402 163.621658579697 148.143367490426
168.560093633607
ABHD14A 372.088129459528 380.821355948507 337.652945958921
386.544913399837 1228.89116158868 297.862653495715 473.60579090007
324.401611136662 334.316711893654 282.47352481715 441.435444003241
434.325609889068
ABHD14B 256.924134409998 284.534369213195 287.334035234496
267.422232051733 321.25076864104 183.993497813128 162.047249964859
176.830879422227 182.698275130489 160.32812671237 326.83982672544
202.343343669937
ABHD15 210.015199832609 203.888365494623 173.773876769683
188.194202408999 485.731287873723 322.956882599715 319.639103463458
492.207833630086 306.773350448741 186.975609955892 336.898462056187
278.737944234055
ABHD2 152.970079142629 148.532339759414 137.361967082029 140.229267364503
204.999796322451 146.470444212748 157.530573508003 183.464897811105
186.185055364718 156.581569063195 173.325724300712 154.537240112989
ABHD3 146.309171902112 135.613240463445 151.382199195953 112.590379042108
186.961638857905 185.071775840607 159.762733951454 329.068463281182
218.08515867102 234.339913501761 234.156713102803 170.67334244323
ABHD4 237.249949461085 247.942904862478 233.809899915394 285.980446338971
218.896760804208 141.666394024363 147.8838487685 151.755205401737
181.453575300661 190.348359536057 187.645390502607 149.303427221111

ABHD5 178.464811133 176.47556448977 149.805955402639 153.922713834296
431.546135503733 446.698665191047 655.431236290512 346.476121089685
306.333710880618 376.962937292973 362.899103657252 375.958347293549
ABHD6 313.322307299463 269.379272725008 308.148212607944 310.23294370018
310.426513884979 233.394506254045 220.791884858764 216.805365763069
267.800523478098 251.011148303246 250.003965480779 198.15257384667
ABHD7 182.357065848298 173.592875508663 216.582862850259 164.253219197128
186.563913311757 334.772648743153 181.622689471026 214.413674287972
197.326259802349 160.469645948225 160.026178505749 162.54008293513
ABHD8 361.904370514991 370.555627307623 377.27961559752 544.669441360594
707.715412321304 280.848426993226 340.728888109597 368.711718874343
245.082816415008 215.532132200821 244.054469051058 271.713695056313
ABHD9 133.71965790848 147.436408919939 120.460433297007 121.04331582508
584.548578914846 165.851677392967 535.758943850734 179.358460170348
353.774297577869 136.71484425521 239.474937674583 220.169099923628
ABI1 333.270203737291 307.363302726074 360.756455184184 302.219514290905
324.346723939622 425.708048519408 316.722160860531 378.308562999445
334.350011399462 356.699466789374 440.657747083721 431.799795646414
ABI2 208.363905749882 186.359770878343 202.44778603643 190.22021453133
183.853381322135 189.142295015168 181.098584949344 185.896304740844
179.376968970326 185.984938124891 170.035994634371 189.434597063058
ABI3 133.427561208907 167.586897846525 125.547399456656 141.181931072161
166.592366578462 139.508029141287 127.663578933727 160.290104492275
181.79418355897 126.216519704249 174.690052785405 127.382266838772
ABI3BP 1658.78888936164 1505.90883940934 979.761537450366
1610.38263368771 132.661036963636 147.630900093277 130.143953880391
192.418895349837 238.124340832815 133.499361793303 148.720181793879
152.28262533486
ABL1 837.603727062008 976.360310504918 778.846928848361 1340.79810963983
364.022018936132 203.693188114912 199.776140037612 222.118666699301
251.268669431283 274.316982298975 371.280926727395 176.912121142015
ABL2 149.721351307304 156.597497458992 142.119327380686 141.233642252649
142.276853170778 147.450681897766 140.085359251038 141.913001976093
137.975369568585 247.145700155364 141.36525744062 141.710076413441
ABLIM1 814.759191652981 743.510369438587 762.8576747113
736.963699775724 268.568824794145 371.369895979832 321.977549744483
373.656487297238 333.270203737291 524.640879446426 833.430847243676
468.569725051459
ABLIM2 153.488903967311 148.054367166566 144.81077501446
165.752558888845 127.00963790814 139.39442529119 120.935863853101
138.712529531951 128.262195551255 140.856864690669 140.552143090027
131.228779762508
ABLIM3 276.434192872962 291.914408947328 175.437651670246
280.656087497806 262.691387377392 135.699583036042 204.851374931284
148.844916975586 173.751615672365 182.624130582754 510.972389503051
174.820064644203
ABO 117.740022708444 114.249891266241 118.04359489354 116.983869766097
122.452541100946 115.002176041974 116.787791109549 118.770061426689
112.742895297124 113.582654469735 133.587927144452 115.224011128183
ABP1 122.903428611669 128.236840746655 124.329980024986 128.150801169886
128.314990390649 125.739946257697 128.783262618114 135.188445928844
129.476648077532 125.280736217267 129.222714748528 139.444577046509

ABR 442.493108530222 455.21285026588 399.248044997427 506.562512021352
307.264586007015 407.442337662943 324.713347085998 641.12919339861
307.083179338165 325.43977732888 468.170972478717 463.187551361986
ABRA 117.834484996559 111.349642316073 119.355650892262 115.587874563785
112.936472036608 115.884253217495 114.902673141786 116.252227301161
119.810421687953 117.958297137758 134.811625739766 118.233024867228
ABT1 183.764215745794 201.507971833902 181.229308386808 192.153420834889
187.30908753142 202.97197145851 225.130011390856 195.831574717135
207.598754515294 170.505553609318 210.64483507528 180.599150102184
ABTB1 414.114051645565 514.463945278314 552.662610888399 600.430996779799
394.186683910377 182.861900982333 237.734385188542 228.524222740306
256.964577037118 245.153736298067 286.230656780335 274.736124442229
ABTB2 160.486513643798 160.573172150801 149.763038696374 155.147962740503
238.375823012603 153.574068503306 139.825088162247 158.890522392445
154.500326135559 162.624786423084 143.127795310829 142.22515098612
ACAA1 404.726926716133 412.344820823968 539.471166362469 426.649095732979
1932.39418448203 827.863789600981 1153.5175534971 1022.55215097491
980.67040349547 746.296354515617 678.365575489532 1468.50184641042
ACAA2 482.048928546145 517.23393716516 552.167699318911 574.399909181723
269.100400678077 399.364160668615 271.638179116269 633.762608454521
274.092048989484 157.035739033122 313.708197191587 313.536451935268
ACACA 533.279122416818 612.758976723556 704.152296359463 867.369315017411
467.187222441283 315.689865927834 571.950858678221 826.771587455122
828.276436883306 479.053300726301 512.895335481876 559.899430596368
ACACB 2124.12760841901 2446.31228564539 2437.63566094439 4016.35381420598
151.334463063946 174.840923772532 167.933231844271 236.052008824688
205.705962405954 163.215507313506 298.573180660083 201.748876422401
ACAD10 247.971366020963 254.359737178714 252.056131165397
308.877027622691 207.400490077106 233.272000923172 313.248029961291
320.154891655397 382.186095123659 211.34069504665 347.411500022053
293.12968694683
ACAD11 803.133619199479 584.199252444401 980.67040349547
616.014975366684 198.815448428294 387.403502892897 438.03648998391
484.658140651032 544.107739610054 591.89966781883 256.538195034956
436.982960786839
ACAD8 271.087125858775 226.287196839644 287.195290301363 266.842778974988
408.60092780174 223.453776362112 272.41397350779 264.628012152168
269.673122990604 182.861900982333 344.247544510881 271.638179116269
ACAD9 342.935878735854 432.726040933545 398.512947526406 518.478990211753
640.90860274227 316.409887372431 610.759447723026 364.964572587289
759.058176862524 418.772484676858 290.754210152709 473.529519031797
ACADL 154.324955232468 140.933444607646 163.290050020756 134.89031458837
138.106499370074 150.856917125736 139.13396769999 148.582022536314
146.916683989962 193.124599671798 122.125746529725 134.013748718539
ACADM 737.42784787426 737.843412273272 935.784743413185 745.153336165802
859.113541787323 800.013123376748 1031.59140059071 1252.28642463619
919.165331064155 703.587139938769 818.225731093346 930.704688414069
ACADS 167.521700144143 185.17273221366 193.04138438685 213.966288689715
351.934158805882 154.001258677199 192.737713851863 223.058699622386
188.567747377006 187.993907528473 200.603836348642 176.579096613849
ACADSB 151.826103919919 167.087850057037 142.625414022199
157.5032461329 139.648187461906 170.370934381883 157.643461899964
164.1572198075 155.737364431382 152.702847643082 159.874161198304
165.523150623132

ACADVL 1722.26954281876 1622.17491781572 1818.92700816112
1918.14260920544 1550.61855325662 1695.26858186096 986.972987865318
1948.24432908972 1276.14915305515 1550.61855325662 1167.26555140613
1164.22995893381
ACAN 105.132185249959 119.701313125748 105.606964521964 106.876902604214
109.266533255266 116.889373249017 113.163651806803 108.631224817901
107.395559299582 109.454483612299 112.922918499398 112.476111928742
ACAP1 173.577723566555 152.265624144393 128.105527466559 134.176106740247
135.146767090951 121.031695489398 136.83883055114 170.306241656131
285.46340356157 159.159106382346 159.276726310525 126.583342262489
ACAP2 171.561940747015 163.616115299926 249.029098902357 170.024833610695
174.928155956396 324.203537490975 228.499052416183 303.483515328621
316.409887372431 282.282663608264 197.848577663238 288.728608411903
ACAP3 144.544001385895 145.00699763203 136.781417098789 138.942901099402
130.657342262492 155.330215726109 113.927168436775 131.142841840284
142.819669471009 157.848456517169 133.642815510945 166.182850621447
ACAT1 1607.52029676299 1754.99663827472 2921.59274577877 1788.35884039549
565.313436777992 939.213447046823 487.799854351205 675.596864001032
576.873245313776 491.635435224507 238.724909152512 475.213525346293
ACAT2 193.674681547989 201.235056368151 223.765583953184 200.468804559127
252.655315526015 359.162549472301 341.761516859319 370.390323116113
384.051170099325 1010.05884008623 249.171307797736 384.365878895439
ACBD3 653.264945856916 662.781145703653 590.142817130684 509.702881232139
727.114956464515 793.891277937782 849.389818300305 589.953707188378
911.782783698439 866.413995755699 811.362219801362 904.211195986553
ACBD4 215.365809536522 209.44506543433 196.955723006838 245.240006198482
202.278323489515 153.154592981962 178.06221214103 150.091854035356
161.577683729655 145.041430440608 221.491633607126 165.192945734166
ACBD5 124.3501719829 114.556542069712 119.968081249475 117.231243105897
134.310685502031 141.141047405344 134.575671434471 132.418902141838
149.987571884748 125.855451679929 137.215025712057 136.124385992905
ACBD6 267.839640412797 274.092048989484 274.120234926832 348.98762709818
267.970798709017 220.816812192169 246.237243726748 228.797912452429
263.321559283966 280.990656342284 285.402072939954 348.506289278042
ACBD7 138.058404474114 141.075421936212 128.645334554749 129.618097261902
207.467591256549 236.707875629511 145.941674563997 167.212960569538
142.474762834848 124.652072110334 130.445920648705 164.917470819096
ACCN1 135.809383370957 127.928195199945 123.926293128246 134.537123332992
134.838366830668 128.79607493422 124.895023786422 133.382099379709
137.994334667126 132.541420804792 139.853333847856 141.630183595813
ACCN2 182.130970182242 193.444080047879 146.056723380149 205.821101661022
143.791908191551 128.306607216705 140.487887891414 131.095431530473
137.022366035333 131.007779278656 130.75707278606 133.911650164508
ACCN3 141.689210867136 138.414916506716 138.086270564406 132.793020446043
135.313148180961 135.807494668545 126.494665743087 147.297163685914
125.67874617392 127.171331062578 139.501279970508 128.351235762192
ACCN4 133.058788697693 123.336074100984 124.69564501518 123.563243360247
127.179420421981 120.485138937899 121.416038813102 126.501648842366
118.970693476152 126.662329971897 138.230197299262 130.355969431473
ACCN5 138.289361041588 122.165046954625 152.72717701452 129.051642626091
140.515996180923 142.281657284178 129.295956874012 128.077692981235
138.383183017683 153.435218661228 134.657787366869 141.597692730835

ACCS 363.539983343859 432.969886744613 509.512410867348 480.593729033799
436.482580413523 222.156471143857 589.722914993267 238.588762326319
509.400965222318 219.351080515508 465.926873285563 291.369577397743
ACCSL 128.637470206958 119.418866166776 123.766373146588 125.863969666806
125.655823846115 129.925548182204 165.767629164724 128.798413084891
119.669441891401 125.563646710294 131.190238038695 123.853588042292
ACD 278.428310548805 277.726756691133 271.823243396395 357.928880740814
437.770841893227 204.851374931284 264.563741039382 209.070372320593
223.128108720503 248.010317392179 224.67853219142 274.092048989484
ACE 136.468434653208 125.328704545727 126.370252616961 135.758133410577
137.161065320948 142.321476446203 138.586620636835 125.315736755156
133.152523054305 126.705446263769 129.346063085291 132.815753681623
ACE2 145.728370703165 158.325554470875 150.346002924933 150.536178574986
134.809348917858 143.458937009197 156.280701500421 159.508223180752
156.895079878556 150.46669393448 165.619206216088 149.8035588048
ACER1 128.245734702114 130.06068534054 128.669887479835 130.321133400634
126.22472822166 136.537117054998 131.880438747579 139.429008129291
127.753065449769 126.536245975137 129.132523684894 117.335748689217
ACER2 174.657352858049 182.478971050701 185.374745525698 155.056545639862
430.649188209901 543.584413650467 835.834013018438 469.930860750773
415.113340335205 203.852316712377 446.262895953004 323.667189206453
ACER3 201.480444219547 179.950904921667 194.998142472864 188.593378315122
162.879707592164 198.191669030952 186.722842989058 155.550532734718
140.002249873656 159.06002608158 188.593378315122 172.774914399844
ACHE 137.904786382385 134.606126134531 143.215828010442 133.994376985194
131.074021609161 137.584340154725 141.990639998829 133.111791289725
144.516274148451 145.311964807443 140.326012404526 141.094578556895
ACIN1 650.263737836685 650.679096367632 662.58366387795 822.88742008931
738.579262579782 422.338508598153 468.170972478717 564.588706307423
568.935113269368 542.544172683021 1244.67453201339 674.571475335107
ACLY 795.720196538039 728.176339659082 596.54904286299 757.406530000489
941.936830901588 1167.26555140613 634.774041584043 1778.73916100529
1241.59946353857 1022.11537745428 480.923468806193 989.77058497525
ACMSD 139.470557140547 119.263824982583 126.557869929234 146.231937458616
126.158204790885 119.866898356501 133.645044060866 111.515588041012
130.890314257211 124.568534983277 126.649739818781 124.436549041911
ACN9 234.182164952936 237.01460308334 274.343238288436 269.673122990604
528.650794915616 483.312894744803 380.310795921752 603.061485415867
405.130899553871 380.146996799549 290.037325939883 549.047065540118
ACO1 1793.75125318113 1777.29626660109 2282.94927629821 2363.50330960038
944.47112348734 552.494327858264 839.662936279727 875.7694907383
757.877066703516 1235.02626456572 592.957842630046 646.117381960716
ACO2 460.24741151155 479.761165832339 737.843412273272 551.206833852215
612.61438059983 571.838717184447 442.493108530222 497.269957926657
576.451964662024 485.576566972696 612.61438059983 579.678635803225
ACOT1 203.652936507291 205.728673153914 234.738241125899 301.49286558088
190.371287639453 138.047487233711 132.823333905679 180.561514455864
155.456077670662 147.176801913145 172.698181201849 152.857278986877
ACOT11 211.891710626953 232.641965649602 210.550995170071
224.335742510577 797.73902873919 339.379250332987 477.87583927675
515.929937825133 465.683139139622 211.438615793742 542.267204956846
551.086785904249
ACOT12 134.081308836364 122.447154928139 124.690141443832
121.661141741782 119.655162437465 117.231243105897 127.31283918221

	125.172189715764	118.122186617611	133.980792743661	150.132420238581
	125.732389000341			
ACOT2	212.78038645414	222.086635504858	283.807827536147	199.980312703646
	170.951009118578	147.211769989922	140.538020869886	178.835679834487
	139.47828235176	140.227863970629	164.181915898563	169.466129742085
ACOT4	171.071893074072	168.588120398433	153.126111237668	155.545904088857
	428.340060893467	179.55905250703	206.650760711745	335.47484353322
	313.248029961291	178.445384902129	281.570685561526	217.670330817769
ACOT6	143.814798097425	149.016767620428	146.007311764212	194.112534914308
	165.480489678822	144.026432356249	160.72962443421	161.234304237838
	150.933724024651	141.831596320865	141.235290792352	143.59105195427
ACOT7	229.017256104437	235.291035302211	211.395593720534	282.797367565702
	423.966625351913	311.848379951974	221.297127685676	373.361263985383
	326.325210657599	386.461076717316	208.869189492052	313.025250337353
ACOT8	143.086872363116	172.657668514226	163.764600217347	187.73896437823
	343.048904374136	214.100698312272	331.018914429561	190.255797389524
	200.536814571322	204.378245089766	173.635827398273	203.616580991715
ACOT9	446.194196064909	318.690043546203	372.94852645418	363.539983343859
	202.369905673351	196.777776509371	189.83433085782	231.439252571168
	239.827648772906	400.353795948162	198.681190016247	207.338645756456
ACOX1	151.961811748754	151.543678860049	164.529828080312	135.380208104863
	247.622351589081	254.133112176024	324.067396271281	631.653707589221
	546.755305058958	194.312315077985	304.985269874607	295.678097392123
ACOX2	595.120528902501	619.487800014979	991.611982265221	818.445501500731
	142.719216118914	130.616175018332	138.814930200619	152.14012587515
	172.345201438148	182.028675726969	128.435400481403	160.085325256991
ACOX3	434.176007498693	614.094097408035	374.033009122079	1085.38819961412
	408.444629429553	362.445813697676	642.934824227327	409.073045356073
	1650.77281891596	538.647888236878	541.460843251764	415.642099109855
ACOXL	121.965131419551	117.324368138398	142.603436693587	128.585911203394
	120.065876525792	147.865114383296	132.256047602234	130.625825024195
	132.210100574787	139.880733465536	168.867373925372	165.062129636008
ACP1	520.120193442648	507.236601481067	837.023434916281	418.319283832879
	895.463355957918	1350.87073284396	1666.80650105101	1870.52504853779
	1707.09830463606	1635.35544761095	911.782783698439	1527.87148052149
ACP2	322.028360309592	335.059844742031	297.071121022183	359.024299091145
	461.143361255467	215.698924670416	352.121800955822	344.298510200653
	319.812293134357	293.4021207271	344.45331938267	285.214585324341
ACP5	153.78045746564	148.452947427498	125.029516472944	131.263641389894
	360.185005480216	145.748174062159	167.724233915361	162.629257046871
	164.327140372968	231.287362732197	187.73896437823	132.277920991504
ACP6	201.517356610591	245.553138677866	234.399431238026	242.365480246106
	806.290662664708	288.88486628661	437.770841893227	526.970555181851
	383.802494922918	185.801540278924	543.710330214344	524.855915371246
ACPL2	187.407526616724	170.522853717911	174.881557472722	169.527424356913
	270.727749014046	439.928008927647	327.566026396726	518.17401959981
	323.841518852386	196.189101941489	237.825857080173	322.956882599715
ACPP	187.183762762259	164.274491958161	229.284931844047	138.712529531951
	211.369442150136	178.347708009405	179.687464522203	171.202766078337
	172.251953149975	217.531179216305	150.48236051337	158.610249594568
ACPT	147.248053518914	142.288110423886	146.698766621345	143.473523258521
	168.588120398433	159.06002608158	139.97636570613	131.696834332724
	148.838294770082	147.722646851008	168.491878435329	139.527577649638

ACR 125.187617901961 116.780639688465 119.11586630281 117.682034050478
142.607578275494 120.188885479773 124.012439490264 120.880547922392
130.235407859542 124.533543270311 150.380814008952 109.719356131043
ACRBP 143.458937009197 133.778595695007 131.110018165002 130.430917518283
140.142884879106 119.822587087968 125.060387518072 188.924084292126
168.560093633607 137.177271162749 143.116451998409 120.577995272053
ACRC 153.734347607222 148.790239150298 147.485960802692 142.440344960504
149.133055368147 136.116468624715 138.570150372729 141.264096014725
136.722636695506 133.697364081712 182.873752254411 144.779146672553
ACRV1 127.874934513708 129.01094835739 133.550008967009 126.629012619208
125.390139830237 116.625518828978 124.635917999969 124.833659452957
120.524859681034 131.468537312778 143.327730271099 125.467668442298
ACSBG1 147.87699690647 142.185903088074 141.541355091514
138.523677181131 140.234485088845 141.18490443471 147.088665779406
1148.80141486692 157.484541464879 146.812248482145 155.76568091161
367.732191251547
ACSBG2 118.708644971927 122.494779505958 108.768730405582
137.840396641618 120.241528853563 108.226917034591 117.119533897652
126.155486659114 113.769815606191 115.194730717797 112.133567992833
118.727279917776
ACSF2 316.955298948771 385.135014083686 292.366934378643 287.242697383558
890.507257588957 738.087203930249 1275.43768532088 670.347447480331
894.46029531926 173.315097944835 861.119262963015 377.462793982812
ACSF3 139.22543724535 161.700258550244 148.861141988806 149.217191121377
182.608712782878 141.537578081123 151.511808301993 143.756242843438
160.923897048848 150.971453664961 141.308299074832 144.685707282641
ACSL1 603.906237227931 525.138476928759 454.732029358748 455.392133846517
618.437282203498 454.64556253724 808.55123510297 1555.28458491244
1390.95426299323 802.017244099902 1484.32547474275 450.220658078322
ACSL3 319.868227988842 264.936739264566 348.506289278042 229.660838239474
394.810886122555 271.989060206794 682.075583857856 277.946395491073
664.025683218037 506.165100641525 774.442392607873 585.721610434501
ACSL4 259.575461599189 227.715558644326 241.675251479835 197.740912476527
169.544515294662 236.676029292922 160.281830914755 223.638850960288
580.859914041323 197.489903263216 210.316431648335 178.512206531256
ACSL5 188.231614694297 248.791254839869 202.943987922418 155.731897985566
285.507315916663 153.104072845043 473.73182675469 704.653073777277
934.173273294189 153.136863548105 1186.23169583595 730.042712083388
ACSL6 136.044670179122 120.606354515072 122.392159083328 123.586359806158
121.141432480683 127.398873370484 130.288547755599 127.310437599803
128.490341173553 122.300069045533 129.228874611916 121.45175516842
ACSM1 118.432307370605 114.308806726044 119.455615461113 108.760780994582
110.355493122568 124.39748786803 113.214390213004 125.027095330593
113.388450168062 108.404733392914 125.598828334465 115.613348109569
ACSM2A 124.980683327416 132.634538733205 126.841836614341
154.876638004094 117.455432751161 136.126542897364 133.988494850477
136.293084080479 134.932416370825 128.179293878252 128.702882993588
147.309245779031
ACSM2B 139.082897743357 136.236441543681 149.72025962797
144.40361834862 134.479828866339 150.343825300998 141.236939351298
136.55090238808 144.040342300793 147.255680306221 126.595668758744
152.432477504097

ACSM3 143.036006001419 148.816369706133 139.805930686563 137.045772853855
190.889832855403 148.825790628891 199.933148219784 547.367381020623
277.129985959184 145.056914913573 190.787437782456 157.776321544382
ACSM4 139.038389244329 134.42983422342 163.346402397924 136.267313223808
124.873289279767 148.627264102939 125.146558509674 137.367390177293
129.831076624826 145.120347147471 119.112063328364 124.277983696239
ACSM5 135.889764019762 149.974080577906 139.811873260553 155.558839375761
140.196653553673 145.892876412762 133.387065801837 136.188868917386
136.969124414576 137.803749343493 138.086270564406 132.433109154079
ACSS1 293.080122239022 290.914381066573 279.159925913207 312.845219991659
197.298982267459 196.470718183575 169.16155608071 215.931781291835
252.473612525884 177.802767772786 230.015981563049 219.318938396989
ACSS2 469.206346008875 526.726224327409 502.063888359427 638.287221212947
346.566241319814 382.630113452429 474.750156829195 367.732191251547
333.049612399093 583.938727139698 282.561381978659 314.292700455545
ACSS3 151.984619204523 137.234384760487 134.664652044646 159.52833930625
129.151171025294 160.997179739138 125.863969666806 131.755881982599
157.891350650633 145.560161803925 159.365070384451 131.447941902406
ACTA1 151.04846635538 213.674150322581 163.919426088318 294.653534388707
127.57284871013 126.061141885666 126.845048064014 124.170242736875
116.780639688465 109.156349683299 120.757288533244 123.884643288534
ACTA2 29868.1819370827 27501.6698286039 30573.5859783923 27501.6698286039
1420.27222765071 2649.39312662326 541.642309122976 3829.87905458534
5240.40795364202 4465.66734676111 1362.1486006778 2568.95060767776
ACTB 23427.4695172122 22750.0246358215 24377.1316448061 24731.0629964468
18953.8127741918 10075.6573898939 14228.7550296952 10953.6229702446
12654.3779830648 18562.671630167 11918.214537866 13217.7001749493
ACTBL2 134.758222337831 128.165775629664 128.0805889548
147.407093814154 147.829502959865 134.635645106042 119.472410874778
130.450930089722 139.526241501184 139.966813133326 134.576622528874
130.839508306116
ACTC1 7786.96143641382 9994.54490550759 7838.92787031423 14755.8570847826
129.754376995879 124.546520456419 136.220324225816 131.500591219916
129.529372508883 133.077760160486 129.819820730239 127.715766031466
ACTG1 8881.86480594052 10836.7380675792 9099.13956219312 9560.32907282993
15745.699731183 18760.3823237058 16925.9167115963 15745.699731183
13324.7465248375 20381.3859535625 10250.645112753 18146.8167991873
ACTG2 26522.0142925211 25295.5071561111 29532.7890259497 25808.3502428915
358.654569870431 1265.88446775977 151.364528898537 768.67030514096
1804.63762008613 938.499337889075 212.933062534244 585.234286698678
ACTL6A 369.974231530826 380.888728347647 502.499539192864
302.601458409102 676.570119607984 1461.64901688125 743.023225859813
1411.90426595229 1298.60552708737 915.015154168627 766.420275252501
1151.89087953837
ACTL6B 129.971670681199 130.753793881071 162.961792330623
138.215708514725 135.143532936828 116.566190779365 149.219602109121
152.56002779985 134.648780131578 131.001996509052 153.630125822178
136.042982529628
ACTL7A 114.835386789236 104.36042819601 105.859916438163
113.840813583258 111.373854978307 112.771141378159 105.164790181452
98.1433670546536 114.977135060317 122.828060462887 124.003914369077
113.018985316061
ACTL7B 140.260053206523 143.025570700585 149.839827383681
158.414776465951 156.704278982517 163.170983091116 145.273209648677

137.824985884649 132.899305777714 163.799748670842 152.188770978528
146.539196738195
ACTL8 120.714981493593 128.563180697606 109.851507510788 117.348231147283
239.31180635241 132.28931085483 132.864018034342 130.0140921487
112.509731206295 130.316282003711 148.530422089937 128.666652639862
ACTN1 7104.65816621275 7167.29945083872 7771.28016059011 9272.37780151073
1141.13648305827 580.859914041323 707.173691257322 731.848844821402
1038.93733638848 2473.22803972431 246.049729366987 705.737228226014
ACTN2 182.919385608842 148.255769084026 162.857083430123 133.186678625229
134.596391166462 131.056011738962 142.041992878641 128.217078449064
138.18056202179 153.721460633166 127.814109293214 144.077080621909
ACTN3 124.672483602517 113.077691687266 119.972337070176 117.992729826121
117.097159106043 120.456817031716 121.95061962467 109.46255295836
111.949644842971 103.36057518781 147.710252756854 108.362609680556
ACTN4 1800.25771851544 2203.82462320864 1007.41624313399 2882.51330563735
2915.72823797537 1024.95957828641 1125.28599595312 715.638378711433
1140.40904196731 853.535700321202 1205.45751308367 731.848844821402
ACTR10 738.579262579782 628.485648053393 828.683748921915
609.100385482325 558.810095726517 587.54075434249 679.557656724243
837.413261433363 568.555967950896 969.360864006371 685.354305381323
754.97177863997
ACTR1A 855.415038498392 1183.82018168635 1069.25265476373
1603.87451959083 597.952909577121 471.15333189869 495.655170027296
470.868288641048 514.213675499369 493.209107049493 653.749043828462
617.474181543858
ACTR1B 697.97116981445 669.731546951281 648.823677010146
676.570119607984 629.97342429259 419.763812610671 312.961981998512
391.185365567603 443.853458865556 387.908774438461 424.495400604335
406.54518900348
ACTR2 1796.26091584668 1515.38811502277 2233.00694575483 1104.61581891596
1530.45813438919 3883.05620256963 2856.14739820295 4465.66734676111
2080.47567326037 2956.63335018838 3304.82581034038 3253.76017336684
ACTR3 1119.71228054585 913.934874451961 1160.78730097715 989.396074034945
457.650982600462 1125.95664591686 895.114975754684 922.50743596248
726.731270955732 1165.95924778817 1281.1883621512 1213.06007305892
ACTR3B 149.019120812629 143.270153772245 155.964242240771
150.734560844391 166.570606752678 160.76044995335 148.866229889479
152.122141650397 146.75924072364 154.499313851756 151.855884026072
159.838626443912
ACTR5 176.355556618289 191.322056430728 187.352655464866 215.81995779019
222.565515020501 248.010317392179 233.103476996669 250.626556981376
228.926077117474 221.231455446574 274.595218795127 227.64196401771
ACTR6 421.459312180809 463.622673539254 472.030150842569 415.322560616847
617.185364180303 562.089999265955 667.892385395063 659.106229917309
558.810095726517 442.698000781597 123.344450694117 494.875547886209
ACTR8 193.008902902654 170.202597821861 198.925776755081 184.611146047556
180.941090529551 200.342786450248 205.4648759901 197.308952757609
227.587590278412 207.764456828813 194.388383297922 213.634295832535
ACTRT1 165.149464497921 184.801068553988 167.806420483187
194.686527131656 187.955617187544 194.112534914308 183.545741265706
178.819486867379 175.452986514346 188.924084292126 192.59376129133
163.463191619941
ACTRT2 119.950579480571 100.04568139926 118.32560379073
112.832122823829 119.131613164405 114.056147008769 132.398041172151

126.401145439668 122.223184326586 128.438401976279 125.050945917101
117.015097017246
ACVR1 1212.55338792557 902.822697602452 833.841469195516 783.373390305885
444.826775824095 533.893360199271 366.636754272029 367.644907862372
384.566987097657 740.973440818582 422.590112942156 571.838717184447
ACVR1B 180.641419603362 183.513101761782 166.014450044695
178.222120802125 235.407064742194 216.05364037271 188.580458613357
198.681190016247 272.16090053595 231.600532144945 212.016247864691
230.424145472916
ACVR1C 143.035245560484 167.521700144143 141.042265614693
128.005043478952 159.920703254935 134.838366830668 143.313605534609
143.336538157639 155.290648078877 171.410912120195 157.547814187442
164.332073056902
ACVR2A 278.555009982029 290.502085667398 359.363523948825
285.402072939954 208.816482659752 440.203336761263 255.945470052489
388.710465457313 289.517820913799 161.841975820372 220.710324965453
288.026656721827
ACVR2B 131.876061826583 133.593569062647 120.060776752215
137.927403204849 154.618685493541 127.789230402245 131.280092370361
117.856184272322 121.286130275542 123.689439578326 146.763046352405
136.067657949844
ACVRL1 476.641032594868 410.954095610904 190.645917992472
358.897128592042 220.047563627558 210.729945718068 180.578528984075
228.968779778407 279.159925913207 232.250434241457 188.348499229585
187.264411519101
ACY1 159.867178630115 207.764456828813 155.119146797226 183.193029399643
1856.05025952611 595.120528902501 400.621427274425 438.340341490622
485.731287873723 394.101052511043 320.877897822963 835.284130042504
ACY3 108.868679899494 114.812254477058 106.900679222135 118.000757530896
123.720229113779 112.949643549398 113.018985316061 115.582048644075
111.457176315964 113.799997627317 111.876915242732 112.252555403121
ACYP1 240.12078342024 216.181417552574 232.778488697337 233.997642139328
240.054164412429 250.037172478288 220.999383789474 332.194163016646
241.353916553991 287.593221911064 239.845747958475 397.985856484851
ACYP2 209.887047474042 224.027605834751 260.787104931135 218.031238893075
240.734451149197 238.548578086943 226.387465899611 227.375773052577
216.903719298575 244.925472495705 214.200081541057 265.61506544085
ADA 242.848821222611 187.183762762259 191.628693083169 245.082816415008
250.647644543963 336.120322555703 272.456310079164 176.579096613849
252.404931238338 590.849222581206 166.988725688589 215.219246394594
ADAD1 146.798444724408 128.594951429021 133.676112304498 132.681475162386
148.794293762142 146.390223079987 150.648444878067 141.895211025017
133.606068125828 124.922004609558 124.447971788029 145.78573175226
ADAD2 116.241531823869 121.619593453259 119.952108688414 115.878861991823
148.49560238679 132.411757518105 129.299810898979 118.430401197079
167.505381046729 115.80656409185 158.799917136119 136.728975205893
ADAL 196.615914367563 187.997940126066 208.095384605737 189.419941381565
155.124158030085 182.074569356713 165.244616507084 166.730974538475
162.466301774088 161.250520154112 160.251996476232 187.73896437823
ADAM10 166.879042308539 160.374491283254 201.931760074628
155.463400179872 178.885625009973 387.355661073935 349.623729591612
312.601510033941 663.668537495345 269.673122990604 353.285905782951
650.494111214312

ADAM11	131.747367613225 124.079745873727 144.529801006864
133.728378162206 142.712218120721 129.003317344469 131.611842804029
135.15791174418 138.776108280637 136.227202534196 170.18781660707
142.294922363188
ADAM12	140.301389592487 146.518478800271 152.204730003016
139.974586762939 140.845958602151 136.946137446743 149.035590153457
138.896595888876 142.605816057276 146.832797779597 141.374831983971
142.33269270703
ADAM15	442.12886967939 716.229989890801 385.888395433066
755.79669622705 4602.81651147558 1219.36024606325 545.362650746876
1496.0924333403 855.112210714686 1258.16519121331 562.232754247201
1102.38234645327
ADAM17	406.54518900348 395.588345893987 326.246702537465
398.571054759451 441.001837510132 445.214621023114 373.096629378654
345.832589037442 283.760131847707 358.217145707718 418.420753332232
309.098519056843
ADAM18	111.336798977475 94.3531468700382 105.952640312359
117.045020667394 102.661440700541 113.40684693417 114.213405341709
107.544568250154 110.651971902576 121.067907968175 105.450273343324
110.651971902576
ADAM19	858.020739710943 664.60530723006 761.89271251455
938.049312229961 143.313605534609 160.769011617127 162.247608931408
154.455641374384 194.584034984339 216.903719298575 164.603403251634
153.95855605751
ADAM2 159.961729971624 144.612549022264 168.744610747563 172.157660228549
172.245089970803 157.627490896581 162.274094333189 173.071892362758
153.200153031275 198.815448428294 160.543465620248 167.645930148254
ADAM20	114.749597426757 129.16337177104 114.589757401741
120.174672871542 132.133979108537 122.706176540192 123.893768254549
129.508990861362 120.702740607924 112.088944448944 119.586157804692
121.347339077474
ADAM21	132.70643154246 123.96665768708 129.502730573463
123.991249190444 125.609542142983 132.541420804792 118.697969266426
123.678365506247 120.413468119484 124.258412895333 147.818956338752
121.580726825432
ADAM21P	133.766199453456 123.959050197189 123.629279569085
110.355493122568 120.337241026338 131.468537312778 116.810827219571
125.729079191033 113.521599852465 118.684077097437 120.238706364491
115.975794003391
ADAM22	130.198147836925 137.468570135972 126.182856027765
140.402944266863 133.439077726411 113.852849454448 116.118745494794
124.511674561183 122.789656787728 116.759526220736 129.044445373107
117.682034050478
ADAM23	148.534143255537 139.714198638713 130.396593013401
148.272252953881 128.702882993588 134.522715664256 126.00304757051
131.899614022767 126.925982415892 177.103802332144 136.02474314035
127.211469322857
ADAM28	126.766964875664 130.092881020271 127.871445879091
118.75237770613 119.774204444899 125.752962155179 140.03443832328
121.508233749444 137.674025476238 118.855500322915 167.640645997754
141.03218895811
ADAM29	147.238896426423 138.707412596757 144.748564682545
149.52880714343 161.543072816738 142.338352884053 158.411846122784

175.437651670246 140.332045911008 160.786907816025 159.286484253487
164.078423844421
ADAM30 131.373012256275 127.657961561231 120.15332480712
136.843164021899 146.125125252096 144.479410859199 151.92638745703
123.972321838378 138.023752854109 136.77260462185 113.521599852465
124.676743480622
ADAM32 142.592455241752 156.47237266917 139.631165400881
144.781438651649 371.06697207449 148.936888662374 140.793616429174
140.385509382606 141.204458186976 130.116771999391 130.66863243733
137.335501759561
ADAM33 173.507835230243 148.353927712013 175.148318011395
154.433679792749 130.814702312493 125.600500146679 127.407418037777
124.324855155055 122.503984297406 138.43301474649 153.222807811478
133.413325516456
ADAM3A 142.268018184723 139.926488041421 136.126542897364
149.471240458766 152.032418787633 145.987228759402 149.37190049932
145.041430440608 151.04846635538 134.519697269443 138.649502040582
146.96155692518
ADAM5 138.875827888646 132.748478264583 127.990453128978 145.212306927438
140.933444607646 146.606442726047 145.291984088491 135.014118238255
129.838790698396 137.006917665541 131.702788057359 136.609461559853
ADAM5P 117.623103460369 109.282814458761 117.480506535399
112.456644814178 110.510191137073 116.700397932007 121.865696440776
124.74291205933 130.043904881142 123.668707648172 119.733311319912
111.73388372572
ADAM6 151.234397206563 134.029984991385 149.786445079456 152.007436762651
129.246066890078 155.824409540332 138.021559586328 146.481298877242
139.480361732772 151.954389699615 136.922282125029 131.17119492692
ADAM7 124.947565043721 122.472909598385 118.45291712775 121.423030266904
123.807693112932 109.74983281634 123.538037290702 120.831070822199
123.291258843371 125.739133257542 137.559204418819 144.220296819311
ADAM8 134.793399942417 136.532168520039 128.144653206398 121.877276961306
143.222342904886 157.528409294029 211.660148497276 214.396920524803
273.402804140541 154.710000519109 321.492900106953 467.04976953755
ADAM9 294.069353373633 228.616558999054 221.18757533703 238.344676327918
265.260411490697 427.390091157726 217.628802427037 177.772019227116
159.738700107615 332.666916002481 178.294972422671 225.820503820866
ADAMDEC1 133.158570377793 140.505338990718 137.500310477851
134.356796881432 144.692900548503 144.568417378353 149.427217345037
272.16090053595 229.660838239474 172.442311733494 149.8035588048
154.985781979984
ADAMTS1 1254.19980463817 1369.89073267996 394.892918166519
631.653707589221 214.739818213164 488.57622466019 358.217145707718
362.899103657252 274.77366169918 277.86338481075 297.228563607097
233.335353447342
ADAMTS10 123.159407368236 119.094868654252 126.097544650407
127.7962242731 117.47631318854 115.033355516602 121.36708642226
119.854401979951 127.921209207879 123.312000381122 124.857461801227
115.305518560239
ADAMTS12 123.361179535626 130.877306095063 117.539640414775
126.853028093579 128.756818133357 120.046892432373 128.36121178972
124.879018642505 123.051777473512 122.75516501062 140.318155161909
129.267923706188

ADAMTS13 128.116394188709 126.401775332213 121.30744400137
129.984214994811 127.015086299437 126.784243009941 130.06441029462
126.310788291075 117.639216182057 125.810756286648 134.262346867514
129.745892720738
ADAMTS14 143.155998311198 137.864637728155 130.272086123019
131.162118170837 125.016608289141 124.97886512783 129.445240570626
128.783262618114 137.625415175495 130.576944864484 136.162938063831
129.854478528455
ADAMTS15 135.664877050226 145.870135196037 159.324816814876
139.051656180599 168.384397488011 170.069291215013 157.328404229451
141.872426752329 142.261639670072 166.655585160657 150.914644894869
149.31422191209
ADAMTS16 115.5765427629 110.081964209973 116.695313319162
111.568329540016 126.401145439668 116.070254821073 110.347631909888
109.680532131035 120.871693555514 105.050093408041 135.155955441604
115.531710316716
ADAMTS17 156.994265858123 148.350919010575 154.947359473187
157.78390301218 137.990806386929 139.420025842675 124.783306383852
154.212927357622 153.770852731422 143.298218989451 153.67215573765
142.736148814019
ADAMTS18 126.809252651352 136.411722212312 132.37450516592
116.844361858869 134.250052775987 136.944378905743 129.505139430366
133.184279519985 124.424565172377 127.629258006335 126.991140761102
130.277107538053
ADAMTS19 140.840895590527 138.938546468136 116.639787832302
112.264005988662 145.511473738224 120.054065939835 114.067790137477
115.389934936253 126.180714796187 120.809659457873 113.460835359833
111.020070405368
ADAMTS2 175.819983804497 161.485353443949 149.513744292932
183.42877315644 138.62994237184 143.818379692331 130.563756903074
134.7552564959 141.486042999328 154.021906399992 128.189685312596
139.490103923544
ADAMTS20 125.746184969983 130.584348445667 131.794065532001
141.225533609907 129.034770823878 136.313623567821 132.440818532628
143.015905927379 132.218907367109 140.762415240824 127.144291519133
133.173541338355
ADAMTS3 146.512045729234 139.501279970508 130.113673389232
139.843416673512 135.296271683847 132.322618637842 134.666505160831
136.587165242663 137.163699337032 148.263293819238 112.661607612444
149.350630572336
ADAMTS4 115.949513659403 110.838647534175 110.003750007057
110.688722702767 121.841080387875 107.365390754133 111.820208385514
112.198629213643 118.533346268154 141.890803125137 131.416923875254
113.163651806803
ADAMTS5 167.243544185778 188.708596785144 158.089922688997
157.713227527385 135.760578586004 138.374092984164 128.340524378217
121.360822445187 140.120051507626 136.470025638057 135.028746262383
127.352386363217
ADAMTS6 179.067275037015 179.854800301573 244.954917633904
235.76573393374 150.613624696604 156.801189586018 141.208106961875
168.205277839471 159.771672778698 144.154217446011 168.801737310947
147.382177739631
ADAMTS7 140.794929983858 137.182323015758 142.530082312562
149.611601613715 154.620194499553 139.776830704087 139.488057371254

140.796641921516 136.633726784926 140.497181450706 139.297276687352
157.672378669483
ADAMTS8 337.585502787016 347.73629731446 154.744065805492
504.617795280805 152.080057231881 175.9510758779 181.747090380435
184.709768457405 155.798048652444 192.022864170934 147.638057399165
172.942666836182
ADAMTS9 214.355086255855 262.259969786927 148.791182443623
269.238332581564 136.357788670568 171.373932115412 125.975257879912
143.546629073997 154.591116158776 131.210866639392 146.6306281408
125.997109913348
ADAMTSL1 139.287871460674 136.846912694371 125.569364049349
132.047328970556 132.164132609317 127.167918433035 124.01558540593
133.954514563283 131.226834630025 124.285969778268 137.271702725819
133.627890571828
ADAMTSL2 410.42088953434 383.503040287678 134.247361216412
168.861884032201 260.195256836955 328.919401925375 163.36682525399
260.489744360233 263.74335003375 295.607161318725 176.194292036542
160.042251279235
ADAMTSL3 416.638529499878 312.339824919124 364.022018936132
363.150472154973 139.811873260553 154.398416018484 144.082452235267
134.646131480502 137.401137659118 149.645548888832 147.086734464001
138.279087298069
ADAMTSL4 206.413554839865 186.430014478919 156.146154578237
210.290500215377 154.591116158776 133.252140649104 136.837430157894
132.115817176834 152.47429737104 181.722373730185 135.358837968905
131.38692240237
ADAMTSL5 171.426334687626 163.607125532236 148.780775862239
185.046826969912 141.539503634005 141.850952191423 165.355764467096
168.588120398433 152.72717701452 202.583967728957 156.392603045231
172.486839924297
ADAP1 181.530767521222 143.455941298415 171.880470629305 137.187640876289
302.809011218879 205.830321788612 145.633647856775 164.295628750438
156.671252606074 154.509924503184 196.269193578525 227.587590278412
ADAP2 269.261595558589 275.543576337502 200.518684601283 331.795832780176
254.607324522225 274.974304050999 250.25959091612 280.948704707655
339.069385816411 588.076436392983 300.401370666633 210.061754644824
ADAR 1320.74943817958 1086.33540858772 835.284130042504 1098.78649254838
1147.96742431386 1149.3250945792 1190.62010668045 1730.30703463073
1341.78081782818 944.92617668298 1373.67062432073 1561.87054049124
ADARB1 1699.8374761666 1727.7144253077 2291.40912229281
2180.31963682546 140.372463604232 149.984657985607 162.786351190659
163.203582580217 184.742960975938 187.920789153124 182.209056248253
127.058373069911
ADARB2 148.454344601562 136.886677060418 150.442821494908
137.861973133557 122.711143209444 144.370811043456 142.02169810999
129.173772079264 142.157512965166 142.874797429568 158.020651857664
151.660152977515
ADAT1 212.016247864691 197.848577663238 251.993821914623 231.180528071672
222.156471143857 277.406445570925 270.231637012624 261.988345506548
304.811116024094 264.476846259384 274.934292929666 328.550286726543
ADAT2 150.610633329908 138.947760022173 145.063062479224 175.98210925643
167.025336068642 156.18389905051 154.886628987246 150.021512237553
140.01151290873 148.993620368781 162.304178370233 184.577511730401

ADAT3 181.966110264509 167.315702949236 156.942156078314 164.48570046081
204.347931209047 215.094633380547 179.950904921667 222.243523614518
180.889946069121 204.237952297239 201.441808401878 184.989027770708
ADC 165.553581848949 149.198753359122 143.45723206497 164.274491958161
134.132312782731 133.794524856909 141.205975588506 130.101422420432
139.515794685984 141.272049404557 139.571752933877 143.137158027738
ADCK1 255.413231082357 275.437030326768 282.282663608264 378.151202933526
352.354687089427 178.966169998125 196.734037460218 219.103510284905
202.458096827378 212.312154721246 192.627266049516 213.652091843896
ADCK2 404.54681330209 438.03648998391 422.973606694449 433.405369327168
1557.00226259883 660.535644289024 946.273229715791 986.441377365124
833.841469195516 491.112594297928 712.94845413251 1018.38836615952
ADCK4 161.803438841859 190.405863264717 158.679903271595 251.787648609546
512.184962338131 271.004741629789 217.748916080172 200.741234443918
184.398672994943 156.073428882485 199.490190715307 171.869472272081
ADCK5 183.397833894087 194.337080066417 162.689415085423 165.82215325714
232.385176936256 172.468440317443 168.018785514306 221.272872023413
237.521746704115 192.580288063603 191.830711714844 183.779065928447
ADCY1 154.792156982219 142.234554325754 144.975347788913 142.416237823488
161.072844903925 144.301577198126 203.206743799353 185.560737176115
159.918778562802 145.483667452136 173.640326140494 162.519642881848
ADCY10 127.233439204926 141.213588194832 140.226586111216
138.196640512429 147.52403424052 144.0534341937 129.80429516198
156.632939064629 147.604213625382 149.907472831817 127.893555451807
135.443856371548
ADCY2 142.063338490772 146.291932367254 155.52533851273 142.299535614113
136.565191271943 131.539170756533 151.282370273189 144.219104742099
142.75592155869 130.642810886161 137.773960847088 132.087183382523
ADCY3 1237.56662467492 1379.26881813339 517.529147171876 961.931130189779
1177.70376288106 1324.56751399862 657.058213650454 831.610501402173
673.824770556885 1012.44277312494 294.323621186916 882.798927428142
ADCY4 331.464977124667 422.840163973614 220.742788129847 336.380724981693
130.927299997874 177.72965427263 128.593112520767 194.488249052694
241.203521822269 156.262515148436 212.107842040568 144.920107068083
ADCY5 228.100112133941 193.95909424044 223.872330412144 242.188218870393
143.795399778897 150.993910190626 143.969566798989 163.298729857831
146.720204808211 143.683545371898 155.004104153679 156.430981244063
ADCY6 284.891415492568 347.866264631778 256.022057262325 356.699466789374
429.922530506572 206.753993163216 391.569187190375 299.851173200352
392.746574862867 188.654966029389 444.374521095495 240.376851873817
ADCY7 172.552923333095 172.222262910052 174.560168983121 187.325542100762
151.958818313561 142.768691899824 155.52533851273 154.71904132098
196.759802659759 302.272304483941 232.621526025603 173.735045816949
ADCY8 142.411117330429 137.597788349982 150.717996520257 157.061602966797
134.216088147701 141.272049404557 135.507247740621 135.670409501435
150.43722766614 145.085091507228 147.140771901106 152.247504238261
ADCY9 359.440639663655 396.988248940183 385.379119927642 431.686900474971
144.436358527414 125.738320262644 180.20999937977 127.368207232723
142.483308497682 144.916193282062 145.826955036113 137.118488157416
ADCYAP1 113.043089777907 116.5272207025 108.955761698524
114.115609163126 115.963144087717 116.46192274217 108.589444216106
115.137475087954 127.731816003312 101.149125916797 121.057111457792
106.576058729493

ADCYAP1R1 115.01682934323 116.082361389405 112.38543517651
111.43827410589 111.152235113722 108.497209666203 109.333276735523
110.273174147374 121.059923601223 112.612099135361 117.178495957357
125.840932898827
ADD1 3528.62443098065 4047.99200599472 3560.23794634993 5347.78127066964
1607.52029676299 1373.67062432073 2131.68478964427 1787.32566525551
1726.15788525017 1490.02976786379 2236.73950603157 1439.37593006882
ADD2 129.086186239653 123.069963218224 127.654130155446 127.155818626031
126.506749037908 124.555847618988 123.813766735115 124.676743480622
127.723052839953 125.234097725749 122.547842148486 125.569364049349
ADD3 612.06231165648 545.760623858473 426.364663722264 464.323065038265
320.812909889014 362.195903246495 517.42417250172 435.183433729519
424.37437028833 296.428342247529 583.697431061521 431.182384100794
ADH1A 2672.77147589342 2028.67873136046 3029.07443443457 1995.6000523628
127.171331062578 295.463118191589 122.297401003756 367.983166440977
454.854528472591 141.568970008164 179.601348561497 126.37572518012
ADH1B 527.226517960073 490.269418483997 454.34729744062 425.13721408756
129.888444399067 157.754622099874 148.844916975586 149.041707216561
158.911404625381 126.288011849541 129.818020166075 135.285224699704
ADH1C 161.813099560175 207.023221559302 161.501062527272 221.152973486208
156.562197068687 192.375251343504 141.865758685796 217.435155984897
173.70035122661 153.222807811478 152.647566393845 154.057771254252
ADH4 153.726935957906 163.146581498065 133.957160768065 165.977852973826
125.143281492089 126.617737577634 126.702705908175 157.739526559807
186.611870261871 130.807596523171 148.382228428693 121.893446546512
ADH5 243.10297424873 228.243668453958 467.512355593598 234.996704304673
158.610249594568 434.509391836336 230.079664410595 169.061364889307
168.687845035401 232.083160165968 232.986366625889 230.723142236623
ADH6 130.450930089722 141.156434225355 131.958760372325 124.300285428221
138.519129384809 124.873289279767 117.65847906412 132.784949538167
359.703969802888 128.611740835252 157.076284096905 143.40955339735
ADH7 135.99579763634 159.424105896749 164.198494818485 170.283532007581
156.262515148436 223.359785518285 165.239214141562 272.456310079164
347.487059316081 214.241635800083 145.517559593811 132.269567096683
ADHFE1 368.0573385048 326.354469134694 727.814681346494
327.480268032814 165.342790388607 171.028432989066 183.83720937589
158.241349852419 159.452527363763 191.445291899108 155.7441762827
163.59704679338
ADI1 507.56875910742 484.903322308422 387.403502892897 552.167699318911
422.590112942156 742.517302567436 501.005898087825 441.958777965408
305.93974940209 319.090371885653 506.562512021352 437.344765185802
ADIG 115.247223837316 120.089228795888 111.908965758799 114.200099628087
111.525984720592 109.770126492743 110.202982683256 104.406314027414
109.113476990309 116.540729581355 132.559118679298 110.382724813928
ADIPOQ 156.548858402702 148.73620012231 162.863781294205
161.655336107432 152.667721542507 146.795608819024 151.84373370805
163.099009810658 161.866405026758 147.459385142695 140.92066676704
152.883970401452
ADIPOR1 320.648966489586 346.356312668445 428.569462312363
295.678097392123 414.200068430184 490.464012303239 721.11578916385
342.315456386729 650.911886508922 909.020708316287 780.734359041347
721.11578916385
ADIPOR2 628.000860165888 659.483452217461 542.740017003369
724.777166238211 386.305638633172 429.595453454621 609.100385482325

693.781708146282 769.958422740085 755.42757464615 600.919152191636
412.202049714695
ADK 184.655641182839 184.655641182839 207.023221559302 172.430211711547
245.153736298067 219.709231012465 285.131244588624 219.792171863015
222.118666699301 357.191675549592 264.628012152168 263.74335003375
ADM 2423.84327263111 1914.96285167893 2078.76377557352 1362.1486006778
1808.26383618901 694.288215755716 2792.13364570755 2222.16226526877
1205.45751308367 12457.6956682374 2086.15449885278 1702.68441407727
ADM2 143.795399778897 141.960809148036 140.36602776806 125.444541266623
154.150568177135 134.273326732349 143.491142564838 159.413535577361
171.384487293154 153.432933930805 142.281657284178 156.375876454394
ADNP 324.671490117952 321.119758814618 376.189276074918 283.874600697211
372.024884481775 673.176084400831 630.473042320288 405.64711132293
495.099443245153 354.656612182522 511.779988437654 425.229044880287
ADNP2 489.022371276542 407.711669482083 441.112782197591 362.502631441716
359.577827010792 546.841563406813 564.200165295159 702.981741565482
459.973764138144 528.767644181119 495.56459361431 631.408955630318
ADO 524.541494680558 485.026081242201 466.4890990178 467.944850290806
432.604462607037 388.573321838751 371.693808540758 340.301980622308
352.945088028205 285.980446338971 521.215655189324 425.13721408756
ADORA1 149.772157318769 151.024201732633 138.411148122407
135.268963938932 144.961095334901 139.54738445816 144.445496145045
136.85323853426 150.985061659285 144.570725886413 150.364511432468
141.181931072161
ADORA2A 165.066555355918 161.704966315183 181.681929801968
178.728925255163 159.592581700976 165.51742391186 166.81024151246
172.310673499572 176.208206282902 176.77183598995 145.522435728191
145.588981165456
ADORA2B 213.338918839446 188.503673684625 156.481871341275
167.100039749329 460.408628542721 447.909277144896 281.694635436938
245.553138677866 160.773510498005 423.489441909617 328.730722366546
267.190228116903
ADORA3 154.316929706697 155.722314592388 155.917655560227
139.334552725255 139.991780068793 151.3362561751 178.835679834487
198.390247605355 171.350620456505 212.710910778949 155.608876496443
179.079061789059
ADPGK 321.794887577351 297.176755638159 315.130912368929 280.913748398876
369.646624431062 458.151323130855 463.187551361986 362.795691170335
502.170430910893 408.037874667173 344.915370555826 429.595453454621
ADPRH 189.678680702271 188.623897093065 183.937484597232 204.103018614608
148.245974323625 161.866405026758 137.736771579507 138.861385127242
165.337956130606 145.551717838423 170.701964358516 138.726894569892
ADPRHL1 137.016495611111 139.989830921287 134.418976976995
137.4552565025 176.167129202773 140.208983238283 129.945543636232
134.737901773547 143.521510363933 128.232091717966 148.237980436128
158.434048174501
ADPRHL2 370.390323116113 425.826693178023 497.916883763059
489.022371276542 686.679573646733 705.020444579319 846.425467119342
684.715981522999 803.840404197117 740.800822095116 495.099443245153
604.737834405874
ADRA1A 132.639070127232 131.861469782134 124.368438691944
126.759554774012 126.720031203392 124.595925483149 129.723501897231
120.735176435195 121.825639714374 126.494665743087 134.405155876727
133.583297305219

ADRA1B	137.543128522665 139.552533656255 136.657107363796 143.305848051593 157.134782748175 136.670253859184 132.541420804792 116.653897702566 138.173547382901 134.084602409011 154.239755912437 125.431735524937
ADRA1D	125.157459724842 111.65024009102 108.850992642446 109.506450285923 114.39161822971 107.48180681159 112.971776964469 114.730641277464 112.936472036608 102.343275379164 127.558034562151 105.050093408041
ADRA2A	209.5184563244 253.463871742303 171.465178761171 237.473927775823 189.985520850855 146.82149406175 165.960176666275 167.893596681867 164.448110885609 173.468958175014 174.576132939661 154.691783046492
ADRA2B	185.374745525698 186.653791045656 178.946928223991 206.502189514795 171.494975393562 187.082082429597 174.027190728107 165.092451754065 168.427124752461 170.266415287865 166.503112149416 176.704617691988
ADRA2C	131.973896985615 131.93683726912 127.277128410056 130.991496243273 135.131890100292 125.800508653231 129.705735906962 124.595925483149 131.534097198718 129.108534267222 150.314462939164 130.980885733161
ADRB1 143.78994470501 139.980504765791 147.595573192579 118.988269692812 168.052288484172 144.565926379402 160.76044995335 164.942652212218 132.681475162386 147.036768856136 171.494975393562 150.754492902512
ADRB2 322.64731764456 325.493177980483 241.019436415703 362.795691170335 221.45275440794 177.549330396268 182.984076352185 252.593574250965 225.803528238368 1460.24985399013 174.274996381853 193.871324880919
ADRB3 190.444387031552 176.317805247749 175.98210925643 246.186104432792 152.925281215593 137.803749343493 141.849319373594 135.175806654971 127.001546144286 130.977864137459 127.941584184869 142.866874321248
ADRBK1	481.933386283351 531.555532787504 515.432065049838 642.255110250493 1455.75693295732 1131.22418479945 797.73902873919 661.826149512811 1144.07896461196 838.93563319979 841.623780229898 816.823443819069
ADRBK2	136.6729514905 131.150252587865 138.836422729663 148.380555606786 152.371569700039 131.442186260672 128.78647242525 139.420025842675 142.411117330429 145.068061371609 148.222477811166 131.917194849285
ADRM1 754.406896156858 816.580880338838 780.461254794127 1084.10984365393 3024.62888503536 1504.15682715484 1230.00744483191 1042.40539020413 1109.79571066685 973.543357809971 960.775251871864 1186.23169583595
ADSL	1198.53117439169 1079.58110075176 1341.78081782818 1364.83036157729 1154.1046856215 1311.34904760947 1061.79595707572 1170.88763035931 1189.11053680293 1558.22238167317 1044.52286986369 1278.30505902957
ADSS	537.982214580518 463.187551361986 657.77730563722 402.824964079515 374.106258053201 902.089561157715 720.077057619735 794.636255515909 601.997464467917 709.303384219887 365.441289363381 1000.05896885496
ADSSL1	154.01988200569 158.371599884762 165.192945734166 164.929445749888 239.845747958475 151.151297821117 185.938983266802 164.284396793834 152.705458417973 240.631264915238 188.818012746123 193.292596471113
AEBP1 3119.93811557461 3019.57419253979 1827.03179366146 2399.88695085752 270.651318838953 247.373571011712 183.236087954054 330.703588385626 533.279122416818 962.559482864549 171.071893074072 180.719868519867

AEBP2 410.216584967666 366.473258467746 505.560841055884 335.938170230765
306.550948766362 306.550948766362 352.180110869664 258.998065427002
278.942695435238 359.363523948825 246.457698183615 262.452869676209
AEN 228.284236550547 207.417884407459 158.374760929273 212.545989139388
239.789994201722 185.100455193182 198.885004378065 199.776140037612
218.134101819445 168.582192754233 172.641322197031 175.874387612235
AES 1470.23096656138 1637.66306286151 1880.35608492553 2159.2992548378
2733.68308784024 1155.4487861163 1405.2134783352 1106.75785702993
1143.0779222425 671.387334588652 1889.05071563459 1228.89116158868
AFAP1 420.446810988909 425.708048519408 434.073729807058 563.648536446918
159.539320132392 162.472783116487 150.19577351885 163.558869203898
166.099940078284 183.472290949588 147.896919205072 181.328458560967
AFAP1L1 162.481362579872 193.008902902654 165.537853089052
190.825209733575 165.673535349675 185.905819051223 151.070654092886
184.505279093688 169.301498852556 238.946506103155 165.977852973826
143.236593995493
AFAP1L2 299.106362530855 291.720204789005 161.581641777014
235.997227173296 165.863118584978 155.172667450053 134.640783050966
156.674411447713 177.60876237572 258.16437138327 245.489266602897
173.599961799334
AFAR3 129.966755942173 155.878493935166 165.570531605115 141.069243738242
145.34041912391 156.399808270033 140.368070973566 139.771342253058
143.112205432731 132.243900762936 150.073816271065 142.390243984362
AFARP1 147.959740692766 138.9183380982 147.367666575705
138.146668191454 143.697931551096 139.886499402506 157.36568754472
155.502363062633 148.380555606786 151.669421563954 138.521952850632
133.883527462382
AFF1 261.541138637729 216.859640534581 297.419034136005 232.151020756625
154.768422926874 257.941367816557 237.940015617254 284.195575802966
289.616527814137 227.467161919443 278.192669077191 270.490971664802
AFF2 124.952026709219 130.270184521521 130.461084106455 123.055210862247
130.433405111592 119.135701239571 121.603608840998 124.176944998906
127.086470811408 124.15094220181 119.882472552083 131.606765174514
AFF3 403.208138816247 401.461825804111 411.71606840872 434.792010353541
122.506058130261 132.568354349687 114.45792163942 118.879931299016
150.176942469551 124.452631766045 120.29173510435 129.421772713718
AFF4 712.699853328955 526.896146537814 1092.74905085695 465.926873285563
208.214764518526 710.531876484467 939.213447046823 691.397238677552
536.46884541183 1185.70008959602 465.683139139622 1194.42925868786
AFG3L1 158.627416752905 158.604431001643 171.254015232189
145.491525555916 141.840858913807 149.277880094957 158.100395482569
157.627490896581 156.695440330379 152.547558436703 167.164675439456
164.991522994763
AFG3L2 893.676600121113 913.22732386079 1004.97723186946
968.548243081111 464.323065038265 1478.09582385838 987.564350211893
1312.44215746801 1086.33540858772 1089.16489859253 961.931130189779
1358.29475573637
AFM 120.944267930809 106.701716475947 107.466184687811 109.625579596864
116.074423050416 111.928204694188 114.867454554206 99.9173294438729
109.398997827329 107.498500136859 125.639355645626 107.221547998974
AFMID 156.389361240637 163.010527914347 162.9279134169 167.594414386475
187.196424491482 188.515336186633 194.430896269275 188.995551740427
180.952017616136 158.8246699332 167.130843160309 210.822216545773

AFP 124.516264338772 144.858656365799 143.227410286571 137.363483527372
127.777945516765 140.482728188987 144.954595802474 122.944699010173
150.058160923921 143.07651558428 129.928047362701 130.701801496564
AFTPH 318.112626495401 310.174932137546 315.786908578669 256.99019407912
348.506289278042 534.276129011237 372.088129459528 502.935749606918
390.809169896009 352.741885295157 380.146996799549 328.486476842582
AGA 250.737252834098 230.469548500304 254.812605074045 222.20526515049
227.689356258276 259.472319994929 385.750371084 347.224999246223
333.822167152866 439.616817413027 391.706404954357 429.351447428661
AGAP1 134.994013668445 144.551233687082 128.186963138555 136.258073395013
140.453219944445 132.70109232982 137.884051774657 135.067056559409
126.378952839132 144.328571856869 134.041098536814 140.158309892319
AGAP11 120.806180547422 121.207040901483 116.288113598488
125.224197852606 127.641370873147 115.920514336615 125.83678485646
115.293844461817 112.90900939264 134.624322761212 121.607527525765
127.247764390727
AGAP2 125.353389826276 137.388190728367 135.082715974111 129.699765645519
129.647514123731 126.720031203392 125.202468372015 122.292692083352
135.151438845363 134.512204051277 157.390513637267 129.794293922951
AGAP3 179.841665686703 205.244843885276 196.942880967705 209.785886171295
333.439929772897 368.134303229682 286.945784387294 243.246632667118
185.691922436569 314.063025950327 227.092319765009 364.191263843897
AGAP6 251.511405838796 250.602978521808 310.8725368426 238.904826940476
210.316431648335 320.390351199433 271.004741629789 347.812754957414
310.301616266762 210.692705153337 248.204358220728 435.037306125887
AGAP7 148.967232828819 138.773042747912 141.508424382665 156.200313064999
152.91729346961 138.681348536008 149.842326869644 152.593934787262
137.415158613413 137.53070284697 150.587882657674 130.143953880391
AGAP8 764.774225930479 736.963699775724 1036.40082234884 692.53483413403
269.468453936818 916.4187683687 753.007562127186 1737.57015396357
1032.4540300024 479.983368253935 838.63350090385 1524.46745239811
AGBL1 118.397482835514 123.505435008269 130.016401638936 115.795989631016
118.388993232718 121.99477069153 115.3611179192 113.23302126254
119.087972087745 125.050945917101 124.990876565715 117.432860005907
AGBL2 153.461396593156 154.136467767927 160.75642485037 135.185283076286
161.744366588564 165.549190801014 162.629257046871 153.541193559658
147.52403424052 160.361025839456 163.227953876329 147.841960346835
AGBL3 132.848926011576 138.834309377225 141.760529549196 138.231715814115
140.264060906597 146.548348470748 140.89666990854 141.65642171417
137.418345713224 132.018833785059 136.130740418851 130.949405011154
AGBL4 121.44038832089 134.512204051277 121.799209715087 119.544004279354
125.04156726504 121.991692204056 117.408465686218 118.93828679356
128.359034755228 117.646963986512 131.340453291188 119.755151432304
AGBL5 396.04031733841 499.321667633972 491.709723745226 749.515603904233
601.640387246951 397.985856484851 447.909277144896 295.047418816148
285.172001915966 300.047728281275 267.869879189095 278.598913464804
AGC1 118.505980204837 130.370405378213 117.555999801887 118.319950396126
142.886493948916 119.463337435512 130.766401858384 120.270397272069
122.243585162452 121.824805374887 143.625188247966 120.433080754232
AGER 142.752536416014 147.310807346272 142.846100831941 159.124592503759
136.369287857303 142.907969322845 154.199534276226 144.708780756278
150.449621353865 141.378381097266 146.805832438977 146.176240539176

AGFG1 285.131244588624 251.787648609546 232.200025766422 241.058691798484
175.989742412417 224.62335524729 262.416137698884 199.96834355685
204.616225498655 269.892633574243 294.48534582475 241.867699338253
AGFG2 172.836730741407 171.602950766191 177.379186278216 172.509081696309
166.964057265095 175.562070915794 197.326259802349 164.352366170527
163.904137395349 176.883702717029 150.584633044901 164.701183249762
AGGF1 249.129943906228 239.73396414153 280.170464338141 228.243668453958
215.897620927751 349.248144552634 476.641032594868 274.14931964455
302.219514290905 368.825190022937 285.659010982301 354.205670284123
AGK 326.525579400607 288.542264120468 390.809169896009 320.774102343889
255.493109705211 470.308610828825 363.343480850598 529.228238133018
394.65807885612 447.579975492958 352.50679573356 574.779350011402
AGL 295.241644408321 301.087947101876 260.224777636733 361.26540006942
210.392662092721 258.109876262342 220.578790067967 217.074639597142
196.615914367563 174.971350923613 207.204293555178 200.72147365413
AGMAT 118.039780360151 108.296042016208 118.833188167696 113.143436184959
220.533113603343 171.36717256867 124.291944151031 213.510783808898
187.112176929485 112.629266772219 159.317429821638 166.803359138942
AGPAT1 199.191065549132 214.888126361789 221.975219056555
245.489266602897 248.531972733475 145.601922576321 171.022976593689
143.567488854011 159.298815559223 142.390243984362 183.406510146725
166.485040297098
AGPAT2 270.727749014046 234.996704304673 252.10470581893
239.762135294506 1027.96306543235 370.066087375401 225.31216514083
309.937351755502 306.193157874683 396.126485121994 724.442411170786
557.405157178952
AGPAT3 127.9575507576 126.214166842097 122.204249636472
121.924036463998 144.551233687082 138.333435927107 169.568915494577
143.193305421206 176.579096613849 158.691830524781 180.456659320226
164.337486856997
AGPAT4 220.446775597113 213.046162847735 157.28429690041
219.619022209985 140.10798818623 129.668506092785 178.606255846041
133.011320549232 170.646233722767 154.949840064881 166.1601847682
132.948096807634
AGPAT5 134.299575239865 127.709638375626 142.51308872439
122.290822230565 155.70645844794 189.174957940401 217.148957110623
277.539110964815 182.886100305943 304.713434036642 193.315380090713
267.589020526148
AGPAT6 174.603285240228 186.103491222924 179.048139035247
178.835679834487 242.848821222611 234.484787292284 198.055877414824
187.607426452073 213.813667557079 194.125840053843 186.296882059398
194.961531388323
AGPAT9 433.491835341321 360.361719991381 421.583441056026
209.464347017443 159.102070980047 140.837360719964 140.301945597122
167.526798465556 161.00635457883 214.229228850802 130.441808139335
158.308558656144
AGPS 258.145539382199 213.914599384797 249.763375041547 236.651601701157
221.961758403614 249.129943906228 231.890885094917 280.558463661137
277.314675225943 452.167674725454 215.115324454215 264.654879741889
AGR2 206.73292907905 480.36420271348 231.239126548656 142.301581642006
2372.26073354008 1074.19589851599 1336.75600604068 1287.3125466789
303.638516587721 172.857643600385 3188.90202907403 4579.62296570302

AGR3 157.08300736206 156.285963197762 143.626857507866 149.42148494796
148.727953739127 162.723310549554 153.681775929182 169.349922114861
142.326319895395 147.978039160763 153.853856339585 144.80318019871
AGRN 278.061498892643 280.204167582951 248.260998007814 301.087947101876
1698.08853832248 681.591028334969 751.077307018319 556.863361789449
628.197839884392 364.331860640225 1139.14730341921 742.751664201272
AGRP 139.962307072428 136.444883411968 135.484360570738 137.830435972191
132.273354705213 133.499361793303 137.024230648861 134.372560968918
134.406692724353 129.633387503618 132.01588294465 133.125084506877
AGT 159.097020087275 135.723554819107 131.007779278656 144.955918499353
161.104993402456 144.92594578882 126.305273697185 175.41089164969
160.98571841915 128.105527466559 155.566065673542 156.704278982517
AGTPBP1 194.337080066417 168.347445148881 180.113222861966
152.91729346961 176.5588805282 156.920867019849 146.29600238749
157.225479764928 165.642728895682 262.488050186425 165.337956130606
132.923237903246
AGTR1 448.260934224454 439.33566689752 666.847934976479 557.868300839263
170.844002412425 170.825161347825 170.768398273919 170.035994634371
167.55209828062 170.040914594182 152.990523860831 160.964435028806
AGTR2 128.02595294499 126.910075365358 124.232144678947 130.272086123019
124.595925483149 129.842252702648 118.764650384561 130.571827557149
126.473768158566 121.567879358508 142.603436693587 115.194730717797
AGTRAP 167.949422301257 174.737828693216 142.736148814019
181.991820141929 651.675427664911 183.081450609516 234.063633889254
181.088268167074 251.324685584062 348.413827101046 179.286187825363
187.112176929485
AGXT 135.773386496434 144.949897872252 145.043494048779 147.975551540256
135.32730674455 139.701504237962 150.63456320644 144.175551691291
140.793616429174 136.68056120003 144.259643132933 160.192540519103
AGXT2 120.71782441569 115.902030217102 118.021169080914 121.360822445187
117.923459503989 116.831725094917 113.40684693417 115.270544506813
126.072053958666 110.081964209973 113.989682635964 114.893462783007
AGXT2L1 116.331428876124 119.446359837504 130.160236228857
131.473001455317 133.42964827086 121.53632327554 120.212294184999
132.958521136 131.31716158922 122.379537202709 132.981346767415
126.133366366356
AGXT2L2 179.730694272916 162.6762533989 166.730974538475
170.866433766203 155.129304059037 146.746669300049 167.796474464069
152.746799687103 171.844483419604 187.686326872667 222.919473675549
178.06221214103
AHCTF1 282.652193666078 282.282663608264 290.867822301134
257.100909006193 205.705962405954 405.469705787631 268.264885392368
386.917038897322 240.054164412429 210.989806933158 195.577739376082
363.594808277506
AHCY 1012.44277312494 891.194779815063 1383.68977267418 912.143424293236
2794.89048827025 1949.60516542965 2398.71483410593 1360.85309203233
1343.2623011534 1392.89422203207 1311.34904760947 1587.84395475807
AHCYL1 2567.4299860517 1923.19560386389 2296.30423509176
1632.84261181074 1284.19937294949 2423.84327263111 1565.64079526732
2328.65814406636 1657.72357544205 1129.76078220457 1730.30703463073
1490.02976786379
AHCYL2 171.528350399075 169.391557085902 167.083758835476
133.21981037185 154.534605635191 170.381897441659 186.222567239198

	170.701964358516	175.767556472012	164.38282966938	181.950658782069
	205.121123131992			
AHDC1	267.342817344746	378.088675670786	195.218005582618	469.714826990063
	238.270501389189	141.499192356469	154.250656489421	132.615324904193
	145.294665838127	193.832033192375	193.292596471113	156.71199811686
AHI1	546.274050902567	476.917496749381	469.794672718173	438.231717096337
	181.558764396201	200.302384864371	276.32528705384	236.108432603479
	249.400820221233	192.61436632814	301.188296465202	261.844708446856
AHNAK	4238.41681589316	3586.00189997341	3024.62888503536	3883.05620256963
	728.176339659082	514.354512083591	685.848203651889	437.931339781992
	754.607039665508	2794.89048827025	1491.05501052706	1304.37930719411
AHNAK2	2521.58055221647	2462.87034563426	1953.88327754946	
	3024.62888503536	134.95415308911	128.601885372662	121.448671447499
	127.694189114288	152.152106138031	3042.88425356552	156.981921830638
	127.447218258785			
AHR	5800.06300826802	5195.2087608308	4511.9116742833	4448.67183518984
	2206.7447886564	6105.64727031051	3215.41238464601	5250.88041666757
	4088.12910138929	2816.29814823646	3855.33040541264	4262.23145131525
AHRR	111.794102015354	114.995889945366	113.321454160244	114.027244455291
	115.884253217495	106.147993332294	122.125746529725	113.091739139626
	116.71331256222	119.366599958278	115.845468537936	115.949513659403
AHSA1	568.555967950896	536.46884541183	731.513306289481	615.254768508691
	730.042712083388	417.615521090665	584.548578914846	916.026216386031
	477.626693328013	620.899414581162	991.611982265221	675.480482223958
AHSA2	509.618968665191	428.959561932229	488.14606875418	451.621888586656
	400.621427274425	471.946284490029	759.058176862524	625.932910814208
	499.321667633972	351.665934915634	731.848844821402	717.886050056576
AHSG	107.622342073167	109.333276735523	110.820329153348	108.730058525654
	114.826501559828	111.716607815635	111.672450681359	107.780763250481
	108.079149792796	115.445219455559	109.282814458761	110.050374895171
AHSP	167.357570666817	165.274189050141	166.182850621447	182.042104888745
	157.367331080003	172.128489882364	174.775676782795	158.522877390859
	179.632562895905	212.78038645414	151.167024833049	165.642728895682
AICDA	157.885229749634	156.173099025259	156.381599544223	152.79367157305
	160.667815093292	185.113098620798	156.576585764311	142.476530302265
	166.272237117441	177.457362387052	151.690538050817	163.773702843544
AIDA	4016.35381420598	3598.30438198858	3833.73632806344	3734.705550862
	1056.43975075255	1751.22580157752	1732.52466021178	1157.02365469464
	1645.05161227372	1800.25771851544	1772.75453735112	1429.25367024696
AIF1	272.854435771465	264.201932544662	219.394500571586	254.812605074045
	196.880135762817	275.696829424061	239.621756857095	347.292438587205
	434.390613381746	308.767036855508	259.338947392465	227.267783818298
AIF1L	1036.40082234884	861.516925304722	385.050282185255	774.997895985917
	347.292438587205	302.735372807236	174.717158790668	261.96528977292
	420.502251256406	267.696284751923	210.803130108618	171.660157220703
AIFM1	237.266455875982	234.5655184349	259.277693314055	294.697622870418
	448.644778906048	397.577760464197	261.469732730752	313.888035511709
	296.915883082902	246.186104432792	383.626469817252	311.848379951974
AIFM2	152.748807709142	166.674240866931	173.690008681514	195.123919692348
	154.932132950843	131.275712196961	140.870849757111	133.088758771353
	150.019181654237	122.793816135585	176.397715825653	160.632809953993
AIFM3	127.720117789235	130.198147836925	131.578042179715	129.818020166075
	219.351080515508	145.172776053	149.17029600091	152.961475450751
	164.1572198075	127.135199316758	198.568180234676	174.928155956396

AIG1 333.868818855397 298.165097953983 362.899103657252 271.568797887976
412.582051585078 376.867306473426 966.731520471976 357.514919818489
307.518881119091 489.022371276542 1098.78649254838 1109.79571066685
AIM1 171.145909843389 171.994330847901 190.371287639453 173.735045816949
253.538171006307 257.29212508324 8 340.42197893246 238.946506103155
292.068293167526 363.445110627483 403.582461109859 222.268494113686
AIM1L 129.216693566478 138.352562277336 128.505296338657 129.088718286391
229.230239609291 224.92725876841 171.042491047256 178.294972422671
190.549084022965 268.264885392368 189.044966519228 226.531255313921
AIM2 157.40255559397 211.545484517339 153.140087873773 161.208713751942
137.597788349982 140.36425603453 311.848379951974 179.54034642468
222.365208637146 244.280971257965 595.882696498804 424.912426797488
AIMP2 634.356322976353 712.067161025165 859.113541787323 705.529158880221
1015.63768413332 921.546033242474 1074.96415574964 1449.33679383001
1050.90881385623 1087.69177809384 937.593129242257 1181.1611856364
AIP 593.821622986351 730.673649872205 638.287221212947 926.670867201327
931.598413783099 403.984761333129 536.609777988581 326.417021307859
431.546135503733 272.314332734711 477.489368828457 449.952497751758
AIPL1 128.190822428484 129.580421464913 123.908458472687 123.775443296174
123.069963218224 119.159078990339 132.675172817752 119.391213033544
119.859508630454 120.460433297007 153.511995286293 131.497454038525
AIRE 6368.23123771343 5846.17594234628 6077.64695330731 6077.64695330731
1638.72366638732 6179.86800320807 3833.73632806344 4641.8793590085
4367.38154535901 3417.22292085668 3737.57754066891 3648.38765465889
AJAP1 143.065258692424 140.336479395866 145.020615552964 137.033502466562
132.122273848715 145.814268094808 136.051070946954 134.024205981268
138.081268025435 137.118488157416 226.936810012475 138.032265227728
AK1 319.685597963267 330.703588385626 293.234972169244 364.331860640225
301.798676825176 233.786136606682 155.214638707046 211.736398352592
182.861900982333 212.239485199343 155.415497832382 174.296028319781
AK2 642.484527246259 714.91842349236 668.286400833485 728.743541467167
1176.01058971561 1131.96129284658 1402.14708471728 931.325266360957
1292.13921040404 1147.96742431386 892.515788744891 1372.63540073207
AK2P2 376.124744806171 362.849380477089 307.020265874666 334.350011399462
199.191065549132 416.475440894731 258.998065427002 653.500279510032
230.367251714289 220.92105843037 282.282663608264 307.866499851733
AK3 1682.64624665931 1683.83803339436 1619.67820476414 1637.66306286151
475.403662030875 896.185223980593 636.781477235579 1322.14570224119
1181.1611856364 1079.58110075176 1222.59895569348 740.527607312565
AK3L1 416.715760038095 374.46180489079 412.277592266475 538.872348151809
154.783699020901 165.320977593677 230.161369375718 161.134322621805
162.98885109088 873.010676463707 166.232856234879 194.161930642068
AK5 147.653573379877 145.797047170492 151.115031304764 156.551925771312
134.91213977595 143.978912435831 132.568354349687 133.962129094978
149.634975068742 140.668597897404 133.180554954396 134.115085057026
AK7 107.72107116838 112.922918499398 113.816165294487 115.400592594965
116.973126196102 109.740801301993 118.983932995126 110.465934808915
114.161540771427 117.296972688814 126.205931638442 119.427342766092
AKAP1 255.156682699288 225.99848688881 284.473085899219 254.517135747445
206.876572583814 207.104548555929 195.019706886336 214.739818213164
186.447049658314 145.56468363155 217.264041781337 178.584378959369
AKAP10 181.453575300661 180.952017616136 238.183096218311
203.436527043293 244.925472495705 200.094610104813 195.230219990395

184.923312649584 172.794570587596 205.121123131992 185.896304740844
173.109461200603
AKAP11 428.462270756201 341.714401872192 528.921893077914
307.020265874666 216.126000558319 413.863345223345 195.384273821324
258.807028561407 298.209907157762 283.326226944158 254.768537214795
397.737702926328
AKAP12 446.07651799079 317.273940108333 356.530530468568
301.871924474014 218.615551704188 185.950131245699 173.260348096238
196.014841240142 181.681929801968 161.704966315183 147.769702509047
159.06002608158
AKAP13 761.726829618132 725.065727257421 820.652972219658
924.312051919404 270.604799126393 289.82072316082 265.068532248682
300.482945704017 349.485727214325 425.59810308048 442.86942574917
274.427235605909
AKAP14 131.148926523383 128.920313557255 128.02595294499
122.786030993629 116.998277297063 125.885715928839 126.245597154052
130.564962937297 133.008987820479 122.644105247178 134.260779370821
124.28313315915
AKAP2 152.445022409208 153.011923016494 156.311009595629 154.932132950843
146.108551647446 146.40904127542 152.699431442817 149.419308407564
146.664285015996 145.560161803925 149.661929885679 150.717996520257
AKAP3 134.319316977485 128.939572714685 119.366599958278 121.952255635669
131.159946917777 118.912989775436 121.010811158088 120.288469716375
123.781002620866 119.399971165857 147.959740692766 117.976107125982
AKAP4 130.677737553727 128.907618218883 125.924219382375 127.093255830978
131.193538235053 125.016608289141 130.890314257211 134.310685502031
129.242093568433 126.63730651721 137.000868503792 130.852932147831
AKAP5 141.735123971775 130.457981404612 144.792035839249 130.179742057015
128.335132315812 140.000880942015 146.460464282676 132.918979842076
128.858923585925 146.851307767958 127.377356715337 129.260551359984
AKAP6 168.818595386221 160.085325256991 189.174957940401 162.358237198517
120.585506729733 123.161953011069 117.101933186269 126.698158279472
125.402714360058 131.292195405993 135.573170189235 116.236538651031
AKAP7 191.040134068942 189.539458686123 189.799091507686 180.610891208146
293.692083555844 166.232856234879 201.139510041141 272.968792195442
146.179543599917 130.914932329297 538.252273740419 205.260673843294
AKAP8 358.355621069787 360.285027176918 363.89527566597 385.135014083686
313.828943866034 443.259619455624 333.592915218163 401.359711029934
291.577778800314 290.326832430372 525.6579615077 405.766055310869
AKAP8L 439.492285515997 544.669441360594 321.750495072759
622.561634660589 483.549698382115 232.465880700217 312.177191402945
334.607533045929 248.403014089812 217.491746961471 258.21341068758
293.448046685909
AKAP9 131.597937570173 130.621860095981 124.840101287634 113.504670764963
125.402714360058 114.488760619745 130.322436253881 138.780488114634
120.241528853563 129.099780410063 157.399880496076 138.780488114634
AKIRIN1 322.099431638246 260.715580924916 331.684298221133
290.420307322101 202.178451227304 356.154842959851 345.494740481759
323.462252156562 283.896516724446 289.859695541999 240.005943851098
344.45331938267
AKIRIN2 586.867772838753 436.891409783298 568.310800941091
429.922530506572 640.029195821436 414.267332328081 633.594811068524
398.971838949075 531.711823481265 570.26564132238 540.946852709312
557.214020485204

AKNA 354.263934274937 347.985315257161 225.820503820866 382.186095123659
316.801725268792 216.680795762761 240.75934760601 317.163768837813
436.891409783298 193.912456093767 272.277433294605 263.204269632206
AKNAD1 159.913028912492 158.658110539162 182.861900982333
176.791758333054 158.937341335707 159.133905701536 144.139062513587
153.723169560972 157.761231106669 177.151218006672 142.639587833858
167.268645224361
AKR1A1 695.321886304133 733.86133557115 698.882871686866
883.702573960415 1615.60472592991 1600.10235468018 984.181272274704
1984.47173096657 1704.25279485563 781.208872924532 969.811933814244
991.611982265221
AKR1B1 1269.67110104843 1285.54492194393 1285.54492194393
1049.52258147141 2288.63508719706 3855.33040541264 2176.59445919725
1528.49457104932 1654.58270183167 692.729789121701 362.65285317327
1756.88092410303
AKR1B10 644.97200337335 511.113083958771 2041.03024136014
568.701661112562 175.28261759639 498.641091757363 300.482945704017
310.533399024851 273.478372861114 5558.51089816921 165.471587934831
131.672014089487
AKR1B15 241.077618798955 207.560911665319 602.86705608228
256.764450748941 148.993620368781 200.506615977654 637.892677173103
152.801801823442 161.430219824313 1730.30703463073 153.062710222618
114.631759839092
AKR1C1 107.520000793184 96.0426636429757 112.509731206295
103.660742045068 114.143874572202 105.771723307783 106.285472845323
109.37466528113 109.044879188971 110.234403581151 111.423140302983
123.703840971923
AKR1C2 343.346755862776 424.285848583977 387.135002880995
234.789040617681 1986.81354260292 5532.93454600454 3515.36256599555
2423.84327263111 320.812909889014 639.011144305614 1176.01058971561
2048.13524244516
AKR1C3 883.702573960415 1775.32632165475 1055.79590411506
837.023434916281 11326.5935775813 20989.6494028551 13645.150201952
10312.8407173696 8322.20177173035 1376.84058291266 5300.67886821231
3949.19206005238
AKR1C4 304.751330890753 385.522443482 369.198837083585
201.167206950576 1622.17491781572 8617.2437024568 4793.22133852447
3775.41389938231 1564.8868894162 970.634736994313 1720.14021303639
2437.63566094439
AKR1CL1 137.570343435498 124.699127988314 140.236618351922
144.81689330206 130.213596986351 138.883710791382 141.970301370404
133.195030751476 131.106613067333 135.24841020592 137.593577427321
132.668344126625
AKR1D1 13324.7465248375 12916.6904389018 14651.3379687477
14348.4009261372 4749.69040198959 13013.8166277495 7930.82679738435
11709.9785619141 9908.59593023839 8211.38792799248 5846.17594234628
9793.92604780228
AKR1E2 153.972471779464 169.158049136321 147.975551540256
163.982415393606 168.124936529582 173.169905329013 143.26896117546
162.900556089071 150.544779197947 162.484777758676 149.857861968652
157.003367685421
AKR7A2 1412.56164938199 1638.72366638732 1594.05763963363
1654.58270183167 4134.02613649483 1846.61430264854 2037.96470181871

2105.87438782831 1895.72330348767 2061.9164323309 2009.21931102311
1544.45026075974
AKR7A3 247.746304138866 264.563741039382 273.252023367028
305.096320805078 613.063954859056 294.323621186916 334.199307788576
323.391456630794 290.241064926886 380.243136469602 328.680312364877
264.429717032654
AKR7L 126.887989471803 130.716570410608 129.794293922951 115.257318321108
129.859331207247 122.498794845416 119.780654293448 130.63591305094
125.35643274458 128.653335099674 133.463464118897 137.632470240792
AKT1 1424.79466712859 1573.03248861784 1416.93831202323 2172.89794460273
2358.46955614953 729.729297413233 1030.97051104597 1133.87328758676
860.688314783077 893.264940736694 1160.25775445193 1374.92806285803
AKT1S1 163.709174813901 186.747375844319 179.265395910119
218.2454853526 377.661589835101 192.140442881209 179.973655632636
192.891507272665 212.272826965935 191.170105307944 192.059255232313
198.411812737082
AKT2 121.696353230886 126.623026026721 128.635684412304 124.892438890234
150.661584243174 123.265810024678 124.859984709406 118.863150637476
130.856249657101 127.718341524417 138.020031233095 130.449669345302
AKT3 146.854523122404 145.10302792449 155.558839375761 151.204487316077
134.325520377207 152.301556397498 145.06736850005 147.841960346835
138.230197299262 138.541779821934 140.338622198206 145.962664629275
AKTIP 723.640113902925 684.428198622716 817.342172321486 736.140249105836
382.485769373364 360.243484271891 516.088776850623 374.337030706199
391.644649726254 698.358285634451 437.049581919144 489.022371276542
ALAD 226.824313642101 205.282221255986 293.17183601265 264.628012152168
188.685460181066 171.042491047256 187.231049280818 189.61702125418
208.214764518526 245.723316636363 193.589480577091 164.008063563113
ALAS1 136.220324225816 127.456181182121 129.497266827284 133.864612785572
268.111403429364 200.065184172871 219.0221768576 166.091736766853
194.617252921731 139.484551562098 161.852665901359 153.039237274992
ALAS2 130.66863243733 138.89354244296 141.398513269219 141.38749436393
141.695537669891 145.507330257603 170.313045875402 135.548042190047
139.023486014525 173.929353266244 134.00481287258 134.288349336742
ALB 160.240052678401 167.384121560438 151.714420758741 161.490941000872
184.602704530362 186.904126552483 175.767556472012 151.276193269421
160.979182561773 155.957570398118 151.463200570499 148.25001336038
ALCAM 986.441377365124 1053.09915077366 830.455793617181 794.165408266062
555.366765340014 847.276523445973 527.498114662967 2116.39371296122
1044.52286986369 487.597040819879 598.287431134037 378.728516779744
ALDH16A1 219.0221768576 235.650688823345 261.592512762969
223.279017022443 1015.10079945997 313.077481033093 352.22815095475
283.760131847707 389.686045618228 218.559622620349 252.761035334279
213.754156680604
ALDH18A1 274.316982298975 288.728608411903 256.795346052751
311.337303524912 563.140382824149 384.733313349438 462.779122987815
356.413190087706 425.471195039676 296.24781576525 359.440639663655
380.664528809678
ALDH1A1 918.162638134305 685.080426814888 522.050619657197
394.65807885612 145.679907578808 181.320895955585 137.508097741526
219.878230463206 215.635313821029 143.147325939994 152.534482748598
129.125709963326
ALDH1A2 255.374689750672 226.203994206714 158.521812954982
229.181725450534 157.419333185365 208.42712014566 275.437030326768

181.681929801968 163.275138097131 164.935120145905 148.638400592682
176.714471790643
ALDH1A3 582.642500194997 494.067878734396 216.991823988763
258.695920967489 323.462252156562 217.435155984897 704.152296359463
323.628636799403 186.496160803789 693.180345649331 376.627801547869
668.286400833485
ALDH1B1 1293.38808483663 1047.78978081493 1481.24705642453
1496.0924333403 221.82340413039 200.017741240426 164.14265547147
193.858884443593 182.397795261147 180.304585602206 177.491294133515
178.717719615544
ALDH1L1 227.52352046188 313.025250337353 303.423450627377
177.182321788711 817.036222521723 153.758353103323 367.644907862372
185.961822133346 174.66966623557 150.359906271672 795.464850398761
680.743052197025
ALDH1L2 184.542232315662 163.331424612891 155.400191634632
185.583997792931 151.998942684339 165.350777445902 142.548833391567
139.432567336814 156.269957530519 167.662761175661 131.72852748835
139.526241501184
ALDH2 1253.05700051015 1237.56662467492 1949.60516542965 1482.15461429206
144.892454033336 148.973277994329 158.884661398415 185.186497037695
1443.68785521063 325.173931553891 174.646321871605 131.657454325126
ALDH3A1 147.086734464001 167.900071375885 141.434699050015
131.117294275244 2666.06582231934 229.511643376643 209.984545596312
250.542405852865 267.901632267999 240.994507285383 116.780639688465
176.680708339236
ALDH3A2 422.973606694449 458.047799748696 597.459920611726
426.769986378276 723.428294037428 418.889972660958 329.779210536849
392.004079531068 282.963958072528 501.166869173253 199.118281632221
228.588693548447
ALDH3B1 150.660106369128 165.549190801014 153.360382235409
175.510160114891 133.99226161886 132.213890220705 135.605541734293
148.160350542849 148.258850340107 133.197002327259 160.361025839456
138.106499370074
ALDH3B2 124.193603137858 131.700854556022 124.977046954694
122.670689386332 331.795832780176 236.651601701157 268.328002606268
296.915883082902 917.188039935737 1447.07989164988 385.584224833836
377.353441970543
ALDH4A1 166.376907377447 193.412827246366 163.256472113745
201.569913451219 591.437752991512 408.758021864761 398.892968579918
206.164058935215 325.173931553891 204.453822958077 384.780426529733
325.43977732888
ALDH5A1 271.468939477052 295.851459742115 295.607161318725
278.335289966805 402.299165610893 448.106077184152 444.479355480138
303.300028849873 358.479218923158 261.639437655633 365.82705202869
321.568127272161
ALDH6A1 552.298965378403 495.099443245153 857.55777793754
384.733313349438 420.95713139384 263.888891822857 344.021696764791
278.335289966805 288.150694843614 224.963277964548 287.889774984754
242.019489058244
ALDH7A1 588.076436392983 608.351527783086 804.362909579005
643.13571432061 1156.19575610918 205.162633740798 2005.76678571689
401.840794789616 1231.37475540937 202.97197145851 169.378422829858
441.112782197591

ALDH8A1	157.192320902906 153.857048476879 169.826068012389
162.531630234824 149.964827055916 156.05583229359 165.267693285882
145.824996954376 156.717086120926 148.219406063533 141.305605155722
151.645636946799
ALDH9A1	4190.38366101529 4836.4008757421 5233.72882607795
4628.04544865227 4022.23000497332 5425.58761099475 2630.78185410861
5479.24427823207 4847.77258830631 4621.99241522677 2921.59274577877
3994.64569045702
ALDOA 1103.81439809121 1221.58522138654 2065.51724858579 1656.02721259517
5981.08862268291 2542.44286642873 4425.66777919789 2159.2992548378
2157.50118108142 5455.91604383389 1488.37146754184 2763.86550710042
ALDOB 162.54008293513 126.162713269943 163.821142892643 131.062360037777
107.126004317323 212.312154721246 161.285587312115 160.664407431261
193.203053225456 143.712450441816 203.436527043293 182.711318507496
ALDOC 383.503040287678 472.669956708398 566.647927867646 468.496972957503
2770.7811954029 312.286788541158 1535.27496251381 1369.89073267996
541.764013317002 2026.85580212422 485.731287873723 932.505095432049
ALG1 131.412979573687 127.949413503055 129.397378057767 127.476819912992
193.589480577091 135.317796804151 153.557991189227 167.447964370395
161.627546190964 172.509081696309 167.077669654786 177.825648107126
ALG10 125.277002586952 154.725484895098 136.378203924435 123.379461430902
183.472290949588 152.970079142629 174.603285240228 163.356993518952
155.561821955048 128.336332748047 133.008120853618 141.691135410429
ALG10B	142.442450622311 150.188128594079 152.296774123599
148.291518446423 188.39062571513 163.487737425232 178.394895649664
161.139253742858 181.193963616037 150.489651880905 171.267907653007
162.586012723045
ALG11 227.499335248402 204.415267938007 274.179232297038 172.909795209733
184.530853446688 245.378747269929 150.933724024651 173.782474972742
183.951404362826 160.293522619371 194.831347257735 263.448958595597
ALG12 194.388383297922 161.084088366981 167.180406126438 191.881157918071
192.914362049985 153.815239954928 156.990325995053 180.994249258457
147.866909109733 147.882530392424 194.724219884752 176.940723174814
ALG13 216.277178481058 185.295480519718 168.906637816046 176.622758931306
310.301616266762 327.480268032814 355.213322760505 258.348298226232
310.917191279571 478.180723959397 241.917371866481 436.586158384666
ALG14 150.72014060286 146.179543599917 152.182968451691 147.502116289881
172.222262910052 229.116153948948 221.512905853031 225.31216514083
199.135932984641 231.547654653783 217.094085829328 256.795346052751
ALG1L 180.923089242255 211.572396862776 193.674681547989 154.459517779315
575.857954264295 821.862271569917 532.394088545421 840.276501350383
603.061485415867 362.252813848151 778.639512538949 770.19255305977
ALG2 131.459400187744 150.259400712685 150.184863108453 142.156488113764
172.486839924297 159.678281513591 154.388776919015 150.746423638097
151.60113116166 160.180961887686 164.816672737691 143.589222264786
ALG3 264.054997398621 283.27806319488 298.028932089399 322.684910198242
641.12919339861 359.256193328366 415.749051861123 415.497783305278
537.982214580518 371.457568934695 378.4377409749 366.007451523992
ALG5 538.156335788156 522.050619657197 476.249239197777 486.197269936713
607.26455470867 836.235700891687 666.570977208535 677.023591987179
800.739422189427 726.731270955732 723.250763797522 1193.82799849555
ALG6 241.527811817146 244.011434605133 188.910933952136 185.396765725228
438.611973005212 490.153210371094 396.459213999636 532.394088545421
393.375554593484 381.121621815163 276.414209660023 318.112626495401

ALG8 245.522497675754 259.900447705311 254.607324522225 247.746304138866
400.924914238052 569.656573915709 428.569462312363 468.170972478717
364.331860640225 295.047418816148 402.965083947376 467.944850290806
ALG9 683.365770196967 480.797281489891 446.819873986024 512.467731561858
440.745866155629 305.577166690215 424.912426797488 449.408793796673
350.58427958432 407.957436380989 383.182507425579 602.703887178113
ALK 159.636846021745 148.478570934155 128.678484452077 170.974759483946
129.501028506432 149.477373469387 161.657344146676 125.642282323003
123.752109790056 162.893396525252 156.733898702331 145.03804642447
ALKBH1 188.995551740427 191.937607311711 217.607230767028
188.277563426829 182.67941458824 185.706228572019 202.797135825668
201.773745532242 170.79303550487 191.740596601895 189.61702125418
202.545147073902
ALKBH2 313.828943866034 331.018914429561 322.302219773624
355.290621188435 451.985108262982 437.836503489425 430.649188209901
342.245182599593 308.208276315292 248.927790738447 282.392419584508
353.285905782951
ALKBH3 331.747622606378 409.883222213537 390.979822976551
374.9555524303 339.598452080917 137.324247703318 496.03509813734
227.806050911154 266.534316786801 463.754869412768 354.579728909336
260.888612454097
ALKBH4 149.31184788671 167.173670781451 143.222342904886
156.224769056915 213.634295832535 147.626071601072 166.600109853461
144.00214898009 156.487049947247 145.25774926819 180.250420976613
182.470538043064
ALKBH5 5932.37714987741 6691.04319670055 6737.97308530049
7104.65816621275 3822.05744774919 3321.1350545346 2708.20624364471
3093.38255333143 3024.62888503536 3677.23129570773 2947.51998516453
2352.01196541874
ALKBH6 307.16835174775 304.402066421813 282.137282466192
382.069677030369 710.003412772888 411.71606840872 412.415549375221
404.876478833713 467.04976953755 361.218491712891 334.056545911557
339.663295617139
ALKBH7 303.858574395969 335.533053221675 343.048904374136
329.068463281182 1092.33328323944 501.863855158062 484.131020584378
358.152044880733 315.828726297864 427.637083424453 530.267835989435
428.340060893467
ALKBH8 185.673549367125 219.103510284905 182.335981256243
155.393326732887 155.626515272084 130.161889021281 145.928643520352
164.198494818485 677.023591987179 154.679076303046 204.914390718161
190.189217962555
ALLC 132.622032754473 133.722739936347 131.563719927798 131.111930163369
133.07218865002 130.939543211439 126.912226185444 128.700516033555
122.619429537799 126.34649262976 147.278236958386 132.521681289019
ALMS1 214.396920524803 235.43707989982 242.614881095485 230.975434289172
218.644953001365 261.671277686467 214.100698312272 231.372316734945
193.744461104128 226.287196839644 204.740183832461 213.710058427654
ALMS1P 168.50246276469 171.373932115412 189.985520850855
160.904946328482 161.090443294391 149.113794439994 171.69834783037
163.102043862117 164.103306492559 154.379788206936 157.254757078837
175.510160114891
ALOX12 143.318291243905 142.882391486465 144.511488474344
137.823152565933 281.119138040097 261.366143735074 140.341589053972

160.882229345156 278.775971122032 242.902900881585 195.645181575647
136.951350782053
ALOX12B 127.911258242431 126.3539479619 119.275061991187
117.324368138398 125.874313058552 116.087696503753 130.820415610578
121.065278765151 125.044092929562 252.015257713669 130.378441113977
115.871151134713
ALOX12P2 130.066177538896 136.384308041001 156.998563448664
166.187099519035 137.846463796994 150.845846206549 138.764834713216
146.975258859244 141.906474266389 141.424508069834 125.701083319929
152.077204721895
ALOX15 144.669509071181 152.729355306724 150.026314061051
149.915251958706 161.632610858756 143.917152377544 142.500537230642
150.117906461237 149.881708604156 146.431166706908 143.703367582367
157.739526559807
ALOX15B 138.426531345939 134.257928609741 141.266129417042
138.639410179404 151.208728458188 153.481752643937 145.606098951179
153.550174378555 158.819151102054 213.046162847735 146.746669300049
137.824985884649
ALOX5 221.719848707318 254.133112176024 245.489266602897 178.445384902129
269.141120401835 362.095074331893 1376.84058291266 990.55038742453
330.944203807675 256.897177822238 1657.72357544205 1431.52979384867
ALOX5AP 651.998433127992 500.246996975 329.48021621087
395.425715632302 304.985269874607 423.966625351913 565.843200661721
793.891277937782 1144.53002508973 1744.58410715956 444.082440002333
386.738154577763
ALOXE3 124.86699236406 148.143367490426 148.8870854102
139.170102961407 156.818197172306 137.846463796994 150.794680375075
142.971972776388 147.164348704012 163.69442149145 156.801189586018
146.854523122404
ALPI 137.710725379804 142.416237823488 164.509354377985 137.44909445642
138.1342592966 149.668587078867 149.925051977052 145.291984088491
159.170943469313 147.922706629104 154.286043402527 137.680019787902
ALPK1 203.244917049745 235.133284525241 200.264756078778 228.100112133941
168.729301938015 172.622816559412 296.721891842563 248.643311972844
259.472319994929 204.078331021637 336.338518973032 249.449151040445
ALPK2 159.476343366991 144.175551691291 127.954231630338 115.678486138934
122.965328766807 117.904366738895 124.359615295938 145.987228759402
206.802308105224 214.566711351899 154.826710617351 132.709845352383
ALPK3 140.860339862967 159.138344477256 139.63811945162 164.092847913322
148.130993521745 142.416237823488 145.751336115646 154.252727576218
144.683260413888 157.927709922368 131.536457148878 134.01566484034
ALPL 172.965746521132 195.078843867124 132.801619479245 129.045189856765
279.113984512619 248.927790738447 239.388546850124 320.525885538523
269.100400678077 225.189991540262 181.876635968785 153.966815534288
ALPP 10836.7380675792 12109.3049135906 7733.95252458705 10533.5868398404
1801.74467005923 11112.4298269818 4857.3155917904 8473.21873478131
7054.92270618408 3744.5666678861 10704.4678928541 5670.70220800096
ALPPL2 154.177340021162 166.135707525987 167.212960569538
154.683723064797 133.9382467268 166.767004180603 150.112549201892
142.440344960504 158.312614511836 154.591116158776 165.200303470106
168.720694813918
ALS2 473.370240289761 381.808809625801 504.617795280805 474.917507160629
252.365946808078 387.224184063662 415.642099109855 606.162976359615
398.796188440256 434.390613381746 300.886032768166 428.782703561238

ALS2CL 130.491742824575 141.86836086844 130.083266044947
137.203813576977 143.833057210822 132.697727983601 139.885054845153
132.84268776659 139.223480216048 170.98312926 160.066390229416
153.358189612307
ALS2CR11 117.467618148107 134.119276589666 124.907788504503
118.098274003405 113.427848757112 125.450077160575 129.462319953515
121.54835308632 122.603226380439 119.131613164405 126.282575548237
114.27112025541
ALS2CR12 140.641308444844 147.537691509156 147.320976507643
126.048382998393 154.598520288855 167.484236948415 114.342980656214
142.003414252064 124.385193388896 148.134642821094 136.149732574763
145.496033735748
ALS2CR14 529.963453165714 590.515758538373 439.492285515997
476.249239197777 232.590542016298 540.797164107185 378.920456227378
452.357898123926 356.05426191171 359.577827010792 534.276129011237
382.485769373364
ALS2CR16 146.64835267642 159.848342125486 176.587559615019
189.589053840976 149.157934946623 167.063762404009 152.961475450751
162.320894555337 156.66832364923 132.681475162386 156.852187451794
157.457468761338
ALS2CR4 309.75140197669 278.942695435238 305.669636871734
236.141982516399 359.162549472301 550.24496459253 510.972389503051
506.80969228563 359.024299091145 684.428198622716 299.170431746759
383.626469817252
ALS2CR8 121.739219824085 129.601234646954 140.372463604232
118.426600479326 117.024039521425 144.490452173336 119.810421687953
128.759751330525 131.426355444136 126.48691003872 145.148058824491
134.05596537871
ALX1 160.247370279802 179.25743545231 160.50439457257 167.791351000743
149.196539425588 139.615706918035 148.215172022129 168.654753237204
185.961822133346 153.318678468245 163.72257517759 151.354187593773
ALX3 144.087004064416 129.701347789845 138.165240772401 147.572660963285
155.861829925386 153.918462601781 132.953762692926 153.563839578065
144.388851371784 151.863048206341 146.738411200698 153.532954370572
ALX4 133.031431118772 108.730058525654 133.545405737639 157.58659226599
140.290598244169 130.471929699923 137.179058645907 125.488511939819
147.893916750136 144.779146672553 126.997614441981 133.63022526522
AMAC1 121.980744551615 136.011474063229 126.398884382537 120.665520347481
125.20712393273 125.390139830237 123.877609932461 129.980354138975
126.614876404146 122.94937342991 130.619624347751 131.789877096016
AMAC1L1 136.682172321984 138.196640512429 143.993611400272
136.321816321193 144.084441938143 161.050438117555 168.813895764991
153.162313098346 154.043604150962 153.711354087243 141.189008902155
152.879041561846
AMAC1L2 140.878935632862 145.1382587433 168.389069732474
147.552635527742 143.1288704752 150.168496148941 160.970946091146
149.241358552148 157.264673452646 145.432667767652 166.485040297098
153.143706194386
AMAC1L3 154.869171648942 155.026276988302 154.892452237027
161.050438117555 126.372374084002 153.295838391099 170.483686891321
152.883970401452 144.161672064561 134.479828866339 157.192320902906
146.880440100898

AMACR 152.633139090274 162.879707592164 174.985831291764 148.844916975586
224.008253365957 371.767182305618 174.059172465573 724.777166238211
190.22021453133 169.933706225406 187.955617187544 174.396061906109
AMBN 147.369767631957 141.668101962049 139.099483279141 136.582137928022
146.541890150631 148.898587505337 142.945392802281 138.59175751301
145.847629576886 141.317113425046 133.353762794271 140.857793157932
AMBP 127.696578575874 141.453255692229 139.502856192876 121.049927973123
133.264245969185 132.555211779043 129.189479305468 126.713610681382
118.492287441192 126.148379057536 133.948997488604 125.157459724842
AMBRA1 157.341422939225 174.812134675989 149.85385474029
156.75308999904 174.526176588454 139.497253870412 170.553940654493
131.156409942604 148.291518446423 153.348110626071 154.136467767927
159.368376366657
AMD1 617.318955611228 461.940616438547 600.919152191636 361.687156919012
399.537363692534 606.38343657878 634.046843336128 560.015499136193
486.047284379068 784.412433571927 371.369895979832 593.639955546556
AMDHD1 126.439948611449 123.643292039274 129.059722322306
140.842305941666 126.462522391125 114.3172128566 114.155592351175
127.000175858468 116.736174147084 166.285052506316 144.295556086504
129.697584673736
AMDHD2 175.617504025145 161.724388995749 146.49022197174
217.287912825619 284.043349407812 195.239829623017 201.800357785022
227.499335248402 215.98963200839 273.75192868583 187.380064367869
204.877041314502
AMELX 127.517446456116 147.313494489487 146.238333981318 136.584199831088
134.449083873514 139.693261291326 130.226993687215 141.794898246882
124.295334523889 137.720747340105 132.883408554592 148.479865869644
AMELY 148.450023054885 146.533374238616 137.352231982942 148.390417679848
132.113317893587 153.267549449395 150.921252152443 153.44628692569
139.470557140547 152.409211647994 148.224587582189 136.553568679965
AMFR 268.034972830233 296.24781576525 305.577166690215 278.498278821829
394.294057009443 292.640251411131 292.28857006885 263.250833727291
255.793823796456 188.062732621031 244.689936990021 303.184785797741
AMH 127.409603645289 115.02681415256 115.545558600232 119.849895979393
176.735168607281 119.052729327147 108.861243071977 119.13963074637
130.557209560361 116.038950886552 125.800508653231 110.774802298005
AMHR2 141.760529549196 128.635684412304 114.740948266517 123.104790590045
111.506345174435 123.173506315166 118.918962530408 124.582577275358
127.028640787946 117.805556408194 125.826111950648 119.539713878832
AMICA1 208.914498369991 186.813888805838 154.623548525522
154.648806105422 159.806221777332 150.841261270382 186.778334749551
254.447650810819 276.138650967632 197.378801636702 184.25384366282
166.311421907434
AMIGO1 172.457165477145 167.526798465556 157.768329108254
192.059255232313 175.638328370133 160.156127781701 176.019082929386
150.122035262873 160.20843950503 148.416483022749 159.932941342403
157.322594990954
AMIGO2 133.459221120979 124.757229519631 126.404523759643
120.422401793885 136.903588075134 123.940632366675 126.499257771192
120.085340927108 116.142650095476 121.168550902281 154.528396813382
129.684916104674
AMIGO3 146.068360606981 120.015436934157 145.815454490804
137.769431537236 147.572660963285 131.159946917777 138.645156942894

137.244552699212 142.42442972908 140.603933807629 133.749271961438
128.711308958929
AMMECR1 193.945494721138 192.548337508559 194.240372898704
190.125009931628 412.202049714695 339.069385816411 288.88486628661
273.106088088825 344.604699562938 486.047284379068 240.511797905018
295.367125961621
AMMECR1L 333.676803661366 376.54010486682 306.716682293251
351.188086838653 256.202924421798 387.305348482856 316.281469585496
313.248029961291 311.10841853984 276.32528705384 516.088776850623
376.627801547869
AMN 150.868712184357 145.374923175872 166.447510782568 172.138554350738
162.895923745031 159.870967387108 149.250205265163 152.418315087218
157.269049853539 161.021740537372 138.381508741146 147.958483355783
AMN1 195.043838688467 163.988099963018 206.715020025609 183.83720937589
150.297422529071 165.926301648161 195.906137340918 172.376544221034
174.854570705 140.82351325907 204.990512488181 169.975329631607
AMOT 408.510567121592 400.676876982655 381.323457262383 412.801213309591
315.689865927834 285.102778078056 197.543089645432 268.034972830233
324.79327985156 157.635531050489 247.053215951505 145.889336010424
AMOTL1 147.319360896679 144.288714958892 156.748330788367
172.926288703434 159.257221624668 163.904137395349 144.28597792625
140.091378604453 149.615585907229 150.058160923921 136.861019517134
153.244758030666
AMOTL2 207.023221559302 192.891507272665 171.629605754643
234.432954093705 173.395370400371 211.104243881644 190.291185322482
177.428161690048 169.674931517111 186.591941221632 203.777944180067
179.100368312505
AMPD1 127.011594171986 129.366567668463 123.709293439934 126.734835642553
121.886886059038 133.412452607155 120.138014822486 140.471953101887
131.93683726912 120.827732270626 130.639974588163 115.775525016883
AMPD2 116.280157890082 114.771585411255 114.255733119076 114.400185930969
107.863971340407 112.219585614955 115.545558600232 112.428707152237
124.042153387391 114.405811161631 122.368581795931 108.118551275778
AMPD3 135.325617701062 130.856249657101 128.827129738041 133.66893163718
134.46179453106 134.753783025994 136.240503841228 135.784481523923
135.212715912271 147.715160308036 136.68056120003 128.356453959987
AMPH 188.39062571513 172.46311371589 181.803275243281 139.450788315602
129.767878283811 139.204218271711 124.033766796333 138.136901441896
142.596113491002 142.191569406599 140.692586715322 134.381042390464
AMT 575.060457772307 549.162299481184 826.771587455122 590.849222581206
225.75155298452 367.439588680092 382.630113452429 277.000882284125
417.731815310276 306.387898121409 681.810927901806 627.066031322234
AMTN 118.731960910224 124.815853677553 117.436119533535 116.029201658798
130.92186531563 121.803676441059 111.152235113722 121.824805374887
130.402496960735 118.442856984861 132.780055030907 117.909050015516
AMY1A 251.472556189141 262.671819506189 203.19299227665 257.706904467657
467.90388853336 511.374117402707 758.517101659762 411.968307579588
295.893482074241 817.529344685717 1028.80076860235 1181.97868824758
AMY1B 185.31769184805 170.430478598594 178.955938825556 172.578252260002
212.545989139388 233.71520737817 257.573614085223 203.298764374262
178.488874074222 274.179232297038 305.325519265387 266.395569467956
AMY1C 3504.77617298452 4425.66777919789 3298.73393277409 4246.19983955771
7406.86318549013 6384.66090426598 7292.0342195035 5759.5586948816
5032.66681917478 7122.07276863122 6028.67062754577 5543.71998558526

AMY2A 198.681190016247 173.459008248696 179.198164545975 178.496344474999
140.155861157819 151.852294315212 165.947514372877 186.268881719287
177.204172894028 150.509710470354 195.185025611945 142.501655618849
AMY2B 187.784865705837 168.538117820213 210.218440444631 204.877041314502
150.117906461237 159.41078351918 174.498521167568 203.436527043293
173.839053227554 179.286187825363 192.848268768579 162.308297940279
AMZ1 110.068122112671 115.933805625814 121.492896642899 122.337240239463
115.863243613121 121.898921780569 111.178024950204 117.893918180502
115.76177719261 116.061356450855 121.107979534361 109.416512252532
AMZ2 741.689442253375 661.390308090982 921.546033242474 668.127527385348
685.080426814888 1071.83063260499 1280.57297272231 1044.00217726486
847.276523445973 606.38343657878 710.228463443265 998.157643452741
ANAPC1 262.488050186425 232.801257700571 272.01602406899
275.888299685521 244.5675215931 348.300300174924 262.452869676209
283.701987275608 250.911817769829 276.434192872962 245.042781391047
330.005950459958
ANAPC10 246.389203940944 246.95071325085 290.954496559897
237.492603094091 319.812293134357 404.618867159141 374.268489263941
307.264586007015 324.25775261173 466.777454704543 271.004741629789
402.366797969833
ANAPC11 280.558463661137 294.033003779363 266.347965690851
351.934158805882 600.264940641426 520.761305892651 565.97975389646
619.380514697732 609.939982655706 447.182566890086 303.184785797741
560.602047253306
ANAPC13 1615.60472592991 1646.76016031831 2622.87486749984
1089.16489859253 1190.62010668045 1724.22121078776 1660.70040058102
1752.50728312967 3168.27825058989 1381.07447204233 1990.94596595493
2330.60389526686
ANAPC2 151.059401217656 151.511808301993 146.328876892698
161.419873271162 184.462286724102 150.449621353865 151.411708598688
157.148880624381 149.410729709855 147.777050372032 142.419777428399
148.470120335761
ANAPC4 770.632068590053 707.048754700047 850.882404546093
765.333633142088 402.473870771741 641.774812292709 644.97200337335
573.244735292944 687.494132125365 553.615252472149 1182.50261323518
678.937151822411
ANAPC5 1211.94273385872 1201.82026045778 1124.67710585154
1273.6393529108 1279.15873231449 2096.25803773108 1309.7660860424
1392.89422203207 1863.96986643676 1204.32784721885 1629.32740709526
1555.28458491244
ANAPC7 146.586879656174 134.094231940612 165.274189050141
136.461182044855 148.185056724262 251.347519777204 197.02284475852
219.619022209985 216.24497587588 212.147155923729 166.842970309527
227.144441241869
ANG 694.429144766435 597.459920611726 846.425467119342 541.460843251764
198.954254778846 194.374817778819 261.89195025254 200.84691030169
204.470661830972 803.133619199479 189.070238523683 171.216275057884
ANGEL1 255.435582426271 274.01377986185 249.292766428035
281.49247863022 455.21285026588 206.845306095447 213.652091843896
230.546762514966 203.616580991715 181.578228610334 249.737588031219
209.887047474042
ANGEL2 751.077307018319 901.197085099854 743.510369438587
846.084919104262 348.98762709818 987.564350211893 873.256632365737

525.920873434688 665.602699607102 391.706404954357 740.973440818582
983.777608766079
ANGPT1 268.328002606268 254.517135747445 284.940858938629
238.823244541016 164.647634120888 180.096239155058 159.094117857743
213.847316974717 179.841665686703 224.824362819477 163.059196519365
192.640717683236
ANGPT2 165.904887259203 195.123919692348 133.905162024888
172.446482096058 153.600132368924 223.84997313236 195.474525582555
197.47340234886 184.148058872585 315.615758089471 165.085010975035
192.848268768579
ANGPT4 122.94737290641 115.277302351501 103.826308328446
103.240215392215 113.963798749897 117.264555636952 117.856184272322
105.221735747452 105.859916438163 108.697696080014 112.198629213643
106.491867793742
ANGPTL1 139.767216030715 150.015696417249 137.907709799074
150.822586904738 126.606163970149 127.699226067847 135.673080643346
125.915689078001 126.748906552086 128.466244127634 123.728481007459
128.580625868379
ANGPTL2 2799.0487754899 1904.39962279504 2346.47361943533
2114.9754128463 229.443064589306 250.885949055834 216.290279815413
250.376178742476 616.014975366684 680.509421043347 214.714726648332
198.191669030952
ANGPTL3 152.461930328923 142.722227179256 158.541906931082
151.183847738922 158.521812954982 155.906054212876 149.725422365342
143.172744119235 148.478570934155 155.824409540332 130.211247943634
158.058346079359
ANGPTL4 194.029961195972 274.281532397691 177.96452209396
191.365014963729 587.654093308465 453.282973743379 1261.61611961272
639.011144305614 383.182507425579 516.577357935963 199.53455266342
408.510567121592
ANGPTL5 137.342348963288 148.204194003593 176.980780681976
128.575364985954 140.765231611948 150.70383460714 126.53466470733
147.751554743466 146.425476034368 137.342348963288 125.982423431253
141.908791151061
ANGPTL6 136.011474063229 171.314681729657 144.019824700027
146.923382271905 149.511776997149 141.632147679743 137.352231982942
142.142633050256 143.836637098747 130.073005698714 129.86336717605
149.668587078867
ANGPTL7 250.84781675544 234.980382243116 279.070493043898
305.16668987495 116.744048998494 117.829228895897 124.178483414968
116.902574164498 130.392575279055 119.29304762171 125.136745288071
119.097521397712
ANK1 121.723964989551 123.22295112139 128.266906202941 123.072413274454
115.639101191444 125.40172813004 123.219903763265 121.632494500096
118.983932995126 121.752481757054 137.025625133986 122.460829835477
ANK2 211.22652563815 176.27969404442 188.685460181066 220.896363324634
124.503016568677 124.528206512938 119.640086587188 124.35721327773
135.227424713702 131.414690782328 129.707421699091 127.159947766922
ANK3 178.551434222154 175.679405338399 191.830711714844 165.607095120227
167.030667499538 182.833961304079 212.33360547376 171.759102794108
164.048326497989 158.263657373911 245.317436234586 214.8478629277
ANKAR 169.132693907633 156.213421099137 176.214950733324 184.294172429054
159.534017722536 142.836229345273 147.169765031208 142.153830345183
146.609485664113 139.664055668127 168.763640193219 166.187099519035

ANKDD1A 1227.36706427754 948.734577648136 755.79669622705
1195.07608384189 274.934292929666 193.261343922604 507.236601481067
314.486603134656 313.351441953859 189.708396835236 436.834825721843
390.501797367707
ANKFN1 111.137519376499 126.832182961671 122.287535209629
129.049273337631 124.646093292382 122.65547320922 124.451718955086
125.166940410459 123.612648219993 130.066177538896 143.036006001419
129.777173431404
ANKFY1 438.03648998391 434.176007498693 442.12886967939
438.611973005212 221.512905853031 286.376638135463 231.981501596284
287.084253886676 268.930428617695 301.546624239855 322.778260588465
410.216584967666
ANKH 129.365004129784 145.690634269858 119.594962686756 123.638001112969
129.145457341484 139.435000959919 121.400652285172 113.644993086829
124.313901870563 127.908872828095 140.855078775719 120.26749529985
ANKHD1 202.568071395884 184.071078414327 208.914498369991
173.790848780011 165.411052799773 220.9384439772 232.918330930051
211.118308738617 168.437049123012 260.888612454097 213.123453925578
224.547982731402
ANKHD1-EIF4EBP3 174.615694779386 177.500110974116 150.660106369128
179.914070416793 173.10229478279 162.140112952434 170.951009118578
175.891947478891 160.32812671237 177.812335593522 187.389039221193
207.076364103755
ANKIB1 684.428198622716 630.176145684641 705.529158880221
475.833242673151 579.493790910006 930.704688414069 880.140852200235
754.97177863997 714.052063877292 867.369315017411 451.718912714608
1295.27257578314
ANKK1 134.648780131578 132.01588294465 130.907879150229 130.979513420888
161.972922129453 132.796961054534 130.268322464805 145.534520042669
137.934850241054 127.372242892513 187.15740398852 132.151765465534
ANKLE1 134.233014993992 134.125038192486 140.857793157932
127.086470811408 125.160161268474 140.726084514407 121.919799968293
138.896595888876 145.967685593635 134.896349513392 133.075748695526
136.101501973431
ANKLE2 371.552970811499 302.809011218879 380.514553097613
276.830814061474 371.693808540758 382.562403424855 431.974037914233
260.278595572575 451.985108262982 422.23268864343 310.426513884979
431.861762635568
ANKMY1 154.420795931034 163.18585933431 160.449911844008
144.30519737569 158.157097243342 142.722227179256 181.54273463264
190.098509544816 165.932628858503 190.138730960643 238.745953695925
232.778488697337
ANKMY2 526.896146537814 463.488995330457 526.182262872235
390.372738176917 359.099750692694 294.069353373633 476.917496749381
456.201998988891 450.120868656638 269.831616158113 610.070236234299
537.06835963726
ANKRA2 477.626693328013 430.94199035855 498.812823949508
358.654569870431 322.099431638246 477.489368828457 865.435843446989
391.500696444149 412.202049714695 326.417021307859 660.535644289024
730.988069115919
ANKRD1 154.2433882385 148.932696684374 148.034899103241
169.417764098848 154.555255038453 173.260348096238 144.914015211747
138.178987367473 156.043994260248 194.500222559978 141.652000792271
152.789338329505

ANKRD10 619.380514697732 547.202354339933 735.8141982568
451.210172051464 263.183010631644 839.662936279727 351.395760651605
498.334266735594 466.909037277853 353.078842299761 543.001249586573
528.650794915616
ANKRD11 449.408793796673 513.428413503031 463.754869412768
600.264940641426 408.993744895434 303.986544460218 512.895335481876
392.430873293903 651.675427664911 513.292377117011 506.659740456829
564.056592672374
ANKRD12 581.54874141672 569.656573915709 680.743052197025
469.333357789149 278.274343962656 378.920456227378 373.189809737885
472.793332506369 390.897464546943 367.567681162661 430.94199035855
436.834825721843
ANKRD13A 1632.84261181074 1268.73280473728 1497.32522786098
1726.15788525017 455.124190541055 632.565736446505 641.645855499704
486.524284354103 1064.12835214671 815.045148478814 616.701037274509
574.6506349984
ANKRD13B 170.646233722767 167.976680048356 152.390371614602
202.357989894268 175.101284960252 165.41729888767 163.552608805894
180.619956937489 163.175801793143 174.787077606165 151.054834307689
147.772490977392
ANKRD13C 179.46936343225 184.451469623426 220.362946606131
193.336246844237 212.78038645414 248.643311972844 206.77605957523
195.333029746339 223.941946219233 248.085349787686 169.003310163058
212.396522888943
ANKRD13D 170.05799929575 180.070209705044 154.528396813382
182.77693508164 205.260673843294 239.277801396834 183.280853265083
163.104822161892 162.096065738375 169.257035030405 185.570062118736
186.951061159734
ANKRD16 214.38058301395 214.200081541057 210.245551279259
236.548439331855 337.22646304739 315.615758089471 238.509970054862
259.316557834724 197.72068061775 170.602603155092 200.342786450248
253.186749111526
ANKRD17 269.981476129439 274.371523790341 285.172001915966
242.269784551092 300.150235383065 263.058972886829 294.871848467326
292.366934378643 307.020265874666 368.466518140043 350.46078795627
326.640168906375
ANKRD18A 147.036768856136 139.306122448857 136.384308041001
136.406132339187 138.230197299262 134.582098893327 141.5739152922
148.532339759414 132.423043364573 146.792951584868 143.063345350253
142.01454627498
ANKRD18B 126.127630885864 129.777173431404 138.981712781568
133.875667250136 135.503338987971 135.774569629738 133.555814326103
127.935614145702 132.813199199389 135.227424713702 138.83304645915
132.283682066898
ANKRD19 109.625579596864 114.818898581401 110.794219964997
115.053039576143 144.992993261203 161.514651272858 105.771723307783
504.275299381356 235.822266856967 158.425950902309 134.325520377207
109.044879188971
ANKRD2 148.327340543504 156.96934803556 155.303903514061
179.30729544595 157.57914268719 159.49930229951 146.261164125255
152.101695555105 151.2639801322 151.543678860049 140.398923642686
150.137061170632
ANKRD20A1 250.406775889932 241.320049298228 219.940167815376
251.546290552828 161.451253146578 295.330307669482 364.678403892354

280.798003990849 195.447966116819 158.186816609641 224.118739978257
212.545989139388
ANKRD20A2 118.276198592775 109.176852761745 120.042717248207
124.033766796333 114.048136176142 125.290320836648 124.256773437346
125.193729067011 111.299115402008 121.36708642226 126.75720720361
122.226875707253
ANKRD20A3 132.570225520163 138.899292894875 136.904851056919
134.102929804129 128.05898686833 140.113334247294 144.110544384466
135.25734629146 126.762116571216 127.706499722854 129.329521415816
137.853137973756
ANKRD20A4 119.275061991187 154.296467085914 145.954700424725
150.303703193967 149.77596374205 125.189588215659 144.505072819693
138.546549217819 128.994977935861 143.812863334832 133.857996630836
141.449119281888
ANKRD20B 129.707421699091 131.221105247771 132.137634927459
133.12904935533 126.34649262976 117.897556445952 118.091125088778
121.556331561879 127.466779212194 123.031945101045 125.94880312094
121.141432480683
ANKRD21 138.142193489481 142.822594215168 151.632801137039
145.997380100999 158.819151102054 140.878935632862 150.126627487419
141.306418675404 138.625680450198 152.660373241584 140.510889880872
132.645196037302
ANKRD22 175.363223521128 186.975609955892 194.702974020747
174.375388013694 199.423120300557 474.378918787006 464.812091712746
393.264726588522 280.085692727562 354.705553237406 389.853534237748
393.796356067403
ANKRD23 156.911538642765 173.97085982677 171.426334687626
161.795378938402 171.465178761171 158.034418043685 170.376620730856
175.084729757407 162.431674335726 158.275283892632 153.494456800685
158.967160833334
ANKRD24 137.840396641618 145.029298288869 138.30326146769
153.140087873773 139.299062352035 143.939522733945 143.628147237159
142.52234368939 147.870549240703 128.009286101341 137.757902615328
130.423465505321
ANKRD26 185.591700082272 170.892319361167 191.507657960386
157.541796722309 172.774914399844 176.456539071735 182.300628120658
155.728609337723 166.263781040251 193.753062958207 178.584378959369
189.758013811338
ANKRD27 316.524115715585 306.43742010208 346.996310517446
273.668871896558 274.202831856221 247.111032124864 251.09230266334
228.100112133941 261.065986251886 239.572670410818 207.467591256549
199.756584044664
ANKRD28 211.059870865354 208.507486041165 273.106088088825
208.628626112623 179.687464522203 300.314088267108 210.392662092721
280.007569817323 220.098071755353 200.831877950718 237.01460308334
227.715558644326
ANKRD29 237.473927775823 215.967319486874 252.138168292964
250.626556981376 131.374680878107 130.378441113977 135.697550878438
128.705587172572 145.666712006532 212.531329857909 139.826273596452
122.932745723044
ANKRD30A 130.878694881324 119.083957999725 123.905462079972
125.081880303992 115.473431881597 117.590393673861 121.464383202986
125.347442446239 116.043095688871 125.367386907897 129.57551086282
120.122719226116

ANKRD30B 7815.98936145067 6793.99737496637 7969.0710242842
7016.12801650552 2000.47497636965 7656.97151002785 4367.38154535901
7144.3257417116 5947.89821087362 3807.16206340771 5382.31371568106
4793.22133852447
ANKRD31 152.935963398921 142.51308872439 149.070750686003
142.942021904533 149.968749911496 145.401101974736 158.226547375305
132.70442458261 140.295307451188 142.363309192458 130.57903858574
157.221008210698
ANKRD32 155.91493956251 143.0235772247 152.486212022518
140.57567768682 170.212679845194 166.277960843095 153.545086287929
186.430014478919 163.661196423902 164.708712337156 154.774977482202
168.783725505491
ANKRD33 283.27806319488 314.708587131145 317.904396212359
317.600728351694 1649.46398639795 369.906476027936 875.7694907383
379.102958448288 437.601814037248 707.173691257322 995.812184456562
369.78624104057
ANKRD34A 169.417764098848 194.161930642068 153.829636228764
216.805365763069 145.569870279379 147.257773507186 133.487774861252
140.377179372804 172.222262910052 150.261107471642 160.446191353364
147.51773051441
ANKRD34B 107.74537523456 117.824694297846 105.348008821162
108.818976813708 116.773599713111 103.36057518781 107.013012418356
108.167670899938 115.264104392064 108.669141498627 117.137027732123
103.594872791657
ANKRD34C 148.789295862953 137.412952822912 164.084737224884
131.626173473789 160.019067307733 150.593928635647 146.198331189991
168.813895764991 169.158049136321 154.455641374384 145.52979050795
165.545136704242
ANKRD35 603.569635276204 597.651682540611 629.97342429259
695.595965352335 173.919504730673 175.186435016433 213.432084172541
164.991522994763 205.305702209672 248.887522059485 186.975609955892
189.693218759349
ANKRD36 139.446914318877 145.235087732482 153.848852170776
133.103403333542 130.735223763219 132.566074764755 124.452631766045
158.07640654075 132.890396520305 146.479009988445 137.698423468762
136.899405404096
ANKRD36B 220.633692394229 200.342786450248 225.64288602828
151.621024295494 143.637018777217 162.494429648551 148.096551269076
169.488652117125 159.374355553219 190.291185322482 164.172084711099
141.310253828965
ANKRD37 259.252807304272 291.167223094542 296.657844888195
244.486311003619 571.64723305848 133.810472192646 441.578261054602
311.157764305033 306.716682293251 2240.33212242003 368.134303229682
286.856346704286
ANKRD38 199.53455266342 209.417052464583 154.096542063701
166.932757191847 127.972708116366 134.134810805413 132.287326702468
151.573920235798 151.345630073141 417.191960489528 160.411218029114
160.297609596399
ANKRD39 251.062649517773 257.075088169589 295.507504000509
288.481981529558 316.955298948771 356.84281623052 277.946395491073
264.955939320296 190.520750756082 263.352118305612 205.011514211101
328.680312364877
ANKRD40 243.308625792448 256.424415389574 277.314675225943
264.158648784101 281.618175110153 238.037110997575 291.683896942007

396.212732639272 304.636618563775 280.595515386655 289.257002046658
265.43897208356
ANKRD41 130.056727701826 131.910285685889 131.08389186558
147.079157199448 135.474834181228 136.19083681188 131.436176224725
159.282348516052 130.097543328456 137.306031826007 141.082569618216
130.231160879338
ANKRD42 127.146101490884 115.718600552187 129.058085094891
124.093412630817 126.969504644414 115.247223837316 118.924317138158
122.506058130261 123.833219705499 120.702740607924 136.381058433385
138.675023495021
ANKRD43 143.530190096012 140.603933807629 143.188958538221
141.964796033176 155.319717753912 140.971253689199 147.653573379877
146.785478478701 167.599784887933 135.809383370957 164.571974509529
134.517774302213
ANKRD44 317.353318759931 294.871848467326 306.193157874683
284.043349407812 131.228779762508 313.828943866034 193.704510912321
229.116153948948 193.275162354467 170.910998705968 285.798047501616
221.359331823286
ANKRD45 160.265389854694 150.868712184357 165.257737836487
163.298729857831 137.491366243256 140.005968571997 135.663734178125
148.977202433511 151.308872564827 171.465178761171 145.423022762916
135.275284142536
ANKRD46 558.934004505074 499.544050752036 580.474775712828
400.472835504879 403.657727266403 421.384700184923 416.861099188252
345.732364434125 487.358720934063 294.697622870418 393.375554593484
363.445110627483
ANKRD47 158.573561336755 161.657344146676 158.221595000183
159.182533998843 173.334154083555 155.952902855994 165.225475655526
166.784804508647 160.17208299932 160.822287037366 154.539367933684
151.032389358279
ANKRD49 303.483515328621 272.193578770314 293.692083555844
215.931781291835 265.068532248682 272.927830312746 260.715580924916
313.351441953859 254.812605074045 206.469040492609 344.379733302418
255.945470052489
ANKRD5 125.475308885588 128.914885412263 130.150998843383
128.662680062294 212.443407602682 140.177708471199 137.902953501634
137.253971514177 141.378381097266 139.868714816245 135.455283504668
137.002473710189
ANKRD50 476.383294794912 463.754869412768 367.983166440977
396.212732639272 211.692743811486 401.189904619902 546.841563406813
232.621526025603 213.754156680604 312.961981998512 705.311256840178
1075.3328563531
ANKRD52 195.033792280119 195.831574717135 165.757295630254
255.198862778405 200.08007665693 180.060096787617 152.054874792646
181.467708718758 182.560363283264 155.960886801091 187.65716654049
161.408318705503
ANKRD53 141.404832197192 150.031863363697 145.968884703737
139.687574432387 132.446406243531 151.663203772586 150.596350682411
153.263883774915 143.987296454585 141.668101962049 155.780657091484
150.534608517044
ANKRD54 387.711471687237 436.982960786839 357.006610037663
613.846166856328 418.772484676858 277.946395491073 245.426639190893
237.312766830206 236.933960698783 227.806050911154 314.610979086485
252.851164967726

ANKRD55 111.7529190597 108.646136431909 112.645720918926
114.213405341709 113.791585365477 106.900679222135 118.731960910224
118.9803314875 118.025603140924 113.347609066395 144.544001385895
103.09089100768
ANKRD56 129.307025798583 143.103779196037 147.995991912855
166.958831664154 186.430014478919 161.510497517852 161.979914539039
173.017662861015 169.999692055871 151.26137860684 139.435000959919
164.423960071908
ANKRD57 1044.52286986369 1085.92360670051 1116.95795024994
855.415038498392 1296.10827265334 3282.73711809465 1430.20038077595
961.931130189779 1136.76110352875 969.811933814244 875.7694907383
1328.38230265655
ANKRD58 144.762006548375 135.105147647223 156.375876454394
179.880812358974 153.853856339585 148.67584969749 155.896065329825
150.367927843529 149.91705328815 167.961390966538 148.216605879958
144.32655838488
ANKRD6 258.348298226232 242.019489058244 267.222762759781
280.007569817323 193.945494721138 175.350734537491 321.049000153127
183.397833894087 177.72965427263 247.528753010193 180.315825600347
177.708263037199
ANKRD7 150.224472335504 146.867740388881 137.440295223749
148.231928963878 147.606311227037 146.29600238749 149.744358230709
147.236728188605 140.916876460413 147.400734637481 136.575784685895
138.401406375804
ANKRD9 435.796737132969 478.699172220804 430.833137328233
558.051041108282 1149.3250945792 389.381039495553 719.628501451187
547.681206272188 397.861172312706 1497.32522786098 717.419388886784
738.579262579782
ANKS1A 1583.79384467611 1821.08523647593 2645.72125268628
2482.27419621925 2850.98525554116 625.544719251096 1379.95968831696
2273.12164779441 1563.71477584676 520.962651968997 1010.23703166498
555.607185132941
ANKS1B 224.724262742427 243.080229016932 226.565823278767
273.550544349798 135.05209575317 150.942078067308 144.868337748544
143.48729871068 144.895601314903 142.806728419652 140.934844668435
157.126460242995
ANKS3 213.301947003462 201.913709602894 251.665539319612 218.773153183388
190.809584998617 218.000351930307 198.522123898437 212.016247864691
179.060189557496 183.302479730653 179.687464522203 189.37832342197
ANKS4B 136.421163870578 141.253030949701 141.340452225686
147.306549450597 136.176688025337 136.755975832051 135.937066152894
136.211694310501 140.42579083092 131.724061631389 124.193603137858
160.861929142249
ANKS6 150.250193889942 139.984102798711 140.152177370089 138.175538905334
128.867710054234 132.535722328813 128.482976013813 142.073529553425
125.165254272421 142.981967333546 133.99226161886 138.709991561675
ANKZF1 182.02250582556 191.170105307944 206.845306095447
192.69687837782 176.183165494563 160.880152014981 224.62335524729
178.106013202731 189.070238523683 205.821101661022 222.051005238856
192.655899720909
ANLN 128.0805889548 140.829927714838 121.476730561812 135.548042190047
408.201232787978 355.14024238296 183.64389891975 309.5988292104
297.332272659995 334.443733461841 197.378801636702 417.528353577952

ANO1 169.815053752464 214.355086255855 175.585857393085 168.314966639178
467.04976953755 314.780899041937 141.21138348075 164.886944426276
162.063689419737 187.586093248437 177.001637712889 204.453822958077
ANO10 261.286548990435 289.667302610069 249.263194321516 271.858059712406
495.769802520637 321.17798246832 253.867947800583 198.659595851481
244.219585315024 324.067396271281 225.142823691625 226.701637237203
ANO2 141.777089987975 145.112399373761 145.414563914801 139.917662338546
135.286686011087 164.223990197735 150.471558219667 149.836491640473
145.428297145967 138.041411135053 156.916784141484 156.018200674475
ANO3 117.824694297846 128.354163132392 112.026113831896 129.420716653358
135.875595614483 129.246884010589 183.115511373872 118.480344364831
125.746184969983 120.413468119484 131.523538461751 130.928718312512
ANO4 143.455941298415 123.781002620866 131.099004325598 124.597605631582
124.847325289068 130.40517506216 120.006982591236 122.714122084817
123.019430618632 114.822700007686 132.018833785059 120.488694213288
ANO5 110.1278204598 110.273174147374 115.840266274696 113.644993086829
112.10312035128 127.052618800004 104.248226402465 108.589444216106
119.598216955485 121.45175516842 125.621467040793 120.470801825675
ANO6 1364.1224589383 1040.73829397913 1680.25851465727 788.244353085369
352.303782547593 729.202002368627 805.120147618984 883.702573960415
897.958748317315 606.162976359615 817.342172321486 1025.27246494218
ANO7 139.178245419517 135.348086420636 132.508133846896 141.469818222503
139.545451634208 135.832454000859 132.762755202326 127.633362302159
134.48825480809 136.77666893627 144.182891591399 139.125340284813
ANO8 154.455641374384 172.002600588279 144.796089646941 152.042744996136
165.659978250378 158.911404625381 190.682392100564 187.494677180331
167.713268780664 220.959843573708 219.564603776774 196.378888436237
ANO9 140.833666276568 151.330035086652 152.479402912801 143.175483349968
239.941641600769 171.229729917612 168.427124752461 174.953232190285
177.752497164503 204.1974438819 220.876114717642 192.244413515864
ANP32A 250.25959091612 215.698924670416 244.280971257965
206.571433405844 267.800523478098 228.820894086914 290.090495047722
189.2642795052 200.146843808423 247.340470321453 271.989060206794
274.371523790341
ANP32B 5314.0635307876 6077.64695330731 5932.37714987741
5670.70220800096 5149.78271096158 2708.20624364471 2850.98525554116
3862.57980488881 4437.40805296517 5947.89821087362 4246.19983955771
3251.62784659994
ANP32C 155.478383896934 159.946124520038 147.8838487685
145.180346905484 174.09267813338 139.636177212128 171.884800118791
146.081940883446 151.360575274272 147.373174160478 181.215046876308
183.247396324425
ANP32D 153.667355714857 135.638156623519 149.390481900424
143.521510363933 143.937726155116 145.226278521493 138.829793136954
133.465593684519 155.762890407496 153.095594614683 118.115015846489
155.598586699376
ANP32E 157.997750997487 152.397984799517 151.535177950367
162.208028489943 155.683727621475 141.132694466739 153.916313595581
146.887506793665 139.297276687352 140.395662148924 154.431051053529
156.680734054531
ANPEP 232.519482087835 210.590774758661 180.290887733427 187.978627462391
163.718595974283 168.958694643382 159.922627970233 166.674240866931
170.169402943662 205.728673153914 144.78971417658 155.04585737344

ANRIL 149.714982846365 140.975056186954 143.325710305008 176.267796612066
156.732595223778 151.148295679242 173.599961799334 157.535792192753
210.043556816529 161.498785105514 138.601616246182 159.393410854649
ANTXR1 1455.75693295732 1071.00507864299 957.54149804446
852.436775264873 478.38361571213 290.037325939883 348.111872425949
278.219561902233 384.641319423853 688.915534194306 379.476768908492
250.085444708918
ANTXR2 4397.32311216672 4000.45196171119 4892.17875938832
4022.23000497332 192.07791693203 309.0578543424 385.379119927642
281.792602269388 471.315730378641 330.850098426464 301.087947101876
434.943371634824
ANTXRL 145.442914818456 154.645353009618 148.067562465748
124.466986821169 117.995551728392 136.344248873826 141.277227483198
130.682112236817 147.322622473251 141.752031758589 126.279119798177
143.438417529799
ANUBL1 131.436176224725 132.387242907281 137.360220389719
124.160433356046 147.019460117987 123.887740029279 125.488511939819
129.548491595432 122.928863588006 119.539713878832 134.758222337831
129.01094835739
ANXA1 2242.73511149559 2081.45603839306 1750.21700780511 1699.8374761666
2267.0818302379 824.070046083713 1177.01310527577 711.166793546075
959.897744425251 13247.3528304243 1345.8970795036 624.825216056495
ANXA10 162.728630453745 201.676984786688 180.610891208146
151.382199195953 154.173439471404 794.165408266062 2399.88695085752
1231.37475540937 165.249658565871 145.314204192565 3403.37530051435
7617.316805798
ANXA11 715.638378711433 641.12919339861 707.715412321304
652.429025114523 259.426050921239 364.191263843897 387.908774438461
264.429717032654 468.496972957503 260.955030428944 585.721610434501
592.613689704907
ANXA13 137.339511714364 139.271829906127 136.743659350798
132.504264154139 131.619951110473 130.179742057015 126.042731009527
129.697584673736 128.775477649887 130.571827557149 136.020600085547
125.777193592751
ANXA2 2068.04582900231 1934.30779703747 1464.04124596264 1875.21979956596
1301.18893681695 1141.13648305827 1219.36024606325 1031.59140059071
1789.51979173209 7342.79622458743 1548.09554827056 3073.40543048878
ANXA2P1 1776.38646235135 1883.74008471233 915.579581760396
1447.07989164988 913.22732386079 623.888568444132 809.362464219294
651.998433127992 1091.69584249713 4866.98027226798 2596.43650409044
1789.51979173209
ANXA2P2 28530.8435957612 31402.6950316226 27667.4034204988
27028.8951412615 26082.8089351205 29532.7890259497 27501.6698286039
28095.1627766565 31402.6950316226 28193.3833726673 18220.6383250956
29868.1819370827
ANXA2P3 184.306452616482 160.180961887686 156.316333575406
191.740596601895 160.055242985219 152.382558543399 158.157097243342
156.830156083795 158.48793705795 355.14024238296 176.486551181451
201.913709602894
ANXA3 146.160188002433 170.257439890277 174.737828693216 156.191659799142
269.495247190598 226.315173823168 211.438615793742 146.056723380149
188.557113298055 2462.87034563426 237.266455875982 172.491604217899

ANXA4 175.210232663606 183.779065928447 167.941671074236 138.377628971966
357.431640917696 307.81941988503 263.994460303088 372.94852645418
1054.47292895417 317.244256751418 345.38424761963 181.364325529405
ANXA5 2723.55722258794 2313.56743052616 2760.57475805426 2779.89882032092
309.318403417731 511.547907091166 323.149792143481 542.883453307
1206.59866455529 3455.20639616159 331.464977124667 519.564563632951
ANXA6 301.369680426563 286.376638135463 339.252515920242 433.11536069928
148.049570580734 144.30519737569 148.482530949134 156.210423316722
166.411219327671 149.182972268104 144.853868519575 144.402160347818
ANXA7 695.595965352335 623.409211766224 659.673052713372 475.403662030875
321.627969147543 339.54582149284 381.971539806271 433.11536069928
407.244564912365 418.163219634152 292.524552445564 447.779854598556
ANXA8 148.00952002903 227.736106796551 165.67554738735 142.61573330083
633.401624753368 811.564396886535 1076.09385014053 360.053590374552
537.510539142248 2358.46955614953 1402.14708471728 1057.1528284934
ANXA8L1 146.235832275574 135.229197100212 131.144774362059
134.483162806498 175.442400672352 237.854774190873 246.552713031445
158.325554470875 176.121563444364 480.22496056277 244.162496497466
299.29004204677
ANXA8L2 128.692917766138 138.98468042464 123.482626777491
117.928456234796 190.871696848118 435.037306125887 336.5543056115
224.92725876841 263.204269632206 1074.96415574964 273.911741951291
652.654673756838
ANXA9 114.999628959921 122.607404698857 115.427201752401 124.381674909742
262.954856857756 138.452698593232 257.989863290786 213.178648382852
348.171901445232 116.456170659686 249.379605914827 178.619580818758
AOAH 140.543382412084 139.841822983821 120.626155234319 131.097598299707
130.027227278931 137.183566882805 137.536053775531 145.540872377507
162.285199080777 131.12953085854 163.480722123193 131.136073942692
AOC2 163.170983091116 151.234397206563 149.883237874886 144.240470871638
157.932243807202 164.509354377985 155.233830396052 190.534176839652
159.994376562272 153.649235507812 151.98710761363 157.809081184874
AOC3 1366.06309834426 1858.12855367119 1136.76110352875 2979.27692778783
154.193086881801 193.084818760857 149.133055368147 218.559622620349
177.802767772786 148.547259731346 158.058346079359 147.463822782303
AOF2 615.254768508691 758.517101659762 558.621429366984 681.810927901806
1314.62071412501 1306.20183851747 1260.50387106816 1311.34904760947
885.927972780392 775.382397816601 1566.65825409044 1123.93087912174
AOX1 322.837446618323 204.596753034535 394.73732350607 176.312626750427
109.209241676429 113.905364405553 117.667611937195 116.280157890082
137.006917665541 121.771891473027 125.525241361472 118.783904603368
AOX2P 142.720769329396 142.752536416014 128.96181458192 124.86699236406
147.1476865809 131.999299893526 139.380610974147 135.285224699704
135.841309719081 142.01454627498 150.907594445048 143.543607779991
AP1B1 271.196028302639 345.494740481759 279.969975697164 383.033242143031
838.93563319979 617.833194119379 588.334329592604 422.973606694449
685.080426814888 414.888512708716 462.888171502285 624.978351618077
AP1G1 337.271784551586 275.203046536524 357.191675549592 251.09230266334
254.9636051363 431.304147578425 317.493387040816 449.598181155897
503.285430318683 390.573484082597 458.218913259336 458.782864873657
AP1G2 184.900027626401 192.465510013851 180.392337212428 167.67952650068
496.697565640162 248.761038207278 437.049581919144 331.999693230194
298.706655333472 355.879841671975 873.879064183441 516.177596584059

AP1GBP1 136.762273376313 137.752238510249 136.181791404331
135.508803488048 154.728941096647 159.012011848781 158.989652405732
176.410016510249 167.822075935474 157.631978594005 160.265389854694
186.420541099902
AP1M1 824.070046083713 936.560290736127 846.084919104262 1328.38230265655
759.058176862524 619.635734567779 691.397238677552 739.085574858917
625.374595058488 860.688314783077 837.872187194784 696.650617523899
AP1M2 155.176714087209 174.624446035895 149.007288765628 146.753212022975
798.1490651671 364.265100349672 281.168898910065 404.618867159141
258.4424008891 237.249949461085 459.068582584181 308.621688157016
AP1S1 198.135240691225 201.931760074628 193.689806171113 204.031081842809
802.304294140514 535.861738240441 500.848273432862 349.485727214325
292.746711619565 436.222568287601 625.699971780258 890.121325723286
AP1S2 809.729160605727 834.537908087672 885.713964813911 849.158029646233
189.816900281053 421.459312180809 216.485113690708 326.703488388966
389.260166168847 438.431160975498 262.019779752857 212.073472189229
AP1S3 145.632060621034 140.711699577809 124.857461801227 137.990806386929
133.216489794592 148.082026641671 161.035985824654 164.820007934887
181.865456674829 141.524239210761 149.400244837133 145.229637715814
AP2A1 186.794165469219 188.981391266203 173.272062328378 221.424451092316
403.208138816247 239.941641600769 163.175801793143 223.128108720503
237.358925391616 182.798526632723 230.770036998466 207.488522933448
AP2A2 307.363302726074 317.244256751418 308.208276315292 369.78624104057
283.918434443669 188.797908601851 178.270849389719 193.148369507405
168.124936529582 200.790119570144 206.376365685112 164.231281729415
AP2B1 201.676984786688 234.484787292284 162.240414467004 308.679189788554
293.018282386003 237.692959394487 167.746786642066 144.474814266475
149.029275579026 140.738977175997 344.529927973082 166.440530119785
AP2M1 922.877217016704 1098.20033709043 941.065508539108 1566.65825409044
1440.14449872047 771.694853062033 818.761934233826 609.665323786904
861.119262963015 795.720196538039 1017.09497861942 1011.14472068314
AP2S1 2712.07480130502 3038.02573182546 2328.65814406636 3876.04966263281
14798.1358474998 5175.17279574945 4276.70912292877 4190.38366101529
6384.66090426598 5281.353685715 2642.24374855334 3354.38704439295
AP3B1 955.604404066769 969.811933814244 1172.96121022579 1053.09915077366
687.269774217064 1587.84395475807 1272.14273680177 927.137881369911
1041.58583268313 1432.93880448974 1119.23362648056 1466.08446530283
AP3B2 156.642441706727 134.703474191011 130.613760017864 133.077760160486
201.748876422401 137.747088378131 155.237180851323 140.242272149424
134.553754121862 140.326012404526 122.746185691827 135.6149724596
AP3D1 290.541184606565 313.773476049783 303.09499683953 408.361368902627
424.665056900526 314.486603134656 320.097510212422 276.284517459626
306.387898121409 303.555333002419 309.0578543424 335.790909130243
AP3M1 284.595024875896 262.53374754047 322.778260588465 268.148673257455
237.456245064363 292.185246573643 346.566241319814 334.377352542623
369.275635424092 269.072221443403 466.691191261997 364.022018936132
AP3M2 495.655170027296 505.560841055884 362.849380477089 501.863855158062
376.007938436483 825.034504959272 563.490027149224 337.728737393381
387.305348482856 325.80154888448 792.634423781309 570.916936074747
AP3S1 574.779350011402 505.404271675438 719.766799746895 404.445038343721
278.555009982029 375.407747753058 925.009157785129 583.314509855875
421.583441056026 1871.91894530722 495.769802520637 613.279465441319

AP3S2 177.652022895288 193.064450357153 208.983138060317 212.443407602682
173.681078272467 153.67215573765 170.129558603697 168.982086590306
166.040918339519 153.256208333775 184.709768457405 143.447768653042
AP4B1 417.36454228858 429.812058546782 440.579262120451 484.658140651032
338.129250886594 454.34729744062 374.56251861444 432.088303693555
497.53605530806 436.891409783298 320.812909889014 311.966971734249
AP4E1 303.555333002419 312.601510033941 324.56043349882 338.326304736048
251.704037951295 244.401472859026 234.626574390973 243.65518489122
245.240006198482 313.888035511709 260.787104931135 281.369059516242
AP4M1 158.344330353326 157.419333185365 151.406384541297 162.10411532202
196.076386020815 125.347442446239 144.636277619886 145.076068630259
144.231539210695 130.495294897642 170.326474367867 155.158028043823
AP4S1 141.59046197203 147.562757301251 147.238896426423 162.439052954676
140.145470073979 131.865164933746 156.62031503525 141.521742442341
149.348231806916 147.743331160624 144.008961720971 132.554328715753
APAF1 207.467591256549 235.704824490397 242.299252896647 206.042395282286
205.226635237992 197.24751449599 301.087947101876 242.019489058244
244.990374822652 238.588762326319 352.945088028205 301.139737613279
APBA1 122.320966091375 125.579970155301 136.159825652148 128.708956947928
140.796641921516 133.216489794592 131.665421461826 130.12638520846
128.69455713173 126.372374084002 125.170874841845 132.934955542743
APBA2 124.263901299025 119.9414421871 120.09801164252 123.084040312047
143.078022283871 116.519527499611 121.855752708908 114.704831217037
118.841699953612 114.755442050481 144.675182563174 119.711070285785
APBA2BP 152.495540563792 161.027590226123 165.048922660581
150.560305000601 196.638594797353 132.832556359483 151.40067812803
154.558968826495 148.09868976776 144.83363420705 162.484777758676
146.431166706908
APBA3 285.214585324341 282.101714660307 298.085365348496 325.387193207414
423.152420192284 276.184421402511 428.959561932229 378.24336892367
314.4408959517 360.90109922539 270.568660560796 307.665281863258
APBB1 138.354300118735 138.455086062782 129.299810898979 141.486042999328
129.185514320138 122.742891063864 128.066043751951 130.818181991958
123.446714388901 130.219888837397 128.867710054234 127.174074003712
APBB1IP 305.096320805078 244.440857688518 246.428746807313
271.287767448621 225.96618659877 1397.84878368705 182.152956182443
463.488995330457 475.403662030875 184.542232315662 183.302479730653
146.513643172496
APBB2 130.545285774056 133.617501101389 136.824357247954 133.895547281411
123.538037290702 131.839890565869 142.709768829207 133.391421497441
131.963419175907 151.243948028303 146.457939801041 133.733674016447
APBB3 542.121375354644 483.845891531326 677.802147190757 559.899430596368
311.04252785157 239.24587884234 587.54075434249 546.274050902567
363.89527566597 1222.014210447 445.348044448953 482.466736153026
APC 152.137669424501 139.755211300398 138.110317032494 158.263657373911
132.751406897891 133.608461289137 148.263293819238 144.96021415188
138.764834713216 134.11753210296 146.166710487448 138.055909969804
APC2 118.955314417914 124.33863635868 119.32696696175 123.817878562971
127.003974141317 120.276236447255 116.031631038289 117.873333897609
116.572001618531 123.081299769162 123.949411804458 124.952026709219
APCDD1 584.813665097407 535.20662670885 345.897250519387
358.152044880733 211.505504834207 285.594100976705 239.845747958475
326.83982672544 400.924914238052 625.374595058488 218.529160053398
244.18714778846

APCDD1L 1181.97868824758 756.390518977556 499.321667633972
526.182262872235 131.986590029726 125.890941441352 119.466200987489
118.724348027528 133.123444023892 171.373932115412 152.095584118603
123.635470154191
APCS 124.652072110334 116.412881235587 127.893555451807 112.817015523662
134.341489592504 119.221976571036 121.219323788324 122.414781975236
111.010889276873 124.977046954694 139.244051199144 133.069347559741
APEG1 616.995114856023 690.246312542011 642.934824227327 1230.00744483191
165.244616507084 145.958718601763 159.909121784808 156.702300398105
145.770601609392 151.522526161299 163.93199635496 203.524150518033
APEH 800.739422189427 928.672013047007 760.272612245092 1080.95725397659
4945.87677849967 1548.09554827056 1394.31467757456 862.669120338424
1566.65825409044 1189.11053680293 1849.21906428944 1551.90775589919
APEX1 704.448123154868 715.93836911426 1171.62361359992 694.644744547412
1226.10881264804 975.315904185938 1626.34607278613 1601.19401366685
1001.35912007916 990.206369590236 1372.63540073207 1722.26954281876
APEX2 260.52593414721 279.879377933144 240.465165615601 291.720204789005
1421.72388964898 489.260272645085 958.005138077899 1140.40904196731
601.640387246951 574.262112643403 399.963640581639 679.191060100076
APH1A 460.089380701982 492.534183006883 392.13175568099 450.99430272315
2944.63971819193 1005.62130301653 924.630951485299 795.720196538039
659.900771633223 571.136519973808 968.548243081111 1008.43888926584
APH1B 224.851680313689 216.05364037271 193.744461104128 221.719848707318
496.218605366298 214.968589907686 290.582957824549 201.383739902873
240.814860403562 290.954496559897 243.732074437237 253.049869714368
API5 724.777166238211 671.134356672693 884.248568386012 605.21685916883
741.433093896472 544.580311371212 484.568420044471 558.051041108282
653.047570485109 724.777166238211 981.313778685019 412.415549375221
APIP 286.431013744652 269.495247190598 532.235193634196 239.109163320417
235.933451977398 291.167223094542 265.148061007894 310.598930734244
402.965083947376 273.197814995543 305.768201359278 233.496804016764
APITD1 176.912121142015 178.939268046323 234.432954093705
189.324370290702 281.570685561526 223.666832044186 250.139061165593
402.213774592085 327.480268032814 225.005311517365 236.651601701157
264.476846259384
APLF 133.505261458701 133.311212623667 144.767233774942 141.37694311902
144.199054675488 151.64806573742 128.189685312596 138.950197595669
143.388028226208 126.557869929234 119.511962218143 144.411176964424
APLN 144.346988809708 138.145677924612 128.213678179359 142.007066214472
159.824012085212 131.866599432062 150.046300135261 135.618562468776
135.116985816353 214.331491507489 157.891350650633 149.160808104789
APLNR 223.184064606222 381.4054004349 213.674150322581 493.715108157171
247.454326301069 175.158180187883 138.548983773386 188.070213212057
340.728888109597 159.166059518117 171.39347085745 201.731223995887
APLP1 153.711354087243 148.649619242172 152.990523860831 133.031431118772
147.191114491117 142.285486339143 145.929907161493 139.850892808592
142.478528530514 131.085965122946 144.273011517032 150.0674092434
APLP2 527.294087043601 539.008286968106 564.749082802433 531.555532787504
589.409235971915 298.519371102278 535.354724252178 454.34729744062
367.518273773904 360.756455184184 605.040026490154 389.381039495553
APM-1 181.715992480635 197.112749959833 154.270034951209 179.230909178397
360.812953749077 321.627969147543 237.989479738133 540.190808200264
547.202354339933 175.313689069815 472.793332506369 581.37957221282

APOA1 155.55288776049 150.945167884077 155.379116566097 159.730925830278
199.149959995133 143.780803045394 169.534469894464 145.597134236583
149.280480796544 163.181323768362 144.092864444802 167.125830254039
APOA1BP   871.528156184927 933.408902924063 975.315904185938
1129.76078220457 4198.61471038267 1277.0921553877 1600.10235468018
2269.44503773126 824.070046083713 1312.75601392984 1143.0779222425
1903.13326109546
APOA2 130.590195477461 124.853659050216 125.013839352291 162.828844182372
143.262810933054 133.864612785572 147.777050372032 143.574584852426
133.272540012108 128.429007921588 133.014551645069 134.086301127594
APOA4 138.953926269905 148.57301274812 148.431101051574 147.400082178491
129.835713633923 129.92319548307 139.752845127584 133.355353808577
130.651062724029 135.810476921749 139.408234219133 142.988500173397
APOA5 126.925982415892 147.52403424052 125.843089077365 126.083599163604
150.655359765209 133.397734409167 133.095245865663 133.944538335319
125.435268901131 129.797601100832 102.932013031511 124.602474178438
APOB 124.460064642088 123.843823256462 124.717079082023 120.365602682311
115.157321350379 115.639101191444 118.230408871812 121.334882368464
119.603565559838 118.816986517698 138.768904143023 110.662552491706
APOB48R   134.087875547168 123.383334044683 130.822330736013
119.275061991187 144.413210364148 154.947359473187 146.879019367939
126.075528093376 164.811120992602 128.36121178972 215.744552410805
158.312614511836
APOBEC1   121.924036463998 125.761205967344 121.381365873873
120.039031290299 124.095340478326 124.267394371656 126.941650500751
121.406758670435 118.859745315302 122.362585067453 145.267225993057
128.182419321978
APOBEC2   125.517076778344 136.513942889781 122.466276478613
133.143695166498 119.103219740679 119.658703392877 116.514299377365
113.163651806803 117.386273592501 130.204571771396 126.470239371932
116.241531823869
APOBEC3A   147.572660963285 112.528908277828 135.25734629146
131.114995025516 136.74788009263 146.035731780312 138.202872275661
184.569063297206 150.046300135261 216.427103365727 147.994386096925
141.863234980537
APOBEC3B   128.96181458192 121.341177395499 122.447154928139
133.707285280448 341.43511159996 199.408907117764 138.897393621114
478.180723959397 262.296456281666 144.986655090517 171.595724119779
137.232722233042
APOBEC3C   227.774354299962 265.43897208356 255.527764534636
283.527656956105 295.463118191589 332.599191067433 177.567833691547
250.481442411876 214.229228850802 197.795275907042 233.272000923172
270.679613997675
APOBEC3D   205.162633740798 192.140442881209 144.896960245309
157.233463720262 142.403121638265 156.040073232376 155.410704148925
222.848935978648 182.588697551677 152.032418787633 185.046826969912
133.35096649325
APOBEC3F   212.571491543077 192.418895349837 198.205113187281
219.687151891777 221.390817970859 219.422260093722 248.204358220728
244.668813440638 243.861834405092 212.396522888943 253.124449721731
251.472556189141
APOBEC3G   185.727947276958 192.848268768579 188.836327151669
173.10229478279 236.082185670774 362.019824026781 371.819911874273

763.533962249076 630.894382644903 190.281570660806 398.971838949075
282.652193666078
APOBEC3H 116.784024112826 114.062099086818 120.822923434486
124.844481048039 162.484777758676 126.991140761102 130.949405011154
154.912576362196 177.468210189888 114.200099628087 164.836381332774
137.779607093226
APOBEC4 134.655842662899 127.215777044942 146.218767446679
142.848438163322 139.263602290256 125.124011798043 145.704057360697
121.146511496024 140.833666276568 147.946236579299 130.204571771396
152.915692742755
APOC1 199.776140037612 220.567951704044 174.383193885933 170.140610481718
806.591035100794 378.728516779744 354.840474468573 740.527607312565
864.908449769749 233.572055978465 333.049612399093 392.6758818959
APOC2 142.288110423886 132.811368246405 146.323776429268 158.330403004116
138.972789382679 152.935963398921 148.031899566208 176.155413809391
155.243643210534 168.302168350095 140.818413826758 163.319882002763
APOC3 129.916677638819 131.800533523123 133.932594124721 121.640681916546
130.29531841096 131.08389186558 124.992439957206 130.288547755599
128.920313557255 132.200648560899 125.685852196342 128.455670598648
APOC4 146.136820062093 138.850077456103 150.52625734893 146.343230597246
136.333928675403 138.69392148885 158.795335941084 136.874135348635
154.861507590906 134.335309757663 125.488511939819 141.640292723776
APOD 4103.06565237121 2856.14739820295 2427.21100330388 2508.50428529255
274.831898505989 248.03801051527 455.486248388968 1091.69584249713
195.160353691349 117.718893827092 940.008438825765 135.29954024755
APOE 626.857289492802 597.952909577121 211.736398352592 465.809961695043
1513.28088592885 254.722836822616 375.539588956213 790.561585641193
1545.71251527145 276.138650967632 407.760503937821 271.287767448621
APOF 133.42964827086 138.237030685795 124.555847618988 126.686404772601
116.819107878692 130.770335966929 117.829228895897 122.35832958054
124.258412895333 128.901707304582 152.155760355248 111.127245394321
APOH 106.341910466588 107.498500136859 101.149125916797 103.826308328446
110.61165787102 110.858201415824 100.04568139926 104.483909687253
111.810575716678 108.868679899494 116.989409891761 117.15886922795
APOL1 129.013667813486 139.406427549733 132.313884627836 132.346363545288
207.298994298453 141.090356426567 411.359106929985 144.988803993714
213.556015518863 131.930814868433 196.363005912532 312.177191402945
APOL2 186.961638857905 181.202396068658 166.998311158378 187.296479395462
301.188296465202 204.82244206816 746.777330152729 167.640645997754
272.571092944711 142.019263564073 950.818917078515 315.912220767901
APOL3 201.139510041141 191.708350637714 186.089988569287 205.966111721195
162.748836971539 146.26936366452 157.425924587516 198.166270627544
285.83626978316 168.729301938015 189.758013811338 200.591916768948
APOL4 139.080142195118 150.632724038845 135.509927917168 130.255689301211
146.306467130749 273.432712475089 241.019436415703 155.513572243604
166.430881793156 139.316189199729 156.441368290572 185.950131245699
APOL5 119.297561749232 108.362609680556 102.725375719353 114.302645757446
118.673325109944 106.982655360194 118.871735074285 113.636570490048
115.066084565112 110.325803953516 136.394920983439 119.952108688414
APOL6 125.868080230094 125.746184969983 125.493004972076 120.521509788705
134.239416179248 126.338676765473 142.553175839278 151.554352209634
150.547096071626 131.972669163481 168.384397488011 146.9554120076
APOLD1 353.571106279836 525.6579615077 174.820064644203
302.1846598234 205.026176598298 702.049571167356 190.077302802065

260.554343693588 211.966139902562 208.476367897256 178.166289723365
229.881164774875
APOM 152.987939791009 141.640292723776 129.483116877682 147.610616423185
147.556482119673 143.096097305398 136.601220755282 123.547821639269
148.82892305156 137.790718305537 155.04585737344 164.509354377985
APOO 267.696284751923 276.057101476947 309.75140197669 360.285027176918
392.824840252824 780.461254794127 435.037306125887 604.237898104299
468.339735327273 367.644907862372 431.546135503733 387.827718022473
APOOL 172.370108210895 164.652795899742 208.816482659752 169.933706225406
231.819341041105 195.303469330682 196.053808536366 206.89716102989
168.911840239685 197.859546367676 182.810581377888 200.668298229815
APP 584.548578914846 436.834825721843 440.495339027491 522.664475688419
325.252108402192 249.449151040445 277.596303701405 293.600165830839
318.690043546203 334.607533045929 419.690542859168 231.698163424706
APPBP2 569.236322997211 518.17401959981 619.380514697732
560.481802403627 239.503369457867 601.164127925014 576.10291008626
527.139476504986 455.21285026588 270.679613997675 435.69811966429
682.538709448692
APPL1 408.60092780174 373.760551592844 418.319283832879 484.131020584378
343.617417488962 359.493075382333 441.958777965408 344.992791638415
281.168898910065 491.290438575428 373.189809737885 338.510045856515
APPL2 442.220572301795 367.732191251547 447.521645706162 380.393153331219
423.396271159345 526.416479408056 526.896146537814 637.695341427976
446.509863498357 412.048097766066 563.490027149224 422.434778100827
APRIN 134.613036378054 146.353239460684 143.865717051998 143.844153373367
127.213640193364 139.329016874967 142.266177608209 127.947547987882
144.523620416439 164.164463389212 116.844361858869 114.28381541078
APRT 400.550044186372 398.971838949075 369.592501881153 378.920456227378
2325.02143920169 638.393099392849 912.143424293236 635.37511798786
974.544773560873 1217.29687282094 703.587139938769 907.904780399957
APTX 398.272713014637 394.35596596181 359.703969802888 415.388760988459
347.557792829093 342.245182599593 410.645457106122 386.679822751367
420.160939829121 455.486248388968 475.213525346293 269.141120401835
AQP1 336.898462056187 278.651369597422 310.533399024851 292.037861000784
159.495272062735 153.191840013692 143.60570420943 157.295602588814
176.449549075475 165.139423820186 166.024236370752 153.731233276485
AQP10 139.310309748986 138.694701349366 138.3792224631 130.639974588163
141.38612990166 129.616142780279 134.181819177347 136.041605158822
132.047328970556 132.996622574111 130.584348445667 122.99993610249
AQP11 155.960886801091 144.049503774298 131.226834630025 159.26101905957
208.932692585748 137.15288362126 161.250520154112 136.133538781918
142.472724215037 132.855812035005 133.511698282195 126.816402300701
AQP12A 147.292241873554 136.769073515537 135.168792286819
133.727447520255 170.091913791176 143.787766055379 142.896723827753
133.869867161978 139.995623005154 136.967408633308 143.449303333495
253.164596735793
AQP12B 135.10155936286 163.245806772716 161.80922869659
149.188357651896 153.758353103323 170.951009118578 181.823450464129
154.061101136889 145.286983999628 193.466377788318 176.289693941489
198.023465629218
AQP2 143.123983359386 157.449891798638 139.253721683078 139.654648891206
153.661632990172 136.513942889781 126.217702283708 134.970609100459
126.939543057414 151.132586978278 145.968884703737 127.478474451121

AQP3 149.941056059544 145.250076958551 133.590552542806 121.464383202986
316.081825144651 254.517135747445 165.491193869938 236.899421827427
193.95909424044 904.211195986553 293.018282386003 501.946140974776
AQP4 121.583096465567 123.235136639469 122.706176540192 118.708644971927
115.607031940137 109.29279076901 117.923459503989 118.521075843163
116.360144963753 131.84454376953 139.049085468313 116.998277297063
AQP5 110.212739445 107.170981307304 114.835386789236 116.746018509166
118.21084414402 116.20967792121 117.883635746379 111.299115402008
115.120702589487 120.507930710099 114.217867810848 113.680875214845
AQP6 127.646032547296 121.425298866613 124.460064642088 124.940836122365
138.21083121895 120.447140228396 122.777544465711 130.938642792468
128.972276851419 126.76986764971 139.008854444223 129.116354997747
AQP7 126.919867899174 139.332164436099 123.019430618632 128.421242037748
197.191648960563 124.329980024986 132.192775993078 131.430921437192
127.712004247155 133.768419410758 125.519298131694 124.375348764151
AQP7P1 185.961822133346 174.979501466228 198.701991021316
253.327404004524 153.78045746564 160.407690552027 159.368376366657
212.473610495213 181.635664531851 126.678746008577 158.039920243116
154.670631900834
AQP7P2 148.350919010575 147.345558455805 142.046082566177
150.2214516849 188.20675818131 139.566124411099 137.555253894825
155.60532199001 143.423661752881 134.256243410916 156.248684477916
135.188445928844
AQP8 136.664500207966 118.850495684547 134.821778017915 125.18424162353
132.413527125434 142.066903301882 133.388533738482 144.748564682545
135.259226552938 144.375109087466 124.025104622631 137.165966740473
AQP9 115.457121859064 122.133292722932 120.85013281321 113.192313147111
125.535877109652 151.0189104086 130.571827557149 182.958991847218
212.996456841785 268.034972830233 143.705475208192 126.295856316628
AQR 256.795346052751 252.733973271584 280.414161197286 275.996693627151
201.383739902873 284.638865013916 245.378747269929 249.823314327355
260.356834114611 261.39435936661 266.534316786801 281.570685561526
AR 212.607094117882 207.467591256549 197.191648960563 205.305702209672
174.727439318223 142.178467668037 137.320792454356 142.728311130348
139.577443171489 136.528123792599 144.649656552 213.26158748941
ARAF 647.955521647635 756.618969995355 696.976036382401 844.462124058351
1163.13845502757 631.653707589221 713.832153334389 909.020708316287
764.137504481329 721.966539760341 894.100792722707 756.618969995355
ARAP1 265.61506544085 352.121800955822 266.80383076887 424.025588636796
402.564148020948 223.408447580188 283.235844022826 212.545989139388
225.61275910008 235.063474370925 344.737964092526 239.621756857095
ARAP2 153.202157381228 146.20679320119 144.907922949811 133.59686364883
161.603317039244 195.561296046507 240.306287921209 173.599961799334
177.401771322485 215.365809536522 266.316485802808 220.242344043437
ARAP3 325.493177980483 379.292531644085 271.638179116269 314.646369385896
434.61711290583 546.274050902567 693.781708146282 364.678403892354
397.307515234646 315.752980521266 387.305348482856 1102.73689840572
ARC 161.83241639654 141.751174078324 125.396523818173 137.096785940897
131.380387461901 138.510722755317 134.045938361221 121.344724993789
133.26167785455 129.953801496113 149.108582827136 145.306369509684
ARCN1 1542.35486950856 1266.53539318831 1584.81089248914 1243.96721244424
1065.36715400306 847.863177169335 990.814111681408 1370.61156801632
1283.68791110988 837.023434916281 1944.15673951528 1437.5568316065

ARD1A 449.304281526081 371.767182305618 455.778776920897 502.063888359427
1127.89414462606 526.896146537814 411.285747588622 424.495400604335
458.047799748696 628.710901107436 337.652945958921 450.327710374141
ARD1B 153.19516365535 148.732027660387 163.616115299926 135.829273398594
156.290018816692 141.882859092658 154.256214901103 147.348400730286
152.197327387252 152.79367157305 139.691470247318 153.626180103404
AREG 109.003365273337 104.248226402465 107.365390754133 109.209241676429
124.01558540593 102.522925993233 112.046615825307 103.307703547649
106.629065516249 109.988237089025 114.27112025541 103.772221923105
AREGB 128.105527466559 128.867710054234 114.664782541261 137.522792377933
123.491930765117 131.302225305148 127.377356715337 123.145830746127
125.974463216884 142.848438163322 118.239637776941 115.491262364277
ARF1 819.887322757281 985.087150371964 909.388774224614 1057.1528284934
1682.64624665931 1220.30347076968 1181.97868824758 832.622843175217
1640.99641023476 1110.43791444289 1044.00217726486 1652.77814259264
ARF3 538.872348151809 562.711562649704 527.498114662967 419.4017188518
858.742139354587 583.619362840814 412.801213309591 507.342877360289
691.205738835917 617.474181543858 436.731545612902 692.53483413403
ARF4 1444.45946291517 1116.95795024994 1223.68146968716 997.460212152591
1639.76556136383 1800.25771851544 1665.14066952917 1535.27496251381
2630.78185410861 1629.32740709526 1549.30900090605 1738.96798001895
ARF5 776.030689773414 909.388774224614 571.950858678221 923.130629121442
3152.64817044581 1103.81439809121 1255.02720867812 1273.6393529108
765.911113050543 1259.75918248468 1370.61156801632 1279.9555644913
ARF6 142.763157232147 153.391895732807 141.587109176431 159.218886890035
162.739253457398 179.321622114583 146.968599872798 154.881797585583
159.166059518117 166.232856234879 185.002106079042 152.199095472082
ARFGAP1 377.593087606046 458.282682131641 275.972447693982
569.236322997211 633.762608454521 317.904396212359 434.855262284265
203.370353613423 266.61343130101 247.08460296705 260.955030428944
219.537335167901
ARFGAP2 344.604699562938 395.927222009721 466.777454704543
458.953009417683 338.260976633501 245.153736298067 328.550286726543
332.599191067433 371.06697207449 471.816954259146 466.141717595301
252.015257713669
ARFGAP3 269.567704345075 231.146155346291 276.79778275734
201.059635671301 271.568797887976 281.49247863022 294.280238648737
281.238656291587 263.183010631644 338.223462327622 201.913709602894
247.622351589081
ARFGEF1 305.669636871734 322.778260588465 363.594808277506
266.918302307596 291.994364783888 616.339439383796 620.371912523332
563.648536446918 652.826023701545 528.098472417251 780.105135529728
541.764013317002
ARFGEF2 139.000870898001 139.219476681919 142.75592155869
127.215777044942 223.0506375638 208.095384605737 313.773476049783
168.210416363286 186.321879879561 197.100205890532 191.961269585431
200.405532717305
ARFIP1 255.793823796456 231.044588483625 291.883157643902
253.186749111526 201.517356610591 310.640107228956 298.209907157762
331.747622606378 369.434158443561 307.363302726074 313.322307299463
357.972320648217
ARFIP2 138.558300898079 169.043206258073 173.440596296333
166.521472376759 160.090914414578 162.096065738375 161.895435127054

164.553511697702 153.920180168328 149.774162138829 163.863677822465 152.265624144393
ARFRP1 132.01588294465 145.832751296646 136.893392154262 141.123272165499 197.12840030662 135.313148180961 175.41089164969 142.082257952004 123.689439578326 129.210356735226 156.572774198042 130.052593375075
ARG1 144.931901042924 153.485731788293 175.040086358446 141.264096014725 133.446934298801 145.414563914801 143.292552892073 162.100547394235 139.361120875198 150.244284323504 125.822346524903 144.259643132933
ARG2 176.070695522397 180.680595239651 196.189101941489 177.214422668922 153.311705636864 215.453915231774 235.933451977398 166.272237117441 192.153420834889 165.890492422031 170.370934381883 247.746304138866
ARG99 161.382041789666 152.796423361971 139.096536887753 155.158028043823 128.975785280465 160.713790305656 136.271412363498 171.093611712143 133.886458291672 142.51308872439 155.511086289504 128.402511355513
ARGFX 116.828333047376 117.040953580545 118.070716076005 122.547842148486 135.744202879162 127.845259467015 111.020070405368 118.298444816712 112.922918499398 118.05386204863 118.034262386827 120.565485864499
ARGFXP2 158.325554470875 158.49220347186 152.791390145148 148.94765984808 144.400004730049 165.424516708502 154.140863428961 153.795665190439 161.655336107432 162.98885109088 152.179281925254 143.477670915091
ARGLU1 2877.92845389611 1810.68433539154 2586.87941889443 1402.14708471728 1274.39152410752 2418.66999907188 1830.56003081769 1479.69309189156 2330.60389526686 1377.75266942574 2728.04758874748 3348.7622174821
ARHGAP1 3115.11499025766 3474.0924656878 3468.14455177277 4690.93553472125 1120.3009282887 719.369561410372 1775.32632165475 975.315904185938 1067.30106203503 1391.89900758685 1713.93035637146 845.369108604819
ARHGAP10 1406.73270115373 1459.31031099055 1268.73280473728 1771.39155164347 250.737252834098 236.899421827427 239.109163320417 244.849563563729 255.3451781862 254.065327837176 357.140791982825 210.218440444631
ARHGAP11A 134.307941199626 129.031338314175 125.542301916791 136.229159638766 130.009322405768 141.937611082276 134.062423178775 138.976386995124 134.000363767958 127.193553054979 139.823017208731 134.441142158135
ARHGAP11B 112.590379042108 114.970485002776 124.176944998906 118.727279917776 115.734230176652 166.285052506316 137.908991199407 142.201672822989 146.165632086981 148.853877151493 156.396579029788 163.315865817864
ARHGAP12 274.047586063773 227.375773052577 244.18714778846 223.408447580188 240.631264915238 273.369722814068 276.184421402511 217.576547690525 216.9491329483 194.430896269275 266.652805624826 274.934292929666
ARHGAP15 388.416668785633 374.215345412947 327.251620296271 364.265100349672 176.874265462986 244.849563563729 180.047231265069 278.428310548805 389.800620731411 229.116153948948 217.074639597142 189.872997882033
ARHGAP17 374.337030706199 389.96566674501 345.135289739599 434.509391836336 302.113807121396 285.355815566148 261.988345506548 286.506419296679 326.45984882427 341.2887754025 270.727749014046 263.123584372086

ARHGAP18 177.438968923426 190.871696848118 138.546549217819
175.186435016433 167.42476096854 181.193963616037 155.952902855994
165.723126764006 164.458565398812 151.548865569913 190.754349388901
165.549190801014
ARHGAP19 291.720204789005 281.996583337694 235.997227173296
321.750495072759 280.170464338141 231.314944217099 247.971366020963
278.469340236003 299.170431746759 190.204960255989 358.654569870431
263.295012964476
ARHGAP20 264.429717032654 256.237185590901 229.483578414375
238.946506103155 129.044445373107 150.275163835924 148.216605879958
145.929907161493 135.9097041056 139.420025842675 147.946236579299
147.022594294963
ARHGAP21 2656.38138490746 2165.41403521012 1808.26383618901
2036.43904731226 920.840069938767 807.604205890244 582.062758687489
620.163512570453 457.905741500454 833.430847243676 630.894382644903
566.228690652783
ARHGAP22 196.583548193097 170.423881865022 131.474485888864
151.06209123698 174.383193885933 120.967523586161 144.210698143215
158.374760929273 163.686535888943 141.719556176029 165.41729888767
144.643770374264
ARHGAP23 410.645457106122 391.400480065 351.395760651605
383.503040287678 747.322544683874 1215.96648642927 1352.14737073174
1478.09582385838 1355.07170485397 417.615521090665 610.591049634222
1226.74043569231
ARHGAP24 229.805500520256 238.946506103155 246.676401619881
229.729195963667 136.899405404096 148.401698825687 149.964827055916
134.946677624454 168.232843307077 188.995551740427 142.810564324354
139.803568564059
ARHGAP25 154.140863428961 159.13662975347 143.122278808091
166.788336808924 144.129889080946 143.629760301493 141.484730939562
155.358918578397 187.407526616724 152.14012587515 160.353649786837
149.759516805355
ARHGAP26 153.189490068047 148.803188053248 128.147484804104
135.958932745982 126.324771800638 132.595360509902 133.807992913171
144.944095553004 147.238896426423 128.458132030921 157.499803444188
129.431497372577
ARHGAP27 110.925532078514 134.304545258148 107.498500136859
110.624328647003 285.63245179313 176.486551181451 218.031238893075
235.961605948276 235.091443641056 189.61702125418 299.616447269111
231.547654653783
ARHGAP28 180.63469438043 180.923089242255 151.855884026072
145.06205326107 137.933122094638 142.612035986517 134.76586363177
143.497909064641 136.790457887057 155.762890407496 138.813126946007
143.184697704389
ARHGAP29 135.028746262383 133.642815510945 117.32734978289
113.716741872499 124.555847618988 123.725998173975 118.283346939564
125.513881890201 115.834056091815 112.75167046116 113.077691687266
117.166509072455
ARHGAP30 180.747527895349 164.96631670799 163.432972512188
183.744959461078 173.463983140514 310.142754559096 173.189465201573
238.095599711942 275.39986964837 195.532022060219 176.516540713888
241.383657414248
ARHGAP4 244.990374822652 222.332748220525 254.188830191845
240.511797905018 287.455622143233 239.541516080716 191.38608906576

275.169415154893 329.660368915848 195.795728497916 207.104548555929 243.402781509365
ARHGAP5 197.308952757609 187.247062073247 184.844915350411 237.045634416039 137.446745269821 205.728673153914 162.702767346393 162.57736365619 164.332073056902 180.952017616136 164.000772606739 173.964378226631
ARHGAP6 164.99860393383 154.804753043168 166.8846964604 153.066630483602 158.282899758551 157.078563342652 157.385256442734 155.837866295709 152.377056383547 168.095061700814 142.043121538885 156.497059936271
ARHGAP8 134.883708339914 125.296240086615 121.327397317398 128.42287610189 142.206016362038 130.973504803813 128.628637585951 141.963297887448 145.096707533824 143.0235772247 132.300508985399 125.290320836648
ARHGAP9 167.250667092211 146.775403409562 135.664877050226 145.751336115646 149.25240100689 148.109847175216 153.488903967311 233.161848094769 389.051174954505 176.088449983147 176.355556618289 135.647660756548
ARHGDIA 1050.40488466179 1524.46745239811 611.772512110915 1858.12855367119 2900.55309635211 966.731520471976 1254.19980463817 1061.29067124163 970.166036472486 1309.01193284992 1551.90775589919 1500.16816750361
ARHGDIB 1628.24528041404 2708.20624364471 1007.7803477458 1948.24432908972 3850.41246770812 3168.27825058989 5702.11995823367 4095.76073123843 5479.24427823207 2032.50052162637 4663.03253705034 4183.06379632713
ARHGDIG 134.93814894432 139.97636570613 129.675420096163 140.840895590527 140.020294940544 137.597788349982 131.657454325126 126.049144107897 126.22472822166 136.70715656782 134.946677624454 123.841366760069
ARHGEF1 210.061754644824 196.031541155674 213.178648382852 213.028568789661 271.287767448621 184.056274452194 183.988537525989 181.898985277176 196.819022688128 203.436527043293 200.741234443918 163.267148547147
ARHGEF10 936.957730361063 654.305724263754 742.751664201272 636.382696018633 186.999459152483 219.597808863947 311.449205777595 190.002630382394 212.545989139388 543.001249586573 249.884250040189 266.135477775641
ARHGEF10L 157.688982037448 172.631499322646 162.500485773812 184.577511730401 179.923966067226 185.743212726702 161.552475294424 164.587657424569 161.510497517852 149.066522533956 198.489278156437 165.491193869938
ARHGEF11 171.190796424612 186.866029149035 152.948391119096 212.016247864691 205.57572471694 162.739253457398 162.063689419737 175.717830026564 157.394035451151 146.937052029619 185.432587490792 172.175584707792
ARHGEF12 359.099750692694 351.515059400947 314.337680575998 359.776068595698 199.96834355685 221.930333726352 223.107391048316 224.135228192655 191.651818696629 169.306573880401 233.59297160203 249.953902454701
ARHGEF15 124.595925483149 150.064179085845 121.423030266904 130.112882549637 122.274656361883 116.248026336995 117.907840952685 115.18875509198 123.557722415957 124.70219935334 115.939620871703 117.688941284427

ARHGEF16 172.355215322173 225.189991540262 145.172776053
153.028658323116 711.729857540712 344.247544510881 362.445813697676
355.14024238296 510.659785046811 374.56251861444 1081.47077865393
814.110601393539
ARHGEF17 306.111172289632 353.674560414317 255.59042517603
482.048928546145 182.833961304079 115.305518560239 122.600274773736
140.238423894192 141.649911527714 133.638664342783 158.224122084011
120.876732151192
ARHGEF18 748.703265448403 938.918431240228 817.529344685717
1421.72388964898 1948.24432908972 1195.6335460045 1821.08523647593
1759.9632914516 1337.38160266788 1041.58583268313 1635.35544761095
1314.62071412501
ARHGEF19 192.878416717298 183.50553222647 169.695658738618
158.425950902309 635.819650434542 426.995700632434 363.963951509433
548.686094748117 452.357898123926 224.089220507176 372.757091748175
892.515788744891
ARHGEF2 1861.19763103381 1996.65760745757 1312.75601392984
2149.13404212591 1385.28044421961 1042.40539020413 714.91842349236
1240.4863200811 1024.95957828641 951.772948602094 930.25842384208
1918.14260920544
ARHGEF3 916.836022395768 1045.49947183547 583.455617065556
817.342172321486 489.022371276542 452.357898123926 468.339735327273
468.060528109338 691.397238677552 787.615195903993 654.108318810728
447.297590650141
ARHGEF4 248.465457421469 244.5675215931 195.776369840059
368.548135194271 121.61143371132 109.274221164316 114.412739085949
107.727848546225 110.586147059226 301.638918685657 112.649012857276
127.997280639609
ARHGEF5 129.075693654369 130.70867409848 126.323649734972
119.366599958278 316.590387560909 197.815264620906 217.576547690525
122.087107663865 168.116928722035 344.992791638415 261.844708446856
365.046815163035
ARHGEF5L 174.928155956396 210.266075382636 166.298554732581
167.049197726715 532.786041960586 325.859917318516 494.538861626895
229.835576260403 736.140249105836 928.672013047007 486.425387717472
748.015452242839
ARHGEF6 990.206369590236 912.483177705931 736.332743195416
866.765054377214 317.244256751418 418.420753332232 456.990425618007
403.720036930931 636.970243888917 294.368406238222 354.783364481955
810.396770151757
ARHGEF7 344.786541451395 337.728737393381 295.851459742115
331.018914429561 279.704940529618 471.510683045651 265.919002666885
282.47352481715 313.773476049783 314.90123632547 150.599425944486
382.960724128105
ARHGEF9 236.651601701157 222.394280571312 262.577672828611
269.424055734143 169.238325933605 161.906823008445 179.96623248843
178.209994522672 169.475714234887 165.602175302848 182.77693508164
167.491399614986
ARID1A 485.136339836897 521.30110104669 496.328898534802
541.764013317002 761.32108609503 439.207122335648 526.625936680738
760.070180572621 678.365575489532 492.637241687561 575.546802138889
723.428294037428
ARID1B 141.599211622756 138.731957926247 147.635761512135
145.606098951179 139.81502919163 144.331409179705 138.104527851969

140.481148289418 136.188868917386 132.109267393571 137.981446468611 131.81975648701
ARID2 213.966288689715 222.508914765788 211.609763150578 211.135364208221 165.638672456873 263.509066170434 218.70412656621 220.959843573708 214.261611197599 205.886398618369 229.729195963667 251.959823608766
ARID3A 280.127735578097 391.644649726254 266.652805624826 346.303862540751 554.800688991977 246.912159676595 215.47323777134 226.086733606951 277.596303701405 403.208138816247 253.385592447339 182.130970182242
ARID3B 196.974807677659 207.940596890723 227.847614397311 209.417052464583 212.147155923729 239.20206002 297.970735665595 231.180528071672 261.286548990435 296.375305136807 228.820894086914 453.875259610173
ARID3C 151.05681004513 143.030777754448 137.867263869936 165.570531605115 148.549257493128 145.28524062436 148.450023054885 145.756455015309 146.84403879162 133.043503979161 134.458958352588 151.064422836695
ARID4A 176.735168607281 174.332913952768 199.001860912128 173.816408146725 144.117964288763 163.500384982242 187.461448376593 193.843978773764 162.397078614596 189.971144220643 196.014841240142 216.485113690708
ARID4B 597.952909577121 535.758943850734 546.029974898225 449.523996620257 234.980382243116 564.41151411028 579.678635803225 582.527835746927 523.073926509865 327.951753740503 448.858580689107 696.976036382401
ARID5A 159.090982014161 136.126542897364 135.170925092209 139.306122448857 144.096624787142 135.056517225198 133.420267697961 133.378374710498 135.548042190047 133.274292647041 131.351303131574 125.765399556488
ARID5B 2974.04694562902 1659.80909859514 1563.71477584676 991.611982265221 379.983165400941 676.570119607984 526.726224327409 539.853394831917 1164.61390835016 492.780880796036 852.436775264873 842.567990276734
ARIH1 286.085833802132 234.925852373127 313.18989508207 235.407064742194 215.585089158905 206.35895739543 301.908667220208 200.242239130026 256.795346052751 397.985856484851 240.915496717526 259.426050921239
ARIH2 812.298381693378 749.220616713093 1076.09385014053 837.413261433363 545.026293696209 649.052806726591 748.703265448403 565.164961255054 967.123240442957 623.508710830895 444.584594376357 651.235249028924
ARL1 1191.88706134921 1086.8615735568 1262.96801072965 1007.7803477458 477.275332763887 1029.75852676191 997.460212152591 1141.49294161587 1161.39230511792 623.050854309724 692.729789121701 790.561585641193
ARL10 119.432201417811 111.985567835684 120.177385114315 116.615250922316 114.213405341709 124.300285428221 124.561511833959 122.312652834289 118.474291525263 109.416512252532 107.283814651497 116.831725094917
ARL11 134.614988219821 154.959217895243 158.335952927257 144.822943770596 140.447933562667 148.096551269076 143.272669418277 130.736466113571 142.922549076496 157.759149893048 147.057116881846 132.873213182775
ARL13A 149.37190049932 134.631638658586 154.021906399992 146.779054605199 150.328995030071 139.711696905287 134.78059811237 141.490911535087 131.138322527536 147.066582834257 143.582187466814 130.632096412204
ARL13B 158.98666068453 154.193086881801 146.233195164295 140.28485470716 138.981712781568 146.332425559689 144.904945833116

154.916023131856 152.418315087218 158.751241730465 138.625185173046
138.374092984164
ARL14 136.77260462185 155.7441762827 139.119638136874 122.572634128442
438.231717096337 134.604565335211 173.70035122661 990.814111681408
127.137447808551 205.495823410422 762.182213353032 794.165408266062
ARL15 181.412790232996 189.770558810153 190.098509544816 177.874821397216
199.170488640762 175.774461934758 198.90533452253 194.257024100927
167.67272339144 193.336246844237 181.681929801968 184.937088280378
ARL16 12818.0509395717 10360.162704111 12368.1423473066 11150.5512437377
1154.86609813021 7573.69742026367 3615.76740804718 6244.58472720999
5582.64254130978 3765.25491426198 4892.17875938832 4690.93553472125
ARL17B 228.560346647306 170.091913791176 195.160353691349
164.337486856997 169.642188114285 203.475080163804 173.182013165174
169.975329631607 214.4829383264 157.663460168247 183.081450609516
194.055394488452
ARL17P1 409.743899750699 236.401834189942 305.542978609337
241.105817192695 152.79367157305 302.272304483941 201.702688815038
181.400788152988 346.691690520054 167.190857452131 220.242344043437
203.693188114912
ARL2 2444.835635949 2910.00269086567 2523.91287654539 3727.70199176703
3277.24948092771 1270.76282064793 1701.43479420427 1011.9675459648
748.940715378564 1164.22995893381 776.411045078049 1058.55850972326
ARL2BP 1344.87842417496 1332.82617944131 1480.52638217173
1260.50387106816 727.814681346494 607.805002786409 912.483177705931
780.734359041347 622.561634660589 1309.7660860424 694.907086612929
798.943242964857
ARL3 231.0814970959 202.819949307954 231.287362732197 213.990575186237
138.546549217819 151.506013510204 128.191959554459 149.696766215866
161.968835903764 129.382242021964 204.103018614608 134.629996026323
ARL4A 211.869300324471 188.53221484746 234.041863839123 167.093409692227
166.681646517413 172.197077149985 202.178451227304 201.016287924499
167.284724396943 212.854068610681 189.784608429624 145.985223908092
ARL4C 151.976432805924 149.020824600931 122.115925891088 117.024039521425
141.436235043864 123.077592992679 136.022614387461 135.068883036882
143.569894807386 148.04206955657 193.336246844237 140.709981878311
ARL4D 251.993821914623 273.911741951291 246.912159676595 315.828726297864
196.974807677659 192.69687837782 243.441938832251 180.680595239651
180.315825600347 357.514919818489 232.276667029184 178.096998157452
ARL5A 2091.73580292332 2051.45788386541 1916.46158204806 1782.2498613913
1316.31088187364 2010.57142784228 1085.92360670051 1126.46241487062
1191.88706134921 1531.54387233661 1062.749459378 1227.94401191112
ARL5B 163.753434774656 166.526875572556 149.574353706911 159.775479686193
235.680462167404 190.66473033826 192.96586114861 178.542247857251
175.030373805031 201.81649023518 166.289763097913 210.620764703468
ARL5C 144.271920003815 157.615017085961 171.051948060796 165.537853089052
156.813992534584 158.411846122784 146.411873503163 134.46743016543
142.34956863109 157.108043150259 155.798048652444 143.105974876327
ARL6 129.289216846128 126.178476831007 120.479270037091 129.029151164978
123.30960025689 120.351217130679 129.808590871788 127.640525889316
124.851025681014 133.902707525069 124.786029449452 115.485515429446
ARL6IP1 682.315322994357 886.157279090932 847.863177169335
575.722713461982 1854.11328962023 4579.62296570302 3188.90202907403
4592.14655702906 2944.63971819193 1671.82101590797 2127.22258112242
2736.98692493933

ARL6IP4 683.655205499779 675.596864001032 578.947889414387
791.840531191783 922.009043194023 364.113665301598 360.475989559226
288.481981529558 404.54681330209 354.263934274937 329.604568691377
352.354687089427
ARL6IP5 3413.39994653641 3744.5666678861 3810.98043907046
2910.00269086567 1102.73689840572 2426.2098937241 2900.55309635211
1562.9779683714 3712.67025366957 1006.03677177164 3106.94181270197
3024.62888503536
ARL6IP6 182.545896049446 183.545741265706 228.184535093641
197.947433984294 280.798003990849 346.917113326564 385.888395433066
271.945851739635 314.708587131145 382.806570442504 265.572253407219
435.796737132969
ARL8A 135.161667673191 129.637555509848 121.039678049579 117.279162323013
132.885515958081 124.959873714884 129.483116877682 131.406366653787
136.660600727111 127.167918433035 142.211357324461 133.340152756136
ARL8B 1010.05884008623 1001.65849130836 1317.1258783404 909.020708316287
1025.88559245013 738.892511276134 1258.16519121331 808.300067805897
1423.02563792293 1767.70023790753 1359.63626195826 1038.93733638848
ARL9 141.150016212967 138.861385127242 131.31716158922 124.138036007288
114.970485002776 127.681662711369 125.217733034761 119.486895914055
243.10297424873 131.326536951329 122.763138007612 137.163699337032
ARMC1 1117.77397620221 1174.0922389136 869.937761943249 1139.76755449089
475.686805714298 769.009371389327 699.800360892516 673.824770556885
773.330948077036 590.515758538373 624.825216056495 532.235193634196
ARMC10 274.534901548678 266.61343130101 371.941204080246
240.07282016658 707.173691257322 397.2528907311 591.189034068137
458.047799748696 461.722481634933 637.457322054293 474.750156829195
844.462124058351
ARMC2 135.186880148352 128.10679682685 130.847215795313 140.659276788384
129.567701626404 126.05295868521 133.45690661612 128.309602266886
130.613760017864 145.259981479761 133.028789808654 122.65547320922
ARMC3 104.633603136988 113.78644620959 113.133798430215 113.694351010121
119.780654293448 118.45291712775 123.766373146588 126.25011972597
122.634741242406 114.39161822971 128.184708916755 123.395897439193
ARMC4 146.565265415908 131.963419175907 144.89062965996 113.857477830423
112.916256784817 113.083678884076 114.528407572496 108.21185649774
114.441223528256 122.287535209629 133.850733997442 117.546508214236
ARMC5 170.748911334847 167.817232948801 127.462293158337 185.961822133346
238.482863150124 160.6823824476 141.914966520049 136.952914053358
156.277567608825 153.795665190439 161.708130705995 130.666781754635
ARMC6 156.605568633665 172.175584707792 150.498954408708 183.023316857537
300.83667045718 231.572677616684 221.916075811355 198.783034238774
208.713555471888 179.973655632636 213.046162847735 201.49630027392
ARMC7 221.594737497504 238.654367531982 188.53221484746 293.692083555844
317.550000625189 237.492603094091 252.138168292964 216.903719298575
332.138346958671 196.287131027947 298.301504581296 218.735620596634
ARMC8 166.600109853461 160.769011617127 177.204172894028 152.767228183775
152.945550129539 191.3765085801 170.410704499804 178.976116597291
185.984938124891 170.585366369563 164.870860592068 180.619956937489
ARMC9 156.998563448664 147.341597963918 158.439409064812 151.59613008133
143.180247878507 136.130027137459 143.340685579645 140.457002126576
134.176925491721 140.613151527886 152.346098251128 143.750656408155
ARMCX1 447.579975492958 359.776068595698 815.388288623756
345.565843875296 145.801228425692 818.761934233826 143.363002903815

161.817609920327 299.986758612161 183.294526267593 154.740395921562
155.699649538664
ARMCX2 764.137504481329 699.800360892516 888.371107987343
546.399972543938 575.060457772307 385.678901612324 1014.63777151551
204.959164579011 782.746481112316 369.78624104057 244.219585315024
312.224309341171
ARMCX3 215.81995779019 205.047145898929 265.260411490697
213.510783808898 180.501409705275 246.049729366987 235.822266856967
174.116797337377 243.336550161566 166.958831664154 233.635876830148
188.864388750448
ARMCX4 235.48876511325 216.077955163344 295.153786338687
206.164058935215 178.339211170442 230.424145472916 198.659595851481
161.559920910487 179.923966067226 171.202766078337 206.845306095447
183.545741265706
ARMCX5 186.257814752354 204.584643964409 202.97197145851
188.924084292126 275.972447693982 327.164773632634 253.049869714368
258.109876262342 232.276667029184 234.5655184349 274.343238288436
277.561376169965
ARMCX6 278.385151185788 262.691387377392 313.708197191587
266.756647182704 386.16563750478 529.854965483609 433.928818077527
256.022057262325 444.479355480138 314.810001770583 382.701467158304
420.248838397618
ARMET 1285.54492194393 1043.14253624883 826.314416168552 1002.44383771056
4913.55892033682 2376.52728883131 3122.71790981947 2718.440149645
3237.94149351627 1518.13775377335 2567.4299860517 3298.73393277409
ARMS2 121.067907968175 112.904318310632 120.721341479615 130.941645638407
124.883575464443 121.628827418997 117.634514818946 119.899649497032
117.467618148107 129.148634038268 154.462766419353 117.889524670597
ARNT 284.351529744475 260.921177985439 218.644953001365 296.24781576525
167.365850007748 178.222120802125 182.67941458824 170.243136294537
170.590817818414 177.257355909681 232.321454436688 180.008152341531
ARNT2 353.385210690169 339.379250332987 413.398641688413 518.17401959981
209.623583937962 238.482863150124 230.893665210918 276.714748212739
291.369577397743 148.08870546717 163.248980088946 214.595639477696
ARNTL 187.686326872667 145.43470695949 172.065593177758 137.627338682823
136.278361222532 129.455347273068 131.380387461901 139.00736252716
145.608087633951 151.747595672228 171.784981874184 137.728163622673
ARNTL2 132.128418409996 139.741862925156 125.859158049343
121.792573534134 124.106971897779 131.249240371529 132.987389721592
124.840101287634 122.160459795959 140.805322576253 148.808928892047
141.362627556137
ARP11 146.231937458616 155.756074127526 141.858928084825 150.272153646712
172.667770359278 276.284517459626 201.152639979358 174.775676782795
212.363151537303 171.759102794108 169.238325933605 290.090495047722
ARPC1A 1092.33328323944 1294.18114559128 1089.58643309156
1508.24281455057 2856.14739820295 1339.83170225337 2153.17165155392
2154.00589005104 1980.4589291261 1411.2586496281 1493.90578573732
2273.12164779441
ARPC1B 186.007710175103 202.524668687686 211.183499580683
187.710185880162 353.078842299761 356.05426191171 385.978867521533
536.609777988581 427.084392965651 506.562512021352 268.660078500783
618.437282203498

ARPC2 1788.35884039549 1910.08485008041 1718.80498208397 1663.71411325131
1899.94714976387 1596.33832632698 2139.42448145954 1534.21175984308
2124.12760841901 2845.0377634896 3073.40543048878 2988.45928881468
ARPC3 740.527607312565 657.058213650454 1062.749459378 549.047065540118
1288.3346537369 2926.06746795504 3359.62015747752 3222.23985545506
3173.14004739767 3688.68740405322 2532.82545752082 3963.40575974446
ARPC4 499.683188662742 517.993093601127 514.922742487521 586.642581067137
1871.91894530722 809.729160605727 892.868661511516 877.598505913096
952.724512915813 817.036222521723 845.369108604819 971.170460915918
ARPC5 2715.55326746591 2500.07677977147 1662.51399707484 2277.60839052397
3798.89743803968 2330.60389526686 2433.83421295012 3712.67025366957
3954.24763752814 3575.60827968852 3328.99028463277 4441.92485662082
ARPC5L 482.226889092547 428.850337327605 401.461825804111
535.20662670885 901.644194512187 433.928818077527 414.888512708716
510.659785046811 689.271009631393 774.180202250076 553.615252472149
462.437471715008
ARPM1 123.727239584489 138.587686193555 132.245217686793 125.247402280272
112.943630133093 125.539199863559 137.267307036807 148.347642424885
143.745594527046 122.149756212115 142.313725116274 147.138814313885
ARPM2 153.315309202016 160.006185904123 153.647343267295 164.032302455558
159.683695366575 155.078035506545 195.185025611945 157.013200713587
160.066390229416 152.340010735322 137.726769003181 148.317958829244
ARPP-21 131.470883854661 134.53861441495 132.941478943584
122.706176540192 127.144291519133 129.231455282441 134.241917425247
131.310537960214 137.907709799074 135.071400668179 134.501410126169
126.895669261503
ARPP19 1341.78081782818 1176.01058971561 1398.60850048351
1113.59310757948 643.468308027006 993.774175699873 927.478050257292
656.009451448101 965.673699120049 859.547169028149 1193.82799849555
1072.37500318471
ARR3 134.469653093466 149.073650082048 154.537240112989 128.486896700405
135.9097041056 147.085006263446 141.038721816572 159.98205335759
139.097764484641 151.690538050817 130.249430984172 161.031148038478
ARRB1 300.765518378297 353.243400332397 258.145539382199 367.229293432041
178.819486867379 184.278213908263 206.972440734038 168.873282919232
192.891507272665 155.07594431078 217.327543190687 140.286870147975
ARRB2 126.340955733062 115.435942397626 124.802639338573 120.682485578097
134.972933635542 137.00397800873 126.902441495413 124.526220300907
131.651851665521 130.388530183353 151.092066533546 124.958287100852
ARRDC1 128.009286101341 123.219903763265 121.274997609378
127.500754400224 278.061498892643 120.724926760252 138.899292894875
145.906601611338 148.700297908071 156.040073232376 216.859640534581
128.781123194219
ARRDC2 190.716424123036 176.874265462986 159.317429821638
180.733673680261 215.143973502566 194.753955100069 220.010399552402
199.714647839712 202.849544035436 191.861598059182 194.979795724712
190.471158323247
ARRDC3 177.478925091583 184.041062308263 155.710272937386
158.631298154353 150.489651880905 190.825209733575 173.929353266244
154.722895261876 168.000247148058 405.766055310869 161.906823008445
224.528190554963
ARRDC4 296.24781576525 253.327404004524 214.229228850802
258.964323941464 156.957578703389 167.817232948801 184.437810305792

175.121069571371 148.585224298375 147.314091141313 229.511643376643
233.07015384759
ARRDC5 153.348110626071 145.819198235878 146.9069812014
157.745805838008 144.148833164952 172.28937151007 147.500009766324
164.423960071908 173.225064625094 158.514079887374 146.863611516518
144.997833436737
ARS2 896.625415670455 871.913035811066 925.205399154409 916.026216386031
1451.64055568759 1343.2623011534 1217.29687282094 1573.03248861784
1414.95226752155 930.704688414069 1144.53002508973 1561.10217536347
ARSA 220.959843573708 193.704510912321 202.129153259445 233.635876830148
288.313931067158 211.483693299981 226.989201461085 237.641190133427
225.890195278753 255.374689750672 277.285526811736 266.032134528055
ARSB 191.405362404386 172.599654598668 170.844002412425 172.175584707792
147.893916750136 153.860039535598 148.594322264158 154.273953018148
157.032746784217 160.073510173041 169.713585775118 169.649251828199
ARSD 234.301520483864 274.564508105177 248.965394617795 281.278843600876
400.783945471037 543.170902795966 301.188296465202 521.30110104669
343.767393640683 253.833756857339 390.752579014611 322.684910198242
ARSE 107.655624022312 109.851507510788 110.068122112671 111.895279866421
109.947300629824 103.660742045068 102.343275379164 109.067239220744
110.764586138261 107.07609243233 114.740948266517 105.450273343324
ARSF 123.689439578326 114.942849575931 106.066897223031 111.962852412774
119.427342766092 121.636361281148 125.45936959544 124.563594928763
120.174672871542 114.334059060506 122.615852682436 117.532063783977
ARSG 137.310014841869 158.181316170382 145.286983999628 143.063345350253
146.619846538124 152.386986818959 149.582406567077 144.621077658674
149.717739166461 144.373226011578 153.366677608026 137.513315427454
ARSH 140.462651395483 135.395071593072 134.671840308041 130.628967781841
129.394359810841 114.711310011993 118.902823494379 140.833666276568
131.717160947964 123.7421178083 125.724143659093 125.150706047419
ARSI 119.746227888415 127.247764390727 123.182903611854 116.784024112826
106.341910466588 112.936472036608 115.496108000499 99.26400442869
115.624167024067 132.398041172151 131.688038068545 110.408301129053
ARSJ 206.257167235126 180.501409705275 177.874821397216 174.832495287192
164.708712337156 149.888719320368 151.340728221261 151.561151654372
151.803707178383 193.609985124825 141.541355091514 149.010477279209
ARSK 169.963805688413 145.938780249776 166.135707525987 149.133055368147
147.958483355783 147.694098231655 163.116334434429 145.709172732805
153.913513669361 170.079723856247 136.814736808725 136.852403978525
ART1 171.784981874184 171.880470629305 165.619206216088 176.390014020936
166.97270256697 152.099431258333 162.9279134169 170.775283924087
148.245974323625 155.313873664923 191.616483214712 161.937384157098
ART3 147.584176709986 149.717739166461 157.512561489621 123.905462079972
119.387459594521 114.234369421536 125.77343813277 119.886433564236
117.05536677976 114.644722005728 130.935922915902 114.042914237667
ART4 132.712573198794 136.251820364823 134.61114877396 145.043494048779
160.655929233978 133.473140653666 141.683298500736 152.317890573534
144.258128917228 140.191954630636 150.833523101057 147.428180649939
ART5 115.619116556625 120.727504500018 122.924017262559 114.927420605185
144.364319564075 111.7529190597 117.172431931323 113.432757764419
108.362609680556 111.010889276873 122.830376253922 123.968639555673
ARTN 122.789656787728 124.267394371656 115.242526386551 120.231559540009
132.881693683384 127.863583325139 118.666654333368 134.725434838052
126.439948611449 132.291759474331 134.769926504916 122.075440298074

ARV1 341.761516859319 318.297819854586 417.191960489528 305.343879847672
309.658122422202 592.957842630046 468.93543246394 396.459213999636
551.823767223086 414.333324610267 391.326200093611 811.362219801362
ARVCF 136.967408633308 145.08246303652 146.709967739596 135.797170561599
156.073428882485 154.2228051929 123.625734357908 138.580264357741
133.120273569229 123.603972078897 155.590197324062 155.371772547395
ARVP6125 139.459975305985 130.304743083321 129.566451901256
129.802596741962 129.92319548307 146.290334865039 137.048292841566
136.575784685895 134.514251897045 140.083495854322 149.962143642332
134.239416179248
ARX 137.728163622673 133.680691168661 122.89752183991 122.295222990408
131.319783710908 119.480037559953 123.035401501279 121.006569103388
123.015371659847 122.438438464097 145.641373616866 122.809241232029
AS3MT 189.0608798771 201.365457496683 173.782474972742 212.420616436137
167.190857452131 168.538117820213 164.601022396674 150.479320779862
155.124158030085 177.549330396268 141.440563159897 161.972922129453
ASAH1 189.136114953249 193.609985124825 218.470365730283 191.018168651394
204.862387642573 251.414717011661 411.160709628865 211.269041187154
213.914599384797 253.124449721731 190.281570660806 346.118765910093
ASAH2 147.570734399004 133.406312157375 140.922678400415 136.635413632094
143.239666389592 146.851307767958 153.829636228764 143.868962478182
138.062345982865 143.59105195427 144.266864839344 145.256660345252
ASAH2B 126.5929421833 124.873289279767 128.168460083895
121.448671447499 126.934059693116 131.872583529419 125.029516472944
134.002424083826 131.970514300585 130.455560026617 144.411176964424
126.555972138115
ASAH2C 171.161297672182 159.374355553219 172.008994980259
162.342112922504 157.08300736206 178.425137262105 201.320381706496
168.560093633607 164.96631670799 189.37832342197 194.430896269275
168.232843307077
ASAM 436.331333134628 270.360397503276 223.666832044186 243.038392457584
127.990453128978 150.690143057308 118.410091682499 137.974323288786
150.860090477342 152.89366585726 133.985582992986 124.720004905439
ASAP1 1103.13439876128 1137.42879398351 776.807656744828 861.516925304722
582.062758687489 820.249910349431 429.457884032977 783.755776589969
520.470790410856 557.868300839263 407.615019710707 333.533043832976
ASAP1IT1 163.824878791966 179.048139035247 160.293522619371
152.906352463546 129.532122459128 298.028932089399 149.786445079456
202.483493595704 159.495272062735 152.906352463546 135.784481523923
158.712241098927
ASAP2 362.65285317327 307.518881119091 324.713347085998 314.221257518059
402.026356201632 442.12886967939 267.747633122387 398.354212009426
319.223127076041 363.025385707747 425.229044880287 354.132752995385
ASAP3 818.761934233826 687.269774217064 508.572981581855 639.011144305614
792.101559967381 360.285027176918 425.708048519408 339.32415558339
314.182403961681 323.95355217044 644.11603826131 286.703712311781
ASB1 610.591049634222 620.371912523332 601.018867455333 873.879064183441
240.147082836599 190.614236149496 238.482863150124 247.307128520272
247.622351589081 249.737588031219 397.985856484851 251.039117540764
ASB10 145.858537779106 146.025781495177 141.284962125757 134.891736344801
132.390330703084 163.621658579697 130.630504666374 143.818379692331
158.138862054715 151.473239782301 144.096624787142 155.483400816946

ASB11	132.762755202326	130.084202182014	129.018088749395	131.470883854661
	123.643292039274	126.308622415913	126.176505541577	133.392934239554
	132.393669306489	134.115085057026	137.824985884649	124.959873714884
ASB12	111.603221942108	122.329290563769	118.988269692812	127.558034562151
	128.634431242405	112.78323842342	119.125002207396	115.988180787152
	107.863971340407	112.987890650593	121.294366964074	127.299853592344
ASB13	210.33390115739	207.440700355457	198.659595851481	207.338645756456
	330.758879244143	344.604699562938	243.95334982553	329.722135361491
	238.183096218311	159.120172969378	371.127480221666	270.945325305492
ASB14	128.588822671032	127.678512375099	124.238319768621	126.872912960681
	128.064189008549	120.979417552159	128.244549276386	126.080780033301
	122.959518532165	124.777895136212	129.838790698396	129.108534267222
ASB15	107.316786994369	113.611651901331	110.419222236698	100.216750525986
	112.081127853476	112.198629213643	120.043811428091	114.133473556359
	103.99234480919	117.67638166062	114.524341239889	106.854721604889
ASB16	140.391753835434	151.367402326399	163.996649606636	143.756242843438
	143.365976841902	156.06439656393	149.490064894136	164.601022396674
	167.040031488406	159.344044864557	158.573561336755	139.619076126267
ASB17	145.409722106953	147.761965925044	147.763017438098	126.796253980623
	151.081339000609	130.555405606993	149.166635189875	138.079494235456
	143.461785113446	141.102274192049	149.133055368147	153.257097727888
ASB18	126.100454068838	134.410706628655	134.060214479265	126.862274244977
	126.5764774133	136.987403767185	133.964163727962	123.76900858347
	131.444734367396	127.802645313091	126.134393447943	129.511465164539
ASB2	236.032288046282	224.851680313689	287.041585684111	286.085833802132
	132.47775411384	137.251513843314	135.582230137515	135.268963938932
	133.243679173608	135.920830568556	153.636804025106	140.616811333466
ASB3	313.077481033093	332.343558229873	335.533053221675	303.638516587721
	258.695920967489	344.992791638415	275.584858082096	282.101714660307
	277.685488726472	310.301616266762	307.982260548106	237.078644686891
ASB4	141.453255692229	137.514952067562	134.579005092929	147.236728188605
	143.152993168137	143.207187847583	139.638617433372	139.092075947067
	138.246963115014	144.2753860947	144.41489969974	149.31020447459
ASB5	700.601917911532	830.684999498776	773.330948077036	864.161191021297
	135.488155152648	140.127379332918	151.020903727566	142.763157232147
	144.368355737187	139.39442529119	130.085138325818	137.200433808907
ASB6	166.88833169342	185.961822133346	145.665155500862	214.785381549181
	229.116153948948	132.589411973628	131.434121613176	133.486155360266
	149.831566330214	129.875986080057	158.565676210997	132.597481743242
ASB7	125.055545087871	134.691038011706	126.349403754775	123.318763391117
	149.505323932806	136.316003989725	144.266864839344	135.349868925415
	141.369723386494	135.47852126603	150.792065744902	132.539585717666
ASB8	402.366797969833	388.002365247498	520.470790410856	440.404188514231
	219.996410908721	293.17183601265	377.083379021079	383.939800422489
	377.593087606046	321.84712975195	450.688800547981	365.541609015117
ASB9	146.398454169593	147.450681897766	139.739448838436	134.78059811237
	149.016767620428	168.10131060526	142.282861913548	177.379186278216
	188.952730860801	147.635761512135	131.089811472017	166.576768836782
ASCC1	589.722914993267	627.335757721489	630.473042320288	720.214044399108
	345.291295192574	483.845891531326	605.040026490154	390.372738176917
	394.35596596181	421.836020491624	401.102586700919	593.990687449346
ASCC2	1052.553421812	1083.26599217847	1183.82018168635	1146.3535752748
	2574.74962955426	1929.52146517699	1908.81864728547	4671.10203385528
	3219.27851139047	1379.95968831696	4222.55224241399	2518.88171283899

ASCC3 202.230920499592 193.641927801805 188.708596785144 200.790119570144
220.362946606131 235.933451977398 261.775269058607 214.546528079149
233.300864925399 318.850765099332 252.197271817794 281.996583337694
ASCL1 114.644722005728 159.784369379077 120.276236447255 112.036753245281
119.949050292223 118.126133973555 116.723991721647 108.131097605872
117.740022708444 122.777544465711 131.730758460802 190.66473033826
ASCL2 174.881557472722 219.729169245025 154.38209765671 156.34272729153
797.28465621772 303.795701960506 736.603605876572 374.56251861444
682.075583857856 199.555406967021 572.262848718437 159.138344477256
ASCL3 135.237042337721 138.446492794924 148.94765984808 151.226242696197
136.907453440141 142.563762845657 138.13987378399 141.917318809044
148.842322753916 150.856917125736 144.235298492095 134.793399942417
ASCL4 114.062099086818 106.182551369456 111.949644842971 105.179281694336
108.007762863766 108.868679899494 113.432757764419 112.75167046116
106.701716475947 105.193775204124 116.046983032621 110.966212705086
ASCL5 136.249621724093 122.00079209554 144.70348930646 128.16147857336
125.929743286163 142.892198239879 132.796961054534 124.551603326682
127.486697506397 146.785478478701 137.237089899964 131.086904662241
ASF1A 230.855806094267 226.020769172424 280.453895029319 196.922837906592
185.627515956848 252.514569129392 275.794847140407 265.61506544085
216.615694348945 217.307872538386 244.251315474587 274.974304050999
ASF1B 149.127963327999 170.505553609318 139.162076054072 200.065184172871
755.79669622705 369.018856220394 268.370347292603 420.502251256406
296.20443113482 220.010399552402 178.445384902129 321.177982468322
ASFMR1 146.805832438977 150.343825300998 138.295019395552
134.851757252167 153.97869518398 133.800111596565 136.508969220268
133.143695166498 135.697550878438 153.613110881454 158.249743182741
150.820808770853
ASGR1 151.538112896642 156.763364462534 130.231160879338 140.254665008195
142.819669471009 172.774914399844 121.425298866613 148.825790628891
175.313689069815 151.503307004675 155.694213876736 135.701041557713
ASGR2 132.634538733205 128.99859423584 132.375905857023 129.596851280135
139.117776552573 131.347213356457 133.201157825808 130.392575279055
131.121343131082 129.79093244374 135.381131982323 121.107979534361
ASH1L 151.663203772586 178.804669910283 151.377932495104 143.533725135227
154.161892545061 153.346972639134 184.937088280378 153.348110626071
138.938546468136 156.290018816692 168.805630890928 146.828099826146
ASH2L 611.36406319509 633.762608454521 821.862271569917 696.131296166255
3391.3005983725 1387.90327883679 723.428294037428 842.22767901391
830.455793617181 964.093668483033 625.544719251096 825.781627776577
ASIP 109.128394735532 115.35376992547 102.851152421943 119.673343767067
111.260486045985 110.594755513247 110.003750007057 97.8058879269165
117.423704591303 106.746545626069 112.179019393947 110.935688075738
ASL 139.293775885106 150.746423638097 140.626181314061 146.975258859244
186.758038665014 146.858292465699 148.074494928651 175.055634340766
150.19577351885 146.929717840755 163.02140155858 169.246302492166
ASMT 117.084984190461 120.060776752215 111.794102015354 123.743971147401
112.252555403121 114.942849575931 111.7529190597 124.783306383852
116.751145098777 113.852849454448 125.547399456656 105.952640312359
ASMTL 354.840474468573 353.826834642154 387.908774438461 402.213774592085
386.544913399837 295.935625475189 162.876684781936 244.280971257965
207.960802948119 220.791884858764 280.798003990849 262.60377731258

ASNA1 172.222262910052 148.624135810148 149.226700202481 174.526176588454
198.191669030952 143.098246338022 164.981665344298 142.126620107418
139.00736252716 154.388776919015 165.186696781985 150.802312408483
ASNS 609.100385482325 561.609211243027 513.292377117011 699.270931701696
343.12166164987 683.019502027271 621.354393918348 771.059329972125
460.941286124613 972.468110850466 472.669956708398 417.36454228858
ASNSD1 751.896334334052 595.270107963842 1033.03719406038
521.625146077176 411.71606840872 1202.74869573717 685.620637807134
654.108318810728 714.91842349236 823.297606185721 822.88742008931
669.330078861673
ASPA 232.444867422559 177.450674276588 145.632060621034 153.267549449395
136.939603341076 150.394483608039 132.402255281009 150.423586547273
165.283700641161 147.823552813852 144.523620416439 159.182533998843
ASPG 163.922353493317 170.169402943662 173.182013165174 173.964378226631
139.334552725255 125.27625242915 131.662585510218 151.360575274272
141.950778601653 349.99912485533 163.407360509803 131.533378871062
ASPH 147.702785358046 157.067264221207 172.80265044721 154.881797585583
233.914433380121 208.080089376299 243.10297424873 176.725347194807
179.273936200852 166.400162299835 165.757295630254 217.45700166961
ASPHD1 131.879526177089 128.324383749931 136.380523452336
122.615852682436 172.008994980259 141.079317977197 150.557982810339
129.231455282441 131.38692240237 130.782296492278 126.908123260394
148.927040248436
ASPHD2 121.668166378914 137.27827836407 132.300508985399
124.88118767481 235.905788497166 182.470538043064 140.02337095267
146.666759322506 158.946177055546 137.194467539969 182.292947734054
166.001279428607
ASPM 151.364528898537 201.865955150869 157.800913051929 160.970946091146
426.364663722264 525.287367366192 324.177924877233 737.650963292668
363.963951509433 415.388760988459 220.633692394229 603.061485415867
ASPN 114.987285062108 101.713609383766 106.772581387455 108.807891863679
99.0767996209245 106.667175621971 117.74682797375 110.003750007057
108.910588012315 109.506450285923 116.625518828978 107.544568250154
ASPRV1 138.508015596135 147.530719472217 128.122197525844
156.339564110766 152.247504238261 140.639012372681 127.3079781139
156.71199811686 137.303701514064 126.711037295629 126.067296703905
133.318665755015
ASPSCR1 359.162549472301 372.81255844018 417.262521411065
466.392151902286 899.336817124312 612.06231165648 584.919615953457
1035.0797237176 664.343553341563 393.98210915852 411.451612988791
617.833194119379
ASRGL1 149.642946071295 155.064761390765 154.156272284591
158.854659281803 145.432667767652 159.227123133854 157.77047742735
220.896363324634 165.752558888845 142.765490244223 146.351622020606
143.596530151289
ASS1 468.82816089485 680.250735532287 428.782703561238 572.913714991136
2200.94974896771 306.232249422327 1165.05055932899 864.547109080675
1023.29995420518 416.715760038095 1403.09138858507 216.903719298575
ASTE1 213.324226314713 215.057917795456 190.889832855403 224.805404798696
189.324370290702 232.250434241457 250.647644543963 290.037325939883
243.589099648895 232.960832410426 273.303240462047 261.251965523684
ASTL 128.381433897602 142.318709347082 130.450930089722 125.689884251847
132.84666796678 125.008209861774 127.786141874218 137.5954358221
130.083266044947 139.606654715326 153.178934750919 125.83678485646

ASTN1 145.56468363155 133.228074579352 128.346598756853 139.783725419023
131.599370671279 131.202090507576 136.84840505336 132.227561632637
133.054453517834 140.714455864886 137.627338682823 133.76515446791
ASTN2 151.739221728872 168.882806817001 158.507624756588 160.778296470857
266.057472924341 143.370698101204 141.596078062845 159.102070980047
139.432567336814 137.353149614081 182.275998840116 150.452467115669
ASXL1 326.570967321756 318.196134631178 414.114051645565 315.963526790659
282.282663608264 348.067061666237 354.132752995385 242.787656836745
280.453895029319 264.955939320296 277.726756691133 267.483886037099
ASXL2 497.916883763059 453.17378995737 543.001249586573 417.731815310276
436.982960786839 725.443026984982 509.618968665191 811.362219801362
523.591472915151 718.449150898903 642.484527246259 547.202354339933
ASXL3 149.912481263284 146.626517077288 131.774882989257 140.938442055097
130.038936315246 131.267177634269 137.198654302648 115.285392699073
118.256000539093 149.190528937822 127.949413503055 119.774204444899
ASZ1 128.499303407596 114.999628959921 121.212521750566 128.975785280465
118.572988141773 127.302714624905 117.084984190461 130.205764567934
124.924794564647 122.452541100946 121.311836944721 132.257989841046
ATAD1 368.711718874343 342.315456386729 431.093544964576 270.33685210971
220.772387104223 309.5988292104   350.308420518351 271.287767448621
303.762967437478 348.640185950469 367.229293432041 458.047799748696
ATAD2 162.333158524811 147.502116289881 187.461448376593 145.610045243277
266.534316786801 425.001043358831 257.5097052677   451.985108262982
459.068582584181 283.163770658619 195.946096353894 300.602422224047
ATAD2B   176.622758931306 187.097765940131 169.45559708224
198.226525986857 175.601912488918 230.263678910356 154.056660680872
189.160454951382 158.679903271595 186.257814752354 170.818182800759
207.467591256549
ATAD3A   323.462252156562 250.139061165593 276.184421402511
321.794887577351 761.89271251455 413.983821839274 318.423602671236
251.511405838796 307.945884504629 362.849380477089 232.344545356395
269.831616158113
ATAD3B   131.755881982599 126.573673878042 148.356726939122
140.820807603291 154.302619274576 154.534605635191 135.175806654971
149.000760379976 156.576585764311 165.055636629118 118.988269692812
145.566861062922
ATAD3C   137.429832103492 144.690880401712 167.958792571511
157.507108643575 146.604519441812 148.744599622165 158.344330353326
167.949422301257 150.115482378938 171.278489808694 137.546487338984
150.809693773343
ATAD5 138.785538575745 116.186846885411 143.247977741752 123.259322051795
130.827726862159 138.89354244296 153.93249158209 142.783159341229
134.474466007553 146.016920431468 140.772074031028 155.026276988302
ATCAY 122.793816135585 125.048770476506 129.3513576591   118.514618614167
126.148379057536 116.335864439545 121.094060100325 115.524441144063
122.809241232029 107.714294216913 137.132095345234 117.270320709161
ATE1 137.907709799074 131.153457976248 132.7284396337   133.606068125828
133.52271411034 131.123149002831 141.263207601393 138.717541782317
132.952355710395 143.242308032336 141.571389983164 141.194900058799
ATF1 226.086733606951 217.074639597142 234.996704304673 180.549865708041
204.219197797869 185.525760847281 218.209523110843 222.285940575778
206.391403266202 219.129781831192 194.734255772365 250.376178742476

ATF2 144.082452235267 133.857996630836 185.627515956848 130.892302357045
131.992186668019 159.513465644268 142.351705613006 149.754842928462
161.019770036957 164.824914851636 167.025336068642 175.442400672352
ATF3 768.053694275398 356.770158430601 395.049127927637 229.594081405784
148.19212125742l 399.620724969771 133.408354896584 153.620767586182
148.195611015135 534.083703564903 145.45768403614 191.708350637714
ATF4 7446.59106452583 6105.64727031051 6091.9840719327 7802.25987814138
2406.67187918735 1309.01193284992 2853.14759681683 2023.92614092273
2822.48671794913 2962.11407134162 2183.18692530644 1881.90353127141
ATF5 635.37511798786 558.051041108282 409.743899750699 492.05680879106
1597.53010317504 676.293399523316 1133.22524345111 986.972987865318
903.503768217033 986.441377365124 463.488995330457 771.694853062033
ATF6 373.033658917608 359.024299091145 349.248144552634 321.977549744483
476.076903428247 744.523059417351 655.094843900848 589.722914993267
496.560127152436 662.781145703653 433.491835341321 962.559482864549
ATF6B 154.764283765316 163.956894843504 122.355409068882 170.18781660707
270.945325305492 143.050004021764 151.68523937819 149.076146942797
157.216793735479 148.980330875743 223.154771092064 166.346572488391
ATF7 158.608479354755 153.54898879562 161.26917182967 161.475694690038
147.511569289425 152.155760355248 150.303703193967 162.077455145261
155.140371265225 147.491439949088 152.074017988748 175.529441666151
ATF7IP 159.479962316199 172.355215322173 193.336246844237
196.990867962546 174.866036336378 159.166059518117 173.624709850889
169.257035030405 163.612347715773 189.324370290702 165.642728895682
161.903016201041
ATF7IP2 211.995890899864 213.145460679531 245.978723809154
183.130891975122 185.485858719975 421.140525880287 275.252966071473
327.010503005631 398.673582680257 173.624709850889 535.861738240441
233.300864925399
ATG10 673.824770556885 690.582550537485 529.854965483609 732.040299742444
237.734385188542 443.444024128516 358.654569870431 359.329520507158
335.858047332641 352.897241809959 452.517134370928 342.403428822464
ATG12 1131.22418479945 1244.67453201339 873.010676463707 1387.90327883679
242.253995801882 400.924914238052 577.33849844574 544.669441360594
395.588345893987 819.151876775691 438.231717096337 506.659740456829
ATG16L1 588.334329592604 601.164127925014 650.0804221227
775.781735108886 356.530530468568 411.160709628865 339.012225830929
310.690317681243 422.12964420709 438.340341490622 425.59810308048
370.678040372273
ATG16L2 269.468453936818 270.847923557176 212.210383398591
244.440857688518 191.023813502294 192.627266049516 180.265418365803
189.662974464847 191.445291899108 226.565823278767 196.908612360694
232.127996106421
ATG2A 271.638179116269 304.636618563775 276.284517459626 380.821355948507
787.615195903993 295.367125961621 335.013713757031 283.46492196692
257.643855420381 304.152390210727 303.380479700334 267.451611731669
ATG2B 183.657525723012 180.798671424395 225.438177833064 177.246890307643
169.31618330657 161.740466814881 174.09267813338 171.086559024882
165.808660192901 192.737713851863 171.022976593689 169.355185349431
ATG3 280.848426993226 305.096320805078 317.659548748735 313.077481033093
319.352940751688 389.177193459 353.385210690169 433.019478861638
479.138177792954 575.857954264295 322.837446618323 425.471195039676

ATG4A	231.698163424706	242.657224881824	252.197271817794	255.71113398292
	207.764456828813	251.588748783592	221.171478972931	210.392662092721
	250.30085097088	230.893665210918	207.850956786183	236.899421827427
ATG4B	149.586651393588	141.809577413906	190.22021453133	129.637555509848
	190.66473033826	232.641965649602	264.8472508692	210.218440444631
	219.597808863947	305.669636871734	192.580288063603	325.252108402192
ATG4C	166.46473789699	166.411219327671	181.758545723241	157.099868633958
	147.325222091872	167.491399614986	175.350734537491	169.147351642004
	186.420541099902	180.142758394321	183.583432296209	161.305860691151
ATG4D	159.257221624668	159.806221777332	153.500478301079	175.111460651956
	220.458449424062	186.263348153627	184.414609874199	176.183165494563
	164.423960071908	176.420075374686	196.169988112042	182.410688592378
ATG5	455.930204471585	485.309521583972	522.17701271254	461.822629852953
	227.236255752375	328.200548902445	314.486603134656	285.931444518474
	269.345125874858	289.09614335372	515.158921349383	322.263925912805
ATG7	300.482945704017	335.698400607132	300.443499140896	397.861172312706
	804.362909579005	303.483515328621	365.650071660806	535.354724252178
	507.040326298457	550.434931878586	277.817786943873	373.033658917608
ATG9A	298.403224729528	350.166837086936	282.719121652073	473.529519031797
	360.124745133393	223.339132121796	257.740674565975	185.876046259727
	225.238369884803	265.712482648121	265.470314691221	229.076507182239
ATG9B	113.879459563492	117.343165720552	109.044879188971	117.652693591865
	115.713909155043	118.04359489354	118.93828679356	120.36718185387
	119.802832736435	112.666812944192	117.910259090745	120.85013281321
ATHL1	284.534369213195	227.64196401771	166.570606752678	180.022646625687
	160.853480692082	130.005909892804	144.763700813057	194.014764659513
	220.742788129847	167.993851183401	278.598913464804	318.850765099332
ATIC	1656.02721259517	1516.97921112926	1628.24528041404	1727.7144253077
	1727.7144253077	1776.38646235135	1054.47292895417	1222.59895569348
	2141.855356411	1191.26028342929	830.455793617181	1720.14021303639
ATL1	286.035428568826	233.496804016764	313.773476049783	267.069665000047
	141.054587977578	153.118492567137	151.463200570499	178.035700298977
	178.304914997023	177.524818593325	202.129153259445	208.72873686638
ATL2	232.344545356395	234.661864595717	362.795691170335	220.258804437828
	234.484787292284	212.78038645414	243.65518489122	237.396466740419
	210.316431648335	389.686045618228	193.16411905957	202.324795686879
ATL3	279.790257881254	310.465070184707	241.557810100265	283.416246133873
	625.374595058488	304.985269874607	507.710604451713	277.75958501062
	250.406775889932	2470.86113150126	315.565349831797	336.5543056115
ATM	294.323621186916	254.036619126409	387.305348482856	233.690524419327
	168.790464803746	231.955750250711	239.363511706064	318.930960243303
	297.466255000163	205.987280015028	315.912220767901	244.766289564524
ATMIN	289.956528242564	299.851173200352	271.113617350982	264.741946816665
	219.228720772236	269.495247190598	219.519896184851	270.679613997675
	276.184421402511	195.438353051306	280.170464338141	292.28857006885
ATN1	277.129985959184	296.20443113482	266.756647182704	391.782120640025
	249.737588031219	173.552535486766	182.495117800073	192.680681636018
	207.417884407459	173.063002566542	212.531329857909	167.941671074236
ATOH1	122.120307446003	109.947300629824	113.38168751499	108.955761698524
	128.675527226161	119.26921157552	112.736231367296	109.613208767992
	106.982655360194	104.544792793756	115.927914533359	111.381427322188
ATOH7	155.268650584431	158.085935267091	162.660027294291	164.264079172779
	156.546769952772	165.673535349675	155.313873664923	159.327764602722
	143.125454748996	152.001155788544	157.200582422961	168.000247148058

ATOH8 277.459328083162 369.726867521935 236.326943078666 262.171775165261
1042.40539020413 207.785320401354 411.451612988791 147.882530392424
167.380798617981 147.079157199448 408.263886786123 201.893722965557
ATOX1 967.123240442957 971.170460915918 798.1490651671 1471.01897242089
959.532977061027 1003.36623972843 824.070046083713 1001.92763014266
935.423045210635 1408.08614963928 681.429327723794 846.425467119342
ATP10A 170.18781660707 158.23884088767 147.491439949088
152.265624144393 145.217261514886 153.879427186342 170.784229026842
157.058912028958 153.903993884123 175.236826554583 182.152956182443
154.499313851756
ATP10B 172.604689611475 178.045931314857 192.353768499582
163.586549438955 239.541516080716 223.872330412144 214.86995782548
333.745543022749 357.259935567823 808.837225580351 481.86545778383
464.577839898252
ATP10D 128.886008923268 129.487458179949 119.475462713424
110.093565974873 105.551878332786 122.659174808512 112.832122823829
129.819820730239 118.438942370745 137.881776548665 149.585616718523
114.805810485989
ATP11A 132.164132609317 137.222125237171 128.099484678519
128.032141013681 123.817878562971 133.585425721984 139.00736252716
140.341589053972 133.027592711375 121.71538073735 125.915689078001
140.490023670841
ATP11B 289.517820913799 305.577166690215 301.428024926428
247.942904862478 215.698924670416 325.082620845425 295.778154263981
307.16835174775 259.796092536601 681.810927901806 539.008286968106
344.604699562938
ATP11C 238.375823012603 241.527811817146 229.046153362151
233.71520737817 271.858059712406 307.480644393018 280.990656342284
210.925203743958 242.037132958656 172.002600588279 248.965394617795
243.080229016932
ATP12A 127.712004247155 159.660564323984 165.926301648161
145.517559593811 145.27890423835 145.632060621034 148.062245676711
141.987635042691 134.551054564417 144.102872001089 154.627270742193
141.86836086844
ATP13A1 223.918931440768 251.09230266334 240.591548171052
295.935625475189 683.655205499779 300.482945704017 495.89837457901
320.936905584424 279.325065941252 224.766155406229 315.47473326786
302.601458409102
ATP13A2 205.362816362477 217.013533525724 175.7330682766
249.633252091596 681.591028334969 266.756647182704 242.410260751835
253.833756857339 210.448904052352 277.75958501062 330.321113875647
239.363511706064
ATP13A3 142.747793303346 147.547944397578 140.435724944447
138.758688249557 152.188770978528 152.957297648595 154.617462666847
149.110300099889 155.655771857361 157.309058982117 148.209164900319
156.519196335454
ATP13A4 135.879975973882 133.875667250136 126.617737577634
123.865424155099 151.198928680018 155.603263242813 156.026806913759
146.370084152094 145.42029807396 140.772074031028 134.533551900843
161.019770036957
ATP13A5 142.664793323255 117.423704591303 101.713609383766
104.633603136988 119.01990099776 107.221547998974 115.845468537936
113.924310293916 122.578170988039 111.708970469189 120.964862799634
118.419311800417

ATP1A1 989.77058497525 1071.47374315822 748.38293089716
891.637685856145 3424.65126200697 1715.51071085304 1737.57015396357
1132.27988339924 1690.96371604294 2986.6208659209 977.227184625781
1271.46608195833
ATP1A2 523.216537391852 472.030150842569 703.587139938769
653.264945856916 121.116206210402 124.811760828557 119.594962686756
121.871105084095 122.670689386332 120.329105003959 125.091038614268
122.35832958054
ATP1A3 162.57736365619 171.545695383454 156.511202150773
202.300293733922 171.426334687626 190.204960255989 188.973595445056
195.674128236589 167.212960569538 156.865049786218 163.558869203898
181.840985962045
ATP1A4 116.029201658798 112.999642731096 125.885715928839
113.22850386845 144.526140751381 123.219903763265 152.821735300697
162.649092834422 121.71538073735 113.22850386845 160.268754786532
175.111460651956
ATP1B1 594.92419264579 765.532997390972 710.228463443265
481.453770150269 1144.53002508973 693.781708146282 1083.26599217847
1297.25632532536 2698.5455305009 799.132592914065 2847.42635118026
2648.41012401723
ATP1B2 177.151218006672 173.507835230243 141.249687936867
149.435684626505 124.641892391159 134.094231940612 141.513636348978
148.096551269076 128.463982896081 124.842426851438 129.155804800869
138.389976620532
ATP1B3 713.832153334389 826.771587455122 490.269418483997
719.766799746895 745.482863218223 1057.1528284934 956.153239481189
736.140249105836 768.334658920971 1704.25279485563 913.684449305965
978.389902096994
ATP1B4 131.051711775229 127.095347480109 120.135349166633
122.484351696828 136.90636489391 114.182798672347 115.450352881048
111.722827315911 117.008277388399 120.565485864499 122.914680091926
118.226036680442
ATP2A1 134.968848574511 130.970694919879 139.606654715326
132.782967735893 140.728126380228 126.127630885864 142.64161581553
136.446877465751 126.326609619996 134.394503574535 128.135216461862
135.583556015955
ATP2A2 1289.56069670699 1377.75266942574 1565.64079526732
1868.82736251524 825.345092964844 624.978351618077 644.11603826131
712.433345978749 877.255923473532 864.908449769749 636.970243888917
713.832153334389
ATP2A3 166.917552275021 185.140211988926 154.455641374384
210.882890077598 152.898148619937 135.915960609269 137.615027659126
139.927773713324 172.120772549791 125.830917527594 165.67554738735
179.663258610881
ATP2B1 234.041863839123 212.798133015556 179.96623248843
206.113290588613 257.032174266905 273.75192868583 214.662648581622
512.895335481876 173.387549650592 174.873893094923 211.395593720534
181.771654391653
ATP2B2 126.209426865327 128.626051185037 125.556444423391
127.685469299406 124.507958339495 128.568463697992 126.48691003872
127.427014611349 126.153806511702 125.08580835258 130.928718312512
119.168045349254
ATP2B3 130.754320787628 126.978144285534 129.221482161409
125.822346524903 138.953926269905 125.761205967344 136.478114537536

128.0366252162 132.119370332298 127.736712231221 138.684070773064
130.905135502296
ATP2B4 2558.91648313228 1797.65684760558 2733.68308784024
2213.1043022626 315.306271141071 259.868701878844 393.375554593484
179.048139035247 272.639413442579 464.907454552364 332.343558229873
506.430593289427
ATP2C1 190.057030596611 184.672330560677 232.492398623889
164.96631670799 270.787490294555 345.627266482686 311.715351215263
329.535023880129 376.276954313674 337.077153882632 275.343591424586
366.961565952239
ATP2C2 141.743873995642 147.335403843539 167.003917876371
140.217243202557 263.426870776148 194.072260528037 399.364160668615
158.085935267091 214.082408290757 244.797792477941 324.177924877233
266.057472924341
ATP4A 127.871445879091 119.48998115497 118.117957479382 114.790717658188
125.256339476772 142.633121727277 114.27112025541 110.304102941202
137.240806191881 121.871105084095 140.815807483203 119.870661213608
ATP4B 131.965184111199 132.94603323912 133.94206203396 135.421804561804
136.674553275255 127.615631240096 127.464485396039 128.36121178972
126.262349005661 127.440987506769 130.677737553727 131.970514300585
ATP5A1 4743.19967685595 5213.40342339233 6500.1854485891
5981.08862268291 3757.39261544326 6233.78489807054 4359.98094976179
7656.97151002785 6670.51138785163 5714.36902486392 6851.54963413886
8649.07367196998
ATP5B 11360.2921551609 12171.1997841553 12493.2940668467 13944.7649147227
12577.0915543468 14454.6736124756 11809.4804843301 18061.615487998
16230.8346205808 12577.0915543468 14544.3732040823 16230.8346205808
ATP5C1 967.797017108447 945.830852603704 1641.70484603137
918.162638134305 1527.87148052149 4759.17973319588 1780.97169244555
2166.68174726155 1146.84239258543 1405.2134783352 1834.08657010335
2462.87034563426
ATP5D 1374.92806285803 1537.71464618832 1596.33832632698 2050.34883263932
2334.97297889593 1267.5716872154 1139.14730341921 1125.95664591686
684.715981522999 1248.69137283095 1504.15682715484 1184.88559645917
ATP5E 1408.08614963928 1626.34607278613 1925.60246708149 1710.60409578293
7733.95252458705 2915.72823797537 3822.05744774919 2206.7447886564
1728.86115695979 2753.89649259242 2316.49886419499 2539.87833317526
ATP5EP2 8292.04796307133 8864.70614223459 8649.07367196998
9636.32818474581 23190.7842325067 13645.150201952 13013.8166277495
11360.2921551609 11360.2921551609 10632.4185569238 14417.2696849334
10988.2836796736
ATP5F1 1071.83063260499 1163.13845502757 1798.5819962261
1038.34611017219 1466.08446530283 2677.17579532816 2741.71904421456
3066.69261114251 2328.65814406636 2715.55326746591 1986.81354260292
3136.27991502603
ATP5G1 737.650963292668 730.988069115919 592.822841077599
992.744093752678 801.702878226247 1035.98574557331 504.617795280805
974.007008568051 907.904780399957 723.250763797522 616.339439383796
774.180202250076
ATP5G2 1099.80705776268 1102.38234645327 1461.64901688125
1181.1611856364 967.797017108447 1248.69137283095 1948.24432908972
1797.65684760558 1838.18547839898 1434.1439428394 1356.43255022363
1865.44709751822

ATP5G3 139.714198638713 153.82440233533 150.129367314696
140.899799480834 143.514848892447 212.836093887552 145.652246303142
200.944865673409 158.716575777014 171.16807372519 177.333305785248
295.099840299681
ATP5H 4875.22637286719 5032.66681917478 5589.21471380445 5688.84918819457
2174.62979843528 5759.5586948816 4938.42484385053 7681.48027081818
5314.0635307876 5965.21815409254 3843.07094386605 7190.89891723932
ATP5I 2970.34597432588 3090.52810501797 2770.7811954029 3615.76740804718
2350.01764791897 3463.23822434026 3334.16277752222 3140.51157972795
3727.70199176703 2653.47752512393 2449.76376608893 3463.23822434026
ATP5J 3173.14004739767 2947.51998516453 5955.10816224456 2965.38280248519
1195.6335460045 3468.14455177277 2819.92361643968 4318.50925204407
4682.62607750437 2799.0487754899 2255.92551080483 4009.22206756187
ATP5J2 1386.56788941677 1669.97250023755 1297.25632532536
1880.35608492553 5073.27433952351 3237.94149351627 2877.92845389611
3807.16206340771 3246.86605825129 3103.4192907748 3481.90072759183
4404.79209330918
ATP5L 1894.14699530855 2012.36542989953 2811.84191700516 1996.65760745757
2845.0377634896 2171.00457877604 2346.47361943533 2660.39990175415
1975.83062789692 1640.99641023476 1766.15001427132 3608.10730106542
ATP5O 3501.86788138647 3515.36256599555 4592.14655702906 3052.86038628748
1552.69879081022 4965.25728333142 3757.39261544326 4901.41175228982
4811.88424516272 3954.24763752814 4073.21075791737 4134.02613649483
ATP5S 212.473610495213 187.112176929485 248.16594076495 185.876046259727
306.111172289632 181.747090380435 309.861048237742 225.209315195163
200.518684601283 361.082957133644 310.174932137546 351.934158805882
ATP5SL 710.003412772888 921.140932799223 949.789710063337
1347.75629878765 788.693133735893 536.067857235731 705.529158880221
646.117381960716 775.781735108886 661.545179083009 589.240581642145
486.653672099699
ATP6AP1 2553.92770795014 2913.2685312165 2482.27419621925
2615.07927836881 13902.1067272762 5932.37714987741 5382.31371568106
3989.14883668905 6244.58472720999 2938.06458269406 4621.99241522677
5094.53287522452
ATP6AP1L 211.692743811486 157.619627711954 215.506175859782
201.954167006591 129.672284417918 166.157713072213 284.272977025491
159.701028026285 181.889592946523 154.302619274576 201.865955150869
155.198503964295
ATP6AP2 2642.24374855334 2770.7811954029 2804.99820187198
2129.84680802619 3211.2719052157 4491.96934862425 3712.67025366957
6358.5018930218 2736.98692493933 3008.43964505208 2527.99279063127
3744.5666678861
ATP6V0A1 1606.01395227661 1423.02563792293 1485.776977235
1708.87666406131 1286.55103838368 844.778755052304 966.281849197568
1198.53117439169 1029.75852676191 966.731520471976 588.863382995875
832.911633923496
ATP6V0A2 234.020419732787 236.326943078666 230.91747428831
247.643123415644 297.862653495715 259.064601204239 218.896760804208
229.373686092654 262.093405224922 217.778414825515 221.82340413039
245.850813387241
ATP6V0A4 128.247871411825 135.455283504668 137.144001915831
131.785745489382 145.681968019851 132.737464610403 129.147246809319
128.499303407596 151.604093626944 129.86336717605 129.384531576943
131.536457148878

ATP6V0B 605.583144451322 688.464980326689 509.292779821787 584.813665097407 5396.55955971617 2240.33212242003 1350.87073284396 1427.70292953827 1651.68171717875 1106.75785702993 885.713964813911 1255.62991601665
ATP6V0C 1431.52979384867 1649.46398639795 1175.27165286568 1580.27059464289 4377.59943975549 1493.90578573732 1806.24732793591 1925.60246708149 2321.41228376157 2255.92551080483 2537.90501983425 1980.4589291261
ATP6V0D1 472.793332506369 553.368945998847 500.132469062228 632.565736446505 1576.21727854603 410.731869154912 444.663812851378 341.015631528464 464.128022170285 762.584280908728 493.492348317616 538.156335788156
ATP6V0D2 184.414609874199 165.602175302848 178.878444404192 169.052101570616 172.438141471784 181.412790232996 206.321272734099 172.652645920958 165.692508737757 185.525760847281 169.391557085902 174.136806313142
ATP6V0E1 2811.84191700516 2801.66917828364 2615.07927836881 2770.7811954029 1996.65760745757 2285.86163259073 4141.8682669995 2395.75453574049 2653.47752512393 4081.13406129846 3655.63669142687 3510.24464112094
ATP6V0E2 662.373189001788 848.154015576436 1066.25208226214 1135.49876948815 1305.2232532959 1634.20135156938 1785.92695250894 1789.51979173209 1503.2049371911 348.300300174924 340.966515437272 1460.24985399013
ATP6V1A 728.176339659082 789.043948009585 725.216684507759 648.38049615541 698.086166782221 1762.09874961415 1273.6393529108 1566.65825409044 1731.62376348283 1846.61430264854 1026.61404215739 1717.09434498876
ATP6V1B1 150.380814008952 139.054140295282 127.048605625519 160.143443392509 338.46332239699 423.607299503704 344.379733302418 142.587032407457 152.987939791009 137.13615301204 141.040862279633 133.032905765836
ATP6V1B2 1356.43255022363 1385.28044421961 1459.31031099055 1525.24196488688 684.866405921987 1091.69584249713 1093.21579738952 1129.15018335354 1481.24705642453 2437.63566094439 1100.81588201714 1237.56662467492
ATP6V1C1 252.404931238338 242.871915060884 209.464347017443 239.079352337243 175.562070915794 218.862705238592 237.312766830206 180.231462528702 226.449705559354 192.113217321485 232.465880700217 204.137195524755
ATP6V1C2 120.538535752185 120.633217745524 131.464229237662 130.416957431512 122.038534506506 122.610529927104 127.018746030517 125.334995649504 133.327778635672 126.242935839683 117.193620642373 128.800907223807
ATP6V1D 1248.69137283095 1458.13354165836 1868.82736251524 1495.04587558545 1257.00798908017 698.086166782221 1164.22995893381 969.360864006371 1131.96129284658 2637.62596741408 1272.14273680177 1440.14449872047
ATP6V1E1 2525.90466050731 2459.05558915812 3211.2719052157 2819.92361643968 1011.9675459648 2080.47567326037 1720.14021303639 1592.29734618345 2051.45788386541 2388.03256067711 1437.5568316065 1685.33737186733
ATP6V1E2 149.460660195293 161.408318705503 162.050520530297 151.384762644292 196.245297362955 171.629605754643 271.615621030842

202.718428167217 157.468934439452 148.916981164664 192.418895349837
159.133905701536
ATP6V1F 2114.9754128463 1992.71224011906 2168.58813300343
2007.0121037284 3441.9805896226 3294.92439159154 3807.16206340771
3618.9385126441 2905.12297639092 2801.66917828364 1570.39362935859
4503.01240138314
ATP6V1G1 1671.82101590797 1840.14350831655 1503.2049371911
1329.90419210779 1846.61430264854 2430.70635781622 2642.24374855334
1792.35343044443 3757.39261544326 2970.34597432588 3518.84173951107
2403.42261883515
ATP6V1G2 155.386226046894 151.406384541297 167.164675439456
141.416706868124 142.833025633146 128.492142236807 129.596851280135
136.03581843125 122.153996665065 123.804039963953 128.936628289974
131.119810851017
ATP6V1G3 151.76341973891 149.747347428419 143.976395954528
138.600017737884 137.183566882805 160.423982411027 148.953720017484
146.830752309354 149.613701531197 141.006540481119 142.116311513299
139.423432468886
ATP6V1H 350.803378042804 333.349203784021 438.533112511098
373.545629693667 509.988617641229 431.861762635568 357.140791982825
304.479999301061 476.168679880357 433.82566459288 405.183882455109
340.608165077422
ATP7A 141.644521617419 145.61758790738 141.296574431203 121.468398802591
170.561596645385 183.513101761782 161.456727894848 148.585224298375
156.548858402702 169.496129800444 185.984938124891 147.040759979063
ATP7B 154.998011079046 161.636411303968 181.889592946523 153.888212689508
167.057202756618 153.54898879562 146.846301957637 146.484364264513
147.868721324602 145.843639385175 171.093611712143 133.392934239554
ATP8A1 155.108146847158 151.71942719041 162.547569833931
143.733505359106 141.937611082276 163.290050020756 148.96493117884
141.717466442384 145.03221570575 143.397823965608 122.729918525032
145.813437992703
ATP8A2 149.974080577906 152.221694141976 142.64161581553
150.142724640067 131.700854556022 136.065134838221 141.259186690966
138.458816023289 141.462379822312 134.801507994667 155.974463324915
143.331929080227
ATP8B1 167.365850007748 174.526176588454 161.849023343715
151.87495966414 325.859917318516 263.005303710717 322.576886792502
290.688850193983 243.402781509365 197.565122821625 363.539983343859
239.419200107721
ATP8B2 298.664864578349 245.351459157365 242.996248524203
283.048002376743 132.611864316181 145.844961942581 143.990328432869
161.951996906162 178.425137262105 152.277810621542 157.108043150259
152.842777535666
ATP8B3 125.45936959544 112.699674279523 115.491262364277
112.357261386644 111.497241941203 119.613985826308 107.365390754133
115.184395637553 116.902574164498 112.3786222545 167.653412908384
113.22850386845
ATP8B4 405.183882455109 401.631377313065 457.779218916808
360.605223912114 182.152956182443 223.89854167651 178.035700298977
230.399207047948 212.460476372263 184.437810305792 205.495823410422
193.37618381669

ATP9A 1100.17618574297 1182.50261323518 1037.00025848491 1085.92360670051
1016.18143428327 837.413261433363 1455.75693295732 521.83794739314
507.342877360289 618.140160251927 1270.76282064793 673.176084400831
ATP9B 196.117609477292 195.474525582555 181.747090380435 201.256649338315
184.724071110756 167.304721591124 200.424975825325 233.36028792331
173.204163917896 190.754349388901 216.522992914538 181.803275243281
ATPAF1 417.731815310276 390.098830270926 491.906642849373
449.859227376918 588.863382995875 397.153801851621 508.302878624289
520.761305892651 367.898258897888 362.742120043509 447.182566890086
408.361368902627
ATPAF2 165.103398617203 169.815053752464 173.082167545676
209.913460781088 203.586310608403 144.206259414981 150.746423638097
141.461435494985 145.491525555916 156.490654498766 167.139257044999
133.978783899572
ATPBD1B 654.881990232975 670.097523566652 607.499206410372
774.180202250076 361.620362526358 583.697431061521 583.619362840814
447.779854598556 447.059120541009 314.845965872164 639.184296110069
382.233826557803
ATPBD3 152.737177514149 155.172667450053 129.075693654369
169.607072855818 255.945470052489 204.031081842809 185.493554506958
181.229308386808 207.940596890723 155.129304059037 204.299375966441
166.825501664231
ATPBD4 181.110403721353 189.509062639279 163.315865817864
183.513101761782 170.684848540493 155.942609847585 201.555964610849
166.257275241937 174.357336296554 167.658560306884 174.680918805036
193.315380090713
ATPGD1 151.210814209064 147.164348704012 149.31422191209
153.230304322351 142.744045151046 145.597134236583 138.733717670748
149.119815471009 134.145771195572 134.865577264797 138.706520278166
146.830752309354
ATPIF1 487.358720934063 486.047284379068 477.741216640575
491.906642849373 916.4187683687 999.658013198849 732.352357321273
767.861763134136 564.41151411028 643.660739343159 995.263014856158
791.402660066193
ATR 131.000186898437 138.361149460093 128.771367080039 129.906744036052
134.590666885938 132.492444216154 141.353084959289 134.838366830668
131.805798804895 142.56910547147 139.657182434153 136.907453440141
ATRIP 215.094633380547 248.129640673859 229.689010178622 290.090495047722
337.87475687838 246.250251445835 232.519482087835 235.530986541549
255.12306184224 193.084818760857 204.113356572343 193.892810132985
ATRN 182.929326147069 184.332299740724 178.394895649664 179.338652960181
239.827648772906 253.789045068884 222.332748220525 209.596792486876
202.061887608293 214.396920524803 210.751430486845 212.915949933033
ATRNL1 149.339492984504 131.041996482401 158.10628865476
148.126745515891 142.970256312313 157.919709478746 147.108661317945
142.613654158833 139.959629641771 159.48526814715 136.635413632094
141.278654291193
ATRX 143.397823965608 135.463385553776 143.393174544776 145.206796089128
135.968346417524 156.433603187722 145.07503221134 142.301581642006
148.556161762978 142.221542344035 157.759149893048 144.231539210695
ATXN1 680.509421043347 660.535644289024 658.746224115551 664.19083888799
190.031339775209 212.396522888943 301.369680426563 263.426870776148
236.651601701157 392.13175568099 271.154246147262 229.483578414375

ATXN10 207.581907737176 200.633009710701 241.799709160815
190.993590849624 178.035700298977 227.715558644326 202.369905673351
202.162432624505 192.331142672469 210.550995170071 268.224669588188
244.73233791067
ATXN1L 224.89911221799 233.103476996669 226.640323471375
220.816812192169 248.927790738447 185.717123241832 191.418673767345
201.365457496683 252.138168292964 208.016730103629 242.614881095485
195.16856673253
ATXN2 1908.81864728547 2470.86113150126 1989.38201206555 2712.07480130502
1047.2693539616 1253.05700051015 1218.05434595121 1013.60354455825
1614.00098175383 1103.81439809121 854.224454791713 1392.89422203207
ATXN2L 155.899547877596 151.488973553441 146.65668813596
146.996442729408 181.966110264509 170.490099282436 153.731233276485
157.848456517169 149.471240458766 147.668990552874 160.254101852355
156.61494867164
ATXN3 360.991542429346 461.314247041387 331.464977124667 521.512936372701
210.960900130785 178.997098204457 210.085329463347 195.547320133122
191.554396977588 312.406286993239 234.339913501761 207.440700355457
ATXN3L 132.021249712589 120.020781939389 150.384559054133
130.827726862159 120.906553066296 143.383697128285 130.661695937728
144.743281367797 142.825203517688 159.887898257218 115.789536577324
135.390226050803
ATXN7 135.335107344583 132.909653744628 127.49352469919 129.589056071463
124.857461801227 130.724642809645 133.004765428575 111.010889276873
115.194730717797 136.368258257435 125.41744801003 126.103561089901
ATXN7L1 132.133226620105 122.414781975236 127.233439204926
114.452922529209 108.061315311823 126.37749112514 106.393497890486
116.241531823869 123.884643288534 120.493082096235 120.944267930809
134.327229861635
ATXN7L2 154.277670551775 162.606273401226 133.56263739203
161.548130965486 231.796421262015 198.681190016247 200.915806094193
201.893722965557 196.734037460218 141.91067228913 201.954167006591
177.874821397216
ATXN7L3 381.253673759781 403.405544395163 356.268960813032
542.379137999196 759.802694618208 538.647888236878 550.566402986082
540.359249705821 581.191693771646 316.524115715585 440.495339027491
465.29290512169
ATXN8OS 108.446988282853 116.378552206652 116.031631038289
112.916256784817 105.816457443844 111.253743009588 121.472885562857
106.147993332294 123.671944323239 118.636694749194 113.422559344178
116.445184223804
AUH 229.907632927358 204.337844243869 238.344676327918 198.815448428294
234.214191437687 219.351080515508 223.89854167651 227.736106796551
259.184713471619 371.224296589448 309.5988292104 219.753039699136
AUP1 290.389345090634 266.057472924341 262.832376198119 315.615758089471
540.946852709312 369.198837083585 403.208138816247 407.152764702613
333.676803661366 420.871349359404 411.359106929985 361.218491712891
AURKA 141.468076901305 178.696562301918 129.360773522498 183.280853265083
916.836022395768 749.859130465356 448.858580689107 578.084277076692
481.453770150269 390.501797367707 203.223604138192 414.267332328081
AURKAIP1 2767.77385345718 3371.24667467952 2913.2685312165
4847.77258830631 9470.58919937464 4081.13406129846 3450.46728258139
3905.35300751401 3618.9385126441 3581.36347643133 2511.04500638697
3378.126661761

AURKAPS1 130.98982984989 136.506347083926 146.934990457666
162.825380100343 138.285325970867 146.146800933028 146.345451252228
138.756638791139 141.914966520049 125.065813262416 146.840735518984
151.592787806315
AURKB 111.196610704726 125.768053724055 107.930325780047 123.643292039274
681.810927901806 495.769802520637 173.520662003159 459.388390392518
291.476113017794 274.891033696257 192.441337304606 251.20133703465
AURKC 123.100949494673 128.864822736402 121.246967460178 125.243735617266
120.384210318 120.571074646092 131.268454024753 118.047978285706
125.309861084957 121.164978896057 124.654146040634 130.542451540402
AUTS2 895.114975754684 1017.09497861942 751.269009484639 1159.44907165386
521.625146077176 442.590987372759 661.390308090982 866.413995755699
295.607161318725 223.551813819581 355.990908506973 421.836020491624
AVEN 319.966785546419 290.090495047722 285.258824662946 329.406649846151
245.850813387241 206.376365685112 191.59400638652 163.873355370767
188.641397688261 395.716879127149 179.711363255355 214.86995782548
AVIL 145.368059425439 169.920799931309 134.125038192486 160.549597620339
158.670835881902 165.932628858503 160.923897048848 173.147311624838
177.011776399483 160.419358656559 172.251953149975 134.255021727555
AVL9 160.338146803754 139.866536599877 151.134466830828 142.348246621217
290.914381066573 137.674025476238 188.847480174391 174.396061906109
167.206513789165 237.113813706224 174.820064644203 239.762135294506
AVP 152.593934787262 143.815682887538 165.890492422031 141.360125377949
164.777719508421 152.317890573534 167.173670781451 149.141033677244
155.376656335974 153.88383201628 150.148902196995 148.692602243778
AVPI1 1195.07608384189 1168.3174645384 1012.44277312494 1125.95664591686
570.130804179186 843.936924659514 1159.44907165386 368.220523234399
536.805979562807 747.804958601763 442.590987372759 775.5664718389
AVPR1A 121.44564199541 111.366283149236 117.962188157317
117.270320709161 123.195738824794 117.137027732123 108.318510762416
118.527464364564 110.068122112671 117.980052938167 136.220324225816
118.951526834915
AVPR1B 123.814841047589 114.01599035448 107.655624022312
116.552626146967 105.521320791331 106.341910466588 115.242526386551
120.14201542563 117.343165720552 105.900294919664 136.589250394236
111.568329540016
AVPR2 204.942500423413 184.135651836627 200.810204162831 220.242344043437
184.900027626401 178.12073430094 218.487334022128 201.987081022654
177.306794546105 196.615914367563 166.803359138942 190.787437782456
AWAT2 105.976284291356 101.530900217921 110.190708230962 117.539640414775
105.450273343324 112.365278258185 112.476111928742 99.5148073333731
108.530158480237 111.217595631623 103.448244268961 108.697696080014
AXIN1 282.244214637625 339.32415558339 336.898462056187 386.598037190663
553.123179808258 312.961981998512 405.322847383739 367.289651112346
240.915496717526 370.877260088621 337.077153882632 337.271784551586
AXIN2 606.38343657878 880.140852200235 763.263858769038 994.213397985121
186.482547532546 403.405544395163 208.385295246155 225.803528238368
1449.33679383001 232.046814892685 215.802713097166 220.047563627558
AXL 1208.9240215109 1243.11900823259 1034.2217095753 1389.93393245455
201.676984786688 199.96834355685 185.140211988926 244.162496497466
241.418016203544 253.495214569632 205.630948393225 178.464811133
AXUD1 1564.8868894162 1778.73916100529 645.438835606933 846.985164747386
911.340266389071 1222.014210447 481.630922092112 308.803322140241
475.686805714298 785.290353402444 518.17401959981 469.714826990063

AYP1p1 418.889972660958 453.593585032712 433.594928641214
552.790706181938 1037.97124386242 390.897464546943 526.970555181851
291.369577397743 305.16668987495 331.464977124667 244.486311003619
404.618867159141
AZGP1 120.857635836068 135.417519318028 127.289269745461 119.029186503752
166.214167530158 134.270043564053 119.288554466773 118.095936088242
143.061415008831 116.029201658798 144.283716416188 150.544779197947
AZI1 226.387465899611 256.202924421798 230.284216427158 297.757657398146
589.240581642145 356.770158430601 196.269193578525 387.068276379114
359.991823789356 228.125521373477 238.482863150124 1022.11537745428
AZI2 347.081036934975 287.889774984754 333.745543022749 290.288001750744
210.061754644824 241.734804013256 249.339449789861 242.871915060884
286.774606295106 274.736124442229 304.058158731957 243.622678182687
AZIN1 651.235249028924 570.916936074747 899.667912542118 558.810095726517
421.836020491624 749.220616713093 668.286400833485 789.971871940893
953.099915501575 702.460234202652 608.959089074987 582.062758687489
AZU1 124.288632450364 144.970383956581 149.808222023726 157.615017085961
155.097300548684 142.379120254855 151.604093626944 139.805930686563
145.503718833974 163.81485578613 132.431566491955 165.424516708502
B2M 10930.6720259935 9349.76443528011 8617.2437024568 6324.87600301793
6560.30885074315 9852.51412359574 8581.41617729997 15561.8923517816
22035.2646000952 13324.7465248375 22182.4877079778 16925.9167115963
B3GALNT1 215.247472744419 244.544473246593 197.231408726321
208.164674386141 376.791876040886 613.846166856328 364.443097338814
345.627266482686 206.469040492609 182.608712782878 760.572409199179
799.423640530777
B3GALNT2 195.414464905232 204.103018614608 192.709473588165
198.191669030952 185.830435830545 164.571974509529 173.082167545676
190.96184674871 177.267145265362 233.953994677015 168.116928722035
221.171478972931
B3GALT1 122.696382705233 157.623092996114 130.520275539672
131.604121223902 130.832462487806 126.430816528764 131.377361030122
113.521599852465 120.812738312059 133.161552914595 167.293320986716
127.511638455985
B3GALT2 141.076652697698 181.968650082343 143.165150890885
156.806407903626 138.796257513868 176.75095377028 141.392661314737
158.610249594568 146.431166706908 136.783974483005 157.631978594005
144.736213465651
B3GALT3 126.029431525943 131.996032643928 128.232091717966
124.113451627593 141.033948839613 178.445384902129 140.36425603453
157.524049610272 157.729732484131 127.152744243038 140.545351041252
213.556015518863
B3GALT4 225.095016600703 229.046153362151 189.389457481768
237.191866335979 373.033658917608 201.320381706496 345.291295192574
188.231614694297 257.168308311848 191.961269585431 251.61451602376
277.86338481075
B3GALT5 115.559551514777 118.871735074285 112.365278258185
107.976233194926 119.249084967945 114.977135060317 112.987890650593
116.934457270701 118.226036680442 112.449662536973 119.525701528869
116.810827219571
B3GALT6 254.552034511333 306.632103708321 215.115324454215
351.079435946154 733.195136251155 209.090788790449 248.260998007814
230.079664410595 343.833659987063 174.854570705 372.54091392596
312.705458415152

B3GALTL 195.513703253634 177.19138801548 182.478971050701
143.625188247966 169.705236302448 202.60339529874 169.590101694098
165.553581848949 168.183542846746 295.778154263981 212.171281020103
198.374163029174
B3GAT1 140.785337524953 139.465935832583 129.750835425023
134.307336063739 205.403820910921 125.870730238298 135.48250324478
136.77666893627 134.27783583528 142.924854393401 224.298067319406
189.971144220643
B3GAT2 120.964862799634 126.394032757479 121.942777234608
111.743461522689 126.391083453973 126.00744446299 101.149125916797
113.557349524174 114.099623939954 119.01236045062 129.485710719291
129.675420096163
B3GAT3 189.575681012578 183.64389891975 148.680794815584
241.058691798484 257.292125083248 166.47851613068 176.650953513413
136.837430157894 141.616920429839 134.673861365626 183.545741265706
138.207553349985
B3Gn-T6 139.039276904833 140.997594159186 137.731390340708
124.840101287634 142.995274641765 150.294432623474 146.110545700163
141.589470439145 153.801971958265 147.443560923128 158.221595000183
141.344034069373
B3GNT1 785.599211190431 677.9972761887 759.802694618208
800.739422189427 323.667189206453 280.127735578097 410.002817143111
258.632372536866 206.68694753419 222.943046618742 338.934501259414
254.517135747445
B3GNT2 169.631386764054 164.841156516242 152.356731704094
162.192879711747 175.236826554583 290.802509012089 201.517356610591
246.061496821699 202.483493595704 326.176058036757 180.1204762193
260.591336959281
B3GNT3 138.21083121895 149.430396727944 157.406969627689
174.177944561595 387.908774438461 213.891435492914 330.321113875647
219.2695037071 179.198164545975 153.602842380788 388.002365247498
296.088938872643
B3GNT4 142.480203661104 140.750004067571 136.016912147651
135.088149158147 175.891947478891 140.301945597122 152.910433120981
146.90847051471 155.516697616637 221.253717494488 151.736824723671
145.783320434143
B3GNT5 106.36673326724 125.979152567959 107.48180681159
104.091301211096 146.59366188125 160.382971629272 133.889858322181
139.685629122498 136.916286605302 222.646371492347 188.322853571195
150.28508305615
B3GNT6 279.921244070981 288.913660405191 348.808646570544
290.638110977807 217.327543190687 187.993907528473 199.597685421675
133.584361509362 161.490941000872 187.325542100762 186.420541099902
154.170007083026
B3GNT7 137.419835194075 125.331215990597 148.245974323625
151.998942684339 145.106362864355 155.358918578397 151.794243108455
156.448355269561 143.650172124086 157.215331288102 132.245217686793
135.015161451899
B3GNT8 172.568852318369 184.819937046061 148.215172022129
213.382179917193 229.805500520256 150.059862107155 186.089988569287
131.785053503951 147.469101571149 298.926727289387 199.597685421675
184.873459141947
B3GNT9 149.207481665265 157.309058982117 137.513315427454
141.236939351298 143.888318202599 130.066177538896 155.078035506545

157.293090577042 149.274901871663 173.082167545676 153.505155981348
149.861776884844
B3GNTL1 143.41350503793 175.774461934758 158.049286838471
199.423120300557 157.528409294029 160.085325256991 171.722839172783
166.842970309527 172.888455552446 164.661445411833 168.389069732474
232.046814892685
B4GALNT1 152.265624144393 141.488910091973 128.023203858837
139.251886500377 129.928047362701 120.693808603805 125.962178569021
196.955723006838 127.997280639609 118.646119379542 140.487887891414
122.284431229917
B4GALNT2 106.719519490874 117.48427100606 110.624328647003
115.524441144063 111.837468699281 103.307703547649 114.078271807864
109.599589420575 113.025793645043 116.939471420419 119.993313087183
110.735199684144
B4GALNT3 143.984222627806 131.081793789906 156.756159786366
133.050798832332 141.884995734267 127.727311123825 147.091280396135
144.683260413888 144.892454033336 127.121068682846 120.07404952486
131.397850276558
B4GALNT4 148.274122690409 140.477307701027 115.995309578182
127.481689955317 382.744523022686 225.890195278753 197.068795788491
201.530921073488 154.71904132098 155.080900277147 184.056274452194
170.825161347825
B4GALT1 325.751369001105 378.4377409749 226.763252784108
340.193863653875 541.764013317002 415.497783305278 424.855168311509
285.05846406041 405.322847383739 287.967349876429 404.172535442156
336.490076158135
B4GALT2 179.220691178402 184.278213908263 161.408318705503
195.69447353356 213.484986945295 177.401771322485 150.218643705925
157.907190910755 149.196539425588 200.264756078778 157.14512525321
147.41591291423
B4GALT3 322.684910198242 428.462270756201 386.917038897322
391.252450588331 1484.32547474275 1169.43742964112 830.455793617181
1103.13439876128 1053.09915077366 515.929937825133 625.374595058488
1033.03719406038
B4GALT4 261.251965523684 280.798003990849 239.20206002
231.338258067616 563.011988279542 544.30845362168 448.95671696823
438.03648998391 583.314509855875 243.038392457584 497.916883763059
600.635914090365
B4GALT5 1169.43742964112 1142.09977159958 1388.91423772335
1137.42879398351 767.377380800066 2316.49886419499 3396.40593844715
1174.69701182094 1632.84261181074 734.71562355687 1223.68146968716
1344.1288416533
B4GALT6 136.508969220268 157.863585655333 145.339287846271
144.019824700027 147.799806976046 165.698527470382 131.582231577364
195.755450324985 220.791884858764 141.758827837244 136.882429810263
155.603263242813
B4GALT7 369.156887999872 451.718912714608 388.638095539186
525.138476928759 674.571475335107 300.985037429772 1112.4055726239
565.059609779245 437.049581919144 716.420861490714 425.708048519408
597.795856268827
B9D1 182.335981256243 169.984859264412 173.939159471022 186.215602572358
350.741303038926 162.642344529383 159.74220366735 185.974598267418
171.238423077347 147.338615809214 145.980929136129 142.071492722749

B9D2	193.190059954084	249.524956682907	223.551813819581	256.614964511846
	700.601917911532	381.121621815163	451.621888586656	421.836020491624
	452.077434390612	205.200459980187	336.120322555703	279.495192828873
BAALC	155.346805606742	148.927040248436	144.879364072868	136.707951572916
	136.865340099645	132.227561632637	136.952914053358	135.90207451581
	147.943844184702	135.489000274316	150.313058722346	146.743260100643
BAAT	114.3172128566	123.98775110588	120.322292418128	109.140708667012
	119.583378101838	116.810827219571	115.871151134713	107.961322525982
	113.460835359833	118.144109564626	100.966979609222	109.760525674874
BACE1	184.135651836627	170.298291784762	159.961729971624	152.821735300697
	141.117659724704	123.275246170603	123.524952603672	127.588907178652
	133.70941349665	137.920724602098	141.066106476109	134.991379445799
BACE2	384.883635849576	400.180574613078	322.614169214764	467.681480781925
	367.518273773904	304.811116024094	344.604699562938	308.679189788554
	387.068276379114	710.999354960979	289.09614335372	475.686805714298
BACH1	123.379461430902	128.135216461862	129.055874691136	126.288011849541
	133.871685084945	125.868080230094	126.715300671036	133.284873295487
	133.411087000196	144.81077501446	143.242308032336	130.138945493943
BACH2	150.021512237553	148.632332840817	148.344941331885	145.190896872008
	134.202497785854	132.913891550054	137.731390340708	137.024230648861
	146.669620721254	133.065366727222	138.940564112156	142.093180304661
BAD	237.216435972201	232.231236745251	227.668046559074	256.174286655796
	437.489929507567	289.164969943385	290.135914275115	201.256649338315
	198.356420768703	297.466255000163	260.314440448486	239.924227955313
BAG1	145.654559623732	141.093532330708	123.413596815384	122.629146433216
	140.190234214948	166.730974538475	170.108500366942	133.640832782576
	144.994931561779	140.876577029209	198.244854812728	154.066939372379
BAG2	1186.23169583595	840.276501350383	733.86133557115	1024.59845685519
	173.182013165174	160.70138318726	148.635174393031	167.087850057037
	163.632148441668	185.41739522833	145.358431402568	171.433001048839
BAG3	1038.34611017219	849.784051720996	1064.85152398919	858.524630425687
	446.819873986024	398.971838949075	692.359779743985	569.353661326724
	665.936368667321	1054.47292895417	504.617795280805	718.449150898903
BAG4	251.869781014029	246.061496821699	203.456296907031	221.975219056555
	653.047570485109	361.26540006942	265.754141836026	219.8511043213
	192.258863431747	215.931781291835	230.749601687217	220.710324965453
BAG5	374.9555524303	328.550286726543	320.525885538523	324.177924877233
	284.574888587832	254.83131041241	310.026522549372	341.564749445905
	267.622579012917	301.591430919567	311.157764305033	395.645016679312
BAGE	152.47429737104	160.628894266843	152.741229289112	153.308956040034
	156.16485596016	166.89884215297	151.770791549511	132.825240485239
	156.090871391873	165.703810120915	151.484420979498	150.059862107155
BAGE2	154.286043402527	152.082927731045	180.047231265069	163.632148441668
	156.525347260458	170.198508633417	150.835044630002	156.191659799142
	159.466972602203	149.202628825354	143.631345956176	164.836381332774
BAGE3	130.586509996354	136.632837717704	134.189023892662	121.681261213422
	127.440987506769	159.289148392284	121.084348994747	141.191500203423
	124.738217731488	122.816049839521	162.312313953026	127.150222504237
BAGE4	126.430816528764	137.513315427454	155.516697616637	151.081339000609
	135.736764623155	137.701271351806	148.049570580734	130.326980619676
	137.871870950302	139.166537591866	107.949834974369	142.889911562811
BAGE5	229.373686092654	223.918931440768	287.145105895235	189.0608798771
	176.497706091676	314.126706052299	238.183096218311	213.791379190359
	169.713585775118	198.885004378065	223.573080955095	238.724909152512

BAHCC1 140.730085305185 144.96670085147 138.899292894875
135.253713075341 146.290334865039 126.98588570464 126.259029676691
131.162118170837 128.434301921433 150.843975804117 124.625603721372
139.667714422309
BAHD1 149.518870320398 168.460095116205 137.668179813327 153.731233276485
198.568180234676 133.345143691828 169.482586449402 144.731299077209
173.497575259833 138.21083121895 199.191065549132 164.61921356441
BAI1 143.752697178913 144.685707282641 148.005500924157 149.3567486207
157.672378669483 155.883386742315 145.698637908482 145.349023185855
138.735156909846 145.090639086338 138.42216862811 147.81143020317
BAI2 118.186426364551 129.466612376702 117.928456234796 131.597937570173
140.201501710276 118.797046082278 120.800764735081 120.193580405229
123.281489592543 123.45004246675 118.486195373059 129.493805009651
BAI3 214.241635800083 193.629237856758 264.306927596371 234.399431238026
135.078198678379 142.019263564073 141.791001213681 144.623222072851
157.759149893048 145.686092190716 137.00397800873 145.637807142732
BAIAP2 193.37618381669 192.046442148183 207.269530621176
223.722742062251 233.690524419327 162.9279134169 173.951605889009
174.149718758884 224.92725876841 305.768201359278 161.700258550244
203.475080163804
BAIAP2L1 145.850605495564 190.754349388901 144.30519737569
124.391225837909 818.445501500731 308.088967616175 928.151632070573
881.15414246017 453.457361487577 1126.46241487062 653.879734066271
1224.49891389348
BAIAP2L2 128.905042692431 158.784190231286 146.361128670362
175.28261759639 680.509421043347 160.082087242964 135.259226552938
195.618369827396 393.115606440229 120.65473121078 168.000247148058
476.249239197777
BAIAP3 143.733505359106 125.634045420702 129.343440464729
169.257035030405 138.345393524161 144.328571856869 152.337631025368
152.204730003016 142.29309119138 134.086301127594 169.293852989203
168.27980269501
BAK1 176.397715825653 141.941814923705 142.14116448528 147.463822782303
345.433025456954 232.519482087835 151.826103919919 164.063104872636
178.429953283621 219.753039699136 307.81941988503 256.614964511846
BAMBI 4246.19983955771 2867.63319136936 2344.11548437508 1879.08108720938
8633.71708020651 4299.06288109563 4651.384971332 5233.72882607795
8368.88320476021 988.297682407351 1744.58410715956 1450.44557173645
BANF1 728.930140593418 729.499296070289 987.564350211893 797.28465621772
1427.27262061653 1390.95426299323 803.840404197117 825.034504959272
925.205399154409 823.658996207582 734.97763567765 1105.10063745656
BANF2 170.866433766203 160.290104492275 167.900071375885 164.548505132213
141.432461374482 172.491604217899 182.633773540249 159.820341130737
164.641508485461 171.298701141684 161.837272617767 185.691922436569
BANK1 134.168381235167 133.305679931132 132.683594640696 143.376817049362
149.390481900424 127.107223387937 160.695913747601 162.609829456486
175.176693060261 127.977782260866 204.031081842809 149.910521500364
BANP 660.315285709317 735.165830487162 603.569635276204 739.251974981981
756.618969995355 482.677635089583 790.321331435096 856.74270641707
906.886423608706 642.255110250493 415.178470246749 662.04757187541
BAP1 222.794196053536 253.098839084408 229.017256104437 317.457588721567
382.701467158304 241.418016203544 281.119138040097 234.996704304673
272.277433294605 302.768870633398 257.24481414803 226.871028543248

BAPX1 137.500310477851 158.933097712363 171.373932115412 175.774461934758
135.897498591391 156.626742111441 190.371287639453 161.285587312115
153.985529457122 183.397833894087 135.422822065599 136.884799902348
BARD1 406.900412510895 410.845034795716 231.314944217099 438.958573676953
258.532432007328 305.93974940209 168.861884032201 261.922485502971
219.103510284905 177.32104138164 180.733673680261 254.812605074045
BARHL1 114.452922529209 127.055165613236 106.36673326724
112.114933398547 119.044902525459 103.772221923105 115.834056091815
118.9428715545 115.384304932365 114.133473556359 113.492900106468
109.886197655915
BARHL2 113.064645688715 135.830453690107 113.891385601095
118.505980204837 121.893446546512 117.455432751161 128.860744370967
123.135918415235 109.886197655915 126.067296703905 124.88118767481
125.658396221949
BARX1 143.082880435412 143.59105195427 138.114938404796 130.150998843383
154.24500773656 138.434347257063 128.074531806621 136.638141963514
138.56436658053 141.693224578298 152.760855359297 155.741507341843
BARX2 113.256481034259 110.807605625125 114.479886899622 113.620070198869
132.141918502025 132.011199659803 115.944280729914 119.29304762171
113.301733735994 133.659475650376 129.408282688425 124.103321614841
BASE 133.92485496581 119.558626216818 122.494779505958 121.031695489398
131.601823550269 131.523538461751 125.783265980169 125.997109913348
126.635105491524 135.518752716008 128.651081092914 126.090985925439
BASP1 2493.5484556802 2583.46653460894 2649.39312662326 1381.07447204233
6021.68798558746 2230.45015969936 4124.03779111916 2236.73950603157
1349.84215478798 932.862030475849 1918.14260920544 1619.67820476414
BAT1 843.545962045705 913.684449305965 722.399669963921 1087.69177809384
739.085574858917 789.971871940893 716.229989890801 769.009371389327
732.352357321273 594.38228123675 793.501244347257 818.225731093346
BAT2 375.407747753058 501.738490253982 283.874600697211 607.979208454176
899.667912542118 285.83626978316 306.838596239511 254.936706344174
265.536429548886 294.653534388707 406.638419179213 279.026742408164
BAT2D1 651.759589129833 567.143102792772 614.775559038663
508.572981581855 723.807658768693 1172.4103691525 779.340880980422
1190.62010668045 1213.06007305892 744.808064061459 837.413261433363
1698.08853832248
BAT2L 393.914413372142 386.85874184746 411.285747588622 424.37437028833
330.143103381406 240.814860403562 242.440966395613 235.091443641056
316.895545640437 222.709720471134 508.449904285771 227.926444925478
BAT3 419.4017188518 496.03509813734 438.03648998391 663.214972254236
686.445635754196 325.493177980483 371.224296589448 275.509805464276
400.353795948162 396.04031733841 411.505580458401 362.581768268116
BAT4 155.641860356683 162.28074297013 156.176537862398 142.142633050256
186.103491222924 126.732121894377 159.113259830623 136.265972909993
156.866867371872 129.707421699091 171.731395791818 136.832494876249
BAT5 361.516275756449 358.52459123416 329.023836559229 430.412897698085
640.255747986724 357.259935567823 357.835334661098 301.087947101876
379.788696204016 319.018213637357 368.825190022937 370.798852361374
BATF 151.144521344129 164.223990197735 123.518170356322 134.501410126169
298.776176246196 199.637257550762 288.370985514978 237.045634416039
312.845219991659 143.340685579645 622.834462068316 493.715108157171
BATF2 152.526785074182 154.812587513407 128.282999719959 168.878645908397
146.20679320119 129.023206862713 147.428180649939 129.353536603449
149.774162138829 146.160188002433 221.768507175814 132.442771000976

BATF3	142.744045151046	158.216790540415	129.92319548307	124.582577275358
	133.443807124822	131.296518221093	133.486155360266	145.572363722788
	175.9219991997	142.41737138634	142.133238886052	128.521380879209
BAX	193.95909424044	187.30908753142	211.135364208221	182.919385608842
	448.260934224454	253.098839084408	263.379982742085	245.997705723007
	288.072720517427	223.279017022443	224.335742510577	262.671819506189
BAZ1A	254.668502187945	224.707455448421	209.5184563244	184.085616342099
	332.978350226738	892.515788744891	455.313553278155	488.249967208882
	363.025385707747	392.824840252824	472.961857076681	655.094843900848
BAZ1B	297.805768288377	298.573180660083	277.561376169965	357.191675549592
	312.129861040709	242.948136060281	264.805236346895	254.83131041241
	270.33685210971	208.045210163121	215.967319486874	284.13865992802
BAZ2A	151.115031304764	158.528301072565	160.53152291979	169.403422185018
	164.534790315992	184.530853446688	168.071171406779	181.783275804424
	162.716712951629	185.342353172151	154.443360208399	171.545695383454
BAZ2B	859.113541787323	905.233241047678	875.38834441246	1116.19403476786
	279.598585549004	444.826775824095	433.405369327168	440.404188514231
	439.122658719747	499.436680354749	696.650617523899	699.270931701696
BBC3	229.542259738438	231.915272112361	229.660838239474	223.184064606222
	272.500572765647	236.164692035094	251.138401278632	191.405362404386
	202.524668687686	227.177100762395	229.483578414375	241.987100712218
BBOX1	122.682761393215	119.312201620247	117.386273592501	114.314425748312
	119.180033382937	151.829217512763	117.532063783977	127.233439204926
	116.321559000405	153.059432055089	168.682307349713	133.290168428696
BBS1	452.843213944986	385.584224833836	489.403061697031	401.960630197091
	250.542405852865	232.200025766422	234.738241125899	235.595194918187
	232.344545356395	214.968589907686	365.362177671146	252.514569129392
BBS10	163.73339229802	158.463444189819	205.765822478178	142.683117062711
	140.40754434058	187.893420010787	202.849544035436	169.642188114285
	189.044966519228	174.526176588454	188.952730860801	176.874265462986
BBS12	148.644800418517	141.741892403029	145.556318940043	153.563839578065
	139.157088843803	125.951927536494	131.722397988741	127.881498149714
	135.800997202323	129.143168240589	172.451128796055	128.635684412304
BBS2	481.86545778383	471.315730378641	539.720389702148	433.314881387431
	270.069634610818	250.718986968076	469.714826990063	328.14373061894
	339.490955270138	487.696347726467	351.665934915634	397.577760464197
BBS4	472.108893832845	438.146919555296	566.417354841707	420.743637858421
	180.599150102184	360.124745133393	314.4408959517	300.443499140896
	328.826483579278	380.514553097613	251.787648609546	327.062810389635
BBS5	150.235026770931	145.304050333309	142.000874773182	140.18668846839
	148.463566046951	144.856801451884	155.622257285651	156.437949042181
	141.015441854424	147.799806976046	142.661504601445	152.957297648595
BBS7	217.684410907748	198.023465629218	206.391403266202	209.840035045167
	164.011683602245	197.72068061775	174.840923772532	163.262893278578
	181.215046876308	171.51411661213	157.162523271165	157.874624979775
BBS9	187.443940151794	165.985885671398	176.440625367948	178.717719615544
	151.159145294127	144.78971417658	178.404791189835	144.032779435658
	157.697168349028	166.182850621447	172.521737547052	159.588575526536
BBX	1490.02976786379	1400.81554054664	1685.33737186733	1232.66885974069
	397.861172312706	919.165331064155	1021.25947613827	723.250763797522
	892.038018567941	787.425941491281	677.265576269103	924.040923247227
BCAM	130.900049359243	131.968781256081	127.211469322857	135.792966926642
	146.586879656174	133.117701703454	131.196196319523	121.080413308265
	131.460388562433	126.992697289075	160.644747532952	142.774449777424

BCAN 137.018521754443 144.824087631654 143.129945647647 138.541779821934
138.807031587657 148.649619242172 146.434716985396 154.546460872415
143.501380161409 135.449044827893 157.58224826673 154.847114601403
BCAP29 220.92105843037 199.746775121317 213.412498914309
193.48293264444 232.735767435327 228.025383952578 236.617896560938
232.023739004791 181.261666651301 305.451247405518 183.979513272793
246.369521698487
BCAP31 2037.96470181871 2395.75453574049 1845.87311313269
2500.07677977147 11874.9808592281 4465.66734676111 3810.98043907046
3359.62015747752 3983.37592132018 2018.81730267601 2833.30537635103
4047.99200599472
BCAR1 257.941367816557 299.463563393179 187.15740398852 277.539110964815
596.076764072945 171.809717138613 234.693846527867 205.341121539273
234.020419732787 244.401472859026 275.755038921952 231.981501596284
BCAR3 302.219514290905 288.631429069336 398.354212009426 275.642882589307
562.387692458382 412.648926259488 582.527835746927 557.702374840532
370.617422894068 276.089623957012 323.04019262387 217.847954914851
BCAR4 154.94587631386 175.96841318924 147.991572632747 171.660157220703
4175.70090899755 142.368601917778 156.519196335454 171.190796424612
162.317084707395 153.534763364558 130.143953880391 167.871703133182
BCAS1 153.028658323116 168.323141675824 202.741211613885 125.029516472944
784.412433571927 231.698163424706 1044.00217726486 417.615521090665
417.442660073269 145.566861062922 1620.7954553764 649.438995824208
BCAS2 161.371477511362 172.438141471784 167.243544185778 158.565676210997
183.513101761782 160.010501226517 194.941454173896 192.532222258033
185.896304740844 196.470718183575 201.383739902873 179.203783335674
BCAS3 129.061316880624 126.086991456747 113.112450527382 118.991494185841
122.258432948151 117.420765321056 122.989642938474 122.636980845207
131.177396785633 117.760045671142 166.64535048582 123.055210862247
BCAS4 309.5988292104 276.79778275734 307.518881119091 344.45331938267
708.534025308731 283.326226944158 431.413030534188 270.181005097697
342.487952136318 184.223623297663 268.52833723726 165.031678768564
BCAT1 248.643311972844 237.854774190873 194.86492119218 183.998458233995
134.397255719047 182.02250582556 134.265479018173 163.582678113215
185.405774238391 250.577945492981 163.413365060063 133.892661981648
BCAT2 363.025385707747 421.303580698397 425.471195039676 446.948699651916
8401.40370474392 2069.70335195314 2141.855356411 1896.69856822105
4222.55224241399 574.496860164135 1145.35733270036 907.306121257236
BCCIP 348.067061666237 323.462252156562 430.728137105601 330.908743070095
281.740495452842 375.651196211048 485.026081242201 399.364160668615
389.588346573823 379.911829623463 409.380002417794 511.996883507889
BCDIN3D 314.221257518059 315.912220767901 379.016207754533
341.2887754025 353.98839683842 301.49286558088 506.898635822094
506.430593289427 601.800326895295 241.353916553991 386.679822751367
374.033009122079
BCHE 572.049540360134 583.455617065556 551.206833852215 458.282682131641
140.11211854056 140.617663657037 143.802883430376 167.451782810394
142.697827839203 152.447978675887 137.742393475755 159.544710238172
BCKDHA 356.376890469558 419.532420763449 412.582051585078
458.33221423434 1247.02514044778 532.063437013967 527.597948979568
546.181615768667 710.003412772888 314.386320310898 417.262521411065
332.464176231219
BCKDHB 182.897563195019 178.647726085678 213.194154171149
161.813099560175 241.105817192695 383.503040287678 337.22646304739

217.165036362034 214.803167803712 187.443940151794 263.295012964476
289.21739941764
BCKDK 976.782815449208 1347.10650016064 1140.40904196731 2026.85580212422
2421.87041217217 1027.96306543235 1724.22121078776 1453.88161495484
1227.94401191112 1496.0924333403 976.782815449208 1207.11788506001
BCL10 140.497181450706 146.079727388849 146.580787463084 137.423810400996
160.374491283254 180.392337212428 164.89597889259 157.990467804353
156.731291756066 177.759729226623 180.986305037222 165.200303470106
BCL11A 206.502189514795 305.200911653347 257.075088169589
393.375554593484 149.374282007673 140.453219944445 206.68694753419
414.333324610267 283.123157828849 351.934158805882 315.752980521266
207.940596890723
BCL11B 140.605866597575 136.284283129801 124.272447276746
122.35832958054 163.451063547847 161.424105316969 149.589901372799
143.030777754448 174.068974937318 249.680729921602 210.245551279259
255.493109705211
BCL2 264.8472508692 278.737944234055 249.858562350415 254.936706344174
151.164936050256 164.000772606739 155.694213876736 171.022976593689
179.854800301573 155.018009545475 170.111653122193 158.023626712181
BCL2A1 138.570150372729 152.095584118603 138.640919060725
145.934643000545 148.501654210579 127.740057874561 139.293775885106
129.151767489346 150.971453664961 163.175801793143 151.727603285205
151.72544764489
BCL2L1 509.14726076246 691.205738835917 436.586158384666
763.880957432979 1505.09908072606 919.626220838414 1283.68791110988
717.270096456387 1536.52218968817 763.263858769038 702.049571167356
749.515603904233
BCL2L10 148.560326703328 145.131215503044 143.587562550004
135.830453690107 180.113222861966 144.665869508406 123.745824514261
133.269658133558 155.368204597287 157.688982037448 159.773576221107
154.207656926108
BCL2L11 140.914966429342 141.304791640723 127.139903623331
136.643481414354 146.554017952963 152.296774123599 148.660440219589
138.035272644139 151.226242696197 160.960599932898 144.534143943252
137.324247703318
BCL2L12 234.550692915716 245.426639190893 230.469548500304
292.145553580042 1822.14935116363 449.408793796673 544.222458349696
820.249910349431 824.427857809495 477.275332763887 252.851164967726
360.185005480216
BCL2L13 1146.84239258543 1021.81988990874 1323.05745869565
1115.53524128833 912.483177705931 543.710330214344 740.196934996626
1013.03054571763 812.298381693378 812.75075757351 1288.3346537369
710.76653239801
BCL2L14 144.850211439338 143.739231124536 146.425476034368
140.190234214948 138.199559519849 144.428776560885 138.529160075977
146.937052029619 145.852430082228 145.650356702429 153.601918768668
148.941194457652
BCL2L15 126.235567899449 118.215641556163 114.785989868306
120.148189532583 120.220156455352 125.063596569451 118.256000539093
126.128597459065 259.935831886014 123.019430618632 148.911306526869
122.514826802737
BCL2L2 1944.15673951528 1989.38201206555 1931.16906839143
2677.17579532816 379.862305342147 349.128741707506 489.022371276542

366.788087591332 375.131728190867 640.255747986724 754.97177863997
715.93836911426
BCL3 308.564935340635 450.851711160136 210.41046499545 350.46078795627
950.144166422001 461.482735533799 312.286788541158 266.71318061066
528.921893077914 518.478990211753 343.121661664987 301.955019831714
BCL6 1000.48723872058 977.999660785113 688.247581763352 802.643357549193
611.088604425595 527.930077857945 933.408902924063 1023.94850651125
748.703265448403 427.98782239715 790.561585641193 622.692834629555
BCL6B 261.592512762969 302.847274046748 149.380733719756 257.989863290786
176.245698601389 197.378801636702 139.748733350375 179.122374556365
207.977817300376 168.45157400976 162.457778455279 148.012353232918
BCL7A 148.670562837021 141.002002397727 143.984222627806 146.735616237082
148.25001336038 135.159590925066 140.912921321622 152.067897082883
166.9499656083 146.416318806523 146.830752309354 140.752616434418
BCL7B 361.768552564027 310.640107228956 456.530337997907 372.024884481775
315.3930052781 232.621526025603 295.935625475189 306.716682293251
251.665539319612 307.906312092805 284.715092831843 389.588346573823
BCL7C 372.475112554793 344.379733302418 424.495400604335 372.894376776771
588.197697799448 455.21285026588 408.201232787978 330.908743070095
274.235001739215 405.570281788901 340.860684371038 466.083451075744
BCL8 124.327827104244 111.127245394321 140.245786278057 156.173099025259
121.30744400137 131.464229237662 157.454558821261 143.644862018676
122.176811388389 133.647637744785 144.011792962785 143.88649412841
BCL9 438.146919555296 489.118864124059 449.103432410618 602.141151173094
406.998800177456 392.430873293903 359.577827010792 415.178470246749
288.968909550533 277.539110964815 303.858574395969 496.426739836535
BCL9L 263.352118305612 289.911635155481 261.775269058607 455.124190541055
413.502150181875 204.802810257516 206.825575257029 184.62149494522
168.838723365068 188.891032176388 231.796421262015 223.601312199935
BCLAF1 624.106546150735 674.571475335107 655.665164535648
491.034395489684 320.390351199433 691.916554922896 707.048754700047
685.354305381323 573.074116810032 580.347496467173 800.373159084181
889.6617510875
BCMO1 128.23910347716 131.045970236632 136.214432257698 151.004738508108
135.641355114263 138.016275878607 153.667355714857 227.689356258276
264.273245283343 134.066948803697 159.701028026285 121.492896642899
BCO2 119.573635447638 127.740057874561 127.816596414779 122.120307446003
121.441901286752 119.511962218143 119.492844191924 112.357261386644
116.576896337786 118.029258839619 136.973804637915 118.743966166637
BCOR 244.219585315024 251.20133703465 202.504840299605 224.824362819477
278.142283306934 253.186749111526 208.164674386141 222.415704486709
212.119922561453 165.813771791917 281.071129128589 259.146673652222
BCORL1 179.663258610881 202.343343669937 171.329984719612
235.162439885207 170.931788192825 142.681626890547 156.373163373309
143.780803045394 156.173099025259 168.753660955229 161.441613587571
153.263883774915
BCORL2 110.935688075738 124.982744176925 110.925532078514
136.039076665712 113.112450527382 130.37544152572 123.551225884748
124.436549041911 133.058788697693 134.70848693317 122.351186755611
118.095936088242
BCR 151.524197474057 153.597557478169 147.053391763304 156.856149172225
174.491551019128 149.039132756817 151.766607453172 149.147411595596
157.490138425943 146.880440100898 156.901209828741 156.986053534635

BCS1L 275.888299685521 272.456310079164 264.654879741889 303.762967437478
248.965394617795 232.023739004791 251.546290552828 237.754067231793
290.420307322101 323.276514394119 295.420691959792 253.754869975548
BCYRN1 3608.10730106542 4246.19983955771 3391.3005983725
4749.69040198959 2601.89405525276 3042.88425356552 1250.58586679251
1932.39418448203 1367.19652160532 1632.07671492041 1995.6000523628
1319.3522488218
BDH1 132.680140469504 132.721469335671 138.094719587545 134.08270035062
180.80811429888 164.935120145905 155.979380606594 152.074017988748
161.338215647108 191.241000498118 140.474338177948 148.437676259828
BDH2 153.705394500855 136.751654856807 120.089228795888 118.662030856474
120.006982591236 131.676609824921 119.618176258524 133.619092072157
133.810472192646 125.889860278265 123.615802924067 126.856038484436
BDKRB1 172.599654598668 168.027892518772 130.01207836961
126.497472087036 134.441142158135 125.331215990597 120.003095000297
126.902441495413 122.659174808512 144.004831360797 142.664793323255
123.692366307662
BDKRB2 237.906142045719 201.517356610591 178.347708009405
162.638805580226 178.939268046323 156.830156083795 143.893751699551
156.361037318986 174.406779990708 173.176900454904 153.044682186978
174.149718758884
BDNF 197.699100346967 144.227440632196 148.00952002903 135.416008633632
127.874934513708 134.688504981603 134.312156667838 130.749763860615
134.541606996118 134.819406064011 145.483667452136 136.047032834863
BDNFOS 128.620852653765 134.943005383277 134.096283448464
128.556280049403 131.342562516383 131.659187927328 140.837360719964
140.401508808129 126.25011972597 130.131800537628 144.900785401228
135.229197100212
BDP1 217.910445051307 194.352807599399 221.171478972931 226.960024551778
168.878645908397 215.769986055534 198.374163029174 173.995889755792
177.716742595321 186.904126552483 189.082778234831 198.088834624665
BEAN 144.145612985703 149.968749911496 151.484420979498 160.43939815269
137.85552865361 146.526744516344 144.54895913821 148.821942578396
137.704257049203 138.227082687797 141.560424984281 148.499207368959
BECN1 547.681206272188 412.941907828617 824.070046083713 449.745016106912
493.492348317616 892.868661511516 1016.18143428327 1468.50184641042
1131.22418479945 807.604205890244 849.158029646233 875.7694907383
BECN1L1 141.649911527714 142.461941376108 125.250900940748
162.574338133241 121.939618902165 137.445344116574 145.045633612194
134.130913969327 134.688504981603 140.626181314061 141.035566928475
141.143572141958
BEGAIN 129.404017329613 134.85590586532 121.462385497795
125.588401196232 184.954673605577 129.467792559297 129.971670681199
132.369872013432 133.796029401157 133.42964827086 190.947996918605
132.118118271944
BEND2 143.120621855946 146.06054700771 130.355969431473 143.370698101204
146.692827583525 152.385014030957 137.485252316816 124.998601194756
140.14062775887 150.602976963975 138.525131957094 137.677396262152
BEND3 142.200427602461 136.931476050688 134.491232148834 135.134213292186
180.34788837703 176.070695522397 170.361237529247 161.877378749103
160.75642485037 147.738146185884 163.042083189818 152.241060990474
BEND4 146.686192012988 138.625680450198 139.718721422663 144.496429994489
136.051070946954 136.480458837869 145.597134236583 140.541925660368
135.613240463445 133.707285280448 142.505050582588 137.09814291566

BEND5	565.97975389646	665.341859712345	792.247301083075	689.821785472506
	293.746733527346	424.751154004399	516.755267232127	572.049540360134
	467.187222441283	224.167511918105	595.120528902501	609.665323786904
BEND6	167.416417895159	175.717830026564	160.261708910517	175.543924200429
	136.489566741917	138.35738548449	131.732416355002	133.814720785534
	134.109051944736	154.617462666847	144.968459820523	128.858923585925
BEND7	216.929969034881	211.916602665089	239.789994201722	235.183590602209
	218.735620596634	433.189221987242	232.986366625889	172.245089970803
	154.159124458167	172.120772549791	145.686092190716	172.521737547052
BEST1	182.397795261147	176.371404525626	161.614426723521	154.312374306932
	144.510062568989	166.330001480033	153.853856339585	152.435416154284
	151.801558435863	142.829099538363	149.106615763242	129.873110494826
BEST2	140.694993778394	130.09050436943	137.264095379365	139.657182434153
	140.632573759426	138.239251358245	143.879597098414	138.161690144518
	134.161664663192	138.620617643924	141.006540481119	139.993960054847
BEST3	123.318763391117	126.897804011992	123.195738824794	124.275741099764
	132.699715693605	126.800718471804	127.814109293214	122.007909794484
	122.982686879394	114.913534674899	122.955724972547	125.945916345628
BEST4	177.401771322485	183.013090662119	161.956032597813	181.098584949344
	177.793860516896	192.709473588165	160.473772347376	167.219062918573
	203.206743799353	160.117742879282	156.632939064629	189.575681012578
BET1	219.2695037071	234.063633889254	224.361277033882	210.912629986133
	224.027605834751	204.695311233073	381.740838644647	305.398934391579
	315.912220767901	277.946395491073	271.468939477052	239.24587884234
BET1L	210.729945718068	219.192900411543	228.901579068997	197.91325268683
	204.770742504612	203.983168778488	176.088449983147	175.573400981771
	197.605208310432	186.951061159734	219.2695037071	180.304585602206
BET3L	132.502153153826	141.577373924378	136.254966566424	147.284005815059
	142.399253818882	128.213678179359	154.725484895098	146.157459765796
	135.645439440707	139.912325578837	123.132178371802	147.619451225526
BEX1	248.993716339029	282.137282466192	304.590636814607	226.824313642101
	453.17378995737	241.168936008174	366.007451523992	232.830038701628
	321.568127272161	185.544411160039	235.407064742194	453.926991868451
BEX2	259.900447705311	255.793823796456	309.423768054614	207.545424360094
	1929.52146517699	1119.23362648056	866.147379099606	501.005898087825
	703.587139938769	299.986758612161	222.365208637146	504.617795280805
BEX4	519.112018353676	448.95671696823	825.034504959272	402.366797969833
	1067.80176606977	499.683188662742	691.916554922896	315.786908578669
	395.521241316058	195.218005582618	246.533813165981	536.805979562807
BEX5	150.000345076364	167.330860677012	157.142101889337	134.302726684539
	144.826861308997	132.989846726946	152.142909854773	148.44261900795
	162.453586139402	150.065275447769	132.639070127232	160.482734190577
BEXL1	1076.62597781124	1009.42265467472	1550.61855325662	1045.49947183547
	1518.13775377335	732.877169346979	1050.40488466179	459.171226689178
	578.297031096292	261.171723011396	393.013539328766	622.834462068316
BEYLA	138.938546468136	138.281188275319	143.974551393951	143.920134762902
	139.236217474885	141.054587977578	136.332399354517	131.882887826338
	142.272117655292	141.652000792271	131.373012256275	158.408494909763
BFAR	456.990425618007	402.965083947376	515.929937825133	423.966625351913
	383.407365952689	431.799795646414	559.0867397858	445.214621023114
	349.99912485533	379.476768908492	442.220572301795	428.569462312363
BFSP1	145.861217875066	147.559983285261	141.927204640355	149.758215028408
	149.422357654247	157.288308753975	135.715745768179	128.411138850655
	129.151767489346	143.360311218423	147.069488043503	153.647343267295

BFSP2 124.001295849091 116.225440873023 125.593595066736 110.804248212067
124.327827104244 120.29173510435 116.408126010926 115.427201752401
132.671227062948 123.406144497577 133.827296071709 112.558241320112
BGLAP 180.968659614238 167.713268780664 180.658930225735 183.152005135504
173.132747018514 168.813895764991 158.48793705795 179.220691178402
182.633773540249 165.424516708502 156.748330788367 167.858490180471
BGN 5655.12135336698 6720.10473881011 1863.96986643676 8514.94395408164
3598.30438198858 2456.36776585765 738.892511276134 2828.97255380291
4377.59943975549 6670.51138785163 1168.3174645384 1474.70984232307
BHLHA15 128.280225530201 140.38936578649 125.945916345628
126.626904958599 131.473001455317 127.548070199148 124.646093292382
134.152586955217 119.040642659337 122.125746529725 137.225659453984
141.420747583126
BHLHB2 1554.16120123599 2309.63504858248 1321.17236442448
1845.87311313269 1255.02720867812 1421.72388964898 1484.32547474275
861.786094902013 1931.16906839143 3504.77617298452 910.982557417978
2863.51286169094
BHLHB3 233.757965816721 351.188086838653 202.778539118009
470.308610828825 236.191384383802 407.052451784773 367.983166440977
335.059844742031 147.608094270078 123.09149124559 402.694564608582
508.127470400078
BHLHB4 149.133055368147 133.165893710176 131.397850276558
130.898067924686 132.369872013432 147.713967317159 131.800533523123
131.092140169861 127.58382536792 137.731390340708 121.438875373878
126.185560760543
BHLHB9 256.590289281076 255.156682699288 253.754869975548
228.45211887819 307.665281863258 207.764456828813 271.989060206794
193.028341755594 252.697160031025 178.563665283267 225.275049011909
183.168629351124
BHLHE22 218.644953001365 198.113398073968 170.046856179083
198.113398073968 150.580133889656 157.907190910755 168.582192754233
182.515302191173 173.761036260869 175.044940252247 127.560308850342
160.990868498842
BHMT 185.706228572019 299.568830444875 279.879377933144 183.056707813352
1136.76110352875 1524.46745239811 270.181005097697 1383.68977267418
3287.37172778474 181.098584949344 178.542247857251 360.812953749077
BHMT2 247.307128520272 204.770742504612 295.678097392123 277.501071684448
136.13751015238 140.839056652898 131.255028381669 144.193756031386
143.349486755648 140.907889482107 132.767474034305 145.777778096701
BICC1 145.203083375014 131.340453291188 139.023486014525 139.6492947529
137.639342727211 121.377666938052 130.306478656159 124.885950473434
131.067958183644 138.654301345398 133.964163727962 127.585851003779
BICD1 150.3259737066 157.570298634172 148.155692596378 161.134322621805
149.135934269866 154.347332766547 151.976432805924 150.303703193967
163.432972512188 146.482652762374 141.722817732641 143.67880977119
BICD2 487.901310664037 495.89837457901 601.997464467917 421.056268094661
377.27961559752 296.657844888195 440.579262120451 388.573321838751
371.168752104821 1540.71556891815 651.43207839949 362.195903246495
BID 168.720694813918 166.085469368498 169.124088593549 146.6886556805
255.3451781862 350.46078795627 266.918302307596 279.113984512619
392.539157974899 192.314484368435 440.203336761263 384.296190598736
BIK 103.99234480919 106.102093734748 116.764795914545 123.02546895475
235.372592051464 130.736466113571 137.897243684023 145.599152743156
144.81320329947 159.487615985875 165.602175302848 130.411849432351

BIN1	3073.40543048878	4009.22206756187	2915.72823797537	6339.16392696068
	373.656487297238	197.262062021667	174.698724064671	238.310077556167
	346.118765910093	274.01377986185	214.050918459805	225.96618659877
BIN2	103.94864861172	105.49491265382	110.113844434493	119.735807506447
	116.112013454335	113.536570797896	115.305518560239	108.715328458556
	126.097544650407	103.550817366724	123.058261039164	109.176852761745
BIN3	209.304654633004	234.531140131763	184.306452616482	229.26243866226
	222.134519361117	230.284216427158	164.520247586053	163.091280469234
	178.563665283267	180.113222861966	248.729653566598	214.785381549181
BIRC1	177.831718771025	148.125665833072	158.098055917452	166.396160626179
	146.721207920553	166.692386207143	146.052153515494	144.214589452164
	168.861884032201	165.267693285882	134.147586627141	139.939863187181
BIRC2	507.710604451713	492.377642058095	406.998800177456	455.778776920897
	534.083703564903	449.859227376918	441.435444003241	533.893360199271
	406.638419179213	482.344514972063	601.800326895295	486.047284379068
BIRC3	1390.95426299323	1424.79466712859	1099.80705776268	1313.89440013246
	302.945860276233	1427.70292953827	783.755776589969	959.897744425251
	932.076509242794	566.813343200268	1120.3009282887	660.315285709317
BIRC5	137.71280215369	160.203521879896	132.628358688595	140.377179372804
	471.510683045651	266.468621734455	219.661502916874	413.555310697751
	428.09583118349	241.019436415703	157.982146236108	216.680795762761
BIRC6	360.185005480216	342.611951034707	378.891597719939	333.868818855397
	275.755038921952	289.911635155481	260.098788125373	283.807827536147
	255.677315946354	263.123584372086	351.854828413334	302.272304483941
BIRC7	118.797046082278	119.352995485911	124.940836122365	126.501648842366
	121.979059173287	118.537378726617	116.305011582033	121.116206210402
	122.35832958054	113.672605838627	131.794065532001	120.672291867109
BIRC8	113.740942665269	121.603608840998	120.272964182307	111.794102015354
	115.409568875238	123.762963668211	122.430140354831	116.294180301039
	118.75237770613	118.249148086655	118.104763610751	121.087514445827
BIVM	479.761165832339	439.852826905352	532.596772298766	389.800620731411
	228.125521373477	527.139476504986	512.184962338131	301.304268871606
	340.301980622308	379.670492165938	509.01330998156	550.24496459253
BLCAP	1067.30106203503	1364.1224589383	924.312051919404	1528.49457104932
	1890.86868383409	1000.48723872058	4009.22206756187	555.259469428492
	462.144334719146	512.312880441098	1607.52029676299	1273.6393529108
BLID	159.055956234445	154.539367933684	167.298438019561	147.716353308549
	140.429462488271	142.059921012561	143.874735808114	136.937327692084
	143.795399778897	154.847114601403	153.850588492326	154.637740117457
BLK	118.486195373059	121.48867035121	115.142111396055	111.041656398985
	110.966212705086	110.475453268997	108.266328505986	148.96493117884
	219.080383154432	126.948090173876	119.720132373023	111.7529190597
BLM	118.465111174212	113.644993086829	107.605526304445	107.221547998974
	169.246302492166	195.438353051306	139.159510392614	195.353512458837
	180.418707740532	130.005909892804	137.20509387214	167.701284424049
BLMH	363.343480850598	372.088129459528	336.434368527549	456.201998988891
	354.922901990615	274.01377986185	343.617417488962	410.533157400023
	289.82072316082	390.979822976551	313.412131722189	288.370985514978
BLNK	139.505864135281	154.869171648942	152.73588666971	173.751615672365
	157.77047742735	178.014057622594	177.851689709807	133.975526667571
	146.96155692518	154.47187360425	148.641780441227	177.910104644497
BLOC1S1	859.860994525775	1189.69730508119	808.300067805897	
	1336.75600604068	1255.62991601665	821.265710686098	595.593867616527

1063.5903770389 712.433345978749 601.164127925014 697.97116981445
786.160002434066
BLOC1S2 159.544710238172 157.733042033549 163.799748670842
162.397078614596 150.815117344434 153.767488352037 176.214950733324
156.248684477916 163.672213020803 156.508428796592 152.520828479026
164.870860592068
BLOC1S3 115.142111396055 117.166509072455 114.533677697026
115.485515429446 118.060781901686 114.084543168359 117.32734978289
120.757288533244 116.647291878845 112.624137732184 131.705912986393
114.673517800886
BLR1 112.166192839859 113.301733735994 112.851805868467 105.771723307783
115.30786313391 119.98864381644 117.045020667394 121.85247363239
108.43334699667 113.256481034259 116.378552206652 102.661440700541
BLVRA 1116.95795024994 1129.76078220457 1313.89440013246 1251.15912264225
395.049127927637 325.525450750788 986.441377365124 745.153336165802
607.805002786409 1223.68146968716 706.759905439674 856.74270641707
BLVRB 599.208370337812 609.665323786904 582.382786876656 769.34961339184
683.179460779062 522.664475688419 534.745066397679 361.768552564027
305.252666831522 454.492780042353 823.297606185721 328.200548902445
BLZF1 8496.2506227676 8401.40370474392 7491.93659373205 8581.41617729997
1098.78649254838 6601.004284244 3328.99028463277 5776.63686968234
4043.28299602858 2895.71836177599 5965.21815409254 3954.24763752814
BMF 149.078664825533 153.494456800685 149.661929885679 161.948033313443
138.235127932016 142.081013617671 165.523150623132 140.42579083092
154.564478911791 133.666244052469 178.976116597291 137.919253591105
BMI1 1032.11574332305 1035.0797237176 1288.3346537369 919.165331064155
1318.3633082502 1184.88559645917 992.320202251161 817.342172321486
787.425941491281 669.124699103132 1045.49947183547 1085.92360670051
BMP1 170.731358903019 173.488272345038 168.773340961473 177.393812005949
161.229621453539 149.710337205986 151.70035428516 151.674143983846
152.769931978291 196.429685815196 159.58125728432 153.891667472876
BMP10 138.810236692956 145.830916005206 158.723133891017 147.939682463235
149.609501725707 139.238936770274 130.363516180139 156.402578892869
149.453103369806 133.120273569229 134.452938571435 143.245660951425
BMP15 121.834248129933 118.064858133264 118.757973008463 115.392989580183
115.933805625814 113.177330690612 114.182798672347 118.708644971927
115.16087297104 110.977904932371 126.877293296185 116.5272207025
BMP2 202.324795686879 303.986544460218 215.057917795456 164.38282966938
1189.11053680293 345.494740481759 519.564563632951 307.866499851733
182.68965110437 622.142195892701 821.020339593525 317.949646885539
BMP2K 146.20028051948 137.675597243706 132.772085211553 127.806138997958
136.942583515174 142.706708823143 132.973551954067 145.165443106042
152.331011756244 267.747633122387 154.400124981573 142.524413202292
BMP2KL 139.921002429353 152.099431258333 141.74792000689
171.145909843389 133.570075399424 155.511086289504 129.088718286391
132.86879723151 136.413745926388 154.153249796248 163.373180928181
143.327234875417
BMP3 118.689408983786 117.30724482798 122.982686879394 122.600274773736
131.099004325598 205.65398844108 174.624446035895 246.355720496468
118.601228886344 118.07991087231 216.836094088131 137.017882995262
BMP4 506.029788151779 451.380376296813 226.989201461085 233.36028792331
137.258429777402 144.219104742099 149.77596374205 178.045931314857
1123.93087912174 187.365807314802 163.284853420896 172.045843691642

BMP5 223.013753785002 240.12078342024 146.823157886228 169.158049136321
136.240503841228 266.842778974988 168.702991603656 194.212177449077
170.602603155092 147.858339902055 173.010799217261 225.423403713718
BMP6 194.724219884752 166.489436134841 138.950197595669 128.402511355513
121.668166378914 129.798965780918 120.740986472733 163.51024840263
150.173766182317 135.085039737228 138.015301767961 127.04953162892
BMP7 147.043228493047 208.321765801227 128.695126347193 125.666013736767
233.71520737817 274.736124442229 309.0578543424 146.649944669876
277.026538720417 135.151438845363 728.176339659082 688.684644604218
BMP8A 124.119104345119 133.879079395215 127.407418037777 119.755151432304
142.043121538885 136.362761829761 120.35956894661 135.730331297139
128.075865243385 130.240366272285 134.85809559975 120.371374172279
BMP8B 296.088938872643 288.182388699147 258.964323941464 278.687780705367
181.425012248539 277.86338481075 238.847091900666 251.869781014029
219.151144724168 181.215046876308 246.850599569231 222.526610842047
BMPER 137.361967082029 126.893023101449 118.533346268154 126.462522391125
117.642441794442 123.598210440312 122.821563586744 117.024039521425
126.839037497031 131.782007636511 147.476805635744 124.22833964472
BMPR1A 392.365008240567 267.028096234702 330.641230715217
253.933478547515 168.254689624696 190.125009931628 216.9491329483
184.294172429054 185.886737029845 190.76537287329 194.86492119218
235.32095278846
BMPR1B 147.009325721272 148.991357883449 149.693559841189
141.512008928271 158.604431001643 130.804850476451 146.698766621345
135.732616463819 138.89579816122 154.252727576218 126.848211027661
145.875240619257
BMPR2 674.905270154355 719.13080264227 653.500279510032 546.274050902567
258.311739685895 942.41077071058 451.887564294551 484.028175202673
371.00603789759 262.452869676209 398.193129265416 658.202765061937
BMS1 522.050619657197 458.33221423434 576.636077770998 477.275332763887
310.465070184707 497.759125486158 558.810095726517 495.769802520637
522.899203071911 481.037647470809 601.640387246951 827.157920721418
BMS1P5 13809.2057928642 14108.0461617941 12046.9345159347
12818.0509395717 1561.10217536347 11190.1778174002 4635.42002659719
9450.52572756597 7406.86318549013 4592.14655702906 8016.1652884418
6427.33783376005
BMX 158.770502070553 160.469645948225 166.964057265095 158.446840764515
152.021576565132 160.261708910517 155.507030276749 152.945550129539
153.010339525816 144.842687993057 152.175423676063 151.360575274272
BNC1 142.368601917778 150.035890574132 147.588213090285 153.108902978819
140.184082384548 142.043121538885 147.978039160763 143.320787131532
130.766401858384 364.766154056243 148.173215617922 146.075794515889
BNC2 571.838717184447 502.935749606918 640.029195821436 550.566402986082
123.418266047461 132.219652920061 124.17231659571 118.641431192759
130.749763860615 139.096536887753 116.179625689647 117.962188157317
BNIP1 174.635853531194 179.44023315251 172.259627777465 191.961269585431
163.926432479673 152.528571776589 182.118544602205 194.475822533231
159.036647992998 208.42712014566 171.561940747015 191.59400638652
BNIP2 531.217306770605 523.591472915151 529.700356666006 450.566031840493
348.575690346536 475.403662030875 664.343553341563 497.629319581786
525.287367366192 534.276129011237 643.660739343159 744.225336494008
BNIP3 993.774175699873 907.610179738644 1182.50261323518 887.147199303294
657.231047829537 348.111872425949 2715.55326746591 373.760551592844
523.717949503639 4491.96934862425 858.742139354587 689.271009631393

BNIP3L 511.779988437654 520.962651968997 598.449359425056
389.96566674501 309.0578543424 504.617795280805 834.788058321257
294.746622369436 336.279209908669 903.503768217033 913.934874451961
497.110852407681
BNIPL 146.97334315047 162.18174589792 150.971453664961 142.184249919621
145.391496022306 146.621996129313 177.023536553193 232.556667737827
326.379855547052 396.526590512521 335.790909130243 186.077477713775
BOAT 146.775403409562 147.939682463235 158.902676384853 157.756885980217
156.157567405927 130.42746492878 147.633793743254 141.045379969014
152.772177215856 193.190059954084 145.924751649542 147.818956338752
BOC 221.961758403614 224.335742510577 215.931781291835 256.648540685098
124.802639338573 115.847063825599 115.120702589487 119.730174479044
116.5272207025 113.249267122926 126.858621735156 125.609542142983
BOK 502.731019454502 526.182262872235 443.444024128516 821.465895280454
495.655170027296 252.810636571566 285.172001915966 207.254220247481
223.453776362112 370.798852361374 283.048002376743 319.018213637357
BOLA1 147.452986308926 165.103398617203 165.143417235314 172.707821624271
283.377663208926 172.297698667311 172.050718136571 220.742788129847
149.288034848931 150.468392290279 159.344044864557 166.863589702453
BOLA2 416.861099188252 536.805979562807 379.292531644085 568.040362658955
2782.94647782764 3441.9805896226 1292.13921040404 1577.15895478896
1671.82101590797 888.371107987343 998.157643452741 1084.95095717937
BOLA2B 136.94525817342 139.268942501979 135.880955433228
138.116568200293 134.364838071696 143.628147237159 155.70645844794
147.150063462728 129.334217858282 126.87882572904 134.354215877283
141.261150586614
BOLA3 546.841563406813 544.462032960964 571.64723305848 648.161646065464
1092.74905085695 1228.89116158868 1227.36706427754 1630.1630053946
842.22767901391 1231.37475540937 547.367381020623 1264.90442763788
BOLL 130.760835441776 129.728266017061 136.742206779136 124.977046954694
131.907246423067 137.163699337032 132.553445658347 125.549968985241
130.607857562345 130.191876914166 127.097605337434 132.91094012928
BOP1 650.679096367632 660.907502776003 698.358285634451 1039.92160906839
1903.13326109546 886.157279090932 695.086203058575 1087.69177809384
1169.43742964112 1235.75946952752 604.453803994875 785.599211190431
BP75 167.341759854203 162.056622258216 167.658560306884 150.019181654237
177.955579729463 166.061891147234 184.611146047556 154.408875182219
204.862387642573 216.559563381808 183.115511373872 196.839016616283
BPESC1 160.368226341458 148.907136936731 164.641508485461
141.579159431339 152.993555372242 170.892319361167 154.814400491704
135.32160992961 148.863297062269 133.113581756557 140.158309892319
156.626742111441
BPGM 155.362503575715 154.537240112989 158.467572658719 146.050714008913
164.299585578035 165.411052799773 424.201556056759 320.097510212422
383.259887828605 173.863373934746 269.892633574243 187.796440205485
BPHL 382.744523022686 382.233826557803 380.821355948507 392.198487606534
541.258721278882 461.088572554864 389.051174954505 533.784151495549
543.389747823877 383.182507425579 413.398641688413 583.938727139698
BPI 140.42579083092 146.343230597246 148.927040248436 137.260359343758
139.2454524913 150.799400717425 142.524413202292 137.464914919148
132.651922794567 162.117224868654 123.554769032715 145.733828447934
BPIL1 130.306478656159 135.659271030454 138.028813480968 149.696766215866
168.33854702953 140.416247564103 163.726898336284 152.543005423742
146.00443946108 155.180238551547 121.30744400137 143.114384950467

BPIL2	124.611144621071	119.842470751496	129.031338314175	132.937942776777
	124.372963874507	129.450089922519	126.401145439668	122.408016372747
	124.446264376282	133.269658133558	125.318166919844	136.297963196301
BPIL3	130.211247943634	135.068883036882	138.313519292093	143.31590446076
	155.785933672918	150.650926518959	143.354858193994	139.271829906127
	130.317604643904	133.794524856909	132.404879616886	134.142423026264
BPNT1	175.891947478891	151.081339000609	248.927790738447	136.948039394935
	161.335677383485	173.708509414674	169.417764098848	182.235490620172
	226.25694727283	247.899467998443	201.235056368151	247.845708587624
BPTF	192.224242967539	176.802886129341	207.488522933448	161.968835903764
	153.159425380743	153.853856339585	160.707290343231	154.170007083026
	153.219954133957	163.385727569154	175.010849510711	152.148784410501
BPY2	140.177708471199	137.367390177293	156.695440330379	133.804489975068
	141.137459291428	146.493927564338	142.910616700526	134.541606996118
	137.210805338701	139.914034708144	132.461730873857	140.45860949168
BPY2B	150.126627487419	139.045162550699	140.946368538661	189.434597063058
	158.445124076906	153.276938815536	178.027417594263	140.615078497305
	160.70138318726	140.52997588229	134.487476333464	139.101471810467
BPY2C	112.219585614955	116.793408204625	131.514114515895	119.859508630454
	108.683531606889	133.106005881815	134.362834437771	116.425301253959
	114.350596695469	107.802792435066	120.527518013376	121.297234504922
BRAF	124.086902599543	151.440732942734	113.192313147111	128.2409378561
	273.252023367028	155.878493935166	177.874821397216	160.277755404001
	174.035835206078	149.204816953404	161.997058981786	141.356127724777
BRAP	105.952640312359	130.535859816562	125.704264033102	125.208803405105
	120.819163462986	113.597215383696	113.769815606191	123.02879683948
	119.643417329571	125.313515459103	135.654614839107	124.057369016694
BRCA1	159.891562221587	167.237705290905	151.224289879776	174.941551089279
	179.30729544595	208.125844007298	195.414464905232	333.822167152866
	202.44778603643	178.819486867379	172.888455552446	210.103974340881
BRCA2	109.978693735206	113.891385601095	104.091301211096	113.479382235297
	123.202829470888	123.513588004371	112.999642731096	123.271452939936
	119.06600048679	123.716400094852	130.526673128214	117.104945989023
BRCC3	152.849810441802	183.255119298602	179.460777888284	170.290455446376
	199.662592246931	195.269099451599	139.476234663488	198.94325874493
	175.810411923013	221.82340413039	217.607230767028	160.742365719929
BRD1	204.078331021637	225.4575611466	254.9636051363	265.754141836026
	238.124340832815	254.63506290698	228.616558999054	227.065194522692
	232.105867345662	269.141120401835	229.157389498775	266.286483421453
BRD2	2358.46955614953	2736.98692493933	2189.53914329263	3015.70954394585
	2547.53445585552	1569.13457493554	1846.61430264854	1753.58770316818
	2414.22821062457	1752.50728312967	3184.98944679038	1586.00600118477
BRD3	978.389902096994	1050.90881385623	839.324324662619	1296.10827265334
	1159.44907165386	383.10686624119	402.824964079515	398.512947526406
	443.259619455624	437.049581919144	658.068932688816	302.462763120177
BRD4	135.628411578541	138.296933822299	134.239416179248	138.576978828316
	167.73288535728	137.342348963288	136.931476050688	127.690860707933
	137.832597613423	147.510848198689	175.717830026564	139.841039854404
BRD7	516.755267232127	510.061974830525	483.699436940387	650.494111214312
	509.01330998156	370.798852361374	450.220658078322	436.834825721843
	620.371912523332	572.548647437438	717.270096456387	506.80969228563
BRD7P2	260.41082304175	258.998065427002	259.767151797583	
	267.869879189095	319.090371885653	235.742017851879	238.430350258686

221.975219056555 300.443499140896 302.408563830315 284.043349407812 258.109876262342
BRD7P3 140.36602776806 139.205887721493 145.633647856775 140.92066676704 133.277326912499 145.219057535246 153.368006275096 137.335501759561 147.150063462728 143.303309656177 151.627496656808 134.606126134531
BRD8 308.621688157016 304.713434036642 363.706789137022 278.555009982029 221.093229309411 252.249994170536 325.387193207414 305.16668987495 246.552713031445 341.501397478593 321.627969147543 253.867947800583
BRD9 636.970243888917 776.807656744828 572.548647437438 958.005138077899 624.638115878703 707.048754700047 737.193295872614 843.075879568455 562.089999265955 546.399972543938 635.038752622494 902.394410348792
BRDG1 142.236565028336 118.718048764985 129.150574563997 130.66863243733 122.03508430361 134.381042390464 124.86699236406 154.076216899447 141.216869032251 133.191797029676 126.056493244568 124.904518221898
BRDT 109.29279076901 109.454483612299 117.628564992187 115.113000553093 115.619116556625 109.806338826826 105.900294919664 112.449662536973 107.622342073167 115.594489123676 119.472410874778 113.064645688715
BRE 348.711663415777 372.54091392596 423.966625351913 427.98782239715 465.029298672645 336.067655676466 308.441120735095 680.743052197025 417.103816140912 358.355621069787 331.32397534437 379.862305342147
BREA2 124.275741099764 137.11719450122 119.330439721904 138.584190903303 122.333765477428 113.427848757112 136.413745926388 131.741688265764 134.237133546276 115.34945441214 129.455347273068 128.878051599968
BRF1 149.584582050615 153.734347607222 140.487887891414 154.79534193124 185.30916081621 130.316282003711 136.575784685895 141.216205703466 135.583556015955 137.4552565025 161.04173806448 136.670253859184
BRF2 252.249994170536 297.12411625104 303.638516587721 342.878522959461 1415.67109281133 383.74357557363 266.468621734455 215.866644799363 203.370353613423 239.789994201722 248.761038207278 233.029655807246
BRI3 1046.65224316733 953.861218000678 1022.55215097491 1041.58583268313 1581.06548216423 851.633522004342 1220.30347076968 663.668537495345 976.360310504918 1442.11349373764 784.170983714588 1663.71411325131
BRI3BP 179.601348561497 190.45739385453 168.412162365467 180.315825600347 462.144334719146 363.777212750779 358.52459123416 341.973002478932 314.949772661046 329.48021621087 284.473085899219 355.443758121675
BRI3P1 850.125434374005 904.733257358133 770.19255305977 936.221322010828 1208.25171673432 561.609211243027 836.235700891687 486.197269936713 700.601917911532 1142.09977159958 1085.38819961412 1100.17618574297
BRIP1 130.033927910392 133.236084190243 135.057847490193 140.381386314817 139.823017208731 142.570182389682 133.106005881815 133.271506838359 139.214589768245 163.234966251618 125.400741907779 129.666957539624
BRIX1 484.658140651032 459.171226689178 606.851072672402 340.239348689005 720.077057619735 1285.54492194393 845.369108604819 1890.86868383409 1033.48252432603 759.534249620343 908.352265585374 1229.47460924638
BRMS1 512.723592291378 524.855915371246 478.909308632726 477.056706273413 1908.81864728547 1067.30106203503 793.071192619058 733.195136251155 686.679573646733 665.602699607102 714.052063877292 656.009451448101
BRMS1L 203.389294254141 204.509884908059 226.796825544749 198.055877414824 168.183542846746 238.847091900666 180.902950045351 184.387409077972 158.770502070553 212.749668083641 188.891032176388 182.608712782878

BRP44 601.997464467917 658.968318349334 654.663838918786 585.721610434501
1724.22121078776 1151.89087953837 1000.05896885496 1931.16906839143
717.270096456387 732.877169346979 1254.19980463817 1033.48252432603
BRP44L 1342.29111510713 1171.62361359992 1545.71251527145
1014.63777151551 593.320132696649 902.822697602452 718.142991571468
699.550347374567 605.040026490154 864.547109080675 860.688314783077
1050.90881385623
BRPF1 671.134356672693 787.874585290601 605.583144451322 850.562379837947
1081.47077865393 759.802694618208 776.030689773414 732.352357321273
986.004777675332 708.779490017407 581.54874141672 674.742955681908
BRPF3 498.05778289794 464.128022170285 506.430593289427 478.847650837714
401.584032365337 355.048838683854 533.37609065123 386.067573158514
486.947380524463 467.512355593598 576.636077770998 390.021666378804
BRS3 122.494779505958 117.969576809288 115.400592594965 116.19219007097
120.628904050564 117.412956162665 113.209569103727 113.249267122926
116.196676877996 123.02167644694 119.312201620247 115.738984899365
BRSK1 439.023181761356 479.469659712246 407.152764702613 661.826149512811
330.505633088656 234.5655184349 171.247946863662 177.96452209396
249.09278446472 166.558080305926 156.365413649149 263.707476735938
BRSK2 262.691387377392 261.922485502971 299.170431746759 298.625127845168
175.874387612235 160.865949438448 151.106858426994 141.308299074832
122.646850766733 135.028746262383 140.82351325907 136.142380560596
BRUNOL4 152.778732614944 146.388285560372 143.807102980397
143.056098955473 140.971253689199 138.171858105728 144.850211439338
147.925744210234 146.579930401696 157.761231106669 144.627784383455
148.263293819238
BRUNOL5 164.787637367264 158.726115143818 149.949425756405
154.996829706812 154.764283765316 168.37043566813 152.495540563792
172.973175988757 153.209409960324 155.147962740503 147.26204590693
139.15175079191
BRUNOL6 186.939168482155 178.563665283267 238.310077556167
189.840366340622 155.069048442532 148.274122690409 161.585555680663
142.579346058088 151.423936838934 162.411156818437 179.026308021985
155.191446350891
BRWD1 212.33360547376 220.345184229077 239.148333781084 223.389795643722
179.551640851353 304.292209676095 338.223462327622 446.601751145647
262.913054904154 207.400490077106 196.144420005021 282.926325589238
BRWD2 271.858059712406 247.340470321453 271.791126011275 273.369722814068
163.726898336284 201.748876422401 300.247304779062 232.778488697337
256.820546409632 199.916301659113 324.203537490975 297.419034136005
BRWD3 124.297848065172 137.423810400996 122.111982327877 123.122705969332
144.850211439338 136.978738825445 127.745801270205 131.628021008753
126.497472087036 140.086978441723 161.543072816738 148.390417679848
BSCL2 739.52947559669 801.52891824571 837.413261433363 972.468110850466
833.198511430615 1443.68785521063 1174.69701182094 1114.10687039922
1187.62490367696 848.620986404914 839.662936279727 1168.3174645384
BSDC1 1134.64039256323 1242.61637859159 1001.65849130836 1293.70593826709
998.631321857182 1331.18605673705 944.92617668298 868.86246684338
947.766127685083 595.593867616527 661.545179083009 646.704226815099
BSG 1074.19589851599 1141.13648305827 849.784051720996 1357.48791782399
3688.68740405322 1071.00507864299 799.423640530777 664.60530723006
613.279465441319 625.152889992949 887.471553448641 768.334658920971

BSN 119.972337070176 136.248506185974 118.360186663758 128.184708916755
155.545904088857 125.470172259885 144.192623511374 121.164978896057
110.865668451601 113.740942665269 138.424464248567 121.116206210402
BSND 117.503848306325 123.791515424464 119.213217733213 123.21732068638
127.305306470493 115.834056091815 116.934457270701 111.43827410589
126.542112891953 127.451155926679 146.882746173951 114.209249333221
BSPH1 147.502116289881 142.586051173329 144.961976523279 155.91493956251
135.041687549769 140.402944266863 144.895601314903 140.067809125008
155.828962183804 143.951756384507 152.823566129571 164.603403251634
BSPRY 144.898770838913 173.484307104751 148.665627690915 177.371157649894
278.651369597422 200.558971047462 188.605328108654 193.744461104128
188.378996128521 221.635333304767 198.863347487219 165.919497521143
BST1 151.540744107423 142.329137800558 137.800512046163 134.458958352588
258.075566417241 118.465111174212 132.591496435822 140.244052740397
125.079330440615 126.969504644414 136.587165242663 128.363331337379
BST2 894.100792722707 932.076509242794 245.675725760577 448.95671696823
2114.9754128463 467.681480781925 2684.69013589621 361.26540006942
1641.70484603137 600.430996779799 312.845219991659 2786.35558271661
BSX 137.220718873614 150.534608517044 141.36525744062 125.861564323476
144.32655838488 137.890006487904 147.161720770972 130.669539169739
138.760800146167 134.178733595096 148.437676259828 134.655842662899
BTAF1 935.423045210635 736.603605876572 775.5664718389 761.097992055119
280.595515386655 471.598175637494 603.764427348016 747.077451281453
410.731869154912 406.369889501135 553.198321853202 655.431236290512
BTBD1 252.432175363972 233.029655807246 322.889296658955 243.159062832085
319.927209914181 391.644649726254 408.444629429553 307.316241247336
339.448524400708 399.724529998085 332.666916002481 417.191960489528
BTBD10 466.777454704543 392.282956362735 589.953707188378
430.728137105601 261.671277686467 447.372026268156 395.521241316058
438.146919555296 380.888728347647 645.183207984114 497.53605530806
456.530337997907
BTBD11 123.551225884748 135.353539458856 121.154389729409
128.280225530201 282.742833166843 173.879952136931 202.909188070184
176.198555355383 185.063870458561 428.959561932229 194.125840053843
173.919504730673
BTBD12 266.347965690851 308.767036855508 317.802633831405
397.064325578854 282.561381978659 263.74335003375 394.73732350607
374.033009122079 345.732364434125 319.417555901733 328.486476842582
266.252875242286
BTBD15 366.240941980786 363.343480850598 355.879841671975
342.717252404581 209.417052464583 207.920724788669 304.844121431627
241.251507026982 237.492603094091 190.939726271153 451.887564294551
277.406445570925
BTBD16 265.791342287571 295.241644408321 256.590289281076
122.557981210434 363.963951509433 1301.18893681695 2374.1382412432
909.727616721285 362.795691170335 119.062611098235 2233.00694575483
6560.30885074315
BTBD17 136.089888639987 146.378522944864 138.875827888646
134.041098536814 140.588811000948 145.131215503044 148.695212949639
144.610341215903 149.579428189895 151.635752404534 141.929924728222
165.074983746785
BTBD2 1620.7954553764 2183.18692530644 2132.90676194993 3255.91703674439
4800.99572384569 2468.47687203968 2567.4299860517 2426.2098937241
1796.26091584668 2340.90990677253 2043.73482086765 2157.50118108142

BTBD3 392.430873293903 386.679822751367 464.577839898252 346.566241319814
270.181005097697 578.084277076692 315.130912368929 320.597495751519
281.846246549202 235.961605948276 647.955521647635 368.711718874343
BTBD6 1002.44383771056 981.781076110568 928.672013047007 1000.91520225219
1281.9073569335 588.076436392983 880.719593734606 908.352265585374
950.144166422001 1103.13439876128 1099.37392456694 974.007008568051
BTBD7 212.710910778949 226.841838641151 213.833894542292 229.284931844047
180.523458795412 170.350891928203 200.518684601283 211.419445018736
183.416437587938 184.577511730401 307.768302846647 197.341423434688
BTBD8 138.150616787218 135.675570204709 135.622888091037 140.051904905695
136.783974483005 133.820936825552 150.203281104753 134.45677772672
147.457193565877 143.349486755648 129.294026995663 136.135223482208
BTBD9 177.023536553193 159.0794075765 155.997210529742 157.919709478746
164.48570046081 154.104321553472 157.353493980339 163.004844049672
155.158028043823 174.807363753172 158.679903271595 167.325227483198
BTC 279.879377933144 285.172001915966 205.728673153914 364.766154056243
408.10780961711 186.04763660516 209.74583569369 190.809584998617
198.390247605355 168.197255166255 195.740661051834 188.490807652188
BTD 138.528083984177 184.25384366282 207.384408045502 166.335294339013
186.813888805838 157.604224004016 143.298218989451 210.043556816529
152.612116997575 138.952082680577 215.897620927751 172.686798187142
BTF3 3757.39261544326 3522.61692270848 5361.3600315322 2867.63319136936
2804.99820187198 3474.0924656878 6560.30885074315 3455.20639616159
3222.23985545506 5361.3600315322 4009.22206756187 5859.56559124843
BTF3L1 132.671227062948 140.482728188987 133.62358240645
138.706520278166 124.740700441589 140.36278240974 141.436235043864
132.363645381743 128.939572714685 140.04612725812 150.513804134588
142.475646565816
BTF3L4 276.867974638972 280.246711704036 393.375554593484
230.053292316517 243.732074437237 913.934874451961 444.082440002333
569.866002248352 404.172535442156 576.997804865103 615.058712877418
552.790706181938
BTG1 3188.90202907403 2995.42172749615 2698.5455305009 1756.88092410303
1219.36024606325 2684.69013589621 3136.27991502603 3784.59114921916
4569.44477073589 5589.21471380445 2756.22069438696 1832.35076992253
BTG2 379.983165400941 604.047164856689 640.90860274227 343.767393640683
671.134356672693 1191.26028342929 2627.12900283627 827.400420888645
739.251974981981 358.897128592042 979.761537450366 1455.75693295732
BTG3 393.707031354025 337.22646304739 302.768870633398 290.213039891361
274.627303114477 346.862322263052 580.474775712828 593.990687449346
681.591028334969 853.314313182949 338.406266073063 415.178470246749
BTG4 113.680875214845 130.59670974887 142.888281750232 131.249240371529
130.582210755516 143.188958538221 176.420075374686 122.97487285065
133.473140653666 138.274057264688 124.964977322158 133.355353808577
BTK 211.012142461725 185.673549367125 175.363223521128 155.938283930571
131.894919149666 140.688570032068 148.04206955657 173.726420791056
282.926325589238 142.372470063444 161.490941000872 152.439608473915
BTLA 138.263361518905 129.063617408979 133.372231702721 135.015161451899
140.615078497305 135.565256045133 147.44463861207 145.768857553634
181.157738347957 128.284564251279 143.21411610625 129.029151164978
BTN1A1 152.511331178337 122.84779238795 134.516390697561
154.493246900289 136.423161205425 141.323417580648 127.650229552084
147.985843605585 133.165893710176 137.736771579507 131.326536951329
123.58727715816

BTN2A1 372.894376776771 367.130155304037 439.727046291381
467.90388853336 217.560691345072 360.684900316265 299.669394721682
297.022984365795 329.333236839082 284.272977025491 348.241471067257
376.124744806171
BTN2A2 128.705587172572 140.073208365817 124.541257225826
132.786500984297 127.269672881591 127.729912390394 124.15094220181
132.496547953595 146.840735518984 134.719531559829 152.422690241301
135.527445907621
BTN2A3 165.767629164724 170.87247413028 162.178410412809
167.040031488406 159.470818285679 155.896065329825 159.41705570719
153.018378403144 148.332750352364 167.43630698726 123.156154667242
154.001258677199
BTN3A1 206.376365685112 196.345065354654 167.100039749329
190.506201184419 151.971337856271 144.36033726557 176.121563444364
185.088667707155 247.454326301069 177.11682975342 459.388390392518
172.077522197357
BTN3A2 239.20206002 199.813528853649 182.009471368798
209.130260450131 152.045391611927 150.971453664961 213.240181616274
241.706645643141 438.533112511098 252.514569129392 370.945254716657
283.191303666907
BTN3A3 180.986305037222 178.920528149177 164.448110885609
153.171822092714 132.66318177913 130.807596523171 157.760190496427
179.94333102877 319.966785546419 155.694213876736 264.563741039382
219.0221768576
BTNL2 124.857461801227 130.43775024291 129.672284417918 120.721341479615
123.45004246675 134.493062923443 116.919109599361 125.879198238067
132.310147451479 123.629279569085 126.784243009941 120.204469421484
BTNL3 137.469177072831 135.509365701442 143.558088035758 140.976675511847
144.19473076362 145.363692072132 145.368059425439 141.079317977197
142.236565028336 142.768691899824 143.695901115673 142.633121727277
BTNL8 121.624218243474 133.871685084945 125.435268901131 126.619475852751
130.601241656346 120.887938517877 125.08580835258 121.483610947845
129.180990946063 138.712529531951 113.064645688715 122.881793230233
BTNL9 129.358112503486 128.606360476298 127.777945516765 108.715328458556
112.832122823829 128.354163132392 118.850495684547 133.096984735092
108.589444216106 126.798976470472 131.681566829159 121.736056336879
BTRC 153.219954133957 166.12184890921 177.055023182887 171.552010365305
137.048292841566 130.224324464992 141.606501859513 141.811563725942
146.524138043215 129.246066890078 163.104822161892 163.004844049672
BUB1 115.713909155043 131.215202712283 117.274977920103 118.81294782091
379.911829623463 493.715108157171 208.932692585748 489.403061697031
259.064601204239 283.191303666907 227.52352046188 314.610979086485
BUB1B 136.815411937352 136.984172424718 136.049167597016 139.229351251973
201.969002368045 248.594000664466 167.011846920792 195.218005582618
216.53417734069 171.433001048839 193.261343922604 204.89842561202
BUB3 640.029195821436 636.781477235579 739.251974981981 656.32782132983
655.094843900848 699.06539333455 808.300067805897 778.639512538949
819.488546995594 677.023591987179 1039.46136549795 950.144166422001
BUD13 290.288001750744 283.326226944158 322.263925912805 292.23287968517
429.992169596618 266.217879616686 298.664864578349 301.369680426563
263.295012964476 226.43414383055 369.974231530826 379.476768908492
BUD31 1093.21579738952 1191.26028342929 1015.63768413332 1318.3633082502
1787.32566525551 1323.05745869565 1986.81354260292 1859.88000417483
1862.88548451154 1582.0221231161 1645.05161227372 2141.855356411

BVES 260.999345837027 257.880331012233 313.322307299463 301.250189051376
137.911816180518 137.550607284001 140.760983862166 134.203949774763
142.56910547147 134.623264303227 139.746733428525 140.307931763353
BYSL 228.968779778407 228.797912452429 186.309367281625 270.973600107882
550.24496459253 459.53652431464 337.39574303968 295.678097392123
299.80615152845 280.033528476199 315.689865927834 361.687156919012
BZRAP1 272.500572765647 254.393421265604 217.969738417693
302.905840432998 153.072429774613 148.996601285688 157.944355564411
144.333945716394 130.672586991186 122.917915232153 132.566074764755
144.975882336334
BZRPL1 161.182901898379 163.284853420896 141.315230997077
173.176900454904 148.782907912893 157.306022722324 151.369387642458
145.072924262206 132.541420804792 167.719651771357 154.681604647652
141.25089277145
BZW1 135.501192996538 132.442771000976 133.944538335319 133.069347559741
144.876300850115 140.374644951491 145.491525555916 153.095594614683
136.185953087697 181.803275243281 158.503284955019 161.979914539039
BZW2 886.517088614968 922.50743596248 1149.3250945792 1070.4597488302
1617.44377452783 1790.53556140959 1722.26954281876 1557.00226259883
912.878077940014 774.997895985917 1286.55103838368 2258.30916111153
C10orf10 344.737964092526 788.693133735893 400.98290237416
447.182566890086 177.001637712889 477.626693328013 303.051124835907
196.16056297158 363.777212750779 167.226663395855 188.53221484746
292.911761762431
C10orf104 485.731287873723 460.807117177285 513.518424350159
413.330906392915 204.1974438819 248.129640673859 318.348505551265
224.062024091915 305.854543429221 186.551385094902 420.871349359404
342.037040881785
C10orf105 144.00214898009 132.390330703084 139.338384535905
148.560326703328 130.784802403934 152.654938328424 153.165872773973
154.675189522146 139.856251508283 143.560065338939 152.142909854773
142.637421204193
C10orf107 134.640783050966 103.307703547649 123.170639180926
102.255408120852 117.805556408194 115.107214937219 105.839106819841
110.033746435328 111.328185454541 112.292129754508 121.643036773773
111.43827410589
C10orf108 160.72009357092 170.684848540493 148.424970450231
172.783430414523 142.916274989816 180.047231265069 146.934990457666
148.590732065459 149.211214631561 340.668473351494 172.677083810424
172.20451139581
C10orf11 154.973990421257 170.164668347783 145.371273337639
178.606255846041 202.643624039869 182.608712782878 386.679822751367
180.113222861966 290.867822301134 183.556662533396 278.88183549759
435.332573825425
C10orf110 145.036719629525 131.300342835696 129.846685899183
135.68605915552 145.733828447934 149.675363177375 159.646538408414
149.188357651896 139.864176113337 143.434571922656 145.906601611338
139.706279455371
C10orf111 114.43491928161 117.718893827092 102.166449349339
115.120702589487 116.258629237662 115.914842445934 124.35374445763
107.48180681159 108.21185649774 113.938636269013 128.466244127634
118.521075843163
C10orf112 135.839123867443 144.071652839025 130.020743385543
125.83521710721 116.769106223472 133.97321873161 142.221542344035

131.734429913106 129.394359810841 125.587057468028 143.760519659724 117.740022708444
C10orf113 123.798513338021 111.232274546928 109.599589420575 109.760525674874 110.266032635568 117.585207821853 116.746018509166 108.036254606246 115.933805625814 126.37749112514 103.550817366724 123.145830746127
C10orf114 151.81754291846 154.079355899056 146.764908249803 143.703367582367 195.10410545445 148.435419356331 143.449303333495 148.668081483434 156.254944793402 141.063156289206 169.544515294662 141.626504154989
C10orf115 122.35832958054 122.317877997527 127.548070199148 125.777193592751 132.383542877819 124.720004905439 126.820834043224 130.689272423595 120.53428198263 136.72715888684 143.718210650338 119.767154020462
C10orf116 3490.77593331727 5094.53287522452 3359.62015747752 2539.87833317526 15194.4972103372 20381.3859535625 16642.71853486 17496.0674176329 27284.7333124039 2037.96470181871 18665.5217411582 18720.0460735814
C10orf118 177.874821397216 170.024833610695 211.505504834207 171.636121363019 145.440471538068 174.136806313142 192.224242967539 197.651165059354 203.071186284148 195.776369840059 189.83433085782 208.348515119045
C10orf119 169.089896796513 173.161152247499 134.160100276452 153.713298074917 154.524588318927 192.113217321485 138.230197299262 136.91078498126 143.419034484087 131.497454038525 184.591789011195 179.905809470907
C10orf12 185.382647297259 193.753062958207 198.753297290444 220.578790067967 205.765822478178 208.666760987764 205.546628064296 208.690347128273 188.062732621031 225.095016600703 186.185055364718 235.216589534371
C10orf120 131.482553251516 125.77343813277 129.428950259722 153.202157381228 121.031695489398 144.741063866547 129.618097261902 134.451423371791 124.174140125309 147.473875763319 144.018198451676 135.286686011087
C10orf122 150.854580568572 151.627496656808 147.54950981909 132.140547119679 159.7197697044 140.885012530186 136.11588777718 134.233014993992 146.915247627846 151.727603285205 127.440987506769 146.311966562883
C10orf125 143.52474218953 141.536073061736 125.558877611499 118.186426364551 279.790257881254 128.72727523654 148.821942578396 129.417232210341 185.46843333826 120.270397272069 143.481112776388 153.477432230141
C10orf128 172.973175988757 167.612577768782 170.731358903019 186.653791045656 163.275138097131 168.763640193219 161.234304237838 203.17525411575 156.562197068687 159.660564323984 174.515815532892 178.179238056905
C10orf129 128.407968177242 136.067657949844 129.785078660064 131.77099316122 127.350368333088 137.601181150371 137.881776548665 138.428527640896 130.804850476451 124.106971897779 153.811165696928 128.144653206398
C10orf131 138.687013437904 156.638070437044 140.782522936146 146.047948193303 154.13120950189 160.17208299932 145.374923175872 138.655815405894 131.965184111199 141.882859092658 143.812863334832 139.147327302581

C10orf136 157.81327663167 167.389632521521 156.237282338037
158.002906093899 243.246632667118 204.999796322451 177.990274115348
152.534482748598 162.173506492562 161.402322405903 248.16594076495
234.693846527867
C10orf137 348.171901445232 276.925017614147 350.926679856514
318.561557474749 215.84044779962 313.025250337353 368.548135194271
272.500572765647 411.603820288919 251.347519777204 291.839070398338
383.033242143031
C10orf140 226.936810012475 292.60467054075 256.093363520707
359.991823789356 134.522715664256 133.080437161283 135.562700479444
141.528115502473 133.465593684519 131.162118170837 148.646699255797
130.867819112143
C10orf141 131.328478053136 125.642282323003 120.485138937899
126.798976470472 118.81294782091 129.301839663122 118.406487008528
126.972399784537 124.375348764151 112.321396616213 132.105929230517
124.066239570648
C10orf18 150.065275447769 159.951697830252 157.841520434044
146.854523122404 144.783770949511 169.568915494577 160.227729155903
147.761374187766 146.633627492419 147.925744210234 149.76931674417
135.989816139218
C10orf2 195.66091457697 194.941454173896 186.167877791105
214.888126361789 264.936739264566 176.397715825653 171.86363382331
183.013090662119 204.438365139666 166.192990554427 154.533544838819
260.443814523694
C10orf21 158.020651857664 146.861981788662 144.769824608083
160.136209944279 149.836491640473 148.216605879958 151.72544764489
134.748177388398 171.528350399075 148.773558380167 139.963969716386
145.281074767624
C10orf25 133.559082256772 138.078214837785 123.607412609763
126.159880038854 136.297963196301 118.180919555585 117.123891785437
116.576896337786 116.742079521048 127.416912237613 157.15947600423
125.746184969983
C10orf26 217.148957110623 270.679613997675 260.52593414721
278.651369597422 236.676029292922 187.325542100762 216.05364037271
192.07791693203 257.292125083248 255.793823796456 410.645457106122
252.761035334279
C10orf27 121.468398802591 126.948090173876 123.921903967493
121.010811158088 113.154880173167 115.337592860132 121.838802846453
117.980052938167 124.783306383852 124.466986821169 134.32174235533
126.274585491044
C10orf28 207.815226999953 233.047541132048 194.22713184875
248.729653566598 202.041958613989 219.441959146815 237.825857080173
200.591916768948 217.684410907748 185.295480519718 260.715580924916
217.050602253281
C10orf30 119.673343767067 134.452938571435 135.023594109361
132.162948517239 122.917915232153 113.163651806803 125.331215990597
115.624167024067 124.821581853501 129.073750290924 149.548900791507
121.770043509503
C10orf31 125.859158049343 153.700168798494 142.478528530514
144.388851371784 140.510889880872 133.363883851093 134.95842832877
156.423687034312 134.005402534879 134.089308635893 134.936025073531
149.717739166461
C10orf32 334.52685347521 276.138650967632 403.812520074723
275.509805464276 188.74403596953 323.04019262387 400.845137156678

247.145700155364 361.768552564027 343.121661664987 515.597523880328
404.21236980164
C10orf33 321.84712975195 307.866499851733 342.878522959461
423.045329000756 259.511314588868 140.892250639524 217.560691345072
175.476184387338 193.522811257085 125.861564323476 364.443097338814
263.005303710717
C10orf35 299.568830444875 318.487401106209 347.3483417873
319.868227988842 383.74357557363 379.102958448288 455.665927568395
427.151797273226 357.006610037663 179.297628357211 343.417041721907
519.829743770022
C10orf37 142.011019998327 143.426053070219 134.880138942756
139.642457979496 154.433679792749 142.290253129837 140.815807483203
132.057808599068 150.231063426302 141.302647488314 134.897958140444
149.193437559422
C10orf39 207.453490385508 212.33360547376 244.251315474587
268.034972830233 179.55905250703 164.773489952844 166.305219348008
152.129092774005 162.161282071736 156.961070790639 173.260348096238
178.684992551017
C10orf4 136.882429810263 135.535020220081 149.061464748604
133.800111596565 140.82351325907 141.653917856148 142.222833574043
136.679405220947 138.121905080822 142.039943511086 150.555125135002
139.153897381147
C10orf40 126.310788291075 134.698857213208 144.408426565541
138.873925577739 125.104229604098 131.975248488461 143.519786615253
129.135985676046 136.319655432311 135.458682745495 127.581332110857
135.264074941678
C10orf41 154.153249796248 147.405050592353 157.237147919803
161.325409589612 134.399228996542 142.635015113687 198.597176657619
148.47415004128 185.801540278924 175.452986514346 165.870621884073
145.862497313025
C10orf46 131.173355686001 140.664303738113 143.252983617945
130.322436253881 123.547821639269 146.681183890181 137.748443205818
152.60462513339 133.032905765836 140.009593108693 152.052084712417
138.521952850632
C10orf47 152.26903721278 166.77329045424 150.929236917651
141.383975990479 222.118666699301 269.567704345075 201.221839281282
199.191065549132 173.10229478279 194.112534914308 180.219813240126
187.572517928061
C10orf50 129.935532080325 131.367075440559 132.257989841046
135.978826529736 115.137475087954 135.196797030404 137.687480242109
132.807755600683 133.773427881651 143.543607779991 110.148555045276
127.453866279181
C10orf51 166.47851613068 145.950701448912 165.443069511115
189.539458686123 159.29430824799 171.869472272081 177.065616871886
173.526005872207 149.595375008252 162.739253457398 129.732352495012
171.155660467037
C10orf53 132.106673891288 134.237133546276 131.157702791998
123.096995744616 122.679628839976 123.82555184043 136.735930829008
132.450954427876 136.878656455606 137.478556403046 128.200531810126
137.681280789305
C10orf54 346.118765910093 323.276514394119 174.66966623557
277.073510273425 217.491746961471 149.4401812 179.230909178397
288.542264120468 371.693808540758 187.700723296295 336.212224495877
220.312301059818

C10orf55 126.217702283708 115.002176041974 119.785062256421
122.083357581665 130.156775178934 129.904942554979 127.84589484404
122.846912279427 117.348231147283 128.84676695859 146.781221728543
124.242539075734
C10orf57 323.841518852386 363.963951509433 493.492348317616
331.62124160152 447.779854598556 517.993093601127 698.358285634451
724.607317443904 1332.82617944131 501.600329334497 446.601751145647
592.387650564803
C10orf58 7753.61594300904 8666.41250683965 8163.29201165065
7038.48958943724 2398.71483410593 9514.74328718935 5748.57645553386
7601.58151657937 7433.03226979876 4520.84412831414 6500.1854485891
12046.9345159347
C10orf59 157.631978594005 159.476343366991 153.354921961854
152.511331178337 155.7441762827 145.304050333309 161.481227197715
181.758545723241 195.69447353356 170.998961353556 178.751706380632
193.777175793218
C10orf6 779.022508413529 902.089561157715 719.369561410372
1151.30600388403 205.821101661022 271.791126011275 350.077206368839
350.46078795627 364.840751972169 249.029098902357 504.436992949763
380.754569467715
C10orf61 189.693218759349 179.896659891106 169.408892048115
162.223175359454 194.281839133887 209.464347017443 237.078644686891
183.385868234361 192.680681636018 200.810204162831 237.492603094091
212.210383398591
C10orf62 128.099484678519 125.157459724842 125.566982723725
119.937665469629 121.522634801124 130.985360700521 119.308345676905
126.134393447943 129.801342433836 117.324368138398 124.422548323449
116.881212516879
C10orf64 166.754995454382 165.634117939571 172.531836863525
186.813888805838 158.829144564361 154.445840992357 161.525131363333
164.625407872446 160.180961887686 167.206513789165 165.274189050141
182.042104888745
C10orf65 155.890403934674 140.790450703097 176.312626750427
157.032746784217 169.541411677993 201.041792159394 240.376851873817
174.192898940601 183.181125586276 147.545667896282 126.640141760199
212.890259409988
C10orf67 147.553953240464 140.855078775719 137.522792377933
159.452527363763 145.909456876796 149.899789105328 157.259599894923
156.05583229359 146.879019367939 148.216605879958 141.855655600443
162.289781945582
C10orf68 140.281512754823 157.604224004016 138.752600540409
148.470120335761 149.88598449818 154.632563476364 142.77817647952
154.744065805492 145.821544099585 150.75878820215 140.616811333466
156.748330788367
C10orf71 114.265920150451 108.543795408713 118.148476979319
115.420810993676 114.77964341895 111.616460084889 103.09089100768
119.449118660481 116.412881235587 108.910588012315 125.029516472944
107.316786994369
C10orf72 174.323881548679 173.260348096238 154.366071635971
187.132496910208 133.070896773384 145.393431956588 140.964208731587
141.533138525284 148.47676851933 148.560326703328 134.582098893327
145.112399373761
C10orf73 166.872332652008 211.160781061336 201.059635671301
178.035700298977 144.568417378353 169.466129742085 191.754626760061

199.756584044664 155.63671938031 137.435736170779 275.437030326768
262.259969786927
C10orf75 164.870860592068 143.548858367002 172.657668514226
142.002145315543 124.940836122365 425.59810308048 143.653452869664
130.99564079691 131.470883854661 138.552423514249 157.419333185365
146.944628763232
C10orf76 280.085692727562 280.595515386655 330.908743070095
360.756455184184 187.784865705837 190.889832855403 211.22652563815
178.488874074222 208.164674386141 232.321454436688 205.765822478178
246.428746807313
C10orf78 148.859129298141 158.670835881902 186.925138700818
152.667721542507 147.28750335168 158.665476708054 155.798048652444
168.007337847195 174.149718758884 174.890624803452 163.22059466037
199.191065549132
C10orf79 153.528429414138 138.242983316259 155.176714087209
151.338765231607 160.399059317919 149.726968520487 150.374573177474
147.547944397578 148.707332454527 144.114177076352 130.728465509213
144.292679144059
C10orf81 131.093917654245 155.317754378283 135.571239294086
138.230197299262 157.668307146391 130.909388246758 195.447966116819
159.89988762842 141.609037401299 132.629453540571 215.558688906165
137.77107995457
C10orf82 131.827899207029 124.657687121111 158.14434287956
147.366877428537 138.985778947618 147.666724626127 134.660181775674
139.94624507228 148.825790628891 145.992883153161 136.654902685184
125.224197852606
C10orf84 163.85327708023 163.704616231638 157.66595229182
169.502103689662 128.833756478439 139.065849548762 132.189426012448
136.214432257698 134.208537331186 132.089375920534 153.93249158209
142.678267012725
C10orf85 129.719122724699 132.836513637574 126.561067704708
122.996616773523 119.432201417811 122.068058346443 129.7137422914
127.101752724954 129.434594784981 132.263954832097 150.28169442832
133.486155360266
C10orf88 192.804447792672 210.900056977861 177.862368731138
226.678503956411 157.960172706254 149.181838320838 248.129640673859
166.570606752678 175.044940252247 172.961115357569 223.975677959345
185.295480519718
C10orf90 113.416743649576 117.112667504781 114.567609871292
117.392898601408 115.502679218125 114.880348648147 119.673343767067
111.66210002202 123.974409226349 111.549719888905 123.868848032411
114.598636725763
C10orf91 131.702788057359 114.539671934155 133.040952285855
134.264584077125 147.117286738537 125.674471551099 132.047328970556
128.2409378561 131.794065532001 128.473834314632 127.130769263811
125.990762414974
C10orf92 124.710368327189 134.446758753426 128.763176477983
122.095978653755 122.557981210434 127.806138997958 127.163025822368
121.168550902281 118.269780903508 134.477081392474 130.881629774224
115.687997771921
C10orf93 133.218742606195 141.215542377796 135.434853296628
138.611074796816 134.408846338374 130.095585254401 140.861849838223
129.878330410179 130.584348445667 137.677396262152 127.675206134992
131.76272538453

C10orf95 111.584425166104 110.707755436868 104.406314027414
111.62514398781 118.783904603368 114.10470841717 118.486195373059
120.118873350459 112.520199390427 120.246240734668 152.921328522953
121.556331561879
C10orf96 147.576533768761 150.629932241703 157.130045415943
158.459129254226 154.778726823719 148.692602243778 149.749660071593
150.269618125943 134.803612821519 135.047546966745 124.345320343901
151.243948028303
C10orf99 157.759149893048 270.727749014046 212.190707408572
154.037622223246 831.610501402173 371.00603789759 2736.98692493933
3143.77161008321 4359.98094976179 1346.53215685106 2666.06582231934
2995.42172749615
C11orf1 240.005943851098 247.504198063293 271.196028302639
215.47323777134 405.032674307193 264.109049504585 372.255208814622
331.32397534437 265.919002666885 199.353823724989 382.630113452429
470.308610828825
C11orf10 3403.37530051435 3093.38255333143 3328.99028463277
3000.46298223166 8140.6363797934 7699.37963536892 5800.06300826802
6253.84268792816 7733.95252458705 3608.10730106542 3618.9385126441
6601.004284244
C11orf16 114.567609871292 118.249148086655 112.179019393947
112.502812746669 116.615250922316 115.242526386551 108.021898335735
109.282814458761 119.952108688414 122.456286448252 111.349642316073
109.471542280224
C11orf17 322.443089373063 321.794887577351 349.99912485533
365.650071660806 246.594244983507 208.776711004753 231.600532144945
224.167511918105 258.311739685895 246.80306998812 270.231637012624
218.334273320319
C11orf2 1305.2232532959 1484.32547474275 1681.54485582868
1787.32566525551 2441.05534211996 1002.88224342342 1316.31088187364
809.729160605727 733.86133557115 1114.10687039922 888.04996477314
1025.88559245013
C11orf20 117.017844025726 117.417112053842 119.627380812035
122.711143209444 123.168787977805 120.897228878841 104.505454151012
122.860898886143 117.455432751161 139.499652059557 133.680691168661
116.850851308701
C11orf21 129.668506092785 131.031639119149 116.483432723207
129.707421699091 128.499303407596 126.825785762925 138.424464248567
124.606795061928 130.70867409848 132.17294083844 127.966883271903
130.907879150229
C11orf24 250.626556981376 206.098534554876 191.795952536469
259.72035004624 291.883157643902 226.114369645703 236.35479928448
185.485858719975 185.717123241832 221.127492253636 182.886100305943
210.803130108618
C11orf30 129.150574563997 154.657316209848 166.635366968851
141.18490443471 147.610616423185 135.205434173702 151.266352916239
150.250193889942 138.361149460093 138.706520278166 157.776321544382
137.048292841566
C11orf31 116.746018509166 136.178501890585 127.532665057083
127.075899925865 126.455271988029 140.538020869886 145.301551442388
115.907902010701 142.019263564073 130.052593375075 167.984051648058
146.392657608076
C11orf34 132.365002835934 137.188849160569 134.845202154446
143.62103263895 132.48841450242 142.819669471009 128.368669716591

136.565191271943 126.464893786922 127.7962242731 123.081299769162
137.567384694894
C11orf35 144.896960245309 117.990622156287 125.40832206502
140.216148769999 202.193338797533 150.031863363697 144.122748003206
142.671190279415 140.960907353937 138.259512736404 171.826623289946
150.144977373708
C11orf36 118.836387680022 139.086498524774 135.51506573405
135.479300698192 130.603837917984 131.575356402693 144.120106441052
132.539585717666 131.597937570173 125.493004972076 136.566588903194
131.18368961955
C11orf37 107.976233194926 112.590379042108 128.251087452744
126.086991456747 127.442587366678 119.894737403889 123.281489592543
104.977794653504 126.503135702506 133.138744413911 129.55573546861
126.115828232308
C11orf39 127.560308850342 128.077692981235 143.532014058535
129.201101445773 131.423918669689 132.081286801266 158.76356962428
131.87052926802 145.017264740722 113.083678884076 125.815256068004
148.821942578396
C11orf40 140.702685210277 130.069789939568 133.864612785572
128.972276851419 131.354275252104 129.758303592589 122.300069045533
155.386226046894 141.478253058549 125.358906118533 114.953328013917
133.841519663164
C11orf41 143.258488502799 135.095095470362 143.066552389513
150.215811815246 147.856285290748 160.778296470857 146.632312874102
160.014635964846 161.072844903925 165.436881839632 225.438177833064
160.584760930779
C11orf42 112.666812944192 114.41737808857 120.585506729733
120.806180547422 120.693808603805 120.514672572565 118.631771242606
114.441223528256 119.168045349254 117.470731811595 137.7872481668
119.717408713154
C11orf44 123.654787271088 134.332550221597 126.520909083643
127.323157914914 135.306127055224 129.151767489346 136.580363776109
134.28095064513 128.099484678519 124.469415593643 132.514706165018
128.075865243385
C11orf45 127.56862018147 119.427342766092 123.692366307662
113.734280863323 130.672586991186 113.209569103727 109.924867620862
129.161305053472 116.046983032621 116.678969343492 141.112047881014
136.879615833565
C11orf46 314.292700455545 287.656436278551 415.322560616847
278.737944234055 209.229537873706 280.337475979102 258.21341068758
320.979443123471 314.221257518059 293.600165830839 255.71113398292
203.436527043293
C11orf47 149.160808104789 150.003399000079 155.851264092344
135.399032687551 137.243175880462 141.865758685796 135.807494668545
137.220718873614 137.127587786536 138.609972011765 152.961475450751
147.893916750136
C11orf48 282.963958072528 296.088938872643 296.24781576525
310.142754559096 827.157920721418 667.693216401836 718.670564185576
718.670564185576 539.125423322738 422.688531130043 387.711471687237
616.523853569261
C11orf49 148.73620012231 158.181316170382 157.58224826673
153.339992195184 159.79463502085 163.081050585097 190.002630382394
163.153676643372 169.598493862848 168.919352453652 165.411052799773
163.899808733609

C11orf51   348.300300174924 326.908959531021 445.95114929414
      435.69811966429 394.101052511043 472.405334652317 257.813193543819
      280.337475979102 277.101000223971 303.423450627377 224.008253365957
      405.469705787631
C11orf52   157.130045415943 146.934990457666 152.857278986877
      144.143692745479 231.572677616684 150.417169156755 230.332652534446
      211.595513743111 159.443697452694 157.838771553729 170.05799929575
      186.679199600379
C11orf53   127.476819912992 123.513588004371 125.5449326311
      129.856994052591 144.540174041867 126.222105816959 114.364953312191
      126.209426865327 125.301602026407 117.503848306325 128.966514855818
      129.140978620864
C11orf54   301.638918685657 300.985037429772 382.744523022686
      302.809011218879 225.783771938969 257.44230597145 240.511797905018
      285.172001915966 252.919704128539 235.795627549799 380.754569467715
      237.601064742453
C11orf55   136.824357247954 131.074021609161 141.016596301267
      131.859204862396 132.287326702468 143.258488502799 141.137459291428
      135.208478510375 140.21908493941 125.431735524937 140.983230581181
      131.298129667973
C11orf57   292.693830444839 281.405355165228 356.413190087706
      301.955019831714 254.235762677639 293.281098275134 314.095043876244
      288.88486628661 281.740495452842 250.542405852865 380.888728347647
      283.416246133873
C11orf58   281.278843600876 251.546290552828 332.749676505218
      233.272000923172 194.212177449077 287.145105895235 228.542423012958
      225.095016600703 200.302384864371 241.917371866481 231.769926214655
      188.780089746036
C11orf59   662.213423011533 758.863418917419 684.866405921987
      860.688314783077 2307.82327595071 1147.33034082693 1247.02514044778
      953.549801897112 822.88742008931 1096.10636044885 1128.60314361459
      1159.44907165386
C11orf60   200.22145130696 231.517412929077 217.86728342043
      203.852316712377 272.41397350779 136.415734370763 189.205724390913
      173.353648927895 195.963361515546 174.991941844263 211.720536220806
      173.879952136931
C11orf61   192.789886894821 182.693963066543 213.602504580364
      188.118989395838 159.317429821638 150.166324293431 162.756185479013
      186.640167849568 182.410688592378 164.191906588579 208.080089376299
      197.262062021667
C11orf63   348.457808063307 316.237519859859 298.979106293393
      293.12968694683 173.640326140494 278.687780705367 210.852300463808
      249.590923098575 220.661922683143 226.221973502783 317.839512360245
      217.811099067262
C11orf64   142.907969322845 128.011387527117 157.692519370734
      148.577336081778 144.476157581998 141.145419751161 136.959011188915
      133.830860948014 143.437546532782 142.914863449833 123.591078216224
      154.645353009618
C11orf65   121.322817570375 122.35832958054 110.562310620515
      117.889524670597 126.617737577634 109.680532131035 121.723964989551
      124.22833964472 119.062611098235 117.445284668654 138.909419415137
      125.596067857941
C11orf66   123.777133327569 124.387133895009 122.232732968363
      121.508233749444 118.677203713024 126.471321085803 128.907618218883

127.527378949803 128.348910964448 128.578494940165 149.712320986646 108.830525786872
C11orf67 725.216684507759 636.970243888917 1164.61390835016 611.088604425595 275.696829424061 439.33566689752 622.561634660589 435.69811966429 403.720036930931 614.775559038663 535.758943850734 293.17183601265
C11orf68 481.453770150269 395.386242257877 423.562598841704 609.309339417625 345.38424761963 217.148957110623 235.742017851879 226.841838641151 219.78041403077 237.396466740419 245.582279957584 226.595006724068
C11orf70 191.507657960386 175.767556472012 178.416969664816 188.268774618164 232.105867345662 151.327544435847 146.442734810495 184.873459141947 491.709723745226 159.257221624668 160.031506612501 184.557630172634
C11orf71 160.379168489216 155.147962740503 201.49630027392 179.15284184774 187.625536724681 156.768976123975 182.919385608842 151.747595672228 168.824578400555 151.418316929551 155.496190048502 167.057202756618
C11orf72 121.224389883994 124.405905157157 127.086470811408 123.725998173975 117.264555636952 116.718099363329 115.128667174745 115.834056091815 116.476747183738 120.488694213288 124.757229519631 117.392898601408
C11orf73 317.904396212359 279.413335686746 412.202049714695 263.352118305612 297.921617748468 305.200911653347 365.441289363381 320.648966489586 292.429831186609 411.221560795912 352.22815095475 424.025588636796
C11orf74 456.530337997907 429.595453454621 753.389428590366 454.030083521756 265.43897208356 230.079664410595 238.946506103155 206.010481981919 406.816482069141 377.661589835101 247.971366020963 189.61702125418
C11orf75 313.641656294638 283.123157828849 251.869781014029 335.623336587396 468.569725051459 170.243136294537 253.975577255772 639.38881853171 678.684323057327 388.573321838751 296.333944172375 300.401370666633
C11orf76 152.06044555622 143.635367691412 141.836648910979 139.62718946833 151.554352209634 149.874424976813 152.620244087653 145.245316640391 149.166635189875 170.430478598594 113.76411537952 148.85250180736
C11orf80 156.704278982517 173.761036260869 184.414609874199 150.902548360746 299.616447269111 153.273568880305 239.73396414153 284.321253087512 218.917357155289 674.905270154355 246.389203940944 239.109163320417
C11orf82 178.294972422671 173.063002566542 150.632724038845 155.07594431078 406.54518900348 349.910535555712 229.483578414375 624.825216056495 356.530530468568 271.858059712406 198.437113196596 334.377352542623
C11orf83 202.01798254763 199.579818754014 245.426639190893 232.879578820053 490.464012303239 314.845965872164 307.570405241182 210.605864685448 256.924134409998 234.661864595717 189.603621918298 231.47915227815
C11orf84 174.221939793486 191.59400638652 187.016569445646 229.835576260403 239.871536598083 211.891710626953 172.583615020042 205.765822478178 163.899808733609 160.176567985923 173.440596296333 175.744275464701

C11orf85 133.680691168661 136.01865173754 126.819413901915
117.449306773025 143.234195669431 126.760835666143 120.616979607398
126.931328660501 120.015436934157 123.681353449198 143.672118850295
128.372225487481
C11orf86 137.4552565025 136.823008283157 132.016944935305
139.792313018728 156.040073232376 136.037555727438 133.120273569229
134.703474191011 154.959217895243 140.800226245569 131.062360037777
133.526213647686
C11orf87 117.404906698431 114.638254225216 118.88525116298
117.49013401175 119.562180492359 115.927914533359 133.032905765836
122.472909598385 129.900540030251 126.462522391125 131.404001006643
123.720229113779
C11orf88 139.13396769999 131.556892859203 137.550607284001
140.947852529815 130.425809387444 143.881744063934 137.076766238637
134.033751039071 139.427216913809 146.460464282676 134.996443685544
137.994334667126
C11orf9 129.284384810812 139.229351251973 138.953926269905
123.275246170603 133.738587139139 132.014213378179 132.128418409996
133.800111596565 138.123754096875 122.20752192519 120.006982591236
165.48594766783
C11orf91 145.520832968878 142.883466086309 132.765942975939
137.747088378131 143.752697178913 141.70844587896 138.56436658053
141.725882943927 146.856133875296 134.423260547727 134.250052775987
133.860077101582
C11orf92 147.905406818238 156.774097885381 155.598586699376
142.514794686932 200.322334039458 122.516192906386 159.384480972135
143.727514442914 121.036054511761 114.342980656214 214.331491507489
103.99234480919
C12orf10 872.512754090023 933.723483173584 1282.38953612054
1206.09815575399 1488.37146754184 783.373390305885 1080.95725397659
1055.79590411506 930.25842384208 942.41077071058 699.984140389009
873.879064183441
C12orf11 253.580286414227 254.188830191845 272.666435295288
267.483886037099 198.390247605355 239.827648772906 262.389583658305
226.449705559354 230.770036998466 300.53017771156 253.327404004524
226.824313642101
C12orf12 130.807596523171 124.766436060041 139.166537591866
128.259909378964 165.654773882752 147.841960346835 142.49005761699
131.252368756892 143.144153021901 149.869314204354 121.961568081299
144.785852437305
C12orf23 373.290426793366 297.609793563225 252.733973271584
283.952604729032 175.832474809232 193.052112923573 296.141012087256
210.502556087175 236.401834189942 189.235998474945 272.117599603586
160.92643755328
C12orf24 243.622678182687 244.251315474587 271.522696252995
256.237185590901 185.661496988658 185.984938124891 182.263724979519
144.090047165489 190.787437782456 243.308625792448 150.412929574507
168.269075560636
C12orf26 283.048002376743 259.979459472217 289.037033338975
276.351810999317 224.186573847185 200.114704018645 283.123157828849
599.208370337812 302.696004448559 220.288758600588 367.567681162661
242.81187187788
C12orf27 120.988749021323 123.086596566142 133.517685144186
124.958287100852 126.129564039673 139.268497466017 126.464893786922

202.061887608293 153.332110179758 124.672483602517 128.835216648178
136.506347083926
C12orf28 133.970510683179 126.089217853289 134.32174235533
129.741644654541 118.66946078241 125.98519427755 130.467450968785
177.793860516896 144.458829225668 129.740166478951 110.093565974873
121.85247363239
C12orf29 358.732947927445 342.147414141627 402.473870771741
269.831616158113 286.310923582432 429.457884032977 303.380479700334
579.493790910006 338.866471651692 464.577839898252 303.608918161742
299.463563393179
C12orf30 258.695920967489 264.8472508692 226.919545367682
272.571092944711 196.990867962546 325.751369001105 270.92671642109
257.338950719432 280.494088228226 197.308952757609 189.434597063058
257.685284145848
C12orf31 266.252875242286 230.723142236623 368.825190022937
220.079379312889 203.487137264 1294.74864310494 299.568830444875
999.658013198849 387.711471687237 451.317592236192 386.817736004263
281.53843450593
C12orf32 240.147082836599 285.46340356157 263.509066170434
268.406778092906 386.679822751367 298.301504581296 413.398641688413
689.590642377619 414.713379367927 309.937351755502 364.022018936132
438.431160975498
C12orf33 130.92186531563 130.516632662261 130.716570410608
135.223957492606 118.912989775436 131.328478053136 127.220812636148
121.543207264598 123.033673289024 136.135223482208 131.204616536341
131.095431530473
C12orf34 147.060637536615 141.490911535087 134.534815676852
150.331324340476 176.11287574658 148.907136936731 130.810353054091
164.357373919714 146.029647001623 144.20764300349 133.611588728019
136.927274496451
C12orf35 350.392951395182 310.8725368426 382.327656839936
248.185148749493 271.064405562085 432.726040933545 402.140437036706
561.379619105609 503.967725251906 294.115163083721 359.493075382333
361.516275756449
C12orf36 141.016596301267 155.319717753912 157.328404229451
140.292132023262 181.922907273871 145.906601611338 177.32104138164
166.655585160657 142.049776668507 154.173439471404 150.534608517044
126.497472087036
C12orf37 152.581729298159 148.482530949134 138.469997677004
142.09146795308 151.004738508108 161.790909578234 150.870830294349
156.901209828741 151.81754291846 140.895053453126 161.618479762188
157.058912028958
C12orf39 122.982686879394 115.14883090249 114.504409331018
124.317870455205 112.166192839859 122.160459795959 115.933805625814
124.103321614841 113.283359026655 123.271452939936 128.576310682958
111.538651711074
C12orf4 212.460476372263 223.975677959345 251.704037951295
232.127996106421 171.216275057884 275.543576337502 203.652936507291
227.774354299962 254.235762677639 265.332196877456 232.801257700571
212.291528248457
C12orf40 137.246089511045 150.51537512828 138.02757574324
143.449303333495 138.496366637658 137.110908101966 140.709981878311
148.883263903527 141.760529549196 136.249621724093 132.028327486943
135.663734178125

C12orf41 780.105135529728 765.128667448261 840.623505494489
688.684644604218 465.29290512169 888.371107987343 749.220616713093
968.548243081111 1235.02626456572 744.225336494008 948.150767619765
811.912727084669
C12orf42 107.187035835972 106.685578858702 108.861243071977
113.680875214845 115.830631252469 105.25877906031 101.981823814699
111.820208385514 108.226917034591 110.296425545476 123.312000381122
109.978693735206
C12orf43 232.590542016298 231.39684528494 252.32907388173
263.800363357443 204.415267938007 178.780662099615 219.597808863947
204.740183832461 222.177290763736 218.529160053398 194.413759761549
184.742960975938
C12orf44 508.449904285771 541.351092873522 434.176007498693
501.166869173253 907.904780399957 467.512355593598 468.712260193746
557.868300839263 620.614820117452 454.64556253724 380.821355948507
500.343476678562
C12orf45 231.547654653783 249.489170536758 306.486275677467
279.495192828873 317.353318759931 316.237519859859 305.398934391579
462.025126897013 256.590289281076 331.164053277361 232.105867345662
309.538690289233
C12orf47 897.958748317315 959.897744425251 908.352265585374
1111.88018487186 490.631757904175 849.389818300305 922.877217016704
787.017214032692 1172.96121022579 650.679096367632 1006.24899131914
701.847905027147
C12orf48 157.5032461329 163.262893278578 141.064086298948
142.948238172973 344.298510200653 309.098519056843 240.836979012241
336.212224495877 242.752995772995 192.340135076104 192.314484368435
230.447203571825
C12orf49 132.75673287656 144.522461545204 123.654787271088
122.914680091926 217.227441947056 193.801858215025 186.127543877065
162.173506492562 247.779718065751 173.979001863717 210.165098572428
202.300293733922
C12orf5 193.858884443593 191.25724234927 218.2454853526
205.871133967588 186.077477713775 228.284236550547 222.243523614518
221.471811658459 182.851203073653 497.916883763059 249.09278446472
272.885527139137
C12orf50 123.111192189217 128.655549652719 134.169645445889
132.263954832097 126.233395051706 131.22473774325 123.632031112507
138.698178321535 124.876226351169 143.594003495859 136.353051841792
127.833653828539
C12orf51 526.625936680738 469.407481002198 550.24496459253
640.90860274227 285.172001915966 340.800632878807 390.897464546943
336.434368527549 362.65285317327 293.080122239022 380.393153331219
340.860684371038
C12orf52 266.032134528055 293.746733527346 291.444155562134
364.191263843897 669.731546951281 456.656123023446 478.606567465979
456.817057099146 453.17378995737 375.767528676937 278.651369597422
369.198837083585
C12orf53 134.992686224944 133.299599411025 116.087696503753
128.354163132392 136.470025638057 125.350567304651 129.904942554979
125.899200544217 119.613985826308 119.717408713154 144.490452173336
122.051473363557
C12orf54 145.424827607383 158.495606141226 141.631165634373
148.492328039604 141.494666823639 150.822586904738 143.08841562593

148.594322264158 139.875260384886 170.725486814469 163.704616231638
134.744175117719
C12orf55 152.68344738009 144.032779435658 139.837616209536
145.61758790738 152.459041674768 144.102872001089 140.844007965202
156.373163373309 140.689631427359 146.188193301627 144.873034900013
153.913513669361
C12orf56 125.315736755156 113.184495948936 138.645156942894
121.356731684516 210.353341004865 145.635453468646 216.929969034881
260.370285504512 244.689936990021 339.663295617139 122.531675048414
229.74418058711
C12orf57 3052.86038628748 2988.45928881468 3253.76017336684
2653.47752512393 2330.60389526686 2137.44704613378 2620.17683549005
2172.89794460273 2135.04382065446 2390.41825815872 2334.97297889593
2982.80580616089
C12orf59 136.158508521357 140.222121251146 134.624322761212
141.528115502473 144.210698143215 138.848119273081 135.931715324468
141.362627556137 133.264245969185 161.305860691151 136.978738825445
153.369334953677
C12orf60 131.17119492692 130.975606477621 142.467552395104
131.590180562108 133.975526667571 132.950480329605 133.213697351807
151.256546638599 142.815817738837 140.608266389167 147.196687432745
136.737964237109
C12orf61 117.093818506721 111.260486045985 111.722827315911
123.554769032715 122.751674866182 104.977794653504 112.699674279523
110.266032635568 110.636713168363 112.41270361848 113.799997627317
114.470670941078
C12orf62 768.67030514096 611.36406319509 828.276436883306
662.213423011533 579.678635803225 935.423045210635 843.545962045705
841.944755849006 863.738809424958 400.783945471037 922.50743596248
775.781735108886
C12orf63 127.644315207178 145.766684470184 132.283682066898
131.601823550269 159.443697452694 134.56763097313 149.151791028837
171.689722533849 147.299872426498 143.950279111815 136.907453440141
127.802645313091
C12orf64 150.093684245826 142.305484770249 132.211379936941
155.53992663898 139.59941545613 143.340685579645 141.059033576475
161.543072816738 147.064543281269 151.449690439424 143.692305478191
139.374278310041
C12orf65 287.967349876429 336.067655676466 265.402310598892
318.487401106209 246.457698183615 182.711318507496 298.827660296715
197.146035305296 283.807827536147 238.617228632505 287.967349876429
193.629237856758
C12orf66 140.357427912361 136.625136565562 145.437544062449
140.127379332918 244.440857688518 143.279331050175 151.403431105471
350.22221782412 173.109461200603 131.856313046545 185.295480519718
147.305679046765
C12orf67 158.445124076906 130.619624347751 128.597272557033
143.351341716363 145.223648967115 148.16526158216 150.021512237553
156.852187451794 130.394687030464 151.981708806765 136.841506794489
121.863062638551
C12orf68 174.02138265758 183.879114762264 151.449690439424
189.472541628897 140.775447163954 136.087702679172 145.754504160436
146.882746173951 143.616189776965 155.162310001164 177.039190810529
167.07092218037

C12orf69 144.428776560885 139.715836473047 132.43999768718
143.635367691412 131.49254872524 145.070927449969 146.361128670362
140.983230581181 132.737464610403 128.521380879209 147.872625576731
151.663203772586
C12orf71 146.944628763232 141.378381097266 144.270828498856
143.125454748996 134.387256336429 149.000760379976 150.998881139061
141.474583706011 144.89062965996 141.653917856148 128.539969915702
135.400054238597
C12orf72 115.507474401239 111.867464669155 111.366283149236
119.998260435095 111.196610704726 115.420810993676 119.112063328364
112.133567992833 112.14768481333 119.590540166384 118.740346254406
118.9428715545
C12orf73 151.946691918521 166.515199732076 160.459100056689
156.589829166493 156.224769056915 169.800006803362 145.987228759402
161.678137139371 185.31769184805 169.246302492166 156.61494867164
167.063762404009
C12orf74 144.421572284568 122.251178920649 128.381433897602
131.880438747579 136.384308041001 141.947478902603 152.808771946509
131.318472643519 138.657241641263 128.72727523654 129.368045611894
129.623601211364
C12orf76 192.992546726522 179.663258610881 213.531307836195
188.818012746123 203.983168778488 241.944581086483 244.689936990021
195.630643168176 268.148673257455 243.936797286495 205.781486117242
260.489744360233
C12orf77 163.474261351901 154.229914729772 161.988082494464
194.072260528037 147.75625228531 152.972779110762 155.851264092344
152.371569700039 146.416318806523 163.926432479673 165.634117939571
176.245698601389
C12orf8 146.983646662811 121.483610947845 130.35985690876
132.450954427876 123.977467396003 127.019978195283 127.337833551369
136.142380560596 139.22543724535 124.042153387391 119.899649497032
134.064206964444
C13orf1 291.105673222859 229.373686092654 326.83982672544
230.546762514966 216.209052148435 311.337303524912 184.577511730401
214.100698312272 236.082185670774 386.598037190663 216.615694348945
365.362177671146
C13orf15 837.413261433363 742.751664201272 657.231047829537
539.720389702148 431.413030534188 1854.11328962023 628.710901107436
947.425912541091 821.020339593525 1701.43479420427 569.133295913677
619.045921382959
C13orf16 144.116298946718 146.351622020606 142.153830345183
152.20173979854 138.393565473857 140.593510265137 141.359142214228
167.536828038178 140.687016530974 151.27882940428 133.254663058352
151.305201022012
C13orf18 160.766121558583 158.221595000183 144.329979284786
143.21411610625 152.778732614944 151.064422836695 139.404820141366
137.902953501634 176.680708339236 145.799530970042 148.145136066071
141.294094833197
C13orf23 195.711047893396 195.438353051306 184.557630172634
171.835438335216 213.028568789661 387.068276379114 246.912159676595
270.33685210971 362.445813697676 251.511405838796 315.130912368929
405.409668978569
C13orf25 153.681775929182 142.41737138634 166.872332652008
164.288645768576 180.610891208146 184.157941055948 186.57665470145

208.363905749882 172.387657419201 173.577723566555 171.410912120195
187.216245895253
C13orf26 149.255405438966 149.52880714343 150.098708715617
155.578524031078 169.631386764054 164.801505601099 146.493927564338
155.410704148925 162.10411532202 151.092066533546 139.128751917566
152.6969498938
C13orf27 240.663883443935 219.151144724168 273.75192868583
220.362946606131 314.845965872164 444.584594376357 290.184801898828
354.067771806542 378.24336892367 317.206754773681 216.559563381808
264.306927596371
C13orf28 120.853961156534 104.896224175106 115.827175143972
121.47058227778 110.437728036347 109.495342943278 118.500956349352
108.970432124412 117.203783530266 122.180428703208 122.176811388389
124.381674909742
C13orf29 156.798431595608 142.719216118914 143.512348693556
140.083495854322 152.56002779985 156.65842876856 147.274945970072
146.40033464957 161.543072816738 160.156127781701 141.980728956046
170.007845523415
C13orf3 129.89329138222 123.601413134287 137.101280421516
139.871928127715 187.389039221193 163.245806772716 152.987939791009
206.027235767232 183.201926323188 167.093409692227 177.84188738734
206.650760711745
C13orf30 113.214390213004 107.07609243233 117.49013401175
114.45792163942 114.062099086818 115.725323816619 106.772581387455
116.871350997346 110.707755436868 106.701716475947 106.41329572636
113.529619135543
C13orf31 250.443147156029 201.415695076278 199.555406967021
213.602504580364 183.416437587938 178.200678423426 180.008152341531
175.79375235478 192.314484368435 232.986366625889 166.879042308539
237.01460308334
C13orf33 231.146155346291 198.597176657619 156.502051001988
260.888612454097 137.609831343223 149.41564356447 133.627389970495
138.936872956065 142.046082566177 140.279222720709 145.466538006138
139.161483596756
C13orf34 176.940723174814 171.714973501563 166.433933349193
178.863722055499 411.285747588622 408.263886786123 268.832884350157
314.063025950327 317.659548748735 255.493109705211 230.469548500304
364.597798667541
C13orf35 128.202619705343 122.287535209629 129.165440930558
127.036976462046 122.243585162452 125.060387518072 126.199219102601
121.952255635669 122.733870240098 122.142323014287 140.518206716196
134.253399256648
C13orf36 142.170705049763 136.835889180879 137.091932621041
140.857793157932 129.818020166075 141.521742442341 134.719531559829
144.018198451676 136.897888440409 138.361149460093 152.368485416992
149.742140955184
C13orf37 167.500410857353 161.866405026758 189.283071231248
177.60876237572 320.597495751519 367.229293432041 355.290621188435
380.664528809678 456.038981546175 391.500696444149 261.89195025254
645.806954296487
C13orf39 109.384112020063 112.509731206295 116.300028266671
113.913337223716 109.398997827329 107.02909139647 109.087574814292
108.79453730066 102.166449349339 115.507474401239 123.089110282465
109.932577444858

C13orf7 279.000687630846 290.389345090634 276.224613560582
242.871915060884 204.183472655276 327.062810389635 220.487665103019
243.95334982553 246.288681426405 218.615551704188 288.436880408624
283.46492196692
C14orf1 112.476111928742 120.688909943167 124.528206512938
121.843992693814 125.426018910517 121.583096465567 127.485734862624
108.101686347192 111.826509343353 117.034228068173 135.467354817201
112.977469350139
C14orf100 302.272304483941 310.598930734244 312.286788541158
263.183010631644 339.32415558339 579.318912497928 487.19625805337
365.82705202869 337.585502787016 494.347792360629 411.039234404499
565.509446660206
C14orf101 163.066051106092 171.795878750365 178.606255846041
175.38376293575 173.995889755792 245.489266602897 188.36361073603
200.468804559127 166.214167530158 148.562200783472 189.461090469712
202.129153259445
C14orf102 549.692164962653 644.449861831749 483.549698382115
654.663838918786 252.280435525505 324.009703043876 413.172954892025
356.770158430601 279.202256032476 380.664528809678 447.727229248315
440.404188514231
C14orf104 168.03501792841 163.059196519365 171.202766078337
144.767233774942 325.96632528242 186.231346659144 233.953994677015
295.571900619972 205.08372943071 205.45079491218 227.774354299962
240.511797905018
C14orf105 152.011551837951 140.78902818186 141.640292723776
141.299677389827 140.597877909094 143.446317006075 150.99622088333
261.322374889497 249.292766428035 138.221749464651 211.679799388351
133.887927451362
C14orf106 292.366934378643 372.255208814622 262.629961694573
307.570405241182 414.713379367927 586.867772838753 406.816482069141
628.000860165888 435.901794650816 341.901565692251 448.371946119074
502.731019454502
C14orf109 354.705553237406 317.309949792707 530.267835989435
306.716682293251 409.477576085245 366.073387075385 385.522443482
466.909037277853 378.862741410359 448.767690095033 401.750302347978
586.366632966978
C14orf11 139.535490288426 148.549257493128 126.285743744314
124.3501719829 153.432933930805 118.410091682499 127.137447808551
128.816610738922 139.289968250444 144.456324230938 190.645917992472
135.938590242744
C14orf112 524.855915371246 583.697431061521 574.262112643403
448.54309632232 1436.57771711084 851.221199352646 1098.78649254838
1109.79571066685 874.816697884109 1184.88559645917 976.050097371579
1375.76915021882
C14orf115 143.338628928675 153.545086287929 159.710615852435
143.662865541299 134.674554546711 142.122556679441 149.818888178632
136.986022901377 150.279529635193 164.063104872636 144.562038493733
142.394644827434
C14orf118 143.307838525991 136.015561289357 134.04235176608
127.941584184869 127.336648342283 138.9878987332 141.168198348093
146.861981788662 124.944563166658 133.717451195844 145.07503221134
146.129805607031
C14orf119 132.926799887017 148.911306526869 133.820936825552
143.080742311914 111.886543737378 120.524859681034 122.333765477428

113.78323485587 113.773684331489 123.25201993575 131.081793789906
130.124601660028
C14orf121 178.665552649698 165.500088159872 153.136863548105
190.76537287329 166.70212615715 157.560117350562 162.728630453745
151.538112896642 157.240466990977 179.601348561497 167.194306682927
165.638672456873
C14orf124 149.541917326637 153.776361241587 189.49408026215
147.036768856136 160.752581271996 156.783896946556 198.753297290444
177.457362387052 160.073510173041 155.091135297162 157.446725405803
220.772387104223
C14orf125 163.422877959747 167.645930148254 146.582858169664
160.72962443421 168.890506147594 147.949560045212 154.623548525522
171.504939477949 154.462766419353 152.520828479026 144.361871207081
141.037217406848
C14orf126 141.901382169334 148.272252953881 150.751386127903
134.614988219821 154.826710617351 162.013164369593 141.735123971775
160.469645948225 145.114382599368 157.419333185365 154.449058407217
146.025781495177
C14orf128 155.263513633703 144.4421922847   151.619329363527
147.983937675946 146.227452269648 148.454344601562 148.919726992353
132.402255281009 143.26896117546 132.956860864455 160.290104492275
141.513636348978
C14orf129 241.203521822269 225.238369884803 296.75326294587
226.144012966486 259.472319994929 313.18989508207 284.195575802966
312.339824919124 265.110264596425 441.435444003241 251.665539319612
400.845137156678
C14orf131 350.983969007868 410.138738410101 401.840794789616
458.607367472574 327.749926183823 319.560819487616 441.001837510132
554.800688991977 348.98762709818 504.275299381356 386.960778432157
562.711562649704
C14orf132 594.713185954954 482.466736153026 408.60092780174
430.833137328233 191.912670416334 189.678680702271 150.029313080668
165.731179108649 192.399146702036 176.256840061919 160.240052678401
161.22761502968
C14orf133 162.786351190659 182.67941458824 203.943245360496
165.504532455205 221.796935051107 175.638328370133 213.097664319741
224.981439707657 195.269099451599 200.603836348642 235.063474370925
220.010399552402
C14orf135 459.388390392518 429.096075419528 450.688800547981
411.71606840872 230.855806094267 315.565349831797 363.150472154973
346.635777358159 308.088967616175 505.302263971007 386.598037190663
410.533157400023
C14orf138 202.583967728957 182.32514927211 183.04531185589
166.540134051424 150.191645194625 175.908645445479 196.819022688128
193.641927801805 173.850390626782 220.330477277988 257.941367816557
220.113261580385
C14orf139 164.608496326181 155.235505614649 140.911080286243
171.36717256867 131.910285685889 132.110906510601 131.373846564542
133.249792744053 147.108661317945 130.049854901235 151.201263631883
124.197397333928
C14orf142 232.151020756625 232.879578820053 240.497413647633
218.811490883852 251.61451602376 257.643855420381 208.080089376299
345.433025456954 294.115163083721 248.643311972844 274.047586063773
249.129943906228

C14orf143 185.507663455634 180.008152341531 184.451469623426
185.027902485379 258.4424008891   195.447966116819 227.491667075987
312.224309341171 202.643624039869 256.590289281076 211.505504834207
218.997165156081
C14orf144 147.49720952988 144.064214573005 135.544048708523
139.706279455371 144.665235198936 141.630183595813 136.649743237542
150.893280574091 136.373761435722 153.46645509759 131.221105247771
162.683665945044
C14orf145 178.43642939806 163.799748670842 178.270849389719
199.947823264085 181.530767521222 147.743331160624 158.528301072565
174.615694779386 173.829219279719 155.114892891737 149.188357651896
145.843639385175
C14orf147 448.715938510132 423.966625351913 417.528353577952
385.678901612324 235.32095278846 559.236012019639 337.077153882632
246.748589597262 246.492735271771 633.401624753368 309.538690289233
325.320888625297
C14orf148 118.757973008463 127.824005374202 117.264555636952
121.961568081299 115.718600552187 131.074021609161 118.294324274616
123.21112178671 116.95195122318 118.419311800417 130.792692240592
117.642441794442
C14orf149 235.32095278846 192.022864170934 234.094722315007
226.25694727283 190.825209733575 181.294497402507 151.308872564827
169.87591929982 154.821960325589 224.547982731402 166.027988561421
175.269675831824
C14orf153 4198.61471038267 4016.35381420598 4175.70090899755
3581.36347643133 553.757847979529 3481.90072759183 1609.54149931375
2542.44286642873 1984.47173096657 1526.66232186013 2328.65814406636
1948.24432908972
C14orf156 3046.47354587949 3140.51157972795 4166.43837922103
3294.92439159154 6585.34962362923 4150.8511719558 3850.41246770812
6810.50193989514 4866.98027226798 5195.2087608308 4602.81651147558
6021.68798558746
C14orf159 975.685771692095 1014.23822508445 1327.65573168233
1414.95226752155 367.983166440977 274.047586063773 613.279465441319
258.695920967489 429.595453454621 321.177982468322 1021.25947613827
715.039905439621
C14orf162 147.405050592353 147.975551540256 165.430811083128
143.537785553911 146.770267005869 147.995991912855 146.871145679651
132.637443692683 148.660440219589 144.997250967317 139.127442770828
133.284873295487
C14orf165 148.029259633449 137.185243517766 126.766964875664
157.6790197503   134.501410126169 142.440344960504 146.431166706908
121.049927973123 137.473779155388 139.654648891206 143.574584852426
130.198147836925
C14orf166 1065.36715400306 1104.61581891596 1482.15461429206
1133.87328758676 1379.95968831696 1013.03054571763 1680.25851465727
1856.05025952611 1028.80076860235 1782.2498613913 1466.08446530283
1600.10235468018
C14orf166B 128.84676695859 128.344679872625 129.289216846128
126.368170959337 136.532168520039 129.466612376702 137.089392202166
124.682396766302 132.213890220705 124.602474178438 139.276102596591
126.88066987372
C14orf167 264.306927596371 274.427235605909 269.62298406345
274.092048989484 263.321559283966 301.591430919567 252.056131165397

235.795627549799 239.050379187643 227.050629701762 326.354469134694 372.594990024312
C14orf169 283.874600697211 292.037861000784 248.562843471699 310.465070184707 412.801213309591 215.167086358143 276.582734590058 375.856200488667 207.204293555178 250.443147156029 335.239364345057 313.077481033093
C14orf173 673.502723625773 928.151632070573 721.31849915135 1165.05055932899 2072.01578063404 628.939201051801 470.42403724799 761.726829618132 845.369108604819 979.019125719101 1482.92498205315 1381.07447204233
C14orf174 196.470718183575 188.40383184521 192.286192329464 214.904634494921 198.583407008317 184.758754197435 238.230110356671 221.424451092316 200.443940449586 225.702629835215 201.139510041141 222.848935978648
C14orf176 130.354641020141 143.618454531431 136.557466007228 124.752198904184 140.724541696229 137.96452133771 130.516632662261 128.793186785667 128.209771391087 119.459625817735 128.108066199718 137.179058645907
C14orf177 116.388079393669 110.562310620515 115.417648857825 109.29279076901 108.497209666203 121.843992693814 113.845770994796 116.647291878845 105.771723307783 111.577400298092 126.741498575874 113.963798749897
C14orf178 131.081793789906 136.739754726714 136.708746582636 146.069543494352 135.229197100212 142.351705613006 145.915129713165 133.411087000196 129.43892498257 126.083599163604 124.22833964472 129.580421464913
C14orf179 705.737228226014 616.014975366684 735.449040059761 763.263858769038 435.901794650816 422.23268864343 485.576566972696 604.737834405874 459.388390392518 406.028724866233 535.20662670885 558.621429366984
C14orf180 126.494665743087 159.504327477065 147.77916610545 148.030579593944 136.092086452096 141.294094833197 128.689465747173 134.776714949136 147.515088047628 140.946008063839 143.25481667885 142.234554325754
C14orf181 119.83679981791 133.203064891923 134.642898234003 130.368933537243 154.201375471487 138.737332306848 136.131453703981 139.782152941027 140.330400168187 127.928195199945 171.444558905575 148.81406900745
C14orf182 141.992490695142 145.395883026207 142.798248249144 136.86685787001 135.368203841678 141.560424984281 135.897498591391 147.915337223934 126.484819755399 145.112399373761 131.747367613225 147.638057399165
C14orf183 122.841135364088 108.404733392914 114.498610080481 116.780639688465 102.786517901308 104.714872508838 112.943630133093 112.851805868467 114.528407572496 106.393497890486 116.87586240208 105.712302595131
C14orf184 142.637421204193 135.244303669342 137.613233533453 150.476228905372 169.16155608071 165.607095120227 164.794626785115 158.224122084011 140.110258415883 161.825134481279 139.955389033983 148.361088792223
C14orf19 166.360005474414 179.996590340102 214.86995782548 143.896324094984 144.904945833116 160.399059317919 215.673878830563 154.088770236213 150.927628184034 179.060189557496 274.736124442229 181.54273463264

C14orf2   412.941907828617 386.817736004263 489.118864124059
      501.005898087825 690.246312542011 416.546844844021 509.01330998156
      476.917496749381 421.836020491624 398.796188440256 512.467731561858
      893.676600121113
C14orf21   151.66733314947 143.197131668608 141.321547895734
      151.971337856271 178.696562301918 149.615585907229 181.966110264509
      168.37043566813 137.170666175895 174.027190728107 166.076692502428
      155.358918578397
C14orf23   135.268963938932 122.777544465711 148.562200783472
      129.677874035481 127.966883271903 136.173596381385 127.625252349231
      137.639342727211 126.512613967438 133.233826283531 135.351344868436
      127.476819912992
C14orf28   449.103432410618 448.54309632232 594.38228123675
      481.630922092112 205.742386129597 214.662648581622 244.766289564524
      224.824362819477 184.085616342099 173.182013165174 190.057030596611
      217.969738417693
C14orf32   565.313436777992 461.581199513891 485.309521583972
      424.665056900526 334.772648743153 982.367000148189 383.182507425579
      591.763423878737 439.207122335648 381.4054004349   452.167674725454
      599.52440136406
C14orf37   168.854819201403 164.820007934887 169.927157878764
      168.295044843056 192.737713851863 166.88833169342 143.336538157639
      221.889331385023 195.353512458837 167.871703133182 172.961115357569
      199.01458214661
C14orf38   118.095936088242 115.409568875238 141.568970008164
      120.035437167539 118.173752075833 118.26427946535 122.232732968363
      130.808911605271 134.449083873514 128.83022039037 118.764650384561
      119.387459594521
C14orf39   134.683553037185 133.832462719784 142.669207969715
      133.711821318079 121.239461514704 132.610618885717 135.028746262383
      122.494779505958 134.128061214081 122.010855318111 132.584454429269
      137.669753723768
C14orf4   2730.50913686613 3099.71798305764 1890.86868383409
      4353.66854914351 4246.19983955771 1573.03248861784 1245.47421607111
      1416.93831202323 862.669120338424 705.737228226014 2012.36542989953
      1145.35733270036
C14orf43   328.14373061894 325.320888625297 292.839392578318
      328.269845211173 211.22652563815 205.679992893049 207.298994298453
      200.633009710701 183.779065928447 277.459328083162 234.063633889254
      246.492735271771
C14orf45   193.472317111841 185.432587490792 239.924227955313
      189.816900281053 167.976680048356 141.939368369872 148.967232828819
      199.305626149918 150.993910190626 151.102431192559 161.072844903925
      147.051399102374
C14orf48   133.617501101389 133.602870982718 129.859331207247
      126.780096523484 127.723052839953 136.520811697544 123.096995744616
      130.513071750123 129.594440309419 124.802639338573 155.683727621475
      133.369314179341
C14orf49   130.887769728442 119.819082491115 117.611606270124
      125.301602026407 122.095978653755 127.369456504277 125.328704545727
      117.962188157317 121.624218243474 122.460829835477 138.829793136954
      123.467227007155
C14orf50   104.826135297161 118.122186617611 110.804248212067
      112.488125161387 108.362609680556 118.816986517698 110.093565974873

118.578205738502 114.488760619745 116.437403962939 154.552605305297 123.822571859503
C14orf53 142.390243984362 129.7137422914 138.805103589625 140.040420521312 140.02337095267 128.26423663004 145.296229550382 136.653140842194 136.570817174009 148.326282600684 136.959011188915 137.801899270172
C14orf56 121.774697402171 133.281505040209 133.816601264067 120.006982591236 120.93965087814 132.596941171401 122.392159083328 115.863243613121 131.261903118177 119.890260409414 115.524441144063 110.794219964997
C14orf64 122.379537202709 125.840932898827 123.29908883119 141.949098898957 137.803749343493 120.379393989882 142.729945734027 119.842470751496 131.17119492692 127.419227292862 114.421383025971 124.735542880811
C14orf65 131.617027772841 118.192302821514 124.443643568527 130.559515534303 126.941650500751 127.524310543779 134.011746666371 125.596067857941 130.316282003711 122.629146433216 129.763063900493 128.207440197765
C14orf68 139.782152941027 143.403396831929 130.609596267168 168.323141675824 143.442361602626 141.442825487243 161.115842628581 164.332073056902 163.552608805894 178.680902599163 144.570725886413 143.250386570155
C14orf70 136.065134838221 136.489566741917 136.995197143668 134.514251897045 123.629279569085 135.122770943076 159.874161198304 145.852430082228 139.067516394141 138.846543789882 126.893023101449 136.381593416533
C14orf72 173.109461200603 161.299920355406 165.055636629118 179.753400474369 174.775676782795 189.840366340622 176.465559710285 181.003812824014 189.887788314879 177.235586767256 172.722177339418 182.644319628554
C14orf73 138.8603234411 137.493436771898 127.130769263811 139.301435614165 135.009205742175 126.737230874367 117.847744476387 127.382266838772 131.773304233929 130.176734524308 141.453255692229 127.736712231221
C14orf78 1602.63977096867 1461.64901688125 1184.3199328099 2018.81730267601 143.080742311914 152.358795162474 145.556318940043 151.137791721752 153.776361241587 2139.42448145954 161.331326682743 154.7326161316
C14orf79 192.627266049516 214.082408290757 199.275716402358 219.597808863947 233.029655807246 173.986750195954 255.039661999732 207.368252249039 202.421990203844 229.284931844047 255.225145197422 246.112720011613
C14orf80 196.777776509371 197.443711199904 155.255279503782 166.47851613068 519.564563632951 173.044636575826 222.820391338687 388.326080754063 234.588584245938 231.955750250711 246.263259852067 284.236970342827
C14orf82 605.21685916883 507.040326298457 603.338538383128 517.993093601127 192.493439659184 494.604716660887 288.150694843614 446.393631025049 288.823376348944 341.761516859319 522.17701271254 455.313553278155
C14orf85 15851.5830405813 17141.9934540963 15704.7314387339 15851.5830405813 2116.39371296122 15463.4998494462 6691.04319670055 12022.5515181682 11112.4298269818 7406.86318549013 13276.1713779481 8918.58940386786

C14orf93 259.625943627223 346.566241319814 340.800632878807
399.071041584646 321.910803473854 221.768507175814 336.002793042439
326.908959531021 249.590923098575 265.29629764006 400.783945471037
297.228563607097
C15orf17 519.450899933049 452.357898123926 498.334266735594
456.656123023446 329.535023880129 376.124744806171 567.465201338735
340.728888109597 534.745066397679 451.380376296813 656.660582895703
653.264945856916
C15orf2 145.318539677981 152.142909854773 132.079255703621
144.425214066833 142.694748293332 151.676232556697 131.246704975084
145.82344959902 121.53632327554 144.738871562895 152.877058628512
153.492072037266
C15orf20 135.148723192316 141.137459291428 163.280786875496
144.476157581998 155.114892891737 157.697168349028 131.089811472017
134.178733595096 157.551854647702 140.16233428205 146.775403409562
172.816477996646
C15orf21 149.77596374205 151.9441336438 142.548833391567
142.135495724286 147.294408903227 148.632332840817 148.697019570015
142.431979387165 142.51308872439 140.092648878007 158.92538634742
143.967197319361
C15orf23 151.88244935494 168.594235675558 152.902458879101
162.453586139402 216.154203111101 194.921021761976 234.432954093705
211.289598823191 221.635333304767 248.613373429151 179.923966067226
233.426170535227
C15orf24 1209.56018864779 1063.5903770389 1607.52029676299
921.140932799223 943.677679525947 1315.66699943181 1675.35431076885
1115.34267967366 1721.20676889116 1713.93035637146 1204.7397215229
1656.02721259517
C15orf26 147.697973457776 147.519991961858 126.359440164601
149.772157318769 148.082026641671 154.159124458167 131.269526665478
124.873289279767 146.025781495177 135.155955441604 151.511808301993
117.995551728392
C15orf27 144.361871207081 143.349486755648 136.599305161841
146.370084152094 141.233642252649 144.316475814221 147.412608079997
137.012646671173 128.686425820835 152.729355306724 117.672645717508
139.013404849037
C15orf28 185.128661141884 202.44778603643 197.262062021667
190.348359536057 174.357336296554 229.157389498775 217.684410907748
202.369905673351 245.582279957584 174.454754564111 185.639696270808
225.702629835215
C15orf29 324.25775261173 286.587945301875 246.748589597262
275.343591424586 221.869191666931 224.963277964548 244.486311003619
249.554443204445 223.638850960288 270.604799126393 239.003718699156
230.227432807772
C15orf32 158.774910841987 133.065366727222 133.36168342199
134.323365608463 153.918462601781 150.570540340126 144.4421922847
166.360005474414 156.830156083795 149.196539425588 134.94189148503
149.634975068742
C15orf33 152.230489051339 148.16526158216 172.230528631803
158.302977853133 141.449119281888 147.284005815059 137.760712896718
150.582568022489 140.73456875677 158.053654524927 128.429007921588
167.011846920792
C15orf34 150.468392290279 140.083495854322 143.301399292539
195.795728497916 137.575688681319 150.599425944486 148.530422089937

147.270369342438 154.354658146438 156.32137627012 160.399059317919
145.540872377507
C15orf37 103.398929051742 108.9291713877 112.817015523662
112.714282429822 119.442114850181 104.544792793756 108.483284007316
116.445184223804 112.875136585606 118.81294782091 132.446406243531
116.331428876124
C15orf38 223.042575796603 214.968589907686 218.269847477919
238.461248099002 368.220523234399 164.458565398812 195.282045838381
152.8810245209 188.891032176388 272.927830312746 179.191401472241
181.247766545135
C15orf39 313.18989508207 463.298781071364 259.643957326575
658.068932688816 419.763812610671 213.602504580364 342.611951034707
222.028626209166 229.157389498775 233.192751618176 221.050093964575
231.462907786174
C15orf40 193.275162354467 201.837695818264 272.770616373687
214.968589907686 180.219813240126 211.844563155792 207.764456828813
177.073245863872 212.239485199343 189.726075394359 247.340470321453
206.446584387595
C15orf41 412.129826478453 328.14373061894 400.783945471037
322.443089373063 158.278496385543 201.593025256991 182.969563278473
210.266075382636 178.130223302924 213.791379190359 230.263678910356
189.082778234831
C15orf42 131.237720549768 158.07640654075 125.353389826276
147.205511952997 202.145180994821 166.606522485953 181.508873226705
176.735168607281 166.285052506316 147.307419859572 143.475725571943
157.768329108254
C15orf43 141.370340242063 133.775716376056 128.739313105907
129.916677638819 136.689064885984 138.414916506716 131.534097198718
128.265571409546 136.821228481217 132.86879723151 144.203716525289
140.138870925001
C15orf44 174.454754564111 168.634534857593 178.166289723365
176.300743660299 185.650862663633 237.01460308334 282.511614929917
223.429738319185 242.570588364106 217.715143869654 221.471811658459
254.393421265604
C15orf48 141.416706868124 150.674189235105 139.962307072428
131.310537960214 314.610979086485 175.251238431716 217.715143869654
294.323621186916 618.058496806334 469.540421642173 236.707875629511
191.112009690591
C15orf49 127.202039384041 135.457871301652 157.668307146391
140.201501710276 128.29486937905 140.055272520317 142.705504445895
140.232244264861 141.432461374482 140.987694585232 132.137634927459
133.150660840801
C15orf5 202.421990203844 182.384190694142 145.63870792125
148.242794464181 118.319950396126 144.20764300349 145.126091016414
123.478896502015 128.001109846227 132.978746105877 165.51742391186
127.938245853896
C15orf51 145.36249226764 135.875595614483 137.330207626732
121.583096465567 140.586497829504 127.206177583275 128.882160435252
127.189310473419 130.504023268824 128.584707181857 134.044789094272
140.829927714838
C15orf52 1731.62376348283 1796.26091584668 2084.44431604433
2828.97255380291 632.565736446505 286.310923582432 427.345809255426
306.632103708321 1226.10881264804 262.416137698884 2486.24762158293
1953.88327754946

C15orf53 160.518055978242 130.131800537628 147.79341726211
129.369968549738 131.575356402693 141.639216893245 137.070864654394
131.287142490322 134.024205981268 145.950701448912 131.556892859203
134.361064431414
C15orf54 138.446492794924 146.666759322506 127.681662711369
141.132694466739 149.387467833684 143.056098955473 143.197131668608
133.396403868645 164.558875058199 152.274957846518 174.873893094923
164.905467905433
C15orf55 120.003095000297 117.060696904303 119.184780023269
117.628564992187 114.43491928161 122.646850766733 117.428152859221
122.309041354217 109.599589420575 122.109009629395 125.5449326311
113.840813583258
C15orf56 124.362182111222 119.301746097298 119.436922168376
110.486191378104 113.077691687266 114.561836290349 123.738223146912
116.46192274217 112.464951599416 113.370762349233 116.053859681399
111.041656398985
C15orf57 403.043257175264 407.518324776453 457.130051460676
475.686805714298 273.478372861114 367.382105696814 607.126514742951
401.631377313065 439.394914369983 351.448985413528 411.160709628865
541.896444696137
C15orf58 128.116394188709 122.436106757578 136.916286605302
133.604705630582 125.227513212286 120.322292418128 131.741688265764
135.244303669342 127.43866970659 115.775525016883 154.150568177135
142.973444931373
C15orf59 318.244342364572 293.234972169244 249.171307797736
377.083379021079 172.552923333095 167.586897846525 155.23152084421
172.707821624271 163.203582580217 193.394225360968 150.476228905372
179.914070416793
C15orf60 139.51836763472 146.479009988445 138.596430973865
139.776830704087 146.781221728543 131.458411820485 136.60514429355
136.202953030465 135.271290872348 137.052507691254 148.274122690409
129.964666668421
C15orf62 164.773489952844 175.656204695951 147.100947801261
152.713528089347 147.706402141169 171.602950766191 169.777032044409
141.767311448808 152.429970809069 142.14116448528 172.376544221034
160.399059317919
C15orf63 5425.58761099475 5175.17279574945 5115.14161559446
4635.42002659719 767.861763134136 4875.22637286719 2154.00589005104
3099.71798305764 2418.66999907188 1822.14935116363 4465.66734676111
2639.60135568
C16orf10 111.568329540016 109.69466049307 123.140027043775
129.171589574238 119.469328303796 140.379874810685 137.370670726978
119.686859562393 110.753661013519 121.93549478261 122.619429537799
120.882166798648
C16orf11 150.160004473338 135.4702891855 132.744261207073
142.394644827434 140.946729014405 138.956057921718 142.745838888446
149.237422245619 144.059368898969 137.249505738539 145.043494048779
152.743680985848
C16orf13 360.39998507367 378.24336892367 396.861835715943
416.475440894731 976.782815449208 522.400055538966 609.665323786904
442.590987372759 401.542072222692 463.880793292701 357.928880740814
402.213774592085
C16orf3 123.968639555673 140.730085305185 126.51808376464
124.518818352113 129.272835188209 123.557722415957 123.225792525733

129.765440519589 114.191245210321 124.174140125309 146.629335531037 117.517649866653
C16orf30 274.371523790341 331.795832780176 170.758319048911 250.82359383789 237.01460308334 294.280238648737 139.469148541078 195.447966116819 185.639696270808 198.954254778846 205.4648759901 190.389158930835
C16orf33 558.251384152474 634.046843336128 562.089999265955 809.545702751155 1275.43768532088 907.904780399957 717.886050056576 925.656701707763 938.499337889075 698.882871686866 154.025625092599 598.287431134037
C16orf35 261.028644858795 247.407442038488 225.505978789388 294.48534582475 532.950400370668 295.153786338687 392.6758818959 339.54582149284 321.977549744483 312.903508201275 290.420307322101 281.618175110153
C16orf38 146.59366188125 136.124385992905 126.708817316134 138.166546598846 134.413962468434 131.377361030122 141.221162787167 140.914966429342 135.792966926642 129.281564491007 140.578378782903 140.903091584884
C16orf42 277.073510273425 303.005912224195 269.673122990604 381.121621815163 413.172954892025 225.189991540262 274.371523790341 242.971958493453 232.492398623889 284.638865013916 229.62164568989 259.316557834724
C16orf45 356.268960813032 371.552970811499 416.307629596091 496.991561192565 116.087696503753 127.139903623331 146.455182336561 137.20253329376 129.547309629132 133.545405737639 144.743281367797 133.775716376056
C16orf46 145.989546968117 142.826898321575 145.432667767652 153.097557560333 152.629680152219 166.825501664231 149.110300099889 144.071652839025 146.311966562883 162.100547394235 121.423030266904 142.616603901398
C16orf47 147.829502959865 141.082569618216 150.582568022489 134.397255719047 149.343930487315 153.538565948453 148.794293762142 133.654232991228 154.598520288855 156.511202150773 150.122035262873 139.969155812688
C16orf48 972.902937649492 1029.43177809276 1151.89087953837 1257.00798908017 735.8141982568 420.647394044431 486.047284379068 458.705688226305 677.503643398297 332.400086578723 702.460234202652 542.740017003369
C16orf5 204.877041314502 207.376329989942 220.098071755353 276.582734590058 149.175458427847 144.858656365799 129.360773522498 151.885992695789 146.890877830248 147.728018766726 133.233826283531 226.203994206714
C16orf50 134.361064431414 138.864844664017 156.293025312901 133.138744413911 136.364830851128 133.308383825604 144.217621961908 142.928920246687 139.178245419517 127.780567080732 143.13304460184 135.730331297139
C16orf52 162.599466021255 159.362293777146 185.405774238391 172.091884817207 168.150270765792 189.0608798771 216.24497587588 172.544504923991 176.410016510249 170.835513021958 186.831266888028 234.841813258798
C16orf53 520.962651968997 367.983166440977 471.598175637494 332.749676505218 974.544773560873 418.062540009856 635.234445318087 494.347792360629 432.088303693555 338.665115108128 256.237185590901 353.912654262209

C16orf54   162.004803385891 163.794436953711 135.039901725369
      140.504486195539 134.483162806498 157.81831741292 149.225754384299
      153.891667472876 157.013200713587 146.681183890181 157.809081184874
      152.563532872898
C16orf55   158.819151102054 146.531692460121 157.553632802599
      157.121142139431 150.263578317272 175.832474809232 165.813771791917
      133.469323412904 138.988919994625 142.959890982885 125.556444423391
      135.571239294086
C16orf56   454.133372808458 457.650982600462 432.174413144791
      560.757496975779 384.780426529733 265.028512407839 259.742175498038
      279.232290927348 278.88183549759 313.412131722189 312.129861040709
      311.375563161772
C16orf57   608.816123736392 664.809419789814 572.913714991136
      882.429823267754 1172.4103691525 742.751664201272 421.056268094661
      617.185364180303 901.197085099854 541.764013317002 1228.89116158868
      607.499206410372
C16orf58   1194.42925868786 1286.55103838368 1535.27496251381
      1373.67062432073 2055.27692947656 1130.32966481315 1509.69760873558
      1850.98311401165 1873.84654908146 1031.59140059071 1332.82617944131
      1118.28423222707
C16orf59   109.342230674869 123.807693112932 110.465934808915
      124.821581853501 444.374521095495 208.321765801227 164.458565398812
      286.376638135463 204.260800563197 185.432587490792 161.055911120534
      195.078843867124
C16orf61   1556.41573079892 1297.25632532536 1109.08288440559
      1411.90426595229 2162.46602203822 1571.87128697931 1285.02292965374
      1470.23096656138 1897.92061704124 1167.72023351098 1034.68475089075
      2096.25803773108
C16orf62   195.816317534388 195.595275594235 182.938824846376
      210.448904052352 203.071186284148 154.908975734985 131.025725443485
      154.798287249541 159.203584692924 172.591885279428 186.925138700818
      158.536178747273
C16orf63   546.755305058958 610.337659723539 614.957397883514
      470.868288641048 442.220572301795 762.8576747113   796.699879495384
      754.607039665508 554.652979820908 593.639955546556 626.857289492802
      696.475639532595
C16orf65   104.861024325857 107.187035835972 109.167312436827
      103.594872791657 103.012774960061 104.826135297161 113.740942665269
      105.450273343324 105.450273343324 108.179513318734 115.473431881597
      111.336798977475
C16orf67   143.712450441816 155.129304059037 148.868265867275
      158.795335941084 168.854819201403 182.275998840116 172.783430414523
      166.754995454382 151.102431192559 141.968949222821 152.662446724781
      175.656204695951
C16orf68   363.255517622046 437.601814037248 497.629319581786
      622.692834629555 225.850439131864 232.363656167375 253.164596735793
      249.129943906228 246.616749748028 358.732947927445 201.221839281282
      227.375773052577
C16orf69   128.014092519305 141.478253058549 120.257886394616
      130.590195477461 133.94667806086 141.784881206191 147.681723355535
      132.300508985399 141.423474583567 134.005402534879 154.64077390011
      130.043904881142
C16orf7   264.355693070937 336.70893120135 225.480366837274
      403.043257175264 317.600728351694 179.828494168009 220.047563627558

182.907444581093 253.580286414227 233.047541132048 286.506419296679
256.174286655796
C16orf70 161.390489603929 160.010501226517 175.617504025145
169.681582570965 169.095270479344 149.460660195293 179.060189557496
180.265418365803 167.850516495529 194.388383297922 183.358807104988
161.390489603929
C16orf71 109.719356131043 114.143874572202 112.943630133093
111.086170834553 113.40684693417 118.239637776941 116.576896337786
107.187035835972 108.288420807873 111.632811186988 105.712302595131
108.760780994582
C16orf72 558.051041108282 492.207833630086 596.934339406636
505.704957182936 279.26508919212 376.54010486682 412.863887464545
386.544913399837 402.473870771741 380.310795921752 475.686805714298
379.016207754533
C16orf73 131.49254872524 142.285486339143 151.243948028303
135.641355114263 157.541796722309 134.449083873514 156.763364462534
137.180793826675 137.58025011566 138.934516083124 135.5237675623
143.262288751967
C16orf74 138.792521681771 150.235026770931 138.276865331434
126.895669261503 265.754141836026 145.123008539369 287.656436278551
165.827405735044 135.68605915552 382.701467158304 282.719121652073
349.382889326613
C16orf75 217.227441947056 218.334273320319 206.010481981919
178.997098204457 677.9972761887 450.327710374141 268.698146407497
491.496499481595 474.14051635531 289.616527814137 340.366016191232
427.766660764042
C16orf78 135.400054238597 154.218967720305 149.628421263062
142.85695042665 170.099045774947 144.013615296685 142.274440538785
143.481112776388 132.268445799639 143.331929080227 120.816130267055
155.615655460277
C16orf79 184.591789011195 195.645181575647 182.066922756289
176.364184489318 163.769959129611 214.546528079149 130.085138325818
279.879377933144 177.143397572907 133.78629612147 180.968659614238
190.809584998617
C16orf80 474.917507160629 488.505191823403 423.740836541835
542.009873308344 772.399017166118 569.353661326724 517.529147171876
495.438672949441 733.195136251155 545.566596665916 558.621429366984
664.60530723006
C16orf81 141.778060508776 122.716563069312 144.662545137915
149.437212090587 125.587057468028 140.52059487958 145.886420087036
139.449895159273 145.783320434143 129.294026995663 135.182524652868
137.047288508371
C16orf82 112.10312035128 120.031976318697 125.753852031496
119.152881350581 112.046615825307 113.125349503734 97.8058879269165
115.981530322377 113.268379270385 114.673517800886 132.227561632637
110.003750007057
C16orf85 160.584760930779 156.053517438255 152.805525883571
160.543465620248 148.317958829244 152.689610196981 159.784369379077
156.043994260248 162.994537815938 142.285486339143 136.378203924435
144.302925948596
C16orf86 173.979001863717 188.39062571513 206.68694753419
216.903719298575 173.306283668215 133.480196904786 117.312094804205
132.5377752987 130.304743083321 123.609527892227 178.872635061826
124.657687121111

C16orf87   212.836093887552 195.50039162477 262.954856857756
196.136080427522 226.35854874278 488.14606875418 349.722963659175
353.621264641814 755.79669622705 297.176755638159 524.725307261999
335.858047332641
C16orf88   127.213640193364 127.675206134992 122.333765477428
124.342911165267 148.069725041021 126.809252651352 153.989839987975
168.942165573542 141.722817732641 132.891375982227 161.891809608511
152.599052194143
C16orf89   179.55905250703 165.919497521143 121.807661901776
121.965131419551 122.561285351587 125.141185839817 115.734230176652
120.235652849894 130.0140921487   122.002822875994 144.013615296685
115.834056091815
C16orf90   138.371873773284 143.331929080227 144.794806650252
149.078664825533 142.167918879613 140.165450892713 140.490023670841
140.245786278057 153.217287503221 136.837430157894 138.573829193205
138.173547382901
C16orf91   228.499052416183 267.069665000047 219.814706416471
273.478372861114 357.672950588403 307.570405241182 317.121202656311
298.519371102278 304.111808763793 302.532985701607 341.096072280715
422.840163973614
C16orf93   170.390200730789 164.276668889213 169.306573880401
166.104915784875 280.246711704036 343.617417488962 295.241644408321
407.518324776453 331.251075774227 195.400963839097 217.646777099512
302.362619893594
C17orf100   169.999692055871 173.959556466614 170.05799929575
157.227118680396 193.148369507405 196.536086443884 193.641927801805
184.278213908263 178.320732186309 181.832218001701 241.527811817146
185.165469121763
C17orf101   210.030846572444 203.87884605779 234.020419732787
214.991170762327 255.374689750672 235.216589534371 302.788940260961
221.390817970859 217.670330817769 205.852731236069 215.40037757604
323.51769984082
C17orf102   131.085965122946 149.863063160222 155.942609847585
148.003041725027 141.196795654442 150.547096071626 154.402315635227
150.301540078433 151.144521344129 154.445840992357 141.739299634007
148.194450105682
C17orf103   140.554684822646 121.197702744078 135.518752716008
139.578603805852 161.657344146676 120.733009784454 128.736591902501
137.790718305537 140.895053453126 157.841520434044 116.89487516867
126.511314417328
C17orf106   150.364511432468 159.7197697044   126.230887413874
159.49930229951 241.494710905339 183.779065928447 211.289598823191
180.096239155058 189.250846546817 187.978627462391 185.765000029242
231.915272112361
C17orf108   172.591885279428 163.422877959747 153.075594260208
213.484986945295 157.58224826673 161.393953325115 132.407736899761
168.6016897585   150.302621632309 145.621384219688 175.28261759639
149.437212090587
C17orf28   155.319717753912 151.905534823403 139.159510392614
169.238325933605 459.53652431464 301.695991929957 295.153786338687
284.13865992802 348.36178568457 155.283003653612 275.169415154893
302.905840432998
C17orf37   166.430881793156 179.191401472241 144.023798119335
167.373416675264 192.353768499582 176.689683169567 197.72068061775

397.449905380349 211.183499580683 190.22021453133 194.60309399944
204.057019122032
C17orf38 174.491551019128 139.730999352036 146.96155692518
136.570817174009 157.278791893435 142.505050582588 169.661107080542
139.687574432387 148.22974445263 164.472980733552 139.991780068793
152.524106827617
C17orf39 249.449151040445 210.620764703468 333.822167152866
263.426870776148 174.749832388791 187.057683141644 175.882911801581
189.53945868612 3180.667897257797 199.118281632221 190.993590849624
183.294526267593
C17orf41 157.180095329946 164.429483677715 152.755535408382
163.487737425232 175.543924200429 178.676812740925 163.040823730286
190.56172211779 162.531630234824 163.099009810658 153.097557560333
164.302998010829
C17orf42 184.724071110756 186.733394375839 215.365809536522
164.811120992602 277.75958501062 199.149959995133 236.899421827427
277.256341592701 245.811312606484 207.902853533132 230.447203571825
234.339913501761
C17orf44 188.580458613357 195.20398666139 150.663062131717
196.429685815196 167.049197726715 190.787437782456 248.791254839869
206.73292907905 221.594737497504 188.242091666446 261.721726664029
234.550692915716
C17orf45 4477.54806526552 4367.38154535901 5175.17279574945
3304.82581034038 2728.04758874748 1967.82064011967 4246.19983955771
2915.72823797537 2171.00457877604 5437.78386044638 9514.74328718935
4836.4008757421
C17orf46 148.197029306137 148.361088792223 128.257292171015
132.081286801266 128.194171809998 137.981446468611 140.238423894192
134.354215877283 130.290454258898 137.141081365469 145.599152743156
129.278833426752
C17orf47 155.187900101727 148.751755543817 143.488761023636
156.94531513373 144.935571704834 141.497125111619 139.734784859495
143.974551393951 127.344210958898 148.149216812602 157.765771942689
133.413325516456
C17orf48 230.770036998466 179.46936343225 169.022351348819
158.396149470397 138.13987378399 157.878110410517 154.108689872903
167.817232948801 159.040409182674 178.329946332806 172.836730741407
139.361120875198
C17orf49 396.988248940183 397.449905380349 398.423396126075
544.956712516196 948.150767619765 817.529344685717 555.758485277905
782.448692528135 553.615252472149 565.509446660206 611.36406319509
475.927798702033
C17orf50 149.589901372799 139.223480216048 157.016659994185
142.25795237339 153.336395746742 150.500676225569 140.356164189622
130.266601299194 153.875760529011 173.364586678954 152.957297648595
148.029259633449
C17orf51 150.878323882503 148.905860943967 153.734347607222
153.723169560972 148.16526158216 142.596113491002 144.964084792568
145.463105638655 151.698385134218 142.763157232147 139.334552725255
145.660417208595
C17orf53 142.175432631861 193.912456093767 162.122250419992
182.786177632757 433.594928641214 510.285766295993 279.113984512619
557.405157178952 375.958347293549 267.422232051733 164.548505132213
338.866471651692

C17orf54   131.85737847837 137.465603365433 130.621860095981
133.353762794271 134.904123162692 138.773042747912 134.158454639998
128.112595697836 133.499361793303 135.956116718089 144.991442620483
139.909719416938
C17orf55   138.712529531951 139.790098895306 137.504147221623
126.230887413874 133.866011732878 134.57472034679 141.850952191423
136.216577685046 141.418737063013 156.822477235844 169.612203020686
158.708054164813
C17orf56   166.335294339013 168.180063344887 171.954184094677
153.93249158209 180.240305360865 185.432587490792 194.112534914308
197.962159288376 178.235281981508 156.349030617265 167.357570666817
196.014841240142
C17orf57   135.583556015955 122.937281123348 120.599440204711
120.452958447555 125.892022613725 115.400592594965 119.176139463015
121.661141741782 120.78129870199 131.566892082703 158.8054811093
125.2051300372
C17orf58   493.887026009442 366.961565952239 413.983821839274
448.644778906048 279.202256032476 385.463209972694 462.888171502285
435.796737132969 413.502150181875 430.94199035855 275.39986964837
352.121800955822
C17orf59   205.630948393225 245.110761598207 202.214495168688
338.739180103373 217.86728342043 155.198503964295 141.143572141958
173.38031514498 156.301214161102 166.14867551435 183.64389891975
154.886628987246
C17orf60   161.788118050564 153.258616843783 157.615017085961
172.491604217899 166.89884215297 157.15947600423 152.853860043701
147.022594294963 179.286187825363 145.09280363189 160.050872656883
136.423161205425
C17orf61   1774.22769195939 2072.01578063404 1848.27927621606
1965.13870083236 4055.40322213638 2441.05534211996 2372.26073354008
2605.13680290987 1424.79466712859 1969.67552562117 2215.43744802035
1006.69598892631
C17orf62   316.281469585496 329.023836559229 225.335753745598
345.649572073924 844.187489702587 508.861268738074 838.267069860172
680.94167651697 589.409235971915 328.269845211173 413.913442080187
591.89966781883
C17orf63   322.576886792502 289.434186747703 350.803378042804
289.09614335372 409.206158277764 359.776068595698 313.641656294638
546.611519125021 415.011847890313 253.65959805538 369.646624431062
397.660202059209
C17orf64   141.717466442384 129.645982978155 136.988519762443
117.598778415488 126.750850861235 137.310014841869 124.028748209411
117.986939058467 156.890299350025 132.765942975939 151.902352951251
141.444958186793
C17orf65   141.272049404557 149.432744784716 155.550532734718
144.772914483212 145.872258115708 133.026879831536 142.898851434337
164.608496326181 156.293025312901 123.545404885618 176.761123480566
146.59366188125
C17orf66   122.309041354217 117.227635924674 143.716377201615
122.685360126599 115.048402956574 129.874235646816 128.972276851419
147.082068348591 127.874934513708 125.77343813277 110.368392735494
133.113581756557
C17orf67   173.563363508314 195.303469330682 192.399146702036
225.480366837274 170.992074907355 171.180188911669 135.188445928844

268.885346595453 160.010501226517 153.843250142472 142.230934617475
150.617039206311
C17orf68 209.960795065047 225.927425596831 208.164674386141
270.92671642109 164.063104872636 161.657344146676 159.749576166342
199.423120300557 199.516043142784 238.978845152474 207.785320401354
151.234397206563
C17orf69 171.410912120195 172.28937151007 166.085469368498
181.758545723241 138.748941735079 179.932404140099 150.513804134588
203.704734790602 191.146276657897 152.465176508162 150.261107471642
168.841313096346
C17orf70 839.324324662619 994.213397985121 683.470866056244
1246.2895253224 1432.93880448974 909.388774224614 1420.27222765071
1434.1439428394 1139.14730341921 1195.6335460045 519.564563632951
1479.69309189156
C17orf71 242.253995801882 250.515579402334 274.77366169918
253.65959805538 281.996583337694 345.565843875296 419.126814511305
362.502631441716 442.590987372759 360.361719991381 369.198837083585
364.895113721763
C17orf72 117.417112053842 137.779607093226 130.142263689536
132.211379936941 153.181097279473 134.53215156472 135.643341446691
146.097201498811 124.530660958725 123.692366307662 139.889264227467
130.593846004132
C17orf73 129.108534267222 129.880696706381 123.798513338021
144.096048197835 148.751755543817 137.945878061978 134.000363767958
130.112882549637 136.409137464692 146.692827583525 137.549175595441
146.404727168045
C17orf74 150.540599489506 160.001115445038 179.687464522203
150.531753446398 158.302977853133 179.74065086312 156.130224537288
157.273309095198 156.433603187722 155.023934989559 176.603760647035
167.206513789165
C17orf75 209.385358804928 245.997705723007 235.650688823345
264.355693070937 165.627852733455 246.88857694249 157.277051544704
203.317264638828 234.661864595717 165.731179108649 159.452527363763
135.654614839107
C17orf76 148.861141988806 143.654677238911 139.796168741191
148.986058473609 131.715579653796 141.494666823639 137.144001915831
134.831550401848 131.412979573687 131.839206336057 124.650711299942
142.58227004035
C17orf77 114.613368640356 112.156010486085 114.067790137477
113.924310293916 108.646136431909 109.947300629824 112.015423658372
106.854721604889 117.089207321783 111.336798977475 104.091301211096
109.74983281634
C17orf78 153.358189612307 154.71904132098 139.432567336814
154.847114601403 140.005968571997 136.764881711137 145.34332740863
124.217337143269 143.212739422069 139.646026806298 168.139351579682
159.762733951454
C17orf79 1223.06438074895 1076.62597781124 1157.02365469464
1164.61390835016 4386.83308119416 764.774225930479 971.99484515987
1254.19980463817 873.408431189194 929.69741581954 558.810095726517
611.36406319509
C17orf80 175.626096716758 164.922193471904 197.873787638411
163.284853420896 149.856041300199 200.712182818203 219.709231012465
200.146843808423 193.974325897472 190.489530928259 196.889520085412
232.465880700217

C17orf81   177.457362387052 176.497706091676 173.624709850889
171.896929562022 169.722974413341 151.899063970777 163.37518739397
170.585366369563 158.411846122784 151.411708598688 190.018871923219
158.396149470397
C17orf82   170.541689758579 176.183165494563 158.98007855379
176.008711524598 307.570405241182 202.01798254763 246.263259852067
174.657352858049 197.034073391478 154.277670551775 221.512905853031
253.580286414227
C17orf85   357.35857422585 317.949646885539 278.469340236003
325.493177980483 198.285073991956 233.572055978465 163.48462232122
225.438177833064 192.032080013216 212.571491543077 318.112626495401
289.037033338975
C17orf86   122.595766303833 154.928072022777 119.562180492359
132.620474788855 138.651419539729 131.636779618185 133.422190038356
129.846685899183 130.06068534054 123.129785564258 125.431735524937
126.121516564343
C17orf87   154.695060071104 154.87316176388 160.411218029114
152.049243916141 146.83577460518 154.030432279825 145.734680574583
150.295611829265 186.794165469219 146.428534132236 158.933097712363
153.468933239223
C17orf88   151.237088659409 168.552631639996 168.394330931524
156.336425973696 151.690538050817 154.683723064797 146.210814568357
153.350690419636 152.317890573534 169.434164890542 154.108689872903
157.663460168247
C17orf89   158.735283017367 163.773702843544 141.440563159897
146.233195164295 180.392337212428 157.316941011117 194.36669779004
186.496160803789 248.299149406938 157.607445509297 157.316941011117
249.858562350415
C17orf90   293.350292227077 327.480268032814 362.019824026781
370.227329649269 896.625415670455 501.738490253982 616.701037274509
561.858563298512 574.779350011402 376.189276074918 427.390091157726
692.359779743985
C17orf91   366.857139377277 271.638179116269 175.186435016433
190.255797389524 159.665989587555 482.857203798212 236.985483155452
211.395593720534 233.426170535227 367.327273578513 428.237210115667
450.99430272315
C17orf93   136.130027137459 145.604066669916 148.641780441227
147.208176455806 146.162330544539 137.727466311162 130.543453631154
129.894339927457 135.805294188016 140.541925660368 145.730054487572
141.129013595331
C17orf95   217.408824020423 222.943046618742 261.39435936661
205.121123131992 253.009376944901 250.928804390353 361.327436394941
340.668473351494 278.469340236003 347.487059316081 260.195256836955
362.252813848151
C17orf96   151.3362561751   189.567241599215 145.048033586779
134.447878635716 1160.25775445193 586.642581067137 303.762967437478
523.717949503639 316.623219008156 152.142909854773 296.553550946307
239.73396414153
C17orf97   263.845733455915 207.73395131954 311.481739745423
217.435155984897 214.933911087009 241.890850719625 174.873893094923
360.475989559226 304.713434036642 149.147411595596 435.183433729519
383.939800422489
C17orf98   114.691842968597 130.729989463558 113.416743649576
120.574820369421 123.427366316096 130.205168168299 124.079109459437

140.790450703097 138.301397056664 170.941404165185 163.579282884905
129.705735906962
C18orf1 144.900785401228 138.717541782317 140.137435303984
137.286331849749 118.134614195753 130.433405111592 112.841406363391
125.780294378066 119.525701528869 115.646023142125 145.681968019851
123.964042357475
C18orf10 302.069236363626 281.570685561526 292.911761762431
322.64731764456 642.255110250493 438.7446527797 329.263397240111
456.038981546175 479.053300726301 372.689712716797 366.473258467746
568.555967950896
C18orf12 123.225792525733 127.177422395113 128.375390373307
138.316043447788 126.848211027661 135.125414452178 126.141462782039
121.6801373043 124.001295849091 141.052322220012 126.383238335161
140.260053206523
C18orf16 133.056659202188 120.246240734668 127.269672881591
129.680406696779 126.893023101449 122.300069045533 125.768053724055
125.176747824384 127.245190474707 120.744994574029 123.108694517244
150.460618402778
C18orf18 137.15288362126 153.551359970644 147.079157199448
137.838096997956 124.077104101209 130.238231520218 114.428050500096
121.895778599116 132.253450751554 139.067516394141 129.543069836687
117.760045671142
C18orf19 141.346722137248 134.623264303227 138.654301345398
129.372522073479 127.6516565653 153.474566767304 145.383354200298
154.634289463743 152.321890796527 411.451612988791 147.882530392424
181.97372140367
C18orf2 140.559553697317 140.288612271406 139.913180140881
145.090639086338 163.566535828036 145.880661952783 138.446492794924
154.140863428961 140.60741459333 146.808519947394 142.103519373191
138.428527640896
C18orf20 160.870390779084 147.442483242063 153.162313098346
156.525347260458 143.905680376268 137.911816180518 149.042693712098
149.505323932806 134.909230280708 148.434497641549 138.386391726042
143.694412162756
C18orf21 268.600782558985 245.811312606484 251.307020289291
274.627303114477 282.604890044452 514.799179506754 499.964000319181
414.403321220432 450.851711160136 451.985108262982 248.465457421469
428.66614441422
C18orf22 248.360540656183 250.626556981376 222.990586491219
325.96632528242 268.930428617695 228.879434668972 240.182016388745
217.628802427037 206.042395282286 206.650760711745 207.514489132778
226.506852362359
C18orf25 463.091387717305 423.396271159345 451.496617003797
411.603820288919 266.80383076887 531.421912541822 421.836020491624
605.437395034282 503.59466398882 450.327710374141 426.649095732979
416.861099188252
C18orf26 177.204172894028 176.612866969431 177.696849418064
192.353768499582 155.014930599769 173.347463632976 170.05799929575
168.358323243918 155.794309652357 162.57736365619 175.686248503777
174.761105961773
C18orf32 388.573321838751 352.741885295157 320.390351199433
283.377663208926 363.195371577097 276.867974638972 456.530337997907
553.198321853202 482.592481827722 412.941907828617 703.815551883635
492.534183006883

C18orf34 194.403848868583 203.852316712377 167.850516495529
180.850779069931 157.328404229451 163.753434774656 131.736694577111
170.602603155092 157.192320902906 141.292421722594 136.423161205425
216.880896769751
C18orf45 197.947433984294 189.90406457741 136.660600727111
182.747079625619 247.307128520272 133.065366727222 134.504873813959
152.044068298273 141.356127724777 172.877947895827 180.01471631205
155.651798065702
C18orf51 135.364039983762 133.032905765836 135.182524652868
135.283742166077 123.804039963953 136.77260462185 126.601395870533
131.72852748835 134.53215156472 135.367261617271 129.490800177429
129.802596741962
C18orf54 172.698181201849 179.015669199358 170.23937234395
189.74261433647 152.942824158634 228.243668453958 163.748692741999
243.336550161566 203.752540576935 160.78368986036 159.170943469313
171.791030104358
C18orf55 703.587139938769 652.201948082657 960.775251871864
754.607039665508 379.200576951933 644.97200337335 590.646248924596
867.601542930533 707.173691257322 780.961416212309 607.979208454176
776.030689773414
C18orf56 151.392024228289 176.761123480566 132.179747646893
136.03420235737 738.37559871635 349.99912485533 232.105867345662
244.954917633904 313.077481033093 168.818595386221 189.389457481768
290.541184606565
C18orf58 157.592607345742 150.015696417249 150.374573177474
139.157088843803 141.712693059287 152.122141650397 152.321890796527
134.56134674893 122.514826802737 145.588981165456 156.654450436728
161.061119536632
C18orf62 126.705446263769 133.900005368973 127.827096314406
111.253743009588 123.13812550459 130.491742824575 123.007103885415
128.689465747173 128.557216790939 119.427342766092 133.692996980809
126.146213417225
C18orf8 427.766660764042 383.10686624119 460.651156484562
346.476121089685 388.192984832638 528.275591281419 419.763812610671
630.473042320288 501.301694626942 444.663812851378 420.502251256406
452.167674725454
C19orf10 445.214621023114 421.056268094661 423.045329000756
464.907454552364 429.812058546782 1717.09434498876 1236.56870367797
1160.78730097715 985.637230639964 671.134356672693 513.292377117011
1103.81439809121
C19orf12 326.417021307859 293.528601172819 262.913054904154
320.214614455231 363.445110627483 326.525579400607 269.770777921703
257.643855420381 320.214614455231 257.168308311848 268.264885392368
267.372241661758
C19orf15 146.984888309556 137.890006487904 151.461857714097
142.52234368939 158.845174721328 163.380402251758 164.008063563113
139.717662557602 132.249906289203 152.566490804809 135.356010814496
154.229914729772
C19orf16 139.263602290256 163.600888221193 155.287837763675
155.83143112621 137.914673796317 153.941193682697 134.145771195572
145.962664629275 149.524040913256 137.991642076355 120.235652849894
157.841520434044
C19orf18 140.007937555694 146.462544135666 138.206065640158
151.976432805924 114.067790137477 121.919799968293 110.148555045276

150.962828147611 159.724824188037 114.042914237667 172.345201438148
113.955459033122
C19orf2 1246.2895253224 1196.34528225417 1400.81554054664
1027.96306543235 1796.26091584668 1965.13870083236 1475.41920838384
1471.01897242089 1376.84058291266 1227.94401191112 1666.80650105101
1418.60088387728
C19orf20 138.304793826214 142.579346058088 131.268454024753
144.159316670264 196.189101941489 124.295334523889 129.03753569157
130.135721123253 127.604087759266 123.195738824794 165.406452831375
137.874370839801
C19orf21 166.70212615715 175.832474809232 197.651165059354
204.710203150526 217.778414825515 190.534176839652 187.645390502607
178.854873582873 189.872997882033 177.666582096539 235.795627549799
197.205792984885
C19orf22 698.358285634451 785.008529077795 576.298671053238
975.685771692095 2321.41228376157 737.42784787426 1080.42884514937
932.505095432049 846.084919104262 1255.02720867812 1206.59866455529
1122.92917004037
C19orf23 131.308384127079 128.725044326566 114.906396746556
125.617143363441 145.193296081527 126.089217853289 127.368207232723
125.26492723752 123.237851647499 124.668658660052 150.053948736789
128.440024330942
C19orf24 215.585089158905 255.527764534636 242.538036654192
296.428342247529 518.281245498542 509.618968665191 392.539157974899
353.078842299761 335.3366819308 282.561381978659 311.337303524912
366.857139377277
C19orf25 189.971144220643 201.105520537151 170.941404165185
219.228720772236 232.444867422559 195.16856673253 197.427788552936
207.624240598841 205.765822478178 198.506786870804 234.182164952936
168.71216652064
C19orf26 121.997885377703 131.102996816222 132.085538520551
155.542691937539 138.456465793474 136.144068118165 158.788210937339
148.227581116046 148.927040248436 139.973692361771 146.290334865039
129.246066890078
C19orf28 148.692602243778 160.449911844008 145.146797807275
164.063104872636 324.79327985156 174.205940911242 184.813094907887
175.510160114891 177.772019227116 200.944865673409 189.324370290702
187.881334544018
C19orf29 209.54573978355 220.772387104223 194.979795724712
259.252807304272 263.64863646012 213.301947003462 208.095384605737
218.000351930307 205.560952959537 223.918931440768 211.118308738617
207.531908382266
C19orf29OS 128.580625868379 130.906507319074 127.368207232723
130.085138325818 133.713275043978 127.661634554996 128.002822538671
127.42914358124 133.441783287131 146.690308698414 139.987681290389
139.936661244361
C19orf30 186.127543877065 166.681646517413 165.131463797217
197.762169504041 177.94501172269 185.295480519718 176.912121142015
156.748330788367 177.582314734704 182.997980582218 166.671563123047
176.612866969431
C19orf31 13645.150201952 12457.6956682374 14544.3732040823
15354.74541236 13324.7465248375 14181.1182110192 11360.2921551609
13563.1694195379 13276.1713779481 11479.7406150358 6368.23123771343
11809.4804843301

C19orf33 294.808980631295 435.983803453746 328.019503002384
168.818595386221 1315.66699943181 1407.43503866211 644.741046498743
1687.85201395597 708.534025308731 2841.96460830166 2187.3010896903
1648.22531304741
C19orf34 115.76177719261 115.157321350379 111.286141277084
120.883785696583 114.734439905052 122.187031504466 124.370381674692
124.119104345119 127.146101490884 108.362609680556 131.601823550269
122.944699010173
C19orf35 122.452541100946 114.428050500096 120.333487171575
120.135349166633 117.017844025726 110.718864165172 127.130769263811
126.845048064014 123.126240234396 119.915330116696 134.73970457131
116.65880728655
C19orf36 144.339819009283 145.814268094808 150.513804134588
146.011694647614 149.110300099889 140.347131743987 149.63291071048
137.13615301204 136.468434653208 163.390929975591 147.348400730286
134.880138942756
C19orf38 152.563532872898 141.652000792271 140.995418231627
162.54567071663 138.800986934663 159.099495828517 154.586825710624
163.661196423902 159.487615985875 139.932849289354 151.884221015031
138.218392984717
C19orf39 134.94189148503 141.706292773073 133.998381302146
134.646131480502 131.265594391542 140.655002540298 135.54708602175
151.41546939633 137.938310376106 115.750690887988 157.729732484131
144.046646189442
C19orf4 122.54127694717 112.520199390427 111.608386387842
113.549098821204 100.637271902591 120.628904050564 123.839006667113
115.766358387923 120.031976318697 124.563594928763 127.42914358124
119.060186838239
C19orf40 173.240598306749 180.060096787617 167.474790447134
164.553511697702 190.614236149496 205.036081947168 216.974834077975
178.153527134452 217.89028736436 180.240305360865 173.315097944835
189.83433085782
C19orf41 119.968081249475 124.247680699048 119.631847624882
116.639787832302 112.036753245281 116.987471433258 112.404562115306
107.431455226979 118.589392612801 124.253582884351 127.473812381059
119.562180492359
C19orf42 708.056533282983 705.987531074737 675.480482223958
722.399669963921 320.097510212422 420.248838397618 529.963453165714
524.973621024573 583.938727139698 514.213675499369 542.544172683021
575.060457772307
C19orf43 1145.96710556468 1165.95924778817 1190.62010668045
1138.00644332379 1442.11349373764 1312.44215746801 1029.43177809276
959.126054626181 915.579581760396 895.821578104681 1093.85316636801
1069.93503700632
C19orf44 184.365354978005 209.023926358109 250.647644543963
256.326661031361 153.068898842283 138.005168756932 187.852455291319
191.616483214712 155.134796468704 138.444427173667 190.255797389524
149.831566330214
C19orf45 128.0171233156 137.844113743956 137.607767467172
147.570734399004 183.998458233995 197.848577663238 165.553581848949
222.177290763736 165.066555355918 137.534079082878 151.110467345727
204.959164579011
C19orf46 142.238908711402 154.912576362196 166.173429365582
160.353649786837 1040.29664343743 499.048754087189 505.704957182936

543.810043433181 191.018168651394 169.844913799159 170.179304745674
164.593531655305
C19orf47 166.330001480033 165.964183460825 181.294497402507
201.661941695238 219.638478100747 151.965103021638 175.262884960324
165.890492422031 160.584760930779 150.132420238581 147.208176455806
146.164093136588
C19orf48 183.804849355341 223.220997990627 186.007710175103
246.77831953556 934.173273294189 329.333236839082 630.670530516275
461.088572554864 506.659740456829 371.552970811499 265.829122816166
370.877260088621
C19orf50 734.139557942719 944.47112348734 695.321886304133
814.110601393539 906.886423608706 743.915675090182 748.940715378564
824.746488539942 628.485648053393 617.318955611228 648.549890890375
588.334329592604
C19orf51 136.869117921313 136.330636532576 130.890314257211
158.411846122784 163.81485578613 136.44218532882 129.594440309419
151.002218995005 143.937726155116 168.996965070202 137.949215501559
139.268052431476
C19orf52 217.86728342043 209.960795065047 259.796092536601
249.590923098575 301.871924474014 279.113984512619 278.428310548805
291.056913909578 239.789994201722 262.061371466369 220.136131941508
312.903508201275
C19orf53 900.039744829725 1012.44277312494 835.834013018438
1200.03423010236 1248.69137283095 1358.29475573637 1750.21700780511
1153.5175534971 1038.34611017219 915.579581760396 912.878077940014
1013.60354455825
C19orf54 182.384190694142 204.137195524755 201.969002368045
291.369577397743 261.426617866925 226.221973502783 230.053292316517
207.338645756456 212.691702227442 159.287816317316 178.984409575632
167.906766146651
C19orf55 123.501843029993 117.089207321783 109.574386044714
105.25877906031 131.01107240791 114.755442050481 106.701716475947
120.333487171575 122.914680091926 116.983869766097 123.668707648172
115.417648857825
C19orf56 1813.79641422879 1978.95750795951 1861.19763103381
2135.04382065446 2882.51330563735 2956.63335018838 2395.75453574049
2470.86113150126 1881.90353127141 1534.21175984308 3905.35300751401
2267.0818302379
C19orf57 142.440344960504 141.398513269219 142.639587833858
142.521260841187 155.883386742315 145.86855878099 141.071184297124
135.814893730549 141.269923271838 139.684141478459 141.994082714379
132.128418409996
C19orf59 130.365804613342 149.247514768446 125.262445870212
134.753783025994 140.435724944447 119.403831071089 140.502667595362
137.728163622673 139.005788243703 196.226732738526 125.902015309754
129.487458179949
C19orf6 440.495339027491 430.728137105601 386.16563750478
500.469781477166 583.938727139698 441.684169663552 469.333357789149
466.777454704543 388.638095539186 421.303580698397 434.325609889068
497.411169898778
C19orf60 867.601542930533 991.611982265221 819.151876775691
925.656701707763 1728.86115695979 778.846928848361 776.411045078049
1092.33328323944 819.887322757281 602.86705608228 642.647554580301
1121.87292339787

C19orf61   185.154876233931 202.458096827378 189.83433085782
190.572712684622 380.562212796792 257.075088169589 273.073938260793
257.602370597316 307.316241247336 179.048139035247 238.068308163643
196.974807677659
C19orf62   572.913714991136 616.188923504376 523.717949503639
770.632068590053 855.415038498392 659.27672123763 526.416479408056
552.298965378403 541.896444696137 511.779988437654 362.376153348015
453.282973743379
C19orf63   152.837739128546 161.096914295309 159.470818285679
174.42996813062 254.879772480457 175.21512930507 194.831347257735
162.373508183992 177.246890307643 157.58659226599 191.692830869809
181.37137053478
C19orf64   137.239088768176 149.987571884748 131.503936441405
139.835983835267 156.763364462534 169.583355171423 137.689158155552
160.98571841915 149.361498483705 147.04481509758 147.155738185574
151.216992571364
C19orf66   393.264726588522 307.597733293218 293.4021207271
338.406266073063 200.98533822317 173.233357775898 281.278843600876
223.918931440768 240.878804911613 256.873090834376 413.084646927035
254.552034511333
C19orf67   146.025781495177 154.16370087233 149.44788347071
169.168869363848 168.925131238831 155.349728277626 150.756776447358
165.430811083128 143.050004021764 151.40067812803 142.351705613006
134.717258604899
C19orf69   122.201293962463 136.553568679965 149.117224130576
139.633941983246 137.736771579507 132.973551954067 133.14988092185
135.531326977319 125.655823846115 134.642898234003 134.7552564959
134.169645445889
C19orf70   1931.16906839143 2099.85702995194 2166.68174726155
2583.46653460894 3455.20639616159 2151.9395095819 2639.60135568
2358.46955614953 1658.78888936164 2321.41228376157 1798.5819962261
1995.6000523628
C19orf71   129.537345537086 129.794293922951 134.024205981268
129.964666668421 147.174977926293 134.11753210296 140.736051985112
133.556805738801 127.665162524824 139.927773713324 158.906481816035
134.132312782731
C19orf73   117.662611650515 114.342980656214 116.59404046487
115.085961282227 187.035957486928 130.874283538818 132.094446606227
127.555954288973 138.925802905636 112.314595956147 139.041005010106
118.895095992424
C19orf75   133.353762794271 132.825240485239 125.165254272421
141.738647095861 136.175268696202 129.307025798583 128.348910964448
127.911258242431 133.930997975106 120.041623078295 134.331420680628
127.333963230749
C1D   125.324187741972 130.721987260546 121.580726825432 123.891115326934
136.089888639987 128.186963138555 125.935071837868 128.593112520767
118.553226856337 125.672285485643 133.486155360266 126.033673708286
C1GALT1   192.493439659184 180.113222861966 164.880911221767
200.094610104813 299.754985162237 192.767155566493 404.172535442156
214.132445321197 238.270501389189 234.484787292284 272.994195639694
265.829122816166
C1GALT1C1   208.816482659752 229.85833221434 236.745054217055
189.90406457741 401.461825804111 502.281584081805 334.350011399462

362.019824026781 360.475989559226 272.238922498583 374.033009122079
421.056268094661
C1orf100 134.796088225931 152.104170622003 142.058086679048
142.058086679048 142.953661611418 139.404820141366 151.102431192559
142.025834438974 139.051656180599 136.218750083407 130.637567221512
155.52533851273
C1orf101 169.888264971936 148.874533472738 166.007913607742
140.475688390415 145.34533633425 173.121618125421 166.346572488391
157.166379887259 161.37617568158 181.622689471026 183.482640529727
156.126009063311
C1orf102 135.609239096236 143.447768653042 154.025625092599
143.410917415691 156.119899067238 121.177681858671 142.939578759097
133.82390829608 140.557871626905 127.820096020782 124.768399055809
122.494779505958
C1orf103 139.62718946833 143.523051024982 139.22776505842
122.501785735146 131.106613067333 146.927825526831 147.93088503956
148.638400592682 145.154323430995 130.574019477669 157.895460674072
135.212715912271
C1orf104 167.083758835476 163.513162036251 155.846106540378
160.254101852355 156.516250236679 144.975882336334 161.94312376726
157.368974632451 154.804753043168 147.620511911043 175.158180187883
169.607072855818
C1orf105 166.071816427719 148.603077434646 166.784804508647
148.484823275343 176.953099943185 182.222567053422 170.67334244323
150.792065744902 152.470980074365 169.705236302448 158.8246699332
183.018203688404
C1orf106 220.9384439772 362.95684683529 274.974304050999
196.716209382446 1690.96371604294 1470.23096656138 1747.33558205825
1658.78888936164 1377.75266942574 851.633522004342 1923.19560386389
2430.70635781622
C1orf107 163.904137395349 157.293090577042 168.398366575579
154.737117718424 148.785977829275 191.912670416334 189.887788314879
155.207839370162 186.591941221632 150.552186000149 188.654966029389
156.353914172996
C1orf108 158.319248599976 137.995313285692 172.403953571339
157.449891798638 139.654648891206 153.871893011106 137.074564265915
154.630837508249 167.373416675264 149.303427221111 152.50843414723
156.191659799142
C1orf109 180.392337212428 200.127994198329 263.606930507829
166.289763097913 246.912159676595 362.502631441716 366.240941980786
343.833659987063 318.06081614151 265.536429548886 214.295260811046
265.366632264085
C1orf110 163.104822161892 173.995889755792 135.078198678379
130.808911605271 100.477911152746 116.043095688871 113.857477830423
115.785084044427 122.095978653755 116.814269969508 140.227863970629
108.007762863766
C1orf111 114.65036208773 127.876894098781 123.463861015933
128.066043751951 124.484589975207 119.631847624882 114.048136176142
121.021776527531 124.036907709461 117.193620642373 130.902319266342
125.18424162353
C1orf112 189.235998474945 186.482547532546 206.488530785667
225.423403713718 340.366016191232 555.366765340014 232.667968049771
374.657816575897 233.161848094769 236.292053621233 183.056707813352
312.645778869254

C1orf113 146.479009988445 128.890685255595 136.444883411968
133.71610564207 137.312488161187 142.05571112803 162.155834067128
143.993611400272 129.896711442418 166.036553680319 152.733409144267
124.217337143269
C1orf114 132.695992860853 132.53122878527 130.738915341653
126.893023101449 128.438401976279 137.757902615328 123.843823256462
133.459221120979 120.39039576721 131.722397988741 129.095469955392
125.870730238298
C1orf115 388.002365247498 440.319777672273 214.413674287972
381.808809625801 292.317998405609 208.955110569558 204.942500423413
205.821101661022 361.969355316032 221.74200490054 144.779146672553
323.628636799403
C1orf116 162.15259972225 196.777776509371 174.593548891693
163.102043862117 422.973606694449 190.700515036197 369.513420606401
370.945254716657 532.235193634196 553.368945998847 683.655205499779
881.15414246017
C1orf117 139.427216913809 160.060406629689 160.48993837044
145.296229550382 134.19455223935 151.479748626813 136.692495869097
161.456727894848 146.559502785264 141.313482534472 134.929915732246
146.565265415908
C1orf118 123.96665768708 120.948246352846 117.335748689217
127.088916295784 121.144174389418 104.442909315944 125.693321471759
118.26427946535 114.498610080481 110.678680817471 120.447140228396
115.646023142125
C1orf120 125.016608289141 128.773129597793 161.456727894848
136.824357247954 129.375772737722 135.102827224896 138.637735867167
136.274199465472 141.245471037222 136.454911192129 125.331215990597
134.485835304676
C1orf122 403.907009699589 377.951523644306 399.071041584646
407.615019710707 833.841469195516 445.505512764045 534.608644989102
509.400965222318 463.99922423125 461.822629852953 511.547907091166
476.917496749381
C1orf123 1211.39534395597 1162.8644618472 1250.58586679251
1752.50728312967 359.329520507158 603.569635276204 744.225336494008
610.759447723026 666.204695328096 566.647927867646 559.0867397858
494.604716660887
C1orf124 148.624135810148 159.563866056798 155.731897985566
150.160004473338 159.166059518117 189.758013811338 168.763640193219
196.189101941489 171.014432075829 175.946843919065 165.370800097602
191.3765085801
C1orf125 137.938310376106 155.214638707046 157.759149893048
137.697452180659 315.786908578669 138.728765780512 147.195127201893
258.16437138327 150.134337895481 152.344248425416 158.550259547851
160.695913747601
C1orf126 135.532820436394 125.189588215659 142.288110423886
128.878051599968 129.878330410179 143.864876796403 146.506263412584
137.120973494952 142.01454627498 122.746185691827 130.491742824575
156.373163373309
C1orf127 173.09379121032 162.267841475739 138.416716745934
142.566028652989 148.461172387171 160.79631681714 146.197678850188
161.354014292373 150.820808770853 147.113541493157 143.284230032303
159.130076733711
C1orf128 1010.73988405469 965.031655021246 1482.92498205315
1001.35912007916 798.943242964857 1255.02720867812 1175.27165286568

1501.18820017008 1281.1883621512 1505.90883940934 1200.03423010236
1003.93455977232
C1orf129   117.862532836494 123.998343902602 123.700076467648
124.738217731488 125.687688416588 119.469328303796 132.146027935802
125.645986634914 118.206034087145 129.642952083513 135.412051450659
120.93965087814
C1orf130   132.518394343751 122.763138007612 132.122273848715
141.038721816572 137.248465324584 129.534721138679 143.791908191551
150.573523216151 147.301909062399 124.116400777472 169.642188114285
140.992801405088
C1orf131   289.037033338975 277.101000223971 265.791342287571
279.413335686746 392.13175568099 617.318955611228 449.598181155897
549.359675306309 488.14606875418 430.590533290315 416.307629596091
498.965895763801
C1orf132   128.800907223807 131.630132952441 160.589575894314
135.386650538345 154.889079187405 148.060086496148 157.702540365469
149.76931674417 146.872432596594 140.149064156135 126.897804011992
144.233097828078
C1orf133   457.130051460676 367.229293432041 628.344122779625
369.373283422151 139.934460900409 243.441938832251 167.500410857353
142.493119780028 152.538048490265 155.535222310709 140.434086004204
185.46843333826
C1orf135   115.531710316716 122.793816135585 114.860437706855
115.816156193574 361.969355316032 384.051170099325 154.479733759712
246.457698183615 200.790119570144 159.199045715212 163.014903707857
162.778480318754
C1orf137   144.068734533254 136.051070946954 138.813126946007
163.245806772716 141.855655600443 140.242272149424 138.409284761652
147.576533768761 116.106772298898 149.964827055916 140.579839043912
139.537865511933
C1orf138   123.190757763412 131.287142490322 149.687372584136
127.800634041606 125.990762414974 146.112388101621 119.735807506447
129.780648518219 131.583841535781 134.231634410324 126.029431525943
146.390223079987
C1orf14   117.992729826121 135.603160096973 110.16174213285
116.572001618531 130.235407859542 121.810767302322 123.442970398288
128.751758311249 121.912927816178 120.355844117573 125.065813262416
126.925982415892
C1orf140   121.524679741065 124.291944151031 122.770033312519
119.242880574306 130.440164900487 114.122395647516 111.949644842971
122.773736159743 111.886543737378 121.057111457792 148.844916975586
113.542803491215
C1orf141   146.353239460684 143.287237534352 150.610633329908
137.326796995017 139.082897743357 131.727533821438 143.931400351525
135.81863662958 134.347898745664 147.056182807232 130.630504666374
145.412368252632
C1orf144   817.342172321486 894.734451279225 809.362464219294
1044.52286986369 970.634736994313 625.152889992949 904.733257358133
677.265576269103 1079.58110075176 907.904780399957 662.781145703653
870.887539668967
C1orf146   147.395335979341 130.374753524012 140.067809125008
144.084441938143 133.581450824362 124.942701123337 132.154131623051
139.825088162247 145.298109334141 141.597692730835 136.745596788338
135.738699497293

C1orf147   134.062423178775 151.372219350291 138.1693725329
    145.71535210212 131.298129667973 149.280480796544 143.160281902098
    154.426605816862 150.549883779193 155.715728894101 129.792548588929
    150.082561726876
C1orf149   247.145700155364 240.005943851098 326.525579400607
    253.124449721731 154.993784224548 198.15257384667 203.456296907031
    185.507663455634 187.997940126066 226.595006724068 193.008902902654
    182.515302191173
C1orf150   155.683727621475 143.327730271099 146.873719524815
    148.260672564835 141.931274901572 123.908458472687 126.917669470999
    125.227513212286 131.780134634616 150.084909674721 124.436549041911
    138.317443162096
C1orf151   201.555964610849 197.12840030662 181.364325529405
    207.902853533132 192.804447792672 194.352807599399 192.258863431747
    169.408892048115 185.186497037695 210.165098572428 179.996590340102
    188.767121505943
C1orf152   168.061346742356 181.950658782069 167.51207060064
    155.091135297162 137.404525565336 151.245520459264 144.490452173336
    152.350537364705 140.888439624918 137.299908075811 145.261662166215
    157.40255559397
C1orf156   152.660373241584 154.197254746757 151.126252768792
    136.341358916638 164.417770955805 167.057202756618 162.411156818437
    179.25743545231 161.096914295309 144.496429994489 167.719651771357
    161.585555680663
C1orf157   133.308383825604 143.011042973347 118.426600479326
    145.024148458133 139.328340242696 140.061045085545 161.506431005203
    133.315888375614 136.051070946954 151.421099926743 148.206529935876
    125.176747824384
C1orf158   147.748142622566 139.685629122498 143.655901618594
    150.401509081265 141.004467676795 154.336009472285 136.669003646622
    144.895601314903 141.657914155491 146.633627492419 143.250386570155
    135.957972463728
C1orf159   149.228977421003 162.850128623401 131.212705319485
    164.236417852165 451.887564294551 227.065194522692 233.786136606682
    209.229537873706 241.782750858091 183.348764012358 251.09230266334
    227.350260241585
C1orf161   150.421116058494 168.521601064374 175.626096716758
    152.845126167802 161.24755274554 174.02138265758 194.903531690239
    170.370934381883 159.074001164836 175.562070915794 158.48793705795
    168.139351579682
C1orf162   388.192984832638 400.353795948162 366.317263094585
    364.443097338814 195.384273821324 402.425130776131 308.679189788554
    690.903797251841 988.788192022196 460.24741151155 277.359422005775
    235.905788497166
C1orf163   189.61702125418 202.849544035436 198.456392063513
    183.115511373872 258.964323941464 245.01029221002 214.434355263652
    304.713434036642 209.112959748917 233.335353447342 220.330477277988
    243.793465720264
C1orf165   221.491633607126 262.019779752857 264.383922859446
    225.142823691625 182.300628120658 194.584034984339 195.674128236589
    212.607094117882 193.428733288774 150.102853150151 202.927700504034
    203.265559787292
C1orf166   329.406649846151 365.590174280114 298.706655333472
    418.645775576811 400.98290237416 261.541138637729 300.937118739654

253.327404004524 243.807038920733 300.401370666633 466.58966852405
280.948704707655
C1orf167 138.664401460739 152.309460630038 149.980924447339
149.41357273704 147.972383728506 154.143331594812 134.978785580693
142.167918879613 128.50733429744 134.824147038515 145.800379695397
151.201263631883
C1orf168 152.419714400591 131.975248488461 135.041687549769
128.594951429021 143.047617455953 152.26903721278 163.231460026317
149.541917326637 155.868109161417 126.960777504772 121.556331561879
130.440164900487
C1orf170 151.377932495104 147.910699446264 138.311892121437
155.801832826848 142.079184950618 140.040420521312 151.946691918521
155.820194280153 145.745466612541 147.505345299689 150.231063426302
143.554726863556
C1orf172 162.223175359454 167.484236948415 169.590101694098
159.913028912492 353.50447714719 258.532432007328 265.110264596425
259.025042304848 205.226635237992 220.458449424062 213.914599384797
277.652851236546
C1orf173 124.253582884351 130.423465505321 121.021776527531
135.261561433405 126.676875319815 141.613898853625 129.816385177336
123.952494134263 125.025064237439 125.639355645626 141.752031758589
122.891946370542
C1orf174 480.923468806193 397.861172312706 420.567884340705
437.489929507567 602.86705608228 581.828502821435 683.365770196967
671.134356672693 680.509421043347 550.124359517322 538.872348151809
636.781477235579
C1orf175 216.645552082667 222.919473675549 403.657727266403
326.83982672544 130.879745244903 152.210867113591 154.932132950843
156.040073232376 148.382228428693 159.534017722536 279.232290927348
147.299872426498
C1orf177 136.37481047902 134.341489592504 145.378779757104
147.056182807232 138.89354244296 150.269618125943 146.309171902112
153.07993334035 149.190528937822 160.272632590118 158.872962797815
138.113751487484
C1orf180 142.284050423299 144.419657674797 126.162713269943
171.180188911669 175.372355078713 160.979182561773 137.803749343493
153.929167381674 142.780100181736 138.03766128249 161.335677383485
131.067958183644
C1orf182 141.425393615228 129.982236484842 151.705219298578
140.242272149424 140.004456934031 152.748807709142 149.839827383681
153.299196905367 154.264142444882 128.580625868379 125.976052547953
144.487533992157
C1orf183 146.40904127542 158.319248599976 159.085639414089
172.077522197357 141.880044424342 138.074645385322 143.759171254761
147.922706629104 147.450681897766 143.982561564661 133.348510114432
140.628614910068
C1orf185 141.241717979079 171.345989796519 140.981666934622
163.672213020803 165.041296694491 139.823017208731 143.951756384507
156.241637687344 136.108500467418 156.546769952772 139.962307072428
150.155154998981
C1orf186 225.209315195163 180.710636205653 155.218336968191
185.186497037695 138.883710791382 193.303987945242 158.459129254226
159.346890686719 186.551385094902 135.247018354192 168.882806817001
408.758021864761

C1orf187 131.709341771599 129.132523684894 141.719556176029
129.511465164539 133.805991920905 139.818998514364 129.054577477882
139.793683692104 133.728378162206 135.508803488048 130.924141812334
133.614973477764
C1orf188 168.906637816046 161.475694690038 210.989806933158
141.710076413441 149.557833322995 174.854570705 161.735609436394
182.357065848298 201.931760074628 131.780134634616 328.019503002384
281.740495452842
C1orf189 121.164978896057 128.630613404354 122.535728427528
131.421663453164 129.278833426752 131.414690782328 126.991140761102
127.177422395113 120.304037458526 118.351607868932 141.735123971775
128.864822736402
C1orf19 342.878522959461 321.049000153127 365.950612890149
327.635520861099 295.571900619972 375.038561820137 648.161646065464
618.140160251927 443.343806055858 421.690849282483 259.900447705311
644.97200337335
C1orf190 181.550597645633 202.483493595704 182.588697551677
233.757965816721 254.188830191845 158.371599884762 175.030373805031
170.052320640686 171.329984719612 152.778732614944 164.160608039304
153.289258540668
C1orf192 157.381401974279 143.368327095618 132.799355149191
159.752644678596 142.809717801796 150.328995030071 151.536645416399
128.41549156443 154.838407529853 146.278921849776 126.544685937984
139.260868989409
C1orf194 149.548900791507 155.368204597287 138.021559586328
166.835922279372 144.185406500473 127.871445879091 129.625046507677
146.763046352405 140.313216623427 140.081280065913 133.604705630582
134.231634410324
C1orf198 971.99484515987 753.007562127186 766.420275252501
868.066644155313 585.075322115635 542.740017003369 368.0573385048
589.070196307574 451.210172051464 435.796737132969 510.659785046811
449.745016106912
C1orf200 140.687016530974 135.233957017664 135.385133545332
142.646570636529 155.259089547817 137.361967082029 136.227202534196
135.717491814078 136.85323853426 144.145612985703 152.077204721895
135.33941967076
C1orf201 150.599425944486 162.683665945044 147.055213172078
179.96623248843 166.360005474414 156.01406297447 162.240414467004
155.201655653645 166.14867551435 152.829437376835 141.865758685796
154.695060071104
C1orf203 208.125844007298 222.820391338687 212.996456841785
219.462274506333 249.554443204445 137.782648137869 186.640167849568
183.302479730653 187.550461418888 174.635853531194 215.272800347166
171.561940747015
C1orf204 130.810353054091 136.937327692084 115.920514336615
123.494412056415 140.047749881567 138.082870754768 114.798573331978
116.86635524597 146.363991934916 141.256915527472 123.168787977805
119.862037840843
C1orf21 511.547907091166 499.776233241535 571.273512452931
505.143815448613 141.685620602226 226.43414383055 224.92725876841
209.33028918341 153.44628692569 164.160608039304 247.194197143562
173.325724300712
C1orf210 146.185931276053 137.968932080074 143.331929080227
129.190900922265 151.747595672228 145.491525555916 134.203949774763

139.161483596756 144.83282018024 149.548900791507 152.109178223495
146.318052064937
C1orf212 225.61275910008 207.864808684047 177.508603290389
251.039117540764 213.194154171149 283.499120551905 193.629237856758
235.905788497166 231.180528071672 280.204167582951 218.433524126584
296.20443113482
C1orf213 148.323729699672 153.041407358576 137.310014841869
147.710252756854 158.503284955019 149.622695843398 147.943844184702
146.470444212748 141.316172207927 140.903091584884 130.217791888656
156.237282338037
C1orf215 135.273184185121 123.227904580467 125.806943645484
135.634385846692 131.97938329944 130.882463936853 132.776236872246
126.748906552086 130.411849432351 127.831950458406 146.179543599917
125.021217009254
C1orf216 267.285998701386 275.696829424061 297.805768288377
326.758278851698 196.419891542139 192.441337304606 178.464811133
183.280853265083 192.353768499582 172.152570321727 175.537039794925
180.561514455864
C1orf218 499.048754087189 566.813343200268 374.9555524303
463.091387717305 509.14726076246 774.997895985917 562.232754247201
846.985164747386 535.612000577004 438.870464403215 718.449150898903
634.356322976353
C1orf220 122.397727522442 123.498540254464 159.29430824799
129.541049979717 132.197346228417 122.251178920649 116.363556872071
122.153996665065 153.762995131216 128.890685255595 123.439897913722
145.424827607383
C1orf222 167.629008083436 170.313045875402 159.166059518117
167.526798465556 168.813895764991 151.979221390032 158.660859266099
144.145612985703 151.070654092886 161.73079180403 143.67880977119
155.747694056319
C1orf226 148.448349327493 147.407093814154 160.026178505749
150.936747236094 154.043604150962 149.103778494524 146.025781495177
163.470025318612 150.235026770931 144.595393134144 145.608087633951
158.98666068453
C1orf227 123.946808975704 134.307336063739 137.163699337032
138.667081004369 138.792521681771 139.162076054072 132.639070127232
124.164523121103 119.213217733213 133.229571269367 145.139966629363
125.897190440417
C1orf229 168.773340961473 160.466187961848 164.032302455558
165.232545721733 146.824420102008 153.818325601312 151.379761514526
149.675363177375 159.572851489665 148.155692596378 148.938822951404
135.638156623519
C1orf24 8342.70113690382 6571.35528952186 10836.7380675792
7753.61594300904 202.662466141301 268.148673257455 300.709410518382
611.208083466988 369.592501881153 819.887322757281 260.52593414721
275.39986964837
C1orf25 211.505504834207 208.067404132325 270.421697521366
214.763232658841 223.339132121796 214.413674287972 275.298642145005
259.643957326575 215.143973502566 208.776711004753 216.974834077975
255.56301534833
C1orf26 176.516540713888 176.355556618289 183.314276033531
184.591789011195 179.460777888284 164.615113709166 171.449326065013
197.827534617557 161.937384157098 170.342828808493 228.766847429517
163.146581498065

C1orf27   180.304585602206 162.634670715627 152.217243924816
184.414609874199 153.241806795134 156.7940590796   174.615694779386
161.481227197715 165.411052799773 178.984409575632 173.147311624838
151.316421810192
C1orf31   144.667638908504 146.500312499353 139.073785024092
153.468933239223 158.809865241567 172.909795209733 201.913709602894
206.303338709554 197.003657786247 235.133284525241 266.135477775641
224.118739978257
C1orf35   276.79778275734 299.106362530855 291.720204789005
328.826483579278 511.547907091166 376.627801547869 362.306819797508
275.343591424586 336.898462056187 296.051639873628 367.289651112346
454.984562256248
C1orf38   134.135748976546 150.815117344434 150.321059418799
148.224587582189 158.756424011719 130.786835186349 143.774841336279
137.77107995457 136.335679863251 143.281050499332 136.891612156271
140.806935772116
C1orf41   295.507504000509 281.740495452842 411.221560795912
227.32463765717 415.113340335205 683.470866056244 514.799179506754
463.99922423125 313.18989508207 539.853394831917 346.566241319814
438.817248820137
C1orf43   839.901173743755 770.19255305977 837.872187194784
789.971871940893 891.637685856145 1096.61874892572 1454.94889750152
1502.3444541735 1462.31151845458 934.173273294189 1301.61742965939
1566.65825409044
C1orf49   147.966224814966 158.322477506517 181.70730090195
147.211769989922 153.249834179388 155.561821955048 166.587100972754
158.10628865476 150.596350682411 159.102070980047 146.185931276053
159.074001164836
C1orf50   443.125195744202 457.292146759215 450.327710374141
504.275299381356 453.593585032712 406.736165197168 554.950092776254
506.029788151779 429.922530506572 626.857289492802 395.425715632302
446.601751145647
C1orf51   158.536178747273 222.848935978648 175.128935809466
274.77366169918 128.207440197765 137.835936352525 141.490911535087
129.794293922951 132.913891550054 128.634431242405 132.813199199389
132.683594640696
C1orf52   474.216057978384 455.564394647943 449.408793796673
477.87583927675 414.596948083067 587.026042400878 552.662610888399
497.110852407681 489.75389978732 472.279492678374 452.606433325387
517.993093601127
C1orf53   147.232015056035 155.376656335974 150.227629690236
134.074425424613 561.858563298512 279.495192828873 319.417555901733
356.323554000768 263.800363357443 522.77407098992 265.148061007894
534.083703564903
C1orf54   1177.70376288106 1022.55215097491 1200.03423010236
1217.29687282094 328.969023478451 487.07860019179 293.17183601265
352.418229893699 548.288640768746 488.379057110543 363.963951509433
341.128917971124
C1orf55   300.150235383065 269.238332581564 321.25076864104
249.507062968243 230.399207047948 613.631311147328 342.935878735854
277.86338481075 364.766154056243 353.243400332397 259.643957326575
551.622587349813
C1orf56   158.890522392445 155.249688219254 159.728097053356
143.506243892155 165.686655824416 220.876114717642 180.34788837703

234.399431238026 191.58185405868 167.817232948801 167.594414386475 221.82340413039
C1orf57 385.379119927642 452.843213944986 412.648926259488 407.760503937821 549.359675306309 388.966303073519 519.450899933049 524.245387973163 449.220056843562 634.774041584043 542.740017003369 659.900771633223
C1orf58 121.54835308632 107.126004317323 120.003095000297 112.57049236673 139.067516394141 124.439917584199 124.989313193779 117.953453145685 125.124011798043 129.992015202357 139.621016978072 126.217702283708
C1orf59 221.027562527572 273.353254857377 293.873921048642 202.193338797533 1107.29687889378 1377.75266942574 816.266384662324 1218.64951314553 905.751410113646 371.127480221666 560.481802403627 532.063437013967
C1orf61 147.096559685764 136.641140715439 138.990971069382 142.376921410427 133.12641302705 139.871928127715 142.101865506094 142.45047744931 153.974384951787 138.552423514249 141.746032519746 122.679628839976
C1orf62 114.805810485989 116.288113598488 125.390139830237 114.618608815885 122.176811388389 133.096984735092 125.386545354096 130.240366272285 116.625518828978 127.818137805569 120.644987579644 124.313901870563
C1orf63 399.537363692534 271.087125858775 396.04031733841 384.780426529733 225.820503820866 341.197826517049 495.438672949441 406.028724866233 501.600329334497 365.590174280114 679.191060100076 558.440840319738
C1orf64 129.507297276862 125.404043810022 125.189588215659 128.883828419932 145.2572048057 149.485497080739 158.943005736645 146.388285560372 136.059884933876 144.612549022264 120.998665308561 138.227082687797
C1orf65 154.401220304515 153.031479923324 186.563913311757 165.808660192901 143.071933285651 171.449326065013 170.08485436731 163.753434774656 140.965498009244 144.295556086504 149.166635189875 161.417207801991
C1orf66 259.742175498038 283.416246133873 264.741946816665 268.328002606268 408.811415529922 200.964306455257 291.994364783888 359.518199449791 333.970844203158 236.052008824688 293.482512212153 304.590636814607
C1orf68 133.001613667656 124.39748786803 138.62994237184 136.734131465433 139.642457979496 128.150801169886 133.35096649325 135.897498591391 129.851616807075 139.890519860289 130.452929012459 133.725177339262
C1orf69 159.115391667901 157.697168349028 164.231281729415 165.919497521143 179.273936200852 167.315702949236 157.590017103385 181.277683429417 177.742616962629 175.9510758779 163.326102597955 158.127523362251
C1orf70 137.82918941689 122.809241232029 133.592691897675 143.444109491884 127.941584184869 135.664877050226 132.514706165018 126.435928518593 146.188193301627 126.241307445165 149.876687693565 129.189479305468
C1orf71 1383.68977267418 1362.75971646355 1282.84159765715 1054.47292895417 560.180656219785 858.524630425687 904.211195986553 895.114975754684 584.919615953457 416.475440894731 658.202765061937 459.30568829087

C1orf74 148.993620368781 162.481362579872 164.253219197128
162.312313953026 203.317264638828 265.712482648121 217.847954914851
197.068795788491 220.959843573708 242.410260751835 246.95071325085
234.156713102803
C1orf75 116.360144963753 113.832885222354 116.969799327195
103.691075663721 112.063713561549 116.053859681399 115.085961282227
116.501174707117 110.428616140857 119.288554466773 110.688722702767
114.927420605185
C1orf77 552.962676093514 557.026028810367 643.13571432061
666.204695328096 905.233241047678 897.51782613323 726.346459754217
924.630951485299 1018.80178630497 748.703265448403 539.125423322738
927.137881369911
C1orf81 146.693573104487 160.122274989224 149.416411285088
154.288075172115 197.51109998167 174.717158790668 173.001985500448
361.390981655795 279.834616502591 148.895841777421 535.49374437073
210.900056977861
C1orf83 234.980382243116 232.590542016298 258.075566417241
235.650688823345 202.300293733922 217.165036362034 206.428618912264
219.78041403077 186.6902304653 213.028568789661 231.146155346291
202.01798254763
C1orf84 141.703410265546 150.993910190626 140.211016178402
149.704586651832 145.787468865642 149.393022408213 145.608087633951
153.443397882028 148.126745515891 142.96594961303 146.058477355527
145.389721946046
C1orf85 576.636077770998 668.570984110088 549.162299481184
712.433345978749 2337.64145466514 605.21685916883 1431.52979384867
1412.56164938199 854.224454791713 1295.27257578314 771.294254795752
1203.47834989609
C1orf86 456.126096817454 550.434931878586 398.673582680257
795.464850398761 1170.88763035931 523.306451464643 442.86942574917
366.857139377277 364.964572587289 370.617422894068 447.995213144892
496.697565640162
C1orf87 140.798706399757 140.903091584884 133.745413210496
145.267225993057 134.283467987991 145.426171336045 143.080742311914
132.680140469504 136.543603346919 141.037217406848 145.432667767652
145.027741086645
C1orf88 180.528296177577 160.419358656559 181.783275804424
167.057202756618 198.338416756432 157.406969627689 177.478925091583
187.955617187544 170.052320640686 138.054470878471 231.239126548656
156.585665091824
C1orf89 146.431166706908 156.720090761933 145.152605166094
153.051143478997 237.191866335979 110.113844434493 156.053517438255
164.313238416498 129.717701654435 142.047828639169 156.779004465552
143.987296454585
C1orf9 150.084909674721 153.354921961854 142.203367260558
146.215459988012 167.43630698726 150.817922696097 155.883386742315
150.208809981642 159.611551480901 155.496190048502 167.057202756618
168.047136398816
C1orf91 154.789187466057 159.005459875338 182.042104888745
189.37832342197 176.890596690798 154.24500773656 199.01458214661
189.552385508772 182.470538043064 190.736796322054 179.973655632636
225.850439131864
C1orf92 136.948039394935 138.880168623766 127.67121559907
135.252082574955 133.43362126395 118.604395992281 120.060776752215

136.44218532882 131.590180562108 135.823975527501 142.663334893424
123.902047880095
C1orf93 221.253717494488 233.809899915394 201.041792159394
231.044588483625 585.371892598422 336.279209908669 252.548942594125
217.24573005272 231.018059960001 208.932692585748 426.995700632434
452.961307006622
C1orf94 121.168550902281 139.061072394426 119.13963074637
121.75662526451 126.209426865327 128.536721595589 115.53736269794
115.101971719657 126.330382425318 114.638254225216 135.122770943076
117.539640414775
C1orf95 141.130462286785 123.467227007155 160.72962443421
141.971207380159 141.052322220012 137.120973494952 153.374296511216
145.862497313025 133.827296071709 139.158299612941 142.274440538785
126.494665743087
C1orf96 126.814332513301 152.500670025483 133.608461289137
146.40904127542 158.558471586662 152.99686989829 147.009325721272
151.436802637988 157.675974692761 154.542190113901 146.90847051471
144.538528198113
C1orf97 208.348515119045 227.267783818298 189.092674335503
227.065194522692 285.659010982301 215.718714189719 283.163770658619
214.461773810376 230.447203571825 249.339449789861 385.135014083686
732.630306104841
C1orf99 137.210805338701 135.807494668545 140.258615682066
131.367075440559 142.712218120721 157.779441363536 137.812249316763
137.947606003247 131.257901340773 141.750316403249 130.590195477461
156.737219981981
C1QA 195.906137340918 181.783275804424 170.622615783608 164.000772606739
192.224242967539 236.855293143293 178.416969664816 271.430915699592
272.41397350779 194.921021761976 249.489170536758 153.93249158209
C1QB 675.480482223958 437.049581919144 345.062426573519 522.486866458783
797.501324589697 616.188923504376 378.38874489611 1355.66237447026
1630.1630053946 644.449861831749 843.075879568455 435.095843349921
C1QBP 708.779490017407 692.53483413403 943.23069404564 738.37559871635
1349.84215478798 2500.07677977147 1361.54107116915 2316.49886419499
1413.37062765856 1974.49672149862 1848.27927621606 2605.13680290987
C1QC 452.077434390612 383.802494922918 328.33387481068 456.038981546175
644.449861831749 572.049540360134 309.098519056843 814.471143753335
994.213397985121 586.366632966978 479.395948662331 447.182566890086
C1QL1 148.000706956532 166.001279428607 129.111259771449 128.785472881136
143.157807632147 126.268418132 129.694178995061 120.786630750686
124.933738341123 133.459221120979 137.476047500852 128.232091717966
C1QL2 153.891667472876 150.737888487925 150.642465826166 153.998849486363
155.272264997151 151.702809524494 150.936747236094 140.487887891414
147.001408450583 150.831554010122 144.892454033336 152.61583482402
C1QL3 127.765987130281 134.867806860993 125.398875527365 132.776236872246
139.195281937545 127.895989232696 128.29486937905 132.864861511517
130.321133400634 140.696326952077 138.159966250659 140.78902818186
C1QL4 114.726808450831 119.894737403889 114.76337651891 103.012774960061
118.983932995126 108.362609680556 111.196610704726 112.729141678141
109.654666772771 110.148555045276 128.521380879209 106.829040075772
C1QTNF1 204.299375966441 307.16835174775 194.186994486095
287.967349876429 166.135707525987 169.920799931309 156.119899067238
191.814634229865 208.816482659752 246.112720011613 157.038121210505
153.13384838492

C1QTNF2   154.431051053529 155.924471321076 142.960875909369
      140.664303738113 152.31268334586 144.724040494873 140.13515064896
      147.345558455805 144.762006548375 156.328454726363 146.512045729234
      153.068898842283
C1QTNF3   169.895672129542 151.88244935494 165.370800097602
      154.475735552851 139.999298193212 140.968351978909 132.021249712589
      138.370241517025 135.51506573405 139.708303388546 145.128919984856
      134.512204051277
C1QTNF4   123.518170356322 122.295222990408 114.880348648147
      110.033746435328 102.255408120852 116.934457270701 124.113451627593
      125.048770476506 106.876902604214 114.170354748872 126.256716138724
      121.215558351076
C1QTNF5   747.322544683874 824.746488539942 256.370052241046
      445.348044448953 342.037040881785 243.425632941131 158.285003899913
      286.703712311781 367.382105696814 271.568797887976 194.80575419836
      226.763252784108
C1QTNF6   154.661456780865 159.390531348676 154.695060071104
      156.759600627542 358.415933397099 188.910933952136 185.743212726702
      165.370800097602 171.722839172783 253.385592447339 168.854819201403
      166.830529779692
C1QTNF7   346.635777358159 247.971366020963 262.416137698884
      181.889592946523 136.171728950893 156.437949042181 137.7872481668
      161.390489603929 158.814407805803 165.149464497921 130.323754933844
      168.753660955229
C1QTNF8   120.212294184999 133.336063137966 128.368669716591
      130.698850298016 124.746457912284 116.273000405449 123.361179535626
      119.395894479869 126.931328660501 124.42974326791 123.420635380886
      126.988118823204
C1QTNF9   131.257901340773 151.751963310212 135.100687929821
      160.28767775843 129.987548571478 137.717859035645 140.051904905695
      136.821228481217 150.003399000079 134.458958352588 120.110473925082
      144.892454033336
C1QTNF9B   138.800986934663 143.569894807386 139.028620375206
      132.439176846818 133.771691580031 137.720747340105 135.873325130921
      135.391788614828 138.397152876585 140.36278240974 135.596007994731
      135.539516564142
C1R   489.909407178594 311.481739745423 599.307072499899 218.917357155289
      176.340265963032 196.802641002079 224.361277033882 260.443814523694
      358.897128592042 337.119524477446 154.300058278533 195.645181575647
C1RL 317.802633831405 266.80383076887 277.501071684448 238.149614104568
      223.84997313236 244.251315474587 275.013838208239 339.012225830929
      351.337287690674 231.180528071672 360.90109922539 296.721891842563
C1S   1836.22560264874 1272.14273680177 1301.18893681695 927.137881369911
      203.524150518033 234.063633889254 174.149718758884 353.674560414317
      510.811470658575 412.863887464545 182.439945229388 201.415695076278
C2   181.914505612128 171.740347129234 166.506602328929 191.912670416334
      168.166938045454 174.406779990708 161.142723532061 163.781674803361
      299.29004204677 168.547575047205 195.755450324985 161.683243808528
C20orf100   1294.18114559128 1615.60472592991 1100.81588201714
      1100.81588201714 233.047541132048 427.766660764042 303.986544460218
      549.569374872629 653.879734066271 796.052115217208 217.227441947056
      275.908856196757
C20orf103   125.107483348755 130.812309202831 116.871350997346
      132.059507616101 117.585207821853 123.042372128864 115.090463537716

120.36402353147 147.962721373177 243.402781509365 121.926377140115
122.636980845207
C20orf106 136.480458837869 135.028746262383 156.020996568814
142.493119780028 137.497145594629 136.957209426823 155.080900277147
138.586620636835 136.162938063831 151.024201732633 130.551216910933
150.468392290279
C20orf107 163.024215671479 154.705630035232 162.828844182372
162.212442593001 164.423960071908 177.333305785248 160.676606760965
166.932757191847 156.396579029788 154.524588318927 160.117742879282
170.05799929575
C20orf108 959.897744425251 869.937761943249 1020.19774509086
791.402660066193 769.586135629964 1087.69177809384 1178.08490218853
305.720812071583 441.331937921872 459.388390392518 1100.17618574297
806.88003660673
C20orf11   888.371107987343 999.658013198849 718.670564185576
787.425941491281 1748.77270039774 1946.45172007314 2271.82656399253
1065.36715400306 1189.69730508119 532.394088545421 1665.14066952917
1029.75852676191
C20orf111 722.399669963921 557.214020485204 1107.99131702522
495.438672949441 500.469781477166 1179.42590997099 1525.24196488688
546.399972543938 716.229989890801 761.89271251455 550.901056837273
411.221560795912
C20orf112 137.85150144918 127.346646117375 132.858424396011
128.224742304889 126.796253980623 132.131378074469 131.759422682853
144.266864839344 134.664652044646 130.726640485925 128.202619705343
129.869363181125
C20orf114 154.161892545061 152.467764730004 151.543678860049
159.629232808787 202.524668687686 127.407418037777 189.589053840976
143.46919253255 148.04206955657 135.395071593072 165.964183460825
158.390337945372
C20orf117 335.47484353322 337.585502787016 287.924891622962
452.517134370928 209.398724613377 189.283071231248 207.785320401354
166.472068379965 159.13662975347 158.322477506517 173.979001863717
160.32812671237
C20orf118 126.282575548237 117.445284668654 122.501785735146
128.082956839709 113.184495948936 125.134516902118 111.11656427738
107.395559299582 113.143436184959 124.062863592295 131.013719535992
102.474380813907
C20orf12   127.512733134453 136.852403978525 126.626904958599
128.040201586267 129.489867764242 155.608876496443 139.06923394504
126.253319664664 138.167369090026 124.246363878094 149.910521500364
154.406369231962
C20orf123 145.962664629275 135.351344868436 141.597692730835
149.836491640473 142.863268359757 157.881569297409 172.045843691642
145.643752002787 135.427985445559 143.294217840279 138.938546468136
137.968932080074
C20orf127 334.101619029105 374.56251861444 284.831817899986
333.91440669951 156.834059922255 226.055406842613 179.828494168009
167.559703634539 193.173703015873 772.061917233344 176.849238577762
169.434164890542
C20orf132 145.106362864355 142.046082566177 133.900005368973
134.033751039071 135.433229702209 126.398884382537 128.213678179359
130.820415610578 127.31790396929 131.142841840284 133.274292647041
120.387275261633

C20orf133 172.430211711547 166.4983332269 182.235490620172
150.079372712689 147.805819921429 153.031479923324 145.852430082228
163.799748670842 150.038722708606 167.397758092427 148.598920567714
149.513744292932
C20orf134 150.058160923921 164.68600028429 153.184575380093
148.96493117884 197.795275907042 142.154646805122 182.292947734054
168.783725505491 156.978585870854 153.911165277809 167.243544185778
176.019082929386
C20orf135 135.542079867325 132.220398477217 148.466658640201
161.498785105514 128.773129597793 138.706520278166 129.684916104674
140.255768023269 142.173279109959 146.378522944864 136.468434653208
136.923876486522
C20orf141 169.349922114861 148.574661934068 167.791351000743
162.500485773812 174.761105961773 164.448110885609 163.521095869383
170.553940654493 160.72009357092 173.647320180498 156.053517438255
168.111124820968
C20orf144 130.342425029134 123.434963905308 128.81022558263
114.999628959921 129.386300987308 131.260111979219 137.58025011566
126.980375035189 116.65635246873 123.084040312047 129.878330410179
111.137519376499
C20orf151 119.029186503752 131.267177634269 115.594489123676
116.445184223804 470.868288641048 185.507663455634 206.845306095447
271.356289141017 275.929414239664 153.159425380743 234.980382243116
217.307872538386
C20orf152 129.956519535525 146.526744516344 156.346014084316
136.005756600948 127.765987130281 154.218967720305 147.971492964243
136.794860440446 147.502116289881 146.293735120772 142.991135981933
144.581747568524
C20orf160 193.502988612307 221.768507175814 138.42029331811
224.724262742427 167.301579775842 147.532747354296 117.716094828779
147.282520596449 171.212749388553 128.486896700405 139.543551482943
120.473038066927
C20orf165 149.402679897813 120.252164075308 143.354858193994
143.219722736891 138.12651823109 127.507557925713 140.622130978368
130.854818615966 134.367115858307 132.598022317288 120.78129870199
157.431972439046
C20orf166 123.809866652834 134.541606996118 119.418866166776
133.423376063284 114.785989868306 115.619116556625 126.88329158544
117.15886922795 123.687439699754 133.022848457361 135.283742166077
120.094156375538
C20orf173 129.513590304528 127.903393519351 115.68358702028
115.914842445934 129.995417218564 127.9928491829 121.843992693814
135.766466392056 123.256613702254 125.143281492089 117.965208293925
131.722397988741
C20orf175 138.940564112156 145.505223177269 145.34332740863
161.142723532061 129.966755942173 135.976837152799 148.916981164664
140.999877170481 145.449099891256 139.442091671938 161.412427590994
149.547635666621
C20orf177 439.122658719747 384.464383569801 644.301665020526
374.406855727422 210.822216545773 557.513306351027 513.20896845398
277.314675225943 328.33387481068 332.749676505218 229.373686092654
365.199125315964
C20orf179 103.826308328446 118.201092260839 118.372047967248
124.606795061928 115.041324824728 109.832757716464 120.076742232995

111.867464669155 123.96665768708 111.743461522689 131.606765174514
110.501341211051
C20orf185 132.832556359483 132.799355149191 130.786835186349
139.220877923507 136.874135348635 129.819820730239 132.99989565368
126.294085960617 140.763592237598 145.208597795717 114.960460615926
132.751406897891
C20orf186 120.263659727964 125.798453479983 128.304919825754
127.407418037777 122.355409068882 136.290486122369 141.70844587896
123.351112976814 123.522738813733 119.934231033302 116.547306374097
160.979182561773
C20orf191 398.354212009426 416.215253990273 550.124359517322
297.022984365795 451.496617003797 551.823767223086 611.540759532294
418.062540009856 444.584594376357 563.011988279542 490.269418483997
326.325210657599
C20orf194 190.318957029666 182.209056248253 211.035617136177
202.024504592763 147.73141408795 179.46936343225 165.731179108649
151.423936838934 156.490654498766 159.89988762842 195.239829623017
180.798671424395
C20orf195 131.2360427855   138.497931380734 123.108694517244
133.778595695007 152.673212084312 167.858490180471 150.942078067308
148.927040248436 145.003900788534 130.965418847276 142.99844601139
136.2727333616
C20orf196 156.233573126063 161.543072816738 148.605816748229
191.170105307944 160.72962443421 175.551853899221 151.645636946799
152.993555372242 162.909468312298 143.802883430376 163.215507313506
156.169405925094
C20orf197 111.992738490468 110.925532078514 109.29279076901
109.274221164316 117.773743603829 115.16087297104 116.802432258299
121.402905161896 116.884397420467 134.76586363177 131.322008851059
112.14768481333
C20orf199 702.460234202652 698.086166782221 948.150767619765
409.691865300362 639.184296110069 948.150767619765 4592.14655702906
1059.80292026589 974.007008568051 1524.46745239811 1673.5387396582
1845.87311313269
C20orf20   622.142195892701 823.297606185721 575.060457772307
839.901173743755 1028.39666838369 920.840069938767 1879.08108720938
615.88518704181 633.401624753368 662.373189001788 696.277780670166
906.359365544713
C20orf200 131.554055357245 136.337076023706 158.420821486138
153.474566767304 130.872296869843 122.941711366641 120.948246352846
125.882246459692 114.585818507336 126.205931638442 137.208796872026
126.730314982553
C20orf201 128.007359083768 120.644987579644 123.294321716108
121.121686280797 134.32174235533 146.482652762374 122.634741242406
126.17133877948 116.540729581355 123.703840971923 132.86879723151
132.245217686793
C20orf24   1179.42590997099 1080.02576143033 1539.80266129509
910.982557417978 4579.62296570302 4800.99572384569 5759.5586948816
3188.90202907403 6091.9840719327 3501.86788138647 1580.27059464289
4276.70912292877
C20orf26   163.153676643372 158.185593294878 159.52833930625
141.733840643889 148.951632781748 137.127587786536 148.655508303794
133.929311295884 152.415752253205 149.975892236985 124.313901870563
143.941358917251

C20orf27   666.570977208535 975.685771692095 1143.0779222425
1245.47421607111 1353.61552261489 745.844395215451 461.581199513891
490.464012303239 605.795439342384 426.896707161414 470.308610828825
528.921893077914
C20orf29   198.737421468412 191.029458520006 195.963361515546
239.649306804266 306.838596239511 208.476367897256 247.407442038488
212.33360547376 225.950750084197 306.716682293251 194.819597436776
184.426874913406
C20orf3   343.488356185437 357.431640917696 295.893482074241
331.747622606378 802.643357549193 1145.35733270036 1071.47374315822
658.068932688816 629.97342429259 385.522443482   947.425912541091
575.857954264295
C20orf30   1552.69879081022 1652.77814259264 2032.50052162637
1373.08023518911 2105.87438782831 1756.88092410303 1263.64725236472
1643.08061372695 2151.9395095819 1454.94889750152 2021.92898855142
1889.05071563459
C20orf38   155.393326732887 145.628239642187 128.329985147034
153.432933930805 152.064982697394 193.843978773764 158.726115143818
176.465559710285 156.218834695891 142.183154756458 142.599815022061
164.098880989592
C20orf4   426.032234135861 558.621429366984 562.711562649704
698.086166782221 430.94199035855 514.213675499369 525.789451405743
508.861268738074 489.403061697031 349.485727214325 474.14051635531
330.590986934704
C20orf43   2758.01526530661 3232.96611074938 2962.11407134162
3552.78712528922 2979.27692778783 2486.24762158293 3093.38255333143
1694.24232555141 2479.34567973462 1848.27927621606 1203.47834989609
1673.5387396582
C20orf45   224.528190554963 218.433524126584 253.385592447339
210.729945718068 828.276436883306 1178.83933381069 736.332743195416
529.529314022043 496.823662262772 535.354724252178 420.743637858421
566.647927867646
C20orf46   142.951829221947 163.319882002763 122.996616773523
177.666582096539 1029.75852676191 641.645855499704 413.330906392915
207.73395131954 266.101158071711 446.698665191047 280.723379809959
771.294254795752
C20orf51   135.879975973882 128.668672935791 172.108795190633
133.028191258668 161.12121879514 137.303701514064 164.120602897751
162.383584508358 150.456500446175 149.752188104803 161.534421340752
156.043994260248
C20orf52   2583.46653460894 2505.8565544797 1939.1525084845
2877.92845389611 4811.88424516272 4901.41175228982 8768.32269645116
6138.10904830456 4327.90939653054 5032.66681917478 3450.46728258139
4592.14655702906
C20orf54   146.184624424737 162.923734210933 144.565926379402
144.154217446011 449.220056843562 186.215602572358 167.304721591124
172.965746521132 209.362806125315 149.696766215866 291.369577397743
201.865955150869
C20orf55   246.77831953556 280.398987330256 295.727275232995
295.047418816148 1534.21175984308 815.779445689417 506.430593289427
466.58966852405 479.395948662331 592.079898463345 639.38881853171
624.638115878703
C20orf56   136.499228715856 134.840285218383 146.612602970963
128.306607216705 131.649830934459 135.173464753848 126.153806511702

129.957292351968 141.613898853625 128.848008246639 129.029151164978
129.127563278016
C20orf62 173.497575259833 171.552010365305 211.269041187154
182.929326147069 156.077604146143 190.489530928259 169.368090266817
180.54209603289 153.600132368924 180.63469438043 169.067064352955
170.390200730789
C20orf66 134.441142158135 142.818122106598 141.035566928475
145.758423590784 137.79714923769 133.449527627723 155.741507341843
139.055892787717 147.799806976046 125.911259629489 139.184647003326
156.328454726363
C20orf7 289.434186747703 270.33685210971 270.201322204641
311.670576741448 212.420616436137 295.187978994932 220.661922683143
229.881164774875 235.563592256037 225.31216514083 234.550692915716
314.063025950327
C20orf70 142.833025633146 136.519697160267 157.070500065037
165.890492422031 160.361025839456 151.81754291846 151.473239782301
151.09974848374 151.758970249544 145.967685593635 133.0349214478
154.978243057971
C20orf71 122.449574852865 116.305011582033 114.466368742403
119.375727296235 117.682034050478 120.857635836068 119.263824982583
119.442114850181 117.943511615792 119.870661213608 121.267153348313
117.47631318854
C20orf72 250.515579402334 268.111403429364 324.25775261173
302.570932942847 333.868818855397 746.777330152729 538.252273740419
554.652979820908 628.939201051801 411.160709628865 574.779350011402
705.987531074737
C20orf75 150.936747236094 149.202628825354 143.477670915091
150.396462763607 165.232545721733 147.2233876988 152.599052194143
132.320536722841 140.091378604453 166.936975782665 144.143692745479
143.063345350253
C20orf77 108.557097186828 109.167312436827 102.563722570171
116.695313319162 107.714294216913 105.450273343324 107.976233194926
111.608386387842 110.148555045276 109.113476990309 139.541759919059
107.07609243233
C20orf79 116.925863450165 107.828378413233 113.680875214845
112.41270361848 112.520199390427 112.747067417774 114.85602874727
114.223991452083 118.761737418652 119.658703392877 145.660417208595
113.362825181676
C20orf85 123.446714388901 141.283042601932 131.119810851017
139.08460576146 134.768694020981 120.345546077894 134.352155462211
141.194900058799 132.475414208533 137.029726467523 129.075693654369
132.681475162386
C20orf86 137.961504178836 150.333572271902 158.869463014801
149.219602109121 138.259512736404 146.335409183715 130.991496243273
146.146800933028 134.95842832877 165.103398617203 157.612067634423
138.802633518223
C20orf91 144.664600892248 150.812739240232 131.273916869754
167.536828038178 145.648810303756 144.829773937102 157.627490896581
138.640919060725 142.551161883518 155.511086289504 147.624372080117
148.139376772567
C20orf94 350.22221782412 403.47026424469 248.690121805116
341.973002478932 258.632372536866 307.410175748955 277.180850774168
282.797367565702 257.477501542521 231.47915227815 322.956882599715
300.675907594637

C20orf95 137.966987037709 146.508628678304 150.449621353865
141.244589488591 137.282038401275 135.526248253492 172.698181201849
164.078423844421 138.972789382679 148.340380102097 141.86370253437
150.498954408708
C20orf96 145.560904207183 147.085006263446 148.373866613126
148.609721587632 165.84822009824 153.005366371425 165.977852973826
142.097126256137 138.1774127311 159.524235332529 146.557058433232
144.433543176593
C21orf100 116.67463130093 121.065278765151 120.628904050564
112.949643549398 119.301746097298 119.594962686756 118.060781901686
107.976233194926 121.45175516842 105.952640312359 126.839037497031
115.048402956574
C21orf114 133.365793958118 142.419777428399 125.958139354095
137.981446468611 139.919332373983 132.07268641595 127.918847837351
130.430917518283 139.505864135281 150.173766182317 134.415163103911
139.469148541078
C21orf119 129.524056217383 143.365976841902 153.149523560021
143.660679324057 158.216790540415 198.489278156437 147.75625228531
135.127041919869 191.937607311711 129.386300987308 149.487167787687
152.454414703534
C21orf121 129.491732597331 146.094951139804 145.072924262206
136.790457887057 165.731179108649 134.441142158135 139.925201225224
132.661036963636 130.095585254401 140.569391362701 141.317113425046
136.841506794489
C21orf122 178.835679834487 252.015257713669 210.822216545773
296.20443113482 139.78795317086 154.094285855743 162.072695554798
133.18744841514 156.349030617265 140.968351978909 129.23899614612
150.52625734893
C21orf123 110.16174213285 115.963144087717 111.09179957013
107.580425848009 111.188410935711 115.409568875238 120.752194675024
130.651062724029 113.927168436775 122.013040475143 128.551335256123
120.020781939389
C21orf124 145.882793872634 153.231965895778 164.21464602746
172.03919618445 171.80440912191 142.882391486465 203.87884605779
305.854543429221 223.601312199935 241.867699338253 185.30916081621
172.478461106269
C21orf125 125.556444423391 144.70348930646 139.817493158675
144.262612328341 155.379116566097 141.964796033176 157.519633258844
151.852294315212 153.485731788293 154.197254746757 155.722314592388
154.227433553131
C21orf126 148.525224975266 151.445254181497 151.191822057706
140.418010366997 157.971293294367 153.972471779464 158.541906931082
152.599052194143 145.999823250463 165.898288304503 157.58659226599
155.134796468704
C21orf128 153.505155981348 148.069725041021 140.474338177948
151.548865569913 158.429761055869 136.587165242663 165.337956130606
153.389279691913 133.014551645069 146.781221728543 161.744366588564
153.136863548105
C21orf129 122.176811388389 114.099623939954 110.033746435328
114.078271807864 118.725813963602 119.693318959661 112.714282429822
127.91637627901 118.07991087231 119.866898356501 129.794293922951
137.451687756052
C21orf130 150.303703193967 146.231937458616 140.92377681377
154.296467085914 140.656633546501 136.241569739318 139.790098895306

136.418105816557 135.609239096236 171.145909843389 126.992697289075
136.575784685895
C21orf131 118.855500322915 135.067056559409 122.679628839976
125.130703953031 128.907618218883 130.165488538277 119.849895979393
117.619337212037 125.410876867635 123.463861015933 123.657246099004
121.613684281664
C21orf136 131.519455635415 119.590540166384 130.983136465761
129.756642179083 136.113421708277 130.900049359243 131.988591391373
126.647643493986 137.623019849539 128.659393756477 139.253721683078
126.242935839683
C21orf2 355.990908506973 384.051170099325 339.753059801136
399.537363692534 187.167255451988 156.238610152902 188.695527267631
172.478461106269 258.384059994446 158.505363728431 177.919885012006
146.557058433232
C21orf24 6271.34208268488 7292.0342195035 6296.24208225227
6601.004284244 1561.87054049124 7245.25086222102 3518.84173951107
5688.84918819457 4882.16892310839 2926.06746795504 3566.89107958971
3968.38723079061
C21orf29 164.601022396674 150.449621353865 150.817922696097
143.460361054254 139.096536887753 137.565050253948 152.925281215593
139.760425990939 140.037379695961 138.53954980556 129.953801496113
136.582137928022
C21orf30 167.993851183401 169.101676908756 170.140610481718
189.461090469712 170.12010526854 200.618080312379 194.125840053843
177.851689709807 164.352366170527 237.078644686891 186.515784641119
220.999383789474
C21orf33 747.587723573212 781.790858580895 784.72162514331
862.669120338424 489.909407178594 621.354393918348 850.562379837947
757.066786599446 811.912727084669 650.263737836685 916.4187683687
729.729297413233
C21orf34 349.722963659175 313.077481033093 301.139737613279
393.115606440229 151.479748626813 146.268073519544 138.206065640158
137.42213762011 141.440563159897 148.199249855894 140.117644705946
152.550031868259
C21orf37 180.219813240126 164.302998010829 156.895079878556
179.973655632636 177.538262133568 157.519633258844 177.204172894028
165.333608076984 177.562561297777 161.129770392464 184.029968090084
179.411426984263
C21orf41 137.134190411005 135.945030816689 132.970515991525
136.22416508552 133.203064891923 134.076865693417 139.706279455371
136.83470470035 135.419628087842 136.724298878331 138.102654893864
142.139331321085
C21orf42 142.230934617475 152.840202260007 143.464614171516
179.509758601636 146.639556437847 144.159316670264 192.286192329464
154.466614452651 154.978243057971 147.452986308926 140.965498009244
169.992657041967
C21orf45 188.118989395838 218.794112567823 189.160454951382
226.763252784108 225.927425596831 395.716879127149 260.787104931135
439.727046291381 445.348044448953 248.729653566598 224.062024091915
259.252807304272
C21orf49 138.802633518223 137.068717031383 137.778033050658
125.687688416588 127.681662711369 125.914267820741 130.564962937297
126.462522391125 135.982797108848 122.670689386332 134.528971384442
121.71538073735

C21orf51 237.940015617254 208.75656341402 292.23287968517
222.709720471134 177.619101765848 261.251965523684 267.285998701386
210.973303650385 247.971366020963 177.598422362298 209.5184563244
260.157440518033
C21orf54 152.169087619827 149.31020447459 144.785852437305
142.041992878641 144.45968532565 162.364266549463 157.776321544382
147.604213625382 154.843607752473 151.351938656508 151.224289879776
155.011931456736
C21orf55 23318.4614981217 21754.9904891312 22354.1731362334
21650.9263365935 7468.70895916615 24731.0629964468 13360.9483836861
20099.9833491654 19496.8914296296 11235.4970815873 15463.4998494462
15354.74541236
C21orf56 186.611870261871 234.550692915716 165.062129636008
262.832376198119 245.915473236403 156.96934803556 236.032288046282
156.62031503525 225.31216514083 157.519633258844 163.245806772716
146.486480663438
C21orf57 199.288100834476 247.08460296705 180.192015483309
263.058972886829 194.22713184875 217.670330817769 203.410932496844
208.914498369991 355.990908506973 230.589110372454 272.666435295288
196.536086443884
C21orf58 1466.82170436229 1255.02720867812 1309.01193284992
1527.87148052149 428.569462312363 1279.9555644913 908.352265585374
1050.40488466179 840.623505494489 654.881990232975 1046.65224316733
1052.553421812
C21orf59 234.063633889254 246.152798696993 275.468194644053
218.369954323686 232.151020756625 439.616817413027 376.276954313674
403.292651623365 492.930906036297 298.519371102278 278.385151185788
347.73629731446
C21orf62 135.668768914964 114.242056888286 130.601241656346
118.201092260839 131.646121568612 134.064206964444 122.385508330707
119.795945889846 122.355409068882 130.795318552623 135.36523178082
131.001996509052
C21orf63 438.611973005212 439.122658719747 577.250493023431
318.423602671236 173.816408146725 212.210383398591 216.522992914538
180.80811429888 178.153527134452 251.09230266334 151.459397907666
155.007516124803
C21orf66 275.696829424061 239.970202560698 299.390443444504
250.25959091612 199.209279169496 422.12964420709 305.720812071583
409.380002417794 335.389274425025 228.926077117474 263.606930507829
301.250189051376
C21orf67 135.168792286819 144.546597366629 120.288469716375
138.199559519849 124.910390240995 120.757288533244 131.532660547328
111.1002996982 118.93828679356 128.504026473695 135.416008633632
114.790717658188
C21orf69 148.742216703684 155.383166865192 139.898996725236
138.885230982475 136.27621600731 125.475308885588 148.923977949304
152.404186073033 147.664505309713 134.89415027205 146.025781495177
147.937679308903
C21orf7 217.778414825515 272.01602406899 259.229153662504
274.70018535436 162.664254855131 193.892810132985 215.585089158905
295.727275232995 275.609716327452 318.423602671236 226.334672545273
310.426513884979
C21orf70 220.047563627558 205.886398618369 230.227432807772
250.647644543963 229.62164568989 176.641899861006 216.427103365727

178.304914997023 197.24751449599 202.622747952763 166.360005474414
191.029458520006
C21orf71 132.524377835171 153.039237274992 127.646032547296
171.022976593689 122.664353374047 144.482364671273 141.533138525284
143.438417529799 141.919263493165 137.73313146889 152.385014030957
125.863969666806
C21orf74 126.94941821459 147.805819921429 127.632566406664
147.127908355066 131.811011266156 128.329985147034 125.342580368417
142.163834995141 132.887701822875 139.962307072428 125.879198238067
135.294664456623
C21orf77 129.27634490676 145.70726782469 142.13191463827
133.699547652179 136.545742688573 145.311964807443 132.641236788053
119.780654293448 124.403662787819 134.160100276452 146.431166706908
136.779180530712
C21orf81 190.152824885328 215.335795337978 216.290279815413
179.923966067226 161.817609920327 400.550044186372 651.235249028924
527.707841428677 237.01460308334 155.323822771755 278.142283306934
232.879578820053
C21orf84 174.66966623557 168.161117444474 172.002600588279
198.94325874493 165.565284723155 168.091278015885 175.199369463703
188.543051108601 184.278213908263 183.168629351124 164.327140372968
196.329602378007
C21orf86 140.002249873656 144.579725208643 169.077657790409
130.836079676477 146.257923971069 140.160349667726 137.77107995457
126.924014801186 136.116468624715 158.758500675009 134.762169062357
136.957209426823
C21orf87 124.391225837909 129.179188863839 119.511962218143
117.303671445328 119.759350702482 113.23302126254 116.092815232176
115.277302351501 117.992729826121 124.571807093748 136.207420489201
119.968081249475
C21orf88 117.470731811595 129.891492052232 126.912226185444
130.410584413031 131.662585510218 127.342017026873 125.398875527365
123.275246170603 134.640783050966 124.74291205933 153.761061653202
125.034243098087
C21orf89 121.559477523123 132.383542877819 136.128092230238
129.041254463346 129.104869596932 123.281489592543 140.84880167881
125.500790447338 124.624046191085 132.815753681623 142.051259785804
121.459780480915
C21orf90 129.1829592305 127.193553054979 120.35956894661
127.259038241163 120.944267930809 130.483765953389 133.638664342783
122.766778018066 131.934680869554 125.926774314737 145.229637715814
140.004456934031
C21orf91 162.531630234824 157.385256442734 147.841960346835
161.543072816738 155.933739693912 174.08222895937 172.222262910052
186.420541099902 189.784608429624 175.874387612235 160.876228450608
161.069535896864
C21orf93 142.906227759701 158.911404625381 164.948785167149
164.753886197136 155.680330654611 148.000706956532 144.235298492095
160.353649786837 151.535177950367 149.166635189875 145.737361356678
161.310855691717
C21orf94 122.860898886143 114.358099210532 125.964335876018
112.884916986215 118.725813963602 115.821231468312 124.995087711332
113.031636180748 114.122395647516 121.124574985464 126.402405227897
114.658668711121

C21orf96 125.481506166304 134.256243410916 128.872496485403 146.176240539176 137.084488743415 141.50968031684 143.881744063934 131.000186898437 138.161690144518 140.855078775719 143.35902319003 145.635453468646
C21orf99 107.91011371422 123.615802924067 122.389557272277 114.734439905052 125.481506166304 110.333339548402 135.559535347364 119.608334527504 133.872561340464 117.552229262461 131.076562651831 123.22295112139
C22orf13 1292.13921040404 1570.39362935859 1512.28202818467 2496.17211592866 1245.47421607111 1053.65938651514 812.75075757351 894.734451279225 1003.93455977232 943.23069404564 917.188039935737 928.672013047007
C22orf15 132.034701177759 114.39161822971 126.323649734972 127.182067293944 113.557349524174 116.031631038289 112.332130647215 121.286130275542 110.794219964997 117.883635746379 116.043095688871 110.820329153348
C22orf23 141.344034069373 138.197500836501 158.610249594568 125.710199961297 109.416512252532 141.069243738242 137.445344116574 130.219888837397 143.229240978574 116.485180988801 131.617027772841 136.256456726841
C22orf24 128.124456615525 136.19083681188 142.133238886052 124.59097574481 139.687574432387 126.124937863679 135.803599969904 143.04129238495 118.679664392666 115.687997771921 144.364319564075 114.704831217037
C22orf25 230.284216427158 246.77831953556 179.854800301573 266.135477775641 296.24781576525 185.396765725228 188.623897093065 186.167877791105 162.58152435515 146.71164737466 205.162633740798 182.747079625619
C22orf26 132.17294083844 159.572851489665 125.9888344762 146.035731780312 130.944610173285 122.914680091926 158.163599942547 137.426226808356 127.361974492821 139.136845089275 133.865312257398 131.312580984876
C22orf27 154.591116158776 123.82555184043 153.377182895472 132.195666100325 179.785198362326 247.407442038488 179.048139035247 269.006221210929 222.593554194453 171.813279175663 173.424541543058 224.167511918105
C22orf28 503.285430318683 467.187222441283 569.236322997211 487.19625805337 646.933504790891 862.132582849511 511.25179427158 767.377380800066 678.937151822411 680.743052197025 678.937151822411 628.197839884392
C22orf29 195.20398666139 227.092319765009 220.345184229077 266.61343130101 315.615758089471 274.534901548678 249.633252091596 242.848821222611 238.375823012603 170.622615783608 309.861048237742 236.326943078666
C22orf30 398.796188440256 383.407365952689 444.374521095495 394.892918166519 489.118864124059 580.164288826741 718.449150898903 824.427857809495 742.517302567436 507.95331185029 615.485984065221 719.766799746895
C22orf31 106.876902604214 119.746227888415 117.05536677976 117.77708734157 111.549719888905 109.700513642061 112.298254005045 117.461198576063 114.43491928161 119.057157701097 108.984922760455 109.098105822556
C22orf32 215.931781291835 209.93789387724 239.73396414153 240.465165615601 169.815053752464 178.245841646175 154.118601977256

150.831554010122 176.100691355682 154.703521794209 200.22145130696
175.7330682766
C22orf33 146.247825342634 150.321059418799 161.877378749103
163.400576719974 171.644710031098 154.395261192114 167.55209828062
143.163083976105 157.04599638368 153.541193559658 145.28524062436
146.929717840755
C22orf34 115.37736249419 127.83967243279 121.150371270451
134.384963577069 117.231243105897 123.582509531804 106.603674195422
114.093935026467 137.612446150193 108.288420807873 120.918190840083
119.349376960211
C22orf36 339.448524400708 347.292438587205 243.893774595317
440.916522871266 149.361498483705 123.807693112932 130.172041917023
127.109798012351 119.498312996987 187.48055574295 154.124057382276
136.180063437699
C22orf37 126.934059693116 129.725706669166 129.305455204291
136.522590104535 128.086493618022 111.837468699281 120.371374172279
128.634431242405 121.053766591551 117.166509072455 142.543569032033
120.971313945446
C22orf39 203.487137264 174.663845688179 225.005311517365
214.082408290757 188.36361073603 149.630481387506 175.585857393085
167.906766146651 171.671895682052 166.944701842349 162.58152435515
172.370108210895
C22orf40 179.297628357211 174.866036336378 192.07791693203
195.608619642038 178.606255846041 160.562164904057 165.074983746785
186.866029149035 169.089896796513 159.074001164836 175.372355078713
204.654489493854
C22orf42 110.148555045276 103.826308328446 107.170981307304
114.099623939954 112.675715371087 108.167670899938 110.419222236698
103.202873534814 109.128394735532 107.431455226979 109.333276735523
118.173752075833
C22orf43 138.698178321535 134.21227169867 134.331420680628
151.796986456254 138.820797333519 159.241573314833 154.248226423862
158.312614511836 154.190469538568 144.288714958892 173.577723566555
148.670562837021
C22orf9 159.508223180752 157.454558821261 180.327051100611
182.42088387266 144.604714612091 147.416991272497 147.457193565877
153.156264091925 153.784923783551 183.50553222647 161.359609192977
156.632939064629
C2CD2 569.353661326724 585.721610434501 546.181615768667 574.035124314622
433.928818077527 567.317223907142 913.22732386079 615.485984065221
866.765054377214 360.551560710852 746.296354515617 614.514126276981
C2CD2L 140.538020869886 134.772545694636 127.097605337434
142.537484065487 171.69834783037 135.961724275921 120.26749529985
120.649467083125 129.737882221259 125.256339476772 132.357517495246
138.9878987332
C2CD3 129.47419698487 128.572388808833 125.236926572282 115.409568875238
121.188761627967 122.379537202709 120.702740607924 123.170639180926
130.973504803813 117.212164526913 137.933122094638 118.945264994404
C2CD4A 137.647282354299 118.597739444445 139.002932985183
140.316066297536 145.176141415244 136.020600085547 158.085935267091
137.58025011566 149.25240100689 141.494666823639 129.527271401014
140.669947387892
C2CD4B 225.050796356289 235.407064742194 144.968459820523
325.320888625297 189.250846546817 144.578454791104 157.838771553729

160.994837128661 196.880135762817 158.23884088767 197.51109998167
140.571143847885
C2CD4C 139.907840403521 152.767228183775 131.391434131813
140.914966429342 146.077082896858 144.39135701666 147.751554743466
132.801619479245 139.138329217724 144.89403947454 157.484541464879
155.346805606742
C2CD4D 129.148634038268 129.780648518219 125.787353491408
135.311980936088 140.543382412084 132.963761182049 136.16560274503
141.284002360584 130.476322267589 135.860777386274 149.219602109121
139.54738445816
C2orf14 135.998087745992 134.371254010574 144.107029869771
152.927956172463 126.132339293132 146.150048478173 149.675363177375
129.852833137493 124.88118767481 133.080437161283 133.541333581593
128.594951429021
C2orf15 151.367402326399 148.444479022559 150.300108097623
154.695060071104 166.187099519035 371.224296589448 257.24481414803
207.692077301142 226.334672545273 181.715992480635 210.973303650385
237.249949461085
C2orf16 103.448244268961 116.71331256222 131.029333093048
128.570067783535 119.407293854922 117.49013401175 128.963962067821
132.183250916482 110.024815508703 117.343165720552 142.75592155869
112.755143719255
C2orf18 184.758754197435 169.520544329439 179.49202902995
192.314484368435 347.985315257161 211.720536220806 200.179796117115
169.089896796513 179.026308021985 198.631354372924 213.813667557079
198.979744266277
C2orf19 142.540433565262 129.785078660064 127.116637906391
128.986795394842 128.054394154801 133.43194756868 135.894512504725
160.082087242964 146.959154938248 143.92831903543 126.51808376464
134.918496279185
C2orf21 160.180961887686 153.511995286293 161.640122360448
148.376194044247 152.11446058397 155.478383896934 154.070450565144
160.903342063542 150.952264779196 163.824878791966 158.584934514596
155.043017644694
C2orf24 462.553267274302 549.359675306309 522.486866458783
680.509421043347 610.070236234299 278.335289966805 283.014222473749
227.288015854809 357.928880740814 336.70893120135 771.694853062033
305.343879847672
C2orf25 760.572409199179 675.480482223958 1402.14708471728
540.359249705821 1040.73829397913 2208.49325118803 1286.55103838368
1677.93424842267 1516.97921112926 1984.47173096657 1199.35864276583
1790.53556140959
C2orf27A 140.226586111216 139.326694135427 130.797498958616
129.973380117841 133.957160768065 120.611442477736 130.513071750123
128.927103442679 130.543453631154 130.705091244939 128.455670598648
141.4587810296
C2orf27B 148.130993521745 136.654902685184 150.176942469551
147.809934374849 133.387065801837 142.639587833858 146.539196738195
130.501010489883 140.630844296177 139.993960054847 136.05610450926
140.025668085978
C2orf28 2056.90127387648 1746.30519466572 2135.04382065446
1443.68785521063 3876.04966263281 2360.87760349888 3222.23985545506
2230.45015969936 1582.0221231161 1576.21727854603 2309.63504858248
2410.32095582515

C2orf29 1138.00644332379 1276.14915305515 1168.68074786473
1571.87128697931 1669.97250023755 2056.90127387648 1530.45813438919
2203.82462320864 2005.76678571689 1166.86593572716 1937.83242524439
2388.03256067711
C2orf3 157.350979071365 141.350135458015 142.157512965166
149.260811184287 137.012646671173 125.533538277681 138.864844664017
142.278523641008 138.62994237184 141.865758685796 152.217243924816
150.815117344434
C2orf30 658.202765061937 677.023591987179 635.819650434542
670.097523566652 675.083275037052 805.875557314697 756.618969995355
870.887539668967 790.790236361922 579.049608568926 622.348802511009
692.095005197252
C2orf32 329.82030842286 279.834616502591 288.182388699147
242.299252896647 144.89403947454 147.004172510641 138.145677924612
159.177303894223 175.139231359014 145.67389337654 152.030182228463
138.48552149031
C2orf34 203.298764374262 185.591700082272 267.589020526148
215.950811436427 237.420951138877 200.890547965585 183.033044475752
212.443407602682 184.813094907887 275.755038921952 197.827534617557
223.666832044186
C2orf37 168.734244316967 163.470025318612 164.841156516242
188.836327151669 190.238332933452 178.665552649698 175.593421867771
185.691922436569 176.650953513413 161.688125121941 150.367927843529
161.172420368335
C2orf39 110.475453268997 121.764933633063 103.99234480919
116.046983032621 106.701716475947 109.760525674874 112.321396616213
110.935688075738 110.774802298005 108.768730405582 117.274977920103
101.149125916797
C2orf40 1367.19652160532 1195.07608384189 1290.39741199701
1050.90881385623 144.313841064968 126.839037497031 127.9575507576
128.56000638844 140.171749368266 121.322817570375 131.067958183644
140.995418231627
C2orf42 432.515970951769 405.469705787631 425.969575503375
461.581199513891 395.645016679312 325.011269531407 378.24336892367
485.373169494941 319.639103463458 309.181150342727 389.177193459
315.786908578669
C2orf43 222.028626209166 178.394895649664 219.151144724168
179.478756495687 195.740661051834 377.148528768679 240.814860403562
195.114012321877 258.552964322998 248.360540656183 247.08460296705
222.051005238856
C2orf44 362.019824026781 348.457808063307 400.353795948162
458.047799748696 312.601510033941 329.779210536849 384.365878895439
420.743637858421 312.484289357493 309.318403417731 351.395760651605
249.925640256322
C2orf47 305.050933898691 314.751799003704 461.413469623811
275.543576337502 463.99922423125 814.110601393539 614.094097408035
1110.43791444289 782.448692528135 622.348802511009 395.927222009721
827.400420888645
C2orf48 126.159880038854 132.23149173224 122.05954649985
115.545558600232 122.841135364088 142.675752131935 143.071933285651
125.535877109652 133.777238071968 113.529619135543 138.017249996128
125.402714360058
C2orf49 236.108432603479 205.765822478178 254.447650810819
199.235895708128 265.866465528654 222.484058975856 255.56301534833

220.510248260182 208.363905749882 333.970844203158 198.840533461525
276.32528705384
C2orf50 129.614692778261 126.05295868521 114.39161822971
138.48552149031 131.963419175907 121.322817570375 128.324383749931
128.501189288377 130.4631522533 132.175368142549 138.720489866687
131.151578665754
C2orf51 141.56459720769 143.853643511287 135.018710687313
146.309171902112 155.91493956251 136.846912694371 139.912325578837
143.496669740707 129.980354138975 138.814930200619 105.606964521964
148.597346678768
C2orf52 142.643027992419 130.056727701826 144.338069855334
133.849237689264 129.1829592305 139.862403685817 143.347066447019
137.25054616038 142.194507957661 138.376176600064 130.641217515008
142.579346058088
C2orf53 141.683298500736 126.970952206224 127.507557925713
132.714532888989 125.358906118533 129.102182717009 123.010614051218
127.476819912992 118.470400686096 121.391637838137 131.753135000312
112.675715371087
C2orf54 152.254519859308 126.305273697185 148.938822951404
150.434376975922 144.339819009283 147.905406818238 140.809956497396
147.407093814154 147.676279102364 147.108661317945 156.863232221623
160.870390779084
C2orf55 152.699431442817 175.121069571371 129.612536218578
145.847629576886 322.36247485445 366.857139377277 251.307020289291
360.812953749077 306.111172289632 283.701987275608 347.866264631778
256.471672362567
C2orf56 208.507486041165 216.559563381808 216.755681508811
222.624150073858 284.195575802966 334.316711893654 311.10841853984
269.770777921703 247.373571011712 237.146972312448 222.990586491219
273.806452412737
C2orf57 133.327778635672 147.585987099202 147.103340218488
149.430396727944 141.104761546577 142.013370954791 163.704616231638
157.702540365469 144.834448238435 149.274901871663 141.259186690966
153.767488352037
C2orf58 133.12641302705 133.012006684865 137.045772853855
132.076865522593 136.799138736925 122.619429537799 116.806720503431
135.499195435803 124.178483414968 128.689465747173 135.607537832704
126.133366366356
C2orf60 120.816130267055 113.416743649576 123.247698282967
114.539671934155 113.832885222354 125.243735617266 122.320966091375
121.30744400137 123.361179535626 119.305917403956 132.807755600683
139.048016900762
C2orf61 124.446264376282 130.268322464805 122.300069045533
137.974323288786 120.13869466889 114.567609871292 141.503351545458
121.464383202986 117.834484996559 128.771367080039 122.187031504466
125.077260389449
C2orf62 130.147848418265 119.233300586525 121.010811158088
128.336332748047 125.034243098087 125.645986634914 131.050255325564
117.995551728392 124.621381699241 131.934680869554 118.414329128347
126.796253980623
C2orf63 178.358003030064 200.486683704179 184.426874913406
196.676479140094 175.529441666151 179.060189557496 204.453822958077
193.704510912321 187.183762762259 216.929969034881 244.766289564524
213.274106157675

C2orf64 600.264940641426 660.315285709317 794.165408266062
641.338900241674 507.95331185029 653.749043828462 491.35949123223
385.584224833836 644.97200337335 363.195371577097 654.663838918786
482.344514972063
C2orf65 135.952202055637 150.452467115669 137.058578192268
144.339819009283 141.982743538528 158.420821486138 140.460927375243
122.158160927149 132.529247880813 146.975258859244 144.120106441052
136.678188851101
C2orf66 120.148189532583 118.490642232641 110.475453268997
118.677203713024 107.303126530947 121.344724993789 121.188761627967
116.273000405449 122.989642938474 117.238968848218 127.284885819903
114.607138204495
C2orf67 170.202597821861 159.286484253487 211.966139902562
162.018860183534 138.59175751301 161.335677383485 158.130933580414
142.617474507282 150.929236917651 153.389279691913 171.80440912191
151.794243108455
C2orf68 212.420616436137 274.934292929666 269.530151236658
283.556453980797 154.113228966411 228.766847429517 195.724873069362
182.165681034082 198.885004378065 156.572774198042 252.32907388173
200.767743455226
C2orf69 13142.4763966378 14228.7550296952 10704.4678928541
11479.7406150358 2403.42261883515 13563.1694195379 6427.33783376005
11326.5935775813 8958.34797715349 4929.26915002209 11432.8213906428
7992.84111657183
C2orf7 379.862305342147 415.749051861123 448.715938510132
450.327710374141 610.915977337787 430.505037012814 257.602370597316
364.022018936132 280.127735578097 290.582957824549 261.922485502971
358.298768623212
C2orf70 126.713610681382 136.052640679977 125.136745288071
140.341589053972 187.965294229013 145.855671447779 143.558088035758
136.067657949844 139.912325578837 142.086300138469 141.369723386494
174.991941844263
C2orf71 141.882859092658 141.412317125776 140.603933807629
145.612695657243 130.570050159191 126.245597154052 141.040862279633
134.339535632463 130.363516180139 136.563471322998 134.239416179248
135.15791174418
C2orf73 133.143695166498 150.573523216151 138.723149134933
140.53597287752 134.682319733908 143.06806126472 129.549673572516
141.209958892647 133.724656321495 137.485252316816 131.992186668019
130.576944864484
C2orf76 253.495214569632 255.74096237558 243.308625792448
253.098839084408 298.827660296715 274.092048989484 216.705908017858
244.925472495705 255.462706428149 293.317880637519 206.127493726541
220.876114717642
C2orf77 158.104336050835 155.62770968777 171.022976593689
155.211976328553 164.185346950306 162.427505889157 138.544053540799
157.350979071365 157.13859713415 153.165872773973 164.38282966938
154.327065859051
C2orf78 152.22701178148 153.02549014611 152.327247552986
181.354279096644 140.216148769999 140.277228495746 147.502116289881
153.230304322351 154.352518161283 147.717748449017 142.960875909369
160.748082658758
C2orf79 210.085329463347 218.96845208513 191.861598059182
204.438365139666 550.671679318681 402.473870771741 450.120868656638

461.482735533799 299.513950278952 372.81255844018 485.309521583972
480.923468806193
C2orf80 136.522590104535 129.485710719291 130.12032144763
137.871870950302 128.793186785667 125.353389826276 135.713266118447
139.82050388626 123.260800590266 125.600500146679 141.647300896529
129.797601100832
C2orf82 145.247717878099 154.479733759712 134.076865693417
136.960656707275 219.394500571586 140.678336884951 158.335952927257
155.078035506545 166.346572488391 167.139257044999 147.870549240703
154.153249796248
C2orf83 105.665621968915 114.43491928161 106.701716475947
115.033355516602 118.902823494379 112.57049236673 110.501341211051
113.222264855561 119.094868654252 107.780763250481 128.339161042799
100.04568139926
C2orf86 124.815853677553 123.732299841696 128.501189288377
129.725706669166 126.177491182442 124.019707408094 131.141668650877
116.664031217378 122.490271802207 132.539585717666 133.743708220112
118.816986517698
C2orf88 137.372131049785 140.277228495746 153.181097279473
133.571962238112 141.170937695272 134.506216147934 143.895399273595
159.879600154928 140.347131743987 121.781562010645 139.367493349339
144.54895913821
C2orf89 125.367386907897 130.939543211439 135.982797108848
133.44592405762 139.22543724535 144.292679144059 139.727248583626
153.028658323116 151.142149775806 148.291518446423 130.443360119099
126.569482909992
C2orf90 119.083957999725 132.937942776777 126.845048064014
132.275953817076 118.348153875356 118.93828679356 138.341918073941
121.672811608791 130.797498958616 122.624828884731 130.822330736013
124.710368327189
C3 210.64483507528 167.746786642066 199.353823724989 153.703682621695
127.712004247155 123.453048609213 306.773350448741 140.78902818186
194.002075848131 136.653140842194 156.144934427609 150.211719786127
C3AR1 156.856149172225 147.966224814966 154.771737131348 152.350537364705
155.07594431078 180.113222861966 163.970187373891 191.58185405868
183.804849355341 170.553940654493 143.629760301493 154.716750150891
C3orf1 178.628885995439 194.702974020747 209.196695368783
205.546628064296 155.971045744096 172.794570587596 183.294526267593
162.072695554798 193.569894919176 168.611652451329 171.145909843389
176.75095377028
C3orf10 750.10819423982 680.743052197025 981.313778685019
553.123179808258 586.366632966978 1093.85316636801 1084.10984365393
1078.41947691758 1105.10063745656 741.433093896472 1124.67710585154
943.677679525947
C3orf14 227.64196401771 211.483693299981 260.370285504512
234.841813258798 955.143610867351 423.740836541835 425.969575503375
263.74335003375 820.249910349431 368.637819994033 580.474775712828
821.862271569917
C3orf15 229.157389498775 243.979769719186 240.631264915238
245.426639190893 157.809081184874 206.391403266202 163.566535828036
159.166059518117 258.807028561407 149.369418844044 140.347131743987
140.16233428205
C3orf16 121.61143371132 116.881212516879 128.354163132392
133.889858322181 126.365153497568 150.769819282075 133.969054754299

124.469415593643 133.173541338355 126.978144285534 114.308806726044
123.391298926957
C3orf17 164.717809928624 157.635531050489 168.27980269501
150.645487532549 139.188234030711 166.289763097913 170.596937075493
142.066903301882 142.047828639169 141.059033576475 129.450937831864
169.844913799159
C3orf18 227.144441241869 216.805365763069 235.019573994855
341.015631528464 178.657325741035 129.487458179949 121.846349358116
132.131378074469 132.799355149191 125.384706864406 139.654648891206
132.48841450242
C3orf19 510.555184032017 547.516635695872 637.892677173103
620.760947818536 374.033009122079 548.014025722956 571.439520427295
796.439819579938 694.02978779693 348.848550937417 715.039905439621
726.731270955732
C3orf20 117.646963986512 114.561836290349 109.274221164316
115.366496551426 138.598167973067 118.115015846489 123.75974844367
116.90832995093 117.517649866653 132.576755070703 131.997666258618
112.348094614855
C3orf21 423.740836541835 454.854528472591 366.473258467746
487.597040819879 486.947380524463 548.455251483009 365.950612890149
484.903322308422 452.167674725454 742.128229715198 476.168679880357
494.747087458483
C3orf22 185.113098620798 204.078331021637 176.236140830355
206.027235767232 156.543267949167 213.531307836195 222.565515020501
189.647009287524 164.634694036993 192.375251343504 158.092775850886
201.837695818264
C3orf23 152.182968451691 153.675939908241 154.648806105422
150.170712508944 162.208028489943 168.996965070202 201.256649338315
171.791030104358 189.799091507686 183.071150926068 166.46473789699
193.052112923573
C3orf24 148.842322753916 136.085524091635 151.128838696891
135.682971958096 152.09245741643 146.49022197174 137.913497715424
148.42845062376 141.329559699056 149.88598449818 129.802596741962
140.56526023628
C3orf25 133.305679931132 137.022366035333 126.455271988029
121.336932276232 126.539877052899 122.337240239463 124.961859983596
126.555972138115 126.609398779757 130.761385822314 153.435218661228
126.635105491524
C3orf26 304.713434036642 347.620001353026 426.995700632434
292.893332413185 360.361719991381 399.537363692534 352.945088028205
451.210172051464 392.824840252824 549.359675306309 300.047728281275
327.384671423687
C3orf27 151.449690439424 136.357788670568 156.233573126063
159.89988762842 156.375876454394 163.926432479673 149.734395134321
165.634117939571 162.982557003317 145.915129713165 141.42859050659
156.030794481734
C3orf30 146.470444212748 141.204458186976 136.648306684974
141.755954088275 148.263293819238 147.059096208832 151.459397907666
145.741465098455 147.87699690647 157.600193849783 129.467792559297
145.172776053
C3orf31 219.996410908721 235.563592256037 241.944581086483
192.418895349837 334.056545911557 366.240941980786 382.233826557803
686.028214419557 350.077206368839 353.50447714719 230.589110372454
387.224184063662

C3orf32   142.466067016788 143.574584852426 125.429085234515
137.539385624206 140.443415669628 128.580625868379 120.031976318697
117.078720392441 139.631165400881 119.233300586525 119.480037559953
130.242779079309
C3orf33   168.161117444474 155.058852833902 149.405118010893
153.43000129811 176.77183598995 151.730862403472 132.069175779393
157.604224004016 141.359142214228 121.018722403628 137.801205656422
147.761965925044
C3orf34   5894.79176478976 5382.31371568106 5981.08862268291
5624.32062666666 1000.48723872058 4562.75659565027 1944.15673951528
3463.23822434026 2608.95227456417 2180.31963682546 2639.60135568
2468.47687203968
C3orf35   137.391141254051 130.461084106455 130.883516197483
136.993102884269 132.5377752987   137.383211680459 135.624881949214
128.864822736402 135.857403020659 133.533718725309 145.544996009633
137.336914480226
C3orf36   158.679903271595 165.380225715852 169.607072855818
196.819022688128 145.904866987074 198.94325874493 198.135240691225
172.002600588279 157.929976848515 163.951459935477 152.93058803161
179.923966067226
C3orf37   299.463563393179 301.304268871606 359.776068595698
310.174932137546 464.812091712746 394.576038252334 317.659548748735
434.509391836336 406.458130553679 398.512947526406 286.469773081481
315.47473326786
C3orf38   179.358460170348 176.603760647035 224.92725876841
161.17923997423 244.627091189503 281.694635436938 292.893332413185
284.534369213195 322.956882599715 296.497100375888 213.097664319741
338.934501259414
C3orf39   425.229044880287 491.496499481595 374.718289636553
569.866002248352 1172.96121022579 297.970735665595 388.002365247498
293.825372123877 311.449205777595 229.347135478142 289.037033338975
239.541516080716
C3orf41   215.897620927751 143.577865527891 184.493681387276
169.147351642004 214.331491507489 187.871017629994 156.01406297447
159.620467672898 164.991522994763 151.815211341628 135.99579763634
139.571752933877
C3orf42   142.729945734027 140.826051635712 143.405079593778
163.380402251758 132.793020446043 144.458829225668 152.713528089347
145.744039709418 147.153129531937 147.397591251955 148.600270795407
132.721469335671
C3orf43   148.423303776817 141.696969082758 132.184217000689
158.550259547851 139.765361891899 139.999298193212 148.553324405123
138.068164255305 145.437544062449 143.403396831929 143.58555556787
138.02757574324
C3orf45   152.733409144267 138.461113585594 149.848740870148
136.271412363498 133.452032750397 143.517327774456 151.563917522864
147.441650320053 159.837102036755 143.707084981291 120.735176435195
131.173355686001
C3orf46   134.56314960832 155.810034593382 153.095594614683
148.532339759414 145.414563914801 144.604714612091 142.305484770249
126.649739818781 136.537117054998 144.667638908504 148.725162101789
164.545596179262
C3orf47   134.288349336742 123.389038274913 137.7724100594
122.561285351587 115.00776048097 129.121598974852 136.337076023706

126.511314417328 139.303747021408 121.619593453259 117.251233255966
137.59017685842
C3orf48 126.085039271526 139.856251508283 122.682761393215
120.01190102634 126.186758919929 122.089332300031 119.052729327147
126.682913361149 123.582509531804 120.304037458526 130.300169316346
131.583841535781
C3orf49 138.079494235456 137.736771579507 126.711037295629
129.242093568433 140.079516848788 147.745780820004 141.289009185018
133.892661981648 138.594435323838 134.102929804129 143.643259003393
129.205783382977
C3orf50 226.35854874278 235.795627549799 234.063633889254
227.558441474918 147.932875151331 292.893332413185 186.257814752354
204.337844243869 186.276450732044 203.071186284148 257.622317771472
228.926077117474
C3orf51 136.040524807653 134.616051224668 140.763592237598
132.271437329873 132.461730873857 162.743192424569 128.705587172572
130.465221782084 148.47415004128 133.875667250136 122.042136913991
131.785745489382
C3orf52 160.778296470857 158.503284955019 169.417764098848
157.353493980339 261.366143735074 212.987082617173 362.502631441716
182.984076352185 198.863347487219 165.932628858503 367.644907862372
237.961826989344
C3orf54 223.551813819581 260.195256836955 211.844563155792
239.31180635241 551.622587349813 272.770616373687 791.402660066193
359.440639663655 306.011283364888 317.949646885539 766.710479603963
605.21685916883
C3orf55 155.752117896424 147.697973457776 150.324132011201
161.24755274554 144.258128917228 157.12360611723 140.288612271406
148.334425146129 152.303524109428 157.627490896581 131.269526665478
144.143692745479
C3orf56 140.762415240824 127.551486042435 124.13945833494
143.589222264786 134.907397302852 145.329128512407 119.937665469629
170.784229026842 133.788197693571 136.698189351878 132.306569548052
142.663334893424
C3orf57 159.184075293158 167.751604259111 148.482530949134
153.853856339585 378.797952580504 299.170431746759 314.708587131145
296.497100375888 201.81649023518 158.841505957036 433.314881387431
316.237519859859
C3orf58 273.478372861114 302.1846598234 234.550692915716
212.643559782754 552.298965378403 557.026028810367 398.571054759451
437.601814037248 227.806050911154 307.410175748955 357.35857422585
235.133284525241
C3orf59 417.615521090665 412.648926259488 358.975346234854
385.050282185255 336.212224495877 356.699466789374 251.665539319612
286.996908547553 419.047108206042 212.291528248457 359.329520507158
333.91440669951
C3orf60 157.259599894923 149.250205265163 146.402352419692
172.020235319192 138.352562277336 134.628807679002 141.565599104245
135.691862612951 149.082061203661 145.97330701968 149.898614923436
141.102274192049
C3orf62 163.416011199431 161.104993402456 186.447049658314
181.070689877681 179.411426984263 199.579818754014 185.332215198617
181.79418355897 202.178451227304 136.16560274503 205.728673153914
167.526798465556

C3orf63 193.444080047879 166.780319723055 210.77489002666
163.824878791966 158.617209794418 214.785381549181 160.748082658758
167.125830254039 191.3765085801 185.783374935551 206.469040492609
196.399050990244
C3orf64 335.389274425025 286.996908547553 353.437466689101
266.71318061066 189.114794029657 270.421697521366 241.293046687076
216.582862850259 227.483999162038 248.299149406938 344.604699562938
300.709410518382
C3orf65 139.140647941752 144.935571704834 155.049051256459
141.634246722965 124.710368327189 137.107621189522 138.685330935707
145.150191397375 145.902572804249 136.959011188915 116.723991721647
130.686857648764
C3orf67 126.640141760199 124.672483602517 119.103219740679
128.438401976279 126.925982415892 130.925881698601 130.213596986351
148.112717810117 133.76515446791 126.780096523484 135.799692347002
119.897193425305
C3orf70 992.320202251161 856.424155671931 1329.3013039618
1149.3250945792 209.362806125315 217.227441947056 144.534143943252
181.148062201932 196.16056297158 163.102043862117 153.289258540668
161.983987604
C3orf71 137.919253591105 146.882746173951 131.965184111199
137.054278745724 144.542320520523 142.886493948916 141.805028495425
130.66863243733 139.623194916971 132.799355149191 127.242627779136
145.858537779106
C3orf72 155.400191634632 165.647471565959 161.510497517852
160.799950553311 168.310680227156 183.406510146725 145.648810303756
176.100691355682 156.768976123975 178.179238056905 150.096558906597
147.338615809214
C3orf74 144.785852437305 130.588481497083 140.784853736558
127.545397354821 129.051642626091 128.967855397644 132.328948598599
119.184780023269 150.378690719669 126.435928518593 119.455615461113
136.944378905743
C3orf75 361.390981655795 340.903961204211 393.579552385074
386.379891865411 506.430593289427 412.582051585078 425.59810308048
338.326304736048 445.505512764045 351.998551165704 351.585604465402
496.991561192565
C4A 154.459517779315 167.91761500242 172.583615020042 172.827288328708
157.739526559807 142.910616700526 169.764014031526 151.766607453172
150.687819649072 152.415752253205 172.552923333095 173.552535486766
C4B 130.924141812334 129.007924852801 139.271829906127 148.297939240474
143.716377201615 147.454902803231 150.35767254363 132.579323148839
133.998381302146 136.964780183468 132.336431473522 125.193729067011
C4BPA 109.74983281634 115.366496551426 115.277302351501 107.239841465534
115.128667174745 113.199690522854 111.078930787656 114.191245210321
110.988514718572 116.718099363329 109.209241676429 118.379914726645
C4BPB 138.913591568111 118.572988141773 132.50027119027 126.061141885666
120.288469716375 136.507961715591 126.796253980623 144.287786573389
139.170102961407 133.789738760547 152.709021529522 132.425596223397
C4orf10 169.206723147505 189.678680702271 182.109092947203
183.50553222647 169.713585775118 155.228605554943 145.574725784632
165.977852973826 162.739253457398 129.680406696779 159.870967387108
150.346002924933
C4orf11 157.659824861915 137.201149245092 158.56996905631
148.673763368264 191.056705282378 375.131728190867 183.464897811105

181.313333665614 169.834078550805 159.771672778698 159.218886890035
194.186994486095
C4orf12 133.9382467268 144.476157581998 135.068883036882
135.982797108848 128.879953205511 141.623851511993 140.882766779119
135.400054238597 146.632312874102 138.62308708151 148.658678900932
155.376656335974
C4orf14 434.716530273383 428.569462312363 702.779943547386
436.891409783298 647.273409499313 416.861099188252 805.600599570258
605.795439342384 641.338900241674 699.270931701696 967.797017108447
650.911886508922
C4orf15 129.263794008537 137.372131049785 126.63730651721
123.219903763265 129.792548588929 121.080413308265 153.051143478997
141.781482381513 137.328370598183 122.557981210434 128.812729239283
134.474466007553
C4orf16 166.904990069484 161.678137139371 153.804419092743
140.443415669628 150.657800679032 185.382647297259 181.453575300661
181.070689877681 172.552923333095 155.801832826848 174.767697866801
156.630366766924
C4orf17 107.48180681159 114.488760619745 107.816312357067
113.326628149066 113.734280863323 105.62620788923 111.608386387842
112.916256784817 108.631224817901 106.263441906404 107.655624022312
108.199628274798
C4orf18 410.731869154912 322.684910198242 206.35895739543
278.598913464804 196.819022688128 247.053215951505 186.961638857905
248.850686917567 320.525885538523 246.263259852067 295.047418816148
230.770036998466
C4orf19 147.15999706579 149.984657985607 148.400915576999
144.736213465651 228.713818522476 177.001637712889 324.25775261173
215.115324454215 241.917371866481 214.100698312272 286.901115411673
177.123497519231
C4orf21 127.749992538985 127.291712212051 116.858439910319
116.276003190171 114.44610607807 125.342580368417 109.482330324734
116.288113598488 107.32960937124 125.579970155301 131.255028381669
124.001295849091
C4orf22 142.036037957345 130.768746032834 137.792806301902
143.912136782374 137.981446468611 150.896076207161 152.274957846518
165.103398617203 151.615675144083 172.03919618445 153.031479923324
132.047328970556
C4orf23 155.166786601813 151.497680466119 151.144521344129
162.756185479013 154.94587631386 159.696443182844 150.79785758452
149.225754384299 180.327051100611 161.852665901359 164.21464602746
160.518055978242
C4orf26 118.446703299916 115.999443701343 127.05677680446
124.35374445763 128.150801169886 130.75707278606 133.180554954396
126.349403754775 123.598210440312 121.188761627967 131.497454038525
129.067538010904
C4orf27 289.859695541999 312.406286993239 316.409887372431
278.095939961034 237.825857080173 242.971958493453 304.058158731957
268.698146407497 344.529927973082 327.566026396726 276.756415094228
241.105817192695
C4orf28 140.28485470716 124.469415593643 133.559082256772
156.938149948126 115.43068422162 128.936628289974 132.560292119968
129.369968549738 123.991249190444 130.255689301211 117.913545753709
128.007359083768

C4orf29 208.713555471888 199.776140037612 263.058972886829
214.8478629277 117.570749340011 258.552964322998 183.371103136697
205.036081947168 199.288100834476 157.126460242995 211.183499580683
223.496400038284
C4orf3 139.754028208983 138.777120638896 145.799530970042
135.388750095746 146.174492025087 138.643442050615 139.737748157901
143.129945647647 153.587737758431 131.878155417065 145.885577577352
150.798629148998
C4orf31 364.964572587289 384.97980473847 151.78607484924
166.162656500954 127.927232724978 156.733898702331 127.972708116366
149.659505956629 139.188234030711 144.930066324767 126.952836653857
127.104868352186
C4orf32 215.84044779962 221.253717494488 197.762169504041
220.288758600588 181.036375971015 428.340060893467 262.691387377392
216.783051958345 188.543051108601 210.803130108618 224.6588173737
163.833372505148
C4orf33 170.465177938235 165.797170648964 142.121399383259
151.174536186463 179.389797867379 251.138401278632 286.587945301875
230.10433160008 234.996704304673 200.360518276784 369.78624104057
327.749926183823
C4orf34 2334.97297889593 2149.13404212591 2018.81730267601
1910.08485008041 1164.61390835016 1492.15137418791 1266.53539318831
2118.73410871718 2320.41293992992 1322.14570224119 2406.67187918735
984.695710317818
C4orf35 135.973752893694 144.062079886084 136.84488044614
167.505381046729 136.659134200122 163.873355370767 155.368204597287
143.669490888725 155.078035506545 139.482462221798 134.490327004118
141.104761546577
C4orf36 147.519991961858 161.548130965486 182.292947734054
176.420075374686 165.866987051333 173.624709850889 162.642344529383
148.614214326995 148.038291195414 168.314966639178 156.502051001988
165.904887259203
C4orf37 161.799743586569 140.588811000948 148.364138636002
141.689210867136 152.08574341962 141.218832234405 148.124586158123
152.219906297607 150.587882657674 146.716053772448 153.869144282319
151.939710544553
C4orf38 154.367285732964 144.769824608083 149.956759038516
141.616920429839 129.372522073479 141.329559699056 132.71633377967
140.347131743987 134.7552564959 134.686251400642 161.16688895179
137.698423468762
C4orf39 166.001279428607 174.840923772532 193.218266574099
193.522811257085 158.062853087619 190.921283434627 153.964765831852
173.050039480459 159.74220366735 171.254015232189 151.833386901811
160.221974600577
C4orf40 145.950701448912 130.465221782084 136.526706711584
130.476322267589 134.463094391766 145.573544748919 130.760835441776
132.928639483854 124.611144621071 137.556552701816 145.333097940759
127.065518421996
C4orf41 479.469659712246 422.23268864343 500.469781477166
387.135002880995 253.186749111526 447.995213144892 494.175437345451
433.491835341321 570.771089597761 498.534168389928 453.457361487577
516.429067667758
C4orf42 173.520662003159 152.654938328424 164.68600028429
160.608674893418 198.437113196596 163.988099963018 178.045931314857

157.58659226599 168.460095116205 165.836534881802 164.109321257638 174.873893094923
C4orf43 151.153466809603 161.412427590994 163.495175710216 161.557558028531 150.601874392926 147.736397115265 175.902751522861 170.95650329595 178.766654213173 138.416716745934 158.467572658719 169.691392833277
C4orf44 119.50754902958 126.017158064032 122.260548647869 125.212404794262 122.327353563917 121.203149121743 122.158160927149 119.301746097298 118.21084414402 122.284431229917 133.768419410758 125.107483348755
C4orf45 118.945264994404 124.547559624623 129.03753569157 112.578934972566 103.520748827831 119.709032462308 120.231559540009 120.727504500018 118.820154030256 115.789536577324 118.492287441192 118.871735074285
C4orf46 159.235289396705 171.229729917612 186.377571176623 191.861598059182 170.531713392258 302.809011218879 178.52221226882 220.258804437828 216.077955163344 171.11282933168 179.821008257902 209.090788790449
C4orf47 123.703840971923 134.805239981342 136.366798516444 135.864853829421 126.614876404146 120.688909943167 152.585572167313 136.135223482208 143.333211390741 159.090982014161 109.52020660772 124.88118767481
C4orf48 137.874370839801 148.309872393 139.316189199729 116.52455193626 433.82566459288 258.489608621438 253.65959805538 241.453456707114 216.126000558319 260.224777636733 171.06385200256 279.969975697164
C4orf49 155.255279503782 176.410016510249 180.219813240126 209.33028918341 134.025385181979 127.792241991414 121.687187898743 123.089110282465 132.154131623051 120.210439163369 116.773599713111 125.379842077431
C4orf50 150.804810443235 159.874161198304 172.338509138759 166.674240866931 165.45479542657 168.05687638693 153.136863548105 151.043967734248 152.29434111068 141.533138525284 147.980827857138 160.311096717492
C4orf51 146.606442726047 127.377356715337 144.281217246178 154.309257657136 152.81544746536 156.4687904356 150.547096071626 140.071879210324 156.4687904356 143.149424892482 144.559193410932 136.948039394935
C4orf6 111.497241941203 108.697696080014 121.311836944721 109.495342943278 115.845468537936 127.291712212051 112.142263331903 118.05386204863 100.216750525986 126.037968297611 119.049388179929 118.81294782091
C4orf7 104.223476853877 118.397482835514 121.294366964074 117.897556445952 107.74537523456 110.368392735494 114.589757401741 136.807118099948 243.691004557778 193.723953003899 112.248047602652 112.142263331903
C5 333.822167152866 329.722135361491 419.846584903512 341.128917971124 183.280853265083 168.269075560636 133.075748695526 171.660157220703 170.758319048911 128.519448608754 213.833894542292 141.401480567426
C5AR1 174.068974937318 178.632720545904 181.715992480635 171.06385200256 166.863589702453 180.710636205653 174.663845688179 188.181647474317 185.128661141884 185.128661141884 161.217964573491 156.852187451794
C5orf13 691.91655492289 6 648.823677010146 505.704957182936 1142.09977159958 205.886398618369 269.238332581564 132.066488589395

247.053215951505 269.053237303244 1918.14260920544 177.955579729463
210.448904052352
C5orf15 991.611982265221 1022.11537745428 869.245621105614
990.814111681408 780.961416212309 1161.39230511792 1690.96371604294
1744.58410715956 2132.90676194993 1873.84654908146 1728.86115695979
2198.4268389426
C5orf20 171.97578836712 161.359609192977 173.839053227554
159.655362710407 171.202766078337 143.491142564838 176.11287574658
162.599466021255 175.989742412417 155.595651896155 158.699778625671
154.187420367647
C5orf21 1244.67453201339 1133.87328758676 1466.82170436229
1136.76110352875 437.836503489425 506.80969228563 956.76879975749
440.038896046559 420.743637858421 409.743899750699 927.478050257292
527.707841428677
C5orf22 155.402421581195 151.833386901811 168.124936529582
134.29036009792 143.475725571943 194.60309399944 174.23955831109
243.772412032046 180.3779211444 204.35801867216 173.334154083555
173.995889755792
C5orf23 140.518206716196 140.40754434058 109.266533255266
120.089228795888 115.914842445934 110.355493122568 106.746545626069
109.832757716464 104.977794653504 107.450945262092 108.955761698524
106.393497890486
C5orf24 182.292947734054 181.036375971015 176.964088090133
169.667751894226 142.027930009598 191.170105307944 193.660159482867
164.870860592068 180.240305360865 235.43707989982 174.775676782795
170.350891928203
C5orf25 175.9510758779 166.533191390639 224.981439707657
174.775676782795 165.134931755194 162.52512327258 187.561298131124
200.72147365413 154.695060071104 186.66469040433 148.272252953881
179.54034642468
C5orf27 125.942455135541 130.886967265639 139.691470247318
129.32466533596 184.223623297663 124.436549041911 127.245190474707
142.62707251786 162.574338133241 146.882746173951 139.974586762939
143.839170216353
C5orf28 3103.4192907748 2811.84191700516 3363.51782842264
2558.91648313228 884.248568386012 4103.06565237121 2446.31228564539
2491.33490046283 2753.89649259242 1789.51979173209 3664.38955037715
2127.22258112242
C5orf30 186.089988569287 193.871324880919 210.14307111352
209.60319248212 200.964306455257 237.113813706224 196.096936985479
200.518684601283 181.364325529405 239.20206002 158.845174721328
186.886677174192
C5orf32 414.333324610267 472.793332506369 439.122658719747
441.578261054602 634.356322976353 687.494132125365 870.887539668967
600.430996779799 666.570977208535 369.646624431062 732.352357321273
374.169744342352
C5orf33 309.798246248863 325.252108402192 555.366765340014
310.8725368426 237.045634416039 263.250833727291 208.838523729744
395.588345893987 219.96819201774 199.53455266342 187.607426452073
194.819597436776
C5orf34 166.23609765059 162.627021719615 159.549936351002
153.323666159436 163.125317704446 171.552010365305 151.660152977515
214.050918459805 159.781313090489 134.640783050966 144.324313048777
162.484777758676

C5orf35 176.497706091676 165.992542962911 240.706548931192 186.420541099902 237.473927775823 267.028096234702 213.26158748941 280.246711704036 167.645930148254 239.31180635241 225.75155298452 245.240006198482
C5orf36 168.405786851399 152.702847643082 155.346805606742 146.029647001623 155.575152212736 157.967824935915 162.237172404557 166.404164069728 150.778292242313 163.495175710216 138.201164395948 159.446705611995
C5orf37 234.588584245938 223.184064606222 276.714748212739 211.869300324471 204.509884908059 344.298510200653 312.224309341171 315.828726297864 245.640193659159 259.72035004624 269.261595558589 402.140437036706
C5orf38 107.02909139647 106.772581387455 123.513588004371 106.204350278634 109.719356131043 102.851152421943 114.084543168359 109.242622591321 108.007762863766 110.966212705086 124.96926662367 110.068122112671
C5orf39 248.43491649623 250.647644543963 220.633692394229 248.465457421469 167.817232948801 180.408639020724 212.691702227442 229.443064589306 273.106088088825 230.367251714289 206.027235767232 213.382179917193
C5orf4 210.711890471222 187.4351946569 231.462907786174 232.083160165968 128.449200385351 132.404879616886 137.920724602098 129.386300987308 137.768666157176 134.679882781788 142.91818218442 144.873034900013
C5orf40 148.700297908071 146.96472350529 136.386031133644 128.140333650496 143.421288665418 149.04685599998 128.618422306397 124.710368327189 136.87712607887 114.598636725763 114.880348648147 139.340699808517
C5orf41 709.303384219887 708.534025308731 845.369108604819 544.30845362168 193.813717161291 266.316485802808 364.265100349672 277.817786943873 227.144441241869 264.233381434738 331.215872600406 208.914498369991
C5orf42 161.874184077257 156.396579029788 158.179586948369 147.077695919758 137.365567527202 143.303309656177 152.368485416992 155.545904088857 131.536457148878 150.354401669138 129.76141737109 129.547309629132
C5orf43 128.897493103888 125.547399456656 136.762273376313 125.929743286163 127.665162524824 123.98775110588 135.453399741892 112.891050337488 143.614075503775 131.773304233929 143.601057437842 150.152613183213
C5orf44 172.20451139581 176.650953513413 259.374392296079 162.595632367022 158.205235596076 256.648540685098 197.687858915757 186.794165469219 196.922837906592 201.731223995887 183.168629351124 272.456310079164
C5orf45 154.080778100852 145.142331981651 159.561431866706 148.139376772567 137.038866196785 150.587882657674 143.440420261133 149.661929885679 150.942078067308 143.667207351415 144.388851371784 148.190696520521
C5orf46 132.105184573944 133.886458291672 135.5668267936 143.868962478182 134.275773172492 129.966755942173 146.483508510944 129.198366503027 130.066177538896 158.799917136119 109.454483612299 129.018088749395
C5orf48 125.347442446239 129.935532080325 119.922676683808 115.072622367144 107.727848546225 116.310166003024 112.252555403121

111.041656398985 127.786141874218 116.723991721647 138.594435323838
117.178495957357
C5orf49 140.84880167881 146.772611341214 136.097571072206
140.244919506549 129.616142780279 131.668163693171 158.295583769612
142.988500173397 141.010588053284 133.18744841514 148.176995739142
138.263361518905
C5orf5 1252.28642463619 1100.17618574297 1267.5716872154
1099.80705776268 278.687780705367 415.011847890313 512.723592291378
451.317592236192 384.780426529733 394.73732350607 886.517088614968
548.138527647594
C5orf51 566.914448800423 544.107739610054 655.431236290512
445.905756082569 410.645457106122 714.052063877292 649.438995824208
869.937761943249 469.794672718173 521.512936372701 532.394088545421
535.354724252178
C5orf53 838.267069860172 820.652972219658 796.052115217208
931.325266360957 202.314814594735 258.998065427002 278.385151185788
227.668046559074 262.832376198119 408.201232787978 414.333324610267
275.755038921952
C5orf54 226.678503956411 245.01029221002 288.823376348944
262.79667156266 180.364089931995 187.586093248437 224.027605834751
195.438353051306 185.432587490792 158.120079903425 199.746775121317
203.642739476708
C5orf56 165.411052799773 156.040073232376 148.835389500419
171.768618884215 152.60462513339 140.446530307169 147.182394557583
147.409936250284 151.411708598688 164.1572198075 149.516743790585
152.095584118603
C5orf60 115.502679218125 119.795945889846 148.065080664185
137.419835194075 126.670227417269 131.007779278656 114.002925521932
123.841366760069 122.285983209924 124.905889975958 130.450930089722
123.327238774943
C5orf62 220.312301059818 192.113217321485 193.245087824217
179.985468369202 169.385340991747 178.745112514526 165.4474699807
173.766372029062 182.929326147069 206.164058935215 154.34872021008
167.194306682927
C6 169.975329631607 149.783542909965 159.368376366657 164.593531655305
149.585616718523 121.472885562857 139.570313872946 139.440415574681
165.177036955944 152.185397218918 149.691340727994 152.222732770706
C6orf1 148.242794464181 162.997720993338 160.131256419524
165.003646188775 193.95909424044 160.882229345156 168.483273292464
185.997327421952 172.403953571339 171.080373870373 169.33926580273
164.084737224884
C6orf10 126.98588570464 116.445184223804 122.182695593404
115.450352881048 114.373370072994 110.586147059226 108.296042016208
113.970633751086 120.55570440025 125.528638246486 114.278113876915
112.635430248416
C6orf100 147.454902803231 174.357336296554 150.126627487419
158.841505957036 160.640160107693 145.146797807275 140.486376016494
150.023791544584 147.425856984368 157.113536717393 168.538117820213
165.570531605115
C6orf103 170.12010526854 159.413535577361 178.027417594263
192.046442148183 155.801832826848 148.840533233712 170.892319361167
173.001985500448 150.863099494798 168.04198447141 151.237088659409
155.566065673542

C6orf105 121.455843337023 115.184395637553 112.428707152237
124.001295849091 113.799997627317 117.316790136526 116.653897702566
136.755975832051 227.033342447929 136.541973790157 175.29178007783
127.365142833355
C6orf106 325.320888625297 324.871248300476 207.104548555929
410.845034795716 291.537725613819 195.724873069362 250.515579402334
242.752995772995 185.233210060342 177.619101765848 195.831574717135
164.784346867158
C6orf107 146.619846538124 139.684141478459 143.167719135403
151.234397206563 172.568852318369 155.762890407496 155.475804754732
142.521260841187 138.717541782317 162.500485773812 144.568417378353
153.505155981348
C6orf108 453.926991868451 411.878556907186 428.959561932229
528.275591281419 843.936924659514 430.649188209901 414.267332328081
332.400086578723 352.897241809959 469.64693728427 373.545629693667
438.340341490622
C6orf111 777.901979189395 563.490027149224 739.874432767734
484.363052437459 247.407442038488 308.767036855508 490.464012303239
325.173931553891 410.42088953434 384.051170099325 620.760947818536
673.502723625773
C6orf114 125.783265980169 131.001996509052 120.880547922392
120.365602682311 120.740986472733 126.667650990284 116.969799327195
122.339819585312 108.266328505986 130.521731606987 135.849839822066
111.992738490468
C6orf115 304.357050745605 323.841518852386 351.585604465402
351.759100605991 1307.96033038529 1316.31088187364 1608.40826380713
1364.83036157729 1110.43791444289 935.042067053226 1741.0131951509
1526.66232186013
C6orf117 134.628807679002 147.343712869579 157.399880496076
141.112047881014 213.338918839446 168.560093633607 185.661496988658
155.319717753912 143.60570420943 157.103246263523 238.88138498771
161.046418048832
C6orf118 166.263781040251 146.058477355527 125.789509802098
143.349486755648 138.728765780512 139.923187410488 145.360466216324
144.718507303105 144.068734533254 158.275283892632 127.125906877098
141.901382169334
C6orf12 129.964666668421 142.984243805285 142.597110130872
132.952355710395 133.783379558598 144.6567095695 130.624702758052
136.459509901229 134.285191872099 129.825824640378 132.132474135959
131.709341771599
C6orf120 193.744461104128 172.509081696309 196.001089662818
161.189717452004 163.899808733609 168.437049123012 233.71520737817
179.624208193114 178.179238056905 139.038389244329 176.506227858258
235.019573994855
C6orf122 134.872654203921 158.509885816997 152.166586496729
147.325222091872 139.361120875198 152.961475450751 157.172970235254
148.739888930189 144.89403947454 151.848131897899 154.569432344392
145.093913515812
C6orf123 135.085039737228 129.902965506953 154.183537565624
157.776321544382 152.052084712417 140.010730572075 136.55090238808
131.132508349222 130.535859816562 156.046085830566 118.578205738502
159.055956234445
C6orf124 157.684694038023 145.576880854443 144.555718186987
179.411426984263 156.001983149983 147.584176709986 126.155486659114

154.483828924645 160.105903942674 138.062345982865 121.248923222807
145.584615439295
C6orf125 711.166793546075 643.905401805862 595.752426322468
673.824770556885 1235.02626456572 970.166036472486 1215.96648642927
849.389818300305 863.086199215353 1192.47480377375 621.354393918348
1346.53215685106
C6orf126 115.656801309856 103.012774960061 114.987285062108
108.66227817548 149.554174907798 116.588138374632 110.355493122568
121.18490040448 113.840813583258 108.604763499678 108.530158480237
121.945541820091
C6orf127 125.280736217267 120.493082096235 144.55710963567
120.910703698292 131.330974614437 155.023934989559 138.692057950694
127.155818626031 131.419663282022 116.796995589816 125.728300338988
136.603035996281
C6orf129 210.605864685448 227.065194522692 183.894417745549
324.009703043876 615.485984065221 525.789451405743 502.063888359427
453.875259610173 500.469781477166 441.222346452864 300.314088267108
452.24100098898
C6orf130 432.372995789404 421.459312180809 617.67819271245
390.979822976551 296.141012087256 242.471259857978 329.88790758321
376.692422468905 458.607367472574 365.541609015117 410.954095610904
326.908959531021
C6orf132 126.359440164601 124.913645688325 125.892022613725
132.074571281938 147.619451225526 152.629680152219 133.499361793303
134.128061214081 167.604959895451 256.394563537658 147.006482557682
145.126091016414
C6orf134 134.744175117719 136.589250394236 139.585373400347
136.589250394236 150.166324293431 149.857861968652 144.592987736094
144.339819009283 155.23922239449 133.272540012108 170.661808797506
157.328404229451
C6orf136 435.48544637769 474.14051635531 531.217306770605
567.869601934138 601.164127925014 600.635914090365 586.867772838753
522.664475688419 651.43207839949 459.30568829087 464.196512015649
833.198511430615
C6orf138 154.755544586846 177.94501172269 194.753955100069
181.976252906929 158.579717282881 187.955617187544 166.20327698517
164.15288821931 159.561431866706 152.182968451691 166.784804508647
165.103398617203
C6orf140 158.859389972316 148.484823275343 140.271557921345
143.650172124086 157.754622099874 164.066839397676 156.525347260458
148.688413944868 139.715836473047 148.624135810148 149.871960981882
158.8054811093
C6orf141 149.551030995489 159.824012085212 160.78368986036
190.66473033826 154.623548525522 151.885992695789 167.42476096854
151.716042884808 158.545435407585 367.732191251547 129.813090345837
174.417259650516
C6orf142 126.788749235867 120.325985681299 122.223184326586
119.034003473459 129.158212237299 130.041462255766 141.622592268163
129.138035235415 123.431969470387 136.486261473545 137.286331849749
135.166677520468
C6orf145 211.609763150578 225.890195278753 174.568253693395
210.245551279259 199.516043142784 139.213953275623 188.878770168595
149.350630572336 165.169920299969 183.937484597232 178.496344474999
167.298438019561

C6orf146 128.200531810126 126.730314982553 124.036907709461
121.410606617535 126.439948611449 132.795064859079 130.465221782084
120.15332480712 126.285743744314 120.560056224145 133.009854792992
119.730174479044
C6orf148 144.820843146628 149.805955402639 151.64806573742
178.200678423426 121.267153348313 152.483240622367 144.883597913037
132.955473649746 147.094123051131 148.750284842503 139.708303388546
121.752481757054
C6orf15 136.408314486465 142.948238172973 129.096939267543
134.495938988321 129.543069836687 130.744788360182 135.68605915552
146.384051525624 134.968848574511 123.237851647499 140.195820033314
129.92319548307
C6orf150 146.574656147816 126.553693803748 133.229571269367
112.314595956147 138.959040014432 123.981053866137 155.945838519586
129.257947818865 158.660859266099 183.545741265706 172.604689611475
129.765440519589
C6orf153 856.74270641707 842.87865208079 768.334658920971
820.249910349431 719.628501451187 906.359365544713 1040.73829397913
948.150767619765 826.771587455122 1175.27165286568 1027.96306543235
1367.19652160532
C6orf154 121.360822445187 117.652693591865 123.977467396003
116.161473967382 144.727773039491 108.646136431909 121.705065573911
116.89487516867 110.486191378104 114.812254477058 116.46192274217
116.507315786826
C6orf155 168.254689624696 158.380964047483 163.416011199431
189.633348499854 156.373163373309 145.97512573326 149.684922983176
144.643770374264 146.70298187005 154.675189522146 136.016912147651
129.601234646954
C6orf156 112.78323842342 107.802792435066 114.441223528256
122.595766303833 115.450352881048 114.691842968597 105.976284291356
118.912989775436 110.22624111837 116.168270198375 121.571579572958
129.537345537086
C6orf160 4061.24537355697 4520.84412831414 3222.23985545506
5410.50736002544 600.430996779799 1503.2049371911 5636.26097921507
965.673699120049 1849.21906428944 1270.76282064793 4641.8793590085
1180.7055699511
C6orf162 163.794436953711 163.446813963119 177.851689709807
140.83570584033 124.995087711332 167.130843160309 138.313519292093
135.367261617271 153.992763544073 132.813199199389 173.484307104751
188.878770168595
C6orf163 164.529828080312 150.193479279784 153.169450192935
146.151639797173 156.130224537288 164.74234322574 207.218779485342
157.394035451151 148.883263903527 157.713227527385 155.868109161417
154.288075172115
C6orf165 129.210356735226 134.683553037185 139.117776552573
141.717466442384 139.175421159925 133.133892264995 163.534117903837
137.717859035645 137.253971514177 133.757741295765 164.1572198075
137.672798701643
C6orf167 144.259643132933 136.618643817686 141.289009185018
139.333282400639 152.024383072265 148.00952002903 145.358431402568
151.26137860684 146.084461430546 145.737361356678 148.499207368959
150.401509081265
C6orf168 245.489266602897 196.287131027947 304.292209676095
304.013547190343 114.849872458647 132.64813248505 125.634045420702

129.583916648999 128.176779923348 130.056727701826 139.140647941752
120.530092530622
C6orf170 740.973440818582 786.160002434066 588.863382995875
685.080426814888 243.772412032046 664.343553341563 399.537363692534
505.143815448613 394.892918166519 365.864755316163 807.92851898782
445.661863626419
C6orf173 245.399847987183 272.712242893937 260.314440448486
308.441120735095 533.279122416818 584.199252444401 425.001043358831
629.381791489871 469.64693728427 620.163512570453 267.839640412797
523.435696794756
C6orf174 128.438401976279 118.694037994926 115.949513659403
124.480385254916 115.53736269794 116.850851308701 117.81168010976
110.952108124186 123.501843029993 112.90900939264 126.359440164601
119.218909479403
C6orf176 138.649502040582 132.963761182049 140.321675137237
138.440663763957 142.723175448765 149.25240100689 136.016912147651
135.054931772725 141.639216893245 143.061415008831 132.605662484463
137.033870931133
C6orf182 173.70035122661 151.722405598425 146.75924072364
161.359609192977 166.872332652008 155.397301207102 150.82596027483
146.188193301627 140.206268652318 142.886493948916 162.500485773812
157.6811998968
C6orf184 134.264584077125 112.729141678141 128.163613794257
134.623264303227 133.541333581593 134.637520371454 127.242627779136
133.343243441383 137.944260488223 128.348910964448 137.767900781368
135.43119649883
C6orf185 135.5237675623 123.689439578326 118.773864607553
122.631814400575 146.210814568357 144.004831360797 141.148849622103
154.774977482202 131.104534164394 152.429970809069 141.778060508776
141.067419336609
C6orf186 149.076146942797 154.227433553131 158.312614511836
147.670425834523 122.673596593654 123.403099206878 123.960761343425
134.964619235561 129.995417218564 128.952986088886 131.568898109933
114.421383025971
C6orf188 150.384559054133 152.791390145148 156.324643994552
142.372470063444 146.623313753608 145.934643000545 130.396593013401
134.062423178775 143.290642777702 140.52059487958 135.281686411685
129.969400693456
C6orf190 125.932540615682 121.545189875973 117.186546702764
111.484426481816 121.846349358116 128.599254558082 120.633217745524
117.992729826121 133.847494826806 125.704264033102 141.923103467306
111.683074830042
C6orf191 164.726382689707 144.720594512581 149.272219727411
136.468434653208 157.838771553729 141.763072120718 148.85250180736
155.683727621475 154.627270742193 147.051399102374 141.368078865065
152.021576565132
C6orf192 306.716682293251 341.2887754025 238.400565506288
368.948300262634 233.635876830148 216.353502371227 314.126706052299
347.73629731446 423.826189747546 353.005548289786 511.670320118232
533.893360199271
C6orf195 133.892661981648 132.047328970556 122.878790127038
121.406758670435 130.935922915902 117.682034050478 126.746388125956
120.883785696583 125.010266083832 118.770061426689 148.356726939122
126.388778582602

C6orf199 144.889019483712 125.422348389595 120.196595293401
126.919867899174 132.085538520551 119.598216955485 134.673861365626
120.669452856028 130.611233072509 102.094370876743 142.715449859191
125.892022613725
C6orf201 123.541709359809 140.239948346946 120.71782441569
136.423161205425 138.304793826214 119.767154020462 127.949413503055
131.49254872524 130.808911605271 138.252656818036 131.774882989257
121.668166378914
C6orf203 236.809829701921 260.787104931135 263.448958595597
263.123584372086 228.212753830905 183.314276033531 226.893147371644
243.10297424873 210.692705153337 204.990512488181 303.762967437478
229.542259738438
C6orf204 289.297370738382 192.331142672469 281.119138040097
166.320461361356 143.839170216353 149.734395134321 185.063870458561
146.227452269648 146.672270660899 132.714532888989 160.035282578603
145.276745572405
C6orf205 140.958277301476 141.1596293382 140.812334019209
130.080728694308 143.727514442914 136.562662050767 132.057808599068
130.836079676477 135.651829752462 128.22735290389 134.599629045981
129.743808480368
C6orf208 129.467202466655 141.963297887448 137.31708798874
131.688038068545 129.703791330416 121.215558351076 124.075077123156
120.054065939835 136.355319997318 123.946808975704 139.693261291326
124.193603137858
C6orf211 177.752497164503 172.276968413879 176.029791617989
167.180406126438 212.363151537303 189.092674335503 254.909076658804
227.441436540742 193.544035570799 220.217745732407 205.428900832926
220.578790067967
C6orf218 155.837866295709 137.314968268692 215.558688906165
151.858466721347 160.031506612501 140.323425587628 157.878110410517
146.416318806523 153.613110881454 144.902807664593 168.418529261977
183.969897305028
C6orf221 130.479845410716 126.34649262976 126.980375035189
118.9803314875 131.84454376953 124.539006083936 127.030113960477
131.067958183644 118.366859032979 122.309041354217 141.340452225686
120.831070822199
C6orf222 140.898380345452 145.34041912391 172.077522197357
156.037150880384 146.229239731545 165.647471565959 153.072429774613
172.794570587596 158.041568121766 155.343619434375 149.25240100689
164.15288821931
C6orf223 149.764447995426 133.895547281411 130.92186531563
132.514706165018 137.641136471239 128.386309075038 137.493436771898
125.293640452686 131.578042179715 137.907709799074 112.208950290962
143.272669418277
C6orf224 144.526499066594 139.238936770274 152.685860451112
149.322537247388 149.651553512698 142.13191463827 158.425950902309
144.380485359951 149.480971898888 138.213742418402 139.493025452991
139.609751976657
C6orf225 333.592915218163 334.350011399462 322.443089373063
314.337680575998 123.794432024417 219.2695037071 163.456245724169
179.509758601636 166.681646517413 145.39060898147 241.039766283972
201.016287924499
C6orf25 129.86336717605 131.13101959543 131.177396785633
133.962129094978 129.653283053893 135.403206634483 127.0631494857

123.413596815384 136.539849534341 133.004765428575 145.020615552964 141.857434100065
C6orf26 135.802258952006 142.411117330429 133.238296113721 137.388190728367 116.994216357989 121.607527525765 138.088712695622 163.805257266374 140.471953101887 136.987403767185 152.91729346961 136.316003989725
C6orf27 123.629279569085 123.418266047461 131.832311911703 152.377056383547 151.299425811105 146.081940883446 134.835962366555 146.257923971069 140.754706171596 151.442463705271 134.384963577069 130.249430984172
C6orf35 149.437212090587 138.325950428197 142.853399942089 137.27506604142 128.901707304582 119.094868654252 119.049388179929 124.395047385025 127.845259467015 133.160197638474 118.567548265638 120.485138937899
C6orf47 535.758943850734 559.559921312181 542.883453307 732.630306104841 537.216291942143 337.87475687838 566.813343200268 350.741303038926 400.180574613078 340.800632878807 425.969575503375 546.181615768667
C6orf48 1484.32547474275 1136.14069801696 1347.10650016064 838.63350090385 394.35596596181 469.206346008875 1798.5819962261 559.668195903429 541.258721278882 929.198774489695 1086.33540858772 1251.15912264225
C6orf52 134.058035351684 147.955701866515 140.616811333466 143.050004021764 189.758013811338 234.399431238026 163.256472113745 176.704617691988 200.934618257908 141.079317977197 130.43775024291 159.693019552703
C6orf54 169.077657790409 157.374537572779 145.051794777243 171.028432989066 154.368499839507 147.199961777192 151.979221390032 146.421195733989 150.255511248598 160.078122267577 126.81140158672 150.328995030071
C6orf57 205.804144075374 176.839917795004 217.380892198811 183.294526267593 353.385210690169 193.028341755594 221.050093964575 224.186573847185 242.440966395613 232.519482087835 224.707455448421 256.394563537658
C6orf58 139.101471810467 133.847494826806 140.602170750673 136.68056120003 160.001115445038 140.938442055097 140.617663657037 142.052454741558 139.762998983487 147.145932651151 130.506708993193 135.159590925066
C6orf59 151.206697943841 165.314290446922 133.037138438863 179.730694272916 135.565256045133 124.844481048039 130.166741978483 125.000876313893 147.668990552874 134.619470709414 134.495938988321 128.883828419932
C6orf61 210.550995170071 202.504840299605 209.130260450131 198.568180234676 165.424516708502 246.492735271771 219.940167815376 196.144420005021 201.041792159394 184.915526626619 248.850686917567 222.134519361117
C6orf62 524.725307261999 509.400965222318 471.946284490029 341.251360936154 643.13571432061 694.907086612929 1006.03677177164 586.246004571452 662.781145703653 518.17401959981 643.468308027006 608.816123736392
C6orf64 238.037110997575 271.064405562085 244.849563563729 274.179232297038 251.959823608766 241.599271777806 308.281798808746 278.061498892643 265.260411490697 271.154246147262 233.496804016764 285.258824662946

C6orf66 224.361277033882 206.825575257029 238.654367531982
197.565122821625 262.832376198119 259.672238210968 288.481981529558
304.900910626137 242.971958493453 265.260411490697 233.536023295039
363.777212750779
C6orf68 127.50624009248 126.259029676691 129.884064654761
137.85618381155 153.228048041467 140.486376016494 135.571239294086
129.213523302268 131.22473774325 146.999370190363 115.587874563785
136.589250394236
C6orf70 268.52833723726 285.882780066861 332.464176231219
280.414161197286 302.570932942847 253.124449721731 398.075524915003
346.173614351499 269.981476129439 252.10470581893 456.201998988891
290.914381066573
C6orf72 260.098788125373 195.724873069362 360.475989559226
199.378179817518 195.364709354814 241.917371866481 356.770158430601
232.641965649602 206.605699708924 420.446810988909 261.203388718689
289.09614335372
C6orf78 145.89801576671 149.035590153457 159.74220366735
154.302619274576 191.754626760061 150.52625734893 141.954467306745
140.574129695953 156.779004465552 142.406236920136 135.987450822209
171.304896264805
C6orf79 128.071379991958 143.307838525991 145.45768403614
131.872583529419 129.910866236901 139.21825391822 129.758303592589
132.161764435769 133.900005368973 122.176811388389 133.805991920905
136.154522071917
C6orf81 147.18948282156 147.673016541108 133.663595328149
148.680794815584 146.411873503163 141.393814611166 139.593950393555
135.412051450659 132.891375982227 141.453255692229 129.771073986855
139.636177212128
C6orf85 205.058210447711 224.008253365957 208.257420634745
209.867189613436 472.030150842569 402.366797969833 551.206833852215
309.0578543424 433.491835341321 1355.07170485397 449.523996620257
303.483515328621
C6orf89 202.545147073902 225.142823691625 187.769988580599
213.274106157675 266.135477775641 132.677272839165 191.628693083169
157.521230460545 171.161297672182 193.892810132985 175.874387612235
186.460034745907
C6orf94 131.77642590781 130.579954890389 136.356895672212
119.655162437465 126.381288663037 116.969799327195 129.233595525585
130.738915341653 140.840895590527 131.041996482401 128.322884322951
131.615462296871
C6orf97 137.745887988474 149.17029600091 157.901967487277
154.406369231962 145.812607895325 142.68640844948 137.464914919148
148.030579593944 140.750004067571 144.358535632993 144.087004064416
146.112388101621
C7 4055.40322213638 2681.48138915107 2441.05534211996 2856.14739820295
119.696656494403 127.00963790814 126.50870982427 150.394483608039
268.406778092906 118.825948323992 117.706795566317 114.067790137477
C7orf10 255.039661999732 205.987280015028 274.564508105177
190.5930909277 151.379761514526 138.338280468272 144.510062568989
132.864861511517 155.259089547817 420.567884340705 171.373932115412
136.390047634643
C7orf11 246.327991259259 223.778708427552 266.99947481587
217.380892198811 442.766704003254 255.305647212135 359.099750692694

284.891415492568 292.640251411131 409.380002417794 341.819090221654
448.767690095033
C7orf13 150.063082731931 145.453460204097 134.02083946631
149.659505956629 204.89842561202 155.04585737344 148.344941331885
139.217113811817 139.727248583626 175.902751522861 162.970595558845
159.811778680604
C7orf16 131.674409480919 122.517559025268 114.561836290349
120.793974839773 126.667650990284 116.176068923513 119.168045349254
120.78129870199 129.728266017061 121.599883734552 120.53428198263
134.190511945944
C7orf20 156.662150993832 170.446156541317 154.935741493562
162.026757124868 392.909481887174 182.335981256243 224.67853219142
194.388383297922 189.434597063058 169.332207863684 163.451063547847
279.202256032476
C7orf23 391.644649726254 362.899103657252 368.220523234399
331.32397534437 992.320202251161 516.088776850623 773.803350315426
574.035124314622 666.344430142655 350.46078795627 538.605085454393
941.572186137467
C7orf25 253.42022722564 241.917371866481 369.018856220394
281.659653592151 294.368406238222 292.145553580042 341.501397478593
344.915370555826 296.721891842563 310.598930734244 327.566026396726
353.50447714719
C7orf26 601.521068811063 596.076764072945 519.564563632951
675.281131488524 987.564350211893 455.08515350006 512.467731561858
567.869601934138 424.912426797488 403.984761333129 596.934339406636
564.200165295159
C7orf27 681.677841333095 879.809795251142 754.97177863997
942.900963522948 1836.22560264874 619.045921382959 757.066786599446
753.997560938003 649.052806726591 713.087392708048 780.461254794127
1219.36024606325
C7orf28A 197.47340234886 188.378996128521 249.354198174438
174.012972901793 332.400086578723 263.845733455915 285.882780066861
312.557093674533 278.192669077191 289.82072316082 289.474517971859
385.050282185255
C7orf28B 248.010317392179 288.313931067158 245.997705723007
272.854435771465 506.80969228563 291.50783830983 256.964577037118
618.437282203498 403.582461109859 441.001837510132 538.156335788156
476.076903428247
C7orf29 185.627515956848 182.665357598121 168.491878435329
176.062665550409 172.186384820044 197.231408726321 291.020789939476
186.215602572358 186.268881719287 177.379186278216 189.509062639279
188.0434100445
C7orf30 949.789710063337 1002.88224342342 1048.85768964657
921.546033242474 1744.58410715956 1063.5903770389 1885.93146154931
1646.76016031831 1440.14449872047 1419.4638331856 1099.80705776268
1808.26383618901
C7orf31 153.952788086758 138.084801633774 142.607578275494
163.119211741255 168.018785514306 156.75308999904 137.823152565933
144.098578955023 155.207839370162 146.846301957637 145.289151849318
139.308325848216
C7orf33 106.551076380988 115.247223837316 106.204350278634
115.473431881597 110.678680817471 110.138220982557 115.468921844902
115.520278378747 108.34997967774 120.988749021323 128.449200385351
113.756115251188

C7orf34 122.543903508753 134.316859658338 137.127587786536
148.678869698203 134.231634410324 132.834433153076 132.411757518105
143.370698101204 115.072622367144 132.038012025708 132.518394343751
131.705912986393
C7orf36 281.570685561526 264.476846259384 289.001004912528
234.322989368622 215.037122365095 288.823376348944 363.89527566597
280.453895029319 318.004190156313 284.764628939128 274.14931964455
374.268489263941
C7orf38 391.185365567603 341.015631528464 376.791876040886
347.932135290714 197.443711199904 333.049612399093 279.026742408164
318.487401106209 249.763375041547 294.410833087913 310.989043012841
282.076744753348
C7orf4 128.988736837863 125.870730238298 142.997365080299
132.820927638053 129.656088836537 141.567425892004 132.155877196474
124.842426851438 133.829889099442 143.370698101204 130.043904881142
134.176925491721
C7orf40 352.811351813364 364.527677995774 358.780920910909
298.519371102278 756.246712133116 625.699971780258 609.504365141529
669.124699103132 429.268202514543 707.048754700047 455.08515350006
709.303384219887
C7orf41 3226.53426370323 3282.73711809465 4043.28299602858
3655.63669142687 389.177193459 578.947889414387 429.268202514543
325.082620845425 491.034395489684 350.658722333252 1006.69598892631
524.973621024573
C7orf42 339.753059801136 280.494088228226 270.360397503276
263.321559283966 543.001249586573 260.85776723575 439.394914369983
486.047284379068 394.294057009443 352.665030560239 405.975221091252
513.292377117011
C7orf43 177.94501172269 206.113290588613 199.96834355685
212.933062534244 347.812754957414 191.881157918071 226.623172906957
254.722836822616 255.198862778405 232.023739004791 221.961758403614
249.193270428348
C7orf44 440.579262120451 354.656612182522 461.581199513891
319.490670412394 888.371107987343 688.247581763352 1091.12493937368
576.451964662024 798.1490651671 769.586135629964 550.24496459253
1031.38207659547
C7orf45 130.641217515008 123.994188725172 124.873289279767
123.02167644694 111.389873428517 123.665983073461 114.927420605185
114.805810485989 118.360186663758 114.364953312191 135.634385846692
115.923424095859
C7orf46 128.803632055969 130.124601660028 137.194467539969
121.113186311514 154.170007083026 157.385256442734 175.810411923013
149.217191121377 185.830435830545 115.827175143972 169.574755162815
208.257420634745
C7orf47 320.097510212422 358.355621069787 421.303580698397
351.02823853348 846.425467119342 526.625936680738 642.647554580301
624.106546150735 331.795832780176 408.444629429553 539.853394831917
866.413995755699
C7orf49 249.171307797736 266.842778974988 260.715580924916
283.235844022826 358.975346234854 230.399207047948 600.264940641426
382.233826557803 332.543652462234 420.647394044431 409.073045356073
590.3584324045
C7orf50 3246.86605825129 3354.38704439295 2794.89048827025
4323.76731419005 3793.02341602102 1236.56870367797 1355.66237447026

1466.08446530283 1590.87426068638 1694.24232555141 1137.42879398351 2515.7035557541
C7orf51 146.11669387238 130.808911605271 143.46919253255 146.96472350529 132.568354349687 150.126627487419 146.14494790114 144.917911601441 142.405569257901 129.284384810812 140.668597897404 142.819669471009
C7orf52 112.420505354139 126.539877052899 116.621735695217 119.974853329265 115.907902010701 117.575009754326 118.397482835514 118.04359489354 117.303671445328 125.215194276842 130.423465505321 118.486195373059
C7orf53 151.440732942734 149.453103369806 159.082447644277 122.780453116176 118.882591201241 116.764795914545 121.124574985464 147.804642251855 132.17211158061 118.527464364564 130.960045345375 140.002249873656
C7orf54 382.701467158304 363.706789137022 244.401472859026 336.338518973032 191.679217737051 476.249239197777 304.013547190343 428.569462312363 328.730722366546 225.238369884803 272.927830312746 371.369895979832
C7orf55 659.27672123763 599.307072499899 877.255923473532 652.201948082657 658.746224115551 740.800822095116 431.093544964576 433.189221987242 773.057095787974 385.978867521533 523.937128789616 712.067161025165
C7orf57 135.399032687551 146.846301957637 143.543607779991 165.257737836487 150.241585041178 162.629257046871 164.848970268132 149.300089212177 145.39813290003 160.221974600577 154.999192460283 166.071816427719
C7orf58 157.419333185365 156.307551403822 151.029785584386 170.046856179083 133.423376063284 141.59046197203 143.434571922656 149.331805754859 147.679233228201 138.336667434109 149.057388078138 141.408545519654
C7orf59 1434.1439428394 1480.52638217173 1368.91287156398 1673.5387396582 3677.23129570773 1540.71556891815 1551.90775589919 1549.30900090605 1302.54547114701 875.38834441246 984.695710317818 1797.65684760558
C7orf60 109.182623414389 103.691075663721 104.714872508838 99.5148073333731 112.015423658372 115.718600552187 109.471542280224 107.103635692135 110.718864165172 109.044879188971 125.402714360058 117.829228895897
C7orf61 148.960077949157 138.114938404796 172.622816559412 146.515240633175 139.449895159273 143.065258692424 158.8054811093 136.846912694371 165.586397236489 141.971207380159 117.045020667394 136.249063953892
C7orf63 205.341121539273 207.831299397177 289.667302610069 219.49354518392 164.455111983634 214.595639477696 185.113098620798 257.24481414803 213.028568789661 184.862871179765 161.720405760536 179.432235153344
C7orf64 115.157321350379 128.090665893885 131.460388562433 149.000760379976 142.419777428399 134.909230280708 129.589056071463 165.118167086792 141.994082714379 143.816567683093 135.65700435306 131.336902822702
C7orf65 136.718598487513 116.328330266803 123.56917054142 129.111259771449 145.692644807799 134.20988291379 137.457453776513 120.711150688559 133.382099379709 145.812607895325 146.406344338757 132.02702068341

C7orf66 139.223480216048 112.126521532091 109.719356131043
113.656820701744 115.25307661968 111.820208385514 107.559685467789
121.076652800015 125.563646710294 110.602406076036 120.530092530622
108.807891863679
C7orf68 237.113813706224 332.885488115858 206.89716102989
197.667299074218 597.459920611726 564.948126039762 2012.36542989953
1401.67503392145 788.244353085369 1849.21906428944 605.583144451322
966.731520471976
C7orf69 106.829040075772 108.2359175332 111.603221942108
117.028963434266 109.793568518462 99.5148073333731 98.4947439453387
108.415715678948 102.932013031511 113.756115251188 132.359319280352
105.583474813289
C7orf70 388.887050777137 344.298510200653 374.657816575897
320.360917595936 344.487982154705 252.851164967726 374.46180489079
353.385210690169 312.557093674533 369.513420606401 272.770616373687
310.815067427296
C8A 116.742079521048 119.449118660481 118.66946078241 114.65036208773
117.590393673861 111.608386387842 118.258886189283 118.279772712167
114.841964705981 106.982655360194 109.318922864507 110.033746435328
C8B 178.245841646175 161.126686827391 156.674411447713 169.541411677993
165.752558888845 158.483006541613 156.085949215738 147.169765031208
160.343551497512 158.56996905631 153.606787306076 157.885229749634
C8G 149.068713925139 145.728370703165 154.591116158776 150.494260282384
148.758316681306 131.454955038299 146.805832438977 144.246749835693
148.272252953881 145.049387327924 129.365004129784 154.395261192114
C8orf12 135.443856371548 141.052322220012 129.099780410063
134.247361216412 135.428592324173 135.067056559409 130.812309202831
129.511465164539 131.839890565869 146.274284557421 159.479962316199
140.763592237598
C8orf13 129.846685899183 138.082870754768 126.934059693116
119.854401979951 134.354215877283 130.95176570982 115.299986138153
128.440024330942 129.966755942173 139.858812885649 120.329105003959
130.385747820089
C8orf15 138.976386995124 154.161892545061 145.880661952783
161.997058981786 146.378522944864 155.615655460277 155.140371265225
142.459230639888 157.399880496076 144.331409179705 164.413893089607
151.965103021638
C8orf16 183.247396324425 188.864388750448 200.322334039458
208.914498369991 141.895211025017 318.244342364572 225.664432111551
185.591700082272 186.430014478919 136.381593416533 211.300733064951
254.607324522225
C8orf17 136.940275156257 143.292552892073 141.272049404557
140.672863685822 140.960907353937 149.133055368147 154.354658146438
146.16821907742 157.435995560423 146.018097597373 127.389942368953
148.832461329713
C8orf22 130.204571771396 147.270369342438 133.369314179341
147.294408903227 133.685254707686 147.826950636822 138.338280468272
121.47058227778 135.005591691192 135.335107344583 115.450352881048
129.703791330416
C8orf30A 138.728765780512 133.583297305219 142.248293068033
141.241717979079 139.828781354865 134.371254010574 146.153528461264
143.710449544388 139.654648891206 140.965498009244 143.107570838895
147.366877428537

C8orf30B 143.322199505844 140.63666629522 171.180188911669
149.199800895684 187.73896437823 184.672330560677 182.074569356713
181.340312140534 189.01309885917 157.058912028958 170.931788192825
218.896760804208
C8orf31 158.200211812067 161.402322405903 155.164238647284
165.459993078103 150.333572271902 170.18781660707 137.988325215021
140.726084514407 138.715208319278 145.78573175226 144.80318019871
146.97203173446
C8orf33 459.652708187549 488.884511065882 456.656123023446
527.498114662967 601.800326895295 444.192734921566 456.298408568636
411.221560795912 893.676600121113 447.059120541009 379.200576951933
447.579975492958
C8orf34 143.488761023636 140.028169667777 134.149446827982
130.532559074698 122.850678471084 121.174618503873 124.699127988314
125.259164818744 125.701083319929 125.897190440417 170.866433766203
129.150574563997
C8orf37 9970.08052783908 9403.76665152182 9994.54490550759
11509.9289491201 2500.07677977147 10329.1999331936 4995.96906061198
7786.96143641382 6810.50193989514 4749.69040198959 5073.27433952351
6214.77579017084
C8orf38 188.695527267631 209.74583569369 193.428733288774
184.672330560677 288.150694843614 330.241479004885 263.707476735938
282.652193666078 321.84712975195 272.41397350779 233.426170535227
210.085329463347
C8orf4 828.683748921915 866.147379099606 615.88518704181
423.562598841704 1054.47292895417 4190.38366101529 683.019502027271
349.99912485533 160.454243375479 270.847923557176 537.982214580518
193.786889748038
C8orf40 724.103864917969 664.19083888799 940.008438825765
634.774041584043 2219.71846304201 1453.88161495484 705.987531074737
1266.53539318831 696.976036382401 686.445635754196 1263.64725236472
897.51782613323
C8orf41 327.951753740503 337.935183275743 340.239348689005
341.901565692251 312.961981998512 352.354687089427 250.911817769829
419.846584903512 368.276805218658 394.294057009443 286.818166898922
319.417555901733
C8orf42 167.278879415171 166.232856234879 164.736294372006
152.56002779985 172.120772549791 142.394644827434 168.668125438154
151.629999903069 163.93199635496 136.60514429355 149.915251958706
174.807363753172
C8orf44 188.53221484746 183.795632040455 240.814860403562
169.332207863684 122.603226380439 146.775403409562 148.1283706199
143.103779196037 180.408639020724 140.74208906156 173.169905329013
147.666724626127
C8orf45 7389.95365139168 7218.21759302518 6508.71638048514
5932.37714987741 1151.30600388403 7555.47017983216 3570.21370790206
6628.17856159198 5609.58233117244 3338.13980198211 5240.40795364202
4280.66857663502
C8orf46 128.554574566655 112.90900939264 125.489777118532
123.756349408641 114.223991452083 120.517691208358 119.26921157552
112.661607612444 114.812254477058 112.509731206295 129.493805009651
115.954828435072
C8orf47 139.311816781874 145.937733795336 139.406427549733
145.453460204097 163.915489303508 173.532300173223 201.530921073488

217.607230767028 202.357989894268 122.922631510846 173.773876769683 187.978627462391
C8orf48 206.825575257029 191.348260799746 229.511643376643 235.453139389486 190.700515036197 175.98210925643 253.933478547515 201.954167006591 215.272800347166 213.891435492914 198.583407008317 190.031339775209
C8orf49 118.300797467864 120.371374172279 121.846349358116 123.225792525733 113.422559344178 124.585675323305 116.943791343147 123.357205547181 127.935614145702 129.305455204291 124.297848065172 118.07991087231
C8orf51 155.111346336412 149.760883745928 155.27707167384 134.824147038515 203.17525411575 153.051143478997 190.843795579234 177.500110974116 166.767004180603 151.899063970777 169.590101694098 167.373416675264
C8orf54 151.07661060324 169.349922114861 148.342114514468 161.338215647108 142.059921012561 134.744175117719 138.152641686151 146.854523122404 137.666849842576 137.890006487904 142.774449777424 150.624740697168
C8orf55 417.191960489528 534.083703564903 699.06539333455 479.761165832339 5032.66681917478 4503.01240138314 3545.8833678963 1525.24196488688 4410.68280125001 659.483452217461 2938.06458269406 4437.40805296517
C8orf58 166.20327698517 173.681078272467 152.06044555622 195.513703253634 170.335930486215 139.430808017891 184.775001243453 163.600888221193 155.365673279823 190.427022049167 174.587558727209 179.753400474369
C8orf59 1625.55754007235 1448.64875525408 1706.06615968622 1142.44180956604 3363.51782842264 3436.32495553246 2799.0487754899 3258.07532987071 2952.67114077688 2285.86163259073 2280.20302441765 2449.76376608893
C8orf62 119.213217733213 115.587874563785 147.570734399004 131.419663282022 120.027996187915 129.625046507677 110.804248212067 128.663320241092 128.649329515033 125.547399456656 138.562576783507 133.545405737639
C8orf71 142.802965008073 144.387375194186 134.086301127594 133.21981037185 151.744902566627 126.110426731941 141.075421936212 156.34272729153 131.181537219479 146.763046352405 144.974813243462 136.788269716383
C8orf73 125.894755032594 150.023791544584 130.128585442593 144.986655090517 199.397633942623 145.34332740863 147.064543281269 131.22473774325 137.398748156909 176.183165494563 151.442463705271 122.387532784748
C8orf74 120.035437167539 123.696942540314 122.868393258957 128.766289767459 115.3611179192 128.402511355513 121.177681858671 117.824694297846 126.680992733621 130.75707278606 123.557722415957 127.239282651919
C8orf76 445.089930641391 427.249733213787 517.42417250172 411.359106929985 644.97200337335 1371.56635975955 1057.1528284934 1067.30106203503 1403.90487313049 672.862678078975 715.638378711433 956.153239481189
C8orf77 149.039132756817 135.289246539398 168.634534857593 165.67554738735 155.417694869593 152.692483731643 158.053654524927 153.440952021924 161.530878860883 154.250656489421 166.70212615715 174.255919030841

C8orf78 141.368078865065 137.956295878287 142.032814623366
144.065554025603 128.79607493422 148.953720017484 142.528480882047
144.056644468886 143.552343056702 143.098246338022 140.110258415883
127.895989232696
C8orf79 142.348246621217 149.117224130576 148.334425146129
148.504996616452 147.103340218488 149.070750686003 152.705458417973
144.808774137749 129.928047362701 137.365567527202 141.786962606666
136.826606228323
C8orf80 141.221162787167 134.089308635893 138.49309476462
132.492444216154 133.519359066907 152.207601283271 135.682971958096
152.479402912801 143.703367582367 147.734708927825 141.799236109474
138.370241517025
C8orf81 133.573744633212 131.919387743094 146.110545700163
136.846912694371 128.870079563075 146.997906452598 129.845344493418
124.183297351614 141.115539663314 132.848926011576 129.031338314175
143.884263862698
C8orf83 203.791916347213 214.684538620841 234.322989368622
186.886677174192 172.345201438148 237.266455875982 260.888612454097
204.031081842809 232.127996106421 184.377923161698 238.904826940476
204.031081842809
C8orf86 160.073510173041 148.22974445263 162.18174589792
147.68047828662 151.965103021638 146.303959493672 144.70348930646
138.988919994625 144.779146672553 166.001279428607 127.395094952914
152.348210524663
C8ORFK29 149.464962540085 152.853860043701 153.767488352037
160.870390779084 138.810236692956 162.762473039474 150.751386127903
163.636550341231 153.492072037266 161.581641777014 148.597346678768
168.289492873475
C8ORFK32 133.201157825808 122.142323014287 117.203783530266
119.237802375552 128.548201588898 121.067907968175 118.540620928268
124.837187124441 123.498540254464 115.37736249419 126.969504644414
118.109991440011
C8ORFK36 128.118223792086 134.501410126169 128.274326990056
134.002424083826 134.273326732349 128.11006485185 129.216693566478
130.949405011154 124.786029449452 132.28931085483 139.052898232393
135.420780282429
C9 111.217595631623 110.718864165172 120.55570440025 115.624167024067
111.895279866421 113.071787850611 110.475453268997 107.605526304445
107.697601478064 106.603674195422 114.835386789236 113.427848757112
C9orf100 148.853877151493 149.138678142327 143.324235640446
141.892214695902 179.400220328552 153.143706194386 148.49560238679
155.741507341843 154.993784224548 146.894793774087 142.680136733947
161.916155040522
C9orf102 341.564749445905 348.300300174924 289.474517971859
362.742120043509 169.036409026328 152.20173979854 169.619338456208
164.066839397676 179.30729544595 192.767155566493 279.704940529618
153.697775290962
C9orf103 333.745543022749 295.678097392123 356.84281623052
287.593221911064 249.292766428035 225.423403713718 217.287912825619
316.364342983155 228.560346647306 269.053237303244 216.32785564297
189.235998474945
C9orf106 138.42216862811 158.160604786093 150.874014332213
147.091280396135 181.364325529405 155.572357799741 158.404154056472

169.101676908756 156.328454726363 147.082068348591 149.6730835169
155.911387663464
C9orf109 152.961475450751 138.611074796816 140.548371692813
154.768422926874 160.261708910517 136.515951031895 150.907594445048
142.29309119138 144.654765322941 146.536055773073 139.147327302581
133.672964925321
C9orf10OS 328.60529053804 318.244342364572 312.845219991659
323.788197265954 387.477198908304 164.824914851636 111.389873428517
247.72190598581 251.546290552828 241.077618798955 412.415549375221
239.20206002
C9orf11 122.527517633626 131.724061631389 125.052770277549
121.942777234608 130.001889950186 127.694189114288 135.344498171119
129.658488766089 121.414076245292 119.974853329265 153.085975710144
127.601379088239
C9orf110 135.141067086648 143.685368799972 150.016550610991
152.199095472082 134.028626989933 138.343232981485 137.769431537236
139.331046480528 143.48729871068 134.633286312653 128.488598467312
150.261107471642
C9orf114 302.532985701607 303.724140156439 319.490670412394
379.476768908492 511.25179427158 415.388760988459 276.434192872962
313.480307434343 524.973621024573 297.970735665595 371.457568934695
250.82359383789
C9orf116 142.338352884053 146.164093136588 143.589222264786
147.15999706579 516.892850913889 227.64196401771 220.510248260182
232.414763435965 284.13865992802 147.910699446264 280.531844086775
179.026308021985
C9orf117 147.638057399165 151.885992695789 148.592330216136
148.179398287068 156.427158441352 158.594064857041 140.946008063839
146.577524520274 153.326420485822 145.168031449425 147.502116289881
153.996429870564
C9orf119 577.33849844574 575.546802138889 548.869880723909
553.757847979529 783.059427015955 377.951523644306 504.141861790903
578.811908030805 834.537908087672 576.873245313776 407.442337662943
401.189904619902
C9orf122 132.151765465534 110.688722702767 120.767950825369
127.520134825082 112.464951599416 122.323876787207 130.416957431512
130.128585442593 137.636900130922 134.69274746407 140.088094614168
125.110064579814
C9orf123 483.312894744803 508.735412486964 584.199252444401
647.439372562271 230.672056810885 304.713434036642 190.850639994777
251.324685584062 297.921617748468 203.693188114912 302.847274046748
178.12073430094
C9orf125 166.061891147234 171.161297672182 182.02250582556
162.904897605929 160.322541355391 130.632096412204 146.366013190845
133.872561340464 157.78390301218 179.632562895905 226.796825544749
195.906137340918
C9orf126 126.837151457845 122.711143209444 127.500754400224
128.675527226161 124.811760828557 123.22295112139 120.78129870199
121.564626579011 120.257886394616 124.629877200816 126.072053958666
124.334450753704
C9orf127 464.128022170285 431.093544964576 433.491835341321
571.950858678221 284.940858938629 263.352118305612 258.109876262342
131.85737847837 172.03919618445 215.698924670416 225.335753745598
243.038392457584

C9orf128 123.24533179875 115.678486138934 127.551486042435
120.944267930809 116.961391747202 118.029258839619 117.467618148107
126.718380351644 113.416743649576 117.703025026759 133.079097536801
123.728481007459
C9orf129 159.032710656826 154.390764802264 161.744366588564
141.327299595068 165.82215325714 148.072816925413 134.750046135498
149.039132756817 146.778156465138 137.570343435498 150.489651880905
131.693923403533
C9orf130 1202.74869573717 1348.80530206238 979.019125719101
1160.78730097715 342.717252404581 859.860994525775 589.070196307574
753.654626233604 686.923164688096 544.580311371212 834.788058321257
584.199252444401
C9orf131 112.875136585606 115.639101191444 116.363556872071
118.593717189375 116.474771810965 120.871693555514 119.455615461113
119.415822905251 108.04922729993 116.126923911804 120.418709541104
115.277302351501
C9orf135 140.616811333466 145.522435728191 147.78095608696
137.416133786828 132.581499870494 131.630132952441 141.797497681512
134.381042390464 144.96021415188 153.393651104539 136.560048419149
155.890403934674
C9orf139 111.716607815635 115.137475087954 99.26400442869
116.456170659686 108.768730405582 108.2359175332 104.03442843396
112.896360244636 110.448948495323 108.970432124412 110.242638217587
109.564709878576
C9orf14 147.818956338752 133.911650164508 136.44218532882
145.131215503044 141.645906784244 145.378779757104 136.760382409185
136.301442794928 140.356164189622 132.287326702468 140.13515064896
126.976353967127
C9orf140 136.478114537536 138.903450375532 129.956519535525
146.357214078158 410.954095610904 226.565823278767 150.232470359841
208.406980186223 226.114369645703 169.378422829858 188.079188251085
203.777944180067
C9orf142 717.686676823785 798.389385197471 650.911886508922
896.625415670455 2253.7522632414 652.429025114523 654.52788411248
784.412433571927 808.55123510297 682.730262408017 689.590642377619
798.389385197471
C9orf144 144.159316670264 146.920496801962 140.844007965202
162.411156818437 145.060085149395 149.17029600091 151.171716392043
139.811873260553 134.222453538421 149.4401812 144.930066324767
142.431979387165
C9orf144B 140.713537140957 118.161192318422 125.447209413787
118.841699953612 131.892151111901 120.308260557986 122.679628839976
127.869038038293 116.819107878692 113.568261995787 131.722397988741
136.689064885984
C9orf150 158.639313207233 184.989027770708 179.191401472241
150.19577351885 137.28323311107 130.557209560361 134.464053009101
210.960900130785 129.524056217383 149.687372584136 144.924212639731
124.591873637605
C9orf152 114.255733119076 130.14600240687 116.67463130093
108.199628274798 227.092319765009 127.562924931536 114.00959999268
136.981043383326 200.506615977654 110.820329153348 193.704510912321
314.014654357569
C9orf153 129.161305053472 124.558026853954 121.564626579011
126.426212216646 123.603972078897 120.094156375538 127.575660581922

127.631770516132 124.595925483149 123.29908883119 145.560904207183
124.507958339495
C9orf156 264.563741039382 258.915802108562 228.058870735016
224.008253365957 192.680681636018 184.085616342099 227.689356258276
226.763252784108 247.280717385562 326.525579400607 340.608165077422
205.403820910921
C9orf16 220.710324965453 225.565045396166 200.486683704179
240.994507285383 779.922653392712 253.30325545013 238.310077556167
203.004815689972 274.281532397691 224.724262742427 479.761165832339
178.766654213173
C9orf163 119.931415360373 120.122719226116 122.992700222262
124.124076599417 126.887989471803 120.441732791854 112.825399620524
119.48998115497 117.048846466839 125.141185839817 154.362571105094
116.346181133756
C9orf164 143.296580629431 146.959154938248 120.110473925082
124.306049555272 163.181323768362 118.366859032979 123.327238774943
135.993598471576 146.569958732206 125.787353491408 149.432744784716
117.499697378893
C9orf167 149.813838004575 139.380610974147 192.532222258033
171.994330847901 172.84784588171 176.714471790643 183.168629351124
167.73288535728 173.790848780011 238.037110997575 211.809434609469
181.3859695951
C9orf169 143.954908055924 163.20714296121 198.15257384667
159.046016068252 313.581986704048 191.59400638652 314.337680575998
204.1974438819 267.149525812834 1894.14699530855 331.937313503755
422.02217814979
C9orf171 161.992141305029 134.208537331186 144.916193282062
158.495606141226 148.742216703684 162.592306630033 146.249951130016
158.92254744379 162.900556089071 156.907788377794 152.657649988502
185.016336026382
C9orf172 149.752188104803 152.746799687103 161.096914295309
166.784804508647 170.13453211897 173.001985500448 140.225329742578
142.655287071175 144.765051967884 136.826606228323 149.020824600931
155.449724554217
C9orf173 146.824420102008 137.72327986309 156.351734527745
161.139253742858 179.985468369202 148.364138636002 151.641486303453
152.247504238261 136.850940500212 144.389805078873 147.868721324602
143.231806900648
C9orf21 253.867947800583 264.383922859446 198.113398073968
205.381840116054 165.376358421533 193.303987945242 167.842122798757
177.055023182887 191.814634229865 455.08515350006 204.078331021637
157.423242850568
C9orf23 212.531329857909 240.306287921209 275.169415154893
253.30325545013 462.779122987815 293.794745907663 286.642323413104
261.671277686467 284.595024875896 237.734385188542 367.439588680092
260.370285504512
C9orf24 151.690538050817 162.909468312298 152.906352463546
183.358807104988 144.432181150583 157.729732484131 136.614620534169
144.736213465651 148.463566046951 137.048292841566 140.377179372804
151.079500558067
C9orf25 238.847091900666 237.396466740419 214.261611197599
258.21341068758 211.300733064951 172.403953571339 191.912670416334
201.837695818264 171.287186905764 188.20675818131 181.467708718758
178.130223302924

C9orf27 139.56332664295 144.5368823731 129.313576009092
145.885577577352 135.388750095746 131.49254872524 139.025598691696
134.944488136297 139.140647941752 144.934103671894 132.629453540571
149.091925032548
C9orf29 119.305917403956 116.196676877996 128.773129597793
121.492896642899 150.468392290279 133.713275043978 152.422690241301
128.655549652719 152.689610196981 123.042372128864 167.645930148254
125.902015309754
C9orf3 484.131020584378 397.307515234646 552.790706181938
446.393631025049 226.763252784108 191.692830869809 114.812254477058
196.536086443884 281.881563164961 229.181725450534 200.618080312379
162.351212914573
C9orf30 372.54091392596 290.541184606565 317.206754773681
263.250833727291 307.906312092805 281.168898910065 254.607324522225
269.448201975645 248.850686917567 675.480482223958 269.853718252574
273.550544349798
C9orf31 172.412059174046 156.349030617265 135.954961086609
160.361025839456 148.029259633449 152.435416154284 152.327247552986
146.740694608146 134.10070984606 131.349297554112 133.638664342783
165.131463797217
C9orf36 161.636411303968 142.049776668507 151.989476683722
147.338615809214 145.625205055601 149.500457158616 142.916274989816
144.889019483712 151.863048206341 158.049286838471 147.15764785567
149.876687693565
C9orf37 199.88618355278 199.058972781223 160.466187961848
271.064405562085 202.397972518715 150.267298097687 161.866405026758
154.865107093653 174.549929320587 154.789187466057 179.985468369202
174.045229095003
C9orf38 145.418696807842 147.743331160624 171.636121363019
141.064086298948 152.077204721895 176.53912635694 174.971350923613
216.974834077975 168.991551886143 179.091917512822 146.808519947394
159.79463502085
C9orf4 123.084040312047 110.636713168363 122.724429060184
128.276471459777 122.624828884731 119.308345676905 126.856038484436
117.735411259153 122.996616773523 123.646558103253 125.513881890201
121.645021899034
C9orf40 219.661502916874 202.583967728957 184.724071110756
194.388383297922 192.532222258033 139.962307072428 130.479845410716
144.501995579502 185.359195899057 167.315702949236 147.412608079997
144.634118305223
C9orf41 140.14062775887 134.084602409011 138.730409717578
144.551233687082 143.728569494708 152.030182228463 146.787927492706
149.405118010893 148.242794464181 148.304863957659 140.356164189622
158.653161314597
C9orf43 131.659187927328 130.068063785009 127.434873844923
128.244549276386 143.114384950467 128.244549276386 122.456286448252
126.162713269943 130.449669345302 119.802832736435 135.968346417524
126.713610681382
C9orf45 171.751149502172 202.887417145119 184.437810305792
215.635313821029 191.708350637714 207.960802948119 162.519642881848
255.3451781862 237.473927775823 198.722993687455 188.543051108601
166.223079895236
C9orf46 373.656487297238 359.493075382333 521.512936372701
347.985315257161 324.871248300476 442.493108530222 349.382889326613

1318.3633082502 488.249967208882 1108.61301774472 270.787490294555
417.731815310276
C9orf47   120.915108114673 116.363556872071 137.114604159523
123.841366760069 122.941711366641 116.998277297063 113.083678884076
104.03442843396 109.156349683299 110.900309203531 121.039678049579
111.506345174435
C9orf5   1160.78730097715 1020.19774509086 2089.82894186824
843.075879568455 340.028737478082 646.461274173693 554.511983790164
474.629173435475 1251.56853099362 445.214621023114 765.333633142088
540.190808200264
C9orf50   149.121894111871 157.259599894923 144.311620064675
181.328458560967 168.394330931524 157.898632788579 156.611727364072
161.603317039244 133.203064891923 163.794436953711 134.700543462375
164.58158284856
C9orf53   133.490805791504 139.159510392614 141.88777975766
141.213588194832 138.407207709066 152.418315087218 143.868962478182
133.061016674211 131.892151111901 150.531753446398 137.756209496729
141.623851511993
C9orf57   135.449919220607 130.898067924686 140.63666629522
151.040909999868 144.946362772133 152.080057231881 142.964043764953
143.725497952919 142.995274641765 140.814009587096 152.409211647994
149.827791077797
C9orf58   230.367251714289 193.974325897472 138.376176600064
184.377923161698 137.68634745111 128.578494940165 120.686906898624
115.755585907264 141.121364213272 125.296240086615 143.752697178913
110.347631909888
C9orf6   213.652091843896 207.850956786183 227.926444925478
194.569325586241 253.098839084408 228.675099004411 238.654367531982
266.101158071711 258.759507964221 359.991823789356 302.696004448559
249.400820221233
C9orf61   357.773949920377 361.160881334174 500.848273432862
417.36454228858 173.532300173223 148.376194044247 155.15575204653
144.592987736094 153.893753448473 144.54895913821 164.438724387992
147.153129531937
C9orf62   136.271412363498 153.169450192935 136.593346330215
160.290104492275 129.931923954109 156.540165645256 142.194507957661
151.167024833049 158.353171576491 144.927731613526 170.590817818414
141.226901872221
C9orf64   343.617417488962 309.937351755502 332.343558229873
285.659010982301 227.144441241869 218.031238893075 246.676401619881
243.691004557778 258.145539382199 270.231637012624 278.555009982029
233.71520737817
C9orf66   132.965467229606 146.608010450782 144.826861308997
141.622592268163 181.681929801968 159.806221777332 163.280786875496
212.959246855759 198.244854812728 178.680902599163 204.959164579011
176.66175597182
C9orf68   143.5709409749   153.492072037266 159.040409182674
148.574661934068 142.780100181736 159.504327477065 139.356478232855
147.428180649939 144.970383956581 143.13304460184 157.423242850568
153.414185387669
C9orf69   696.277780670166 831.08287594363 519.450899933049
1344.87842417496 1509.69760873558 329.82030842286 416.715760038095
328.730722366546 525.138476928759 580.859914041323 332.798130757632
340.301980622308

C9orf7 204.681956301306 235.742017851879 212.272826965935
267.372241661758 588.678767007017 248.360540656183 227.209630859635
190.736796322054 211.160781061336 181.123571246557 325.493177980483
202.622747952763
C9orf70 144.355348009294 138.828562311512 144.392522947899
143.896324094984 136.494339004002 137.984988819967 125.280736217267
137.497145594629 133.324531689254 135.973752893694 141.683298500736
142.968008233583
C9orf71 118.628559951472 129.415078042622 119.01559016005
121.54835308632 117.79844873842 124.466986821169 127.480717762966
126.995207465164 114.072600760808 121.414076245292 127.79057041268
125.637138636558
C9orf72 167.049197726715 151.788278519057 165.327972973978
146.590495255969 145.245316640391 173.010799217261 143.198622036573
151.790240553272 148.57301274812 172.552923333095 159.576585660511
150.409919207059
C9orf75 197.326259802349 209.090788790449 212.591357254196
200.867515935703 560.602047253306 234.738241125899 293.646275396425
235.216589534371 205.45079491218 219.462274506333 378.509324619452
219.78041403077
C9orf78 1479.69309189156 1597.53010317504 1806.24732793591
1683.83803339436 830.684999498776 1082.00448164658 828.683748921915
1396.4168898623 1559.26487922551 1617.44377452783 1564.8868894162
967.123240442957
C9orf79 135.457059862669 141.5311434224 126.045231525329
134.746175914762 142.978818998012 134.323365608463 128.702882993588
125.364821583438 129.819820730239 138.267726309873 153.907737500104
130.669539169739
C9orf80 9636.32818474581 8617.2437024568 8768.32269645116
9514.74328718935 2004.19532096239 9820.93899217345 4448.67183518984
7717.01422791837 6114.33638002734 5115.14161559446 6830.46932926077
6188.04738762584
C9orf82 216.24497587588 205.821101661022 164.61921356441
227.715558644326 161.37617568158 196.716209382446 145.773932754923
181.566951620202 166.433933349193 198.470718196139 235.162439885207
159.009211991511
C9orf84 153.415901281564 152.274957846518 145.005991300977
158.325554470875 155.410704148925 155.690773461824 150.295611829265
161.463042036376 155.878493935166 150.596350682411 150.176942469551
145.587386656619
C9orf85 165.523150623132 166.192990554427 189.205724390913
155.268650584431 203.475080163804 168.180063344887 232.321454436688
183.979513272793 169.184111483933 280.398987330256 284.891415492568
185.432587490792
C9orf86 291.779980648289 298.706655333472 266.468621734455
408.7151485563 701.208391798006 257.706904467657 200.371280127668
225.4575611466 301.428024926428 272.01602406899 396.345382600445
252.789376168324
C9orf89 290.502085667398 259.742175498038 272.994195639694
260.129513331703 391.911768848267 205.305702209672 124.180511991605
219.422260093722 206.825575257029 338.822464893411 249.524956682907
261.366143735074
C9orf9 175.21512930507 212.27282696593 5 267.970798709017
260.41082304175 188.308588359317 143.152993168137 150.227629690236

166.135707525987 201.059635671301 142.266177608209 148.919726992353
132.788102310611
C9orf90 249.858562350415 253.789045068884 257.44230597145
311.449205777595 551.823767223086 230.855806094267 300.985037429772
279.921244070981 303.483515328621 271.989060206794 257.075088169589
320.097510212422
C9orf91 237.456245064363 223.601312199935 243.35880641566
284.940858938629 228.820894086914 153.371065247493 183.04531185589
180.437648146588 228.270158363878 195.816317534388 200.915806094193
179.369845168822
C9orf93 141.71400004456 146.96155692518 145.431393142782
139.621016978072 125.081880303992 132.211379936941 136.103088234442
139.314945862113 142.446514275674 131.489715975987 144.736213465651
133.601133679159
C9orf95 282.101714660307 316.524115715585 357.431640917696
253.164596735793 269.530151236658 269.673122990604 261.814227719605
302.637859140061 244.162496497466 244.544473246593 613.279465441319
232.023739004791
C9orf96 149.929454280702 146.574656147816 154.273953018148
187.645390502607 143.021435512861 145.383354200298 169.33926580273
152.11956441072 162.168246402027 139.178245419517 144.66675420575
159.41078351918
C9orf98 145.793794049657 164.000772606739 159.021304119265
135.52861375119 163.632148441668 143.836637098747 131.193538235053
141.327299595068 153.261002653668 130.478834977547 150.542478166361
151.266352916239
CA1 122.153996665065 108.118551275778 114.14858855653 119.188446525733
132.94603323912 112.999642731096 119.899649497032 344.128576341892
116.335864439545 111.43827410589 132.744261207073 109.274221164316
CA10 138.967080709927 120.14201542563 121.771891473027 143.227410286571
177.32104138164 142.804411455413 140.10798818623 151.717558541683
129.985797334276 138.304793826214 143.451080466172 150.922279808832
CA11 180.761100930677 172.8993416872 176.11287574658 160.865949438448
164.268280525424 146.863611516518 145.369791572077 130.657342262492
145.272073426254 121.722412245175 139.750281709938 129.242093568433
CA12 308.767036855508 324.56043349882 362.95684683529 284.831817899986
715.039905439621 1307.19490564658 639.011144305614 366.636754272029
430.505037012814 2149.13404212591 735.165830487162 1508.24281455057
CA13 132.313884627836 145.058110060639 142.234554325754 147.183141990315
129.822926691091 153.363629311799 148.317958829244 162.466301774088
199.53455266342 152.175423676063 178.083311435916 173.782474972742
CA14 138.114938404796 133.796029401157 133.911650164508 167.521700144143
117.555999801887 117.417112053842 118.691723466789 129.515390475589
106.147993332294 126.388778582602 148.038291195414 117.48427100606
CA2 139.071908528327 153.941193682697 181.941224633917 196.226732738526
336.434368527549 351.292682758926 377.89330639683 1885.93146154931
200.867515935703 3798.89743803968 415.113340335205 434.073729807058
CA3 126.3539479619 137.172030225913 137.823152565933 172.961115357569
125.250900940748 124.889829874126 129.127563278016 127.524310543779
129.179188863839 132.184217000689 121.991692204056 121.619593453259
CA4 220.113261580385 261.366143735074 200.712182818203 260.356834114611
161.694109087893 285.214585324341 188.941900997781 1184.3199328099
408.10780961711 186.222567239198 163.086539217826 196.001089662818

```
CA5A  121.336932276232 104.780306305981 121.134305707234 112.904318310632
      112.495783269169 111.568329540016 115.310953814885 105.859916438163
      112.642429076777 116.5272207025   121.505178996279 108.530158480237
CA5B  234.484787292284 202.622747952763 171.595724119779 190.204960255989
      161.094103878416 173.408784502027 166.335294339013 169.940177050294
      147.590189238316 187.73896437823  172.310673499572 165.703810120915
CA5BP 139.166537591866 142.543569032033 145.241301006518 149.490064894136
      151.183847738922 139.440415574681 145.217261514886 142.474762834848
      149.703317565769 133.681703350531 124.291944151031 141.062226285596
CA6   122.868393258957 125.107483348755 115.933805625814 115.316430915446
      122.708102546276 128.0171233156   130.309092946921 119.112063328364
      120.74273444647  129.743808480368 150.856917125736 136.511669406292
CA7   111.086170834553 112.875136585606 108.007762863766 110.977904932371
      118.924317138158 114.874358180801 117.969576809288 108.604763499678
      115.656801309856 114.849872458647 115.949513659403 108.179513318734
CA8   118.9952230535   119.315932907373 113.756115251188 113.529619135543
      135.283742166077 113.913337223716 104.780306305981 112.742895297124
      107.91011371422  126.544685937984 114.512028664352 107.385565665491
CA9   111.962852412774 122.735376540421 119.135701239571 118.918962530408
      144.631407964232 123.657246099004 462.025126897013 194.240372898704
      129.299810898979 811.144838166905 231.728984785324 388.573321838751
CAB39 2344.11548437508 2093.67403119512 2853.14759681683 1671.82101590797
      910.982557417978 1279.15873231449 1643.08061372695 1840.14350831655
      1792.35343044443 2369.233048505   2249.8123426309  1936.88813111969
CAB39L      205.930921729192 208.42712014566  223.408447580188
      163.319882002763 210.218440444631 354.336462793628 186.925138700818
      263.058972886829 459.652708187549 178.245841646175 278.775971122032
      438.958573676953
CABC1 1106.21439150501 1586.00600118477 1589.70261092241 2311.05587511258
      951.371815352591 517.020259129974 473.529519031797 487.07860019179
      612.61438059983  236.592839463278 453.760162338163 343.121661664987
CABIN1      394.73732350607  388.096183735126 282.392419584508
      578.084277076692 382.401290201025 298.403224729528 228.100112133941
      240.306287921209 241.856820205666 199.170488640762 325.859917318516
      234.126500054945
CABLES1     262.389583658305 284.236970342827 201.555964610849
      200.944865673409 457.779218916808 151.669421563954 188.131876231735
      282.511614929917 140.82351325907  166.341564850299 285.83626978316
      134.55255098289
CABLES2     174.364798143068 185.639696270808 167.526798465556
      193.098116087792 316.237519859859 205.058210447711 309.36704220687
      141.622592268163 183.937484597232 148.525224975266 168.27980269501
      182.066922756289
CABP1 139.113675690483 152.247504238261 131.345038923753 160.060406629689
      137.85552865361  130.479845410716 131.774882989257 134.044789094272
      130.892302357045 123.256613702254 125.321968563987 127.167918433035
CABP2 146.318052064937 144.751413348459 153.206468539619 157.623092996114
      144.142765177298 151.573920235798 138.687013437904 155.908951665928
      140.03443832328  148.450023054885 142.095289977576 148.562200783472
CABP4 160.853480692082 179.810507975486 149.244970309131 182.109092947203
      189.61702125418  193.974325897472 182.736067431579 179.648479201164
      175.251238431716 185.258354905314 178.425137262105 201.256649338315
```

CABP5 138.639410179404 155.452842883109 156.88782735706 170.361237529247
146.398454169593 148.80452641061 132.253450751554 149.353072778508
149.057388078138 154.562761981868 140.89390566467 145.326803807448
CABP7 145.821544099585 160.632809953993 154.938900278679 133.085476994246
133.753999983813 144.043818335414 134.044789094272 136.694143268855
222.605195861218 136.80943340993 173.468958175014 163.239195865198
CABYR 141.597692730835 145.539731152955 130.501010489883 140.010730572075
174.985831291764 142.079184950618 167.100039749329 273.303240462047
176.128369308989 152.207601283271 179.551640851353 173.586160103539
CACHD1 198.583407008317 176.5588805282 150.920224503051
141.541355091514 132.28931085483 133.12641302705 135.588132669957
125.04327844782 129.854478528455 132.606735652984 146.194467350201
125.04327844782
CACNA1A 145.025703051661 135.497033143331 135.028746262383
157.607445509297 144.942289590793 148.302775746627 137.049677918041
134.275773172492 136.440671408022 158.325554470875 134.038213827843
152.035992124554
CACNA1B 145.392463986225 130.659631155616 135.114900669781
129.222714748528 156.007630568494 131.514114515895 129.067538010904
143.728569494708 140.784853736558 152.890239929335 138.444427173667
147.491439949088
CACNA1C 342.998459706966 377.083379021079 413.913442080187
580.859914041323 136.563471322998 139.00736252716 145.020615552964
145.351662473858 147.281035392817 157.466686487471 144.387375194186
139.034940762344
CACNA1D 152.56002779985 135.32730674455 137.463764193883
157.960172706254 166.289763097913 147.113541493157 153.423579724911
157.113536717393 156.957578703389 146.96155692518 176.317805247749
165.333608076984
CACNA1E 142.700311964178 138.333435927107 135.391788614828
125.899200544217 152.719997757291 151.406384541297 168.323141675824
140.334331111538 147.459385142695 136.638141963514 135.190698960212
129.83316008718
CACNA1F 111.032156655273 110.024815508703 102.932013031511
101.597648528389 111.949644842971 107.884870799936 99.0767996209245
111.10737497653 104.248226402465 114.41737808857 111.538651711074
107.638492587723
CACNA1G 129.165440930558 126.362418476129 129.466612376702
128.297879410629 130.25146185271 127.377356715337 127.344210958898
127.291712212051 124.672483602517 126.743579586052 116.769106223472
127.856634523494
CACNA1H 1421.04485772969 1879.08108720938 2061.9164323309
2992.18604022433 163.982415393606 152.547558436703 126.151017944639
144.195705502443 161.520362399369 182.588697551677 417.987533397449
443.259619455624
CACNA1I 145.159572111039 142.132576760619 132.297716337104
138.041411135053 218.350166753833 191.322056430728 137.012646671173
183.764215745794 138.69392148885 146.569958732206 143.945039636839
325.751369001105
CACNA1S 155.997210529742 160.331940529151 140.845958602151
174.284268998257 152.805525883571 143.141852982371 164.227134687088
147.463822782303 155.669470674985 160.537464426971 183.033044475752
139.130560988108

CACNA2D1 138.609972011765 147.879286704 131.138322527536
134.182902752156 132.677272839165 141.434699050015 134.32174235533
133.018406625714 126.784243009941 141.412317125776 137.014698467096
115.734230176652
CACNA2D2 142.956870642583 140.009593108693 142.734103971267
146.223880979601 163.558869203898 143.256590560776 143.461785113446
141.492458250787 139.08460576146 134.683553037185 139.22543724535
143.497909064641
CACNA2D3 132.834433153076 121.85247363239 119.192893408873
114.062099086818 112.825399620524 120.95428135683 108.895922800601
125.160161268474 113.716741872499 149.717739166461 136.3615279557
122.682761393215
CACNA2D4 141.168198348093 144.102872001089 139.163958829511
135.098343717085 139.268052431476 136.472404812817 134.89415027205
137.838096997956 131.827899207029 146.128066033346 143.114384950467
134.961156870831
CACNB1 141.196795654442 143.401971479167 141.996233801459
142.939578759097 133.951322753098 133.805991920905 136.028011201077
131.460388562433 139.706279455371 136.093473732174 137.916872642522
126.029431525943
CACNB2 192.69687837782 180.994249258457 221.82340413039
201.865955150869 131.305785417254 132.74579603362 129.765440519589
126.292433523959 125.04156726504 127.333963230749 126.308622415913
133.590552542806
CACNB3 539.853394831917 580.996071040561 395.521241316058
444.945078908489 636.56692204969 860.315938621256 675.083275037052
891.194779815063 600.162650426961 604.453803994875 970.634736994313
1277.0921553877
CACNB4 133.085476994246 136.147810636915 127.030113960477
142.331050242704 130.429076594441 131.400031203603 123.182903611854
125.377377116057 128.179293878252 140.138870925001 135.273694626342
124.075077123156
CACNG1 129.728266017061 150.290025009187 123.446714388901
138.824082857747 150.093684245826 134.534815676852 133.564350740049
109.398997827329 129.719122724699 126.924014801186 147.364301916733
136.241569739318
CACNG2 120.951365231971 117.207266754941 114.115609163126
106.102093734748 111.743461522689 123.796657561057 121.353233207209
121.181654892305 121.88070274109 117.101933186269 135.708804956764
113.199690522854
CACNG3 136.13751015238 133.11040099808 128.939572714685
127.760995691556 136.66358267095 137.171348199209 124.347680638586
128.528510920423 129.956519535525 122.102644097132 158.359440014674
126.515480612924
CACNG4 154.688685142904 150.741291526753 144.604714612091
179.211502448703 157.353493980339 155.143752422033 157.885229749634
161.44729973777 142.865320802784 164.126980102525 143.370698101204
138.618761382177
CACNG5 121.643036773773 131.141668650877 127.006746526023
136.044670179122 128.890685255595 123.58727715816 125.600500146679
128.116795372698 123.665983073461 127.588907178652 150.78145177853
130.512057032369
CACNG6 143.292552892073 138.724959677368 134.475296383752
134.517774302213 138.760800146167 132.937942776777 135.711590400394

139.061072394426 132.340183565771 131.87052926802 143.061415008831
140.818413826758
CACNG7 115.242526386551 119.034003473459 109.700513642061
119.645983822002 113.487357534546 119.608334527504 115.463150857002
122.460829835477 114.290611267606 119.636334259263 124.059778432167
119.305917403956
CACNG8 126.37572518012 125.404043810022 129.020356016653
122.944699010173 122.051473363557 128.451788292588 138.279087298069
124.889829874126 131.595868226775 120.957197551997 132.197346228417
138.582427155109
CACYBP 178.476483428474 182.77693508164 265.366632264085
146.649944669876 265.670643951648 334.056545911557 279.790257881254
491.112594297928 451.317592236192 399.364160668615 226.824313642101
394.35596596181
CAD 196.802641002079 195.561296046507 204.396576475753 301.188296465202
616.701037274509 273.197814995543 215.84044779962 279.541294184609
218.2454853526 242.695286989672 204.183472655276 203.510106134882
CADM1 527.930077857945 598.924339094496 640.255747986724 523.216537391852
179.421854100287 161.968835903764 274.281532397691 263.448958595597
176.953099943185 229.443064589306 258.807028561407 408.811415529922
CADM2 153.920180168328 152.91729346961 166.917552275021 141.380276932556
122.042136913991 123.547821639269 117.910259090745 137.463764193883
153.46645509759 131.92059356875 127.453866279181 131.863018503922
CADM3 307.480644393018 261.541138637729 214.546528079149 258.25658838015
152.910433120981 143.562164660825 149.545278163222 138.409284761652
157.841520434044 143.094799968981 138.825961991209 145.077517845584
CADM4 226.315173823168 236.191384383802 239.277801396834 311.848379951974
436.331333134628 284.438411011954 271.770138565186 235.183590602209
193.336246844237 311.806908624308 162.056622258216 211.34069504665
CADPS 133.737573120711 150.384559054133 156.806407903626 139.941724115063
135.877646659023 138.086270564406 131.540436531741 144.134485888258
143.922878410438 139.503902205317 148.8870854102 145.241301006518
CADPS2 255.225145197422 247.307128520272 229.85833221434
217.013533525724 319.128582107184 242.269784551092 212.460476372263
188.40383184521 264.936739264566 162.090585987836 169.110693867431
153.752502094283
CAGE1 135.233957017664 144.914015211747 145.041430440608 157.225479764928
143.410917415691 141.721829342179 150.724359913994 145.514859606031
131.800533523123 142.81401345781 148.913181799692 150.25322837689
CALB1 119.894737403889 117.682034050478 111.837468699281 111.949644842971
114.721282552636 115.995309578182 103.202873534814 146.70774472499
110.875112640101 111.484426481816 122.053867525393 110.952108124186
CALB2 164.593531655305 159.629232808787 158.928949614338 161.126686827391
195.963361515546 144.210698143215 162.904897605929 139.938739652347
160.72962443421 165.863118584978 192.140442881209 142.14116448528
CALCA 131.796941315992 140.299459596491 131.849442776642 138.701871179502
137.47514052942 139.589612144962 146.114159600635 146.608010450782
137.675597243706 143.607579034109 127.678512375099 141.797497681512
CALCB 124.121555438466 143.354858193994 117.77708734157 123.601413134287
111.161283080299 126.573673878042 128.659393756477 127.2369420721
134.867806860993 124.933738341123 134.986030185477 107.126004317323
CALCOCO1 367.567681162661 379.476768908492 483.429488817674
449.220056843562 181.991820141929 152.445022409208 153.398587738216

195.595275594235 193.412827246366 164.377227860031 291.167223094542
160.390564881326
CALCOCO2 293.600165830839 271.897511879509 218.209523110843
243.979769719186 209.840035045167 196.363005912532 289.257002046658
239.827648772906 262.577672828611 213.240181616274 346.917113326564
221.152973486208
CALCR 118.655954393594 112.693431140524 112.684334910243 116.625518828978
120.470801825675 115.871151134713 123.545404885618 111.788481822686
110.286187184448 114.122395647516 122.410340121549 119.26921157552
CALCRL 162.274094333189 134.345387934995 125.008209861774
126.539877052899 129.031338314175 258.830885826237 126.839037497031
150.587882657674 177.083095120017 219.151144724168 151.159145294127
133.141683141696
CALD1 14938.5975217303 13190.4826009741 13809.2057928642 14499.7866852901
202.778539118009 694.02978779693 233.884660128222 481.933386283351
541.642309122976 1259.38111177521 232.414763435965 272.712242893937
CALHM1 130.410584413031 132.100026388604 138.145677924612
138.532916327009 126.910075365358 126.925982415892 140.770705453805
127.808579455383 128.635684412304 124.295334523889 119.548432046443
130.40517506216
CALHM2 285.63245179313 358.843277491684 320.979443123471
365.046815163035 193.858884443593 177.831718771025 175.148318011395
196.450308368198 220.079379312889 207.977817300376 187.145134088286
230.263678910356
CALHM3 124.578552584603 112.404562115306 132.99989565368
147.844734028193 155.535222310709 130.890314257211 135.792966926642
115.619116556625 126.755389668637 114.902673141786 120.120796272896
129.930888107958
CALM1 6990.13809515289 7433.03226979876 7815.98936145067 9881.69734721233
1050.40488466179 1792.35343044443 1197.00004043747 1699.17470788219
2525.90466050731 1018.80178630497 1875.21979956596 2041.03024136014
CALM2 9433.53967760357 9793.92604780228 9671.66667662331 10988.2836796736
6720.10473881011 9323.53099359448 3560.23794634993 6044.78942014135
6919.4226838505 4682.62607750437 3909.47437917608 5759.5586948816
CALM3 7717.01422791837 8532.46059476152 9070.86397918429 11281.201625198
7433.03226979876 5776.63686968234 4956.21683075884 6830.46932926077
8342.70113690382 3321.1350545346 5636.26097921507 5558.51089816921
CALML3 140.648918459179 140.227863970629 123.45004246675
190.174729017089 151.78607484924 819.151876775691 211.085778902367
125.803854387512 230.053292316517 3241.23835624706 182.055718892894
130.2867846895
CALML4 163.451063547847 168.091278015885 187.833356349429
161.911468506919 152.812142230375 169.852769679312 149.52880714343
149.582406567077 161.359609192977 157.04599638368 163.134784474437
167.993851183401
CALML5 177.182321788711 155.043017644694 173.907933001446
159.282348516052 203.87884605779 175.704031930103 178.200678423426
160.937256625847 196.378888436237 1418.60088387728 161.061119536632
167.984051648058
CALML6 120.721341479615 136.109975252514 119.815258991448
124.530660958725 106.900679222135 118.432307370605 113.905364405553
116.810827219571 122.664353374047 109.30275288389 143.725497952919
118.006338536085

CALN1 181.261666651301 189.985520850855 180.747527895349 199.70041209487
177.428161690048 174.540209145695 189.74261433647 179.389797867379
167.500410857353 191.628693083169 169.527424356913 182.095387488796
CALR 809.545702751155 864.547109080675 498.965895763801 1076.09385014053
3712.67025366957 648.823677010146 1741.0131951509 1473.70433292382
1120.3009282887 1001.65849130836 1348.80530206238 1080.42884514937
CALR3 112.41270361848 119.845508281414 121.613684281664 114.68345964966
122.543903508753 119.252737868132 118.492287441192 111.260486045985
117.672645717508 111.691973758042 128.666652639862 116.621735695217
CALU 1694.24232555141 1113.59310757948 1371.56635975955 1274.39152410752
455.08515350006 418.574045635945 475.927798702033 439.928008927647
496.426739836535 635.234445318087 489.528040775004 477.626693328013
CALY 119.9414421871 132.359319280352 142.934869757362 138.047487233711
136.68056120003 122.909152047474 122.917915232153 126.089217853289
134.885883559391 141.099781722697 130.425809387444 135.899595115146
CAMK1 384.780426529733 404.073802241516 366.007451523992 472.793332506369
313.248029961291 196.226732738526 181.003812824014 242.614881095485
183.358807104988 203.642739476708 150.694215349678 169.332207863684
CAMK1D 185.027902485379 166.452651396293 203.524150518033
175.019580368717 168.398366575579 221.849661343124 177.752497164503
167.100039749329 173.879952136931 171.791030104358 162.134726710448
204.219197797869
CAMK1G 146.274284557421 159.98636352484 137.322392546784
137.634298014431 157.848456517169 153.008756051525 145.200638964782
421.384700184923 350.392951395182 157.643461899964 163.799748670842
182.698275130489
CAMK2A 220.010399552402 242.141362101262 223.253794809571
332.599191067433 129.090955637629 116.87586240208 110.575582352222
116.700397932007 120.220156455352 128.798413084891 122.716563069312
131.690417323838
CAMK2B 129.52578899011 139.414912158809 132.883408554592
138.97776257708 130.443360119099 136.188868917386 133.911650164508
129.925548182204 136.846912694371 134.61114877396 139.023486014525
145.387562372871
CAMK2D 200.065184172871 173.38031514498 217.353396632732
175.476184387338 155.211976328553 144.023798119335 154.38209765671
149.952762225731 153.734347607222 253.975577255772 144.064884297748
137.996291911198
CAMK2G 899.336817124312 916.836022395768 1280.57297272231
1331.18605673705 250.481442411876 443.964363178742 431.861762635568
363.343480850598 365.82705202869 222.565515020501 604.687265229902
498.180196203245
CAMK2N1 1632.07671492041 1848.27927621606 2208.49325118803
1257.32413345748 14983.8256502293 11709.9785619141 9908.59593023839
4124.03779111916 6652.21610967461 1387.90327883679 4206.37667440908
7753.61594300904
CAMK2N2 108.697696080014 107.697601478064 111.299115402008
106.854721604889 117.718893827092 111.409447323674 110.885714048075
94.3531468700382 105.839106819841 109.586955365611 128.458132030921
116.969799327195
CAMK4 130.143953880391 132.887701822875 134.455623159793 142.116311513299
135.325617701062 135.455283504668 133.425151103622 142.639587833858
144.940119680152 144.920107068083 141.363863974586 143.881744063934

CAMKK1 133.491963477649 148.758316681306 142.495333453346
132.557945249016 132.697727983601 136.81406168343 132.508133846896
130.091692689423 128.755697083014 140.349431258812 143.393174544776
138.494266722799
CAMKK2 284.638865013916 236.141982516399 281.740495452842
317.00629920068 216.353502371227 189.94130405123 161.359609192977
213.412498914309 164.429483677715 177.16711492983 179.753400474369
157.881569297409
CAMKV 112.977469350139 122.373400988227 112.776764215618 111.506345174435
110.594755513247 111.876915242732 127.356622898971 113.66416989856
118.514618614167 121.248923222807 116.252227301161 113.334665910428
CAMLG 3552.78712528922 3896.49785254733 4602.81651147558 3463.23822434026
1444.45946291517 1536.52218968817 3195.71436046397 2014.48411650613
1314.62071412501 2639.60135568 2518.88171283899 1535.27496251381
CAMP 108.984922760455 114.314425748312 107.961322525982 112.891050337488
110.847680175669 121.353233207209 108.730058525654 109.978693735206
107.961322525982 116.501174707117 130.720097323382 105.348008821162
CAMSAP1 388.326080754063 328.826483579278 379.102958448288
356.770158430601 277.219796733134 253.933478547515 245.454945056317
286.310923582432 281.238656291587 417.103816140912 325.907037744936
269.927694984528
CAMSAP1L1 1082.75419087929 1013.60354455825 738.37559871635
835.284130042504 401.840794789616 618.058496806334 513.518424350159
479.983368253935 373.189809737885 378.151202933526 563.339837352678
503.285430318683
CAMTA1 182.066922756289 195.776369840059 161.129770392464
160.277755404001 216.427103365727 186.831266888028 183.744959461078
155.7441762827 135.096321258167 182.118544602205 149.747347428419
153.222807811478
CAMTA2 137.22727238555 139.537865511933 135.106072664488
140.711699577809 125.682297697017 114.812254477058 124.334450753704
126.585679159459 125.166940410459 117.976107125982 147.400082178491
129.89329138222
CAND1 341.819090221654 347.812754957414 362.195903246495 300.094509257518
357.063270015318 4222.55224241399 528.650794915616 2200.94974896771
425.826693178023 400.621427274425 437.601814037248 424.134483971176
CAND2 328.431289733169 273.303240462047 298.573180660083 336.279209908669
167.301579775842 139.642457979496 124.873289279767 141.990639998829
151.998942684339 140.02337095267 130.665947823977 140.051904905695
CANT1 279.495192828873 309.36704220687 247.942904862478 256.370052241046
756.068695265118 471.069295685718 658.202765061937 413.983821839274
558.934004505074 446.07651799079 486.947380524463 542.379137999196
CANX 415.642099109855 356.413190087706 452.732079010502 370.390323116113
236.141982516399 302.847274046748 351.854828413334 462.553267274302
364.113665301598 353.187829498591 357.063270015318 403.984761333129
CAP1 4965.25728333142 4793.22133852447 5558.51089816921 4811.88424516272
3413.39994653641 5955.10816224456 4222.55224241399 5373.2173152906
4836.4008757421 6358.5018930218 4214.83058308503 4938.42484385053
CAP2 1932.39418448203 1603.87451959083 2479.34567973462 1818.92700816112
194.941454173896 215.40037757604 272.770616373687 159.994376562272
211.34069504665 526.182262872235 222.544542998356 228.212753830905
CAPG 228.125521373477 262.954856857756 206.340478393262 195.160353691349
832.335328912437 256.370052241046 481.037647470809 334.709049890042
339.98018551524 605.795439342384 659.900771633223 310.989043012841

CAPN1 734.469481390593 836.613819700658 812.75075757351 1010.23703166498
2639.60135568 2736.98692493933 2583.46653460894 1532.47920327769
1492.83354014907 2703.76111776248 3976.40140405628 2965.38280248519
CAPN10 138.685330935707 141.306418675404 144.898770838913
142.456527359961 148.764956861822 141.059033576475 147.125005489393
134.325520377207 138.483436753942 137.09814291566 134.932416370825
144.321757243275
CAPN11 166.730974538475 170.154539416255 157.172970235254
170.350891928203 147.225911875196 155.513572243604 145.165443106042
159.399804351771 163.153676643372 160.277755404001 197.191648960563
149.200848439601
CAPN12 155.820194280153 164.991522994763 146.666759322506
147.436408919939 368.418553384965 157.646948327818 174.66966623557
212.798133015556 191.754626760061 169.568915494577 575.722713461982
271.468939477052
CAPN13 125.220416203903 154.938900278679 125.894755032594
115.76177719261 146.099555861577 234.301520483864 153.059432055089
802.643357549193 128.229729571899 109.128394735532 943.677679525947
142.528480882047
CAPN14 142.558109812638 150.445647133244 139.268052431476
129.272835188209 146.865904106038 159.393410854649 231.338258067616
189.887788314879 152.925281215593 145.404469340821 182.907444581093
159.683695366575
CAPN2 302.735372807236 261.39435936661 231.39684528494 299.106362530855
145.684294398911 158.973872644973 201.016287924499 155.578524031078
175.704031930103 308.517561840596 212.78038645414 184.148058872585
CAPN3 157.713227527385 151.619329363527 174.012972901793 143.814798097425
132.024244263352 131.015519445322 145.831833648039 138.934516083124
149.131218007756 169.113439729353 161.172420368335 149.17029600091
CAPN5 580.859914041323 629.97342429259 435.983803453746 507.040326298457
1261.61611961272 837.023434916281 388.638095539186 1720.14021303639
1834.08657010335 195.019706886336 2282.94927629821 1126.46241487062
CAPN6 162.841318222066 156.095520355029 161.02075528415 192.224242967539
155.828962183804 136.90421956457 144.662545137915 138.088712695622
150.655359765209 157.256812907353 173.189465201573 147.606311227037
CAPN7 184.505279093688 178.863722055499 251.588748783592 164.491207767654
196.486499238623 255.066583469964 214.175691239597 256.873090834376
204.637887777803 218.997165156081 197.169703621888 207.036293322882
CAPN8 129.471713415228 128.054394154801 115.520278378747 114.818898581401
129.621296567128 114.987285062108 123.756349408641 127.242627779136
139.117776552573 123.524952603672 136.253264628863 161.769370009823
CAPN9 139.576852399535 131.288785100733 138.633019006784 130.803561448083
195.333029746339 134.27783583528 214.200081541057 149.622695843398
186.222567239198 121.344724993789 317.600728351694 140.225329742578
CAPNS1 1549.30900090605 1782.2498613913 1472.54829263217
2089.82894186824 4773.62220024088 1435.4236709581 1367.92869625376
1226.74043569231 1478.09582385838 1708.87666406131 1453.88161495484
1355.07170485397
CAPNS2 144.065554025603 187.407526616724 153.485731788293
135.704986104812 199.53455266342 563.011988279542 617.185364180303
284.351529744475 145.941674563997 337.434882771953 850.882404546093
681.810927901806
CAPRIN1 298.827660296715 270.894153548841 311.157764305033
283.123157828849 333.676803661366 330.505633088656 327.749926183823

281.53843450593 337.39574303968 373.545629693667 263.845733455915
265.174306663185
CAPRIN2   352.303782547593 258.489608621438 411.784922368377
277.314675225943 184.542232315662 307.264586007015 315.912220767901
311.449205777595 214.162670073541 522.17701271254 574.496860164135
275.101766414328
CAPS 244.797792477941 309.229066204308 347.620001353026 295.893482074241
2390.41825815872 1914.96285167893 1936.88813111969 1602.63977096867
941.936830901588 184.148058872585 909.727616721285 1160.78730097715
CAPS2 262.671819506189 255.992494620859 248.761038207278 263.204269632206
178.235281981508 231.239126548656 211.966139902562 214.499670852615
193.098116087792 191.297539967707 201.555964610849 226.678503956411
CAPSL 147.270369342438 143.432889871079 137.974323288786 151.532859494021
144.2753860947   145.493977576106 145.636906369786 153.056369304663
144.68114486527 143.125454748996 136.142380560596 143.082880435412
CAPZA1   212.749668083641 206.571433405844 190.506201184419
187.852455291319 235.595194918187 319.639103463458 299.170431746759
271.638179116269 268.186156177622 325.011269531407 549.569374872629
306.632103708321
CAPZA2   3277.24948092771 3342.8737952486 2841.96460830166
4061.24537355697 2069.70335195314 1742.84803427086 1513.28088592885
1524.46745239811 1286.55103838368 1093.85316636801 1299.4782626583
1812.07394646463
CAPZA3   142.630563791102 144.155591092387 132.976977898242
139.238936770274 134.639151701327 125.796438432905 130.616175018332
128.753623886188 124.15094220181 132.461730873857 136.769073515537
139.388484543482
CAPZB 1091.69584249713 1259.75918248468 1027.11849332495 1934.30779703747
1660.70040058102 1100.81588201714 952.724512915813 839.662936279727
783.991448771818 932.076509242794 1366.06309834426 877.598505913096
CARD10   202.024504592763 271.430915699592 226.173501275026
276.653753970476 430.590533290315 301.304268871606 223.722742062251
182.861900982333 206.845306095447 588.678767007017 266.71318061066
454.235121917464
CARD11   213.123453925578 196.802641002079 213.028568789661
194.212177449077 1004.6207350158 478.847650837714 747.804958601763
523.591472915151 426.132037075477 216.582862850259 793.071192619058
1742.84803427086
CARD14   175.686248503777 165.627852733455 175.832474809232
142.215285988753 195.185025611945 177.011776399483 203.983168778488
175.832474809232 174.006516433854 186.112835637689 205.08372943071
198.701991021316
CARD16   149.570242115956 152.404186073033 141.468076901305
139.576261630082 146.838547724148 137.539385624206 143.209289043824
147.378228560979 150.148902196995 156.808992920564 156.239937979051
146.421195733989
CARD17   138.730409717578 142.64421300687 138.307574579062
137.660051943656 162.863781294205 154.428779374984 151.839306445341
137.258429777402 154.479733759712 166.662801900754 129.867246424399
135.697550878438
CARD18   121.59661598908 117.361778263056 113.726931741236
118.167740643871 117.953453145685 114.960460615926 120.957197551997
120.060776752215 114.002925521932 122.098850053347 124.486701452062
124.00806841872

CARD6 149.394952856085 149.689071018094 157.353493980339 140.058153633802
156.638070437044 153.500478301079 162.664254855131 150.283447275981
141.284962125757 183.152005135504 156.119899067238 151.76341973891
CARD8 193.394225360968 216.154203111101 169.434164890542 232.250434241457
151.210814209064 234.339913501761 205.509515144263 265.028512407839
286.282132382462 169.649251828199 213.274106157675 171.373932115412
CARD9 164.48570046081 157.390513637267 142.663334893424 185.627515956848
364.895113721763 126.409995188106 232.879578820053 212.915949933033
271.397087928135 150.885460975199 340.301980622308 148.79958121087
CARHSP1 554.950092776254 566.543765584009 391.644649726254
422.590112942156 1473.70433292382 776.030689773414 1125.95664591686
1115.53524128833 1021.25947613827 3757.39261544326 730.673649872205
1265.88446775977
CARKD 585.552940765069 566.647927867646 657.562129888446 619.75986112089
348.808646570544 600.919152191636 444.945078908489 379.292531644085
615.662993833692 475.833242673151 660.907502776003 639.688482258192
CARM1 1772.75453735112 2200.94974896771 2080.47567326037 2986.6208659209
1207.11788506001 1460.24985399013 816.823443819069 1196.34528225417
778.639512538949 1482.92498205315 656.867138022837 1101.66868138797
CARS 524.640879446426 441.684169663552 411.968307579588 592.284071247642
266.563990988678 303.423450627377 282.47352481715 289.956528242564
305.669636871734 379.788696204016 294.368406238222 276.089623957012
CARS2 288.261250589513 273.668871896558 294.922183826144 309.283897272661
278.385151185788 328.550286726543 234.243977361232 246.710233781784
218.96845208513 323.04019262387 302.381946749711 355.14024238296
CART1 143.574584852426 125.687688416588 137.808307749725 150.521719129181
138.677282903963 136.108500467418 130.100501333725 134.519697269443
136.448045428081 129.3513576591 132.466796864599 131.552276774901
CARTPT 128.765369834041 131.237720549768 127.229854957588
137.314968268692 126.957335244119 129.541049979717 120.839256811295
127.6078497227 114.217867810848 131.693923403533 147.905406818238
123.622533094122
CASC1 141.655169779627 168.73918684069 136.423161205425 137.565050253948
110.148555045276 122.002822875994 126.832182961671 126.182856027765
116.814269969508 120.452958447555 130.309092946921 114.755442050481
CASC2 130.680629893396 133.814720785534 134.504873813959 141.056924917269
118.743966166637 126.828097770667 135.005591691192 136.201103852785
120.799317509529 130.985360700521 113.840813583258 132.475414208533
CASC3 831.289724465877 884.747476952289 1000.05896885496 1103.13439876128
675.281131488524 489.022371276542 378.088675670786 629.97342429259
535.758943850734 386.305638633172 377.951523644306 319.685597963267
CASC4 186.04763660516 181.722373730185 223.058699622386 151.064422836695
153.217287503221 232.960832410426 260.129513331703 207.023221559302
260.999345837027 191.179662650057 224.766155406229 260.41082304175
CASC5 132.937942776777 137.731390340708 124.248997533959 116.497714896018
143.851670859917 131.84454376953 124.507958339495 136.649743237542
136.317978146216 128.26423663004 132.998223400178 134.427601777639
CASD1 181.425012248539 184.923312649584 192.752224665459 184.071078414327
161.102731941183 206.303338709554 162.199381540369 160.618586213311
168.295044843056 179.250373572781 202.797135825668 259.868701878844
CASK 203.510106134882 194.617252921731 209.60319248212 230.424145472916
246.369521698487 406.133430120039 322.263925912805 266.286483421453
261.028644858795 362.445813697676 173.761036260869 393.579552385074

CASKIN1 125.172189715764 121.796519488793 124.387133895009
122.804934452095 118.988269692812 118.424520312293 136.617105945904
135.596007994731 121.607527525765 125.000876313893 130.402496960735
128.383541329939
CASKIN2 146.301300103564 146.580787463084 122.699993989189
136.762273376313 133.728378162206 118.655954393594 139.963969716386
119.180033382937 116.787791109549 124.35721327773 128.699441450952
143.036006001419
CASP1 232.250434241457 218.70412656621 165.186696781985 156.727153398506
136.327014189932 180.952017616136 196.450308368198 270.973600107882
385.888395433066 633.104379362137 360.852730026133 435.48544637769
CASP10 138.883710791382 133.446429177254 134.29036009792
133.984078167807 142.137885203233 140.420815852109 143.123131081201
141.666394024363 130.182320154521 152.961475450751 150.378690719669
136.459509901229
CASP12 148.930073905413 141.97908047949 140.456277147131
148.511059836029 138.576978828316 141.043668963717 126.652197216347
139.168307371516 124.561511833959 140.074024949157 146.06717772919
125.707228291312
CASP14 128.611740835252 133.324531689254 122.002822875994
137.850437814788 118.792612926619 124.859984709406 128.517315006019
130.105800581241 130.383001462828 205.08372943071 130.374065525934
129.946925636595
CASP2 306.193157874683 383.259887828605 274.047586063773 415.113340335205
463.187551361986 917.646958427824 607.26455470867 783.991448771818
735.8141982568 554.115756730498 448.54309632232 930.25842384208
CASP3 314.845965872164 335.3366819308 337.935183275743 316.590387560909
612.758976723556 1018.38836615952 814.110601393539 700.353187874339
890.507257588957 500.132469062228 653.264945856916 525.515169794593
CASP4 190.165771406362 196.269193578525 163.582678113215 158.07640654075
160.429643729685 192.891507272665 233.536023295039 210.900056977861
249.737588031219 679.822276398569 398.673582680257 355.990908506973
CASP5 113.051381310122 111.837468699281 116.86635524597 106.629065516249
122.789656787728 117.47631318854 120.524859681034 117.47631318854
127.180743851077 123.379461430902 133.773427881651 105.521320791331
CASP6 217.811099067262 203.831047867731 278.88183549759 217.307872538386
201.616302398819 545.566596665916 438.611973005212 725.216684507759
442.766704003254 346.635777358159 363.150472154973 378.24336892367
CASP7 143.349486755648 149.911501378622 141.130462286785 142.633121727277
145.051794777243 141.752031758589 157.003367685421 154.350107666084
176.070695522397 147.064543281269 202.524668687686 190.489530928259
CASP8 117.969576809288 120.672291867109 113.76411537952 118.08607083874
115.992487254175 134.569883788949 125.725832564087 140.978511668091
135.186880148352 135.634385846692 142.613654158833 140.796641921516
CASP8AP2 118.122186617611 129.192326574697 113.822580779824
119.436922168376 122.180428703208 120.082391311921 117.003205777245
121.624218243474 110.266032635568 117.432860005907 126.673587174832
116.983869766097
CASP9 200.890547965585 205.546628064296 212.545989139388 207.718619151226
258.605188272761 260.356834114611 254.83131041241 241.203521822269
212.915949933033 180.456659320226 206.042395282286 220.415211013843
CASQ1 168.801737310947 198.135240691225 174.364798143068 248.531972733475
184.056274452194 140.171749368266 215.219246394594 328.019503002384
165.570531605115 152.648490300617 262.233918187791 170.622615783608

CASQ2 2794.89048827025 2350.01764791897 3463.23822434026 2580.98143004019
140.036463888037 147.51773051441 153.639559509292 148.22974445263
155.70645844794 143.320787131532 137.536053775531 148.124586158123
CASR 110.764586138261 110.553759711075 96.0426636429757 106.772581387455
112.436972452838 108.631224817901 113.78644620959 108.655415285806
98.4947439453387 102.522925993233 120.551603446364 122.005340508177
CASRL1 159.611551480901 149.262900833177 147.875855077738
148.284412570438 139.089444892187 126.230887413874 145.158039427157
144.472969298036 141.023875058322 141.255406288178 145.168031449425
152.401204710292
CASS4 109.176852761745 115.631414599991 117.47631318854 118.206034087145
113.339155126202 115.587874563785 119.612288460361 99.7487411813724
113.582654469735 105.49491265382 126.924014801186 109.425878885118
CAST 1006.24899131914 916.026216386031 1259.75918248468 812.75075757351
223.279017022443 381.4054004349 530.267835989435 357.773949920377
280.749681050778 774.442392607873 704.653073777277 628.344122779625
CASZ1 473.947538805454 555.109005518622 493.209107049493 854.224454791713
442.698000781597 256.687384001447 219.422260093722 301.428024926428
241.648044508699 311.715351215263 264.054997398621 193.609985124825
CAT 524.392279804734 616.995114856023 945.830852603704 463.802270256246
840.276501350383 814.471143753335 605.21685916883 1159.44907165386
1214.16777512885 605.583144451322 1079.58110075176 617.318955611228
CATSPER1 138.343232981485 147.818956338752 128.56495182759
130.257242615762 201.800357785022 153.778409339975 162.723310549554
143.028618831382 135.024750597761 125.315736755156 150.0512732245
333.049612399093
CATSPER2 6508.71638048514 5981.08862268291 6281.01687932005
5521.74673982411 720.907992182476 5396.55955971617 2300.9407349072
4043.28299602858 3235.38149926838 2608.95227456417 4956.21683075884
2905.12297639092
CATSPER2P1 184.398672994943 183.314276033531 185.016336026382
199.077893147154 150.105503384881 210.448904052352 222.118666699301
192.804447792672 190.045156046541 171.880470629305 145.152605166094
211.357321758564
CATSPER3 135.81863662958 137.68634745111 118.838332117453
127.213640193364 119.943408501009 132.257989841046 114.242056888286
118.45291712775 128.389494971858 132.610618885717 145.677477231455
116.412881235587
CATSPER4 145.120347147471 149.725422365342 142.45047744931
144.895601314903 149.560505078257 136.099663831028 164.352366170527
143.804903119745 148.702473828167 149.570242115956 147.754489751457
139.223480216048
CATSPERB 111.708970469189 117.104945989023 104.798333931019
102.725375719353 121.85247363239 120.711150688559 107.580425848009
118.08607083874 116.465213348716 106.551076380988 130.30561086685
122.046901607713
CAV1 5040.35379681644 4037.15442268836 5058.57872936027 4663.03253705034
239.363511706064 440.319777672273 236.470206707351 363.594808277506
427.452114719877 2670.9151276786 342.769825986756 318.690043546203
CAV2 507.827473844576 462.437471715008 562.387692458382 412.736636437651
174.149718758884 230.053292316517 205.630948393225 246.989526028884
218.896760804208 1314.62071412501 431.304147578425 263.321559283966

CAV3 139.201122725213 126.912226185444 125.450077160575 129.428950259722
119.702874945404 117.040953580545 122.891946370542 130.477824552202
112.166192839859 129.493805009651 129.305455204291 128.460750472464
CBARA1 426.649095732979 433.594928641214 505.404271675438
659.483452217461 190.572712684622 179.624208193114 219.638478100747
186.831266888028 229.62164568989 182.02250582556 271.238736772408
215.094633380547
CBFA2T2 181.135519188216 183.416437587938 171.093611712143
199.118281632221 163.919426088318 159.110614190088 192.61436632814
176.256840061919 155.43699805497 152.609055890822 186.722842989058
161.26917182967
CBFA2T3 137.754373055094 133.834423222601 138.962649407431
143.338628928675 132.282903926191 133.028789808654 141.016596301267
139.278366709094 134.192409943723 130.450930089722 145.997380100999
143.103779196037
CBFB 240.706548931192 244.280971257965 227.689356258276 193.394225360968
251.993821914623 376.082040863747 298.403224729528 263.123584372086
335.142319387164 317.802633831405 375.958347293549 405.975221091252
CBL 307.124331101976 266.918302307596 244.219585315024 282.47352481715
207.440700355457 209.684346388892 163.41031443893 241.917371866481
219.192900411543 186.06425658259 216.645552082667 240.147082836599
CBLB 1084.95095717937 1255.62991601665 1544.45026075974 1168.68074786473
348.300300174924 596.934339406636 583.192225756596 656.660582895703
633.762608454521 435.183433729519 877.598505913096 422.338508598153
CBLC 146.795608819024 156.194996994993 142.14654303591 127.101752724954
1212.55338792557 232.830038701628 284.891415492568 425.708048519408
490.022369664024 330.553659265085 558.051041108282 273.984715370774
CBLL1 241.944581086483 248.229468372363 250.217137496422 219.441959146815
323.086368767924 253.601378730911 297.368197598038 301.695991929957
279.413335686746 268.148673257455 280.204167582951 322.099431638246
CBLN1 146.531692460121 157.58224826673 144.038859705973 171.85890372225
135.237042337721 152.467764730004 156.466728833063 156.460374293022
144.419657674797 136.460345970481 132.928639483854 129.878330410179
CBLN2 142.594553057288 128.478724185042 134.831550401848 129.623601211364
151.037282417145 188.580458613357 117.417112053842 1827.03179366146
133.233826283531 118.505980204837 138.687013437904 137.260359343758
CBLN3 275.794847140407 217.748916080172 224.298067319406 223.107391048316
190.534176839652 150.489651880905 217.491746961471 181.550597645633
189.403934838648 183.033044475752 206.548878646452 191.830711714844
CBLN4 130.195234920895 131.144774362059 122.842463675109 126.800718471804
127.478474451121 126.877293296185 115.072622367144 122.079685222733
109.318922864507 115.524441144063 131.551085224112 122.89752183991
CBR1 177.355210393749 204.1974438819 183.138955445765 219.246271924313
194.819597436776 222.646371492347 261.639437655633 190.152824885328
184.056274452194 262.53374754047 167.505381046729 172.707821624271
CBR3 402.866491417369 514.002313819685 396.781827940009 688.464980326689
170.040914594182 158.325554470875 306.896732729554 272.351487980415
234.925852373127 217.607230767028 214.413674287972 164.520247586053
CBR4 274.494074082359 323.149792143481 330.457534783492 265.972387419683
164.513371267476 486.947380524463 340.903961204211 385.678901612324
303.241375487319 181.491120129045 302.735372807236 355.609033348692
CBS 272.41397350779 248.330154701293 246.327991259259 243.336550161566
308.088967616175 317.353318759931 212.037224737798 223.220997990627
796.936154737659 225.050796356289 199.681103910705 300.150235383065

CBWD1 148.797226586765 150.346002924933 157.378317015602 149.057388078138
143.30977386038 147.772490977392 152.348210524663 152.197327387252
148.649619242172 141.474583706011 158.911404625381 147.371905597494
CBWD2 129.294026995663 145.000523037022 123.042372128864 122.917915232153
129.900540030251 127.676859244344 119.297561749232 136.108500467418
119.436922168376 131.419663282022 111.328185454541 120.649467083125
CBWD3 155.410704148925 137.328370598183 153.435218661228 143.893751699551
176.382377784553 225.850439131864 178.984409575632 196.207631868755
185.220017451419 152.587331888423 163.726898336284 147.526701500887
CBWD5 203.553696099031 187.205674045893 285.214585324341 190.754349388901
223.220997990627 331.937313503755 350.22221782412 381.971539806271
302.069236363626 328.826483579278 383.407365952689 354.531898366438
CBWD6 133.125084506877 126.991140761102 131.597937570173 128.194171809998
133.579067514064 122.079685222733 132.584454429269 121.826474059574
116.46192274217 125.025064237439 110.501341211051 123.571641517019
CBWD7 141.999730335668 130.073005698714 134.808485735121 139.505864135281
136.841506794489 170.483686891321 140.743917481548 145.562772839517
166.104915784875 137.125637347852 145.621384219688 180.418707740532
CBX1 318.73799597811 241.039766283972 354.579728909336 215.967319486874
232.023739004791 150.573523216151 194.529045934216 240.497413647633
249.489170536758 265.572253407219 195.160353691349 229.347135478142
CBX2 128.318545300038 140.074841537258 136.801338583441 140.206268652318
455.486248388968 245.522497675754 139.898996725236 201.913709602894
172.667770359278 169.927157878764 153.875760529011 159.379420001197
CBX3 351.02823853348 386.379891865411 311.715351215263 370.066087375401
447.297590650141 425.229044880287 395.425715632302 384.296190598736
410.533157400023 453.593585032712 487.537691131703 664.809419789814
CBX4 270.231637012624 258.532432007328 251.833571288967 207.581907737176
470.308610828825 796.936154737659 360.285027176918 434.792010353541
578.465753476561 688.464980326689 445.089930641391 525.789451405743
CBX5 340.860684371038 400.98290237416 274.01377986185 272.314332734711
375.255445179952 200.486683704179 220.567951704044 270.462865174106
295.935625475189 266.395569467956 288.728608411903 419.4017188518
CBX6 1726.15788525017 2426.2098937241 2033.67956299225 3510.24464112094
2399.88695085752 895.114975754684 449.220056843562 1302.54547114701
837.413261433363 283.048002376743 1677.93424842267 483.429488817674
CBX7 1982.3154578939 2065.51724858579 2285.86163259073 2328.65814406636
410.216584967666 338.46332239699 336.490076158135 378.797952580504
343.488356185437 208.095384605737 507.56875910742 257.706904467657
CBX8 138.611074796816 128.736591902501 130.659631155616 121.18490040448
161.997058981786 140.474338177948 160.903342063542 160.322541355391
145.106362864355 135.715745768179 164.337486856997 152.633139090274
CBY1 377.538276846051 372.94852645418 442.334886941795 442.86942574917
262.296456281666 292.28857006885 223.184064606222 206.845306095447
238.482863150124 228.212753830905 240.306287921209 266.842778974988
CBY3 166.23609765059 157.967824935915 151.369387642458 172.677083810424
156.114825514646 131.846160346403 167.219062918573 165.186696781985
153.408890091225 143.602039774312 158.127523362251 171.444558905575
CC2D1A 178.270849389719 213.178648382852 187.903298116031
233.964929444112 325.751369001105 236.35479928448 245.351459157365
198.701991021316 180.747527895349 215.585089158905 285.30221551106
215.167086358143
CC2D1B 193.218266574099 157.126460242995 190.331589656379
177.123497519231 141.272049404557 168.790464803746 185.544411160039

168.790464803746 164.092847913322 159.368376366657 167.350290326189 184.832531598928
CC2D2A 276.476790719416 286.901115411673 364.191263843897 307.363302726074 181.695203523798 162.212442593001 163.805257266374 155.080900277147 188.36361073603 157.779441363536 139.834535091869 171.924364939807
CC2D2B 123.991249190444 126.970952206224 121.118933682515 119.249084967945 109.355444907633 117.060696904303 104.714872508838 115.053039576143 118.708644971927 106.522641798274 112.999642731096 106.667175621971
CCAR1 167.219062918573 157.368974632451 172.084184539157 173.272062328378 161.683243808528 154.133723492173 229.511643376643 160.35668299062 173.250350363688 178.464811133 181.081050726043 185.560737176115
CCBE1 4030.42725798136 3575.60827968852 4068.18567686753 3354.38704439295 441.958777965408 5213.40342339233 1892.8338612507 2525.90466050731 2305.11856249143 1702.68441407727 4198.61471038267 2269.44503773126
CCBL1 240.915496717526 229.77428612216 291.261765585473 280.204167582951 194.724219884752 188.45479937455 206.340478393262 188.981391266203 251.833571288967 222.243523614518 290.867822301134 187.48055574295
CCBL2 218.314136992677 217.435155984897 237.940015617254 201.458970969685 257.477501542521 352.180110869664 274.534901548678 323.149792143481 233.20900339048 287.503594820801 332.543652462234 294.746622369436
CCBP2 181.048789688798 181.876635968785 148.704917490429 155.441190892146 971.170460915918 142.570182389682 601.521068811063 158.188039933865 176.497706091676 145.830916005206 143.572668991313 135.930107381894
CCDC101 247.779718065751 272.41397350779 274.316982298975 328.019503002384 334.916875239616 245.607130404306 219.832904615582 233.103476996669 198.90533452253 179.551640851353 218.997165156081 210.015199832609
CCDC102A 654.663838918786 763.880957432979 927.478050257292 1080.02576143033 236.855293143293 170.430478598594 182.897563195019 164.660269016695 174.593548891693 239.079352337243 169.089896796513 150.765477999064
CCDC102B 133.666244052469 138.852212944492 140.190234214948 132.395993254065 129.216693566478 125.729079191033 118.988269692812 144.559193410932 120.456817031716 116.947370388179 133.161552914595 127.028640787946
CCDC103 163.400576719974 147.710252756854 141.444958186793 161.090443294391 135.134213292186 169.956827727468 182.335981256243 150.42705284743 141.473218014605 144.629350188283 144.277263594387 125.443242860371
CCDC104 309.098519056843 309.658122422202 360.285027176918 275.203046536524 217.910445051307 280.558463661137 317.244256751418 298.827660296715 221.930333726352 252.280435525505 308.088967616175 246.95071325085
CCDC105 122.174036435094 128.251087452744 127.79057041268 141.444958186793 131.973896985615 135.745821200254 120.843429388102 120.024235522682 123.389038274913 116.378552206652 143.913625824599 135.07697911271
CCDC106 544.669441360594 563.907454700224 616.995114856023 692.359779743985 1401.67503392145 468.93543246394 569.236322997211 324.713347085998 660.096727566678 331.32397534437 571.136519973808 440.916522871266

CCDC107 405.843083758177 343.121661664987 458.440488401029
410.42088953434 174.680918805036 127.6078497227 138.98468042464
160.713790305656 153.38494705081 186.359770878343 172.750926094884
140.311843829601
CCDC108 119.005230164124 121.154389729409 119.669441891401
115.954828435072 130.047353911148 129.711973013336 126.875483714734
120.846789922177 118.622346984257 125.444541266623 125.744408397808
122.817695619508
CCDC109A 342.555640222963 341.251360936154 371.369895979832
426.896707161414 192.022864170934 161.708130705995 215.300064529551
192.565733545715 214.461773810376 228.766847429517 473.260534978556
235.530986541549
CCDC109B 473.151324002277 446.698665191047 462.779122987815
397.577760464197 358.780920910909 452.606433325387 313.322307299463
743.915675090182 811.144838166905 1975.83062789692 375.407747753058
257.477501542521
CCDC11 119.312201620247 134.991379445799 119.052729327147
119.890260409414 135.776154891006 127.215777044942 131.755881982599
139.948097704949 123.843823256462 122.68719599986 139.537865511933
125.999595008166
CCDC110 164.136551371866 152.422690241301 179.663258610881
140.678336884951 135.017196494069 137.666849842576 128.44311762955
135.781190598068 125.199837162105 158.872962797815 141.463324155943
121.472885562857
CCDC111 183.371103136697 190.549084022965 212.78038645414
168.180063344887 168.570133927038 202.909188070184 195.645181575647
175.974598536074 177.508603290389 175.28261759639 177.582314734704
161.849023343715
CCDC112 191.445291899108 188.45479937455 204.802810257516
191.725728813959 152.238845270993 181.914505612128 163.215507313506
194.724219884752 174.761105961773 227.288015854809 179.624208193114
162.970595558845
CCDC113 138.409284761652 154.916023131856 180.304585602206
143.68613742378 159.90466743683 168.552631639996 160.811051590991
153.02127620577 157.259599894923 144.798664281991 154.420795931034
164.092847913322
CCDC114 107.283814651497 119.387459594521 108.631224817901
114.143874572202 106.876902604214 117.856184272322 108.199628274798
114.740948266517 109.586955365611 109.318922864507 119.596589810052
106.088807114602
CCDC115 444.082440002333 454.235121917464 609.504365141529
429.992169596618 481.453770150269 683.861021234927 486.334840298921
493.492348317616 463.802270256246 399.537363692534 736.332743195416
837.023434916281
CCDC116 153.658747612827 157.237147919803 140.169200413643
142.38097037888 128.99859423584 133.281505040209 160.511688319771
137.510586346207 158.931023649818 162.885054245809 133.310323593945
169.502103689662
CCDC117 368.276805218658 339.448524400708 339.32415558339
270.421697521366 230.367251714289 279.75914135192 196.990867962546
322.153713097778 305.200911653347 247.015574724901 238.400565506288
175.711836112986
CCDC12 698.590134238302 803.840404197117 702.049571167356
948.734577648136 991.081498002429 818.225731093346 699.270931701696

575.23272138912 991.081498002429 747.077451281453 790.321331435096
928.151632070573
CCDC120   199.746775121317 211.786963444117 231.439252571168
216.859640534581 369.974231530826 335.3366819308   299.80615152845
321.25076864104 231.547654653783 306.333710880618 419.846584903512
324.949988230276
CCDC121   182.833961304079 171.967974673053 177.500110974116
166.368623023308 186.733394375839 167.526798465556 174.510523238374
177.562561297777 169.771005675296 153.461396593156 183.764215745794
166.385492585683
CCDC122   138.090158433822 134.399228996542 135.071400668179
136.450062043587 136.627904756283 134.748177388398 136.705326991088
139.646026806298 136.140327952231 143.618454531431 144.917911601441
137.910618360623
CCDC123   215.037122365095 212.223906015932 241.734804013256
229.62164568989 206.68694753419 196.990867962546 224.981439707657
209.811693771836 187.247062073247 190.389158930835 165.581217879395
197.003657786247
CCDC124   222.315451077459 209.727089546377 222.394280571312
288.166541335652 360.39998507367 178.542247857251 190.716424123036
193.660159482867 172.457165477145 217.24573005272 220.113261580385
175.313689069815
CCDC125   4923.36698713285 5489.19362780613 4457.64479507833
4291.1724030909 815.045148478814 4892.17875938832 2482.27419621925
4280.66857663502 2965.38280248519 2074.29945034697 3789.1316515671
2645.72125268628
CCDC126   148.717545103261 151.55925331302 158.747029092329
118.622346984257 174.603285240228 199.798533722581 163.290050020756
164.865846124876 180.164353124488 176.810979494826 189.971144220643
249.234065130083
CCDC127   215.866644799363 257.573614085223 231.955750250711
282.137282466192 241.599271777806 160.567934923347 225.423403713718
300.047728281275 194.212177449077 159.461778054423 214.639374741553
207.204293555178
CCDC128   749.220616713093 695.595965352335 676.293399523316
796.936154737659 306.277823025572 419.4017188518   360.551560710852
398.892968579918 333.745543022749 419.763812610671 230.161369375718
376.692422468905
CCDC129   119.459625817735 124.899019467557 129.976540483758
126.693235227579 133.63668145135 128.994977935861 125.313515459103
123.410467384598 113.249267122926 125.976052547953 146.619846538124
125.162662242842
CCDC13   131.188208693139 158.584934514596 138.104527851969
161.37617568158 147.098474227445 143.100436913184 138.730409717578
153.787447416111 147.236728188605 160.0697586671   145.396723478249
146.979988781462
CCDC130   1172.4103691525 1303.33810980467 1552.69879081022
1406.73270115373 945.403379196643 1582.0221231161 1194.42925868786
1589.70261092241 1157.02365469464 1243.96721244424 1102.38234645327
1505.09908072606
CCDC132   163.010527914347 164.008063563113 163.982415393606
150.088034336851 155.803946849495 153.318678468245 160.035282578603
165.896420029269 157.463122989559 175.128935809466 176.176392369178
179.250373572781

CCDC134 107.559685467789 130.93033679281 120.957197551997
115.5765427629 191.937607311711 129.150574563997 130.266601299194
126.580227722115 125.951927536494 126.987002259013 133.892661981648
137.834158814913
CCDC135 159.154102271517 140.372463604232 159.487615985875
147.145932651151 160.73807125195 157.256812907353 161.12121879514
160.648791273795 148.953720017484 163.773702843544 148.818562911616
147.213616261735
CCDC136 985.087150371964 815.388288623756 1493.90578573732
1155.4487861163 171.161297672182 233.335353447342 220.772387104223
237.456245064363 185.650862663633 183.863365305103 169.368090266817
243.65518489122
CCDC137 160.039086963419 173.892380305166 150.544779197947
188.417078622503 172.7330526128 210.803130108618 215.744552410805
325.859917318516 217.778414825515 188.724274062294 176.587559615019
207.138680369683
CCDC138 151.889644436875 159.335990863027 154.683723064797
145.176141415244 196.777776509371 250.18189549573 158.735283017367
212.531329857909 184.102171963542 187.784865705837 161.143782746501
164.801505601099
CCDC14 681.429327723794 659.900771633223 616.701037274509
572.407760064901 470.746477818201 1084.95095717937 739.085574858917
1333.73334006986 1220.30347076968 587.654093308465 756.390518977556
888.706978062337
CCDC140 156.808992920564 162.741222929067 137.286331849749
137.360220389719 149.495077381649 138.376176600064 132.913891550054
149.339492984504 137.642858036384 132.23149173224 137.27506604142
140.550044737476
CCDC141 123.586359806158 123.588194516972 111.639177843969
119.023688551496 121.113186311514 126.50870982427 123.240482782331
120.050548624975 123.657246099004 117.567991653999 129.033433971346
124.76274408368
CCDC142 139.305220220411 121.350491314441 123.161953011069
139.150209111606 124.561511833959 129.452826518288 156.923675862035
147.994386096925 130.746465595759 122.873661677648 132.748478264583
135.907702243922
CCDC144A 123.156154667242 117.724353829377 125.798453479983
122.144209450757 122.412141495264 125.45936959544 125.569364049349
122.333765477428 120.957197551997 126.967212770393 139.421753451936
129.891492052232
CCDC144B 132.50027119027 128.303046695142 124.516264338772
131.861469782134 126.442999559539 138.412022634224 129.34905533265
136.378203924435 116.566190779365 131.809235171629 146.764908249803
127.398873370484
CCDC144C 136.654902685184 125.00254973945 129.930888107958
149.505323932806 137.675597243706 137.683228179974 129.099780410063
136.585541662028 118.558673085303 127.648224668964 144.4421922847
139.628966306048
CCDC144NL 125.019064287385 145.371926271034 151.930565620382
133.004765428575 142.13191463827 162.056622258216 150.809693773343
140.531789506024 147.697973457776 142.29309119138 137.663038788415
134.161664663192
CCDC146 226.221973502783 216.974834077975 304.713434036642
212.5119818331 149.879951192667 161.189717452004 188.924084292126

180.578528984075 180.456659320226 194.702974020747 260.489744360233
207.815226999953
CCDC147 149.143087870963 150.503278880269 138.972789382679
149.77596374205 140.946008063839 145.054873480806 151.788278519057
141.698329968978 136.984172424718 146.900087470856 145.237221169839
138.701871179502
CCDC148 106.437660157036 111.794102015354 118.176124626025
108.079149792796 117.900892627473 110.014935095391 114.960460615926
116.106772298898 118.918962530408 113.38168751499 108.868679899494
111.484426481816
CCDC149 249.823314327355 222.134519361117 220.816812192169
227.499335248402 156.502051001988 204.1974438819 171.80440912191
181.3859695951 164.773489952844 141.784881206191 157.337193457189
148.883263903527
CCDC15 163.668476670167 169.541411677993 181.966110264509
168.594235675558 211.208937901871 219.044271073282 179.15284184774
204.415267938007 178.657325741035 176.631604815669 191.179662650057
204.318316638312
CCDC150 139.826273596452 130.880795616911 135.046003135933
123.386622160181 148.188717941032 167.887047631073 149.717739166461
164.678646890649 141.442825487243 139.97636570613 133.699547652179
141.281116867082
CCDC151 138.192550945411 139.667714422309 153.072429774613
134.756377802151 210.245551279259 144.518132543976 155.673532440475
143.969566798989 135.303322290834 115.619116556625 139.685629122498
138.651419539729
CCDC152 167.842122798757 155.219860096943 153.273568880305
169.107948050094 142.334338599752 142.937182720493 145.096707533824
136.071856104688 156.534634574244 155.906054212876 156.511202150773
137.540275229889
CCDC153 116.787791109549 118.859745315302 113.734280863323
124.904518221898 121.212521750566 125.093376753408 120.148189532583
120.114423236962 125.682297697017 119.466200987489 134.418976976995
123.470830748348
CCDC155 148.922537778634 138.990971069382 142.810564324354
144.975882336334 162.117224868654 133.067237748129 134.395879639746
139.353080958675 142.798248249144 146.720204808211 149.525115175915
145.726651022813
CCDC158 117.069094451283 110.829107724132 107.976233194926
116.031631038289 130.513071750123 106.450154495663 112.547907748842
100.841352336505 111.788481822686 118.239637776941 111.232274546928
107.239841465534
CCDC16 568.701661112562 519.829743770022 521.30110104669
442.493108530222 363.343480850598 402.866491417369 573.509780150018
643.468308027006 635.038752622494 428.340060893467 460.089380701982
427.345809255426
CCDC17 134.949572471421 136.039076665712 134.356796881432
130.119313219923 123.896578371318 134.216088147701 118.859745315302
151.367402326399 138.745560049251 143.783078837501 143.390971600678
119.049388179929
CCDC18 148.461172387171 148.594322264158 142.379120254855
140.798706399757 144.393688888553 171.924364939807 163.51024840263
214.888126361789 169.293852989203 157.742948919213 155.405519023053
162.188509438986

CCDC19 132.854945224697 128.014092519305 128.339161042799
123.45581374935 159.334165651951 142.481897141275 144.464071397369
149.995272585012 153.579955370444 139.82050388626 140.321675137237
140.668597897404
CCDC21 147.543411823657 174.347451984066 161.12121879514
179.358460170348 269.379272725008 268.454310218604 239.079352337243
430.728137105601 225.142823691625 283.918434443669 201.616302398819
209.5184563244
CCDC22 178.200678423426 171.36717256867 181.110403721353
199.490190715307 450.851711160136 213.914599384797 169.984859264412
204.564770370164 179.220691178402 152.864765895508 223.638850960288
181.97372140367
CCDC23 1000.91520225219 929.69741581954 1071.47374315822
771.294254795752 1145.35733270036 1238.11700377838 1082.00448164658
975.685771692095 860.315938621256 760.272612245092 556.31484498921
1170.88763035931
CCDC24 226.334672545273 278.192669077191 306.333710880618
266.101158071711 780.105135529728 585.721610434501 520.120193442648
517.42417250172 405.843083758177 259.575461599189 473.529519031797
456.656123023446
CCDC25 491.635435224507 481.751102333172 490.022369664024
578.811908030805 285.131244588624 408.811415529922 445.905756082569
300.401370666633 308.767036855508 446.819873986024 457.292146759215
422.590112942156
CCDC26 134.717258604899 149.939035476611 145.304050333309
149.6730835169 141.350135458015 164.593531655305 139.864176113337
152.987939791009 142.600685998678 150.227629690236 135.139676303901
136.939603341076
CCDC27 142.815817738837 135.390226050803 164.072443259668
150.238582112636 157.374537572779 140.471953101887 152.331011756244
160.096507818256 155.813740201987 142.064680109047 155.43577761798
151.423936838934
CCDC28A 542.883453307 667.156197045748 732.877169346979
651.998433127992 379.615659443164 341.901565692251 590.142817130684
517.529147171876 669.330078861673 516.429067667758 791.402660066193
301.695991929957
CCDC28B 238.230110356671 232.127996106421 241.845941562418
283.191303666907 242.787656836745 232.699786290509 201.81649023518
205.546628064296 200.127994198329 207.73395131954 176.138621462315
230.508733145583
CCDC29 131.019345449516 138.150616787218 138.221749464651
134.728594388428 135.592128081677 147.491439949088 140.687016530974
128.563180697606 130.253372129786 125.18424162353 128.040201586267
132.297716337104
CCDC3 491.496499481595 469.107624227456 450.120868656638 352.121800955822
141.056924917269 146.658048787833 147.463822782303 165.964183460825
198.840533461525 218.269847477919 262.861105654051 142.690911343704
CCDC33 150.536178574986 175.019580368717 165.647471565959
163.600888221193 158.859389972316 145.034179171835 152.188770978528
152.390371614602 156.110073009771 152.952479729682 155.592219571564
152.393260140571
CCDC34 278.942695435238 313.708197191587 243.468673768141
305.906480933354 349.65350886897 333.439929772897 370.301819403832

409.477576085245 469.107624227456 465.029298672645 484.903322308422
309.75140197669
CCDC35 171.49930976135 163.542848861552 166.305219348008
150.182173149394 159.48526814715 143.903216264503 168.897620634337
156.990325995053 156.674411447713 163.432972512188 167.536828038178
150.856917125736
CCDC36 160.493314658788 149.990378213915 178.707066209511
155.515134922269 150.711857623087 165.558963147657 187.216245895253
151.522526161299 162.308297940279 136.627904756283 120.379393989882
134.867806860993
CCDC37 112.428707152237 125.549968985241 126.019246614944
114.768639931659 118.486195373059 115.120702589487 120.786630750686
118.258886189283 119.198789189881 113.339155126202 124.443643568527
123.357205547181
CCDC38 131.330974614437 157.83121828793 139.131857948357
146.291932367254 141.968949222821 149.712320986646 147.029988235786
152.382558543399 144.5368823731 149.343930487315 136.095753586495
145.107068919893
CCDC39 137.222125237171 125.021217009254 132.469190523971
144.36575644625 147.928629511539 135.305147371641 134.340512608931
135.707354953722 127.959474148217 129.278833426752 131.692626563078
140.726084514407
CCDC4 156.437949042181 153.636804025106 144.32655838488 161.956032597813
156.254944793402 149.480971898888 156.61494867164 154.954906872501
154.528396813382 145.909456876796 141.919263493165 146.986891588172
CCDC40 141.611712747368 136.386031133644 140.578378782903
152.980843102933 154.634289463743 142.948238172973 144.36033726557
147.543411823657 135.744202879162 134.946677624454 141.86836086844
149.432744784716
CCDC41 174.136806313142 179.794718712964 164.587657424569
157.195967361907 188.53221484746 206.321272734099 159.046016068252
173.773876769683 188.780089746036 169.502103689662 182.478971050701
164.541417588646
CCDC42 136.66358267095 127.827096314406 139.205887721493
139.805930686563 128.447393268611 140.264060906597 134.08270035062
139.87715485885 123.561016079801 156.994265858123 135.296271683847
124.858723248944
CCDC43 295.367125961621 307.728083981504 318.850765099332
304.636618563775 259.643957326575 352.811351813364 481.238450505207
442.334886941795 385.798784913201 432.604462607037 344.298510200653
322.443089373063
CCDC45 561.251860964966 506.898635822094 647.666334416707
541.642309122976 245.351459157365 523.216537391852 564.948126039762
700.877661166739 578.084277076692 392.909481887174 626.298707790229
868.066644155313
CCDC46 178.707066209511 166.835922279372 169.541411677993
145.500011956699 115.766358387923 130.022574966461 124.302581985661
121.003207135942 124.474221706022 126.359440164601 126.115828232308
117.131720092783
CCDC47 247.236004734567 226.43414383055 271.897511879509
212.987082617173 253.933478547515 319.927209914181 359.822784070765
344.786541451395 262.019779752857 260.443814523694 379.862305342147
454.133372808458

CCDC48 223.601312199935 255.903775525554 186.831266888028
203.004815689972 163.582678113215 155.964242240771 161.922462797736
181.695203523798 160.839277476397 156.949439724914 135.713266118447
150.203281104753
CCDC49 293.986292438714 300.443499140896 311.670576741448
258.998065427002 186.231346659144 318.850765099332 339.917319653382
616.523853569261 396.345382600445 256.964577037118 297.176755638159
329.333236839082
CCDC5 173.233357775898 156.727153398506 193.261343922604 166.360005474414
171.145909843389 262.061371466369 197.443711199904 230.469548500304
216.783051958345 234.895245871534 187.461448376593 206.715020025609
CCDC50 342.147414141627 286.673016219195 385.750371084
341.361299896997 273.402804140541 221.390817970859 274.564508105177
325.751369001105 366.073387075385 493.492348317616 242.695286989672
254.133112176024
CCDC51 219.96819201774 243.283375388645 267.342817344746
273.959220003188 309.36704220687 255.56301534833 280.170464338141
258.21341068758 320.877897822963 308.877027622691 247.015574724901
288.631429069336
CCDC52 151.855884026072 128.247871411825 149.393022408213
144.71955090408 141.547112973262 142.339740800356 155.463400179872
121.084348994747 144.321757243275 131.630132952441 135.372516179244
146.019622621809
CCDC53 787.615195903993 856.039380523678 917.188039935737
871.528156184927 631.653707589221 594.713185954954 648.549890890375
729.202002368627 762.8576747113 783.755776589969 674.571475335107
498.334266735594
CCDC54 131.897352210959 140.323425587628 153.962758393332
163.07139884152 153.464360995071 146.388285560372 157.067264221207
148.840533233712 148.501654210579 131.984467471242 137.556552701816
139.607944722497
CCDC55 296.051639873628 248.299149406938 339.172031360524
267.67093646012 208.476367897256 211.609763150578 259.979459472217
342.611951034707 289.911635155481 271.004741629789 250.718986968076
230.546762514966
CCDC56 878.167149313285 970.166036472486 839.901173743755
1158.88528996131 1379.26881813339 1081.47077865393 1161.39230511792
1581.06548216423 1290.39741199701 984.695710317818 961.355625338687
977.999660785113
CCDC57 142.179197020805 164.043866642712 155.6299578128
168.6016897585 157.089650890944 158.175138008678 151.992112109698
155.371772547395 153.248199842194 153.066630483602 150.974364125849
166.46473789699
CCDC58 173.741466458636 182.042104888745 165.085010975035
157.646948327818 355.80042210486 551.622587349813 448.260934224454
231.239126548656 373.452370692334 472.405334652317 324.481229901658
604.047164856689
CCDC59 732.040299742444 593.639955546556 900.039744829725
546.841563406813 708.268962600305 917.188039935737 1231.37475540937
2421.87041217217 1100.81588201714 977.227184625781 907.610179738644
807.92851898782
CCDC6 1920.16520804856 2618.64973582507 2334.97297889593 2763.86550710042
927.478050257292 1436.57771711084 1527.87148052149 1178.83933381069
977.227184625781 845.150147618462 1840.14350831655 1599.12034906314

CCDC60 286.996908547553 218.997165156081 356.05426191171
282.244214637625 209.196695368783 177.083095120017 154.170007083026
144.78971417658 146.402352419692 145.997380100999 176.830879422227
154.503958858173
CCDC61 157.541796722309 149.763038696374 147.659772694732
194.789918629905 135.892248893607 157.244026506342 163.086539217826
139.524233960073 171.589889474122 149.941056059544 145.449099891256
180.035652063942
CCDC62 112.916256784817 115.920514336615 120.565485864499
123.22295112139 127.585851003779 132.772085211553 118.773864607553
121.045367784918 125.008209861774 123.53212786736 120.606354515072
130.792692240592
CCDC63 117.112667504781 117.523834738903 115.718600552187
118.965055790274 110.419222236698 102.094370876743 109.274221164316
117.930665298163 121.850967084709 116.95195122318 116.802432258299
118.488997046932
CCDC64 135.041687549769 135.437227744668 139.582054825147
140.706490845379 136.258073395013 115.053039576143 131.967245387861
129.411734209444 132.623563631011 132.251093122058 130.114464233633
148.065080664185
CCDC64B 151.59613008133 130.644937322186 132.496547953595
131.099004325598 241.845941562418 164.185346950306 220.330477277988
322.263925912805 266.842778974988 192.465510013851 346.303862540751
244.849563563729
CCDC65 144.402160347818 133.731500722599 143.774841336279
127.779401352802 126.686404772601 127.918847837351 119.036965883266
139.765361891899 148.895841777421 114.77964341895 132.681475162386
140.254665008195
CCDC66 231.955750250711 201.085046871333 271.087125858775
216.705908017858 158.23884088767 207.204293555178 213.028568789661
197.775397831129 224.466829629468 197.191648960563 160.906550609416
180.986305037222
CCDC67 105.712302595131 119.168045349254 115.914842445934
114.494394548044 114.161540771427 110.081964209973 117.48427100606
114.200099628087 110.510191137073 117.706795566317 120.045351920348
112.509731206295
CCDC68 162.890650672747 147.70193890965 146.885404321622
153.187148832063 151.007812189859 163.205362761005 162.923734210933
146.402352419692 149.839827383681 152.163489631563 156.448355269561
160.949030247406
CCDC69 588.458314503034 525.287367366192 538.252273740419
832.622843175217 135.607537832704 143.572668991313 133.070896773384
144.384287335249 171.51411661213 144.654765322941 172.973175988757
144.868337748544
CCDC7 154.033776287398 153.088710298368 148.644800418517 146.156634581799
148.504996616452 154.645353009618 158.594064857041 153.574068503306
146.798444724408 148.686975104375 148.219406063533 164.848970268132
CCDC70 128.583503171594 128.803632055969 119.849895979393
111.908965758799 122.54127694717 119.442114850181 121.942777234608
118.379914726645 125.800508653231 123.762963668211 137.550607284001
117.172431931323
CCDC71 133.478829770103 142.792294709664 130.069789939568
135.993598471576 588.076436392983 129.055874691136 158.299765118221

144.103622373381 154.33224999163 144.342905860503 271.196028302639
130.582210755516
CCDC72 2828.97255380291 2334.97297889593 2567.4299860517
1932.39418448203 5894.79176478976 7433.03226979876 6271.34208268488
4819.85672511343 7468.70895916615 4030.42725798136 5207.37974585707
6691.04319670055
CCDC73 141.803250679131 144.368355737187 167.604959895451
179.648479201164 139.044150521333 140.441521267218 153.305679563467
138.359423363142 150.218643705925 141.828740140733 141.239354296152
152.921328522953
CCDC74A 302.905840432998 256.795346052751 365.199125315964
303.986544460218 146.728881202697 182.938824846376 141.834582245454
157.844617425219 162.916978850245 141.746032519746 134.364838071696
175.955932037011
CCDC74B 190.871696848118 186.309367281625 228.45211887819
163.239195865198 139.503902205317 124.347680638586 134.675247731364
137.923852688695 143.53550972177 129.495680232567 141.497125111619
138.399665664034
CCDC75 134.490327004118 143.541810469447 129.151767489346
135.345616014558 136.843164021899 133.638664342783 131.734429913106
129.564482772168 137.867263869936 141.950778601653 153.261002653668
143.442361602626
CCDC76 263.058972886829 252.697160031025 251.787648609546
177.759729226623 196.566937667522 365.590174280114 332.138346958671
324.79327985156 296.290104133844 197.298982267459 253.42022722564
312.129861040709
CCDC77 311.157764305033 310.989043012841 353.826834642154
348.300300174924 312.406286993239 434.716530273383 365.286234166621
576.873245313776 526.970555181851 376.54010486682 361.327436394941
311.449205777595
CCDC78 114.880348648147 111.559325333192 112.565341690522
120.379393989882 125.095999326598 125.021217009254 117.097159106043
120.452958447555 119.795945889846 121.515872228716 129.658488766089
114.3172128566
CCDC79 115.605206174867 122.115925891088 125.405373274081
115.417648857825 121.065278765151 117.15886922795 135.348086420636
125.621467040793 129.058085094891 122.133292722932 115.194730717797
126.63730651721
CCDC8 145.195065312551 148.074494928651 116.939471420419 138.581345752206
117.296972688814 113.031636180748 119.720132373023 115.366496551426
122.327353563917 117.428152859221 116.787791109549 120.138014822486
CCDC80 288.182388699147 202.662466141301 217.684410907748
253.42022722564 127.646032547296 122.503984297406 119.023688551496
122.111982327877 127.299853592344 129.034770823878 139.065849548762
112.015423658372
CCDC81 181.313333665614 156.69025356689 144.084441938143
163.805257266374 133.79868477192 123.960761343425 128.895325544109
138.566954604414 127.150222504237 127.206177583275 135.842090777339
128.5581535393
CCDC82 123.30960025689 122.389557272277 126.245597154052
113.005774989833 111.312348806957 118.578205738502 123.491930765117
119.83679981791 121.54835308632 120.387275261633 136.884799902348
117.199217938531

CCDC83 110.286187184448 111.188410935711 98.1433670546536
112.771141378159 107.385565665491 107.559685467789 102.421174406474
106.437660157036 103.202873534814 124.712789674509 115.180659017144
112.088944448944
CCDC84 393.796356067403 319.560819487616 461.143361255467
285.214585324341 245.269004485826 352.562711030848 471.069295685718
355.372836460906 369.513420606401 224.118739978257 478.847650837714
371.280926727395
CCDC85A 140.127379332918 149.004343851423 129.153882779488
140.865990571023 137.151210946281 141.094578556895 131.310537960214
130.009322405768 124.717079082023 121.356731684516 131.244759170642
130.98982984989
CCDC85B 246.369521698487 277.459328083162 277.073510273425
307.316241247336 264.805236346895 217.24573005272 209.146021798649
183.556662533396 191.507657960386 239.503369457867 186.377571176623
201.702688815038
CCDC86 347.147961254829 380.310795921752 340.728888109597
368.418553384965 661.826149512811 918.162638134305 429.595453454621
331.523536427527 746.523071806534 391.326200093611 283.807827536147
657.058213650454
CCDC87 162.850128623401 154.270034951209 192.113217321485
173.161152247499 162.364266549463 182.958991847218 173.986750195954
148.145136066071 157.172970235254 171.844483419604 142.700311964178
179.265395910119
CCDC88A 169.541411677993 162.140112952434 138.230197299262
188.995551740427 115.858924428037 119.806933383138 119.502758763438
121.353233207209 117.900892627473 134.449083873514 148.484823275343
133.99226161886
CCDC88B 167.751604259111 136.659134200122 150.471558219667
149.747347428419 191.445291899108 150.790155351217 156.906285133852
154.340042518983 161.234304237838 179.841665686703 164.231281729415
161.382041789666
CCDC88C 236.141982516399 295.153786338687 274.891033696257
328.919401925375 243.979769719186 163.010527914347 178.751706380632
223.778708427552 191.861598059182 228.616558999054 206.620708230452
195.303469330682
CCDC89 168.223865801948 169.598493862848 189.575681012578
189.575681012578 143.231806900648 145.465589138345 211.369442150136
159.23414970379 143.594003495859 178.313238745665 146.531692460121
186.321879879561
CCDC9 134.364838071696 136.380523452336 117.356389952799 163.02140155858
217.45700166961 131.290698895743 133.590552542806 120.626155234319
130.396593013401 120.606354515072 156.123528587917 119.188446525733
CCDC90A 339.917319653382 368.548135194271 317.273940108333
418.062540009856 505.704957182936 328.14373061894 392.430873293903
383.74357557363 374.718289636553 615.254768508691 495.438672949441
326.354469134694
CCDC90B 547.202354339933 535.49374437073 555.758485277905
562.089999265955 766.420275252501 419.902534806131 820.249910349431
646.704226815099 632.565736446505 681.591028334969 725.621153262924
875.38834441246
CCDC91 290.688850193983 262.233918187791 383.182507425579
252.138168292964 189.74261433647 170.335930486215 213.412498914309

229.933577323411 217.811099067262 265.43897208356 188.268774618164
182.453722105813
CCDC92 1031.38207659547 1025.88559245013 1011.9675459648
1286.55103838368 438.958573676953 417.36454228858 597.651682540611
568.040362658955 445.95114929414 818.761934233826 590.515758538373
643.660739343159
CCDC93 692.095005197252 637.892677173103 644.97200337335
893.264940736694 342.611951034707 475.833242673151 359.703969802888
500.246996975 446.819873986024 515.432065049838 380.310795921752
358.60777927888
CCDC94 161.021740537372 173.586160103539 153.76202838917
171.16807372519 190.645917992472 172.750926094884 143.554726863556
143.040254576088 175.222531098958 147.182394557583 153.008756051525
166.173429365582
CCDC96 150.573523216151 143.979760385364 172.387657419201
136.290486122369 167.526798465556 155.926739769177 158.92254744379
154.051705030348 130.770335966929 152.152106138031 145.070927449969
158.95714101556
CCDC97 399.476401949891 431.019896339435 335.938170230765
395.588345893987 777.901979189395 377.538276846051 466.909037277853
487.537691131703 448.54309632232 298.625127845168 503.466550870577
272.351487980415
CCDC99 147.378228560979 161.781529659549 137.652358744527
140.496058408953 165.757295630254 160.748082658758 206.937770272939
313.708197191587 187.264411519101 244.514230664694 196.536086443884
206.89716102989
CCHCR1 168.150270765792 176.289693941489 147.612454763666
216.974834077975 199.459711023907 140.096717188376 171.629605754643
168.144811084102 163.54669668396 152.57905274438 141.872426752329
158.459129254226
CCIN 143.252983617945 145.259981479761 126.27024495428 155.405519023053
143.653452869664 132.897554980037 136.396579126676 153.432933930805
141.331397551086 140.163936299053 121.815538475678 122.274656361883
CCK 145.730054487572 133.125084506877 142.294922363188 131.071614211431
191.297539967707 156.949439724914 139.414912158809 140.281512754823
187.729071402782 150.933724024651 125.364821583438 175.989742412417
CCKAR 140.232244264861 140.138870925001 137.235469802064 143.945039636839
147.368455727098 159.2488784104 156.994265858123 138.601616246182
146.599636794606 145.980929136129 155.343619434375 136.041605158822
CCKBR 157.521230460545 155.061749542085 144.064214573005 155.964242240771
152.492163303139 158.660859266099 149.202628825354 134.57472034679
147.570734399004 144.170287726456 130.795318552623 162.397078614596
CCL1 145.650356702429 128.312170913496 161.142723532061 150.73248190848
146.176240539176 129.852833137493 141.013392458859 134.606126134531
135.766466392056 135.797170561599 144.713108022551 133.358701657264
CCL11 169.752525994991 186.679199600379 156.353914172996 165.158465031363
151.285933600303 177.223953859764 161.182901898379 147.024759173313
139.238936770274 161.475694690038 127.305306470493 140.826051635712
CCL13 231.846509449829 201.289402228512 122.531675048414 128.990911924499
240.439368352772 189.509062639279 315.689865927834 293.317880637519
430.728137105601 389.588346573823 343.307036883726 188.891032176388
CCL14 528.478292835214 694.288215755716 257.24481414803 524.973621024573
140.806935772116 158.07640654075 140.123348569709 200.322334039458
325.999687273862 140.634047988278 382.562403424855 134.206518622834

CCL15	287.503594820801	323.788197265954	178.43642939806	254.424207773566
	141.150016212967	144.771457221413	151.327544435847	162.774716879847
	190.506201184419	137.974323288786	208.914498369991	140.722297244564
CCL16	145.235087732482	135.29954024755	135.309431186017	136.832494876249
	129.446928016147	138.743505629497	140.730085305185	129.707421699091
	138.20899000519	131.218141814078	165.041296694491	152.254519859308
CCL17	106.667175621971	121.54835308632	102.421174406474	112.424606178394
	132.137634927459	130.937742379689	168.180063344887	127.218362452043
	152.459041674768	121.303696898208	164.652795899742	125.055545087871
CCL18	128.421242037748	134.821778017915	144.227440632196	147.333142209628
	145.276745572405	133.59686364883	156.801189586018	153.500478301079
	314.610979086485	137.988325215021	183.226294747874	144.968459820523
CCL19	164.763269377973	172.364618542922	137.754373055094	135.43939472868
	145.526941881413	145.064773625365	151.833386901811	417.528353577952
	1565.64079526732	135.704067249674	187.824032557194	161.212598761378
CCL2	635.234445318087	1889.05071563459	193.472317111841	354.579728909336
	221.322724563472	364.597798667541	255.527764534636	253.789045068884
	474.517750753745	761.32108609503	208.64349688412	206.042395282286
CCL20	112.867662941811	130.408519735001	121.006569103388	121.157614971438
	148.538400348149	147.260260687975	129.069366041625	152.500670025483
	233.36028792331	912.483177705931	240.410292351169	155.951509643078
CCL21	284.995098167431	192.153420834889	250.718986968076	257.132618311227
	131.820842728171	130.199581370385	127.361974492821	166.97270256697
	367.130155304037	126.617737577634	150.936747236094	127.171331062578
CCL22	114.504409331018	145.311964807443	115.605206174867	127.135199316758
	251.787648609546	111.204065131669	225.480366837274	162.464201058576
	223.975677959345	117.710502693638	316.590387560909	127.884750925048
CCL23	152.916493104088	196.189101941489	171.410912120195	309.5988292104
	134.877046981678	125.687688416588	127.7962242731	140.689100728713
	150.639235242268	138.530236176135	155.30926640293	127.03153185998
CCL24	128.089328553242	134.418976976995	129.732352495012	120.967523586161
	136.935498266723	137.229871801676	139.987681290389	141.789840190632
	138.469997677004	135.880955433228	123.277867938275	132.317972465689
CCL25	138.325950428197	122.969215709433	130.418739793498	132.361106053517
	124.757229519631	121.207040901483	132.269567096683	133.519359066907
	126.501648842366	130.697999379259	132.481710681317	131.946302885243
CCL26	159.29430824799	164.1572198075	162.739253457398	195.474525582555
	144.20764300349	143.550908565401	156.077604146143	146.696029753123
	148.299945894738	156.296046600921	140.724541696229	128.775477649887
CCL27	140.429462488271	127.75729795293	125.596067857941	147.512290383685
	140.248610391566	135.802258952006	137.620843137286	138.197500836501
	152.77616981167	134.769926504916	113.568261995787	133.467092274663
CCL28	131.089811472017	131.907246423067	140.669947387892	135.803599969904
	135.122770943076	133.689255383855	127.560308850342	147.405050592353
	135.508803488048	145.245316640391	145.416733388652	140.443415669628
CCL3	168.840018225742	170.05799929575	146.838547724148	178.542247857251
	205.509515144263	331.795832780176	182.77693508164	180.747527895349
	193.930569647995	395.927222009721	162.47662918768	247.550312458738
CCL3L1	138.818492907643	145.524305076788	146.014790698087	
	137.663941989772	150.778292242313	212.730163981978	136.811620595243
	146.59366188125	163.558869203898	258.695920967489	156.114825514646
	193.16411905957			
CCL3L3	141.86370253437	157.468934439452	160.140509175287	
	167.782882015229	210.043556816529	438.533112511098	166.076692502428

144.281217246178 225.189991540262 502.170430910893 184.542232315662
282.561381978659
CCL4 132.808559892154 132.322618637842 118.432307370605 141.831596320865
145.183463058696 130.630504666374 135.264074941678 133.327778635672
142.180927190578 127.661634554996 129.127563278016 129.846685899183
CCL4L1 174.941551089279 166.540134051424 149.831566330214
152.581729298159 181.941224633917 299.986758612161 164.665907233149
168.018785514306 303.724140156439 420.248838397618 165.972090075204
236.219981978324
CCL4L2 146.777258330572 150.95033946042 140.890227770353
133.231066635063 151.705219298578 255.866847225916 154.698312173556
154.686025022972 212.037224737798 241.383657414248 161.735609436394
178.166289723365
CCL5 563.490027149224 531.217306770605 291.056913909578 289.576706584644
285.594100976705 329.023836559229 363.195371577097 682.315322994357
1197.58205444449 524.725307261999 436.155731212592 327.480268032814
CCL7 111.041656398985 106.603674195422 110.670057121283 106.685578858702
118.604395992281 116.007006375993 112.773952761845 113.451566510208
118.442856984861 118.220555783444 118.039780360151 112.005939063045
CCL8 194.584034984339 254.768537214795 135.93352186143 157.50873249647
216.9491329483 161.499923812379 149.707357499252 150.132420238581
313.536451935268 292.429831186609 213.26158748941 155.335777680002
CCM2 426.249411635589 509.880640462 416.215253990273 617.474181543858
705.311256840178 333.131139447511 445.763740779039 413.757630469808
406.224345292389 595.120528902501 384.641319423853 615.88518704181
CCNA1 123.61956613951 123.02546895475 110.61165787102 120.748910864064
109.228587997052 122.158160927149 115.766358387923 114.249891266241
117.980052938167 125.058165131184 114.412739085949 107.283814651497
CCNA2 185.396765725228 196.486499238623 203.999142031297 194.724219884752
441.112782197591 372.54091392596 279.598585549004 443.343806055858
529.963453165714 462.025126897013 232.735767435327 377.593087606046
CCNB1 157.328404229451 166.182850621447 163.22480684222 166.46473789699
391.782120640025 448.95671696823 309.798246248863 520.470790410856
284.321253087512 324.346723939622 210.41046499545 330.281193038198
CCNB1IP1 502.281584081805 401.267425721838 472.108893832845
383.802494922918 213.990575186237 255.225145197422 316.237519859859
264.054997398621 196.519125510368 316.409887372431 455.486248388968
348.808646570544
CCNB2 126.155486659114 175.485672103348 138.83304645915 140.02337095267
748.015452242839 551.350758868377 380.562212796792 896.185223980593
771.512332113075 658.746224115551 341.251360936154 665.602699607102
CCNB3 121.197702744078 127.026168862919 120.843429388102 119.312201620247
124.045745662173 133.07218865002 117.207266754941 128.429007921588
124.897463208595 118.122186617611 118.220555783444 123.182903611854
CCNC 180.850779069931 192.353768499582 248.010317392179 162.427505889157
310.640107228956 422.688531130043 587.388038484307 405.409668978569
348.111872425949 572.913714991136 254.393421265604 392.282956362735
CCND1 3055.80298721424 3083.98169220838 2691.41389079777 3789.1316515671
5846.17594234628 2627.12900283627 1840.14350831655 1459.31031099055
18562.671630167 1184.3199328099 5032.66681917478 7771.28016059011
CCND2 4520.84412831414 3989.14883668905 5195.2087608308 5589.21471380445
189.589053840976 310.917191279571 198.338416756432 395.261210769232
715.638378711433 1584.81089248914 1646.76016031831 209.93789387724

CCND3 1397.84878368705 1554.16120123599 1305.2232532959 1692.01248523526
2099.85702995194 1388.91423772335 1251.15912264225 1201.82026045778
1744.58410715956 938.049312229961 955.604404066769 1206.59866455529
CCNDBP1 514.799179506754 494.347792360629 729.499296070289
435.983803453746 543.710330214344 793.071192619058 1120.3009282887
782.746481112316 1048.85768964657 841.944755849006 936.560290736127
980.67040349547
CCNE1 161.424105316969 164.917470819096 164.50490221903 170.342828808493
422.23268864343 552.962676093514 216.582862850259 450.327710374141
205.871133967588 172.866517097198 195.123919692348 261.39435936661
CCNE2 113.529619135543 116.609122768341 113.58962177275 122.664353374047
143.747664482225 165.177036955944 137.022366035333 184.989027770708
182.109092947203 168.323141675824 147.017958916908 177.333305785248
CCNF 198.338416756432 213.813667557079 196.615914367563 202.583967728957
1502.3444541735 645.806954296487 469.107624227456 736.332743195416
621.825622897512 421.384700184923 378.967410725775 583.022090879298
CCNG1 628.485648053393 612.06231165648 840.276501350383 443.732580517751
610.591049634222 780.105135529728 1436.57771711084 790.321331435096
582.062758687489 717.686676823785 826.08372791138 1093.85316636801
CCNG2 218.433524126584 225.96618659877 189.816900281053 178.878444404192
334.443733461841 258.348298226232 626.104755496369 345.291295192574
326.703488388966 371.693808540758 491.034395489684 277.314675225943
CCNH 226.25694727283 233.192751618176 241.527811817146 218.269847477919
262.171775165261 292.746711619565 286.194197552489 206.753993163216
227.375773052577 349.054741747798 289.517820913799 248.927790738447
CCNI 12221.7819812315 11150.5512437377 13090.0615116619 8918.58940386786
5106.27623905309 5138.28186306948 7656.97151002785 6021.68798558746
9070.86397918429 6960.46610781696 11971.1664283024 7218.21759302518
CCNI2 150.914644894869 137.266420259287 132.342063356763 151.958818313561
160.039086963419 150.653030597808 137.378528742953 156.293025312901
163.315865817864 138.833677916752 132.845116298605 175.462290891421
CCNJ 161.557558028531 150.019181654237 140.581943144813 147.314687795555
152.780908985916 170.439725847568 160.293522619371 167.226663395855
160.042251279235 153.528429414138 161.17923997423 204.695311233073
CCNJL 159.801315111886 139.378915474473 141.473218014605 136.988519762443
201.415695076278 138.936872956065 195.929328710358 188.475801713801
150.166324293431 151.181422205549 279.070493043898 207.581907737176
CCNK 672.862678078975 697.97116981445 481.630922092112 648.823677010146
977.227184625781 414.200068430184 608.611927946607 596.934339406636
575.722713461982 628.197839884392 714.6859666834 601.640387246951
CCNL1 421.140525880287 315.3930052781 352.945088028205 258.807028561407
270.462865174106 453.457361487577 633.104379362137 571.950858678221
498.534168389928 680.250735532287 505.302263971007 661.096668635469
CCNL2 139.856251508283 138.494266722799 155.741507341843 136.995866452787
146.29600238749 159.932941342403 192.580288063603 152.733409144267
165.496065029161 155.295464263911 160.695913747601 180.336623146341
CCNO 174.161146689117 169.771005675296 170.028047334267 136.480458837869
370.678040372273 256.471672362567 461.413469623811 1239.13016938378
876.783710734594 241.494710905339 303.241375487319 251.138401278632
CCNT1 212.959246855759 203.276353321185 180.710636205653 174.576132939661
163.521095869383 204.787016443123 185.128661141884 166.223079895236
175.462290891421 154.883309503689 176.953099943185 193.753062958207

CCNT2 187.881334544018 191.160340745501 194.388383297922 156.010027635627
170.465177938235 233.536023295039 171.844483419604 234.432954093705
207.104548555929 204.959164579011 219.103510284905 226.086733606951
CCNY 732.630306104841 619.045921382959 738.892511276134 562.543347322063
699.270931701696 574.399909181723 497.411169898778 367.025117016472
422.840163973614 408.361368902627 447.909277144896 560.180656219785
CCNYL1 151.508738608925 161.144841967903 181.467708718758
159.138344477256 158.167384416968 296.333944172375 167.357570666817
227.884445686928 170.878962027347 388.477382640003 154.388776919015
182.209056248253
CCNYL2 141.772724512502 137.789611340925 132.698721834882
135.673080643346 135.509927917168 148.57301274812 153.114065147266
130.754320787628 135.810476921749 138.471894596606 129.007924852801
135.028746262383
CCNYL3 148.74881415573 139.679681813932 135.781190598068
132.998223400178 135.368203841678 130.457981404612 120.406577236405
135.311980936088 140.06989960847 142.821131835602 135.592128081677
134.434198188045
CCPG1 451.496617003797 378.695635790714 481.933386283351 400.180574613078
184.085616342099 208.690347128273 175.805472275887 188.654966029389
186.140970908638 198.456392063513 196.839016616283 151.073257114127
CCR1 129.680406696779 137.720747340105 155.114892891737 149.88598449818
131.138322527536 138.916830356763 161.564505054217 134.140533647509
167.933231844271 159.59942312149 163.165374495635 149.899789105328
CCR10 159.424105896749 157.631978594005 148.713747597311 148.100508271232
148.47415004128 140.826051635712 154.288075172115 160.164327420125
143.528208105237 173.440596296333 142.097126256137 167.341759854203
CCR2 139.063209125209 138.62308708151 140.239948346946 137.76307316083
138.652735298338 140.110258415883 135.221786448517 142.452409969639
157.358696886078 139.023486014525 144.769824608083 138.792521681771
CCR3 119.915330116696 121.51208055933 119.122305582754 114.749597426757
121.997885377703 113.716741872499 118.718048764985 117.879058520659
112.747067417774 118.93828679356 130.532559074698 110.61165787102
CCR4 120.182787241888 118.604395992281 109.342230674869 141.139737175648
135.878996521596 136.654902685184 128.108066199718 129.127563278016
142.38097037888 115.072622367144 144.603401904061 134.814082968113
CCR5 140.036463888037 144.231539210695 131.87404363664 134.405155876727
129.434594784981 131.362128083159 122.412141495264 134.247361216412
124.124076599417 142.869595099569 102.522925993233 128.720517906732
CCR6 9994.54490550759 9852.51412359574 11598.65499235 11071.0448447818
5965.21815409254 11598.65499235 7491.93659373205 10329.1999331936
10106.8225600705 7771.28016059011 4732.31591164752 8140.6363797934
CCR7 125.371128588617 130.698850298016 108.21185649774 115.34945441214
460.742037437298 218.96845208513 245.522497675754 351.934158805882
1610.38263368771 141.180647782041 224.135228192655 185.447093292348
CCR8 176.964088090133 135.961724275921 164.665907233149 175.245611094434
165.122737166322 158.845174721328 176.964088090133 172.438141471784
157.944355564411 149.659505956629 150.968998243223 169.321832528939
CCR9 132.100026388604 136.584199831088 137.968932080074 140.251168248117
127.445199722147 137.669753723768 135.17827963343 131.159946917777
133.524698103972 138.215708514725 141.950778601653 137.639342727211
CCRK 380.888728347647 370.390323116113 493.071213662125 500.643499051523
286.818166898922 221.889331385023 214.684538620841 213.833894542292
245.454945056317 214.824908522393 211.844563155792 206.21848253717

CCRL1 135.915252483475 133.31378749951 135.085039737228 143.881744063934
137.902953501634 132.908367372426 136.400959554698 147.535225305685
161.104993402456 160.766121558583 134.298521475727 143.644862018676
CCRL2 143.969566798989 142.043121538885 143.705475208192 143.839170216353
191.170105307944 179.863096463897 156.543267949167 173.225064625094
189.027594735456 167.796474464069 209.398724613377 158.104336050835
CCRN4L 150.371716899336 156.399808270033 152.230489051339
155.150904036206 288.728608411903 240.591548171052 222.005022189667
172.7330526128 185.17273221366 340.301980622308 168.634534857593
193.466377788318
CCS 284.574888587832 273.106088088825 318.971509860209 329.926864732606
306.946103334814 269.424055734143 255.677315946354 238.375823012603
218.735620596634 276.830814061474 204.584643964409 236.548439331855
CCT2 769.34961339184 648.38049615541 970.166036472486 618.058496806334
814.110601393539 16533.5738137081 1127.89414462606 9139.73212514992
1774.22769195939 1439.37593006882 1131.96129284658 1605.2454230722
CCT3 773.330948077036 724.777166238211 1024.59845685519 922.50743596248
900.839073800181 1362.75971646355 1074.19589851599 1300.40889834984
1165.95924778817 1051.98943987926 1243.96721244424 1504.15682715484
CCT4 173.260348096238 173.611295051326 189.082778234831 169.136919982294
215.47323777134 239.12905945808 205.362816362477 226.173501275026
170.212679845194 210.117033165026 236.809829701921 164.181915898563
CCT5 240.054164412429 264.109049504585 237.01460308334 236.933960698783
304.713434036642 499.176926371542 192.680681636018 400.676876982655
220.92105843037 158.429761055869 230.723142236623 246.850599569231
CCT6A 529.04238100948 494.604716660887 652.201948082657 494.175437345451
422.338508598153 583.455617065556 627.526008812413 872.320823993033
626.298707790229 773.803350315426 655.094843900848 865.800709006332
CCT6B 202.887417145119 192.188662949149 211.679799388351 204.161375686761
261.469732730752 195.711047893396 226.936810012475 219.814706416471
189.985520850855 193.629237856758 224.466829629468 192.375251343504
CCT6P1 413.687046868111 408.7151485563 637.30300219673
352.945088028205 394.576038252334 413.330906392915 482.048928546145
562.711562649704 408.7151485563 588.197697799448 550.434931878586
648.549890890375
CCT7 1024.59845685519 1139.76755449089 1301.61742965939 1163.13845502757
2300.9407349072 2139.42448145954 1514.53784631689 2063.42824094932
1293.38808483663 2101.93705983383 1255.02720867812 1472.54829263217
CCT8 1401.67503392145 1323.91781040143 1592.29734618345 986.65554605455
1143.0779222425 3270.88476148156 2956.63335018838 3890.14358014877
4457.64479507833 2414.22821062457 2433.83421295012 2941.69058596077
CCT8L1 141.085111670206 152.080057231881 122.978576290038
128.446229320855 141.94585838352 145.204870282027 124.558026853954
126.333814565085 138.407207709066 143.857385585662 142.806728419652
144.829773937102
CCT8L2 159.820341130737 159.148717478277 159.7197697044
142.487424651482 147.040759979063 139.109712542702 150.073816271065
130.760835441776 152.204730003016 144.087004064416 158.854659281803
142.475646565816
CD109 121.23469201559 141.119491147925 125.642282323003 127.310437599803
124.876226351169 118.076139318165 113.913337223716 112.962180108363
114.174153178166 146.014790698087 136.366798516444 118.342067845126

```
CD14  999.159652508318 838.93563319979  601.800326895295 1022.55215097491
      902.822697602452 672.348723983535 461.143361255467 688.464980326689
      1520.59210521859 2305.11856249143 867.601542930533 710.228463443265
CD151 3251.62784659994 3850.41246770812 2891.03258768937 5846.17594234628
      3643.11229751348 1637.66306286151 1563.71477584676 1738.96798001895
      1830.56003081769 2261.31473132315 2684.69013589621 1519.38642390142
CD160 125.241484840139 133.031431118772 121.103620738719 127.905616521853
      122.517559025268 121.553107861517 106.182551369456 119.746227888415
      124.705614182596 111.596690019694 143.47200560038  122.042136913991
CD163 180.902950045351 174.313718712035 168.314966639178 172.310673499572
      198.840533461525 301.087947101876 167.474790447134 365.362177671146
      401.750302347978 214.434355263652 306.632103708321 167.817232948801
CD163L1     146.828099826146 140.936204899093 128.287127680554
      157.248117633013 170.516743115762 154.831307586538 143.953043041292
      193.008902902654 167.620915011868 168.384397488011 177.103802332144
      182.222567053422
CD164 375.651196211048 311.157764305033 325.320888625297 256.590289281076
      311.375563161772 433.314881387431 471.720496000839 298.347201056937
      271.004741629789 341.714401872192 772.776364492813 434.716530273383
CD164L2     160.315029612814 161.530878860883 157.83121828793
      157.067264221207 176.27969404442  141.548272427617 196.889520085412
      167.416417895159 146.318052064937 170.212679845194 126.100454068838
      153.088710298368
CD177 125.058165131184 116.806720503431 126.428284589412 119.346574301289
      118.117957479382 130.653971083035 126.766964875664 121.743701509981
      127.26820735867  136.630678899513 122.793816135585 129.322481607056
CD180 162.592306630033 162.022232949254 163.02140155858  175.562070915794
      152.341361646984 160.773510498005 150.235026770931 164.227134687088
      150.091854035356 166.014450044695 156.920867019849 159.49930229951
CD19  119.498312996987 129.297217956843 117.724353829377 130.156775178934
      126.185560760543 119.131613164405 141.874188629438 161.877378749103
      354.579728909336 116.507315786826 123.135918415235 113.582654469735
CD1A  131.256325361974 141.085111670206 159.686279002456 141.686945908315
      148.510023599641 179.985468369202 278.335289966805 176.506227858258
      168.818595386221 169.321832528939 163.203582580217 161.441613587571
CD1B  157.108043150259 160.395053149199 147.661748633455 163.340435089911
      150.866204945762 159.848342125486 160.975741751317 187.389039221193
      172.276968413879 179.432235153344 138.244585395561 155.507030276749
CD1C  153.126111237668 153.38494705081  125.861564323476 148.641780441227
      128.599254558082 144.968459820523 141.558927907067 154.574826922632
      161.050438117555 149.520911664673 143.49969914174  145.630497368137
CD1D  127.320624058583 125.899200544217 124.452631766045 137.519771130707
      134.712040258989 131.136073942692 133.141683141696 144.159316670264
      145.090639086338 140.545351041252 133.581450824362 136.267313223808
CD1E  138.116568200293 140.014167357286 141.431172407792 134.489421865495
      138.386391726042 150.439715066903 148.797226586765 175.7330682766
      148.286700314107 149.547635666621 155.290648078877 135.409613764459
CD2   185.17273221366  197.947433984294 169.895672129542 173.714940254592
      186.640167849568 185.41739522833  256.590289281076 331.795832780176
      565.313436777992 278.005620372937 300.247304779062 204.337844243869
CD200 196.363005912532 163.234966251618 177.182321788711 160.272632590118
      134.166297738657 130.607857562345 134.544368736184 132.786500984297
      140.74035966401  143.574584852426 136.623658204558 132.622032754473
```

CD200R1 168.000247148058 151.655854407186 148.883263903527
158.429761055869 168.000247148058 145.07503221134 163.741411769369
150.601874392926 151.846610656503 174.107661206415 141.216869032251
161.886342770305
CD200R1L 138.852212944492 129.946925636595 147.057116881846
153.579955370444 131.067958183644 139.316189199729 140.881084272745
136.19725093985 139.423432468886 142.238908711402 132.081286801266
141.59046197203
CD207 146.6306281408 149.294728629784 154.499313851756 150.931114848235
188.231614694297 147.681723355535 187.425086057782 154.999192460283
147.585081901818 435.983803453746 146.362705590778 145.492751560845
CD209 164.241874094897 170.198508633417 143.262288751967 203.244917049745
178.262619516994 160.035282578603 124.88118767481 179.663258610881
195.400963839097 158.660859266099 227.350260241585 154.935741493562
CD22 149.744358230709 161.948033313443 171.795878750365 157.436513275737
162.923734210933 161.77611826709 141.597692730835 147.006482557682
166.12184890921 148.729753117861 164.281196089736 154.818414925242
CD226 113.122185375734 111.161283080299 111.253743009588 129.307025798583
120.257886394616 117.628564992187 121.505178996279 113.184495948936
123.267817390796 123.488418655586 124.275741099764 114.68345964966
CD24 508.127470400078 642.647554580301 319.812293134357 226.020769172424
8787.57716309558 7144.3257417116 6470.73189429877 4335.76831372031
6358.5018930218 5800.06300826802 5138.28186306948 2093.67403119512
CD244 135.840528665314 124.121555438466 168.289492873475 127.099995420363
132.17294083844 128.582520754296 121.215558351076 134.86940314824
138.773042747912 129.213523302268 158.809865241567 143.873048967535
CD247 201.256649338315 194.998142472864 155.842195370853 186.529656751005
365.199125315964 159.693019552703 194.063827325017 327.324711245289
546.399972543938 167.273378118452 269.100400678077 177.524818593325
CD248 2311.05587511258 1801.74467005923 769.958422740085 2444.835635949
305.93974940209 215.365809536522 157.499803444188 195.185025611945
428.569462312363 592.822841077599 185.560737176115 227.884445686928
CD27 142.960875909369 142.914863449833 129.658488766089 139.318078285343
160.010501226517 125.887854850942 133.507865919719 202.278323489515
347.411500022053 126.702705908175 158.650226303456 132.074571281938
CD274 119.000436602325 103.594872791657 107.02909139647 105.179281694336
120.853961156534 122.083357581665 115.649568330705 110.753661013519
115.672307128514 118.470400686096 136.472404812817 121.320750074882
CD276 277.101000223971 270.973600107882 239.24587884234 366.564822549834
434.073729807058 299.851173200352 266.347965690851 233.029655807246
232.465880700217 242.971958493453 287.195290301363 250.037172478288
CD28 142.988500173397 150.484823814674 149.927773276383 139.367493349339
134.06996703302 152.077204721895 156.390982134534 140.275942067561
186.854553034865 148.02460124587 152.021576565132 145.852430082228
CD2AP 172.8993416872 189.662974464847 164.548505132213 141.950778601653
315.513423195489 682.538709448692 763.533962249076 590.849222581206
589.541347392215 328.60529053804 618.140160251927 802.304294140514
CD2BP2 1332.11815966955 1420.27222765071 1405.2134783352
1312.75601392984 1445.72064343302 1581.06548216423 1912.2369374258
1168.3174645384 1355.66237447026 1323.91781040143 1367.19652160532
1830.56003081769
CD300A 137.744318243173 121.657744897668 109.087574814292
131.159946917777 123.554769032715 132.196506161702 132.783958633333

150.297422529071 152.834894011142 141.505120938773 150.102853150151
123.347836436966
CD300C 144.007071548045 142.584504912189 133.336063137966
163.504122327429 150.911524755251 153.713298074917 142.461941376108
145.459919416288 156.187809481625 149.712320986646 147.422656909557
139.577443171489
CD300E 180.549865708041 143.937726155116 149.149858208397
152.182968451691 121.455843337023 134.919972250554 126.413124995943
124.818436949131 142.079184950618 132.125545886181 119.466200987489
125.533538277681
CD300LB 145.487783396345 154.011708696954 130.820415610578
125.79193748535 130.191876914166 130.068063785009 136.195555442748
128.848008246639 129.856994052591 123.809866652834 128.329985147034
137.071402810983
CD300LD 140.199334790854 135.409613764459 126.652197216347
135.840528665314 141.141047405344 139.875260384886 137.890006487904
135.961724275921 145.958718601763 143.925616978401 139.219476681919
158.645402461996
CD300LF 114.042914237667 124.207200622684 113.043089777907
119.920097823022 135.950465168457 114.749597426757 130.520275539672
125.661697165943 134.084602409011 152.54039461337 135.767818903328
129.496473527496
CD300LG 160.724319010751 168.244523461915 148.614214326995
178.636555178682 141.297629157305 151.09974848374 151.142149775806
135.965779958403 142.07664288788 132.311344653106 140.779962389412
143.942810095867
CD302 244.897869154624 219.597808863947 233.786136606682 179.177884962513
138.021559586328 161.548130965486 150.989202517615 172.46311371589
156.146154578237 157.547814187442 144.448503894295 153.07993334035
CD320 254.812605074045 274.120234926832 218.896760804208 279.452819132461
1142.44180956604 520.218545108099 342.096409626768 285.214585324341
208.555506929113 216.991823988763 165.919497521143 197.049616858296
CD33 152.459041674768 139.334552725255 135.878996521596 139.3465236423
120.679474100322 122.885879294532 133.367085445965 142.963036923104
144.924212639731 133.489068665353 131.481679396553 128.473834314632
CD34 948.734577648136 866.765054377214 735.165830487162 1030.56836625083
201.004491067972 249.400820221233 201.593025256991 216.645552082667
257.813193543819 229.74418058711 179.35113214277 183.115511373872
CD36 185.519000961898 231.47915227815 156.934589811742 284.13865992802
130.231160879338 174.461487057542 161.534421340752 198.244854812728
141.092486112278 276.925017614147 136.184427467391 116.14662237541
CD37 150.843975804117 139.931092990984 141.308299074832 137.052507691254
165.169920299969 137.398748156909 147.768191157163 171.051948060796
316.663128947813 161.655336107432 158.751241730465 139.128751917566
CD38 168.437049123012 162.71020036416 156.144934427609 144.985280401441
141.457040042276 142.406904585501 157.28429690041 187.016569445646
230.855806094267 148.73620012231 159.966729941991 144.636277619886
CD3D 173.169905329013 169.681582570965 142.071492722749 133.932594124721
161.031148038478 171.130215858803 200.618080312379 339.843776738468
572.548647437438 225.275049011909 299.935166870211 173.766372029062
CD3E 152.14012587515 141.225533609907 135.589211439847 142.101865506094
142.625414022199 134.831550401848 138.497931380734 154.094285855743
187.93380542729 138.161690144518 179.61374889626 144.845726048937

CD3EAP 129.246066890078 141.904041228678 127.509583634884
140.692586715322 171.212749388553 145.646191500321 142.747793303346
127.349190705235 147.178598908199 147.942495862714 154.096542063701
141.746032519746
CD3G 134.330234316896 141.482344048665 144.546597366629 147.202510900548
148.840533233712 136.532168520039 154.620194499553 151.083551412109
178.476483428474 140.216148769999 160.525939340745 142.217762896203
CD4 126.910075365358 142.354731066206 123.379461430902 131.141668650877
135.753430034183 121.492896642899 136.285410546751 144.946362772133
165.009696356892 128.69455713173 163.868129628544 137.138266859156
CD40 196.839016616283 174.603285240228 169.733427590141 163.558869203898
227.418630852215 166.305219348008 393.013539328766 182.181756507598
206.303338709554 155.243643210534 147.642693777383 217.684410907748
CD40LG 146.693573104487 147.096559685764 125.624661345169
123.625734357908 145.015434711984 130.776213987522 131.202090507576
133.702845956639 142.968008233583 121.201267751612 136.357788670568
135.842090777339
CD44 1994.00516809361 1561.10217536347 2055.27692947656 1144.07896461196
302.532985701607 701.481003619067 551.350758868377 485.731287873723
993.774175699873 1359.63626195826 699.550347374567 784.170983714588
CD46 437.344765185802 400.621427274425 527.597948979568 264.936739264566
624.106546150735 1803.06322760249 1796.26091584668 742.751664201272
1047.78978081493 712.067161025165 1650.77281891596 2116.39371296122
CD47 569.967953318205 564.588706307423 551.350758868377 496.03509813734
191.525291860022 554.800688991977 408.263886786123 270.021093158885
347.081036934975 682.075583857856 354.205670284123 319.090371885653
CD48 202.129153259445 147.720061897962 123.132178371802 119.612288460361
125.689884251847 165.003646188775 155.121309543738 286.774606295106
508.127470400078 136.269445455239 206.113290588613 125.234097725749
CD5 111.515588041012 108.557097186828 99.9173294438729 104.36042819601
111.867464669155 117.760045671142 116.678969343492 117.760045671142
131.185836673529 102.786517901308 142.03837243998 107.76497424356
CD52 237.71636970719 226.678503956411 239.762135294506 226.701637237203
194.529045934216 284.798325549665 269.673122990604 551.086785904249
1170.07851727046 291.261765585473 305.343879847672 293.482512212153
CD53 157.519633258844 157.435477846812 140.554684822646 141.40690581885
145.267225993057 167.451782810394 148.874533472738 201.517356610591
254.607324522225 180.54209603289 179.389797867379 152.648490300617
CD55 196.096936985479 218.862705238592 278.274343962656 160.243405030742
361.429760858441 208.611557959861 323.20628717764 221.18757533703
239.388546850124 305.096320805078 687.494132125365 175.417320301614
CD58 201.569913451219 182.181756507598 206.071709262227 162.15259972225
256.873090834376 379.200576951933 335.142319387164 294.17588071297
265.829122816166 461.722481634933 343.200277800027 386.817736004263
CD59 260.443814523694 271.468939477052 244.440857688518 227.267783818298
174.375388013694 225.480366837274 234.041863839123 232.892635502424
217.491746961471 347.081036934975 361.620362526358 255.225145197422
CD5L 113.644993086829 118.650091706226 150.173766182317 140.288612271406
156.001983149983 132.84666796678 140.212048180435 132.478801067837
139.711696905287 120.530092530622 132.419898122309 136.425263391752
CD6 151.831627236638 149.661929885679 120.329105003959 117.619337212037
188.242091666446 129.058085094891 136.524640445539 196.839016616283
330.080428155378 130.022574966461 284.638865013916 134.226711030456

CD63	4457.64479507833	3757.39261544326	3693.4058156307	4457.64479507833
	4465.66734676111	3149.26998719015	3455.20639616159	3237.94149351627
	3905.35300751401	2172.89794460273	4377.59943975549	3570.21370790206
CD68	1175.27165286568	1088.25132365974	897.0125616892	1109.08288440559
	537.828979447378	1196.34528225417	1809.58702081877	1099.37392456694
	1277.0921553877	2217.82628612929	1018.80178630497	783.373390305885
CD69	182.439945229388	196.014841240142	151.374665021513	166.341564850299
	181.003812824014	225.99848688881	146.606442726047	187.978627462391
	219.288597751645	197.427788552936	156.453108485249	143.207187847583
CD7	156.698856643638	160.042251279235	136.722636695506	146.204122329465
	197.651165059354	162.203570429542	152.503159091556	206.669783013221
	379.476768908492	170.18781660707	164.253219197128	157.546123603057
CD70	152.146033353926	144.311620064675	152.796423361971	153.214696479001
	139.943915452851	154.64077390011	147.52178512946	148.504996616452
	155.752117896424	144.710613442383	163.396651801661	145.333097940759
CD72	135.493053871272	124.368438691944	131.449370465834	125.463718280745
	150.311274161073	127.712004247155	125.26492723752	146.345451252228
	183.482640529727	125.299513989334	165.350777445902	118.047978285706
CD74	1011.9675459648	1324.56751399862	436.834825721843	972.902937649492
	1189.69730508119	541.351092873522	848.620986404914	1973.20689165831
	3765.25491426198	721.11578916385	1373.08023518911	942.900963522948
CD79A	140.603933807629	143.222342904886	133.080437161283	139.39112068213
	149.508432746895	147.49720952988	143.442361602626	210.103974340881
	354.263934274937	150.297422529071	153.792925101459	142.07664288788
CD79B	196.759802659759	214.241635800083	177.919885012006	215.897620927751
	181.308852106289	227.236255752375	176.874265462986	201.337186768645
	440.916522871266	164.525784478026	193.052112923573	194.445463745418
CD80	143.385915173392	150.72835989269	146.032456391441	142.427198128376
	166.232856234879	153.633095853017	147.870549240703	150.376234759097
	169.192327500032	174.059172465573	147.401387099359	182.77693508164
CD81	17917.7303120499	18338.3001930708	19087.9532735841	21110.9013462062
	23053.5660790052	10048.4260790604	9715.20127757161	12109.3049135906
	15990.893941344	8368.88320476021	12690.801964606	9514.74328718935
CD82	223.722742062251	204.990512488181	235.563592256037	232.667968049771
	340.903961204211	214.132445321197	353.285905782951	225.99848688881
	268.52833723726	460.651156484562	306.068269136821	215.585089158905
CD83	224.186573847185	234.301520483864	189.708396835236	210.852300463808
	201.105520537151	248.729653566598	240.005943851098	232.083160165968
	301.695991929957	223.601312199935	204.057019122032	286.818166898922
CD84	157.21132653949	155.417694869593	162.127697884504	184.25384366282
	133.482237631013	146.57274511202	158.064409011013	173.408784502027
	172.030687707578	152.272198017355	149.364498870469	147.694098231655
CD86	141.925012927873	142.759452994928	140.096717188376	142.156488113764
	165.39758732	177.666582096539	165.613274361543	184.954673605577
	208.914498369991	209.887047474042	171.722839172783	153.653453808799
CD8A	172.909795209733	165.82215325714	144.621077658674	134.41693612969
	134.061318824472	147.096559685764	138.30402764483	200.890547965585
	255.74096237558	171.751149502172	149.730467498266	145.539731152955
CD8B	129.910866236901	124.025104622631	133.14988092185	129.023206862713
	133.022848457361	122.985117824212	124.66386961898	128.972276851419
	132.66318177913	126.37572518012	130.352674121108	131.572485018063
CD9	762.8576747113	692.095005197252	951.772948602094	526.625936680738
	1329.3013039618	2153.17165155392	1794.82283439196	3474.0924656878
	2639.60135568	1337.38160266788	2124.12760841901	2026.85580212422

CD93 560.180656219785 779.340880980422 302.113807121396 551.350758868377
288.68376542946 771.059329972125 206.376365685112 613.279465441319
774.774214696342 451.210172051464 517.881547706391 381.971539806271
CD96 175.148318011395 183.26554404668 142.61573330083 134.759283147919
184.029968090084 160.090914414578 238.823244541016 292.746711619565
413.757630469808 177.696849418064 482.592481827722 453.058639550475
CD97 832.335328912437 889.054356462993 930.704688414069 949.260897408911
352.562711030848 410.337491313251 305.499194075747 383.033242143031
396.781827940009 282.392419584508 217.491746961471 394.892918166519
CD99 1856.05025952611 1491.05501052706 1582.0221231161 1231.92253664482
1775.32632165475 756.068695265118 673.824770556885 328.078808625906
834.187014201026 650.911886508922 372.689712716797 676.570119607984
CD99L2 1584.81089248914 1850.98311401165 1558.22238167317
2154.00589005104 402.425130776131 306.632103708321 394.576038252334
398.423396126075 388.966303073519 388.638095539186 277.406445570925
304.237479317271
CDA 149.294728629784 152.601626526117 146.503431281242 182.810581377888
160.441879289883 183.963578322984 161.803438841859 157.878110410517
158.104336050835 198.15257384667 158.735283017367 143.484004107893
CDADC1 159.276726310525 160.399059317919 184.682478530334
171.465178761171 117.003205777245 144.818492604488 144.188147197178
168.347445148881 149.927773276383 147.471494294543 136.44218532882
134.455623159793
CDAN1 5912.48904507462 5688.84918819457 5479.24427823207 5373.2173152906
1569.13457493554 5601.24254062491 3536.14527317085 4602.81651147558
4150.8511719558 3173.14004739767 4061.24537355697 3784.59114921916
CDC123 324.401611136662 305.398934391579 322.36247485445
359.493075382333 569.656573915709 1508.24281455057 471.946284490029
414.200068430184 511.779988437654 451.147418314412 747.077451281453
681.677841333095
CDC14A 138.733717670748 152.748807709142 141.466287523861
134.072912593456 147.630900093277 149.233023751582 142.974541687789
156.642441706727 171.982071800592 135.800997202323 145.387562372871
138.325950428197
CDC14B 243.225815954365 222.415704486709 283.048002376743
241.168936008174 202.741211613885 201.913709602894 239.452559981542
225.238369884803 202.145180994821 191.897449038297 316.409887372431
211.160781061336
CDC14C 155.777281781161 148.967232828819 172.214433016685
149.563405421366 141.557420268382 139.706279455371 137.702802090925
166.12184890921 159.970332650101 160.482734190577 165.673535349675
151.433634564939
CDC16 1505.09908072606 1421.72388964898 1665.14066952917 1408.08614963928
691.916554922896 1559.26487922551 1242.61637859159 949.789710063337
1313.89440013246 1411.90426595229 1611.58245311122 2137.44704613378
CDC2 126.908123260394 148.022287939066 136.08040874432 135.923022674367
423.740836541835 453.926991868451 282.101714660307 421.303580698397
408.361368902627 264.526189799049 206.098534554876 537.982214580518
CDC20 131.566892082703 259.826625866106 167.121623035375 219.080383154432
4621.99241522677 1889.05071563459 750.494286476465 2819.92361643968
1577.15895478896 1931.16906839143 337.87475687838 1206.09815575399
CDC20B 142.656762407774 150.292858985795 136.522590104535
146.181764812924 142.661504601445 139.186416741431 149.113794439994

141.995158253845 142.036037957345 137.832597613423 131.009425832936
139.652974659584
CDC23 572.262848718437 570.533103778482 566.914448800423 576.873245313776
392.746574862867 445.661863626419 518.925188630246 624.638115878703
465.29290512169 757.406530000489 657.77730563722 427.98782239715
CDC25A 127.132970760907 135.556943919872 133.561188057423
137.652358744527 284.595024875896 218.917357155289 144.283716416188
170.561596645385 210.925203743958 159.177303894223 141.815571093975
208.895657507883
CDC25B 389.260166168847 358.092809491843 307.597733293218
354.45785837703 547.042461070351 326.354469134694 179.297628357211
217.45700166961 307.363302726074 837.413261433363 207.123420880185
214.4829383264
CDC25C 134.966883985611 137.244552699212 134.479828866339
126.523201566273 189.01309885917 218.134101819445 163.843143947012
197.859546367676 176.791758333054 178.416969664816 164.509354377985
176.839917795004
CDC26 1388.91423772335 1218.05434595121 1209.56018864779 1084.95095717937
1106.75785702993 814.759191652981 945.830852603704 951.772948602094
1201.82026045778 883.140984728615 1538.78070281348 831.610501402173
CDC27 110.794219964997 123.76900858347 121.764933633063 123.294321716108
113.913337223716 136.201103852785 127.699226067847 111.860763079995
118.871735074285 115.545558600232 146.349773982488 119.735807506447
CDC2L1 183.50553222647 175.044940252247 229.542259738438
210.692705153337 189.0608798771 230.620456579897 199.353823724989
180.968659614238 196.076386020815 187.472028627511 180.060096787617
209.702683803624
CDC2L2 522.17701271254 493.715108157171 445.214621023114
545.760623858473 249.129943906228 304.058158731957 271.897511879509
268.111403429364 289.001004912528 212.822328466993 184.267958772058
282.742833166843
CDC2L5 370.555627307623 351.395760651605 347.932135290714
353.912654262209 336.981561928411 298.979106293393 377.225452025984
322.506898833351 342.611951034707 302.219514290905 374.169744342352
412.582051585078
CDC2L6 680.94167651697 765.911113050543 767.377380800066
818.761934233826 668.127527385348 520.470790410856 694.644744547412
573.074116810032 544.852019338212 561.858563298512 1106.21439150501
500.848273432862
CDC34 468.496972957503 523.717949503639 413.863345223345 839.324324662619
894.100792722707 522.050619657197 406.638419179213 373.803772983872
323.391456630794 369.838429521168 493.209107049493 370.945254716657
CDC37 4956.21683075884 4956.21683075884 5624.32062666666 5489.19362780613
6776.11712291246 5122.60899285635 4159.96851384358 5955.10816224456
6427.33783376005 5789.71932579854 5094.53287522452 7245.25086222102
CDC37L1 129.431497372577 125.386545354096 122.072091994548
126.180714796187 127.174074003712 133.604705630582 124.815853677553
121.267153348313 125.455347031878 130.467450968785 150.507308901391
117.580122770251
CDC40 420.361583975644 411.221560795912 477.275332763887 375.856200488667
322.684910198242 547.817028243811 634.556887178869 504.838017072725
485.026081242201 469.206346008875 123.25201993575 662.958637409597

CDC42 216.32785564297 261.96528977292 235.372592051464 228.797912452429
463.754869412768 364.527677995774 394.414934831535 349.054741747798
312.177191402945 360.852730026133 702.981741565482 329.535023880129
CDC42BPA 387.355661073935 369.513420606401 406.54518900348
490.022369664024 176.008711524598 174.229093061024 160.53152291979
168.418529261977 175.417320301614 177.371157649894 203.888365494623
204.564770370164
CDC42BPB 469.930860750773 536.253405705975 359.099750692694
683.019502027271 419.126814511305 204.237952297239 246.369521698487
241.383657414248 229.076507182239 333.439929772897 338.260976633501
232.276667029184
CDC42BPG 125.318166919844 122.883806494491 131.825172822888
135.928428484011 169.475714234887 151.349739492368 135.137615432923
131.595868226775 133.640832782576 127.449028221682 144.007071548045
127.545397354821
CDC42EP1 172.622816559412 153.62335478774 145.513333676569
161.683243808528 255.56301534833 171.216275057884 155.295464263911
142.228042097379 162.629257046871 191.679217737051 172.214433016685
170.553940654493
CDC42EP2 342.403428822464 325.570839576032 229.62164568989
369.018856220394 171.247946863662 163.758054869675 171.212749388553
163.69172577831 254.065327837176 189.74261433647 211.135364208221
167.130843160309
CDC42EP3 149.791777262424 164.58158284856 235.350819745387
146.96155692518 120.748910864064 145.107068919893 149.693559841189
155.762890407496 130.172041917023 130.201635099678 143.962842383778
129.171589574238
CDC42EP4 1551.90775589919 1932.39418448203 1336.15622439522
1600.10235468018 2033.67956299225 2065.51724858579 2365.99940149374
1762.09874961415 2782.94647782764 1378.40627876546 1439.37593006882
3115.11499025766
CDC42EP5 765.911113050543 894.100792722707 719.628501451187
565.843200661721 1927.467713716 865.800709006332 4866.98027226798
385.798784913201 426.464641742092 339.598452080917 6571.35528952186
2166.68174726155
CDC42SE1 191.725728813959 183.556662533396 165.355764467096
142.830950036054 284.351529744475 427.542390397986 284.764628939128
279.495192828873 282.335582153109 236.401834189942 401.461825804111
418.889972660958
CDC42SE2 278.142283306934 246.676401619881 240.971675534641
206.236303011662 183.130891975122 281.570685561526 483.429488817674
329.023836559229 328.14373061894 354.783364481955 462.144334719146
360.605223912114
CDC45L 113.356070669308 134.203949774763 113.184495948936
120.638330368388 606.679284487803 677.023591987179 249.029098902357
574.399909181723 746.296354515617 425.348619281876 206.257167235126
489.260272645085
CDC5L 367.289651112346 278.469340236003 331.62124160152 270.201322204641
247.504198063293 313.536451935268 408.7151485563 330.590986934704
310.598930734244 392.004079531068 378.088675670786 330.241479004885
CDC6 150.333572271902 157.979749139651 136.300195727908 149.247514768446
156.392603045231 153.02127620577 141.194900058799 164.981665344298
157.148880624381 140.692586715322 147.060637536615 148.060086496148

CDC7 166.97270256697 177.065616871886 154.354658146438 151.0189104086
305.906480933354 315.430399077266 196.076386020815 357.431640917696
388.326080754063 244.73233791067 190.152824885328 279.704940529618
CDC73 128.05898686833 148.450023054885 139.902610126952 123.981053866137
128.626051185037 130.742940708861 126.355699811531 127.159947766922
136.561852783331 135.317796804151 154.657316209848 121.531401500498
CDCA1 100.477911152746 109.932577444858 123.582509531804 108.861243071977
118.07991087231 257.622317771472 120.041623078295 161.017267304159
142.853399942089 123.582509531804 121.653754668804 175.073595611435
CDCA2 114.790717658188 124.317870455205 112.133567992833 111.137519376499
187.673748059596 147.970243979273 133.079097536801 152.11446058397
168.963976596839 174.168079247768 149.117224130576 155.988447182637
CDCA3 118.480344364831 137.765042156434 114.093935026467 132.697727983601
542.009873308344 208.793650722271 175.222531098958 259.146673652222
292.802762636861 184.029968090084 145.762724663455 202.458096827378
CDCA4 168.156002330441 178.209994522672 168.310680227156 187.183762762259
337.973433871323 212.037224737798 229.443064589306 244.011434605133
231.890885094917 370.227329649269 226.531255313921 334.316711893654
CDCA5 153.494456800685 222.675370226786 133.348510114432 203.223604138192
1249.89496565284 995.263014856158 484.363052437459 868.40984928812
635.37511798786 563.779294315901 267.747633122387 594.180861801958
CDCA7 162.096065738375 183.201926323188 170.866433766203 195.724873069362
326.758278851698 421.056268094661 242.787656836745 327.062810389635
458.705688226305 591.763423878737 247.622351589081 406.133430120039
CDCA7L 151.72544764489 175.711836112986 182.725987056391
178.684992551017 263.250833727291 158.36796888959 153.774548290902
174.820064644203 182.77693508164 180.913502162011 149.842326869644
192.032080013216
CDCA8 138.081268025435 171.39347085745 144.189175238177 154.248226423862
541.351092873522 392.909481887174 227.715558644326 397.153801851621
277.75958501062 242.382534384155 171.130215858803 278.192669077191
CDCP1 156.239937979051 164.231281729415 159.544710238172 157.142101889337
234.370850809531 218.209523110843 242.188218870393 190.843795579234
216.522992914538 345.135289739599 179.460777888284 229.157389498775
CDCP2 144.727773039491 165.803549113373 159.23414970379 148.927040248436
156.942156078314 148.291518446423 152.344248425416 151.421099926743
142.999526950651 162.678619800767 157.341422939225 156.942156078314
CDGAP 139.925201225224 148.702473828167 134.403789206552 134.698857213208
122.793816135585 126.362418476129 140.191264837331 125.789509802098
121.838802846453 121.271403807802 116.597352307016 120.590654346066
CDH1 186.496160803789 478.180723959397 320.648966489586 146.256076936456
4847.77258830631 2698.5455305009 4198.61471038267 4866.98027226798
4857.3155917904 2586.87941889443 2325.02143920169 4612.81760689869
CDH10 152.309460630038 133.296200563657 157.390513637267 151.137791721752
149.213990424575 147.684875777977 151.345630073141 152.942824158634
144.011792962785 134.441142158135 122.578170988039 140.812334019209
CDH11 1235.02626456572 1169.43742964112 679.191060100076 1118.28423222707
170.720018572735 260.715580924916 156.437949042181 316.081825144651
392.004079531068 514.002313819685 162.427505889157 215.98963200839
CDH12 152.78638215326 141.255406288178 149.925051977052 139.557059245431
139.938739652347 155.108146847158 127.03153185998 170.725486814469
141.996233801459 147.939682463235 149.423230365631 144.456324230938
CDH12P 132.324630056168 129.561012565141 169.147351642004
139.595986615703 136.858037332222 155.086559474139 132.661036963636

	145.42392518235	137.15288362126	139.113675690483	125.666013736767
	150.191645194625			
CDH13	185.485858719975	166.12695134858	147.164348704012	141.340452225686
	111.398760606544	130.213596986351	118.039780360151	133.866011732878
	122.466276478613	235.407064742194	123.524952603672	120.304037458526
CDH15	133.891337645936	141.198692540628	132.933035878334	127.578272480621
	156.702300398105	132.249906289203	130.24527031028	127.665162524824
	133.090474303043	133.125084506877	150.566842106244	141.186913979346
CDH16	119.422996538444	119.530214079037	124.942701123337	140.05012958679
	127.507557925713	137.58025011566	134.943005383277	126.305273697185
	131.723229807439	145.522435728191	164.641508485461	129.654234837398
CDH17	164.917470819096	149.808222023726	161.04173806448	161.430219824313
	164.357373919714	155.43577761798	175.784250947874	151.362686497226
	155.899547877596	165.092451754065	127.745801270205	153.276938815536
CDH18	165.682617337317	145.711007027894	152.56002779985	148.340380102097
	150.981208776163	145.741465098455	141.822974252935	150.112549201892
	145.912712700067	150.920224503051	138.275371291639	148.82034800623
CDH19	194.60309399944	172.973175988757	197.405472374722	176.245698601389
	135.485867373592	149.153827025981	139.679681813932	142.49005761699
	155.820194280153	138.466136108202	137.973211667003	147.838660813476
CDH2	332.666916002481	282.742833166843	704.653073777277	203.317264638828
	127.354826381656	139.380610974147	134.877046981678	124.503016568677
	139.358218798638	180.80811429888	156.043994260248	155.542691937539
CDH20	147.169765031208	155.04585737344	166.1677526	164.1572198075
	147.292241873554	140.460927375243	156.262515148436	130.201635099678
	149.110300099889	171.449326065013	163.380402251758	154.177340021162
CDH22	122.051473363557	120.126700838056	122.323876787207	120.300291760091
	120.170269310738	131.241878941749	120.835207882788	126.056493244568
	123.072413274454	122.860898886143	141.486042999328	124.080382291282
CDH23	147.216303852902	150.134337895481	133.419239158866	144.746134643761
	183.815810733176	162.233096422572	187.586093248437	145.889336010424
	166.735962853075	129.828896822571	332.296938254329	194.637233028442
CDH24	133.757741295765	133.095245865663	129.408282688425	128.626051185037
	140.074841537258	133.877857711552	139.981905225845	137.198654302648
	135.63604256516	129.549673572516	141.043668963717	134.57472034679
CDH26	146.457939801041	161.072844903925	145.635453468646	146.526744516344
	356.05426191171	351.02823853348	612.61438059983	407.442337662943
	196.676479140094	138.56812029378	311.04252785157	192.130214856642
CDH29	123.173506315166	134.04235176608	136.596127228365	121.977373818244
	119.735807506447	133.238296113721	136.659134200122	128.113966507586
	132.297716337104	143.647714966406	136.205702793983	116.118745494794
CDH3	148.16526158216	175.199369463703	131.907246423067	133.365793958118
	705.987531074737	235.183590602209	403.582461109859	201.850932289186
	177.955579729463	944.47112348734	203.475080163804	421.929674562786
CDH4	135.455283504668	132.647152200128	123.916497557628	116.331428876124
	131.873313581009	138.880168623766	115.002176041974	128.591292689996
	115.309408464043	123.56917054142	115.51493368527	129.935532080325
CDH5	271.238736772408	373.656487297238	182.736067431579	473.057488824363
	176.883702717029	210.41046499545	134.751916364581	206.275885047321
	225.99848688881	179.711363255355	193.428733288774	150.851504454247
CDH6	182.633773540249	175.372355078713	177.267145265362	165.262738484387
	158.536178747273	163.495175710216	139.573371281569	165.500088159872
	151.971337856271	164.816672737691	143.349486755648	147.457193565877

CDH7 120.894583222813 130.454496708632 125.371128588617 127.484178603011
122.027632589515 124.574323079707 125.519298131694 122.751674866182
126.856038484436 118.589392612801 136.762273376313 123.375592272781
CDH8 126.806537241253 137.326796995017 138.902097203056 143.793506250131
137.734887788912 132.5377752987 148.288798428149 164.098880989592
130.135721123253 138.316043447788 154.746081871718 133.78629612147
CDH9 145.887262601586 125.416113646128 149.99203906203 162.096065738375
151.497680466119 173.306283668215 144.159316670264 149.712320986646
127.398873370484 157.546123603057 139.131857948357 131.146604635155
CDIPT 303.300028849873 315.47473326786 337.271784551586 309.937351755502
476.495282784602 381.182514249587 330.080428155378 289.911635155481
288.261250589513 213.754156680604 256.093363520707 261.426617866925
CDK10 255.3451781862 251.138401278632 284.748764120614 282.282663608264
316.125364442611 219.96819201774 269.194172034595 235.453139389486
320.360917595936 310.426513884979 248.594000664466 363.963951509433
CDK2 284.831817899986 272.238922498583 231.670761847089 221.45275440794
593.090436323333 681.810927901806 350.926679856514 538.156335788156
735.449040059761 377.27961559752 323.51769984082 349.722963659175
CDK2AP1 4280.66857663502 4124.03779111916 3290.27308365921
3784.59114921916 7144.3257417116 5149.78271096158 5479.24427823207
2708.20624364471 6691.04319670055 4134.02613649483 2277.60839052397
5800.06300826802
CDK2AP2 216.102897582821 246.710233781784 222.20526515049
351.998551165704 659.900771633223 358.780920910909 222.544542998356
230.672056810885 242.996248524203 184.591789011195 223.311817085871
258.998065427002
CDK3 154.483828924645 155.780657091484 134.93814894432 140.604900199281
149.691340727994 152.348210524663 144.718507303105 172.583615020042
157.021010130169 185.140211988926 164.766295057217 182.397795261147
CDK4 981.781076110568 1076.09385014053 942.41077071058 1166.86593572716
2482.27419621925 1832.35076992253 1903.13326109546 3575.60827968852
2261.31473132315 1174.0922389136 1374.92806285803 1611.58245311122
CDK5 191.128539630621 206.257167235126 192.640717683236 216.836094088131
460.514672966125 369.513420606401 327.164773632634 282.335582153109
236.985483155452 304.402066421813 256.394563537658 320.774102343889
CDK5R1 127.833653828539 150.042173991463 137.561067667054
131.200042525339 249.953902454701 171.350620456505 157.14512525321
237.78482574225 204.453822958077 357.431640917696 149.127963327999
140.890227770353
CDK5R2 120.906553066296 157.227118680396 128.533900744619
134.628807679002 150.924948809354 118.540620928268 145.485453268383
139.923187410488 130.443360119099 135.628982370682 124.310436914027
149.353072778508
CDK5RAP1 256.495848662843 259.868701878844 270.973600107882
297.419034136005 322.576886792502 359.363523948825 370.140588087649
351.665934915634 298.165097953983 278.168897504985 307.728083981504
269.770777921703
CDK5RAP2 441.684169663552 422.688531130043 386.738154577763
477.489368828457 282.836518011311 233.161848094769 197.341423434688
255.945470052489 304.402066421813 293.12968694683 289.368511254335
199.70041209487
CDK5RAP3 512.895335481876 531.711823481265 618.058496806334
503.093655556038 497.916883763059 765.911113050543 765.532997390972

591.641325402032 862.132582849511 534.608644989102 758.863418917419
777.651238374269
CDK6 490.153210371094 433.189221987242 570.26564132238 470.746477818201
139.010652831798 159.633987751501 262.913054904154 269.673122990604
907.306121257236 504.617795280805 279.541294184609 148.723165059122
CDK7 404.21236980164 431.974037914233 535.49374437073 378.4377409749
471.720496000839 565.97975389646 943.23069404564 550.434931878586
468.82816089485 1183.1601379136 738.087203930249 776.807656744828
CDK8 187.082082429597 138.265155903051 178.863722055499 142.750432197016
134.420446702459 164.87433908877 136.497295707149 139.733149319736
132.059507616101 168.674809329231 162.997720993338 188.45479937455
CDK9 479.638653311025 537.510539142248 507.040326298457 498.05778289794
536.067857235731 282.604890044452 241.599271777806 324.615093103582
312.019603761673 442.334886941795 469.107624227456 307.363302726074
CDKAL1 145.416733388652 133.891337645936 148.158062155247
131.255028381669 125.304045138994 142.294922363188 136.415734370763
134.835962366555 154.594339065082 150.059862107155 147.202510900548
162.373508183992
CDKL1 151.679534447228 140.856864690669 155.673532440475 148.120968141789
130.445920648705 134.35958389775 129.558589108727 132.005753222789
130.772410329685 126.421148348261 216.353502371227 134.376870769669
CDKL2 128.056668224393 162.689415085423 165.500088159872 190.291185322482
142.89427871125 135.508803488048 146.785478478701 151.76341973891
142.440344960504 146.164093136588 128.4702079007 151.679534447228
CDKL3 204.89842561202 190.189217962555 216.929969034881 169.619338456208
162.914301705151 171.816841286562 180.968659614238 188.027848008991
172.992936821292 161.434331154045 175.908645445479 155.777281781161
CDKL4 115.855948612544 134.178733595096 129.315412536491 124.749727939593
135.291264978088 120.355844117573 137.719257499747 131.933026690056
123.442970398288 139.271829906127 147.508817393901 125.935071837868
CDKL5 123.351112976814 110.501341211051 115.184395637553 121.231939494619
119.873844587252 117.692662275393 118.195512252508 119.75069850668
114.143874572202 119.655162437465 122.243585162452 114.539671934155
CDKN1A 879.301932542031 1456.76683429755 653.047570485109
515.597523880328 1359.63626195826 1564.8868894162 2426.2098937241
1007.41624313399 2828.97255380291 3232.96611074938 1949.60516542965
2891.03258768937
CDKN1B 514.463945278314 654.881990232975 697.774882173237
572.049540360134 485.309521583972 445.089930641391 471.598175637494
608.611927946607 477.741216640575 321.910803473854 442.12886967939
508.735412486964
CDKN1C 400.924914238052 430.100415399881 486.947380524463
400.676876982655 182.028675726969 188.941900997781 187.183762762259
181.914505612128 263.888891822857 314.751799003704 186.021181691517
181.813812621767
CDKN2A 127.6078497227 139.480361732772 129.642952083513
128.689465747173 156.66832364923 148.286700314107 128.351235762192
167.139257044999 201.269380130546 137.915773215037 163.143613079037
132.367155914392
CDKN2AIP 306.896732729554 309.997909440664 386.221302830248
313.828943866034 218.156162474715 336.338518973032 336.639473916174
292.068293167526 264.456926651554 329.660368915848 242.971958493453
308.517561840596

CDKN2AIPNL 13526.3170858629 13423.8921651885 14499.7866852901
13902.1067272762 1848.27927621606 14592.6308255978 6571.35528952186
12283.9915232122 10900.1783397368 7082.28969354712 5572.23400804004
9044.54885739666
CDKN2B 181.288519308219 177.023536553193 174.680918805036
165.39758732 167.67952650068 170.108500366942 153.992763544073
174.187510325385 285.131244588624 170.335930486215 185.886737029845
163.516075721791
CDKN2BAS 123.615802924067 120.581554457723 116.961391747202
129.362793087439 115.247223837316 110.212739445 119.242880574306
119.480037559953 140.839056652898 121.531401500498 150.911524755251
124.031916322066
CDKN2C 168.327600230929 165.808660192901 272.885527139137
150.685245572093 125.9888344762 130.242779079309 142.573662441786
136.612903661685 141.344034069373 145.08853313195 131.12953085854
181.288519308219
CDKN2D 200.424975825325 193.315380090713 199.597685421675
207.993943398697 199.597685421675 235.76573393374 173.735045816949
181.215046876308 194.488249052694 205.026176598298 170.646233722767
191.365014963729
CDKN3 135.928428484011 151.839306445341 126.37749112514 141.198692540628
440.657747083721 611.772512110915 195.303469330682 425.826693178023
269.468453936818 337.728737393381 162.100547394235 316.663128947813
CDNF 140.617663657037 152.721146962124 138.724959677368 134.91697121091
143.351341716363 157.494596757446 157.736101691222 135.379284233708
148.498352529396 142.510745106328 150.775805918537 135.934976183762
CDO1 326.246702537465 270.945325305492 397.153801851621 228.086256222312
136.735930829008 139.469148541078 130.772410329685 137.846463796994
141.855655600443 139.375386456759 151.248365605631 127.859873416515
CDON 140.77762487941 127.424848301217 128.413213851142 134.292099546804
118.918962530408 118.095936088242 121.316836274255 124.530660958725
123.709293439934 112.348094614855 126.143886553557 119.391213033544
CDR1 115.80656409185 113.708683868028 116.746018509166 118.457573670556
109.586955365611 117.392898601408 116.700397932007 115.468921844902
116.615250922316 119.603565559838 124.66386961898 110.286187184448
CDR2 2004.19532096239 927.137881369911 1279.15873231449 830.455793617181
493.715108157171 689.590642377619 813.034056288861 1095.52349352502
619.635734567779 318.73799597811 711.491528818004 478.909308632726
CDR2L 746.296354515617 711.729857540712 600.430996779799 745.482863218223
1311.34904760947 1207.11788506001 800.013123376748 1379.95968831696
2491.33490046283 483.174262289559 574.6506349984 783.991448771818
CDRT1 148.112717810117 160.892163220381 145.276745572405 145.789840511876
150.498954408708 147.870549240703 153.200153031275 175.372355078713
160.006185904123 151.930565620382 155.140371265225 144.935571704834
CDRT15 147.268006631005 127.661634554996 129.127563278016
133.743708220112 147.022594294963 142.637421204193 152.654938328424
150.35767254363 150.626826756019 145.079450753969 140.646946425036
147.230113868203
CDRT15L2 141.037217406848 117.77708734157 128.451788292588
116.90832995093 120.733009784454 139.835983835267 131.930814868433
140.152177370089 125.172189715764 141.822974252935 126.588382665802
138.836422729663
CDRT15P 109.30275288389 109.760525674874 115.3611179192
110.024815508703 111.794102015354 109.098105822556 108.768730405582

106.000943456483 111.584425166104 124.248997533959 118.45291712775
118.474291525263
CDRT4 216.209052148435 228.675099004411 231.63127072986 167.811412309356
157.86653024793 217.89028736436 202.421990203844 228.125521373477
194.500222559978 203.317264638828 303.483515328621 153.649235507812
CDS1 193.786889748038 242.695286989672 154.990319019521 132.091532692048
485.87138934376 786.62709337246 625.544719251096 751.568940762011
838.267069860172 757.877066703516 899.667912542118 479.638653311025
CDS2 361.160881334174 315.752980521266 289.368511254335 269.892633574243
307.982260548106 287.455622143233 262.488050186425 235.350819745387
231.47915227815 254.607324522225 185.220017451419 296.603054615145
CDSN 124.075077123156 115.559551514777 130.617505834747 124.837187124441
122.706176540192 122.194493542073 116.604371744082 127.792241991414
125.637138636558 163.778790431064 137.483830932387 124.933738341123
CDT1 102.851152421943 154.055550115498 122.989642938474 142.06907380533
602.480822390671 341.2887754025 184.682478530334 338.822464893411
304.152390210727 239.762135294506 169.422015793848 331.747622606378
CDV3 463.754869412768 416.546844844021 542.121375354644 404.073802241516
327.480268032814 398.673582680257 424.751154004399 473.853999833395
479.261755380872 532.950400370668 393.115606440229 437.489929507567
CDX1 119.52150378372 152.088488171996 130.368933537243 134.825699448987
147.118987674809 147.153129531937 149.88598449818 138.548983773386
140.66266106824 162.996129396867 134.241917425247 144.940119680152
CDX2 119.95666716163 120.862516074358 122.149756212115 124.02280670421
125.701083319929 207.298994298453 119.709032462308 123.857527555048
118.220555783444 112.809629077555 141.457040042276 128.725044326566
CDX4 114.902673141786 114.526374388145 107.07609243233 108.79453730066
120.272964182307 116.263405190786 120.325985681299 109.793568518462
113.919529047225 122.087107663865 107.780763250481 107.013012418356
CDY1 141.152406037213 126.310788291075 132.20764558027 135.741404460897
133.301527400458 130.982011094627 140.113334247294 128.23910347716
133.634886800037 137.848546040474 133.71965790848 137.555903296788
CDY1B 148.739888930189 147.875855077738 143.867013358737 147.73141408795
146.550421651378 147.73141408795 145.666712006532 152.514455788489
144.165277164871 147.446381217887 145.873038603155 152.990523860831
CDY2A 107.239841465534 108.061315311823 114.818898581401 114.161540771427
106.953841653951 123.141928612324 121.79022464086 116.20008794813
114.129044806825 109.760525674874 125.114303270024 117.137027732123
CDY2B 120.337241026338 116.572001618531 117.65847906412 125.093376753408
125.798453479983 120.507930710099 121.968807253639 118.737244693366
114.452922529209 114.874358180801 141.424950841838 120.050548624975
CDYL 162.488819873513 164.319378349317 160.478519202775 163.306853098724
183.61611813765 145.534520042669 173.761036260869 157.142101889337
167.365850007748 158.356964958885 218.031238893075 179.94333102877
CDYL2 170.439725847568 173.782474972742 159.973758408655 172.521737547052
191.241000498118 183.951404362826 211.269041187154 184.518666956721
167.43630698726 183.115511373872 153.315309202016 190.825209733575
CEACAM1 151.431513447721 176.208206282902 139.172437503442
142.898851434337 382.630113452429 437.931339781992 691.205738835917
285.980446338971 961.931130189779 277.219796733134 715.43295523922
521.512936372701
CEACAM16 143.16930809425 140.762415240824 133.554822920765
148.503282415207 150.880818951088 133.370488470007 142.248293068033

142.230934617475 132.941478943584 127.488853386792 132.628358688595
152.805525883571
CEACAM18 123.336074100984 127.344210958898 115.299986138153
117.773743603829 130.867819112143 124.885950473434 115.321501844663
115.687997771921 114.874358180801 119.01559016005 139.331046480528
120.384210318
CEACAM19 130.949405011154 139.130560988108 118.930792960643
129.636135486593 134.572552391307 124.408996713299 131.367075440559
132.011199659803 126.796253980623 138.438792881135 131.797936395014
126.606163970149
CEACAM20 115.25307661968 115.775525016883 110.651971902576
107.07609243233 108.247365868673 103.826308328446 109.113476990309
112.90900939264 104.714872508838 107.126004317323 119.01559016005
103.734303632543
CEACAM21 156.516250236679 170.18781660707 162.453586139402
162.769643886845 154.347332766547 155.63328143658 171.982071800592
185.140211988926 163.262893278578 146.135269553731 157.78390301218
167.330860677012
CEACAM3 114.561836290349 122.174036435094 123.893768254549
117.60787142216 116.282988576774 116.449064666763 114.302645757446
121.599883734552 123.463861015933 117.986939058467 138.054470878471
112.38543517651
CEACAM4 160.382971629272 158.020651857664 150.831554010122
165.737618983611 162.997720993338 142.299535614113 131.069515666204
136.60514429355 148.704917490429 137.493436771898 150.931114848235
149.233023751582
CEACAM5 135.521802085177 134.455623159793 152.315376022976
147.491439949088 240.283918204202 139.953572663277 143.712450441816
168.398366575579 158.390337945372 222.544542998356 172.491604217899
134.060214479265
CEACAM6 156.576585764311 314.4408959517 133.717451195844
147.77916610545 922.50743596248 143.407371444437 268.930428617695
549.047065540118 156.114825514646 985.637230639964 1090.20254511452
155.383166865192
CEACAM7 113.620070198869 127.712004247155 118.650091706226
122.10489926804 137.176144667253 128.205022859019 104.861024325857
114.598636725763 122.84603217721 111.788481822686 230.079664410595
114.902673141786
CEACAM8 118.694037994926 125.950365319029 133.103403333542
125.025064237439 127.669334230458 114.452922529209 119.334967856721
125.765399556488 128.785472881136 121.003207135942 125.865974180252
132.446406243531
CEBPA 317.273940108333 424.912426797488 303.005912224195 304.937584086529
9596.10781313429 3592.7878705048 2605.13680290987 2249.8123426309
4386.83308119416 455.930204471585 1464.04124596264 802.017244099902
CEBPB 3294.92439159154 4979.31150268741 2124.12760841901 2031.01186679713
5521.74673982411 3712.67025366957 3734.705550862 1639.76556136383
6156.29203791697 4628.04544865227 3253.76017336684 2399.88695085752
CEBPD 5240.40795364202 5965.21815409254 1873.84654908146 1436.57771711084
3989.14883668905 1896.69856822105 1573.03248861784 1362.75971646355
2406.67187918735 3693.4058156307 1871.91894530722 1896.69856822105
CEBPE 105.096927149239 115.907902010701 120.543881800775 112.248047602652
110.304102941202 112.005939063045 113.268379270385 117.231243105897
122.144209450757 128.591292689996 120.538535752185 112.081127853476

CEBPG 866.147379099606 685.848203651889 562.543347322063 539.27766384282
688.915534194306 1175.27165286568 792.634423781309 948.734577648136
964.473774310687 675.596864001032 745.153336165802 624.264994653057
CEBPZ 1818.92700816112 1875.21979956596 1788.35884039549 1634.20135156938
1222.014210447 1510.77099418802 1792.35343044443 2131.68478964427
1357.48791782399 1477.10828981572 1865.44709751822 2295.16438420175
CECR1 244.011434605133 220.288758600588 166.595529296463 147.138814313885
1047.78978081493 159.282348516052 196.136080427522 440.495339027491
293.080122239022 138.903450375532 528.650794915616 1118.86225415732
CECR2 143.818379692331 128.986795394842 127.50624009248 151.61049450647
130.408519735001 153.415901281564 130.998637545686 129.845344493418
127.065518421996 122.262664384198 137.086936444421 140.597877909094
CECR4 141.990639998829 159.990561741675 172.916234073981 158.282899758551
155.734509841719 226.623172906957 150.570540340126 151.96843908074
154.248226423862 138.308965237491 159.438084699061 155.612543356905
CECR5 374.872358131797 358.217145707718 410.42088953434 396.781827940009
1153.5175534971 704.448123154868 580.164288826741 642.484527246259
464.436380568216 510.061974830525 450.220658078322 546.399972543938
CECR6 145.112399373761 131.335344547051 124.395047385025 131.773304233929
142.201672822989 126.205931638442 122.587545171646 140.812334019209
129.201101445773 128.40650280096 147.297163685914 140.842305941666
CECR7 630.473042320288 740.800822095116 668.127527385348 1182.50261323518
249.171307797736 324.949988230276 271.397087928135 272.41397350779
260.045018348182 209.196695368783 270.360397503276 293.794745907663
CEL 143.765257998859 198.979744266277 178.636555178682 152.72717701452
325.999687273862 172.276968413879 268.600782558985 272.666435295288
211.869300324471 182.084333814175 206.446584387595 171.11282933168
CELA2B 123.004137408444 123.427366316096 115.224011128183
122.03508430361 121.760488406107 127.202039384041 127.086470811408
119.150626141327 125.290320836648 121.886886059038 130.112882549637
125.761205967344
CELA3B 147.52178512946 136.27621600731 151.507376053438
160.261708910517 152.47429737104 147.854171854456 159.327764602722
148.764956861822 147.4182675362 152.993555372242 152.935963398921
150.580133889656
CELP 151.992112109698 132.973551954067 127.404415477384 148.975112606364
130.798254041597 134.336279655683 147.87699690647 154.475735552851
135.52861375119 129.914077170794 123.122705969332 138.544053540799
CELSR1 135.56878810365 139.400521576978 140.248610391566
142.12808103815 154.079355899056 145.873819094778 155.962564511907
130.257242615762 134.522715664256 139.615706918035 127.261349543394
133.12904935533
CELSR2 189.647009287524 208.214764518526 237.906142045719
280.595515386655 355.990908506973 200.468804559127 261.541138637729
210.5689532432 198.285073991956 398.272713014637 182.698275130489
335.623336587396
CELSR3 146.709967739596 179.525651423361 125.506466437764
137.199718376452 882.037055216609 517.23393716516 653.749043828462
147.70836747108 369.198837083585 204.787016443123 767.662367310672
211.995890899864
CEMP1 120.606354515072 133.599098708818 124.193603137858 124.095340478326
144.077080621909 117.343165720552 121.665157726433 120.07404952486
116.977202125093 121.526724715417 165.947514372877 123.02167644694

CEND1 168.052288484172 157.948814637726 177.802767772786 182.665357598121
183.081450609516 176.347535860374 151.010493695217 173.964378226631
174.635853531194 162.61434899634 148.953720017484 157.519633258844
CENPA 117.078720392441 121.497645719165 113.627435441258 124.374156313613
348.241471067257 180.20999937977 155.558839375761 255.59042517603
211.505504834207 220.379186481273 144.660095230819 201.969002368045
CENPB 2227.70654174379 2274.75133434566 2355.29862638453 2792.13364570755
4663.03253705034 1995.6000523628 1327.65573168233 1033.03719406038
1430.20038077595 967.123240442957 1726.15788525017 1759.9632914516
CENPBD1 182.810581377888 203.816305810343 180.478294414245
200.342786450248 206.669783013221 140.014167357286 207.940596890723
181.876635968785 158.072347847051 184.672330560677 176.440625367948
169.881866987359
CENPC1 147.351826045609 158.665476708054 141.964796033176
138.763250836766 159.524235332529 135.758133410577 140.579839043912
156.737219981981 158.185593294878 142.192934622228 172.395805302715
177.582314734704
CENPE 149.94715601106 161.035985824654 162.850128623401 147.932875151331
204.161375686761 394.35596596181 229.660838239474 394.73732350607
246.88857694249 289.037033338975 195.16856673253 291.537725613819
CENPF 151.354187593773 165.213327555428 153.610428214886 186.140970908638
335.858047332641 302.637859140061 181.048789688798 312.484289357493
451.496617003797 250.18189549573 176.300743660299 378.308562999445
CENPH 150.931114848235 140.804008584006 145.320945010364 156.361037318986
205.560952959537 160.20843950503 182.515302191173 152.131804134092
169.107948050094 185.396765725228 150.28508305615 182.181756507598
CENPI 128.756818133357 147.552635527742 127.539091409962 128.910261453632
146.70298187005 147.561362873855 135.27739087376 152.601626526117
149.941056059544 143.429394097222 133.62358240645 142.924854393401
CENPJ 297.332272659995 243.807038920733 316.663128947813 254.552034511333
197.848577663238 403.812520074723 302.601458409102 313.099028594073
239.704459251571 239.474937674583 274.77366169918 282.836518011311
CENPK 153.882081780197 168.969180646603 154.2228051929 162.748836971539
180.070209705044 223.154771092064 196.378888436237 215.300064529551
243.772412032046 216.53417734069 185.46843333826 271.004741629789
CENPL 178.584378959369 163.593106951255 210.803130108618 161.155280539113
178.542247857251 241.706645643141 204.438365139666 281.881563164961
223.81987303579 207.440700355457 189.403934838648 227.065194522692
CENPM 119.252737868132 130.991496243273 121.965131419551 133.040952285855
514.124012546829 274.343238288436 183.764215745794 308.621688157016
333.131139447511 211.766884719761 173.347463632976 269.495247190598
CENPN 183.83720937589 178.496344474999 171.221972336784 183.397833894087
452.077434390612 391.782120640025 340.608165077422 390.573484082597
297.12411625104 456.038981546175 255.903775525554 400.353795948162
CENPO 135.711590400394 134.464053009101 129.155804800869 133.74741832303
149.230226445583 187.920789153124 139.730999352036 164.901810642726
155.108146847158 147.457193565877 151.954389699615 147.183889426843
CENPP 123.330142002791 129.015422924366 130.807596523171 132.712573198794
156.277567608825 143.444109491884 147.821639002833 135.928428484011
136.400959554698 150.674189235105 145.739537463907 126.872912960681
CENPQ 130.024546949158 138.616719975373 135.616911926164 136.865340099645
212.669156800241 169.725534829981 161.160974998803 173.520662003159
173.690008681514 171.49930976135 167.260319361746 139.615706918035

CENPT 406.028724866233 416.861099188252 338.46332239699 530.267835989435
368.276805218658 258.012234458166 230.947339143332 275.203046536524
253.009376944901 316.281469585496 286.703712311781 243.936797286495
CENPV 332.138346958671 339.54582149284 319.768792196774 426.464641742092
687.700356531038 874.348941257592 314.610979086485 502.063888359427
236.833653794223 170.596937075493 668.127527385348 208.321765801227
CENTA1 735.8141982568 464.577839898252 988.788192022196
461.413469623811 1865.44709751822 897.0125616892 1231.92253664482
696.277780670166 867.601542930533 469.714826990063 799.132592914065
2203.82462320864
CENTB2 215.698924670416 206.605699708924 238.617228632505
192.009562635198 184.451469623426 329.604568691377 243.732074437237
304.547738011742 298.664864578349 297.022984365795 255.12306184224
256.022057262325
CENTD1 132.681475162386 120.696557306298 125.655823846115
125.071387239411 119.213217733213 130.355969431473 142.617474507282
129.397378057767 134.8745127454 139.418394104487 131.17119492692
128.556280049403
CENTD2 210.218440444631 209.398724613377 153.541193559658
234.156713102803 245.607130404306 151.338765231607 194.724219884752
174.840923772532 167.817232948801 158.73040336393 208.270622752385
173.839053227554
CENTG2 542.544172683021 460.742037437298 478.040234382488
635.37511798786 390.573484082597 313.641656294638 458.953009417683
294.922183826144 332.749676505218 468.712260193746 266.162244070031
395.521241316058
CENTG3 412.736636437651 387.355661073935 325.751369001105
430.220363263561 644.741046498743 535.758943850734 470.154677948419
449.523996620257 327.749926183823 272.854435771465 272.712242893937
631.106334754678
CEP110 188.40383184521 192.565733545715 205.403820910921
219.940167815376 183.905926269285 206.73292907905 179.273936200852
231.517412929077 217.435155984897 202.662466141301 288.823376348944
207.864808684047
CEP120 134.509830749072 131.48517604564 147.626071601072
138.880168623766 126.766964875664 151.431513447721 143.985850833974
183.247396324425 158.322477506517 173.387549650592 149.227646026658
151.479748626813
CEP135 251.731327363027 229.181725450534 228.926077117474
265.260411490697 221.561252012695 271.823243396395 303.724140156439
508.127470400078 294.17588071297 343.617417488962 258.384059994446
352.418229893699
CEP152 158.193008411967 156.460374293022 169.764014031526
147.968104627262 160.496585125233 170.818182800759 178.440907093913
207.104548555929 205.162633740798 187.561298131124 176.86489466512
196.908612360694
CEP164 286.774606295106 340.193863653875 222.484058975856
444.192734921566 282.392419584508 146.979988781462 188.836327151669
188.36361073603 176.167129202773 165.100811808026 245.351459157365
172.502036705114
CEP170 149.331805754859 146.447745446317 157.765771942689
153.184575380093 151.148295679242 152.395272857974 159.424105896749
146.84891312893 153.664503112778 157.507108643575 141.083966525597
157.203304477454

CEP170L 136.587165242663 132.793020446043 139.860093278582 138.401406375804 134.487476333464 134.81595608984 138.758688249557 140.641956393993 141.541355091514 140.752616434418 122.305514440939 140.142884879106
CEP192 239.541516080716 222.990586491219 289.297370738382 242.221324619171 177.073245863872 450.120868656638 324.346723939622 431.019896339435 322.889296658955 280.798003990849 331.62124160152 392.430873293903
CEP250 139.502856192876 135.085039737228 140.608266389167 140.772074031028 160.786907816025 148.605816748229 162.762473039474 146.054535585464 148.104173238606 153.171822092714 149.606576415873 136.149732574763
CEP27 2496.17211592866 2639.60135568 2618.64973582507 2398.71483410593 522.400055538966 2446.31228564539 1505.90883940934 1828.66165670455 1519.38642390142 1280.57297272231 1946.45172007314 1307.96033038529
CEP290 214.4829383264 196.566937667522 218.156162474715 198.620777452192 167.46432961654 167.106870400592 242.269784551092 323.51769984082 175.543924200429 141.778060508776 239.704459251571 208.611557959861
CEP350 587.54075434249 595.593867616527 626.104755496369 436.731545612902 549.96567374713 903.824331691969 789.401526545277 583.697431061521 408.811415529922 441.785666261936 956.76879975749 730.673649872205
CEP55 129.938723074546 161.16688895179 125.379842077431 132.213890220705 423.045329000756 426.548994267836 217.670330817769 479.761165832339 336.786227017391 277.726756691133 242.096692715029 439.492285515997
CEP57 193.522811257085 201.49630027392 206.164058935215 151.345630073141 193.064450357153 156.75001042701 161.581641777014 193.052112923573 177.639351778834 157.468934439452 328.200548902445 176.631604815669
CEP63 216.181417552574 215.272800347166 247.145700155364 204.842791081358 185.843639043466 202.300293733922 252.432175363972 234.947745514175 244.668813440638 199.275716402358 230.508733145583 249.823314327355
CEP68 180.008152341531 199.209279169496 227.884445686928 186.66469040433 155.752117896424 139.199446543251 127.540848073484 165.244616507084 131.423918669689 146.035731780312 158.080749926756 148.996601285688
CEP70 218.2454853526 184.542232315662 199.947823264085 187.352655464866 224.424524191566 205.08372943071 179.794718712964 312.406286993239 245.01029221002 138.358404420064 358.52459123416 228.499052416183
CEP72 177.825648107126 175.948959885759 180.493406236326 182.02250582556 217.748916080172 226.086733606951 205.026176598298 182.57496767012 172.686798187142 196.802641002079 150.333572271902 193.641927801805
CEP76 141.028377381019 154.384766720753 153.974384951787 145.365935017728 135.689889081912 157.944355564411 145.404469340821 159.495272062735 131.081793789906 161.965270932582 130.393631150485 155.957570398118
CEP78 183.385868234361 172.020235319192 167.46432961654 170.661808797506 271.196028302639 276.224613560582 191.961269585431 247.407442038488 204.113356572343 188.952730860801 190.389158930835 185.706228572019
CEP97 153.843250142472 144.364319564075 157.881569297409 151.933078262284 147.959740692766 160.876228450608 153.655778377717 141.29868389128 146.362705590778 142.071492722749 112.126521532091 139.073785024092
CEPT1 359.256193328366 291.537725613819 368.637819994033 254.065327837176 365.140886302762 347.620001353026 375.407747753058 318.778978094743 424.751154004399 344.737964092526 374.46180489079 293.234972169244

CER1 126.595668758744 125.98519427755 137.237089899964 123.926293128246
138.088712695622 146.359038260378 131.519455635415 140.316066297536
132.200648560899 127.080149144234 145.841028932318 125.9888344762
CERCAM 400.23671843715 382.069677030369 278.651369597422
485.373169494941 380.243136469602 156.336425973696 172.568852318369
157.841520434044 162.850128623401 353.826834642154 163.262893278578
157.348475799643
CERK 458.218913259336 421.255522475862 370.390323116113 429.268202514543
163.053121879323 210.989806933158 167.599784887933 200.405532717305
251.869781014029 286.194197552489 217.013533525724 274.281532397691
CERKL 133.866011732878 126.217702283708 134.369532556726 141.466287523861
135.526248253492 136.705326991088 131.510494315469 134.978785580693
134.915900729865 138.050999864689 144.181359740481 138.233799607706
CES1 701.481003619067 753.654626233604 463.880793292701 929.198774489695
132.439176846818 141.808122700968 158.73040336393 155.911387663464
158.92538634742 144.210698143215 131.850952248545 137.662135592983
CES2 378.151202933526 462.553267274302 447.779854598556 492.377642058095
348.711663415777 372.81255844018 457.292146759215 341.501397478593
378.695635790714 1099.37392456694 513.428413503031 550.434931878586
CES3 138.510722755317 147.728018766726 140.06989960847 135.458682745495
160.150336334888 144.419657674797 151.632801137039 145.881727908814
153.39738748803 141.097257687941 148.816369706133 151.522526161299
CES4 142.997365080299 140.115154069602 144.848839185565 143.030777754448
135.886859004208 135.766466392056 139.073785024092 131.023892409073
133.45690661612 142.358754646258 123.182903611854 133.337863400762
CES7 129.976540483758 142.311108110185 158.531514776098 135.798387504219
130.66511389864 161.930142040623 135.810476921749 161.755290291637
149.089067389266 147.278236958386 129.281564491007 144.311620064675
CES8 150.786618560854 159.781313090489 136.249621724093 147.488635834397
221.411372022459 175.656204695951 176.317805247749 187.472028627511
181.3859695951 169.043206258073 202.061887608293 217.89028736436
CETN1 151.104891644466 147.195127201893 143.347066447019 154.507721014473
151.252137441251 138.242983316259 145.326803807448 150.105503384881
138.22823004719 146.750774496317 143.260448301109 150.914644894869
CETN2 2853.14759681683 2782.94647782764 3570.21370790206 3298.73393277409
1758.7809492929 2217.82628612929 2165.41403521012 1841.63015706195
1978.12229412176 1727.7144253077 1505.09908072606 1667.86825735893
CETN3 471.069295685718 504.502047826003 401.631377313065 455.486248388968
350.166837086936 343.488356185437 534.083703564903 264.563741039382
311.715351215263 282.244214637625 560.757496975779 403.720036930931
CETP 148.272252953881 149.230226445583 144.345080865349 151.178586065066
157.227118680396 161.079169854907 153.658747612827 149.019120812629
162.61434899634 156.373163373309 155.824409540332 163.697117248984
CFB 612.374364731095 442.493108530222 369.78624104057 391.569187190375
345.897250519387 163.265020898999 296.830999357172 265.110264596425
1867.55140010155 203.913123559942 435.983803453746 262.954856857756
CFC1 145.320945010364 145.924751649542 154.104321553472 177.825648107126
150.35767254363 153.393651104539 157.891350650633 155.176714087209
142.392860474555 147.78847662997 134.819406064011 157.466686487471
CFC1B 137.194467539969 144.402160347818 137.570343435498 164.914114315732
149.357617406983 142.296446144179 137.53070284697 144.595393134144
157.990467804353 143.526983440589 154.600966693059 135.057847490193

CFD	21956.7541751958	15561.8923517816	14592.6308255978	13142.4763966378
	392.6758818959	1021.81988990874	416.215253990273	1046.13353853089
	1680.25851465727	494.747087458483	394.414934831535	630.473042320288
CFDP1	861.516925304722	783.755776589969	898.877732360841	863.738809424958
	513.292377117011	481.751102333172	801.051513639714	290.582957824549
	479.983368253935	886.157279090932	782.139151423572	856.039380523678
CFH	1070.4597488302	667.421612441201	746.777330152729	342.555640222963
	139.936661244361	231.146155346291	479.469659712246	378.695635790714
	312.601510033941	255.59042517603	445.505512764045	434.390613381746
CFHR1	127.811923445503	119.876835248732	119.229322984564	109.092840191351
	127.979574243743	120.862516074358	124.641892391159	129.969400693456
	114.067790137477	125.199837162105	156.923675862035	116.011005504201
CFHR2	165.692508737757	166.355086055404	174.515815532892	170.701964358516
	153.776361241587	179.250373572781	161.021740537372	163.778790431064
	168.73918684069	159.110614190088	166.22013573342	157.293090577042
CFHR3	117.261443445891	120.521509788705	130.466571969657	114.373370072994
	120.590654346066	125.364821583438	138.275371291639	119.583378101838
	126.235567899449	119.492844191924	123.427366316096	150.019181654237
CFHR4	115.440152891339	110.624328647003	112.239647575661	105.583474813289
	118.438942370745	105.606964521964	114.43491928161	128.635684412304
	118.500956349352	103.307703547649	124.395047385025	130.314247368361
CFHR5	126.606163970149	125.324187741972	131.275712196961	125.704264033102
	130.902319266342	122.176811388389	123.45956380046	122.262664384198
	121.942777234608	129.23899614612	144.029891756677	120.809659457873
CFI	270.490971664802	249.925640256322	185.088667707155	240.410292351169
	163.833372505148	198.023465629218	178.676812740925	216.154203111101
	249.524956682907	542.121375354644	233.975864722289	180.867208892996
CFL1	2863.51286169094	3706.41283595794	2932.64120183406	4892.17875938832
	12690.801964606	6793.99737496637	3989.14883668905	5022.15374558514
	4711.21389812942	5058.57872936027	7555.47017983216	5846.17594234628
CFL2	179.794718712964	185.950131245699	199.837938317725	206.257167235126
	144.206259414981	148.09868976776	139.646026806298	140.646946425036
	140.279222720709	151.019907064794	131.148926523383	138.887159625058
CFLAR	1808.26383618901	1589.70261092241	1562.9779683714	1645.05161227372
	272.666435295288	967.123240442957	573.846138124455	1004.6207350158
	913.22732386079	681.172359997071	1098.20033709043	537.510539142248
CFP	147.2233876988	137.161065320948	147.805819921429	162.337707154123
	153.762995131216	152.08574341962	151.130401750721	157.765771942689
	150.261107471642	136.390047634643	151.679534447228	134.493062923443
CFTR	145.03221570575	135.129656281091	139.336891718408	139.340699808517
	163.643554173995	149.204816953404	134.378951325825	150.629932241703
	145.414563914801	134.881854036527	158.408494909763	152.538048490265
CGA	138.102654893864	135.141067086648	129.740166478951	127.218362452043
	145.154323430995	142.272117655292	138.753419730571	127.612859839494
	111.65024009102	159.633987751501	141.389330839189	144.148833164952
CGB	164.423960071908	151.293676705634	163.81485578613	153.577349107512
	153.448882783807	165.108876136738	155.801832826848	162.447903830131
	159.070355781276	163.54669668396	153.363629311799	171.62327142765
CGB1	138.345393524161	133.998381302146	131.393633337276	144.216394153095
	142.243866937888	153.846723846716	146.286319248569	139.462804363695
	146.271180025157	145.34041912391	133.032905765836	138.367321366804
CGB2	165.686655824416	156.208747691032	139.422592957883	159.203584692924
	164.15288821931	128.545699891734	152.842777535666	146.00443946108
	150.380814008952	171.967974673053	141.91067228913	161.781529659549

CGB5 134.787564568352 138.328508662816 130.854818615966 157.103246263523
160.98571841915 142.863268359757 138.091795130567 142.101865506094
133.206919058442 127.161975533812 143.06806126472 142.928920246687
CGB7 138.308965237491 132.245217686793 138.667081004369 137.961504178836
142.058086679048 140.238423894192 134.20988291379 128.39171386085
138.870636101334 131.848213704003 148.28101912019 132.657106732366
CGB8 139.957560460935 152.954944775793 138.173547382901 145.870135196037
152.251128595777 146.595540577994 142.594553057288 160.695913747601
157.035739033122 132.004294377032 135.212715912271 136.995197143668
CGGBP1 515.158921349383 513.816017871024 539.008286968106
416.638529499878 469.407481002198 678.937151822411 645.183207984114
635.234445318087 696.475639532595 616.339439383796 583.938727139698
615.485984065221
CGI-96 149.147411595596 150.442821494908 152.129092774005
163.451063547847 255.74096237558 151.635752404534 181.340312140534
186.778334749551 179.067275037015 188.515336186633 162.464201058576
205.08372943071
CGN 189.389457481768 302.570932942847 226.623172906957 136.103088234442
1492.83354014907 1198.53117439169 1955.96772733047 2320.41293992992
1421.72388964898 206.391403266202 2101.93705983383 2300.9407349072
CGNL1 1834.08657010335 2078.76377557352 2491.33490046283 4228.5490138553
149.792368068107 193.777175793218 204.82244206816 179.109090120497
314.810001770583 250.602978521808 260.278595572575 160.415807880532
CGREF1 145.117574928189 137.478556403046 143.061415008831
138.071335383596 136.020600085547 129.385416279101 139.104796184094
128.626051185037 130.548611758921 139.423432468886 139.59941545613
130.883516197483
CGRRF1 374.46180489079 301.139737613279 418.968095159939
279.325065941252 271.615621030842 542.121375354644 320.774102343889
316.457274749526 254.991678321253 408.993744895434 296.375305136807
353.437466689101
CH25H 249.524956682907 285.980446338971 163.889522525848 193.218266574099
151.363607695078 158.756424011719 148.704917490429 181.202396068658
169.527424356913 171.304896264805 135.589211439847 167.237705290905
CHAC1 129.452826518288 121.322817570375 113.919529047225 114.528407572496
126.503135702506 123.090300758271 112.800733398209 113.572326615571
126.752999657665 139.241145027974 128.748663215466 121.212521750566
CHAC2 131.421663453164 122.5651974076 121.88070274109 119.972337070176
134.842877075983 123.865424155099 127.171331062578 153.226604317177
140.019540869992 140.063142443074 116.369395555451 134.27783583528
CHAD 112.943630133093 110.753661013519 131.362128083159 108.333135424315
110.465934808915 111.11656427738 109.947300629824 120.272964182307
111.632811186988 112.520199390427 131.828902700702 122.804934452095
CHADL 170.602603155092 173.552535486766 151.051672692659 171.610742435469
297.022984365795 164.120602897751 166.988725688589 141.015441854424
154.417731984391 142.367003292264 181.193963616037 163.053121879323
CHAF1A 124.697283474333 129.573520898314 127.075899925865
115.507474401239 291.167223094542 157.932243807202 136.821228481217
203.960838492793 179.250373572781 148.052222389368 167.817232948801
161.142723532061
CHAF1B 137.367390177293 149.666293193219 132.937942776777
134.294287947795 232.986366625889 282.282663608264 205.058210447711
363.89527566597 275.298642145005 230.424145472916 190.331589656379
204.113356572343

CHAT 141.040862279633 140.2533871084 137.13615301204 143.873048967535
138.289361041588 148.927040248436 141.561769570516 144.700955682048
141.902711692778 146.579073345319 140.527521345097 143.877226672663
CHCHD1 210.666799498141 197.565122821625 204.183472655276
223.89854167651 307.480644393018 264.563741039382 375.255445179952
271.713695056313 268.600782558985 257.602370597316 394.186683910377
329.023836559229
CHCHD10 1904.39962279504 2246.89876694944 1999.10320742823
2698.5455305009 1503.2049371911 830.455793617181 593.639955546556
1270.76282064793 527.498114662967 509.988617641229 1764.53168329081
2355.29862638453
CHCHD2 5291.83393775761 6427.33783376005 5300.67886821231
6233.78489807054 10790.3738999898 7802.25987814138 8739.02966610274
7082.28969354712 8558.70496234991 6296.24208225227 6460.4291301245
9596.10781313429
CHCHD3 354.132752995385 317.719381113816 425.708048519408
357.431640917696 291.616195651001 253.463871742303 290.802509012089
341.761516859319 314.386320310898 341.973002478932 195.69447353356
343.946831770916
CHCHD4 719.13080264227 775.5664718389 1144.07896461196
949.789710063337 626.298707790229 524.973621024573 432.372995789404
897.0125616892 658.202765061937 616.995114856023 589.953707188378
690.903797251841
CHCHD5 306.838596239511 310.718449468465 349.128741707506
294.595972099366 784.170983714588 444.374521095495 379.862305342147
355.609033348692 403.104188866804 399.476401949891 318.778978094743
623.050854309724
CHCHD6 203.693188114912 249.292766428035 267.149525812834
292.693830444839 491.034395489684 297.022984365795 383.583234619781
264.741946816665 300.358738353443 287.83303360511 213.178648382852
239.970202560698
CHCHD7 323.737610506003 283.014222473749 374.268489263941
325.642275889649 211.626970008102 329.660368915848 280.085692727562
318.297819854586 317.719381113816 266.442565636226 279.000687630846
378.037190077449
CHCHD8 181.247766545135 171.071893074072 181.247766545135
234.693846527867 244.596233138747 276.351810999317 309.580750386386
248.204358220728 209.146021798649 229.230239609291 272.01602406899
251.347519777204
CHCHD9 1466.08446530283 1403.90487313049 1897.92061704124
1401.67503392145 2874.43919426772 2921.59274577877 3143.77161008321
2332.98074920207 2385.5958396359 2452.1304336603 2426.2098937241
3363.51782842264
CHD1 646.933504790891 532.394088545421 535.354724252178 491.709723745226
269.072221443403 573.644925180854 659.900771633223 516.177596584059
370.798852361374 594.180861801958 487.696347726467 687.700356531038
CHD1L 280.007569817323 266.563990988678 202.909188070184 277.256341592701
314.90123632547 290.135914275115 238.617228632505 262.60377731258
249.633252091596 306.111172289632 301.747788416496 298.979106293393
CHD2 141.202092246048 147.713967317159 136.420062669039 130.253372129786
146.6886556805 149.200848439601 154.645353009618 147.469101571149
147.78847662997 168.27980269501 150.308048500142 161.762768590193

CHD3 162.351212914573 183.83720937589 142.049776668507 174.246705740225
153.435218661228 165.370800097602 149.341736870692 155.249688219254
158.049286838471 159.966729941991 219.192900411543 151.625248091441
CHD4 1063.5903770389 1199.35864276583 976.360310504918 1284.19937294949
1061.79595707572 950.144166422001 827.157920721418 785.290353402444
1172.4103691525 598.449359425056 1239.66725147648 900.839073800181
CHD5 195.364709354814 189.693218759349 203.17525411575 186.460034745907
148.045391886623 168.394330931524 156.986053534635 155.162310001164
154.201375471487 146.819830256127 146.782830477983 171.287186905764
CHD6 264.741946816665 252.32907388173 302.847274046748 255.3451781862
233.300864925399 355.443758121675 393.500781010617 273.651114834013
265.470314691221 217.560691345072 294.410833087913 236.855293143293
CHD7 231.018059960001 233.464856303761 226.701637237203 242.253995801882
487.597040819879 321.367338145787 371.168752104821 404.54681330209
397.064325578854 266.681711962236 465.29290512169 366.240941980786
CHD8 1183.1601379136 1432.93880448974 846.985164747386 1753.58770316818
764.137504481329 607.979208454176 729.729297413233 596.076764072945
498.812823949508 508.861268738074 309.580750386386 642.484527246259
CHD9 1701.43479420427 1731.62376348283 1348.80530206238 1507.39761667421
638.393099392849 964.473774310687 856.424155671931 479.638653311025
594.38228123675 751.896334334052 1753.58770316818 1096.61874892572
CHDH 127.887666721519 140.462651395483 131.121343131082 139.138329217724
156.511202150773 142.930903395131 138.746987455826 150.687819649072
163.104822161892 136.566588903194 131.719227272527 144.110544384466
CHEK1 132.273354705213 134.260779370821 130.224324464992 129.256138453675
266.316485802808 185.46843333826 165.30072618497 276.5177779623
215.426918011158 221.411372022459 184.923312649584 268.698146407497
CHEK2 157.474913177177 148.755559144241 138.207553349985 146.432468585736
206.042395282286 264.526189799049 158.285003899913 224.869569455052
192.827534610486 169.552424961465 178.045931314857 205.852731236069
CHERP 239.277801396834 277.219796733134 261.366143735074 311.481739745423
408.263886786123 255.839878446312 268.961643716282 271.823243396395
261.89195025254 197.827534617557 350.658722333252 242.5085895186
CHES1 4386.83308119416 4022.23000497332 3671.86460903374 4486.35687688252
716.420861490714 637.30300219673 862.669120338424 938.499337889075
706.292905820227 789.971871940893 833.841469195516 520.218545108099
CHFR 659.483452217461 686.801358367968 654.881990232975 897.958748317315
1109.08288440559 686.923164688096 852.926365088632 853.314313182949
1102.38234645327 1078.41947691758 1112.4055726239 903.503768217033
CHGA 162.945789604505 175.210232663606 179.582086214179 163.010527914347
147.70836747108 154.904424749528 158.104336050835 171.61704153463
161.724388995749 165.30072618497 151.221783750989 214.739818213164
CHGB 116.214857935694 119.288554466773 117.218616752346 107.91011371422
112.624137732184 118.312891459156 116.007006375993 110.486191378104
117.766156390814 112.133567992833 115.775525016883 132.028327486943
CHI3L1 168.753660955229 159.592581700976 159.724824188037
163.880402141404 160.353649786837 138.395943168158 137.559204418819
192.827534610486 369.906476027936 199.459711023907 143.307838525991
139.779754416105
CHI3L2 129.925548182204 118.930792960643 116.425301253959
116.969799327195 120.297848408451 120.114423236962 122.736882859231
133.229571269367 148.350919010575 123.247698282967 138.299629744589
115.002176041974

CHIA 144.991442620483 126.94941821459 141.412317125776 133.181855719499
134.89031458837 158.859389972316 129.992015202357 127.687299576139
138.078214837785 135.622888091037 144.145612985703 137.916872642522
CHIC1 151.305201022012 136.450062043587 141.202092246048 147.245636620228
147.278236958386 130.240366272285 137.514952067562 138.808591995741
144.931901042924 132.883408554592 149.836491640473 132.975603230163
CHIC2 605.040026490154 606.38343657878 480.923468806193 462.833644033427
816.266384662324 649.438995824208 806.290662664708 663.214972254236
913.684449305965 1225.33666840349 503.093655556038 864.908449769749
CHID1 115.097376525707 151.823845684041 134.52098220054 130.619624347751
121.908945872994 141.289009185018 122.793816135585 134.238642042323
127.251594986725 137.146961654597 158.483006541613 129.895388481159
CHIT1 132.719730606922 131.792339956719 127.125906877098 139.372441366224
129.077660633761 134.250052775987 134.772545694636 129.360773522498
126.214166842097 130.282212339065 125.287738115754 129.436712663487
CHKA 232.621526025603 222.365208637146 267.696284751923 249.953902454701
328.33387481068 250.737252834098 281.659653592151 342.555640222963
275.468194644053 153.420392741659 207.298994298453 161.704966315183
CHKB 286.703712311781 274.77366169918 313.025250337353 316.457274749526
199.980312703646 222.332748220525 245.553138677866 232.879578820053
282.190861594303 321.524921332256 241.890850719625 327.164773632634
CHL1 148.275939659102 131.252368756892 142.487424651482 134.970609100459
131.200042525339 132.206133020786 115.923424095859 136.569250038372
134.483162806498 142.846100831941 135.071400668179 126.969504644414
CHM 237.78482574225 244.054469051058 248.204358220728 230.079664410595
198.023465629218 249.064573038094 206.275885047321 181.813812621767
224.118739978257 261.96528977292 203.510106134882 259.643957326575
CHML 153.500478301079 172.152570321727 153.189490068047 183.406510146725
180.1204762193 164.661445411833 204.057019122032 172.764511709731
156.745051321143 162.134726710448 177.132361355899 170.577057395536
CHMP1A 693.339946866196 885.713964813911 510.811470658575
1157.02365469464 1357.48791782399 599.735180191091 542.740017003369
495.099443245153 633.594811068524 536.805979562807 786.160002434066
540.005877322817
CHMP1B 397.153801851621 434.073729807058 581.37957221282
321.524921332256 322.889296658955 866.147379099606 650.679096367632
900.233783407148 998.157643452741 1615.60472592991 602.371843434907
721.966539760341
CHMP2A 572.548647437438 583.619362840814 666.570977208535
605.795439342384 999.159652508318 725.216684507759 934.546444634669
1002.88224342342 936.957730361063 1059.80292026589 737.650963292668
877.255923473532
CHMP2B 389.96566674501 348.640185950469 448.858580689107
263.005303710717 892.515788744891 753.007562127186 940.008438825765
970.166036472486 792.101559967381 749.859130465356 924.312051919404
779.340880980422
CHMP4A 1213.06007305892 1092.74905085695 1331.18605673705
1133.22524345111 702.265884752474 495.655170027296 556.863361789449
845.646455991008 646.704226815099 618.058496806334 984.181272274704
834.537908087672
CHMP4B 1442.11349373764 1223.06438074895 1650.77281891596
1383.68977267418 812.75075757351 1003.93455977232 1186.23169583595
926.670867201327 847.564628337955 527.597948979568 1146.84239258543
867.601542930533

CHMP4C 158.554429311951 185.661496988658 152.8810245209
146.824420102008 354.205670284123 444.945078908489 601.800326895295
485.87138934376 563.140382824149 506.80969228563 846.084919104262
454.492780042353
CHMP5 707.404709303506 606.003956839826 887.147199303294 490.849163737598
932.862030475849 1746.30519466572 1214.77298621465 1100.81588201714
1576.21727854603 1416.93831202323 1029.75852676191 830.684999498776
CHMP6 203.524150518033 205.185206972064 175.176693060261 236.833653794223
335.3366819308 169.16155608071 293.825372123877 263.379982742085
222.315451077459 208.456472621593 212.073472189229 298.347201056937
CHMP7 220.136131941508 226.387465899611 195.608619642038 251.268669431283
175.084729757407 139.363571392476 184.655641182839 140.802033706122
168.582192754233 143.356999170877 217.408824020423 163.794436953711
CHN1 210.77489002666 199.001860912128 163.373180928181 164.816672737691
125.038427667173 144.082452235267 117.461198576063 157.940873610797
181.941224633917 279.325065941252 142.432995657729 174.006516433854
CHN2 261.203388718689 313.480307434343 301.087947101876 435.037306125887
159.784369379077 223.094496588657 286.856346704286 169.744100665038
193.777175793218 155.3016514732 183.193029399643 291.683896942007
CHODL 168.674809329231 151.351938656508 189.61702125418 148.567630572897
137.685104040843 134.698857213208 126.793778645808 145.483667452136
132.712573198794 147.118987674809 138.060245284993 131.573729430926
CHORDC1 176.579096613849 161.821372157067 195.447966116819
171.287186905764 221.050093964575 183.472290949588 237.692959394487
223.107391048316 214.019190624184 217.202541167691 186.033093285894
213.028568789661
CHP 873.256632365737 775.781735108886 1127.07858210156 609.665323786904
1012.44277312494 791.840531191783 1753.58770316818 1074.19589851599
1405.2134783352 1119.71228054585 1822.14935116363 1294.18114559128
CHP2 136.811620595243 152.640683776403 149.625528206375 145.7202993771
422.12964420709 589.953707188378 1332.82617944131 529.700356666006
129.642952083513 134.307336063739 178.854873582873 136.162938063831
CHPF 391.706404954357 465.41233536979 343.571296297462 476.917496749381
778.639512538949 301.955019831714 387.224184063662 304.590636814607
274.14931964455 311.208703262497 367.983166440977 299.170431746759
CHPF2 163.284853420896 189.633348499854 174.761105961773 204.584643964409
364.265100349672 192.848268768579 244.73233791067 187.920789153124
192.244413515864 213.26158748941 197.740912476527 237.71636970719
CHPT1 631.408955630318 537.06835963726 761.097992055119 420.160939829121
382.233826557803 828.683748921915 410.216584967666 1002.44383771056
855.415038498392 242.188218870393 623.050854309724 345.209128382239
CHR415SYT 121.344724993789 145.498372398845 151.089328533476
145.658251059524 158.726115143818 127.800634041606 164.347168837655
131.690417323838 137.581659385375 168.013310840048 128.141725976839
122.265071206163
CHRAC1 188.45479937455 183.937484597232 195.532022060219
209.112959748917 131.981505236332 149.551030995489 144.30519737569
158.625060605976 189.136114953249 180.641419603362 204.616225498655
186.961638857905
CHRD 152.643087608513 155.762890407496 132.399633003253 131.761564745495
119.525701528869 116.305011582033 112.90900939264 114.479886899622
112.758617084341 124.574323079707 121.884067622971 119.308345676905
CHRDL1 142.745838888446 135.434853296628 142.081013617671
129.95138839192 117.967392531385 122.144209450757 122.213445520365

124.207200622684 123.853588042292 118.773864607553 120.039031290299
129.76141737109
CHRDL2 226.43414383055 226.454107654372 283.377663208926
320.097510212422 164.448110885609 143.101750222256 161.951996906162
156.228039404029 264.476846259384 159.926719599391 157.826457203706
128.066043751951
CHRFAM7A 139.779754416105 132.514706165018 138.308965237491
142.19015013243 139.744142223177 135.943252855457 143.038248313305
131.604121223902 142.211357324461 144.845726048937 127.320624058583
133.278923716119
CHRM1 127.900901613784 116.621735695217 121.04331582508 111.837468699281
115.366496551426 119.036965883266 120.182787241888 115.734230176652
121.599883734552 114.830474086443 127.779401352802 108.43334699667
CHRM2 139.926488041421 143.051871940423 143.830319259804 140.439698763808
138.864844664017 136.403353223002 136.348252715447 135.434853296628
129.77374228644 138.778496466408 129.477908941439 131.004145546364
CHRM3 637.457322054293 560.602047253306 541.764013317002 720.907992182476
135.17827963343 174.635853531194 129.661073814998 122.962219297463
122.063996556575 192.032080013216 132.887701822875 110.486191378104
CHRM4 145.374923175872 148.196320158939 166.068489027866 144.806478328053
142.316441155937 148.320310040998 140.496058408953 151.836705989155
151.486362260537 150.45404183949 138.442378791712 135.866980620144
CHRM5 122.498794845416 111.111969531957 119.218909479403 107.544568250154
117.766156390814 112.714282429822 115.048402956574 113.209569103727
109.552501987529 112.825399620524 111.299115402008 108.061315311823
CHRNA1 126.695387748732 123.935072913078 125.010266083832
132.137634927459 128.574419295906 130.738915341653 128.545699891734
129.767878283811 130.788557333561 138.850077456103 129.636845496276
132.395993254065
CHRNA10 141.547112973262 139.166537591866 134.46743016543
128.650205300992 138.93292293598 139.17480484652 148.720181793879
138.953926269905 142.338352884053 142.428578698775 153.865702787334
141.872426752329
CHRNA2 181.783275804424 168.861884032201 201.893722965557
175.040086358446 181.823450464129 170.602603155092 180.798671424395
194.22713184875 165.055636629118 191.84511431253 186.496160803789
165.467462247066
CHRNA3 141.97908047949 163.134784474437 172.245089970803
187.30908753142 166.879042308539 161.651643789597 147.454902803231
152.713528089347 157.28429690041 162.586012723045 144.224287382131
157.619627711954
CHRNA4 120.345546077894 116.14662237541 115.981530322377
117.008277388399 131.447941902406 113.184495948936 124.207200622684
118.631771242606 115.25307661968 116.186846885411 131.023892409073
116.316845829267
CHRNA5 2539.87833317526 2303.0998502001 2379.15291239548
2116.39371296122 358.092809491843 1969.67552562117 986.004777675332
1455.75693295732 1145.96710556468 877.598505913096 1003.93455977232
1230.63920848575
CHRNA6 140.327799236005 139.880733465536 135.379284233708
143.262288751967 131.540436531741 139.97127025207 132.976977898242
131.989955864584 126.531502877517 133.206919058442 153.748035612441
136.786462547129

CHRNA7 164.274491958161 155.548684011748 129.452826518288
131.260111979219 134.066948803697 136.585541662028 160.331940529151
121.803676441059 131.347213356457 142.959890982885 134.751916364581
131.816989019024
CHRNA9 135.429199205506 127.422129210789 134.064206964444
135.304167695152 132.994716261577 132.634538733205 142.678267012725
134.936025073531 129.247701136266 141.232464220879 130.953237161257
133.792673129743
CHRNB1 201.105520537151 204.959164579011 221.869191666931
220.458449424062 218.314136992677 207.890408761692 191.507657960386
202.583967728957 189.307823427362 210.973303650385 230.947339143332
192.827534610486
CHRNB2 107.993707827557 113.38168751499 116.507315786826
114.849872458647 116.850851308701 104.483909687253 125.9713489569
112.404562115306 109.680532131035 116.118745494794 141.261150586614
108.604763499678
CHRNB3 119.442114850181 126.877293296185 136.071856104688
124.446264376282 124.291944151031 116.445184223804 129.123522999226
120.015436934157 124.1819046638 132.363645381743 123.893768254549
140.014167357286
CHRNB4 128.346598756853 134.060214479265 118.597739444445
141.119491147925 156.774097885381 136.278361222532 143.514848892447
125.498081339586 161.456727894848 138.159966250659 155.625320865565
124.546520456419
CHRND 110.113844434493 114.325967923393 101.530900217921 116.947370388179
121.356731684516 110.651971902576 114.170354748872 108.318510762416
112.41270361848 114.488760619745 137.141081365469 115.848659135231
CHRNE 162.678619800767 151.41546939633 146.937052029619 197.827534617557
140.743917481548 158.347972566681 152.035992124554 147.579672274205
149.461747964498 167.858490180471 160.127254444487 161.559920910487
CHRNG 127.595574823053 121.580726825432 114.624259793353 128.113966507586
122.460829835477 144.612549022264 125.461701282922 124.190161132628
116.236538651031 123.190757763412 140.171749368266 118.841699953612
CHST1 170.622615783608 167.698883940638 148.776470607859 163.039564280484
181.747090380435 175.93459327917 137.567384694894 166.001279428607
162.664254855131 174.615694779386 159.856190281237 157.313737338264
CHST10 204.453822958077 261.775269058607 206.571433405844
298.706655333472 149.538429805372 213.710058427654 145.692644807799
119.161972571516 131.330974614437 130.189958197332 135.143532936828
148.949829934611
CHST11 135.621035941292 114.479886899622 134.499588539919
127.494672751434 142.326319895395 121.150371270451 143.477670915091
131.028469461315 135.93352186143 141.763072120718 145.610045243277
144.262612328341
CHST12 359.363523948825 343.946831770916 321.367338145787
389.051174954505 275.169415154893 205.630948393225 260.642170838161
289.667302610069 217.607230767028 234.156713102803 201.661941695238
185.661496988658
CHST13 160.060406629689 143.614075503775 189.784608429624
149.361498483705 161.755290291637 157.963634535239 146.245915271213
133.524698103972 188.242091666446 158.138862054715 170.079723856247
162.47662918768
CHST14 224.981439707657 237.473927775823 178.142393629606
235.091443641056 201.837695818264 153.585849286113 214.763232658841

162.54008293513 166.526875572556 147.208176455806 241.648044508699
201.167206950576
CHST15 346.065746911798 490.153210371094 183.863365305103
750.10819423982 215.365809536522 176.670752486376 172.03919618445
233.671878221507 291.261765585473 487.19625805337 159.390531348676
161.891809608511
CHST2 150.0674092434 160.941804737367 154.159124458167 144.526499066594
269.194172034595 128.16147857336 266.563990988678 178.89423690001
160.592404712197 219.814706416471 213.364858759772 153.613110881454
CHST3 493.622989939072 399.664121956178 395.588345893987 555.366765340014
220.345184229077 195.618369827396 183.50553222647 224.027605834751
229.729195963667 219.441959146815 237.312766830206 226.25694727283
CHST4 123.431969470387 134.584076788979 138.224383646894 139.246945739764
157.742948919213 128.952986088886 137.853137973756 129.891492052232
133.962129094978 147.351826045609 141.132694466739 135.253713075341
CHST5 125.150706047419 127.969833368917 126.741498575874 142.043121538885
119.26921157552 135.068883036882 132.8310947234 132.591496435822
122.371715108957 124.857461801227 133.067237748129 131.757695616207
CHST6 119.854401979951 138.444427173667 133.599098708818 125.439033142431
135.227424713702 134.623264303227 128.2409378561 126.446675008883
126.711037295629 140.890227770353 139.877953680967 138.53403492914
CHST7 369.838429521168 339.753059801136 291.50783830983 370.466403181536
226.701637237203 198.205113187281 196.16056297158 269.261595558589
292.037861000784 188.308588359317 202.483493595704 172.230528631803
CHST8 151.613134875009 148.253612913109 144.669509071181 133.929311295884
163.714231827866 144.762006548375 137.427338867944 139.286592412216
154.291717784335 149.117224130576 145.360466216324 156.84104238878
CHST9 137.861973133557 139.987681290389 132.224831869802 127.003974141317
123.240482782331 123.681353449198 118.646119379542 128.436499050769
125.130703953031 122.418378964982 137.047288508371 125.398875527365
CHSY1 1069.25265476373 1301.18893681695 470.95418656881 776.807656744828
566.417354841707 965.673699120049 770.632068590053 680.509421043347
688.247581763352 1042.40539020413 676.570119607984 690.246312542011
CHSY3 205.861932396193 268.660078500783 134.216088147701 163.704616231638
133.94206203396 133.939847853073 121.696353230886 144.0534341937
149.427217345037 199.991077347491 124.924794564647 126.995207465164
CHTF18 187.389039221193 179.821008257902 198.257497903131
214.355086255855 403.47026424469 205.804144075374 220.533113603343
321.910803473854 178.946928223991 216.9491329483 169.956827727468
218.559622620349
CHTF8 137.324247703318 141.645906784244 153.784923783551 146.539196738195
161.310855691717 148.684855678535 154.515091049526 145.03804642447
155.734509841719 152.72717701452 167.106870400592 158.224122084011
CHUK 360.361719991381 286.230656780335 295.047418816148 319.639103463458
247.053215951505 317.802633831405 344.529927973082 307.363302726074
317.457588721567 278.219561902233 316.722160860531 315.3930052781
CHURC1 762.182213353032 596.934339406636 900.233783407148
399.248044997427 365.046815163035 693.339946866196 901.644194512187
312.019603761673 608.351527783086 378.797952580504 572.913714991136
1284.19937294949
CHX10 119.693318959661 117.862532836494 128.548201588898 135.800997202323
120.504814127237 112.891050337488 129.391376518208 106.204350278634
115.816156193574 116.58452862953 135.310766630401 124.992439957206

CIAO1 887.471553448641 871.528156184927 932.862030475849 930.25842384208
338.739180103373 1108.61301774472 611.208083466988 907.610179738644
795.720196538039 410.954095610904 889.054356462993 894.100792722707
CIAPIN1 213.364858759772 211.736398352592 191.365014963729
205.612579504495 279.452819132461 306.068269136821 238.149614104568
229.77428612216 258.868119227979 252.138168292964 238.095599711942
292.524552445564
CIB1 2041.03024136014 2449.76376608893 1971.42527720714 2684.69013589621
6793.99737496637 3130.30693451802 4979.31150268741 5058.57872936027
4134.02613649483 3876.04966263281 4318.50925204407 4995.96906061198
CIB2 306.59090554099 308.330044958513 198.489278156437 354.783364481955
494.175437345451 183.676020826291 199.101099226668 156.557792402376
142.416237823488 139.921002429353 231.890885094917 210.392662092721
CIB3 113.222264855561 117.149781437983 111.467722538327 109.851507510788
107.48180681159 110.910693353871 108.604763499678 108.543795408713
117.74682797375 116.046983032621 115.545558600232 114.378420394004
CIB4 152.97753810644 149.223102493474 163.181323768362 139.127442770828
132.379706531581 146.531692460121 150.914644894869 145.821544099585
136.01865173754 142.073529553425 128.29486937905 144.031305845388
CIC 277.652851236546 344.021696764791 238.588762326319 479.395948662331
450.688800547981 190.255797389524 272.712242893937 202.622747952763
244.897869154624 188.39062571513 263.800363357443 203.727455875381
CICE 203.265559787292 196.450308368198 289.164969943385 206.203717450138
298.209907157762 304.013547190343 385.463209972694 326.570967321756
365.140886302762 415.957832362051 270.069634610818 364.766154056243
CICK0721Q.1 170.129558603697 158.330403004116 174.192898940601
161.066226957782 158.882535865069 148.493435517221 148.219406063533
142.984243805285 149.52880714343 161.37617568158 167.965981130329
150.272153646712
CIDEA 153.485731788293 153.519207482423 146.539196738195 153.832132196284
143.766249538352 143.870849001074 146.495845282846 151.148295679242
144.972694558182 153.261002653668 139.238936770274 152.346098251128
CIDEB 145.245316640391 151.27882940428 157.463122989559 146.768631086088
112.371497988132 170.784229026842 118.034262386827 156.06439656393
148.224587582189 260.129513331703 230.399207047948 146.95272087759
CIDEC 169.475714234887 147.958483355783 201.507971833902 183.604011732066
132.179747646893 132.413527125434 140.543382412084 138.907036194384
134.642898234003 140.213080190065 145.650356702429 130.191876914166
CIDECP 217.45700166961 194.775234425687 237.641190133427
222.675370226786 190.291185322482 194.445463745418 171.909358721737
183.32467147543 177.343586047942 189.90406457741 130.033927910392
198.753297290444
CIITA 135.079944196457 142.166650343224 160.322541355391 149.799681364551
145.338156577438 135.135834717193 167.713268780664 175.647878042257
171.795878750365 124.985249190127 145.690634269858 161.035985824654
CILP 1973.20689165831 1841.63015706195 1980.4589291261 3111.50508060882
142.851432926979 152.315376022976 135.166677520468 149.227646026658
163.099009810658 211.183499580683 150.048431572712 144.007071548045
CILP2 125.130703953031 134.436423298225 139.979025013908 137.864637728155
240.497413647633 145.331858938535 130.657342262492 117.523834738903
135.945030816689 129.899391127479 108.404733392914 130.083266044947
CINP 146.421195733989 152.738468369498 130.444766583917 144.606963457243
173.408784502027 129.933228671483 131.273916869754 135.061176512365
136.071856104688 278.88183549759 157.466686487471 182.810581377888

CIP29 1673.5387396582 1715.51071085304 1699.17470788219 1665.14066952917
1586.00600118477 2246.89876694944 1554.16120123599 2018.81730267601
1903.13326109546 1532.47920327769 2198.4268389426 2217.82628612929
CIR1 363.445110627483 374.718289636553 456.038981546175 326.703488388966
174.568253693395 281.341452463344 364.766154056243 278.775971122032
325.011269531407 285.882780066861 412.648926259488 307.597733293218
CIRBP 3258.07532987071 3655.63669142687 6358.5018930218 2666.06582231934
4009.22206756187 2288.63508719706 4299.06288109563 3810.98043907046
1682.64624665931 2618.64973582507 4441.92485662082 4410.68280125001
CIRH1A 934.173273294189 821.862271569917 1107.29687889378
731.284098301795 898.877732360841 1362.1486006778 999.159652508318
1876.64079464928 2423.84327263111 957.54149804446 852.926365088632
1366.06309834426
CISD1 2099.85702995194 2041.03024136014 2470.86113150126 2427.21100330388
826.08372791138 1095.52349352502 1301.18893681695 1200.03423010236
875.7694907383 1035.98574557331 1139.76755449089 1200.03423010236
CISD2 195.981555981238 223.638850960288 199.980312703646 195.20398666139
189.633348499854 309.36704220687 293.350292227077 340.608165077422
287.041585684111 297.609793563225 330.590986934704 235.183590602209
CISD3 144.958176005711 140.842305941666 125.631073476381 136.261907928796
156.783896946556 138.700301754711 156.830156083795 143.001661958814
141.453255692229 148.758316681306 128.211076776316 148.412895185127
CISH 144.240470871638 167.29332098671 6 149.899789105328 151.196713920345
194.475822533231 168.805630890928 171.650247980576 144.278830378899
153.181097279473 136.069631519097 150.904618960507 149.689071018094
CIT 111.1002996982 110.662552491706 117.399753685854 114.691842968597
116.126923911804 106.393497890486 115.058061620379 123.400143662218
117.602986362773 113.479382235297 121.908945872994 128.574419295906
CITED1 115.995309578182 111.336798977475 114.77964341895
111.312348806957 113.005774989833 121.743701509981 109.067239220744
114.574362093931 104.36042819601 114.65036208773 139.576261630082
112.916256784817
CITED2 766.710479603963 605.795439342384 786.160002434066
499.048754087189 1751.22580157752 655.665164535648 909.727616721285
1179.42590997099 683.861021234927 960.775251871864 824.427857809495
900.039744829725
CITED4 323.788197265954 302.219514290905 213.652091843896
265.536429548886 1627.02882286283 529.04238100948 404.95944990868
196.638594797353 244.440857688518 598.095370768587 281.846246549202
242.382534384155
CIZ1 172.758230728437 165.682617337317 160.644747532952 206.186324475847
183.013090662119 127.445199722147 123.561016079801 135.144220487117
132.224831869802 146.70298187005 163.899808733609 131.913786256215
CKAP2 169.720414035325 168.314966639178 180.408639020724 189.362958041175
319.018213637357 422.590112942156 203.244917049745 320.812909889014
274.974304050999 302.847274046748 295.330307669482 351.759100605991
CKAP2L 107.498500136859 134.403789206552 111.178024950204
110.475453268997 245.640193659159 388.002365247498 177.132361355899
412.582051585078 309.5988292104 337.87475687838 175.593421867771
334.607533045929
CKAP4 1264.90442763788 1164.61390835016 936.221322010828 1283.68791110988
2452.1304336603 1297.25632532536 1929.52146517699 823.297606185721
1203.47834989609 1187.62490367696 1810.68433539154 1218.64951314553

CKAP5 490.464012303239 533.893360199271 573.244735292944 577.801009921199
433.751675703425 657.77730563722 572.262848718437 566.417354841707
618.829716818725 627.335757721489 311.208703262497 425.826693178023
CKB 4527.53010866974 6585.34962362923 5347.78127066964 10048.4260790604
1601.19401366685 191.740596601895 192.980781927122 661.096668635469
192.655899720909 266.918302307596 1147.33034082693 137.803749343493
CKLF 377.353441970543 383.74357557363 396.126485121994 313.351441953859
685.620637807134 1046.13353853089 869.245621105614 986.65554605455
1255.02720867812 724.442411170786 1142.44180956604 1426.56252600395
CKM 122.190734676247 183.693300680495 134.378951325825 285.594100976705
166.59781955922 115.142111396055 120.853961156534 119.905127545336
156.880120259304 111.020070405368 136.783974483005 116.572001618531
CKMT1A 146.321713521846 168.124936529582 167.173670781451
152.832573288735 476.383294794912 470.746477818201 422.434778100827
282.442528230519 619.487800014979 704.653073777277 258.532432007328
1125.95664591686
CKMT1B 140.684066288019 146.044279076949 140.870849757111
119.843989506832 395.786295710815 261.844708446856 273.369722814068
196.117609477292 365.362177671146 426.649095732979 206.650760711745
614.957397883514
CKMT2 256.237185590901 259.575461599189 340.366016191232 307.81941988503
139.826273596452 169.992657041967 152.845126167802 165.808660192901
167.063762404009 141.553465481789 149.808222023726 176.650953513413
CKS1B 366.705048374078 351.759100605991 378.585058475083 309.098519056843
1347.10650016064 1744.58410715956 638.77182631975 2500.07677977147
1441.29676998365 983.777608766079 664.809419789814 1699.17470788219
CKS2 211.660148497276 274.494074082359 209.867189613436 200.767743455226
2074.29945034697 1243.11900823259 502.731019454502 1578.87371296418
1010.05884008623 1071.83063260499 770.632068590053 716.229989890801
CLASP1 826.08372791138 918.742625270041 678.684323057327
956.153239481189 321.492900106953 406.224345292389 325.80154888448
345.686640885419 312.406286993239 353.385210690169 489.022371276542
349.773289724658
CLASP2 172.631499322646 159.655362710407 215.453915231774
155.924471321076 184.041062308263 275.437030326768 187.997940126066
175.989742412417 252.789376168324 249.263194321516 187.997940126066
227.418630852215
CLC 146.411873503163 141.064086298948 147.818956338752 153.719751724358
174.396061906109 157.099868633958 166.1677526 241.782750858091
219.129781831192 149.188357651896 152.73588666971 156.843997723699
CLCA1 131.543439103033 146.090291252325 157.517955771507 136.049167597016
140.895053453126 135.48250324478 122.10489926804 135.433229702209
150.885460975199 144.763700813057 124.927989289122 134.66169946771
CLCA2 109.140708667012 362.581768268116 108.296042016208 133.345143691828
358.355621069787 401.267425721838 1783.8031212092 111.448871835514
113.995301093609 5846.17594234628 216.485113690708 219.878230463206
CLCA3 120.883785696583 135.049657594221 121.387799881297 119.005230164124
125.574201687659 114.200099628087 126.162713269943 120.493082096235
121.492896642899 117.870230005125 119.079680805247 119.819082491115
CLCA4 174.100921524023 256.924134409998 268.698146407497 157.277051544704
348.111872425949 2508.50428529255 3938.94050658589 383.259887828605
241.856820205666 377.538276846051 1662.51399707484 631.819659701092

CLCC1 185.661496988658 183.42877315644 174.587558727209 194.921021761976
245.042781391047 214.229228850802 201.480444219547 273.668871896558
228.713818522476 427.766660764042 182.028675726969 236.985483155452
CLCF1 177.123497519231 180.658930225735 141.264096014725 172.197077149985
203.276353321185 194.257024100927 122.742891063864 123.61956613951
133.266524623338 272.994195639694 161.451253146578 148.776470607859
CLCN1 150.775805918537 142.534195262988 130.416957431512 148.326282600684
136.301442794928 126.948090173876 140.274655651173 147.8838487685
135.862790395984 133.507865919719 155.63671938031 141.775100066311
CLCN2 144.787836177147 159.479962316199 143.741676966126 147.666724626127
189.90406457741 180.354899434843 170.111653122193 159.138344477256
136.781417098789 148.470120335761 148.492328039604 159.98205335759
CLCN3 240.07282016658 254.552034511333 261.251965523684 236.052008824688
285.768837438859 349.623729591612 447.297590650141 377.083379021079
499.048754087189 400.23671843715 427.452114719877 657.77730563722
CLCN4 143.129945647647 117.883635746379 162.267841475739 130.694506122253
123.275246170603 130.838350486331 133.617501101389 134.53861441495
140.509950286228 146.484364264513 123.198873421356 142.707913210557
CLCN5 166.355086055404 157.028963417138 171.006582229921 204.137195524755
180.590215659031 161.172420368335 185.727947276958 180.035652063942
145.97330701968 188.623897093065 146.26304624648 161.948033313443
CLCN6 289.257002046658 276.714748212739 319.685597963267 313.967636827811
205.65398844108 200.944865673409 208.983138060317 220.242344043437
214.739818213164 195.906137340918 208.611557959861 191.467626362851
CLCN7 1327.65573168233 1699.17470788219 1255.62991601665 2222.16226526877
4291.1724030909 1181.1611856364 1452.63736288691 1600.10235468018
2068.04582900231 1605.2454230722 1301.18893681695 929.198774489695
CLCNKA 139.980504765791 164.525784478026 139.404820141366
141.66312605042 176.128369308989 136.489566741917 133.423376063284
131.342562516383 136.290486122369 127.859873416515 142.675752131935
155.759861336991
CLCNKB 135.790955294639 144.902807664593 124.001295849091
124.652072110334 142.221542344035 127.336648342283 119.866898356501
128.63301490285 121.231939494619 120.465934391246 144.511488474344
127.874934513708
CLDN1 184.187847236508 349.330629649093 273.353254857377 131.832311911703
775.382397816601 3055.80298721424 1374.92806285803 3046.47354587949
1116.95795024994 1240.4863200811 2583.46653460894 423.562598841704
CLDN10 128.102787239877 134.412129843957 138.079494235456
120.551603446364 119.612288460361 139.123489349026 124.269446398587
123.086596566142 130.033927910392 132.079255703621 139.793683692104
139.687574432387
CLDN11 1171.62361359992 837.603727062008 687.987647193563
384.051170099325 179.764260117544 247.622351589081 235.091443641056
936.221322010828 284.831817899986 144.725650040212 154.816213491233
175.767556472012
CLDN12 367.382105696814 368.276805218658 338.822464893411
330.457534783492 337.487980551006 459.652708187549 525.138476928759
369.646624431062 359.493075382333 723.807658768693 478.38361571213
706.628675634893
CLDN14 235.372592051464 242.614881095485 224.062024091915
274.281532397691 222.484058975856 209.44506543433 621.489906442001
259.796092536601 288.596513753617 211.844563155792 319.868227988842
296.24781576525

CLDN15 196.703794742237 190.534176839652 178.728925255163
175.902751522861 175.199369463703 190.45739385453 205.908052642222
311.848379951974 257.100909006193 161.744366588564 204.438365139666
287.751093664085
CLDN16 151.178586065066 136.332399354517 134.371254010574
134.730275384832 148.288798428149 145.760577027524 143.070170783764
150.648444878067 139.387445141015 128.147484804104 137.539385624206
138.700301754711
CLDN17 112.714282429822 107.67395123577 119.112063328364
108.415715678948 117.22284971665 117.024039521425 110.707755436868
108.34997967774 111.152235113722 110.501341211051 136.511669406292
112.565341690522
CLDN18 132.5130209643 129.205783382977 128.134246199718
133.801549026691 128.717719117128 132.164132609317 133.284873295487
150.058160923921 134.487476333464 130.850419527271 134.658664100744
134.623264303227
CLDN19 159.223980639045 146.136820062093 161.769370009823
147.60284257326 148.789295862953 145.346970289157 160.562164904057
155.129304059037 148.577336081778 154.703521794209 141.670426206169
144.896960245309
CLDN2 143.061415008831 149.565725707356 151.532859494021 144.145612985703
138.020031233095 158.434048174501 142.013370954791 140.616811333466
140.456277147131 122.664353374047 133.069347559741 148.126745515891
CLDN20 165.932628858503 164.603403251634 152.680068580858
151.739221728872 140.26206122305 151.914252332792 148.678869698203
139.693261291326 153.815239954928 151.92638745703 166.097952462607
141.730912072471
CLDN22 155.30926640293 153.592819860747 158.163599942547
158.446840764515 144.662545137915 156.481871341275 149.593135821045
165.880071298733 163.516075721791 145.671099346237 154.515091049526
145.835240952992
CLDN23 216.154203111101 221.45275440794 179.973655632636
146.624631389743 1910.08485008041 1652.77814259264 760.272612245092
1332.82617944131 522.400055538966 258.384059994446 1738.96798001895
2172.89794460273
CLDN24 149.645548888832 145.152605166094 136.528123792599
131.000186898437 132.74579603362 145.163084967499 142.179197020805
136.355319997318 134.273326732349 133.587927144452 139.195281937545
141.59474425685
CLDN3 124.526220300907 133.420267697961 126.737230874367 124.29420915051
404.618867159141 143.245660951425 117.602986362773 226.458509834965
268.454310218604 138.021559586328 184.602704530362 131.12775424877
CLDN4 135.800997202323 148.227581116046 146.940545711162 160.932913244423
305.615423200097 194.655710454472 186.185055364718 163.024215671479
182.275998840116 150.294432623474 180.761100930677 169.220974415101
CLDN5 471.15333189869 1111.47772523331 282.511614929917 787.615195903993
167.782882015229 207.977817300376 141.722817732641 264.383922859446
272.077877821339 119.972337070176 147.459385142695 132.956860864455
CLDN6 108.850992642446 109.425878885118 103.888971448792 105.408689610638
116.784024112826 107.466184687811 103.826308328446 111.632811186988
115.427201752401 103.826308328446 114.774530966446 111.985567835684
CLDN7 158.175138008678 249.763375041547 174.075670633438 134.329334422886
2760.57475805426 1395.63393859192 1084.5920943349 978.389902096994
906.359365544713 309.75140197669 3140.51157972795 825.345092964844

CLDN8 130.912137250515 149.772157318769 130.935922915902 129.884064654761
137.46978401237 136.701400398129 135.155955441604 136.042982529628
132.769455423385 136.432109029368 136.904851056919 138.645156942894
CLDN9 136.330636532576 130.433405111592 120.679474100322 131.179786925029
147.604213625382 124.821581853501 136.135223482208 128.950308413612
130.790872334222 128.2409378561 157.709832297666 130.562422910426
CLDND1 1284.19937294949 1114.85422895959 1609.54149931375
777.651238374269 392.004079531068 955.604404066769 729.499296070289
689.821785472506 977.999660785113 1287.3125466789 679.822276398569
964.093668483033
CLDND2 301.695991929957 294.280238648737 587.388038484307
298.827660296715 261.286548990435 296.375305136807 177.524818593325
191.651818696629 239.277801396834 154.143331594812 206.340478393262
184.611146047556
CLEC10A 154.086510608016 140.840895590527 149.032116392457
146.495845282846 145.754504160436 140.573285987781 144.772914483212
149.026243159195 152.071081175788 147.688664545959 135.641355114263
143.537785553911
CLEC11A 404.073802241516 404.95944990868 244.117082987416
419.047108206042 410.138738410101 349.330629649093 201.085046871333
195.282045838381 212.147155923729 339.172031360524 155.023934989559
225.505978789388
CLEC12A 143.713962738562 143.867013358737 135.603160096973
144.92594578882 138.62308708151 146.894793774087 137.225659453984
142.819669471009 157.252701277198 146.11669387238 135.797170561599
143.587562550004
CLEC12B 124.924794564647 132.924341641217 118.24368469845
120.799317509529 113.845770994796 118.283346939564 121.508233749444
112.649012857276 124.248997533959 121.113186311514 130.509415570889
127.720117789235
CLEC14A 457.905741500454 649.052806726591 289.82072316082
691.205738835917 234.947745514175 424.578659172414 201.987081022654
400.472835504879 412.941907828617 265.791342287571 330.080428155378
221.45275440794
CLEC16A 525.789451405743 507.342877360289 540.005877322817
655.665164535648 372.255208814622 402.824964079515 553.198321853202
566.914448800423 569.236322997211 555.109005518622 547.817028243811
571.64723305848
CLEC17A 157.821491577808 162.026757124868 151.877035446575
168.21856603538 144.853868519575 149.18603646016 170.734355440231
161.17923997423 156.65842876856 156.61494867164 146.64835267642
148.130993521745
CLEC18C 115.002176041974 126.848211027661 117.040953580545
120.922042875444 117.443721637362 115.194730717797 119.315932907373
109.087574814292 136.72715888684 135.758133410577 142.709768829207
121.330383384978
CLEC1A 166.85671415475 169.147351642004 140.202762690515
151.318320077986 127.829001155781 162.808270845998 123.689439578326
160.407690552027 164.625407872446 127.278117740813 139.781351410748
127.781732819297
CLEC1B 122.610529927104 111.568329540016 124.285969778268
117.244895482991 128.678484452077 112.488125161387 115.631414599991
116.305011582033 117.770032157393 115.299986138153 116.828333047376
112.268518171041

CLEC2A 121.871105084095 136.817517693076 139.039276904833
147.192815700248 132.720599968449 129.338551879984 150.602976963975
137.744318243173 128.922269289649 136.790457887057 123.889427666612
146.039797334426
CLEC2B 200.668298229815 189.575681012578 155.438218501542
196.860228537112 147.301909062399 143.62315472858 131.917194849285
148.030579593944 163.004844049672 308.088967616175 182.495117800073
144.179880084235
CLEC2D 1643.08061372695 1614.00098175383 2365.99940149374
1307.96033038529 954.488185198006 1500.16816750361 2099.85702995194
1614.00098175383 1531.54387233661 2605.13680290987 1628.24528041404
2014.48411650613
CLEC2L 122.989642938474 138.152641686151 138.858138379065
135.946808893146 138.647667867725 132.089375920534 150.247639459773
149.262900833177 113.504670764963 127.398873370484 139.755211300398
135.399032687551
CLEC3A 168.091278015885 197.938447651153 220.217745732407
189.971144220643 138.43301474649 134.09920571607 134.10707191601
131.164778405297 131.83430682495 129.485710719291 135.605541734293
126.470239371932
CLEC3B 3205.74787769881 1706.06615968622 1226.74043569231
1762.09874961415 163.661196423902 188.53221484746 151.755205401737
154.616279878492 180.658930225735 145.463105638655 166.767004180603
150.48236051337
CLEC4A 149.636887782574 151.81754291846 163.119211741255
152.885721101687 146.392657608076 146.692827583525 154.076216899447
171.410912120195 201.152639979358 233.975864722289 154.218967720305
158.292581724934
CLEC4C 149.740404686695 138.873925577739 136.888915439473
155.587018319981 133.351623142435 145.338156577438 144.581747568524
140.980119908597 146.574656147816 139.070571230254 138.789908033669
154.831307586538
CLEC4D 134.58933109001 133.16973309664 129.985797334276
135.665577776938 145.043494048779 131.17119492692 119.449118660481
121.778756075848 140.232244264861 152.81544746536 147.910699446264
147.962721373177
CLEC4E 151.484420979498 170.035994634371 178.284294783418
153.379118228796 186.268881719287 157.81327663167 170.079723856247
157.216793735479 169.403422185018 164.415832011273 155.018009545475
170.54511631708
CLEC4F 131.210866639392 130.231160879338 122.174036435094
138.664401460739 138.424464248567 141.695537669891 124.297848065172
127.675206134992 125.884565343075 115.605206174867 134.504873813959
128.322884322951
CLEC4G 161.189717452004 136.415734370763 159.099495828517
149.929454280702 159.245344068037 154.466614452651 141.685620602226
160.010501226517 154.2228051929 151.167024833049 132.425596223397
149.772157318769
CLEC4GP1 135.647660756548 142.768691899824 130.110413621436
135.031430487047 124.215466523555 120.387275261633 130.004329307977
122.405469833024 128.978197156274 119.192893408873 134.022324496729
133.94667806086
CLEC4M 144.792035839249 135.516856254532 150.566842106244
129.573520898314 169.963805688413 147.565485281003 135.781190598068

150.639235242268 147.169765031208 153.658747612827 136.2336179571 154.449058407217
CLEC5A 109.044879188971 107.841100741858 113.370762349233 94.3531468700382 104.934718065476 109.342230674869 119.312201620247 122.165046954625 123.518170356322 141.082569618216 136.508969220268 113.177330690612
CLEC6A 171.816841286562 151.010493695217 170.290455446376 150.694215349678 169.022351348819 153.085975710144 149.726968520487 158.784190231286 160.435789207464 168.773340961473 163.170983091116 165.262738484387
CLEC7A 118.593717189375 118.673325109944 113.315182274675 109.91537150286 118.366859032979 119.590540166384 111.802672864815 121.150371270451 126.37572518012 134.986030185477 125.439033142431 117.404906698431
CLEC9A 150.754492902512 147.034021626893 150.906106695444 142.017169259522 143.416816860089 150.663062131717 138.455775926409 142.151209745109 163.915489303508 142.255657518926 162.147989253357 149.511776997149
CLECL1 107.344825308293 116.839796680107 119.717408713154 129.221482161409 111.785821053048 112.661607612444 111.876915242732 130.797498958616 153.507553306723 119.48998115497 128.669887479835 114.072600760808
CLGN 119.172965618216 124.69564501518 124.012439490264 124.592771536872 122.531675048414 126.450300907283 129.369968549738 126.910075365358 120.135349166633 123.974409226349 113.879459563492 126.086991456747
CLIC1 2032.50052162637 2217.82628612929 1918.14260920544 1937.83242524439 3468.14455177277 3253.76017336684 3408.6535437109 1843.76920134139 2574.74962955426 3235.38149926838 6179.86800320807 3905.35300751401
CLIC2 179.451637129672 189.027594735456 143.978912435831 158.25554353787 122.005340508177 134.318290950377 132.297716337104 144.199054675488 145.695402948337 119.859508630454 150.508851767517 127.787890405862
CLIC3 195.595275594235 183.894417745549 134.633286312653 147.760782452857 1564.8868894162 130.073005698714 297.862653495715 241.019436415703 716.420861490714 2932.64120183406 687.700356531038 178.885625009973
CLIC4 2046.64499507352 1593.49895307361 2129.84680802619 1601.19401366685 216.805365763069 399.963640581639 223.638850960288 251.939696394174 287.374706071354 822.129234065933 185.783374935551 195.776369840059
CLIC5 135.304167695152 154.046550866249 132.034701177759 131.552276774901 133.004765428575 112.977469350139 113.78323485587 114.734439905052 125.895972730616 113.636570490048 110.024815508703 114.441223528256
CLIC6 212.730163981978 371.941204080246 294.997671630798 174.471304281502 151.865924163231 165.496065029161 132.164132609317 1883.74008471233 224.062024091915 193.945494721138 1573.03248861784 260.787104931135
CLINT1 874.816697884109 653.047570485109 1130.32966481315 637.695341427976 459.30568829087 653.264945856916 1253.05700051015 1018.80178630497 693.339946866196 1388.91423772335 932.862030475849 878.520681466946
CLIP1 873.879064183441 701.481003619067 871.528156184927 736.603605876572 355.714202328378 390.284689962661 441.873247266342 410.645457106122 504.141861790903 670.856008549143 416.215253990273 362.376153348015
CLIP2 266.71318061066 258.807028561407 201.616302398819 326.45984882427 199.077893147154 158.353171576491 195.595275594235 172.698181201849 165.576758218571 151.852294315212 174.027190728107 192.980781927122

CLIP3 5207.37974585707 5410.50736002544 6214.77579017084 7342.79622458743
208.385295246155 185.002106079042 183.130891975122 251.993821914623
324.481229901658 307.16835174775 166.097952462607 155.938283930571
CLIP4 354.067771806542 308.281798808746 696.475639532595 255.225145197422
156.296046600921 223.918931440768 246.88857694249 184.260835471542
177.896726792666 413.000072500071 147.362120584524 218.917357155289
CLK1 857.55777793754 759.534249620343 1311.34904760947 621.173315178959
255.462706428149 498.812823949508 472.030150842569 522.400055538966
521.215655189324 745.153336165802 552.494327858264 502.281584081805
CLK2 238.654367531982 254.424207773566 239.871536598083 259.472319994929
316.409887372431 283.048002376743 306.632103708321 345.649572073924
278.005620372937 259.316557834724 355.714202328378 350.983969007868
CLK2P 151.619329363527 151.083551412109 148.094036534405 163.926432479673
151.167024833049 152.353494489233 153.687536747147 150.247639459773
150.412929574507 141.857434100065 153.653453808799 155.91493956251
CLK3 286.282132382462 320.597495751519 326.758278851698 410.216584967666
331.937313503755 256.729891423908 334.916875239616 252.697160031025
321.31562287169 365.727197088581 284.831817899986 388.477382640003
CLK4 230.546762514966 239.050379187643 316.524115715585 228.968779778407
136.691865286093 161.084088366981 178.854873582873 181.530767521222
164.865846124876 196.566937667522 217.45700166961 160.227729155903
CLLU1 136.466367853188 163.22480684222 152.54039461337 162.464201058576
140.254665008195 150.933724024651 173.97085982677 141.512008928271
137.070326499918 151.632801137039 137.899291413431 147.77916610545
CLLU1OS 114.242056888286 100.216750525986 109.680532131035
112.747067417774 115.920514336615 117.069094451283 107.949834974369
115.80245224388 111.876915242732 111.886543737378 109.274221164316
122.549274599774
CLMN 150.156865995098 149.471240458766 141.292421722594 131.973896985615
185.583997792931 140.583804906052 157.301617257941 137.745887988474
147.638057399165 135.773386496434 165.890492422031 137.877756077011
CLN3 226.960024551778 270.490971664802 206.825575257029 305.768201359278
599.52440136406 306.677271064305 353.243400332397 296.088938872643
273.61337724143 223.638850960288 417.442660073269 229.567444213626
CLN5 328.969023478451 338.510045856515 320.812909889014 281.44159425243
215.019239034819 391.185365567603 307.124331101976 287.889774984754
273.432712475089 275.509805464276 486.334840298921 334.52685347521
CLN6 148.25001336038 140.678336884951 125.500790447338 140.099069486005
330.850098426464 163.432972512188 174.075670633438 154.190469538568
174.461487057542 170.164668347783 188.973595445056 164.202554380274
CLN8 158.263657373911 157.997750997487 159.41078351918 138.936872956065
140.323425587628 139.025598691696 188.952730860801 170.452989060697
144.675182563174 157.410205523901 171.69834783037 155.608876496443
CLNK 139.260868989409 137.006917665541 138.810236692956 149.483826392464
134.172033051676 121.85247363239 144.007071548045 143.093156971372
133.419753427423 129.780648518219 151.546908501054 137.570343435498
CLNS1A 866.413995755699 921.546033242474 952.357976050712
825.034504959272 1426.56252600395 1120.87534972745 1923.19560386389
1072.37500318471 1361.54107116915 1326.95332435394 1569.13457493554
1456.76683429755
CLOCK 240.75934760601 272.500572765647 266.964673763497 262.671819506189
186.167877791105 231.600532144945 252.015257713669 261.251965523684
254.036619126409 274.235001739215 261.117845544884 257.100909006193

CLP1 197.618757939729 201.441808401878 217.940575516933 219.129781831192
228.414049417352 292.965109701719 260.921177985439 228.713818522476
240.341031437493 251.869781014029 222.005022189667 258.727309442475
CLPB 143.093156971372 131.263641389894 116.038950886552 127.150222504237
125.528638246486 131.904624845305 134.27783583528 141.463324155943
124.796876920269 136.2727333616 122.679628839976 131.879526177089
CLPP 328.486476842582 381.323457262383 326.570967321756 372.81255844018
1084.95095717937 634.774041584043 468.82816089485 514.799179506754
337.271784551586 460.408628542721 341.973002478932 426.649095732979
CLPS 149.004343851423 157.248117633013 146.775403409562 135.038195181839
135.596007994731 146.440630638891 157.901967487277 144.321757243275
139.572562105377 145.988387859158 135.313148180961 130.33205883588
CLPTM1 512.184962338131 585.371892598422 365.82705202869
756.390518977556 3008.43964505208 858.020739710943 708.056533282983
665.602699607102 717.025533065694 499.683188662742 584.433155497378
572.049540360134
CLPTM1L 965.673699120049 990.55038742453 868.066644155313
1013.03054571763 2596.43650409044 1307.96033038529 1448.64875525408
1465.2419150392 883.702573960415 838.267069860172 169.077657790409
985.637230639964
CLPX 235.091443641056 255.198862778405 257.813193543819 232.105867345662
288.370985514978 354.067771806542 376.791876040886 300.83667045718
418.163219634152 436.482580413523 254.552034511333 350.22221782412
CLRN1 151.780459290205 153.350690419636 165.480489678822 161.12121879514
162.274094333189 163.41031443893 159.346890686719 149.467626202395
160.91894600916 165.274189050141 162.574338133241 171.254015232189
CLRN1OS 139.991780068793 119.75069850668 140.904883114871
146.306467130749 141.606501859513 129.165440930558 135.725202462502
141.691135410429 129.421772713718 138.557464672111 121.44564199541
126.127630885864
CLRN2 127.183924911179 139.932849289354 140.123348569709 135.003236040032
124.164523121103 125.609542142983 134.331420680628 137.850437814788
135.776154891006 137.79714923769 132.186120302011 128.788443476759
CLRN3 135.651829752462 131.532660547328 120.979417552159 119.352995485911
122.996616773523 129.334217858282 121.53632327554 124.533543270311
117.923459503989 118.724348027528 141.059033576475 124.772219140795
CLSPN 107.930325780047 107.239841465534 118.875640081461 117.22284971665
120.727504500018 122.68719599986 106.341910466588 111.409447323674
113.822580779824 115.097376525707 117.598778415488 122.773736159743
CLSTN1 723.250763797522 962.559482864549 576.10291008626
941.572186137467 826.314416168552 517.881547706391 591.641325402032
448.260934224454 362.095074331893 816.266384662324 523.717949503639
708.056533282983
CLSTN2 141.719556176029 146.16821907742 122.320966091375
118.836387680022 127.077668562103 116.839796680107 116.421771593211
118.558673085303 118.126133973555 147.285764611006 148.090431893307
123.058261039164
CLSTN3 160.482734190577 146.306467130749 162.519642881848
167.468390483999 189.915720614467 163.67719444924 199.87003105816
157.5032461329 159.245344068037 147.309245779031 156.594337089784
202.024504592763
CLTA 607.499206410372 614.514126276981 671.387334588652 642.934824227327
1399.41653736746 636.382696018633 683.655205499779 722.399669963921
708.779490017407 1224.49891389348 1096.10636044885 549.460612060753

CLTB 1445.72064343302 1331.18605673705 1231.37475540937 1724.22121078776
1562.9779683714 660.096727566678 821.862271569917 502.731019454502
484.568420044471 3073.40543048878 825.034504959272 1257.00798908017
CLTC 228.45211887819 266.99947481587 188.277563426829 205.244843885276
228.675099004411 337.119524477446 396.212732639272 318.06081614151
336.380724981693 303.762967437478 279.495192828873 370.140588087649
CLTCL1 146.800324349321 148.49560238679 144.839170491334
151.267897610115 155.397301207102 164.057147728625 166.085469368498
132.890396520305 152.826356673175 162.841318222066 135.175806654971
155.104697146664
CLU 134.031960707639 133.401620574886 135.033601686007 129.34905533265
129.689855810051 133.906867716171 129.822926691091 130.659631155616
131.212705319485 126.520909083643 131.736694577111 131.97938329944
CLUAP1 5558.51089816921 5300.67886821231 6919.4226838505
5912.48904507462 4690.93553472125 6114.33638002734 4882.16892310839
4749.69040198959 5195.2087608308 4690.93553472125 3093.38255333143
4819.85672511343
CLUL1 134.517774302213 130.669085802748 134.992686224944 134.555873832434
130.016401638936 131.961236721149 130.052593375075 135.348086420636
130.185134787768 136.398521726896 140.682757521394 135.940629737073
CLVS2 150.870830294349 169.000137586852 137.835936352525 157.797222258336
141.131578372349 153.566080603874 150.754492902512 150.531753446398
160.19942770666 135.915252483475 134.592495777159 157.878110410517
CLYBL 297.862653495715 341.973002478932 325.96632528242 443.623904480087
166.640325848102 152.017655364287 167.330860677012 192.59376129133
163.41031443893 320.097510212422 190.138730960643 212.987082617173
CMA1 130.173734963758 142.848438163322 165.314290446922 137.398748156909
142.478528530514 137.894482933806 132.637443692683 135.938590242744
143.572668991313 141.373059612034 129.358112503486 138.514356748249
CMAH 188.952730860801 164.066839397676 192.059255232313 165.992542962911
139.949563856936 160.543465620248 145.351662473858 172.355215322173
177.065616871886 154.049004176595 142.187694439611 152.898148619937
CMAS 553.198321853202 542.009873308344 624.264994653057 508.735412486964
647.955521647635 737.843412273272 929.69741581954 1037.46485756166
818.225731093346 1125.28599595312 687.987647193563 1109.08288440559
CMBL 655.431236290512 487.696347726467 617.474181543858 585.371892598422
211.891710626953 239.33642662112 273.303240462047 284.574888587832
266.652805624826 243.979769719186 357.514919818489 285.659010982301
CMC1 208.476367897256 205.830321788612 236.508498739509 221.635333304767
509.618968665191 367.289651112346 330.281193038198 323.244664586314
552.662610888399 465.809961695043 248.993716339029 316.281469585496
CMIP 505.704957182936 675.083275037052 394.962978677736 600.162650426961
607.979208454176 466.777454704543 618.140160251927 588.076436392983
827.157920721418 1312.44215746801 759.802694618208 685.620637807134
CMKLR1 138.909419415137 127.392877191785 112.219585614955
121.865696440776 124.222529964098 114.768639931659 114.913534674899
117.445284668654 136.659134200122 118.60878652724 133.778595695007
112.054379249781
CMPK1 1110.43791444289 922.877217016704 1253.05700051015 649.906677394861
740.196934996626 937.375361806971 1032.11574332305 1038.34611017219
925.656701707763 922.009043194023 736.140249105836 621.173315178959
CMPK2 148.396148091332 124.359615295938 145.217261514886 156.402578892869
152.717642489582 143.1288704752 176.236140830355 154.843607752473
159.570288565855 134.73970457131 133.236084190243 146.462544135666

CMTM1	128.570067783535	134.30624644595	137.280933631075	139.568610307888
	136.152827218905	145.578721432709	135.504526903482	133.365793958118
	142.191569406599	139.449895159273	139.39442529119	135.56878810365
CMTM2	124.436549041911	130.890314257211	119.739204799544	128.303046695142
	121.099290122973	149.007288765628	126.068445507098	152.863035012564
	133.223310036542	150.52625734893	120.333487171575	130.639974588163
CMTM3	345.565843875296	335.142319387164	211.357321758564	287.924891622962
	195.929328710358	225.142823691625	266.681711962236	233.71520737817
	256.687384001447	254.133112176024	416.546844844021	284.891415492568
CMTM4	184.260835471542	181.79418355897	181.081050726043	188.910933952136
	270.520229426015	221.45275440794	242.096692715029	300.602422224047
	361.26540006942	244.054469051058	272.193578770314	230.015981563049
CMTM5	158.390337945372	148.665627690915	181.976252906929	167.620915011868
	134.719531559829	133.777238071968	150.893280574091	158.23562423217
	158.039920243116	135.261561433405	168.477388641982	143.019052979243
CMTM6	455.313553278155	489.528040775004	415.113340335205	321.049000153127
	664.809419789814	1144.07896461196	879.809795251142	950.818917078515
	1316.31088187364	741.689442253375	1424.79466712859	858.742139354587
CMTM7	312.406286993239	334.916875239616	268.406778092906	261.96528977292
	1992.71224011906	550.566402986082	1411.2586496281	760.572409199179
	1597.53010317504	370.945254716657	896.185223980593	625.932910814208
CMTM8	188.605328108654	216.427103365727	141.753560518393	165.530397739051
	992.744093752678	610.915977337787	715.93836911426	293.925995360234
	1537.71464618832	345.649572073924	194.961531388323	302.381946749711
CMYA3	109.740801301993	124.02280670421	144.217621961908	117.30724482798
	126.912226185444	132.647152200128	133.146347243168	131.473001455317
	126.561067704708	141.147683040881	132.570225520163	130.452929012459
CMYA5	230.975434289172	205.560952959537	215.40037757604	183.314276033531
	183.795632040455	161.525131363333	533.893360199271	165.149464497921
	366.473258467746	121.799209715087	195.776369840059	164.458565398812
CNBD1	138.017249996128	129.564482772168	135.959893035019	135.452340998039
	139.109712542702	133.744560712587	142.05571112803	130.091692689423
	132.637443692683	131.849442776642	134.149446827982	133.195030751476
CNBP	1129.76078220457	932.862030475849	1713.93035637146	685.848203651889
	574.035124314622	882.037055216609	1206.59866455529	930.704688414069
	1467.50846948144	1608.40826380713	818.761934233826	1093.21579738952
CNDP1	128.375390373307	121.441901286752	115.137475087954	124.203250865069
	134.457932303561	127.424848301217	108.118551275778	120.971313945446
	126.782805974059	127.3079781139	139.47828235176	118.572988141773
CNDP2	1153.5175534971	1623.92991386706	1239.13016938378	1106.75785702993
	3149.26998719015	2979.27692778783	4166.43837922103	6522.27232911897
	8163.29201165065	4404.79209330918	10862.3952192141	3103.4192907748
CNFN	165.493629431627	197.762169504041	148.421611987655	156.674411447713
	818.225731093346	305.16668987495	249.064573038094	229.483578414375
	458.953009417683	4276.70912292877	198.456392063513	197.526492324088
CNGA1	114.334059060506	132.873213182775	119.9414421871	110.1278204598
	139.125340284813	121.278346729876	213.791379190359	387.908774438461
	236.592839463278	144.896960245309	212.591357254196	206.571433405844
CNGA2	126.089217853289	126.670227417269	119.308345676905	118.488997046932
	125.470172259885	128.436499050769	129.213523302268	126.279119798177
	126.895669261503	122.751674866182	150.778292242313	120.748910864064
CNGA3	158.10628865476	151.342902631318	159.029406517237	159.446705611995
	150.939877261936	157.172970235254	149.207481665265	154.552605305297
	146.877475544399	162.683665945044	159.082447644277	157.684694038023

CNGA4 141.248526020456 140.655002540298 147.036768856136 152.134895623504
135.665577776938 152.752870674108 139.607944722497 142.143709425075
147.882530392424 136.013989349192 131.261903118177 145.810213943313
CNGB1 135.391788614828 143.519786615253 130.75707278606 140.009593108693
132.175368142549 139.697477263244 146.298733090986 139.497253870412
142.703116372162 135.375268329248 154.818414925242 135.634385846692
CNGB3 132.221351364892 136.859366444351 115.992487254175 143.277771142501
141.649911527714 126.501648842366 133.396403868645 148.219406063533
127.159947766922 150.911524755251 129.547309629132 122.673596593654
CNIH 511.996883507889 442.766704003254 949.260897408911 327.251620296271
568.040362658955 1097.60805874165 942.900963522948 631.106334754678
706.759905439674 1144.92109214621 731.284098301795 872.512754090023
CNIH2 138.93292293598 141.150016212967 140.132816040735 141.870640394206
133.773427881651 144.908551199481 136.607783800741 141.862767428244
142.367003292264 145.150191397375 141.222884136243 144.853868519575
CNIH3 153.653453808799 147.395335979341 148.353927712013 169.583355171423
178.847858667374 132.005753222789 128.168460083895 139.503902205317
141.793216038882 144.619374612317 147.659772694732 142.305484770249
CNIH4 1204.7397215229 978.389902096994 1037.97124386242 765.532997390972
3122.71790981947 2410.32095582515 2325.02143920169 1389.93393245455
2007.0121037284 2544.58522979036 455.778776920897 2452.1304336603
CNKSR1 117.367470441908 122.821563586744 126.185560760543
122.773736159743 171.39347085745 155.004104153679 165.085010975035
191.310372695208 159.349719495214 166.714793209109 192.96586114861
153.579955370444
CNKSR2 159.120172969378 143.451080466172 147.684875777977
161.331326682743 144.019824700027 147.15999706579 162.841318222066
156.68541512214 158.665476708054 134.329334422886 157.130045415943
140.650987394155
CNKSR3 1247.02514044778 521.714719320355 593.462634352052
465.029298672645 145.875240619257 243.772412032046 342.878522959461
275.929414239664 171.924364939807 299.513950278952 261.469732730752
238.548578086943
CNN1 15667.6576521981 14983.8256502293 10862.3952192141 24377.1316448061
119.412461116903 131.02157879562 103.550817366724 120.501697624977
159.620467672898 133.226542641174 118.850495684547 119.573635447638
CNN2 463.880793292701 512.467731561858 536.609777988581 581.37957221282
934.546444634669 787.017214032692 771.294254795752 446.819873986024
495.655170027296 1033.03719406038 537.216291942143 599.735180191091
CNN3 4913.55892033682 3822.05744774919 2736.98692493933 3029.07443443457
1548.09554827056 1632.07671492041 891.194779815063 2578.85391480563
1183.82018168635 1370.61156801632 1181.1611856364 775.382397816601
CNNM1 206.753993163216 240.814860403562 206.620708230452 244.849563563729
115.927914533359 113.862790983655 128.597272557033 116.043095688871
125.612670947405 121.246967460178 139.286592412216 116.196676877996
CNNM2 140.550044737476 128.83022039037 137.485252316816 138.872536475067
136.121873179928 131.268454024753 141.860850603561 133.453702113571
133.722739936347 133.695284981677 142.00857631537 129.938723074546
CNNM3 263.321559283966 363.539983343859 279.325065941252 412.344820823968
451.147418314412 264.905947922753 250.003965480779 286.194197552489
229.907632927358 204.489837323989 287.503594820801 229.406012088782
CNNM4 154.057771254252 144.361871207081 136.040524807653 148.406031732776
223.373877165342 169.733427590141 159.961729971624 145.203083375014
146.040995022257 141.139737175648 179.764260117544 147.982280671798

CNO 324.009703043876 373.096629378654 300.199170426996 381.253673759781
769.958422740085 368.466518140043 646.117381960716 426.464641742092
536.9324595176 416.638529499878 612.06231165648 556.863361789449
CNOT1 535.041545916303 568.935113269368 537.982214580518 535.354724252178
544.107739610054 526.726224327409 638.287221212947 583.022090879298
518.281245498542 498.05778289794 885.104778820041 737.42784787426
CNOT10 177.371157649894 181.479652131104 187.097765940131
191.405362404386 331.164053277361 290.184801898828 194.775234425687
251.138401278632 356.62074247786 201.289402228512 237.601064742453
233.953994677015
CNOT2 557.026028810367 620.614820117452 743.915675090182 452.843213944986
352.811351813364 781.461780481171 747.077451281453 3184.98944679038
871.268905562551 1029.75852676191 823.658996207582 779.022508413529
CNOT3 224.027605834751 231.728984785324 179.321622114583 232.444867422559
693.180345649331 183.201926323188 247.643123415644 215.426918011158
274.316982298975 183.613051401168 253.463871742303 180.437648146588
CNOT4 303.762967437478 266.032134528055 284.574888587832 306.838596239511
260.016906472407 206.521761031181 259.472319994929 253.186749111526
274.371523790341 267.800523478098 265.028512407839 280.170464338141
CNOT6 411.039234404499 445.089930641391 455.930204471585 482.344514972063
216.929969034881 427.345809255426 395.927222009721 386.379891865411
285.768837438859 584.199252444401 484.028175202673 480.101253056873
CNOT6L 182.851203073653 174.221939793486 166.22013573342
169.87591929982 196.470718183575 176.890596690798 193.190059954084
174.136806313142 172.657668514226 178.657325741035 162.895923745031
173.082167545676
CNOT7 318.196134631178 277.685488726472 377.225452025984 236.676029292922
279.070493043898 551.92688162618 561.609211243027 575.060457772307
473.529519031797 484.028175202673 517.42417250172 569.133295913677
CNOT8 314.646369385896 320.707687986324 344.128576341892 262.093405224922
236.833653794223 434.073729807058 504.436992949763 445.905756082569
334.101619029105 474.14051635531 649.906677394861 542.544172683021
CNP 162.364266549463 164.084737224884 147.955701866515 164.910911528819
139.399195840117 140.217243202557 132.425596223397 162.560758971445
140.301945597122 139.931092990984 129.497266827284 139.510764003291
CNPY1 142.876069462608 143.544139742002 148.539881077647 152.657649988502
145.385816618728 145.711007027894 136.176688025337 136.092086452096
141.127519700052 130.198147836925 136.041605158822 142.948238172973
CNPY2 742.517302567436 861.786094902013 653.264945856916 706.292905820227
1870.52504853779 1592.29734618345 2144.52984392647 1949.60516542965
1948.24432908972 888.706978062337 1838.18547839898 1316.31088187364
CNPY3 181.85552254233 192.475309217696 186.625452120269 212.190707408572
535.20662670885 271.638179116269 373.452370692334 240.631264915238
237.045634416039 185.896304740844 271.858059712406 295.420691959792
CNPY4 287.145105895235 246.389203940944 249.489170536758 279.159925913207
201.059635671301 249.09278446472 177.306794546105 204.283030911897
186.296882059398 188.490807652188 165.70840218273 195.88263399968
CNR1 167.893596681867 190.152824885328 197.775397831129 170.910998705968
175.810411923013 174.663845688179 191.25724234927 152.286545050749
170.95650329595 151.382199195953 259.767151797583 166.360005474414
CNR2 129.132523684894 131.185836673529 128.963962067821 120.816130267055
124.897463208595 124.847325289068 126.730314982553 124.927989289122
123.410467384598 115.520278378747 131.282765307273 121.239461514704

CNRIP1 496.426739836535 466.4890990178 432.372995789404
428.462270756201 148.247826830556 173.611295051326 134.519697269443
165.851677392967 190.682392100564 166.910561772689 134.066948803697
149.12669444179
CNTD1 124.116400777472 134.460035654795 135.281686411685 130.905135502296
128.052753068991 122.290822230565 137.75060150486 127.887666721519
129.785078660064 137.744318243173 123.908458472687 139.180086017033
CNTD2 127.224081416987 143.08841562593 132.220398477217 133.108094159933
127.229854957588 125.296240086615 118.312891459156 134.257928609741
128.383541329939 126.619475852751 145.463105638655 124.723519607109
CNTF 120.39039576721 114.265920150451 110.810963139914 110.286187184448
120.371374172279 112.096627288813 111.243322115126 117.343165720552
106.470524594239 112.054379249781 127.709638375626 104.321352818065
CNTFR 138.760800146167 141.191500203423 130.564962937297 141.016596301267
132.75673287656 131.849442776642 125.35643274458 130.586509996354
122.460829835477 127.951403055054 129.621296567128 127.07186140748
CNTLN 148.01816554072 146.875733210901 148.936888662374 159.012011848781
139.073785024092 122.472909598385 128.927103442679 120.07034081564
123.07559417607 133.032905765836 156.01406297447 127.314089756429
CNTN1 188.0434100445 185.342353172151 289.859695541999 183.443006525616
128.344679872625 123.059971161711 121.099290122973 127.681662711369
121.640681916546 251.731327363027 132.595360509902 122.883806494491
CNTN2 141.923103467306 153.962758393332 145.459919416288 186.007710175103
144.806478328053 143.950279111815 146.654925456132 148.779043570204
140.197487078987 143.984222627806 128.994977935861 170.844002412425
CNTN3 194.569325586241 174.717158790668 198.470718196139 156.47237266917
185.063870458561 147.264690436042 192.273528391588 185.128661141884
193.871324880919 142.000874773182 228.317527475322 167.532512839891
CNTN4 114.673517800886 120.843429388102 122.928863588006 117.770032157393
115.692324946633 123.857527555048 112.875136585606 110.651971902576
111.286141277084 118.372047967248 127.903393519351 116.425301253959
CNTN5 145.700589723224 141.541355091514 142.635015113687 145.754504160436
136.390047634643 138.213742418402 149.8035588048 142.750432197016
147.051399102374 148.408616955694 147.635761512135 144.400004730049
CNTN6 146.506263412584 158.58863949084 155.603263242813 138.956057921718
180.132104305583 128.191959554459 177.379186278216 157.874624979775
140.216148769999 163.946569217706 142.305484770249 155.214638707046
CNTNAP1 338.739180103373 281.44159425243 298.460273458181
356.376890469558 150.843975804117 164.274491958161 159.455596750304
202.761449711661 215.167086358143 200.558971047462 192.258863431747
176.761123480566
CNTNAP2 123.347836436966 138.728765780512 109.37466528113
140.861849838223 108.79453730066 105.665621968915 106.522641798274
101.530900217921 135.722938102035 351.079435946154 137.962542261072
161.77611826709
CNTNAP3 119.01559016005 129.524056217383 133.627890571828
117.05536677976 122.985117824212 124.025104622631 129.828896822571
135.930107381894 142.866874321248 127.50624009248 127.6078497227
114.255733119076
CNTNAP3B 147.019460117987 143.802883430376 148.327340543504
155.319717753912 136.83883055114 150.508851767517 134.799648755296
151.511808301993 141.86836086844 142.674671804361 132.539585717666
154.661456780865

CNTNAP4 124.513891912986 126.260691770282 140.704317015307
130.161889021281 141.811563725942 136.91779948054 135.417519318028
136.572645314012 129.368045611894 123.482626777491 136.799138736925
131.265594391542
CNTNAP5 135.920830568556 148.446949168794 131.662585510218
134.883708339914 139.016274575854 127.585851003779 127.512733134453
136.871568211946 137.248465324584 135.771423315597 156.187809481625
138.45137517137
CNTROB 145.076793236112 178.270849389719 149.827791077797
162.914301705151 149.6730835169 130.288547755599 133.760183242137
154.993784224548 136.032310569127 126.404523759643 163.773702843544
116.288113598488
COASY 422.434778100827 490.631757904175 386.067573158514 466.257158794525
903.824331691969 765.532997390972 775.382397816601 1107.29687889378
1166.86593572716 642.057106241761 603.061485415867 544.30845362168
COBL 264.936739264566 245.454945056317 188.695527267631 290.754210152709
171.504939477949 171.121834829333 142.858554460651 167.149578610266
177.94501172269 174.979501466228 139.979025013908 145.787468865642
COBLL1 1550.61855325662 1667.86825735893 1127.89414462606
1444.45946291517 1181.97868824758 705.311256840178 1556.41573079892
1129.76078220457 3921.67860557329 806.290662664708 1746.30519466572
1096.10636044885
COBRA1 366.317263094585 385.379119927642 321.794887577351
492.780880796036 794.165408266062 256.924134409998 237.358925391616
242.695286989672 273.252023367028 370.678040372273 358.298768623212
221.594737497504
COCH 149.387467833684 157.341422939225 145.835240952992 149.925051977052
146.153528461264 147.77916610545 162.743192424569 194.998142472864
165.389652763451 136.939603341076 138.259512736404 143.828909773599
COG1 210.41046499545 207.902853533132 249.449151040445 200.22145130696
203.616580991715 194.212177449077 212.987082617173 217.86728342043
232.385176936256 190.825209733575 210.061754644824 238.310077556167
COG2 373.760551592844 398.354212009426 403.292651623365 465.41233536979
331.464977124667 411.603820288919 470.95418656881 486.653672099699
402.299165610893 349.382889326613 321.119758814618 468.170972478717
COG3 263.707476735938 230.179200974631 248.330154701293 222.332748220525
174.657352858049 264.936739264566 166.741762854246 211.966139902562
252.10470581893 246.457698183615 278.942695435238 295.607161318725
COG4 290.754210152709 317.600728351694 311.337303524912 378.797952580504
323.149792143481 304.636618563775 335.47484353322 276.79778275734
343.571296297462 279.26508919212 258.964323941464 276.224613560582
COG5 221.45275440794 181.364325529405 220.9384439772 171.537040561599
193.912456093767 248.260998007814 271.196028302639 244.251315474587
224.766155406229 256.924134409998 188.708596785144 266.217879616686
COG6 294.595972099366 297.708113541379 290.213039891361 246.594244983507
250.085444708918 389.686045618228 306.011283364888 241.494710905339
351.079435946154 392.282956362735 326.570967321756 366.788087591332
COG7 239.148333781084 193.892810132985 218.298791348295 212.531329857909
186.653791045656 194.702974020747 264.201932544662 228.270158363878
204.959164579011 203.456296907031 171.304896264805 198.356420768703
COG8 180.867208892996 167.500410857353 174.615694779386 180.219813240126
186.778334749551 186.66469040433 184.187847236508 170.169402943662
193.028341755594 181.695203523798 169.136919982294 176.27969404442

COIL 442.027630433527 493.071213662125 429.096075419528 435.332573825425
402.824964079515 644.868034665476 781.790858580895 705.737228226014
646.933504790891 485.87138934376 594.38228123675 634.556887178869
COL10A1   109.471542280224 108.751654061536 109.924867620862
118.510960607141 105.010952544907 107.828378413233 111.260486045985
111.848472437543 113.347609066395 834.788058321257 116.902574164498
115.3611179192
COL11A1   128.44311762955 128.888149939072 133.99046926032
133.883527462382 132.071099697667 126.029431525943 126.134393447943
127.215777044942 126.875483714734 306.277823025572 132.70888569469
135.966929064294
COL11A2   150.590611246086 144.073739611496 141.115539663314
156.756159786366 144.102872001089 146.602252563413 137.966987037709
140.84880167881 141.42859050659 145.793794049657 149.14863489698
139.47828235176
COL12A1   356.154842959851 209.60319248212 180.667897257797
199.87003105816 198.411812737082 199.170488640762 155.355051932357
198.285073991956 197.068795788491 613.846166856328 187.625536724681
166.232856234879
COL13A1   266.681711962236 194.143649677822 145.117574928189
152.680068580858 147.196687432745 150.163801964104 137.562157262928
150.138643811727 153.38260557666 203.206743799353 160.562164904057
142.964043764953
COL14A1   429.457884032977 247.643123415644 310.815067427296
223.84997313236 150.902548360746 187.700723296295 151.26137860684
173.892380305166 217.125327913156 147.661748633455 160.469645948225
152.767228183775
COL15A1   815.045148478814 549.96567374713 379.670492165938
623.888568444132 192.032080013216 194.998142472864 124.897463208595
206.413554839865 388.782774926005 231.670761847089 191.207048581306
146.402352419692
COL16A1   2171.00457877604 1704.25279485563 1442.11349373764
2091.73580292332 201.731223995887 238.461248099002 214.38058301395
269.567704345075 315.615758089471 763.880957432979 176.901042102323
236.933960698783
COL17A1   167.645930148254 239.109163320417 204.470661830972
146.136820062093 163.758054869675 145.002309193867 209.229537873706
203.19299227665 167.173670781451 4246.19983955771 1737.57015396357
872.320823993033
COL18A1   890.507257588957 816.823443819069 662.781145703653
1067.80176606977 200.486683704179 144.342905860503 127.612859839494
184.021010234322 279.668007824988 393.198813167839 189.633348499854
139.188234030711
COL19A1   144.954595802474 153.557991189227 150.756776447358
156.768976123975 143.188958538221 140.343790129071 129.375772737722
152.657649988502 133.680691168661 141.115539663314 128.833756478439
133.329392986316
COL1A1   2801.66917828364 2874.43919426772 1114.10687039922
4425.66777919789 1514.53784631689 664.60530723006 317.353318759931
721.603421985572 1273.6393529108 9139.73212514992 408.10780961711
870.409935772423
COL1A2   5636.26097921507 3528.62443098065 2539.87833317526
3200.61067185412 981.781076110568 1136.76110352875 450.688800547981

1607.52029676299 2311.05587511258 9192.8966763778 579.493790910006
1382.12750708801
COL20A1 170.23937234395 166.485040297098 180.060096787617
190.427022049167 179.100368312505 185.128661141884 193.589480577091
167.776363541939 161.948033313443 177.246890307643 166.422360032182
166.027988561421
COL21A1 226.595006724068 223.747350951099 238.270501389189
179.421854100287 136.265972909993 148.045391886623 156.4687904356
147.049287475127 140.903091584884 143.383697128285 149.513744292932
132.695992860853
COL22A1 144.619374612317 146.188193301627 127.787890405862
137.805895248174 200.72147365413 319.090371885653 899.336817124312
139.356478232855 126.566165822427 147.153129531937 619.045921382959
622.142195892701
COL23A1 145.513333676569 143.776637809106 149.103778494524
146.00443946108 144.405191855684 151.71942719041 138.76667538162
150.72835989269 151.054834307689 147.310807346272 173.526005872207
153.0141888126
COL24A1 211.844563155792 203.791916347213 245.240006198482
153.085975710144 150.419668144913 165.71382076724 137.524968609776
153.56082876276 142.80027343095 156.411563412665 148.544887355857
161.735609436394
COL25A1 124.062863592295 129.958065173006 127.610304071204
121.734873711651 119.603565559838 122.351186755611 122.65547320922
126.383238335161 125.107483348755 120.883785696583 133.959208066964
132.796961054534
COL27A1 148.367132982373 144.834448238435 125.914267820741
139.089444892187 145.770601609392 112.578934972566 124.103321614841
129.391376518208 120.460433297007 196.889520085412 178.676812740925
162.411156818437
COL28 146.986891588172 144.40361834862 127.517446456116 148.334425146129
153.358189612307 136.220324225816 140.628614910068 139.138329217724
160.73807125195 149.070750686003 131.202090507576 135.664877050226
COL28A1 178.3652866131 165.337956130606 170.342828808493
185.544411160039 162.47662918768 136.216577685046 145.542888937635
175.537039794925 149.227646026658 171.014432075829 153.563839578065
155.023934989559
COL29A1 184.989027770708 158.396149470397 127.055165613236
144.585120811896 131.345038923753 162.606273401226 144.32655838488
142.288110423886 127.302714624905 140.238423894192 141.264096014725
139.273651128094
COL2A1 136.317978146216 132.119370332298 143.427721577372
134.034970203523 140.043256690184 139.926488041421 128.137646789236
126.479964493857 137.024230648861 137.764057655114 128.221548195595
132.642931022024
COL3A1 4819.85672511343 4255.59773503364 2263.73444150082
4569.44477073589 744.225336494008 1657.72357544205 398.796188440256
1485.776977235 1978.95750795951 7601.58151657937 586.867772838753
1023.29995420518
COL4A1 7656.97151002785 5609.58233117244 5122.60899285635
7930.82679738435 1704.25279485563 1658.78888936164 548.014025722956
1899.94714976387 2023.92614092273 3481.90072759183 903.144845227364
1355.66237447026

COL4A2 844.462124058351 833.198511430615 759.058176862524
1413.37062765856 299.669394721682 219.0221768576 152.692483731643
281.694635436938 328.200548902445 374.761007556839 155.490897312915
188.593378315122
COL4A3 135.159590925066 128.451788292588 134.35958389775
130.370405378213 123.665983073461 124.119104345119 133.802898895457
123.745824514261 126.690066353698 129.906744036052 130.380766252137
120.337241026338
COL4A3BP 625.544719251096 500.469781477166 526.726224327409
341.501397478593 308.208276315292 401.584032365337 332.798130757632
291.221427744817 284.272977025491 380.053166305637 440.579262120451
306.59090554099
COL4A4 219.564603776774 189.0608798771 152.074017988748
208.713555471888 112.133567992833 109.128394735532 123.207402172696
120.835207882788 124.899019467557 120.922042875444 118.161192318422
123.762963668211
COL4A5 1513.28088592885 1343.2623011534 2010.57142784228
1312.44215746801 448.371946119074 624.638115878703 689.821785472506
208.713555471888 224.724262742427 530.387315639095 646.461274173693
940.716682221364
COL4A6 571.64723305848 538.156335788156 821.020339593525
593.090436323333 151.301484852387 132.926799887017 284.351529744475
131.950295185477 134.748177388398 184.530853446688 183.472290949588
383.682951666155
COL5A1 1699.17470788219 1774.22769195939 776.411045078049
2300.9407349072 522.77407098992 357.972320648217 260.554343693588
530.122419830951 713.525134497596 3592.7878705048 247.145700155364
356.05426191171
COL5A2 1312.75601392984 1233.27155759407 733.49038042129
1154.1046856215 372.374101464689 335.47484353322 164.558875058199
473.260534978556 533.140740560189 2965.38280248519 267.970798709017
339.843776738468
COL5A3 143.94902198632 187.700723296295 147.43089976575
246.710233781784 141.760529549196 149.756512110538 136.420062669039
136.207420489201 144.505072819693 226.796825544749 136.032310569127
133.396403868645
COL6A1 5489.19362780613 5670.70220800096 3706.41283595794
7446.59106452583 268.224669588188 264.604297630839 225.61275910008
349.816819166182 398.892968579918 1748.77270039774 194.143649677822
229.230239609291
COL6A2 2132.90676194993 2116.39371296122 1597.53010317504
2385.5958396359 198.722993687455 196.117609477292 161.17923997423
239.541516080716 250.647644543963 691.632061537999 157.010145291837
159.524235332529
COL6A3 5226.05969701201 4732.31591164752 3093.38255333143
6571.35528952186 367.439588680092 574.262112643403 326.325210657599
926.283018664897 1439.37593006882 3177.80599774525 237.575788579215
508.572981581855
COL6A6 124.904518221898 119.52150378372 139.684141478459
129.697584673736 122.236134282757 135.647660756548 119.984301761327
127.942759794556 134.624322761212 150.263578317272 112.714282429822
119.926169902322
COL7A1 559.236012019639 550.671679318681 233.997642139328
366.636754272029 604.237898104299 765.128667448261 858.524630425687

410.42088953434 470.95418656881 3522.61692270848 723.640113902925
1932.39418448203
COL8A1 772.399017166118 735.8141982568 361.429760858441
433.82566459288 206.113290588613 234.925852373127 211.572396862776
355.048838683854 487.19625805337 493.715108157171 190.572712684622
257.408058436941
COL8A2 529.854965483609 450.566031840493 483.067471841953
547.367381020623 197.02284475852 189.434597063058 182.747079625619
199.149959995133 187.352655464866 258.727309442475 185.485858719975
191.200215655114
COL9A1 126.775180133301 132.07457128193 8 123.609527892227
121.884067622971 138.365589511693 133.037138438863 130.128585442593
123.687439699754 127.030113960477 126.355699811531 141.090356426567
142.358754646258
COL9A2 195.795728497916 193.843978773764 165.424516708502
155.397301207102 241.293046687076 120.944267930809 162.638805580226
177.825648107126 389.109250187902 123.998343902602 267.451611731669
248.993716339029
COL9A3 159.420533375725 142.22515098612 147.077695919758
147.15764785567 145.924751649542 133.633281583673 152.823566129571
149.066522533956 136.935498266723 136.678188851101 132.261161069329
177.780479906048
COLEC10 114.470670941078 112.365278258185 137.239088768176
127.107223387937 107.949834974369 123.660836177932 147.382177739631
120.65473121078 117.710502693638 128.536721595589 130.742940708861
119.407293854922
COLEC11 280.170464338141 207.581907737176 169.33926580273
218.134101819445 146.64560446484 151.015243921984 161.667782505656
173.532300173223 177.592497020798 136.156548919515 129.499283650632
143.399877326927
COLEC12 583.619362840814 427.98782239715 514.213675499369
376.374248850987 132.873213182775 154.051705030348 140.05419835434
146.011694647614 178.222120802125 277.685488726472 167.887047631073
142.459230639888
COLQ 178.007364708903 173.121618125421 157.076284096905 180.599150102184
158.287108069245 151.877035446575 167.157420848604 155.883386742315
167.73288535728 157.77047742735 166.239339129505 148.987672562273
COMMD1 1008.875250028 1143.0779222425 906.886423608706
1497.32522786098 920.083835296898 716.229989890801 957.54149804446
901.644194512187 708.056533282983 1329.3013039618 614.775559038663
748.703265448403
COMMD10 265.174306663185 266.19823384117 346.065746911798
251.472556189141 212.223906015932 288.913660405191 324.79327985156
300.709410518382 235.76573393374 581.828502821435 227.144441241869
398.512947526406
COMMD2 229.26243866226 199.88618355278 198.597176657619
173.532300173223 340.098306132634 230.770036998466 293.482512212153
348.171901445232 385.522443482 553.123179808258 240.971675534641
244.401472859026
COMMD3 1567.89763050645 1419.4638331856 1992.71224011906
1418.60088387728 3328.99028463277 2388.03256067711 1932.39418448203
1790.53556140959 1957.48508632607 1174.69701182094 1978.95750795951
2051.45788386541

COMMD4 345.135289739599 427.542390397986 334.377352542623
477.741216640575 800.373159084181 657.058213650454 675.596864001032
428.237210115667 634.046843336128 731.513306289481 365.590174280114
528.767644181119
COMMD5 267.069665000047 242.070630946726 232.363656167375
259.868701878844 357.191675549592 271.522696252995 239.31180635241
293.873921048642 327.324711245289 279.026742408164 313.248029961291
284.831817899986
COMMD6 2299.23805014176 2473.22803972431 3417.22292085668
2039.55802107872 2046.64499507352 2553.92770795014 2847.42635118026
1771.39155164347 2018.81730267601 2352.01196541874 2151.9395095819
3338.13980198211
COMMD7 1596.33832632698 1600.10235468018 1652.77814259264
1793.75125318113 2180.31963682546 2459.05558915812 3693.4058156307
1863.96986643676 2346.47361943533 1385.28044421961 1955.96772733047
1846.61430264854
COMMD8 181.607253339427 194.500222559978 218.08515867102
208.363905749882 251.011148303246 330.758879244143 359.329520507158
364.527677995774 362.306819797508 484.903322308422 297.862653495715
361.904370514991
COMMD9 365.864755316163 346.996310517446 388.710465457313
421.303580698397 433.019478861638 391.039114500903 349.540558521616
369.275635424092 430.220363263561 598.287431134037 314.557323400201
277.129985959184
COMP 200.094610104813 177.562561297777 130.867819112143 146.316145173343
156.901209828741 120.201521341263 116.925863450165 154.236652966294
179.15284184774 137.599810249536 126.845048064014 126.732121894377
COMT 545.026293696209 491.906642849373 567.715714233608 450.468410589415
2193.376951552 1419.4638331856 1698.08853832248 1066.25208226214
1336.15622439522 924.312051919404 955.143610867351 2309.63504858248
COMTD1 240.306287921209 235.216589534371 187.893420010787
245.675725760577 516.755267232127 250.217137496422 243.772412032046
232.735767435327 316.524115715585 346.691690520054 243.425632941131
343.666488527549
COPA 1727.7144253077 1505.09908072606 2039.55802107872 1306.20183851747
1623.92991386706 3200.61067185412 2508.50428529255 2947.51998516453
3737.57754066891 2537.90501983425 2139.42448145954 3119.93811557461
COPB1 884.747476952289 780.961416212309 885.104778820041 678.684323057327
734.139557942719 1211.94273385872 861.786094902013 906.886423608706
1184.3199328099 1072.82323999638 687.789976218334 763.533962249076
COPB2 510.061974830525 492.05680879106 514.799179506754 472.669956708398
719.766799746895 807.3257139219 1013.03054571763 1213.61170930345
1459.31031099055 995.263014856158 558.251384152474 866.147379099606
COPE 554.511983790164 583.022090879298 513.816017871024 704.152296359463
1916.46158204806 1026.61404215739 819.151876775691 945.830852603704
781.208872924532 974.007008568051 487.07860019179 852.099019968701
COPG 565.843200661721 522.17701271254 394.576038252334 523.937128789616
1024.95957828641 521.83794739314 712.94845413251 562.089999265955
953.549801897112 511.113083958771 881.15414246017 484.363052437459
COPG2 248.204358220728 243.691004557778 193.723953003899 205.45079491218
378.509324619452 236.745054217055 178.790758444269 204.396576475753
167.180406126438 195.674128236589 231.819341041105 260.921177985439
COPG2IT1 147.617354114584 140.708683675555 144.592987736094
150.456500446175 153.714902281332 153.310330832285 152.872139211415

137.519771130707 152.045391611927 132.249906289203 153.412469512967
176.029791617989
COPS2 205.950347620158 189.915720614467 258.830885826237 169.568915494577
213.833894542292 174.953232190285 242.019489058244 164.955263076598
191.418673767345 246.369521698487 277.459328083162 194.921021761976
COPS3 1286.55103838368 1123.93087912174 1344.87842417496 1097.60805874165
636.970243888917 1586.00600118477 629.97342429259 1203.47834989609
789.401526545277 1331.18605673705 941.572186137467 938.499337889075
COPS4 461.088572554864 466.392151902286 634.906566690042 411.221560795912
620.371912523332 1004.97723186946 657.231047829537 566.813343200268
512.541205235935 552.298965378403 615.662993833692 574.496860164135
COPS5 1044.91923772265 1055.79590411506 1362.1486006778 963.343120586863
996.999238316991 1687.85201395597 1378.40627876546 1849.21906428944
1995.6000523628 1437.5568316065 1544.45026075974 1348.80530206238
COPS6 542.267204956846 559.236012019639 642.647554580301 658.47864958685
569.47446215213 405.570281788901 513.428413503031 599.52440136406
414.114051645565 425.969575503375 283.377663208926 714.052063877292
COPS7A 1605.2454230722 2076.51786803222 1843.76920134139
2642.24374855334 1403.90487313049 1100.17618574297 1827.03179366146
2234.85920641661 2255.92551080483 1899.94714976387 1813.79641422879
2091.73580292332
COPS7B 395.386242257877 420.871349359404 551.823767223086
543.584413650467 344.786541451395 377.661589835101 407.875393458322
291.994364783888 512.723592291378 359.099750692694 408.037874667173
591.437752991512
COPS8 381.808809625801 385.678901612324 381.867665449509 316.722160860531
238.724909152512 275.996693627151 308.330044958513 229.284931844047
326.417021307859 462.948429969719 291.444155562134 345.897250519387
COPZ1 487.19625805337 527.226517960073 454.64556253724 555.607185132941
618.140160251927 957.54149804446 704.448123154868 836.613819700658
1162.8644618472 896.625415670455 684.428198622716 737.193295872614
COPZ2 345.38424761963 337.434882771953 262.756237406794 360.684900316265
153.871893011106 145.372579207362 128.400741751903 151.054834307689
165.451132663092 250.060734219351 153.690189121681 150.300108097623
COQ10A 302.637859140061 270.568660560796 301.955019831714
278.775971122032 209.051768656607 225.275049011909 184.709768457405
214.162670073541 238.095599711942 235.530986541549 246.186104432792
199.077893147154
COQ10B 1245.47421607111 1032.4540300024 1162.8644618472
989.77058497525 552.790706181938 1317.1258783404 711.166793546075
1082.75419087929 809.362464219294 993.34112647221 1113.59310757948
1046.65224316733
COQ2 272.571092944711 253.186749111526 265.972387419683 230.227432807772
482.344514972063 403.47026424469 491.112594297928 362.252813848151
538.605085454393 778.639512538949 482.965800108538 380.146996799549
COQ3 424.025588636796 399.248044997427 468.339735327273 493.492348317616
367.025117016472 330.457534783492 423.396271159345 450.120868656638
426.548994267836 570.916936074747 378.695635790714 454.030083521756
COQ4 144.4421922847 153.187148832063 139.674001912099 157.58224826673
145.212306927438 129.801342433836 128.580625868379 165.286724415145
135.916668738753 136.526706711584 216.783051958345 144.237691418911
COQ5 627.335757721489 625.699971780258 763.880957432979 604.047164856689
741.875751347917 663.214972254236 795.037968409957 1003.36623972843
1116.19403476786 657.058213650454 356.154842959851 729.202002368627

COQ6 245.378747269929 239.452559981542 243.732074437237 275.101766414328
240.253944604431 213.274106157675 231.239126548656 229.31486399741
185.727947276958 224.027605834751 211.966139902562 259.229153662504
COQ7 226.506852362359 217.969738417693 223.703371558666 232.986366625889
174.593548891693 178.946928223991 235.350819745387 211.208937901871
199.149959995133 178.358003030064 180.719868519867 252.733973271584
COQ9 1274.39152410752 1435.4236709581 1707.09830463606 2171.00457877604
688.464980326689 526.285114043212 713.23381913235 647.273409499313
745.153336165802 761.586663689579 943.23069404564 811.144838166905
CORIN 164.429483677715 138.883097077879 119.651470884046 111.716607815635
115.572088632106 110.662552491706 109.318922864507 120.53428198263
105.62620788923 114.400185930969 113.301733735994 116.082361389405
CORO1A 237.989479738133 179.914070416793 156.448355269561
186.222567239198 449.952497751758 630.473042320288 229.200570583629
458.33221423434 903.824331691969 232.918330930051 347.147961254829
237.575788579215
CORO1B 285.659010982301 324.177924877233 279.000687630846
388.966303073519 2273.12164779441 611.088604425595 720.364080505785
328.919401925375 433.594928641214 346.173614351499 743.510369438587
621.489906442001
CORO1C 3106.94181270197 2637.62596741408 2154.00589005104
3490.77593331727 290.241064926886 275.203046536524 344.575967665039
247.845708587624 307.410175748955 663.668537495345 614.957397883514
266.918302307596
CORO2A 163.876218743322 164.61921356441 148.388093331273
151.043967734248 237.88097138936 257.032174266905 397.2528907311
348.921877633633 375.189005729903 368.0573385048 387.908774438461
242.996248524203
CORO2B 185.258354905314 172.073751088968 156.043994260248
157.729732484131 140.091378604453 141.950778601653 141.444958186793
129.894339927457 145.793794049657 138.301397056664 134.349043119219
149.574353706911
CORO6 359.577827010792 311.375563161772 411.451612988791 460.408628542721
192.168634727505 169.695658738618 153.258616843783 181.54273463264
156.285963197762 158.208998509937 143.669490888725 135.393901125889
CORO7 238.270501389189 239.621756857095 207.920724788669 281.881563164961
423.607299503704 257.477501542521 204.842791081358 263.183010631644
234.738241125899 213.432084172541 254.287838845201 277.817786943873
CORT 131.023892409073 140.083495854322 137.393871430276 141.508424382665
132.295937828888 138.145677924612 134.423260547727 136.614620534169
123.891115326934 129.315412536491 124.837187124441 138.956057921718
COTL1 217.125327913156 212.730163981978 173.520662003159 250.139061165593
387.305348482856 153.346972639134 217.513118676535 180.923089242255
174.364798143068 171.784981874184 210.620764703468 238.847091900666
COX10 484.028175202673 572.262848718437 652.654673756838 746.777330152729
307.945884504629 498.180196203245 406.458130553679 496.426739836535
423.562598841704 472.961857076681 256.687384001447 376.867306473426
COX11 150.261107471642 149.609501725707 152.395272857974 130.242779079309
114.191245210321 117.965208293925 128.816610738922 128.967855397644
139.781351410748 131.045970236632 143.341711274401 115.35376992547
COX11P 142.613654158833 150.434376975922 154.816213491233
136.214432257698 132.941478943584 162.122250419992 155.669470674985
159.724824188037 154.239755912437 160.349557404154 136.111569529393
146.366013190845

COX15 219.171591031864 205.305702209672 269.567704345075 282.836518011311
184.832531598928 166.2468445883 210.882890077598 188.166168428752
186.951061159734 157.361421272816 177.32104138164 214.763232658841
COX16 144.587253120884 129.391376518208 138.842935439006 140.238423894192
170.79303550487 140.357427912361 133.71610564207 136.077039612526
131.338853426561 140.471953101887 135.531326977319 154.011708696954
COX17 2009.21931102311 1949.60516542965 2144.52984392647 2023.92614092273
2567.4299860517 2574.74962955426 2905.12297639092 2618.64973582507
5670.70220800096 3083.98169220838 1207.11788506001 2660.39990175415
COX18 126.195222228248 116.252227301161 122.646850766733 124.28033876033
131.967245387861 124.310436914027 118.783904603368 121.912927816178
127.202039384041 121.157614971438 140.440610012557 118.902823494379
COX19 825.781627776577 741.255013151687 613.631311147328 792.101559967381
263.707476735938 562.232754247201 397.064325578854 745.482863218223
476.641032594868 341.128917971124 659.483452217461 497.53605530806
COX4I1 8532.46059476152 9881.69734721233 8958.34797715349
11971.1664283024 10632.4185569238 7585.47289520004 8182.20106181946
6877.52553567598 8401.40370474392 7585.47289520004 9016.04641569485
10953.6229702446
COX4I2 166.981212790122 187.796440205485 127.511638455985
203.456296907031 163.463191619941 168.594235675558 130.311471059267
148.1652615821 6 169.285901212492 134.064206964444 138.758688249557
125.876114993687
COX4NB 256.424415389574 219.171591031864 323.086368767924
282.926325589238 277.685488726472 269.892633574243 244.797792477941
232.801257700571 234.484787292284 233.914433380121 244.350562866593
308.974786799114
COX5A 2021.92898855142 2083.02482378721 2388.03256067711 2236.73950603157
5240.40795364202 4367.38154535901 4373.91897463938 4353.66854914351
4527.53010866974 4055.40322213638 3688.68740405322 4929.26915002209
COX5B 5609.58233117244 5008.4888270731 6105.64727031051 5250.88041666757
8416.19700892758 8918.58940386786 6091.9840719327 6691.04319670055
5894.79176478976 7733.95252458705 7873.0525548049 8581.41617729997
COX6A1 1293.70593826709 1527.87148052149 1442.94175124818
1532.47920327769 4404.79209330918 2527.99279063127 2403.42261883515
2753.89649259242 2666.06582231934 2288.63508719706 2574.74962955426
2649.39312662326
COX6A2 145.864902319111 148.763098715961 137.832597613423
140.885012530186 144.501995579502 154.209689403188 146.709967739596
127.799190369808 142.625414022199 163.039564280484 152.026310884327
137.666849842576
COX6B1 3219.27851139047 3309.66193624859 3677.23129570773
3684.05513498098 9070.86397918429 8581.41617729997 5992.92384160188
8096.28093399619 9403.76665152182 4548.5418978622 4628.04544865227
6522.27232911897
COX6B2 135.143532936828 144.329979284786 134.686251400642
135.713266118447 118.966992191892 129.869363181125 132.602336133471
130.892302357045 159.936703616817 128.255141263975 140.949367846425
144.559193410932
COX6BP1 173.432960318793 161.585555680663 162.216377983292
144.534143943252 156.557792402376 154.281674854552 159.775479686193
177.306794546105 153.092864798642 137.024230648861 140.702685210277
168.594235675558

COX6C 6877.52553567598 7815.98936145067 8581.41617729997 8416.19700892758
7902.9189607838 7617.316805798 6368.23123771343 9759.87403504619
11190.1778174002 6214.77579017084 6296.24208225227 8261.82420896892
COX7A1 3989.14883668905 3403.37530051435 4251.82554412383
3938.94050658589 190.348359536057 335.790909130243 175.165305179665
252.249994170536 261.426617866925 289.474517971859 189.192494970145
183.863365305103
COX7A2 5165.90865799172 4901.41175228982 5532.93454600454
4511.9116742833 10417.5218406801 9970.08052783908 8030.19731415448
8666.41250683965 9560.32907282993 7992.84111657183 8982.75408120297
10765.0336035748
COX7A2L 1461.64901688125 1224.49891389348 1671.82101590797
990.55038742453 1667.86825735893 2016.52142068599 2388.03256067711
1908.81864728547 1475.41920838384 1364.83036157729 2046.64499507352
1427.70292953827
COX7B 1742.84803427086 1939.1525084845 2000.47497636965 1854.11328962023
2841.96460830166 3298.73393277409 2076.51786803222 1774.22769195939
2014.48411650613 2525.90466050731 3122.71790981947 2583.46653460894
COX7B2 134.553754121862 135.210887148305 135.798387504219
122.063996556575 136.506347083926 189.082778234831 124.461997520222
267.589020526148 448.106077184152 125.2051300372 135.031430487047
142.677386111317
COX7C 9323.53099359448 9044.54885739666 11709.9785619141 8401.40370474392
9820.93899217345 9099.13956219312 11071.0448447818 9908.59593023839
9272.37780151073 10397.3656932566 12457.6956682374 12654.3779830648
COX8A 3810.98043907046 4214.83058308503 4183.06379632713 4491.96934862425
14499.7866852901 9403.76665152182 6894.63087357693 6652.21610967461
5532.93454600454 6652.21610967461 4000.45196171119 9219.38828280399
COX8C 123.296382913131 132.450954427876 116.428373788459 117.820996523337
125.513881890201 113.292688210897 126.435928518593 118.426600479326
119.281300456243 120.085340927108 123.860747215706 127.407418037777
CP 119.150626141327 126.516782182087 122.167669805597 109.342230674869
108.118551275778 112.509731206295 143.318291243905 108.751654061536
115.524441144063 118.076139318165 143.999000471746 117.710502693638
CP110 606.851072672402 580.474775712828 542.009873308344 521.215655189324
435.603911474926 1344.1288416533 589.953707188378 590.646248924596
477.489368828457 390.194764817385 373.893428804488 869.937761943249
CPA1 136.783974483005 126.470239371932 125.628281846465 122.941711366641
128.202619705343 130.93033679281 135.531326977319 115.789536577324
119.176139463015 118.802030418792 132.043196381009 124.003914369077
CPA2 131.806645427543 119.480037559953 140.983230581181 133.252140649104
144.940119680152 131.421663453164 126.834926242799 124.661088039427
135.803599969904 146.574656147816 117.461198576063 143.188958538221
CPA3 1083.26599217847 515.597523880328 712.699853328955 387.305348482856
186.185055364718 658.47864958685 171.221972336784 382.744523022686
654.108318810728 216.05364037271 174.491551019128 205.495823410422
CPA4 136.398521726896 130.672586991186 154.205877662428 149.193437559422
150.610633329908 147.253919952267 144.423509403938 166.143084832472
140.319851351329 228.852495818211 157.57914268719 146.225422797118
CPA5 134.533551900843 149.125425566378 144.619374612317 143.917152377544
151.891318080151 149.977924522553 134.52098220054 129.111259771449
131.662585510218 151.461857714097 138.192550945411 140.831508792119

CPA6 137.675597243706 136.44218532882 135.725202462502 149.615585907229
147.79501373062 136.295523520203 149.068713925139 121.322817570375
145.938780249776 137.725329029129 128.678484452077 144.333945716394
CPAMD8 186.999459152483 184.844915350411 180.837420957945
187.824032557194 176.940723174814 186.794165469219 176.284693922048
173.919504730673 152.954944775793 159.354364289526 173.024402954772
197.667299074218
CPB1 162.127697884504 137.561067667054 144.165277164871 154.264142444882
140.682757521394 146.266350809492 161.348562186667 140.160349667726
132.918979842076 141.123272165499 149.867541008113 149.749660071593
CPB2 121.130837254861 138.440663763957 129.449242018727 137.462720784077
143.481112776388 134.47218436552 143.635367691412 140.738977175997
133.552141086987 132.767474034305 127.646032547296 131.958760372325
CPD 424.285848583977 462.144334719146 361.160881334174 383.407365952689
1479.69309189156 868.066644155313 1702.68441407727 1467.50846948144
892.868661511516 543.810043433181 652.826023701545 522.050619657197
CPE 603.061485415867 718.670564185576 297.466255000163 474.629173435475
207.384408045502 413.687046868111 243.080229016932 260.715580924916
257.775569674531 541.258721278882 166.311421907434 242.141362101262
CPEB1 210.900056977861 203.17525411575 261.721726664029 212.607094117882
124.597605631582 134.641840638331 137.418345713224 135.10155936286
135.296271683847 131.099004325598 119.031594964239 126.65748100631
CPEB2 278.168897504985 219.422260093722 225.4575611466 220.010399552402
149.734395134321 185.727947276958 156.756159786366 173.761036260869
150.648444878067 273.959220003188 162.466301774088 140.717348834454
CPEB3 360.475989559226 376.443086293235 377.89330639683 424.201556056759
154.395261192114 158.127523362251 183.853381322135 160.315029612814
161.825134481279 157.575902297677 183.719279448844 164.673579538023
CPEB4 255.992494620859 215.673878830563 265.536429548886 207.150354734189
148.356726939122 189.2642795052 167.532512839891 173.552535486766
159.301581158613 233.997642139328 163.951459935477 225.803528238368
CPLX1 176.410016510249 190.975263192006 146.31980311926 157.81327663167
254.936706344174 152.746799687103 133.810472192646 159.752644678596
160.395053149199 121.646971898731 137.974323288786 195.513703253634
CPLX2 143.839170216353 137.93067182632 155.46017572591 140.120051507626
149.300089212177 164.442400539653 149.303427221111 148.665627690915
150.265385257579 151.540744107423 145.259981479761 154.737117718424
CPLX3 124.809739073169 117.728410015076 116.310166003024 116.155949676272
113.740942665269 122.365693072105 125.048770476506 114.774530966446
107.283814651497 112.268518171041 122.885879294532 113.440171712791
CPLX4 122.554760526767 136.718598487513 136.285410546751 143.840979923821
147.450681897766 133.056659202188 129.858162624661 122.746185691827
144.170287726456 132.361106053517 133.736252068443 139.318078285343
CPM 149.457761307441 141.898948675823 142.986412837634 143.421288665418
173.109461200603 152.158269344693 155.249688219254 150.645487532549
156.077604146143 157.572446865749 160.511688319771 150.269618125943
CPN1 143.185372109165 136.468434653208 141.76964678852 146.619846538124
162.212442593001 153.315309202016 148.564655847496 152.44293820056
145.768857553634 159.856190281237 135.391788614828 147.668990552874
CPN2 163.77183097588 176.783978625103 155.830196650117 161.748547772524
163.67719444924 179.996590340102 173.611295051326 159.097020087275
161.148734808267 190.993590849624 171.869472272081 167.662761175661

CPNE1 365.286234166621 365.140886302762 336.786227017391 471.069295685718
670.571106793204 738.579262579782 783.991448771818 408.10780961711
299.568830444875 346.411586524655 331.684298221133 449.408793796673
CPNE2 147.066582834257 144.863617174741 155.801832826848 152.842777535666
163.336324451735 135.695576019185 125.882246459692 153.869144282319
155.326276840738 164.423960071908 114.00959999268 142.41737138634
CPNE3 1239.13016938378 1212.55338792557 1475.41920838384 857.090339943417
1005.62130301653 3164.55070502403 2135.04382065446 2285.86163259073
1912.2369374258 1766.15001427132 1858.12855367119 1671.82101590797
CPNE4 122.480000773859 117.503848306325 115.343251487944 111.467722538327
173.599961799334 118.918962530408 126.025161261042 119.658703392877
123.566522075398 113.051381310122 134.166297738657 110.636713168363
CPNE5 124.342911165267 119.052729327147 115.468921844902 117.923459503989
111.922007318017 122.572634128442 121.027465953657 121.45175516842
121.06219555903 119.806933383138 121.197702744078 119.150626141327
CPNE6 134.983884722607 150.494260282384 131.643633737316 163.910041674646
126.017158064032 116.501174707117 123.711828013052 115.157321350379
109.859878974527 120.551603446364 117.207266754941 120.148189532583
CPNE7 146.370084152094 141.066106476109 140.460927375243 141.278654291193
140.624437508178 138.407207709066 144.958176005711 136.438788088263
143.643259003393 141.466287523861 137.194467539969 139.127442770828
CPNE8 351.585604465402 320.154891655397 259.426050921239 259.229153662504
229.907632927358 200.08007665693 150.606329295069 158.665476708054
215.365809536522 696.475639532595 156.866867371872 148.49288177738
CPNE9 121.825639714374 118.025603140924 113.799997627317 112.664210248256
114.607138204495 109.886197655915 109.003365273337 120.606354515072
118.060781901686 115.321501844663 110.465934808915 112.993452506946
CPO 151.625248091441 122.921245774755 164.164463389212 146.488402397597
135.65700435306 156.740412695351 145.726651022813 138.584190903303
126.245597154052 142.68640844948 106.701716475947 139.432567336814
CPOX 306.550948766362 354.705553237406 335.301103690781 367.327273578513
380.754569467715 312.177191402945 384.216141843863 336.639473916174
295.367125961621 576.636077770998 444.945078908489 332.666916002481
CPPED1 185.165469121763 169.992657041967 175.744275464701
176.587559615019 161.354014292373 168.04198447141 156.349030617265
159.806221777332 162.347140552869 178.790758444269 145.732814287289
161.26201213997
CPS1 200.915806094193 167.250667092211 155.759861336991 172.877947895827
128.331867822629 142.609678462615 132.061436165754 126.049144107897
172.370108210895 395.261210769232 138.62308708151 137.000868503792
CPSF1 472.514017604645 408.201232787978 550.434931878586 561.379619105609
507.342877360289 420.017044147123 443.444024128516 507.827473844576
702.779943547386 392.582612044755 388.416668785633 383.10686624119
CPSF2 227.884445686928 244.219585315024 269.927694984528 265.61506544085
219.441959146815 193.412827246366 223.128108720503 239.050379187643
220.010399552402 274.494074082359 266.347965690851 222.243523614518
CPSF3 642.057106241761 707.715412321304 623.050854309724 788.693133735893
2808.78353621111 1583.79384467611 903.144845227364 1398.60850048351
858.742139354587 751.077307018319 1465.2419150392 897.958748317315
CPSF3L 357.514919818489 355.519647649551 383.939800422489
449.408793796673 455.392133846517 328.826483579278 313.967636827811
268.832884350157 302.532985701607 271.770138565186 354.705553237406
260.314440448486

CPSF4 520.218545108099 565.843200661721 473.60579090007 655.431236290512
1217.29687282094 685.354305381323 746.523071806534 874.348941257592
644.868034665476 522.050619657197 501.005898087825 907.610179738644
CPSF4L 149.013011292165 139.909719416938 134.81595608984
182.861900982333 138.487657776553 152.258389962 162.026757124868
161.475694690038 149.758215028408 152.356731704094 152.673212084312
146.533374238616
CPSF6 132.381685434931 137.586032396204 130.049854901235 127.361974492821
142.922549076496 188.242091666446 146.235832275574 159.491388998564
145.063062479224 129.625046507677 146.044279076949 139.369815367465
CPT1A 160.11539252042 164.708712337156 169.725534829981 171.573342518254
146.129805607031 161.027590226123 153.498332327444 144.738871562895
149.576563410584 148.160350542849 154.96616957771 157.58224826673
CPT1B 163.175801793143 157.570298634172 132.611864316181 134.364838071696
153.681775929182 133.552141086987 159.539320132392 184.682478530334
169.095270479344 145.195065312551 253.009376944901 166.099940078284
CPT1C 173.773876769683 195.740661051834 136.755975832051 168.838723365068
165.942761337382 128.879953205511 126.617737577634 140.288612271406
153.658747612827 123.434963905308 128.445065383646 129.902965506953
CPT2 320.774102343889 339.252515920242 407.442337662943 367.825328832492
660.535644289024 705.987531074737 714.274201838067 964.093668483033
525.6579615077 583.314509855875 548.686094748117 824.070046083713
CPVL 583.314509855875 396.459213999636 540.078145038497 260.85776723575
171.414869677333 305.615423200097 243.589099648895 378.728516779744
558.621429366984 580.996071040561 311.715351215263 181.823450464129
CPXCR1 148.94765984808 148.424970450231 148.189707227474
139.736465430177 149.76931674417 163.858222964048 148.109847175216
146.164093136588 138.098165515898 153.507553306723 142.101865506094
158.503284955019
CPXM1 196.226732738526 215.532132200821 179.54034642468 256.795346052751
208.611557959861 167.157420848604 228.766847429517 208.793650722271
221.471811658459 289.517820913799 265.219444616245 303.795701960506
CPXM2 1825.10168716051 1825.10168716051 2816.29814823646 2741.71904421456
154.710000519109 149.941056059544 153.879427186342 196.096936985479
255.435582426271 187.425086057782 166.17959304455 133.599098708818
CPZ 301.591430919567 203.317264638828 204.019377262081 184.937088280378
471.946284490029 146.374656140032 247.145700155364 150.899664887945
153.626180103404 172.442311733494 167.226663395855 134.664652044646
CR1 137.476047500852 130.803561448083 140.197487078987 137.353149614081
136.526706711584 135.735535533738 132.958521136 138.42029331811
131.406366653787 131.498890334548 131.330974614437 135.814893730549
CR1L 159.452527363763 144.767233774942 167.629008083436 163.108825245937
170.818182800759 153.044682186978 163.668476670167 158.509885816997
158.10628865476 149.432744784716 143.484004107893 160.395053149199
CR2 151.428283279424 154.483828924645 161.237544868191 160.975741751317
163.504122327429 144.494401248678 155.255279503782 159.218886890035
249.064573038094 154.630837508249 148.527528612375 158.085935267091
CRABP1 175.93459327917 137.365567527202 135.465076459043
146.192761134077 131.429247528936 143.105974876327 137.742393475755
158.153875553171 146.897528834303 138.887159625058 128.495276191943
154.316929706697
CRABP2 273.61337724143 586.642581067137 274.494074082359
343.270642449751 3949.19206005238 1074.96415574964 479.866450988091

358.843277491684 4047.99200599472 1429.25367024696 889.26314145856 1177.70376288106
CRADD 324.346723939622 279.159925913207 349.65350886897 359.256193328366 264.383922859446 320.979443123471 401.750302347978 317.904396212359 345.565843875296 382.902151239988 234.243977361232 377.538276846051
CRAMP1L 263.64863646012 317.802633831405 293.746733527346 367.518273773904 272.314332734711 240.631264915238 272.01602406899 302.905840432998 261.639437655633 240.561105692347 248.43491649623 322.36247485445
CRAT 173.532300173223 181.425012248539 169.201608999937 198.338416756432 287.195290301363 162.147989253357 174.406779990708 166.503112149416 164.534790315992 146.321713521846 182.997980582218 153.448882783807
CRB1 142.027930009598 138.986877479278 147.937679308903 139.168307371516 150.596350682411 148.107004870598 157.134782748175 147.088665779406 148.21588894931 165.022029012505 151.839306445341 147.23376282233
CRB2 115.624167024067 123.557722415957 118.414329128347 121.556331561879 137.75060150486 133.807992913171 121.036054511761 113.083678884076 118.424520312293 123.260800590266 129.180990946063 135.511902377219
CRB3 138.745560049251 144.318556353673 142.573662441786 145.039617860588 961.355625338687 521.30110104669 625.374595058488 389.109250187902 264.805236346895 323.841518852386 316.895545640437 505.143815448613
CRBN 741.875751347917 722.725186727796 1161.39230511792 650.263737836685 462.948429969719 560.913732951675 612.374364731095 417.103816140912 562.232754247201 591.641325402032 491.496499481595 471.720496000839
CRCP 2605.13680290987 2187.3010896903 3106.94181270197 2586.87941889443 713.087392708048 3618.9385126441 1421.04485772969 2274.75133434566 1994.00516809361 1472.54829263217 2234.85920641661 1635.35544761095
CRCT1 148.609721587632 145.559419404453 177.151218006672 141.362627556137 185.332215198617 132.721469335671 133.34207595093 208.587188476069 144.883597913037 1751.22580157752 209.623583937962 144.604714612091
CREB1 6427.33783376005 6324.87600301793 5947.89821087362 6358.5018930218 1482.92498205315 6628.17856159198 3391.3005983725 5382.31371568106 4612.81760689869 3354.38704439295 2867.63319136936 3883.05620256963
CREB3 178.976116597291 152.948391119096 148.118705147238 173.782474972742 165.84822009824 136.468434653208 154.947359473187 122.914680091926 152.124910935545 130.839508306116 202.369905673351 119.998260435095
CREB3L1 143.234195669431 140.036463888037 135.336754997434 164.865846124876 163.988099963018 147.397591251955 164.948785167149 168.753660955229 143.222342904886 152.834894011142 142.409003426775 138.395943168158
CREB3L2 1589.70261092241 1127.89414462606 1432.93880448974 1110.43791444289 819.488546995594 994.802763887989 1012.44277312494 1120.87534972745 1317.1258783404 634.046843336128 1421.72388964898 823.297606185721
CREB3L3 112.239647575661 109.832757716464 133.469323412904 126.676875319815 113.87024833117 113.170014668758 102.563722570171 114.342980656214 121.877276961306 106.285472845323 110.061459744714 123.442970398288
CREB3L4 111.949644842971 134.06834346533 129.966755942173 125.321968563987 197.91325268683 156.813992534584 161.83241639654 152.842777535666 166.582931148846 126.583342262489 178.339211170442 179.996590340102

CREB5 217.847954914851 178.147960295054 138.1774127311 151.65364467801
126.080780033301 130.363516180139 131.099004325598 124.946064096174
125.879198238067 139.87715485885 138.104527851969 125.806943645484
CREBBP 378.862741410359 455.665927568395 443.125195744202
478.180723959397 334.607533045929 330.553659265085 291.313681141442
361.429760858441 387.355661073935 355.443758121675 324.009703043876
360.756455184184
CREBL1 167.018380617832 165.100811808026 142.151209745109
169.124088593549 206.446584387595 153.379118228796 169.31618330657
149.599523779175 167.077669654786 180.327051100611 244.849563563729
142.079184950618
CREBL2 228.001046173405 208.932692585748 187.247062073247
200.712182818203 144.007071548045 163.977562404508 196.942880967705
193.203053225456 169.31618330657 184.655641182839 299.986758612161
157.86653024793
CREBZF 230.749601687217 223.013753785002 265.29629764006
181.37137053478 225.4575611466 221.322724563472 324.56043349882
212.210383398591 219.519896184851 276.714748212739 371.693808540758
397.387054218873
CREG1 1174.0922389136 903.503768217033 1813.79641422879 761.32108609503
1355.66237447026 2271.82656399253 1858.12855367119 1912.2369374258
1389.93393245455 1271.46608195833 979.019125719101 1409.42016970226
CREG2 132.611864316181 152.516485296644 124.288632450364 134.669763149268
143.574584852426 129.953801496113 125.450077160575 134.10070984606
122.68719599986 134.814082968113 152.853860043701 155.669470674985
CRELD1 333.200230327106 290.241064926886 368.711718874343
330.321113875647 302.219514290905 163.205362761005 310.640107228956
249.737588031219 199.597685421675 209.727089546377 246.80306998812
217.560691345072
CRELD2 716.229989890801 759.058176862524 561.062594540005
975.315904185938 1192.72383754851 909.020708316287 585.234286698678
1058.55850972326 931.325266360957 736.140249105836 477.626693328013
1168.68074786473
CREM 145.354740501503 141.168198348093 132.382614153117 124.992439957206
171.298701141684 134.237133546276 128.504026473695 132.703034017237
136.718598487513 128.050677475339 118.134614195753 126.700147643
CRH 145.944809232038 140.149064156135 147.121049266117 140.762415240824
1504.15682715484 5226.05969701201 3798.89743803968 601.521068811063
274.343238288436 124.638075441482 442.766704003254 138.186616955926
CRHBP 159.06002608158 145.150191397375 139.000248033373 141.350135458015
153.293125258215 145.610045243277 140.946729014405 145.449099891256
142.19015013243 140.682757521394 142.625414022199 138.411148122407
CRHR1 134.166297738657 140.943732564849 145.198638080646 132.435409278702
136.862764950361 133.254663058352 137.322392546784 131.666137651694
133.213697351807 132.16600019449 150.685245572093 134.378951325825
CRHR2 131.050255325564 130.844272373448 131.261903118177 126.580227722115
124.396267620543 125.861564323476 124.542879928448 136.394920983439
134.93814894432 141.649911527714 138.688881393504 144.986655090517
CRIM1 325.011269531407 200.405532717305 255.74096237558 155.575152212736
160.180961887686 410.138738410101 289.761702484888 351.292682758926
226.203994206714 207.173653740239 247.72190598581 232.830038701628
CRIM2 132.135448831967 148.943735606315 141.071184297124 140.36278240974
133.370488470007 118.636694749194 135.892248893607 124.897463208595
136.575784685895 133.280418227922 142.669207969715 117.380281099932

CRIP1 2859.94396089224 2833.30537635103 1098.78649254838 2804.99820187198
536.609777988581 353.243400332397 336.898462056187 621.354393918348
1036.40082234884 433.928818077527 773.057095787974 519.672796004691
CRIP2 1429.25367024696 1625.55754007235 1057.1528284934 1967.82064011967
642.484527246259 355.213322760505 163.326102597955 360.124745133393
504.275299381356 312.286788541158 794.165408266062 1061.29067124163
CRIP3 128.626051185037 145.514859606031 130.63591305094 117.104945989023
125.396523818173 136.951350782053 138.993251266382 147.75625228531
126.780096523484 135.679481480408 112.371497988132 128.492142236807
CRIPAK 267.622579012917 251.869781014029 510.555184032017
318.196134631178 214.461773810376 291.369577397743 320.812909889014
287.558897878375 364.265100349672 291.839070398338 251.731327363027
236.052008824688
CRIPT 365.650071660806 355.879841671975 565.509446660206 354.531898366438
278.335289966805 292.60467054075 453.760162338163 418.889972660958
499.436680354749 525.6579615077 313.18989508207 336.434368527549
CRISP1 118.820154030256 116.850851308701 119.755151432304
132.069175779393 117.820996523337 116.421771593211 123.510413258172
120.308260557986 119.207503310618 114.888157310977 122.989642938474
121.519309043513
CRISP2 136.84840505336 138.525131957094 147.066582834257
131.911579676047 129.487458179949 130.697148466042 147.004172510641
137.597788349982 147.751220590539 134.00361847288 166.489436134841
130.119313219923
CRISP3 115.613348109569 104.861024325857 108.868679899494
109.96017424123 123.833219705499 108.079149792796 117.367470441908
110.315549501241 100.746407116143 112.547907748842 342.096409626768
105.900294919664
CRISPLD1 130.374065525934 130.99288239869 149.79295887612
129.940297696849 122.955724972547 129.372522073479 124.095340478326
130.814702312493 127.524310543779 147.679233228201 130.642810886161
126.381288663037
CRISPLD2 3949.19206005238 3119.93811557461 1206.59866455529
1398.60850048351 263.606930507829 587.654093308465 217.778414825515
562.232754247201 639.38881853171 416.933146258428 236.855293143293
353.98839683842
CRK 487.537691131703 411.71606840872 580.996071040561 397.660202059209
218.862705238592 563.648536446918 315.786908578669 430.94199035855
376.692422468905 600.919152191636 365.950612890149 526.970555181851
CRKL 1361.54107116915 1418.60088387728 1254.19980463817 1450.44557173645
977.999660785113 1472.54829263217 1295.27257578314 984.181272274704
1662.51399707484 1310.60610806797 1452.63736288691 1251.56853099362
CRKRS 178.920528149177 178.707066209511 152.853860043701 154.661456780865
254.393421265604 263.606930507829 196.955723006838 565.97975389646
418.889972660958 208.587188476069 264.8472508692 220.487665103019
CRLF1 169.36029946801 170.725486814469 183.963578322984 177.491294133515
163.54669668396 197.12840030662 169.321832528939 157.010145291837
174.873893094923 185.507663455634 167.273378118452 183.50553222647
CRLF2 141.38749436393 143.265532486512 145.09909498449 140.556631658219
138.659387475119 137.192570825063 150.25322837689 132.01588294465
150.593928635647 141.691135410429 147.485960802692 141.649911527714
CRLF3 185.493554506958 176.088449983147 184.157941055948 177.001637712889
221.685824197352 230.697077917675 220.345184229077 286.431013744652
259.338947392465 287.242697383558 204.161375686761 216.805365763069

CRLS1 589.070196307574 612.374364731095 816.823443819069 509.14726076246
7082.28969354712 2018.81730267601 1331.18605673705 1197.58205444449
2086.15449885278 1360.24752587939 1065.74382494535 1518.13775377335
CRMP1 144.219104742099 142.763157232147 126.88066987372 147.174977926293
142.434356144925 128.009286101341 123.535803027153 123.650702461773
119.612288460361 128.314990390649 133.249792744053 132.04032925495
CRNKL1 164.466332705334 154.428779374984 152.021576565132
135.148723192316 127.83967243279 188.654966029389 141.784881206191
143.275056599274 153.255318944823 132.28010415054 153.756213924949
128.76720970745
CRNN 126.649739818781 125.924219382375 133.917283965036 141.649911527714
140.397184813186 135.185283076286 134.584076788979 136.858037332222
140.856864690669 405.975221091252 152.612116997575 135.603160096973
CROCC 250.003965480779 279.704940529618 216.903719298575 312.601510033941
220.816812192169 184.954673605577 169.101676908756 172.364618542922
155.686889874806 167.46432961654 210.666799498141 178.878444404192
CROCCL1 139.450788315602 151.663203772586 148.337793323867
134.57472034679 143.347066447019 131.68571824487 132.263954832097
147.804642251855 139.530196462344 134.491232148834 146.854523122404
162.134726710448
CROCCL2 163.014903707857 169.927157878764 227.587590278412
183.556662533396 132.275953817076 165.654773882752 167.830629616121
153.38260557666 161.525131363333 149.726968520487 155.15575204653
155.417694869593
CROP 750.494286476465 593.320132696649 725.065727257421 519.564563632951
273.959220003188 411.878556907186 524.640879446426 563.339837352678
538.647888236878 305.720812071583 560.015499136193 547.516635695872
CROT 397.2528907311 543.584413650467 434.716530273383 314.557323400201
856.74270641707 1578.87371296418 2332.98074920207 1031.38207659547
868.86246684338 215.967319486874 1843.76920134139 780.461254794127
CRP 129.045934344719 122.365693072105 126.383238335161 119.535267326862
140.694993778394 141.143572141958 129.108534267222 127.987449338817
133.142689150296 124.35374445763 131.395959781477 131.12953085854
CRSP2 151.064422836695 158.06129717954 170.748911334847 162.519642881848
145.983627592197 148.404000406948 142.772680460689 147.9084767638
147.89884142817 151.234397206563 136.328447182666 153.601918768668
CRSP6 130.579954890389 116.86635524597 124.340773743603 125.531371673679
134.475296383752 127.765987130281 128.623302491798 142.063338490772
126.583342262489 121.915043935246 154.634289463743 128.886008923268
CRSP9 200.790119570144 198.94325874493 198.795112039249 177.84188738734
216.457866890673 193.522811257085 213.301947003462 211.995890899864
164.917470819096 240.410292351169 274.281532397691 205.149094703318
CRTAC1 321.492900106953 559.0867397858 519.672796004691
145.395042579024 430.833137328233 481.238450505207 3630.80752744347
223.474531516972 665.341859712345 121.815538475678 18421.818380318
4979.31150268741
CRTAM 120.696557306298 139.488057371254 139.984102798711 135.839123867443
116.082361389405 127.161975533812 143.182399285928 127.146101490884
136.450062043587 130.924141812334 126.118745937997 132.675172817752
CRTAP 487.696347726467 419.126814511305 780.105135529728 466.777454704543
193.275162354467 209.258707976348 309.318403417731 191.170105307944
236.235744374476 313.581986704048 169.933706225406 241.039766283972

CRTC1 168.942165573542 166.2468445883 143.760519659724 225.189991540262
182.009471368798 176.912121142015 184.029968090084 176.849238577762
161.12121879514 154.443360208399 158.509885816997 180.164353124488
CRTC2 258.384059994446 279.026742408164 211.419445018736 269.194172034595
432.088303693555 240.497413647633 188.079188251085 267.696284751923
212.749668083641 181.229308386808 290.326832430372 258.868119227979
CRTC3 1415.67109281133 1530.45813438919 1559.26487922551 2105.87438782831
510.226374558703 524.855915371246 597.189355986293 560.757496975779
585.933822585718 456.817057099146 521.512936372701 580.996071040561
CRX 120.979417552159 123.260800590266 114.302645757446 115.128667174745
128.779829252045 130.917269984571 119.341806810441 123.077592992679
120.827732270626 108.895922800601 134.432461986523 113.627435441258
CRY1 520.761305892651 274.343238288436 299.513950278952 272.238922498583
278.737944234055 283.952604729032 219.228720772236 256.237185590901
266.982073722644 233.36028792331 186.167877791105 261.171723011396
CRY2 771.059329972125 980.120729515821 671.821418586421 1379.26881813339
347.866264631778 231.257543784864 338.075238647592 239.621756857095
232.641965649602 269.448201975645 390.194764817385 188.444498848767
CRYAA 155.699649538664 156.543267949167 170.251197850968 163.280786875496
141.528115502473 157.003367685421 160.127254444487 166.214167530158
165.347173283335 168.634534857593 146.04584032214 170.335930486215
CRYAB 3518.84173951107 2756.22069438696 2385.5958396359 3136.27991502603
149.198753359122 160.311096717492 144.892454033336 159.19183334563
201.530921073488 351.854828413334 142.948238172973 125.889860278265
CRYBA1 126.750850861235 157.932243807202 152.317890573534
155.287837763675 144.277263594387 151.500282404318 159.110614190088
158.514079887374 137.086936444421 150.544779197947 123.306499698566
136.463867598256
CRYBA2 154.760697311395 149.393022408213 148.698658730008
159.820341130737 133.599098708818 148.038291195414 126.037968297611
164.777719508421 148.534143255537 152.131804134092 154.390764802264
143.804903119745
CRYBA4 126.124937863679 152.173055053184 136.649743237542
137.235469802064 132.626349611404 124.418295003824 142.650451413991
130.962368929833 141.941814923705 140.469508410073 138.412897151567
118.991494185841
CRYBB1 132.651922794567 126.077945699709 118.816986517698
117.423704591303 109.495342943278 128.41549156443 116.825172441325
117.539640414775 120.822923434486 114.078271807864 130.257242615762
111.810575716678
CRYBB2 158.167384416968 170.079723856247 149.089067389266
148.065080664185 164.313238416498 168.942165573542 144.574566623882
148.448349327493 150.203281104753 145.144401160102 201.059635671301
180.354899434843
CRYBB3 125.672285485643 114.607138204495 136.959011188915
140.323425587628 150.283447275981 135.603160096973 151.431513447721
140.687016530974 130.10852948682 137.011595584638 123.974409226349
134.425483661043
CRYBG3 132.469190523971 155.865540894526 131.999299893526
131.498890334548 149.808222023726 153.996429870564 159.102070980047
137.784270510433 143.874735808114 146.817659734182 137.884051774657
144.988803993714

CRYGA 118.426600479326 142.047828639169 127.720117789235 120.901639342731
143.528208105237 138.544053540799 139.166537591866 141.059033576475
128.112595697836 133.975526667571 158.953705937936 133.502176289023
CRYGB 163.758054869675 161.229621453539 151.148295679242 145.793794049657
147.345558455805 143.675696734051 146.476960694197 145.812607895325
147.10214400501 156.301214161102 160.042251279235 163.170983091116
CRYGC 126.620889938532 118.497920969001 137.830435972191 137.773960847088
150.82596027483 142.765490244223 133.452032750397 135.571239294086
134.256243410916 139.291775075915 142.215285988753 140.688570032068
CRYGD 129.645982978155 151.625248091441 141.463324155943 136.573928520456
132.373302367869 124.922004609558 134.9404536551 130.031303483631
129.654234837398 129.051642626091 135.501192996538 133.490805791504
CRYGN 126.635105491524 146.110545700163 110.718864165172 119.305917403956
125.794788766462 111.082550752119 133.995928435022 146.06054700771
120.633217745524 126.755389668637 138.348160604288 139.717662557602
CRYGS 177.468210189888 176.347535860374 177.281197832647 150.896076207161
146.063554646851 166.472068379965 170.052320640686 194.775234425687
185.673549367125 143.446317006075 189.726075394359 155.55288776049
CRYL1 362.445813697676 302.069236363626 371.00603789759 342.611951034707
341.2887754025 321.910803473854 255.793823796456 247.194197143562
202.114480291084 285.594100976705 204.415267938007 224.466829629468
CRYM 203.704734790602 212.497773003032 271.397087928135 250.928804390353
141.600833670693 136.2336179571 138.968690339299 161.534421340752
141.518491890811 141.137459291428 168.830344627025 133.544149685358
CRYZ 882.429823267754 800.013123376748 1193.82799849555 585.075322115635
398.61164689194 378.509324619452 576.298671053238 651.675427664911
691.916554922896 642.484527246259 1934.30779703747 1521.61996270758
CRYZL1 416.307629596091 378.862741410359 512.312880441098
331.523536427527 171.528350399075 395.521241316058 271.087125858775
359.493075382333 265.366632264085 248.330154701293 297.419034136005
306.277823025572
CS 1044.00217726486 1080.95725397659 1347.75629878765 1429.25367024696
989.396074034945 1239.66725147648 930.25842384208 1561.87054049124
1248.69137283095 964.473774310687 1111.47772523331 1407.43503866211
CSAD 312.080864996592 223.84997313236 361.327436394941 232.735767435327
182.929326147069 266.395569467956 322.443089373063 343.346755862776
397.577760464197 367.025117016472 285.931444518474 281.119138040097
CSAG1 124.310436914027 132.560292119968 131.470883854661 132.220398477217
149.260811184287 478.699172220804 129.226815062589 167.871703133182
134.786678978467 121.884067622971 137.815541222457 144.460541430705
CSAG2 110.977904932371 117.335748689217 118.655954393594 109.167312436827
183.26554404668 153.703682621695 116.045039344469 115.445219455559
111.837468699281 109.242622591321 123.833219705499 121.919799968293
CSAG3 114.325967923393 120.616979607398 121.480455781173 125.227513212286
135.52861375119 153.140087873773 117.386273592501 122.777544465711
120.497796762933 126.17133877948 116.373841472226 120.922042875444
CSAG3A 132.514706165018 140.055272520317 133.370488470007
141.019494314354 221.916075811355 250.003965480779 129.788395150404
162.439052954676 131.144774362059 145.423022762916 145.749887769807
134.292099546804
CSAG3B 117.270320709161 129.570960882942 117.051541123239
119.666650919579 189.887788314879 287.656436278551 114.880348648147
140.603933807629 128.729687597908 132.881693683384 124.59937620719
130.274288258599

CSDA 6894.63087357693 6214.77579017084 7122.07276863122 6188.04738762584
4190.38366101529 3615.76740804718 4682.62607750437 3671.86460903374
3784.59114921916 8709.2221371432 4437.40805296517 4945.87677849967
CSDC2 421.056268094661 368.134303229682 338.129250886594 400.353795948162
127.618913664155 146.559502785264 131.486917325752 129.005211657936
149.55916919466 146.332425559689 125.45936959544 136.543603346919
CSDE1 294.697622870418 319.090371885653 310.718449468465 301.798676825176
185.275787562938 298.664864578349 260.52593414721 237.171424222756
218.000351930307 239.109163320417 288.313931067158 280.558463661137
CSE1L 663.214972254236 721.603421985572 777.410297605252 717.886050056576
1087.69177809384 1973.20689165831 1445.72064343302 1015.63768413332
1003.36623972843 1072.37500318471 707.048754700047 838.93563319979
CSF1 138.093181144173 136.965972077733 137.089392202166 144.649656552
124.132215738502 130.63591305094 133.504078347324 132.899305777714
129.195962576953 135.137615432923 125.780294378066 131.373012256275
CSF1R 816.823443819069 762.8576747113 559.559921312181 859.547169028149
416.075109522039 444.712990986138 327.951753740503 710.531876484467
922.009043194023 402.824964079515 675.281131488524 287.195290301363
CSF2 136.897888440409 137.691870502653 135.102827224896 149.586651393588
168.618667871117 148.306990501663 161.002880798225 152.910433120981
154.24500773656 197.873787638411 146.606442726047 197.112749959833
CSF2RA 2572.01353724341 2590.80821234237 2162.46602203822
2187.3010896903 676.293399523316 2900.55309635211 1467.50846948144
2258.30916111153 1581.06548216423 1482.15461429206 2649.39312662326
1689.00127592755
CSF2RB 157.032746784217 147.799806976046 143.275056599274
144.040342300793 172.03919618445 136.855445270531 135.455283504668
146.247825342634 155.572357799741 144.937697857147 126.630787911053
130.788557333561
CSF3 124.749727939593 131.464229237662 122.160459795959 119.511962218143
120.691810909148 121.699702849509 124.955676894776 122.396214045451
127.135199316758 122.888626394154 133.927844489731 122.083357581665
CSF3R 143.245660951425 139.128751917566 136.754116864655 155.926739769177
137.916872642522 152.526785074182 147.248053518914 231.338258067616
206.321272734099 249.064573038094 142.68640844948 132.437234970375
CSGALNACT1 316.004332524937 302.362619893594 294.280238648737
216.53417734069 137.356539213081 159.724824188037 126.84966022925
135.465076459043 142.215285988753 154.402315635227 139.258468432364
130.607857562345
CSGALNACT2 136.267313223808 134.514251897045 144.423509403938
115.858924428037 115.053039576143 148.479865869644 122.531675048414
130.066177538896 142.589312139207 146.197678850188 138.409284761652
137.020473156363
CSH1 148.11536596061 136.949403128211 156.151075965844 147.074807267102
139.925844631843 156.243337414127 144.94824685435 140.588811000948
150.421116058494 141.332402615602 126.112672816035 131.500591219916
CSH2 130.128585442593 125.915689078001 130.119313219923 126.045231525329
129.57551086282 133.408354896584 140.018786803502 132.809364188497
136.16560274503 135.726760147904 133.532323053215 129.24482916531
CSHL1 153.391895732807 147.185378560761 147.006482557682 157.639688257892
150.962828147611 143.833057210822 147.701092466105 153.008756051525
150.650926518959 140.571143847885 157.668307146391 144.345080865349

CSK 458.875979229339 551.086785904249 377.951523644306 480.712219116621
2656.38138490746 876.288248962843 1138.00644332379 1011.14472068314
1638.72366638732 900.839073800181 1219.36024606325 1198.53117439169
CSMD1 138.464793389999 151.921235758457 149.682150633146 145.502055020513
137.15571320152 149.219602109121 140.398923642686 139.56332664295
133.106005881815 135.188445928844 149.17815859943 133.376631703373
CSMD2 150.016550610991 143.262810933054 154.207656926108 152.08574341962
155.290648078877 146.070871543153 152.222213455455 155.595651896155
172.214433016685 207.488522933448 159.706443636973 172.578252260002
CSMD3 130.63591305094 121.937556824952 130.354641020141 130.147848418265
122.287535209629 125.542301916791 123.501843029993 141.363863974586
132.245217686793 132.063395162855 146.719445835497 130.865693081945
CSN1S1 130.004329307977 135.108420614547 138.461113585594
138.144085131733 146.056723380149 135.99579763634 134.371254010574
130.66511389864 149.070750686003 141.715307041887 132.109267393571
140.719388544476
CSN1S2A 144.400004730049 137.555253894825 124.217337143269
134.166297738657 133.839554874862 142.589312139207 128.463982896081
138.371873773284 137.406420101257 143.681220324074 131.356437247149
131.649830934459
CSN1S2B 138.67221078031 127.954231630338 131.142841840284
131.322008851059 138.224383646894 135.5668267936 139.798940929781
132.956860864455 127.000175858468 127.41463780496 134.47218436552
129.898242234869
CSN2 150.542478166361 130.778164962583 135.817028661429 122.512587751135
150.794680375075 135.377118045038 127.895989232696 132.813199199389
131.001996509052 125.259164818744 131.641435734821 128.717719117128
CSN3 122.287535209629 125.381865040574 110.382724813928 119.822587087968
117.089207321783 113.644993086829 124.901759819007 124.378096177801
112.298254005045 120.447140228396 135.508803488048 127.55397077248
CSNK1A1 301.428024926428 284.891415492568 337.728737393381
231.728984785324 175.437651670246 291.221427744817 294.410833087913
248.927790738447 225.169110687004 599.926717145115 291.105673222859
380.821355948507
CSNK1A1L 144.934103671894 143.410917415691 166.024236370752
141.908791151061 161.83241639654 148.534143255537 145.029298288869
169.391557085902 145.889336010424 173.109461200603 150.711857623087
153.850588492326
CSNK1A1P 129.065491477375 121.094060100325 132.021249712589
131.423918669689 133.704706615071 139.805930686563 136.491140756702
133.404671629495 131.562345881038 142.013370954791 126.690066353698
123.219903763265
CSNK1D 299.80615152845 354.405235378302 271.004741629789
528.098472417251 867.601542930533 558.621429366984 917.188039935737
824.070046083713 591.641325402032 668.286400833485 559.899430596368
809.068563907145
CSNK1E 696.475639532595 1038.34611017219 769.009371389327
1017.09497861942 1272.14273680177 718.142991571468 765.333633142088
561.062594540005 696.131296166255 783.373390305885 779.623566217277
606.851072672402
CSNK1G1 216.427103365727 220.079379312889 218.134101819445
225.803528238368 220.791884858764 212.571491543077 277.073510273425
226.796825544749 253.789045068884 216.680795762761 297.12411625104
247.194197143562

CSNK1G2 1951.69651954627 2168.58813300343 1362.75971646355
2120.14463347544 6339.16392696068 3930.2841381915 3552.78712528922
3334.16277752222 2811.84191700516 2502.72913594103 3334.16277752222
3552.78712528922
CSNK1G3 176.631604815669 176.830879422227 184.135651836627
158.747029092329 165.031678768564 191.297539967707 252.789376168324
188.503673684625 176.830879422227 266.468621734455 202.397972518715
216.991823988763
CSNK2A1 185.602093266907 175.030373805031 213.364858759772
177.065616871886 347.73629731446 368.220523234399 268.454310218604
259.511314588868 341.361299896997 300.314088267108 230.201464318433
308.679189788554
CSNK2A1P 208.237011955037 239.827648772906 197.795275907042
272.885527139137 266.964673763497 262.691387377392 243.501083338279
215.81995779019 248.887522059485 279.834616502591 204.470661830972
250.515579402334
CSNK2A2 583.192225756596 653.879734066271 857.090339943417
716.420861490714 437.122373082112 381.253673759781 530.561387049511
326.962330523237 436.586158384666 349.722963659175 438.340341490622
294.595972099366
CSNK2B 944.47112348734 1028.39666838369 983.094485868275
1032.11574332305 2222.16226526877 1053.09915077366 1129.15018335354
838.93563319979 1212.55338792557 1581.06548216423 1015.63768413332
1187.62490367696
CSPG4 1396.4168898623 1548.09554827056 1089.16489859253 2246.89876694944
197.699100346967 191.365014963729 130.628967781841 142.485427762372
182.152956182443 169.179630363359 144.258128917228 141.450800126786
CSPG4LYP1 135.728336465002 134.449083873514 139.633941983246
144.227440632196 141.453255692229 127.412539650291 146.268073519544
143.576177625265 153.318678468245 158.455783755247 134.527165454543
143.539741029895
CSPG4LYP2 144.338069855334 129.098408596418 153.044682186978
141.067419336609 155.221833416673 153.075594260208 149.223102493474
166.485040297098 143.579660352652 144.155591092387 140.971253689199
159.52147764862
CSPG5 154.865107093653 125.889860278265 148.797226586765 150.655359765209
156.923675862035 145.428297145967 158.650226303456 140.590802695412
151.073257114127 156.602240897907 140.837360719964 145.758423590784
CSPP1 235.867149024041 224.274329367512 268.52833723726 216.731618532814
205.244843885276 288.728608411903 247.236004734567 270.293807247372
278.274343962656 218.369954323686 210.960900130785 245.082816415008
CSRNP2 318.850765099332 268.224669588188 296.375305136807
262.954856857756 250.30085097088 298.165097953983 317.163768837813
440.579262120451 390.573484082597 236.899421827427 327.951753740503
321.25076864104
CSRNP3 169.434164890542 179.753400474369 171.304896264805
227.177100762395 127.604087759266 132.941478943584 125.315736755156
135.638156623519 128.699441450952 142.331050242704 138.088712695622
139.860093278582
CSRP1 23878.0022344804 24731.0629964468 21409.4415798676 29532.7890259497
1086.33540858772 720.907992182476 409.691865300362 424.37437028833
1027.11849332495 1192.72383754851 810.769136227155 1252.28642463619

CSRP2 723.807658768693 987.564350211893 802.017244099902 900.839073800181
500.132469062228 843.545962045705 325.173931553891 1052.553421812
895.463355957918 1876.64079464928 228.243668453958 302.021033686056
CSRP2BP 217.646777099512 215.365809536522 239.970202560698
215.98963200839 223.154771092064 243.893774595317 282.282663608264
210.353341004865 199.275716402358 293.825372123877 214.396920524803
231.287362732197
CSRP3 112.509731206295 114.466368742403 103.550817366724 104.714872508838
106.36673326724 109.255470590389 114.696935316053 114.078271807864
116.973126196102 113.400050190315 124.905889975958 111.860763079995
CST1 114.498610080481 134.860896462638 109.128394735532 110.501341211051
172.836730741407 122.436106757578 134.475296383752 221.411372022459
1063.5903770389 910.982557417978 146.063554646851 137.734887788912
CST11 131.859204862396 136.925645302362 142.206016362038 127.890438577754
132.657106732366 128.588822671032 133.957160768065 129.966755942173
128.044165393924 139.672652347877 137.803749343493 126.750850861235
CST2 105.375878824562 107.76497424356 115.559551514777 116.186846885411
115.878861991823 107.841100741858 110.988514718572 112.800733398209
109.038182799871 112.248047602652 145.383354200298 101.597648528389
CST3 13090.0615116619 13142.4763966378 12406.7143043992 14300.8562978211
17324.7697569598 12615.8332290932 10506.1271023809 8787.57716309558
7717.01422791837 6006.01682776552 9970.08052783908 8633.71708020651
CST4 137.079479050963 143.572668991313 148.16526158216 161.552475294424
138.261506562904 160.72009357092 137.756209496729 145.280485286937
150.508080332477 149.223102493474 145.09280363189 149.795655754301
CST5 139.420025842675 138.049846456876 129.365004129784 146.105844205919
120.880547922392 148.654099017805 125.405373274081 157.410205523901
129.83316008718 143.250386570155 149.879951192667 142.605816057276
CST6 144.964084792568 128.115993005977 138.121905080822 131.143808097612
262.233918187791 169.089896796513 171.093611712143 157.484541464879
217.628802427037 578.465753476561 183.657525723012 263.183010631644
CST7 196.536086443884 173.986750195954 188.767121505943 199.991077347491
160.628894266843 172.758230728437 192.640717683236 199.353823724989
226.173501275026 198.004357069446 180.035652063942 153.974384951787
CST8 123.182903611854 127.048605625519 133.760183242137 117.692662275393
118.034262386827 128.396326753926 116.045039344469 113.832885222354
115.830631252469 112.015423658372 144.079935036119 113.268379270385
CST9 120.660349211375 130.99564079691 147.032210333966 144.492756323647
132.418902141838 152.323927317575 138.12651823109 141.96771965623
132.748478264583 146.499065641095 128.331867822629 133.419239158866
CST9L 108.910588012315 109.52020660772 113.388450168062 115.631414599991
111.785821053048 107.949834974369 118.527464364564 103.594872791657
123.797585446062 111.475394003575 121.462385497795 110.586147059226
CSTA 124.518818352113 154.091431691072 130.98982984989 128.499303407596
266.681711962236 145.478233255862 196.839016616283 165.590892262736
136.751654856807 1037.00025848491 190.534176839652 136.001505582342
CSTB 2234.85920641661 2376.52728883131 2300.9407349072 1732.52466021178
15354.74541236 9349.76443528011 29162.1093037754 14181.1182110192
21409.4415798676 30573.5859783923 17404.1167486753 8211.38792799248
CSTF1 132.751406897891 131.232417654705 134.344021502031 130.89594032962
133.862203806529 136.384308041001 149.331805754859 140.52997588229
132.973551954067 134.294287947795 139.101471810467 137.729777643977

CSTF2 210.942865876533 255.839878446312 210.925203743958 246.912159676595 645.438835606933 436.586158384666 336.338518973032 348.111872425949 402.366797969833 455.392133846517 402.866491417369 305.669636871734
CSTF2T 145.108281984556 173.09379121032 154.47187360425 147.271220791722 144.741063866547 149.730467498266 138.4303531022 118.117957479382 155.542691937539 133.651203764842 222.268494113686 153.231965895778
CSTF3 303.380479700334 271.238736772408 274.595218795127 259.767151797583 304.479999301061 481.453770150269 340.098306132634 396.781827940009 382.233826557803 278.142283306934 419.593558001947 372.54091392596
CSTL1 120.481712066062 122.978576290038 119.872252889863 125.114303270024 131.548634017902 128.312170913496 121.915043935246 137.701271351806 117.399753685854 121.810767302322 134.264584077125 115.037340101655
CT45-2 125.215194276842 130.420753247797 120.433080754232 130.247700541716 138.997524526643 131.593705080542 132.881693683384 127.723052839953 124.682396766302 117.943511615792 117.781804727205 118.126133973555
CT45A1 123.671944323239 118.655954393594 130.161889021281 124.048926099805 132.855812035005 127.358616953147 134.530516701271 124.93629294955 125.685852196342 129.361783301027 129.80429516198 127.977782260866
CT45A4 134.777979170126 134.696571019896 139.437073790935 145.372579207362 145.126091016414 157.848456517169 137.981446468611 132.851791525098 147.027904536486 144.736213465651 131.029333093048 144.331409179705
CT45A5 137.225659453984 128.76720970745 135.668768914964 136.716554749722 128.862699779871 135.75568942028 128.585911203394 132.550022967297 131.741688265764 129.969400693456 125.121517583946 129.045934344719
CT45A6 147.138814313885 144.117964288763 135.349868925415 149.791777262424 152.783534456473 148.913181799692 142.889911562811 141.380276932556 128.783262618114 149.560505078257 154.408875182219 140.548371692813
CT47A1 132.088335186716 138.098165515898 149.678629612616 137.182323015758 127.040027125755 135.991630745082 135.598709185334 135.343380336913 131.000186898437 127.453866279181 127.277128410056 132.848926011576
CT47A6 137.045772853855 130.410584413031 130.782296492278 133.131953362455 125.839101611277 136.333928675403 120.465934391246 121.57069706238 128.363331337379 129.436712663487 134.922488718676 126.5764774133
CT47A7 167.830629616121 146.621996129313 157.717145014769 166.600109853461 153.748035612441 153.709230037306 162.54567071663 157.244026506342 146.062050819539 151.923356524322 142.650451413991 159.282348516052
CTAG1A 144.86271068967 143.212739422069 148.486946087388 152.017655364287 1378.40627876546 153.907737500104 145.389721946046 150.346002924933 138.889999702313 154.904424749528 142.514794686932 147.333142209628
CTAG1B 143.277771142501 145.86855878099 137.16842164453 132.781725233271 172.888455552446 133.324531689254 125.815256068004 128.738746025289 128.803632055969 137.702802090925 137.575688681319 137.25679451567

CTAG2 137.891372981851 141.628203045085 134.187456260969 135.918590725404
1058.55850972326 136.724298878331 135.013075032671 138.733717670748
131.198284945008 133.392934239554 129.195047484888 135.893380694453
CTAGE1 113.262357892613 118.547605249191 128.765369834041
126.305273697185 118.480344364831 124.101111442605 124.345320343901
130.63390061855 127.374600506681 126.383238335161 123.484987952263
119.535267326862
CTAGE4 121.854113159619 127.997280639609 128.179293878252
134.675247731364 128.002822538671 128.407235487011 139.555322603323
118.497920969001 124.02280670421 126.695387748732 118.838332117453
126.413124995943
CTAGE5 142.474762834848 150.73248190848 143.147325939994
146.812248482145 138.530236176135 142.348246621217 135.057847490193
142.191569406599 136.86685787001 152.628220216104 161.419873271162
134.968848574511
CTAGE6 182.529167529936 168.398366575579 162.6762533989
157.565633456909 193.502988612307 191.467626362851 225.169110687004
198.15257384667 203.852316712377 185.639696270808 194.352807599399
217.353396632732
CTBP1 433.314881387431 462.686385881492 421.255522475862 583.938727139698
643.660739343159 329.48021621087 446.509863498357 400.924914238052
483.312894744803 411.603820288919 541.642309122976 399.872401268958
CTBP2 216.033564783918 220.791884858764 222.508914765788 215.769986055534
172.544504923991 190.291185322482 176.167129202773 157.659824861915
157.374537572779 178.734634888773 193.466377788318 190.427022049167
CTBS 264.628012152168 273.959220003188 251.011148303246 208.348515119045
348.413827101046 258.25658838015 351.585604465402 302.601458409102
263.084557246752 318.487401106209 283.527656956105 314.182403961681
CTCF 264.805236346895 278.775971122032 351.079435946154 273.402804140541
296.915883082902 302.462763120177 279.413335686746 268.264885392368
439.852826905352 222.415704486709 292.037861000784 370.066087375401
CTCFL 120.975542702338 131.150252587865 115.967513057229 123.25201993575
121.837571435384 113.568261995787 125.185929751363 123.498540254464
111.928204694188 125.396523818173 118.622346984257 116.793408204625
CTDP1 192.980781927122 201.800357785022 199.756584044664 233.964929444112
243.807038920733 181.37137053478 183.676020826291 205.162633740798
195.256498208339 188.444498848767 219.080383154432 187.389039221193
CTDSP1 748.38293089716 897.958748317315 544.462032960964
1112.4055726239 748.940715378564 336.639473916174 616.523853569261
348.640185950469 540.584471920486 492.207833630086 915.015154168627
623.508710830895
CTDSP2 1936.88813111969 2291.40912229281 1929.52146517699
2639.60135568 725.443026984982 681.429327723794 1196.34528225417
1231.92253664482 1386.56788941677 734.139557942719 1392.89422203207
886.517088614968
CTDSPL 1493.90578573732 1772.75453735112 1453.88161495484
2462.87034563426 596.54904286299 563.339837352678 663.668537495345
595.752426322468 915.015154168627 801.051513639714 680.94167651697
791.840531191783
CTDSPL2 218.209523110843 205.162633740798 236.707875629511
197.034073391478 195.269099451599 285.562276640039 330.553659265085
230.723142236623 241.944581086483 328.919401925375 245.153736298067
285.768837438859

CTF1 173.714940254592 167.724233915361 174.274996381853 200.045606111354
174.338440780636 149.980924447339 161.498785105514 135.562700479444
136.167141767484 132.908367372426 170.390200730789 151.998942684339
CTGF 8261.82420896892 3321.1350545346 1812.07394646463 1806.24732793591
339.069385816411 2118.73410871718 365.864755316163 605.21685916883
425.229044880287 3749.80462365748 264.273245283343 536.9324595176
CTGLF1 158.829144564361 157.423242850568 171.545695383454
133.628391175037 135.015161451899 145.126091016414 153.541193559658
200.127994198329 159.798024189461 141.463324155943 181.635664531851
184.322250984457
CTGLF3 987.564350211893 1044.52286986369 1197.58205444449
1204.32784721885 319.490670412394 705.737228226014 755.79669622705
1164.61390835016 914.654262296682 617.67819271245 1146.3535752748
1427.27262061653
CTGLF5 160.565049887783 153.320911882529 144.129889080946
180.164353124488 172.002600588279 159.588575526536 148.446949168794
159.122382721225 142.09146795308 136.591299462104 135.638156623519
165.085010975035
CTGLF6 114.099623939954 116.070254821073 121.575942603856
129.505139430366 117.048846466839 137.73313146889 133.915830295107
127.453866279181 127.284885819903 125.410876867635 110.16174213285
124.528206512938
CTGLF7 1139.76755449089 1214.77298621465 964.473774310687
1213.06007305892 860.688314783077 1274.39152410752 3103.4192907748
1442.94175124818 1706.06615968622 913.684449305965 1821.08523647593
1309.7660860424
CTH 150.51056917809 145.500011956699 154.316929706697 145.371273337639
155.516697616637 154.849264978968 146.660826209767 140.515996180923
159.09825795308 166.382301680189 146.370084152094 140.03202338666
CTHRC1 710.76653239801 466.909037277853 659.900771633223
490.269418483997 288.182388699147 221.18757533703 217.531179216305
387.827718022473 479.866450988091 1493.90578573732 180.336623146341
242.221324619171
CTLA4 131.074021609161 129.295956874012 122.295222990408 120.470801825675
129.422854634791 140.256871047018 136.960656707275 186.185055364718
253.833756857339 137.161065320948 163.885645883066 135.356010814496
CTNNA1 2850.98525554116 2553.92770795014 2775.84742592713
2733.68308784024 1512.28202818467 1681.54485582868 2255.92551080483
2363.50330960038 1865.44709751822 3983.37592132018 2988.45928881468
2505.8565544797
CTNNA2 153.51550202829 143.823368276815 121.957155610892
125.157459724842 137.782648137869 142.579346058088 131.483427112288
141.603532965962 140.804008584006 132.7284396337 154.037622223246
129.583916648999
CTNNA3 128.251087452744 145.08853313195 135.894512504725
135.159590925066 120.935863853101 134.008118774075 136.913957413224
133.97321873161 136.547868436335 133.537552829999 133.70941349665
126.862274244977
CTNNAL1 561.379619105609 428.782703561238 558.810095726517
446.194196064909 249.884250040189 216.381643581705 209.960795065047
269.379272725008 644.449861831749 233.426170535227 202.97197145851
199.714647839712
CTNNB1 176.714471790643 178.404791189835 190.809584998617
160.186527465164 179.61374889626 327.324711245289 245.640193659159

225.75155298452 232.519482087835 219.878230463206 208.690347128273 241.168936008174
CTNNBIP1 181.088268167074 181.922907273871 156.477249817844 209.54573978355 324.25775261173 276.756415094228 241.648044508699 210.822216545773 176.347535860374 222.005022189667 308.148212607944 178.790758444269
CTNNBL1 632.278924237678 675.281131488524 793.891277937782 731.513306289481 595.593867616527 891.194779815063 778.639512538949 571.838717184447 607.26455470867 581.37957221282 626.104755496369 517.529147171876
CTNND1 185.233210060342 181.088268167074 201.913709602894 179.996590340102 171.035391211596 243.501083338279 183.023316857537 170.878962027347 173.024402954772 217.435155984897 186.6902304653 213.324226314713
CTNND2 110.138220982557 119.682747854629 115.053039576143 113.209569103727 116.736174147084 119.144128641393 115.738984899365 167.764352429391 108.697696080014 114.528407572496 132.140547119679 110.496084922302
CTNS 226.531255313921 225.130011390856 200.902123076244 254.63506290698 287.287337931982 209.213115976788 200.360518276784 237.249949461085 224.335742510577 298.573180660083 240.341031437493 211.844563155792
CTPS 992.744093752678 646.933504790891 866.147379099606 810.396770151757 599.735180191091 566.228690652783 260.016906472407 365.286234166621 394.962978677736 373.096629378654 267.422232051733 414.538237455116
CTPS2 349.054741747798 342.717252404581 392.06522028189 334.056545911557 229.660838239474 403.582461109859 336.786227017391 485.136339836897 283.499120551905 202.797135825668 421.583441056026 369.156887999872
CTR9 185.843639043466 176.912121142015 179.079061789059 158.126480180017 170.941404165185 175.585857393085 172.877947895827 205.011514211101 214.132445321197 221.849661343124 242.948136060281 184.900027626401
CTRB1 127.959474148217 111.217595631623 126.134393447943 126.656077572961 125.444541266623 131.789877096016 122.514826802737 125.603448542611 128.097206000942 127.358616953147 152.650867743471 116.844361858869
CTRB2 138.775095929764 142.383723965616 137.752238510249 143.94902198632 127.527378949803 132.526286141997 129.785078660064 139.3465236423 143.881744063934 136.831280469398 140.538813908652 132.287326702468
CTRC 156.802353238128 160.168654167074 163.141010815086 185.843639043466 157.003367685421 206.571433405844 146.353239460684 189.915720614467 163.306853098724 184.056274452194 129.529372508883 157.049773524382
CTRL 145.608087633951 138.30326146769 135.892248893607 132.74579603362 128.327528039933 133.99046926032 125.374764771516 145.855671447779 152.57040089034 131.85737847837 170.941404165185 140.10798818623
CTSA 413.757630469808 483.549698382115 429.992169596618 544.107739610054 873.256632365737 478.38361571213 923.383014892221 419.047108206042 509.618968665191 716.229989890801 277.219796733134 287.656436278551
CTSB 2505.8565544797 2493.5484556802 2580.98143004019 2271.82656399253 847.863177169335 1561.87054049124 1897.92061704124 1328.38230265655 2139.42448145954 2596.43650409044 2346.47361943533 1266.53539318831
CTSC 240.561105692347 229.200570583629 222.134519361117 213.215996114827 227.441436540742 1062.749459378 1123.93087912174 1279.9555644913 524.855915371246 1562.9779683714 571.439520427295 541.258721278882
CTSD 519.672796004691 522.899203071911 638.393099392849 407.711669482083 4520.84412831414 717.270096456387 1583.79384467611 690.582550537485 956.76879975749 1168.3174645384 3342.8737952486 682.730262408017

CTSE 152.28262533486 171.504939477949 152.215146681655 158.675960205792
1052.553421812 199.714647839712 233.426170535227 195.230219990395
1904.39962279504 179.582086214179 5058.57872936027 1975.83062789692
CTSF 1029.75852676191 972.468110850466 1029.09818399518 705.987531074737
1322.14570224119 527.294087043601 799.132592914065 204.681956301306
199.037998955485 227.144441241869 194.161930642068 170.655648004461
CTSG 793.501244347257 658.202765061937 701.208391798006 1488.37146754184
129.099780410063 241.494710905339 141.523470346891 205.950347620158
510.434895296759 142.839077978518 127.442587366678 141.482344048665
CTSH 610.759447723026 1062.03157932144 693.516351173476 408.510567121592
4410.68280125001 5233.72882607795 7802.25987814138 3384.19521857242
4536.46344538492 569.353661326724 4866.98027226798 6409.23524786095
CTSK 2222.16226526877 1514.53784631689 2009.21931102311 1028.39666838369
207.417884407459 497.53605530806 250.626556981376 632.031410234577
1066.25208226214 2165.41403521012 298.028932089399 284.473085899219
CTSL1 2045.24382334506 1965.13870083236 3119.93811557461 2708.20624364471
1317.1258783404 793.501244347257 1209.56018864779 1548.09554827056
2176.59445919725 4811.88424516272 1391.89900758685 419.272193677273
CTSL2 123.881658891576 136.742206779136 111.765812595638 120.324139035544
176.001771318766 160.553214127047 192.095235288818 426.995700632434
161.310855691717 1424.04009914668 198.15257384667 137.607767467172
CTSL3 158.483006541613 144.396774242135 131.984467471242 140.004456934031
143.94902198632 150.46669393448 145.34533633425 139.293775885106
154.816213491233 137.938310376106 168.03501792841 159.213940274232
CTSO 361.26540006942 399.071041584646 323.225475312396 275.972447693982
240.878804911613 303.555333002419 339.663295617139 381.57978490093
415.642099109855 299.33156125224 603.569635276204 242.72424280444
CTSS 126.601395870533 143.931400351525 112.208950290962 117.65847906412
143.763292219833 131.050255325564 175.327597807738 225.362253409428
194.430896269275 115.884253217495 265.332196877456 161.027590226123
CTSW 186.529656751005 182.958991847218 169.992657041967 315.306271141071
199.637257550762 123.494412056415 135.349868925415 135.028746262383
188.20675818131 134.289354713568 195.547320133122 168.73918684069
CTSZ 430.220363263561 298.625127845168 253.164596735793 257.685284145848
969.811933814244 315.306271141071 467.777995124655 245.042781391047
225.664432111551 204.019377262081 397.387054218873 460.941286124613
CTTN 625.152889992949 548.014025722956 580.859914041323 590.142817130684
896.185223980593 838.63350090385 1034.68475089075 365.199125315964
623.409211766224 835.284130042504 1115.34267967366 1135.49876948815
CTTNBP2 143.552343056702 127.859873416515 179.211502448703
163.346402397924 129.719122724699 128.734694496357 122.909152047474
136.931476050688 145.773932754923 140.101511236793 128.368669716591
141.249687936867
CTTNBP2NL 153.664503112778 155.826394389322 143.614075503775
126.839037497031 136.869117921313 223.058699622386 210.620764703468
184.085616342099 183.545741265706 213.077884261943 193.744461104128
226.387465899611
CTU2 178.12073430094 163.407360509803 167.568776552757 178.166289723365
228.879434668972 177.103802332144 172.364618542922 171.671895682052
165.103398617203 167.776363541939 177.011776399483 161.44729973777
CTXN1 1578.87371296418 1473.70433292382 1484.32547474275 2267.0818302379
908.352265585374 504.275299381356 221.869191666931 292.468585109951
407.957436380989 198.311821616128 255.305647212135 438.03648998391

CTXN2 137.085712588455 137.071402810983 139.515794685984 149.595375008252
146.271180025157 125.634045420702 153.474566767304 150.964506212368
147.351826045609 140.464476678448 139.372441366224 141.29868389128
CTXN3 122.022611139895 131.657454325126 124.66386961898 116.678969343492
121.197702744078 124.297848065172 127.476819912992 120.547683754604
123.434963905308 122.268560536152 117.335748689217 115.299986138153
CUBN 148.054367166566 139.310309748986 157.57914268719 141.289009185018
139.748733350375 131.933026690056 144.224287382131 143.810050834214
144.754401731628 158.7912155022 140.706490845379 138.546549217819
CUEDC1 1887.82245931444 2421.87041217217 1901.97972168836
3222.23985545506 988.788192022196 396.861835715943 614.775559038663
570.130804179186 731.848844821402 357.835334661098 634.046843336128
457.292146759215
CUEDC2 646.117381960716 690.010326122574 836.235700891687
904.733257358133 1007.41624313399 553.198321853202 585.552940765069
413.330906392915 591.763423878737 365.650071660806 499.436680354749
544.852019338212
CUGBP1 209.623583937962 181.550597645633 195.724873069362
188.40383184521 204.283030911897 173.147311624838 198.437113196596
190.444387031552 177.103802332144 206.548878646452 202.162432624505
198.113398073968
CUGBP2 207.651142295123 192.932285494022 269.379272725008
189.662974464847 129.34905533265 128.045931477827 132.928639483854
151.650777127117 158.919340819357 130.212422459695 136.421163870578
126.482660154852
CUL1 819.488546995594 683.365770196967 834.187014201026 803.60413590137
489.75389978732 617.185364180303 583.938727139698 539.125423322738
616.523853569261 607.805002786409 350.926679856514 741.255013151687
CUL2 631.819659701092 558.934004505074 697.299604049748 543.810043433181
567.143102792772 693.180345649331 495.769802520637 493.071213662125
524.725307261999 458.047799748696 648.38049615541 541.642309122976
CUL3 148.927040248436 163.885645883066 158.536178747273 155.878493935166
135.033601686007 149.805955402639 124.985249190127 151.567693140302
152.600339354701 122.007909794484 136.888915439473 134.675247731364
CUL4A 547.817028243811 481.86545778383 644.449861831749 478.606567465979
349.816819166182 495.241179224412 358.897128592042 396.708183244581
490.153210371094 616.014975366684 511.25179427158 677.265576269103
CUL4B 249.400820221233 264.805236346895 286.901115411673 244.990374822652
195.981555981238 267.800523478098 210.590774758661 176.008711524598
185.396765725228 189.092674335503 241.675251479835 204.509884908059
CUL5 679.557656724243 682.315322994357 758.517101659762 545.200687301057
496.991561192565 634.906566690042 708.268962600305 754.406896156858
648.161646065464 489.909407178594 641.645855499704 915.579581760396
CUL7 163.504122327429 156.540165645256 144.016137459279 157.77398996415
237.146972312448 174.587558727209 234.301520483864 143.837402372008
181.003812824014 139.834535091869 212.223906015932 176.547853494057
CUL9 234.626574390973 285.768837438859 269.141120401835 312.645778869254
240.12078342024 196.519125510368 243.979769719186 224.335742510577
230.975434289172 258.807028561407 256.495848662843 247.08460296705
CUTA 1033.48252432603 1002.44383771056 1102.38234645327 971.99484515987
2819.92361643968 1601.19401366685 1271.46608195833 877.255923473532
1114.10687039922 769.34961339184 1064.12835214671 1415.67109281133

CUTC 568.935113269368 491.709723745226 523.435696794756 352.741885295157
166.81024151246 227.614623963331 267.028096234702 203.125568980238
235.680462167404 257.880331012233 250.602978521808 230.672056810885
CUTL1 781.208872924532 923.130629121442 793.501244347257 955.604404066769
466.691191261997 378.088675670786 543.001249586573 441.785666261936
388.096183735126 624.978351618077 303.986544460218 588.863382995875
CUX1 155.119146797226 156.779004465552 160.713790305656 155.515134922269
161.788118050564 142.264536484668 152.743680985848 136.256456726841
138.618761382177 151.340728221261 156.037150880384 155.488346781292
CUX2 162.624786423084 145.572363722788 142.469353808346 131.749403497912
129.668506092785 141.374831983971 122.550707067805 136.321816321193
142.757327296852 126.734835642553 123.586359806158 138.158400190671
CUZD1 147.856285290748 129.073750290924 143.397823965608 151.783090997855
151.819220292189 140.051904905695 159.115391667901 154.533544838819
159.43317827517 138.098165515898 147.572660963285 162.9279134169
CWC15 648.161646065464 662.373189001788 895.463355957918 692.945420109927
489.260272645085 743.023225859813 927.137881369911 838.63350090385
790.561585641193 1208.9240215109 806.591035100794 956.76879975749
CWC22 203.19299227665 187.784865705837 192.418895349837 156.698856643638
146.632312874102 170.01557699913 152.748807709142 175.908645445479
158.541906931082 176.704617691988 175.704031930103 158.604431001643
CWF19L1 719.766799746895 850.882404546093 748.015452242839
806.290662664708 836.613819700658 733.86133557115 807.604205890244
524.392279804734 894.100792722707 699.984140389009 693.781708146282
668.853969513382
CWF19L2 390.809169896009 387.711471687237 461.088572554864
344.070947256963 257.989863290786 228.713818522476 232.778488697337
219.753039699136 212.171281020103 236.164692035094 302.532985701607
224.361277033882
CX3CL1 1126.46241487062 1518.13775377335 1488.37146754184
2410.32095582515 225.095016600703 250.30085097088 185.886737029845
200.179796117115 464.323065038265 210.960900130785 232.519482087835
176.347535860374
CX3CR1 142.41737138634 148.823805841381 130.201635099678
138.74117178479 142.510745106328 131.089811472017 140.934144636302
154.710000519109 150.584633044901 137.701271351806 163.570018900148
141.779801021888
CXADR 214.499670852615 493.347308943133 288.728608411903 131.381189141974
1584.81089248914 3829.87905458534 2662.43181510326 2355.29862638453
8864.70614223459 1548.09554827056 2525.90466050731 1978.95750795951
CXADRP2 145.365935017728 123.150299476109 129.265788005518
126.690066353698 146.590495255969 126.468167800414 132.811368246405
138.924820359912 129.828896822571 121.347339077474 123.720229113779
132.998223400178
CXADRP3 133.788197693571 146.798444724408 141.492458250787
151.431513447721 155.40832015652 128.597272557033 151.893573927407
136.268379335353 128.289680999565 134.81595608984 144.829773937102
141.334394187941
CXCL1 139.959629641771 177.549330396268 126.259029676691 153.165872773973
181.202396068658 151.024201732633 244.849563563729 293.692083555844
622.692834629555 827.863789600981 200.944865673409 221.74200490054
CXCL10 169.067064352955 217.670330817769 142.617474507282
163.099009810658 236.401834189942 256.538195034956 329.660368915848

669.731546951281 1487.12201643008 404.21236980164 598.095370768587
317.061815822659
CXCL11 117.445284668654 135.193659028591 123.306499698566
124.63978985808 122.284431229917 129.589056071463 131.560971848628
139.104796184094 134.350054065624 124.791221329502 130.163574198827
123.833219705499
CXCL12 810.396770151757 756.246712133116 575.857954264295
735.449040059761 157.994474909749 153.639559509292 137.20253329376
172.502036705114 231.146155346291 154.162796706044 161.89906072679
152.826356673175
CXCL13 142.080099281203 135.87026484074 142.777235931165
147.808262170269 160.939530665541 140.518206716196 160.180961887686
378.967410725775 643.306565600931 160.72009357092 130.266601299194
150.112549201892
CXCL14 517.020259129974 391.569187190375 416.075109522039
548.686094748117 217.013533525724 518.478990211753 392.909481887174
796.699879495384 443.964363178742 2166.68174726155 787.874585290601
476.641032594868
CXCL16 255.527764534636 264.273245283343 163.607125532236
231.314944217099 535.041545916303 385.050282185255 340.966515437272
419.126814511305 301.188296465202 356.530530468568 260.045018348182
330.908743070095
CXCL17 168.139351579682 170.12010526854 162.28074297013
150.870830294349 164.491207767654 172.364618542922 231.846509449829
169.568915494577 420.647394044431 158.07640654075 402.140437036706
182.009471368798
CXCL2 187.519053043173 209.196695368783 171.835438335216 214.241635800083
151.836705989155 166.068489027866 137.72327986309 141.410953064267
160.786907816025 243.691004557778 141.5068665919 157.36568754472
CXCL3 124.794260959228 147.854171854456 150.489651880905 141.858928084825
122.182695593404 142.354731066206 142.736148814019 137.843244642235
143.351341716363 132.70888569469 151.503307004675 129.21974206851
CXCL5 131.926504315744 145.123845983625 137.77107995457 141.08625682411
168.27980269501 136.356895672212 144.507230362869 145.026722065573
213.364858759772 203.616580991715 131.463081749448 133.192757350144
CXCL6 135.381131982323 138.478911439144 145.85414203374 134.947657801444
147.561362873855 141.730912072471 192.188662949149 171.444558905575
189.726075394359 183.236087954054 175.350734537491 155.878493935166
CXCL9 124.424565172377 147.238896426423 127.512733134453 112.332130647215
189.283071231248 159.620467672898 112.642429076777 433.751675703425
523.306451464643 179.55905250703 239.24587884234 182.861900982333
CXCR1 148.751755543817 150.294432623474 140.347131743987 149.300089212177
152.334669286305 148.209164900319 145.34041912391 142.416237823488
151.663203772586 202.718428167217 158.670835881902 146.982584024102
CXCR3 127.096476403758 126.333814565085 119.931415360373 114.405811161631
145.675032688915 109.988237089025 118.945264994404 142.669207969715
181.294497402507 126.292433523959 178.946928223991 129.026732674277
CXCR4 161.657344146676 147.208176455806 118.912989775436 127.177422395113
128.011387527117 184.260835471542 134.4380641709 239.871536598083
329.214958614925 209.684346388892 133.613988293895 155.23152084421
CXCR5 133.929311295884 131.956481591804 136.685430908977 135.407536491819
127.951403055054 124.551603326682 139.583514773196 138.980079440205
181.747090380435 138.588751758469 146.616041788595 120.342513393659

CXCR6 122.535728427528 112.547907748842 123.344450694117 115.242526386551
126.601395870533 119.774204444899 120.337241026338 145.804420322848
173.001985500448 119.107992692097 173.766372029062 128.152956417755
CXCR7 2261.31473132315 1665.14066952917 1759.9632914516 1302.54547114701
856.039380523678 402.965083947376 217.408824020423 438.231717096337
244.251315474587 478.699172220804 327.89660021611 460.24741151155
CXorf1 133.274292647041 145.169804325334 135.286686011087
131.643633737316 140.466612569756 139.732074331751 136.545742688573
129.483116877682 138.308965237491 145.437544062449 139.505864135281
138.97776257708
CXorf12 179.779007442837 207.624240598841 224.67853219142
179.624208193114 497.110852407681 232.667968049771 192.752224665459
222.20526515049 217.094085829328 233.496804016764 228.616558999054
222.605195861218
CXorf15 117.943511615792 122.575357101038 129.116354997747
115.463150857002 128.697647007046 141.611712747368 131.473743669996
137.702802090925 125.9888344762 142.495333453346 160.914009756867
151.619329363527
CXorf18 123.786227856015 135.467354817201 135.141067086648
131.02157879562 131.573729430926 134.491232148834 120.831070822199
134.041098536814 134.717258604899 129.267923706188 131.239986488033
122.699993989189
CXorf20 132.177829138863 131.568898109933 132.109267393571
126.013957128603 126.680992733621 126.497472087036 133.054453517834
137.123016784819 132.300508985399 142.299535614113 128.705587172572
128.850480964163
CXorf21 132.739435456445 139.399195840117 116.685023927951
129.750835425023 143.62103263895 127.797666392002 135.884635448277
138.76667538162 127.595574823053 126.141462782039 136.022614387461
130.374065525934
CXorf22 121.027465953657 122.236134282757 131.617027772841
140.36425603453 119.346574301289 130.824958143557 136.737964237109
136.444883411968 127.310437599803 136.614620534169 126.777633512781
130.402496960735
CXorf23 175.019580368717 161.102731941183 171.579123521304
155.183210738478 166.606522485953 148.791182443623 185.717123241832
205.560952959537 154.273953018148 152.393260140571 186.999459152483
160.625367261779
CXorf24 138.023752854109 145.612695657243 124.003914369077
130.969085716681 136.540568915609 129.38021642675 143.694412162756
131.601823550269 142.179197020805 128.02595294499 126.298187538812
134.842877075983
CXorf26 822.88742008931 736.332743195416 657.058213650454
620.899414581162 537.698489904622 616.014975366684 876.783710734594
667.892385395063 690.903797251841 1080.42884514937 1190.62010668045
1010.73988405469
CXorf27 144.2753860947 145.621384219688 152.872139211415
146.792951584868 139.000248033373 155.140371265225 147.420580715112
154.725484895098 152.915692742755 151.72544764489 138.970853057174
144.492756323647
CXorf30 139.153897381147 132.407736899761 134.58799530734
124.253582884351 131.215202712283 143.897248922317 151.104891644466
137.029726467523 155.249688219254 117.539640414775 137.435736170779
127.595574823053

CXorf36 146.192761134077 125.439033142431 132.464205461991
136.903588075134 141.198692540628 135.800997202323 140.307931763353
135.310766630401 126.314233529267 139.155456340488 132.875314170399
141.393814611166
CXorf38 198.456392063513 199.637257550762 195.69447353356
139.517413585211 300.886032768166 490.022369664024 440.203336761263
663.782083028745 446.601751145647 335.790909130243 416.475440894731
600.919152191636
CXorf39 159.845890841215 157.77398996415 156.863232221623
159.324816814876 359.991823789356 181.202396068658 215.506175859782
217.89028736436 203.149001616967 218.839681873515 196.777776509371
225.362253409428
CXorf40A 248.129640673859 221.424451092316 223.89854167651
231.372316734945 583.314509855875 406.900412510895 521.83794739314
366.317263094585 421.459312180809 294.746622369436 393.500781010617
393.198813167839
CXorf40B 241.105817192695 193.084818760857 205.509515144263
169.940177050294 451.380376296813 357.140791982825 471.15333189869
328.33387481068 377.225452025984 287.334035234496 385.522443482
339.252515920242
CXorf41 113.238466329843 127.694189114288 113.262357892613
109.74983281634 111.928204694188 121.141432480683 125.787353491408
112.239647575661 116.263405190786 109.552501987529 119.98864381644
117.261443445891
CXorf42 136.265972909993 145.733828447934 139.579764449867
147.385714746214 134.301187092088 147.414834563852 148.711943433557
139.153897381147 139.041005010106 135.985358106767 155.957570398118
131.712745388563
CXorf45 194.637233028442 171.97578836712 233.029655807246
161.960392594363 177.598422362298 188.062732621031 183.951404362826
175.236826554583 209.984545596312 205.341121539273 193.786889748038
179.49202902995
CXorf48 138.18056202179 137.550607284001 143.673778816953
144.599578905334 137.790718305537 134.972933635542 140.699287635171
152.071081175788 143.031909545902 146.286319248569 133.298480558419
140.922678400415
CXorf50 136.627904756283 141.059033576475 146.83577460518
137.068717031383 143.119405329718 140.073208365817 125.181327386534
111.253743009588 144.193756031386 124.168162885295 126.017158064032
137.187640876289
CXorf52 125.876114993687 129.733935614802 123.383334044683
134.331420680628 131.282091618102 115.080736217256 118.679664392666
137.495288670441 129.649043291997 119.176139463015 118.424520312293
125.617143363441
CXorf55 145.658251059524 132.651922794567 142.466067016788
130.37544152572 132.671227062948 138.215708514725 138.005168756932
137.77601089414 111.398760606544 117.119533897652 131.267177634269
121.722412245175
CXorf56 144.095471610836 131.519455635415 160.122274989224
127.116637906391 226.936810012475 190.520750756082 146.512045729234
181.607253339427 158.87755965229 167.500410857353 128.513011425662
143.004090696463
CXorf57 159.461778054423 229.116153948948 160.853480692082
137.586032396204 966.731520471976 2482.27419621925 826.314416168552

801.051513639714 1257.00798908017 364.678403892354 920.342429282889 389.500591134279
CXorf58 122.329290563769 130.165488538277 118.544870925791 125.2051300372 124.469415593643 136.08297876405 116.685023927951 126.132339293132 128.413213851142 121.425298866613 126.05295868521 119.9414421871
CXorf59 140.475688390415 142.981967333546 132.854078420045 126.775180133301 164.172084711099 181.022635001204 148.435419356331 137.372131049785 151.796986456254 156.098240321674 141.535381351126 153.219954133957
CXorf61 118.838332117453 113.071787850611 116.335864439545 110.16174213285 103.240215392215 113.356070669308 115.097376525707 112.464951599416 116.445184223804 111.497241941203 138.476953078729 106.147993332294
CXorf64 141.555366493541 131.748753064488 147.932875151331 150.746423638097 191.924177620803 186.563913311757 226.919545367682 182.384190694142 199.620978536397 245.522497675754 160.261708910517 225.783771938969
CXorf65 135.873325130921 138.759744193844 116.331428876124 126.826941761528 123.265810024678 128.685065364693 133.805991920905 126.763282018991 129.11843197744 122.382304966782 141.935435502163 135.336754997434
CXorf67 148.932696684374 142.85695042665 143.26896117546 153.38260557666 140.043256690184 135.168792286819 145.385816618728 126.288011849541 148.367132982373 140.802033706122 117.270320709161 142.635015113687
CXXC1 787.425941491281 868.86246684338 922.009043194023 987.564350211893 2468.47687203968 1001.65849130836 1992.71224011906 1747.33558205825 1797.65684760558 1106.21439150501 1300.40889834984 1099.80705776268
CXXC4 145.180346905484 167.121623035375 158.63432604978 151.492236411757 139.593950393555 127.773292289242 138.600017737884 156.411563412665 134.29036009792 124.270946828609 185.801540278924 142.163834995141
CXXC5 1637.66306286151 1450.44557173645 765.333633142088 1512.28202818467 2473.22803972431 921.140932799223 2072.01578063404 864.161191021297 1505.90883940934 1495.04587558545 2751.75479578587 794.636255515909
CXXC6 165.48594766783 161.382041789666 152.224281847792 191.445291899108 147.094123051131 146.404727168045 164.763269377973 164.520247586053 173.035013611377 172.677083810424 145.919359461744 162.702767346393
CXYorf2 129.190900922265 120.711150688559 189.603621918298 129.89329138222 134.381042390464 144.64136816491 140.688570032068 154.701413581915 148.791182443623 147.751220590539 125.661697165943 146.832797779597
CYB561 224.62335524729 231.180528071672 219.044271073282 237.88097138936 393.375554593484 379.292531644085 423.152420192284 454.854528472591 490.631757904175 320.024259346646 383.033242143031 615.662993833692
CYB561D1 337.119524477446 331.999693230194 372.088129459528 404.366325262322 593.462634352052 409.206158277764 439.023181761356 554.950092776254 577.250493023431 322.837446618323 559.559921312181 363.89527566597
CYB561D2 184.709768457405 196.16056297158 142.187694439611 150.311274161073 1282.38953612054 304.152390210727 424.578659172414 376.54010486682 521.512936372701 257.372740535895 310.917191279571 367.327273578513

CYB5A 1100.81588201714 1051.98943987926 1549.30900090605 992.320202251161
2444.835635949 4183.06379632713 2691.41389079777 6384.66090426598
4938.42484385053 1081.47077865393 4255.59773503364 2553.92770795014
CYB5B 1622.17491781572 1856.05025952611 1411.2586496281 1620.7954553764
1522.87703817058 2578.85391480563 3575.60827968852 3111.50508060882
2756.22069438696 1769.6386910047 2444.835635949 2433.83421295012
CYB5D1 355.372836460906 327.062810389635 409.380002417794
416.392874553797 147.100947801261 447.579975492958 205.781486117242
402.425130776131 199.459711023907 315.963526790659 207.977817300376
187.132496910208
CYB5D2 269.770777921703 258.21341068758 269.981476129439
243.95334982553 256.174286655796 310.758803815671 266.61343130101
358.298768623212 246.031568259337 181.37137053478 369.275635424092
305.200911653347
CYB5R1 922.50743596248 1027.96306543235 910.541650889451
1019.627634011 936.957730361063 790.321331435096 2225.59207490574
367.229293432041 737.193295872614 1569.13457493554 1149.90248188644
1282.84159765715
CYB5R2 227.558441474918 224.724262742427 163.074919710259
157.878110410517 486.047284379068 402.564148020948 274.77366169918
248.993716339029 278.335289966805 426.769986378276 290.288001750744
400.621427274425
CYB5R3 4929.26915002209 5582.64254130978 5008.4888270731
6628.17856159198 3775.41389938231 2986.6208659209 2340.90990677253
2012.36542989953 2926.06746795504 2476.51542420593 2493.5484556802
2466.36457617864
CYB5R4 219.78041403077 180.231462528702 255.305647212135
208.067404132325 269.238332581564 335.623336587396 384.733313349438
349.910535555712 294.48534582475 398.571054759451 371.168752104821
255.770193180874
CYB5RL 142.674671804361 149.207481665265 138.489443610941
152.470980074365 138.639410179404 148.484823275343 140.842305941666
126.077945699709 145.954700424725 132.774127230452 136.066396388185
132.801619479245
CYBA 692.359779743985 630.670530516275 401.189904619902 350.166837086936
14938.5975217303 1557.00226259883 2046.64499507352 2441.05534211996
3833.73632806344 2744.77453865893 4110.76546218806 5992.92384160188
CYBASC3 298.706655333472 324.79327985156 309.997909440664
402.564148020948 753.007562127186 416.307629596091 552.962676093514
360.852730026133 389.500591134279 331.848516303011 400.621427274425
372.024884481775
CYBB 176.289693941489 172.916234073981 157.381401974279 176.155413809391
159.438084699061 189.603621918298 178.636555178682 229.542259738438
346.476121089685 172.909795209733 227.033342447929 183.3378528327
CYBRD1 3237.94149351627 2240.33212242003 3338.13980198211
1350.87073284396 167.505381046729 309.423768054614 197.205792984885
260.67310907387 373.361263985383 570.130804179186 163.298729857831
169.132693907633
CYC1 1687.06111969368 1708.87666406131 1809.58702081877 2590.80821234237
5226.05969701201 1880.35608492553 1810.68433539154 3215.41238464601
3012.49794349837 2225.59207490574 2217.82628612929 2444.835635949
CYCS 379.670492165938 338.260976633501 486.334840298921 363.025385707747
190.506201184419 303.005912224195 170.007845523415 189.450316943845
210.353341004865 190.850639994777 273.353254857377 423.489441909617

CYCSL1 970.166036472486 937.375361806971 1120.3009282887
933.408902924063 368.134303229682 1144.92109214621 688.915534194306
1134.64039256323 945.403379196643 1014.63777151551 1051.98943987926
1382.95068105649
CYCSP52 136.913957413224 150.566842106244 133.106005881815
149.589901372799 133.889858322181 146.075794515889 142.892198239879
135.395071593072 142.878258542775 143.175483349968 162.586012723045
154.928072022777
CYFIP1 230.015981563049 213.468420853218 249.953902454701
203.265559787292 383.939800422489 229.805500520256 314.063025950327
237.734385188542 330.005950459958 428.462270756201 330.758879244143
322.778260588465
CYFIP2 644.449861831749 787.425941491281 400.472835504879
1578.87371296418 205.871133967588 311.966971734249 360.185005480216
316.663128947813 287.703569453502 187.461448376593 241.867699338253
294.323621186916
CYGB 1072.82323999638 1177.01310527577 431.546135503733 631.819659701092
317.309949792707 329.146025181674 230.508733145583 274.736124442229
363.706789137022 261.775269058607 231.572677616684 321.367338145787
CYHR1 210.353341004865 199.191065549132 224.963277964548 217.607230767028
225.065765924246 167.373416675264 191.777253122672 158.359440014674
164.043866642712 159.124592503759 190.318957029666 165.932628858503
CYLC1 142.452409969639 136.263658288289 130.433405111592 134.239416179248
126.931328660501 121.053766591551 133.439077726411 130.083266044947
134.361949431678 130.720097323382 147.313494489487 124.17231659571
CYLC2 116.328330266803 110.602406076036 118.80458302894 113.773684331489
112.114933398547 118.006338536085 119.285026330891 102.166449349339
104.739307327595 109.482330324734 114.589757401741 106.437660157036
CYLD 195.547320133122 186.708059183893 189.324370290702 173.010799217261
136.631948656268 180.610891208146 159.399804351771 161.108249802467
171.267907653007 187.167255451988 177.538262133568 167.713268780664
CYLN2 396.708183244581 446.948699651916 236.401834189942 421.255522475862
272.277433294605 148.192121257421 242.902900881585 179.067275037015
169.355185349431 160.592404712197 184.157941055948 159.696443182844
CYMP 134.275773172492 141.115539663314 138.701871179502 124.679444852938
126.421148348261 130.803561448083 123.168787977805 141.603532965962
122.010855318111 129.405749374776 137.128849888863 134.268075583733
CYorf14 190.939726271153 167.395369670065 216.381643581705
175.847256673734 170.676752248138 138.805103589625 148.388093331273
153.104072845043 157.248117633013 156.243337414127 168.753660955229
228.284236550547
CYorf15A 246.112720011613 221.491633607126 224.588225855066
172.741890316331 362.252813848151 128.616121042106 153.308956040034
573.509780150018 383.10686624119 140.77762487941 422.688531130043
748.38293089716
CYorf15B 199.135932984641 164.202554380274 175.686248503777
167.961390966538 180.867208892996 141.362627556137 151.619329363527
255.793823796456 209.130260450131 155.615655460277 210.761398461329
350.741303038926
CYP11A1 165.118167086792 167.850516495529 168.261511797218
183.619184925353 220.312301059818 177.379186278216 168.173803952157
173.21291920566 162.769643886845 167.91761500242 160.786907816025
180.994249258457

CYP11B1 121.472885562857 117.436119533535 136.344248873826
124.180511991605 128.458132030921 121.294366964074 108.939608619532
131.141668650877 126.100454068838 120.867408760075 122.498794845416
130.42746492878
CYP11B2 106.797707723826 118.32560379073 118.60878652724
117.284724854784 125.00254973945 119.645983822002 119.802832736435
110.202982683256 118.465111174212 114.01599035448 147.040759979063
119.297561749232
CYP17A1 112.534864678892 112.813322240155 116.497714896018
111.515588041012 121.657744897668 106.102093734748 107.103635692135
118.9952230535 126.578035793406 115.224011128183 119.502758763438
111.908965758799
CYP19A1 127.108510693625 129.493805009651 128.197031700084
129.835713633923 130.611233072509 124.568534983277 128.17223916536
130.838350486331 120.711150688559 133.455138380277 133.921012946195
134.541606996118
CYP1A1 170.801761175916 188.166168428752 585.933822585718
135.58042404479 111.253743009588 120.288469716375 116.688949603376
133.906867716171 109.182623414389 162.769643886845 143.461785113446
119.281300456243
CYP1A2 127.271423859953 135.079944196457 123.881658891576
125.720102558499 133.985582992986 126.899985772157 118.154953746492
138.57506931811 133.278923716119 119.275061991187 135.343380336913
125.940081665592
CYP1B1 641.774812292709 503.967725251906 696.003998372271
343.666488527549 157.643461899964 143.427721577372 166.910561772689
185.359195899057 167.07092218037 284.748764120614 148.139376772567
135.976837152799
CYP20A1 250.30085097088 213.028568789661 250.085444708918
202.01798254763 220.010399552402 340.487850009685 183.937484597232
321.794887577351 208.793650722271 286.541028896473 226.144012966486
279.598585549004
CYP21A2 154.634289463743 158.56996905631 139.310309748986
185.088667707155 132.324630056168 133.249792744053 128.179293878252
140.719388544476 138.117916412819 142.609678462615 150.021512237553
143.449303333495
CYP24A1 112.809629077555 150.590611246086 114.428050500096
111.276543583667 930.704688414069 130.429076594441 339.172031360524
125.147444382537 1288.3346537369 127.535214313797 461.413469623811
121.207040901483
CYP26A1 136.79732903972 133.805991920905 138.588751758469
137.402567669086 134.922488718676 132.518394343751 136.251820364823
132.891375982227 127.442587366678 131.811011266156 143.090886224647
130.262411213764
CYP26B1 257.372740535895 248.643311972844 222.764568757453
296.051639873628 150.841261270382 149.72025962797 163.356993518952
173.250350363688 178.847858667374 260.715580924916 150.642465826166
142.914863449833
CYP26C1 121.508233749444 128.611740835252 118.974727968283
107.205247133938 132.168821720799 125.976052547953 115.999443701343
128.005043478952 117.442158626871 120.020781939389 146.518478800271
125.780294378066
CYP27A1 696.650617523899 702.981741565482 774.180202250076
588.334329592604 203.816305810343 199.837938317725 157.742948919213

200.558971047462 443.623904480087 197.526492324088 176.328994356625
187.336349390012
CYP27B1 141.73248778957 159.59942312149 169.441789096441
143.085022888434 203.125568980238 238.654367531982 168.911840239685
249.680729921602 205.57572471694 256.99019407912 172.741890316331
179.985468369202
CYP27C1 190.045156046541 191.507657960386 167.077669654786
168.116928722035 155.942609847585 159.970332650101 156.487049947247
145.206796089128 146.31468639383 192.512661648653 269.981476129439
159.154102271517
CYP2A13 157.499803444188 151.519845657755 158.092775850886
143.383697128285 150.677852949794 137.453705355364 145.967685593635
145.143366567189 139.650726173186 148.31140879454 146.421195733989
137.773960847088
CYP2A6 109.91537150286 113.845770994796 109.067239220744
109.156349683299 103.202873534814 112.404562115306 111.559325333192
103.09089100768 104.321352818065 119.67745324922 114.696935316053
115.02681415256
CYP2A7 126.722212054469 126.242935839683 133.762141373091
135.325617701062 123.338921824167 128.548201588898 131.181537219479
135.787323859105 134.78059811237 132.906201428703 127.310437599803
138.003440266411
CYP2B6 136.543603346919 131.04482088126 120.706421084476
129.210356735226 133.307149027384 137.048292841566 128.788443476759
138.005168756932 133.665206343648 137.792150225149 132.320536722841
144.421572284568
CYP2B7P1 119.993313087183 130.005909892804 127.121068682846
120.599440204711 126.265475477399 115.43068422162 128.730649233876
106.066897223031 115.858924428037 115.080736217256 117.754181884939
114.501509669035
CYP2C18 141.971207380159 150.034029031033 140.754706171596
137.157492354617 288.542264120468 278.219561902233 193.753062958207
283.191303666907 512.895335481876 281.926552464214 213.556015518863
297.022984365795
CYP2C19 136.324020406359 143.438417529799 143.931400351525
126.719205774829 143.626857507866 142.318709347082 133.994376985194
133.384498169568 132.916608405637 137.103879676652 138.880168623766
137.383211680459
CYP2C8 131.48517604564 129.32466533596 124.661088039427
118.553226856337 125.887854850942 132.146027935802 143.552343056702
151.026905311756 135.894512504725 116.433239116749 235.019573994855
226.640323471375
CYP2C9 108.386545220984 97.8058879269165 110.234403581151
125.963257217901 137.893630842968 117.303671445328 122.095978653755
140.123348569709 291.779980648289 115.435942397626 182.095387488796
132.918979842076
CYP2D6 136.008118769513 142.372470063444 135.330241638301
139.111604268732 134.299575239865 138.319321806028 133.606068125828
148.057700860759 134.584076788979 142.788848338402 154.252727576218
131.829906202014
CYP2D7P1 151.917359928516 139.293775885106 137.91121726927
153.02549014611 150.863099494798 150.484823814674 146.349773982488
151.889644436875 152.032418787633 138.023752854109 162.199381540369
158.092775850886

CYP2E1 178.676812740925 177.478925091583 211.289598823191
189.482046688226 255.839878446312 174.338440780636 157.600193849783
143.488761023636 153.661632990172 137.269267022826 174.313718712035
155.049051256459
CYP2F1 130.621860095981 126.643229085068 131.747367613225
136.300195727908 132.381685434931 128.570067783535 129.687743133943
123.865424155099 126.492058851464 124.077104101209 115.107214937219
127.251594986725
CYP2J2 202.357989894268 313.641656294638 323.462252156562
199.53455266342 1410.58825009446 861.786094902013 2863.51286169094
1032.11574332305 2213.1043022626 404.876478833713 2944.63971819193
1638.72366638732
CYP2R1 281.881563164961 238.270501389189 234.484787292284
250.911817769829 184.414609874199 213.26158748941 230.855806094267
215.635313821029 182.950456188966 250.737252834098 315.828726297864
152.890239929335
CYP2S1 125.584202037776 141.278654291193 122.339819585312
124.00806841872 127.146101490884 119.103219740679 124.528206512938
133.680691168661 320.467139255533 127.690860707933 151.939710544553
131.339653356438
CYP2U1 232.960832410426 214.566711351899 254.101184686036
215.453915231774 153.528429414138 144.318556353673 213.145460679531
173.260348096238 178.284294783418 151.604093626944 152.088488171996
155.622257285651
CYP2W1 134.053685488055 121.255814891176 121.526724715417
127.006746526023 121.71538073735 117.335748689217 127.723052839953
121.747622617011 124.253582884351 116.519527499611 119.894737403889
118.963119420175
CYP39A1 155.295464263911 162.728630453745 190.921283434627
135.810476921749 133.093234861388 141.685620602226 145.180346905484
173.142436025281 136.301442794928 154.861507590906 137.697452180659
151.815211341628
CYP3A4 140.903091584884 134.066948803697 136.353051841792
130.110413621436 122.821563586744 125.321968563987 139.652974659584
129.865581506307 131.383113302567 147.420580715112 151.492236411757
137.697452180659
CYP3A43 130.316282003711 130.217791888656 126.484819755399
132.699715693605 133.722739936347 137.408247668043 127.601379088239
133.52271411034 134.356796881432 127.327765407459 130.656206845965
127.488853386792
CYP3A5 278.005620372937 304.811116024094 331.062744782933
210.605864685448 153.888212689508 207.488522933448 920.083835296898
803.133619199479 517.42417250172 157.78390301218 3090.52810501797
2053.71550326011
CYP3A7 134.819406064011 126.620889938532 135.296271683847
149.6036366066 135.038195181839 138.391203321266 174.795822188504
167.976680048356 149.990378213915 134.018379799826 123.418266047461
132.454025879177
CYP46A1 165.789846154721 156.102877632001 141.980728956046
178.476483428474 133.36168342199 140.047749881567 141.306418675404
148.803188053248 144.665869508406 130.967476533254 142.036037957345
133.99046926032
CYP4A11 117.580122770251 124.408996713299 121.334882368464
111.260486045985 137.324247703318 124.800221241981 125.526744092256

121.316836274255 125.606269267903 112.684334910243 154.947359473187
122.517559025268
CYP4A22 117.907840952685 120.465934391246 118.836387680022
125.013839352291 120.094156375538 120.679474100322 126.830269821364
122.863612588759 124.079109459437 120.835207882788 132.695992860853
124.985249190127
CYP4B1 193.084818760857 218.298791348295 174.483233557264
151.508738608925 1788.35884039549 1505.90883940934 1381.07447204233
2053.71550326011 2219.71846304201 192.059255232313 1846.61430264854
499.776233241535
CYP4F11 129.265788005518 140.383646018409 133.682715540066
130.579954890389 308.330044958513 305.906480933354 696.277780670166
393.198813167839 137.331864485838 145.165443106042 323.788197265954
152.56002779985
CYP4F12 440.203336761263 374.337030706199 403.720036930931
270.231637012624 694.429144766435 1181.97868824758 522.77407098992
814.110601393539 332.798130757632 147.123477579978 1978.12229412176
932.076509242794
CYP4F2 146.257923971069 143.897248922317 163.566535828036
150.707113487334 172.142411008084 155.243643210534 178.745112514526
272.770616373687 152.840202260007 155.253032078086 150.606329295069
160.001115445038
CYP4F22 149.848740870148 185.583997792931 189.250846546817
117.662611650515 1803.06322760249 616.995114856023 181.695203523798
929.198774489695 570.533103778482 207.204293555178 713.087392708048
767.861763134136
CYP4F3 154.360558958914 155.651798065702 145.872258115708
153.415901281564 172.214433016685 283.123157828849 227.774354299962
232.699786290509 183.115511373872 151.70035428516 208.194788326109
189.509062639279
CYP4F8 128.990911924499 139.785767470943 125.589744938813
119.876835248732 196.908612360694 193.753062958207 437.344765185802
250.003965480779 185.088667707155 133.452032750397 132.113317893587
146.97334315047
CYP4V2 753.007562127186 692.359779743985 690.582550537485
434.855262284265 201.893722965557 432.969886744613 396.345382600445
1226.10881264804 481.630922092112 292.839392578318 345.291295192574
247.971366020963
CYP4X1 166.558080305926 180.719868519867 139.757191608836
155.333294650676 150.324132011201 234.020419732787 214.355086255855
135.521802085177 163.889522525848 130.445920648705 226.595006724068
194.36669779004
CYP4Z1 124.883575464443 125.060387518072 120.006982591236
122.789656787728 123.98775110588 135.913632939299 134.465488237877
124.982744176925 124.246363878094 124.313901870563 151.669421563954
127.135199316758
CYP4Z2P 131.693923403533 138.4303531022 138.030842581932
132.683594640696 145.741465098455 154.539367933684 150.599425944486
143.21411610625 139.890519860289 136.028011201077 156.010027635627
142.097126256137
CYP51A1 306.43742010208 280.848426993226 220.896363324634
233.300864925399 329.660368915848 191.029458520006 333.131139447511
353.285905782951 246.989526028884 519.564563632951 348.921877633633
333.349203784021

CYP7A1 102.786517901308 108.515401509039 110.347631909888
101.981823814699 104.896224175106 109.016991399605 103.888971448792
102.024765473404 107.816312357067 113.470356909765 112.858669660245
102.255408120852
CYP7B1 137.53070284697 141.332402615602 154.691783046492
153.293125258215 150.46224324459 137.745887988474 137.79714923769
147.829502959865 148.63131331279 149.250205265163 151.115031304764
137.358139652612
CYP8B1 119.355650892262 127.302714624905 116.061356450855
126.176505541577 126.191057198995 118.148476979319 118.677203713024
133.027236270978 116.126923911804 127.856634523494 128.232091717966
122.258432948151
CYR61 2321.41228376157 2311.05587511258 967.797017108447 922.009043194023
306.387898121409 1115.34267967366 266.652805624826 346.917113326564
231.257543784864 2505.8565544797 168.295044843056 246.552713031445
CYS1 124.39748786803 125.846029991447 122.469113037809 122.005340508177
120.054065939835 124.190161132628 120.551603446364 120.204469421484
126.629012619208 129.415078042622 141.84653748835 122.449574852865
CYSLTR1 196.990867962546 215.40037757604 175.055634340766
188.708596785144 156.293025312901 203.276353321185 171.345989796519
180.599150102184 206.669783013221 160.997179739138 184.813094907887
184.387409077972
CYSLTR2 136.104965836123 122.243585162452 121.113186311514
130.083266044947 126.182856027765 139.201122725213 123.336074100984
119.01990099776 119.036965883266 124.222529964098 146.276706674761
124.807244018373
CYTH1 264.233381434738 275.841929188802 169.661107080542 268.568824794145
223.872330412144 280.531844086775 247.683819429859 290.090495047722
367.025117016472 347.557792829093 498.965895763801 392.13175568099
CYTH2 184.672330560677 257.477501542521 174.635853531194 175.946843919065
551.92688162618 792.247301083075 473.057488824363 1706.06615968622
1085.92360670051 201.349209185128 544.956712516196 302.113807121396
CYTH3 274.120234926832 273.478372861114 227.065194522692 273.514200733343
151.043967734248 167.806420483187 153.363629311799 168.095061700814
171.791030104358 204.740183832461 171.465178761171 172.297698667311
CYTH4 170.590817818414 166.071816427719 164.39557148282 160.20843950503
178.455966482206 149.959767051801 153.348110626071 163.066051106092
218.997165156081 168.027892518772 157.313737338264 148.906498938982
CYTIP 137.927403204849 139.644701550661 127.8514513762 133.465593684519
125.717328918165 133.340152756136 122.423561732526 138.116568200293
144.664600892248 126.908123260394 131.099004325598 137.744318243173
CYTL1 185.997327421952 199.305626149918 152.972779110762 166.746037983859
131.568898109933 197.087015414039 129.854478528455 179.369845168822
171.006582229921 157.463122989559 141.874188629438 137.644942697483
CYTSA 1021.81988990874 1129.15018335354 924.630951485299 1364.1224589383
517.020259129974 407.711669482083 364.895113721763 467.512355593598
479.638653311025 442.220572301795 435.037306125887 382.562403424855
CYTSB 146.550421651378 141.587109176431 135.767818903328 143.759171254761
306.716682293251 147.009325721272 139.675292022085 136.935498266723
148.212445624699 166.22013573342 148.534143255537 139.782152941027
CYYR1 274.092048989484 310.815067427296 174.941551089279 241.453456707114
158.414776465951 235.32095278846 147.512290383685 183.482640529727
195.618369827396 150.28169442832 181.898985277176 148.450023054885

D21S2056E 114.585818507336 131.736694577111 147.679233228201
130.874283538818 110.368392735494 129.075693654369 118.75237770613
123.913203953936 127.026168862919 131.271979461703 142.612035986517
126.972399784537
D2HGDH 844.187489702587 811.564396886535 879.809795251142
923.383014892221 799.423640530777 346.78158577526 1129.76078220457
549.162299481184 734.139557942719 446.601751145647 753.007562127186
1272.14273680177
D4S234E 175.767556472012 204.161375686761 172.750926094884
142.628296442123 157.990467804353 348.711663415777 426.995700632434
181.261666651301 197.795275907042 663.782083028745 941.065508539108
1442.11349373764
DAAM1 813.772804994191 789.401526545277 532.063437013967 595.409419899436
330.405026390912 362.581768268116 441.684169663552 338.510045856515
270.181005097697 771.059329972125 532.063437013967 426.249411635589
DAAM2 338.822464893411 238.461248099002 297.285037835812 224.027605834751
158.98007855379 175.767556472012 158.31771109146 156.994265858123
152.454414703534 189.307823427362 144.746134643761 142.942021904533
DAB1 118.740346254406 124.505487429568 115.624167024067 113.432757764419
119.122305582754 116.695313319162 115.211731367278 122.854722097864
126.428284589412 118.909153405102 129.735862775199 107.450945262092
DAB2 1304.37930719411 963.343120586863 1023.29995420518 879.301932542031
534.920870202582 413.000072500071 283.084102918359 1053.65938651514
1539.80266129509 490.849163737598 483.312894744803 289.297370738382
DAB2IP 150.279529635193 153.620767586182 132.548259643258
187.494677180331 158.622993108149 129.508990861362 140.251168248117
127.996535708833 132.203310946961 127.562924931536 203.370353613423
132.793020446043
DACH1 188.093450717857 219.8511043213 143.127795310829 159.374355553219
152.11956441072 154.564478911791 133.728378162206 143.464614171516
149.073650082048 131.706721548422 143.816567683093 139.801108744526
DACH2 148.773558380167 143.05872558805 148.760280320258 178.819486867379
155.548684011748 140.702685210277 195.863843860549 165.545136704242
150.188128594079 160.459100056689 165.932628858503 156.671252606074
DACT1 280.398987330256 233.20900339048 216.033564783918 215.143973502566
143.703367582367 155.945838519586 143.265532486512 145.493977576106
152.148784410501 202.849544035436 149.874424976813 144.386968241512
DACT2 153.088710298368 157.739526559807 129.771073986855 154.33224999163
126.199219102601 124.93629294955 123.905462079972 128.533900744619
124.857461801227 120.839256811295 158.89305345139 122.535728427528
DACT3 3211.2719052157 3338.13980198211 2744.77453865893 5532.93454600454
182.181756507598 157.709832297666 139.353080958675 146.890877830248
205.121123131992 194.819597436776 180.430425314885 159.362293777146
DAD1 2476.51542420593 2069.70335195314 2833.30537635103 1890.86868383409
2921.59274577877 2105.87438782831 3237.94149351627 4276.70912292877
3862.57980488881 3822.05744774919 2320.41293992992 3684.05513498098
DAD1L 127.706499722854 132.62551190584 125.794788766462 128.324383749931
121.118933682515 129.971670681199 124.924794564647 122.75516501062
127.799190369808 117.399753685854 119.802832736435 115.858924428037
DAG1 433.928818077527 499.683188662742 410.954095610904 645.438835606933
1382.95068105649 618.437282203498 600.635914090365 379.200576951933
544.222458349696 323.737610506003 439.394914369983 376.276954313674

DAGLA 152.238845270993 159.046016068252 181.048789688798 167.456481678813
254.991678321253 184.148058872585 162.342112922504 160.799950553311
150.324132011201 149.556644928013 145.270140096768 193.134414137433
DAGLB 304.844121431627 327.164773632634 318.73799597811 367.289651112346
539.125423322738 436.834825721843 442.12886967939 444.945078908489
575.546802138889 509.880640462 375.651196211048 512.467731561858
DAK 134.768694020981 158.753832849946 142.365009028839 139.350622585937
518.478990211753 202.162432624505 225.64288602828 171.775741648309
187.425086057782 140.45860949168 182.608712782878 197.191648960563
DALRD3 142.42442972908 146.64560446484 147.396463611334
145.237221169839 151.889644436875 144.368355737187 157.189041157784
146.155809402461 145.599152743156 180.192015483309 148.723165059122
160.822287037366
DAND5 116.95195122318 118.319950396126 112.635430248416 116.793408204625
119.355650892262 113.326628149066 116.736174147084 125.951927536494
114.927420605185 109.274221164316 126.862274244977 113.311132067152
DAO 141.115539663314 156.65842876856 149.231475480618 145.917220112846
146.362705590778 143.275056599274 130.069789939568 150.184863108453
147.75625228531 153.345834660643 160.478519202775 123.551225884748
DAOA 113.274668934773 128.695695565175 112.809629077555 116.328330266803
131.062360037777 114.726808450831 125.609542142983 120.394220715157
113.043089777907 112.142263331903 130.842257229979 117.770032157393
DAP 354.45785837703 379.615659443164 294.697622870418 376.627801547869
722.974310297586 608.129959758538 636.970243888917 682.075583857856
654.663838918786 257.477501542521 1092.33328323944 441.435444003241
DAP3 640.568342662067 717.686676823785 891.194779815063 656.009451448101
929.198774489695 1111.47772523331 910.087388930485 1497.32522786098
776.030689773414 856.74270641707 467.04976953755 1622.17491781572
DAPK1 263.204269632206 255.56301534833 342.998459706966 255.677315946354
520.962651968997 429.351447428661 451.985108262982 616.014975366684
561.062594540005 226.25694727283 642.934824227327 329.779210536849
DAPK2 180.035652063942 153.393651104539 152.071081175788 161.335677383485
145.777778096701 141.901382169334 147.52403424052 144.883597913037
147.635761512135 154.869171648942 152.042744996136 153.643157329902
DAPK3 466.691191261997 397.387054218873 265.712482648121 533.279122416818
316.364342983155 196.053808536366 225.362253409428 178.872635061826
166.622070620588 186.640167849568 193.134414137433 156.920867019849
DAPL1 128.137646789236 140.232244264861 130.642810886161 111.935554828717
109.471542280224 114.512028664352 113.862790983655 114.559189149447
114.200099628087 107.385565665491 114.624259793353 158.882535865069
DAPP1 8840.92490099301 8261.82420896892 7786.96143641382 8211.38792799248
1859.88000417483 8121.54750071211 4527.53010866974 6156.29203791697
5572.23400804004 4280.66857663502 6585.34962362923 5734.76405709934
DARC 822.545007054668 1408.08614963928 451.718912714608 1161.39230511792
125.443242860371 228.184535093641 132.113317893587 404.21236980164
817.913210743023 114.234369421536 210.529782898493 121.505178996279
DARS 4486.35687688252 5106.27623905309 5080.63091497522 4938.42484385053
2101.93705983383 4068.18567686753 3371.24667467952 2385.5958396359
3042.88425356552 3712.67025366957 3298.73393277409 3855.33040541264
DARS2 146.418147447541 174.471304281502 130.226993687215 166.12695134858
439.122658719747 321.25076864104 258.384059994446 316.004332524937
242.096692715029 225.927425596831 276.830814061474 336.002793042439

DAXX 230.672056810885 258.552964322998 189.589053840976 275.169415154893
362.849380477089 211.660148497276 210.529782898493 185.31769184805
183.081450609516 205.381840116054 204.710203150526 204.914390718161
DAZ1 113.05681175258 122.160459795959 109.384112020063 118.076139318165
127.430408113725 106.000943456483 110.586147059226 112.565341690522
111.10737497653 117.580122770251 114.68345964966 113.422559344178
DAZ2 137.94246602811 143.093156971372 142.900756181761 146.70774472499
142.749568012703 143.07651558428 142.711117470317 144.940119680152
147.04481509758 143.20823844185 139.383528389668 143.759171254761
DAZ3 162.122250419992 152.401204710292 146.470444212748 155.960886801091
140.496058408953 140.954264409241 150.769819282075 152.650867743471
140.722297244564 153.681775929182 144.201723591119 155.326276840738
DAZ4 149.31020447459 148.216605879958 147.230113868203 148.012353232918
153.633095853017 149.760883745928 153.118492567137 149.786445079456
168.365262340217 148.715065348779 142.819669471009 148.304863957659
DAZAP1 359.991823789356 367.289651112346 338.026161865853
401.267425721838 652.654673756838 413.084646927035 461.088572554864
374.106258053201 432.92947962962 399.620724969771 413.687046868111
460.742037437298
DAZAP2 3000.46298223166 2915.72823797537 3615.76740804718
1978.95750795951 2751.75479578587 3384.19521857242 5314.0635307876
6409.23524786095 7342.79622458743 4159.96851384358 5789.71932579854
4425.66777919789
DAZL 129.425699141995 137.976533193418 141.25089277145 137.412952822912
132.675172817752 137.618447592489 140.603933807629 135.907702243922
148.1283706199 131.097598299707 127.416912237613 142.236565028336
DBC1 143.692305478191 133.561188057423 130.233769058693 138.677282903963
135.928428484011 135.675570204709 142.719216118914 126.88329158544
131.111930163369 149.211214631561 229.284931844047 149.113794439994
DBF4 155.43577761798 164.981665344298 152.346098251128 151.09670688533
160.368226341458 158.87755965229 145.938780249776 157.976810829398
151.518116154483 160.557818466874 146.503431281242 163.788418732315
DBF4B 116.126923911804 117.517649866653 121.024572716324 122.607404698857
122.013040475143 114.638254225216 115.755585907264 122.063996556575
121.194138394375 118.366859032979 139.168307371516 116.029201658798
DBH 132.118118271944 133.252140649104 126.662329971897 118.176124626025
121.792573534134 114.191245210321 131.601823550269 116.126923911804
121.103620738719 118.342067845126 131.582231577364 111.975027473284
DBI 1147.33034082693 1360.85309203233 1082.75419087929 1029.09818399518
4457.64479507833 6006.01682776552 3869.12225821096 5894.79176478976
3883.05620256963 3552.78712528922 2116.39371296122 3983.37592132018
DBN1 731.284098301795 673.502723625773 571.136519973808 917.188039935737
190.270819689158 185.186497037695 147.208176455806 165.752558888845
173.919504730673 326.908959531021 170.844002412425 242.752995772995
DBNDD1 198.244854812728 182.827395663312 224.547982731402
204.237952297239 725.065727257421 378.24336892367 296.24781576525
348.457808063307 419.763812610671 737.42784787426 273.152907354565
417.103816140912
DBNDD2 2915.72823797537 3173.14004739767 3232.96611074938
5049.57929757135 398.971838949075 424.495400604335 292.640251411131
366.473258467746 543.170902795966 516.177596584059 724.103864917969
1153.5175534971

DBNL 871.913035811066 996.240188311002 803.133619199479 1545.71251527145
1366.06309834426 426.132037075477 960.491731797061 759.058176862524
761.726829618132 991.611982265221 859.547169028149 1584.81089248914
DBP 171.504939477949 289.001004912528 243.807038920733 402.425130776131
286.194197552489 180.132104305583 210.692705153337 162.056622258216
157.792868983503 282.076744753348 172.84784588171 152.837739128546
DBR1 195.831574717135 196.470718183575 189.872997882033 186.296882059398
219.129781831192 209.840035045167 210.711890471222 256.174286655796
225.702629835215 208.270622752385 211.438615793742 217.287912825619
DBT 602.86705608228 607.126514742951 789.752781067888 584.433155497378
237.754067231793 801.702878226247 595.270107963842 839.324324662619
558.051041108282 495.438672949441 531.555532787504 396.212732639272
DBX1 119.168045349254 118.527464364564 118.486195373059 118.161192318422
122.093290585838 108.386545220984 116.609122768341 123.081299769162
111.232274546928 115.305518560239 130.317604643904 113.913337223716
DBX2 139.021552573529 133.070896773384 137.955289723046 138.42029331811
122.736882859231 134.703474191011 127.070677267233 125.195549796301
130.410584413031 125.683334853039 128.967855397644 118.32560379073
DC36 143.094799968981 129.775280076365 143.368327095618 137.984988819967
133.816601264067 172.214433016685 153.408890091225 153.187148832063
170.283532007581 153.217287503221 181.622689471026 181.183277133224
DCAF10 353.774297577869 313.18989508207 337.585502787016
366.857139377277 206.813941353924 211.035617136177 220.458449424062
193.98814423456 269.892633574243 267.372241661758 228.560346647306
184.482849019679
DCAF12L2 105.284300851422 109.067239220744 118.312891459156
112.776764215618 120.984641102879 118.298444816712 115.457121859064
115.507474401239 126.370252616961 119.873844587252 142.469353808346
117.445284668654
DCAF15 192.418895349837 195.513703253634 194.312315077985
248.887522059485 323.737610506003 232.590542016298 181.607253339427
216.277178481058 186.679199600379 208.793650722271 179.177884962513
215.335795337978
DCAF16 246.912159676595 200.322334039458 335.389274425025
162.254062646631 164.661445411833 207.023221559302 197.827534617557
178.3652866131 243.732074437237 199.837938317725 183.385868234361
195.218005582618
DCAF4L1 137.033870931133 147.826950636822 135.837733533444
134.955823960889 143.141852982371 137.853137973756 146.129805607031
149.100294463597 139.914034708144 132.686915065196 158.260676113325
124.3501719829
DCAF6 639.184296110069 526.285114043212 693.180345649331 528.767644181119
593.990687449346 418.968095159939 487.993796425035 572.753294955207
344.298510200653 519.672796004691 581.828502821435 370.555627307623
DCAF7 897.51782613323 844.778755052304 715.43295523922 920.083835296898
682.538709448692 1085.92360670051 1179.42590997099 1191.88706134921
1436.57771711084 603.569635276204 1175.27165286568 1538.78070281348
DCAF8L1 122.389557272277 111.708970469189 116.604371744082
119.598216955485 108.021898335735 121.760488406107 126.013957128603
123.538037290702 114.14858855653 111.196610704726 110.355493122568
117.692662275393
DCAKD 1144.92109214621 1222.014210447 1251.56853099362 1534.21175984308
1565.64079526732 825.781627776577 802.017244099902 1139.14730341921
1010.23703166498 356.413190087706 805.600599570258 782.139151423572

DCAMKL3 136.742206779136 133.346626315812 130.588481497083
132.428675012593 136.411722212312 129.553282780541 127.859873416515
117.386273592501 138.376176600064 135.58042404479 133.610143526954
132.885515958081
DCBLD1 203.675655444583 223.666832044186 140.447933562667
156.375876454394 198.311821616128 184.505279093688 245.269004485826
168.384397488011 174.702891494074 361.768552564027 195.256498208339
247.942904862478
DCBLD2 937.375361806971 909.020708316287 806.88003660673
937.375361806971 372.594990024312 662.213423011533 263.568160216149
175.593421867771 398.512947526406 608.351527783086 164.905467905433
186.156006020985
DCC 119.810421687953 111.086170834553 111.1002996982 119.01990099776
122.115925891088 124.507958339495 111.286141277084 106.982655360194
119.11586630281 119.709032462308 129.515390475589 118.372047967248
DCD 139.954480845683 169.590101694098 154.250656489421 162.422173095045
141.418737063013 160.331940529151 152.516485296644 151.676232556697
138.584190903303 144.562038493733 138.709991561675 298.165097953983
DCDC1 162.289781945582 144.393688888553 139.963969716386 150.49183794045
147.60284257326 156.62031503525 140.29803170157 171.262412981612
142.379120254855 127.898533598679 119.097521397712 136.013989349192
DCDC2 137.844113743956 140.77762487941 142.179197020805 143.59105195427
139.297276687352 152.709021529522 144.934103671894 157.733042033549
146.413845130977 137.734887788912 125.410876867635 159.102070980047
DCDC2B 144.079935036119 131.294930860752 168.982086590306
140.142884879106 120.031976318697 131.903632821793 145.193296081527
141.292421722594 156.126009063311 168.10131060526 123.100949494673
147.640100078933
DCDC5 145.00699763203 135.041687549769 126.326609619996 143.215828010442
142.007066214472 133.357385559881 132.224831869802 143.258488502799
140.443415669628 136.299079457533 136.05434446747 133.074027423549
DCHS1 290.867822301134 408.263886786123 215.950811436427 634.906566690042
152.933230159216 130.698850298016 123.646558103253 129.52578899011
151.921235758457 141.666394024363 143.407371444437 123.940632366675
DCHS2 125.426018910517 125.810756286648 149.196539425588 132.823333905679
132.555211779043 134.178733595096 126.895669261503 138.506569544482
127.488853386792 135.281686411685 133.213697351807 147.745780820004
DCI 379.911829623463 557.868300839263 557.868300839263 693.180345649331
523.216537391852 512.895335481876 386.067573158514 848.620986404914
491.35949123223 460.514672966125 677.023591987179 416.638529499878
DCK 162.975988989106 149.714982846365 194.637233028442 158.87755965229
317.719381113816 279.834616502591 232.621526025603 264.456926651554
277.817786943873 267.069665000047 180.142758394321 234.094722315007
DCLK1 246.186104432792 222.243523614518 251.546290552828 282.561381978659
178.06221214103 169.674931517111 165.257737836487 161.525131363333
180.070209705044 311.481739745423 154.548286860883 160.066390229416
DCLK2 227.418630852215 198.681190016247 177.306794546105 242.614881095485
117.251233255966 125.220416203903 116.355027746922 121.031695489398
141.37694311902 109.495342943278 125.256339476772 115.914842445934
DCLK3 118.743966166637 124.76274408368 142.324077852852 121.657744897668
124.048926099805 130.661695937728 149.532459719175 139.691470247318
142.090009728666 146.256076936456 139.650726173186 148.87690497281
DCLRE1A 174.406779990708 178.186273279101 179.810507975486
194.529045934216 184.398672994943 163.106823691633 188.431244390961

205.260673843294 204.544266331066 159.334165651951 218.70412656621
200.964306455257
DCLRE1B 125.433502200592 128.082956839709 117.965208293925
122.347696497767 142.772680460689 158.58863949084 109.182623414389
126.784243009941 128.886008923268 138.093181144173 140.24988931401
147.187681416171
DCLRE1C 618.140160251927 591.189034068137 538.647888236878
592.613689704907 238.037110997575 682.730262408017 385.050282185255
465.41233536979 348.457808063307 305.499194075747 510.285766295993
328.14373061894
DCN 10862.3952192141 7969.0710242842 11112.4298269818 7433.03226979876
281.071129128589 1974.49672149862 445.089930641391 1207.11788506001
2392.93562797606 3671.86460903374 334.709049890042 391.400480065
DCP1A 381.484062427466 373.361263985383 352.811351813364 367.130155304037
247.971366020963 345.897250519387 240.439368352772 202.887417145119
302.905840432998 234.399431238026 266.681711962236 265.791342287571
DCP1B 231.600532144945 232.699786290509 245.582279957584 222.315451077459
183.937484597232 166.780319723055 195.816317534388 190.787437782456
204.842791081358 155.069048442532 151.219051767056 209.7558663154
DCP2 496.697565640162 512.895335481876 415.642099109855 408.758021864761
376.443086293235 485.026081242201 563.011988279542 551.823767223086
551.92688162618 484.230510636533 645.183207984114 511.891269255193
DCPS 221.664431984861 212.545989139388 200.146843808423 286.945784387294
536.253405705975 262.389583658305 264.685862877779 276.653753970476
211.809434609469 201.167206950576 259.511314588868 260.195256836955
DCST1 155.6299578128 139.904952662852 148.396148091332 151.823845684041
153.132559737356 150.696963699371 147.83240038699 167.011846920792
143.127795310829 151.273261581765 159.113259830623 153.018378403144
DCST2 127.866992108488 123.488418655586 119.920097823022 127.869038038293
109.988237089025 119.415822905251 118.601228886344 138.149037534929
133.082433951922 123.822571859503 129.932576311158 123.893768254549
DCT 136.489566741917 144.166567293421 138.050999864689 125.353389826276
117.335748689217 122.115925891088 125.940081665592 131.748753064488
128.381433897602 123.845504986626 148.592330216136 108.970432124412
DCTD 525.287367366192 534.745066397679 499.683188662742 551.823767223086
584.919615953457 642.255110250493 654.881990232975 517.020259129974
891.194779815063 665.341859712345 910.541650889451 723.250763797522
DCTN1 698.086166782221 762.182213353032 702.460234202652 968.157701932137
623.409211766224 480.22496056277 319.090371885653 305.669636871734
322.213266293148 603.338538383128 439.023181761356 293.692083555844
DCTN2 2515.7035557541 2648.41012401723 2532.82545752082 2801.66917828364
2246.89876694944 2198.4268389426 2217.82628612929 2111.32236867229
2063.42824094932 2728.04758874748 1996.65760745757 2036.43904731226
DCTN3 554.800688991977 554.652979820908 654.108318810728 683.655205499779
556.725550806207 622.348802511009 396.126485121994 376.962937292973
448.644778906048 702.265884752474 343.946831770916 392.06522028189
DCTN4 593.090436323333 595.752426322468 474.14051635531 499.964000319181
276.79778275734 342.147414141627 429.096075419528 409.691865300362
309.658122422202 578.650614747598 571.950858678221 432.174413144791
DCTN5 833.430847243676 819.488546995594 858.742139354587 947.425912541091
578.811908030805 855.415038498392 731.284098301795 580.347496467173
686.198351821711 615.88518704181 694.429144766435 586.246004571452

DCTN6 380.562212796792 364.597798667541 473.529519031797 356.530530468568
223.253794809571 333.533043832976 421.303580698397 275.609716327452
392.282956362735 554.511983790164 543.810043433181 558.810095726517
DCTPP1 505.560841055884 628.939201051801 569.353661326724
681.591028334969 3093.38255333143 3789.1316515671 1586.00600118477
2382.87910783088 1629.32740709526 1048.85768964657 1547.16936835284
1780.97169244555
DCUN1D1 175.537039794925 168.729301938015 218.559622620349
165.972090075204 180.798671424395 228.9901869793 179.841665686703
213.652091843896 195.561296046507 198.088834624665 166.521472376759
205.4648759901
DCUN1D2 151.228823957828 144.757068610657 159.005459875338
139.038389244329 133.737573120711 167.830629616121 151.277511331109
146.361128670362 145.324499089416 147.174977926293 148.574661934068
154.030432279825
DCUN1D3 643.905401805862 390.501797367707 332.032371697186
360.124745133393 262.53374754047 316.457274749526 286.376638135463
324.25775261173 276.830814061474 468.569725051459 300.83667045718
269.673122990604
DCUN1D4 827.400420888645 746.777330152729 774.442392607873
798.943242964857 252.056131165397 284.675990197899 339.32415558339
301.051775938853 261.39435936661 348.575690346536 314.126706052299
255.12306184224
DCUN1D5 292.746711619565 250.060734219351 285.63245179313
250.085444708918 565.059609779245 381.014521577291 572.407760064901
541.351092873522 542.379137999196 811.362219801362 406.224345292389
623.409211766224
DCX 124.646093292382 133.389930962407 124.222529964098 129.231455282441
137.85150144918 127.419227292862 127.83967243279 123.554769032715
135.489000274316 132.554328715753 132.624674205566 138.030842581932
DCXR 373.189809737885 427.390091157726 509.880640462 356.05426191171
1169.43742964112 750.10819423982 1032.4540300024 1278.30505902957
933.723483173584 641.774812292709 712.433345978749 1092.74905085695
DDA1 786.62709337246 896.185223980593 721.603421985572 1114.85422895959
1994.00516809361 1645.05161227372 1505.09908072606 1456.76683429755
1167.26555140613 928.151632070573 1158.88528996131 1514.53784631689
DDAH1 617.833194119379 601.800326895295 732.630306104841 605.583144451322
577.98969050409 688.684644604218 405.570281788901 887.471553448641
647.273409499313 184.414609874199 314.949772661046 342.878522959461
DDAH2 650.494111214312 625.932910814208 426.769986378276 712.067161025165
253.65959805538 188.36361073603 228.058870735016 183.181125586276
230.10433160008 287.455622143233 177.065616871886 143.31117188259
DDB1 1017.6969123529 1069.93503700632 1097.60805874165 1015.10079945997
1873.84654908146 1712.47877322331 1503.2049371911 1080.42884514937
949.260897408911 1092.33328323944 1278.30505902957 1214.16777512885
DDB2 213.240181616274 193.522811257085 179.100368312505 175.647878042257
142.739686751422 143.533725135227 187.824032557194 166.77329045424
186.886677174192 146.04584032214 253.30325545013 202.421990203844
DDC 96.8261937365062 112.867662941811 107.187035835972 107.884870799936
109.770126492743 103.09089100768 106.36673326724 107.344825308293
109.333276735523 103.09089100768 126.698158279472 111.497241941203
DDEF2 460.807117177285 441.331937921872 344.604699562938 400.924914238052
549.460612060753 551.206833852215 332.978350226738 466.4890990178
381.625983354158 444.945078908489 542.379137999196 463.622673539254

DDHD1 137.037299675471 157.821491577808 142.726324048628 146.940545711162
144.801071221985 146.460464282676 160.374491283254 152.291176493005
151.81251450855 136.284283129801 150.601874392926 143.727514442914
DDHD2 245.675725760577 240.94056877904 308.679189788554 244.401472859026
674.101599637653 600.264940641426 359.935451429371 326.246702537465
246.912159676595 342.555640222963 233.394506254045 250.342080183479
DDI1 118.000757530896 130.74794199972 132.825240485239 135.676854553162
138.023752854109 123.766373146588 140.53597287752 120.09801164252
138.3792224631 122.431337063053 140.05012958679 141.81391138301
DDI2 127.354826381656 132.70888569469 128.367370419533 128.812729239283
128.568463697992 123.431969470387 124.958287100852 133.301527400458
121.668166378914 134.897958140444 132.074571281938 134.61114877396
DDIT3 466.141717595301 372.374101464689 399.762451559805 294.535979194434
181.607253339427 158.272071464923 456.817057099146 264.158648784101
252.851164967726 452.732079010502 190.371287639453 206.303338709554
DDIT4 1051.98943987926 2363.50330960038 939.213447046823 582.382786876656
1321.17236442448 581.54874141672 2947.51998516453 1951.69651954627
1285.02292965374 4651.384971332 3447.19587767281 4743.19967685595
DDIT4L 245.317436234586 320.525885538523 289.517820913799
393.264726588522 156.307551403822 136.569250038372 159.26101905957
252.514569129392 178.551434222154 173.726420791056 181.508873226705
166.315369318355
DDN 144.708780756278 140.03443832328 143.980608339892 136.858037332222
147.483379646393 155.917655560227 143.290642777702 140.655002540298
141.500790455788 136.978738825445 140.145470073979 147.925744210234
DDO 151.094682146074 157.481645415121 158.053654524927 158.863516051439
149.369418844044 151.461857714097 157.028963417138 144.191491000257
160.6823824476 145.517559593811 132.057808599068 154.918405633244
DDOST 1512.28202818467 1609.54149931375 1291.12004949379 1570.39362935859
4491.96934862425 4175.70090899755 4103.06565237121 3391.3005983725
2962.11407134162 1912.2369374258 2615.07927836881 3618.9385126441
DDR1 187.871017629994 236.219981978324 220.258804437828 206.413554839865
790.561585641193 435.48544637769 747.322544683874 338.260976633501
428.66614441422 383.033242143031 593.090436323333 523.216537391852
DDR2 696.131296166255 568.310800941091 400.060972573122 528.650794915616
142.508525378171 154.183537565624 145.628239642187 147.485960802692
161.788118050564 162.595632367022 149.25705328406 144.964084792568
DDRGK1 322.36247485445 355.990908506973 441.873247266342
317.719381113816 1528.49457104932 476.782624160552 623.888568444132
612.374364731095 542.544172683021 647.439372562271 776.98412594767
603.764427348016
DDT 1980.4589291261 2271.82656399253 2681.48138915107 2372.26073354008
5361.3600315322 4592.14655702906 3403.37530051435 3592.7878705048
2542.44286642873 3570.21370790206 3700.75770419386 3765.25491426198
DDTL 148.466658640201 161.496700767073 142.199182392836 160.773510498005
160.042251279235 146.550421651378 152.064982697394 161.965270932582
124.533543270311 148.868265867275 168.52952604693 164.032302455558
DDX1 1785.92695250894 1783.8031212092 2403.42261883515 1750.21700780511
2296.30423509176 2618.64973582507 1392.89422203207 1929.52146517699
1611.58245311122 2127.22258112242 1349.84215478798 1806.24732793591
DDX10 444.945078908489 447.059120541009 459.652708187549 439.928008927647
449.859227376918 345.209128382239 335.059844742031 488.57622466019
403.984761333129 281.49247863022 405.130899553871 442.493108530222

DDX11 137.212568398657 140.010730572075 145.651301499721 136.459509901229
185.374745525698 142.866874321248 148.391885541349 168.958694643382
160.742365719929 150.639235242268 157.81831741292 162.629257046871
DDX12 147.607202745865 145.128919984856 144.059368898969 156.146154578237
152.173055053184 158.23884088767 148.468389477892 158.738932073809
151.316421810192 147.385714746214 144.599578905334 149.292552957665
DDX17 1221.58522138654 1178.83933381069 945.403379196643 1151.89087953837
536.9324595176 759.058176862524 833.430847243676 541.896444696137
680.94167651697 684.180060287047 1076.62597781124 817.036222521723
DDX18 990.814111681408 1006.69598892631 1178.83933381069 905.751410113646
560.362502519639 1646.76016031831 882.037055216609 1104.61581891596
922.50743596248 1170.07851727046 822.129234065933 1475.41920838384
DDX19-DDX19L 140.35175858015 163.661196423902 190.947996918605
165.283700641161 169.101676908756 149.082061203661 163.516075721791
172.631499322646 174.012972901793 177.500110974116 184.694741621812
191.543371466252
DDX19A 514.354512083591 481.933386283351 507.95331185029
579.923640505245 263.888891822857 289.368511254335 307.597733293218
239.503369457867 331.32397534437 311.966971734249 297.022984365795
305.499194075747
DDX19B 272.712242893937 277.359422005775 327.566026396726
323.391456630794 293.794745907663 296.553550946307 323.841518852386
337.585502787016 333.868818855397 459.771019162573 338.46332239699
408.60092780174
DDX20 150.948076715456 140.595496714752 132.751406897891 146.070871543153
146.451918215469 133.99226161886 130.786835186349 135.057847490193
137.805895248174 126.290840923683 183.863365305103 144.461999591223
DDX21 327.324711245289 259.511314588868 356.770158430601 257.408058436941
391.400480065 777.901979189395 499.321667633972 594.180861801958
656.009451448101 599.52440136406 476.782624160552 1001.92763014266
DDX23 462.948429969719 520.120193442648 522.400055538966 471.720496000839
570.533103778482 495.438672949441 523.591472915151 628.344122779625
705.529158880221 397.449905380349 542.883453307 553.123179808258
DDX24 1190.62010668045 1176.62418139379 1411.90426595229 1189.11053680293
501.863855158062 444.479355480138 500.469781477166 552.790706181938
484.131020584378 564.41151411028 424.751154004399 580.474775712828
DDX25 142.56910547147 135.003236040032 136.507961715591 140.447933562667
144.333945716394 139.099483279141 147.191114491117 125.962178569021
131.451173463405 140.675094228846 132.550022967297 137.704257049203
DDX26B 166.411219327671 158.675960205792 152.22701178148
176.506227858258 162.140112952434 168.37850726499 204.219197797869
164.39910609636 152.386986818959 154.159124458167 190.56172211779
161.817609920327
DDX27 677.9972761887 712.433345978749 482.857203798212 663.782083028745
450.566031840493 546.181615768667 741.255013151687 365.046815163035
480.593729033799 440.319777672273 403.984761333129 492.207833630086
DDX28 223.253794809571 243.772412032046 264.526189799049 264.306927596371
353.674560414317 299.234482744038 329.406649846151 284.104619768635
264.383922859446 212.223906015932 365.286234166621 309.36704220687
DDX31 163.146581498065 164.558875058199 166.709393547104 167.817232948801
195.595275594235 170.111653122193 168.183542846746 168.295044843056
165.954266127697 176.940723174814 170.892319361167 172.794570587596

DDX39	434.792010353541	479.866450988091	348.413827101046	482.739666411407
	1356.43255022363	1192.47480377375	855.415038498392	1032.4540300024
	803.133619199479	750.10819423982	598.647226284738	874.816697884109
DDX3X	766.176882605175	790.790236361922	790.790236361922	523.717949503639
	573.644925180854	1367.92869625376	839.324324662619	843.545962045705
	741.255013151687	938.918431240228	992.744093752678	793.891277937782
DDX3Y	195.88263399968	182.275998840116	159.563866056798	185.519000961898
	186.939168482155	167.442950045855	233.82434521462	203.265559787292
	168.05687638693	132.86711707789	172.138554350738	222.565515020501
DDX4	137.464914919148	134.442947822188	146.554017952963	135.215356532498
	135.899595115146	130.74794199972	146.999370190363	137.144001915831
	148.179398287068	123.700076467648	142.724123724574	160.364644321965
DDX41	306.946103334814	396.345382600445	284.675990197899	414.797416270335
	419.532420763449	258.695920967489	391.326200093611	317.949646885539
	255.273375146278	694.02978779693	389.588346573823	379.102958448288
DDX42	707.173691257322	740.527607312565	971.170460915918	773.803350315426
	381.57978490093	612.61438059983	623.508710830895	459.771019162573
	614.094097408035	345.686640885419	548.288640768746	887.471553448641
DDX43	174.116797337377	166.754995454382	193.704510912321	201.337186768645
	167.293320986716	147.769702509047	183.226294747874	225.927425596831
	144.924212639731	181.453575300661	122.82388804774	156.402578892869
DDX46	339.252515920242	324.671490117952	359.162549472301	314.095043876244
	167.640645997754	356.530530468568	316.801725268792	293.448046685909
	287.656436278551	274.934292929666	270.490971664802	462.779122987815
DDX47	911.782783698439	803.60413590137	1100.17618574297	710.228463443265
	929.69741581954	1069.93503700632	1320.74943817958	1361.54107116915
	1243.11900823259	846.084919104262	1375.76915021882	1394.31467757456
DDX49	169.764014031526	198.737421468412	215.897620927751	258.759507964221
	386.85874184746	262.196293231028	268.660078500783	213.26158748941
	198.681190016247	191.418673767345	213.674150322581	181.593291870136
DDX5	4892.17875938832	3481.90072759183	3630.80752744347	3042.88425356552
	1897.92061704124	6427.33783376005	3677.23129570773	4425.66777919789
	5396.55955971617	3024.62888503536	3528.62443098065	6548.65498784806
DDX50	753.389428590366	650.911886508922	1003.93455977232	622.348802511009
	296.962992349662	438.870464403215	524.245387973163	365.541609015117
	388.192984832638	483.699436940387	631.819659701092	478.040234382488
DDX51	5233.72882607795	4938.42484385053	5327.13051940508	4759.17973319588
	696.976036382401	5748.57645553386	2511.04500638697	4068.18567686753
	3359.62015747752	2599.421026133	3822.05744774919	2921.59274577877
DDX52	273.959220003188	291.50783830983	231.018059960001	303.858574395969
	283.123157828849	310.174932137546	347.292438587205	619.995008098949
	361.516275756449	326.640168906375	405.409668978569	332.749676505218
DDX53	150.132420238581	154.740395921562	141.436235043864	159.495272062735
	149.432744784716	155.02097482487	146.229239731545	142.536450265886
	154.684874039602	170.807514224412	138.60849268758	150.639235242268
DDX54	141.770977101211	142.910616700526	123.748788684337	147.52403424052
	266.468621734455	207.417884407459	156.47237266917	272.927830312746
	233.786136606682	149.60803906364	191.040134068942	207.368252249039
DDX55	542.009873308344	545.200687301057	807.604205890244	588.458314503034
	473.370240289761	635.536499281829	822.129234065933	846.425467119342
	1000.05896885496	728.176339659082	564.41151411028	977.227184625781
DDX56	463.298781071364	507.56875910742	392.282956362735	579.493790910006
	1003.36623972843	610.070236234299	584.813665097407	601.401329244185
	568.701661112562	599.735180191091	290.389345090634	824.427857809495

DDX58 240.836979012241 176.791758333054 210.590774758661 179.241385747152
141.609037401299 153.911165277809 199.288100834476 172.992936821292
176.138621462315 182.130970182242 242.070630946726 168.161117444474
DDX59 214.684538620841 200.558971047462 266.19823384117 211.160781061336
189.2642795052 212.016247864691 227.884445686928 222.820391338687
212.669156800241 236.326943078666 220.415211013843 284.748764120614
DDX6 158.244741207036 172.370108210895 155.056545639862 160.598186999524
186.831266888028 141.37694311902 179.730694272916 151.488973553441
154.999192460283 142.13191463827 194.112534914308 180.850779069931
DDX60 211.555248754731 187.035957486928 218.031238893075 217.050602253281
179.996590340102 208.067404132325 259.025042304848 233.536023295039
305.577166690215 227.64196401771 288.370985514978 211.438615793742
DDX60L 167.260319361746 134.076865693417 146.019622621809
129.421772713718 131.899614022767 136.689064885984 204.89842561202
180.667897257797 186.57665470145 165.500088159872 167.92409473064
234.063633889254
DEADC1 136.959011188915 126.045231525329 118.465111174212
114.174153178166 119.787925356307 125.732389000341 123.094324479352
128.72727523654 124.777895136212 120.322292418128 151.148295679242
121.271403807802
DEAF1 193.544035570799 177.742616962629 174.498521167568 221.322724563472
198.15257384667 171.221972336784 162.140112952434 159.784369379077
166.187099519035 179.54034642468 189.0608798771 156.119899067238
DEC1 146.359038260378 161.17923997423 154.949840064881 144.662545137915
139.410739260343 158.047112652223 129.452826518288 134.730275384832
143.976395954528 151.629999903069 133.789738760547 145.409722106953
DECR1 1303.33810980467 1497.32522786098 1306.20183851747 1491.05501052706
1840.14350831655 2608.95227456417 1689.00127592755 4118.58682247781
3668.19307488953 2016.52142068599 3575.60827968852 4214.83058308503
DECR2 270.92671642109 291.883157643902 318.778978094743 330.405026390912
461.722481634933 309.181150342727 315.863232876382 432.515970951769
283.048002376743 360.39998507367 378.037190077449 445.089930641391
DEDD 291.476113017794 298.347201056937 259.979459472217 310.917191279571
308.803322140241 310.465070184707 241.890850719625 265.260411490697
245.675725760577 208.164674386141 320.024259346646 295.571900619972
DEDD2 498.812823949508 611.540759532294 618.437282203498 566.417354841707
1500.16816750361 727.814681346494 1383.68977267418 1060.24125879951
1427.27262061653 745.844395215451 1230.63920848575 725.621153262924
DEF6 200.179796117115 186.231346659144 166.20327698517 190.031339775209
370.390323116113 221.411372022459 427.390091157726 368.825190022937
379.200576951933 403.582461109859 833.198511430615 317.719381113816
DEF8 400.060972573122 380.562212796792 391.400480065 517.676093149981
442.493108530222 332.082749517913 379.016207754533 270.520229426015
345.062426573519 452.517134370928 239.924227955313 361.082957133644
DEFA1 108.079149792796 120.880547922392 108.118551275778 122.317877997527
115.174678745401 121.334882368464 113.989682635964 111.137519376499
114.704831217037 120.547683754604 112.635430248416 216.077955163344
DEFA1B 137.971108694323 135.601916560736 136.90636489391
143.307838525991 142.783159341229 146.094951139804 149.730467498266
135.697550878438 141.106863165059 131.260111979219 136.760382409185
185.639696270808
DEFA3 106.263441906404 103.09089100768 102.522925993233 106.030441145334
119.711070285785 101.316813921703 108.226917034591 110.093565974873
120.565485864499 114.798573331978 130.388530183353 174.471304281502

DEFA4 120.992440100307 129.428950259722 122.396214045451 120.53428198263
124.15094220181 117.716094828779 118.510960607141 131.015519445322
130.143953880391 126.045231525329 146.709967739596 131.269526665478
DEFA5 144.840218312168 135.521802085177 143.473523258521 139.693261291326
128.714459781294 155.187900101727 144.417333931582 124.654146040634
130.124601660028 141.775100066311 145.709172732805 156.423687034312
DEFA6 134.4380641709 133.978783899572 120.325985681299 148.309872393
129.4807689399 149.833346575446 155.05329654089 133.048720362039
127.622397233692 122.517559025268 114.255733119076 131.666853845458
DEFB1 311.10841853984 194.86492119218 185.886737029845 155.756074127526
174.059172465573 458.282682131641 446.698665191047 203.831047867731
183.152005135504 3396.40593844715 346.476121089685 191.651818696629
DEFB103A 142.719216118914 140.435724944447 135.728336465002
131.025725443485 135.346501914337 133.026094667212 129.495680232567
136.2336179571 133.301527400458 1313.89440013246 181.783275804424
133.720904256137
DEFB103B 155.490897312915 137.824985884649 159.110614190088
163.552608805894 140.875923123766 147.225911875196 160.850840925561
139.067516394141 141.999730335668 8445.36695108418 242.410260751835
153.636804025106
DEFB104A 124.899019467557 148.391885541349 158.937341335707
147.362120584524 136.728067043351 122.696382705233 124.375348764151
135.381131982323 127.520134825082 141.294094833197 145.732814287289
141.170937695272
DEFB104B 152.142909854773 146.097201498811 145.314204192565
153.07993334035 146.390223079987 139.242481781927 147.78847662997
141.5739152922 141.906474266389 182.969563278473 140.03202338666
160.159630699959
DEFB105A 101.316813921703 116.331428876124 116.437403962939
107.780763250481 113.283359026655 108.101686347192 110.61165787102
106.701716475947 113.87024833117 111.608386387842 120.871693555514
105.132185249959
DEFB105B 108.970432124412 126.211525910631 119.845508281414
120.74273444647 120.29173510435 134.341489592504 123.671944323239
113.199690522854 113.492900106468 134.367115858307 107.697601478064
126.159880038854
DEFB106A 163.942131559267 144.754401731628 161.651643789597
152.566490804809 132.535722328813 153.762995131216 151.169283505727
153.600132368924 137.605244950708 152.20173979854 129.83316008718
141.91067228913
DEFB106B 167.698883940638 150.845846206549 157.58659226599
141.431172407792 178.476483428474 150.590611246086 145.085091507228
152.104170622003 139.211746200839 147.592770523507 158.459129254226
154.686025022972
DEFB107A 119.859508630454 114.691842968597 124.666487633365
114.182798672347 115.257318321108 123.45956380046 126.828097770667
118.230408871812 115.366496551426 140.127379332918 127.123123583856
118.663847950696
DEFB107B 120.160652344221 131.428466882762 121.991692204056
127.490889148422 142.22515098612 135.666278507269 124.145492642453
121.728711587014 118.974727968283 136.556183079815 113.78323485587
131.354275252104
DEFB108B 126.629012619208 125.334995649504 127.488853386792
130.238231520218 132.211379936941 126.48691003872 124.555847618988

131.973896985615 126.791714564926 128.576310682958 144.505072819693
122.387532784748
DEFB109 115.821231468312 114.200099628087 101.981823814699
111.876915242732 117.60787142216 107.780763250481 105.816457443844
107.07609243233 103.307703547649 118.820154030256 119.107992692097
113.740942665269
DEFB109P1 126.868970616955 119.198789189881 131.878155417065
128.992837936606 129.305455204291 128.748663215466 112.558241320112
117.611606270124 122.716563069312 115.963144087717 127.490889148422
119.845508281414
DEFB110 125.589744938813 125.67874617392 124.638075441482
134.691038011706 130.095585254401 129.297217956843 126.17133877948
135.832454000859 125.379842077431 129.537345537086 137.840396641618
135.723554819107
DEFB112 129.269919529415 128.282999719959 154.496005175117
140.085359251038 135.1951749488 156.32137627012 146.929717840755
133.602002328114 141.311749168934 127.745801270205 118.276198592775
155.153328022432
DEFB113 131.04482088126 133.9382467268 137.593577427321
124.641892391159 131.717160947964 128.368669716591 132.383542877819
140.149064156135 142.674671804361 125.572299527276 126.333814565085
123.159407368236
DEFB114 122.089332300031 125.513881890201 136.591299462104
127.827096314406 126.37749112514 136.378203924435 126.159880038854
131.426355444136 129.931923954109 128.941490662452 121.021776527531
128.895325544109
DEFB115 129.360773522498 130.914932329297 132.087183382523
124.959873714884 116.507315786826 120.325985681299 125.911259629489
126.557869929234 127.352386363217 120.276236447255 128.287127680554
125.461701282922
DEFB116 151.226242696197 159.41705570719 140.021640936288
167.568776552757 146.912751127571 141.555366493541 147.60284257326
159.572851489665 157.530573508003 152.612116997575 162.879707592164
149.17815859943
DEFB117 128.835216648178 133.59686364883 136.881724888516
129.507297276862 122.659174808512 114.358099210532 130.92093805204
133.617501101389 129.098408596418 124.180511991605 116.653897702566
130.872296869843
DEFB118 112.198629213643 114.574362093931 118.56332995149
110.113844434493 111.802672864815 121.815538475678 114.41737808857
113.852849454448 122.917915232153 116.070254821073 121.048673869836
116.046983032621
DEFB119 128.36121178972 130.960045345375 131.910285685889
142.412731964179 129.653283053893 131.83430682495 122.888626394154
134.337249560706 138.555443236183 134.805239981342 117.773743603829
129.808590871788
DEFB121 121.103620738719 113.125349503734 113.440171712791
106.341910466588 116.65880728655 111.62514398781 115.172427482285
109.988237089025 100.841352336505 115.954828435072 116.136849683574
114.995889945366
DEFB122 155.945838519586 169.168869363848 159.633987751501
137.997756995882 156.411563412665 170.326474367867 161.874184077257
163.22059466037 160.469645948225 153.989839987975 151.92638745703
147.885769870843

DEFB123 157.293090577042 160.338146803754 173.995889755792
150.046300135261 159.286484253487 162.188509438986 163.045605069267
147.274945970072 152.563532872898 150.809693773343 140.345652629553
163.72257517759
DEFB124 134.06996703302 127.478474451121 128.011387527117
130.268322464805 125.321968563987 121.734873711651 123.07559417607
115.113000553093 117.270320709161 131.93683726912 146.151639797173
121.915043935246
DEFB125 161.646807806182 154.962835391567 154.2433882385
141.830432745537 143.10996074311 166.611809859059 175.551853899221
164.000772606739 153.969493052468 162.294114485312 157.170136439095
154.98147307591
DEFB126 110.465934808915 110.202982683256 116.092815232176
106.491867793742 157.348475799643 140.123348569709 115.389934936253
161.126686827391 133.012692824038 118.276198592775 125.606269267903
112.317996234709
DEFB127 144.12742465348 151.174536186463 157.797222258336
169.441789096441 166.061891147234 150.082561726876 156.346014084316
152.89366585726 158.396149470397 137.27298184582 148.812015298003
141.229805348717
DEFB128 159.824012085212 154.507721014473 157.12360611723
163.991378703958 155.603263242813 178.766654213173 158.7912155022
176.77183598995 155.964242240771 161.451253146578 142.334338599752
169.417764098848
DEFB129 123.141928612324 131.924162435354 134.260779370821
123.291258843371 140.567565996849 148.551340840517 133.003743285746
131.148926523383 143.342736976496 116.416896654975 136.71278398686
119.092335265325
DEFB130 143.473523258521 136.897888440409 128.131324375789
130.786835186349 145.496033735748 137.190593489949 131.615462296871
123.235136639469 132.256047602234 128.840466579373 113.025793645043
124.731056918418
DEFB131 137.331864485838 128.411138850655 142.063338490772
135.194416986571 129.990534497026 135.553183871663 139.128751917566
132.051614319023 130.773945967285 134.136687154241 134.666505160831
141.765004355609
DEFB132 121.248923222807 130.205764567934 129.532122459128
130.754320787628 121.36708642226 110.325803953516 124.842426851438
133.984078167807 135.659271030454 130.991496243273 119.172965618216
141.70123350351
DEFB133 124.261531006131 130.742940708861 149.538429805372
123.807693112932 124.393008692801 135.929267930361 123.065355919378
130.991496243273 135.285224699704 136.596127228365 142.379120254855
136.829137120584
DEFB134 137.706893763747 127.869038038293 148.821942578396
128.19878174316 138.119788738226 150.471558219667 137.232722233042
129.208429861964 131.025725443485 145.526941881413 134.062423178775
125.815256068004
DEFB135 118.342067845126 121.826474059574 109.398997827329
118.442856984861 125.584202037776 115.235444833856 113.440171712791
114.158566522557 112.858669660245 116.71331256222 115.830631252469
113.938636269013
DEFB137 101.713609383766 102.166449349339 113.077691687266
98.1433670546536 112.41270361848 113.470356909765 111.691973758042

117.05536677976 112.227434965199 106.923686318112 121.598249850839
105.164790181452
DEFB32 137.668179813327 131.76272538453 123.323528039848
136.173596381385 118.308102542942 131.732416355002 129.095469955392
127.876894098781 132.168821720799 129.971670681199 126.724973604103
128.093945009882
DEFB4 150.460618402778 139.490103923544 155.569733738768 160.099731578122
128.853089272901 146.132506246152 169.355185349431 148.124586158123
122.226875707253 1023.29995420518 134.378951325825 130.195234920895
DEGS1 1638.72366638732 1232.66885974069 1338.83705469394 1399.41653736746
230.975434289172 577.98969050409 397.307515234646 381.121621815163
1024.59845685519 902.822697602452 243.225815954365 539.471166362469
DEGS2 120.530092530622 127.401551376191 126.279119798177 111.525984720592
160.251996476232 126.452500728601 128.314990390649 139.577443171489
123.857527555048 130.621860095981 206.715020025609 165.386056999339
DEK 1001.65849130836 1296.10827265334 1275.43768532088 914.654262296682
1041.58583268313 995.812184456562 951.371815352591 837.872187194784
1130.32966481315 1086.33540858772 940.716682221364 1120.3009282887
DEM1 1984.47173096657 1651.68171717875 2001.92441724356 1607.52029676299
311.612118247493 1766.15001427132 740.800822095116 1260.50387106816
918.162638134305 764.774225930479 1248.69137283095 883.702573960415
DENND1A 143.045416067967 148.317958829244 142.318709347082
151.387150218354 232.556667737827 126.94941821459 133.164566694394
134.992686224944 154.183537565624 149.31020447459 148.888698484378
133.491963477649
DENND1B 140.699287635171 134.06996703302 119.415822905251
132.499182058275 115.34945441214 136.261907928796 125.165254272421
122.554760526767 131.809235171629 140.906421938377 117.580122770251
141.66312605042
DENND1C 167.542332146706 159.997580819443 188.580458613357
161.397788731595 170.473956720945 163.165374495635 155.699649538664
139.650726173186 157.57914268719 152.452246595937 135.544048708523
140.949367846425
DENND2A 907.610179738644 887.471553448641 642.255110250493
932.862030475849 277.314675225943 189.770558810153 165.511561121012
187.407526616724 186.529656751005 201.49630027392 120.177385114315
157.792868983503
DENND2C 172.707821624271 189.758013811338 170.326474367867
153.893753448473 176.214950733324 167.114291248266 232.363656167375
199.854728619468 175.562070915794 454.984562256248 215.98963200839
149.029275579026
DENND2D 183.863365305103 183.397833894087 232.385176936256
173.850390626782 771.059329972125 615.662993833692 909.020708316287
781.790858580895 713.832153334389 338.510045856515 2895.71836177599
1120.87534972745
DENND3 164.337486856997 155.837866295709 150.215811815246
158.320863045015 131.036225888592 137.990806386929 127.121068682846
148.302775746627 152.28262533486 116.346181133756 153.326420485822
139.847813838017
DENND4A 184.530853446688 170.568719982587 181.991820141929
183.988537525989 165.027938535393 189.90406457741 199.058972781223
165.131463797217 185.016336026382 177.862368731138 208.214764518526
188.567747377006

DENND4B 579.049608568926 585.933822585718 367.130155304037
751.077307018319 713.23381913235 261.775269058607 366.961565952239
360.185005480216 422.434778100827 268.111403429364 463.880793292701
406.900412510895
DENND4C 901.644194512187 941.065508539108 850.562379837947
1006.69598892631 292.429831186609 591.763423878737 701.208391798006
594.713185954954 574.262112643403 488.14606875418 631.408955630318
405.64711132293
DENND5A 1527.87148052149 1119.71228054585 1144.92109214621
1431.52979384867 257.643855420381 283.874600697211 286.506419296679
359.256193328366 522.486866458783 326.962330523237 407.875393458322
194.903531690239
DENND5B 271.823243396395 239.419200107721 339.490955270138
240.341031437493 153.308956040034 187.686326872667 155.957570398118
169.933706225406 173.552535486766 130.613760017864 150.981208776163
147.001408450583
DENR 2086.15449885278 1953.88327754946 1774.22769195939 1759.9632914516
761.097992055119 1937.83242524439 1482.15461429206 1936.88813111969
1622.17491781572 1231.92253664482 1470.23096656138 1197.00004043747
DEPDC1 126.355699811531 133.307149027384 122.631814400575
130.657342262492 173.010799217261 160.050872656883 138.970853057174
188.145154995617 153.128027266281 145.431393142782 127.824005374202
157.858267778665
DEPDC1B 147.657151780506 138.924820359912 140.865990571023
137.904786382385 211.809434609469 220.510248260182 178.946928223991
206.340478393262 179.048139035247 183.708917501519 145.304050333309
224.497348027469
DEPDC4 143.427721577372 124.491884305455 145.889336010424
140.132816040735 133.333067546558 145.750611940928 140.581943144813
133.584361509362 139.048016900762 141.585172896047 138.962649407431
134.160100276452
DEPDC5 155.475804754732 159.227123133854 150.46669393448
169.33926580273 154.207656926108 144.904021527125 149.25705328406
151.351938656508 151.201263631883 144.882207209439 161.581641777014
149.722442994599
DEPDC6 208.164674386141 168.873282919232 181.439775614781
168.763640193219 226.796825544749 205.149094703318 221.74200490054
259.72035004624 165.634117939571 153.008756051525 264.233381434738
176.810979494826
DEPDC7 133.998381302146 138.227082687797 140.668597897404
137.944260488223 133.175265384038 134.624322761212 136.779180530712
138.882483367088 141.8268805463 155.211976328553 127.601379088239
139.907840403521
DERA 733.49038042129 713.087392708048 907.610179738644 747.077451281453
824.427857809495 709.778171909457 1089.16489859253 1131.96129284658
1099.80705776268 1879.08108720938 981.781076110568 785.008529077795
DERL1 634.046843336128 498.812823949508 710.003412772888 392.6758818959
498.812823949508 1064.12835214671 594.713185954954 774.997895985917
1119.23362648056 502.499539192864 341.2887754025 729.499296070289
DERL2 297.419034136005 252.249994170536 265.43897208356 202.129153259445
415.957832362051 460.514672966125 393.707031354025 431.413030534188
309.538690289233 353.621264641814 399.537363692534 513.036729214095

DERL3 133.146347243168 159.515698265424 138.156430188312 145.958718601763
142.013370954791 149.836491640473 140.306808081269 151.641486303453
160.234704406302 157.446725405803 143.496669740707 146.039797334426
DERPC 115.085961282227 120.549643584553 119.95959059725 113.980716384814
145.698637908482 126.837151457845 142.324077852852 122.846912279427
139.089444892187 125.170874841845 136.638141963514 132.550022967297
DES 14417.2696849334 17009.4268910772 9908.59593023839 25295.5071561111
125.879198238067 128.678484452077 127.990453128978 114.638254225216
139.042034301544 114.841964705981 109.482330324734 117.790593990682
DET1 285.102778078056 244.18714778846 318.112626495401 293.825372123877
239.762135294506 180.747527895349 280.453895029319 167.599784887933
227.715558644326 161.225608630791 184.62149494522 203.149001616967
DEXI 931.598413783099 1046.65224316733 902.089561157715 1465.2419150392
947.425912541091 617.474181543858 737.650963292668 598.287431134037
702.049571167356 720.907992182476 772.061917233344 948.734577648136
DFFA 453.760162338163 437.836503489425 529.228238133018 501.738490253982
355.290621188435 462.779122987815 518.281245498542 514.922742487521
381.57978490093 357.584458526365 381.014521577291 355.80042210486
DFFB 177.083095120017 155.243643210534 178.696562301918 168.248982292489
172.877947895827 199.327939161502 181.215046876308 210.666799498141
163.538862303362 157.166379887259 179.593817076317 196.734037460218
DFNA5 857.090339943417 795.720196538039 572.262848718437 726.556553101727
162.850128623401 245.110761598207 727.425595270342 214.229228850802
204.031081842809 702.779943547386 239.277801396834 1106.75785702993
DFNB31 206.236303011662 191.38608906576 188.181647474317
217.125327913156 146.879019367939 182.143506470859 158.260676113325
167.049197726715 167.42476096854 153.256208333775 162.317084707395
158.400572147671
DFNB59 178.955938825556 168.33854702953 217.227441947056
181.277683429417 161.678137139371 191.418673767345 150.624740697168
154.792156982219 173.656241217331 149.355389396683 184.832531598928
164.513371267476
DGAT1 273.984715370774 259.094411953361 246.88857694249 351.515059400947
501.600329334497 254.235762677639 329.214958614925 277.406445570925
282.604890044452 326.83982672544 309.75140197669 324.79327985156
DGAT2 116.179625689647 129.289216846128 118.184571821176 136.357788670568
313.708197191587 159.452527363763 194.337080066417 140.579839043912
156.448355269561 128.351235762192 299.851173200352 206.071709262227
DGAT2L3 117.65847906412 106.285472845323 119.937665469629
119.297561749232 119.32696696175 113.726931741236 115.624167024067
100.746407116143 113.886420801388 136.016912147651 119.603565559838
114.888157310977
DGAT2L6 136.930523377107 140.616811333466 133.16868415814
130.265055842999 138.006973261364 128.137646789236 123.357205547181
124.28313315915 130.022574966461 127.187020441742 145.155989928336
124.238319768621
DGCR10 121.219323788324 139.503902205317 126.494665743087
128.79607493422 131.312580984876 127.8514513762 142.920939571001
136.864106181375 134.811625739766 137.871870950302 133.871685084945
145.677477231455
DGCR11 202.643624039869 210.061754644824 211.160781061336
234.895245871534 215.635313821029 210.529782898493 214.499670852615
203.943245360496 214.86995782548 254.9636051363 229.130679600654
212.239485199343

DGCR14	209.785886171295 224.805404798696 228.100112133941
222.943046618742 227.32463765717 198.390247605355 184.974347306539
196.486499238623 187.700723296295 191.310372695208 160.143443392509
195.384273821324
DGCR2 408.10780961711 577.250493023431 465.54817048856 628.710901107436
664.19083888799 382.744523022686 415.388760988459 306.277823025572
387.224184063662 349.128741707506 628.710901107436 408.444629429553
DGCR5 138.188522429661 120.846789922177 140.639012372681 127.58382536792
116.609122768341 153.214696479001 139.959629641771 143.100436913184
133.215151497536 129.072173761566 131.015519445322 142.858554460651
DGCR6 418.772484676858 470.868288641048 404.21236980164 617.67819271245
986.65554605455 1018.80178630497 553.615252472149 498.812823949508
451.621888586656 762.182213353032 394.810886122555 537.828979447378
DGCR6L	564.200165295159 632.843164522867 503.466550870577
815.779445689417 935.423045210635 972.468110850466 537.828979447378
479.395948662331 362.899103657252 628.939201051801 376.54010486682
448.644778906048
DGCR8 373.545629693667 507.95331185029 270.33685210971 621.946994961864
326.525579400607 377.353441970543 311.966971734249 381.625983354158
395.133831206474 454.854528472591 560.180656219785 399.248044997427
DGCR9 132.555211779043 124.63978985808 134.57472034679 123.344450694117
143.692305478191 134.725434838052 134.628807679002 131.244759170642
131.635641515901 125.865974180252 147.75625228531 149.619141707962
DGKA 150.653030597808 157.759149893048 146.814457904682 138.624689897665
162.494429648551 209.568066932367 255.273375146278 196.657475905674
221.719848707318 241.039766283972 320.390351199433 341.714401872192
DGKB 162.802392115386 167.260319361746 143.04129238495 183.82450756022
132.653795458388 136.18698517676 126.398884382537 128.066043751951
133.495243957927 134.952584272187 132.439176846818 126.603552416008
DGKD 195.218005582618 202.545147073902 169.067064352955 251.307020289291
226.531255313921 168.061346742356 160.349557404154 165.885072907195
180.304585602206 142.452409969639 190.939726271153 164.822461375001
DGKE 149.393022408213 151.188412354358 131.897352210959 139.762998983487
155.826394389322 133.965220820813 146.64560446484 153.267549449395
146.660826209767 135.325617701062 140.073208365817 126.523201566273
DGKG 192.752224665459 199.854728619468 191.018168651394 237.940015617254
132.271437329873 125.358906118533 125.455347031878 138.213742418402
134.078506829054 131.266386010519 141.123272165499 152.194337248455
DGKH 119.375727296235 135.576975868985 131.838522109796 128.155080148958
144.694679571377 150.247639459773 134.56134674893 135.693520183014
130.941645638407 137.500310477851 149.41357273704 153.801971958265
DGKI 109.266533255266 113.356070669308 111.788481822686 119.658703392877
121.480455781173 125.509221735447 108.655415285806 122.072091994548
120.394220715157 111.935554828717 123.383334044683 108.850992642446
DGKK 110.688722702767 108.266328505986 112.332130647215 114.249891266241
105.62620788923 107.863971340407 125.093376753408 113.22850386845
118.757973008463 117.084984190461 114.28381541078 120.581554457723
DGKQ 296.290104133844 336.639473916174 203.816305810343 361.516275756449
678.937151822411 394.73732350607 418.645775576811 685.080426814888
457.650982600462 449.220056843562 546.611519125021 697.299604049748
DGKZ 131.409796639443 133.008120853618 131.442186260672 136.284283129801
131.111930163369 136.539130156863 136.612903661685 128.335132315812
132.518394343751 129.192326574697 139.204218271711 138.343232981485

DGUOK 605.437395034282 581.54874141672 539.125423322738 581.191693771646
1244.67453201339 1054.47292895417 1035.98574557331 1093.85316636801
911.340266389071 677.9972761887 785.008529077795 1147.33034082693
DHCR24 161.80922869659 255.039661999732 145.864902319111
225.31216514083 1011.14472068314 241.675251479835 936.221322010828
337.973433871323 603.338538383128 1100.81588201714 395.716879127149
367.025117016472
DHCR7 220.999383789474 262.171775165261 197.740912476527 252.761035334279
1141.49294161587 327.89660021611 331.937313503755 335.698400607132
343.270642449751 527.226517960073 232.641965649602 321.31562287169
DHDDS 239.503369457867 264.779154931102 258.552964322998 266.19823384117
405.843083758177 216.559563381808 232.845268785472 249.925640256322
284.236970342827 240.147082836599 268.328002606268 205.930921729192
DHDH 127.699226067847 139.703304553486 124.847325289068 118.521075843163
335.623336587396 147.955701866515 131.676609824921 351.079435946154
191.38608906576 546.274050902567 143.868962478182 114.585818507336
DHFR 157.615017085961 152.22701178148 194.125840053843 143.271346378958
155.790759250672 197.049616858296 153.554159360954 166.722369066673
147.813170436139 155.590197324062 144.096624787142 205.742386129597
DHFRL1 242.096692715029 298.979106293393 277.359422005775
280.531844086775 174.812134675989 179.49202902995 182.810581377888
211.809434609469 270.894153548841 165.041296694491 198.257497903131
189.250846546817
DHH 125.861564323476 117.244895482991 117.847744476387 137.350851760173
114.174153178166 133.353762794271 127.068313862984 139.175421159925
116.769106223472 119.555954372381 122.508761687823 123.865424155099
DHODH 146.557058433232 142.126620107418 168.235729347406 159.926719599391
158.356964958885 136.092086452096 153.966815534288 152.488480322526
148.816369706133 151.459397907666 141.657914155491 157.838771553729
DHPS 639.38881853171 613.279465441319 769.586135629964 660.315285709317
318.778978094743 539.125423322738 525.6579615077 498.965895763801
526.726224327409 636.970243888917 482.048928546145 761.097992055119
DHRS1 633.762608454521 664.025683218037 915.015154168627 780.105135529728
907.610179738644 523.591472915151 493.071213662125 474.917507160629
460.514672966125 2758.01526530661 1027.11849332495 876.288248962843
DHRS11 219.441959146815 238.482863150124 247.08460296705
247.550312458738 544.956712516196 429.812058546782 287.145105895235
996.240188311002 423.396271159345 325.642275889649 320.154891655397
547.681206272188
DHRS12 174.549929320587 170.590817818414 186.496160803789
185.31769184805 161.72730923536 172.827288328708 166.001279428607
164.82904652634 169.777032044409 193.37618381669 177.752497164503
193.428733288774
DHRS13 157.225479764928 183.168629351124 154.515091049526
156.392603045231 1036.40082234884 279.790257881254 566.228690652783
406.816482069141 448.371946119074 154.449058407217 386.544913399837
297.12411625104
DHRS2 715.93836911426 1146.84239258543 1277.0921553877 245.378747269929
6522.27232911897 10862.3952192141 8053.22070239424 10836.7380675792
12283.9915232122 339.98018551524 10135.7800994367 3789.1316515671
DHRS3 1014.23822508445 932.505095432049 763.533962249076 888.371107987343
1362.75971646355 761.097992055119 2801.66917828364 1211.94273385872
679.557656724243 844.778755052304 1302.54547114701 1324.56751399862

DHRS4 721.966539760341 785.290353402444 711.166793546075 850.125434374005
999.658013198849 371.941204080246 488.746614111382 307.518881119091
412.801213309591 674.742955681908 574.896304593109 496.328898534802
DHRS4L1 156.420501464224 147.535225305685 168.132172570809
154.528396813382 158.039920243116 147.108661317945 156.328454726363
162.825380100343 150.670783991982 151.423936838934 157.986640251147
156.759600627542
DHRS4L2 224.224264678645 236.108432603479 269.726397478364
174.26780796802 440.495339027491 178.928873342925 233.671878221507
181.578228610334 200.094610104813 328.550286726543 257.477501542521
244.307627077001
DHRS7 1214.16777512885 1174.69701182094 1447.07989164988 813.034056288861
763.533962249076 947.425912541091 1355.07170485397 1699.8374761666
871.913035811066 2871.34694522381 2114.9754128463 2193.376951552
DHRS7B 257.168308311848 304.590636814607 290.090495047722
295.507504000509 1023.29995420518 452.732079010502 275.642882589307
483.845891531326 251.959823608766 308.621688157016 392.004079531068
270.293807247372
DHRS7C 161.385691765316 140.020294940544 133.733674016447
135.108420614547 147.387754442307 137.20253329376 132.989846726946
128.101371225192 156.179501567984 146.105844205919 134.770852936106
132.752401872376
DHRS8 155.333294650676 148.406031732776 163.967616022079 150.507308901391
141.681266095578 184.462286724102 145.536260846052 159.276726310525
158.374760929273 166.173429365582 162.223175359454 152.640683776403
DHRS9 137.522792377933 146.743260100643 132.755256423863 154.025625092599
129.701347789845 128.08497128067 127.818137805569 137.416133786828
145.136075215082 386.221302830248 136.169525758215 121.076652800015
DHRSX 208.587188476069 186.778334749551 192.827534610486 202.00431685587
206.488530785667 196.676479140094 191.335616210093 174.881557472722
194.029961195972 163.858222964048 169.744100665038 249.263194321516
DHTKD1 177.708263037199 169.909886347082 170.381897441659
178.358003030064 277.406445570925 388.326080754063 175.121069571371
170.646233722767 189.74261433647 163.422877959747 225.480366837274
220.079379312889
DHX15 1509.69760873558 1200.94425675913 1513.28088592885 1039.46136549795
1614.00098175383 2837.36293710422 2288.63508719706 2482.27419621925
2427.21100330388 1602.63977096867 1421.04485772969 2032.50052162637
DHX16 645.438835606933 519.450899933049 684.715981522999 624.467319041884
661.096668635469 628.485648053393 642.255110250493 647.955521647635
629.707646271678 602.703887178113 503.285430318683 505.302263971007
DHX29 514.213675499369 449.745016106912 649.052806726591 432.92947962962
287.503594820801 445.348044448953 461.822629852953 801.702878226247
408.993744895434 523.435696794756 447.579975492958 563.907454700224
DHX30 325.907037744936 395.425715632302 308.726328440723 469.64693728427
951.772948602094 331.32397534437 310.533399024851 260.41082304175
373.096629378654 246.389203940944 315.411701623527 310.075815009404
DHX32 506.659740456829 464.323065038265 585.552940765069 484.480643763982
385.798784913201 598.449359425056 787.017214032692 467.187222441283
473.947538805454 648.161646065464 537.06835963726 994.213397985121
DHX33 309.47429862515 315.786908578669 343.946831770916 342.096409626768
241.917371866481 486.425387717472 280.749681050778 386.461076717316
262.691387377392 347.224999246223 366.705048374078 433.928818077527

DHX34 194.903531690239 191.795952536469 215.167086358143 206.68694753419
341.819090221654 237.641190133427 256.093363520707 259.064601204239
222.675370226786 175.810411923013 195.66091457697 221.231455446574
DHX35 397.064325578854 307.982260548106 392.909481887174 395.645016679312
167.330860677012 269.448201975645 224.497348027469 213.584269089596
184.414609874199 191.018168651394 213.145460679531 245.378747269929
DHX36 299.669394721682 270.021093158885 320.774102343889 287.656436278551
367.567681162661 332.032371697186 347.932135290714 437.836503489425
378.797952580504 337.935183275743 353.187829498591 373.189809737885
DHX37 214.162670073541 250.82359383789 203.004815689972 284.351529744475
601.018867455333 280.246711704036 335.938170230765 273.073938260793
294.368406238222 268.186156177622 323.462252156562 285.30221551106
DHX38 317.457588721567 303.762967437478 307.906312092805 349.382889326613
301.250189051376 221.253717494488 260.443814523694 247.643123415644
232.556667737827 218.334273320319 262.954856857756 237.989479738133
DHX40 266.057472924341 273.864293250688 365.441289363381 277.86338481075
242.019489058244 438.146919555296 379.102958448288 402.866491417369
429.680602294138 336.639473916174 263.352118305612 317.493387040816
DHX40P 118.383317097375 119.144128641393 127.527378949803
125.277002586952 136.351332391315 120.231559540009 129.486584446672
127.001546144286 134.70636200986 132.094446606227 139.318078285343
137.670655293096
DHX57 156.460374293022 145.104173410801 154.051705030348 138.098165515898
147.211769989922 169.043206258073 153.505155981348 162.756185479013
144.351763882823 149.736837985314 147.978039160763 156.000732564221
DHX58 203.727455875381 174.690052785405 143.893751699551 185.493554506958
172.451128796055 145.780927034516 313.412131722189 289.164969943385
293.794745907663 146.266350809492 202.504840299605 175.245611094434
DHX8 163.67719444924 183.3378528327 154.900676756492 202.324795686879
169.301498852556 190.018871923219 153.929167381674 195.946096353894
193.801858215025 172.310673499572 201.931760074628 165.797170648964
DHX9 171.080373870373 164.659092629962 208.080089376299 185.485858719975
187.586093248437 228.739556788924 210.117033165026 239.12905945808
205.908052642222 206.203717450138 192.209344004015 266.534316786801
DIABLO 692.53483413403 733.648333788712 587.893636997642
713.23381913235 908.704610877319 837.872187194784 866.413995755699
749.220616713093 1075.3328563531 624.825216056495 734.139557942719
837.413261433363
DIAPH1 413.172954892025 421.140525880287 266.217879616686
353.243400332397 430.329437928013 410.645457106122 408.993744895434
458.875979229339 368.0573385048 798.389385197471 600.162650426961
607.979208454176
DIAPH2 374.718289636553 389.051174954505 259.472319994929
280.990656342284 186.388067641269 359.991823789356 147.297163685914
168.091278015885 167.900071375885 202.483493595704 178.43642939806
172.965746521132
DIAPH3 138.74117178479 142.321476446203 134.434198188045
138.003440266411 170.706932460836 172.120772549791 143.444109491884
158.845174721328 168.132172570809 160.010501226517 148.297939240474
167.284724396943
DICER1 260.045018348182 236.548439331855 273.369722814068
232.699786290509 190.138730960643 222.051005238856 225.4575611466
273.044064077 221.272872023413 279.879377933144 353.385210690169
336.380724981693

DIDO1 195.724873069362 182.851203073653 226.506852362359 190.489530928259
159.848342125486 243.936797286495 316.125364442611 166.447510782568
202.691507273819 160.992494552269 221.18757533703 257.880331012233
DIMT1L 1426.56252600395 1208.25171673432 1407.43503866211
1361.54107116915 696.475639532595 930.25842384208 1051.45401327176
722.974310297586 800.373159084181 1025.88559245013 740.527607312565
1329.90419210779
DIO1 150.042173991463 140.067809125008 140.605866597575 141.533138525284
138.111242936657 141.522701487198 148.609721587632 151.577450119302
140.374644951491 137.014698467096 129.195047484888 144.979749941424
DIO2 188.131876231735 176.714471790643 186.215602572358 202.761449711661
188.981391266203 157.313737338264 136.630678899513 152.429970809069
162.923734210933 238.461248099002 175.073595611435 168.047136398816
DIO3 178.885625009973 160.215396651606 201.81649023518 178.304914997023
150.208809981642 148.510023599641 156.511202150773 145.71535210212
155.172667450053 149.173121214851 144.240470871638 155.61996871093
DIO3OS 293.925995360234 248.360540656183 291.577778800314
195.981555981238 146.201445857452 139.503902205317 142.963036923104
145.432667767652 147.368455727098 124.275741099764 135.782158269978
137.524968609776
DIP2A 147.106355591496 146.112388101621 137.613233533453 143.728569494708
158.33974431499 140.552143090027 151.891318080151 161.640122360448
154.156272284591 179.828494168009 176.631604815669 151.019907064794
DIP2B 321.049000153127 355.80042210486 276.414209660023 362.899103657252
397.660202059209 484.764806127211 438.870464403215 525.138476928759
502.063888359427 571.273512452931 481.453770150269 409.743899750699
DIP2C 497.759125486158 546.029974898225 719.13080264227 705.311256840178
218.470365730283 269.530151236658 184.482849019679 185.396765725228
194.637233028442 159.683695366575 178.284294783418 229.97640195632
DIPAS 134.04235176608 129.461160777216 133.004765428575 134.565199667396
139.231340020014 134.161664663192 126.402405227897 136.249621724093
136.120813399579 143.697931551096 130.355969431473 135.200049257744
DIRAS1 141.693224578298 134.808485735121 120.184782039538
134.399228996542 117.404906698431 120.78129870199 107.884870799936
116.403106568357 124.359615295938 128.844026422327 105.952640312359
118.591554881375
DIRAS2 153.600132368924 139.078162119733 153.996429870564
159.094117857743 170.069291215013 141.873307688149 164.066839397676
173.010799217261 171.629605754643 145.998177732093 169.963805688413
154.426605816862
DIRAS3 169.834078550805 150.831554010122 164.690739760426
154.212927357622 147.125005489393 154.491373296392 145.906601611338
142.930903395131 151.506013510204 174.471304281502 147.441650320053
145.476377346532
DIRC1 148.444479022559 127.855167644203 147.829502959865 144.692900548503
144.00214898009 140.782522936146 146.609485664113 126.026938396257
137.823152565933 155.278984760268 141.622592268163 161.564505054217
DIRC2 297.609793563225 309.0578543424 320.305852961452 257.292125083248
726.731270955732 573.244735292944 897.958748317315 523.937128789616
641.645855499704 537.216291942143 683.861021234927 470.868288641048
DIS3 161.010206405916 152.864765895508 167.612577768782 162.61434899634
163.516075721791 206.548878646452 162.895923745031 169.095270479344
175.573400981771 154.291717784335 158.295583769612 245.01029221002

DIS3L 911.340266389071 1023.29995420518 1119.71228054585 1095.52349352502
503.757797957538 622.834462068316 763.263858769038 600.919152191636
508.449904285771 550.671679318681 720.710754387733 797.73902873919
DIS3L2 221.171478972931 230.079664410595 238.685014863143
238.310077556167 193.832033192375 220.079379312889 214.8478629277
193.134414137433 205.036081947168 238.183096218311 191.777253122672
219.792171863015
DISC1 133.792673129743 132.695992860853 133.875667250136 132.475414208533
131.665421461826 125.617143363441 123.505435008269 122.583461806645
131.856313046545 125.780294378066 139.162076054072 129.52578899011
DISC2 161.979914539039 155.031272695335 167.575968906119 188.724274062294
153.577349107512 145.965900441953 138.529160075977 152.342712570625
148.448349327493 150.105503384881 141.147683040881 148.44261900795
DISP1 499.544050752036 705.737228226014 672.862678078975 929.69741581954
186.625452120269 214.739818213164 247.942904862478 191.284275676256
220.136131941508 199.933148219784 314.063025950327 266.71318061066
DISP2 144.349602838518 149.290007708698 139.048016900762 136.704018446388
206.502189514795 133.537552829999 146.557058433232 139.119638136874
132.014213378179 131.215202712283 174.155599133789 145.983627592197
DIXDC1 330.553659265085 298.085365348496 556.863361789449
265.219444616245 118.239637776941 136.649743237542 120.649467083125
132.751406897891 138.891642304194 144.062079886084 134.121026382437
132.697727983601
dJ222E13.2 129.549673572516 141.486042999328 130.902319266342
124.449679223202 130.874283538818 131.257901340773 135.654614839107
138.79473329684 158.604431001643 120.727504500018 154.722895261876
128.071379991958
dJ341D10.1 227.267783818298 215.094633380547 233.192751618176
192.375251343504 174.202069525223 195.532022060219 194.014764659513
185.984938124891 170.784229026842 229.62164568989 200.790119570144
176.890596690798
DKC1 825.345092964844 882.798927428142 837.603727062008 768.67030514096
2269.44503773126 2086.15449885278 1410.58825009446 1717.09434498876
1327.65573168233 1213.61170930345 1581.06548216423 1582.0221231161
DKFZP434A062 128.002822538671 130.142263689536 125.117333434527
134.425483661043 128.76720970745 124.998601194756 117.567991653999
141.585172896047 129.7137422914 136.001505582342 121.905741525277
139.602168254761
DKFZP434B061 135.760578586004 139.912325578837 143.185372109165
142.326319895395 138.114938404796 126.349403754775 148.060086496148
134.897958140444 140.021640936288 144.769824608083 140.447933562667
140.457727109762
DKFZp434E1119 138.215708514725 135.693520183014 143.028618831382
149.630481387506 137.788504385206 148.715065348779 131.383113302567
128.583503171594 131.523538461751 138.746987455826 136.497295707149
134.029984991385
DKFZP434I0714 161.761125440228 178.130223302924 154.066939372379
180.34788837703 155.30926640293 152.837739128546 178.245841646175
172.562162872242 181.37137053478 153.56082876276 144.458829225668
171.06385200256
DKFZp434I1020 150.822586904738 135.767818903328 140.936204899093
151.416893156246 142.470799054068 153.485731788293 141.374831983971
144.669509071181 138.776108280637 151.282370273189 148.922537778634
143.669490888725

DKFZp434J1015 125.342580368417 126.563876945046 155.355051932357
150.267298097687 157.659824861915 129.015422924366 151.392024228289
147.905406818238 142.268018184723 147.370836610849 128.620852653765
148.932696684374
DKFZp434K191 586.642581067137 561.858563298512 459.171226689178
519.672796004691 329.926864732606 688.464980326689 521.512936372701
1649.46398639795 483.067471841953 339.490955270138 443.964363178742
611.540759532294
DKFZP434L187 144.474814266475 142.09146795308 152.321890796527
140.16233428205 148.605816748229 136.906909165944 139.217113811817
138.940564112156 148.376194044247 141.205975588506 147.803464591663
138.261506562904
DKFZp434M131 142.732625557628 145.272073426254 146.160188002433
141.050384368733 148.132450240784 142.928920246687 143.543607779991
161.084088366981 140.01151290873 153.655778377717 138.349773531218
152.042744996136
DKFZp434N035 142.709768829207 145.574725784632 145.873819094778
144.136328845967 163.470025318612 160.742365719929 150.452467115669
154.924190468067 152.247504238261 157.619627711954 149.657951204528
159.365070384451
DKFZP434P211 148.590732065459 153.056369304663 153.972471779464
159.990561741675 162.033909093358 149.100294463597 138.266328639467
152.760855359297 145.139966629363 144.708780756278 142.833025633146
154.771737131348
DKFZp451A211 159.166059518117 172.961115357569 151.563917522864
177.073245863872 133.692996980809 140.833666276568 134.819406064011
135.232878188098 139.696266471271 140.482728188987 129.321346185566
135.1951749488
DKFZp451M2119 157.221008210698 154.661456780865 161.922462797736
153.095594614683 123.61956613951 129.295956874012 158.14434287956
153.589626253969 149.740404686695 163.37518739397 147.079157199448
151.758970249544
DKFZp547K054 154.838407529853 150.661584243174 158.36796888959
160.449911844008 180.923089242255 141.505120938773 159.716066475442
136.515951031895 132.582062079623 134.572552391307 146.287161912291
153.843250142472
DKFZP547L112 134.140533647509 144.954595802474 155.957570398118
140.667415746111 146.528945049588 164.820007934887 133.113581756557
132.820927638053 129.203236898198 124.391225837909 127.095347480109
140.156797128045
DKFZP564C196 132.282125790062 143.078022283871 154.027862576708
131.655316312379 154.091431691072 130.51901676405 128.79724400425
138.541779821934 140.818413826758 142.469353808346 130.836079676477
132.817645625115
DKFZP564J102 212.171281020103 223.429738319185 301.188296465202
292.429831186609 129.642952083513 159.835577644136 148.302775746627
222.315451077459 266.057472924341 142.796371223204 170.394088019518
133.849237689264
DKFZp564N2472 127.171331062578 126.997614441981 120.220156455352
129.647514123731 134.76586363177 140.190234214948 132.23149173224
128.517315006019 118.000757530896 127.620377473963 164.659092629962
124.176944998906
DKFZP564O0523 169.544515294662 146.395248696835 160.608674893418
151.72544764489 161.841975820372 153.258616843783 169.826068012389

178.058616028026 186.033093285894 170.158189150422 163.846242743377
163.474261351901
DKFZP586I1420 213.097664319741 226.531255313921 226.824313642101
213.194154171149 170.596937075493 282.392419584508 298.165097953983
265.791342287571 203.791916347213 315.064477782052 406.736165197168
320.597495751519
DKFZp667F0711 132.934955542743 123.410467384598 140.089210795507
136.625136565562 134.46743016543 129.088718286391 135.275284142536
120.931585398914 129.318409053566 128.020911959353 119.459625817735
131.648308760913
DKFZp667M2411 167.984051648058 163.159571059471 263.74335003375
176.995049477843 181.340312140534 210.822216545773 217.013533525724
211.609763150578 169.583355171423 188.166168428752 180.354899434843
194.734255772365
DKFZp686A1627 126.416290818296 120.065876525792 127.084267631845
126.635105491524 144.152325539824 126.557869929234 145.487783396345
133.780423932326 136.670253859184 148.844916975586 134.507558495307
124.578552584603
DKFZP686C2281 139.306122448857 150.122035262873 159.730925830278
121.531401500498 116.465213348716 157.58659226599 150.286277647569
130.189958197332 138.820797333519 140.665332203898 131.734429913106
144.104372749581
DKFZp686D0972 113.947948541258 109.113476990309 113.249267122926
114.77964341895 113.980716384814 111.010889276873 115.582048644075
120.521509788705 122.631814400575 121.807661901776 111.765812595638
112.714282429822
DKFZP686E2158 133.465593684519 126.338676765473 123.571641517019
123.285059856341 132.440818532628 133.951322753098 139.237360556305
143.88649412841 128.545699891734 126.394032757479 150.168496148941
149.642946071295
DKFZp686E2433 123.860747215706 153.43000129811 151.936543085044
131.349297554112 161.799743586569 163.521095869383 155.731897985566
148.090431893307 131.497454038525 124.01558540593 173.907933001446
162.808270845998
DKFZp686I15217 182.698275130489 187.63609695026 258.489608621438
202.178451227304 163.748692741999 185.31769184805 182.275998840116
174.587558727209 171.145909843389 179.49202902995 177.549330396268
195.269099451599
DKFZp686J0529 162.06940121135 148.347642424885 146.481298877242
145.646191500321 147.449186980143 149.364498870469 154.183537565624
164.057147728625 142.024171830924 145.615291241733 143.521510363933
141.473218014605
DKFZp686K1684 164.525784478026 162.439052954676 164.836381332774
148.660440219589 157.108043150259 148.720181793879 152.112177721714
155.441190892146 146.286319248569 172.169129806643 153.885582272269
152.772177215856
DKFZp686O1327 135.596007994731 152.758653228708 123.998343902602
153.143706194386 133.666244052469 151.293676705634 148.585224298375
151.59613008133 152.074017988748 181.783275804424 134.30624644595
158.232100962615
DKFZp686O24166 217.940575516933 219.441959146815 260.016906472407
211.289598823191 175.010849510711 172.008994980259 202.397972518715
186.975609955892 189.2642795052 212.33360547376 170.257439890277
180.837420957945

DKFZp761E198 161.402322405903 130.262411213764 132.059507616101
121.855752708908 129.290542121353 126.439948611449 127.731816003312
161.514651272858 139.503902205317 137.419835194075 139.380610974147
143.470181156799
DKFZp761O2018 162.494429648551 167.243544185778 151.669421563954
161.441613587571 149.647926859169 172.521737547052 164.302998010829
174.646321871605 159.758196845781 148.112717810117 135.627840788802
147.994386096925
DKFZp761P0423 177.716742595321 171.444558905575 171.216275057884
150.629932241703 364.191263843897 266.71318061066 233.047541132048
240.915496717526 348.921877633633 511.374117402707 233.232319881243
336.639473916174
DKFZp779B1634 145.870135196037 149.65417821994 138.301397056664
139.564898998489 136.966690353637 230.749601687217 154.027862576708
188.864388750448 163.326102597955 145.728370703165 133.601133679159
126.310788291075
DKFZP779L1853 154.127346849182 146.720204808211 137.742393475755
132.725232102483 144.321757243275 128.978197156274 142.508525378171
135.980886879513 141.071184297124 129.226815062589 140.78902818186
136.311902195562
DKFZp779M0652 142.38097037888 143.381277901138 160.429643729685
155.483400816946 127.361974492821 145.250076958551 152.812142230375
142.89427871125 138.455086062782 155.358918578397 152.713528089347
143.521510363933
DKFZP781G0119 148.785977829275 143.458937009197 147.125005489393
156.481871341275 153.46645509759 156.136962310077 146.527844778835
150.155154998981 156.381599544223 145.924751649542 145.52979050795
150.339554736141
DKFZp781N1041 156.866867371872 167.640645997754 160.423982411027
155.608876496443 158.233862587586 158.308558656144 154.804753043168
157.507108643575 157.142101889337 153.128027266281 140.245786278057
151.121293928309
DKK1 148.686975104375 175.902751522861 141.879359965317 135.153588830407
706.628675634893 233.953994677015 151.305201022012 169.733427590141
163.066051106092 159.561431866706 232.778488697337 162.61434899634
DKK2 168.302168350095 158.704425428694 170.4583554058 173.02970820174
173.810080280732 167.70368494182 165.932628858503 158.6953558198
159.765585531442 166.988725688589 137.75060150486 173.299643109576
DKK3 1720.14021303639 1205.45751308367 699.800360892516 414.267332328081
270.231637012624 463.091387717305 273.651114834013 439.852826905352
505.560841055884 1095.52349352502 311.966971734249 350.58427958432
DKK4 124.726035027951 113.582654469735 115.002176041974 121.619593453259
122.878790127038 124.365185583133 154.618685493541 111.178024950204
601.401329244185 122.587545171646 111.949644842971 143.868962478182
DKKL1 135.550882872661 135.286686011087 130.539632182729 146.681183890181
146.309171902112 147.333142209628 137.172030225913 141.099781722697
131.556892859203 127.6516565653 162.244691888031 132.966873990501
DLAT 271.522696252995 257.602370597316 330.703588385626 265.572253407219
264.905947922753 283.46492196692 221.272872023413 297.636316709322
264.233381434738 241.418016203544 233.953994677015 275.013838208239
DLC1 484.903322308422 555.758485277905 276.476790719416 367.644907862372
172.783430414523 289.09614335372 530.819778703194 134.503552797411
186.06425658259 136.884799902348 307.410175748955 125.384706864406

DLD 468.170972478717 487.537691131703 715.039905439621 468.93543246394
628.000860165888 606.162976359615 651.998433127992 835.284130042504
527.930077857945 602.371843434907 610.759447723026 1189.11053680293
DLEC1 136.333928675403 140.42579083092 135.025907096066 140.704317015307
142.00857631537 134.477081392474 140.074841537258 137.581659385375
138.909419415137 152.323927317575 144.104372749581 137.832597613423
DLEU1 173.408784502027 157.927709922368 154.417731984391 148.79958121087
144.310362660942 141.794898246882 124.153063838817 141.052322220012
190.947996918605 161.534421340752 146.146800933028 270.92671642109
DLEU2 169.933706225406 171.320653882424 177.393812005949 175.29178007783
181.722373730185 264.983729290122 169.206723147505 202.343343669937
178.394895649664 195.711047893396 172.951567541592 234.947745514175
DLEU2L 119.578240680666 116.89487516867 108.895922800601
111.409447323674 103.307703547649 115.605206174867 112.198629213643
109.003365273337 104.861024325857 112.565341690522 130.110413621436
115.80656409185
DLEU7 139.817493158675 141.779801021888 149.000760379976 143.973464003036
136.582137928022 143.839170216353 132.197346228417 146.530318748416
156.543267949167 149.99203906203 137.229871801676 154.683723064797
DLG1 174.461487057542 182.798526632723 191.58185405868 178.464811133
149.630481387506 158.76356962428 187.48055574295 182.410688592378
211.609763150578 220.113261580385 178.707066209511 171.589889474122
DLG2 164.541417588646 171.212749388553 141.062226285596 158.8246699332
127.382266838772 129.672284417918 121.168550902281 122.456286448252
129.105655010455 141.999730335668 132.69067627743 116.902574164498
DLG3 194.686527131656 211.844563155792 188.070213212057 230.447203571825
176.155413809391 196.169988112042 166.489436134841 152.957297648595
164.455111983634 139.642457979496 202.024504592763 195.10410545445
DLG4 277.539110964815 474.216057978384 212.915949933033 650.679096367632
366.788087591332 176.312626750427 141.474583706011 147.245636620228
168.007337847195 135.894512504725 159.099495828517 175.585857393085
DLG5 216.05364037271 218.644953001365 199.776140037612 213.7326050342
210.751430486845 173.299643109576 237.88097138936 167.293320986716
210.729945718068 188.291563792748 260.224777636733 196.076386020815
DLGAP1 126.66432941518 129.462319953515 127.951403055054
121.774697402171 128.131324375789 141.719556176029 125.005019889478
127.545397354821 120.160652344221 128.069504716124 136.222421297872
137.018521754443
DLGAP2 122.295222990408 126.798976470472 119.096195018596
125.672285485643 112.456644814178 112.239647575661 132.22974656114
135.383585408897 134.045938361221 112.710573586525 140.624437508178
124.551603326682
DLGAP3 147.891442774921 129.329521415816 124.647992480694
129.992015202357 145.090639086338 129.854478528455 144.096624787142
142.175432631861 150.51537512828 134.599629045981 126.732121894377
135.098343717085
DLGAP4 324.713347085998 364.766154056243 250.406775889932
515.432065049838 284.995098167431 201.81649023518 220.92105843037
182.633773540249 211.208937901871 161.930142040623 197.341423434688
178.28963352311
DLGAP5 127.736712231221 152.504620068655 131.169769031768
137.044079235724 335.47484353322 721.966539760341 186.321879879561
261.065986251886 257.685284145848 314.337680575998 184.989027770708
341.973002478932

DLK1 109.425878885118 125.997109913348 113.791585365477 116.397855001959
118.540620928268 113.879459563492 112.684334910243 110.234403581151
114.524341239889 116.533361070234 131.593705080542 158.183045411298
DLK2 144.393688888553 154.420795931034 144.926838698422 142.108805047729
159.307394610466 143.801139839773 162.351212914573 146.586879656174
136.755975832051 262.233918187791 149.315684447787 140.870849757111
DLL1 342.315456386729 329.926864732606 175.327597807738 225.730739636442
156.445294524594 315.47473326786 306.277823025572 232.492398623889
210.14307111352 288.770330002807 277.539110964815 224.766155406229
DLL3 125.882246459692 122.97229333908 123.643292039274 130.955161943331
152.373931758229 135.918590725404 139.22543724535 116.969799327195
120.894583222813 131.892151111901 121.150371270451 140.081280065913
DLL4 137.025625133986 137.725329029129 126.022145004164 137.225659453984
142.259196792767 141.486042999328 134.516390697561 133.412452607155
140.245786278057 138.354300118735 143.209289043824 141.997165490755
DLST 279.070493043898 321.977549744483 192.980781927122 386.305638633172
309.47429862515 142.575382559357 161.16688895179 163.988099963018
158.653161314597 162.06940121135 327.696359071012 170.290455446376
DLX1 153.311705636864 145.958718601763 153.492072037266 157.423242850568
148.022287939066 151.285933600303 146.748572532267 148.408616955694
142.082257952004 172.245089970803 156.085949215738 139.494962965774
DLX2 150.117906461237 162.55521875551 134.834641868502 149.4401812
126.515480612924 134.420446702459 172.774914399844 158.200211812067
140.985432994867 143.082880435412 164.74234322574 167.55209828062
DLX3 152.346098251128 146.792951584868 161.755290291637 144.287786573389
248.690121805116 205.560952959537 191.924177620803 165.491193869938
199.408907117764 246.710233781784 170.531713392258 184.306452616482
DLX4 139.723117427543 139.872976072847 151.567693140302 143.403396831929
138.216926722025 137.368211928541 140.644800545854 138.789908033669
140.497181450706 153.191840013692 141.666394024363 143.501380161409
DLX5 134.125038192486 141.112047881014 132.91094012928 121.271403807802
243.10297424873 170.233084517898 253.42022722564 333.676803661366
545.362650746876 754.97177863997 411.71606840872 170.974759483946
DLX6 143.554726863556 135.968346417524 121.159593382017 125.528638246486
152.364417528975 129.79093244374 131.924162435354 129.692578787988
130.593846004132 113.154880173167 141.513636348978 127.144291519133
DLX6AS 133.496922906874 134.688504981603 134.616051224668
140.374644951491 221.471811658459 142.688809469455 189.633348499854
154.01988200569 159.854639082837 156.126009063311 178.416969664816
150.593928635647
DMAP1 523.591472915151 631.653707589221 466.257158794525 773.330948077036
632.843164522867 497.629319581786 550.671679318681 540.078145038497
544.30845362168 473.260534978556 620.899414581162 544.669441360594
DMBT1 129.051642626091 145.919359461744 139.491769969541 130.844272373448
138.142193489481 131.510494315469 142.440344960504 132.135448831967
137.926193455157 127.466779212194 143.182399285928 141.628203045085
DMBX1 134.036236880951 142.589312139207 140.655002540298 149.604507483606
143.861970001207 158.726115143818 136.91779948054 174.767697866801
139.851678406637 152.545261723881 162.026757124868 153.46645509759
DMC1 8958.34797715349 10506.1271023809 8261.82420896892 9671.66667662331
1659.80909859514 10135.7800994367 4697.94415026712 7902.9189607838
6203.08085790474 3810.98043907046 8292.04796307133 5636.26097921507

DMD 203.852316712377 184.557630172634 214.684538620841 195.316077537716
132.224831869802 130.955161943331 134.033751039071 125.942455135541
130.198147836925 126.601395870533 134.624322761212 124.45782375705
DMGDH 132.851791525098 120.235652849894 135.68605915552 133.613988293895
111.708970469189 124.975454733084 126.279119798177 125.717328918165
127.749992538985 126.097544650407 139.342430217926 132.439176846818
DMKN 168.643026828823 175.874387612235 155.98413216883 151.083551412109
277.652851236546 2952.67114077688 177.333305785248 224.67853219142
188.268774618164 1762.09874961415 308.281798808746 421.255522475862
DMP1 132.845116298605 120.450111018432 133.028789808654 119.583378101838
124.869732867761 120.691810909148 126.216519704249 117.682034050478
125.247402280272 123.571641517019 119.040642659337 131.633827974141
DMPK 285.30221551106 320.467139255533 250.963601187362 561.609211243027
137.767900781368 135.673080643346 126.837151457845 127.247764390727
142.25795237339 126.294085960617 161.371477511362 130.729989463558
DMRT1 126.589865262427 113.274668934773 115.293844461817 147.740657356905
130.770335966929 141.990639998829 147.449186980143 137.971108694323
128.917399281607 133.834423222601 139.180086017033 129.23899614612
DMRT2 123.391298926957 135.823975527501 134.000363767958 131.483427112288
128.729687597908 137.008110557038 128.738746025289 132.975603230163
136.584199831088 132.871179509995 182.02250582556 121.316836274255
DMRT3 104.896224175106 113.970633751086 109.471542280224 112.977469350139
121.567879358508 120.106270603148 111.985567835684 122.102644097132
103.36057518781 114.749597426757 110.003750007057 110.093565974873
DMRTA1 137.581659385375 139.297276687352 146.624631389743
130.009322405768 125.243735617266 135.978826529736 125.752962155179
128.087991226561 122.098850053347 120.967523586161 154.426605816862
155.233830396052
DMRTA2 142.991135981933 145.983627592197 140.975056186954
145.000523037022 166.958831664154 147.26204590693 143.260448301109
137.054278745724 143.546629073997 161.740466814881 154.783699020901
141.550553034455
DMRTB1 132.113317893587 127.800634041606 116.403106568357
115.118170902045 127.097605337434 125.065813262416 125.478878240473
122.075440298074 119.830261862805 125.362754088168 164.319378349317
122.94937342991
DMRTC1 150.874014332213 136.621173927187 129.89329138222
132.478801067837 127.295532954648 132.354503055131 125.443242860371
135.6149724596 126.381288663037 138.858138379065 109.69466049307
128.0366252162
DMRTC2 104.739307327595 121.752481757054 110.392617179163
130.339214643083 120.280241193135 112.675715371087 113.400050190315
112.776764215618 112.07238018581 122.187031504466 124.130599753093
118.176124626025
DMTF1 529.529314022043 448.260934224454 531.421912541822 421.384700184923
292.145553580042 383.802494922918 357.773949920377 437.248311551717
338.026161865853 303.300028849873 414.403321220432 426.995700632434
DMWD 464.436380568216 666.570977208535 372.255208814622 934.546444634669
474.917507160629 365.541609015117 262.954856857756 304.058158731957
355.714202328378 265.219444616245 234.484787292284 277.073510273425
DMXL1 164.777719508421 182.725987056391 183.005535466221 166.780319723055
138.861385127242 161.463042036376 180.569583206488 159.738700107615
137.677396262152 171.504939477949 171.589889474122 134.432461986523

DMXL2 159.399804351771 152.910433120981 151.282370273189 167.698883940638
187.112176929485 224.186573847185 253.580286414227 191.754626760061
238.149614104568 199.597685421675 186.951061159734 222.820391338687
DNA2 134.637520371454 138.5833090264 136.922282125029 135.079944196457
158.374760929273 171.768618884215 149.029275579026 151.831627236638
158.064409011013 136.83883055114 168.71216652064 160.331940529151
DNAH1 192.441337304606 201.850932289186 202.369905673351 248.43491649623
144.373226011578 194.500222559978 194.655710454472 204.89842561202
238.745953695925 188.567747377006 186.653791045656 156.239937979051
DNAH10 130.161889021281 136.908861280856 138.687013437904
143.909501365162 137.290687951485 137.243175880462 138.810236692956
139.430808017891 142.664793323255 129.7536873083 138.044751228397
139.089444892187
DNAH11 146.227452269648 134.160100276452 154.854538639035
146.303959493672 140.120051507626 161.225608630791 159.199045715212
157.425924587516 151.87495966414 146.336371857755 121.834248129933
144.408426565541
DNAH12L 126.532293165932 113.083678884076 115.734230176652
119.221976571036 111.639177843969 112.227434965199 116.46192274217
111.497241941203 118.991494185841 122.232732968363 112.943630133093
115.5765427629
DNAH14 138.370241517025 139.517413585211 141.200676699805
137.00532429734 137.683228179974 137.607767467172 143.085022888434
141.640292723776 136.067657949844 133.738587139139 137.016495611111
141.968949222821
DNAH17 104.798333931019 111.078930787656 113.656820701744
117.907840952685 123.004137408444 115.608857734241 113.773684331489
116.118745494794 115.118170902045 175.663960113118 127.269672881591
131.40880014672
DNAH2 150.098708715617 158.788210937339 144.685707282641 148.294870328642
178.647726085678 230.367251714289 146.808519947394 153.02549014611
155.468848105615 148.582022536314 147.216303852902 152.435416154284
DNAH3 130.856249657101 156.611727364072 131.200042525339 149.515244034237
156.732595223778 132.200648560899 142.843882404269 141.722817732641
142.034369454643 140.184082384548 141.490911535087 142.110469428644
DNAH5 112.348094614855 146.416318806523 127.709638375626 110.212739445
119.636334259263 122.418378964982 120.77691423934 119.544004279354
113.845770994796 117.312094804205 141.018087546273 112.747067417774
DNAH6 134.676039394438 126.00304757051 130.497405221273 141.644521617419
141.169908353507 128.806964077824 143.185372109165 132.822351718257
141.840858913807 125.914267820741 136.823008283157 132.450954427876
DNAH7 140.892250639524 122.053867525393 123.551225884748 127.372242892513
133.56263739203 131.336902822702 121.771891473027 124.072512083237
133.374199939042 132.332128706656 144.468333012278 129.090955637629
DNAH8 129.687743133943 141.858928084825 149.541917326637 146.832797779597
150.831554010122 158.770502070553 147.019460117987 146.290334865039
139.768555796264 141.797497681512 150.371716899336 164.120602897751
DNAH9 135.531326977319 130.0140921487 123.98384416057 133.122174596886
148.074494928651 136.337076023706 161.632610858756 128.383541329939
125.358906118533 128.368669716591 126.553693803748 129.320210774045
DNAHL1 134.05596537871 139.161483596756 122.873661677648
123.237851647499 137.623019849539 142.358754646258 135.196797030404
149.863063160222 130.680629893396 129.208429861964 109.654666772771
137.410687900298

DNAI1 160.001115445038 130.288547755599 141.793216038882 141.15458637181
136.03420235737 138.436029489092 143.733505359106 141.749118200005
151.619329363527 132.755256423863 125.396523818173 144.008961720971
DNAI2 136.348252715447 150.056508971921 119.517021355598 150.399313562031
152.146033353926 152.832573288735 135.491412261604 129.103133449097
129.661073814998 119.125002207396 128.116795372698 143.672118850295
DNAJA1 1508.24281455057 1197.00004043747 1385.28044421961
1128.60314361459 2449.76376608893 3111.50508060882 2681.48138915107
2586.87941889443 4819.85672511343 2511.04500638697 3560.23794634993
1879.08108720938
DNAJA2 2240.33212242003 2215.43744802035 1456.76683429755
2547.53445585552 1474.70984232307 1482.15461429206 1226.10881264804
1561.10217536347 1592.29734618345 1281.1883621512 1582.0221231161
1520.59210521859
DNAJA3 1016.18143428327 1055.22233969473 1233.27155759407
1271.46608195833 1273.6393529108 1084.5920943349 1362.1486006778
1648.22531304741 1871.91894530722 1176.01058971561 1378.40627876546
1623.92991386706
DNAJA4 494.747087458483 420.743637858421 439.928008927647
355.80042210486 771.694853062033 931.598413783099 1641.70484603137
1542.35486950856 1908.81864728547 748.940715378564 1584.81089248914
2165.41403521012
DNAJB1 704.653073777277 573.074116810032 689.590642377619
516.088776850623 701.847905027147 779.340880980422 964.093668483033
932.076509242794 604.237898104299 1681.54485582868 818.445501500731
476.383294794912
DNAJB11 925.656701707763 878.520681466946 673.824770556885
762.584280908728 2233.00694575483 1322.14570224119 1429.25367024696
1727.7144253077 2215.43744802035 1307.96033038529 321.84712975195
1994.00516809361
DNAJB12 196.486499238623 210.392662092721 216.880896769751
217.074639597142 201.269380130546 189.348690353874 206.502189514795
197.947433984294 196.990867962546 184.709768457405 220.533113603343
218.592402813219
DNAJB13 121.613684281664 125.010266083832 122.69021100699
126.324771800638 121.838802846453 125.019064287385 120.384210318
125.234097725749 125.101995710829 119.475462713424 140.24988931401
122.503984297406
DNAJB14 236.427507270325 199.135932984641 237.146972312448
175.93459327917 167.893596681867 304.479999301061 263.295012964476
242.570588364106 230.947339143332 257.740674565975 197.618757939729
325.173931553891
DNAJB2 901.197085099854 951.772948602094 797.501324589697
1006.03677177164 577.56891124468 480.36420271348 708.534025308731
504.502047826003 600.757699463777 776.030689773414 1329.90419210779
587.54075434249
DNAJB3 123.403099206878 119.278231442871 129.121598974852
125.843089077365 125.308318672456 137.729777643977 128.460750472464
133.571962238112 119.249084967945 112.10312035128 150.645487532549
131.676609824921
DNAJB4 306.632103708321 227.236255752375 244.307627077001
200.127994198329 159.14477373952 171.835438335216 169.061364889307
190.921283434627 182.644319628554 207.718619151226 163.36682525399
152.910433120981

DNAJB5 517.881547706391 405.032674307193 326.176058036757
642.057106241761 143.787766055379 145.463105638655 137.858371717399
140.421945574721 143.827500301207 163.20714296121 145.280485286937
138.553151063218
DNAJB6 1025.88559245013 1004.97723186946 1504.15682715484
1289.56069670699 546.755305058958 796.699879495384 650.911886508922
1027.96306543235 801.52891824571 1040.73829397913 514.61297221467
663.782083028745
DNAJB7 121.810767302322 118.792612926619 108.199628274798
116.987471433258 117.946664604751 120.988749021323 126.209426865327
116.884397420467 121.539958110688 120.29173510435 128.50733429744
120.039031290299
DNAJB8 116.196676877996 127.419227292862 119.312201620247
128.449200385351 119.233300586525 117.149781437983 110.829107724132
127.484178603011 123.303143302526 124.075077123156 139.170794914815
122.90702157852
DNAJB9 368.637819994033 331.684298221133 308.564935340635
306.486275677467 333.592915218163 288.505502880424 381.808809625801
349.65350886897 399.963640581639 374.268489263941 388.966303073519
251.61451602376
DNAJC1 247.454326301069 247.845708587624 313.099028594073
233.335353447342 209.385358804928 174.690052785405 204.237952297239
190.238332933452 178.106013202731 212.050953977485 194.847305068008
196.676479140094
DNAJC10 375.038561820137 353.571106279836 347.985315257161
327.811191081135 229.567444213626 537.828979447378 291.50783830983
412.415549375221 451.718912714608 397.861172312706 431.019896339435
368.220523234399
DNAJC11 128.785472881136 129.933228671483 120.270397272069
121.381365873873 188.40383184521 172.175584707792 156.144934427609
151.751963310212 166.111496598928 150.053948736789 149.213990424575
155.666943703002
DNAJC12 172.175584707792 173.147311624838 184.167124143272
188.973595445056 130.647944268116 137.956975047909 138.158400190671
142.090009728666 147.213616261735 192.399146702036 135.461112284848
136.886677060418
DNAJC13 360.243484271891 306.232249422327 427.249733213787
435.796737132969 254.63506290698 264.476846259384 299.616447269111
240.591548171052 336.067655676466 334.52685347521 401.960630197091
382.186095123659
DNAJC14 212.5119818331 206.68694753419 183.226294747874
186.077477713775 283.235844022826 220.896363324634 253.124449721731
222.456810024193 260.098788125373 224.135228192655 303.423450627377
212.312154721246
DNAJC15 563.648536446918 578.650614747598 626.582864105324
793.501244347257 155.820194280153 189.816900281053 165.108876136738
335.533053221675 386.221302830248 166.735962853075 170.614129599775
451.985108262982
DNAJC16 130.031303483631 135.260393988134 131.026665636006
140.978511668091 141.632147679743 134.528971384442 141.002002397727
135.279197125157 134.963301368391 127.503849690009 175.442400672352
137.792806301902
DNAJC17 196.703794742237 187.016569445646 160.861929142249
195.963361515546 183.744959461078 218.2454853526 258.868119227979

219.288597751645 210.590774758661 230.546762514966 256.202924421798
265.712482648121
DNAJC18 229.729195963667 223.941946219233 284.764628939128
214.050918459805 170.394088019518 190.189217962555 223.042575796603
162.337707154123 169.612203020686 178.142393629606 150.384559054133
168.963976596839
DNAJC19 182.665357598121 176.198555355383 193.843978773764
152.429970809069 171.677654793775 191.335616210093 222.943046618742
222.544542998356 247.942904862478 178.055019987648 222.8766610624
165.51742391186
DNAJC2 150.685245572093 147.354535468933 126.330382425318
132.045595972712 162.247608931408 124.157302365291 153.22516060649
160.113042196058 149.518870320398 151.761369162723 179.15284184774
156.34272729153
DNAJC21 172.214433016685 174.42996813062 201.569913451219
173.907933001446 147.491439949088 193.641927801805 163.165374495635
186.406439948242 165.530397739051 151.073257114127 180.478294414245
158.531514776098
DNAJC22 133.322752419739 155.734509841719 122.786030993629
135.465076459043 135.695576019185 125.393848851951 131.249240371529
137.528342105233 131.119810851017 132.828179725129 144.339819009283
135.68605915552
DNAJC24 221.561252012695 210.478088018501 253.833756857339
202.343343669937 147.479390172796 148.709795687391 204.103018614608
173.939159471022 180.501409705275 227.441436540742 165.225475655526
144.36033726557
DNAJC25 296.497100375888 305.16668987495 324.481229901658
246.616749748028 231.728984785324 193.930569647995 181.771654391653
238.724909152512 209.887047474042 249.234065130083 303.724140156439
169.211708954329
DNAJC25-GNG10 135.959893035019 136.645091356305 135.904163043783
144.873034900013 136.077039612526 143.296580629431 140.590802695412
150.421116058494 146.194467350201 153.922713834296 160.690442125281
142.737537206204
DNAJC27 407.711669482083 382.562403424855 430.329437928013
372.594990024312 189.482046688226 365.362177671146 295.678097392123
303.724140156439 223.279017022443 172.84784588171 246.031568259337
304.844121431627
DNAJC28 137.20253329376 139.188234030711 134.901659808633
137.53070284697 131.528729642144 130.975606477621 137.867263869936
132.651922794567 128.336332748047 138.394923932916 143.747664482225
136.290486122369
DNAJC3 161.564505054217 151.573920235798 145.498372398845
138.416716745934 183.693300680495 171.093611712143 178.928873342925
214.824908522393 175.121069571371 211.609763150578 151.316421810192
190.357437485436
DNAJC30 177.132361355899 198.437113196596 203.752540576935
190.057030596611 204.137195524755 206.715020025609 227.092319765009
201.221839281282 181.550597645633 144.507230362869 181.971189935628
216.582862850259
DNAJC4 287.656436278551 319.639103463458 350.308420518351
405.183882455109 404.366325262322 227.033342447929 304.590636814607
245.489266602897 232.801257700571 263.250833727291 386.379891865411
296.830999357172

DNAJC5 189.90406457741 174.417259650516 169.642188114285
193.98814423456 425.348619281876 235.063474370925 357.063270015318
161.704966315183 209.684346388892 220.60173086462 230.053292316517
196.802641002079
DNAJC5B 133.849237689264 140.354508398385 163.977562404508
139.757191608836 147.405050592353 140.978511668091 153.811165696928
145.56468363155 158.49220347186 144.724040494873 124.153063838817
159.346890686719
DNAJC5G 156.986053534635 155.041173289759 142.480203661104
140.451465188624 150.038722708606 137.563246867433 173.741466458636
159.148717478277 146.418147447541 161.397788731595 142.734103971267
138.359423363142
DNAJC6 144.570725886413 133.123444023892 148.1283706199
134.389518389888 143.396264354814 150.011603347578 135.353539458856
144.258128917228 138.068164255305 150.845846206549 129.884064654761
150.034029031033
DNAJC7 449.745016106912 452.517134370928 411.505580458401
508.302878624289 393.115606440229 521.512936372701 444.374521095495
448.106077184152 537.350891960682 432.372995789404 473.370240289761
404.54681330209
DNAJC8 2856.14739820295 2847.42635118026 3301.98381607571
3246.86605825129 2129.84680802619 2532.82545752082 1727.7144253077
2084.44431604433 1561.10217536347 1149.3250945792 1897.92061704124
2242.73511149559
DNAJC9 475.686805714298 565.97975389646 554.800688991977
494.604716660887 544.30845362168 778.639512538949 723.640113902925
775.781735108886 1040.73829397913 869.542306259337 879.144005147183
1300.40889834984
DNAL1 190.238332933452 176.516540713888 175.350734537491 205.495823410422
215.167086358143 184.62149494522 165.647471565959 171.768618884215
169.482586449402 184.387409077972 174.568253693395 173.773876769683
DNAL4 503.967725251906 635.038752622494 540.946852709312 603.338538383128
1632.84261181074 1015.63768413332 1072.37500318471 964.473774310687
1249.89496565284 694.907086612929 924.040923247227 969.360864006371
DNALI1 412.582051585078 387.617166711971 467.187222441283
431.019896339435 147.366877428537 363.343480850598 147.272072245929
162.9279134169 251.138401278632 146.446922196868 140.644800545854
162.267841475739
DNASE1 150.650926518959 138.471894596606 126.00304757051
128.115993005977 163.119211741255 132.651922794567 161.195562296834
159.79463502085 140.35175858015 138.216926722025 146.002585729701
142.650451413991
DNASE1L1 144.631407964232 147.691011072898 152.169087619827
151.905534823403 202.061887608293 167.100039749329 223.573080955095
178.347708009405 175.019580368717 196.519125510368 179.067275037015
190.002630382394
DNASE1L2 108.955761698524 115.473431881597 109.69466049307
121.730973193225 126.386738502885 123.687439699754 118.930792960643
141.874188629438 142.669207969715 113.78644620959 137.640239596303
113.326628149066
DNASE1L3 151.655854407186 145.692644807799 126.090985925439
115.649568330705 130.557209560361 123.875478428652 136.19083681188
177.679330300381 195.438353051306 130.258087965663 165.213327555428
166.04965258862

DNASE2 641.645855499704 674.742955681908 681.172359997071
689.04773343291 659.483452217461 1133.87328758676 1438.33289891548
1023.29995420518 658.068932688816 641.12919339861 1196.34528225417
522.77407098992
DNASE2B 116.670491972948 120.995697049378 121.53632327554
123.813766735115 121.968807253639 122.410340121549 118.963119420175
120.177385114315 113.609215169397 119.845508281414 126.069594320759
114.749597426757
DNCL1 6522.27232911897 6059.74270502037 5291.83393775761 6560.30885074315
11235.4970815873 4913.55892033682 5894.79176478976 6548.65498784806
7332.15849011291 7969.0710242842 7054.92270618408 5396.55955971617
DNCL2A 144.019824700027 126.796253980623 141.599211622756
172.794570587596 149.091925032548 163.37518739397 146.731584143625
174.274996381853 130.024546949158 157.269049853539 125.08580835258
142.796371223204
DND1 164.509354377985 151.606932937644 159.997580819443 151.538112896642
163.385727569154 160.848201202361 143.812863334832 163.141010815086
150.623182458747 164.870860592068 129.869363181125 141.703410265546
DNER 183.556662533396 154.277670551775 175.452986514346 147.734708927825
186.886677174192 123.344450694117 120.329105003959 140.345652629553
128.820757822782 186.529656751005 133.368199807997 131.379640556636
DNHD1 153.545086287929 167.049197726715 155.091135297162 157.474913177177
119.184780023269 129.260551359984 114.524341239889 163.045605069267
142.058086679048 122.005340508177 145.636906369786 129.413729619216
DNHD2 136.162938063831 133.902707525069 133.587927144452 127.442587366678
150.799400717425 124.395047385025 145.97512573326 137.593577427321
119.849895979393 132.562090247233 125.044092929562 131.521311288114
DNHL1 209.7558663154 234.738241125899 192.767155566493 209.004374703564
158.477017294725 176.138621462315 169.800006803362 165.480489678822
155.379116566097 146.00443946108 171.320653882424 160.435789207464
DNLZ 486.524284354103 473.370240289761 798.758496171493 504.141861790903
1218.05434595121 407.760503937821 289.517820913799 779.623566217277
527.597948979568 417.528353577952 375.539588956213 373.545629693667
DNM1 177.598422362298 166.382301680189 145.487783396345 178.696562301918
119.237802375552 126.025161261042 111.366283149236 112.987890650593
122.408016372747 130.672586991186 136.218750083407 119.583378101838
DNM1L 342.769825986756 292.640251411131 513.662594875431 325.252108402192
260.888612454097 413.555310697751 442.590987372759 480.712219116621
406.736165197168 426.364663722264 397.737702926328 410.42088953434
DNM1P35 136.180063437699 132.399633003253 148.638400592682
137.832597613423 134.964619235561 141.536764775726 138.121905080822
131.856313046545 119.229322984564 123.522738813733 137.446745269821
136.473948855498
DNM2 273.432712475089 306.011283364888 259.146673652222 329.779210536849
977.450397042213 364.443097338814 430.505037012814 330.850098426464
306.550948766362 242.570588364106 508.127470400078 280.337475979102
DNM3 161.683243808528 176.670752486376 174.979501466228 164.009873572691
165.333608076984 165.022029012505 165.619206216088 168.897620634337
156.053517438255 155.035727630756 149.267019532847 157.77047742735
DNMBP 169.301498852556 165.355764467096 189.509062639279 195.481680273686
168.801737310947 159.990561741675 191.040134068942 186.257814752354
177.478925091583 165.545136704242 153.181097279473 168.248982292489

DNMT1 1084.5920943349 986.65554605455 859.547169028149 1044.00217726486
2249.8123426309 2055.27692947656 1174.0922389136 2459.05558915812
2365.99940149374 1925.60246708149 951.772948602094 1937.83242524439
DNMT3A 137.524968609776 136.195555442748 138.777120638896
146.278921849776 140.802033706122 156.603904756947 135.315732654507
135.539516564142 137.471563342956 129.356401843185 138.777120638896
142.143709425075
DNMT3B 147.034021626893 153.191840013692 149.193437559422
154.98147307591 187.607426452073 199.813528853649 205.381840116054
180.867208892996 180.867208892996 168.437049123012 203.389294254141
164.442400539653
DNMT3L 135.417519318028 138.159966250659 140.960907353937
136.161633283029 136.448045428081 141.689210867136 131.328478053136
127.949413503055 128.165775629664 138.299629744589 141.939368369872
130.140958819402
DNPEP 334.377352542623 301.955019831714 360.852730026133 355.519647649551
415.497783305278 276.5177779623 288.770330002807 268.224669588188
277.539110964815 376.374248850987 351.292682758926 358.732947927445
DNTT 132.820927638053 129.737882221259 126.682913361149 132.69067627743
137.307786147787 124.638075441482 125.885715928839 129.001192144163
133.125084506877 125.67874617392 128.400741751903 130.226993687215
DNTTIP1 518.478990211753 520.218545108099 726.000622725287
549.692164962653 637.695341427976 752.585744322608 742.128229715198
404.95944990868 522.664475688419 466.392151902286 407.052451784773
463.802270256246
DNTTIP2 166.32555285213 166.430881793156 141.369723386494
136.580363776109 136.743659350798 146.97203173446 175.210232663606
145.980929136129 148.450023054885 153.551359970644 208.406980186223
164.365630106697
DOC2A 151.833386901811 145.015434711984 150.623182458747 149.904691068054
143.730966588794 161.997058981786 153.345834660643 142.810564324354
146.486480663438 150.0084730766 152.28262533486 142.194507957661
DOC2B 147.017958916908 149.386285571937 142.272117655292 151.629999903069
162.373508183992 158.716575777014 131.507618414676 133.517685144186
141.261150586614 162.304178370233 142.464394555901 138.57506931811
DOCK1 391.911768848267 320.979443123471 364.964572587289 308.974786799114
155.368204597287 158.767439363085 198.506786870804 164.61921356441
173.306283668215 192.69687837782 180.008152341531 223.107391048316
DOCK10 244.054469051058 229.933577323411 215.094633380547
225.387674139093 183.988537525989 237.71636970719 225.702629835215
285.83626978316 367.983166440977 276.5177779623 205.341121539273
230.947339143332
DOCK11 597.459920611726 528.767644181119 697.581541438891
508.127470400078 258.348298226232 213.077884261943 166.223079895236
211.720536220806 324.346723939622 271.791126011275 184.102171963542
147.088665779406
DOCK2 265.366632264085 218.917357155289 173.424541543058 214.739818213164
183.583432296209 198.583407008317 207.453490385508 306.011283364888
512.467731561858 235.680462167404 219.753039699136 234.980382243116
DOCK3 204.031081842809 200.810204162831 259.316557834724 262.53374754047
212.739915809291 187.65716654049 184.832531598928 207.960802948119
202.369905673351 195.645181575647 194.475822533231 146.274284557421

DOCK4 131.200042525339 127.966883271903 154.016622581204 134.797624902585
118.909153405102 137.176144667253 154.537240112989 126.958366599622
136.978738825445 133.921012946195 116.839796680107 126.426212216646
DOCK5 146.105009838122 147.725396196054 147.925744210234 150.632724038845
151.655854407186 140.216148769999 146.227452269648 138.438792881135
135.289246539398 159.688862680139 164.562302265155 135.472135601859
DOCK6 262.832376198119 310.23294370018 213.93763684072 348.36178568457
454.030083521756 281.846246549202 248.729653566598 258.552964322998
226.678503956411 291.369577397743 299.80615152845 251.161789722751
DOCK7 260.715580924916 219.878230463206 326.354469134694 236.35479928448
253.049869714368 509.988617641229 315.014639682046 326.525579400607
292.60467054075 284.831817899986 273.197814995543 389.177193459
DOCK8 164.748676009072 156.497059936271 137.53234136613 133.583297305219
168.460095116205 207.545424360094 166.917552275021 300.886032768166
272.238922498583 168.161117444474 379.911829623463 254.879772480457
DOCK9 158.400572147671 185.650862663633 166.272237117441 130.63390061855
137.927403204849 243.35880641566 174.985831291764 161.596086502946
151.293676705634 208.690347128273 210.14307111352 209.023926358109
DOHH 208.793650722271 197.543089645432 200.98533822317 221.297127685676
222.544542998356 196.860228537112 179.400220328552 213.215996114827
185.240692860387 183.879114762264 173.161152247499 190.736796322054
DOK1 190.309453030884 151.717558541683 147.553953240464 150.942078067308
164.886944426276 155.979380606594 134.304545258148 147.530719472217
135.413840160615 165.283700641161 137.650917262528 149.88598449818
DOK2 130.226993687215 126.501648842366 123.706910388724 119.455615461113
130.772410329685 124.15094220181 124.857461801227 133.64938083537
139.607944722497 128.886008923268 134.397255719047 120.574820369421
DOK3 119.686859562393 121.057111457792 111.935554828717 112.971776964469
123.513588004371 116.403106568357 113.184495948936 117.404906698431
131.915864620669 139.997245010492 122.97229333908 119.603565559838
DOK4 291.167223094542 287.084253886676 221.635333304767 320.597495751519
308.281798808746 240.915496717526 160.865949438448 145.407517107023
166.376907377447 204.637887777803 225.890195278753 208.838523729744
DOK5 140.968351978909 166.868688936939 143.209289043824 167.993851183401
134.253399256648 152.426527124644 139.406427549733 139.558611792069
151.671024612136 145.758423590784 142.872683795338 149.557833322995
DOK6 242.221324619171 280.033528476199 219.901025447571 335.3366819308
147.155738185574 147.508817393901 162.494429648551 152.334669286305
143.954908055924 167.893596681867 140.898380345452 133.367085445965
DOK7 127.2369420721 133.917283965036 122.075440298074 136.720837676326
200.831877950718 137.627338682823 152.657649988502 139.894559834783
132.807755600683 125.463718280745 172.370108210895 150.255511248598
DOLK 194.374817778819 226.763252784108 170.18781660707 212.915949933033
414.797416270335 203.370353613423 214.295260811046 204.453822958077
196.429685815196 201.480444219547 232.892635502424 170.941404165185
DOLPP1 175.400802387403 192.61436632814 162.656951764783
214.396920524803 411.039234404499 261.721726664029 216.859640534581
275.101766414328 340.860684371038 314.610979086485 289.616527814137
229.62164568989
DOM3Z 207.368252249039 206.548878646452 201.016287924499 220.567951704044
314.4408959517 180.590215659031 262.60377731258 189.603621918298
189.915720614467 202.145180994821 231.600532144945 239.12905945808
DONSON 149.857861968652 132.431566491955 142.02169810999
126.435928518593 163.396651801661 239.845747958475 182.873752254411

287.83303360511 249.449151040445 221.050093964575 150.536178574986
177.011776399483
DOPEY1 233.272000923172 200.668298229815 228.9901869793
185.765000029242 134.474466007553 180.54209603289 190.389158930835
161.470611189347 171.455216889348 163.741411769369 208.628626112623
199.991077347491
DOPEY2 646.704226815099 682.075583857856 578.650614747598
610.070236234299 351.188086838653 947.766127685083 554.282040414368
692.729789121701 560.757496975779 504.9864660689 615.88518704181
439.928008927647
DOT1L 147.103340218488 138.156430188312 131.378893655618 153.44628692569
168.238615437246 165.257737836487 183.193029399643 145.517559593811
156.654450436728 152.950227635138 174.323881548679 151.632801137039
DPAGT1 369.513420606401 337.271784551586 395.645016679312
345.291295192574 700.877661166739 454.492780042353 510.061974830525
558.621429366984 495.89837457901 342.611951034707 398.354212009426
620.163512570453
DPCR1 160.949030247406 143.207187847583 163.125317704446 168.358323243918
145.522435728191 159.406462604758 152.173055053184 130.67342529187
146.384051525624 161.325409589612 155.518807026156 159.848342125486
DPEP1 124.251699375036 115.097376525707 125.67874617392 123.467227007155
114.888157310977 121.939618902165 124.121555438466 124.786029449452
130.293492553638 122.093290585838 140.626181314061 123.696942540314
DPEP2 164.545596179262 164.438724387992 157.43824048982 161.00635457883
159.572851489665 187.247062073247 174.727439318223 179.96623248843
186.309367281625 155.664126780859 756.618969995355 149.867541008113
DPEP3 119.469328303796 125.166940410459 120.669452856028 116.742079521048
119.815258991448 122.763138007612 114.479886899622 111.992738490468
115.53736269794 116.764795914545 142.964043764953 117.873333897609
DPF1 146.198331189991 145.640156204495 156.961070790639 155.383166865192
155.298546821687 160.026178505749 154.924190468067 156.205127163263
142.81401345781 146.090291252325 153.108902978819 147.236728188605
DPF2 761.586663689579 915.579581760396 922.877217016704 1067.30106203503
808.837225580351 647.273409499313 579.923640505245 475.038389500056
717.686676823785 670.347447480331 791.840531191783 646.461274173693
DPF3 158.92254744379 144.700955682048 146.075794515889 153.78045746564
139.437073790935 145.459919416288 143.512348693556 144.243598691782
143.015905927379 143.733505359106 151.406384541297 139.29087166025
DPH1 122.00079209554 115.619116556625 121.419301042072 117.199217938531
129.116354997747 136.341358916638 139.892371997205 147.064543281269
139.429008129291 128.39171386085 146.857213166531 128.680762033378
DPH2 218.298791348295 211.720536220806 222.285940575778 242.019489058244
519.829743770022 469.794672718173 328.431289733169 435.603911474926
372.81255844018 263.64863646012 307.480644393018 283.377663208926
DPH3 187.586093248437 179.551640851353 187.4351946569 172.667770359278
301.747788416496 224.824362819477 280.246711704036 266.217879616686
218.672831303767 312.601510033941 188.475801713801 288.150694843614
DPH5 264.654879741889 259.146673652222 332.543652462234 305.577166690215
215.40037757604 262.171775165261 377.27961559752 404.366325262322
351.188086838653 278.651369597422 261.96528977292 301.747788416496
DPM1 644.301665020526 564.056592672374 832.911633923496 515.327420453564
1034.68475089075 1764.53168329081 2491.33490046283 920.083835296898
1127.07858210156 1055.79590411506 1071.83063260499 1449.33679383001

DPM2 187.529556428609 192.209344004015 196.908612360694 195.353512458837
409.592855687405 194.055394488452 222.675370226786 214.52087460757
225.820503820866 260.52593414721 203.675655444583 193.48293264444
DPM3 590.142817130684 610.070236234299 723.428294037428 566.813343200268
3164.55070502403 1821.08523647593 709.303384219887 1156.19575610918
1138.00644332379 413.330906392915 621.825622897512 1090.20254511452
DPP10 143.962842383778 150.471558219667 156.365413649149 152.166586496729
142.892198239879 146.204122329465 142.123716536843 151.68523937819
144.741063866547 147.476805635744 146.559502785264 147.294408903227
DPP3 157.50873249647 160.319687451789 151.796986456254 191.830711714844
649.052806726591 361.904370514991 276.057101476947 274.564508105177
247.504198063293 255.435582426271 263.058972886829 326.246702537465
DPP4 171.262412981612 172.430211711547 183.50553222647 174.448910958935
168.702991603656 165.139423820186 158.760577365464 165.926301648161
180.641419603362 187.997940126066 157.431972439046 168.890506147594
DPP6 149.05280915667 141.5068665919 147.314687795555 157.619627711954
145.185902174023 149.881708604156 153.585849286113 150.0512732245
141.879359965317 136.982719190537 145.123845983625 145.385816618728
DPP7 388.710465457313 340.366016191232 426.464641742092 344.786541451395
870.887539668967 352.121800955822 342.487952136318 361.082957133644
364.678403892354 390.098830270926 254.936706344174 359.822784070765
DPP8 245.153736298067 247.280717385562 277.129985959184 212.33360547376
170.590817818414 265.068532248682 251.472556189141 212.073472189229
235.961605948276 243.538374440134 202.00431685587 250.443147156029
DPP9 536.805979562807 646.117381960716 344.992791638415 871.268905562551
640.568342662067 414.888512708716 442.027630433527 499.891000749138
323.244664586314 361.429760858441 389.109250187902 379.788696204016
DPPA2 142.759452994928 128.069504716124 129.346063085291 128.312170913496
139.108656684059 128.112595697836 132.983739057264 130.207485841284
134.013748718539 139.660641694206 139.807421433384 117.990622156287
DPPA3 147.393178410954 137.685104040843 135.735535533738 148.779043570204
143.451080466172 146.515240633175 141.88072888667 147.863198012238
152.673212084312 149.851792558427 140.588811000948 148.090431893307
DPPA4 111.448871835514 114.902673141786 118.308102542942 115.224011128183
109.564709878576 110.688722702767 110.368392735494 120.131839791515
114.209249333221 104.861024325857 131.503936441405 108.655415285806
DPPA5 110.347631909888 118.308102542942 111.928204694188 110.273174147374
130.204571771396 114.574362093931 128.594951429021 115.914842445934
113.879459563492 112.252555403121 118.770061426689 119.943408501009
DPRX 138.62994237184 138.859261763075 131.371129237005 132.926799887017
130.114464233633 121.476730561812 129.320210774045 124.899019467557
131.85737847837 129.777173431404 118.05386204863 143.305848051593
DPRXP4 135.573170189235 155.388752614336 146.443313598482
155.207839370162 174.840923772532 144.896960245309 159.227123133854
151.88244935494 142.26998944788 142.799260836457 154.954906872501
138.773042747912
DPT 2691.41389079777 1863.96986643676 1986.81354260292 2144.52984392647
139.82050388626 148.012353232918 133.631676386591 132.140547119679
151.171716392043 194.475822533231 143.141852982371 130.816301920872
DPY19L1 516.088776850623 422.973606694449 491.290438575428
419.126814511305 302.696004448559 442.220572301795 434.792010353541
442.12886967939 357.063270015318 466.083451075744 637.892677173103
919.165331064155

DPY19L2 191.525291860022 168.139351579682 180.54598082867
138.655815405894 121.294366964074 127.229854957588 114.452922529209
127.023543953724 121.341177395499 125.98519427755 108.386545220984
120.517691208358
DPY19L2P1 124.203250865069 129.299810898979 157.182954894643
144.329979284786 125.472342351466 145.948772123142 134.367115858307
136.513942889781 134.682319733908 142.470799054068 127.947547987882
123.9209907098
DPY19L2P2 160.979182561773 155.694213876736 146.927825526831
135.503338987971 144.629350188283 172.26834556696 156.75308999904
148.396148091332 152.262147284452 145.968884703737 153.613110881454
177.223953859764
DPY19L2P4 140.229267364503 154.94587631386 146.26304624648
154.969503835593 126.00744446299 141.913001976093 146.763046352405
128.521380879209 143.990328432869 170.590817818414 133.460655803085
144.787836177147
DPY19L3 134.58933109001 114.44610607807 116.850851308701
115.208555742308 137.445344116574 171.521579011287 159.182533998843
140.595496714752 179.509758601636 129.233595525585 138.883710791382
144.949897872252
DPY19L4 224.167511918105 237.734385188542 212.710910778949
191.418673767345 332.749676505218 227.587590278412 336.434368527549
244.5675215931 297.708113541379 255.56301534833 261.541138637729
306.946103334814
DPY30 510.285766295993 461.482735533799 733.648333788712 391.911768848267
597.651682540611 1164.61390835016 1050.90881385623 1145.35733270036
878.167149313285 887.471553448641 584.199252444401 733.648333788712
DPYD 268.148673257455 221.635333304767 279.790257881254 199.001860912128
174.313718712035 157.895460674072 193.16411905957 194.847305068008
181.277683429417 215.115324454215 190.444387031552 199.620978536397
DPYS 113.184495948936 118.514618614167 119.704436785439 118.636694749194
123.153925912632 106.263441906404 112.675715371087 111.055738573603
112.987890650593 118.438942370745 127.342017026873 114.913534674899
DPYSL2 4206.37667440908 3921.67860557329 3195.71436046397
3744.5666678861 326.1109406486 742.128229715198 386.305638633172
621.489906442001 1039.92160906839 878.167149313285 476.076903428247
347.557792829093
DPYSL3 11071.0448447818 11709.9785619141 9636.32818474581
16818.1954169657 234.550692915716 301.428024926428 202.253560413436
315.615758089471 551.086785904249 783.991448771818 200.831877950718
316.457274749526
DPYSL4 125.682297697017 217.24573005272 140.146894533044
454.854528472591 904.733257358133 108.557097186828 428.782703561238
251.731327363027 110.190708230962 115.766358387923 390.021666378804
520.120193442648
DPYSL5 140.347131743987 134.591581328442 132.47775411384
141.557420268382 128.174382934295 134.503552797411 126.324771800638
117.417112053842 139.285128128996 141.695537669891 158.049286838471
141.917318809044
DQX1 137.885506916098 155.660302399877 142.45411401883 160.73807125195
171.373932115412 169.422015793848 272.16090053595 187.046760972666
150.792065744902 277.596303701405 219.228720772236 158.6953558198

DR1 275.929414239664 258.036064876416 271.154246147262 204.318316638312
249.763375041547 544.462032960964 373.860920943049 283.499120551905
367.644907862372 378.088675670786 318.487401106209 376.007938436483
DRAM1 507.137104111742 429.351447428661 340.193863653875 384.641319423853
206.391403266202 294.48534582475 293.794745907663 280.292616101827
492.377642058095 263.321559283966 296.290104133844 205.728673153914
DRAP1 1090.20254511452 1337.38160266788 1021.25947613827 1789.51979173209
3253.76017336684 1328.38230265655 984.695710317818 801.52891824571
772.061917233344 2189.53914329263 730.988069115919 1031.59140059071
DRD1 118.88525116298 131.22421475185 126.260691770282 131.065633574494
127.473812381059 166.89285636895 137.647282354299 126.386738502885
124.3501719829 125.682297697017 151.354187593773 124.611144621071
DRD1IP 129.564482772168 123.391298926957 129.208429861964
114.191245210321 131.440195723963 131.208112906919 114.421383025971
122.817695619508 123.145830746127 122.042136913991 144.252481420079
126.686404772601
DRD2 145.517559593811 138.976386995124 134.830021570666 132.357517495246
131.185836673529 136.625136565562 132.885515958081 133.012692824038
131.312580984876 127.605805815475 137.683228179974 127.818137805569
DRD3 143.46919253255 149.308475163534 127.91637627901 138.813126946007
138.136901441896 133.41548225025 127.681662711369 132.733821031511
124.449679223202 137.215025712057 160.176567985923 129.70974867852
DRD4 188.503673684625 288.436880408624 345.627266482686 368.134303229682
159.476343366991 153.929167381674 146.550421651378 213.877482604185
185.071775840607 160.648791273795 217.89028736436 146.450806121927
DRD5 150.724359913994 128.020374599828 143.370698101204 138.628169506372
143.589222264786 121.865696440776 129.733935614802 141.775100066311
136.111569529393 125.489777118532 123.227904580467 128.284564251279
DRG1 627.526008812413 576.451964662024 814.110601393539 525.287367366192
484.568420044471 916.026216386031 645.438835606933 683.655205499779
690.246312542011 880.140852200235 669.602939159192 669.731546951281
DRG2 338.510045856515 429.992169596618 330.944203807675 488.249967208882
339.753059801136 309.47429862515 236.855293143293 291.839070398338
245.042781391047 279.000687630846 248.299149406938 245.522497675754
DRGX 143.287237534352 150.048431572712 143.737021989907 137.03111066852
145.843639385175 129.190900922265 155.76860362365 129.517511177852
128.440024330942 153.500478301079 147.913148020331 140.174087049513
DRP2 114.364953312191 117.32734978289 113.716741872499 106.066897223031
118.731960910224 108.970432124412 116.011005504201 116.742079521048
114.953328013917 121.668166378914 134.955823960889 106.982655360194
DSC1 133.54741488531 149.964827055916 152.748807709142 143.821269460523
138.401406375804 137.045772853855 133.26167785455 149.387467833684
137.987255649072 136.22416508552 139.907840403521 149.467626202395
DSC2 147.123477579978 156.437949042181 145.950701448912 159.133905701536
158.650226303456 225.005311517365 152.109178223495 204.318316638312
163.621658579697 4875.22637286719 340.239348689005 153.464360995071
DSC3 185.570062118736 177.223953859764 184.900027626401 185.258354905314
175.210232663606 223.81987303579 162.06940121135 221.171478972931
181.654319266685 1410.58825009446 207.920724788669 176.267796612066
DSCAM 141.652000792271 139.430808017891 134.569883788949 145.369791572077
143.497909064641 137.734887788912 140.150884219813 128.372225487481
140.946729014405 141.156434225355 156.642441706727 138.237030685795
DSCAML1 121.076652800015 145.656767712617 158.278496385543
141.592180897595 136.758262322199 148.492328039604 133.444865587023

139.817493158675 137.683228179974 144.181359740481 117.602986362773
130.485988201308
DSCC1 197.357728220717 197.72068061775 156.130224537288 206.488530785667
287.374706071354 277.101000223971 203.004815689972 351.585604465402
341.973002478932 217.910445051307 208.555506929113 257.24481414803
DSCR10 175.341310423897 181.003812824014 206.303338709554
163.873355370767 156.050585687098 176.810979494826 210.77489002666
187.035957486928 167.965981130329 173.260348096238 161.475694690038
173.050039480459
DSCR1L1 155.979380606594 147.228812647235 138.43190460314
128.950308413612 175.41089164969 137.185243517766 143.523051024982
148.717545103261 206.753993163216 147.745780820004 176.650953513413
140.29803170157
DSCR3 398.512947526406 379.200576951933 529.04238100948 414.333324610267
257.573614085223 491.112594297928 458.218913259336 390.752579014611
566.228690652783 461.143361255467 523.435696794756 457.548150688248
DSCR4 118.308102542942 142.807799847935 139.418394104487 140.177708471199
140.83570584033 133.74051512341 129.583916648999 130.425809387444
148.160350542849 140.281512754823 117.815045605383 129.072173761566
DSCR5 143.378998368344 153.882081780197 146.639556437847 134.30624644595
128.645334554749 130.559515534303 136.563471322998 140.081280065913
131.260111979219 135.872060352476 162.481362579872 128.191959554459
DSCR6 132.047328970556 158.36796888959 120.686906898624 129.900540030251
666.204695328096 1111.88018487186 244.219585315024 356.530530468568
498.05778289794 132.593747375239 395.327097512479 687.987647193563
DSCR8 119.184780023269 127.144291519133 120.465934391246 121.497645719165
128.692917766138 367.644907862372 122.676368520743 123.354093930164
121.843992693814 119.285026330891 140.826051635712 125.029516472944
DSCR9 151.181422205549 140.49969612403 156.528649383827 139.943915452851
153.108902978819 153.610428214886 173.563363508314 158.434048174501
141.102274192049 168.911840239685 149.280480796544 150.798629148998
DSE 238.548578086943 202.162432624505 183.657525723012 181.202396068658
151.627496656808 142.980393157113 142.900756181761 152.983678871668
178.780662099615 265.148061007894 152.772177215856 142.03837243998
DSEL 216.582862850259 195.447966116819 160.256877369493 186.1961391235
141.880044424342 140.511988097197 133.340152756136 140.839056652898
138.204611862656 147.971492964243 164.757907224537 137.77601089414
DSG1 114.441223528256 128.992837936606 137.871870950302 118.567548265638
127.881498149714 133.94206203396 117.873333897609 156.638070437044
127.507557925713 1906.81344236287 157.70581824787 116.615250922316
DSG2 196.657475905674 356.699466789374 285.798047501616 156.316333575406
649.906677394861 1133.22524345111 485.373169494941 1530.45813438919
552.167699318911 1315.66699943181 351.337287690674 1274.39152410752
DSG3 141.934351226006 131.166819048309 161.874184077257 134.053685488055
142.705504445895 138.30326146769 138.831297375659 127.114560076444
132.786500984297 7245.25086222102 170.684848540493 139.08460576146
DSG4 118.144109564626 126.492058851464 142.474762834848 145.466538006138
122.201293962463 130.374065525934 142.851432926979 155.119146797226
128.158605637751 133.271506838359 125.296240086615 132.484079454476
DSN1 207.298994298453 208.776711004753 269.238332581564 218.350166753833
225.209315195163 347.147961254829 315.430399077266 390.670702155192
284.534369213195 282.926325589238 224.547982731402 220.742788129847

DSP	160.598186999524	159.082447644277	167.341759854203	154.675189522146
	209.785886171295	254.287838845201	241.494710905339	187.097765940131
	208.095384605737	3119.93811557461	234.661864595717	200.065184172871
DSPP	137.09814291566	131.836036241459	137.408247668043	130.187833435052
	115.293844461817	133.70941349665	126.090985925439	131.271979461703
	126.222105816959	131.911579676047	124.317870455205	139.943915452851
DST	188.378996128521	171.626438561923	171.086559024882	152.601626526117
	139.974586762939	192.860161535869	227.288015854809	152.492163303139
	145.562772839517	209.7558663154	279.921244070981	153.59485367224
DSTN	8416.19700892758	8182.20106181946	8558.70496234991	9192.8966763778
	1566.65825409044	2172.89794460273	2127.22258112242	1662.51399707484
	2016.52142068599	2215.43744802035	2192.17997650999	1484.32547474275
DSTYK	299.29004204677	316.663128947813	282.101714660307	335.013713757031
	199.620978536397	207.651142295123	232.590542016298	191.335616210093
	234.768591315095	238.270501389189	208.587188476069	254.768537214795
DTD1	253.186749111526	248.085349787686	226.144012966486	289.761702484888
	371.280926727395	578.465753476561	339.98018551524	327.566026396726
	312.339824919124	599.307072499899	259.672238210968	394.101052511043
DTL	126.755389668637	137.961504178836	138.16337207876	131.434121613176
	185.220017451419	243.691004557778	148.938822951404	173.879952136931
	213.194154171149	225.065765924246	179.220691178402	230.179200974631
DTNA	300.401370666633	258.4424008891	411.160709628865	288.166541335652
	145.221594295772	157.348475799643	154.675189522146	152.840202260007
	157.182954894643	286.945784387294	135.441550832737	150.891358590974
DTNB	122.343979874508	140.557871626905	123.991249190444	121.065278765151
	155.343619434375	117.051541123239	125.696434307671	140.411200873743
	135.268963938932	123.341568893224	186.222567239198	117.119533897652
DTNBP1	176.802886129341	165.327972973978	188.864388750448	
	169.860061216981	169.434164890542	153.787447416111	171.660157220703
	155.734509841719	157.723341862468	153.075594260208	149.098381037466
	156.94531513373			
DTWD1	215.272800347166	219.044271073282	293.482512212153	203.752540576935
	224.274329367512	160.604195264968	195.050834479424	172.120772549791
	204.378245089766	207.023221559302	163.256472113745	184.204036882642
DTWD2	4690.93553472125	4913.55892033682	3909.47437917608	3921.67860557329
	668.853969513382	3833.73632806344	1797.65684760558	2853.14759681683
	2153.17165155392	1577.15895478896	3363.51782842264	2208.49325118803
DTX1	158.928949614338	166.315369318355	134.669763149268	143.582187466814
	145.204870282027	127.144291519133	128.300838927918	138.675023495021
	139.87715485885	137.344198938623	140.079516848788	134.81595608984
DTX2	289.164969943385	347.487059316081	316.895545640437	378.38874489611
	686.028214419557	295.678097392123	506.290241222253	524.640879446426
	449.523996620257	368.276805218658	428.340060893467	389.96566674501
DTX3	475.540659315339	476.495282784602	476.168679880357	743.915675090182
	207.940596890723	145.374923175872	189.2642795052	349.722963659175
	229.230239609291	140.36602776806	157.385256442734	233.842749683689
DTX3L	149.322537247388	151.237088659409	163.091280469234	165.333608076984
	160.695913747601	197.489903263216	159.970332650101	190.975263192006
	197.962159288376	170.257439890277	197.605208310432	231.0814970959
DTX4	161.968835903764	128.007359083768	130.839508306116	144.546597366629
	136.432109029368	124.486701452062	146.70774472499	145.034179171835
	133.423376063284	133.556805738801	136.91078498126	140.374644951491

DTYMK 132.587392944892 115.656801309856 143.745594527046 158.872962797815
169.417764098848 145.349023185855 150.072005848234 147.651620353593
137.950955473086 151.492236411757 120.397683153445 145.311964807443
DUB3 126.421148348261 136.37481047902 125.497317360782 137.282038401275
125.621467040793 117.374025543149 121.438875373878 119.704436785439
132.446406243531 122.343979874508 117.962188157317 128.87498876738
DUB4 136.689064885984 122.020405846301 134.748177388398 133.160197638474
134.374551571013 124.69564501518 119.696656494403 128.601885372662
132.359319280352 143.015905927379 129.263794008537 128.714459781294
DULLARD 294.746622369436 346.691690520054 252.894371238554
416.546844844021 338.866471651692 276.925017614147 253.754869975548
235.48876511325 232.892635502424 240.341031437493 319.018213637357
236.164692035094
DUOX1 146.672270660899 164.690739760426 148.01816554072 132.600208090551
188.277563426829 151.690538050817 224.062024091915 170.233084517898
152.89366585726 352.180110869664 244.440857688518 193.358728044565
DUOX2 109.728809191027 114.704831217037 111.328185454541 105.952640312359
111.935554828717 119.845508281414 130.450930089722 110.942138645171
123.896578371318 119.432201417811 219.246271924313 115.235444833856
DUOXA1 158.723133891017 145.274345879987 166.803359138942
149.155345369055 167.125830254039 162.244691888031 250.928804390353
223.094496588657 154.8975607055 337.342034950908 345.565843875296
175.199369463703
DUOXA2 151.41546939633 157.891350650633 167.662761175661
146.740694608146 152.395272857974 173.647320180498 152.007436762651
161.930142040623 179.674744176904 187.231049280818 192.009562635198
152.219906297607
DUPD1 159.320692108734 128.669887479835 145.999823250463 154.218967720305
131.148926523383 148.120968141789 142.785631812039 142.180927190578
126.515480612924 147.995991912855 126.678746008577 145.005991300977
DUS1L 254.133112176024 289.576706584644 200.468804559127 264.476846259384
492.377642058095 402.140437036706 350.983969007868 558.810095726517
391.185365567603 244.011434605133 329.82030842286 503.093655556038
DUS2L 275.39986964837 261.814227719605 250.060734219351 329.660368915848
537.350891960682 369.156887999872 382.960724128105 403.907009699589
455.665927568395 334.857690525582 435.901794650816 361.390981655795
DUS3L 299.717935831288 316.364342983155 333.868818855397 369.434158443561
594.38228123675 447.182566890086 413.084646927035 482.466736153026
292.185246573643 293.986292438714 329.779210536849 382.401290201025
DUS4L 216.290279815413 209.130260450131 239.109163320417 227.418630852215
295.153786338687 271.238736772408 455.21285026588 379.476768908492
295.571900619972 246.850599569231 257.685284145848 381.740838644647
DUSP1 4009.22206756187 6044.78942014135 3424.65126200697 2578.85391480563
381.867665449509 3757.39261544326 451.317592236192 1339.83170225337
795.464850398761 9070.86397918429 618.829716818725 1000.48723872058
DUSP10 211.766884719761 175.341310423897 179.593817076317
173.306283668215 160.870390779084 204.637887777803 251.039117540764
162.351212914573 172.764511709731 261.671277686467 203.335012796883
186.033093285894
DUSP11 286.194197552489 262.389583658305 280.246711704036
216.381643581705 417.36454228858 570.26564132238 533.54045706916
536.805979562807 400.783945471037 623.888568444132 460.742037437298
449.304281526081

DUSP12	554.652979820908 500.848273432862 637.695341427976
412.277592266475 654.108318810728 1206.59866455529 840.276501350383
815.388288623756 772.399017166118 647.273409499313 692.945420109927
1214.77298621465
DUSP13	143.298218989451 135.872692740227 149.902420337518
143.68613742378 144.188147197178 145.233055765001 146.265518481456
144.781438651649 140.014167357286 140.949367846425 148.166340306996
139.741862925156
DUSP14	616.523853569261 397.153801851621 423.607299503704
398.075524915003 271.522696252995 339.012225830929 261.671277686467
423.152420192284 257.032174266905 589.240581642145 195.414464905232
218.314136992677
DUSP15	144.690880401712 154.778726823719 151.694193636313
142.41737138634 138.613373798613 139.545451634208 147.060637536615
140.599584289987 137.354782768068 141.015441854424 151.712097221291
143.864036545717
DUSP16	212.669156800241 194.637233028442 190.66473033826
199.477350542491 222.268494113686 224.851680313689 331.888150158628
319.223127076041 268.78904911703 213.194154171149 357.006610037663
250.406775889932
DUSP18	213.93763684072 237.420951138877 225.702629835215
273.984715370774 249.234065130083 276.653753970476 271.430915699592
293.234972169244 323.95355217044 199.250680260804 306.896732729554
255.839878446312
DUSP19	11554.7330886956 10608.0671430066 9937.36968397141
10280.5093368677 1144.07896461196 8140.6363797934 3474.0924656878
6484.36742084268 5425.58761099475 4009.22206756187 4811.88424516272
4353.66854914351
DUSP2 171.384487293154 189.94130405123 328.200548902445 163.326102597955
468.339735327273 374.872358131797 308.517561840596 193.723953003899
237.420951138877 201.059635671301 434.176007498693 315.828726297864
DUSP21	149.492550066785 144.203716525289 128.23432652395
154.321219471319 144.487533992157 134.69274746407 140.976675511847
158.56996905631 147.973274498131 142.791015845309 140.456277147131
146.050714008913
DUSP22	924.630951485299 888.706978062337 824.427857809495
959.897744425251 381.971539806271 445.95114929414 639.688482258192
318.850765099332 424.201556056759 827.400420888645 414.538237455116
496.560127152436
DUSP23	786.160002434066 1001.92763014266 718.449150898903
847.863177169335 4000.45196171119 2135.04382065446 1488.37146754184
2051.45788386541 1464.04124596264 1445.72064343302 1410.58825009446
1775.32632165475
DUSP26	175.236826554583 166.396160626179 145.212306927438
167.273378118452 131.023892409073 134.372560968918 137.440295223749
158.499787920522 167.093409692227 144.116298946718 137.537197575152
146.735616237082
DUSP27	140.385509382606 135.730331297139 139.621016978072
131.397850276558 135.999030570565 132.855812035005 139.171486871664
126.205931638442 124.961859983596 139.739448838436 154.758831302515
138.199559519849
DUSP28	261.322374889497 260.591336959281 309.861048237742
281.304989265608 245.317436234586 281.037762232619 267.622579012917

274.316982298975 286.194197552489 243.732074437237 317.493387040816
413.172954892025
DUSP3 7371.9761952537 6628.17856159198 4923.36698713285 9527.86367804896
982.367000148189 1273.6393529108 1087.69177809384 1167.26555140613
1257.32413345748 1862.88548451154 908.704610877319 722.725186727796
DUSP4 133.670967970136 138.22823004719 124.766436060041 121.939618902165
160.882229345156 156.748330788367 137.510586346207 132.54577436954
125.720102558499 128.680762033378 158.188039933865 140.74208906156
DUSP5 1035.98574557331 853.314313182949 419.763812610671 625.152889992949
463.091387717305 1008.875250028 535.041545916303 291.261765585473
339.843776738468 817.342172321486 1176.62418139379 480.22496056277
DUSP5P 122.68719599986 126.899985772157 117.856184272322
110.820329153348 119.952108688414 110.266032635568 114.308806726044
111.794102015354 112.649012857276 120.767950825369 138.383183017683
119.922676683808
DUSP6 446.948699651916 621.354393918348 283.163770658619 465.102234374389
594.713185954954 1193.82799849555 624.638115878703 1181.1611856364
332.296938254329 1456.76683429755 864.908449769749 602.480822390671
DUSP7 136.80943340993 153.922713834296 156.808992920564 147.736397115265
164.914114315732 141.296574431203 145.254692995502 141.391508027714
153.78976320015 227.499335248402 133.800111596565 156.061579024549
DUSP8 527.498114662967 402.564148020948 351.188086838653 433.491835341321
192.340135076104 243.038392457584 182.938824846376 170.748911334847
199.70041209487 155.255279503782 201.969002368045 205.45079491218
DUSP9 143.35902319003 145.133151090252 175.245611094434 154.395261192114
177.896726792666 138.152641686151 147.294408903227 157.856019979644
152.088488171996 149.160808104789 124.723519607109 160.648791273795
DUT 240.283918204202 227.847614397311 245.082816415008 197.068795788491
349.722963659175 354.783364481955 327.207408852474 289.474517971859
430.100415399881 304.844121431627 447.372026268156 378.695635790714
DUX1 140.50127034921 138.094719587545 164.991522994763 142.37457375182
153.545086287929 143.301399292539 134.631638658586 150.063082731931
138.616719975373 139.195281937545 142.232918684259 141.828740140733
DUX2 121.154389729409 111.1002996982 111.525984720592 109.333276735523
127.736712231221 119.06600048679 122.527517633626 117.642441794442
111.078930787656 132.375905857023 146.59366188125 122.187031504466
DUX3 155.737364431382 164.929445749888 148.320310040998 161.874184077257
138.716375045891 159.05213637758 149.040720727554 159.479962316199
149.262900833177 154.064184525311 145.954700424725 168.697565122105
DUX4 142.866874321248 151.040909999868 147.449186980143 145.320945010364
152.418315087218 146.879019367939 173.790848780011 153.320911882529
149.343930487315 154.916023131856 153.56082876276 151.503307004675
DUX4C 155.608876496443 150.531753446398 163.899808733609 181.110403721353
148.846121873401 166.570606752678 157.108043150259 153.488903967311
144.785852437305 147.875855077738 153.832132196284 157.646948327818
DUX5 104.780306305981 108.910588012315 106.667175621971 104.15418293001 8
108.404733392914 124.895023786422 107.91011371422 110.148555045276
113.963798749897 111.703224727793 122.667593730152 110.988514718572
DUXA 138.254479653425 123.122705969332 116.491342107103 130.334622987505
137.77107995457 146.528945049588 162.870317933909 136.691234705997
136.411722212312 132.94603323912 163.758054869675 163.48462232122
DUXAP3 8633.71708020651 9671.66667662331 7266.3946293904
8368.88320476021 1634.20135156938 8864.70614223459 4335.76831372031

6312.36452724398 5467.84611694564 3468.14455177277 6441.13602696535
5022.15374558514
DVL1 160.748082658758 157.524049610272 160.446191353364 158.647814364392
197.277310409328 161.951996906162 155.576838112772 160.6823824476
151.914252332792 180.250420976613 158.598999414864 154.688685142904
DVL2 292.145553580042 272.117599603586 222.315451077459 306.111172289632
276.057101476947 163.910041674646 218.672831303767 213.966288689715
176.029791617989 213.710058427654 215.167086358143 172.073751088968
DVL3 535.354724252178 427.345809255426 647.439372562271 524.392279804734
360.991542429346 411.784922368377 470.868288641048 445.95114929414
462.888171502285 517.020259129974 364.113665301598 563.648536446918
DYDC1 141.681266095578 123.482626777491 154.443360208399 148.112717810117
156.534634574244 142.126620107418 135.52861375119 134.420446702459
132.811368246405 137.999190557332 125.299513989334 141.67550758321
DYDC2 164.553511697702 156.813992534584 157.507108643575 162.237172404557
151.751963310212 187.710185880162 168.996965070202 171.059849459102
160.031506612501 186.231346659144 165.71382076724 159.307394610466
DYM 620.614820117452 598.449359425056 803.840404197117 638.77182631975
414.267332328081 384.97980473847 426.896707161414 406.369889501135
414.200068430184 672.637760135254 578.297031096292 399.963640581639
DYNC1H1 1803.06322760249 1654.58270183167 1605.2454230722
1946.45172007314 1332.82617944131 846.084919104262 911.782783698439
1172.96121022579 969.811933814244 1169.43742964112 896.625415670455
890.507257588957
DYNC1I1 689.271009631393 594.38228123675 1244.67453201339
746.296354515617 182.42088387266 255.12306184224 198.390247605355
218.997165156081 262.980079074599 158.319248599976 258.4424008891
195.239829623017
DYNC1I2 1045.49947183547 989.77058497525 1531.54387233661
1072.37500318471 732.877169346979 1727.7144253077 753.654626233604
911.782783698439 944.92617668298 1512.28202818467 1242.61637859159
1297.25632532536
DYNC1LI1 147.357032438442 166.330001480033 144.610341215903
125.712260481294 148.791182443623 142.720769329396 133.239703270008
142.981967333546 140.694993778394 158.963726206466 172.578252260002
124.251699375036
DYNC1LI2 5138.28186306948 4923.36698713285 5250.88041666757
7122.07276863122 1477.10828981572 2068.04582900231 2344.11548437508
2311.05587511258 3371.24667467952 2398.71483410593 3949.19206005238
2574.74962955426
DYNC2H1 295.935625475189 253.867947800583 324.401611136662
266.892524131545 170.753615127089 180.437648146588 183.3378528327
154.98147307591 201.676984786688 253.327404004524 219.044271073282
190.238332933452
DYNC2LI1 154.728941096647 157.50873249647 164.365630106697
159.344044864557 175.693873820963 214.068044241507 236.164692035094
253.098839084408 183.130891975122 196.759802659759 227.418630852215
223.872330412144
DYNLL1 2952.67114077688 2733.68308784024 3012.49794349837
2947.51998516453 6006.01682776552 2365.99940149374 3015.70954394585
2910.00269086567 4175.70090899755 4923.36698713285 4291.1724030909
2789.67986601883
DYNLL2 264.685862877779 286.469773081481 235.48876511325
275.013838208239 667.892385395063 482.226889092547 582.382786876656

584.199252444401 565.164961255054 347.620001353026 223.918931440768
806.290662664708
DYNLRB1 1115.53524128833 1204.32784721885 1492.15137418791
1314.62071412501 958.63560158343 1223.06438074895 1397.84878368705
992.744093752678 1280.57297272231 1002.44383771056 927.137881369911
910.087388930485
DYNLRB2 137.138266859156 177.379186278216 137.669753723768
138.265155903051 167.575968906119 156.638070437044 190.625250757654
136.623658204558 150.471558219667 126.647643493986 150.500676225569
126.529763042574
DYNLT1 1160.25775445193 1046.13353853089 1016.18143428327
1032.4540300024 1162.8644618472 1788.35884039549 1369.89073267996
1341.78081782818 1259.75918248468 1093.21579738952 1537.71464618832
1682.64624665931
DYNLT3 268.724338967108 293.925995360234 295.278972023002
242.141362101262 403.043257175264 397.307515234646 270.604799126393
603.338538383128 327.696359071012 420.502251256406 251.546290552828
258.695920967489
DYRK1A 347.292438587205 299.616447269111 447.297590650141
253.601378730911 198.055877414824 452.167674725454 332.599191067433
391.706404954357 442.698000781597 322.028360309592 278.498278821829
390.284689962661
DYRK1B 151.87495966414 180.392337212428 158.594064857041
194.903531690239 264.604297630839 187.824032557194 171.731395791818
171.784981874184 174.820064644203 144.448503894295 194.584034984339
152.404186073033
DYRK2 501.946140974776 429.680602294138 329.214958614925 356.323554000768
390.809169896009 1094.67740173529 417.442660073269 1806.24732793591
362.195903246495 353.285905782951 408.993744895434 332.082749517913
DYRK3 142.914863449833 141.73248778957 140.958277301476 136.366798516444
132.087183382523 131.719227272527 137.907709799074 137.801899270172
129.514490386931 156.704278982517 138.731957926247 137.944260488223
DYRK4 598.095370768587 548.869880723909 699.984140389009 654.108318810728
760.070180572621 240.253944604431 148.423303776817 485.309521583972
376.54010486682 565.97975389646 179.663258610881 412.648926259488
DYSF 688.684644604218 672.13164205724 605.040026490154 1092.74905085695
253.463871742303 212.959246855759 153.899573902499 150.434376975922
227.065194522692 218.773153183388 187.352655464866 162.047249964859
DYSFIP1 145.503718833974 153.423579724911 175.28261759639
152.197327387252 162.422173095045 148.176995739142 140.145470073979
158.841505957036 140.794929983858 148.930073905413 151.453985465369
143.401971479167
DYTN 139.993960054847 152.194337248455 140.202762690515 156.259214645427
134.994013668445 138.166546598846 131.185836673529 128.462674862751
136.563471322998 136.287909668938 121.252042151856 138.760800146167
DYX1C1 144.046646189442 152.315376022976 141.191500203423
157.809081184874 156.585665091824 129.413729619216 149.601145171769
148.00952002903 148.74881415573 149.283495832731 133.647637744785
128.436499050769
DZIP1 179.25743545231 165.71382076724 184.041062308263 170.028047334267
153.690189121681 148.027464692171 147.854171854456 143.207187847583
155.502363062633 144.233097828078 136.077039612526 148.510023599641
DZIP1L 177.93138555339 183.988537525989 188.268774618164
174.979501466228 197.582402276079 187.494677180331 172.120772549791

	193.218266574099	157.049773524382	184.989027770708	173.233357775898
	164.757907224537			
DZIP3	168.27980269501	149.899789105328	161.079169854907	165.108876136738
	138.256765189241	156.662150993832	146.132506246152	148.838294770082
	154.764283765316	156.141123407905	145.329128512407	151.823845684041
E2F1	166.662801900754	172.091884817207	176.641899861006	169.534469894464
	188.818012746123	177.023536553193	209.54573978355	198.257497903131
	182.118544602205	204.654489493854	101.597648528389	231.63127072986
E2F2	173.450023117394	186.625452120269	149.18603646016	174.787077606165
	592.613689704907	513.518424350159	233.914433380121	640.255747986724
	533.893360199271	281.618175110153	235.742017851879	454.34729744062
E2F3	345.897250519387	389.500591134279	291.683896942007	381.740838644647
	519.940347384877	717.025533065694	524.973621024573	577.801009921199
	489.528040775004	320.707687986324	537.350891960682	1049.52258147141
E2F4	507.236601481067	524.640879446426	558.051041108282	603.906237227931
	624.264994653057	582.642500194997	583.697431061521	511.891269255193
	632.031410234577	634.356322976353	766.176882605175	894.734451279225
E2F5	184.832531598928	189.37832342197	214.52087460757	205.781486117242
	297.466255000163	267.869879189095	228.739556788924	333.131139447511
	437.836503489425	208.628626112623	215.769986055534	327.010503005631
E2F6	230.620456579897	212.119922561453	224.008253365957	238.770586150777
	336.120322555703	298.625127845168	263.058972886829	241.320049298228
	239.24587884234	201.637540930213	210.882890077598	319.490670412394
E2F7	146.885404321622	160.590990297027	169.784837251279	155.187900101727
	174.807363753172	198.863347487219	188.062732621031	178.12073430094
	202.397972518715	229.017256104437	192.565733545715	244.486311003619
E2F8	108.939608619532	144.63774603725	122.673596593654	118.909153405102
	165.642728895682	158.92254744379	137.093170378295	159.42746786085
	161.618479762188	136.478114537536	149.939035476611	147.483379646393
E4F1	663.668537495345	655.431236290512	779.623566217277	821.862271569917
	884.747476952289	926.283018664897	796.936154737659	1014.23822508445
	829.126529857044	801.52891824571	646.704226815099	861.119262963015
EAF1	280.531844086775	276.5177779623	294.368406238222	251.869781014029
	354.705553237406	325.320888625297	356.84281623052	476.641032594868
	447.995213144892	434.855262284265	274.120234926832	348.300300174924
EAF2	166.868688936939	171.954184094677	144.801071221985	179.674744176904
	194.789918629905	227.467161919443	198.285073991956	235.76573393374
	246.186104432792	198.583407008317	212.119922561453	179.687464522203
EAPP	455.486248388968	451.147418314412	565.609400092543	380.146996799549
	474.750156829195	268.186156177622	426.548994267836	513.428413503031
	410.645457106122	606.679284487803	666.344430142655	503.757797957538
EARS2	243.191238594142	215.931781291835	272.854435771465	239.148333781084
	247.528753010193	281.792602269388	343.666488527549	375.539588956213
	283.27806319488	243.893774595317	292.317998405609	304.811116024094
EBAG9	414.596948083067	367.567681162661	463.622673539254	337.119524477446
	364.443097338814	310.598930734244	484.764806127211	397.387054218873
	520.218545108099	464.436380568216	542.121375354644	394.576038252334
EBF1	617.185364180303	825.034504959272	511.996883507889	751.896334334052
	125.926774314737	141.263207601393	111.928204694188	142.456527359961
	187.336349390012	151.829217512763	128.0171233156	119.184780023269
EBF2	154.24500773656	151.705219298578	147.029988235786	142.367003292264
	136.169525758215	149.181838320838	143.035245560484	140.779962389412
	131.058360514673	146.70594914192	136.821228481217	148.888698484378

EBF3 233.59297160203 273.044064077 241.706645643141 341.096072280715
143.242308032336 156.221773628832 141.176376265078 139.025598691696
137.109541758085 129.532122459128 145.36249226764 138.279775054156
EBF4 218.269847477919 229.157389498775 193.912456093767 253.463871742303
148.556161762978 155.530415929059 150.082561726876 138.970853057174
148.052222389368 143.211127738941 148.635174393031 161.720405760536
EBI2 228.086256222312 238.617228632505 265.068532248682 249.680729921602
141.602233757588 249.234065130083 161.548130965486 210.030846572444
253.867947800583 212.037224737798 195.608619642038 172.544504923991
EBI3 109.947300629824 118.966992191892 118.338713846941 117.48427100606
120.460433297007 125.689884251847 139.765361891899 169.132693907633
172.951567541592 114.479886899622 190.921283434627 133.177460728749
EBNA1BP2 994.802763887989 970.634736994313 1202.74869573717
1078.41947691758 2427.21100330388 2982.80580616089 2116.39371296122
2468.47687203968 1687.85201395597 2379.15291239548 1292.13921040404
2050.34883263932
EBP 167.114291248266 204.544266331066 194.002075848131 192.475309217696
569.236322997211 604.453803994875 354.922901990615 652.201948082657
411.505580458401 684.866405921987 318.196134631178 369.838429521168
EBPL 252.810636571566 264.456926651554 333.270203737291 208.456472621593
580.645924062301 501.434722063309 326.908959531021 456.990425618007
720.710754387733 956.76879975749 457.548150688248 841.944755849006
ECAT1 148.149216812602 148.577336081778 130.582210755516 151.676232556697
149.929454280702 142.566028652989 143.330025840618 130.49338418515
145.329128512407 151.821324922628 140.916876460413 147.826950636822
ECAT8 159.344044864557 152.342712570625 161.534421340752 152.743680985848
136.149732574763 133.659475650376 141.035566928475 177.065616871886
150.445647133244 163.936973279176 146.904354404095 173.21291920566
ECD 236.326943078666 218.735620596634 278.095939961034 234.661864595717
203.370353613423 278.88183549759 275.072079692264 251.161789722751
284.104619768635 232.845268785472 307.906312092805 342.096409626768
ECE1 159.854639082837 179.96623248843 141.380276932556 174.635853531194
159.331110936668 151.086688327023 136.767307268217 155.104697146664
153.140087873773 140.774304412579 138.090158433822 148.000706956532
ECE2 137.499290547619 132.133979108537 135.221786448517 130.351302840602
285.931444518474 155.162310001164 164.652795899742 165.695518076741
161.229621453539 162.013164369593 157.713227527385 141.130462286785
ECEL1 117.231243105897 118.393203921859 110.900309203531 117.546508214236
125.707228291312 115.072622367144 112.000309477972 111.975027473284
115.457121859064 108.34997967774 124.876226351169 138.603793282123
ECGF1 601.164127925014 562.387692458382 209.051768656607 411.968307579588
1618.64901092072 491.496499481595 787.425941491281 1009.42265467472
1444.45946291517 4190.38366101529 844.778755052304 549.692164962653
ECH1 1741.0131951509 2295.16438420175 2525.90466050731 2530.00219297102
4166.43837922103 1659.80909859514 1606.01395227661 2456.36776585765
2280.20302441765 1218.05434595121 1708.87666406131 1583.79384467611
ECHDC1 249.953902454701 269.853718252574 330.505633088656
215.84044779962 185.639696270808 250.542405852865 316.004332524937
237.71636970719 202.761449711661 245.110761598207 322.576886792502
267.222762759781
ECHDC2 3536.14527317085 3000.46298223166 3490.77593331727
2767.77385345718 2642.24374855334 2547.53445585552 2648.41012401723
2715.55326746591 2059.09598012857 1436.57771711084 3468.14455177277
2081.45603839306

ECHDC3 233.232319881243 255.59042517603 276.653753970476
220.098071755353 331.251075774227 124.503016568677 128.152956417755
141.168198348093 123.179986410723 128.488598467312 140.870849757111
137.20253329376
ECHS1 997.460212152591 1168.68074786473 1191.26028342929 1305.2232532959
2328.65814406636 1210.40302822121 1555.28458491244 1547.16936835284
2421.87041217217 1134.64039256323 1750.21700780511 2521.58055221647
ECM1 394.962978677736 412.863887464545 243.793465720264 445.505512764045
191.348260799746 177.457362387052 153.159425380743 184.348894461271
203.642739476708 1502.3444541735 182.130970182242 169.948277715908
ECM2 353.187829498591 271.356289141017 281.659653592151 226.287196839644
131.114995025516 149.35848619832 127.195619567384 139.282741110499
138.708304921088 191.881157918071 127.339018771488 131.796941315992
ECOP 193.871324880919 170.692146255416 213.877482604185 176.397715825653
239.970202560698 198.795112039249 170.725486814469 199.001860912128
223.765583953184 175.79375235478 202.778539118009 290.326832430372
ECSCR 459.771019162573 506.80969228563 259.094411953361 534.505132658177
175.585857393085 243.807038920733 200.831877950718 220.458449424062
295.678097392123 184.25384366282 204.283030911897 183.853381322135
ECSIT 237.078644686891 278.385151185788 250.30085097088 346.356312668445
252.957489543915 194.569325586241 208.214764518526 198.205113187281
167.025336068642 194.655710454472 216.154203111101 189.01309885917
ECT2 201.865955150869 236.082185670774 186.06425658259 251.20133703465
581.828502821435 770.19255305977 280.127735578097 674.742955681908
607.979208454176 431.861762635568 243.893774595317 401.750302347978
ECT2L 160.520677267863 154.908975734985 152.348210524663 149.267019532847
138.905709709063 160.415807880532 167.850516495529 151.327544435847
149.388974859453 163.582678113215 146.446922196868 153.083451626042
EDA 138.020031233095 143.160281902098 127.172702525749 137.440295223749
140.36602776806 131.973896985615 129.469949305913 127.137447808551
130.917269984571 131.034010150362 127.855167644203 128.292627005614
EDA2R 142.514794686932 141.585172896047 160.082087242964 132.86711707789
134.442947822188 144.787836177147 162.168246402027 153.871893011106
153.829636228764 152.56002779985 136.408314486465 137.120973494952
EDAR 195.963361515546 181.607253339427 177.355210393749 172.108795190633
148.503282415207 180.837420957945 161.079169854907 170.731358903019
133.969054754299 156.695440330379 140.57567768682 154.849264978968
EDARADD 159.177303894223 200.964306455257 147.285764611006
147.460432992291 248.260998007814 466.141717595301 760.070180572621
302.362619893594 290.688850193983 177.780479906048 473.73182675469
504.275299381356
EDC3 191.486717579847 183.023316857537 165.108876136738 231.819341041105
202.887417145119 156.233573126063 188.277563426829 146.771439168861
183.979513272793 162.919656039331 206.802308105224 168.477388641982
EDC4 767.662367310672 821.265710686098 567.143102792772 1016.18143428327
1397.84878368705 467.777995124655 581.191693771646 703.815551883635
690.010326122574 596.076764072945 938.918431240228 616.701037274509
EDEM1 251.307020289291 218.106150021482 216.05364037271 190.809584998617
448.715938510132 497.269957926657 531.711823481265 309.720081933252
617.833194119379 529.529314022043 710.999354960979 554.950092776254
EDEM2 265.402310598892 282.190861594303 227.736106796551 334.377352542623
663.214972254236 543.933989965705 666.204695328096 533.37609065123
347.147961254829 296.20443113482 385.750371084 317.273940108333

EDEM3 213.077884261943 210.751430486845 189.853136769155 216.783051958345
210.502556087175 216.645552082667 211.104243881644 234.895245871534
206.376365685112 178.394895649664 253.697419495959 241.494710905339
EDF1 2459.05558915812 2703.76111776248 2360.87760349888 2841.96460830166
4068.18567686753 1830.56003081769 1904.39962279504 1868.82736251524
2288.63508719706 2583.46653460894 2470.86113150126 1732.52466021178
EDG1 201.320381706496 253.697419495959 192.860161535869 210.529782898493
194.430896269275 222.526610842047 173.353648927895 182.644319628554
196.598810934837 190.357437485436 218.672831303767 168.668125438154
EDG4 202.622747952763 235.595194918187 173.071892362758 168.801737310947
1495.04587558545 1103.13439876128 949.260897408911 923.383014892221
556.725550806207 464.812091712746 868.066644155313 628.710901107436
EDG7 115.270544506813 117.499697378893 108.167670899938 107.283814651497
206.915239638443 116.552626146967 116.844361858869 126.620889938532
124.548598801497 254.393421265604 159.513465644268 120.060776752215
EDIL3 151.043967734248 138.620617643924 154.108689872903 152.670691262738
128.186963138555 178.872635061826 141.229805348717 154.537240112989
142.064680109047 143.007237184254 130.191876914166 128.51523581959
EDN1 195.057830521304 329.779210536849 141.168198348093 244.054469051058
153.916313595581 385.978867521533 265.402310598892 245.766885283797
786.160002434066 425.826693178023 299.669394721682 139.56332664295
EDN2 176.839917795004 169.022351348819 169.844913799159 155.376656335974
202.99342484328 184.451469623426 159.374355553219 159.49930229951
172.531836863525 215.335795337978 173.959556466614 187.494677180331
EDN3 110.61165787102 115.120702589487 111.078930787656 118.230408871812
114.658668711121 106.772581387455 106.854721604889 115.692324946633
111.935554828717 114.027244455291 129.84933846313 112.817015523662
EDNRA 1149.3250945792 1487.12201643008 751.077307018319 2069.70335195314
130.260174841484 179.674744176904 115.531710316716 137.666849842576
170.918584418153 282.870785313621 159.040409182674 136.794860440446
EDNRB 167.741556663724 161.129770392464 151.295855278218 139.094167748687
133.101674466013 203.125568980238 130.551216910933 132.780055030907
144.724040494873 135.521802085177 133.906867716171 133.266524623338
EEA1 166.182850621447 153.846723846716 151.342902631318 131.272948162154
153.044682186978 148.463566046951 233.394506254045 153.371065247493
171.740347129234 157.399880496076 161.499923812379 168.773340961473
EED 217.165036362034 192.69687837782 198.549047189499 187.082082429597
231.287362732197 182.209056248253 210.666799498141 213.046162847735
201.221839281282 196.839016616283 226.712995229189 238.183096218311
EEF1A1 21754.9904891312 21409.4415798676 25558.9005130606
19356.3155476146 9099.13956219312 20887.0399135573 18862.7632438112
19496.8914296296 18146.8167991873 19889.8233042542 12615.8332290932
23427.4695172122
EEF1A2 145.431393142782 260.67310907387 205.011514211101
154.499313851756 5714.36902486392 3184.98944679038 1045.49947183547
2195.41077320434 324.177924877233 334.772648743153 559.668195903429
2970.34597432588
EEF1AL7 18338.3001930708 17067.0086403248 20099.9833491654
13758.4768543075 6358.5018930218 9044.54885739666 15463.4998494462
9070.86397918429 8840.92490099301 15194.4972103372 15116.95436573
14651.3379687477
EEF1B2 2263.73444150082 2344.11548437508 2599.421026133
2141.855356411 1225.33666840349 3222.23985545506 3008.43964505208

2786.35558271661 1953.88327754946 4141.8682669995 3671.86460903374
3501.86788138647
EEF1D 2139.42448145954 1920.16520804856 2325.02143920169 1925.60246708149
2016.52142068599 1040.73829397913 1571.87128697931 1047.78978081493
1482.92498205315 1790.53556140959 1095.04571025381 1401.67503392145
EEF1E1 231.517412929077 249.737588031219 247.340470321453
186.377571176623 536.805979562807 587.388038484307 428.09583118349
445.348044448953 557.868300839263 379.983165400941 398.512947526406
581.54874141672
EEF1G 4651.384971332 5543.71998558526 5022.15374558514 4397.32311216672
9192.8966763778 7308.57540715512 9514.74328718935 6091.9840719327
4491.96934862425 10741.3064142855 8401.40370474392 6776.11712291246
EEF2 11281.201625198 11235.4970815873 9514.74328718935 12334.7921691489
12457.6956682374 4246.19983955771 8514.94395408164 5300.67886821231
4763.89426388268 8514.94395408164 8121.54750071211 5572.23400804004
EEF2K 1600.10235468018 1601.19401366685 1708.87666406131 2146.4265920569
524.640879446426 546.399972543938 453.926991868451 571.273512452931
420.95713139384 523.937128789616 528.098472417251 491.635435224507
EEFSEC 127.453866279181 137.803749343493 120.094156375538
129.508990861362 172.20451139581 110.885714048075 123.067278658698
125.687688416588 133.820936825552 120.724926760252 127.132970760907
117.071585083051
EEPD1 208.932692585748 190.22021453133 183.556662533396 174.66966623557
148.258850340107 127.224081416987 123.323528039848 140.928627507036
139.885054845153 110.942138645171 161.385691765316 136.133538781918
EFCAB1 179.582086214179 183.82450756022 174.221939793486
139.963969716386 168.365262340217 145.852430082228 162.466301774088
146.482652762374 150.36160186175 155.815785551964 144.117964288763
157.765771942689
EFCAB2 164.659092629962 148.635174393031 140.010730572075
139.880733465536 132.421863261746 105.348008821162 130.656206845965
145.068061371609 128.563180697606 122.651702760414 135.218648235324
132.411757518105
EFCAB3 126.800718471804 118.56332995149 120.77691423934
118.05386204863 118.636694749194 124.381674909742 129.013667813486
141.147683040881 128.05898686833 137.360220389719 153.305679563467
128.262195551255
EFCAB4A 202.041958613989 211.941623411369 181.3859695951
194.655710454472 416.638529499878 204.654489493854 180.354899434843
547.042461070351 309.181150342727 168.470685171939 507.342877360289
286.774606295106
EFCAB4B 128.356453959987 143.376817049362 131.395959781477
135.86149691566 135.067056559409 128.513011425662 128.966514855818
117.60787142216 127.898533598679 127.551486042435 134.531334130512
130.63591305094
EFCAB5 137.990806386929 131.246704975084 127.663578933727
134.640783050966 130.829825154842 126.049905221996 129.665507668047
131.700854556022 132.608622131443 132.608622131443 134.463094391766
139.566124411099
EFCAB6 151.930565620382 149.402679897813 160.277755404001
150.468392290279 141.592180897595 135.987450822209 136.682172321984
148.179398287068 150.283447275981 140.334331111538 148.983182193411
138.764834713216

EFCAB7	152.54039461337	158.130933580414 178.684992551017
157.952018720438 153.606787306076 153.541193559658 180.437648146588
151.78607484924	157.736101691222 162.766038656637 164.268280525424
167.653412908384
EFCBP1	159.012011848781 148.326282600684 211.438615793742
151.466689967666 118.816986517698 117.084984190461 120.255081121925
116.342242990651 122.900675062181 121.739219824085 110.437728036347
121.499802749899
EFEMP1	1315.66699943181 624.106546150735 888.706978062337
638.129080224049 144.170287726456 172.169129806643 137.318442197233
200.902123076244 219.878230463206 195.043838688467 139.768555796264
151.747595672228
EFEMP2	1302.54547114701 1279.15873231449 908.704610877319
1535.27496251381 856.424155671931 212.050953977485 266.964673763497
288.182388699147 356.376890469558 464.323065038265 204.82244206816
326.525579400607
EFHA1 601.640387246951 604.737834405874 523.591472915151 517.23393716516
552.494327858264 582.527835746927 552.298965378403 528.098472417251
654.305724263754 797.501324589697 613.631311147328 913.934874451961
EFHA2 164.520247586053 160.833948531272 167.125830254039 169.447827110558
126.714455673392 130.052593375075 130.788557333561 118.167740643871
129.111259771449 131.198284945008 145.806935833967 130.06068534054
EFHB	153.700168798494 150.587882657674 132.149864626575 156.043994260248
142.211357324461 161.781529659549 135.010937737018 156.285963197762
143.05872558805	143.234195669431 143.175483349968 137.52653395793
EFHC1 191.830711714844 174.396061906109 188.995551740427 144.266108481571
181.098584949344 208.406980186223 204.787016443123 184.008405576875
194.445463745418 166.440530119785 210.199788806189 191.207048581306
EFHC2 161.73079180403	199.991077347491 139.209080672396 171.071893074072
133.760183242137 124.955676894776 120.342513393659 269.853718252574
130.812309202831 121.764933633063 131.001996509052 126.620889938532
EFHD1 516.892850913889 662.958637409597 488.379057110543 634.556887178869
276.925017614147 182.545896049446 129.699765645519 175.51767355303
232.083160165968 216.755681508811 169.052101570616 159.887898257218
EFHD2 1127.07858210156 1438.33289891548 972.468110850466 1391.89900758685
8322.20177173035 2808.78353621111 2841.96460830166 3328.99028463277
2274.75133434566 1032.4540300024	3384.19521857242 4246.19983955771
EFNA1 385.978867521533 562.891241563711 342.403428822464 318.778978094743
1411.90426595229 1177.01310527577 1713.93035637146 654.663838918786
1265.88446775977 1083.26599217847 619.223672253014 408.201232787978
EFNA2 143.365976841902 131.676609824921 139.044150521333 133.026879831536
146.351622020606 136.730623822061 131.338853426561 147.508817393901
138.846543789882 135.852591930882 135.271290872348 127.50624009248
EFNA3 147.968104627262 143.211127738941 140.593510265137 137.42213762011
150.49183794045	136.207420489201 133.345885001759 148.874533472738
144.065554025603 156.646803208934 124.873289279767 140.214475962177
EFNA4 154.637740117457 168.71216652064	144.242034773232 148.057700860759
581.37957221282	214.434355263652 275.203046536524 318.561557474749
250.376178742476 230.723142236623 250.30085097088	245.399847987183
EFNA5 126.647643493986 121.18490040448	105.050093408041 119.29304762171
120.184782039538 109.113476990309 113.955459033122 115.605206174867
117.965208293925 112.635430248416 113.862790983655 113.077691687266

EFNB1 254.991678321253 319.927209914181 183.676020826291 347.292438587205
621.825622897512 321.977549744483 277.101000223971 195.513703253634
192.340135076104 308.441120735095 403.812520074723 396.708183244581
EFNB2 336.845908630493 319.812293134357 151.615675144083 241.944581086483
321.119758814618 627.526008812413 232.127996106421 538.647888236878
273.303240462047 703.29965727829 373.656487297238 783.059427015955
EFNB3 126.523201566273 124.411213807179 114.032874859062 122.708102546276
138.476953078729 184.387409077972 122.20752192519 123.752109790056
122.042136913991 245.240006198482 126.416290818296 134.266373965186
EFR3A 1948.24432908972 1861.19763103381 2246.89876694944 1889.05071563459
553.90008440429 956.153239481189 817.529344685717 897.51782613323
1174.0922389136 951.371815352591 1060.24125879951 834.788058321257
EFR3B 162.620593985693 151.563917522864 146.763046352405 130.860938463815
163.773702843544 136.643481414354 124.652072110334 132.568354349687
158.6067091347 134.835962366555 152.356731704094 147.44463861207
EFS 242.657224881824 241.734804013256 183.406510146725 276.756415094228
408.037874667173 169.844913799159 189.872997882033 171.426334687626
211.438615793742 195.033792280119 220.258804437828 219.192900411543
EFTUD1 213.531307836195 218.2454853526 254.879772480457
240.182016388745 209.867189613436 224.766155406229 213.556015518863
179.015669199358 207.05325577114 259.900447705311 242.440966395613
228.852495818211
EFTUD2 505.83860711693 524.245387973163 416.933146258428
593.462634352052 1481.24705642453 1020.19774509086 980.67040349547
1008.875250028 1098.78649254838 644.741046498743 835.284130042504
739.874432767734
EGF 126.141462782039 137.419835194075 127.740057874561 125.621467040793
119.005230164124 116.664031217378 137.410687900298 127.04953162892
122.170662903259 116.670491972948 137.356539213081 122.423561732526
EGFL10 139.701504237962 127.389942368953 132.466796864599
137.16842164453 139.768555796264 133.82548013342 135.950465168457
141.318353848615 132.338144393918 139.750281709938 146.162330544539
133.689255383855
EGFL6 186.57665470145 189.971144220643 198.285073991956 218.70412656621
161.530878860883 178.734634888773 132.871179509995 478.040234382488
212.643559782754 256.174286655796 207.718619151226 205.908052642222
EGFL7 176.019082929386 190.572712684622 144.402160347818 185.374745525698
179.220691178402 160.822287037366 143.331929080227 147.009325721272
148.538400348149 154.865107093653 156.488852212628 147.909588100857
EGFL8 140.481148289418 142.780100181736 147.274945970072 128.504026473695
135.164117176127 122.333765477428 142.281657284178 123.98384416057
146.937052029619 128.182419321978 127.152744243038 132.560292119968
EGFLAM 193.064450357153 206.071709262227 193.444080047879
263.52595855321 159.765585531442 157.568150431884 152.074017988748
170.401653214867 198.191669030952 249.953902454701 155.911387663464
174.075670633438
EGFR 185.876046259727 168.897620634337 175.400802387403 158.404154056472
174.635853531194 142.266177608209 164.276668889213 149.283495832731
140.490023670841 502.598518192729 178.394895649664 159.021304119265
EGLN1 174.690052785405 159.52147764862 171.941887376462 166.89285636895
291.476113017794 171.16807372519 248.531972733475 193.569894919176
194.388383297922 302.809011218879 172.03919618445 225.095016600703

EGLN2 1111.88018487186 1305.2232532959 1339.83170225337 2012.36542989953
1640.99641023476 823.658996207582 824.746488539942 871.528156184927
1167.72023351098 597.795856268827 672.348723983535 628.939201051801
EGLN3 337.652945958921 342.096409626768 273.651114834013 398.193129265416
321.977549744483 147.238896426423 326.570967321756 164.276668889213
135.817028661429 411.968307579588 279.325065941252 178.28963352311
EGOT 153.532954370572 148.989286668424 142.321476446203 144.188147197178
152.385014030957 139.872976072847 152.270617606866 140.419686138586
161.202844994203 155.91493956251 194.337080066417 161.299920355406
EGR1 4000.45196171119 4118.58682247781 2093.67403119512 3177.80599774525
351.292682758926 1650.77281891596 222.484058975856 1292.13921040404
357.35857422585 3334.16277752222 585.933822585718 351.079435946154
EGR2 190.96184674871 232.519482087835 141.069243738242 201.105520537151
155.846106540378 203.308014296113 139.776830704087 164.784346867158
178.262619516994 337.822337721287 156.061579024549 134.762169062357
EGR3 172.097502416776 151.16261275916 132.478801067837 138.928287624746
129.360773522498 160.632809953993 126.678746008577 131.246704975084
129.375772737722 164.562302265155 148.803188053248 127.893555451807
EGR4 120.36718185387 114.10470841717 122.644105247178 127.453866279181
129.813090345837 115.3611179192 131.766114156138 126.372374084002
116.919109599361 127.811923445503 115.785084044427 112.3786222545
EHBP1 1497.32522786098 1213.61170930345 1561.10217536347 1222.014210447
336.002793042439 522.17701271254 463.754869412768 547.817028243811
408.037874667173 1092.74905085695 408.7151485563 371.224296589448
EHBP1L1 2421.87041217217 2808.78353621111 2350.01764791897
3700.75770419386 1075.3328563531 428.569462312363 465.41233536979
324.671490117952 515.597523880328 1808.26383618901 1459.31031099055
936.560290736127
EHD1 1816.3719976564 2416.49806112207 1477.10828981572 3149.26998719015
1439.37593006882 960.775251871864 846.985164747386 698.882871686866
1155.4487861163 855.112210714686 914.654262296682 850.882404546093
EHD2 879.809795251142 1006.24899131914 491.112594297928 1536.52218968817
395.133831206474 181.467708718758 240.792557915987 170.046856179083
197.02284475852 385.135014083686 297.071121022183 160.026178505749
EHD3 165.262738484387 167.542332146706 182.508415915215 160.557818466874
195.019706886336 183.115511373872 226.678503956411 160.806678614325
158.598999414864 146.376149543438 175.327597807738 182.300628120658
EHD4 372.255208814622 502.281584081805 311.612118247493 369.592501881153
551.350758868377 680.509421043347 710.999354960979 528.767644181119
710.228463443265 588.863382995875 682.538709448692 540.946852709312
EHF 127.824005374202 156.883255634126 141.97478198531 127.080149144234
257.44230597145 576.873245313776 596.076764072945 683.179460779062
837.023434916281 558.051041108282 727.814681346494 595.409419899436
EHHADH 149.383429241903 135.99579763634 160.353649786837
158.294082740156 162.841318222066 189.575681012578 162.18174589792
285.882780066861 353.285905782951 236.108432603479 153.795665190439
213.93763684072
EHMT1 172.478461106269 161.997058981786 162.683665945044 158.335952927257
157.83460319212 160.28767775843 151.855884026072 156.490654498766
159.588575526536 148.816369706133 157.821491577808 153.551359970644
EHMT2 165.993897306927 178.717719615544 157.446725405803 180.63469438043
229.77428612216 145.791950392049 168.969180646603 155.014930599769
157.940873610797 152.08574341962 184.530853446688 158.053654524927

EI24 864.161191021297 885.104778820041 955.604404066769 843.545962045705
1293.70593826709 1264.90442763788 1485.776977235 1776.38646235135
1394.31467757456 1102.38234645327 2550.08769415478 1858.12855367119
EID1 204.544266331066 233.997642139328 252.514569129392 227.483999162038
174.795822188504 166.595529296463 198.470718196139 179.230909178397
171.449326065013 207.920724788669 179.358460170348 194.702974020747
EID2 167.157420848604 164.018470368389 160.806678614325 170.505553609318
258.21341068758 262.019779752857 215.81995779019 209.568066932367
191.322056430728 187.769988580599 223.359785518285 176.783978625103
EID2B 7771.28016059011 7016.12801650552 6203.08085790474 7585.47289520004
933.723483173584 6409.23524786095 2837.36293710422 4651.384971332
4166.43837922103 3195.71436046397 4793.22133852447 3455.20639616159
EID3 234.768591315095 184.021010234322 270.12941437975 181.735072060023
120.688909943167 121.588054040961 127.979574243743 138.064951932825
127.763186950161 157.990467804353 122.634741242406 129.4807689399
EIF1 6281.01687932005 5233.72882607795 5912.48904507462 4030.42725798136
1969.67552562117 4280.66857663502 3232.96611074938 5624.32062666666
6253.84268792816 4043.28299602858 4141.8682669995 3188.90202907403
EIF1AD 130.185134787768 147.679233228201 150.396462763607
150.072005848234 166.422360032182 138.456465793474 160.090914414578
144.077080621909 159.368376366657 151.379761514526 174.698724064671
145.185902174023
EIF1AX 318.778978094743 315.863232876382 301.695991929957
249.858562350415 301.087947101876 854.224454791713 374.106258053201
774.180202250076 322.684910198242 435.037306125887 364.766154056243
369.373283422151
EIF1AY 451.147418314412 419.902534806131 683.365770196967
431.093544964576 445.95114929414 149.520911664673 181.803275243281
679.191060100076 572.407760064901 211.572396862776 459.171226689178
748.161769171818
EIF1B 1055.22233969473 878.167149313285 1144.53002508973 732.352357321273
1071.47374315822 853.314313182949 970.166036472486 773.330948077036
1042.40539020413 1047.2693539616 831.08287594363 937.593129242257
EIF2A 1049.52258147141 737.193295872614 1326.95332435394 809.729160605727
484.131020584378 1017.09497861942 1235.75946952752 902.822697602452
951.371815352591 1329.90419210779 1183.82018168635 1349.84215478798
EIF2AK1 732.352357321273 838.63350090385 739.52947559669
779.922653392712 1391.89900758685 992.744093752678 1870.52504853779
1518.13775377335 1742.84803427086 1091.69584249713 1179.42590997099
1627.02882286283
EIF2AK2 2466.36457617864 1776.38646235135 1185.70008959602
1832.35076992253 878.986106116952 1337.38160266788 1427.70292953827
1174.0922389136 844.462124058351 826.314416168552 1634.20135156938
1073.40463823132
EIF2AK3 195.230219990395 183.181125586276 168.687845035401
201.837695818264 263.448958595597 269.141120401835 259.900447705311
288.770330002807 256.471672362567 186.794165469219 250.963601187362
201.569913451219
EIF2AK4 2269.44503773126 2437.63566094439 1567.89763050645
2072.01578063404 427.345809255426 1793.75125318113 1027.11849332495
1484.32547474275 1078.41947691758 953.549801897112 1417.76295714364
1163.13845502757
EIF2B1 413.502150181875 410.533157400023 568.701661112562
491.635435224507 375.038561820137 453.760162338163 524.392279804734

355.714202328378 549.460612060753 526.896146537814 390.979822976551
488.746614111382
EIF2B2 521.512936372701 413.757630469808 527.294087043601
370.877260088621 384.051170099325 443.343806055858 661.826149512811
544.222458349696 495.438672949441 655.431236290512 351.02823853348
572.913714991136
EIF2B3 381.740838644647 442.698000781597 382.960724128105
493.347308943133 337.585502787016 393.98210915852 266.135477775641
363.255517622046 376.189276074918 303.724140156439 336.380724981693
282.137282466192
EIF2B4 833.198511430615 802.304294140514 916.836022395768
894.100792722707 874.348941257592 941.936830901588 737.843412273272
961.355625338687 618.140160251927 866.765054377214 782.448692528135
645.183207984114
EIF2B5 474.378918787006 498.05778289794 453.593585032712
534.083703564903 513.816017871024 505.560841055884 552.167699318911
638.393099392849 665.106631364961 438.611973005212 605.437395034282
671.134356672693
EIF2C1 286.376638135463 338.866471651692 292.746711619565
383.259887828605 324.615093103582 323.667189206453 257.032174266905
250.139061165593 308.679189788554 238.149614104568 260.591336959281
224.724262742427
EIF2C2 1257.00798908017 1251.15912264225 911.340266389071
1377.75266942574 1037.46485756166 1098.20033709043 671.821418586421
823.658996207582 1031.59140059071 598.924339094496 587.181357555703
653.500279510032
EIF2C3 149.675363177375 144.266864839344 148.192121257421
127.040027125755 171.086559024882 148.527528612375 133.206919058442
142.694748293332 137.854873498783 141.66312605042 135.659271030454
142.96594961303
EIF2C4 185.199972151831 191.543371466252 185.525760847281
223.253794809571 146.264686158155 140.021640936288 164.413893089607
137.539385624206 153.488903967311 145.628239642187 189.142295015168
147.808262170269
EIF2S1 458.782864873657 483.174262289559 517.23393716516
509.618968665191 381.484062427466 400.783945471037 348.36178568457
360.90109922539 305.615423200097 265.972387419683 459.973764138144
347.081036934975
EIF2S2 213.674150322581 232.276667029184 206.605699708924
266.316485802808 169.482586449402 264.158648784101 237.420951138877
185.199972151831 235.997227173296 168.882806817001 208.776711004753
193.07463429041
EIF2S3 1505.90883940934 1301.61742965939 2399.88695085752
1126.46241487062 726.556553101727 2107.99357083999 2043.73482086765
1223.68146968716 1117.77397620221 1561.10217536347 1161.39230511792
1166.86593572716
EIF3A 1660.70040058102 1540.71556891815 1320.74943817958 1309.7660860424
1006.69598892631 714.274201838067 1086.8615735568 871.268905562551
1209.56018864779 868.40984928812 1752.50728312967 1212.55338792557
EIF3B 1748.77270039774 2195.41077320434 2063.42824094932 2850.98525554116
3749.80462365748 1336.75600604068 1526.66232186013 1550.61855325662
1990.94596595493 1657.72357544205 1224.49891389348 2236.73950603157

EIF3C 212.643559782754 228.901579068997 226.25694727283 247.845708587624
208.690347128273 154.648806105422 230.723142236623 155.76568091161
164.02617650236 220.578790067967 199.933148219784 200.84691030169
EIF3CL 163.407360509803 180.667897257797 233.335353447342
171.014432075829 197.762169504041 205.930921729192 262.019779752857
202.849544035436 172.599654598668 279.070493043898 187.196424491482
305.768201359278
EIF3D 1875.21979956596 1971.42527720714 2127.22258112242 2225.59207490574
1783.8031212092 1704.25279485563 1708.87666406131 1571.87128697931
1660.70040058102 1707.09830463606 2135.04382065446 1718.80498208397
EIF3E 10790.3738999898 10075.6573898939 10280.5093368677 9251.21629832018
9349.76443528011 7038.48958943724 11874.9808592281 8292.04796307133
9715.20127757161 10417.5218406801 9560.32907282993 8840.92490099301
EIF3F 1608.40826380713 1867.55140010155 2392.93562797606 1675.35431076885
2081.45603839306 1304.37930719411 1752.50728312967 1538.78070281348
1710.60409578293 3149.26998719015 2149.13404212591 1192.47480377375
EIF3G 2096.25803773108 2197.44189747261 2293.05058904656 2185.46850787298
2165.41403521012 1542.35486950856 2151.9395095819 1721.20676889116
1312.44215746801 1863.96986643676 1627.02882286283 1416.93831202323
EIF3H 1724.22121078776 1522.87703817058 2200.94974896771 1701.43479420427
704.653073777277 1034.68475089075 2104.59597571915 1503.2049371911
1798.5819962261 1724.22121078776 1754.99663827472 1643.08061372695
EIF3I 1319.3522488218 1208.9240215109 1618.64901092072 1267.5716872154
1103.13439876128 2406.67187918735 1901.97972168836 2176.59445919725
2166.68174726155 2118.73410871718 1198.53117439169 1940.42283070543
EIF3IP1 146.174492025087 153.761061653202 137.044079235724
137.683228179974 140.750004067571 144.501995579502 142.13436730069
158.8054811093 140.52997588229 137.401137659118 149.366554238043
150.173766182317
EIF3J 267.535469518664 226.35854874278 307.866499851733 227.736106796551
215.094633380547 274.77366169918 313.18989508207 259.672238210968
314.292700455545 388.096183735126 239.73396414153 315.430399077266
EIF3K 2662.43181510326 2979.27692778783 2708.20624364471 3481.90072759183
3417.22292085668 2941.69058596077 2770.7811954029 3012.49794349837
2730.50913686613 2530.00219297102 2741.71904421456 2792.13364570755
EIF3L 6793.99737496637 6138.10904830456 7038.48958943724 5947.89821087362
3436.32495553246 4612.81760689869 4892.17875938832 4527.53010866974
3938.94050658589 4763.89426388268 4749.69040198959 5175.17279574945
EIF3M 941.065508539108 807.92851898782 1302.54547114701 644.868034665476
1203.47834989609 1183.82018168635 1742.84803427086 1258.16519121331
1027.48490028905 1958.96627327952 2041.03024136014 1142.44180956604
EIF4A1 3168.27825058989 3396.40593844715 2611.61260614408
2988.45928881468 4088.12910138929 6585.34962362923 4511.9116742833
5032.66681917478 4214.83058308503 6244.58472720999 5373.2173152906
3810.98043907046
EIF4A2 9908.59593023839 9099.13956219312 9852.51412359574
10704.4678928541 2605.13680290987 4651.384971332 7539.6945050098
3949.19206005238 5489.19362780613 4198.61471038267 7406.86318549013
5437.78386044638
EIF4A3 1038.93733638848 1131.96129284658 1021.81988990874
907.306121257236 2542.44286642873 2720.83642320419 2105.87438782831
2452.1304336603 2913.2685312165 2630.78185410861 1801.74467005923
2246.89876694944

EIF4B 4711.21389812942 4150.8511719558 6810.50193989514 3688.68740405322
1100.17618574297 1732.52466021178 2728.04758874748 2208.49325118803
2938.06458269406 3793.02341602102 4134.02613649483 2744.77453865893
EIF4E 144.87013280941 143.175483349968 134.751916364581 146.763046352405
142.263088069997 147.055213172078 140.52997588229 149.818888178632
146.278921849776 153.39738748803 159.89988762842 145.789840511876
EIF4E1B 134.986030185477 149.066522533956 155.463400179872
156.053517438255 139.699189528879 131.773304233929 157.221008210698
157.182954894643 158.400572147671 135.594186066942 146.40904127542
131.578042179715
EIF4E2 523.937128789616 559.668195903429 534.083703564903
569.656573915709 796.699879495384 882.798927428142 680.743052197025
681.429327723794 896.625415670455 1401.67503392145 897.958748317315
910.982557417978
EIF4E3 694.644744547412 545.026293696209 839.662936279727
488.884511065882 205.322733011516 284.195575802966 286.085833802132
228.675099004411 301.087947101876 401.267425721838 264.905947922753
350.077206368839
EIF4EBP1 171.121834829333 161.916155040522 178.045931314857
189.985520850855 571.950858678221 237.601064742453 186.758038665014
156.179501567984 166.803359138942 253.697419495959 174.540209145695
152.864765895508
EIF4EBP2 592.957842630046 600.162650426961 711.491528818004
509.400965222318 412.415549375221 250.577945492981 401.960630197091
339.448524400708 504.709613434391 284.236970342827 665.602699607102
458.440488401029
EIF4EBP3 170.878962027347 177.759729226623 162.168246402027
202.369905673351 159.515698265424 135.594186066942 156.654450436728
160.297609596399 151.459397907666 176.874265462986 185.961822133346
153.606787306076
EIF4ENIF1 392.746574862867 334.140757209353 523.073926509865
419.272193677273 332.464176231219 330.080428155378 252.957489543915
280.033528476199 251.011148303246 295.153786338687 335.389274425025
354.336462793628
EIF4G1 686.198351821711 640.255747986724 567.465201338735
902.394410348792 1470.23096656138 646.117381960716 525.287367366192
597.189355986293 799.759250503507 702.049571167356 583.455617065556
596.54904286299
EIF4G2 3807.16206340771 3712.67025366957 3954.24763752814
3474.0924656878 3968.38723079061 4793.22133852447 4190.38366101529
4457.64479507833 4945.87677849967 5601.24254062491 5800.06300826802
3441.9805896226
EIF4G3 807.92851898782 776.411045078049 819.887322757281
720.364080505785 720.364080505785 716.420861490714 672.637760135254
788.244353085369 756.390518977556 698.086166782221 921.140932799223
611.088604425595
EIF4H 1566.65825409044 1509.69760873558 2159.2992548378 1531.54387233661
2031.01186679713 1293.38808483663 1787.32566525551 1766.15001427132
1677.93424842267 2337.64145466514 1435.4236709581 2344.11548437508
EIF5 239.845747958475 240.410292351169 367.439588680092 201.555964610849
224.062024091915 342.315456386729 436.331333134628 417.262521411065
270.651318838953 387.305348482856 402.299165610893 371.767182305618

EIF5A 768.334658920971 1121.87292339787 1212.55338792557 1285.54492194393
3664.38955037715 1625.55754007235 1133.87328758676 1475.41920838384
1037.97124386242 3078.76695443407 3241.23835624706 1461.64901688125
EIF5A2 239.388546850124 217.327543190687 201.773745532242
209.258707976348 164.50490221903 187.833356349429 137.740497848238
155.842195370853 158.147502554814 182.478971050701 131.249240371529
141.232464220879
EIF5AL1 158.73040336393 162.732574014686 171.455216889348
162.634670715627 153.38260557666 160.78368986036 165.537853089052
169.752525994991 160.816803370151 152.780908985916 146.609485664113
158.120079903425
EIF5B 482.965800108538 438.7446527797 558.934004505074 498.812823949508
304.402066421813 364.964572587289 359.493075382333 369.838429521168
395.786295710815 494.604716660887 478.180723959397 487.993796425035
EIF6 472.961857076681 529.04238100948 491.034395489684 555.259469428492
1200.94425675913 1608.40826380713 1536.52218968817 885.713964813911
1121.87292339787 802.304294140514 1053.65938651514 1269.67110104843
ELA1 110.392617179163 114.155592351175 112.3786222545 100.637271902591
111.603221942108 104.03442843396 111.992738490468 106.393497890486
102.851152421943 104.03442843396 117.49013401175 114.953328013917
ELA2A 139.099483279141 145.395883026207 179.297628357211 149.647926859169
126.734835642553 142.796371223204 147.581827861592 133.211362932569
140.354508398385 154.00005407707 153.464360995071 128.978197156274
ELA3A 126.064311640223 129.265788005518 132.804216633852 129.859331207247
127.783892576352 130.532559074698 125.874313058552 132.23149173224
130.552037435735 137.77107995457 147.241136598626 122.799487123057
ELAC1 167.92409473064 137.497145594629 158.411846122784 172.329225247019
173.484307104751 172.531836863525 147.921826551905 174.635853531194
177.151218006672 174.075670633438 161.084088366981 173.751615672365
ELAC2 274.627303114477 271.823243396395 296.290104133844 360.243484271891
213.710058427654 361.429760858441 182.545896049446 292.893332413185
200.98533822317 228.901579068997 223.551813819581 177.742616962629
ELANE 194.212177449077 161.94312376726 150.093684245826 147.78847662997
140.692586715322 148.589330990191 136.855445270531 131.534097198718
127.209043595332 140.844007965202 130.674263597931 162.007384919302
ELAVL1 194.789918629905 208.456472621593 187.784865705837
239.836698194959 140.518206716196 287.751093664085 212.190707408572
223.184064606222 153.377182895472 154.792156982219 202.324795686879
229.835576260403
ELAVL2 119.205234864031 132.291759474331 118.500956349352
122.5651974076 125.780294378066 129.636135486593 123.470830748348
127.080149144234 123.207402172696 126.94941821459 133.832462719784
128.289680999565
ELAVL3 141.655169779627 136.962442874268 145.387562372871
138.704839659727 140.898380345452 124.68729078792 126.762116571216
125.287738115754 134.509830749072 137.826458479398 134.519697269443
130.812309202831
ELAVL4 118.724348027528 115.949513659403 115.600308687778
115.646023142125 123.881658891576 114.373370072994 117.718893827092
113.177330690612 108.604763499678 116.161473967382 133.75142055959
134.105941085621
ELF1 783.755776589969 763.263858769038 761.32108609503 599.735180191091
525.920873434688 1105.10063745656 617.318955611228 1080.02576143033
1332.11815966955 1102.73689840572 1957.48508632607 1841.63015706195

```
ELF2  392.198487606534 393.264726588522 426.132037075477 398.423396126075
      226.678503956411 372.757091748175 305.200911653347 305.325519265387
      287.924891622962 289.956528242564 347.73629731446  345.565843875296
ELF3  286.133496092533 424.201556056759 468.82816089485  160.150336334888
      2938.06458269406 1728.86115695979 2048.13524244516 1671.82101590797
      1949.60516542965 940.716682221364 1999.10320742823 2153.17165155392
ELF4  233.786136606682 257.706904467657 216.126000558319 229.933577323411
      1482.15461429206 899.667912542118 1172.4103691525  791.402660066193
      601.164127925014 369.592501881153 475.927798702033 959.532977061027
ELF5  133.70941349665  146.690308698414 142.481897141275 119.036965883266
      169.321832528939 322.263925912805 148.908758741034 336.981561928411
      156.050585687098 130.584348445667 214.617852048151 235.795627549799
ELFN1 138.5270206057   153.920180168328 130.894384800009 126.077945699709
      119.279765939686 143.229240978574 140.980119908597 129.015422924366
      134.403789206552 138.986877479278 134.479828866339 122.109009629395
ELFN2 120.638330368388 112.825399620524 101.597648528389 116.810827219571
      128.662039886682 110.61165787102  119.498312996987 115.37736249419
      104.826135297161 109.087574814292 122.937281123348 111.000303209442
ELK1  486.334840298921 527.707841428677 325.082620845425 517.42417250172
      1253.05700051015 659.900771633223 471.315730378641 726.346459754217
      540.005877322817 467.681480781925 401.542072222692 410.002817143111
ELK3  168.244523461915 150.255511248598 131.797936395014 162.876684781936
      173.272062328378 139.155456340488 140.092648878007 144.167857433516
      153.311705636864 177.478925091583 151.548865569913 141.964796033176
ELK4  133.99226161886  128.659393756477 135.054931772725 131.132508349222
      141.739299634007 135.291264978088 141.088456756806 133.414198431469
      134.307336063739 148.260672564835 150.088034336851 143.290642777702
ELL   282.137282466192 293.17183601265  306.550948766362 318.73799597811
      349.054741747798 305.252666831522 285.931444518474 285.768837438859
      207.023221559302 259.796092536601 345.977609212137 287.041585684111
ELL2  376.443086293235 223.453776362112 205.08372943071  226.712995229189
      205.305702209672 254.036619126409 310.718449468465 222.134519361117
      226.595006724068 821.265710686098 255.435582426271 225.050796356289
ELL3  189.362958041175 165.480489678822 174.491551019128 192.532222258033
      149.089067389266 167.397758092427 155.187900101727 147.991572632747
      178.200678423426 200.688465653117 151.020903727566 177.500110974116
ELMO1 167.304721591124 164.417770955805 143.523051024982 152.454414703534
      172.934156790775 162.531630234824 152.680068580858 171.16807372519
      173.810080280732 139.510764003291 159.98636352484  151.026905311756
ELMO2 257.740674565975 237.641190133427 242.5085895186   268.370347292603
      219.687151891777 256.897177822238 239.541516080716 200.387623661458
      209.74583569369  214.803167803712 207.254220247481 218.470365730283
ELMO3 165.131463797217 177.582314734704 191.348260799746 155.664126780859
      481.751102333172 277.685488726472 427.084392965651 321.977549744483
      347.73629731446  476.495282784602 406.133430120039 370.390323116113
ELMOD1      138.285325970867 157.765771942689 151.829217512763
      154.740395921562 151.312853764675 162.756185479013 150.042173991463
      155.097300548684 159.289148392284 156.330571337475 135.407536491819
      161.202844994203
ELMOD2      175.752091217351 177.001637712889 159.765585531442
      220.330477277988 304.058158731957 195.577739376082 264.054997398621
      212.710910778949 275.509805464276 263.295012964476 222.848935978648
      252.628516068724
```

ELMOD3 177.851689709807 157.328404229451 154.778726823719 130.616175018332 160.949030247406 150.602976963975 156.448355269561 170.007845523415 147.553953240464 149.103778494524 174.749832388791 157.81327663167
ELN 244.307627077001 212.987082617173 161.916155040522 297.176755638159 153.412469512967 172.599654598668 152.73588666971 155.061749542085 156.280701500421 156.723502568908 146.845170370268 149.37190049932
ELOF1 364.527677995774 377.148528768679 379.788696204016 438.817248820137 444.663812851378 479.866450988091 374.033009122079 331.062744782933 315.513423195489 445.089930641391 306.550948766362 343.617417488962
ELOVL1 246.63579788514 252.10470581893 233.07015384759 233.572055978465 941.572186137467 661.826149512811 486.425387717472 503.967725251906 396.04031733841 415.497783305278 565.164961255054 626.857289492802
ELOVL2 123.413596815384 129.85040048805 119.636334259263 119.122305582754 123.323528039848 134.686251400642 132.622032754473 126.956303896995 122.449574852865 123.615802924067 135.758133410577 127.265211069837
ELOVL3 136.660600727111 168.37043566813 158.895584550655 153.178934750919 151.034392433934 156.487049947247 140.845958602151 157.322594990954 163.821142892643 161.903016201041 131.945667475797 162.161282071736
ELOVL4 295.678097392123 303.051124835907 283.084102918359 271.522696252995 162.599466021255 252.365946808078 193.472317111841 133.318665755015 166.335294339013 384.296190598736 157.555410977564 139.883360543042
ELOVL5 377.462793982812 287.558897878375 335.858047332641 285.30221551106 431.686900474971 341.361299896997 408.361368902627 420.647394044431 319.768792196774 341.43511159996 234.399431238026 351.854828413334
ELOVL6 216.805365763069 248.887522059485 226.893147371644 221.471811658459 375.131728190867 467.347509383673 456.038981546175 659.900771633223 567.317223907142 393.707031354025 1091.69584249713 725.443026984982
ELOVL7 150.927628184034 152.395272857974 134.928967716193 151.459397907666 152.733409144267 161.735609436394 158.120079903425 147.872625576731 158.859389972316 207.150354734189 152.415752253205 169.168869363848
ELP2 570.916936074747 476.249239197777 793.300636780035 517.529147171876 482.592481827722 609.504365141529 734.139557942719 637.892677173103 726.346459754217 614.094097408035 1008.875250028 662.58366387795
ELP2P 135.098343717085 158.287108069245 135.441550832737 133.470643135354 154.455641374384 136.603035996281 127.6516565653 154.627270742193 130.14600240687 158.039920243116 142.124876403709 128.147484804104
ELP3 804.800251415824 757.066786599446 717.686676823785 979.019125719101 385.584224833836 427.151797273226 502.170430910893 311.544429285897 1022.55215097491 509.01330998156 601.018867455333 536.253405705975
ELP4 179.460777888284 206.164058935215 195.906137340918 236.470206707351 215.81995779019 140.82351325907 164.043866642712 156.385100395531 170.179304745674 219.901025447571 205.57572471694 133.906867716171
ELSPBP1 115.299986138153 117.05536677976 119.894737403889 110.17590675298 110.829107724132 118.712730897987 117.513523498725 113.627435441258 107.802792435066 103.398929051742 106.551076380988 118.625070307695

ELTD1 222.118666699301 194.281839133887 161.485353443949 166.70212615715
140.446530307169 331.164053277361 153.836520485018 200.22145130696
204.137195524755 177.94501172269 150.763810844645 194.352807599399
EMB 121.402905161896 129.845344493418 132.576755070703 126.391083453973
144.032779435658 128.202619705343 145.718019736542 135.417519318028
129.072173761566 133.827296071709 127.407418037777 140.343790129071
EMCN 193.134414137433 201.748876422401 145.700589723224 185.41739522833
147.710252756854 244.011434605133 124.00806841872 153.577349107512
170.298291784762 127.093255830978 154.812587513407 132.580937663749
EMD 2309.63504858248 2114.9754128463 2511.04500638697 1971.42527720714
2718.440149645 1321.17236442448 1142.44180956604 1029.09818399518
1109.08288440559 1375.76915021882 861.516925304722 1071.00507864299
EME1 148.847472886691 140.999877170481 158.999305963902 137.123016784819
173.761036260869 182.055718892894 149.664506761117 200.506615977654
171.759102794108 162.002221893617 152.495540563792 151.248365605631
EME2 139.273651128094 141.196795654442 137.374784875444 135.784481523923
129.788395150404 138.331509117817 144.112401069818 130.863753083296
131.911579676047 129.023206862713 145.612695657243 135.764392116216
EMG1 393.013539328766 467.777995124655 434.855262284265 389.177193459
563.490027149224 501.005898087825 883.140984728615 977.999660785113
707.048754700047 777.410297605252 745.482863218223 737.843412273272
EMID1 145.971656491073 144.937697857147 124.646093292382 121.110498703017
151.453985465369 139.161483596756 141.152406037213 139.921002429353
147.512290383685 115.371180170291 123.179986410723 124.901759819007
EMID2 121.181654892305 120.757288533244 126.199219102601 125.839101611277
151.508738608925 121.723964989551 129.185514320138 153.219954133957
117.193620642373 122.941711366641 140.093412995935 121.377666938052
EMILIN1 724.607317443904 1082.75419087929 650.494111214312
1969.67552562117 142.498280938736 120.594874820899 123.868848032411
144.120106441052 158.562631157512 167.751604259111 138.436029489092
116.547306374097
EMILIN2 418.163219634152 358.897128592042 243.772412032046
455.21285026588 214.904634494921 461.822629852953 556.725550806207
412.863887464545 518.760142491104 249.292766428035 226.678503956411
469.64693728427
EMILIN3 123.150299476109 113.694351010121 121.47058227778
117.706795566317 112.666812944192 115.277302351501 103.168665937677
108.296042016208 117.642441794442 109.003365273337 96.8261937365062
109.242622591321
EML1 617.474181543858 554.115756730498 667.693216401836 694.907086612929
223.128108720503 170.910998705968 146.683594365673 150.191645194625
190.389158930835 248.562843471699 153.992763544073 151.372219350291
EML2 238.724909152512 211.438615793742 248.643311972844 243.772412032046
362.445813697676 195.863843860549 228.384686248604 234.661864595717
180.20999937977 220.999383789474 304.292209676095 255.74096237558
EML3 797.73902873919 849.389818300305 586.867772838753 859.113541787323
1323.05745869565 748.940715378564 1037.00025848491 509.988617641229
685.848203651889 648.38049615541 983.777608766079 844.778755052304
EML4 647.439372562271 576.636077770998 633.104379362137 504.436992949763
509.512410867348 1185.70008959602 1072.82323999638 1102.73689840572
784.170983714588 576.298671053238 1001.65849130836 981.781076110568
EML5 131.347213356457 127.229854957588 138.887159625058 121.438875373878
130.844272373448 132.021249712589 132.198536906752 142.859971805867
133.446934298801 154.778726823719 140.750004067571 129.176532377543

EMP1	1558.22238167317	1559.26487922551	647.955521647635	1390.95426299323
	303.005912224195	1314.62071412501	367.518273773904	494.875547886209
	525.920873434688	3046.47354587949	359.256193328366	396.781827940009
EMP2	109.333276735523	108.895922800601	108.830525786872	115.445219455559
	130.473842500245	121.027465953657	114.498610080481	116.19219007097
	100.966979609222	130.878694881324	126.737230874367	126.305273697185
EMP3	2845.0377634896	2261.31473132315	2112.84348677276	2197.44189747261
	444.712990986138	316.081825144651	265.536429548886	431.974037914233
	899.336817124312	796.439819579938	299.29004204677	280.414161197286
EMR1	112.268518171041	112.758617084341	114.334059060506	107.993707827557
	118.091125088778	108.779204939177	121.381365873873	115.072622367144
	111.62514398781	132.902437860565	119.949050292223	120.436408685057
EMR2	169.619338456208	147.696506705909	151.092066533546	152.486212022518
	144.930066324767	165.436881839632	150.409919207059	218.350166753833
	240.706548931192	223.765583953184	190.357437485436	172.652645920958
EMR3	118.215641556163	115.975794003391	116.373841472226	115.766358387923
	121.865696440776	121.464383202986	119.636334259263	122.941711366641
	131.641435734821	122.89752183991	129.896711442418	119.455615461113
EMR4	131.532660547328	135.037385686705	146.550421651378	137.911816180518
	128.864822736402	138.399665664034	140.36278240974	140.605866597575
	134.993349945063	141.836648910979	133.490805791504	143.061415008831
EMR4P	129.228874611916	139.530196462344	126.573673878042	121.526724715417
	124.001295849091	130.792692240592	146.231937458616	121.294366964074
	118.783904603368	124.130599753093	135.323341505875	116.816688899055
EMX1	135.849839822066	144.00214898009	146.755803731358	139.989830921287
	134.226711030456	158.610249594568	161.441613587571	147.476805635744
	151.702809524494	144.836902182737	130.410584413031	154.615097099186
EMX2	227.467161919443	208.816482659752	238.149614104568	183.081450609516
	302.768870633398	174.068974937318	185.639696270808	258.964323941464
	179.55905250703	155.813740201987	290.582957824549	149.657951204528
EMX2OS	244.766289564524	256.093363520707	334.607533045929	
	294.746622369436	225.169110687004	222.943046618742	239.12905945808
	281.846246549202	201.235056368151	234.370850809531	247.504198063293
	221.635333304767			
EN1	123.545404885618	119.029186503752	116.670491972948	122.284431229917
	123.551225884748	121.360822445187	121.830137285032	124.405905157157
	120.897228878841	118.991494185841	139.291775075915	136.446877465751
EN2	118.180919555585	120.979417552159	118.689408983786	125.000876313893
	114.598636725763	132.115817176834	138.508015596135	125.489777118532
	130.073005698714	124.362182111222	138.152641686151	122.714122084817
ENAH	892.868661511516	700.877661166739	730.988069115919	811.564396886535
	155.83143112621	274.14931964455	182.453722105813	147.143407224469
	161.852665901359	163.451063547847	196.583548193097	193.813717161291
ENAM	123.875478428652	141.954467306745	138.572054821151	165.813771791917
	176.47556448977	171.39347085745	177.652022895288	154.277670551775
	155.511086289504	165.71382076724	135.862790395984	148.578688042147
ENC1	426.548994267836	465.029298672645	186.033093285894	201.123529130035
	305.542978609337	347.557792829093	312.903508201275	323.95355217044
	671.821418586421	315.828726297864	464.128022170285	359.493075382333
ENDOD1	339.663295617139	384.365878895439	337.434882771953	
	274.534901548678	165.406452831375	197.526492324088	341.361299896997
	212.119922561453	180.690409026809	743.023225859813	207.815226999953
	206.203717450138			

ENDOG	172.338509138759	189.647009287524	192.009562635198	198.925776755081
	720.710754387733	237.906142045719	164.587657424569	208.270622752385
	233.671878221507	264.201932544662	382.233826557803	253.697419495959
ENG	457.779218916808	574.896304593109	247.280717385562	700.353187874339
	564.749082802433	290.288001750744	210.043556816529	274.202831856221
	357.835334661098	275.609716327452	242.570588364106	264.233381434738
ENHO	154.834679562341	157.66595229182	140.03443832328	170.516743115762
	168.552631639996	155.522274777451	156.609370182062	153.448882783807
	150.645487532549	149.678629612616	163.053121879323	155.76860362365
ENKUR	153.519207482423	146.856133875296	137.717859035645	132.425596223397
	136.436762791912	141.356127724777	140.012295249756	131.804952187685
	140.40754434058	144.748564682545	137.53152210411	140.222121251146
ENO1	4882.16892310839	6179.86800320807	4875.22637286719	7815.98936145067
	18287.6015702456	9470.58919937464	9820.93899217345	11017.1478502767
	11392.4189359453	13700.8527522612	6244.58472720999	9099.13956219312
ENO2	932.076509242794	850.125434374005	843.545962045705	1001.92763014266
	326.325210657599	186.021181691517	550.434931878586	166.422360032182
	221.231455446574	918.162638134305	222.709720471134	298.209907157762
ENO3	239.109163320417	238.344676327918	217.050602253281	315.064477782052
	155.896065329825	192.96586114861	153.589626253969	164.525784478026
	161.035985824654	154.834679562341	178.476483428474	165.602175302848
ENOPH1	411.968307579588	373.860920943049	442.766704003254	
	357.063270015318	687.494132125365	501.946140974776	888.706978062337
	767.054576874115	1030.97051104597	745.482863218223	650.263737836685
	516.577357935963			
ENOSF1	385.888395433066	419.4017188518	722.725186727796	
	333.349203784021	410.337491313251	607.499206410372	654.305724263754
	910.541650889451	635.234445318087	1018.38836615952	468.569725051459
	765.128667448261			
ENOX1	160.105903942674	141.831596320865	144.804829253992	157.736101691222
	117.65847906412	106.576058729493	116.397855001959	119.202966460612
	120.862516074358	115.830631252469	135.855243915966	104.544792793756
ENOX2	159.005459875338	163.915489303508	171.038941092587	162.620593985693
	153.647343267295	173.839053227554	149.390481900424	164.717809928624
	161.77611826709	142.548833391567	145.369791572077	145.813437992703
ENPEP	164.299585578035	182.42088387266	164.748676009072	202.114480291084
	168.911840239685	148.695212949639	166.582931148846	153.893753448473
	158.282899758551	221.685824197352	185.071775840607	157.77398996415
ENPP1	199.70041209487	201.637540930213	207.902853533132	206.585387130993
	140.381386314817	144.252481420079	144.377501600001	130.495294897642
	132.579323148839	144.73389393695	167.67952650068	130.09958025354
ENPP2	454.492780042353	365.286234166621	193.832033192375	252.655315526015
	167.559703634539	224.528190554963	491.709723745226	310.426513884979
	274.595218795127	175.693873820963	286.945784387294	164.126980102525
ENPP3	141.503351545458	127.263061874385	128.591292689996	118.388993232718
	132.236355533277	123.711828013052	118.578205738502	119.094868654252
	118.777105073561	118.039780360151	138.261506562904	120.13937451914
ENPP4	181.54273463264	173.334154083555	210.33390115739	149.919344933814
	197.554105926359	205.546628064296	292.965109701719	249.489170536758
	197.740912476527	224.335742510577	213.652091843896	249.680729921602
ENPP5	399.664121956178	354.067771806542	360.991542429346	360.185005480216
	180.231462528702	157.055133233709	373.925939492544	254.133112176024
	221.297127685676	195.740661051834	242.221324619171	223.747350951099

ENPP6 149.467626202395 169.124088593549 175.693873820963 144.214589452164
127.088916295784 113.78323485587 136.83470470035 123.943080151532
146.722211039754 129.724604278515 123.267817390796 120.571074646092
ENPP7 141.645906784244 147.500009766324 143.096097305398 160.035282578603
149.339492984504 143.086872363116 158.691830524781 149.498020676126
123.847186739627 156.813992534584 157.885229749634 164.284396793834
ENSA 251.61451602376 252.514569129392 311.375563161772 254.607324522225
511.374117402707 624.825216056495 492.534183006883 542.740017003369
416.392874553797 362.899103657252 551.92688162618 621.354393918348
ENTHD1 114.528407572496 122.079685222733 118.011195631598
115.702900810606 107.828378413233 114.441223528256 120.910703698292
118.731960910224 117.186546702764 108.461141538047 127.926270257252
120.006982591236
ENTPD1 383.682951666155 367.644907862372 410.533157400023
405.570281788901 142.956870642583 174.526176588454 154.24500773656
239.941641600769 270.727749014046 229.130679600654 175.363223521128
174.441287575034
ENTPD2 129.025000034273 132.983739057264 126.640141760199
127.274768398849 136.893392154262 140.784853736558 124.126715773068
133.44592405762 128.765369834041 131.338853426561 143.728569494708
131.076562651831
ENTPD3 205.149094703318 237.113813706224 245.153736298067
201.365457496683 510.811470658575 358.152044880733 1193.82799849555
252.32907388173 294.323621186916 307.316241247336 2007.0121037284
711.729857540712
ENTPD4 169.705236302448 172.783430414523 174.332913952768
184.204036882642 170.266415287865 227.774354299962 322.302219773624
165.972090075204 210.266075382636 220.446775597113 273.402804140541
301.638918685657
ENTPD5 187.264411519101 175.28261759639 193.974325897472
173.063002566542 191.335616210093 180.008152341531 242.971958493453
326.176058036757 187.494677180331 178.425137262105 218.350166753833
192.752224665459
ENTPD6 244.280971257965 246.616749748028 244.117082987416
253.580286414227 403.984761333129 443.732580517751 357.35857422585
282.870785313621 294.808980631295 269.567704345075 228.001046173405
310.917191279571
ENTPD7 180.231462528702 169.642188114285 152.439608473915
142.436624546651 203.19299227665 235.563592256037 296.428342247529
179.250373572781 252.514569129392 209.130260450131 277.75958501062
177.379186278216
ENTPD8 128.463982896081 130.34939602913 121.260355515095
125.683334853039 124.749727939593 122.568743478314 123.908458472687
121.71538073735 120.050548624975 123.45956380046 128.419406507915
125.199837162105
ENY2 1025.27246494218 1000.05896885496 1029.43177809276 873.408431189194
1940.42283070543 1648.22531304741 1547.16936835284 2096.25803773108
2050.34883263932 1706.06615968622 1184.3199328099 1417.76295714364
EOMES 153.132559737356 186.515784641119 126.129564039673 114.785989868306
136.556183079815 133.736252068443 119.097521397712 172.857643600385
199.798533722581 138.307574579062 140.242272149424 122.297401003756
EP300 244.596233138747 246.186104432792 299.851173200352 258.075566417241
229.31486399741 304.402066421813 233.300864925399 245.522497675754
249.354198174438 264.456926651554 337.973433871323 274.595218795127

EP400 574.399909181723 589.070196307574 530.819778703194 608.351527783086
304.844121431627 486.334840298921 447.579975492958 418.420753332232
516.892850913889 341.197826517049 432.604462607037 493.887026009442
EP400NL 133.654232991228 130.949405011154 127.358616953147
130.095585254401 126.237900397603 134.30624644595 123.974409226349
134.85809559975 123.875478428652 130.270184521521 133.944538335319
134.553754121862
EPAS1 1373.08023518911 1690.96371604294 953.861218000678 1106.21439150501
3103.4192907748 3536.14527317085 1458.13354165836 1492.15137418791
1434.1439428394 1475.41920838384 1285.02292965374 1133.87328758676
EPB41 136.591299462104 131.336902822702 138.519129384809 126.00744446299
139.913180140881 170.95650329595 157.11867820027 158.779261405573
166.637846390031 141.567425892004 158.205235596076 163.36682525399
EPB41L1 173.66499836293 179.923966067226 176.056306041601
174.909258852845 206.071709262227 171.014432075829 229.116153948948
174.603285240228 153.505155981348 156.144934427609 190.077302802065
168.660907237558
EPB41L2 487.07860019179 426.649095732979 382.069677030369
365.362177671146 198.549047189499 182.346834032145 201.676984786688
192.992546726522 194.143649677822 195.269099451599 224.869569455052
217.227441947056
EPB41L3 257.132618311227 219.687151891777 201.517356610591
200.668298229815 171.650247980576 163.239195865198 141.725882943927
162.656951764783 208.257420634745 461.581199513891 174.603285240228
133.048720362039
EPB41L4A 180.315825600347 193.052112923573 225.130011390856
202.397972518715 191.25724234927 189.662974464847 229.62164568989
192.96586114861 196.031541155674 222.285940575778 202.357989894268
192.891507272665
EPB41L4B 133.190147746722 134.299575239865 140.89390566467
130.644937322186 169.656238760589 143.750656408155 150.974364125849
165.467462247066 171.909358721737 143.686906051698 166.47851613068
140.794929983858
EPB41L5 209.60319248212 210.550995170071 245.522497675754
191.628693083169 159.862252588012 183.26554404668 169.720414035325
204.470661830972 162.723310549554 143.942810095867 171.121834829333
162.244691888031
EPB42 126.388778582602 109.770126492743 117.48427100606 132.762755202326
124.053190169486 118.802030418792 121.113186311514 119.297561749232
121.699702849509 128.883828419932 115.101971719657 115.639101191444
EPB49 144.55710963567 166.47851613068 150.075738752092 144.731299077209
205.52620827767 130.763957515652 164.942652212218 136.734131465433
123.111192189217 139.312636530887 168.347445148881 183.181125586276
EPC1 224.707455448421 180.276082632155 177.772019227116 131.704192327287
164.520247586053 151.891318080151 137.21916902421 126.315423596685
137.540275229889 126.84966022925 167.011846920792 145.900272803108
EPC2 182.222567053422 183.193029399643 163.102043862117 201.702688815038
143.555987405898 131.261903118177 140.634047988278 148.454344601562
130.491742824575 131.563719927798 181.088268167074 138.861385127242
EPCAM 145.152605166094 233.71520737817 164.603403251634 139.59941545613
2036.43904731226 1870.52504853779 864.547109080675 1507.39761667421
1718.80498208397 588.458314503034 832.014011481807 2183.18692530644

EPDR1 1123.93087912174 800.373159084181 1003.36623972843 865.800709006332
321.750495072759 142.33269270703 145.551717838423 662.958637409597
454.984562256248 287.084253886676 185.41739522833 138.201164395948
EPGN 139.709959820327 111.032156655273 128.327528039933 130.760835441776
127.806138997958 125.598828334465 129.161305053472 126.830269821364
136.022614387461 404.54681330209 139.444577046509 126.899985772157
EPHA1 162.513107496558 177.457362387052 172.128489882364 153.453969224725
878.520681466946 983.777608766079 504.709613434391 356.921627358141
399.071041584646 612.06231165648 213.531307836195 763.880957432979
EPHA10 137.958714945718 140.890227770353 140.026918871291
137.263202517233 163.248980088946 149.869314204354 147.017958916908
143.096097305398 141.36525744062 148.794293762142 164.914114315732
138.842935439006
EPHA2 163.809967446414 203.298764374262 156.265168005937 179.067275037015
486.524284354103 421.836020491624 205.403820910921 220.633692394229
320.305852961452 422.520372346993 319.090371885653 331.684298221133
EPHA3 229.567444213626 243.246632667118 167.887047631073 218.839681873515
143.655901618594 154.701413581915 135.726760147904 147.174977926293
140.946008063839 133.985582992986 129.417232210341 140.898380345452
EPHA4 187.035957486928 192.914362049985 206.502189514795 189.192494970145
174.587558727209 435.183433729519 237.601064742453 188.959655155629
219.171591031864 299.669394721682 292.429831186609 286.587945301875
EPHA5 121.952255635669 127.174074003712 114.405811161631 119.135701239571
115.840266274696 121.846349358116 114.893462783007 116.019123495537
110.475453268997 115.923424095859 132.956860864455 120.948246352846
EPHA6 138.48552149031 133.78629612147 133.259321087534 136.274199465472
134.888404433315 136.645091356305 139.099483279141 137.748443205818
133.478829770103 134.66169946771 130.083266044947 134.418976976995
EPHA7 117.442158626871 117.218616752346 128.463982896081 113.370762349233
117.77708734157 114.849872458647 120.944267930809 128.36533737469
123.086596566142 119.463337435512 125.013839352291 119.631847624882
EPHA8 145.588981165456 145.929907161493 154.996829706812 163.946569217706
141.329559699056 144.840218312168 161.144841967903 143.494511683426
150.72835989269 149.016767620428 154.160508495401 140.319851351329
EPHB1 374.106258053201 307.945884504629 132.024244263352 135.676854553162
125.800508653231 119.701313125748 131.617027772841 124.328903559955
122.955724972547 122.317877997527 136.522590104535 121.327397317398
EPHB2 149.423230365631 134.932416370825 130.962368929833 142.690911343704
199.947823264085 150.215811815246 147.606311227037 141.239354296152
140.251168248117 134.087875547168 166.635366968851 134.312156667838
EPHB3 157.809081184874 135.465076459043 142.765490244223 163.858222964048
247.111032124864 135.860057860699 143.167719135403 135.338077911839
150.096558906597 210.015199832609 149.574353706911 115.821231468312
EPHB4 292.23287968517 281.119138040097 175.562070915794 229.74418058711
1061.29067124163 225.4575611466 286.282132382462 289.761702484888
170.410704499804 344.298510200653 297.285037835812 196.363005912532
EPHB6 219.687151891777 240.994507285383 179.648479201164 251.011148303246
501.301694626942 558.934004505074 335.239364345057 290.802509012089
178.083311435916 142.61573330083 387.827718022473 1205.45751308367
EPHX1 706.292905820227 839.662936279727 712.433345978749 902.089561157715
791.402660066193 261.426617866925 258.915802108562 298.028932089399
372.475112554793 210.882890077598 250.25959091612 201.041792159394

EPHX2 552.662610888399 532.063437013967 1035.98574557331 689.590642377619
258.552964322998 196.615914367563 253.327404004524 158.7912155022
221.18757533703 232.385176936256 169.534469894464 479.866450988091
EPM2A 277.596303701405 280.656087497806 323.628636799403 318.348505551265
134.828360289676 142.825203517688 147.893916750136 168.027892518772
150.272153646712 153.02549014611 168.05687638693 157.099868633958
EPM2AIP1 588.678767007017 650.0804221227 619.635734567779
606.679284487803 271.989060206794 570.533103778482 398.354212009426
292.524552445564 399.664121956178 252.655315526015 509.292779821787
414.403321220432
EPN1 1853.20812924694 2635.31067044245 1923.19560386389 3396.40593844715
6203.08085790474 2141.855356411 2859.94396089224 1623.92991386706
3111.50508060882 2508.50428529255 1698.08853832248 2192.17997650999
EPN2 279.202256032476 319.283603277754 292.317998405609 374.215345412947
203.510106134882 195.316077537716 185.830435830545 214.968589907686
176.735168607281 234.996704304673 206.68694753419 170.283532007581
EPN3 136.962442874268 146.537204621419 126.611663427876 138.279087298069
558.051041108282 352.50679573356 487.901310664037 882.037055216609
772.776364492813 368.418553384965 570.771089597761 474.517750753745
EPO 119.403831071089 121.459780480915 114.314425748312 110.333339548402
113.529619135543 122.494779505958 109.859878974527 119.739204799544
106.204350278634 114.084543168359 116.241531823869 115.29099102455
EPOR 155.358918578397 155.942609847585 130.928718312512 138.573829193205
170.497804969996 143.263333116046 134.265479018173 147.635761512135
130.883516197483 124.461997520222 178.186273279101 117.467618148107
EPPB9 144.340734361996 157.474913177177 140.648918459179 132.978746105877
305.768201359278 127.55397077248 132.848926011576 159.74220366735
140.971253689199 131.792339956719 150.902548360746 123.916497557628
EPPK1 144.352987324713 141.639216893245 142.804411455413 192.032080013216
147.691011072898 152.72717701452 134.268075583733 146.326067008478
149.300089212177 179.950904921667 142.278523641008 151.440732942734
EPR1 122.621839614189 134.803612821519 117.461198576063 121.815538475678
139.792313018728 131.218141814078 144.316475814221 144.464071397369
137.466291815166 144.801071221985 131.911579676047 147.330897105911
EPRS 985.637230639964 998.157643452741 1192.72383754851 868.40984928812
749.220616713093 1590.87426068638 1213.61170930345 1227.94401191112
1580.27059464289 1806.24732793591 1385.28044421961 1509.69760873558
EPS15 330.005950459958 303.138041758745 316.165926273381 338.223462327622
189.027594735456 346.118765910093 246.95071325085 236.651601701157
243.225815954365 316.081825144651 335.623336587396 276.961847241646
EPS15L1 153.059432055089 151.585818981801 183.181125586276
154.892452237027 181.668090319293 193.064450357153 166.311421907434
182.851203073653 156.62031503525 147.416991272497 157.768329108254
158.87755965229
EPS8 556.31484498921 503.093655556038 486.653672099699 415.497783305278
264.456926651554 450.851711160136 565.313436777992 439.616817413027
310.174932137546 178.166289723365 423.256021602613 323.462252156562
EPS8L1 121.320750074882 132.440818532628 125.18424162353
126.589865262427 240.376851873817 131.90169441925 176.631604815669
138.760800146167 171.784981874184 159.973758408655 168.244523461915
144.298011270428
EPS8L2 153.263883774915 160.73807125195 157.264673452646
165.163490032007 158.975786937923 155.018009545475 152.397984799517

171.97578836712 152.158269344693 178.606255846041 145.928643520352
186.185055364718
EPS8L3 151.338765231607 147.28750335168 153.440952021924
146.994103804515 144.107029869771 159.438084699061 151.051672692659
155.855564068891 159.06002608158 147.18948282156 177.343586047942
149.339492984504
EPSTI1 628.344122779625 250.443147156029 156.259214645427
187.519053043173 218.839681873515 168.007337847195 272.854435771465
439.394914369983 1119.71228054585 523.306451464643 481.933386283351
385.888395433066
EPX 114.174153178166 112.179019393947 114.913534674899 117.261443445891
107.655624022312 106.315801001506 113.091739139626 115.211731367278
115.435942397626 116.053859681399 155.438218501542 109.384112020063
EPYC 118.558673085303 108.04922729993 113.609215169397 107.520000793184
108.868679899494 111.515588041012 108.515401509039 108.955761698524
108.415715678948 117.361778263056 144.752418965039 116.609122768341
ERAL1 443.732580517751 577.56891124468 472.669956708398 735.8141982568
1939.1525084845 938.049312229961 1195.6335460045 1673.5387396582
1120.87534972745 798.943242964857 473.60579090007 717.025533065694
ERAP1 149.725422365342 151.07661060324 150.682964448325 146.781221728543
136.02474314035 152.729355306724 172.457165477145 139.273651128094
138.916830356763 164.092847913322 161.514651272858 171.350620456505
ERAP2 396.781827940009 341.64890727921 200.668298229815 284.272977025491
194.374817778819 358.298768623212 186.406439948242 511.374117402707
346.996310517446 597.651682540611 721.966539760341 1259.75918248468
ERAS 129.033433971346 126.009358591395 150.224472335504 163.459718635161
141.536764775726 144.4421922847 141.102274192049 138.796257513868
130.062015525637 138.158400190671 127.361974492821 140.531789506024
ERBB2 308.679189788554 379.016207754533 296.497100375888 320.467139255533
1254.19980463817 879.809795251142 920.840069938767 2372.26073354008
1153.5175534971 433.405369327168 1002.44383771056 515.929937825133
ERBB2IP 147.178598908199 144.699084515687 168.071171406779
131.460388562433 136.818557078622 169.441789096441 158.371599884762
150.799400717425 156.420501464224 150.031863363697 161.85913459364
164.146345008398
ERBB3 201.167206950576 219.709231012465 198.979744266277 183.493455947795
648.823677010146 974.544773560873 1184.88559645917 954.488185198006
1445.72064343302 398.423396126075 1109.79571066685 642.057106241761
ERBB4 122.095978653755 122.503984297406 114.249891266241 117.097159106043
100.216750525986 134.629996026323 125.143281492089 108.446988282853
126.298187538812 132.994716261577 124.378096177801 114.01599035448
ERC1 208.385295246155 235.822266856967 237.692959394487 256.394563537658
148.533241504738 143.063345350253 148.16526158216 153.711354087243
143.589222264786 173.552535486766 156.961070790639 146.157459765796
ERC2 103.36057518781 109.182623414389 109.228587997052 116.685023927951
125.498081339586 123.692366307662 111.639177843969 106.576058729493
122.405469833024 124.980683327416 114.805810485989 111.962852412774
ERCC-00002 124.635917999969 108.199628274798 112.348094614855
115.371180170291 109.506450285923 113.799997627317 121.85247363239
105.712302595131 117.101933186269 114.561836290349 121.919799968293
111.559325333192
ERCC-00003 116.540729581355 120.241528853563 116.280157890082
117.728410015076 115.058061620379 111.802672864815 118.021169080914

110.392617179163 115.440152891339 112.394223360492 131.345038923753
110.575582352222
ERCC-00004 111.810575716678 111.860763079995 114.68345964966
101.431474941041 105.193775204124 112.904318310632 107.466184687811
113.726931741236 113.077691687266 106.41329572636 114.888157310977
107.884870799936
ERCC-00009 127.718341524417 119.608334527504 120.672291867109
131.210866639392 107.02909139647 123.478896502015 122.719785082196
118.951526834915 121.997885377703 116.566190779365 136.578058206829
120.78129870199
ERCC-00012 103.772221923105 106.41329572636 113.209569103727
101.713609383766 111.616460084889 108.061315311823 108.079149792796
114.45792163942 105.665621968915 107.816312357067 113.773684331489
104.091301211096
ERCC-00013 124.003914369077 115.51493368527 113.521599852465
114.350596695469 119.057157701097 104.223476853877 123.372689985035
110.014935095391 120.114423236962 120.688909943167 119.974853329265
104.223476853877
ERCC-00014 142.191569406599 129.405749374776 126.158204790885
138.56812029378 137.134190411005 125.945916345628 128.019837242559
124.851025681014 136.08297876405 130.674263597931 146.077082896858
125.429085234515
ERCC-00016 125.902015309754 131.302225305148 127.604087759266
113.816165294487 147.790720062226 125.964335876018 119.237802375552
125.170874841845 122.816049839521 138.634814756987 145.372579207362
127.941584184869
ERCC-00017 111.639177843969 114.373370072994 122.719785082196
121.330383384978 116.46192274217 119.915330116696 116.987471433258
111.467722538327 112.332130647215 120.36402353147 113.726931741236
123.004137408444
ERCC-00019 123.571641517019 123.004137408444 121.898921780569
109.30275288389 127.42914358124 125.215194276842 126.129564039673
117.22284971665 122.027632589515 124.825103464238 142.442450622311
118.650091706226
ERCC-00022 116.825172441325 108.970432124412 111.783160346742
112.90900939264 117.897556445952 113.572326615571 105.551878332786
109.700513642061 113.479382235297 112.962180108363 120.456817031716
108.515401509039
ERCC-00024 100.966979609222 109.087574814292 113.238466329843
113.810645407837 112.07238018581 119.870661213608 115.594489123676
111.127245394321 106.285472845323 111.086170834553 108.036254606246
112.891050337488
ERCC-00025 110.651971902576 113.995301093609 105.521320791331
109.176852761745 113.568261995787 108.333135424315 112.449662536973
114.129044806825 108.830525786872 107.156457065853 108.131097605872
110.688722702767
ERCC-00028 116.007006375993 117.79844873842 131.426355444136
134.653131708903 125.405373274081 117.928456234796 133.050798832332
127.558034562151 118.712730897987 137.406420101257 124.145492642453
122.796362813922
ERCC-00031 108.101686347192 107.559685467789 101.431474941041
115.848659135231 109.016991399605 108.861243071977 121.583096465567
102.343275379164 118.891276329399 105.132185249959 114.10470841717
104.681530703922

ERCC-00033 108.266328505986 100.637271902591 111.538651711074
110.544044723728 106.470524594239 121.926377140115 109.96017424123
96.0426636429757 105.010952544907 110.636713168363 112.565341690522
114.664782541261
ERCC-00034 113.38168751499 118.372047967248 113.274668934773
116.204882835742 123.389038274913 115.321501844663 105.375878824562
105.551878332786 117.930665298163 132.066488589395 120.897228878841
117.60787142216
ERCC-00035 132.686915065196 116.282988576774 133.509538648848
111.989153105684 120.873985900666 118.625070307695 112.464951599416
115.949513659403 117.420765321056 127.972708116366 133.707285280448
114.032874859062
ERCC-00039 105.816457443844 117.289037817013 108.79453730066
107.365390754133 94.3531468700382 107.32960937124 111.616460084889
110.368392735494 119.375727296235 111.000303209442 118.957512510094
116.305011582033
ERCC-00040 128.197031700084 112.114933398547 118.578205738502
125.195549796301 114.867454554206 110.17590675298 118.148476979319
121.580726825432 118.684077097437 115.180659017144 135.33941967076
113.091739139626
ERCC-00041 133.192757350144 134.374551571013 129.32466533596
129.353536603449 146.185931276053 131.543439103033 144.46549577902
132.99989565368 123.420635380886 129.301839663122 138.730409717578
121.843992693814
ERCC-00042 115.608857734241 121.294366964074 112.90900939264
116.425301253959 111.722827315911 115.878861991823 110.910693353871
115.858924428037 115.392989580183 100.637271902591 138.231715814115
108.779204939177
ERCC-00043 99.5148073333731 110.437728036347 103.36057518781
111.922007318017 104.861024325857 99.26400442869 105.467761348787
106.41329572636 103.734303632543 102.255408120852 118.902823494379
116.588138374632
ERCC-00044 119.998260435095 120.786630750686 127.466779212194
120.100672968415 115.440152891339 116.437403962939 128.568463697992
120.241528853563 123.801380911257 136.05610450926 114.995889945366
119.315932907373
ERCC-00046 119.122305582754 118.833188167696 120.786630750686
115.235444833856 123.638001112969 126.526489290128 118.820154030256
112.520199390427 112.993452506946 111.603221942108 139.157088843803
112.78323842342
ERCC-00048 109.282814458761 106.876902604214 110.437728036347
98.6941019230576 113.927168436775 112.321396616213 109.209241676429
101.761615925097 116.604371744082 104.681530703922 112.675715371087
106.36673326724
ERCC-00051 130.820415610578 117.532063783977 120.126700838056
136.410841327254 134.498439965049 125.301602026407 134.102929804129
126.233395051706 127.189310473419 132.119370332298 141.141047405344
116.540729581355
ERCC-00053 116.074423050416 119.346574301289 115.834056091815
109.740801301993 126.786434897401 122.05954649985 115.47716627378
113.018985316061 111.639177843969 118.21084414402 113.536570797896
119.806933383138
ERCC-00054 123.273349540679 121.905741525277 116.958052905168
115.963144087717 122.84603217721 118.025603140924 107.07609243233

112.282839763467 116.305011582033 114.10470841717 125.908576249439
110.304102941202
ERCC-00057 109.66209172615 102.725375719353 105.284300851422
105.221735747452 103.772221923105 116.397855001959 102.166449349339
112.65454329872 106.066897223031 110.113844434493 115.68358702028
109.016991399605
ERCC-00058 144.904021527125 126.115828232308 125.674471551099
122.153996665065 129.779076484273 123.051777473512 128.258600768314
122.711143209444 122.770033312519 134.731687541347 138.778496466408
129.054577477882
ERCC-00059 111.055738573603 118.679664392666 121.014538677616
118.578205738502 136.511669406292 117.296972688814 116.793408204625
121.53632327554 112.904318310632 125.163958250922 136.348252715447
122.201293962463
ERCC-00060 125.937660544107 125.091038614268 134.952584272187
120.276236447255 123.943080151532 119.530214079037 121.567879358508
133.559082256772 129.933228671483 127.524310543779 142.765490244223
129.32466533596
ERCC-00061 119.658703392877 110.1278204598 132.049224908868
126.837151457845 122.220327032799 129.462319953515 129.121598974852
118.712730897987 113.440171712791 118.070716076005 112.642429076777
128.375390373307
ERCC-00062 113.177330690612 116.784024112826 113.177330690612
110.935688075738 116.647291878845 109.96017424123 121.860428893248
106.900679222135 109.167312436827 117.380281099932 124.019707408094
109.29279076901
ERCC-00067 112.371497988132 113.734280863323 109.669517182289
121.679013405559 114.906396746556 108.79453730066 116.20967792121
118.544870925791 120.184782039538 117.289037817013 129.272835188209
111.078930787656
ERCC-00069 115.981530322377 108.669141498627 110.296425545476
115.68358702028 111.065596151792 111.082550752119 100.477911152746
117.207266754941 106.797707723826 110.510191137073 116.397855001959
112.292129754508
ERCC-00071 140.370626016794 131.326536951329 123.732299841696
145.632060621034 134.73970457131 126.87882572904 136.381593416533
138.684070773064 131.679566454851 135.120878931851 138.712529531951
142.713318779614
ERCC-00073 124.684937344502 113.620070198869 111.506345174435
124.106971897779 117.399753685854 123.813766735115 120.387275261633
117.511162285879 127.095347480109 134.499588539919 148.326282600684
132.86711707789
ERCC-00074 128.837901660985 115.16087297104 117.580122770251
126.750850861235 116.328330266803 112.699674279523 129.725706669166
120.80221197797 120.027996187915 128.96181458192 138.867555269076
122.412141495264
ERCC-00075 110.17590675298 107.103635692135 102.661440700541
112.000309477972 113.549098821204 105.49491265382 104.091301211096
102.094370876743 102.094370876743 125.809027384863 122.682761393215
96.0426636429757
ERCC-00076 123.839006667113 117.101933186269 118.480344364831
125.497317360782 121.406758670435 118.724348027528 113.708683868028
99.0767996209245 109.016991399605 103.240215392215 126.585679159459
124.61344300674

ERCC-00077 116.810827219571 114.618608815885 108.43334699667
116.225440873023 108.483284007316 103.012774960061 117.069094451283
106.522641798274 116.136849683574 105.284300851422 111.055738573603
116.604371744082
ERCC-00078 107.260023460914 105.665621968915 116.136849683574
113.512670856029 105.284300851422 111.743461522689 109.282814458761
109.886197655915 112.081127853476 106.030441145334 113.822580779824
115.321501844663
ERCC-00079 109.438965262717 106.854721604889 108.984922760455
116.112013454335 119.135701239571 113.154880173167 120.365602682311
112.026113831896 114.849872458647 104.248226402465 110.694256186941
110.925532078514
ERCC-00081 129.865581506307 124.974113630014 114.91722828113
125.937660544107 136.330636532576 126.401775332213 123.055210862247
133.576812299149 113.163651806803 120.543881800775 126.435928518593
124.42974326791
ERCC-00083 103.594872791657 108.288420807873 130.186484104417
109.564709878576 107.961322525982 110.966212705086 108.2359175332
112.036753245281 98.6941019230576 116.998277297063 137.443463471483
123.231397809701
ERCC-00084 118.521075843163 107.580425848009 123.22295112139
119.192893408873 131.719227272527 111.895279866421 111.497241941203
112.675715371087 122.621839614189 120.054065939835 126.680992733621
122.878790127038
ERCC-00085 115.827175143972 111.253743009588 112.309528593741
112.987890650593 111.1002996982 106.470524594239 107.802792435066
117.593226427794 112.771141378159 113.071787850611 112.502812746669
99.9173294438729
ERCC-00086 129.208429861964 117.696262292883 117.585207821853
119.806933383138 112.340161674529 118.689408983786 122.68719599986
122.139832263188 112.896360244636 117.480506535399 128.713131994442
124.786029449452
ERCC-00092 124.995087711332 128.023203858837 113.886420801388
117.408465686218 116.844361858869 111.055738573603 108.43334699667
125.426018910517 119.459625817735 117.623103460369 115.890431424899
111.178024950204
ERCC-00095 109.113476990309 110.694256186941 96.8261937365062
103.734303632543 103.09089100768 98.4947439453387 117.619337212037
109.274221164316 109.471542280224 104.934718065476 110.068122112671
115.907902010701
ERCC-00096 122.575357101038 112.629266772219 118.663847950696
117.930665298163 118.797046082278 125.720102558499 119.135701239571
122.719785082196 115.337592860132 112.404562115306 127.404415477384
117.264555636952
ERCC-00097 116.514299377365 113.492900106468 121.059923601223
119.613985826308 118.537378726617 124.641892391159 141.38749436393
129.148634038268 117.546508214236 118.117957479382 139.103395748894
124.418295003824
ERCC-00098 120.235652849894 114.867454554206 112.264005988662
111.559325333192 121.021776527531 118.414329128347 116.378552206652
112.629266772219 110.242638217587 114.452922529209 134.034970203523
120.131839791515
ERCC-00099 132.066488589395 119.261305304165 126.042731009527
120.085340927108 115.194730717797 113.492900106468 129.888444399067

125.848597767733 120.126700838056 131.897352210959 123.654787271088
118.011195631598
ERCC-00104 107.580425848009 102.786517901308 109.978693735206
118.348153875356 112.747067417774 103.594872791657 102.474380813907
109.654666772771 107.655624022312 113.125349503734 135.058525398122
113.970633751086
ERCC-00108 115.863243613121 111.703224727793 107.303126530947
114.906396746556 118.201092260839 114.278113876915 110.925532078514
123.336074100984 116.90832995093 112.790552379447 110.753661013519
107.580425848009
ERCC-00109 124.130599753093 127.006746526023 120.727504500018
116.736174147084 116.236538651031 117.67638166062 111.810575716678
123.922817231917 125.027095330593 122.139832263188 119.418866166776
126.780096523484
ERCC-00111 121.640681916546 125.696434307671 114.768639931659
118.474291525263 121.99477069153 123.804039963953 130.119313219923
129.971670681199 126.279119798177 116.89487516867 131.027605835275
122.167669805597
ERCC-00112 129.049273337631 130.684569384626 116.456170659686
124.501262458393 116.225440873023 120.822923434486 120.85013281321
134.21363751173 115.468921844902 136.02474314035 151.293676705634
123.574319348191
ERCC-00113 119.036965883266 124.705614182596 110.935688075738
114.334059060506 116.0999658586 109.69466049307 119.382151435379
110.496084922302 105.221735747452 118.201092260839 142.826898321575
108.543795408713
ERCC-00116 114.078271807864 117.015097017246 117.467618148107
122.843792000493 120.216999784094 110.148555045276 116.597352307016
109.342230674869 119.029186503752 118.514618614167 131.136073942692
117.186546702764
ERCC-00117 110.942138645171 107.714294216913 119.795945889846
119.159078990339 115.463150857002 111.975027473284 103.240215392215
109.91537150286 109.176852761745 101.713609383766 100.477911152746
102.786517901308
ERCC-00120 118.294324274616 106.204350278634 104.154182930018
108.850992642446 107.450945262092 111.577400298092 118.505980204837
109.817705421158 106.685578858702 111.584425166104 113.154880173167
114.812254477058
ERCC-00123 105.467761348787 99.9173294438729 109.113476990309
116.021343227054 110.764586138261 110.694256186941 109.988237089025
108.818976813708 106.126202301438 112.851805868467 106.923686318112
106.551076380988
ERCC-00126 115.939620871703 137.700110923212 132.204721976343
139.429008129291 145.847629576886 126.865622462379 121.57069706238
123.059971161711 121.991692204056 124.998601194756 154.021906399992
136.418105816557
ERCC-00130 118.126133973555 120.270397272069 118.708644971927
118.126133973555 109.91537150286 111.956783714784 131.454955038299
112.081127853476 108.730058525654 130.718729445329 113.61408868553
114.161540771427
ERCC-00131 115.293844461817 122.051473363557 106.719519490874
107.02909139647 131.672014089487 117.270320709161 103.448244268961
114.290611267606 99.7487411813724 109.924867620862 122.94937342991
110.536316697732

ERCC-00134 118.816986517698 124.365185583133 119.144128641393
116.723991721647 122.676368520743 115.734230176652 125.034243098087
123.182903611854 122.714122084817 106.36673326724 141.524239210761
115.270544506813
ERCC-00136 121.903115540516 117.590393673861 127.675206134992
122.158160927149 117.141089194822 127.900901613784 119.603565559838
127.83654093961 120.255081121925 119.079680805247 138.076501233454
119.886433564236
ERCC-00137 126.737230874367 136.779180530712 132.295937828888
124.947565043721 127.769405736754 127.535214313797 129.171589574238
135.516856254532 122.396214045451 129.069366041625 154.509924503184
126.828097770667
ERCC-00138 128.798413084891 135.48250324478 119.998260435095
126.544685937984 135.208478510375 119.355650892262 129.208429861964
123.781002620866 124.031916322066 133.681703350531 130.760835441776
127.918847837351
ERCC-00142 110.858201415824 107.930325780047 113.018985316061
107.638492587723 121.274997609378 107.205247133938 102.024765473404
106.603674195422 115.085961282227 105.450273343324 112.046615825307
116.981243525714
ERCC-00143 119.06600048679 119.01236045062 121.643036773773
117.356389952799 113.334665910428 118.697969266426 124.327827104244
118.56332995149 113.857477830423 118.480344364831 138.264258708067
115.68358702028
ERCC-00144 122.631814400575 121.138461509096 113.334665910428
120.679474100322 115.400592594965 125.621467040793 116.225440873023
125.010266083832 119.360478719424 118.875640081461 131.924162435354
127.972708116366
ERCC-00145 111.09179957013 114.027244455291 108.148715413122
116.5272207025 116.839796680107 107.520000793184 119.517021355598
113.315182274675 114.790717658188 115.310953814885 114.504409331018
108.404733392914
ERCC-00147 105.859916438163 105.450273343324 109.728809191027
107.303126530947 118.841699953612 116.65880728655 106.876902604214
109.52020660772 111.703224727793 105.375878824562 120.208584170364
125.802001118545
ERCC-00148 112.081127853476 107.884870799936 109.355444907633
105.467761348787 108.939608619532 110.735199684144 115.400592594965
107.520000793184 100.04568139926 107.187035835972 113.888903174188
107.816312357067
ERCC-00150 119.366599958278 114.470670941078 117.662611650515
108.497209666203 123.696942540314 119.492844191924 112.314595956147
113.891385601095 125.215194276842 124.530660958725 119.746227888415
116.043095688871
ERCC-00154 116.939471420419 112.340161674529 119.862037840843
116.136849683574 125.940081665592 119.330439721904 118.011195631598
123.368137868951 115.496108000499 123.24533179875 128.372225487481
115.827175143972
ERCC-00156 115.316430915446 113.672605838627 117.623103460369
112.742895297124 110.810963139914 118.951526834915 120.441732791854
113.416743649576 102.786517901308 115.3611179192 103.772221923105
112.340161674529
ERCC-00157 128.126354569418 126.820834043224 121.297234504922
125.9888344762 137.264814431548 123.843823256462 125.584202037776

119.9652990395   119.934231033302 117.071585083051 160.766121558583
132.110906510601
ERCC-00158 106.953841653951 111.73388372572 112.624137732184
112.675715371087 111.848472437543 117.251233255966 118.81294782091
114.76337651891 106.102093734748 109.654666772771 115.53736269794
110.448948495323
ERCC-00160 118.148476979319 132.249906289203 127.182067293944
109.471542280224 114.936648783324 118.154953746492 113.822580779824
118.32560379073 110.315549501241 109.067239220744 132.471014832851
111.603221942108
ERCC-00162 113.955459033122 111.66210002202 123.822571859503
105.284300851422 116.67463130093 110.501341211051 115.778736387131
116.483432723207 116.947370388179 110.865668451601 112.14768481333
114.740948266517
ERCC-00163 124.551603326682 121.381365873873 123.716400094852
116.825172441325 113.09980008288 125.195549796301 121.150371270451
120.410307377977 124.68729078792 120.216999784094 139.220877923507
129.330592465123
ERCC-00164 134.255021727555 120.418709541104 123.88619164923
126.63730651721 133.517685144186 132.395993254065 137.246089511045
119.352995485911 121.18490040448 123.551225884748 115.848659135231
125.293640452686
ERCC-00165 109.700513642061 113.840813583258 114.798573331978
115.264104392064 123.364999283696 119.802832736435 111.708970469189
104.798333931019 120.283706459738 125.371128588617 108.646136431909
111.448871835514
ERCC-00168 113.091739139626 122.072091994548 105.408689610638
109.128394735532 121.519309043513 107.74537523456 107.714294216913
104.223476853877 124.116400777472 114.85602874727 121.792573534134
113.568261995787
ERCC-00170 115.871151134713 118.186426364551 124.989313193779
114.364953312191 119.198789189881 123.190757763412 118.655954393594
116.897299691798 126.544685937984 130.09958025354 140.307931763353
107.780763250481
ERCC-00171 120.511896155396 121.871105084095 126.606163970149
114.067790137477 117.790593990682 121.497645719165 120.473038066927
122.190734676247 128.020911959353 121.441901286752 136.72715888684
109.680532131035
ERCC1 314.386320310898 370.678040372273 271.858059712406 450.220658078322
731.513306289481 256.614964511846 433.751675703425 288.823376348944
298.573180660083 302.381946749711 245.607130404306 333.868818855397
ERCC2 260.129513331703 283.527656956105 221.471811658459 375.539588956213
308.974786799114 191.056705282378 250.037172478288 196.287131027947
214.662648581622 209.984545596312 205.381840116054 223.359785518285
ERCC3 361.969355316032 340.800632878807 429.268202514543 341.761516859319
303.09499683953 405.130899553871 295.047418816148 298.085365348496
330.321113875647 301.250189051376 435.095843349921 296.553550946307
ERCC4 138.000714797563 137.448412532999 162.778480318754 137.27298184582
131.566892082703 130.497405221273 142.543569032033 144.417333931582
143.180247878507 142.124876403709 144.935571704834 142.266177608209
ERCC5 845.150147618462 935.423045210635 855.415038498392 910.541650889451
280.337475979102 614.957397883514 570.916936074747 597.651682540611
592.957842630046 647.666334416707 1031.59140059071 889.054356462993

ERCC6 138.428527640896 169.132693907633 168.223865801948 196.329602378007
170.150889760372 145.281664250703 151.705219298578 146.455182336561
141.198692540628 152.755535408382 142.336164180947 158.125437004665
ERCC6L 141.577373924378 149.032116392457 148.390417679848
173.919504730673 188.605328108654 183.804849355341 171.644710031098
172.980441569522 164.50490221903 152.204730003016 148.247826830556
156.411563412665
ERCC8 131.193538235053 135.895945142328 128.434301921433 134.391386851101
144.699084515687 134.06996703302 142.385902610309 137.401137659118
130.270184521521 160.319687451789 135.51506573405 146.501559368223
EREG 161.099805358232 137.198654302648 151.770791549511 147.135640862542
154.205877662428 172.866517097198 150.924948809354 143.383697128285
147.401387099359 267.535469518664 161.979914539039 150.494260282384
ERF 365.199125315964 445.661863626419 253.697419495959 369.513420606401
950.818917078515 415.178470246749 344.128576341892 232.363656167375
260.224777636733 319.927209914181 372.088129459528 274.14931964455
ERG 136.403353223002 135.582230137515 145.766684470184 139.2454524913
136.003277940379 126.378952839132 136.173596381385 135.862790395984
134.961156870831 146.994103804515 126.260691770282 135.790168628803
ERGIC1 426.364663722264 439.928008927647 374.761007556839
463.99922423125 279.879377933144 271.713695056313 229.933577323411
227.418630852215 199.933148219784 254.909076658804 548.455251483009
270.421697521366
ERGIC2 502.063888359427 466.141717595301 459.973764138144
348.921877633633 366.961565952239 354.840474468573 595.752426322468
526.896146537814 436.155731212592 506.430593289427 597.189355986293
612.61438059983
ERGIC3 1189.69730508119 1239.13016938378 1220.30347076968
1299.4782626583 3342.8737952486 2863.51286169094 3430.76532174701
1867.55140010155 1619.67820476414 1389.93393245455 1261.61611961272
2131.68478964427
ERH 1099.37392456694 1336.75600604068 1281.9073569335 1023.94850651125
760.572409199179 1484.32547474275 1298.60552708737 1420.27222765071
997.460212152591 925.009157785129 1362.75971646355 1565.64079526732
ERI1 277.561376169965 283.499120551905 287.967349876429 335.858047332641
244.73233791067 358.654569870431 304.237479317271 414.797416270335
352.811351813364 297.757657398146 321.31562287169 316.623219008156
ERI2 165.781667492391 154.634289463743 178.245841646175 140.434086004204
193.261343922604 188.708596785144 187.389039221193 190.549084022965
179.94333102877 175.302832015761 189.90406457741 195.755450324985
ERI3 234.895245871534 248.403014089812 215.300064529551 314.810001770583
427.542390397986 314.610979086485 332.885488115858 283.27806319488
282.870785313621 233.757965816721 239.621756857095 235.997227173296
ERICH1 275.101766414328 256.471672362567 308.621688157016
237.601064742453 238.461248099002 272.456310079164 292.60467054075
344.487982154705 329.068463281182 388.887050777137 273.668871896558
357.835334661098
ERLIN1 172.980441569522 179.593817076317 149.138678142327
181.635664531851 205.630948393225 163.889522525848 211.419445018736
180.549865708041 209.596792486876 163.982415393606 349.485727214325
226.623172906957
ERLIN2 218.592402813219 226.086733606951 209.004374703564
228.588693548447 1259.38111177521 414.596948083067 285.30221551106

266.252875242286 191.207048581306 217.094085829328 208.983138060317
297.708113541379
ERMAP 182.68965110437 181.508873226705 195.239829623017 158.06915048955
201.256649338315 167.43630698726 176.839917795004 210.803130108618
210.761398461329 166.730974538475 263.47760086159 227.467161919443
ERMN 120.65473121078 117.735411259153 111.312348806957 117.703025026759
122.937281123348 121.837571435384 116.373841472226 114.960460615926
116.59404046487 117.642441794442 133.76515446791 118.401963042662
ERMP1 307.945884504629 459.068582584181 318.487401106209 326.379855547052
900.233783407148 727.425595270342 944.47112348734 900.039744829725
1151.89087953837 779.623566217277 2193.376951552 482.592481827722
ERN1 188.079188251085 209.867189613436 166.12695134858 178.429953283621
206.303338709554 244.440857688518 220.169099923628 187.686326872667
260.591336959281 213.966288689715 173.544556426164 225.546125484563
ERN2 151.16261275916 133.593569062647 135.073449743301 143.802883430376
143.496669740707 149.94715601106 163.876218743322 248.965394617795
165.985885671398 145.169804325334 298.926727289387 288.770330002807
ERO1L 196.053808536366 249.884250040189 214.803167803712 191.795952536469
355.609033348692 266.057472924341 826.08372791138 515.597523880328
291.839070398338 1838.18547839898 522.899203071911 704.653073777277
ERO1LB 143.650172124086 132.958521136 137.4552565025
132.533234335153 142.64421300687 140.349431258812 149.398118078302
140.36602776806 151.716042884808 124.375348764151 139.314945862113
133.307149027384
ERP27 183.988537525989 320.024259346646 248.791254839869 152.93058803161
408.510567121592 234.550692915716 1204.7397215229 889.054356462993
1746.30519466572 147.370836610849 1419.4638331856 378.4377409749
ERP29 1407.43503866211 1569.13457493554 1527.26678152732 1458.13354165836
2051.45788386541 1996.65760745757 2677.17579532816 2476.51542420593
2956.63335018838 1804.63762008613 2392.93562797606 1145.96710556468
ERP44 183.348764012358 172.128489882364 192.140442881209 175.363223521128
154.661456780865 179.048139035247 150.875392181869 164.429483677715
172.157660228549 168.963976596839 177.802767772786 154.755544586846
ERRFI1 1574.65731564294 1030.97051104597 1155.4487861163
688.915534194306 1594.05763963363 2111.32236867229 1385.28044421961
762.8576747113 623.68308750251 1030.97051104597 1213.61170930345
757.066786599446
ERV3 117.917454674671 119.517021355598 117.112667504781 123.959050197189
145.553219709768 154.21623304309 142.032814623366 242.902900881585
188.74403596953 146.215459988012 204.318316638312 137.391141254051
ERVK6 130.176734524308 130.955161943331 154.64077390011 132.243900762936
139.369815367465 127.959474148217 127.310437599803 125.517076778344
137.927403204849 123.588194516972 121.130837254861 136.254966566424
ERVWE1 163.704616231638 172.457165477145 150.945167884077
152.28262533486 171.97578836712 147.309245779031 144.064214573005
151.178586065066 161.992141305029 128.872496485403 165.45479542657
145.193296081527
ESAM 305.398934391579 401.750302347978 208.194788326109 371.941204080246
356.376890469558 275.841929188802 230.91747428831 266.756647182704
292.145553580042 268.660078500783 257.775569674531 196.598810934837
ESCO1 199.714647839712 180.035652063942 195.513703253634 184.482849019679
187.686326872667 241.019436415703 215.897620927751 247.806371601734
189.61702125418 214.739818213164 233.997642139328 209.662262708551

ESCO2 122.719785082196 115.01682934323 119.01990099776 120.571074646092
125.005019889478 131.823019555685 125.052770277549 130.169291786693
133.236084190243 120.160652344221 130.894384800009 136.71484425521
ESD 1362.1486006778 1250.58586679251 2037.96470181871 1122.92917004037
458.705688226305 958.005138077899 501.166869173253 677.802147190757
823.297606185721 1619.67820476414 633.104379362137 1309.01193284992
ESF1 137.760712896718 133.82390829608 135.536810684197 128.781123194219
147.891442774921 132.966873990501 131.792339956719 128.888149939072
134.06996703302 131.290698895743 128.314990390649 119.152881350581
ESM1 146.162330544539 153.360382235409 145.237221169839 152.213746577124
223.638850960288 393.707031354025 263.204269632206 209.417052464583
185.905819051223 443.444024128516 267.149525812834 403.104188866804
ESP33 125.905785653648 131.683942368268 121.400652285172 133.52971984184
135.357424384319 126.237900397603 127.961153369579 128.877218650072
133.984078167807 135.889764019762 137.089392202166 137.393871430276
ESPL1 140.099069486005 149.704586651832 138.323567041191 143.011042973347
299.513950278952 250.481442411876 186.1961391235 193.912456093767
180.022646625687 173.416999972133 144.019824700027 173.281506912652
ESPN 130.988294699995 120.41188773836 123.501843029993 121.628827418997
158.347972566681 118.383317097375 120.998665308561 137.973211667003
161.402322405903 281.304989265608 126.899985772157 132.146027935802
ESPNL 180.478294414245 283.048002376743 175.989742412417 365.864755316163
152.528571776589 141.229805348717 143.576177625265 150.626826756019
158.089922688997 147.547944397578 135.799692347002 159.289148392284
ESPNP 129.322481607056 130.209752198964 133.872561340464 136.788269716383
140.678336884951 141.884995734267 119.395894479869 140.964208731587
132.216453078716 126.676875319815 124.749727939593 120.862516074358
ESR1 240.465165615601 250.676092725829 203.586310608403 221.127492253636
230.201464318433 178.358003030064 176.001771318766 272.277433294605
147.433388299999 147.959740692766 140.938442055097 148.302775746627
ESR2 137.650917262528 145.802944788548 136.029898048127 137.033502466562
145.335830265052 133.892661981648 141.576177844986 132.458764637407
132.664379033058 129.171589574238 118.091125088778 129.233595525585
ESRP2 106.746545626069 106.576058729493 112.922918499398 110.325803953516
116.382348831504 103.550817366724 111.041656398985 115.033355516602
114.084543168359 111.568329540016 121.419301042072 109.067239220744
ESRRA 270.421697521366 280.292616101827 307.570405241182 381.867665449509
615.058712877418 360.053590374552 401.189904619902 286.469773081481
260.489744360233 329.023836559229 462.265785715695 398.354212009426
ESRRAP2 378.308562999445 376.627801547869 435.603911474926
550.901056837273 822.88742008931 507.342877360289 595.882696498804
401.542072222692 287.83303360511 435.603911474926 769.34961339184
606.162976359615
ESRRB 117.706795566317 138.685330935707 115.975794003391 121.294366964074
114.704831217037 131.597937570173 135.200049257744 119.229322984564
120.951365231971 121.846349358116 140.667415746111 128.462674862751
ESRRG 159.946124520038 155.965426559332 158.434048174501 149.269417496936
145.169804325334 151.043967734248 141.164985468046 141.797497681512
144.384287335249 145.932170561687 147.483379646393 172.251953149975
ESX1 124.837187124441 118.060781901686 118.497920969001 113.708683868028
119.709032462308 121.942777234608 112.026113831896 115.409568875238
106.315801001506 116.919109599361 114.115609163126 114.936648783324

ESYT1	2479.34567973462	2748.51360874099	1871.91894530722	3528.62443098065
	3188.90202907403	946.273229715791	1813.79641422879	1021.25947613827
	1643.08061372695	981.781076110568	1243.11900823259	1092.33328323944
ETAA1	132.371165851333	141.66312605042	129.580421464913	123.692366307662
	122.644105247178	127.333963230749	142.183154756458	138.333435927107
	115.639101191444	128.383541329939	169.561657768387	126.372374084002
ETF1	1170.88763035931	981.313778685019	1009.42265467472	1036.40082234884
	508.449904285771	706.628675634893	790.561585641193	664.809419789814
	633.104379362137	1033.48252432603	936.957730361063	909.020708316287
ETFA	802.017244099902	781.208872924532	1104.61581891596	705.737228226014
	1059.19317548869	2521.58055221647	1989.38201206555	1879.08108720938
	1374.92806285803	1612.79779117655	935.784743413185	1961.26255930192
ETFB	590.515758538373	708.268962600305	872.512754090023	827.863789600981
	1746.30519466572	626.298707790229	604.737834405874	678.684323057327
	1015.63768413332	733.86133557115	571.273512452931	684.715981522999
ETFDH	361.429760858441	345.897250519387	469.540421642173	327.324711245289
	194.88319590729	295.607161318725	350.392951395182	454.492780042353
	367.439588680092	283.235844022826	238.430350258686	394.294057009443
ETHE1	140.477307701027	136.438788088263	128.069504716124	142.934869757362
	235.997227173296	151.561151654372	152.303524109428	161.019770036957
	173.088291472857	160.085325256991	228.524222740306	180.070209705044
ETNK1	140.624437508178	141.999730335668	137.591469499374	133.292854943218
	216.24497587588	172.552923333095	170.522853717911	182.495117800073
	202.504840299605	174.08222895937	162.117224868654	161.510497517852
ETNK2	148.02460124587	173.024402954772	139.801108744526	162.975988989106
	310.815067427296	168.702991603656	207.581907737176	145.146797807275
	159.113259830623	163.643554173995	178.000672046849	225.438177833064
ETS1	972.468110850466	1159.44907165386	605.795439342384	939.213447046823
	705.737228226014	797.73902873919	959.897744425251	1238.11700377838
	1230.00744483191	753.997560938003	2068.04582900231	949.260897408911
ETS2	703.815551883635	813.034056288861	474.629173435475	487.901310664037
	332.082749517913	1567.89763050645	767.861763134136	752.142287661845
	1061.79595707572	541.351092873522	530.122419830951	659.27672123763
ETV1	113.748724650063	115.180659017144	112.578934972566	100.966979609222
	106.576058729493	111.691973758042	105.25877906031	108.570463496052
	103.012774960061	110.624328647003	104.154182930018	102.094370876743
ETV2	128.368669716591	138.889999702313	132.865704994046	128.144653206398
	177.825648107126	148.143367490426	150.508851767517	148.597346678768
	162.505305538911	188.070213212057	192.286192329464	166.396160626179
ETV3	175.395900648455	159.2488784104	148.85250180736	152.052084712417
	169.072574423443	246.152798696993	231.202047619476	306.838596239511
	190.871696848118	184.322250984457	210.605864685448	259.900447705311
ETV3L	116.653897702566	129.290542121353	122.317877997527	128.23432652395
	137.58025011566	119.281300456243	121.138461509096	127.095347480109
	120.644987579644	110.1278204598	138.096262211809	116.635455849571
ETV4	139.328340242696	147.748142622566	140.671406418004	159.148717478277
	337.521614253267	210.218440444631	193.04138438685	272.117599603586
	204.283030911897	156.466728833063	168.013310840048	179.509758601636
ETV5	206.972440734038	237.940015617254	167.637638693195	213.833894542292
	309.720081933252	241.293046687076	184.758754197435	149.202628825354
	163.215507313506	158.87755965229	171.426334687626	177.688568435599
ETV6	624.467319041884	751.896334334052	597.651682540611	852.649997188692
	445.763740779039	520.120193442648	809.545702751155	1332.11815966955
	829.992212133165	256.326661031361	1233.99503576308	776.98412594767

ETV7	136.032310569127	144.760229351772	137.745887988474	151.2639801322
	151.577450119302	137.962542261072	212.443407602682	152.548795147468
	165.376358421533	133.532323053215	181.803275243281	156.731291756066
EVC	219.878230463206	197.277310409328	211.692743811486	211.085778902367
	131.539170756533	134.914830257315	133.666244052469	125.974463216884
	128.745737514912	150.711857623087	144.772914483212	136.633726784926
EVC2	128.184708916755	126.87100632595	124.533543270311	125.868080230094
	121.438875373878	111.423140302983	109.898406285895	113.716741872499
	123.541709359809	114.631759839092	111.596690019694	128.591292689996
EVI1	563.779294315901	870.409935772423	469.407481002198	392.582612044755
	1407.43503866211	3019.57419253979	2084.44431604433	1657.72357544205
	2033.67956299225	201.865955150869	2344.11548437508	2313.56743052616
EVI2A	131.367075440559	130.953237161257	127.987449338817	133.443807124822
	131.72852748835	139.205887721493	133.337863400762	149.457761307441
	160.01685162095	133.841519663164	161.569793476847	132.07268641595
EVI2B	158.07640654075	156.420501464224	141.170937695272	152.038902807701
	132.693707848508	151.178586065066	156.003233745771	206.203717450138
	227.614623963331	175.767556472012	159.199045715212	155.129304059037
EVI5	1325.77498031227	1247.02514044778	1118.28423222707	1147.33034082693
	349.330629649093	1106.75785702993	702.460234202652	1061.79595707572
	719.369561410372	562.711562649704	787.425941491281	725.216684507759
EVI5L	484.764806127211	534.276129011237	580.164288826741	858.742139354587
	750.494286476465	347.411500022053	544.580311371212	392.824840252824
	272.712242893937	593.990687449346	514.213675499369	457.411248351795
EVL	2926.06746795504	2779.89882032092	2986.6208659209	3807.16206340771
	902.394410348792	942.900963522948	1474.70984232307	1822.14935116363
	2369.233048505	1607.52029676299	1856.05025952611	1771.39155164347
EVPL	167.55209828062	212.571491543077	205.52620827767	159.473580802408
	861.786094902013	992.320202251161	924.312051919404	1474.70984232307
	1089.58643309156	1840.14350831655	850.125434374005	1268.73280473728
EVX1	136.529820456592	128.455670598648	143.107570838895	128.179293878252
	142.303654405005	128.116795372698	115.507474401239	128.265571409546
	121.747622617011	131.850952248545	141.758827837244	138.936872956065
EVX2	137.28323311107	130.772410329685	138.328508662816	123.069963218224
	130.677737553727	125.851611268218	140.509950286228	132.634538733205
	124.160433356046	130.380766252137	123.077592992679	138.058404474114
EWSR1	960.491731797061	1029.09818399518	976.782815449208	1190.62010668045
	1009.42265467472	1082.75419087929	771.512332113075	911.340266389071
	1156.19575610918	851.221199352646	1061.29067124163	1051.98943987926
EXD1	133.11040099808	158.249743182741	143.712450441816	150.748298569951
	152.265624144393	147.393178410954	154.475735552851	145.485453268383
	141.278654291193	121.278346729876	122.592960588642	136.996535765175
EXD2	446.819873986024	368.879825610754	461.940616438547	348.808646570544
	305.577166690215	271.430915699592	419.593558001947	372.594990024312
	302.768870633398	427.452114719877	384.296190598736	397.153801851621
EXD3	189.082778234831	188.605328108654	205.679992893049	213.364858759772
	152.961475450751	147.463822782303	141.125248464806	133.345885001759
	145.03804642447	140.628614910068	203.149001616967	139.534014289993
EXO1	127.545397354821	139.147327302581	131.848213704003	133.239703270008
	168.116928722035	168.963976596839	145.783320434143	215.247472744419
	183.138955445765	163.125317704446	147.87699690647	184.451469623426
EXOC1	194.734255772365	196.615914367563	222.086635504858	173.424541543058
	194.979795724712	253.754869975548	298.519371102278	283.556453980797
	296.497100375888	324.56043349882	367.732191251547	295.778154263981

EXOC2 324.79327985156 264.233381434738 302.637859140061 262.691387377392
202.324795686879 219.171591031864 245.082816415008 226.678503956411
255.225145197422 294.323621186916 201.987081022654 275.696829424061
EXOC3 415.82312615045 449.523996620257 514.354512083591 491.35949123223
472.108893832845 323.788197265954 393.264726588522 321.627969147543
309.229066204308 379.862305342147 307.518881119091 304.751330890753
EXOC3L 152.585572167313 163.432972512188 177.11682975342
162.494429648551 170.381897441659 138.780488114634 146.432468585736
158.770502070553 170.198508633417 152.088488171996 145.079450753969
161.657344146676
EXOC3L2 128.539969915702 142.924854393401 139.85010721496
128.5581535393 137.091932621041 131.04767483614 138.093181144173
125.121517583946 119.83679981791 131.185836673529 129.831076624826
125.091038614268
EXOC4 358.975346234854 435.095843349921 357.928880740814 523.306451464643
310.174932137546 335.389274425025 401.87674893993 410.845034795716
369.373283422151 340.966515437272 348.711663415777 402.866491417369
EXOC5 205.821101661022 202.97197145851 222.268494113686 226.640323471375
182.397795261147 213.877482604185 207.204293555178 185.717123241832
183.863365305103 271.945851739635 188.847480174391 226.334672545273
EXOC6 222.456810024193 208.363905749882 231.698163424706 218.592402813219
170.320777845496 175.128935809466 168.783725505491 176.390014020936
185.046826969912 165.027938535393 207.902853533132 194.403848868583
EXOC6B 126.110426731941 130.150998843383 124.378096177801
136.030538642815 120.131839791515 130.473842500245 135.783125948784
127.350368333088 135.991630745082 142.259196792767 155.371772547395
128.074531806621
EXOC7 791.402660066193 847.564628337955 772.061917233344 1145.35733270036
765.128667448261 819.887322757281 932.505095432049 821.465895280454
920.083835296898 425.471195039676 829.992212133165 1127.89414462606
EXOC8 148.688413944868 147.78095608696 139.555322603323 125.384706864406
149.34605615699 146.181764812924 137.381470768186 140.559553697317
135.351344868436 160.773510498005 183.294526267593 132.562090247233
EXOD1 153.56082876276 153.126111237668 170.223509007961 193.832033192375
147.882530392424 180.493406236326 137.462720784077 196.942880967705
160.975741751317 153.45050352117 150.191645194625 177.802767772786
EXOG 146.873719524815 143.865717051998 144.822943770596 122.850678471084
139.799467834109 144.139062513587 150.439715066903 142.272117655292
143.259468398603 140.730085305185 143.899201949317 141.422441104854
EXOSC1 371.369895979832 348.711663415777 449.598181155897
363.445110627483 328.269845211173 420.502251256406 528.478292835214
458.782864873657 409.883222213537 423.826189747546 475.540659315339
593.320132696649
EXOSC10 635.536499281829 639.688482258192 707.173691257322
668.570984110088 542.740017003369 745.153336165802 894.46029531926
766.420275252501 921.140932799223 894.46029531926 802.643357549193
829.126529857044
EXOSC2 269.448201975645 253.495214569632 302.1846598234
236.899421827427 277.073510273425 242.538036654192 237.249949461085
298.664864578349 251.993821914623 264.273245283343 180.34788837703
271.087125858775
EXOSC3 431.019896339435 412.415549375221 452.077434390612
382.806570442504 712.94845413251 560.362502519639 576.636077770998

674.905270154355 703.29965727829 775.5664718389 664.60530723006
502.170430910893
EXOSC4 214.434355263652 219.246271924313 207.960802948119
257.5097052677 832.911633923496 455.564394647943 407.518324776453
340.193863653875 447.909277144896 297.805768288377 322.614169214764
337.935183275743
EXOSC5 220.896363324634 232.344545356395 289.911635155481
299.463563393179 998.157643452741 564.056592672374 588.863382995875
511.779988437654 563.907454700224 323.244664586314 290.184801898828
267.342817344746
EXOSC6 398.892968579918 405.183882455109 284.534369213195
377.951523644306 790.790236361922 314.90123632547 424.025588636796
297.285037835812 448.260934224454 714.052063877292 472.279492678374
483.549698382115
EXOSC7 456.656123023446 430.505037012814 587.026042400878
477.160464512705 972.468110850466 630.670530516275 784.72162514331
376.189276074918 661.545179083009 479.761165832339 402.213774592085
575.722713461982
EXOSC8 372.81255844018 386.598037190663 390.098830270926
376.189276074918 246.355720496468 579.678635803225 394.810886122555
445.763740779039 433.405369327168 502.281584081805 573.846138124455
632.031410234577
EXOSC9 347.620001353026 330.193132765826 340.42197893246
344.128576341892 288.88486628661 449.745016106912 413.000072500071
470.95418656881 502.935749606918 558.621429366984 399.872401268958
443.623904480087
EXPH5 141.857434100065 128.56495182759 132.71633377967 126.700147643
146.320758317435 132.409582164007 122.773736159743 151.248365605631
130.150998843383 132.357517495246 148.199249855894 124.183297351614
EXT1 550.671679318681 446.07651799079 429.595453454621 445.95114929414
309.181150342727 259.625943627223 337.434882771953 272.571092944711
313.412131722189 376.082040863747 282.742833166843 354.840474468573
EXT2 347.411500022053 304.679197422439 251.511405838796 286.774606295106
527.294087043601 259.146673652222 450.851711160136 234.182164952936
234.531140131763 292.640251411131 248.03801051527 300.482945704017
EXTL1 136.504438803108 159.218886890035 138.514356748249 142.812521349963
154.183537565624 119.755151432304 129.1829592305 135.092226300609
123.994188725172 122.079685222733 141.145419751161 128.23432652395
EXTL2 308.330044958513 289.09614335372 313.077481033093 279.921244070981
187.50236021149 374.657816575897 255.435582426271 237.692959394487
234.041863839123 244.897869154624 329.660368915848 284.715092831843
EXTL3 420.248838397618 496.426739836535 369.434158443561 690.010326122574
346.065746911798 272.01602406899 342.315456386729 264.526189799049
237.78482574225 273.478372861114 358.897128592042 261.721726664029
EYA1 140.447933562667 137.46978401237 130.257242615762 130.543453631154
126.476866025397 127.416912237613 129.114161128113 133.554822920765
123.735827460404 130.365804613342 129.980354138975 132.490323093932
EYA2 132.119370332298 136.118702626862 138.032265227728 127.295532954648
203.410932496844 162.828844182372 337.728737393381 189.37832342197
163.764600217347 128.517315006019 180.518621542868 131.08389186558
EYA3 135.461112284848 130.226993687215 132.615324904193 129.930888107958
133.883527462382 143.658600740177 140.48372620406 142.866874321248
128.700516033555 141.74792000689 132.421863261746 148.143367490426

EYA4	143.879597098414	142.397433929092	136.585541662028	133.611588728019
	123.725998173975	134.885883559391	128.090665893885	128.723261477217
	123.038646914863	128.720517906732	129.088718286391	115.545558600232
EYS	188.166168428752	191.335616210093	172.965746521132	207.674249122047
	151.051672692659	201.517356610591	174.953232190285	189.678680702271
	180.561514455864	158.109836032481	196.759802659759	176.964088090133
EZH1	164.072443259668	155.02097482487	163.248980088946	158.089922688997
	149.419308407564	161.73079180403	153.018378403144	150.45404183949
	149.586651393588	166.788336808924	165.307808075027	161.463042036376
EZH2	141.784881206191	137.449776383224	138.336667434109	149.795655754301
	184.819937046061	229.97640195632	173.204163917896	237.940015617254
	233.975864722289	221.424451092316	174.441287575034	236.707875629511
EZR	4811.88424516272	5955.10816224456	5382.31371568106	4527.53010866974
	16974.4822983201	6877.52553567598	10741.3064142855	8016.1652884418
	8211.38792799248	7038.48958943724	2185.46850787298	6670.51138785163
F10	440.657747083721	301.250189051376	236.108432603479	210.117033165026
	108.715328458556	120.809659457873	113.886420801388	118.201092260839
	124.238319768621	111.985567835684	118.206034087145	114.143874572202
F11	159.735182155088	163.863677822465	163.407360509803	186.321879879561
	161.046418048832	167.456481678813	156.866867371872	161.614426723521
	159.781313090489	155.014930599769	149.912481263284	164.690739760426
F11R	221.849661343124	283.556453980797	199.991077347491	173.281506912652
	532.235193634196	694.644744547412	852.436775264873	832.014011481807
	437.122373082112	718.871732206362	1367.92869625376	892.868661511516
F12	135.916668738753	139.835983835267	144.235298492095	157.295602588814
	296.497100375888	259.252807304272	214.595639477696	183.130891975122
	163.82704877497	1282.38953612054	214.684538620841	499.048754087189
F13A1	237.312766830206	216.457866890673	198.954254778846	223.778708427552
	172.980441569522	323.391456630794	148.868265867275	316.895545640437
	277.561376169965	274.77366169918	312.224309341171	205.047145898929
F13B	120.682485578097	108.79453730066	109.209241676429	99.26400442869
	107.013012418356	113.321454160244	117.048846466839	114.39161822971
	112.088944448944	115.389934936253	112.420505354139	113.249267122926
F2	134.70636200986	135.750578960284	124.033766796333	144.924212639731
	131.928871107722	145.058110060639	128.723261477217	121.792573534134
	139.609751976657	117.251233255966	140.149064156135	134.522715664256
F2R	15354.74541236	15053.7547401937	18953.8127741918	17917.7303120499
	14108.0461617941	17975.1602090863	12690.801964606	14798.1358474998
	15354.74541236	12368.1423473066	9715.20127757161	14544.3732040823
F2RL1	147.595573192579	180.80811429888	180.952017616136	140.864196804152
	194.388383297922	172.509081696309	480.712219116621	190.281570660806
	162.047249964859	708.268962600305	512.723592291378	414.333324610267
F2RL2	138.082870754768	119.072213573568	126.776406817106	126.784243009941
	133.345143691828	133.771691580031	134.961156870831	131.416923875254
	127.265211069837	125.077260389449	129.43892498257	127.242627779136
F2RL3	133.702845956639	141.801472885127	129.578488688293	137.778033050658
	136.891612156271	150.272153646712	132.620474788855	131.83430682495
	131.268454024753	138.489443610941	132.526286141997	141.13343407335
F3	409.883222213537	393.707031354025	178.320732186309	201.773745532242
	160.537464426971	306.333710880618	159.207567680134	245.01029221002
	205.509515144263	1116.95795024994	193.275162354467	232.250434241457
F5	140.434086004204	143.926968000575	139.557059245431	122.65547320922
	151.687402863648	147.722646851008	126.88066987372	177.955579729463
	157.410205523901	140.83570584033	150.468392290279	130.076926011271

F7 145.698637908482 136.394920983439 144.985280401441 142.19728670565
127.975441922402 137.687480242109 144.043818335414 139.871928127715
142.783159341229 138.469997677004 153.821728151219 138.903450375532
F8 137.354782768068 138.963864375073 140.29803170157 138.988919994625
127.927232724978 137.650917262528 126.728375196449 128.019837242559
130.385747820089 140.377179372804 131.132508349222 133.587927144452
F8A1 232.105867345662 220.379186481273 260.888612454097 287.455622143233
569.133295913677 226.960024551778 229.130679600654 263.005303710717
207.254220247481 305.577166690215 262.061371466369 292.429831186609
F8A2 130.430917518283 144.515002122324 136.908157358689 139.779754416105
154.2433882385 139.984102798711 126.178476831007 149.860490620507
132.17211158061 156.75308999904 135.959893035019 141.038721816572
F8A3 143.464614171516 154.954906872501 154.861507590906 152.925281215593
159.801315111886 144.863617174741 140.804008584006 190.520750756082
158.528301072565 161.979914539039 154.843607752473 177.752497164503
F9 155.756074127526 140.861849838223 139.628966306048 157.40255559397
143.1288704752 152.56002779985 150.687819649072 159.199045715212
139.708303388546 150.109192774079 147.89884142817 149.331805754859
FA2H 113.656820701744 108.589444216106 114.927420605185 119.746227888415
201.458970969685 117.390049487174 119.32696696175 123.964042357475
128.400741751903 129.083756379376 208.016730103629 125.887854850942
FAAH 156.68541512214 177.567833691547 160.035282578603 156.998563448664
574.262112643403 262.671819506189 217.380892198811 202.60339529874
242.848821222611 223.408447580188 332.032371697186 359.329520507158
FAAH2 158.163599942547 151.219051767056 169.940177050294 155.259089547817
189.678680702271 173.432960318793 213.382179917193 203.777944180067
194.072260528037 195.481680273686 227.965783945702 240.224826736571
FABP1 138.363553671822 119.005230164124 151.020903727566 151.554352209634
139.15175079191 144.589482298525 145.841028932318 149.280480796544
141.186913979346 155.516697616637 124.140880678888 131.346126135605
FABP12 130.962368929833 119.176139463015 129.176532377543
128.407968177242 116.346181133756 136.162285671867 122.268560536152
118.486195373059 124.501262458393 129.45936538019 125.26492723752
135.304167695152
FABP2 134.310685502031 124.555847618988 126.086991456747 136.164270397912
127.093255830978 127.030113960477 127.167918433035 123.991249190444
120.470801825675 129.365004129784 146.229239731545 125.800508653231
FABP3 207.204293555178 222.736706411035 171.722839172783 310.533399024851
211.916602665089 121.157614971438 129.873110494826 123.434963905308
123.439897913722 112.78323842342 142.77817647952 126.752999657665
FABP4 1007.7803477458 1744.58410715956 1136.14069801696 1427.70292953827
178.313238745665 340.193863653875 270.069634610818 7733.95252458705
226.871028543248 260.746952459282 15745.699731183 12171.1997841553
FABP5 223.107391048316 307.083179338165 254.83131041241 250.676092725829
827.863789600981 1291.12004949379 2423.84327263111 2867.63319136936
363.195371577097 9293.35991367953 2730.50913686613 2637.62596741408
FABP5L2 515.432065049838 958.005138077899 584.919615953457
634.356322976353 3262.24877138481 3504.77617298452 6324.87600301793
7948.88765120294 1093.85316636801 18146.8167991873 5981.08862268291
7144.3257417116
FABP5L3 119.603565559838 128.341887728118 120.773552653073
128.870079563075 127.560308850342 126.728375196449 129.153882779488
137.365567527202 119.315932907373 224.274329367512 161.002880798225
128.50733429744

FABP5L7 135.975265059269 132.416618775337 160.584760930779
149.408530395745 137.534079082878 138.846543789882 148.490236128772
133.367085445965 135.509927917168 134.074425424613 146.247825342634
143.313605534609
FABP6 117.77708734157 126.995207465164 119.161972571516 113.582654469735
202.024504592763 374.718289636553 301.638918685657 576.997804865103
169.067064352955 189.174957940401 465.54817048856 683.365770196967
FABP7 129.45936538019 128.032141013681 137.157492354617 134.772545694636
122.442590463781 130.306478656159 131.528729642144 122.254824223167
122.03508430361 119.525701528869 150.73248190848 138.76667538162
FABP9 127.099995420363 132.47775411384 128.671626815639 131.218141814078
123.259322051795 132.99989565368 134.301187092088 126.812867041543
131.200042525339 120.325985681299 122.959518532165 130.054105892394
FADD 324.615093103582 367.439588680092 370.617422894068 344.856199123543
805.875557314697 512.312880441098 688.464980326689 446.948699651916
475.213525346293 431.546135503733 671.821418586421 735.165830487162
FADS1 317.719381113816 300.314088267108 231.372316734945 271.397087928135
490.269418483997 187.016569445646 231.314944217099 254.036619126409
269.495247190598 208.555506929113 183.779065928447 189.985520850855
FADS2 165.154844613899 152.334669286305 149.308475163534 189.092674335503
172.583615020042 150.498954408708 146.311966562883 172.412059174046
178.751706380632 167.629008083436 160.454243375479 153.169450192935
FADS3 159.603586227897 161.708130705995 152.890239929335 168.668125438154
157.631978594005 153.033582154882 166.998311158378 150.489651880905
154.088770236213 147.512290383685 136.534812980964 158.23884088767
FADS6 155.566065673542 114.3172128566 121.336932276232 127.422129210789
130.052593375075 134.024205981268 116.58452862953 117.667611937195
160.724319010751 119.229322984564 151.226242696197 164.986006226427
FAF1 406.133430120039 450.99430272315 447.182566890086 576.451964662024
342.555640222963 358.975346234854 378.797952580504 364.840751972169
385.678901612324 412.582051585078 370.301819403832 472.279492678374
FAF2 947.425912541091 975.315904185938 904.733257358133 1033.03719406038
580.347496467173 817.036222521723 1172.96121022579 1076.62597781124
755.42757464615 1097.18236659974 1534.21175984308 1239.66725147648
FAH 251.161789722751 253.164596735793 233.426170535227 247.528753010193
329.406649846151 433.491835341321 336.120322555703 185.493554506958
243.839402991061 230.399207047948 197.357728220717 313.322307299463
FAHD1 396.526590512521 350.308420518351 427.766660764042 374.761007556839
281.037762232619 283.645434184168 529.368609163949 379.016207754533
372.757091748175 516.892850913889 432.088303693555 378.088675670786
FAHD2A 189.708396835236 191.912670416334 195.050834479424
219.537335167901 225.480366837274 173.334154083555 166.692386207143
151.231685705236 184.482849019679 211.104243881644 196.450308368198
174.332913952768
FAHD2B 481.238450505207 471.946284490029 381.57978490093
585.234286698678 694.02978779693 296.603054615145 269.100400678077
401.189904619902 297.022984365795 311.544429285897 225.950750084197
272.927830312746
FAIM 159.495272062735 148.1283706199 160.742365719929 149.364498870469
169.888264971936 179.376968970326 194.686527131656 196.734037460218
189.603621918298 193.275162354467 221.74200490054 207.920724788669
FAIM2 350.077206368839 279.495192828873 263.352118305612 345.897250519387
136.633726784926 165.836534881802 162.013164369590 134.750046135498
156.808992920564 135.063259138429 159.2488784104 146.34029698108

FAIM3 113.726931741236 131.22421475185 108.446988282853 119.730174479044
124.76274408368 124.851025681014 123.940632366675 210.478088018501
422.23268864343 153.846723846716 213.791379190359 181.79418355897
FALZ 143.877226672663 122.297401003756 134.887143990464 118.60878652724
118.597739444445 152.791390145148 136.801338583441 140.222121251146
131.335344547051 125.034243098087 136.987403767185 165.686655824416
FAM100A 308.031816782343 336.279209908669 278.942695435238
436.982960786839 439.492285515997 220.258804437828 247.111032124864
221.253717494488 195.414464905232 190.152824885328 266.918302307596
183.071150926068
FAM100B 170.335930486215 198.390247605355 204.851374931284
210.502556087175 493.347308943133 377.462793982812 338.822464893411
317.659548748735 414.596948083067 200.302384864371 442.027630433527
356.699466789374
FAM101A 115.600308687778 132.540503258053 122.514826802737
128.994977935861 124.090268443064 123.21732068638 120.55570440025
143.942810095867 115.041324824728 124.666487633365 132.739435456445
169.583355171423
FAM101B 162.6762533989 163.290050020756 178.872635061826
171.924364939807 156.396579029788 160.833948531272 149.639835429332
158.98007855379 167.806420483187 161.713311969291 167.698883940638
154.193086881801
FAM102A 318.561557474749 314.182403961681 228.243668453958
350.926679856514 290.090495047722 177.246890307643 184.204036882642
211.135364208221 239.109163320417 193.052112923573 241.353916553991
152.81544746536
FAM102B 373.925939492544 379.911829623463 312.961981998512
315.47473326786 196.922837906592 231.338258067616 338.866471651692
213.710058427654 185.583997792931 214.068044241507 266.286483421453
289.82072316082
FAM103A1 319.417555901733 345.686640885419 280.558463661137
291.167223094542 350.983969007868 415.113340335205 517.676093149981
359.822784070765 457.548150688248 461.314247041387 409.282410265733
398.892968579918
FAM104A 805.875557314697 835.284130042504 656.32782132983
773.057095787974 542.121375354644 756.246712133116 837.413261433363
890.507257588957 813.454826901794 754.406896156858 808.55123510297
1051.45401327176
FAM104B 369.373283422151 388.710465457313 314.063025950327
463.298781071364 241.782750858091 308.679189788554 251.915179734134
302.735372807236 236.35479928448 182.042104888745 317.659548748735
243.246632667118
FAM105A 188.864388750448 182.57496767012 171.896929562022
162.735609734946 174.205940911242 217.45700166961 220.379186481273
307.945884504629 186.460034745907 194.488249052694 246.061496821699
223.765583953184
FAM105B 203.983168778488 203.616580991715 215.219246394594
206.098534554876 301.369680426563 263.936023499588 330.908743070095
351.448985413528 281.996583337694 256.538195034956 255.493109705211
241.599271777806
FAM106A 131.12775424877 135.689889081912 157.293090577042
135.607537832704 141.076652697698 130.782296492278 132.780890129463
134.155655670103 140.234485088845 144.931901042924 130.047353911148
123.58727715816

FAM107A 1311.34904760947 1091.69584249713 312.339824919124 290.954496559897 128.517315006019 325.12056698785 143.681220324074 290.754210152709 207.368252249039 115.949513659403 139.583514773196 118.388993232718
FAM107B 694.288215755716 958.63560158343 402.140437036706 392.13175568099 1185.70008959602 1451.64055568759 1205.45751308367 524.541494680558 1046.13353853089 329.214958614925 1223.06438074895 589.722914993267
FAM108A1 121.679013405559 130.564962937297 127.997280639609 126.00304757051 142.566028652989 130.445343615035 135.609239096236 136.473948855498 122.362585067453 131.328478053136 125.846029991447 130.009322405768
FAM108A2 362.376153348015 359.256193328366 411.878556907186 407.957436380989 803.60413590137 568.555967950896 430.833137328233 359.577827010792 390.021666378804 500.643499051523 385.463209972694 407.518324776453
FAM108A3 177.306794546105 182.608712782878 178.628885995439 172.251953149975 304.937584086529 228.901579068997 219.832904615582 213.432084172541 203.335012796883 244.954917633904 202.718428167217 239.789994201722
FAM108B1 161.640122360448 195.906137340918 177.752497164503 158.320863045015 269.448201975645 267.696284751923 242.141362101262 306.946103334814 224.805404798696 200.456087759904 388.638095539186 201.441808401878
FAM108C1 567.869601934138 1147.96742431386 610.337659723539 1223.68146968716 1648.22531304741 1258.16519121331 2267.0818302379 1230.00744483191 1712.47877322331 1118.28423222707 2111.32236867229 2043.73482086765
FAM109A 199.378179817518 187.216245895253 210.392662092721 212.749668083641 279.834616502591 229.835576260403 201.167206950576 205.226635237992 184.294172429054 202.99342484328 183.708917501519 210.756414415156
FAM109B 203.752540576935 206.469040492609 146.978110148492 236.707875629511 242.996248524203 155.686889874806 118.348153875356 129.499283650632 142.892198239879 158.946177055546 146.040995022257 173.497575259833
FAM10A4 498.641091757363 514.213675499369 661.826149512811 460.24741151155 322.213266293148 324.25775261173 421.140525880287 395.645016679312 392.909481887174 531.421912541822 496.426739836535 457.905741500454
FAM10A6 108.362609680556 121.505178996279 114.155592351175 108.318510762416 113.504670764963 120.177385114315 116.161473967382 132.471014832851 125.212404794262 120.345546077894 131.839890565869 112.07238018581
FAM10A7 220.816812192169 216.705908017858 340.860684371038 205.162633740798 189.567241599215 279.159925913207 232.960832410426 254.909076658804 246.676401619881 285.562276640039 239.789994201722 293.317880637519
FAM110A 158.795335941084 178.222120802125 154.086510608016 155.710272937386 894.734451279225 446.07651799079 348.241471067257 312.645778869254 281.304989265608 330.281193038198 299.717935831288 349.330629649093
FAM110B 419.047108206042 346.862322263052 299.717935831288 367.025117016472 134.201392031199 149.560505078257 129.461160777216

132.782967735893 145.799530970042 119.922676683808 129.086186239653
126.233395051706
FAM110C 138.388749930671 148.983182193411 145.329128512407
145.591320521493 236.772907994543 169.238325933605 185.560737176115
147.125005489393 167.764352429391 205.630948393225 159.539320132392
185.233210060342
FAM111A 574.035124314622 539.720389702148 414.713379367927
423.826189747546 542.544172683021 799.132592914065 675.480482223958
681.810927901806 849.158029646233 559.236012019639 862.669120338424
792.247301083075
FAM111B 120.413468119484 112.239647575661 125.484453841158
118.731960910224 139.372441366224 121.895778599116 115.656801309856
130.666781754635 125.672285485643 133.621325902558 140.271557921345
127.486216183602
FAM113A 246.457698183615 242.538036654192 223.601312199935
188.797908601851 322.614169214764 209.385358804928 304.292209676095
217.013533525724 297.757657398146 227.774354299962 311.449205777595
314.557323400201
FAM113B 343.833659987063 351.934158805882 378.797952580504
332.138346958671 443.732580517751 243.468673768141 361.26540006942
500.848273432862 547.681206272188 333.592915218163 602.480822390671
660.096727566678
FAM114A1 191.897449038297 159.735182155088 210.043556816529
154.051705030348 131.102996816222 127.028640787946 139.708303388546
152.038902807701 172.057586741925 210.14307111352 155.63328143658
132.712573198794
FAM114A2 146.900087470856 139.696266471271 145.104173410801
133.860077101582 121.402905161896 130.749763860615 129.946925636595
145.941674563997 129.527271401014 162.652929339533 164.423960071908
115.755585907264
FAM115A 16123.3486890049 15809.9561036761 18862.7632438112
19087.9532735841 6500.1854485891 16742.6286866851 10360.162704111
15238.4097431619 13700.8527522612 10135.7800994367 8182.20106181946
12406.7143043992
FAM115B 139.63811945162 136.520811697544 127.961153369579
139.623194916971 144.159316670264 140.194414869595 135.622888091037
135.281686411685 127.401551376191 141.730912072471 140.49969612403
146.728881202697
FAM115C 150.529257416176 147.307419859572 133.742490937191
139.959629641771 144.266864839344 142.053649707364 129.70974867852
137.206961927035 139.316189199729 142.155463269751 142.757327296852
135.621035941292
FAM116A 729.202002368627 726.731270955732 692.359779743985
684.866405921987 736.332743195416 623.050854309724 558.934004505074
507.710604451713 460.651156484562 571.64723305848 172.421637564661
437.931339781992
FAM116B 259.935831886014 255.770193180874 240.511797905018
286.703712311781 317.206754773681 252.056131165397 251.731327363027
295.187978994932 287.558897878375 374.718289636553 288.481981529558
491.709723745226
FAM117A 171.967974673053 196.245297362955 181.098584949344
232.590542016298 142.643027992419 144.018198451676 150.434376975922
137.546487338984 166.157713072213 139.537865511933 145.095023408224
125.974463216884

FAM117B 399.963640581639 527.498114662967 428.237210115667
430.590533290315 320.648966489586 874.816697884109 422.590112942156
705.311256840178 585.371892598422 300.765518378297 714.91842349236
469.930860750773
FAM118A 162.411156818437 180.986305037222 279.452819132461
150.72014060286 165.570531605115 175.341310423897 172.020235319192
219.129781831192 225.480366837274 145.875240619257 720.907992182476
183.998458233995
FAM118B 188.444498848767 192.580288063603 204.89842561202
208.793650722271 162.039394839415 173.66499836293 180.913502162011
168.427124752461 177.182321788711 171.287186905764 176.612866969431
173.97085982677
FAM119A 3195.71436046397 2921.59274577877 3536.14527317085
3115.11499025766 505.83860711693 2756.22069438696 1443.68785521063
2437.63566094439 1759.9632914516 1687.85201395597 1904.39962279504
1706.06615968622
FAM119B 294.033003779363 316.457274749526 336.212224495877
327.566026396726 287.084253886676 261.322374889497 377.024120035829
345.38424761963 265.754141836026 284.715092831843 346.173614351499
344.128576341892
FAM120A 2905.12297639092 3052.86038628748 2756.22069438696
3994.64569045702 2313.56743052616 1466.82170436229 193.098116087792
2532.82545752082 2816.29814823646 2786.35558271661 2263.73444150082
1992.71224011906
FAM120AOS 896.185223980593 823.658996207582 827.863789600981
830.684999498776 779.022508413529 319.128582107184 140.911080286243
471.15333189869 567.143102792772 459.068582584181 635.536499281829
456.298408568636
FAM120B 796.936154737659 888.04996477314 1044.52286986369
1042.40539020413 684.428198622716 594.38228123675 1191.88706134921
776.411045078049 1175.27165286568 912.878077940014 1340.79810963983
1127.07858210156
FAM120C 138.889999702313 146.803549606737 139.844984829379
150.751386127903 133.226542641174 124.59937620719 129.142073426086
140.350144438462 134.750046135498 130.666781754635 114.860437706855
134.301187092088
FAM122A 199.980312703646 188.070213212057 177.16711492983
181.558764396201 159.977528879208 177.355210393749 174.202069525223
165.70840218273 191.628693083169 182.074569356713 188.671807443641
163.686535888943
FAM122B 311.04252785157 271.004741629789 275.252966071473
244.925472495705 441.684169663552 735.8141982568 339.252515920242
394.962978677736 354.45785837703 251.787648609546 445.214621023114
388.416668785633
FAM122C 153.918462601781 154.542190113901 176.397715825653
166.692386207143 145.862497313025 157.006278815282 161.031148038478
174.593548891693 154.792156982219 161.44729973777 157.635531050489
154.433679792749
FAM123A 142.61573330083 142.45411401883 135.779516940893
138.474429223902 135.301844382418 145.619570216377 134.087875547168
144.259643132933 135.400054238597 145.444483891989 137.540275229889
139.55065372881
FAM123B 150.981208776163 153.83891040723 163.227953876329
142.33269270703 133.329392986316 147.760782452857 138.701871179502

142.993401965852 125.556444423391 126.832182961671 137.801205656422
139.648187461906
FAM123C 152.042744996136 153.165872773973 154.804753043168
144.523620416439 161.585555680663 147.013598868335 143.53550972177
161.708130705995 157.155105022743 163.942131559267 146.112388101621
151.196713920345
FAM124A 161.490941000872 164.442400539653 156.336425973696
176.714471790643 132.081286801266 143.942810095867 127.488853386792
154.091431691072 147.863198012238 128.244549276386 115.624167024067
135.679481480408
FAM124B 146.698766621345 159.90466743683 127.979574243743
173.640326140494 117.672645717508 123.756349408641 122.247130087475
125.666013736767 136.826606228323 123.330142002791 135.723554819107
127.769405736754
FAM125A 513.428413503031 556.863361789449 599.52440136406
604.737834405874 1307.19490564658 623.508710830895 988.297682407351
859.113541787323 545.760623858473 1013.60354455825 812.298381693378
753.389428590366
FAM125B 211.916602665089 239.474937674583 191.708350637714
272.077877821339 235.133284525241 156.69025356689 182.084333814175
181.747090380435 167.91761500242 148.960077949157 229.443064589306
184.923312649584
FAM126A 145.068754246476 141.166605868578 151.132586978278
139.13396769999 135.311980936088 141.321547895734 157.195967361907
143.752697178913 147.648020819059 165.851677392967 129.806104966617
154.710000519109
FAM126B 190.843795579234 181.37137053478 200.127994198329
176.670752486376 138.055190422262 220.578790067967 189.142295015168
229.200570583629 191.467626362851 244.132645171301 199.714647839712
186.591941221632
FAM127A 1481.24705642453 1681.54485582868 1632.84261181074
1999.10320742823 1297.25632532536 727.114956464515 856.039380523678
466.257158794525 758.863418917419 1105.10063745656 528.921893077914
685.354305381323
FAM127B 341.251360936154 371.369895979832 363.963951509433
456.990425618007 476.641032594868 396.345382600445 334.709049890042
282.190861594303 279.070493043898 330.405026390912 222.736706411035
308.031816782343
FAM127C 206.669783013221 202.741211613885 234.182164952936
196.450308368198 213.364858759772 220.242344043437 191.3765085801
208.385295246155 193.37618381669 231.698163424706 174.301952147167
192.465510013851
FAM128A 194.413759761549 236.508498739509 186.112835637689
219.792171863015 286.703712311781 214.162670073541 235.650688823345
153.774548290902 171.69834783037 333.868818855397 190.975263192006
181.238505183667
FAM128B 119.13963074637 130.383001462828 115.871151134713
124.486701452062 145.09909498449 129.171589574238 113.656820701744
118.537378726617 117.513523498725 125.488511939819 115.992487254175
119.036965883266
FAM129A 6460.4291301245 4866.98027226798 6691.04319670055
5080.63091497522 187.824032557194 176.317805247749 259.064601204239
467.04976953755 310.075815009404 668.127527385348 213.847316974717
222.365208637146

FAM129B   912.483177705931 862.132582849511 1010.05884008623
      767.662367310672 3019.57419253979 1165.05055932899 1312.44215746801
      1082.00448164658 2544.58522979036 4527.53010866974 1416.93831202323
      1065.36715400306
FAM129C   139.60494712939 149.848740870148 146.660826209767
      152.971429120739 149.08527298577 147.213616261735 144.951973735763
      144.107029869771 174.898012759712 169.184111483933 143.537785553911
      153.500478301079
FAM12A   130.570050159191 127.073045558761 121.110498703017
      121.723964989551 124.093412630817 128.363331337379 123.413596815384
      130.434497375903 133.789738760547 118.871735074285 153.159425380743
      131.356437247149
FAM12B   113.568261995787 117.392898601408 118.895095992424
      132.510116371195 130.541449457342 117.203783530266 124.503016568677
      121.06219555903 118.034262386827 113.879459563492 139.237360556305
      120.135349166633
FAM131A   614.775559038663 475.213525346293 420.502251256406
      598.449359425056 150.845846206549 278.061498892643 278.192669077191
      264.654879741889 192.286192329464 436.834825721843 258.552964322998
      296.333944172375
FAM131B   157.66595229182 138.13987378399 124.985249190127
      145.815454490804 130.639974588163 142.815817738837 139.163958829511
      130.406895174038 128.557216790939 154.598520288855 123.662678397678
      118.974727968283
FAM131C   141.25089277145 134.011746666371 150.691815494794
      149.419308407564 155.035727630756 140.137435303984 140.292132023262
      131.338853426561 145.619570216377 147.54950981909 138.377628971966
      176.183165494563
FAM132A   132.647152200128 127.781732819297 132.472505035111
      122.447154928139 141.021723049782 136.392389842232 125.304045138994
      138.887159625058 132.263954832097 130.565474957655 151.694193636313
      200.915806094193
FAM132B   118.117957479382 136.951350782053 121.406758670435
      138.42216862811 137.146961654597 131.552276774901 135.457059862669
      136.03420235737 130.905135502296 133.889858322181 144.715167331434
      126.48691003872
FAM133A   136.540568915609 138.269955723817 129.558589108727
      128.788443476759 136.62213567467 132.983739057264 120.922042875444
      127.57284871013 127.961153369579 128.407968177242 124.35374445763
      138.171858105728
FAM133B   779.623566217277 751.077307018319 543.933989965705
      687.700356531038 322.263925912805 438.431160975498 617.833194119379
      554.282040414368 385.463209972694 411.71606840872 553.757847979529
      584.813665097407
FAM134A   195.316077537716 226.506852362359 226.020769172424
      237.692959394487 284.891415492568 197.341423434688 241.251507026982
      197.034073391478 196.716209382446 219.422260093722 266.101158071711
      211.535720730612
FAM134B   185.41739522833 171.254015232189 168.210416363286
      161.972922129453 145.024925752814 170.079723856247 160.567934923347
      138.341918073941 146.65668813596 143.860092956392 132.848926011576
      137.03111066852
FAM134C   169.385340991747 228.649073045499 234.243977361232
      230.672056810885 224.724262742427 215.057917795456 285.980446338971

239.109163320417 231.819341041105 163.922353493317 285.46340356157
198.338416756432
FAM135A 187.145134088286 197.341423434688 202.061887608293
179.35113214277 176.704617691988 186.430014478919 226.25694727283
218.672831303767 168.763640193219 317.457588721567 198.815448428294
221.975219056555
FAM135B 185.071775840607 163.67719444924 153.178934750919
152.834894011142 153.0141888126 147.066582834257 148.767517078508
151.846610656503 136.387904287903 146.590495255969 148.02460124587
151.356975600962
FAM136A 562.089999265955 573.644925180854 660.907502776003
494.067878734396 1539.80266129509 1229.47460924638 694.429144766435
1232.66885974069 741.689442253375 696.003998372271 1147.96742431386
658.746224115551
FAM136B 163.091280469234 148.435419356331 155.043017644694
139.957560460935 306.632103708321 225.065765924246 203.17525411575
243.861834405092 193.641927801805 211.22652563815 199.662592246931
188.654966029389
FAM139A 122.160459795959 125.796438432905 118.547605249191
118.294324274616 138.250288796456 133.165893710176 130.49338418515
132.257989841046 130.260174841484 157.512561489621 132.685098573183
138.677282903963
FAM13A 217.513118676535 238.068308163643 247.683819429859
242.971958493453 225.583966894864 325.999687273862 386.917038897322
275.841929188802 221.027562527572 172.370108210895 588.678767007017
246.533813165981
FAM13AOS 139.246945739764 147.103340218488 145.208597795717
151.397068235685 124.59937620719 161.744366588564 152.755535408382
148.776470607859 146.20679320119 156.030794481734 181.328458560967
170.198508633417
FAM13B 2274.75133434566 2124.12760841901 2153.17165155392
2340.90990677253 359.776068595698 632.843164522867 725.216684507759
625.152889992949 605.21685916883 558.251384152474 994.213397985121
815.388288623756
FAM13C1 141.232464220879 139.286592412216 130.800427334056
146.563078818674 137.926193455157 135.268963938932 133.006192351471
143.526983440589 134.002424083826 130.058902161953 134.950905279775
129.730685860688
FAM149A 394.65807885612 410.731869154912 386.461076717316
624.825216056495 154.691783046492 201.931760074628 191.679217737051
356.154842959851 441.958777965408 154.193086881801 195.019706886336
185.199972151831
FAM149B1 395.261210769232 375.856200488667 434.792010353541
381.484062427466 226.43414383055 205.226635237992 404.21236980164
257.775569674531 338.406266073063 217.670330817769 451.380376296813
354.922901990615
FAM14B 155.893018702615 151.96843908074 173.112439658454
172.222262910052 158.639313207233 142.381669157145 141.972403120632
148.632332840817 155.710272937386 178.665552649698 176.579096613849
143.764266466204
FAM150A 155.612543356905 160.454243375479 193.544035570799
147.920946479942 199.327939161502 242.382534384155 279.969975697164
288.370985514978 342.555640222963 159.040409182674 176.038123840296
330.758879244143

FAM150B 285.355815566148 275.013838208239 154.309257657136
153.159425380743 156.630366766924 174.787077606165 156.745051321143
173.063002566542 139.278366709094 145.25198093836 142.047828639169
134.008118774075
FAM151A 127.715766031466 128.554574566655 126.905235161069
123.922817231917 134.140533647509 125.908576249439 130.768746032834
126.872912960681 134.57472034679 132.168821720799 140.232244264861
137.345542507421
FAM151B 115.43068422162 120.255081121925 120.246240734668
119.172965618216 136.492892469272 118.497920969001 114.191245210321
119.594962686756 126.394032757479 122.543903508753 132.062415660672
115.725323816619
FAM153A 128.455670598648 119.586157804692 120.577995272053
138.530236176135 133.334314509183 131.986590029726 127.155818626031
141.076652697698 120.786630750686 127.930072902878 140.656633546501
130.450930089722
FAM153B 595.882696498804 635.536499281829 511.547907091166
540.190808200264 248.330154701293 560.481802403627 391.185365567603
457.905741500454 417.528353577952 363.89527566597 577.250493023431
406.736165197168
FAM153C 119.278231442871 118.945264994404 127.745801270205
127.458099438957 128.289680999565 129.976540483758 119.558626216818
124.242539075734 123.413596815384 124.210840121525 128.182419321978
131.169769031768
FAM154A 138.936872956065 151.123104810793 129.368045611894
133.441783287131 133.505261458701 134.927386619223 127.898533598679
126.708817316134 125.293640452686 128.029736384348 139.720343780228
127.369456504277
FAM154B 129.672284417918 160.887121295848 165.496065029161
161.402322405903 166.335294339013 157.609756554916 142.46680970401
139.295597388725 142.007066214472 145.371273337639 141.537578081123
140.05419835434
FAM155A 116.70650322987 119.972337070176 118.122186617611
121.274997609378 116.355027746922 127.036976462046 117.251233255966
112.936472036608 124.625603721372 128.29486937905 121.723964989551
111.956783714784
FAM155B 143.305848051593 143.434571922656 139.055892787717
130.355969431473 147.255680306221 138.186616955926 143.528208105237
144.182891591399 135.32730674455 135.708804956764 166.910561772689
150.962828147611
FAM156A 271.989060206794 232.363656167375 289.434186747703
243.861834405092 251.731327363027 304.937584086529 305.050933898691
368.0573385048 433.928818077527 255.3451781862 326.1109406486
264.805236346895
FAM156B 504.436992949763 446.819873986024 501.301694626942
449.304281526081 517.23393716516 632.031410234577 686.198351821711
844.187489702587 937.375361806971 506.659740456829 649.052806726591
565.164961255054
FAM158A 253.30325545013 245.640193659159 237.312766830206
339.379250332987 469.107624227456 211.916602665089 248.643311972844
242.657224881824 196.486499238623 223.107391048316 300.094509257518
250.84781675544
FAM159A 117.81168010976 110.061459744714 110.202982683256
112.198629213643 120.473038066927 108.697696080014 104.321352818065

116.176068923513 128.643539098937 108.515401509039 120.80221197797
112.156010486085
FAM159B 141.84490608317 124.215466523555 132.002835547397
123.198873421356 128.0805889548 137.068717031383 141.580944960818
132.930042559647 130.37185420347 136.805379739087 132.412642318813
133.425151103622
FAM160A1 117.585207821853 137.192570825063 129.842252702648
130.804850476451 120.883785696583 134.130913969327 123.122705969332
137.042097702398 137.618447592489 124.110446144763 146.176240539176
133.985582992986
FAM160A2 216.522992914538 253.754869975548 222.794196053536
299.33156125224 259.252807304272 157.521230460545 178.186273279101
172.065593177758 201.555964610849 319.223127076041 240.75934760601
161.956032597813
FAM160B1 583.455617065556 635.819650434542 630.670530516275
717.270096456387 210.729945718068 267.451611731669 285.562276640039
222.593554194453 246.327991259259 200.668298229815 337.935183275743
319.868227988842
FAM160B2 398.571054759451 385.317560758502 360.90109922539
506.029788151779 336.380724981693 239.762135294506 335.389274425025
192.709473588165 256.99019407912 327.635520861099 424.37437028833
322.576886792502
FAM161A 156.423687034312 149.6036366066 160.839277476397
135.585648980469 147.006482557682 149.016767620428 151.922296137689
152.767228183775 155.558839375761 157.792868983503 151.836705989155
147.04481509758
FAM161B 123.977467396003 129.6505724783 138.4303531022
132.066488589395 129.307025798583 136.147810636915 154.959217895243
121.734873711651 130.009322405768 136.359189516452 119.463337435512
149.760883745928
FAM162A 443.853458865556 488.746614111382 455.486248388968
374.169744342352 1348.80530206238 781.790858580895 1479.69309189156
839.901173743755 454.492780042353 861.119262963015 692.095005197252
514.61297221467
FAM162B 493.347308943133 546.181615768667 191.335616210093
281.740495452842 230.546762514966 282.926325589238 170.825161347825
270.651318838953 224.6588173737 223.429738319185 178.153527134452
218.96845208513
FAM163A 125.143281492089 117.261443445891 134.517774302213
119.198789189881 130.336466131215 121.984179437382 123.801380911257
121.930362099901 125.280736217267 123.004137408444 121.799209715087
128.032141013681
FAM163B 145.61758790738 139.186416741431 136.923876486522
147.473875763319 141.801472885127 143.468123097363 137.634298014431
133.699547652179 126.423739360894 139.158299612941 145.96012463103
139.831964368637
FAM164A 563.339837352678 580.347496467173 494.604716660887
440.319777672273 302.408563830315 867.369315017411 457.650982600462
409.883222213537 339.663295617139 290.184801898828 416.075109522039
323.896313343796
FAM164C 158.179586948369 174.92026613834 155.259089547817
174.08222895937 162.774716879847 137.897243684023 154.209689403188
168.412162365467 152.409211647994 130.290454258898 164.164463389212
186.66469040433

FAM165B 296.428342247529 245.522497675754 482.739666411407
241.675251479835 185.169100632101 537.350891960682 375.316610221279
322.028360309592 461.940616438547 236.082185670774 299.390443444504
495.655170027296
FAM166A 159.68134949161 147.359279533039 154.978243057971
150.786618560854 163.868129628544 156.269957530519 166.688771186254
171.994330847901 154.229914729772 157.944355564411 144.825231501747
143.025570700585
FAM166B 129.725706669166 142.888281750232 142.545823714168
131.747367613225 131.076562651831 125.876114993687 130.038936315246
158.536178747273 135.06498850776 130.563756903074 149.520911664673
135.784481523923
FAM167A 132.122273848715 126.737230874367 151.164936050256
111.66210002202 138.50032459657 118.544870925791 130.485988201308
122.883806494491 115.033355516602 125.224197852606 118.912989775436
120.979417552159
FAM167B 158.788210937339 148.538400348149 132.596941171401
135.915252483475 137.890689733185 136.438788088263 129.984214994811
136.131453703981 124.807244018373 129.150574563997 157.592607345742
143.426053070219
FAM168A 115.734230176652 116.769106223472 110.688722702767
125.055545087871 128.803632055969 120.501697624977 115.01682934323
116.112013454335 116.871350997346 129.27634490676 135.078198678379
117.937856116492
FAM168B 568.310800941091 585.552940765069 617.318955611228
829.740755449333 336.490076158135 287.967349876429 236.745054217055
296.375305136807 274.831898505989 270.945325305492 273.252023367028
236.251507822415
FAM169A 143.383697128285 147.541437871615 128.118223792086
158.8054811093 144.104372749581 151.325068155715 149.327794670124
141.348548071751 140.782522936146 141.204458186976 148.096551269076
155.243643210534
FAM169B 119.759350702482 114.830474086443 109.564709878576
110.586147059226 120.857635836068 118.100611964852 116.497714896018
120.585506729733 113.031636180748 113.656820701744 135.52861375119
107.91011371422
FAM170A 156.32137627012 154.01988200569 137.037299675471
151.176697752603 146.434716985396 146.256076936456 136.557466007228
144.513730107393 148.194450105682 149.226700202481 133.804489975068
149.915251958706
FAM171A1 878.986106116952 926.283018664897 666.204695328096
821.020339593525 160.997179739138 231.846509449829 152.939759795631
302.570932942847 298.827660296715 188.40383184521 188.252507246148
212.473610495213
FAM171B 117.238968848218 125.724143659093 121.59661598908
117.623103460369 126.555972138115 133.699547652179 122.139832263188
129.194132399305 122.038534506506 116.639787832302 120.131839791515
115.559551514777
FAM172A 382.630113452429 377.538276846051 466.58966852405
342.769825986756 184.482849019679 209.74583569369 315.963526790659
190.716424123036 180.528296177577 216.485113690708 251.939696394174
199.88618355278
FAM173A 390.098830270926 414.403321220432 422.23268864343
522.050619657197 944.92617668298 334.709049890042 423.826189747546

611.540759532294 437.489929507567 375.189005729903 430.505037012814
555.758485277905
FAM173B 194.475822533231 201.731223995887 253.098839084408
181.941224633917 237.492603094091 317.273940108333 254.768537214795
340.860684371038 222.134519361117 201.913709602894 289.859695541999
279.75914135192
FAM174A 194.941454173896 206.010481981919 264.685862877779
189.235998474945 296.830999357172 231.462907786174 354.783364481955
267.342817344746 225.730739636442 461.229315272354 274.235001739215
505.560841055884
FAM174B 269.238332581564 342.998459706966 348.300300174924
216.615694348945 1625.55754007235 603.906237227931 2253.7522632414
531.555532787504 587.654093308465 130.169291786693 1510.77099418802
1567.89763050645
FAM175A 9070.86397918429 9272.37780151073 7446.59106452583
8649.07367196998 808.300067805897 7190.89891723932 3000.46298223166
6324.87600301793 4335.76831372031 2915.72823797537 5688.84918819457
3876.04966263281
FAM175B 495.89837457901 512.723592291378 519.829743770022
431.974037914233 244.18714778846 359.577827010792 567.143102792772
419.4017188518 489.909407178594 373.925939492544 578.947889414387
565.059609779245
FAM176A 134.084602409011 136.944378905743 130.541449457342
135.9489027281 135.59027155522 130.621860095981 135.098343717085
127.866992108488 138.544053540799 140.885915460425 141.350135458015
124.858723248944
FAM176B 259.672238210968 284.638865013916 200.342786450248
255.992494620859 333.533043832976 225.31216514083 221.18757533703
210.41046499545 214.175691239597 243.95334982553 172.80265044721
218.134101819445
FAM177A1 10075.6573898939 10024.4662743012 11918.214537866
11918.214537866 7873.0525548049 11071.0448447818 8140.6363797934
9793.92604780228 10741.3064142855 8633.71708020651 5601.24254062491
9671.66667662331
FAM177B 134.28095064513 129.507297276862 157.394035451151
149.556644928013 159.241573314833 133.526213647686 131.395959781477
152.685860451112 152.06044555622 141.935435502163 138.136901441896
138.418604660734
FAM178A 268.660078500783 239.941641600769 272.238922498583
295.851459742115 151.09974848374 186.103491222924 162.879707592164
183.853381322135 179.079061789059 182.786177632757 215.335795337978
197.146035305296
FAM178B 168.560093633607 185.002106079042 205.4648759901
267.622579012917 390.284689962661 149.402679897813 155.655771857361
135.627840788802 140.719388544476 147.221425204534 146.090291252325
142.812521349963
FAM179A 150.144977373708 173.647320180498 163.203582580217
185.359195899057 111.041656398985 125.029516472944 125.754741914111
140.762415240824 157.713227527385 132.801619479245 134.299575239865
130.700175060255
FAM179B 463.488995330457 447.579975492958 394.35596596181
445.214621023114 255.638433159437 229.31486399741 294.997671630798
293.17183601265 212.890259409988 259.935831886014 680.509421043347
416.475440894731

FAM180A 206.845306095447 168.783725505491 221.424451092316
178.43642939806 131.917194849285 124.080382291282 123.30960025689
124.140880678888 125.121517583946 123.660836177932 125.937660544107
121.723964989551
FAM181A 116.839796680107 123.687439699754 120.100672968415
114.048136176142 117.040953580545 111.66210002202 118.9428715545
133.725177339262 116.019123495537 111.204065131669 114.893462783007
116.449064666763
FAM181B 173.790848780011 208.64349688412 150.737888487925
161.799743586569 900.039744829725 134.751916364581 864.908449769749
324.481229901658 285.355815566148 141.879359965317 210.015199832609
162.994537815938
FAM183A 129.867246424399 143.481112776388 147.1476865809
147.103340218488 125.785388817819 173.387549650592 147.620511911043
203.586310608403 150.359906271672 157.316941011117 156.110073009771
154.760697311395
FAM183B 145.928643520352 140.145470073979 151.758970249544
142.088243118644 138.780488114634 123.601413134287 132.70888569469
128.486896700405 157.494596757446 141.063156289206 128.599254558082
131.029333093048
FAM184A 248.791254839869 250.003965480779 322.099431638246
296.290104133844 143.895399273595 154.695060071104 194.734255772365
187.965294229013 180.837420957945 156.037150880384 153.146646647258
174.909258852845
FAM184B 169.806644442071 146.122816653035 129.228874611916
147.034021626893 138.35738548449 157.844617425219 151.758970249544
129.449242018727 150.841261270382 149.6036366066 137.462720784077
151.453985465369
FAM186B 106.576058729493 114.91722828113 114.078271807864
116.961391747202 108.850992642446 110.764586138261 109.454483612299
113.913337223716 114.893462783007 107.605526304445 120.342513393659
109.781334128478
FAM187B 142.412731964179 133.333067546558 149.984657985607
146.863611516518 137.950955473086 143.180247878507 147.476805635744
141.956996308304 150.029313080668 139.925201225224 144.377501600001
142.007066214472
FAM188A 743.915675090182 757.877066703516 783.059427015955
643.306565600931 438.7446527797 974.007008568051 328.200548902445
301.250189051376 378.920456227378 335.301103690781 418.163219634152
458.33221423434
FAM188B 259.979459472217 310.690317681243 242.657224881824
339.069385816411 219.044271073282 173.088291472857 180.698727334322
224.274329367512 188.708596785144 178.584378959369 191.486717579847
211.609763150578
FAM189A1 127.79057041268 145.745466612541 135.367261617271
137.00397800873 202.691507273819 139.166537591866 129.176532377543
124.110446144763 142.37457375182 135.975265059269 138.586620636835
126.423739360894
FAM189B 416.546844844021 418.889972660958 544.580311371212
536.609777988581 695.086203058575 487.901310664037 571.64723305848
477.741216640575 406.133430120039 436.331333134628 444.712990986138
554.115756730498
FAM18A 140.085359251038 116.939471420419 120.188885479773
136.85323853426 129.775280076365 121.118933682515 136.211694310501

134.509830749072 140.746062850603 129.029151164978 119.442114850181 127.727311123825
FAM18B 210.245551279259 229.230239609291 306.068269136821 222.593554194453 232.641965649602 621.825622897512 356.921627358141 488.746614111382 412.863887464545 482.466736153026 310.365203383647 409.691865300362
FAM18B2 158.915858403237 159.570288565855 164.433462115832 148.452947427498 137.401137659118 218.70412656621 194.702974020747 185.41739522833 196.470718183575 231.517412929077 174.100921524023 180.814610631281
FAM190B 624.638115878703 566.914448800423 1095.04571025381 497.411169898778 143.974551393951 233.426170535227 260.85776723575 222.156471143857 234.5655184349 285.30221551106 288.913660405191 243.691004557778
FAM193A 338.585771590234 316.590387560909 346.303862540751 373.189809737885 302.637859140061 222.005022189667 320.979443123471 299.754985162237 347.224999246223 277.129985959184 393.198813167839 284.940858938629
FAM193B 182.997980582218 174.549929320587 170.878962027347 200.360518276784 169.408892048115 165.70840218273 198.088834624665 177.083095120017 167.629008083436 191.128539630621 166.411219327671 176.256840061919
FAM194A 109.898406285895 110.900309203531 119.535267326862 106.450154495663 113.572326615571 122.99993610249 112.904318310632 115.066084565112 100.637271902591 113.76411537952 117.051541123239 113.071787850611
FAM195A 330.405026390912 303.907003535357 423.489441909617 343.307036883726 1188.08712008638 348.413827101046 571.838717184447 432.969886744613 376.007938436483 357.006610037663 392.13175568099 427.084392965651
FAM195B 561.761160223609 592.079898463345 499.048754087189 811.362219801362 941.065508539108 400.23671843715 480.22496056277 424.285848583977 326.758278851698 245.082816415008 338.822464893411 321.049000153127
FAM197Y2 121.207040901483 122.659174808512 149.34605615699 139.54738445816 145.25198093836 132.37450516592 142.229488350074 128.492142236807 147.216303852902 133.686850910378 128.692917766138 144.122748003206
FAM19A1 108.021898335735 106.088807114602 105.193775204124 100.477911152746 102.094370876743 109.067239220744 113.995301093609 105.132185249959 110.61165787102 99.5148073333731 121.271403807802 111.949644842971
FAM19A2 136.653140842194 144.195705502443 133.008120853618 127.440987506769 133.495243957927 146.252974508529 137.801899270172 139.59941545613 132.817645625115 126.182856027765 132.291759474331 128.398711525305
FAM19A3 134.728594388428 139.587192809431 129.728266017061 142.217762896203 140.768926302035 137.641136471239 132.336431473522 136.151754558226 138.649502040582 141.162443312437 133.365793958118 142.066903301882
FAM19A4 141.360125377949 137.045772853855 139.038389244329 145.42029807396 142.124876403709 144.417333931582 136.357788670568 138.889999702313 138.363553671822 134.237133546276 149.450921195838 150.769819282075

FAM19A5 134.881854036527 125.016608289141 125.297877027282
118.650091706226 124.491884305455 120.910703698292 138.159966250659
133.720904256137 118.115015846489 133.152523054305 136.625136565562
146.197678850188
FAM20A 167.201570648434 150.409919207059 153.843250142472
158.708054164813 156.010027635627 177.582314734704 144.116298946718
132.524377835171 163.607125532236 147.211769989922 154.87316176388
146.633627492419
FAM20B 1599.12034906314 1478.09582385838 1973.20689165831
1682.64624665931 732.630306104841 1050.40488466179 1305.2232532959
1772.75453735112 1488.37146754184 850.562379837947 1055.22233969473
1904.39962279504
FAM20C 242.382534384155 273.252023367028 177.688568435599
300.793542873709 150.829439012542 133.857996630836 134.755817147859
149.213990424575 173.520662003159 157.130045415943 151.776232847515
135.5668267936
FAM21A 136.599305161841 139.711696905287 143.607579034109
124.796876920269 141.577373924378 134.668233738635 128.23910347716
133.220878146063 148.410647472149 134.93814894432 156.265168005937
139.280151859534
FAM21B 162.347140552869 150.821697835175 165.972090075204
160.664407431261 164.146345008398 168.166938045454 166.788336808924
155.759861336991 160.625367261779 167.298438019561 159.346890686719
167.91761500242
FAM21C 153.985529457122 153.643157329902 156.787932134605
153.568321662387 146.71868686671 151.06209123698 155.836471680447
147.171090285223 149.821848101995 161.788118050564 146.755803731358
157.280532261425
FAM21D 278.498278821829 208.270622752385 253.933478547515
214.461773810376 189.403934838648 202.129153259445 204.564770370164
188.45479937455 176.704617691988 194.998142472864 215.931781291835
201.458970969685
FAM22A 168.958694643382 166.655585160657 169.777032044409
174.749832388791 157.457468761338 174.075670633438 147.469101571149
150.038722708606 161.160974998803 161.960392594363 154.080778100852
149.25705328406
FAM22B 146.157459765796 155.686889874806 152.152106138031
157.768329108254 159.133905701536 156.957578703389 172.304496150036
146.079727388849 161.144841967903 156.095520355029 155.615655460277
165.74458564887
FAM22C 157.126460242995 157.971293294367 160.945886507456
156.098240321674 150.227629690236 144.826861308997 151.338765231607
154.329176514501 146.824420102008 148.143367490426 151.879398444495
157.113536717393
FAM22D 148.415036326153 161.720405760536 151.858466721347
133.891337645936 132.50027119027 133.804489975068 119.229322984564
154.354658146438 147.057116881846 135.088149158147 143.224678017244
131.943014970855
FAM22E 136.438788088263 105.712302595131 129.384531576943
132.294365151614 121.68422452 119.278231442871 130.808911605271
134.266373965186 123.563243360247 129.477908941439 122.452541100946
135.039901725369
FAM22F 129.015422924366 138.89579816122 134.711144967036
148.450023054885 161.525131363333 156.704278982517 154.969503835593

131.657454325126 147.191965093225 136.101501973431 157.221008210698
156.487049947247
FAM22G 112.208950290962 119.920097823022 112.675715371087
112.096627288813 109.454483612299 116.029201658798 119.785062256421
120.138014822486 120.473038066927 109.37466528113 121.924036463998
109.700513642061
FAM23A 131.853183159267 128.42287610189 122.260548647869
125.746184969983 121.381365873873 139.502068079466 129.561012565141
132.739435456445 121.320750074882 129.596851280135 107.930325780047
136.44218532882
FAM23B 145.358431402568 133.412452607155 127.723052839953
142.17773831901 152.552277008504 161.627546190964 158.322477506517
142.524413202292 147.949560045212 141.035566928475 154.209689403188
148.898587505337
FAM24A 145.938780249776 138.428527640896 142.560797028716
140.655002540298 145.133151090252 136.965972077733 140.309394542299
143.351341716363 135.393901125889 150.580133889656 129.765440519589
147.238896426423
FAM24B 156.316333575406 180.418707740532 166.440530119785
164.018470368389 175.537039794925 166.388683552371 216.615694348945
187.645390502607 184.008405576875 151.877035446575 193.203053225456
273.709160508205
FAM25A 130.814702312493 151.151297821117 142.583098605144
132.028327486943 146.460464282676 153.903993884123 177.267145265362
145.335830265052 152.600339354701 519.829743770022 142.64161581553
146.798444724408
FAM25C 157.237147919803 155.569733738768 151.950883677297
157.15947600423 137.779607093226 164.541417588646 149.141033677244
151.958818313561 154.080778100852 183.247396324425 166.595529296463
147.595573192579
FAM25G 158.726115143818 157.182954894643 136.499228715856
139.080142195118 144.290286088611 153.285830139349 142.307520290692
137.642858036384 139.783725419023 159.42746786085 147.389892239448
128.689465747173
FAM26A 151.676232556697 134.756377802151 131.615462296871
159.932941342403 175.111460651956 167.776363541939 175.395900648455
159.245344068037 143.974551393951 156.916784141484 141.550553034455
185.359195899057
FAM26C 138.021559586328 121.353233207209 126.935731176024
138.246963115014 122.490271802207 151.26137860684 134.994013668445
129.507297276862 140.873210629865 134.072912593456 135.25734629146
128.029736384348
FAM26D 150.521719129181 174.787077606165 179.109090120497
177.500110974116 175.476184387338 154.509924503184 164.231281729415
160.676606760965 143.165150890885 158.704425428694 139.404820141366
170.490099282436
FAM26E 194.014764659513 194.686527131656 163.873355370767
248.03801051527 147.015427944761 129.70974867852 141.18490443471
132.179747646893 137.165966740473 138.149037534929 153.962758393332
141.565599104245
FAM26F 158.06915048955 152.344248425416 164.247251507109
156.71199811686 353.285905782951 163.256472113745 193.723953003899
255.374689750672 249.80160363144 179.821008257902 228.901579068997
230.10433160008

FAM27A 135.105147647223 145.261662166215 138.154799037807
153.798367953373 143.16930809425 141.949098898957 138.204611862656
145.294665838127 140.105675520432 132.37450516592 131.093917654245
149.086836205064
FAM27L 150.423586547273 151.377932495104 166.722369066673
154.469132478321 139.888008605915 145.105268133449 146.517645037071
151.070654092886 167.408314067262 152.913814113632 144.298011270428
135.37120759813
FAM30A 152.217243924816 154.455641374384 157.15729049829
169.733427590141 162.304178370233 159.674396591041 155.360711066715
176.612866969431 170.67334244323 147.185378560761 148.11536596061
167.764352429391
FAM32A 668.127527385348 778.846928848361 900.839073800181
986.441377365124 547.516635695872 1286.55103838368 924.040923247227
1146.3535752748 743.915675090182 839.662936279727 805.120147618984
767.662367310672
FAM35A 233.635876830148 217.940575516933 257.477501542521
211.22652563815 190.45739385453 233.671878221507 203.357914242777
214.763232658841 247.08460296705 226.763252784108 286.194197552489
251.665539319612
FAM36A 811.564396886535 783.373390305885 946.640841062802
832.911633923496 228.616558999054 540.078145038497 343.121661664987
523.435696794756 332.599191067433 326.45984882427 579.678635803225
594.713185954954
FAM38A 1897.92061704124 2558.91648313228 1830.56003081769
3706.41283595794 1982.3154578939 499.436680354749 634.906566690042
800.373159084181 535.20662670885 1443.68785521063 1041.58583268313
1209.56018864779
FAM38B 378.38874489611 267.222762759781 274.534901548678
195.10410545445 166.944701842349 212.996456841785 182.439945229388
165.496065029161 188.145154995617 154.999192460283 345.494740481759
436.222568287601
FAM39DP 778.639512538949 761.89271251455 771.694853062033
895.821578104681 947.766127685083 1992.71224011906 493.209107049493
1887.82245931444 631.819659701092 549.460612060753 605.795439342384
595.593867616527
FAM39E 3408.6535437109 3450.46728258139 3905.35300751401
4159.96851384358 3789.1316515671 8739.02966610274 2558.91648313228
9099.13956219312 3822.05744774919 2748.51360874099 3226.53426370323
3237.94149351627
FAM3A 1797.65684760558 1916.46158204806 1752.50728312967 2486.24762158293
4073.21075791737 1288.3346537369 1971.42527720714 1394.31467757456
1199.35864276583 1097.60805874165 1525.24196488688 1298.60552708737
FAM3B 148.938822951404 190.825209733575 174.059172465573 148.486946087388
490.022369664024 1045.49947183547 1195.07608384189 1092.74905085695
1008.43888926584 146.731584143625 1202.74869573717 1131.22418479945
FAM3C 178.780662099615 184.742960975938 169.496129800444 154.079355899056
200.915806094193 413.172954892025 502.935749606918 334.857690525582
289.037033338975 301.428024926428 338.585771590234 383.583234619781
FAM3D 132.976977898242 142.510745106328 130.304743083321 122.051473363557
1109.79571066685 128.949103967865 135.114056458478 127.758381206968
293.018282386003 147.93442757931 1966.09691567857 294.871848467326
FAM40A 223.339132121796 232.492398623889 242.382534384155
248.403014089812 280.453895029319 207.902853533132 228.901579068997

192.340135076104 231.239126548656 216.077955163344 183.201926323188
241.293046687076
FAM40B 1131.96129284658 1006.03677177164 808.837225580351
951.371815352591 274.14931964455 922.009043194023 528.921893077914
690.246312542011 522.050619657197 443.125195744202 982.367000148189
487.537691131703
FAM41AY2 120.910703698292 140.914966429342 140.354508398385
137.504147221623 144.246749835693 129.205783382977 135.648906382608
129.428950259722 131.782007636511 127.509583634884 143.028618831382
135.30767047671
FAM41C 135.503338987971 132.829401072883 137.584340154725
144.562038493733 126.301178976594 147.971492964243 130.142263689536
135.767818903328 137.748443205818 135.433229702209 118.572988141773
128.168460083895
FAM43A 749.859130465356 878.986106116952 234.461229199142
344.247544510881 173.70035122661 193.336246844237 133.231066635063
182.084333814175 219.537335167901 443.964363178742 170.439725847568
134.819406064011
FAM43B 165.493629431627 188.891032176388 133.495243957927
210.316431648335 163.704616231638 142.667173697017 139.010652831798
154.737117718424 143.849399031223 149.912481263284 150.091854035356
162.285199080777
FAM44A 146.1289358176 143.730966588794 142.470799054068
150.144977373708 146.748572532267 141.348548071751 133.866011732878
140.898380345452 132.815753681623 137.260359343758 162.9279134169
133.198832503062
FAM44B 1000.05896885496 915.015154168627 797.28465621772
983.777608766079 777.651238374269 641.12919339861 1018.38836615952
602.141151173094 508.302878624289 843.075879568455 933.408902924063
947.425912541091
FAM45A 523.717949503639 529.228238133018 591.89966781883
496.218605366298 371.552970811499 496.697565640162 841.623780229898
584.548578914846 550.901056837273 685.620637807134 609.665323786904
641.774812292709
FAM45B 130.965418847276 154.564478911791 152.861304149219
152.660373241584 144.068734533254 141.261150586614 149.879951192667
162.565576110247 158.171302275984 202.568071395884 154.617462666847
146.443892388756
FAM46A 3304.82581034038 2944.63971819193 2505.8565544797
2358.46955614953 948.734577648136 1480.52638217173 1182.50261323518
852.926365088632 576.636077770998 1205.45751308367 1097.60805874165
597.459920611726
FAM46B 1159.44907165386 1152.50523614436 747.322544683874
1625.55754007235 182.633773540249 136.473948855498 135.952202055637
126.549876718792 136.222421297872 1099.80705776268 140.906421938377
113.479382235297
FAM46C 538.252273740419 446.509863498357 357.140791982825
474.14051635531 276.582734590058 194.734255772365 181.364325529405
665.936368667321 659.106229917309 179.421854100287 422.23268864343
225.142823691625
FAM46D 152.074017988748 169.583355171423 142.62707251786
151.617989938924 164.365630106697 133.020551937677 165.350777445902
138.652077417473 153.09926854747 175.647878042257 162.793214727475
152.052084712417

FAM47A 127.377356715337 134.572552391307 133.113581756557
119.95666716163 118.379914726645 138.685330935707 124.455340203012
126.806537241253 122.508761687823 125.287738115754 147.354535468933
122.5651974076
FAM47B 138.449057377027 129.108534267222 134.719531559829
134.658664100744 139.291775075915 137.151210946281 143.036006001419
143.281050499332 130.154756165574 126.614876404146 130.957441406855
138.466136108202
FAM47C 124.334450753704 137.861973133557 142.930903395131
137.288653308858 141.503351545458 133.369314179341 137.451687756052
137.586032396204 120.538535752185 132.788102310611 127.299853592344
143.481112776388
FAM47E 151.351938656508 138.386391726042 149.73250641246
138.986877479278 151.459397907666 147.863198012238 138.211845785985
198.023465629218 184.862871179765 134.924082270115 154.71904132098
132.772085211553
FAM48A 170.636731478608 162.297999435613 189.685949591531
165.803549113373 152.908358615016 184.332299740724 175.040086358446
165.570531605115 187.586093248437 171.610742435469 178.377461551319
216.731618532814
FAM48B1 149.477373469387 144.258128917228 124.536322123442
139.768555796264 151.833386901811 135.628982370682 145.09909498449
145.10302792449 143.401971479167 139.936661244361 148.185056724262
143.215828010442
FAM48B2 152.045391611927 143.378998368344 128.141725976839
110.858201415824 127.115598987172 114.170354748872 143.294217840279
132.950480329605 133.286702819953 138.383183017683 138.802633518223
135.54708602175
FAM49A 204.842791081358 197.068795788491 171.080373870373
194.60309399944 283.163770658619 190.405863264717 159.706443636973
185.602093266907 199.378179817518 253.580286414227 177.759729226623
163.612347715773
FAM49B 186.831266888028 193.744461104128 181.412790232996
169.220974415101 266.71318061066 358.732947927445 367.732191251547
407.760503937821 391.706404954357 307.768302846647 415.388760988459
378.797952580504
FAM50A 1927.467713716 2135.04382065446 2236.73950603157
2550.08769415478 2877.92845389611 1394.31467757456 1671.82101590797
1418.60088387728 1596.33832632698 2033.67956299225 1432.93880448974
1291.12004949379
FAM50B 273.550544349798 257.740674565975 354.336462793628
268.698146407497 164.347168837655 130.260174841484 139.974586762939
122.490271802207 138.643442050615 174.795822188504 191.128539630621
200.242239130026
FAM53A 144.507230362869 149.408530395745 155.456077670662
184.493681387276 304.152390210727 147.595573192579 206.715020025609
192.752224665459 148.570769470645 161.099805358232 164.057147728625
137.864637728155
FAM53B 293.794745907663 317.550000625189 262.452869676209
427.452114719877 497.53605530806 202.253560413436 299.33156125224
260.157440518033 300.53017771156 232.444867422559 563.011988279542
280.453895029319
FAM53C 868.86246684338 617.833194119379 730.673649872205
753.997560938003 526.285114043212 450.468410589415 726.556553101727

640.029195821436 531.217306770605 697.97116981445 494.747087458483 533.54045706916
FAM54A 154.049004176595 158.819151102054 139.293775885106 159.588575526536 202.943987922418 194.413759761549 160.499355902773 213.301947003462 175.819983804497 175.847256673734 150.610633329908 173.810080280732
FAM54B 347.557792829093 357.35857422585 408.201232787978 452.732079010502 197.378801636702 190.357437485436 254.359737178714 199.813528853649 198.311821616128 187.754968096521 214.175691239597 231.819341041105
FAM55A 132.981346767415 139.646026806298 127.419227292862 132.291759474331 116.397855001959 127.720117789235 122.751674866182 117.965208293925 132.985645792864 122.423561732526 117.412956162665 118.308102542942
FAM55B 121.239461514704 118.712730897987 128.112595697836 117.034228068173 109.282814458761 117.123891785437 117.227635924674 121.977373818244 610.759447723026 127.445199722147 124.16920280673 126.553693803748
FAM55C 175.874387612235 144.12472003202 225.546125484563 137.31708798874 122.634741242406 124.324855155055 125.682297697017 136.859366444351 128.432188632218 119.640086587188 125.0723702213 133.161552914595
FAM55D 117.628564992187 119.50754902958 123.004137408444 118.070716076005 123.507963359911 114.174153178166 117.404906698431 116.225440873023 116.501174707117 124.297848065172 128.36533737469 117.251233255966
FAM57A 275.072079692264 374.268489263941 213.382179917193 272.41397350779 508.302878624289 375.856200488667 463.298781071364 397.861172312706 511.891269255193 502.731019454502 536.46884541183 400.783945471037
FAM57B 138.605661097886 144.603401904061 137.248465324584 131.560971848628 130.357913155625 138.504398858454 150.560305000601 139.399195840117 137.639342727211 143.954908055924 158.950169226637 143.62315472858
FAM58A 397.985856484851 383.939800422489 405.183882455109 398.673582680257 871.268905562551 563.907454700224 482.677635089583 387.617166711971 396.708183244581 449.103432410618 335.47484353322 509.988617641229
FAM58B 184.071078414327 149.999838961906 170.390200730789 161.720405760536 152.199095472082 168.744610747563 165.757295630254 154.778726823719 153.545086287929 143.730966588794 128.497571151343 151.181422205549
FAM59A 175.7330682766 187.769988580599 162.994537815938 174.301952147167 229.406012088782 278.651369597422 311.208703262497 378.151202933526 241.453456707114 201.850932289186 337.521614253267 399.537363692534
FAM59B 169.940177050294 180.20999937977 190.489530928259 188.062732621031 220.92105843037 181.400788152988 179.896659891106 180.315825600347 174.795822188504 195.929328710358 142.524413202292 215.718714189719
FAM5B 126.168223112165 127.449028221682 131.310537960214 118.537378726617 136.256456726841 128.255141263975 134.755817147859 130.980885733161 127.123123583856 133.491963477649 142.825203517688 150.767158077219

FAM5C 155.211976328553 160.281830914755 168.552631639996 162.082166316002
157.316941011117 166.20327698517 148.624135810148 162.304178370233
153.643157329902 154.417731984391 144.528360135884 147.681723355535
FAM60A 327.010503005631 341.361299896997 287.503594820801
254.9636051363 426.896707161414 1022.55215097491 462.948429969719
627.066031322234 837.872187194784 467.90388853336 519.112018353676
631.653707589221
FAM62B 5811.97916044218 4882.1692310839 4562.75659565027
5636.26097921507 772.061917233344 2053.71550326011 2069.70335195314
1329.3013039618 1420.27222765071 1508.24281455057 2299.23805014176
1973.20689165831
FAM62C 169.574755162815 150.91730768674 171.671895682052
143.198622036573 147.633793743254 147.543411823657 147.257773507186
124.699127988314 133.99226161886 156.176537862398 131.104534164394
144.718507303105
FAM63A 1208.25171673432 1144.53002508973 1217.29687282094
1091.12493937368 369.275635424092 959.897744425251 581.828502821435
737.843412273272 680.250735532287 499.321667633972 698.086166782221
695.595965352335
FAM63B 251.915179734134 266.468621734455 220.330477277988
246.676401619881 149.516743790585 182.357065848298 201.931760074628
173.678096732903 194.055394488452 192.095235288818 226.701637237203
216.705908017858
FAM64A 124.164523121103 130.863753083296 124.291944151031
125.475308885588 261.844708446856 323.086368767924 140.081280065913
311.612118247493 202.214495168688 175.857952663383 158.353171576491
202.869364962231
FAM65A 977.999660785113 1057.80846320642 881.15414246017
1214.16777512885 765.911113050543 438.340341490622 507.827473844576
369.156887999872 384.97980473847 562.543347322063 562.711562649704
664.025683218037
FAM65B 194.831347257735 180.889946069121 180.761100930677
152.021576565132 134.569883788949 170.858049313581 136.463867598256
207.920724788669 266.918302307596 152.301556397498 165.163490032007
168.327600230929
FAM65C 217.969738417693 188.952730860801 155.041173289759
147.713967317159 141.678529125136 129.669484303621 127.756214708077
133.320865155329 150.809693773343 153.818325601312 149.225754384299
129.735862775199
FAM66E 161.891809608511 165.752558888845 152.158269344693
153.371065247493 162.728630453745 143.007237184254 150.134337895481
160.30784625946 165.857032207991 157.754622099874 138.651419539729
151.971337856271
FAM69A 528.275591281419 469.333357789149 457.905741500454
497.629319581786 351.585604465402 187.729071402782 312.080864996592
199.477350542491 183.071150926068 214.617852048151 164.986006226427
173.867252292026
FAM69B 174.107661206415 214.546528079149 156.402578892869
250.376178742476 158.459129254226 148.347642424885 122.955724972547
133.647637744785 148.373866613126 123.9209907098 145.804420322848
154.398416018484
FAM70A 186.904126552483 161.506431005203 164.406564594296
146.654925456132 137.350851760173 141.760529549196 140.659276788384

143.712450441816 142.744045151046 139.969155812688 147.83240038699
137.848546040474
FAM70B 160.843325877861 149.490064894136 160.096507818256
159.79463502085 162.267841475739 165.085010975035 171.410912120195
167.491399614986 154.949840064881 159.43317827517 153.263883774915
159.159106382346
FAM71A 139.72868421672 144.019824700027 141.233642252649
131.841436867483 159.867178630115 150.741291526753 160.79631681714
159.446705611995 142.342811880173 147.348400730286 140.479321382192
156.307551403822
FAM71B 142.607578275494 147.813170436139 129.895388481159
145.139966629363 146.750774496317 143.558088035758 142.143709425075
134.21363751173 134.711144967036 137.567384694894 142.373521903746
143.127795310829
FAM71C 157.800913051929 129.45936538019 160.520677267863
158.639313207233 139.246945739764 142.706708823143 150.48744233923
134.47218436552 147.86104992164 153.044682186978 144.342905860503
140.762415240824
FAM71D 123.02879683948 107.863971340407 109.625579596864
122.793816135585 111.373854978307 117.12833256636 119.725487374119
111.949644842971 114.032874859062 108.543795408713 129.508990861362
108.955761698524
FAM71E1 112.790552379447 128.816610738922 126.121516564343
124.859984709406 346.996310517446 149.416411285088 265.174306663185
154.637740117457 192.992546726522 133.414198431469 167.764352429391
136.781417098789
FAM71E2 144.769824608083 145.070927449969 136.645091356305
151.992881963981 131.48517604564 133.511698282195 130.721987260546
139.338384535905 134.169645445889 142.594553057288 133.672964925321
144.520435611318
FAM71F1 135.434853296628 124.28033876033 132.764150075465
130.832462487806 146.150048478173 135.778099658349 125.439033142431
145.877943006533 136.824357247954 136.162938063831 132.291157461345
138.416716745934
FAM71F2 111.549719888905 112.565341690522 120.975542702338
116.814269969508 107.884870799936 118.88525116298 105.49491265382
117.008277388399 112.635430248416 116.929398630733 120.085340927108
108.939608619532
FAM72A 170.918584418153 163.718595974283 155.91493956251
163.829218786716 184.294172429054 200.603836348642 164.986006226427
174.059172465573 218.433524126584 166.521472376759 161.627546190964
185.525760847281
FAM72B 171.031912064945 159.236429097777 154.408875182219
182.384190694142 172.387657419201 183.056707813352 151.655854407186
217.531179216305 199.756584044664 188.145154995617 167.822075935474
173.440596296333
FAM72D 137.345542507421 138.496366637658 127.449028221682
122.816049839521 254.447650810819 175.902751522861 145.09280363189
245.640193659159 256.495848662843 135.795717307603 144.69686018715
188.242091666446
FAM73A 1535.27496251381 1577.15895478896 1637.66306286151
1565.64079526732 267.622579012917 1918.14260920544 878.167149313285
1375.76915021882 1072.37500318471 795.464850398761 1481.24705642453
1036.40082234884

FAM73B 290.420307322101 301.908667220208 256.326661031361
438.03648998391 497.269957926657 194.22713184875 186.679199600379
233.300864925399 242.614881095485 198.783034238774 293.4021207271
170.099045774947
FAM74A3 132.268445799639 117.923459503989 140.377179372804
125.882246459692 132.703034017237 131.305785417254 131.716370298507
126.839037497031 126.890841187249 152.77616981167 117.958297137758
125.435268901131
FAM74A4 125.600500146679 121.821868944354 133.962129094978
123.364999283696 133.74741832303 115.473431881597 122.460829835477
137.625415175495 123.30960025689 125.929743286163 143.94902198632
112.547907748842
FAM75A1 148.364138636002 148.835389500419 150.508851767517
145.210787864362 153.962758393332 142.470799054068 155.42165869714
147.81143020317 166.32555285213 151.755205401737 153.033582154882
152.780908985916
FAM75A2 146.105009838122 128.941490662452 154.483828924645
145.25774926819 135.071400668179 133.52271411034 155.043017644694
151.635752404534 144.175551691291 152.650867743471 128.738746025289
146.795608819024
FAM75A3 154.38209765671 152.347154384235 177.438968923426
155.835077077665 145.241301006518 174.962380276394 172.46311371589
137.386308389265 148.253612913109 138.384797779855 137.100207190052
147.364301916733
FAM75A4 126.025161261042 139.611455791957 144.031305845388
130.052593375075 130.957441406855 133.762141373091 117.757113741542
129.875986080057 135.338077911839 141.362627556137 121.580726825432
132.421863261746
FAM75A5 134.00481287258 125.859158049343 131.38881091292
142.063338490772 167.698883940638 133.53523683945 154.820187615267
140.451465188624 128.482976013813 124.995087711332 142.780100181736
129.541049979717
FAM75A6 142.167918879613 146.528945049588 140.760983862166
141.695537669891 129.737882221259 155.091135297162 144.807626228351
142.157512965166 141.927204640355 145.498372398845 144.601370216162
147.077695919758
FAM75A7 128.229729571899 117.770032157393 134.91697121091
121.036054511761 122.829218352947 124.752198904184 132.277920991504
122.399241018147 126.322527679273 130.557209560361 112.495783269169
137.971108694323
FAM75B 153.626180103404 160.960599932898 143.951756384507
161.979914539039 162.606273401226 149.394952856085 158.863516051439
194.281839133887 161.83241639654 159.815932985135 153.136863548105
163.181323768362
FAM75C1 146.96472350529 153.219954133957 153.238678313147
148.094036534405 155.268650584431 132.140547119679 168.697565122105
152.832573288735 157.639688257892 168.180063344887 148.941194457652
148.879605388902
FAM76A 183.583432296209 177.598422362298 146.231937458616
149.500457158616 168.897620634337 156.430981244063 161.85913459364
167.653412908384 148.986058473609 158.023626712181 160.001115445038
141.874188629438
FAM76B 239.789994201722 220.113261580385 236.032288046282
173.979001863717 223.107391048316 270.181005097697 181.37137053478

216.859640534581 195.608619642038 211.208937901871 235.530986541549 240.306287921209
FAM78A 128.883828419932 141.343125513329 160.975741751317 156.123528587917 170.251197850968 148.367132982373 168.150270765792 158.6067091347 163.248980088946 124.477586539576 143.803893271515 160.76044995335
FAM78B 158.208998509937 165.118167086792 148.492328039604 188.623897093065 245.240006198482 286.818166898922 150.71435300188 181.294497402507 165.342790388607 164.736294372006 160.211460008777 156.803516898875
FAM7A1 152.957297648595 148.930073905413 152.009494286376 161.764411756848 140.102266035804 139.353080958675 150.549883779193 167.822075935474 145.787468865642 145.641373616866 147.558603709566 150.19577351885
FAM7A2 141.876237297525 140.553613300431 150.434376975922 139.972797966319 143.546629073997 146.04584032214 144.101376910581 137.976533193418 152.741229289112 140.522470775478 140.174087049513 146.303959493672
FAM7A3 145.386689493179 146.660826209767 162.513107496558 142.910616700526 138.873925577739 155.651798065702 151.079500558067 140.895053453126 150.731093808891 157.172970235254 145.684294398911 151.379761514526
FAM80A 121.807661901776 134.356796881432 133.12904935533 144.510062568989 156.926617030752 132.985645792864 210.973303650385 133.976997391944 131.630132952441 142.063338490772 134.821778017915 138.940564112156
FAM80B 181.431827312784 165.504532455205 179.94333102877 161.382041789666 117.81168010976 114.28381541078 118.336654504789 130.411849432351 117.149781437983 136.103088234442 114.249891266241 124.833659452957
FAM81A 120.679474100322 123.577138762427 111.886543737378 129.425699141995 133.402515225624 115.090463537716 117.585207821853 122.042136913991 121.188761627967 122.54127694717 129.116354997747 116.230993734675
FAM81B 153.320911882529 142.348246621217 158.631298154353 156.961070790639 146.281429685059 146.052153515494 164.857465272302 148.709795687391 136.099663831028 151.221783750989 153.969493052468 149.710337205986
FAM82A1 128.950308413612 125.077260389449 126.992697289075 131.77642590781 129.79093244374 124.00806841872 124.546520456419 125.481506166304 113.112450527382 128.247871411825 122.05954649985 125.328704545727
FAM82A2 571.439520427295 484.764806127211 651.675427664911 496.560127152436 415.749051861123 421.583441056026 659.27672123763 511.547907091166 926.670867201327 732.630306104841 622.142195892701 738.892511276134
FAM82B 142.531683761072 143.028618831382 174.02138265758 136.952914053358 129.343440464729 209.887047474042 146.861981788662 164.472980733552 176.27969404442 167.746786642066 160.520677267863 176.431477850092
FAM83A 127.612859839494 128.460750472464 146.432468585736 125.682297697017 173.001985500448 329.88790758321 217.607230767028 121.170340423391 208.776711004753 676.293399523316 189.49408026215 151.285933600303

FAM83B 141.730912072471 150.602976963975 131.430921437192
132.956860864455 164.558875058199 192.168634727505 282.836518011311
196.378888436237 156.934589811742 449.859227376918 268.370347292603
172.591885279428
FAM83C 150.902548360746 152.60462513339 161.618479762188
159.120172969378 227.483999162038 224.089220507176 278.555009982029
143.473523258521 159.085639414089 499.544050752036 277.000882284125
244.689936990021
FAM83D 1442.94175124818 384.641319423853 582.062758687489
409.883222213537 743.023225859813 388.477382640003 222.134519361117
384.641319423853 341.901565692251 304.479999301061 172.430211711547
277.946395491073
FAM83E 116.814269969508 129.369968549738 116.236538651031
118.32560379073 133.544149685358 119.01990099776 149.019120812629
138.990971069382 125.666013736767 303.241375487319 169.791616021987
123.400143662218
FAM83F 182.055718892894 186.033093285894 215.532132200821
217.811099067262 345.209128382239 226.919545367682 288.313931067158
205.322733011516 207.890408761692 295.851459742115 221.359331823286
230.749601687217
FAM83G 154.843607752473 133.403409882362 132.071099697667
138.970853057174 144.421572284568 134.776714949136 147.805819921429
134.119276589666 131.580226088647 140.909875639658 140.981666934622
128.312170913496
FAM83H 149.211214631561 210.803130108618 150.593928635647
129.888444399067 1834.08657010335 755.42757464615 778.28014337938
692.095005197252 964.093668483033 1069.25265476373 924.630951485299
881.561688713557
FAM84A 130.116771999391 130.406895174038 117.420765321056
140.372463604232 358.843277491684 171.636121363019 271.356289141017
170.430478598594 166.741762854246 146.70774472499 178.329946332806
140.569391362701
FAM84B 174.347451984066 211.679799388351 149.180704382192
151.029785584386 535.354724252178 619.995008098949 742.751664201272
706.292905820227 843.936924659514 288.370985514978 317.550000625189
535.612000577004
FAM86A 178.657325741035 179.230909178397 182.608712782878
188.878770168595 216.645552082667 188.695527267631 187.965294229013
214.38058301395 182.439945229388 182.747079625619 193.723953003899
195.194505906435
FAM86B1 180.096239155058 160.557818466874 183.83720937589
195.303469330682 185.405774238391 186.460034745907 196.310746973798
202.819949307954 182.919385608842 173.70035122661 164.513371267476
178.696562301918
FAM86B2 120.871693555514 137.103879676652 144.259643132933
133.408354896584 133.028789808654 141.29868389128 131.636779618185
135.661131815375 130.571827557149 130.93033679281 130.697148466042
122.583461806645
FAM86C 143.662865541299 171.035391211596 165.682617337317
205.728673153914 226.387465899611 156.008829097457 159.524235332529
175.711836112986 162.895923745031 134.451423371791 179.841665686703
145.08853313195
FAM86D 172.562162872242 192.878416717298 203.11015591813
169.881866987359 221.594737497504 198.506786870804 203.510106134882

199.077893147154 172.486839924297 168.422826952385 212.643559782754
174.383193885933
FAM87A 167.268645224361 143.899201949317 165.017178571218
159.824012085212 126.94941821459 133.988494850477 161.424105316969
155.343619434375 138.304793826214 152.254519859308 135.484360570738
134.051756243089
FAM87B 138.83304645915 144.55710963567 121.224389883994
152.385014030957 149.18603646016 146.056723380149 138.712529531951
141.408545519654 133.133892264995 149.390481900424 133.324531689254
155.588607813902
FAM89A 202.114480291084 230.770036998466 158.455783755247
153.885582272269 289.297370738382 369.838429521168 690.010326122574
212.363151537303 176.300743660299 651.759589129833 169.844913799159
247.643123415644
FAM89B 851.633522004342 1024.95957828641 774.774214696342
1615.60472592991 1681.54485582868 886.517088614968 601.997464467917
425.59810308048 442.493108530222 789.043948009585 450.120868656638
534.745066397679
FAM8A1 363.195371577097 332.138346958671 475.403662030875
284.321253087512 180.814610631281 218.298791348295 242.614881095485
195.019706886336 235.350819745387 253.009376944901 288.88486628661
221.390817970859
FAM90A1 126.34649262976 132.885515958081 112.005939063045
125.331215990597 182.810581377888 143.733505359106 127.395094952914
123.323528039848 136.151754558226 117.990622156287 116.20008794813
133.008120853618
FAM90A10 152.599052194143 153.891667472876 136.598215718877
155.427282703527 140.169200413643 138.442378791712 146.101547300741
146.065491816894 145.793794049657 136.712083407769 157.061602966797
138.449057377027
FAM90A11P 131.319783710908 144.004831360797 141.269923271838
133.619092072157 166.868688936939 141.78867917709 140.223599881961
133.951322753098 147.772490977392 131.095431530473 133.384498169568
157.919709478746
FAM90A12 149.952762225731 134.591581328442 129.247701136266
172.057586741925 126.5929421833 139.3864057463 125.884565343075
171.813279175663 136.831280469398 149.326216726716 126.503135702506
136.858037332222
FAM90A13 118.015175851934 114.785989868306 121.356731684516
130.646956516128 123.129785564258 110.286187184448 115.789536577324
125.833649377492 119.558626216818 128.895325544109 113.199690522854
121.170340423391
FAM90A14 131.025725443485 124.477586539576 118.510960607141
116.558752619296 124.116400777472 125.374764771516 131.285279061742
124.859984709406 116.408126010926 126.308622415913 142.431979387165
117.112667504781
FAM90A15 118.9803314875 114.960460615926 147.476805635744
127.758381206968 130.674263597931 127.475316138156 136.764881711137
128.637470206958 129.153882779488 121.632494500096 141.180647782041
118.249148086655
FAM90A16 149.984657985607 148.597346678768 126.969504644414
141.730912072471 130.430917518283 144.713108022551 139.263602290256
128.490341173553 148.412895185127 138.474429223902 140.713537140957
142.937182720493

FAM90A17 122.619429537799 124.892438890234 133.473140653666
127.354826381656 138.79473329684 129.614692778261 146.782830477983
132.686915065196 140.747967724586 127.492376657284 142.272117655292
133.864612785572
FAM90A18 139.217113811817 133.788197693571 140.338622198206
135.054931772725 133.296200563657 151.567693140302 123.817878562971
157.649902282769 137.702802090925 141.334394187941 144.520435611318
150.985061659285
FAM90A19 132.958521136 128.922269289649 122.84779238795
138.633019006784 113.170014668758 150.657800679032 123.798513338021
125.331215990597 134.074425424613 116.421771593211 121.903115540516
119.32696696175
FAM90A20 130.574019477669 145.077517845584 133.215151497536
128.0171233156 127.333963230749 139.543551482943 144.752418965039
138.947760022173 136.68056120003 151.61049450647 131.741688265764
145.581755438161
FAM90A2P 145.574725784632 152.407614042236 141.976051648478
148.435419356331 160.099731578122 153.238678313147 141.553465481789
142.590631181358 158.425950902309 151.188412354358 145.241301006518
152.964940071133
FAM90A3 219.462274506333 221.719848707318 203.335012796883
222.544542998356 238.183096218311 222.315451077459 197.981112645168
189.307823427362 210.061754644824 195.946096353894 199.423120300557
214.229228850802
FAM90A5 118.527464364564 129.942594772751 138.352562277336
125.124011798043 111.538651711074 132.493888285566 121.973581817041
118.895095992424 129.23899614612 125.154784284706 136.764881711137
123.756349408641
FAM90A6P 136.965972077733 145.517559593811 143.59105195427
154.928072022777 149.645548888832 146.739552899981 147.446381217887
139.328340242696 141.670426206169 143.416816860089 135.760578586004
146.26936366452
FAM90A7 115.738984899365 132.895500657638 132.458764637407
136.022614387461 124.625603721372 127.977782260866 132.84268776659
140.295307451188 106.182551369456 122.97229333908 143.071052031994
125.189588215659
FAM90A8 140.980119908597 126.473768158566 137.358139652612
138.359423363142 140.936204899093 159.337816095012 145.768857553634
148.697019570015 133.416938451465 139.981905225845 122.414781975236
145.371273337639
FAM90A9 131.759422682853 126.988118823204 135.173464753848
137.760712896718 128.359034755228 130.546471770588 117.480506535399
132.642931022024 131.876061826583 132.845116298605 138.233799607706
141.317113425046
FAM91A1 138.717541782317 144.460541430705 149.666293193219
162.403942351933 217.125327913156 213.754156680604 208.713555471888
186.904126552483 212.210383398591 215.47323777134 165.066555355918
183.951404362826
FAM91A2 154.990319019521 143.101750222256 164.309588213547
165.686655824416 132.823333905679 140.251168248117 141.836648910979
146.64835267642 141.348548071751 139.372441366224 128.029736384348
149.255405438966
FAM92A1 177.60876237572 172.604689611475 257.372740535895
152.547558436703 167.357570666817 195.50039162477 192.69687837782

177.281197832647 199.058972781223 166.760758231104 129.964666668421 214.52087460757
FAM92A3 117.561878567749 128.306607216705 127.637319802339 117.205525129664 142.228042097379 122.265071206163 124.905889975958 122.786030993629 105.164790181452 121.924036463998 115.907902010701 126.948090173876
FAM92B 147.734708927825 151.682386885888 150.708102506794 153.687536747147 136.472404812817 150.694215349678 164.791545858875 150.384559054133 140.78582131501 151.625248091441 145.487783396345 146.11669387238
FAM96A 292.965109701719 311.337303524912 336.120322555703 271.004741629789 1093.85316636801 1167.72023351098 1373.67062432073 1105.80735904293 1509.69760873558 963.343120586863 893.676600121113 1810.68433539154
FAM96B 723.428294037428 700.353187874339 670.571106793204 667.892385395063 1046.13353853089 959.532977061027 789.752781067888 626.582864105324 1318.3633082502 986.65554605455 664.19083888799 932.862030475849
FAM98A 1704.25279485563 1718.80498208397 1226.10881264804 1980.4589291261 1138.00644332379 1142.44180956604 885.104778820041 1163.13845502757 1076.62597781124 1025.27246494218 1016.18143428327 931.325266360957
FAM98B 152.591091223395 140.805322576253 152.93058803161 154.548286860883 143.011042973347 163.704616231638 146.370084152094 149.689071018094 153.440952021924 147.52178512946 145.751336115646 159.005459875338
FAM98C 228.926077117474 222.484058975856 210.882890077598 280.848426993226 411.160709628865 293.350292227077 262.79667156266 257.706904467657 259.511314588868 207.076364103755 220.458449424062 244.954917633904
FAM99A 151.191822057706 129.672284417918 133.067237748129 139.966813133326 125.506466437764 158.604431001643 120.01190102634 148.492328039604 139.997245010492 129.38021642675 145.217261514886 139.866536599877
FAM9A 135.458682745495 128.381433897602 156.890299350025 145.45097990828 147.694098231655 140.132816040735 155.587018319981 153.853856339585 152.137669424501 135.750578960284 120.413468119484 132.875314170399
FAM9B 124.66386961898 130.317604643904 115.963144087717 127.245190474707 117.567991653999 119.184780023269 121.898921780569 116.939471420419 117.12833256636 119.704436785439 115.923424095859 121.252042151856
FAM9C 139.089444892187 123.527903217158 141.378381097266 140.680326850085 133.211362932569 137.82918941689 138.340652133783 128.872496485403 142.747793303346 145.250076958551 137.260359343758 151.712097221291
FANCA 149.560505078257 141.5739152922 149.788558681761 147.784078315091 162.52512327258 143.705475208192 129.719122724699 142.480203661104 142.745838888446 151.902352951251 146.565265415908 160.446191353364
FANCB 140.750004067571 144.988803993714 129.880696706381 135.991630745082 179.432235153344 161.00635457883 141.08625682411 201.167206950576 146.755803731358 145.544996009633 153.697775290962 150.244284323504
FANCC 173.001985500448 159.133905701536 127.540848073484 142.988500173397 179.137734914428 144.466914388684 148.956326355381 151.794243108455 163.566535828036 159.26101905957 178.966169998125 137.195906276069
FANCD2 125.822346524903 138.97443063851 118.294324274616 124.851025681014 251.268669431283 213.447886742681 158.56996905631

228.560346647306 215.300064529551 184.365354978005 185.602093266907
219.394500571586
FANCE 281.405355165228 369.373283422151 290.135914275115 418.420753332232
535.612000577004 287.889774984754 359.024299091145 213.123453925578
321.794887577351 924.630951485299 249.925640256322 276.284517459626
FANCF 104.934718065476 107.303126530947 115.639101191444 100.325215581101
119.48998115497 127.706499722854 125.67874617392 118.064858133264
118.825948323992 104.714872508838 135.923022674367 122.379537202709
FANCG 267.800523478098 332.749676505218 282.963958072528 342.147414141627
761.726829618132 463.880793292701 293.281098275134 594.92419264579
459.53652431464 461.088572554864 285.980446338971 287.703569453502
FANCI 121.843992693814 136.140327952231 128.195916531413 123.722744366103
325.43977732888 372.088129459528 286.541028896473 335.389274425025
411.968307579588 290.688850193983 212.669156800241 386.067573158514
FANCL 167.430767074847 169.705236302448 183.056707813352 165.30072618497
199.813528853649 242.657224881824 255.638433159437 247.942904862478
221.512905853031 214.4829383264 286.774606295106 331.018914429561
FANCM 124.975454733084 120.131839791515 121.194138394375 120.270397272069
115.16087297104 108.760780994582 119.590540166384 113.816165294487
110.113844434493 114.953328013917 131.085965122946 114.999628959921
FANK1 138.02757574324 120.748910864064 120.862516074358 123.518170356322
130.749763860615 120.93965087814 132.960708384399 129.603952706231
133.890597982015 130.49338418515 159.670815631794 122.309041354217
FAP 174.383193885933 172.934156790775 172.816477996646 220.379186481273
167.842122798757 146.16821907742 172.345201438148 180.710636205653
194.921021761976 578.811908030805 148.616543842015 183.556662533396
FAR1 613.846166856328 739.52947559669 744.808064061459 649.052806726591
308.877027622691 915.015154168627 355.609033348692 547.516635695872
617.67819271245 362.502631441716 1304.37930719411 390.809169896009
FAR2 173.892380305166 157.742948919213 166.232856234879 160.50439457257
211.289598823191 321.119758814618 158.573561336755 479.261755380872
245.850813387241 180.814610631281 183.82450756022 211.736398352592
FARP1 372.94852645418 369.974231530826 280.656087497806 478.040234382488
209.74583569369 275.343591424586 209.568066932367 355.443758121675
375.539588956213 221.796935051107 369.838429521168 328.826483579278
FARP2 186.747375844319 195.141733146452 209.090788790449 213.791379190359
161.911468506919 166.099940078284 180.590215659031 171.629605754643
190.318957029666 173.951605889009 343.048904374136 197.47340234886
FARS2 403.47026424469 438.533112511098 482.226889092547 542.267204956846
300.199170426996 394.294057009443 355.990908506973 350.926679856514
373.290426793366 372.894376776771 229.046153362151 418.319283832879
FARSA 206.605699708924 187.978627462391 176.208206282902 261.988345506548
478.180723959397 256.99019407912 240.591548171052 252.789376168324
202.01798254763 204.802810257516 238.124340832815 228.184535093641
FARSB 141.466287523861 136.935498266723 129.640303191006 128.729687597908
114.405811161631 127.001546144286 122.351186755611 124.168162885295
121.113186311514 126.259029676691 127.000175858468 127.979574243743
FARSLB 29532.7890259497 30573.5859783923 31402.6950316226
29868.1819370827 17830.1333276624 29868.1819370827 21110.9013462062
30573.5859783923 30573.5859783923 20887.0399135573 28530.8435957612
25808.3502428915
FAS 172.774914399844 155.951509643078 141.518491890811 142.122556679441
125.79193748535 144.81320329947 136.541973790157 146.11669387238
149.8919086368 166.472068379965 156.695440330379 151.577450119302

FASLG 114.818898581401 104.091301211096 124.889829874126 114.421383025971
110.875112640101 114.704831217037 112.488125161387 113.756115251188
118.100611964852 111.794102015354 125.962178569021 117.003205777245
FASN 463.622673539254 593.090436323333 722.974310297586 785.290353402444
15809.9561036761 2004.19532096239 3510.24464112094 5195.2087608308
5373.2173152906 2249.8123426309 993.774175699873 3093.38255333143
FASTK 459.53652431464 488.379057110543 456.298408568636 656.867138022837
823.297606185721 565.509446660206 748.38293089716 696.003998372271
579.923640505245 501.946140974776 674.338740875512 766.176882605175
FASTKD1 202.869364962231 183.302479730653 189.482046688226
205.65398844108 255.677315946354 258.964323941464 204.161375686761
225.546125484563 204.237952297239 252.789376168324 343.571296297462
210.852300463808
FASTKD2 175.679405338399 186.406439948242 243.283375388645
198.737421468412 166.472068379965 362.899103657252 200.668298229815
267.285998701386 222.243523614518 196.734037460218 213.93763684072
258.830885826237
FASTKD3 171.660157220703 164.03801002919 193.502988612307
175.601912488918 188.322853571195 325.907037744936 253.463871742303
351.02823853348 245.317436234586 232.465880700217 187.796440205485
260.52593414721
FASTKD5 278.469340236003 265.572253407219 255.039661999732
272.639413442579 341.361299896997 567.869601934138 372.894376776771
457.292146759215 462.948429969719 333.533043832976 368.466518140043
410.337491313251
FAT1 716.420861490714 766.420275252501 563.648536446918 656.660582895703
852.436775264873 1345.8970795036 1502.3444541735 1165.95924778817
1287.3125466789 1465.2419150392 2078.76377557352 1492.15137418791
FAT2 145.122171099946 156.964080041261 124.408996713299 129.83316008718
138.634814756987 131.351303131574 179.191401472241 141.476041451832
141.550553034455 1080.95725397659 191.297539967707 255.71113398292
FAT3 578.084277076692 497.269957926657 558.251384152474 603.061485415867
167.791351000743 169.158049136321 174.657352858049 181.715992480635
157.594038046897 178.262619516994 192.737713851863 164.048326497989
FAT4 223.573080955095 170.646233722767 165.45479542657 188.685460181066
141.644521617419 153.162313098346 141.097257687941 138.188522429661
139.524233960073 148.168587884369 141.139737175648 141.727639388452
FATE1 131.204616536341 139.369815367465 124.892438890234 129.733935614802
140.36425603453 136.799138736925 137.586032396204 130.597786455581
135.691862612951 127.696578575874 131.369177333568 146.875733210901
FAU 10106.8225600705 9433.53967760357 10661.2216586049 8121.54750071211
11281.201625198 10474.0047946187 8982.75408120297 8532.46059476152
9016.04641569485 8864.70614223459 5265.74259211695 10704.4678928541
FBF1 150.176942469551 146.428534132236 133.775716376056 137.793462381778
143.322199505844 138.428527640896 149.933919274985 138.746987455826
136.461182044855 129.13353785479 140.493975416335 143.373932371335
FBL 1611.58245311122 1753.58770316818 1530.45813438919 1695.26858186096
4793.22133852447 2131.68478964427 2712.07480130502 2004.19532096239
1899.94714976387 1552.69879081022 2792.13364570755 1944.15673951528
FBLIM1 162.229052648595 171.086559024882 162.955366577418
160.713790305656 147.330897105911 191.241000498118 136.93893152919
148.624135810148 143.028618831382 234.28146549561 153.972471779464
179.704370382771

FBLN1 7003.06505884735 4875.22637286719 3700.75770419386 3712.67025366957
5250.88041666757 3408.6535437109 3413.39994653641 2962.11407134162
4073.21075791737 249.400820221233 1706.06615968622 396.04031733841
FBLN2 3130.30693451802 2104.59597571915 1109.79571066685 2421.87041217217
1371.56635975955 178.984409575632 265.470314691221 179.009510246709
306.59090554099 575.23272138912 196.990867962546 172.245089970803
FBLN5 1504.15682715484 890.810315261602 1001.92763014266 895.463355957918
164.21464602746 203.704734790602 156.852187451794 215.673878830563
220.362946606131 182.42088387266 164.901810642726 170.158189150422
FBLN7 159.973758408655 181.400788152988 125.576103876855 142.919910421524
128.667662783862 131.265594391542 127.466779212194 135.088149158147
140.907889482107 137.244552699212 165.277935422113 131.273916869754
FBN1 204.237952297239 187.519053043173 148.25001336038 260.921177985439
116.983869766097 113.38168751499 120.644987579644 123.929180327423
136.923079303455 140.309394542299 141.724442944322 122.133292722932
FBN2 165.880071298733 174.389706515542 174.576132939661 155.896065329825
380.146996799549 167.421776383056 146.919110530891 217.408824020423
214.200081541057 1144.07896461196 167.325227483198 176.77183598995
FBN3 146.927825526831 147.319360896679 149.821848101995 142.311108110185
149.824791474186 144.961976523279 159.2488784104 147.038700869156
137.17370871536 154.996829706812 145.741465098455 145.407517107023
FBP1 336.70893120135 552.962676093514 599.926717145115 188.641397688261
4043.28299602858 2309.63504858248 168.521601064374 2352.01196541874
1732.52466021178 296.088938872643 1736.03342040646 1696.69982157582
FBP2 136.788269716383 147.218415065692 128.782192901724 133.387065801837
119.418866166776 145.799530970042 143.137158027738 151.826103919919
142.372470063444 148.08870546717 146.070871543153 173.577723566555
FBRS 397.737702926328 457.130051460676 434.509391836336 566.914448800423
697.97116981445 541.460843251764 484.658140651032 478.847650837714
642.057106241761 445.348044448953 532.786041960586 443.444024128516
FBS1 186.722842989058 207.218779485342 181.036375971015 204.260800563197
220.242344043437 178.606255846041 200.264756078778 187.296479395462
192.640717683236 192.627266049516 222.156471143857 182.929326147069
FBXL10 578.465753476561 624.467319041884 472.793332506369
643.468308027006 907.306121257236 643.306565600931 563.648536446918
648.38049615541 743.510369438587 504.141861790903 302.570932942847
600.757699463777
FBXL11 664.343553341563 651.998433127992 472.279492678374
889.6617510875 634.046843336128 542.883453307 472.279492678374
401.461825804111 564.588706307423 399.664121956178 137.519771130707
428.09583118349
FBXL12 359.493075382333 336.380724981693 333.200230327106
339.172031360524 277.129985959184 365.864755316163 357.972320648217
319.966785546419 269.831616158113 343.346755862776 370.140588087649
419.126814511305
FBXL13 153.551359970644 155.502363062633 145.640156204495
158.098055917452 148.215172022129 150.405715534245 148.176995739142
145.662818023154 139.217113811817 141.557420268382 147.526701500887
142.616603901398
FBXL14 187.65716654049 176.155413809391 212.420616436137
181.79418355897 174.962380276394 187.30908753142 183.998458233995
154.371946675862 159.696443182844 177.508603290389 155.904257649751
176.11287574658

FBXL15 361.082957133644 391.185365567603 338.866471651692
486.653672099699 359.935451429371 201.637540930213 249.449151040445
207.940596890723 226.712995229189 209.960795065047 330.241479004885
320.467139255533
FBXL16 142.939578759097 139.936661244361 145.726651022813
139.008854444223 147.841960346835 148.11536596061 137.391141254051
156.873097743717 167.67952650068 155.368204597287 150.218643705925
168.210416363286
FBXL17 164.022021048626 141.033948839613 125.416113646128
135.4702891855 142.866874321248 165.249658565871 155.243643210534
125.27625242915 132.011199659803 171.350620456505 171.358746749715
165.003646188775
FBXL18 229.200570583629 283.235844022826 278.168897504985
374.337030706199 546.181615768667 212.531329857909 256.291984914927
387.224184063662 324.009703043876 297.419034136005 223.474531516972
262.577672828611
FBXL19 132.19734622841 7 147.766043861937 150.231063426302
129.116354997747 143.298218989451 153.626180103404 157.04599638368
153.457715729768 153.719751724358 137.685104040843 141.628203045085
135.493053871272
FBXL2 252.957489543915 260.098788125373 257.408058436941 255.770193180874
155.837866295709 199.854728619468 181.491120129045 169.720414035325
171.190796424612 200.867515935703 201.349209185128 240.915496717526
FBXL20 299.616447269111 263.005303710717 318.348505551265
332.082749517913 275.584858082096 229.230239609291 407.442337662943
601.800326895295 366.857139377277 307.480644393018 210.925203743958
245.489266602897
FBXL21 176.214950733324 142.663334893424 148.454344601562
146.782830477983 153.519207482423 140.092648878007 141.302075343586
154.618685493541 130.161889021281 129.884064654761 138.875827888646
155.673532440475
FBXL22 421.690849282483 607.805002786409 505.83860711693
837.872187194784 142.745838888446 156.737219981981 166.036553680319
122.624828884731 149.73250641246 129.737882221259 128.077692981235
149.070750686003
FBXL3 153.350690419636 177.88534558205 201.593025256991 149.651553512698
152.585572167313 177.491294133515 151.330035086652 160.640160107693
169.332207863684 196.245297362955 180.528296177577 189.853136769155
FBXL4 104.544792793756 111.111969531957 115.409568875238 111.577400298092
108.861243071977 112.07238018581 110.718864165172 113.905364405553
116.839796680107 107.40922161073 117.12833256636 116.294180301039
FBXL5 317.659548748735 267.622579012917 424.855168311509 293.350292227077
212.190707408572 402.299165610893 299.106362530855 282.742833166843
297.544216175512 289.667302610069 322.64731764456 319.560819487616
FBXL6 189.37832342197 193.569894919176 208.555506929113 215.426918011158
243.159062832085 246.63579788514 181.183277133224 276.184421402511
272.117599603586 294.280238648737 281.369059516242 276.582734590058
FBXL7 395.716879127149 333.592915218163 288.68376542946 433.751675703425
130.042683562719 121.260355515095 119.06600048679 133.891337645936
148.89180107448 130.735223763219 161.841975820372 133.43362126395
FBXL8 121.384582834956 129.171589574238 125.661697165943 140.579839043912
140.779962389412 120.602818832247 117.841844605128 134.448481253264
118.144109564626 123.7615105541 142.588013648337 125.208803405105

FBXO10 139.847813838017 142.674671804361 151.451443480833
158.126480180017 138.104527851969 133.496922906874 134.619470709414
131.525916493834 129.320210774045 136.13751015238 119.9414421871
126.77232818113
FBXO11 537.698489904622 516.577357935963 523.216537391852
502.499539192864 396.781827940009 489.528040775004 439.33566689752
463.802270256246 370.390323116113 457.779218916808 444.826775824095
443.853458865556
FBXO15 131.988591391373 144.214589452164 144.853868519575
130.613760017864 171.287186905764 138.994054501048 137.388190728367
162.208028489943 155.247452081206 127.887666721519 132.236355533277
142.324077852852
FBXO16 137.779607093226 141.25089277145 124.738217731488
156.602240897907 154.037622223246 167.811412309356 134.949572471421
139.736465430177 154.197254746757 146.637799077812 143.419034484087
138.571102593669
FBXO17 174.168079247768 182.066922756289 176.725347194807
187.280334341504 130.049854901235 134.432461986523 136.126542897364
121.841080387875 145.801228425692 146.077082896858 133.544777710022
133.9382467268
FBXO18 582.062758687489 587.388038484307 464.323065038265
650.911886508922 636.781477235579 594.92419264579 443.853458865556
464.812091712746 435.183433729519 358.780920910909 572.548647437438
435.69811966429
FBXO2 150.968998243223 173.10229478279 139.403087367992 140.52997588229
167.149578610266 222.8766610624 194.961531388323 219.228720772236
204.802810257516 212.987082617173 236.401834189942 200.424975825325
FBXO21 787.017214032692 846.985164747386 965.031655021246
769.009371389327 665.602699607102 1055.79590411506 907.904780399957
888.706978062337 1053.65938651514 674.571475335107 976.360310504918
917.188039935737
FBXO22 153.655778377717 155.702330867056 165.158465031363
159.918778562802 166.368623023308 169.895672129542 210.015199832609
183.081450609516 183.371103136697 237.01460308334 186.077477713775
213.602504580364
FBXO22OS 147.243263182654 142.06907380533 187.978627462391
160.066390229416 137.018521754443 144.81689330206 153.832132196284
145.787468865642 148.294870328642 150.613624696604 143.180247878507
142.444072809204
FBXO24 122.501785735146 123.111192189217 117.716094828779
117.856184272322 121.483610947845 117.969576809288 124.794260959228
113.479382235297 124.190161132628 114.664782541261 132.952355710395
116.718099363329
FBXO25 154.003862874241 135.381131982323 150.021512237553
151.194282143046 148.120968141789 143.140502042489 138.220016828327
137.884051774657 143.653452869664 164.109321257638 143.791908191551
148.227581116046
FBXO27 125.768053724055 121.116206210402 111.62514398781
131.532660547328 182.292947734054 136.058527150792 161.870389635266
172.073751088968 155.648782851696 133.846275669922 169.87591929982
118.631771242606
FBXO28 285.83626978316 283.807827536147 229.805500520256
239.970202560698 269.006221210929 462.025126897013 333.676803661366

259.338947392465 359.329520507158 428.66614441422 246.63579788514
463.754869412768
FBXO3 191.937607311711 180.590215659031 243.402781509365 166.095964870713
160.876228450608 194.979795724712 197.91325268683 177.151218006672
182.665357598121 259.146673652222 229.347135478142 177.415756020166
FBXO30 579.923640505245 510.434895296759 706.628675634893
470.154677948419 247.194197143562 256.237185590901 254.552034511333
270.490971664802 186.127543877065 269.379272725008 201.085046871333
211.595513743111
FBXO31 1370.61156801632 1607.52029676299 1413.37062765856
2061.9164323309 447.059120541009 297.071121022183 336.279209908669
394.294057009443 328.431289733169 335.142319387164 337.487980551006
330.641230715217
FBXO32 1116.19403476786 1145.35733270036 2430.70635781622
1420.27222765071 251.665539319612 206.257167235126 254.036619126409
310.075815009404 245.351459157365 338.585771590234 225.96618659877
164.281196089736
FBXO33 392.909481887174 466.691191261997 414.333324610267
441.958777965408 372.149771075286 464.196512015649 534.920870202582
454.030083521756 383.682951666155 372.54091392596 331.747622606378
440.319777672273
FBXO34 291.683896942007 306.946103334814 303.138041758745
246.327991259259 264.685862877779 446.509863498357 418.244376024741
334.101619029105 316.364342983155 398.673582680257 513.816017871024
350.658722333252
FBXO36 110.501341211051 117.312094804205 107.385565665491
110.368392735494 102.725375719353 108.850992642446 110.602406076036
112.005939063045 106.854721604889 124.387133895009 131.670601860412
112.987890650593
FBXO38 272.770616373687 265.754141836026 303.184785797741
274.120234926832 227.52352046188 310.365203383647 378.037190077449
283.377663208926 267.372241661758 367.229293432041 348.111872425949
354.656612182522
FBXO39 110.304102941202 116.759526220736 103.202873534814
102.661440700541 106.147993332294 106.629065516249 114.249891266241
112.166192839859 117.028963434266 104.977794653504 99.9173294438729
123.227904580467
FBXO4 167.51207060064 165.262738484387 171.775741648309 161.021740537372
211.085778902367 235.650688823345 174.42996813062 245.378747269929
179.785198362326 149.584582050615 182.074569356713 193.394225360968
FBXO40 107.780763250481 98.6941019230576 111.672450681359
109.806338826826 113.083678884076 102.421174406474 112.809629077555
109.781334128478 99.5148073333731 112.896360244636 120.003095000297
112.026113831896
FBXO41 141.859889340936 160.265389854694 159.668402591441
137.280933631075 180.179981672095 210.316431648335 197.947433984294
203.675655444583 158.325554470875 149.959767051801 156.609370182062
174.454754564111
FBXO42 201.441808401878 205.4648759901 166.001279428607
212.710910778949 210.711890471222 141.114148695402 177.11682975342
182.248178961707 188.252507246148 194.257024100927 214.991170762327
179.137734914428
FBXO43 130.416957431512 136.682172321984 129.719122724699
130.346150829949 136.085524091635 137.885506916098 133.122174596886

142.577102697682 135.743159681223 139.771342253058 145.274345879987
137.592523459311
FBXO44 123.692366307662 124.885950473434 122.834017529045
129.935532080325 124.415058391979 135.007473769551 140.234485088845
137.443463471483 140.864196804152 125.019064287385 151.730862403472
138.53179773391
FBXO45 185.617936412633 160.949030247406 190.572712684622
188.515336186633 179.905809470907 200.902123076244 196.329602378007
204.35801867216 271.356289141017 190.5930909277 173.432960318793
179.286187825363
FBXO46 249.633252091596 311.04252785157 273.303240462047
344.575967665039 662.958637409597 458.218913259336 372.374101464689
351.188086838653 431.182384100794 266.652805624826 377.89330639683
275.437030326768
FBXO47 130.548611758921 159.994376562272 145.948772123142
148.597346678768 149.902420337518 151.32179802686 133.834423222601
147.595573192579 154.552605305297 141.683298500736 132.240470270795
135.10155936286
FBXO48 162.259941098076 152.79367157305 153.426409060413
155.018009545475 141.54943189147 157.067264221207 140.892250639524
157.643461899964 156.930119977809 143.537785553911 144.682202635712
154.681604647652
FBXO5 174.749832388791 185.396765725228 153.165872773973 173.87113073582
224.167511918105 240.283918204202 235.162439885207 281.49247863022
236.745054217055 219.394500571586 167.278879415171 296.75326294587
FBXO6 183.482640529727 158.356964958885 194.819597436776 182.633773540249
711.491528818004 334.377352542623 500.246996975 541.258721278882
417.262521411065 318.930960243303 382.069677030369 271.858059712406
FBXO7 344.915370555826 273.651114834013 334.316711893654 310.989043012841
332.194163016646 338.934501259414 279.75914135192 279.452819132461
296.141012087256 323.628636799403 350.166837086936 334.916875239616
FBXO8 499.176926371542 409.380002417794 817.913210743023 434.716530273383
259.094411953361 347.985315257161 767.662367310672 371.224296589448
611.772512110915 662.58366387795 468.339735327273 459.53652431464
FBXO9 138.28374062778 133.766199453456 132.769455423385 129.322481607056
139.886499402506 130.642810886161 142.566028652989 137.340844554597
142.752536416014 143.423661752881 150.620603731907 145.039617860588
FBXW10 140.149064156135 141.968949222821 141.949098898957
139.951275828765 161.096914295309 139.843416673512 163.799748670842
152.486212022518 148.204194003593 148.235495611248 135.403206634483
149.844677055508
FBXW11 346.78158577526 353.005548289786 409.282410265733
318.930960243303 229.74418058711 261.500728878974 328.60529053804
279.75914135192 254.133112176024 374.337030706199 286.642323413104
401.840794789616
FBXW12 132.987389721592 147.357032438442 133.122174596886
140.590802695412 152.222732770706 154.928072022777 145.187490179166
149.035590153457 134.096283448464 140.194414869595 142.14116448528
146.666759322506
FBXW2 203.317264638828 208.72873686638 198.023465629218 235.595194918187
215.607567127195 167.900071375885 194.979795724712 170.655648004461
195.230219990395 218.896760804208 188.941900997781 173.044636575826

FBXW4 729.729297413233 858.524630425687 968.157701932137 935.784743413185
573.846138124455 347.812754957414 560.015499136193 327.384671423687
360.852730026133 422.02217814979 675.083275037052 620.899414581162
FBXW5 267.869879189095 278.555009982029 263.845733455915 359.329520507158
403.292651623365 179.241385747152 179.593817076317 174.928155956396
208.628626112623 226.458509834965 288.026656721827 193.008902902654
FBXW7 216.126000558319 220.896363324634 186.733394375839 212.291528248457
209.702683803624 223.429738319185 223.389795643722 211.160781061336
224.439293045835 255.039661999732 221.869191666931 242.657224881824
FBXW8 145.020615552964 158.157097243342 150.927628184034 151.885992695789
161.019770036957 148.386249316173 144.273011517032 158.020651857664
160.32111439724 152.28262533486 158.915858403237 159.324816814876
FBXW9 215.453915231774 213.93763684072 211.300733064951 235.795627549799
331.062744782933 470.868288641048 341.973002478932 287.593221911064
339.012225830929 347.812754957414 282.652193666078 304.402066421813
FCAMR 141.561769570516 148.859129298141 133.177950880393 139.092075947067
134.580526467375 135.501192996538 144.182891591399 141.652959320966
152.972779110762 132.6175371563 137.033134002983 134.648780131578
FCAR 1989.38201206555 1508.24281455057 1638.72366638732 1722.26954281876
265.61506544085 756.618969995355 463.99922423125 520.962651968997
411.784922368377 409.073045356073 547.202354339933 448.715938510132
FCER1A 351.934158805882 269.981476129439 184.021010234322
208.869189492052 131.525916493834 268.034972830233 340.028737478082
193.16411905957 147.937679308903 161.552475294424 166.526875572556
163.042083189818
FCER1G 233.572055978465 222.118666699301 179.932404140099
217.576547690525 194.702974020747 266.286483421453 203.777944180067
391.400480065 648.549890890375 402.299165610893 258.695920967489
231.202047619476
FCER2 109.167312436827 111.243322115126 124.992439957206 119.107992692097
111.09179957013 118.553226856337 121.248923222807 121.472885562857
184.694741621812 115.878861991823 142.248293068033 115.184395637553
FCF1 319.639103463458 266.756647182704 446.393631025049 238.588762326319
204.695311233073 195.66091457697 212.239485199343 300.358738353443
201.123529130035 336.898462056187 199.250680260804 229.85833221434
FCGBP 141.236939351298 167.55209828062 131.816989019024 151.121293928309
718.449150898903 146.238333981318 263.845733455915 160.791355483048
246.457698183615 280.414161197286 280.990656342284 184.260835471542
FCGR1A 117.048846466839 131.077814710839 117.829228895897
117.12833256636 134.460035654795 124.05019986924 132.804216633852
129.318409053566 144.610341215903 134.678445085174 162.333158524811
117.227635924674
FCGR1B 122.615852682436 117.688941284427 117.270320709161
118.833188167696 128.488598467312 116.118745494794 121.121686280797
138.175538905334 157.130045415943 157.560117350562 156.540165645256
126.533083459283
FCGR1C 133.473140653666 138.654301345398 136.647239621513
144.769824608083 140.010730572075 150.531753446398 139.936661244361
152.22701178148 168.384397488011 161.585555680663 161.870389635266
139.205887721493
FCGR2A 159.563866056798 159.032710656826 154.79534193124
148.934593102452 166.452651396293 164.991522994763 168.890506147594
199.555406967021 256.897177822238 307.597733293218 200.322334039458
167.442950045855

FCGR2B 194.775234425687 165.619206216088 178.572212478544
176.725347194807 172.338509138759 158.554429311951 180.952017616136
188.836327151669 180.733673680261 185.275787562938 155.904257649751
161.581641777014
FCGR2C 109.770126492743 109.574386044714 115.208555742308
104.58424134098 107.316786994369 118.303150165804 111.448871835514
110.735199684144 112.867662941811 110.753661013519 144.961976523279
115.30786313391
FCGR3A 227.736106796551 202.778539118009 223.638850960288
227.689356258276 140.332045911008 215.143973502566 168.289492873475
187.65716654049 175.601912488918 174.284268998257 169.598493862848
182.439945229388
FCGR3B 187.754968096521 182.810581377888 183.397833894087
191.486717579847 189.603621918298 182.263724979519 207.890408761692
195.88263399968 215.506175859782 249.171307797736 200.424975825325
168.687845035401
FCGRT 1197.00004043747 1105.10063745656 815.045148478814 976.782815449208
2720.83642320419 977.227184625781 941.065508539108 676.293399523316
1010.73988405469 364.964572587289 363.445110627483 738.37559871635
FCHO1 119.819082491115 120.387275261633 115.332409360957 120.252164075308
578.084277076692 246.80306998812 160.060406629689 154.834679562341
153.13384838492 120.050548624975 190.809584998617 187.754968096521
FCHO2 262.756237406794 309.861048237742 262.861105654051 250.406775889932
241.418016203544 251.731327363027 242.382534384155 192.512661648653
181.758545723241 266.80383076887 217.125327913156 251.833571288967
FCHSD1 156.650388509656 162.955366577418 155.272264997151
174.646321871605 173.017662861015 172.338509138759 174.941551089279
159.182533998843 147.436408919939 159.678281513591 154.236652966294
169.695658738618
FCHSD2 383.10686624119 366.007451523992 267.939624375137
375.255445179952 225.803528238368 220.169099923628 220.959843573708
219.441959146815 254.909076658804 175.757588503244 256.614964511846
197.2893692747
FCN1 199.305626149918 201.059635671301 173.459008248696 205.830321788612
166.330001480033 247.746304138866 216.880896769751 237.358925391616
411.878556907186 235.76573393374 156.200313064999 194.60309399944
FCN2 130.485988201308 117.199217938531 126.828097770667 127.104097294238
127.797666392002 125.937660544107 120.163286072512 127.325087202483
125.528638246486 134.558435781672 133.467092274663 114.182798672347
FCN3 130.766401858384 142.024171830924 131.554055357245 130.647944268116
179.191401472241 178.429953283621 160.543465620248 176.11287574658
168.594235675558 166.850279817976 187.132496910208 147.624372080117
FCRL1 125.674471551099 136.970505971671 134.285191872099 137.834158814913
137.793462381778 135.513494853742 146.276706674761 135.419628087842
132.595360509902 138.79473329684 122.149756212115 131.22473774325
FCRL2 136.366798516444 130.824958143557 142.173279109959 139.155456340488
140.782522936146 142.939578759097 148.166340306996 141.102274192049
145.212306927438 130.89594032962 134.835962366555 141.248526020456
FCRL3 142.671190279415 139.458529687684 138.440663763957 140.782085233573
138.951140134927 131.48517604564 137.59017685842 146.595540577994
181.110403721353 139.016274575854 137.640239596303 137.861973133557
FCRL4 118.011195631598 137.307786147787 143.455941298415 133.384498169568
122.900675062181 131.212705319485 136.113421708277 125.826111950648
132.069175779393 122.368581795931 117.420765321056 127.665162524824

FCRL5 156.880120259304 169.301498852556 164.048326497989 172.568852318369
156.417000271727 174.795822188504 167.719651771357 158.767439363085
178.12073430094 179.905809470907 151.606932937644 168.188793271919
FCRL6 148.027464692171 154.904424749528 140.779962389412 154.24500773656
162.096065738375 145.733828447934 130.532559074698 147.681723355535
173.056715623574 170.05799929575 144.92594578882 142.259196792767
FCRLA 113.891385601095 118.039780360151 108.386545220984 108.543795408713
299.80615152845 124.327827104244 275.468194644053 161.079169854907
330.850098426464 114.249891266241 134.11753210296 120.276236447255
FCRLB 169.293852989203 212.107842040568 165.545136704242 178.657325741035
7555.47017983216 817.913210743023 2992.18604022433 453.593585032712
948.734577648136 251.61451602376 1885.93146154931 598.924339094496
FDFT1 948.150767619765 1042.40539020413 1215.96648642927 896.185223980593
2225.59207490574 1432.93880448974 2756.22069438696 4892.17875938832
2107.99357083999 2036.43904731226 2845.0377634896 2171.00457877604
FDPS 341.714401872192 356.530530468568 310.174932137546 374.657816575897
409.073045356073 441.435444003241 359.518199449791 484.230510636533
422.688531130043 478.040234382488 483.549698382115 440.657747083721
FDPSL2A 157.454558821261 166.285052506316 177.630246034993
185.830435830545 137.03111066852 171.650247980576 178.707066209511
171.521579011287 153.996429870564 163.396651801661 166.8846964604
173.526005872207
FDX1 164.848970268132 182.439945229388 190.625250757654 169.012926546641
196.329602378007 182.248178961707 196.657475905674 183.619184925353
164.185346950306 174.364798143068 180.747527895349 160.039086963419
FDX1L 344.070947256963 370.301819403832 331.888150158628 462.144334719146
403.812520074723 379.387095129725 380.754569467715 287.287337931982
213.531307836195 347.3483417873 200.633009710701 293.018282386003
FDXACB1 138.054470878471 145.416733388652 143.272669418277
148.618327213374 169.206723147505 156.923675862035 142.953661611418
143.446317006075 152.017655364287 154.150568177135 163.504122327429
169.568915494577
FDXR 129.394359810841 142.039943511086 131.347213356457 139.265915951162
139.002213373439 138.079494235456 115.68358702028 134.576622528874
134.008118774075 149.65417821994 137.157492354617 126.659859384257
FECH 232.845268785472 228.349507726225 269.495247190598 258.012234458166
154.985781979984 177.742616962629 152.454414703534 199.916301659113
143.373932371335 181.747090380435 197.231408726321 173.892380305166
FEM1A 647.273409499313 725.621153262924 611.540759532294 834.537908087672
869.937761943249 631.819659701092 815.388288623756 741.255013151687
766.420275252501 611.286068355939 721.31849915135 601.401329244185
FEM1B 161.552475294424 171.465178761171 131.013719535992 138.745560049251
167.586897846525 154.066939372379 189.853136769155 147.294408903227
147.823552813852 284.995098167431 158.287108069245 161.988082494464
FEM1C 929.69741581954 911.340266389071 808.55123510297 784.72162514331
261.065986251886 610.591049634222 505.143815448613 590.3584324045
313.480307434343 558.440840319738 510.555184032017 468.060528109338
FEN1 225.783771938969 265.670643951648 237.266455875982 275.929414239664
683.470866056244 668.853969513382 410.731869154912 691.632061537999
572.049540360134 429.812058546782 314.4408959517 569.656573915709
FER 215.40037757604 208.476367897256 243.691004557778 191.348260799746
165.459993078103 171.98820121502 172.502036705114 151.895142552067
162.056622258216 507.040326298457 163.463191619941 177.11682975342

```
FER1L3      941.572186137467 760.572409199179 1084.95095717937
      831.08287594363  650.263737836685 586.246004571452 1243.11900823259
      623.68308750251  1178.08490218853 664.809419789814 1025.27246494218
      756.068695265118
FER1L4      204.183472655276 250.718986968076 267.869879189095
      179.46936343225  595.120528902501 1887.82245931444 3184.98944679038
      2045.24382334506 943.677679525947 166.88833169342  1595.22997714906
      1969.67552562117
FER1L5      147.633793743254 145.146797807275 146.04584032214
      158.452089329714 139.677109265241 140.311843829601 137.826458479398
      140.78582131501  147.387754442307 154.558968826495 158.171302275984
      155.641860356683
FER1L6      137.048292841566 125.653011880654 130.59670974887
      135.627840788802 107.580425848009 128.276471459777 106.126202301438
      144.845726048937 126.365861978893 136.497295707149 151.761369162723
      119.040642659337
FERD3L      128.005043478952 134.891736344801 131.330974614437
      136.19725093985  131.367075440559 119.382151435379 115.440152891339
      122.603226380439 123.913203953936 126.980375035189 157.675974692761
      121.838802846453
FERMT1      158.509885816997 169.417764098848 160.799950553311
      149.565725707356 1289.56069670699 433.11536069928  504.275299381356
      386.85874184746  504.502047826003 462.437471715008 370.227329649269
      501.301694626942
FERMT2      9099.13956219312 7406.86318549013 6670.51138785163
      9820.93899217345 460.651156484562 459.973764138144 247.550312458738
      325.387193207414 431.413030534188 473.057488824363 244.596233138747
      246.676401619881
FERMT3      161.408318705503 151.214631373344 131.626173473789
      145.615291241733 207.173653740239 153.440078723527 147.866909109733
      201.123529130035 263.936023499588 148.905860943967 195.481680273686
      142.480203661104
FES   291.020789939476 279.790257881254 243.10297424873  264.233381434738
      468.170972478717 228.270158363878 252.365946808078 450.851711160136
      250.542405852865 163.143613079037 240.376851873817 293.281098275134
FETUB 131.739418639854 145.7202993771   170.505553609318 128.882160435252
      158.95714101556  132.059507616101 139.421753451936 143.426053070219
      137.933122094638 141.710076413441 157.216793735479 146.384051525624
FEV   140.792400101142 155.187900101727 145.193296081527 178.939268046323
      145.783320434143 166.182850621447 175.372355078713 153.405052909585
      159.470818285679 164.247251507109 156.336425973696 136.087702679172
FEZ1  485.309521583972 417.442660073269 448.767690095033 443.444024128516
      267.372241661758 156.852187451794 184.377923161698 195.561296046507
      172.604689611475 209.279164243868 183.023316857537 148.086979061158
FEZ2  1350.87073284396 1344.1288416533  1229.47460924638 1319.3522488218
      809.729160605727 701.847905027147 795.464850398761 802.304294140514
      752.585744322608 1130.32966481315 909.388774224614 820.249910349431
FEZF1 159.515698265424 139.804518460303 156.707737817443 183.779065928447
      138.637735867167 149.586651393588 149.049931380459 142.886493948916
      144.905870145004 154.854538639035 140.864196804152 157.761231106669
FEZF2 137.876234091056 145.904866987074 132.966873990501 127.385776388242
      140.767270136561 120.041623078295 136.454911192129 145.281664250703
      140.427188241455 129.70974867852  144.450810504499 151.403431105471
```

FFAR1	126.76986764971	149.160808104789	138.855454645579	156.740412695351
	126.561067704708	134.565199667396	130.314247368361	139.595986615703
	137.919253591105	135.503338987971	156.385100395531	131.071614211431
FFAR2	118.988269692812	111.196610704726	114.512028664352	107.049865500702
	116.007006375993	116.221426977629	108.007762863766	112.693431140524
	125.940081665592	149.898614923436	121.113186311514	109.613208767992
FFAR3	123.650702461773	127.818137805569	130.689272423595	134.436423298225
	119.475462713424	128.282999719959	127.740057874561	125.576103876855
	128.56000638844	128.523703756493	151.106858426994	133.610143526954
FGA	131.456697863268	132.385096888962	127.019978195283	135.823975527501
	131.479997342133	128.341887728118	133.42964827086	130.055416790504
	130.004329307977	128.99859423584	136.349603320553	130.074786642758
FGB	137.415158613413	135.208478510375	140.545351041252	128.717719117128
	137.901317305531	139.97636570613	140.000880942015	141.819762045169
	135.229197100212	145.815454490804	140.590802695412	142.003414252064
FGD1	213.990575186237	228.542423012958	203.791916347213	252.473612525884
	357.584458526365	249.171307797736	206.620708230452	227.558441474918
	153.464360995071	212.933062534244	114.607138204495	179.969944022261
FGD2	196.676479140094	180.968659614238	170.807514224412	172.951567541592
	136.587165242663	162.786351190659	185.016336026382	220.415211013843
	332.400086578723	162.494429648551	245.240006198482	292.468585109951
FGD3	270.568660560796	269.770777921703	286.703712311781	204.787016443123
	1176.62418139379	498.05778289794	196.566937667522	904.733257358133
	1754.99663827472	549.047065540118	1862.88548451154	386.461076717316
FGD4	118.859745315302	120.212294184999	109.586955365611	113.557349524174
	107.559685467789	119.920097823022	103.398929051742	114.631759839092
	114.658668711121	124.844481048039	129.125709963326	112.710573586525
FGD5	371.767182305618	394.962978677736	182.084333814175	338.129250886594
	757.406530000489	199.288100834476	687.269774217064	152.528571776589
	234.895245871534	139.282741110499	635.37511798786	315.615758089471
FGD6	133.131953362455	144.940119680152	130.751522895053	126.097544650407
	131.734429913106	136.754116864655	134.679882781788	147.706402141169
	133.601133679159	177.772019227116	131.950295185477	120.413468119484
FGF1	127.395094952914	133.040952285855	133.175265384038	132.626349611404
	138.233799607706	136.511669406292	139.606654715326	133.731500722599
	138.72219653692	127.943935415045	140.960907353937	129.25124636593
FGF10	125.653011880654	117.296972688814	123.122705969332	107.622342073167
	114.805810485989	114.099623939954	116.019123495537	108.021898335735
	116.533361070234	118.192302821514	112.599970121166	113.460835359833
FGF11	118.572988141773	117.907840952685	103.168665937677	118.773864607553
	236.251507822415	147.808262170269	284.473085899219	144.876300850115
	129.591396165575	407.589300080181	290.213039891361	124.258412895333
FGF12	145.07503221134	133.994376985194	137.09814291566	131.148926523383
	138.370241517025	129.775280076365	130.939543211439	162.274094333189
	150.570540340126	136.475652722007	131.2360427855	123.709293439934
FGF13	153.405052909585	152.152106138031	148.541832125061	142.598106777707
	132.712573198794	129.603952706231	131.282091618102	121.591962855513
	131.532660547328	134.728594388428	133.42964827086	125.299513989334
FGF14	116.136849683574	115.389934936253	110.885714048075	114.129044806825
	116.547306374097	117.255496552581	113.301733735994	108.697696080014
	109.625579596864	122.320966091375	116.258629237662	116.126923911804
FGF16	129.366567668463	147.844734028193	133.913578037255	139.981905225845
	131.001996509052	143.541810469447	140.290598244169	140.856864690669
	128.539969915702	122.636980845207	154.113228966411	141.855655600443

FGF17 136.506347083926 160.039086963419 144.377501600001 139.442091671938
147.962721373177 157.863585655333 147.562757301251 150.244284323504
150.899664887945 141.721829342179 149.190528937822 154.865107093653
FGF18 162.825380100343 158.963726206466 153.19516365535 159.74220366735
149.997047695778 172.457165477145 155.326276840738 154.286043402527
143.544671705984 174.274996381853 149.628421263062 153.181097279473
FGF19 143.874735808114 134.607501073433 132.014213378179 122.937281123348
137.907709799074 120.413468119484 132.257989841046 133.727447520255
130.355969431473 126.185560760543 145.844961942581 134.027006076154
FGF2 295.973370766318 267.869879189095 299.29004204677 251.704037951295
134.66169946771 134.952584272187 129.318409053566 138.028813480968
139.611455791957 125.107483348755 134.524644163286 127.259038241163
FGF20 135.122770943076 129.189479305468 132.66318177913 129.794293922951
138.444427173667 130.59886317117 133.978783899572 131.230501496659
164.008063563113 136.428826558493 134.57472034679 131.364187047583
FGF21 132.955473649746 136.387904287903 140.583804906052 138.679545821015
145.669033804967 143.390971600678 136.444883411968 129.47419698487
131.951649373072 135.624881949214 144.270828498856 136.044670179122
FGF22 115.224011128183 133.303201646987 117.348231147283 118.144109564626
123.45956380046 119.391213033544 112.07238018581 112.832122823829
116.363556872071 116.310166003024 126.512613967438 117.754181884939
FGF23 154.705630035232 152.251128595777 157.256812907353 136.19083681188
134.897958140444 145.208597795717 138.39424470172 149.962143642332
140.154925193842 134.045938361221 164.497445195378 148.497497694754
FGF3 109.680532131035 120.984641102879 114.812254477058 113.832885222354
118.679664392666 113.66416989856 111.66210002202 110.838647534175
105.467761348787 110.784682137658 120.714981493593 110.392617179163
FGF4 123.943080151532 123.827771590213 116.112013454335 120.740986472733
116.552626146967 118.117957479382 124.003914369077 124.411213807179
122.133292722932 136.649743237542 125.739946257697 122.985117824212
FGF5 131.753135000312 138.275371291639 138.220016828327 134.235164622221
131.510494315469 132.611864316181 138.381508741146 136.925645302362
142.528480882047 133.040952285855 146.11669387238 132.978746105877
FGF6 125.863969666806 129.816385177336 118.47752077032 120.530092530622
125.010266083832 119.730174479044 117.348231147283 122.371715108957
127.774702462567 131.796941315992 137.154298404093 118.764650384561
FGF7 189.589053840976 241.453456707114 229.200570583629 166.917552275021
146.814457904682 147.121049266117 130.092881020271 145.999823250463
157.306022722324 128.549768412962 136.670253859184 131.404001006643
FGF8 122.418378964982 110.465934808915 111.975027473284 110.419222236698
111.584425166104 108.415715678948 108.830525786872 111.985567835684
131.525916493834 109.932577444858 122.519746599387 109.003365273337
FGF9 654.305724263754 567.869601934138 476.641032594868 294.871848467326
162.100547394235 214.763232658841 220.098071755353 182.32514927211
182.786177632757 192.980781927122 163.22480684222 177.091809538824
FGFBP1 135.497033143331 163.004844049672 121.961568081299
142.864294577585 137.80710149367 139.034940762344 149.073650082048
140.115154069602 135.980886879513 4397.32311216672 171.880470629305
132.206133020786
FGFBP2 152.609055890822 153.273568880305 150.058160923921
137.386308389265 120.196595293401 116.369395555451 115.30786313391
108.910588012315 113.321454160244 114.512028664352 120.864962392459
109.438965262717

FGFBP3 157.170136439095 132.113317893587 157.374537572779
152.371569700039 153.568321662387 164.276668889213 152.563532872898
124.611144621071 147.865114383296 148.87242524521 148.082026641671
180.456659320226
FGFR1 150.845846206549 147.745780820004 158.73040336393 136.518409978222
133.74051512341 129.566451901256 133.074027423549 119.815258991448
127.125906877098 126.262349005661 120.748910864064 129.034770823878
FGFR1OP 132.602336133471 136.607783800741 137.426226808356
123.738223146912 193.428733288774 147.024759173313 152.983678871668
132.062415660672 179.551640851353 174.962380276394 193.522811257085
158.193008411967
FGFR1OP2 338.129250886594 269.831616158113 351.759100605991
260.157440518033 243.589099648895 275.929414239664 274.627303114477
256.614964511846 280.656087497806 365.82705202869 293.17183601265
305.93974940209
FGFR2 115.649568330705 112.884916986215 120.724926760252 106.470524594239
114.01599035448 112.436972452838 116.670491972948 126.561067704708
117.65847906412 116.397855001959 135.715745768179 107.40922161073
FGFR3 313.967636827811 568.701661112562 407.518324776453 225.362253409428
1152.50523614436 1329.90419210779 5361.3600315322 1875.21979956596
3734.705550862 6233.78489807054 4068.18567686753 5811.97916044218
FGFR4 160.469645948225 155.773639092819 118.567548265638 125.887854850942
127.758381206968 121.572462089942 122.005340508177 118.100611964852
121.316836274255 117.261443445891 128.781123194219 119.355650892262
FGFRL1 245.723316636363 210.353341004865 200.633009710701
209.279164243868 292.60467054075 166.071816427719 197.191648960563
201.186963432715 200.810204162831 174.417259650516 160.945886507456
175.010849510711
FGG 122.438438464097 117.15886922795 129.216693566478 124.883575464443
122.211000410541 121.886886059038 129.257947818865 138.348160604288
123.315362903961 114.613368640356 117.820996523337 121.260355515095
FGGY 205.966111721195 202.691507273819 225.850439131864 170.553940654493
295.330307669482 376.276954313674 243.468673768141 395.521241316058
352.562711030848 252.548942594125 307.124331101976 362.306819797508
FGL1 152.872139211415 151.007812189859 150.132420238581 159.611551480901
160.903342063542 155.194521954405 149.419308407564 159.207567680134
151.089328533476 158.653161314597 159.854639082837 153.78976320015
FGL2 2107.99357083999 1643.08061372695 2486.24762158293 1060.24125879951
192.140442881209 245.317436234586 256.424415389574 330.193132765826
410.138738410101 254.63506290698 366.961565952239 249.80160363144
FGR 185.432587490792 187.881334544018 150.218643705925 165.908756485356
184.008405576875 201.865955150869 216.680795762761 295.330307669482
261.988345506548 218.134101819445 215.84044779962 304.547738011742
FH 1381.07447204233 1521.61996270758 1325.77498031227 1870.52504853779
878.167149313285 1881.90353127141 1000.91520225219 1659.80909859514
1142.09977159958 1233.99503576308 949.260897408911 1681.54485582868
FHAD1 152.385014030957 145.079450753969 150.549883779193 145.756455015309
147.866011743792 143.681220324074 151.231685705236 137.286331849749
148.859129298141 160.887121295848 146.184624424737 148.423303776817
FHDC1 162.895923745031 164.160608039304 131.319783710908 162.6762533989
128.936628289974 191.937607311711 133.935910746769 172.686798187142
191.725728813959 169.201608999937 184.204036882642 133.039045348692

FHIT 154.71904132098 175.693873820963 188.252507246148 161.424105316969
223.765583953184 156.061579024549 168.124936529582 137.874370839801
142.431979387165 133.602870982718 157.702540365469 160.265389854694
FHL1 8445.36695108418 5811.97916044218 5965.21815409254 5992.92384160188
138.688881393504 205.966111721195 161.331326682743 195.645181575647
260.642170838161 162.732574014686 150.439715066903 143.78994470501
FHL2 1111.47772523331 1078.41947691758 820.249910349431 862.132582849511
1078.41947691758 725.065727257421 1094.67740173529 1334.69145271749
3744.5666678861 976.360310504918 1816.3719976564 570.130804179186
FHL3 590.3584324045 667.892385395063 448.95671696823 759.802694618208
280.033528476199 157.901967487277 169.852769679312 162.427505889157
164.295628750438 250.676092725829 138.504398858454 212.147155923729
FHL5 126.87100632595 147.428180649939 118.505980204837 132.568354349687
121.564626579011 122.305514440939 114.378420394004 132.243900762936
133.502176289023 143.114384950467 128.191959554459 131.570358580547
FHOD1 282.076744753348 256.326661031361 187.167255451988 340.301980622308
341.714401872192 202.369905673351 138.605661097886 171.229729917612
181.578228610334 306.486275677467 194.903531690239 162.879707592164
FHOD3 607.26455470867 582.382786876656 480.484639351046 1034.68475089075
153.929167381674 208.628626112623 283.048002376743 284.831817899986
151.148295679242 156.976539870288 220.60173086462 227.614623963331
FIBCD1 138.242983316259 161.713311969291 168.254689624696
199.798533722581 157.189041157784 145.193296081527 147.322622473251
161.37617568158 148.490236128772 172.387657419201 155.431833398023
159.801315111886
FIBIN 179.338652960181 153.850588492326 152.789338329505 150.555125135002
126.256716138724 147.354535468933 140.125575976708 147.014513403704
147.441650320053 142.997365080299 137.79714923769 148.32201986048
FIBP 300.83667045718 335.301103690781 261.988345506548 435.095843349921
1010.23703166498 577.250493023431 500.643499051523 405.322847383739
374.761007556839 488.505191823403 430.728137105601 433.405369327168
FICD 254.315783394932 235.063474370925 178.717719615544 252.171420358437
202.343343669937 189.726075394359 199.980312703646 237.420951138877
250.25959091612 204.057019122032 169.752525994991 178.606255846041
FIG4 319.223127076041 322.956882599715 332.296938254329 430.94199035855
324.56043349882 365.650071660806 435.983803453746 323.667189206453
381.808809625801 194.961531388323 394.35596596181 358.217145707718
FIGF 239.924227955313 201.337186768645 278.598913464804 259.826625866106
137.824985884649 141.797497681512 136.248506185974 152.329129642988
133.849237689264 147.272072245929 169.834078550805 128.608401533371
FIGLA 118.07991087231 122.603226380439 120.255081121925 114.325967923393
108.669141498627 114.39161822971 114.400185930969 107.303126530947
110.678680817471 110.428616140857 123.336074100984 120.054065939835
FIGN 122.992700222262 137.163699337032 147.400082178491 125.4408430419
117.12833256636 124.63978985808 115.332409360957 130.953237161257
112.661607612444 117.244895482991 139.209080672396 124.972772541334
FIGNL1 143.195338329759 132.149015697532 148.840533233712
132.297716337104 175.161742647546 204.161375686761 166.360005474414
215.967319486874 189.174957940401 185.240692860387 151.449690439424
195.630643168176
FIGNL2 159.683695366575 138.667081004369 175.417320301614
134.676039394438 159.994376562272 180.986305037222 138.374092984164
140.236618351922 161.331326682743 157.180095329946 169.552424961465
161.806144472744

FILIP1 1635.35544761095 769.34961339184 811.564396886535
809.362464219294 139.159510392614 136.937327692084 131.525916493834
153.068898842283 140.293431620804 150.815117344434 158.130933580414
146.094951139804
FILIP1L 1812.07394646463 703.587139938769 727.425595270342
635.038752622494 153.088710298368 238.978845152474 147.079157199448
188.252507246148 226.144012966486 299.80615152845 159.504327477065
169.806644442071
FIP1L1 261.500728878974 238.685014863143 253.538171006307
226.919545367682 234.182164952936 213.145460679531 246.748589597262
263.47760086159 277.000882284125 231.63127072986 185.396765725228
256.291984914927
FIS 144.472969298036 146.321713521846 165.763459146818 144.110544384466
142.527422462824 157.507108643575 146.40904127542 142.401333414583
142.679201870274 138.783017264895 134.190511945944 166.095964870713
FIS1 1250.58586679251 1192.72383754851 1528.49457104932 1394.31467757456
1867.55140010155 948.734577648136 1081.47077865393 651.235249028924
762.584280908728 799.423640530777 761.097992055119 1471.01897242089
FIT1 169.681582570965 171.537040561599 185.382647297259 182.275998840116
158.092775850886 158.396149470397 172.138554350738 157.761231106669
168.588120398433 167.083758835476 178.745112514526 163.529157756197
FITM2 120.724926760252 123.231397809701 117.943511615792 107.531907287727
148.624135810148 106.204350278634 119.952108688414 121.239461514704
109.416512252532 116.449064666763 131.354275252104 125.484453841158
FIZ1 239.363511706064 282.335582153109 259.672238210968 324.067396271281
376.627801547869 225.950750084197 241.134385745554 206.620708230452
265.332196877456 224.805404798696 262.671819506189 249.292766428035
FJX1 168.111124820968 181.715992480635 170.568719982587 186.309367281625
274.494074082359 210.392662092721 276.756415094228 183.708917501519
238.724909152512 375.958347293549 253.933478547515 197.795275907042
FKBP10 167.756465178715 171.358746749715 124.958287100852
225.783771938969 226.919545367682 118.139487760542 126.585679159459
126.705446263769 127.954231630338 125.789509802098 151.96843908074
131.074021609161
FKBP11 379.016207754533 329.660368915848 224.186573847185
266.563990988678 298.403224729528 436.155731212592 403.47026424469
713.832153334389 1039.46136549795 941.936830901588 570.916936074747
566.813343200268
FKBP14 5094.53287522452 4266.92715355342 5314.0635307876
3671.86460903374 648.161646065464 4749.69040198959 1957.48508632607
3363.51782842264 2056.90127387648 1673.5387396582 2801.66917828364
2154.00589005104
FKBP15 228.9901869793 247.194197143562 263.994460303088
280.085692727562 215.247472744419 186.747375844319 221.594737497504
194.569325586241 211.844563155792 312.484289357493 267.800523478098
175.040086358446
FKBP1A 438.533112511098 456.990425618007 482.965800108538
514.61297221467 1387.90327883679 961.355625338687 615.662993833692
662.213423011533 930.704688414069 860.315938621256 432.174413144791
949.789710063337
FKBP1B 164.74234322574 169.806644442071 171.338371572269
149.462835741621 147.454902803231 156.144934427609 124.025104622631
140.479321382192 173.573412346985 173.761036260869 136.580363776109
204.596753034535

FKBP1P1 222.177290763736 242.752995772995 220.242344043437
267.149525812834 144.700955682048 221.127492253636 175.79375235478
175.882911801581 170.251197850968 165.134931755194 179.25743545231
187.625536724681
FKBP2 354.405235378302 310.758803815671 371.457568934695 356.921627358141
525.138476928759 429.992169596618 360.605223912114 299.616447269111
344.128576341892 397.064325578854 360.361719991381 503.967725251906
FKBP3 428.959561932229 431.799795646414 487.993796425035 408.444629429553
388.887050777137 386.679822751367 560.362502519639 569.47446215213
365.286234166621 759.058176862524 699.06539333455 618.829716818725
FKBP4 373.860920943049 362.095074331893 425.59810308048 417.262521411065
942.41077071058 529.700356666006 658.968318349334 875.38834441246
983.777608766079 543.933989965705 662.958637409597 513.662594875431
FKBP5 661.545179083009 714.494228450456 1145.35733270036 691.632061537999
333.049612399093 841.285351463894 380.664528809678 1142.44180956604
499.176926371542 1823.53870218167 439.492285515997 346.303862540751
FKBP6 118.646119379542 109.156349683299 119.9652990395 114.27112025541
127.512733134453 123.031945101045 111.409447323674 116.751145098777
124.749727939593 118.792612926619 118.597739444445 127.524310543779
FKBP7 125.617143363441 123.809866652834 134.978785580693 127.729912390394
125.865974180252 124.491884305455 126.588382665802 129.806104966617
122.517559025268 119.451099896048 117.517649866653 122.472909598385
FKBP8 183.193029399643 226.989201461085 170.198508633417 244.514230664694
235.063474370925 165.062129636008 162.536009333787 153.756213924949
161.646807806182 162.536009333787 249.234065130083 173.035013611377
FKBP9 147.15764785567 131.319783710908 137.000868503792 143.705475208192
158.06915048955 147.442483242063 141.275667354541 141.970301370404
137.773960847088 136.450062043587 138.150616787218 142.336164180947
FKBP9L 1084.10984365393 867.369315017411 783.991448771818
881.561688713557 375.767528676937 417.987533397449 631.653707589221
397.577760464197 335.623336587396 717.025533065694 524.245387973163
621.946994961864
FKBPL 143.31117188259 154.546460872415 157.723341862468 155.478383896934
193.218266574099 171.731395791818 167.370030272847 173.056715623574
155.494095135266 162.127697884504 167.206513789165 175.93459327917
FKRP 215.950811436427 250.406775889932 190.152824885328 293.528601172819
249.379605914827 177.235586767256 203.089661928125 185.617936412633
223.359785518285 210.218440444631 202.909188070184 159.620467672898
FKSG2 109.635825046436 110.847680175669 118.239637776941 99.0767996209245
131.12775424877 114.841964705981 108.461141538047 102.563722570171
102.725375719353 112.07238018581 108.939608619532 116.142650095476
FKSG29 151.451443480833 147.978039160763 152.042744996136
155.379116566097 145.601922576321 134.239416179248 140.934144636302
141.048882618651 151.188412354358 154.774977482202 141.970301370404
138.820797333519
FKSG30 3309.66193624859 3195.71436046397 4929.26915002209
4081.13406129846 1914.96285167893 1038.34611017219 2249.8123426309
1405.2134783352 1245.47421607111 4477.54806526552 1827.03179366146
2018.81730267601
FKSG44 113.470356909765 113.521599852465 116.074423050416
112.142263331903 109.140708667012 104.739307327595 105.741092375243
113.963798749897 107.580425848009 109.947300629824 116.881212516879
108.333135424315

FKSG49 134.010031592003 129.665507668047 132.155877196474 144.825231501747 133.82390829608 134.109051944736 140.453219944445 132.923237903246 117.598778415488 151.848131897899 130.41429837092 126.599418127637
FKSG83 150.031863363697 152.045391611927 135.395071593072 171.714973501563 138.274057264688 138.596430973865 162.100547394235 143.004090696463 151.990794390998 136.720837676326 151.790240553272 141.018087546273
FKTN 10900.1783397368 11432.8213906428 11554.7330886956 10953.6229702446 3575.60827968852 11971.1664283024 6628.17856159198 10024.4662743012 9139.73212514992 6156.29203791697 7838.92787031423 8163.29201165065
FLAD1 259.826625866106 290.037325939883 257.775569674531 347.224999246223 596.743397794872 620.760947818536 341.096072280715 854.815394943219 490.849163737598 301.304268871606 376.962937292973 589.953707188378
FLCN 153.366677608026 156.911538642765 153.893753448473 150.960096893517 174.23955831109 152.890239929335 152.080057231881 156.75308999904 138.216926722025 154.87316176388 151.615675144083 153.675939908241
FLG 110.273174147374 106.315801001506 108.655415285806 110.753661013519 110.138220982557 108.199628274798 107.049865500702 104.544792793756 96.0426636429757 223.872330412144 125.058165131184 103.36057518781
FLG2 118.871735074285 118.963119420175 128.29486937905 125.35643274458 125.848597767733 130.880795616911 134.194555223935 128.304919825754 141.685620602226 125.136745288071 137.165966740473 133.305679931132
FLI1 206.999289979742 212.363151537303 204.990512488181 231.547654653783 140.800226245569 148.700297908071 158.946177055546 209.180298459605 219.0221768576 159.730925830278 192.532222258033 166.558080305926
FLII 577.98969050409 502.063888359427 410.845034795716 611.540759532294 431.304147578425 336.981561928411 218.334273320319 336.338518973032 228.100112133941 440.745866155629 456.298408568636 270.520229426015
FLJ00312 135.741404460897 141.533138525284 144.224287382131 144.760229351772 145.551717838423 140.018786803502 143.307838525991 145.556318940043 141.391508027714 139.853333847856 142.739686751422 146.885404321622
FLJ10081 890.810315261602 783.059427015955 961.931130189779 771.694853062033 321.696082249696 513.816017871024 469.794672718173 432.372995789404 442.12886967939 459.53652431464 561.251860964966 460.651156484562
FLJ10088 148.618327213374 163.198834359304 136.338683078123 148.87242524521 140.13515064896 138.196640512429 140.195820033314 145.594378536072 130.983136465761 131.800533523123 148.863297062269 147.882530392424
FLJ10120 178.339211170442 177.214422668922 166.415922313464 166.627372924295 141.434699050015 176.86489466512 162.10411532202 137.578516107502 153.804419092743 175.882911801581 151.059401217656 164.811120992602
FLJ10213 348.413827101046 348.36178568457 340.487850009685 280.494088228226 256.764450748941 480.593729033799 448.371946119074 311.966971734249 340.668473351494 299.234482744038 541.764013317002 408.993744895434
FLJ10246 181.771654391653 162.212442593001 163.396651801661 133.215151497536 167.741556663724 214.714726648332 144.942289590793 212.78038645414 171.247946863662 164.352366170527 161.424105316969 152.99686989829

FLJ10324 199.662592246931 209.051768656607 206.098534554876
179.273936200852 132.683594640696 144.669509071181 143.21411610625
137.391141254051 147.60284257326 131.104534164394 145.938780249776
137.15571320152
FLJ10357 170.568719982587 201.81649023518 174.454754564111
184.426874913406 159.227123133854 158.963726206466 159.133905701536
148.082026641671 153.850588492326 159.245344068037 171.602950766191
144.603401904061
FLJ10374 306.677271064305 388.573321838751 419.690542859168
416.715760038095 378.862741410359 389.853534237748 349.330629649093
420.95713139384 395.425715632302 367.382105696814 342.998459706966
397.064325578854
FLJ10489 139.613193302406 139.140647941752 120.387275261633
131.820842728171 125.75980494927 121.619593453259 116.91470505098
120.488694213288 135.118955626451 139.691470247318 120.020781939389
132.714532888989
FLJ10661 141.840253068921 139.582054825147 138.661548678191
138.35738548449 143.279331050175 131.563719927798 137.460936879873
141.839647226623 135.313148180961 140.870849757111 126.578035793406
130.501010489883
FLJ10781 339.490955270138 200.712182818203 232.960832410426
260.045018348182 184.322250984457 144.834448238435 128.753623886188
122.634741242406 136.293084080479 135.682971958096 127.777945516765
136.783974483005
FLJ10803 140.277228495746 134.814082968113 134.786678978467
130.176734524308 126.814332513301 138.541779821934 146.937052029619
149.944351491364 145.758423590784 138.638573020758 143.665359343809
133.104560685558
FLJ10916 643.468308027006 762.584280908728 514.002313819685
674.338740875512 181.354279096644 239.572670410818 157.70581824787
206.391403266202 550.124359517322 237.521746704115 874.348941257592
389.686045618228
FLJ10986 297.757657398146 307.316241247336 372.475112554793
227.774354299962 499.964000319181 871.528156184927 478.699172220804
595.270107963842 600.635914090365 329.779210536849 540.190808200264
754.406896156858
FLJ10996 169.488652117125 165.545136704242 174.962380276394
200.810204162831 157.499803444188 186.140970908638 164.497445195378
194.753955100069 164.61921356441 206.915239638443 155.669470674985
165.118167086792
FLJ11235 164.910911528819 150.082561726876 143.284230032303
146.094951139804 229.130679600654 149.547635666621 162.364266549463
138.017249996128 170.079723856247 167.937451406243 149.247514768446
164.816672737691
FLJ11292 202.691507273819 185.743212726702 209.180298459605
195.674128236589 187.519053043173 195.630643168176 207.044774373302
223.253794809571 178.072538529634 193.203053225456 184.148058872585
178.488874074222
FLJ11783 204.564770370164 216.880896769751 164.48570046081
195.776369840059 212.073472189229 184.974347306539 184.742960975938
174.761105961773 163.280786875496 167.100039749329 182.470538043064
186.939168482155
FLJ11795 116.977202125093 120.406577236405 115.435942397626
115.02681415256 113.726931741236 109.817705421158 120.488694213288

129.940297696849 128.620852653765 123.118724072534 121.526724715417
114.400185930969
FLJ11827 144.916193282062 136.5680632044 139.995623005154
143.296580629431 154.055550115498 148.757112903673 156.585665091824
148.938822951404 135.598709185334 155.134796468704 145.34332740863
132.807755600683
FLJ12078 389.686045618228 478.040234382488 309.318403417731
651.759589129833 199.88618355278 299.568830444875 374.761007556839
263.509066170434 220.242344043437 206.303338709554 334.52685347521
256.687384001447
FLJ12331 131.04767483614 147.230113868203 154.924190468067
157.979749139651 117.829228895897 143.252983617945 147.679233228201
146.366013190845 128.090665893885 121.61143371132 134.383024487323
144.188147197178
FLJ12334 151.276193269421 148.376194044247 145.428297145967
154.489459651566 147.238896426423 135.583556015955 139.696266471271
138.28745132277 144.450810504499 151.259426521768 115.097376525707
162.212442593001
FLJ12355 177.88534558205 177.955579729463 164.057147728625
205.886398618369 469.64693728427 159.41705570719 310.23294370018
214.355086255855 236.191384383802 151.043967734248 195.303469330682
175.51767355303
FLJ12529 139.372441366224 148.222477811166 131.601823550269
139.482462221798 133.41548225025 143.737021989907 141.66312605042
137.320792454356 143.070170783764 137.994334667126 164.820007934887
141.42859050659
FLJ12684 116.497714896018 112.420505354139 106.470524594239
103.36057518781 171.314681729657 144.405191855684 195.400963839097
163.575300618666 204.877041314502 209.180298459605 196.691380885508
123.427366316096
FLJ12688 152.296774123599 143.834501071806 178.676812740925
150.042173991463 156.976539870288 160.055242985219 172.128489882364
156.46322298175 143.814798097425 170.223509007961 146.018097597373
155.463400179872
FLJ12949 217.560691345072 233.635876830148 226.203994206714
251.347519777204 238.847091900666 226.712995229189 241.557810100265
249.354198174438 188.7348696188 238.654367531982 219.519896184851
232.892635502424
FLJ13197 196.860228537112 215.247472744419 283.326226944158
212.239485199343 142.785631812039 132.891375982227 128.23432652395
137.687480242109 123.609527892227 142.383723965616 154.352518161283
189.362958041175
FLJ13224 144.523620416439 137.212568398657 138.1342592966
141.97478198531 143.340685579645 138.224383646894 147.292241873554
149.593135821045 143.787766055379 141.363863974586 134.86940314824
146.072585331002
FLJ13305 194.617252921731 223.81987303579 187.380064367869
191.241000498118 153.703682621695 158.10628865476 149.520911664673
158.232100962615 160.76044995335 196.136080427522 153.891667472876
139.617608585463
FLJ13611 130.452929012459 123.766373146588 116.71331256222
113.09980008288 120.060776752215 137.704257049203 122.852700267837
115.944280729914 125.060387518072 115.468921844902 137.052507691254
125.535877109652

FLJ13614 110.496084922302 122.410340121549 122.773736159743
116.486929280635 109.128394735532 109.038182799871 126.168223112165
123.035401501279 117.32734978289 121.826474059574 135.899595115146
119.373287155621
FLJ14082 135.375268329248 144.848839185565 164.497445195378
150.927628184034 135.783125948784 144.131743978192 135.736764623155
157.264673452646 140.779962389412 145.368059425439 146.225422797118
176.145076825955
FLJ14100 139.526241501184 142.639587833858 139.358218798638
137.782648137869 139.77436722364 138.201164395948 142.771243742561
146.990660831392 142.851432926979 151.796986456254 140.047749881567
137.954283575143
FLJ14107 139.073785024092 149.039132756817 150.822586904738
150.854580568572 164.625407872446 158.390337945372 147.301909062399
147.28750335168 142.970256312313 169.488652117125 147.815673580657
153.062710222618
FLJ14166 148.991357883449 142.071492722749 160.923897048848
144.70348930646 157.907190910755 147.23376282233 139.834535091869
177.103802332144 152.11446058397 194.202977693583 151.0189104086
152.163489631563
FLJ14186 136.745596788338 155.873969001251 149.31184788671
148.603077434646 164.726382689707 158.617209794418 151.687402863648
154.256214901103 151.245520459264 149.863063160222 144.839170491334
154.003862874241
FLJ14213 191.881157918071 176.382377784553 121.207040901483
126.924014801186 131.267177634269 121.327397317398 122.619429537799
125.889860278265 162.411156818437 174.707059022891 168.124936529582
127.712004247155
FLJ14712 121.699702849509 130.444766583917 147.407093814154
146.474228901408 139.22776505842 135.726760147904 128.647790058795
156.546769952772 137.267307036807 159.213940274232 126.94941821459
137.891372981851
FLJ14816 122.136861827049 127.642215862573 124.526220300907
129.96280577492 124.347680638586 123.839006667113 129.786941565218
125.964335876018 135.930911350803 122.449574852865 134.626371781978
123.132178371802
FLJ16124 113.192313147111 114.734439905052 111.10737497653
111.448871835514 122.472909598385 122.84603217721 122.27785621724
113.460835359833 118.08607083874 106.685578858702 108.760780994582
106.953841653951
FLJ16126 145.274345879987 129.061316880624 133.389930962407
129.125709963326 134.298521475727 124.815853677553 122.506058130261
121.771891473027 116.52455193626 122.115925891088 128.765369834041
128.901707304582
FLJ16165 138.341918073941 122.469113037809 130.836079676477
140.82351325907 125.724143659093 142.737537206204 152.951353678265
145.952759497544 167.811412309356 165.4474699807 125.783265980169
140.408989128575
FLJ16171 151.486362260537 144.944095553004 159.838626443912
142.126620107418 157.615017085961 172.251953149975 166.714793209109
155.272264997151 152.393260140571 160.970946091146 108.318510762416
155.625320865565
FLJ16323 158.308558656144 148.757112903673 162.47662918768
153.996429870564 169.368090266817 152.28262533486 156.895079878556

146.181764812924 141.560424984281 160.6823824476 161.916155040522 161.208713751942
FLJ16331 146.787927492706 141.647300896529 151.507376053438 140.397184813186 148.919726992353 147.16778072296 159.932941342403 155.582036890436 156.243337414127 133.363883851093 157.463122989559 156.895079878556
FLJ16369 146.222195387635 122.568743478314 143.281050499332 132.37450516592 137.429832103492 127.464485396039 147.588213090285 134.227796103219 149.198753359122 133.65688823581 123.935072913078 133.216489794592
FLJ16686 153.289258540668 133.337863400762 143.437546532782 132.197346228417 146.079727388849 134.608734423113 151.086688327023 139.517413585211 135.730331297139 152.552277008504 138.071335383596 157.484541464879
FLJ16734 136.258073395013 135.2979041294 135.027715094704 132.119370332298 117.367470441908 181.607253339427 160.19942770666 137.897243684023 145.07503221134 132.074571281938 190.098509544816 145.578721432709
FLJ16779 132.306569548052 139.706279455371 125.073353210914 138.733717670748 150.929236917651 131.102996816222 131.744454088995 144.350683356627 126.713610681382 137.982667022604 124.174140125309 122.153996665065
FLJ16793 166.521472376759 137.876234091056 175.529441666151 165.177036955944 147.195127201893 160.140509175287 173.892380305166 185.113098620798 189.678680702271 137.318442197233 171.629605754643 176.735168607281
FLJ20021 300.937118739654 313.828943866034 339.069385816411 440.657747083721 350.22221782412 200.652195873026 312.339824919124 216.522992914538 199.980312703646 234.214191437687 242.848821222611 181.500222423634
FLJ20125 196.429685815196 185.46843333826 221.594737497504 188.242091666446 168.763640193219 153.354921961854 163.529157756197 181.803275243281 163.102043862117 200.017741240426 185.691922436569 170.858049313581
FLJ20184 145.335830265052 147.75188889715 144.201723591119 129.645982978155 151.409290231274 148.977202433511 130.147848418265 151.409290231274 155.988447182637 148.683761948082 153.620767586182 140.089210795507
FLJ20209 142.705504445895 155.798048652444 144.774621469452 165.723126764006 179.593817076317 195.608619642038 148.42845062376 143.381277901138 164.109321257638 147.52403424052 186.231346659144 162.737431585974
FLJ20254 378.4377409749 395.049127927637 303.380479700334 479.983368253935 598.647226284738 304.111808763793 310.8725368426 243.283375388645 261.775269058607 241.168936008174 307.570405241182 223.81987303579
FLJ20273 138.659387475119 177.831718771025 135.075759558051 126.988118823204 443.343806055858 401.631377313065 482.466736153026 581.828502821435 663.782083028745 226.387465899611 674.905270154355 261.814227719605
FLJ20297 140.801129972945 142.750432197016 160.055242985219 140.802033706122 154.854538639035 167.993851183401 137.914673796317 148.278828579424 156.873097743717 172.128489882364 128.860744370967 151.308872564827

FLJ20309 145.352802493486 137.15571320152 136.149732574763
137.641136471239 140.286870147975 138.944595164363 133.495243957927
134.545983176488 125.609542142983 124.145492642453 153.687536747147
135.803599969904
FLJ20397 137.159781458748 145.117574928189 142.93332947113
139.430808017891 139.430808017891 144.00214898009 184.267958772058
138.119788738226 153.636804025106 148.764956861822 142.222833574043
191.056705282378
FLJ20444 193.912456093767 191.961269585431 185.30916081621
193.843978773764 165.789846154721 226.841838641151 231.819341041105
190.489530928259 203.410932496844 160.136209944279 183.348764012358
170.706932460836
FLJ20464 146.858292465699 142.577102697682 138.986877479278
143.185372109165 148.668081483434 135.32160992961 146.00443946108
134.907397302852 136.124385992905 145.103600666515 137.331864485838
132.969187044132
FLJ20489 296.962992349662 314.646369385896 354.783364481955
332.543652462234 592.284071247642 185.199972151831 247.528753010193
175.9510758779 194.961531388323 191.040134068942 280.414161197286
242.269784551092
FLJ20518 149.272219727411 160.140509175287 140.007937555694
151.829217512763 143.831294912255 155.639666182376 156.301214161102
147.734708927825 144.346988809708 146.411873503163 143.618454531431
151.921235758457
FLJ20581 147.228812647235 151.153466809603 154.003862874241
159.576585660511 167.620915011868 177.065616871886 152.935963398921
155.444668113276 148.757112903673 146.948261640482 165.504532455205
170.040914594182
FLJ20628 211.369442150136 197.191648960563 168.33854702953
190.45739385453 236.809829701921 297.12411625104 284.13865992802
238.509970054862 245.522497675754 206.845306095447 260.999345837027
266.61343130101
FLJ20674 166.503112149416 162.885054245809 159.874161198304
165.995251661994 222.764568757453 260.045018348182 277.256341592701
162.15259972225 233.572055978465 151.833386901811 182.545896049446
217.380892198811
FLJ20699 328.019503002384 338.406266073063 399.724529998085
479.866450988091 262.756237406794 275.755038921952 231.146155346291
222.624150073858 230.508733145583 273.550544349798 214.763232658841
226.960024551778
FLJ20712 139.342430217926 160.30784625946 149.815494316469
162.560758971445 157.096799934494 154.978243057971 140.717348834454
156.083002868004 154.118601977256 161.569793476847 149.055493714692
133.259321087534
FLJ20718 294.280238648737 259.767151797583 293.018282386003
256.964577037118 484.480643763982 509.400965222318 365.199125315964
323.086368767924 320.390351199433 439.122658719747 287.145105895235
449.859227376918
FLJ20850 153.477432230141 144.507230362869 146.731584143625
155.237180851323 180.968659614238 161.870389635266 179.624208193114
168.611652451329 155.386226046894 148.386249316173 146.57274511202
160.903342063542
FLJ20920 243.732074437237 254.991678321253 221.74200490054
232.892635502424 498.05778289794 515.729905361375 687.700356531038

393.115606440229 642.647554580301 187.196424491482 452.077434390612
305.252666831522
FLJ20972 145.291984088491 138.734437288431 142.436624546651
117.017844025726 145.704057360697 129.661073814998 150.677852949794
149.198753359122 137.076766238637 143.327730271099 126.233395051706
145.331858938535
FLJ21511 125.658396221949 115.090463537716 120.212294184999
124.148194572004 139.166537591866 116.07902130325 108.362609680556
131.362128083159 152.221694141976 246.031568259337 140.975056186954
114.826501559828
FLJ21687 159.196987090991 168.841313096346 190.843795579234
158.080749926756 264.201932544662 287.242697383558 275.972447693982
217.491746961471 371.369895979832 173.09379121032 282.926325589238
241.353916553991
FLJ21767 151.067819234238 148.185056724262 162.312313953026
147.89884142817 155.191446350891 132.32633643811 151.423936838934
148.962593686337 140.875923123766 137.846463796994 133.619092072157
153.687536747147
FLJ21839 123.273349540679 130.526673128214 143.899201949317
136.436762791912 148.983182193411 130.272086123019 153.597557478169
144.112401069818 129.045934344719 121.289159587394 143.541810469447
121.406758670435
FLJ21865 212.591357254196 211.891710626953 182.300628120658
192.954269340616 304.292209676095 193.098116087792 249.80160363144
334.199307788576 462.686385881492 228.879434668972 417.191960489528
240.706548931192
FLJ21986 2210.67250954072 2206.7447886564 1927.467713716
2369.233048505 163.873355370767 249.489170536758 136.829137120584
190.947996918605 199.101099226668 150.343825300998 130.150998843383
160.17208299932
FLJ22184 138.993251266382 171.080373870373 150.417169156755
154.443360208399 370.945254716657 511.996883507889 295.507504000509
300.675907594637 169.888264971936 141.870640394206 325.43977732888
252.894371238554
FLJ22222 212.691702227442 202.061887608293 178.329946332806
230.947339143332 183.32467147543 176.256840061919 195.20398666139
201.661941695238 243.080229016932 155.660302399877 208.507486041165
196.860228537112
FLJ22269 155.064761390765 136.841506794489 143.449303333495
124.895023786422 142.586051173329 143.216903218928 118.625070307695
124.942701123337 145.468654174037 140.711699577809 137.239088768176
128.551335256123
FLJ22447 138.525131957094 144.568417378353 159.406462604758
149.274901871663 140.242272149424 136.734131465433 147.856285290748
147.338615809214 136.420062669039 143.141852982371 128.39171386085
145.003900788534
FLJ22531 163.262893278578 164.497445195378 171.494975393562
165.380225715852 162.022232949254 160.644747532952 174.928155956396
166.70212615715 162.695319777455 185.027902485379 158.244741207036
178.235281981508
FLJ22536 274.595218795127 216.102897582821 248.085349787686
194.257024100927 200.424975825325 192.059255232313 133.615958668897
199.170488640762 226.796825544749 188.797908601851 139.400521576978
147.734708927825

FLJ22639 197.962159288376 133.004765428575 149.668587078867
131.001996509052 118.891276329399 148.074494928651 126.191057198995
129.741644654541 131.540436531741 124.918089223417 139.949563856936
141.712693059287
FLJ22662 667.693216401836 523.937128789616 521.625146077176
453.926991868451 940.716682221364 740.527607312565 900.233783407148
1846.61430264854 842.87865208079 1279.15873231449 4022.23000497332
746.296354515617
FLJ22675 139.061072394426 122.578170988039 136.669003646622
151.81251450855 141.36525744062 136.05610450926 142.06162974139
129.665507668047 129.477908941439 130.300169316346 126.268418132
139.171486871664
FLJ22795 252.593574250965 270.787490294555 242.410260751835
284.764628939128 388.573321838751 233.757965816721 401.631377313065
360.991542429346 431.304147578425 582.642500194997 647.273409499313
437.122373082112
FLJ23152 160.150336334888 140.242272149424 157.14512525321
151.245520459264 159.584350289413 140.793616429174 144.731299077209
150.874014332213 159.909121784808 169.903088388196 152.301556397498
150.290025009187
FLJ23172 129.859331207247 121.23469201559 126.362418476129
132.965467229606 142.461941376108 121.084348994747 140.765231611948
118.294324274616 114.412739085949 117.824694297846 128.0805889548
115.113000553093
FLJ23356 145.643752002787 152.821735300697 146.291932367254
146.528945049588 142.678267012725 154.462766419353 157.237147919803
139.837616209536 139.150209111606 151.224289879776 122.223184326586
153.658747612827
FLJ23584 192.848268768579 239.363511706064 215.037122365095
265.174306663185 173.635827398273 158.98666068453 167.237705290905
174.100921524023 169.681582570965 167.421776383056 193.48293264444
166.842970309527
FLJ23754 143.861970001207 168.611652451329 191.093283411655
181.865456674829 178.751706380632 166.311421907434 169.052101570616
184.493681387276 171.202766078337 163.066051106092 146.384051525624
168.261511797218
FLJ23834 137.858371717399 110.003750007057 129.562747657036
130.642810886161 122.746185691827 113.980716384814 114.255733119076
117.803508098322 119.319884675074 121.429270259367 128.504661404607
127.898533598679
FLJ23865 129.76141737109 122.109009629395 136.229159638766
120.316950428535 139.459975305985 139.213953275623 141.189008902155
137.565050253948 143.873048967535 138.525131957094 134.036236880951
128.920313557255
FLJ25006 164.603403251634 167.858490180471 154.362571105094
147.985843605585 163.799748670842 202.741211613885 179.456207450779
393.796356067403 174.168079247768 136.24103678923 175.055634340766
203.544294328395
FLJ25037 140.156797128045 137.742393475755 168.365262340217
144.46549577902 131.853183159267 146.450806121927 170.028047334267
145.89593390936 140.300833590056 129.442111153737 124.439917584199
159.286484253487
FLJ25076 150.35767254363 144.185406500473 138.452698593232
147.121049266117 138.336667434109 142.249715102513 134.218867540274

143.603022117503 140.496058408953 146.421195733989 135.5668267936
134.066948803697
FLJ25169 138.537763477627 137.940675746397 134.949572471421
145.079450753969 126.069594320759 137.416133786828 137.549175595441
133.879079395215 146.192761134077 140.041977349982 141.137459291428
143.818379692331
FLJ25328 113.521599852465 120.590654346066 109.988237089025
109.266533255266 116.90832995093 110.230322274208 112.3786222545
112.971776964469 106.576058729493 114.161540771427 107.520000793184
109.654666772771
FLJ25363 1730.30703463073 1617.44377452783 1349.84215478798
1423.02563792293 399.664121956178 1867.55140010155 900.039744829725
1263.64725236472 941.065508539108 837.603727062008 1558.22238167317
990.206369590236
FLJ25404 135.682971958096 147.891442774921 143.047617455953
133.355353808577 149.31184788671 178.083311435916 143.685368799972
141.494666823639 147.28750335168 133.297361715205 145.350342823866
156.866867371872
FLJ25439 159.241573314833 157.654040469774 158.779261405573
169.31618330657 152.957297648595 168.173803952157 140.381386314817
160.039086963419 155.550532734718 159.879600154928 167.776363541939
144.30699572795
FLJ25715 142.459230639888 149.997047695778 144.724040494873
166.209190082084 142.791015845309 142.658562927154 156.187809481625
154.449058407217 149.589901372799 170.198508633417 136.446877465751
139.411792975474
FLJ25758 143.88649412841 143.552343056702 127.325087202483
142.997365080299 123.678365506247 132.439176846818 146.252974508529
138.956057921718 141.821550320574 139.557059245431 137.947606003247
148.543359732604
FLJ25996 133.087166272749 123.470830748348 125.997109913348
115.47716627378 140.616811333466 126.305273697185 159.942971344216
233.07015384759 169.147351642004 111.065596151792 221.231455446574
185.801540278924
FLJ26056 130.564962937297 140.28485470716 130.616175018332
136.181791404331 137.994334667126 133.365793958118 160.664407431261
138.331509117817 124.35374445763 140.595496714752 140.608266389167
129.390398431836
FLJ26850 155.143752422033 135.860057860699 137.172030225913
143.108765786014 137.235469802064 145.036719629525 135.923022674367
141.619432083003 144.507230362869 146.033591220134 141.431172407792
143.04129238495
FLJ27243 127.03153185998 133.238296113721 130.770335966929
126.87100632595 133.875667250136 123.847186739627 126.396385337235
135.936021164312 108.333135424315 132.760567403706 117.071585083051
119.598216955485
FLJ27255 143.00521196918 157.787233338636 152.001155788544
149.138678142327 149.801573181126 158.919340819357 137.042097702398
145.221594295772 135.346501914337 165.225475655526 132.23149173224
153.320911882529
FLJ27354 159.446705611995 165.972090075204 185.743212726702
159.300198352916 156.259214645427 166.12184890921 164.593531655305
143.360311218423 153.178934750919 175.350734537491 150.517832812714
165.041296694491

FLJ27365 172.345201438148 170.866433766203 160.628894266843
169.695658738618 190.057030596611 237.754067231793 161.208713751942
161.424105316969 177.371157649894 197.357728220717 163.62582285753
163.400576719974
FLJ27465 145.980929136129 135.592128081677 142.508525378171
135.544048708523 148.286700314107 147.155738185574 144.056644468886
142.185903088074 135.976051103762 153.834326325003 159.74220366735
150.193479279784
FLJ30058 121.580726825432 133.93015463284 136.973804637915
134.650161382949 129.502730573463 128.755697083014 134.459497002613
121.448671447499 131.340453291188 130.418739793498 131.48517604564
149.108582827136
FLJ30092 192.009562635198 178.416969664816 195.569517538477
207.298994298453 167.077669654786 184.414609874199 162.58152435515
161.530878860883 164.223990197735 163.336324451735 159.110614190088
177.696849418064
FLJ30375 130.991496243273 159.611551480901 145.036719629525
160.604195264968 149.754842928462 155.6299578128 169.428195354763
153.836520485018 150.606329295069 148.704917490429 146.081940883446
143.00521196918
FLJ30428 150.431052959951 143.874735808114 135.607537832704
135.609239096236 161.208713751942 148.614214326995 167.91761500242
135.56878810365 134.620896927473 122.619429537799 125.884565343075
130.150998843383
FLJ30430 159.633987751501 154.827746791634 180.850779069931
175.585857393085 157.006278815282 156.759600627542 162.652929339533
152.321890796527 163.030275835038 236.427507270325 165.613274361543
154.798287249541
FLJ30594 129.684916104674 139.54021659275 134.555873832434
137.685725744573 153.571517567657 129.099780410063 131.155117105954
125.876114993687 128.077692981235 135.664877050226 144.572485418264
134.487476333464
FLJ30679 176.267796612066 148.408616955694 149.348231806916
171.099675679944 172.421637564661 159.301581158613 153.972471779464
146.20679320119 151.059401217656 147.598602877625 135.928428484011
171.644710031098
FLJ30719 150.367927843529 153.616692588396 161.694109087893
138.684070773064 139.476234663488 156.567519536997 141.181931072161
147.584176709986 147.407093814154 135.950465168457 155.641860356683
136.08297876405
FLJ30851 124.306049555272 122.94937342991 134.362834437771
132.256047602234 156.187809481625 125.884565343075 140.904883114871
128.44311762955 123.45956380046 120.901639342731 124.408996713299
129.058085094891
FLJ31132 145.70726782469 154.760697311395 127.295532954648
128.398711525305 159.41705570719 161.89906072679 166.232856234879
159.344044864557 142.334338599752 128.292627005614 162.622690190878
154.277670551775
FLJ31222 117.15886922795 139.595986615703 130.009322405768
145.549126188292 122.829218352947 146.355229778997 127.773292289242
110.678680817471 135.010937737018 118.383317097375 133.399632102473
147.172440755724
FLJ31306 405.322847383739 314.014654357569 341.197826517049
304.811116024094 219.96819201774 248.690121805116 328.486476842582

411.71606840872 310.8725368426   250.963601187362 758.517101659762
431.304147578425
FLJ31401   126.997614441981 127.560308850342 136.565191271943
128.002822538671 121.130837254861 138.246963115014 140.300833590056
137.811304134207 128.232091717966 121.072192130078 113.644993086829
143.161623642178
FLJ31568   145.549126188292 146.709967739596 138.466136108202
137.232722233042 144.80318019871 141.401480567426 175.704031930103
139.969155812688 136.381593416533 153.140087873773 151.151297821117
133.638664342783
FLJ31813   149.366554238043 138.215708514725 153.02127620577
132.832556359483 146.858292465699 146.077082896858 144.820843146628
145.885577577352 151.65364467801 143.562164660825 120.894583222813
147.357032438442
FLJ31945   146.481298877242 131.507618414676 149.813838004575
142.421913396368 143.594003495859 142.365009028839 142.545823714168
132.251093122058 144.917911601441 132.84268776659 139.966813133326
144.513730107393
FLJ32011   156.157567405927 146.824420102008 178.209994522672
142.589312139207 145.150191397375 151.002218995005 169.311264420133
144.450810504499 160.778296470857 163.686535888943 144.338069855334
151.051672692659
FLJ32063   137.114604159523 130.041462255766 140.336479395866
143.074187168031 137.306031826007 137.882914156968 125.346526762316
126.66432941518 144.69686018715 141.215542377796 140.605866597575
140.13515064896
FLJ32065   146.675159409376 142.428578698775 138.367321366804
153.039237274992 165.213327555428 150.184863108453 142.326319895395
142.729945734027 141.644521617419 158.967160833334 146.101547300741
149.492550066785
FLJ32154   154.627270742193 119.472410874778 133.191797029676
145.968884703737 130.698850298016 135.573170189235 130.905135502296
147.098474227445 148.82034800623 147.029988235786 132.213890220705
139.138329217724
FLJ32214   132.709845352383 122.312652834289 121.607527525765
132.903959880979 138.405215507471 117.015097017246 138.538656638714
135.425518707018 118.902823494379 127.368207232723 115.058061620379
133.74741832303
FLJ32252   133.238296113721 146.128066033346 125.682297697017
135.212715912271 129.806104966617 138.4303531022   137.632470240792
154.816213491233 141.733840643889 141.198692540628 146.664285015996
340.728888109597
FLJ32255   144.499680685194 179.079061789059 141.659197251282
179.376968970326 164.665907233149 146.582858169664 203.791916347213
157.041481397151 197.443711199904 203.727455875381 174.635853531194
199.662592246931
FLJ32310   129.104084188187 136.244995503799 134.140533647509
140.264060906597 138.076501233454 145.256660345252 147.85133493803
121.939618902165 158.200211812067 159.693019552703 120.433080754232
154.627270742193
FLJ32569   146.652641667543 141.544454931788 157.368974632451
144.461999591223 204.654489493854 316.004332524937 543.584413650467
499.683188662742 184.591789011195 146.199305851486 286.310923582432
206.669783013221

FLJ32575   125.201152760148 141.026045918091 132.553445658347
124.825103464238 168.173803952157 131.045970236632 142.06162974139
131.202090507576 128.959487155628 116.248026336995 131.412979573687
139.727248583626
FLJ32658   171.042491047256 153.405052909585 150.496616601699
168.03501792841 171.835438335216 141.992490695142 152.237183848896
149.634975068742 160.525939340745 161.079169854907 135.254996386778
163.143613079037
FLJ32679   142.421913396368 137.379558348782 137.709120153505
140.281512754823 136.120813399579 142.047828639169 157.264673452646
134.022324496729 128.83022039037 146.222195387635 138.478911439144
146.033591220134
FLJ32682   120.473038066927 121.583096465567 124.415058391979
129.195962576953 129.366567668463 126.013957128603 136.214432257698
128.314990390649 122.636980845207 127.233439204926 123.943080151532
133.611588728019
FLJ32784   145.78573175226 142.869595099569 150.863099494798
157.425924587516 150.023791544584 143.635367691412 156.930119977809
138.959040014432 140.113334247294 141.236939351298 144.02511523177
152.861304149219
FLJ32790   135.582230137515 130.119313219923 145.566861062922
141.004467676795 148.945466917496 137.794858639838 127.532665057083
130.165488538277 127.104482822627 129.049273337631 144.496429994489
129.515390475589
FLJ32810   145.889336010424 150.870830294349 148.076841184769
154.373739162815 156.609370182062 158.517778100137 144.233097828078
154.086510608016 131.723229807439 146.395248696835 153.998849486363
157.805871400914
FLJ32867   138.367321366804 152.979190595761 150.792065744902
148.686975104375 149.061464748604 124.961859983596 115.14883090249
132.653795458388 147.140771901106 154.046550866249 148.406031732776
149.390481900424
FLJ32955   149.842326869644 149.548900791507 142.390243984362
162.484777758676 166.936975782665 158.414776465951 150.819365726571
148.446949168794 150.371716899336 160.828718858381 161.83241639654
157.358696886078
FLJ33360   142.385902610309 135.369146072643 124.74291205933
142.586051173329 133.594446233378 121.632494500096 123.375592272781
123.722744366103 122.75516501062 110.935688075738 124.17231659571
121.021776527531
FLJ33387   137.350851760173 129.842252702648 144.032779435658
141.753560518393 138.670077127324 143.837402372008 143.804903119745
160.461846976268 138.044751228397 140.05419835434 130.980885733161
137.381470768186
FLJ33534   156.046085830566 164.198494818485 151.226242696197
153.485731788293 190.357437485436 153.219954133957 138.399665664034
170.179304745674 228.125521373477 148.046961712162 144.482364671273
167.250667092211
FLJ33544   180.590215659031 164.608496326181 148.789295862953
189.528686948872 170.05799929575 145.177195093247 184.819937046061
176.328994356625 168.660907237558 160.066390229416 151.858466721347
189.887788314879
FLJ33590   175.158180187883 160.459100056689 154.524588318927
180.392337212428 138.643442050615 167.756465178715 147.505345299689

157.723341862468 163.146581498065 166.007913607742 161.285587312115
148.582022536314
FLJ33630 256.326661031361 235.019573994855 240.792557915987
259.277693314055 171.954184094677 187.472028627511 189.575681012578
168.890506147594 172.169129806643 157.259599894923 177.976550224863
184.530853446688
FLJ33651 114.200099628087 137.09814291566 124.616629956864
131.486917325752 142.802965008073 121.965131419551 141.541355091514
120.043811428091 117.356389952799 128.007359083768 120.26749529985
126.488299049827
FLJ33706 136.074837228096 154.768422926874 146.326067008478
138.643442050615 139.00736252716 144.107029869771 131.789877096016
148.027464692171 137.73313146889 150.51660396548 133.849237689264
121.640681916546
FLJ33708 116.369395555451 124.752198904184 149.147411595596
134.918496279185 117.432860005907 127.824005374202 124.800221241981
133.707285280448 140.831508792119 122.165046954625 120.288469716375
138.592943322694
FLJ33996 164.352366170527 161.960392594363 173.850390626782
171.086559024882 148.437676259828 171.278489808694 160.415807880532
204.616225498655 157.067264221207 153.762995131216 172.657668514226
164.571974509529
FLJ34047 319.352940751688 364.113665301598 312.019603761673
304.985269874607 180.641419603362 307.316241247336 210.448904052352
234.339913501761 187.784865705837 195.69447353356 246.88857694249
271.064405562085
FLJ34306 135.017196494069 127.525844737562 155.007516124803
114.470670941078 126.547883999721 134.407776033032 127.842177770276
132.464205461991 138.818492907643 117.513523498725 142.093180304661
133.088758771353
FLJ34503 145.998177732093 140.775447163954 149.435684626505
135.745821200254 138.783017264895 145.968884703737 131.08389186558
148.79958121087 144.36575644625 135.25734629146 130.920010795017
145.89593390936
FLJ34690 168.925131238831 153.952788086758 180.276082632155
152.461930328923 143.984222627806 174.727439318223 166.533191390639
148.732027660387 152.555437272666 140.659276788384 130.661695937728
163.326102597955
FLJ34870 142.088243118644 130.473842500245 137.024230648861
128.856640014006 138.661548678191 122.928863588006 132.498092935232
129.058085094891 115.5765427629 154.087640417973 114.520066232155
129.614692778261
FLJ34969 166.640325848102 133.685254707686 139.340699808517
154.539367933684 154.959217895243 157.180095329946 143.279331050175
154.13120950189 148.832461329713 150.331324340476 127.282374513433
154.856345114916
FLJ35024 155.981989040279 146.155809402461 141.218832234405
151.690538050817 126.499257771192 148.770315933249 139.872976072847
138.446492794924 137.307786147787 153.43000129811 152.057315195367
141.825162897153
FLJ35220 224.424524191566 181.157738347957 173.573412346985
215.057917795456 135.51506573405 120.560056224145 123.100949494673
128.840466579373 129.397378057767 135.803599969904 147.585987099202
121.792573534134

FLJ35258 1140.40904196731 1223.68146968716 669.330078861673
1555.28458491244 135.409613764459 139.080142195118 164.433462115832
146.96155692518 182.907444581093 179.914070416793 144.516274148451
190.549084022965
FLJ35282 176.167129202773 169.482586449402 191.987301954927
169.039807608035 139.804518460303 160.078122267577 141.004467676795
143.514848892447 143.799113486767 146.669620721254 145.177195093247
220.510248260182
FLJ35379 134.182902752156 124.288632450364 127.442587366678
141.86836086844 127.209043595332 127.475316138156 117.343165720552
128.725044326566 122.834017529045 126.158204790885 125.005019889478
124.699127988314
FLJ35390 6670.51138785163 7371.9761952537 6441.13602696535
6271.34208268488 1396.4168898623 6508.71638048514 3501.86788138647
4965.25728333142 4228.5490138553 3188.90202907403 5776.63686968234
3712.67025366957
FLJ35409 126.279119798177 117.706795566317 120.80221197797
121.501959818928 111.559325333192 120.027996187915 128.26957712082
119.119693776276 120.272964182307 120.975542702338 134.189023892662
117.585207821853
FLJ35424 133.011320549232 135.005591691192 135.723554819107
139.711696905287 133.610143526954 136.271412363498 147.106355591496
141.685620602226 120.984641102879 133.962129094978 130.449669345302
137.356539213081
FLJ35429 171.093611712143 161.77611826709 144.905870145004
168.911840239685 131.362128083159 157.301617257941 153.402227428787
149.577995793381 149.848740870148 163.788418732315 129.336402686549
147.949560045212
FLJ35740 137.450774903611 125.576103876855 148.660440219589
141.727639388452 141.719556176029 140.067809125008 121.410606617535
138.216926722025 137.185243517766 125.707228291312 148.100508271232
129.689855810051
FLJ35767 163.039564280484 153.600132368924 165.122737166322
180.3779211444 174.26780796802 166.411219327671 151.790240553272
153.545086287929 169.441789096441 165.009696356892 144.384287335249
190.572712684622
FLJ35776 157.12360611723 166.606522485953 173.299643109576
156.904781904311 166.311421907434 145.481279504679 157.874624979775
160.176567985923 166.162656500954 152.769931978291 167.521700144143
157.507108643575
FLJ35785 141.079317977197 156.000732564221 153.202157381228
151.604093626944 141.56459720769 151.409290231274 157.960172706254
148.401698825687 149.554174907798 157.142101889337 139.310309748986
154.267020640428
FLJ35801 143.085022888434 151.629999903069 189.539458686123
142.26998944788 189.348690353874 255.793823796456 197.938447651153
298.776176246196 229.835576260403 186.276450732044 216.277178481058
235.704824490397
FLJ35816 128.72727523654 139.56332664295 129.544594679212
146.501559368223 142.734103971267 161.911468506919 180.850779069931
140.486376016494 142.623106544789 147.387754442307 128.008322588928
166.988725688589
FLJ35848 142.527422462824 144.181359740481 155.072752529178
138.106499370074 144.774621469452 145.870135196037 153.85854399897

146.779054605199 134.036236880951 137.363483527372 138.119788738226
142.268018184723
FLJ35880 179.163748161358 147.043228493047 138.79473329684
142.339046840513 153.326420485822 155.413300826229 156.731291756066
133.627389970495 136.903588075134 138.106499370074 153.934872807607
129.558589108727
FLJ35894 113.283359026655 116.871350997346 114.494394548044
108.404733392914 103.36057518781 118.47752077032 106.102093734748
106.746545626069 113.177330690612 113.995301093609 135.111066946207
115.058061620379
FLJ35934 152.713528089347 139.204218271711 154.080778100852
146.746669300049 138.411148122407 147.70836747108 133.378374710498
161.172420368335 144.145612985703 155.400191634632 123.094324479352
136.901107766796
FLJ35946 136.181791404331 116.483432723207 125.277752749246
133.319765450636 126.355699811531 131.360310497851 138.216926722025
124.568534983277 128.22735290389 119.346574301289 106.204350278634
119.418866166776
FLJ36031 136.618643817686 142.036037957345 129.338551879984
126.549876718792 119.297561749232 147.422656909557 137.846463796994
134.504873813959 140.439698763808 136.762273376313 111.286141277084
135.691862612951
FLJ36032 147.121049266117 145.730054487572 141.418737063013
142.89427871125 132.680140469504 139.001493765421 148.901723851925
151.84373370805 138.112168847027 144.110544384466 146.164093136588
140.244052740397
FLJ36070 162.464201058576 180.837420957945 168.861884032201
251.324685584062 257.813193543819 142.088243118644 194.092089507551
154.760697311395 158.863516051439 166.027988561421 145.549126188292
170.08485436731
FLJ36116 143.356999170877 145.971656491073 130.004329307977
139.001493765421 138.227082687797 146.781221728543 132.576755070703
133.550008967009 144.958176005711 156.399808270033 138.106499370074
155.397301207102
FLJ36131 4425.66777919789 4276.70912292877 4697.94415026712
3905.35300751401 454.854528472591 3648.38765465889 1449.33679383001
2574.74962955426 2031.01186679713 1669.97250023755 2703.76111776248
1890.86868383409
FLJ36144 139.541759919059 150.311274161073 143.652009342522
137.022366035333 149.010477279209 131.748753064488 144.649656552
140.586497829504 150.423586547273 142.945392802281 153.974384951787
143.766249538352
FLJ36157 130.12032144763 122.204249636472 123.7421178083
130.917269984571 131.587987756056 128.22735290389 134.914830257315
134.840285218383 124.213104788426 136.263658288289 139.671302796695
130.176734524308
FLJ36166 157.006278815282 138.132390735567 149.939035476611
121.699702849509 155.639666182376 143.052799804962 140.321675137237
128.324383749931 119.658703392877 124.697283474333 108.604763499678
131.041996482401
FLJ36492 142.810564324354 160.014635964846 161.490941000872
142.851432926979 131.702788057359 157.40255559397 134.828360289676
145.159572111039 138.341918073941 138.603793282123 122.333765477428
147.268006631005

FLJ36644 148.941194457652 166.521472376759 182.384190694142 149.143087870963 149.547635666621 139.579764449867 169.136919982294 162.748836971539 159.49930229951 169.072574423443 155.842195370853 155.548684011748
FLJ36701 146.238333981318 148.908758741034 135.279197125157 152.175423676063 139.462804363695 163.22480684222 144.853868519575 153.4392054301 143.036006001419 141.016596301267 127.58382536792 159.337816095012
FLJ36777 131.215202712283 134.521848929606 139.677109265241 122.624828884731 131.065633574494 133.62358240645 126.580227722115 125.20712393273 129.143168240589 125.130703953031 135.360087349634 111.161283080299
FLJ36848 206.68694753419 181.635664531851 297.12411625104 196.014841240142 156.846991384413 207.150354734189 186.591941221632 199.378179817518 184.915526626619 178.728925255163 163.416011199431 171.320653882424
FLJ37078 157.604224004016 166.422360032182 122.251178920649 141.738647095861 289.667302610069 182.130970182242 192.709473588165 134.255021727555 164.955263076598 139.075537402658 162.565576110247 259.374392296079
FLJ37201 144.96670085147 133.054453517834 176.038123840296 148.466658640201 155.280897870265 168.682307349713 145.594378536072 153.613110881454 132.395993254065 140.117644705946 124.424565172377 132.171282327983
FLJ37228 149.087951792991 155.101850847148 125.08580835258 172.857643600385 142.431979387165 162.664254855131 144.65238608866 138.4303531022 149.013011292165 128.800907223807 149.813838004575 140.258615682066
FLJ37307 158.120079903425 143.560065338939 184.25384366282 160.022481227931 130.824958143557 168.974155750959 144.68114486527 162.327039624723 158.297624312257 146.891471907784 149.791777262424 163.203582580217
FLJ37396 191.987301954927 199.327939161502 225.96618659877 198.753297290444 155.988447182637 188.981391266203 176.066680490625 163.053121879323 161.285587312115 176.890596690798 158.886786960196 161.094103878416
FLJ37453 166.472068379965 166.091736766853 167.746786642066 183.371103136697 186.722842989058 156.46322298175 137.25679451567 147.910699446264 166.8846964604 142.567737460006 136.91512200431 128.686425820835
FLJ37512 150.602976963975 132.346363545288 148.423303776817 145.86855878099 136.823008283157 124.995087711332 140.40754434058 124.555847618988 134.527165454543 127.769405736754 134.825699448987 135.452340998039
FLJ37543 134.653131708903 131.798931481549 138.414916506716 133.061016674211 148.284412570438 130.2867846895 142.331050242704 135.802258952006 135.105147647223 154.256214901103 146.777258330572 134.711144967036
FLJ37587 117.131720092783 149.586651393588 137.992477770842 135.15791174418 124.449679223202 134.033751039071 135.25734629146 128.666652639862 145.236154447244 135.43119649883 104.285022032604 127.9575507576
FLJ37644 164.38282966938 142.658562927154 164.717809928624 150.279529635193 153.328585916625 162.294114485312 144.627784383455

150.034029031033 136.19481894871 133.257477196196 155.058852833902
141.082569618216
FLJ37673 165.908756485356 150.011603347578 168.323141675824
149.213990424575 149.004343851423 160.402220755071 159.443697452694
171.080373870373 157.668307146391 159.570288565855 142.50631960137
162.55521875551
FLJ37786 138.903450375532 134.251843621261 126.603552416008
133.733674016447 135.961724275921 135.054931772725 155.8871985642
135.86149691566 127.816596414779 139.658932672049 132.94603323912
146.660826209767
FLJ38377 148.780775862239 144.746134643761 140.35175858015
143.953043041292 133.895547281411 148.288798428149 143.093156971372
131.693923403533 152.054874792646 140.421945574721 121.984179437382
143.976395954528
FLJ38379 141.426756696799 144.78971417658 148.863297062269
152.337631025368 143.576177625265 143.710449544388 138.53403492914
130.363516180139 143.271346378958 139.488057371254 131.726540162023
142.726324048628
FLJ38482 197.815264620906 181.654319266685 245.640193659159
141.884995734267 200.618080312379 260.224777636733 381.182514249587
289.82072316082 404.95944990868 250.25959091612 254.63506290698
326.962330523237
FLJ38576 162.519642881848 145.544996009633 145.315875226867
173.507835230243 127.023543953724 140.372463604232 161.397788731595
127.642215862573 149.551030995489 148.245974323625 119.544004279354
147.379741260871
FLJ38717 506.562512021352 487.597040819879 416.546844844021
497.110852407681 203.999142031297 344.786541451395 329.604568691377
286.541028896473 317.163768837813 230.079664410595 319.128582107184
278.555009982029
FLJ38723 121.968807253639 123.752109790056 123.159407368236
115.299986138153 113.38168751499 114.470670941078 122.578170988039
122.708102546276 118.527464364564 130.277107538053 127.954231630338
124.327827104244
FLJ38773 144.456324230938 146.633627492419 148.654099017805
135.005591691192 136.561852783331 143.756242843438 166.784804508647
139.404820141366 147.70836747108 153.532954370572 135.458682745495
154.291717784335
FLJ38969 151.747595672228 157.960172706254 149.77596374205
162.919656039331 167.858490180471 172.478461106269 142.737537206204
156.365413649149 166.447510782568 170.298291784762 158.100395482569
155.535222310709
FLJ38973 184.008405576875 183.853381322135 152.768580075051
167.087850057037 160.209949749785 216.783051958345 171.561940747015
192.130214856642 139.484551562098 171.573342518254 198.113398073968
160.338146803754
FLJ39061 136.674553275255 140.765231611948 145.358431402568
138.868641301709 137.537197575152 139.181131600753 137.629855378016
136.657107363796 143.519786615253 139.59941545613 134.381042390464
136.946137446743
FLJ39080 157.273309095198 147.784078315091 144.863617174741
138.121905080822 142.826898321575 135.728336465002 152.624623171797
137.476047500852 145.168031449425 144.757068610657 137.920724602098
151.059401217656

FLJ39632 139.532508621268 156.557792402376 151.032389358279
149.013011292165 262.79667156266 302.021033686056 624.825216056495
161.112906953532 315.430399077266 207.651142295123 175.139231359014
773.803350315426
FLJ39639 172.108795190633 189.61702125418 186.515784641119
229.443064589306 145.455411983653 152.801801823442 166.767004180603
167.830629616121 163.582678113215 160.861929142249 167.51207060064
159.629232808787
FLJ39653 223.638850960288 188.013181859196 269.053237303244
226.449705559354 147.854171854456 198.631354372924 208.793650722271
297.12411625104 212.959246855759 152.219906297607 215.673878830563
199.209279169496
FLJ39660 148.211354898758 167.629008083436 146.637799077812
145.581755438161 146.190750749851 225.335753745598 161.960392594363
166.558080305926 170.87247413028 155.214638707046 153.568321662387
163.843143947012
FLJ39739 143.639894909386 146.781221728543 147.118987674809
144.071652839025 146.451918215469 140.975056186954 146.98903251354
146.31980311926 149.511776997149 139.099483279141 143.410917415691
142.321476446203
FLJ39827 351.079435946154 417.191960489528 360.39998507367
451.985108262982 288.261250589513 292.068293167526 245.195406638979
294.595972099366 247.528753010193 237.492603094091 232.151020756625
213.215996114827
FLJ40039 133.921012946195 130.858419819734 135.866980620144
123.706910388724 127.323157914914 129.543069836687 128.755697083014
129.775280076365 130.838350486331 128.736591902501 139.06923394504
136.104965836123
FLJ40113 152.861304149219 151.208728458188 160.254101852355
158.316173597874 153.051143478997 156.445294524594 153.738794649373
147.182394557583 158.23884088767 173.87113073582 157.155105022743
157.976810829398
FLJ40125 154.908975734985 158.193008411967 163.718595974283
132.615324904193 164.274491958161 165.244616507084 411.221560795912
166.803359138942 182.67941458824 162.308297940279 187.729071402782
184.085616342099
FLJ40194 152.783534456473 137.81819274774 171.39347085745
151.645636946799 155.330215726109 147.679233228201 146.554017952963
152.152106138031 151.993651822163 144.954595802474 139.005788243703
154.679076303046
FLJ40244 115.174678745401 110.820329153348 112.884916986215
100.04568139926 113.886420801388 109.176852761745 110.838647534175
112.590379042108 101.981823814699 115.107214937219 116.825172441325
100.746407116143
FLJ40288 117.889524670597 109.564709878576 114.999628959921
121.997885377703 112.57049236673 118.220555783444 115.678486138934
116.335864439545 114.00959999268 120.174672871542 140.194414869595
119.334967856721
FLJ40292 143.118188813831 141.834582245454 163.010527914347
149.019120812629 148.807422049327 149.066522533956 135.594186066942
155.211976328553 149.437212090587 135.494894933583 124.606795061928
161.603317039244
FLJ40296 141.779801021888 139.844984829379 146.252974508529
145.566861062922 143.821269460523 140.981666934622 131.500591219916

142.945392802281 142.321476446203 138.333435927107 134.163388748526
145.724294862079
FLJ40330 154.452964510369 158.147502554814 152.077204721895
160.315029612814 155.400191634632 169.806644442071 162.002221893617
153.633095853017 155.19452195405 161.441613587571 150.208809981642
162.895923745031
FLJ40411 147.045944019838 145.226278521493 127.597601325212
141.272049404557 143.331929080227 145.117574928189 143.431389389919
140.192644427334 141.611712747368 138.50252234048 143.50323531501
127.373421694142
FLJ40434 155.317754378283 150.557982810339 126.401145439668
141.553465481789 124.682396766302 137.920724602098 136.049167597016
141.838206951224 143.021435512861 134.509830749072 117.829228895897
130.379603677874
FLJ40448 124.740700441589 120.106270603148 125.208803405105
131.342562516383 124.657687121111 131.77099316122 117.193620642373
120.355844117573 120.906553066296 127.174074003712 131.648308760913
130.99564079691
FLJ40453 148.592330216136 141.580944960818 137.812249316763
149.480971898888 160.446191353364 141.21138348075 159.68134949161
149.133055368147 153.005366371425 159.563866056798 140.319851351329
135.592128081677
FLJ40473 160.79631681714 179.091917512822 166.32555285213
149.666293193219 148.868265867275 171.051948060796 159.674396591041
154.080778100852 145.409722106953 153.051143478997 146.261164125255
163.824878791966
FLJ40504 192.046442148183 372.757091748175 203.87884605779
150.99622088333 4255.59773503364 1501.18820017008 1332.11815966955
1176.01058971561 2921.59274577877 316.623219008156 2542.44286642873
1211.94273385872
FLJ40606 139.034940762344 144.595393134144 141.730912072471
146.580787463084 159.968531285904 130.854818615966 124.261531006131
120.857635836068 135.504526903482 125.220416203903 138.248708129528
124.942701123337
FLJ40672 121.926377140115 125.277002586952 132.387242907281
130.029719192412 127.447218258785 136.712083407769 127.551486042435
121.347339077474 133.412452607155 121.646971898731 129.189479305468
132.871179509995
FLJ40712 127.918847837351 145.855671447779 141.023875058322
148.201649039381 134.89415027205 126.17342715006 136.366798516444
130.521731606987 129.353536603449 129.247701136266 140.311843829601
139.878752507646
FLJ40722 1162.8644618472 1139.14730341921 944.92617668298
971.170460915918 300.793542873709 1259.75918248468 670.571106793204
970.634736994313 622.142195892701 559.0867397858 1850.98311401165
825.034504959272
FLJ40852 176.680708339236 170.998961353556 182.851203073653
178.06221214103 157.273309095198 175.711836112986 191.897449038297
172.877947895827 158.95714101556 192.140442881209 173.225064625094
168.205277839471
FLJ41046 125.885715928839 122.706176540192 112.464951599416
118.256000539093 128.967855397644 116.476747183738 121.459780480915
124.160433356046 126.50870982427 127.107223387937 146.450806121927
136.408314486465

FLJ41047 135.508025612102 150.799400717425 144.30699572795 143.961467163406 122.903428611669 147.461480849333 153.679479224178 142.198234546084 141.241717979079 145.505223177269 148.945466917496 123.100949494673
FLJ41170 167.906766146651 138.657241641263 150.507308901391 138.467624450885 132.183250916482 154.665212863369 134.432461986523 149.18603646016 142.221542344035 152.19583231051 115.417648857825 147.373174160478
FLJ41200 143.099341621411 137.047288508371 150.815117344434 161.849023343715 140.318155161909 148.94765984808 157.394035451151 146.395248696835 140.359796538887 142.33269270703 172.412059174046 152.29434111068
FLJ41327 140.40754434058 139.14544832776 159.374355553219 152.401204710292 155.355051932357 151.770791549511 149.004343851423 144.165277164871 141.739299634007 156.061579024549 135.16316100494 150.28169442832
FLJ41352 131.878155417065 135.643341446691 150.939877261936 135.535020220081 136.625136565562 138.562576783507 148.245974323625 138.196640512429 133.814720785534 136.231747456996 137.821010169859 138.267726309873
FLJ41423 146.803549606737 127.43866970659 132.151765465534 122.018200592563 120.822923434486 131.034010150362 130.325073627151 138.015301767961 135.292532783991 123.877609932461 123.051777473512 123.998343902602
FLJ41481 161.397788731595 154.499313851756 143.810050834214 146.090291252325 164.824914851636 156.210423316722 141.079317977197 171.449326065013 145.917220112846 161.485353443949 146.875733210901 141.966490100288
FLJ41484 169.403422185018 178.835679834487 143.256590560776 236.592839463278 138.175538905334 163.416011199431 204.113356572343 161.397788731595 206.972440734038 132.721469335671 219.49354518392 401.359711029934
FLJ41562 135.59027155522 137.658183450715 134.109051944736 133.689255383855 120.711150688559 131.956481591804 118.628559951472 134.335309757663 135.964630862223 133.615958668897 117.144778357339 136.156548919515
FLJ41603 433.019478861638 565.164961255054 560.602047253306 679.557656724243 187.529556428609 201.221839281282 191.170105307944 179.46936343225 214.068044241507 402.366797969833 166.872332652008 209.684346388892
FLJ41649 118.351607868932 112.10312035128 111.243322115126 122.603226380439 114.953328013917 123.135918415235 113.321454160244 114.00959999268 112.624137732184 119.407293854922 127.068313862984 119.842470751496
FLJ41733 142.121399383259 156.632939064629 144.443844205426 133.631676386591 131.85737847837 126.823886603295 133.311212623667 129.606271613463 133.452032750397 142.21279866857 148.065080664185 147.78095608696
FLJ41766 184.482849019679 188.268774618164 247.899467998443 199.305626149918 142.919910421524 143.864876796403 187.796440205485 163.529157756197 137.408247668043 146.404727168045 163.456245724169 145.987228759402
FLJ41821 153.261002653668 154.312374306932 176.506227858258 148.516653846017 154.166668493152 144.436358527414 141.755954088275

161.764411756848 163.331424612891 154.722895261876 143.737021989907 159.592581700976
FLJ41841 150.290025009187 141.67277923437 138.553151063218 136.618643817686 151.473239782301 153.173746756225 139.39112068213 146.229239731545 141.259186690966 142.64161581553 141.281116867082 148.326282600684
FLJ41856 154.856345114916 132.492444216154 133.269658133558 143.31117188259 150.549883779193 141.604832186257 154.520935683104 147.15764785567 139.981905225845 168.111124820968 140.38686070363 143.094799968981
FLJ41941 148.907136936731 142.744045151046 152.219906297607 168.365262340217 162.06940121135 137.016495611111 135.666278507269 147.121049266117 149.772157318769 152.964940071133 146.871145679651 149.320667417893
FLJ42102 108.79453730066 112.684334910243 114.488760619745 116.796995589816 107.365390754133 112.624137732184 115.840266274696 104.681530703922 118.792612926619 102.166449349339 125.374764771516 110.602406076036
FLJ42133 133.160197638474 137.408247668043 136.946137446743 125.908576249439 118.757973008463 125.717328918165 132.137634927459 121.668166378914 129.386300987308 125.163958250922 119.366599958278 119.122305582754
FLJ42177 121.330383384978 125.463718280745 117.172431931323 127.287538993899 111.032156655273 118.438942370745 127.289269745461 110.061459744714 126.162713269943 122.868393258957 137.243864288111 122.300069045533
FLJ42220 160.608674893418 153.603765998461 142.282861913548 148.823805841381 135.594186066942 157.77047742735 150.566842106244 152.780908985916 165.597236278435 142.126620107418 128.986795394842 138.523677181131
FLJ42258 156.296046600921 166.85671415475 181.971189935628 173.863373934746 162.018860183534 163.331424612891 179.265395910119 173.299643109576 163.726898336284 173.810080280732 160.311096717492 163.153676643372
FLJ42280 142.22515098612 141.076652697698 146.464937541891 143.999000471746 135.18026749895 134.394503574535 138.956057921718 144.579725208643 125.739946257697 135.513494853742 130.613760017864 139.000248033373
FLJ42289 152.612116997575 141.239354296152 141.480793979634 133.757741295765 158.708054164813 140.531789506024 139.767216030715 136.176688025337 141.980728956046 133.645044060866 135.778099658349 141.719556176029
FLJ42291 145.832751296646 140.89390566467 174.127444395862 144.064214573005 150.817922696097 152.511331178337 150.629932241703 147.52403424052 159.106269972346 152.44293820056 150.464468573079 148.02460124587
FLJ42393 179.96623248843 160.240052678401 172.20451139581 155.981989040279 134.805239981342 178.235281981508 152.342712570625 151.625248091441 136.485034823945 148.534143255537 130.037840598918 146.70774472499
FLJ42418 129.601234646954 114.213405341709 121.599883734552 118.641431192759 122.702175900246 128.87498876738 127.04217189075 126.800718471804 129.493805009651 134.992686224944 125.362754088168 129.618097261902

FLJ42562 135.90207451581 139.119638136874 133.935910746769
139.47828235176 153.891667472876 135.433229702209 167.542332146706
143.589222264786 146.616041788595 131.601823550269 170.452989060697
134.848210768603
FLJ42627 205.987280015028 209.362806125315 164.241874094897
262.233918187791 216.506914782226 151.159145294127 181.668090319293
201.480444219547 241.557810100265 173.839053227554 304.900910626137
179.854800301573
FLJ42709 126.830269821364 143.844153373367 133.281505040209
129.553282780541 128.736591902501 118.694037994926 125.489777118532
120.280241193135 132.733821031511 143.287237534352 121.154389729409
126.84966022925
FLJ42842 149.687372584136 134.686251400642 166.162656500954
145.887262601586 150.144977373708 149.34605615699 143.787766055379
141.91067228913 132.320536722841 145.387562372871 134.408846338374
154.600966693059
FLJ42875 177.592497020798 165.249658565871 169.428195354763
185.46843333826 165.665796569389 166.1677526 172.152570321727
154.600966693059 154.371946675862 156.053517438255 157.927709922368
165.70840218273
FLJ42953 116.273000405449 114.598636725763 119.000436602325
122.262664384198 123.827771590213 117.706795566317 112.729141678141
112.07238018581 117.580122770251 112.578934972566 121.895778599116
115.343251487944
FLJ42957 126.748906552086 156.296046600921 130.674263597931
148.195611015135 118.372047967248 134.883708339914 134.176925491721
141.216205703466 126.398884382537 140.688570032068 117.48427100606
129.47419698487
FLJ42986 114.589757401741 118.677203713024 130.584348445667
113.025793645043 116.89487516867 121.764933633063 119.631847624882
124.76274408368 125.861564323476 132.317972465689 121.400652285172
127.019978195283
FLJ43080 123.849651946683 133.190147746722 146.204122329465
135.930107381894 126.143886553557 125.655823846115 142.878258542775
133.634886800037 133.838032323605 140.071879210324 146.67875644027
141.739299634007
FLJ43093 161.724388995749 161.83241639654 146.421195733989
174.694388371236 145.671099346237 143.074187168031 137.788504385206
134.696571019896 152.077204721895 153.679479224178 138.062345982865
156.768976123975
FLJ43276 136.643481414354 135.699583036042 135.388750095746
140.604900199281 138.880168623766 135.367261617271 140.875923123766
132.823333905679 134.292099546804 141.393814611166 135.151438845363
137.499290547619
FLJ43390 132.582062079623 121.414076245292 145.68886453261
129.176532377543 115.785084044427 141.028377381019 131.921799405427
129.044445373107 131.364187047583 122.351186755611 137.961504178836
138.771088752839
FLJ43663 186.276450732044 161.142723532061 216.457866890673
154.627270742193 130.12032144763 165.471587934831 150.952264779196
140.343790129071 165.866987051333 127.274768398849 149.831566330214
138.499293816467
FLJ43681 6296.24208225227 6114.33638002734 5776.63686968234
3630.80752744347 9908.59593023839 4318.50925204407 11554.7330886956

12690.801964606 8261.82420896892 4979.31150268741 8532.46059476152 7389.95365139168
FLJ43752 132.966873990501 129.353536603449 122.327353563917 126.139188586523 123.434963905308 121.539958110688 130.326980619676 126.567824355343 129.964666668421 129.033433971346 148.556161762978 128.56000638844
FLJ43763 119.725487374119 113.119021336236 123.762963668211 120.998665308561 136.543603346919 119.057157701097 120.204469421484 119.168045349254 120.252164075308 119.682747854629 128.895325544109 118.015175851934
FLJ43806 160.303394115537 149.866592541235 126.656077572961 128.478724185042 151.676232556697 150.998881139061 144.292679144059 142.358754646258 128.645334554749 143.778327403708 143.354858193994 136.639637503571
FLJ43826 145.063062479224 153.865702787334 152.601626526117 151.102431192559 143.467053670148 141.84490608317 126.899985772157 126.939543057414 132.54577436954 129.802596741962 113.66416989856 147.302953100995
FLJ43859 134.719531559829 134.46179453106 126.523201566273 133.337863400762 127.235190626458 125.794788766462 121.588054040961 134.91213977595 130.795318552623 155.673532440475 122.382304966782 142.763157232147
FLJ43860 153.402227428787 140.397184813186 142.759452994928 143.491142564838 143.710449544388 144.964084792568 146.59366188125 154.783699020901 137.973211667003 151.032389358279 141.019494314354 151.126252768792
FLJ43870 152.479402912801 146.030484284194 135.294664456623 174.107661206415 150.265385257579 140.857328923536 150.691815494794 142.865320802784 142.590631181358 151.463200570499 149.759516805355 162.716712951629
FLJ43879 140.66266106824 145.247717878099 148.370331097467 131.288785100733 139.783725419023 132.450954427876 161.046418048832 140.545351041252 137.940675746397 137.020473156363 143.506243892155 134.728594388428
FLJ43950 144.190203286506 136.511669406292 145.784526088216 146.128066033346 148.493435517221 142.007066214472 146.690308698414 138.076501233454 146.218767446679 148.479865869644 142.024171830924 143.951756384507
FLJ43980 108.34997967774 111.765812595638 113.362825181676 119.415822905251 121.080413308265 119.451099896048 116.769106223472 108.266328505986 108.061315311823 114.217867810848 120.14201542563 113.822580779824
FLJ43987 134.23001942966 145.432667767652 127.144291519133 128.905042692431 149.763038696374 136.095753586495 135.233417601805 128.482976013813 132.450954427876 150.896076207161 139.313456284725 122.629146433216
FLJ44005 167.484236948415 160.78368986036 182.810581377888 173.09379121032 165.67554738735 169.541411677993 181.865456674829 168.013310840048 172.980441569522 155.522274777451 156.073428882485 158.869463014801
FLJ44006 141.284962125757 140.36425603453 135.017196494069 139.244051199144 129.072173761566 138.328508662816 148.861141988806 134.983884722607 133.581450824362 138.478911439144 123.247698282967 151.702809524494

FLJ44048 138.462826342765 134.711144967036 130.430917518283
146.790059505709 143.207187847583 177.93138555339 137.404525565336
171.267907653007 135.699583036042 140.868946922293 190.204960255989
175.093007163166
FLJ44054 210.620764703468 204.740183832461 231.600532144945
216.427103365727 189.83433085782 237.940015617254 197.100205890532
213.274106157675 198.470718196139 203.586310608403 180.680595239651
187.63609695026
FLJ44060 114.00959999268 122.418378964982 120.78129870199
121.915043935246 119.107992692097 122.201293962463 118.32560379073
117.051541123239 120.384210318 119.418866166776 120.846789922177
116.280157890082
FLJ44076 125.924219382375 127.409603645289 119.780654293448
112.282839763467 112.599970121166 127.31283918221 118.115015846489
121.138461509096 122.00079209554 107.559685467789 129.442111153737
120.992440100307
FLJ44082 142.041992878641 122.587545171646 143.287237534352
143.401971479167 127.938245853896 127.05677680446 136.986713332541
116.678969343492 118.677203713024 138.972789382679 136.294479720968
135.971507823633
FLJ44112 155.76568091161 158.975786937923 156.098240321674
159.678281513591 144.63774603725 145.630497368137 152.377056383547
142.487424651482 134.160100276452 158.536178747273 138.771088752839
149.42148494796
FLJ44124 15924.2167735021 17658.0552091534 14228.7550296952
14983.8256502293 3309.66193624859 18061.615487998 7681.48027081818
14037.2575989329 11432.8213906428 6628.17856159198 12654.3779830648
9970.08052783908
FLJ44186 132.850601603567 136.948039394935 152.11956441072
140.806935772116 136.294479720968 136.158508521357 145.503718833974
140.759006929593 152.024383072265 140.641308444844 131.263641389894
144.212231875922
FLJ44216 151.515460040167 169.441789096441 154.98147307591
139.178245419517 154.912576362196 153.467694163404 154.496005175117
161.806144472744 170.522853717911 153.687536747147 173.810080280732
149.714982846365
FLJ44290 155.365673279823 154.821960325589 138.743505629497
141.968949222821 132.69067627743 161.139253742858 143.943676099522
132.158817539201 136.214432257698 135.738699497293 138.322109975426
156.733898702331
FLJ44313 145.695402948337 156.748330788367 163.991378703958
153.816782770382 125.606269267903 110.875112640101 134.013748718539
136.470025638057 139.356478232855 146.231937458616 154.373739162815
146.495845282846
FLJ44342 190.5930909277 181.668090319293 206.446584387595
181.467708718758 130.576944864484 290.867822301134 181.840985962045
288.596513753617 200.045606111354 179.94333102877 224.396933003452
215.019239034819
FLJ44379 190.348359536057 169.466129742085 182.873752254411
162.100547394235 162.919656039331 150.542478166361 154.003862874241
157.635531050489 156.026806913759 148.89180107448 134.051756243089
175.774461934758
FLJ44385 158.299765118221 148.993620368781 139.943915452851
150.576660798865 191.200215655114 170.655648004461 169.422015793848

165.062129636008 154.01988200569 143.823368276815 153.176268095608
159.820341130737
FLJ44450   148.656917603142 138.807031587657 136.432109029368
134.224781719944 139.576261630082 139.81502919163 156.602240897907
136.280653079814 144.988803993714 137.188849160569 146.832797779597
156.622993107052
FLJ44451   144.46549577902 124.15094220181 149.760883745928
144.675182563174 142.707913210557 144.977958320895 149.272219727411
146.21362402936 141.350135458015 136.74788009263 148.027464692171
148.076841184769
FLJ44477   147.027904536486 138.144085131733 158.085935267091
165.080825814849 149.125425566378 131.878155417065 143.421288665418
136.705326991088 143.368327095618 156.589829166493 155.444668113276
157.555410977564
FLJ44606   156.895079878556 160.243405030742 158.738932073809
166.936975782665 175.840639511966 164.82904652634 207.400490077106
170.768398273919 155.507030276749 184.900027626401 156.890299350025
214.888126361789
FLJ44635   147.164348704012 127.70306883967 146.119700458464
141.342216963124 139.714198638713 135.705904966172 137.593577427321
135.803599969904 142.785631812039 130.242779079309 149.974080577906
148.983182193411
FLJ44653   127.036976462046 133.783379558598 135.27739087376
130.782296492278 124.210840121525 131.526532700651 128.685065364693
121.905741525277 124.551603326682 129.116354997747 137.334548525575
130.022574966461
FLJ44674   138.755173333171 150.405715534245 146.101547300741
168.982086590306 135.90207451581 174.576132939661 157.381401974279
143.094799968981 157.516278302035 169.206723147505 177.562561297777
151.519845657755
FLJ44790   163.331424612891 167.822075935474 160.724319010751
162.364266549463 152.071081175788 166.111496598928 171.051948060796
147.301909062399 146.986891588172 145.212306927438 167.442950045855
149.63291071048
FLJ44817   124.103321614841 140.796641921516 141.370957100324
142.737537206204 136.9834458057   146.536055773073 146.616041788595
147.128805198619 145.36249226764 135.864853829421 136.264815594226
130.898067924686
FLJ44861   152.179281925254 145.82344959902 172.46311371589
152.68344738009 118.930792960643 143.874735808114 137.369033684705
147.060637536615 147.257773507186 155.569733738768 129.265788005518
154.091431691072
FLJ44881   130.027227278931 146.97334315047 130.89594032962
134.253399256648 128.040201586267 126.722212054469 142.253826696208
137.21916902421 136.764881711137 161.548130965486 127.016845640004
130.836079676477
FLJ44955   113.112450527382 116.819107878692 111.448871835514
115.871151134713 118.492287441192 118.430401197079 119.373287155621
105.839106819841 115.463150857002 130.354641020141 117.703025026759
117.883635746379
FLJ45032   165.857032207991 157.81831741292 173.635827398273
175.350734537491 198.737421468412 161.44729973777 163.20714296121
163.996649606636 162.592306630033 183.988537525989 152.379441401583
167.958792571511

FLJ45079 134.619470709414 150.303703193967 146.64835267642
166.192990554427 133.236084190243 147.552635527742 132.487362867399
143.260448301109 152.337631025368 163.02140155858 134.485835304676
149.211214631561
FLJ45121 111.260486045985 109.988237089025 112.790552379447
117.893918180502 113.78323485587 109.564709878576 111.962852412774
106.923686318112 119.164791441052 113.40684693417 125.243735617266
116.597352307016
FLJ45139 115.400592594965 126.00744446299 114.696935316053
108.04922729993 115.224011128183 122.97487285065 125.334995649504
116.321559000405 109.760525674874 116.258629237662 121.668166378914
108.557097186828
FLJ45202 139.771342253058 153.206468539619 172.774914399844
139.444577046509 162.732574014686 145.606098951179 143.191095552619
162.472783116487 142.656762407774 141.21138348075 166.315369318355
152.60462513339
FLJ45244 206.113290588613 233.786136606682 256.394563537658
267.901632267999 181.622689471026 160.419358656559 216.991823988763
199.837938317725 166.637846390031 157.541796722309 183.988537525989
174.698724064671
FLJ45248 151.766607453172 150.073816271065 129.436712663487
146.101547300741 140.533704857854 135.05209575317 138.090158433822
144.046646189442 140.199334790854 146.481298877242 147.416991272497
133.592691897675
FLJ45256 189.603621918298 177.11682975342 174.107661206415
191.3765085801 144.043818335414 158.831813796522 154.499313851756
164.103306492559 157.600193849783 156.365413649149 158.863516051439
166.162656500954
FLJ45300 148.600270795407 131.179786925029 128.398711525305
140.195820033314 141.174971007547 129.073750290924 123.629279569085
131.750053934548 124.370381674692 161.035985824654 136.541973790157
137.765042156434
FLJ45337 198.568180234676 176.456539071735 197.02284475852
238.400565506288 171.768618884215 176.631604815669 209.090788790449
234.370850809531 200.652195873026 414.267332328081 182.633773540249
209.180298459605
FLJ45340 146.351622020606 144.991442620483 147.16778072296
142.745838888446 129.140978620864 141.879359965317 139.746733428525
135.05209575317 141.180647782041 133.365793958118 161.338215647108
164.39733878009
FLJ45422 142.090009728666 150.376234759097 162.411156818437
156.508428796592 147.784078315091 145.904866987074 153.787447416111
155.530415929059 138.202872275661 150.924948809354 147.691011072898
148.585224298375
FLJ45445 111.596690019694 116.95195122318 118.091125088778
112.624137732184 117.990622156287 120.918190840083 120.160652344221
119.717408713154 111.467722538327 125.04156726504 121.402905161896
117.149781437983
FLJ45455 124.291944151031 131.46193427125 120.227386958188
137.217104547797 145.971656491073 137.149156133537 131.015519445322
133.141683141696 123.625734357908 134.978785580693 140.464476678448
126.640141760199
FLJ45513 155.413300826229 130.116771999391 126.635105491524
143.177915754998 141.555366493541 138.35538364303 140.010161839229

228.820894086914 140.527521345097 136.355319997318 155.035727630756
145.226278521493
FLJ45537 141.302647488314 130.42746492878 132.642931022024
131.12775424877 135.943252855457 124.452631766045 135.521802085177
132.440818532628 137.710725379804 125.398875527365 154.615097099186
133.003743285746
FLJ45684 163.004844049672 148.130993521745 139.374278310041
155.933739693912 164.991522994763 128.523703756493 162.422173095045
163.647306551474 142.848438163322 135.267015629083 113.810645407837
153.423579724911
FLJ45721 128.259909378964 141.422441104854 136.734131465433
139.715836473047 122.312652834289 149.831566330214 183.416437587938
128.986795394842 131.528729642144 123.02879683948 126.433146708506
126.51808376464
FLJ45831 118.95971064289 114.405811161631 116.476747183738
118.104763610751 122.662260070778 128.738746025289 124.539006083936
116.95195122318 117.803508098322 110.419222236698 120.744994574029
121.468398802591
FLJ45832 130.217791888656 131.292195405993 140.750004067571
136.313623567821 134.292099546804 143.521510363933 126.205931638442
119.693318959661 132.294365151614 132.548259643258 141.693224578298
132.897554980037
FLJ45872 143.326739481448 129.483116877682 135.618562468776
135.880955433228 128.583503171594 137.435736170779 154.493246900289
144.992993261203 135.59027155522 136.015561289357 131.498890334548
140.09417711803
FLJ45964 119.795945889846 125.525241361472 124.310436914027
117.962188157317 120.918190840083 116.858439910319 118.841699953612
116.161473967382 119.355650892262 114.539671934155 147.171765518924
119.636334259263
FLJ45966 156.191659799142 151.78607484924 146.181764812924
152.738468369498 144.101376910581 156.179501567984 161.530878860883
156.806407903626 142.836229345273 154.316929706697 156.723502568908
142.566028652989
FLJ45974 149.239390385906 143.284230032303 147.868721324602
154.076216899447 135.16316100494 152.368485416992 141.469818222503
151.971337856271 154.938900278679 157.612067634423 134.033751039071
158.404154056472
FLJ45983 137.381470768186 136.826606228323 124.922004609558
133.022848457361 177.716742595321 134.483162806498 135.28448343086
128.765369834041 129.561012565141 140.574129695953 181.308852106289
137.689158155552
FLJ45994 144.510062568989 148.85250180736 127.486697506397
125.146558509674 143.993611400272 143.685368799972 129.194132399305
143.917152377544 135.05209575317 136.106596229215 138.158400190671
125.236926572282
FLJ46010 151.528377719344 137.27506604142 135.643341446691
150.213657393257 142.633121727277 145.259981479761 155.865540894526
131.552276774901 139.397282021664 154.051705030348 124.190161132628
131.261903118177
FLJ46020 156.030794481734 154.177340021162 130.211247943634
140.78902818186 133.780423932326 125.73560975196 149.91705328815
160.192540519103 147.526701500887 147.537691509156 150.962828147611
150.931114848235

FLJ46026 124.731056918418 134.191460941478 132.425596223397
138.506569544482 126.3539479619 122.27785621724 135.81863662958
139.856251508283 141.363863974586 134.222453538421 113.734280863323
119.412461116903
FLJ46109 125.121517583946 135.346501914337 137.73313146889
161.559920910487 134.527165454543 143.730966588794 136.08297876405
128.245734702114 134.489421865495 129.408282688425 125.929743286163
135.446072698039
FLJ46154 126.585679159459 132.02259465879 157.990467804353
144.564250008106 153.085975710144 139.494962965774 139.664055668127
126.315423596685 137.198654302648 127.302714624905 145.255676667046
143.035245560484
FLJ46156 132.283682066898 138.434347257063 142.697827839203
158.536178747273 164.922193471904 141.233642252649 148.754725272846
137.549175595441 149.609501725707 141.809577413906 145.437544062449
142.527422462824
FLJ46210 116.989409891761 121.072192130078 125.091038614268
132.976977898242 126.832182961671 131.633827974141 132.767474034305
124.215466523555 136.520811697544 127.709638375626 129.063617408979
121.834248129933
FLJ46230 157.337193457189 147.284885210407 165.48594766783
179.297628357211 131.092140169861 140.964208731587 145.734680574583
144.206259414981 144.377501600001 148.466658640201 133.419239158866
153.031479923324
FLJ46257 140.456277147131 121.572462089942 151.65364467801
135.317796804151 127.995790782392 148.358847576281 147.51773051441
132.631819830347 141.565599104245 138.825961991209 138.471894596606
129.904942554979
FLJ46284 158.882535865069 140.307931763353 128.992837936606
132.596941171401 154.804753043168 150.364511432468 130.443360119099
159.603586227897 120.688909943167 135.559535347364 145.505223177269
138.336667434109
FLJ46300 141.457040042276 146.223880979601 138.161690144518
157.838771553729 164.02617650236 128.322884322951 140.82351325907
151.181422205549 126.308622415913 152.748807709142 157.745805838008
147.145932651151
FLJ46309 8581.41617729997 9293.35991367953 8982.75408120297
9139.73212514992 1472.54829263217 9378.87287299048 4022.23000497332
6960.46610781696 6077.64695330731 4175.70090899755 6960.46610781696
5314.0635307876
FLJ46321 122.937281123348 119.651470884046 127.088916295784
118.115015846489 125.967294930279 124.113451627593 114.143874572202
123.974409226349 122.013040475143 128.23432652395 133.829889099442
122.736882859231
FLJ46347 105.925737068775 115.264104392064 119.567911704298
116.360144963753 107.103635692135 117.766156390814 106.066897223031
111.381427322188 111.66210002202 118.269780903508 127.167918433035
114.818898581401
FLJ46358 141.36525744062 149.869314204354 144.090047165489
134.251843621261 147.688664545959 146.128066033346 161.299920355406
147.057116881846 129.704763615045 152.169087619827 137.971108694323
157.394035451151
FLJ46361 152.337631025368 142.434356144925 140.793616429174
146.880440100898 141.259186690966 149.294728629784 155.417694869593

140.652086055776 142.331050242704 141.931274901572 138.558300898079 149.419308407564
FLJ46363 131.528729642144 150.075738752092 155.737364431382 138.751781355083 164.126980102525 150.460618402778 134.132312782731 147.2233876988 135.719702250709 132.310147451479 138.76667538162 135.515960991334
FLJ46380 135.807494668545 134.7552564959 138.434347257063 131.540436531741 146.235832275574 152.432477504097 145.815454490804 169.466129742085 144.079935036119 149.995272585012 171.85890372225 181.022635001204
FLJ46446 120.246240734668 142.558109812638 133.056659202188 210.043556816529 113.680875214845 113.005774989833 127.74861041569 123.657246099004 126.058167594519 118.360186663758 120.283706459738 122.706176540192
FLJ46552 171.006582229921 150.724359913994 144.277263594387 150.409919207059 152.350537364705 155.134796468704 162.812665683603 168.3523504179 144.114177076352 186.103491222924 221.272872023413 179.376968970326
FLJ46836 138.962649407431 131.159946917777 147.416991272497 127.675206134992 128.501189288377 133.704706615071 134.498439965049 141.799236109474 136.044670179122 128.850480964163 140.343790129071 136.434045183143
FLJ46838 135.139676303901 120.628904050564 139.097764484641 123.403099206878 129.750835425023 134.158454639998 121.039678049579 114.28381541078 117.575009754326 113.687931899298 127.104097294238 118.393203921859
FLJ46906 181.202396068658 172.214433016685 135.916668738753 166.655585160657 211.012142461725 162.560758971445 170.046856179083 193.609985124825 197.262062021667 142.234554325754 167.087850057037 166.061891147234
FLJ77644 125.498081339586 122.438438464097 131.933026690056 116.053859681399 133.830860948014 125.250900940748 126.146213417225 134.034970203523 131.140263788783 113.09980008288 111.691973758042 130.467450968785
FLJ90036 132.998223400178 141.971207380159 147.970243979273 133.869867161978 137.812249316763 149.361498483705 146.112388101621 147.679233228201 159.633987751501 147.053391763304 130.609596267168 145.154323430995
FLJ90086 199.46853058821 184.260835471542 183.416437587938 183.735201125198 282.511614929917 225.583966894864 209.051768656607 220.136131941508 237.396466740419 245.915473236403 269.62298406345 233.59297160203
FLJ90231 142.924854393401 139.817493158675 152.520828479026 147.550385608773 139.62718946833 146.772611341214 145.150191397375 158.882535865069 137.586032396204 153.5256657638 134.885883559391 146.792951584868
FLJ90650 178.790758444269 173.044636575826 165.139423820186 168.310680227156 169.391557085902 143.655901618594 180.468984099647 163.119211741255 144.352987324713 193.689806171113 150.653030597808 152.097609803404
FLJ90680 148.868265867275 159.384480972135 149.096182775507 138.525131957094 132.593747375239 133.360017767635 141.632147679743 142.709768829207 139.828781354865 145.372579207362 128.645334554749 157.021010130169

FLJ90757 188.847480174391 194.584034984339 193.609985124825
188.348499229585 164.000772606739 158.56996905631 186.904126552483
179.411426984263 174.510523238374 174.338440780636 156.78124246844
242.971958493453
FLNA 3024.62888503536 3789.1316515671 2874.43919426772 5165.90865799172
185.507663455634 156.420501464224 215.802713097166 160.887121295848
222.086635504858 438.231717096337 188.557113298055 170.298291784762
FLNB 2473.22803972431 2132.90676194993 2433.83421295012 2703.76111776248
1920.16520804856 1444.45946291517 1301.61742965939 999.159652508318
1470.23096656138 1787.32566525551 3024.62888503536 1650.77281891596
FLNC 19189.4835303597 15704.7314387339 16230.8346205808 22543.625434682
140.413503801467 252.761035334279 132.024244263352 186.156006020985
239.649306804266 190.002630382394 135.90207451581 189.915720614467
FLOT1 790.561585641193 917.646958427824 734.71562355687 1145.96710556468
842.87865208079 417.528353577952 640.029195821436 358.355621069787
501.738490253982 675.083275037052 426.132037075477 513.20896845398
FLOT2 2666.06582231934 3869.12225821096 3143.77161008321 3843.07094386605
13360.9483836861 3430.76532174701 3168.27825058989 7003.06505884735
4749.69040198959 1491.05501052706 2758.01526530661 3727.70199176703
FLRT1 146.541890150631 145.365935017728 142.42442972908 142.843006238215
139.06923394504 164.288645768576 139.2454524913 153.597557478169
138.000714797563 161.573975848093 137.495288670441 143.197131668608
FLRT2 292.317998405609 309.798246248863 309.0578543424 403.812520074723
128.554574566655 153.159425380743 156.540165645256 147.113541493157
169.87591929982 151.019907064794 192.188662949149 156.589829166493
FLRT3 247.08460296705 266.162244070031 296.333944172375 263.295012964476
560.757496975779 711.166793546075 515.158921349383 246.428746807313
477.275332763887 247.683819429859 749.515603904233 276.351810999317
FLT1 113.334665910428 101.431474941041 109.932577444858 108.118551275778
114.691842968597 124.526220300907 116.52455193626 110.437728036347
121.157614971438 111.232274546928 143.440420261133 107.303126530947
FLT3 114.342980656214 120.118873350459 114.048136176142 110.952108124186
118.619176257622 136.222421297872 133.849237689264 126.00304757051
118.731960910224 119.759350702482 141.209958892647 123.413596815384
FLT3LG 222.508914765788 199.170488640762 148.742216703684
187.264411519101 171.265160295274 115.468921844902 140.45860949168
126.88066987372 165.092451754065 128.755697083014 151.826103919919
133.280418227922
FLT4 116.987471433258 118.360186663758 121.939618902165 128.282999719959
121.619593453259 121.146511496024 123.959050197189 119.603565559838
127.512733134453 119.172965618216 119.057157701097 125.393848851951
FLVCR1 160.234704406302 157.535792192753 157.649902282769
134.149446827982 175.744275464701 186.496160803789 148.034899103241
157.838771553729 152.552277008504 148.648159241815 144.064884297748
148.185056724262
FLVCR2 231.890885094917 230.332652534446 250.602978521808
271.989060206794 298.165097953983 229.26243866226 268.148673257455
289.859695541999 297.176755638159 359.935451429371 267.67093646012
282.511614929917
FLYWCH1 160.975741751317 170.966588957686 166.835922279372
201.004491067972 174.441287575034 144.738871562895 133.008987820479
143.877226672663 130.588481497083 150.399313562031 155.690773461824
137.522792377933

FLYWCH2 200.08007665693 205.908052642222 194.724219884752
248.791254839869 421.056268094661 207.254220247481 239.941641600769
265.148061007894 216.805365763069 236.985483155452 189.01309885917
188.070213212057
FMN1 131.732416355002 129.436712663487 128.777337552038 136.685430908977
128.878051599968 122.557981210434 126.862274244977 131.093917654245
130.541449457342 128.666652639862 120.71782441569 131.774882989257
FMN2 139.048016900762 119.870661213608 147.056182807232 136.823008283157
135.348086420636 155.648782851696 122.418378964982 128.473834314632
142.750432197016 141.329559699056 126.791714564926 142.878258542775
FMNL1 140.041977349982 127.05677680446 143.739231124536 137.44909445642
136.534812980964 133.75142055959 130.336466131215 148.046961712162
138.970853057174 153.4392054301 131.328478053136 129.85040048805
FMNL2 250.481442411876 193.689806171113 242.902900881585 192.640717683236
154.288075172115 163.02140155858 143.402684153777 145.000523037022
164.438724387992 328.730722366546 171.161297672182 132.352268262631
FMNL3 142.025834438974 128.280225530201 129.867246424399 133.195030751476
135.781190598068 136.83470470035 132.310147451479 139.737748157901
140.067809125008 135.65700435306 139.388484543482 133.62358240645
FMO1 176.761123480566 163.390929975591 183.853381322135 175.989742412417
151.243948028303 159.752644678596 156.043994260248 161.441613587571
171.414869677333 374.9555524303 140.462651395483 171.896929562022
FMO2 208.270622752385 232.892635502424 267.839640412797 296.603054615145
156.085949215738 180.478294414245 157.08300736206 171.238423077347
165.613274361543 165.808660192901 172.091884817207 165.992542962911
FMO3 153.047317528007 150.354401669138 136.470025638057 130.539632182729
119.9652990395 134.17351030102 128.030938693369 145.440471538068
151.266352916239 145.03221570575 136.313623567821 145.172776053
FMO4 143.333211390741 154.594339065082 132.70109232982 145.442914818456
148.635174393031 128.054394154801 144.413210364148 147.891442774921
163.709174813901 144.119035360928 165.103398617203 157.192320902906
FMO5 145.542888937635 160.752581271996 142.354731066206 143.438417529799
179.067275037015 182.929326147069 186.377571176623 173.044636575826
199.991077347491 146.604519441812 162.127697884504 153.528429414138
FMO6P 131.866599432062 146.084461430546 135.499195435803 137.58025011566
135.116985816353 140.922678400415 134.335309757663 136.682172321984
133.52271411034 131.633827974141 155.993849743656 129.420716653358
FMO9P 158.670835881902 286.818166898922 226.712995229189 192.59376129133
1032.4540300024 1033.48252432603 836.613819700658 4711.21389812942
1812.07394646463 189.840366340622 623.508710830895 344.247544510881
FMOD 643.13571432061 449.859227376918 296.428342247529 394.73732350607
173.463983140514 173.109461200603 148.23392074197 174.214747421898
229.046153362151 258.868119227979 165.938009275308 163.922353493317
FMR1 118.355532305846 129.233595525585 110.138220982557 121.429270259367
114.696935316053 108.036254606246 127.213640193364 121.067907968175
129.372522073479 109.680532131035 138.058404474114 121.121686280797
FMR1NB 119.525701528869 117.467618148107 120.93965087814
115.840266274696 124.791221329502 123.510413258172 118.9952230535
116.796995589816 116.053859681399 117.47631318854 116.621735695217
115.16087297104
FN1 153.748035612441 137.345542507421 165.789846154721 141.408545519654
130.973504803813 131.252368756892 132.629453540571 133.257477196196
130.314247368361 147.135640862542 129.664016819143 119.666650919579

FN3K 134.825699448987 133.504078347324 134.71514094826 127.706499722854
123.9209907098 120.53428198263 123.129785564258 127.962546712222
128.662680062294 133.711821318079 161.417207801991 133.439077726411
FN3KRP 774.442392607873 744.225336494008 1222.59895569348
764.493610798958 684.180060287047 1080.02576143033 1248.69137283095
1296.10827265334 1214.77298621465 1896.69856822105 663.214972254236
1249.89496565284
FNBP1 6138.10904830456 6919.4226838505 6253.84268792816 8982.75408120297
539.853394831917 268.568824794145 420.160939829121 644.301665020526
907.610179738644 691.397238677552 550.124359517322 303.638516587721
FNBP1L 249.554443204445 284.13865992802 213.484986945295
234.301520483864 421.384700184923 850.882404546093 667.421612441201
815.779445689417 608.611927946607 388.966303073519 632.565736446505
529.368609163949
FNBP4 1344.1288416533 941.936830901588 1373.67062432073 900.039744829725
409.691865300362 1117.77397620221 1480.52638217173 2255.92551080483
1093.21579738952 894.734451279225 1447.07989164988 955.604404066769
FNDC1 140.57567768682 137.354782768068 115.180659017144 121.480455781173
121.434713764246 116.736174147084 119.511962218143 117.144778357339
132.369872013432 164.665907233149 113.87024833117 113.845770994796
FNDC3A 276.184421402511 277.314675225943 309.658122422202
244.011434605133 186.309367281625 294.115163083721 147.07272598888
228.542423012958 245.723316636363 227.668046559074 306.43742010208
390.979822976551
FNDC3B 861.119262963015 773.330948077036 675.281131488524
574.779350011402 613.631311147328 787.874585290601 1324.56751399862
786.160002434066 868.066644155313 692.095005197252 882.037055216609
1085.38819961412
FNDC4 204.584643964409 217.353396632732 201.105520537151 204.990512488181
192.932285494022 198.257497903131 213.531307836195 181.85552254233
186.345823604119 193.008902902654 153.336395746742 176.198555355383
FNDC5 411.451612988791 316.801725268792 357.514919818489 554.950092776254
299.935166870211 158.92538634742 158.049286838471 171.528350399075
141.189008902155 139.089444892187 144.046646189442 151.989476683722
FNDC7 144.342905860503 151.613134875009 139.792313018728 149.995272585012
129.143168240589 146.434716985396 149.173121214851 123.603972078897
150.163801964104 150.601874392926 146.087596132093 135.670409501435
FNDC8 118.201092260839 113.66416989856 116.20008794813 115.890431424899
116.597352307016 117.511162285879 115.559551514777 117.837894438834
114.421383025971 115.587874563785 131.038758046782 108.101686347192
FNIP1 240.878804911613 249.823314327355 318.930960243303 237.216435972201
167.521700144143 208.507486041165 269.853718252574 193.084818760857
175.908645445479 363.777212750779 177.091809538824 245.723316636363
FNIP2 242.269784551092 220.330477277988 249.823314327355 207.960802948119
141.850952191423 141.758827837244 178.684992551017 179.810507975486
170.54511631708 210.751430486845 177.812335593522 179.358460170348
FNTA 460.408628542721 422.12964420709 616.014975366684 401.102586700919
660.096727566678 1245.47421607111 717.686676823785 647.439372562271
401.584032365337 901.644194512187 698.590134238302 658.47864958685
FNTB 347.73629731446 315.963526790659 392.824840252824 374.872358131797
311.269750643358 257.408058436941 363.777212750779 309.658122422202
328.60529053804 357.773949920377 309.423768054614 274.235001739215

FOLH1 127.358616953147 127.856634523494 117.045020667394 115.944280729914
117.112667504781 119.562180492359 121.360822445187 118.457573670556
119.060186838239 127.144291519133 138.733717670748 119.95959059725
FOLH1B 134.664652044646 137.252742673245 146.050714008913
146.787927492706 162.244691888031 150.079372712689 140.796641921516
145.928643520352 151.727603285205 153.440952021924 133.882126180703
160.113042196058
FOLR1 131.715579653796 140.105675520432 134.963301368391 136.60514429355
137.581659385375 130.539632182729 137.017882995262 128.377928524315
133.692996980809 134.410706628655 132.283682066898 129.033433971346
FOLR2 197.687858915757 169.408892048115 136.194082458655 174.491551019128
204.942500423413 160.127254444487 144.675182563174 166.396160626179
222.624150073858 158.758500675009 199.579818754014 166.9499656083
FOLR3 130.105800581241 127.500754400224 121.71538073735 117.212164526913
132.780055030907 129.077660633761 115.343251487944 122.351186755611
115.594489123676 242.848821222611 152.272198017355 129.806104966617
FOLR4 136.93893152919 138.29167712246 142.403121638265 136.603035996281
139.138329217724 137.093170378295 136.507961715591 143.188958538221
137.391141254051 145.832751296646 134.436423298225 139.679681813932
FOS 1710.60409578293 3954.24763752814 3976.40140405628 1622.17491781572
674.742955681908 6188.04738762584 780.105135529728 2932.64120183406
437.248311551717 6830.46932926077 1762.09874961415 2244.6916226146
FOSB 1125.95664591686 2004.19532096239 976.050097371579 559.0867397858
282.244214637625 6253.84268792816 142.42442972908 1295.27257578314
231.981501596284 2242.73511149559 179.94333102877 433.594928641214
FOSL1 166.533191390639 153.758353103323 181.308852106289 167.941671074236
222.508914765788 217.491746961471 160.382971629272 169.158049136321
164.962201718762 369.070376276471 163.125317704446 190.45739385453
FOSL2 233.842749683689 213.097664319741 204.942500423413 215.037122365095
400.23671843715 306.111172289632 264.526189799049 257.292125083248
221.070448906398 355.372836460906 274.316982298975 199.96834355685
FOXA1 144.051468970593 374.9555524303 188.348499229585 124.368438691944
3737.57754066891 2530.00219297102 2416.49806112207 2639.60135568
2532.82545752082 179.321622114583 2515.7035557541 3000.46298223166
FOXA2 124.49814598066 123.841366760069 120.106270603148 118.034262386827
119.06600048679 130.563756903074 124.625603721372 121.124574985464
119.873844587252 119.830261862805 119.192893408873 127.373421694142
FOXA3 120.177385114315 131.87404363664 117.097159106043 120.14201542563
145.534520042669 108.483284007316 116.831725094917 112.252555403121
122.329290563769 118.761737418652 119.968081249475 120.397683153445
FOXB1 122.578170988039 131.573729430926 132.028327486943 119.83679981791
126.938068475032 127.997280639609 127.239282651919 128.155080148958
123.330142002791 134.56314960832 120.511896155396 130.06441029462
FOXB2 118.727279917776 125.892022613725 126.473768158566 119.32696696175
125.932540615682 114.818898581401 117.992729826121 122.97229333908
118.544870925791 115.713909155043 123.752109790056 118.601228886344
FOXC1 430.728137105601 934.546444634669 325.387193207414 467.04976953755
1095.52349352502 951.371815352591 2203.82462320864 2240.33212242003
717.886050056576 1177.01310527577 1336.75600604068 2390.41825815872
FOXC2 193.04138438685 181.238505183667 203.389294254141 217.513118676535
139.313456284725 134.243435358729 127.913463809158 143.519786615253
139.948097704949 131.241878941749 142.993401965852 126.290840923683

FOXD1 135.504526903482 144.892454033336 110.368392735494 140.911080286243
150.031863363697 153.457715729768 133.610143526954 146.672270660899
140.664303738113 308.974786799114 132.634538733205 134.17351030102
FOXD2 166.741762854246 170.807514224412 148.130993521745 155.40832015652
156.456332386608 165.896420029269 220.9384439772 183.879114762264
155.237180851323 158.886786960196 183.83720937589 192.61436632814
FOXD3 122.472909598385 129.532122459128 118.712730897987 130.031303483631
116.540729581355 113.018985316061 120.873985900666 113.427848757112
114.265920150451 124.96926662367 111.1002996982 111.577400298092
FOXD4 141.329559699056 139.674001912099 136.135223482208 129.758303592589
146.198331189991 171.426334687626 145.335830265052 197.112749959833
211.941623411369 137.084488743415 185.332215198617 155.061749542085
FOXD4L1 153.176268095608 152.628220216104 152.461930328923
146.418147447541 164.066839397676 202.849544035436 161.412427590994
215.98963200839 243.246632667118 143.512348693556 177.666582096539
158.963726206466
FOXD4L2 140.923227606022 146.016920431468 149.907472831817
140.441521267218 139.720343780228 147.027904536486 146.824420102008
152.573063322741 146.885404321622 142.655287071175 134.642898234003
144.335732650795
FOXD4L3 118.100611964852 123.929180327423 103.398929051742
109.282814458761 108.167670899938 119.567911704298 111.716607815635
126.0944894315 121.294366964074 112.675715371087 148.431101051574
113.734280863323
FOXD4L4 166.714793209109 180.941090529551 214.904634494921
194.029961195972 148.190696520521 205.765822478178 183.583432296209
177.752497164503 164.701183249762 164.241874094897 171.714973501563
158.928949614338
FOXD4L5 127.6516565653 123.81177057082 154.617462666847
127.727311123825 130.521731606987 134.262346867514 141.478253058549
137.000868503792 139.205887721493 135.673080643346 147.420580715112
131.556892859203
FOXD4L6 131.892151111901 138.127899706873 131.558842131826
121.874296836854 131.2360427855 125.899200544217 150.070213543579
126.732121894377 136.425263391752 125.631073476381 122.405469833024
130.705091244939
FOXE1 118.81294782091 125.486324819682 122.109009629395 121.926377140115
129.455347273068 129.47419698487 111.467722538327 135.651829752462
135.952202055637 133.473140653666 129.083756379376 120.241528853563
FOXE3 121.515872228716 124.847325289068 120.300291760091 123.260800590266
120.831070822199 125.367386907897 125.247402280272 139.488057371254
127.818137805569 121.377666938052 131.709341771599 126.784243009941
FOXF1 1265.88446775977 1389.93393245455 889.054356462993 1191.26028342929
141.941814923705 177.696849418064 124.738217731488 155.603263242813
167.536828038178 141.305605155722 172.667770359278 150.399313562031
FOXF2 288.596513753617 261.592512762969 167.447964370395 131.023892409073
128.651081092914 165.131463797217 207.269530621176 160.898061905021
131.850952248545 143.766249538352 160.861929142249 235.453139389486
FOXG1 168.323141675824 133.692025039613 145.924751649542 131.298129667973
142.900756181761 171.916201289104 134.648780131578 134.096283448464
119.370219299491 148.763098715961 143.825062714273 145.610045243277
FOXH1 126.614876404146 142.301581642006 134.237133546276 144.219104742099
142.37457375182 126.81140158672 145.724294862079 138.206065640158
140.145470073979 142.724123724574 113.504670764963 130.370405378213

FOXI1	129.242093568433	129.213523302268	145.463105638655	135.852591930882
	133.864612785572	130.430917518283	128.541715437188	136.611670766233
	126.988118823204	131.500591219916	143.53550972177	132.881693683384
FOXI2	143.204058393718	147.863198012238	129.96280577492	148.551340840517
	151.169283505727	141.561769570516	133.775716376056	135.899595115146
	151.132586978278	136.948039394935	134.160100276452	160.880152014981
FOXI3	161.115842628581	155.46017572591	170.646233722767	148.356726939122
	164.529828080312	150.557982810339	156.720090761933	163.542848861552
	145.233055765001	161.046418048832	172.583615020042	170.306241656131
FOXJ1	118.833188167696	132.197941566244	126.780096523484	125.176747824384
	498.641091757363	315.3930052781	115.16087297104	145.136075215082
	132.883408554592	119.931415360373	139.036978052242	127.713286292101
FOXJ2	1581.06548216423	1812.07394646463	1569.13457493554	2244.6916226146
	566.914448800423	667.421612441201	719.766799746895	952.357976050712
	598.095370768587	728.485195792194	549.460612060753	630.894382644903
FOXJ3	1091.12493937368	1070.4597488302	805.875557314697	1080.42884514937
	743.510369438587	732.630306104841	896.185223980593	778.846928848361
	775.382397816601	690.582550537485	767.054576874115	750.10819423982
FOXK1	284.321253087512	358.298768623212	358.298768623212	473.73182675469
	418.889972660958	199.135932984641	281.037762232619	271.196028302639
	249.263194321516	245.607130404306	336.490076158135	332.978350226738
FOXK2	143.265532486512	133.41548225025	135.16316100494	139.536677895126
	138.246963115014	152.725025539491	157.446725405803	157.457468761338
	142.579346058088	149.595375008252	141.275667354541	156.62031503525
FOXL1	147.882530392424	139.273651128094	139.034940762344	137.339511714364
	129.247701136266	144.895601314903	141.091421265404	141.649911527714
	128.245734702114	143.799113486767	144.876300850115	152.274957846518
FOXL2	134.700543462375	136.03117924052	149.44520146627	146.162330544539
	139.923187410488	142.771243742561	139.057572927874	140.397184813186
	150.098708715617	142.12808103815	133.594446233378	141.907307422968
FOXM1	164.959415532174	152.360670942951	149.350630572336	171.689722533849
	399.071041584646	224.497348027469	190.645917992472	240.465165615601
	243.65518489122	186.563913311757	167.55209828062	202.357989894268
FOXN1	121.497645719165	141.216869032251	133.672964925321	137.546487338984
	117.186546702764	128.947211021681	122.053867525393	137.936605651724
	128.478724185042	134.10707191601	117.716094828779	131.305785417254
FOXN2	638.129080224049	631.408955630318	658.202765061937	560.602047253306
	487.993796425035	606.851072672402	437.248311551717	544.462032960964
	349.540558521616	587.893636997642	378.862741410359	372.088129459528
FOXN3	140.416247564103	134.222453538421	139.650726173186	133.839554874862
	130.083266044947	142.334338599752	143.195338329759	130.646956516128
	142.376921410427	138.250288796456	134.102929804129	136.861019517134
FOXN4	123.877609932461	134.268075583733	124.121555438466	127.139903623331
	126.737230874367	130.520275539672	131.447941902406	160.778296470857
	121.239461514704	121.968807253639	125.828998007608	117.696262292883
FOXO1	674.101599637653	646.461274173693	669.602939159192	764.137504481329
	289.517820913799	422.973606694449	332.082749517913	419.690542859168
	329.023836559229	560.602047253306	529.963453165714	571.273512452931
FOXO3	1040.73829397913	1096.10636044885	867.601542930533	795.720196538039
	428.66614441422	687.789976218334	730.988069115919	600.264940641426
	428.237210115667	1003.93455977232	532.950400370668	358.975346234854
FOXO4	1109.79571066685	1179.42590997099	1487.12201643008	1382.12750708801
	506.165100641525	532.786041960586	518.478990211753	374.718289636553
	784.7216251433	291.616195651001	429.992169596618	279.325065941252

FOXO6 146.748572532267 143.149424892482 159.016247993816 153.19516365535
129.442111153737 146.912751127571 140.120051507626 141.038721816572
130.249430984172 142.169951083733 134.477081392474 126.080780033301
FOXP1 318.690043546203 292.693830444839 248.594000664466 329.023836559229
197.815264620906 195.414464905232 199.077893147154 166.795671029174
184.923312649584 155.70645844794 215.194909819764 184.462286724102
FOXP2 177.639351778834 154.300058278533 192.095235288818 149.788558681761
153.206468539619 135.274205069488 149.552704503066 139.955389033983
143.452992974333 138.456465793474 133.427561208907 139.234549949916
FOXP3 119.608334527504 124.83124879272 133.143695166498 123.07559417607
139.31967261371 142.700311964178 137.199718376452 134.4380641709
129.502730573463 141.213588194832 137.340844554597 115.01682934323
FOXP4 132.626349611404 131.604121223902 136.394920983439 133.037138438863
136.973804637915 146.34029698108 144.861804210272 136.349603320553
139.60494712939 135.531326977319 147.799806976046 145.210787864362
FOXQ1 538.484170364629 1346.53215685106 965.673699120049 194.979795724712
870.409935772423 3664.38955037715 4929.26915002209 4206.37667440908
2886.92690692502 871.913035811066 4030.42725798136 6470.73189429877
FOXR1 144.64757514198 144.972694558182 142.421913396368 147.1476865809
138.934516083124 142.581441480371 149.147411595596 152.407614042236
146.624631389743 134.296645422941 137.220718873614 139.491769969541
FOXR2 119.062611098235 114.520066232155 115.855948612544 122.27785621724
105.348008821162 111.484426481816 120.565485864499 105.952640312359
115.582048644075 110.774802298005 123.260800590266 125.500790447338
FOXRED1 337.728737393381 413.398641688413 338.665115108128
553.90008440429 890.810315261602 324.871248300476 274.14931964455
516.088776850623 297.609793563225 252.628516068724 238.978845152474
305.096320805078
FOXRED2 231.47915227815 228.852495818211 226.114369645703
230.855806094267 389.500591134279 298.706655333472 269.981476129439
250.963601187362 228.184535093641 178.035700298977 190.291185322482
183.795632040455
FOXS1 150.924948809354 162.638805580226 146.919110530891 181.340312140534
158.953705937936 127.199124606827 121.330383384978 138.003440266411
131.031639119149 132.596941171401 148.376194044247 137.823152565933
FPGS 128.213678179359 128.576310682958 131.287142490322 128.389494971858
140.981666934622 128.814967091609 125.154784284706 127.961153369579
132.8615886934 130.09050436943 143.814798097425 140.435724944447
FPGT 118.578205738502 132.475414208533 125.887854850942 119.125002207396
130.555405606993 112.729141678141 137.065315360321 125.371128588617
122.863612588759 124.263901299025 114.223991452083 118.276198592775
FPR1 156.859539612504 153.062710222618 165.860075368566 153.776361241587
165.389652763451 212.607094117882 166.925695810781 240.005943851098
231.955750250711 254.424207773566 182.32514927211 149.964827055916
FPR2 138.240874738649 131.436176224725 131.40880014672 124.22833964472
134.130913969327 130.592061896237 127.003974141317 127.333963230749
139.036978052242 134.803612821519 131.168294031745 122.232732968363
FPR3 166.014450044695 160.695913747601 138.150616787218 152.158269344693
159.2488784104 140.946729014405 145.10302792449 176.382377784553
223.573080955095 184.286192995915 179.54034642468 148.242794464181
FRAG1 488.884511065882 545.362650746876 549.460612060753 579.049608568926
774.774214696342 540.946852709312 549.047065540118 847.276523445973
850.562379837947 998.631321857182 1217.29687282094 584.919615953457

FRAP1 565.509446660206 491.112594297928 536.805979562807 536.805979562807
405.570281788901 578.297031096292 459.652708187549 590.515758538373
570.26564132238 590.142817130684 400.676876982655 509.880640462
FRAS1 141.5068665919 157.792868983503 150.084909674721 152.173055053184
197.947433984294 201.800357785022 207.815226999953 204.637887777803
177.073245863872 167.769114519774 289.21739941764 186.611870261871
FRAT1 139.130560988108 137.914085754617 139.408234219133 141.177522313723
182.042104888745 139.423432468886 145.78573175226 145.049387327924
158.080749926756 142.688809469455 170.251197850968 147.192815700248
FRAT2 256.538195034956 345.832589037442 367.732191251547 312.903508201275
1165.05055932899 599.926717145115 651.43207839949 670.571106793204
796.699879495384 329.333236839082 1007.41624313399 518.281245498542
FREM1 228.414049417352 261.469732730752 209.417052464583 483.845891531326
158.208998509937 140.553613300431 188.242091666446 157.565633456909
165.370800097602 163.438043894189 163.346402397924 156.907788377794
FREM2 145.476377346532 144.190203286506 131.917194849285 138.242983316259
174.615694779386 194.529045934216 142.474762834848 169.881866987359
202.300293733922 134.381042390464 184.462286724102 144.258128917228
FREM3 135.784481523923 123.571641517019 122.837807905774 131.267177634269
128.580625868379 115.664551127497 121.803676441059 118.015175851934
120.577995272053 119.221976571036 110.113844434493 118.900338956203
FREQ 2350.01764791897 2596.43650409044 1984.47173096657 4503.01240138314
468.712260193746 260.52593414721 271.287767448621 246.389203940944
309.75140197669 300.793542873709 379.983165400941 263.47760086159
FRG1 921.546033242474 1028.80076860235 822.129234065933 1185.70008959602
374.46180489079 736.603605876572 587.181357555703 654.305724263754
797.73902873919 953.099915501575 1022.11537745428 633.762608454521
FRG1B 147.288282361079 142.524413202292 133.252140649104 126.452500728601
144.822943770596 140.146894533044 135.664877050226 142.137885203233
135.753430034183 144.78971417658 127.964855970099 134.633286312653
FRG2 115.896463925125 129.203236898198 123.61956613951 128.368669716591
122.226875707253 128.002822538671 128.551335256123 126.042731009527
124.752198904184 122.953869661977 143.31590446076 124.940836122365
FRG2B 139.484551562098 137.975369568585 133.862203806529 144.316475814221
143.837402372008 146.920496801962 127.893555451807 138.038446102921
136.131453703981 138.836422729663 130.182320154521 143.954908055924
FRG2C 147.994386096925 129.642952083513 150.367927843529 133.399632102473
132.433109154079 132.171282327983 133.067237748129 132.610618885717
132.70888569469 130.960045345375 130.161889021281 129.818020166075
FRK 168.6016897585 164.377227860031 152.657649988502 161.310855691717
190.22021453133 159.902277514765 230.546762514966 202.927700504034
163.500384982242 183.900171917391 217.86728342043 190.534176839652
FRMD1 94.3531468700382 104.798333931019 112.832122823829 105.976284291356
109.700513642061 111.810575716678 111.860763079995 109.760525674874
108.818976813708 105.408689610638 119.530214079037 113.470356909765
FRMD3 245.766885283797 249.263194321516 203.888365494623 206.73292907905
187.920789153124 219.753039699136 159.344044864557 162.634670715627
168.289492873475 180.881464829981 637.695341427976 174.646321871605
FRMD4A 233.536023295039 271.154246147262 189.94130405123
271.238736772408 172.8993416872 282.836518011311 160.402220755071
219.687151891777 198.701991021316 169.31618330657 304.679197422439
199.477350542491
FRMD4B 132.891375982227 130.838350486331 142.577102697682
125.136745288071 123.53212786736 134.810212106122 132.446406243531

134.891736344801 135.554736716321 131.583841535781 129.140978620864
139.375386456759
FRMD5 127.389942368953 139.574895021783 123.809866652834 135.883119645931
131.486917325752 125.815256068004 126.931328660501 124.964977322158
122.749182465923 127.824005374202 128.78647242525 131.973896985615
FRMD6 497.53605530806 418.163219634152 480.36420271348 445.763740779039
230.947339143332 337.652945958921 336.981561928411 282.963958072528
196.144420005021 725.065727257421 446.509863498357 474.917507160629
FRMD7 137.11719450122 123.129785564258 145.749887769807 135.046003135933
126.656077572961 133.509538648848 133.382099379709 131.606765174514
125.5109819304 149.541917326637 125.720102558499 143.397823965608
FRMD8 343.200277800027 410.216584967666 385.135014083686 457.905741500454
1187.62490367696 911.782783698439 1000.48723872058 738.37559871635
706.628675634893 1658.78888936164 1323.05745869565 1626.34607278613
FRMPD1 109.242622591321 110.315549501241 106.450154495663
113.521599852465 112.590379042108 114.00959999268 122.469113037809
112.156010486085 114.01599035448 109.038182799871 123.323528039848
120.793974839773
FRMPD2 125.410876867635 120.549643584553 124.602474178438
126.715300671036 129.222714748528 128.300838927918 128.986795394842
125.202468372015 126.103561089901 128.113966507586 127.781732819297
131.35955406222
FRMPD2L1 121.138461509096 130.882463936853 141.588478913204
136.008118769513 122.362585067453 129.77374228644 130.34939602913
130.471929699923 133.028789808654 127.829001155781 140.882766779119
136.106596229215
FRMPD2L2 119.451099896048 126.277165320165 126.762116571216
121.687187898743 125.172189715764 121.059923601223 122.646850766733
126.921925660996 122.216479103528 130.526673128214 133.667852983262
125.587057468028
FRMPD3 151.356975600962 148.387171320858 138.889999702313
131.261903118177 190.427022049167 164.784346867158 187.057683141644
145.606098951179 151.325068155715 159.466972602203 162.022232949254
138.496366637658
FRMPD4 125.489777118532 126.191057198995 127.562924931536
127.473812381059 132.005753222789 126.279119798177 124.717079082023
123.327238774943 125.531371673679 125.531371673679 153.310330832285
126.061141885666
FRRS1 142.958906063186 156.351734527745 158.495606141226 154.951136632863
170.410704499804 169.271175460047 212.591357254196 189.250846546817
157.751477529076 172.237115475147 150.677852949794 163.880402141404
FRS2 133.841519663164 131.17119492692 131.580226088647 138.000714797563
133.550008967009 147.920946479942 136.152827218905 152.097609803404
137.899291413431 136.529820456592 139.165248204716 133.379872189405
FRS3 291.313681141442 323.04019262387 479.469659712246 410.954095610904
250.676092725829 261.592512762969 298.979106293393 289.517820913799
240.734451149197 293.482512212153 282.335582153109 348.413827101046
FRY 191.467626362851 178.89423690001 232.414763435965 154.993784224548
161.720405760536 181.428419748662 169.332207863684 156.046085830566
159.549936351002 132.047328970556 222.646371492347 188.033890834418
FRYL 222.20526515049 189.142295015168 210.5689532432 206.446584387595
177.16711492983 244.596233138747 251.268669431283 270.568660560796
234.063633889254 233.59297160203 203.206743799353 252.404931238338

FRZB	432.604462607037	605.040026490154	268.111403429364	330.505633088656
	221.253717494488	368.418553384965	157.75304980664	266.652805624826
	560.481802403627	213.382179917193	332.400086578723	218.773153183388
FSBP	158.053654524927	168.547575047205	197.687858915757	166.671563123047
	166.64535048582	161.499923812379	169.920799931309	155.114892891737
	172.8993416872	139.310309748986	155.488346781292	166.611809859059
FSCB	164.89597889259	159.730925830278	195.384273821324	171.602950766191
	151.845172175463	158.841505957036	154.079355899056	140.477307701027
	147.872625576731	157.717145014769	142.970256312313	148.861141988806
FSCN1	1382.95068105649	1632.84261181074	639.184296110069	1561.10217536347
	1309.7660860424	2567.4299860517	3684.05513498098	1871.91894530722
	1783.8031212092	10280.5093368677	3391.3005983725	8787.57716309558
FSCN2	130.824958143557	132.177829138863	116.751145098777	119.567911704298
	127.255966406144	123.048352070915	119.188446525733	113.582654469735
	118.593717189375	123.104790590045	118.26427946535	111.086170834553
FSCN3	136.85323853426	151.532859494021	133.483766053954	152.379441401583
	154.746081871718	145.71535210212	136.657107363796	165.342790388607
	151.289464845626	149.437212090587	139.324006489527	150.123784990646
FSD1	134.449083873514	137.537197575152	138.12651823109	139.949563856936
	196.583548193097	153.731233276485	146.639556437847	146.072585331002
	142.363309192458	142.409003426775	140.796641921516	147.43089976575
FSD1CL	114.849872458647	120.660349211375	113.636570490048	
	119.590540166384	114.749597426757	111.381427322188	106.030441145334
	110.113844434493	117.428152859221	126.00304757051	130.340819826224
	111.596690019694			
FSD1L	129.327645260099	137.546487338984	137.398748156909	131.093917654245
	130.128585442593	133.191797029676	140.856864690669	144.699084515687
	144.116298946718	137.681280789305	142.45411401883	153.308956040034
FSD2	126.853028093579	120.220156455352	131.85737847837	118.66946078241
	122.80785489118	124.944563166658	120.322292418128	121.23469201559
	125.761205967344	111.765812595638	123.56917054142	115.427201752401
FSHB	124.222529964098	138.349773531218	117.766156390814	118.694037994926
	126.834926242799	126.404523759643	123.351112976814	121.107979534361
	120.406577236405	124.070171664904	126.492058851464	118.75237770613
FSHR	112.65454329872	110.355493122568	115.687997771921	108.461141538047
	112.476111928742	113.769815606191	113.644993086829	108.779204939177
	118.117957479382	115.473431881597	116.65880728655	107.828378413233
FSIP1	174.680918805036	150.211719786127	158.573561336755	161.010206405916
	157.295602588814	154.969503835593	194.312315077985	184.267958772058
	150.888616811881	170.007845523415	192.680681636018	163.346402397924
FSIP2	139.510764003291	136.265972909993	146.29600238749	134.793399942417
	123.21732068638	150.763810844645	155.330215726109	159.771672778698
	147.485960802692	153.571517567657	141.023875058322	149.508432746895
FST	200.387623661458	252.404931238338	137.700110923212	145.476377346532
	133.353762794271	150.105503384881	141.70123350351	136.303510503231
	136.648306684974	305.906480933354	176.890596690798	145.318539677981
FSTL1	2760.57475805426	2162.46602203822	2217.82628612929	2756.22069438696
	398.571054759451	498.965895763801	270.973600107882	384.216141843863
	773.803350315426	728.930140593418	243.35880641566	273.651114834013
FSTL3	621.173315178959	348.808646570544	219.246271924313	345.832589037442
	177.93138555339	125.35643274458	180.63469438043	155.228605554943
	175.679405338399	469.107624227456	207.864808684047	172.992936821292

FSTL4 143.546629073997 147.446381217887 146.26936366452 149.160808104789
150.255511248598 148.417929733447 172.157660228549 155.972754525145
151.55925331302 131.928871107722 138.899292894875 150.870830294349
FSTL5 120.333487171575 115.988180787152 113.091739139626 113.071787850611
109.669517182289 115.128667174745 122.075440298074 104.826135297161
135.125414452178 114.174153178166 121.225996958339 103.888971448792
FTCD 145.481279504679 151.043967734248 136.162938063831 154.400124981573
131.794065532001 125.048770476506 134.769926504916 141.870640394206
130.238231520218 133.412452607155 124.053190169486 122.405469833024
FTH1 1353.61552261489 1200.03423010236 1846.61430264854 1113.05943954513
550.566402986082 692.359779743985 2468.47687203968 1175.27165286568
795.037968409957 1332.11815966955 1160.78730097715 889.26314145856
FTHL11 3765.25491426198 3855.33040541264 4016.35381420598
3417.22292085668 4979.31150268741 3177.80599774525 6522.27232911897
3315.21586182496 2767.77385345718 3721.86725798812 4448.67183518984
3033.50962589321
FTHL12 6944.90824302953 7573.69742026367 8292.04796307133
6470.73189429877 11777.9172529698 6296.24208225227 13976.0448927093
7389.95365139168 6760.37324850804 8053.22070239424 9251.21629832018
6760.37324850804
FTHL16 7539.6945050098 8163.29201165065 9970.08052783908
7491.93659373205 13190.4826009741 9793.92604780228 12406.7143043992
11150.5512437377 10250.645112753 10474.0047946187 5734.76405709934
9881.69734721233
FTHL17 151.372219350291 126.743579586052 137.920724602098
146.29600238749 232.892635502424 128.753623886188 142.495333453346
126.578035793406 134.850260739685 147.15999706579 132.686915065196
126.704076078563
FTHL19 132.310147451479 113.740942665269 118.646119379542
120.473038066927 125.293640452686 127.075899925865 120.15332480712
117.740022708444 118.655954393594 105.583474813289 119.972337070176
115.678486138934
FTHL2 2882.51330563735 2684.69013589621 3149.26998719015 2459.05558915812
4150.8511719558 2466.36457617864 5040.35379681644 2344.11548437508
2048.13524244516 2819.92361643968 2992.18604022433 2311.05587511258
FTHL3 2093.67403119512 2141.855356411 2305.11856249143 1800.25771851544
3481.90072759183 1466.08446530283 3727.70199176703 1477.10828981572
1307.19490564658 1695.26858186096 1704.25279485563 1452.63736288691
FTHL7 5776.63686968234 5314.0635307876 7573.69742026367 5233.72882607795
10360.162704111 7815.98936145067 10862.3952192141 8416.19700892758
7354.94071109106 8401.40370474392 5281.353685715 7539.6945050098
FTHL8 3481.90072759183 3560.23794634993 4291.1724030909 2672.77147589342
4866.98027226798 3424.65126200697 7446.59106452583 3447.19587767281
3195.71436046397 4612.81760689869 3850.41246770812 3447.19587767281
FTL 20381.3859535625 19661.6741558107 21754.9904891312 20887.0399135573
29868.1819370827 15851.5830405813 29532.7890259497 21551.9994556128
29162.1093037754 28730.9248954575 13423.8921651885 9560.32907282993
FTMT 154.904424749528 139.927773713324 140.696326952077 142.480203661104
145.114382599368 156.411563412665 169.107948050094 150.156865995098
149.813838004575 142.296446144179 135.556943919872 141.191500203423
FTO 281.926552464214 300.401370666633 299.106362530855 310.026522549372
189.647009287524 155.890403934674 199.135932984641 152.791390145148
171.028432989066 185.519000961898 183.937484597232 182.235490620172

FTSJ1 183.493455947795 190.444387031552 194.445463745418 168.33854702953
234.841813258798 293.317880637519 229.046153362151 290.502085667398
216.485113690708 294.069353373633 263.204269632206 229.200570583629
FTSJ2 177.630246034993 173.624709850889 165.954266127697 177.401771322485
207.560911665319 175.236826554583 218.058543664561 225.130011390856
228.879434668972 174.012972901793 247.280717385562 268.961643716282
FTSJ3 214.8478629277 227.441436540742 237.216435972201 254.393421265604
257.408058436941 227.884445686928 251.011148303246 280.595515386655
246.263259852067 214.019190624184 231.338258067616 245.915473236403
FTSJD1 176.689683169567 173.416999972133 178.766654213173
149.974080577906 203.389294254141 279.879377933144 249.379605914827
259.625943627223 250.481442411876 228.243668453958 276.32528705384
272.994195639694
FTSJD2 452.606433325387 451.621888586656 414.538237455116
554.652979820908 432.174413144791 382.806570442504 399.476401949891
282.392419584508 346.356312668445 342.769825986756 374.215345412947
299.390443444504
FUBP1 138.882483367088 138.944595164363 126.168223112165 131.414690782328
181.85552254233 145.148058824491 151.178586065066 174.66966623557
169.927157878764 164.057147728625 175.693873820963 177.72965427263
FUBP3 444.374521095495 395.327097512479 535.758943850734 392.282956362735
310.8725368426 316.801725268792 320.390351199433 334.916875239616
444.374521095495 472.030150842569 301.304268871606 355.714202328378
FUCA1 562.543347322063 591.437752991512 635.038752622494 415.749051861123
1360.24752587939 1364.1224589383 2430.70635781622 2337.64145466514
1562.9779683714 772.399017166118 3354.38704439295 2427.21100330388
FUCA2 215.143973502566 208.983138060317 222.177290763736 188.166168428752
407.244564912365 323.20628717764 305.451247405518 299.039192744355
280.170464338141 274.371523790341 356.323554000768 378.920456227378
FUK 316.125364442611 328.680312364877 334.443733461841 394.414934831535
1277.0921553877 326.379855547052 447.182566890086 346.411586524655
463.298781071364 321.492900106953 472.030150842569 309.997909440664
FUNDC1 248.594000664466 266.252875242286 290.541184606565
279.704940529618 490.153210371094 527.226517960073 544.852019338212
671.821418586421 377.353441970543 353.674560414317 537.510539142248
621.825622897512
FUNDC2 132.454025879177 130.790872334222 129.949894903048
133.601133679159 136.695790688466 125.013839352291 121.231939494619
116.421771593211 143.94902198632 130.624702758052 152.241060990474
118.757973008463
FURIN 190.45739385453 201.480444219547 170.54511631708 202.662466141301
401.359711029934 186.6902304653 289.911635155481 168.547575047205
216.929969034881 203.335012796883 254.83131041241 221.961758403614
FUS 166.635366968851 184.915526626619 160.395053149199 179.785198362326
178.377461551319 153.784923783551 176.641899861006 151.540744107423
164.244562778996 178.130223302924 194.637233028442 189.192494970145
FUSSEL18 153.328585916625 131.782007636511 152.624623171797
143.801139839773 144.902807664593 127.542288885184 156.324643994552
125.280736217267 133.602870982718 132.454025879177 137.854873498783
132.692027897739
FUT1 109.781334128478 116.011005504201 111.497241941203 128.074531806621
135.864853829421 128.505296338657 114.504409331018 107.930325780047
125.321968563987 125.674471551099 126.17342715006 119.198789189881

FUT10	136.730623822061	136.692495869097	130.844272373448	130.270184521521
	127.631770516132	136.258073395013	131.22473774325	130.721987260546
	127.259038241163	142.082257952004	133.502176289023	141.801472885127
FUT11	151.546908501054	140.005968571997	157.233463720262	146.332425559689
	146.706846930708	149.949425756405	194.569325586241	157.070500065037
	166.071816427719	250.406775889932	199.305626149918	157.361421272816
FUT2	150.538541347453	132.016944935305	150.741291526753	144.013615296685
	130.142263689536	139.969155812688	153.231965895778	134.849235750248
	170.069291215013	226.824313642101	147.943844184702	128.860744370967
FUT3	137.601181150371	154.816213491233	120.473038066927	131.838522109796
	338.406266073063	153.741061283101	210.14307111352	173.690008681514
	203.704734790602	386.738154577763	226.221973502783	488.57622466019
FUT4	188.070213212057	188.027848008991	190.031339775209	159.384480972135
	182.439945229388	142.719216118914	135.968346417524	161.021740537372
	176.725347194807	135.68605915552	135.399032687551	147.579672274205
FUT5	122.170662903259	112.38543517651	111.820208385514	112.809629077555
	123.089110282465	102.563722570171	109.416512252532	114.042914237667
	114.428050500096	119.391213033544	133.507865919719	127.58382536792
FUT6	257.292125083248	256.687384001447	249.80160363144	269.072221443403
	145.06736850005	212.107842040568	167.278879415171	193.261343922604
	184.709768457405	178.696562301918	200.365899129972	257.740674565975
FUT7	118.047978285706	123.53212786736	126.332299789266	133.063422581428
	140.002249873656	127.622397233692	116.136849683574	118.308102542942
	121.855752708908	136.879615833565	158.374760929273	117.461198576063
FUT8	218.839681873515	183.583432296209	163.463191619941	147.019460117987
	169.909886347082	163.106823691633	157.709832297666	170.720018572735
	139.153897381147	134.97514790642	152.194337248455	147.915337223934
FUT9	130.262411213764	137.473779155388	150.606329295069	138.434347257063
	157.028963417138	178.847858667374	151.613134875009	163.438043894189
	171.221972336784	135.92712081285	164.116142541038	153.041407358576
FUZ	166.22013573342	181.578228610334	171.36717256867	184.873459141947
	222.675370226786	161.688125121941	195.532022060219	171.11282933168
	190.572712684622	149.684922983176	157.944355564411	153.82440233533
FVT1	1185.70008959602	1095.52349352502	1303.33810980467	823.658996207582
	493.887026009442	654.663838918786	683.470866056244	622.834462068316
	624.638115878703	754.607039665508	768.334658920971	644.449861831749
FXC1	192.827534610486	209.662262708551	271.238736772408	194.961531388323
	183.764215745794	180.179981672095	212.291528248457	181.971189935628
	195.033792280119	206.340478393262	191.029458520006	212.607094117882
FXN	127.74861041569	136.280653079814	136.428826558493	131.693274981709
	129.094667159621	131.364187047583	125.908576249439	137.940675746397
	125.738320262644	147.405050592353	135.192181609947	141.670426206169
FXR1	583.938727139698	507.137104111742	715.93836911426	468.82816089485
	296.051639873628	407.615019710707	548.288640768746	506.659740456829
	480.797281489891	586.246004571452	461.940616438547	412.277592266475
FXR2	279.232290927348	316.722160860531	315.752980521266	312.286788541158
	298.706655333472	284.473085899219	202.622747952763	223.89854167651
	190.645917992472	252.894371238554	372.374101464689	189.2642795052
FXYD1	301.908667220208	287.83303360511	235.961605948276	277.026538720417
	129.294026995663	139.049085468313	132.645196037302	128.525769531728
	148.012353232918	131.356437247149	152.746799687103	137.360220389719
FXYD2	128.729687597908	123.386622160181	134.367115858307	129.918038866006
	130.169291786693	127.938245853896	123.24533179875	124.33863635868
	123.395897439193	125.746184969983	134.407776033032	130.829825154842

FXYD3	138.252656818036	146.506263412584	131.436176224725	127.471479301983
	311.449205777595	437.489929507567	910.982557417978	789.752781067888
	722.725186727796	348.067061666237	1025.88559245013	637.695341427976
FXYD4	121.215558351076	126.724973604103	108.818976813708	114.209249333221
	180.035652063942	131.037491961571	122.770033312519	721.31849915135
	333.439929772897	120.85013281321	174.296028319781	132.155877196474
FXYD5	629.381791489871	672.862678078975	363.150472154973	599.926717145115
	389.051174954505	673.502723625773	518.17401959981	452.961307006622
	674.101599637653	1122.92917004037	1038.93733638848	1410.58825009446
FXYD6	1762.09874961415	2127.22258112242	2508.50428529255	2837.36293710422
	121.157614971438	131.255028381669	120.402155551818	122.828060462887
	173.647320180498	130.43775024291	133.412452607155	141.043668963717
FXYD7	128.052753068991	126.931328660501	113.995301093609	125.027095330593
	120.193580405229	116.828333047376	129.806104966617	124.210840121525
	135.173464753848	118.974727968283	139.736465430177	118.351607868932
FYB	142.534195262988	137.503405573817	123.875478428652	121.645021899034
	124.387133895009	127.171331062578	126.017158064032	182.310140388705
	230.806950763402	152.467764730004	181.558764396201	129.049273337631
FYCO1	1801.74467005923	1789.51979173209	3042.88425356552	2059.09598012857
	480.484639351046	379.788696204016	497.110852407681	529.854965483609
	494.747087458483	580.164288826741	512.541205235935	398.673582680257
FYN	519.940347384877	459.30568829087	515.729905361375	566.543765584009
	174.767697866801	234.461229199142	182.950456188966	189.044966519228
	260.41082304175	172.562162872242	166.89884215297	158.774910841987
FYTTD1	569.47446215213	506.290241222253	596.743397794872	
	554.282040414368	420.361583975644	518.760142491104	388.887050777137
	421.140525880287	425.001043358831	473.853999833395	377.024120035829
	403.582461109859			
FZD1	184.25384366282	189.552385508772	174.624446035895	156.407166112409
	219.661502916874	213.028568789661	164.309588213547	171.602950766191
	159.356121576029	175.040086358446	189.887788314879	162.237172404557
FZD10	145.333097940759	142.540433565262	142.185345091219	144.988803993714
	135.168792286819	159.016247993816	144.384287335249	136.621173927187
	146.087596132093	142.45047744931	152.538048490265	150.374573177474
FZD2	205.52620827767	210.960900130785	126.76986764971	162.178410412809
	557.513306351027	150.948076715456	295.727275232995	144.487533992157
	202.193338797533	188.767121505943	160.53152291979	121.61143371132
FZD3	238.978845152474	209.568066932367	317.309949792707	274.831898505989
	208.666760987764	400.621427274425	286.818166898922	246.80306998812
	271.087125858775	245.378747269929	202.583967728957	298.403224729528
FZD4	667.421612441201	761.586663689579	489.260272645085	1003.93455977232
	568.555967950896	1201.82026045778	943.677679525947	225.890195278753
	324.203537490975	195.88263399968	284.272977025491	216.353502371227
FZD5	155.835077077665	147.642693777383	153.655778377717	171.304896264805
	306.333710880618	191.725728813959	219.351080515508	194.529045934216
	174.454754564111	151.801558435863	179.810507975486	180.468984099647
FZD6	312.286788541158	302.381946749711	325.011269531407	261.922485502971
	405.409668978569	563.490027149224	668.570984110088	361.327436394941
	559.899430596368	447.909277144896	498.05778289794	443.732580517751
FZD7	733.648333788712	623.68308750251	593.990687449346	641.12919339861
	219.246271924313	179.96623248843	187.196424491482	195.740661051834
	185.938983266802	174.680918805036	198.94325874493	177.592497020798

FZD8 169.771005675296 160.923897048848 153.700168798494 164.562302265155
154.296467085914 153.807525239478 176.506227858258 157.901967487277
144.818492604488 163.198834359304 132.57219914253 154.43808450305
FZD9 124.585675323305 133.827296071709 124.116400777472 135.975265059269
412.736636437651 129.033433971346 119.530214079037 129.491732597331
123.059971161711 120.616979607398 128.745737514912 123.046069309901
FZR1 150.584633044901 157.55776414649 149.423230365631 169.844913799159
172.108795190633 142.851432926979 130.408519735001 136.998259410887
156.698856643638 141.038721816572 153.703682621695 133.446934298801
G0S2 177.281197832647 193.098116087792 136.728975205893 191.074428905553
139.353080958675 141.164985468046 143.402684153777 141.630183595813
144.474814266475 206.010481981919 130.651062724029 139.59941545613
G3BP1 350.658722333252 289.517820913799 249.064573038094 246.850599569231
382.902151239988 326.962330523237 399.620724969771 511.670320118232
311.848379951974 636.048536814356 479.983368253935 418.968095159939
G3BP2 433.594928641214 384.566987097657 530.122419830951 346.173614351499
433.491835341321 703.29965727829 559.559921312181 642.057106241761
592.284071247642 467.777995124655 453.282973743379 543.001249586573
G6PC 153.075594260208 140.129882333528 149.895214204184 142.623106544789
154.938900278679 154.267020640428 141.840858913807 141.229805348717
138.53179773391 146.425476034368 149.609501725707 133.131953362455
G6PC2 134.601565799073 138.911993471119 132.994716261577 134.469653093466
137.190593489949 135.127041919869 135.022438246099 134.73970457131
139.113675690483 137.25679451567 136.879615833565 136.895650862562
G6PC3 295.099840299681 345.38424761963 233.161848094769 354.336462793628
1342.29111510713 286.133496092533 384.883635849576 380.310795921752
396.212732639272 190.45739385453 357.431640917696 270.727749014046
G6PD 365.441289363381 399.872401268958 363.343480850598 591.763423878737
1096.10636044885 354.45785837703 625.932910814208 259.252807304272
290.638110977807 345.732364434125 260.921177985439 255.59042517603
GAA 238.430350258686 291.261765585473 229.881164774875 261.203388718689
739.874432767734 157.248117633013 345.732364434125 304.402066421813
178.416969664816 148.057700860759 369.513420606401 151.054834307689
GAB1 148.823805841381 153.238678313147 144.178210824181 142.613654158833
126.019246614944 130.290454258898 143.281050499332 132.937942776777
137.06192448185 149.902420337518 133.158570377793 131.730758460802
GAB2 274.14931964455 262.832376198119 231.819341041105 281.792602269388
155.104697146664 156.026806913759 159.738700107615 181.558764396201
159.58125728432 183.998458233995 162.560758971445 144.683260413888
GAB3 160.861929142249 157.768329108254 143.785769607842 146.604519441812
139.752845127584 133.647637744785 141.758827837244 135.341892490559
143.980608339892 143.971152352313 120.733009784454 133.537552829999
GAB4 137.237089899964 140.381386314817 152.654938328424 153.941193682697
138.071335383596 129.346063085291 135.210887148305 134.631638658586
135.571239294086 147.085006263446 113.687931899298 150.874014332213
GABARAP 214.991170762327 229.406012088782 205.705962405954
251.161789722751 211.555248754731 244.162496497466 198.979744266277
213.145460679531 157.203304477454 243.246632667118 247.307128520272
188.671807443641
GABARAPL1 1577.15895478896 1321.17236442448 1603.87451959083
1388.91423772335 329.722135361491 350.22221782412 478.909308632726
431.861762635568 494.175437345451 346.065746911798 374.872358131797
244.668813440638

GABARAPL2 903.824331691969 754.406896156858 1129.15018335354
701.481003619067 852.099019968701 891.637685856145 1009.42265467472
759.534249620343 1304.37930719411 1111.47772523331 939.213447046823
1059.19317548869
GABBR1 266.892524131545 227.288015854809 244.897869154624
235.063474370925 128.382487609446 148.353927712013 144.794806650252
140.774304412579 135.904163043783 169.271175460047 145.82344959902
235.063474370925
GABBR2 148.641780441227 135.061176512365 154.264142444882
142.984243805285 195.630643168176 134.631638658586 153.875760529011
156.481871341275 156.05583229359 151.206697943841 205.930921729192
139.875260384886
GABPA 145.392463986225 149.710337205986 147.879286704 135.722321387765
129.55573546861 197.443711199904 166.70212615715 178.657325741035
173.766372029062 160.140509175287 162.54008293513 193.472317111841
GABPAP 147.024759173313 139.157088843803 144.476157581998
140.630844296177 148.484823275343 151.88244935494 147.138814313885
146.202611204712 143.675696734051 147.301909062399 141.836648910979
139.951275828765
GABPB1 159.870967387108 149.350630572336 160.402220755071
150.445647133244 163.407360509803 166.746037983859 176.617812881115
166.835922279372 165.327972973978 194.22713184875 172.157660228549
164.14265547147
GABPB2 7631.6124232898 7468.70895916615 8072.83367137971
8072.83367137971 3052.86038628748 8666.41250683965 5058.57872936027
6990.13809515289 6585.34962362923 5233.72882607795 3921.67860557329
5932.37714987741
GABRA1 132.253450751554 126.085039271526 119.159078990339
113.292688210897 125.117333434527 114.242056888286 122.624828884731
115.30786313391 113.199690522854 113.549098821204 129.846685899183
108.570463496052
GABRA2 129.57551086282 125.345611085082 136.438788088263
118.604395992281 125.914267820741 131.436176224725 123.662678397678
116.566190779365 117.953453145685 116.514299377365 141.755954088275
120.686906898624
GABRA3 144.96021415188 135.797170561599 158.645402461996
136.935498266723 156.068308349225 162.55521875551 138.706520278166
150.182173149394 168.254689624696 152.709924336348 139.339542167402
159.98636352484
GABRA4 127.483420376792 111.632811186988 113.22850386845
125.802001118545 115.923424095859 116.134550788966 119.795945889846
107.580425848009 115.755585907264 140.89390566467 128.297879410629
110.202982683256
GABRA5 122.969215709433 146.160188002433 151.629999903069
138.436029489092 142.487424651482 148.74881415573 135.472135601859
140.439698763808 139.847813838017 139.055892787717 128.83022039037
143.180247878507
GABRA6 108.861243071977 118.176124626025 119.373287155621
107.40922161073 120.602818832247 110.753661013519 116.87586240208
118.24368469845 117.555999801887 116.685023927951 131.393633337276
118.718048764985
GABRB1 130.71040889651 120.574820369421 132.582062079623
136.169525758215 127.132970760907 138.154799037807 130.110413621436

116.925863450165 133.268239762624 133.526213647686 133.720904256137
132.088335186716
GABRB2 112.599970121166 133.274292647041 134.503552797411
138.952082680577 126.700147643 128.488598467312 132.74579603362
135.495964034239 121.434713764246 128.773129597793 132.103084472404
125.060387518072
GABRB3 145.176141415244 144.898770838913 138.116568200293
143.182399285928 170.283532007581 142.099541115973 156.572774198042
147.191114491117 131.215202712283 136.623658204558 159.646538408414
137.244552699212
GABRD 116.89487516867 145.212306927438 119.395894479869 125.631073476381
145.99474597064 132.334666750573 121.455843337023 119.578240680666
129.910866236901 114.999628959921 143.138613573764 129.272835188209
GABRE 192.737713851863 194.257024100927 168.095061700814 149.25240100689
182.109092947203 273.651114834013 467.387427864242 138.657241641263
201.289402228512 229.77428612216 161.77611826709 140.401508808129
GABRG1 109.454483612299 106.719519490874 105.665621968915
119.418866166776 106.923686318112 113.051381310122 115.137475087954
104.36042819601 106.36673326724 112.436972452838 115.211731367278
111.253743009588
GABRG2 129.589056071463 130.425809387444 134.774591679724
142.581441480371 135.154441560264 131.292195405993 134.122776198036
142.846100831941 124.668658660052 133.951322753098 132.878638378328
126.435928518593
GABRG3 123.29908883119 131.013719535992 129.995417218564
126.199219102601 134.811625739766 119.502758763438 116.176068923513
118.95971064289 113.334665910428 111.672450681359 129.108534267222
127.990453128978
GABRP 149.262900833177 145.801228425692 142.825203517688 186.388067641269
164.241874094897 140.53597287752 140.395662148924 160.549597620339
143.056098955473 155.548684011748 145.428297145967 149.980924447339
GABRQ 140.120051507626 138.692057950694 138.023752854109 148.645749834125
130.207485841284 143.438417529799 134.932416370825 131.177396785633
136.184427467391 125.765399556488 132.322618637842 130.029719192412
GABRR1 144.271920003815 155.176714087209 146.67875644027
137.844113743956 173.790848780011 155.496190048502 163.422877959747
156.280701500421 158.699778625671 143.635367691412 134.490327004118
143.431389389919
GABRR2 122.592960588642 122.083357581665 120.497796762933
123.240482782331 125.982423431253 125.272796155561 120.975542702338
122.789656787728 125.785388817819 128.119556520151 117.112667504781
119.67745324922
GABRR3 145.566861062922 151.3362561751 145.885577577352
145.542888937635 139.519321690754 141.015441854424 148.600270795407
133.691053105482 140.688570032068 175.960788330149 116.635455849571
134.56134674893
GAD1 138.033668840392 144.683260413888 139.06923394504 138.244585395561
139.265915951162 148.029259633449 134.340512608931 180.085975596327
161.066226957782 155.298546821687 159.413535577361 232.105867345662
GAD2 122.629146433216 140.833666276568 129.356401843185 121.30744400137
135.976837152799 158.007926822094 119.202966460612 124.466986821169
121.36708642226 131.521311288114 144.059368898969 128.44311762955
GADD45A 655.665164535648 511.996883507889 541.642309122976
311.375563161772 518.17401959981 537.06835963726 916.836022395768

1210.40302822121 437.931339781992 1252.28642463619 977.999660785113
1022.55215097491
GADD45B 592.079898463345 479.638653311025 277.285526811736
363.195371577097 211.34069504665 527.597948979568 256.924134409998
198.583407008317 201.139510041141 540.078145038497 199.620978536397
239.704459251571
GADD45G 380.243136469602 361.768552564027 446.601751145647
376.962937292973 297.285037835812 149.057388078138 166.746037983859
203.223604138192 173.147311624838 207.036293322882 166.850279817976
146.731584143625
GADD45GIP1 175.908645445479 174.576132939661 157.410205523901
212.959246855759 245.426639190893 202.343343669937 183.693300680495
172.230528631803 164.313238416498 190.921283434627 197.565122821625
158.523941833883
GADL1 115.845468537936 106.629065516249 111.43827410589 113.791585365477
114.441223528256 102.725375719353 106.088807114602 116.958052905168
124.647992480694 111.72835538405 111.820208385514 122.561285351587
GAFA1 138.032265227728 136.541973790157 132.881693683384 137.987255649072
133.670967970136 136.418105816557 135.168792286819 140.949367846425
137.788504385206 146.363991934916 139.199446543251 134.29036009792
GAFA2 149.829363505519 149.492550066785 135.244303669342 153.044682186978
134.451423371791 152.237183848896 139.39442529119 144.20764300349
144.64136816491 144.355348009294 141.488910091973 141.870640394206
GAFA3 149.712320986646 151.993651822163 138.603793282123 132.582062079623
134.48825480809 148.065080664185 144.99221793877 134.931263946202
125.513881890201 142.272117655292 142.973444931373 145.352802493486
GAGE1 126.532293165932 136.599305161841 138.354300118735 133.507865919719
132.631819830347 145.352802493486 132.50027119027 137.161065320948
132.948096807634 131.151578665754 130.909388246758 134.507558495307
GAGE10 128.723261477217 113.542803491215 117.503848306325
125.467668442298 127.866992108488 116.746018509166 124.811760828557
131.181537219479 128.205022859019 130.069789939568 122.007909794484
117.953453145685
GAGE12B 131.092140169861 145.861217875066 137.94246602811
128.523703756493 185.128661141884 648.161646065464 137.778033050658
135.353539458856 131.981505236332 132.99989565368 132.587392944892
125.589744938813
GAGE12C 119.774204444899 146.698766621345 136.967408633308
126.935731176024 217.89028736436 808.300067805897 128.840466579373
152.088488171996 124.883575464443 122.804934452095 124.077104101209
133.834423222601
GAGE12D 115.664551127497 128.194171809998 125.431735524937
133.976997391944 128.122197525844 156.585665091824 149.696766215866
136.282608531191 124.959873714884 123.344450694117 135.548042190047
120.724926760252
GAGE12E 129.400895994387 142.253826696208 121.263939656119
130.795318552623 150.694215349678 224.424524191566 140.63666629522
136.66358267095 132.930042559647 138.708304921088 130.543453631154
154.38977085744
GAGE12F 131.560971848628 141.448017858785 131.510494315469
143.899201949317 211.118308738617 572.913714991136 149.797850183848
136.862764950361 125.689884251847 126.87100632595 128.96181458192
138.717541782317

GAGE12G 146.179543599917 135.800997202323 126.825785762925
119.11586630281 218.269847477919 1319.3522488218 139.984102798711
149.44788347071 136.582137928022 148.065080664185 120.507930710099
149.85385474029
GAGE12H 141.484730939562 159.811778680604 131.717160947964
135.781190598068 195.776369840059 761.32108609503 136.2336179571
149.604507483606 144.853868519575 135.196797030404 141.277227483198
147.256726902984
GAGE12I 136.572645314012 135.338077911839 126.526489290128
143.045416067967 201.81649023518 747.804958601763 125.079330440615
134.533551900843 129.189479305468 131.872583529419 125.048770476506
123.777133327569
GAGE12J 143.440420261133 155.622257285651 151.0189104086
131.87404363664 200.712182818203 890.507257588957 160.272632590118
150.837717923505 149.628421263062 145.636906369786 121.027465953657
155.086559474139
GAGE2A 128.519448608754 137.426226808356 136.515951031895
129.586337068199 138.399665664034 345.732364434125 123.338921824167
128.182419321978 130.59886317117 116.958052905168 110.718864165172
128.620852653765
GAGE2B 142.973444931373 159.606633817645 133.148442269517
129.615417777242 291.914408947328 1375.76915021882 153.187148832063
166.611809859059 151.950883677297 144.490452173336 144.368355737187
156.460374293022
GAGE2C 134.712935556892 122.392159083328 125.683334853039
120.160652344221 119.608334527504 124.624046191085 122.615852682436
125.212404794262 117.962188157317 128.944295118239 123.678365506247
119.432201417811
GAGE2E 123.804039963953 120.871693555514 123.662678397678
118.492287441192 150.137061170632 397.449905380349 124.533543270311
129.431497372577 127.449028221682 125.785388817819 122.087107663865
112.179019393947
GAGE4 139.898996725236 154.548286860883 140.665332203898 131.440195723963
225.189991540262 1349.84215478798 146.694318629238 149.929454280702
133.65688823581 132.411757518105 146.501559368223 145.959421614703
GAGE5 142.331050242704 143.754105312957 157.231166272318 145.070927449969
230.227432807772 915.579581760396 133.97321873161 150.241585041178
133.97321873161 138.117916412819 136.695790688466 140.500483234415
GAGE6 125.243735617266 131.970514300585 124.518818352113 135.446072698039
162.155834067128 408.10780961711 135.068883036882 135.679481480408
124.574323079707 119.763020707899 123.646558103253 130.902319266342
GAGE7 136.957209426823 140.634047988278 138.775095929764 127.011594171986
186.515784641119 206.669783013221 133.0349214478 129.997181739652
141.535381351126 137.893630842968 137.065315360321 132.748478264583
GAGE8 100.216750525986 110.382724813928 114.673517800886 115.048402956574
114.325967923393 231.981501596284 111.515588041012 113.422559344178
115.750690887988 114.721282552636 139.174188535843 116.925863450165
GAK 377.083379021079 402.694564608582 400.550044186372 466.58966852405
692.729789121701 384.16579891756 502.598518192729 418.772484676858
590.849222581206 431.413030534188 468.82816089485 460.514672966125
GAL 102.024765473404 111.467722538327 100.966979609222 108.036254606246
174.832495287192 121.679013405559 110.875112640101 109.574386044714
109.255470590389 119.352995485911 105.49491265382 112.904318310632

GAL3ST1 138.322109975426 135.275284142536 110.486191378104
117.436119533535 118.631771242606 116.14662237541 122.924017262559
120.13937451914 128.628637585951 127.350368333088 133.666244052469
122.247130087475
GAL3ST2 136.621173927187 124.528206512938 141.758827837244
144.203716525289 147.815673580657 133.061016674211 137.260359343758
124.153063838817 125.802001118545 140.557871626905 125.658396221949
117.567991653999
GAL3ST3 137.636900130922 142.097126256137 145.308548379215
136.504438803108 131.915864620669 137.642858036384 145.924751649542
135.603160096973 126.741498575874 126.566165822427 140.091378604453
134.668233738635
GAL3ST4 355.290621188435 349.910535555712 278.192669077191
336.639473916174 211.160781061336 201.773745532242 194.445463745418
202.214495168688 248.791254839869 267.222762759781 185.186497037695
222.285940575778
GALC 276.057101476947 309.155070674445 282.836518011311 241.251507026982
323.244664586314 330.005950459958 400.924914238052 319.639103463458
250.718986968076 311.337303524912 443.343806055858 433.314881387431
GALE 150.046300135261 145.980929136129 145.412368252632 137.369033684705
353.243400332397 178.339211170442 355.714202328378 300.765518378297
223.058699622386 206.620708230452 196.378888436237 309.720081933252
GALIG 201.221839281282 171.384487293154 188.847480174391 162.870317933909
160.608674893418 315.064477782052 212.545989139388 211.786963444117
174.903798946103 217.811099067262 183.804849355341 183.472290949588
GALK1 642.934824227327 626.104755496369 589.070196307574 771.512332113075
1880.35608492553 653.879734066271 833.841469195516 1037.97124386242
723.428294037428 2382.87910783088 556.863361789449 756.246712133116
GALK2 203.335012796883 203.071186284148 198.390247605355 206.845306095447
169.403422185018 242.096692715029 232.641965649602 205.781486117242
245.766885283797 239.827648772906 192.640717683236 241.077618798955
GALM 242.81187187788 247.454326301069 269.831616158113 307.945884504629
285.562276640039 371.06697207449 376.962937292973 382.562403424855
369.726867521935 159.082447644277 207.545424360094 213.301947003462
GALNS 195.674128236589 183.969897305028 209.258707976348 195.057830521304
243.225815954365 252.611044555689 218.156162474715 196.777776509371
197.308952757609 275.468194644053 270.520229426015 253.789045068884
GALNT1 179.109090120497 185.507663455634 172.578252260002
153.6855881767 355.519647649551 555.259469428492 489.260272645085
514.213675499369 293.528601172819 330.505633088656 596.3549500121
492.780880796036
GALNT10 173.395370400371 152.624623171797 175.476184387338
170.12010526854 146.31468639383 139.793683692104 152.350537364705
159.539320132392 152.14012587515 204.103018614608 165.82215325714
146.67875644027
GALNT11 1095.04571025381 952.357976050712 1091.69584249713
953.861218000678 798.758496171493 1342.29111510713 1564.8868894162
941.065508539108 718.670564185576 792.101559967381 1011.14472068314
1385.28044421961
GALNT12 282.742833166843 191.861598059182 301.250189051376
209.5184563244 192.789886894821 139.437073790935 207.173653740239
216.033564783918 160.122274989224 247.373571011712 339.663295617139
297.466255000163

GALNT13 164.03801002919 147.056182807232 158.799917136119
155.129304059037 145.34332740863 142.002145315543 138.322109975426
135.511902377219 146.861981788662 143.090886224647 145.054873480806
163.20714296121
GALNT14 120.29173510435 125.785388817819 114.325967923393
117.953453145685 202.458096827378 138.393565473857 141.698329968978
197.2893692747 130.450930089722 129.487458179949 230.227432807772
147.905406818238
GALNT17 114.223991452083 112.14768481333 121.980744551615
118.424520312293 105.236768436069 118.185499089226 116.230993734675
112.394223360492 116.437403962939 108.807891863679 126.823886603295
111.743461522689
GALNT2 163.181323768362 161.463042036376 187.112176929485
173.513108409372 205.728673153914 188.252507246148 172.248521526206
169.246302492166 156.466728833063 185.193234472203 243.589099648895
209.867189613436
GALNT3 527.597948979568 560.757496975779 446.698665191047
503.967725251906 213.602504580364 689.821785472506 330.405026390912
466.691191261997 331.383533814347 286.818166898922 498.534168389928
354.45785837703
GALNT4 171.718906292137 175.585857393085 179.122374556365
119.926169902322 135.479300698192 214.52087460757 243.35880641566
1025.27246494218 218.350166753833 324.79327985156 300.675907594637
234.996704304673
GALNT5 138.546549217819 139.318078285343 133.613988293895
139.708303388546 121.877276961306 133.327778635672 155.147962740503
134.817665153925 140.429462488271 142.029398672217 141.67550758321
132.602336133471
GALNT6 153.44628692569 150.231063426302 135.639558187703
132.245217686793 130.890314257211 131.354275252104 125.898195488305
135.040794634617 135.434853296628 187.082082429597 149.645548888832
125.416113646128
GALNT7 111.484426481816 135.144908040903 110.727742629085
112.07238018581 131.051711775229 124.048926099805 125.413619056436
121.22760405399 120.485138937899 113.845770994796 155.147962740503
136.494339004002
GALNT8 124.961859983596 128.663320241092 124.522974214212
114.056147008769 121.926377140115 123.004137408444 142.211357324461
123.111192189217 130.732397679661 135.717491814078 142.367003292264
132.328948598599
GALNT9 151.751963310212 143.272669418277 148.003041725027
142.095289977576 146.675159409376 143.151478156897 139.828781354865
151.330035086652 143.681220324074 141.753560518393 149.464962540085
135.033601686007
GALNTL1 219.537335167901 213.240181616274 191.665827345888
306.677271064305 151.214631373344 141.368078865065 142.680136733947
154.496005175117 152.93058803161 148.327340543504 143.607579034109
143.625188247966
GALNTL2 233.496804016764 185.100455193182 326.1109406486
130.939543211439 108.589444216106 126.820834043224 107.638492587723
114.818898581401 114.182798672347 120.394220715157 133.099175110023
114.278113876915
GALNTL4 382.327656839936 269.261595558589 256.924134409998
257.477501542521 214.684538620841 164.630705813584 142.095289977576

176.874265462986 163.946569217706 370.066087375401 176.53912635694
140.418010366997
GALNTL5 159.758196845781 160.251996476232 148.81406900745
143.405079593778 152.617507769717 152.06044555622 146.893429874574
134.799648755296 158.477017294725 161.663893432522 122.527517633626
143.338628928675
GALNTL6 172.03919618445 158.943005736645 148.049570580734
148.227581116046 151.05681004513 146.425476034368 147.882530392424
141.432461374482 140.290598244169 153.092864798642 142.084294087481
156.720090761933
GALP 128.033383607545 124.183297351614 124.563594928763 126.77232818113
127.58382536792 134.218867540274 127.987449338817 124.045745662173
132.47775411384 124.539006083936 142.411117330429 128.368669716591
GALR1 123.400143662218 118.783904603368 120.402155551818 124.072512083237
129.942594772751 127.822440239962 129.610980807708 123.696942540314
123.491930765117 130.33205883588 122.362585067453 136.990610254852
GALR2 120.450111018432 118.614641349402 106.629065516249 110.408301129053
128.69455713173 122.339819585312 105.450273343324 137.629855378016
122.99993610249 118.88525116298 115.687997771921 135.753430034183
GALR3 121.874296836854 125.815256068004 123.745824514261 123.153925912632
143.195338329759 129.940297696849 125.050945917101 120.571074646092
120.517691208358 128.134246199718 138.67221078031 121.603608840998
GALT 220.415211013843 213.382179917193 242.348427307994 232.621526025603
227.587590278412 172.073751088968 254.879772480457 222.943046618742
250.139061165593 273.152907354565 194.702974020747 213.7326050342
GAMT 242.299252896647 261.322374889497 285.83626978316 300.247304779062
161.614426723521 145.043494048779 145.847629576886 160.026178505749
148.996601285688 259.868701878844 143.200939506995 230.697077917675
GAN 138.221749464651 136.572645314012 133.951322753098 139.282741110499
152.286545050749 131.747367613225 139.429908070687 143.114384950467
156.144934427609 125.393848851951 151.9441336438 139.008854444223
GANAB 626.582864105324 625.544719251096 645.806954296487 766.176882605175
990.206369590236 750.494286476465 844.187489702587 564.41151411028
575.23272138912 649.052806726591 544.852019338212 713.525134497596
GANC 119.159078990339 129.987548571478 113.810645407837 118.614641349402
118.195512252508 125.958139354095 116.814269969508 109.669517182289
105.132185249959 114.942849575931 113.487357534546 122.941711366641
GAP43 167.395369670065 165.880071298733 145.615291241733 192.848268768579
155.979380606594 137.6160344044 147.552635527742 150.029313080668
148.717545103261 151.334463063946 147.745780820004 151.130401750721
GAPDH 5300.67886821231 5636.26097921507 6585.34962362923 8016.1652884418
8918.58940386786 4262.23145131525 7601.58151657937 7555.47017983216
6737.97308530049 15924.2167735021 6990.13809515289 8416.19700892758
GAPDHL6 234.094722315007 170.901599088282 153.366677608026
149.055493714692 230.508733145583 309.318403417731 325.43977732888
143.265532486512 151.451443480833 158.708054164813 161.799743586569
338.46332239699
GAPDHS 132.641236788053 125.707228291312 128.878051599968
121.991692204056 140.63666629522 135.16316100494 141.765004355609
134.160882467542 131.863018503922 125.628281846465 130.101422420432
133.964163727962
GAPT 145.968884703737 136.344248873826 133.749271961438 121.980744551615
121.632494500096 125.481506166304 140.226586111216 151.224289879776
181.701252112196 128.122197525844 130.231160879338 132.733821031511

GAPVD1 759.534249620343 678.684323057327 696.277780670166
661.096668635469 481.630922092112 421.929674562786 479.053300726301
470.308610828825 514.002313819685 612.374364731095 547.681206272188
333.200230327106
GAR1 386.817736004263 405.130899553871 404.366325262322 411.878556907186
556.568221094639 599.208370337812 558.251384152474 621.946994961864
797.28465621772 545.200687301057 428.959561932229 522.17701271254
GARNL3 275.996693627151 262.29645628166 376.276954313674
322.614169214764 164.791545858875 166.688771186254 162.077455145261
149.606576415873 163.113457178357 152.358795162474 178.130223302924
157.180095329946
GARNL4 152.386986818959 152.011551837951 144.237691418911
146.692827583525 216.705908017858 181.530767521222 165.597236278435
277.073510273425 182.833961304079 247.454326301069 249.064573038094
226.315173823168
GARS 1452.63736288691 1386.56788941677 964.093668483033 1472.54829263217
1333.73334006986 981.781076110568 1333.73334006986 1285.02292965374
1652.77814259264 2672.77147589342 1115.53524128833 1862.88548451154
GART 247.340470321453 226.173501275026 274.627303114477 225.095016600703
294.595972099366 597.459920611726 366.705048374078 389.588346573823
515.729905361375 399.762451559805 428.850337327605 434.509391836336
GAS1 938.918431240228 910.541650889451 1505.90883940934 1052.553421812
166.85671415475 179.794718712964 148.773558380167 175.964600718395
200.890547965585 522.300029971277 147.657151780506 144.256029338053
GAS2 142.726324048628 133.367085445965 138.897393621114 136.254966566424
144.011792962785 164.529828080312 134.786678978467 261.814227719605
138.962649407431 144.338069855334 152.768580075051 140.182150393054
GAS2L1 144.375109087466 142.798248249144 141.840858913807
141.170937695272 168.412162365467 130.666781754635 134.074425424613
143.561114996045 123.288028185949 131.138322527536 146.035731780312
130.977864137459
GAS2L2 117.829228895897 138.240874738649 125.830917527594
158.244741207036 128.40650280096 129.647514123731 131.2360427855
127.028640787946 141.226901872221 128.655549652719 135.790955294639
138.322109975426
GAS2L3 149.103778494524 151.972509608956 130.140958819402
140.626181314061 157.301617257941 165.286724415145 134.250052775987
187.15740398852 155.465250172768 140.256871047018 247.236004734567
143.816567683093
GAS5 155.666943703002 151.515460040167 146.720204808211 152.291176493005
148.81406900745 141.468076901305 156.311009595629 159.895303363094
164.820007934887 145.773932754923 165.896420029269 172.46311371589
GAS6 2388.03256067711 2586.87941889443 1646.76016031831 2970.34597432588
177.919885012006 196.839016616283 150.610633329908 212.291528248457
306.232249422327 305.252666831522 195.10410545445 249.354198174438
GAS7 287.084253886676 301.369680426563 210.316431648335 383.939800422489
129.505139430366 128.014092519305 125.704264033102 123.518170356322
134.239029110227 128.814967091609 130.25146185271 118.703108284564
GAS8 221.869191666931 278.142283306934 292.037861000784 302.735372807236
310.142754559096 270.33685210971 199.149959995133 244.054469051058
255.374689750672 218.156162474715 244.5675215931 302.696004448559
GAST 107.961322525982 113.214390213004 115.587874563785 120.280241193135
120.96098661114 119.525701528869 129.222714748528 121.961568081299
124.697283474333 116.881212516879 140.258615682066 127.055165613236

GATA1 131.911579676047 151.794243108455 140.165450892713 148.691132137188
133.762141373091 166.257275241937 134.691038011706 147.161720770972
129.763063900493 161.155280539113 131.99470265134 133.286702819953
GATA2 180.085975596327 190.255797389524 174.953232190285 137.636900130922
926.670867201327 279.668007824988 333.970844203158 171.589889474122
1163.13845502757 131.926504315744 511.891269255193 387.068276379114
GATA3 181.654319266685 244.766289564524 213.510783808898 146.099555861577
872.320823993033 989.77058497525 940.716682221364 958.63560158343
153.511995286293 159.301581158613 1051.45401327176 1074.19589851599
GATA4 115.954828435072 121.952255635669 111.7529190597 122.022611139895
113.620070198869 121.021776527531 122.317877997527 119.341806810441
115.566303449363 110.17590675298 108.118551275778 116.977202125093
GATA5 503.757797957538 725.443026984982 543.170902795966 1301.18893681695
114.528407572496 124.783306383852 116.273000405449 132.828179725129
125.396523818173 112.476111928742 131.887778588005 119.669441891401
GATA6 156.949439724914 172.304496150036 159.775479686193 156.798431595608
140.347131743987 138.640919060725 140.968351978909 124.451718955086
138.877999126533 150.0674092434 149.898614923436 135.283742166077
GATAD1 241.734804013256 232.667968049771 295.099840299681
204.616225498655 193.689806171113 167.640645997754 276.653753970476
177.132361355899 179.985468369202 167.67952650068 313.828943866034
286.996908547553
GATAD2A 847.564628337955 793.071192619058 698.086166782221
965.031655021246 1752.50728312967 767.377380800066 879.301932542031
765.911113050543 754.97177863997 1067.30106203503 1125.28599595312
767.054576874115
GATAD2B 340.193863653875 322.889296658955 378.967410725775
288.370985514978 243.861834405092 251.307020289291 194.403848868583
246.237243726748 218.592402813219 213.274106157675 227.065194522692
271.196028302639
GATC 158.041568121766 142.866874321248 150.748298569951 136.216577685046
201.221839281282 167.018380617832 212.710910778949 171.473538842796
195.84531580224 228.100112133941 144.763700813057 177.851689709807
GATM 214.050918459805 190.716424123036 358.654569870431 184.22362329766
198.783034238774 126.288011849541 337.521614253267 297.757657398146
191.074428905553 117.078720392441 148.25001336038 108.9291713877
GATS 553.90008440429 604.453803994875 577.33849844574 748.703265448403
262.671819506189 381.57978490093 289.09614335372 226.936810012475
543.001249586573 137.936605651724 240.283918204202 256.495848662843
GBA 326.640168906375 324.401611136662 307.768302846647 308.517561840596
741.689442253375 326.055727207966 473.853999833395 622.142195892701
360.285027176918 347.411500022053 295.099840299681 432.92947962962
GBA2 198.023465629218 196.691380885508 194.488249052694 284.748764120614
168.071171406779 164.103306492559 152.617507769717 177.652022895288
174.540209145695 171.130215858803 198.954254778846 172.26834556696
GBA3 128.853089272901 130.224324464992 130.173734963758 132.550022967297
399.620724969771 131.526532700651 132.671227062948 135.639558187703
130.965418847276 119.502758763438 134.853253781256 133.604705630582
GBAP 162.134726710448 169.061364889307 176.420075374686 180.493406236326
171.140106467064 162.945789604505 156.978585870854 161.911468506919
158.027935395289 178.186273279101 155.233830396052 175.955932037011
GBAS 546.029974898225 618.661249163439 713.832153334389 662.781145703653
375.651196211048 399.762451559805 529.854965483609 658.746224115551
355.443758121675 382.069677030369 670.097523566652 647.439372562271

GBE1	298.926727289387	296.428342247529	357.584458526365	297.805768288377
	294.280238648737	282.190861594303	412.344820823968	331.251075774227
	345.494740481759	729.202002368627	321.696082249696	399.071041584646
GBF1	241.383657414248	273.402804140541	194.029961195972	368.374002925137
	241.987100712218	145.681968019851	164.696616863082	156.003233745771
	190.111477641308	141.855655600443	252.697160031025	190.625250757654
GBGT1	204.637887777803	219.49354518392	170.684848540493	169.349922114861
	217.607230767028	167.604959895451	179.674744176904	184.332299740724
	222.919473675549	237.854774190873	159.005459875338	218.058543664561
GBP1	398.075524915003	289.859695541999	275.929414239664	250.84781675544
	250.376178742476	312.406286993239	416.638529499878	578.465753476561
	940.716682221364	518.281245498542	781.461780481171	332.400086578723
GBP2	3008.43964505208	3290.27308365921	3334.16277752222	4150.8511719558
	2198.4268389426	2203.82462320864	4251.82554412383	1937.83242524439
	3608.10730106542	1048.26872286983	2974.04694562902	1801.74467005923
GBP3	174.274996381853	178.606255846041	139.268942501979	142.759452994928
	175.417320301614	134.136687154241	226.841838641151	283.982575660968
	281.570685561526	129.391376518208	581.37957221282	181.85552254233
GBP4	340.028737478082	382.186095123659	261.065986251886	429.457884032977
	204.82244206816	247.971366020963	396.861835715943	685.848203651889
	1612.79779117655	265.992741398183	842.22767901391	331.62124160152
GBP5	145.97330701968	141.537578081123	130.297203474319	124.28313315915
	145.200638964782	151.194282143046	169.246302492166	227.715558644326
	349.054741747798	412.801213309591	223.253794809571	135.025907096066
GBP6	127.913463809158	133.022848457361	125.905785653648	138.720489866687
	134.648780131578	125.088717405087	134.21227169867	143.250386570155
	135.648906382608	953.861218000678	134.623264303227	138.897393621114
GBP7	148.499207368959	116.46192274217	139.708303388546	138.02757574324
	141.623851511993	128.611740835252	141.132694466739	139.484551562098
	130.48231275063	148.888698484378	130.941645638407	142.086300138469
GBX1	131.846160346403	136.545742688573	132.820927638053	126.089217853289
	129.405749374776	131.288785100733	125.231476412101	130.582210755516
	130.420753247797	146.464937541891	154.686025022972	133.416938451465
GBX2	118.457573670556	115.834056091815	121.252042151856	133.527906709491
	131.876061826583	126.086991456747	126.301178976594	127.781732819297
	121.778756075848	120.193580405229	136.610566158577	118.139487760542
GC	137.434039024162	141.917318809044	154.277670551775	131.086904662241
	137.450774903611	140.704317015307	138.834309377225	150.769819282075
	155.099575681229	136.733225604928	149.65417821994	139.771342253058
GCA	216.903719298575	214.803167803712	197.827534617557	175.752091217351
	427.084392965651	428.09583118349	480.484639351046	728.485195792194
	539.27766384282	393.264726588522	448.95671696823	347.866264631778
GCAT	204.318316638312	255.225145197422	223.778708427552	316.237519859859
	438.146919555296	401.359711029934	314.292700455545	311.208703262497
	282.797367565702	419.272193677273	189.647009287524	188.641397688261
GCC1	392.282956362735	404.445038343721	324.067396271281	378.695635790714
	354.656612182522	320.936905584424	486.778046640624	478.606567465979
	382.069677030369	429.922530506572	540.078145038497	499.321667633972
GCC2	158.658110539162	156.407166112409	154.33224999163	145.971656491073
	146.054535585464	171.080373870373	172.531836863525	159.365070384451
	155.883386742315	157.649902282769	170.410704499804	161.501062527272
GCDH	209.44506543433	228.384686248604	267.622579012917	286.996908547553
	357.259935567823	293.746733527346	279.070493043898	235.933451977398
	271.287767448621	247.971366020963	200.767743455226	252.056131165397

GCET2 156.611727364072 147.299872426498 152.660373241584 170.720018572735
145.873819094778 147.241136598626 156.423687034312 162.168246402027
156.083002868004 170.046856179083 171.221972336784 157.826457203706
GCG 140.296609941747 143.756242843438 131.516100334597 155.494095135266
142.175432631861 143.699398679866 152.951353678265 154.239755912437
134.241917425247 144.694679571377 130.716570410608 145.612695657243
GCGR 109.209241676429 115.058061620379 102.474380813907 121.648921929686
104.544792793756 112.340161674529 116.282988576774 112.977469350139
116.20008794813 105.712302595131 113.919529047225 104.739307327595
GCH1 122.90702157852 126.775180133301 124.403662787819 113.339155126202
135.582230137515 199.477350542491 140.146894533044 151.908841481413
141.522701487198 144.445496145045 186.04763660516 154.094285855743
GCHFR 215.300064529551 227.467161919443 221.889331385023 188.033890834418
702.981741565482 385.379119927642 351.448985413528 290.954496559897
382.562403424855 185.447093292348 292.60467054075 531.421912541822
GCK 129.733935614802 126.484819755399 122.053867525393 121.076652800015
114.099623939954 122.460829835477 128.56495182759 125.579970155301
122.843792000493 117.992729826121 123.117923525539 124.503016568677
GCKR 140.326012404526 160.050872656883 150.870830294349 161.592528958127
143.00932329728 142.301581642006 162.732574014686 148.539881077647
156.540165645256 150.166324293431 149.103778494524 150.070213543579
GCLC 269.673122990604 325.12056698785 428.09583118349 282.511614929917
315.130912368929 625.932910814208 508.572981581855 852.436775264873
303.555333002419 322.684910198242 763.880957432979 325.387193207414
GCLM 332.032371697186 317.659548748735 396.212732639272 281.341452463344
340.301980622308 490.269418483997 352.665030560239 482.857203798212
436.731545612902 280.494088228226 366.636754272029 305.577166690215
GCM1 161.603317039244 168.295044843056 176.631604815669 177.143397572907
168.156002330441 171.740347129234 176.810979494826 198.795112039249
159.660564323984 172.065593177758 167.421776383056 198.863347487219
GCM2 159.838626443912 144.352987324713 182.197625235191 138.842935439006
161.870389635266 154.854538639035 142.446514275674 140.804008584006
129.495680232567 160.159630699959 139.287871460674 141.842919268688
GCN1L1 1376.84058291266 1358.29475573637 1159.44907165386
1681.54485582868 2293.05058904656 1612.79779117655 1853.20812924694
1612.79779117655 2677.17579532816 1037.97124386242 1663.71411325131
1580.27059464289
GCNT1 134.85590586532 135.023594109361 130.406895174038 146.381147992719
130.219888837397 135.036576196425 144.388851371784 145.442914818456
139.374278310041 157.010145291837 158.658110539162 135.073449743301
GCNT2 149.355389396683 148.567630572897 141.865758685796 143.653452869664
134.830021570666 141.259186690966 139.804518460303 137.984988819967
139.77436722364 134.955823960889 146.264686158155 129.831076624826
GCNT3 113.852849454448 117.937856116492 122.813441348997 111.848472437543
112.962180108363 113.347609066395 118.351607868932 135.079944196457
118.945264994404 128.411138850655 121.110498703017 122.129348411763
GCNT4 127.987449338817 121.286130275542 125.955431094455 146.904354404095
135.395071593072 142.64161581553 142.285486339143 134.475296383752
129.906744036052 139.028620375206 144.038859705973 128.543460982378
GCNT6 119.349376960211 134.425483661043 122.90702157852 136.929570710155
132.232444196227 129.018088749395 156.622993107052 139.121639005366
131.608580511282 134.23001942966 118.226036680442 124.895023786422

GCNT7 120.252164075308 139.097764484641 124.484589975207 117.834484996559
126.702705908175 129.995417218564 123.968639555673 135.306127055224
136.489566741917 132.566074764755 122.828060462887 127.327765407459
Gcom1 228.058870735016 213.274106157675 463.488995330457 226.387465899611
153.738794649373 149.666293193219 171.473538842796 159.997580819443
163.51024840263 151.144521344129 154.2433882385 163.487737425232
GCSH 195.333029746339 187.145134088286 212.749668083641 191.093283411655
303.423450627377 272.854435771465 233.997642139328 220.362946606131
193.689806171113 256.370052241046 185.140211988926 215.802713097166
GDA 123.81177057082 148.302775746627 130.440164900487 135.023594109361
142.883466086309 139.377027955744 129.847481170604 137.483830932387
148.13657736444 129.57551086282 127.990453128978 134.840285218383
GDAP1 143.2438241778 140.304810288752 146.983646662811 144.482364671273
142.822594215168 143.376817049362 155.259089547817 164.458565398812
148.211354898758 151.191822057706 153.655778377717 161.992141305029
GDAP1L1 132.466796864599 125.672285485643 127.941584184869
128.440024330942 134.592495777159 128.077692981235 124.76274408368
126.737230874367 121.419301042072 117.602986362773 140.765231611948
122.659174808512
GDAP2 155.587018319981 167.337624316172 154.564478911791 157.6811998968
208.456472621593 153.492072037266 166.192990554427 153.5256657638
171.644710031098 148.364138636002 232.127996106421 171.035391211596
GDE1 426.896707161414 464.812091712746 342.717252404581 691.397238677552
306.677271064305 491.034395489684 431.019896339435 374.215345412947
300.314088267108 252.056131165397 424.855168311509 498.641091757363
GDEP 154.884821436554 136.540568915609 146.286319248569 139.010652831798
157.073850948746 174.357336296554 164.959415532174 182.736067431579
146.135269553731 172.686798187142 171.740347129234 154.384766720753
GDF1 129.831076624826 129.138035235415 133.155925118245 133.692996980809
140.149064156135 130.818181991958 131.628021008753 133.106005881815
127.04953162892 135.238301951626 140.426489534449 124.15094220181
GDF10 161.841975820372 169.671341667693 154.16370087233 191.679217737051
146.725123303782 137.079479050963 148.82892305156 141.204458186976
142.195764444916 141.937611082276 134.226711030456 152.908358615016
GDF11 172.057586741925 164.548505132213 158.175138008678 161.608941774391
163.104822161892 193.173703015873 155.143752422033 152.185397218918
160.861929142249 152.845126167802 162.756185479013 162.748836971539
GDF15 392.582612044755 589.722914993267 424.025588636796 151.641486303453
4763.89426388268 1399.41653736746 2532.82545752082 2645.72125268628
1228.89116158868 238.230110356671 582.062758687489 832.622843175217
GDF2 111.389873428517 119.366599958278 115.646023142125 116.416896654975
100.966979609222 115.491262364277 110.437728036347 113.347609066395
113.989682635964 108.386545220984 116.273000405449 104.58424134098
GDF3 114.867454554206 115.285392699073 115.896463925125 129.013667813486
113.315182274675 111.848472437543 113.05681175258 122.022611139895
121.099290122973 126.939543057414 130.59670974887 123.312000381122
GDF5 114.539671934155 111.041656398985 119.640086587188 115.725323816619
112.096627288813 113.479382235297 115.600308687778 113.840813583258
116.897299691798 115.613348109569 131.599370671279 123.843823256462
GDF5OS 137.195906276069 140.672863685822 129.835713633923
134.914830257315 132.953762692926 136.578058206829 134.768694020981
139.522018789485 125.493004972076 136.152827218905 147.78095608696
140.21908493941

GDF6 121.930362099901 132.089375920534 125.979152567959 120.447140228396
125.277752749246 121.905741525277 116.342242990651 125.98519427755
126.242935839683 120.39039576721 132.786500984297 108.318510762416
GDF7 111.152235113722 122.27785621724 116.276003190171 123.185652476312
121.45175516842 112.248047602652 120.436408685057 120.660349211375
114.826501559828 121.792573534134 143.793506250131 115.649568330705
GDF9 158.297624312257 140.326012404526 162.55521875551 135.013075032671
141.213588194832 144.380485359951 147.808262170269 158.160604786093
158.095373931016 140.264060906597 148.594322264158 145.830916005206
GDI1 720.077057619735 797.28465621772 487.07860019179 1055.79590411506
647.439372562271 288.631429069336 414.596948083067 333.970844203158
402.564148020948 465.102234374389 503.757797957538 343.417041721907
GDI2 2544.58522979036 2414.22821062457 2642.24374855334 1785.92695250894
3042.88425356552 4343.46317176151 2132.90676194993 3078.76695443407
2244.6916226146 1634.20135156938 2941.69058596077 2227.70654174379
GDNF 126.905235161069 135.281686411685 126.471321085803 133.146347243168
127.419227292862 134.673861365626 132.697727983601 130.258933321051
130.416957431512 132.580937663749 133.289175078053 127.964855970099
GDPD1 591.641325402032 523.435696794756 655.094843900848 464.812091712746
162.58152435515 388.096183735126 234.895245871534 357.006610037663
248.360540656183 242.221324619171 312.286788541158 287.455622143233
GDPD2 156.208747691032 154.493246900289 143.795399778897 146.838547724148
161.061119536632 156.077604146143 158.645402461996 151.04846635538
143.655901618594 159.185616602396 237.171424222756 201.81649023518
GDPD3 168.897620634337 175.476184387338 138.13987378399 145.594378536072
616.995114856023 829.740755449333 216.077955163344 945.403379196643
589.240581642145 349.540558521616 199.275716402358 142.215285988753
GDPD4 176.138621462315 171.022976593689 161.525131363333 171.671895682052
165.763459146818 162.085813676373 163.198834359304 156.357126871243
162.756185479013 160.022481227931 162.629257046871 162.096065738375
GDPD5 360.756455184184 308.679189788554 227.806050911154 263.47760086159
148.490236128772 130.700175060255 133.796029401157 119.759350702482
137.042097702398 129.771073986855 140.675094228846 144.898770838913
GEFT 137.17370871536 169.184111483933 135.423839577038 217.86728342043
125.393848851951 122.35832958054 121.327397317398 121.784166691545
125.114303270024 129.103133449097 131.300342835696 125.104229604098
GEM 449.952497751758 377.89330639683 661.096668635469 305.200911653347
143.973464003036 181.865456674829 144.492756323647 149.31422191209
163.976361576383 203.244917049745 144.358535632993 152.769931978291
GEMIN4 437.049581919144 526.625936680738 580.347496467173
597.459920611726 839.662936279727 606.003956839826 522.664475688419
732.040299742444 539.471166362469 574.779350011402 516.429067667758
529.04238100948
GEMIN5 162.173506492562 170.720018572735 202.357989894268
179.079061789059 175.79375235478 173.507835230243 220.079379312889
173.315097944835 180.430425314885 217.86728342043 174.717158790668
205.804144075374
GEMIN6 289.911635155481 286.431013744652 382.806570442504
313.099028594073 642.934824227327 642.057106241761 626.857289492802
562.891241563711 421.303580698397 431.019896339435 395.588345893987
391.706404954357
GEMIN7 145.660417208595 140.651536723893 137.734887788912
135.817028661429 129.301839663122 149.995272585012 143.720044122451

133.834423222601 140.061045085545 137.50266393001 146.286319248569
142.675752131935
GEMIN8 206.010481981919 201.349209185128 214.331491507489
208.214764518526 214.200081541057 293.482512212153 185.186497037695
233.496804016764 194.831347257735 164.791545858875 226.449705559354
196.889520085412
GEN1 147.619451225526 141.480793979634 136.118702626862 146.235832275574
153.613110881454 147.357032438442 145.610045243277 161.16688895179
146.637799077812 145.039617860588 156.277567608825 150.075738752092
GFAP 119.135701239571 111.389873428517 109.91537150286 113.315182274675
119.795945889846 114.14858855653 122.868393258957 117.045020667394
115.285392699073 120.894583222813 126.964621107029 118.500956349352
GFER 142.566028652989 142.677386111317 117.324368138398 145.060085149395
153.171822092714 141.336866031027 148.512096079648 158.347972566681
130.935922915902 150.763810844645 152.798927898416 137.049677918041
GFI1 161.618479762188 157.288308753975 147.696506705909 141.947478902603
142.688809469455 145.864902319111 137.372131049785 159.470818285679
173.372949622338 159.115391667901 162.536009333787 152.798927898416
GFI1B 160.311096717492 156.423687034312 156.000732564221 170.430478598594
148.012353232918 185.783374935551 155.349728277626 158.175138008678
153.934872807607 145.395042579024 147.539226876636 151.581593330224
GFM1 794.636255515909 895.463355957918 705.987531074737 781.790858580895
747.077451281453 635.37511798786 622.142195892701 755.42757464615
631.106334754678 462.888171502285 517.020259129974 724.103864917969
GFM2 165.737618983611 158.408494909763 163.805257266374 166.533191390639
166.850279817976 198.753297290444 240.994507285383 171.644710031098
184.387409077972 202.819949307954 164.373697447004 207.692077301142
GFOD1 974.544773560873 1157.02365469464 662.958637409597 1248.69137283095
178.43642939806 494.347792360629 203.675655444583 280.913748398876
232.735767435327 573.074116810032 172.20451139581 211.626970008102
GFOD2 193.336246844237 207.985880193245 197.427788552936 209.684346388892
310.598930734244 262.349315496653 297.544216175512 275.972447693982
378.088675670786 306.232249422327 277.596303701405 329.88790758321
GFPT1 272.193578770314 238.183096218311 223.531323159808 219.8511043213
836.235700891687 375.767528676937 440.745866155629 578.297031096292
595.593867616527 426.464641742092 416.933146258428 313.888035511709
GFPT2 260.642170838161 225.850439131864 172.376544221034 175.670738308809
120.263659727964 132.994716261577 116.082361389405 114.512028664352
122.554760526767 151.992112109698 119.937665469629 108.131097605872
GFRA1 146.110545700163 148.794293762142 134.010031592003 134.445893291422
135.934249020651 131.540436531741 130.754320787628 131.255028381669
137.30143217822 136.031744903648 137.114604159523 132.53122878527
GFRA2 305.615423200097 382.960724128105 239.363511706064 505.404271675438
254.287838845201 166.936975782665 153.184575380093 202.691507273819
188.995551740427 152.188770978528 207.674249122047 172.531836863525
GFRA3 219.151144724168 236.707875629511 195.831574717135 319.560819487616
167.961390966538 130.370405378213 128.984613946021 131.702788057359
142.709768829207 126.868970616955 125.326393776938 143.774841336279
GFRA4 133.504078347324 135.75568942028 122.568743478314 137.605244950708
136.971869448818 132.606735652984 130.74794199972 130.551216910933
122.799487123057 126.214166842097 147.78847662997 127.797666392002
GFRAL 131.18368961955 132.657106732366 130.590195477461 129.446084290637
140.013231300392 128.0366252162 130.829825154842 123.219903763265
131.968781256081 118.95971064289 133.198832503062 129.940297696849

GGA1 5543.71998558526 5040.35379681644 6601.004284244 6944.90824302953
3200.61067185412 6203.08085790474 5532.93454600454 5207.37974585707
5846.17594234628 4901.41175228982 2733.68308784024 5532.93454600454
GGA2 445.348044448953 476.168679880357 346.862322263052 442.766704003254
258.16437138327 278.555009982029 489.403061697031 397.985856484851
335.938170230765 259.742175498038 553.123179808258 326.703488388966
GGA3 218.811490883852 207.545424360094 208.270622752385 207.05325577114
200.242239130026 193.609985124825 216.783051958345 225.664432111551
219.228720772236 211.505504834207 233.335353447342 276.434192872962
GGCT 323.086368767924 412.277592266475 408.811415529922 335.239364345057
1071.83063260499 595.270107963842 604.687265229902 896.625415670455
832.911633923496 1172.96121022579 690.010326122574 1032.4540300024
GGCX 181.238505183667 186.321879879561 166.868688936939 148.454344601562
405.322847383739 199.235895708128 317.273940108333 296.553550946307
300.247304779062 236.470206707351 238.344676327918 219.171591031864
GGH 185.447093292348 202.145180994821 175.974598536074 192.580288063603
665.936368667321 1357.48791782399 422.688531130043 1366.06309834426
796.439819579938 635.37511798786 251.138401278632 325.525450750788
GGN 116.300028266671 118.622346984257 120.853961156534 110.347631909888
114.350596695469 118.338713846941 114.830474086443 115.963144087717
120.696557306298 119.83679981791 107.714294216913 116.408126010926
GGNBP1 130.01207836961 139.168307371516 140.868946922293
152.921328522953 147.056182807232 136.607783800741 130.114464233633
132.819726980184 149.556644928013 155.798048652444 132.582062079623
152.413389525982
GGNBP2 782.448692528135 734.97763567765 821.265710686098
657.562129888446 454.984562256248 805.600599570258 884.747476952289
1257.32413345748 1528.49457104932 737.193295872614 946.640841062802
843.545962045705
GGPS1 422.688531130043 403.043257175264 432.515970951769 369.070376276471
242.188218870393 291.839070398338 357.431640917696 499.544050752036
377.083379021079 435.095843349921 434.855262284265 495.438672949441
GGT1 141.950778601653 138.618761382177 140.42579083092 136.026302491961
142.656762407774 144.386561289985 138.59175751301 223.339132121796
143.626857507866 137.473779155388 135.346501914337 140.096717188376
GGT2 141.162443312437 145.241301006518 147.017958916908 137.701271351806
135.883119645931 138.409284761652 136.522590104535 179.996590340102
139.136845089275 138.938546468136 147.488635834397 142.630563791102
GGT3 148.683761948082 151.382199195953 151.88244935494 158.6953558198
162.961792330623 158.691830524781 138.395943168158 178.606255846041
147.07272598888 140.611071681667 132.576755070703 127.346646117375
GGT3P 150.784262878145 136.981043383326 150.639235242268 133.106005881815
137.185243517766 140.149064156135 150.981208776163 177.60876237572
155.283003653612 136.171728950893 173.09379121032 135.925813154268
GGT5 173.353648927895 149.41564356447 119.943408501009 147.605262422483
128.196474114536 121.968807253639 121.278346729876 126.678746008577
127.945144368504 122.226875707253 131.381189141974 123.178049594968
GGT6 130.33205883588 160.441879289883 149.233023751582 110.594755513247
568.935113269368 384.296190598736 334.140757209353 507.236601481067
344.575967665039 150.584633044901 711.166793546075 551.92688162618
GGT7 147.038700869156 142.179197020805 129.148634038268 134.367115858307
113.536570797896 128.890685255595 136.093473732174 127.052618800004
138.386391726042 122.472909598385 137.493436771898 148.370331097467

GGT8P 121.059923601223 135.971507823633 117.917454674671 120.308260557986
127.07186140748 125.208803405105 117.137027732123 123.198873421356
122.423561732526 124.012439490264 136.209796259197 128.827129738041
GGTA1 158.135321157995 143.035625780446 132.624674205566 147.928629511539
128.257292171015 145.06736850005 154.834679562341 126.365861978893
141.401480567426 138.537763477627 145.836613334985 143.455941298415
GGTL3 166.526875572556 163.248980088946 158.960488862727 159.592581700976
141.294094833197 169.447827110558 172.599654598668 152.30599498619
152.327247552986 145.07503221134 132.383542877819 182.827395663312
GGTLC1 135.553183871663 147.643985046683 133.801549026691
135.774569629738 130.980199575227 135.453399741892 131.503936441405
130.321133400634 134.678445085174 138.096262211809 152.768580075051
132.741859397511
GGTLC2 133.022848457361 123.865424155099 122.379537202709
128.922269289649 124.627339426244 114.161540771427 112.624137732184
119.530214079037 122.136861827049 127.540848073484 128.386309075038
113.521599852465
GH1 128.76720970745 131.592172917738 122.274656361883 128.126354569418
132.456945758174 122.373400988227 125.367386907897 118.875640081461
131.12953085854 126.825785762925 128.671626815639 135.281686411685
GH2 143.512348693556 135.618562468776 137.181558419086 150.731093808891
138.936872956065 135.975265059269 141.248526020456 148.199249855894
134.10707191601 141.770977101211 140.123348569709 144.035936160694
GHDC 294.22752955801 331.937313503755 295.935625475189 340.487850009685
526.970555181851 221.093229309411 277.459328083162 299.80615152845
266.347965690851 258.964323941464 325.96632528242 223.918931440768
GHITM 3592.7878705048 3504.77617298452 4536.46344538492 3342.8737952486
2316.49886419499 2325.02143920169 2670.9151276786 2794.89048827025
2808.78353621111 2811.84191700516 2712.07480130502 3417.22292085668
GHR 628.939201051801 517.529147171876 441.958777965408 487.07860019179
107.498500136859 142.238908711402 104.826135297161 168.582192754233
113.170014668758 148.503282415207 125.609542142983 110.753661013519
GHRH 120.514672572565 118.540620928268 116.723991721647 115.778736387131
115.896463925125 109.728809191027 116.221426977629 109.30275288389
117.856184272322 111.65024009102 120.345546077894 129.023206862713
GHRHR 135.966929064294 138.519129384809 131.648308760913 132.928639483854
128.074531806621 135.210887148305 140.132816040735 141.143572141958
134.182902752156 131.823019555685 131.208112906919 128.486023696636
GHRL 141.537578081123 118.45291712775 126.295856316628 122.667593730152
123.609527892227 116.20008794813 115.664551127497 122.232732968363
129.733935614802 110.014935095391 147.463822782303 124.977046954694
GHRLOS 128.510903178542 126.952836653857 127.605805815475
124.288632450364 149.904691068054 121.980744551615 122.258432948151
151.325068155715 132.659196898439 141.499192356469 151.089328533476
121.952255635669
GHSR 124.480385254916 129.153882779488 130.957441406855 129.365004129784
127.245190474707 128.634431242405 127.490889148422 128.458132030921
131.49254872524 127.412539650291 129.70974867852 124.539006083936
GIF 120.543881800775 129.35944300615 123.782746527199 119.13963074637
111.608386387842 124.077104101209 116.751145098777 124.546520456419
787.874585290601 131.051711775229 132.7284396337 130.48231275063
GIGYF1 197.91325268683 201.954167006591 203.563098303937
203.370353613423 194.686527131656 178.584378959369 202.643624039869

163.858222964048 179.015669199358 173.635827398273 175.96841318924 192.580288063603
GIGYF2 149.927773276383 146.539196738195 143.394704760983 147.44463861207 147.863198012238 138.02757574324 138.150616787218 138.925802905636 146.04584032214 159.52833930625 143.405079593778 137.504147221623
GIMAP1 156.88782735706 199.798533722581 125.134516902118 236.508498739509 121.049927973123 121.567879358508 121.926377140115 153.111226896983 186.758038665014 110.575582352222 168.21856603538 123.513588004371
GIMAP2 169.285901212492 167.505381046729 155.064761390765 170.13453211897 152.317890573534 161.090443294391 189.61702125418 184.672330560677 227.926444925478 149.387467833684 191.160340745501 180.902950045351
GIMAP4 435.69811966429 675.978379566859 357.972320648217 595.882696498804 228.542423012958 413.398641688413 256.471672362567 474.750156829195 727.814681346494 263.707476735938 338.129250886594 263.568160216149
GIMAP5 202.819949307954 226.640323471375 148.211354898758 245.01029221002 186.156006020985 190.716424123036 150.364511432468 204.113356572343 308.726328440723 141.397046998678 228.926077117474 144.278830378899
GIMAP6 150.831554010122 159.966729941991 142.024171830924 141.882859092658 147.171090285223 158.704425428694 130.753266976637 166.850279817976 198.94325874493 125.04327844782 173.513108409372 135.013075032671
GIMAP7 317.600728351694 390.284689962661 250.737252834098 373.096629378654 141.971207380159 235.961605948276 163.398614249033 280.531844086775 402.824964079515 146.897528834303 239.109163320417 167.173670781451
GIMAP8 311.670576741448 518.281245498542 275.298642145005 520.218545108099 205.036081947168 213.123453925578 223.496400038284 251.09230266334 355.609033348692 188.74403596953 234.895245871534 187.472028627511
GIN1 235.216589534371 285.63245179313 254.359737178714 423.256021602613 140.78902818186 157.733042033549 182.725987056391 148.400132332444 156.803516898875 160.724319010751 220.791884858764 146.658048787833
GINS1 139.242481781927 135.336754997434 165.590892262736 187.561298131124 152.593934787262 166.22013573342 136.118702626862 158.869463014801 144.782706468281 152.601626526117 158.147502554814 130.776213987522
GINS2 130.729989463558 174.680918805036 134.932416370825 164.164463389212 623.68308750251 418.319283832879 300.047728281275 260.045018348182 359.518199449791 446.393631025049 199.135932984641 450.566031840493
GINS3 149.987571884748 153.528429414138 144.507230362869 163.18585933431 286.431013744652 283.084102918359 240.12078342024 262.171775165261 237.456245064363 232.830038701628 273.709160508205 256.127197303751
GINS4 129.675420096163 149.42148494796 115.664551127497 129.095469955392 208.72873686638 209.93789387724 141.169908353507 194.055394488452 149.88598449818 137.03111066852 152.207601283271 136.071856104688
GIP 119.144128641393 136.716554749722 115.157321350379 111.020070405368 119.330439721904 115.502679218125 122.170662903259 117.303671445328 130.445920648705 115.128667174745 136.70715656782 121.770043509503

GIPC1	601.800326895295	829.740755449333	601.401329244185	987.78377957731
	3515.36256599555	1424.04009914668	1223.68146968716	987.78377957731
	776.807656744828	1186.96351738518	1420.27222765071	1371.56635975955
GIPC2	137.714430743204	132.05513812578	124.213104788426	118.855500322915
	139.640918572569	131.369177333568	136.074837228096	136.975775196224
	137.296790373528	129.995417218564	119.023688551496	138.452698593232
GIPC3	126.176505541577	128.683044953833	124.348926304504	117.688941284427
	124.012439490264	121.543207264598	115.118170902045	108.984922760455
	119.415822905251	112.264005988662	110.562310620515	116.268259618103
GIPR	106.701716475947	110.727742629085	114.226028611171	106.667175621971
	117.412956162665	112.282839763467	112.858669660245	107.02909139647
	111.895279866421	110.355493122568	128.485150698799	108.589444216106
GIT1	232.046814892685	258.109876262342	175.882911801581	295.778154263981
	827.400420888645	222.624150073858	231.572677616684	275.996693627151
	208.016730103629	174.903798946103	239.20206002	216.154203111101
GIT2	176.256840061919	154.710000519109	176.27969404442	143.830319259804
	144.937697857147	175.744275464701	160.042251279235	152.191393042132
	187.296479395462	200.72147365413	159.320692108734	162.10411532202
GIYD1	171.85890372225	180.528296177577	153.846723846716	184.102171963542
	363.150472154973	466.58966852405	309.937351755502	289.21739941764
	272.351487980415	213.674150322581	344.021696764791	217.748916080172
GIYD2	123.827771590213	125.114303270024	124.511674561183	118.777105073561
	178.955938825556	206.042395282286	148.514806562804	176.890596690798
	163.438043894189	153.206468539619	165.590892262736	148.525224975266
GJA1	717.270096456387	668.853969513382	583.192225756596	388.477382640003
	331.747622606378	1235.75946952752	865.800709006332	1610.38263368771
	528.275591281419	2886.92690692502	180.192015483309	237.266455875982
GJA10	127.028640787946	151.092066533546	144.994931561779	129.485710719291
	140.477307701027	164.753886197136	152.346098251128	143.943676099522
	153.412469512967	144.610341215903	132.484079454476	148.100508271232
GJA3	126.877293296185	118.631771242606	123.959050197189	126.314233529267
	123.260800590266	126.103561089901	129.436712663487	121.61143371132
	121.336932276232	150.642465826166	133.44592405762	127.551486042435
GJA4	227.177100762395	351.998551165704	173.829219279719	255.156682699288
	176.839917795004	195.963361515546	145.505223177269	173.97085982677
	196.974807677659	172.794570587596	175.974598536074	156.702300398105
GJA5	162.774716879847	174.08222895937	165.960176666275	178.072538529634
	141.849319373594	161.874184077257	164.50490221903	174.347451984066
	169.238325933605	159.640027194076	160.277755404001	165.942761337382
GJA8	116.428373788459	121.476730561812	123.635470154191	119.517021355598
	127.773292289242	130.018928906449	118.206034087145	114.226028611171
	117.034228068173	126.839037497031	145.902572804249	113.451566510208
GJA9	116.053859681399	129.257947818865	124.186557690235	114.638254225216
	116.501174707117	109.898406285895	113.283359026655	105.236768436069
	117.907840952685	119.780654293448	135.116985816353	110.977904932371
GJB1	121.327397317398	126.806537241253	125.195549796301	122.216479103528
	134.412129843957	131.140263788783	111.895279866421	147.598602877625
	147.803464591663	120.964862799634	157.178048352017	126.715300671036
GJB2	149.505323932806	194.22713184875	146.011084331925	177.428161690048
	538.872348151809	904.211195986553	691.632061537999	386.305638633172
	271.154246147262	10506.1271023809	1250.58586679251	3630.80752744347
GJB3	130.749763860615	148.060086496148	149.801573181126	135.884635448277
	206.999289979742	176.008711524598	146.743260100643	138.052032298683
	155.803946849495	373.860920943049	191.3765085801	169.541411677993

GJB4	146.132506246152	181.491120129045	156.330571337475	137.996291911198
	199.235895708128	249.925640256322	143.589222264786	153.075594260208
	159.856190281237	578.084277076692	276.224613560582	439.727046291381
GJB5	133.486155360266	124.451718955086	113.311132067152	119.815258991448
	197.087015414039	174.603285240228	184.157941055948	138.880168623766
	169.574755162815	464.196512015649	208.164674386141	296.141012087256
GJB6	152.566490804809	188.079188251085	156.576585764311	158.249743182741
	801.52891824571	1044.52286986369	509.988617641229	405.570281788901
	319.417555901733	4569.44477073589	1427.70292953827	1563.71477584676
GJB7	127.101752724954	132.809364188497	151.05681004513	154.8975607055
	138.67221078031	146.418147447541	198.411812737082	135.100687929821
	161.748547772524	129.135985676046	200.668298229815	154.821960325589
GJC1	15194.4972103372	14798.1358474998	16588.0599171804	15603.3490258675
	5138.28186306948	15924.2167735021	9323.53099359448	13423.8921651885
	12493.2940668467	7930.82679738435	8633.71708020651	10741.3064142855
GJC2	526.182262872235	544.222458349696	518.281245498542	757.877066703516
	156.973511952828	159.207567680134	125.579970155301	142.505050582588
	158.712241098927	196.676479140094	167.575968906119	169.520544329439
GJC3	132.637443692683	147.494937826597	148.302775746627	147.863198012238
	140.025668085978	129.130212375815	145.168031449425	128.543460982378
	142.185624089373	131.221105247771	146.920496801962	144.351763882823
GJD2	127.344210958898	126.763282018991	115.097376525707	121.53632327554
	117.494422663086	116.615250922316	117.316790136526	124.186557690235
	122.80785489118	116.87586240208	142.863268359757	134.97514790642
GJD3	140.890227770353	141.448017858785	121.031695489398	127.842177770276
	213.652091843896	373.290426793366	383.939800422489	134.222453538421
	192.954269340616	127.019978195283	659.106229917309	168.37850726499
GJD4	115.128667174745	115.605206174867	115.848659135231	114.774530966446
	127.982696242304	119.207503310618	119.67745324922	126.730314982553
	122.830376253922	115.975794003391	119.551741097918	112.282839763467
GK	139.878752507646	148.067562465748	144.175551691291	130.863753083296
	227.715558644326	282.797367565702	246.428746807313	374.169744342352
	349.248144552634	222.848935978648	198.885004378065	217.940575516933
GK2	126.233395051706	117.238968848218	121.197702744078	132.642931022024
	123.344450694117	118.866073031304	125.381865040574	143.340685579645
	119.9414421871	129.940297696849	123.275246170603	117.718893827092
GK5	230.91747428831	205.011514211101	294.323621186916	186.06425658259
	167.421776383056	280.948704707655	236.933960698783	285.507315916663
	184.041062308263	206.446584387595	211.786963444117	280.398987330256
GKAP1	265.028512407839	234.895245871534	348.067061666237	247.145700155364
	163.36682525399	142.390243984362	157.565633456909	152.017655364287
	158.554429311951	142.211357324461	155.257184514112	156.18389905051
GKN1	143.956726538569	151.276193269421	185.447093292348	152.207601283271
	139.733149319736	142.34956863109	137.910618360623	198.885004378065
	158.937341335707	136.378203924435	5437.78386044638	145.152605166094
GKN2	120.935863853101	122.672142981381	116.831725094917	119.759350702482
	121.723964989551	117.386273592501	128.477342430713	116.7299185256
	118.406487008528	119.583378101838	201.837695818264	127.562924931536
GLA	358.217145707718	393.500781010617	325.999687273862	322.506898833351
	1549.30900090605	2154.00589005104	542.121375354644	635.536499281829
	991.611982265221	424.201556056759	769.586135629964	565.843200661721
GLB1	393.98210915852	390.670702155192	344.45331938267	350.58427958432
	1409.42016970226	604.237898104299	976.050097371579	713.23381913235
	657.058213650454	568.310800941091	585.371892598422	629.197022551069

GLB1L 170.350891928203 161.769370009823 172.355215322173 141.082569618216
151.397068235685 126.964621107029 134.076865693417 137.634298014431
159.23414970379 133.559082256772 159.665989587555 145.768857553634
GLB1L2 156.806407903626 182.984076352185 124.959873714884
226.086733606951 367.289651112346 155.837866295709 209.33028918341
127.492376657284 144.498245098592 110.475453268997 152.925281215593
149.593135821045
GLB1L3 126.037968297611 128.174382934295 143.314754993075
129.061316880624 153.426409060413 116.136849683574 135.281686411685
129.964666668421 123.190757763412 133.771691580031 120.089228795888
137.804977813144
GLCCI1 122.010855318111 117.270320709161 120.857635836068
112.3786222545 200.302384864371 142.91818218442 168.116928722035
170.212679845194 123.418266047461 133.94206203396 154.368499839507
131.759422682853
GLCE 422.338508598153 432.174413144791 331.795832780176 335.389274425025
344.45331938267 288.150694843614 638.129080224049 275.013838208239
377.148528768679 427.249733213787 213.028568789661 316.895545640437
GLDC 133.331037738063 124.00806841872 119.733311319912 124.546520456419
513.036729214095 774.442392607873 851.633522004342 143.577865527891
120.039031290299 115.30786313391 153.39738748803 107.655624022312
GLDN 179.704370382771 188.671807443641 136.355319997318 167.46432961654
124.558026853954 127.604087759266 130.763957515652 166.099940078284
122.382304966782 174.383193885933 186.244764337256 142.403121638265
GLE1 541.351092873522 668.127527385348 624.825216056495 767.377380800066
477.489368828457 480.101253056873 353.774297577869 585.933822585718
391.569187190375 639.38881853171 707.715412321304 310.533399024851
GLG1 3184.98944679038 3111.50508060882 2786.35558271661 3501.86788138647
1995.6000523628 1065.36715400306 1825.10168716051 1397.84878368705
1398.60850048351 2258.30916111153 1694.24232555141 1963.23068846837
GLI1 207.764456828813 188.580458613357 169.713585775118 155.23922239449
171.826623289946 167.594414386475 155.728609337723 151.833386901811
133.913578037255 154.768422926874 137.912656945408 147.697973457776
GLI2 200.342786450248 171.338371572269 137.850437814788 151.362686497226
120.530092530622 110.230322274208 117.580122770251 111.336798977475
116.378552206652 110.804248212067 112.977469350139 113.927168436775
GLI3 229.230239609291 237.989479738133 196.734037460218 234.925852373127
182.950456188966 182.42088387266 184.638141004171 171.254015232189
164.696616863082 189.292740365891 175.711836112986 182.950456188966
GLI4 158.608479354755 159.320692108734 154.400124981573 198.15257384667
168.483273292464 133.886458291672 128.118223792086 182.698275130489
164.553511697702 131.750053934548 168.6016897585 135.497033143331
GLIPR1 238.782040902727 205.679992893049 214.763232658841
165.277935422113 136.859366444351 173.714940254592 165.985885671398
170.326474367867 204.990512488181 256.237185590901 146.885404321622
158.809865241567
GLIPR1L1 112.38543517651 129.386300987308 117.455432751161
120.606354515072 120.721341479615 117.847744476387 132.359319280352
123.668707648172 114.470670941078 113.726931741236 144.446903878863
122.281263517691
GLIPR1L2 131.464229237662 145.754504160436 138.136901441896
139.631165400881 129.985797334276 158.448557470725 132.50676380992
137.872742675806 148.530422089937 153.879427186342 134.52098220054
136.109975252514

GLIPR2 3798.89743803968 3734.705550862 2550.08769415478
5543.71998558526 206.521761031181 236.108432603479 217.050602253281
322.956882599715 608.959089074987 441.222346452864 230.672056810885
186.57665470145
GLIS1 124.913645688325 137.079479050963 122.620634570073 146.476960694197
137.528342105233 143.122278808091 132.906201428703 128.580625868379
145.267225993057 157.874624979775 117.990622156287 124.484589975207
GLIS2 141.378381097266 136.359189516452 146.501559368223 148.742216703684
148.076841184769 156.731291756066 142.995274641765 141.508424382665
145.559419404453 141.706292773073 133.286702819953 144.944095553004
GLIS3 162.634670715627 167.003917876371 150.109192774079 158.59135215074
139.652974659584 141.67550758321 145.827592345054 164.406564594296
160.419358656559 148.130993521745 143.399877326927 144.692900548503
GLMN 247.280717385562 205.930921729192 237.575788579215 243.283375388645
311.10841853984 357.773949920377 248.16594076495 343.488356185437
233.842749683689 243.425632941131 280.749681050778 269.300548648755
GLO1 734.71562355687 773.057095787974 1199.35864276583 642.484527246259
1332.11815966955 1414.95226752155 2296.30423509176 1356.43255022363
1412.56164938199 2096.25803773108 697.581541438891 1326.95332435394
GLOD4 867.369315017411 925.009157785129 882.429823267754 956.76879975749
611.208083466988 920.083835296898 690.246312542011 856.424155671931
675.281131488524 814.110601393539 1048.85768964657 1095.52349352502
GLOD5 120.050548624975 126.939543057414 124.13945833494 119.052729327147
106.854721604889 123.561016079801 119.873844587252 119.278231442871
126.132339293132 127.401551376191 145.010108826238 120.479270037091
GLP1R 112.896360244636 119.360478719424 112.916256784817 115.51493368527
116.061356450855 112.046615825307 122.780453116176 111.549719888905
116.074423050416 112.904318310632 124.752198904184 114.217867810848
GLP2R 144.743281367797 143.837402372008 141.750316403249 123.615802924067
146.499065641095 139.628966306048 142.96594961303 135.527445907621
139.498452959831 140.051904905695 137.350851760173 134.58799530734
GLRA1 126.00304757051 137.089392202166 127.490889148422 126.643229085068
137.347554578171 124.650711299942 123.809866652834 128.669887479835
119.567911704298 124.958287100852 120.196595293401 119.882472552083
GLRA2 133.239703270008 133.449527627723 139.574895021783 136.251820364823
142.290253129837 131.782007636511 130.590195477461 118.773864607553
132.850601603567 142.717436384151 118.544870925791 136.470025638057
GLRA3 138.047487233711 140.260053206523 134.947657801444 132.941478943584
136.229159638766 148.859129298141 145.739537463907 146.355229778997
143.421288665418 139.032275257901 137.578516107502 143.772376623996
GLRA4 137.298549014192 117.766156390814 133.88072491369 127.289269745461
120.649467083125 115.775525016883 123.7421178083 122.631814400575
116.118745494794 128.071379991958 117.923459503989 117.060696904303
GLRB 140.552143090027 136.077039612526 154.170007083026 138.088712695622
125.828998007608 128.075865243385 120.394220715157 110.081964209973
174.568253693395 127.822440239962 121.377666938052 117.361778263056
GLRX 475.927798702033 408.361368902627 401.87674893993 362.376153348015
279.413335686746 555.959424692051 494.604716660887 476.383294794912
787.017214032692 400.550044186372 1454.94889750152 553.615252472149
GLRX2 219.753039699136 224.67853219142 208.932692585748 209.487240895674
238.068308163643 209.004374703564 270.33685210971 275.755038921952
209.54573978355 303.907003535357 214.434355263652 277.219796733134

GLRX3 297.708113541379 302.462763120177 435.901794650816 246.989526028884
418.319283832879 888.706978062337 818.445501500731 711.491528818004
682.315322994357 884.747476952289 546.274050902567 1034.2217095753
GLRX5 3441.9805896226 3055.80298721424 3938.94050658589 3338.13980198211
5080.63091497522 2116.39371296122 3287.37172778474 3518.84173951107
2877.92845389611 3055.80298721424 2748.51360874099 3655.63669142687
GLS 682.538709448692 491.034395489684 710.999354960979 541.896444696137
194.617252921731 416.392874553797 219.246271924313 438.958573676953
251.224369831275 346.476121089685 227.668046559074 514.922742487521
GLS2 146.326067008478 147.028946382445 127.650229552084 141.488910091973
160.865949438448 170.040914594182 173.726420791056 171.795878750365
143.915205639368 145.000523037022 147.679233228201 158.799917136119
GLT1D1 139.997245010492 123.887740029279 100.216750525986
115.718600552187 118.457573670556 117.494422663086 115.473431881597
124.712789674509 107.841100741858 116.136849683574 138.783017264895
120.39039576721
GLT25D1 1097.18236659974 1192.47480377375 947.766127685083
1816.3719976564 1646.76016031831 954.488185198006 863.086199215353
1106.21439150501 644.301665020526 1138.44837250274 716.229989890801
543.170902795966
GLT25D2 153.104072845043 157.231166272318 159.668402591441
162.812665683603 142.736148814019 124.095340478326 159.14477373952
165.219393390909 130.721987260546 133.82548013342 127.631770516132
139.871928127715
GLT6D1 157.58224826673 141.315230997077 139.717662557602
150.998881139061 156.490654498766 143.123983359386 188.641397688261
145.143366567189 147.199961777192 142.937182720493 142.14654303591
156.551925771312
GLT8D1 233.192751618176 229.26243866226 213.447886742681
199.756584044664 288.505502880424 286.085833802132 432.969886744613
270.727749014046 263.448958595597 237.045634416039 338.665115108128
262.019779752857
GLT8D2 500.848273432862 384.296190598736 394.101052511043
337.822337721287 162.994537815938 227.558441474918 173.38031514498
222.086635504858 238.88138498771 361.390981655795 160.949030247406
197.034073391478
GLTP 1571.87128697931 2033.67956299225 1792.35343044443 1608.40826380713
3090.52810501797 5347.78127066964 4118.58682247781 3664.38955037715
7446.59106452583 6044.78942014135 4743.19967685595 4030.42725798136
GLTPD1 147.032210333966 179.61374889626 155.592219571564
185.100455193182 355.14024238296 189.37832342197 199.037998955485
181.247766545135 193.134414137433 180.501409705275 197.034073391478
180.80811429888
GLTPD2 143.363002903815 138.255457081996 136.368258257435
134.703474191011 145.581755438161 132.128418409996 147.441650320053
147.526701500887 150.494260282384 136.633726784926 125.75980494927
138.402890124039
GLTSCR1 227.847614397311 238.310077556167 292.28857006885
323.628636799403 739.52947559669 215.506175859782 341.43511159996
264.983729290122 305.325519265387 242.299252896647 240.734451149197
261.117845544884
GLTSCR2 11598.65499235 13013.8166277495 11360.2921551609
12654.3779830648 10222.4435857673 3693.4058156307 9636.32818474581

6427.33783376005 7555.47017983216 4711.21389812942 10953.6229702446
8096.28093399619
GLUD1 2508.50428529255 2433.83421295012 2462.87034563426 2441.05534211996
967.123240442957 1667.86825735893 1386.56788941677 934.546444634669
2648.41012401723 1142.44180956604 2141.855356411 1554.16120123599
GLUD2 152.379441401583 126.282575548237 142.305484770249 127.874934513708
125.055545087871 134.494167613686 146.981521393075 143.909501365162
132.353385654164 128.197031700084 159.990561741675 136.641140715439
GLUL 146.014790698087 155.933739693912 151.613134875009 148.197029306137
140.730085305185 153.679479224178 150.775805918537 149.577995793381
150.155154998981 142.027930009598 148.757112903673 152.54039461337
GLYAT 125.470172259885 132.28010415054 130.548611758921 123.929180327423
124.246363878094 121.93549478261 125.318166919844 126.832182961671
134.01566484034 121.146511496024 136.461182044855 120.345546077894
GLYATL1 147.75625228531 142.573662441786 159.491388998564
160.050872656883 151.879398444495 152.643087608513 158.658110539162
144.288714958892 139.001493765421 157.675974692761 149.233023751582
138.719480482945
GLYATL2 121.53632327554 130.06441029462 107.32960937124
116.263405190786 108.530158480237 104.36042819601 107.40922161073
107.239841465534 118.572988141773 100.325215581101 113.769815606191
121.201267751612
GlyBP 155.680330654611 153.457715729768 173.577723566555 166.563411635721
169.852769679312 158.943005736645 154.569432344392 163.331424612891
163.575300618666 154.65319127051 149.833346575446 155.798048652444
GLYCAM1 135.013075032671 134.191460941478 139.530196462344
143.320787131532 131.395959781477 139.42531690926 139.260868989409
137.509100088226 132.74579603362 135.159590925066 133.874468336025
142.652939807341
GLYCTK 157.826457203706 164.769320792025 147.04481509758
155.035727630756 303.300028849873 160.105903942674 186.611870261871
163.764600217347 205.987280015028 151.216992571364 177.990274115348
189.461090469712
gm127 174.375388013694 156.176537862398 184.348894461271 156.626742111441
147.658462231804 164.347168837655 167.003917876371 157.805871400914
168.261511797218 168.511517285223 144.954595802474 168.33854702953
GM2A 262.093405224922 257.5097052677 343.767393640683 222.134519361117
210.085329463347 243.308625792448 314.014654357569 297.419034136005
288.68376542946 1171.62361359992 243.839402991061 226.144012966486
GMCL1 210.529782898493 233.536023295039 250.443147156029 191.310372695208
432.515970951769 514.922742487521 375.131728190867 343.617417488962
345.135289739599 357.259935567823 278.168897504985 318.561557474749
GMCL1L 129.093864368842 121.176150171592 127.543729713161
128.539969915702 127.401551376191 131.530256606799 125.500790447338
121.926377140115 120.948246352846 130.325073627151 134.132312782731
130.746465595759
GMDS 317.949646885539 376.374248850987 291.221427744817 402.694564608582
1097.18236659974 553.90008440429 889.054356462993 643.13571432061
699.800360892516 803.60413590137 789.752781067888 1106.21439150501
GMEB1 190.993590849624 191.924177620803 175.543924200429 165.118167086792
221.719848707318 225.050796356289 161.80922869659 195.577739376082
188.767121505943 212.073472189229 210.33390115739 202.041958613989

GMEB2	450.99430272315	571.273512452931	446.509863498357	722.974310297586
	970.166036472486	603.061485415867	653.879734066271	469.794672718173
	569.47446215213	367.898258897888	470.578335285993	434.176007498693
GMFB	1165.05055932899	1031.38207659547	832.014011481807	878.167149313285
	904.211195986553	1692.01248523526	666.344430142655	922.009043194023
	765.532997390972	1136.76110352875	1318.3633082502	851.221199352646
GMFG	325.642275889649	375.407747753058	273.668871896558	307.866499851733
	206.376365685112	370.678040372273	412.736636437651	479.053300726301
	589.953707188378	309.997909440664	837.023434916281	1629.32740709526
GMIP	147.327675057481	142.80027343095	108.570463496052	119.627380812035
	569.967953318205	235.453139389486	217.264041781337	295.778154263981
	200.831877950718	135.223957492606	209.984545596312	209.487240895674
GML	119.586157804692	114.644722005728	110.081964209973	108.62191665589
	104.03442843396	111.935554828717	110.081964209973	112.57049236673
	116.416896654975	110.266032635568	123.389038274913	108.461141538047
GMNN	160.322541355391	177.793860516896	165.686655824416	130.483765953389
	287.967349876429	836.613819700658	424.285848583977	777.410297605252
	602.703887178113	451.496617003797	478.699172220804	456.126096817454
GMPPA	279.541294184609	286.856346704286	250.18189549573	438.7446527797
	441.578261054602	197.277310409328	252.10470581893	252.197271817794
	304.985269874607	302.905840432998	238.823244541016	229.046153362151
GMPPB	178.512206531256	173.810080280732	184.398672994943	187.50236021149
	296.657844888195	195.906137340918	227.965783945702	274.047586063773
	220.258804437828	235.091443641056	191.220064414876	280.085692727562
GMPR	197.582402276079	203.983168778488	220.533113603343	229.97640195632
	169.124088593549	141.075421936212	171.916201289104	158.495606141226
	159.110614190088	164.231281729415	157.86653024793	173.708509414674
GMPR2	426.464641742092	426.548994267836	501.863855158062	422.338508598153
	323.276514394119	318.930960243303	618.829716818725	510.972389503051
	402.140437036706	547.042461070351	657.058213650454	707.048754700047
GMPS	300.047728281275	284.798325549665	407.711669482083	240.878804911613
	543.584413650467	554.115756730498	572.753294955207	925.205399154409
	737.650963292668	632.278924237678	439.616817413027	574.896304593109
GNA11	1065.74382494535	1241.59946353857	997.460212152591	1731.62376348283
	980.67040349547	561.761160223609	441.785666261936	436.982960786839
	398.272713014637	384.641319423853	396.40172043456	412.048097766066
GNA12	328.269845211173	343.666488527549	310.640107228956	451.496617003797
	209.54573978355	175.7330682766	172.376544221034	194.584034984339
	164.491207767654	170.775283924087	170.108500366942	204.396576475753
GNA13	487.597040819879	538.484170364629	592.957842630046	382.701467158304
	378.967410725775	671.134356672693	710.228463443265	710.003412772888
	582.527835746927	395.386242257877	565.97975389646	561.858563298512
GNA14	197.087015414039	182.118544602205	237.358925391616	197.112749959833
	186.215602572358	165.586397236489	165.71382076724	218.334273320319
	162.192879711747	161.632610858756	173.38031514498	158.072347847051
GNA15	179.896659891106	245.042781391047	159.820341130737	156.859539612504
	1174.0922389136	845.646455991008	908.704610877319	665.106631364961
	694.644744547412	2374.1382412432	1325.77498031227	1064.85152398919
GNAI1	304.013547190343	271.713695056313	304.479999301061	227.965783945702
	178.222120802125	187.046760972666	207.674249122047	165.364935913232
	143.956726538569	262.671819506189	211.160781061336	197.169703621888
GNAI2	1782.2498613913	2021.92898855142	1690.96371604294	2332.98074920207
	4030.42725798136	1137.42879398351	872.320823993033	832.911633923496
	1274.39152410752	1301.18893681695	2026.85580212422	1435.4236709581

GNAI3 307.518881119091 257.775569674531 240.182016388745 261.89195025254
292.366934378643 265.866465528654 315.182134426778 395.927222009721
260.85776723575 546.841563406813 259.229153662504 305.906480933354
GNAL 167.373416675264 164.673579538023 156.269957530519 170.410704499804
149.995272585012 146.484364264513 143.144153021901 145.026722065573
146.867740388881 154.329176514501 149.180704382192 135.120878931851
GNAO1 209.487240895674 239.541516080716 182.275998840116 359.099750692694
136.48748813417 130.947003916049 136.90636489391 130.532559074698
139.336891718408 134.038213827843 118.727279917776 136.749771286979
GNAQ 203.357914242777 189.01309885917 388.782774926005 162.188509438986
200.741234443918 192.353768499582 177.582314734704 186.591941221632
190.405863264717 199.70041209487 173.741466458636 209.398724613377
GNAS 3384.19521857242 3636.59530016939 3598.30438198858 3757.39261544326
6214.77579017084 4682.62607750437 3843.07094386605 2891.03258768937
3468.14455177277 3294.92439159154 2789.67986601883 3436.32495553246
GNASAS 134.584076788979 141.03218895811 119.432201417811
140.462651395483 139.008854444223 136.097571072206 140.545351041252
135.68481384332 136.770515182612 137.070326499918 131.846160346403
115.142111396055
GNAT1 146.840735518984 136.781417098789 154.452964510369 146.242897296397
146.318052064937 175.41089164969 147.16778072296 156.65842876856
133.085476994246 146.608010450782 150.578023008958 151.761369162723
GNAT2 118.154953746492 127.975441922402 130.285187693932 117.532063783977
126.446675008883 128.975785280465 118.514618614167 122.20752192519
117.503848306325 118.100611964852 122.153996665065 121.334882368464
GNAT3 125.189588215659 128.963962067821 141.537578081123 140.925705285169
137.000868503792 140.579839043912 131.943014970855 140.782522936146
131.811011266156 127.926270257252 109.29279076901 137.81819274774
GNAZ 181.876635968785 198.795112039249 160.272632590118 233.884660128222
137.459095066272 127.964855970099 127.57284871013 128.730649233876
124.362182111222 119.558626216818 143.589222264786 115.37736249419
GNB1 4448.67183518984 4929.26915002209 4569.44477073589 5800.06300826802
3119.93811557461 3807.16206340771 3436.32495553246 4262.23145131525
4800.99572384569 4255.59773503364 3211.2719052157 4397.32311216672
GNB1L 176.382377784553 173.520662003159 130.306478656159 224.981439707657
429.096075419528 185.938983266802 177.371157649894 154.267020640428
181.722373730185 143.638456836103 179.828494168009 178.946928223991
GNB2 262.629961694573 319.352940751688 187.673748059596 272.277433294605
1139.14730341921 244.925472495705 284.438411011954 243.936797286495
218.811490883852 321.119758814618 488.249967208882 310.301616266762
GNB2L1 21225.306360484 23318.4614981217 20764.6732139063
21315.9767866977 20217.6497214386 16925.9167115963 28095.1627766565
23318.4614981217 18421.818380318 28930.8933474342 26010.7684336005
23190.7842325067
GNB3 168.867373925372 127.453866279181 146.023710941284 130.784802403934
113.891385601095 119.11586630281 108.697696080014 125.754741914111
109.495342943278 112.208950290962 117.561878567749 113.87024833117
GNB4 239.24587884234 211.626970008102 206.321272734099 197.582402276079
138.049846456876 185.016336026382 159.452527363763 175.485672103348
168.173803952157 201.337186768645 193.609985124825 168.570133927038
GNB5 252.733973271584 238.904826940476 290.754210152709 223.058699622386
168.594235675558 191.200215655114 196.860228537112 158.505363728431
180.814610631281 298.301504581296 161.73079180403 182.873752254411

GNE	602.371843434907	646.704226815099	628.197839884392	593.320132696649
	297.970735665595	353.774297577869	363.445110627483	660.315285709317
	938.049312229961	330.321113875647	582.642500194997	253.327404004524
GNG10	302.381946749711	256.897177822238	418.244376024741	193.938032040991
	239.277801396834	473.60579090007	398.193129265416	380.514553097613
	425.708048519408	791.402660066193	479.053300726301	407.244564912365
GNG11	1958.96627327952	1531.54387233661	2120.14463347544	1812.07394646463
	277.256341592701	802.643357549193	261.922485502971	492.05680879106
	515.158921349383	519.450899933049	303.907003535357	349.128741707506
GNG12	196.819022688128	169.052101570616	315.182134426778	146.347213904792
	114.65036208773	165.995251661994	189.49408026215	183.033044475752
	210.103974340881	536.9324595176	171.610742435469	295.851459742115
GNG13	159.811778680604	176.312626750427	164.678646890649	174.068974937318
	148.216605879958	168.547575047205	182.624130582754	165.471587934831
	154.710000519109	154.691783046492	145.472859089272	161.195562296834
GNG2	123.794432024417	152.204730003016	120.514672572565	132.018833785059
	114.027244455291	127.633362302159	119.144128641393	130.298686386894
	148.962593686337	131.027605835275	148.347642424885	118.988269692812
GNG3	132.149864626575	131.662585510218	130.408519735001	137.601181150371
	123.22295112139	144.520435611318	116.759526220736	134.494167613686
	125.231476412101	126.775180133301	139.336891718408	130.773945967285
GNG4	165.665796569389	161.295030274012	162.638805580226	156.437949042181
	144.54895913821	149.410729709855	151.561151654372	146.875733210901
	143.47200560038	149.377153449536	141.772724512502	149.231475480618
GNG5	221.322724563472	242.902900881585	222.624150073858	222.268494113686
	669.330078861673	648.38049615541	536.067857235731	525.789451405743
	450.99430272315	511.996883507889	658.968318349334	557.702374840532
GNG7	505.302263971007	557.405157178952	717.270096456387	552.298965378403
	183.963578322984	174.928155956396	178.455966482206	164.726382689707
	188.623897093065	139.573371281569	168.611652451329	151.889644436875
GNG8	168.805630890928	164.886944426276	172.992936821292	196.419891542139
	179.211502448703	186.420541099902	181.479652131104	164.58158284856
	162.728630453745	165.471587934831	152.643087608513	169.815053752464
GNGT1	153.46645509759	146.099555861577	123.632031112507	140.36602776806
	144.991442620483	119.603565559838	194.637233028442	170.446156541317
	132.762755202326	181.898985277176	160.979182561773	262.196293231028
GNGT2	141.696969082758	145.393431956588	173.599961799334	158.704425428694
	124.717079082023	155.180238551547	175.93459327917	173.656241217331
	170.335930486215	151.961811748754	166.263781040251	176.194292036542
GNL1	428.66614441422	514.354512083591	396.708183244581	467.387427864242
	770.632068590053	465.41233536979	552.494327858264	396.40172043456
	388.002365247498	477.626693328013	752.142287661845	554.652979820908
GNL2	1413.37062765856	1340.79810963983	1379.26881813339	1179.42590997099
	1111.47772523331	2681.48138915107	2080.47567326037	2374.1382412432
	2486.24762158293	2114.9754128463	1477.10828981572	2369.233048505
GNL3	355.714202328378	320.305852961452	356.921627358141	286.506419296679
	776.807656744828	1978.12229412176	2093.67403119512	1357.48791782399
	1525.24196488688	577.56891124468	957.54149804446	1424.04009914668
GNL3L	417.103816140912	439.207122335648	375.189005729903	387.617166711971
	528.556049506759	655.431236290512	490.631757904175	686.679573646733
	467.512355593598	369.018856220394	406.900412510895	458.705688226305
GNLY	152.274957846518	152.581729298159	137.082234284426	141.645906784244
	148.179398287068	133.789738760547	143.229240978574	160.949030247406
	182.997980582218	170.290455446376	167.850516495529	141.941814923705

GNMT 122.673596593654 114.032874859062 116.552626146967 112.005939063045
125.269726165403 118.743966166637 109.654666772771 112.858669660245
123.545404885618 115.343251487944 118.66946078241 119.733311319912
GNPAT 515.729905361375 563.011988279542 603.764427348016 565.609400092543
306.59090554099 598.647226284738 476.782624160552 576.298671053238
603.569635276204 410.645457106122 478.606567465979 569.353661326724
GNPDA1 804.362909579005 1015.10079945997 994.802763887989
1212.55338792557 1129.76078220457 903.144845227364 3905.35300751401
1461.64901688125 1118.28423222707 1198.53117439169 1953.88327754946
1750.21700780511
GNPDA2 241.293046687076 218.470365730283 278.555009982029
229.31486399741 181.3859695951 162.337707154123 222.8766610624
190.270819689158 194.552533397944 233.536023295039 229.689010178622
206.753993163216
GNPNAT1 406.369889501135 338.934501259414 338.510045856515
369.906476027936 376.082040863747 329.068463281182 386.16563750478
372.894376776771 319.868227988842 346.118765910093 466.777454704543
302.637859140061
GNPTAB 200.242239130026 177.091809538824 243.936797286495
189.01309885917 205.4648759901 253.538171006307 225.75155298452
265.332196877456 235.183590602209 237.754067231793 258.348298226232
263.426870776148
GNPTG 643.660739343159 581.828502821435 720.907992182476 629.197022551069
401.267425721838 502.598518192729 431.182384100794 587.388038484307
550.24496459253 621.489906442001 411.878556907186 692.729789121701
GNRH1 277.285526811736 251.472556189141 283.760131847707 259.643957326575
161.992141305029 230.447203571825 190.904614772409 170.577057395536
196.014841240142 172.980441569522 211.035617136177 187.183762762259
GNRH2 129.421772713718 131.840663714409 123.330142002791 122.924017262559
121.628827418997 119.682747854629 126.391083453973 121.696353230886
116.294180301039 121.657744897668 132.641236788053 124.449679223202
GNRHR 108.333135424315 113.568261995787 122.238996815745 109.886197655915
119.492844191924 111.43827410589 110.966212705086 106.667175621971
96.8261937365062 135.747939020471 115.878861991823 106.746545626069
GNRHR2 112.957395064957 119.968081249475 108.715328458556
120.971313945446 116.421771593211 117.990622156287 109.635825046436
113.238466329843 107.559685467789 115.718600552187 110.212739445
111.196610704726
GNS 4047.99200599472 4291.1724030909 3855.33040541264 4009.22206756187
3963.40575974446 4386.83308119416 7555.47017983216 14699.3902364192
5912.48904507462 3270.88476148156 5479.24427823207 5149.78271096158
GOLGA1 269.495247190598 278.005620372937 312.645778869254
347.11449748201 194.584034984339 220.010399552402 188.475801713801
214.200081541057 217.227441947056 207.890408761692 358.843277491684
172.065593177758
GOLGA2 365.046815163035 288.370985514978 326.325210657599
349.540558521616 298.625127845168 252.894371238554 247.280717385562
221.635333304767 253.164596735793 310.174932137546 268.111403429364
230.620456579897
GOLGA2L1 132.930042559647 142.211357324461 121.815538475678
118.619176257622 126.662329971897 133.838032323605 124.690141443832
123.007103885415 137.20509387214 123.738223146912 155.756074127526
121.146511496024

GOLGA2LY1 113.822580779824 127.999820474468 124.062863592295
113.845770994796 119.830261862805 114.421383025971 118.249148086655
115.305518560239 112.809629077555 105.010952544907 118.655954393594
116.947370388179
GOLGA2LY2 141.480793979634 121.402905161896 147.962721373177
150.922279808832 139.161483596756 132.109267393571 145.279694760493
143.245660951425 138.560399473164 131.76272538453 137.858371717399
128.336332748047
GOLGA3 2203.82462320864 2048.13524244516 1822.14935116363
2728.04758874748 1521.61996270758 1089.58643309156 1601.19401366685
1250.58586679251 2069.70335195314 1029.43177809276 1259.38111177521
1182.50261323518
GOLGA4 297.12411625104 246.457698183615 264.8472508692
240.12078342024 224.89911221799 219.537335167901 267.696284751923
199.327939161502 348.067061666237 272.456310079164 330.505633088656
261.322374889497
GOLGA5 473.853999833395 463.802270256246 516.892850913889
443.853458865556 439.33566689752 435.69811966429 556.31484498921
543.584413650467 454.64556253724 664.19083888799 796.052115217208
591.641325402032
GOLGA6 137.757902615328 138.233799607706 143.650172124086
139.703304553486 140.288612271406 142.528480882047 138.859261763075
141.043668963717 146.094951139804 133.608461289137 139.699189528879
145.079450753969
GOLGA6A 147.920946479942 128.087991226561 152.91729346961
126.730314982553 128.860744370967 143.686906051698 157.528409294029
150.667001746475 151.436802637988 142.62707251786 145.768857553634
140.878935632862
GOLGA6B 163.446813963119 136.60514429355 170.490099282436
133.346626315812 131.119810851017 152.609055890822 181.914505612128
170.892319361167 170.099045774947 212.669156800241 170.091913791176
200.342786450248
GOLGA7 560.913732951675 576.298671053238 641.338900241674
474.378918787006 635.234445318087 880.719593734606 452.357898123926
486.947380524463 444.192734921566 677.265576269103 569.47446215213
406.224345292389
GOLGA7B 136.764881711137 130.471929699923 149.119815471009
134.029984991385 136.202953030465 140.762415240824 136.306727083526
139.013404849037 130.738915341653 135.736764623155 125.008209861774
163.41031443893
GOLGA8A 410.954095610904 340.608165077422 596.3549500121
260.67310907387 179.880812358974 238.230110356671 353.437466689101
207.204293555178 206.340478393262 501.434722063309 178.027417594263
300.83667045718
GOLGA8B 1031.59140059071 716.420861490714 1029.75852676191
646.117381960716 319.560819487616 690.582550537485 1046.65224316733
564.749082802433 657.77730563722 1117.77397620221 484.764806127211
915.015154168627
GOLGA8E 137.460936879873 142.999526950651 142.385902610309
143.823368276815 145.122171099946 133.334314509183 125.843089077365
129.522339467532 144.295556086504 143.025570700585 128.643539098937
136.480458837869
GOLGA8F 164.21464602746 149.386285571937 145.997380100999
154.496005175117 138.684070773064 145.573544748919 163.718595974283

136.948039394935 167.125830254039 158.92538634742 148.288798428149
164.256764783784
GOLGA8G 151.96843908074 144.339819009283 156.62031503525
154.390764802264 141.518491890811 153.494456800685 147.129702047639
136.278361222532 136.862764950361 141.794898246882 140.548371692813
145.904866987074
GOLGA9P 205.886398618369 211.419445018736 201.530921073488
211.468099571442 136.313623567821 157.449891798638 164.917470819096
174.075670633438 155.810034593382 142.348246621217 187.439567353342
145.826955036113
GOLGB1 608.129959758538 615.254768508691 562.891241563711
592.822841077599 492.534183006883 950.818917078515 1053.65938651514
965.031655021246 702.981741565482 817.913210743023 1344.1288416533
869.542306259337
GOLIM4 206.585387130993 249.680729921602 201.289402228512
330.005950459958 239.003718699156 179.663258610881 176.5588805282
202.797135825668 192.475309217696 194.072260528037 289.297370738382
214.714726648332
GOLM1 162.140112952434 177.630246034993 162.110943457969 144.361104234288
191.38608906576 164.848970268132 185.17273221366 171.869472272081
169.844913799159 195.364709354814 266.842778974988 147.891442774921
GOLPH3 855.112210714686 723.250763797522 781.790858580895
615.662993833692 631.819659701092 812.298381693378 628.000860165888
1246.2895253224 732.040299742444 617.833194119379 975.315904185938
736.963699775724
GOLPH3L 245.110761598207 238.978845152474 264.476846259384
241.648044508699 277.101000223971 226.387465899611 349.816819166182
412.801213309591 318.73799597811 224.89911221799 302.637859140061
259.72035004624
GOLPH4 341.197826517049 330.553659265085 265.866465528654
328.431289733169 267.696284751923 278.469340236003 191.348260799746
228.384686248604 293.4021207271 211.483693299981 282.076744753348
212.107842040568
GOLSYN 187.978627462391 199.837938317725 172.03919618445
180.641419603362 111.810575716678 121.821868944354 118.95971064289
160.57963709333 121.472885562857 144.139062513587 171.62327142765
111.065596151792
GOLT1A 193.777175793218 226.893147371644 192.891507272665
162.447903830131 617.474181543858 327.207408852474 809.068563907145
335.142319387164 677.9972761887 147.439303942026 646.117381960716
1502.3444541735
GOLT1B 311.806908624308 295.099840299681 302.272304483941
234.041863839123 294.808980631295 351.079435946154 321.31562287169
373.189809737885 397.387054218873 349.330629649093 307.16835174775
275.642882589307
GON4L 250.18189549573 275.972447693982 253.975577255772 330.193132765826
217.050602253281 277.817786943873 278.095939961034 320.214614455231
275.642882589307 237.456245064363 177.257355909681 276.653753970476
GOPC 334.140757209353 284.574888587832 387.711471687237 281.926552464214
203.244917049745 238.88138498771 277.285526811736 224.6588173737
197.651165059354 256.093363520707 364.895113721763 259.338947392465
GORASP1 642.255110250493 634.774041584043 541.896444696137
801.051513639714 872.512754090023 511.25179427158 577.98969050409

465.683139139622 555.366765340014 356.05426191171 824.070046083713
497.916883763059
GORASP2 629.97342429259 637.457322054293 598.287431134037
732.877169346979 965.031655021246 686.445635754196 577.250493023431
528.275591281419 736.332743195416 583.022090879298 799.423640530777
701.481003619067
GOSR1 177.223953859764 164.905467905433 187.035957486928 173.635827398273
169.784837251279 180.994249258457 173.798554769204 230.697077917675
210.620764703468 150.951302116738 177.428161690048 176.167129202773
GOSR2 159.539320132392 157.216793735479 147.129702047639 151.765013587671
171.449326065013 182.181756507598 205.45079491218 199.637257550762
222.820391338687 155.911387663464 164.970438494223 179.674744176904
GOT1 569.866002248352 581.37957221282 568.555967950896 660.535644289024
454.492780042353 487.993796425035 452.732079010502 537.510539142248
615.254768508691 630.894382644903 610.337659723539 707.715412321304
GOT1L1 140.154925193842 126.941650500751 148.551340840517
139.858812885649 130.317604643904 149.260811184287 143.045416067967
131.709341771599 127.855167644203 138.248708129528 144.035936160694
121.75662526451
GOT2 1645.05161227372 1618.64901092072 1635.35544761095 1822.14935116363
3046.47354587949 1675.35431076885 1883.74008471233 1544.45026075974
1605.2454230722 1168.68074786473 1561.10217536347 1894.14699530855
GP1BA 194.312315077985 170.67334244323 147.638057399165 173.364586678954
216.485113690708 211.679799388351 224.424524191566 197.357728220717
209.229537873706 187.035957486928 197.859546367676 184.591789011195
GP2 142.363309192458 158.631298154353 137.702802090925 148.47415004128
144.073739611496 135.206755687105 148.803188053248 143.07651558428
142.495333453346 140.393624985807 139.117053855066 145.640156204495
GP5 145.493977576106 129.362793087439 149.962143642332 159.113259830623
149.99203906203 142.324077852852 153.97869518398 138.576978828316
139.63811945162 160.880152014981 127.640525889316 138.1342592966
GP6 165.239214141562 166.89285636895 154.783699020901 166.376907377447
148.986058473609 170.167035629256 170.661808797506 163.704616231638
152.379441401583 144.266864839344 153.293125258215 157.344758285153
GP9 141.544454931788 141.038721816572 146.586879656174 131.212705319485
131.954310449801 133.044969965784 138.773042747912 138.952082680577
143.078022283871 137.927403204849 133.675029107803 143.206100064821
GPA33 144.738871562895 151.863048206341 191.754626760061 172.245089970803
176.431477850092 171.759102794108 151.846610656503 157.982146236108
155.702330867056 150.751386127903 158.463444189819 170.661808797506
GPAA1 369.726867521935 336.786227017391 347.224999246223 380.243136469602
1577.15895478896 483.549698382115 464.577839898252 524.725307261999
591.189034068137 432.088303693555 385.678901612324 586.867772838753
GPAM 265.29629764006 246.850599569231 277.946395491073 265.29629764006
207.076364103755 322.153713097778 251.833571288967 657.058213650454
217.513118676535 186.515784641119 213.510783808898 184.602704530362
GPAT2 153.38260557666 157.528409294029 152.344248425416 177.562561297777
431.974037914233 172.961115357569 155.756074127526 153.222807811478
148.310640591781 137.784270510433 144.66675420575 140.943732564849
GPATC4 129.977863589097 146.326067008478 156.453108485249
153.191840013692 134.772545694636 143.618454531431 142.342811880173
150.46224324459 137.693410133625 146.184624424737 123.035401501279
142.397433929092

GPATCH1 155.147962740503 168.327600230929 161.402322405903 157.635531050489 131.628021008753 131.432729794636 157.568150431884 149.615585907229 162.237172404557 155.223806761489 143.971152352313 147.62157260418
GPATCH2 166.440530119785 161.44729973777 167.559703634539 150.065275447769 183.100286716683 228.797912452429 208.237011955037 148.82892305156 214.050918459805 224.707455448421 252.249994170536 254.909076658804
GPATCH3 187.247062073247 227.668046559074 211.660148497276 250.060734219351 334.52685347521 211.289598823191 244.596233138747 224.851680313689 182.42088387266 188.252507246148 186.611870261871 169.744100665038
GPATCH4 260.787104931135 253.30325545013 247.622351589081 265.712482648121 261.721726664029 288.968909550533 212.531329857909 341.096072280715 298.301504581296 238.509970054862 255.56301534833 330.850098426464
GPATCH8 178.153527134452 172.065593177758 170.129558603697 180.15338103 158.602687876661 149.535008475427 192.860161535869 160.482734190577 154.205877662428 173.459008248696 188.145154995617 163.863677822465
GPBAR1 283.701987275608 251.161789722751 165.977852973826 306.632103708321 139.94624507228 139.527577649638 137.562157262928 149.048057509964 164.389447533163 148.139376772567 147.52403424052 144.361871207081
GPBP1 375.958347293549 365.199125315964 439.33566689752 303.051124835907 263.123584372086 359.024299091145 502.281584081805 455.486248388968 414.403321220432 423.396271159345 566.647927867646 499.544050752036
GPBP1L1 314.708587131145 346.78158577526 315.3930052781 350.22221782412 264.654879741889 303.762967437478 291.616195651001 330.005950459958 316.125364442611 312.177191402945 437.122373082112 285.507315916663
GPC1 177.793860516896 227.177100762395 188.973595445056 210.912629986133 621.946994961864 237.191866335979 301.871924474014 193.098116087792 232.444867422559 326.703488388966 413.502150181875 550.124359517322
GPC2 164.373697447004 158.445124076906 166.850279817976 170.376620730856 214.068044241507 215.744552410805 177.19138801548 242.141362101262 209.258707976348 197.169703621888 155.964242240771 328.431289733169
GPC3 265.829122816166 267.285998701386 161.683243808528 159.257221624668 909.727616721285 2662.43181510326 730.673649872205 243.979769719186 183.056707813352 125.467668442298 410.42088953434 127.7962242731
GPC4 630.176145684641 447.779854598556 765.911113050543 428.569462312363 308.031816782343 296.141012087256 317.550000625189 177.874821397216 234.182164952936 173.790848780011 250.139061165593 200.387623661458
GPC5 146.915965807149 150.25322837689 156.540165645256 166.558080305926 149.918199106603 145.105268133449 157.66470622511 138.722672835109 135.708804956764 143.891725249061 150.166324293431 146.975258859244
GPC6 379.476768908492 514.799179506754 396.492900824907 469.540421642173 214.355086255855 205.495823410422 222.315451077459 196.031541155674 236.052008824688 267.483886037099 220.487665103019 224.851680313689
GPD1 149.087951792991 174.136806313142 161.072844903925 170.748911334847 146.007311764212 143.00521196918 144.026432356249 129.561012565141 161.636411303968 147.740657356905 146.694318629238 153.563839578065

GPD1L 3111.50508060882 3468.14455177277 4009.22206756187 3618.9385126441
3744.5666678861 2891.03258768937 3954.24763752814 2799.0487754899
866.413995755699 424.134483971176 1388.91423772335 1230.00744483191
GPD2 143.942810095867 147.135640862542 151.645636946799 142.864294577585
181.400788152988 203.17525411575 178.3652866131 182.263724979519
163.919426088318 155.427282703527 159.957371011316 213.990575186237
GPER 382.186095123659 518.478990211753 344.915370555826 703.815551883635
130.240366272285 140.368070973566 136.355319997318 161.83241639654
145.78573175226 156.548858402702 142.652939807341 143.324235640446
GPHA2 135.164117176127 139.041519654872 127.31283918221 130.513071750123
129.03753569157 129.52578899011 131.271979461703 131.674409480919
118.60878652724 125.806943645484 155.108146847158 135.904163043783
GPHB5 162.723310549554 158.946177055546 160.70138318726 166.714793209109
160.91894600916 164.202554380274 186.447049658314 162.904897605929
159.399804351771 182.292947734054 167.201570648434 167.542332146706
GPHN 228.542423012958 221.272872023413 270.568660560796 211.183499580683
174.707059022891 205.987280015028 200.045606111354 272.994195639694
179.648479201164 203.544294328395 202.193338797533 263.800363357443
GPI 929.198774489695 1037.46485756166 1172.4103691525 1742.84803427086
3205.74787769881 819.488546995594 1838.18547839898 1160.25775445193
1200.94425675913 1690.96371604294 522.664475688419 1007.41624313399
GPIHBP1 171.350620456505 206.845306095447 151.379761514526
163.967616022079 142.960875909369 148.26621607986 145.837985729893
133.483766053954 135.943252855457 133.829889099442 150.809693773343
129.835713633923
GPKOW 314.751799003704 306.896732729554 354.45785837703 329.88790758321
523.937128789616 438.231717096337 547.367381020623 393.98210915852
561.251860964966 386.067573158514 481.037647470809 376.374248850987
GPLD1 138.281188275319 128.09266452749 132.782967735893 134.629996026323
128.336332748047 186.167877791105 135.06498850776 127.482662155082
125.413619056436 121.483610947845 125.060387518072 130.858419819734
GPM6A 132.780055030907 125.783265980169 136.48748813417 132.303506517431
142.183154756458 138.952082680577 136.149732574763 130.473842500245
137.188849160569 126.673587174832 160.567934923347 130.644937322186
GPM6B 313.536451935268 299.986758612161 269.261595558589 264.983729290122
137.593577427321 162.274094333189 131.477589792769 134.166297738657
136.341358916638 198.549047189499 140.781647532361 127.959474148217
GPN1 406.736165197168 445.905756082569 497.110852407681 427.084392965651
647.666334416707 1075.3328563531 780.461254794127 966.731520471976
751.269009484639 793.300636780035 607.26455470867 683.470866056244
GPN2 369.434158443561 387.068276379114 306.946103334814 447.059120541009
1019.627634011 536.9324595176 893.676600121113 780.961416212309
423.045329000756 767.054576874115 472.108893832845 677.023591987179
GPN3 236.082185670774 220.876114717642 282.076744753348 193.008902902654
233.572055978465 329.535023880129 295.851459742115 297.970735665595
318.196134631178 305.200911653347 224.89911221799 268.186156177622
GPNMB 1423.02563792293 1037.00025848491 1455.75693295732 873.256632365737
544.852019338212 322.64731764456 225.96618659877 1144.07896461196
889.6617510875 1887.82245931444 719.766799746895 162.155834067128
GPR1 1352.14737073174 1849.21906428944 1387.90327883679 1188.08712008638
327.062810389635 1509.69760873558 773.057095787974 957.54149804446
873.879064183441 736.963699775724 763.263858769038 666.570977208535
GPR101 127.209043595332 129.940297696849 128.217078449064
142.369661737453 156.402578892869 141.319594283071 135.467354817201

146.332425559689 129.114161128113 127.265211069837 147.530719472217
135.205434173702
GPR103 185.295480519718 160.903342063542 167.737220954468
199.579818754014 174.255919030841 196.974807677659 169.844913799159
164.309588213547 173.281506912652 167.637638693195 138.405215507471
163.039564280484
GPR107 142.737537206204 129.973380117841 143.823368276815
136.698600389364 142.650451413991 138.483436753942 135.151438845363
145.737361356678 155.058852833902 158.226547375305 138.440663763957
131.643633737316
GPR108 265.260411490697 314.292700455545 369.726867521935
316.524115715585 452.843213944986 340.366016191232 307.480644393018
307.020265874666 256.093363520707 336.212224495877 240.631264915238
321.84712975195
GPR109A 128.929244741296 142.476530302265 133.439077726411
126.488299049827 137.074564265915 139.029404397979 187.871017629994
144.774621469452 142.013370954791 234.841813258798 134.93455083142
138.530236176135
GPR109B 133.008120853618 137.120973494952 143.947729578484
142.749568012703 155.906054212876 137.358139652612 162.505305538911
147.376482709004 151.573920235798 158.49220347186 134.20988291379
144.715167331434
GPR110 137.493436771898 169.975329631607 136.261907928796
129.848276446896 212.933062534244 136.348252715447 520.585319342817
213.077884261943 165.763459146818 146.609485664113 292.468585109951
564.588706307423
GPR111 120.054065939835 120.931585398914 127.195619567384
127.263061874385 123.059971161711 123.02167644694 128.363331337379
120.304037458526 121.387799881297 119.608334527504 135.067056559409
124.142882936234
GPR112 184.915526626619 157.176001400747 179.674744176904
135.360087349634 147.391535316043 171.954184094677 131.17119492692
137.812249316763 145.540872377507 153.178934750919 145.526941881413
136.758262322199
GPR113 132.213890220705 120.384210318 133.423376063284
125.269726165403 147.373174160478 137.410687900298 135.839826264562
136.499228715856 128.497571151343 141.164985468046 142.053649707364
130.506708993193
GPR114 155.438218501542 142.536450265886 136.957209426823
157.756885980217 146.716053772448 142.169951083733 158.562631157512
188.878770168595 209.279164243868 167.456481678813 166.192990554427
143.419034484087
GPR115 117.923459503989 116.007006375993 115.217608555334
121.248923222807 122.552733780528 144.384287335249 199.854728619468
134.045938361221 118.355532305846 166.360005474414 205.65398844108
236.592839463278
GPR116 612.758976723556 883.140984728615 343.121661664987
622.834462068316 302.381946749711 683.655205499779 364.191263843897
529.368609163949 510.226374558703 449.408793796673 347.3483417873
330.143103381406
GPR119 108.669141498627 109.924867620862 111.457176315964
114.556542069712 114.488760619745 106.923686318112 116.236538651031
105.467761348787 112.063713561549 115.582048644075 116.540729581355
117.766156390814

GPR12 122.268560536152 130.521731606987 125.068362001087 116.142650095476
130.818181991958 117.261443445891 116.621735695217 123.294321716108
119.651470884046 125.347442446239 134.983884722607 116.328330266803
GPR120 116.321559000405 125.929743286163 107.40922161073
110.190708230962 117.270320709161 113.064645688715 120.767950825369
110.17590675298 104.934718065476 116.576896337786 119.436922168376
114.91722828113
GPR123 127.382266838772 125.154784284706 133.065366727222
132.344296085487 123.141928612324 128.445065383646 148.347642424885
140.28485470716 133.585425721984 142.440344960504 129.338551879984
134.620896927473
GPR124 730.988069115919 936.221322010828 575.23272138912
1582.0221231161 184.611146047556 133.640832782576 137.513315427454
157.36568754472 168.611652451329 173.334154083555 177.246890307643
147.706402141169
GPR125 181.508873226705 165.789846154721 176.689683169567
185.650862663633 160.992494552269 200.322334039458 178.920528149177
186.529656751005 175.891947478891 153.011923016494 147.54950981909
151.214631373344
GPR126 158.933097712363 186.276450732044 174.045229095003
178.872635061826 286.587945301875 306.193157874683 429.922530506572
395.425715632302 193.358728044565 186.1961391235 527.139476504986
659.483452217461
GPR128 146.252974508529 149.457761307441 144.818492604488
145.23242365333 138.734437288431 1271.46608195833 142.047828639169
146.101547300741 166.192990554427 146.235832275574 152.609055890822
137.128849888863
GPR132 125.929743286163 117.264555636952 114.384296185446
108.557097186828 108.807891863679 112.314595956147 113.597215383696
135.18026749895 134.703474191011 108.296042016208 139.886499402506
124.395047385025
GPR133 120.925930409474 116.176068923513 116.769106223472
116.134550788966 128.141725976839 122.575357101038 117.770032157393
121.289159587394 116.507315786826 119.886433564236 137.191582153943
122.466276478613
GPR135 134.119276589666 138.600017737884 129.608034744581
141.817230824364 148.614214326995 129.1829592305 127.713286292101
133.921012946195 132.86711707789 134.904123162692 134.238642042323
129.664016819143
GPR137 676.293399523316 805.600599570258 765.532997390972
1131.96129284658 2120.14463347544 1109.79571066685 1116.95795024994
749.859130465356 828.683748921915 495.099443245153 688.684644604218
839.901173743755
GPR137B 289.667302610069 289.297370738382 318.561557474749
292.640251411131 314.063025950327 333.349203784021 527.226517960073
295.420691959792 478.38361571213 361.160881334174 340.903961204211
384.566987097657
GPR137C 140.680326850085 144.448503894295 140.481148289418
158.735283017367 150.460618402778 178.045931314857 171.376976179465
144.212231875922 152.628220216104 149.007288765628 143.961467163406
155.6299578128
GPR139 138.418604660734 116.700397932007 131.968781256081
142.071492722749 132.807755600683 131.106613067333 132.744261207073

126.791714564926 137.545063966957 142.142633050256 129.436712663487
127.018746030517
GPR141 139.567367353959 134.516390697561 144.743281367797
143.603022117503 132.981346767415 130.092881020271 130.182320154521
149.045654499682 132.175368142549 142.466067016788 114.350596695469
138.670077127324
GPR142 151.216992571364 147.069488043503 137.901317305531
129.005211657936 148.084844579881 129.493805009651 129.165440930558
132.297716337104 142.232918684259 134.226711030456 139.577443171489
165.480489678822
GPR143 143.053727675518 142.342811880173 144.154217446011
143.195338329759 178.863722055499 158.799917136119 157.907190910755
163.18585933431 158.68792959603 164.295628750438 178.647726085678
167.273378118452
GPR144 129.994160770036 146.813353189258 141.543147273205
163.362182585182 129.7642522046 149.485497080739 136.981043383326
133.666244052469 154.011708696954 147.973274498131 156.160899295182
132.518394343751
GPR146 164.302998010829 190.002630382394 171.784981874184
189.678680702271 182.074569356713 154.737117718424 149.936366672592
152.548795147468 154.537240112989 160.853480692082 152.910433120981
160.543465620248
GPR148 126.316613675316 117.60787142216 124.277983696239
112.922918499398 122.216479103528 129.21974206851 113.694351010121
118.21084414402 120.649467083125 117.123891785437 139.941724115063
115.785084044427
GPR149 127.727311123825 121.846349358116 118.75237770613
116.437403962939 130.383001462828 121.849460555655 117.331280455759
113.292688210897 118.104763610751 116.043095688871 125.41744801003
123.835583753941
GPR15 153.181097279473 150.038722708606 138.033668840392 146.26304624648
143.980608339892 160.240052678401 164.608496326181 152.386986818959
150.629932241703 153.620767586182 147.091280396135 163.647306551474
GPR150 123.145830746127 114.68345964966 112.65454329872
122.452541100946 129.828896822571 132.448602065824 119.427342766092
114.867454554206 132.14412512622 116.20008794813 139.792313018728
113.222264855561
GPR151 136.561852783331 144.787836177147 146.264686158155
147.303997146992 140.492715555674 141.018087546273 149.795655754301
147.973274498131 145.183463058696 153.95855605751 128.882160435252
143.937726155116
GPR152 114.226028611171 119.643417329571 126.178476831007
108.768730405582 117.962188157317 120.633217745524 111.381427322188
109.167312436827 113.799997627317 110.810963139914 116.925863450165
123.259322051795
GPR153 171.869472272081 170.326474367867 171.573342518254
168.687845035401 177.582314734704 156.745051321143 170.866433766203
160.735428753783 167.958792571511 158.584934514596 166.272237117441
159.870967387108
GPR155 138.201164395948 136.66358267095 138.123754096875
134.95842832877 134.29036009792 147.359279533039 141.127519700052
139.888008605915 132.303506517431 133.122174596886 140.688570032068
157.639688257892

GPR156 164.043866642712 169.211708954329 168.974155750959
169.211708954329 158.967160833334 156.353914172996 174.707059022891
169.999692055871 160.243405030742 168.03501792841 164.541417588646
184.62149494522
GPR157 124.885950473434 134.086301127594 138.866469244936
126.106777865128 118.718048764985 150.513804134588 139.067516394141
135.585648980469 131.468537312778 149.668587078867 128.482976013813
135.735535533738
GPR158 141.939368369872 137.500310477851 136.807118099948
129.977863589097 148.773558380167 134.060214479265 124.657687121111
145.360466216324 126.359440164601 133.403409882362 147.77916610545
138.731957926247
GPR158L1 139.168307371516 156.916784141484 152.122141650397
153.420392741659 151.403431105471 163.821142892643 146.67875644027
157.155105022743 145.407517107023 146.873719524815 143.562164660825
152.14012587515
GPR160 136.94525817342 138.48552149031 111.484426481816
121.146511496024 404.21236980164 188.567747377006 487.358720934063
485.026081242201 419.126814511305 166.64535048582 400.353795948162
188.818012746123
GPR161 150.42705284743 155.979380606594 152.705458417973
160.265389854694 142.495333453346 141.108383815109 135.025907096066
137.821010169859 126.928328902507 155.530415929059 143.470181156799
131.839890565869
GPR162 199.854728619468 206.127493726541 202.114480291084
230.723142236623 142.96594961303 155.158028043823 145.010108826238
155.041173289759 152.217243924816 145.787468865642 134.465488237877
147.225911875196
GPR17 150.311274161073 139.276102596591 146.787927492706 147.140771901106
142.598106777707 141.222884136243 138.110317032494 142.282861913548
137.499290547619 146.34029698108 158.499787920522 151.450566957592
GPR171 131.146604635155 150.672985449063 142.139331321085
152.131804134092 154.8975607055 142.736148814019 141.119491147925
148.511059836029 134.427601777639 125.486324819682 124.306049555272
140.48372620406
GPR172A 310.365203383647 317.904396212359 225.583966894864
338.510045856515 1862.88548451154 719.766799746895 496.426739836535
695.086203058575 1125.95664591686 669.602939159192 479.866450988091
745.153336165802
GPR172B 139.54021659275 146.536055773073 146.658048787833
157.590017103385 162.237172404557 210.960900130785 152.395272857974
167.250667092211 133.44592405762 134.285191872099 142.837688062688
163.668476670167
GPR173 139.421753451936 133.944538335319 132.240470270795
136.537117054998 137.546487338984 128.771367080039 124.682396766302
135.645439440707 129.608034744581 128.692917766138 142.978818998012
135.457059862669
GPR174 126.667650990284 130.555405606993 130.66511389864
126.614876404146 133.369314179341 132.01588294465 135.837733533444
132.098421212796 129.867246424399 125.293640452686 125.04327844782
128.79607493422
GPR175 299.234482744038 319.490670412394 304.237479317271
311.04252785157 704.152296359463 276.089623957012 320.024259346646

282.561381978659 350.308420518351 312.808044035117 279.879377933144
271.397087928135
GPR176 152.104170622003 156.808992920564 151.794243108455
144.464071397369 151.946691918521 152.327247552986 167.637638693195
145.306369509684 152.51547053919 145.03804642447 138.902097203056
155.846106540378
GPR177 784.72162514331 681.810927901806 1135.49876948815
591.013515828247 305.252666831522 188.39062571513 447.674610082872
281.926552464214 524.541494680558 509.400965222318 179.091917512822
172.568852318369
GPR179 153.39738748803 142.863268359757 139.071908528327
136.519697160267 147.473875763319 143.647714966406 143.218850583533
139.714198638713 138.116568200293 136.195555442748 142.551161883518
147.461480849333
GPR18 120.998665308561 130.49338418515 107.828378413233 117.943511615792
121.874296836854 132.551989498706 120.208584170364 117.946664604751
164.630705813584 123.317063135818 139.395743826525 124.918089223417
GPR180 290.24106492688 6 260.999345837027 260.554343693588
259.146673652222 288.481981529558 396.781827940009 310.598930734244
311.10841853984 301.369680426563 318.778978094743 250.376178742476
281.304989265608
GPR182 159.110614190088 136.560048419149 134.401733295755
132.269567096683 126.067296703905 141.102274192049 139.582054825147
159.58125728432 132.956860864455 128.753623886188 157.374537572779
131.464229237662
GPR183 151.208728458188 152.680068580858 151.073257114127
160.486513643798 140.89666990854 166.001279428607 141.368078865065
146.867740388881 160.399059317919 139.086498524774 141.241717979079
159.788143847569
GPR19 136.124385992905 143.512348693556 125.5109819304 140.74035966401
139.061072394426 140.918728758975 129.253638326633 163.456245724169
141.750316403249 145.148058824491 131.590180562108 150.180037731272
GPR20 163.69172577831 174.149718758884 172.951567541592 188.0434100445
153.418095627352 183.033044475752 142.664793323255 152.222732770706
146.805832438977 176.245698601389 155.731897985566 179.841665686703
GPR21 134.483162806498 138.883097077879 132.198536906752 152.097609803404
125.025064237439 139.3465236423 121.480455781173 134.604565335211
156.942156078314 139.54021659275 123.789035728194 131.148926523383
GPR22 146.978110148492 152.11956441072 141.401480567426 157.259599894923
154.750512277909 143.167719135403 144.324313048777 147.591479875268
149.867541008113 134.42983422342 150.063082731931 149.004343851423
GPR25 122.484351696828 126.056493244568 130.191876914166 114.479886899622
133.372231702721 126.137627769256 124.449679223202 114.849872458647
112.693431140524 121.22760405399 122.170662903259 120.085340927108
GPR26 105.221735747452 118.740346254406 118.740346254406 114.226028611171
119.072213573568 116.023563001039 103.012774960061 104.633603136988
103.888971448792 108.43334699667 112.528908277828 116.155949676272
GPR27 141.539503634005 139.564898998489 137.141081365469 158.814407805803
166.320461361356 143.575381236637 167.063762404009 145.560904207183
144.342905860503 141.104761546577 130.396593013401 161.22761502968
GPR3 153.363629311799 156.907788377794 152.197327387252 161.04173806448
229.933577323411 191.348260799746 167.958792571511 156.154079736651
152.498222035534 175.158180187883 145.485453268383 173.907933001446

GPR31	152.635792342068	126.741498575874	136.175268696202	147.369767631957
	131.988591391373	137.15571320152	147.183889426843	144.954595802474
	143.215828010442	156.06439656393	136.306727083526	125.117333434527
GPR32	129.898242234869	139.345434097957	148.23392074197	148.695212949639
	137.830435972191	156.964080041261	133.254663058352	154.691783046492
	129.864474336459	133.742490937191	138.370241517025	129.740166478951
GPR34	154.220886444667	145.276745572405	132.454025879177	141.283042601932
	146.303959493672	171.338371572269	150.2214516849	157.809081184874
	156.033897758869	143.844153373367	144.073739611496	144.774621469452
GPR35	150.476228905372	151.604093626944	151.783090997855	154.515091049526
	162.629257046871	150.809693773343	128.773129597793	142.77817647952
	137.352231982942	133.966277922005	153.474566767304	136.130027137459
GPR37	161.37617568158	163.336324451735	206.73292907905	168.52952604693
	119.144128641393	119.092335265325	110.753661013519	111.525984720592
	123.277867938275	166.767004180603	145.029298288869	106.450154495663
GPR37L1	125.955431094455	120.894583222813	131.04767483614	
	121.141432480683	123.668707648172	125.666013736767	125.637138636558
	124.625603721372	115.00776048097	122.153996665065	124.474221706022
	109.37466528113			
GPR39	128.582520754296	147.796610216375	143.363002903815	139.650726173186
	148.85250180736	153.882081780197	159.177303894223	156.973511952828
	141.209958892647	140.460927375243	124.711578994973	141.233642252649
GPR4	138.393565473857	200.915806094193	119.096195018596	137.666849842576
	179.177884962513	223.722742062251	121.021776527531	149.410729709855
	167.568776552757	141.431172407792	162.634670715627	144.612549022264
GPR42	133.932594124721	133.499361793303	130.546471770588	144.30699572795
	128.112595697836	144.346988809708	136.645091356305	143.766249538352
	136.480458837869	156.083002868004	133.94667806086	161.079169854907
GPR44	117.900892627473	123.285059856341	103.594872791657	110.774802298005
	112.851805868467	104.091301211096	109.932577444858	113.78644620959
	122.702175900246	104.780306305981	109.947300629824	110.355493122568
GPR45	165.055636629118	161.079169854907	147.530719472217	182.938824846376
	168.091278015885	165.30072618497	148.439740062804	160.215396651606
	159.539320132392	160.335043636423	147.469101571149	136.593346330215
GPR50	123.7421178083	126.65748100631	117.131720092783	120.714981493593
	125.644134465307	112.742895297124	127.58382536792	117.264555636952
	116.179625689647	127.921209207879	157.607445509297	111.820208385514
GPR52	139.316189199729	139.353080958675	148.096551269076	139.297276687352
	133.697364081712	140.650987394155	144.090047165489	145.937733795336
	131.237720549768	143.093156971372	141.013392458859	141.954467306745
GPR55	156.567519536997	148.01816554072	142.945392802281	136.561852783331
	144.013615296685	138.548983773386	145.444483891989	153.468933239223
	142.428578698775	145.298109334141	141.5311434224	144.12742465348
GPR56	264.476846259384	392.746574862867	272.456310079164	205.476230849391
	1858.12855367119	1537.71464618832	1706.06615968622	1168.68074786473
	1659.80909859514	1618.64901092072	1608.40826380713	1951.69651954627
GPR6	124.654146040634	131.498890334548	119.176139463015	120.227386958188
	126.899985772157	124.222529964098	123.849651946683	125.253637835638
	123.773753287854	113.87024833117	143.650172124086	113.748724650063
GPR61	147.310807346272	133.348510114432	139.303747021408	139.320621744553
	135.866980620144	136.995197143668	135.375268329248	117.348231147283
	136.643481414354	130.238231520218	143.667207351415	137.183566882805

GPR62 112.90900939264 128.949103967865 115.939620871703 114.860437706855
132.553445658347 114.067790137477 109.613208767992 117.069094451283
112.282839763467 125.79193748535 118.011195631598 116.934457270701
GPR63 135.247018354192 125.9713489569 119.686859562393 121.760488406107
122.696382705233 117.781804727205 114.265920150451 115.608857734241
113.356070669308 142.053649707364 139.082897743357 113.283359026655
GPR64 145.814268094808 132.034701177759 139.583514773196 133.541333581593
191.112009690591 127.430408113725 131.498890334548 129.290542121353
134.28095064513 133.749271961438 129.253638326633 143.298218989451
GPR65 195.532022060219 190.809584998617 176.53912635694 177.619101765848
176.062665550409 167.521700144143 173.325724300712 173.810080280732
203.276353321185 174.483233557264 174.389706515542 153.162313098346
GPR68 249.925640256322 206.236303011662 140.569391362701 143.436675541053
127.485734862624 127.7962242731 120.514672572565 136.86685787001
172.002600588279 563.140382824149 151.002218995005 112.624137732184
GPR75 147.556482119673 149.821848101995 130.937742379689 127.171331062578
130.265055842999 147.553953240464 120.227386958188 135.056517225198
148.789295862953 128.862699779871 124.401083237195 144.708780756278
GPR77 130.166741978483 143.766249538352 133.602870982718 138.154799037807
139.522018789485 143.378998368344 127.302714624905 127.365142833355
132.103084472404 125.739133257542 124.3501719829 135.417519318028
GPR78 125.945916345628 128.618422306397 122.158160927149 115.845468537936
134.389518389888 124.263901299025 130.617505834747 133.281505040209
126.81140158672 131.659187927328 150.301540078433 127.699226067847
GPR81 121.877276961306 140.256871047018 137.801899270172 136.566588903194
411.784922368377 201.480444219547 226.458509834965 173.850390626782
300.150235383065 128.986795394842 231.981501596284 165.71382076724
GPR82 128.572388808833 130.520275539672 132.681475162386 120.329105003959
123.582509531804 129.442111153737 132.490323093932 129.859331207247
124.811760828557 124.661088039427 132.850601603567 130.768746032834
GPR83 134.049094181433 133.63668145135 126.619475852751 135.028746262383
146.334446516009 136.85323853426 135.43119649883 139.117776552573
134.361949431678 123.720229113779 127.8514513762 123.338921824167
GPR84 163.463191619941 166.958831664154 163.926432479673 181.431827312784
151.445254181497 155.468848105615 173.675115244522 169.168869363848
165.803549113373 166.135707525987 193.444080047879 175.437651670246
GPR85 120.126700838056 112.624137732184 114.734439905052 112.875136585606
111.65024009102 106.854721604889 122.368581795931 116.961391747202
120.170269310738 111.188410935711 124.910390240995 108.021898335735
GPR87 135.025907096066 155.207839370162 138.700301754711 126.87882572904
263.936023499588 154.412506915569 310.758803815671 208.72873686638
183.513101761782 322.889296658955 314.751799003704 217.264041781337
GPR88 118.983932995126 133.682715540066 120.748910864064 118.838332117453
121.675007387533 124.541257225826 139.568610307888 129.637555509848
121.930362099901 123.657246099004 127.712004247155 119.079680805247
GPR89A 134.537123332992 137.832597613423 146.506263412584
124.76274408368 159.749576166342 339.490955270138 242.037132958656
262.913054904154 201.969002368045 233.671878221507 156.339564110766
309.423768054614
GPR89B 213.178648382852 197.049616858296 246.186104432792
192.095235288818 232.321454436688 514.61297221467 317.839512360245
439.023181761356 304.751330890753 315.912220767901 201.773745532242
377.27961559752

GPR89C 164.736294372006 187.461448376593 207.036293322882
160.01685162095 183.181125586276 415.322560616847 297.921617748468
336.70893120135 252.222481637238 237.71636970719 216.381643581705
382.744523022686
GPR97 139.47828235176 140.878935632862 141.306418675404 139.353080958675
142.180927190578 139.660641694206 139.146387811999 133.88072491369
136.241569739318 143.791908191551 139.034940762344 138.467624450885
GPR98 124.197397333928 143.508657511633 132.061436165754 116.751145098777
213.26158748941 316.125364442611 360.361719991381 227.587590278412
168.347445148881 257.941367816557 568.310800941091 365.286234166621
GPRASP1 204.616225498655 193.261343922604 256.291984914927
193.704510912321 188.995551740427 147.313494489487 165.857032207991
179.177884962513 158.872962797815 163.141010815086 152.629680152219
169.800006803362
GPRASP2 288.481981529558 265.829122816166 291.369577397743
247.015574724901 188.671807443641 167.949422301257 177.457362387052
163.679104694139 169.541411677993 184.157941055948 171.795878750365
154.021906399992
GPRC5A 165.003646188775 166.46473789699 139.841822983821
115.231121959397 380.393153331219 193.569894919176 153.767488352037
155.40832015652 189.450316943845 270.973600107882 238.770586150777
132.271437329873
GPRC5B 158.531514776098 165.654773882752 197.489903263216
154.681604647652 143.363002903815 188.7348696188 151.819220292189
164.634694036993 137.961504178836 174.149718758884 171.16807372519
177.96452209396
GPRC5C 206.620708230452 282.076744753348 200.371280127668
203.816305810343 1326.95332435394 845.369108604819 995.812184456562
384.16579891756 944.47112348734 213.914599384797 626.582864105324
848.620986404914
GPRC5D 124.285969778268 112.675715371087 120.633217745524
111.10737497653 130.062015525637 121.57069706238 121.661141741782
119.720132373023 121.014538677616 119.349376960211 137.020473156363
120.611442477736
GPRC6A 160.707290343231 141.494666823639 145.131215503044
154.701413581915 146.909471248864 158.125437004665 166.440530119785
160.941804737367 156.883255634126 173.681078272467 172.152570321727
146.20679320119
GPRIN1 113.970633751086 116.514299377365 115.678486138934
117.193620642373 128.800907223807 118.909153405102 133.155925118245
120.257886394616 110.033746435328 138.667081004369 142.329137800558
128.96181458192
GPRIN2 144.031305845388 124.629877200816 113.119021336236
123.406144497577 161.569793476847 125.528638246486 142.826050917117
134.356796881432 125.224197852606 130.779805222363 139.473459411633
127.560308850342
GPRIN3 108.2359175332 118.419311800417 122.554760526767
114.170354748872 108.21185649774 110.636713168363 109.625579596864
123.30960025689 125.826111950648 107.67395123577 131.427686241226
124.49814598066
GPS1 597.189355986293 652.826023701545 622.561634660589 738.087203930249
1430.20038077595 743.510369438587 705.020444579319 935.784743413185
702.460234202652 564.056592672374 387.355661073935 741.875751347917

GPS2 459.854279050269 441.001837510132 476.495282784602 522.300029971277
306.486275677467 318.06081614151 142.024171830924 382.630113452429
259.826625866106 403.47026424469 308.877027622691 224.981439707657
GPSM1 379.200576951933 351.448985413528 285.30221551106 443.964363178742
340.860684371038 242.848821222611 196.226732738526 238.149614104568
214.763232658841 319.868227988842 168.588120398433 232.699786290509
GPSM2 175.41089164969 180.814610631281 166.760758231104 159.584350289413
243.501083338279 263.707476735938 223.551813819581 459.068582584181
253.186749111526 909.388774224614 197.91325268683 318.244342364572
GPSM3 166.404164069728 164.777719508421 139.648187461906 129.448507622171
208.045210163121 144.871396308673 233.842749683689 243.538374440134
342.998459706966 182.453722105813 317.309949792707 409.282410265733
GPT 130.40770745199 121.599883734552 131.21740780848 139.597493526257
146.291932367254 129.366567668463 122.932745723044 125.563646710294
153.149523560021 120.793974839773 154.604363252526 127.473812381059
GPT2 256.202924421798 310.142754559096 254.552034511333 340.098306132634
1358.29475573637 988.297682407351 478.180723959397 786.62709337246
1722.26954281876 366.857139377277 532.235193634196 310.718449468465
GPX1 894.46029531926 976.782815449208 864.547109080675 1000.05896885496
4323.76731419005 2340.90990677253 2753.89649259242 1508.24281455057
2000.47497636965 2316.49886419499 1487.12201643008 1632.07671492041
GPX2 458.151323130855 1299.4782626583 986.65554605455 194.161930642068
4159.96851384358 5636.26097921507 9937.36968397141 5094.53287522452
10608.0671430066 301.087947101876 11190.1778174002 6652.21610967461
GPX3 4569.44477073589 3348.7622174821 3829.87905458534 1754.99663827472
370.798852361374 235.867149024041 183.619184925353 276.961847241646
436.331333134628 213.877482604185 176.088449983147 220.288758600588
GPX4 4037.15442268836 4159.96851384358 5049.57929757135 4183.06379632713
9560.32907282993 5702.11995823367 5240.40795364202 5049.57929757135
2871.34694522381 3441.9805896226 2956.63335018838 3246.86605825129
GPX5 147.581827861592 138.1342592966 146.171094379173 147.178598908199
134.346754381858 138.570150372729 139.690093593646 137.376602654929
139.333282400639 134.10070984606 136.952914053358 136.929570710155
GPX6 149.881708604156 148.224587582189 138.639410179404 148.155692596378
142.796371223204 155.319717753912 182.410688592378 160.72009357092
152.179281925254 128.421242037748 162.723310549554 142.588013648337
GPX7 312.019603761673 300.150235383065 305.768201359278 269.530151236658
340.800632878807 461.413469623811 320.648966489586 329.333236839082
219.792171863015 343.946831770916 201.004491067972 466.141717595301
GPX8 906.886423608706 806.290662664708 516.577357935963 804.362909579005
183.168629351124 244.668813440638 203.524150518033 227.32463765717
303.423450627377 477.741216640575 221.849661343124 227.806050911154
GRAMD1A 542.740017003369 573.509780150018 648.549890890375
738.579262579782 242.471259857978 344.070947256963 454.235121917464
376.791876040886 1129.15018335354 328.486476842582 521.83794739314
269.194172034595
GRAMD1B 131.750053934548 131.688038068545 134.56134674893
125.318166919844 132.897554980037 132.132474135959 127.019978195283
125.932540615682 140.933444607646 113.170014668758 137.288653308858
135.624881949214
GRAMD1C 121.821868944354 123.161953011069 116.678969343492
117.862532836494 131.873313581009 141.550553034455 202.278323489515
171.86363382331 153.952788086758 132.011199659803 178.055019987648
173.416999972133

GRAMD2 137.066799877341 143.373932371335 161.382041789666
166.223079895236 185.154876233931 142.288110423886 147.022594294963
157.099868633958 162.013164369593 205.226635237992 157.976810829398
156.441368290572
GRAMD3 291.261765585473 285.102778078056 305.325519265387
230.589110372454 330.944203807675 462.888171502285 732.630306104841
652.654673756838 316.457274749526 326.1109406486 382.485769373364
647.666334416707
GRAMD4 212.798133015556 223.253794809571 195.10410545445
223.339132121796 203.791916347213 213.194154171149 273.432712475089
185.830435830545 215.558688906165 310.075815009404 255.462706428149
323.086368767924
GRAP 154.774977482202 157.076284096905 144.352987324713 165.954266127697
144.555718186987 155.542691937539 148.87242524521 152.044068298273
174.107661206415 169.791616021987 147.07272598888 149.427217345037
GRAP2 118.410091682499 122.610529927104 108.318510762416 115.293844461817
122.170662903259 125.612670947405 123.488418655586 128.668672935791
126.853028093579 109.384112020063 142.099541115973 114.524341239889
GRASP 180.569583206488 217.45700166961 131.377361030122 168.27980269501
120.894583222813 136.372712400493 132.303506517431 140.447933562667
150.573523216151 150.456500446175 162.904897605929 136.807118099948
GRB10 298.256131536504 265.29629764006 321.492900106953 308.088967616175
168.05687638693 199.53455266342 157.979749139651 190.331589656379
181.467708718758 160.28767775843 244.18714778846 199.746775121317
GRB14 152.465176508162 151.936543085044 142.920939571001 149.464962540085
148.547259731346 151.330035086652 149.300089212177 158.100395482569
142.991135981933 166.70212615715 144.621077658674 152.504620068655
GRB2 299.33156125224 331.888150158628 324.346723939622 397.387054218873
366.007451523992 323.896313343796 361.768552564027 368.879825610754
444.826775824095 333.200230327106 333.533043832976 356.268960813032
GRB7 130.399501479248 142.365009028839 130.628967781841 135.434853296628
452.24100098898 270.945325305492 301.139737613279 704.152296359463
325.525450750788 195.185025611945 335.938170230765 234.895245871534
GREB1 142.964043764953 142.617474507282 129.57551086282 152.498222035534
143.647714966406 144.927731613526 141.193081790037 142.095289977576
135.940629737073 135.535020220081 135.701041557713 138.440663763957
GREM1 167.713268780664 161.761125440228 143.662865541299 138.783017264895
158.098055917452 152.796423361971 131.747367613225 160.203521879896
466.083451075744 577.98969050409 144.988803993714 129.707421699091
GREM2 179.369845168822 183.531156021887 153.726935957906 150.328995030071
138.967080709927 155.731897985566 141.686945908315 136.639637503571
140.451465188624 143.937726155116 127.804392143587 151.9565369062
GRHL1 135.153588830407 143.147325939994 138.538656638714 126.058167594519
252.628516068724 239.704459251571 221.027562527572 182.77693508164
180.968659614238 265.919002666885 199.149959995133 246.989526028884
GRHL2 145.28524062436 155.582036890436 134.541606996118 120.413468119484
235.822266856967 298.256131536504 172.888455552446 206.071709262227
244.797792477941 161.354014292373 341.761516859319 311.10841853984
GRHL3 133.443807124822 145.773932754923 139.399195840117 133.599098708818
442.12886967939 392.746574862867 285.46340356157 520.120193442648
663.214972254236 425.229044880287 415.322560616847 398.796188440256
GRHPR 348.848550937417 350.58427958432 460.742037437298 425.229044880287
437.248311551717 286.469773081481 332.24505665756 288.68376542946
296.962992349662 512.723592291378 401.584032365337 262.233918187791

GRIA1 120.204469421484 113.504670764963 108.589444216106 102.024765473404
112.054379249781 113.192313147111 109.29279076901 114.999628959921
109.806338826826 107.303126530947 116.981243525714 121.416038813102
GRIA2 129.580421464913 125.12823219142 134.28095064513 134.208537331186
147.369767631957 124.758906471654 127.91637627901 132.695992860853
139.125340284813 125.172189715764 123.119524624735 146.777258330572
GRIA3 154.665212863369 142.112120308118 146.753212022975 166.730974538475
141.84490608317 163.919426088318 151.488973553441 155.471795004237
154.350107666084 170.410704499804 144.071652839025 157.454558821261
GRIA4 115.35376992547 120.039031290299 112.088944448944 114.084543168359
119.261305304165 109.471542280224 113.479382235297 109.333276735523
114.056147008769 104.285022032604 114.032874859062 113.416743649576
GRID1 119.75069850668 124.329980024986 113.570294287496 113.726931741236
121.965131419551 111.286141277084 118.544870925791 114.658668711121
120.998665308561 124.963418643156 126.446675008883 111.312348806957
GRID2 153.577349107512 150.610633329908 162.327039624723 159.286484253487
136.238646974312 151.687402863648 141.432461374482 133.857996630836
158.287108069245 129.501028506432 149.327794670124 126.148379057536
GRIK1 148.155692596378 145.027741086645 148.87242524521 127.669334230458
131.789877096016 138.014046365103 133.087166272749 136.048100211752
135.576975868985 131.479997342133 133.628391175037 133.602870982718
GRIK2 143.073060222403 140.870849757111 137.018521754443 133.645044060866
140.941447663862 138.064951932825 138.719480482945 138.158400190671
137.984988819967 141.455841055881 140.18668846839 137.161065320948
GRIK3 144.540174041867 151.029785584386 150.014842228372 154.607214750531
144.620226132989 135.784481523923 154.327065859051 152.845126167802
141.426756696799 156.077604146143 145.744039709418 143.756242843438
GRIK4 121.297234504922 111.935554828717 111.260486045985 121.88070274109
119.854401979951 119.446359837504 119.535267326862 115.371180170291
109.96017424123 108.415715678948 124.539006083936 111.608386387842
GRIK5 172.722177339418 176.440625367948 161.514651272858 238.88138498771
149.255405438966 146.151639797173 153.255318944823 154.7326161316
147.983937675946 136.949403128211 159.182533998843 162.769643886845
GRIN1 132.595360509902 148.373866613126 134.273326732349 139.042034301544
138.206065640158 135.217240511902 142.234554325754 140.539606951892
148.096551269076 142.431979387165 139.350622585937 150.387906232396
GRIN2A 148.760280320258 149.85385474029 165.347173283335
182.67941458824 151.557004418682 165.2045030333 189.235998474945
166.12695134858 149.639835429332 164.332073056902 159.887898257218
162.447903830131
GRIN2B 119.704436785439 122.144209450757 121.801291293249
115.967513057229 126.724973604103 119.301746097298 125.509221735447
128.651081092914 116.397855001959 121.260355515095 139.817493158675
115.502679218125
GRIN2C 141.356127724777 136.238646974312 135.48250324478
144.217621961908 141.904041228678 142.34471764492 121.849460555655
132.399633003253 144.599578905334 128.324383749931 124.913645688325
142.551161883518
GRIN2D 116.19219007097 127.822440239962 130.59886317117
119.040642659337 134.038213827843 125.287738115754 124.501262458393
120.433080754232 115.963144087717 112.599970121166 134.060214479265
114.302645757446
GRIN3A 168.289492873475 168.801737310947 156.221773628832
163.170983091116 166.4983332269 149.241358552148 174.127444395862

155.55288776049 168.166938045454 156.745051321143 157.468934439452
182.495117800073
GRIN3B 129.664016819143 132.86711707789 135.425518707018
140.78582131501 156.890299350025 138.586620636835 131.959998540928
139.140647941752 120.082391311921 197.443711199904 138.864844664017
126.877293296185
GRINA 455.564394647943 477.489368828457 513.20896845398 479.053300726301
1347.75629878765 467.387427864242 712.699853328955 632.565736446505
682.730262408017 519.112018353676 779.340880980422 504.9864660689
GRINL1A 142.537484065487 132.642931022024 141.819762045169
129.477908941439 142.592455241752 132.50027119027 136.944378905743
132.626349611404 131.996032643928 149.219602109121 129.067538010904
135.959893035019
GRIP1 126.290840923683 129.093864368842 126.089217853289 126.952836653857
131.046822533615 129.446928016147 129.400895994387 151.067819234238
121.648921929686 129.165440930558 123.256613702254 138.444427173667
GRIP2 139.008854444223 136.518409978222 140.225329742578 136.428826558493
138.848119273081 135.148723192316 134.703474191011 132.623563631011
147.838660813476 144.380485359951 134.579005092929 139.321570881862
GRIPAP1 2330.60389526686 2086.15449885278 2258.30916111153
1989.38201206555 378.920456227378 1741.0131951509 776.807656744828
1118.28423222707 940.008438825765 834.187014201026 1387.90327883679
958.63560158343
GRK1 117.166509072455 127.001546144286 131.249240371529 132.419400131137
119.176139463015 121.103620738719 135.695576019185 127.490889148422
125.400741907779 115.608857734241 135.264074941678 135.722321387765
GRK4 145.604066669916 140.912921321622 142.719216118914 140.774304412579
147.93088503956 126.730314982553 130.257242615762 141.383975990479
128.019837242559 137.246089511045 129.916677638819 134.4380641709
GRK5 799.759250503507 681.591028334969 615.662993833692 759.534249620343
210.605864685448 237.473927775823 237.78482574225 243.225815954365
385.135014083686 224.588225855066 308.679189788554 210.942865876533
GRK6 152.840202260007 159.932941342403 140.579839043912 165.074983746785
245.723316636363 147.327675057481 165.406452831375 169.33926580273
164.373697447004 174.624446035895 171.202766078337 170.818182800759
GRK7 149.408530395745 144.474814266475 142.342811880173 133.499361793303
127.385776388242 155.535222310709 133.069347559741 165.619206216088
135.335107344583 137.06192448185 140.199334790854 150.888616811881
GRLF1 129.079917151186 134.465488237877 137.00397800873 133.953303801604
134.85809559975 124.103321614841 133.576812299149 130.233769058693
131.798931481549 129.813090345837 137.11719450122 127.116637906391
GRM1 118.091125088778 132.834433153076 117.692662275393 113.334665910428
128.44311762955 121.024572716324 118.104763610751 118.206034087145
126.800718471804 118.944068268432 129.366567668463 116.421771593211
GRM2 157.70581824787 185.30916081621 174.161146689117 147.085006263446
173.082167545676 194.847305068008 168.477388641982 199.88618355278
189.235998474945 168.477388641982 172.030687707578 166.104915784875
GRM3 131.212705319485 140.223599881961 135.131890100292 144.302925948596
136.957209426823 258.311739685895 163.495175710216 262.416137698884
146.222195387635 130.975606477621 155.785933672918 155.413300826229
GRM4 133.378374710498 136.952914053358 143.853643511287 134.376870769669
137.495288670441 154.264142444882 156.981921830638 137.575688681319
141.596078062845 148.448349327493 130.122672154144 149.995272585012

GRM5 153.675939908241 138.5833090264 162.297999435613 143.120621855946
155.908951665928 137.210805338701 138.950197595669 144.178210824181
141.544454931788 153.156264091925 148.692602243778 149.308475163534
GRM6 120.157438564977 117.611606270124 131.095431530473 116.943791343147
119.822587087968 121.414076245292 106.263441906404 112.348094614855
109.988237089025 118.32560379073 115.066084565112 120.283706459738
GRM7 143.951756384507 144.738871562895 147.28750335168 140.26206122305
140.086978441723 139.517413585211 136.529820456592 128.057827541114
138.582427155109 141.135840314665 137.070326499918 140.916876460413
GRM8 162.050520530297 149.833346575446 166.382301680189 117.238968848218
115.890431424899 122.426568507144 124.258412895333 123.609527892227
153.293125258215 121.888886996534 138.656528521745 133.71610564207
GRN 538.369868115137 610.591049634222 503.093655556038 675.978379566859
2527.99279063127 857.090339943417 2050.34883263932 994.213397985121
1344.87842417496 1865.44709751822 1539.80266129509 945.830852603704
GRP 109.988237089025 284.595024875896 116.0999658586 155.317754378283
118.589392612801 120.196595293401 110.061459744714 109.454483612299
119.469328303796 147.463822782303 121.36708642226 752.142287661845
GRPEL1 314.126706052299 311.715351215263 293.646275396425
309.861048237742 435.183433729519 528.767644181119 544.956712516196
508.302878624289 571.950858678221 442.027630433527 259.277693314055
634.774041584043
GRPEL2 362.849380477089 333.822167152866 367.518273773904
364.964572587289 325.320888625297 432.174413144791 575.857954264295
435.901794650816 310.026522549372 660.096727566678 348.300300174924
445.905756082569
GRPR 145.371273337639 144.619374612317 137.367390177293 154.412506915569
149.055493714692 143.993611400272 147.942495862714 149.294728629784
124.676743480622 148.863297062269 129.861693025157 146.740694608146
GRRP1 211.104243881644 259.252807304272 161.046418048832 226.315173823168
181.193963616037 275.642882589307 148.547259731346 173.109461200603
192.932285494022 138.276865331434 157.350979071365 159.624341769644
GRSF1 626.104755496369 645.438835606933 720.214044399108 622.142195892701
1747.33558205825 757.877066703516 937.593129242257 798.389385197471
1148.80141486692 787.874585290601 1329.3013039618 842.22767901391
GRTP1 174.761105961773 164.865846124876 178.083311435916 149.997047695778
205.08372943071 216.077955163344 208.257420634745 238.124340832815
221.424451092316 225.546125484563 223.666832044186 393.264726588522
GRWD1 386.917038897322 515.729905361375 289.09614335372 491.112594297928
1520.59210521859 395.049127927637 388.192984832638 403.47026424469
469.206346008875 424.665056900526 389.96566674501 385.678901612324
GRXCR1 150.168496148941 146.281429685059 128.184708916755
139.508029141287 128.628637585951 118.650091706226 131.58537948771
149.200848439601 127.226937898225 148.79958121087 119.221976571036
136.489566741917
GRXCR2 135.242328667085 127.251594986725 130.761936205169
131.749403497912 120.300291760091 129.111259771449 129.735862775199
128.195916531413 117.703025026759 129.621296567128 132.871179509995
120.89590604359
GS85 132.024244263352 112.208950290962 137.134190411005 128.568463697992
133.281505040209 140.815807483203 135.966929064294 125.08580835258
130.49338418515 139.057572927874 115.713909155043 120.669452856028

GSC	170.140610481718	145.464640276742	166.330001480033	173.044636575826
	184.473198008234	164.03801002919	147.561362873855	167.42476096854
	166.027988561421	160.006185904123	165.813771791917	157.032746784217
GSC2	123.868848032411	115.550587451456	144.199054675488	150.945167884077
	127.04217189075	133.213697351807	117.47631318854	123.344450694117
	116.994216357989	135.591199815271	141.583936153499	141.38612990166
GSDM1	124.324855155055	125.143281492089	137.782648137869	130.584348445667
	197.357728220717	129.385416279101	146.75924072364	131.814528687104
	144.784835438567	164.136551371866	146.65668813596	135.067056559409
GSDMA	149.113794439994	145.989546968117	150.626826756019	144.101376910581
	152.829437376835	153.566080603874	160.168654167074	158.946177055546
	161.632610858756	215.143973502566	155.228605554943	185.591700082272
GSDMB	335.790909130243	339.663295617139	340.903961204211	270.520229426015
	206.972440734038	457.292146759215	620.614820117452	1995.6000523628
	636.56692204969	267.285998701386	964.473774310687	1116.95795024994
GSDMC	136.5680632044	146.215459988012	134.310685502031	123.45004246675
	151.431513447721	153.498332327444	159.487615985875	150.25322837689
	157.5032461329	770.632068590053	160.482734190577	146.689482187129
GSDMD	608.959089074987	577.801009921199	620.899414581162	631.408955630318
	671.563789496889	723.250763797522	501.434722063309	803.840404197117
	841.623780229898	505.704957182936	664.343553341563	555.259469428492
GSG1	133.691053105482	148.844916975586	147.004172510641	143.939522733945
	142.589312139207	139.502068079466	143.432889871079	150.198869833614
	138.252656818036	144.339819009283	152.832573288735	148.80452641061
GSG1L	125.958139354095	112.371497988132	121.146511496024	116.300028266671
	109.599589420575	115.988180787152	114.734439905052	127.494672751434
	109.69466049307	106.797707723826	120.822923434486	110.875112640101
GSG2	99.26400442869	114.888157310977	115.80656409185	122.010855318111
	136.061992660698	134.244658505187	111.632811186988	136.978738825445
	118.383317097375	119.550086560732	111.837468699281	127.527378949803
GSK3A	136.016912147651	131.202090507576	126.630787911053	130.98982984989
	145.476377346532	128.69455713173	146.105844205919	137.954283575143
	128.708956947928	120.308260557986	127.931887647108	150.367927843529
GSK3B	344.298510200653	328.269845211173	265.219444616245	385.584224833836
	585.552940765069	456.990425618007	395.588345893987	458.151323130855
	492.534183006883	665.106631364961	292.185246573643	547.042461070351
GSN	2563.02488807613	2010.57142784228	2527.99279063127	2900.55309635211
	344.737964092526	167.984051648058	160.322541355391	176.456539071735
	197.169703621888	264.604297630839	238.782040902727	182.886100305943
GSPT1	1974.49672149862	1899.94714976387	1883.74008471233	1777.29626660109
	6028.67062754577	2856.14739820295	3258.07532987071	3298.73393277409
	3149.26998719015	3090.52810501797	2033.67956299225	2974.04694562902
GSPT2	362.095074331893	321.426100243434	437.836503489425	338.866471651692
	274.047586063773	200.424975825325	176.312626750427	190.371287639453
	213.602504580364	183.443006525616	203.276353321185	344.529927973082
GSR	201.616302398819	193.930569647995	237.045634416039	226.221973502783
	261.96528977292	228.616558999054	306.387898121409	252.655315526015
	219.49354518392	282.797367565702	229.406012088782	256.538195034956
GSS	226.458509834965	239.572670410818	219.792171863015	293.646275396425
	548.869880723909	539.853394831917	505.404271675438	475.213525346293
	311.481739745423	458.218913259336	329.88790758321	401.461825804111
GSTA1	126.473768158566	138.891642304194	124.768399055809	133.850733997442
	167.201570648434	249.029098902357	245.723316636363	241.987100712218
	159.926719599391	136.306727083526	179.950904921667	141.634246722965

GSTA2 134.226711030456 129.001192144163 138.67221078031 141.021723049782
142.139331321085 160.203521879896 174.454754564111 216.457866890673
146.395248696835 137.267307036807 156.638070437044 136.238646974312
GSTA3 160.131256419524 147.545667896282 150.552186000149 148.046961712162
144.529801006864 147.607202745865 149.4401812 160.105903942674
140.616811333466 143.897248922317 133.631676386591 148.13657736444
GSTA4 448.767690095033 469.64693728427 567.869601934138 439.023181761356
190.549084022965 290.754210152709 173.459008248696 305.906480933354
209.051768656607 515.729905361375 175.111460651956 223.389795643722
GSTA5 110.678680817471 119.103219740679 114.658668711121 114.428050500096
117.706795566317 108.570463496052 112.661607612444 127.3079781139
110.575582352222 114.607138204495 137.109541758085 116.273000405449
GSTCD 144.972694558182 152.987939791009 165.932628858503 146.666759322506
148.853877151493 157.83121828793 154.483828924645 158.420821486138
157.15947600423 141.420747583126 144.287786573389 151.61049450647
GSTK1 1243.11900823259 1210.40302822121 1168.3174645384 1240.4863200811
1375.76915021882 2660.39990175415 2208.49325118803 2244.6916226146
2154.00589005104 1750.21700780511 2539.87833317526 4037.15442268836
GSTM1 1838.18547839898 2120.14463347544 2372.26073354008 1438.33289891548
270.360397503276 24098.0251582624 17496.0674176329 18220.6383250956
756.068695265118 194.734255772365 604.237898104299 15924.2167735021
GSTM1L 143.222342904886 137.176144667253 136.152827218905
141.919263493165 140.57567768682 132.817645625115 143.152993168137
155.42165869714 142.577102697682 150.566842106244 129.452826518288
136.444883411968
GSTM2 2346.47361943533 2799.0487754899 2952.67114077688 1776.38646235135
185.984938124891 17830.1333276624 12968.2945749 16328.5991894287
1007.41624313399 208.385295246155 336.067655676466 13360.9483836861
GSTM3 1883.74008471233 1367.19652160532 1284.19937294949 974.544773560873
167.395369670065 1110.43791444289 192.209344004015 148.511059836029
408.263886786123 149.292552957665 397.660202059209 815.779445689417
GSTM4 197.034073391478 185.727947276958 218.839681873515 193.190059954084
145.493977576106 257.813193543819 453.282973743379 268.52833723726
182.32514927211 134.648780131578 202.869364962231 294.410833087913
GSTM5 589.953707188378 539.853394831917 484.764806127211 711.729857540712
140.083495854322 152.054874792646 121.207040901483 141.368078865065
144.298011270428 141.619432083003 133.900005368973 156.208747691032
GSTO1 1863.96986643676 1832.35076992253 1978.12229412176 1559.26487922551
1137.42879398351 1690.96371604294 2166.68174726155 1505.09908072606
1738.96798001895 2068.04582900231 2601.89405525276 4882.16892310839
GSTO2 212.037224737798 325.859917318516 207.831299397177 155.715728894101
722.399669963921 680.250735532287 887.471553448641 699.984140389009
666.847934976479 786.62709337246 1153.5175534971 1327.65573168233
GSTP1 4373.91897463938 5361.3600315322 3949.19206005238 6670.51138785163
11598.65499235 7902.9189607838 5776.63686968234 5714.36902486392
7771.28016059011 19087.9532735841 10765.0336035748 9527.86367804896
GSTT1 633.594811068524 591.013515828247 1329.90419210779 139.324006489527
167.822075935474 1334.69145271749 878.986106116952 141.15458637181
796.052115217208 161.514651272858 122.262664384198 714.274201838067
GSTT2 168.071171406779 178.235281981508 151.839306445341 206.149539851803
145.00699763203 163.07139884152 151.973681370675 141.739299634007
138.323567041191 189.027594735456 140.903091584884 156.456332386608
GSTT2B 164.327140372968 144.470642732973 127.177422395113
169.999692055871 137.416133786828 128.155080148958 137.954283575143

135.75568942028 119.48998115497 196.974807677659 124.595925483149
148.353927712013
GSTTP1 148.649619242172 134.291229819546 131.649830934459
132.834433153076 141.064086298948 146.108551647446 141.587109176431
138.962649407431 125.707228291312 129.856994052591 130.772410329685
139.191746593825
GSTTP2 312.645778869254 267.535469518664 285.46340356157
287.287337931982 167.645930148254 328.33387481068 218.529160053398
245.082816415008 211.572396862776 201.954167006591 280.913748398876
224.6588173737
GSTZ1 152.371569700039 157.269049853539 138.317443162096 176.355556618289
187.881334544018 147.691011072898 156.114825514646 218.70412656621
164.890716656336 185.560737176115 179.163748161358 184.844915350411
GSX1 134.964619235561 126.359440164601 116.07902130325 119.469328303796
123.952494134263 141.533138525284 127.354826381656 116.977202125093
130.351302840602 122.583461806645 133.04222812639 116.263405190786
GSX2 116.437403962939 125.079330440615 112.036753245281 107.316786994369
99.7487411813724 107.260023460914 103.594872791657 115.194730717797
104.406314027414 112.219585614955 103.520748827831 115.392989580183
GTDC1 180.437648146588 166.692386207143 179.177884962513 163.426663985855
182.143506470859 168.830344627025 172.84784588171 171.314681729657
167.186780161168 206.802308105224 172.562162872242 172.509081696309
GTF2A1 134.46743016543 134.70848693317 128.097206000942
139.287871460674 125.739946257697 131.342562516383 135.023594109361
132.322618637842 134.42983422342 127.339018771488 133.115543233699
150.909559587354
GTF2A1L 181.500222423634 164.665907233149 136.115306932124
160.415807880532 120.767950825369 133.686850910378 130.66863243733
122.176811388389 125.04327844782 130.822330736013 134.06996703302
134.741781820234
GTF2A2 946.273229715791 937.593129242257 1230.00744483191
781.208872924532 1151.89087953837 1296.10827265334 1778.73916100529
1682.64624665931 2039.55802107872 1630.1630053946 1238.11700377838
1821.08523647593
GTF2B 302.1846598234 281.792602269388 361.082957133644 232.276667029184
324.401611136662 704.653073777277 511.113083958771 751.077307018319
650.0804221227 520.585319342817 462.779122987815 516.755267232127
GTF2E1 171.731395791818 180.733673680261 161.172420368335
160.17208299932 196.429685815196 277.219796733134 208.816482659752
244.401472859026 212.854068610681 174.761105961773 219.814706416471
197.938447651153
GTF2E2 1176.62418139379 1067.30106203503 1273.6393529108
1199.35864276583 1045.49947183547 1025.27246494218 1424.04009914668
703.29965727829 1387.90327883679 1492.83354014907 942.41077071058
1492.83354014907
GTF2F1 375.131728190867 499.964000319181 437.344765185802
567.143102792772 617.67819271245 565.112283062121 448.106077184152
452.606433325387 449.408793796673 544.30845362168 698.358285634451
518.925188630246
GTF2F2 783.373390305885 723.807658768693 1047.78978081493
825.781627776577 407.442337662943 619.75986112089 290.213039891361
580.474775712828 776.411045078049 994.213397985121 421.303580698397
919.626220838414

GTF2H1 266.563990988678 242.096692715029 259.826625866106
249.064573038094 170.98312926 183.937484597232 219.792171863015
195.078843867124 198.522123898437 211.736398352592 238.654367531982
178.43642939806
GTF2H2 117.499697378893 119.469328303796 117.445284668654
107.930325780047 116.011005504201 106.285472845323 136.238646974312
119.859508630454 115.35376992547 120.665520347481 130.209752198964
127.535214313797
GTF2H2B 234.947745514175 183.100286716683 231.728984785324
200.204064462384 186.951061159734 217.576547690525 299.717935831288
230.284216427158 229.130679600654 287.402339322061 262.488050186425
357.140791982825
GTF2H2D 120.633217745524 139.038389244329 137.72327986309
129.419280058541 135.10155936286 113.609215169397 132.651922794567
118.091125088778 133.425151103622 129.705735906962 117.312094804205
128.23910347716
GTF2H3 198.659595851481 178.684992551017 228.025383952578
214.200081541057 226.506852362359 306.59090554099 352.741885295157
332.978350226738 327.010503005631 394.892918166519 234.461229199142
315.064477782052
GTF2H4 282.926325589238 323.628636799403 305.252666831522
469.206346008875 598.449359425056 251.161789722751 318.06081614151
244.897869154624 229.85833221434 275.888299685521 309.937351755502
286.469773081481
GTF2H5 1228.89116158868 1219.36024606325 1640.99641023476
1243.11900823259 1178.83933381069 1303.33810980467 2379.15291239548
1946.45172007314 1395.63393859192 1628.24528041404 2004.19532096239
1958.96627327952
GTF2I 163.362182585182 153.574068503306 158.058346079359 146.713861499546
190.5930909277 190.850639994777 209.362806125315 228.616558999054
194.186994486095 187.572517928061 185.905819051223 287.242697383558
GTF2IP1 401.542072222692 376.791876040886 537.510539142248
353.826834642154 621.354393918348 754.97177863997 867.369315017411
1050.90881385623 1028.39666838369 517.993093601127 687.269774217064
1040.73829397913
GTF2IRD1 209.702683803624 208.237011955037 180.881464829981
221.390817970859 304.751330890753 202.145180994821 243.246632667118
209.662262708551 210.605864685448 224.424524191566 258.036064876416
240.631264915238
GTF2IRD2 176.431477850092 158.25554353787 228.675099004411
158.221595000183 157.915292037378 157.619627711954 164.529828080312
186.653791045656 173.121618125421 172.827288328708 153.418095627352
172.604689611475
GTF2IRD2B 233.884660128222 224.766155406229 353.674560414317
223.918931440768 463.622673539254 309.580750386386 455.08515350006
274.627303114477 266.71318061066 420.361583975644 264.306927596371
651.43207839949
GTF2IRD2P 212.545989139388 192.399146702036 321.177982468322
251.915179734134 169.201608999937 158.653161314597 163.284853420896
174.323881548679 171.080373870373 141.858928084825 176.689683169567
171.080373870373
GTF3A 1752.50728312967 1961.26255930192 1500.16816750361 2648.41012401723
1978.95750795951 1944.15673951528 1500.16816750361 997.460212152591
1607.52029676299 1249.89496565284 1323.91781040143 1306.20183851747

GTF3C1 292.185246573643 339.172031360524 252.473612525884
407.152764702613 580.164288826741 287.374706071354 263.183010631644
257.941367816557 306.896732729554 228.820894086914 206.605699708924
229.511643376643
GTF3C2 357.835334661098 394.576038252334 397.737702926328
365.286234166621 865.435843446989 785.008529077795 669.731546951281
813.034056288861 611.208083466988 491.496499481595 733.49038042129
698.086166782221
GTF3C3 370.466403181536 360.991542429346 469.714826990063
339.917319653382 346.476121089685 771.512332113075 544.462032960964
734.97763567765 533.54045706916 692.359779743985 749.220616713093
346.476121089685
GTF3C4 157.078563342652 131.587987756056 140.181179402328
128.525769531728 163.736324784234 145.301551442388 144.346988809708
134.134810805413 145.27890423835 147.530719472217 133.874468336025
143.2438241778
GTF3C5 296.915883082902 370.617422894068 302.601458409102
407.518324776453 706.292905820227 370.301819403832 249.354198174438
290.638110977807 336.212224495877 318.628084728596 290.241064926886
202.943987922418
GTF3C6 243.65518489122 249.449151040445 294.22752955801
255.435582426271 320.979443123471 370.877260088621 259.252807304272
385.135014083686 286.541028896473 465.683139139622 356.84281623052
543.710330214344
GTPBP1 142.434356144925 152.552277008504 134.599629045981
163.045605069267 173.544556426164 120.727504500018 145.42029807396
137.049677918041 137.80436357695 137.908991199407 152.353494489233
133.36168342199
GTPBP10 155.61996871093 159.443697452694 187.264411519101
137.5954358221 141.218832234405 163.566535828036 156.269957530519
177.524818593325 169.791616021987 156.210423316722 159.311949668403
206.620708230452
GTPBP2 117.024039521425 118.708644971927 118.540620928268
128.753623886188 149.712320986646 127.961153369579 152.385014030957
122.226875707253 135.513494853742 141.117659724704 182.747079625619
129.751916520201
GTPBP3 238.400565506288 236.933960698783 230.447203571825
260.642170838161 317.457588721567 297.636316709322 258.759507964221
286.085833802132 246.594244983507 303.005912224195 204.770742504612
307.316241247336
GTPBP4 920.342429282889 804.800251415824 917.646958427824
716.229989890801 1694.24232555141 3008.43964505208 1279.9555644913
1793.75125318113 1329.3013039618 945.830852603704 1260.50387106816
1942.08859727404
GTPBP5 141.066106476109 160.496585125233 130.998637545686
115.217608555334 141.426756696799 124.401083237195 125.025064237439
126.124937863679 127.593764897583 129.020356016653 143.870849001074
125.712260481294
GTPBP6 1728.86115695979 1682.64624665931 2004.19532096239
2418.66999907188 3843.07094386605 2041.03024136014 1617.44377452783
1751.22580157752 1626.34607278613 1013.03054571763 1601.19401366685
2493.5484556802
GTPBP8 197.740912476527 198.055877414824 212.836093887552
196.076386020815 190.152824885328 217.327543190687 237.906142045719

231.915272112361 241.105817192695 338.739180103373 243.402781509365
228.879434668972
GTSCR1 161.866405026758 144.114177076352 153.744962185987
150.51056917809 163.134784474437 183.302479730653 169.691392833277
163.081050585097 157.43824048982 163.809967446414 146.197678850188
170.342828808493
GTSE1 122.733870240098 125.253637835638 117.696262292883 129.838790698396
203.298764374262 174.127444395862 160.35668299062 191.961269585431
179.663258610881 209.146021798649 170.361237529247 199.353823724989
GTSF1 145.799530970042 179.286187825363 158.120079903425 147.772490977392
547.202354339933 168.223865801948 150.52625734893 242.5085895186
159.199045715212 170.825161347825 146.093387013881 161.573975848093
GTSF1L 119.696656494403 119.498312996987 115.14883090249
123.716400094852 106.088807114602 112.993452506946 125.742631850732
118.593717189375 117.480506535399 113.644993086829 138.365589511693
122.410340121549
GUCA1A 143.787766055379 146.359038260378 147.670425834523
152.122141650397 141.91067228913 144.63774603725 130.321133400634
130.445920648705 143.707084981291 136.104965836123 157.113536717393
140.684066288019
GUCA1B 159.082447644277 163.340435089911 160.001115445038
157.189041157784 139.162076054072 144.358535632993 142.452409969639
162.289781945582 149.824791474186 150.000345076364 150.403702679274
141.53717142784
GUCA1C 179.905809470907 227.32463765717 124.561511833959
121.57069706238 114.400185930969 119.558626216818 124.418295003824
114.470670941078 112.314595956147 113.460835359833 142.034369454643
120.003095000297
GUCA2A 143.965049742993 151.134466830828 148.711943433557
141.436235043864 480.593729033799 188.444498848767 185.627515956848
155.612543356905 167.397758092427 150.746423638097 150.655359765209
155.056545639862
GUCA2B 144.143692745479 167.114291248266 133.443807124822
152.57040089034 198.470718196139 153.748035612441 164.357373919714
166.640325848102 149.483826392464 183.795632040455 124.031916322066
157.736101691222
GUCY1A2 132.894049694258 132.550022967297 139.699189528879
166.12184890921 135.790955294639 143.532014058535 153.818325601312
135.943252855457 160.261708910517 153.675939908241 150.336115779606
148.463566046951
GUCY1A3 257.685284145848 287.503594820801 173.647320180498
275.584858082096 159.815932985135 278.775971122032 178.072538529634
192.475309217696 222.764568757453 344.786541451395 151.869928694006
147.676279102364
GUCY1B2 136.229159638766 160.303394115537 135.195985987169
155.247452081206 149.200848439601 164.545596179262 140.732618320154
165.169920299969 150.578023008958 148.421611987655 135.771423315597
183.416437587938
GUCY1B3 157.530573508003 160.293522619371 167.139257044999
161.16688895179 142.635015113687 157.821491577808 137.438984175012
153.279026396412 137.172030225913 159.68134949161 153.752502094283
139.408234219133
GUCY2C 140.338622198206 136.378203924435 131.202090507576
132.308358487671 123.98384416057 147.079157199448 124.222529964098

131.497454038525 125.892022613725 133.318665755015 124.959873714884 132.216453078716
GUCY2D 151.730862403472 145.159572111039 156.548858402702 161.640122360448 156.698856643638 157.126460242995 142.004558968904 163.904137395349 164.274491958161 174.155599133789 149.331805754859 161.17923997423
GUCY2E 136.335679863251 134.327229861635 137.674025476238 171.706534562905 139.42531690926 127.26820735867 143.764266466204 156.311009595629 146.740694608146 143.616189776965 146.157459765796 137.432492568789
GUCY2F 143.06806126472 153.051143478997 169.87591929982 179.632562895905 155.358918578397 187.132496910208 159.324816814876 169.574755162815 164.136551371866 168.878645908397 140.301945597122 165.022029012505
GUCY2G 141.121364213272 141.602233757588 145.389721946046 132.122273848715 119.370219299491 135.504526903482 140.646946425036 138.35738548449 136.256456726841 134.465488237877 140.909875639658 143.785769607842
GUF1 191.692830869809 205.495823410422 259.72035004624 210.085329463347 231.338258067616 250.82359383789 213.813667557079 223.359785518285 280.558463661137 379.102958448288 197.895094054577 244.5675215931
GUK1 2374.1382412432 2320.41293992992 1828.66165670455 2352.01196541874 3490.77593331727 2129.84680802619 1790.53556140959 1025.88559245013 2076.51786803222 2992.18604022433 1609.54149931375 2952.67114077688
GULP1 164.281196089736 157.81327663167 170.825161347825 131.364187047583 120.325985681299 123.591078216224 114.826501559828 123.247698282967 122.254824223167 139.907840403521 112.693431140524 123.25201993575
GUSB 457.548150688248 434.325609889068 489.528040775004 438.533112511098 1034.2217095753 461.143361255467 782.448692528135 836.235700891687 609.100385482325 609.665323786904 661.826149512811 781.790858580895
GUSBL1 458.705688226305 360.124745133393 833.198511430615 279.070493043898 314.646369385896 551.086785904249 402.694564608582 500.132469062228 451.147418314412 481.86545778383 461.482735533799 612.758976723556
GUSBL2 294.997671630798 269.726397478364 252.222481637238 373.860920943049 295.778154263981 329.406649846151 326.45984882427 341.43511159996 226.960024551778 261.366143735074 236.191384383802 271.770138565186
GVIN1 344.487982154705 390.752579014611 212.5119818331 282.963958072528 202.819949307954 392.582612044755 222.848935978648 410.138738410101 493.209107049493 190.238332933452 207.73395131954 166.958831664154
GXYLT1 252.10470581893 249.80160363144 254.424207773566 250.718986968076 193.522811257085 273.353254857377 314.386320310898 315.752980521266 266.19823384117 186.268881719287 287.287337931982 278.687780705367
GXYLT2 152.199095472082 149.419308407564 162.351212914573 136.514946957145 115.566303449363 135.600312863552 160.493314658788 133.348510114432 136.432109029368 134.094231940612 136.104965836123 145.048033586779
GYG1 2962.11407134162 2305.11856249143 4276.70912292877 2601.89405525276 1162.09529660662 844.187489702587 873.879064183441 1029.75852676191 889.054356462993 1017.6969123529 986.65554605455 994.802763887989

GYG2 205.476230849391 193.98814423456 206.469040492609 224.062024091915
177.592497020798 465.926873285563 177.151218006672 146.215459988012
178.955938825556 130.342425029134 135.68605915552 140.525013926642
GYLTL1B 147.371905597494 119.207503310618 140.493975416335
142.179197020805 146.164093136588 146.366013190845 159.701028026285
142.052454741558 148.866229889479 140.016122996485 138.184717352015
146.686192012988
GYPA 122.002822875994 112.476111928742 115.863243613121 110.256771079797
107.841100741858 109.318922864507 115.293844461817 116.282988576774
106.088807114602 113.091739139626 114.143874572202 117.303671445328
GYPB 119.072213573568 128.488598467312 121.45175516842 122.676368520743
111.448871835514 111.137519376499 121.065278765151 117.862532836494
106.341910466588 116.884397420467 120.660349211375 125.121517583946
GYPC 1093.85316636801 1080.42884514937 1026.61404215739 1367.92869625376
161.94312376726 195.400963839097 148.211354898758 203.475080163804
312.286788541158 187.046760972666 172.502036705114 152.532083511961
GYPE 146.1289358176 139.59941545613 146.235832275574 153.726935957906
140.714455864886 146.619846538124 135.1951749488 140.327799236005
139.917662338546 136.278361222532 134.327229861635 137.068717031383
GYS1 166.315369318355 168.773340961473 155.298546821687 181.135519188216
357.835334661098 166.335294339013 207.076364103755 165.642728895682
173.395370400371 186.167877791105 174.161146689117 193.569894919176
GYS2 124.944563166658 137.430389116025 122.751674866182 115.327402810793
121.04331582508 119.198789189881 118.572988141773 116.186846885411
120.201521341263 128.118223792086 138.213742418402 130.317604643904
GZF1 128.927103442679 145.540872377507 143.519786615253 138.661548678191
144.513730107393 184.813094907887 129.95138839192 139.200284631709
135.134213292186 120.076742232995 136.251820364823 138.394923932916
GZMA 177.450674276588 177.679330300381 130.268322464805 131.017355402204
145.296229550382 169.349922114861 136.353051841792 220.113261580385
334.772648743153 190.444387031552 393.264726588522 152.635792342068
GZMB 123.981053866137 143.234195669431 114.378420394004 121.177681858671
137.654833215154 139.997245010492 132.926799887017 200.536814571322
523.435696794756 201.507971833902 179.048139035247 132.649409216699
GZMH 183.226294747874 160.045715314465 138.685330935707 154.783699020901
138.158400190671 155.70645844794 146.242897296397 157.787233338636
201.415695076278 155.741507341843 134.9404536551 152.356731704094
GZMK 187.046760972666 158.316173597874 127.137447808551 143.658600740177
117.692662275393 202.99342484328 162.122250419992 345.135289739599
305.499194075747 152.488480322526 189.575681012578 158.521812954982
GZMM 164.288645768576 154.98147307591 144.903414594588 154.569432344392
159.909121784808 168.824578400555 167.268645224361 164.690739760426
177.812335593522 149.831566330214 143.830319259804 164.000772606739
H19 2816.29814823646 3615.76740804718 1969.67552562117 7054.92270618408
462.686385881492 6156.29203791697 269.62298406345 302.272304483941
248.229468372363 20099.9833491654 691.205738835917 250.25959091612
H1F0 1399.41653736746 1966.09691567857 1106.75785702993 2291.40912229281
5811.97916044218 2168.58813300343 1566.65825409044 2871.34694522381
2449.76376608893 1567.89763050645 1129.15018335354 1978.12229412176
H1FNT 167.468390483999 154.371946675862 184.029968090084 168.415345783635
175.313689069815 161.390489603929 162.484777758676 157.891350650633
166.452651396293 152.317890573534 149.541917326637 171.49930976135

H1FOO 137.53234136613 144.41489969974 154.831307586538 150.737888487925
165.547163740218 157.792868983503 148.614214326995 160.382971629272
151.342902631318 162.505305538911 138.652735298338 169.293852989203
H1FX 627.803400871547 761.726829618132 481.037647470809 803.840404197117
1726.15788525017 414.713379367927 454.133372808458 422.23268864343
750.494286476465 646.704226815099 499.891000749138 459.652708187549
H2AFB1 130.799009128937 131.730758460802 148.832461329713
145.172776053 141.139737175648 134.89415027205 131.707530115415
134.56314960832 123.129785564258 134.635645106042 136.805379739087
141.65642171417
H2AFB2 113.886420801388 113.879459563492 109.30275288389
123.782746527199 131.04482088126 115.713909155043 109.700513642061
134.530516701271 120.089228795888 117.790593990682 115.384304932365
128.840466579373
H2AFB3 123.119524624735 132.329892644992 145.741465098455
130.40517506216 147.889486635135 158.675960205792 148.975112606364
139.593950393555 121.675007387533 128.914885412263 116.71331256222
142.090009728666
H2AFJ 620.760947818536 742.128229715198 632.031410234577 596.076764072945
4266.92715355342 1391.89900758685 1596.33832632698 1582.0221231161
2515.7035557541 1066.25208226214 1281.9073569335 1195.6335460045
H2AFV 168.682307349713 161.310855691717 180.265418365803 171.51411661213
194.352807599399 165.48594766783 172.631499322646 172.319604146201
170.775283924087 149.675363177375 145.165443106042 172.387657419201
H2AFX 157.358696886078 184.493681387276 167.421776383056 175.908645445479
660.315285709317 253.697419495959 212.571491543077 306.333710880618
398.423396126075 219.78041403077 239.541516080716 300.358738353443
H2AFY 1154.1046856215 1202.74869573717 1153.5175534971 935.423045210635
1780.97169244555 2242.73511149559 2527.99279063127 2649.39312662326
2200.94974896771 3403.37530051435 2061.9164323309 2979.27692778783
H2AFY2 824.746488539942 799.132592914065 745.482863218223
881.15414246017 1258.16519121331 934.546444634669 961.931130189779
1008.43888926584 1149.3250945792 657.562129888446 1460.24985399013
2005.76678571689
H2AFZ 3693.4058156307 3566.89107958971 4995.96906061198 3255.91703674439
6830.46932926077 10280.5093368677 5250.88041666757 10222.4435857673
9852.51412359574 5932.37714987741 2910.00269086567 6508.71638048514
H2BFM 153.603765998461 141.473218014605 128.65678873939 138.113751487484
149.113794439994 140.92377681377 144.167857433516 145.641373616866
138.726894569892 139.888008605915 142.495333453346 179.321622114583
H2BFWT 117.207266754941 117.847744476387 113.672605838627
105.450273343324 115.545558600232 101.431474941041 113.529619135543
127.451155926679 112.495783269169 102.563722570171 108.779204939177
123.484987952263
H2BFXP 147.93088503956 126.793778645808 142.49005761699
139.81502919163 127.699226067847 126.330382425318 137.757902615328
134.966883985611 130.661695937728 134.85590586532 149.508432746895
134.616051224668
H3F3A 6044.78942014135 7491.93659373205 6179.86800320807 6793.99737496637
17067.0086403248 15809.9561036761 9852.51412359574 9596.10781313429
16077.1585881362 7656.97151002785 10048.4260790604 15809.9561036761
H3F3B 1885.93146154931 2741.71904421456 1426.56252600395 1973.20689165831
416.546844844021 3455.20639616159 976.360310504918 1149.3250945792
1226.74043569231 655.094843900848 343.833659987063 1593.49895307361

H6PD	165.386056999339	159.801315111886	171.61704153463	167.640645997754
	146.019622621809	141.980728956046	162.37762946664	156.157567405927
	144.774621469452	135.036576196425	157.639688257892	154.38977085744
HAAO	177.267145265362	197.100205890532	194.847305068008	171.791030104358
	175.757588503244	163.72257517759	171.791030104358	173.233357775898
	162.55521875551	165.459993078103	168.173803952157	168.061346742356
HABP2	109.355444907633	110.486191378104	98.6941019230576	123.538037290702
	111.962852412774	116.236538651031	104.58424134098	119.334967856721
	109.564709878576	120.300291760091	116.507315786826	113.388450168062
HABP4	453.282973743379	416.307629596091	478.479988875208	461.088572554864
	199.933148219784	150.962828147611	158.747029092329	166.4983332269
	172.237115475147	169.16155608071	172.108795190633	137.188849160569
HACE1	167.536828038178	155.259089547817	181.622689471026	154.808798024399
	173.850390626782	167.658560306884	179.985468369202	166.04965258862
	164.89597889259	181.431827312784	197.205792984885	184.241640200823
HACL1	421.836020491624	430.329437928013	465.809961695043	392.004079531068
	628.485648053393	574.6506349984	510.659785046811	1247.02514044778
	814.759191652981	436.731545612902	496.328898534802	465.029298672645
HADH	1650.77281891596	1590.87426068638	2114.9754128463	1629.32740709526
	1364.1224589383	1490.02976786379	3277.24948092771	2293.05058904656
	5382.31371568106	887.147199303294	1969.67552562117	1287.3125466789
HADH2	228.852495818211	206.669783013221	168.71216652064	261.426617866925
	459.388390392518	358.897128592042	262.53374754047	367.898258897888
	310.690317681243	364.113665301598	227.467161919443	330.553659265085
HADHA	444.584594376357	476.383294794912	399.664121956178	573.644925180854
	391.500696444149	360.605223912114	360.243484271891	444.479355480138
	273.073938260793	363.343480850598	240.561105692347	318.73799597811
HADHB	470.746477818201	463.99922423125	716.420861490714	536.067857235731
	669.124699103132	928.151632070573	692.945420109927	1464.04124596264
	694.907086612929	458.151323130855	867.369315017411	765.333633142088
HAGH	581.828502821435	548.288640768746	740.196934996626	640.568342662067
	732.040299742444	597.651682540611	602.86705608228	586.366632966978
	522.300029971277	479.261755380872	413.000072500071	398.272713014637
HAGHL	144.101376910581	145.560904207183	145.830916005206	135.313148180961
	152.738468369498	160.019067307733	147.576533768761	148.034899103241
	149.108582827136	163.764600217347	150.452467115669	151.148295679242
HAL	147.592770523507	148.544887355857	144.242034773232	164.185346950306
	155.502363062633	150.924948809354	153.100979553727	154.648806105422
	158.625060605976	150.904618960507	129.705735906962	145.265512354674
HAMP	145.261662166215	143.526983440589	138.625680450198	164.347168837655
	161.331326682743	170.918584418153	167.408314067262	157.915292037378
	168.3523504179	169.124088593549	196.638594797353	164.678646890649
HAND1	920.083835296898	996.999238316991	1801.74467005923	1887.82245931444
	119.087972087745	110.273174147374	118.875640081461	118.505980204837
	120.297848408451	128.742617750403	127.935614145702	116.52455193626
HAND2	259.064601204239	275.298642145005	393.707031354025	309.997909440664
	144.114177076352	148.635174393031	143.567488854011	151.169283505727
	152.733409144267	153.363629311799	151.742938010671	145.841028932318
HAO1	137.794858639838	146.392657608076	142.01454627498	140.52059487958
	131.722397988741	143.107570838895	148.054367166566	142.724123724574
	145.804420322848	154.076216899447	136.969124414576	139.522018789485
HAO2	135.281686411685	137.027334042585	140.73456875677	150.642465826166
	134.415163103911	144.946362772133	127.43866970659	133.651203764842
	141.353084959289	138.698178321535	126.085039271526	140.232244264861

HAP1 120.322292418128 118.855500322915 126.578035793406 128.65678873939
130.912137250515 129.664016819143 122.696382705233 127.980776299586
129.618097261902 125.443242860371 142.744045151046 127.12451522286
HAPLN1 146.266350809492 142.792294709664 141.626504154989
133.209211811498 142.964043764953 127.242627779136 153.485731788293
159.334165651951 168.729301938015 156.934589811742 142.163834995141
149.874424976813
HAPLN2 174.177944561595 167.194306682927 158.371599884762
190.165771406362 160.35668299062 189.362958041175 179.091917512822
170.622615783608 146.332425559689 172.836730741407 160.75642485037
183.905926269285
HAPLN3 125.379842077431 144.725650040212 121.945541820091
130.75707278606 164.276668889213 123.949411804458 125.189588215659
131.373012256275 198.226525986857 139.191746593825 159.094117857743
134.964619235561
HAPLN4 122.254824223167 133.017537400068 114.143874572202
122.182695593404 131.430921437192 120.925930409474 116.087696503753
128.075865243385 122.281263517691 122.281263517691 127.202039384041
131.326536951329
HAR1A 135.491412261604 156.445294524594 155.993849743656 125.979152567959
136.284283129801 149.625528206375 148.47415004128 142.650451413991
130.659631155616 126.902441495413 136.420062669039 143.931400351525
HAR1B 122.561285351587 146.484364264513 135.047546966745 152.074017988748
132.098421212796 129.846685899183 141.772724512502 134.534815676852
149.921414617149 142.690911343704 159.724824188037 135.840528665314
HARBI1 143.444109491884 141.88777975766 147.919058170753
132.741859397511 141.482344048665 142.605816057276 148.782907912893
140.955772094739 146.501559368223 132.554328715753 146.402352419692
128.957236136715
HARS 964.473774310687 1027.11849332495 871.913035811066 1493.90578573732
485.576566972696 730.673649872205 844.778755052304 637.457322054293
512.184962338131 1200.03423010236 657.231047829537 700.353187874339
HARS2 669.330078861673 647.439372562271 643.468308027006 675.480482223958
361.082957133644 590.142817130684 1003.93455977232 924.312051919404
475.927798702033 992.320202251161 1010.05884008623 1112.4055726239
HAS1 122.724429060184 119.943408501009 130.326980619676 115.194730717797
112.612099135361 117.873333897609 117.178495957357 115.00776048097
118.924317138158 114.734439905052 130.268322464805 119.551741097918
HAS2 141.842919268688 137.666849842576 135.659271030454 140.267351731167
122.436106757578 138.930413256469 136.929570710155 130.920010795017
136.817517693076 138.388749930671 143.626857507866 137.782648137869
HAS2AS 166.143084832472 153.159425380743 133.625358030199
160.374491283254 179.009510246709 150.866204945762 129.476648077532
151.418316929551 149.286184588967 142.461941376108 150.244284323504
164.615113709166
HAS3 157.874624979775 245.766885283797 135.693520183014 130.233769058693
852.926365088632 1168.68074786473 1022.55215097491 1520.59210521859
768.053694275398 399.872401268958 1577.15895478896 761.726829618132
HAT1 330.241479004885 334.316711893654 341.2887754025 258.384059994446
631.106334754678 636.781477235579 346.476121089685 491.635435224507
384.296190598736 434.509391836336 455.313553278155 443.005424069211
HAUS4 383.802494922918 410.002817143111 504.9864660689 429.351447428661
315.014639682046 218.269847477919 280.595515386655 348.711663415777
236.676029292922 338.934501259414 288.968909550533 381.253673759781

HAUS5 137.614020921217 133.517685144186 127.976612086284 125.855451679929
141.420747583126 132.475414208533 143.508657511633 139.574895021783
149.103778494524 125.517076778344 145.210787864362 141.311749168934
HAUS6 127.640525889316 125.79193748535 132.622032754473 124.786029449452
131.349297554112 125.073353210914 129.16337177104 127.107223387937
127.787890405862 139.287871460674 127.687299576139 123.11447940203
HAUS8 159.775479686193 174.749832388791 170.212679845194 154.831307586538
221.424451092316 273.073938260793 171.190796424612 281.037762232619
192.848268768579 187.796440205485 196.955723006838 218.811490883852
HAVCR1 131.447941902406 119.720132373023 125.617143363441
118.154953746492 143.874735808114 125.160161268474 117.303671445328
178.551434222154 127.090219286772 112.590379042108 180.113222861966
125.818432070199
HAVCR2 271.430915699592 308.208276315292 262.488050186425
323.04019262387 232.465880700217 318.690043546203 321.627969147543
495.89837457901 822.545007054668 402.965083947376 320.214614455231
287.084253886676
HAX1 382.233826557803 332.082749517913 453.17378995737 362.306819797508
373.803772983872 402.213774592085 390.021666378804 464.128022170285
387.617166711971 358.092809491843 297.805768288377 444.945078908489
HBA1 3342.8737952486 1211.39534395597 675.083275037052 981.313778685019
477.056706273413 1285.02292965374 2414.22821062457 1152.50523614436
4759.17973319588 3371.24667467952 243.501083338279 578.084277076692
HBA2 8401.40370474392 4386.83308119416 1419.4638331856 5425.58761099475
1243.11900823259 2828.97255380291 5073.27433952351 2521.58055221647
10329.1999331936 7292.0342195035 357.773949920377 1246.2895253224
HBB 11809.4804843301 4359.98094976179 2225.59207490574 3403.37530051435
767.662367310672 5437.78386044638 9378.87287299048 4511.9116742833
13013.8166277495 12022.5515181682 550.566402986082 1856.05025952611
HBBP1 157.195967361907 170.401653214867 152.411545237204 150.36160186175
146.101547300741 155.587018319981 164.991522994763 157.203304477454
171.022976593689 166.681646517413 154.856345114916 153.414185387669
HBD 151.606932937644 142.541728149687 163.21153580549 159.765585531442
137.702036719238 134.801507994667 142.45411401883 134.994013668445
139.328340242696 158.080749926756 125.558877611499 150.160004473338
HBE1 108.543795408713 127.239282651919 112.114933398547 111.475394003575
104.505454151012 103.168665937677 107.32960937124 103.660742045068
108.655415285806 103.94864861172 107.126004317323 112.605287278882
HBEGF 297.228563607097 296.553550946307 163.331424612891 372.54091392596
184.591789011195 764.493610798958 153.4392054301 248.531972733475
215.037122365095 946.273229715791 204.396576475753 213.145460679531
HBG1 168.594235675558 132.782967735893 134.828360289676 143.962842383778
126.98588570464 144.842687993057 146.649944669876 145.139112683819
165.244616507084 147.121049266117 116.897299691798 137.332729889295
HBG2 212.987082617173 168.418529261977 162.808270845998 167.226663395855
163.380402251758 147.439303942026 163.612347715773 144.685707282641
201.569913451219 175.819983804497 141.604832186257 153.524100996368
HBII-52-45 127.593764897583 127.112296857923 150.51056917809
136.438788088263 122.35832958054 134.455623159793 132.526286141997
126.143886553557 137.7872481668 140.150884219813 127.352386363217
141.18490443471
HBM 118.879931299016 118.9803314875 112.893705259844 111.639177843969
110.651971902576 113.78644620959 116.994216357989 119.787925356307
111.722827315911 110.594755513247 108.861243071977 105.193775204124

HBP1 466.58966852405 554.800688991977 684.180060287047 530.122419830951
383.503040287678 386.960778432157 428.340060893467 380.754569467715
461.314247041387 521.714719320355 557.214020485204 320.936905584424
HBQ1 151.877035446575 132.608622131443 158.622993108149 161.852665901359
156.336425973696 147.805819921429 137.240806191881 144.361871207081
160.290104492275 156.085949215738 131.058360514673 157.6790197503
HBS1L 146.366013190845 155.523806637546 166.70212615715 153.571517567657
134.514251897045 150.751386127903 162.61434899634 157.654040469774
157.038121210505 140.11211854056 161.596086502946 161.73079180403
HBXIP 789.401526545277 769.009371389327 805.120147618984 650.0804221227
1184.3199328099 1912.2369374258 1777.29626660109 1966.09691567857
1934.30779703747 1547.16936835284 1087.69177809384 1730.30703463073
HBZ 121.71538073735 124.952026709219 115.507474401239 104.826135297161
118.521075843163 106.066897223031 105.521320791331 118.727279917776
113.832885222354 107.828378413233 107.961322525982 105.408689610638
HCCA2 619.223672253014 717.886050056576 709.136137559152 1044.91923772265
360.475989559226 283.556453980797 284.995098167431 263.295012964476
272.927830312746 282.137282466192 258.16437138327 230.079664410595
HCCS 228.766847429517 201.221839281282 223.042575796603 200.591916768948
193.315380090713 245.978723809154 205.871133967588 295.367125961621
258.727309442475 221.664431984861 231.846509449829 219.996410908721
HCFC1 1692.01248523526 1845.87311313269 1580.27059464289 2382.87910783088
3282.73711809465 1525.24196488688 1377.75266942574 1590.87426068638
2061.9164323309 1004.97723186946 1343.2623011534 1557.00226259883
HCFC1R1 2197.44189747261 2144.52984392647 1272.14273680177
2760.57475805426 773.330948077036 521.215655189324 731.848844821402
532.786041960586 511.113083958771 1002.88224342342 638.393099392849
376.54010486682
HCFC2 253.049869714368 231.769926214655 279.413335686746 230.367251714289
134.657787366869 177.182321788711 205.185206972064 181.110403721353
179.354796119133 210.590774758661 168.897620634337 172.304496150036
HCG18 160.592404712197 163.373180928181 149.547635666621 163.758054869675
158.044938495884 168.919352453652 185.673549367125 173.325724300712
165.870621884073 172.568852318369 137.303701514064 176.364184489318
HCG22 133.195030751476 149.687372584136 145.027741086645 151.451443480833
138.221749464651 138.903450375532 131.190238038695 139.591231478841
133.743708220112 133.885725703056 136.261907928796 138.352562277336
HCG26 129.127563278016 138.289361041588 138.358404420064 141.970301370404
140.54700821058 144.649656552 145.442914818456 131.391434131813
155.933739693912 133.465593684519 160.473772347376 143.50323531501
HCG27 152.821735300697 165.773929204209 155.253032078086 166.803359138942
138.915185051749 128.284564251279 161.700258550244 143.307838525991
152.99686989829 129.059722322306 138.841385034636 153.511995286293
HCG2P7 11326.5935775813 10135.7800994367 9596.10781313429
11809.4804843301 1738.96798001895 9560.32907282993 4965.25728333142
7753.61594300904 6894.63087357693 4318.50925204407 5425.58761099475
5601.24254062491
HCG3 175.128935809466 145.383354200298 139.153897381147 139.699189528879
134.896349513392 160.640160107693 167.250667092211 120.944267930809
145.308548379215 142.274440538785 124.975454733084 137.220718873614
HCG4 355.879841671975 414.114051645565 309.720081933252 406.998800177456
418.968095159939 299.463563393179 452.517134370928 443.125195744202
937.593129242257 678.937151822411 773.803350315426 1149.90248188644

HCG4P6 143.891725249061 148.644800418517 157.277051544704
121.255814891176 152.834894011142 146.20028051948 158.679903271595
146.271180025157 135.353539458856 144.63774603725 136.779180530712
153.761061653202
HCG8 125.974463216884 137.00397800873 136.566588903194 138.747964592012
151.936543085044 129.391376518208 129.471713415228 131.104534164394
127.433203229267 123.427366316096 135.697550878438 133.229571269367
HCG9 116.087696503753 137.641136471239 112.666812944192 107.466184687811
119.000436602325 128.257292171015 121.402905161896 115.545558600232
115.738984899365 110.586147059226 135.507247740621 127.289269745461
HCK 191.179662650057 188.543051108601 205.45079491218 199.037998955485
200.114704018645 203.563098303937 201.773745532242 200.204064462384
242.948136060281 212.591357254196 196.031541155674 197.859546367676
HCLS1 229.689010178622 226.960024551778 149.518870320398 174.898012759712
262.093405224922 245.454945056317 1343.2623011534 1438.33289891548
1049.52258147141 709.778171909457 494.175437345451 214.050918459805
HCN1 145.791950392049 162.007384919302 158.064409011013 144.477267427347
182.066922756289 141.147683040881 153.649235507812 143.10996074311
158.157097243342 183.100286716683 138.69392148885 138.654301345398
HCN2 131.696834332724 139.776830704087 133.012006684865 136.372712400493
123.75974844367 139.987681290389 128.944295118239 139.157088843803
134.549628985766 129.409899416034 124.990876565715 137.899291413431
HCN3 179.57046217355 181.813812621767 178.976116597291 193.028341755594
220.113261580385 239.419200107721 171.610742435469 192.493439659184
197.565122821625 189.662974464847 205.950347620158 223.778708427552
HCN4 154.512619892977 161.365443930448 152.913814113632 164.39910609636
185.113098620798 160.290104492275 169.45559708224 154.261443597434
155.896065329825 175.543924200429 167.370030272847 156.508428796592
HCP5 401.87674893993 583.938727139698 199.459711023907 439.207122335648
671.387334588652 268.961643716282 245.978723809154 636.781477235579
2642.24374855334 924.040923247227 1818.92700816112 1596.33832632698
HCRP1 130.624702758052 147.46607538135 153.415901281564 129.532122459128
118.289405191861 160.618586213311 147.579672274205 146.894793774087
162.199381540369 164.701183249762 143.038248313305 183.201926323188
HCRT 147.717748449017 153.916313595581 159.223980639045 169.661107080542
153.857048476879 148.594322264158 159.620467672898 151.198928680018
151.976432805924 153.111226896983 142.722227179256 146.06717772919
HCRTR1 164.284396793834 163.042083189818 158.185593294878
144.446903878863 149.464962540085 157.195967361907 153.709230037306
160.70138318726 149.815494316469 160.625367261779 153.985529457122
167.030667499538
HCRTR2 131.644877647087 131.434121613176 133.724135305758
135.453399741892 137.086936444421 126.915338864323 134.805239981342
134.950905279775 134.759283147919 140.999877170481 153.457715729768
135.618562468776
HCST 274.235001739215 219.318938396989 182.074569356713 193.674681547989
253.164596735793 266.563990988678 227.736106796551 345.977609212137
616.188923504376 259.643957326575 254.036619126409 193.522811257085
HDAC1 1978.12229412176 2372.26073354008 2635.31067044245 2193.376951552
4503.01240138314 3062.22499714914 3883.05620256963 4520.84412831414
4183.06379632713 2622.87486749984 3287.37172778474 4628.04544865227
HDAC10 114.032874859062 114.170354748872 115.47716627378
109.113476990309 128.372225487481 130.069789939568 114.027244455291

126.788749235867 121.065278765151 123.833219705499 157.11867820027
130.524630778991
HDAC11 184.056274452194 182.009471368798 157.457468761338
200.618080312379 357.928880740814 202.024504592763 195.10410545445
187.529556428609 144.273011517032 194.80575419836 230.284216427158
191.018168651394
HDAC2 748.015452242839 620.899414581162 768.67030514096 530.561387049511
1089.16489859253 1011.9675459648 1154.1046856215 1047.2693539616
1080.95725397659 765.911113050543 1107.99131702522 1320.74943817958
HDAC3 543.584413650467 591.763423878737 498.05778289794 682.730262408017
638.129080224049 600.162650426961 745.844395215451 652.429025114523
423.489441909617 1032.11574332305 945.403379196643 747.587723573212
HDAC4 611.540759532294 551.622587349813 631.408955630318 739.52947559669
208.291714758548 146.301300103564 167.180406126438 162.606273401226
176.145076825955 178.885625009973 268.454310218604 140.292132023262
HDAC5 183.406510146725 174.971350923613 179.996590340102 209.398724613377
141.750316403249 132.155877196474 137.478556403046 137.641136471239
142.274440538785 145.665155500862 154.524588318927 137.654833215154
HDAC6 316.663128947813 315.615758089471 281.238656291587 405.130899553871
361.829819621662 254.879772480457 221.471811658459 230.015981563049
218.529160053398 220.136131941508 302.945860276233 206.972440734038
HDAC7 201.702688815038 203.960838492793 173.97085982677 219.394500571586
259.72035004624 153.146646647258 184.322250984457 158.829144564361
184.260835471542 166.12695134858 229.181725450534 196.777776509371
HDAC7A 202.60339529874 216.033564783918 185.876046259727
247.779718065751 228.349507726225 166.104915784875 174.690052785405
166.709393547104 175.847256673734 171.677654793775 223.84997313236
182.109092947203
HDAC8 169.136919982294 149.888719320368 191.112009690591 198.90533452253
140.687016530974 177.19138801548 164.708712337156 163.19340939648
174.274996381853 163.108825245937 199.756584044664 174.161146689117
HDAC9 125.639355645626 127.416912237613 127.220812636148 125.967294930279
123.960761343425 117.992729826121 126.256716138724 121.903115540516
126.029431525943 129.013667813486 133.909219998872 130.457981404612
HDC 400.180574613078 308.564935340635 252.365946808078 356.268960813032
152.877058628512 166.863589702453 138.846543789882 168.248982292489
243.936797286495 131.757695616207 142.246864759311 137.058578192268
HDDC2 1946.45172007314 2210.67250954072 1787.32566525551 2282.94927629821
1082.75419087929 1336.15622439522 1548.09554827056 1100.17618574297
1406.73270115373 2344.11548437508 2010.57142784228 1875.21979956596
HDDC3 193.974325897472 203.913123559942 197.087015414039 209.727089546377
512.541205235935 249.80160363144 437.931339781992 274.934292929666
340.487850009685 252.404931238338 241.494710905339 320.707687986324
HDGF 422.02217814979 473.260534978556 389.109250187902 471.315730378641
2104.59597571915 908.352265585374 674.905270154355 867.369315017411
761.32108609503 744.523059417351 894.46029531926 899.667912542118
HDGF2 232.127996106421 262.913054904154 245.269004485826 365.199125315964
384.216141843863 217.408824020423 185.783374935551 198.90533452253
178.542247857251 207.453490385508 205.560952959537 201.383739902873
HDGFL1 157.83121828793 136.858037332222 155.018009545475
143.071933285651 139.295597388725 140.626181314061 133.391421497441
138.633019006784 137.782648137869 144.792035839249 130.758707848676
145.892876412762

HDGFRP3 432.174413144791 411.039234404499 390.021666378804 337.22646304739 230.053292316517 234.947745514175 439.928008927647 327.696359071012 360.185005480216 262.861105654051 361.26540006942 221.297127685676
HDHD1A 217.531179216305 247.72190598581 258.4424008891 263.379982742085 273.369722814068 464.323065038265 375.958347293549 442.766704003254 320.979443123471 264.306927596371 469.64693728427 484.568420044471
HDHD2 766.420275252501 887.147199303294 832.335328912437 970.634736994313 372.54091392596 443.853458865556 537.06835963726 622.692834629555 714.274201838067 510.555184032017 1257.32413345748 686.445635754196
HDHD3 364.597798667541 352.354687089427 518.478990211753 505.83860711693 763.880957432979 239.050379187643 407.711669482083 452.732079010502 484.903322308422 295.420691959792 786.62709337246 284.321253087512
HDLBP 135.385133545332 134.013748718539 138.657241641263 133.959208066964 137.971108694323 134.193482579542 129.798965780918 128.152956417755 135.16316100494 142.051259785804 155.981989040279 136.682172321984
HDX 148.304863957659 142.802965008073 136.406132339187 142.594553057288 141.943910304145 134.762169062357 141.198692540628 149.108582827136 133.491963477649 144.39135701666 155.6299578128 129.534721138679
HEATR1 306.011283364888 337.822337721287 379.387095129725 343.571296297462 374.56251861444 751.077307018319 472.669956708398 610.915977337787 529.04238100948 654.52788411248 368.948300262634 852.649997188692
HEATR2 259.72035004624 248.613373429151 339.598452080917 304.402066421813 562.089999265955 263.568160216149 285.798047501616 339.069385816411 296.088938872643 330.241479004885 357.672950588403 395.716879127149
HEATR3 181.277683429417 192.827534610486 196.053808536366 176.145076825955 204.489837323989 196.691380885508 173.432960318793 167.186780161168 172.108795190633 196.990867962546 164.959415532174 181.508873226705
HEATR4 105.839106819841 113.060728652796 117.870230005125 115.321501844663 119.164791441052 109.355444907633 113.924310293916 112.309528593741 112.891050337488 112.239647575661 129.446928016147 110.518806964366
HEATR5A 234.738241125899 185.046826969912 264.233381434738 162.140112952434 164.181915898563 184.682478530334 172.214433016685 217.287912825619 178.676812740925 209.702683803624 188.641397688261 175.543924200429
HEATR5B 356.921627358141 337.973433871323 406.736165197168 345.135289739599 292.911761762431 609.100385482325 404.366325262322 487.597040819879 361.218491712891 386.917038897322 464.323065038265 447.521645706162
HEATR6 273.044064077 259.643957326575 345.649572073924 371.224296589448 184.241640200823 209.362806125315 312.406286993239 319.812293134357 246.428746807313 300.886032768166 301.139737613279 340.193863653875
HEATR7A 139.476234663488 132.661036963636 132.32633643811 137.954283575143 204.596753034535 136.185190275407 134.91213977595 145.369791572077 157.986640251147 136.041605158822 139.690093593646 148.327340543504
HEATR7B2 135.511902377219 134.378951325825 130.872296869843 136.91078498126 145.219057535246 139.995623005154 132.085538520551

139.834535091869 132.346363545288 137.830435972191 145.773932754923
143.643259003393
HEBP1 1537.71464618832 1439.37593006882 1417.76295714364 1300.40889834984
1148.80141486692 1368.91287156398 2180.31963682546 1454.94889750152
852.436775264873 919.626220838414 1557.00226259883 811.564396886535
HEBP2 2026.85580212422 2172.89794460273 2183.18692530644 2189.53914329263
5265.74259211695 2379.15291239548 5688.84918819457 1804.63762008613
3968.38723079061 3829.87905458534 5624.32062666666 4763.89426388268
HECA 300.985037429772 296.141012087256 314.014654357569 307.665281863258
246.428746807313 179.810507975486 409.073045356073 281.278843600876
303.051124835907 288.313931067158 446.698665191047 279.113984512619
HECTD1 1233.99503576308 974.007008568051 1189.69730508119
965.673699120049 778.28014337938 826.314416168552 859.860994525775
1084.5920943349 799.423640530777 1196.34528225417 1253.05700051015
1161.39230511792
HECTD2 195.929328710358 185.382647297259 197.582402276079
170.08485436731 127.07186140748 180.761100930677 147.362120584524
157.061602966797 148.016402654687 149.157934946623 171.884800118791
151.089328533476
HECTD3 261.117845544884 280.558463661137 293.448046685909
347.557792829093 341.564749445905 285.30221551106 287.751093664085
346.691690520054 303.907003535357 331.795832780176 430.220363263561
242.440966395613
HECW1 145.7202993771 130.441808139335 138.883710791382 142.650451413991
162.55521875551 136.566588903194 151.318320077986 130.173734963758
132.16600019449 158.06129717954 153.299196905367 146.536055773073
HECW2 152.550031868259 165.286724415145 122.456286448252 132.589411973628
163.456245724169 245.582279957584 133.571962238112 186.388067641269
153.228048041467 172.468440317443 158.98007855379 150.843975804117
HEG1 574.496860164135 369.838429521168 356.62074247786 378.037190077449
219.878230463206 252.222481637238 173.468958175014 259.767151797583
270.679613997675 211.626970008102 254.668502187945 217.491746961471
HELB 125.908576249439 133.439077726411 140.726084514407 116.449064666763
111.484426481816 143.176699547317 123.354093930164 150.997551005337
155.176714087209 129.001192144163 157.195967361907 115.958740595234
HELLS 165.642728895682 158.854659281803 140.954264409241 161.781529659549
206.203717450138 201.256649338315 188.348499229585 221.796935051107
203.17525411575 192.441337304606 162.342112922504 211.572396862776
HELQ 204.82244206816 187.920789153124 191.651818696629 186.406439948242
163.487737425232 188.20675818131 179.648479201164 204.103018614608
192.627266049516 163.19340939648 169.826068012389 179.525651423361
HELT 166.485040297098 164.755896698569 162.732574014686 154.562761981868
159.942971344216 168.847039069786 159.588575526536 155.592219571564
164.406564594296 154.343424387068 158.7912155022 162.6762533989
HELZ 501.863855158062 570.771089597761 508.861268738074 500.132469062228
235.091443641056 589.070196307574 369.838429521168 334.350011399462
430.649188209901 280.292616101827 303.555333002419 417.987533397449
HEMGN 125.139029579691 119.669441891401 122.351186755611 121.624218243474
121.289159587394 116.989409891761 118.88525116298 122.592960588642
124.546520456419 120.36718185387 121.239461514704 116.806720503431
HEMK1 198.954254778846 181.803275243281 183.201926323188 234.461229199142
141.208106961875 162.15259972225 198.15257384667 191.59400638652
196.657475905674 135.491412261604 171.314681729657 152.555437272666

HEPACAM	123.716400094852 129.992015202357 123.185652476312
129.628859533303 122.11049596959 129.910866236901 132.322618637842
134.537123332992 129.514490386931 119.95666716163 128.168460083895
129.546119539687
HEPACAM2	120.524859681034 120.280241193135 117.22284971665
120.272964182307 123.247698282967 122.051473363557 122.592960588642
118.446703299916 124.486701452062 113.980716384814 134.292099546804
117.034228068173
HEPH	302.696004448559 307.768302846647 260.921177985439 414.114051645565
133.074027423549 127.595574823053 124.403662787819 136.332399354517
143.763292219833 130.272086123019 128.556280049403 123.700076467648
HEPHL1	129.656088836537 130.4631522533 122.142323014287
120.786630750686 129.741644654541 128.060612106762 130.701801496564
139.986119754908 135.301844382418 130.088408942008 133.154004369076
129.780648518219
HEPN1	137.572574328004 138.279431175685 163.910041674646 140.525013926642
168.37043566813 132.748478264583 141.294094833197 135.185283076286
139.127442770828 159.266921692363 135.250379276888 149.851792558427
HERC1	395.521241316058 360.39998507367 534.920870202582 400.060972573122
238.904826940476 275.013838208239 322.36247485445 267.839640412797
350.803378042804 331.062744782933 278.095939961034 316.801725268792
HERC2	299.986758612161 296.962992349662 306.677271064305 351.665934915634
430.412897698085 230.91747428831 279.834616502591 204.82244206816
308.877027622691 274.831898505989 307.316241247336 244.054469051058
HERC2P2	155.198503964295 139.524233960073 152.492163303139
157.162523271165 146.873719524815 159.036647992998 161.365443930448
153.100979553727 151.259426521768 170.483686891321 158.712241098927
148.364138636002
HERC2P4	127.018746030517 133.854760285591 133.310323593945
127.41463780496 148.948744887393 129.802596741962 133.133892264995
130.470034945295 124.132215738502 136.18698517676 127.349190705235
123.179986410723
HERC3	187.833356349429 175.847256673734 230.975434289172 137.680019787902
144.754401731628 193.554028681854 177.592497020798 170.320777845496
166.298554732581 274.281532397691 166.173429365582 181.668090319293
HERC4	248.085349787686 233.161848094769 266.534316786801 199.714647839712
191.777253122672 196.880135762817 253.538171006307 223.601312199935
233.335353447342 235.019573994855 265.29629764006 309.937351755502
HERC5	1186.96351738518 796.936154737659 728.743541467167 688.247581763352
188.864388750448 229.406012088782 858.020739710943 253.124449721731
445.763740779039 231.981501596284 169.777032044409 164.935120145905
HERC6	628.710901107436 256.614964511846 277.685488726472 238.068308163643
175.186435016433 203.544294328395 511.779988437654 288.505502880424
398.61164689194 335.533053221675 277.256341592701 278.061498892643
HERPUD1	632.565736446505 588.334329592604 663.214972254236
551.622587349813 670.856008549143 984.695710317818 751.896334334052
1101.66868138797 1223.68146968716 750.494286476465 600.635914090365
593.462634352052
HERPUD2	241.867699338253 213.324226314713 230.015981563049
209.7558663154 196.207631868755 206.428618912264 229.157389498775
218.08515867102 196.536086443884 247.622351589081 242.72424280444
262.488050186425
HERV-FRD	127.818137805569 121.771891473027 129.158212237299
135.499195435803 112.661607612444 122.072091994548 115.51493368527

116.360144963753 112.357261386644 112.420505354139 121.124574985464
113.512670856029
HES1 244.401472859026 297.970735665595 257.5097052677 222.456810024193
501.434722063309 811.362219801362 535.20662670885 775.382397816601
469.407481002198 670.097523566652 1113.05943954513 935.423045210635
HES2 132.192775993078 132.146027935802 131.734429913106 134.776714949136
136.699011428085 131.741688265764 140.174087049513 140.002249873656
189.94130405123 588.334329592604 134.85590586532 144.615156175885
HES3 139.067516394141 137.881776548665 152.699431442817 153.38494705081
147.462651811169 148.507598818039 143.793506250131 152.030182228463
139.241145027974 132.825240485239 150.160004473338 140.154925193842
HES4 1251.56853099362 1030.56836625083 958.63560158343 1116.95795024994
1099.37392456694 4811.88424516272 3909.47437917608 2261.31473132315
2580.98143004019 2010.57142784228 7519.94306346326 2255.92551080483
HES5 134.024205981268 168.188793271919 210.061754644824 174.338440780636
149.408530395745 216.9491329483 167.243544185778 183.583432296209
170.541689758579 180.179981672095 293.448046685909 207.05325577114
HES6 188.033890834418 239.277801396834 166.563411635721 255.56301534833
683.861021234927 513.20896845398 306.068269136821 253.049869714368
270.973600107882 261.426617866925 222.243523614518 531.711823481265
HES7 109.067239220744 107.48180681159 103.307703547649 109.038182799871
106.437660157036 111.000303209442 111.065596151792 120.208584170364
110.273174147374 116.445184223804 109.242622591321 109.832757716464
HESX1 115.231121959397 114.078271807864 112.742895297124 107.74537523456
116.91470505098 114.314425748312 124.132215738502 121.834248129933
129.054577477882 116.497714896018 118.015175851934 118.446703299916
HEXA 171.826623289946 150.072005848234 145.435794613203 146.97203173446
126.33556674738 113.549098821204 155.058852833902 121.121686280797
136.737964237109 127.855167644203 149.535008475427 145.063062479224
HEXB 3015.70954394585 2662.43181510326 3038.02573182546 1823.53870218167
2012.36542989953 1438.33289891548 2748.51360874099 1881.90353127141
2074.29945034697 3019.57419253979 2104.59597571915 2479.34567973462
HEXDC 263.74335003375 231.257543784864 304.058158731957 252.894371238554
250.060734219351 185.830435830545 246.533813165981 353.50447714719
329.535023880129 214.050918459805 281.405355165228 331.251075774227
HEXIM1 192.96586114861 197.873787638411 173.281506912652
170.069291215013 173.782474972742 153.875760529011 190.56172211779
182.919385608842 172.142411008084 145.455411983653 253.164596735793
163.967616022079
HEXIM2 176.328994356625 197.169703621888 225.927425596831
221.512905853031 256.127197303751 160.676606760965 232.151020756625
176.312626750427 221.253717494488 173.850390626782 226.458509834965
186.06425658259
HEY1 209.362806125315 304.357050745605 197.357728220717 205.185206972064
261.592512762969 269.853718252574 313.708197191587 317.802633831405
183.735201125198 198.390247605355 412.863887464545 192.96586114861
HEY2 153.795665190439 203.223604138192 131.173355686001 167.500410857353
142.637421204193 142.711117470317 116.268259618103 142.09146795308
159.012011848781 235.216589534371 129.583916648999 140.574129695953
HEYL 368.220523234399 465.683139139622 230.947339143332 393.013539328766
274.77366169918 267.097999873387 173.281506912652 222.394280571312
263.994460303088 375.651196211048 171.216275057884 205.612579504495

HFE 143.50323531501 134.896349513392 126.442999559539 130.092881020271
140.793616429174 132.765942975939 133.567630962178 127.993841618875
141.763072120718 133.613988293895 135.810476921749 139.286592412216
HFE2 116.929398630733 118.336654504789 119.261305304165 123.868848032411
116.831725094917 119.739204799544 114.638254225216 127.991651150332
114.644722005728 121.219323788324 125.413619056436 117.480506535399
HFM1 150.896076207161 138.071335383596 141.348548071751 146.932655562613
149.524040913256 147.052395429464 142.669207969715 131.794065532001
151.364528898537 169.631386764054 138.516066340784 145.42029807396
HGD 154.716750150891 149.876687693565 154.818414925242 164.634694036993
156.179501567984 149.738621325385 149.231475480618 167.192582058634
156.262515148436 161.506431005203 157.293090577042 148.729753117861
HGF 127.576966524587 127.884750925048 140.038295509875 126.378952839132
123.687439699754 123.814841047589 129.260551359984 124.256773437346
129.669484303621 120.157438564977 135.651829752462 118.875640081461
HGFAC 128.029736384348 147.373174160478 120.138014822486 119.492844191924
120.524859681034 121.834248129933 133.07218865002 124.342911165267
126.917669470999 122.724429060184 133.012692824038 123.82555184043
HGS 1080.42884514937 1303.94157493616 1367.92869625376 1550.61855325662
2032.50052162637 1934.30779703747 2061.9164323309 3115.11499025766
1927.467713716 1965.13870083236 1206.09815575399 2376.52728883131
HGSNAT 159.203584692924 148.570769470645 170.483686891321
158.627416752905 174.454754564111 228.588693548447 196.287131027947
168.116928722035 157.350979071365 182.907444581093 171.660157220703
172.468440317443
HHAT 134.389518389888 175.989742412417 140.160349667726 145.628239642187
141.398513269219 133.463464118897 157.697168349028 137.555253894825
170.636731478608 126.433146708506 195.043838688467 160.882229345156
HHATL 161.027590226123 151.766607453172 164.857465272302 155.974463324915
164.39910609636 153.066630483602 173.109461200603 133.886091996863
161.295030274012 142.737537206204 156.911538642765 189.74261433647
HHCM 137.48217464385 127.816596414779 136.639637503571 135.286686011087
127.112296857923 136.430232926793 128.274326990056 142.528480882047
136.732319750425 135.212715912271 145.034179171835 134.113464449375
HHEX 279.704940529618 273.197814995543 226.458509834965 277.685488726472
171.06385200256 179.067275037015 141.801472885127 142.861359851088
194.202977693583 136.009969058857 159.801315111886 137.984988819967
HHIP 171.994330847901 176.953099943185 143.330025840618 158.809865241567
132.428675012593 131.466580723837 136.327014189932 141.296574431203
131.354275252104 146.29600238749 142.902838068479 143.284230032303
HHIPL1 139.730999352036 131.481679396553 137.125637347852
151.635752404534 136.691865286093 139.365595511181 128.303046695142
132.024244263352 138.934516083124 132.851791525098 137.77107995457
150.026314061051
HHIPL2 116.412881235587 124.126715773068 136.524640445539
112.736231367296 119.517021355598 112.90900939264 120.07034081564
115.337592860132 115.775525016883 111.152235113722 116.425301253959
120.01190102634
HHLA1 127.419227292862 140.992801405088 139.932849289354 150.241585041178
129.436712663487 138.334626498686 138.020031233095 144.087004064416
141.497125111619 128.933672074497 139.684141478459 135.741404460897
HHLA2 157.512561489621 121.087514445827 150.405715534245 140.750004067571
137.728163622673 136.436762791912 139.611455791957 127.478474451121
135.930911350803 136.923876486522 139.805930686563 152.823566129571

HHLA3 138.030842581932 134.981272056795 147.720061897962 154.489459651566 157.572446865749 139.338384535905 142.259196792767 151.243948028303 143.710449544388 147.86104992164 141.644521617419 142.157512965166
HIAT1 240.182016388745 216.929969034881 287.889774984754 233.20900339048 228.766847429517 400.676876982655 356.05426191171 325.570839576032 354.336462793628 320.979443123471 275.509805464276 406.816482069141
HIATL1 229.483578414375 218.592402813219 280.033528476199 202.622747952763 349.382889326613 339.172031360524 212.749668083641 242.440966395613 288.505502880424 344.856199123543 255.066583469964 307.264586007015
HIATL2 11741.7473778842 11971.1664283024 9378.87287299048 10765.0336035748 1715.51071085304 10900.1783397368 4309.86375945008 7771.28016059011 5811.97916044218 3528.62443098065 5825.9968524214 5291.83393775761
HIBADH 1402.14708471728 1170.88763035931 1578.87371296418 1344.1288416533 894.46029531926 674.338740875512 720.907992182476 909.388774224614 701.481003619067 498.965895763801 792.247301083075 974.544773560873
HIBCH 427.872407051254 452.732079010502 569.656573915709 511.374117402707 441.435444003241 858.742139354587 565.164961255054 734.469481390593 417.865461351681 539.125423322738 716.731984660463 900.233783407148
HIC1 175.165305179665 198.088834624665 184.482849019679 234.814109965002 147.309245779031 153.519207482423 134.728594388428 141.302647488314 169.520544329439 159.227123133854 152.591091223395 147.919058170753
HIC2 223.419092696074 223.094496588657 201.837695818264 220.533113603343 262.577672828611 684.428198622716 197.582402276079 302.768870633398 634.556887178869 178.404791189835 360.991542429346 512.895335481876
HIF1A 274.974304050999 303.09499683953 191.616483214712 209.229537873706 264.158648784101 298.347201056937 316.663128947813 230.770036998466 267.222762759781 742.517302567436 350.741303038926 251.588748783592
HIF1AN 310.690317681243 342.245182599593 286.085833802132 447.995213144892 268.52833723726 203.149001616967 267.869879189095 250.602978521808 230.893665210918 223.84997313236 253.049869714368 214.100698312272
HIF3A 139.783725419023 146.274284557421 137.438984175012 137.241991031057 131.863018503922 123.722744366103 126.260691770282 138.525131957094 129.920586100555 131.885529813944 136.867987890996 130.182320154521
HIGD1A 968.548243081111 986.972987865318 1074.96415574964 844.778755052304 3038.02573182546 2639.60135568 1715.51071085304 1421.72388964898 1762.09874961415 2093.67403119512 1478.09582385838 2756.22069438696
HIGD1B 152.291176493005 162.766038656637 116.382348831504 137.853137973756 123.288028185949 184.398672994943 112.635430248416 142.183154756458 126.76986764971 122.849235421041 112.817015523662 110.465934808915
HIGD1C 126.413124995943 124.869732867761 142.599815022061 126.705446263769 130.626965797365 138.43301474649 128.933672074497 131.065633574494 135.906115546805 128.574419295906 137.491366243256 137.320792454356
HIGD2A 2635.31067044245 2938.06458269406 3019.57419253979 3168.27825058989 1390.95426299323 1532.47920327769 2586.87941889443 1989.38201206555 1465.2419150392 3447.19587767281 4651.384971332 2944.63971819193

HIGD2B	135.217240511902 138.399665664034 147.81143020317
137.011595584638 141.954467306745 142.29309119138 144.215491799806
135.129656281091 145.812607895325 138.601616246182 142.02169810999
146.994103804515
HILS1 118.338713846941 113.913337223716 118.438942370745 115.37736249419
116.373841472226 106.953841653951 122.452541100946 112.046615825307
115.128667174745 127.9928491829 137.430946130817 120.300291760091
HINFP 185.088667707155 181.70730090195 199.798533722581 192.548337508559
190.471158323247 146.240060512152 162.147989253357 195.84531580224
183.214922844221 155.699649538664 206.77605957523 156.13331481937
HINT1 2886.92690692502 2622.87486749984 4055.40322213638 2063.42824094932
4732.31591164752 6851.54963413886 9470.58919937464 4773.62220024088
3793.02341602102 9759.87403504619 4262.23145131525 7082.28969354712
HINT2 617.67819271245 730.042712083388 746.523071806534 851.221199352646
1364.83036157729 516.429067667758 398.512947526406 723.807658768693
641.774812292709 476.641032594868 560.913732951675 420.361583975644
HINT3 210.290500215377 178.339211170442 394.810886122555 187.625536724681
214.52087460757 188.878770168595 208.067404132325 198.135240691225
217.074639597142 256.495848662843 175.98210925643 174.898012759712
HIP1 197.895094054577 203.586310608403 179.241385747152 197.191648960563
191.897449038297 206.937770272939 155.263513633703 189.708396835236
206.89716102989 174.23955831109 170.12010526854 207.104548555929
HIP1R 133.625358030199 145.601922576321 137.094814176566 133.762141373091
228.184535093641 168.052288484172 165.169920299969 143.116451998409
206.275885047321 152.868787961804 196.053808536366 173.929353266244
HIP2 211.395593720534 195.863843860549 195.618369827396 207.173653740239
248.594000664466 240.306287921209 297.466255000163 338.223462327622
289.164969943385 309.580750386386 279.026742408164 273.959220003188
HIPK1 137.344198938623 127.887666721519 132.665590728755 128.939572714685
137.212568398657 130.829825154842 134.863223806176 129.502730573463
137.229871801676 140.007937555694 130.991496243273 133.403409882362
HIPK2 455.778776920897 535.354724252178 373.361263985383 473.151324002277
331.383533814347 291.577778800314 264.355693070937 355.290621188435
286.469773081481 358.60777927888 299.568830444875 344.856199123543
HIPK3 120.060776752215 122.554760526767 124.621381699241 120.15332480712
115.656801309856 127.818137805569 118.749046585251 113.734280863323
121.588054040961 157.147002927578 134.096283448464 115.902030217102
HIPK4 106.204350278634 111.276543583667 101.915858150866 111.867464669155
115.775525016883 115.18875509198 110.774802298005 114.504409331018
111.178024950204 108.818976813708 108.557097186828 119.802832736435
HIRA 150.696963699371 149.77596374205 140.256871047018 139.377027955744
159.854639082837 138.783017264895 164.717809928624 147.439303942026
152.503159091556 151.026905311756 144.642042956057 132.322618637842
HIRIP3	456.298408568636 469.930860750773 634.556887178869
637.457322054293 305.343879847672 428.237210115667 357.672950588403
387.403502892897 379.615659443164 356.84281623052 295.893482074241
326.055727207966
HISPPD1	110.575582352222 129.315412536491 115.485515429446
119.830261862805 116.342242990651 114.226028611171 107.450945262092
115.097376525707 113.274668934773 120.897228878841 135.096321258167
113.125349503734
HISPPD2A	186.854553034865 185.923374274306 206.236303011662
204.914390718161 168.095061700814 175.417320301614 171.97578836712

179.525651423361 187.057683141644 177.065616871886 191.708350637714 197.687858915757
HIST1H1A 170.775283924087 153.108902978819 171.267907653007 164.202554380274 160.773510498005 188.475801713801 172.175584707792 184.557630172634 155.680330654611 159.52147764862 161.348562186667 163.07139884152
HIST1H1B 154.496005175117 144.454141873387 145.724294862079 151.09974848374 155.007516124803 174.26780796802 144.301577198126 158.356964958885 142.64421300687 148.380555606786 135.892248893607 156.351734527745
HIST1H1C 350.46078795627 407.052451784773 276.138650967632 293.234972169244 798.389385197471 1856.05025952611 384.641319423853 652.826023701545 3789.1316515671 1535.27496251381 868.40984928812 458.875979229339
HIST1H1D 139.711696905287 141.457040042276 125.27625242915 132.70643154246 149.317146997809 139.834535091869 138.28745132277 132.203310946961 140.850680024381 158.98666068453 137.968932080074 145.108281984556
HIST1H1E 129.949894903048 131.106613067333 122.373400988227 124.61344300674 136.261907928796 112.612099135361 118.457573670556 118.235640920527 115.734230176652 145.766684470184 116.388079393669 112.063713561549
HIST1H1T 114.021546689428 113.209569103727 112.142263331903 106.315801001506 111.826509343353 119.785062256421 108.955761698524 114.68345964966 122.69021100699 111.448871835514 124.558026853954 100.966979609222
HIST1H2AA 125.384706864406 131.882887826338 115.392989580183 124.232144678947 132.294365151614 123.035401501279 125.990762414974 125.979152567959 128.2409378561 138.872536475067 141.750316403249 121.04331582508
HIST1H2AB 128.495276191943 134.85809559975 141.359142214228 132.411757518105 157.590017103385 162.900556089071 124.907788504503 123.629279569085 146.508628678304 150.931114848235 136.121873179928 137.957654220875
HIST1H2AC 182.109092947203 180.437648146588 189.419941381565 130.935922915902 175.93459327917 356.323554000768 153.871893011106 186.359770878343 1132.27988339924 1843.76920134139 198.631354372924 228.560346647306
HIST1H2AD 153.774548290902 151.92638745703 163.951459935477 149.698664700146 143.318291243905 161.359609192977 149.628421263062 153.759707372298 168.45157400976 170.844002412425 149.799681364551 159.273225917177
HIST1H2AE 152.340010735322 136.338683078123 160.227729155903 139.432567336814 157.898632788579 151.073257114127 141.712693059287 139.660641694206 184.306452616482 270.201322204641 130.838350486331 148.231928963878
HIST1H2AG 142.793573585472 140.051904905695 134.961156870831 141.929924728222 144.004831360797 143.294217840279 151.385956426616 137.47514052942 141.484730939562 150.272153646712 146.335409183715 147.140771901106
HIST1H2AH 113.301733735994 102.343275379164 110.212739445 114.76337651891 113.970633751086 118.974727968283 112.268518171041 112.456644814178 105.976284291356 111.559325333192 111.041656398985 106.238539254093

HIST1H2AI 126.319103548711 130.726640485925 133.533718725309
142.780100181736 137.719257499747 138.12651823109 131.142841840284
135.892248893607 142.427198128376 153.996429870564 143.548858367002
134.860896462638
HIST1H2AJ 126.446675008883 126.595668758744 120.082391311921
117.227635924674 135.377118045038 121.014538677616 109.680532131035
117.442158626871 112.565341690522 122.027632589515 138.548983773386
118.88525116298
HIST1H2AK 124.090268443064 116.230993734675 130.131800537628
118.419311800417 114.880348648147 118.372047967248 111.328185454541
113.680875214845 116.934457270701 113.955459033122 130.925881698601
123.561016079801
HIST1H2AL 138.647667867725 120.319621393685 119.060186838239
139.365595511181 125.093376753408 130.682112236817 124.378096177801
124.922004609558 134.458958352588 121.99477069153 131.638954862848
145.780927034516
HIST1H2AM 166.655585160657 151.639515017015 149.066522533956
170.531713392258 165.30072618497 160.992494552269 189.552385508772
145.478233255862 155.298546821687 139.904952662852 144.796089646941
154.555255038453
HIST1H2BA 147.174977926293 146.276706674761 160.778296470857
131.798931481549 140.196653553673 148.04206955657 149.353072778508
148.656917603142 148.124586158123 160.742365719929 147.04481509758
141.253030949701
HIST1H2BB 142.446514275674 155.035727630756 149.912481263284
141.584554523423 170.257439890277 148.725162101789 150.056508971921
158.025633316671 133.63022526522 150.334844020375 130.748852926994
141.947478902603
HIST1H2BC 140.665332203898 145.968884703737 137.838096997956
160.742365719929 161.748547772524 174.767697866801 140.03202338666
157.436513275737 236.235744374476 290.420307322101 154.143331594812
146.526744516344
HIST1H2BD 173.225064625094 206.937770272939 144.368355737187
141.537578081123 308.148212607944 213.833894542292 165.545136704242
208.983138060317 498.965895763801 906.886423608706 229.230239609291
202.909188070184
HIST1H2BE 110.510191137073 123.156154667242 115.107214937219
123.315362903961 143.038248313305 136.862764950361 124.899019467557
125.386545354096 177.333305785248 208.321765801227 144.154217446011
117.436119533535
HIST1H2BF 119.663182347566 120.238706364491 107.91011371422
114.01599035448 140.462651395483 125.979152567959 132.502153153826
120.691810909148 129.001192144163 152.067897082883 128.978197156274
120.995697049378
HIST1H2BG 131.338853426561 141.152406037213 137.999190557332
132.895500657638 159.19183334563 201.269380130546 137.709120153505
142.674671804361 198.113398073968 500.848273432862 145.320945010364
143.086872363116
HIST1H2BH 154.281674854552 140.630844296177 163.516075721791
187.754968096521 179.711363255355 259.426050921239 167.397758092427
151.004738508108 189.840366340622 213.584269089596 151.211710155053
243.159062832085
HIST1H2BI 110.694256186941 114.631759839092 114.242056888286
113.184495948936 110.325803953516 118.419311800417 110.847680175669

111.703224727793 117.611606270124 122.959518532165 118.457573670556 124.815853677553
HIST1H2BJ 138.250288796456 138.467624450885 143.08841562593 137.508097741526 181.467708718758 159.115391667901 129.397378057767 132.218907367109 199.579818754014 194.88319590729 125.677019190255 138.516066340784
HIST1H2BK 619.045921382959 859.860994525775 547.202354339933 550.24496459253 1718.80498208397 1989.38201206555 675.281131488524 648.549890890375 4628.04544865227 1946.45172007314 1110.43791444289 628.485648053393
HIST1H2BL 123.059971161711 111.000303209442 117.178495957357 113.862790983655 124.251699375036 121.855752708908 114.129044806825 123.439897913722 110.562310620515 125.634045420702 128.729687597908 118.331237160995
HIST1H2BM 131.176413983785 141.308299074832 152.14012587515 149.103778494524 147.544539855657 131.87404363664 128.530540860266 147.029988235786 130.326980619676 146.361128670362 140.714455864886 144.384287335249
HIST1H2BN 133.507865919719 131.878155417065 149.91705328815 141.453255692229 125.065813262416 130.506708993193 142.919910421524 138.367321366804 154.818414925242 126.468167800414 125.593595066736 124.863832760607
HIST1H2BO 171.636121363019 146.681183890181 174.26780796802 173.147311624838 157.592607345742 168.935348409102 180.923089242255 164.962201718762 144.555718186987 171.426334687626 153.072429774613 169.113439729353
HIST1H3A 127.247764390727 134.905760222839 117.30724482798 119.252737868132 128.029736384348 130.800427334056 129.221482161409 137.805895248174 122.414781975236 145.89801576671 141.819762045169 122.909152047474
HIST1H3B 145.306369509684 147.980827857138 151.718492863169 152.778732614944 154.431051053529 148.022287939066 142.046082566177 139.049085468313 156.61494867164 158.205235596076 132.402255281009 136.584199831088
HIST1H3C 113.23302126254 109.700513642061 108.515401509039 112.371497988132 126.81140158672 138.89579816122 110.315549501241 112.943630133093 118.312891459156 119.000436602325 121.553107861517 113.656820701744
HIST1H3D 146.114159600635 134.14147833356 133.213697351807 140.117644705946 181.840985962045 145.815454490804 145.985223908092 127.144291519133 193.190059954084 296.915883082902 151.486362260537 140.434086004204
HIST1H3E 159.7197697044 142.505050582588 136.62213567467 154.205877662428 183.138955445765 150.815117344434 168.007337847195 168.674809329231 162.216377983292 193.801858215025 178.619580818758 159.276726310525
HIST1H3F 140.379874810685 141.568970008164 152.088488171996 150.28169442832 178.3652866131 205.52620827767 170.4583554058 145.314204192565 265.148061007894 214.8478629277 160.713790305656 178.083311435916
HIST1H3G 112.962180108363 116.58452862953 123.400143662218 110.885714048075 158.928949614338 130.988294699995 118.622346984257 116.092815232176 142.68640844948 120.951365231971 130.983136465761 128.137646789236

HIST1H3H 125.269726165403 129.96280577492 131.454955038299
128.597272557033 141.913001976093 130.562422910426 118.303150165804
138.69392148885 198.023465629218 168.183542846746 135.294664456623
125.016608289141
HIST1H3I 126.573673878042 125.621467040793 121.44564199541
120.238706364491 125.27625242915 126.191057198995 124.59937620719
121.980744551615 127.422129210789 132.560292119968 145.700589723224
125.868080230094
HIST1H3J 147.513689209024 144.669509071181 152.673212084312
141.725882943927 153.767488352037 152.224281847792 146.624631389743
139.858812885649 150.788386945666 145.662818023154 149.595375008252
139.97127025207
HIST1H4A 114.624259793353 107.316786994369 106.126202301438
108.895922800601 111.672450681359 113.283359026655 111.506345174435
115.496108000499 115.02681415256 123.294321716108 94.3531468700382
99.7487411813724
HIST1H4B 148.49560238679 141.249687936867 146.30781951018
146.119700458464 152.741229289112 148.42845062376 160.164327420125
141.088456756806 143.780803045394 140.357427912361 116.186846885411
137.620843137286
HIST1H4C 8053.22070239424 8881.86480594052 7104.65816621275
7902.9189607838 29162.1093037754 20217.6497214386 9881.69734721233
14108.0461617941 12334.7921691489 6585.34962362923 7389.95365139168
13190.4826009741
HIST1H4D 147.879286704 152.317890573534 132.428675012593
151.821324922628 145.985223908092 150.536178574986 138.060245284993
160.655929233978 174.775676782795 136.022614387461 115.954828435072
147.49720952988
HIST1H4E 132.504264154139 142.331050242704 153.690189121681
155.63328143658 185.876046259727 142.919910421524 160.898061905021
163.668476670167 170.326474367867 212.497773003032 150.292858985795
147.07272598888
HIST1H4F 166.998311158378 152.54413356964 153.865702787334
183.795632040455 148.512096079648 171.40549153673 157.66470622511
148.955023180732 151.010493695217 149.270818605598 124.452631766045
175.341310423897
HIST1H4G 137.314968268692 145.411264691728 143.423661752881
142.468453098878 135.946808893146 142.137885203233 154.954906872501
142.130797378448 117.069094451283 143.05872558805 133.984078167807
138.231715814115
HIST1H4H 150.231063426302 158.545435407585 139.123489349026
130.278980631435 199.746775121317 148.808928892047 125.796438432905
138.393565473857 350.46078795627 171.414869677333 152.191393042132
138.175538905334
HIST1H4I 143.909501365162 130.272086123019 132.446406243531
135.732616463819 135.061176512365 130.454496708632 142.363309192458
130.40517506216 139.843416673512 148.492328039604 124.530660958725
120.422401793885
HIST1H4J 151.243948028303 145.041430440608 156.901209828741
143.434571922656 231.698163424706 192.789886894821 137.471563342956
154.362571105094 202.99342484328 191.924177620803 222.593554194453
192.627266049516
HIST1H4K 163.315865817864 172.097502416776 196.450308368198
170.01557699913 499.544050752036 468.496972957503 164.185346950306

155.130745957603 434.61711290583 392.746574862867 339.843776738468
353.078842299761
HIST1H4L 127.771225410866 124.372963874507 154.179891883612
144.713108022551 154.462766419353 167.599784887933 154.296467085914
130.555405606993 139.925201225224 149.764447995426 129.138035235415
157.572446865749
HIST2H2AA3 356.62074247786 423.045329000756 313.581986704048
213.813667557079 762.182213353032 760.070180572621 228.560346647306
463.622673539254 2758.01526530661 1885.93146154931 555.607185132941
386.544913399837
HIST2H2AA4 409.206158277764 400.924914238052 331.383533814347
239.388546850124 1066.25208226214 958.63560158343 221.889331385023
567.465201338735 2995.42172749615 2072.01578063404 570.130804179186
419.763812610671
HIST2H2AB 122.909152047474 134.064206964444 118.446703299916
121.36708642226 182.084333814175 146.735616237082 116.540729581355
130.579954890389 228.212753830905 193.064450357153 154.932132950843
133.44592405762
HIST2H2AC 378.797952580504 414.713379367927 261.286548990435
276.138650967632 1306.20183851747 777.410297605252 238.588762326319
426.769986378276 1648.22531304741 1336.15622439522 428.782703561238
366.408565514304
HIST2H2BE 435.901794650816 369.070376276471 443.964363178742
331.684298221133 401.87674893993 349.722963659175 152.578091976678
199.980312703646 1522.87703817058 569.236322997211 389.686045618228
267.622579012917
HIST2H2BF 171.267907653007 183.963578322984 166.681646517413
172.478461106269 164.377227860031 169.285901212492 175.717830026564
146.900087470856 176.883702717029 254.036619126409 168.873282919232
200.810204162831
HIST2H3A 115.594489123676 112.578934972566 123.190757763412
131.811011266156 148.400132332444 151.384762644292 135.323341505875
115.316430915446 125.345611085082 131.305785417254 106.491867793742
123.796657561057
HIST2H3C 112.547907748842 119.202966460612 110.544044723728
120.871693555514 135.548042190047 118.514618614167 114.613368640356
125.019064287385 124.477586539576 135.976837152799 136.850940500212
112.629266772219
HIST2H3D 126.285743744314 129.38021642675 126.103561089901
142.697827839203 144.448503894295 138.902097203056 139.897320032723
151.10837100314 145.864902319111 151.639515017015 151.436802637988
144.199054675488
HIST2H4A 168.460095116205 167.219062918573 184.974347306539
171.545695383454 201.167206950576 172.612152484322 184.260835471542
177.039190810529 420.248838397618 181.479652131104 163.474261351901
175.784250947874
HIST2H4B 135.86149691566 142.956870642583 132.659196898439
134.427601777639 136.952914053358 150.049852391879 149.752188104803
155.873969001251 251.787648609546 157.751477529076 166.404164069728
161.755290291637
HIST3H2A 196.734037460218 192.804447792672 149.910521500364
158.59135215074 457.292146759215 639.688482258192 436.731545612902
214.904634494921 1223.06438074895 409.691865300362 349.816819166182
289.956528242564

HIST3H2BB 138.709991561675 141.743873995642 129.450937831864
127.398873370484 155.598586699376 129.546119539687 126.63730651721
128.118223792086 132.628358688595 156.123528587917 166.533191390639
128.163613794257
HIST3H3 132.416618775337 127.814109293214 136.005756600948
131.892151111901 122.727385599754 143.951756384507 141.901382169334
143.555987405898 131.623527073985 142.412731964179 124.777895136212
149.272219727411
HIST4H4 140.820807603291 130.156775178934 148.512096079648
133.333067546558 129.601234646954 132.521681289019 143.827500301207
139.21331678591 156.365413649149 146.122816653035 145.107068919893
146.256076936456
HIT-40 169.447827110558 161.557558028531 204.260800563197
157.273309095198 155.295464263911 243.402781509365 184.530853446688
225.730739636442 171.093611712143 142.329137800558 165.003646188775
164.68600028429
HIVEP1 206.915239638443 199.077893147154 183.464897811105
220.446775597113 147.285764611006 156.460374293022 146.574656147816
156.704278982517 173.424541543058 157.81831741292 174.727439318223
157.333615322941
HIVEP2 335.301103690781 312.177191402945 240.306287921209
274.736124442229 195.863843860549 245.240006198482 402.965083947376
248.010317392179 231.698163424706 240.497413647633 441.001837510132
369.726867521935
HIVEP3 145.748174062159 152.124910935545 148.484823275343
139.811873260553 151.96843908074 163.066051106092 133.277326912499
151.449690439424 156.01406297447 122.292692083352 168.412162365467
159.106269972346
HJURP 121.993231438082 148.288798428149 127.879723100698 148.327340543504
376.374248850987 226.893147371644 184.306452616482 242.538036654192
298.625127845168 210.529782898493 185.974598267418 302.735372807236
HK1 1127.89414462606 1349.84215478798 1337.38160266788 1780.97169244555
924.630951485299 699.800360892516 1085.38819961412 615.662993833692
793.300636780035 816.823443819069 1094.67740173529 1042.40539020413
HK2 169.052101570616 206.21848253717 185.843639043466 167.637638693195
505.302263971007 282.870785313621 653.047570485109 460.514672966125
234.980382243116 1654.58270183167 873.256632365737 756.390518977556
HK3 164.811120992602 149.322537247388 144.505072819693 171.320653882424
153.457715729768 159.146745596683 135.904163043783 150.079372712689
162.6762533989 168.50246276469 151.891318080151 141.019494314354
HKDC1 136.413745926388 133.138744413911 153.405052909585 125.685852196342
139.524233960073 136.841506794489 129.564482772168 154.539367933684
151.219051767056 140.804008584006 135.594186066942 150.072005848234
HKR1 242.538036654192 207.376329989942 204.031081842809 222.285940575778
169.568915494577 170.410704499804 205.728673153914 203.276353321185
189.589053840976 175.417320301614 378.920456227378 217.013533525724
HLA-A 5195.2087608308 5655.12135336698 4255.59773503364 4309.86375945008
5175.17279574945 2965.38280248519 4095.76073123843 6188.04738762584
13479.8546809758 7699.37963536892 9596.10781313429 10048.4260790604
HLA-A29.1 775.382397816601 870.887539668967 818.761934233826
587.893636997642 133.834423222601 342.611951034707 1603.87451959083
2198.4268389426 2859.94396089224 2863.51286169094 4404.79209330918
2828.97255380291

HLA-B 2001.92441724356 2005.76678571689 772.776364492813 1102.73689840572
4335.76831372031 1065.74382494535 2547.53445585552 4175.70090899755
11741.7473778842 4417.98524179686 8053.22070239424 4222.55224241399
HLA-C 185.220017451419 163.960242712692 143.070170783764 174.483233557264
839.324324662619 207.993943398697 338.406266073063 319.417555901733
418.968095159939 212.996456841785 899.336817124312 309.155070674445
HLA-DMA 891.637685856145 908.704610877319 444.584594376357
669.602939159192 1507.39761667421 599.52440136406 1071.00507864299
1967.82064011967 3152.64817044581 790.321331435096 731.513306289481
573.846138124455
HLA-DMB 718.142991571468 828.276436883306 397.387054218873
615.88518704181 442.334886941795 516.577357935963 547.516635695872
1462.31151845458 2882.51330563735 589.541347392215 819.887322757281
441.785666261936
HLA-DOA 197.277310409328 215.769986055534 147.225911875196
200.603836348642 228.588693548447 169.332207863684 157.176001400747
240.07282016658 319.018213637357 169.881866987359 202.568071395884
155.176714087209
HLA-DOB 162.018860183534 145.599152743156 153.592819860747
151.831627236638 165.613274361543 142.402227523617 140.429462488271
184.148058872585 276.756415094228 153.38260557666 150.939877261936
135.809383370957
HLA-DPA1 1172.96121022579 903.824331691969 478.293220151258
612.374364731095 597.189355986293 1002.44383771056 912.878077940014
2770.7811954029 4465.66734676111 1053.09915077366 1101.66868138797
466.691191261997
HLA-DPB1 136.307646393792 146.686192012988 121.164978896057
122.841135364088 120.638330368388 148.916981164664 127.146101490884
155.861829925386 192.465510013851 122.796362813922 165.995251661994
114.048136176142
HLA-DPB2 140.401508808129 141.520125030042 141.544454931788
145.374923175872 146.238333981318 140.10798818623 135.360087349634
122.644105247178 147.268006631005 143.868962478182 129.522339467532
136.843164021899
HLA-DQA1 247.194197143562 215.143973502566 147.643985046683
207.368252249039 191.507657960386 322.576886792502 311.481739745423
1184.88559645917 2026.85580212422 256.729891423908 383.802494922918
328.60529053804
HLA-DQA2 137.280933631075 141.121364213272 129.049273337631
134.796088225931 144.792035839249 130.176734524308 130.189958197332
129.054577477882 123.170639180926 131.712745388563 139.62718946833
136.184427467391
HLA-DQB1 195.256498208339 213.077884261943 148.326282600684
166.8846964604 151.345630073141 184.923312649584 215.635313821029
154.548286860883 331.62124160152 299.851173200352 139.353080958675
181.425012248539
HLA-DQB2 157.166379887259 150.138643811727 165.565284723155
143.62315472858 186.1961391235 139.276102596591 329.926864732606
171.221972336784 217.531179216305 164.50490221903 326.525579400607
177.235586767256
HLA-DRA 2244.6916226146 2139.42448145954 1065.74382494535
1141.49294161587 1010.73988405469 2630.78185410861 2063.42824094932
5981.08862268291 10533.5868398404 2346.47361943533 2105.87438782831
1914.96285167893

HLA-DRB1 283.760131847707 247.746304138866 286.856346704286 172.612152484322 928.151632070573 401.960630197091 151.0189104086 761.097992055119 1810.68433539154 164.659092629962 134.06834346533 544.956712516196
HLA-DRB3 573.074116810032 547.681206272188 432.726040933545 452.167674725454 668.570984110088 424.025588636796 451.210172051464 667.693216401836 980.120729515821 414.114051645565 545.200687301057 439.023181761356
HLA-DRB4 353.243400332397 380.393153331219 286.194197552489 268.832884350157 303.762967437478 510.972389503051 298.573180660083 994.802763887989 1914.96285167893 415.113340335205 253.754869975548 353.005548289786
HLA-DRB5 338.326304736048 312.080864996592 117.513523498725 141.560424984281 825.781627776577 134.796088225931 128.50733429744 1545.71251527145 3636.59530016939 149.772157318769 131.575356402693 564.41151411028
HLA-DRB6 417.987533397449 332.194163016646 217.202541167691 333.822167152866 416.215253990273 325.252108402192 553.757847979529 1055.22233969473 1004.6207350158 482.965800108538 583.022090879298 360.124745133393
HLA-E 2316.49886419499 2744.77453865893 2104.59597571915 1881.90353127141 5008.4888270731 2093.67403119512 2611.61260614408 4548.5418978622 6470.73189429877 3775.41389938231 5195.2087608308 3090.52810501797
HLA-F 692.729789121701 792.247301083075 516.755267232127 441.001837510132 975.315904185938 427.637083424453 606.851072672402 917.188039935737 3188.90202907403 1682.64624665931 4088.12910138929 1949.60516542965
HLA-G 402.564148020948 389.381039495553 249.737588031219 265.470314691221 377.951523644306 212.171281020103 387.068276379114 351.854828413334 679.191060100076 559.668195903429 2547.53445585552 718.670564185576
HLA-H 1500.16816750361 1662.51399707484 1013.60354455825 864.908449769749 2515.7035557541 752.142287661845 1477.10828981572 2141.855356411 6720.10473881011 3436.32495553246 7681.48027081818 3757.39261544326
HLCS 182.736067431579 171.941887376462 167.536828038178 177.862368731138 158.455783755247 225.505978789388 194.998142472864 190.077302802065 213.93763684072 171.051948060796 216.53417734069 192.314484368435
HLF 320.877897822963 374.761007556839 423.256021602613 366.961565952239 138.755173333171 148.278828579424 153.171822092714 159.476343366991 156.609370182062 146.287161912291 162.57736365619 160.122274989224
HLTF 326.379855547052 280.723379809959 398.61164689194 300.765518378297 201.016287924499 412.736636437651 431.686900474971 366.961565952239 421.255522475862 312.645778869254 328.550286726543 405.032674307193
HLX 220.533113603343 267.589020526148 195.141733146452 337.585502787016 182.209056248253 150.084909674721 145.68886453261 161.585555680663 177.001637712889 166.835922279372 156.243337414127 162.383584508358
HLXB9 140.923227606022 142.282861913548 140.04612725812 131.300342835696 130.09958025354 152.974593669153 136.560048419149 146.238333981318 138.530236176135 163.315865817864 154.321219471319 148.993620368781
HM13 164.103306492559 165.67554738735 193.008902902654 149.199800895684 211.438615793742 257.24481414803 259.796092536601 215.167086358143 231.439252571168 243.336550161566 175.847256673734 207.624240598841
HMBOX1 457.650982600462 386.16563750478 373.925939492544 303.423450627377 219.2695037071 351.395760651605 303.608918161742 259.575461599189 311.208703262497 324.671490117952 381.182514249587 301.908667220208

HMBS 188.973595445056 198.522123898437 174.323881548679 219.192900411543
498.334266735594 222.394280571312 213.178648382852 246.492735271771
252.280435525505 238.068308163643 191.525291860022 314.095043876244
HMCN1 124.042153387391 126.433146708506 113.542803491215 107.32960937124
119.207503310618 118.192302821514 114.65036208773 121.356731684516
110.325803953516 122.702175900246 108.007762863766 106.126202301438
HMG20A 248.229468372363 254.668502187945 296.141012087256
264.779154931102 200.405532717305 228.542423012958 244.990374822652
218.269847477919 209.702683803624 242.037132958656 235.48876511325
268.406778092906
HMG20B 1069.93503700632 1240.4863200811 1037.46485756166
1794.82283439196 2712.07480130502 1481.24705642453 1029.09818399518
1399.41653736746 774.442392607873 958.005138077899 1293.70593826709
976.360310504918
HMGA1 158.138862054715 171.869472272081 150.560305000601 158.130933580414
845.150147618462 278.005620372937 334.772648743153 364.331860640225
300.793542873709 316.457274749526 355.879841671975 485.731287873723
HMGA2 139.21825391822 136.135223482208 136.80943340993 134.8745127454
136.071856104688 131.643633737316 140.434086004204 138.156430188312
131.655316312379 132.328948598599 143.787766055379 134.624322761212
HMGB1 338.075238647592 404.618867159141 372.689712716797 338.46332239699
265.29629764006 565.313436777992 251.939696394174 278.598913464804
269.194172034595 186.244764337256 284.321253087512 375.131728190867
HMGB1L1 1055.79590411506 1103.13439876128 952.724512915813
664.809419789814 553.368945998847 1327.65573168233 785.290353402444
838.267069860172 924.630951485299 898.877732360841 1513.28088592885
1607.52029676299
HMGB2 604.687265229902 615.662993833692 492.377642058095 507.342877360289
662.58366387795 1031.59140059071 633.762608454521 1388.91423772335
1323.91781040143 797.73902873919 438.533112511098 987.564350211893
HMGB3 147.471494294543 171.304896264805 151.846610656503 136.244995503799
544.669441360594 548.869880723909 354.45785837703 447.579975492958
317.121202656311 161.992141305029 353.005548289786 304.937584086529
HMGB3L1 162.982557003317 150.198869833614 149.964827055916
161.195562296834 139.123489349026 150.888616811881 141.290905146249
141.418737063013 137.330207626732 136.611670766233 140.009593108693
168.460095116205
HMGB4 116.87586240208 115.821231468312 113.557349524174 110.496084922302
113.76411537952 119.636334259263 119.161972571516 114.217867810848
118.095936088242 108.333135424315 117.724353829377 118.777105073561
HMGCL 261.426617866925 276.476790719416 310.301616266762 352.665030560239
553.615252472149 310.426513884979 305.325519265387 371.767182305618
389.96566674501 254.188830191845 426.364663722264 240.994507285383
HMGCLL1 143.865717051998 140.590802695412 141.600833670693
138.1342592966 142.553175839278 140.370626016794 138.639410179404
145.155989928336 126.482660154852 145.63870792125 138.0249543452
134.877046981678
HMGCR 348.111872425949 436.731545612902 315.513423195489 437.836503489425
436.155731212592 576.451964662024 782.746481112316 543.170902795966
650.263737836685 1291.12004949379 824.746488539942 728.930140593418
HMGCS1 798.943242964857 873.879064183441 589.240581642145
841.285351463894 959.897744425251 683.179460779062 668.853969513382
2466.36457617864 2770.7811954029 2299.23805014176 845.150147618462
711.491528818004

HMGCS2 225.99848688881 644.97200337335 398.796188440256
207.940596890723 8649.07367196998 13526.3170858629 4327.90939653054
17749.5685495445 14037.2575989329 396.345382600445 7631.6124232898
7733.95252458705
HMGN1 1563.71477584676 1696.69982157582 1838.18547839898 1492.83354014907
2084.44431604433 4417.98524179686 4417.98524179686 5106.27623905309
8140.6363797934 4238.41681589316 3545.8833678963 7104.65816621275
HMGN2 1082.00448164658 987.78377957731 1315.66699943181 807.3257139219
1813.79641422879 1682.64624665931 1282.84159765715 2410.32095582515
2388.03256067711 1792.35343044443 1174.69701182094 1778.73916100529
HMGN3 156.519196335454 152.067897082883 146.393953146723 157.04599638368
163.764600217347 155.280897870265 154.47187360425 160.454243375479
154.368499839507 170.179304745674 163.960242712692 155.873969001251
HMGN4 1432.93880448974 1360.24752587939 1645.05161227372 937.593129242257
672.13164205724 1897.92061704124 1372.63540073207 1536.52218968817
1300.40889834984 1135.49876948815 1887.82245931444 2056.90127387648
HMGXB4 183.693300680495 179.648479201164 199.933148219784
187.871017629994 181.157738347957 230.469548500304 198.023465629218
203.999142031297 198.088834624665 210.085329463347 184.294172429054
222.456810024193
HMHA1 298.776176246196 262.452869676209 290.420307322101 277.129985959184
291.577778800314 239.31180635241 244.132645171301 221.961758403614
201.661941695238 164.14265547147 204.219197797869 179.593817076317
HMHB1 146.425476034368 134.14147833356 145.677477231455 146.226437529862
146.443892388756 139.228558152937 136.428826558493 152.158269344693
118.338713846941 140.899799480834 150.822586904738 153.941193682697
HMMR 151.312853764675 151.917359928516 133.158570377793 138.186616955926
312.224309341171 419.846584903512 235.453139389486 583.619362840814
332.978350226738 249.823314327355 218.470365730283 469.333357789149
HMOX1 189.853136769155 196.226732738526 157.83460319212 183.719279448844
430.505037012814 208.456472621593 165.938009275308 315.513423195489
244.5675215931 252.957489543915 196.001089662818 150.822586904738
HMOX2 182.827395663312 175.327597807738 169.475714234887 232.465880700217
151.736824723671 147.598602877625 162.620593985693 135.481300250507
173.939159471022 161.142723532061 180.902950045351 151.289464845626
HMP19 149.609501725707 162.06940121135 155.343619434375 165.581217879395
140.38936578649 152.780908985916 162.774716879847 143.533725135227
152.397984799517 152.071081175788 164.791545858875 154.205877662428
HMSD 140.976675511847 140.648918459179 128.05898686833 136.381593416533
130.732397679661 141.453255692229 138.023752854109 134.648780131578
139.793683692104 139.517413585211 133.579067514064 131.239986488033
HMX1 122.476178620771 117.494422663086 113.214390213004 116.718099363329
122.399241018147 114.533677697026 120.13937451914 111.876915242732
111.559325333192 114.155592351175 131.597937570173 115.778736387131
HMX2 148.570769470645 149.502132524093 159.994376562272 162.007384919302
135.931715324468 155.933739693912 146.087596132093 145.267225993057
147.049287475127 138.829793136954 129.994160770036 148.461172387171
HMX3 135.991630745082 134.519697269443 129.46430369977 131.930814868433
156.678686363755 146.462544135666 144.364319564075 137.587917564413
135.170925092209 135.860777386274 118.792612926619 151.70035428516
HN1 162.254062646631 184.937088280378 145.161116298118 155.488346781292
437.344765185802 873.879064183441 623.050854309724 776.807656744828
866.147379099606 618.829716818725 401.359711029934 1097.18236659974

HN1L 229.77428612216 239.12905945808 266.563990988678 302.381946749711
204.113356572343 293.925995360234 256.237185590901 279.704940529618
247.194197143562 226.173501275026 302.021033686056 245.900840732668
HNF1A 131.095431530473 120.649467083125 135.203507726561 126.141462782039
125.677019190255 148.255769084026 152.514455788489 137.448412532999
130.778164962583 131.568898109933 136.087702679172 134.48825480809
HNF1B 155.801832826848 148.423303776817 141.353084959289 134.725434838052
320.024259346646 252.171420358437 248.850686917567 402.824964079515
385.238961394234 133.743708220112 289.001004912528 258.16437138327
HNF4A 145.389721946046 143.920134762902 137.383211680459 130.390722272955
131.582231577364 129.958065173006 136.814736808725 136.745596788338
129.788395150404 126.199219102601 131.792339956719 133.617501101389
HNF4G 119.511962218143 122.002822875994 125.280736217267 118.220555783444
120.13869466889 119.312201620247 118.461037435114 131.566892082703
129.108534267222 119.562180492359 127.26820735867 128.545699891734
HNMT 246.049729366987 284.438411011954 273.514200733343 243.936797286495
220.446775597113 390.098830270926 543.933989965705 290.389345090634
297.805768288377 177.214422668922 448.644778906048 425.59810308048
HNRNPA0 862.132582849511 942.900963522948 1013.03054571763
723.428294037428 694.288215755716 1037.00025848491 977.999660785113
1271.46608195833 882.429823267754 1000.05896885496 1068.63350031223
1197.58205444449
HNRNPA1 170.725486814469 175.960788330149 185.332215198617
153.22516060649 154.496005175117 177.759729226623 220.710324965453
256.471672362567 218.314136992677 275.543576337502 175.601912488918
217.327543190687
HNRNPA1L2 167.298438019561 156.901209828741 185.617936412633
148.688413944868 136.937327692084 155.3394927753 163.385727569154
153.568321662387 161.673818303367 140.177708471199 166.305219348008
176.47556448977
HNRNPA2B1 1275.43768532088 859.113541787323 1086.8615735568
748.940715378564 388.326080754063 598.287431134037 746.296354515617
1144.53002508973 780.961416212309 549.569374872629 817.036222521723
1491.05501052706
HNRNPA3 187.903298116031 175.38376293575 173.189465201573
212.210383398591 315.752980521266 285.768837438859 210.5689532432
254.235762677639 306.068269136821 192.565733545715 422.12964420709
280.656087497806
HNRNPA3P1 169.984859264412 185.717123241832 161.299920355406
179.593817076317 191.987301954927 181.578228610334 184.387409077972
167.192582058634 183.443006525616 173.959556466614 253.833756857339
176.289693941489
HNRNPAB 1480.52638217173 1248.69137283095 1606.01395227661
1333.73334006986 2493.5484556802 2112.84348677276 1828.66165670455
3029.07443443457 1823.53870218167 2311.05587511258 1442.11349373764
2542.44286642873
HNRNPC 414.888512708716 455.392133846517 402.965083947376
357.35857422585 307.866499851733 815.388288623756 492.207833630086
581.37957221282 529.854965483609 533.784151495549 622.692834629555
809.729160605727
HNRNPCL1 144.927731613526 134.57472034679 140.615078497305
133.64938083537 146.06717772919 139.302591312993 144.20764300349
144.806478328053 147.951996191313 140.556631658219 138.293242905081
147.796610216375

HNRNPD   4763.89426388268 4299.06288109563 4690.93553472125
      3833.73632806344 4309.86375945008 3232.96611074938 4047.99200599472
      4228.5490138553 6508.71638048514 3157.98451877581 3636.59530016939
      3581.36347643133
HNRNPF   156.349030617265 157.751477529076 144.501995579502
      152.503889578356 187.561298131124 202.483493595704 193.203053225456
      176.029791617989 175.537039794925 137.717859035645 194.655710454472
      177.679330300381
HNRNPH1   1206.09815575399 852.649997188692 1258.7730046784
      628.344122779625 728.930140593418 2037.96470181871 1296.10827265334
      1750.21700780511 1618.64901092072 1914.96285167893 1502.3444541735
      2662.43181510326
HNRNPH2   264.456926651554 224.224264678645 314.708587131145
      212.272826965935 315.430399077266 457.779218916808 291.720204789005
      224.724262742427 354.783364481955 348.36178568457 322.302219773624
      342.315456386729
HNRNPH3   741.433093896472 618.140160251927 933.408902924063
      518.925188630246 260.554343693588 390.372738176917 460.941286124613
      537.350891960682 543.584413650467 340.42197893246 430.833137328233
      381.182514249587
HNRNPK   544.462032960964 407.589300080181 704.448123154868
      364.597798667541 681.677841333095 938.918431240228 623.68308750251
      748.940715378564 834.788058321257 882.037055216609 797.501324589697
      819.887322757281
HNRNPL   366.788087591332 332.24505665756 300.886032768166
      378.585058475083 698.358285634451 474.216057978384 514.002313819685
      291.914408947328 429.457884032977 386.960778432157 322.36247485445
      343.833659987063
HNRNPM   1629.32740709526 1595.22997714906 1771.39155164347
      1651.68171717875 3246.86605825129 2233.00694575483 1978.12229412176
      2822.48671794913 2720.83642320419 1854.11328962023 1870.52504853779
      2346.47361943533
HNRNPR   780.461254794127 624.264994653057 766.710479603963
      569.133295913677 823.658996207582 1521.61996270758 974.544773560873
      1229.47460924638 1176.62418139379 1006.69598892631 1141.49294161587
      1282.38953612054
HNRNPU   2390.41825815872 2285.86163259073 2910.00269086567
      1920.16520804856 286.901115411673 1747.33558205825 733.648333788712
      1253.05700051015 945.830852603704 753.007562127186 1227.94401191112
      898.877732360841
HNRNPUL1   159.470818285679 148.779043570204 142.255657518926
      156.423687034312 168.882806817001 148.408616955694 177.011776399483
      153.415901281564 150.724359913994 147.8838487685   149.552704503066
      163.045605069267
HNRNPUL2   1098.20033709043 1181.1611856364 1087.69177809384
      1539.80266129509 815.388288623756 681.172359997071 803.133619199479
      510.811470658575 681.677841333095 683.019502027271 892.868661511516
      797.28465621772
HNRPA1L-2 1066.25208226214 964.473774310687 1213.61170930345
      845.369108604819 786.62709337246 1275.43768532088 1707.09830463606
      1810.68433539154 1231.92253664482 1211.39534395597 1472.54829263217
      1726.15788525017
HNRPA1P4   1707.09830463606 1503.2049371911 2174.62979843528
      1518.13775377335 1280.57297272231 2363.50330960038 2622.87486749984

3570.21370790206 2316.49886419499 2307.82327595071 1589.70261092241
3219.27851139047
HNRPA2B1 3869.12225821096 3430.76532174701 3581.36347643133
3143.77161008321 5022.15374558514 5080.63091497522 4641.8793590085
5992.92384160188 7802.25987814138 3668.19307488953 5506.81944079182
7003.06505884735
HNRPC 533.140740560189 554.950092776254 629.381791489871 396.40172043456
555.259469428492 734.71562355687 619.75986112089 716.731984660463
754.406896156858 919.165331064155 1298.60552708737 924.312051919404
HNRPDL 767.861763134136 679.191060100076 1000.48723872058
597.189355986293 212.119922561453 426.464641742092 452.167674725454
593.462634352052 670.856008549143 660.907502776003 538.369868115137
596.934339406636
HNRPH1 280.798003990849 203.704734790602 360.684900316265
198.659595851481 252.197271817794 363.150472154973 342.245182599593
343.200277800027 337.822337721287 375.131728190867 464.436380568216
521.83794739314
HNRPH3 620.371912523332 486.425387717472 607.126514742951
396.861835715943 228.9901869793 305.542978609337 395.261210769232
370.301819403832 383.033242143031 274.427235605909 321.750495072759
349.910535555712
HNRPK 728.485195792194 662.04757187541 981.781076110568 509.292779821787
669.602939159192 1072.37500318471 728.176339659082 1282.38953612054
1271.46608195833 1423.02563792293 1211.94273385872 1143.0779222425
HNRPLL 246.710233781784 284.473085899219 261.541138637729
243.308625792448 413.000072500071 504.141861790903 325.011269531407
307.570405241182 315.565349831797 318.297819854586 316.004332524937
325.011269531407
HNRPM 1669.97250023755 1650.77281891596 1853.20812924694 1846.61430264854
2992.18604022433 1963.23068846837 2021.92898855142 2877.92845389611
2662.43181510326 1973.20689165831 1980.4589291261 2210.67250954072
HNRPR 1758.7809492929 1629.32740709526 1648.22531304741 1295.27257578314
1599.12034906314 2799.0487754899 2146.4265920569 2672.77147589342
2332.98074920207 1728.86115695979 1800.25771851544 3055.80298721424
HNRPUL1 1187.62490367696 1440.14449872047 1115.34267967366
1662.51399707484 2374.1382412432 1381.07447204233 1464.04124596264
1279.15873231449 1698.08853832248 971.170460915918 1151.30600388403
1361.54107116915
HNRPUL2 814.471143753335 711.491528818004 737.193295872614
888.04996477314 295.278972023002 556.568221094639 567.715714233608
456.530337997907 364.597798667541 466.4890990178 480.36420271348
480.484639351046
HOM-TES-103 146.750774496317 130.524630778991 142.56910547147
141.600833670693 127.962546712222 120.241528853563 124.548598801497
128.922269289649 132.760567403706 142.121399383259 124.521130036013
118.622346984257
HOMER1 135.91276813517 133.659475650376 113.879459563492
123.30960025689 146.042460762767 172.446482096058 163.298729857831
130.592061896237 137.012646671173 168.269075560636 150.331324340476
156.597497458992
HOMER2 137.118488157416 137.017882995262 143.378998368344
143.967197319361 247.015574724901 162.656951764783 145.455411983653
138.883710791382 268.885346595453 466.691191261997 142.719216118914
148.448349327493

HOMER3 207.488522933448 194.88319590729 205.950347620158
268.454310218604 379.546197364027 304.844121431627 223.84997313236
243.080229016932 173.315097944835 356.376890469558 292.839392578318
279.879377933144
HOMEZ 141.03218895811 128.152956417755 142.483308497682 153.610428214886
157.28429690041 148.406031732776 139.367493349339 147.83240038699
161.667782505656 156.654450436728 185.727947276958 179.30729544595
HOOK1 147.354535468933 200.603836348642 146.932655562613 119.952108688414
422.688531130043 695.595965352335 436.891409783298 551.350758868377
386.817736004263 870.409935772423 300.709410518382 427.542390397986
HOOK2 245.997705723007 249.029098902357 190.002630382394 242.5085895186
582.527835746927 324.56043349882 562.711562649704 414.888512708716
334.443733461841 360.185005480216 536.805979562807 357.514919818489
HOOK3 179.94333102877 179.163748161358 174.795822188504 181.898985277176
197.034073391478 246.95071325085 182.209056248253 174.26780796802
162.304178370233 207.269530621176 167.456481678813 163.534117903837
HOPX 351.854828413334 302.905840432998 361.390981655795 370.555627307623
623.888568444132 348.457808063307 414.797416270335 808.837225580351
535.861738240441 653.264945856916 309.47429862515 169.368090266817
HORMAD1 131.324822348301 121.736056336879 125.472342351466
135.582230137515 131.608580511282 167.532512839891 146.010474018786
173.497575259833 136.55090238808 178.045931314857 123.195738824794
139.174188535843
HORMAD2 145.873819094778 133.692025039613 148.598920567714
167.243544185778 139.837616209536 147.614293127043 150.682964448325
158.089922688997 146.526744516344 134.565199667396 147.995991912855
153.67215573765
HOTAIR 156.630366766924 138.43301474649 130.452929012459
134.109051944736 120.043811428091 131.071614211431 110.242638217587
124.952026709219 131.619951110473 134.316859658338 132.84268776659
139.804518460303
HOXA1 144.892454033336 144.871396308673 148.695212949639 140.419686138586
150.456500446175 139.400521576978 151.981708806765 136.601220755282
140.005968571997 132.697727983601 148.714406471586 144.450810504499
HOXA10 330.281193038198 409.691865300362 444.826775824095
406.900412510895 310.026522549372 127.104097294238 208.456472621593
298.706655333472 282.836518011311 180.354899434843 273.651114834013
452.732079010502
HOXA11 141.943910304145 173.979001863717 149.545278163222
167.900071375885 120.551603446364 131.919387743094 110.014935095391
112.054379249781 114.860437706855 116.011005504201 132.917794118568
113.40684693417
HOXA11AS 329.88790758321 464.436380568216 431.019896339435
623.050854309724 173.741466458636 144.214589452164 141.777089987975
141.626504154989 169.903088388196 143.521510363933 157.643461899964
196.207631868755
HOXA13 256.614964511846 253.975577255772 440.038896046559
283.084102918359 276.184421402511 174.698724064671 196.363005912532
289.368511254335 161.983987604 143.436675541053 177.793860516896
202.849544035436
HOXA2 191.554396977588 180.304585602206 227.614623963331 181.654319266685
178.284294783418 163.315865817864 164.955263076598 176.300743660299
221.45275440794 161.139253742858 183.255119298602 187.4351946569

HOXA3 153.574068503306 149.826291268485 147.024759173313 150.364511432468
136.832494876249 133.579067514064 127.123123583856 135.844983721014
141.397046998678 129.121598974852 147.955701866515 136.19725093985
HOXA4 173.115418167551 163.275138097131 179.74065086312 183.905926269285
181.566951620202 147.86104992164 163.570018900148 169.909886347082
166.981212790122 174.246705740225 150.42705284743 162.732574014686
HOXA5 759.802694618208 1172.4103691525 1316.31088187364 967.123240442957
502.281584081805 592.284071247642 775.5664718389 531.711823481265
1370.61156801632 438.7446527797 1141.13648305827 1259.38111177521
HOXA6 286.945784387294 344.247544510881 380.754569467715 404.726926716133
214.38058301395 253.833756857339 192.009562635198 240.410292351169
253.124449721731 225.96618659877 297.368197598038 267.028096234702
HOXA7 123.247698282967 139.607944722497 118.900338956203 128.618422306397
124.961859983596 122.00079209554 132.821639676246 133.59269189767 5
133.86835278231 122.339819585312 131.198284945008 126.982340580909
HOXA9 301.051775938853 386.738154577763 393.796356067403 424.495400604335
134.333679772065 129.228874611916 124.35721327773 134.241917425247
149.048057509964 141.644521617419 163.141010815086 153.366677608026
HOXB1 128.899712906082 126.404523759643 121.61143371132 128.647790058795
135.036576196425 143.727514442914 114.749597426757 142.26998944788
129.327645260099 129.511465164539 138.393565473857 127.532665057083
HOXB13 134.455623159793 147.858339902055 159.345467769285
144.858656365799 145.835240952992 155.902461107328 147.113541493157
141.457040042276 141.262319193649 139.205887721493 108.730058525654
138.213742418402
HOXB2 191.25724234927 178.166289723365 199.477350542491 196.399050990244
132.674061422332 197.112749959833 174.448910958935 302.809011218879
264.604297630839 293.018282386003 543.584413650467 500.469781477166
HOXB3 129.297217956843 147.425856984368 138.391203321266 145.889336010424
144.064214573005 153.169450192935 150.292858985795 182.833961304079
164.472980733552 144.829773937102 234.996704304673 238.745953695925
HOXB4 151.734683227196 161.26917182967 135.323341505875 141.094578556895
196.716209382446 159.065612005442 175.946843919065 153.985529457122
193.084818760857 172.758230728437 225.238369884803 163.718595974283
HOXB5 242.614881095485 297.071121022183 297.636316709322 224.963277964548
287.703569453502 311.157764305033 377.800067476868 916.4187683687
653.500279510032 218.298791348295 2213.1043022626 1724.22121078776
HOXB6 150.287472248483 153.156264091925 138.85328409337 145.672496354689
146.548348470748 143.313605534609 154.466614452651 173.182013165174
152.783534456473 167.380798617981 292.965109701719 192.932285494022
HOXB7 190.204960255989 233.36028792331 172.961115357569 196.245297362955
650.494111214312 525.515169794593 760.572409199179 510.061974830525
856.039380523678 509.14726076246 848.154015576436 876.783710734594
HOXB8 142.095289977576 138.036876466521 129.522339467532 134.013748718539
124.726035027951 148.079107235035 123.706910388724 187.673748059596
152.107025120177 123.364999283696 1360.85309203233 683.861021234927
HOXB9 123.927736719427 113.557349524174 120.793974839773 112.664210248256
104.798333931019 107.385565665491 125.589744938813 116.825172441325
104.505454151012 108.79453730066 117.980052938167 109.52020660772
HOXC10 141.958902715369 139.545451634208 125.911259629489
150.394483608039 141.028377381019 136.923876486522 126.143886553557
141.905641114703 132.021249712589 135.388750095746 111.826509343353
138.269955723817

HOXC11 130.396593013401 105.551878332786 114.704831217037
130.766401858384 164.935120145905 119.002833359088 128.606360476298
126.856038484436 132.387242907281 103.691075663721 113.133798430215
131.471942650725
HOXC12 120.196595293401 102.661440700541 122.281263517691
118.07991087231 118.689408983786 104.505454151012 109.167312436827
114.174153178166 105.375878824562 111.949644842971 115.738984899365
109.586955365611
HOXC13 147.9084767638 152.11446058397 152.495540563792
143.427721577372 370.617422894068 269.981476129439 334.101619029105
182.209056248253 257.740674565975 216.457866890673 190.700515036197
213.097664319741
HOXC4 148.215172022129 146.500312499353 138.628169506372 154.324955232468
232.879578820053 197.873787638411 205.560952959537 221.869191666931
201.256649338315 170.052320640686 147.70836747108 248.129640673859
HOXC5 109.495342943278 108.830525786872 128.075198523268 131.652854369619
120.967523586161 127.047679628867 119.972337070176 115.613348109569
118.816986517698 114.624259793353 131.592172917738 117.255496552581
HOXC6 160.415807880532 163.926432479673 148.224587582189 181.288519308219
178.665552649698 211.135364208221 237.521746704115 253.975577255772
206.071709262227 208.125844007298 158.631298154353 257.132618311227
HOXC8 163.616115299926 193.394225360968 139.295597388725 161.182901898379
150.896076207161 196.429685815196 169.028519464989 159.504327477065
127.610304071204 212.190707408572 146.014790698087 241.890850719625
HOXC9 121.760488406107 120.436408685057 123.30960025689 128.207440197765
160.567934923347 176.5588805282 135.976837152799 148.02460124587
150.799400717425 174.214747421898 145.956477465634 243.336550161566
HOXD1 133.241744350369 161.694109087893 133.180554954396 126.05295868521
190.975263192006 322.684910198242 756.068695265118 159.05213637758
157.787233338636 128.445065383646 465.102234374389 189.027594735456
HOXD10 172.376544221034 169.355185349431 211.626970008102
175.711836112986 145.45768403614 166.741762854246 186.854553034865
162.127697884504 164.562302265155 462.265785715695 132.709845352383
161.877378749103
HOXD11 157.187894260746 148.530422089937 167.756465178715
131.514114515895 167.756465178715 144.456324230938 144.170287726456
136.526706711584 159.19183334563 711.166793546075 154.630837508249
142.693337885596
HOXD12 147.978039160763 141.616920429839 126.659859384257
131.975248488461 142.383723965616 137.736771579507 146.996442729408
127.575660581922 143.440420261133 147.241136598626 110.670057121283
138.116568200293
HOXD13 139.214589768245 129.3513576591 136.430232926793
121.796519488793 151.826103919919 120.790536146461 133.9382467268
125.598828334465 136.959011188915 124.190161132628 130.973504803813
123.905462079972
HOXD3 155.664126780859 161.419873271162 173.024402954772 157.765771942689
152.54039461337 175.874387612235 159.798024189461 178.339211170442
156.417000271727 177.281197832647 181.412790232996 192.353768499582
HOXD4 151.885992695789 140.213080190065 146.122816653035 152.47429737104
204.347931209047 198.004357069446 199.96834355685 152.155760355248
154.798287249541 140.09417711803 189.348690353874 147.284005815059

HOXD8 141.602233757588 134.853253781256 142.299535614113 128.635684412304
149.97218338771 153.047317528007 138.188522429661 150.391286215189
128.468978498871 176.622758931306 148.67584969749 124.086902599543
HOXD9 136.428826558493 154.683723064797 120.752194675024 128.329985147034
205.830321788612 158.374760929273 162.825380100343 172.446482096058
154.954906872501 158.380964047483 147.174977926293 130.682112236817
HP 134.529744040637 132.699715693605 147.971492964243 143.388028226208
144.417333931582 140.861849838223 122.216479103528 145.758423590784
148.454344601562 131.786437478446 148.504996616452 131.230501496659
HP1BP3 198.760049629983 183.472290949588 212.607094117882
191.554396977588 163.821142892643 255.435582426271 210.266075382636
226.114369645703 234.339913501761 199.001860912128 148.930073905413
201.637540930213
HPCA 140.82351325907 136.735930829008 144.738871562895 143.641777655752
157.200582422961 152.503159091556 158.041568121766 148.466658640201
153.574068503306 145.185902174023 152.463553409903 140.441521267218
HPCAL1 282.282663608264 330.908743070095 264.158648784101
355.609033348692 923.130629121442 334.350011399462 263.005303710717
321.524921332256 376.082040863747 296.721891842563 220.896363324634
392.539157974899
HPCAL4 140.36425603453 141.426756696799 154.698312173556
155.873969001251 168.611652451329 163.516075721791 160.769011617127
158.639313207233 155.6299578128 142.34471764492 155.086559474139
157.748641658052
HPD 180.501409705275 179.674744176904 204.637887777803 193.609985124825
154.657316209848 145.96012463103 154.528396813382 166.925695810781
155.55288776049 153.219954133957 149.696766215866 162.358237198517
HPDL 122.804934452095 114.805810485989 120.964862799634 119.795945889846
157.176001400747 132.398041172151 120.606354515072 119.612288460361
120.714981493593 119.746227888415 138.859261763075 120.177385114315
HPGD 284.473085899219 455.124190541055 450.220658078322 283.27806319488
1108.61301774472 4404.79209330918 2149.13404212591 6441.13602696535
1906.81344236287 189.647009287524 3810.98043907046 2253.7522632414
HPGDS 145.079450753969 156.609370182062 129.511465164539 152.717642489582
135.841309719081 154.758831302515 138.464793389999 146.915247627846
141.97478198531 158.085935267091 135.237042337721 155.62770968777
HPN 125.326393776938 121.855752708908 117.207266754941 123.762963668211
123.347836436966 120.835207882788 122.174036435094 127.011594171986
125.170874841845 119.312201620247 128.806964077824 115.51493368527
HPR 142.342811880173 147.132625797946 149.865644080359 141.393814611166
136.249621724093 143.794453011397 133.324531689254 143.572668991313
135.666278507269 146.007311764212 152.134895623504 149.712320986646
HPRT1 355.519647649551 365.541609015117 352.897241809959 300.83667045718
1070.4597488302 831.08287594363 681.172359997071 935.423045210635
596.076764072945 1181.1611856364 487.597040819879 912.143424293236
HPS1 172.667770359278 157.547814187442 165.181215117323 162.047249964859
209.180298459605 178.3652866131 168.552631639996 198.522123898437
179.091917512822 202.230920499592 153.111226896983 191.692830869809
HPS3 155.974463324915 174.375388013694 221.070448906398 187.145134088286
211.135364208221 221.869191666931 249.858562350415 251.062649517773
220.379186481273 210.729945718068 201.676984786688 222.736706411035
HPS4 138.317443162096 135.589211439847 142.356747337127 138.325950428197
129.344834474578 140.943732564849 139.287871460674 140.613151527886
140.892250639524 149.91705328815 154.537240112989 138.129281196474

HPS5 200.127994198329 188.836327151669 191.38608906576 193.938032040991
      142.558109812638 185.997327421952 167.887047631073 203.206743799353
      208.385295246155 214.714726648332 184.187847236508 164.562302265155
HPS6 424.134483971176 518.925188630246 561.251860964966 497.269957926657
      1628.24528041404 664.19083888799 1411.90426595229 798.1490651671
      876.288248962843 666.344430142655 975.685771692095 1286.55103838368
HPSE 154.47187360425 142.853399942089 161.960392594363 152.57905274438
      166.904990069484 172.091884817207 154.539367933684 163.042083189818
      169.428195354763 210.605864685448 138.363553671822 152.79367157305
HPSE2 187.63609695026 169.895672129542 175.537039794925 196.470718183575
      184.923312649584 153.187148832063 160.589575894314 168.521601064374
      141.724442944322 159.58125728432 125.631073476381 166.944701842349
HPVC1 133.517685144186 132.002835547397 128.495276191943 136.919702354166
      129.130212375815 138.397152876585 134.288349336742 139.526241501184
      142.365009028839 143.776637809106 150.360754064321 139.646026806298
HPX   165.41729888767 142.19728670565 163.143613079037 177.72965427263
      139.825088162247 128.63301490285 141.882859092658 140.683411903185
      138.212860360467 140.876577029209 139.378915474473 139.72868421672
HPYR1 124.176944998906 131.879526177089 130.342425029134 132.223321299997
      137.7724100594   130.590195477461 129.935532080325 123.347836436966
      128.447393268611 125.202468372015 137.054278745724 125.926774314737
HR   136.614620534169 145.378779757104 134.524644163286 138.566954604414
      131.773304233929 128.071379991958 141.842919268688 138.695481214267
      150.314462939164 135.349868925415 146.669620721254 133.948997488604
HRAS 233.997642139328 275.929414239664 221.719848707318 282.392419584508
      420.017044147123 251.869781014029 296.657844888195 252.919704128539
      258.489608621438 495.655170027296 377.800067476868 254.722836822616
HRASLS   142.051259785804 150.417169156755 151.409290231274
      166.825501664231 311.966971734249 194.686527131656 152.99686989829
      145.085091507228 154.491373296392 184.742960975938 126.704076078563
      153.338193960419
HRASLS2   154.291717784335 147.870549240703 177.246890307643
      157.096799934494 167.07092218037 153.756213924949 168.720694813918
      164.256764783784 174.491551019128 160.435789207464 151.004738508108
      158.477017294725
HRASLS3   930.704688414069 985.637230639964 1023.94850651125
      1077.95929298567 355.443758121675 428.66614441422 1010.05884008623
      368.374002925137 340.098306132634 178.304914997023 221.930333726352
      267.901632267999
HRASLS5   139.948097704949 148.005500924157 144.035936160694
      126.37749112514 112.771141378159 148.970251898527 127.424848301217
      151.27882940428 139.72868421672 156.179501567984 119.370219299491
      139.941724115063
HRB   177.508603290389 191.616483214712 201.167206950576 178.984409575632
      187.205674045893 178.106013202731 201.954167006591 164.236417852165
      185.876046259727 220.633692394229 183.951404362826 204.770742504612
HRC   158.63432604978 220.362946606131 151.625248091441 207.624240598841
      162.900556089071 158.179586948369 134.11753210296 139.885054845153
      145.048710455772 148.361088792223 153.187148832063 147.384050169545
HRCT1 154.187420367647 139.631165400881 127.3079781139   144.393688888553
      129.161305053472 111.276543583667 126.800718471804 117.834484996559
      131.148926523383 114.115609163126 140.611071681667 111.922007318017

HRES1 159.615233521257 157.344758285153 159.758196845781 145.932170561687
154.420795931034 161.683243808528 153.551359970644 145.017264740722
144.606963457243 163.709174813901 161.988082494464 152.024383072265
HRG 125.693321471759 143.912136782374 131.552276774901 127.593764897583
128.300838927918 123.07559417607 131.652854369619 141.40690581885
128.113966507586 146.264686158155 129.808590871788 136.351332391315
HRH1 198.411812737082 180.240305360865 166.819234137224 165.192945734166
174.909258852845 156.763364462534 154.187420367647 182.588697551677
163.407360509803 201.221839281282 185.447093292348 187.082082429597
HRH2 112.054379249781 109.30275288389 114.556542069712 106.238539254093
107.466184687811 106.522641798274 111.055738573603 103.168665937677
109.74983281634 107.949834974369 109.574386044714 112.10312035128
HRH3 118.08607083874 102.474380813907 110.977904932371 115.277302351501
115.120702589487 106.030441145334 106.629065516249 108.288420807873
109.740801301993 113.311132067152 122.522978939399 121.110498703017
HRH4 124.705614182596 124.749727939593 123.336074100984 129.527271401014
128.020911959353 121.194138394375 134.668233738635 120.481712066062
131.269526665478 124.63978985808 136.649743237542 129.920586100555
HRIHFB2122 172.276968413879 169.107948050094 158.150949554785
153.992763544073 193.203053225456 185.961822133346 207.514489132778
185.801540278924 163.495175710216 176.317805247749 168.365262340217
203.831047867731
HRK 131.415807324048 128.06233429201 122.031894952046 148.370331097467
133.59686364883 130.112882549637 133.604705630582 129.745892720738
133.645044060866 160.989242461839 121.95061962467 123.198873421356
HRNBP3 131.069515666204 141.830432745537 141.278654291193
151.792921211607 132.466796864599 132.8310947234 138.436029489092
129.854478528455 121.462385497795 124.501262458393 129.945543636232
127.442587366678
HRNR 108.9291713877 114.711310011993 108.021898335735 120.994068563884
113.177330690612 108.715328458556 116.491342107103 111.196610704726
116.20967792121 125.749024723383 138.199559519849 114.644722005728
HRSP12 182.57496767012 184.008405576875 224.224264678645
188.654966029389 252.404931238338 248.791254839869 246.355720496468
282.719121652073 290.502085667398 243.159062832085 229.660838239474
168.358323243918
HS1BP3 618.437282203498 732.877169346979 653.879734066271
1008.43888926584 610.337659723539 241.039766283972 278.942695435238
368.637819994033 221.127492253636 364.895113721763 399.071041584646
171.384487293154
HS2ST1 219.422260093722 241.987100712218 237.754067231793
217.531179216305 240.341031437493 386.16563750478 322.956882599715
296.603054615145 245.399847987183 180.719868519867 333.270203737291
243.468673768141
HS3ST1 178.404791189835 190.077302802065 152.66513184083
160.6823824476 226.565823278767 264.456926651554 386.379891865411
265.068532248682 362.581768268116 260.85776723575 549.96567374713
512.312880441098
HS3ST2 119.060186838239 121.807661901776 114.209249333221
121.945541820091 122.268560536152 118.393203921859 121.679013405559
116.615250922316 134.614988219821 111.328185454541 128.163613794257
118.442856984861
HS3ST3A1 192.032080013216 201.569913451219 177.450674276588
175.717830026564 148.457872807838 149.589901372799 190.018871923219

150.79785758452 173.176900454904 360.812953749077 152.262147284452
166.754995454382
HS3ST3B1 122.339819585312 140.206268652318 143.967197319361
137.102353661381 135.461112284848 149.068713925139 140.802033706122
128.649329515033 144.0534341937 139.736465430177 122.431337063053
144.528360135884
HS3ST4 124.477586539576 129.666957539624 124.955676894776
123.159407368236 134.147586627141 117.639216182057 149.651553512698
116.433239116749 126.899985772157 125.269726165403 147.450681897766
130.465221782084
HS3ST5 129.906744036052 124.602474178438 126.87882572904
121.103620738719 145.351662473858 136.62213567467 137.222125237171
129.334217858282 118.351607868932 116.288113598488 234.5655184349
138.349773531218
HS3ST6 150.180037731272 152.952479729682 153.231965895778
136.130027137459 140.831508792119 143.554726863556 150.412929574507
162.695319777455 153.500478301079 152.532083511961 152.107025120177
141.552315273863
HS6ST1 153.579955370444 145.956477465634 177.123497519231
180.569583206488 159.213940274232 179.841665686703 171.809717138613
182.725987056391 189.770558810153 161.115842628581 169.771005675296
212.223906015932
HS6ST2 122.900675062181 131.430921437192 123.877609932461
119.049388179929 366.073387075385 462.686385881492 246.63579788514
223.722742062251 151.128838696891 154.362571105094 214.803167803712
310.690317681243
HS6ST3 108.830525786872 115.337592860132 114.021546689428
113.356070669308 103.99234480919 111.584425166104 110.138220982557
119.67745324922 106.450154495663 109.69466049307 131.879526177089
117.623103460369
HSBP1 1398.60850048351 1427.70292953827 1293.38808483663 1549.30900090605
995.812184456562 3106.94181270197 1920.16520804856 1923.19560386389
1646.76016031831 1201.82026045778 1974.49672149862 2007.0121037284
HSCB 720.907992182476 687.789976218334 619.75986112089 683.470866056244
270.679613997675 636.048536814356 421.255522475862 471.598175637494
476.917496749381 400.924914238052 529.700356666006 458.151323130855
HSD11B1 228.649073045499 180.096239155058 164.962201718762
160.496585125233 125.843089077365 133.161552914595 123.601413134287
142.722227179256 160.409454280874 126.658670189701 136.390047634643
123.065355919378
HSD11B1L 203.888365494623 213.891435492914 196.536086443884
228.713818522476 227.614623963331 180.968659614238 186.708059183893
171.320653882424 170.4583554058 189.482046688226 160.989242461839
194.143649677822
HSD11B2 151.397068235685 140.485102148597 126.294085960617
148.320310040998 290.037325939883 139.530196462344 139.631165400881
141.331397551086 126.528589411462 152.049243916141 182.300628120658
129.507297276862
HSD17B1 137.899291413431 129.055874691136 116.736174147084
129.45936538019 314.557323400201 309.720081933252 132.521681289019
159.961729971624 237.312766830206 503.59466398882 153.114065147266
145.329128512407
HSD17B10 240.341031437493 247.550312458738 201.676984786688
245.153736298067 529.368609163949 533.784151495549 296.962992349662

518.760142491104 313.888035511709 491.35949123223 227.499335248402
396.459213999636
HSD17B11 302.809011218879 270.293807247372 602.703887178113
221.74200490054 218.559622620349 1302.54547114701 622.348802511009
1576.21727854603 590.142817130684 350.077206368839 1154.1046856215
613.846166856328
HSD17B12 1561.10217536347 1485.776977235 1117.77397620221
1064.12835214671 1341.78081782818 971.99484515987 2309.63504858248
1259.38111177521 1004.97723186946 2233.00694575483 1214.16777512885
923.130629121442
HSD17B13 139.693261291326 143.204058393718 157.301617257941
140.125575976708 140.556631658219 140.806935772116 142.066903301882
134.487476333464 142.406904585501 134.339535632463 132.591496435822
134.608734423113
HSD17B14 214.019190624184 211.104243881644 213.324226314713
157.83460319212 1171.62361359992 321.750495072759 417.615521090665
224.251136332123 233.029655807246 130.309092946921 223.453776362112
147.056649843798
HSD17B2 491.034395489684 511.25179427158 195.84531580224
131.507618414676 446.393631025049 2007.0121037284 1466.08446530283
1395.63393859192 1396.4168898623 306.011283364888 3237.94149351627
3029.07443443457
HSD17B3 140.101511236793 144.627784383455 171.155660467037
129.522339467532 144.243598691782 139.715836473047 143.611930851914
164.253219197128 132.551989498706 137.871870950302 122.841135364088
160.592404712197
HSD17B4 964.093668483033 910.087388930485 1139.14730341921
780.734359041347 968.157701932137 1748.77270039774 1462.31151845458
1111.88018487186 1152.50523614436 1468.50184641042 1825.10168716051
1641.70484603137
HSD17B6 945.403379196643 707.404709303506 2056.90127387648
544.462032960964 161.906823008445 188.079188251085 195.50039162477
176.355556618289 166.825501664231 147.103340218488 137.265533487496
141.38749436393
HSD17B7 9715.20127757161 9820.93899217345 8322.20177173035
9349.76443528011 1559.26487922551 8840.92490099301 4183.06379632713
7342.79622458743 6312.36452724398 3615.76740804718 6427.33783376005
4875.22637286719
HSD17B7P2 156.324643994552 138.858138379065 160.469645948225
161.667782505656 155.303903514061 164.084737224884 155.561821955048
182.950456188966 164.529828080312 161.408318705503 165.232545721733
153.028658323116
HSD17B8 284.675990197899 247.053215951505 409.206158277764
300.985037429772 478.293220151258 284.321253087512 499.436680354749
569.967953318205 253.697419495959 1011.14472068314 443.125195744202
459.973764138144
HSD3B1 157.848456517169 137.243175880462 142.052454741558
136.925645302362 243.080229016932 139.595986615703 209.596792486876
171.376976179465 148.057700860759 135.791961106915 151.973681370675
145.801228425692
HSD3B2 125.443242860371 114.826501559828 126.569482909992
116.507315786826 126.262349005661 131.326536951329 108.807891863679
122.320966091375 121.010811158088 118.047978285706 133.396403868645
122.115925891088

HSD3B7 261.469732730752 257.941367816557 172.486839924297
313.025250337353 249.09278446472 221.685824197352 161.112906953532
161.408318705503 168.161117444474 281.168898910065 183.998458233995
139.113675690483
HSDL1 264.905947922753 231.439252571168 245.454945056317 233.786136606682
157.649902282769 184.694741621812 184.923312649584 188.567747377006
188.959655155629 154.491373296392 220.169099923628 185.485858719975
HSDL2 581.37957221282 643.468308027006 683.861021234927 502.935749606918
313.322307299463 506.430593289427 299.986758612161 880.719593734606
844.187489702587 314.126706052299 980.67040349547 238.375823012603
HSF1 136.891612156271 123.706910388724 119.854401979951 143.167719135403
153.415901281564 127.802645313091 134.682319733908 139.799467834109
146.476960694197 131.310537960214 183.815810733176 137.339511714364
HSF2 264.273245283343 262.60377731258 347.147961254829 273.152907354565
203.943245360496 289.434186747703 244.514230664694 268.568824794145
206.999289979742 213.833894542292 194.88319590729 210.266075382636
HSF2BP 109.924867620862 123.595133376092 121.48867035121
105.050093408041 150.384559054133 124.74291205933 143.901267937876
122.90702157852 132.975603230163 124.025104622631 131.473001455317
120.998665308561
HSF4 188.308588359317 167.73288535728 230.589110372454 178.572212478544
145.67389337654 143.62103263895 133.369314179341 159.832405510505
158.943005736645 149.41357273704 154.402315635227 136.317978146216
HSF5 135.536810684197 136.16560274503 131.356437247149 141.166605868578
141.255406288178 124.561511833959 131.290698895743 140.290598244169
135.86149691566 137.201149245092 121.628827418997 142.169951083733
HSFX1 133.216489794592 128.425838805072 140.279222720709 141.685620602226
128.377928524315 137.4552565025 138.889999702313 134.66169946771
132.428675012593 136.452338158252 136.903588075134 135.129656281091
HSFY1 161.916155040522 142.858554460651 139.720343780228 150.617039206311
149.756512110538 144.5368823731 143.365976841902 154.886628987246
144.313841064968 142.995274641765 139.54021659275 134.626371781978
HSFY2 139.834535091869 132.216453078716 140.413503801467 140.286870147975
144.868337748544 143.812863334832 142.493119780028 159.977528879208
160.898061905021 131.820842728171 127.323157914914 143.281050499332
HSFYP1 153.815239954928 153.311705636864 152.465176508162
154.7326161316 162.940190224943 156.808992920564 173.656241217331
153.474566767304 145.658251059524 154.637740117457 153.913513669361
169.022351348819
HSGT1 176.927500850631 181.048789688798 227.236255752375 184.989027770708
149.881708604156 190.56172211779 189.509062639279 171.967974673053
203.586310608403 183.963578322984 215.532132200821 243.191238594142
HSH2D 113.963798749897 115.257318321108 113.427848757112 106.797707723826
139.987681290389 129.767878283811 233.496804016764 221.050093964575
189.348690353874 109.425878885118 271.064405562085 181.193963616037
HSN2 181.813812621767 166.024236370752 188.599353117244 163.331424612891
142.858554460651 201.004491067972 165.565284723155 145.185902174023
155.280897870265 204.564770370164 136.001505582342 194.86492119218
HSP90AA1 3896.49785254733 4134.02613649483 4110.76546218806
3648.38765465889 4923.36698713285 4945.87677849967 5149.78271096158
9378.87287299048 6628.17856159198 5688.84918819457 4150.8511719558
6281.01687932005
HSP90AB1 3122.71790981947 3188.90202907403 3648.38765465889
2956.63335018838 6156.29203791697 3417.22292085668 3655.63669142687

3387.5366256957 3501.86788138647 2532.82545752082 4965.25728333142
4043.28299602858
HSP90AB2P 149.797850183848 141.874188629438 138.968690339299
160.096507818256 145.56468363155 133.372231702721 133.954514563283
138.82269002406 147.059096208832 130.898067924686 157.878110410517
142.379120254855
HSP90AB4P 129.007924852801 142.68640844948 132.664379033058
124.433114779288 133.903934769355 159.629232808787 147.545667896282
144.185406500473 145.968884703737 124.031916322066 165.131463797217
156.779004465552
HSP90AB6P 133.53523683945 141.8268805463 140.085359251038
126.566165822427 135.997144927956 158.477017294725 153.46645509759
134.401733295755 124.582577275358 126.658670189701 101.530900217921
138.182987173284
HSP90B1 3298.73393277409 2792.13364570755 3184.98944679038
2775.84742592713 4299.06288109563 4141.8682669995 6114.33638002734
7122.07276863122 7003.06505884735 3890.14358014877 4373.91897463938
6006.01682776552
HSP90B3P 122.142323014287 117.203783530266 108.807891863679
103.09089100768 112.776764215618 106.603674195422 120.887938517877
114.255733119076 116.773599713111 111.922007318017 117.662611650515
110.670057121283
HSPA12A 425.59810308048 371.280926727395 270.021093158885
398.971838949075 137.826458479398 138.202872275661 142.299535614113
138.442378791712 145.636906369786 138.000714797563 126.936899820148
131.956481591804
HSPA12B 314.90123632547 377.800067476868 205.630948393225
491.290438575428 135.57577294564 150.324132011201 122.327353563917
137.908991199407 150.073816271065 118.393203921859 145.172776053
123.577138762427
HSPA13 161.359609192977 150.874014332213 159.455596750304
143.313605534609 133.108094159933 164.158913914661 155.98413216883
145.250076958551 144.858656365799 205.011514211101 150.180037731272
150.134337895481
HSPA14 241.648044508699 249.554443204445 266.61343130101
224.497348027469 813.772804994191 1487.12201643008 380.821355948507
339.172031360524 318.487401106209 276.961847241646 329.146025181674
451.210172051464
HSPA1A 3522.61692270848 3294.92439159154 5702.11995823367
3152.64817044581 1516.97921112926 1602.63977096867 224.089220507176
2539.87833317526 721.31849915135 3545.8833678963 748.940715378564
1171.62361359992
HSPA1B 3235.38149926838 3688.68740405322 6409.23524786095
3073.40543048878 5558.51089816921 2213.1043022626 4291.1724030909
1767.70023790753 4103.06565237121 2365.99940149374 4335.76831372031
1737.57015396357
HSPA1L 186.708059183893 184.611146047556 201.458970969685
197.526492324088 156.173099025259 132.508133846896 165.314290446922
146.129805607031 164.413893089607 129.845344493418 198.191669030952
134.76397845723
HSPA2 1174.69701182094 638.129080224049 748.161769171818 500.848273432862
248.129640673859 159.286484253487 150.213657393257 424.751154004399
257.622317771472 223.975677959345 316.955298948771 221.127492253636

HSPA4 416.475440894731 393.115606440229 417.731815310276 416.075109522039
479.638653311025 542.009873308344 724.607317443904 771.294254795752
465.809961695043 773.057095787974 617.185364180303 762.8576747113
HSPA4L 131.241878941749 137.4552565025 161.764411756848
118.375739674144 134.109051944736 140.174087049513 127.494672751434
145.690634269858 149.734395134321 223.359785518285 149.164469334038
140.295307451188
HSPA5 517.23393716516 454.492780042353 271.356289141017 457.130051460676
348.848550937417 484.363052437459 333.91440669951 499.048754087189
625.932910814208 255.945470052489 529.228238133018 600.162650426961
HSPA6 195.384273821324 185.088667707155 220.60173086462 200.98533822317
256.924134409998 217.778414825515 199.681103910705 187.264411519101
303.795701960506 283.499120551905 160.293522619371 192.69687837782
HSPA7 182.32514927211 157.717145014769 167.408314067262 189.136114953249
206.257167235126 208.214764518526 172.973175988757 144.777043589925
216.353502371227 225.75155298452 179.122374556365 175.752091217351
HSPA8 3430.76532174701 3130.30693451802 3807.16206340771 3424.65126200697
7332.15849011291 7292.0342195035 6105.64727031051 8958.34797715349
7992.84111657183 5467.84611694564 8368.88320476021 6919.4226838505
HSPA9 1561.87054049124 1594.05763963363 1657.72357544205 1400.81554054664
1213.06007305892 1610.38263368771 2001.92441724356 2253.7522632414
1415.67109281133 2350.01764791897 1696.69982157582 2328.65814406636
HSPB1 22035.2646000952 23656.0669757593 20887.0399135573 28095.1627766565
21110.9013462062 15745.699731183 18953.8127741918 22182.4877079778
19087.9532735841 25002.6870134094 13944.7649147227 18287.6015702456
HSPB2 314.4408959517 341.096072280715 252.789376168324 312.808044035117
117.480506535399 133.594446233378 131.739418639854 136.511669406292
141.547112973262 133.264245969185 129.647514123731 120.201521341263
HSPB3 447.909277144896 288.261250589513 282.797367565702 256.471672362567
159.735182155088 148.680794815584 150.048431572712 164.478006648035
163.85327708023 157.390513637267 163.794436953711 172.259627777465
HSPB6 4068.18567686753 4819.85672511343 3566.89107958971 9323.53099359448
139.348494759948 155.323822771755 127.6078497227 133.749271961438
225.387674139093 123.561016079801 147.230113868203 121.053766591551
HSPB7 1859.88000417483 2282.94927629821 860.688314783077 4562.75659565027
119.899649497032 137.360220389719 118.239637776941 130.812309202831
148.370331097467 119.263824982583 134.972933635542 126.793778645808
HSPB8 5396.55955971617 4641.8793590085 5149.78271096158 7371.9761952537
643.905401805862 336.212224495877 696.976036382401 149.131218007756
487.901310664037 163.262893278578 300.358738353443 123.431969470387
HSPB9 116.621735695217 120.876732151192 123.964042357475 124.915772594451
130.905135502296 127.758381206968 129.003317344469 141.587109176431
128.530540860266 124.83124879272 114.785989868306 136.524640445539
HSPBAP1 223.747350951099 224.981439707657 229.74418058711
260.591336959281 212.497773003032 209.304654633004 225.505978789388
242.348427307994 249.953902454701 209.93789387724 238.149614104568
176.355556618289
HSPBL2 1472.54829263217 1785.92695250894 1364.83036157729
2392.93562797606 1685.33737186733 1000.05896885496 1793.75125318113
1513.28088592885 1014.63777151551 3560.23794634993 1867.55140010155
1556.41573079892
HSPBP1 147.79341726211 162.494429648551 152.030182228463
163.647306551474 316.895545640437 202.178451227304 174.168079247768

175.065781438801 204.942500423413 192.286192329464 163.37518739397
205.509515144263
HSPC047 201.186963432715 228.713818522476 228.524222740306
202.214495168688 156.248684477916 281.071129128589 184.694741621812
149.007288765628 176.761123480566 179.109090120497 163.438043894189
216.277178481058
HSPC072 108.631224817901 118.81294782091 119.75069850668
123.9209907098 128.382487609446 108.34997967774 110.050374895171
114.607138204495 118.930792960643 119.415822905251 126.235567899449
114.249891266241
HSPC111 191.38608906576 205.65398844108 218.811490883852
233.029655807246 259.672238210968 355.609033348692 370.066087375401
340.966515437272 274.891033696257 391.911768848267 245.997705723007
418.420753332232
HSPC157 256.394563537658 215.167086358143 303.762967437478
204.183472655276 266.61343130101 224.135228192655 225.850439131864
259.184713471619 192.580288063603 159.413535577361 173.816408146725
226.221973502783
HSPC159 165.985885671398 154.600966693059 162.274094333189
167.70368494182 167.769114519774 218.18756955522 210.803130108618
185.843639043466 157.623092996114 2253.7522632414 172.774914399844
186.653791045656
HSPC171 343.417041721907 361.969355316032 311.208703262497
330.553659265085 699.800360892516 417.024088698182 593.990687449346
504.9864660689 871.528156184927 756.618969995355 827.400420888645
638.393099392849
HSPC268 5073.27433952351 5149.78271096158 4836.4008757421
5022.15374558514 522.664475688419 3921.67860557329 1789.51979173209
2979.27692778783 2195.41077320434 1776.38646235135 2311.05587511258
1999.10320742823
HSPCAL3 164.865846124876 162.397078614596 163.51024840263
163.37518739397 143.754105312957 148.707332454527 165.480489678822
170.350891928203 154.288075172115 158.034418043685 173.281506912652
160.281830914755
HSPD1 1300.40889834984 1368.91287156398 1396.4168898623 1264.90442763788
1608.40826380713 2518.88171283899 1969.67552562117 2988.45928881468
1861.19763103381 1514.53784631689 1440.14449872047 2120.14463347544
HSPE1 842.87865208079 780.105135529728 807.92851898782 540.005877322817
1793.75125318113 2728.04758874748 1881.90353127141 3441.9805896226
1845.87311313269 1561.87054049124 3436.32495553246 2720.83642320419
HSPG2 134.805239981342 154.509924503184 131.265594391542 197.605208310432
162.547569833931 122.629146433216 126.806537241253 129.061316880624
128.202619705343 138.700301754711 147.49720952988 124.548598801497
HSPH1 1142.44180956604 899.667912542118 1370.61156801632 708.534025308731
1431.52979384867 2642.24374855334 1619.67820476414 2596.43650409044
2208.49325118803 2580.98143004019 1769.6386910047 3184.98944679038
HSZFP36 275.609716327452 299.935166870211 261.469732730752
280.246711704036 646.461274173693 697.97116981445 842.22767901391
880.140852200235 505.83860711693 228.058870735016 832.911633923496
453.17378995737
HTA 143.30977386038 134.97514790642 145.326803807448 139.809114250476
136.118702626862 131.907246423067 145.005991300977 146.979988781462
139.652974659584 130.457981404612 132.615324904193 129.404017329613

HTATIP2 410.337491313251 426.995700632434 527.930077857945
424.285848583977 434.716530273383 478.909308632726 568.310800941091
590.142817130684 731.284098301795 892.868661511516 662.04757187541
635.038752622494
HTATSF1 135.86924353735 125.945916345628 132.100026388604
139.013404849037 136.654902685184 123.072413274454 139.125340284813
147.77916610545 128.878051599968 137.747088378131 142.984243805285
143.62103263895
HTN1 110.486191378104 100.841352336505 109.46255295836 118.215641556163
125.379842077431 103.520748827831 104.977794653504 103.734303632543
108.101686347192 110.437728036347 117.199217938531 109.30275288389
HTN3 146.08320115156 148.610537244399 137.290687951485 137.982667022604
133.478829770103 149.657951204528 136.209796259197 140.427188241455
146.64835267642 142.858554460651 144.944095553004 133.836390935612
HTR1A 109.37466528113 119.663182347566 113.025793645043 115.566303449363
122.514826802737 117.428152859221 110.510191137073 114.520066232155
117.238968848218 101.981823814699 124.119104345119 114.223991452083
HTR1B 130.779805222363 133.550008967009 170.108500366942 131.670601860412
124.317870455205 137.012646671173 140.038295509875 150.613624696604
142.829099538363 127.966883271903 124.961859983596 138.28745132277
HTR1D 120.493082096235 115.914842445934 123.031945101045 108.34997967774
118.470400686096 119.551741097918 109.255470590389 122.426568507144
119.387459594521 112.282839763467 146.00443946108 124.585675323305
HTR1E 169.408892048115 155.253032078086 141.416706868124 154.533544838819
208.348515119045 173.592875508663 185.876046259727 186.321879879561
164.160608039304 152.898148619937 182.665357598121 173.010799217261
HTR1F 128.354163132392 138.411148122407 122.543903508753 129.150574563997
154.273953018148 128.224742304889 125.195549796301 131.261903118177
146.252974508529 138.17454314053 122.512587751135 135.377118045038
HTR2A 168.52952604693 158.95714101556 139.858812885649 148.493435517221
128.643539098937 152.538048490265 130.074786642758 133.374199939042
146.557058433232 143.47200560038 141.033948839613 148.334425146129
HTR2B 234.339913501761 190.291185322482 190.682392100564 166.640325848102
149.563405421366 147.314687795555 148.82034800623 150.936747236094
156.407166112409 141.513636348978 166.341564850299 145.770601609392
HTR2C 134.909230280708 121.865696440776 119.859508630454 125.293640452686
135.725202462502 125.512431901925 133.008987820479 133.608461289137
120.581554457723 133.713275043978 130.351302840602 125.181327386534
HTR3A 141.890803125137 148.922537778634 142.729945734027 133.352279794853
139.736465430177 135.638156623519 138.566954604414 133.812639120892
139.457084084368 132.342063356763 141.008604646743 133.185908839766
HTR3B 138.592943322694 147.835936450126 138.231715814115 146.010474018786
141.565599104245 152.635792342068 136.194082458655 122.038534506506
149.995272585012 145.912712700067 138.659387475119 160.79631681714
HTR3C 152.295451313545 137.102353661381 178.304914997023 147.835936450126
160.914009756867 155.233830396052 153.943227610765 153.93927752488
158.109836032481 143.874735808114 153.169450192935 143.52474218953
HTR3D 142.510745106328 122.924017262559 143.836637098747 135.154441560264
132.484079454476 143.796699867588 128.184708916755 122.924017262559
129.808590871788 138.831297375659 130.965418847276 137.176144667253
HTR3E 107.828378413233 111.506345174435 115.29099102455 117.316790136526
116.936964318685 119.578240680666 118.432307370605 113.58962177275
111.506345174435 124.723519607109 119.866898356501 114.191245210321

HTR4	126.825785762925	125.801254883675	126.673587174832	130.293492553638
	121.429270259367	128.473834314632	125.73560975196	122.917915232153
	126.938068475032	128.606360476298	139.897320032723	123.129785564258
HTR5A	125.526744092256	149.740404686695	147.642693777383	150.471558219667
	133.592691897675	129.236471271181	127.622397233692	142.656762407774
	142.388076341254	140.702685210277	133.775716376056	137.876234091056
HTR6	115.692324946633	127.372242892513	116.793408204625	111.381427322188
	107.816312357067	125.826111950648	115.435942397626	119.937665469629
	118.154953746492	138.196640512429	133.329392986316	127.358616953147
HTR7	127.447218258785	127.218362452043	112.10312035128	118.047978285706
	167.662761175661	127.551486042435	138.903450375532	133.792673129743
	116.718099363329	130.944610173285	179.009510246709	147.858339902055
HTR7P	144.774621469452	152.024383072265	156.073428882485	157.891350650633
	146.851307767958	158.041568121766	145.452220050902	138.192550945411
	140.466612569756	150.301540078433	151.273261581765	136.90636489391
HTRA1	3338.13980198211	3387.5366256957	2261.31473132315	2715.55326746591
	344.128576341892	432.604462607037	278.168897504985	464.577839898252
	547.516635695872	1525.24196488688	295.278972023002	453.760162338163
HTRA2	229.181725450534	212.016247864691	235.704824490397	236.082185670774
	275.996693627151	224.466829629468	256.729891423908	229.511643376643
	197.815264620906	226.960024551778	209.60319248212	216.033564783918
HTRA3	183.905926269285	162.002221893617	147.272072245929	178.347708009405
	122.583461806645	118.684077097437	118.56332995149	118.636694749194
	117.760045671142	132.008330288281	130.850419527271	122.708102546276
HTRA4	159.43317827517	153.996429870564	166.104915784875	153.943227610765
	159.646538408414	165.480489678822	144.767233774942	161.061119536632
	154.64077390011	149.18603646016	165.885072907195	165.370800097602
HTT	213.710058427654	252.222481637238	215.967319486874	349.485727214325
	195.057830521304	195.384273821324	195.316077537716	162.013164369593
	207.150354734189	180.761100930677	202.278323489515	171.494975393562
HULC	149.742140955184	122.153996665065	125.065813262416	134.69274746407
	146.013430937168	137.552979154943	137.519771130707	137.306031826007
	152.796423361971	133.074027423549	125.025064237439	132.595360509902
HUNK	148.204194003593	145.180346905484	140.385509382606	151.770791549511
	129.618097261902	135.254996386778	138.081268025435	126.058167594519
	138.206065640158	154.066939372379	141.996233801459	138.994857740356
HUS1	136.589250394236	128.675527226161	133.208065430037	138.384797779855
	140.292132023262	129.896711442418	136.886677060418	131.523538461751
	141.38612990166	133.511698282195	151.87495966414	128.955241553429
HUS1B	197.003657786247	175.010849510711	193.813717161291	176.75095377028
	181.500222423634	205.705962405954	205.322733011516	179.35113214277
	176.486551181451	202.343343669937	177.598422362298	182.42088387266
HUWE1	371.457568934695	376.189276074918	393.013539328766	493.071213662125
	321.84712975195	293.646275396425	263.606930507829	312.961981998512
	291.616195651001	275.072079692264	502.935749606918	236.191384383802
HVCN1	157.113536717393	155.890403934674	137.232722233042	151.945412775776
	147.978039160763	147.129702047639	147.79341726211	138.848119273081
	169.649251828199	145.440471538068	151.60113116166	145.754504160436
HYAL1	178.166289723365	193.358728044565	157.553632802599	219.171591031864
	131.868557613642	141.750316403249	132.357517495246	145.256660345252
	160.459100056689	136.712083407769	149.717739166461	139.348494759948
HYAL2	175.593421867771	174.761105961773	170.423881865022	165.797170648964
	471.15333189869	223.373877165342	202.99342484328	197.762169504041
	201.49630027392	184.071078414327	173.714940254592	200.127994198329

HYAL3 162.077455145261 157.952018720438 113.487357534546 150.168496148941
432.726040933545 188.291563792748 149.108582827136 165.602175302848
161.212598761378 137.459095066272 151.479748626813 126.997614441981
HYAL4 177.011776399483 156.007630568494 184.542232315662 171.298701141684
135.226240774124 168.156002330441 150.079372712689 143.912136782374
143.04129238495 145.810213943313 162.397078614596 161.614426723521
HYALP1 153.964765831852 170.622615783608 151.27882940428
133.724135305758 180.569583206488 167.121623035375 205.476230849391
162.122250419992 152.26903721278 177.716742595321 190.66473033826
163.306853098724
HYDIN 145.812607895325 142.232918684259 151.911494020123 145.748174062159
145.076068630259 147.272072245929 143.669490888725 142.30928744202
146.981521393075 143.188958538221 141.334394187941 142.986412837634
HYI 198.701991021316 209.146021798649 205.781486117242 212.473610495213
208.895657507883 139.723117427543 170.516743115762 146.035731780312
154.518641757391 122.220327032799 178.819486867379 164.822461375001
HYLS1 192.59376129133 189.324370290702 219.619022209985 185.063870458561
335.790909130243 248.465457421469 200.810204162831 301.546624239855
227.288015854809 161.700258550244 224.089220507176 216.381643581705
HYMAI 173.816408146725 167.958792571511 158.157097243342 166.173429365582
146.345451252228 146.198331189991 133.103403333542 150.109192774079
140.274655651173 168.37043566813 129.421772713718 135.388750095746
HYOU1 231.462907786174 269.673122990604 194.500222559978 276.925017614147
656.32782132983 218.644953001365 229.181725450534 253.218123990428
259.252807304272 195.963361515546 328.919401925375 268.600782558985
HYPB 139.367493349339 144.597060118959 139.466683069609 128.517315006019
144.41489969974 147.75188889715 148.89180107448 142.772680460689
153.616692588396 137.929082565223 132.141918502025 155.30926640293
IAH1 700.353187874339 597.189355986293 852.436775264873 590.515758538373
1086.8615735568 864.161191021297 734.97763567765 704.448123154868
574.399909181723 863.738809424958 443.444024128516 776.411045078049
IAPP 117.950058826379 153.966815534288 149.675363177375 140.311843829601
130.535859816562 163.422877959747 145.880661952783 137.093170378295
137.282038401275 136.409960447884 130.411849432351 151.219051767056
IARS 228.713818522476 234.28146549561 298.664864578349 236.032288046282
293.234972169244 309.658122422202 279.921244070981 262.954856857756
330.405026390912 444.192734921566 362.195903246495 248.204358220728
IARS2 924.040923247227 968.548243081111 1050.40488466179 784.412433571927
960.491731797061 1660.70040058102 1371.56635975955 883.140984728615
1247.91041567746 1364.1224589383 1231.37475540937 1895.72330348767
IBSP 158.364031234356 143.008280236963 160.618586213311 159.570288565855
140.909875639658 164.808139478109 146.421195733989 147.553953240464
136.393655406964 149.019120812629 153.126111237668 163.885645883066
IBTK 808.55123510297 738.579262579782 613.063954859056 786.160002434066
686.923164688096 462.437471715008 774.997895985917 571.439520427295
500.643499051523 573.244735292944 796.439819579938 686.198351821711
ICA1 895.821578104681 806.88003660673 941.572186137467 717.025533065694
412.941907828617 736.140249105836 598.647226284738 708.534025308731
542.883453307 403.907009699589 842.567990276734 777.410297605252
ICA1L 149.508432746895 151.113342302947 171.212749388553 146.49022197174
136.211694310501 144.227440632196 131.298129667973 145.401101974736
141.70123350351 128.186963138555 144.678895821726 133.211362932569

ICAM1 141.21138348075 150.203281104753 123.857527555048 121.803676441059
144.954595802474 140.427188241455 128.680762033378 135.501192996538
172.652645920958 168.854819201403 131.724061631389 122.010855318111
ICAM2 362.742120043509 462.948429969719 236.141982516399 425.59810308048
202.568071395884 222.118666699301 221.768507175814 282.604890044452
413.983821839274 178.872635061826 327.207408852474 985.087150371964
ICAM3 288.823376348944 287.402339322061 271.945851739635 295.099840299681
436.222568287601 581.191693771646 393.198813167839 633.594811068524
618.437282203498 300.675907594637 429.812058546782 338.326304736048
ICAM4 135.513494853742 143.587562550004 141.891508908764 136.812841133892
138.82269002406 138.990971069382 138.050999864689 132.555211779043
144.425214066833 131.528729642144 147.640100078933 146.442734810495
ICAM5 160.17208299932 140.182150393054 149.829363505519 157.337193457189
148.052222389368 147.132625797946 146.225422797118 138.521952850632
147.93088503956 148.323729699672 136.040524807653 151.037282417145
ICF45 163.203582580217 172.008994980259 184.241640200823 166.868688936939
140.348718082786 143.744075931566 158.171302275984 148.538400348149
153.4392054301 162.289781945582 163.074919710259 151.727603285205
ICK 205.65398844108 219.080383154432 243.622678182687 213.584269089596
173.416999972133 188.378996128521 207.850956786183 180.658930225735
163.636550341231 172.531836863525 200.72147365413 176.214950733324
ICMT 330.758879244143 324.25775261173 299.754985162237 391.400480065
522.050619657197 362.95684683529 290.914381066573 267.901632267999
356.699466789374 304.111808763793 288.542264120468 308.564935340635
ICOS 171.880470629305 165.553581848949 162.919656039331 160.676606760965
171.40549153673 161.700258550244 215.335795337978 204.438365139666
256.648540685098 170.858049313581 219.709231012465 166.209190082084
ICOSLG 163.582678113215 160.192540519103 158.208998509937
149.897440750742 167.350290326189 166.533191390639 171.384487293154
182.152956182443 153.154592981962 146.091822904705 166.209190082084
165.169920299969
ICT1 373.452370692334 415.011847890313 483.845891531326 530.387315639095
467.347509383673 795.720196538039 595.409419899436 696.475639532595
778.28014337938 473.73182675469 530.969825236996 709.136137559152
ID1 554.115756730498 746.523071806534 764.493610798958 246.492735271771
5347.78127066964 4251.82554412383 6281.01687932005 2189.53914329263
3251.62784659994 1698.08853832248 3876.04966263281 3598.30438198858
ID2 831.08287594363 817.342172321486 708.534025308731 479.638653311025
2479.34567973462 2421.87041217217 2174.62979843528 696.131296166255
1159.44907165386 281.037762232619 1747.33558205825 1713.93035637146
ID2B 172.583615020042 173.735045816949 170.725486814469 180.889946069121
172.491604217899 166.830529779692 174.898012759712 170.335930486215
174.26780796802 157.909530751723 145.724294862079 184.102171963542
ID3 1080.02576143033 1399.41653736746 1038.34611017219 481.86545778383
6894.63087357693 2225.59207490574 7122.07276863122 822.129234065933
2285.86163259073 784.72162514331 4536.46344538492 1769.6386910047
ID4 342.245182599593 277.539110964815 213.791379190359 176.874265462986
187.893420010787 122.777544465711 211.059870865354 129.090955637629
147.393178410954 122.00079209554 347.292438587205 140.388212037661
IDE 152.209234189672 157.519633258844 192.69687837782 153.541193559658
214.295260811046 270.021093158885 203.913123559942 256.127197303751
229.805500520256 204.89842561202 202.114480291084 328.401537339876

IDH1	447.372026268156	613.846166856328	738.087203930249	436.586158384666
	1942.08859727404	11326.5935775813	3348.7622174821	6460.4291301245
	4773.62220024088	1057.1528284934	1659.80909859514	2197.44189747261
IDH2	737.193295872614	830.455793617181	685.620637807134	1524.46745239811
	1271.46608195833	611.540759532294	693.180345649331	591.437752991512
	835.834013018438	868.066644155313	473.057488824363	654.52788411248
IDH3A	312.177191402945	295.330307669482	411.039234404499	326.354469134694
	226.449705559354	350.58427958432	266.057472924341	243.038392457584
	262.139211406599	250.30085097088	217.380892198811	335.389274425025
IDH3B	863.086199215353	890.121325723286	1123.93087912174	895.114975754684
	1089.58643309156	1199.35864276583	681.429327723794	777.651238374269
	800.013123376748	1144.53002508973	683.019502027271	1076.09385014053
IDH3G	328.826483579278	371.127480221666	374.215345412947	403.104188866804
	575.546802138889	299.616447269111	310.174932137546	265.866465528654
	289.576706584644	269.927694984528	262.53374754047	338.075238647592
IDI1	283.416246133873	301.798676825176	249.680729921602	278.88183549759
	367.644907862372	406.458130553679	303.300028849873	380.821355948507
	361.390981655795	497.411169898778	370.798852361374	326.83982672544
IDI2	146.838547724148	155.111346336412	171.238423077347	149.668587078867
	180.952017616136	168.027892518772	162.127697884504	166.024236370752
	155.603263242813	168.124936529582	156.585665091824	175.805472275887
IDO1	151.858466721347	136.214432257698	132.069175779393	139.457084084368
	176.355556618289	173.132747018514	227.668046559074	255.71113398292
	299.935166870211	161.490941000872	173.675115244522	154.199534276226
IDO2	148.317958829244	151.002218995005	145.730927308023	156.492488946383
	149.141033677244	156.200313064999	148.065080664185	164.986006226427
	150.292858985795	147.138814313885	149.14863489698	148.67584969749
IDS	400.621427274425	300.358738353443	383.802494922918	336.845908630493
	394.892918166519	330.590986934704	349.773289724658	250.342080183479
	270.360397503276	252.697160031025	293.12968694683	242.948136060281
IDUA	257.775569674531	269.300548648755	199.490190715307	230.697077917675
	733.648333788712	201.530921073488	439.207122335648	313.581986704048
	256.764450748941	178.000672046849	320.305852961452	340.487850009685
IER2	231.796421262015	288.150694843614	224.396933003452	254.768537214795
	412.048097766066	426.649095732979	274.343238288436	256.795346052751
	216.154203111101	401.750302347978	292.746711619565	210.756414415156
IER3	565.609400092543	1316.31088187364	744.225336494008	553.198321853202
	1095.04571025381	1816.3719976564	671.134356672693	509.512410867348
	540.946852709312	4327.90939653054	155.62770968777	935.042067053226
IER3IP1	331.523536427527	270.462865174106	245.042781391047	
	208.14618424983	425.826693178023	473.151324002277	569.133295913677
	605.583144451322	659.483452217461	497.629319581786	396.708183244581
	513.816017871024			
IER5	217.264041781337	236.676029292922	177.759729226623	184.260835471542
	410.42088953434	445.763740779039	387.403502892897	275.696829424061
	356.154842959851	809.729160605727	569.866002248352	345.649572073924
IER5L	208.955110569558	246.355720496468	169.791616021987	210.5689532432
	994.213397985121	278.598913464804	205.428900832926	173.161152247499
	313.641656294638	265.670643951648	390.284689962661	166.014450044695
IFFO1	580.164288826741	507.827473844576	525.138476928759	634.046843336128
	180.690409026809	195.981555981238	170.145574727175	219.080383154432
	278.428310548805	233.272000923172	282.797367565702	183.988537525989

IFFO2 316.237519859859 283.760131847707 256.648540685098 358.732947927445
314.949772661046 554.282040414368 447.059120541009 228.926077117474
348.413827101046 506.290241222253 215.718714189719 226.449705559354
IFI16 388.966303073519 404.876478833713 338.585771590234 298.926727289387
153.468933239223 279.704940529618 515.432065049838 313.18989508207
408.60092780174 947.766127685083 386.461076717316 572.262848718437
IFI27 2159.2992548378 1391.89900758685 513.428413503031 1071.83063260499
8739.02966610274 726.556553101727 2350.01764791897 513.036729214095
1260.50387106816 6138.10904830456 449.220056843562 6460.4291301245
IFI27L1 307.866499851733 355.048838683854 334.772648743153
432.726040933545 227.499335248402 240.994507285383 192.475309217696
349.382889326613 276.79778275734 474.216057978384 209.44506543433
238.400565506288
IFI27L2 563.907454700224 622.142195892701 408.758021864761
672.637760135254 200.767743455226 291.537725613819 346.173614351499
260.356834114611 318.244342364572 779.340880980422 222.134519361117
800.013123376748
IFI30 153.857048476879 167.451782810394 140.597877909094 162.774716879847
184.775001243453 177.619101765848 197.378801636702 300.150235383065
409.206158277764 317.904396212359 233.029655807246 203.19299227665
IFI35 594.180861801958 466.083451075744 355.714202328378 475.927798702033
271.004741629789 248.16594076495 656.660582895703 639.184296110069
698.882871686866 362.795691170335 573.244735292944 350.926679856514
IFI44 850.562379837947 299.29004204677 233.884660128222 291.313681141442
186.377571176623 234.895245871534 811.362219801362 275.39986964837
445.905756082569 384.883635849576 278.335289966805 664.343553341563
IFI44L 1285.02292965374 259.025042304848 181.566951620202
242.070630946726 129.497266827284 216.755681508811 639.184296110069
264.805236346895 413.687046868111 141.972403120632 223.872330412144
174.680918805036
IFI6 3749.80462365748 1262.96801072965 726.731270955732 1119.71228054585
2285.86163259073 593.639955546556 4000.45196171119 977.227184625781
1447.07989164988 830.455793617181 1479.69309189156 690.582550537485
IFIH1 281.618175110153 195.577739376082 172.438141471784 182.997980582218
204.438365139666 155.166786601813 244.280971257965 230.161369375718
339.917319653382 278.192669077191 318.561557474749 193.689806171113
IFIT1 600.757699463777 319.768792196774 347.292438587205 328.550286726543
152.221694141976 182.633773540249 377.951523644306 174.338440780636
199.170488640762 185.691922436569 199.980312703646 164.48570046081
IFIT1L 113.832885222354 112.015423658372 111.559325333192
116.321559000405 116.897299691798 112.264005988662 114.466368742403
114.314425748312 117.841844605128 105.839106819841 126.837151457845
122.716563069312
IFIT2 467.387427864242 280.990656342284 315.565349831797 297.636316709322
200.065184172871 142.178467668037 290.037325939883 202.397972518715
288.182388699147 203.704734790602 363.594808277506 199.597685421675
IFIT3 294.410833087913 172.750926094884 192.96586114861 185.128661141884
146.323776429268 141.181931072161 231.044588483625 161.906823008445
244.596233138747 168.37850726499 200.045606111354 202.741211613885
IFIT5 172.934156790775 163.416011199431 152.838970689314 139.493025452991
134.936025073531 140.522470775478 149.606576415873 131.01107240791
144.916193282062 131.503936441405 180.549865708041 157.844617425219
IFITM1 2340.90990677253 1312.75601392984 499.964000319181
1046.13353853089 384.464383569801 399.724529998085 529.04238100948

617.833194119379 1102.73689840572 624.467319041884 373.760551592844 227.32463765717
IFITM2 7354.94071109106 5734.76405709934 4238.41681589316 6203.08085790474 1673.5387396582 1840.14350831655 1285.54492194393 2021.92898855142 3348.7622174821 3424.65126200697 872.512754090023 1055.22233969473
IFITM3 7733.95252458705 6006.01682776552 3608.10730106542 5609.58233117244 1570.39362935859 1254.19980463817 1109.08288440559 1345.8970795036 2267.0818302379 2662.43181510326 633.401624753368 654.663838918786
IFITM4P 132.54577436954 137.962542261072 124.251699375036 124.197397333928 132.850601603567 124.96926662367 130.784802403934 120.925930409474 138.743505629497 139.092075947067 136.311902195562 120.027996187915
IFITM5 108.807891863679 116.635455849571 113.083678884076 113.283359026655 121.679013405559 121.483610947845 120.880547922392 112.714282429822 114.39161822971 118.737244693366 122.996616773523 124.132215738502
IFLTD1 135.862790395984 130.388530183353 175.21512930507 153.807525239478 149.450921195838 164.264079172779 167.858490180471 156.646803208934 150.552186000149 155.608876496443 143.144153021901 150.968998243223
IFNA1 127.511638455985 151.374665021513 157.166379887259 145.684294398911 168.223865801948 164.268280525424 161.790909578234 148.823805841381 148.567630572897 134.676039394438 138.070353990356 151.245520459264
IFNA10 155.444668113276 140.593510265137 151.617989938924 156.448355269561 148.594322264158 130.593846004132 144.201723591119 141.152406037213 137.902953501634 149.113794439994 128.533900744619 137.170666175895
IFNA13 145.428297145967 126.165220332132 132.213890220705 138.442378791712 127.9928491829 132.553445658347 138.5270206057 129.421772713718 129.583916648999 140.678336884951 135.980886879513 132.125545886181
IFNA14 131.198284945008 120.196595293401 128.604270382206 126.381288663037 132.834433153076 131.215202712283 133.526213647686 125.593595066736 124.297848065172 135.776154891006 127.654130155446 126.37749112514
IFNA16 133.34207595093 148.678869698203 135.215356532498 135.99579763634 130.546471770588 135.29954024755 133.12641302705 122.167669805597 128.850480964163 140.689631427359 136.128092230238 142.42442972908
IFNA17 100.325215581101 98.1433670546536 106.41329572636 110.829107724132 96.8261937365062 103.398929051742 110.428616140857 104.321352818065 105.583474813289 102.421174406474 102.094370876743 94.3531468700382
IFNA2 160.085325256991 150.374573177474 156.307551403822 166.111496598928 160.75642485037 168.991551886143 170.775283924087 138.471894596606 151.248365605631 155.064761390765 137.27827836407 139.757191608836
IFNA21 154.518641757391 155.335777680002 162.289781945582 154.384766720753 138.376176600064 171.80440912191 152.217243924816 161.289708941062 167.278879415171 161.359609192977 159.688862680139 191.58185405868

IFNA4 131.181537219479 135.013075032671 131.373012256275 137.639342727211
133.268239762624 143.643259003393 128.914885412263 127.729912390394
132.50027119027 131.68571824487 137.074564265915 138.950197595669
IFNA5 103.520748827831 104.154182930018 108.079149792796 101.530900217921
116.95195122318 114.85602874727 101.915858150866 105.606964521964
110.014935095391 108.118551275778 122.662260070778 122.072091994548
IFNA6 117.32734978289 126.364445020215 127.814109293214 128.575364985954
121.648921929686 118.703108284564 114.704831217037 126.62990026202
119.427342766092 128.777337552038 133.228074579352 118.863150637476
IFNA7 136.461182044855 120.702740607924 125.768053724055 125.342580368417
133.500750738061 139.515794685984 132.118118271944 128.671626815639
127.685469299406 132.509125105338 115.310953814885 137.298549014192
IFNA8 145.128919984856 141.356127724777 134.514251897045 152.104170622003
136.453821861379 141.422441104854 144.578454791104 148.216605879958
138.50032459657 138.064951932825 133.101674466013 138.076501233454
IFNAR1 372.757091748175 422.02217814979 418.062540009856
495.89837457901 249.029098902357 301.638918685657 329.068463281182
308.441120735095 422.590112942156 228.086256222312 392.06522028189
277.000882284125
IFNAR2 183.619184925353 199.281908522212 172.251953149975
182.545896049446 214.785381549181 204.544266331066 239.827648772906
227.926444925478 226.315173823168 170.531713392258 387.403502892897
234.041863839123
IFNB1 118.703108284564 107.520000793184 117.017844025726 108.167670899938
114.942849575931 113.512670856029 104.798333931019 114.14858855653
109.506450285923 110.333339548402 113.177330690612 110.315549501241
IFNE 158.902676384853 156.430981244063 135.097332483841 129.257947818865
140.581943144813 136.627904756283 144.736213465651 141.374831983971
148.167464091421 140.146894533044 299.234482744038 168.37043566813
IFNE1 147.341597963918 148.412895185127 149.387467833684 140.431591727649
127.887666721519 135.724378638305 138.907036194384 144.292679144059
144.219104742099 141.301503201175 210.711890471222 147.600825146067
IFNG 132.786500984297 121.957155610892 125.809027384863 125.955431094455
128.200531810126 119.972337070176 122.180428703208 130.729989463558
160.92643755328 125.311403516443 151.577450119302 124.272447276746
IFNGR1 895.463355957918 851.633522004342 790.321331435096
582.527835746927 964.093668483033 1477.10828981572 2328.65814406636
1170.07851727046 1333.73334006986 1758.7809492929 2271.82656399253
1774.22769195939
IFNGR2 1526.66232186013 1501.18820017008 1205.45751308367
1470.23096656138 2166.68174726155 2215.43744802035 2236.73950603157
2048.13524244516 3581.36347643133 2334.97297889593 2775.84742592713
2672.77147589342
IFNK 132.963761182049 127.484178603011 137.966987037709 132.407736899761
139.174188535843 132.113317893587 132.75673287656 129.687743133943
134.058035351684 141.03218895811 106.147993332294 118.833188167696
IFNW1 134.32174235533 138.829793136954 147.301909062399 139.524233960073
140.481148289418 142.472724215037 139.991780068793 141.426756696799
135.416008633632 143.229240978574 155.598586699376 152.733409144267
IFP38 744.808064061459 767.861763134136 892.868661511516 874.348941257592
875.7694907383 474.517750753745 729.202002368627 643.905401805862
543.933989965705 991.081498002429 966.281849197568 386.85874184746

IFRD1 344.379733302418 274.179232297038 306.277823025572 241.134385745554
233.671878221507 215.607567127195 210.103974340881 240.147082836599
208.611557959861 273.514200733343 192.914362049985 224.424524191566
IFRD2 259.184713471619 212.291528248457 261.322374889497 300.314088267108
734.71562355687 308.517561840596 261.251965523684 251.61451602376
311.04252785157 382.233826557803 269.567704345075 305.854543429221
IFRG15 137.192570825063 112.252555403121 118.249148086655
111.820208385514 134.485835304676 120.565485864499 131.672014089487
134.633286312653 126.230887413874 169.920799931309 125.12823219142
123.361179535626
IFT122 202.797135825668 216.903719298575 213.754156680604
255.462706428149 183.226294747874 163.681014961332 169.556358586336
160.865949438448 178.854873582873 175.752091217351 159.891562221587
159.655362710407
IFT140 154.201375471487 154.309257657136 154.760697311395
134.335309757663 227.177100762395 141.244589488591 143.494511683426
152.752870674108 144.861804210272 129.570960882942 408.60092780174
197.91325268683
IFT172 207.123420880185 226.565823278767 234.895245871534
234.020419732787 200.371280127668 163.538862303362 226.763252784108
157.172970235254 166.746037983859 158.8054811093 167.42476096854
156.361037318986
IFT20 294.368406238222 265.260411490697 329.722135361491 244.251315474587
663.782083028745 373.361263985383 555.959424692051 521.714719320355
599.926717145115 396.708183244581 438.431160975498 346.691690520054
IFT52 183.214922844221 184.377923161698 213.813667557079 196.908612360694
200.204064462384 232.986366625889 287.195290301363 187.430140289193
199.01458214661 207.960802948119 200.688465653117 214.639374741553
IFT57 344.529927973082 354.132752995385 298.403224729528 345.062426573519
160.520677267863 300.709410518382 186.388067641269 177.491294133515
212.710910778949 158.795335941084 174.549929320587 176.371404525626
IFT74 563.011988279542 577.33849844574 680.509421043347 554.800688991977
292.965109701719 391.252450588331 324.949988230276 445.661863626419
380.146996799549 378.728516779744 513.518424350159 283.27806319488
IFT80 147.461480849333 137.814391189269 144.942289590793 143.092021593505
140.421945574721 139.103395748894 145.25774926819 139.442091671938
142.858554460651 143.301399292539 149.856041300199 143.647714966406
IFT81 150.911524755251 139.117776552573 149.597396040729 130.354641020141
117.028963434266 130.385747820089 129.566451901256 129.413729619216
137.504147221623 121.416038813102 147.319360896679 137.79714923769
IFT88 193.660159482867 195.384273821324 193.801858215025 155.413300826229
162.909468312298 170.024833610695 155.30926640293 148.1283706199
174.749832388791 144.886005815977 172.338509138759 186.747375844319
IGBP1 705.987531074737 583.314509855875 821.465895280454 459.53652431464
440.916522871266 580.474775712828 1239.66725147648 519.450899933049
574.6506349984 624.264994653057 1406.73270115373 627.526008812413
IGDCC3 125.08580835258 115.400592594965 121.539958110688
127.382266838772 202.545147073902 139.844984829379 127.104868352186
118.553226856337 141.067419336609 128.359034755228 125.710199961297
120.891023626809
IGDCC4 351.337287690674 268.78904911703 210.751430486845
225.890195278753 131.972669163481 135.693520183014 124.735542880811
151.07661060324 181.715992480635 173.142436025281 126.462522391125
137.27506604142

IGF1 258.16437138327 224.497348027469 176.735168607281 215.300064529551
136.504438803108 132.198536906752 137.752238510249 129.400895994387
161.217964573491 137.17370871536 137.082234284426 133.727447520255
IGF1R 137.253971514177 135.127041919869 122.94937342991 141.150016212967
140.299459596491 100.841352336505 117.652693591865 104.248226402465
123.9209907098 124.892438890234 147.471494294543 110.190708230962
IGF2 155.494095135266 155.280897870265 131.711193516482 134.880138942756
133.198832503062 173.863373934746 134.70636200986 134.89031458837
136.353051841792 136.338683078123 377.462793982812 1336.15622439522
IGF2AS 144.490452173336 129.176532377543 146.347213904792
138.546549217819 130.370405378213 137.823152565933 138.494266722799
152.646642492666 134.415163103911 151.788278519057 163.686535888943
153.379118228796
IGF2BP1 122.368581795931 138.997524526643 121.278346729876
132.50676380992 106.000943456483 135.9097041056 133.922855125616
121.391637838137 131.805798804895 129.931923954109 166.12184890921
130.322436253881
IGF2BP2 540.797164107185 626.857289492802 337.119524477446
485.309521583972 204.318316638312 621.946994961864 410.42088953434
428.782703561238 322.099431638246 976.050097371579 362.795691170335
379.983165400941
IGF2BP3 137.176144667253 129.508990861362 134.081308836364
129.422854634791 129.86336717605 121.311836944721 147.074807267102
182.624130582754 122.204249636472 291.779980648289 141.639216893245
120.74273444647
IGF2R 1349.84215478798 1325.77498031227 1240.4863200811 1441.29676998365
1497.32522786098 690.010326122574 1225.33666840349 966.281849197568
1264.90442763788 1448.64875525408 1136.76110352875 1352.14737073174
IGFALS 124.494727558127 137.973211667003 136.425263391752
129.228874611916 129.971670681199 139.607944722497 136.671602668186
136.687813884132 123.467227007155 139.103395748894 127.786141874218
131.324822348301
IGFBP1 124.563594928763 125.628281846465 124.63978985808
123.227904580467 132.502153153826 145.591320521493 124.606795061928
127.075899925865 132.642931022024 127.687299576139 130.839508306116
119.739204799544
IGFBP2 394.810886122555 527.294087043601 364.265100349672
252.222481637238 461.088572554864 200.387623661458 236.219981978324
588.197697799448 200.558971047462 609.939982655706 2048.13524244516
317.206754773681
IGFBP3 762.584280908728 1722.26954281876 441.578261054602
996.999238316991 10988.2836796736 4511.9116742833 5022.15374558514
3636.59530016939 7873.0525548049 1732.52466021178 9908.59593023839
2476.51542420593
IGFBP4 1555.28458491244 2166.68174726155 1076.62597781124
3205.74787769881 2210.67250954072 1754.99663827472 1342.29111510713
1282.84159765715 1969.67552562117 393.115606440229 1480.52638217173
1293.70593826709
IGFBP5 5115.14161559446 5800.06300826802 3348.7622174821
5455.91604383389 1699.17470788219 314.292700455545 221.796935051107
657.562129888446 1699.8374761666 523.073926509865 2586.87941889443
256.202924421798
IGFBP6 6059.74270502037 4198.61471038267 4299.06288109563
4682.62607750437 241.168936008174 165.813771791917 139.325478877749

143.215828010442 263.352118305612 789.401526545277 184.569063297206 968.157701932137
IGFBP7 6253.84268792816 4773.62220024088 6156.29203791697 5265.74259211695 1229.47460924638 2072.01578063404 667.693216401836 2215.43744802035 2656.38138490746 2982.80580616089 1133.22524345111 1130.32966481315
IGFBPL1 135.373824773008 130.653971083035 141.194900058799 138.855454645579 148.347642424885 136.425263391752 130.161889021281 126.072053958666 127.070677267233 153.93927752488 156.71199811686 132.994716261577
IGFL1 152.8810245209 180.913502162011 132.247611790269 130.166741978483 380.053166305637 1148.80141486692 150.731093808891 311.715351215263 196.638594797353 4110.76546218806 227.32463765717 160.67285689491
IGFL2 138.104527851969 139.106442824079 136.638141963514 144.134485888258 139.326694135427 136.916286605302 129.413729619216 131.892151111901 146.378522944864 223.154771092064 124.072512083237 157.891350650633
IGFL3 145.383354200298 145.726651022813 133.736252068443 135.736764623155 125.259164818744 151.619329363527 136.732319750425 151.299425811105 135.068883036882 142.385902610309 164.357373919714 148.104173238606
IGFL4 138.197070673796 139.048016900762 124.863832760607 154.924190468067 132.591496435822 148.139376772567 140.574129695953 140.434086004204 141.342216963124 138.731957926247 117.367470441908 151.524197474057
IGFN1 104.406314027414 116.576896337786 114.585818507336 122.362585067453 106.030441145334 120.208584170364 103.520748827831 104.091301211096 109.209241676429 142.024171830924 114.226028611171 122.619429537799
IGHMBP2 162.237172404557 171.278489808694 162.58152435515 175.222531098958 198.979744266277 140.619606039582 144.825231501747 133.204793127029 146.398454169593 145.904866987074 172.934156790775 145.058110060639
IGJ 170.169402943662 160.937256625847 129.189479305468 127.464485396039 167.114291248266 337.342034950908 212.854068610681 2567.4299860517 1424.04009914668 172.276968413879 141.568970008164 219.246271924313
IGLL1 353.437466689101 574.399909181723 134.626371781978 228.879434668972 1168.3174645384 427.872407051254 690.582550537485 9293.35991367953 8666.41250683965 891.194779815063 169.211708954329 859.113541787323
IGLL3 136.457424070709 133.027236270978 126.865622462379 126.228513847262 232.363656167375 141.876237297525 136.128092230238 1197.00004043747 842.567990276734 139.62718946833 133.392934239554 140.581943144813
IGSF1 124.408996713299 126.858621735156 118.945264994404 122.265071206163 124.197397333928 121.170340423391 126.777633512781 123.22295112139 119.608334527504 119.422996538444 136.258073395013 118.505980204837
IGSF10 206.876572583814 185.974598267418 186.975609955892 179.794718712964 144.972694558182 140.888439624918 150.314462939164 148.130993521745 153.97869518398 138.877999126533 124.347680638586 150.667001746475
IGSF11 161.134322621805 154.586825710624 159.44089105118 159.13662975347 152.680068580858 167.206513789165 155.686889874806 148.996601285688 165.972090075204 152.371569700039 161.365443930448 159.257221624668
IGSF2 114.785989868306 116.074423050416 119.663182347566 129.223872979789 124.217337143269 122.370148442415 115.619116556625 125.990762414974 115.107214937219 122.817695619508 131.437847958077 114.977135060317
IGSF21 219.792171863015 213.531307836195 207.368252249039 252.056131165397 113.905364405553 94.3531468700382 117.199217938531

101.149125916797 112.239647575661 124.484589975207 109.128394735532
123.104790590045
IGSF22 110.242638217587 134.613036378054 118.951526834915
112.190904058889 133.71610564207 128.593112520767 118.636694749194
121.942777234608 117.716094828779 126.603552416008 145.989546968117
119.005230164124
IGSF3 186.975609955892 261.286548990435 205.305702209672 214.617852048151
338.822464893411 769.586135629964 596.743397794872 1033.48252432603
695.595965352335 787.017214032692 739.251974981981 647.955521647635
IGSF5 121.689416823766 139.108656684059 124.676743480622 131.539170756533
150.011603347578 116.282988576774 135.956116718089 112.578934972566
117.928456234796 112.943630133093 174.396061906109 115.128667174745
IGSF6 127.723052839953 129.389420352857 120.62139061084 129.334217858282
134.13812854554 130.278980631435 135.907702243922 172.816477996646
206.585387130993 140.383646018409 159.832405510505 129.007924852801
IGSF8 165.350777445902 173.726420791056 193.522811257085 196.638594797353
267.869879189095 204.470661830972 196.638594797353 194.637233028442
175.186435016433 180.164353124488 193.858884443593 225.890195278753
IGSF9 120.452958447555 142.906227759701 122.822725811743 124.003914369077
398.423396126075 393.013539328766 265.992741398183 374.268489263941
276.351810999317 170.446156541317 177.60876237572 321.794887577351
IGSF9B 385.317560758502 456.817057099146 438.7446527797
640.029195821436 189.225893411944 167.093409692227 176.579096613849
149.879951192667 150.833523101057 188.013181859196 155.498580125421
154.634289463743
IHH 131.744454088995 138.723149134933 136.169525758215 124.791221329502
148.373866613126 127.566369352079 129.43892498257 143.538763288573
157.170136439095 133.988494850477 130.59886317117 143.541810469447
IHPK3 157.709832297666 160.150336334888 154.727212986222 180.336623146341
140.306808081269 151.234397206563 145.496033735748 157.994474909749
132.348603553752 157.83121828793 177.630246034993 139.781351410748
IIP45 139.232893342069 133.117701703454 131.354275252104 119.785062256421
139.484551562098 138.659387475119 121.419301042072 138.921608664788
130.399501479248 133.345143691828 135.335107344583 134.076865693417
IK 793.300636780035 647.666334416707 992.320202251161 779.022508413529
316.281469585496 456.126096817454 641.774812292709 607.126514742951
498.641091757363 844.187489702587 941.936830901588 623.68308750251
IKBIP 164.886944426276 163.239195865198 146.861981788662 152.340010735322
150.521719129181 169.385340991747 161.799743586569 161.035985824654
160.941804737367 217.264041781337 171.671895682052 171.145909843389
IKBKAP 139.252804088704 132.738450029766 144.346988809708
129.966755942173 130.773945967285 132.313884627836 137.522792377933
134.376870769669 141.577373924378 146.10417547509 151.239879652761
131.310537960214
IKBKB 303.423450627377 304.900910626137 288.436880408624 307.768302846647
388.416668785633 501.301694626942 328.969023478451 337.39574303968
291.683896942007 282.511614929917 353.078842299761 359.024299091145
IKBKE 182.042104888745 191.651818696629 167.442950045855 175.452986514346
280.749681050778 171.775741648309 213.602504580364 183.613051401168
204.654489493854 176.062665550409 221.411372022459 233.103476996669
IKBKG 448.95671696823 427.872407051254 511.25179427158 509.988617641229
1201.82026045778 616.523853569261 592.822841077599 447.182566890086
599.52440136406 610.759447723026 522.050619657197 616.014975366684

IKZF1 185.063870458561 170.099045774947 147.488635834397 149.048057509964
144.597060118959 159.154102271517 153.741061283101 186.077477713775
226.763252784108 184.758754197435 167.724233915361 143.712450441816
IKZF2 147.085006263446 147.937679308903 152.194337248455 138.618761382177
156.324643994552 180.501409705275 174.962380276394 158.528301072565
158.244741207036 158.6067091347 188.605328108654 179.950904921667
IKZF3 136.524640445539 134.918496279185 132.43999768718 128.221548195595
131.757695616207 133.602870982718 129.580421464913 201.49630027392
157.273309095198 139.106442824079 162.188509438986 164.548505132213
IKZF4 237.641190133427 256.495848662843 195.078843867124 262.577672828611
143.537785553911 153.962758393332 157.021010130169 140.74035966401
173.907933001446 142.715449859191 189.307823427362 135.007473769551
IKZF5 145.665155500862 142.013370954791 155.3016514732 134.936025073531
159.738700107615 167.751604259111 144.092864444802 153.899573902499
147.73141408795 157.437376880409 169.192327500032 149.583494304891
IL10 6188.04738762584 5373.2173152906 5543.71998558526 4612.81760689869
1223.68146968716 6281.01687932005 3055.80298721424 4663.03253705034
3630.80752744347 2568.95060767776 6409.23524786095 3749.80462365748
IL10RA 166.104915784875 160.493314658788 145.349023185855
161.951996906162 131.436176224725 130.221351857959 127.951403055054
178.429953283621 223.496400038284 131.649830934459 167.756465178715
117.773743603829
IL10RB 517.676093149981 478.38361571213 485.136339836897
448.260934224454 373.545629693667 571.950858678221 725.065727257421
741.433093896472 748.38293089716 748.161769171818 1319.3522488218
799.132592914065
IL11 146.355229778997 146.595540577994 136.008118769513 138.818492907643
159.23414970379 149.250205265163 148.327340543504 156.077604146143
157.344758285153 146.108551647446 138.885230982475 144.48495176385
IL11RA 747.804958601763 734.469481390593 682.730262408017
958.63560158343 169.933706225406 237.734385188542 161.104993402456
199.87003105816 189.971144220643 173.21291920566 188.181647474317
158.565676210997
IL12A 139.508029141287 120.135349166633 114.805810485989 106.437660157036
113.356070669308 120.479270037091 125.157459724842 124.679444852938
121.438875373878 119.739204799544 140.373554273624 125.549968985241
IL12B 105.741092375243 121.799209715087 109.156349683299 112.635430248416
121.076652800015 110.033746435328 107.48180681159 112.528908277828
116.796995589816 106.667175621971 148.380555606786 113.005774989833
IL12RB1 125.065813262416 123.786227856015 133.101674466013
124.359615295938 117.803508098322 127.83967243279 122.535728427528
134.076865693417 135.36523178082 124.796876920269 135.779516940893
132.149864626575
IL12RB2 144.938908763591 132.817645625115 134.477081392474
142.900756181761 145.378779757104 152.898148619937 145.190896872008
145.393431956588 153.114065147266 158.179586948369 155.747694056319
145.505223177269
IL13 120.300291760091 115.242526386551 106.263441906404 112.520199390427
115.316430915446 122.830376253922 130.033927910392 117.089207321783
111.820208385514 111.276543583667 123.072413274454 115.101971719657
IL13RA1 705.020444579319 796.439819579938 650.679096367632
562.891241563711 1077.33874251157 1205.45751308367 1290.39741199701
1034.68475089075 1353.61552261489 1622.17491781572 850.562379837947
849.158029646233

IL13RA2 141.075421936212 145.632060621034 137.354782768068
133.449527627723 148.235495611248 158.241349852419 160.27511502306
159.43317827517 182.263724979519 159.798024189461 112.036753245281
158.287108069245
IL15 165.613274361543 159.926719599391 152.543005423742 153.241806795134
148.922537778634 139.818998514364 152.225646808517 157.927709922368
159.461778054423 179.35113214277 141.587109176431 149.959767051801
IL15RA 137.975369568585 143.281050499332 131.679566454851
126.887989471803 124.362182111222 124.02280670421 121.492896642899
125.435268901131 128.720517906732 139.234549949916 132.579323148839
129.875986080057
IL16 146.223880979601 135.880955433228 129.808590871788 137.047288508371
134.512204051277 121.164978896057 128.082956839709 128.5581535393
140.955772094739 126.910075365358 135.25612528874 128.229729571899
IL17A 148.507598818039 158.919340819357 136.846912694371 149.964827055916
149.467626202395 154.798287249541 131.911579676047 161.00635457883
170.243136294537 150.820808770853 154.990319019521 163.396651801661
IL17B 133.845056524142 139.934460900409 139.607944722497 159.327764602722
122.881793230233 117.790593990682 118.180919555585 112.78323842342
119.275061991187 109.978693735206 120.883785696583 120.355844117573
IL17C 142.379120254855 156.368487784719 143.080742311914 155.813740201987
130.962368929833 122.180428703208 142.036037957345 134.888404433315
163.647306551474 141.604832186257 131.934680869554 134.931263946202
IL17D 445.95114929414 324.203537490975 327.89660021611 317.163768837813
187.48055574295 263.448958595597 136.678188851101 144.498245098592
163.380402251758 174.698724064671 167.003917876371 135.27739087376
IL17F 115.118170902045 117.028963434266 116.346181133756 122.436106757578
111.217595631623 115.371180170291 122.133292722932 131.724061631389
122.535728427528 121.134305707234 146.770267005869 128.251087452744
IL17RA 203.223604138192 186.296882059398 159.203584692924
197.02284475852 211.966139902562 192.565733545715 172.552923333095
175.452986514346 192.565733545715 193.292596471113 191.170105307944
217.202541167691
IL17RB 146.378522944864 145.363692072132 155.648782851696
154.900676756492 181.288519308219 176.901042102323 150.313058722346
194.941454173896 235.372592051464 137.700110923212 138.576978828316
169.927157878764
IL17RC 166.305219348008 176.167129202773 153.092864798642
185.673549367125 200.001438979007 147.164348704012 159.576585660511
152.812142230375 147.26204590693 156.010027635627 161.761125440228
160.140509175287
IL17RD 2076.51786803222 2328.65814406636 1710.60409578293
1957.48508632607 426.464641742092 2059.09598012857 925.656701707763
1323.05745869565 1040.29664343743 885.927972780392 1731.62376348283
1111.47772523331
IL17RE 127.090219286772 128.367370419533 125.217733034761
124.602474178438 131.827899207029 129.114161128113 126.314233529267
129.190900922265 124.563594928763 128.837901660985 133.802898895457
140.125575976708
IL17REL 118.510960607141 131.261903118177 119.459625817735
130.022574966461 126.629012619208 125.199837162105 121.274997609378
122.696382705233 115.914842445934 121.583096465567 136.19083681188
114.860437706855

IL18 24098.0251582624 25002.6870134094 23427.4695172122 23427.4695172122
7753.61594300904 25558.9005130606 12916.6904389018 20510.096303537
20887.0399135573 11392.4189359453 16588.0599171804 16742.6286866851
IL18BP 164.753886197136 171.722839172783 154.679076303046
165.565284723155 147.581827861592 153.934872807607 163.340435089911
189.027594735456 189.708396835236 163.873355370767 177.696849418064
157.909530751723
IL18R1 184.819937046061 166.988725688589 130.778164962583
180.881464829981 139.308325848216 151.259426521768 143.040254576088
236.292053621233 186.388067641269 168.365262340217 170.052320640686
141.180647782041
IL18RAP 152.769931978291 138.316043447788 134.537123332992
137.731390340708 139.404820141366 167.850516495529 136.647239621513
184.029968090084 177.990274115348 161.849023343715 157.04599638368
143.603022117503
IL19 119.01236045062 127.808579455383 125.982423431253 117.575009754326
124.650711299942 116.973126196102 128.421242037748 126.019246614944
127.471479301983 122.290822230565 138.274057264688 126.578035793406
IL1A 123.577138762427 140.487887891414 134.025385181979 129.260551359984
165.896420029269 175.428000742222 153.457715729768 194.413759761549
215.866644799363 1978.95750795951 485.87138934376 473.853999833395
IL1B 110.829107724132 126.589865262427 110.553759711075 115.827175143972
117.015097017246 142.752536416014 171.579123521304 132.20887307183
985.087150371964 3384.19521857242 175.044940252247 239.003718699156
IL1F10 123.042372128864 122.197625748146 124.927989289122
130.576944864484 122.682761393215 129.532122459128 130.878000486351
127.251594986725 127.490889148422 173.520662003159 116.810827219571
123.952494134263
IL1F5 121.410606617535 130.33205883588 124.466986821169 125.253637835638
123.062390088305 118.258886189283 123.45004246675 122.770033312519
130.564962937297 363.150472154973 122.005340508177 118.850495684547
IL1F6 142.153830345183 145.076068630259 144.131743978192 151.210814209064
146.320758317435 129.471713415228 149.350630572336 138.386391726042
128.280225530201 144.994931561779 134.413962468434 132.233396667074
IL1F7 131.865164933746 130.63490683087 141.767311448808 134.888404433315
129.320210774045 135.803599969904 135.628982370682 132.714532888989
134.13812854554 138.480633738745 126.98588570464 137.353149614081
IL1F8 144.148833164952 151.423936838934 141.33919297402 146.042460762767
152.166586496729 154.584367902971 145.107068919893 145.686092190716
147.341597963918 160.50439457257 147.973274498131 145.296229550382
IL1F9 117.773743603829 134.275773172492 116.664031217378 126.322527679273
108.760780994582 114.734439905052 123.069963218224 121.336932276232
126.978144285534 862.132582849511 124.74291205933 110.885714048075
IL1R1 324.871248300476 259.625943627223 171.97578836712 177.919885012006
224.251136332123 219.661502916874 155.878493935166 252.473612525884
285.63245179313 208.257420634745 385.050282185255 138.67221078031
IL1R2 136.171728950893 139.955389033983 136.77666893627 135.463385553776
146.755803731358 152.624623171797 132.91094012928 184.819937046061
158.380964047483 535.49374437073 155.582036890436 145.576880854443
IL1RAP 135.697550878438 130.854818615966 133.274292647041
124.062863592295 135.835518477299 145.010108826238 135.08927101395
150.2214516849 143.047617455953 268.52833723726 184.322250984457
147.086734464001

IL1RAPL1 406.816482069141 403.208138816247 296.088938872643
276.476790719416 148.673763368264 172.065593177758 155.569733738768
150.046300135261 153.000232171457 167.021858307031 139.515794685984
157.089650890944
IL1RAPL2 155.313873664923 139.388484543482 133.807992913171
139.027836356854 135.598709185334 137.599810249536 142.157512965166
135.889764019762 149.636887782574 136.705326991088 134.387256336429
154.533544838819
IL1RL1 141.494666823639 145.665155500862 142.793573585472
141.555366493541 138.891642304194 141.914966520049 143.962842383778
146.750774496317 142.480203661104 419.690542859168 132.674061422332
141.905641114703
IL1RL2 127.496885890349 129.195962576953 139.195281937545
141.850952191423 157.83121828793 144.2753860947 160.628894266843
148.423303776817 147.378228560979 149.628421263062 159.611551480901
147.679233228201
IL1RN 127.585851003779 139.859453080651 125.863969666806 124.327827104244
200.790119570144 155.595651896155 193.930569647995 275.468194644053
341.714401872192 1133.87328758676 315.306271141071 340.800632878807
IL2 137.075665247855 138.825961991209 150.690143057308 139.256416415149
151.239879652761 130.416957431512 151.961811748754 122.187031504466
129.802596741962 132.739435456445 142.736148814019 137.53070284697
IL20 165.581217879395 183.152005135504 154.143331594812 173.287859527767
156.986053534635 175.199369463703 166.64535048582 144.470642732973
144.708780756278 200.934618257908 144.992993261203 134.810212106122
IL20RA 134.044789094272 142.436624546651 135.937066152894
129.640303191006 183.545741265706 219.519896184851 292.185246573643
194.337080066417 251.307020289291 190.520750756082 364.265100349672
255.866847225916
IL20RB 184.462286724102 178.976116597291 154.856345114916
203.616580991715 157.58659226599 152.148784410501 174.02138265758
160.343551497512 156.630366766924 1120.3009282887 168.443386067427
164.50490221903
IL21 135.343380336913 136.767307268217 137.423810400996 140.904883114871
174.698724064671 134.025385181979 143.814798097425 152.329129642988
145.971656491073 150.648444878067 143.040254576088 126.766964875664
IL21R 132.657106732366 141.689210867136 132.306569548052 137.323320121918
142.12808103815 126.388778582602 129.568951363607 134.330234316896
151.121293928309 139.699189528879 150.401509081265 133.890597982015
IL22 130.215290990406 136.22416508552 126.791714564926 135.518752716008
126.402405227897 120.812738312059 144.278830378899 129.382242021964
123.720229113779 135.32730674455 129.290542121353 132.628358688595
IL22RA1 124.641892391159 125.160161268474 120.436408685057
113.627435441258 122.619429537799 128.093945009882 123.577138762427
126.877293296185 147.808262170269 202.00431685587 142.907969322845
139.6492947529
IL22RA2 145.562772839517 140.446530307169 131.70505265403
137.593577427321 139.14544832776 134.397255719047 136.101501973431
135.141067086648 136.047032834863 139.871928127715 136.335679863251
135.303322290834
IL23A 137.773960847088 136.507961715591 142.368601917778 121.61143371132
131.941069239017 134.122776198036 225.387674139093 140.672863685822
182.608712782878 144.895601314903 139.841822983821 175.693873820963

IL23R 120.706421084476 123.535803027153 138.557464672111 132.668344126625
120.55570440025 122.903428611669 115.981530322377 131.086434891752
116.221426977629 123.007103885415 139.452773383624 129.365004129784
IL24 150.629932241703 146.376149543438 145.773932754923 144.387375194186
150.672985449063 145.553219709768 159.311949668403 138.237030685795
166.415922313464 186.377571176623 144.738871562895 148.329635131296
IL25 169.368090266817 166.007913607742 170.531713392258 178.628885995439
204.031081842809 165.737618983611 198.244854812728 175.262884960324
176.267796612066 186.747375844319 180.265418365803 187.15740398852
IL26 124.59937620719 122.281263517691 117.980052938167 121.888886996534
123.09149124559 117.65847906412 115.520278378747 121.072192130078
133.069347559741 110.212739445 125.452973441746 113.609215169397
IL27 126.086991456747 123.117923525539 122.519746599387 118.372047967248
127.052618800004 124.876226351169 121.30744400137 119.95666716163
124.710368327189 113.542803491215 113.440171712791 125.405373274081
IL27RA 176.347535860374 177.182321788711 148.012353232918
181.771654391653 177.742616962629 169.744100665038 170.748911334847
197.049616858296 232.699786290509 237.171424222756 182.958991847218
161.608941774391
IL28A 130.758707848676 122.512587751135 116.802432258299 110.304102941202
123.21112178671 115.29099102455 116.572001618531 113.529619135543
126.913782515341 117.408465686218 142.688809469455 115.327402810793
IL28B 137.42213762011 121.181654892305 138.708304921088 132.399633003253
139.785767470943 134.319316977485 125.506466437764 139.232893342069
134.796088225931 130.110413621436 125.390139830237 145.372579207362
IL28RA 123.835583753941 141.186913979346 127.799190369808
134.607501073433 194.240372898704 185.765000029242 206.413554839865
187.812436252228 154.487546030444 314.182403961681 223.89854167651
250.718986968076
IL29 130.06068534054 130.613760017864 124.321503368855 149.135934269866
126.619475852751 132.024244263352 138.280481662932 124.080382291282
126.699152957332 136.904851056919 125.132610413051 129.642952083513
IL2RA 149.980924447339 154.250656489421 149.874424976813 160.825408895349
150.51056917809 164.836381332774 154.400124981573 170.858049313581
179.163748161358 175.111460651956 157.240466990977 155.715728894101
IL2RB 137.947606003247 162.127697884504 119.701313125748 167.776363541939
188.118989395838 192.532222258033 148.28101912019 242.221324619171
435.983803453746 239.277801396834 286.996908547553 185.332215198617
IL2RG 154.645353009618 164.553511697702 148.816369706133 166.440530119785
139.057572927874 168.911840239685 156.911538642765 154.947359473187
159.895303363094 165.960176666275 154.43808450305 162.216377983292
IL3 138.12651823109 152.61583482402 132.492444216154 146.775403409562
141.25089277145 156.173099025259 151.744902566627 151.632801137039
152.660373241584 138.582427155109 137.029726467523 146.099555861577
IL31 121.722412245175 111.09179957013 122.089332300031 105.606964521964
121.95061962467 107.156457065853 111.596690019694 108.768730405582
122.292692083352 106.576058729493 124.918089223417 109.255470590389
IL31RA 123.598210440312 126.132339293132 130.097543328456
128.653335099674 127.514381389109 124.321503368855 133.685254707686
128.0805889548 125.126100642395 128.984613946021 142.888281750232
122.120307446003
IL32 206.321272734099 207.254220247481 144.219104742099 214.803167803712
249.590923098575 167.260319361746 215.607567127195 238.344676327918
606.162976359615 328.33387481068 289.956528242564 195.019706886336

IL33	261.844708446856	234.789040617681	300.675907594637	245.454945056317
	119.094868654252	135.719702250709	137.947606003247	131.050255325564
	157.997750997487	152.129092774005	152.373931758229	118.838332117453
IL34	166.040918339519	153.505155981348	151.40067812803	221.889331385023
	138.667081004369	139.256416415149	141.191500203423	140.518206716196
	152.729355306724	151.051672692659	139.872976072847	141.805028495425
IL3RA	147.476805635744	167.46432961654	125.563646710294	161.299920355406
	133.063422581428	140.756898474665	140.548371692813	134.423260547727
	174.881557472722	141.391508027714	173.204163917896	153.694557276814
IL4	130.304743083321	140.003353399494	137.497145594629	132.54355871539
	130.311471059267	123.732299841696	139.374278310041	143.13304460184
	129.869363181125	127.424848301217	132.02259465879	132.529247880813
IL4I1	141.266129417042	146.923382271905	142.537484065487	159.455596750304
	158.147502554814	151.403431105471	137.466291815166	161.799743586569
	178.684992551017	142.771243742561	156.494323415504	149.453103369806
IL4R	614.957397883514	814.110601393539	581.828502821435	786.62709337246
	287.242697383558	255.198862778405	665.341859712345	287.145105895235
	394.73732350607	401.189904619902	579.923640505245	295.893482074241
IL5	137.416133786828	125.234097725749	127.883124527039	131.676609824921
	124.679444852938	132.760567403706	135.542079867325	134.664652044646
	136.603035996281	121.88070274109	132.038012025708	128.135216461862
IL5RA	178.878444404192	175.948959885759	172.050718136571	189.49408026215
	172.329225247019	162.536009333787	184.085616342099	172.197077149985
	178.885625009973	189.816900281053	172.142411008084	161.285587312115
IL6	137.658183450715	331.747622606378	163.54669668396	204.396576475753
	160.240052678401	244.766289564524	148.272252953881	145.881727908814
	156.273445355976	222.990586491219	142.865320802784	173.315097944835
IL6R	163.996649606636	161.510497517852	147.889486635135	154.988494760267
	147.868721324602	150.875392181869	210.030846572444	167.900071375885
	150.417169156755	184.260835471542	147.875855077738	148.94765984808
IL6ST	136.432109029368	133.897397228615	129.549673572516	128.56495182759
	123.681353449198	132.688531077861	128.893208935783	125.345611085082
	131.87404363664	126.22472822166	123.031945101045	123.526427901605
IL7	133.836390935612	136.308565710257	134.058035351684	130.220620345623
	128.888149939072	137.070326499918	130.282212339065	132.683594640696
	139.282741110499	134.361064431414	148.73620012231	150.188128594079
IL7R	182.410688592378	177.039190810529	131.879526177089	142.49005761699
	153.062710222618	308.281798808746	248.562843471699	455.392133846517
	635.536499281829	392.6758818959	339.32415558339	212.272826965935
IL8	172.073751088968	265.470314691221	162.586012723045	156.430981244063
	533.54045706916	472.669956708398	397.449905380349	228.499052416183
	769.009371389327	5624.32062666666	303.300028849873	429.812058546782
IL8RB	125.416113646128	123.27145293936	128.611740835252	136.207420489201
	135.049657594221	141.382096896508	160.979182561773	125.9713489569
	141.050384368733	146.174492025087	135.982797108848	153.377182895472
IL8RBP	134.580526467375	126.749878702933	130.420753247797	
	138.609972011765	131.08389186558	136.059884933876	136.278361222532
	137.706893763747	153.044682186978	162.422173095045	134.896349513392
	140.802033706122			
IL9	136.355319997318	143.096097305398	151.110467345727	149.518870320398
	142.804411455413	151.794243108455	142.91818218442	140.482728188987
	147.314687795555	162.077455145261	140.736051985112	155.572357799741

IL9R 170.483686891321 159.561431866706 173.468958175014 163.091280469234
165.100811808026 174.820064644203 153.711354087243 134.329334422886
158.356964958885 174.690052785405 146.948261640482 185.968210090666
ILDR1 249.193270428348 254.936706344174 259.868701878844 264.383922859446
463.802270256246 331.684298221133 395.716879127149 457.548150688248
432.726040933545 254.936706344174 413.172954892025 373.096629378654
ILDR2 143.844153373367 150.173766182317 125.701083319929 144.876300850115
136.871568211946 139.690093593646 147.604213625382 139.698333393438
142.948238172973 142.339740800356 141.278654291193 135.776154891006
ILF2 875.7694907383 851.221199352646 863.086199215353 696.475639532595
1853.20812924694 3226.53426370323 1772.75453735112 4255.59773503364
2476.51542420593 1753.58770316818 1658.78888936164 2859.94396089224
ILF3 353.285905782951 419.047108206042 342.555640222963 526.896146537814
423.562598841704 553.123179808258 423.045329000756 463.091387717305
415.497783305278 369.156887999872 433.751675703425 434.792010353541
ILK 3905.35300751401 4353.66854914351 4477.54806526552 6059.74270502037
742.751664201272 534.608644989102 546.029974898225 537.828979447378
594.180861801958 950.144166422001 1029.43177809276 419.593558001947
ILKAP 345.433025456954 285.05846406041 348.98762709818 375.958347293549
250.515579402334 248.887522059485 249.737588031219 205.495823410422
228.243668453958 266.252875242286 249.633252091596 247.504198063293
ILVBL 695.086203058575 663.782083028745 724.442411170786 520.120193442648
4037.15442268836 895.821578104681 1168.3174645384 1195.6335460045
594.713185954954 878.986106116952 1144.07896461196 1070.4597488302
IMAA 8812.91092406563 8709.2221371432 10632.4185569238 10312.8407173696
8864.70614223459 11017.1478502767 7753.61594300904 8918.58940386786
9192.8966763778 7717.01422791837 4417.98524179686 8596.90650022148
IMMP1L 157.316941011117 132.439176846818 163.73339229802
127.756214708077 178.12073430094 136.612903661685 153.756213924949
138.405215507471 147.688664545959 163.668476670167 162.678619800767
154.479733759712
IMMP2L 315.565349831797 271.945851739635 427.084392965651
270.787490294555 381.121621815163 197.357728220717 350.166837086936
305.451247405518 224.167511918105 258.25658838015 263.509066170434
448.54309632232
IMMT 1389.93393245455 1344.87842417496 1350.87073284396 1733.96382719909
1227.36706427754 1361.54107116915 736.963699775724 1495.04587558545
971.99484515987 1121.87292339787 1135.49876948815 1281.1883621512
IMP3 1268.73280473728 1451.64055568759 1319.3522488218 1102.38234645327
2611.61260614408 2718.440149645 3896.49785254733 2446.31228564539
4000.45196171119 3684.05513498098 3677.23129570773 3693.4058156307
IMP4 1138.44837250274 1374.92806285803 1218.05434595121 1986.81354260292
1782.2498613913 1437.5568316065 1170.88763035931 1798.5819962261
1253.05700051015 1286.55103838368 830.684999498776 1411.2586496281
IMP5 129.356401843185 124.612293808606 130.842257229979 129.615417777242
142.160941592768 121.657744897668 127.905616521853 138.055909969804
126.277165320165 124.217337143269 150.156865995098 125.563646710294
IMPA1 235.680462167404 271.522696252995 233.690524419327 223.013753785002
242.221324619171 479.469659712246 295.463118191589 395.049127927637
447.297590650141 447.995213144892 585.075322115635 444.192734921566
IMPA2 1646.76016031831 1542.35486950856 2352.01196541874 1775.32632165475
479.983368253935 405.64711132293 243.861834405092 865.435843446989
322.576886792502 1574.65731564294 185.938983266802 232.231236745251

IMPACT 143.982561564661 144.482364671273 162.879707592164
157.986640251147 140.123348569709 154.507721014473 146.692827583525
156.534634574244 144.750407738866 162.026757124868 144.178210824181
146.052153515494
IMPAD1 256.764450748941 237.71636970719 277.75958501062
274.371523790341 540.359249705821 346.996310517446 368.948300262634
380.393153331219 379.387095129725 279.598585549004 201.49630027392
306.232249422327
IMPDH1 205.4648759901 215.019239034819 195.674128236589
249.507062968243 357.773949920377 194.961531388323 157.225479764928
220.791884858764 217.646777099512 288.823376348944 185.31769184805
227.092319765009
IMPDH2 1359.63626195826 1656.02721259517 1720.14021303639
1515.38811502277 2974.04694562902 1622.17491781572 3917.05893989806
2444.835635949 1620.7954553764 2660.39990175415 3177.80599774525
2681.48138915107
IMPG1 137.448412532999 136.977435235496 143.40955339735 134.11753210296
135.678138913772 130.37185420347 136.109975252514 135.229197100212
136.859366444351 149.108582827136 122.860898886143 141.681266095578
IMPG2 151.445254181497 154.336009472285 168.897620634337 157.528409294029
149.657951204528 141.099781722697 141.719556176029 151.70035428516
150.138643811727 142.934869757362 157.003367685421 153.267549449395
INA 135.518752716008 157.982146236108 138.778496466408 126.702705908175
845.369108604819 1019.627634011 581.37957221282 580.859914041323
1362.75971646355 189.250846546817 1499.07462479938 584.433155497378
INADL 224.963277964548 237.249949461085 257.168308311848 210.33390115739
173.063002566542 437.122373082112 368.276805218658 276.925017614147
211.995890899864 274.14931964455 304.357050745605 359.577827010792
INCA1 160.735428753783 165.283700641161 189.348690353874 192.737713851863
179.704370382771 164.429483677715 164.448110885609 160.19942770666
160.806678614325 154.826710617351 139.490103923544 145.308548379215
INCENP 135.27739087376 147.457193565877 135.420780282429
142.324077852852 290.389345090634 171.006582229921 155.902461107328
157.986640251147 160.045715314465 158.625060605976 159.241573314833
157.967824935915
INDO 126.460390022632 134.865577264797 119.281300456243 128.914885412263
159.218886890035 146.609485664113 191.310372695208 223.975677959345
286.035428568826 146.626517077288 174.812134675989 136.48748813417
INE1 152.251128595777 130.680629893396 167.653412908384 169.077657790409
141.634246722965 149.286184588967 140.826051635712 153.097557560333
146.231937458616 156.169405925094 121.472885562857 155.166786601813
INE2 131.548634017902 142.412731964179 136.351332391315 138.263361518905
131.269526665478 132.839431368337 137.537197575152 136.116468624715
147.508817393901 124.132215738502 134.534815676852 135.659271030454
INF2 212.854068610681 240.878804911613 196.598810934837 326.640168906375
524.392279804734 197.895094054577 173.347463632976 201.289402228512
224.274329367512 205.362816362477 348.848550937417 299.986758612161
ING1 132.887701822875 149.756512110538 151.546908501054 135.589211439847
160.17208299932 151.210814209064 131.312580984876 146.223880979601
152.129092774005 140.759006929593 152.221694141976 161.234304237838
ING2 275.584858082096 271.615621030842 360.605223912114 258.915802108562
318.423602671236 291.476113017794 454.34729744062 335.938170230765
378.308562999445 696.277780670166 367.327273578513 463.99922423125

ING3 188.818012746123 174.323881548679 203.983168778488 145.161116298118
149.364498870469 152.710827148511 174.761105961773 181.308852106289
158.150949554785 173.161152247499 206.876572583814 200.486683704179
ING4 184.148058872585 186.06425658259 222.028626209166 228.001046173405
176.725347194807 193.37618381669 218.944052733757 179.61374889626
203.298764374262 184.813094907887 257.132618311227 180.761100930677
ING5 150.998881139061 146.486480663438 150.267298097687 139.472008268541
141.987635042691 149.65417821994 145.03221570575 142.427198128376
152.545261723881 143.722135315998 151.958818313561 154.675189522146
INGX 132.688531077861 128.593112520767 126.910075365358 134.168381235167
138.818492907643 132.031693838834 118.424520312293 135.040794634617
125.905785653648 120.316950428535 147.127908355066 134.970609100459
INHA 145.100964170053 135.484360570738 142.534195262988 150.952264779196
142.153830345183 146.639556437847 136.934313990609 151.007812189859
138.855454645579 132.283682066898 129.822926691091 158.167384416968
INHBA 122.768405654503 116.248026336995 123.865424155099 123.338921824167
123.341568893224 115.559551514777 121.067907968175 124.464941711781
129.675420096163 128.152956417755 127.657961561231 118.679664392666
INHBB 170.676752248138 265.866465528654 152.099431258333 157.631978594005
151.70035428516 185.856755885891 154.555255038453 164.164463389212
150.788386945666 188.53221484746 305.252666831522 204.299375966441
INHBC 158.911404625381 155.8871985642 154.675189522146 147.52178512946
141.018087546273 148.15920634463 153.719751724358 167.350290326189
141.953114738413 150.452467115669 136.585541662028 155.592219571564
INHBE 139.121639005366 157.702540365469 161.799743586569 155.853414065788
149.076146942797 154.637740117457 128.827129738041 145.524305076788
156.622993107052 138.424464248567 158.185593294878 157.717145014769
INMT 1026.61404215739 651.759589129833 399.872401268958 950.818917078515
143.517327774456 124.818436949131 136.37481047902 157.358696886078
153.6855881767 120.371374172279 140.60741459333 132.161764435769
INO80 290.802509012089 297.834209533939 306.387898121409 339.663295617139
212.107842040568 217.050602253281 239.704459251571 241.418016203544
244.401472859026 250.626556981376 255.74096237558 264.741946816665
INO80B 172.329225247019 181.976252906929 195.946096353894
214.068044241507 344.529927973082 196.014841240142 264.955939320296
205.026176598298 181.971189935628 181.247766545135 221.127492253636
231.180528071672
INO80C 215.769986055534 250.911817769829 259.900447705311
228.560346647306 381.014521577291 533.140740560189 532.950400370668
471.816954259146 515.929937825133 565.609400092543 667.693216401836
632.843164522867
INO80D 592.613689704907 590.3584324045 686.445635754196
598.924339094496 246.616749748028 811.912727084669 341.64890727921
482.592481827722 369.838429521168 564.200165295159 221.685824197352
545.760623858473
INO80E 435.983803453746 433.314881387431 513.036729214095
588.197697799448 589.541347392215 672.13164205724 379.983165400941
570.916936074747 417.024088698182 453.875259610173 440.319777672273
445.214621023114
INPP1 571.950858678221 554.511983790164 493.715108157171 480.484639351046
354.45785837703 623.409211766224 522.17701271254 755.79669622705
539.008286968106 808.55123510297 1028.39666838369 1359.63626195826
INPP4A 134.646131480502 126.503135702506 132.823333905679
130.441808139335 151.201263631883 135.503338987971 130.031303483631

125.533538277681 140.318155161909 124.235958498969 138.22823004719
127.931887647108
INPP4B 193.554028681854 240.224826736571 191.029458520006
189.61702125418 369.018856220394 383.939800422489 651.675427664911
417.865461351681 481.751102333172 312.339824919124 569.353661326724
345.732364434125
INPP5A 484.568420044471 592.387650564803 451.147418314412
808.55123510297 268.264885392368 205.428900832926 229.406012088782
201.637540930213 210.41046499545 203.17525411575 234.041863839123
270.651318838953
INPP5B 239.941641600769 229.542259738438 284.351529744475
224.118739978257 192.399146702036 162.347140552869 148.544887355857
159.009211991511 159.806221777332 165.504532455205 160.256877369493
152.89366585726
INPP5D 198.840533461525 187.15740398852 148.692602243778
145.85414203374 181.313333665614 133.083877012766 256.687384001447
176.533058719908 287.751093664085 219.564603776774 362.095074331893
391.911768848267
INPP5E 274.831898505989 267.097999873387 339.843776738468
286.282132382462 284.236970342827 173.176900454904 175.363223521128
261.426617866925 276.961847241646 298.347201056937 292.145553580042
197.068795788491
INPP5F 159.74220366735 159.346890686719 166.521472376759
160.79631681714 165.885072907195 167.139257044999 150.837717923505
158.344330353326 160.117742879282 155.228605554943 177.19138801548
162.743192424569
INPP5J 146.818744991143 155.091135297162 146.418147447541
139.534014289993 156.856149172225 128.803632055969 151.522526161299
144.665869508406 138.267726309873 135.762603711262 124.640841120186
135.699583036042
INPP5K 252.32907388173 219.129781831192 231.572677616684
273.106088088825 271.945851739635 221.070448906398 157.293090577042
216.615694348945 171.099675679944 201.616302398819 220.999383789474
170.725486814469
INPPL1 977.227184625781 1239.66725147648 954.488185198006
1766.15001427132 969.360864006371 576.636077770998 677.9972761887
482.965800108538 463.187551361986 448.715938510132 531.711823481265
627.803400871547
INS 157.940873610797 155.140371265225 152.368485416992 164.231281729415
155.582036890436 147.056182807232 175.010849510711 163.552608805894
185.525760847281 140.646946425036 157.963634535239 154.423801637411
INS-IGF2 118.21084414402 123.748788684337 113.708683868028
119.060186838239 115.090463537716 111.178024950204 112.264005988662
111.7529190597 110.355493122568 113.326628149066 276.138650967632
464.196512015649
INSC 161.084088366981 149.962143642332 158.014017648839 149.125425566378
139.497253870412 144.270828498856 164.641508485461 143.59105195427
156.221773628832 165.737618983611 137.368211928541 145.139966629363
INSIG1 311.337303524912 332.599191067433 295.463118191589
240.631264915238 294.997671630798 419.047108206042 313.536451935268
482.048928546145 353.621264641814 722.974310297586 333.349203784021
399.476401949891
INSIG2 352.180110869664 341.128917971124 384.051170099325
296.962992349662 251.414717011661 477.87583927675 409.743899750699

288.261250589513 390.284689962661 701.208391798006 426.464641742092
407.589300080181
INSL3 122.167669805597 114.65036208773 112.314595956147 123.640123613529
118.584773788416 116.793408204625 108.543795408713 108.868679899494
121.632494500096 117.688941284427 133.289175078053 116.0999658586
INSL4 134.222453538421 144.408426565541 136.612287212568 152.191393042132
159.185616602396 135.497033143331 145.315875226867 148.294870328642
144.623222072851 141.927204640355 151.27882940428 151.191822057706
INSL5 123.267817390796 126.976353967127 133.606068125828 134.731687541347
147.983937675946 135.164117176127 135.596007994731 135.190698960212
126.205931638442 123.400143662218 117.503848306325 134.372560968918
INSL6 132.895500657638 134.457932303561 120.337241026338 133.236084190243
132.146027935802 131.196196319523 129.187574143637 126.033673708286
123.195738824794 133.504078347324 123.748788684337 126.022145004164
INSM1 142.208987267497 157.607445509297 148.925418133902 155.826394389322
153.492072037266 136.040524807653 129.051642626091 145.476377346532
138.440663763957 152.479402912801 164.433462115832 165.376358421533
INSM2 101.149125916797 105.193775204124 112.502812746669 110.448948495323
106.772581387455 109.851507510788 120.74273444647 112.999642731096
110.900309203531 114.726808450831 121.917596225576 112.971776964469
INSR 129.267923706188 123.265810024678 117.511162285879 119.150626141327
149.725422365342 139.847813838017 142.01454627498 132.943957535655
145.99474597064 124.869732867761 166.662801900754 125.598828334465
INSRR 146.713861499546 135.159590925066 154.686025022972 140.38936578649
177.772019227116 155.62770968777 140.372463604232 156.720090761933
148.132450240784 137.429832103492 143.004090696463 145.259981479761
INTS1 393.198813167839 486.334840298921 417.442660073269 613.631311147328
1198.53117439169 290.241064926886 446.194196064909 388.638095539186
492.05680879106 490.022369664024 404.21236980164 487.696347726467
INTS10 776.98412594767 600.919152191636 896.185223980593
782.139151423572 452.517134370928 808.837225580351 734.469481390593
494.538861626895 676.570119607984 672.13164205724 857.090339943417
747.804958601763
INTS12 354.579728909336 366.408565514304 436.331333134628
441.331937921872 276.284517459626 512.541205235935 464.436380568216
455.124190541055 539.720389702148 523.591472915151 323.667189206453
401.87674893993
INTS2 280.246711704036 270.231637012624 257.338950719432 270.293807247372
199.275716402358 331.215872600406 380.393153331219 395.133831206474
301.139737613279 315.014639682046 254.133112176024 348.171901445232
INTS3 1168.68074786473 1081.47077865393 1096.61874892572 1307.19490564658
747.587723573212 767.861763134136 991.611982265221 629.707646271678
457.411248351795 467.387427864242 906.886423608706 1014.63777151551
INTS4 233.690524419327 223.531323159808 266.135477775641 278.942695435238
224.528190554963 252.32907388173 234.28146549561 283.416246133873
252.761035334279 290.754210152709 213.338918839446 251.062649517773
INTS5 199.408907117764 239.003718699156 183.604011732066 255.527764534636
519.112018353676 228.926077117474 257.44230597145 199.53455266342
220.487665103019 200.405532717305 235.650688823345 199.305626149918
INTS6 369.275635424092 393.796356067403 332.138346958671 356.413190087706
246.989526028884 297.757657398146 227.52352046188 287.503594820801
331.215872600406 340.860684371038 381.867665449509 395.927222009721

INTS7	172.387657419201	175.805472275887	158.507624756588	166.842970309527
	155.61996871093	211.269041187154	177.696849418064	193.173703015873
	223.220997990627	281.369059516242	202.230920499592	263.936023499588
INTS8	424.912426797488	438.870464403215	367.382105696814	391.644649726254
	484.363052437459	393.796356067403	432.088303693555	535.861738240441
	514.463945278314	342.403428822464	368.276805218658	353.774297577869
INTS9	544.580311371212	609.309339417625	664.19083888799	683.365770196967
	592.957842630046	592.822841077599	715.638378711433	466.083451075744
	593.320132696649	581.54874141672	804.362909579005	649.906677394861
INTU	178.647726085678	155.247452081206	194.961531388323	164.332073056902
	160.493314658788	150.038722708606	183.32467147543	165.386056999339
	191.740596601895	189.450316943845	185.743212726702	195.795728497916
INVS	160.833948531272	164.801505601099	159.515698265424	168.394330931524
	176.267796612066	144.492756323647	152.601626526117	193.629237856758
	174.441287575034	202.691507273819	176.183165494563	158.425950902309
IP6K1	456.817057099146	573.846138124455	456.201998988891	715.93836911426
	841.285351463894	358.52459123416	447.521645706162	353.912654262209
	538.369868115137	377.024120035829	376.791876040886	335.3366819308
IP6K2	146.474228901408	154.412506915569	177.073245863872	139.411792975474
	408.758021864761	256.820546409632	273.514200733343	243.441938832251
	293.646275396425	222.028626209166	277.685488726472	364.964572587289
IP6K3	134.682319733908	128.217078449064	138.528083984177	153.315309202016
	130.545285774056	133.722739936347	127.231647068635	126.724973604103
	147.241136598626	140.380630560717	126.670227417269	141.360125377949
IPF1	142.825203517688	149.317146997809	129.852833137493	134.512204051277
	133.171854425031	131.444734367396	124.530660958725	120.688909943167
	133.099175110023	122.821563586744	144.798664281991	124.170242736875
IPMK	123.094324479352	118.521075843163	125.107483348755	124.443643568527
	125.111993560078	130.865693081945	122.236134282757	144.433543176593
	129.442111153737	135.200049257744	143.559076683944	122.10489926804
IPO11	709.778171909457	717.419388886784	524.541494680558	734.139557942719
	361.327436394941	679.557656724243	510.434895296759	352.121800955822
	404.876478833713	580.645924062301	436.586158384666	559.668195903429
IPO13	478.699172220804	540.797164107185	431.799795646414	687.269774217064
	434.855262284265	318.112626495401	276.925017614147	257.5097052677
	258.830885826237	257.032174266905	253.601378730911	237.171424222756
IPO4	209.229537873706	210.711890471222	213.274106157675	282.190861594303
	538.605085454393	222.177290763736	249.234065130083	235.704824490397
	190.843795579234	282.652193666078	234.947745514175	301.188296465202
IPO5	414.538237455116	350.741303038926	537.698489904622	325.751369001105
	183.385868234361	408.037874667173	230.975434289172	199.681103910705
	215.219246394594	312.019603761673	220.567951704044	291.476113017794
IPO7	175.462290891421	146.119700458464	216.522992914538	130.957441406855
	140.553613300431	150.034029031033	145.172776053	144.871396308673
	145.606098951179	157.006278815282	192.789886894821	136.003277940379
IPO8	425.969575503375	417.731815310276	503.967725251906	495.769802520637
	267.839640412797	309.937351755502	248.43491649623	250.060734219351
	434.792010353541	459.973764138144	248.260998007814	204.470661830972
IPO9	297.970735665595	269.100400678077	284.104619768635	233.161848094769
	247.845708587624	380.053166305637	293.986292438714	238.685014863143
	317.839512360245	321.627969147543	249.263194321516	364.265100349672
IPP	701.847905027147	568.040362658955	795.464850398761	561.251860964966
	242.070630946726	449.304281526081	318.487401106209	414.596948083067
	358.298768623212	309.5988292104	360.124745133393	353.243400332397

IPPK 137.065315360321 132.828179725129 133.866011732878 150.034029031033
154.701413581915 119.125002207396 130.452929012459 131.853183159267
165.350777445902 338.129250886594 170.95650329595 128.501189288377
IPW 186.215602572358 164.406564594296 200.387623661458 176.456539071735
128.354163132392 121.212521750566 142.700311964178 131.641435734821
133.353762794271 145.45768403614 166.592366578462 127.164076119599
IQCA1 145.08246303652 141.565599104245 137.890006487904 142.090009728666
155.587018319981 137.102353661381 138.97776257708 137.504147221623
149.492550066785 161.735609436394 125.342580368417 218.615551704188
IQCB1 209.74583569369 200.065184172871 236.35479928448 173.690008681514
250.25959091612 506.898635822094 312.019603761673 379.788696204016
392.6758818959 298.776176246196 249.193270428348 337.585502787016
IQCC 215.426918011158 255.945470052489 251.062649517773 298.301504581296
299.29004204677 245.850813387241 272.117599603586 413.687046868111
266.316485802808 259.672238210968 256.291984914927 292.965109701719
IQCD 111.876915242732 118.294324274616 124.998601194756 121.144174389418
114.798573331978 113.938636269013 118.909153405102 117.238968848218
116.0999658586 118.924317138158 116.031631038289 119.415822905251
IQCE 141.659197251282 136.364830851128 123.484987952263 145.378779757104
122.629146433216 114.995889945366 131.287142490322 128.168460083895
118.660213790078 125.693321471759 135.535020220081 129.044445373107
IQCF1 112.675715371087 120.035437167539 112.825399620524 127.271423859953
122.578170988039 112.156010486085 122.274656361883 106.450154495663
115.400592594965 106.238539254093 117.532063783977 119.612288460361
IQCF2 115.53736269794 105.839106819841 113.989682635964 120.212294184999
111.683074830042 106.238539254093 111.423140302983 115.485515429446
111.196610704726 114.835386789236 124.358414281035 105.551878332786
IQCF3 135.884635448277 136.151754558226 128.748663215466 131.593705080542
136.108500467418 133.111791289725 131.176413983785 134.614988219821
141.822974252935 134.062423178775 131.342562516383 124.422548323449
IQCG 204.509884908059 193.173703015873 181.425012248539 201.676984786688
180.77686098195 156.346014084316 211.995890899864 207.417884407459
202.583967728957 210.925203743958 217.670330817769 183.963578322984
IQCH 133.303201646987 135.125414452178 140.370626016794 123.383334044683
119.730174479044 128.375390373307 125.942455135541 131.726540162023
134.595090783504 132.899305777714 127.206177583275 128.41549156443
IQCJ 131.065633574494 128.824243581806 134.803612821519 140.49145570631
129.198366503027 131.12775424877 142.596113491002 142.122556679441
138.237030685795 143.931400351525 131.142841840284 129.914077170794
IQCK 316.590387560909 301.638918685657 319.283603277754 276.057101476947
206.845306095447 278.274343962656 294.17588071297 339.98018551524
284.473085899219 291.313681141442 351.759100605991 291.313681141442
IQGAP1 969.360864006371 883.702573960415 1332.82617944131
799.132592914065 368.466518140043 728.743541467167 587.893636997642
493.209107049493 536.067857235731 1141.13648305827 1189.11053680293
664.19083888799
IQGAP2 165.30072618497 162.188509438986 141.901382169334
174.603285240228 121.915043935246 149.541917326637 117.611606270124
154.555255038453 154.127346849182 131.613738869093 145.574725784632
121.23469201559
IQGAP3 146.448568700393 144.400004730049 148.590732065459
158.364031234356 150.25322837689 184.071078414327 156.200313064999
145.108281984556 152.990523860831 152.633139090274 159.443697452694
149.79295887612

IQSEC1 411.784922368377 418.968095159939 320.214614455231
396.708183244581 303.858574395969 241.799709160815 218.134101819445
246.533813165981 293.986292438714 226.315173823168 204.35801867216
252.222481637238
IQSEC2 171.835438335216 173.204163917896 174.471304281502
183.013090662119 204.584643964409 193.858884443593 176.689683169567
193.786889748038 175.101284960252 171.982071800592 170.998961353556
163.582678113215
IQSEC3 137.790718305537 133.830860948014 130.572923512809
129.898242234869 136.908861280856 127.282374513433 142.007066214472
136.406132339187 131.02157879562 143.839170216353 149.460660195293
134.56763097313
IQUB 127.931887647108 145.267225993057 128.87498876738 133.254663058352
136.612903661685 114.91722828113 146.541890150631 137.663941989772
147.412608079997 133.046435968561 126.936899820148 143.565597315289
IRAK1 864.908449769749 857.55777793754 672.637760135254 1035.98574557331
3636.59530016939 1376.84058291266 1297.25632532536 1323.91781040143
1218.64951314553 980.120729515821 1036.40082234884 929.69741581954
IRAK1BP1 206.446584387595 259.796092536601 178.984409575632
200.017741240426 174.155599133789 186.222567239198 178.819486867379
172.509081696309 171.552010365305 197.775397831129 147.034021626893
188.39062571513
IRAK2 249.029098902357 347.081036934975 336.5543056115 259.575461599189
667.693216401836 575.386048896223 950.818917078515 890.121325723286
936.221322010828 293.528601172819 1172.96121022579 822.545007054668
IRAK3 197.981112645168 218.209523110843 183.005535466221 166.526875572556
153.998849486363 226.144012966486 158.774910841987 214.082408290757
182.143506470859 206.605699708924 152.049243916141 159.660564323984
IRAK4 168.996965070202 137.572574328004 163.72257517759 138.719480482945
134.558435781672 160.446191353364 161.027590226123 207.718619151226
170.108500366942 142.505050582588 217.969738417693 175.044940252247
IREB2 168.830344627025 148.145136066071 175.757588503244 157.021010130169
152.566490804809 262.577672828611 252.197271817794 214.595639477696
244.18714778846 242.752995772995 163.586549438955 259.979459472217
IRF1 412.648926259488 602.141151173094 416.638529499878 382.327656839936
522.17701271254 326.758278851698 662.781145703653 861.119262963015
1747.33558205825 904.733257358133 1612.79779117655 1646.76016031831
IRF2 161.673818303367 165.703810120915 168.027892518772 174.795822188504
150.741291526753 151.936543085044 168.660907237558 155.694213876736
170.585366369563 158.224122084011 197.100205890532 163.385727569154
IRF2BP1 182.275998840116 211.809434609469 167.491399614986
285.355815566148 478.606567465979 145.472859089272 157.952018720438
141.255406288178 177.88534558205 139.744142223177 187.710185880162
159.539320132392
IRF2BP2 623.508710830895 648.161646065464 776.030689773414
585.933822585718 379.476768908492 596.3549500121 408.037874667173
239.704459251571 583.022090879298 444.584594376357 280.337475979102
421.690849282483
IRF3 358.52459123416 408.037874667173 327.749926183823 461.482735533799
1403.09138858507 374.337030706199 498.534168389928 472.669956708398
527.294087043601 344.45331938267 399.248044997427 466.4890990178
IRF4 151.418316929551 146.675159409376 145.756455015309 182.886100305943
155.626515272084 140.158309892319 163.315865817864 186.999459152483
207.815226999953 166.503112149416 161.886342770305 151.228823957828

IRF5	151.965103021638	156.218834695891	165.118167086792	172.7330526128
	325.525450750788	147.896919205072	243.402781509365	332.666916002481
	165.863118584978	202.545147073902	200.536814571322	179.785198362326
IRF6	152.524106827617	176.927500850631	145.45097990828	143.783078837501
	439.727046291381	408.444629429553	532.596772298766	226.623172906957
	308.621688157016	769.958422740085	499.544050752036	414.114051645565
IRF7	243.468673768141	166.788336808924	144.181359740481	163.579282884905
	156.269957530519	170.992074907355	284.321253087512	220.567951704044
	252.733973271584	382.186095123659	230.332652534446	174.881557472722
IRF8	218.917357155289	198.338416756432	173.766372029062	207.104548555929
	181.062188174871	204.219197797869	228.797912452429	372.757091748175
	840.276501350383	225.209315195163	345.686640885419	225.927425596831
IRF9	1841.63015706195	1041.58583268313	928.151632070573	938.918431240228
	871.528156184927	587.181357555703	1867.55140010155	1348.80530206238
	2045.24382334506	1320.74943817958	2913.2685312165	1396.4168898623
IRG1	124.345320343901	126.711037295629	123.296382913131	136.607783800741
	135.849839822066	129.95138839192	132.175368142549	142.240333104987
	125.506466437764	135.423839577038	139.847813838017	133.346626315812
IRGC	137.363483527372	138.154799037807	138.916830356763	127.908872828095
	119.544004279354	142.726324048628	127.527378949803	124.441699369614
	136.390047634643	149.524040913256	148.362613706275	153.371065247493
IRGM	132.440818532628	132.760567403706	129.680406696779	142.757327296852
	124.269446398587	117.642441794442	122.804934452095	127.449028221682
	129.336402686549	128.779829252045	140.671406418004	128.83022039037
IRGQ	146.528945049588	129.838790698396	134.352155462211	146.912751127571
	142.793573585472	148.539881077647	165.232545721733	141.830432745537
	137.97889661868	143.442361602626	128.941490662452	144.182891591399
IRS1	310.23294370018	378.797952580504	375.767528676937	365.950612890149
	285.05846406041	213.634295832535	349.65350886897	215.718714189719
	493.347308943133	241.527811817146	401.631377313065	544.462032960964
IRS2	383.939800422489	378.308562999445	572.049540360134	312.224309341171
	222.943046618742	226.796825544749	227.491667075987	192.188662949149
	162.997720993338	302.021033686056	222.20526515049	218.433524126584
IRS4	147.545667896282	156.243337414127	137.260359343758	149.147411595596
	145.578721432709	149.511776997149	156.901209828741	150.287472248483
	145.335830265052	154.978243057971	151.440732942734	161.866405026758
IRX1	255.198862778405	259.229153662504	299.463563393179	293.018282386003
	260.591336959281	237.358925391616	312.845219991659	267.190228116903
	256.424415389574	280.246711704036	305.499194075747	267.839640412797
IRX2	137.923852688695	124.197397333928	139.065849548762	139.191746593825
	141.706292773073	136.324020406359	134.69274746407	140.29803170157
	132.725232102483	124.321503368855	144.55710963567	131.592172917738
IRX3	295.893482074241	307.124331101976	146.826292449698	158.160604786093
	130.865693081945	829.126529857044	915.579581760396	509.702881232139
	173.640326140494	2236.73950603157	524.392279804734	722.399669963921
IRX4	120.740986472733	138.010385979692	123.981053866137	142.34471764492
	126.404523759643	287.084253886676	138.047487233711	137.282635754873
	135.661131815375	140.553613300431	128.635684412304	123.038646914863
IRX5	150.500676225569	152.185397218918	155.824409540332	151.660152977515
	146.495845282846	152.364417528975	177.88534558205	155.960886801091
	150.431052959951	162.778480318754	155.548684011748	192.188662949149
IRX6	147.1476865809	141.557420268382	137.627338682823	135.663734178125
	140.281512754823	142.89427871125	138.576978828316	141.758827837244
	144.129889080946	143.347066447019	147.556482119673	127.642215862573

ISCA1 328.919401925375 317.00629920068 446.948699651916 288.728608411903
294.697622870418 340.860684371038 282.719121652073 326.354469134694
304.357050745605 441.873247266342 282.190861594303 248.260998007814
ISCA1L 183.735201125198 208.406980186223 248.03801051527
195.218005582618 246.061496821699 223.279017022443 215.558688906165
226.960024551778 216.974834077975 324.009703043876 244.668813440638
196.136080427522
ISCA2 166.415922313464 173.829219279719 163.748692741999 163.769959129611
209.130260450131 137.629855378016 190.809584998617 187.183762762259
161.596086502946 154.118601977256 191.507657960386 197.24751449599
ISCU 1539.80266129509 1355.07170485397 1816.3719976564 1434.1439428394
709.778171909457 1017.6969123529 891.637685856145 914.654262296682
1477.10828981572 804.800251415824 1001.35912007916 1257.32413345748
ISG15 2372.26073354008 701.847905027147 408.037874667173 840.623505494489
6244.58472720999 2944.63971819193 2137.44704613378 2938.06458269406
2390.41825815872 1119.23362648056 1531.54387233661 2083.02482378721
ISG20 310.718449468465 263.379982742085 188.74403596953 191.897449038297
449.103432410618 339.98018551524 430.329437928013 892.868661511516
1096.10636044885 530.267835989435 408.811415529922 235.742017851879
ISG20L1 346.476121089685 350.658722333252 277.000882284125
266.252875242286 413.687046868111 305.325519265387 364.597798667541
287.374706071354 395.386242257877 291.537725613819 306.677271064305
385.463209972694
ISG20L2 448.858580689107 458.705688226305 436.482580413523
474.517750753745 454.732029358748 595.752426322468 474.517750753745
525.515169794593 472.405334652317 462.686385881492 499.321667633972
567.465201338735
ISL1 205.509515144263 311.10841853984 156.179501567984 186.640167849568
155.335777680002 169.888264971936 153.562334163034 151.902352951251
169.211708954329 307.665281863258 169.311264420133 312.484289357493
ISL2 115.16087297104 112.449662536973 113.840813583258 117.81168010976
134.08270035062 131.679566454851 126.759554774012 130.10852948682
150.28169442832 119.862037840843 167.599784887933 139.459975305985
ISLR 537.419316887523 479.983368253935 362.252813848151 460.807117177285
144.139062513587 128.692917766138 120.300291760091 131.356437247149
141.781482381513 176.364184489318 135.159590925066 127.377356715337
ISLR2 148.247826830556 152.983678871668 136.109975252514 178.339211170442
130.886164807755 164.063104872636 129.46430369977 136.335679863251
146.828099826146 137.991642076355 131.876061826583 125.742631850732
ISM1 152.538048490265 165.108876136738 154.057771254252 156.787932134605
149.869314204354 153.711354087243 151.70917316194 162.090585987836
166.936975782665 163.053121879323 158.160604786093 151.532859494021
ISM2 142.675752131935 139.023486014525 146.129805607031 144.450810504499
124.885950473434 136.728975205893 153.83891040723 148.744599622165
149.55916919466 149.477373469387 149.707357499252 144.518132543976
ISOC1 440.745866155629 504.617795280805 503.59466398882 411.784922368377
656.867138022837 985.087150371964 1023.94850651125 1139.76755449089
627.335757721489 1080.02576143033 500.246996975 901.644194512187
ISOC2 317.244256751418 397.737702926328 219.996410908721 669.124699103132
1424.79466712859 381.625983354158 420.567884340705 259.935831886014
334.607533045929 199.490190715307 461.229315272354 309.5988292104
ISX 116.552626146967 115.840266274696 125.741289047045 110.296425545476
118.025603140924 122.204249636472 112.054379249781 119.459625817735
110.392617179163 114.43491928161 125.0723702213 127.945144368504

ISY1 348.98762709818 363.195371577097 458.151323130855 394.294057009443
273.550544349798 380.562212796792 400.180574613078 379.911829623463
405.766055310869 482.048928546145 391.911768848267 415.113340335205
ISYNA1 164.455111983634 202.00431685587 144.464783586439
153.393651104539 275.543576337502 405.843083758177 229.729195963667
223.453776362112 192.767155566493 133.733674016447 214.229228850802
305.325519265387
ITCH 322.506898833351 314.337680575998 320.597495751519 327.062810389635
248.03801051527 488.249967208882 362.095074331893 261.117845544884
212.933062534244 242.269784551092 295.935625475189 298.085365348496
ITFG1 331.848516303011 274.627303114477 343.488356185437 249.80160363144
357.514919818489 353.187829498591 428.850337327605 328.431289733169
358.732947927445 447.779854598556 355.80042210486 473.947538805454
ITFG2 375.856200488667 426.464641742092 437.489929507567 464.196512015649
415.642099109855 233.047541132048 354.205670284123 241.648044508699
559.559921312181 220.362946606131 477.275332763887 484.658140651032
ITFG3 919.165331064155 1282.84159765715 822.88742008931 2183.18692530644
1675.35431076885 509.01330998156 714.052063877292 682.538709448692
539.853394831917 410.731869154912 499.176926371542 448.260934224454
ITGA1 438.870464403215 372.024884481775 451.985108262982 495.655170027296
127.279107079261 137.460936879873 117.399753685854 143.303309656177
160.53152291979 187.812436252228 162.093325839949 132.419898122309
ITGA10 205.244843885276 214.261611197599 205.185206972064
231.572677616684 170.646233722767 190.348359536057 158.503284955019
196.245297362955 176.714471790643 158.33974431499 157.454558821261
182.263724979519
ITGA11 218.96845208513 199.53455266342 173.919504730673
199.662592246931 147.049287475127 143.539741029895 133.599098708818
149.927773276383 143.161623642178 195.230219990395 146.40033464957
141.246352591355
ITGA2 198.113398073968 215.585089158905 209.785886171295 170.825161347825
192.465510013851 273.402804140541 570.771089597761 338.585771590234
287.084253886676 869.245621105614 770.19255305977 924.630951485299
ITGA2B 134.060214479265 135.022438246099 154.324955232468
155.98413216883 146.672270660899 169.257035030405 160.243405030742
151.531667383273 147.592770523507 172.214433016685 142.752536416014
156.026806913759
ITGA3 304.937584086529 379.546197364027 302.408563830315 399.963640581639
437.931339781992 866.765054377214 656.32782132983 293.646275396425
481.86545778383 1008.43888926584 797.73902873919 988.297682407351
ITGA4 153.966815534288 140.774304412579 135.437227744668 148.776470607859
184.306452616482 178.790758444269 149.541917326637 162.488819873513
159.874161198304 161.338215647108 158.928949614338 166.97270256697
ITGA5 4800.99572384569 4486.35687688252 3073.40543048878 7969.0710242842
367.732191251547 325.570839576032 185.801540278924 219.192900411543
265.219444616245 980.67040349547 181.715992480635 244.162496497466
ITGA6 158.23884088767 175.469375624274 147.150063462728 144.501995579502
170.202597821861 209.811693771836 193.084818760857 209.398724613377
174.832495287192 303.986544460218 454.64556253724 170.111653122193
ITGA7 239.621756857095 227.418630852215 210.085329463347 332.219608862536
157.948814637726 167.547551072373 192.130214856642 171.494975393562
165.896420029269 184.801068553988 160.76044995335 188.378996128521

ITGA8 1218.05434595121 1034.2217095753 256.99019407912 774.442392607873
157.986640251147 223.638850960288 178.429953283621 184.862871179765
196.777776509371 157.221008210698 193.945494721138 173.513108409372
ITGA9 234.28146549561 204.942500423413 189.307823427362 280.398987330256
163.362182585182 201.49630027392 185.275787562938 184.322250984457
181.308852106289 190.057030596611 163.778790431064 171.521579011287
ITGAD 112.046615825307 117.703025026759 110.325803953516 114.358099210532
125.796438432905 103.448244268961 126.921925660996 116.486929280635
124.982744176925 128.168460083895 141.73799456072 116.89487516867
ITGAE 566.647927867646 522.77407098992 586.246004571452 461.229315272354
450.220658078322 789.401526545277 445.348044448953 850.562379837947
530.387315639095 537.06835963726 497.629319581786 739.52947559669
ITGAL 147.761965925044 145.556318940043 138.29167712246 131.846160346403
143.458937009197 146.944628763232 132.034701177759 168.974155750959
216.033564783918 132.50027119027 143.191095552619 137.070326499918
ITGAM 200.146843808423 188.36361073603 179.501659253141 181.3859695951
185.186497037695 156.096880332427 164.820007934887 166.592366578462
183.693300680495 176.267796612066 145.444483891989 136.227202534196
ITGAV 557.214020485204 528.014268424539 671.563789496889 441.873247266342
366.473258467746 1107.29687889378 564.749082802433 771.512332113075
497.916883763059 956.153239481189 1001.92763014266 724.607317443904
ITGAX 126.27024495428 124.452631766045 116.780639688465 121.643036773773
133.402515225624 125.299513989334 122.153996665065 132.233396667074
157.195967361907 149.480971898888 125.876114993687 126.359440164601
ITGB1 2307.82327595071 1951.69651954627 2036.43904731226 2112.84348677276
1687.85201395597 1576.21727854603 1418.60088387728 1067.80176606977
1077.33874251157 1474.70984232307 1733.96382719909 1634.20135156938
ITGB1BP1 548.686094748117 486.778046640624 646.117381960716
479.138177792954 507.56875910742 573.846138124455 624.467319041884
535.758943850734 473.260534978556 809.068563907145 467.512355593598
530.122419830951
ITGB1BP2 161.485353443949 179.358460170348 144.008961720971
173.017662861015 127.137447808551 135.842090777339 129.25124636593
138.316043447788 135.920830568556 136.824357247954 144.417333931582
151.538112896642
ITGB1BP3 134.774591679724 130.872296869843 122.452541100946
124.621381699241 129.958065173006 125.558877611499 136.728975205893
[truncated: 436,921 more chars]
