# Supplementary material for: Novel robust biomarkers for human bladder cancer based on activation of intracellular signaling pathways
Source: Oncotarget. 2014 Sep 16;5(19):9022–32. doi: 10.18632/oncotarget.2493 (PMC4253415; doi:10.18632/oncotarget.2493)
Supplement: Supplementary file 2 [file oncotarget-05-9022-s002.pdf]

SYMBOL	Tumour_X1.2.AVG_Signal	Tumour_X2.AVG_Signal	Tumour_X3.AVG_Signal	Tumour_X4.AVG_Signal	Tumour_X5.AVG_Signal	Tumour_X6.AVG_Signal	Tumour_X7.AVG_Signal	Tumour_X8.AVG_Signal	Tumour_X2.2.AVG_Signal	Normal_X10.AVG_Signal	Normal_X11.AVG_Signal	Normal_X12.AVG_Signal
7A5	42.2455707947223	37.2810767789306	39.6451483836089	35.314469458379	36.08956004634	37.9951997351435	36.9355798143571	36.3405039629059	35.6139301508335	36.4651112441576	41.0808257270221	42.1537476443423
A1BG	41.5421429082231	45.6948619492056	44.5134109184888	41.4727452533295	45.6895223823001	41.554942319225	40.8681871019812	43.0390857698452	45.2421183842044	42.2614524730856	41.7627910693483	46.4952206446229
A1CF	45.3872406432794	50.4872122867759	42.6981470312295	40.2427205947092	40.7176735294868	40.800866389165	42.7431359850339	41.599960574499	38.2795150354677	38.9271399577966	41.1367478979484	40.4639532172338
A26A1	41.483412492426	41.8848959280916	43.8153459269057	44.7401523707775	40.8873907150245	43.3269087731399	42.9803717121865	44.5658219748173	38.0213051227859	39.6719190054379	41.7272875295469	43.1805016690297
A26B1	40.0557788909923	35.9305494063137	44.9758339877851	38.3166554965434	38.9496515344837	43.1826328292558	40.1235656101329	44.0390019242862	38.9834959368496	43.1839109702714	37.6484782516569	39.1553169409075
A26C1B	45.3872406432794	40.148780650831	43.7630587657205	49.1786207920603	45.1137938810047	39.7394181011603	46.8613212048981	42.357910063876	40.4997186069494	43.9450023643394	42.5426057453993	45.8546719310325
A26C3	44.6231541342	41.5061991514748	44.7829632472754	48.7860245405122	46.2560238004757	44.8142634024197	45.8204185866107	44.0687697278114	50.9493788151501	46.0119912782855	42.2455707947223	45.3763275588341
A2BP1	40.018129854	41.433180957815	38.741573807882	39.2336402665402	39.8278057109141	39.1685416657025	41.2927600802729	37.3925854497082	36.4747777548802	42.447226754834	42.4580504587581	40.8277078176464
A2M	239.023368693467	227.567182783564	313.233036704221	270.202959012185	182.06733830953	396.167219131922	57.8870110456492	608.886783362316	56.7991853808475	369.717462266442	448.923538083456	300.412043023998
A2ML1	193.262615637464	45.1694246393676	45.8844325913247	44.1483976313959	43.004943518025	40.650742261428	199.404836798715	170.475212920685	81.7566533443175	48.8623729374733	58.7389869173495	43.3744503319109
A3GALT2	37.1485601150873	39.3667161654772	38.35720098134	37.8129870676592	41.3387790895716	40.5281496800812	43.9450023643394	39.0620487644635	40.8420925848403	39.8455060654531	39.7576136507593	41.9083581529276
A4GNT	33.2552842693684	41.7337506166619	40.4070046183108	32.9046688061235	36.8059561571675	44.6524308640784	37.0564573649223	41.0381860307445	42.5406888281114	40.6022003765153	38.5668610487033	48.1627097465883
AAAS	66.6547247910487	60.3093790894864	54.6820069269589	63.6334154351075	65.0605897122361	58.7404123614695	68.2708120569221	74.383456365584	71.787754116845	71.5032996449402	64.2174369755376	57.0524725494273
AACS	91.6826894933058	86.4898752526885	91.8533850418261	61.2213001749372	80.7321212028516	64.7905331739366	113.322929203297	78.9900928241723	145.607100979838	74.2379710965903	82.5225075943931	66.8026147385682
AACSL	42.1537476443423	41.159706131351	36.769073068743	49.9014504704021	38.9496515344837	43.4681646416585	44.4639405506967	44.9977782988116	36.5393857306744	41.5435611403119	42.8263096141654	42.1537476443423

AADAC 81.4269715766354 46.6996902348633 47.2962360458401 42.357910063876
348.65230953701 66.4015820814547 43.4544762830742 72.4834467838798
66.603126065489 45.7911932481722 65.5386366902998 49.2064066614865
AADACL1 95.1037791424727 80.5412889315011 70.6065558064758
165.121380058529 98.0143500562821 71.0197205018137 127.49459343016
136.190823415283 109.029196594263 117.586275152761 97.5534379346899
81.6458112867541
AADACL2 41.8409927585705 39.6995529770239 38.7745734037492
44.0451487108213 52.4370718282084 52.7156971154405 43.6753843488695
46.695848687694 45.5779686360641 47.7180816682562 42.7275315121163
60.5948704279864
AADACL3 42.0532901689036 39.7835513496873 42.0621425894624
46.4555908533915 40.3452320167286 37.1317006162938 43.6753843488695
43.844855336215 45.6582729799009 45.7911932481722 41.6336636285875
47.7962548556026
AADACL4 41.5421429082231 41.159706131351 37.8386378737561
51.3851157261451 32.8704945047196 41.6590564937735 33.2425023465738
32.229154316188 35.1155486926999 43.0919221375514 32.775958132716
37.5556299264699
AADAT 112.591529656786 90.1284518302601 63.0670482657086 43.3497340062963
91.1674392930267 55.9351706773613 80.7168944828052 56.6135586098886
64.5598765457963 66.3230625217466 56.8056093987178 56.1404433602127
AAK1 44.7044140979108 51.7495306734657 52.6275131390249 46.1148643659988
53.4109882045447 47.428201097025 43.0177448037924 63.9851732498913
44.0383630714326 45.3872406432794 54.4825201506931 54.1090536870282
AAMP 122.495387796233 95.39134217438 104.037949992458 101.250422688816
125.052841200703 115.044790975532 101.716697533604 128.996351661564
116.949813289388 116.311176407088 133.549684980003 118.696127260286
AANAT 41.1523487516364 43.3458769998118 39.7411455670172 37.7469621225553
36.4434672195785 33.6859433472087 37.9430145836712 37.1317006162938
38.1169703906207 40.5090849993014 31.9682164860619 36.064718696392
AARS 799.803676957263 883.06773454117 1279.02702402372 996.520340280883
742.726253630865 1060.64261592822 1212.61529575798 863.082385199922
1457.72260892651 1043.26325848938 1068.10627239579 886.75789054784
AARS2 97.3929166450379 86.277692569799 65.9476039189301 89.4504034432321
107.294098632695 76.0240426902567 84.7782034884529 76.4637616585578
101.81885073944 89.6670947876322 92.7667038784226 82.4362183543935
AARSD1 189.267281390459 135.836577310513 179.694413858411
169.144145421022 237.161912299581 203.178221073902 222.01145815859
227.791956085471 227.439319520245 249.679512440891 272.604402990069
210.255784687052
AASDH 58.4920021605316 90.8550873130713 91.3890908971311 66.0397471691747
126.088821850066 85.6038071277319 86.0796981361148 71.6202385170491
77.1767494799711 120.692330556005 108.407179111715 90.0942781313264
AASDHPPT 97.9401338178996 108.806649898122 127.789065386943
72.1401612718245 129.179537795204 102.282066142307 89.9929484069346
96.7280766051052 95.8446272966902 84.5411149237799 106.654782265295
115.861269747317
AASS 49.9718538204798 47.6914221838216 51.5822637582617 44.3638402537972
61.3851695159988 63.1091746640396 46.0817481577316 49.5185112777773
69.2685201884906 54.4825201506931 58.0295642638519 55.1109784383525
AATF 336.737571879156 298.848001894468 304.718140992378 377.221676837377
357.507822825765 404.599730419192 644.924175130155 399.027079798213
486.044304392285 351.439404852971 335.881117053472 244.529657015078

AATK 44.4581200816365 43.8163035242859 41.0705134231814 41.5464765641928
38.8964578286293 40.9506062495231 44.6032218589199 45.7030835309976
40.2669497398702 41.7877325563821 39.8617019260946 40.2250934110735
ABAT 38.385865183581 45.7562276914441 46.9040704638073 43.2427390263564
43.4812166409346 39.5637454855233 42.6981470312295 41.2551966132486
44.7058233759912 47.7779356299965 45.5106666933411 41.800198520267
ABCA1 420.761600667193 281.452208033175 158.702003835578 752.837247653926
306.115856873599 582.511224532378 263.706801812277 440.728435508514
240.139560726101 619.950241162173 361.861166927682 315.186937216422
ABCA10 44.1737502492846 46.6598879466655 49.3000844711116
41.3567204694281 44.0977148249302 41.5132248356707 44.7255207589606
41.5934628038429 45.2897806651929 37.2686490268265 49.1067959632109
43.4709415392696
ABCA11 42.0532901689036 50.4872122867759 41.9666541498638
37.5382094267692 51.3511088648386 54.5952754712007 38.8417151851134
41.3506832276112 49.5539622682049 48.1392888470269 49.1067959632109
44.7401523707775
ABCA12 45.7356815244513 41.8073910693411 38.8775560911841
40.0322420615846 45.1137938810047 38.385865183581 38.5474297403025
41.0844534776541 43.7078399718247 42.8414096678708 38.0365987358005
40.2237056928605
ABCA13 41.8409927585705 43.2649781354792 41.1367478979484
42.0102671859606 46.6397391251762 40.244271403831 41.8315602975157
43.6434576713809 53.4604353973381 42.1622837416589 39.3667161654772
34.9138625012006
ABCA2 49.9419897484381 51.909968572392 45.2640606138546 49.6479308322777
41.7929491793697 43.5752393599169 39.5496902678788 44.5029690493211
41.074824727477 46.6598879466655 50.1649089793069 48.9088046212958
ABCA3 41.0608773166058 46.9919960628953 38.6727842796202 46.1673159982664
40.2449343247539 52.3187919317561 41.706879587091 48.87875862356
38.7298222155532 55.4037482627222 54.6089451252049 50.379587451669
ABCA4 69.3418521417182 66.0147097736612 70.9377012403023 71.5249259772583
79.1039287828151 60.877229406755 79.7564155881026 65.3019328668633
65.1424206085551 63.9129423266349 53.2083147523758 59.7999569757275
ABCA5 39.5676051939342 41.5061991514748 40.7620472083159 39.3513480332078
36.8428706077385 40.800866389165 39.5496902678788 43.5738557773903
42.7150227340364 49.5839230147011 46.4753015651202 45.1735152121056
ABCA6 45.2875173643252 68.3864191351179 139.328628553223 65.524821699432
52.2089306840125 188.008141649466 61.7572675712557 44.2527566343661
48.0163377492228 120.943323740458 130.524308471577 229.379918828848
ABCA7 64.3502363726659 52.3660712483179 58.7992572743257 74.9661773375536
75.2977438265751 44.931941140132 65.7873990226533 72.7934987008399
65.6810122794645 60.391458857108 59.8070012006708 59.2659299592508
ABCA8 40.6062943725118 46.6314009405034 44.7829632472754 46.546116650292
133.244526666651 200.41782363596 43.8206425661746 59.9682734755814
45.1569741609597 116.891649452816 139.294987640071 98.5663841503803
ABCA9 39.3709357615493 51.0175677516029 66.2167164788023 43.6726153508978
55.6069214673486 97.8915084053153 40.6750540874299 40.5158867964016
44.7771939752086 49.5265949137207 55.1099824417989 54.2746621762462
ABCB1 41.9918605927928 54.9821263631962 55.5567699051046 75.1163398247735
44.5092684716474 57.8944021095265 45.0283997415478 58.676230030835
42.846832824953 54.3554233228353 66.7106881373359 54.5921287593554
ABCB10 57.0006212637829 86.1924491308234 126.578693258328
71.9062462001972 92.5135737873857 89.9789975503195 98.0023132593711

61.016921238986 77.9978216366573 89.3011777476649 82.2519263268941
118.044483475719
ABCB11 48.8941062980393 41.9894412416874 41.6977706798234
45.7397504535169 47.5647821785529 44.6524308640784 44.4639405506967
42.2577689927563 44.5303781239209 49.3000844711116 44.7926323492519
43.5738557773903
ABCB4 40.4639532172338 42.8542642792862 41.8751578329365 48.1662933382789
37.1565984182084 40.5281496800812 38.4005795891396 41.5464765641928
42.8036731479932 53.9412154719864 41.9083581529276 41.7575097298927
ABCB5 41.2424637338424 37.5469247328848 37.2838080935695 36.0481934423077
41.0885562160946 45.6129956328415 35.3362091871364 37.33030683349
42.6366765186182 37.5343260962758 35.3362091871364 38.3291713594599
ABCB6 100.663054866186 141.319488024794 136.125236142909 72.0694688774804
195.127419251015 127.701556328274 105.945216149549 133.211237782894
75.4572860692649 312.154320305976 232.628747594691 167.639975497722
ABCB7 86.6655289455628 69.4820178305563 61.356483470053 92.6230008876347
62.1836961153924 87.6507489853626 74.0039764902057 106.78552222475
80.2680308979464 99.548445157506 112.881899194726 101.627551729492
ABCB8 47.0940613507321 48.5905518770847 44.6032218589199 50.2163892855092
49.1928206164702 43.2805590023412 42.7431359850339 44.4031868028216
58.2401724760744 41.1222709786153 47.2134341420411 45.0985088502363
ABCB9 109.832638804839 71.599955759893 95.2367177680328 66.224559122062
79.2185250088688 55.1037284831588 100.499763255098 74.2517888452553
141.594428857706 58.0218262176065 52.8190882379316 58.174555206394
ABCC1 59.6008106247114 53.3974326903004 66.4079972657486 52.7787010032051
60.3768221891131 53.6858208094027 48.4099370357307 52.6960621424156
67.8688083153991 49.8393069675192 55.7868713984479 54.1440452292081
ABCC10 70.9419509323007 56.3560498705517 60.9621846036092
56.9299697426319 63.5932443357808 49.9699131196797 60.7992483126292
53.9465584609317 56.0496524902806 68.6788460999238 66.8842530423095
55.3563533836982
ABCC11 41.8890826572422 44.3236524597291 47.834436078111
46.1148643659988 42.9775348174342 46.0867976914262 43.1696495767273
45.7476095696859 44.8997235283254 44.7958122466628 40.4057097917487
42.357910063876
ABCC12 48.1952521496489 52.0268699306396 47.0055981216687
44.550322123994 60.5290966885203 50.9724155054821 49.3854238365325
47.7889447429963 51.3511088648386 52.5522545249206 43.5710871617107
49.6629259325705
ABCC13 44.7915315085948 48.8842909466002 47.5673419710251
45.0798408927912 47.0533794594844 42.157627240486 46.0905079010237
44.3550497419584 45.8803291099765 43.4885245598939 48.0901896064056
45.4799270968969
ABCC2 47.1962846565261 45.4143854024453 46.3411353377615 64.0903425729408
43.3332476888305 48.3686935595826 54.8074655158941 45.1714698794331
43.8482802038826 47.0055981216687 45.2265319826548 40.5703581862752
ABCC3 384.280434953152 574.467486140819 1226.43929953115 129.005213737493
268.309382865636 61.9432904657734 113.198141130994 270.662475158799
234.778900405354 647.868022835702 595.790464730847 911.661341702443
ABCC4 71.2963721541702 113.866742186841 59.1533197797534 90.1632837282491
81.8837844326824 83.6351558609087 66.8475050031365 81.3978207813439
92.0711167984454 162.955976144315 148.32293918881 312.015358620043

ABCC5	159.554916039519	238.893145911331	522.125691279772	82.5279746006378
	253.527553472516	94.8556191290544	120.721482177994	167.47269732721
	239.569514501529	570.824732318332	349.90835241442	252.870708814507
ABCC6	42.6575535390785	38.861780967827	38.3541523698926	39.5558610526094
	39.3133871964036	41.8871259674102	40.1572444265517	42.0847226073592
	39.3592387366314	40.4488698263442	41.6336636285875	39.6938066205531
ABCC8	39.5676051939342	34.7135536792489	41.6079958749402	38.6414316877471
	39.1650142708257	43.5752393599169	35.9894133432376	43.4544762830742
	39.4762546236449	41.7405682794166	40.547835538778	34.806104801006
ABCC9	43.3085358214154	42.7303139017786	40.8860076320216	40.9157352260901
	40.0266778690352	48.6621527194963	39.6222000738266	41.599960574499
	41.3481355128089	44.3046896365956	44.4688999098904	42.6758568421347
ABCD1	102.25509551881	64.5347406608537	67.895735332714	100.168016285684
	79.5175547131776	80.3805507705979	92.8579489575841	113.444217634165
	71.1894129030602	94.8742116922003	84.607833108004	62.8485724004124
ABCD2	42.7150227340364	39.3667161654772	39.8656165405284	39.6292788039793
	37.8343741244069	40.4359177791566	38.7337086062875	39.7509165247419
	41.863927676552	37.920964499903	43.9802831041664	39.5748670524951
ABCD3	78.8357122967692	87.1434190022564	83.8679756709676	62.3936376596431
	90.6661301391451	67.9306669919176	108.263706890599	102.380096190518
	101.954354107731	63.6197695952215	82.0206083292981	78.3467096982408
ABCD4	45.8676005470521	45.2960223240174	42.02667925031	42.5321207892864
	41.1575777497576	41.4219820984507	40.9607305725196	41.989786971805
	41.4435352858263	44.619512785899	42.5864346233886	41.40680865944
ABCE1	120.96329303892	257.050603883498	176.785214225094	144.427680369554
	374.427492246826	187.422036538629	144.711001798454	183.887843429281
	238.120731107691	136.140128421585	143.913007288353	191.298525555624
ABCF1	572.19538884395	370.027120412227	373.802562320876	364.962649416567
	546.80979198725	346.116601612353	416.40156274382	354.015808474189
	373.330920144642	347.750020791938	458.47341225284	380.015934356432
ABCF2	76.1417635449123	89.8808683919332	82.0149542477384	69.8314582428552
	148.758736522703	92.2447473381285	84.8569316561572	69.1973371867031
	104.754762973117	69.8941497035747	85.2598847559951	80.2289541115146
ABCF3	80.0113001016496	66.7256057011273	95.7820879892969	67.1961800572759
	124.22494803053	66.8632294055679	86.7968607218397	62.5528124447176
	83.8679756709676	106.317388820963	80.4895102121889	72.5346469941833
ABCG1	49.4878464533902	55.6904617926772	89.6115806694241	71.4473248804357
	48.0901896064056	48.6957875504748	49.8281348927244	54.571866945312
	51.7098181032862	61.2153068100932	55.3415844848806	59.1570467228834
ABCG2	36.4564050256915	45.3486182278177	51.6159542017038	39.066284740632
	45.1137938810047	44.6524308640784	36.8369172900902	34.8949555140527
	38.5252456306656	44.7958122466628	53.4921736109774	47.6040671292013
ABCG4	43.2724556187214	40.3384318592985	39.1799070031819	39.7411455670172
	44.3557408297309	44.3648993568405	53.8687221233237	45.5581356216957
	46.4164127487052	51.0055825931636	42.7275315121163	43.1805016690297
ABCG5	47.0940613507321	50.3586212130973	43.9450023643394	50.8567345921473
	66.5187917407182	43.6670755115848	48.6395795163502	48.7814131120945
	51.9545473262296	55.6865353018811	47.0940613507321	52.2832912331043
ABCG8	41.3387790895716	46.8954576545929	45.4865173433646	45.5869891570251
	39.1650142708257	38.5252456306656	43.9450023643394	45.7476095696859
	38.3418933808844	45.9004543328976	40.9157352260901	44.6484005362281
ABHD1	41.4508241771315	34.5023764006976	35.8265993915844	40.1804147506786
	48.0234896130991	39.7394181011603	37.0564573649223	54.1731035633439
	42.5406888281114	42.785429206422	43.0016969050339	40.2669497398702

ABHD10 108.022329297132 89.3342384381684 74.3523005665671
94.5501397951415 122.824055084598 111.096799408676 99.9896394343368
90.500550952567 79.2824265044758 131.973056760893 102.706563820232
103.458867136383
ABHD11 81.6233038764401 76.6094442444074 74.5654028199926
47.9915414951747 89.8957070711076 64.9498081060676 78.7320333941149
60.747393219527 105.914573205761 47.2299678280724 53.5453975444568
53.0216110762751
ABHD12 259.481036467571 400.633619994399 155.392743674792
187.002948435359 177.111143479957 110.487281983469 451.820169649522
247.074756460224 199.776860110557 294.7772562683 221.325534445978
178.4681248137
ABHD12B 46.263826584899 41.9894412416874 40.4412246793572
42.5054997771887 41.6285300488826 41.5146360101556 43.607701594929
41.2040352443154 40.6957787921451 41.3752447964111 40.0440633413412
40.3330033416496
ABHD13 38.6213341190836 41.1212129940704 43.0602678767669
41.0219290963778 43.3896803974186 40.8091178323174 43.6025443656008
39.4710743516517 44.7752571196612 40.2615278292875 39.4410515050885
43.1805016690297
ABHD14A 58.651329686676 179.605537867711 160.883501367581
103.530430972911 319.092568066532 202.708232693973 128.391264770998
106.292775688671 225.589127792587 167.319747725488 181.545493080363
112.944044862693
ABHD14B 84.2728210732532 113.929486370331 105.814959179397
95.1423940686828 94.952392592003 109.943883439481 122.118080859526
122.143519601825 165.093395508014 219.186221756668 186.195424728755
126.115065200084
ABHD2 53.9612654850046 50.0425298550559 53.4413436510999 65.2107270580361
49.5210706664792 50.0352509660599 45.7452456977857 60.1444334141838
50.6750785584772 48.9826868640753 48.2050207071554 50.9339938653926
ABHD3 77.9057333780847 111.522812019895 116.949813289388 142.048367760597
86.8871118411414 72.5482509922085 78.3082773071531 64.8336014343435
149.231260844854 55.9283039581662 68.9745034946434 99.3956523785983
ABHD4 168.036319777283 53.812632592983 53.3127618245672 83.014860007187
46.3150221024046 84.1922656126304 88.0664425843955 89.5130163419167
78.6048653630165 102.294142438313 93.2484615907966 107.799236230743
ABHD5 80.5437011468815 164.735191308776 122.748811531184 332.659496278775
159.540626405 93.2630594602634 114.526825145076 150.649377863375
111.291087721139 71.7464249053513 77.2834482217973 101.699631771662
ABHD6 50.6473771879221 63.8399092891728 68.4720753153522 93.7782490229256
71.1607965028029 73.1169003714219 74.1353964055485 66.211494935811
89.7862835647016 75.0665411373616 84.2960453806012 73.408861359425
ABHD7 43.4626049853157 43.5182502375207 42.3536549114165 42.1273928203021
39.7568367479301 46.8954576545929 37.2892641289258 47.2864663693787
41.3803269429468 41.8613406887824 40.3452320167286 37.9607839934735
ABHD8 147.187118389181 107.094663242259 114.611557317665 164.917385239962
195.512986297442 170.223657280542 168.91210897306 204.862161704394
132.57180783668 169.96123729479 186.477689350702 142.251426773121
ABHD9 86.8262463672891 71.4197504261453 102.607924735378 39.3750969348308
229.881790983135 69.0530953932111 101.716697533604 48.1591264215184
43.8482802038826 56.057906531592 54.7883681830995 52.6922486991742

ABI1 63.6222532132037 67.4984616497574 74.2769881586739 75.9099205260748
58.8188963227059 65.3096524773304 58.8361533901971 62.0553450433433
71.4864243594056 55.9283039581662 70.1597323687098 80.1809075683154
ABI2 62.3042205863409 58.7992572743257 74.3523005665671 51.5913529948887
78.258486366912 64.5187138759372 65.5800543140431 77.720889005668
64.4837166864084 58.5831897269778 58.3912728421768 67.3779879510397
ABI3 55.1306642449627 63.2804984998739 55.6865353018811 90.5216273844682
49.9493230064613 49.9699131196797 41.8315602975157 49.2019743609873
48.5792058061372 57.4665857273093 57.5430365699386 42.1537476443423
ABI3BP 62.7669079605627 66.7256057011273 49.8344973836834
155.564141715819 35.6434124419782 347.196922795214 37.0564573649223
136.650986373497 42.5406888281114 177.047048502104 99.3744103955071
188.513581321162
ABL1 188.943404504867 134.537040608687 81.7967230859562 116.434267173815
128.844013736935 294.090159716249 140.866324039667 138.618449365819
111.438294720953 143.822560762049 168.17913316957 166.036790614812
ABL2 70.4732736918246 49.2050538150106 52.1132472786412 92.526038865874
50.1213497293778 61.8757975011721 45.5683029249676 63.8694991450134
47.0027453663546 46.3838120599769 50.3745056312199 51.5437850416773
ABLIM1 93.8968899420039 402.978328664331 398.43592055172
134.17280153232 179.158053533144 536.290060852815 302.20349084931
175.652989203497 286.927016665685 551.304967553891 559.296669575666
493.705301673753
ABLIM2 47.2862299990938 47.087835967892 45.7968648044613
51.949834638817 49.1928206164702 53.6858208094027 44.4639405506967
57.2350830635912 37.6383530120621 51.5855515705917 76.8713035584472
53.5643281930979
ABLIM3 51.6558970571712 54.0461131287999 94.3953345963359
74.2429851177794 54.0126916508417 98.2843999216669 60.2399886747451
65.4714272652857 70.9462008788871 131.471030729546 97.2615918872914
101.156215957297
ABO 42.7150227340364 43.0227387991135 46.5650556481721 38.4208429103799
39.7568367479301 37.3799542961839 39.5496902678788 35.3608605269276
39.1790517315259 43.1839109702714 42.911855821602 44.8372404608604
ABP1 44.7044140979108 44.9370339275041 45.2313723727673 58.3475983630509
46.3150221024046 42.9695585740813 44.0687697278114 44.293922970134
46.4861258030402 42.1622837416589 36.7283081422707 39.6352613956998
ABR 129.605603092944 181.705809767061 203.53267380432 179.214713709599
139.472956453414 113.179313022693 101.16781803884 110.379403044901
99.7361599442168 197.52820443061 176.963666283432 171.360248172138
ABRA 41.3387790895716 44.6918600441328 43.0016969050339 46.614092935749
44.3557408297309 42.357910063876 50.3519150282938 34.1806289878747
41.074824727477 45.0155543555707 42.5426057453993 40.1700117452801
ABT1 59.8248482438562 54.0052948715814 55.4502804236424 47.3574589211166
75.4652763126449 56.1671440556142 108.147548794301 51.6660481331154
46.4164127487052 49.7259371799014 52.104880481326 46.2625470410195
ABTB1 77.6056319020116 127.741016067967 91.7844162703214 216.063990451376
64.5531442955004 133.074560812703 107.951009163316 80.7067505750848
71.3197554517106 125.661351053514 129.810477623628 97.3929166450379
ABTB2 50.1533437545239 37.4540772310118 46.2625470410195 46.0228732507872
44.8897737954573 47.7556758818053 43.1931731665131 56.1404433602127
47.5564423692142 50.8826620053386 52.5930185400362 58.112348043634

ACAA1 150.031554835542 494.162646055732 581.706970365754 226.057278804077
1127.96214301065 297.701126167694 365.517719684028 231.685652845234
376.289659542649 339.767107648884 329.120125961774 403.92758879201
ACAA2 86.8262463672891 127.043177339038 61.5824518349507 116.122324921267
46.5557478175976 124.751020763508 212.664574366477 148.595294991115
70.0801020070064 89.9284109077375 92.2772139468085 90.8140983242733
ACACA 241.31366702947 180.844663304872 162.447872391923 86.8596934326046
295.69493803986 205.449627031002 320.13475752314 249.776078532596
357.182869779274 234.615033303459 223.040734249612 255.365088438518
ACACB 83.2737524877952 79.271901834289 99.4546022538476 81.1189042503645
83.2652824564032 452.883337614277 75.4053820110486 104.950400251642
72.8653205184014 370.779448406233 348.206465194205 151.641746442323
ACAD10 69.0975200641705 94.1289383735162 82.5344501505114
65.084620579901 128.38299874379 70.2553917409773 108.007227281411
59.220967994972 128.70380102034 100.413031488199 112.644426507762
74.4365426051588
ACAD11 205.086228002776 151.936097714553 226.926786963011
152.545682001987 187.580695643466 177.688415930665 77.4046818339539
152.76005226611 102.706563820232 209.214729757636 176.557322629245
206.146732318529
ACAD8 114.888828460086 86.0630164348952 132.036058401203 88.6717761948382
121.552188287725 81.9983163893373 75.5034623354897 97.8764110456132
86.4975836583091 91.9185612536409 85.4635831413678 100.386011517899
ACAD9 211.926421068313 295.473526251997 259.456935785521 183.613579501306
377.221676837377 202.01900875263 358.63899765183 263.44529683749
232.591262307238 211.253227661874 236.536058101486 172.95999061341
ACADL 42.9104454767916 41.2598458726002 37.6557714476853 42.4580504587581
39.7568367479301 46.263826584899 44.3440839686558 42.5435283762933
54.0192060888782 43.4885245598939 42.0750889188255 42.1537476443423
ACADM 193.853749625939 307.550936236018 302.667538432677 258.375785708747
195.018775118528 351.439404852971 343.990395610093 250.41086379231
153.059039745191 299.74710723735 311.901423694475 400.093769705765
ACADS 81.8104679118753 86.7710020879725 95.7820879892969 76.0975301977715
83.764882413379 105.197692490363 100.289042948128 82.9322141700549
125.383837024985 114.796237914478 121.936597605894 92.8006484232438
ACADSB 38.2083710281545 66.8693882433359 55.4502804236424
46.8954576545929 46.3150221024046 47.8636003588181 44.4639405506967
46.4672637826582 43.8482802038826 56.8201201798712 52.5930185400362
49.4786466722535
ACADVL 861.427193617946 1149.90692951795 1676.22158135012
550.861615347663 808.857027942253 638.305455110267 900.517711546397
720.552354470517 687.011162822211 1357.41745370754 1704.0522304387
1169.14891282789
ACAN 37.8386378737561 41.554942319225 41.9666541498638 40.9607305725196
36.6354013629791 33.2552842693684 40.1235656101329 40.6171774724171
40.9901805728485 40.1179210534118 36.8282383070068 38.4330755397458
ACAP1 78.0020909283414 98.9988668602468 120.289145237975 305.619340338331
43.1252272136667 60.2083550387718 65.1041577962151 75.3462586192013
52.7512110769042 64.3383155834968 94.5111909776371 49.9692385479389
ACAP3 50.379587451669 77.0927030014721 63.2475738480323 41.1886975241176
64.4999048115671 49.8472708021525 51.4349390827895 59.9968592852122
54.2825877218965 50.5252048155459 48.8744644207821 47.6938628797168

ACAT1	224.202855268674	212.187855950414	144.609635554528	301.198565023795
	277.284338888467	966.803172864162	264.600870859012	423.608694638779
	232.911951245618	339.767107648884	266.250887288858	363.324816966828
ACAT2	109.795084617739	133.040933717958	186.655552987116	156.476627024647
	131.57403028894	94.952392592003	190.647389246848	106.140215438654
	207.957319374194	70.2888664894537	78.59966540743	106.99628036922
ACBD3	344.061882582662	280.622184239624	313.233036704221	311.985010273921
	281.602712448925	206.072527867605	280.563427715895	293.755623192857
	252.583117397109	268.072653271299	188.042978085327	270.78959225422
ACBD4	40.6559419616644	46.0023068797812	41.528370959196	41.3027031037343
	42.1650127805175	44.1737502492846	36.9355798143571	43.844855336215
	36.4476883897928	50.7855742231932	45.390192977585	41.2437378488025
ACBD5	40.1145011765998	43.9852350008246	39.8299340037009	44.1144663373989
	45.1137938810047	43.7218212419823	40.2154497375715	38.745552403895
	41.9635525160083	36.9102644736882	39.0156305603334	42.3214282169749
ACBD6	131.483899499125	132.098298410135	156.770274308736	112.957927208627
	176.670909065664	170.560304063093	170.501116810593	162.833785414988
	176.816741656659	147.945429978177	131.216899549608	129.938114164087
ACBD7	40.2543341073001	38.1811246394237	41.785676764591	38.9074189954657
	40.6723334103791	40.9506062495231	74.7534423194688	63.9110569727542
	61.7559532093027	47.9056710878325	44.3763859390287	44.2527566343661
ACCN1	43.4100411978366	43.896638326678	40.5386195997545	41.5464765641928
	38.7004513165111	38.5707420487455	39.3585350448719	44.6560353921013
	40.9460396298042	41.9097689475185	40.6373400186325	43.8575940017119
ACCN2	44.2350046179299	43.826317180678	42.4890076400051	44.7401523707775
	70.1238785863433	51.9421032049606	44.1407835440612	43.0390857698452
	49.2879849674517	49.461728580617	48.6494451061637	43.5420658412177
ACCN3	47.1216490839161	51.1740855632044	42.9104454767916	47.0874209746031
	55.3100442506299	52.0299937917925	46.4388841354122	50.9099078696349
	54.1745577382276	51.0055825931636	49.2879849674517	54.6659845741133
ACCN4	42.2651653674882	43.0829701603126	40.8860076320216	39.853175885492
	38.0184648502986	36.8059561571675	41.8887298788489	42.157627240486
	43.4125951053517	36.9795961564828	39.9519547348106	38.3757040629343
ACCN5	39.8662795144616	38.1312950900467	40.4070046183108	39.2780515621926
	36.6354013629791	32.771863438151	35.1307934330047	38.4540849632254
	40.4997186069494	37.2089297656361	34.7308008643142	42.7782216342806
ACD	122.849949163123	119.512426152446	145.607100979838	124.803597244786
	99.4458090623294	136.711122812062	151.135296427249	141.48637125542
	115.328304312071	135.824520574227	127.49459343016	107.693162310852
ACE	40.0124413244394	39.3681232503406	38.6005272730664	45.4634652319753
	40.148780650831	41.628211174823	40.9157352260901	38.9229928131225
	38.9444001301141	39.9948390976317	39.2542963855807	39.8497227447888
ACE2	66.6547247910487	48.3077814647455	50.0748292497578	38.9534635879195
	41.3755760434934	42.5660695551615	49.2546642180676	43.2551508830106
	58.6431245059655	51.4454142248566	49.3901004211918	49.904979214379
ACHE	42.668311948721	45.1694246393676	42.2577689927563	43.3554905202712
	39.5317722761104	44.6085311049673	42.0016830644258	45.3872406432794
	42.1594905198929	39.6333888530279	44.4408504417009	42.4322338033273
ACIN1	444.858305070415	312.923326422701	264.161390133378	270.394857925086
	268.683528469759	220.79051545138	279.841798764418	308.393270347486
	324.994911416877	435.478616005848	444.471597593258	285.56460901579
ACLY	700.477686061791	584.724083441601	548.601467852543	370.495645380436
	841.316771679857	324.126628444078	593.345148568451	595.012422454829
	719.815240381415	319.871452890512	385.827237353675	456.467726693355

ACMSD 61.7551416187554 54.7083331985678 69.4047473173218 57.8277957325085
65.3019328668633 58.4435847520801 53.3111230705754 59.220967994972
70.4262478188233 72.6549171886783 64.9959712888709 64.4156641487994
ACN9 77.441946514938 129.925137664962 103.233768723364 114.979124960177
243.843941346211 120.789309915619 123.674164545425 112.506613718654
208.915145509698 83.946822476147 85.6989061660348 128.717988447086
ACO1 790.094848284547 480.386431986343 352.246108891022 595.012422454829
408.991719312986 775.931494648677 661.718946652592 625.435083064752
509.879213331349 577.276184555955 823.933615182634 690.568858101531
ACO2 123.138085708441 185.72957253374 146.42128243217 156.476627024647
158.853244260329 169.18132209076 159.835690229505 157.543887960599
194.644164648125 125.512567429299 166.349012388957 164.780216383719
ACOT1 74.3135387703106 51.2776048338 54.0536858212253 59.3618843678775
46.572043843509 76.889690218685 51.8667932650345 40.831207953647
93.3718038663074 47.9056710878325 60.2509851471485 60.5548506034677
ACOT11 164.097531361519 183.286063142727 217.39432336811
64.8154690917175 82.2348885628237 53.63657997649 173.08677862231
141.423857667761 151.641746442323 230.975122271137 244.678323059428
186.161177110898
ACOT12 40.6559419616644 40.716553247236 38.6727842796202
40.4359177791566 43.3332476888305 42.2577689927563 37.0564573649223
43.1567024035183 44.0519245668737 51.4454142248566 50.0303076717704
41.2437378488025
ACOT2 109.137372445043 71.0645521832919 88.9928368134295 54.2886483704949
67.0163226819964 112.039388952208 66.6118713143653 45.1151758109609
84.6158251687884 52.6879797806876 56.8436081694537 78.1623301184313
ACOT4 252.170412162149 43.9421822132391 53.2046867327331 103.961956810572
117.260454792593 59.0686629877982 139.177720567217 60.419911425109
157.334378544568 55.6069214673486 57.7786197537899 66.6118713143653
ACOT6 45.7356815244513 47.087835967892 39.4710743516517 40.9607305725196
36.6354013629791 38.5252456306656 39.3585350448719 38.6454098044474
38.0213051227859 44.4652605017595 32.9879066304034 38.3291713594599
ACOT7 132.3192754634 102.706563820232 85.4960014582244 113.026772174726
106.049736669661 89.6115806694241 159.485978728 226.804294585045
80.3142559621726 99.7361599442168 92.7254735358203 96.9051967257119
ACOT8 45.8108990837624 64.8903340853337 70.7238101239421 63.4531374125233
54.4586176399381 52.2089306840125 77.9028721785469 57.9713749600418
75.4572860692649 47.8207974930031 62.105802634526 60.5948704279864
ACOT9 93.2557602398938 70.5534237141767 77.2048580540589 157.095926797414
76.8393340617473 122.099297248392 97.4515987591398 87.275548276476
72.0334593545916 62.4530276414691 63.8294437579951 96.3232257371077
ACOX1 53.8184424596644 73.1640951333742 76.4116336410131 52.8502823653368
64.0752803796842 53.3185097662527 67.0455170947389 99.5192591352063
72.6888636455541 62.7669079605627 70.2565873027242 75.4317740345967
ACOX2 70.3757673501097 48.9899488302637 46.2625470410195 80.5537984388921
50.8875036861067 300.121875447445 36.5352572191451 54.0507720792049
41.4636047919453 158.853244260329 135.012695962236 116.475941571644
ACOX3 124.22494803053 225.794026573235 269.284584971871 150.082905944963
177.620987812591 147.557451530784 344.133384411423 190.61997068475
198.497745548245 188.533240079423 238.893145911331 156.311985997533
ACOXL 44.2350046179299 49.4982025424177 41.785676764591 41.1886975241176
45.4751076037511 36.0306975524309 50.5252048155459 59.8760452220989
45.4799270968969 59.5785089386245 48.7502645382856 66.0786923699684

ACP1 712.80113322987 494.733185937057 422.237766234202 351.365159217524
793.570993808328 443.863346367342 304.41727337972 380.541502795438
458.47341225284 148.71330612986 197.617554897454 376.289659542649
ACP2 118.614027673431 91.9238405195899 123.729687262037 172.102568448455
83.9387217886961 104.587564056763 91.0154871536701 104.232196493024
134.152059562322 106.219480656782 144.928534157065 116.434267173815
ACP5 141.649461526745 115.519896726673 79.7733480606334 794.806085783439
97.1271615802556 85.0275638433103 110.589130460258 139.177720567217
59.2566572428546 63.7002949745101 62.0404348860702 64.4156641487994
ACP6 71.1521292570086 132.946528291039 239.385229462309 80.5537984388921
174.64729344609 80.7067505750848 214.873872837015 643.218735985767
298.200087139598 173.5606219477 158.590601962928 123.536432289877
ACPL2 132.684895918889 97.4709987978999 131.632115558243 74.4365426051588
204.774090707986 113.287380238064 125.997088252817 153.812414753676
219.771890732457 86.4114854104675 99.0647786948132 182.885934774318
ACPP 49.2595762436995 52.502055890816 43.7630587657205 40.4359177791566
40.9950639693255 41.9543948167375 51.0013344286851 39.6474008369935
50.2223403889892 45.4614234442527 46.4753015651202 41.863927676552
ACPT 62.928986708012 54.437526296816 53.7792717770978 68.4261608261522
60.2276476399612 60.877229406755 61.1022920882065 60.5571721986885
55.4030910454028 59.0630473216622 55.7868713984479 54.400905818898
ACR 43.4626049853157 42.6939394537076 37.8386378737561 40.5484717678957
40.6723334103791 48.4758590019384 40.1235656101329 42.4580504587581
46.581796165231 39.3698811593364 42.0750889188255 37.5556299264699
ACRBP 42.1537476443423 48.4980626712175 40.4070046183108 61.3747706295648
44.7829632472754 44.6524308640784 60.5125774827425 44.1311654985682
41.4636047919453 49.0841460667818 45.9104478407175 46.7182294568603
ACRC 57.0006212637829 46.0023068797812 49.3780606970538 55.2214187655851
48.6224651100733 50.2163892855092 40.4359177791566 45.1714698794331
51.1055541075029 53.3059058105836 46.0905079010237 46.917149434588
ACRV1 46.0398513158469 42.9020321355927 43.0971787292711 40.4967168818294
65.7471714715797 44.6024422254257 42.4939802311136 59.3923286524971
47.27543600687 49.0841460667818 40.7725311293918 41.7575097298927
ACSBG1 41.0608773166058 51.6869155634183 71.3527183280827
37.8442841690502 44.147557390293 49.4157684139858 41.0381860307445
39.7509165247419 51.0062122023297 66.274247400793 50.7159667633757
42.8864415494484
ACSBG2 39.266565827316 41.4508241771315 36.0306975524309
35.8472302065546 40.8873907150245 38.3418933808844 42.357910063876
42.7431359850339 43.3744503319109 43.6033221838149 44.553780984244
39.6352613956998
ACSF3 40.7609952437622 39.6123051184545 46.8267540220952 43.5627649099623
41.7929491793697 40.8634043028528 44.9863726205428 42.5435283762933
45.1899271235974 45.0155543555707 45.8180710486329 44.2527566343661
ACSL1 90.8236750745974 131.67159377585 75.9174504780598 434.841521967958
111.501058276821 105.814959179397 176.839214683647 114.719721626067
189.650905999631 82.8275497073451 76.7063975550116 80.8366627364071
ACSL3 146.543232229227 240.6614822416 229.130963999337 214.586915093492
368.390023942835 154.132233967972 148.887819162926 156.95090716929
392.409679550956 127.708268637068 166.951916348418 300.762511215441
ACSL4 73.4122412764173 85.7390214986603 75.4317740345967 169.889618506937
83.877343750194 103.080153415433 151.99110418225 131.67159377585
93.8042295944812 74.9236800269025 71.4414688202373 94.7718338968252

ACSL5 94.3674661847143 251.4927207704 218.917221711155 138.401109242778
171.427692959758 72.0674952635945 307.445116836976 112.375316615894
239.316215255944 462.362735276132 402.08877403329 543.268021785502
ACSL6 42.3620028137457 41.3277288245157 39.9101908547397 40.5061122751948
41.1486675205552 39.9718514851461 39.6158343667921 41.989786971805
40.741722894262 38.5782171290591 41.9908237694988 38.5279745308203
ACSM1 45.2875173643252 38.4330755397458 35.3649449469275 36.9912468186322
43.5544347011646 45.0882356578404 42.8684958525042 38.4540849632254
41.863927676552 41.5435611403119 41.9908237694988 38.9496515344837
ACSM2A 31.8228237796461 37.6383530120621 37.5597671487621
44.1483976313959 40.148780650831 38.6414316877471 41.5817737564592
37.7612949798895 40.2274522635576 40.5090849993014 38.745552403895
38.745552403895
ACSM2B 40.0124413244394 42.5269793679335 44.1976966800856
40.1321264723693 40.4261913313642 40.9506062495231 41.8315602975157
39.2672712041774 42.2577689927563 38.4187686307607 37.9540590751706
41.0657892043496
ACSM3 56.5319193881205 55.3932018462158 53.6334631215581 41.9635525160083
55.3100442506299 56.9320717885002 83.9647518349407 287.247399926139
71.9771425105735 71.2409890549638 71.3868929454853 69.5544840164738
ACSM4 43.0930293831497 36.89524015739 39.5450079747895 36.1562979552173
38.9496515344837 39.6251842352088 39.4600924554505 41.9315419791594
35.0305278582179 41.5435611403119 38.745552403895 41.4508241771315
ACSM5 41.1523487516364 43.1560065801875 42.157627240486 44.8813851439455
42.046575509735 48.8744644207821 44.4074592653814 42.0494116081998
44.0519245668737 44.3046896365956 45.452834313916 42.3047762223294
ACSS1 68.6627996695828 148.458171821142 62.3936376596431 98.9560329133205
67.9032387662129 65.6914955077809 157.843337785472 104.089998273812
105.247736284747 106.527144354592 127.345094323724 90.9361642101537
ACSS2 167.16650846037 152.132756414024 181.2179249806 252.649651285136
182.976132230487 177.133999361532 166.168385855406 203.373397813051
320.13475752314 254.639522735592 210.958578914607 180.562005428669
ACSS3 38.8561375693335 38.8336427948187 36.8730514568925 34.7266486944713
34.1686574241133 41.0582900711341 40.9157352260901 43.7396619462033
44.4395580493118 41.3321861371202 33.2774014136617 41.863927676552
ACTA1 81.8104679118753 44.0409178714232 43.5710871617107 42.6913548400127
54.1225249245036 44.2607789091176 45.4799270968969 43.9366081640576
41.5635499110307 46.325232414659 46.4753015651202 57.5372732453319
ACTA2 10996.2779403491 4482.5285743878 3999.32180022337 2816.7754199004
3966.51586153125 16661.6081002448 215.800662077432 2640.06306087033
461.123856163975 14226.3743910711 13230.9863405074 14226.3743910711
ACTB 10351.5373412096 7980.71309230447 8349.59691588869 8827.37298329149
6258.22561934986 8630.67463560037 5776.17749026501 6258.22561934986
7627.46814033012 5479.94045861143 6555.04068292972 7476.94487791412
ACTC1 56.2508140932179 42.466333667839 53.3791548006794 42.8848887336829
44.0451487108213 1900.96666834569 44.2166118829877 47.5439485437351
39.853866204927 1310.03307063841 918.083610591772 1190.10462185567
ACTG1 8545.48005557056 13656.2581082905 9488.06491634484 6819.74871209328
10996.2779403491 6186.45345364665 9060.10165509099 8703.02873382565
10235.3288951482 5438.34345794521 5733.54417656207 5177.04105968809
ACTG2 3653.59181300011 1247.50618540769 981.039763059681 702.737741114234
1806.22468459769 15358.8899487375 60.9648845690177 1891.37245670852
90.4957279507369 15358.8899487375 12456.731302703 16661.6081002448

ACTL6A 311.901423694475 383.853296408061 280.402165030951
177.82359682336 491.8684831079 249.246628143531 442.380936516984
284.795064481553 451.820169649522 151.601402122351 179.779145453083
289.975375372371
ACTL6B 39.0620487644635 39.0894172982439 44.3344900481112
41.3027031037343 40.8873907150245 45.0882356578404 40.5585676679482
42.5435283762933 41.6616444147804 48.6124127998952 36.0481934423077
44.9070250604284
ACTL7A 41.1523487516364 45.6533175604486 41.8751578329365
37.4223289687135 37.4470183315826 35.0970060242246 45.3597029614942
44.2159020605784 43.7630587657205 37.0320432433314 38.8507804383261
41.9430737035925
ACTL7B 48.6621527194963 49.2050538150106 51.3268475761593
47.6687908290818 48.1473938886239 46.1513019317765 42.8684958525042
45.7476095696859 45.2897806651929 51.3351387089044 51.2318935933116
47.5025821041537
ACTL8 37.2474303302343 44.7829632472754 42.5406888281114 33.9645433044281
71.7263663921367 49.7458652239071 43.9450023643394 40.7311497914871
34.8552366193358 41.1222709786153 39.4410515050885 42.2577689927563
ACTN1 1159.77262479002 458.66739585187 918.083610591772 1985.3999418878
957.682898193927 2138.23653387331 612.441960343995 413.067407579649
670.023570141464 933.968759959712 1418.94307844471 1342.10592868516
ACTN2 55.2182004838121 51.4413099192899 46.6588655218735 44.265834798964
61.3077641493507 50.0748292497578 45.6042625176633 57.445796273665
46.581796165231 63.3872214856708 55.4694986901287 65.2771529943605
ACTN3 35.5179217192467 38.7344853699576 34.4716904283753 37.7469621225553
34.3633615460307 37.3799542961839 42.629412460409 38.956280281655
36.0741672064298 40.3235655331796 41.0808257270221 38.3291713594599
ACTN4 2365.73772015255 644.368005872231 669.776067547468 441.090368426661
624.097858948785 636.701499995107 1266.41536472956 939.453799186397
796.359355922952 858.975085087497 1120.83891847181 762.855719373733
ACTR10 231.333080775773 245.489660195494 265.570814613305
226.057278804077 226.262409390222 355.92328557644 190.960237766104
190.375732394043 204.490115029568 166.847053797281 231.060688044554
263.937966521917
ACTR1A 350.584135047249 345.876877384089 364.337811621248
393.944898808828 300.299201489787 527.439095170064 444.858305070415
356.565801074289 311.149068752273 473.223156200183 401.202348352506
430.618167106529
ACTR1B 191.59357986481 206.103296228683 210.843643231855
142.840631375936 185.843333258735 190.442443946259 198.257381933305
153.462055740758 235.101442092497 291.327030793085 293.883582116334
250.585851778133
ACTR2 1012.0373748933 1093.04440713629 1058.61208471961 1483.40077183826
981.039763059681 1183.62228577053 655.389317052689 1001.99175009124
758.462554576949 385.064309855006 571.170643523692 912.757335169259
ACTR3 107.637618676176 149.397632479523 133.576489624155 246.869486382264
110.729504563799 172.433936159136 109.14984236229 121.510087314197
145.791398829369 109.844156801015 105.406282523992 171.043935516593
ACTR3B 42.3620028137457 46.0713306971594 49.33855351691
41.2507419031133 56.2305155589239 47.5216423686268 49.1928206164702
42.1874057736633 52.5380506348717 44.0409178714232 43.1724740315182
45.7979032939211

ACTR5 99.9752275166514 71.2370255410834 73.967017874248 65.084620579901
84.4230484528318 69.5645254789371 79.4485571328293 78.5375244257332
90.3485862002091 86.6246917308185 79.4832790698673 72.5346469941833
ACTR6 129.938114164087 164.205608279417 181.516519907011 171.238235624104
176.861690566909 178.688550924272 254.112875195524 214.369365247268
230.09336914921 252.82316545981 201.180226922613 298.670959394883
ACTR8 42.3433931248155 73.6855877240541 64.9072331703985 63.1604223919703
75.1674447943072 58.1798515131249 62.5506324864207 52.5098795466615
53.3932110698117 46.8954576545929 52.3761286517985 55.3563533836982
ACTRT1 69.7442393277127 48.7847528954724 52.2132783757422
50.2163892855092 52.4370718282084 53.4466111440611 66.9239565096247
83.0497574039035 62.0967404944031 61.1851254827343 53.4921736109774
51.4968903591058
ACVR1 293.755623192857 206.559526386642 193.897678586782 320.504100222566
179.629622763381 195.654315886452 95.2218627805387 195.168348967505
147.023857419777 274.445985892762 256.766675299448 252.313870846326
ACVR1B 80.5794942236641 81.909815795953 94.1627883101274
107.391994714261 91.6649478634836 69.4214927857472 105.45765455249
105.53472751588 155.239308003808 169.838827072973 158.17598244564
129.909461876076
ACVR1C 37.6397652536077 40.5400384479766 44.2405728317107
42.2496337895968 40.2449343247539 35.7034203372528 44.0687697278114
44.7044140979108 42.6366765186182 37.3179578037963 38.8507804383261
42.7782216342806
ACVR2A 76.6094442444074 89.3342384381684 78.258486366912
59.897987870592 87.8681250426295 91.0847547974748 60.5125774827425
53.2808392130566 85.4790745407113 98.2843999216669 104.076098789396
91.4786089554886
ACVR2B 37.1485601150873 36.1689925825846 38.5045333213784
35.4355497822498 38.2496050953547 33.6248915845234 40.1235656101329
38.132587347852 33.7574445386307 37.5597671487621 37.2559756339764
32.4951076826876
ACVRL1 74.9006142438341 73.4899394858239 72.7987593815919
171.238235624104 62.9553576299033 149.856848496725 46.4388841354122
67.6082563445866 41.1742244762318 75.3333024462557 108.779357041421
76.3824005526731
ACY1 160.320045581768 708.005870621064 607.042450917813 119.253559091088
660.295993198711 163.742560819575 354.634819362978 203.109765192983
1136.88710104691 239.590143292142 301.139592414061 227.567182783564
ACY3 37.6397652536077 42.6022652963326 40.5734458834617 41.8778873678551
41.6806984441849 38.7340969861754 36.197424053556 40.1474543172332
41.7627910693483 35.4990901121893 41.0050831223107 33.6902293666089
ACYP1 106.418690759491 62.6984594468418 76.3583555666815 59.3549213146532
106.418690759491 72.6824533717231 121.487591828005 69.5380697712471
85.7111041637416 77.0927030014721 86.7076796107636 152.47776741936
ACYP2 55.0078788655089 95.3222932230888 86.6798536307388 67.474556589181
83.555058970129 89.680151549258 77.8220670093392 93.299474822664
87.404437522542 73.2445837559745 73.5788549535062 99.8754776648876
ADA 133.229859346689 131.045615546105 188.513581321162 263.765914589758
205.854666205308 234.727123301682 89.2913927776683 164.62751527755
182.31306890697 82.475816811779 80.8366627364071 90.5732028806631
ADAD1 38.0089018744739 35.9305494063137 40.4882741869718 37.7469621225553
35.2163900400329 40.650742261428 38.941855825269 34.4896350146096
36.3649677956953 43.9450023643394 36.5359455830388 35.9604262434141

ADAD2 41.1523487516364 42.8542642792862 41.1367478979484 41.7614975630362
46.3150221024046 35.8227939208521 48.8319884367456 42.2577689927563
55.4030910454028 51.3351387089044 41.9908237694988 36.7762265039864
ADAL 47.9175508887533 43.0227387991135 50.1860191642359 41.6564075217517
57.2960245538881 56.1671440556142 42.357910063876 49.5185112777773
48.3954967265007 54.8979897528792 52.022479439889 53.3059058105836
ADAM10 76.8864157610323 147.017279035061 71.7997882389171
69.9499565182502 130.336599857526 66.5234828659154 85.3930226794591
77.2834482217973 107.741669334989 47.3791873993182 71.5249259772583
79.9447969637409
ADAM11 39.8662795144616 44.9370339275041 41.9666541498638
39.5050383901884 62.9553576299033 51.1621379498014 50.8388984085636
56.4779645313958 41.863927676552 48.8623729374733 39.4410515050885
44.1452943804044
ADAM12 45.9356781508849 40.6806219262998 39.4710743516517
40.7941759414114 37.3730111198605 41.800198520267 37.4440365247457
41.0920120134247 39.1433524273815 36.2802866890489 37.3377031071126
40.9243125941198
ADAM15 501.817654561915 385.36587480414 823.933615182634
380.541502795438 740.994425128952 381.426926151034 471.046851874447
894.486674059951 642.211963248041 189.984886903595 245.888237156849
201.800572658224
ADAM17 263.21493129287 74.2653431904071 88.0368652545541
123.190887747662 122.160438114932 74.3187437598836 152.17335005192
372.02379581293 114.230358276599 128.657014567195 110.440072569721
100.962000280931
ADAM18 40.5654775358427 42.2577689927563 44.1452943804044
41.3027031037343 47.4548471240051 42.8598805316863 42.0605906240248
44.293922970134 40.0403898620771 37.4302011516587 39.4410515050885
39.6352613956998
ADAM19 234.043251639296 70.9567983915276 69.727028012962
188.582370265555 95.8149149211251 143.536318305185 76.8788592884109
79.4593310742125 45.7226406704445 109.25950270396 94.5111909776371
156.822939275312
ADAM2 43.0930293831497 46.7591817286632 45.1569741609597 39.3750969348308
40.5710637884029 47.8636003588181 38.8417151851134 47.3791873993182
50.2223403889892 36.8059561571675 43.5710871617107 44.2527566343661
ADAM20 40.2543341073001 38.3322849790657 42.7303139017786
41.4273122914951 36.2048942610546 36.6271061451613 31.5556621809114
37.0390589468421 44.5303781239209 41.9666541498638 40.9157352260901
32.0636251428594
ADAM21 48.1125722136997 44.8529259387308 49.7350276169552
44.7980740332174 43.6587492470348 42.4580504587581 46.8613212048981
40.1474543172332 44.3440839686558 42.785429206422 44.1148826235374
45.3763275588341
ADAM22 36.7631292849091 35.6027791404152 34.806104801006
36.7128997214853 36.7356360093603 39.8455060654531 35.1714932603619
48.5681478665796 37.9849928059771 37.2089297656361 36.0947367757232
40.1179210534118
ADAM23 55.4004611309636 51.2405397507224 40.931294690669
59.4674205670657 63.3638417360105 44.3648993568405 49.1067959632109
55.1109784383525 49.3780606970538 45.1569741609597 42.7275315121163
36.7762265039864

ADAM28 43.4626049853157 45.7023929486654 42.381482941987
44.7520317855773 41.2457941494573 39.3638967048812 42.57175366709
38.2125616761557 44.2952004276566 48.5330664799905 51.1483696410762
57.5941849631773
ADAM29 44.2350046179299 39.6123051184545 36.3604951341921
37.29566077996 35.4484652445078 40.5281496800812 35.7055410599146
45.9604707098953 47.7086093893038 37.4302011516587 45.7328341667151
39.9519547348106
ADAM30 42.9104454767916 42.0709558258414 43.0016969050339
42.357910063876 44.0451487108213 37.3799542961839 41.0381860307445
46.695848687694 42.0621425894624 37.1275334308172 43.7585538437696
41.863927676552
ADAM32 37.3463875295726 38.5252456306656 40.3397581924196
39.7840818430159 41.7525817198982 37.2039944420316 38.1793598983615
40.5640952422779 39.6413313949444 38.957687788868 41.4959139197962
42.3152792870779
ADAM33 45.0985088502363 44.7829632472754 41.6550823405646
48.4916117779992 45.2556760873335 55.3202238943712 44.0137388752186
45.8108990837624 44.1211971585961 43.0177448037924 45.9725715538987
49.7336769474446
ADAM3A 38.0508336668122 38.0226905622357 37.1175717254729
38.745552403895 37.5556299264699 39.779935291659 39.8390535343912
41.8792703207442 38.401850731664 36.1550293353654 36.9060997041453
38.1945786164609
ADAM5 40.6957787921451 39.932537801639 40.148780650831 40.5484717678957
38.6213341190836 39.2985791324641 46.7100796279384 44.0390019242862
39.4762546236449 41.2276963324981 40.547835538778 39.4830039788612
ADAM5P 39.0620487644635 42.1607322031054 46.4569566520107
36.890925044696 39.1650142708257 40.8634043028528 40.2154497375715
38.2782436497441 40.8970401536578 40.2112371512838 42.170702295259
37.4554876386402
ADAM6 38.3418933808844 33.4316681623175 41.1367478979484 42.4061338151892
36.8400213077707 37.9430145836712 40.8681871019812 45.0972184964705
40.5912213523345 36.2885860224741 44.4329005595031 40.1235656101329
ADAM7 46.8141313516997 40.5400384479766 46.7508829977587 71.5249259772583
46.3150221024046 45.0882356578404 37.6036146841933 47.3791873993182
40.5912213523345 37.1275334308172 44.6286581923295 46.7182294568603
ADAM8 114.404099081799 210.919385184575 215.15351875081 409.554706384667
72.9154861179393 60.5803031036931 175.820979889343 132.52749757795
61.5807110546718 72.961444366037 73.3321469094819 77.8620267163666
ADAM9 73.6362523983338 87.275548276476 101.494432845825 80.8728961146267
79.0039595317374 97.4062608315831 68.878562323012 82.0925058822176
64.0541263553251 73.0394191668858 72.3822545261582 95.491982090349
ADAMDEC1 97.9806802695685 97.9305595485282 53.0630927250439
341.078728185917 81.0259717888094 43.5416683728269 60.8857255846432
74.8270640933572 37.6630259432398 37.2686490268265 40.2649309204478
48.27609330316
ADAMTS1 59.1311136551108 147.017279035061 152.132756414024
115.913030448537 128.38299874379 775.515071756239 47.7390230243778
134.952392466784 100.178375869002 110.785049776763 168.482495410546
137.378222527303
ADAMTS10 43.4626049853157 42.6022652963326 35.9305494063137
44.550322123994 41.467005949568 45.4882438975269 38.4005795891396

39.7509165247419 40.137818555383 42.1622837416589 45.0589947942306
38.745552403895
ADAMTS12 38.6454098044474 41.7337506166619 34.8956628705329
40.8681871019812 39.9697332660833 38.8336427948187 40.4359177791566
39.5466975353131 35.3227485563945 41.5435611403119 37.2892641289258
45.3763275588341
ADAMTS13 39.2192721306168 43.3030298816484 39.3344387353654
38.3684202018524 35.1872005076088 37.4782441311626 40.0668875818227
42.5435283762933 42.2331334096358 38.957687788868 41.1367478979484
38.2795150354677
ADAMTS14 41.7780833969533 39.0894172982439 40.0599807618105
41.6186264038868 40.9450021938518 41.9543948167375 37.6527901109005
40.3662560853635 40.8351751854504 43.401782064778 40.7776803630095
39.0536056496869
ADAMTS15 41.3387790895716 41.6466169548846 43.4709415392696
44.9370339275041 42.4864576619427 33.5578650644246 42.7431359850339
40.931294690669 46.7760357462635 39.7835513496873 35.860430241131
45.0035308952466
ADAMTS16 47.2862299990938 44.0409178714232 44.8872784170036
42.6913548400127 43.6587492470348 40.8634043028528 39.8695929627783
48.7814131120945 40.6957787921451 40.7063426028871 43.401782064778
41.863927676552
ADAMTS17 44.6231541342 40.8880530359822 42.9104454767916
57.0629653735694 41.5668884168669 47.0940613507321 41.8315602975157
42.357910063876 44.8003345643334 46.8954576545929 43.0016969050339
36.9976231206067
ADAMTS18 36.9660447385262 35.860430241131 41.9666541498638
42.281606728072 40.7779767565889 39.8455060654531 40.2601451506332
36.6968906669565 42.0126083977597 42.9866214102525 39.3789825717286
40.9683275553869
ADAMTS19 60.562215295948 50.6774883370457 60.3155766445629
57.2856683017619 57.5040500497076 59.0686629877982 68.673864919178
59.6720198281342 54.7033747046992 55.5206840401643 56.3884556031093
52.9609836781499
ADAMTS2 62.8422809723679 41.0226232127351 41.2679739649179
49.3654837541353 45.8669209716694 55.0009147092272 42.9803717121865
42.357910063876 40.8970401536578 46.325232414659 37.5343260962758
51.1897992768608
ADAMTS20 40.0270326792985 44.9370339275041 39.6894175734463
41.0057887014368 40.7369744663513 36.4448748739506 39.8390535343912
37.9837233601316 44.7058233759912 41.8613406887824 47.9376640244611
39.3931655934497
ADAMTS3 39.1590964332473 37.8217989841918 42.640829914102
42.8235313007746 34.0927410219282 38.6414316877471 38.6243997507646
38.079222289614 42.9020321355927 38.745552403895 37.3687900733549
39.5418280570561
ADAMTS4 40.3478825593224 40.3384318592985 46.4569566520107
41.3027031037343 45.2556760873335 48.5835484742456 49.3854238365325
46.7980335276155 41.074824727477 38.6454098044474 41.8168470823451
39.0536056496869
ADAMTS5 52.2236479204029 49.83247261979 43.5710871617107
51.3851157261451 39.3667161654772 64.7905331739366 40.2154497375715
42.357910063876 43.2761454096265 55.4037482627222 38.956280281655
49.904979214379

ADAMTS6 47.6397712007919 43.9421822132391 44.3344900481112 56.0794100106654 40.8873907150245 45.1873487463472 35.8227939208521 45.2806199505029 51.0062122023297 44.7958122466628 42.0750889188255 42.57175366709
ADAMTS7 54.1745577382276 55.0009147092272 64.6111899743678 38.5252456306656 75.2391243965793 43.1826328292558 47.7390230243778 47.6952179922944 70.178974380507 50.3388505346542 43.9450023643394 47.5025821041537
ADAMTS8 36.7356360093603 39.1982470626863 47.2055133646385 38.3166554965434 43.2484996523549 65.4643755537228 35.4623691645629 58.0892402609917 33.3643994200057 40.8049408996274 47.0110068985119 61.8529316708431
ADAMTS9 77.9057333780847 73.9376772899278 140.504336039225 134.567947469561 90.385018479478 123.674164545425 47.8947595316832 129.605603092944 40.6957787921451 58.115198353247 63.6057995724852 111.691813916343
ADAMTSL1 43.8678829064872 39.5050383901884 41.7397731376692 43.8485914240791 41.7929491793697 43.0860383229288 38.7718834335368 42.384269090877 39.5404280901064 44.5276078676423 38.1207627353276 41.5937402943136
ADAMTSL2 210.255784687052 151.255948388415 260.656475394736 102.786429761295 109.922909644299 79.0940380276977 52.0942589368478 92.4953883194488 54.7966692984192 145.193498675059 223.397586938079 139.472956453414
ADAMTSL3 38.385865183581 36.7976562409506 37.7384958256497 35.6928955245309 42.4864576619427 51.6053353177789 31.9682164860619 35.7973685350741 42.2577689927563 50.3388505346542 54.8532558909227 44.2527566343661
ADAMTSL4 49.7336769474446 47.7233409198209 44.2771355310435 62.7377155597666 47.6884372645373 74.4851052263387 43.6753843488695 41.2551966132486 37.0478905185041 57.9713749600418 50.4022543872993 53.4175306626669
ADAMTSL5 71.1521292570086 46.0023068797812 61.356483470053 39.1706564265495 53.1884505015411 52.4351670989268 46.7100796279384 72.121833189378 54.998510746958 66.7299966077909 63.6057995724852 60.7495043977707
ADAP2 159.071016200059 147.017279035061 101.804757613639 312.589169184897 131.365512154718 201.486224052899 94.6952414258466 147.187118389181 57.7865070833351 71.5032996449402 74.5654028199926 98.9017950719795
ADAR 522.377154567257 446.162602155964 551.915864019927 501.817654561915 535.313561995599 369.584648534533 449.243439865944 492.519023728038 382.744696166534 394.073743195755 458.861461526841 328.548716133094
ADARB1 118.714977712617 72.983338488605 79.4528447794527 92.1455386560941 94.717335243904 502.205366225368 84.2104160850167 88.5140510969737 72.9274964092369 275.222714101589 308.393270347486 339.52415679299
ADARB2 44.0328992213727 35.7393138893354 34.9849896310235 39.5050383901884 35.1411239266238 35.9305494063137 41.5817737564592 39.9576138799997 41.4636047919453 42.3606390470387 39.6403089864678 36.9976231206067
ADAT1 156.636360703756 211.196171061328 253.980053738171 137.176719695561 139.712472369259 119.333788465158 144.928534157065 208.189151365575 207.600413958886 138.637058021129 165.107387190373 167.920131968611

ADAT3 111.248876636045 78.1623301184313 70.6592421895338 77.2218991550552
132.400225103653 56.0477607422828 68.2708120569221 85.6170780640867
93.8042295944812 62.8201270538435 71.9658486648999 57.0524725494273
ADC 67.2437599548936 45.3486182278177 43.5710871617107 55.0604256312495
51.5888395924843 54.2503023961401 41.0381860307445 65.1637149462661
48.6494451061637 61.1851254827343 57.0154839296649 49.7336769474446
ADCK1 92.6545309624315 76.4439037944547 112.977333058979 89.9284109077375
77.7140342228764 78.1952428431942 72.1401612718245 93.9880852748373
107.961407367823 115.414481087246 136.482957391332 83.0460206754856
ADCK2 370.917477324773 368.913147706435 382.425576479893 294.155188719236
491.468862309918 209.551557135809 423.896735383118 356.565801074289
678.684833360104 425.776214512634 426.276230164353 316.660303090646
ADCK4 62.3042205863409 66.3904351698776 56.2441895183416 41.1886975241176
54.0126916508417 74.6375749739389 61.8571521441451 74.7794253940974
63.6222532132037 78.59966540743 62.6048727240291 51.9447450229707
ADCK5 97.0254711947754 95.5627648703794 84.4443931765378 64.898351831341
87.9297641099558 64.6835308827804 74.0039764902057 67.2714646016644
94.3537052300317 85.5439309325541 77.9085946827079 60.0940570991178
ADCY1 49.7827734675743 47.4076235350059 54.1157382341041 42.281606728072
60.4684603480369 53.63657997649 46.5650556481721 59.4674205670657
46.2027193115397 49.461728580617 43.6629145624937 47.3661873039785
ADCY2 56.1912096308988 44.2105195734073 51.223859150798 71.3932787152381
54.3116862772198 57.6034762511847 62.5506324864207 49.7309743973184
56.2441895183416 55.1089864632456 51.0492389286271 56.1875519519366
ADCY3 264.116434279258 606.761670821739 563.065549077627 554.163106145921
558.215290347282 510.232992765054 480.386431986343 823.933615182634
330.096171705212 388.661268904002 496.163796158189 448.383843915421
ADCY4 61.6544855697851 87.9152471471338 115.256208610595 140.662969373546
70.18594926052 131.319040057576 40.1235656101329 79.6133633483649
34.8552366193358 70.0907269565408 110.818741525514 72.2211523775459
ADCY5 38.957687788868 38.4330755397458 40.7620472083159 34.2080003860523
35.3327761997046 51.8103411703261 34.9344532055397 40.6171774724171
28.5895665533366 56.4553747079294 49.4593570700221 42.357910063876
ADCY6 104.796373568883 102.605473054584 160.033150948641 67.3439996689706
92.9136587752031 88.1068080491008 122.824055084598 234.946245049134
92.3901719424121 133.720493104769 113.444217634165 115.441414762268
ADCY7 62.0553450433433 56.9312835667828 45.7968648044613 78.9479233685889
46.0977687311222 51.9421032049606 51.0013344286851 56.9299697426319
45.5779686360641 57.3562426592112 49.8393069675192 51.0647893605839
ADCY8 41.2424637338424 42.7775752471711 44.2405728317107 39.7411455670172
41.9842695818285 41.159706131351 46.9929836306816 35.1738399826814
42.6366765186182 41.8613406887824 39.0550139257658 37.4554876386402
ADCY9 54.7786516309154 60.3093790894864 46.4569566520107 53.8086134112366
46.6397391251762 135.985408824299 39.0458123429605 47.6952179922944
63.2753814090696 81.6233038764401 85.3930226794591 98.1220820761255
ADCYAP1 56.921728372453 48.9899488302637 50.1860191642359
68.6379135603075 52.2089306840125 159.416887501941 46.7100796279384
45.1714698794331 43.1805016690297 47.3791873993182 47.0110068985119
49.5539622682049
ADCYAP1R1 42.7150227340364 46.0023068797812 41.3366779545815
45.4634652319753 38.0598374201407 42.2577689927563 45.7452456977857
47.7889447429963 38.7298222155532 43.6033221838149 39.9899430628542
40.6614700944853

ADD1 508.474794786176 830.295872113419 942.247437385029 588.736435715365
509.671528514181 1345.37091574015 683.75696650704 541.631969808467
744.080606078086 1718.44944271916 1921.48678333837 1093.04440713629
ADD2 39.2076937845359 40.0230205296014 40.6312687664188 42.9969462198633
43.5544347011646 35.2067831864574 41.3594868055879 39.9091538088603
40.0870341983328 39.1269447164792 40.3180310664156 39.9519547348106
ADD3 85.0065217565876 135.106756607914 116.461730749198 94.7283425282671
68.534940191185 215.831300220615 98.2074490157865 120.426087248804
83.7019398654324 175.960838580876 157.312247745825 171.270750760376
ADFP 1001.99175009124 437.542517888395 327.024556075244 1217.21440297138
1136.88710104691 567.978225839768 539.188951045594 309.102633242573
575.863848923457 421.971171686029 427.818758455214 1539.35554229258
ADH1A 46.263826584899 108.019575675653 47.7737810047679 41.0726336841311
51.5888395924843 2672.94421680196 607.431621448599 64.964255000378
358.979854534484 1114.15989325456 844.431085166353 422.58866112053
ADH1B 40.5654775358427 44.5152708955623 35.4484652445078 42.357910063876
42.4864576619427 372.982648892131 42.629412460409 42.2577689927563
42.0621425894624 235.925374041038 178.012676342712 84.7947249929138
ADH1C 40.0557788909923 66.5864907685351 40.931294690669 39.3750969348308
37.6527901109005 126.319731178054 619.950241162173 46.4672637826582
301.025314855652 62.8201270538435 61.4121672148248 53.4735821194294
ADH4 42.2618615571566 39.5050383901884 39.9101908547397 40.646702696033
40.8873907150245 39.3565586789274 41.0381860307445 45.4757965005055
39.7537274655115 38.8507804383261 39.5792604499113 40.7704587752083
ADH5 47.3791873993182 46.4385386401326 34.1806289878747 37.0732977341185
53.3086645804786 95.0026207727863 47.2927436772465 42.9301944191913
58.6431245059655 48.0211752672327 54.113533179847 67.1675878355908
ADH6 37.9387791625384 41.9894412416874 42.5406888281114 46.0228732507872
44.5668141895992 44.0804700754786 62.9165938167938 43.1567024035183
42.6366765186182 43.401782064778 44.553780984244 40.5703581862752
ADH7 37.7384958256497 80.0502531022254 43.8495248991589 38.4208429103799
41.6806984441849 38.6414316877471 42.7431359850339 45.642304356051
137.10896135739 42.0621425894624 44.553780984244 39.0536056496869
ADHFE1 44.3355304829989 67.2649356062263 129.501808300251
56.1780366332082 59.135659721231 95.0026207727863 42.4939802311136
69.4009439491061 40.7460990419215 122.15017220566 92.0711167984454
105.05842887748
ADI1 43.6706764143908 52.1062000766427 43.5710871617107 36.264823544143
50.2163892855092 49.4157684139858 39.6535880152834 41.5464765641928
54.0192060888782 59.6863210839828 51.7538628098347 48.6037684272115
ADIG 37.8386378737561 40.148780650831 49.1067959632109 41.6564075217517
40.5710637884029 43.0860383229288 46.0228732507872 46.3685439301234
43.3744503319109 46.7646877499666 43.844855336215 38.8495095797881
ADIPOQ 40.9558083259517 43.5873188565519 41.0356368374708
48.665578118898 37.5556299264699 39.3841861812262 38.8417151851134
46.7980335276155 40.6957787921451 48.2596564318071 48.4717951775049
38.8495095797881
ADIPOR1 278.140612054333 312.245547906683 472.159979716705
411.254448823371 289.716911324484 217.533011278692 236.910422092851
247.676607756929 217.721787448311 265.667557678228 283.484056482174
355.288272452203
ADIPOR2 320.086549121732 257.851199206959 355.821515437298
356.226114718307 282.328238928883 298.607482741423 268.990075898188

311.502922515027 544.946796643478 307.754078087321 309.994784298282
303.818507395434
ADK 71.1521292570086 93.5358142196619 105.247736284747 67.5489259940822
114.445779948228 71.4792239075993 93.3216420803293 69.6272083256793
199.57367025082 80.5794942236641 70.9319024596628 98.6401096175932
ADM 2575.86919902296 2921.68921750799 1127.96214301065 7323.77427153966
850.656711617458 1199.29782389306 2892.02001412369 788.232434009173
3043.12489526581 505.156485179198 1021.71286017481 1269.36837611919
ADM2 69.9431447778061 68.307513091369 67.8010199320754 97.3870880869036
80.428712610358 73.2751502965172 70.5534237141767 65.813547096222
90.7037473522607 67.3308332069908 60.394307846869 63.4685821260797
ADNP 98.9988668602468 99.0647786948132 122.099297248392 78.6344248102932
122.824055084598 79.4916415027015 107.668663185697 100.696072546592
106.907589984092 107.242476674629 118.795369922438 98.5339473137891
ADNP2 144.266359480086 188.552900887757 193.468773556057 227.567182783564
122.099297248392 138.916915463074 174.117816184497 113.179313022693
281.452208033175 197.868575583033 173.08677862231 144.250839367023
ADO 243.763402918148 117.165519915808 92.3034609409756 155.928417606775
103.968071466212 129.434864701718 115.861269747317 95.491982090349
219.676249419297 151.451107406752 120.418406532204 128.230543301378
ADORA1 60.1258784595219 41.6972107715212 38.4428081526233
42.357910063876 41.3440240757217 39.4953494583071 43.0398514059634
44.8643898647322 44.2222841572272 37.6623366050401 42.6913548400127
43.9541718254769
ADORA2A 66.3095900379311 77.0927030014721 75.4317740345967
280.622184239624 51.4527545197672 51.294537308199 90.2915974511786
61.5824518349507 48.9931786790166 56.8201201798712 58.4816190076338
57.445796273665
ADORA2B 80.0866955521632 132.588447812638 269.184000351658
116.694275999729 125.333208461509 63.9650465690339 59.9792284253187
86.7710020879725 227.686495419561 86.3374157614394 82.5225075943931
189.80672643731
ADORA3 87.2394571967316 91.6777754418002 69.4583814833055
211.451463038315 64.3003533494489 65.9014252111112 86.7968607218397
74.5173894803763 65.1129635608639 56.8875898013449 56.5896227808363
100.774098314706
ADPGK 235.888544042065 174.275409358652 143.706880936186 452.351360652434
244.089971761037 135.829530518199 203.808195515747 214.369365247268
172.39907939484 172.83517237888 153.103233619506 156.864177272437
ADPRH 53.0290605707162 37.920964499903 42.7303139017786 34.9221408381038
37.7576900881741 43.1826328292558 40.0052661202207 37.0390589468421
33.0463640202858 35.3849221682845 37.5583886316038 36.5474283500601
ADPRHL1 47.7737810047679 46.1835531253984 50.3950981988762
43.0001432913058 54.2301964914419 46.263826584899 57.6075177910677
42.6975474602554 51.9247097592613 48.1907683998167 54.2999306485758
43.2776458914333
ADPRHL2 100.289042948128 116.094881058504 106.058751335133
90.0942781313264 142.124053129543 97.2865629929716 163.62024333166
120.447568925399 143.122201568705 90.6805895192707 101.434947182387
121.969082013079
ADRA1A 40.5265498315468 39.2907645923217 40.7273974755431
39.2524372699287 39.7312050662551 42.6200095806555 36.9355798143571
39.5259298883656 39.8067641711565 41.5685493982342 38.3684202018524
41.7575097298927

ADRA1B	54.3554233228353 43.8495248991589 52.6275131390249
44.265834798964 39.1650142708257 62.9688525319822 46.1634822993009
47.5950227477807 47.27543600687 42.785429206422 46.4205654140754
43.2776458914333
ADRA1D	41.7397731376692 43.7644428186265 48.1997158668729
61.7502624458447 44.4489662507893 45.7300501624017 39.7576136507593
43.6434576713809 44.2607789091176 47.611018474151 50.8826620053386
47.1060577218993
ADRA2A	48.2910530887214 51.524711857679 43.9450023643394
70.0402992940342 41.8806343997257 47.7556758818053 44.3440839686558
54.5125550937898 42.6366765186182 52.7972156492746 45.8180710486329
47.2131144508464
ADRA2B	40.4639532172338 40.9731067463382 44.9758339877851
41.7614975630362 41.5668884168669 41.0582900711341 45.8980298854198
48.272324215504 44.619512785899 38.528621456378 40.547835538778
50.656598108034
ADRA2C	40.4179476219497 40.5852276210998 42.5406888281114
38.0958223740111 47.4548471240051 40.5281496800812 43.9450023643394
42.7431359850339 38.0213051227859 42.4594149893798 40.040778349137
38.2350720635382
ADRB1 36.8072833828297 38.6414316877471 48.2883694323339 39.5050383901884
37.3508690072156 42.750861693377 43.3262148375525 36.9355798143571
37.3600014814388 37.2089297656361 41.9908237694988 38.0452862258803
ADRB2 44.9896851005259 96.981661155293 118.619819027443 81.8727530460686
57.1935729219207 96.9739786719591 56.0515443021293 101.681822254799
95.65872032796 135.973016473297 96.3495443565738 97.3929166450379
ADRB3 41.8409927585705 43.8495248991589 46.6588655218735 47.9915414951747
33.6330903323612 45.4882438975269 38.7337086062875 38.956280281655
38.9006788676407 43.9450023643394 46.7386599624801 41.863927676552
ADRBK1	38.8561375693335 36.4002279956519 38.4043916290561
39.6292788039793 40.4639532172338 42.157627240486 45.6042625176633
37.6557714476853 35.860430241131 38.745552403895 40.4504608195666
42.357910063876
ADRBK2	41.0608773166058 41.3610369241157 46.9040704638073
37.7469621225553 42.0709558258414 37.0180934800573 40.4359177791566
40.831207953647 39.7537274655115 34.8137085420423 36.5359455830388
40.3691324547049
ADRM1 309.102633242573 484.218958528411 408.739953606815 363.527881831453
427.199940706169 377.847116683974 583.690350347288 422.104447912999
583.421166842011 314.091389958069 356.025084823326 247.676607756929
ADSL	539.720977763663 467.884559162462 423.608694638779 523.25106120389
402.794855141389 565.72800641089 635.050535522135 512.821381202441
469.30167430316 490.652353306749 429.983084929605 456.874884453965
ADSS	241.455752070163 311.055487966495 357.254500791202 270.724111544154
404.175196605897 293.300203370043 293.086409068972 222.985386635178
305.684635013927 274.445985892762 269.618616631857 357.111253129662
ADSSL1	74.3766944386886 49.1041309537036 134.271727318694
49.6314545328943 56.7776874358774 56.5500409758451 48.2748123114598
54.2445399731644 126.930102684077 69.2406343120143 60.8976148193383
67.1675878355908
AEBP1 2138.23653387331 219.582785177134 112.977333058979 432.945510073269
129.270792739554 675.832363405287 56.0515443021293 269.546586017659
55.8510296341925 442.380936516984 354.861943266984 179.342338803631

AEBP2 71.0340758556049 64.643220133046 70.4794854241035 66.9218519793499
79.7808217577226 96.9739786719591 82.7853677822046 57.7805922926828
98.1495112518979 89.8035133597498 102.87193290903 107.799236230743
AES 311.055487966495 738.059797123491 875.139073253298 302.50850157207
582.017297217368 797.019657306322 571.790034754076 638.848775860866
689.228901128857 1154.6386362 965.530928317269 687.862452802453
AFAP1 97.1131393750315 54.6538849155354 57.070382426461 64.5347406608537
57.5245761633468 88.9321928608078 56.1336444195175 63.4889805083459
50.7216807571528 117.293778127059 79.7389069715403 81.0169370911764
AFAP1L1 53.0290605707162 57.5941849631773 82.8205269728224
65.3191216270241 65.1757400078647 59.4949177415847 44.0687697278114
57.037639245179 49.9951674107458 60.7495043977707 59.4188882103773
50.8442982601142
AFAP1L2 75.9236575152151 81.4621222177298 100.645556491457
79.1000897258245 75.7464604972652 71.6050174762551 58.6431245059655
115.155021677978 62.900814964804 104.068748231847 99.432142513601
88.6207486236976
AFAR3 41.1523487516364 36.2103905554984 42.5406888281114 31.8431345631453
36.4434672195785 36.9060997041453 34.1889222434768 37.1317006162938
31.1401844809132 35.4990901121893 42.357910063876 37.5556299264699
AFARP1 39.4250105372135 40.0952076421149 37.8002829036779
38.6835649080278 38.6454098044474 43.5752393599169 39.9559575274857
42.7431359850339 38.8271346375425 40.848280311228 42.8263096141654
39.5418280570561
AFF1 68.0700069295224 112.02980664709 93.0410625954471 119.39177524986
79.9971782971285 109.221269056557 97.4515987591398 105.308648028552
105.197692490363 86.6516906104905 108.12482131423 107.585504340548
AFF2 46.5549623118323 46.5650556481721 39.9101908547397 46.0228732507872
41.0885562160946 46.3715909653832 38.8417151851134 45.7476095696859
34.226171989766 37.3179578037963 42.4580504587581 41.4508241771315
AFF3 39.8234920704779 42.9887069977696 39.9519547348106 47.2009420656854
38.7004513165111 73.1169003714219 41.3094485574845 43.1931731665131
35.6970528713709 64.1097859982912 69.3645322033576 73.7986685173579
AFF4 85.4713284900688 79.271901834289 79.4998723172096 87.6763065012524
60.9710783023013 110.202401893789 64.5722284870633 65.3715655467882
87.4335749014848 83.1874704625702 97.712117589297 113.008760911729
AFG3L1 53.8278927154175 68.7227011958449 93.2402285327166
61.9753438182501 51.524711857679 50.2957175518356 67.9599395822006
52.407151732257 58.2879293130296 63.6197695952215 63.3544794716167
66.0224755625662
AFG3L2 255.241295723095 293.086409068972 231.085533860683
169.99300253813 397.520960156896 209.794541970107 496.163796158189
307.002236094206 369.877521477669 300.186402341399 336.390195295291
314.458148321064
AFM 39.3709357615493 37.1685072112618 36.769073068743 38.6414316877471
37.2567512265363 35.314469458379 29.0303154053123 38.1835938174625
42.9020321355927 34.4875013487777 33.8558232866797 37.8490162439785
AFMID 61.2691385007595 65.1291617069903 57.4622430729629 53.1124375923907
75.3580625318157 44.0804700754786 62.1448806673973 62.9364422691302
63.4531374125233 47.9707112435212 50.4740079109854 64.3383155834968
AFP 42.2455707947223 40.2493681589104 42.7303139017786 48.4916117779992
39.6535880152834 42.8598805316863 40.3302268293707 33.8558232866797
41.3803269429468 40.8970401536578 41.0050831223107 38.4330755397458

AFTPH	98.8719561245756	68.9603421415748	67.0120793767211	77.9085946827079
	72.1591028956359	69.64983895075	61.7695376560802	119.609614885616
	76.6027293497456	65.5800543140431	82.8770530537113	79.8878785326568
AGA	91.4786089554886	113.853553531089	164.62751527755	150.649377863375
	66.0411740221276	58.2531879680192	102.626604917608	74.6572522248684
	114.829148878375	119.634256037186	107.971806573919	84.4349803529132
AGAP1	42.0532901689036	41.4223563635976	38.0057289062111	41.7144525110048
	40.1151953732637	39.7394181011603	44.5390641547628	39.6474008369935
	37.5469247328848	33.9967481174035	36.8730514568925	39.9941474864076
AGAP2	41.2424637338424	48.109747794187	40.6737160001291	34.9221408381038
	44.8897737954573	38.8336427948187	37.9430145836712	39.3709357615493
	43.5669306063196	37.3179578037963	36.6298807161755	37.8490162439785
AGAP3	53.0290605707162	91.0952554578659	73.7682148056628	76.5411379733694
	85.0852996855365	56.1115976952096	58.1591117988945	51.3907321092333
	76.1869593072029	56.7482794147852	71.3527183280827	51.6660481331154
AGAP7	43.9309845324767	44.0409178714232	46.3411353377615	50.8567345921473
	44.5668141895992	46.1513019317765	42.629412460409	43.5544347011646
	48.8067321016961	49.8393069675192	47.1477056711335	51.0013344286851
AGBL1	39.5676051939342	37.5469247328848	36.8124076923959	37.5764485234171
	36.6354013629791	38.8336427948187	37.3799542961839	35.5179217192467
	36.4816715148518	36.3612409063792	38.3684202018524	36.9060997041453
AGBL2	41.2424637338424	41.8848959280916	51.0539624358342	40.5484717678957
	45.5809132232681	47.6803619768589	40.1235656101329	40.3357777530419
	40.2274522635576	45.2556760873335	44.2925355984056	43.1805016690297
AGBL3	37.4554876386402	43.7644428186265	41.3366779545815	36.6501141438378
	39.4693871570028	43.8582747295766	40.3302268293707	43.9366081640576
	37.5469247328848	42.5664883002833	38.6662194645633	44.9070250604284
AGBL4	35.4287975493553	42.8542642792862	40.7620472083159	44.1483976313959
	40.7779767565889	42.157627240486	38.6243997507646	38.2782436497441
	42.0621425894624	39.1621898366355	37.4642807402741	39.6352613956998
AGBL5	124.041740442038	114.161435210562	98.1968987868987	57.165971310746
	178.249889756228	151.831767769522	193.703214707909	192.058030454066
	120.26537515666	169.527304857688	148.130140385248	116.374125502825
AGC1	53.4921736109774	48.9259041798949	47.2055133646385	43.6726153508978
	44.4489662507893	46.574682061365	46.1634822993009	45.0972184964705
	44.8997235283254	48.4890615340603	50.0808824383821	45.8546719310325
AGER	44.2760426402717	41.0269624684418	46.3057385979341	40.6857324478814
	41.7405682794166	39.3841861812262	38.6243997507646	40.5158867964016
	43.1235886043549	42.8482181313226	41.8168470823451	41.863927676552
AGFG2	43.781101409552	47.3010040326346	46.7058093820839	47.8947595316832
	56.2937283136959	50.6360662655392	40.968974769473	48.3077814647455
	64.4837166864084	61.3529667465225	61.3733228199803	52.3896785600751
AGGF1	81.1350396749303	129.653830303132	123.013017662425	117.539037885384
	107.994880298791	95.3754438202863	107.251974803232	76.6094442444074
	147.023857419777	94.1575209300592	94.0777119695783	109.714619050525
AGK	159.603518169388	197.325473090277	165.867229890877	127.986477405999
	223.316311787046	144.954155897343	182.885934774318	130.533514998688
	187.147252401703	103.968071466212	124.041740442038	143.749408328062
AGL	54.720204938924	57.4989424388426	54.106779254225	55.307880834418
	49.904979214379	62.7924038489274	60.1837189855762	68.0796261751272
	65.5349012839562	102.655050997714	79.6452419769253	81.929796716054
AGMAT	53.0290605707162	130.848965290163	64.2404293863468	51.949834638817
	245.960009147234	49.1013967533186	145.267770598988	216.03402309102
	126.485649460666	62.1035858593388	64.2174369755376	64.650642183463

AGPAT1 71.1737934325596 56.7272804480684 60.8635925649592
59.1771672022928 64.4999048115671 57.5685357492202 61.5000955267432
63.5738862549306 61.5807110546718 77.0226403968683 64.1829732120822
61.3216070188812
AGPAT2 308.393270347486 400.633619994399 88.7801816370065
131.738693662183 76.9535968101411 118.638732417836 137.410781124396
130.716532316693 136.935255842208 129.820909990346 138.728648830528
97.095992167029
AGPAT3 41.7397731376692 72.93977835851 63.5896512396512
45.6677978788306 63.994439164071 45.2421183842044 61.3620062177524
44.6107027295193 53.0145811822111 42.1414432370095 44.4688999098904
45.9693232070002
AGPAT4 65.7899239985344 47.2299678280724 47.6040671292013
88.3459116251233 43.5544347011646 59.4309046716701 49.2546642180676
51.3472428383325 51.2066664465185 77.0703146713865 56.9320717885002
56.2804354745795
AGPAT5 59.7179445506128 70.4933405598359 91.1834104205004
57.8277957325085 78.7320333941149 72.4651758215622 85.5027511448958
48.0582855095143 67.3230935444252 46.441301934328 55.6972661824064
66.9239565096247
AGPAT6 48.1952521496489 54.7855575216687 44.0451487108213
46.1673159982664 41.5668884168669 45.9266745571724 40.2154497375715
44.1311654985682 38.0213051227859 35.8304004211528 50.7159667633757
45.3763275588341
AGPAT9 47.8249696958403 45.3486182278177 51.7138050344355
96.7799695976296 40.5710637884029 83.5274372175811 51.7714254834195
63.7002949745101 49.3780606970538 66.0224755625662 57.8991270166934
78.1623301184313
AGPS 209.138751343454 146.432425205891 140.719373122712 230.197573134703
244.226756210656 198.754967214875 138.923143963742 191.116764060948
117.678385591938 107.610858427836 115.052948380817 179.522572239758
AGR2 67.7831020218081 117.706922397135 103.64431747876 34.4382958501338
53.4109882045447 45.3882138838137 52.4111687156893 43.6434576713809
92.4839055864605 67.1278109419819 73.6362523983338 78.5108643908344
AGR3 93.3605294458382 41.5132248356707 41.9287023391451 40.8084239472012
45.6895223823001 39.5581166303303 44.9094465483072 47.4439407315335
43.1805016690297 40.8049408996274 43.1931731665131 50.2471139112912
AGRN 180.592281750722 227.791956085471 218.773587021224 66.224559122062
112.391188787919 58.9676332048764 147.115466503902 372.421330252979
239.385229462309 166.349012388957 219.771890732457 127.345094323724
AGRP 40.8681871019812 40.5400384479766 40.7222199182322 92.1725548553384
41.3387790895716 38.5252456306656 37.7136011474903 40.4359177791566
44.293922970134 38.4829727716328 39.2542963855807 38.2350720635382
AGT 40.5654775358427 51.0175677516029 41.528370959196 75.2475238554276
63.3638417360105 46.8954576545929 38.1793598983615 48.272324215504
40.0403898620771 39.5637454855233 44.8872784170036 43.6753843488695
AGTPBP1 92.9894133685392 64.2621389350588 71.2246788429078
174.332103484796 66.3075095149783 82.2838944641385 94.312912693545
73.0186209472046 57.3005868295668 67.6733764807973 63.7598547294225
72.1687283197901
AGTR1 54.0170817319522 43.3016395799961 40.5734458834617 41.2445895434274
43.2907292669418 153.028210322286 37.0564573649223 42.1860813954352
45.4799270968969 86.500678864429 76.5505585423421 58.4598072919688

AGTR2 36.2496364442264 42.5269793679335 42.5406888281114 33.6816578730693
40.8873907150245 41.9543948167375 47.4439407315335 45.9604707098953
40.3316153483289 40.7063426028871 45.4799270968969 37.5556299264699
AGTRAP 91.7437837246405 68.1040271620925 74.3239491140189
102.365615739399 74.6833250738044 72.7213787264217 88.3954618645976
60.7942162859036 77.4769634299218 52.4887473044594 62.1397042662581
65.2670315837785
AGXT 52.4179224871914 35.7393138893354 41.6977706798234 38.745552403895
39.0564222526273 44.3648993568405 39.0458123429605 40.047314525032
43.8482802038826 39.1621898366355 37.6484782516569 37.6557714476853
AGXT2 34.6977013306396 42.5269793679335 35.5220586859301 43.8002456102021
50.7615314686244 35.4142595025086 40.4359177791566 40.7311497914871
39.5806465938526 46.0119912782855 37.7384958256497 40.9683275553869
AGXT2L1 42.6036491382658 41.8534933577029 39.5118967430322
38.8495095797881 41.0476065156912 39.066284740632 40.3302268293707
40.047314525032 44.8636979668258 40.9558083259517 43.0016969050339
40.5703581862752
AGXT2L2 79.1369056968656 76.2193772627406 103.189199090363
117.408885431274 72.2016884438365 97.4967204298499 62.6236991757723
80.4967881959046 57.0690683036183 91.3418781023824 91.0847547974748
91.4786089554886
AHCTF1 103.080153415433 106.595042669127 107.844799332742
130.615540145155 150.529917336161 124.56803502087 133.41667178516
135.757061334489 97.9586375307737 109.922909644299 114.657635596229
109.832638804839
AHCY 629.763102939071 755.852629128252 432.945510073269 436.35723605534
1509.04753111016 640.596557352885 785.79878416775 633.819901764391
811.142611128461 569.244038644604 560.954913956997 579.038638774301
AHCYL1 526.146757036152 488.649780652311 379.79657860938
348.165210494253 365.459728613746 506.75912743556 367.685570437603
716.754656773542 684.238999553691 688.612748336336 785.79878416775
874.419252731128
AHCYL2 75.9909523932333 82.904037973319 104.135574209392
76.0975301977715 66.0411740221276 70.8674195845729 57.8870110456492
71.5062409407193 90.1430468048409 98.1835264890769 83.5895816521251
79.5898953726467
AHDC1 68.3234331335073 78.258486366912 72.9051985681184 63.6334154351075
69.2787430208317 72.3523300799007 65.0031119608637 51.555506218204
63.8740412329753 60.4837400912239 72.3822545261582 56.1875519519366
AHI1 98.0307965290686 92.5391033814713 112.532906407663 155.928417606775
84.7782034884529 152.17335005192 102.913039837094 181.093943398293
67.1278109419819 168.926743047741 143.042664581646 132.036058401203
AHNAK 433.447319956981 256.55408314817 278.850071205987 351.114115662108
188.30834153268 711.773459577386 168.70463124443 245.960009147234
314.992390390165 757.833254346899 653.880422442079 567.675954219815
AHR 1366.59129560817 1369.13346566895 1149.90692951795 1511.33844030783
1752.11152588494 732.454837219705 1664.00821059181 2545.03032507597
1317.29255644089 1686.40194722823 1855.0119962662 1375.14388134842
AHRR 42.7150227340364 44.2105195734073 43.0016969050339 43.9282059368202
45.8051846088552 40.4359177791566 49.3854238365325 44.1311654985682
45.2897806651929 34.9138625012006 47.0940613507321 44.0451487108213
AHSA1 530.710127703061 259.332340491331 295.371694979522 289.975375372371
433.320698413679 483.882804472844 377.679662622526 260.581111456578
390.906200053021 273.593213694396 325.949742917126 341.020604260602

AHSA2	89.2913927776683	65.9098543816057	94.717335243904	39.1706564265495
	134.099684731177	78.6402083787251	58.5087296620514	68.8058577447202
	57.8772482723463	124.362644216178	80.0113001016496	101.403739410832
AHSG	41.8409927585705	40.6380495994768	37.7384958256497	39.6292788039793
	38.4540849632254	39.4953494583071	39.4600924554505	41.4508241771315
	46.6944238390932	37.1275334308172	37.6484782516569	41.7575097298927
AICDA	38.304249186058	36.3110701990588	43.8495248991589	44.7980740332174
	40.9950639693255	37.0180934800573	38.1793598983615	33.8558232866797
	42.6366765186182	40.5090849993014	36.43924066428	35.7451401627836
AIF1	167.352766082239	119.947688643528	125.221639689345	662.041552795049
	94.8408264309872	249.729778471447	119.905655993777	188.196662406725
	49.4786466722535	80.9907498621309	57.6171094774963	119.39177524986
AIFM1	103.631449539809	130.177232923004	94.3450965454031	96.1825551158198
	165.29964739949	103.955842514553	106.367841843476	114.445779948228
	130.925724760241	87.7918574811458	86.1633564934019	104.611421732227
AIFM2	57.1046180136491	68.6074516459969	45.1569741609597	39.7411455670172
	48.3814421939888	64.7905331739366	83.6935756500468	56.8094693588238
	62.370559099758	59.3618843678775	54.8532558909227	62.4743608775767
AIFM3	40.7609952437622	34.6306468196142	35.2838283525874	35.6928955245309
	38.0598374201407	44.2607789091176	43.1931731665131	37.4484310268909
	36.8127605061228	42.0621425894624	40.1742127891824	38.4330755397458
AIG1	188.386331007465	276.360065404067	207.632489510045	100.001150612051
	111.77505926669	92.1151486847283	108.263706890599	144.034489386072
	103.934830132715	145.819204020818	178.525751043592	267.565744773928
AIM1	93.4355842714864	58.9128486419884	49.4573631027613	53.3967242431461
	46.3150221024046	48.5835484742456	52.7002314230604	78.8250642061439
	51.8824384192988	86.3374157614394	75.3969721155153	68.6047087267554
AIM1L	50.8826620053386	57.6831182451733	54.3554233228353	43.9798942439645
	48.0901896064056	41.0057887014368	44.0687697278114	71.6849360818041
	68.3157692917711	54.8439819445945	59.1002829627368	51.3709954257843
AIM2	87.6861665523251	123.219313750907	230.242190204243	113.783910320605
	46.5557478175976	49.3172204533818	50.1185085888209	44.293922970134
	173.343091136741	43.781101409552	53.3408430956148	52.3896785600751
AIP	294.443061798191	364.337811621248	411.047310950296	422.702925141293
	320.13475752314	494.300858189605	530.314249832491	286.68367978144
	285.12034141287	511.164722585602	487.261132702908	330.946598275585
AIPL1	43.8582747295766	46.4569566520107	44.8872784170036	46.3203995372658
	46.5557478175976	41.4508241771315	45.4799270968969	45.9604707098953
	44.2142581488029	43.7303813515864	46.5308571957384	44.3521099857368
AIRE	1468.75291542154	658.282562295783	717.997180920384	1221.31634960736
	577.993850127555	427.972184856393	913.704049709264	1999.21832626916
	561.2769907009	1652.85734308754	1588.70376317303	1180.7204565402
AJAP1	36.6002100641539	42.9034257147996	45.8409758448533	43.8524422305633
	41.4273122914951	41.3553369544097	43.6753843488695	42.0778399888414
	41.7117861328546	46.0727230219512	42.0424641526302	44.9070250604284
AK1	86.6655289455628	69.5544840164738	57.0427273538849	63.7909104347097
	119.41355955168	95.6783002876222	67.3976855661302	101.39385858723
	62.5542376788358	71.5948944013406	69.0663624160288	67.5047089908724
AK2	256.55408314817	265.790391813663	343.990395610093	241.51647940043
	387.039093527273	265.520773157991	457.097617649604	540.037371261388
	506.173054520807	304.658728492827	297.701126167694	361.524083649962
AK3	324.936044300546	540.721068846198	353.531051185171	347.003621791919
	595.012422454829	923.470702344816	527.439095170064	783.039774594242
	342.475405815033	1333.01876209753	746.252816568691	849.412637017524

AK3L1 112.146987452306 45.0115870036541 55.9459886217809 98.1701560122734
48.3814421939888 93.4494731509092 57.3286965007828 46.9354126752815
94.2987879109502 68.3324949971284 57.6511495792664 102.288104112092

AK5 45.7071930619474 38.2795150354677 36.8086106563517 40.3180310664156
40.188873541274 40.6994262313917 43.3744503319109 38.8495095797881
42.5406888281114 43.1447142498924 42.170702295259 40.9683275553869

AK7 38.2083710281545 44.0409178714232 37.7384958256497 43.1847628331565
41.9842695818285 45.1873487463472 43.9450023643394 39.4710743516517
43.1805016690297 42.4594149893798 38.6662194645633 32.7800533388945

AKAP1 50.2203578628217 57.1595768494068 50.4662238169527 47.1332119496722
54.3554233228353 58.4984917725442 63.0842071602791 55.3122077514658
61.6839343510302 56.4553747079294 61.0979269700412 57.1021912253879

AKAP10 67.9531693869426 73.9376772899278 88.878414939392
83.1614238528477 67.1278109419819 76.331185787832 85.8154958329408
55.1933004802607 65.644767142472 70.0907269565408 76.111908977613
61.9882398986333

AKAP11 89.680151549258 91.1363393944977 98.8595497262968
93.9805435116156 78.4428909886495 102.380096190518 83.014860007187
76.6868184563568 88.2639730639822 115.643361390258 120.123385036416
112.17141283151

AKAP12 43.1993673617091 45.620219291723 44.3648993568405
48.3749160953344 43.3982712035296 73.1640951333742 46.6267962186599
57.9713749600418 51.1596992013828 51.7343702554597 53.6578934390465
58.3164247463092

AKAP13 173.357715186701 120.679433440203 132.730723037077
370.64147085217 97.8825824657468 336.679355848349 95.9298109273155
120.240956797301 104.049367545498 170.015373751824 201.027801320306
180.902423012987

AKAP14 43.6706764143908 37.1685072112618 40.301483914446
37.2575268352423 36.8457174858615 39.5637454855233 40.0557788909923
39.6910816302609 36.5675023356094 39.6719190054379 37.6908810658759
36.9976231206067

AKAP2 45.5342598239058 44.5491926807637 43.3438593008468 49.239733414453
42.7107928951766 46.8954576545929 44.797320243931 46.4301247577801
43.2239171482126 44.571915452417 45.5936699846945 42.4322338033273

AKAP3 52.8427643366617 42.7775752471711 44.7829632472754 60.1837189855762
51.940083408061 42.8598805316863 44.3440839686558 60.7942162859036
51.8824384192988 54.3554233228353 53.9517841438975 47.7962548556026

AKAP4 42.7671420440641 41.554942319225 40.3452320167286 40.9157352260901
43.7517118200398 42.2189674040934 42.7431359850339 44.6484005362281
39.044540595942 48.9259041798949 41.1367478979484 43.3276027198418

AKAP5 35.1307934330047 40.148780650831 32.1218888714537 36.0481934423077
35.7973685350741 33.6859433472087 43.5627649099623 32.229154316188
31.2688290305385 38.0078357808393 39.0550139257658 30.6242215267208

AKAP6 41.3387790895716 38.5252456306656 42.2577689927563 37.651298468202
42.9090346219023 64.5187138759372 38.4005795891396 48.0582855095143
37.5469247328848 48.7414853386615 43.0016969050339 40.4639532172338

AKAP7 46.1513019317765 170.450752247109 83.616030092144 60.2674188885735
45.9725715538987 64.8903340853337 70.1845639720491 50.4463723277824
51.4003427453474 92.526038865874 70.4482288143151 70.7987307549177

AKAP8 145.883224917856 239.117228656704 216.782658096982 172.39907939484
206.963315276491 178.092166249096 138.748233664345 131.808634405319
181.340089705052 277.204818876581 204.944787289897 182.31306890697

AKAP8L 228.933483421328 202.325629133577 319.043862813161
115.63300104804 338.475097952023 161.817972012879 256.941243394182
271.436000380202 233.468474830926 271.529471399941 384.522115820048
165.684719727205
AKAP9 39.9697332660833 41.7614975630362 44.1070537445005 40.3691324547049
38.9496515344837 39.9397419279057 40.369768755239 40.6171774724171
42.3138951003676 37.6637152940566 40.723990462047 44.1452943804044
AKIRIN1 111.760213205992 200.483228109704 216.03402309102
136.293724800658 222.336600180634 198.979794834746 166.752205770965
117.074650076861 401.202348352506 77.8620267163666 93.1850674428555
127.78061946174
AKIRIN2 226.090904721424 401.202348352506 317.406465928295
1012.91803181784 178.525751043592 263.21493129287 243.27954361539
234.495626550751 236.565670642447 165.121380058529 179.974187861013
255.197732321307
AKNA 126.404988409795 254.639522735592 154.248946683126 389.583938820073
108.168107241759 191.141778714144 235.613437037284 134.085457101528
69.0037087543684 188.236842521312 197.421622315466 136.456679728935
AKR1A1 767.489657259345 691.94603958547 429.983084929605
757.833254346899 435.632247999054 410.257694738546 788.232434009173
687.862452802453 721.266942608349 465.595459259015 354.38923772101
445.421601813221
AKR1B1 785.79878416775 320.452418537108 378.795484311455
814.294297312057 4128.92158369026 1058.61208471961 888.56944380788
809.577874019028 450.139987981805 471.297503316471 442.711822532078
638.064750551015
AKR1B10 79.1369056968656 85.4420599474339 644.733901442573
40.646702696033 65.3019328668633 316.745819100367 55.4004611309636
110.191860661108 107.808744445896 128.616776019243 204.944787289897
110.731862140996
AKR1C1 34.1336988442837 37.920964499903 33.553634633839
38.2041059863839 32.9756367838345 35.314469458379 35.9894133432376
36.1375121409002 41.5635499110307 42.2614524730856 35.9563099719284
34.4570158133896
AKR1C2 47.6019996326339 437.84407370064 1726.90584784425
59.2241547536843 681.474524031866 251.803102904399 377.679662622526
82.2838944641385 966.803172864162 1120.83891847181 1369.13346566895
866.712228831149
AKR1C3 293.649670545233 714.075528895169 2878.82618761804
214.51120218554 2575.86919902296 818.927157206447 3478.67753301901
465.430886956232 4617.45430247751 3021.24285327836 2528.6392272518
3428.74022048394
AKR1C4 58.1027665533767 364.141443387516 1294.42501356365
66.1082848714279 750.151825182993 252.583117397109 364.795587956819
81.2228688683149 789.511653564341 212.353676518748 437.84407370064
761.341795853104
AKR1CL1 38.957687788868 40.7952353240579 43.0016969050339
53.593974682612 44.3557408297309 44.8872784170036 42.357910063876
42.157627240486 44.619512785899 40.8970401536578 42.4580504587581
39.6352613956998
AKR1CL2 40.8681871019812 32.2133093774284 42.4580504587581
40.3180310664156 41.1868052124344 46.4648868771662 43.0930293831497
48.6441988290952 44.1519466943185 37.7315666632629 49.3901004211918
37.9607839934735

AKR1D1 4277.93019582362 1900.96666834569 2374.1766901821
3124.84336032646 1673.60994518271 1756.86714809925 3001.25236839028
4015.88651424568 1771.43541011426 3786.90274075023 3726.73487426505
3070.83113612925
AKR7A2 295.841196113053 534.881442644054 662.041552795049
412.880824530845 740.423874031773 790.440637272613 821.013687795434
651.795139128843 849.412637017524 718.832367050609 586.281182112872
514.70012608304
AKR7A3 94.4618437533343 104.409397223301 120.149188463323
83.1614238528477 97.0813208326187 113.979504140326 122.720768249799
97.0589984189239 131.330480963914 96.9880759408512 86.1633564934019
78.2393202422664
AKT1 766.027617496754 555.044139654886 808.857027942253 440.728435508514
628.403814060213 603.737787922337 678.684833360104 641.462632112151
686.154904023493 547.072513229674 562.258108316474 491.468862309918
AKT1S1 52.109723558511 51.3800036046133 51.223859150798
48.4916117779992 54.4586176399381 51.702461863489 54.4934782594218
57.3286965007828 68.75304552302 56.3441077510744 55.6972661824064
47.9566297250148
AKT2 41.8409927585705 57.5197017161209 52.1242102345408 43.4410741327254
60.2886873195135 46.7800033479223 39.5496902678788 43.0390857698452
52.3579564953125 41.1222709786153 46.8954576545929 42.0424641526302
AKT3 41.8982199176791 40.5933302721015 39.1480645318984 41.7064916467908
41.7525817198982 48.3119523820103 41.1886975241176 39.8016018354453
39.6333888530279 40.3548989788016 41.2226607554583 46.0207973898744
AKTIP 148.871702513875 164.262531918679 192.112101000423 109.503117647487
104.599492214295 219.582785177134 199.152637378893 187.656257079035
177.935411895372 212.436948565474 189.398959817735 198.729330487352
ALAD 67.6767845246581 61.6878176260761 62.9967097752591 69.4501305462809
57.4622430729629 87.9871827215149 58.7585623394335 53.0338164320907
53.6982043633833 72.9051985681184 72.4834467838798 79.0918252621521
ALAS1 43.2239171482126 58.8281931439093 57.0032851500427 55.7418331108915
55.421880601441 48.1167207436826 102.063594231253 104.232196493024
70.0133734659853 51.1621379498014 48.8744644207821 70.25295977615
ALAS2 42.3433931248155 40.148780650831 44.6484005362281 42.7613994022988
44.9467652377494 46.4308160577459 53.3932110698117 39.9955307208157
39.6345710603047 48.1392888470269 42.4098506918871 40.5281496800812
ALB 42.0833588105728 41.7614975630362 40.244271403831 41.4636047919453
41.7929491793697 41.9069736908705 43.1602899171656 43.2890595186135
42.5406888281114 89.8035133597498 43.0016969050339 40.1700117452801
ALCAM 157.906415100288 116.596605311488 65.8455652907556 215.907790093087
75.5510570035251 92.47006193987 117.706922397135 137.302487233881
127.596368103014 172.83517237888 137.484063876244 218.354538624978
ALDH16A1 87.3342335690393 158.557256842416 122.024487499513
117.948047796034 220.597255695837 94.6167194649276 124.44575078108
125.484233748017 216.976116788572 85.6763929745253 114.629316822574
92.5011103881887
ALDH18A1 190.053043382767 180.756651647731 143.526781144716
183.613579501306 226.262409390222 139.883681090758 237.864776225441
157.082769443568 149.040814842403 150.396608064863 133.401306624347
182.188883821722
ALDH1A1 78.5216977073842 59.5534127075228 61.62834757456
134.519244495139 43.4885245598939 331.390452289344 111.496552406657

73.0394191668858 67.2022549641647 124.738024302128 138.486962379511
99.7529382840954
ALDH1A2 54.1437245703767 45.3756389170606 45.7968648044613
193.395549316561 55.4919197421746 86.4725057696307 220.36831243882
79.2701322339022 48.8524897151151 98.0967511777819 173.476817564711
75.5861488664745
ALDH1A3 118.316810638061 118.49449420132 58.2986825661503
104.672760332135 207.943044411392 184.898658656843 195.376772135023
201.941251308732 436.216696884703 229.697141930047 270.303708972854
393.001025840896
ALDH1B1 102.443884864631 62.7743500747415 46.7058093820839
48.055683217958 67.2779942308321 139.121494956408 54.5772630900888
52.3068458655445 48.8067321016961 84.0691608193405 108.779357041421
192.398575159246
ALDH1L1 39.4710743516517 56.6927078228789 973.363245512859
49.9014504704021 204.370291548631 83.2083460752986 131.097521229971
145.526972410673 356.318517117856 1117.26060212107 1035.67547271468
1183.62228577053
ALDH1L2 50.5252048155459 43.5627649099623 39.7411455670172
48.9944486068347 38.0598374201407 46.4329203134841 44.3440839686558
37.33030683349 46.6491671626143 41.3321861371202 39.6833800000587
40.1700117452801
ALDH2 89.646647563364 175.28322146764 297.950627905767 536.973599140459
406.104588997462 618.293140877956 172.285394357607 130.960165577212
107.808744445896 389.026128346842 382.312820492386 538.716947884528
ALDH3A1 41.7397731376692 83.4951332849212 448.290058854438
40.5484717678957 74.8013460023603 46.1513019317765 291.086512261106
48.4670130605028 173.212430860273 193.951097560903 120.418406532204
1509.04753111016
ALDH3A2 136.456679728935 107.264443219718 145.607100979838
136.461760992832 95.9750691854349 237.039707901886 355.7118155732
100.289042948128 142.545268664218 238.746837986347 194.536619596804
309.39599482851
ALDH3B1 74.6277165793069 58.8281931439093 52.5353664902258
108.928854330744 49.5666074434383 65.1291617069903 55.5578334877425
51.8347877274018 73.2607982527105 89.9615463738575 81.1350396749303
79.5270065657539
ALDH3B2 268.097966292089 87.7881690937563 103.356071080819
43.1131704517186 51.7456939295414 43.3356152004262 203.334053441063
374.026381773215 99.2898960399974 96.4072955983042 84.575384445856
58.0852316663986
ALDH4A1 59.2628513357357 124.646386399592 209.038077542654
47.8910351649754 135.29225447532 62.8131992098474 221.383645621978
98.5554785420459 275.549982777518 364.628602969366 388.74199767336
307.445116836976
ALDH5A1 105.308648028552 108.27606179864 102.394035383591
56.4578213622965 422.931891494195 105.267519627444 319.092568066532
212.062417120966 188.13862794196 184.123415143164 164.96500173066
168.492959515167
ALDH6A1 74.3135387703106 63.3948709726117 103.883220270094
60.2877434874622 90.2652818919579 124.338989399317 91.3992526702001
93.3718038663074 86.5616422570166 91.7227477250701 111.501058276821
99.2898960399974

ALDH7A1 145.561345203804 178.4681248137 425.776214512634
143.383133346874 647.297964303385 303.139550962377 498.608204176238
104.516141143655 43.3262148375525 425.522158941437 457.502917254598
641.462632112151
ALDH8A1 42.7671420440641 41.0177881453725 40.3452320167286
40.6907735219854 44.9936360641969 42.8181679217665 41.1818632392526
42.3180724671488 42.5406888281114 43.1839109702714 42.6913548400127
43.4709415392696
ALDH9A1 1237.93130196998 1598.78859278229 1969.8871504719
1182.10714525377 1914.9556343048 1985.3999418878 1483.40077183826
1127.96214301065 1142.67599037881 1886.05449875658 2061.66570137425
1473.52943897204
ALDOA 1249.41227924272 1640.74027400791 3099.21690111993 1021.71286017481
2002.0536042686 1046.42391243532 1130.2486883492 837.959914510114
1900.96666834569 572.667024472672 738.059797123491 1182.10714525377
ALDOB 53.7507429765038 52.1062000766427 59.2778887402357 51.223859150798
49.8304479381623 48.4758590019384 49.6872467499977 67.6082563445866
50.1603795873336 63.6197695952215 55.4694986901287 47.8931773060789
ALDOC 341.302185430613 206.931452638325 411.73316510051 247.193935816149
289.05213385509 197.569816397993 576.367042159347 251.33720343548
1125.31394383117 226.35806628102 174.584330660466 248.599530435666
ALG1 63.3717459766798 74.8071157013401 102.500172212095 61.2213001749372
96.5030525416431 67.9306669919176 74.7534423194688 52.1788489555942
82.2694707335812 52.6879797806876 53.4921736109774 61.2685222381296
ALG10B 46.0920218698606 47.7962548556026 42.5864346233886
46.3203995372658 45.1899271235974 41.8613406887824 45.8311458106942
43.9604529016594 61.1851254827343 48.5374448344297 44.7114813656401
47.2862299990938
ALG11 46.8141313516997 50.0425298550559 62.8362064074814 60.7148442408147
57.6791750431251 51.9421032049606 44.2753808209563 47.6388115824631
49.6078549993388 41.1222709786153 52.104880481326 51.7833333848627
ALG12 49.4151347850687 56.3560498705517 59.1040441894478 51.223859150798
58.5450868286167 51.1621379498014 47.4439407315335 60.2691630545162
64.3425836997629 61.6670071424359 55.3942006156899 49.9692385479389
ALG13 79.3539393480885 117.071280682786 106.679905803492 121.281573820819
152.187430924861 108.04376659067 113.866742186841 91.5258329186709
136.004535901075 69.805066579191 67.3269632644926 115.283739065312
ALG14 74.4062547641529 65.1291617069903 63.0670482657086 65.084620579901
65.3019328668633 65.6382617782876 76.9234119135629 55.2412344988347
91.6978282652998 55.3153799233744 65.4137646609535 64.1080166181228
ALG2 51.413025495745 56.1355483199198 49.8344973836834 52.5307002738469
59.0965219753805 50.6915708372926 56.4809146784765 53.0409330844067
48.9482359914659 51.4454142248566 46.29198852834 47.3992733452821
ALG3 126.775432305478 119.647202975116 161.205086002542 127.148417780972
245.853111206859 112.340869310085 146.840603547323 127.051878502711
163.766548456924 138.027432399991 120.679433440203 120.571680725698
ALG5 175.271140689255 262.579533452771 227.439319520245 306.578554914328
163.809567262808 213.799316520531 246.09270373893 161.563526062761
348.206465194205 221.015209694864 211.701234819986 214.537530518962
ALG6 224.976641879383 145.193498675059 163.028918580618 130.515102593803
167.746168796779 85.8059456975413 120.231435193839 155.099281008688
363.602775933416 128.749209040345 118.57174795047 152.36389833079

ALG8 136.155022329495 109.714619050525 192.922622120507 131.068763088422
192.509049577236 113.518333340953 146.299417934358 182.601427407211
188.457593309678 136.998739859195 149.832238573027 161.817972012879
ALG9 106.219480656782 106.8866235778 148.582198572949 83.5895816521251
114.611557317665 167.760135663101 92.0880591999243 93.7090094630558
161.326580647514 124.44575078108 118.720812623965 191.896416838806
ALK 45.9090659324223 44.5884811528117 44.3344900481112 48.4916117779992
42.3685136297603 43.4681646416585 41.4742337714895 47.0940613507321
44.2607789091176 41.2276963324981 49.6706535876636 42.357910063876
ALKBH1 69.5766550328677 59.9174456081704 69.11736279512
74.8071157013401 74.2769881586739 67.4018925617901 60.3928833351886
50.4511067853644 72.828269171954 61.5635554619635 68.0751111916102
71.4197504261453
ALKBH2 170.850089909832 136.67168846573 205.789533350801
99.2432911346944 532.196768917735 246.500690127803 361.524083649962
163.827112371305 201.875082892488 207.600413958886 199.937572174194
134.185571685143
ALKBH3 158.702003835578 225.654863797316 172.479002170227
228.615793354146 175.018819532801 165.322633260592 138.82907142482
156.844010597219 182.836805462079 227.817928402227 229.188779190649
161.739131587013
ALKBH4 73.8374823243007 52.502055890816 71.8706847571669
44.550322123994 73.9456720099807 52.1024135605001 55.4004611309636
50.7364620410977 85.5095013644374 67.6983931011126 69.1414574152997
54.5921287593554
ALKBH5 1573.61108703981 1591.51486001004 1611.35913846618
1478.26733379357 1969.8871504719 2191.09005731751 1354.50256889331
1149.90692951795 1762.93413846062 1783.87583818834 2054.64068255575
1573.61108703981
ALKBH6 88.0979122006842 127.437627356956 153.711194034581
95.9391851122771 200.698372627272 92.9820860411485 173.536155357688
125.786923817078 121.562769658314 96.0236731791095 135.92805172353
102.163854544231
ALKBH7 145.694779669933 169.970634268117 214.774035015293
203.559653641339 321.686871145301 213.981743647681 156.56745342836
99.2432911346944 218.013913036142 174.469283513638 159.268245287939
152.272399644814
ALKBH8 41.8409927585705 51.7495306734657 45.1569741609597
46.1673159982664 61.1216330559316 53.0290605707162 48.6395795163502
46.4672637826582 45.1899271235974 49.9493230064613 55.3942006156899
46.7182294568603
ALLC 39.8184668132023 40.6380495994768 43.0697338561114 42.5054997771887
40.4261913313642 39.8873706855774 41.8695090436685 41.3506832276112
40.8351751854504 39.0325254636537 40.0436653463863 36.7762265039864
ALMS1 76.6868184563568 101.699631771662 72.0243286940589 70.506090074243
81.7765447802116 96.143327683221 134.271727318694 127.596368103014
86.2563919366781 86.2118355769367 101.187156681366 62.3470274683312
ALOX12 40.2543341073001 140.369714831352 43.5710871617107
45.334106390212 149.792346524401 46.7800033479223 140.41738844565
81.1284422173774 43.7630587657205 37.5343260962758 42.2618615571566
42.9887069977696
ALOX12B 45.3872406432794 40.6380495994768 34.806104801006
42.2496337895968 40.2449343247539 37.8102105794655 42.4939802311136

43.3262148375525 47.27543600687 44.8997235283254 45.7328341667151
43.5738557773903
ALOX12P2 45.0985088502363 48.5905518770847 55.3122077514658
47.2740099216972 70.506090074243 42.157627240486 41.3094485574845
43.7396619462033 54.9426456410692 53.1309801459151 50.4009921377716
45.3763275588341
ALOX15 42.3620028137457 42.1218529375265 38.9790184198145
40.7481155008596 46.0566822875389 44.7804080899431 48.2768592317085
43.6753843488695 44.0141223586787 41.6373771565199 40.8681871019812
41.5961238651523
ALOX15B 42.668311948721 39.7509165247419 49.0629169775674
56.7351380543324 39.7568367479301 44.0804700754786 39.721757876562
38.956280281655 40.7847110517594 46.7646877499666 41.4784106456949
43.5367669381526
ALOX5 288.065258507995 557.455966274117 442.080188140864 833.136660528149
203.651890041185 207.087723777954 626.390544175817 313.660064080622
212.622278823808 588.247843264181 770.715044136717 645.408557927175
ALOX5AP 505.018127833201 233.850715388018 311.901423694475
5895.03230740197 133.541341560868 651.621028098039 589.912813291296
156.844010597219 96.1710806345503 195.543760781509 211.451463038315
433.447319956981
ALOXE3 40.148780650831 45.4143854024453 46.3411353377615
36.0481934423077 38.8456942580061 39.8455060654531 37.830876898596
63.0748373122119 33.4698406117406 44.4652605017595 43.3085358214154
40.7704587752083
ALPI 41.4508241771315 46.6996902348633 35.8265993915844 44.550322123994
46.4343327861696 43.9724628401189 40.5585676679482 44.4031868028216
44.3440839686558 45.4614234442527 43.401782064778 42.57175366709
ALPK1 127.072685504735 125.154933118907 140.030757545662 241.384699095468
110.009834478125 116.35075921851 106.317388820963 88.1861828863404
109.654559065946 178.219279777774 116.793627324217 170.327588764719
ALPK2 102.25509551881 53.4735821194294 42.0621425894624 81.6780581904253
48.8381507187986 42.750861693377 56.2181240696479 51.9447450229707
43.2761454096265 34.4875013487777 40.4504608195666 40.4639532172338
ALPK3 44.436995101921 40.6380495994768 38.7745734037492 43.4410741327254
46.6397391251762 41.4508241771315 35.8227939208521 41.8417024632349
36.0741672064298 45.9004543328976 45.5707650534259 39.6352613956998
ALPL 69.1641374421455 126.14245679817 129.810477623628 708.005870621064
130.293915348934 100.017263238916 253.237824005249 85.6673237797982
63.1944558106132 60.8730416435467 122.15017220566 66.2305843819029
ALPP 2018.28984520892 825.558929673308 1260.45604038487 1554.42895572029
767.921811623919 621.012072500314 1521.20630233299 2844.5506310828
712.80113322987 2456.86796652313 2306.14613042834 1526.94101719128
ALPPL2 61.2130609867477 46.1569135229922 40.8420925848403
51.5913529948887 46.0043807840705 49.7458652239071 46.5650556481721
45.2806199505029 40.9901805728485 41.4397539024976 43.4709415392696
43.9541718254769
ALS2 188.272588632909 151.686513723244 147.34358398245 133.365282773876
187.21383075601 156.259762538052 128.966814822678 146.719419166639
145.607100979838 168.522052983892 135.308001652191 141.255712367708
ALS2CL 44.7915315085948 51.9601694822199 73.3881007284998
40.7941759414114 49.1928206164702 41.9097689475185 46.9929836306816
43.781101409552 45.7226406704445 59.4309046716701 54.7883681830995
64.7703246910536

ALS2CR11 40.8681871019812 38.4330755397458 40.301483914446
43.074472921841 46.5557478175976 39.4953494583071 45.6042625176633
48.4670130605028 46.6944238390932 40.3235655331796 40.9157352260901
43.9541718254769
ALS2CR12 37.6397652536077 35.6434124419782 37.6557714476853
32.2212308728305 31.4686555405126 35.9305494063137 33.1392460259336
43.844855336215 33.9456837661864 37.0320432433314 40.723990462047
35.3525469364579
ALS2CR13 265.320447667174 240.934550408797 206.559526386642
175.271140689255 262.215263489321 142.03031230486 231.060688044554
412.162238678684 318.920760335454 551.304967553891 498.958550919515
450.759762047652
ALS2CR14 134.113913870501 83.1308709614708 92.1646038105091
143.822560762049 78.5141640924736 76.5888466260562 111.025105784166
227.642500471069 74.1282220615085 149.27230040094 121.729442469889
106.912316023598
ALS2CR16 46.441301934328 46.0713306971594 52.0742951366864
39.1706564265495 44.5668141895992 44.2607789091176 46.8613212048981
41.1575777497576 43.0930293831497 59.8130420783374 49.5923712603197
46.5650556481721
ALS2CR4 186.522518256229 178.675242612997 235.925374041038
86.1703662107384 367.081499539744 154.355197155161 200.002687565519
256.843419520398 249.729778471447 117.293778127059 128.873145026756
153.940127525369
ALS2CR8 45.3872406432794 46.8954576545929 55.094385444315
47.8164193292842 43.4544762830742 44.2607789091176 43.8206425661746
46.0633179231318 47.7086093893038 46.5650556481721 38.3684202018524
45.3763275588341
ALX3 41.2424637338424 43.2649781354792 41.8389336728199 39.9084621723809
41.2740822675592 39.7394181011603 41.8799523546812 38.5457716679071
38.2215591930186 37.3179578037963 36.1812049498986 40.2237056928605
ALX4 37.4554876386402 42.7775752471711 36.8730514568925 35.3829784221072
36.6354013629791 32.2133093774284 38.2933870238743 35.3608605269276
40.0403898620771 43.2988017098387 34.9428206949566 37.9607839934735
AMAC1 38.8561375693335 43.1805016690297 37.9482157381008 41.0726336841311
37.3508690072156 40.1428591998404 36.8369172900902 39.9576138799997
36.0741672064298 45.0155543555707 35.6041926503637 39.7411455670172
AMAC1L1 35.8208542506595 40.7952353240579 40.7620472083159
49.7350276169552 44.147557390293 42.9695585740813 46.4388841354122
43.6434576713809 45.0017158586743 41.1222709786153 39.3667161654772
41.3481355128089
AMAC1L2 39.5068753428772 40.3180310664156 40.9861485852278
43.1131704517186 39.5317722761104 45.8325181856483 42.1607322031054
43.844855336215 43.2100129360085 42.4594149893798 40.1742127891824
44.1211971585961
AMACR 61.356483470053 135.19133529995 75.9768176242642 47.0033848638449
83.5069968129733 77.7481370715949 148.570371099987 189.650905999631
95.1224420992193 51.909968572392 57.2008272301399 61.0025829061063
AMBN 39.8334299607129 41.9390340055841 43.7630587657205 45.4912838971528
39.5357194705749 44.0430338499507 42.7431359850339 41.1975417470633
44.9645354363967 41.5852317867787 38.2293008890613 41.5262906399874
AMBP 49.085741080241 35.5310164682026 45.3974838013476 39.9697332660833
41.5668884168669 41.4508241771315 43.9450023643394 43.2551508830106
45.2897806651929 46.5650556481721 44.7114813656401 45.5800840647989

AMBRA1 42.3433931248155 44.5884811528117 40.6737160001291
45.5869891570251 43.1252272136667 38.5252456306656 39.6535880152834
43.1567024035183 48.2123120250343 49.9493230064613 43.0016969050339
50.8999542937724
AMD1 398.43592055172 176.963666283432 203.178221073902 220.454785391748
246.725921565571 325.275883498219 176.839214683647 487.065758294354
160.100408020768 123.918925337643 143.693373312575 209.497482517298
AMDHD1 41.8409927585705 44.5152708955623 40.5734458834617
65.7321440516177 43.844855336215 43.7630587657205 47.7390230243778
51.3472428383325 44.4395580493118 44.3648993568405 42.5426057453993
43.8575940017119
AMDHD2 165.606015336587 114.965999573704 114.84900130932
298.924873508403 90.385018479478 155.270252661048 175.165302282522
249.195312452368 82.1974756449763 83.0700092537618 109.076358658606
59.3082373468217
AMELX 39.8662795144616 40.2493681589104 34.5718473203083 41.5464765641928
42.7982502551634 39.2985791324641 40.3302268293707 44.797320243931
41.074824727477 40.6022003765153 47.8284278211109 41.863927676552
AMELY 39.0620487644635 46.8954576545929 40.931294690669 41.7614975630362
45.2556760873335 41.6590564937735 40.7910160811527 45.1714698794331
47.3791873993182 41.7405682794166 44.0409178714232 48.6037684272115
AMFR 172.990533342251 111.492046718579 149.330073823683 74.9236800269025
147.017279035061 151.641746442323 112.362505628749 105.965906809429
83.105117076029 106.486195938039 62.3990310986805 119.243515595981
AMH 69.3418521417182 40.2493681589104 41.528370959196 38.4208429103799
51.940083408061 42.0574848021044 76.0041771381018 59.5830868881343
127.82585786161 42.6793753550854 38.5668610487033 39.7411455670172
AMHR2 40.5654775358427 40.5400384479766 44.2405728317107 43.6726153508978
45.4751076037511 57.8005007012205 38.2933870238743 41.4508241771315
37.1820543575123 48.7414853386615 46.6442692119997 36.064718696392
AMICA1 94.8260360398356 160.973602503707 117.689172019617
940.781370938596 92.3034609409756 120.123385036416 144.227861902786
95.2292899846287 54.5952754712007 95.0026207727863 104.626873082138
81.6458112867541
AMIGO1 38.6454098044474 33.3069622728735 39.3709357615493
37.0732977341185 43.844855336215 49.2050538150106 48.1747307200218
39.853866204927 40.6957787921451 45.5776159432079 39.7342718293432
43.7672102682067
AMIGO2 42.9104454767916 49.4982025424177 45.989212296035
66.0397471691747 42.7107928951766 41.159706131351 52.2476631126873
46.3685439301234 44.8003345643334 42.3606390470387 46.0095710163378
46.5650556481721
AMIGO3 43.844855336215 46.8954576545929 44.9758339877851
51.5913529948887 43.7517118200398 46.0364000143537 46.1634822993009
39.3709357615493 48.8067321016961 43.844855336215 46.2560238004757
49.1200355938198
AMMECR1 56.0787402018234 83.2873728545705 94.9785931907153
46.0860140689254 108.063001800611 66.2167164788023 73.0433648756396
96.6485200415333 136.766779297051 62.3470274683312 63.3872214856708
76.0240426902567
AMMECR1L 113.298540312696 93.3718038663074 77.0301627655229
72.6824533717231 87.8170592981737 74.0995052892511 63.2726414544553
101.39385858723 77.6496445815897 106.641283957692 87.4273245333478
93.1067450332473

AMN 45.6517320730042 38.7344853699576 50.0748292497578 38.3166554965434
39.5595233162192 46.574682061365 42.357910063876 42.7431359850339
41.3803269429468 46.325232414659 41.3553369544097 40.0557788909923
AMN1 51.1740855632044 48.109747794187 37.3687900733549 41.1886975241176
39.1650142708257 46.3715909653832 44.2166118829877 37.6557714476853
41.1742244762318 44.2580151149956 55.6972661824064 48.6037684272115
AMOT 72.4966502412724 51.6869155634183 42.3536549114165 48.856176220627
55.9283039581662 135.361059757705 98.0023132593711 104.089998273812
77.9978216366573 103.264100180823 78.6402083787251 73.408861359425
AMOTL1 45.9090659324223 51.6869155634183 48.0191002461099
55.8709511400674 50.7615314686244 51.9421032049606 49.2546642180676
48.4670130605028 49.1940901905636 59.8130420783374 46.2560238004757
49.8253396161069
AMOTL2 33.5994525227062 36.89524015739 40.301483914446
38.5252456306656 43.004943518025 40.9506062495231 34.4570158133896
45.8669209716694 34.3842972343227 40.5090849993014 32.6463318577756
42.7782216342806
AMPD1 36.9060997041453 45.3486182278177 37.7384958256497 87.9098670472958
38.745552403895 37.7000400506818 37.7136011474903 42.357910063876
37.1820543575123 41.3321861371202 40.547835538778 40.2669497398702
AMPD2 39.6609238036815 39.7509165247419 39.3344387353654 39.4469765850607
36.6354013629791 38.0698669928229 40.6750540874299 36.5919488248006
35.9083761938612 35.3362091871364 37.6122808321894 37.3600014814388
AMPD3 42.2455707947223 42.6471103236957 45.0985088502363 53.290742997456
41.3755760434934 40.5703581862752 42.9214252283214 40.6171774724171
44.3780249232864 45.0885830154678 44.1148826235374 45.7958263385498
AMPH 61.5112659162073 44.2630383225933 42.108130037421 51.949834638817
73.1215732923523 61.8571521441451 45.4799270968969 46.0106098064475
46.6491671626143 49.3000844711116 49.0361180089431 54.0733528420802
AMT 58.1868359012515 227.206974078547 343.591727067749 71.7167037224069
241.202687910704 102.163854544231 183.584085676174 87.9468208580228
66.2552876795581 273.349866592589 178.012676342712 177.088290547513
AMTN 40.5654775358427 39.8695929627783 36.2683886571585 36.890925044696
38.2496050953547 38.1169703906207 39.4600924554505 36.43924066428
36.0741672064298 38.1042454705119 39.9899430628542 37.4554876386402
AMY1A 65.5495324730507 96.3918711895208 67.3469281984384 67.2968280876013
87.4623442823265 54.9460876400605 88.2201332015312 86.5316479840325
84.7503257101143 55.7930787690756 59.9876575158981 56.6146179789815
AMY1B 55.0823430906161 57.5197017161209 58.4137028673494 56.0794100106654
63.7818442618767 47.1389972732223 58.7239345900248 61.8665316900291
56.9733113918431 51.0506441018166 49.1653186595732 47.6040671292013
AMY1C 1928.53890809223 4344.25847766877 2951.88559311416 2306.14613042834
3854.42494497831 1981.03929621057 3296.66056024983 3421.24021192326
3653.59181300011 2237.08790397818 2337.7738990994 2164.51925341969
AMY2A 43.2135791259317 38.8336427948187 39.6080759138364 41.1426570149164
39.118426249677 45.7812172258509 42.6913548400127 43.6753843488695
38.1725197931105 55.5588970907417 53.1221282279739 46.9939712192224
AMY2B 44.1294459089314 44.8529259387308 42.4890076400051 43.6726153508978
44.3166120733789 47.9077889806053 42.0605906240248 42.3110002461296
39.6413313949444 47.9056710878325 50.8373208590626 46.6397391251762
AMZ1 42.2455707947223 37.7384958256497 42.9104454767916 42.2496337895968
46.1804647841331 40.9506062495231 41.9393838138203 38.5457716679071
44.5303781239209 47.7180816682562 40.723990462047 47.3172462673426

AMZ2 266.717224821545 325.909358171616 421.611594456877 299.524171071613
331.001195453225 350.584135047249 303.93332785291 294.048088688381
384.727543700024 281.906398109158 268.265540230814 384.280434953152
ANAPC1 104.642326714249 135.344261822716 117.732966326465
83.014860007187 209.138751343454 115.954295099681 115.016486106706
154.551927302005 126.319731178054 116.817127851913 123.658435242998
106.641283957692
ANAPC10 88.8760662992208 124.324905704041 112.865496075486
127.596368103014 145.193498675059 131.727983826985 111.202737864873
98.8478655411492 148.016994658777 107.906865732219 93.5045354990096
143.053774297331
ANAPC11 122.904323796484 101.987604729928 104.773064067069
110.757899996422 149.27230040094 107.087870999702 176.991120397205
137.831070407907 168.58183910352 95.8075612515186 99.9752275166514
101.857595722021
ANAPC13 504.281506941044 779.996851709716 560.878675769733
605.338871441599 1260.45604038487 802.036670929858 413.067407579649
381.198152331624 450.139987981805 539.471011648536 562.531738167208
1018.35211991691
ANAPC2 50.0802438185974 55.8046062733709 47.7737810047679
44.0451487108213 56.432280646473 49.2871583830897 54.339958888486
46.2286164776607 54.7033747046992 55.1804535845496 57.9864701726379
46.8840006833906
ANAPC4 214.586915093492 216.063990451376 281.774690337889
260.581111456578 219.555240189863 226.235985918654 267.107863045114
262.624624672404 245.853111206859 525.16535463798 344.133384411423
435.325038193179
ANAPC5 687.011162822211 831.578043520215 864.302844775203
613.740813446728 708.005870621064 539.471011648536 683.099079617304
557.094756796515 860.265067346622 635.906934423716 642.946369683508
530.314249832491
ANAPC7 77.1900613562529 107.836867810972 107.413918714848
56.0794100106654 110.411217828763 106.590506599473 83.5149142053137
56.2441895183416 110.312681023811 55.6865353018811 58.6464918752765
65.6213260281822
ANG 159.306689057291 56.8017497008794 57.0775902639215 161.65846687852
50.0580547772867 273.593213694396 77.9493708990494 99.5958921252583
47.945438269111 263.636675573982 214.281036491237 242.966185793816
ANGEL1 123.963659641243 88.3636380348802 72.9051985681184
88.5511408870489 116.099186392258 83.0554837248934 83.6935756500468
90.6347108049639 65.644767142472 86.500678864429 79.5743379965984
81.1189042503645
ANGEL2 254.756894822368 276.633536223159 459.737628144571
222.985386635178 204.490115029568 269.681925095165 263.706801812277
235.169335305672 224.450710664296 396.167219131922 275.673080691335
196.623560874725
ANGPT1 66.1650385535549 49.2934904960601 49.1589534780597
43.2551508830106 53.1884505015411 98.0473811574767 44.7255207589606
61.2343203799193 75.891637602914 64.6835308827804 56.3884556031093
55.2886892579203
ANGPT2 271.601892365742 221.450737998571 589.685739519627
93.7782490229256 220.167309646966 144.17470770145 82.2650787041549
81.6529991377083 95.5920087912438 50.7855742231932 63.1566669782858
87.2175634803271

ANGPT4 41.4508241771315 42.5269793679335 37.6557714476853
38.3166554965434 38.4540849632254 36.8059561571675 44.4639405506967
38.4540849632254 40.0403898620771 39.6719190054379 44.4688999098904
35.9604262434141
ANGPTL1 41.187562301961 42.2900457798748 40.301483914446
38.6835649080278 39.3667161654772 49.2871583830897 40.369768755239
40.0965255448873 40.7460990419215 44.0719548209593 40.3452320167286
48.6447083765118
ANGPTL2 514.261598258395 94.2182728193924 197.608255265624
196.760671234837 118.10944558223 439.558287621096 91.983638239817
125.013317684129 46.3203995372658 171.043935516593 211.829374574045
252.712633314274
ANGPTL3 35.4287975493553 37.958111840848 39.1480645318984
39.9697332660833 41.7419877611072 39.8785903668981 41.5464765641928
40.9861485852278 38.2215591930186 39.3113207075992 36.8282383070068
41.9741368971524
ANGPTL4 52.6960621424156 51.8315727494672 203.010958724293
71.5249259772583 66.224559122062 66.9340967279385 52.761508834671
70.7238101239421 227.791956085471 54.8439819445945 62.0404348860702
200.615897175992
ANGPTL5 36.1232327599945 39.5050383901884 35.2101597814352
39.3750969348308 41.467005949568 80.0243450227647 42.0605906240248
39.7509165247419 38.9006788676407 46.2286164776607 47.7535797917441
42.2577689927563
ANGPTL6 43.2724556187214 45.2556760873335 38.6727842796202
46.0228732507872 46.9906120932727 45.1873487463472 42.0605906240248
51.2623287419357 40.5912213523345 44.571915452417 46.0095710163378
42.2577689927563
ANGPTL7 41.2424637338424 37.1685072112618 38.5045333213784
39.7411455670172 41.467005949568 53.7442594289333 34.5633623842083
36.5393857306744 38.7298222155532 50.6486940895674 44.7114813656401
41.863927676552
ANK1 41.6354649482853 55.0009147092272 43.4709415392696 39.9084621723809
40.6723334103791 38.9378717994178 37.2892641289258 42.9887069977696
45.5779686360641 41.0057887014368 40.1362682639916 41.9083581529276
ANK2 41.5421429082231 36.7976562409506 38.2280071951674 40.591923288644
39.7210115145672 59.0162231611996 38.941855825269 42.1860813954352
42.0126083977597 41.7405682794166 42.5023941254529 40.2237056928605
ANK3 50.0697497247754 47.0967801720364 51.4308501933893 40.0698728584484
51.0683600511812 45.338588656043 41.706879587091 52.9813204449021
61.4329643389309 42.9866214102525 50.4022543872993 48.9892593408116
ANKAR 40.8091178323174 43.0227387991135 47.7052132264218 37.3600014814388
40.7779767565889 45.3233134695905 39.2533668167483 42.2577689927563
39.5806465938526 61.7502624458447 48.7502645382856 45.4799270968969
ANKDD1A 80.6434122355417 106.398825125486 111.880226272362
126.14245679817 132.81898450496 203.178221073902 113.322929203297
114.533239859288 155.617122020913 521.029507993615 344.314901683001
431.353025968649
ANKFN1 32.6285977114039 33.3069622728735 40.301483914446
38.5252456306656 39.3667161654772 32.3796054199032 35.8227939208521
44.4031868028216 35.6139301508335 34.1648535095605 33.1645364749371
39.7411455670172
ANKFY1 98.7215951303498 88.3636380348802 97.1884078242617
77.6056319020116 74.4722810710524 83.1392079778036 84.7782034884529

66.5774376622485 74.1282220615085 115.342852277396 116.253417059012 105.34055372228
ANKH 49.2091117967683 43.7410507935043 41.3366779545815 38.9074189954657 34.0927410219282 44.6524308640784 38.2933870238743 38.8159908342801 40.1738310955128 40.1613888954735 43.5258090162298 37.7315666632629
ANKHD1 80.1549028765037 79.8701900696361 76.5053813427296 92.0269919454947 87.6178717341967 96.7799695976296 71.8466937395628 74.8377761440265 69.3645322033576 72.0443425395404 70.7045582269595 72.909783842776
ANKHD1-EIF4EBP3 56.921728372453 64.9959712888709 69.9179689232341 49.9014504704021 37.8679241954337 52.5330333302271 59.6780848951767 73.9624143741222 82.4467808843629 84.7642634532073 71.6502000742477 68.9258122619781
ANKK1 36.2496364442264 53.4735821194294 41.2365089689635 37.1894304543751 37.4470183315826 34.3887641786747 48.4859552186772 38.2782436497441 33.1602385985469 51.5855515705917 42.357910063876 39.1553169409075
ANKMY1 63.1291723738553 73.3395284923099 73.4122412764173 49.9506025936211 49.3970231087231 46.7442228855095 51.5338331947847 85.9970657980042 71.9771425105735 79.262977183608 76.2876332597671 61.6702707724448
ANKMY2 152.585423208022 168.78407943637 178.738800467958 106.8866235778 167.319747725488 183.584085676174 209.467874193952 187.711309781257 178.4681248137 240.217589414972 175.009248614892 252.134800295199
ANKRA2 116.685453817071 221.22852405053 250.585851778133 131.955454394903 149.24022100428 144.046404221564 115.314880288079 114.113460279317 164.343309332109 299.74710723735 213.118335449552 237.100802227545
ANKRD1 38.304249186058 35.9305494063137 41.0356368374708 43.5627649099623 37.6527901109005 36.9060997041453 38.0598374201407 40.4359177791566 39.853866204927 38.2069499294002 41.7272875295469 58.2251348002272
ANKRD10 224.030034866938 195.108184885541 149.09682467711 143.399636653204 166.064317271991 155.123252685926 97.7620313090597 142.545268664218 128.126336084436 139.543015878846 140.976643799742 133.244526666651
ANKRD11 157.493875535302 212.622278823808 226.672191323896 155.287591646469 209.860757709908 145.170590045628 207.632489510045 105.220978559157 195.635797217403 122.419178597878 150.925721934159 177.958475301951
ANKRD12 143.265312025262 133.558028920419 129.091447141381 235.544416908354 111.357508398822 186.818097299665 134.713725350463 128.827672077002 155.148913816397 174.860894067053 173.819948756127 161.38599012777
ANKRD13A 401.347734852083 265.367588517131 196.760671234837 353.213447404132 218.245471601353 445.016226732679 425.174170332175 230.879083362388 237.401487995882 347.908556198025 295.014183084118 296.664472126617
ANKRD13B 44.436995101921 47.5647821785529 43.9450023643394 41.1886975241176 50.3713170502557 48.8744644207821 47.8947595316832 60.9009676344258 49.0986627051784 60.0372015630618 47.3791873993182 41.9430737035925
ANKRD13C 94.8260360398356 88.6663961027926 74.2769881586739 77.4676205299631 76.301438841111 87.0685571351947 80.7563265626648

69.4677195088043 61.3733228199803 66.274247400793 75.0704619826557
85.0113628886031
ANKRD13D 47.3289285415289 61.9036392303558 63.5738862549306
44.6905691366696 54.6111272572767 43.9724628401189 54.8860931820001
60.5571721986885 47.2134341420411 56.6135586098886 58.4139484810943
52.8144580580955
ANKRD16 74.9179396623258 73.5743059777365 55.3812135024656
62.6048727240291 110.125787064192 63.8740412329753 95.8002081462966
69.6693950681855 93.9387650094061 58.2297667026887 64.863894209343
56.0477607422828
ANKRD17 101.144615206297 95.614885914917 96.8945937999801
98.7215951303498 88.5140510969737 97.4967204298499 85.7013212907541
77.3640580819535 95.4942268742467 86.9626166234066 86.9329220905001
117.228320608065
ANKRD19 38.7537364570873 52.5380506348717 41.0356368374708
39.1706564265495 221.22852405053 45.7300501624017 59.1774824918359
49.7309743973184 39.5806465938526 47.4767851750188 40.1742127891824
42.6758568421347
ANKRD2 37.7384958256497 43.5873188565519 43.3085358214154
42.8235313007746 40.148780650831 43.2805590023412 44.2166118829877
44.8997235283254 42.2577689927563 40.2112371512838 41.7272875295469
39.7411455670172
ANKRD20A1 44.9370339275041 73.7133884699889 66.1185883826035
63.3612993417558 43.7517118200398 50.5148383400703 45.5629520869561
47.3102170376006 50.378948785456 117.835389983392 91.2769094348604
93.1668080716761
ANKRD20A3 39.7320564340365 39.5573415356949 38.741573807882
39.853175885492 43.2484996523549 35.1307934330047 40.968974769473
33.7279747699292 41.3468610075772 37.4642807402741 36.7892997857376
41.1868052124344
ANKRD20B 37.3463875295726 36.6968906669565 36.8196632231462
37.361552364697 35.5942749164671 37.6484782516569 39.9397419279057
39.5173442227948 36.4476883897928 39.3698811593364 38.8034431562381
37.7357214460683
ANKRD21 35.1307934330047 37.10170285405 41.0356368374708
41.5464765641928 38.5512429290626 39.7394181011603 41.1507980856436
34.6977013306396 41.3803269429468 39.8873706855774 38.956280281655
37.2810767789306
ANKRD22 116.852483297949 128.952325188088 81.2359429678503
168.798543634965 56.1186281675573 51.1081003576577 73.9192243586276
64.4156641487994 103.577201248705 149.386774957393 163.585662735512
164.435300801872
ANKRD23 44.9896851005259 48.0296332183129 48.8100349749783
46.8249967544573 43.9994592139885 41.9069736908705 41.3094485574845
48.1591264215184 44.3440839686558 46.1390887492649 43.1931731665131
46.5905131500956
ANKRD24 42.6575535390785 48.275452803061 42.9104454767916
40.8681871019812 51.1915474019836 48.6621527194963 39.7576136507593
48.9826868640753 43.5669306063196 55.8510296341925 55.898892031444
55.216338306812
ANKRD26 60.4593810615882 54.2445399731644 74.0913177754682
51.3851157261451 81.0259717888094 58.8605811695948 70.8835636550472
56.4779645313958 78.2393202422664 62.3997407955361 60.5608545741456
66.0224755625662

ANKRD27 53.593974682612 51.524711857679 48.3847843498476
39.066284740632 71.4183281327686 63.8740412329753 66.5422130374814
61.130286531713 71.3932787152381 61.6670071424359 67.0361353184222
61.2108152457954
ANKRD28 71.2246788429078 71.9539136431119 72.5288683768049
83.764882413379 78.3542840447759 79.8026267036553 79.6581393791238
66.9340967279385 64.0903425729408 84.1639779987105 72.6731815563159
79.7808217577226
ANKRD29 40.8681871019812 43.5873188565519 44.2733573199954
85.9799968870368 42.9655396732962 103.569150754726 38.6492234339227
41.0844534776541 38.1169703906207 57.0581769379589 51.0492389286271
52.2832912331043
ANKRD30A 38.7270975453573 35.8618428755446 37.4642807402741
40.7941759414114 37.7115233202949 38.9378717994178 39.2809305246091
36.43924066428 37.8319142847141 37.267270263961 41.3553369544097
35.2115468000258
ANKRD30B 1311.88650141004 743.094853186365 868.899376368472
1205.08559206317 597.996273468244 470.676006277762 976.566026758227
2096.51999792183 529.210992541911 1578.32969650385 1673.60994518271
1234.59589464045
ANKRD31 37.4554876386402 42.1607322031054 34.8956628705329
32.9046688061235 41.8806343997257 33.6859433472087 36.8369172900902
36.1375121409002 36.8127605061228 36.4651112441576 41.6336636285875
34.4570158133896
ANKRD32 46.3884811643547 49.6479308322777 48.7039705048129
47.0874209746031 48.5000946421718 42.808889470973 46.0228732507872
54.720204938924 54.8439819445945 41.0057887014368 48.1552989524108
44.9734311311394
ANKRD33 320.086549121732 187.347386400244 279.782392390288
823.933615182634 197.52820443061 150.801729138762 193.01447006167
233.539376791695 452.2453761868 282.115786635657 260.824949424185
288.552986300755
ANKRD34A 45.1912496459517 44.0409178714232 40.1985790927248
42.9358255168086 55.3100442506299 44.8872784170036 51.3064929551674
42.8482181313226 42.7150227340364 56.057906531592 50.0303076717704
47.3992733452821
ANKRD34B 45.1912496459517 36.1232327599945 43.3085358214154
42.8235313007746 39.7568367479301 43.5752393599169 44.2166118829877
39.4710743516517 42.6366765186182 41.8613406887824 39.6403089864678
33.3192849177028
ANKRD34C 40.148780650831 41.159706131351 45.7968648044613
51.5913529948887 40.4639532172338 44.6524308640784 45.223075311113
43.4544762830742 46.2027193115397 42.6793753550854 43.3085358214154
40.8681871019812
ANKRD35 83.2083460752986 51.3084273211359 77.3864518736634
76.5411379733694 53.7554360529143 227.598514023545 40.6750540874299
71.7624419474395 56.3737927112624 197.52820443061 169.970634268117
98.4699679900651
ANKRD36 43.0568990939473 38.2688405721389 43.0555549979484
46.839133315864 38.4135023167287 40.244271403831 47.091779053479
45.505885458784 43.6670755115848 40.5537267820608 40.1428591998404
40.3295323726475
ANKRD36B 56.8534370691413 48.743491202539 49.8899020044306
68.8167148906225 49.5136713986758 53.6858208094027 74.0039764902057

67.2714646016644 53.2735797144627 51.5238121536228 56.9628808664342 56.5914743235484
ANKRD37 263.53897660224 364.628602969366 99.406307365363 336.795797976236 91.9685593100867 186.965367827758 295.848968688724 153.940127525369 539.471011648536 111.056599389048 96.3495443565738 104.089998273812
ANKRD38 60.2547250212462 39.7835513496873 48.7039705048129 41.1886975241176 40.148780650831 43.4681646416585 45.4799270968969 42.0494116081998 43.7630587657205 48.1392888470269 46.0905079010237 39.1553169409075
ANKRD39 128.923092916744 110.559251404688 112.007118067348 99.2432911346944 136.736085245679 109.832638804839 131.097521229971 100.989056102358 90.9202199678627 102.607924735378 91.5541137821803 85.0113628886031
ANKRD40 44.8914371498528 48.7084357480922 51.9214790114499 55.6481366056639 41.8806343997257 46.4648868771662 38.1793598983615 43.7396619462033 49.3780606970538 49.7259371799014 50.3876754565799 51.8562635270111
ANKRD41 49.085741080241 49.2050538150106 44.8872784170036 51.2973486610884 88.9038123445083 49.1718303518849 49.1928206164702 47.4740726032707 50.7619345314571 45.2556760873335 46.29198852834 46.0106098064475
ANKRD42 44.3355304829989 43.1805016690297 38.6727842796202 44.1483976313959 35.6434124419782 38.2293008890613 44.0687697278114 41.6466169548846 39.4762546236449 44.571915452417 41.8168470823451 44.6484005362281
ANKRD43 47.5647821785529 43.3458769998118 44.7829632472754 40.8681871019812 42.7107928951766 47.3791873993182 73.6855877240541 69.0819394834919 72.3095457386378 54.4825201506931 56.7765567227989 70.6903168772326
ANKRD44 70.88236913251 52.4665168142681 51.8636455065553 63.3717459766798 48.6780871992396 45.5758521267508 57.0262601962425 85.9474373829081 52.6922486991742 80.7986033188909 85.8277891453447 70.25295977615
ANKRD45 39.8662795144616 45.3486182278177 51.223859150798 43.6726153508978 43.2484996523549 41.7627910693483 37.9430145836712 40.1474543172332 45.7452456977857 40.8970401536578 39.8184668132023 41.9430737035925
ANKRD46 176.866350130563 173.856274004533 169.874602999057 109.851796671234 266.702252514277 240.696431900526 317.406465928295 133.314819245044 532.581631305218 235.764999941441 219.838542502016 225.875798191473
ANKRD47 49.3463416794995 62.0220558452834 64.0162246399972 57.2856683017619 51.5888395924843 62.5542376788358 43.9450023643394 60.5571721986885 51.6731332961917 56.685925255591 64.9959712888709 51.0013344286851
ANKRD49 170.850089909832 109.23113111292 137.696331574552 129.190555260332 149.139710097196 166.168385855406 117.811967515956 108.57858133896 119.253559091088 129.336439341458 118.251739134889 139.731620245421
ANKRD5 38.957687788868 40.8420925848403 41.0705134231814 36.833813533944 45.5051950394186 39.2623423164488 40.0052661202207 38.9207612933107 40.5491080069964 41.4606714407332 45.4304452450628 38.3880780363593

ANKRD50 107.176733647834 119.263603432127 852.769974690777
159.440571032045 208.3497732496 195.168348967505 225.50098418159
86.0944261788554 768.888528247493 266.687281047486 310.358667418222
501.452602126588
ANKRD53 38.6454098044474 41.8073910693411 45.3974838013476
42.357910063876 48.2748123114598 42.357910063876 46.5650556481721
43.3262148375525 47.1744296124496 41.7405682794166 45.5707650534259
40.0557788909923
ANKRD54 161.910748720932 116.74427460886 111.735319884512
96.7799695976296 144.976378707447 146.814825143817 178.675242612997
160.917024416961 155.541828028535 139.999709303614 149.629940171074
124.559758809231
ANKRD55 39.5676051939342 34.0382693188794 37.6557714476853
53.8086134112366 45.1137938810047 40.1428591998404 38.8417151851134
40.2382908228517 39.4762546236449 39.2623423164488 37.2018803984555
35.7451401627836
ANKRD56 33.2552842693684 41.8848959280916 42.0621425894624
42.1273928203021 50.3713170502557 37.3799542961839 40.4359177791566
36.2539950294089 38.5252456306656 37.920964499903 44.0409178714232
39.6352613956998
ANKRD57 274.887090359131 1120.83891847181 894.486674059951
338.586852166449 445.875994521817 502.67939538729 395.298033031033
1061.61186520958 734.143790759359 1366.59129560817 789.511653564341
1021.71286017481
ANKRD58 42.2455707947223 38.2263227947189 35.6139301508335
44.265834798964 37.2567512265363 36.2015763253919 37.830876898596
36.9355798143571 39.853866204927 43.844855336215 42.170702295259
37.0953622406687
ANKRD6 69.493532577364 51.9635228411223 58.2986825661503
70.8288090024328 56.6951078698291 87.539553421006 78.212704825539
112.506613718654 66.5461118812206 91.5825612510669 84.4349803529132
81.4465496915693
ANKRD7 40.8681871019812 41.3610369241157 43.7630587657205
40.2455972565968 43.9994592139885 41.6590564937735 40.968974769473
41.4812544772644 39.8873706855774 41.4812544772644 39.9070785037411
41.0226232127351
ANKRD9 259.769260154253 232.879882989322 299.334388262225
141.79653024085 294.443061798191 167.229755109155 211.485592971588
172.777241940677 343.418685340038 149.59360999995 131.68956899407
159.12163104195
ANKS1A 437.280601907944 946.081492716653 250.176074707634
169.425386468707 419.652892824029 484.218958528411 974.296760034984
174.584330660466 403.92758879201 572.19538884395 597.191426276771
524.451156145427
ANKS1B 42.668311948721 43.3030298816484 40.6216891610774
42.7431359850339 41.6383327669122 67.2397076992564 43.1931731665131
43.781101409552 42.8036731479932 49.3557747406709 49.0816063338953
49.0048900199083
ANKS3 68.2614370871382 66.1863819168127 77.7907484334847 45.4634652319753
78.0077300979911 63.5738862549306 77.2154078285418 55.5578334877425
76.7100878053735 90.0767501615808 94.2275161759277 59.7999569757275
ANKS4B 42.1537476443423 40.5400384479766 39.9101908547397
42.4580504587581 42.1650127805175 40.9506062495231 35.8227939208521

35.3608605269276 36.6312676076142 38.2069499294002 43.1931731665131
39.2469742642337
ANKS6 44.7915315085948 43.0971787292711 41.3366779545815 37.29566077996
48.3814421939888 42.2577689927563 40.5585676679482 41.6466169548846
41.5635499110307 38.8507804383261 42.911855821602 46.5650556481721
ANKZF1 87.1735705257803 66.3404989659221 79.3066495980045
66.9997306355227 92.9747592911324 61.0506827768127 70.8386539994956
77.441946514938 80.4176406820326 96.7799695976296 80.1419822448876
75.6428949408034
ANLN 67.8774399765179 85.6038071277319 64.1080166181228 48.856176220627
105.317399386109 50.2163892855092 90.4957279507369 89.2385568686014
137.551493752877 40.3235655331796 37.4642807402741 40.3691324547049
ANO10 99.3956523785983 133.798096986107 146.249568587351 118.911578268073
165.974848305379 119.993761317523 167.229755109155 113.179313022693
139.590367312743 138.189579100482 140.35042464015 113.277043026153
ANO4 42.7150227340364 42.1607322031054 39.8299340037009 45.5869891570251
41.6806984441849 41.0582900711341 48.8319884367456 43.7396619462033
48.4869909888679 42.8936990772838 47.6884372645373 47.2131144508464
ANO5 37.9387791625384 40.2493681589104 36.2683886571585 35.0448343468967
35.0181575202066 41.8613406887824 36.634024014558 43.0390857698452
37.7384958256497 39.9948390976317 41.7272875295469 40.4639532172338
ANO6 277.936167684143 322.678762360476 240.731386634957 174.559216176175
220.79051545138 408.991719312986 190.77754486426 190.940906139713
339.679549262298 304.33749254654 339.313853828248 488.908231529696
ANO7 41.9823787224629 43.4396866157171 41.3366779545815 39.3513480332078
46.567903751033 38.4330755397458 42.7982502551634 42.0494116081998
42.0621425894624 45.3872406432794 42.8710836704219 39.3002993140611
ANO8 67.7831020218081 134.099684731177 76.1493261430901 47.481302504066
99.8699868526053 49.641159251878 63.5738862549306 73.4820932957859
77.1767494799711 56.685925255591 63.3544794716167 51.5741598315033
ANO9 53.0290605707162 102.926945888985 139.218017862207 60.843426120801
72.1591028956359 43.4681646416585 64.4127389689053 57.8111054584154
66.0350709576651 82.1809804715696 80.0243450227647 70.6138659908885
ANP32A 40.8681871019812 41.554942319225 42.4580504587581
40.646702696033 47.6884372645373 45.1873487463472 41.253645578517
46.695848687694 48.2123120250343 37.920964499903 45.390192977585
49.2064066614865
ANP32B 2215.01815680371 2206.44541412808 1423.29307392266
1580.58484708669 1932.2647526188 2865.25344854768 2513.15722262176
1801.44003951248 2266.34088015654 2024.4791883541 2191.09005731751
2621.65272077828
ANP32C 39.6609238036815 43.0227387991135 41.2365089689635
41.8291484190524 41.2424637338424 43.6427759607073 45.223075311113
46.3685439301234 43.9450023643394 46.441301934328 36.7892997857376
45.8546719310325
ANP32D 40.148780650831 37.920964499903 37.7384958256497
36.0481934423077 41.8806343997257 37.0180934800573 41.253645578517
39.6474008369935 43.1805016690297 38.528621456378 33.8558232866797
37.6557714476853
ANP32E 36.8072833828297 43.9421822132391 34.806104801006
33.9645433044281 34.0927410219282 37.8102105794655 36.08956004634
40.1474543172332 37.7384958256497 35.8304004211528 37.2892641289258
41.2437378488025

ANPEP 101.103458852775 69.4820178305563 85.9692990407066 630.53864514662
60.4122350478242 109.607667469015 103.118579484715 121.945468001236
41.4636047919453 79.9810256029665 104.398842509776 63.8558858499025
ANRIL 45.2875173643252 40.2493681589104 41.785676764591 42.1273928203021
39.859345745831 40.244271403831 52.4111687156893 41.0381860307445
48.2123120250343 43.6033221838149 39.7342718293432 45.7562276914441
ANTXR1 615.838713743621 129.143252341926 99.3448096967314
219.64979849407 194.570513601724 285.826380698286 89.847560289665
221.450737998571 109.155021443311 283.88971280023 230.664926508522
291.94914449218
ANTXR2 664.453262499828 139.311807081199 214.537530518962
581.396808977873 128.04037427867 893.185203928127 89.9284109077375
233.539376791695 130.039674722115 815.810781070699 756.801598119483
622.964590694346
ANTXRL 36.9060997041453 38.2263227947189 34.6977013306396
43.9282059368202 38.4540849632254 33.0463640202858 36.3194282084339
36.6452480995946 36.197424053556 39.4710743516517 43.3085358214154
40.4639532172338
ANUBL1 45.1912496459517 42.9358255168086 38.1421709520616
46.3203995372658 42.1650127805175 41.3553369544097 43.4544762830742
45.2806199505029 37.7384958256497 40.7063426028871 34.8383995317036
34.2141875904985
ANXA1 2133.78836857697 1094.91235708087 949.278493776928 3478.67753301901
738.66912772732 2697.96877833185 838.974043046676 1864.63057767867
1037.51323185348 1468.75291542154 1199.29782389306 1981.03929621057
ANXA10 36.4564050256915 1595.69503047506 2844.5506310828
47.0874209746031 73.4535007027931 67.0290776715331 36.4098694708483
151.438409623226 1698.18614771363 129.951091959262 397.199620114138
735.751447981069
ANXA11 88.1861828863404 160.733526836282 135.377859777529
170.327588764719 91.8533850418261 130.192692323611 151.663341350505
105.914573205761 311.502922515027 156.65747550889 227.317566600324
186.984157187427
ANXA13 39.3709357615493 39.9339434067497 41.2747736315207
38.2516470858206 38.4113893955425 38.6414316877471 41.0995198212729
38.745552403895 38.0658216688259 40.650742261428 36.7837318601824
38.9074189954657
ANXA2 2771.14077845128 1096.1705171588 953.143041145545 2243.24648907474
947.656220635965 1421.44237064813 1349.09482344633 1271.62760896761
1621.89443989657 963.224016480187 1097.92519921873 1480.95754707554
ANXA2P1 476.421959423835 176.871009816977 134.394743648475
395.457351443921 121.023370640285 211.164957509884 204.089652936303
272.198774144152 271.275703732022 212.822978676596 214.051824405993
303.139550962377
ANXA2P3 69.4501305462809 46.2286164776607 49.9256753636167
80.1809075683154 42.7107928951766 49.641159251878 54.6414321010929
49.9332280449549 55.8510296341925 43.2988017098387 47.5439485437351
48.0350549043063
ANXA3 52.5792653122861 38.2263227947189 33.4240466670776 39.7411455670172
41.6806984441849 49.9699131196797 34.806104801006 33.5578650644246
37.10170285405 37.8217989841918 37.4642807402741 36.9976231206067
ANXA4 61.1022920882065 50.0425298550559 45.7968648044613 54.6089451252049
50.8875036861067 46.8954576545929 48.1747307200218 50.9933795450314
50.1603795873336 43.2988017098387 49.1067959632109 44.3521099857368

ANXA5 1416.47763911039 527.238018264393 830.295872113419 2424.0414102425
448.446274966018 1506.33570635551 579.038638774301 644.733901442573
492.324859107777 759.566075437072 540.170798097714 924.316085565917
ANXA6 68.6095266659765 53.2046867327331 55.5567699051046 83.555058970129
55.9283039581662 135.226924329183 37.830876898596 44.9631458622215
43.5669306063196 70.9702772728923 68.878562323012 68.3799828461475
ANXA7 251.268123356533 234.408769393378 278.562093102424 319.092568066532
235.075347885884 401.45848063233 289.533968432846 228.499788774214
915.936211119242 320.555790243104 342.475405815033 397.850286751468
ANXA8 73.7133884699889 283.257142524687 1588.70376317303 88.4036642243438
153.139323479953 69.3468840908665 80.2620230510528 160.056757080676
569.244038644604 260.656475394736 332.060941890092 272.405201392009
ANXA8L2 57.50915811428 187.957702651679 461.83420090894
68.0471049167726 70.0133734659853 52.7156971154405 75.4053820110486
59.4674205670657 214.484877083166 79.0249585610565 101.888001006345
104.704892803169
ANXA9 52.109723558511 51.8610060995363 57.2960245538881 34.9221408381038
73.6965494979349 37.3799542961839 62.0404348860702 385.064309855006
106.243765443714 39.6719190054379 50.7903824729818 40.0557788909923
AOAH 44.6231541342 60.4646208319121 56.0477607422828 136.236041346288
57.6791750431251 51.8103411703261 62.5506324864207 54.8412943008635
44.8997235283254 49.7259371799014 47.299647867533 45.8546719310325
AOC2 40.7063426028871 41.2176232897704 47.4356803320662 42.5054997771887
43.6880562748566 42.0574848021044 41.8887298788489 46.1081155967151
43.0342930070984 45.0155543555707 45.0997987980157 47.9566297250148
AOC3 43.844855336215 47.9665737016961 45.310660704531 36.3604951341921
47.2074332636566 63.1091746640396 34.4570158133896 42.7431359850339
31.372171135652 52.7972156492746 41.8168470823451 43.6753843488695
AOF1 40.4639532172338 40.6380495994768 39.4710743516517 38.745552403895
35.2163900400329 39.6251842352088 48.3411413510851 41.4508241771315
40.0403898620771 37.2089297656361 41.4508241771315 37.5556299264699
AOF2 841.850722284484 456.757277476069 467.351570171652 230.401917528056
803.548811322583 277.5073518536 839.850453078119 938.151864971251
621.489785652602 577.993850127555 508.081726674689 442.711822532078
AOX1 45.4799270968969 45.4143854024453 36.8730514568925 91.5202521578181
36.9060997041453 365.792144095604 40.0052661202207 38.5457716679071
38.4344387289956 102.044838876428 100.663054866186 106.020559220488
AOX2P 42.442231667357 43.1805016690297 45.6989664108554 39.5050383901884
35.2163900400329 45.3882138838137 40.2154497375715 42.0494116081998
37.7384958256497 39.4710743516517 41.9083581529276 41.0657892043496
AP1B1 104.462230406058 183.526640731647 154.166621635012 116.122324921267
141.180682718758 99.7807691901691 178.789064142469 168.70463124443
163.62024333166 140.698550434486 193.758357282411 149.118265845458
AP1G1 134.407474121421 139.758805241617 178.323615832679 123.768417863139
103.133138547093 107.391994714261 112.519759295176 124.18058128276
152.649165330093 95.1513370156467 135.644934816665 139.142835448881
AP1G2 202.795613942391 229.659465419141 334.349204174886 83.6935756500468
142.957149812186 59.2012779686869 134.567947469561 125.061012833314
180.029671376384 214.774035015293 208.410194427484 201.208357379329
AP1GBP1 45.1569741609597 57.9941756686635 57.8715555065641
56.9299697426319 46.7508829977587 41.9543948167375 44.6809101024392
50.857522488428 54.7033747046992 50.5734308316823 48.5835484742456
49.904979214379

AP1M1	227.170944398338	260.824949424185	369.952313383216	322.774093041134
	358.498678171092	309.802432824498	231.472806936915	182.976132230487
	262.277293680018	256.364301298918	300.412043023998	273.705329906513
AP1M2	36.1232327599945	50.0425298550559	53.5698357689852	32.554751989205
	49.5666074434383	39.9397419279057	50.8388984085636	50.7364620410977
	62.0272989884264	46.7646877499666	62.2200723764909	51.6660481331154
AP1S1	166.644251737703	317.560970617018	177.261319004133	118.044483475719
	174.416924448198	89.7862835647016	210.958578914607	183.286063142727
	386.00975855841	100.756165194192	89.3228209926236	183.918927595056
AP1S2	233.232472394727	135.836577310513	226.567000755017	1039.06999658544
	98.2280780221631	536.73426937255	114.396745444915	138.436822676252
	65.8306182796781	173.5606219477	150.009352890706	218.552143403657
AP1S3	38.0089018744739	37.7384958256497	39.0775870687758	36.3604951341921
	40.0599807618105	38.8336427948187	32.6039814489621	39.6474008369935
	37.10170285405	43.4885245598939	40.8225507267584	40.7704587752083
AP2A1	52.7466456080101	67.6169355376864	58.4591497641998	57.2856683017619
	67.222434502867	60.2554326890045	56.154679970047	69.3883443059504
	73.8800799416166	59.7240416362194	67.3439996689706	53.5966673719267
AP2A2	92.6545309624315	63.6287257620165	74.9113162056974	74.6159297822533
	65.1757400078647	126.485649460666	69.6301237585571	64.5257016285499
	92.1817062395346	95.92436282414	132.555169949054	80.4404044229341
AP2B1	97.3188285021124	82.9322141700549	79.1257499539205	96.4913003384143
	126.204310123186	89.4111455826308	133.151333210366	161.500218853268
	123.933814581281	116.17375251898	115.299308625328	105.807556324486
AP2M1	478.296779356592	385.064309855006	486.911782180966	407.336051853854
	597.191426276771	500.168833003599	428.307428028806	382.872356030213
	538.716947884528	534.629009719855	535.585999498708	454.106740411779
AP2S1	3966.51586153125	1845.34855798442	1921.48678333837	2488.32876359853
	2803.11698341125	1864.63057767867	2417.02418518222	2146.1548633579
	2803.11698341125	1442.72006787259	1626.71747158994	1351.49940447914
AP3B1	422.474427987338	727.788854407888	621.980260052085	580.109585240053
	437.411540294212	419.741944629785	459.182372959714	402.794855141389
	651.078746026341	475.159378115819	477.263677441074	616.912306849151
AP3B2	56.6086202928268	37.6383530120621	36.8730514568925	45.5869891570251
	42.269054475455	47.0940613507321	49.8281348927244	66.211494935811
	37.8319142847141	67.3308332069908	51.3103617600342	47.0130468198828
AP3D1	162.447872391923	217.875223856197	188.900125060451	192.922622120507
	276.86542239106	185.191228931155	169.527304857688	188.692678542986
	197.325473090277	160.733526836282	160.868571611805	146.432425205891
AP3M1	141.255712367708	189.245501343165	190.099949303905	149.747000657513
	163.809567262808	180.562005428669	158.464244518317	105.686346729469
	359.185856400148	125.052841200703	147.841274204532	200.318682162912
AP3M2	282.68881112438	193.730784033221	172.789150115627	129.190555260332
	230.344359535334	139.445008123768	201.657389063895	294.7772562683
	177.494605794577	437.280601907944	325.224254365274	386.98657211459
AP3S1	136.786772972738	139.022985518859	182.31306890697	247.04098109003
	150.925721934159	415.759723627205	148.570371099987	156.433762355183
	233.07404514708	149.076166432567	139.543015878846	196.096147690942
AP3S2	76.3933911546385	59.1938299291358	50.6004078448405	62.6048727240291
	67.8233625051884	88.7605288058249	87.3508610599789	79.9597944271831
	87.7881690937563	93.5511826850067	88.5511408870489	87.5759422500374
AP4B1	186.450759425396	136.140128421585	160.566474370004	172.789150115627
	167.653939596625	134.239606718101	155.406719968961	168.522052983892
	157.020685801527	161.326580647514	164.44912535816	148.043320937247

AP4E1 107.637618676176 110.559251404688 91.0443726041992 130.10148014895
96.6128615107975 112.721024337362 121.050369804136 135.829530518199
124.22494803053 180.255771684507 191.811190401876 143.749408328062
AP4M1 47.9175508887533 54.6793272901524 56.1434024065079 41.1886975241176
57.9161791762225 47.9503724044453 50.8388984085636 74.0831311663524
56.2441895183416 60.7495043977707 52.022479439889 49.8253396161069
AP4S1 54.7786516309154 54.4190527512508 53.5698357689852 57.4595416719581
50.2163892855092 64.0985088513857 52.8496345990125 53.838484549134
56.6079099547057 47.3791873993182 55.3147456054175 50.656598108034
APAF1 96.8098282020349 124.803597244786 115.610095595509 123.608921653229
87.1109435144663 93.5045354990096 153.436974478757 91.7679182632783
126.088821850066 101.020328028769 96.6969760559963 107.211374033204
APBA1 40.9558083259517 38.2835812177457 39.9101908547397 39.2780515621926
39.2608760193011 41.2567470068038 38.8775560911841 42.5435283762933
40.2615278292875 43.8710182120571 40.4094901248527 37.8490162439785
APBA2 37.9387791625384 36.7976562409506 38.8775560911841 41.0726336841311
36.4434672195785 41.7627910693483 42.0605906240248 39.6474008369935
39.7537274655115 40.5090849993014 39.9070785037411 38.5279745308203
APBA2BP 38.7537364570873 40.0984333134909 39.779935291659
38.042028333747 39.2114264935239 38.1699123824764 41.9393838138203
37.5511560880902 35.0033376705042 36.7439906400209 39.4410515050885
40.4192415967414
APBA3 173.035987237848 142.221867895111 190.403032965372 222.617854741466
288.284384035759 153.42135363926 158.590601962928 135.706144105357
161.817972012879 124.56803502087 124.204392146484 117.052403700291
APBB1 41.9823787224629 42.4715575796089 41.7442113152211 39.0266821712099
40.8351751854504 36.3419131924389 38.0598374201407 38.7229679986571
39.0470836345045 39.8511044247748 40.0394833064005 41.8982199176791
APBB1IP 115.10044062133 119.362992523422 115.256208610595
861.729405234231 244.089971761037 184.105396912056 144.456117600129
234.615033303459 59.5929655877036 101.954354107731 114.78317743597
86.1433529921684
APBB2 57.1046180136491 50.1027491117756 55.7764940944098 43.074472921841
50.7615314686244 60.7488007939604 43.8206425661746 44.7044140979108
47.1744296124496 44.8997235283254 52.022479439889 47.3992733452821
APBB3 273.231509963261 274.493399403322 247.723546145775 234.043251639296
257.662291702982 189.915336406793 173.603999265239 205.051252996628
148.016994658777 251.210603834628 249.451753363306 168.307676704652
APC 36.3649677956953 39.7835513496873 36.3604951341921 37.5382094267692
37.6527901109005 37.4782441311626 40.7910160811527 36.7460922671619
36.2733735494625 35.8304004211528 41.7272875295469 37.2810767789306
APC2 39.0620487644635 43.5873188565519 38.2280071951674 40.0698728584484
44.0451487108213 37.3799542961839 45.1017494688414 47.7889447429963
40.137818555383 40.1179210534118 41.0808257270221 44.4475838947656
APCDD1 109.5145190042 141.994123068328 380.988918630713
127.986477405999 182.204124363356 302.79352975792 53.3111230705754
137.555275829874 72.4220552950926 306.115856873599 532.581631305218
161.109600577001
APCDD1L 97.2535496140648 41.554942319225 43.8495248991589
87.114638423256 46.3150221024046 89.5597629049747 39.4600924554505
46.9986083378817 37.10170285405 112.281286038716 144.098339910117
75.1674447943072

APCS 53.2046867327331 46.9919960628953 46.6588655218735 47.3574589211166
52.6922486991742 47.0940613507321 53.0277416531043 50.628152951493
50.6750785584772 49.0841460667818 49.9338905397767 43.4709415392696
APEG1 60.1705241170421 45.8669209716694 43.0016969050339 39.7411455670172
51.1368583672022 75.5561597374812 43.5627649099623 46.695848687694
40.7847110517594 52.2256177934134 65.9273663561152 48.1627097465883
APEH 229.159869771704 427.972184856393 484.050852319927 297.187417426993
491.100053064936 266.905855859261 487.540800793233 325.769076169162
419.741944629785 446.37035118289 455.774407260725 381.6928205836
APEX1 1142.67599037881 856.790359133679 720.552354470517 448.446274966018
855.602941621804 661.269882993465 809.577874019028 553.517839467557
934.48498309932 467.351570171652 546.94113683381 587.385368995676
APEX2 331.984279378726 277.665554240228 338.000998124097 216.744244428166
283.563531065269 197.103673938152 556.728676214581 317.892699374852
325.363156416947 218.056839407813 254.874064929741 197.660480001139
APH1A 374.167501183341 330.550018306903 371.709014310561 216.976116788572
426.015657750123 164.96500173066 273.231509963261 477.023373302787
357.839241226513 255.724996015069 303.670126704469 239.316215255944
APH1B 130.703767898434 84.5572894783679 114.488672372067 301.79813401066
146.701131209299 91.640286556353 113.866742186841 229.745018977185
82.7229468743536 119.301672388541 116.304054038726 103.458867136383
API5 251.268123356533 326.500804513133 305.048508698231 297.701126167694
195.864827360933 410.808988840343 266.717224821545 364.534123862511
274.731304343034 351.951456642829 357.839241226513 412.421643885499
APIP 84.7222416138393 139.862070204656 131.097521229971 165.029434090892
122.576931376426 188.713487905549 140.199798713912 181.814283147675
133.344625514809 99.3956523785983 108.881703882906 185.667983376361
APITD1 82.0292880742189 106.809028844031 91.4581948567397
60.8581462216607 82.3181867658154 72.6182747525443 136.293724800658
97.2750076101755 101.375030043104 60.6779753362314 78.5325880060762
101.337735124049
APLN 55.3153799233744 43.0971787292711 60.6567691532561 48.9944486068347
44.6834648302383 40.7616966228404 40.1235656101329 40.6171774724171
54.0192060888782 41.2276963324981 42.5426057453993 43.7672102682067
APLNR 77.1900613562529 103.071727621257 96.2739664178142 249.148159759128
60.5290966885203 107.148000219752 54.339958888486 74.7794253940974
43.7630587657205 45.4614234442527 55.1099824417989 42.7782216342806
APLP1 39.266565827316 39.8695929627783 38.6414316877471 36.7892997857376
38.5512429290626 39.7394181011603 37.3799542961839 41.9315419791594
38.4970433581463 33.8440826703183 37.6159995071541 41.6550823405646
APLP2 198.682422442825 193.094756585577 313.594031411352 226.785831708938
178.060342847557 230.197573134703 179.07886477603 259.456935785521
331.001195453225 293.755623192857 281.602712448925 210.843643231855
APM-1 99.0545942554244 186.195424728755 202.360546931172 52.5307002738469
60.0736861987141 61.8187341713197 165.606015336587 111.573111498905
248.258290059808 187.836567614419 132.931052537195 102.49785018292
APOA1 36.6480005877129 39.0894172982439 29.9932610852132 34.8137085420423
39.2608760193011 44.5544727111028 44.0687697278114 40.3357777530419
35.2143190355414 43.4885245598939 41.0050831223107 40.2669497398702
APOA1BP 508.688028777582 446.37035118289 796.359355922952
338.415128695208 741.908952472992 475.881757842864 478.296779356592
505.294880430281 593.345148568451 391.83348187444 445.644244692294
420.197648653758

APOA2 42.7150227340364 46.5650556481721 45.4865173433646 44.550322123994
42.269054475455 39.8455060654531 40.6750540874299 41.3506832276112
48.8842909466002 48.7414853386615 44.7926323492519 42.4607795638552
APOA4 37.2474303302343 32.396635964353 36.0306975524309 36.9912468186322
35.1411239266238 33.2552842693684 37.4772922422734 37.6557714476853
37.2810767789306 38.2069499294002 33.6902293666089 35.5523662009011
APOA5 43.4626049853157 36.9994058279362 39.5450079747895 39.1706564265495
37.1565984182084 34.8956628705329 40.2154497375715 42.157627240486
40.7847110517594 37.2089297656361 41.5421429082231 35.5523662009011
APOB 35.0202257459026 41.159706131351 38.4043916290561 37.8442841690502
38.9496515344837 42.9695585740813 39.0458123429605 35.0510338774593
39.6609238036815 37.7315666632629 39.4410515050885 37.5556299264699
APOB48R 49.8472708021525 49.0697565324841 76.0240426902567
196.065114892186 41.1868052124344 53.3185097662527 58.5087296620514
55.1109784383525 35.7973685350741 101.58345410371 86.1055862202537
73.1447139654971
APOBEC1 47.6397712007919 42.7775752471711 43.6670755115848
37.651298468202 40.4639532172338 47.9503724044453 40.4359177791566
41.6466169548846 41.1742244762318 41.3321861371202 43.4709415392696
41.2437378488025
APOBEC2 45.6517320730042 40.9731067463382 36.8730514568925
38.2041059863839 39.6535880152834 40.1428591998404 39.5496902678788
38.8495095797881 38.8271346375425 41.5435611403119 42.357910063876
45.0035308952466
APOBEC3A 39.4710743516517 36.3110701990588 36.769073068743
43.074472921841 34.9062568194127 35.8227939208521 41.8315602975157
46.8954576545929 33.8558232866797 37.3179578037963 34.174642681781
36.8690774823319
APOBEC3B 76.5505585423421 95.8446272966902 51.6159542017038
51.949834638817 86.8871118411414 42.8598805316863 311.301613620475
189.650905999631 113.026772174726 36.2435874904689 42.357910063876
46.5650556481721
APOBEC3C 64.4415447415281 60.9311907846259 69.9179689232341
63.1604223919703 57.4163094213712 53.0290605707162 102.993620677265
205.154378853199 75.9909523932333 112.02980664709 82.1659654020827
78.1623301184313
APOBEC3D 48.8941062980393 38.3322849790657 39.0775870687758
39.2780515621926 43.004943518025 39.2248354955045 40.7910160811527
39.4710743516517 40.4220196471124 41.1222709786153 40.9157352260901
42.8864415494484
APOBEC3F 82.1509530759594 85.9799968870368 72.1591028956359
79.7061415085215 76.0496166059449 72.9154861179393 162.000275136397
155.564141715819 78.055431384988 75.6471706262645 73.5527788467294
73.6036945547683
APOBEC3G 98.6401096175932 145.459578481501 125.690995228055
160.187478921145 164.312946712409 115.143810463938 704.15545332905
726.42773300581 151.266092912661 72.9126349246129 99.9752275166514
97.8764110456132
APOBEC3H 42.6036491382658 49.4067286971348 69.7855392178808
49.3654837541353 39.1650142708257 41.6590564937735 53.1884505015411
55.1933004802607 50.7619345314571 48.0211752672327 46.4205654140754
45.0035308952466
APOBEC4 39.5676051939342 37.1685072112618 34.806104801006
43.4410741327254 39.2608760193011 45.3882138838137 43.4544762830742

38.2782436497441 43.0930293831497 48.6124127998952 35.3362091871364
38.2350720635382
APOC1 345.876877384089 130.121040883877 126.171040948196 815.810781070699
157.250548147161 89.0495215957248 296.463473585355 446.613670519745
103.423003544792 132.851109034738 115.204421753719 67.1675878355908
APOC2 44.0328992213727 43.9421822132391 41.528370959196 41.8778873678551
40.6723334103791 41.7627910693483 34.2773859804062 39.9576138799997
37.3600014814388 34.3674468322242 44.1148826235374 35.9604262434141
APOC3 38.7537364570873 41.2598458726002 36.3604951341921 45.4634652319753
42.269054475455 42.0574848021044 41.5817737564592 38.1835938174625
41.1742244762318 43.4885245598939 43.844855336215 37.8490162439785
APOC4 39.9697332660833 42.2577689927563 36.8730514568925 44.4367387274922
39.1650142708257 39.6251842352088 44.6032218589199 43.4544762830742
43.1805016690297 42.0621425894624 40.8225507267584 41.3481355128089
APOD 390.331295238889 89.0898346470643 53.2046867327331 181.488068017883
211.485592971588 3190.0108307463 41.4742337714895 174.021676013865
55.2114969675545 1575.71609894038 1225.62057881626 2551.57660786358
APOE 2266.34088015654 580.109585240053 411.981987305512 3618.78958408675
766.647626178442 918.083610591772 1004.28134410536 1554.42895572029
146.117756310709 137.294289316915 100.883604376864 94.8036856848998
APOF 41.3387790895716 39.9697332660833 39.7411455670172 40.5484717678957
41.8806343997257 37.7000400506818 41.5817737564592 38.6454098044474
42.2577689927563 39.0648733099061 44.2925355984056 41.4508241771315
APOH 38.6454098044474 37.2810767789306 38.3166554965434 37.29566077996
36.6354013629791 39.2985791324641 42.357910063876 41.4508241771315
40.2274522635576 41.6373771565199 40.6373400186325 40.7704587752083
APOL1 50.0466349801009 109.23113111292 94.1627883101274 46.8249967544573
47.6884372645373 47.0504142893738 96.1825551158198 55.6904617926772
76.6389264422388 46.8954576545929 61.130286531713 55.9927099883616
APOL2 59.5612330871274 90.9010016862442 105.15507796881 43.4877740913713
48.3814421939888 49.2677835329543 83.0207998537207 76.7063975550116
79.8913276294876 73.5743059777365 64.3312319756553 59.5370927657967
APOL3 58.7065473526185 68.3604212141462 70.380703483689 77.5889289318438
42.9627566278041 95.2956103624468 61.4121672148248 62.3470274683312
73.0403099404 57.6791750431251 67.8067315230738 63.8558858499025
APOL4 45.334106390212 52.0770287673248 48.7646976789783 43.367124590178
51.1368583672022 42.808889470973 54.5772630900888 63.1291723738553
55.1804535845496 41.5435611403119 41.6336636285875 39.9519547348106
APOL5 36.0144420013819 46.5650556481721 43.0016969050339 36.890925044696
43.844855336215 41.7627910693483 41.8315602975157 39.7509165247419
40.8970401536578 44.6918600441328 40.8225507267584 35.4484652445078
APOL6 40.4639532172338 47.9665737016961 39.7411455670172 58.0218262176065
40.4639532172338 46.8954576545929 40.7910160811527 46.1673159982664
53.2046867327331 37.7315666632629 42.7275315121163 47.6938628797168
APOLD1 90.1056480599534 169.838827072973 360.784460384046
198.324467851365 117.033529761587 393.001025840896 59.3549213146532
213.768352764473 57.3385421837183 71.7464249053513 112.048972076936
104.02653369229
APOM 49.2595762436995 46.0713306971594 61.1076362129586 66.4047895961325
64.193536444915 58.0910843932181 56.9320717885002 49.3880946790306
56.702421372165 48.7414853386615 55.6151308605445 54.1090536870282
APOO 60.8357760968871 76.7710944291287 73.967017874248 72.6824533717231
79.5175547131776 76.7791768896805 97.29969628425 85.0308877626746
91.4121563150184 80.7338782543351 56.3106156221316 76.7791768896805

APOOL 52.310270746917 50.9643363406241 55.8810837854139 53.9517841438975
43.2484996523549 48.8744644207821 69.9214859673839 66.1288889125299
58.8201021345225 44.3648993568405 43.6629145624937 47.1060577218993
APP 439.376432049616 615.011590811277 680.837950890159 316.327885002562
690.568858101531 929.862002014478 388.336994961328 233.710655978992
425.60854194674 299.74710723735 516.349618186681 633.819901764391
APPBP2 114.888828460086 140.465247047676 100.057155418659
88.2314365506494 121.552188287725 148.529482776555 113.539485042978
76.8964050504238 133.344625514809 144.239350177363 132.425058995617
158.532307373857
APPL1 73.8374823243007 73.0385284042351 62.7605715573218 66.9218519793499
65.6003544901554 69.2685201884906 102.00080367858 86.3237770387663
70.7238101239421 74.8170892324966 68.3799828461475 66.4626619269287
APPL2 310.461601607662 289.213144287085 236.488376031891 166.304210248623
161.573327074375 193.395549316561 205.510539955583 161.422918681934
326.250872625748 179.953662481428 169.75692324104 184.421617360366
APRIN 41.5421429082231 39.6995529770239 45.063792968927 42.1273928203021
42.4864576619427 39.7394181011603 38.5066108855005 51.7538628098347
41.9635525160083 46.0119912782855 43.3085358214154 40.3691324547049
APRT 310.120875196999 465.266372824212 821.013687795434 313.660064080622
560.802447943839 309.022069786139 490.301992866742 339.854688605186
512.691506909762 396.355023788839 340.662853333208 329.282615395786
APTX 130.925724760241 104.900627862215 83.2240772671045 87.4850071876743
111.096799408676 98.2843999216669 176.318618244066 138.26207427245
109.565260080683 137.547711779867 142.714450804595 149.747000657513
AQP1 48.9906383294199 49.32293280121 47.0474493061198 55.2214187655851
42.3536549114165 86.3185927557402 40.4359177791566 45.5581356216957
43.6039443285137 53.2160608018435 77.6052681514721 75.9136854087044
AQP10 34.8099089553046 59.4253780950488 37.5597671487621 47.9915414951747
43.7517118200398 50.3245489035833 51.3064929551674 44.6107027295193
44.8003345643334 41.4397539024976 51.7538628098347 44.0451487108213
AQP11 59.9232969481163 44.3529919625344 42.3536549114165 66.5921967967793
44.147557390293 42.357910063876 54.6414321010929 47.8947595316832
50.0526763693329 49.8393069675192 46.3370338074996 38.745552403895
AQP12A 47.5647821785529 91.9238405195899 107.251974803232
61.0845101400379 40.7779767565889 49.7458652239071 45.1017494688414
48.4670130605028 42.8036731479932 41.3321861371202 38.8507804383261
39.3575468494941
AQP2 41.4508241771315 40.3384318592985 43.3085358214154 48.1662933382789
36.9702228618069 37.9003288112261 40.6750540874299 46.1673159982664
42.992884918158 37.6383530120621 39.9899430628542 45.3763275588341
AQP3 39.1590964332473 45.2556760873335 43.7630587657205 40.1804147506786
37.6527901109005 40.7616966228404 32.4466419672036 39.6474008369935
46.3203995372658 46.441301934328 44.0409178714232 53.655233033193
AQP4 38.6454098044474 40.2093230376545 39.6451483836089 37.1894304543751
37.956732373465 40.3357777530419 39.8299340037009 35.3608605269276
38.1658452457336 40.5571841955033 43.2551508830106 39.9941474864076
AQP5 41.2424637338424 39.0894172982439 43.7630587657205 36.5393857306744
36.7356360093603 36.3845892764084 39.1539892144371 38.745552403895
45.1899271235974 37.6383530120621 38.5668610487033 38.6454098044474
AQP6 44.7520317855773 45.1390218677367 44.014441886238 40.9141850646578
49.7235523248653 43.3269087731399 46.0228732507872 49.8344973836834
49.3780606970538 49.5635427311883 46.1673159982664 46.917149434588

AQP7 39.4710743516517 39.2907645923217 44.3344900481112 39.2780515621926
40.5710637884029 39.2985791324641 40.4359177791566 44.293922970134
42.357910063876 55.4037482627222 51.7538628098347 48.5123041297835
AQP7P2 40.4133123405898 42.7240538244549 41.7419877611072
43.9282059368202 38.9856269395186 43.8427403434964 39.0894172982439
49.6769349856548 38.2981083038519 71.5484567134601 67.0078363401212
63.6254894053045
AQP8 35.3227485563945 41.0678614026819 33.6816578730693 40.0698728584484
42.0709558258414 41.0582900711341 36.8369172900902 51.555506218204
41.4636047919453 38.528621456378 40.2649309204478 37.6557714476853
AQP9 135.106756607914 58.1027665533767 114.039396407323 3021.24285327836
85.2849531543957 171.597378768542 371.393488010768 68.2309359831273
40.9901805728485 42.6793753550854 56.1379957867124 58.7382738968248
AQR 133.541341560868 123.841740274811 92.0606634977068 130.02102880365
155.378768637562 138.486962379511 128.766242896259 122.23209417744
138.618449365819 132.242070552057 125.027865666372 118.115557136662
AR 35.8758508994673 45.7589758958741 46.4569566520107 43.074472921841
41.8407088454059 40.3840175122961 58.037539503209 45.0478115350159
45.2448074369659 41.9219580093938 46.9893284247299 48.9155368657318
ARAF 179.756604242873 203.373397813051 279.485290822376 190.960237766104
151.99110418225 173.745808259419 204.251854342995 218.868344683409
252.134800295199 234.333935191237 236.139267415604 193.395549316561
ARC 43.5710871617107 53.8499082625091 43.9450023643394 48.1662933382789
43.7517118200398 43.8582747295766 44.9863726205428 47.4740726032707
40.4997186069494 41.4397539024976 43.5710871617107 49.5539622682049
ARCN1 650.328627263899 688.168272082335 641.103199748891 484.567462838523
731.071171171638 637.565156571985 504.281506941044 490.301992866742
472.159979716705 414.17770012536 500.794458547464 651.078746026341
ARD1A 161.109600577001 197.720854621428 220.573371581491 195.893183546434
457.860665614779 215.68113524702 205.627744150936 177.535502927617
262.37779288036 154.355197155161 157.7623351008 171.899182876869
AREG 40.7609952437622 37.1685072112618 38.9790184198145 39.3750969348308
38.8456942580061 38.1169703906207 35.9894133432376 44.6107027295193
44.7058233759912 39.4710743516517 42.0750889188255 43.0930293831497
ARF1 448.196293409768 573.832332097619 577.276184555955 564.468049419762
557.455966274117 488.089090043145 667.837116137964 368.005081137665
550.861615347663 395.875593488613 530.314249832491 396.63268714181
ARF3 320.684862879691 232.113067048781 279.901217752341 209.004669589169
277.837785971587 227.739219647972 201.800572658224 317.406465928295
273.108672916862 235.33716196909 260.15500351311 266.581466060604
ARF4 634.226367147025 665.122091380755 524.972936438175 806.827205415602
708.839262759372 714.075528895169 550.642253702646 422.931891494195
492.324859107777 309.593721653575 355.7118155732 645.408557927175
ARF5 748.913487947791 551.490493022416 653.880422442079 534.292112344761
452.351360652434 411.254448823371 623.574706535278 597.593714375156
1031.4414268329 799.399983719702 683.75696650704 440.282299601096
ARF6 29.745266251498 38.3322849790657 39.9101908547397 40.3180310664156
37.8679241954337 40.5281496800812 43.8206425661746 36.8400213077707
39.1790517315259 45.5776159432079 40.3452320167286 40.0557788909923
ARFGAP1 266.985013788569 124.064628168507 132.84476910529
170.462982145147 166.488589768213 104.687705245581 168.145003400654
208.804071011518 110.909109947659 132.851109034738 149.076166432567
96.8141712830545

ARFGAP2 90.9521112485099 125.446628066221 125.383837024985
97.6774247176874 127.908440924429 102.020047144427 83.2162112994735
91.9919654450652 133.64996113791 136.766779297051 147.875578127733
86.6854406823303
ARFGAP3 230.286815921497 110.960501850409 179.581456201363
358.30596676183 99.9841766494502 187.836567614419 101.250422688816
148.458171821142 159.727829541783 90.1696131791736 125.62281563325
190.487609390723
ARFGEF1 162.160380044249 232.553783060082 195.893183546434
129.909461876076 150.649377863375 102.863599315528 138.027432399991
103.631449539809 302.978457293429 151.325962700025 180.029671376384
188.670053901101
ARFGEF2 60.1705241170421 87.6763065012524 81.961632059749
89.2503940049313 69.493532577364 54.5952754712007 84.9182861692273
126.620595729562 103.199200862158 72.3297091475814 67.7280831060202
67.9850517608624
ARFIP1 69.2001494999588 92.8951148347802 79.0442418260005
97.2865629929716 81.7765447802116 84.1922656126304 96.3563214065014
88.4439480366515 100.654701845984 88.5601339449828 95.3145142003966
111.874193363836
ARFIP2 35.9305494063137 35.4484652445078 33.0260566875355
32.6817359372544 45.9104478407175 31.3901365951213 36.4098694708483
33.0038344711883 32.396635964353 36.1550293353654 35.4386677371439
32.4951076826876
ARFRP1 70.8288090024328 63.3667304749164 65.49167373382
80.7168944828052 69.7413896580273 62.0660509811111 81.454335582503
68.2309359831273 65.2881198932621 83.6524523974491 76.5505585423421
60.8662233098324
ARG1 39.5676051939342 41.8073910693411 44.696023817801 51.0175677516029
34.3633615460307 44.7804080899431 42.7431359850339 38.3684202018524
43.8482802038826 49.1928206164702 38.2795150354677 41.6550823405646
ARG2 50.2203578628217 64.2621389350588 51.5292723244077 37.7469621225553
65.8319449883221 57.0775902639215 49.540449125788 73.5950442915143
63.6222532132037 51.5855515705917 47.6884372645373 86.9924799927707
ARG99 42.9104454767916 45.1694246393676 39.4710743516517 46.7577982595088
44.2607789091176 53.0290605707162 45.4799270968969 42.8482181313226
45.0997987980157 37.8217989841918 44.7926323492519 45.9693232070002
ARGFX 30.9241129241985 36.5219488315447 34.6977013306396 36.0481934423077
35.6434124419782 34.3887641786747 38.6243997507646 37.2237132058632
36.5393857306744 35.8304004211528 33.1645364749371 37.6557714476853
ARGFXP2 38.5007204246293 37.4772922422734 43.4544762830742
41.9620007529243 40.2449343247539 44.812853905056 41.3094485574845
39.3709357615493 42.2577689927563 42.2313175006715 39.7576136507593
39.4490233720848
ARGLU1 798.515089763944 843.333981146512 1014.6400720037
591.192020333181 1123.99110499679 721.887210480162 868.899376368472
725.680256063016 657.031980628955 1163.0370991733 962.079396754147
823.933615182634
ARHGAP1 643.9329225331 782.639346040155 1125.31394383117
388.831358461844 460.162024551004 965.530928317269 335.249352041342
306.895449954881 675.832363405287 1171.207937488 1058.61208471961
806.827205415602
ARHGAP10 91.4786089554886 148.879760620316 129.609377870182
126.14245679817 134.981483569602 379.098697147575 197.895987726243

183.469819903584 166.834334710682 412.880824530845 492.519023728038
271.275703732022
ARHGAP11A 56.3947991135032 53.4336038235373 53.1124375923907
67.2714646016644 57.4163094213712 47.8636003588181 57.7805922926828
47.4740726032707 59.1716224344299 48.1392888470269 49.2206713037152
53.1182952086284
ARHGAP11B 37.4554876386402 49.6203214143464 53.3127618245672
42.6913548400127 79.9161139112432 37.8102105794655 37.7136011474903
42.0494116081998 43.5669306063196 41.2276963324981 43.7585538437696
43.5738557773903
ARHGAP12 53.0290605707162 58.9128486419884 55.0280380566685
46.3203995372658 49.1263312877155 56.6871913719063 51.5967098638347
53.5271601314404 75.4572860692649 62.2025996860055 62.7743500747415
81.8983491901699
ARHGAP15 77.9057333780847 109.654559065946 114.979124960177
473.223156200183 61.8471691369569 166.910579215196 89.2913927776683
355.319959025243 50.5785738734431 77.268323196327 69.4877749654481
64.7963220317334
ARHGAP17 134.743068051122 116.882934971483 141.942507674009
171.468580016846 165.349426587915 132.144567805599 110.223625398261
171.829798001437 101.81885073944 120.980558571109 111.071209057746
105.05842887748
ARHGAP18 44.436995101921 45.9266745571724 41.785676764591
65.7321440516177 37.7576900881741 37.9951997351435 43.9450023643394
51.0724630072723 40.2274522635576 44.8997235283254 40.2649309204478
43.1805016690297
ARHGAP19 66.5500089527674 141.055218279065 96.6895594961001
146.268519891864 111.501058276821 97.152024864444 113.866742186841
76.8393340617473 74.5694657001572 122.702789229752 118.316810638061
105.515662220161
ARHGAP20 58.4920021605316 40.7952353240579 38.7745734037492
54.8412943008635 44.6834648302383 88.8760662992208 42.9803717121865
40.7311497914871 53.838484549134 70.8749669449099 61.7191344476295
55.6351010690528
ARHGAP21 430.868309653765 225.918246061369 156.008696165877
330.737070535671 368.913147706435 487.261132702908 346.200944964577
538.716947884528 369.451882513347 670.958499409833 373.399443929227
507.620619345969
ARHGAP22 70.3757673501097 57.6831182451733 69.0503036666628
104.99788782377 62.8270556619309 67.4846787600629 48.9826868640753
43.6434576713809 56.7991853808475 73.2445837559745 65.8220821345163
56.0477607422828
ARHGAP23 146.543232229227 417.4609354305 663.904584382358
97.1271615802556 670.958499409833 190.27040993548 475.621740714165
265.026710493466 477.741319532507 376.289659542649 274.887090359131
394.437597863822
ARHGAP24 57.2364979699541 50.7774722329703 52.5799250974039
61.6395445916212 48.3182672138682 99.9619062444102 40.4359177791566
43.7396619462033 41.0375488957099 56.045633409935 56.0183341691645
56.4750145384091
ARHGAP25 52.6631075917563 55.012605207172 56.2937283136959
182.06733830953 45.8051846088552 53.3185097662527 44.2688720583707
47.9269108813022 45.1899271235974 60.5548506034677 58.9941076318768
51.1055541075029

ARHGAP26 54.0170817319522 44.9370339275041 78.7103847663605
75.4466520363487 42.4864576619427 51.1621379498014 46.3057385979341
56.5914743235484 54.1745577382276 76.3583555666815 75.6883778954949
54.9919060580109
ARHGAP27 43.4626049853157 42.9358255168086 47.0055981216687
48.4916117779992 45.8051846088552 41.554942319225 42.4939802311136
43.4544762830742 48.7358147030082 41.2276963324981 47.5439485437351
50.3118072462443
ARHGAP28 50.9836157087562 44.8935103222402 42.3536549114165
40.0322420615846 54.7447380922582 43.9366081640576 38.0598374201407
51.6159542017038 46.2718838939055 47.9056710878325 55.0823430906161
44.9734311311394
ARHGAP29 43.2724556187214 37.920964499903 40.1985790927248
43.8002456102021 44.147557390293 38.4330755397458 40.3302268293707
41.0381860307445 44.7058233759912 36.1550293353654 41.2598458726002
40.9683275553869
ARHGAP30 94.4999269932618 113.417671029461 96.7948977485109
286.11425202743 64.2551943403496 104.305801811472 89.3680268066234
120.660602388292 83.616030092144 59.8916740212935 69.1641374421455
62.046151104685
ARHGAP4 133.229859346689 266.9457657143 150.114520857436
326.055799445169 291.232601825013 164.097531361519 116.74427460886
220.054040993848 56.7991853808475 77.3367966395554 107.154549409927
66.6118713143653
ARHGAP5 48.7646976789783 34.7135536792489 43.3085358214154
46.4555908533915 36.4434672195785 46.3715909653832 45.223075311113
38.5457716679071 46.0106098064475 47.611018474151 49.0361180089431
47.3992733452821
ARHGAP6 37.0320432433314 39.1539892144371 39.5185194094434
38.9534635879195 38.5512429290626 43.3785777889265 45.4040191352687
41.8417024632349 41.3487726034097 40.45773165894 40.2298112543679
41.2437378488025
ARHGAP8 55.8579198774476 46.4555908533915 53.5698357689852
41.5937402943136 72.2665537421443 42.4580504587581 49.540449125788
45.3530683538086 46.4164127487052 40.3235655331796 43.6629145624937
43.0456011922275
ARHGAP9 65.5029051840465 125.768826615813 96.213791081731
454.701322158431 59.526516100518 95.9391851122771 104.573463811298
75.4272782742534 47.945438269111 51.173096357454 64.3032703931013
49.1855323567224
ARHGDIA 563.750309421406 619.950241162173 502.67939538729
565.72800641089 500.431738940003 464.99944459917 768.354209323884
565.72800641089 537.841677481011 552.480471244789 651.969296681551
432.001201940414
ARHGDIB 2013.08489754172 2844.5506310828 3217.83121827744
4784.36905964712 2067.66516256598 1829.30985071028 3681.41904661708
3099.21690111993 3690.00752647453 2225.65559724391 1789.07186722972
2150.8715161597
ARHGDIG 50.379587451669 44.3187284857534 41.1367478979484
40.8084239472012 42.9090346219023 42.0016830644258 48.8319884367456
43.6434576713809 42.846832824953 43.401782064778 41.0476065156912
44.5432192783161
ARHGEF1 56.4729864553319 60.9987978554263 65.3843877368233
79.6770200338268 59.9749158217504 53.2775641275138 56.9320717885002

64.6566383383512 64.5875183163009 59.5785089386245 65.342842101283
55.1037284831588
ARHGEF10 213.028412890553 160.868571611805 244.907057340556
189.137593177772 210.282066967005 368.005081137665 43.1931731665131
127.213248611204 93.5665536754775 258.079468517805 279.632025097952
215.175765091556
ARHGEF10L 44.9896851005259 43.4396866157171 48.8584917918986
43.3744503319109 40.7779767565889 42.6575535390785 44.0687697278114
47.6361499000296 48.9614101792105 47.0627306565247 50.8276808181086
47.1060577218993
ARHGEF11 62.4317959077388 57.2871072823547 66.4079972657486
65.3191216270241 71.5345397102823 58.7404123614695 60.0601788842942
58.3608287133675 59.3580517977711 57.0248378289195 57.5430365699386
60.1555629233618
ARHGEF12 67.8774399765179 67.9697948557876 79.0962508551502
50.2163892855092 63.4753448572245 111.009050621499 71.9474405671289
69.1973371867031 78.5174639327946 163.788056447509 108.512469038407
131.645714321903
ARHGEF15 36.5393857306744 41.8073910693411 42.9104454767916
36.264823544143 36.5393857306744 42.0574848021044 41.706879587091
37.33030683349 42.6366765186182 35.93950183586 49.3901004211918
42.0424641526302
ARHGEF16 61.5882375235292 60.0601788842942 61.2153068100932
34.9221408381038 59.3611824459962 34.3887641786747 54.1707158323527
51.8347877274018 71.3932787152381 66.0224755625662 66.0282565447846
75.3271272345679
ARHGEF17 93.9756797434207 60.808065997635 75.9174504780598
68.1992221684165 70.2565873027242 108.961145389101 51.4349390827895
62.105802634526 44.8997235283254 84.4349803529132 80.9821967801845
65.524821699432
ARHGEF18 414.539381761279 810.021622878441 1117.26060212107
429.983084929605 927.61341837276 362.268767190535 486.911782180966
461.394007153992 590.709061039001 735.60603239712 660.295993198711
628.403814060213
ARHGEF19 163.280931300437 188.13862794196 208.189151365575
71.2572485987604 423.804472820231 95.5022104475922 306.391154574276
217.78290286894 291.086512261106 201.301705082976 129.091447141381
120.090807153941
ARHGEF2 452.351360652434 372.525928228911 563.573152902421
637.035929429013 292.389429869034 416.149496747947 372.421330252979
330.814531248695 162.106596527489 347.196922795214 302.978457293429
407.949645788215
ARHGEF3 254.280873627155 205.449627031002 274.493399403322
557.979646421047 195.340273658052 337.51861578231 206.514263103112
296.080833914967 267.565744773928 648.885062676884 567.675954219815
524.972936438175
ARHGEF4 46.8954576545929 48.1627097465883 40.931294690669
38.745552403895 51.940083408061 61.2387559102636 41.1886975241176
49.7309743973184 47.9035532886869 48.1829653238565 60.8201366212589
50.7975577422986
ARHGEF5 87.8746766762445 106.595042669127 103.43536623122
63.6334154351075 93.9708162269401 59.4949177415847 74.7534423194688
63.0748373122119 121.142787642824 109.432338567044 76.111908977613
92.9600461444587

ARHGEF6 189.737472782071 428.729935028911 337.013669560645
593.345148568451 146.840603547323 535.313561995599 210.880198736298
229.47693624489 337.627557996504 521.874349042344 500.549865625701
393.464673405634
ARHGEF7 123.433224929087 118.044483475719 129.2860308084
112.469702446962 121.737071786167 94.5261917871909 166.717256080341
128.908350338087 106.70211644918 135.377859777529 134.884412058457
121.851515410237
ARHGEF9 49.9419897484381 47.4076235350059 47.646800205189
39.7411455670172 44.0451487108213 71.5249259772583 47.4439407315335
43.844855336215 42.0621425894624 63.7836577144372 58.8188963227059
58.0218262176065
ARID1A 174.559216176175 238.510952165358 280.563427715895
207.741669973713 389.481124074714 194.435776102991 282.764445883135
310.461601607662 311.055487966495 257.325282051981 241.24267509333
218.819478568299
ARID1B 41.3785995857154 46.8954576545929 38.0958223740111
45.6706505553383 45.7452456977857 43.2312848495114 45.1694246393676
42.0494116081998 43.8482802038826 44.571915452417 45.9725715538987
40.1700117452801
ARID2 72.1066584199167 72.8472187581193 60.2528550551807 63.5195937348531
57.8715555065641 74.8170892324966 72.2099571536717 56.6341453289967
98.9113424606303 68.1674425673171 67.495246759946 74.2180011215707
ARID3A 418.01691355558 222.617854741466 88.9928368134295
265.667557678228 334.813163079302 106.951862149262 96.2959923858457
305.684635013927 79.1074681877535 99.3956523785983 101.634888749356
112.316483867372
ARID3B 84.7642634532073 279.448591201269 96.7948977485109
163.995560004001 233.539376791695 95.6783002876222 107.325456368022
108.838406710326 101.01459505339 74.1228645930185 78.3542840447759
89.0727613846343
ARID3C 43.3638918705225 42.5269793679335 42.4580504587581
40.4359177791566 42.4864576619427 39.4953494583071 43.1931731665131
37.9837233601316 47.798305839406 40.6022003765153 41.8168470823451
46.6397391251762
ARID4A 46.6862836031114 57.0504111569238 66.9665871090772
59.5573227689649 52.0042367882776 68.009873198833 48.2748123114598
52.1788489555942 60.5608545741456 50.9673994772567 56.0642694832108
67.6983931011126
ARID4B 72.613397507717 97.2316181542823 91.6713614282714
158.225884127529 84.7275805640731 94.4999269932618 94.8260360398356
119.088986733238 75.5681913475287 80.8593396441328 96.0539709962851
102.49785018292
ARID5A 47.6153629331146 44.6402215707065 45.1846041469628
55.9692669442536 48.9980022280763 64.388373296782 41.5421429082231
42.4864576619427 42.5743069255835 44.8522313252892 48.5848226464449
45.338588656043
ARID5B 1630.25992078321 457.202737754842 332.933704863586
624.097858948785 410.808988840343 889.638287212686 166.009268520425
396.167219131922 746.847753035399 452.351360652434 530.710127703061
706.087945508248
ARIH1 102.655050997714 88.0720840588632 68.6095266659765 125.934747056908
115.132600341395 106.186051982612 122.316617618658 138.55578310538
98.9712341137316 84.2960453806012 96.4278951689874 134.869029698594

ARIH2 179.756604242873 246.20973320244 289.909646207746 269.284584971871
312.245547906683 293.086409068972 235.222472959869 129.810477623628
289.625425434103 412.29192088057 291.596692248591 274.994470779348
ARL1 254.280873627155 576.140066807763 447.448900931272 268.745928429069
309.293546994308 705.283979182062 324.936044300546 264.495576211074
356.565801074289 461.123856163975 559.9670724278 421.971171686029
ARL10 46.6348258763289 43.4396866157171 38.7745734037492 40.4359177791566
43.1252272136667 40.7616966228404 41.1507980856436 41.1575777497576
35.6139301508335 42.8936990772838 43.0971787292711 42.1537476443423
ARL11 41.2424637338424 41.9894412416874 37.4642807402741 39.853175885492
39.859345745831 40.040778349137 48.1747307200218 38.956280281655
37.7384958256497 39.4710743516517 40.1742127891824 39.0536056496869
ARL13A 38.8561375693335 41.8073910693411 42.4580504587581
46.8954576545929 39.2608760193011 39.4953494583071 40.4359177791566
38.1835938174625 45.5779686360641 45.2556760873335 42.8263096141654
37.9607839934735
ARL13B 41.1523487516364 40.4359177791566 40.439898672334
39.853175885492 43.2907292669418 37.1894304543751 36.08956004634
37.3875631460917 39.4762546236449 40.3235655331796 39.3667161654772
44.2527566343661
ARL14 155.928417606775 195.968119243879 87.3634135323569 67.7121534934164
77.9085946827079 50.3245489035833 51.7714254834195 228.907776306695
92.3388129097482 167.578081645481 130.048910817957 88.3636380348802
ARL15 49.4786466722535 48.551327455445 49.7827734675743 52.9156540211793
48.0234896130991 49.8472708021525 43.6025443656008 50.7364620410977
50.1603795873336 49.1928206164702 50.0564735608363 52.0359304386476
ARL16 511.792908607692 315.186937216422 356.079406408042 411.981987305512
294.090159716249 267.107863045114 412.694325761831 828.403901345618
291.232601825013 515.856807472321 813.242005949542 675.079525434102
ARL17P1 61.7551416187554 98.0023132593711 66.0411740221276
117.699959435983 161.890859629246 55.1987351219665 65.644767142472
79.9597944271831 61.8824218975538 87.9871827215149 67.0361353184222
147.137598111239
ARL2 519.563402444472 421.146145642412 406.104588997462 348.551545320445
935.815928529014 925.486088550061 783.440408023061 448.508714709248
410.329983506955 900.517711546397 855.602941621804 818.290793784784
ARL2BP 472.982369155078 380.541502795438 448.290058854438
400.973553604192 441.247306467645 527.037018015766 333.122044191102
269.857104259782 418.927102569912 433.194113859912 392.622487173359
410.716248834784
ARL3 56.2508140932179 65.5367689605146 47.7737810047679 46.614092935749
56.2305155589239 101.693848821246 61.6544855697851 54.3820590274321
56.6079099547057 75.0665411373616 74.7669841575348 71.5327770882433
ARL4A 55.4590083528414 48.2220547806679 56.2843540044585 90.6347108049639
48.7664156229426 63.2804984998739 46.6318564520584 47.4740726032707
57.8772482723463 61.3919023984003 54.9765783642973 61.5300817099814
ARL4C 55.5276281380084 40.148780650831 38.6005272730664 66.4047895961325
40.148780650831 47.0027453663546 44.2166118829877 40.3357777530419
45.4799270968969 45.7911932481722 47.8284278211109 45.4799270968969
ARL4D 51.1740855632044 49.9085372049438 69.11736279512 40.9607305725196
62.046151104685 62.7515429046888 44.8643898647322 58.2043532960091
82.1974756449763 81.915829566737 79.8701900696361 67.0331844416938

ARL5A 329.763197973922 405.148979342648 322.509777895373 546.80979198725
439.955651727755 521.029507993615 320.038347979923 453.652548085387
211.596652798395 581.396808977873 532.981045084589 572.968592371501
ARL5B 65.2295773603116 67.3779879510397 67.235655687817 155.564141715819
66.5187917407182 66.4568741702152 102.711934290382 92.227126770051
71.7408778060956 48.9826868640753 51.0013344286851 60.4872675915085
ARL5C 44.7044140979108 43.683792219729 41.785676764591 40.64670269603
42.9090346219023 45.0882356578404 43.6753843488695 40.3357777530419
40.4220196471124 40.8049408996274 44.4688999098904 41.863927676552
ARL6 38.0089018744739 40.0527893189096 39.5118967430322 44.3440839686558
40.6251465440216 36.2376693877628 36.7800683777849 39.9576138799997
41.0476065156912 39.6719190054379 40.7786237231186 39.6682560919759
ARL6IP1 727.788854407888 1221.31634960736 917.179747195874
583.690350347288 795.381998233245 548.815225941887 1245.45371155527
904.122237964854 1606.26712101449 785.79878416775 722.514380928291
854.712488657987
ARL6IP4 84.4349803529132 78.8741966117641 88.2959958615748
81.2269477373795 80.3768086728545 105.621640443267 104.504444994123
82.2266112813039 100.499763255098 111.703187941409 115.016486106706
91.2330127140591
ARL6IP5 583.915973546284 622.699896083816 675.832363405287
1031.4414268329 317.835330028579 786.57695312967 266.717224821545
403.428042132639 595.012422454829 1430.73068989193 1069.3975598703
1023.81381775221
ARL6IP6 82.6246523811721 145.607100979838 152.555556133364
107.413918714848 104.840865638204 78.1952428431942 102.457537266105
65.9476039189301 109.005343769033 68.1040271620925 87.036020750455
136.081259669601
ARL8A 40.3478825593224 37.4540772310118 38.3166554965434 41.7614975630362
41.6806984441849 39.0266821712099 39.1539892144371 40.6171774724171
42.7150227340364 40.8049408996274 39.5399188536544 37.8490162439785
ARL8B 296.664472126617 246.313146942736 255.915599666591 308.280127837858
233.539376791695 266.178061771978 208.7739716139 366.326747630465
301.941928552396 322.509777895373 345.876877384089 375.354746298695
ARL9 45.5758521267508 93.6717518147993 45.989212296035 43.074472921841
145.972133104142 66.8632294055679 151.135296427249 89.7184505543918
69.4009439491061 47.7180816682562 43.3085358214154 42.1537476443423
ARMC1 158.862722884436 181.844123841452 117.689172019617 134.394743648475
171.073367662623 193.262615637464 155.018308238778 156.220155161711
145.676955166566 148.570371099987 164.847648206552 148.944852825695
ARMC10 241.175454742029 273.320144423733 352.600793906035
186.477689350702 294.966924715506 270.947794977829 183.2003374677
194.951677338012 277.588692595406 100.499763255098 135.757061334489
232.911951245618
ARMC2 42.1537476443423 41.554942319225 39.6833800000587 43.0016969050339
42.551356054931 40.8634043028528 39.3585350448719 46.695848687694
38.7578265341627 38.9207612933107 42.0750889188255 44.5432192783161
ARMC4 39.5676051939342 39.9697332660833 38.7745734037492 33.6816578730693
40.5710637884029 70.1074782747874 36.5352572191451 46.1673159982664
35.0305278582179 33.4316681623175 40.8225507267584 45.0985088502363
ARMC5 44.9896851005259 56.9312835667828 47.2055133646385 43.1847628331565
33.0463640202858 51.1621379498014 38.7337086062875 52.1788489555942
44.4395580493118 46.1390887492649 59.1443775199516 43.2776458914333

ARMC6 59.046385957117 72.4325984501758 78.8357122967692 62.3936376596431
143.773311381452 76.7063975550116 82.1144251872199 87.799979434508
96.5175130622216 92.563449632567 76.9454624478854 70.9377012403023
ARMC7 104.232196493024 113.669790961194 137.294289316915 135.669367786875
115.408418935236 75.7502062965698 146.117756310709 188.567635000971
209.524518082089 259.812381486915 214.562221385406 157.856158440506
ARMC8 57.1046180136491 62.0008474085033 74.2122914028677 62.2878942799819
76.6160788176402 97.8052968915826 68.5174271063894 62.6100476086964
66.9119713241466 54.691568065544 59.5612330871274 66.7256057011273
ARMC9 44.436995101921 36.0366673493555 41.1367478979484 37.9875302985336
38.745552403895 48.6621527194963 37.4772922422734 39.4710743516517
38.4344387289956 37.1275334308172 42.2618615571566 42.1537476443423
ARMCX1 79.3889841821749 201.360008430353 63.2475738480323
86.2488614616838 50.2953638261991 268.54298415337 124.687212034276
89.19837608959   64.4837166864084 99.4546022538476 111.803238196297
113.692418102755
ARMCX2 131.277614500948 189.47090669378 160.733526836282
116.122324921267 311.502922515027 210.958578914607 245.691073028699
75.5830548489892 187.733278812798 224.480311257437 227.791956085471
294.567137562864
ARMCX3 70.8667493706048 67.222434502867 57.3697604614404
58.4476706144086 66.9340967279385 89.4380336458796 58.9656737189764
55.2412344988347 62.1796792097014 55.2214187655851 62.0946663000834
77.3260618801358
ARMCX4 49.8472708021525 64.6970918899831 53.0630927250439
42.8235313007746 57.4163094213712 46.1513019317765 69.6301237585571
48.7814131120945 41.7627910693483 103.385447205588 62.8408273986904
57.2350830635912
ARMCX5 58.0139597055449 79.271901834289 60.7530213660442
67.1961800572759 66.4047895961325 57.678188678075 71.5249259772583
77.7615826856723 80.6434122355417 73.7133884699889 84.7058085717914
75.7991766454243
ARMCX6 83.877343750194 134.817234321847 145.405415337402
98.1433318215702 111.096799408676 87.6018870099686 110.422857502267
123.618276821013 111.153589230866 104.288073521816 86.4195039096886
99.4546022538476
ARMET 1180.7204565402 1789.07186722972 1578.32969650385 1749.06320703181
2032.18819581205 838.974043046676 1618.92099084622 892.323580265478
1282.9181829891 562.394906600222 637.035929429013 965.530928317269
ARMETL1 39.6609238036815 45.4882438975269 51.0539624358342
47.2009420656854 48.9627346956003 39.2985791324641 43.6753843488695
47.2009420656854 47.7086093893038 46.441301934328 48.7502645382856
42.8864415494484
ARNT 42.2618615571566 42.6450179841129 44.7255207589606 35.49696651597
41.6340461942423 45.0283997415478 37.8877750141557 39.8965727231863
43.3262148375525 46.7182294568603 48.3570240922825 50.0159415986047
ARNT2 54.1745577382276 52.7493349111555 34.4716904283753 41.0726336841311
47.4548471240051 82.5956705829173 79.1432909353319 112.021971795593
46.3203995372658 60.6266092582071 70.9319024596628 55.5712957590038
ARNTL 70.2541961995753 59.4253780950488 57.5666238219027 93.5358142196619
50.0580547772867 70.380703483689 46.4388841354122 50.1833483241864
57.8772482723463 56.3441077510744 53.8441980932644 83.0460206754856
ARNTL2 40.9558083259517 35.9305494063137 36.1042012504758
40.9607305725196 35.7973685350741 39.4953494583071 35.1307934330047

38.745552403895 33.6902293666089 37.0320432433314 37.2892641289258
41.6550823405646
ARPC1A 1161.71671016964 908.328776521114 1052.16097214718
519.775555680262 1117.26060212107 851.108027634251 922.047328788961
949.923102632043 1107.96086673001 778.723771259404 898.114119553014
752.837247653926
ARPC1B 311.739149980904 303.458085392002 274.259417498569
459.021889198824 191.298525555624 198.324467851365 212.187855950414
130.62791061956 266.07996221256 60.4837400912239 82.9256976863272
124.18058128276
ARPC2 868.899376368472 1418.94307844471 1425.37639004849 1604.80031304031
976.566026758227 955.088515235485 1149.02954135912 640.78586776574
1137.99307039386 851.108027634251 989.0602710707   1199.29782389306
ARPC3 2096.51999792183 2313.21212353993 2559.659041589   2951.88559311416
1864.63057767867 1453.77801653969 2138.23653387331 1174.92419086759
1939.66920192372 708.005870621064 766.027617496754 1664.00821059181
ARPC4 379.473739290565 611.538630852232 469.30167430316 516.076634990003
421.013324906569 376.749116251503 460.162024551004 506.75912743556
444.618035487523 233.263058459431 350.451704069255 310.188313944313
ARPC5 736.846933148564 625.435083064752 804.592753722875 1291.71781703042
387.749285019292 414.794323433931 802.650020720921 504.676944739425
571.170643523692 515.409979010663 384.919835924792 659.396637478356
ARPC5L 657.936229435025 713.685405035516 312.015358620043
345.811673759417 450.271658272512 276.321098534247 877.673009984357
610.149769483374 483.416873464931 289.112248695929 319.248898194029
341.020604260602
ARPM1 42.7150227340364 40.7952353240579 50.6004078448405 39.5050383901884
39.6535880152834 44.3648993568405 47.1345949868029 40.5158867964016
44.1519466943185 45.1569741609597 48.9754340235127 38.6454098044474
ARPM2 43.1805016690297 40.2493681589104 40.7620472083159 40.9607305725196
39.859345745831 41.554942319225 39.2533668167483 41.0381860307445
43.1805016690297 38.4187686307607 39.7342718293432 41.4508241771315
ARPP-19 92.5903918620825 150.875092086496 96.143327683221
130.02102880365 134.554839076558 198.54718783437 157.843337785472
117.948047796034 155.795095438102 168.897476166126 146.311363191836
178.789064142469
ARPP-21 39.4355966872342 38.5252456306656 47.8241765062483
38.0334163721268 37.7088333099214 38.6773650691783 38.6558726824244
36.9865203259851 40.2274522635576 39.5637454855233 36.3390947880186
40.3691324547049
ARR3 34.6977013306396 40.716553247236 39.2707964550441 40.0698728584484
45.1137938810047 40.7616966228404 41.706879587091 38.956280281655
38.5252456306656 42.0621425894624 39.6403089864678 43.3744503319109
ARRB1 93.787204465284 82.9449579428926 70.2141759405882 126.485649460666
70.18594926052   129.565589054551 212.530138323284 241.706783147271
67.3230935444252 116.530589514041 139.49198349194 90.1430468048409
ARRB2 43.9518547354262 45.2556760873335 44.014441886238 96.9944911507111
46.4343327861696 50.8533737532786 47.209353240759 48.7039705048129
40.8420925848403 44.571915452417 40.2649309204478 40.8681871019812
ARRDC1 87.6178717341967 99.7361599442168 71.3527183280827
61.0845101400379 62.9553576299033 47.2009420656854 89.6056978251991
74.8893925448138 70.8606919488782 74.1228645930185 73.4853298204744
70.6903168772326

ARRDC2 54.6738135880328 69.4347592040783 90.500550952567
136.644759501653 71.7263663921367 60.4065050472082 40.7340599105623
63.9851732498913 59.9770720847728 71.4183281327686 69.9214859673839
57.0178321687063
ARRDC3 73.0970339739187 67.3269632644926 85.33395043174
77.9028721785469 73.7741438283854 83.2925226467532 70.1845639720491
59.6291051806275 90.8109585999257 55.0016608333828 59.9682734755814
68.0751111916102
ARRDC4 66.3095900379311 59.7406133873058 50.6004078448405
73.4788569136441 50.1185085888209 108.589036223189 39.9519547348106
46.0633179231318 52.6922486991742 61.776442749921 67.9141812398066
80.4404044229341
ARRDC5 46.0023068797812 50.2696827764943 39.1799070031819
38.5252456306656 38.745552403895 35.8227939208521 40.4359177791566
38.8495095797881 38.7298222155532 43.401782064778 44.7926323492519
39.8497227447888
ARS2 375.095491465679 571.992675891226 498.958550919515 299.052630634189
666.816487335088 341.666968456707 423.608694638779 491.468862309918
440.728435508514 427.199940706169 410.623529765253 355.7118155732
ARSA 88.9589979655541 76.3228890963999 73.2144205844199 111.522812019895
91.8935361535002 64.1777305915695 75.9236575152151 94.6432868887401
47.5057905459278 65.4784797364469 62.5528124447176 76.3334723210842
ARSB 53.593974682612 46.7353388797135 46.4150464549477 60.620259884953
45.6348103033479 46.0001907122425 42.4386427013314 46.9354126752815
43.3322911155321 41.3782540398445 45.3597029614942 44.9734311311394
ARSD 146.066596263955 188.734299563005 194.079940066252 73.5025309968566
74.4379676234684 94.3953345963359 231.685652845234 417.4609354305
204.862161704394 292.47671022688 240.139560726101 144.609635554528
ARSE 40.8681871019812 40.0527893189096 38.3166554965434 41.7614975630362
39.859345745831 37.1317006162938 36.4098694708483 41.5464765641928
38.2215591930186 48.1392888470269 36.2435874904689 40.1700117452801
ARSF 36.9060997041453 38.6414316877471 42.640829914102 38.5252456306656
39.3667161654772 38.6414316877471 40.0052661202207 44.5029690493211
40.3316153483289 35.4990901121893 40.2649309204478 40.6614700944853
ARSG 43.6706764143908 53.565717255469 49.1067959632109 68.0471049167726
62.5106969730855 42.357910063876 43.6753843488695 64.8336014343435
56.8622704143595 46.6598879466655 50.6473771879221 49.904979214379
ARSH 43.4626049853157 41.4508241771315 40.931294690669 38.5252456306656
39.5595233162192 36.8059561571675 42.629412460409 37.2237132058632
47.3791873993182 41.5435611403119 40.9157352260901 41.6550823405646
ARSI 43.4626049853157 39.8695929627783 43.8495248991589 38.3166554965434
43.9450023643394 47.8636003588181 33.7574445386307 37.33030683349
45.1899271235974 37.1275334308172 35.4386677371439 40.8681871019812
ARSJ 48.6621527194963 46.5650556481721 39.9101908547397 43.8002456102021
51.0107745412152 43.5752393599169 48.1747307200218 41.9315419791594
44.8003345643334 50.8826620053386 48.8744644207821 58.8188963227059
ARSK 48.7646976789783 47.3536182445315 52.7005092555102 53.9517841438975
61.8471691369569 61.3620062177524 53.7423898357583 45.642304356051
51.2066664465185 49.9493230064613 51.1483696410762 58.8188963227059
ART1 38.2083710281545 37.10170285405 38.9790184198145 37.29566077996
45.5809132232681 32.771863438151 44.4639405506967 38.745552403895
38.9006788676407 39.1621898366355 43.0971787292711 47.3172462673426

ART3 38.5648843914189 41.554942319225 41.0356368374708 43.1847628331565
38.3561682218911 48.5835484742456 38.4005795891396 39.5466975353131
42.7150227340364 47.4767851750188 41.2598458726002 40.3691324547049
ART4 50.379587451669 42.5269793679335 49.7350276169552 39.5050383901884
41.0885562160946 52.6226745117396 46.3057385979341 45.4757965005055
48.8842909466002 57.4665857273093 50.7903824729818 47.3172462673426
ART5 39.9697332660833 43.4396866157171 39.7411455670172 37.8129870676592
47.5647821785529 40.650742261428 38.8417151851134 40.1474543172332
37.7805366040928 41.7932258301997 49.0361180089431 42.6758568421347
ARTN 47.9796120205361 47.5203201376024 39.5450079747895 40.5829008199411
43.0723817756832 39.6789412274485 50.1185085888209 39.9576138799997
59.010844825874 47.1001757441025 44.1148826235374 45.0693869004277
ARV1 120.837993273922 187.439021508049 264.718875269207 155.018308238778
231.417150220669 154.891160328731 141.261334415286 175.099998331811
188.750770811584 176.07173250529 174.584330660466 196.034087004464
ARVCF 57.2960245538881 44.5152708955623 37.4642807402741 35.7674462337723
45.5809132232681 43.0860383229288 41.9393838138203 37.2237132058632
38.1169703906207 33.2987269789328 41.8168470823451 46.8129509799753
ARVP6125 42.1537476443423 40.6380495994768 38.4043916290561
45.5869891570251 41.1868052124344 40.1428591998404 41.5817737564592
45.4757965005055 42.0621425894624 42.5664883002833 43.844855336215
39.7411455670172
ARX 48.9906383294199 38.9338521051669 39.6451483836089 47.6687908290818
41.5668884168669 43.8582747295766 37.2892641289258 50.1833483241864
36.2733735494625 43.6033221838149 37.5583886316038 41.4508241771315
AS3MT 40.3478825593224 40.7952353240579 36.5474283500601 49.1786207920603
40.2449343247539 53.8461734834857 48.3411413510851 51.8347877274018
41.7627910693483 45.9004543328976 43.3085358214154 48.0350549043063
ASAH1 55.7246872330059 69.0663624160288 78.7103847663605 80.8728961146267
55.0031529299037 73.2144205844199 54.4313108506244 48.1591264215184
62.5542376788358 49.015238049903 51.3560869192313 64.2164483533411
ASAH2 45.2372944462326 42.9358255168086 44.696023817801 41.8778873678551
42.7475279082279 43.9421822132391 44.0687697278114 42.4580504587581
42.5750559787101 43.5599890586394 43.917817324545 42.4607795638552
ASAH2B 45.5758521267508 44.1533327883807 40.931294690669
44.1483976313959 42.9655396732962 43.8582747295766 43.0930293831497
41.7877325563821 46.6944238390932 52.7972156492746 43.0016969050339
44.4067063477243
ASAH3 44.9896851005259 41.8848959280916 39.6451483836089 44.550322123994
38.745552403895 35.5158505219112 38.4005795891396 36.9355798143571
42.4540072551568 42.6793753550854 43.0971787292711 40.5703581862752
ASAH3L 45.2875173643252 202.872083191114 95.7820879892969
43.074472921841 189.678033532012 57.4777075615651 69.9214859673839
227.642500471069 111.397578350396 79.4158467034017 83.3729277601518
66.6118713143653
ASAM 159.797219482154 56.3560498705517 46.8267540220952 169.144145421022
60.1730663840476 211.227389226633 53.4921736109774 108.463445585898
45.4799270968969 82.966263655525 138.859992681388 117.052403700291
ASAP3 132.985150338484 53.565717255469 83.0959169307948 68.6379135603075
83.3905516293346 136.875335055866 78.080034922779 186.570670850819
113.218291314173 132.107655569343 202.795613942391 103.376225272425
ASB1 83.7215159198897 59.5534127075228 77.0301627655229 63.3006977162657
53.1884505015411 112.551747307723 69.9214859673839 62.4219686957673
50.9010149857308 92.9894133685392 89.0684909049451 93.2319962015465

ASB10	38.8561375693335	46.8236022402545	44.5134109184888	42.5664883002833
	40.3452320167286	41.0582900711341	44.2166118829877	45.7476095696859
	55.3122077514658	46.0119912782855	43.0016969050339	42.357910063876
ASB11	39.8662795144616	44.0409178714232	39.4987030972173	44.7210190486327
	39.9697332660833	39.673290670647	43.0177448037924	39.0102066700839
	41.0522938990031	41.9666541498638	40.547835538778	44.7857390222342
ASB12	41.9407041937973	39.2907645923217	51.6159542017038	43.5627649099623
	36.8059561571675	45.1873487463472	42.629412460409	41.3506832276112
	42.4540072551568	56.057906531592	53.8260140375214	50.0571060701893
ASB13	101.448989278761	224.509915754308	168.522052983892	97.0813208326187
	277.284338888467	149.076166432567	141.572530741416	126.10194284238
	206.190699642477	241.792833275833	194.046072415847	165.311139930529
ASB14	44.9896851005259	40.716553247236	39.6451483836089	39.7411455670172
	41.7929491793697	36.1190722948669	36.634024014558	42.357910063876
	40.7847110517594	48.3693338695392	30.802355637173	40.3691324547049
ASB15	41.4508241771315	43.9421822132391	40.4882741869718	38.3166554965434
	39.5595233162192	43.9724628401189	40.5585676679482	45.1714698794331
	39.6609238036815	39.9948390976317	40.8225507267584	41.4508241771315
ASB16	37.0320432433314	46.0713306971594	39.0775870687758	85.7983996395757
	41.3755760434934	42.5660695551615	36.7356360093603	39.6474008369935
	43.0930293831497	46.325232414659	39.7342718293432	39.9519547348106
ASB17	40.6062943725118	39.0894172982439	40.0557788909923	39.4569929845912
	42.4864576619427	40.1428591998404	38.2280071951674	39.6474008369935
	36.5393857306744	41.5804111566887	38.956280281655	38.3834435509221
ASB2	43.844855336215	43.8495248991589	43.0016969050339	53.9517841438975
	40.9950639693255	58.8605811695948	44.6032218589199	45.2806199505029
	44.4395580493118	50.6486940895674	47.8988096575648	42.8864415494484
ASB3	127.437627356956	91.0327980861321	120.825113658705	123.444247442399
	93.6591201235745	101.577767481269	96.1181720777683	119.157952832454
	100.591821617326	130.230482445573	104.773064067069	108.855682928578
ASB4	41.3848620620968	35.7725167297364	36.0306975524309	36.7128997214853
	35.5833299694641	43.2219358930127	39.9620349773712	38.9229928131225
	39.3435164409386	45.4953384153622	41.4918397757335	40.8254641406268
ASB5	42.9641759692926	34.9262464079777	32.9756367838345	37.1894304543751
	36.3853022034874	160.080366694799	37.8877750141557	41.4508241771315
	37.1820543575123	107.987729161223	75.6883778954949	183.77850568836
ASB6	65.8959431201418	67.0402749076279	51.3268475761593	59.5177293266879
	53.207589413582	60.8357760968871	84.1327646296491	64.0752803796842
	64.0096843640535	79.3775933954493	69.1605201046373	58.7625374942126
ASB7	46.2190348814788	46.9670128044811	42.9734677356549	41.5464765641928
	47.965492554043	41.0582900711341	42.7982502551634	41.5817737564592
	41.863927676552	41.5435611403119	44.8278961893983	41.1914475733743
ASB8	89.9580440276828	80.0502531022254	79.7733480606334	73.7986685173579
	64.428683021978	102.471939526704	67.3359520423391	67.3976855661302
	95.0604882262201	78.3509756704423	93.5982028088194	79.2525964926483
ASB9	56.8534370691413	41.6466169548846	48.4890615340603	46.9919960628953
	55.3718910891548	52.9482338979681	68.0157730844432	54.9071823326024
	80.1505160355175	44.3303034026662	45.4799270968969	42.2577689927563
ASCC1	279.901217752341	232.879882989322	394.437597863822	354.086949676663
	363.527881831453	334.438091900861	293.258858033133	298.420882349382
	720.552354470517	328.788681417522	275.928723831899	313.528012643479
ASCC2	585.237875919074	963.224016480187	1712.55038816005	423.43141595962
	634.226367147025	507.239294307089	1296.28036613191	2903.00880209344
	792.880882528835	1226.43929953115	1343.66876893729	1026.51177853708

ASCC3 67.1108025189303 102.605473054584 102.77259665192 75.6883778954949
63.7417623042104 66.5774376622485 82.5506190124216 70.18107651423
85.9596109950132 71.2963721541702 79.4832790698673 86.8596934326046
ASCC3L1 62.7669079605627 53.565717255469 57.9279989359711
54.2886483704949 49.32293280121 106.067766766889 58.0139597055449
59.1107691694766 67.235655687817 131.645714321903 134.504159974855
96.1710806345503
ASCL1 50.0466349801009 39.0894172982439 45.063792968927 45.1944222548351
42.7107928951766 42.9695585740813 43.3262148375525 41.4508241771315
43.8482802038826 39.6719190054379 45.310660704531 46.7182294568603
ASCL2 382.251727747377 118.010166045155 120.881290860109 154.019005732316
106.951862149262 61.1022920882065 161.929470244265 204.68542226859
561.509463439533 486.487366153374 327.283431584541 263.096895937991
ASCL3 36.0144420013819 39.0102066700839 40.8420925848403 37.1894304543751
39.4693871570028 40.040778349137 40.6750540874299 47.4740726032707
34.8552366193358 39.5637454855233 44.2142581488029 35.8429598823818
ASCL4 39.1590964332473 38.2263227947189 39.991353369336 47.8164193292842
37.8679241954337 41.2567470068038 36.3194282084339 36.1375121409002
43.1805016690297 39.4710743516517 40.1742127891824 36.1647506311964
ASF1A 116.042856605255 149.933827818309 121.86893260878 97.8309399470621
85.7842344710572 155.795095438102 102.380096190518 163.476051754305
113.477848709768 127.937436596431 125.661351053514 114.809299879058
ASF1B 91.3967044940546 94.3229189690855 79.8773095098789 55.6481366056639
152.792457231813 52.9482338979681 150.985239447761 131.258204302177
204.009492151839 40.3235655331796 44.553780984244 53.0446815750355
ASGR1 53.8856501960117 45.8669209716694 46.2625470410195 74.6159297822533
56.9360840563854 53.8461734834857 57.6643188499117 57.8765910878975
49.4786466722535 48.3693338695392 46.8954576545929 48.1627097465883
ASGR2 38.9152309941518 44.4031868028216 41.437237454134 77.7973642829228
38.1096479110295 42.4058515827212 41.3594868055879 40.6171774724171
38.6322728994674 41.9666541498638 39.5847121472157 42.6208563330129
ASH1L 44.1294459089314 41.159706131351 43.5710871617107 43.9282059368202
40.148780650831 40.5281496800812 41.4742337714895 41.6466169548846
47.7086093893038 40.8970401536578 43.0971787292711 47.3172462673426
ASH2L 254.519518210024 408.216955625565 456.612479133179 298.367217540986
342.387761195952 462.562743021008 467.351570171652 321.359569119182
214.873872837015 272.315378390681 362.123479430887 279.448591201269
ASIP 47.7267190613906 38.2263227947189 40.6737160001291 65.524821699432
38.2496050953547 59.1570467228834 46.4388841354122 43.1567024035183
46.2027193115397 42.1622837416589 46.0095710163378 40.3691324547049
ASL 51.5888395924843 55.5712957590038 64.4512696384839 49.4786466722535
58.4383854340181 47.2009420656854 61.746195120737 49.6733735285073
75.7257660897685 48.1392888470269 50.3221380889396 46.1980780531506
ASMT 45.6517320730042 38.3322849790657 41.437237454134 44.550322123994
38.8456942580061 39.0266821712099 38.941855825269 39.9576138799997
40.7847110517594 41.0057887014368 40.8225507267584 36.8690774823319
ASMTL 135.567012693522 85.0653700751969 95.6731932757489 132.973097496421
76.2591822114739 166.821616593685 72.2873593966596 116.490154128114
58.1180488026629 119.157952832454 130.35828139117 93.8630393470857
ASNA1 36.9060997041453 45.0115870036541 37.6557714476853 41.3027031037343
41.9842695818285 36.3845892764084 37.830876898596 39.0620487644635
40.7847110517594 35.93950183586 43.5710871617107 37.0953622406687

ASNS 205.187554095744 165.905008152959 158.321459945365 186.477689350702
195.740109330843 195.512986297442 260.766438659762 300.412043023998
339.313853828248 168.67163443217 160.152738034528 162.02498480452
ASNSD1 367.808375988242 308.64836180781 295.5163490537
334.093449847539 386.271174243357 576.830397705584 217.39432336811
280.034472186873 280.446302631438 186.229678647062 237.532800487032
353.076989769287
ASPA 35.0202257459026 38.0226905622357 48.4890615340603 41.6564075217517
43.4544762830742 76.7791768896805 39.2533668167483 38.5457716679071
37.4540772310118 57.165971310746 62.2965257278236 64.863894209343
ASPH 66.4079972657486 87.1109435144663 75.8779316255794 63.0153175111747
63.4249771379563 69.3368205576982 62.2982003555312 60.1156117108741
73.5025309968566 48.1907683998167 49.4157684139858 57.4054069675016
ASPHD1 57.7877627794725 40.2973086894872 42.0960619209699
44.9370339275041 68.1674425673171 42.2577689927563 45.8204185866107
43.0390857698452 40.8503192605024 38.1472552468971 41.5638908543693
41.5083179498332
ASPHD2 56.121460370892 75.5775406974686 56.607199625498
71.7167037224069 50.5110359971301 44.9896851005259 68.0157730844432
58.2043532960091 68.3157692917711 45.4614234442527 58.3586374083245
62.5447504571181
ASPM 140.063595285844 113.26670675749 125.137803364368 68.6379135603075
205.672652429972 53.8461734834857 226.553946570328 242.450054864856
184.123415143164 49.0841460667818 38.956280281655 49.3984571719129
ASPN 42.9104454767916 44.3529919625344 46.8267540220952 55.4180794172794
43.844855336215 43.5752393599169 41.4742337714895 37.9837233601316
45.8325181856483 41.2276963324981 44.7114813656401 47.3172462673426
ASPRV1 41.4508241771315 47.6914221838216 46.2625470410195
83.1614238528477 55.1563703466328 48.3686935595826 50.0088611244948
45.5581356216957 41.863927676552 55.4037482627222 47.9942520395434
46.917149434588
ASPSCR1 169.251790128723 235.888544042065 197.011675186182
160.283895105835 298.313562383092 129.143252341926 227.94699237549
175.529493306927 350.917244438732 166.304210248623 183.526640731647
142.012216533823
ASRGL1 43.2724556187214 46.5650556481721 37.6557714476853
45.334106390212 38.1644569189523 42.5660695551615 54.8074655158941
62.4219686957673 35.6970528713709 38.1042454705119 36.7283081422707
42.0424641526302
ASS1 307.445116836976 119.747995520476 107.741669334989 98.4763347474813
186.64040927334 153.797431763616 704.601607617837 331.239629083778
798.033975337132 306.578554914328 408.216955625565 266.865951970965
ASTE1 56.4553747079294 74.6847594297153 74.0913177754682 63.3006977162657
87.8170592981737 65.6914955077809 73.3042538589134 54.720204938924
82.0993943936197 82.2431666775699 73.7060605978536 68.9885481504003
ASTL 39.5676051939342 44.2105195734073 43.0016969050339 39.5050383901884
46.5557478175976 46.3715909653832 56.7780520416533 45.642304356051
43.7630587657205 47.9056710878325 41.9083581529276 39.9519547348106
ASTN1 39.6609238036815 38.5921548841176 39.5450079747895 47.3574589211166
35.9894133432376 43.8308097066396 41.4186558780356 41.6916508480321
38.7298222155532 42.9866214102525 41.0050831223107 39.1553169409075
ASTN2 46.1673159982664 56.1838945110653 41.6564075217517 41.5464765641928
46.5557478175976 49.2731519807085 41.4742337714895 51.3240729268978
42.6861857638473 67.2047715601721 57.115361349315 58.8856060454401

ASXL1 129.336439341458 105.204861495017 76.1493261430901 110.112236384679
141.960773054307 113.377818029985 149.156754860718 76.4637616585578
105.914573205761 108.788705797019 102.87193290903 85.8154958329408
ASXL2 139.712472369259 201.764909400617 253.072560237983 290.316757790733
247.356027979377 204.477547372897 190.465025329717 299.890705180422
103.720371491639 148.925503526873 193.897678586782 221.65810716537
ASXL3 39.0620487644635 38.3322849790657 41.8751578329365 39.3750969348308
39.7568367479301 40.1428591998404 42.629412460409 39.853866204927
39.853866204927 38.1042454705119 38.8507804383261 40.0557788909923
ASZ1 39.6609238036815 38.861780967827 35.6765064335425 42.5193208629123
35.7098133359722 34.3473914754963 38.8775560911841 38.1835938174625
38.7578265341627 35.8914040545805 37.9553529562144 40.7704587752083
ATAD1 100.663054866186 129.984065394671 111.077646506425 105.422823184744
124.295423292168 176.991120397205 115.496195377983 81.2228688683149
134.581057139589 105.046407769128 136.081259669601 173.328468320432
ATAD2 137.360931076154 197.617554897454 115.840734064724 75.9099205260748
175.60601369033 70.7238101239421 161.439879456216 90.9010016862442
221.049503614433 56.3441077510744 54.4140381300469 76.5411379733694
ATAD3A 105.739423435604 97.4709987978999 94.5055588176321
86.7823828209946 189.137593177772 103.569150754726 170.87055409699
148.514056973195 177.379924616924 88.4385631861141 94.5111909776371
85.9286114777649
ATAD3B 48.5835484742456 53.3683962805281 57.0427273538849
43.1847628331565 75.4652763126449 50.6360662655392 56.4809146784765
45.7476095696859 62.0272989884264 38.745552403895 52.1788489555942
46.2625470410195
ATAD3C 45.6517320730042 39.8695929627783 40.931294690669
46.0228732507872 47.4548471240051 51.702461863489 46.9929836306816
45.5581356216957 41.9635525160083 43.401782064778 43.0971787292711
48.0350549043063
ATAD4 284.013140015092 128.70380102034 137.424708183613 73.7124988616836
110.869341159607 80.3805507705979 234.172977976583 139.645717741863
221.71759246183 112.760972238512 123.032006903803 83.9236799134394
ATAD5 41.1523487516364 39.6123051184545 40.301483914446 42.1273928203021
43.844855336215 43.7630587657205 43.3262148375525 37.0390589468421
37.7384958256497 40.5090849993014 41.2598458726002 38.6454098044474
ATCAY 41.7397731376692 44.8529259387308 38.6005272730664 43.9282059368202
44.2607789091176 42.750861693377 36.7356360093603 42.4580504587581
45.4799270968969 40.8970401536578 41.0808257270221 40.3691324547049
ATE1 40.1909200785004 58.2924877745079 49.5424425850549 48.0673507506626
46.9058511192313 50.1663232705412 43.1931731665131 44.293922970134
44.2237010679985 49.7259371799014 46.8954576545929 49.2064066614865
ATF1 47.4678477186801 44.7829632472754 57.1226931773006 49.3654837541353
38.3561682218911 44.9896851005259 48.8319884367456 48.7814131120945
57.4777075615651 49.4006068471301 46.0095710163378 53.3904307221902
ATF2 64.1882546112044 58.2571968311834 81.1249338842464 74.8706881113318
79.0039595317374 79.6895408438974 65.7873990226533 57.5640428227111
72.5993974280876 53.5498255316009 60.3431719501866 84.5354986509039
ATF3 164.941191767024 728.257836227637 98.9988668602468 192.112101000423
77.7852875052734 584.724083441601 303.139550962377 551.490493022416
120.679433440203 152.679927202674 360.900649927623 164.96500173066
ATF4 626.390544175817 513.891389190131 548.047774435177 693.056793637199
400.506633704584 834.14766574977 713.685405035516 751.953794043451
636.137048640939 1377.24483375075 990.431472174705 1068.10627239579

ATF5 164.146682514925 236.910422092851 172.574996256912 354.634819362978
249.308868215205 218.958199087236 155.855834721836 185.395460281034
232.147792636393 135.973016473297 158.901175732866 125.383837024985
ATF6 188.042978085327 290.761706869722 386.787221140343 361.091144969609
311.901423694475 232.740032714891 214.245832611737 177.379924616924
254.239142284878 186.372887375058 211.654245727798 253.267901237295
ATF7 45.3872406432794 47.4076235350059 44.696023817801 42.2496337895968
45.4751076037511 37.0180934800573 51.0013344286851 45.0972184964705
43.4656637536244 38.745552403895 45.310660704531 40.3691324547049
ATF7IP 50.2203578628217 48.3699741879723 48.1997158668729
51.0175677516029 42.1650127805175 44.5544727111028 44.7255207589606
42.0494116081998 46.4164127487052 38.528621456378 40.6373400186325
46.4499258373233
ATF7IP2 82.9467575626263 256.026804821501 338.475097952023
100.344513700137 177.82359682336 70.2553917409773 86.7968607218397
153.901083585889 216.063990451376 403.243939813779 434.523449093691
371.167168940365
ATG10 100.057155418659 79.1432909353319 106.443777149496 124.490684428024
66.5694839794116 111.691813916343 100.597578643877 128.70380102034
81.3858672064773 128.291287011686 141.370796803908 104.860298453006
ATG12 238.647490689325 186.450759425396 206.481424743576 349.096725048776
164.709142211696 217.189489936251 171.722423740869 429.725397949276
135.043488274939 248.797398220982 273.645674189546 264.600870859012
ATG16L1 277.069127740648 161.65846687852 186.54041506387
176.492987902471 209.705575423447 221.972553229362 136.883475451494
184.519582091623 191.59357986481 200.958421322871 225.760096749053
201.208357379329
ATG16L2 70.6065558064758 69.734208465775 133.893108529325
145.526972410673 96.0418367475772 85.5027511448958 109.901105821696
68.3372228877569 67.6983931011126 157.312247745825 116.080897948094
111.227016020927
ATG2A 97.8141040140486 159.835690229505 180.844663304872 267.024267633341
229.792906003602 129.675613712326 164.580141651883 134.185571685143
127.863441809606 139.45393201022 129.236597599908 83.3729277601518
ATG2B 57.934905173625 55.5294887653184 62.9313387640547 82.2766822827861
58.6481644777552 56.4819802857598 61.5000955267432 63.0748373122119
71.9062462001972 55.6865353018811 68.3157692917711 66.2305843819029
ATG3 142.309369995384 204.68542226859 233.692490031492 535.585999498708
241.455752070163 244.089971761037 221.598637828404 165.905008152959
223.040734249612 122.099297248392 112.721024337362 157.639656300073
ATG4A 61.4416707689893 59.2643906275025 76.474194677956 83.877343750194
68.8362790228976 66.7278011183421 56.9320717885002 69.845169494661
86.9274372004388 59.0630473216622 63.4610447573417 53.7820518773759
ATG4B 90.1056480599534 103.289476128549 118.251739134889 62.6048727240291
94.7604013126314 82.1974756449763 90.2915974511786 65.6914955077809
104.17651993553 43.4885245598939 52.022479439889 64.4156641487994
ATG4C 72.0334593545916 63.4570909617664 61.8701572127817 92.084526929319
72.0482026084702 77.6574950328663 66.1940959364976 57.037639245179
84.4111182389028 56.284748518655 60.7516146716018 68.3234331335073
ATG4D 49.7827734675743 70.9970523709176 57.6371756925687 42.2496337895968
62.7488501311848 51.3886035574416 48.9088046212958 50.0802438185974
66.7357366223349 53.2340637435567 61.7572675712557 54.7953298048942

ATG5 164.262531918679 72.1401612718245 85.8945498067934 81.6780581904253
63.955655956484 107.440379066528 76.0041771381018 159.99819920335
67.591570323687 138.637058021129 127.051878502711 128.098188757701
ATG7 356.826869112433 212.353676518748 225.960701908311 361.945511835039
156.433762355183 175.876518898179 196.034087004464 345.084002073846
128.923092916744 181.873969432903 186.301404367238 134.869029698594
ATG9A 136.613915120582 86.7823828209946 117.149897485203 99.126949393699
127.908440924429 97.5962784863854 101.634888749356 97.3870880869036
132.098298410135 135.169625016001 115.052948380817 95.6511488323697
ATG9B 47.0940613507321 42.0709558258414 44.0451487108213 46.0228732507872
38.4540849632254 42.8598805316863 43.8206425661746 46.2779564481158
59.7483827511172 40.1179210534118 41.9083581529276 47.0130468198828
ATHL1 143.084029849218 377.847116683974 294.849830545881 264.263104842911
100.386011517899 85.0275638433103 87.0685571351947 91.3607028449916
62.6386437936516 70.7003843117511 85.8277891453447 84.4349803529132
ATIC 929.862002014478 706.087945508248 710.579735673002 419.993482471924
807.620534408416 479.079946537291 567.978225839768 879.708199333257
659.126408705362 808.857027942253 801.351998609551 625.435083064752
ATL2 244.343381048643 107.176733647834 134.725028732282 111.522812019895
163.028918580618 179.522572239758 245.960009147234 207.928770428477
131.451150955309 86.9626166234066 98.9560329133205 122.368552598681
ATL3 245.691073028699 93.3718038663074 181.966839676473 267.70610731202
180.255771684507 113.979504140326 184.269633116351 394.877241043728
116.388069891158 142.846665249354 104.873452487222 170.184914156265
ATM 72.5559426064896 64.7318794662958 71.2867274976738 128.542999559673
91.4786089554886 109.659686701319 60.4492532011622 59.9770720847728
70.4933405598359 74.9236800269025 78.8741966117641 83.2240772671045
ATMIN 114.404099081799 72.5397346298724 79.8196996067124 57.9459232180399
77.2007992738116 71.7585486695516 69.6301237585571 66.8364940429355
95.5325488969517 59.8130420783374 91.6253858792439 66.9239565096247
ATN1 75.7140064167134 72.1090339831747 62.4317959077388 54.1240583139871
68.6199296680241 88.5207664670319 91.4514026506253 89.3868257841517
74.0831311663524 110.909109947659 99.6288131333575 81.382229980722
ATOH1 42.5193208629123 38.4330755397458 43.3085358214154 37.9875302985336
33.6330903323612 38.7340969861754 47.7390230243778 45.1714698794331
37.6383530120621 38.745552403895 33.9478220100351 38.8495095797881
ATOH7 39.266565827316 38.2835812177457 44.0451487108213 47.420942961753
44.0451487108213 42.1046072075724 47.6810611485413 41.1575777497576
40.2739700860114 39.528971131769 41.5668884168669 39.4830039788612
ATOH8 37.8386378737561 63.1780854659428 95.7820879892969 45.1944222548351
70.4607501403122 51.3886035574416 54.0170817319522 47.4740726032707
82.9256976863272 206.08791147394 222.8744321245 69.1099722732115
ATOX1 590.709061039001 268.745928429069 500.241231327789 785.79878416775
391.397318564183 442.711822532078 404.356143472409 761.922486104216
351.290929267326 604.357590751951 532.35007819883 562.531738167208
ATP10A 45.4799270968969 39.7835513496873 37.5597671487621
47.481302504066 37.1565984182084 39.8455060654531 48.9826868640753
42.8482181313226 43.2761454096265 41.0057887014368 35.1470994647364
37.7526485343328
ATP10B 534.881442644054 137.696331574552 174.275409358652
53.9517841438975 45.2556760873335 43.9724628401189 71.8466937395628
398.229956085523 53.7442594289333 49.8393069675192 51.524711857679
49.0655215909632

ATP10D 32.406093465115 44.5884811528117 41.528370959196
44.1483976313959 42.269054475455 40.7616966228404 38.5066108855005
37.0390589468421 47.798305839406 44.2580151149956 47.1477056711335
44.7401523707775
ATP11A 38.1169703906207 37.3179578037963 40.5734458834617
40.3180310664156 37.8679241954337 40.8970401536578 38.7337086062875
39.8016018354453 39.6413313949444 36.6893251782922 35.5179217192467
40.8098117292322
ATP11B 68.6627996695828 66.6332042895961 91.7844162703214
86.500678864429 58.7460488758053 81.2314452281031 94.6952414258466
86.033725825968 75.9298650598599 81.7967230859562 86.3054012048636
97.8764110456132
ATP11C 49.7827734675743 55.4848084120513 54.0536858212253
57.7057442885013 70.2565873027242 55.4409373780772 63.5738862549306
74.2517888452553 70.7760293129408 59.9792284253187 66.5285423077808
94.1844708285913
ATP12A 38.2083710281545 44.1239711172417 40.1985790927248
35.6928955245309 41.3755760434934 48.0350549043063 42.8684958525042
66.6320017520888 43.5669306063196 40.8049408996274 41.5421429082231
42.4607795638552
ATP13A1 101.688066199665 132.307107503819 122.988056802699
271.070078611015 172.349874473605 75.1674447943072 127.232160404726
323.957165066178 140.586248661832 105.212030988223 113.612874206329
97.6552689677199
ATP13A2 111.760213205992 111.071209057746 107.637618676176
77.3864518736634 111.033548392096 61.2387559102636 124.803597244786
134.952392466784 96.9051967257119 73.0394191668858 75.8976034525022
78.4023698691285
ATP13A3 46.263826584899 49.015238049903 45.3974838013476
59.0965219753805 45.402658694824 44.6524308640784 43.6025443656008
43.9604529016594 39.7537274655115 39.4710743516517 43.6919270353542
48.6795178347248
ATP13A4 50.5670783769975 36.1232327599945 47.2962360458401
35.3829784221072 39.0564222526273 34.3887641786747 38.4005795891396
50.3972033014227 35.3227485563945 38.745552403895 39.3667161654772
40.9683275553869
ATP13A5 40.7609952437622 36.0366673493555 39.0775870687758
35.1451084093692 39.0564222526273 41.0582900711341 40.4359177791566
42.6450179841129 40.5912213523345 41.1222709786153 40.0766543123257
36.064718696392
ATP1A1 882.105892529265 454.02808608679 657.031980628955
674.270050283701 570.085422388476 555.727708387209 698.595302020108
745.219025960188 977.776395810338 603.737787922337 858.975085087497
821.922096305069
ATP1A2 29.633229264005 40.2493681589104 39.3709357615493
38.745552403895 27.4352294118391 90.2790229242453 42.0605906240248
40.831207953647 41.9635525160083 77.0226403968683 65.7587943905649
40.7704587752083
ATP1A3 37.0320432433314 42.4493032770053 42.5406888281114
48.4916117779992 41.6806984441849 50.6360662655392 40.4359177791566
45.5581356216957 42.0621425894624 41.3321861371202 36.9060997041453
41.5536168238478
ATP1A4 39.9182914210537 43.3458769998118 63.6334154351075
41.8778873678551 46.7058093820839 41.3094485574845 40.7910160811527

38.7864161493321 70.6784633355742 48.665578118898 52.8297102061403
64.3571664944642
ATP1B1 679.810781641519 627.914461428624 614.603743904512
120.571680725698 213.768352764473 201.875082892488 559.296669575666
376.289659542649 427.363555286404 227.94699237549 300.057362423939
271.478148993789
ATP1B2 65.8306182796781 55.9269493191353 51.223859150798
50.378948785456 80.428712610358 59.3618843678775 75.287377665837
76.4637616585578 73.7873098190297 65.0928075335306 78.3542840447759
72.5346469941833
ATP1B3 212.062417120966 207.768377497439 368.390023942835
442.080188140864 347.247950836937 190.403032965372 246.680724959444
359.628703006773 439.106576928682 238.584514797019 237.039707901886
379.280296622892
ATP1B4 38.5007204246293 41.8073910693411 37.9482157381008
45.5869891570251 42.6022652963326 42.0574848021044 34.806104801006
45.3872406432794 40.5912213523345 33.7883338848419 39.2542963855807
42.357910063876
ATP2A1 43.4215162178364 40.2093230376545 38.0958223740111
41.0219290963778 41.3387790895716 40.2851212785573 39.4600924554505
43.7839209303388 41.1349567921107 41.0657892043496 41.8606936669687
42.6187506958386
ATP2A2 465.595459259015 441.872082665398 487.749060801066
343.892794667047 659.666977039793 472.522115719366 360.523229691604
278.629995328901 667.583623414832 434.841521967958 519.351335801817
471.59454368268
ATP2A3 41.4508241771315 50.7119825096136 49.3780606970538
65.4068498877665 40.2449343247539 42.808889470973 41.706879587091
43.1567024035183 39.3506426665347 42.1622837416589 49.6314545328943
37.3600014814388
ATP2B1 42.6561897411324 43.0227387991135 44.9174294707972
52.6040246568056 50.3713170502557 42.4263859458031 40.0668875818227
44.293922970134 49.0986627051784 48.4365720634326 48.6561813834854
46.4499258373233
ATP2B2 40.9201978689462 38.2795150354677 37.0174299366022
40.7941759414114 39.9042726867255 41.6590564937735 42.2143023923639
38.9229928131225 39.7107284579423 41.2276963324981 41.0554874392252
42.4322338033273
ATP2B3 41.9823787224629 39.0894172982439 40.8833572570383
42.6111832024096 39.3667161654772 38.2293008890613 42.357910063876
39.7509165247419 39.853866204927 36.6452480995946 39.6403089864678
38.2795150354677
ATP2B4 181.200738629391 124.683201596193 469.553640187203
180.349984862124 74.5758811681995 446.162602155964 66.0979829658877
108.616118600231 80.3805507705979 370.779448406233 313.233036704221
357.44124276077
ATP2C1 120.240956797301 155.795095438102 144.02257553612
115.913030448537 179.214713709599 111.071209057746 129.605603092944
109.326279686847 142.945182805248 74.4393926690587 90.1964013788257
110.074666148474
ATP2C2 50.0466349801009 144.954155897343 128.146059846414
47.6687908290818 53.3086645804786 41.159706131351 67.3976855661302
54.5125550937898 82.0081006332668 61.8529316708431 65.7587943905649
65.7853960266587

ATP4A 34.3473914754963 37.5469247328848 37.1643693306382 41.8778873678551
40.0599807618105 45.2852528185486 45.6042625176633 37.1317006162938
37.5469247328848 40.3235655331796 34.8383995317036 38.8495095797881
ATP4B 41.0608773166058 43.3972849968032 39.7847448502731 42.5321207892864
41.0441816173391 42.9695585740813 44.2753808209563 40.6750540874299
42.3004033054354 40.5620233728036 41.3945375181463 39.7844133452634
ATP5A1 1652.85734308754 3800.19142219719 4048.33132153903
1801.44003951248 1907.47560253019 2733.80542760533 2337.7738990994
2488.32876359853 5479.94045861143 2435.70120719503 2755.23573327276
3263.05269150231
ATP5B 3618.78958408675 5636.93532853955 5566.52516420571 2465.65854657837
4752.94498024732 3399.75820494572 4569.48246031279 5479.94045861143
7022.41612496714 4458.76622334251 4277.93019582362 4617.45430247751
ATP5C1 609.243908604949 598.404003732481 619.337575854369
401.347734852083 704.15545332905 572.19538884395 657.031980628955
386.640440174303 949.923102632043 236.374695859748 242.190059393491
531.36214636517
ATP5D 407.818713538416 781.223434214089 596.84269596976 460.340300658834
1137.99307039386 740.994425128952 509.004054793277 563.927521628693
696.946621824188 882.105892529265 975.544591158047 612.014486689487
ATP5EP2 4328.723715755 3361.67247904403 4259.50675863393
5387.08056045279 4503.77675488367 4093.70002166179 3726.73487426505
6356.05807792831 4948.93803183911 3253.86278287661 3575.78813099057
5354.58328030315
ATP5F1 527.97309138633 828.403901345618 955.088515235485
662.848386826617 920.504890206997 713.685405035516 661.269882993465
641.103199748891 1083.50383916482 541.105845300009 611.538630852232
936.978983469829
ATP5G1 387.311743342483 273.764998628636 327.631923082696
285.826380698286 453.652548085387 277.244576031507 382.940842652744
574.14982128922 437.280601907944 459.546279672649 443.269396125834
426.622204035624
ATP5G2 398.229956085523 392.622487173359 608.73319976638
340.553073657744 223.850148640071 419.360641528378 369.258587948416
761.341795853104 565.411298018845 507.375704144286 455.223217772744
585.054570630252
ATP5G3 64.9367707455078 76.4439037944547 60.3973027611091
64.2130157241811 95.2833228196616 74.5405407002217 58.5087296620514
58.3046521633424 60.8635925649592 65.9189023794337 62.6048727240291
82.9467575626263
ATP5H 1792.42490815074 1195.24078948059 1433.1813604069 1287.41527826233
1559.50766982801 1390.12380748446 1689.77066894108 2002.0536042686
2133.78836857697 1645.71527614542 1493.20520979227 2124.40034922306
ATP5I 903.756866967447 1320.7894611262 1089.08276262641 1243.32650314893
1043.26325848938 1372.51401036753 1473.52943897204 1330.19215036764
1480.95754707554 1789.07186722972 1570.45778081591 1607.73526966988
ATP5J 753.976423227915 901.793120584383 1311.88650141004 842.82310025167
1080.43016747082 1366.59129560817 834.14766574977 1086.12171444149
1349.09482344633 910.898566308567 843.078502001733 1384.80615431656
ATP5J2 1171.207937488 861.427193617946 831.578043520215
795.381998233245 1145.59111340228 594.611678976716 993.626865290891
1255.10866316499 1554.42895572029 763.947361966861 798.996494242432
871.238169066998

ATP5L 953.143041145545 771.554891868456 943.73554571528 1055.00793806075
1213.3477481374 1234.59589464045 985.328482076985 1064.35745220225
1313.92053019421 723.210613328966 816.746599467159 1275.78715071615
ATP5O 751.953794043451 1056.57081612661 1513.74711975248 866.712228831149
1450.53108586858 1398.21942794938 812.386878774445 963.74601421037
1105.00321488322 843.333981146512 1087.75682788799 1601.52194699063
ATP5S 64.8254097948745 112.202543937743 116.530589514041 83.4756142147167
73.6965494979349 99.9486407529027 104.076098789396 79.7733480606334
83.2925226467532 75.7427148831879 81.7117547901526 79.4710406831985
ATP5SL 335.002151198999 440.93348620369 357.839241226513
263.587821561589 467.351570171652 509.463928291526 580.444451816014
305.399644617845 543.975237512604 440.93348620369 446.902178094675
468.98857415608
ATP6AP1 1801.44003951248 1428.47642890543 1615.83220398811
1349.09482344633 2294.75523373329 835.171475474151 1645.71527614542
1945.33618761041 1364.06060391538 1585.90718459092 2024.4791883541
1119.11683235081
ATP6AP2 805.590413262364 651.969296681551 814.643009598624
889.638287212686 483.882804472844 591.192020333181 549.67905283016
740.423874031773 780.708896888794 548.217757366495 593.345148568451
730.168814097451
ATP6V0A1 285.826380698286 229.26498093497 369.717462266442
315.880072026714 311.361885152025 286.045253557021 238.510952165358
266.250887288858 231.859167242606 230.197573134703 311.055487966495
219.431561967403
ATP6V0A2 112.551747307723 87.2351169088172 91.0443726041992
130.274451794636 85.2849531543957 97.4062608315831 91.3607028449916
93.2402285327166 124.397048303668 75.2053836356882 92.9820860411485
74.2748495231478
ATP6V0A4 100.114114765033 39.9697332660833 40.4070046183108
41.5464765641928 41.7426502114261 41.399074748665 43.2721716572958
44.0839329808106 40.5497440764264 42.4594149893798 40.5090849993014
41.9159449067072
ATP6V0B 1184.18994044558 814.294297312057 493.322302312578
1485.68317899614 702.737741114234 431.727858902449 936.978983469829
924.316085565917 1007.03747721563 510.232992765054 471.59454368268
397.745727767552
ATP6V0C 1135.43644148537 1223.01485352248 1390.12380748446
1907.47560253019 834.14766574977 766.647626178442 1052.16097214718
731.071171171638 1097.92519921873 1055.69506483576 1335.4111951034
852.769974690777
ATP6V0D1 386.00975855841 366.716775127375 539.32996290794
887.118866402149 279.063292799015 383.190278161083 320.555790243104
300.803955139552 406.417924060562 296.463473585355 400.8670859408
265.728967648382
ATP6V0D2 44.0328992213727 39.2907645923217 39.0775870687758
44.1483976313959 40.7779767565889 45.0882356578404 39.1539892144371
35.2642250366721 44.3440839686558 40.6022003765153 36.7283081422707
37.9607839934735
ATP6V0E1 1638.16317738035 696.113365766871 983.972164685474
1762.93413846062 796.359355922952 902.691776563423 802.036670929858
1217.21440297138 924.316085565917 1045.20336057833 1062.93431683428
1241.21734508421

ATP6V0E2 352.917938567734 216.1413998895 121.650910373947
212.813224717262 1132.19026676901 305.279475274196 1057.44729389649
605.338871441599 536.013150313097 182.905368976291 211.137455821181
166.304210248623
ATP6V1A 615.442484911026 645.702744916884 759.184121753305
1325.2479053325 636.367246129079 441.5479786095 469.553640187203
448.508714709248 501.319993127353 459.92905629125 589.685739519627
509.463928291526
ATP6V1B1 182.411383422586 46.8236022402545 73.2814174739782
54.4586176399381 74.9113162056974 49.2050538150106 60.0981687048269
124.18058128276 42.9020321355927 53.9412154719864 46.1673159982664
50.0571060701893
ATP6V1B2 1202.11455437028 501.817654561915 855.157599239267
3465.3013599062 371.167168940365 985.328482076985 542.467997217663
583.915973546284 432.001201940414 764.571579067138 779.996851709716
659.396637478356
ATP6V1C1 102.952512767439 87.5002066150725 64.4588422949237
142.48688219605 48.1473938886239 73.9456720099807 64.1777305915695
57.2350830635912 74.9741052063425 100.849815617918 88.4594321382477
76.6094442444074
ATP6V1C2 82.7351935657355 38.3322849790657 41.2365089689635
38.3668692419741 38.514058966346 35.7034203372528 41.5817737564592
44.0390019242862 39.4433784795209 43.6670755115848 34.9023759742338
40.2237056928605
ATP6V1D 874.419252731128 288.869882644391 669.419736252343
931.401929277001 303.239819890428 512.255695437272 401.403103922907
510.056072375132 417.4609354305 558.451033789799 549.117045813593
875.139073253298
ATP6V1E1 1557.42399344934 1052.16097214718 1263.76838179117
1640.74027400791 612.441960343995 1236.32289322872 1137.99307039386
1073.84987604662 891.080174768895 1549.93897368117 1239.68399088145
1466.88103496067
ATP6V1E2 95.7571966348067 67.4018925617901 80.0183739952818
51.3851157261451 67.7155610106537 54.4753345912615 57.1070449050465
74.383456365584 59.9108736364178 71.9124897002253 67.3976855661302
60.3816817102326
ATP6V1F 1252.94510628089 789.511653564341 856.790359133679
2071.90741263566 1058.61208471961 962.079396754147 950.740991182127
801.694261678224 809.577874019028 841.850722284484 884.036280777358
974.296760034984
ATP6V1G1 1634.61795848352 1570.45778081591 701.168333137671
1382.25775340179 763.947361966861 862.031722874406 1731.19220270448
1109.17675353971 975.02086340962 1250.15619347362 1379.51119430451
1271.62760896761
ATP6V1G2 51.8781065691038 44.6360645306497 43.3465699185405
38.5707420487455 46.6397391251762 57.013135787334 42.0605906240248
40.0965255448873 40.2274522635576 45.4882438975269 46.201352759435
48.7814131120945
ATP6V1G3 45.6517320730042 43.9783383422823 42.0621425894624
41.8778873678551 42.7489468897708 42.1046072075724 46.1634822993009
43.4544762830742 40.4518431978241 42.5193208629123 43.9802831041664
40.9683275553869
ATP6V1H 171.270750760376 301.457545514208 156.69976952483
205.021644155954 142.532754436212 172.433936159136 373.626843592567

112.930164222877 172.506486240017 137.294289316915 138.859992681388 150.562062400737
ATP7A 59.7179445506128 53.9275079525238 81.5782824513693 65.084620579901 48.6224651100733 63.2343637481705 76.7483302168542 66.295597720304 84.0899854321388 92.3388129097482 87.8967913380047 105.699748702315
ATP7B 54.5921287593554 45.9732108120834 57.0427273538849 45.9441733841041 56.5933259268408 53.2874286456249 57.7096546601208 52.6654109976514 78.1448136847078 58.6652401079158 55.2828387400799 63.0733654065132
ATP8A1 34.2180669723984 44.8529259387308 40.7620472083159 41.8778873678551 44.5668141895992 40.9506062495231 41.253645578517 39.9576138799997 39.853866204927 40.8049408996274 49.1868644496737 44.0451487108213
ATP8A2 54.3554233228353 49.83247261979 48.6037684272115 47.8164193292842 50.6470461863549 44.1737502492846 46.4388841354122 46.3685439301234 41.9635525160083 42.785429206422 47.3791873993182 45.7562276914441
ATP8B1 50.6473771879221 137.955007835039 127.836645375421 41.8778873678551 88.1199908664065 59.8102324370568 142.789889238338 63.287067794195 167.678403380953 119.747995520476 145.267770598988 119.700522283289
ATP8B2 47.9175508887533 48.675318481211 42.4939802311136 44.7520317855773 41.5421429082231 67.2968280876013 42.57175366709 44.2580151149956 43.802800826921 43.4598349902811 46.3563690926644 53.5819170097952
ATP8B3 44.2350046179299 47.8400694856726 50.5089777244781 50.5310598656998 39.9697332660833 47.0027453663546 42.1607322031054 52.5098795466615 52.8502823653368 42.0621425894624 47.0940613507321 46.0633179231318
ATP8B4 98.6766028183598 72.0792554308917 58.637422084759 220.977092747428 55.9283039581662 230.879083362388 66.6894494536346 88.2568398791731 52.1788489555942 113.090530993447 100.883604376864 95.7383730980717
ATP9A 455.774407260725 255.68068369366 263.387877739079 106.15531508764 154.811704749343 228.08291792003 330.096171705212 544.946796643478 250.65667565007 737.604036996153 594.611678976716 631.632335640448
ATP9B 53.8856501960117 48.8584917918986 56.607199625498 62.8201270538435 46.6397391251762 68.4323624797175 49.540449125788 45.2806199505029 69.8899273527731 82.966263655525 76.153346954349 56.2804354745795
ATPAF1 144.711001798454 128.596547789044 142.124053129543 98.6825522152097 177.757247913754 157.9970294734 162.32721608943 146.681796324406 187.601223003233 180.15711251649 119.528720998614 187.331614826395
ATPAF2 55.5276281380084 56.1355483199198 52.0742951366864 43.5627649099623 58.4383854340181 53.1052228246467 53.4921736109774 54.720204938924 64.9030399249327 49.8393069675192 65.8748459696377 79.5898953726467
ATPBD1B 155.982503792091 117.239774193934 101.494432845825 110.666686474986 151.451107406752 116.51037006692 168.561260174501 176.866350130563 144.098339910117 271.529471399941 205.187554095744 208.878921620914
ATPBD1C 39.0620487644635 34.5023764006976 39.2707964550441 60.620259884953 40.7779767565889 35.314469458379 42.9803717121865 40.6171774724171 34.9428206949566 36.4651112441576 40.723990462047 37.3600014814388

ATPBD3 56.6951078698291 46.8954576545929 45.1569741609597
44.1483976313959 63.3638417360105 43.1826328292558 59.0457625163577
44.797320243931 61.3733228199803 48.3693338695392 48.8088340735921
50.379587451669
ATPBD4 51.6159542017038 47.5203201376024 40.4070046183108
42.9358255168086 64.9072331703985 53.63657997649 55.4004611309636
44.4031868028216 59.3164315859919 54.0917531668054 57.2674335547631
64.4766262198475
ATPIF1 115.357402077859 140.076084766156 171.746499373501
143.973063517706 193.897678586782 109.137372445043 157.9970294734
212.712025285843 200.174103125152 135.540738841602 129.320408155239
131.90520406914
ATR 40.2216612339451 52.2759825608835 44.2698656253406 40.7910160811527
42.5428186846763 37.0747059730919 39.8695929627783 37.4484310268909
43.2047465154928 40.2579040125416 38.6355088942179 37.2810767789306
ATRIP 100.057155418659 88.0720840588632 96.0126406688619 113.179313022693
183.702709922933 105.45765455249 152.824869071575 103.302837652919
108.525500270474 127.61938370689 106.340844838265 82.9467575626263
ATRN 63.8273035273651 71.501028931626 80.4554147910354 72.149478755262
83.6935756500468 55.5789922793469 66.7475567712242 58.0892402609917
70.4262478188233 70.3629652777935 71.8887764487177 70.1184116246724
ATRNL1 40.8681871019812 44.6918600441328 43.7630587657205
40.3180310664156 42.9090346219023 46.0364000143537 43.5627649099623
43.3262148375525 39.6609238036815 40.4136535208406 44.4688999098904
41.2437378488025
ATRX 38.9152309941518 43.3458769998118 38.738305958734 38.2516470858206
41.7503755215516 42.1095407186921 40.9157352260901 38.3194261431961
36.1537607600257 36.9948667320377 38.6662194645633 40.2669497398702
ATXN1 68.8005532048281 47.5870162399764 60.7530213660442 77.5091723127198
48.3814421939888 88.2639730639822 43.1931731665131 60.9801094225351
74.6110659745477 92.1908585323292 82.2519263268941 85.7908542452355
ATXN10 53.4921736109774 55.2485665256307 55.4502804236424
44.3638402537972 67.7155610106537 71.3272569463294 55.4004611309636
62.3524254253348 57.1401776589964 70.6138659908885 64.7598303406649
71.7937709257351
ATXN2 498.958550919515 547.888464549389 544.946796643478 358.779372054826
562.798580332885 446.757901018137 551.915864019927 430.868309653765
612.843402058384 943.73554571528 814.991871217268 765.145138238955
ATXN2L 50.6937447392501 52.1062000766427 63.104563489528
51.7074776656716 63.8810437279875 45.9972262774766 47.6773473969653
59.9968592852122 49.6011834770324 52.0237462570423 52.4212131710421
48.5544398725959
ATXN3 90.2721521466478 65.8082111065882 99.2100734209486 116.927619635255
76.5375674421131 89.3168921945222 94.233414682323 66.4207938873541
80.1505160355175 78.6402083787251 87.069143362676 106.5477041881
ATXN3L 39.6609238036815 38.2263227947189 45.063792968927
39.066284740632 35.7973685350741 36.2768299545794 38.0598374201407
40.1474543172332 38.5252456306656 46.325232414659 43.401782064778
36.9976231206067
ATXN7 41.3387790895716 39.0102066700839 34.1806289878747 33.3231576795423
40.3452320167286 39.4953494583071 43.4544762830742 38.2782436497441
41.5635499110307 35.6208308220005 38.6662194645633 37.6557714476853
ATXN7L1 48.9906383294199 43.0971787292711 52.9111155236961
59.8102324370568 43.4544762830742 44.8872784170036 43.3262148375525

44.293922970134 47.1744296124496 42.1622837416589 50.3221380889396
36.8690774823319
ATXN7L2 68.5457752893587 86.8262463672891 63.1450855271137
45.1944222548351 98.0143500562821 48.9826868640753 70.2635892414678
60.2691630545162 70.3613168347492 65.813547096222 64.898351831341
47.7962548556026
ATXN7L3 375.697071471585 411.981987305512 329.677454924062
260.824949424185 432.512515345015 208.189151365575 393.001025840896
284.105466865264 463.527266972118 253.527553472516 290.316757790733
207.087723777954
ATXN8OS 42.442231667357 41.2598458726002 42.157627240486
40.3180310664156 43.844855336215 37.9003288112261 40.1235656101329
37.6557714476853 36.2733735494625 37.8217989841918 41.4508241771315
39.9519547348106
AUH 90.4512650366645 153.139323479953 95.0738959950301 96.2385885003071
75.8306078651291 116.253417059012 101.716697533604 85.3842287274022
100.774098314706 74.9113162056974 107.538934056558 139.472956453414
AUP1 99.2432911346944 97.3813557754832 112.811643697986 111.771533072688
154.869301720717 80.8333046859586 117.260454792593 135.829530518199
115.271200532229 117.921615219634 99.2575888644764 97.1271615802556
AURKA 201.301705082976 231.824347524066 145.459578481501 122.316617618658
452.139416552986 98.5609311953771 370.917477324773 179.779145453083
290.261616334371 66.3956936248447 53.4921736109774 58.3882937676176
AURKAIP1 1480.95754707554 1387.28483887704 1408.59798540643
1232.7632290973 1985.3999418878 1188.3486464341 2067.66516256598
2294.75523373329 1537.76265834076 1478.26733379357 1483.40077183826
1125.31394383117
AURKAPS1 41.0608773166058 43.683792219729 46.3411353377615
38.745552403895 47.8075998240396 41.2567470068038 53.1884505015411
44.0390019242862 49.0986627051784 45.4614234442527 45.5707650534259
42.4607795638552
AURKB 172.89937021234 321.171736253617 214.537530518962 79.7937658787441
361.57814355509 73.0247140074919 597.996273468244 237.532800487032
348.757322819568 48.4890615340603 48.9754340235127 52.3896785600751
AURKC 40.7609952437622 52.3432130135266 40.641503836284 40.7941759414114
85.7842344710572 46.3715909653832 38.9733632798532 38.0240760521689
41.9180247412837 38.957687788868 43.4370048647278 40.5281496800812
AUTS2 304.777565078155 115.221134337342 220.398778650109 163.718576695806
272.545033107746 262.9072085191 219.134220280612 244.736442646499
437.015713629277 522.377154567257 399.027079798213 282.196234758364
AVEN 68.8698243753339 60.1102859845739 48.1331309338997 70.2541961995753
80.428712610358 77.8490019489375 55.5578334877425 55.421880601441
61.5807110546718 61.1851254827343 60.1466326805616 72.6182747525443
AVIL 52.109723558511 52.4351670989268 48.1997158668729 52.6947911480086
47.3172462673426 42.6575535390785 53.7423898357583 42.157627240486
48.9931786790166 55.0016608333828 46.7386599624801 42.9887069977696
AVP 48.0201377454632 55.5867487811643 57.9279989359711 54.5125550937898
45.5198200633333 44.5917081663148 50.5252048155459 47.5439485437351
48.7646976789783 49.0841460667818 49.8135461150818 44.9734311311394
AVPI1 345.443895623911 402.221227674218 398.229956085523 635.52621743309
302.465485470899 356.133736281036 279.521995263196 858.975085087497
332.830805115112 2528.6392272518 1122.44489338159 1266.41536472956
AVPR1A 42.1537476443423 43.1805016690297 43.0971787292711
36.0481934423077 44.3557408297309 43.9724628401189 44.0687697278114

40.5158867964016 32.8704945047196 46.5650556481721 46.7386599624801
40.3691324547049
AVPR1B 35.6111578834671 33.4967065724734 31.1853651446075
42.1273928203021 34.0045980663271 34.0045980663271 37.1643693306382
38.079222289614 40.7847110517594 38.745552403895 40.4504608195666
37.1747156131306
AVPR2 38.304249186058 35.6434124419782 36.769073068743 35.1451084093692
42.7107928951766 39.6251842352088 38.5066108855005 41.5464765641928
36.4476883897928 37.7315666632629 41.6336636285875 37.9607839934735
AXIN1 206.514263103112 233.263058459431 304.133852957499 144.998604924513
226.804294585045 203.808195515747 207.957319374194 106.398825125486
218.819478568299 162.628603963754 176.224645007435 148.781721594419
AXIN2 148.595294991115 476.653723741096 287.423909789368 89.7554896946341
81.0259717888094 255.087214364926 248.194954741095 67.0904616289706
85.8154958329408 341.206585871289 364.439669236186 188.099292617901
AXL 248.797398220982 105.257627491307 94.3953345963359 162.721587349434
98.6553805168141 276.795797048951 52.0942589368478 208.97126697486
50.6346949248834 303.818507395434 220.426780242132 210.485586903711
AXUD1 478.939577875017 762.855719373733 518.074074000609 3253.86278287661
438.22469176123 2745.8277126123 672.515393219419 1545.76489731307
291.785618741057 675.832363405287 688.168272082335 639.391575249793
AYP1p1 78.9028182143923 71.3015974749703 91.2769094348604
118.911578268073 118.49449420132 176.670909065664 140.264270771578
156.636360703756 76.2591822114739 109.654559065946 99.1952492360239
100.567309348016
AZGP1 42.0532901689036 50.0425298550559 43.4709415392696 37.8442841690502
70.6903168772326 49.7458652239071 42.7431359850339 42.8482181313226
48.2123120250343 58.5087296620514 54.8532558909227 54.0374522273907
AZI1 141.043326064913 249.451753363306 147.498355916596 82.7394574977114
248.321641540666 95.2367177680328 200.986069552443 280.239058266018
111.291087721139 172.990533342251 129.925137664962 86.0735583144514
AZI2 67.9531693869426 74.2653431904071 63.5107036952143 137.555275829874
61.954674965065 141.594428857706 63.3924515518667 55.421880601441
68.2372031645261 86.7076796107636 76.4389426517528 85.3431175579775
AZIN1 400.973553604192 291.42149037871 212.664574366477 310.706520144173
233.96534264787 323.780380243759 251.299662213686 156.02592319461
324.320137371059 210.133037772969 193.806701229141 284.575587310164
AZU1 36.3649677956953 37.4540772310118 39.9101908547397 51.223859150798
47.0954207417646 43.7630587657205 32.9962272670005 40.047314525032
40.4997186069494 40.4136535208406 41.6336636285875 38.6454098044474
B2M 4299.4451139256 3761.48462220262 3263.05269150231 5636.93532853955
1339.29022988209 2323.81110653289 2146.1548633579 2417.02418518222
2745.8277126123 1618.92099084622 2506.05102956991 2611.32405374107
B3GALNT1 157.658760470424 104.361353204648 274.134201700259
53.2046867327331 102.655050997714 77.9820921829024 83.8811735017279
118.259459346012 164.735191308776 144.427680369554 109.758297200083
175.652989203497
B3GALNT2 65.0721641331632 56.9312835667828 65.49167373382
56.4578213622965 82.6157566292355 87.4239220649478 54.4934782594218
57.3286965007828 65.644767142472 71.5032996449402 67.3976855661302
76.9953921834156
B3GALT1 41.3387790895716 46.8236022402545 42.2577689927563
48.856176220627 44.6834648302383 45.9266745571724 40.1235656101329

45.3872406432794 44.8003345643334 48.8623729374733 40.0766543123257
40.3691324547049
B3GALT2 36.0144420013819 36.89524015739 37.8386378737561
45.0798408927912 38.9496515344837 39.8455060654531 38.2933870238743
34.3865306339645 39.3750969348308 39.9948390976317 38.6662194645633
47.2131144508464
B3GALT3 46.2231598401576 47.9665737016961 70.4014100046391
36.5393857306744 48.4724651632766 39.6251842352088 41.0871827071116
37.9837233601316 50.9612733880858 40.0557788909923 38.5252456306656
51.2776048338
B3GALT4 150.801729138762 116.295564219236 126.017900743748
128.635811501506 55.4919197421746 76.1019439127881 125.184689092836
123.506372870621 152.605418131968 131.645714321903 115.88156897199
85.1114448808779
B3GALT5 39.3303857231606 40.3180310664156 38.6727842796202
39.7411455670172 39.8238654968211 40.5281496800812 40.4995052849302
41.0920120134247 42.8414096678708 41.4606714407332 41.9083581529276
37.2810767789306
B3GALT6 171.501864800548 106.8866235778 124.94083215058
120.436827608155 87.6178717341967 93.3718038663074 146.840603547323
135.540738841602 136.644759501653 170.150808455621 158.408644964483
93.7924763988692
B3GALTL 51.1740855632044 55.0897019602305 56.4779645313958
63.4531374125233 61.3077641493507 61.3620062177524 88.9430141071732
52.0503062610092 69.0037087543684 69.2963913574682 53.1505787220552
68.2309359831273
B3GAT1 45.6517320730042 40.6380495994768 41.9335763813389
43.8002456102021 43.1833429589719 43.2427390263564 54.8946088631931
50.9933795450314 41.809704036355 47.4439407315335 44.2925355984056
50.7487159427973
B3GAT2 41.4508241771315 41.0678614026819 40.1985790927248
36.5393857306744 39.2608760193011 38.2293008890613 39.2533668167483
36.2539950294089 37.8319142847141 44.3648993568405 41.4508241771315
42.8864415494484
B3GAT3 112.865496075486 75.6614221167993 126.578693258328
125.114613377133 117.893268074013 92.9820860411485 133.344625514809
130.184962393833 78.2393202422664 138.765621737736 147.579873410455
91.7906797118637
B3Gn-T6 35.2267149016596 37.3634897565502 39.1799070031819
26.6357008041456 34.5301949364147 48.7781962164988 33.5578650644246
33.0038344711883 39.853866204927 42.3606390470387 34.7308008643142
34.1509644696334
B3GNT2 83.5646728968172 101.634888749356 117.355761277966
85.9087280306456 94.6432868887401 76.5888466260562 84.9182861692273
66.295597720304 86.0735583144514 54.0374522273907 55.3147456054175
61.356483470053
B3GNT3 43.9309845324767 47.2770121962655 45.7968648044613
33.8111782249716 44.3557408297309 36.2015763253919 38.4005795891396
37.4484310268909 47.6195009345747 43.6033221838149 45.6348103033479
47.2131144508464
B3GNT4 42.7150227340364 39.7835513496873 41.6977706798234
38.8495095797881 34.7018263647985 44.6524308640784 39.5496902678788
39.3709357615493 45.2897806651929 37.920964499903 41.6336636285875
38.5279745308203

B3GNT5 45.6517320730042 40.716553247236 45.7968648044613
65.524821699432 39.9697332660833 40.9506062495231 53.0277416531043
69.3468840908665 49.2879849674517 40.3235655331796 38.4689132737495
44.6484005362281
B3GNT6 77.6056319020116 80.173718023183 86.7823828209946
72.2577390063259 108.607805039005 130.336599857526 78.080034922779
75.0377156190383 79.4528447794527 117.502489192855 107.792406701643
88.8330881965986
B3GNT7 38.1169703906207 31.3542159583213 36.5474283500601
38.2041059863839 36.08956004634 37.9951997351435 34.5633623842083
35.7973685350741 43.4656637536244 38.3092241935352 35.2339574411711
32.7043743065388
B3GNT8 96.0907205856398 52.683711208052 51.223859150798
57.4595416719581 44.4489662507893 59.9876575158981 48.6395795163502
62.4219686957673 72.5027069303875 67.5503668962444 71.1607965028029
60.4872675915085
B3GNT9 40.0557788909923 41.159706131351 39.8299340037009
44.550322123994 43.1252272136667 38.8336427948187 39.4600924554505
41.7419877611072 44.8003345643334 42.5664883002833 47.7535797917441
38.0452862258803
B3GNTL1 58.0139597055449 59.2643906275025 47.7737810047679
56.2508140932179 54.5683518971261 48.1591264215184 54.339958888486
56.2441895183416 67.3230935444252 60.4837400912239 62.8408273986904
54.7953298048942
B4GALNT1 43.2724556187214 41.159706131351 37.3687900733549
42.4580504587581 46.4343327861696 40.3357777530419 39.6535880152834
42.0494116081998 34.3842972343227 36.8059561571675 38.956280281655
41.2437378488025
B4GALNT2 39.3709357615493 40.6380495994768 35.7105247506212
40.8681871019812 36.6354013629791 36.3845892764084 35.0305278582179
38.4540849632254 41.3803269429468 36.8059561571675 34.266241549578
40.0557788909923
B4GALNT3 36.1232327599945 39.2907645923217 38.5045333213784
36.0481934423077 38.1644569189523 39.2248354955045 37.2892641289258
34.8949555140527 32.2133093774284 41.1222709786153 35.1470994647364
36.449101775775
B4GALNT4 86.9816932158186 65.2001674269291 33.0260566875355
34.7266486944713 130.184962393833 40.3357777530419 59.5205055437765
56.9299697426319 52.2631308081333 41.5435611403119 36.5359455830388
40.4639532172338
B4GALT1 189.737472782071 101.351907645804 78.450972383987
110.25582862843 101.738441694376 89.3168921945222 162.160380044249
368.246708866999 115.001179779307 124.986782737446 175.099998331811
106.5477041881
B4GALT2 39.3709357615493 43.5873188565519 40.3384318592985
42.1273928203021 45.1846041469628 40.5710637884029 44.2637911785804
42.6975474602554 43.6177016821274 40.3519145017156 42.5153507543837
42.3047762223294
B4GALT3 509.463928291526 460.340300658834 501.234159111283
197.720854621428 1030.31910195147 232.147792636393 473.688869614777
374.427492246826 1252.94510628089 313.921236120658 263.937966521917
257.584248252408
B4GALT4 130.313381811168 184.989940464859 100.305262467668
89.0684909049451 211.485592971588 91.8295472317386 104.932630862885

174.905877478501 176.02137177982 110.729504563799 122.203872796773
111.357508398822
B4GALT5 402.412173952792 364.962649416567 349.030389220324
755.852629128252 504.847507464269 509.879213331349 407.564154455615
455.223217772744 400.093769705765 424.709002690579 573.613497159192
702.737741114234
B4GALT6 43.5710871617107 34.8137085420423 41.0356368374708
35.8472302065546 44.147557390293 39.132725377815 54.4934782594218
97.1271615802556 40.9901805728485 46.325232414659 40.9157352260901
44.2527566343661
B4GALT7 239.838643553139 155.099281008688 250.176074707634
178.156332261759 181.200738629391 156.445000453282 178.012676342712
229.035497774884 260.698359835873 216.744244428166 336.737571879156
202.833844963097
B9D2 105.739423435604 92.4953883194488 76.6914642534934 103.961956810572
90.0157406552551 71.3932787152381 120.401643722713 99.1438953564062
173.212430860273 103.808640431626 87.8967913380047 92.084526929319
BAALC 43.632365631458 48.4980626712175 44.8872784170036 45.9441733841041
38.745552403895 43.3785777889265 40.9157352260901 41.8417024632349
44.293922970134 49.2317401696588 46.824299585607 44.9070250604284
BAAT 39.0620487644635 36.6002100641539 37.1643693306382 33.8111782249716
44.5668141895992 35.9305494063137 35.3362091871364 41.4508241771315
39.0775870687758 37.0320432433314 37.0180934800573 37.2810767789306
BACE1 49.6241203903354 42.6939394537076 43.401782064778 48.2465811839557
40.9416717019827 45.7300501624017 37.6502409810897 47.2368301203382
41.8197029429821 48.0756651489012 48.9184531207247 46.6681271693877
BACE2 82.0081006332668 78.5174639327946 132.036058401203 65.9703614596867
64.8367546135053 77.4812665677338 99.7529382840954 99.9896394343368
88.4594321382477 61.8529316708431 63.664265793911 69.1743062945144
BACH1 42.5633441928261 41.9548604205448 40.2382908228517 40.5018032884134
41.3331561144087 39.2985791324641 39.0831492773955 41.0381860307445
40.0766543123257 40.162771030532 39.0775870687758 39.8845726397282
BACH2 43.1180113655693 42.3138951003676 41.4534762662066 41.6564075217517
42.5534565507139 42.4058515827212 36.7356360093603 39.4987030972173
39.1790517315259 39.4012855594431 42.7762820332795 41.863927676552
BAD 92.4449920876332 97.822911929574 88.5907320965949 81.8242150474253
185.94424664238 152.545682001987 159.76714232068 112.328675926999
131.57403028894 118.410983614453 114.215602320391 87.275548276476
BAG1 44.436995101921 61.7572675712557 52.2132783757422 46.4555908533915
57.2960245538881 41.8613406887824 44.6032218589199 51.9447450229707
56.7991853808475 56.1912096308988 52.5930185400362 59.5875857071693
BAG3 301.025314855652 305.339554034294 364.337811621248 254.066741436612
324.582288788326 749.825513359683 241.202687910704 235.764999941441
305.786520619504 222.453547273598 274.328990210853 439.955651727755
BAG4 48.2910530887214 51.0612189196487 54.3554233228353 46.614092935749
51.7983110178095 46.8319561350724 44.5390641547628 48.1591264215184
46.1101825640108 51.6778366958973 50.5810338885769 51.9447450229707
BAG5 202.957014793417 95.3507664364654 132.931052537195 103.276116332402
124.738024302128 129.268600210689 131.537639104397 148.059385111127
160.499410146402 157.9970294734 144.609635554528 130.498680522614
BAGE 35.9305494063137 44.3529919625344 44.7829632472754 33.6816578730693
48.8381507187986 45.7300501624017 40.6750540874299 43.9366081640576
46.2027193115397 42.8936990772838 46.4205654140754 47.5025821041537

BAGE2 38.385865183581 45.4143854024453 41.528370959196 40.1804147506786
38.6454098044474 37.0180934800573 38.1793598983615 38.5457716679071
39.6609238036815 42.785429206422 41.2598458726002 39.4490233720848
BAGE3 42.1537476443423 39.0894172982439 41.1367478979484 40.8681871019812
39.9697332660833 38.4330755397458 39.6535880152834 36.5393857306744
41.1742244762318 38.6454098044474 45.0589947942306 37.7526485343328
BAGE4 39.9220998751503 37.3179578037963 40.0557788909923 38.4208429103799
37.6119337673806 35.3502070248613 39.0759686770589 37.9837233601316
38.0702079388879 37.4302011516587 36.2885860224741 37.0953622406687
BAGE5 48.6621527194963 51.4413099192899 42.157627240486 51.223859150798
51.1368583672022 49.8472708021525 45.4799270968969 48.0582855095143
42.0621425894624 44.1379638573455 49.319499137678 47.9566297250148
BAHD1 37.7384958256497 39.7835513496873 34.9849896310235 35.49696651597
41.6806984441849 33.7818132446042 42.8684958525042 38.079222289614
34.3842972343227 35.4990901121893 35.6041926503637 40.2669497398702
BAI1 41.483412492426 40.7623977968067 43.1660110696573 40.9165100646088
41.2507419031133 44.0804700754786 42.6758568421347 42.4026480597025
42.4540072551568 41.5852317867787 40.5090849993014 41.0136069709699
BAI2 33.6902293666089 48.9259041798949 41.1367478979484 42.0102671859606
35.3327761997046 39.2985791324641 38.5066108855005 41.8417024632349
45.9227107476843 45.4614234442527 40.723990462047 41.4508241771315
BAI3 38.7537364570873 40.6380495994768 38.7745734037492 39.853175885492
38.2496050953547 63.8740412329753 34.1889222434768 36.43924066428
41.1742244762318 54.4825201506931 49.3901004211918 46.0633179231318
BAIAP2   72.4492911262373 63.9650465690339 67.6373573669424
59.5232818903622 73.7525516589171 64.2848885182563 70.7987307549177
76.9373289454707 105.74849082793 73.6855877240541 77.9287386697519
66.3188075271379
BAIAP2L1   359.296873000994 255.047622225942 522.811631783729
48.665578118898 270.036719921343 80.5478617156852 274.648263378054
250.244199415895 570.824732318332 266.687281047486 222.985386635178
392.796524588309
BAIAP2L2   51.1740855632044 69.640968387662 71.6573554353691
55.2214187655851 92.6736029146947 55.7197525213035 128.966814822678
110.287699369482 136.004535901075 61.2604442862522 63.3544794716167
48.8670821164567
BAIAP3   40.148780650831 45.1694246393676 39.8299340037009
35.7674462337723 43.1252272136667 35.9305494063137 39.7576136507593
38.079222289614 46.1101825640108 48.8623729374733 43.4709415392696
42.8864415494484
BAK1 72.0334593545916 57.4538469832241 69.3468840908665 59.8102324370568
62.5106969730855 55.540888910486 46.7100796279384 58.8022152133979
81.552417816742 59.2628513357357 56.4567728276775 54.4313108506244
BAMBI 233.435884567494 1478.26733379357 439.955651727755 92.999328769924
994.404707955031 349.90835241442 1152.55315876935 2306.14613042834
1448.17151189672 1007.03747721563 1201.00042684883 1031.4414268329
BANF1 663.372296297652 552.321665894163 613.27814206548 422.931891494195
1024.97546870349 645.702744916884 1014.6400720037 637.565156571985
814.294297312057 481.905052451245 625.435083064752 660.295993198711
BANF2 41.7397731376692 42.2577689927563 47.2962360458401 41.6564075217517
45.5809132232681 47.0940613507321 43.8206425661746 41.9315419791594
41.1742244762318 43.7303813515864 44.9713479185144 37.6557714476853

BANK1 52.7570461169969 63.6966761077037 100.205124008648 135.669367786875
64.193536444915 44.0804700754786 63.0119737420787 91.9919654450652
103.513421757522 68.0556717366572 64.6446465846487 73.3321469094819
BANP 362.506098778318 304.718140992378 331.306005726359 401.202348352506
403.243939813779 235.127539195661 361.776841675393 239.385229462309
287.363928615517 272.663785805238 316.419421649196 267.427827956058
BAP1 88.5140510969737 85.1950626054282 95.7820879892969 93.6212502203405
110.411217828763 108.022329297132 105.802035886293 81.3978207813439
94.9831937714438 110.909109947659 97.1271615802556 87.6598268114592
BAPX1 48.6621527194963 40.716553247236 40.301483914446 44.9370339275041
44.7829632472754 42.2577689927563 42.629412460409 39.853866204927
56.2441895183416 47.7180816682562 44.2142581488029 38.0452862258803
BARD1 162.993316022946 97.5849495690144 101.935321521672 148.333778341005
109.017269529278 168.492959515167 187.331614826395 293.463475974405
143.607457611143 157.7623351008 204.200566808626 121.449647909665
BARHL1 36.3649677956953 36.89524015739 37.2838080935695
43.5627649099623 39.859345745831 40.5281496800812 41.8315602975157
42.157627240486 39.6609238036815 41.1222709786153 41.6336636285875
38.3291713594599
BARHL2 36.8072833828297 35.4484652445078 37.5597671487621
39.5050383901884 40.8873907150245 42.8598805316863 43.4544762830742
41.8417024632349 39.7537274655115 37.8217989841918 47.5439485437351
38.5279745308203
BARX1 44.9896851005259 38.4330755397458 41.1367478979484 43.5627649099623
41.8806343997257 42.2577689927563 36.08956004634 40.831207953647
42.9020321355927 38.957687788868 42.0750889188255 40.2669497398702
BARX2 46.7293238583888 42.7775752471711 43.401782064778 53.3967242431461
45.2556760873335 41.0582900711341 55.8019623191927 45.0972184964705
50.1603795873336 43.1839109702714 37.7384958256497 42.57175366709
BASP1 1017.1803754111 1019.67015337432 1033.99699402259 3818.71752181269
363.002913525646 1317.29255644089 3329.68037676265 3726.73487426505
1134.52018899688 562.394906600222 804.070613101101 663.372296297652
BAT1 618.293140877956 514.70012608304 454.939614583134 351.114115662108
425.174170332175 394.437597863822 572.968592371501 468.381233506231
383.853296408061 651.078746026341 598.970109654466 414.062259640915
BAT2 382.872356030213 230.763782802004 210.255784687052 198.103081445734
323.295794446334 203.729732499418 306.115856873599 252.870708814507
268.54298415337 238.120731107691 250.308866488702 166.304210248623
BAT2D1 411.431488342465 332.137622104526 396.63268714181
319.248898194029 556.092573451208 291.713831398096 287.39391763763
270.662475158799 363.947777340854 278.773982275525 421.791344753995
382.872356030213
BAT3 163.62024333166 123.247207157645 132.52749757795 103.233768723364
115.277469628296 112.086648546139 158.156217755244 108.57858133896
117.869393694564 141.266956686625 133.673536028938 110.860844149372
BAT4 45.0985088502363 50.2163892855092 43.8495248991589 62.1776700757427
49.32293280121 54.5952754712007 55.695361644141 44.9977782988116
57.1016072897358 56.057906531592 42.911855821602 42.9887069977696
BAT5 357.762505796238 307.922754395377 217.6996233059 266.439343650315
171.722423740869 215.955919625615 187.086662645246 135.829530518199
263.096895937991 249.002758392739 188.432979046693 203.53267380432
BATF 146.333318667921 131.99898435227 236.661422875826 310.255767356827
108.607805039005 175.271140689255 272.257070024902 226.168990555083
227.843903680287 177.55994726993 195.456604920944 197.515728627482

BATF2	56.6951078698291	55.3477556906605	46.4569566520107	73.3725472767369
	42.9090346219023	46.0364000143537	40.2154497375715	47.9942520395434
	44.4395580493118	63.3872214856708	61.4121672148248	48.9733758107788
BATF3	63.3717459766798	59.5534127075228	67.0421310720934	144.813029178278
	59.4830424460714	128.873145026756	55.2088411137193	47.7889447429963
	41.7627910693483	42.5664883002833	48.8744644207821	59.7999569757275
BAX	176.75369241505	200.384444538476	261.421333472097	167.920131968611
	307.133157118667	107.808744445896	216.303036873073	128.732177437756
	186.229678647062	96.1596075221747	115.155021677978	126.404988409795
BAZ1A	92.0136417064806	186.504623165619	161.65846687852	322.109212713082
	109.14984236229	116.51037006692	160.499410146402	154.959129917681
	194.278792348966	114.617965877564	119.872031126474	144.757886965525
BAZ1B	137.785462971848	106.691530592115	112.727277807495	138.189579100482
	132.997204273031	156.822939275312	134.024691118762	137.302487233881
	181.950503247343	165.121380058529	162.23769718358	112.113501370522
BAZ2A	44.5282181324797	43.2649781354792	41.3366779545815	42.0102671859606
	46.4343327861696	40.650742261428	43.0930293831497	44.0390019242862
	41.9635525160083	42.1622837416589	43.4709415392696	40.0557788909923
BAZ2B	188.156490868789	144.098339910117	188.979306100283	213.590067519021
	130.758455807465	192.666532389331	147.010700944686	223.749512763927
	112.131738588405	413.31638457585	336.159430045964	257.325282051981
BBC3	40.9558083259517	42.5269793679335	39.9101908547397	36.5393857306744
	38.745552403895	36.3845892764084	35.9894133432376	32.6039814489621
	41.863927676552	45.7911932481722	39.3667161654772	42.7782216342806
BBOX1	42.9104454767916	40.6380495994768	42.7303139017786	37.29566077996
	46.3150221024046	37.8102105794655	36.9355798143571	45.7476095696859
	62.0272989884264	41.8613406887824	37.5583886316038	34.5633623842083
BBS1	103.945335792682	114.526825145076	122.748811531184	91.9185612536409
	152.942332679485	215.109108265052	136.751431410199	122.482560090133
	114.755820699611	208.3497732496	208.915145509698	182.188883821722
BBS10	37.8386378737561	38.5252456306656	40.5734458834617	33.0463640202858
	34.0045980663271	51.0506441018166	30.8360322325222	36.5393857306744
	48.2123120250343	38.1042454705119	42.911855821602	45.2783024981315
BBS12	60.7371790125714	58.6249721386149	55.8810837854139	53.593974682612
	55.6991707857987	56.1671440556142	51.4349390827895	51.3472428383325
	58.2401724760744	60.1466326805616	55.9604109457703	53.840976437122
BBS2	164.487183006292	192.356440003154	303.189681281366	131.57403028894
	190.422737436225	218.675464231277	130.506891299903	129.525395445221
	157.109085253328	228.21982974423	251.905799624693	166.208731611481
BBS4	106.356074933606	89.5887648181505	96.0654949568324	98.3948200992292
	77.1287755867582	128.559660070271	98.2843999216669	123.506372870621
	101.81885073944	139.543015878846	119.993761317523	131.645714321903
BBS5	43.0394685862018	43.0751697224728	43.3085358214154	40.9157352260901
	44.8248621498677	38.2898960213646	42.7431359850339	39.1236645052911
	41.6285300488826	39.9576138799997	43.0016969050339	43.6285248402465
BBS7	44.0527419856743	49.7235523248653	49.0701538857974	48.3996768970487
	58.7157078555305	60.3351200884626	50.420938814754	46.4379857641858
	48.0428788647292	56.9463858443402	51.3103617600342	55.6904617926772
BBS9	57.5685357492202	47.5647821785529	57.8111054584154	57.165971310746
	47.6153629331146	66.0411740221276	44.8643898647322	54.9919060580109
	60.2886873195135	73.4447529556358	66.4568741702152	63.3974067065125
BBX	155.174579191838	262.865950409375	257.851199206959	217.293240552946
	173.035987237848	306.578554914328	234.615033303459	166.734730009916
	161.890859629246	243.640796499438	376.931257615162	292.247095517604

BCAM 55.6781016051988 54.113533179847 68.4229528414965 44.7520317855773
53.3683962805281 42.2857372794461 48.2439143986124 48.7213586071409
54.0536858212253 57.0581769379589 50.4022543872993 46.9919960628953
BCAN 42.5506742787391 44.1634313979547 45.0017158586743 50.6141827957642
44.2607789091176 43.8378717575959 45.8381398279472 43.0390857698452
43.3165374444543 44.1956990708089 43.6875139416756 46.581796165231
BCAP29 68.1506072825075 67.156827317798 78.3740506001325
64.9959712888709 70.1238785863433 77.2048580540589 65.7873990226533
63.7491070301764 86.4114854104675 58.7389869173495 60.7103211409901
79.8878785326568
BCAP31 1448.17151189672 1545.76489731307 1379.51119430451
888.56944380788 2191.09005731751 824.946988188872 1621.89443989657
1839.09068171097 2071.90741263566 1969.8871504719 2232.61002288121
1642.75555340648
BCAR1 315.799449362526 152.66454549157 177.958475301951 87.9098670472958
147.043419185015 106.590506599473 131.645714321903 175.099998331811
183.690616959682 115.235144612428 118.251739134889 110.626479311082
BCAR3 181.738902391483 159.747484721905 365.620745180605 97.4967204298499
203.82788889213 155.693953208351 203.127486721124 121.270627995568
272.828896268482 183.165559564289 205.510539955583 317.560970617018
BCAS1 68.9258122619781 661.494376716475 624.954583057431 51.223859150798
89.4111455826308 67.1067574196203 70.4794854241035 133.38594323309
107.610858427836 151.030500297499 145.741469970609 184.808179948172
BCAS2 46.1673159982664 40.7952353240579 46.3150221024046 39.7840818430159
40.2449343247539 43.7078399718247 44.2166118829877 42.0494116081998
41.7233690251839 38.0078357808393 42.170702295259 41.7072675309997
BCAS3 47.6397712007919 52.1967540852276 50.6987358483181 37.1894304543751
52.0583273941813 48.4758590019384 42.8684958525042 42.8482181313226
64.830448408529 60.2877434874622 52.8489752025797 46.1592873342534
BCAS4 219.737294408079 75.0873720488265 159.618353114269 70.3871361660046
93.4093169676983 95.0026207727863 172.381808887528 266.07996221256
116.663501139305 208.097209050425 156.401873737061 136.766779297051
BCAT1 111.397578350396 39.6123051184545 48.0191002461099 196.513433958685
85.0017995689256 134.922819713509 81.0331497830643 51.9447450229707
74.6955454743937 47.7180816682562 54.113533179847 64.1607001437806
BCAT2 107.836867810972 674.270050283701 559.559234461244 139.101561420631
975.544591158047 329.282615395786 1171.207937488 579.482255191456
1243.32650314893 826.024359447649 667.583623414832 684.238999553691
BCCIP 128.542999559673 136.843285454801 167.430013807712 142.251426773121
259.626847216881 186.265538054701 172.506486240017 150.925721934159
146.432425205891 91.1057573288203 111.397578350396 161.283979969295
BCDIN3D 119.993761317523 116.882934971483 112.469702446962
122.024487499513 104.914715637173 113.377818029985 163.458716079887
191.523447800488 197.540681021755 139.645717741863 161.228738711412
112.944044862693
BCHE 46.0398513158469 43.1805016690297 42.281606728072 49.9672547361866
38.3085139808968 112.659254685552 39.4600924554505 43.9366081640576
92.8951148347802 69.6511571901941 55.0031529299037 48.3648494350286
BCKDHA 328.341232263338 263.937966521917 337.156068385541
276.86542239106 415.05124917015 239.590143292142 445.307046237791
427.199940706169 567.393665550133 457.860665614779 401.403103922907
320.74875942191
BCKDHB 55.7246872330059 112.421316052392 92.563449632567
44.6246431218094 76.4588249117885 73.438931050354 77.4812665677338

65.9143782252686 73.7873098190297 87.0187282933132 91.31756001203
84.5998418020787
BCKDK 666.816487335088 440.728435508514 838.974043046676 848.079114196986
1143.51046585232 538.290200196057 644.368005872231 1249.41227924272
389.131545459708 529.430648638878 459.182372959714 450.271658272512
BCL10 52.109723558511 61.8824218975538 56.2441895183416 61.3747706295648
50.7116312517035 55.0009147092272 52.8496345990125 50.563951934942
44.476519804187 42.3606390470387 47.8676131893619 55.8775622338124
BCL11A 42.5534565507139 63.5693267269002 44.5658219748173
106.276392548097 45.9811664221893 66.7475567712242 87.3213916666808
84.6811991332281 79.0918252621521 156.311985997533 79.9567256807679
84.1401040507557
BCL11B 43.9309845324767 88.6905706130505 105.376705821141
74.7047606031323 63.0383605052305 46.8319561350724 61.8571521441451
56.9299697426319 101.549779766678 70.0907269565408 57.0514418438652
53.5292049088661
BCL2 55.7979038616211 54.1437245703767 47.3435572390933 90.8188865732032
44.3292391134969 61.9787117490846 50.2428460389837 50.3972033014227
56.8436081694537 114.5696064637 89.680151549258 72.2211523775459
BCL2A1 38.304249186058 39.8695929627783 41.3366779545815
92.8026641003757 38.3561682218911 47.5733603903505 41.3594868055879
40.047314525032 44.0519245668737 41.7405682794166 38.8507804383261
45.2783024981315
BCL2L1 886.397061577531 592.010988342149 481.492404948275
590.139974503603 583.915973546284 383.853296408061 779.996851709716
444.858305070415 522.377154567257 486.757854744053 601.611039798626
391.292537565032
BCL2L10 38.7537364570873 40.2493681589104 48.0191002461099
40.4359177791566 59.4830424460714 46.3715909653832 40.3302268293707
43.0390857698452 60.0736861987141 49.0841460667818 50.7903824729818
52.4992300612807
BCL2L11 44.8446015771755 46.1204054852871 48.2274642570366
56.0794100106654 49.8966674037224 39.8873706855774 47.8179475230844
43.4812166409346 48.0563448184655 40.0514069727124 43.1931731665131
42.3214282169749
BCL2L12 113.218291314173 139.758805241617 121.782045054657
91.9185612536409 454.185408362583 125.091427687365 232.048938339214
183.393353108744 220.217797955745 89.8957070711076 81.2992152187529
82.0124493733776
BCL2L13 488.183286350725 578.456468865003 640.0926391761
438.681666410245 434.223722811041 666.816487335088 371.513106715598
392.622487173359 534.755211286733 803.548811322583 768.888528247493
634.226367147025
BCL2L14 41.8409927585705 41.3366779545815 38.2502942836481
38.1421709520616 40.7376840028647 39.4551617322991 39.2010256871265
41.3945375181463 39.1400590089945 42.9358255168086 40.9157352260901
40.6308525754377
BCL2L15 75.8122107270212 41.5132248356707 37.0642268327439
41.6564075217517 41.8806343997257 37.842873463785 56.1463616087682
43.0930293831497 35.8265993915844 37.7598032934773 36.9768361759916
40.2669497398702
BCL2L2 213.933347509147 152.880772824839 112.328675926999
104.158950889938 126.088821850066 402.978328664331 86.6798536307388

112.40706320177 79.6198202883864 233.96534264787 205.051252996628
364.439669236186
BCL3 335.943994595615 208.097209050425 197.103673938152 416.844848063269
270.89711217076 422.702925141293 316.548339196872 506.173054520807
497.591040004541 108.905276555106 128.300809076274 239.792011765323
BCL6 267.232315058148 305.786520619504 299.334388262225 731.670315613547
385.36587480414 633.819901764391 347.552309199761 173.694418385132
530.710127703061 400.416888798951 405.852280880924 535.313561995599
BCL6B 48.8941062980393 46.4385386401326 44.8872784170036 42.0102671859606
44.2607789091176 42.5660695551615 33.1392460259336 49.2019743609873
39.4762546236449 40.2112371512838 45.1432412071268 44.2527566343661
BCL7A 39.2958230580497 40.8503192605024 40.7620472083159 39.7742005651286
44.7114813656401 44.0328992213727 38.0598374201407 39.3086414047188
42.6861857638473 41.9666541498638 39.9899430628542 43.8291226513066
BCL7B 83.5646728968172 116.51037006692 126.420070148631 121.281573820819
133.074560812703 182.601427407211 101.065203693106 82.7269382676336
162.658589763207 113.008760911729 112.007118067348 127.2070318269
BCL7C 195.740109330843 179.897685693038 185.378190201718 146.931674964658
215.399634344016 298.771150048863 157.677866955988 219.64979849407
143.607457611143 135.436347750159 149.45630414346 115.001179779307
BCL8 42.9104454767916 39.6995529770239 38.3166554965434 39.2780515621926
37.8679241954337 36.2015763253919 43.1931731665131 39.7509165247419
38.5252456306656 45.0155543555707 39.6403089864678 40.0557788909923
BCL9 304.658728492827 116.094881058504 138.362914843774 62.0404348860702
154.355197155161 100.650129064849 142.465925522042 129.179537795204
155.541828028535 118.726647822102 103.199200862158 92.4350913779886
BCL9L 92.1908585323292 70.1238785863433 70.9377012403023 48.9944486068347
66.5187917407182 63.2343637481705 78.3082773071531 78.6911549149983
64.9030399249327 72.2443746456494 72.6162848018047 47.2131144508464
BCLAF1 213.590067519021 240.520228413182 190.548265163011
185.165420581989 194.435776102991 241.736113354506 236.374695859748
185.924923239764 199.264520680174 265.320447667174 270.600852806342
244.837105178705
BCMO1 61.8529316708431 43.5873188565519 43.0016969050339 38.745552403895
37.6527901109005 39.4953494583071 82.6381941930783 56.1404433602127
48.7358147030082 46.441301934328 44.9713479185144 36.8690774823319
BCO2 37.55264801907 43.3458769998118 45.3974838013476 39.6292788039793
39.0564222526273 41.9543948167375 37.830876898596 43.2551508830106
36.197424053556 40.7063426028871 38.4689132737495 38.5279745308203
BCOR 67.3136079922956 99.1565755621214 94.6432868887401 56.3525213736952
63.785471218558 68.5174271063894 79.856662992182 103.447730395424
112.048972076936 96.5102825310977 87.1651984640976 80.2070411865083
BCORL1 51.413025495745 56.8373628733077 50.7502704822135
51.7739270955254 60.7495043977707 62.6839349907803 73.8313212063649
46.3685439301234 49.7336769474446 60.7495043977707 59.9682734755814
54.7953298048942
BCORL2 45.3872406432794 44.5152708955623 39.991353369336
41.8778873678551 43.6587492470348 40.8634043028528 40.7910160811527
41.3506832276112 39.7537274655115 43.2988017098387 43.401782064778
41.9430737035925
BCR 54.4384603094793 57.3562426592112 54.4825201506931 47.4119039314844
49.5172323138783 45.9989204975182 50.7568454312473 52.1788489555942
53.8052648457439 47.9056710878325 52.4665168142681 48.8165026569686

BCS1L 116.311176407088 106.951862149262 168.883366756191 81.0057400164851
220.426780242132 113.612874206329 97.712117589297 103.513421757522
121.36340610316 120.381190859636 113.791456064084 125.92333271598
BCYRN1 413.918050849658 192.98559983091 183.584085676174
265.520773157991 144.290966198687 279.175841990291 212.822978676596
877.673009984357 139.328628553223 477.558242266308 608.579654909865
426.015657750123
BDH1 41.181169807632 60.8857255846432 48.2274642570366 39.2780515621926
69.3468840908665 44.5085634580805 63.1137861754989 38.7023886970298
47.6667014835128 56.2508140932179 44.1148826235374 46.1592873342534
BDH2 38.8561375693335 39.1982470626863 48.7039705048129 35.8472302065546
46.0043807840705 46.3715909653832 39.5496902678788 38.1835938174625
44.0519245668737 40.8049408996274 41.5421429082231 39.5418280570561
BDKRB1 88.0186381834147 51.524711857679 54.6820069269589
59.987045425047 65.8319449883221 152.86270446968 47.8947595316832
73.2037966614621 43.9450023643394 59.5785089386245 67.3976855661302
57.445796273665
BDKRB2 44.2350046179299 42.3550738181144 39.9101908547397
55.6481366056639 44.5668141895992 52.1024135605001 44.4639405506967
64.964255000378 43.2761454096265 49.4006068471301 58.8961770381033
53.5643281930979
BDNF 40.4239925664876 43.5182502375207 39.9538181787289 39.5766049255681
44.4122940018742 39.3467744082997 40.4999319300923 42.2869243641471
39.439415116651 44.2580151149956 42.5932477401361 72.2544131004774
BDP1 45.2875173643252 49.83247261979 48.6037684272115 43.6726153508978
37.956732373465 41.6590564937735 47.4439407315335 44.6107027295193
41.6616444147804 42.785429206422 56.1379957867124 49.3984571719129
BEAN 57.2960245538881 42.7775752471711 46.1312573416873 48.4916117779992
41.467005949568 43.7078399718247 45.6706505553383 53.1613811648611
42.6762317063737 41.5435611403119 41.2598458726002 39.7411455670172
BECN1 117.074650076861 203.373397813051 198.010793115588 153.153205095999
144.799432299339 159.835690229505 126.253578620746 120.142759111608
178.379117285008 91.5417574979683 109.386655134289 141.784776736918
BEGAIN 58.0139597055449 47.087835967892 45.6989664108554
52.5307002738469 54.0126916508417 49.641159251878 59.6780848951767
52.407151732257 46.6944238390932 61.7502624458447 49.3901004211918
50.3118072462443
BEST1 43.4626049853157 42.0709558258414 51.3268475761593 157.677866955988
47.0954207417646 59.7009992986607 41.5817737564592 42.0494116081998
41.4636047919453 41.7405682794166 48.8744644207821 49.5539622682049
BEST2 32.406093465115 40.9731067463382 37.9482157381008 43.6726153508978
31.3142073726046 42.6575535390785 43.4544762830742 34.6977013306396
36.8127605061228 39.9948390976317 36.7283081422707 35.9604262434141
BEST3 35.2267149016596 40.5400384479766 40.5734458834617 43.5627649099623
37.8679241954337 37.9951997351435 49.6872467499977 40.831207953647
42.1594905198929 37.8217989841918 37.9230440150891 40.5703581862752
BEST4 44.1294459089314 48.7084357480922 47.0940613507321 42.357910063876
41.8806343997257 41.159706131351 53.7423898357583 42.7431359850339
41.4636047919453 47.8207974930031 42.911855821602 41.4508241771315
BET1 100.403196213407 84.5572894783679 86.6910280940418 95.7571966348067
104.072423445726 100.151116279734 83.5149142053137 71.8314470868658
125.676172266736 72.1090339831747 114.918205718649 122.084249177093

BET1L 54.0990839134272 56.5319193881205 54.9944485073259 50.7046706880325
51.940083408061 57.6034762511847 35.6056062164318 40.4359177791566
48.4869909888679 74.5694657001572 64.2174369755376 56.6888826857797
BEX1 66.7299966077909 58.3475983630509 109.029196594263 61.8293674397954
78.5141640924736 154.891160328731 81.5582217750731 57.824185116862
78.3962885374388 58.5087296620514 64.7023558190673 72.1090339831747
BEX2 130.848965290163 109.525921548011 289.533968432846 124.976348531594
225.589127792587 180.63535336811 483.20917825594 558.451033789799
428.729935028911 209.068725760522 146.087016079575 161.04166688561
BEX4 163.946447772618 148.544012961815 296.598410253206 169.144145421022
172.039289618908 313.046160403661 221.049503614433 162.658589763207
353.810269076023 175.28322146764 172.676819138152 346.285308869994
BEX5 38.2083710281545 43.4396866157171 44.696023817801 42.6913548400127
39.6535880152834 61.1022920882065 43.6753843488695 62.2982003555312
56.2441895183416 43.844855336215 47.2134341420411 52.3896785600751
BEXL1 212.152751915521 156.061711961514 393.648308810931 198.729330487352
197.895987726243 325.769076169162 276.86542239106 235.613437037284
331.762920362224 288.687391623935 264.88963894526 400.327164003217
BFAR 95.4841914842335 154.612425659555 167.746168796779 131.955454394903
134.981483569602 149.352994264999 143.152895211088 135.11808699414
103.139459009593 112.17141283151 127.811684080184 161.156612172544
BFSP1 49.8472708021525 38.5252456306656 47.9035532886869 35.49696651597
41.7929491793697 46.3715909653832 43.6753843488695 54.9919060580109
39.1790517315259 52.8928296360463 42.170702295259 40.9683275553869
BFSP2 39.0620487644635 38.8336427948187 38.2280071951674 39.2780515621926
36.7356360093603 45.1873487463472 41.0381860307445 43.7396619462033
37.6383530120621 44.0409178714232 42.8263096141654 44.7401523707775
BGLAP 62.9952378379206 59.0709442090059 73.2814174739782 49.5424425850549
106.804269453898 70.380703483689 60.5125774827425 52.5098795466615
69.0037087543684 58.3813211109331 63.0043412964216 58.2251348002272
BGN 9709.60940482862 2697.96877833185 3465.3013599062 4015.88651424568
2113.73880184695 1043.26325848938 343.418685340038 2384.00264993279
268.936151471133 1058.61208471961 991.630429625437 766.027617496754
BHLHB2 2903.00880209344 1333.01876209753 2318.50555842521
2697.96877833185 706.087945508248 1497.73669971925 1106.57048989685
1829.30985071028 1731.19220270448 2337.7738990994 2133.78836857697
4550.56817942699
BHLHB3 65.0721641331632 139.758805241617 169.557757933574
101.708164294697 58.8961770381033 60.1042270393382 66.0350709576651
102.818879165949 272.663785805238 175.136856246782 118.720812623965
167.920131968611
BHLHB4 33.0463640202858 35.4484652445078 44.4311655729487
40.646702696033 39.1650142708257 40.7616966228404 43.1931731665131
34.3865306339645 42.2577689927563 39.0648733099061 44.4688999098904
39.4490233720848
BHLHB5 80.9663216993544 57.2871072823547 47.9035532886869
130.673639258681 45.1137938810047 110.540953771795 39.3585350448719
43.0390857698452 44.1519466943185 51.6778366958973 61.1801493883012
43.0930293831497
BHLHB8 43.1805016690297 41.159706131351 38.8775560911841
41.5464765641928 42.4864576619427 49.9699131196797 35.9894133432376
46.2779564481158 40.6957787921451 41.2276963324981 45.7328341667151
47.6040671292013

BHLHB9 54.3554233228353 60.9311907846259 54.0536858212253
57.0629653735694 65.6824363141962 75.353039684456 85.6236091398006
67.6082563445866 65.9254098858352 109.045804556923 92.3901719424121
99.7529382840954
BHMT 33.9561816279577 1939.66920192372 56.607199625498 38.9534635879195
480.386431986343 87.2437977005922 2306.14613042834 144.898757916905
225.50098418159 507.375704144286 433.447319956981 154.793640473854
BHMT2 42.1537476443423 42.9358255168086 36.5201388311834 46.1074305700679
40.3021896270845 78.812106589086 38.0598374201407 46.7508829977587
35.7973685350741 63.0748373122119 61.940656536312 63.4427357028556
BICC1 40.148780650831 35.8227939208521 43.8495248991589 38.6414316877471
36.9702228618069 38.8336427948187 41.706879587091 41.6466169548846
37.1820543575123 42.9866214102525 37.0180934800573 38.745552403895
BICD1 47.1962846565261 44.6918600441328 48.8941062980393 44.4912617551615
47.3791873993182 43.8308097066396 44.5390641547628 48.7039705048129
45.1899271235974 45.0864985243943 45.5955029316007 46.1592873342534
BICD2 107.951009163316 173.11353027932 128.436305192918 163.20801480093
163.43197105141 121.441763452598 123.739272396278 138.027432399991
105.247736284747 150.114520857436 174.392758289374 139.218017862207
BID 65.7002710293248 99.6392844274488 73.2144205844199 137.335224748204
97.7153995525346 72.4094549259929 104.932630862885 92.4251917286941
90.7037473522607 56.8201201798712 57.0427273538849 62.0967404944031
BIK 72.3388488936832 53.0383634135393 76.889690218685 41.1886975241176
45.2556760873335 34.9849896310235 102.150058925164 86.2118355769367
102.457537266105 47.1001757441025 48.1352470945488 43.2776458914333
BIN1 373.330920144642 128.885933852704 123.908152883436 320.684862879691
91.4121563150184 1161.71671016964 114.611557317665 197.325473090277
126.753713093485 572.968592371501 826.490051619826 346.438778257772
BIN2 39.9697332660833 35.6434124419782 40.5734458834617 38.5252456306656
42.9090346219023 40.1428591998404 39.5496902678788 40.931294690669
39.2809305246091 37.2089297656361 40.8225507267584 44.5432192783161
BIN3 96.3232257371077 51.524711857679 51.8122261766744 50.8567345921473
49.0828761839116 48.3686935595826 63.928488869459 45.1714698794331
49.3780606970538 71.9124897002253 74.466989900636 66.8026147385682
BIRC1 42.3433931248155 41.2598458726002 43.0971787292711 41.4273122914951
38.6454098044474 38.4330755397458 34.2773859804062 32.8834169462007
41.9635525160083 42.5664883002833 37.1059647239814 29.633229264005
BIRC2 213.140641182603 191.394143505573 162.708058265337 269.438510636306
172.676819138152 163.20801480093 259.481036467571 201.800572658224
190.921576470336 299.240215762404 225.760096749053 240.696431900526
BIRC3 256.116479208201 185.360921731157 230.554867472663 527.037018015766
141.266956686625 255.946758662074 244.529657015078 462.762837284748
151.815294281745 318.527516338791 294.372319595103 279.632025097952
BIRC5 130.455608913636 86.5926014007562 76.2533853403813 52.5307002738469
155.378768637562 54.7083331985678 194.951677338012 139.177720567217
219.676249419297 46.9598474201212 41.2267153672559 40.5703581862752
BIRC6 74.7955767483841 60.0601788842942 71.5484567134601 51.3851157261451
65.3019328668633 72.9154861179393 69.3468840908665 89.9227845135785
67.9176876913772 91.2016867646161 71.5249259772583 77.3260618801358
BIRC7 47.7779356299965 41.1212129940704 39.8299340037009 67.1209520838357
43.004943518025 43.9366081640576 41.8315602975157 39.0170123539232
39.6609238036815 37.9661004970253 38.2795150354677 38.0089018744739

BIRC8 44.5282181324797 43.2649781354792 35.9305494063137 40.3180310664156
46.0043807840705 39.6251842352088 42.7431359850339 40.5158867964016
40.0403898620771 41.8613406887824 41.6336636285875 38.5279745308203
BIVM 145.459578481501 138.568626520133 132.679840262941 95.2218627805387
141.410183156424 149.173801572239 119.124583755587 97.9497090233081
121.270627995568 156.08027400493 148.608392563633 150.011814577972
BLCAP 375.529704925121 244.089971761037 389.481124074714 143.624756920887
286.996922972683 151.709689636448 232.320396482547 248.527031538874
558.451033789799 1109.17675353971 797.019657306322 626.749986744506
BLID 43.3638918705225 38.6414316877471 41.2365089689635 36.890925044696
41.467005949568 40.040778349137 44.4639405506967 41.0381860307445
44.1519466943185 41.3321861371202 38.5668610487033 42.1537476443423
BLK 42.442231667357 55.8678615468603 52.3432130135266 134.952392466784
37.7576900881741 38.9378717994178 47.1345949868029 55.1109784383525
45.4799270968969 38.4187686307607 39.9070785037411 38.6454098044474
BLM 86.3054012048636 64.4837166864084 56.939860285007 85.7983996395757
110.411217828763 49.4157684139858 129.143252341926 130.960165577212
75.891637602914 50.8826620053386 44.1148826235374 40.9683275553869
BLMH 80.3730667493235 78.7282247242035 80.531334073336 60.1837189855762
75.8306078651291 73.5743059777365 88.5907320965949 78.1448136847078
132.369828264246 107.015362116555 87.8967913380047 96.5813769696577
BLNK 35.1307934330047 40.4359177791566 36.2683886571585 37.651298468202
41.6806984441849 39.2985791324641 39.3585350448719 41.0381860307445
35.9645119596869 36.4651112441576 37.2018803984555 36.3454320848591
BLOC1S1 159.485978728 182.411383422586 230.117814622646
301.79813401066 108.607805039005 310.763904671301 220.03226547675
269.546586017659 283.036694859397 250.308866488702 233.263058459431
284.942182200489
BLOC1S2 46.1513019317765 47.0981389355244 44.5544727111028
44.6905691366696 44.0977148249302 54.7083331985678 42.22763273471
44.0390019242862 42.992884918158 47.1785588364488 44.3440839686558
48.8266530503117
BLOC1S3 44.6231541342 43.1805016690297 45.063792968927
47.0874209746031 41.3755760434934 46.0364000143537 44.0687697278114
44.4031868028216 46.4861258030402 45.3530683538086 40.3452320167286
42.0424641526302
BLR1 41.4508241771315 37.2810767789306 41.2365089689635 34.9221408381038
38.2496050953547 39.2248354955045 35.8227939208521 39.4710743516517
37.2810767789306 36.4651112441576 37.2892641289258 39.8497227447888
BLVRA 177.088290547513 81.4621222177298 150.801729138762 201.782740241526
67.222434502867 183.690616959682 116.74427460886 136.332339821167
113.026772174726 159.359051158123 140.976643799742 193.676161965973
BLVRB 92.084526929319 57.0082102559228 84.4443931765378 69.8314582428552
71.7263663921367 136.517445122438 102.294142438313 116.967446594767
107.222176166001 120.60052672409 99.2749350936362 108.082240435027
BLZF1 1050.05355467301 462.762837284748 670.958499409833 835.171475474151
388.336994961328 374.723141438448 689.738256133922 1564.55598228713
481.492404948275 1561.83892117664 1693.5377672895 952.16345035163
BMF 63.1291723738553 45.9732108120834 46.8934754723474 62.5106969730855
43.1252272136667 44.0399598874356 51.6784647420677 52.9240098541574
42.589641010388 48.3119523820103 42.1454750198208 40.9149601380325
BMI1 166.281651016457 318.369837408344 323.855522995794 259.812381486915
366.515210930763 419.182443507848 245.691073028699 307.550936236018
362.387413555537 241.486113826383 234.043251639296 387.867128019374

BMP1 124.866058211181 56.9463858443402 58.0852316663986 63.5107036952143
55.3951994031724 54.0865464112237 45.9368273382327 46.1159021593282
53.6446045619513 69.8397773950218 58.6176747515895 59.7118480874431
BMP10 38.6454098044474 37.6383530120621 40.1985790927248 46.1673159982664
44.9936360641969 42.157627240486 40.2154497375715 38.956280281655
41.1742244762318 43.4885245598939 40.8225507267584 42.2577689927563
BMP15 39.9697332660833 40.0527893189096 42.2577689927563 42.357910063876
39.1650142708257 43.7630587657205 38.941855825269 50.3972033014227
36.7267557061938 42.9866214102525 37.7384958256497 41.2437378488025
BMP2 61.356483470053 260.279110870904 263.53897660224 83.877343750194
78.1275793755619 79.2701322339022 70.2635892414678 111.291087721139
66.0350709576651 325.275883498219 358.979854534484 141.572530741416
BMP2K 48.2324547902378 54.8133029594319 51.0188883041845 84.7503257101143
51.940083408061 57.0775902639215 52.4898616460704 49.6534108863792
48.0163377492228 44.1765254182701 46.1960490865583 46.1592873342534
BMP2KL 40.8681871019812 38.9338521051669 37.6557714476853
47.9915414951747 48.1473938886239 46.574682061365 41.3594868055879
40.6171774724171 43.6670755115848 40.6022003765153 39.7342718293432
42.6758568421347
BMP3 59.220967994972 48.3699741879723 60.4809187893259 32.1218888714537
41.2740822675592 38.8336427948187 44.8643898647322 31.8228237796461
41.863927676552 38.957687788868 42.6366765186182 48.5123041297835
BMP4 77.1008255162781 72.4978364649174 47.5673419710251 52.0470560622094
66.9260611060819 110.223625398261 148.312100828662 76.011923854897
64.9890714157316 98.1433318215702 114.464626286414 95.0513978212968
BMP5 40.6559419616644 53.8499082625091 41.3366779545815 43.8002456102021
54.2301964914419 55.3202238943712 42.357910063876 99.6392844274488
47.27543600687 58.7198210787055 65.7587943905649 53.840976437122
BMP6 54.5358897781765 57.8876850634922 42.8270038153139 67.8978240198575
43.004943518025 46.574682061365 42.629412460409 39.5466975353131
36.8127605061228 40.7063426028871 46.7386599624801 59.0894921452893
BMP7 41.5421429082231 209.31241212773 148.027258506301 48.1662933382789
147.388500451427 54.1440452292081 34.4570158133896 61.2343203799193
76.5444845463963 131.645714321903 127.345094323724 116.793627324217
BMP8A 33.0463640202858 37.5469247328848 39.991353369336 40.4359177791566
39.9697332660833 35.5158505219112 42.629412460409 37.4484310268909
38.3418933808844 38.6454098044474 33.1645364749371 41.5536168238478
BMP8B 61.1022920882065 48.1627097465883 53.4743535852344 44.4367387274922
48.8381507187986 42.6575535390785 60.6548851977964 64.6316818494785
66.8632294055679 51.1169368198437 54.8532558909227 55.4635297192076
BMPER 43.0001432913058 38.8336427948187 36.5474283500601 44.6905691366696
39.5595233162192 38.7340969861754 39.6535880152834 36.1375121409002
34.226171989766 35.0668743708816 29.6891949090828 35.4484652445078
BMPR1A 62.5968484440453 80.882176929636 89.3868257841517
50.7774722329703 68.8362790228976 140.853812988562 75.8779316255794
64.7838753386185 78.6602721596257 92.4534854832186 89.4111455826308
117.191735492199
BMPR1B 32.742439449766 36.1232327599945 45.063792968927
37.4223289687135 43.5544347011646 45.1873487463472 34.0777108441756
40.4359177791566 39.1790517315259 39.9948390976317 38.1835938174625
40.4639532172338
BMPR2 177.584394977912 174.368595478868 157.927114480316 170.804822846738
133.893108529325 221.598637828404 98.5663841503803 170.475212920685
204.273912273411 177.80178351875 164.547318765586 195.018775118528

BMS1	174.021676013865	133.192618821838	150.545989010484	120.284321694275
	165.322633260592	143.973063517706	217.065944053174	160.638833695464
	204.63343271995	152.792457231813	160.203825526096	155.252915611651
BMX	39.6279144647521	43.4031723677362	38.2523289394497	39.9182914210537
	38.8964578286293	45.0241191707651	41.7656868106485	41.7419877611072
	39.3592387366314	39.7397636748413	41.0554874392252	42.5294887665766
BNC1	39.4987030972173	38.855011124887	40.6451121071568	44.2142581488029
	39.4693871570028	41.9543948167375	40.4359177791566	46.6301159152913
	40.1730675449465	41.057460518552	44.528828405681	40.6312687664188
BNC2	108.649424858333	51.0175677516029	41.8751578329365	64.6945454069792
	46.4343327861696	166.910579215196	67.0455170947389	40.2382908228517
	36.5393857306744	107.122319086614	98.5609311953771	88.4819048592858
BNIP1	66.4399491500833	56.0642694832108	74.6514558411477	56.9299697426319
	66.6118713143653	75.0310858627394	82.0174591986046	64.2512089157122
	97.8764110456132	68.8167148906225	64.830448408529	74.5173894803763
BNIP2	204.130491885587	292.247095517604	171.746499373501	455.774407260725
	297.187417426993	279.632025097952	288.597205372736	224.749607192071
	305.279475274196	243.763402918148	262.024495926094	263.44529683749
BNIP3	931.401929277001	123.281175151414	170.105455298636	379.865108456536
	295.127975044241	389.994289973076	593.801086643125	301.525915314179
	53.9287524861617	358.194667053419	292.93036571522	562.258108316474
BNIP3L	257.761541860837	123.597489107285	217.456127221867	
	395.298033031033	86.1633564934019	277.367257933324	120.231435193839
	87.1183334887694	221.22852405053	186.965367827758	180.663636520073
	280.202284365004			
BNIPL	134.407474121421	78.1761025629963	103.14577985944	42.357910063876
	49.0322894371656	45.2852528185486	47.3661873039785	227.94699237549
	49.9547657591437	161.405959689541	123.247207157645	81.7566533443175
BOAT	45.8574226904273	45.2098033805679	43.917817324545	51.1313686723929
	45.5198200633333	43.6427759607073	51.8667932650345	57.8765910878975
	41.8197029429821	45.3894119568143	45.3597029614942	44.293922970134
BOC	53.1309801459151	41.6466169548846	36.6522278218553	42.1273928203021
	44.147557390293	90.8335894699136	33.8558232866797	40.931294690669
	40.6957787921451	57.165971310746	55.6972661824064	43.5738557773903
BOK	208.915145509698	140.853812988562	162.833785414988	184.087380444203
	91.2681347227088	136.103246130094	139.855023065402	118.341308125578
	157.677866955988	156.844010597219	133.401306624347	128.635811501506
BOLA1	54.1745577382276	49.6203214143464	60.4809187893259	38.2041059863839
	58.9875400392427	49.8472708021525	63.0119737420787	55.1109784383525
	52.7512110769042	51.1169368198437	50.8826620053386	54.8630527326985
BOLA2	821.922096305069	429.725397949276	632.577797921655	476.421959423835
	1120.83891847181	354.634819362978	573.832332097619	1652.85734308754
	320.555790243104	307.445116836976	270.78959225422	314.609371608151
BOLA3	561.509463439533	414.062259640915	427.199940706169	363.108973029004
	781.923984640049	417.4609354305	607.042450917813	577.276184555955
	607.431621448599	198.54718783437	244.872078761743	458.861461526841
BOLL	38.7537364570873	43.1567024035183	41.0356368374708	40.2406186868086
	40.5158867964016	40.5703581862752	41.0808257270221	40.1797228736088
	40.1738310955128	38.8274796955507	39.3770402804901	42.0798970785317
BOP1	575.863848923457	494.300858189605	278.453446231317	257.194689822426
	850.656711617458	289.472303440614	342.146013966482	362.196116025799
	513.891389190131	272.930242946726	344.985739524681	205.806939441015

BP75	59.8248482438562	63.1780854659428	91.9919654450652	47.9915414951747
	55.8173045043348	67.6817814091697	68.5725871332774	66.5234828659154
	63.785471218558	58.6439719071099	49.5923712603197	86.0230765383602
BPESC1	44.7044140979108	38.8336427948187	42.5406888281114	
	38.4208429103799	49.32293280121	40.4359177791566	36.5352572191451
	44.7044140979108	43.7630587657205	44.4652605017595	40.3452320167286
	43.2776458914333			
BPGM	50.7903824729818	51.1621379498014	59.5907693818143	69.9652162720742
	61.5922826482868	57.678188678075	92.1817062395346	111.071209057746
	62.0660509811111	55.0016608333828	48.1682125052975	56.7139808194115
BPHL	390.647843814517	302.978457293429	356.948763043851	128.157032116902
	578.820199675035	278.140612054333	402.412173952792	275.975192937951
	507.375704144286	266.9457657143	326.805803266857	285.254985583609
BPI	38.304249186058	42.7775752471711	35.2101597814352	42.5664883002833
	43.1252272136667	39.4953494583071	39.7576136507593	37.7612949798895
	38.9006788676407	40.8049408996274	44.4688999098904	38.6454098044474
BPIL1	36.2496364442264	44.1239711172417	38.2280071951674	42.357910063876
	39.4693871570028	37.5887129572848	40.0052661202207	33.4534810696886
	39.0775870687758	42.4594149893798	37.5583886316038	36.7762265039864
BPIL2	35.0202257459026	35.7393138893354	40.6737160001291	39.9697332660833
	42.4864576619427	36.2015763253919	40.0052661202207	43.844855336215
	44.2607789091176	38.528621456378	38.1835938174625	37.8490162439785
BPIL3	34.8099089553046	39.5050383901884	36.2683886571585	43.8002456102021
	44.0451487108213	35.6125439901743	43.4544762830742	38.745552403895
	40.6957787921451	39.8873706855774	43.0971787292711	46.0633179231318
BPNT1	90.7551885299639	177.29096218424	209.860757709908	145.676955166566
	154.355197155161	117.907440794926	286.11425202743	181.488068017883
	116.576093937704	85.9910479975356	145.01315947031	100.188736523729
BPTF	55.9563971339766	51.909968572392	50.6987358483181	63.092106057211
	52.6226745117396	61.5824518349507	48.6395795163502	48.381918148032
	57.5430365699386	46.9598474201212	53.655233033193	55.5276281380084
BPY2	38.8336427948187	43.3458769998118	40.4882741869718	44.3440839686558
	41.0885562160946	35.4484652445078	39.8695929627783	38.3269307390572
	43.5140141743856	41.4812544772644	40.7725311293918	40.4192415967414
BPY2B	41.7397731376692	40.8880530359822	44.1452943804044	40.9607305725196
	43.9450023643394	42.0574848021044	54.8074655158941	40.4359177791566
	51.2776048338	40.5090849993014	43.1931731665131	40.8681871019812
BRAF	53.3059058105836	79.1039287828151	50.8303921211581	54.8412943008635
	88.3954618645976	46.7800033479223	74.1353964055485	70.5322939116406
	58.2401724760744	65.5349012839562	63.3544794716167	64.4156641487994
BRAP	37.6397652536077	40.7952353240579	36.6522278218553	30.9241129241985
	39.0564222526273	38.9378717994178	30.9403229851671	37.0390589468421
	38.2215591930186	43.2988017098387	36.6298807161755	34.9138625012006
BRCA1	53.9517841438975	51.9595173266422	42.2061277250364	42.4061338151892
	63.6172860741922	42.9083290030575	54.4934782594218	47.0940613507321
	54.0626629538146	41.9226500021333	39.6403089864678	48.27609330316
BRCA2	48.1952521496489	40.9731067463382	40.301483914446	41.1886975241176
	42.0709558258414	40.5281496800812	44.3440839686558	43.9366081640576
	46.0106098064475	40.8049408996274	39.6403089864678	41.9430737035925
BRCC3	80.8943070653517	103.139459009593	77.1614179978243	81.1189042503645
	137.239944506217	89.9789975503195	103.447730395424	126.360723656793
	89.2056902297352	84.0691608193405	73.408861359425	85.8154958329408

BRD1 84.1254258487497 100.066535982734 110.973625869849 75.0377156190383
130.002385558757 92.9820860411485 98.9988668602468 73.2037966614621
113.367264677544 79.262977183608 87.6689055777294 75.6428949408034
BRD2 1019.67015337432 923.470702344816 616.912306849151 877.673009984357
751.150916058018 602.726022292973 790.786577596971 915.936211119242
810.201903561366 1062.93431683428 1040.97380790412 764.571579067138
BRD3 378.964387825252 366.915943380617 148.906660152784 173.013258797349
228.882072078733 280.680953068339 498.958550919515 280.968337220318
224.266559865201 354.158105175308 378.964387825252 305.554059609771
BRD4 41.6354649482853 46.857923273572 46.8928145400696 43.074472921841
47.5647821785529 41.7309289572934 47.2927436772465 44.5544727111028
45.0997987980157 40.4544974307172 45.3649611783915 43.551658853562
BRD7 229.792906003602 158.637047990511 236.488376031891 151.01326330819
104.072423445726 165.029434090892 222.16475904496 196.465477936749
209.004669589169 158.685201053359 168.225699005719 228.499788774214
BRD8 107.232979387172 116.51037006692 91.0443726041992 85.1847490113446
88.3212200388592 101.177486898065 72.2577390063259 113.336158464071
119.051596183968 91.8251275769679 102.24202987437 93.0474674148534
BRD9 366.129480083279 269.745248423773 251.027270265657 178.289913291111
576.140066807763 243.672418126374 427.972184856393 340.553073657744
235.222472959869 298.14782785085 353.868749867979 219.805214090875
BRDG1 39.266565827316 39.1982470626863 38.2280071951674 41.6564075217517
45.8051846088552 35.4142595025086 42.7431359850339 37.1317006162938
36.7267557061938 41.0057887014368 35.6041926503637 39.9519547348106
BRDT 38.4278323739711 37.03486806852 39.3344387353654 40.5991006167568
40.3124262005404 39.3638967048812 35.3362091871364 37.4484310268909
38.8507804383261 39.6719190054379 37.9607839934735 38.6454098044474
BRE 200.93077689667 214.484877083166 212.967855232105 175.632783805966
110.729504563799 212.117653688186 308.568617575909 234.113622230498
167.626012561905 175.632783805966 167.999042735556 169.251790128723
BRF1 49.4151347850687 50.0748292497578 46.7508829977587 42.4580504587581
54.8630527326985 48.1324253195354 57.4508537244497 63.6476735543021
61.0845101400379 61.1851254827343 54.2683296643972 56.233217375698
BRF2 169.75692324104 81.2182827624857 120.401643722713 101.144615206297
169.561107057293 150.801729138762 136.062864107443 173.944080519006
114.304598397252 224.635749257133 211.137455821181 136.541379502691
BRI3 744.080606078086 653.089523541803 668.584452504579 1792.42490815074
600.34379987143 1149.02954135912 705.283979182062 453.260719561292
590.139974503603 469.78210773245 536.73426937255 578.311131274653
BRI3BP 73.4853298204744 83.555058970129 66.4079972657486
61.4518104624768 83.2652824564032 50.6774883370457 97.095992167029
62.9364422691302 97.4709987978999 53.7507429765038 53.7423898357583
65.564930068976
BRIP1 41.9407041937973 43.4396866157171 44.8872784170036 38.2041059863839
43.4544762830742 42.8598805316863 45.223075311113 43.4544762830742
38.7298222155532 46.5650556481721 41.9083581529276 39.5418280570561
BRMS1 506.75912743556 351.188432232165 439.770250139925 460.340300658834
494.231747291283 267.261896105785 609.243908604949 1012.0373748933
461.83420090894 406.104588997462 431.353025968649 268.936151471133
BRMS1L 50.2663821500171 66.0479079234982 67.1299518044849
51.2973486610884 57.2647835956181 110.036754099036 56.0515443021293
46.8482302725294 65.9900865113645 60.3816817102326 62.0404348860702
66.53831442217

BRP44 262.98784416804 319.871452890512 897.198663109723 316.185342210304
537.213035625492 439.862941165528 721.887210480162 275.314994786888
488.277500837251 281.527450183603 270.303708972854 387.631477822638
BRP44L 197.040881690021 328.855843739232 286.832539203237
375.818861583151 184.808179948172 438.50123471613 183.584085676174
214.823948126294 267.170081805168 294.908371819124 261.51893439249
409.554706384667
BRPF1 374.026381773215 420.401914084346 334.016152807947 323.095468495074
572.19538884395 289.716911324484 552.162906190653 715.662312404643
289.975375372371 374.096934824025 402.571146447946 260.383969219887
BRPF3 189.111092737256 151.088035451997 113.18951586976 96.5813769696577
120.309345127552 119.015431849218 123.455270940015 69.6473843718254
129.37368123079 109.386655134289 103.376225272425 94.8036856848998
BRS3 39.7576136507593 41.3610369241157 44.0451487108213 47.6687908290818
49.1928206164702 41.0582900711341 40.3302268293707 38.5457716679071
37.1820543575123 40.4136535208406 41.0808257270221 38.2350720635382
BRSK1 141.255712367708 125.027865666372 141.577762652792 484.968116511768
281.262083731954 194.666769955149 85.9692990407066 264.659866487253
57.2716485658185 127.093495914275 150.221698290502 94.4769283676944
BRSK2 41.5421429082231 35.2143190355414 40.7620472083159 37.651298468202
38.1644569189523 39.8455060654531 44.4639405506967 43.5544347011646
36.7267557061938 33.7883338848419 42.170702295259 40.7704587752083
BRUNOL4 43.2135791259317 41.2283968464263 41.3366779545815
40.8084239472012 39.2010256871265 42.712908390565 42.4386427013314
43.1567024035183 39.2809305246091 40.6500508575994 41.2324382727265
42.9285286409526
BRUNOL5 39.266565827316 45.7317208802318 38.5045333213784
41.4273122914951 50.7615314686244 40.4359177791566 39.5496902678788
44.293922970134 42.6366765186182 38.1042454705119 41.9083581529276
41.5536168238478
BRUNOL6 40.2543341073001 45.7979032939211 49.6347864008749
58.3475983630509 43.1252272136667 49.4157684139858 40.2154497375715
46.2779564481158 47.1744296124496 53.0357938671646 49.5923712603197
56.1875519519366
BRWD1 52.4570161184697 78.9563955412096 83.3322777515023 56.6458456890715
76.4339818310247 77.4307558516809 79.4158467034017 83.1702007661831
63.4753448572245 65.6260887743331 66.9494774452406 67.2500663500334
BRWD2 84.2104160850167 98.4763347474813 132.223745961048 95.6834075721066
92.3901719424121 87.6960277122561 99.5844079573552 84.1639779987105
83.7829283976292 98.0967511777819 97.7965639721152 102.899135663993
BRWD3 38.385865183581 40.0527893189096 43.5710871617107 47.0874209746031
49.7235523248653 34.0045980663271 42.357910063876 43.0390857698452
51.8083208406279 43.0919221375514 40.723990462047 47.5025821041537
BSCL2 352.132645260048 323.048740324895 345.942093303061 402.794855141389
519.351335801817 328.46798329618 577.276184555955 714.075528895169
409.554706384667 547.442608452594 468.98857415608 336.737571879156
BSDC1 185.563590013028 295.856741468603 206.337508331566 186.866378606003
251.105933757167 207.513604688444 263.492132556601 460.602626145229
163.458716079887 298.848001894468 308.931677425404 266.9457657143
BSG 1047.33749100342 1111.89793143647 692.630044242985 544.946796643478
1029.12917222294 854.712488657987 1055.69506483576 848.079114196986
993.626865290891 788.232434009173 911.661341702443 537.471287449696

BSN 45.9090659324223 41.8848959280916 37.7384958256497 42.8235313007746
49.9493230064613 42.9695585740813 41.4742337714895 50.9933795450314
37.4540772310118 44.0409178714232 45.6348103033479 39.2469742642337
BSND 41.5421429082231 36.5219488315447 48.1331309338997 39.7411455670172
43.844855336215 42.4580504587581 41.8315602975157 41.7419877611072
43.3744503319109 41.1222709786153 48.2910530887214 41.5536168238478
BST1 48.4849194706125 44.8529259387308 61.356483470053 82.5279746006378
52.8190882379316 99.4846339370133 92.4534854832186 46.7980335276155
109.374838860558 53.7196173866021 48.4717951775049 47.6040671292013
BST2 315.696895080135 368.660803187338 667.583623414832 300.486048273148
516.349618186681 211.701234819986 79.4832790698673 342.475405815033
641.103199748891 113.655306804539 640.407302868904 148.944852825695
BTAF1 202.459251553516 292.86576686019 266.250887288858 303.139550962377
216.943972794603 225.794026573235 233.201890340549 298.848001894468
242.83357283464 441.247306467645 324.767804809056 325.769076169162
BTBD1 72.3388488936832 100.535211780514 74.9113162056974 79.7937658787441
125.052841200703 110.637910297739 75.0092868910417 58.4920021605316
76.2591822114739 58.5831897269778 55.854474649572 108.213380368446
BTBD10 95.042308285667 125.290426098227 136.236041346288
100.001150612051 96.5030525416431 140.629562742418 81.9504925867759
64.0476795495651 151.245804544502 117.502489192855 120.679433440203
191.116764060948
BTBD11 49.5666074434383 58.0655276913048 102.483081702338
49.0881325319702 60.7770994863828 41.399074748665 42.1046072075724
60.1796637353976 82.9995913411894 98.3733183587283 71.5836731059913
75.8331683850176
BTBD12 81.7117547901526 87.9963662401769 93.9472621843566
71.7167037224069 75.3580625318157 63.8740412329753 75.5830548489892
57.2350830635912 75.2337946941103 64.428683021978 73.0970339739187
67.4197263416701
BTBD14A 139.311807081199 69.2082505043031 65.6620676514157
98.5663841503803 104.486555491331 64.6835308827804 89.7242042805798
93.0474674148534 74.3394153125605 49.1928206164702 53.5698357689852
56.9379721393904
BTBD14B 53.1309801459151 46.6314009405034 46.7508829977587
42.1273928203021 47.4548471240051 53.2046867327331 43.5627649099623
52.0503062610092 57.8772482723463 45.3530683538086 51.6414075056938
38.745552403895
BTBD15 39.9697332660833 50.4022543872993 47.0940613507321
49.7350276169552 51.3511088648386 52.2089306840125 50.5252048155459
52.7787010032051 59.9770720847728 81.915829566737 63.1566669782858
62.3470274683312
BTBD16 43.2724556187214 1301.07436212673 2164.51925341969
58.0218262176065 74.5758811681995 73.0247140074919 65.644767142472
100.114114765033 563.573152902421 1950.93175239958 1509.04753111016
1845.34855798442
BTBD17 39.3709357615493 33.3643994200057 37.9482157381008
34.5425384901279 40.148780650831 44.9896851005259 38.1793598983615
36.3405039629059 40.4997186069494 34.064698871541 35.6041926503637
42.1537476443423
BTBD3 97.6205705034407 137.016387648703 75.8201286658785 63.1604223919703
103.513421757522 107.808744445896 73.5700402711904 60.3481762331447
75.949657568749 98.5746416750459 97.3756238014731 108.138395958121

BTBD6 263.832968124343 211.701234819986 273.070365538675 181.488068017883
258.757703729905 262.37779288036 232.320396482547 195.58977358589
229.881790983135 296.463473585355 268.38649000825 219.616289279562
BTBD7 77.1287755867582 62.5528124447176 77.518381991736 72.3388488936832
65.1129635608639 68.2428802339852 59.6105564219031 61.3864512710242
92.9894133685392 104.964345794911 87.3508610599789 81.8426140611297
BTBD8 37.6817357354748 37.8685585043426 41.1367478979484 47.0874209746031
42.0709558258414 45.0283997415478 41.0871827071116 40.8833572570383
43.423558862426 44.3303034026662 43.1931731665131 44.3521099857368
BTBD9 49.7188990489616 41.554942319225 46.2625470410195 48.665578118898
51.5888395924843 59.9174456081704 47.1345949868029 39.1652965788406
52.0583273941813 55.2214187655851 50.7903824729818 52.1918130336397
BTC 42.3433931248155 49.1283590719461 49.6347864008749 44.9370339275041
50.2163892855092 61.3620062177524 53.3111230705754 77.3999378505514
56.702421372165 53.6429962450784 55.9269493191353 50.379587451669
BTD 72.9154861179393 98.7446830481164 93.9472621843566 89.1290937615998
94.4129246851651 100.305262467668 95.3754438202863 73.8374823243007
88.8772406115484 116.311176407088 86.8330533522204 114.016824404095
BTF3 1445.73674428909 3202.8848520816 3385.65959808808 1468.75291542154
1824.79010714035 2306.14613042834 1387.28483887704 1375.14388134842
2294.75523373329 2206.44541412808 2150.8715161597 3166.48568397766
BTF3L4 184.808179948172 209.860757709908 179.186381381821
159.925087917307 230.787791652978 242.899629257173 167.05554959607
115.801367301965 184.50283792606 104.504444994123 103.491711140137
187.767803297395
BTG1 892.754288149486 1249.41227924272 1071.64675274806 2678.43227619157
607.431621448599 1401.46625360427 947.656220635965 1058.61208471961
1442.72006787259 742.317490569544 1136.88710104691 970.092441721494
BTG2 150.262748333939 2133.78836857697 1180.7204565402 1125.81685334899
735.083155673644 2435.70120719503 463.022816882856 729.342532316666
724.881002237334 1792.42490815074 1039.06999658544 838.974043046676
BTG3 188.767243497644 227.439319520245 331.762920362224 332.830805115112
234.477015135311 228.845881709556 382.050584680669 187.923050034941
273.032063533642 156.36242271528 179.285390561592 318.234214341679
BTG4 41.8409927585705 40.4359177791566 41.2365089689635 39.6292788039793
38.8456942580061 45.4882438975269 34.2773859804062 38.1835938174625
43.9450023643394 40.8970401536578 37.3687900733549 41.6550823405646
BTK 53.3059058105836 74.4696354388511 47.2055133646385 156.476627024647
48.0234896130991 89.3168921945222 48.6395795163502 52.1788489555942
38.4344387289956 45.0155543555707 42.0750889188255 50.4847465816184
BTLA 49.540449125788 50.2163892855092 45.3675390992851 83.014860007187
43.4885245598939 43.8308097066396 43.2721716572958 48.381918148032
43.2761454096265 44.4652605017595 46.9246510824681 41.4508241771315
BTN1A1 33.8558232866797 33.5924201549249 40.6737160001291
40.3180310664156 33.0463640202858 32.9046688061235 34.9344532055397
34.8949555140527 33.3643994200057 35.3849221682845 34.6366195121669
32.7800533388945
BTN2A1 94.4618437533343 101.192523227755 84.5998418020787
96.4913003384143 95.2833228196616 109.607667469015 213.073369426342
146.892027926888 70.9767460064692 78.1683130800805 102.170521765252
92.3388129097482
BTN2A2 45.0283997415478 49.5384557467329 49.6978906361694
52.403135056701 44.2607789091176 40.4359177791566 77.5813459454823

51.2189089780361 51.9545473262296 55.3477556906605 46.2560238004757
44.3521099857368
BTN2A3 43.5710871617107 37.1685072112618 40.301483914446
39.9697332660833 40.9950639693255 36.1190722948669 42.2577689927563
40.831207953647 38.8271346375425 35.195364621675 39.2542963855807
42.0424641526302
BTN3A1 69.4501305462809 97.4327882336954 89.4111455826308
98.8296358083144 53.4751250621694 60.4837400912239 100.891869710611
108.138395958121 68.0796261751272 84.2434917159728 91.9375312869437
62.8485724004124
BTN3A2 70.6784633355742 158.156217755244 216.526205739151
151.99110418225 77.1287755867582 102.980622026276 169.251790128723
131.57403028894 100.234661597218 79.4916415027015 99.5125674355716
94.1128427766432
BTN3A3 86.0796981361148 88.1413320312949 75.9174504780598
76.9373289454707 61.5236837309467 70.5699797830702 91.0154871536701
78.9900928241723 58.174555206394 62.2025996860055 58.4816190076338
52.5752569429057
BTNL2 46.0920218698606 40.716553247236 59.3549213146532 54.2886483704949
41.6806984441849 52.8421046802739 45.7452456977857 40.7311497914871
47.9035532886869 51.0055825931636 38.6662194645633 45.2783024981315
BTNL3 40.5320714850602 44.714975193517 41.7419877611072 42.6322698334662
39.9220998751503 40.5940318808889 42.7431359850339 41.9315419791594
42.7522261624826 42.4247468951743 42.5805189064329 41.0920120134247
BTNL8 37.3875631460917 35.7393138893354 37.4302011516587 39.7411455670172
35.8429598823818 37.2686490268265 39.7576136507593 35.8345448206824
35.7973685350741 33.2987269789328 34.9428206949566 36.9162349182365
BTNL9 39.0620487644635 44.9370339275041 45.4865173433646 46.614092935749
44.9936360641969 50.8533737532786 46.7100796279384 50.9933795450314
41.4636047919453 37.920964499903 42.8263096141654 42.357910063876
BTRC 45.0985088502363 50.2163892855092 49.1718303518849 44.4367387274922
38.7864161493321 49.3654837541353 45.3597029614942 42.5435283762933
49.0986627051784 51.0055825931636 47.4964755141472 49.9419897484381
BUB1 112.122619608668 147.471179343348 89.4747568145717 63.9570596163747
190.921576470336 52.8421046802739 142.614006543733 153.35286239647
153.165464817911 49.5265949137207 39.153226699662 44.9070250604284
BUB1B 41.7397731376692 44.1239711172417 44.1452943804044 37.0732977341185
58.4383854340181 43.1826328292558 43.0930293831497 47.4740726032707
43.4656637536244 32.9003644440608 45.9104478407175 41.863927676552
BUB3 229.881790983135 413.516548092483 348.206465194205 273.320144423733
334.016152807947 209.61080762285 424.539111965342 332.830805115112
288.642294976 358.194667053419 278.850071205987 283.111920665176
BUD13 77.9057333780847 83.1308709614708 96.317642888143 85.3754356809649
107.094663242259 68.3234331335073 89.9284109077375 92.804679821289
76.7820250461545 72.6549171886783 79.4832790698673 74.3442318693865
BUD31 837.959914510114 581.2449195499 427.540236966999 596.84269596976
483.20917825594 598.970109654466 598.970109654466 648.380417557148
711.773459577386 359.889643461047 422.237766234202 452.351360652434
BVES 55.1306642449627 45.3486182278177 41.6079958749402 46.1673159982664
43.7517118200398 94.3953345963359 57.8205747266529 49.1303869398773
47.2106324363708 73.0070079786149 63.0383605052305 68.0388928513985
BXDC1 593.345148568451 284.942182200489 344.133384411423 352.019218177371
278.507764368929 357.762505796238 210.348939191359 1789.07186722972
207.726011653345 366.998712124711 258.913621521866 360.900649927623

BXDC2 309.994784298282 444.320714312359 298.771150048863 183.364949777567
456.023620287811 280.968337220318 770.715044136717 1379.51119430451
345.615640348066 183.613579501306 204.944787289897 332.276510973163
BXDC5 123.868848519181 120.221914344372 103.43536623122 92.6230008876347
75.6471706262645 96.2490896573253 116.74427460886 115.052948380817
85.1920126487447 99.1438953564062 75.8056934060866 130.293915348934
BYSL 377.409970666607 162.628603963754 169.459466601559 114.363816429963
351.700618429121 153.992331037033 238.697159169213 191.955732544426
210.605371352247 152.187430924861 144.546897317106 150.562062400737
BZRAP1 52.8427643366617 52.0268699306396 48.0191002461099
59.4674205670657 45.2556760873335 57.1752846228778 51.5967098638347
50.0526763693329 69.8899273527731 78.5141640924736 75.0237373605584
61.7502624458447
BZRPL1 43.1805016690297 37.920964499903 36.9795961564828
35.314469458379 32.5318884330468 39.8455060654531 37.0564573649223
38.8495095797881 38.7298222155532 34.2638875325204 36.6298807161755
35.0222940937513
BZW1 45.7700386532539 45.8966572750941 45.5616307514223 49.8231861631652
46.0405632631442 45.7300501624017 44.4004151500264 45.6948619492056
48.6064806408096 41.9219580093938 42.5153507543837 46.0201052072883
BZW2 608.886783362316 558.451033789799 542.901822331329 213.204385896543
798.996494242432 465.266372824212 629.301646370726 829.152040996817
814.991871217268 428.206419080492 464.99944459917 683.099079617304
C10orf10 242.546821017091 114.161435210562 126.826715174564
116.852483297949 106.125117937458 250.040637073537 171.853856533964
134.83544047809 62.9922928277769 82.8275497073451 157.493875535302
286.611037700479
C10orf104 103.561100886469 110.666686474986 90.954592973767
132.52749757795 104.80882886573 193.703214707909 138.436822676252
83.0497574039035 337.363827785838 175.960838580876 187.689343320587
203.228211195516
C10orf107 47.4678477186801 44.9370339275041 36.769073068743
38.4208429103799 38.8456942580061 37.8102105794655 41.253645578517
41.9315419791594 44.0519245668737 41.0057887014368 46.4205654140754
37.1747156131306
C10orf108 41.9407041937973 41.4508241771315 40.301483914446
38.0958223740111 36.08956004634 38.3418933808844 38.4005795891396
42.357910063876 39.0775870687758 42.5664883002833 46.0095710163378
42.4607795638552
C10orf109 38.385865183581 36.4002279956519 34.1806289878747
34.8137085420423 33.4316681623175 37.9951997351435 40.4359177791566
40.2382908228517 31.5151915411476 37.8217989841918 35.4386677371439
31.036133494654
C10orf11 50.9381343674699 104.964345794911 92.0880591999243
89.2503940049313 80.9160262677505 81.3978207813439 141.261334415286
105.308648028552 331.474920376907 107.242476674629 87.2316853416374
95.491982090349
C10orf110 42.1537476443423 40.148780650831 47.646800205189
44.4367387274922 40.4639532172338 40.9506062495231 46.3057385979341
39.5466975353131 46.4164127487052 45.0155543555707 47.6125796208753
44.2527566343661
C10orf111 42.6036491382658 41.0678614026819 34.3633615460307
41.5464765641928 39.5595233162192 42.9695585740813 39.7576136507593

39.6474008369935 46.581796165231 40.1179210534118 37.1059647239814
42.2577689927563
C10orf113 36.4564050256915 40.6380495994768 40.4882741869718
38.8495095797881 40.148780650831 37.9951997351435 38.0598374201407
41.6466169548846 38.2215591930186 37.7315666632629 41.5421429082231
38.1421709520616
C10orf114 41.2424637338424 41.8073910693411 37.8386378737561
42.9358255168086 46.5557478175976 33.7818132446042 39.2533668167483
35.5798430594271 50.9010149857308 46.441301934328 47.8988096575648
42.6758568421347
C10orf115 39.1590964332473 33.0553208959996 32.3881195727383
32.554751989205 33.6330903323612 32.4301586830653 30.7315474127249
33.4534810696886 39.0775870687758 32.2133093774284 31.7413650507468
32.0636251428594
C10orf116 85.8694579123452 13230.9863405074 8144.52133665388
412.162238678684 6673.00322263275 2779.37660132136 7785.77078492993
5895.03230740197 5932.92478996609 6413.42384145372 6027.02412617194
4632.08655821299
C10orf118 41.1523487516364 44.9370339275041 42.4580504587581
39.1706564265495 44.2607789091176 39.0266821712099 35.1307934330047
37.1317006162938 34.8552366193358 33.6188027862739 41.0050831223107
37.9607839934735
C10orf119 52.5792653122861 60.7495043977707 64.3312319756553
77.7973642829228 50.0580547772867 51.6053353177789 88.8039637390786
58.3046521633424 51.6731332961917 73.6240672213673 59.8509516999438
57.5372732453319
C10orf12 36.8072833828297 48.8584917918986 44.1452943804044
46.8954576545929 56.1186281675573 50.8533737532786 65.3694267397114
53.5271601314404 48.3119523820103 58.115198353247 53.6895401581575
62.58750768974
C10orf120 37.9387791625384 36.3110701990588 42.3536549114165
41.0726336841311 37.0526522140242 33.895835691032 35.2163900400329
44.5029690493211 34.5633623842083 38.2069499294002 36.0481934423077
36.5474283500601
C10orf122 47.8249696958403 43.0227387991135 48.1331309338997
46.4555908533915 45.6895223823001 50.4022543872993 42.4939802311136
45.4757965005055 42.9020321355927 39.2623423164488 44.3763859390287
44.4475838947656
C10orf125 75.3761360028971 75.9802606133139 54.7855575216687
113.388372364837 66.0411740221276 64.6835308827804 428.105433953405
49.7309743973184 77.0703146713865 58.7198210787055 64.5347406608537
50.8999542937724
C10orf128 51.9545473262296 43.5182502375207 47.646800205189
71.5249259772583 43.2484996523549 53.9517841438975 37.2892641289258
41.5464765641928 39.9444191873552 44.7958122466628 45.9104478407175
39.3575468494941
C10orf129 38.6454098044474 39.932537801639 37.2838080935695
38.5252456306656 38.3085139808968 39.3841861812262 35.5310164682026
37.2237132058632 39.2497056720346 38.4829727716328 34.266241549578
36.064718696392
C10orf132 46.5549623118323 50.3586212130973 42.4580504587581
43.074472921841 40.0599807618105 38.5252456306656 43.3262148375525
45.5581356216957 45.2897806651929 36.6893251782922 45.4799270968969
46.0633179231318

C10orf136 41.5421429082231 70.1238785863433 58.112348043634
55.4180794172794 52.5645416722441 46.3715909653832 46.9929836306816
51.8347877274018 44.2607789091176 42.2614524730856 41.7272875295469
46.8129509799753
C10orf137 73.6362523983338 106.160416826673 90.1284518302601
71.5249259772583 106.276392548097 76.4439037944547 95.0190813062982
60.6706478075023 73.8800799416166 85.7732262204681 86.2118355769367
78.1623301184313
C10orf141 69.1306000876802 52.0268699306396 48.8100349749783
81.588696805606 52.7288095750893 61.4944752562671 65.4784797364469
61.0831990510269 65.1424206085551 53.6788556779989 52.4393177875001
60.0517078759277
C10orf18 42.2455707947223 42.6939394537076 43.9450023643394
44.265834798964 44.0451487108213 46.4648868771662 44.7255207589606
43.6434576713809 51.8824384192988 39.0648733099061 49.0361180089431
45.8546719310325
C10orf2 46.0920218698606 69.4347592040783 64.0162246399972
49.7350276169552 98.3126451737573 71.0197205018137 49.8281348927244
50.1833483241864 59.9770720847728 54.4825201506931 58.8961770381033
62.3470274683312
C10orf21 45.1912496459517 41.8848959280916 44.6032218589199
40.9607305725196 36.08956004634 44.3648993568405 36.634024014558
39.4710743516517 48.3119523820103 41.9666541498638 40.547835538778
40.3691324547049
C10orf25 37.8386378737561 34.3715326040967 34.1806289878747
36.1562979552173 35.9894133432376 34.7983399096627 37.4772922422734
32.229154316188 29.7878371181841 37.920964499903 37.9230440150891
38.745552403895
C10orf26 149.260740603715 138.12608134713 171.021755568144
178.858202962948 109.758297200083 179.767874494671 108.558977253184
72.4834467838798 84.673375308972 94.8742116922003 105.814959179397
89.4747568145717
C10orf27 39.266565827316 44.6918600441328 46.5650556481721
45.1944222548351 44.7829632472754 45.1873487463472 44.8643898647322
42.8482181313226 43.9450023643394 41.8613406887824 41.4508241771315
44.4475838947656
C10orf28 51.1740855632044 57.0082102559228 53.4088637045445
70.8835636550472 56.8307116636372 67.3469281984384 65.1041577962151
60.419911425109 70.3613168347492 84.8999904158793 66.4251023257203
72.5346469941833
C10orf30 39.9697332660833 42.7775752471711 38.1421709520616
39.7411455670172 42.7107928951766 36.4986986135769 36.7356360093603
43.6434576713809 43.3744503319109 41.0057887014368 39.3667161654772
38.1421709520616
C10orf31 44.1294459089314 47.3536182445315 43.3085358214154
41.3027031037343 45.8051846088552 43.2805590023412 41.706879587091
41.6466169548846 45.8325181856483 42.6793753550854 43.7585538437696
44.6484005362281
C10orf32 110.272925554245 235.962209790392 132.679840262941
199.181938253995 171.205726660714 295.856741468603 171.238235624104
116.793627324217 97.2425832658887 161.075134901675 175.136856246782
203.808195515747
C10orf33 135.889195284785 121.668011907792 170.619111196551
59.987045425047 59.5844022026141 76.5888466260562 39.1539892144371

59.4674205670657 50.9010149857308 265.860397918913 130.35828139117
160.283895105835
C10orf35 286.178856785544 200.002687565519 128.680405667403
54.8412943008635 278.303493506139 125.642081865992 334.726233105118
385.827237353675 500.313640131475 172.65049903872 134.567947469561
188.099292617901
C10orf37 38.0648056183784 42.6450179841129 41.6977706798234
38.2599935139101 40.7273974755431 44.2607789091176 44.6032218589199
43.3838505886856 41.074824727477 45.7911932481722 38.4251036088595
39.7411455670172
C10orf39 63.3717459766798 42.8542642792862 44.696023817801
49.7350276169552 45.3675390992851 56.0477607422828 48.8319884367456
51.1694248620208 43.9450023643394 52.0999467564713 57.8991270166934
42.357910063876
C10orf4 42.2594065417187 41.6825727187466 43.6670755115848
43.3078542962968 43.2882076149301 43.1244350694755 41.982678229701
42.4990747926235 43.1260193724086 42.9311657796657 43.5710871617107
41.9430737035925
C10orf41 64.2690842803282 58.2571968311834 73.2814174739782
59.0965219753805 58.7460488758053 54.2503023961401 88.3335649692467
54.720204938924 141.672695206961 65.2361525412756 59.2106652388061
47.7962548556026
C10orf46 47.3791873993182 61.0144918827511 72.0243286940589
42.8235313007746 88.5564369981649 73.4820932957859 63.5738862549306
44.1311654985682 57.8772482723463 38.2069499294002 40.6373400186325
56.4578213622965
C10orf47 63.1291723738553 59.2643906275025 52.2132783757422
48.1662933382789 78.4023698691285 46.263826584899 82.0174591986046
51.8972117537157 81.8491450876942 83.5069968129733 72.2720428359203
96.2280884890067
C10orf49 40.8681871019812 45.5842286511645 39.4710743516517
41.7614975630362 46.3150221024046 40.650742261428 33.7574445386307
48.272324215504 47.6195009345747 51.2318935933116 36.43924066428
41.7575097298927
C10orf51 40.2543341073001 43.8495248991589 37.8386378737561
43.3078542962968 37.7576900881741 37.9003288112261 34.4570158133896
37.86982638952 43.7630587657205 39.8873706855774 39.9070785037411
41.0657892043496
C10orf53 40.0557788909923 40.213344051206 39.8676052969935
40.1272810917202 42.5428186846763 42.086839811428 45.9635923776719
44.5658219748173 40.3536148015242 39.1121819152043 40.7786237231186
39.2839132447749
C10orf54 57.7877627794725 121.036604456449 106.243765443714
457.202737754842 63.0383605052305 134.239606718101 69.9214859673839
69.4677195088043 145.01315947031 100.413031488199 78.7260936224802
139.142835448881
C10orf55 47.5647821785529 46.0713306971594 35.9305494063137
42.8235313007746 31.8228237796461 42.2577689927563 44.4639405506967
43.2551508830106 49.2879849674517 39.1621898366355 42.2618615571566
44.7401523707775
C10orf57 79.2665546280232 152.972693071526 177.425508756002
86.9274372004388 165.797721197832 101.177486898065 224.412339736769
198.54718783437 612.441960343995 167.896094603299 145.64473441957
126.404988409795

C10orf58 1303.33517942343 2865.25344854768 4482.5285743878
854.712488657987 1084.23003918768 733.358170858378 1448.17151189672
1585.90718459092 5074.07305640514 2816.7754199004 2196.59044510709
2633.34100695103
C10orf59 46.8141313516997 63.0383605052305 51.2796327239617
59.7044210110693 53.1221282279739 60.3481762331447 52.8496345990125
57.6913861717513 67.389417016806 61.9459245072385 59.7483827511172
69.2213838763756
C10orf6 256.766675299448 165.29964739949 320.620320066275
208.30969642805 164.813928846511 290.81245174957 262.215263489321
152.920586451578 188.887559923238 493.513764839058 522.377154567257
260.279110870904
C10orf61 70.6065558064758 90.2721521466478 96.0654949568324
68.3419511055057 93.4093169676983 90.1080159649841 72.4651758215622
69.7242825662275 65.0217598379984 78.0133696753253 84.4349803529132
101.540333720181
C10orf62 38.8561375693335 39.9697332660833 47.2962360458401
33.4819739460148 41.6806984441849 40.8634043028528 38.4005795891396
44.1311654985682 43.8482802038826 39.0648733099061 34.9428206949566
33.2382077236252
C10orf63 41.9407041937973 41.0678614026819 40.3397581924196
45.7397504535169 44.2120774064277 42.8598805316863 40.6750540874299
39.3709357615493 43.7078399718247 39.9948390976317 39.153226699662
46.2625470410195
C10orf64 43.844855336215 47.4076235350059 47.3861758958576
57.8277957325085 41.7929491793697 44.1737502492846 38.1793598983615
47.2009420656854 42.4540072551568 44.1379638573455 40.2649309204478
41.2437378488025
C10orf65 42.2455707947223 43.9421822132391 44.9758339877851
38.9534635879195 52.4370718282084 35.8227939208521 37.7136011474903
44.0390019242862 40.137818555383 48.1392888470269 42.0750889188255
38.4330755397458
C10orf67 42.1537476443423 34.6306468196142 36.4564050256915
40.1804147506786 42.9090346219023 44.2607789091176 40.5585676679482
38.079222289614 41.3803269429468 40.3235655331796 39.6403089864678
35.9604262434141
C10orf68 45.7356815244513 39.6995529770239 36.8730514568925
45.5869891570251 41.0885562160946 37.9951997351435 42.357910063876
40.1474543172332 40.9901805728485 44.4652605017595 38.956280281655
41.0657892043496
C10orf71 36.2496364442264 35.6434124419782 33.553634633839
38.3166554965434 35.7973685350741 35.6125439901743 40.3302268293707
35.2642250366721 34.0382693188794 35.93950183586 40.9157352260901
34.6720534245631
C10orf72 42.8596678196469 43.0227387991135 41.0752399035779
41.0057887014368 43.7828659616842 42.2006337556379 39.9620349773712
39.7509165247419 39.8763736543526 40.162771030532 43.6960408897486
41.5536168238478
C10orf73 45.7356815244513 49.4067286971348 88.4211119834434
74.7732045170597 47.4548471240051 92.3477214133335 42.4939802311136
45.8669209716694 43.0930293831497 72.2443746456494 79.0940380276977
64.2769859135548
C10orf76 83.946822476147 102.863599315528 102.228965899392
99.8063480813138 82.4615433148846 88.6287852712744 108.558977253184

75.7947206223934 94.8408264309872 154.707548614663 112.957927208627
100.623973215137
C10orf78 62.5036823572932 78.4289643729837 77.9085946827079
76.2591822114739 74.4623288220969 97.4062608315831 76.6094442444074
72.5835488194631 74.1031848443392 53.6429962450784 61.940656536312
70.3309641302984
C10orf79 38.7537364570873 39.8695929627783 38.9790184198145
40.646702696033 38.0598374201407 40.5281496800812 44.8643898647322
36.5393857306744 36.6312676076142 42.5664883002833 44.1148826235374
38.2350720635382
C10orf81 51.9545473262296 41.2598458726002 50.1860191642359
38.2041059863839 41.1868052124344 43.9724628401189 81.7434024567356
89.3868257841517 58.4891791448514 42.2614524730856 44.9713479185144
44.9070250604284
C10orf82 37.0320432433314 34.5023764006976 42.3536549114165
33.4240466670776 36.6354013629791 42.8598805316863 37.3799542961839
43.6434576713809 38.1169703906207 34.2638875325204 41.5421429082231
40.5703581862752
C10orf84 48.4849194706125 52.5868047927666 45.5883690965192
61.2213001749372 41.1868052124344 66.0069448981947 56.2181240696479
45.0972184964705 51.2066664465185 51.4454142248566 57.9864701726379
51.6660481331154
C10orf85 70.8288090024328 41.8073910693411 42.4580504587581
39.5050383901884 45.9104478407175 38.9378717994178 46.4388841354122
46.8954576545929 42.0621425894624 42.8936990772838 45.9104478407175
45.2783024981315
C10orf88 45.5758521267508 49.2050538150106 47.7737810047679
39.2780515621926 49.8304479381623 40.9506062495231 37.6036146841933
56.2441895183416 45.6582729799009 51.6778366958973 56.233217375698
50.8442982601142
C10orf90 35.9305494063137 37.920964499903 40.8420925848403
40.3180310664156 40.9950639693255 43.3785777889265 38.2933870238743
43.2551508830106 38.2215591930186 37.920964499903 41.3553369544097
42.0424641526302
C10orf91 42.2455707947223 45.4143854024453 41.785676764591
44.550322123994 41.6806984441849 41.7256940298128 48.3411413510851
46.4672637826582 43.8482802038826 42.9866214102525 42.2618615571566
43.6434576713809
C10orf92 43.1805016690297 39.6995529770239 42.0621425894624
57.0629653735694 44.4489662507893 51.0506441018166 45.223075311113
42.9301944191913 41.1742244762318 39.9948390976317 45.8180710486329
42.0424641526302
C10orf93 40.604930211629 37.6678021168009 35.4089520062205
37.9315270017242 37.6527901109005 37.7441423440745 40.2669497398702
39.1652965788406 38.1548766517774 39.6333888530279 38.932557955217
40.7063426028871
C10orf95 44.5282181324797 51.7495306734657 37.1643693306382
39.3750969348308 42.7982502551634 45.3882138838137 60.9648845690177
47.8947595316832 43.9450023643394 47.9056710878325 41.0808257270221
41.7575097298927
C10orf96 39.9697332660833 37.7384958256497 40.7620472083159
38.745552403895 36.6354013629791 39.2248354955045 43.3262148375525
41.1575777497576 36.197424053556 38.3092241935352 40.4504608195666
38.6454098044474

C10orf97 211.926421068313 126.389908470189 134.725028732282
125.801834682035 94.1575209300592 168.91210897306 105.549249728506
121.510087314197 123.25443049561 184.269633116351 162.993316022946
180.255771684507
C10orf99 39.4710743516517 917.631567606105 1395.33665255999
43.4410741327254 77.0301627655229 59.0686629877982 140.961217055867
88.9038123445083 269.546586017659 443.581460031166 416.844848063269
352.292837622545
C11orf1 102.25509551881 167.319747725488 207.632489510045
122.952515297686 159.288478050818 137.976238593619 142.389246750828
102.24202987437 129.320408155239 130.533514998688 129.965747812294
195.430996540859
C11orf10 1379.51119430451 1046.42391243532 766.027617496754
1199.29782389306 1607.73526966988 894.486674059951 1270.49749036491
1357.41745370754 1440.41726370246 919.542051089664 843.078502001733
1582.45600339543
C11orf16 46.9939712192224 40.2493681589104 42.4580504587581
40.646702696033 39.1650142708257 41.8613406887824 43.4544762830742
38.745552403895 42.992884918158 42.0621425894624 44.9713479185144
42.2577689927563
C11orf17 123.963659641243 145.073599404007 147.602298697211
98.0473811574767 102.597012252626 112.306016029829 107.576033955049
91.7969435808283 152.545682001987 114.674541610026 138.12608134713
139.614715580418
C11orf2 468.573956652904 731.670315613547 878.863554510943
632.577797921655 950.740991182127 1048.98028527123 1081.97502255142
802.650020720921 490.652353306749 1135.43644148537 1167.25317435052
676.672146226382
C11orf24 152.294357984689 80.882176929636 86.2895084312264
164.176142753482 102.597012252626 96.2490896573253 92.6545309624315
103.945335792682 87.3815040456105 94.7110944331075 90.1284518302601
75.6428949408034
C11orf30 43.5710871617107 39.6123051184545 42.157627240486
42.1273928203021 46.0977687311222 47.5733603903505 52.8496345990125
49.7309743973184 37.7384958256497 37.920964499903 48.4717951775049
45.2783024981315
C11orf31 51.5888395924843 60.0601788842942 47.4740726032707
61.3747706295648 67.6011992144113 55.8243694410835 72.1401612718245
48.5681478665796 59.5093038629766 69.5080454202415 61.5392012551041
53.4735821194294
C11orf34 44.7599897211788 42.1607322031054 39.6833800000587
41.0219290963778 44.0977148249302 45.4882438975269 47.3661873039785
41.4812544772644 39.9777866059804 40.4136535208406 42.170702295259
40.6614700944853
C11orf35 51.0225132036905 63.1780854659428 77.9085946827079
53.0284011078097 49.8304479381623 44.5544727111028 43.9450023643394
57.7805922926828 72.7934987008399 80.5388767883646 60.9446532180211
54.1941919612524
C11orf36 36.1232327599945 38.4330755397458 33.4240466670776
42.4580504587581 36.9702228618069 39.4953494583071 39.3585350448719
38.079222289614 42.1594905198929 39.9948390976317 35.6041926503637
41.2437378488025
C11orf37 41.0608773166058 37.1685072112618 33.7737772393677
37.29566077996 40.5710637884029 40.1428591998404 38.6243997507646

43.6434576713809 38.4344387289956 42.2614524730856 41.6336636285875
38.4330755397458
C11orf38 55.2515150764173 61.4242242807301 55.5005760719069
37.4223289687135 51.1915474019836 51.334128582289 61.141483572676
67.1702819938085 53.6837370963602 65.2361525412756 66.6118713143653
64.4156641487994
C11orf39 40.5654775358427 40.148780650831 41.9666541498638
44.1483976313959 45.5809132232681 36.6271061451613 37.0564573649223
41.2551966132486 45.1899271235974 39.2623423164488 38.1835938174625
41.2437378488025
C11orf40 43.844855336215 31.6295801599123 38.9790184198145
39.066284740632 37.6527901109005 36.8059561571675 36.5352572191451
41.7419877611072 37.9273653326866 39.9948390976317 39.4410515050885
36.449101775775
C11orf41 53.0290605707162 44.3529919625344 59.2012779686869
46.3203995372658 51.0785933435539 44.3187284857534 63.0842071602791
49.6629259325705 68.8353800542132 58.3285450269055 47.3791873993182
64.6996384732208
C11orf42 42.442231667357 37.7384958256497 46.5650556481721
37.5382094267692 39.9697332660833 39.7394181011603 41.9393838138203
44.797320243931 41.4636047919453 34.4875013487777 35.2339574411711
41.863927676552
C11orf44 41.3387790895716 43.5873188565519 38.8775560911841
42.1273928203021 40.0599807618105 37.0180934800573 41.4742337714895
39.2672712041774 45.8325181856483 41.3321861371202 38.079222289614
40.9683275553869
C11orf45 53.8856501960117 43.0971787292711 53.4088637045445
58.5406981871153 54.3116862772198 52.8421046802739 62.5506324864207
61.7572675712557 54.2825877218965 54.4825201506931 49.7363778831003
54.1941919612524
C11orf46 62.6702927225656 87.9963662401769 83.0262409967927
113.979504140326 67.4534389579576 156.445000453282 61.2183034191703
57.7805922926828 68.0295963383707 63.6197695952215 82.7309298534989
114.142157259903
C11orf47 41.5421429082231 45.9732108120834 44.2733573199954
43.1847628331565 50.0580547772867 44.8872784170036 41.9918605927928
40.6663433492193 43.423558862426 46.6598879466655 44.4329005595031
45.3763275588341
C11orf48 214.245832611737 274.731304343034 257.761541860837
218.917221711155 424.87896140215 196.592478823849 433.447319956981
313.46376203503 277.069127740648 154.149426842591 160.360785581153
257.584248252408
C11orf49 49.5810174435089 87.4273245333478 65.9476039189301
47.2740099216972 53.6210959113222 54.7806868394478 63.0842071602791
51.3851157261451 76.1205714556592 52.6879797806876 56.1097418153893
56.8534370691413
C11orf51 58.7404123614695 72.5397346298724 80.0183739952818
81.382229980722 74.8013460023603 109.137372445043 85.5027511448958
78.1448136847078 104.80882886573 85.6763929745253 86.6798536307388
78.8968326770219
C11orf52 70.083473392272 57.0082102559228 87.8724689898823
52.1201052574162 60.1730663840476 43.0860383229288 79.3861418060845
63.4570909617664 77.5480089703916 82.8275497073451 91.3723635213821
90.3485862002091

C11orf53 39.3709357615493 40.148780650831 44.3344900481112
46.4555908533915 43.844855336215 43.4681646416585 48.0283573444179
49.7309743973184 51.8824384192988 45.6868589181768 45.2265319826548
43.7672102682067
C11orf54 115.622641633992 163.067962559455 154.752920877816
135.138667957956 124.841811761412 157.9970294734 123.336184278054
80.0213594533301 89.6736229308069 131.779333469955 138.748233664345
169.723009455614
C11orf55 45.2875173643252 35.7393138893354 42.5864346233886
43.4812166409346 43.004943518025 40.209110303983 41.5464765641928
42.9887069977696 36.0741672064298 38.957687788868 42.6366765186182
42.2577689927563
C11orf57 70.5096706089299 67.7189686993694 54.6051538001037
71.9062462001972 68.0414563597154 66.295597720304 65.3694267397114
66.4207938873541 63.1944558106132 62.6984594468418 60.0765286625112
64.2769859135548
C11orf58 45.6517320730042 42.4493032770053 46.5650556481721
39.066284740632 41.467005949568 35.6125439901743 54.339958888486
42.2577689927563 51.2776048338 38.4187686307607 42.6366765186182
47.6938628797168
C11orf59 458.47341225284 659.666977039793 549.117045813593
682.227514643175 546.287195443189 575.268446033282 757.257495810249
358.194667053419 763.947361966861 516.076634990003 418.810270137243
393.944898808828
C11orf60 64.1882546112044 75.2824161164996 101.804757613639
58.1798515131249 91.1057573288203 128.776189744323 107.895235897156
78.4815128101967 111.397578350396 111.607814098243 88.0877175295544
106.356074933606
C11orf61 59.987045425047 58.6249721386149 68.6095266659765
54.4586176399381 72.768998788927 60.9822577063534 56.393737074538
56.1404433602127 58.3564461855595 60.7495043977707 60.5803031036931
66.53831442217
C11orf63 65.2427283850219 52.1379596312084 54.182676064099
65.6165636276827 54.182676064099 47.7556758818053 67.6011992144113
89.0275988550016 60.0070278969795 80.6966079421763 67.4760456862775
67.8774399765179
C11orf65 41.5421429082231 43.683792219729 37.0642268327439
54.8412943008635 40.148780650831 42.750861693377 39.1539892144371
45.642304356051 42.1594905198929 40.6022003765153 41.7272875295469
45.1735152121056
C11orf66 43.0001432913058 47.6229101519945 43.1931731665131
44.265834798964 46.4343327861696 47.8636003588181 42.629412460409
45.0972184964705 37.7384958256497 46.441301934328 49.532546810992
42.9887069977696
C11orf67 160.442808769594 65.8082111065882 93.1349769600388
83.014860007187 95.9075629352341 253.333114749013 163.995560004001
167.746168796779 110.983311402531 178.379117285008 147.151416108234
246.965844426351
C11orf68 93.1759373099875 52.8796369426118 57.1226931773006
64.2130157241811 53.8260140375214 77.0226403968683 66.5422130374814
72.121833189378 74.0614295574729 96.0236731791095 111.97030382494
68.9885481504003
C11orf70 43.844855336215 42.9358255168086 108.12482131423
49.5424425850549 95.3754438202863 80.6550905475507 93.8009890556664

87.0116305167987 92.6608453151206 58.7460488758053 56.6086202928268
71.2963721541702
C11orf71 66.2154717217675 59.3406363431414 54.1637401903523
51.0175677516029 94.8408264309872 67.3469281984384 71.5249259772583
51.4360013601805 54.4912806027381 52.3304586399695 52.7817052791259
51.8562635270111
C11orf72 43.5710871617107 44.3529919625344 42.9104454767916
41.5464765641928 40.6723334103791 37.9003288112261 42.4939802311136
41.4508241771315 41.863927676552 44.2580151149956 36.5359455830388
41.2437378488025
C11orf73 75.7342400123505 67.1108025189303 77.4246856935596
84.7275805640731 93.1238695478498 101.789162379972 74.466989900636
53.7378346199838 79.7061415085215 54.6089451252049 60.8201366212589
91.3723635213821
C11orf74 106.521012342668 104.492750153479 105.052418151358
134.394743648475 145.439350995461 262.624624672404 134.407474121421
175.41042153356 147.269661124015 171.043935516593 132.760054781304
186.866378606003
C11orf75 172.102568448455 79.7061415085215 62.3936376596431
161.48562926756 69.6330393135101 77.7481370715949 268.144525192544
49.2901038327894 92.9388175558241 53.5498255316009 63.7598547294225
92.3388129097482
C11orf76 35.8772145077273 34.8942481719111 37.7384958256497
37.4642807402741 38.0089018744739 35.9851865044027 36.3604951341921
39.3709357615493 36.0741672064298 40.7063426028871 35.6331082899192
35.8429598823818
C11orf77 52.5792653122861 54.113533179847 59.3549213146532
56.0794100106654 56.4685270445642 58.0095518210499 49.6872467499977
48.3590401011623 58.3564461855595 53.5498255316009 56.6884575165011
57.8111054584154
C11orf80 116.9812483023 86.4898752526885 115.256208610595
137.095435683619 58.5450868286167 62.1857038870283 238.697159169213
159.884578124679 103.139459009593 122.024487499513 91.5541137821803
79.3875629814087
C11orf82 83.0534943004588 109.943883439481 69.24946602508
63.3006977162657 221.325534445978 56.6172748471831 146.117756310709
150.829277770546 127.863441809606 43.7303813515864 45.2265319826548
53.0446815750355
C11orf84 41.0608773166058 35.5310164682026 36.3604951341921
39.7411455670172 40.3452320167286 35.8227939208521 36.3194282084339
34.1806289878747 38.3418933808844 41.7405682794166 41.7272875295469
39.9519547348106
C11orf85 40.2543341073001 42.9887069977696 39.6451483836089
46.6849259108754 37.10170285405 41.554942319225 44.9094465483072
39.3709357615493 41.6340461942423 44.7958122466628 39.1878770155069
40.6273137602368
C11orf87 45.0985088502363 43.2649781354792 37.0642268327439
42.6913548400127 40.8873907150245 41.7627910693483 45.3597029614942
43.3262148375525 40.4997186069494 45.2556760873335 47.4630625313159
41.3481355128089
C11orf88 35.2267149016596 36.5219488315447 36.769073068743
38.8495095797881 39.7568367479301 36.8059561571675 43.4544762830742
35.9023742468744 41.6616444147804 35.3849221682845 38.1835938174625
43.0930293831497

C11orf9 44.7044140979108 47.087835967892 40.1985790927248
38.0958223740111 46.8674366115656 38.6414316877471 42.9803717121865
44.1311654985682 76.7100878053735 39.3698811593364 35.9563099719284
39.6352613956998
C12orf10 256.892326800908 292.99497881914 321.31271583925
252.097980496533 296.664472126617 371.393488010768 370.027120412227
412.694325761831 477.741319532507 345.443895623911 350.584135047249
320.555790243104
C12orf11 73.4611178120841 67.9259304928255 72.1154333023225
71.9062462001972 93.0410625954471 73.2144205844199 63.5738862549306
53.1052228246467 101.036327583799 57.2813205536176 60.5125774827425
71.2491135258076
C12orf12 45.8108990837624 31.3542159583213 39.3709357615493
33.4240466670776 41.5668884168669 35.2067831864574 42.357910063876
47.8947595316832 39.853866204927 42.3606390470387 43.7585538437696
38.1421709520616
C12orf23 59.220967994972 54.1773909134233 45.7968648044613
53.593974682612 50.5110359971301 59.9876575158981 53.4921736109774
61.9787117490846 54.2825877218965 63.1091746640396 54.1963147679251
60.4872675915085
C12orf24 59.046385957117 43.7644428186265 48.7039705048129
50.5310598656998 177.620987812591 104.80882886573 72.5956443098684
43.1567024035183 47.1744296124496 49.3000844711116 52.4393177875001
54.9919060580109
C12orf26 80.8285585008048 93.0251525517037 137.696331574552
116.852483297949 86.1633564934019 125.81889994067 94.4618437533343
65.0656365694468 135.19133529995 164.176142753482 146.840603547323
114.935101645207
C12orf28 44.1294459089314 43.8495248991589 44.3344900481112
43.3078542962968 44.6834648302383 42.9695585740813 42.9803717121865
45.0972184964705 52.3579564953125 47.9056710878325 48.4717951775049
44.6484005362281
C12orf29 72.613397507717 104.824641162138 70.0955779023465
83.6935756500468 82.6157566292355 145.170590045628 106.140215438654
56.6910078150745 97.1271615802556 104.763913120468 98.5609311953771
119.512426152446
C12orf30 69.8899273527731 71.6537776654915 70.0567634568557
59.0262390168156 118.251739134889 63.0205913130827 80.1461294346211
54.2877981508636 87.1819433915847 71.8314470868658 78.7260936224802
67.363348155206
C12orf31 122.0305509548 145.391705645319 96.213791081731
103.961956810572 163.809567262808 148.144200968432 165.411942074375
77.8946473414547 194.046072415847 86.0832675838966 91.036114822363
129.268600210689
C12orf32 97.8141040140486 167.05554959607 146.087016079575
117.539037885384 112.506613718654 83.0554837248934 214.073500272341
168.366869831376 342.300139006451 119.015431849218 111.186223357396
124.998771394658
C12orf34 57.0006212637829 48.9259041798949 42.0621425894624
41.8778873678551 41.6806984441849 41.0582900711341 48.3411413510851
47.5950227477807 54.1745577382276 42.785429206422 41.9908237694988
42.357910063876
C12orf35 76.4439037944547 80.6083247572711 65.9098543816057
177.27613997459 71.5345397102823 86.3185927557402 78.9900928241723

59.2720151666582 70.6311537856734 59.9876575158981 59.7044210110693
65.564930068976
C12orf36 178.881586334601 45.5842286511645 41.6977706798234
44.265834798964 48.9627346956003 38.9378717994178 43.5627649099623
49.085741080241 54.8946088631931 38.4187686307607 46.3370338074996
37.0953622406687
C12orf37 41.7397731376692 45.0115870036541 39.0775870687758
42.1273928203021 48.2748123114598 47.8636003588181 44.8643898647322
47.3791873993182 52.3579564953125 43.2988017098387 39.8184668132023
46.7182294568603
C12orf39 40.2543341073001 36.9994058279362 40.4070046183108
37.7469621225553 45.6895223823001 43.7630587657205 39.8695929627783
44.5029690493211 38.4344387289956 39.9948390976317 45.9104478407175
36.5474283500601
C12orf4 78.5216977073842 78.9203273221608 68.4720753153522
125.801834682035 86.033725825968 100.650129064849 84.0691608193405
58.8022152133979 86.5616422570166 66.7299966077909 62.105802634526
74.6734358603424
C12orf40 38.6454098044474 39.8369676404424 37.8386378737561
37.4585919849752 38.5185220021014 39.9871741554388 35.6561235063937
39.8048943625718 39.0775870687758 40.2669497398702 42.4956274276424
42.0424641526302
C12orf41 373.714692628967 238.205365476671 242.751164798133
250.504350952583 320.889609846069 291.486402879057 342.475405815033
414.539381761279 436.757445186987 357.182869779274 294.567137562864
251.374750265492
C12orf42 38.957687788868 35.3307157989724 33.0260566875355
36.6501141438378 32.2133093774284 33.0463640202858 33.9750800030909
34.4896350146096 39.5806465938526 33.8984830800739 31.6063174764616
31.372171135652
C12orf43 79.953657052127 68.456297913777 75.7464604972652
63.25187762908 71.6537776654915 87.1735705257803 82.1144251872199
69.1973371867031 112.900479809091 84.2434917159728 98.4699679900651
87.8967913380047
C12orf44 919.542051089664 310.461601607662 323.453481668415
430.450581874766 322.440680131096 330.241189572237 501.817654561915
762.855719373733 611.538630852232 327.455030020799 344.985739524681
288.065258507995
C12orf45 94.1967497986791 95.1423940686828 134.537040608687
127.225812893692 118.748969134194 99.7445419301229 118.089250727398
116.547937420457 148.844652125095 78.1683130800805 71.8314470868658
105.382126899524
C12orf47 341.302185430613 261.023196824723 248.861100647206
194.395766807005 472.159979716705 258.30549459038 286.927016665685
269.963186760811 339.52415679299 313.233036704221 288.508774004052
326.902009704746
C12orf48 52.8427643366617 76.2876332597671 59.1533197797534
61.3149617644002 92.5135737873857 55.540888910486 74.7534423194688
69.0819394834919 90.1430468048409 42.8936990772838 41.6336636285875
46.9867599298218
C12orf49 74.6776631832355 91.4990733687068 68.8098607236981
80.7168944828052 75.3580625318157 55.3202238943712 65.3694267397114
54.1731035633439 81.2850357499192 55.9283039581662 59.0537479001842
55.4635297192076

C12orf5 78.5216977073842 75.2101993995344 98.9657264176664
106.20479137406 86.5513342734384 70.380703483689 61.0979269700412
74.2517888452553 67.3230935444252 43.844855336215 61.6084234634424
81.0169370911764
C12orf50 43.0462952354436 41.4508241771315 36.4564050256915
38.7879667686732 40.4166536885831 45.1873487463472 40.3302268293707
39.4322247076926 36.1457789072601 39.8873706855774 37.8372168909583
38.8834538955
C12orf52 291.5513298383 136.736085245679 109.374838860558
101.403739410832 337.156068385541 124.112216491975 163.20801480093
119.088986733238 174.443102016472 145.28255999556 117.586275152761
94.1128427766432
C12orf53 40.8681871019812 39.8695929627783 38.4043916290561
43.8002456102021 41.7929491793697 40.5281496800812 45.4799270968969
41.5464765641928 37.8319142847141 39.6719190054379 41.6336636285875
43.2776458914333
C12orf54 40.7609952437622 48.8584917918986 46.0727230219512
35.8472302065546 39.9697332660833 42.5660695551615 44.4639405506967
43.4544762830742 42.9020321355927 59.9354531144375 43.4709415392696
42.0424641526302
C12orf56 51.7607563002005 80.6083247572711 44.696023817801
41.7614975630362 94.5055588176321 41.2567470068038 112.436153468759
73.0394191668858 128.436305192918 44.1379638573455 45.7328341667151
43.3744503319109
C12orf57 942.247437385029 986.40790007743 695.255317861845
852.291461317697 635.050535522135 1975.28674300761 1111.89793143647
884.036280777358 1260.45604038487 1296.28036613191 1305.35453202355
1261.18520360134
C12orf59 44.7044140979108 36.89524015739 46.6588655218735
56.0794100106654 38.8456942580061 41.9543948167375 43.0930293831497
44.4031868028216 45.6582729799009 41.6373771565199 38.3684202018524
43.8575940017119
C12orf60 46.3884811643547 43.0971787292711 43.3085358214154
39.7411455670172 42.7107928951766 39.066284740632 42.9191764437547
46.1159021593282 49.2248095024373 37.7598032934773 42.5426057453993
46.3411353377615
C12orf61 44.6231541342 43.3458769998118 41.6079958749402
46.7577982595088 42.6022652963326 43.4681646416585 34.3633615460307
44.2159020605784 43.6670755115848 43.6033221838149 46.0905079010237
39.8497227447888
C12orf62 204.344278991396 130.524308471577 118.467231612638
161.25239489071 114.674541610026 224.914980996667 235.514830602053
259.127235335036 274.259417498569 164.462951076718 169.970634268117
179.186381381821
C12orf64 41.3785995857154 41.2598458726002 40.4418875035651
41.8161064926185 43.844855336215 41.0582900711341 41.5464765641928
41.8417024632349 41.5358173001885 40.4518431978241 38.738305958734
42.357910063876
C12orf65 67.3816715975056 48.5905518770847 50.0748292497578
58.5406981871153 67.9032387662129 77.1317865202676 71.6890300780549
58.4200819624972 72.3095457386378 72.1090339831747 58.8961770381033
60.2550788540864
C12orf66 53.8856501960117 44.6918600441328 48.3847843498476
44.265834798964 46.6397391251762 44.3648993568405 56.2181240696479

47.9942520395434 65.4784797364469 49.8393069675192 55.6151308605445
53.9431600945419
C12orf67 39.6609238036815 44.1239711172417 41.2365089689635
51.0175677516029 42.0709558258414 38.1169703906207 44.0687697278114
47.2864663693787 43.3744503319109 42.4594149893798 42.5426057453993
41.6550823405646
C12orf69 39.8662795144616 38.6414316877471 42.5406888281114
40.7481155008596 38.3561682218911 32.2133093774284 37.7136011474903
37.7612949798895 37.3600014814388 39.9948390976317 40.2649309204478
40.4639532172338
C12orf71 44.3355304829989 41.8073910693411 47.4740726032707
45.1944222548351 45.3675390992851 42.9695585740813 49.1067959632109
40.4359177791566 44.8997235283254 40.7063426028871 42.4580504587581
42.2577689927563
C12orf72 41.1523487516364 35.6434124419782 31.9895827886879
39.5050383901884 33.2021437980841 42.0574848021044 40.9157352260901
41.1575777497576 40.6957787921451 35.2880714952489 39.2542963855807
34.5633623842083
C12orf8 33.8558232866797 35.2143190355414 32.2133093774284
35.1451084093692 37.2567512265363 34.4896350146096 35.0305278582179
37.86982638952 37.4540772310118 34.7308008643142 38.745552403895
38.6454098044474
C13orf1 65.4561038762234 78.0522475574761 88.4897895318663
58.9168466928565 63.8315840603904 66.7278011183421 48.1747307200218
53.6210959113222 58.0722340887264 71.7464249053513 76.0420039542595
86.7710020879725
C13orf15 966.803172864162 892.323580265478 1103.90449800536
2280.42953098693 475.881757842864 1455.64106630257 328.962069934895
781.923984640049 214.537530518962 361.158558609751 370.64147085217
570.824732318332
C13orf16 52.0299937917925 43.5873188565519 41.6977706798234
49.3654837541353 43.844855336215 45.0882356578404 44.4639405506967
45.642304356051 46.6944238390932 46.7646877499666 39.9070785037411
46.5650556481721
C13orf18 78.1313732870053 46.2677540712845 45.989212296035
139.679585117348 42.551356054931 54.2978574294868 75.0092868910417
45.5581356216957 41.0476065156912 43.1839109702714 40.2649309204478
42.7782216342806
C13orf23 163.360824752796 162.160380044249 103.104022477607
87.114638423256 132.473918053428 82.6709662235238 160.459991480576
105.111902911639 133.720493104769 93.6787442149817 85.6170780640867
101.540333720181
C13orf25 49.8472708021525 54.1773909134233 47.3861758958576
45.8844325913247 59.8102324370568 48.260931742163 58.6431245059655
56.6910078150745 51.5229124652769 45.3530683538086 44.8872784170036
44.5432192783161
C13orf26 38.385865183581 43.4396866157171 38.0057289062111
41.8778873678551 38.2496050953547 37.1317006162938 36.4098694708483
42.3110002461296 44.5678064264717 40.9558083259517 41.6657053704762
41.6550823405646
C13orf27 551.915864019927 586.735744054398 754.903348372161
786.57695312967 637.694633519528 689.738256133922 575.057786209737
810.382224368065 553.328261478032 720.183703120662 606.123659335794
750.151825182993

C13orf28 40.4639532172338 40.2493681589104 34.9849896310235
37.7469621225553 32.771863438151 39.3841861812262 41.8315602975157
38.4540849632254 39.5806465938526 37.920964499903 40.0766543123257
39.8497227447888
C13orf3 41.3387790895716 46.8954576545929 41.8751578329365
41.7614975630362 54.802736966312 44.3648993568405 58.0139597055449
55.8087097725119 60.4837400912239 38.1042454705119 45.9104478407175
35.1451084093692
C13orf30 39.266565827316 36.2103905554984 36.769073068743
35.0448343468967 35.0181575202066 36.6996187567892 38.0598374201407
36.9355798143571 46.8873421271259 40.5090849993014 34.8383995317036
38.4330755397458
C13orf31 42.1537476443423 41.4508241771315 34.1806289878747
43.3078542962968 33.6330903323612 38.9378717994178 37.4772922422734
42.4580504587581 35.9645119596869 32.9981302187884 40.0766543123257
47.5025821041537
C13orf33 56.921728372453 43.2649781354792 51.6159542017038
89.4504034432321 57.6791750431251 244.089971761037 40.5585676679482
60.2691630545162 44.5303781239209 62.8201270538435 52.022479439889
93.523394623588
C13orf34 105.914573205761 97.712117589297 82.2266112813039
84.6435656850129 115.827601697699 61.2387559102636 98.5663841503803
83.2873728545705 124.56803502087 52.3304586399695 52.7817052791259
68.3234331335073
C13orf35 40.5654775358427 42.7775752471711 44.2405728317107
45.7397504535169 46.5557478175976 37.1317006162938 39.3585350448719
43.6434576713809 44.7058233759912 41.4397539024976 45.6348103033479
43.6753843488695
C13orf39 38.385865183581 39.9697332660833 37.2838080935695
50.0526763693329 37.6527901109005 41.2567470068038 38.8417151851134
35.5798430594271 38.6322728994674 37.1275334308172 36.0481934423077
35.7451401627836
C13orf7 63.6767189846426 52.9603588177082 54.9071823326024
57.8277957325085 55.8173045043348 69.8832723783498 56.9320717885002
46.4672637826582 62.6386437936516 70.5704019848974 63.9617548533553
57.290967307936
C14orf1 35.5179217192467 43.9421822132391 37.1643693306382
38.2041059863839 40.3452320167286 51.3886035574416 37.3799542961839
37.33030683349 37.0126425326534 37.4302011516587 43.4709415392696
40.0557788909923
C14orf100 138.82907142482 193.196385709124 213.232693288132
164.435300801872 122.160438114932 110.088243267424 97.299696284253
110.440072569721 145.492831993859 140.063595285844 133.893108529325
205.314410185216
C14orf101 49.6241203903354 52.7493349111555 52.7005092555102
43.1847628331565 54.802736966312 51.294537308199 42.1607322031054
47.0940613507321 49.9951674107458 45.2556760873335 46.7386599624801
55.7067742610401
C14orf102 142.69744253318 97.2535496140648 129.414877519657
171.920449949522 102.49785018292 140.128033028434 153.081027338185
94.8717345601966 133.41667178516 271.275703732022 323.957165066178
172.252848888699
C14orf104 71.1521292570086 75.0873720488265 61.356483470053
46.8954576545929 78.6286416672119 62.6839349907803 75.164719839017

62.105802634526 90.2790229242453 65.5349012839562 56.0477607422828
73.3321469094819
C14orf105 41.7397731376692 44.1239711172417 46.7058093820839
45.8844325913247 42.269054475455 37.8102105794655 46.8613212048981
50.7364620410977 41.9635525160083 42.785429206422 42.0750889188255
46.4952206446229
C14orf106 121.542387231625 211.109957714241 120.123385036416
245.439289067575 122.160438114932 122.284299477218 172.015856827154
153.462055740758 206.031918669687 170.804822846738 126.920579478593
143.031555728753
C14orf108 101.560929859785 113.351710503778 100.708709225818
104.784718169822 86.6854406823303 96.2490896573253 77.5813459454823
95.2915739972533 148.887819162926 91.3418781023824 89.3456573431171
118.115557136662
C14orf11   39.4710743516517 37.8217989841918 42.4580504587581
45.5869891570251 37.4470183315826 42.157627240486 40.1235656101329
40.2382908228517 37.5469247328848 40.7063426028871 40.3452320167286
38.8495095797881
C14orf112 362.03448469738 306.746364062777 296.271895016431
343.139450863356 319.092568066532 279.001128345613 285.901316389161
428.307428028806 385.064309855006 328.788681417522 239.350719871688
419.271533050938
C14orf115 40.3478825593224 30.6802399384205 39.0775870687758
38.2041059863839 48.2748123114598 39.3841861812262 44.0687697278114
39.1652965788406 42.4540072551568 41.2276963324981 40.8225507267584
36.7762265039864
C14orf118 48.8333989709933 48.6795178347248 47.5673419710251
51.1313686723929 51.6433699009012 45.4882438975269 50.5252048155459
49.3217779272599 55.540888910486 42.8936990772838 46.8954576545929
48.4849194706125
C14orf119 43.4626049853157 33.5924201549249 40.0998167098057
34.7266486944713 38.3561682218911 36.2015763253919 35.3362091871364
42.5435283762933 39.7537274655115 39.3698811593364 46.3370338074996
47.8931773060789
C14orf121 47.5647821785529 46.8954576545929 41.9666541498638
57.8277957325085 54.4586176399381 52.7156971154405 52.7002314230604
48.4670130605028 44.3440839686558 52.7972156492746 45.390192977585
45.5800840647989
C14orf124 53.8856501960117 57.8876850634922 62.1893884200745
49.1786207920603 60.7495043977707 57.8944021095265 61.6544855697851
47.3791873993182 64.1267046511632 54.3554233228353 54.4140381300469
66.9239565096247
C14orf125 44.5282181324797 43.2649781354792 44.4311655729487
46.3203995372658 37.6527901109005 39.4953494583071 40.7910160811527
38.6454098044474 38.9006788676407 39.6719190054379 37.9230440150891
43.4709415392696
C14orf126 57.8765910878975 84.0691608193405 52.5353664902258
58.3475983630509 66.6118713143653 68.9562915755919 53.4921736109774
48.5681478665796 71.0211264570699 40.8970401536578 51.1483696410762
51.4968903591058
C14orf128 42.6036491382658 40.6380495994768 38.7745734037492
40.1804147506786 37.956732373465 39.0266821712099 40.9157352260901
39.853866204927 44.3440839686558 38.0078357808393 43.0016969050339
42.2577689927563

C14orf129 157.109085253328 95.7125018118399 140.369714831352
147.010700944686 94.9104200955155 122.904323796484 114.437872597779
113.064858029749 129.042495848264 94.3329263062576 82.2519263268941
123.868848519181
C14orf131 201.180226922613 111.409762290528 255.915599666591
131.57403028894 179.064697219173 144.785836697047 201.208357379329
178.881586334601 175.099998331811 256.766675299448 258.038442651578
141.447763308653
C14orf132 188.887559923238 56.2305155589239 50.7502704822135
94.7283425282671 56.9360840563854 166.648551193168 67.5206810636627
68.6704961638492 47.7086093893038 134.749803376372 139.385367339095
69.6021553604792
C14orf133 123.608921653229 65.6721629145797 123.597489107285
94.3229189690855 82.9322141700549 97.6084681131585 75.7342400123505
97.4421930425128 103.631449539809 112.551747307723 101.187156681366
89.9460400399039
C14orf135 230.286815921497 202.627009037401 188.236842521312
213.933347509147 189.890451486324 203.979798245524 126.711833914667
148.595294991115 158.685201053359 201.800572658224 238.077051775147
206.649471678786
C14orf138 83.7215159198897 92.8006484232438 85.8362180324381
77.5352427498412 71.4792239075993 74.7047606031323 66.8475050031365
79.0249585610565 75.7734167073594 77.9085946827079 72.7071934110997
89.9460400399039
C14orf139 44.9896851005259 48.5905518770847 49.7350276169552
71.5249259772583 44.2607789091176 74.7669841575348 40.0052661202207
52.407151732257 45.6582729799009 51.6778366958973 49.7363778831003
52.8455400026194
C14orf142 93.2557602398938 68.2521461582047 60.9621846036092
114.876346000655 108.779357041421 89.0495215957248 80.4479092568961
65.5820437889838 89.0203733091224 64.6446465846487 66.5063354430003
81.4881766238989
C14orf143 58.9201382292404 59.3406363431414 58.1840322066411
57.5437445697293 46.7508829977587 52.5330333302271 62.4317959077388
57.2878071731043 73.1640951333742 50.3910151654231 45.0985088502363
57.290967307936
C14orf144 40.018129854 37.958111840848 41.528370959196
41.4273122914951 41.1868052124344 44.4311655729487 43.8206425661746
42.5435283762933 43.1805016690297 39.3999056968598 40.3180310664156
41.7575097298927
C14orf145 43.2724556187214 43.2649781354792 42.8270038153139
67.7121534934164 39.9697332660833 41.6590564937735 46.0228732507872
61.3864512710242 43.8482802038826 46.1390887492649 42.170702295259
41.4508241771315
C14orf147 211.109957714241 133.329721547016 231.361506886859
148.964204638494 146.840603547323 350.917244438732 155.406719968961
102.085154509866 279.901217752341 150.46866649779 175.798140574991
207.540972220982
C14orf148 42.0532901689036 41.159706131351 44.3344900481112
40.8681871019812 42.1650127805175 38.3418933808844 36.634024014558
39.3709357615493 40.8970401536578 45.0155543555707 39.7342718293432
39.3575468494941
C14orf149 57.4376528157926 51.4660044978406 46.1987217359283
67.6034960135297 77.7714622826402 80.7563265626648 49.1067959632109

51.4931742685391 40.2615278292875 58.8605811695948 50.7502704822135
52.7156971154405
C14orf151 148.158262886253 74.621822948059 87.104092810486
86.9274372004388 93.7924763988692 62.6839349907803 78.8250642061439
74.5173894803763 97.5394336319916 75.9037617394486 74.1425714439424
73.6512983066046
C14orf153 1673.60994518271 1030.31910195147 1053.67279913417
1171.207937488 705.283979182062 631.632335640448 1311.88650141004
1718.44944271916 666.816487335088 2091.0611913538 2036.72286752819
1519.01001038342
C14orf156 1369.13346566895 645.114504971255 1067.08316477623
913.704049709264 1580.58484708669 1195.24078948059 955.088515235485
1090.86617066541 1601.52194699063 779.996851709716 761.922486104216
1445.73674428909
C14orf159 136.355036262681 303.281551940647 348.450810225807
280.034472186873 232.847812981531 359.381284202484 160.917024416961
123.689895848613 356.948763043851 723.853348464335 587.88126282119
465.266372824212
C14orf162 41.2424637338424 37.1685072112618 39.1799070031819
42.8235313007746 38.0598374201407 46.263826584899 40.5585676679482
39.853866204927 41.6616444147804 41.2276963324981 37.1059647239814
46.6397391251762
C14orf165 45.8108990837624 40.9731067463382 37.2838080935695
42.5664883002833 39.5595233162192 44.4619248757502 36.197424053556
38.1835938174625 39.853866204927 38.745552403895 35.046946054314
34.3409660445447
C14orf166 278.562093102424 266.79157803247 232.553783060082
255.42436389413 262.215263489321 273.032063533642 238.338646661062
314.992390390165 265.026710493466 182.80711310964 190.375732394043
329.763197973922
C14orf166B 44.6231541342 40.2093230376545 40.5386195997545
40.9141850646578 45.9104478407175 42.4580504587581 44.6809101024392
43.1931731665131 42.5864346233886 38.0540928703622 41.0808257270221
41.0657892043496
C14orf169 102.443884864631 47.3536182445315 58.2986825661503
53.9517841438975 65.8319449883221 61.3620062177524 51.1541843174981
70.7805103907761 85.6441969757275 76.1382217104599 61.2529834995512
50.0571060701893
C14orf172 107.538934056558 56.0477607422828 89.3568413748778
53.593974682612 81.552417816742 92.47006193987 94.4618437533343
110.746059249478 81.6508259258669 105.414552529943 113.504745041505
76.6707262000712
C14orf173 395.875593488613 363.002913525646 777.854349637516
300.186402341399 150.114520857436 225.329170139176 315.163147652199
546.287195443189 572.667024472672 324.126628444078 398.43592055172
452.969595001684
C14orf177 37.8386378737561 35.5310164682026 35.2101597814352
39.9697332660833 35.7973685350741 41.2567470068038 40.3302268293707
38.3684202018524 41.9635525160083 40.7063426028871 44.1148826235374
36.449101775775
C14orf178 37.7384958256497 36.6968906669565 35.5220586859301
34.7266486944713 38.0598374201407 34.8956628705329 32.6039814489621
33.6816578730693 38.3418933808844 34.1648535095605 33.5370783156523
30.9241129241985

C14orf179 249.451753363306 147.209004629384 212.530138323284
155.928417606775 145.972133104142 271.857378677296 193.782527748199
163.261867589623 159.306689057291 178.559242961756 218.313812956524
278.378459771943
C14orf180 37.1485601150873 44.9370339275041 38.9790184198145
44.7980740332174 44.3557408297309 43.0860383229288 42.4939802311136
40.6171774724171 40.4997186069494 40.8970401536578 42.7275315121163
43.0930293831497
C14orf181 42.1537476443423 34.9262464079777 45.3974838013476
45.7397504535169 42.269054475455 40.8634043028528 48.6395795163502
33.9687743313429 49.8304479381623 39.2623423164488 45.1432412071268
44.3521099857368
C14orf19 41.678190413467 40.148780650831 40.5734458834617
43.7410507935043 40.831207953647 46.574682061365 38.6243997507646
40.047314525032 43.423558862426 37.3179578037963 41.1638563416981
46.1592873342534
C14orf2 227.317566600324 92.2530518251152 120.401643722713
161.48562926756 247.676607756929 136.293724800658 156.56745342836
162.993316022946 192.861174667738 155.270252661048 141.672695206961
169.920726825854
C14orf21 55.0078788655089 58.2571968311834 54.6820069269589
82.0292880742189 66.1650385535549 40.3357777530419 61.6544855697851
48.272324215504 61.3733228199803 51.9547984751408 52.6960621424156
61.7502624458447
C14orf23 39.8662795144616 41.159706131351 34.1806289878747
40.7481155008596 38.1644569189523 35.0970060242246 43.0930293831497
37.86982638952 33.6288663955758 38.8507804383261 36.6298807161755
38.4330755397458
C14orf28 58.7404123614695 77.1737254584648 75.4317740345967
67.8978240198575 53.1884505015411 139.625593914573 52.2476631126873
53.838484549134 53.7442594289333 103.14577985944 85.6989061660348
104.335120306753
C14orf32 200.019523376661 255.521720396044 193.043344611355
253.980053738171 144.290966198687 196.065114892186 170.150808455621
171.705718494906 218.510816443243 147.945429978177 137.197328985851
211.625447303889
C14orf37 48.3451401499671 83.3729277601518 39.3709357615493
42.0621425894624 45.1899271235974 48.260931742163 47.5963750520409
38.9074189954657 58.7382738968248 41.2700204291848 45.6706505553383
44.8872784170036
C14orf39 47.0940613507321 42.5660695551615 41.9666541498638
43.1275490164761 42.8510579683751 44.9370339275041 42.8684958525042
43.7396619462033 46.824299585607 42.1622837416589 43.0016969050339
42.357910063876
C14orf4 359.465715235071 173.013258797349 176.07173250529
128.38299874379 254.023393889525 281.201604778921 257.325282051981
626.570239685127 274.493399403322 268.872239581507 406.964801954621
336.737571879156
C14orf43 100.83436298853 68.921126710047 83.3826639718546
80.2774079897733 77.0578241280903 95.1224420992193 73.0433648756396
82.1225676987279 84.3526980051279 101.01459505339 93.9708162269401
91.6777754418002
C14orf45 54.5358897781765 50.9895771651972 51.0175677516029
49.5424425850549 41.6340461942423 64.6446465846487 55.6268253440479

50.3972033014227 47.9035532886869 54.0374522273907 49.3592128116865 50.3388505346542
C14orf48 48.8333989709933 42.2577689927563 46.8267540220952 45.1944222548351 42.7982502551634 48.4758590019384 44.4639405506967 44.1311654985682 42.9020321355927 48.0211752672327 40.723990462047 43.2776458914333
C14orf49 40.0557788909923 42.0709558258414 44.4311655729487 47.2009420656854 40.2449343247539 42.157627240486 37.1643693306382 44.7044140979108 46.2027193115397 39.5637454855233 45.310660704531 43.3744503319109
C14orf50 49.4151347850687 37.3634897565502 42.7303139017786 41.4273122914951 39.7568367479301 37.3799542961839 42.0605906240248 36.43924066428 43.2761454096265 39.7835513496873 38.079222289614 39.9519547348106
C14orf68 33.4240466670776 42.1607322031054 36.6522278218553 41.5464765641928 44.6834648302383 40.244271403831 40.5585676679482 43.1567024035183 42.357910063876 40.8970401536578 38.8507804383261 39.0536056496869
C14orf70 46.7293238583888 42.6939394537076 42.7303139017786 42.0102671859606 35.3327761997046 35.4142595025086 42.357910063876 44.4031868028216 41.5635499110307 38.745552403895 41.6336636285875 45.0035308952466
C14orf72 50.1533437545239 48.4980626712175 50.3950981988762 80.0042268333735 42.7107928951766 39.8455060654531 42.1607322031054 54.3820590274321 39.1790517315259 46.2286164776607 47.5439485437351 44.3521099857368
C14orf73 47.1216490839161 42.2114134935898 51.1347974668251 46.614092935749 44.147557390293 40.209110303983 45.7452456977857 49.1399035787209 41.5296458719756 41.3641352453779 43.2551508830106 41.1554481567603
C14orf78 312.530335465784 50.854895889378 60.1555629233618 88.9113618165337 45.1137938810047 284.942182200489 40.8617699346501 57.2856683017619 60.1102859845739 231.990562664196 195.740109330843 120.309345127552
C14orf79 85.5362566454799 80.7198077526005 111.735319884512 55.0604256312495 162.151812425949 76.331185787832 95.096268847843 95.1037791424727 79.856662992182 113.46454061183 104.02653369229 72.9580216367379
C14orf80 112.351686948586 73.845912534353 81.2850357499192 67.7121534934164 106.951862149262 63.2343637481705 120.02765867291 175.60601369033 107.222176166001 64.6446465846487 76.0420039542595 55.2449003905956
C14orf82 108.022329297132 107.836867810972 99.2504397421242 108.991044950132 61.1216330559316 62.2818512646893 93.879589378061 120.046457114882 81.6508259258669 121.45753287862 118.10944558223 96.8141712830545
C14orf85 2689.80835574654 1539.35554229258 1769.18069954113 2393.42629339901 970.379875059112 1136.88710104691 2077.74475301595 2978.46032143413 1384.80615431656 3088.44063817936 3070.83113612925 2323.81110653289
C14orf93 131.277614500948 145.741469970609 128.436305192918 112.368910939752 127.760816230317 123.493308132159 145.267770598988 93.9880852748373 180.189935936245 175.028390974125 155.492099076744 176.138603484209

C14orf94 149.076166432567 83.9760574088975 100.205124008648
114.876346000655 69.7413896580273 108.39424630351 69.0037087543684
65.9143782252686 88.3335649692467 94.7110944331075 78.7260936224802
100.677422836631
C15orf15 640.407302868904 515.216890329893 295.371694979522
771.554891868456 622.435313940734 1021.71286017481 619.114937854476
753.491584605447 673.002926695262 887.118866402149 805.309720893073
957.682898193927
C15orf17 212.688298502868 344.38057912034 166.608353896248
101.008449241109 221.808325312905 205.55797140415 191.631209717054
377.847116683974 165.311139930529 426.015657750123 307.705431227338
275.096637886925
C15orf2 37.0320432433314 40.4359177791566 40.0998167098057
36.890925044696 38.1644569189523 35.9305494063137 34.4570158133896
37.7612949798895 41.1742244762318 40.5090849993014 47.7535797917441
35.1451084093692
C15orf20 33.0463640202858 36.4002279956519 41.785676764591
35.2143190355414 40.0599807618105 37.2686490268265 36.9355798143571
34.064698871541 37.10170285405 34.3674468322242 36.3390947880186
37.0953622406687
C15orf21 44.7463386786906 42.985929467107 44.5622847010474
50.766576799961 37.8305309577781 41.3553369544097 44.6032218589199
44.4031868028216 41.8225292427334 42.22763273471 43.9701242600947
40.4310982248029
C15orf23 66.3095900379311 86.0832675838966 53.0630927250439
48.309866878365 99.8699868526053 64.0354923953233 101.250422688816
67.9667104519753 80.7067505750848 42.233842834629 43.4424616940528
47.2131144508464
C15orf24 853.947671456736 716.754656773542 554.551745559065
1177.44875377686 908.328776521114 670.271164195182 634.023101883136
621.734974486654 651.969296681551 483.20917825594 510.379313524019
736.846933148564
C15orf26 40.4639532172338 40.3384318592985 38.6727842796202
32.2212308728305 41.6806984441849 37.0180934800573 42.2577689927563
38.6454098044474 36.4476883897928 37.1275334308172 42.4580504587581
34.9138625012006
C15orf27 38.957687788868 44.6918600441328 37.7384958256497
44.265834798964 40.4639532172338 39.4953494583071 39.5496902678788
41.7419877611072 43.6670755115848 40.8970401536578 40.1742127891824
41.6550823405646
C15orf28 77.4999637278723 72.0792554308917 64.0162246399972
79.1000897258245 105.739423435604 54.7083331985678 95.3754438202863
76.0709828871754 80.2112932898502 113.759627906335 114.215602320391
96.2739664178142
C15orf29 47.5647821785529 42.9358255168086 37.4642807402741
44.265834798964 44.0451487108213 36.8059561571675 39.6535880152834
43.7396619462033 44.3440839686558 55.2214187655851 45.390192977585
47.7962548556026
C15orf32 42.9104454767916 40.2493681589104 40.6737160001291
40.8681871019812 38.745552403895 35.2067831864574 45.6042625176633
42.9301944191913 49.641159251878 40.8970401536578 38.956280281655
39.7411455670172
C15orf33 38.6454098044474 38.6414316877471 42.9104454767916
36.0481934423077 38.4540849632254 41.2567470068038 46.4388841354122

40.5158867964016 39.4762546236449 37.6383530120621 40.8225507267584
39.3575468494941
C15orf37 40.9558083259517 39.0102066700839 46.6588655218735
41.6564075217517 38.1644569189523 42.6575535390785 40.2154497375715
43.4544762830742 41.863927676552 38.1042454705119 41.9083581529276
44.6484005362281
C15orf38 130.184962393833 81.1189042503645 65.6620676514157
106.20479137406 67.0163226819964 94.7980740999988 160.360785581153
89.0275988550016 78.8968326770219 117.502489192855 100.663054866186
99.6243435693941
C15orf39 189.737472782071 116.967446594767 82.3344892042646
180.012192630556 131.766252431636 151.522338087219 166.652850759559
192.700843009002 153.35286239647 105.118498939628 110.440072569721
87.6598268114592
C15orf40 62.7669079605627 65.084620579901 60.4809187893259
69.3525901554637 69.0975200641705 77.7481370715949 78.6402083787251
65.5820437889838 77.7907484334847 76.5375674421131 73.4853298204744
77.707180044659
C15orf41 68.0700069295224 108.39424630351 78.8357122967692
49.7350276169552 152.792457231813 126.128760255542 96.5030525416431
69.1973371867031 183.336550845401 131.471030729546 141.423857667761
165.311139930529
C15orf42 67.1387898740161 59.0709442090059 40.4882741869718
43.4410741327254 78.5141640924736 46.0364000143537 87.2351169088172
82.2266112813039 66.1448688879431 40.5090849993014 49.2206713037152
40.9683275553869
C15orf43 44.7915315085948 40.7952353240579 43.7085219318425
46.2462906014337 46.0977687311222 46.3715909653832 38.8417151851134
39.8016018354453 41.134180463103 45.4614234442527 44.4688999098904
39.0536056496869
C15orf44 71.6050174762551 94.8556191290544 73.6323065529916
63.9570596163747 125.559679916646 86.3888090084564 99.0815700721606
67.5709261701192 91.6649478634836 60.3768221891131 67.686778662624
80.7227211275429
C15orf48 151.652543512032 70.6467150815052 78.1683130800805
1145.59111340228 118.143444770806 52.5826607585468 260.232090974645
68.6095266659765 100.874437079608 40.2669497398702 39.5399188536544
40.6134262962375
C15orf5 41.4508241771315 42.0709558258414 43.4709415392696
42.2496337895968 46.9906120932727 33.4492509999281 51.0013344286851
44.5029690493211 41.5635499110307 51.2318935933116 38.5668610487033
42.357910063876
C15orf51 34.2180669723984 39.0894172982439 35.2101597814352
36.6501141438378 40.6723334103791 37.1317006162938 38.5066108855005
36.8400213077707 40.5912213523345 33.6902293666089 42.170702295259
40.2669497398702
C15orf52 348.206465194205 845.41973083225 411.254448823371
94.7283425282671 153.139323479953 330.737070535671 60.3928833351886
167.746168796779 215.955919625615 711.08269990525 543.084891192607
461.500892766266
C15orf53 37.4554876386402 34.3715326040967 38.0452862258803
50.2163892855092 37.5556299264699 36.4986986135769 38.8417151851134
40.1474543172332 38.1169703906207 39.2623423164488 39.8184668132023
40.0557788909923

C15orf54 36.1232327599945 34.8137085420423 34.1806289878747
33.0463640202858 34.0927410219282 30.8824580894159 38.5066108855005
39.0620487644635 32.4931776430014 32.0298369224145 31.1853651446075
32.8921051249019
C15orf55 42.6036491382658 40.4359177791566 36.1042012504758
41.4273122914951 37.956732373465 43.2805590023412 40.4359177791566
38.4540849632254 38.6322728994674 36.2435874904689 40.9157352260901
38.8495095797881
C15orf56 39.266565827316 31.7645650530049 40.6737160001291
36.3604951341921 39.5595233162192 40.9506062495231 42.2577689927563
35.4526946152955 40.3316153483289 35.195364621675 35.3362091871364
40.3691324547049
C15orf57 61.356483470053 55.6972661824064 47.0940613507321
76.9373289454707 58.8961770381033 60.877229406755 45.8980298854198
49.7309743973184 65.9254098858352 56.1912096308988 53.8856501960117
78.6048653630165
C15orf58 42.7150227340364 48.4497719907777 47.4740726032707
41.3027031037343 42.9090346219023 47.2009420656854 49.1067959632109
44.4031868028216 47.9035532886869 49.7259371799014 43.4709415392696
40.5703581862752
C15orf59 52.6960621424156 45.2556760873335 41.3366779545815
43.5627649099623 79.1039287828151 94.1128427766432 44.3440839686558
50.9933795450314 60.3977304934451 70.9702772728923 68.1513215633609
60.0940570991178
C15orf60 39.4710743516517 38.3322849790657 38.6727842796202
43.4410741327254 35.7973685350741 34.6977013306396 37.830876898596
36.8400213077707 41.4636047919453 38.528621456378 46.4205654140754
40.4639532172338
C16orf13 118.05982513603 165.220069268347 160.171133984141
114.41703170676 186.697432898147 154.355197155161 179.629622763381
90.3531863862054 162.160380044249 124.56803502087 132.172127966602
107.337113678019
C16orf14 65.0721641331632 77.1737254584648 80.1665291227124
79.7937658787441 117.389308836013 94.1128427766432 79.909124697198
43.9366081640576 96.7422527189063 59.4309046716701 84.8808332740321
80.3500323413132
C16orf3 39.7576136507593 44.7829632472754 43.9450023643394
38.9534635879195 38.6454098044474 39.4953494583071 35.9058393060637
41.6466169548846 38.7298222155532 37.4302011516587 40.3452320167286
39.6352613956998
C16orf30 134.113913870501 121.542387231625 244.498602338731
140.175068871414 117.893268074013 175.74424749223 45.8980298854198
68.8058577447202 51.0062122023297 230.486681581762 282.035361446993
114.224091812993
C16orf33 310.255767356827 299.524171071613 263.765914589758
230.197573134703 408.080620074472 272.663785805238 404.524605612471
172.433936159136 380.988918630713 290.710970844525 251.2365884576
208.379981648601
C16orf35 94.4340798026852 113.26670675749 143.068901273486
94.6432868887401 128.89714160809 82.9449579428926 134.489077146096
82.1659654020827 138.67794356779 122.405413080836 116.663501139305
90.8550873130713
C16orf42 75.2475238554276 73.5534417023453 97.5849495690144
66.7750249658122 88.7928773498158 83.7019398654324 81.0331497830643

54.0507720792049 79.856662992182 95.8075612515186 95.8149149211251
76.4580830432622
C16orf45 49.1868644496737 41.0678614026819 60.6567691532561
41.7614975630362 45.4751076037511 103.569150754726 39.8695929627783
43.3262148375525 44.4395580493118 77.6056319020116 73.845912534353
76.2533853403813
C16orf46 42.6036491382658 40.6380495994768 46.4569566520107
42.6913548400127 42.1650127805175 38.7340969861754 43.3262148375525
44.4031868028216 40.8970401536578 38.8507804383261 39.9070785037411
43.9541718254769
C16orf47 45.1912496459517 46.3635803515159 50.0748292497578
51.0175677516029 47.3172462673426 42.2577689927563 55.4004611309636
41.7419877611072 41.863927676552 46.8954576545929 48.2050207071554
40.8681871019812
C16orf48 93.9756797434207 149.213844580441 211.468527316402
108.168107241759 149.665486488005 203.178221073902 227.059460958349
154.633672616367 172.39907939484 188.13862794196 224.14182624489
176.670909065664
C16orf5 49.6241203903354 49.1283590719461 100.205124008648
61.3747706295648 46.0043807840705 78.5276518966943 40.9157352260901
44.797320243931 45.3756389170606 78.0658258913875 68.6199296680241
68.3234331335073
C16orf50 39.9697332660833 39.5573415356949 43.8495248991589
38.9074189954657 46.2478069975709 38.5252456306656 39.2533668167483
39.4710743516517 40.4518431978241 41.7405682794166 39.0788811357542
41.0657892043496
C16orf52 44.2350046179299 50.6774883370457 47.3861758958576
46.7577982595088 68.4323624797175 51.8103411703261 45.1017494688414
42.0494116081998 50.2223403889892 53.0357938671646 46.0905079010237
53.3059058105836
C16orf53 105.212030988223 85.8945498067934 79.0962508551502
83.6935756500468 247.134338953977 126.659160169574 137.239944506217
197.540681021755 102.771611905219 95.92436282414 97.6552689677199
95.9391851122771
C16orf54 42.1537476443423 41.4508241771315 46.0727230219512
37.29566077996 37.7576900881741 35.2067831864574 42.1607322031054
51.8347877274018 33.6288663955758 42.3606390470387 37.0180934800573
46.5650556481721
C16orf55 42.442231667357 36.3110701990588 44.3344900481112
41.0726336841311 36.7356360093603 39.6251842352088 33.9750800030909
34.2814697823693 37.8319142847141 39.7835513496873 38.3684202018524
42.2577689927563
C16orf56 294.966924715506 270.349279607582 585.421238639853
217.976796656266 254.920345078558 199.660904693786 284.105466865264
342.73665541549 176.43047680293 339.767107648884 323.705254926762
204.774090707986
C16orf57 145.694779669933 120.418406532204 144.281881262975
140.894046694804 104.704892803169 91.4446109489387 132.541333041312
111.714563124738 88.4594321382477 85.2634032339854 83.1874704625702
94.4769283676944
C16orf58 538.153124145211 640.407302868904 789.511653564341
335.249352041342 513.57613902662 431.168903637888 334.170764774986
808.857027942253 308.183340806373 585.054570630252 542.069156178791
495.705224781189

C16orf59 93.3605294458382 96.981661155293 69.6554376880691 42.1273928203021 120.309345127552 45.4882438975269 113.039124313172 68.8058577447202 102.962105111048 40.5090849993014 41.6336636285875 46.917149434588
C16orf61 898.668526777346 576.140066807763 776.670493028756 659.666977039793 721.266942608349 460.162024551004 982.428740433235 666.816487335088 922.047328788961 463.84209540456 496.791391622565 704.601607617837
C16orf62 48.4849194706125 52.1967540852276 70.4794854241035 71.7167037224069 58.6481644777552 63.7818442618767 45.6042625176633 48.6441988290952 59.7483827511172 57.0581769379589 64.898351831341 59.2035499250243
C16orf63 319.596352961751 284.241284057415 357.111253129662 246.274692971423 227.504982571709 353.751797948675 216.606583011866 297.701126167694 298.988745247509 278.140612054333 277.665554240228 302.143385957116
C16orf65 33.4902421268456 38.5252456306656 37.2838080935695 42.4580504587581 39.0564222526273 32.4301586830653 38.7337086062875 33.7818132446042 38.0213051227859 32.406093465115 37.1059647239814 35.8429598823818
C16orf68 139.311807081199 168.426372075344 183.584085676174 171.043935516593 140.309952483162 167.319747725488 144.046404221564 145.050655626219 173.891042920642 129.213574379351 137.302487233881 120.149188463323
C16orf69 40.3478825593224 40.5400384479766 42.157627240486 44.9370339275041 45.2556760873335 47.3791873993182 47.8947595316832 42.4580504587581 43.9450023643394 50.5252048155459 42.2618615571566 41.3481355128089
C16orf7 190.675425748381 131.917451557976 263.765914589758 259.43283734195 81.929796716054 114.769498252688 146.117756310709 119.41355955168 152.441506175141 121.36340610316 114.5696064637 98.6039579639704
C16orf70 125.891602778425 75.885672222265 106.912316023598 80.4007868743886 60.8550020364269 71.2787833369055 86.6798536307388 78.6911549149983 93.2557602398938 77.268323196327 86.4195039096886 69.1099722732115
C16orf71 35.8208542506595 40.5400384479766 34.1806289878747 43.5627649099623 37.956732373465 45.0882356578404 42.629412460409 41.2551966132486 38.0213051227859 41.6373771565199 39.9070785037411 43.2776458914333
C16orf72 143.443713592074 120.001312008518 145.711534348895 235.544416908354 133.765486891316 165.107387190373 93.9805435116156 162.513675180449 139.328628553223 204.710892826638 182.866502637283 220.426780242132
C16orf73 40.3478825593224 41.8848959280916 41.9666541498638 47.3574589211166 37.8679241954337 43.5752393599169 38.5066108855005 39.5466975353131 44.3440839686558 39.2623423164488 39.9899430628542 39.0536056496869
C16orf74 51.5888395924843 91.31756001203 157.463465828715 42.357910063876 67.6011992144113 37.5887129572848 71.0369238830779 52.5909491535773 153.279284223069 169.18132209076 123.190887747662 112.585860083115
C16orf75 164.262531918679 224.412339736769 266.005181829441 100.168016285684 162.30238363798 80.9821967801845 368.972794063004

401.45848063233 259.626847216881 105.414552529943 102.87193290903
136.011269830829
C16orf78 40.2543341073001 36.6002100641539 33.8558232866797
42.0102671859606 44.147557390293 44.3648993568405 36.08956004634
37.6557714476853 43.0930293831497 40.7063426028871 34.9428206949566
39.0536056496869
C16orf79 64.546412747238 86.6854406823303 68.7091708087448
61.2213001749372 60.8550020364269 45.1873487463472 81.454335582503
60.7942162859036 80.3805507705979 69.8941497035747 65.8748459696377
63.2699016184868
C16orf80 188.042978085327 264.161390133378 149.792346524401
145.526972410673 119.088986733238 167.319747725488 200.002687565519
137.720182545143 156.02592319461 189.47090669378 143.973063517706
206.857208722995
C16orf82 33.2552842693684 34.5023764006976 39.9101908547397
37.0732977341185 40.4639532172338 37.3799542961839 46.1634822993009
42.0494116081998 37.4540772310118 41.7405682794166 36.7283081422707
36.064718696392
C16orf84 42.8482181313226 53.6816179316072 45.270814840882
42.7431359850339 51.2024692590155 44.2607789091176 52.2476631126873
46.1265102517879 46.0679399375659 43.6701228397031 45.390192977585
45.7258895124195
C16orf85 36.6480005877129 34.7135536792489 40.6737160001291
37.8442841690502 36.5393857306744 38.2293008890613 42.0605906240248
36.2539950294089 36.2733735494625 39.8873706855774 39.0550139257658
34.806104801006
C16orf86 44.6231541342 52.2501726671381 64.5232087947596
75.4466520363487 49.452985143568 57.3797835448761 58.0139597055449
42.0494116081998 46.4861258030402 65.813547096222 63.9029785089421
55.7067742610401
C16orf87 112.551747307723 178.675242612997 166.734730009916
92.8026641003757 142.251426773121 104.914715637173 187.888403806895
127.596368103014 140.01041112896 95.5156363110528 128.559660070271
145.14768503072
C16orf89 32.9174234020987 44.0409178714232 41.8751578329365
34.8137085420423 33.8558232866797 41.4508241771315 39.0458123429605
39.7509165247419 37.3600014814388 37.6383530120621 41.0808257270221
35.7451401627836
C16orf90 38.4330755397458 43.3972849968032 41.3366779545815
42.5664883002833 40.148780650831 39.6251842352088 40.4359177791566
42.7965563818112 40.7847110517594 40.7620472083159 37.9553529562144
37.5556299264699
C17orf28 116.17375251898 108.649424858333 125.27802197261
59.0965219753805 79.1039287828151 52.2089306840125 119.512426152446
118.854313907627 105.140085606412 107.987729161223 90.9641417408475
69.1099722732115
C17orf37 53.4921736109774 53.2841144999258 58.4137028673494
33.4819739460148 55.6991707857987 50.0748292497578 60.2399886747451
48.4670130605028 86.8463723709337 60.6266092582071 57.6831182451733
59.6757573886627
C17orf38 45.7356815244513 47.765848710116 46.2625470410195
54.6089451252049 41.9842695818285 43.2805590023412 40.1235656101329
42.357910063876 44.0519245668737 43.1839109702714 39.6403089864678
38.8495095797881

C17orf39	54.2648201582657 54.1577182755284 61.8701572127817
61.7502624458447 63.5441268694915 79.953657052127 54.5772630900888
52.9153129566242 53.3932110698117 65.7321440516177 71.6050174762551
70.9377012403023
C17orf41	45.9542131221291 44.1649412077735 42.8270038153139
43.7517118200398 43.2995113692066 43.5752393599169 43.2783959177939
44.4496572515025 40.2649309204478 40.6500508575994 42.253702372605
44.1861590259178
C17orf42	39.0620487644635 44.5152708955623 50.1860191642359
37.4223289687135 44.3557408297309 41.7627910693483 40.5585676679482
38.745552403895 38.9834959368496 39.4710743516517 38.2795150354677
46.8129509799753
C17orf44	53.7507429765038 110.960501850409 111.880226272362
120.807573842238 65.8319449883221 54.7083331985678 73.4820932957859
53.4109882045447 61.3733228199803 74.2379710965903 55.4694986901287
50.0571060701893
C17orf45	1595.69503047506 2581.34849558524 1501.67042548971
1426.87261062373 2091.0611913538 3010.38998829232 1634.61795848352
1231.77360353254 1928.53890809223 3465.3013599062 3263.05269150231
3421.24021192326
C17orf46	45.3872406432794 44.6918600441328 43.1931731665131
39.9697332660833 40.8873907150245 40.3357777530419 38.941855825269
49.085741080241 44.8997235283254 42.8936990772838 45.390192977585
39.8497227447888
C17orf47	43.0001432913058 40.6380495994768 42.0233072093141
37.8129870676592 39.7568367479301 44.2607789091176 40.2154497375715
39.4223544019653 38.2874316439311 40.8970401536578 41.6657053704762
40.1700117452801
C17orf48	58.2902084992082 50.8114699212931 57.2960245538881
54.8412943008635 73.6965494979349 64.0985088513857 71.5249259772583
71.6202385170491 58.3564461855595 89.3989848564067 72.842552380479
106.5477041881
C17orf49	569.244038644604 423.804472820231 440.282299601096
601.611039798626 352.339572553069 291.94914449218 850.205634919459
279.485290822376 305.119894105297 283.484056482174 326.500804513133
347.552309199761
C17orf50	47.1962846565261 43.3458769998118 42.157627240486
51.949834638817 40.4639532172338 41.4508241771315 41.1507980856436
40.931294690669 43.2761454096265 41.7405682794166 38.745552403895
40.9683275553869
C17orf51	46.8954576545929 41.0678614026819 41.9666541498638
40.1804147506786 43.7517118200398 50.560825686187 41.4742337714895
45.063792968927 42.4784375889044 44.3648993568405 46.1390887492649
45.1296099011771
C17orf53	180.475056805817 138.568626520133 96.0654949568324
66.7750249658122 237.754990288051 65.564930068976 236.139267415604
221.049503614433 203.373397813051 60.2877434874622 49.4593570700221
48.5123041297835
C17orf54	44.3355304829989 39.9697332660833 40.4070046183108
41.5464765641928 40.8873907150245 47.6803619768589 39.5496902678788
36.9355798143571 41.863927676552 44.4652605017595 39.2542963855807
41.3481355128089
C17orf55	59.220967994972 47.2055133646385 44.2405728317107
50.2163892855092 51.6869155634183 47.7556758818053 50.8388984085636

47.9942520395434 44.5303781239209 56.685925255591 53.9517841438975
50.5511827666954
C17orf56 61.5882375235292 71.4197504261453 75.1477470681238
59.6153684374766 87.9963662401769 53.4466111440611 64.5722284870633
53.1052228246467 76.2591822114739 61.8529316708431 58.9676332048764
65.1251519077199
C17orf57 43.9309845324767 44.0409178714232 44.3344900481112
39.9697332660833 39.4693871570028 42.157627240486 42.1607322031054
41.1575777497576 40.9901805728485 46.0119912782855 44.9713479185144
43.6753843488695
C17orf58 149.330073823683 195.672836308456 108.779357041421
131.431274187099 232.47874874281 502.435496018532 229.188779190649
106.917042272027 192.112101000423 193.758357282411 149.173801572239
200.203393423652
C17orf59 56.121460370892 56.2851430356168 76.3583555666815
68.4261608261522 69.6330393135101 64.0985088513857 81.0331497830643
63.61404854326 66.1448688879431 99.1438953564062 89.0684909049451
77.4769634299218
C17orf60 63.2753814090696 55.0897019602305 50.3950981988762
118.316810638061 79.0039595317374 67.8208976798364 42.0605906240248
47.6952179922944 57.7865070833351 44.3648993568405 46.0095710163378
49.7336769474446
C17orf61 970.379875059112 475.621740714165 707.4124863603
750.151825182993 762.388959943712 756.801598119483 1214.73798692268
1213.3477481374 572.968592371501 876.959744461441 867.856842601788
901.793120584383
C17orf62 385.36587480414 333.421080658854 338.966238813782
558.451033789799 304.977139992391 233.294970051779 365.792144095604
266.905855859261 395.875593488613 256.843419520398 202.060761076547
186.866378606003
C17orf63 134.285947677624 157.615972646659 135.00141569592
89.2503940049313 214.51120218554 105.197692490363 144.456117600129
144.515737827731 128.749209040345 111.703187941409 130.64028226556
142.012216533823
C17orf64 37.3463875295726 46.6314009405034 41.2365089689635
48.4916117779992 48.1473938886239 40.7616966228404 48.4859552186772
44.293922970134 44.0519245668737 46.1390887492649 40.547835538778
48.6037684272115
C17orf65 97.0254711947754 63.2804984998739 60.3973027611091
44.0451487108213 90.0942781313264 55.7197525213035 83.374777060444
72.5835488194631 73.4853298204744 72.961444366037 79.6198202883864
60.5948704279864
C17orf66 44.5282181324797 36.5219488315447 36.4564050256915
44.6905691366696 42.6022652963326 37.9951997351435 42.7431359850339
47.6952179922944 39.9444191873552 38.528621456378 43.6629145624937
42.357910063876
C17orf67 31.8228237796461 34.7135536792489 34.1806289878747
34.5425384901279 34.806104801006 34.7983399096627 40.0052661202207
34.806104801006 37.8319142847141 29.4322896559759 42.7275315121163
33.1559412791294
C17orf68 52.5307002738469 65.7457567201236 75.3761360028971
88.0827215704624 58.8961770381033 63.0324885262582 78.4937314423253
83.9976136901125 59.7863401969736 90.3041737295567 88.878414939392
74.2473868510319

C17orf69 64.1882546112044 60.3928833351886 67.0421310720934
37.29566077996 71.9737561190706 41.7627910693483 72.1401612718245
61.9787117490846 58.4891791448514 92.9894133685392 67.3976855661302
81.6458112867541
C17orf70 428.307428028806 594.812016966595 418.552186865503
312.045709918301 474.729653426958 252.870708814507 561.393215036856
506.585006700107 419.993482471924 555.727708387209 490.301992866742
356.88781087408
C17orf71 68.5457752893587 54.3554233228353 46.9040704638073
41.3027031037343 71.9737561190706 57.6034762511847 62.5506324864207
47.7889447429963 67.6983931011126 44.571915452417 52.022479439889
53.3059058105836
C17orf73 44.7915315085948 45.3486182278177 55.8810837854139
51.223859150798 46.0043807840705 44.8872784170036 51.5967098638347
49.9332280449549 48.8067321016961 56.8201201798712 52.3761286517985
55.4635297192076
C17orf74 45.5758521267508 41.554942319225 45.063792968927
48.665578118898 44.9936360641969 43.9724628401189 50.0088611244948
53.2046867327331 42.2577689927563 44.6918600441328 47.4630625313159
44.3521099857368
C17orf75 40.2543341073001 49.3581578587582 44.8872784170036
53.9517841438975 47.8075998240396 56.6172748471831 46.7100796279384
47.2009420656854 41.6616444147804 50.6486940895674 48.5292718280498
43.6753843488695
C17orf76 44.8914371498528 42.7775752471711 41.6977706798234
45.334106390212 44.0451487108213 47.5733603903505 38.7337086062875
42.357910063876 45.1899271235974 42.4594149893798 50.3876754565799
47.5025821041537
C17orf77 39.8662795144616 40.0527893189096 38.4043916290561
45.0798408927912 36.9702228618069 38.2293008890613 49.1067959632109
34.9580369221164 37.9273653326866 41.0057887014368 39.3667161654772
41.2437378488025
C17orf78 38.2083710281545 39.1982470626863 37.0642268327439
35.2143190355414 37.2567512265363 38.9378717994178 43.6753843488695
40.6171774724171 42.5406888281114 34.3674468322242 36.43924066428
37.5556299264699
C17orf79 125.997088252817 79.8196996067124 60.8942621888353
87.9098670472958 133.151333210366 134.884412058457 124.965915196815
98.4405692451144 104.103899614521 73.4447529556358 87.129027106167
105.74849082793
C17orf80 66.4399491500833 60.3928833351886 60.8357760968871
64.6945454069792 94.3450965454031 66.8632294055679 63.5738862549306
51.3472428383325 75.891637602914 51.3351387089044 51.6414075056938
66.8026147385682
C17orf81 62.0104160416544 55.8046062733709 68.878562323012
59.217781407734 53.5594683028749 65.1073309230686 62.1397042662581
58.4816190076338 69.6272083256793 59.0965219753805 56.9097893863465
64.7050732790408
C17orf82 51.5888395924843 63.7721358300879 61.2153068100932
44.265834798964 61.3077641493507 48.1591264215184 66.5422130374814
59.8760452220989 70.0801020070064 79.1074681877535 64.7598303406649
45.5800840647989
C17orf85 117.439175394651 99.571118823509 93.5717680539357
87.9098670472958 91.7629175712824 117.907440794926 134.908982962131

109.738537924214 84.2299424587647 139.328628553223 127.596368103014 102.605473054584
C17orf87 51.6558970571712 52.5868047927666 41.6977706798234 142.048367760597 44.2607789091176 44.8872784170036 52.7002314230604 46.0633179231318 39.853866204927 41.8613406887824 43.3085358214154 39.4490233720848
C17orf90 334.349204174886 263.330471155421 350.451704069255 183.286063142727 392.796524588309 181.2179249806 397.745727767552 231.060688044554 327.108892199594 263.832968124343 298.252355588344 193.676161965973
C17orf91 96.6833301748676 255.884444464408 239.023368693467 277.204818876581 111.877209777434 342.058245939495 79.5707751325986 148.906660152784 101.46584765758 86.7076796107636 82.6116645182046 157.979422616649
C17orf92 43.0930293831497 33.1817617228171 42.0621425894624 44.7980740332174 42.269054475455 50.4022543872993 46.7100796279384 48.4670130605028 42.357910063876 254.756894822368 123.689895848613 407.949645788215
C17orf95 58.6352379241827 61.2108152457954 74.9113162056974 67.5518078291428 79.271901834289 67.0577715520007 69.5544840164738 54.000164292461 75.7734167073594 50.4882744710823 54.0374522273907 66.991625911443
C17orf97 99.5578314630436 124.214669663291 103.746508465792 91.8207081349104 56.359578588344 57.9559082408796 119.846620703059 110.399579382196 74.9741052063425 87.7918574811458 98.8537074610939 76.3334723210842
C18orf1 46.7293238583888 46.9919960628953 41.785676764591 40.0698728584484 43.9450023643394 50.5148383400703 35.3362091871364 46.4672637826582 41.074824727477 38.2069499294002 37.7384958256497 38.1421709520616
C18orf10 104.573463811298 288.597205372736 181.381479219186 88.3459116251233 132.007307754378 124.112216491975 227.843903680287 134.83544047809 390.266798496438 144.239350177363 112.881899194726 159.506157112627
C18orf16 40.3478825593224 45.3486182278177 39.0775870687758 36.7892997857376 47.0954207417646 40.244271403831 40.6750540874299 45.2806199505029 40.0403898620771 46.2286164776607 40.723990462047 43.2776458914333
C18orf19 66.1229691315616 65.3694267397114 55.2141529491512 96.3741876853801 84.5572894783679 53.3185097662527 60.3928833351886 48.9826868640753 72.3095457386378 34.9138625012006 45.2265319826548 61.9036392303558
C18orf2 39.3709357615493 41.6466169548846 40.7620472083159 39.3750969348308 48.3814421939888 37.9003288112261 38.2933870238743 43.844855336215 41.6616444147804 39.8873706855774 41.9083581529276 37.4554876386402
C18orf20 38.957687788868 37.8217989841918 41.3366779545815 33.5578650644246 39.9697332660833 46.0364000143537 38.941855825269 40.831207953647 39.9444191873552 38.2069499294002 43.844855336215 39.8497227447888
C18orf21 98.4084364053686 133.401306624347 100.43365828852 91.1834104205004 84.8751572193902 89.8567957512307 108.709630036731 110.440072569721 145.676955166566 81.4621222177298 85.5839487225537 91.6328359149182

C18orf22 72.692416146584 98.1433318215702 101.020328028769
85.9087280306456 73.5541045639348 87.2437977005922 90.4957279507369
61.9787117490846 97.8009303343756 81.915829566737 77.6653462778309
108.082240435027
C18orf24 52.6960621424156 82.214837116747 52.3432130135266
54.1240583139871 57.9161791762225 49.2050538150106 79.1432909353319
56.9299697426319 81.8983491901699 47.0055981216687 41.2598458726002
46.1592873342534
C18orf25 138.027432399991 209.61080762285 187.862483922786
147.689479458785 101.738441694376 138.748233664345 153.544554979202
225.960701908311 276.699609951994 213.520663841371 217.721787448311
205.121208974511
C18orf26 38.1169703906207 34.15544299189 32.7043743065388
37.9875302985336 43.1252272136667 39.6251842352088 33.8558232866797
40.2382908228517 36.8127605061228 36.3612409063792 38.8507804383261
36.9976231206067
C18orf32 48.1952521496489 52.683711208052 50.5089777244781
48.9944486068347 40.4639532172338 46.4648868771662 39.3585350448719
49.9332280449549 61.4842573582507 49.6203214143464 52.2229654768933
52.7530873093837
C18orf34 43.4626049853157 44.9370339275041 56.4779645313958
49.5424425850549 48.5000946421718 59.7009992986607 39.1539892144371
46.7980335276155 39.0775870687758 47.1001757441025 51.0492389286271
51.4968903591058
C18orf45 61.2130609867477 66.5864907685351 67.1299518044849
46.614092935749 77.2608629053098 57.3797835448761 62.801431502597
74.0831311663524 60.2614254907771 54.6089451252049 66.5063354430003
54.293880614303
C18orf51 43.1805016690297 39.2907645923217 37.6356984043643
38.1392356671704 37.8329516992791 40.040778349137 39.8390535343912
39.2262196979245 43.2100129360085 43.881163603004 40.6373400186325
39.9519547348106
C18orf54 50.8875036861067 59.2251746728404 53.4088637045445
48.3772965749984 54.1745577382276 46.0364000143537 55.695361644141
51.0724630072723 70.8606919488782 52.8496345990125 48.4717951775049
44.5432192783161
C18orf55 197.660480001139 241.849566505679 245.004434952963
192.861174667738 240.086349973689 413.31638457585 233.501069644324
126.814751973459 368.793978305607 249.584588371496 256.892326800908
289.843931942051
C18orf56 131.859371655356 111.880226272362 64.8254097948745
54.8412943008635 248.963785838427 73.9456720099807 242.792365320043
155.099281008688 165.867229890877 43.9450023643394 53.4088637045445
51.6660481331154
C18orf58 45.4799270968969 47.4076235350059 42.7303139017786
38.9534635879195 45.2556760873335 44.7804080899431 35.7055410599146
42.6450179841129 43.6670755115848 40.2112371512838 38.8507804383261
41.7575097298927
C18orf8 111.186223357396 215.399634344016 188.670053901101
214.927272114101 152.159270481787 175.009248614892 155.287591646469
168.798543634965 189.80672643731 122.024487499513 146.483908799607
164.435300801872
C19orf10 268.309382865636 329.437593022939 208.97126697486
331.173265739654 510.379313524019 189.267281390459 260.766438659762

243.403622361932 341.666968456707 177.722828589884 167.352766082239
199.007437365676
C19orf12 138.599959012396 102.457537266105 89.9227845135785
105.515662220161 116.951729886784 119.253559091088 113.430943555215
122.824055084598 108.196445499586 115.155021677978 110.559251404688
102.288104112092
C19orf15 60.9710783023013 39.7835513496873 44.696023817801
40.9607305725196 45.5809132232681 45.3882138838137 44.7255207589606
42.4580504587581 43.9450023643394 46.2286164776607 45.7328341667151
45.0985088502363
C19orf16 41.5421429082231 41.6466169548846 39.1799070031819
37.651298468202 45.6895223823001 39.8455060654531 40.5585676679482
38.5457716679071 43.2761454096265 42.4594149893798 39.7342718293432
42.57175366709
C19orf18 43.7585538437696 49.2934904960601 42.640829914102
37.651298468202 38.0598374201407 45.7300501624017 44.6032218589199
41.8417024632349 42.7150227340364 50.8826620053386 40.723990462047
43.8575940017119
C19orf2 472.522115719366 709.730555484551 538.427311162325
326.963277148673 1033.99699402259 642.946369683508 555.356799014777
781.223434214089 589.395944599882 585.421238639853 523.25106120389
626.390544175817
C19orf20 51.413025495745 46.4569566520107 48.3847843498476
57.8277957325085 54.5055246687169 49.641159251878 49.8281348927244
48.8333989709933 60.6505672212119 47.1001757441025 51.1137916805019
48.3648494350286
C19orf21 68.1780382173625 50.4872122867759 54.0536858212253
63.0153175111747 48.8381507187986 49.2050538150106 42.629412460409
65.4714272652857 48.2123120250343 46.6598879466655 50.0808824383821
37.4554876386402
C19orf22 739.104939969224 738.66912772732 581.093069802913
976.566026758227 822.372309550325 503.529187388654 715.662312404643
582.017297217368 1078.86166154061 449.564281713066 576.830397705584
475.75173151481
C19orf23 49.9419897484381 45.9266745571724 43.0971787292711
46.8954576545929 44.8897737954573 44.4619248757502 54.339958888486
51.6660481331154 52.3579564953125 47.4767851750188 46.0905079010237
42.1537476443423
C19orf24 161.109600577001 167.678403380953 148.654594696193
180.648144492464 313.399524593316 129.675613712326 153.736638194572
197.598956071422 145.526972410673 183.311305255895 132.3192754634
103.071727621257
C19orf25 96.4033477961156 76.6936010119768 82.051509943308
96.3741876853801 136.04447103201 97.8052968915826 81.7434024567356
87.1561568256908 92.3388129097482 96.3495443565738 112.281286038716
90.9361642101537
C19orf26 42.7150227340364 35.8227939208521 38.6727842796202
32.554751989205 41.1868052124344 36.9060997041453 39.5496902678788
38.956280281655 36.8127605061228 36.9102644736882 38.079222289614
38.1421709520616
C19orf28 68.2184093305248 64.8903340853337 58.465017287283
92.6736029146947 75.0180570721975 44.6524308640784 57.51482768194
61.0831990510269 61.7559532093027 41.3641352453779 52.5930185400362
44.1861590259178

C19orf29 63.9851732498913 65.6010220593397 78.0013795192762
49.5424425850549 101.681822254799 70.1074782747874 66.3944891131864
67.3410712668476 83.6000059297772 76.1382217104599 79.1922819549042
60.4275887778048
C19orf30 50.4872122867759 46.1569135229922 42.8270038153139
52.1201052574162 46.4343327861696 52.2089306840125 48.3411413510851
48.7814131120945 51.2066664465185 55.6069214673486 47.9942520395434
50.5511827666954
C19orf31 11795.0477101407 6873.84878164654 8545.48005557056
7785.77078492993 7168.83136860899 4858.39551231606 9326.9018948838
9060.10165509099 6258.22561934986 8827.37298329149 8259.38594893529
7075.82842238887
C19orf33 7681.71833395626 701.168333137671 610.502562878788
81.6780581904253 147.388500451427 66.0069448981947 949.923102632043
1566.12726152736 350.584135047249 1698.18614771363 569.966632625157
867.856842601788
C19orf34 37.1485601150873 46.8954576545929 34.1806289878747
37.4223289687135 41.467005949568 41.8613406887824 37.7136011474903
43.6434576713809 37.1820543575123 41.8613406887824 39.8184668132023
35.8429598823818
C19orf35 40.9558083259517 48.8584917918986 44.8872784170036
46.1673159982664 45.9104478407175 45.4882438975269 37.6036146841933
42.2577689927563 42.0621425894624 46.6598879466655 42.4580504587581
42.8864415494484
C19orf36 39.1590964332473 44.9877531846933 43.0589430473426
39.0031581123233 48.7288537840562 39.3483872785093 49.6872467499977
42.0778399888414 44.4812539947185 42.6211340086915 44.6892782664938
46.0201052072883
C19orf39 49.3463416794995 54.6793272901524 52.6275131390249
58.5406981871153 60.7495043977707 41.6590564937735 51.1541843174981
43.844855336215 52.4539007817929 53.3059058105836 56.7765567227989
49.3087627580849
C19orf4 45.7356815244513 44.7829632472754 43.8495248991589
44.9370339275041 48.9627346956003 47.4699236780425 46.163482299300
60.9009676344258 42.1594905198929 45.6868589181768 47.0110068985119
45.4799270968969
C19orf40 57.934905173625 62.5528124447176 65.7670524353661
56.7927450229377 84.3040298294164 59.8102324370568 106.712703356565
72.9752827496503 88.4594321382477 54.1683282066029 53.4088637045445
52.3896785600751
C19orf41 39.266565827316 41.6466169548846 40.4070046183108
41.4273122914951 34.6227169056411 37.5887129572848 38.1793598983615
37.33030683349 41.4636047919453 38.957687788868 38.6662194645633
40.0557788909923
C19orf42 126.897002134472 96.5102825310977 130.313381811168
95.6215575902122 134.83544047809 163.405230398941 112.113501370522
123.219313750907 132.684895918889 166.648551193168 177.460053912279
147.902378647394
C19orf43 276.029709051798 322.509777895373 247.298165265093
233.294970051779 578.820199675035 434.523449093691 378.358853591082
251.210603834628 321.686871145301 186.64040927334 290.316757790733
275.314994786888
C19orf44 51.2851682978581 53.8499082625091 64.0162246399972
53.2046867327331 57.5685357492202 50.5148383400703 45.7452456977857

50.5070012045444 59.1223319112238 71.5032996449402 70.0965466297064
65.1251519077199
C19orf45 59.046385957117 171.360248172138 57.6371756925687
43.1847628331565 76.3790849853724 45.2852528185486 120.231435193839
127.961989181945 77.5480089703916 48.1392888470269 51.524711857679
43.5738557773903
C19orf46 156.636360703756 132.84476910529 152.679927202674
42.8235313007746 195.864827360933 55.7197525213035 220.36831243882
148.255880793905 185.874151272261 51.4454142248566 66.8188271427672
50.3118072462443
C19orf47 50.379587451669 46.0023068797812 48.7039705048129
44.1483976313959 48.3814421939888 50.4022543872993 65.2361525412756
59.7744650739434 65.3672880026114 69.6511571901941 50.3876754565799
51.8562635270111
C19orf48 126.826715174564 157.250548147161 134.152059562322
70.7202372217584 373.914455300185 115.277469628296 248.744491123257
164.96500173066 253.072560237983 166.233911320677 151.111664091983
106.20479137406
C19orf50 150.396608064863 177.29096218424 145.250323683031
177.985573759136 180.916465721523 232.048938339214 155.855834721836
159.52633805025 192.536546957155 169.527304857688 182.37433006793
140.01041112896
C19orf51 69.7442393277127 42.5269793679335 42.640829914102
35.9521941716188 47.6884372645373 39.6251842352088 37.830876898596
54.1731035633439 47.7086093893038 40.3235655331796 42.8263096141654
42.6758568421347
C19orf52 152.054785478928 157.394782571468 210.032782918825
101.403739410832 245.004434952963 122.599723509633 192.003788858976
149.515611848932 164.343309332109 121.557478857883 135.580139597457
159.747484721905
C19orf53 543.640122689871 284.620447030305 319.871452890512
398.332925006466 886.397061577531 456.023620287811 483.882804472844
594.611678976716 444.471597593258 440.282299601096 435.901460958456
483.20917825594
C19orf54 59.987045425047 74.4696354388511 67.0421310720934
58.7338159410728 79.5175547131776 60.4065050472082 107.325456368022
87.1561568256908 69.2001494999588 85.1950626054282 72.6731815563159
58.8188963227059
C19orf55 38.7537364570873 48.109747794187 44.8872784170036
35.49696651597 49.32293280121 45.9266745571724 52.0942589368478
42.6450179841129 48.2123120250343 44.4652605017595 42.357910063876
45.0985088502363
C19orf56 394.00931573564 367.451332073134 490.301992866742
309.698059657254 575.268446033282 431.353025968649 383.578947165143
586.281182112872 480.13416648493 684.238999553691 592.989860385203
476.653723741096
C19orf57 43.8582747295766 44.6360645306497 46.1653991089885
44.9370339275041 45.310660704531 44.2321845867179 43.1931731665131
47.0362584198466 40.8420925848403 43.0919221375514 48.7814131120945
48.1627097465883
C19orf59 58.7404123614695 41.8848959280916 51.7138050344355
1693.5377672895 51.6869155634183 57.0775902639215 149.551650453179
45.4757965005055 37.9273653326866 35.4990901121893 37.5583886316038
45.2783024981315

C19orf6 191.503068870249 368.793978305607 285.053017606956
176.07173250529 555.044139654886 188.647431971957 189.425423538906
212.117653688186 170.475212920685 181.381479219186 252.909964162809
167.49597550111
C19orf61 91.1363393944977 77.8946473414547 69.9179689232341
76.6232527679845 124.559758809231 86.6910280940418 110.223625398261
125.90746674767 123.933814581281 68.6095266659765 78.5325880060762
89.5687388187015
C19orf62 339.854688605186 245.996655628334 302.083293019113
250.359859946258 429.725397949276 283.484056482174 288.284384035759
400.093769705765 319.722393409101 242.282363793567 299.136348099827
220.79051545138
C19orf63 37.5100503181769 51.6527806785838 45.310660704531
48.4916117779992 51.4527545197672 46.263826584899 50.2016912396771
46.9986083378817 57.5430365699386 48.9088046212958 49.4593570700221
43.6753843488695
C19orf66 104.335120306753 123.281175151414 144.858109227889
120.068630086702 100.774098314706 92.47006193987 73.1654226019863
116.184007615285 84.9075858560758 141.960773054307 119.355990535521
119.124583755587
C19orf70 433.637087338002 675.079525434102 744.080606078086
608.886783362316 1626.71747158994 995.397944432196 643.71203221441
612.014486689487 825.558929673308 651.621028098039 887.118866402149
655.03569504267
C1D 38.1672336230186 41.690833180291 41.6977706798234 44.3440839686558
41.1507980856436 42.7107928951766 43.0930293831497 44.8997235283254
40.4220196471124 40.7063426028871 39.0550139257658 42.357910063876
C1GALT1 66.9417866448504 48.0296332183129 51.223859150798
51.5913529948887 48.7288537840562 46.574682061365 45.1017494688414
55.6904617926772 55.7522234735038 54.1683282066029 51.7538628098347
55.7067742610401
C1GALT1C1 58.4394991753791 71.1737934325596 54.8532558909227
71.0211264570699 59.0460742359145 58.0458175620469 60.5713059273145
58.5406981871153 73.3417666566438 56.9463858443402 64.4570366205389
78.5276518966943
C1orf100 35.5179217192467 41.2598458726002 42.8270038153139
45.7397504535169 44.0451487108213 34.5886782974123 37.0564573649223
37.4484310268909 40.8970401536578 44.571915452417 40.6373400186325
41.5536168238478
C1orf101 41.2424637338424 44.4411734611229 49.2041300973417
38.4208429103799 41.8806343997257 42.750861693377 43.6753843488695
42.357910063876 36.6312676076142 59.2628513357357 45.4799270968969
51.138877923954
C1orf102 54.9879584700715 49.8642691497064 53.8333108984467
41.9495387255163 48.3814421939888 55.5847026602643 57.8870110456492
67.0120793767211 48.9614101792105 50.3388505346542 51.524711857679
55.8087097725119
C1orf103 38.0648056183784 35.1224289925572 39.8299340037009
42.6222445667107 38.6454098044474 39.5581166303303 38.5066108855005
38.4540849632254 42.8036731479932 42.9358255168086 39.0550139257658
42.6758568421347
C1orf104 44.5798743103596 46.0023068797812 66.737740728688
40.4967168818294 46.2478069975709 45.9266745571724 46.0817481577316

47.2368301203382 49.1940901905636 55.3477556906605 54.9765783642973
47.5540235065658
C1orf105 42.7150227340364 38.1312950900467 40.1985790927248
40.9607305725196 45.5809132232681 42.8598805316863 38.1793598983615
37.6557714476853 38.4344387289956 37.8217989841918 40.9157352260901
40.3691324547049
C1orf106 569.244038644604 691.132727131746 1566.12726152736
78.1583955490018 320.504100222566 85.1544284060549 440.830948933792
445.875994521817 892.323580265478 600.34379987143 516.704027135707
408.739953606815
C1orf107 47.3289285415289 53.9634127270611 46.9040704638073
39.7411455670172 57.1187536733236 45.3882138838137 59.6201808414962
53.2046867327331 45.4154248354367 51.9247097592613 45.5707650534259
48.9906383294199
C1orf108 37.3463875295726 39.9697332660833 43.0016969050339
38.9534635879195 45.1137938810047 40.1428591998404 39.7576136507593
37.33030683349 44.7058233759912 36.6893251782922 41.8168470823451
37.4554876386402
C1orf109 105.621640443267 97.712117589297 118.854313907627
81.2269477373795 178.323615832679 117.127994102455 102.77259665192
107.971806573919 99.7668527666705 70.0907269565408 88.3086070495983
111.874193363836
C1orf110 46.1673159982664 36.4002279956519 43.401782064778
41.1886975241176 44.6834648302383 45.3882138838137 38.941855825269
44.8997235283254 37.2810767789306 45.6868589181768 41.8168470823451
47.6040671292013
C1orf111 43.0930293831497 43.9421822132391 38.9790184198145
35.6928955245309 36.6354013629791 42.750861693377 40.0052661202207
41.9315419791594 37.10170285405 37.6383530120621 39.3667161654772
39.9519547348106
C1orf112 101.103458852775 68.6804583979297 70.3613168347492
68.6379135603075 159.540626405 69.1523164481201 207.275014827939
170.644636226884 116.949813289388 70.3629652777935 77.0774476767901
75.1674447943072
C1orf113 42.442231667357 43.1805016690297 36.178547300352
38.9534635879195 40.8873907150245 49.9699131196797 35.9058393060637
39.9576138799997 46.1101825640108 55.7930787690756 52.5154971256566
59.3709786862962
C1orf114 45.1912496459517 33.6902293666089 35.6139301508335
46.3203995372658 39.2608760193011 35.8227939208521 31.098971689775
40.1474543172332 35.1155486926999 40.7063426028871 38.6662194645633
31.1150868775826
C1orf115 131.738693662183 95.9750691854349 82.4362183543935
161.25239489071 85.7842344710572 183.393353108744 156.061711961514
210.768576894328 120.001312008518 180.80065212092 175.193752938517
111.874193363836
C1orf116 129.865093249948 692.630044242985 555.727708387209
68.9038222450855 160.080366694799 68.9562915755919 254.756894822368
172.624182951104 249.246628143531 249.195312452368 209.551557135809
166.511915820497
C1orf117 40.2543341073001 40.2093230376545 37.3404647796601
38.8495095797881 39.6123051184545 52.5799250974039 38.7745734037492
39.5466975353131 44.1519466943185 41.7932258301997 41.8168470823451
48.27609330316

C1orf118 27.4352294118391 47.2770121962655 35.8265993915844
37.651298468202 40.6723334103791 34.1806289878747 32.9962272670005
42.4580504587581 38.9006788676407 37.0320432433314 38.5668610487033
38.3291713594599
C1orf120 43.6706764143908 43.2649781354792 41.1367478979484
37.5382094267692 43.1252272136667 40.1428591998404 40.9157352260901
36.9355798143571 39.5806465938526 37.4302011516587 44.3763859390287
36.7762265039864
C1orf122 184.536327776767 182.551901102169 175.619398238068
227.567182783564 302.667538432677 207.464632062656 224.665128374218
228.384073242481 171.191059267941 162.000275136397 150.362006499825
160.755701876741
C1orf123 507.620619345969 260.062749979574 286.340091833711
283.161835288074 241.08817915181 496.791391622565 321.069136463599
304.258586051126 308.480931502803 505.156485179198 379.79657860938
417.4609354305
C1orf124 50.4109031027914 65.1516921033166 73.0464199386528
57.6511495792664 79.1432909353319 53.7848321213704 58.7065473526185
51.1279389775667 65.3672880026114 48.6669787485795 51.2729241080159
54.5358897781765
C1orf125 44.0328992213727 46.4091752823403 40.439898672334
40.3180310664156 38.8456942580061 36.064718696392 65.0031119608637
44.7520317855773 50.2223403889892 59.8130420783374 50.7502704822135
44.1063440402552
C1orf126 41.3387790895716 42.4493032770053 43.7630587657205
41.7614975630362 43.844855336215 39.6251842352088 40.5585676679482
36.7460922671619 37.4540772310118 44.7958122466628 42.7275315121163
36.064718696392
C1orf127 38.5007204246293 31.1853651446075 38.8775560911841
50.5310598656998 37.3508690072156 39.3841861812262 35.8227939208521
36.3405039629059 36.0741672064298 35.4990901121893 40.8225507267584
46.4499258373233
C1orf128 439.770250139925 502.435496018532 538.716947884528
396.63268714181 963.224016480187 615.838713743621 633.385474385877
767.489657259345 543.268021785502 426.933064567744 461.394007153992
523.465208483426
C1orf129 42.442231667357 41.8848959280916 45.063792968927
44.6905691366696 39.0564222526273 42.9695585740813 40.2154497375715
38.4540849632254 38.4344387289956 40.1179210534118 39.2542963855807
42.1537476443423
C1orf131 178.525751043592 247.843927763772 315.276749671783
206.791464145857 231.685652845234 193.043344611355 255.521720396044
154.829574442056 189.245501343165 162.864661742501 178.789064142469
207.380865650247
C1orf132 39.5676051939342 40.3384318592985 40.4070046183108
42.6913548400127 39.3667161654772 35.9305494063137 41.9393838138203
45.5581356216957 38.7298222155532 42.2614524730856 40.8225507267584
39.4490233720848
C1orf135 95.2614267797703 73.1640951333742 50.0050760710506
45.8844325913247 148.99515914482 45.3882138838137 155.406719968961
244.226756210656 122.837001441551 34.8137085420423 33.4071187618571
36.8690774823319
C1orf137 50.2203578628217 46.5650556481721 48.8100349749783
50.7046706880325 44.6834648302383 41.554942319225 45.1017494688414

40.831207953647 43.7630587657205 44.4652605017595 39.9899430628542
47.1060577218993
C1orf138   41.5421429082231 42.3550738181144 43.0971787292711
44.4367387274922 36.9702228618069 47.9503724044453 47.4439407315335
44.7044140979108 38.2215591930186 41.4397539024976 39.4410515050885
39.2469742642337
C1orf14   36.3649677956953 38.9338521051669 36.769073068743
52.5307002738469 44.9936360641969 41.159706131351 37.0564573649223
40.931294690669 38.9006788676407 38.8507804383261 37.3687900733549
43.8575940017119
C1orf140   42.0532901689036 39.3999056968598 42.640829914102
39.1706564265495 42.4864576619427 42.6575535390785 40.7910160811527
40.7311497914871 37.3600014814388 42.8936990772838 43.401782064778
45.1735152121056
C1orf141   43.2724556187214 41.7614975630362 39.991353369336
39.3750969348308 40.148780650831 39.266565827316 43.8835874380262
43.1931731665131 46.5411372437515 45.3894119568143 38.956280281655
42.5365257225985
C1orf142   71.7624419474395 71.5249259772583 111.227016020927
92.999328769924 136.736085245679 123.032006903803 117.556281726503
85.5316122504411 120.679433440203 108.63277045284 88.2314365506494
84.2728210732532
C1orf144   305.199674259628 221.450737998571 219.878589678991
294.7772562683   282.196234758364 276.029709051798 244.070778149931
329.677454924062 287.932410610182 220.36831243882 246.09270373893
306.820897953626
C1orf146   42.6575535390785 41.2598458726002 46.7058093820839
40.4359177791566 37.6527901109005 41.1090481599939 41.706879587091
43.1567024035183 42.9413736814915 41.1222709786153 40.7725311293918
39.5792604499113
C1orf147   40.4639532172338 42.6022652963326 44.3344900481112
35.49696651597   39.3667161654772 43.8582747295766 41.4742337714895
43.6434576713809 50.6750785584772 39.7835513496873 46.6442692119997
41.0657892043496
C1orf149   60.3041738676245 53.9568457310634 62.6532574724345
48.856176220627 62.0967404944031 83.9004792286435 60.4593810615882
61.9787117490846 66.2552876795581 68.9562915755919 62.9336909080086
69.6579723433329
C1orf150   44.9896851005259 38.1312950900467 40.931294690669
44.6905691366696 37.0526522140242 46.3715909653832 35.8227939208521
36.0306975524309 42.8036731479932 32.5178504369282 33.9478220100351
46.7182294568603
C1orf151   45.1648925137409 42.0709558258414 44.5544727111028
44.1483976313959 45.4751076037511 46.852437033077 41.1507980856436
46.5717043290731 45.8325181856483 43.0919221375514 43.363245077835
42.7275315121163
C1orf152   41.9823787224629 43.9063490244748 41.0705134231814
39.1302941526825 44.7114813656401 41.5146360101556 40.9784333688086
54.2445399731644 43.2761454096265 57.8005007012205 53.8033413701278
45.7258895124195
C1orf156   40.6062943725118 39.1982470626863 39.9101908547397
38.6835649080278 36.0439235933921 45.0882356578404 42.22763273471
37.2237132058632 41.2424637338424 39.6719190054379 40.9683275553869
48.6037684272115

C1orf157 39.7576136507593 37.2810767789306 39.1799070031819
37.9875302985336 45.1137938810047 37.9951997351435 39.4600924554505
39.2672712041774 39.0775870687758 39.3698811593364 39.5399188536544
40.8681871019812
C1orf158 44.9896851005259 41.8073910693411 40.8420925848403
44.4367387274922 35.9058393060637 44.0804700754786 42.8684958525042
38.5457716679071 42.0621425894624 33.6902293666089 41.0050831223107
39.6352613956998
C1orf159 109.415404315559 145.741469970609 144.412165495719
69.8314582428552 114.230358276599 64.9498081060676 119.277678423944
149.173801572239 129.042495848264 108.905276555106 101.699631771662
78.6705291023488
C1orf161 46.1673159982664 40.6380495994768 48.4890615340603
37.4223289687135 42.3685136297603 40.244271403831 44.6032218589199
45.642304356051 39.853866204927 44.571915452417 48.6767028205398
50.0571060701893
C1orf162 199.776860110557 143.391384762612 219.616289279562
1062.93431683428 113.747633871556 400.506633704584 191.291480830946
178.98901256658 64.3425836997629 114.136234102574 119.808125185521
250.41086379231
C1orf163 92.3659612653822 71.4197504261453 99.1502352565568
73.1447139654971 118.638732417836 75.4227827818591 82.9431583622036
72.9074911694002 73.5541045639348 61.9882398986333 59.2106652388061
63.7744603935682
C1orf165 66.0303950772736 73.7873098190297 65.8455652907556
46.0228732507872 78.258486366912 89.680151549258 90.7037473522607
68.2309359831273 108.196445499586 65.3377940117969 69.7215372275927
60.8662233098324
C1orf166 70.083473392272 74.7625228047952 96.577572948724
87.4850071876743 80.8238125943267 91.4446109489387 78.3082773071531
85.4437963805637 105.434580236651 104.409397223301 124.350816245276
78.0586153423713
C1orf167 41.5421429082231 41.8073910693411 36.769073068743
35.49696651597 42.4864576619427 36.6271061451613 39.1539892144371
42.2577689927563 39.9444191873552 39.0648733099061 34.174642681781
35.6427313468066
C1orf168 41.0608773166058 40.148780650831 40.0998167098057
41.7614975630362 40.8873907150245 42.4580504587581 39.4600924554505
40.931294690669 44.4395580493118 44.0409178714232 47.3791873993182
38.3291713594599
C1orf170 44.7044140979108 44.1239711172417 50.5089777244781
44.550322123994 45.9725715538987 45.6129956328415 46.4388841354122
42.9887069977696 47.945438269111 41.9097689475185 45.1432412071268
44.1452943804044
C1orf172 92.9323002425821 106.058751335133 107.895235897156
50.7046706880325 122.0305509548 50.8533737532786 158.532307373857
98.3158011081832 144.633447485038 91.9185612536409 87.7844808613265
83.5069968129733
C1orf173 41.8409927585705 43.8495248991589 38.8775560911841
47.0874209746031 45.9104478407175 43.7630587657205 45.7452456977857
41.1575777497576 42.357910063876 41.4397539024976 48.1352470945488
40.2669497398702
C1orf175 40.9558083259517 44.7829632472754 46.4569566520107
39.6292788039793 36.2048942610546 47.6803619768589 35.7055410599146

64.2174369755376 32.8704945047196 92.9043863423147 74.7669841575348
51.0647893605839
C1orf176 378.626656077546 226.843444192483 224.14182624489
331.001195453225 190.993853866124 164.287737381703 301.139592414061
426.015657750123 208.67074469962 425.174170332175 454.939614583134
299.334388262225
C1orf177 44.7044140979108 47.9128504683618 45.2313723727673
44.7980740332174 43.004943518025 46.263826584899 42.7431359850339
40.5158867964016 43.7630587657205 47.611018474151 46.972521499515
47.8931773060789
C1orf180 36.1232327599945 42.6939394537076 40.8420925848403
39.5050383901884 42.7982502551634 41.2567470068038 40.9157352260901
38.1835938174625 39.5806465938526 37.8217989841918 40.4504608195666
39.4490233720848
C1orf181 243.015896514493 276.795797048951 433.637087338002
153.711194034581 324.877187846959 316.548339196872 311.301613620475
272.930242946726 122.504770242427 235.514830602053 250.832808506455
247.905335219826
C1orf182 45.5758521267508 42.3550738181144 42.8270038153139
41.5464765641928 42.9090346219023 39.0266821712099 41.8315602975157
43.4544762830742 44.3440839686558 44.4652605017595 49.1067959632109
42.357910063876
C1orf183 44.3754281580557 46.2691225551294 42.4939802311136
44.8813851439455 46.1606380564009 48.0121351688736 42.57175366709
43.2939209072046 44.8568725629882 42.0621425894624 45.5707650534259
42.57175366709
C1orf185 33.6902293666089 38.0226905622357 39.1799070031819
35.2143190355414 37.956732373465 35.8227939208521 37.4772922422734
34.5886782974123 34.0382693188794 39.1621898366355 38.5668610487033
34.6720534245631
C1orf186 57.934905173625 104.17651993553 196.29493890075
58.7338159410728 68.9766943946607 254.159017331462 114.947362505193
45.3872406432794 43.9450023643394 95.0026207727863 89.377425801133
53.1182952086284
C1orf187 48.5835484742456 39.7835513496873 40.0557788909923
46.0905079010237 45.1899271235974 43.3785777889265 42.3194600586894
52.5909491535773 39.4433784795209 42.8414096678708 42.2510526338368
44.696023817801
C1orf188 105.247736284747 81.2182827624857 172.237164781821
61.8997157694697 176.064476306748 67.9306669919176 72.8653205184014
169.322551237636 121.449647909665 116.311176407088 108.12482131423
123.022511916727
C1orf189 39.0620487644635 40.5400384479766 44.1452943804044
42.1273928203021 38.1644569189523 44.2607789091176 39.3585350448719
38.1835938174625 42.992884918158 42.5664883002833 33.7948557837079
42.8864415494484
C1orf19 161.38599012777 196.267504470029 186.450759425396
162.932479392087 444.544810510611 208.457587834567 206.261432452217
192.058030454066 302.79352975792 129.081183929988 149.792346524401
303.281551940647
C1orf190 44.9896851005259 44.7829632472754 45.310660704531
49.1786207920603 43.4544762830742 49.4157684139858 40.3302268293707
44.2159020605784 49.0986627051784 56.9463858443402 58.2315917325629
50.379587451669

C1orf192 38.7537364570873 44.4411734611229 41.8751578329365
44.4367387274922 38.6454098044474 36.0306975524309 39.2533668167483
45.1714698794331 37.4540772310118 38.745552403895 43.1931731665131
48.8670821164567
C1orf194 34.4021554788966 37.920964499903 41.3366779545815
44.6905691366696 44.2607789091176 37.1317006162938 40.5585676679482
36.43924066428 38.0213051227859 39.4710743516517 44.2142581488029
39.0536056496869
C1orf198 247.843927763772 261.195021649777 240.139560726101
106.582060920252 195.127419251015 264.786938886338 299.706257810523
186.655552987116 234.45840519702 162.864661742501 231.304658157247
324.658458599373
C1orf2 100.802977734227 136.517445122438 212.062417120966
89.2503940049313 259.481036467571 121.744701580607 138.23147386089
140.226992517573 111.845105854608 118.899737794318 106.165518810891
124.998771394658
C1orf200 40.0557788909923 41.4223563635976 42.640829914102
43.9744941494111 40.2449343247539 43.6670755115848 43.2721716572958
44.0390019242862 46.1634822993009 39.4710743516517 38.9240290061433
47.2862299990938
C1orf201 40.0557788909923 39.0102066700839 39.3709357615493
44.0451487108213 40.5710637884029 41.9543948167375 47.4439407315335
42.5435283762933 42.1594905198929 36.8059561571675 42.5426057453993
42.7782216342806
C1orf21 55.0078788655089 52.5868047927666 78.8357122967692
59.0965219753805 59.6922302604696 241.08817915181 51.1541843174981
56.1404433602127 55.5086026637973 182.246578375636 135.308001652191
122.160438114932
C1orf210 46.3884811643547 48.5905518770847 42.157627240486
40.4967168818294 48.3287893866569 40.3946758935842 42.1372626664516
40.5640952422779 48.1578509702472 43.8710182120571 39.3667161654772
44.6484005362281
C1orf212 60.3381680822012 55.9269493191353 70.0955779023465
62.6048727240291 68.0414563597154 49.8472708021525 64.0985088513857
73.8374823243007 64.4588422949237 64.428683021978 58.2941346100716
65.3585701203568
C1orf213 44.0328992213727 45.3022591001932 47.3381845358719
41.6564075217517 44.6231541342 45.2852528185486 42.629412460409
44.0390019242862 41.9635525160083 46.2286164776607 43.1931731665131
42.828737648529
C1orf215 43.0001432913058 44.2105195734073 40.6737160001291
44.550322123994 40.6723334103791 46.3715909653832 41.3594868055879
47.9942520395434 51.5229124652769 42.9866214102525 41.9908237694988
38.3291713594599
C1orf216 83.0534943004588 50.4022543872993 54.2670850492947
76.6232527679845 77.4769634299218 93.3718038663074 72.8653205184014
70.7805103907761 74.0614295574729 64.5347406608537 66.6788758487969
74.2748495231478
C1orf218 299.665413950641 135.540738841602 198.432987946699
293.040690378216 114.78317743597 117.250114037308 175.960838580876
319.043862813161 269.438510636306 301.904682664871 284.013140015092
195.765247956108
C1orf220 44.8914371498528 39.0102066700839 43.3085358214154
36.264823544143 38.2496050953547 42.157627240486 38.7337086062875

36.6452480995946 38.2215591930186 45.9004543328976 41.0808257270221
37.4554876386402
C1orf222 43.4626049853157 45.0905928998779 49.8344973836834
40.0698728584484 40.3452320167286 47.4699236780425 45.6042625176633
47.3791873993182 40.137818555383 44.1379638573455 46.2560238004757
49.5539622682049
C1orf225 65.3531590366085 853.464809072841 345.443895623911
58.0218262176065 90.385018479478 184.218705402964 90.1500004950245
94.3773551105819 131.717764850211 177.845412804098 231.304658157247
330.946598275585
C1orf226 45.8108990837624 41.7614975630362 44.7255207589606
40.3691324547049 41.9842695818285 45.1411314881341 42.357910063876
41.0381860307445 40.6957787921451 39.7835513496873 41.2267153672559
46.9867599298218
C1orf24 131.645714321903 175.74424749223 532.581631305218
966.803172864162 114.533239859288 3088.44063817936 215.452117252205
196.844428284247 106.590506599473 1224.80240464583 1524.01520893763
2101.59244895554
C1orf25 64.1267046511632 70.2288685232877 117.228320608065
79.1000897258245 89.4111455826308 70.8674195845729 72.1401612718245
53.4820309182878 85.6038071277319 75.8280474316969 68.0960719007162
79.7808217577226
C1orf26 35.9305494063137 36.0366673493555 39.4710743516517
37.4223289687135 33.7494761745342 42.9695585740813 36.5352572191451
38.4540849632254 45.0017158586743 41.4397539024976 39.9070785037411
39.8497227447888
C1orf27 37.8386378737561 36.6002100641539 47.7737810047679
44.265834798964 37.5556299264699 40.3357777530419 42.629412460409
41.3506832276112 36.5393857306744 42.0621425894624 38.6662194645633
35.1451084093692
C1orf31 61.5882375235292 62.6984594468418 61.5824518349507
63.0153175111747 76.209239524646 52.6226745117396 70.7238101239421
63.0748373122119 65.4784797364469 44.0409178714232 59.3379918468928
63.9371474698484
C1orf32 31.5599106749655 41.6466169548846 35.7105247506212
48.665578118898 45.2556760873335 39.0266821712099 50.0088611244948
40.5158867964016 39.9444191873552 48.6124127998952 44.4688999098904
43.5738557773903
C1orf35 128.827672077002 167.982626899395 224.60637398191
106.8866235778 184.303357812653 132.059215102854 240.934550408797
192.564048264712 189.737472782071 198.497745548245 200.401133392259
143.112509176873
C1orf38 46.6348258763289 44.1239711172417 42.9104454767916
58.1798515131249 38.1644569189523 43.6670755115848 42.357910063876
45.3872406432794 37.10170285405 40.1179210534118 41.8168470823451
38.2350720635382
C1orf41 120.129857395808 121.936597605894 124.551483147457
154.166621635012 135.737771384104 149.5664106654 159.571799428131
96.0654949568324 198.922575188741 111.248876636045 97.1271615802556
208.097209050425
C1orf43 176.224645007435 190.487609390723 197.923403667051
188.457593309678 186.195424728755 192.240593720489 161.205086002542
227.206974078547 135.106756607914 99.6544408669512 119.355990535521
160.755701876741

C1orf49 47.6397712007919 43.0971787292711 41.1367478979484
33.9645433044281 38.3561682218911 39.6251842352088 41.8315602975157
41.5464765641928 44.8003345643334 45.7911932481722 45.390192977585
48.6795178347248
C1orf50 439.955651727755 211.701234819986 289.716911324484
244.837105178705 334.438091900861 226.926786963011 282.38663225575
401.956364010068 258.913621521866 292.800712773283 278.562093102424
246.680724959444
C1orf51 53.8856501960117 45.9266745571724 59.8273072817267
47.8164193292842 55.9283039581662 53.565717255469 38.5066108855005
53.4109882045447 69.734208465775 61.4569050766419 60.394307846869
48.1627097465883
C1orf52 322.678762360476 195.836475280084 258.517751279605
199.522267292552 208.97126697486 163.048439401341 248.527031538874
214.980684661682 90.3916996175708 318.069132873489 274.081959734368
233.710655978992
C1orf53 191.394143505573 75.4732674021193 179.897685693038
59.2778887402357 188.120766710975 75.5561597374812 117.260454792593
68.2309359831273 182.411383422586 69.1623287497421 69.6097529955737
66.3944891131864
C1orf54 332.830805115112 150.957220787781 220.192552354287
669.776067547468 111.256949641793 547.729200972776 114.526825145076
229.159869771704 68.5599263000733 263.44529683749 260.405228196514
245.540041661003
C1orf55 131.738693662183 179.974187861013 169.790843803064
187.982920458868 175.922685424474 187.16833117416 180.128769312512
160.479699603187 144.500148287951 96.7422527189063 123.868848519181
140.961217055867
C1orf56 48.2910530887214 60.4646208319121 73.967017874248
46.7577982595088 56.0477607422828 47.6803619768589 50.8388984085636
58.3046521633424 61.1022920882065 53.0357938671646 53.2083147523758
61.5300817099814
C1orf57 191.291480830946 275.673080691335 459.182372959714
180.128769312512 189.678033532012 232.147792636393 252.487015579489
274.02972772427 218.165286111397 232.238647125646 208.7739716139
266.323732730581
C1orf58 37.3463875295726 43.2649781354792 45.2313723727673
38.9534635879195 49.5666074434383 39.8455060654531 38.4005795891396
44.8997235283254 43.9450023643394 44.7958122466628 39.4410515050885
47.3992733452821
C1orf59 324.506136847875 246.820301517765 220.192552354287
180.873540853324 413.620123676085 129.190555260332 344.44626908553
258.589426470951 355.00914527669 153.812414753676 143.443713592074
174.148714092395
C1orf61 61.9504984796308 32.8881500803623 34.4716904283753
40.3180310664156 36.6354013629791 35.5158505219112 40.9157352260901
39.7509165247419 42.8036731479932 37.0320432433314 40.3452320167286
30.7315474127249
C1orf62 43.1805016690297 42.6939394537076 41.1742244762318
41.8778873678551 41.6806984441849 41.4508241771315 42.7431359850339
40.6663433492193 38.7298222155532 42.5664883002833 38.5252456306656
46.4499258373233
C1orf63 90.8236750745974 95.6662924228901 135.997802304719
101.403739410832 149.19643034862 67.3469281984384 63.6695826053997

110.666686474986 93.310557793228 70.4895190282848 70.3381994440425
103.376225272425
C1orf64 41.2424637338424 38.9338521051669 47.2055133646385
44.6905691366696 43.4544762830742 37.9951997351435 39.7576136507593
37.9837233601316 42.5406888281114 37.7315666632629 44.553780984244
44.7401523707775
C1orf65 38.2083710281545 34.2618170189676 43.0971787292711
42.4580504587581 40.6723334103791 37.2686490268265 41.8315602975157
42.2577689927563 42.992884918158 39.3698811593364 43.0016969050339
38.9496515344837
C1orf66 188.61489831342 195.765247956108 320.812668695531
127.596368103014 284.575587310164 136.190823415283 226.090904721424
133.060894083935 228.703786640132 236.536058101486 264.88963894526
198.257381933305
C1orf67 43.7585538437696 47.087835967892 44.0451487108213
43.1847628331565 53.6907197364586 46.8954576545929 39.4600924554505
45.7476095696859 55.9423752286714 41.1222709786153 42.911855821602
40.5703581862752
C1orf69 53.4921736109774 54.113533179847 54.9944485073259
58.5406981871153 56.2305155589239 68.9562915755919 47.1345949868029
55.421880601441 52.4539007817929 57.4665857273093 52.7817052791259
51.4308501933893
C1orf70 43.3638918705225 34.15544299189 43.8495248991589
42.6913548400127 41.5668884168669 44.7804080899431 41.253645578517
39.5466975353131 32.4931776430014 42.6793753550854 42.357910063876
42.357910063876
C1orf71 187.733278812798 218.531478946522 264.263104842911
345.084002073846 198.54718783437 276.214079735778 194.570513601724
201.427008153788 210.768576894328 270.922452389109 327.024556075244
283.643027929014
C1orf74 62.9952378379206 55.2893984191264 65.5820437889838
55.0604256312495 75.9909523932333 45.4882438975269 56.9320717885002
67.0904616289706 63.8740412329753 62.8201270538435 51.2318935933116
51.9447450229707
C1orf75 46.8954576545929 38.0226905622357 39.9101908547397
38.5252456306656 36.8059561571675 37.7000400506818 46.0228732507872
46.5717043290731 40.7847110517594 42.4594149893798 46.824299585607
38.1421709520616
C1orf76 45.0985088502363 51.1740855632044 53.6837370963602
61.7502624458447 55.3100442506299 50.9724155054821 50.2016912396771
61.3864512710242 52.7512110769042 52.6879797806876 50.8826620053386
53.655233033193
C1orf77 308.568617575909 368.162822151919 327.631923082696
223.826005691932 335.655540947167 207.245428311682 494.231747291283
378.358853591082 330.241189572237 207.380865650247 246.313146942736
281.452208033175
C1orf83 77.3752541676627 70.1238785863433 62.0008474085033
67.8978240198575 87.1109435144663 75.2735539010032 77.118454956956
62.5528124447176 70.0801020070064 116.441764463618 93.4144098590192
90.9361642101537
C1orf84 41.1928222537953 43.387539499126 43.1746360109207
44.9370339275041 41.2740822675592 44.0399598874356 43.760806942409
42.7431359850339 41.863927676552 45.8471859855979 43.5710871617107
42.1957516560122

C1orf85 614.088027946471 374.167501183341 842.82310025167
777.854349637516 867.856842601788 340.429325650126 514.853892585612
340.812638857273 299.524171071613 445.421601813221 363.602775933416
268.265540230814
C1orf86 171.386815652277 188.943404504867 230.486681581762
189.890451486324 343.262403838579 233.00902546331 271.478148993789
210.880198736298 232.591262307238 179.285390561592 213.261004438127
168.225699005719
C1orf87 41.6354649482853 37.7598032934773 45.4634652319753
46.6849259108754 43.4544762830742 41.9543948167375 46.6267962186599
40.2382908228517 40.3662560853635 42.8936990772838 42.9634662926872
39.9519547348106
C1orf88 44.5798743103596 48.2220547806679 41.2679739649179
45.505885458784 43.2484996523549 48.6621527194963 44.5390641547628
44.7044140979108 41.7627910693483 41.5804111566887 49.1495645678651
62.7669079605627
C1orf89 45.5758521267508 49.6841415268611 48.7039705048129
39.5050383901884 44.7829632472754 49.8472708021525 42.8684958525042
58.676230030835 53.4604353973381 52.3304586399695 69.3645322033576
56.0477607422828
C1orf9 36.0551493501805 42.0546888913836 39.4916414839923
38.4571887275378 40.2062851265091 39.0648733099061 42.9176765336717
37.1781720867518 38.5583217392131 43.0156099182214 40.547835538778
41.5536168238478
C1orf91 56.0836516733215 52.7493349111555 74.9113162056974
45.1151758109609 53.1884505015411 42.2114134935898 53.1884505015411
52.0042367882776 51.2776048338 60.4369223102493 56.233217375698
54.0374522273907
C1orf92 41.8409927585705 42.0709558258414 40.931294690669
39.066284740632 38.3561682218911 43.2805590023412 35.9894133432376
37.86982638952 45.3756389170606 39.2623423164488 43.0016969050339
41.3481355128089
C1orf94 42.8036731479932 40.7952353240579 37.8386378737561
38.8495095797881 40.9950639693255 43.5752393599169 39.7576136507593
42.2577689927563 40.4997186069494 43.2988017098387 41.1638563416981
45.0985088502363
C1orf95 34.5886782974123 36.9994058279362 37.0642268327439
37.1894304543751 36.08956004634 31.1150868775826 32.7676164492555
43.6434576713809 39.3750969348308 38.528621456378 38.079222289614
40.5703581862752
C1orf96 41.4508241771315 41.9894412416874 45.8844325913247
45.0798408927912 44.9936360641969 40.9506062495231 44.3440839686558
54.9919060580109 49.1940901905636 43.844855336215 45.5707650534259
40.1700117452801
C1orf97 67.5361056077111 151.936097714553 249.101015988111
109.144663527001 61.1216330559316 68.3799828461475 88.1413320312949
110.52925781809 102.322851896214 75.7991766454243 83.4465817617201
75.7464604972652
C1orf99 40.9558083259517 39.0894172982439 40.7620472083159
41.0219290963778 43.844855336215 38.2293008890613 35.6056062164318
39.8016018354453 41.7233690251839 40.4136535208406 42.2618615571566
38.9834959368496

C1QA 110.048921728774 96.981661155293 117.556281726503 199.687268345276
87.6178717341967 236.595286890677 66.5422130374814 111.714563124738
51.1055541075029 77.775644509079 51.3103617600342 93.2319962015465
C1QB 633.385474385877 570.997661726825 539.32996290794 1094.91235708087
538.716947884528 1634.61795848352 382.190644764853 494.162646055732
78.2393202422664 192.564048264712 235.544416908354 744.762787024945
C1QBP 569.966632625157 1345.37091574015 826.490051619826 432.355300448283
1050.05355467301 578.311131274653 1016.13142851085 418.410951118141
680.837950890159 328.080879084015 408.739953606815 700.477686061791
C1QC 618.892379887961 466.935292808402 435.901460958456 746.550225537883
392.622487173359 1418.94307844471 346.200944964577 557.979646421047
85.3086977427271 225.389113475883 217.533011278692 603.480973260738
C1QL1 42.6036491382658 51.0175677516029 43.5710871617107 39.3750969348308
53.0419743379342 49.7458652239071 45.4799270968969 44.293922970134
42.0621425894624 48.8623729374733 63.1566669782858 45.0035308952466
C1QL2 36.1232327599945 39.0894172982439 45.989212296035 40.8681871019812
44.147557390293 51.8103411703261 38.1793598983615 42.5435283762933
47.27543600687 42.6793753550854 46.5650556481721 41.9430737035925
C1QL3 43.2135791259317 38.7578265341627 41.785676764591 37.8442841690502
39.4693871570028 44.0399598874356 42.22763273471 39.5954353681031
41.074824727477 40.7063426028871 42.4162492820012 41.863927676552
C1QL4 39.3709357615493 36.7976562409506 40.1985790927248 40.9607305725196
39.9697332660833 37.9951997351435 39.0458123429605 43.9366081640576
35.9645119596869 40.8049408996274 41.7272875295469 37.2810767789306
C1QTNF1 217.575115287224 63.5519501251416 76.5053813427296
163.62024333166 62.588825192561 91.0526311350893 46.7704450730477
138.26207427245 46.0679399375659 50.2016912396771 69.1072548732529
65.9476039189301
C1QTNF2 45.3872406432794 47.087835967892 37.6557714476853
46.8954576545929 40.2449343247539 46.263826584899 42.1607322031054
44.0390019242862 47.0804681460898 39.6719190054379 38.4689132737495
43.7672102682067
C1QTNF3 67.3269632644926 43.4396866157171 43.0971787292711
47.2740099216972 39.8114752099365 83.4465817617201 42.8684958525042
41.4061152929154 37.2810767789306 50.007830364606 45.0589947942306
42.3152792870779
C1QTNF4 43.0930293831497 43.7644428186265 45.989212296035
39.9697332660833 40.5710637884029 63.3612993417558 36.5352572191451
37.9837233601316 57.4777075615651 46.441301934328 47.9942520395434
44.5432192783161
C1QTNF5 482.5816387921 138.418964807714 202.360546931172
183.918927595056 155.050936910986 185.703644892356 65.3694267397114
136.332339821167 59.2566572428546 103.968071466212 178.4681248137
98.9017950719795
C1QTNF6 156.088284999883 66.6199412368667 56.9280216270272
71.2168693013526 68.2585858486846 38.486220880314 50.3519150282938
52.8297102061403 66.3863814740963 54.4825201506931 49.81481191219
49.1786207920603
C1QTNF7 48.9906383294199 49.5443425465542 47.0940613507321
42.6913548400127 46.3150221024046 112.039388952208 42.357910063876
50.0526763693329 36.3649677956953 80.3915591207869 66.8188271427672
55.5712957590038
C1QTNF8 43.2724556187214 40.4359177791566 42.3536549114165
41.1886975241176 34.7018263647985 44.5544727111028 36.8369172900902

38.2782436497441 49.0986627051784 42.3606390470387 42.0750889188255
42.7782216342806
C1QTNF9 43.3638918705225 37.8217989841918 37.5597671487621
34.5425384901279 42.4864576619427 48.0350549043063 39.0458123429605
38.2782436497441 34.5633623842083 34.6720534245631 41.0050831223107
37.1747156131306
C1R 265.667557678228 135.323750661937 70.2141759405882 167.653939596625
150.852183189276 679.309039810384 58.691436315511 89.6670947876322
73.640198455128 91.9185612536409 116.195366065932 153.42135363926
C1RL 152.17335005192 166.847053797281 152.294357984689 214.51120218554
96.8490167966217 134.646159826262 159.571799428131 122.292794686468
173.013258797349 154.707548614663 138.637058021129 200.790001763897
C1S 878.863554510943 190.647389246848 85.3718124658316 422.474427987338
248.691595276298 994.015710537746 53.7423898357583 282.961489041903
54.3458391089652 411.114836576804 415.364456363434 466.454993547103
C2 205.772128732705 118.752208441493 133.981674663494 145.936763882526
59.9326265577462 109.468741823602 114.755820699611 91.5258329186709
157.568088397305 63.197604174872 58.4816190076338 64.531467526747
C20orf100 385.675278463448 182.551901102169 325.083760277008
362.788520130059 229.188779190649 260.15500351311 92.3034609409756
429.576301511638 76.1869593072029 270.600852806342 616.912306849151
287.689260887067
C20orf103 55.6268253440479 48.8584917918986 44.1452943804044
86.6411972262415 42.4864576619427 44.2607789091176 45.223075311113
43.4544762830742 44.3440839686558 44.4652605017595 41.4508241771315
40.3691324547049
C20orf106 43.5710871617107 38.1835938174625 44.1452943804044
46.1074305700679 44.0451487108213 42.8598805316863 49.4573631027613
43.0930293831497 43.3744503319109 43.5627649099623 42.7775752471711
42.6758568421347
C20orf107 44.1294459089314 44.5152708955623 45.1899271235974
57.6511495792664 51.3103617600342 41.7627910693483 45.6042625176633
47.5439485437351 47.3158504988956 43.8943892311541 46.4753015651202
44.9734311311394
C20orf108 242.190059393491 255.884444464408 142.769349424534
126.911056987607 179.064697219173 226.804294585045 234.615033303459
185.312851727212 160.853643241489 282.115786635657 235.544416908354
238.746837986347
C20orf11 651.795139128843 547.888464549389 300.732955139561
349.371668774717 381.613092202209 331.935072199058 600.007191467065
336.390195295291 447.448900931272 683.75696650704 533.468347491982
454.939614583134
C20orf111 244.089971761037 196.17328008185 145.335751135441
532.581631305218 273.070365538675 308.568617575909 293.755623192857
327.238337722366 242.080992016235 277.5073518536 247.04098109003
325.769076169162
C20orf112 36.0144420013819 36.6968906669565 33.6816578730693
33.5578650644246 36.7356360093603 34.7983399096627 33.3231576795423
35.2642250366721 33.6902293666089 31.8890957468737 35.6970528713709
32.0636251428594
C20orf114 43.6706764143908 57.0875538213735 77.3367966395554
39.853175885492 48.0234896130991 45.3882138838137 43.8206425661746
44.5029690493211 52.5772854102326 39.4710743516517 41.8168470823451
47.6938628797168

C20orf116 355.423376744934 446.757901018137 404.86512250958
298.670959394883 575.863848923457 324.320137371059 548.966115135286
203.010958724293 340.311633794489 256.071638089438 357.668448180268
365.459728613746
C20orf117 93.299474822664 48.9800181621986 49.6872467499977
46.6986977330569 57.2960245538881 79.0304119352941 88.2568398791731
88.5706300816555 58.4598072919688 79.1649337367773 72.1090339831747
66.5539062525344
C20orf118 43.9309845324767 46.4385386401326 42.0621425894624
41.0726336841311 44.8897737954573 60.2083550387718 43.8206425661746
45.4757965005055 43.9450023643394 42.8936990772838 47.2134341420411
44.2527566343661
C20orf12 46.0023068797812 51.6060290544385 50.3950981988762
45.8844325913247 55.9283039581662 45.4882438975269 49.1067959632109
51.2623287419357 43.9450023643394 50.0564735608363 52.3761286517985
53.1182952086284
C20orf123 37.1485601150873 44.4411734611229 37.8386378737561
41.5464765641928 39.1650142708257 41.9543948167375 40.9157352260901
38.745552403895 34.5633623842083 38.2069499294002 44.0409178714232
37.0953622406687
C20orf127 58.1868359012515 44.7829632472754 42.640829914102
75.7666827480067 61.954674965065 88.7605288058249 41.253645578517
49.3880946790306 51.6731332961917 52.0999467564713 47.299647867533
51.6660481331154
C20orf132 41.7397731376692 40.4003119241613 43.0016969050339
42.1607322031054 39.3667161654772 41.0582900711341 41.1818632392526
43.3729486804084 41.3803269429468 45.8424887733913 42.4580504587581
40.3330033416496
C20orf133 43.4626049853157 45.0115870036541 47.0940613507321
43.3078542962968 45.4751076037511 38.2293008890613 48.1747307200218
39.1652965788406 43.4656637536244 41.1222709786153 45.6348103033479
43.4709415392696
C20orf134 50.1533437545239 54.8979897528792 52.1242102345408
46.8954576545929 50.3713170502557 40.7616966228404 59.6780848951767
62.9922928277769 96.9051967257119 54.8979897528792 51.4413099192899
46.4499258373233
C20orf135 42.6036491382658 37.8217989841918 37.7384958256497
39.066284740632 38.0598374201407 39.6251842352088 41.0381860307445
40.4359177791566 38.8271346375425 40.8970401536578 39.8184668132023
43.4709415392696
C20orf141 66.8544766980562 40.8880530359822 40.4882741869718
42.9358255168086 48.3814421939888 40.650742261428 45.8980298854198
45.3872406432794 49.8304479381623 36.8059561571675 45.2265319826548
42.2577689927563
C20orf142 41.7397731376692 45.2556760873335 40.5734458834617
46.614092935749 39.4693871570028 45.0882356578404 41.0381860307445
47.3791873993182 42.8036731479932 42.1622837416589 44.7114813656401
38.4330755397458
C20orf144 44.1294459089314 34.3715326040967 39.7411455670172
37.4223289687135 38.1644569189523 39.4953494583071 38.2933870238743
36.5393857306744 35.4332080966594 35.195364621675 39.6403089864678
39.7411455670172
C20orf149 97.3929166450379 155.099281008688 96.577572948724
49.7350276169552 83.6438735616921 125.642081865992 197.325473090277

169.425386468707 166.834334710682 118.726647822102 123.768417863139 127.486023318527
C20orf151 42.8036731479932 41.8848959280916 37.1643693306382 41.8778873678551 39.6535880152834 38.6414316877471 55.2088411137193 36.1375121409002 40.4220196471124 40.5090849993014 45.1432412071268 38.2350720635382
C20orf152 37.55264801907 37.6383530120621 39.1799070031819 39.7411455670172 38.0598374201407 35.314469458379 40.3302268293707 41.9315419791594 34.9428206949566 41.5435611403119 41.7272875295469 43.6753843488695
C20orf160 66.3095900379311 76.6936010119768 114.488672372067 96.9476136220382 58.2780916793348 119.333788465158 51.0013344286851 63.9110569727542 51.0062122023297 58.115198353247 81.5678694264684 61.9036392303558
C20orf165 36.4564050256915 36.3110701990588 39.5450079747895 43.6726153508978 40.3452320167286 44.6524308640784 40.0052661202207 38.956280281655 33.802253812079 42.2614524730856 36.5359455830388 42.8864415494484
C20orf166 43.0001432913058 35.4484652445078 39.5450079747895 40.5484717678957 32.5318884330468 47.2927436772465 32.9962272670005 34.4896350146096 43.8482802038826 37.7315666632629 37.7384958256497 40.0557788909923
C20orf173 42.7150227340364 41.8848959280916 45.4865173433646 55.8709511400674 46.0977687311222 46.3715909653832 46.5650556481721 41.2551966132486 41.3803269429468 50.3388505346542 43.5710871617107 42.4607795638552
C20orf175 46.263826584899 45.0905928998779 50.8303921211581 51.3851157261451 50.3713170502557 48.5835484742456 56.9320717885002 40.3357777530419 41.5635499110307 45.6868589181768 46.7386599624801 46.1592873342534
C20orf177 101.81885073944 230.927097824131 241.222680673439 175.798140574991 150.43149196048 202.325629133577 131.955454394903 88.9038123445083 82.0993943936197 107.122319086614 149.076166432567 104.704892803169
C20orf179 41.0608773166058 40.9731067463382 39.7411455670172 37.29566077996 40.8873907150245 38.9378717994178 46.4388841354122 37.7612949798895 43.8482802038826 36.5806287103157 41.2598458726002 42.7782216342806
C20orf185 40.5654775358427 42.3550738181144 45.6989664108554 37.7469621225553 37.8679241954337 38.7340969861754 41.1507980856436 37.1317006162938 37.9273653326866 41.1222709786153 37.6484782516569 40.9683275553869
C20orf186 47.4678477186801 36.9994058279362 44.2405728317107 39.853175885492 35.3327761997046 34.3887641786747 41.4742337714895 40.3357777530419 44.619512785899 41.3321861371202 38.6662194645633 36.5474283500601
C20orf191 103.252085427319 116.694275999729 159.571799428131 101.560929859785 201.456613927613 121.682085315899 135.997802304719 242.932905246176 97.5394336319916 146.15014824361 114.321083441061 144.079981029616
C20orf194 46.6882473842548 60.8259591514868 53.8441980932644 47.9915414951747 51.8699412145606 61.6637436851405 47.6861474684326 46.3224743127757 44.3440839686558 58.3285450269055 54.9765783642973 57.6406584929274

C20orf195 61.9504984796308 49.6841415268611 48.0191002461099
44.9370339275041 49.5666074434383 42.750861693377 80.354283778818
61.130286531713 53.3932110698117 54.3554233228353 48.5292718280498
57.934622029119
C20orf196 64.546412747238 56.3560498705517 71.3527183280827
62.3936376596431 90.0942781313264 56.7429967489965 77.4046818339539
54.9919060580109 62.4776210012906 106.527144354592 84.1112814779578
67.0331844416938
C20orf197 41.5421429082231 44.7829632472754 37.8386378737561
38.5252456306656 41.0885562160946 39.2248354955045 32.9962272670005
38.6454098044474 42.9020321355927 38.528621456378 45.310660704531
40.9683275553869
C20orf20 614.603743904512 347.908556198025 449.427251674566
310.821299796803 755.852629128252 288.190015621249 523.031300344992
475.008345432596 645.114504971255 461.394007153992 403.428042132639
317.0659599072
C20orf200 40.2543341073001 40.148780650831 41.8751578329365
42.9358255168086 46.3150221024046 50.0748292497578 44.4639405506967
40.931294690669 42.4540072551568 49.0841460667818 46.4753015651202
40.1700117452801
C20orf24 2004.94135236716 1485.68317899614 1319.32406112346
1679.12630849964 2405.8635632802 1055.00793806075 3070.83113612925
1291.71781703042 1723.37649434054 478.296779356592 693.056793637199
929.862002014478
C20orf26 42.3433931248155 40.2493681589104 38.9790184198145
42.8235313007746 45.4751076037511 48.4758590019384 41.8315602975157
42.5435283762933 43.3744503319109 48.2596564318071 45.0589947942306
44.9070250604284
C20orf27 104.335120306753 144.835567449189 130.184962393833
110.112236384679 440.728435508514 281.201604778921 187.086662645246
131.808634405319 122.642481894309 159.844078593375 160.425627898607
116.17375251898
C20orf29 89.9580440276828 82.4362183543935 74.5886835786452
108.991044950132 109.398472684587 78.6402083787251 126.63987648161
54.720204938924 97.5394336319916 112.760972238512 92.6608453151206
67.0331844416938
C20orf3 202.795613942391 169.031144318639 180.502282381416
142.48688219605 241.31366702947 141.594428857706 151.451107406752
164.529367656711 139.191263237202 120.381190859636 162.569762535436
175.529493306927
C20orf30 742.726253630865 955.088515235485 825.558929673308
693.727004855319 1330.19215036764 1173.47285626656 924.316085565917
530.314249832491 554.551745559065 673.522667186915 652.480459437977
1029.12917222294
C20orf38 51.1740855632044 43.5873188565519 41.6977706798234
44.6905691366696 47.9128504683618 45.9266745571724 46.3057385979341
50.9933795450314 44.1519466943185 43.844855336215 44.6286581923295
41.2437378488025
C20orf39 151.175959542368 44.0409178714232 46.0727230219512
56.6458456890715 46.0043807840705 51.1621379498014 43.0930293831497
45.7476095696859 46.3203995372658 57.5713378330876 64.898351831341
46.7182294568603
C20orf4 350.278532614563 200.174103125152 204.774090707986
214.732968482214 256.675896425075 261.326713935356 337.842694716836

243.803668806533 236.727577348109 242.016748817428 235.075347885884 199.501451042641
C20orf43 838.974043046676 665.510487460287 818.290793784784 1111.89793143647 766.027617496754 1090.86617066541 1099.52344340557 997.492549573274 773.781266760336 1351.49940447914 1024.97546870349 942.247437385029
C20orf45 214.823948126294 404.356143472409 266.005181829441 203.152852313746 361.57814355509 238.746837986347 346.530266177867 317.127679512963 280.870472307893 113.994256868581 112.428734515811 251.2365884576
C20orf46 328.908952548641 376.749116251503 225.559405596065 62.6048727240291 166.652850759559 53.9517841438975 94.1289383735162 182.188883821722 138.362914843774 47.4767851750188 50.2203578628217 75.032753683526
C20orf51 45.0985088502363 41.9894412416874 45.3974838013476 51.223859150798 48.3814421939888 42.2577689927563 43.1931731665131 47.4740726032707 46.9739160257217 42.3606390470387 47.1477056711335 43.9541718254769
C20orf52 1119.61164710566 1323.73429724066 1259.2978319548 1089.08276262641 1440.41726370246 1285.49870339222 2474.22199172028 1401.46625360427 1145.59111340228 1134.52018899688 1033.99699402259 1393.24135330226
C20orf54 192.264432082651 59.8102324370568 66.0411740221276 47.481302504066 64.193536444915 41.8613406887824 83.8811735017279 94.9654919877791 57.404059248727 101.759714275976 107.646103699516 74.1282220615085
C20orf55 230.286815921497 199.604105066243 228.660261263355 77.7973642829228 191.979759198021 74.6375749739389 206.261432452217 147.405190988088 193.703214707909 228.499788774214 220.977092747428 199.404836798715
C20orf59 48.2910530887214 45.3486182278177 45.063792968927 79.5838979704243 67.0163226819964 37.3799542961839 39.6535880152834 47.0940613507321 40.9901805728485 38.2069499294002 35.046946054314 40.3691324547049
C20orf62 44.2350046179299 39.8695929627783 40.1985790927248 39.9697332660833 38.1644569189523 39.2985791324641 33.5578650644246 43.4544762830742 42.9020321355927 40.4136535208406 44.7114813656401 40.8681871019812
C20orf7 76.2738693944687 86.4114854104675 75.3580625318157 75.1163398247735 109.758297200083 95.275072356529 98.6706537801886 78.7450149191851 78.839411865787 123.868848519181 109.594402843796 130.293915348934
C20orf70 41.6354649482853 40.0527893189096 52.2132783757422 37.1894304543751 34.0045980663271 42.357910063876 46.1634822993009 44.8997235283254 37.9273653326866 38.745552403895 41.8168470823451 44.0451487108213
C20orf71 40.4261913313642 40.8880530359822 38.4428081526233 42.357910063876 39.8238654968211 38.7658621977098 41.2040352443154 42.1887890806988 40.2274522635576 37.8217989841918 44.7114813656401 37.0390589468421
C20orf72 192.058030454066 472.159979716705 381.31252208432 249.704644191338 641.462632112151 178.608544676872 398.139730642919 186.570670850819 208.410194427484 221.015209694864 244.435346243998 233.577690223567

C20orf74 39.3100256336821 49.2560467081687 38.8417151851134
34.2685957283626 42.4864576619427 39.266565827316 43.6753843488695
47.8947595316832 38.1658452457336 37.3179578037963 38.6335676551747
38.9834959368496
C20orf75 43.7585538437696 43.5182502375207 46.5650556481721
44.265834798964 55.4030910454028 44.9896851005259 47.7390230243778
38.079222289614 46.3203995372658 45.3530683538086 46.6442692119997
41.3481355128089
C20orf77 39.5676051939342 43.1805016690297 36.769073068743
42.0102671859606 38.6454098044474 43.6670755115848 42.0605906240248
42.4580504587581 38.7298222155532 40.2112371512838 45.390192977585
39.4490233720848
C20orf79 45.5758521267508 41.6466169548846 46.1653991089885
44.265834798964 44.5668141895992 44.9896851005259 39.5496902678788
39.853866204927 50.9010149857308 45.4614234442527 44.2925355984056
37.3600014814388
C20orf82 125.62281563325 49.6841415268611 66.737740728688
49.5424425850549 38.5512429290626 49.9699131196797 44.6032218589199
87.1561568256908 50.5785738734431 46.325232414659 50.4740079109854
49.1200355938198
C20orf85 44.7915315085948 42.5269793679335 50.0748292497578
44.6905691366696 44.6834648302383 45.0882356578404 43.3262148375525
49.5185112777773 50.7619345314571 43.1839109702714 43.0971787292711
33.939263369489
C20orf86 44.0328992213727 40.4359177791566 43.401782064778
43.8002456102021 41.7929491793697 40.7616966228404 34.4570158133896
41.7419877611072 45.9227107476843 42.3606390470387 37.6484782516569
32.6203270051744
C20orf91 42.7613994022988 41.0212349917651 41.8751578329365
45.0017158586743 42.7982502551634 39.2985791324641 43.9450023643394
43.1567024035183 42.846832824953 46.1390887492649 42.1537476443423
42.934549734195
C20orf94 55.6268253440479 60.1685144469519 75.4317740345967
72.2577390063259 66.1650385535549 54.9108046955355 71.3527183280827
59.1107691694766 54.4059109100338 84.5913809068614 71.6849360818041
72.3297091475814
C20orf95 49.6241203903354 43.3458769998118 44.5134109184888
48.1662933382789 47.4548471240051 46.4648868771662 59.0457625163577
45.9604707098953 45.1899271235974 45.1569741609597 44.6286581923295
46.2625470410195
C20orf96 69.3418521417182 50.4022543872993 42.4580504587581
41.7614975630362 63.686652234709 36.4986986135769 41.8315602975157
54.720204938924 41.2870505273664 49.4006068471301 51.0492389286271
44.3521099857368
C21orf100 38.5007204246293 37.5469247328848 47.2055133646385
38.2041059863839 48.0234896130991 43.9724628401189 41.9393838138203
52.5098795466615 46.0106098064475 45.6868589181768 41.9908237694988
45.7562276914441
C21orf114 36.2496364442264 41.2598458726002 40.931294690669
42.4580504587581 41.7929491793697 41.6590564937735 41.4742337714895
39.0620487644635 39.5806465938526 44.7958122466628 43.0971787292711
39.3575468494941
C21orf119 64.668912004173 60.6548851977964 66.4079972657486
65.084620579901 74.9113162056974 56.4819802857598 85.1227830573138

57.3286965007828 66.4251023257203 58.3813211109331 51.7538628098347
55.1804535845496
C21orf121 44.8914371498528 41.9894412416874 39.3709357615493
45.5869891570251 40.9950639693255 41.6590564937735 48.6395795163502
53.6210959113222 40.9901805728485 50.22432299342 41.5421429082231
39.9519547348106
C21orf122 43.6706764143908 43.7644428186265 46.3411353377615
46.8954576545929 43.1252272136667 59.7009992986607 40.1235656101329
39.7509165247419 54.0192060888782 56.8201201798712 55.0404566626821
48.27609330316
C21orf123 33.5994525227062 38.5252456306656 34.3633615460307
41.7614975630362 39.4693871570028 34.0045980663271 39.0458123429605
36.2539950294089 37.9273653326866 39.6719190054379 36.3390947880186
40.4639532172338
C21orf124 88.5140510969737 50.9643363406241 65.2670315837785
52.6947911480086 72.828269171954 47.0027453663546 62.0404348860702
55.3122077514658 65.1424206085551 61.1851254827343 63.2684764441521
62.5447504571181
C21orf125 44.1294459089314 45.9266745571724 48.4890615340603
43.5627649099623 43.7517118200398 36.9060997041453 46.8613212048981
44.0390019242862 52.5772854102326 43.401782064778 39.4410515050885
39.8497227447888
C21orf128 46.8141313516997 46.8236022402545 49.2041300973417
43.9282059368202 50.7615314686244 51.4931742685391 53.1884505015411
53.838484549134 49.2879849674517 50.22432299342 45.310660704531
42.6758568421347
C21orf129 42.8036731479932 51.119812302881 61.841867306916
44.265834798964 65.5029051840465 43.2805590023412 44.2166118829877
55.421880601441 52.9482338979681 56.5379973844443 47.9942520395434
47.8931773060789
C21orf2 78.9028182143923 77.4046818339539 104.351256270039
128.157032116902 71.7263663921367 148.467977704907 87.455991111513
140.030757545662 75.9909523932333 187.21383075601 169.561107057293
132.508880040274
C21orf24 3490.63949257581 1999.21832626916 2280.42953098693
2365.73772015255 1529.52516575545 1335.4111951034 2440.77408185413
4235.59743465057 1509.04753111016 3776.54943917367 3653.59181300011
2598.78910128081
C21orf29 36.7356360093603 43.9421822132391 41.3366779545815
37.5382094267692 43.6587492470348 43.6670755115848 43.4544762830742
37.9837233601316 40.4220196471124 43.1839109702714 38.6662194645633
34.1509644696334
C21orf33 214.537530518962 316.185342210304 464.671881584567
263.44529683749 616.912306849151 370.330147221688 407.336051853854
350.978524278048 423.254211470935 401.679080975109 384.280434953152
341.535750991033
C21orf34 51.688369435871 38.2795150354677 42.3962308437248
44.0895090772372 40.5372008011901 90.9010016862442 36.7356360093603
46.0633179231318 39.044540595942 68.9766943946607 58.8559455338355
48.0010635382918
C21orf37 48.1125722136997 46.8954576545929 51.223859150798
48.1662933382789 41.9842695818285 49.3172204533818 40.7910160811527
44.5029690493211 55.2114969675545 46.2286164776607 47.8284278211109
45.5800840647989

C21orf41 39.5234709385294 40.716553247236 41.3366779545815
38.889943614258 40.7376840028647 40.8091178323174 40.745874931373
41.0752399035779 41.627892303206 39.2057863854896 41.134180463103
37.6200174008473
C21orf42 42.1537476443423 41.2598458726002 48.0191002461099
51.0175677516029 45.8051846088552 39.132725377815 40.6750540874299
42.7431359850339 39.2809305246091 43.401782064778 40.6373400186325
43.3744503319109
C21orf45 53.1309801459151 60.4646208319121 50.9519768764673
41.8291484190524 85.0017995689256 43.1826328292558 53.1884505015411
55.6904617926772 66.603126065489 46.325232414659 45.7792335738285
45.0035308952466
C21orf49 41.6354649482853 33.939263369489 42.3536549114165
40.646702696033 33.4316681623175 38.6414316877471 35.4623691645629
40.047314525032 42.0621425894624 39.5637454855233 45.2265319826548
34.0382693188794
C21orf51 68.3928560299063 66.1595900891671 83.0262409967927
66.1448688879431 91.6777754418002 94.4999269932618 67.1209520838357
60.0502421545569 89.3868257841517 72.834275688835 69.7215372275927
91.8935361535002
C21orf55 4916.82166008511 2568.97574985073 3602.30252142918
3428.74022048394 2456.86796652313 1442.72006787259 3502.80810319252
5811.22276358172 2365.73772015255 4898.89844391477 4259.50675863393
3502.80810319252
C21orf56 47.5647821785529 64.0096843640535 137.176719695561
70.0402992940342 211.829374574045 102.471939526704 41.3594868055879
68.1311562787361 41.3803269429468 65.0928075335306 97.7965639721152
46.6397391251762
C21orf57 123.04622719856 108.806649898122 187.189412320761
150.529917336161 186.891912776245 140.866324039667 171.176393131741
107.741669334989 182.643498505575 140.063595285844 114.918205718649
114.623641209561
C21orf58 49.8304479381623 50.5070012045444 60.6080172588224
41.3567204694281 61.0246305235631 45.0882356578404 67.1477341610979
56.4557243125318 62.7605715573218 46.2819623896574 44.2560775855603
44.1861590259178
C21orf59 120.980558571109 162.106596527489 171.992427227088
95.3070802596041 240.58403560014 130.177232923004 203.651890041185
152.679927202674 129.820909990346 90.716460692818 100.663054866186
116.434267173815
C21orf62 40.9558083259517 37.7384958256497 37.4642807402741
39.066284740632 44.5668141895992 43.8582747295766 39.8695929627783
38.956280281655 35.5220586859301 38.8507804383261 40.2649309204478
45.6692241948108
C21orf63 68.0700069295224 50.543526404059 59.7483827511172
87.9098670472958 59.4830424460714 199.294913752704 46.5650556481721
73.746472097049 61.1022920882065 166.572463787763 150.082905944963
161.526970033915
C21orf66 92.5391033814713 115.221134337342 127.986477405999
81.5135916455124 159.485978728 84.5354986509039 135.592113666246
107.348865210074 100.326655761429 95.9075629352341 90.8831233784641
91.6876038082112
C21orf67 41.8409927585705 41.554942319225 38.1421709520616
39.066284740632 43.7517118200398 37.2686490268265 39.8695929627783

42.4580504587581 42.6366765186182 37.5343260962758 38.956280281655
32.1545709776983
C21orf69 36.9060997041453 39.0894172982439 34.4716904283753
37.0732977341185 49.32293280121 37.7000400506818 37.6036146841933
58.4200819624972 40.9901805728485 39.8873706855774 40.8225507267584
39.7411455670172
C21orf7 57.4376528157926 90.3691010312535 95.0738959950301
74.6159297822533 73.5541045639348 45.6129956328415 55.9276266345493
46.4672637826582 70.5699797830702 65.5349012839562 58.7389869173495
65.4435809676217
C21orf70 59.9546218258435 62.9391937505369 67.3469281984384
63.6334154351075 127.246625628593 77.2218991550552 109.811921160228
68.580705761277 73.3042538589134 57.3562426592112 71.136135376296
66.6118713143653
C21orf74 40.6559419616644 41.3610369241157 37.7384958256497
39.7411455670172 39.6535880152834 43.9724628401189 46.4388841354122
41.8417024632349 41.3803269429468 42.1622837416589 40.4504608195666
41.7575097298927
C21orf77 41.9407041937973 36.89524015739 40.4882741869718
44.550322123994 40.2449343247539 39.7394181011603 40.3302268293707
41.1575777497576 40.2274522635576 42.1622837416589 48.2050207071554
43.5738557773903
C21orf81 40.5158867964016 71.8156159183499 89.3168921945222
43.2427390263564 40.6373400186325 40.800866389165 42.629412460409
44.081163104701 55.2336859873958 191.668846959973 135.490875023792
114.755820699611
C21orf84 36.6480005877129 41.2598458726002 33.1559412791294
35.8472302065546 38.5512429290626 39.0266821712099 37.2892641289258
41.6466169548846 39.6609238036815 42.0621425894624 39.9070785037411
37.9607839934735
C21orf86 40.6559419616644 41.6466169548846 34.8956628705329
40.646702696033 40.4639532172338 43.3785777889265 40.5585676679482
36.9355798143571 45.0017158586743 47.3791873993182 40.6373400186325
42.7782216342806
C21orf87 36.1232327599945 33.8237407699472 32.5418171758086
34.9849896310235 34.3633615460307 39.5581166303303 36.4098694708483
35.1224289925572 44.2142581488029 41.0057887014368 37.9230440150891
39.5792604499113
C21orf88 44.3355304829989 39.8695929627783 39.8299340037009
39.9697332660833 39.1650142708257 45.4882438975269 39.7576136507593
36.2539950294089 44.2607789091176 33.8984830800739 44.3763859390287
34.5633623842083
C21orf90 44.7044140979108 43.9421822132391 40.7620472083159
42.5664883002833 59.0965219753805 42.9695585740813 37.830876898596
41.4508241771315 41.7627910693483 43.401782064778 43.4709415392696
36.1647506311964
C21orf91 50.4260283682427 54.0461131287999 58.465017287283
67.1209520838357 55.3718910891548 48.1591264215184 58.4262160879174
57.5640428227111 59.5929655877036 49.2317401696588 47.8988096575648
51.7330492465443
C21orf93 42.7150227340364 50.0425298550559 48.9944486068347
50.2163892855092 43.7517118200398 44.3648993568405 43.5627649099623
50.5070012045444 43.7630587657205 47.3791873993182 46.0905079010237
46.3411353377615

C21orf94 41.3387790895716 41.8848959280916 40.4070046183108
48.665578118898 41.8806343997257 42.157627240486 46.5650556481721
42.5435283762933 38.7298222155532 41.5435611403119 45.310660704531
42.9887069977696
C22orf13 754.700295017212 451.711598030737 466.695081390256
475.621740714165 385.594118191769 562.531738167208 1115.27580736237
529.210992541911 486.487366153374 497.591040004541 476.653723741096
444.169882251299
C22orf15 45.6517320730042 44.5884811528117 48.1997158668729
43.5627649099623 54.6820069269589 49.641159251878 45.223075311113
46.8954576545929 44.619512785899 51.8412522234186 49.8393069675192
39.4490233720848
C22orf23 51.2851682978581 56.463481075522 52.2132783757422
60.4646208319121 51.1368583672022 44.5544727111028 45.8980298854198
57.1362071277099 51.6731332961917 61.4569050766419 53.9517841438975
51.4308501933893
C22orf25 232.440458525975 95.0738959950301 94.3953345963359
134.394743648475 65.3019328668633 114.769498252688 84.260633540656
143.653706909456 78.8250642061439 92.9043863423147 93.9956276432715
79.9447969637409
C22orf26 46.1673159982664 38.2263227947189 40.5734458834617
41.0726336841311 50.3713170502557 40.4359177791566 49.3854238365325
43.6434576713809 44.5303781239209 38.2069499294002 44.2142581488029
48.0350549043063
C22orf28 379.933650669101 294.006023678979 320.038347979923
309.994784298282 255.636379050741 318.768560912915 327.283431584541
318.234214341679 367.451332073134 335.167786298149 272.022473793857
279.782392390288
C22orf29 133.823107212467 108.734392572981 83.0262409967927
66.7750249658122 93.4726264307041 81.5782824513693 68.878562323012
97.7415824971555 85.3718124658316 106.418690759491 83.1184264089873
81.2068428211497
C22orf30 40.7609952437622 39.8695929627783 37.8386378737561
42.357910063876 42.3685136297603 37.8102105794655 42.2577689927563
44.2159020605784 38.3418933808844 42.5664883002833 41.2598458726002
34.9138625012006
C22orf31 39.266565827316 38.5252456306656 36.5474283500601
40.5484717678957 40.4639532172338 40.7616966228404 49.2546642180676
32.8834169462007 48.2123120250343 36.8059561571675 35.860430241131
43.9541718254769
C22orf32 54.2648201582657 69.734208465775 71.3527183280827
75.2475238554276 67.3568864741196 88.3503153240018 75.7342400123505
64.4156641487994 65.8622973953004 124.986782737446 105.277476070937
87.4273245333478
C22orf33 33.0463640202858 42.1607322031054 36.4564050256915
39.9697332660833 36.9702228618069 38.8336427948187 39.1539892144371
39.3709357615493 29.4159269895179 38.528621456378 32.406093465115
38.6454098044474
C22orf34 34.4896350146096 40.7952353240579 40.931294690669
38.4208429103799 71.5345397102823 36.0306975524309 41.1507980856436
37.9837233601316 39.7537274655115 36.4651112441576 41.2598458726002
42.357910063876
C22orf36 80.4479092568961 57.0082102559228 43.6670755115848
62.3936376596431 58.9875400392427 127.789065386943 110.223625398261

59.1107691694766 69.0037087543684 102.136265168978 113.169111095305
72.1090339831747
C22orf39 69.7442393277127 55.0009147092272 72.4202724008052
78.1583955490018 60.6400800519777 103.569150754726 57.8870110456492
54.0507720792049 91.5370872909881 65.5349012839562 62.5036823572932
68.2309359831273
C22orf40 56.8534370691413 56.7826399116207 52.8483158143741
50.1185085888209 61.0468099710416 66.0069448981947 60.4593810615882
47.834436078111 54.4059109100338 61.4569050766419 56.7888691338261
51.9447450229707
C22orf9 44.3754281580557 48.4711252009937 44.696023817801
52.1201052574162 56.1671440556142 50.2513819310656 43.2645805835667
45.8095171770165 44.7804080899431 45.4614234442527 44.6286581923295
52.2216613535546
C2CD2 95.5530572805104 146.658116519622 190.403032965372 89.7554896946341
264.88963894526 128.219615202232 195.836475280084 179.47783529
167.537023543498 247.416016413788 219.918644151214 204.171339006867
C2CD2L 56.8168526942685 61.2108152457954 54.6820069269589
70.506090074243 57.9161791762225 56.8505380943422 61.2183034191703
73.362936710475 47.3791873993182 50.0564735608363 50.4740079109854
43.1805016690297
C2CD3 45.8108990837624 43.5182502375207 47.3861758958576 42.357910063876
50.5110359971301 43.6670755115848 48.9826868640753 57.037639245179
41.3803269429468 41.0057887014368 46.0905079010237 45.9693232070002
C2orf13 44.7915315085948 41.3610369241157 41.6079958749402
46.614092935749 42.269054475455 40.650742261428 41.8315602975157
40.7311497914871 40.4997186069494 46.325232414659 45.0589947942306
40.9683275553869
C2orf15 74.3135387703106 115.321592104747 75.561262816077
47.8164193292842 106.951862149262 45.1873487463472 111.367789538268
118.93749473833 96.0838701986116 57.165971310746 61.2529834995512
75.7140064167134
C2orf16 43.4626049853157 46.2286164776607 38.3166554965434
38.0958223740111 32.9756367838345 37.7000400506818 41.9393838138203
43.4544762830742 38.2215591930186 41.7405682794166 43.4709415392696
38.3291713594599
C2orf18 129.785929766258 84.9182861692273 93.6591201235745
99.2432911346944 92.3034609409756 94.2678678253657 86.9185256838836
107.682738819549 108.27606179864 82.475816811779 95.39134217438
97.3929166450379
C2orf19 35.7012997405508 38.1312950900467 37.3687900733549
37.0732977341185 37.8679241954337 42.4580504587581 38.0598374201407
37.1317006162938 40.3316153483289 33.5412189543102 39.9899430628542
36.2582325396375
C2orf21 39.3709357615493 41.3277288245157 43.917817324545
41.4273122914951 39.8114752099365 40.8970401536578 46.3611042575528
41.6466169548846 43.6670755115848 48.5374448344297 39.8184668132023
40.6614700944853
C2orf24 159.937192675974 90.3691010312535 100.057155418659
143.304938314706 106.454367084321 118.410983614453 117.260454792593
203.759116151774 127.232160404726 292.065366215278 207.18510695093
133.541341560868
C2orf25 523.679443405512 1151.25293766167 747.975233849989
623.574706535278 615.011590811277 614.807633538557 442.99540222483

388.132295362791 487.065758294354 290.524288545599 315.696895080135
621.012072500314
C2orf27 47.7267190613906 42.3550738181144 36.8730514568925
39.6292788039793 38.1644569189523 39.8455060654531 40.2154497375715
36.5393857306744 37.2810767789306 35.6208308220005 36.8282383070068
38.6454098044474
C2orf28 816.746599467159 722.514380928291 788.950470077369
716.754656773542 718.414652618295 982.428740433235 709.730555484551
875.787532301293 716.434968700383 752.837247653926 776.670493028756
884.036280777358
C2orf29 1783.87583818834 981.039763059681 939.453799186397
476.653723741096 1291.71781703042 427.540236966999 792.880882528835
1307.19925493293 999.13039066242 1083.50383916482 900.517711546397
759.184121753305
C2orf3 35.6111578834671 32.7800533388945 37.6557714476853
43.5627649099623 50.7615314686244 37.9951997351435 37.3799542961839
37.86982638952 35.1155486926999 37.1275334308172 37.9230440150891
36.7762265039864
C2orf30 398.049525642349 413.918050849658 384.522115820048
583.053232396803 253.333114749013 321.949943388959 242.58992925232
310.706520144173 267.427827956058 337.013669560645 306.533241954966
394.764369100963
C2orf32 73.7133884699889 59.2643906275025 69.0503036666628
92.8026641003757 63.1443731864055 193.043344611355 48.6395795163502
54.6067620335514 47.27543600687 86.2118355769367 89.5887648181505
92.6545309624315
C2orf33 121.744701580607 140.559370165331 111.735319884512
112.854520785284 124.051109959228 202.627009037401 87.2351169088172
97.2750076101755 140.51841571801 114.796237914478 125.891602778425
160.755701876741
C2orf34 68.8698243753339 82.4362183543935 105.052418151358
50.0526763693329 104.914715637173 84.5913809068614 103.118579484715
181.873969432903 90.8109585999257 88.8568707880669 102.993620677265
98.9017950719795
C2orf37 49.7188990489616 50.6229884142872 55.9459886217809
38.2041059863839 47.5647821785529 48.0350549043063 48.1747307200218
57.5640428227111 41.1742244762318 51.2318935933116 51.1483696410762
55.1804535845496
C2orf39 41.8409927585705 39.7835513496873 36.1042012504758
40.3180310664156 38.2496050953547 40.8634043028528 40.2154497375715
36.7460922671619 44.1519466943185 41.0057887014368 37.3687900733549
41.6550823405646
C2orf40 44.1294459089314 37.8217989841918 39.5450079747895
43.1847628331565 43.4544762830742 635.52621743309 36.8369172900902
47.9942520395434 33.9456837661864 320.889609846069 248.012529897462
225.420624458188
C2orf42 117.074650076861 94.7008981904016 132.369828264246
117.071280682786 103.792424865682 122.758873982957 77.4046818339539
107.427186954956 104.626873082138 138.765621737736 135.706144105357
120.679433440203
C2orf43 84.0388582916959 93.5045354990096 82.1344514651393
82.2766822827861 104.388288863224 75.8976034525022 131.097521229971
96.0654949568324 95.9298109273155 87.7918574811458 83.1874704625702
123.27141431776

C2orf44 103.561100886469 67.1299518044849 62.5610562594275
67.8978240198575 93.1238695478498 75.2735539010032 120.401643722713
145.791398829369 58.8201021345225 94.233414682323 102.121491187437
100.567309348016
C2orf47 298.420882349382 383.371300272181 326.70962514228
208.097209050425 397.798003824167 188.008141649466 270.662475158799
308.280127837858 315.487514440864 131.779333469955 144.546897317106
189.083059081845
C2orf48 53.4921736109774 46.4861258030402 58.112348043634
42.5664883002833 53.8260140375214 46.4648868771662 62.0404348860702
175.193752938517 53.7442594289333 39.2623423164488 43.7585538437696
44.8372404608604
C2orf49 89.4747568145717 69.1414574152997 86.6186787663248
82.5279746006378 117.260454792593 84.7113556042844 91.8733417805775
96.332996264667 96.3232257371077 70.194061829681 63.7963501146829
91.3112603277315
C2orf50 39.266565827316 44.0409178714232 41.8751578329365
38.3166554965434 39.1650142708257 35.4142595025086 41.9393838138203
48.9826868640753 36.3649677956953 41.0057887014368 42.0750889188255
45.4799270968969
C2orf51 42.9104454767916 37.5469247328848 41.437237454134
40.1804147506786 36.7356360093603 37.4782441311626 35.7055410599146
35.9023742468744 34.2973261206803 40.7063426028871 40.3452320167286
42.7782216342806
C2orf52 42.5534565507139 39.6123051184545 41.6079958749402
40.8681871019812 40.7779767565889 42.750861693377 43.7585538437696
44.5029690493211 46.1634822993009 46.7646877499666 40.9683275553869
40.716553247236
C2orf53 42.442231667357 38.3322849790657 37.9482157381008
43.074472921841 44.5668141895992 43.9724628401189 40.2154497375715
48.4670130605028 42.6366765186182 42.4594149893798 44.7114813656401
40.6614700944853
C2orf54 38.385865183581 35.8227939208521 36.4564050256915
38.6414316877471 31.3942305191093 37.8102105794655 37.9430145836712
35.4526946152955 42.1594905198929 34.4875013487777 42.4580504587581
34.2141875904985
C2orf56 94.1289383735162 99.7361599442168 119.681745926474
96.8620230524393 112.990752518897 93.8361140725228 126.500059295217
194.435776102991 99.9896394343368 78.9420136903401 76.375769561992
108.57858133896
C2orf57 43.7585538437696 45.0905928998779 40.6737160001291
40.646702696033 39.859345745831 42.0574848021044 40.7910160811527
48.1591264215184 40.7847110517594 42.0621425894624 38.2795150354677
36.064718696392
C2orf58 39.8662795144616 43.5182502375207 35.0970060242246
40.4359177791566 35.6434124419782 43.2805590023412 40.6750540874299
40.831207953647 36.197424053556 40.8970401536578 42.357910063876
34.2141875904985
C2orf60 38.1169703906207 42.4493032770053 40.301483914446
40.3180310664156 43.3332476888305 37.8102105794655 40.3302268293707
39.0620487644635 41.7627910693483 38.745552403895 37.6484782516569
42.9887069977696
C2orf61 39.7576136507593 38.9338521051669 36.3604951341921
41.4273122914951 37.8679241954337 30.0771630269067 32.9962272670005

39.3709357615493 34.8552366193358 39.2623423164488 43.3085358214154
35.6427313468066
C2orf62 41.8409927585705 38.8336427948187 39.6451483836089
54.2886483704949 39.9697332660833 41.6590564937735 37.7136011474903
42.6450179841129 40.8970401536578 41.7405682794166 37.9230440150891
39.4490233720848
C2orf63 42.9104454767916 49.2050538150106 48.1997158668729
39.5050383901884 38.6454098044474 37.4782441311626 37.6036146841933
54.5125550937898 44.3440839686558 43.2988017098387 54.4825201506931
57.6860752168769
C2orf64 140.598583034034 138.82907142482 121.737071786167
87.4850071876743 148.99515914482 169.723009455614 138.436822676252
110.440072569721 159.973746908596 161.326580647514 160.962078065068
209.986313475002
C2orf65 51.7607563002005 53.2841144999258 46.4569566520107
50.7046706880325 43.9450023643394 54.8265655909623 47.7390230243778
48.7814131120945 44.7058233759912 46.6598879466655 46.7386599624801
44.3521099857368
C2orf66 39.266565827316 43.3458769998118 37.4642807402741
42.5664883002833 36.8059561571675 42.157627240486 38.8417151851134
40.3357777530419 42.0621425894624 43.7303813515864 43.401782064778
40.2669497398702
C2orf67 37.9387791625384 46.0023068797812 39.0775870687758
42.1273928203021 41.467005949568 42.8598805316863 40.9157352260901
39.3709357615493 43.3744503319109 39.0648733099061 46.0095710163378
41.4508241771315
C2orf7 91.3967044940546 69.3106426188075 83.7159102306394
70.2541961995753 93.6591201235745 99.3956523785983 64.232967574797
95.2915739972533 148.467977704907 105.828093778276 103.561100886469
83.7019398654324
C2orf73 39.8662795144616 39.0894172982439 34.3633615460307
35.7674462337723 39.5595233162192 36.6271061451613 38.0598374201407
42.8482181313226 38.9834959368496 38.745552403895 37.1059647239814
36.8690774823319
C2orf76 75.0704619826557 53.7332797903102 57.423873475544
74.2429851177794 51.7983110178095 59.7009992986607 56.2181240696479
70.18107651423 67.4304134449444 66.8026147385682 56.8436081694537
75.4053820110486
C2orf77 37.6397652536077 43.683792219729 38.6005272730664
41.8778873678551 38.5512429290626 41.159706131351 41.4742337714895
40.931294690669 43.5669306063196 41.1222709786153 45.7328341667151
42.1537476443423
C2orf78 40.6559419616644 44.5884811528117 39.7411455670172
50.8567345921473 52.0583273941813 42.4580504587581 43.8206425661746
47.2009420656854 45.3756389170606 47.8207974930031 43.0971787292711
43.4709415392696
C2orf79 84.9756044280631 128.005663945917 105.814959179397
101.708164294697 81.0259717888094 73.688423848106 148.944852825695
113.655306804539 138.23147386089 98.9657264176664 93.5045354990096
96.8555197062122
C2orf83 43.1805016690297 35.5310164682026 43.3085358214154
42.5664883002833 45.9104478407175 37.3799542961839 40.1235656101329
45.9604707098953 38.4344387289956 43.7303813515864 43.0016969050339
45.6692241948108

C3 48.8704059222613 49.015238049903 58.6736679311765 61.6608323085662
44.3557408297309 130.648278937122 40.4941260038116 41.8417024632349
40.6503965580437 53.4109882045447 50.2750065554387 61.9882398986333
C3AR1 64.1882546112044 50.543526404059 49.1067959632109 339.262360833465
40.3452320167286 81.3978207813439 39.8695929627783 50.3082459161777
38.8271346375425 44.8997235283254 38.3684202018524 44.0451487108213
C3orf1 58.1868359012515 57.0082102559228 57.423873475544
70.2541961995753 76.7820250461545 62.0660509811111 56.6537562775715
62.105802634526 62.275808835675 72.42732668079 68.1992221684165
56.6146179789815
C3orf10 273.108672916862 413.067407579649 279.485290822376
286.832539203237 468.98857415608 464.583498621466 298.282957415786
259.127235335036 261.195021649777 293.649670545233 268.683528469759
374.167501183341
C3orf14 121.682085315899 127.2070318269 259.626847216881
110.540953771795 204.318269745079 128.657014567195 278.303493506139
176.057220407246 296.39460008432 87.5074297754031 99.0647786948132
128.436305192918
C3orf15 48.8333989709933 51.2405397507224 41.437237454134
41.5464765641928 42.1650127805175 76.5888466260562 70.7238101239421
104.089998273812 50.2223403889892 69.6511571901941 66.5063354430003
48.5123041297835
C3orf17 82.8095009455738 80.1461294346211 71.940968073473
86.6411972262415 61.954674965065 76.1205714556592 62.801431502597
55.7341433378734 87.6018870099686 67.591570323687 61.6084234634424
80.4404044229341
C3orf18 46.5549623118323 38.6414316877471 49.2041300973417
46.614092935749 41.6806984441849 50.2163892855092 37.7136011474903
39.2672712041774 44.4395580493118 48.7414853386615 51.2318935933116
39.3575468494941
C3orf19 165.775214685061 323.048740324895 267.170081805168
198.497745548245 288.190015621249 233.201890340549 350.365107642944
316.373650015333 245.356135592468 398.43592055172 357.347859577591
223.267781866783
C3orf20 44.7044140979108 32.5482839399637 44.6032218589199
40.3180310664156 43.6587492470348 34.8956628705329 35.0305278582179
37.9837233601316 34.226171989766 37.0320432433314 42.357910063876
36.064718696392
C3orf21 230.879083362388 134.407474121421 160.336517310154
117.3417887148 272.022473793857 93.1067450332473 181.545493080363
170.475212920685 258.30549459038 148.358988653522 119.528720998614
103.75895351937
C3orf22 50.4872122867759 39.8695929627783 40.301483914446
43.6726153508978 45.9104478407175 46.0364000143537 54.0170817319522
39.2672712041774 47.798305839406 40.4136535208406 42.911855821602
44.2527566343661
C3orf23 41.0970259989281 60.8608693323868 58.7065473526185
46.4555908533915 67.5047089908724 59.4153120842154 51.0459299480813
44.64770889737 59.9354531144375 44.5324600042493 46.9886864457762
53.5643281930979
C3orf24 40.8091178323174 42.2900457798748 40.148780650831
40.4967168818294 41.3755760434934 37.842873463785 39.6158343667921
44.9631458622215 37.6383530120621 40.2615278292875 42.3413611855132
39.1553169409075

C3orf25 48.8333989709933 43.4396866157171 43.3085358214154
41.3027031037343 41.0885562160946 39.3841861812262 48.9826868640753
47.2864663693787 41.863927676552 42.785429206422 43.7585538437696
40.4639532172338
C3orf26 122.316617618658 131.766252431636 99.3448096967314
126.69706456173 313.711588658312 206.676666936979 198.922575188741
199.660904693786 152.792457231813 92.4534854832186 105.277476070937
140.961217055867
C3orf27 36.4564050256915 38.9338521051669 37.6557714476853
41.4273122914951 40.0599807618105 43.7630587657205 40.7910160811527
34.9580369221164 37.9273653326866 39.3698811593364 37.8372168909583
42.1537476443423
C3orf30 44.9370339275041 47.2299678280724 43.5710871617107
44.6131679221578 43.7783560914188 43.3269087731399 48.4099370357307
40.8833572570383 45.9227107476843 42.785429206422 43.3085358214154
43.5420658412177
C3orf31 149.260740603715 257.194689822426 209.497482517298
134.394743648475 273.645674189546 153.42135363926 245.960009147234
323.576613035353 208.30969642805 148.781721594419 140.245630405973
176.839214683647
C3orf32 43.2724556187214 45.1694246393676 39.7411455670172
40.8681871019812 40.7779767565889 43.8582747295766 38.4005795891396
45.642304356051 51.1055541075029 38.6454098044474 49.532546810992
45.5800840647989
C3orf33 39.266565827316 39.6123051184545 40.0998167098057
35.7674462337723 45.9104478407175 46.8954576545929 46.9929836306816
43.4544762830742 44.4395580493118 56.8201201798712 44.4688999098904
43.0930293831497
C3orf34 2206.44541412808 1377.24483375075 1541.9572099378
1917.90025587049 987.427672417105 926.61614755153 1864.63057767867
2448.64580552439 1065.70206468134 2978.46032143413 2830.73694055758
2024.4791883541
C3orf35 40.5320714850602 38.7578265341627 42.108130037421
38.6835649080278 45.1137938810047 39.6251842352088 43.5004520034498
41.8813257103492 40.1719219196935 43.128936937037 37.8844698547804
41.863927676552
C3orf36 45.7356815244513 55.0897019602305 57.2107492415265
59.6153684374766 39.3667161654772 43.1826328292558 40.2154497375715
46.9986083378817 46.7760357462635 45.6868589181768 43.5710871617107
45.4799270968969
C3orf37 112.551747307723 100.535211780514 152.395295329633
134.258351436533 173.11353027932 112.768270540409 242.339375770778
133.151333210366 169.061093538895 151.775928080246 133.244526666651
130.775349446374
C3orf38 91.8088250889427 124.803597244786 120.771048750176
119.502912878938 162.569762535436 117.598001907902 97.299696284253
84.1639779987105 99.9057667432209 61.3149617644002 67.5963845975985
85.8154958329408
C3orf39 97.9401338178996 100.616027485975 95.0026207727863
82.5279746006378 197.011675186182 131.451150955309 143.152895211088
111.714563124738 111.096799408676 127.567860003773 114.78317743597
103.607345835114
C3orf41 37.2474303302343 34.6306468196142 42.0621425894624
38.745552403895 43.2484996523549 48.5835484742456 41.0381860307445

38.6454098044474 43.2761454096265 37.920964499903 43.401782064778
38.745552403895
C3orf42 40.6062943725118 44.4411734611229 41.3708420203838
44.3440839686558 47.8697093824642 38.4741160538511 42.1372626664516
42.7965563818112 40.1738310955128 44.6918600441328 41.3234441859498
41.9741368971524
C3orf44 34.6977013306396 37.2810767789306 38.6005272730664
39.6292788039793 38.1644569189523 40.7616966228404 40.3302268293707
40.4359177791566 40.8970401536578 39.4710743516517 34.4716904283753
33.939263369489
C3orf45 35.8208542506595 38.8336427948187 47.3861758958576
35.7674462337723 38.1644569189523 40.9506062495231 38.941855825269
37.33030683349 43.7630587657205 44.7958122466628 41.8168470823451
40.6614700944853
C3orf46 39.7576136507593 45.4143854024453 41.3366779545815
38.9534635879195 38.3561682218911 39.6251842352088 38.4005795891396
39.5466975353131 39.2809305246091 36.6893251782922 37.9946685306274
35.8429598823818
C3orf47 42.1537476443423 36.6968906669565 41.785676764591
48.665578118898 38.745552403895 47.5733603903505 39.2533668167483
44.4031868028216 38.2215591930186 38.745552403895 45.9104478407175
42.357910063876
C3orf48 38.7537364570873 41.4508241771315 41.785676764591
38.4208429103799 45.9104478407175 40.040778349137 51.1541843174981
42.0494116081998 42.5406888281114 39.5637454855233 42.4580504587581
39.4490233720848
C3orf52 72.4094549259929 117.074650076861 103.14577985944
46.8954576545929 106.527144354592 64.5187138759372 96.2959923858457
111.573111498905 96.8141712830545 112.551747307723 174.752830194939
132.37539586883
C3orf54 60.7371790125714 159.99819920335 204.63343271995
79.1000897258245 148.333778341005 94.3953345963359 105.945216149549
45.0972184964705 205.854666205308 267.85990321012 173.150885069619
138.486962379511
C3orf55 39.8662795144616 39.7835513496873 43.3085358214154
46.4555908533915 48.3814421939888 42.357910063876 45.4799270968969
43.3262148375525 36.8127605061228 43.2988017098387 40.6373400186325
39.0536056496869
C3orf56 41.9407041937973 41.9894412416874 37.1643693306382
44.6905691366696 35.9058393060637 42.157627240486 44.7255207589606
41.8417024632349 40.4220196471124 42.3606390470387 38.8507804383261
39.0536056496869
C3orf57 38.6454098044474 48.0010635382918 52.4234283832574
43.1847628331565 85.2849531543957 47.0504142893738 75.2101993995344
210.50519205965 46.4569566520107 47.8207974930031 57.2674335547631
118.806070800059
C3orf58 93.0714382925187 104.740168626646 77.268323196327
107.111886993156 312.648013979436 115.441414762268 160.360785581153
135.829530518199 132.684895918889 165.942795019496 172.433936159136
134.763105070421
C3orf59 87.8746766762445 96.6359641162026 73.4962349716902
188.713487905549 104.072423445726 94.8884063433735 145.526972410673
272.475108279834 71.5327770882433 139.645717741863 125.221639689345
128.635811501506

C3orf60   182.80711310964 263.125290391542 262.9072085191
267.608189207303 236.910422092851 264.263104842911 361.023743912857
247.723546145775 235.101442092497 236.791729190996 224.509915754308
245.996655628334
C3orf62   49.2595762436995 51.6060290544385 56.3471340054074
73.5834042105313 55.6069214673486 50.3245489035833 61.2183034191703
52.1788489555942 57.9713749600418 51.6778366958973 56.233217375698
64.7963220317334
C3orf63   67.8774399765179 73.0385284042351 121.120217132666
89.7554896946341 83.9387217886961 114.965999573704 71.3527183280827
56.4779645313958 69.779139490544 72.42732668079   75.3075812535743
68.7753456118815
C3orf64   66.0069448981947 91.3475300950304 99.5125674355716
69.9214859673839 74.6474346708746 95.1000239210194 65.8986841086198
57.7125861123012 71.0211264570699 97.7620313090597 91.4990733687068
104.796373568883
C3orf65   40.0988483617359 37.8685585043426 36.064718696392
37.1894304543751 37.2567512265363 40.0766543123257 40.0052661202207
41.7419877611072 37.1820543575123 37.8592299544905 39.3086414047188
38.195965820384
C3orf67   43.4626049853157 39.2907645923217 35.7105247506212
36.7892997857376 41.7929491793697 35.6125439901743 36.634024014558
41.3506832276112 60.4837400912239 55.6865353018811 41.9908237694988
40.1700117452801
C3orf70   52.9609836781499 54.7855575216687 51.0539624358342
47.6687908290818 58.9875400392427 118.49449420132 51.7714254834195
40.2382908228517 51.6731332961917 108.709630036731 85.2598847559951
107.799236230743
C3orf72   75.5751205142867 47.1332119496722 42.8270038153139
50.5310598656998 55.1563703466328 48.5835484742456 53.3111230705754
60.6706478075023 47.0804681460898 51.1169368198437 42.5426057453993
51.7833333848627
C4A   30.1388203764747 37.3634897565502 28.5895665533366 35.9521941716188
36.8059561571675 36.4986986135769 34.6720534245631 33.5578650644246
30.8360322325222 36.2435874904689 35.4386677371439 32.3796054199032
C4B   38.0089018744739 42.1607322031054 34.5718473203083 37.1894304543751
45.4751076037511 39.9397419279057 38.4005795891396 37.1317006162938
48.8067321016961 42.8936990772838 40.0766543123257 39.2469742642337
C4BPA 92.4896666046729 41.4508241771315 35.6139301508335 42.6913548400127
42.1650127805175 37.2686490268265 43.0930293831497 44.7044140979108
31.859589438824 44.8997235283254 36.7283081422707 38.0452862258803
C4BPB 48.0570064280814 39.5050383901884 47.5673419710251 40.2455972565968
40.7273974755431 42.0016830644258 46.2448677480802 38.2782436497441
57.5430365699386 38.3501188844251 41.5421429082231 39.0536056496869
C4orf11   36.3649677956953 33.4967065724734 37.9482157381008
42.6913548400127 42.0709558258414 40.9506062495231 38.6243997507646
48.5681478665796 39.5806465938526 38.3092241935352 40.1742127891824
39.7411455670172
C4orf12   36.9060997041453 39.6995529770239 40.4070046183108
45.0798408927912 46.1804647841331 42.0574848021044 37.4772922422734
43.3262148375525 40.3316153483289 39.7835513496873 40.0766543123257
44.3521099857368
C4orf14   184.989940464859 311.985010273921 344.44626908553
203.759116151774 440.93348620369 294.908371819124 229.035497774884

163.097224522154 288.642294976 205.086228002776 216.744244428166
249.220968977184
C4orf15 54.3554233228353 56.3560498705517 52.4485219671976
57.4595416719581 54.6820069269589 59.7009992986607 65.7873990226533
49.9332280449549 59.7483827511172 67.1278109419819 69.7215372275927
58.112348043634
C4orf16 44.7520317855773 47.9665737016961 44.3648993568405
45.9441733841041 50.4463723277824 46.1513019317765 44.5390641547628
49.2019743609873 47.0055981216687 42.1317134987795 45.1778228816327
46.201352759435
C4orf17 43.5710871617107 35.6434124419782 39.4710743516517
34.4382958501338 43.3332476888305 46.0364000143537 42.629412460409
39.853866204927 39.1790517315259 40.8049408996274 38.3684202018524
39.8497227447888
C4orf18 159.440571032045 85.1544284060549 78.7921230549822
179.756604242873 70.3213394542625 153.279284223069 85.3227908896318
65.0234989597222 73.1215732923523 98.5746416750459 88.7461361855771
99.2749350936362
C4orf19 65.2735059202804 96.038271786159 64.7318794662958
41.1886975241176 207.540972220982 64.7905331739366 84.0691608193405
117.215323339475 111.291087721139 87.7918574811458 66.737740728688
67.9850517608624
C4orf21 42.6036491382658 46.29198852834 34.2890785915814
36.9912468186322 38.4540849632254 36.3845892764084 39.5496902678788
37.9837233601316 44.1519466943185 32.1260264272477 38.4689132737495
40.4639532172338
C4orf22 41.7397731376692 40.4359177791566 42.0621425894624
38.745552403895 50.0580547772867 44.2607789091176 42.1607322031054
45.9604707098953 37.6383530120621 43.844855336215 37.6484782516569
41.1554481567603
C4orf23 46.441301934328 51.2405397507224 50.1520209533747
64.6945454069792 53.1884505015411 49.2050538150106 58.6431245059655
48.7814131120945 51.7833333848627 50.7855742231932 46.6442692119997
51.8251888604386
C4orf26 44.1294459089314 37.920964499903 41.6079958749402
38.4208429103799 34.1686574241133 43.3785777889265 39.3585350448719
40.931294690669 37.9273653326866 41.2276963324981 39.8184668132023
39.6352613956998
C4orf27 114.979124960177 87.8681250426295 117.074650076861
82.7394574977114 130.515102593803 135.567012693522 140.610918488396
96.213791081731 127.232160404726 94.4129246851651 85.7732262204681
126.838679504229
C4orf28 42.2455707947223 40.6380495994768 47.9035532886869
40.4359177791566 46.3150221024046 40.040778349137 32.7676164492555
38.745552403895 47.6195009345747 49.0841460667818 41.9083581529276
37.6557714476853
C4orf29 57.2960245538881 77.3367966395554 91.7844162703214
78.0020909283414 84.3040298294164 55.6242009731278 66.0350709576651
58.2043532960091 77.2413564369342 95.4072431786073 110.589130460258
101.699631771662
C4orf30 139.191263237202 219.702703529829 148.906660152784
143.13189461696 203.559653641339 202.957014793417 207.513604688444
165.820230766206 186.450759425396 158.637047990511 200.860376997315
126.115065200084

C4orf31 44.436995101921 43.9421822132391 34.3633615460307
36.4564050256915 56.1186281675573 43.6670755115848 33.4316681623175
80.7067505750848 40.5912213523345 75.0665411373616 184.536327776767
85.6964911093755
C4orf32 47.8249696958403 43.3016395799961 47.646800205189
48.856176220627 50.0173461659833 53.8461734834857 44.1407835440612
41.9820792173615 46.4861258030402 51.7343702554597 54.6603683143597
56.796621176582
C4orf33 48.6621527194963 77.518381991736 79.0962508551502
69.6511571901941 57.0504111569238 53.8461734834857 59.3549213146532
59.7744650739434 97.1271615802556 64.2174369755376 62.1796792097014
53.2046867327331
C4orf34 264.600870859012 545.514314651238 601.611039798626
352.600793906035 146.046179302584 455.223217772744 221.22852405053
566.77872642437 350.192608372742 615.011590811277 734.143790759359
741.908952472992
C4orf35 40.5654775358427 39.3999056968598 41.9666541498638
39.1706564265495 46.4343327861696 42.6575535390785 36.634024014558
38.8495095797881 40.5912213523345 42.1622837416589 41.0808257270221
39.7411455670172
C4orf36 52.109723558511 52.1967540852276 59.5444359507702
51.3851157261451 49.0828761839116 53.4466111440611 62.6885730386156
55.1933004802607 61.8824218975538 52.0999467564713 48.2050207071554
56.1875519519366
C4orf37 42.2455707947223 43.3972849968032 43.7085219318425
38.2599935139101 41.9397336249742 42.5660695551615 39.3999056968598
37.7217193704498 46.0633179231318 40.6022003765153 42.3413611855132
40.9149601380325
C4orf38 46.0920218698606 41.3610369241157 38.1421709520616
40.5484717678957 39.3667161654772 40.1428591998404 42.9803717121865
36.0306975524309 42.4540072551568 37.2089297656361 52.1788489555942
45.7562276914441
C4orf39 40.4639532172338 44.1239711172417 43.9450023643394
48.9944486068347 47.3172462673426 46.263826584899 49.540449125788
40.7311497914871 50.2223403889892 46.0119912782855 45.6348103033479
49.7336769474446
C4orf41 126.528654651607 121.135772395548 136.011269830829
118.587373377593 137.081911344143 137.878417253635 121.300283654541
102.717305041352 110.599131324937 112.469702446962 115.676508349703
127.986477405999
C4orf42 49.540449125788 45.1694246393676 44.6032218589199
45.5869891570251 44.4489662507893 50.3245489035833 42.2577689927563
44.5029690493211 42.4540072551568 44.571915452417 42.7275315121163
46.3411353377615
C4orf43 42.3433931248155 46.8954576545929 41.1367478979484
48.665578118898 38.2496050953547 43.5752393599169 41.253645578517
46.0633179231318 45.8325181856483 40.1179210534118 43.9450023643394
44.1452943804044
C4orf44 42.2618615571566 44.4639405506967 41.2365089689635
43.6726153508978 39.6123051184545 40.8634043028528 41.9741368971524
42.5435283762933 40.1738310955128 42.5193208629123 41.8168470823451
42.1537476443423
C4orf45 36.7356360093603 41.7337506166619 37.5597671487621
46.614092935749 44.147557390293 37.3799542961839 44.8643898647322

39.7509165247419 42.992884918158 36.1550293353654 41.9908237694988
43.1805016690297
C4orf6 45.9090659324223 44.6918600441328 43.0971787292711
45.7397504535169 43.1252272136667 45.0882356578404 48.8319884367456
43.4544762830742 44.3440839686558 43.4885245598939 43.6629145624937
44.8372404608604
C4orf7 35.8208542506595 66.3026572113793 38.9790184198145
225.827961496781 37.5556299264699 37.3799542961839 44.3440839686558
44.8997235283254 42.5406888281114 44.8997235283254 47.299647867533
40.3691324547049
C4orf8 74.3135387703106 94.6300022444815 83.1874704625702
82.2766822827861 78.0077300979911 82.7269382676336 100.650129064849
90.9010016862442 117.457080110677 114.796237914478 126.204310123186
100.049462976601
C5 72.8120269375546 53.7332797903102 76.1493261430901 79.4528447794527
56.359578588344 115.659933682533 87.8746766762445 83.9236799134394
63.3694113050568 108.788705797019 91.8414653633828 72.5346469941833
C5AR1 108.493693862509 75.41408189458 72.6731815563159 1070.52156560795
75.6471706262645 240.139560726101 275.928723831899 109.241121384212
38.6322728994674 43.6033221838149 46.6442692119997 57.445796273665
C5orf13 877.673009984357 191.503068870249 352.843053667822
232.516262874696 236.595286890677 198.15553991394 184.421617360366
330.550018306903 87.7337304906377 190.592556065939 132.090336898032
80.0381580139229
C5orf15 740.423874031773 662.041552795049 957.682898193927
521.546082699431 474.379113836506 423.608694638779 670.958499409833
811.142611128461 742.726253630865 706.087945508248 751.953794043451
583.915973546284
C5orf20 44.8914371498528 59.3406363431414 45.310660704531
88.3459116251233 45.6895223823001 43.6670755115848 40.9157352260901
45.5581356216957 45.0017158586743 40.5090849993014 46.824299585607
41.4508241771315
C5orf21 138.100169316776 240.178571901813 263.125290391542
213.345169940536 149.475318376458 383.190278161083 190.77754486426
182.343696914747 168.883366756191 369.451882513347 282.646286144563
311.1022748407
C5orf22 47.5647821785529 54.0052948715814 55.7764940944098
57.2856683017619 54.4586176399381 53.0290605707162 70.7238101239421
53.838484549134 66.1448688879431 43.6033221838149 51.5822637582617
48.9733758107788
C5orf23 38.6454098044474 50.7487159427973 41.6079958749402
41.4273122914951 43.3332476888305 41.6590564937735 38.4005795891396
45.0972184964705 43.2761454096265 52.2256177934134 39.153226699662
40.6614700944853
C5orf24 54.5358897781765 59.5534127075228 67.8010199320754
42.2496337895968 49.452985143568 67.4846787600629 55.4004611309636
44.9977782988116 61.6839343510302 44.8997235283254 56.8436081694537
62.0967404944031
C5orf25 70.2541961995753 47.4740726032707 53.838484549134
56.1780366332082 44.8351976221735 65.564930068976 58.6431245059655
72.8378863017531 57.2716485658185 67.8233625051884 74.1425714439424
62.58750768974
C5orf28 770.715044136717 391.397318564183 470.231929311877
677.481235695517 335.569876624791 295.127975044241 570.824732318332

1188.3486464341 308.841311505431 1105.00321488322 862.031722874406 746.252816568691
C5orf29 43.4626049853157 59.9682734755814 61.1076362129586 206.791464145857 42.269054475455 76.331185787832 48.0283573444179 47.0940613507321 41.863927676552 44.6918600441328 43.3085358214154 48.4535991392809
C5orf30 56.8168526942685 47.8400694856726 55.6865353018811 51.7739270955254 48.1473938886239 51.4931742685391 92.3034609409756 66.0303950772736 74.9179396623258 55.4037482627222 56.0477607422828 53.840976437122
C5orf32 307.922754395377 198.360449141425 130.925724760241 559.9670724278 164.612971085488 229.926637466766 295.848968688724 293.151956467056 313.660064080622 246.044674996861 376.453560620912 837.959914510114
C5orf33 50.379587451669 53.9275079525238 57.9279989359711 52.3004056941891 62.588825192561 101.909225305064 85.2088054619229 89.0275988550016 68.9038222450855 56.8201201798712 73.7873098190297 61.356483470053
C5orf34 49.9014504704021 52.8455400026194 54.1157382341041 46.7577982595088 111.714563124738 61.2387559102636 91.3144101155547 59.220967994972 61.2070368742244 51.6778366958973 49.6706535876636 47.2862299990938
C5orf35 63.3717459766798 77.4676205299631 86.0550457387351 96.0770202999531 81.3759380182158 98.501431740607 91.116454717205 52.7787010032051 101.708164294697 83.5069968129733 76.8713035584472 70.18107651423
C5orf36 40.018129854 40.2493681589104 39.5893773029164 44.265834798964 39.4693871570028 38.861780967827 42.7431359850339 38.6454098044474 38.3981095229401 42.8936990772838 40.1362682639916 37.0431286518306
C5orf37 86.6655289455628 142.863673914134 105.965906809429 99.4184778430267 95.1131101630937 114.643475335002 111.558107942386 79.2701322339022 124.44575078108 96.5102825310977 99.9752275166514 79.5898953726467
C5orf38 42.6036491382658 37.2810767789306 41.528370959196 40.1804147506786 45.5809132232681 41.4508241771315 50.2016912396771 46.695848687694 45.1899271235974 40.6022003765153 45.4799270968969 46.917149434588
C5orf39 94.1967497986791 98.4175422336425 109.144663527001 155.018308238778 91.5258329186709 96.8783070574131 127.811684080184 72.5835488194631 97.5394336319916 107.242476674629 102.471939526704 89.1771576058145
C5orf4 44.9896851005259 44.4772114267866 51.2776048338 43.3078542962968 42.9090346219023 78.1132840562953 43.3262148375525 43.0895173605825 43.6177016821274 83.8419777255838 68.7091708087448 55.540888910486
C5orf41 82.2266112813039 98.2280780221631 112.532906407663 401.956364010068 77.7852875052734 260.426488908824 99.2341244350412 117.948047796034 73.0970339739187 136.947616119439 185.191228931155 154.386505379733
C5orf42 44.4639405506967 44.066785003302 42.0621425894624 42.1622837416589 42.3685136297603 42.9104454767916 42.8684958525042 45.9604707098953 41.3803269429468 47.1785588364488 42.046575509735 41.0226232127351

C5orf44 56.9360840563854 57.8005007012205 66.6332042895961
43.367124590178 56.1186281675573 54.400905818898 43.5627649099623
43.2551508830106 57.0301170427216 41.3641352453779 52.0583273941813
58.0722340887264
C5orf45 48.8333989709933 54.5027833453824 50.2869791217819
51.849385316267 57.0504111569238 58.512477459583 52.8496345990125
49.9951674107458 53.838484549134 53.3577647219133 52.9567386506107
52.6151169127166
C5orf46 91.31756001203 36.7976562409506 38.3166554965434
52.5307002738469 40.5710637884029 41.159706131351 38.1793598983615
39.0620487644635 40.0403898620771 43.7303813515864 35.860430241131
35.9604262434141
C5orf48 44.0328992213727 40.148780650831 41.1367478979484
39.853175885492 36.9702228618069 38.2293008890613 44.7255207589606
42.4580504587581 38.3418933808844 34.6720534245631 35.6970528713709
41.0657892043496
C5orf5 125.786923817078 137.955007835039 117.074650076861
155.018308238778 115.132600341395 325.563540852801 90.1500004950245
135.11808699414 179.47783529 446.902178094675 327.193250073326
234.333935191237
C6 43.0001432913058 44.5152708955623 36.6522278218553 43.074472921841
37.7576900881741 95.0738959950301 48.4859552186772 41.2551966132486
39.6609238036815 47.8207974930031 47.299647867533 42.57175366709
C6orf1 62.5239209616027 50.3586212130973 54.000164292461
57.0629653735694 55.0618818132967 49.2731519807085 46.3486551193976
48.8467058855408 53.0756537912154 53.5993601965286 50.0577385875347
58.9069025298754
C6orf10 43.0001432913058 36.1232327599945 38.8775560911841
43.8002456102021 41.2740822675592 38.1169703906207 44.2166118829877
41.7419877611072 44.0519245668737 42.6793753550854 42.7275315121163
41.7575097298927
C6orf103 47.3791873993182 41.554942319225 45.2313723727673
45.0798408927912 42.4864576619427 45.2852528185486 46.7100796279384
46.0633179231318 45.2897806651929 40.1179210534118 43.3085358214154
44.1452943804044
C6orf105 169.970634268117 49.9547657591437 61.6878176260761
89.9284109077375 54.2301964914419 59.5979861826984 120.721482177994
67.9103729751746 59.5093038629766 49.8393069675192 56.1379957867124
48.6037684272115
C6orf106 106.620429405618 63.3357696118348 64.3312319756553
90.6516730646251 78.1275793755619 72.0674952635945 100.386011517899
79.8913276294876 83.8679756709676 93.955760127914 85.2598847559951
82.2266112813039
C6orf107 35.8208542506595 41.3610369241157 46.9040704638073
38.0958223740111 55.3100442506299 40.8634043028528 42.7431359850339
44.0390019242862 46.0106098064475 47.0055981216687 46.972521499515
41.3481355128089
C6orf108 511.067537752967 224.249210397247 261.929727465462
254.639522735592 466.074595343541 276.399037769005 399.410299413602
244.907057340556 409.312313055794 212.803471204964 256.215133513379
211.279069257808
C6orf111 204.130491885587 201.616017553422 318.369837408344
219.291627770586 140.199798713912 190.703466372947 125.393232344778

146.133951379664 181.123652339134 234.675357616705 230.117814622646
315.592187396219
C6orf114 29.4486614241988 30.3554137634399 35.2838283525874
37.5382094267692 34.9062568194127 31.4686555405126 34.6720534245631
34.9580369221164 35.7973685350741 35.4990901121893 34.8383995317036
35.7451401627836
C6orf115 215.557210899786 140.648209468568 117.556281726503
110.431464700539 123.433224929087 67.1729762600915 205.789533350801
151.438409623226 99.2341244350412 91.4684013366068 72.842552380479
110.860844149372
C6orf117 44.7044140979108 69.2082505043031 244.736442646499
55.6481366056639 47.2074332636566 41.4508241771315 116.311176407088
57.445796273665 99.5192591352063 81.537796828766 63.0043412964216
73.7335870746827
C6orf118 39.7576136507593 44.2630383225933 41.0356368374708
36.3604951341921 40.4639532172338 44.2607789091176 41.5817737564592
49.6203214143464 40.9901805728485 48.7414853386615 45.9104478407175
40.7704587752083
C6orf12 39.7320564340365 46.3370338074996 45.063792968927
40.3691324547049 40.148780650831 41.6590564937735 47.0874209746031
43.1931731665131 46.4164127487052 50.1649089793069 41.4508241771315
44.4951343330035
C6orf120 49.3463416794995 55.8046062733709 52.3432130135266
61.0845101400379 56.4685270445642 52.4351670989268 44.7255207589606
45.0972184964705 49.641159251878 50.6486940895674 53.4088637045445
58.6481644777552
C6orf122 38.6454098044474 40.2493681589104 41.9666541498638
39.9697332660833 42.269054475455 44.2607789091176 42.629412460409
40.1474543172332 49.0986627051784 47.3791873993182 43.0971787292711
43.5738557773903
C6orf123 39.266565827316 42.6022652963326 41.785676764591
42.1273928203021 38.9496515344837 48.260931742163 45.8980298854198
40.3357777530419 41.863927676552 38.8507804383261 42.4580504587581
39.6352613956998
C6orf125 671.959673037066 319.248898194029 511.067537752967
374.427492246826 788.950470077369 375.354746298695 425.435793468733
516.992846073349 732.454837219705 377.847116683974 374.427492246826
505.294880430281
C6orf126 88.7928773498158 43.5873188565519 38.3166554965434
42.4580504587581 48.9627346956003 40.650742261428 101.549779766678
64.3284163241986 55.5086026637973 41.6373771565199 39.6403089864678
42.1537476443423
C6orf128 40.5235471987417 49.4067286971348 57.3697604614404
55.9563971339766 46.2478069975709 43.5416683728269 41.8751578329365
46.7980335276155 51.4111372893136 44.2580151149956 48.2573234404682
51.8562635270111
C6orf129 266.865951970965 178.323615832679 175.362103430164
280.622184239624 234.477015135311 165.322633260592 408.865817081303
149.899176549323 297.950627905767 109.525921548011 100.057155418659
153.081027338185
C6orf130 165.107387190373 124.002268940785 118.720812623965
150.941470539313 98.5828998914961 188.386331007465 157.095926797414
96.8240908274121 159.973746908596 185.395460281034 155.378768637562
202.459251553516

C6orf134 53.7141746468176 48.1802212380953 51.0902940392974
43.1447142498924 58.8281931439093 43.3234153488091 57.51482768194
55.421880601441 49.2879849674517 51.631710130416 52.8337362018272
47.2797818740024
C6orf136 152.66454549157 176.492987902471 208.097209050425
96.3741876853801 212.187855950414 112.873697337138 200.002687565519
99.3448096967314 193.569399061809 174.584330660466 172.676819138152
165.311139930529
C6orf138 48.5835484742456 47.765848710116 51.6159542017038
47.6687908290818 48.1473938886239 46.574682061365 50.3519150282938
47.2009420656854 55.5751438859404 50.6486940895674 46.824299585607
56.2804354745795
C6orf141 156.636360703756 68.1969125201241 49.2041300973417
41.8778873678551 108.479856042609 61.6219790276408 67.3976855661302
52.0503062610092 51.5229124652769 52.7972156492746 49.6706535876636
50.5511827666954
C6orf142 40.6559419616644 37.6383530120621 39.4710743516517
34.9221408381038 38.5512429290626 43.7630587657205 40.4359177791566
39.9576138799997 42.2577689927563 38.4187686307607 40.9157352260901
38.1421709520616
C6orf145 37.55264801907 36.9994058279362 44.4311655729487
51.949834638817 38.0598374201407 55.401776072578 36.7356360093603
45.7476095696859 50.4974478884077 49.8393069675192 42.8263096141654
47.2131144508464
C6orf146 36.1232327599945 36.4002279956519 43.5710871617107
41.7614975630362 40.8873907150245 40.9506062495231 39.4600924554505
38.6454098044474 45.6582729799009 44.3648993568405 39.153226699662
35.3525469364579
C6orf148 46.441301934328 44.6918600441328 43.7630587657205
39.853175885492 47.9128504683618 46.3715909653832 40.4359177791566
47.2864663693787 42.2577689927563 48.4890615340603 46.7386599624801
45.8546719310325
C6orf15 38.5648843914189 41.159706131351 37.0642268327439
37.5382094267692 32.2133093774284 40.4359177791566 43.3262148375525
38.6454098044474 39.4762546236449 36.9102644736882 37.9946685306274
35.8429598823818
C6orf150 47.7267190613906 70.0470864733496 46.2625470410195
102.177189421378 48.2748123114598 48.9826868640753 119.277678423944
44.6107027295193 56.7991853808475 42.4594149893798 41.8168470823451
52.9609836781499
C6orf151 69.6449298794042 58.2571968311834 53.0104469636221
38.6414316877471 80.9160262677505 62.5542376788358 60.7992483126292
49.6203214143464 81.8983491901699 49.5265949137207 63.4323398447015
74.6734358603424
C6orf153 442.380936516984 301.025314855652 419.563859911367
341.397811775105 530.314249832491 294.372319595103 357.574415292533
252.616382150668 374.601792595646 263.125290391542 242.932905246176
235.148436322043
C6orf154 41.1523487516364 38.6414316877471 39.0775870687758
40.4359177791566 36.8059561571675 36.4986986135769 41.9393838138203
40.7311497914871 39.853866204927 40.3235655331796 36.0481934423077
38.3291713594599
C6orf155 36.6480005877129 38.8336427948187 40.0998167098057
37.8442841690502 37.1565984182084 38.9378717994178 37.3799542961839

43.7396619462033 40.137818555383 37.3179578037963 39.5399188536544 40.4639532172338
C6orf156 44.5282181324797 42.5269793679335 37.4642807402741 36.6501141438378 42.269054475455 32.771863438151 43.5627649099623 39.0620487644635 41.4636047919453 36.3612409063792 42.911855821602 36.7762265039864
C6orf159 39.0237504776589 41.7614975630362 44.5134109184888 45.334106390212 45.8051846088552 49.1718303518849 49.3854238365325 47.3791873993182 45.6080994842498 44.1861590259178 48.042366934301 55.216338306812
C6orf160 354.158105175308 304.977139992391 324.877187846959 221.743315748689 99.3596089438084 779.333013700435 221.743315748689 503.793770799548 174.985336606027 582.511224532378 1137.99307039386 564.929183025034
C6orf162 49.3463416794995 62.1642339365777 48.3458108324478 50.5310598656998 47.0954207417646 77.4812665677338 60.1705241170421 47.0940613507321 53.6446045619513 49.9493230064613 70.9638091288679 61.4366256816323
C6orf163 46.5549623118323 42.0709558258414 39.9101908547397 39.3750969348308 41.1868052124344 45.4882438975269 45.7452456977857 44.2159020605784 42.7150227340364 44.4652605017595 41.8168470823451 43.9541718254769
C6orf165 43.7783560914188 41.3351256327455 44.3662228328134 42.7431359850339 41.6285300488826 41.0582900711341 41.4244721780607 40.5585676679482 40.3704050658025 44.8522313252892 41.0441816173391 42.7782216342806
C6orf167 42.9104454767916 44.7829632472754 38.5045333213784 37.651298468202 45.5809132232681 50.7516773099639 44.2166118829877 52.1788489555942 44.2607789091176 43.401782064778 48.4717951775049 43.5738557773903
C6orf168 42.8036731479932 46.2286164776607 40.931294690669 47.8164193292842 45.4751076037511 98.501431740607 44.0687697278114 60.419911425109 43.1805016690297 70.4895190282848 70.2215218476683 75.7140064167134
C6orf170 113.351710503778 103.631449539809 107.637618676176 115.913030448537 68.3419511055057 78.0586153423713 121.01013827107 188.734299563005 73.2144205844199 151.266092912661 149.19643034862 120.96329303892
C6orf173 121.992700597635 128.885933852704 79.4998723172096 84.2804410361247 73.1654226019863 67.8208976798364 176.839214683647 131.67159377585 148.333778341005 65.813547096222 60.2165699959135 57.2350830635912
C6orf174 39.5676051939342 42.6939394537076 35.3649449469275 43.3078542962968 47.6884372645373 44.1737502492846 42.629412460409 44.0390019242862 45.0997987980157 41.9666541498638 41.7272875295469 44.7401523707775
C6orf182 39.4710743516517 40.0527893189096 43.9450023643394 39.3750969348308 37.4470183315826 46.0364000143537 44.3440839686558 52.1788489555942 41.4636047919453 48.4890615340603 45.390192977585 43.6753843488695
C6orf184 45.5758521267508 43.5182502375207 39.8299340037009 43.0001432913058 40.148780650831 40.9967936671247 43.4933087941017 54.9025626101017 40.1751033251565 42.4301183791899 41.9594153733826 43.3234153488091

C6orf185 34.8099089553046 38.0226905622357 35.2101597814352
35.7674462337723 38.5512429290626 35.8227939208521 32.9962272670005
113.655306804539 40.4997186069494 37.1275334308172 37.7384958256497
32.0636251428594
C6orf188 54.0990839134272 45.7317208802318 44.4311655729487
48.1662933382789 42.0709558258414 54.4753345912615 50.3519150282938
44.797320243931 43.4656637536244 39.0648733099061 52.6960621424156
46.3411353377615
C6orf190 40.0557788909923 47.2770121962655 49.5424425850549
49.9014504704021 40.2449343247539 43.3785777889265 41.4742337714895
43.1567024035183 39.2809305246091 40.6022003765153 41.4508241771315
47.5025821041537
C6orf191 38.5007204246293 38.8336427948187 41.785676764591
41.0726336841311 36.9060997041453 40.1428591998404 38.2933870238743
37.0390589468421 42.1594905198929 37.2089297656361 38.1835938174625
39.4490233720848
C6orf192 136.155022329495 190.30941883755 181.738902391483
179.07886477603 136.429803886927 119.866221751572 171.853856533964
240.11295387591 115.052948380817 215.504612948048 189.267281390459
180.63535336811
C6orf195 43.9309845324767 41.8848959280916 45.5883690965192
42.0102671859606 45.9104478407175 40.5281496800812 42.7431359850339
47.2864663693787 45.1899271235974 46.7646877499666 46.4205654140754
42.9887069977696
C6orf199 41.2424637338424 49.5443425465542 45.7968648044613
40.7481155008596 40.8873907150245 46.6872654833579 51.5967098638347
99.2432911346944 37.10170285405 41.6373771565199 45.310660704531
52.5752569429057
C6orf201 40.148780650831 42.6022652963326 43.0971787292711
46.7577982595088 41.5668884168669 42.2577689927563 40.6750540874299
42.2577689927563 46.581796165231 50.3388505346542 39.8184668132023
42.1537476443423
C6orf203 51.8781065691038 43.5873188565519 38.0452862258803
40.8681871019812 39.5595233162192 45.6129956328415 38.6243997507646
88.9038123445083 42.5406888281114 43.2988017098387 47.8284278211109
49.2064066614865
C6orf204 44.3355304829989 40.931294690669 37.7963032976457
44.6905691366696 36.0439235933921 43.3785777889265 41.7614975630362
40.4359177791566 41.9635525160083 40.6533975461148 45.447310733898
43.7672102682067
C6orf205 45.5758521267508 39.8695929627783 39.991353369336
38.3166554965434 43.9450023643394 53.2046867327331 42.9803717121865
46.325232414659 40.6957787921451 44.4294261786154 46.29198852834
44.293922970134
C6orf206 49.085741080241 40.148780650831 43.0971787292711
45.334106390212 37.5556299264699 41.554942319225 43.1931731665131
41.1575777497576 35.6139301508335 41.2276963324981 36.9060997041453
39.0536056496869
C6orf208 47.4678477186801 43.5182502375207 38.5045333213784
48.309866878365 40.4639532172338 38.9378717994178 46.7100796279384
37.33030683349 40.2274522635576 46.5650556481721 42.8263096141654
44.3521099857368
C6orf211 51.2851682978581 58.5406981871153 71.7336217321663
59.198088069573 68.4961024593552 64.0354923953233 63.0842071602791

43.7731874801003 58.7382738968248 48.9826868640753 53.4088637045445
62.3997407955361
C6orf218 39.4710743516517 42.6939394537076 43.1931731665131
44.0451487108213 40.8873907150245 41.3553369544097 43.1931731665131
39.5466975353131 38.2215591930186 37.5343260962758 40.2649309204478
36.1647506311964
C6orf222 46.7293238583888 39.9697332660833 44.7829632472754
44.550322123994 46.0977687311222 42.4580504587581 43.9450023643394
42.6450179841129 48.0163377492228 41.9666541498638 38.8507804383261
43.4709415392696
C6orf224 44.5282181324797 42.6939394537076 38.1421709520616
42.357910063876 37.4470183315826 40.4359177791566 42.7431359850339
59.8760452220989 38.0213051227859 39.3698811593364 40.9157352260901
39.4490233720848
C6orf25 43.9309845324767 44.8024081259593 43.917817324545
41.1886975241176 38.5512429290626 40.7158901597788 40.6085376283663
41.7877325563821 42.5736687688554 40.1613888954735 39.0550139257658
41.2836317141451
C6orf26 55.3153799233744 50.6229884142872 55.7764940944098
40.4359177791566 59.8102324370568 39.132725377815 58.2315917325629
51.9447450229707 50.7619345314571 59.6863210839828 50.0808824383821
50.0571060701893
C6orf27 43.844855336215 39.5050383901884 43.6670755115848
42.6913548400127 40.0599807618105 43.8582747295766 46.3057385979341
45.2806199505029 46.581796165231 53.6429962450784 46.3370338074996
44.7401523707775
C6orf35 41.5421429082231 40.2093230376545 41.6977706798234
45.9441733841041 41.6806984441849 40.1930209223556 46.4388841354122
37.4484310268909 36.4816715148518 40.7063426028871 42.9634662926872
41.2437378488025
C6orf47 135.567012693522 97.0589984189239 92.3034609409756
85.5959901987745 117.033529761587 96.5287245311078 107.466490458133
88.5511408870489 110.699536638528 118.410983614453 127.49459343016
110.947379383048
C6orf48 451.711598030737 448.196293409768 527.686970237622
368.660803187338 471.874412428677 1174.92419086759 345.294930735691
287.423909789368 460.340300658834 604.951648398924 744.080606078086
560.954913956997
C6orf52 74.2151462073098 47.4740726032707 49.6347864008749
60.620259884953 49.5666074434383 50.7516773099639 111.714563124738
107.427186954956 91.5370872909881 60.6266092582071 53.0798381965427
60.3816817102326
C6orf54 45.4799270968969 43.3458769998118 37.1643693306382
35.1451084093692 41.7929491793697 41.8613406887824 38.0598374201407
44.7044140979108 39.4762546236449 43.6033221838149 40.3452320167286
41.863927676552
C6orf57 69.9431447778061 91.6978282652998 121.423421895502
94.9785931907153 84.7782034884529 55.3202238943712 102.77259665192
75.8976034525022 79.2129308982479 57.9713749600418 57.4622430729629
105.828093778276
C6orf58 39.4710743516517 41.2598458726002 38.1421709520616
36.6501141438378 34.3633615460307 42.357910063876 39.5496902678788
40.4359177791566 44.2607789091176 42.4594149893798 39.3667161654772
35.3525469364579

C6orf59 41.9407041937973 38.1312950900467 44.1452943804044
52.3004056941891 40.8873907150245 46.3715909653832 38.5066108855005
45.7476095696859 39.0775870687758 60.6266092582071 49.7363778831003
46.7182294568603
C6orf61 55.9249770393176 60.8608693323868 73.0128142320238
61.7502624458447 51.4527545197672 41.6590564937735 46.8613212048981
54.8412943008635 48.2123120250343 63.3006977162657 68.4659783447092
76.2533853403813
C6orf62 262.153247969144 153.660685857642 159.727829541783
172.540737848092 183.498228118271 135.323750661937 409.433491782481
494.733185937057 169.168332370229 180.029671376384 171.058650956601
153.864236983777
C6orf64 125.383837024985 66.8026147385682 82.2766822827861
73.1447139654971 79.7808217577226 74.6375749739389 85.6236091398006
72.3822545261582 86.4498396868205 102.771611905219 80.9024704745445
89.4747568145717
C6orf65 94.1967497986791 45.4882438975269 40.7620472083159
56.9299697426319 42.4864576619427 100.650129064849 48.4859552186772
44.5029690493211 45.1899271235974 71.0619469948187 49.9338905397767
54.6659845741133
C6orf66 58.691436315511 70.2288685232877 58.112348043634
55.9563971339766 55.2214187655851 65.813547096222 58.7157078555305
52.9813204449021 62.58750768974 45.3530683538086 47.3791873993182
65.564930068976
C6orf68 39.266565827316 41.2598458726002 41.785676764591
40.7481155008596 40.4639532172338 40.1428591998404 41.0381860307445
41.2551966132486 44.3440839686558 43.401782064778 44.2142581488029
39.3575468494941
C6orf70 121.104663867264 120.129857395808 154.309544187593
136.541379502691 106.527144354592 126.753713093485 142.614006543733
91.3607028449916 108.04376659067 134.601217321316 137.016387648703
111.691813916343
C6orf72 54.3554233228353 57.2231446406982 70.7238101239421
47.6687908290818 56.8307116636372 69.8832723783498 51.5967098638347
42.5435283762933 52.5772854102326 51.3351387089044 48.1352470945488
65.6876778328592
C6orf78 45.1912496459517 46.0713306971594 48.6037684272115
47.2009420656854 48.8381507187986 49.8472708021525 48.3411413510851
42.9301944191913 46.0106098064475 44.1379638573455 47.2134341420411
41.2437378488025
C6orf79 40.7609952437622 36.7976562409506 40.0998167098057
36.6501141438378 42.0709558258414 45.2852528185486 44.0687697278114
39.0620487644635 44.619512785899 38.528621456378 39.0550139257658
34.806104801006
C6orf81 47.5647821785529 48.9259041798949 52.0742951366864
39.1706564265495 43.1252272136667 47.4699236780425 44.0687697278114
41.2551966132486 47.6195009345747 42.2614524730856 44.553780984244
42.7782216342806
C6orf85 167.760135663101 168.926743047741 262.153247969144
98.2447263268855 137.132535402637 77.2834482217973 119.512426152446
314.091389958069 338.530970447766 110.909109947659 140.976643799742
99.7529382840954
C6orf89 99.1565755621214 77.8946473414547 95.1666040574435
105.790224260281 85.7842344710572 101.909225305064 90.1500004950245

100.370937669662 94.7110944331075 86.2118355769367 79.8701900696361
72.6182747525443
C6orf91 41.9407041937973 45.8669209716694 47.0940613507321
41.8778873678551 46.0043807840705 47.9503724044453 44.9863726205428
43.4544762830742 52.9482338979681 46.1390887492649 47.8988096575648
44.0451487108213
C6orf94 37.3463875295726 35.9305494063137 34.1806289878747
36.7892997857376 34.5301949364147 34.9849896310235 36.3194282084339
32.8834169462007 33.2715481910159 37.4302011516587 37.3687900733549
35.5523662009011
C6orf97 46.6348258763289 46.5650556481721 43.1931731665131
51.223859150798 48.6224651100733 55.8243694410835 49.8281348927244
47.0940613507321 48.0163377492228 43.401782064778 44.3763859390287
45.4799270968969
C7 40.9558083259517 35.3307157989724 34.5718473203083 54.1240583139871
34.2638875325204 312.245547906683 90.8691042645133 34.1806289878747
31.5151915411476 64.9959712888709 115.88156897199 98.5339473137891
C7orf10 102.513192587614 55.9269493191353 67.5417980425932
78.7450149191851 66.0411740221276 78.1952428431942 51.7714254834195
50.0526763693329 46.7760357462635 62.2025996860055 58.8188963227059
138.070735985503
C7orf11 109.192770642308 93.3216420803293 81.1249338842464
84.1868287819063 168.798543634965 84.9099930524863 108.806649898122
69.8099797531111 113.026772174726 58.2818491355907 67.6271456814495
86.4195039096886
C7orf13 52.310270746917 47.2055133646385 48.1331309338997
66.4047895961325 67.8233625051884 47.2009420656854 59.1774824918359
54.5125550937898 50.2223403889892 60.7495043977707 63.4323398447015
48.9733758107788
C7orf16 41.6354649482853 40.9731067463382 43.0971787292711
44.1483976313959 42.6022652963326 44.3648993568405 42.8684958525042
46.9986083378817 43.5669306063196 39.9948390976317 41.0808257270221
45.6692241948108
C7orf20 79.4916415027015 87.2351169088172 88.5458450926665
66.4841955249254 139.566023291311 65.3096524773304 110.935994444429
106.99628036922 94.1844708285913 61.9179665598635 66.4015820814547
61.1630754250653
C7orf23 146.658116519622 311.985010273921 361.158558609751
360.668308246913 423.254211470935 259.332340491331 264.116434279258
191.284436365696 372.982648892131 207.380865650247 225.120999402048
299.094486437911
C7orf25 99.5058761858884 91.7629175712824 99.7403508491191
71.9062462001972 103.199200862158 100.802977734227 115.408418935236
83.7019398654324 141.319488024794 77.4046818339539 87.3342335690393
102.035798846699
C7orf26 390.834121304799 289.410651581802 210.255784687052
229.792906003602 356.079406408042 271.104193102849 246.313146942736
293.755623192857 319.248898194029 308.728126648231 331.677243885539
210.524799041662
C7orf27 241.901039109114 201.941251308732 218.313812956524
197.171449112363 419.182443507848 187.422036538629 164.580141651883
152.456314990408 249.37112382902 264.263104842911 234.232348771362
155.464685445566

C7orf28A 68.8195659637992 85.0946882743418 74.3902189072372
62.1776700757427 101.699631771662 79.7564155881026 91.6526163804547
70.4262478188233 107.70358681113 57.5245761633468 60.4991141099239
71.6694991209816
C7orf28B 129.697400781396 196.096147690942 133.893108529325
121.8863522969 174.252441615373 158.449570606242 172.869292380866
124.605379715077 195.430996540859 74.0420372528779 72.2544131004774
136.504157082236
C7orf29 86.6655289455628 51.7983110178095 48.9944486068347
57.6511495792664 55.4919197421746 51.3886035574416 54.8074655158941
47.5950227477807 56.432280646473 52.8928296360463 53.4088637045445
46.3411353377615
C7orf30 726.42773300581 681.156163097495 781.223434214089
482.238595345574 1305.35453202355 855.602941621804 822.822769402853
535.585999498708 845.41973083225 331.591589534469 346.621778258188
602.726022292973
C7orf31 38.6454098044474 44.0409178714232 44.5134109184888
49.9014504704021 43.9450023643394 44.9896851005259 44.0687697278114
41.8417024632349 43.6670755115848 44.2580151149956 40.0766543123257
39.1553169409075
C7orf33 39.4710743516517 38.0226905622357 45.989212296035
38.8495095797881 44.4489662507893 43.4681646416585 38.7337086062875
41.3506832276112 38.9006788676407 38.0078357808393 45.1432412071268
46.3411353377615
C7orf34 41.1523487516364 36.6968906669565 35.2838283525874
38.3166554965434 39.4693871570028 36.3845892764084 40.3302268293707
39.853866204927 36.0741672064298 45.7911932481722 33.8558232866797
39.9519547348106
C7orf36 90.2721521466478 66.7256057011273 69.7855392178808
55.6481366056639 125.891602778425 83.0554837248934 80.0733119200914
82.6246523811721 108.937199348319 69.4131195465674 72.2544131004774
76.6707262000712
C7orf41 80.7462144785541 105.446338599737 159.99819920335
119.253559091088 185.667983376361 725.680256063016 116.311176407088
145.791398829369 87.5074297754031 597.996273468244 545.827286197333
583.237170605534
C7orf42 192.337581451878 219.134220280612 306.294404016622
263.330471155421 270.89711217076 186.67069792963 163.809567262808
313.46376203503 309.022069786139 133.192618821838 151.912182086236
174.718984988254
C7orf43 103.356071080819 103.43536623122 112.865496075486
97.6774247176874 89.8035133597498 66.1082848714279 77.5813459454823
79.8264217848103 106.521012342668 84.6563174894574 100.159565926265
72.7015808995427
C7orf44 365.860501246879 239.466489599238 304.258586051126
241.222680673439 350.106705208362 228.08291792003 297.662970257144
221.972553229362 357.762505796238 226.090904721424 255.598083334598
364.337811621248
C7orf45 46.5549623118323 42.6022652963326 40.5734458834617
46.3203995372658 35.9894133432376 42.9695585740813 38.7337086062875
47.5950227477807 44.8003345643334 48.4890615340603 47.2134341420411
48.3648494350286
C7orf46 51.1740855632044 51.0175677516029 47.646800205189
44.550322123994 59.0965219753805 50.6360662655392 65.2361525412756

67.0904616289706 63.3694113050568 57.4665857273093 58.0800636554122
61.0025829061063
C7orf47 207.710354513208 343.262403838579 310.461601607662
206.41879845205 273.349866592589 159.071016200059 256.766675299448
238.819980744789 343.892794667047 240.476103976894 255.324974349269
224.231862271463
C7orf49 109.029196594263 121.613731804111 123.908152883436
75.6103053700122 121.354667375673 82.4900927793125 112.113501370522
65.1637149462661 144.500148287951 112.375316615894 102.559678338485
91.40570426491
C7orf50 636.367246129079 726.42773300581 517.664764759512
530.710127703061 1039.58636143171 1032.44965920118 962.079396754147
999.13039066242 1239.68399088145 922.047328788961 888.56944380788
487.163435704392
C7orf51 37.8386378737561 43.5182502375207 41.6977706798234
40.1804147506786 38.745552403895 33.0463640202858 35.9058393060637
40.4359177791566 39.1790517315259 36.0481934423077 36.8282383070068
47.8931773060789
C7orf52 41.2424637338424 43.2649781354792 40.8420925848403
37.0732977341185 39.4693871570028 41.2567470068038 38.6243997507646
40.4359177791566 45.5779686360641 43.2988017098387 39.8184668132023
38.9496515344837
C7orf53 55.2182004838121 47.4076235350059 40.7620472083159
84.4422623834916 45.8051846088552 45.4882438975269 46.7100796279384
51.4360013601805 42.9020321355927 56.4553747079294 46.8954576545929
57.6860752168769
C7orf54 155.564141715819 115.40235710164 107.971806573919
235.544416908354 127.60426512782 127.265200736376 101.46584765758
145.170590045628 106.10668428032 174.058845828966 141.261334415286
138.070735985503
C7orf55 120.426087248804 106.160416826673 90.8831233784641
84.8948024208923 148.879760620316 150.009352890706 174.985336606027
73.362936710475 95.5920087912438 138.027432399991 123.190887747662
97.822911929574
C7orf57 39.3709357615493 35.5310164682026 39.6451483836089
39.066284740632 39.7568367479301 42.4580504587581 51.5967098638347
37.5511560880902 43.6670755115848 38.528621456378 43.4709415392696
42.8864415494484
C7orf59 209.967057706137 113.102823481296 207.632489510045
218.446312290024 200.986069552443 255.946758662074 287.883101814563
240.11295387591 239.569514501529 299.094486437911 389.364650157102
374.601792595646
C7orf60 39.266565827316 41.0678614026819 45.4865173433646
41.4273122914951 37.0526522140242 42.6575535390785 41.253645578517
40.831207953647 40.0403898620771 41.7405682794166 40.9157352260901
37.7526485343328
C7orf63 83.5646728968172 63.3667304749164 69.9179689232341
68.1992221684165 52.8190882379316 95.1468654370952 71.9474405671289
163.38302606725 67.0290776715331 94.7110944331075 79.3191219048672
94.7008981904016
C8A 38.385865183581 44.5152708955623 44.1452943804044 41.0726336841311
41.1868052124344 40.650742261428 40.9157352260901 40.4359177791566
39.9444191873552 41.4397539024976 39.153226699662 36.6603338274678

C8B 36.7356360093603 44.6918600441328 40.4070046183108 39.3750969348308 37.4470183315826 41.3553369544097 45.223075311113 38.956280281655 40.4997186069494 42.3606390470387 44.0409178714232 36.9976231206067
C8G 42.8036731479932 47.4076235350059 43.6670755115848 37.29566077996 44.2607789091176 46.3715909653832 42.1607322031054 37.9837233601316 43.5669306063196 41.1222709786153 43.0016969050339 40.4639532172338
C8orf13 55.5276281380084 37.8217989841918 43.9450023643394 65.7321440516177 49.7235523248653 41.2567470068038 43.9450023643394 47.6952179922944 42.5406888281114 44.571915452417 39.4410515050885 36.6603338274678
C8orf15 45.7356815244513 42.8542642792862 44.0451487108213 38.2041059863839 44.5668141895992 42.4580504587581 39.2533668167483 42.8482181313226 42.9020321355927 39.0648733099061 39.8184668132023 46.7182294568603
C8orf16 45.5758521267508 51.2405397507224 61.9293105089291 67.3439996689706 47.0954207417646 44.7804080899431 45.223075311113 47.4740726032707 42.1594905198929 69.1623287497421 84.2960453806012 89.3868257841517
C8orf17 36.9060997041453 39.0102066700839 41.3366779545815 48.665578118898 38.6454098044474 39.7394181011603 38.1793598983615 40.931294690669 39.9444191873552 44.3648993568405 40.4504608195666 38.745552403895
C8orf22 36.0144420013819 44.4411734611229 43.1931731665131 38.9534635879195 40.4639532172338 41.3553369544097 44.3440839686558 37.6557714476853 41.1742244762318 42.2614524730856 40.3452320167286 40.9683275553869
C8orf30A 44.2350046179299 43.2028319630577 43.26160898693 46.5331216396695 51.8636455065553 42.2189674040934 42.3360569471619 39.5466975353131 43.2100129360085 41.0529409675663 43.8093452203209 41.3844657483749
C8orf31 47.5647821785529 45.0905928998779 42.640829914102 42.2496337895968 60.8550020364269 45.0882356578404 41.706879587091 50.628152951493 48.8842909466002 47.4767851750188 53.7475011049549 47.5025821041537
C8orf32 154.730266766226 175.099998331811 111.227016020927 90.8188865732032 124.37984507038 130.02102880365 162.486448421519 101.156215957297 142.066425511614 91.5417574979683 99.1952492360239 141.888282820511
C8orf33 238.510952165358 203.870471031175 170.287619901365 121.851515410237 177.515053183327 192.398575159246 163.809567262808 136.250317107253 270.756849919679 178.060342847557 170.780021121783 149.173801572239
C8orf34 54.4384603094793 56.3560498705517 63.1450855271137 51.223859150798 49.5666074434383 81.8426140611297 62.0404348860702 59.1107691694766 54.8946088631931 71.1607965028029 63.9029785089421 60.8662233098324
C8orf37 1345.37091574015 499.744856832174 704.601607617837 782.639346040155 549.117045813593 440.728435508514 909.843512894628 1762.93413846062 525.35784336478 1393.24135330226 1370.76188322111 1020.75813028358
C8orf38 130.058147569795 105.118498939628 68.8098607236981 44.265834798964 178.44531401852 87.4239220649478 106.486195938039 83.4679576894911 111.643323610073 51.3351387089044 60.394307846869 69.8505620106065

C8orf4 234.615033303459 320.684862879691 484.567462838523
203.759116151774 561.509463439533 932.849256536912 208.7739716139
1269.36837611919 735.751447981069 126.764572234324 245.585026913915
274.328990210853
C8orf40 306.115856873599 386.934057829091 315.799449362526
386.98657211459 358.779372054826 378.964387825252 510.525676243746
439.955651727755 271.138311888031 206.312848708143 250.210134743225
370.917477324773
C8orf41 70.083473392272 59.3406363431414 62.8362064074814
51.3851157261451 54.5683518971261 62.843734579668 54.1707158323527
72.692416146584 74.0614295574729 58.6439719071099 81.8648220673828
89.5687388187015
C8orf42 57.2960245538881 41.8848959280916 50.7502704822135
44.7980740332174 58.037539503209 47.0940613507321 61.2183034191703
54.3820590274321 44.2607789091176 56.1912096308988 62.6885730386156
57.934622029119
C8orf44 52.2236479204029 65.3694267397114 59.4431489877328
43.8002456102021 52.5645416722441 47.8636003588181 41.1507980856436
40.047314525032 48.8842909466002 57.165971310746 59.5830868881343
54.4992295592001
C8orf45 503.793770799548 328.962069934895 435.209988251425
551.1195044975 271.070078611015 216.358862796367 399.80505620721
786.57695312967 230.787791652978 749.206358448939 677.855726232203
507.375704144286
C8orf46 39.3709357615493 43.5182502375207 40.7620472083159
42.8235313007746 39.7568367479301 43.2805590023412 43.5627649099623
40.2382908228517 43.0930293831497 42.8936990772838 44.9713479185144
42.357910063876
C8orf47 42.7613994022988 51.1541843174981 42.9641759692926
42.7489468897708 49.0145368354835 39.8785903668981 115.087963545095
68.6704961638492 56.7991853808475 49.9493230064613 56.4567728276775
63.2936377704897
C8orf48 48.6621527194963 43.7644428186265 47.2962360458401
47.481302504066 45.9104478407175 43.6670755115848 42.7431359850339
41.5464765641928 47.798305839406 51.0055825931636 52.7817052791259
45.5800840647989
C8orf49 36.5393857306744 30.9403229851671 42.7303139017786
40.0698728584484 38.1644569189523 42.157627240486 42.8684958525042
42.2577689927563 39.9444191873552 39.6719190054379 36.8282383070068
39.5418280570561
C8orf51 124.599352191013 53.9275079525238 48.3847843498476
45.4634652319753 71.5345397102823 40.7616966228404 57.8870110456492
67.2714646016644 62.4776210012906 47.1001757441025 51.1483696410762
46.5650556481721
C8orf54 46.0023068797812 36.0366673493555 40.6737160001291
42.4580504587581 30.7606118575469 33.4492509999281 37.830876898596
36.7460922671619 39.2809305246091 41.1222709786153 41.2598458726002
37.7526485343328
C8orf55 563.750309421406 1858.94179312821 924.316085565917
118.762101740563 969.805093523651 175.876518898179 642.211963248041
195.813431189188 1068.10627239579 693.727004855319 719.815240381415
572.667024472672
C8orf58 87.5146535320072 52.3896785600751 57.50915811428
54.2886483704949 42.9090346219023 44.9896851005259 46.5650556481721

45.1714698794331 46.4164127487052 61.8529316708431 57.4078217542368
55.9555622193202
C8orf59 377.76338037467 332.230531176052 274.809186311931
266.717224821545 396.63268714181 263.67173636178 308.336693903042
326.605198138576 347.829279462722 295.061449024268 239.590143292142
418.927102569912
C8orf71 38.8561375693335 43.1805016690297 44.8872784170036
46.8954576545929 44.5668141895992 37.8102105794655 37.9430145836712
41.4508241771315 43.5669306063196 36.3612409063792 43.0016969050339
40.1700117452801
C8orf74 40.9558083259517 41.159706131351 42.9104454767916
41.0219290963778 42.2455707947223 41.3553369544097 43.9450023643394
42.3887531663733 45.4799270968969 41.0057887014368 40.547835538778
44.3521099857368
C8orf76 611.538630852232 477.410937135204 274.962689159729
317.004252313348 373.46798029115 180.065047496273 334.349204174886
182.80711310964 338.164006403 290.81245174957 232.628747594691
256.675896425075
C8orf77 47.1962846565261 34.9262464079777 48.1331309338997
57.0629653735694 44.4489662507893 40.5281496800812 50.2016912396771
38.079222289614 51.6731332961917 40.5090849993014 42.7275315121163
38.8495095797881
C8orf78 39.266565827316 42.8542642792862 37.9482157381008
45.0798408927912 37.0526522140242 38.8336427948187 42.629412460409
36.0306975524309 37.7384958256497 39.6719190054379 42.8263096141654
38.6454098044474
C8orf79 43.7585538437696 40.2493681589104 37.8386378737561
40.646702696033 39.6535880152834 40.9506062495231 40.4941260038116
39.6995529770239 40.0403898620771 40.3235655331796 43.8002456102021
40.9683275553869
C8orf80 45.8108990837624 40.5400384479766 49.5424425850549
40.1804147506786 44.5668141895992 44.7804080899431 43.5627649099623
40.6171774724171 44.5303781239209 42.0621425894624 42.6366765186182
44.0451487108213
C8ORFK29 42.2455707947223 48.4980626712175 35.6139301508335
39.5050383901884 46.6397391251762 41.8613406887824 41.0871827071116
41.8417024632349 46.6944238390932 39.6719190054379 38.1253290586431
40.5235471987417
C8ORFK32 42.442231667357 39.0894172982439 38.7745734037492
47.6687908290818 52.5645416722441 41.6590564937735 46.1634822993009
42.6450179841129 42.1594905198929 39.6719190054379 45.4799270968969
43.8575940017119
C8ORFK36 38.1713217245067 41.159706131351 46.4569566520107
39.9084621723809 41.3387790895716 42.0847226073592 34.3345418156105
39.6474008369935 39.8873706855774 38.8173773425835 41.3945375181463
43.4709415392696
C9 38.5007204246293 40.148780650831 40.1985790927248 39.066284740632
37.7576900881741 46.6872654833579 39.4600924554505 41.5464765641928
39.0775870687758 36.3612409063792 37.7384958256497 35.8429598823818
C9orf100 47.0940613507321 52.8388295105803 46.1907664490247
39.8920832347262 51.2525124735102 45.0882356578404 60.162766751307
50.628152951493 56.6848620589031 40.9506062495231 42.4238335236362
39.2780515621926

C9orf102 82.6983700200815 73.6855877240541 62.5610562594275
66.0397471691747 68.0414563597154 92.681224908389 83.6935756500468
67.2714646016644 82.6157566292355 111.703187941409 115.496195377983
92.1646038105091
C9orf103 96.8783070574131 108.019575675653 111.735319884512
128.786137360755 82.1344514651393 204.251854342995 105.05842887748
105.05842887748 68.8353800542132 122.702789229752 113.403020750953
94.3953345963359
C9orf106 37.7384958256497 43.1805016690297 43.1931731665131
49.1786207920603 41.9842695818285 42.9695585740813 48.0283573444179
46.1673159982664 45.6582729799009 41.4397539024976 47.9942520395434
39.9519547348106
C9orf109 55.4004611309636 46.1569135229922 42.9104454767916
59.4674205670657 46.8674366115656 43.1826328292558 51.7714254834195
49.3880946790306 50.9010149857308 43.401782064778 39.0550139257658
41.0657892043496
C9orf10OS 104.832753086298 94.9831937714438 86.2330249146534
91.9185612536409 97.4137766910365 118.186590679218 93.2133061608764
129.179537795204 100.591821617326 122.170704886985 122.469733727349
119.512426152446
C9orf11 40.3478825593224 40.6380495994768 42.157627240486
43.1847628331565 40.4639532172338 39.0266821712099 41.253645578517
42.4580504587581 35.3227485563945 44.2580151149956 39.2542963855807
40.2669497398702
C9orf110 36.2496364442264 39.3999056968598 38.7745734037492
37.5382094267692 38.9496515344837 40.1428591998404 31.8431345631453
40.5158867964016 40.0403898620771 40.3235655331796 43.0971787292711
34.3409660445447
C9orf114 284.351747562807 327.238337722366 179.779145453083
191.291480830946 384.992066112903 174.392758289374 431.353025968649
166.734730009916 162.467159261789 312.469507672374 267.85990321012
223.749512763927
C9orf116 106.620429405618 128.542999559673 65.6620676514157
48.7646976789783 145.267770598988 55.6617952786921 239.610773859055
95.7263252232278 123.768417863139 57.752606600685 60.0981687048269
65.084620579901
C9orf117 44.2718517563299 39.3999056968598 42.5406888281114
38.5252456306656 43.1847628331565 38.9661430990729 41.8751578329365
43.0930293831497 41.9635525160083 43.0919221375514 37.7384958256497
38.1421709520616
C9orf119 287.363928615517 176.624023915568 194.317275235015
185.924923239764 242.83357283464 210.437554813031 488.277500837251
385.827237353675 276.149150203786 295.5163490537 255.915599666591
310.706520144173
C9orf122 37.0320432433314 35.8227939208521 43.401782064778
33.8111782249716 35.1411239266238 32.6368705146258 39.7576136507593
35.4526946152955 39.1790517315259 38.528621456378 42.170702295259
40.4639532172338
C9orf123 77.6056319020116 93.6717518147993 66.0411740221276
69.5544840164738 90.6661301391451 219.073357087203 77.0003455479124
64.6316818494785 55.3122077514658 160.733526836282 144.771861156788
109.090304050495
C9orf125 41.6354649482853 83.9760574088975 71.8706847571669
41.3027031037343 51.4527545197672 50.7516773099639 56.0515443021293

40.6171774724171 69.0037087543684 106.049736669661 97.1271615802556
111.691813916343
C9orf126 48.3814421939888 48.275452803061 42.3536549114165
47.3574589211166 41.8806343997257 37.3799542961839 47.7390230243778
43.9366081640576 38.7298222155532 48.1392888470269 37.8372168909583
43.6753843488695
C9orf127 101.494432845825 64.0903425729408 84.2385359528743
57.165971310746 78.1275793755619 95.4393274079354 68.5725871332774
88.2568398791731 62.2097408040123 126.470889051684 113.539485042978
103.104022477607
C9orf128 43.0001432913058 36.4002279956519 42.4580504587581
39.1706564265495 36.6354013629791 43.1826328292558 40.2154497375715
52.6960621424156 38.3418933808844 42.8936990772838 42.6366765186182
44.1452943804044
C9orf130 123.868848519181 77.9992448636936 90.2638559113894
151.225738885272 66.3075095149783 74.9006142438341 141.261334415286
207.513604688444 82.8150137756969 184.484466722861 211.335020823243
123.978615472736
C9orf131 36.0144420013819 40.4359177791566 40.148780650831
44.550322123994 40.9416717019827 39.7697110924474 37.1643693306382
39.1128725343972 43.423558862426 45.9004543328976 39.7342718293432
40.3691324547049
C9orf135 43.6706764143908 44.4411734611229 36.178547300352
42.357910063876 44.0451487108213 36.6271061451613 48.8319884367456
46.5717043290731 39.4762546236449 43.4885245598939 38.6662194645633
40.4639532172338
C9orf139 41.1523487516364 35.5310164682026 40.1985790927248
37.5382094267692 40.6723334103791 41.0582900711341 37.9430145836712
38.956280281655 36.3649677956953 36.9102644736882 39.153226699662
44.4475838947656
C9orf14 43.4626049853157 40.4359177791566 37.5597671487621
39.853175885492 43.3332476888305 42.8598805316863 37.7136011474903
38.5457716679071 40.9901805728485 39.0648733099061 37.9946685306274
39.3575468494941
C9orf140 178.643141452509 474.729653426958 72.7987593815919
64.898351831341 276.633536223159 51.8103411703261 228.907776306695
145.791398829369 134.817234321847 41.9666541498638 39.7342718293432
39.7411455670172
C9orf142 853.464809072841 767.489657259345 437.148137705103
378.358853591082 632.019639546131 363.002913525646 547.442608452594
732.454837219705 580.444451816014 564.929183025034 740.994425128952
402.886581458688
C9orf144 42.1537476443423 39.2907645923217 44.0451487108213
42.0102671859606 41.0885562160946 45.4882438975269 39.3585350448719
40.4359177791566 38.9834959368496 37.2089297656361 48.6767028205398
45.0985088502363
C9orf150 68.7436392042714 53.7332797903102 59.1040441894478
43.4410741327254 51.3511088648386 67.1067574196203 41.4742337714895
35.2642250366721 58.0722340887264 52.4351670989268 46.972521499515
52.4992300612807
C9orf152 181.545493080363 129.190555260332 50.5089777244781
55.6481366056639 48.7288537840562 45.1873487463472 53.3111230705754
44.8997235283254 148.333778341005 65.0928075335306 110.214922820817
82.9467575626263

C9orf153 39.266565827316 37.5469247328848 43.3085358214154
41.6564075217517 36.4434672195785 42.5660695551615 39.2533668167483
39.0620487644635 38.4344387289956 43.9450023643394 43.0016969050339
39.3575468494941
C9orf156 103.458867136383 98.6825522152097 81.2136969156029
168.926743047741 74.5758811681995 82.3364971911816 98.5663841503803
85.0308877626746 86.6516906104905 118.115557136662 133.981674663494
106.804269453898
C9orf16 100.057155418659 78.6705291023488 65.7670524353661
49.5424425850549 73.1654226019863 85.2805684630976 137.081911344143
99.9896394343368 106.317388820963 169.18132209076 151.758051124404
169.554408876006
C9orf163 43.5710871617107 47.1332119496722 46.5650556481721
38.0958223740111 47.3172462673426 45.9266745571724 41.8315602975157
45.642304356051 48.5792058061372 43.9450023643394 39.2542963855807
42.6758568421347
C9orf164 83.0534943004588 56.7826399116207 47.2962360458401
79.7937658787441 41.6806984441849 38.7340969861754 45.3597029614942
46.5717043290731 53.2046867327331 60.1466326805616 64.3824529624475
48.27609330316
C9orf165 40.7609952437622 39.0102066700839 36.8730514568925
34.7266486944713 44.6834648302383 41.6590564937735 36.197424053556
50.3972033014227 33.5578650644246 33.8984830800739 37.3687900733549
40.5703581862752
C9orf167 84.511841810844 110.214922820817 57.8111054584154
95.5022104475922 101.383978726419 56.7429967489965 79.5707751325986
52.9813204449021 46.4861258030402 67.0163226819964 54.2999306485758
75.032753683526
C9orf169 107.034189847828 79.7691160370015 74.6901522573522
45.5869891570251 54.0126916508417 37.7000400506818 61.2183034191703
75.5830548489892 57.9713749600418 95.3300728806601 85.5175308034109
67.1675878355908
C9orf21 70.2541961995753 50.4022543872993 49.3780606970538
104.335120306753 48.9627346956003 99.0731740277519 46.4388841354122
54.9919060580109 43.2761454096265 51.8412522234186 48.6767028205398
58.5478485621444
C9orf23 105.027318695305 80.7894026811963 76.6571453659046
77.1767494799711 118.288549974348 74.2690269784394 101.804757613639
87.7295604723036 87.2282539094452 81.5678694264684 101.759714275976
80.1665291227124
C9orf24 39.4196620674248 45.123403685928 45.1037002240382
47.7462490950965 42.65897235979 62.0056315405014 44.2688720583707
44.4031868028216 44.7058233759912 48.2596564318071 44.8872784170036
45.7258895124195
C9orf25 70.2541961995753 54.113533179847 57.1226931773006
60.620259884953 60.2886873195135 57.2767879969284 49.1067959632109
69.845169494661 45.5779686360641 59.5785089386245 60.2165699959135
51.0647893605839
C9orf27 32.5418171758086 38.4330755397458 33.4240466670776
38.0958223740111 38.0598374201407 35.9305494063137 35.3362091871364
32.6039814489621 35.7973685350741 38.745552403895 34.3633615460307
35.4484652445078
C9orf29 40.6559419616644 37.8217989841918 35.6139301508335
37.29566077996 39.9697332660833 34.2773859804062 39.5496902678788

40.6171774724171 44.8997235283254 38.745552403895 38.745552403895 37.1747156131306
C9orf3 78.5216977073842 90.4512650366645 47.9035532886869 50.7046706880325 71.7263663921367 117.250114037308 46.8613212048981 50.9933795450314 72.6675345946258 101.759714275976 65.088713927995 99.3956523785983
C9orf30 116.547937420457 83.014860007187 66.5285423077808 119.39177524986 72.6675345946258 78.812106589086 111.367789538268 90.2915974511786 74.7934385405346 57.7805922926828 62.9336909080086 78.5108643908344
C9orf31 45.3872406432794 41.9894412416874 36.3604951341921 39.1706564265495 42.7982502551634 41.2567470068038 36.634024014558 39.0620487644635 36.8127605061228 39.1621898366355 35.860430241131 42.9887069977696
C9orf36 41.1523487516364 39.1982470626863 42.7881985796277 45.8844325913247 44.0977148249302 45.1411314881341 41.6466169548846 41.1575777497576 40.9901805728485 42.7275315121163 38.4251036088595 39.7844133452634
C9orf37 51.2851682978581 34.2618170189676 45.310660704531 45.8844325913247 52.0583273941813 45.4882438975269 49.540449125788 43.3262148375525 45.0017158586743 67.3934788330567 71.9658486648999 54.4992295592001
C9orf38 42.442231667357 50.3586212130973 49.9256753636167 47.3574589211166 42.0709558258414 45.3882138838137 40.2154497375715 40.831207953647 43.2761454096265 41.2276963324981 41.0808257270221 43.6753843488695
C9orf4 36.3649677956953 38.1312950900467 43.8495248991589 43.3078542962968 40.4639532172338 43.0860383229288 43.3262148375525 42.6450179841129 37.5469247328848 42.5664883002833 44.2142581488029 45.0985088502363
C9orf40 61.356483470053 53.2046867327331 54.3554233228353 54.1240583139871 85.1661952927159 84.3755392205436 73.6855877240541 57.037639245179 107.538934056558 65.0928075335306 68.4659783447092 69.4383198054368
C9orf41 43.9309845324767 43.5182502375207 41.1367478979484 45.5869891570251 45.1137938810047 39.7697110924474 43.4544762830742 43.4544762830742 44.0519245668737 41.6724739851995 39.0550139257658 43.4709415392696
C9orf43 38.957687788868 38.3322849790657 39.7411455670172 33.8111782249716 40.8873907150245 32.5219627196161 35.1307934330047 37.5511560880902 36.197424053556 35.93950183586 37.6484782516569 33.3192849177028
C9orf45 68.2614370871382 72.3297091475814 61.0370242377391 77.2218991550552 57.2960245538881 60.7488007939604 93.0538726751588 78.6911549149983 58.7382738968248 119.979854891563 92.1646038105091 83.2925226467532
C9orf46 255.559793355361 337.948887427898 188.513581321162 455.223217772744 177.912351477808 320.086549121732 255.762638489554 559.559234461244 187.147252401703 145.936763882526 169.061093538895 295.473526251997
C9orf47 37.55264801907 36.5219488315447 39.8299340037009 37.0732977341185 37.3508690072156 44.0804700754786 36.8369172900902 39.7509165247419 44.619512785899 37.6383530120621 42.6366765186182 41.1554481567603

C9orf5 126.404988409795 224.945809325258 131.413578749264
120.284321694275 158.918242816842 236.178265404264 165.606015336587
110.869341159607 119.253559091088 324.320137371059 340.311633794489
416.149496747947
C9orf50 38.385865183581 43.7644428186265 41.2365089689635
41.0726336841311 39.9697332660833 42.9695585740813 40.7910160811527
38.956280281655 48.5792058061372 36.5806287103157 39.3667161654772
43.4709415392696
C9orf53 43.3638918705225 40.716553247236 45.3974838013476
37.4223289687135 40.2449343247539 41.8613406887824 38.8417151851134
35.1738399826814 35.1155486926999 37.1275334308172 38.8507804383261
40.5703581862752
C9orf57 40.8681871019812 41.8848959280916 37.8386378737561
39.066284740632 38.6454098044474 39.9397419279057 41.0381860307445
40.4359177791566 41.9635525160083 40.5090849993014 47.3791873993182
40.8681871019812
C9orf58 84.3850767822327 43.7410507935043 43.0602678767669
40.1804147506786 53.1100587888055 88.0777258947198 55.8510296341925
66.8364940429355 46.824299585607 52.8496345990125 51.8812208789107
49.434568584642
C9orf6 105.371285021629 153.364107550338 97.3756238014731
123.022511916727 135.829530518199 113.46454061183 125.835967514239
104.480361196422 147.165235402905 85.3431175579775 96.593956699892
147.300356318739
C9orf61 60.0793579902362 51.1740855632044 56.1434024065079
53.0284011078097 43.9450023643394 147.782301091454 44.3440839686558
52.3068458655445 57.0301170427216 186.64040927334 183.690616959682
94.4769283676944
C9orf62 54.6235981131919 42.9358255168086 48.6037684272115
56.4578213622965 55.3100442506299 46.6872654833579 46.9929836306816
51.6660481331154 46.8873421271259 49.1928206164702 43.7585538437696
46.6397391251762
C9orf64 59.9232969481163 69.1414574152997 58.9135553403521
86.7823828209946 65.9413235523618 67.6817814091697 60.2399886747451
48.7814131120945 55.7522234735038 56.1912096308988 72.768998788927
67.9850517608624
C9orf66 63.2753814090696 82.6602736441839 64.8254097948745
97.4967204298499 55.6991707857987 60.7488007939604 89.7242042805798
71.8314470868658 67.0290776715331 65.5349012839562 63.5195937348531
63.1443731864055
C9orf68 41.3785995857154 42.0500582486532 46.8267540220952
41.1886975241176 40.831207953647 39.4512099697623 39.8695929627783
36.5933320803035 38.7298222155532 49.7259371799014 43.6919270353542
48.3648494350286
C9orf7 217.575115287224 132.400225103653 98.1433318215702
78.3509756704423 83.6438735616921 60.9822577063534 198.729330487352
228.660261263355 151.344720108793 142.095236399173 152.200107222761
109.017269529278
C9orf71 40.7609952437622 36.1232327599945 37.9482157381008
46.614092935749 38.9496515344837 36.0306975524309 36.08956004634
40.1474543172332 39.2809305246091 35.3849221682845 40.723990462047
41.6550823405646
C9orf72 47.9707112435212 50.7487159427973 43.4456430103334
100.413031488199 44.5134109184888 53.3185097662527 49.6872467499977

49.8638539895028 43.8017789369861 51.0459299480813 51.9490810272761 49.8253396161069
C9orf75 76.309158701514 72.6162848018047 41.6079958749402 40.0698728584484 61.6452971581503 52.8421046802739 56.0515443021293 49.9332280449549 51.3511088648386 78.7543199947688 60.0765286625112 61.0025829061063
C9orf78 612.441960343995 717.997180920384 394.990145259101 428.411504354946 592.989860385203 830.295872113419 738.059797123491 398.641868389511 393.944898808828 500.794458547464 594.184150517102 572.19538884395
C9orf79 44.2350046179299 46.6314009405034 47.0055981216687 46.1673159982664 44.6834648302383 43.4681646416585 46.7100796279384 48.272324215504 44.8997235283254 44.7958122466628 43.4709415392696 41.0657892043496
C9orf80 1756.86714809925 1075.04105984319 1159.77262479002 1453.77801653969 822.372309550325 682.479411898549 1411.12613289991 2365.73772015255 797.019657306322 2054.64068255575 2032.18819581205 1575.71609894038
C9orf82 62.0553450433433 60.3928833351886 53.0630927250439 62.6048727240291 60.5290966885203 59.2643906275025 66.0350709576651 51.0724630072723 66.0350709576651 78.0658258913875 74.9006142438341 77.0262811825953
C9orf84 42.4757115539502 42.3138951003676 38.7429005059745 42.9358255168086 43.2988017098387 40.8956286697415 42.3360569471619 43.0895173605825 40.8351751854504 40.4136535208406 39.9070785037411 43.423558862426
C9orf85 40.8681871019812 53.9634127270611 41.0718387273629 41.7614975630362 45.7407938943718 42.0574848021044 40.1613888954735 45.3872406432794 51.2776048338 39.5637454855233 44.553780984244 54.4644832805303
C9orf86 260.766438659762 264.495576211074 107.034189847828 107.799236230743 149.551650453179 153.992331037033 276.633536223159 157.281394920992 160.425627898607 126.897002134472 119.277678423944 96.8555197062122
C9orf89 148.477784236365 148.781721594419 119.243515595981 274.02972772427 109.758297200083 199.660904693786 202.01900875263 122.877322487518 174.350348527098 130.15891745292 127.596368103014 139.062073456923
C9orf9 55.6268253440479 50.1027491117756 46.1653991089885 46.0228732507872 55.4919197421746 81.6949047529395 84.260633540656 64.3284163241986 49.1940901905636 70.4895190282848 65.5386366902998 58.112348043634
C9orf90 105.308648028552 137.424708183613 112.159199477006 106.582060920252 98.93324263369 95.6783002876222 129.810477623628 111.492046718579 153.812414753676 116.441764463618 103.63788330957 92.8006484232438
C9orf91 99.6265783263109 85.5135159896819 66.8632294055679 118.49449420132 68.1674425673171 64.7905331739366 96.0539709962851 76.3509533017572 98.5609311953771 100.271925566074 95.92436282414 92.3388129097482
C9orf93 47.3791873993182 43.2109153553335 42.5864346233886 49.260242314197 43.1847628331565 46.1974343793412 40.7910160811527 51.555506218204 44.0256815382334 55.073022935193 45.9725715538987 46.7182294568603

C9orf95 63.2753814090696 115.735359157053 112.727277807495
135.323750661937 66.3075095149783 76.0240426902567 56.2181240696479
47.5950227477807 76.2591822114739 173.891042920642 91.5541137821803
206.857208722995
C9orf96 44.2350046179299 38.7344853699576 43.4709415392696
47.9915414951747 42.7982502551634 41.7627910693483 38.8417151851134
41.7419877611072 34.226171989766 45.0155543555707 44.8872784170036
32.4951076826876
C9orf97 66.9417866448504 69.1414574152997 63.6334154351075
66.224559122062 61.7566103867825 75.7502062965698 68.673864919178
51.9447450229707 67.9176876913772 54.7827221235949 60.6505672212119
78.3467096982408
C9orf98 45.2875173643252 38.3322849790657 40.301483914446
43.8002456102021 44.9936360641969 47.4699236780425 43.4544762830742
41.7419877611072 38.9006788676407 36.6893251782922 43.5710871617107
42.9887069977696
CA1 43.3638918705225 38.3322849790657 37.2838080935695 46.3203995372658
42.3685136297603 41.6590564937735 122.118080859526 39.4710743516517
37.6383530120621 45.0155543555707 40.9157352260901 41.1554481567603
CA10 40.9558083259517 42.5269793679335 43.401782064778 41.4273122914951
35.9894133432376 41.7627910693483 46.1634822993009 43.6434576713809
36.6312676076142 40.8049408996274 44.9713479185144 48.6795178347248
CA11 60.7371790125714 45.7979032939211 49.7350276169552 80.4007868743886
64.4999048115671 91.2681347227088 49.3854238365325 61.130286531713
58.9870089759222 75.5171857162768 87.6689055777294 60.7495043977707
CA12 228.809697062726 119.311590524726 534.881442644054 428.105433953405
336.490083577377 241.31366702947 139.08163074096 176.816741656659
851.108027634251 676.672146226382 596.384327208699 662.041552795049
CA13 39.3709357615493 47.1332119496722 47.3861758958576 48.856176220627
41.9842695818285 37.1317006162938 40.7910160811527 43.2551508830106
48.1174861108648 46.8954576545929 45.310660704531 42.57175366709
CA14 38.957687788868 38.8336427948187 43.401782064778 36.3604951341921
39.5595233162192 39.2985791324641 38.0598374201407 38.079222289614
37.1820543575123 38.745552403895 38.1835938174625 37.7526485343328
CA2 235.018922676506 389.364650157102 273.849703944739 424.085665569421
158.464244518317 71.5249259772583 405.148979342648 97.398745552008
85.9970657980042 65.813547096222 71.0369238830779 112.021971795593
CA3 38.8406533304174 42.9358255168086 39.9101908547397 41.8291484190524
36.489383074099 46.9515828775339 42.6913548400127 44.1642484298135
42.8036731479932 44.2580151149956 43.6629145624937 40.5703581862752
CA4 330.892010103508 83.4951332849212 50.8303921211581 82.2766822827861
97.2316181542823 79.4053001255904 274.081959734368 341.535750991033
59.5093038629766 63.197604174872 124.041740442038 52.4992300612807
CA5A 37.0320432433314 37.920964499903 36.178547300352 39.853175885492
38.6454098044474 34.0045980663271 38.8417151851134 36.6452480995946
35.6970528713709 38.6454098044474 38.6662194645633 35.6427313468066
CA5B 71.9062462001972 54.2924642952939 67.6373573669424 64.898351831341
44.4489662507893 69.9819944829311 81.7434024567356 69.7729022157455
52.2631308081333 102.044838876428 81.0562186907518 61.356483470053
CA6 45.1912496459517 44.7829632472754 42.3536549114165 45.1944222548351
41.9842695818285 42.4580504587581 41.1507980856436 42.7431359850339
50.6750785584772 45.1569741609597 48.8088340735921 49.3984571719129

```
CA7   39.3100256336821 38.6704566269103 37.3687900733549 40.1804147506786
      38.6454098044474 35.9851865044027 37.7136011474903 40.0965255448873
      38.0213051227859 39.3698811593364 39.6403089864678 35.5137794453559
CA8   40.7609952437622 45.2556760873335 43.4709415392696 35.8472302065546
      42.6022652963326 49.641159251878  42.357910063876  43.4544762830742
      42.1594905198929 46.441301934328  43.3085358214154 40.8681871019812
CA9   116.253417059012 140.369714831352 129.179537795204 40.7481155008596
      132.946528291039 49.3172204533818 202.983984966874 43.1567024035183
      1545.76489731307 49.5265949137207 51.838002904753  37.9607839934735
CAB39 589.395944599882 563.573152902421 449.059703234741 725.680256063016
      490.301992866742 747.577738135496 432.001201940414 623.574706535278
      478.799250340246 540.721068846198 679.309039810384 817.631505579476
CAB39L      91.8414653633828 155.730834354163 128.860357469791
      61.6702707724448 115.676508349703 101.577767481269 177.779514321673
      165.974848305379 96.4740168904128 87.2532250821857 85.551605908163
      92.9043863423147
CABC1 168.036319777283 334.726233105118 335.655540947167 189.984886903595
      308.728126648231 616.265771322835 400.973553604192 389.422882761332
      176.02137177982  527.686970237622 550.371519625344 225.875798191473
CABIN1      158.042994416158 163.718576695806 173.08677862231
      182.219366179899 106.871269007146 165.662539866309 247.700075839517
      134.664025291418 138.036631806718 182.079641528208 215.609821689043
      131.330480963914
CABLES1     74.2151462073098 182.80711310964  84.1401040507557
      68.8167148906225 70.0133734659853 86.6246917308185 55.5578334877425
      72.5835488194631 75.3761360028971 107.987729161223 84.607833108004
      83.5069968129733
CABLES2     74.4565795414712 62.9904770954043 62.1893884200745
      49.3654837541353 60.0736861987141 60.4065050472082 79.7564155881026
      74.6572522248684 60.164635768614  69.4131195465674 50.0808824383821
      44.1452943804044
CABP1 44.0328992213727 43.8336862998829 42.1860813954352 41.0273093212371
      39.3100256336821 43.5474493120701 40.7910160811527 42.4580504587581
      37.3600014814388 46.325232414659  44.0484280023299 41.6550823405646
CABP2 41.7780833969533 41.6757511223421 38.7429005059745 42.629412460409
      40.4098450813524 45.764546125016  43.1696495767273 42.5435283762933
      41.7269688948274 43.0290260867894 40.8225507267584 40.9243125941198
CABP4 49.7188990489616 49.9085372049438 59.9690744676539 35.3829784221072
      45.8051846088552 44.8872784170036 49.6872467499977 50.628152951493
      44.5303781239209 40.4136535208406 42.357910063876  43.9541718254769
CABP5 43.3638918705225 41.7337506166619 41.2365089689635 37.4223289687135
      34.6227169056411 38.8336427948187 35.9894133432376 41.8417024632349
      36.3649677956953 39.0648733099061 38.079222289614  43.2776458914333
CABP7 47.7267190613906 50.9643363406241 55.4502804236424 39.1706564265495
      40.0599807618105 38.7340969861754 88.8039637390786 55.421880601441
      57.1863195337055 46.2286164776607 47.8284278211109 48.3648494350286
CABYR 40.148780650831  41.1212129940704 49.7350276169552 38.1421709520616
      45.310660704531  42.357910063876  44.7958122466628 66.3558126170575
      88.3335649692467 52.7972156492746 47.0110068985119 50.5165823866945
CACHD1      46.5759625882541 42.2577689927563 49.3780606970538
      42.2496337895968 38.5512429290626 52.6747142992864 40.5585676679482
      50.857522488428  47.3158504988956 64.1724883992867 64.898351831341
      60.3816817102326
```

CACNA1A 43.4626049853157 40.6723334103791 40.301483914446
39.0156305603334 39.4693871570028 37.0180934800573 45.4164642922183
39.9576138799997 39.6413313949444 39.3999056968598 40.7725311293918
39.2469742642337
CACNA1B 63.8626921347231 54.3554233228353 61.841867306916
87.114638423256 62.3233491851723 52.0299937917925 62.3556396662215
61.016921238986 59.8158518516035 48.6124127998952 57.0154839296649
64.650642183463
CACNA1C 44.7044140979108 41.7575097298927 43.5448014872301
40.4359177791566 39.8238654968211 67.4846787600629 38.2197745884174
40.0873854338318 40.2752425854368 47.2299678280724 49.8678783525263
52.8796369426118
CACNA1D 51.413025495745 45.4143854024453 52.9111155236961
42.8235313007746 46.6397391251762 47.6803619768589 51.4349390827895
45.642304356051 49.1940901905636 39.6719190054379 51.0492389286271
46.5650556481721
CACNA1E 42.0532901689036 44.8529259387308 38.1421709520616
55.8709511400674 45.6895223823001 37.0180934800573 43.5627649099623
41.2551966132486 40.4220196471124 43.6033221838149 43.7585538437696
39.3575468494941
CACNA1F 35.1307934330047 41.3610369241157 37.9482157381008
41.0726336841311 39.5595233162192 38.3418933808844 43.1931731665131
39.853866204927 38.9006788676407 43.9450023643394 41.6336636285875
40.2669497398702
CACNA1G 38.0709016691086 40.9291911731327 38.2502942836481
42.4580504587581 42.8596678196469 37.74484750596 41.7469782431906
40.0842340147135 40.8331190350174 38.745552403895 38.5668610487033
38.989836064185
CACNA1H 86.5564881117807 382.538365722661 341.206585871289
58.0218262176065 78.812106589086 561.855286945959 51.0724630072723
65.5820437889838 51.6258604434499 263.765914589758 280.820745089796
197.540681021755
CACNA1I 78.6344248102932 98.8478655411492 105.814959179397
41.6564075217517 54.9249759425252 39.7697110924474 42.3194600586894
63.2125855149045 65.326134385614 42.8414096678708 36.7283081422707
39.9941474864076
CACNA1S 38.304249186058 41.9894412416874 41.0356368374708
38.5252456306656 39.4693871570028 38.1169703906207 36.9355798143571
32.7676164492555 44.8997235283254 35.195364621675 41.0808257270221
40.3691324547049
CACNA2D1 40.3478825593224 38.3322849790657 34.9849896310235
37.4223289687135 40.6723334103791 42.750861693377 35.1307934330047
38.6454098044474 34.5633623842083 41.1222709786153 42.4580504587581
37.6557714476853
CACNA2D2 44.5282181324797 45.9732108120834 42.1901757067073
46.1673159982664 54.7488960681248 50.6915708372926 44.1407835440612
47.5950227477807 42.1089765085639 51.173096357454 47.8988096575648
45.6692241948108
CACNA2D3 51.2851682978581 54.2924642952939 47.7737810047679
58.9168466928565 40.8873907150245 46.8954576545929 41.4742337714895
40.831207953647 46.1101825640108 48.9826868640753 42.8263096141654
50.0571060701893
CACNA2D4 43.7920955808549 43.2649781354792 43.5710871617107
57.8277957325085 44.0977148249302 43.9366081640576 46.3611042575528

41.4061152929154 43.3262148375525 45.0155543555707 42.911855821602
46.3411353377615
CACNB1 44.2807847072108 40.6663433492192 40.7977328778231
43.3772464014781 44.8351976221735 40.6959906307191 43.3925093042325
41.5783566611007 43.9204029310411 42.785429206422 42.2618615571566
43.2102212096561
CACNB2 36.3649677956953 38.0226905622357 40.7237253120485
39.8965727231863 41.3351256327455 70.7702047405718 38.6510172759977
38.5457716679071 37.854877390128 47.965492554043 47.3791873993182
52.3242437351389
CACNB3 420.401914084346 526.146757036152 360.523229691604
100.483113678344 371.709014310561 125.364451149941 531.36214636517
839.850453078119 496.163796158189 213.768352764473 222.05036990665
168.869258524932
CACNB4 40.0557788909923 38.0226905622357 39.3260254992968
37.5680550468481 39.3100256336821 40.713858228386 41.2884376047238
38.5143356726527 37.6383530120621 37.267270263961 38.2350720635382
37.6276092293112
CACNG1 32.2133093774284 36.89524015739 47.646800205189
35.1451084093692 36.3390947880186 36.0306975524309 37.2892641289258
41.3506832276112 36.5393857306744 42.2614524730856 39.0550139257658
35.3525469364579
CACNG2 30.6242215267208 40.7952353240579 40.931294690669
42.0102671859606 49.5666074434383 37.2686490268265 40.7910160811527
39.3709357615493 41.074824727477 38.8507804383261 41.5421429082231
39.2469742642337
CACNG3 51.413025495745 42.0709558258414 37.3687900733549
42.0102671859606 41.1868052124344 43.6670755115848 41.5817737564592
43.9366081640576 45.1899271235974 41.8613406887824 42.357910063876
40.9683275553869
CACNG4 39.8662795144616 43.683792219729 48.0191002461099
37.9875302985336 45.1137938810047 46.574682061365 39.0458123429605
40.3357777530419 40.6957787921451 42.3606390470387 41.4508241771315
36.064718696392
CACNG5 39.0950295411867 42.558457786077 40.8833572570383
40.3850714987723 39.7568367479301 38.2293008890613 43.8973899364093
42.6975474602554 39.7537274655115 38.6933639353772 40.4166536885831
40.4639532172338
CACNG6 43.3138142470517 45.7589758958741 43.9450023643394
44.9936360641969 39.2608760193011 41.2567470068038 43.8664428040913
43.3729486804084 45.8325181856483 42.8304701368556 40.5865217310152
39.7411455670172
CACNG7 45.5758521267508 39.3999056968598 39.2707964550441
44.4367387274922 34.806104801006 38.1169703906207 46.9929836306816
36.8400213077707 37.7384958256497 43.0919221375514 40.1742127891824
39.4490233720848
CACNG8 45.9426591985894 42.6022652963326 45.9227107476843
43.7410507935043 44.6202070855297 42.9104454767916 45.6706505553383
47.0362584198466 46.0106098064475 44.5303781239209 45.0017158586743
40.8098117292322
CACYBP 90.9521112485099 97.1271615802556 127.034476771265
91.8295472317386 105.783933158577 106.8866235778 111.845105854608
70.3935694362568 110.540953771795 60.7985356140269 69.8505620106065
96.221685383385

CAD 112.924613715969 91.3941563889513 104.832753086298 94.3229189690855
160.566474370004 85.8059456975413 124.965915196815 156.220155161711
85.9850306181736 76.011923854897 71.751972433516 72.3297091475814
CADM1 101.58345410371 177.602690452814 113.6408244935 103.961956810572
80.3500323413132 93.2226507128199 75.4053820110486 114.959624673123
43.0930293831497 158.478919789334 102.77259665192 290.174315362911
CADM2 33.4902421268456 33.0553208959996 37.3687900733549 36.6501141438378
38.6454098044474 40.7616966228404 33.9750800030909 39.7509165247419
40.0403898620771 42.0621425894624 39.2542963855807 38.8495095797881
CADM3 41.0608773166058 40.5400384479766 39.4710743516517 51.7739270955254
42.6022652963326 114.526825145076 41.8315602975157 42.4580504587581
37.7384958256497 105.828093778276 92.2772139468085 63.2699016184868
CADM4 67.6767845246581 48.2220547806679 54.6051538001037 39.9697332660833
87.2282539094452 50.5148383400703 66.0350709576651 145.050655626219
78.9521593412581 50.6486940895674 61.940656536312 56.5225014032155
CADPS 36.0674080694129 38.956280281655 38.4428081526233 37.9315270017242
41.7503755215516 40.0746066749942 40.3850714987723 42.6008809597607
40.8503192605024 41.800198520267 40.2649309204478 40.9243125941198
CADPS2 75.1701698483856 57.5941849631773 63.0670482657086
62.7108230507424 52.2089306840125 69.8832723783498 59.7444979429174
67.6767845246581 96.2385885003071 80.9024704745445 72.4325984501758
65.6213260281822
CAGE1 41.3785995857154 42.1372626664516 39.4710743516517 51.4802440299586
43.004943518025 39.266565827316 44.5390641547628 45.9604707098953
45.8325181856483 38.4829727716328 44.6834648302383 45.1735152121056
CALB1 42.0532901689036 43.5182502375207 35.2838283525874 43.4410741327254
37.4470183315826 37.2686490268265 62.801431502597 44.0390019242862
38.1169703906207 39.3698811593364 38.745552403895 39.0536056496869
CALB2 187.16833117416 43.0227387991135 43.917817324545 53.4785152499923
43.1847628331565 105.111902911639 42.22763273471 58.676230030835
49.2248095024373 59.2628513357357 56.3884556031093 48.1125722136997
CALCA 43.0930293831497 41.3610369241157 38.6005272730664 39.7742005651286
43.5880694884799 41.9543948167375 42.0605906240248 41.0808257270221
39.7210115145672 43.0156099182214 43.7924421812682 42.3214282169749
CALCB 41.7397731376692 45.6533175604486 43.0971787292711 53.593974682612
47.5647821785529 39.9397419279057 49.6872467499977 52.8622615027833
38.5252456306656 47.611018474151 45.8180710486329 42.8864415494484
CALCOCO1 71.4276813927272 77.3367966395554 74.8071157013401
88.6717761948382 62.6860949544223 125.036267601897 62.5506324864207
132.3192754634 67.1278109419819 140.837788264535 113.403020750953
111.760213205992
CALCOCO2 94.3674661847143 86.8262463672891 85.5839487225537
92.8026641003757 72.5027069303875 128.126336084436 72.5956443098684
86.7710020879725 111.735319884512 110.334176022265 103.883220270094
95.1224420992193
CALCR 46.9939712192224 36.6968906669565 41.0356368374708 45.8844325913247
37.7576900881741 38.3418933808844 36.197424053556 37.33030683349
40.3316153483289 42.6793753550854 42.2618615571566 36.5474283500601
CALCRL 71.2963721541702 61.8492980174846 89.1115172961797
139.925369282704 62.9553576299033 76.1991031349451 43.4544762830742
42.8482181313226 43.7630587657205 44.3648993568405 63.2684764441521
53.9431600945419

CALD1 996.520340280883 371.167168940365 320.452418537108 402.571146447946
340.553073657744 2830.73694055758 77.518381991736 336.904716156626
120.96329303892 1634.61795848352 1320.7894611262 2083.69812106867
CALHM2 53.8856501960117 54.8439819445945 50.6004078448405
73.3725472767369 55.0031529299037 104.650071846586 47.7390230243778
48.4670130605028 52.8502823653368 75.7140064167134 80.4239296853518
57.6860752168769
CALM1 1453.77801653969 1163.0370991733 927.921867516809 1236.32289322872
502.32041794318 2568.97574985073 766.647626178442 833.136660528149
700.010354583503 2009.75680599899 1611.35913846618 1347.15664445545
CALM2 3055.1209535841 1487.80306986156 1161.71671016964 1642.75555340648
1311.88650141004 2257.78227736778 1591.51486001004 2854.82112538867
1280.77934331703 2215.01815680371 1723.37649434054 2061.66570137425
CALM3 821.013687795434 1881.7281441986 1237.93130196998 610.853753376105
1638.16317738035 2232.61002288121 1364.06060391538 942.991198007376
1539.35554229258 1231.77360353254 1578.32969650385 1585.90718459092
CALML3 43.5710871617107 101.131391476546 51.3268475761593
47.0874209746031 286.243476131448 92.681224908389 46.7100796279384
49.3880946790306 84.0388582916959 50.22432299342 44.8872784170036
39.9519547348106
CALML4 87.3957386451235 93.8968899420039 94.8717345601966
125.052841200703 113.905373770586 141.471672990444 95.4533981420022
96.8240908274121 98.9712341137316 109.654559065946 100.326655761429
117.869393694564
CALML5 38.8561375693335 39.3999056968598 40.8420925848403
32.9879066304034 46.4343327861696 43.1826328292558 43.5627649099623
44.5029690493211 43.9450023643394 40.8049408996274 36.131485826118
37.3600014814388
CALML6 40.2543341073001 47.2770121962655 38.6727842796202
44.550322123994 39.9697332660833 41.2567470068038 36.3194282084339
46.4672637826582 45.8325181856483 42.4594149893798 36.2435874904689
47.5025821041537
CALN1 77.1521355570024 57.3286965007828 58.8936382809165 90.2790229242453
62.7248529539226 62.3497263884167 70.3613168347492 59.7744650739434
69.1641374421455 61.6084234634424 60.2550788540864 64.2084111281705
CALR 234.408769393378 211.926421068313 209.658186168638 314.856937113103
293.649670545233 179.214713709599 269.546586017659 264.407051461506
287.423909789368 205.279698624867 230.833432994593 184.859183550225
CALR3 45.5758521267508 41.8073910693411 39.3709357615493 42.9358255168086
43.5544347011646 40.9506062495231 41.8315602975157 39.6474008369935
39.2809305246091 38.1042454705119 40.1742127891824 44.6484005362281
CALU 641.103199748891 221.049503614433 186.450759425396 433.320698413679
336.490083577377 395.457351443921 150.562062400737 172.221482103025
173.511692216696 324.936044300546 229.26498093497 368.078954546259
CAMK1 103.808640431626 90.6852012808762 74.5886835786452 152.329124189473
137.424708183613 293.300203370043 180.622563149455 143.952224627989
114.483475728117 145.676955166566 136.883475451494 97.6552689677199
CAMK1D 50.8736372975089 49.0986627051784 49.8344973836834
81.2992152187529 44.9384441371181 57.3171834644877 74.2690269784394
62.7950861902067 63.3315266915127 48.4890615340603 55.898892031444
52.4396589505396
CAMK1G 43.0930293831497 102.797268694325 129.2860308084
335.002151198999 40.5710637884029 43.8582747295766 43.5627649099623

47.3791873993182 35.9645119596869 37.0320432433314 42.8263096141654
40.0557788909923
CAMK2A 46.6348258763289 29.8304689114955 32.6121532029976
37.0732977341185 35.5220586859301 47.9503724044453 40.2154497375715
40.4359177791566 34.2973261206803 50.7855742231932 44.7114813656401
37.8490162439785
CAMK2B 41.6900155285862 42.2577689927563 41.5541470778104
42.1594905198929 44.8897737954573 40.1821097335139 42.6968849949058
45.3494477934196 42.9449078135763 42.2265242378645 45.2765291940328
41.8921592969155
CAMK2D 43.0930293831497 44.5884811528117 44.7829632472754
45.0053460050241 42.7982502551634 52.7156971154405 46.9185175407863
47.420942961753 46.824299585607 41.2700204291848 43.2551508830106
54.4992295592001
CAMK2G 86.4937293696262 174.815922224726 174.216025744156
68.3157692917711 104.220256076979 225.918246061369 95.9871941453611
73.4331096065698 306.578554914328 224.871193467078 192.356440003154
163.067962559455
CAMK2N1 906.833938613875 3070.83113612925 2122.39240932934
206.125013129462 802.650020720921 790.440637272613 1453.77801653969
2126.41018877615 1398.21942794938 367.56843259639 865.63777294494
666.265306908449
CAMK2N2 43.3638918705225 39.2907645923217 35.4484652445078
44.265834798964 46.4343327861696 38.8336427948187 38.1793598983615
36.5393857306744 48.6494451061637 39.8873706855774 38.3684202018524
41.6550823405646
CAMK4 44.7915315085948 38.2263227947189 46.3121956889449 42.0102671859606
40.2449343247539 43.3785777889265 45.7452456977857 40.931294690669
38.3981095229401 37.7315666632629 42.7275315121163 42.1860813954352
CAMKK1 47.9901855912362 45.9266745571724 43.7630587657205
47.6687908290818 47.3791873993182 45.0283997415478 43.6753843488695
47.4740726032707 42.8036731479932 45.9426591985894 42.9634662926872
48.27609330316
CAMKK2 35.0510338774593 54.9821263631962 49.1067959632109
51.6869155634183 44.9936360641969 57.1752846228778 44.3440839686558
36.7016645007743 45.4143854024453 37.6637152940566 43.4424616940528
41.7575097298927
CAMKV 33.5994525227062 37.8217989841918 38.6005272730664 34.3595588146954
51.940083408061 34.8956628705329 36.4098694708483 33.8558232866797
38.9006788676407 39.9948390976317 35.2339574411711 38.5279745308203
CAMLG 1106.57048989685 592.399245288911 689.738256133922 860.265067346622
877.673009984357 1263.76838179117 627.914461428624 1186.06822787922
592.694479269186 1493.20520979227 983.972164685474 1114.15989325456
CAMP 51.413025495745 45.4143854024453 43.7630587657205 132.760054781304
41.9842695818285 49.4157684139858 62.6885730386156 51.8347877274018
43.7630587657205 56.685925255591 40.9157352260901 41.9430737035925
CAMSAP1 161.205086002542 238.077051775147 135.106756607914
138.581471125405 112.830467116678 132.518188482163 185.378190201718
133.810601514962 114.230358276599 173.035987237848 156.150710793563
119.947688643528
CAMSAP1L1 234.408769393378 106.160416826673 193.043344611355
142.714450804595 202.154195547644 200.698372627272 158.321459945365
238.616000665585 141.672695206961 301.904682664871 235.544416908354
195.221917652149

CAMTA1 57.4376528157926 49.3581578587582 55.4502804236424
61.8997157694697 49.452985143568 54.4753345912615 42.357910063876
57.445796273665 49.7336769474446 62.1035858593388 60.9446532180211
59.3709786862962
CAMTA2 47.1962846565261 45.0115870036541 47.0940613507321
43.3078542962968 44.3557408297309 42.5660695551615 51.7714254834195
43.1567024035183 38.2215591930186 43.1839109702714 50.5810338885769
45.5800840647989
CAND1 67.2437599548936 70.3629652777935 43.0971787292711 56.6458456890715
55.6991707857987 58.5530199658661 58.3675164086197 51.6660481331154
77.3367966395554 63.9129423266349 80.8366627364071 76.6707262000712
CAND2 43.515290338756 50.2163892855092 48.7039705048129 50.6346949248834
94.0532777456537 70.2553917409773 69.8505620106065 70.9702772728923
63.2753814090696 69.7729022157455 68.7780894982816 64.3383155834968
CANT1 316.917793468502 203.092045210928 241.017227991238 282.603767561794
267.107863045114 134.646159826262 229.992809034971 306.66244801017
293.217518524507 211.701234819986 208.529062113623 161.890859629246
CANX 204.862161704394 158.918242816842 199.522267292552 687.011162822211
139.08163074096 322.371597171026 146.634440537624 144.427680369554
225.875798191473 194.689377887477 173.925818790068 204.423912426477
CAP1 3681.41904661708 2083.69812106867 2243.24648907474 2640.06306087033
1325.2479053325 1792.42490815074 1806.22468459769 1928.53890809223
3941.3269626913 1595.69503047506 1649.78627644882 1900.96666834569
CAP2 96.4971762611189 77.9992448636936 134.068233310299 86.3663084195994
146.268519891864 359.185856400148 301.525915314179 110.287699369482
106.97432416825 250.621261212308 236.536058101486 302.667538432677
CAPG 85.7983996395757 65.6721629145797 69.24946602508 185.677720870442
53.4109882045447 54.5952754712007 88.0664425843955 99.2432911346944
79.7882961549974 101.58345410371 94.7008981904016 82.0869179287671
CAPN1 521.710190052245 1645.71527614542 1801.44003951248 366.915943380617
1656.16581692717 530.996217072759 1442.72006787259 1067.08316477623
1301.07436212673 1280.77934331703 1176.37732422904 908.328776521114
CAPN10 47.8751677727227 50.5165823866945 47.0055981216687
47.1416699964223 47.494999378108 48.1318607461064 47.5963750520409
43.0390857698452 43.5669306063196 46.8231837782006 48.5292718280498
46.4936313298184
CAPN11 45.8108990837624 48.7847528954724 50.3950981988762
60.620259884953 55.3100442506299 54.4753345912615 46.9929836306816
49.2019743609873 38.4344387289956 50.7855742231932 50.2203578628217
43.9541718254769
CAPN12 204.477547372897 91.2107065307656 122.748811531184
49.1786207920603 97.0055689383756 55.8938720883203 103.376225272425
77.2048580540589 85.6441969757275 112.375316615894 66.0664586519214
84.6435656850129
CAPN13 72.2099571536717 67.1926218405833 105.052418151358
37.651298468202 43.3332476888305 42.357910063876 39.6535880152834
653.089523541803 99.5901498757707 86.9626166234066 83.1184264089873
58.112348043634
CAPN14 47.5647821785529 63.6287257620165 60.0856496605043
39.1706564265495 42.3685136297603 44.7804080899431 41.706879587091
48.1591264215184 43.5669306063196 75.2053836356882 53.4921736109774
62.2025996860055

CAPN2 76.309158701514 48.9899488302637 60.3155766445629 83.6935756500468
52.2089306840125 67.4846787600629 46.5650556481721 65.6914955077809
59.198088069573 77.775644509079 66.0282565447846 63.1443731864055
CAPN3 49.1868644496737 57.8759339109109 52.2132783757422 60.1837189855762
48.6224651100733 52.260196361018 42.3194600586894 50.8160430472218
47.3213303943727 60.4347860127923 55.1359824959234 51.1740855632044
CAPN5 128.827672077002 398.229956085523 282.72662597454 72.2577390063259
79.271901834289 146.046179302584 336.390195295291 700.477686061791
229.226876896347 395.298033031033 369.877521477669 216.063990451376
CAPN7 62.1875461262634 78.6705291023488 79.4158467034017 72.2577390063259
82.9322141700549 74.6375749739389 74.0039764902057 64.120219344545
68.4229528414965 57.6791750431251 65.2295773603116 77.947556605547
CAPN9 39.8234920704779 163.280931300437 441.5479786095 41.0219290963778
39.3667161654772 41.9069736908705 46.2448677480802 42.357910063876
42.9413736814915 65.9703614596867 78.0020909283414 58.0722340887264
CAPNS1 727.181489302632 858.228943026906 1325.2479053325
651.969296681551 679.810781641519 863.082385199922 1323.73429724066
875.139073253298 1351.49940447914 1030.31910195147 1213.3477481374
781.923984640049
CAPNS2 50.2203578628217 374.723141438448 210.73006676203
51.223859150798 81.552417816742 51.1621379498014 41.4742337714895
57.2350830635912 154.93193897888 231.198764780818 153.258204588589
148.178364767218
CAPRIN1 107.034189847828 152.608998839745 186.655552987116
123.689895848613 177.29096218424 141.501071047479 141.471672990444
238.616000665585 187.486245101512 160.187478921145 140.01041112896
181.200738629391
CAPRIN2 115.10044062133 158.478919789334 126.911056987607
80.8728961146267 65.6824363141962 79.1257499539205 68.878562323012
73.9624143741222 82.5344501505114 114.617965877564 101.577767481269
99.3956523785983
CAPS 170.527024636624 161.422918681934 457.202737754842 56.6458456890715
141.852330109472 71.2787833369055 183.77850568836 380.862719539633
414.539381761279 341.535750991033 351.188432232165 203.373397813051
CAPS2 36.4564050256915 35.5310164682026 34.806104801006 35.8472302065546
36.7356360093603 38.5252456306656 36.5352572191451 31.3302670771182
32.4931776430014 39.0648733099061 40.723990462047 41.5536168238478
CAPSL 43.5710871617107 39.5595233162192 39.1553169409075 41.8778873678551
40.8270141118472 41.3012916486748 41.7656868106485 42.6450179841129
39.5377896745682 43.9118340045283 42.5426057453993 42.4607795638552
CAPZA1 52.888431709934 55.5867487811643 45.645828394758
72.3607760798558 53.1100587888055 48.0350549043063 51.2417443769676
49.6629259325705 62.0967404944031 50.007830364606 49.4878464533902
56.0787402018234
CAPZA2 827.365231733511 532.35007819883 488.908231529696
1084.23003918768 385.594118191769 1190.10462185567 417.086922265732
960.526372655577 810.201903561366 1191.93354266135 932.849256536912
1016.13142851085
CAPZA3 42.1537476443423 41.3610369241157 43.401782064778
41.0726336841311 41.0885562160946 41.554942319225 37.1643693306382
42.6450179841129 40.9901805728485 43.1839109702714 36.6298807161755
41.5536168238478

CAPZB 393.001025840896 318.234214341679 406.668753694984 422.237766234202
317.406465928295 367.158502379417 323.576613035353 428.729935028911
259.382584049498 304.49707512712 341.601353423389 356.503013319738
CARD10 126.682296930289 149.118265845458 115.530384771495
91.1834104205004 133.38594323309 102.711934290382 75.7342400123505
116.852483297949 204.490115029568 182.80711310964 175.652989203497
185.264053754114
CARD11 148.016994658777 320.812668695531 219.737294408079
91.7068354379889 226.057278804077 93.748984888348 192.470747766298
195.063474879275 239.117228656704 210.958578914607 191.979759198021
162.721587349434
CARD14 44.4669000196164 43.8495248991589 53.4088637045445
39.5748670524951 45.7486514056858 42.157627240486 39.8695929627783
43.4544762830742 55.3122077514658 42.5664883002833 41.6616444147804
44.1915202657512
CARD18 37.4554876386402 37.6383530120621 41.528370959196
41.3027031037343 36.9702228618069 38.8336427948187 43.9450023643394
34.5886782974123 39.4762546236449 39.5637454855233 35.1470994647364
38.6454098044474
CARD6 43.3638918705225 38.0226905622357 44.7829632472754 43.1847628331565
41.467005949568 35.314469458379 39.3585350448719 40.831207953647
38.9834959368496 41.8613406887824 45.1432412071268 43.6753843488695
CARD8 89.1632548302738 66.8026147385682 95.2915739972533 168.288592321667
82.0149542477384 59.9876575158981 41.8315602975157 91.8568043052819
56.0496524902806 84.8999904158793 70.8670844767965 55.3563533836982
CARD9 170.150808455621 253.072560237983 105.204861495017 224.249210397247
123.868848519181 102.499011190932 132.541333041312 145.791398829369
99.2341244350412 93.3897477314201 95.6731932757489 84.9280683258012
CARHSP1 823.933615182634 588.247843264181 589.18407096044
382.872356030213 236.595286890677 340.662853333208 999.13039066242
1075.04105984319 622.964590694346 332.322497133752 287.786165030406
402.886581458688
CARKD 198.288493088076 208.915145509698 208.878921620914 128.635811501506
235.925374041038 211.451463038315 299.136348099827 186.984157187427
187.862483922786 259.456935785521 274.493399403322 227.817928402227
CARM1 689.228901128857 608.579654909865 569.966632625157 461.123856163975
1119.61164710566 783.440408023061 606.378839153932 480.386431986343
448.383843915421 542.268540029548 507.620619345969 323.515041493836
CARS 95.5920087912438 121.036604456449 120.660602388292 139.554519110944
105.018199901228 101.693848821246 104.696298671673 128.827672077002
131.39588569388 128.749209040345 115.853867920678 97.7824843991218
CARS2 68.0700069295224 69.9767092409915 61.9293105089291 61.5515639412892
84.2158827857221 75.7502062965698 69.6301237585571 77.6174893940285
61.6839343510302 63.197604174872 65.342842101283 65.7853960266587
CART1 35.1307934330047 38.4330755397458 33.6816578730693 37.4223289687135
38.1644569189523 39.0266821712099 37.6036146841933 36.1375121409002
49.9162522057712 44.4652605017595 39.2542963855807 37.7526485343328
CARTPT 46.5549623118323 47.4076235350059 45.1569741609597
59.987045425047 45.2556760873335 41.7627910693483 46.0228732507872
44.5029690493211 41.5635499110307 47.9056710878325 45.2265319826548
46.5650556481721
CASC1 38.7537364570873 41.8848959280916 41.8751578329365 41.5464765641928
40.4639532172338 47.9503724044453 41.253645578517 39.4710743516517
46.2027193115397 47.8207974930031 46.824299585607 44.7401523707775

CASC2 50.0802438185974 50.7712194930085 53.0104469636221 46.8249967544573
45.5198200633333 54.8660503290883 44.0687697278114 54.6067620335514
55.9423752286714 60.9905272201951 56.9628808664342 52.1186591838715
CASC3 169.970634268117 122.621100838222 103.746508465792 184.235914591152
159.697488404476 204.63343271995 161.870972980732 170.750057881363
166.097017297742 256.614982558637 200.401133392259 180.255771684507
CASC4 104.573463811298 102.655050997714 83.247335725921 96.2385885003071
111.18019985642 165.496075703734 108.147548794301 85.7013212907541
104.900627862215 66.0786923699684 91.4786089554886 117.439175394651
CASC5 43.9551059970289 46.7279104095328 43.3085358214154 35.8041229658453
49.1928206164702 40.9506062495231 47.0874209746031 48.272324215504
48.7358147030082 37.3687900733549 36.6603338274678 43.5738557773903
CASD1 73.4611178120841 75.5775406974686 65.49167373382 59.6153684374766
69.493532577364 97.9586375307737 55.9276266345493 50.0526763693329
84.5998418020787 46.0119912782855 73.3321469094819 67.6983931011126
CASK 151.740176274202 131.67159377585 284.105466865264 95.3070802596041
127.008218054726 91.4446109489387 162.160380044249 125.197314240529
256.071638089438 79.7389069715403 86.500678864429 121.449647909665
CASKIN1 40.7609952437622 45.0115870036541 39.0775870687758
41.6564075217517 42.1650127805175 47.5733603903505 39.2533668167483
37.4484310268909 39.5806465938526 37.6383530120621 39.9899430628542
43.6753843488695
CASKIN2 41.3387790895716 38.2263227947189 36.769073068743
30.802355637173 34.2638875325204 34.1806289878747 36.3194282084339
46.3685439301234 39.5806465938526 32.9003644440608 41.9083581529276
32.4951076826876
CASP1 117.502489192855 185.264053754114 239.924746743429 547.442608452594
61.8757975011721 144.657263336504 95.5724734464786 115.577618925646
158.843766201769 117.181144429729 123.114162330406 132.81898450496
CASP10 49.8472708021525 46.2677540712845 46.9711270147079
48.9944486068347 44.8351976221735 44.5099734963815 44.7255207589606
46.4329203134841 48.2123120250343 43.4885245598939 44.0527419856743
45.9104478407175
CASP12 34.8099089553046 41.7337506166619 42.7303139017786
37.9875302985336 40.7779767565889 43.4681646416585 38.4005795891396
39.4710743516517 37.6383530120621 40.3235655331796 38.4689132737495
45.1735152121056
CASP14 42.5193208629123 40.3384318592985 47.646800205189
40.5484717678957 44.4489662507893 42.4580504587581 41.3594868055879
37.4484310268909 67.6983931011126 39.3698811593364 45.9104478407175
38.9496515344837
CASP2 376.141477833569 263.832968124343 221.277023931945 146.087016079575
318.846373031568 125.768826615813 243.489225650886 264.924262411772
327.729165976539 211.625447303889 181.705809767061 147.782301091454
CASP3 238.246319539302 246.09270373893 177.584394977912 211.109957714241
193.262615637464 144.412165495719 276.399037769005 121.050369804136
273.460165751162 115.235144612428 125.559679916646 121.696160351886
CASP4 103.199200862158 89.7093249557939 110.909109947659 237.359267889848
55.0767527801658 93.1850674428555 75.353039684456 78.8250642061439
85.5269681075807 77.0578241280903 68.5888253504788 87.4623442823265
CASP5 37.1485601150873 46.7591817286632 38.5045333213784 91.0346886951891
37.8679241954337 48.7781962164988 45.223075311113 41.0381860307445
37.5469247328848 44.3648993568405 37.2892641289258 36.9976231206067

CASP6 58.9201382292404 105.287433456133 63.2779399027463 53.3967242431461
99.1769280484821 70.1831787109201 85.0065217565876 61.0935621637178
89.5777156320178 50.0564735608363 54.8532558909227 70.380703483689
CASP7 57.0430104900107 75.3174199658042 59.0590113926295 66.1364624511164
53.6907197364586 68.7871223112501 80.882176929636 60.0835531094681
70.0335127723592 60.2165699959135 61.8613729054269 69.7095546047257
CASP8 41.8871259674102 52.2330073838342 51.0261383607462 46.9858151779864
45.3115139242709 41.6225742046331 41.2080274755436 42.0726170351604
45.5178748361093 40.7594643110892 36.6298807161755 44.1148826235374
CASP8AP2 39.6609238036815 44.2630383225933 40.0998167098057
40.5484717678957 38.1644569189523 43.1826328292558 39.4600924554505
38.745552403895 39.853866204927 42.0621425894624 39.5399188536544
34.0382693188794
CASP9 61.2691385007595 88.7605288058249 113.051477801522 92.999328769924
84.1639779987105 65.4206801651661 92.8579489575841 153.812414753676
76.0514378922651 114.190951215203 83.6898920876764 96.6957892186886
CASQ1 40.3478825593224 40.4359177791566 85.8945498067934 36.5393857306744
45.3675390992851 49.2050538150106 47.7390230243778 51.555506218204
89.0684909049451 197.52820443061 135.308001652191 73.6512983066046
CASQ2 48.6621527194963 44.5884811528117 38.0452862258803 37.651298468202
38.5512429290626 998.311134235214 40.3302268293707 48.1591264215184
41.9635525160083 407.161973433666 332.830805115112 700.010354583503
CASR 43.6706764143908 44.2630383225933 38.8775560911841 39.6292788039793
41.0885562160946 34.4896350146096 40.3302268293707 44.8997235283254
44.0519245668737 56.685925255591 82.0206083292981 57.8111054584154
CASRL1 39.3709357615493 38.1811246394237 40.0998167098057
39.853175885492 40.3021896270845 41.5132248356707 39.1539892144371
35.7182224415467 39.8873706855774 36.1933006350162 36.2435874904689
36.5219488315447
CASS4 37.55264801907 39.0894172982439 40.301483914446 79.262977183608
41.8806343997257 46.3715909653832 37.6036146841933 37.6557714476853
34.7722083264266 38.3092241935352 41.6336636285875 40.6614700944853
CAST 65.0217598379984 92.6290175610859 156.844010597219 77.0262811825953
63.5932443357808 112.316483867372 63.7002949745101 65.5820437889838
86.9709077763908 121.92772785579 112.340869310085 118.625610664218
CASZ1 123.739272396278 114.740276704399 127.87945182399 64.6111899743678
139.66584446954 114.113460279317 138.666805923912 201.027801320306
109.525921548011 149.231260844854 141.924244643825 93.8968899420039
CAT 124.22494803053 290.261616334371 280.275836993258 235.33716196909
168.54068375757 281.452208033175 238.164418453962 185.677720870442
293.755623192857 180.679129876248 260.740251007349 616.265771322835
CATSPER1 45.0985088502363 48.7084357480922 45.4865173433646
76.6232527679845 38.5512429290626 44.1737502492846 43.1931731665131
43.3262148375525 39.1790517315259 38.1042454705119 39.9070785037411
40.5703581862752
CATSPER2 2384.00264993279 1157.65264077595 1126.31998762007
1991.4199819544 879.708199333257 772.368030410394 1649.78627644882
3707.73750609316 792.022171185649 2721.97551918611 2357.96534075689
1813.65521720537
CATSPER2P1 59.2903915056319 69.7855392178808 50.0050760710506
52.7924213633885 75.8976034525022 61.7566103867825 74.9059650336395
93.9387650094061 70.4933405598359 75.9802606133139 73.2234405348435
65.8959431201418

CATSPER3 41.7397731376692 38.6414316877471 37.1643693306382
40.4359177791566 41.0885562160946 40.4359177791566 41.0381860307445
38.4540849632254 36.8127605061228 36.5806287103157 37.3687900733549
38.3291713594599
CATSPER4 43.0001432913058 37.10170285405 42.2577689927563
39.6292788039793 44.4489662507893 38.8336427948187 33.5578650644246
36.2539950294089 39.0775870687758 40.1179210534118 41.4508241771315
39.3575468494941
CATSPERB 48.7646976789783 44.9370339275041 43.8495248991589
46.3203995372658 47.8075998240396 39.4953494583071 47.4439407315335
65.813547096222 40.5912213523345 46.8954576545929 46.5650556481721
46.2625470410195
CAV1 282.196234758364 181.064239330482 290.956194006554 315.880072026714
322.774093041134 1413.36279821142 62.6236991757723 239.272650332949
92.3901719424121 753.491584605447 656.355333007615 712.80113322987
CAV2 102.513192587614 107.094663242259 218.165286111397 81.4367600457584
107.348865210074 218.958199087236 57.1070449050465 74.4696354388511
109.889322825648 113.518333340953 129.091447141381 157.856158440506
CAV3 41.8833988894167 34.9732824977751 42.108130037421 36.9912468186322
40.7779767565889 36.0306975524309 43.6025443656008 44.293922970134
40.4518431978241 36.7439906400209 37.6862447187338 40.1235656101329
CBARA1 148.758736522703 108.649424858333 145.027715477051
144.813029178278 79.0039595317374 143.152895211088 166.553853424708
121.510087314197 279.521995263196 171.722423740869 145.741469970609
143.391384762612
CBFA2T2 53.5698357689852 52.9875116869966 52.1242102345408
51.0175677516029 58.1027665533767 55.4294204189628 53.4921736109774
56.8622704143595 61.1129808051181 66.8026147385682 61.6966068000136
57.8111054584154
CBFA2T3 38.528621456378 41.8503210829559 39.3709357615493
42.2674195935107 42.9775348174342 41.7627910693483 47.2131144508464
43.2855454938964 44.8568725629882 45.9573418095075 46.060742611045
42.1915582886043
CBFB 110.214922820817 161.422918681934 182.448444305445 134.646159826262
73.2607982527105 101.103458852775 138.618449365819 102.177189421378
156.844010597219 109.045804556923 101.954354107731 124.512081978396
CBL 62.1448806673973 60.0856496605043 61.8701572127817 85.0653700751969
67.8233625051884 62.4450952595811 55.8510296341925 67.9667104519753
46.2027193115397 58.9069025298754 64.9401081242429 67.6138276415458
CBLB 247.547769833089 277.993522959259 276.084235934753 254.322611819303
211.693380728047 321.26586939039 186.891912776245 300.803955139552
163.931827466775 258.973633899103 265.570814613305 225.53019299714
CBLC 64.8813495504917 170.501116810593 150.737482160358 38.4208429103799
231.198764780818 73.3506594385504 230.664926508522 368.005081137665
313.046160403661 188.900125060451 181.983177572371 126.302564098322
CBLL1 80.4967881959046 82.6116645182046 64.9559356780521 103.631449539809
100.645556491457 86.0630164348952 84.3526980051279 102.770627167954
115.544036763985 63.1394716239975 70.8940689219829 84.2728210732532
CBLN1 44.1294459089314 44.0409178714232 40.4882741869718 46.7577982595088
45.1137938810047 39.8455060654531 34.5633623842083 41.6466169548846
46.8873421271259 41.3321861371202 41.9908237694988 46.2625470410195
CBLN2 50.8388984085636 41.7337506166619 38.0452862258803 38.2041059863839
43.2484996523549 82.5956705829173 41.253645578517 42.8482181313226
37.6383530120621 86.3374157614394 68.0414563597154 89.5687388187015

CBLN3	106.219480656782	77.775644509079	58.9135553403521	73.5834042105313
	46.5557478175976	75.5561597374812	47.5963750520409	63.287067794195
	43.7630587657205	91.3941563889513	63.2684764441521	62.0967404944031
CBLN4	44.7915315085948	38.8336427948187	41.8751578329365	42.2496337895968
	40.6723334103791	40.7616966228404	41.5817737564592	40.4359177791566
	47.4699236780425	47.1001757441025	41.5421429082231	39.2469742642337
CBR1	94.9365530062692	75.0873720488265	106.912316023598	82.5279746006378
	136.875335055866	131.727983826985	109.456162739433	69.1973371867031
	101.577767481269	88.4385631861141	86.915098846441	107.585504340548
CBR3	128.281765653795	94.2182728193924	78.080034922779	65.084620579901
	85.7842344710572	218.354538624978	147.758917745955	110.983311402531
	182.757684959879	182.685579297058	107.906865732219	81.4881766238989
CBR4	117.20232751191	289.533968432846	180.663636520073	98.2447263268855
	166.488589768213	145.439350995461	136.461760992832	65.3715655467882
	171.021755568144	244.109166881519	221.598637828404	219.616289279562
CBS	60.4593810615882	903.756866967447	59.7483827511172	131.57403028894
	2440.77408185413	375.757961593064	195.108184885541	113.179313022693
	232.77788697408	210.005571009784	75.0237373605584	133.314819245044
CBWD1	39.8184668132023	40.0195349109823	41.0705134231814	42.2674195935107
	38.3099344193405	40.2889845955035	38.2307941336596	38.8495095797881
	38.6558726824244	40.769767732912	39.0156305603334	39.3575468494941
CBWD2	46.263826584899	36.9994058279362	36.5474283500601	39.066284740632
	38.6454098044474	42.4580504587581	41.5817737564592	39.3709357615493
	39.853866204927	41.8613406887824	41.7272875295469	38.8495095797881
CBWD5	111.954989477689	132.663052961193	106.361958225818	90.696780047049
	91.6253858792439	82.9449579428926	97.8309399470621	91.0354017559834
	98.7623993959996	74.2379710965903	76.0420039542595	134.307645598718
CBWD6	38.5007204246293	41.159706131351	36.5474283500601	41.7614975630362
	33.2021437980841	35.5158505219112	36.5352572191451	36.43924066428
	37.3600014814388	34.6720534245631	35.046946054314	37.4554876386402
CBX1	47.1962846565261	64.8903340853337	59.8273072817267	44.9370339275041
	51.4527545197672	54.8265655909623	48.6395795163502	46.4672637826582
	92.4839055864605	56.057906531592	49.2206713037152	60.7495043977707
CBX2	116.852483297949	56.8760943789568	49.2458645560476	46.2399969315262
	120.19033659661	54.3478800757394	123.162013735234	148.006731522923
	101.337735124049	43.119837808537	43.9701242600947	43.6753843488695
CBX3	97.4515987591398	116.596605311488	131.330480963914	154.633672616367
	139.847976281228	116.35075921851	174.985336606027	112.873697337138
	121.84142967836	81.9727730469029	85.1774859932565	91.1057573288203
CBX4	102.136265168978	224.827414462257	155.492099076744	121.423421895502
	157.927114480316	88.6287852712744	113.039124313172	166.734730009916
	158.071355628454	68.8167148906225	107.391994714261	80.7227211275429
CBX5	74.9006142438341	70.7003843117511	78.6402083787251	81.0057400164851
	75.2391243965793	71.6050174762551	87.0685571351947	73.362936710475
	76.5444845463963	58.115198353247	61.1801493883012	74.5886835786452
CBX6	131.990661474974	155.855834721836	80.0183739952818	206.791464145857
	157.927114480316	420.401914084346	232.048938339214	469.553640187203
	96.3232257371077	183.057478637596	282.802560118785	212.062417120966
CBX7	100.289042948128	142.124053129543	109.251112569215	101.867388894697
	84.8751572193902	411.582299081553	84.9182861692273	101.516595046503
	73.5541045639348	363.67768546508	329.677454924062	229.971492699272
CBX8	55.3153799233744	60.5571721986885	53.9612654850046	46.1673159982664
	78.258486366912	43.8582747295766	69.4677195088043	53.6210959113222
	54.4059109100338	51.0055825931636	53.0177144132449	48.0350549043063

CBY1 132.117013391364 113.169111095305 96.0654949568324 72.1687283197901
118.49449420132 134.853649092952 137.955007835039 128.146059846414
115.001179779307 149.8814624626 136.04447103201 131.010196997585
CC2D1A 48.7646976789783 75.9802606133139 94.3953345963359
44.7980740332174 87.2282539094452 49.2050538150106 67.0455170947389
85.3842287274022 97.0348634204639 79.6133633483649 79.4832790698673
73.7335870746827
CC2D1B 54.4140381300469 53.9634127270611 55.6195455298284
56.4578213622965 52.8190882379316 50.2729836196996 65.644767142472
55.1109784383525 60.7480970026669 62.5528124447176 58.4816190076338
57.404059248727
CC2D2A 61.2130609867477 49.4982025424177 46.9040704638073
62.3936376596431 69.8804970538895 132.364260893831 61.2183034191703
72.9074911694002 70.8606919488782 102.38706554984 73.9126724639466
60.2550788540864
CC2D2B 38.7537364570873 43.5873188565519 48.9944486068347
47.3574589211166 45.5809132232681 40.9506062495231 48.9826868640753
41.8417024632349 41.7627910693483 45.5776159432079 41.1638563416981
42.1537476443423
CCAR1 44.7044140979108 47.6229101519945 42.0621425894624 48.9944486068347
42.3685136297603 47.8636003588181 52.8496345990125 44.8997235283254
51.2776048338 46.1390887492649 46.4205654140754 41.863927676552
CCBE1 189.737472782071 114.755820699611 124.469290554848 184.65212689433
103.302837652919 77.7481370715949 179.241252961119 285.459248157609
111.18019985642 231.990562664196 239.885284409329 182.49650467392
CCBL1 58.5759178381027 84.0691608193405 82.5344501505114 61.2213001749372
58.7460488758053 67.9306669919176 64.8367546135053 49.5185112777773
66.7750249658122 273.891856200567 162.993316022946 101.627551729492
CCBL2 66.0303950772736 77.0578241280903 70.0059240383657 60.3968750318023
73.9986015964321 73.2751502965172 70.3935694362568 93.461049073831
48.8067321016961 59.3923286524971 55.812813573397 75.4935574755043
CCBP2 350.917244438732 53.9275079525238 64.2404293863468 60.4646208319121
91.7629175712824 69.64983895075 61.2183034191703 221.383645621978
49.7336769474446 70.3629652777935 92.8579489575841 135.138667957956
CCDC101 81.0562186907518 69.1414574152997 102.926945888985
78.3509756704423 131.766252431636 112.131738588405 95.6697427870979
91.6033269431304 112.565968152932 133.127645982507 110.865092573085
85.3431175579775
CCDC102A 140.226992517573 86.1924491308234 74.8071157013401
73.3725472767369 80.5437011468815 361.524083649962 62.0404348860702
78.0020909283414 90.4957279507369 200.790001763897 207.671418362257
140.648209468568
CCDC102B 41.4508241771315 43.3458769998118 49.4573631027613
37.651298468202 39.9697332660833 38.3418933808844 36.634024014558
47.3791873993182 38.9834959368496 40.8049408996274 39.4410515050885
42.4607795638552
CCDC103 45.8108990837624 53.0383634135393 44.696023817801
44.7980740332174 60.1730663840476 48.1591264215184 59.1774824918359
51.2623287419357 45.3756389170606 49.8393069675192 42.5426057453993
46.917149434588
CCDC104 75.0237373605584 64.3032703931013 81.0295607064541
61.8997157694697 56.7602749462766 100.510815049535 59.9174456081704
91.9919654450652 79.5488891958168 76.6868184563568 73.7873098190297
94.7718338968252

CCDC105 43.4626049853157 41.0678614026819 43.0016969050339
47.0874209746031 45.1137938810047 38.8336427948187 41.3594868055879
40.931294690669 37.8319142847141 38.8507804383261 36.3390947880186
43.4709415392696
CCDC106 75.3761360028971 149.933827818309 173.308366696429
97.3870880869036 327.238337722366 166.734730009916 220.36831243882
100.989056102358 84.5998418020787 189.890451486324 193.395549316561
124.998771394658
CCDC107 66.1229691315616 57.4538469832241 48.7039705048129
57.8277957325085 65.9413235523618 85.8059456975413 52.7002314230604
48.6441988290952 69.1129431075514 75.3333024462557 84.607833108004
73.7335870746827
CCDC108 40.1925952773317 36.7495703772303 35.9714104962197
39.2780515621926 38.2496050953547 38.9647316146867 35.9058393060637
35.6194521200331 38.1548766517774 38.3092241935352 38.5668610487033
35.6111578834671
CCDC109A 126.10194284238 104.072423445726 102.607924735378
71.5249259772583 76.1293960715431 85.0275638433103 92.3034609409756
92.681224908389 306.487935692954 122.099297248392 121.552188287725
156.53223142598
CCDC109B 219.34503297115 313.921236120658 127.949712300256
713.880440315813 86.2836002982515 442.380936516984 326.500804513133
236.178265404264 286.340091833711 178.012676342712 146.691463448295
219.616289279562
CCDC11 40.0557788909923 43.8495248991589 48.3847843498476
44.4367387274922 43.2484996523549 45.8325181856483 38.0598374201407
43.5544347011646 46.1101825640108 44.1379638573455 47.2134341420411
40.5703581862752
CCDC110 41.8409927585705 38.0226905622357 46.4569566520107
48.9944486068347 46.3150221024046 53.0290605707162 44.7255207589606
54.9919060580109 38.9834959368496 42.785429206422 45.390192977585
39.5418280570561
CCDC111 59.1311136551108 55.9269493191353 56.6888826857797
65.524821699432 61.7566103867825 73.9456720099807 64.5722284870633
52.1788489555942 64.2174369755376 51.1169368198437 49.9338905397767
62.6868461044048
CCDC112 60.4593810615882 50.3586212130973 47.7737810047679
72.4834467838798 57.6791750431251 63.2343637481705 59.8273072817267
64.8336014343435 66.9499465764301 53.8260140375214 59.3379918468928
53.9431600945419
CCDC113 40.9558083259517 43.5182502375207 39.1799070031819
36.7892997857376 37.5556299264699 42.157627240486 37.3799542961839
43.5544347011646 42.6366765186182 34.8137085420423 39.9070785037411
41.1554481567603
CCDC114 36.7356360093603 40.9731067463382 41.0356368374708
40.1804147506786 35.9058393060637 42.4580504587581 37.2892641289258
43.2551508830106 40.6957787921451 41.8613406887824 44.553780984244
36.5474283500601
CCDC115 196.322377166278 376.453560620912 435.632247999054
221.163225922749 261.421333472097 324.994911416877 172.748740911831
222.088653823382 294.698849594622 280.490447179545 312.045709918301
289.332274319357
CCDC116 45.2875173643252 43.1805016690297 45.6989664108554
42.8235313007746 44.0451487108213 43.4681646416585 57.4047331041592

44.8997235283254 46.6944238390932 42.0621425894624 45.310660704531
42.0424641526302
CCDC117 132.473918053428 92.8089247593564 84.2880616879945
112.585860083115 91.3723635213821 106.679905803492 109.525921548011
83.2240772671045 117.823678167648 97.8389686233842 110.009834478125
101.156215957297
CCDC12 276.550265506988 314.281125546552 397.94989372796
331.306005726359 500.794458547464 275.611524861938 484.651969259966
341.397811775105 411.431488342465 479.318513108472 522.49156295512
342.387761195952
CCDC120 195.127419251015 126.253578620746 113.6408244935
46.3203995372658 74.2283328056604 54.0382756991549 98.1371527802953
171.957278903005 124.56803502087 100.271925566074 95.5325488969517
100.386011517899
CCDC121 46.9939712192224 43.2109153553335 47.3861758958576
43.6726153508978 43.4544762830742 52.260196361018 43.8206425661746
48.4670130605028 45.0017158586743 47.3791873993182 43.8002456102021
46.7182294568603
CCDC122 41.678190413467 38.7578265341627 45.4865173433646
42.6913548400127 42.7107928951766 41.408887993053 43.2721716572958
46.7508829977587 43.4656637536244 43.401782064778 44.4329005595031
39.9941474864076
CCDC123 61.8529316708431 65.2966707319433 60.4809187893259
57.6511495792664 80.257554904218 67.6817814091697 97.0155195562193
105.743957034577 75.5681913475287 64.428683021978 65.8748459696377
66.6118713143653
CCDC124 93.0714382925187 83.7739049195868 83.955786676926
105.234356571498 152.272399644814 152.95751212223 107.325456368022
120.286733441947 114.136234102574 107.122319086614 109.386655134289
90.5216273844682
CCDC126 50.2203578628217 51.9421032049606 48.9944486068347
46.614092935749 49.8304479381623 48.5835484742456 45.3597029614942
47.2864663693787 48.6494451061637 46.2286164776607 51.838002904753
55.4635297192076
CCDC127 137.897231677665 85.7732262204681 116.949813289388
62.6048727240291 155.174579191838 114.769498252688 143.913007288353
66.295597720304 105.036862798568 82.8275497073451 88.3725577276628
75.9136854087044
CCDC128 131.990661474974 76.7710944291287 79.8773095098789
75.2475238554276 49.9493230064613 120.980558571109 70.0567634568557
143.399636653204 77.7907484334847 153.42135363926 162.993316022946
148.944852825695
CCDC129 48.8941062980393 43.8495248991589 39.3709357615493
35.3829784221072 46.7508829977587 34.4896350146096 33.7574445386307
35.6825474397235 38.1169703906207 38.745552403895 44.8872784170036
38.5279745308203
CCDC13 46.0920218698606 43.9421822132391 38.941855825269
42.8848887336829 45.1137938810047 41.3553369544097 39.4916414839923
43.3262148375525 44.6717213146407 45.0155543555707 43.5258090162298
42.7275315121163
CCDC130 537.841677481011 933.968759959712 1012.91803181784
549.407911286514 1199.29782389306 517.104607715151 484.968116511768
585.771656887036 639.391575249793 920.504890206997 969.805093523651
624.51670158046

CCDC131 72.613397507717 72.6888636455541 86.9032093551446 82.5279746006378 74.6833250738044 58.8605811695948 54.6414321010929 63.9110569727542 108.503081044355 100.344513700137 76.6232527679845 100.677422836631
CCDC132 48.1460386356622 44.2630383225933 45.3688612476977 43.8524422305633 43.9937687851164 43.0860383229288 42.8149277408658 43.5738557773903 43.515290338756 47.1001757441025 47.3941571188342 48.2220547806679
CCDC134 70.5096706089299 48.4497719907777 52.0742951366864 48.4916117779992 53.0419743379342 53.7442594289333 56.4809146784765 54.1731035633439 57.4777075615651 47.7180816682562 50.7159667633757 51.3244198342335
CCDC135 43.9309845324767 46.8954576545929 47.0474493061198 46.3203995372658 45.5809132232681 45.1873487463472 53.1884505015411 47.2368301203382 46.1101825640108 42.5193208629123 45.5707650534259 45.2783024981315
CCDC136 51.413025495745 52.8264142735929 45.989212296035 46.7577982595088 50.2163892855092 202.957014793417 54.0170817319522 79.8264217848103 53.6446045619513 137.658868649032 143.251956814524 100.824660338235
CCDC137 49.7188990489616 64.3312319756553 68.1780382173625 47.2009420656854 66.5187917407182 47.4699236780425 70.2635892414678 46.695848687694 57.1016072897358 46.441301934328 45.390192977585 48.4535991392809
CCDC138 51.9545473262296 70.7003843117511 59.0308764112247 51.949834638817 109.398472684587 56.1671440556142 76.1869593072029 72.2610650652674 64.0541263553251 45.0155543555707 46.5650556481721 53.3059058105836
CCDC14 372.525928228911 283.484056482174 272.746328542895 183.028254350275 529.210992541911 152.630871384615 348.913213159222 333.731080636948 209.986313475002 343.677907141241 269.745248423773 251.803102904399
CCDC140 77.0301627655229 59.4253780950488 64.9839099669442 86.0630164348952 74.8013460023603 64.7905331739366 81.8569832022381 89.5130163419167 60.2614254907771 84.8999904158793 78.3542840447759 72.8306652548968
CCDC141 35.6111578834671 39.1982470626863 44.0451487108213 39.3750969348308 34.2638875325204 44.8872784170036 36.9355798143571 41.1575777497576 44.619512785899 46.441301934328 37.6484782516569 45.7562276914441
CCDC142 40.4639532172338 38.9338521051669 41.1367478979484 41.0726336841311 48.9627346956003 37.3799542961839 41.3594868055879 41.5464765641928 35.6970528713709 42.0621425894624 37.2018803984555 45.5800840647989
CCDC144A 40.9999112795884 36.4505152165662 37.7895883057656 38.3613388187252 37.5044776781216 38.8336427948187 37.762350605077 37.491503408018 37.6685064050907 38.0078357808393 37.5303767522393 41.1914475733743
CCDC146 56.5094373275922 60.8608693323868 125.880019627499 63.4531374125233 72.828269171954 118.745729915256 66.5422130374814 65.813547096222 74.3394153125605 78.59966540743 65.5386366902998 93.3644318958633
CCDC147 46.4732328055735 42.7275315121163 44.4652605017595 50.3127977239083 44.4031868028216 41.554942319225 55.7522234735038

41.8767957887205 45.9227107476843 44.3648993568405 43.5258090162298
41.4508241771315
CCDC148 37.3463875295726 34.3715326040967 45.989212296035
43.6726153508978 41.9842695818285 40.244271403831 35.0305278582179
45.9604707098953 49.1940901905636 38.6454098044474 40.8225507267584
45.8546719310325
CCDC149 41.7397731376692 46.4861258030402 49.6347864008749
44.7980740332174 44.5668141895992 55.401776072578 41.0381860307445
46.1673159982664 41.7627910693483 79.6133633483649 61.6084234634424
52.5752569429057
CCDC15 54.4140381300469 58.174555206394 53.8724557170772
51.7739270955254 66.2366101899356 52.5330333302271 64.6446465846487
71.9369353731849 48.5136862664526 49.461728580617 57.5430365699386
50.2471139112912
CCDC150 39.5676051939342 43.0227387991135 44.6032218589199
39.066284740632 46.0977687311222 42.4580504587581 35.8227939208521
44.1311654985682 44.8003345643334 40.8970401536578 42.5426057453993
43.6753843488695
CCDC153 40.2087556903277 41.2262949117288 39.1799070031819
46.7577982595088 42.9655396732962 38.8336427948187 41.7614975630362
46.7508829977587 37.4937394063612 43.7303813515864 41.862634402486
37.6557714476853
CCDC16 184.235914591152 123.841740274811 113.18951586976
142.840631375936 157.082769443568 156.36242271528 180.349984862124
173.694418385132 190.960237766104 145.509901200987 149.747000657513
159.937192675974
CCDC17 51.8781065691038 52.5868047927666 59.9690744676539
50.378948785456 63.686652234709 51.4931742685391 49.2546642180676
47.2009420656854 57.8772482723463 59.1496467460507 48.8744644207821
56.1875519519366
CCDC18 53.3343962424887 54.437526296816 50.0050760710506
45.334106390212 54.1225249245036 50.3245489035833 52.773844004595
62.5528124447176 62.6386437936516 45.8471859855979 45.310660704531
49.7336769474446
CCDC21 86.8262463672891 75.6614221167993 78.9701459806641
71.5249259772583 129.270792739554 58.9676332048764 87.0685571351947
99.6392844274488 78.4428909886495 55.0016608333828 59.8070012006708
63.0266170942566
CCDC22 44.3355304829989 50.1027491117756 51.1347974668251
41.4273122914951 47.0954207417646 48.5835484742456 47.4439407315335
49.5185112777773 63.955655956484 46.5650556481721 53.5698357689852
57.0524725494273
CCDC23 560.954913956997 542.467997217663 244.089971761037
299.334388262225 389.248080358801 343.016541928592 663.372296297652
451.603052501528 431.727858902449 264.718875269207 334.639325701194
346.029336456779
CCDC24 102.655050997714 127.596368103014 158.958308702808
47.0874209746031 104.599492214295 62.5542376788358 123.247207157645
176.318618244066 113.026772174726 184.964039515715 159.827302305842
169.664184704618
CCDC25 154.509548705054 114.230358276599 214.369365247268
168.67163443217 88.6057390890775 270.253329297593 131.258204302177
87.2068743613955 153.736638194572 254.199076651191 213.520663841371
218.217851814655

CCDC26 38.7537364570873 38.3322849790657 46.5650556481721
47.9915414951747 43.4544762830742 44.1737502492846 34.6720534245631
42.8482181313226 40.0403898620771 43.0919221375514 37.4642807402741
41.9430737035925
CCDC27 47.2862299990938 46.1569135229922 39.8299340037009
38.6414316877471 44.5668141895992 44.1737502492846 53.8687221233237
47.6952179922944 45.0017158586743 46.5650556481721 39.9899430628542
44.1452943804044
CCDC28A 275.061444728555 146.355277438643 173.308366696429
253.140146266702 104.486555491331 202.154195547644 242.339375770778
158.769954040462 150.009352890706 353.213447404132 232.628747594691
328.295200683554
CCDC28B 62.1448806673973 62.0220558452834 68.0649030501515
68.3157692917711 132.555169949054 74.6955454743937 78.4937314423253
64.9114266867808 79.0962508551502 66.9497120104244 57.2008272301399
55.9283039581662
CCDC3 123.00053659939 118.143444770806 154.752920877816 109.144663527001
142.863673914134 169.554408876006 47.5963750520409 111.240804216088
63.7046039657949 150.649377863375 103.491711140137 69.3483108270756
CCDC32 427.199940706169 311.985010273921 192.398575159246
515.007705025879 322.440680131096 424.085665569421 310.763904671301
454.185408362583 362.788520130059 406.417924060562 463.022816882856
446.162602155964
CCDC33 38.8561375693335 41.8073910693411 36.4564050256915
32.6817359372544 38.1644569189523 35.0970060242246 43.4544762830742
43.1567024035183 38.0213051227859 40.6022003765153 39.9070785037411
38.9496515344837
CCDC34 98.5339473137891 279.901217752341 165.029434090892
72.9580216367379 209.794541970107 146.372411304933 326.121479451246
151.55043196879 178.624349589089 133.626390404589 161.603201688692
160.950554451488
CCDC35 36.8072833828297 39.8695929627783 46.6588655218735
41.5464765641928 47.0954207417646 45.1873487463472 42.0605906240248
45.9604707098953 48.3119523820103 47.1001757441025 39.6403089864678
45.0035308952466
CCDC36 39.0620487644635 36.6968906669565 42.7303139017786
38.6414316877471 43.6587492470348 39.0266821712099 42.0605906240248
45.2806199505029 42.7150227340364 37.3179578037963 37.5583886316038
43.1805016690297
CCDC37 37.0320432433314 41.8073910693411 39.5450079747895
34.1051821768421 41.5668884168669 43.4681646416585 49.1067959632109
41.9315419791594 42.0621425894624 39.9948390976317 47.0110068985119
40.8681871019812
CCDC38 38.0089018744739 41.7337506166619 40.7620472083159
40.646702696033 41.6806984441849 39.4953494583071 41.3594868055879
37.4484310268909 41.3803269429468 44.0409178714232 45.390192977585
39.3575468494941
CCDC4 41.4727452533295 44.4581200816365 42.4804257679172 43.2504382247503
44.3166120733789 48.1331309338997 42.5499248634774 47.0362584198466
47.5870162399764 45.7328341667151 45.0589947942306 47.0130468198828
CCDC40 42.0532901689036 45.620219291723 42.3536549114165
47.5733603903505 46.9058511192313 46.574682061365 38.3291713594599
40.047314525032 42.1089765085639 40.1179210534118 40.1362682639916
40.3691324547049

CCDC41 56.4819802857598 50.4872122867759 55.9459886217809
59.2778887402357 64.428683021978 60.6548851977964 59.7744650739434
62.7669079605627 79.0276852011359 52.6040246568056 55.3147456054175
58.5759178381027
CCDC42 33.0463640202858 35.0222940937513 35.5220586859301
35.49696651597 38.0598374201407 40.1428591998404 42.9803717121865
41.6466169548846 34.6720534245631 37.3179578037963 40.0766543123257
41.5536168238478
CCDC43 51.413025495745 52.3896785600751 53.3127618245672
53.2046867327331 65.3933030211114 66.8632294055679 50.6774883370457
57.6684494624163 75.6382797289563 40.8970401536578 46.0095710163378
50.7487159427973
CCDC44 224.266559865201 196.561401686392 227.439319520245
191.771063132796 345.084002073846 199.501451042641 358.194667053419
260.740251007349 325.172633427119 290.261616334371 234.113622230498
233.435884567494
CCDC45 206.378149389396 154.232642598588 149.260740603715
118.762101740563 166.317689650501 142.957149812186 197.171449112363
206.28819203182 183.126129333645 231.733564500645 189.267281390459
206.41879845205
CCDC46 52.3432130135266 67.8208976798364 46.1335462158451
53.7045101264881 45.1846041469628 90.4622684763614 42.106632087165
58.2043532960091 49.84186525659 60.9905272201951 59.3615334058994
62.3822663237992
CCDC47 49.085741080241 65.6721629145797 82.9449579428926
49.7350276169552 99.0647786948132 63.6894484284172 85.5027511448958
73.9624143741222 93.4180535865914 59.8130420783374 62.2200723764909
77.1059855034406
CCDC48 35.0202257459026 42.1607322031054 38.4043916290561
35.0448343468967 43.004943518025 44.4619248757502 36.3194282084339
40.7311497914871 40.3316153483289 40.6022003765153 48.2910530887214
44.5432192783161
CCDC49 102.443884864631 144.239350177363 129.091447141381
214.281036491237 127.908440924429 116.253417059012 141.830305704504
84.3526980051279 122.301290485889 96.6833301748676 100.663054866186
108.855682928578
CCDC5 51.413025495745 59.0322602067702 53.3791548006794 58.5406981871153
51.9885249508283 54.400905818898 61.8571521441451 54.9919060580109
70.3613168347492 50.5252048155459 51.1905212004904 57.5372732453319
CCDC50 107.722626390174 151.255948388415 105.277476070937
143.706880936186 82.4975645442705 155.617122020913 163.360824752796
118.49449420132 103.67004664951 97.6774247176874 99.5531381996521
149.27230040094
CCDC51 81.1350396749303 84.4596873416863 82.6983700200815
72.0694688774804 124.664792638889 81.9983163893373 109.844156801015
52.5098795466615 82.2694707335812 78.6859709780134 67.2575005672188
77.3999378505514
CCDC52 41.3387790895716 39.1982470626863 46.6588655218735
44.7980740332174 46.8674366115656 42.4580504587581 44.8643898647322
44.6107027295193 48.1174861108648 37.5343260962758 42.5426057453993
40.4639532172338
CCDC53 242.080992016235 234.408769393378 308.568617575909
364.439669236186 137.424708183613 369.258587948416 253.841638589385

255.284866561352 467.884559162462 454.820452764822 356.628599887098
384.522115820048
CCDC54 38.6454098044474 40.716553247236 34.0382693188794
40.4359177791566 41.5668884168669 38.8336427948187 37.0564573649223
40.831207953647 38.3418933808844 46.2286164776607 41.1638563416981
34.806104801006
CCDC55 76.5505585423421 68.2521461582047 87.6178717341967
73.3725472767369 74.6833250738044 117.699959435983 64.8367546135053
69.4677195088043 87.6726059613969 70.3629652777935 66.4251023257203
102.49785018292
CCDC56 462.105603187328 435.094968715663 438.681666410245
285.053017606956 414.539381761279 347.003621791919 523.031300344992
698.595302020108 683.099079617304 518.527535205975 529.650395907046
482.238595345574
CCDC57 43.0462952354436 46.4569566520107 42.7303139017786
40.9141850646578 51.4092491522288 42.808889470973 42.8036731479932
49.3880946790306 47.417512242919 45.9004543328976 47.9942520395434
44.6032218589199
CCDC58 113.704541686372 84.6158251687884 76.2533853403813
58.3475983630509 231.824347524066 72.4651758215622 199.404836798715
62.6984594468418 165.311139930529 43.9450023643394 96.5451512198207
119.512426152446
CCDC59 417.086922265732 683.75696650704 462.762837284748
602.441843384617 592.010988342149 635.050535522135 748.913487947791
415.251775687794 646.791777335656 443.863346367342 416.246271724181
534.881442644054
CCDC6 1319.32406112346 583.690350347288 691.94603958547 587.88126282119
376.749116251503 638.848775860866 339.52415679299 537.213035625492
786.57695312967 897.198663109723 741.908952472992 622.435313940734
CCDC60 42.9104454767916 42.8542642792862 44.2405728317107
45.1944222548351 41.9842695818285 69.8832723783498 41.3594868055879
46.2779564481158 48.2123120250343 67.8233625051884 84.5267954034597
53.655233033193
CCDC61 36.4564050256915 40.8880530359822 46.7508829977587
38.2041059863839 40.5710637884029 45.3882138838137 40.0052661202207
40.931294690669 45.3756389170606 32.5178504369282 41.8168470823451
40.3691324547049
CCDC62 41.1523487516364 48.1392888470269 44.3648993568405
42.2496337895968 38.0170438383012 43.1826328292558 37.0564573649223
45.5842286511645 42.7150227340364 44.8536205629296 44.2925355984056
40.3316153483289
CCDC63 42.7150227340364 39.3999056968598 41.2365089689635
41.4273122914951 43.9450023643394 43.5752393599169 40.2154497375715
42.357910063876 40.7847110517594 37.920964499903 37.9946685306274
39.6352613956998
CCDC64 43.844855336215 42.5269793679335 39.8299340037009
49.7350276169552 43.4544762830742 48.5835484742456 44.4639405506967
45.642304356051 45.4799270968969 39.3698811593364 46.0095710163378
42.2577689927563
CCDC65 37.0320432433314 41.554942319225 34.6977013306396
36.7892997857376 36.3390947880186 41.554942319225 37.1643693306382
38.4540849632254 37.1820543575123 39.0648733099061 39.6403089864678
37.4554876386402

CCDC66 50.7390160399184 68.5251167137806 64.3312319756553
53.8086134112366 68.7911855725594 90.8335894699136 67.5206810636627
71.1607965028029 74.4365426051588 59.1496467460507 64.830448408529
71.501028931626
CCDC67 38.0089018744739 42.9358255168086 39.9101908547397
41.3027031037343 41.467005949568 39.4953494583071 44.6032218589199
40.4359177791566 37.8319142847141 38.0078357808393 38.079222289614
36.9976231206067
CCDC68 46.0398513158469 52.8455400026194 43.2721716572958
46.0228732507872 41.3387790895716 70.7003843117511 45.8980298854198
46.9454141780379 51.3084273211359 64.7488184936482 58.6464918752765
74.2180011215707
CCDC69 39.3709357615493 40.0527893189096 33.9435456570176
43.5627649099623 42.0709558258414 84.4433277732938 47.1345949868029
39.0620487644635 37.3600014814388 64.1097859982912 60.4620008899889
62.58750768974
CCDC7 38.1644569189523 40.591923288644 42.3536549114165 40.9022255200169
41.6285300488826 42.9695585740813 41.4244721780607 40.3662560853635
40.3566407936735 43.6033221838149 41.863927676552 37.9607839934735
CCDC70 41.6354649482853 34.0382693188794 35.7105247506212
37.5382094267692 38.8456942580061 40.9506062495231 41.1507980856436
37.0390589468421 40.0403898620771 38.745552403895 39.6403089864678
39.4490233720848
CCDC71 68.8698243753339 74.2653431904071 44.8872784170036
49.3654837541353 60.0736861987141 55.7197525213035 74.1353964055485
41.0381860307445 70.8606919488782 63.5107036952143 62.0404348860702
59.4465684293847
CCDC72 1208.33498233518 1259.2978319548 1119.61164710566
1083.50383916482 1676.22158135012 1214.73798692268 1792.42490815074
1012.91803181784 1850.06063216244 838.974043046676 1075.04105984319
1382.25775340179
CCDC73 44.2350046179299 34.0382693188794 40.0998167098057
40.1804147506786 36.8059561571675 41.159706131351 31.8431345631453
40.931294690669 40.7847110517594 36.6893251782922 41.2598458726002
38.745552403895
CCDC74A 44.436995101921 45.7317208802318 47.646800205189
41.8778873678551 64.193536444915 84.7113556042844 43.4544762830742
43.0390857698452 62.4776210012906 61.3529667465225 65.485076402841
71.9369353731849
CCDC74B 39.4710743516517 40.9731067463382 58.7382738968248
41.5464765641928 46.0043807840705 52.7156971154405 43.9450023643394
46.0633179231318 44.8997235283254 48.9826868640753 55.0404566626821
44.2527566343661
CCDC75 48.1125722136997 46.6314009405034 48.9944486068347
37.8442841690502 48.3814421939888 46.8954576545929 47.8947595316832
45.2806199505029 46.1101825640108 48.6124127998952 39.6403089864678
43.0930293831497
CCDC76 102.863599315528 140.853812988562 120.508644792138
85.5959901987745 128.532373805894 118.745729915256 100.499763255098
86.0944261788554 109.374838860558 115.98265270452 135.580139597457
126.500059295217
CCDC77 168.91210897306 116.805376997045 78.0013795192762
95.3070802596041 165.797721197832 83.5274372175811 158.081235081686

173.262440365651 146.789051265805 120.129857395808 118.467231612638
97.822911929574
CCDC78 42.9641759692926 37.920964499903 42.5406888281114
38.6414316877471 44.0451487108213 40.040778349137 38.1169703906207
38.3269307390572 40.4997186069494 42.6366765186182 38.1835938174625
40.716553247236
CCDC8 42.9104454767916 40.3384318592985 44.6032218589199 65.524821699432
43.7517118200398 47.2009420656854 39.8695929627783 45.0972184964705
36.7267557061938 53.3059058105836 47.2134341420411 46.6397391251762
CCDC80 48.1125722136997 46.1131345131683 42.0621425894624
46.2462906014337 42.6575535390785 136.517445122438 41.9393838138203
42.1860813954352 41.3803269429468 64.5845827915699 50.7159667633757
52.5420632648124
CCDC81 50.1533437545239 44.5884811528117 44.1452943804044
41.0726336841311 51.4527545197672 48.3686935595826 42.629412460409
39.5466975353131 48.7358147030082 52.0999467564713 47.5439485437351
51.7833333848627
CCDC82 41.0608773166058 38.8336427948187 37.4642807402741
39.853175885492 39.4693871570028 37.7000400506818 37.4772922422734
39.2672712041774 34.1294694030979 43.7303813515864 40.4504608195666
40.7704587752083
CCDC83 41.7397731376692 36.6002100641539 36.9795961564828
38.2041059863839 40.3452320167286 35.5158505219112 39.3585350448719
39.0620487644635 38.4344387289956 38.957687788868 43.844855336215
37.7526485343328
CCDC84 153.028210322286 198.15553991394 235.544416908354
130.274451794636 130.002385558757 167.49597550111 153.019365845424
166.304210248623 152.86270446968 252.206029058968 199.23413224268
136.011269830829
CCDC85A 39.5676051939342 39.0102066700839 43.4709415392696
38.5252456306656 38.3085139808968 40.4359177791566 39.2010256871265
39.853866204927 38.8495095797881 39.8455060654531 39.9586075662602
38.8964578286293
CCDC85B 42.1537476443423 45.0115870036541 36.6522278218553
31.3142073726046 43.9450023643394 45.8325181856483 44.9863726205428
40.2382908228517 35.6970528713709 41.1222709786153 47.0110068985119
44.9070250604284
CCDC86 193.730784033221 340.311633794489 261.929727465462
146.573018487871 943.73554571528 276.550265506988 539.879151334921
343.50519530761 252.134800295199 236.178265404264 231.733564500645
219.34503297115
CCDC87 39.7576136507593 39.0102066700839 34.1806289878747
34.8137085420423 37.4470183315826 41.6590564937735 41.5817737564592
37.5511560880902 34.9428206949566 40.2112371512838 39.9899430628542
35.0222940937513
CCDC88A 51.0225132036905 53.3094843669564 62.7605715573218
83.014860007187 48.7288537840562 62.5542376788358 69.3468840908665
69.0819394834919 79.2824265044758 79.1074681877535 62.358854072801
49.5539622682049
CCDC88B 45.6517320730042 52.3187919317561 58.7382738968248
108.246687420741 53.6907197364586 50.0748292497578 76.4889974392545
60.5571721986885 52.0583273941813 53.7196173866021 57.8991270166934
54.7953298048942

CCDC89 68.3234331335073 67.7189686993694 89.8354888041611
69.8314582428552 59.8102324370568 61.8187341713197 60.0981687048269
49.8344973836834 47.0804681460898 72.42732668079 55.9604109457703
61.0025829061063
CCDC9 54.7786516309154 44.2630383225933 45.1569741609597 57.8277957325085
52.5645416722441 43.3785777889265 49.8281348927244 42.7431359850339
41.863927676552 50.7855742231932 51.524711857679 49.3984571719129
CCDC90A 419.182443507848 142.365344693239 170.327588764719
189.769786121649 272.315378390681 200.144817111902 245.0324923457
327.238337722366 250.244199415895 158.308338986864 132.973097496421
170.327588764719
CCDC90B 347.074754731148 320.555790243104 363.67768546508
323.957165066178 193.043344611355 282.262229126907 414.794323433931
376.141477833569 290.546420262364 289.843931942051 223.219262492785
344.44626908553
CCDC91 56.1538818251632 60.4275887778048 54.0536858212253
67.6034960135297 47.6153629331146 103.43536623122 70.0868449397269
50.9099078696349 69.5741225986433 65.1516921033166 58.0295642638519
83.9236799134394
CCDC92 371.709014310561 322.774093041134 335.330937634207
260.062749979574 169.459466601559 522.377154567257 349.371668774717
277.548019244696 177.535502927617 381.198152331624 369.258587948416
301.79813401066
CCDC96 46.9939712192224 47.5647821785529 47.5673419710251
55.0604256312495 44.5668141895992 50.3245489035833 44.4639405506967
52.9813204449021 52.1788489555942 40.1179210534118 46.8954576545929
48.0350549043063
CCDC97 137.156113501125 143.391384762612 98.5746416750459
147.033637977077 182.685579297058 116.51037006692 150.562062400737
160.264217082275 175.619398238068 127.345094323724 121.936597605894
104.335120306753
CCDC99 101.351907645804 95.5627648703794 87.104092810486
68.4261608261522 144.290966198687 82.8218647837178 86.9185256838836
106.51488068372 109.241121384212 53.8260140375214 57.7786197537899
72.4094549259929
CCHCR1 37.2474303302343 35.6434124419782 43.8495248991589
39.3750969348308 33.143488395779 36.3845892764084 35.6056062164318
34.5886782974123 37.4540772310118 35.6208308220005 34.064698871541
42.7782216342806
CCIN 39.6609238036815 40.2493681589104 42.157627240486 48.309866878365
40.4639532172338 42.9695585740813 42.8684958525042 43.7396619462033
42.6366765186182 42.3606390470387 41.3553369544097 43.8575940017119
CCK 40.0557788909923 35.7393138893354 44.0451487108213 36.0481934423077
40.4639532172338 42.0574848021044 43.3262148375525 39.853866204927
41.2870505273664 38.528621456378 34.6366195121669 38.1421709520616
CCKAR 40.9558083259517 34.6306468196142 37.1643693306382 42.8235313007746
42.9090346219023 45.9266745571724 44.2166118829877 40.2382908228517
37.3600014814388 38.0078357808393 43.1931731665131 37.0953622406687
CCKBR 46.263826584899 44.3529919625344 43.1931731665131 40.1804147506786
37.7576900881741 36.1190722948669 41.8315602975157 41.8417024632349
40.6957787921451 38.1042454705119 41.5421429082231 38.6454098044474
CCL1 40.148780650831 43.4396866157171 39.1799070031819 51.0175677516029
41.0885562160946 36.4986986135769 44.3440839686558 40.5158867964016
43.7630587657205 34.3674468322242 38.3684202018524 35.8429598823818

CCL11	80.6434122355417	43.9421822132391	40.0998167098057	63.3006977162657
	47.4548471240051	42.357910063876	39.6535880152834	50.1833483241864
	43.5669306063196	35.7202909649682	35.9563099719284	37.6557714476853
CCL13	55.8218917166321	99.7361599442168	113.051477801522	190.442443946259
	59.4830424460714	89.680151549258	98.7446830481164	75.4272782742534
	47.9035532886869	42.4594149893798	51.3103617600342	47.6040671292013
CCL15	42.0532901689036	44.2630383225933	39.6833800000587	77.5091723127198
	43.5836473788312	165.411942074375	40.9157352260901	54.2301964914419
	40.4220196471124	92.2857885595617	105.406282523992	105.05842887748
CCL16	41.8409927585705	37.10170285405	38.7745734037492	45.1944222548351
	37.2567512265363	38.6414316877471	39.3585350448719	42.4580504587581
	39.0775870687758	43.401782064778	38.1835938174625	45.2783024981315
CCL17	40.5654775358427	50.2696827764943	70.7238101239421	132.205423909251
	43.1252272136667	42.6575535390785	47.7390230243778	43.844855336215
	37.2810767789306	43.6033221838149	38.3684202018524	43.8575940017119
CCL18	40.2543341073001	42.2577689927563	46.4569566520107	124.611407530723
	37.4470183315826	36.0306975524309	50.5252048155459	34.6977013306396
	30.9728654320265	33.1559412791294	32.775958132716	35.0222940937513
CCL19	46.0920218698606	98.0023132593711	75.9174504780598	369.952313383216
	41.1868052124344	56.4819802857598	51.9421032049606	217.658421476562
	46.4164127487052	60.7495043977707	48.2910530887214	43.2776458914333
CCL2	721.266942608349	310.255767356827	97.1271615802556	1704.0522304387
	268.097966292089	2083.69812106867	111.714563124738	962.079396754147
	92.5512757064631	96.6833301748676	198.872002827342	220.743173420176
CCL20	61.4884606677516	45.7317208802318	155.018308238778	895.110715324701
	72.2665537421443	47.7556758818053	193.15756263121	91.8568043052819
	42.9020321355927	40.1179210534118	41.4508241771315	49.3984571719129
CCL21	77.0301627655229	64.8903340853337	47.0055981216687	478.566247444176
	48.2748123114598	389.686780706341	38.2933870238743	69.8941497035747
	46.2027193115397	98.7331384143685	148.514056973195	150.362006499825
CCL22	46.7293238583888	64.5347406608537	70.2577825186943	235.222472959869
	51.1368583672022	42.357910063876	53.3111230705754	58.3046521633424
	53.1249776278067	41.8613406887824	38.956280281655	36.9976231206067
CCL23	57.5685357492202	48.0570064280814	55.7764940944098	133.365282773876
	50.7116312517035	76.5339970774168	60.581035231763	56.9621724830201
	46.824299585607	56.4553747079294	57.6831182451733	61.1022920882065
CCL24	33.4240466670776	40.8880530359822	35.8265993915844	39.2780515621926
	40.8873907150245	38.7340969861754	33.4316681623175	40.6171774724171
	38.2215591930186	38.957687788868	41.3553369544097	38.6454098044474
CCL25	43.7585538437696	41.5132248356707	39.8299340037009	44.265834798964
	40.5235471987417	40.040778349137	43.6753843488695	39.512606833112
	40.6503965580437	42.8936990772838	41.862634402486	41.3803269429468
CCL26	69.6449298794042	40.3384318592985	46.6588655218735	51.5913529948887
	39.7568367479301	55.1987351219665	44.8643898647322	42.9301944191913
	49.4786466722535	50.22432299342	48.8744644207821	42.4607795638552
CCL27	40.3478825593224	38.5252456306656	44.9758339877851	45.1944222548351
	41.3755760434934	41.9543948167375	45.6042625176633	45.1714698794331
	46.1101825640108	42.2614524730856	39.4410515050885	39.5418280570561
CCL28	52.1646670865427	42.4493032770053	47.1719672813096	40.9022255200169
	69.4009439491061	44.5973052166071	48.7338046695859	40.2906799807722
	56.8373628733077	47.611018474151	43.9701242600947	45.9693232070002
CCL3	164.262531918679	85.5135159896819	207.464632062656	2374.1766901821
	71.2787833369055	115.235144612428	595.012422454829	118.736188485344
	59.5929655877036	55.7930787690756	45.8180710486329	50.379587451669

CCL3L1 79.0304119352941 58.9656737189764 95.5724734464786
1810.67209360676 44.7312617135491 59.3618843678775 207.768377497439
68.580705761277 47.30559806758 43.401782064778 44.7699099047043
46.1005394461164
CCL3L3 235.888544042065 153.139323479953 372.131471268346
6555.04068292972 70.5676924620725 146.007510897838 925.486088550061
256.026804821501 73.0403099404 52.4351670989268 53.1505787220552
49.8253396161069
CCL4 40.3478825593224 43.4396866157171 40.931294690669 41.1886975241176
41.3755760434934 42.750861693377 38.6243997507646 39.3709357615493
42.992884918158 42.1622837416589 38.956280281655 42.57175366709
CCL4L1 51.5888395924843 53.6429962450784 64.9072331703985
1180.7204565402 40.8873907150245 56.3525213736952 110.837476359817
88.5511408870489 50.4974478884077 48.2596564318071 46.824299585607
42.8864415494484
CCL4L2 81.7117547901526 60.7495043977707 93.7782490229256
1423.29307392266 47.9707112435212 110.935994444429 157.9970294734
90.9010016862442 48.5136862664526 44.4652605017595 54.113533179847
43.8336862998829
CCL5 312.154320305976 312.795596385258 508.688028777582 1012.0373748933
90.2227706363325 161.603201688692 223.397586938079 282.68881112438
80.8728961146267 317.127679512963 812.386878774445 111.071209057746
CCL7 43.5710871617107 44.6918600441328 42.0621425894624 90.0942781313264
40.8873907150245 44.2607789091176 50.2016912396771 41.4508241771315
44.8003345643334 37.920964499903 45.4799270968969 39.4490233720848
CCL8 456.992521713593 130.775349446374 95.0738959950301 533.820505121067
69.7413896580273 880.992328229414 113.866742186841 225.690879436355
52.4539007817929 53.0357938671646 45.9104478407175 51.9447450229707
CCM2 293.516547661658 174.179617483251 191.141778714144 243.763402918148
165.613176704286 181.064239330482 149.02612008344 175.74424749223
186.350104132737 172.748740911831 188.767243497644 146.763378089693
CCNA1 49.3463416794995 41.4508241771315 45.4865173433646 82.5279746006378
46.0977687311222 50.2163892855092 81.3082994649699 62.8885806455128
52.1186591838715 42.0621425894624 45.0589947942306 46.3411353377615
CCNA2 102.25509551881 224.14182624489 148.906660152784 83.877343750194
355.00914527669 72.3523300799007 226.553946570328 109.241121384212
273.320144423733 43.1839109702714 42.170702295259 50.656598108034
CCNB1 71.4276813927272 75.9802606133139 62.0967404944031 53.593974682612
131.045615546105 47.4699236780425 74.0039764902057 78.0020909283414
82.9256976863272 41.1222709786153 50.6473771879221 43.9541718254769
CCNB1IP1 176.936216428226 137.744037647063 114.901312275861
99.3396385536245 127.160252453685 145.936763882526 115.213198315106
100.370937669662 120.571680725698 161.65846687852 173.035987237848
171.485221601137
CCNB2 411.114836576804 352.917938567734 136.657213529097 110.25582862843
586.281182112872 104.492750153479 612.843402058384 434.523449093691
583.053232396803 43.1839109702714 41.4508241771315 52.1186591838715
CCNB3 37.7766325325437 44.4612332327353 42.4580504587581 41.8347680672208
39.2192721306168 41.5104252657176 39.0735041982358 42.5435283762933
42.357910063876 38.2599935139101 42.5426057453993 40.1249533205858
CCNC 93.6464901357407 143.932614622477 128.937837181433 105.647054678056
78.4692960800668 130.585540374294 87.9638809147835 80.2680308979464
94.3953345963359 62.7777335817742 64.2174369755376 102.365615739399

CCND1 1626.71747158994 4916.82166008511 7022.41612496714 551.490493022416
4482.5285743878 1266.41536472956 309.494842450837 862.031722874406
16661.6081002448 2108.03351084959 2091.0611913538 1188.3486464341
CCND2 492.519023728038 324.936044300546 230.633139180542 701.935442033195
119.965950077259 1539.35554229258 89.3868257841517 335.943994595615
69.5000200266713 1144.34555072807 876.959744461441 743.463635670141
CCND3 817.631505579476 646.323419285676 1114.15989325456 1202.11455437028
637.694633519528 716.434968700383 824.946988188872 597.593714375156
549.67905283016 901.793120584383 828.403901345618 652.480459437977
CCNDBP1 245.356135592468 397.199620114138 235.127539195661
368.853558193357 266.439343650315 317.777971035617 319.871452890512
268.35323266566 283.484056482174 308.841311505431 284.351747562807
461.500892766266
CCNE1 193.15756263121 134.936657884035 108.242021650278 55.8709511400674
261.081844108932 74.2122914028677 301.728842562175 355.495119544081
116.9812483023 58.6652401079158 54.1683282066029 51.7460357029749
CCNE2 64.893664076381 64.0605738099971 52.4234283832574 54.9919060580109
107.693162310852 49.3654837541353 87.3957386451235 50.9099078696349
94.0532777456537 40.7063426028871 40.4094901248527 45.2783024981315
CCNF 234.727123301682 226.456072107079 316.548339196872 94.9785931907153
309.698059657254 74.2690269784394 273.231509963261 173.262440365651
333.122044191102 332.933704863586 183.311305255895 115.441414762268
CCNG1 83.877343750194 91.0526311350893 143.913007288353 65.6260887743331
101.156215957297 165.496075703734 129.2860308084 88.9038123445083
124.310823604006 115.853867920678 101.383978726419 205.510539955583
CCNG2 116.9812483023 150.661852768073 82.0081006332668 70.8835636550472
91.1834104205004 70.7238101239421 82.5050369860039 105.589475270494
131.645714321903 77.707180044659 78.1623301184313 86.6854406823303
CCNH 68.021673481871 66.0282565447846 78.2484708472671 162.782568037255
77.1287755867582 84.7113556042844 78.4023698691285 84.7058085717914
89.9789975503195 70.6138659908885 63.9029785089421 83.6610321130843
CCNI 4898.89844391477 4406.55576617324 3653.59181300011 3234.36469403715
2465.65854657837 4916.82166008511 3190.0108307463 3391.65926812276
2220.90215179682 3490.63949257581 4665.45637589484 3575.78813099057
CCNJ 44.9896851005259 43.683792219729 45.8844325913247 42.0102671859606
44.7829632472754 44.2607789091176 48.0283573444179 45.3872406432794
53.0357938671646 39.0648733099061 46.5650556481721 50.656598108034
CCNK 345.615640348066 230.117814622646 294.51382100172 306.174148874454
230.169916252549 191.291480830946 202.872083191114 335.167786298149
379.933650669101 314.992390390165 248.924819383887 335.943994595615
CCNL1 156.754606475323 387.311743342483 245.660544821992 489.232537791775
280.202284365004 472.159979716705 263.937966521917 243.803668806533
153.626578526803 179.897685693038 206.931452638325 259.43283734195
CCNL2 44.9896851005259 55.0190472891348 56.0800798275077 47.0874209746031
71.0204234759627 41.2567470068038 54.1113282154394 48.6441988290952
62.9922928277769 47.9056710878325 48.1352470945488 46.4136802014079
CCNO 47.5647821785529 92.6169846049935 68.3234331335073 35.0448343468967
73.2607982527105 44.8872784170036 84.0691608193405 76.4637616585578
107.420552630045 89.07557840469 85.2598847559951 98.403191200835
CCNT1 59.046385957117 51.7983110178095 58.637422084759 69.8314582428552
54.2301964914419 67.0290776715331 60.3928833351886 56.9299697426319
56.1733549054832 70.3629652777935 69.1945249877407 63.6254894053045

CCNT2 78.1313732870053 74.1425714439424 76.9671788424922 56.9299697426319
69.6554376880691 63.0119737420787 55.3141112947345 70.1347490781514
97.1634390603131 73.2956678446902 81.0295607064541 81.54661427144
CCNY 115.801367301965 138.82907142482 132.425058995617 116.20672562701
180.916465721523 235.420582508332 142.192315159235 140.992072231913
120.26537515666 114.073853703538 123.536432289877 128.391264770998
CCNYL1 65.8703820581684 63.2475738480323 69.8712342353417
100.271925566074 66.4047895961325 74.4508307047509 74.0039764902057
67.1702819938085 79.7061415085215 53.4785152499923 52.6960621424156
64.6945454069792
CCPG1 177.845412804098 74.8071157013401 89.7661004674344 219.771890732457
60.2276476399612 163.476051754305 65.0031119608637 84.2434917159728
70.7702047405718 146.249568587351 106.340844838265 134.697808298417
CCR1 79.0304119352941 49.4067286971348 45.5883690965192 293.755623192857
48.3814421939888 76.7063975550116 46.5650556481721 45.2806199505029
40.9901805728485 42.6793753550854 48.2910530887214 49.8253396161069
CCR10 46.3390850717989 46.1569135229922 43.4709415392696 41.4273122914951
41.8806343997257 39.6251842352088 51.5967098638347 40.6171774724171
45.1899271235974 46.1390887492649 44.2925355984056 39.0536056496869
CCR2 43.1131704517186 44.0559121083962 43.0602678767669 86.2118355769367
42.2321685526192 39.3841861812262 41.4742337714895 41.7022936121242
40.6440776327492 43.5554751425009 42.152777131319 42.4121858888871
CCR3 45.6517320730042 42.0500582486532 45.0115870036541 59.198088069573
43.1252272136667 44.6524308640784 42.2577689927563 42.0847226073592
40.9460396298042 44.4652605017595 42.2618615571566 40.9683275553869
CCR4 40.7609952437622 42.8542642792862 34.5718473203083 59.0965219753805
36.4434672195785 36.2015763253919 39.5496902678788 37.86982638952
40.7847110517594 37.4302011516587 39.2542963855807 39.6352613956998
CCR5 39.6292788039793 45.6271294527191 38.741573807882 39.6292788039793
42.0709558258414 41.2567470068038 42.6913548400127 38.079222289614
38.7571208777225 36.0144420013819 38.745552403895 42.6758568421347
CCR6 4482.5285743878 2266.34088015654 3143.97163345458 3114.05035137552
2581.34849558524 1780.14552702126 3490.63949257581 4569.48246031279
2150.8715161597 4168.41947709165 3869.93390477952 3329.68037676265
CCR7 43.1805016690297 760.745188431719 105.965906809429 293.649670545233
51.6869155634183 47.8636003588181 156.311985997533 80.5508300225954
41.5635499110307 50.5252048155459 49.1067959632109 42.8864415494484
CCR8 39.6609238036815 42.3550738181144 42.7303139017786 40.1804147506786
43.844855336215 43.0860383229288 37.2892641289258 37.5511560880902
41.4636047919453 38.6454098044474 42.7275315121163 40.1700117452801
CCR9 30.9241129241985 37.1685072112618 38.941855825269 33.5947667322717
33.143488395779 39.0266821712099 35.3362091871364 36.6452480995946
38.2874316439311 31.036133494654 39.1878770155069 35.4484652445078
CCRK 105.914573205761 89.6389968279976 72.3508448479791 61.9976238937207
102.085154509866 110.715042245563 112.60230393988 126.620595729562
64.9418945936106 137.057972437595 179.285390561592 102.035798846699
CCRL1 39.1878770155069 38.0855630164262 40.3458948319669 39.7988965027289
37.5979144071815 42.2114134935898 43.6084517252627 40.8833572570383
40.547835538778 41.0057887014368 40.0766543123257 45.3170039418236
CCRN4L 90.3691010312535 56.8373628733077 64.1777305915695
89.1290937615998 85.7842344710572 60.4991141099239 88.1861828863404
179.47783529 79.6198202883864 56.685925255591 50.8826620053386
63.1443731864055

CCS	88.6663961027926	88.9641595603127	128.436305192918	79.9126192278104	140.264270771578	107.232979387172	83.2925226467532	97.8309399470621	73.3725472767369	106.356074933606	102.24202987437	91.7679182632783
CCT2	950.740991182127	968.405745860353	816.746599467159	542.069156178791	714.075528895169	767.489657259345	1046.42391243532	543.975237512604	765.145138238955	312.154320305976	397.850286751468	606.378839153932
CCT3	466.454993547103	485.310097523194	665.122091380755	288.869882644391	790.094848284547	503.187121806288	616.912306849151	281.602712448925	592.010988342149	310.358667418222	364.962649416567	308.280127837858
CCT4	80.0113001016496	61.3838877877366	72.2016884438365	70.7202372217584	109.017269529278	78.3488426553071	70.0567634568557	83.7019398654324	61.6839343510302	76.826527950236	75.3075812535743	75.6428949408034
CCT5	79.8844295847317	61.4800543360853	56.4779645313958	56.2508140932179	64.193536444915	60.9822577063534	97.5962784863854	66.295597720304	73.4853298204744	54.3554233228353	59.8070012006708	68.5174271063894
CCT6A	228.439524702578	172.285394357607	179.994715581707	153.711194034581	268.097966292089	221.195872577098	184.536327776767	173.49425401442	224.635749257133	123.608921653229	144.300051706447	207.928770428477
CCT6B	55.2182004838121	54.6089451252049	46.9040704638073	52.5307002738469	45.2556760873335	53.8461734834857	74.9059650336395	100.114114765033	66.4251023257203	65.813547096222	54.5711704180349	49.7336769474446
CCT6P1	114.230358276599	107.610858427836	100.756165194192	86.5414905689606	109.255307556049	122.331946672266	101.634888749356	118.651855622915	127.78061946174	96.2385885003071	103.961956810572	112.768270540409
CCT7	779.333013700435	824.946988188872	748.913487947791	499.356036793973	1384.80615431656	1014.6400720037	1136.88710104691	822.822769402853	669.419736252343	327.631923082696	537.213035625492	557.094756796515
CCT8	1247.50618540769	1771.43541011426	1570.45778081591	939.453799186397	2036.72286752819	1024.97546870349	1242.75259587926	1273.52492984569	1649.78627644882	740.423874031773	922.047328788961	1369.13346566895
CCT8L2	44.0328992213727	34.8137085420423	42.4580504587581	35.8472302065546	35.7973685350741	39.8455060654531	45.1017494688414	48.4670130605028	44.1519466943185	35.8304004211528	39.8184668132023	42.6758568421347
CD109	43.7585538437696	39.3999056968598	39.2707964550441	53.8086134112366	38.8456942580061	44.7804080899431	43.3262148375525	42.9301944191913	34.7722083264266	42.785429206422	46.8954576545929	40.5703581862752
CD14	1259.2978319548	860.265067346622	813.242005949542	5156.59948231705	584.724083441601	2575.86919902296	1167.25317435052	834.14766574977	177.584394977912	242.503720442197	277.878824242495	460.340300658834
CD151	1217.21440297138	915.519281747943	1564.55598228713	671.959673037066	688.612748336336	1223.01485352248	751.150916058018	852.769974690777	1275.78715071615	1327.93833415582	1516.33074288338	1152.55315876935
CD160	41.0608773166058	45.0115870036541	42.5406888281114	41.5464765641928	41.7929491793697	46.263826584899	40.5585676679482	41.1575777497576	45.6582729799009	46.325232414659	50.7903824729818	54.8630527326985
CD163	193.094756585577	124.76401857899	195.836475280084	825.558929673308	163.995560004001	476.421959423835	124.551483147457	92.804679821289	46.581796165231	60.6266092582071	56.032814067931	286.11425202743
CD164	49.7188990489616	53.6429962450784	50.5089777244781	48.856176220627	40.148780650831	41.6590564937735	39.8695929627783	80.2680308979464	42.9020321355927	50.6486940895674	55.6972661824064	53.7442594289333

CD164L2 37.0320432433314 40.0527893189096 43.4709415392696
34.5425384901279 57.8039717833131 38.8336427948187 41.5817737564592
49.7309743973184 47.0804681460898 34.8137085420423 40.3452320167286
40.9683275553869
CD177 40.4639532172338 39.1982470626863 38.8775560911841 32.7960913111995
41.6806984441849 40.9506062495231 45.8980298854198 47.2009420656854
41.6616444147804 41.7405682794166 40.6373400186325 42.4607795638552
CD180 42.1537476443423 47.9665737016961 43.1931731665131 42.5664883002833
36.9702228618069 36.2768299545794 40.6750540874299 35.6825474397235
39.6609238036815 46.6598879466655 42.8263096141654 36.8690774823319
CD19 51.7607563002005 110.214922820817 58.0218262176065 252.280105035425
50.8875036861067 47.0940613507321 46.3057385979341 108.945545005203
48.3954967265007 42.3606390470387 45.0589947942306 43.5738557773903
CD1A 46.0920218698606 60.2547250212462 37.1643693306382 89.0203733091224
55.4919197421746 40.3357777530419 64.232967574797 59.220967994972
83.105117076029 38.0078357808393 36.9060997041453 37.1747156131306
CD1B 39.7576136507593 50.6229884142872 40.5734458834617 76.6232527679845
39.2608760193011 35.7034203372528 46.3057385979341 41.4508241771315
37.1820543575123 40.6022003765153 37.8372168909583 36.3454320848591
CD1C 55.0078788655089 53.0383634135393 52.2132783757422 105.376705821141
53.4109882045447 63.3612993417558 47.2927436772465 55.6904617926772
43.2761454096265 45.4614234442527 41.4508241771315 43.9541718254769
CD1D 42.0532901689036 48.1829653238565 39.3303857231606 53.8732900241064
42.0709558258414 44.7804080899431 41.8315602975157 42.4580504587581
42.2248608921879 40.9558083259517 43.0971787292711 45.7562276914441
CD1E 48.3583786497948 47.3010040326346 43.1660110696573 61.9976238937207
41.1868052124344 40.7063426028871 45.1017494688414 45.2806199505029
45.2897806651929 45.2556760873335 43.4424616940528 41.5132248356707
CD2 78.1313732870053 194.189674572424 187.439021508049 355.568857981155
45.1137938810047 50.2163892855092 77.5813459454823 76.2258667419953
44.4395580493118 77.1767494799711 94.0777119695783 46.7182294568603
CD200 48.5123041297835 44.5152708955623 46.3411353377615 50.378948785456
44.915045386675 64.0020613101392 38.8775560911841 44.6560353921013
43.1805016690297 46.0119912782855 50.1649089793069 51.0647893605839
CD200R1 45.0283997415478 44.7200265825016 42.5864346233886
47.0874209746031 40.0145811166794 49.3654837541353 41.8315602975157
45.223075311113 43.3262148375525 40.7063426028871 42.9634662926872
46.1592873342534
CD200R2 39.7576136507593 34.6306468196142 38.0452862258803
46.1673159982664 34.9062568194127 38.5252456306656 41.9393838138203
40.047314525032 35.860430241131 41.6373771565199 35.7953070087288
42.4607795638552
CD207 40.5654775358427 38.9338521051669 44.3344900481112 37.4223289687135
39.2608760193011 40.3357777530419 42.7431359850339 42.5435283762933
46.0106098064475 40.6022003765153 41.9908237694988 38.4330755397458
CD209 71.5484567134601 100.139490974785 111.227016020927 170.901596796384
93.4726264307041 561.2769907009 74.0039764902057 71.6202385170491
45.2897806651929 70.8749669449099 52.5154971256566 72.7502212566295
CD22 41.5421429082231 39.8695929627783 37.4642807402741 47.481302504066
34.4570158133896 42.5660695551615 35.3362091871364 36.9355798143571
33.2715481910159 41.7405682794166 39.0550139257658 40.0557788909923
CD226 37.4554876386402 44.4411734611229 46.7508829977587 70.8835636550472
42.0709558258414 43.4681646416585 37.6036146841933 40.7311497914871
38.3418933808844 39.1621898366355 42.0750889188255 44.9070250604284

CD24 11410.3307451176 3786.90274075023 1642.75555340648 333.566613571224
1490.1549980903 389.364650157102 4427.89837060471 12770.8282719641
1969.8871504719 553.328261478032 792.880882528835 971.856840043688
CD244 39.8662795144616 44.3529919625344 47.7737810047679 44.6905691366696
41.5668884168669 39.8455060654531 37.1643693306382 39.3709357615493
38.4344387289956 46.1390887492649 44.7926323492519 43.6753843488695
CD247 95.7194132679937 213.204385896543 218.446312290024 262.735075993242
75.4272782742534 69.9819944829311 132.903804194161 116.275899706695
59.3580517977711 95.3507664364654 107.682738819549 65.4435809676217
CD248 606.276674926567 274.648263378054 233.468474830926 360.342857415756
348.165210494253 987.427672417105 56.7780520416533 147.704325041249
64.643220133046 647.044821320392 395.875593488613 584.724083441601
CD27 43.4626049853157 67.5417980425932 52.3432130135266 120.068630086702
38.3561682218911 41.8613406887824 43.6753843488695 43.9366081640576
35.5220586859301 52.0999467564713 43.3085358214154 42.9887069977696
CD274 41.5421429082231 41.7337506166619 42.0621425894624 77.3864518736634
44.0451487108213 48.0350549043063 47.8947595316832 57.6684494624163
44.619512785899 42.9866214102525 46.1673159982664 47.6040671292013
CD276 209.705575423447 112.542326463419 89.1632548302738 100.234661597218
126.274897979594 86.1924491308234 161.326580647514 169.889618506937
128.860357469791 87.5967418612698 81.2673471767743 78.5697118992327
CD28 44.436995101921 47.4076235350059 47.9035532886869 59.2778887402357
46.4343327861696 48.0350549043063 43.0930293831497 47.2864663693787
38.3418933808844 39.8873706855774 44.6286581923295 47.1060577218993
CD2AP 77.1900613562529 109.386655134289 95.0738959950301 48.309866878365
62.1836961153924 52.4351670989268 83.6935756500468 54.9919060580109
122.301290485889 58.8022152133979 65.2295773603116 90.8140983242733
CD2BP2 892.754288149486 609.243908604949 1111.13061382818
736.405815842011 1037.51323185348 668.584452504579 497.290172759099
670.271164195182 368.162822151919 646.323419285676 675.832363405287
488.649780652311
CD300A 136.736085245679 83.0574731969818 72.0243286940589
701.168333137671 63.955655956484 95.8075612515186 79.909124697198
65.1637149462661 48.9931786790166 52.5522545249206 49.0361180089431
61.8529316708431
CD300C 44.7044140979108 47.3536182445315 41.9666541498638
111.071209057746 46.5557478175976 50.6360662655392 44.2166118829877
50.0526763693329 41.5635499110307 46.1390887492649 41.3553369544097
42.1537476443423
CD300E 38.7537364570873 40.3384318592985 39.6451483836089
51.5913529948887 42.7107928951766 49.1013967533186 38.5066108855005
38.3684202018524 42.9020321355927 39.9948390976317 39.4410515050885
41.7575097298927
CD300LB 43.5710871617107 38.9338521051669 37.9482157381008
55.2214187655851 38.8456942580061 46.3715909653832 51.1541843174981
43.3262148375525 39.3750969348308 42.3606390470387 37.9230440150891
47.5025821041537
CD300LF 58.8224410806741 50.7487159427973 55.3122077514658
276.550265506988 49.1928206164702 45.6129956328415 44.3440839686558
46.695848687694 40.4997186069494 39.8873706855774 40.9157352260901
46.917149434588
CD300LG 40.7022020432945 46.5952288260503 50.5089777244781
47.5733603903505 43.9994592139885 51.1621379498014 42.9803717121865

48.0582855095143 46.0633179231318 51.0492389286271 46.5308571957384
45.1735152121056
CD302 56.3884556031093 71.6537776654915 65.2670315837785 110.25582862843
87.9963662401769 219.527698657901 66.4258159334241 66.9340967279385
50.5368663784059 69.5853093594169 97.4515987591398 99.8277095030346
CD320 157.189139558365 129.081183929988 107.792406701643 121.281573820819
468.573956652904 174.117816184497 53.4921736109774 136.913434348036
144.37233706688 105.29989739819 120.418406532204 116.793627324217
CD33 50.0466349801009 49.83247261979 49.5424425850549 143.624756920887
48.9627346956003 118.57174795047 44.7255207589606 41.5464765641928
43.3744503319109 46.6598879466655 46.4753015651202 58.5087296620514
CD34 127.986477405999 120.465621774337 179.040571908629 118.614027673431
83.8419777255838 581.093069802913 51.688837858865 94.8260360398356
52.0999467564713 400.093769705765 281.452208033175 232.440458525975
CD36 47.4767851750188 59.9682734755814 52.683711208052 117.8523906126
48.9980022280763 203.651890041185 45.223075311113 49.7748385529588
59.3709786862962 58.0218262176065 49.8393069675192 61.2343203799193
CD37 78.7045070574092 111.689615107055 85.0946882743418 572.19538884395
44.9936360641969 112.519759295176 105.45765455249 81.9218437790485
44.8636979668258 51.4454142248566 54.7061116419212 51.2776048338
CD38 41.7397731376692 57.4538469832241 53.6837370963602 152.21278457652
48.3814421939888 39.3841861812262 39.2533668167483 51.555506218204
33.5578650644246 39.8873706855774 38.3684202018524 37.4554876386402
CD3D 72.2797007105795 98.403191200835 100.139490974785 204.659954879641
48.7906379070015 46.2027193115397 53.593974682612 76.4116336410131
44.5766390180978 67.8774399765179 85.7390214986603 55.2776978524578
CD3E 48.1952521496489 61.7572675712557 67.235655687817 87.4850071876743
47.0954207417646 42.8598805316863 48.1747307200218 57.6684494624163
40.2274522635576 57.3562426592112 69.6097529955737 55.3563533836982
CD3EAP 46.441301934328 48.4497719907777 48.8941062980393
43.074472921841 71.0593419018503 45.0882356578404 42.357910063876
48.6441988290952 52.8502823653368 53.1993028936883 43.9450023643394
49.6629259325705
CD3G 48.7646976789783 52.3896785600751 53.0104469636221 69.6511571901941
38.0598374201407 44.4619248757502 48.6395795163502 47.4740726032707
39.7537274655115 42.8936990772838 45.7328341667151 47.6938628797168
CD4 67.1387898740161 58.9910892923962 67.9850517608624 168.869258524932
50.5110359971301 70.2553917409773 51.1541843174981 69.7729022157455
46.1101825640108 147.945429978177 92.7254735358203 92.3388129097482
CD40 59.6651708873036 58.0218262176065 62.8635815439291 125.052841200703
70.2565873027242 67.9922604709859 46.0228732507872 57.3837827230023
80.5437011468815 66.6788758487969 78.0020909283414 70.2680820278385
CD40LG 39.5676051939342 44.5884811528117 51.9214790114499
46.614092935749 44.6834648302383 41.6590564937735 40.2154497375715
33.4534810696886 37.6383530120621 45.2556760873335 41.2598458726002
44.6484005362281
CD44 447.248719481853 272.022473793857 471.297503316471 1716.82239359769
247.966757890598 735.460645553438 224.914980996667 467.884559162462
314.091389958069 691.132727131746 591.192020333181 673.522667186915
CD46 346.116601612353 617.808073077341 933.968759959712 272.981148492864
413.31638457585 255.284866561352 300.762511215441 370.330147221688
552.860979481958 382.872356030213 439.955651727755 699.488969175268

CD47	102.952512767439	136.67168846573	116.839274984089	170.560304063093
	101.987604729928	175.673196925527	85.2805684630976	87.3634135323569
	138.292681458047	140.698550434486	152.200107222761	139.328628553223
CD48	58.651329686676	56.0477607422828	64.9072331703985	417.738831997557
	43.9450023643394	54.0382756991549	61.3620062177524	53.4109882045447
	39.4762546236449	61.8529316708431	59.0537479001842	53.2046867327331
CD5	45.4799270968969	57.2871072823547	55.3122077514658	70.506090074243
	43.4544762830742	43.5752393599169	52.4111687156893	47.7889447429963
	41.2870505273664	40.6022003765153	44.7114813656401	38.9496515344837
CD52	162.864661742501	212.688298502868	201.330854646158	1372.51401036753
	71.0593419018503	113.218291314173	117.556281726503	237.809876921353
	64.2174369755376	142.515628954457	129.592927998658	113.130838519437
CD53	62.4317959077388	58.7460488758053	58.637422084759	437.84407370064
	45.310660704531	67.6817814091697	57.51482768194	53.6782125131958
	40.1738310955128	48.3077814647455	45.6706505553383	50.6141827957642
CD55	264.116434279258	305.119894105297	280.920208331594	686.154904023493
	180.255771684507	282.035361446993	248.355842321304	444.618035487523
	143.251956814524	279.782392390288	180.994353525666	305.119894105297
CD58	113.632855481549	194.012210675469	198.788202490193	356.826869112433
	97.0055689383756	132.223745961048	122.514153407264	84.9280683258012
	206.190699642477	80.1419822448876	87.6098790075242	178.858202962948
CD59	76.1417635449123	66.7585874672879	112.811643697986	112.506613718654
	84.1639779987105	89.3168921945222	53.7423898357583	74.7794253940974
	58.129051241441	75.2053836356882	84.8808332740321	125.221639689345
CD5L	36.2496364442264	40.4359177791566	36.6522278218553	37.1894304543751
	37.7576900881741	34.7983399096627	37.3799542961839	38.079222289614
	36.0741672064298	43.2988017098387	37.2892641289258	40.6614700944853
CD6	61.9504984796308	124.56803502087	123.597489107285	233.539376791695
	75.1674447943072	53.7442594289333	68.0157730844432	76.6868184563568
	53.7442594289333	66.5422130374814	81.3454589502043	54.0374522273907
CD63	194.924141888391	205.510539955583	219.616289279562	242.282363793567
	120.136330103936	271.857378677296	170.87055409699	193.853749625939
	142.647217549267	201.530434441408	273.108672916862	201.530434441408
CD68	802.036670929858	255.365088438518	478.682734715193	1945.33618761041
	200.986069552443	473.223156200183	270.487135967976	585.421238639853
	137.239944506217	328.548716133094	249.855746700674	475.008345432596
CD69	63.0505463098231	116.17375251898	599.670771796585	1107.96086673001
	44.2607789091176	460.865101118125	136.293724800658	78.5174639327946
	557.094756796515	62.0500352705887	61.0506827768127	49.7336769474446
CD7	69.9431447778061	79.7691160370015	81.7434024567356	197.540681021755
	51.2525124735102	53.9517841438975	61.8571521441451	79.9597944271831
	51.0062122023297	52.3304586399695	95.2490714726663	50.4847465816184
CD70	260.279110870904	49.1283590719461	43.0971787292711	52.1201052574162
	46.4343327861696	45.4882438975269	50.0088611244948	43.0390857698452
	40.7847110517594	47.4767851750188	43.5710871617107	41.6550823405646
CD72	61.2130609867477	51.3084273211359	56.1434024065079	132.205423909251
	48.2748123114598	43.6670755115848	60.9648845690177	55.5578334877425
	39.6609238036815	45.4614234442527	40.547835538778	44.7401523707775
CD74	1226.43929953115	1073.84987604662	960.526372655577	3263.05269150231
	427.818758455214	771.554891868456	706.914069768466	1118.62223628039
	1485.68317899614	500.549865625701	952.16345035163	381.198152331624
CD79A	43.9477262001082	88.8568707880669	73.7060605978536	379.031536537372
	42.9627566278041	41.8890826572422	43.607701594929	60.9009676344258
	42.3060097375421	43.2988017098387	39.5748670524951	42.1915582886043

CD79B 48.6621527194963 78.0730371064675 65.6620676514157 212.353676518748
47.965492554043 65.0391287166255 51.5119005564951 67.4775348162369
46.2657906047993 59.6565330440648 60.5948704279864 55.1037284831588
CD80 40.6559419616644 44.7829632472754 42.2577689927563 61.3747706295648
39.6535880152834 48.1591264215184 45.1017494688414 41.7419877611072
34.8552366193358 42.9866214102525 39.7342718293432 37.5556299264699
CD81 4632.08655821299 6673.00322263275 6606.35767508139 6309.53691418065
5246.60459228386 8259.38594893529 4820.70258471362 3263.05269150231
8050.256095383 8703.02873382565 9326.9018948838 7393.77508374057
CD82 92.2447473381285 91.5464279432216 160.100408020768 183.613579501306
89.4111455826308 100.510815049535 94.4618437533343 83.3322777515023
112.743602566917 73.9192243586276 99.3142021810133 88.8330881965986
CD83 127.072685504735 146.196470375622 84.3755392205436 831.578043520215
237.532800487032 161.268186656743 158.998384690038 303.139550962377
66.8077748120381 69.0530953932111 61.5392012551041 65.8455652907556
CD84 48.5835484742456 53.3175733763272 57.50915811428 138.581471125405
43.9450023643394 61.3309323244567 48.1069139823064 49.3217779272599
45.4154248354367 44.4294261786154 46.0905079010237 46.5905131500956
CD86 72.4325984501758 67.686778662624 88.1068080491008 388.336994961328
56.359578588344 67.6817814091697 71.5836731059913 73.5459854447472
47.7086093893038 44.1956990708089 45.9104478407175 48.1627097465883
CD8A 57.4376528157926 78.3488426553071 101.086264355915 137.831070407907
44.2607789091176 44.5180992653789 49.6872467499977 75.9136854087044
42.8530333223554 74.9491421278749 151.848243044839 61.6311118308635
CD8B 39.7349536408822 39.2542963855807 39.9101908547397 42.2496337895968
39.9697332660833 39.780640540889 44.0687697278114 40.9861485852278
45.0583552831013 40.5620233728036 43.2551508830106 39.2839132447749
CD9 160.015674121695 126.171040948196 219.036267470696 168.11088055793
86.033725825968 221.383645621978 196.200780398883 187.923050034941
478.799250340246 141.567299023381 241.677456498713 310.188313944313
CD93 352.47015903013 519.775555680262 836.472724085903 1886.05449875658
263.636675573982 621.489785652602 125.30588698166 314.856937113103
72.1591028956359 132.959812230068 212.117653688186 242.546821017091
CD96 54.1745577382276 140.226992517573 164.205608279417 143.973063517706
89.9615463738575 50.8056327587768 174.007271403635 184.269633116351
94.5716601751149 130.946383858668 132.242070552057 87.8967913380047
CD97 152.132756414024 194.644164648125 180.029671376384 968.405745860353
297.739286969257 376.931257615162 208.379981648601 258.120500906788
167.319747725488 146.789051265805 190.053043382767 201.875082892488
CD99 457.681756480301 112.146987452306 109.374838860558 226.456072107079
81.2359429678503 439.862941165528 250.621261212308 320.74875942191
132.985150338484 218.675464231277 166.910579215196 186.229678647062
CD99L2 287.689260887067 155.026564617762 156.615248744544
287.363928615517 144.484560429882 532.981045084589 115.98265270452
200.144817111902 107.961407367823 388.336994961328 416.844848063269
290.81245174957
CDA 58.1868359012515 62.2150049214174 78.9701459806641 136.155022329495
48.2748123114598 61.7091923488929 126.079949368634 72.692416146584
55.2114969675545 62.6984594468418 55.2374601141719 58.2879293130296
CDADC1 41.9407041937973 45.4882438975269 52.4485219671976
55.4180794172794 48.1473938886239 47.2009420656854 55.0897019602305
48.0582855095143 40.4997186069494 56.3441077510744 50.0303076717704
48.27609330316

CDAN1 1067.08316477623 529.977491403949 652.480459437977 942.247437385029
551.1195044975 393.648308810931 781.223434214089 1607.73526966988
459.546279672649 1416.47763911039 1234.59589464045 938.802606388076
CDC123 354.015808474189 469.893430869091 438.22469176123
322.109212713082 612.014486689487 352.246108891022 827.365231733511
307.329227013816 585.771656887036 221.163225922749 299.524171071613
267.232315058148
CDC14A 44.9126614290929 45.7023929486654 47.7112511352621
48.267960165858 43.9450023643394 45.6706505553383 44.0137388752186
45.9104478407175 50.379587451669 47.8636003588181 49.2751108739288
49.3533917376456
CDC14B 66.6547247910487 117.556281726503 91.6713614282714
65.8926843080114 83.9387217886961 111.532958750074 92.9894133685392
62.6735174039054 100.294952875307 133.074560812703 146.249568587351
162.447872391923
CDC14C 47.2862299990938 43.7644428186265 48.1997158668729
39.066284740632 40.9950639693255 43.9724628401189 41.1507980856436
43.0390857698452 45.6582729799009 40.2112371512838 39.9899430628542
43.0930293831497
CDC16 484.482971152057 421.971171686029 497.290172759099 341.666968456707
614.603743904512 459.546279672649 351.039814818516 346.438778257772
362.788520130059 634.226367147025 577.993850127555 555.044139654886
CDC2 138.50445813694 153.037055310357 92.0880591999243 48.665578118898
122.988056802699 52.0583273941813 82.8485522398913 77.8946473414547
296.842555066902 41.0057887014368 44.0409178714232 45.7562276914441
CDC20 471.297503316471 562.258108316474 376.453560620912 134.394743648475
1149.90692951795 124.490684428024 1174.92419086759 974.296760034984
826.490051619826 55.6865353018811 54.0374522273907 64.3571664944642
CDC20B 50.315768338317 48.4980626712175 53.9612654850046
49.3654837541353 51.7983110178095 49.9699131196797 45.223075311113
46.7980335276155 46.2027193115397 49.0841460667818 47.8284278211109
46.8129509799753
CDC23 210.667709831149 159.463273263774 197.103673938152 163.718576695806
224.373975368936 157.856158440506 163.809567262808 240.476103976894
268.683528469759 302.79352975792 235.764999941441 243.341575080264
CDC25A 61.7551416187554 68.5251167137806 49.0655215909632
43.0016969050339 93.6591201235745 54.0382756991549 91.8733417805775
75.2950416358859 80.7409247380392 43.6670755115848 43.0016969050339
49.5197902747103
CDC25B 248.527031538874 135.226924329183 135.19133529995
276.267583952979 174.058845828966 143.478533731355 302.978457293429
185.474249522831 161.573327074375 149.59360999995 115.229070906222
96.9476136220382
CDC25C 63.1291723738553 64.232967574797 59.2012779686869
44.7255207589606 70.1597323687098 46.3150221024046 79.3315961732103
90.4160198134865 73.4687357112674 41.9666541498638 39.4736654224088
42.9292230806949
CDC26 369.451882513347 278.850071205987 243.27954361539 477.263677441074
318.302018651677 493.705301673753 433.89783033465 329.120125961774
389.364650157102 452.797096652556 407.564154455615 594.184150517102
CDC27 44.0328992213727 39.8695929627783 39.6451483836089 44.9370339275041
41.0885562160946 47.0027453663546 40.6750540874299 49.5185112777773
37.8319142847141 45.0155543555707 44.6286581923295 40.9683275553869

CDC2L1 63.6767189846426 61.8701572127817 74.7625228047952
82.0292880742189 82.5344501505114 108.847044476692 82.3364971911816
62.4219686957673 71.5836731059913 61.0311116373365 72.768998788927
72.2720428359203
CDC2L2 85.6964911093755 52.9603588177082 65.8455652907556
68.3157692917711 61.3851695159988 63.1604223919703 91.6526163804547
82.966263655525 68.6847047963888 60.0793579902362 74.9006142438341
64.2769859135548
CDC2L5 99.1565755621214 117.934830767297 98.9113424606303
120.3698209682 110.146992754585 91.3418781023824 100.057155418659
117.948047796034 144.578232267116 142.291320727209 122.642481894309
107.337113678019
CDC2L6 662.848386826617 188.734299563005 243.15571937138
324.877187846959 103.199200862158 203.913062066149 304.258586051126
1014.6400720037 224.030034866938 341.078728185917 366.129480083279
255.087214364926
CDC34 137.156113501125 183.165559564289 157.133627517432 93.6212502203405
315.696895080135 214.537530518962 153.901083585889 151.686513723244
197.923403667051 107.840833498938 157.020685801527 119.947688643528
CDC37 2921.68921750799 4148.07163380544 4665.45637589484 2294.75523373329
5047.91205452316 2581.34849558524 3618.78958408675 3282.4852236415
3143.97163345458 2650.09507972593 3124.84336032646 2232.61002288121
CDC37L1 42.9104454767916 37.4540772310118 42.157627240486
45.4634652319753 45.1137938810047 44.3648993568405 50.0088611244948
40.4359177791566 42.2577689927563 44.1379638573455 41.1638563416981
44.4475838947656
CDC40 215.15351875081 288.508774004052 287.247399926139 257.325282051981
122.904323796484 277.665554240228 172.433936159136 514.853892585612
210.059998353905 219.616289279562 240.731386634957 278.697914107208
CDC42 64.7318794662958 76.4796910268368 61.1745030100746 82.1809804715696
58.174555206394 60.1591647295056 69.2322821603564 84.0899854321388
78.1761025629963 68.4409478568282 64.0541263553251 83.105117076029
CDC42BPA 52.6364613846829 57.5197017161209 59.1040441894478
52.9156540211793 51.540723788287 74.0995052892511 55.2998422481855
55.0698932795613 51.0062122023297 69.8941497035747 62.7743500747415
78.5468300294626
CDC42BPB 191.09175268141 107.951009163316 154.633672616367
104.335120306753 107.54932544455 167.626012561905 102.913039837094
181.298709635609 148.333778341005 289.112248695929 218.613795121752
143.749408328062
CDC42BPG 40.8681871019812 48.7084357480922 53.4088637045445
43.6726153508978 39.859345745831 38.4330755397458 42.1607322031054
42.5435283762933 43.1805016690297 39.1621898366355 39.5399188536544
42.9887069977696
CDC42EP1 58.9201382292404 58.174555206394 49.5424425850549
47.7362556248858 55.7657817295432 48.8744644207821 54.2445399731644
48.7814131120945 74.1282220615085 40.0557788909923 44.6286581923295
48.1627097465883
CDC42EP2 70.6065558064758 62.0908175930716 67.1299518044849
144.94134446105 110.214922820817 154.355197155161 142.614006543733
93.879589378061 67.591570323687 130.812376632261 114.093655272795
120.286733441947
CDC42EP3 51.9545473262296 47.3536182445315 55.9459886217809
70.2541961995753 45.5809132232681 77.2834482217973 45.3597029614942

35.6825474397235 39.9444191873552 42.1622837416589 48.1352470945488
53.655233033193
CDC42EP4 592.989860385203 882.105892529265 1485.68317899614
420.401914084346 1269.36837611919 1483.40077183826 928.230419226078
784.268074988084 1780.14552702126 1137.99307039386 1455.64106630257
976.566026758227
CDC42EP5 337.084861453686 1083.50383916482 963.224016480187
202.751918610777 386.271174243357 349.371668774717 223.749512763927
230.117814622646 983.972164685474 2126.41018877615 2138.23653387331
1217.21440297138
CDC42SE1 79.7238173467616 95.913238814854 96.6294773538052
71.7167037224069 79.5175547131776 53.2874286456249 65.3694267397114
140.41738844565 92.0711167984454 58.5831897269778 65.4643755537228
100.326655761429
CDC42SE2 108.57858133896 121.236881661832 131.045615546105
211.685526927495 81.7765447802116 97.8702400145827 77.0774476767901
119.362992523422 143.122201568705 134.646159826262 128.559660070271
181.064239330482
CDC45L 161.38599012777 208.878921620914 113.18951586976
76.9373289454707 248.058310353348 58.3387916574158 314.281125546552
248.058310353348 246.869486382264 45.2556760873335 41.7272875295469
59.8760452220989
CDC5L 136.530734456168 94.8766888888828 92.6672238953636 112.957927208627
118.638732417836 114.526825145076 94.8766888888828 84.9075858560758
114.304598397252 80.0651801266824 86.4195039096886 142.812241748319
CDC6 47.0940613507321 46.4385386401326 37.3687900733549 53.3967242431461
47.4548471240051 45.0882356578404 50.0088611244948 43.6434576713809
47.5564423692142 43.844855336215 43.6629145624937 40.7704587752083
CDC7 60.1705241170421 48.8584917918986 51.9885249508283 37.651298468202
64.3571664944642 40.650742261428 66.1940959364976 63.4570909617664
64.9725753882126 37.7315666632629 39.2542963855807 45.0035308952466
CDC73 49.6241203903354 40.5400384479766 44.0451487108213 53.593974682612
45.2556760873335 40.3357777530419 37.3799542961839 53.2046867327331
41.7627910693483 39.8873706855774 43.6629145624937 48.27609330316
CDCA1 34.1336988442837 41.9894412416874 45.2313723727673 45.4634652319753
51.940083408061 41.2567470068038 41.8315602975157 37.4484310268909
37.9273653326866 35.93950183586 38.8507804383261 42.57175366709
CDCA2 74.0913177754682 59.4253780950488 59.1040441894478 44.4367387274922
66.4047895961325 48.3686935595826 80.7563265626648 67.8061298261394
67.3230935444252 40.4136535208406 47.6125796208753 44.2527566343661
CDCA3 81.9258201510475 71.9162507601624 73.967017874248 44.4367387274922
114.972374827795 50.5148383400703 161.205086002542 86.5926014007562
116.461730749198 40.6022003765153 44.3763859390287 43.9541718254769
CDCA4 101.627551729492 79.5707751325986 82.6983700200815 95.096268847843
132.555169949054 104.914715637173 117.502489192855 86.2413316441255
135.138667957956 98.1433318215702 91.2202310968307 94.233414682323
CDCA5 338.475097952023 303.281551940647 201.782740241526 124.976348531594
1020.75813028358 164.287737381703 901.793120584383 469.78210773245
450.759762047652 49.9493230064613 49.532546810992 64.863894209343
CDCA7 141.79653024085 114.526825145076 69.6554376880691 59.6153684374766
292.735673136799 78.4815128101967 177.602690452814 102.294142438313
172.698530496379 58.115198353247 51.0013344286851 86.6854406823303
CDCA7L 54.0990839134272 50.8114699212931 48.9944486068347
47.481302504066 85.0017995689256 68.5457752893587 82.2650787041549

46.9986083378817 36.2733735494625 50.5252048155459 50.2203578628217
52.6922486991742
CDCA8 141.087571926258 113.624887028422 108.928854330744 63.6334154351075
228.185436206823 58.6439719071099 195.108184885541 287.981727851442
170.150808455621 51.8412522234186 51.0492389286271 54.3587260982091
CDCP1 92.0136417064806 72.2377222744463 69.7855392178808 87.7460859694899
102.717305041352 48.3119523820103 89.7242042805798 97.1884078242617
113.612874206329 71.2409890549638 68.6666478088834 87.1651984640976
CDCP2 36.5393857306744 39.0102066700839 40.8420925848403 41.3027031037343
40.8873907150245 46.263826584899 41.4742337714895 40.4359177791566
39.3750969348308 40.8049408996274 42.0750889188255 40.9683275553869
CDGAP 43.844855336215 40.716553247236 41.6977706798234 76.2591822114739
51.0107745412152 45.9266745571724 38.941855825269 43.7396619462033
40.9901805728485 48.1392888470269 45.1432412071268 42.2577689927563
CDH1 2146.1548633579 2621.65272077828 3070.83113612925 133.720493104769
1104.45371981807 185.563590013028 1226.43929953115 1900.96666834569
2474.22199172028 1024.97546870349 1459.69929955338 968.405745860353
CDH10 31.5599106749655 47.1332119496722 47.7737810047679 41.7614975630362
43.4544762830742 37.0180934800573 39.1539892144371 42.6450179841129
40.3316153483289 34.2638875325204 42.7275315121163 38.1421709520616
CDH11 556.417356025042 193.730784033221 88.4897895318663 286.996922972683
188.670053901101 174.392758289374 45.8980298854198 237.632703283663
65.0810479794187 151.912182086236 179.629622763381 146.117756310709
CDH12 35.7012997405508 43.0971787292711 56.8595344107711 38.4208429103799
40.0599807618105 39.2248354955045 37.6036146841933 40.831207953647
40.137818555383 68.4137459386155 48.2910530887214 48.0350549043063
CDH12P 41.0608773166058 38.5252456306656 38.6727842796202
44.0451487108213 39.4693871570028 43.5752393599169 41.5817737564592
42.5435283762933 42.6366765186182 40.2112371512838 39.7342718293432
39.3575468494941
CDH13 67.8774399765179 58.9910892923962 64.1777305915695 69.5544840164738
52.6922486991742 50.9724155054821 40.7910160811527 49.6203214143464
45.0017158586743 65.3377940117969 50.0808824383821 59.2035499250243
CDH15 52.310270746917 43.7644428186265 38.4043916290561 40.5484717678957
44.8897737954573 36.3845892764084 40.6750540874299 41.1575777497576
42.8036731479932 43.0919221375514 36.2435874904689 40.7704587752083
CDH16 44.5282181324797 47.9128504683618 41.3366779545815 39.2780515621926
38.5512429290626 43.4681646416585 41.8315602975157 46.1673159982664
45.6582729799009 47.611018474151 44.7114813656401 42.357910063876
CDH17 43.6706764143908 41.0678614026819 39.1799070031819 39.5050383901884
43.9450023643394 43.4681646416585 42.7431359850339 42.157627240486
43.2761454096265 42.9866214102525 44.8872784170036 42.6758568421347
CDH18 34.9138625012006 38.1312950900467 38.1421709520616 33.1137701681495
36.7356360093603 37.7000400506818 37.4772922422734 35.9023742468744
35.3227485563945 37.1275334308172 36.2435874904689 30.4639019828888
CDH19 42.6575535390785 37.0390589468421 39.5893773029164 40.3180310664156
39.1087466732192 60.2083550387718 40.4941260038116 47.7889447429963
46.8873421271259 40.5571841955033 48.7814131120945 41.2437378488025
CDH2 148.758736522703 59.6359245937831 64.9072331703985 102.00080367858
69.1862663389904 91.4446109489387 48.8319884367456 59.220967994972
51.7549258776884 64.5347406608537 55.1099824417989 107.91849682084
CDH20 50.8388984085636 44.5152708955623 42.7303139017786 43.074472921841
44.7829632472754 38.2293008890613 40.1235656101329 44.4031868028216
43.4656637536244 43.0919221375514 44.7114813656401 45.9693232070002

CDH22	35.7012997405508	52.5868047927666	40.931294690669	40.3180310664156
	48.6224651100733	43.7630587657205	41.4742337714895	42.5435283762933
	35.2143190355414	43.0919221375514	39.3667161654772	35.7451401627836
CDH23	46.8626801744519	116.685453817071	94.1206342908306	44.3557408297309
	49.452985143568	58.2957814921605	69.5225793065367	78.9088042058577
	59.3353474684952	123.350559552455	150.957220787781	64.9030399249327
CDH24	45.7802253890958	42.985929467107	41.437237454134	41.3027031037343
	40.1874619442128	40.650742261428	40.4882741869718	43.7658269153047
	42.7711437826303	39.2623423164488	43.5367669381526	38.8862294018533
CDH26	45.8546719310325	84.4349803529132	44.1452943804044	41.4812544772644
	44.5668141895992	42.8598805316863	48.1747307200218	42.6808744813778
	55.2635381074136	41.2276963324981	42.2455707947223	44.6946363672948
CDH3	601.829364668202	104.409397223301	373.172504097546	99.0647786948132
	166.572463787763	60.7488007939604	298.14782785085	203.759116151774
	538.427311162325	193.519079768562	77.9085946827079	214.127390534906
CDH4	35.6111578834671	41.159706131351	40.0998167098057	41.3027031037343
	50.2163892855092	41.7627910693483	35.9894133432376	41.2551966132486
	38.5252456306656	43.1839109702714	47.4630625313159	42.2577689927563
CDH5	140.465247047676	195.430996540859	334.639325701194	272.981148492864
	106.691530592115	176.43047680293	46.3057385979341	118.93749473833
	59.5093038629766	90.8188865732032	113.169111095305	88.8330881965986
CDH6	47.611018474151	65.5730163728961	73.4122412764173	49.8304479381623
	55.3100442506299	44.4311655729487	41.0381860307445	53.7378346199838
	46.4164127487052	49.6561029118699	41.1638563416981	50.8442982601142
CDH7	39.5185194094434	41.9397336249742	39.673290670647	42.0605906240248
	44.4954892619459	40.5710637884029	43.5627649099623	38.2083710281545
	39.5806465938526	42.3346523186171	41.7677037290981	40.5207392413115
CDH8	40.5654775358427	39.7835513496873	41.9666541498638	42.1273928203021
	33.3231576795423	42.357910063876	30.9403229851671	34.3865306339645
	41.863927676552	37.4302011516587	36.3390947880186	41.3481355128089
CDH9	43.0930293831497	41.8848959280916	53.2046867327331	47.0874209746031
	44.3557408297309	45.4882438975269	58.7585623394335	51.4360013601805
	48.3954967265007	48.1392888470269	49.9338905397767	49.2064066614865
CDIPT	57.0006212637829	56.1912096308988	56.7272804480684	50.6262443356159
	59.6496385170473	61.9432904657734	51.5967098638347	56.8595344107711
	46.2718838939055	62.4530276414691	57.5955320223035	53.1182952086284
CDK10	209.794541970107	189.650905999631	314.091389958069	106.309161354804
	190.422737436225	90.3439862484238	224.827414462257	143.112509176873
	140.921774805792	228.778506556448	220.573371581491	147.137598111239
CDK2	237.359267889848	209.099378471892	150.925721934159	152.329124189473
	228.571334092678	150.941470539313	375.354746298695	389.422882761332
	243.446420243813	123.219313750907	134.239606718101	221.123510823097
CDK2AP2	64.9367707455078	81.7367617812869	88.2959958615748	
	95.6215575902122	88.1199908664065	70.8674195845729	144.593933058291
	102.717305041352	129.958419679179	86.8691342571214	111.656567824564
	112.944044862693			
CDK3	50.379587451669	54.3554233228353	53.5698357689852	40.9607305725196
	62.1836961153924	45.2852528185486	57.4047331041592	57.445796273665
	57.4777075615651	73.6240672213673	64.4570366205389	52.2832912331043
CDK4	588.736435715365	557.094756796515	287.363928615517	338.000998124097
	611.538630852232	437.280601907944	853.117321142008	701.168333137671
	656.355333007615	321.21879937379	307.445116836976	303.818507395434

CDK5 124.095181002305 87.9963662401769 102.500172212095 100.001150612051
102.372855708929 75.353039684456 92.0880591999243 84.5055910320032
121.142787642824 72.3297091475814 78.806097512943 81.3082994649699
CDK5R1 56.7776874358774 53.4439773326843 41.924725226806
51.223859150798 59.4153120842154 41.408887993053 47.5963750520409
49.2019743609873 55.6991707857987 40.0557788909923 43.0016969050339
40.3691324547049
CDK5R2 41.2424637338424 38.2263227947189 39.3709357615493
39.2780515621926 35.6434124419782 38.6414316877471 40.1235656101329
42.157627240486 43.0930293831497 41.8613406887824 39.5399188536544
38.3291713594599
CDK5RAP1 101.560929859785 85.352285669007 115.408418935236
88.051652677564 133.64996113791 96.143327683221 106.668282281471
122.702789229752 121.696160351886 103.447730395424 112.881899194726
101.935321521672
CDK5RAP2 98.9463403584348 99.9486407529027 69.3181565573028
118.186590679218 94.4501786945034 140.01041112896 150.051297690759
127.283778555702 99.0596863442346 106.590506599473 134.936657884035
95.2614267797703
CDK5RAP3 400.093769705765 308.025419469334 589.18407096044
212.117653688186 527.037018015766 217.875223856197 360.900649927623
343.346644539158 273.645674189546 422.474427987338 326.250872625748
287.932410610182
CDK6 71.2963721541702 67.4018925617901 101.187156681366 127.789065386943
55.4919197421746 113.612874206329 70.2635892414678 64.5257016285499
87.4335749014848 97.4769942713663 85.8277891453447 105.293665242737
CDK7 140.853812988562 128.885933852704 116.839274984089 97.2220312216969
118.885205874725 84.096146903568 88.7029126110977 102.085154509866
127.115038236278 86.9626166234066 102.610376474754 135.718484174661
CDK8 50.8388984085636 61.0144918827511 66.1541420734416 50.7046706880325
79.271901834289 84.5913809068614 73.8313212063649 53.7378346199838
69.3300058073438 48.0211752672327 50.9815930710768 79.9447969637409
CDK9 166.752205770965 177.602690452814 127.694844372277 103.530430972911
144.578232267116 114.389392280707 139.472956453414 123.022511916727
118.862494537893 161.588263691127 157.592292551459 148.42234459694
CDKAL1 67.2437599548936 63.1137861754989 82.9449579428926
56.7927450229377 126.492854172749 75.1674447943072 163.995560004001
63.4570909617664 57.3385421837183 53.7196173866021 64.6446465846487
75.7991766454243
CDKL1 42.8036731479932 49.3581578587582 38.6005272730664 42.4580504587581
49.7235523248653 57.3797835448761 49.1067959632109 50.3972033014227
45.3756389170606 54.6089451252049 54.4825201506931 49.5539622682049
CDKL2 38.0089018744739 35.7818918460549 37.1188989884324 38.0958223740111
39.4693871570028 37.3799542961839 38.6537063231682 37.6557714476853
37.6630259432398 37.3179578037963 36.8282383070068 41.4508241771315
CDKL3 49.1868644496737 45.620219291723 50.4662238169527 63.0153175111747
45.6409129284202 59.5979861826984 47.7390230243778 45.3389970694851
51.3511088648386 60.2877434874622 52.9567386506107 73.4876346170053
CDKL4 36.7356360093603 38.1312950900467 44.3344900481112 43.8002456102021
50.6470461863549 43.0860383229288 40.6750540874299 38.956280281655
39.7537274655115 44.6918600441328 44.7926323492519 42.8864415494484
CDKL5 42.2455707947223 40.3021896270845 44.5622847010474 42.1607322031054
39.5317722761104 39.4469765850607 44.14228771963 42.0494116081998
38.9834959368496 39.6333888530279 38.9087995969091 38.3880780363593

CDKN1A	223.826005691932 667.837116137964 1199.29782389306
920.504890206997 275.975192937951 1239.68399088145 409.433491782481
371.709014310561 576.367042159347 820.147156401172 636.367246129079
564.468049419762
CDKN1B	112.306016029829 177.535502927617 125.690995228055
185.28845113422 147.209004629384 140.393549614927 180.648144492464
194.666769955149 236.910422092851 147.300356318739 129.810477623628
145.391705645319
CDKN1C	80.8943070653517 95.39134217438 84.4443931765378
73.3725472767369 66.0411740221276 185.360921731157 128.024853362102
54.8412943008635 130.925724760241 131.973056760893 151.652543512032
111.071209057746
CDKN2A	70.3757673501097 49.386089018323 45.9240324357072
43.9282059368202 139.945446129591 46.9613376350744 177.912351477808
55.9927099883616 111.939677225005 39.0209841108908 44.7765484590865
39.901614985832
CDKN2AIP	96.0236731791095 93.1067450332473 94.6130003107132
90.9106103192162 108.168107241759 109.352296288717 100.139490974785
102.49785018292 102.063594231253 73.8313212063649 83.5069968129733
108.479856042609
CDKN2AIPNL	4235.59743465057 2779.37660132136 3001.25236839028
3190.0108307463 1876.51621222292 1294.42501356365 2803.11698341125
5566.52516420571 2331.12401106076 4665.45637589484 4597.60989502097
3465.3013599062
CDKN2B	95.9485602132766 56.7668947356134 56.5357495488911
55.9521647240225 59.3611824459962 60.0502421545569 73.7335870746827
61.5429260998857 86.0735583144514 57.4151953663881 54.4140381300469
61.356483470053
CDKN2C	46.581796165231 49.0069391697542 51.8122261766744
53.3094843669564 37.7576900881741 85.8995211302699 42.57175366709
46.1673159982664 95.2956103624468 48.5571567616945 49.81481191219
51.9950964009224
CDKN2D	61.8124171056747 59.4253780950488 57.2107492415265
112.13936276116 70.0133734659853 100.650129064849 106.599578931817
66.7106881373359 73.6965494979349 55.3477556906605 52.9567386506107
72.5918913856713
CDKN3	77.8130233266962 79.1822591641672 73.4122412764173 63.6334154351075
82.5344501505114 50.9724155054821 84.607833108004 109.241121384212
140.01041112896 41.1222709786153 41.6336636285875 44.2527566343661
CDO1	47.4678477186801 38.8336427948187 44.6511073781104 41.6045220683604
69.0975200641705 129.951091959262 40.9157352260901 49.6203214143464
46.2718838939055 70.7003843117511 66.2027948645675 66.274247400793
CDON	41.9823787224629 37.7384958256497 39.8617019260946 40.0337926471478
39.1650142708257 40.9506062495231 38.5442780220289 39.6474008369935
38.5019918517688 40.9506062495231 39.4736654224088 41.5083179498332
CDR1	41.483412492426 39.0578297166485 37.9003288112261 37.29566077996
38.1644569189523 40.7063426028871 38.977631784251 38.5086872173449
38.4344387289956 33.4316681623175 39.7342718293432 39.6833800000587
CDR2	209.524518082089 331.762920362224 178.156332261759 189.47090669378
212.353676518748 263.44529683749 206.814831223864 454.939614583134
169.500205172068 453.115133900701 382.312820492386 416.40156274382
CDR2L	660.295993198711 363.215063520042 264.495576211074 209.004669589169
625.805967352327 304.298036741211 608.579654909865 759.94822128584
401.956364010068 164.642060754649 146.969565088793 187.462631817772

CDRT1 40.2209794935386 48.2220547806679 43.8495248991589 44.604338271769
43.4812166409346 44.2335949843431 38.7337086062875 51.7866317456986
47.8485718564184 40.7063426028871 44.1478805895017 41.0269624684418
CDRT15 41.8409927585705 37.5469247328848 44.4311655729487
41.0726336841311 42.6022652963326 48.6621527194963 43.0930293831497
44.797320243931 47.1744296124496 42.6793753550854 38.2795150354677
44.9070250604284
CDRT15P 38.2083710281545 39.9697332660833 43.9450023643394
33.8111782249716 46.3150221024046 37.4782441311626 41.4742337714895
39.1652965788406 40.8970401536578 41.7405682794166 39.8184668132023
43.0930293831497
CDRT4 89.8567957512307 103.104022477607 138.618449365819 86.1703662107384
73.2607982527105 68.839885767937 77.118454956956 125.333208461509
81.1416376689959 231.060688044554 155.287591646469 130.865421554474
CDS1 104.573463811298 252.134800295199 141.293219914385 62.3936376596431
86.6854406823303 59.1570467228834 188.552900887757 566.318259999405
137.302487233881 262.277293680018 223.562130663956 178.98901256658
CDS2 161.910748720932 170.932645135423 150.011814577972 259.220844287491
213.933347509147 166.064317271991 169.723009455614 156.56745342836
116.17887995398 166.648551193168 165.942795019496 143.6667863386
CDSN 37.4554876386402 45.4882438975269 39.3709357615493 43.3078542962968
43.2484996523549 40.4359177791566 38.4005795891396 39.2672712041774
37.0126425326534 37.5343260962758 40.547835538778 36.3454320848591
CDT1 104.462230406058 107.331284864757 90.807818984128 42.8235313007746
140.369714831352 51.294537308199 268.613247119583 97.5962784863854
168.7696164772 34.3674468322242 36.43924066428 43.8575940017119
CDV3 176.866350130563 137.955007835039 147.782301091454 251.374750265492
202.360546931172 193.569399061809 131.808634405319 151.601402122351
133.765486891316 94.8742116922003 107.453572798098 173.212430860273
CDX1 46.1673159982664 39.7835513496873 43.4709415392696 43.3078542962968
43.7517118200398 38.5252456306656 46.1634822993009 42.5435283762933
42.6366765186182 42.1622837416589 41.0050831223107 42.0424641526302
CDX2 97.2535496140648 62.0220558452834 34.6977013306396 39.7411455670172
44.2607789091176 43.6670755115848 213.622816589927 92.804679821289
53.2046867327331 39.5637454855233 40.3452320167286 43.5738557773903
CDX4 34.3473914754963 36.5219488315447 35.0970060242246 33.4819739460148
40.3452320167286 36.1190722948669 34.4570158133896 38.2782436497441
39.0775870687758 44.571915452417 42.4580504587581 45.7562276914441
CDY1 40.4639532172338 42.3647968647967 41.9335763813389 43.1847628331565
43.0860383229288 40.3969295784597 41.4244721780607 42.2577689927563
39.1433524273815 39.6205203043973 40.8730692351493 41.1914475733743
CDY1B 39.512606833112 34.1910413697549 42.4804257679172 40.8084239472012
37.5079203359641 38.1569957197319 43.4544762830742 38.3269307390572
39.0775870687758 38.3561682218911 41.7608506445769 42.3288275690551
CDY2A 37.7384958256497 39.5050383901884 43.4709415392696 40.646702696033
38.745552403895 44.8872784170036 47.1345949868029 42.157627240486
37.4540772310118 45.9004543328976 37.7384958256497 40.2669497398702
CDY2B 42.1537476443423 41.2598458726002 40.4070046183108 33.8111782249716
38.4540849632254 30.7058829591553 38.1793598983615 46.0633179231318
39.3750969348308 38.745552403895 39.8184668132023 38.6454098044474
CDYL 69.9431447778061 55.9459886217809 57.9279989359711 48.4916117779992
91.3723635213821 57.7463129434768 50.0088611244948 42.8936990772838
103.010279797396 57.0581769379589 55.0823430906161 56.0477607422828

CDYL2 56.0477607422828 43.683792219729 47.9035532886869 45.334106390212
42.9090346219023 47.8636003588181 46.1634822993009 49.2019743609873
49.0986627051784 44.6918600441328 43.844855336215 44.3521099857368
CEACAM1 287.363928615517 148.964204638494 130.792245267896
190.77754486426 98.403191200835 63.5195937348531 70.9462008788871
160.853643241489 222.126944340681 98.8922486048877 99.3744103955071
187.238252376588
CEACAM16 39.8662795144616 39.3999056968598 51.4308501933893
38.6414316877471 40.5710637884029 41.3553369544097 38.8417151851134
42.8482181313226 44.0519245668737 39.7835513496873 43.0016969050339
37.9607839934735
CEACAM19 43.7585538437696 40.9731067463382 41.8751578329365
43.074472921841 43.2484996523549 46.1513019317765 40.1235656101329
51.4360013601805 43.0930293831497 44.3648993568405 47.5439485437351
42.0424641526302
CEACAM20 38.8561375693335 40.5400384479766 35.2101597814352
42.5664883002833 38.4540849632254 38.8336427948187 34.9344532055397
37.1317006162938 41.6616444147804 40.4136535208406 36.5359455830388
37.4554876386402
CEACAM21 48.8941062980393 45.7979032939211 42.0621425894624
49.1786207920603 39.2608760193011 41.4508241771315 42.7431359850339
44.5029690493211 40.5912213523345 44.2580151149956 41.0050831223107
39.2469742642337
CEACAM3 39.1590964332473 37.3634897565502 39.8299340037009
123.281175151414 41.6806984441849 44.1737502492846 49.8281348927244
43.4544762830742 43.0930293831497 38.1042454705119 39.4410515050885
37.3600014814388
CEACAM4 43.3638918705225 45.2556760873335 44.2405728317107
64.2130157241811 48.2748123114598 40.4359177791566 43.8206425661746
37.2237132058632 45.6582729799009 47.7180816682562 41.9908237694988
41.9430737035925
CEACAM5 220.682149473323 83.7019398654324 35.5220586859301
43.9282059368202 46.0043807840705 39.7394181011603 40.2154497375715
42.9301944191913 41.9635525160083 40.3235655331796 41.9083581529276
40.8681871019812
CEACAM6 2237.08790397818 403.92758879201 65.5820437889838
185.812520354849 33.3231576795423 36.2768299545794 72.2577390063259
204.318269745079 57.9713749600418 33.4316681623175 36.43924066428
40.1700117452801
CEACAM7 222.81957100654 50.6229884142872 52.5353664902258
40.7481155008596 41.8806343997257 40.5281496800812 36.197424053556
39.2672712041774 38.1169703906207 43.1839109702714 35.1470994647364
40.9683275553869
CEACAM8 107.637618676176 37.4540772310118 41.9666541498638
36.7892997857376 40.6723334103791 41.2567470068038 42.2577689927563
40.047314525032 42.4540072551568 41.3321861371202 42.170702295259
45.5800840647989
CEBPA 124.095181002305 851.813216452015 276.86542239106 204.502683458676
866.712228831149 240.520228413182 724.881002237334 704.15545332905
644.733901442573 479.079946537291 430.618167106529 198.729330487352
CEBPB 4569.48246031279 1810.67209360676 3562.96193568469 9060.10165509099
2844.5506310828 4632.08655821299 3592.44409869527 2779.37660132136
2844.5506310828 952.16345035163 1810.67209360676 1999.21832626916

CEBPD 701.551782736435 1255.10866316499 1213.3477481374 1975.28674300761
2206.44541412808 5855.6498679729 2498.54878472152 1427.67429455277
994.404707955031 1190.10462185567 2215.01815680371 2004.94135236716
CEBPE 35.2267149016596 46.29198852834 42.7303139017786 57.8277957325085
44.6834648302383 39.3841861812262 45.3597029614942 44.4031868028216
47.5564423692142 39.6719190054379 43.9450023643394 40.1700117452801
CEBPG 160.320045581768 209.178131628825 140.992072231913 137.831070407907
350.278532614563 261.51893439249 359.465715235071 163.995560004001
391.187784616808 209.401109514058 183.2003374677 240.265816943765
CEBPZ 440.93348620369 414.794323433931 324.320137371059 505.018127833201
484.968116511768 588.247843264181 634.023101883136 637.035929429013
324.126628444078 428.8440159628 405.148979342648 541.631969808467
CECR1 171.623830248512 202.103107745135 160.385057525357 726.804513440787
192.318724749487 126.232262861308 296.080833914967 155.872353189452
135.889195284785 172.102568448455 114.5696064637 164.580141651883
CECR2 45.2875173643252 42.0709558258414 43.6670755115848 38.2041059863839
41.9842695818285 40.9506062495231 55.2088411137193 43.0390857698452
45.4799270968969 47.611018474151 44.1148826235374 40.5703581862752
CECR5 268.54298415337 297.739286969257 228.747320301945 232.13042919324
271.138311888031 168.073605090354 317.777971035617 226.672191323896
381.6928205836 227.206974078547 214.691909801388 240.58403560014
CECR6 54.7786516309154 45.9266745571724 45.063792968927 111.703187941409
41.9842695818285 42.6575535390785 53.0277416531043 55.9303347977781
52.4539007817929 42.9866214102525 40.9157352260901 40.9683275553869
CECR7 136.530734456168 84.1922656126304 118.854313907627 211.790532633462
93.1238695478498 191.811190401876 91.0154871536701 164.813928846511
101.954354107731 267.107863045114 185.312851727212 134.307645598718
CEL 69.9431447778061 93.2630594602634 72.7987593815919 43.3078542962968
99.8699868526053 52.2089306840125 77.0003455479124 60.6706478075023
166.717256080341 201.530434441408 86.7730065232892 88.3636380348802
CELP 39.8662795144616 36.3110701990588 40.8420925848403 44.1483976313959
42.269054475455 36.9060997041453 40.1235656101329 38.079222289614
44.8997235283254 40.8049408996274 39.153226699662 39.6352613956998
CELSR1 64.3502363726659 64.1607001437806 58.637422084759
70.0402992940342 66.8733921725051 64.7905331739366 52.2476631126873
48.6441988290952 59.0563795877672 64.2174369755376 58.4816190076338
63.3578893149245
CELSR2 53.3059058105836 44.6918600441328 58.2986825661503
38.4208429103799 67.0163226819964 41.0582900711341 47.8947595316832
66.9340967279385 81.2850357499192 62.6984594468418 53.4088637045445
56.7711602236706
CELSR3 217.976796656266 68.6074516459969 91.6033269431304
58.5406981871153 259.906934432819 55.0009147092272 241.017227991238
131.67159377585 183.526640731647 45.9004543328976 46.8954576545929
41.7575097298927
CEMP1 36.9060997041453 38.3322849790657 35.9305494063137 47.9915414951747
37.7576900881741 39.8455060654531 47.7390230243778 36.8400213077707
37.5469247328848 39.4710743516517 35.4386677371439 39.2469742642337
CEND1 44.9896851005259 41.6466169548846 45.063792968927 41.3027031037343
41.1868052124344 38.8336427948187 44.6032218589199 41.6466169548846
44.8997235283254 50.22432299342 42.170702295259 45.4799270968969
CENPA 119.714551585774 82.1062834830477 62.5194460957899 53.0284011078097
163.299997237276 53.565717255469 134.968128884298 130.230482445573
152.792457231813 44.3648993568405 43.363245077835 44.3521099857368

CENPB 700.010354583503 757.833254346899 672.759115794267 523.25106120389
893.185203928127 1157.65264077595 1048.98028527123 513.891389190131
695.255317861845 886.397061577531 1051.58050264114 839.850453078119
CENPC1 48.8333989709933 43.8495248991589 43.401782064778
40.0698728584484 46.0977687311222 39.3841861812262 44.6032218589199
40.7311497914871 50.0526763693329 45.3530683538086 47.8284278211109
43.3744503319109
CENPE 68.2184093305248 104.305801811472 85.1981126713031 60.7148442408147
114.161435210562 46.1974343793412 115.98265270452 91.0952554578659
110.312681023811 45.5776159432079 44.2925355984056 44.4475838947656
CENPF 291.232601825013 120.825113658705 107.034189847828 59.2778887402357
269.618616631857 56.8505380943422 338.415128695208 428.515605971056
217.39432336811 40.8970401536578 41.6336636285875 45.9693232070002
CENPH 42.1679276329916 43.3016395799961 48.4890615340603 42.4580504587581
44.147557390293 42.7107928951766 44.4045701012801 44.1311654985682
41.9083581529276 44.4652605017595 40.5824352835642 41.2437378488025
CENPI 46.263826584899 47.8947595316832 38.2593044488288 41.3567204694281
52.2089306840125 44.9896851005259 56.9320717885002 56.8595344107711
52.6144123387561 42.5193208629123 37.6484782516569 40.3691324547049
CENPJ 67.2437599548936 60.5571721986885 52.3432130135266 50.5310598656998
67.6011992144113 57.678188678075 72.6959688212342 62.9922928277769
59.9108736364178 77.676210875358 64.830448408529 54.4992295592001
CENPK 86.4195039096886 100.483113678344 74.2769881586739 52.1201052574162
115.408418935236 52.5330333302271 108.007227281411 78.0020909283414
112.014137492029 42.5664883002833 51.2318935933116 56.4578213622965
CENPL 77.0301627655229 60.3928833351886 60.8357760968871 45.4634652319753
83.0497574039035 54.8265655909623 61.6544855697851 47.7889447429963
65.8622973953004 43.9450023643394 41.4508241771315 45.9693232070002
CENPM 108.479856042609 93.146085697072 84.6935029586625 64.4512696384839
110.517563101892 48.5251714843637 172.748740911831 146.046179302584
223.501516374076 40.8970401536578 41.9908237694988 48.4835379173794
CENPN 125.540457221408 100.483113678344 112.328675926999 94.3229189690855
107.54932544455 56.0477607422828 153.643631245787 158.195749606017
207.087723777954 58.2818491355907 41.9908237694988 68.8529879402902
CENPO 50.7774722329703 51.4749724393807 51.4308501933893 46.614092935749
73.7741438283854 47.5733603903505 66.7750249658122 90.1430468048409
59.7961440557352 50.4234835272833 42.9634662926872 41.6031169661721
CENPP 82.8485522398913 74.3729155936862 60.4809187893259 66.9218519793499
95.2833228196616 57.4777075615651 67.5206810636627 54.5125550937898
53.5224822626949 57.2813205536176 51.4413099192899 43.5738557773903
CENPQ 58.7404123614695 55.2182004838121 49.2041300973417 50.7046706880325
52.8190882379316 46.7800033479223 46.1634822993009 46.2779564481158
51.8083208406279 48.4890615340603 48.2050207071554 51.0013344286851
CENTA1 564.929183025034 492.519023728038 293.300203370043
449.701353531924 372.758184542037 290.535354193244 369.717462266442
606.761670821739 271.813877560354 309.06234888929 292.065366215278
401.679080975109
CENTB2 70.9419509323007 77.7481370715949 71.6354627933716
111.186223357396 97.6205705034407 86.2563919366781 93.3897477314201
86.5926014007562 81.6508259258669 76.0532592222027 70.2606858200937
81.2068428211497
CENTD1 49.7827734675743 48.6447083765118 49.8845794776469
56.7351380543324 42.8414096678708 47.4119039314844 44.7915315085948

46.8954576545929 49.5539622682049 46.1390887492649 46.9950086252052
54.761682143135
CENTD2 71.4937262725923 58.0218262176065 43.5710871617107
81.2269477373795 55.6069214673486 61.7091923488929 64.0291286735557
65.1637149462661 59.8509516999438 59.5297504864052 57.3417959180493
56.4873776344986
CENTD3 155.762961582213 269.044011137638 530.314249832491
221.163225922749 192.564048264712 124.490684428024 203.127486721124
193.196385709124 323.855522995794 548.217757366495 425.435793468733
465.981133154896
CENTG2 135.829530518199 139.554519110944 85.7111041637416
56.4578213622965 177.620987812591 136.913434348036 91.6978282652998
159.071016200059 108.27606179864 75.7801512652102 79.9567256807679
96.5813769696577
CENTG3 223.219262492785 378.626656077546 190.403032965372
266.717224821545 239.924746743429 176.871009816977 195.108184885541
171.705718494906 223.88063506365 331.935072199058 235.925374041038
183.702709922933
CEP110 62.6702927225656 98.9988668602468 84.8948024208923
114.520410790135 75.0665411373616 83.0554837248934 101.375030043104
69.3468840908665 76.4796910268368 85.7732262204681 82.8205269728224
79.5898953726467
CEP120 54.931538325692 53.4735821194294 56.607199625498
60.9379216295576 44.8897737954573 59.0686629877982 48.8319884367456
44.293922970134 50.8485682149772 38.2069499294002 46.4205654140754
51.0647893605839
CEP135 124.37984507038 165.606015336587 126.420070148631
156.976072444343 199.54796711653 94.1128427766432 125.052841200703
154.248946683126 139.328628553223 106.897106266912 117.033529761587
106.20479137406
CEP152 51.540723788287 57.4966000742671 50.0050760710506
46.9919960628953 64.9839099669442 50.6915708372926 56.8505380943422
55.6282425338897 53.2046867327331 47.611018474151 49.319499137678
50.6141827957642
CEP164 147.371811804618 95.2833228196616 94.0749342825094
101.008449241109 127.46182304081 108.08526252608 119.015431849218
163.917208464731 90.9810377546009 117.033529761587 135.138667957956
82.0124493733776
CEP170 43.4626049853157 44.5491926807637 41.7364024833895
49.6641575194943 46.0977687311222 44.9411655353314 46.7704450730477
42.8978721487095 47.9525424951159 41.683412864532 42.881371896984
46.0201052072883
CEP170L 38.7277789106124 37.7598032934773 38.6414316877471
40.7481155008596 40.2449343247539 43.1012097821911 40.0603121770344
42.0730322247777 37.8631570409126 38.904137905631 39.7342718293432
41.9741368971524
CEP192 72.8472187581193 112.281286038716 134.896696950857
82.1437429222046 97.7153995525346 75.854888525664 99.5844079573552
95.0026207727863 114.363816429963 83.877343750194 73.3321469094819
86.2974544521879
CEP250 49.3000844711116 49.2934904960601 46.2625470410195
45.4634652319753 49.7526478730976 51.1621379498014 43.6753843488695
47.5950227477807 50.8485682149772 52.2832912331043 47.5733603903505
51.8562635270111

CEP27 289.05213385509 122.576931376426 157.281394920992 293.355303451014
130.936053902005 128.657014567195 255.197732321307 555.356799014777
128.786137360755 374.601792595646 302.083293019113 269.184000351658
CEP290 57.0006212637829 59.0709442090059 64.0162246399972
49.1786207920603 51.3511088648386 68.6424653037264 55.8019623191927
61.8665316900291 61.4842573582507 68.534940191185 57.4622430729629
66.4626619269287
CEP350 90.2721521466478 116.74427460886 113.051477801522
151.740176274202 98.0143500562821 96.5287245311078 97.8309399470621
128.38299874379 84.2299424587647 122.350248266459 126.79509061532
126.115065200084
CEP55 109.738537924214 126.389908470189 86.1563473462864 83.014860007187
164.312946712409 49.8472708021525 217.848539722853 171.705718494906
172.624182951104 41.2276963324981 42.6366765186182 52.1186591838715
CEP57 47.1962846565261 46.5650556481721 53.9612654850046 47.9915414951747
49.9493230064613 49.4157684139858 56.2181240696479 51.8347877274018
61.8445915808329 54.7827221235949 48.3847843498476 47.2131144508464
CEP63 64.9367707455078 69.3525901554637 84.0305689846475 80.8078050043929
79.9719218603993 86.4898752526885 73.2144205844199 78.0020909283414
72.8653205184014 73.6240672213673 69.036463612998 89.7184505543918
CEP68 45.9090659324223 55.7405934254857 48.1997158668729 46.8954576545929
53.3086645804786 72.8472187581193 54.8074655158941 57.1362071277099
48.3119523820103 57.9713749600418 64.2174369755376 66.0786923699684
CEP70 97.1271615802556 55.4106986624448 62.3245608177257 47.0874209746031
107.770004093887 69.0530953932111 119.609614885616 87.7295604723036
119.512426152446 84.0691608193405 78.963270461629 66.6118713143653
CEP72 46.6348258763289 50.2696827764943 45.3974838013476 41.1886975241176
120.825113658705 41.159706131351 73.1654226019863 38.3684202018524
52.7512110769042 42.8936990772838 41.0808257270221 38.0452862258803
CEP76 45.2875173643252 49.6841415268611 48.9944486068347 43.4410741327254
45.5809132232681 45.8325181856483 40.6750540874299 43.4544762830742
54.8946088631931 41.9666541498638 52.7817052791259 45.5800840647989
CEP97 33.8558232866797 37.8217989841918 35.4484652445078 39.7411455670172
36.4434672195785 36.3845892764084 35.8227939208521 34.8949555140527
43.1805016690297 39.7835513496873 32.9879066304034 39.6352613956998
CEPT1 131.126942328593 110.609133094021 125.061012833314 170.644636226884
143.399636653204 124.599352191013 122.877322487518 129.268600210689
182.9407471819 118.501031900986 117.20232751191 169.344094528032
CER1 38.385865183581 39.4629815741023 36.7172300749476 40.8084239472012
40.3021896270845 42.2114134935898 39.3999056968598 39.2248354955045
39.5806465938526 37.2686490268265 41.3234441859498 39.2469742642337
CERCAM 383.371300272181 62.5528124447176 93.310557793228
149.426965431854 101.681822254799 148.255880793905 116.74427460886
138.666805923912 91.6978282652998 115.643361390258 116.839274984089
99.9685666586407
CERK 113.791456064084 91.8207081349104 79.9391673512018 197.070092523652
119.45528139824 149.639066617945 89.0203733091224 86.0944261788554
77.720889005668 104.462230406058 94.284665243755 85.3431175579775
CERKL 36.4564050256915 36.8285908669795 35.5220586859301 37.8153135197325
37.29566077996 39.5581166303303 36.5783930720979 38.6151680924119
38.3867490877227 37.4642807402741 35.3042041383893 38.7012264805355
CES1 61.0278709944136 45.5533570914817 52.7005092555102 125.52123746909
39.3100256336821 110.589130460258 39.3585350448719 62.5528124447176
38.2795150354677 79.4158467034017 80.5237920647059 54.1618170692792

CES2	104.492750153479	199.604105066243	243.403622361932	112.13936276116
	78.6286416672119	147.945429978177	107.668663185697	141.180682718758
	118.089250727398	166.064317271991	161.075134901675	152.942332679485
CES3	42.0833588105728	43.3458769998118	49.0655215909632	48.055683217958
	42.2455707947223	40.8970401536578	44.3440839686558	44.9631458622215
	42.2248608921879	46.0119912782855	44.3763859390287	46.8129509799753
CES4	41.2424637338424	42.1372626664516	42.1901757067073	52.4212131710421
	39.3667161654772	56.2229687620557	39.2533668167483	39.2672712041774
	38.2874316439311	42.9866214102525	39.3086414047188	40.4639532172338
CES7	37.9387791625384	44.9370339275041	41.8751578329365	48.856176220627
	41.6806984441849	39.2248354955045	50.0088611244948	38.4540849632254
	46.2027193115397	41.0057887014368	33.1645364749371	43.7672102682067
CETN1	40.0557788909923	42.9020321355927	40.5386195997545	45.5869891570251
	40.0599807618105	41.2567470068038	40.1235656101329	42.8482181313226
	37.9837233601316	40.4136535208406	42.5023941254529	40.9149601380325
CETN2	969.805093523651	698.595302020108	687.011162822211	513.109067557976
	1541.9572099378	1245.45371155527	1144.34555072807	989.0602710707
	670.023570141464	938.151864971251	810.382224368065	990.431472174705
CETN3	59.3353474684952	73.3950348855139	67.9410473129639	74.4365426051588
	60.1730663840476	71.2787833369055	60.726977722583	70.7087323885819
	65.1911280716126	75.8280474316969	88.2314365506494	103.252085427319
CETP	51.9545473262296	49.5443425465542	62.8131992098474	65.524821699432
	51.4527545197672	51.0071565607171	41.5464765641928	48.7039705048129
	45.1569741609597	48.9088046212958	49.319499137678	49.434568584642
CFB	592.010988342149	139.758805241617	740.994425128952	400.506633704584
	202.154195547644	229.035497774884	286.611037700479	158.493140172253
	202.081933301612	155.225701588645	146.691463448295	224.320261187595
CFC1	40.8681871019812	43.1805016690297	46.614092935749	47.8164193292842
	42.0709558258414	39.7697110924474	45.4799270968969	42.4580504587581
	43.7630587657205	44.3648993568405	40.4094901248527	44.293922970134
CFC1B	53.3967242431461	44.5152708955623	49.4573631027613	40.7481155008596
	50.8875036861067	45.0882356578404	51.5967098638347	51.1694248620208
	47.1744296124496	49.7259371799014	48.7502645382856	44.1452943804044
CFD	223.669853276931	237.237762964658	227.170944398338	776.670493028756
	264.161390133378	9060.10165509099	97.299696284253	256.721281849737
	616.265771322835	3800.19142219719	4617.45430247751	3681.41904661708
CFDP1	87.5146535320072	48.7847528954724	87.455991111513	46.4555908533915
	54.802736966312	81.2314452281031	63.3924515518667	85.1544284060549
	62.0272989884264	61.7502624458447	72.0389007415456	91.6328359149182
CFH	86.0443764319119	84.8808332740321	179.285390561592	86.2488614616838
	60.0736861987141	289.472303440614	46.6267962186599	66.1650385535549
	196.623560874725	167.034910927177	156.884346540644	184.938142193035
CFHR1	38.385865183581	41.9894412416874	41.2365089689635	37.29566077996
	39.4693871570028	39.7394181011603	34.2773859804062	47.5950227477807
	33.9456837661864	40.7063426028871	42.0750889188255	48.5123041297835
CFHR2	42.8036731479932	39.5050383901884	44.2405728317107	43.9282059368202
	50.3713170502557	46.1513019317765	38.0598374201407	48.7814131120945
	46.3203995372658	45.9004543328976	43.9450023643394	46.5650556481721
CFHR3	41.1523487516364	41.8534933577029	47.3861758958576	39.6833800000587
	39.9088079891223	33.8357261052093	41.6466169548846	37.2738321651142
	37.8631570409126	39.7411455670172	37.9946685306274	41.8200132437731
CFHR4	39.0620487644635	42.1607322031054	42.7303139017786	37.0732977341185
	37.5556299264699	42.157627240486	33.8558232866797	40.6171774724171
	36.9060997041453	37.1275334308172	39.6403089864678	35.1451084093692

CFHR5 42.3433931248155 38.8336427948187 32.8745892439217 33.1817617228171
34.9062568194127 34.5886782974123 38.1793598983615 36.0306975524309
37.9273653326866 31.7992509287736 34.5633623842083 35.6427313468066
CFI 62.9952378379206 43.5873188565519 41.9666541498638 48.856176220627
45.2556760873335 44.6524308640784 43.6753843488695 39.1652965788406
76.7100878053735 40.1179210534118 48.8088340735921 73.1447139654971
CFL1 1347.15664445545 1888.71160604243 1497.73669971925 1718.44944271916
1595.69503047506 1047.33749100342 2313.21212353993 2032.18819581205
1801.44003951248 894.486674059951 1080.43016747082 1040.102982885
CFL2 41.3387790895716 43.1464706017434 41.3366779545815 45.2759158146116
39.6535880152834 62.4077024344427 39.7576136507593 40.6171774724171
41.1742244762318 43.5627649099623 48.042366934301 53.7442594289333
CFLAR 199.404836798715 165.336029381509 286.497342437243 680.837950890159
132.172127966602 209.705575423447 153.901083585889 255.724996015069
83.5646728968172 278.773982275525 406.104588997462 325.769076169162
CFP 41.8409927585705 46.0023068797812 40.1985790927248 103.961956810572
43.2484996523549 56.8505380943422 47.2927436772465 42.5435283762933
43.2761454096265 38.4187686307607 38.5668610487033 45.2783024981315
CFTR 109.5145190042 44.2105195734073 41.2365089689635 39.3750969348308
36.2048942610546 45.2852528185486 43.0930293831497 39.5466975353131
36.3649677956953 39.9948390976317 39.7342718293432 40.9683275553869
CGA 39.1590964332473 35.2143190355414 43.3085358214154 60.620259884953
41.6806984441849 40.5281496800812 36.5352572191451 36.7460922671619
40.6957787921451 34.1648535095605 36.131485826118 43.4709415392696
CGB 48.6621527194963 50.543526404059 54.4384603094793 46.4555908533915
42.6022652963326 44.4619248757502 42.629412460409 49.5185112777773
49.7336769474446 47.611018474151 43.1931731665131 46.2625470410195
CGB1 41.8833988894167 48.0010635382918 48.4890615340603 46.1074305700679
56.0477607422828 41.7256940298128 42.7065584433947 41.0844534776541
47.0055981216687 43.9783383422823 43.0016969050339 48.5123041297835
CGB2 38.5648843914189 31.498275130159 40.4070046183108 38.6414316877471
44.147557390293 40.8634043028528 45.1017494688414 40.3357777530419
44.1519466943185 37.5343260962758 38.1835938174625 37.9607839934735
CGB5 50.0802438185974 53.7332797903102 55.1547682957918 39.9084621723809
65.6003544901554 42.9104454767916 51.5229124652769 49.8666125578518
55.4030910454028 55.8510296341925 49.8783924642347 47.8431972374582
CGB7 41.8409927585705 41.1212129940704 40.8420925848403 36.4564050256915
39.8198309498737 41.0012755104374 38.3346870469391 41.4061152929154
37.4937394063612 40.7063426028871 38.5252456306656 42.4229201717624
CGB8 42.5193208629123 50.1027491117756 49.8344973836834 40.7481155008596
52.0583273941813 41.7627910693483 42.8684958525042 52.1788489555942
49.0986627051784 46.441301934328 40.1742127891824 39.9519547348106
CGGBP1 154.067791946816 204.992039590717 246.274692971423
265.930422462959 239.517996511065 223.397586938079 232.911951245618
226.023657887826 223.168185178205 196.267504470029 196.465477936749
252.583117397109
CGI-96 60.8357760968871 65.0567596131122 62.6074601128958
44.3440839686558 58.7065473526185 74.0995052892511 71.193768715137
63.5693267269002 59.9770720847728 42.8936990772838 49.2879849674517
47.4699236780425
CGN 258.726193861248 422.702925141293 798.996494242432 45.334106390212
647.868022835702 91.640286556353 450.271658272512 1638.16317738035
402.978328664331 734.424771383522 638.305455110267 430.868309653765

CGNL1 190.675425748381 62.6984594468418 92.0880591999243 99.5958921252583
47.0954207417646 1000.65752290829 63.5738862549306 212.00247895282
39.0775870687758 373.714692628967 260.618790701505 234.043251639296
CGREF1 37.9387791625384 42.2577689927563 42.5406888281114
44.265834798964 53.5594683028749 46.1513019317765 36.5352572191451
47.4740726032707 39.0775870687758 37.8217989841918 37.7384958256497
40.1700117452801
CGRRF1 87.5146535320072 93.8009890556664 90.209585044076
116.295564219236 64.7488184936482 155.693953208351 70.8835636550472
85.9474373829081 110.219274023648 90.9570747667407 83.5895816521251
127.811684080184
CH25H 85.5362566454799 109.844156801015 66.0411740221276 268.121244731705
131.766252431636 722.514380928291 77.0003455479124 133.453860783605
43.1805016690297 67.0163226819964 60.8662233098324 58.0218262176065
CHAC1 43.6706764143908 45.8669209716694 40.1985790927248 48.309866878365
45.1137938810047 43.7630587657205 41.0381860307445 44.7044140979108
46.2027193115397 41.9666541498638 41.0050831223107 43.2776458914333
CHAC2 33.3307770183871 40.148780650831 47.7737810047679 41.8778873678551
45.1137938810047 39.0266821712099 41.3594868055879 38.745552403895
42.357910063876 39.7835513496873 37.0180934800573 41.1554481567603
CHAD 40.3478825593224 42.3550738181144 38.0452862258803 39.1706564265495
40.6723334103791 43.3785777889265 45.223075311113 41.5464765641928
44.5303781239209 42.0621425894624 41.2598458726002 44.2527566343661
CHAF1A 57.50915811428 68.4278345554565 50.6004078448405
65.084620579901 87.3957386451235 42.6575535390785 99.5844079573552
79.7170181855209 79.4528447794527 52.6879797806876 50.0303076717704
49.2064066614865
CHAF1B 58.1027665533767 64.4837166864084 49.4573631027613
43.3078542962968 92.0958762013613 47.6803619768589 106.486195938039
66.0303950772736 73.6965494979349 47.3791873993182 45.9104478407175
46.8129509799753
CHAT 44.8914371498528 44.2105195734073 41.0356368374708 43.4410741327254
42.9655396732962 43.0038142244237 44.6809101024392 42.1860813954352
43.3118137410238 44.0527419856743 43.9975207836855 45.3763275588341
CHCHD1 103.458867136383 85.1207977556852 81.0295607064541
88.9113618165337 132.400225103653 86.3888090084564 98.403191200835
77.2048580540589 218.165286111397 63.3872214856708 61.2529834995512
95.491982090349
CHCHD10 625.620497724795 1035.67547271468 1393.24135330226
690.568858101531 1423.29307392266 1099.52344340557 787.671342652389
917.179747195874 1466.88103496067 1214.73798692268 1771.43541011426
977.776395810338
CHCHD2 2294.75523373329 1907.47560253019 2621.65272077828
1411.12613289991 1956.75875151802 2474.22199172028 1749.06320703181
1752.11152588494 2323.81110653289 2067.66516256598 2440.77408185413
1634.61795848352
CHCHD3 92.7427427543974 70.7702047405718 62.9967097752591
83.5895816521251 112.506613718654 101.46584765758 97.299696284253
74.0831311663524 102.063594231253 69.5080454202415 66.1229691315616
99.9685666586407
CHCHD4 215.15351875081 202.494545985006 138.81810375553
137.335224748204 285.322305931794 207.726011653345 277.665554240228
508.474794786176 174.021676013865 183.799353414581 184.105396912056
194.862177434028

```
CHCHD5      190.570409327756 276.214079735778 386.787221140343
      255.636379050741 220.79051545138  201.163076338483 259.127235335036
      136.913434348036 394.437597863822 361.861166927682 282.196234758364
      297.475572902965
CHCHD6      130.985178898633 94.9831937714438 96.3741876853801
      162.151812425949 225.827961496781 143.679866958604 147.945429978177
      112.328675926999 134.749803376372 106.049736669661 121.084398596738
      119.124583755587
CHCHD7      88.5638310461364 119.088986733238 85.5839487225537
      62.6048727240291 123.729687262037 135.29225447532  78.9203273221608
      53.6782125131958 93.2557602398938 96.6027330566908 89.0684909049451
      100.883604376864
CHCHD8      71.5484567134601 71.1595608255838 77.9085946827079
      73.7124988616836 122.0305509548   80.2112932898502 110.589130460258
      75.0377156190383 85.5095013644374 57.0581769379589 59.1443775199516
      91.9534828522538
CHCHD9      915.102542160713 917.631567606105 581.706970365754
      678.996864855592 654.682263833024 568.371815312895 746.252816568691
      710.831173303701 886.397061577531 418.693470287372 412.421643885499
      511.792908607692
CHD1  153.310447977094 111.72494102259  104.037949992458 291.021345839327
      125.424626287985 154.355197155161 138.349952332963 143.952224627989
      123.077231445204 164.580141651883 113.322929203297 165.797721197832
CHD1L 115.894467332637 83.5274372175811 105.726287089142 100.483113678344
      105.187381311145 95.2367177680328 98.5663841503803 131.67159377585
      88.0055507173538 92.804679821289  86.8330533522204 92.9600461444587
CHD2  53.4921736109774 53.9275079525238 46.5113110023498 58.3475983630509
      59.9326265577462 56.9320717885002 62.4317959077388 67.8061298261394
      70.178974380507  49.8393069675192 47.299647867533  62.1718158914638
CHD3  45.8574226904273 44.3977035913759 44.5544727111028 54.1952533541951
      47.3791873993182 40.9506062495231 47.3661873039785 50.2391482329046
      43.2163597439147 49.2317401696588 50.3672856609022 50.0571060701893
CHD4  688.612748336336 447.709872889701 592.010988342149 500.168833003599
      518.074074000609 580.109585240053 706.087945508248 670.958499409833
      871.238169066998 640.944514118411 657.59007878546  319.502295093307
CHD5  41.1523487516364 42.2577689927563 44.9758339877851 35.1451084093692
      43.3332476888305 42.157627240486  40.2154497375715 46.0633179231318
      37.6383530120621 43.1839109702714 56.4567728276775 50.5511827666954
CHD6  50.0466349801009 56.6617674371349 55.3275365686503 45.5869891570251
      42.8510579683751 52.1408852421186 68.8005532048281 64.4801713556664
      56.432280646473  70.2565873027242 63.2684764441521 59.0894921452893
CHD7  324.936044300546 210.255784687052 140.992072231913 166.064317271991
      306.294404016622 102.980622026276 198.729330487352 178.249889756228
      214.774035015293 182.37433006793  173.328468320432 159.827302305842
CHD8  522.49156295512  204.903470332387 243.549194928524 206.28819203182
      193.897678586782 194.536619596804 228.907776306695 320.504100222566
      160.425627898607 316.745819100367 411.857557412386 270.487135967976
CHD9  289.213144287085 292.389429869034 379.933650669101 350.917244438732
      135.829530518199 351.700618429121 306.578554914328 483.313014703768
      386.170232791927 1005.45493369789 671.959673037066 545.514314651238
CHDH  37.3463875295726 39.6123051184545 33.1559412791294 44.265834798964
      43.2484996523549 37.9951997351435 46.7100796279384 33.6816578730693
      43.3744503319109 36.1550293353654 36.131485826118  39.0536056496869
```

CHEK1 102.25509551881 114.965999573704 74.6901522573522 71.3932787152381
243.489225650886 72.4651758215622 252.487015579489 156.844010597219
151.047739254277 44.1379638573455 49.1067959632109 56.9379721393904
CHEK2 75.9099205260748 92.3307175266839 80.943990104641 65.3843877368233
112.919063481867 67.9778438150245 109.811921160228 70.5992463788381
118.288549974348 61.5236837309467 59.4465684293847 75.4935574755043
CHERP 82.5344501505114 94.1289383735162 82.6983700200815 71.1583251698218
123.978615472736 80.5478617156852 111.714563124738 86.3237770387663
113.026772174726 98.9657264176664 96.4278951689874 76.2533853403813
CHES1 261.653821192385 106.160416826673 122.405413080836 332.137622104526
130.184962393833 583.915973546284 122.720768249799 127.961989181945
236.565670642447 438.50123471613 555.356799014777 601.139894733857
CHFR 596.84269596976 488.089090043145 452.883337614277 561.855286945959
295.127975044241 281.774690337889 452.139416552986 150.985239447761
432.669787408904 373.172504097546 438.681666410245 258.589426470951
CHGA 39.1590964332473 46.2286164776607 42.8270038153139 42.0102671859606
44.7829632472754 41.6590564937735 45.7452456977857 43.1567024035183
40.8970401536578 43.0919221375514 37.2892641289258 41.7575097298927
CHGB 32.821392987958 38.0226905622357 39.8299340037009 40.646702696033
36.3390947880186 42.0574848021044 41.706879587091 40.5158867964016
38.9834959368496 41.0057887014368 37.9230440150891 36.3454320848591
CHI3L1   39.266565827316 45.0905928998779 46.3411353377615
49.9014504704021 37.0526522140242 38.3418933808844 43.8206425661746
49.3880946790306 37.4540772310118 48.1392888470269 45.2265319826548
41.2437378488025
CHI3L2   54.7786516309154 44.5152708955623 47.0055981216687
165.311139930529 45.9104478407175 55.1547682957918 44.6809101024392
51.2623287419357 44.9645354363967 46.0713306971594 44.9081227425497
46.4499258373233
CHIA 38.9271399577966 40.2493681589104 45.2313723727673 39.6705473876526
43.004943518025 42.2577689927563 40.0052661202207 40.8833572570383
39.1790517315259 42.5664883002833 38.6662194645633 39.3575468494941
CHIC1 40.9558083259517 43.3972849968032 37.9482157381008 50.9493788151501
43.004943518025 43.2805590023412 40.5585676679482 45.8176925260962
42.9413736814915 42.9358255168086 46.9246510824681 45.9104478407175
CHIC2 294.443061798191 379.933650669101 203.82788889213 524.451156145427
339.601844139729 338.053116855608 286.611037700479 277.014526407597
365.792144095604 244.736442646499 250.210134743225 311.668075784386
CHID1 42.8036731479932 39.6123051184545 36.4564050256915 40.1804147506786
37.6527901109005 39.9397419279057 40.0052661202207 43.844855336215
39.9444191873552 42.1622837416589 34.064698871541 40.0557788909923
CHKA 79.6133633483649 53.2398469202731 72.9051985681184 79.8701900696361
93.7260389087192 65.0774755750423 83.1614238528477 75.5510570035251
113.905373770586 118.651855622915 100.535211780514 99.6695996119465
CHKB 142.957149812186 148.016994658777 301.941928552396 157.830518171698
148.817606651444 107.91849682084 82.3328061091245 101.197890058764
88.0877175295544 247.298165265093 188.943404504867 157.133627517432
CHL1 41.7835997867848 37.5469247328848 42.1901757067073 35.6027791404152
39.7162631015511 39.266565827316 41.9918605927928 40.6723334103791
38.0213051227859 40.5090849993014 36.9768361759916 41.5132248356707
CHM   73.3270037834227 93.461049073831 93.5717680539357 121.510087314197
71.193768715137 101.516595046503 97.4515987591398 88.3335649692467
90.0420794458477 117.586275152761 103.423003544792 105.197692490363

CHML 50.0466349801009 48.3699741879723 49.8344973836834 67.8978240198575
59.6922302604696 54.3478800757394 55.2088411137193 62.2982003555312
56.432280646473 55.7930787690756 47.299647867533 54.9919060580109
CHMP1A 332.659496278775 273.032063533642 316.831358204198
190.242383482058 194.703024752864 184.989940464859 451.364230598722
236.759651096748 286.538414026118 380.736562250846 362.615047009897
251.4927207704
CHMP1B 459.546279672649 724.881002237334 470.676006277762
845.41973083225 524.972936438175 370.917477324773 753.491584605447
931.401929277001 413.990148966099 207.600413958886 317.669452297159
428.958127252533
CHMP2A 138.151998239414 182.091945578277 266.439343650315
302.50850157207 184.048982286364 242.673166257518 193.897678586782
215.175765091556 300.762511215441 184.859183550225 198.15553991394
250.176074707634
CHMP2B 171.021755568144 300.560071753181 222.957344929381
244.837105178705 271.50380898418 306.14500148663 308.841311505431
216.194306146777 264.659866487253 127.567860003773 148.781721594419
310.900527198512
CHMP4A 348.450810225807 125.027865666372 203.373397813051
207.741669973713 158.17598244564 216.526205739151 170.87055409699
239.070294068861 210.524799041662 281.016474354764 263.330471155421
289.533968432846
CHMP4B 395.634889855838 367.158502379417 323.002018912848
387.867128019374 341.535750991033 702.737741114234 555.727708387209
411.431488342465 515.007705025879 512.951288394692 605.338871441599
522.651572868385
CHMP4C 55.3153799233744 90.791818798144 50.2869791217819
35.2143190355414 40.9950639693255 35.6125439901743 44.3440839686558
45.5581356216957 54.8946088631931 56.3441077510744 58.7389869173495
67.9141812398066
CHMP5 212.822978676596 214.537530518962 164.847648206552 189.137593177772
177.82359682336 209.31241212773 179.158053533144 184.269633116351
130.65627609817 99.7807691901691 113.726153436343 288.923207993626
CHMP6 75.2923395421694 100.370937669662 85.0342118119783 95.5022104475922
62.1836961153924 87.7460859694899 108.263706890599 76.2258667419953
124.815448107572 100.975810139572 99.3142021810133 67.9850517608624
CHMP7 62.5528124447176 52.1062000766427 55.094385444315 65.3191216270241
46.8674366115656 68.839885767937 59.6780848951767 50.0526763693329
62.5542376788358 70.4895190282848 75.4732674021193 60.7495043977707
CHN1 179.522572239758 89.7554896946341 100.708709225818 160.425627898607
89.6313467455698 68.4906691348625 78.9900928241723 133.893108529325
48.4869909888679 76.2533853403813 119.747995520476 57.0082102559228
CHN2 47.7267190613906 57.2871072823547 42.1204847421254 66.7106881373359
54.3736840324687 83.5274372175811 98.8657527308305 97.1634390603131
90.0157406552551 91.4990733687068 76.7806009547111 65.854213724711
CHODL 59.5000730792772 45.5842286511645 43.7630587657205 42.6913548400127
44.6834648302383 54.3478800757394 46.0228732507872 52.9813204449021
66.0350709576651 61.7502624458447 58.3586374083245 51.8562635270111
CHORDC1 55.2182004838121 57.0575084219334 46.1312573416873
47.5733603903505 56.359578588344 53.63657997649 63.928488869459
64.3669451425581 62.8635815439291 43.6033221838149 54.8532558909227
60.7985356140269

CHP 167.578081645481 160.425627898607 104.704892803169 132.760054781304
167.824386984618 97.152024864444 149.076166432567 188.789206373516
167.198128794195 114.321083441061 128.070047614488 171.360248172138
CHP2 40.3478825593224 42.1607322031054 146.087016079575 42.2496337895968
43.9450023643394 40.1428591998404 44.2166118829877 46.1673159982664
92.8206287414481 142.731630650904 109.076358658606 148.570371099987
CHPF 621.980260052085 154.869301720717 159.046483157634 203.759116151774
216.821078573868 162.932479392087 147.010700944686 279.125471103327
161.439879456216 227.535855856645 201.657389063895 164.642060754649
CHPT1 78.1313732870053 249.101015988111 150.387474375456 128.157032116902
95.042308285667 222.126944340681 74.9059650336395 100.616027485975
128.230543301378 91.3418781023824 144.868987794412 142.124053129543
CHR415SYT 38.6454098044474 38.3322849790657 43.401782064778
56.7927450229377 40.2449343247539 37.4782441311626 41.0381860307445
38.745552403895 43.4656637536244 41.7405682794166 41.9908237694988
39.2469742642337
CHRAC1 91.6253858792439 94.2182728193924 66.5285423077808
70.0402992940342 78.7320333941149 99.2749350936362 63.928488869459
63.61404854326 81.6508259258669 77.4999637278723 80.6550905475507
64.863894209343
CHRD 71.4276813927272 43.3458769998118 44.3344900481112 57.0629653735694
46.4343327861696 62.843734579668 43.6753843488695 37.6557714476853
41.7627910693483 45.1569741609597 49.9338905397767 38.9496515344837
CHRDL1 40.9558083259517 39.2907645923217 42.3887531663733
42.6913548400127 39.1650142708257 54.2978574294868 35.8227939208521
41.5934628038429 38.3418933808844 40.6533975461148 45.9938357766653
42.828737648529
CHRDL2 52.6960621424156 53.0383634135393 40.4882741869718
86.3663084195994 37.8679241954337 80.5478617156852 42.2577689927563
44.4031868028216 38.6322728994674 44.3648993568405 40.4504608195666
62.58750768974
CHRFAM7A 43.8674747059508 40.7623977968067 38.7745734037492
41.1531238436707 37.956732373465 39.8455060654531 38.5519892723521
39.5893773029164 43.3744503319109 39.3866822630357 41.8645741488184
42.4147793282701
CHRM1 35.6111578834671 35.4484652445078 38.6727842796202 40.4359177791566
35.9058393060637 32.771863438151 37.1643693306382 33.9687743313429
36.2733735494625 41.9666541498638 36.2435874904689 43.3744503319109
CHRM2 40.7609952437622 39.3688265853724 38.2593044488288 42.7628396725929
42.8510579683751 41.554942319225 39.2730372355057 41.6466169548846
38.2874316439311 40.1508555768978 42.59352468383 42.1957516560122
CHRM4 43.5710871617107 40.2493681589104 41.528370959196 40.0698728584484
38.745552403895 40.244271403831 40.1235656101329 40.7311497914871
44.8003345643334 36.6893251782922 38.8507804383261 47.7962548556026
CHRM5 41.3387790895716 36.89524015739 39.0775870687758 45.7397504535169
38.9496515344837 38.5252456306656 43.6753843488695 37.33030683349
39.3750969348308 41.5435611403119 38.745552403895 35.8429598823818
CHRNA1 69.7908294064179 46.0364000143537 39.991353369336
51.6974465478564 43.2869475999748 38.9378717994178 40.1572444265517
46.2231598401576 39.8022382792104 39.0325254636537 37.7882601416133
39.5806465938526
CHRNA10 40.7609952437622 51.9421032049606 40.6737160001291
49.3654837541353 36.08956004634 44.7804080899431 38.6243997507646

39.0620487644635 39.9444191873552 44.4652605017595 38.8507804383261
36.1647506311964
CHRNA2 45.4799270968969 39.0102066700839 40.6737160001291
40.7481155008596 39.3667161654772 36.2768299545794 37.2892641289258
40.5158867964016 43.3744503319109 42.5664883002833 38.3684202018524
40.2669497398702
CHRNA3 46.8141313516997 43.2649781354792 44.0451487108213
47.27543600687 42.5449473689701 37.5469247328848 46.1634822993009
40.9861485852278 48.9540527545343 42.3413611855132 41.5685493982342
42.6758568421347
CHRNA4 45.0985088502363 44.2105195734073 47.0055981216687
36.7892997857376 45.8051846088552 40.5281496800812 47.7390230243778
43.6434576713809 38.2215591930186 43.4885245598939 44.3763859390287
45.4799270968969
CHRNA5 1083.50383916482 530.314249832491 734.705859548078
728.257836227637 486.757854744053 401.202348352506 991.630429625437
1320.7894611262 501.585246203512 1450.53108586858 1411.12613289991
900.517711546397
CHRNA6 45.0985088502363 45.7979032939211 46.6588655218735
54.6089451252049 46.3150221024046 49.8472708021525 44.7255207589606
52.7787010032051 45.9227107476843 51.9547984751408 47.2134341420411
41.5536168238478
CHRNA7 42.8036731479932 42.1607322031054 44.3344900481112
42.1273928203021 41.9842695818285 46.263826584899 43.9450023643394
44.797320243931 52.5772854102326 41.5435611403119 39.7342718293432
43.8575940017119
CHRNA9 37.5887129572848 40.3104838541906 39.4250105372135
44.3440839686558 39.3667161654772 40.7616966228404 42.357910063876
40.7311497914871 40.4518431978241 37.0740019672788 37.9230440150891
38.9834959368496
CHRNB1 79.2665546280232 69.4820178305563 85.3718124658316
65.7321440516177 68.4323624797175 71.6050174762551 82.5050369860039
63.7002949745101 64.1267046511632 75.7801512652102 70.6138659908885
82.3254961129575
CHRNB2 36.4564050256915 33.5924201549249 33.6816578730693
42.4580504587581 37.8679241954337 39.6251842352088 35.9894133432376
36.43924066428 36.0741672064298 37.7315666632629 35.3362091871364
37.9607839934735
CHRNB3 38.957687788868 38.1312950900467 38.4043916290561
36.1562979552173 41.9842695818285 44.1737502492846 46.8613212048981
36.1375121409002 34.8552366193358 34.4875013487777 41.7272875295469
36.064718696392
CHRND 43.844855336215 45.1694246393676 46.5650556481721 36.0481934423077
38.5512429290626 38.5252456306656 36.8369172900902 40.831207953647
40.4997186069494 37.1275334308172 37.7384958256497 40.1700117452801
CHRNE 40.9558083259517 41.7337506166619 48.8941062980393 40.3180310664156
45.8051846088552 43.5752393599169 48.3411413510851 47.5950227477807
42.992884918158 47.3791873993182 48.042366934301 40.3691324547049
CHRNG 39.7576136507593 36.2103905554984 40.301483914446 42.0102671859606
44.8897737954573 39.9397419279057 39.5496902678788 41.1575777497576
43.2761454096265 44.7958122466628 43.4709415392696 51.5741598315033
CHST1 54.4384603094793 42.4493032770053 56.0477607422828 59.987045425047
50.3713170502557 40.040778349137 40.6750540874299 38.956280281655
53.2046867327331 45.0155543555707 50.8826620053386 45.7562276914441

CHST10 63.1291723738553 52.8796369426118 47.0055981216687
66.5921967967793 105.018199901228 76.4439037944547 85.0342118119783
96.8945937999801 68.4229528414965 93.3216420803293 88.2314365506494
55.8775622338124
CHST11 35.6111578834671 36.5219488315447 32.5052327840481
35.7674462337723 37.5556299264699 36.1190722948669 35.7055410599146
38.2782436497441 36.0741672064298 39.2623423164488 35.7953070087288
36.9976231206067
CHST12 186.64040927334 93.2630594602634 97.6663462144457
124.350816245276 136.272019225585 91.2681347227088 88.5907320965949
88.4439480366515 119.947688643528 187.923050034941 152.031470139119
127.093495914275
CHST13 47.8249696958403 60.1102859845739 46.3411353377615
80.5537984388921 78.7320333941149 56.6172748471831 43.3262148375525
51.1694248620208 37.7384958256497 43.2988017098387 45.4799270968969
36.5474283500601
CHST14 127.352997770688 149.494335028498 100.305262467668
96.2385885003071 110.729504563799 103.734064904898 127.418173390084
96.213791081731 165.598854278557 221.450737998571 160.360785581153
127.567860003773
CHST2 41.3387790895716 52.7493349111555 41.3366779545815 37.4223289687135
37.7576900881741 47.9503724044453 38.2933870238743 45.2806199505029
43.8482802038826 49.5265949137207 43.0016969050339 45.7562276914441
CHST3 215.033807819308 75.2824161164996 88.2959958615748 90.696780047049
75.6471706262645 107.60291670075 73.4820932957859 108.700838142885
78.6048653630165 121.806692423791 81.7500436717038 100.423344358771
CHST4 108.558977253184 42.8542642792862 40.4882741869718 37.0732977341185
35.1411239266238 36.2768299545794 40.2154497375715 36.2539950294089
39.6609238036815 40.2112371512838 40.6373400186325 36.449101775775
CHST5 38.9229928131225 36.3110701990588 45.9227107476843 40.1804147506786
40.7779767565889 43.1180113655693 38.977631784251 40.4359177791566
44.9645354363967 36.0481934423077 42.4580504587581 38.0452862258803
CHST6 61.6544855697851 42.0709558258414 42.3536549114165 52.5307002738469
39.9697332660833 42.2577689927563 55.695361644141 44.293922970134
47.9035532886869 44.8997235283254 43.401782064778 51.0013344286851
CHST7 118.358996104366 107.513442725922 101.403739410832 374.978401470437
99.6821042055758 276.666571115104 130.72385089567 72.7596094170237
92.4839055864605 146.691463448295 110.440072569721 106.20479137406
CHST8 38.8561375693335 41.554942319225 41.528370959196 40.8681871019812
40.6723334103791 44.3648993568405 38.0598374201407 55.6904617926772
48.2123120250343 38.3092241935352 36.8282383070068 35.0222940937513
CHST9 38.304249186058 34.2618170189676 36.3604951341921 32.7960913111995
42.7982502551634 43.4681646416585 39.2533668167483 40.2382908228517
40.9901805728485 44.2580151149956 45.7328341667151 49.7336769474446
CHSY1 595.790464730847 231.637751095711 208.804071011518 491.8684831079
391.658764737446 311.432395849636 347.145902252055 443.863346367342
235.294634126766 345.876877384089 289.780414673585 306.578554914328
CHSY3 106.356074933606 48.2220547806679 54.9071823326024 61.3747706295648
51.940083408061 57.1752846228778 42.8684958525042 55.3122077514658
55.5086026637973 55.9283039581662 71.751972433516 48.7814131120945
CHTF18 117.439175394651 107.654589391726 100.645556491457
51.949834638817 161.180847265279 63.5738862549306 132.090336898032
80.9092480872499 116.576093937704 70.4895190282848 70.8670844767965
60.0940570991178

CHUK 94.8260360398356 128.146059846414 131.717764850211 234.308728996238
135.41852974791 139.554519110944 135.012695962236 117.488737569957
117.893268074013 82.0149542477384 107.646103699516 142.812241748319
CHURC1 108.649424858333 107.951009163316 152.920586451578
228.809697062726 488.908231529696 407.336051853854 97.0155195562193
80.8333046859586 415.251775687794 336.44013572926 375.697071471585
192.216758313982
CHX10 34.2180669723984 40.3384318592985 40.0998167098057 36.7892997857376
40.8873907150245 38.5252456306656 42.357910063876 38.8495095797881
35.860430241131 38.957687788868 37.2018803984555 40.6614700944853
CIAO1 317.892699374852 371.709014310561 265.414737742846 263.125290391542
277.588692595406 287.247399926139 258.446095955014 272.663785805238
315.276749671783 361.023743912857 359.241360412129 348.65230953701
CIAPIN1 104.832753086298 101.523741547876 99.2504397421242
72.6824533717231 58.7460488758053 70.8674195845729 83.0647477737971
66.9340967279385 142.748812565309 72.2443746456494 69.6097529955737
89.9460400399039
CIB1 4752.94498024732 3021.24285327836 2513.15722262176 2448.64580552439
1718.44944271916 1384.80615431656 2771.14077845128 3800.19142219719
3905.8371644287 2313.21212353993 2405.8635632802 2091.0611913538
CIB2 63.7818442618767 52.683711208052 43.0971787292711 48.309866878365
61.6452971581503 47.7556758818053 75.287377665837 48.6441988290952
58.6431245059655 49.7259371799014 60.4620008899889 54.5921287593554
CIB3 35.0202257459026 43.683792219729 41.0356368374708 42.357910063876
41.7929491793697 42.9695585740813 41.1507980856436 46.2779564481158
42.992884918158 42.9866214102525 45.2265319826548 44.4475838947656
CIB4 34.6977013306396 39.6995529770239 32.5052327840481 34.9221408381038
46.6397391251762 40.7616966228404 34.9344532055397 41.9315419791594
38.7298222155532 37.6383530120621 38.1835938174625 35.9604262434141
CIC 113.444217634165 74.0614295574729 82.6983700200815 84.6435656850129
101.277664841914 99.1667512831715 88.5907320965949 102.49785018292
85.2598847559951 77.6056319020116 98.1495112518979 84.8808332740321
CICE 108.251353392321 117.389308836013 147.557451530784 141.206849814363
136.806769571262 153.515845356859 127.49459343016 116.434267173815
117.072965367702 79.4158467034017 90.6191520659197 131.57403028894
CICK0721Q.1 45.3872406432794 46.4861258030402 42.640829914102
41.5464765641928 41.2740822675592 48.5835484742456 46.9929836306816
41.4508241771315 45.0997987980157 48.7414853386615 46.1673159982664
48.4535991392809
CIDEA 36.1831439734485 42.8971749680871 48.3609527732104 37.1317006162938
44.3292391134969 46.0364000143537 38.0598374201407 37.830876898596
49.0655215909632 56.4553747079294 43.0016969050339 49.3731715216836
CIDEB 50.0466349801009 74.6847594297153 76.1493261430901 102.971363152434
48.6224651100733 65.813547096222 75.164719839017 41.8417024632349
37.10170285405 51.0055825931636 52.1788489555942 67.9850517608624
CIDEC 43.7920955808549 39.5050383901884 40.1985790927248 37.5764485234171
40.4639532172338 103.063302515808 39.7576136507593 42.6450179841129
44.0519245668737 45.9426591985894 40.8681871019812 54.2677073532779
CIDECP 48.9906383294199 47.8400694856726 44.8872784170036
38.6414316877471 47.5647821785529 51.9421032049606 42.4939802311136
51.0724630072723 45.3756389170606 41.6373771565199 46.7386599624801
44.1452943804044

CIITA 36.4564050256915 45.9266745571724 39.6451483836089 54.6089451252049
33.143488395779 43.8582747295766 30.3554137634399 45.2806199505029
44.1519466943185 39.2623423164488 37.1059647239814 40.6614700944853
CILP 271.601892365742 41.8848959280916 42.3887531663733 44.265834798964
43.1252272136667 878.863554510943 37.3508690072156 46.6384636367629
41.1742244762318 426.622204035624 268.072653271299 347.003621791919
CILP2 51.413025495745 36.5219488315447 40.7620472083159 36.264823544143
51.6869155634183 38.8336427948187 35.1307934330047 52.407151732257
39.0775870687758 44.3648993568405 35.6041926503637 42.9887069977696
CINP 66.0303950772736 45.0115870036541 68.8098607236981 64.2130157241811
50.5110359971301 54.1440452292081 51.0013344286851 56.4779645313958
63.6222532132037 49.6203214143464 50.8826620053386 54.720204938924
CIP29 675.832363405287 511.067537752967 443.863346367342 692.630044242985
475.466074293565 585.054570630252 768.354209323884 1208.33498233518
827.365231733511 755.106456358949 628.84789420227 809.577874019028
CIR 68.9745034946434 65.9098543816057 59.5444359507702 102.365615739399
48.8381507187986 65.4643755537228 52.0942589368478 64.4156641487994
65.564930068976 59.9354531144375 63.2684764441521 68.7753456118815
CIRBP 805.590413262364 1566.12726152736 1891.37245670852 784.268074988084
1723.37649434054 2498.54878472152 814.991871217268 1046.42391243532
1081.97502255142 1693.5377672895 2067.66516256598 751.150916058018
CIRH1A 340.105993325165 374.427492246826 529.430648638878
239.792011765323 431.864508795316 382.538365722661 519.775555680262
446.162602155964 389.686780706341 444.937258895152 366.615979176657
358.402309513947
CISD1 501.585246203512 565.72800641089 503.529187388654 549.117045813593
589.685739519627 706.087945508248 251.2365884576 449.701353531924
679.810781641519 617.539783972955 621.489785652602 771.554891868456
CISD2 69.6449298794042 82.2766822827861 81.6486527718661 91.3967044940546
77.4769634299218 87.7460859694899 72.4651758215622 72.121833189378
114.136234102574 63.1091746640396 62.2965257278236 101.017461500409
CISH 52.4179224871914 50.4022543872993 51.4308501933893 136.155022329495
54.802736966312 67.0290776715331 49.3854238365325 51.1694248620208
50.401623258584 60.0372015630618 60.4620008899889 49.1200355938198
CIT 40.2543341073001 41.7337506166619 50.7502704822135 34.9221408381038
42.269054475455 36.4986986135769 36.7356360093603 38.5457716679071
39.0775870687758 33.6188027862739 37.2892641289258 41.863927676552
CITED1 36.9060997041453 38.3322849790657 44.696023817801
36.264823544143 37.5556299264699 37.9003288112261 40.1235656101329
34.6977013306396 38.7298222155532 38.528621456378 37.3687900733549
38.1421709520616
CITED2 349.371668774717 795.381998233245 168.7696164772
265.520773157991 186.54041506387 285.689076149891 267.70610731202
156.337202322452 211.137455821181 148.358988653522 378.626656077546
221.76904201992
CITED4 1858.94179312821 185.563590013028 346.621778258188
78.5141640924736 260.618790701505 187.689343320587 219.036267470696
250.65667565007 155.018308238778 196.427458710993 187.01452011215
186.54041506387
CIZ1 46.0920218698606 51.3084273211359 56.0477607422828 42.0102671859606
49.8304479381623 52.2089306840125 64.0985088513857 57.037639245179
57.0301170427216 58.5087296620514 52.5930185400362 52.4992300612807

CKAP2 65.6556779936059 125.503897988367 76.3583555666815 73.8480604979804
99.406307365363 56.796621176582 98.2843999216669 70.9266654533052
111.291087721139 61.6670071424359 61.0094935318105 57.6860752168769
CKAP2L 88.0979122006842 109.654559065946 85.5839487225537
53.2046867327331 185.843333258735 59.4949177415847 180.128769312512
193.981651711507 116.576093937704 43.7303813515864 41.0050831223107
48.3648494350286
CKAP4 760.745188431719 1135.43644148537 862.031722874406 733.750875664432
632.577797921655 442.080188140864 395.875593488613 438.50123471613
813.242005949542 550.861615347663 538.716947884528 547.729200972776
CKAP5 309.39599482851 365.517719684028 288.816567137193 235.925374041038
441.5479786095 321.550107822925 433.637087338002 302.422475486532
511.067537752967 261.138426747953 292.93036571522 266.178061771978
CKB 313.399524593316 618.892379887961 174.117816184497 60.1837189855762
270.543988412382 1111.13061382818 156.56745342836 104.950400251642
329.978705095448 1337.53997230271 1125.31394383117 365.860501246879
CKLF 381.533380474563 276.699609951994 496.791391622565 908.328776521114
154.775578306203 212.664574366477 422.931891494195 353.868749867979
250.457603010506 131.319040057576 137.10896135739 298.420882349382
CKM 44.3355304829989 50.2163892855092 35.3649449469275 43.4410741327254
48.1473938886239 40.650742261428 55.9276266345493 45.5581356216957
41.9635525160083 44.4652605017595 41.2598458726002 43.1805016690297
CKMT1A 66.4399491500833 162.000275136397 46.6588655218735
46.3203995372658 217.848539722853 64.5187138759372 213.933347509147
224.509915754308 361.57814355509 74.3981029634178 109.23113111292
102.24202987437
CKMT1B 45.4799270968969 85.5135159896819 52.1242102345408
40.4359177791566 120.465621774337 44.6524308640784 98.5663841503803
142.714450804595 188.13862794196 60.6266092582071 67.9599395822006
68.2309359831273
CKMT2 75.6471706262645 37.10170285405 43.3085358214154 42.4580504587581
43.2484996523549 100.423344358771 49.540449125788 45.1714698794331
45.0017158586743 68.9115937148858 65.6010220593397 59.7999569757275
CKS1B 631.110081413363 549.67905283016 524.047008889711 188.887559923238
1068.10627239579 322.888035865453 753.976423227915 443.581460031166
608.579654909865 178.219279777774 138.189579100482 289.17237603899
CKS2 487.065758294354 398.049525642349 167.075682925069 466.074595343541
305.786520619504 112.421316052392 422.237766234202 351.8239141662
415.251775687794 76.7630128194088 84.1922656126304 90.689813277023
CLASP1 230.787791652978 254.023393889525 211.109957714241
211.227389226633 175.362103430164 313.921236120658 191.426587373391
235.169335305672 218.313812956524 365.517719684028 379.189486013898
260.426488908824
CLASP2 63.9851732498913 88.0720840588632 96.3741876853801
78.5141640924736 117.260454792593 109.221269056557 70.3935694362568
67.0904616289706 69.8899273527731 58.9069025298754 67.0361353184222
71.381069076765
CLC 47.0940613507321 89.4111455826308 45.3974838013476 117.8523906126
42.7982502551634 47.2927436772465 50.2016912396771 46.1673159982664
40.3316153483289 42.8936990772838 40.6373400186325 44.2527566343661
CLCA1 37.3463875295726 40.148780650831 38.3166554965434 44.0451487108213
40.7779767565889 42.4580504587581 40.5585676679482 34.9580369221164
46.6944238390932 40.4136535208406 42.8263096141654 46.1592873342534

CLCA2 63.9851732498913 152.456314990408 2978.46032143413 54.8412943008635
40.0599807618105 67.8208976798364 43.0930293831497 101.39385858723
162.895543924742 56.9463858443402 50.6473771879221 53.655233033193
CLCA3 48.5835484742456 42.8542642792862 45.989212296035 43.3078542962968
41.1868052124344 42.157627240486 41.706879587091 47.6952179922944
43.1805016690297 46.6598879466655 40.0766543123257 40.0557788909923
CLCA4 35.6111578834671 363.67768546508 474.729653426958 44.9370339275041
76.6914642534934 53.1052228246467 43.5627649099623 144.324036829708
325.676668250281 320.889609846069 367.451332073134 1078.86166154061
CLCC1 76.1417635449123 73.0070079786149 78.6602721596257 71.5948944013406
68.0414563597154 66.4015820814547 115.98265270452 101.463033319045
115.597550918377 60.5390917151449 69.7215372275927 81.5678694264684
CLCF1 79.7564155881026 76.6346767983285 52.9111155236961 76.450993090133
49.7235523248653 75.2735539010032 72.8653205184014 104.351256270039
66.8632294055679 44.8997235283254 65.8220821345163 56.361708620372
CLCN1 35.8208542506595 42.8542642792862 41.0356368374708 44.7980740332174
41.6806984441849 42.4580504587581 37.0564573649223 34.9580369221164
39.3750969348308 35.6208308220005 41.0050831223107 41.6550823405646
CLCN2 41.4508241771315 50.4022543872993 53.3127618245672 50.378948785456
49.8304479381623 41.159706131351 40.5585676679482 40.2382908228517
44.4395580493118 43.0919221375514 46.0905079010237 39.8497227447888
CLCN3 104.763913120468 163.585662735512 138.189579100482 76.1682394916873
112.311249826645 83.7971204489995 112.805898501551 103.071727621257
126.274897979594 128.365609268093 118.614027673431 168.407377937447
CLCN4 41.5421429082231 35.7393138893354 37.7384958256497 39.6292788039793
40.0599807618105 39.6251842352088 43.5627649099623 42.8482181313226
44.0519245668737 41.5435611403119 41.9083581529276 43.8575940017119
CLCN5 40.6559419616644 43.7644428186265 42.8270038153139 46.614092935749
50.7615314686244 39.2248354955045 41.8315602975157 46.8954576545929
47.798305839406 41.0057887014368 39.9070785037411 41.7575097298927
CLCN6 82.0124493733776 75.7427148831879 78.9701459806641 85.9636350040248
84.2728210732532 84.9970776435852 82.5708447805445 66.9340967279385
61.6353280670242 92.8579489575841 88.7928773498158 84.1030588938732
CLCN7 1060.64261592822 749.49934348018 1337.53997230271 1261.91478863258
790.786577596971 525.917089808641 639.778130093087 532.043503787542
639.778130093087 573.832332097619 766.647626178442 468.573956652904
CLCNKA 54.4825201506931 46.7279104095328 45.1899271235974
42.7489468897708 69.236458110243 45.3882138838137 50.7502704822135
112.328675926999 53.0357938671646 50.294087888475 46.3635803515159
44.2527566343661
CLCNKB 40.148780650831 41.3610369241157 45.4865173433646
40.646702696033 42.9090346219023 41.0582900711341 45.7452456977857
53.6210959113222 41.1742244762318 42.4594149893798 38.4689132737495
41.6550823405646
CLDN1 137.081911344143 1310.03307063841 1123.99110499679 91.9185612536409
2071.90741263566 241.222680673439 377.679662622526 167.999042735556
1683.24873205783 666.816487335088 760.745188431719 862.031722874406
CLDN10 149.185115531381 39.5595233162192 38.9790184198145
42.1679276329916 37.0526522140242 37.9003288112261 39.1539892144371
43.6796077697854 51.1554608265302 44.7672565800508 41.231791494452
42.0424641526302
CLDN11 62.6702927225656 60.9755936952178 63.5107036952143
63.1604223919703 62.1836961153924 123.908152883436 63.1604223919703

72.9074911694002 50.6750785584772 160.973602503707 92.9043863423147 95.1224420992193
CLDN12 118.923419920947 115.735359157053 98.4930941487534 65.084620579901 115.676508349703 81.3978207813439 103.913834330352 69.7729022157455 203.913062066149 129.754481922118 120.20612443357 141.447763308653
CLDN14 62.3862792065884 71.787754116845 67.0120793767211 71.4356132400211 73.8949517122417 65.7753115172195 64.9072331703985 77.2048580540589 78.5375244257332 96.9662967971995 75.8976034525022 88.1733775711738
CLDN15 64.4895418579465 78.59966540743 118.67727980117 88.4439480366515 83.8637731385607 64.863894209343 60.612413730593 72.692416146584 97.9305595485282 69.1623287497421 74.280612516205 65.6213260281822
CLDN16 41.9918605927928 45.9266745571724 46.4150464549477 45.334106390212 45.2556760873335 42.1046072075724 45.2830225385602 41.8417024632349 42.0126083977597 42.0621425894624 41.3553369544097 40.8681871019812
CLDN17 40.4639532172338 37.10170285405 41.1367478979484 40.7481155008596 44.0451487108213 38.4330755397458 43.3262148375525 44.797320243931 40.8970401536578 38.957687788868 44.2925355984056 43.2776458914333
CLDN18 44.3355304829989 50.0642388903563 47.3861758958576 46.3889523358338 45.6895223823001 45.8325181856483 44.1407835440612 45.3389970694851 52.8843091010101 46.2768056702424 46.0095710163378 49.3984571719129
CLDN19 44.7044140979108 39.6123051184545 44.0451487108213 47.481302504066 41.7929491793697 41.9543948167375 45.1017494688414 48.0582855095143 42.4540072551568 45.5776159432079 40.1742127891824 40.9683275553869
CLDN2 41.1523487516364 37.2810767789306 40.5734458834617 37.9875302985336 38.9496515344837 37.9003288112261 42.8684958525042 44.0390019242862 38.6322728994674 42.785429206422 35.5179217192467 43.5738557773903
CLDN20 53.3059058105836 42.7775752471711 49.1067959632109 50.2163892855092 44.5668141895992 46.574682061365 44.3440839686558 50.628152951493 47.27543600687 50.6486940895674 46.5650556481721 45.5800840647989
CLDN22 44.9896851005259 36.0366673493555 40.931294690669 40.0698728584484 36.08956004634 37.0180934800573 43.1931731665131 37.1317006162938 39.6609238036815 37.3179578037963 39.153226699662 39.9519547348106
CLDN23 215.290701336046 395.755223372329 305.786520619504 218.675464231277 133.893108529325 76.331185787832 443.722380814789 528.512401493184 274.731304343034 342.223067809915 252.487015579489 181.064239330482
CLDN3 314.006301513986 39.3999056968598 39.2707964550441 39.2780515621926 45.4751076037511 39.2985791324641 66.1940959364976 66.0303950772736 58.3564461855595 41.4397539024976 44.553780984244 40.7704587752083
CLDN4 48.8333989709933 72.0112676564347 58.7382738968248 40.4359177791566 50.2163892855092 46.4648868771662 60.7992483126292 78.1448136847078 80.8728961146267 55.1089864632456 54.8532558909227 41.0657892043496
CLDN5 61.6544855697851 88.8039637390786 175.798140574991 122.548989126048 51.940083408061 258.657801126493 44.7255207589606 89.3868257841517 42.9020321355927 129.550925624841 165.72797054597 137.897231677665

CLDN6 41.5421429082231 41.4508241771315 36.4564050256915 37.9875302985336
40.7779767565889 35.4142595025086 43.0930293831497 40.3357777530419
46.3203995372658 44.1379638573455 44.0409178714232 37.8490162439785
CLDN7 687.862452802453 1068.10627239579 597.996273468244 112.23915619297
724.881002237334 114.769498252688 1541.9572099378 850.205634919459
1059.63540694644 412.880824530845 627.914461428624 506.75912743556
CLDN8 280.202284365004 42.4493032770053 44.014441886238 38.042028333747
41.0476065156912 38.9378717994178 67.6767845246581 97.0589984189239
41.9635525160083 45.9004543328976 44.2527566343661 43.4709415392696
CLDN9 52.4518619184838 42.3550738181144 39.7697110924474 41.8778873678551
45.5809132232681 41.6178506967511 42.4939802311136 45.063792968927
42.846832824953 41.3641352453779 43.0710577955487 43.6434576713809
CLDND1 328.341232263338 409.724772942357 287.121456559795
464.671881584567 380.4099232546 701.168333137671 435.094968715663
322.594259063038 186.600931545083 413.918050849658 422.702925141293
668.961646543849
CLDND2 71.9062462001972 83.1308709614708 117.355761277966
95.7571966348067 140.309952483162 146.983805232769 112.113501370522
65.9143782252686 77.4186160113063 192.356440003154 202.154195547644
73.408861359425
CLEC10A 37.7935752088916 50.9140366501664 44.5134109184888
71.3132890220731 43.9937687851164 69.4214927857472 43.3889851892323
45.0972184964705 38.7298222155532 44.4652605017595 42.5824910860403
48.5123041297835
CLEC11A 275.407306413402 84.3672289945664 117.869393694564
202.360546931172 341.97050042695 252.870708814507 51.3064929551674
101.681822254799 61.9682048429354 138.521956104698 179.405972075294
86.2974544521879
CLEC12A 41.2773842727759 107.232979387172 40.8420925848403
111.703187941409 40.0273874927069 41.817587685188 37.8815832675582
43.3838505886856 45.6061814448258 40.8049408996274 43.1785624103807
43.0462952354436
CLEC12B 43.3638918705225 40.148780650831 37.1643693306382
47.2009420656854 38.1644569189523 42.357910063876 45.8980298854198
39.7509165247419 39.1790517315259 36.3612409063792 37.1059647239814
39.0536056496869
CLEC14A 104.986694941968 162.513675180449 310.900527198512
158.862722884436 97.8825824657468 159.747484721905 52.4111687156893
93.9880852748373 44.7058233759912 66.8026147385682 124.687212034276
101.505513341316
CLEC16A 202.627009037401 271.478148993789 377.847116683974
196.709620264975 205.187554095744 165.843728663632 190.375732394043
347.552309199761 222.81957100654 197.52820443061 216.358862796367
202.081933301612
CLEC17A 38.5007204246293 38.1312950900467 39.8299340037009
36.5393857306744 39.4693871570028 40.3357777530419 35.8227939208521
39.6474008369935 39.6609238036815 33.8984830800739 41.6336636285875
44.0451487108213
CLEC1A 54.1745577382276 60.6080172588224 104.135574209392
151.047739254277 48.8381507187986 85.8059456975413 37.9430145836712
55.1933004802607 50.2223403889892 47.1001757441025 62.1796792097014
64.4156641487994
CLEC1B 36.0144420013819 37.2810767789306 40.1985790927248
52.3004056941891 38.6454098044474 47.0027453663546 40.3302268293707

37.33030683349 41.9635525160083 37.4302011516587 40.4504608195666
37.4554876386402
CLEC2A 42.442231667357 49.9547657591437 42.5406888281114
42.4580504587581 42.269054475455 42.157627240486 48.4859552186772
40.1474543172332 179.767874494671 42.5664883002833 40.6373400186325
44.6484005362281
CLEC2B 72.613397507717 53.7332797903102 51.5292723244077
109.851796671234 55.9283039581662 73.8105012820769 40.1235656101329
44.7044140979108 166.168385855406 51.0055825931636 61.7191344476295
50.656598108034
CLEC2D 990.431472174705 728.679189702033 905.76958446454
675.832363405287 852.769974690777 782.639346040155 814.294297312057
1134.52018899688 802.650020720921 1012.91803181784 806.827205415602
1134.52018899688
CLEC2L 38.304249186058 38.4330755397458 38.7745734037492
39.7411455670172 43.2484996523549 36.3845892764084 36.7356360093603
40.831207953647 38.8271346375425 37.6383530120621 43.401782064778
38.8495095797881
CLEC3A 44.0707087774725 45.04437764097 43.1660110696573
45.0798408927912 42.4864576619427 117.642468577831 44.0202052410607
41.9315419791594 41.9083581529276 49.1928206164702 75.5103237141193
53.5643281930979
CLEC3B 45.0985088502363 54.0461131287999 84.1401040507557
76.450993090133 43.9450023643394 946.081492716653 40.1235656101329
52.9813204449021 40.137818555383 633.385474385877 582.511224532378
185.378190201718
CLEC4A 71.0593419018503 57.7757446006652 73.4611178120841
1097.92519921873 52.8843091010101 61.6702707724448 96.5615807039306
55.1109784383525 56.0286903335634 55.6069214673486 50.9246396457383
51.6660481331154
CLEC4C 44.3355304829989 45.5587966941116 45.3675390992851
48.055683217958 38.8834538955 49.641159251878 42.8684958525042
41.2551966132486 44.0256815382334 45.2949878966345 44.2527566343661
43.4290452504928
CLEC4D 43.0001432913058 39.3999056968598 40.4882741869718
74.4365426051588 36.6354013629791 38.7340969861754 40.5585676679482
40.5158867964016 41.074824727477 33.4316681623175 36.2435874904689
40.5703581862752
CLEC4E 42.5193208629123 48.4497719907777 46.7508829977587
88.1494891612632 44.0451487108213 41.0582900711341 48.3411413510851
43.6434576713809 45.8325181856483 44.7958122466628 39.3667161654772
44.2527566343661
CLEC4F 42.5193208629123 43.683792219729 41.9666541498638
37.5382094267692 41.2740822675592 39.6251842352088 60.3928833351886
44.0390019242862 36.6312676076142 54.1683282066029 44.3763859390287
42.7782216342806
CLEC4G 38.0089018744739 45.8669209716694 43.6670755115848
57.6511495792664 49.452985143568 82.5956705829173 44.4639405506967
44.0390019242862 39.5806465938526 39.9948390976317 44.2925355984056
50.5511827666954
CLEC4GP1 45.2421183842044 39.5050383901884 39.8656165405284
39.9697332660833 39.3667161654772 47.1477056711335 42.5660695551615
37.7612949798895 40.2739700860114 43.128936937037 41.6657053704762
41.2905987859365

CLEC4M 44.5282181324797 38.0226905622357 39.991353369336
43.3078542962968 39.0564222526273 38.9378717994178 39.7576136507593
37.2237132058632 34.6720534245631 38.4187686307607 40.3452320167286
37.3600014814388
CLEC5A 76.3933911546385 43.0971787292711 45.6989664108554
399.027079798213 42.7982502551634 37.9951997351435 57.8870110456492
48.5681478665796 43.3744503319109 44.2580151149956 40.4504608195666
40.6614700944853
CLEC6A 39.5676051939342 39.0102066700839 39.0775870687758
40.3180310664156 41.5668884168669 41.9543948167375 38.6243997507646
46.0633179231318 44.2607789091176 42.785429206422 35.3362091871364
41.1554481567603
CLEC7A 42.8036731479932 46.2625470410195 45.1912496459517
119.670948133232 44.0451487108213 42.6575535390785 55.0404566626821
42.9301944191913 39.1433524273815 40.1179210534118 40.4504608195666
40.5249349201863
CLEC9A 44.2718517563299 44.8044806324595 46.7058093820839
43.5627649099623 35.5895375758021 42.9695585740813 44.2166118829877
39.9576138799997 42.3138951003676 43.0177448037924 40.6373400186325
40.0557788909923
CLECL1 48.5835484742456 49.0697565324841 39.991353369336
106.582060920252 41.0885562160946 48.5835484742456 44.6032218589199
52.5909491535773 43.5669306063196 48.6124127998952 44.7114813656401
39.4490233720848
CLGN 40.2543341073001 40.5852276210998 40.7620472083159 41.7614975630362
38.4135023167287 40.4359177791566 40.1235656101329 40.7311497914871
39.7537274655115 40.7620472083159 41.5421429082231 38.3291713594599
CLIC1 1855.0119962662 1279.90288378214 1744.56688411113 2146.1548633579
839.412133682574 1342.10592868516 1125.31394383117 1211.8832855325
1455.64106630257 1598.78859278229 1157.65264077595 1645.71527614542
CLIC2 41.8409927585705 49.4982025424177 47.0055981216687 45.8844325913247
42.7982502551634 61.6219790276408 42.4939802311136 49.3880946790306
40.4220196471124 47.2299678280724 53.0177144132449 51.6660481331154
CLIC3 332.322497133752 400.093769705765 361.023743912857 113.528908699366
144.976378707447 79.953657052127 71.3527183280827 127.213248611204
173.013258797349 91.6713614282714 102.121491187437 61.1022920882065
CLIC4 178.060342847557 77.4046818339539 75.2391243965793 174.416924448198
101.250422688816 464.209794165593 96.0126406688619 104.650071846586
95.2956103624468 185.191228931155 172.433936159136 307.656792056993
CLIC5 45.5758521267508 44.1239711172417 41.8751578329365 62.3936376596431
56.2305155589239 46.574682061365 41.9393838138203 43.1567024035183
42.6366765186182 50.3388505346542 51.524711857679 48.7814131120945
CLIC6 122.495387796233 296.842555066902 175.056828374791 188.336316985251
63.8315840603904 50.4022543872993 1154.6386362 621.734974486654
345.36940514835 1297.78424326775 687.862452802453 875.787532301293
CLINT1 517.216393517181 349.589955244523 574.467486140819
444.320714312359 343.591727067749 556.417356025042 332.184073392054
382.312820492386 694.590958116425 369.032450063274 396.93169345515
680.279119780451
CLIP1 117.893268074013 115.048869605875 98.7535408247698 131.659314490437
70.5208169106324 209.967057706137 98.6825522152097 100.948192310983
117.823678167648 167.034910927177 145.561345203804 239.590143292142

CLIP2 80.3730667493235 56.463481075522 59.0308764112247 71.0820747228972
67.0163226819964 88.4695322145032 80.6083247572711 89.140025661759
63.994439164071 83.1874704625702 79.1922819549042 72.4094549259929
CLIP3 334.639325701194 144.633447485038 102.150058925164 246.044674996861
191.298525555624 1459.69929955338 85.6236091398006 221.808325312905
81.8104679118753 1242.75259587926 1377.24483375075 729.342532316666
CLIP4 74.0913177754682 55.6323803479191 69.7855392178808 125.801834682035
99.571118823509 215.452117252205 60.7992483126292 65.3019328668633
84.673375308972 85.6763929745253 112.984042589704 161.228738711412
CLK1 227.243009473121 201.875082892488 192.564048264712 313.763121699885
186.042094228091 293.649670545233 102.053879707074 195.221917652149
153.626578526803 274.887090359131 273.379591993579 253.980053738171
CLK2 123.918925337643 113.735641101347 171.957278903005 128.157032116902
183.584085676174 91.4446109489387 108.39424630351 106.233860458285
93.8968899420039 112.23915619297 106.582060920252 105.317399386109
CLK2P 41.3387790895716 47.1856919329105 47.9035532886869 40.5484717678957
48.3287893866569 47.0027453663546 45.3597029614942 44.5658219748173
46.4569566520107 40.6022003765153 43.0971787292711 46.7182294568603
CLK3 150.875092086496 187.486245101512 131.586389151079 176.057220407246
181.516519907011 155.956315815754 249.584588371496 167.626012561905
190.647389246848 161.25239489071 176.963666283432 170.475212920685
CLK4 65.3531590366085 54.5374188496807 62.7605715573218 87.4850071876743
57.2960245538881 72.6182747525443 51.0013344286851 58.5406981871153
57.5883027758003 86.2118355769367 74.5654028199926 74.9294208313176
CLLU1 40.148780650831 43.683792219729 40.6737160001291 35.2143190355414
39.1650142708257 40.3357777530419 40.4359177791566 35.9023742468744
42.4540072551568 38.1042454705119 41.8168470823451 38.2350720635382
CLLU1OS 33.5994525227062 45.2556760873335 39.4710743516517
67.7121534934164 45.1137938810047 43.6670755115848 38.7337086062875
34.9580369221164 35.4332080966594 43.2988017098387 41.7272875295469
44.6484005362281
CLMN 45.6517320730042 43.4396866157171 46.0727230219512 45.7397504535169
44.0451487108213 36.8059561571675 43.6753843488695 61.3864512710242
41.7627910693483 43.1839109702714 47.2134341420411 39.4490233720848
CLN3 155.123252685926 123.387418707738 275.975192937951 219.838542502016
248.527031538874 109.86466812799 147.875578127733 273.849703944739
188.36429659391 232.944023917808 197.720854621428 154.681233315729
CLN5 114.148080724618 101.020328028769 102.443884864631 158.66112269515
86.8871118411414 157.463465828715 77.118454956956 93.2402285327166
95.2018660650205 238.647490689325 176.492987902471 218.701583105141
CLN6 62.7669079605627 58.174555206394 48.0191002461099 58.9168466928565
70.0133734659853 46.1513019317765 68.2708120569221 54.1731035633439
79.9391673512018 54.4825201506931 53.5698357689852 49.6629259325705
CLN8 44.0707087774725 56.2508140932179 68.3234331335073 69.4677195088043
68.4720753153522 47.7086093893038 53.6294062560285 55.3664856922473
47.5183209236505 64.2848885182563 60.7231725808886 62.1718158914638
CLNS1A 356.133736281036 473.455955645297 541.631969808467
322.774093041134 794.806085783439 366.129480083279 493.705301673753
415.759723627205 469.78210773245 488.183286350725 448.196293409768
583.690350347288
CLOCK 124.866058211181 67.4811066936124 62.0008474085033 97.4967204298499
82.0149542477384 112.873697337138 122.514153407264 162.181462827314
85.9087280306456 122.350248266459 94.7008981904016 76.7791768896805

CLP1 103.727217972304 91.31756001203 100.43365828852 96.2385885003071
156.088284999883 90.407200101342 125.752132815786 93.879589378061
91.9291200887492 82.966263655525 67.1477341610979 62.0967404944031
CLPB 44.7044140979108 43.5873188565519 41.2365089689635 42.8235313007746
43.844855336215 38.2293008890613 44.8643898647322 45.2806199505029
40.8970401536578 40.1179210534118 43.1931731665131 37.7526485343328
CLPP 185.771041814711 230.664926508522 241.31366702947 190.442443946259
462.105603187328 236.851068206869 278.067057781084 242.450054864856
232.47874874281 167.319747725488 161.439879456216 163.917208464731
CLPS 36.3649677956953 37.7384958256497 37.9482157381008 37.7469621225553
40.3452320167286 41.0582900711341 43.9450023643394 40.6171774724171
48.0163377492228 40.6022003765153 39.9899430628542 37.8490162439785
CLPTM1 683.099079617304 637.694633519528 626.390544175817
531.69595054137 990.431472174705 479.696469083953 811.142611128461
813.242005949542 761.922486104216 585.771656887036 693.727004855319
401.202348352506
CLPTM1L 776.670493028756 723.210613328966 690.568858101531
555.727708387209 1347.15664445545 683.099079617304 1260.45604038487
688.390474335958 883.06773454117 534.056256675345 684.865133028228
479.079946537291
CLPX 185.771041814711 207.99040155018 116.709906902201 145.936763882526
165.190373710254 142.444971930298 214.873872837015 219.555240189863
224.14182624489 155.406719968961 139.796374175417 163.643603259911
CLRN1 49.3901004211918 47.7246924920997 48.2883694323339 47.9915414951747
43.5836473788312 55.401776072578 50.0088611244948 53.4109882045447
49.1399035787209 50.3388505346542 46.1673159982664 45.0035308952466
CLRN3 43.0930293831497 36.9994058279362 37.0642268327439 47.8164193292842
41.9842695818285 39.9397419279057 43.1931731665131 41.1575777497576
38.3418933808844 44.0409178714232 42.4580504587581 45.4799270968969
CLSPN 42.9104454767916 45.5842286511645 40.4070046183108 36.0481934423077
38.6454098044474 40.9506062495231 38.1793598983615 39.853866204927
36.2733735494625 37.1275334308172 40.0766543123257 39.0536056496869
CLSTN1 494.162646055732 456.023620287811 631.632335640448
263.21493129287 331.762920362224 259.220844287491 221.847615564378
184.466097348908 572.19538884395 492.519023728038 534.881442644054
433.194113859912
CLSTN2 55.6268253440479 46.7591817286632 46.0727230219512
49.5424425850549 47.6884372645373 66.295597720304 39.8695929627783
47.8947595316832 43.8482802038826 47.1001757441025 42.4580504587581
48.0350549043063
CLSTN3 63.2753814090696 75.5775406974686 66.4079972657486
57.4595416719581 69.1862663389904 45.6129956328415 61.5000955267432
52.3068458655445 72.0655217037557 63.3872214856708 63.7598547294225
52.9609836781499
CLTA 364.628602969366 426.276230164353 260.062749979574 375.529704925121
308.183340806373 449.059703234741 563.573152902421 261.195021649777
390.647843814517 333.421080658854 351.883708151967 403.664724682094
CLTB 612.014486689487 179.158053533144 477.410937135204 377.573838771285
252.206029058968 589.395944599882 204.606913997301 352.292837622545
339.854688605186 421.013324906569 465.595459259015 459.546279672649
CLTC 60.2547250212462 59.7098454699348 50.3950981988762 68.6379135603075
52.2089306840125 52.2089306840125 58.0139597055449 56.0496524902806
72.1591028956359 47.2299678280724 50.4740079109854 56.0477607422828

CLTCL1 41.9918605927928 49.0986627051784 46.3150221024046
38.8964578286293 39.8198309498737 43.9724628401189 38.6537063231682
48.9393675671956 54.7966692984192 39.5637454855233 36.131485826118
38.1421709520616
CLU 36.5000644040258 37.5797639044694 38.0452862258803 39.4469765850607
36.7800683777849 39.2707964550441 37.9974889154201 36.9876272594318
39.8089320768344 38.4979629870086 39.9070785037411 36.6284938772458
CLUAP1 3520.7579823657 3010.38998829232 3253.86278287661
3055.1209535841 3176.96480659196 2243.24648907474 3202.8848520816
3190.0108307463 2513.15722262176 3478.67753301901 3088.44063817936
2944.3433949897
CLUL1 38.6454098044474 40.0870341983328 42.4580504587581 40.0145811166794
42.5428186846763 41.8613406887824 37.9430145836712 38.745552403895
39.4433784795209 38.2550371518309 38.1835938174625 39.6682560919759
CLYBL 50.4109031027914 43.9852350008246 49.5143331736386 61.8437321327938
58.2780916793348 72.1955435994725 60.0981687048269 54.1261903591606
53.8803543493797 103.199200862158 90.0223113095186 93.7260389087192
CMA1 41.4508241771315 43.7644428186265 46.3411353377615 36.5393857306744
43.5544347011646 78.812106589086 47.7390230243778 41.1575777497576
43.2761454096265 40.1179210534118 37.7384958256497 42.357910063876
CMAH 57.8578469574034 57.9509155144056 59.3549213146532 94.4501786945034
42.3685136297603 87.1735705257803 46.4944619663072 50.857522488428
41.1742244762318 51.7594352155304 55.5276281380084 56.1136936405786
CMAS 291.42149037871 532.043503787542 535.449763420086 245.853111206859
265.667557678228 376.453560620912 512.691506909762 284.942182200489
523.679443405512 224.231862271463 262.9072085191 372.330242931581
CMBL 90.8236750745974 135.769551614412 185.474249522831 93.6212502203405
152.608998839745 368.005081137665 196.666585862505 77.0262811825953
293.649670545233 289.625425434103 278.140612054333 327.786619565391
CMC1 86.6655289455628 127.232160404726 148.521769674605 94.080489738662
213.830284761604 102.607924735378 160.917024416961 88.4439480366515
165.662539866309 65.2361525412756 73.408861359425 98.9017950719795
CMIP 271.813877560354 310.706520144173 394.073743195755 369.258587948416
244.907057340556 166.36685654692 178.422506138888 134.307645598718
279.337384456822 295.430709655678 273.231509963261 426.276230164353
CMKLR1 42.3433931248155 48.109747794187 39.1799070031819
61.3747706295648 40.4639532172338 48.8744644207821 43.3262148375525
36.1375121409002 42.0621425894624 39.7835513496873 37.3687900733549
36.9976231206067
CMPK1 134.817234321847 168.426372075344 116.596605311488 171.992427227088
171.722423740869 249.002758392739 175.085270395769 277.878824242495
191.523447800488 175.673196925527 158.843766201769 235.514830602053
CMPK2 44.5282181324797 67.6169355376864 63.3544794716167 42.9358255168086
38.3561682218911 41.8613406887824 45.8980298854198 44.0390019242862
44.7058233759912 43.6033221838149 39.6403089864678 42.9887069977696
CMTM1 60.7556909485011 44.8942011457385 50.6486940895674 55.3345906164297
42.9627566278041 41.159706131351 54.9765783642973 54.7897614560757
44.3440839686558 41.1222709786153 42.4580504587581 40.5703581862752
CMTM2 41.7397731376692 48.5905518770847 50.3950981988762 377.679662622526
39.0564222526273 87.3429006429216 135.737771384104 49.5185112777773
35.3227485563945 42.3606390470387 44.553780984244 37.1747156131306
CMTM3 273.645674189546 137.239944506217 115.93159653774 247.47601939651
92.0958762013613 158.918242816842 242.190059393491 227.059460958349
74.1677748563797 139.08163074096 106.140215438654 134.394743648475

CMTM4	110.818741525514	69.392742408248	63.0383605052305	53.8658229797154
	59.067083526499	54.5358897781765	86.3793067659925	126.882948837851
	61.8529316708431	73.3479915711031	59.4549267937433	77.951185234781
CMTM5	35.9305494063137	44.4411734611229	40.301483914446	41.7614975630362
	36.4434672195785	46.8954576545929	44.6032218589199	40.4359177791566
	38.4344387289956	38.957687788868	42.2618615571566	42.0424641526302
CMTM6	368.727384734032	473.455955645297	441.247306467645	1051.58050264114
	551.490493022416	226.553946570328	355.495119544081	390.489537450621
	542.467997217663	277.12373983597	420.761600667193	466.935292808402
CMTM7	310.706520144173	312.530335465784	361.632211543977	499.744856832174
	1061.61186520958	295.69493803986	795.381998233245	316.185342210304
	459.182372959714	657.031980628955	670.271164195182	665.510487460287
CMTM8	229.697141930047	226.202485756581	252.487015579489	136.786772972738
	319.092568066532	107.60291670075	273.231509963261	212.813224717262
	184.218705402964	159.949298350852	218.19156738004	293.702642091269
CMYA3	41.0608773166058	46.1569135229922	41.528370959196	39.2780515621926
	37.8679241954337	42.5660695551615	38.0598374201407	41.7419877611072
	41.1742244762318	37.3179578037963	40.1742127891824	38.3291713594599
CMYA5	74.6776631832355	138.418964807714	106.175784900392	54.2886483704949
	77.2608629053098	85.2805684630976	105.382126899524	91.1834104205004
	116.094881058504	245.630020408551	178.012676342712	96.6957892186886
CNBD1	42.2455707947223	39.6123051184545	37.9482157381008	42.0102671859606
	41.1868052124344	38.9378717994178	41.8315602975157	42.357910063876
	37.4540772310118	42.3606390470387	46.2560238004757	35.4484652445078
CNBP	370.495645380436	555.727708387209	549.407911286514	552.860979481958
	565.72800641089	903.756866967447	411.73316510051	318.527516338791
	500.168833003599	299.524171071613	375.529704925121	581.093069802913
CNDP1	44.0328992213727	41.8848959280916	43.9450023643394	54.1240583139871
	40.5710637884029	47.2927436772465	41.8315602975157	38.8495095797881
	42.2577689927563	39.4710743516517	39.153226699662	46.0633179231318
CNDP2	1561.83892117664	3313.98659031004	1273.52492984569	1673.60994518271
	489.42739398838	617.271611375695	2232.61002288121	1191.93354266135
	2672.94421680196	2559.659041589	2178.59848996529	1891.37245670852
CNFN	295.127975044241	70.7702047405718	70.8606919488782	57.8277957325085
	168.307676704652	60.4065050472082	247.700075839517	353.338686666875
	147.165235402905	88.5601339449828	91.6826894933058	70.0519247980052
CNGA1	56.6086202928268	58.3627863274274	60.6567691532561	38.2041059863839
	68.9766943946607	46.1513019317765	78.1623301184313	379.031536537372
	57.7865070833351	156.844010597219	79.1922819549042	174.985336606027
CNGA2	36.2496364442264	40.4359177791566	41.6977706798234	40.7481155008596
	35.9894133432376	45.3882138838137	36.634024014558	38.8495095797881
	37.2810767789306	36.8059561571675	40.0766543123257	40.9683275553869
CNGA3	40.9201978689462	39.3585350448719	42.0164252051496	42.1607322031054
	43.5808453440435	39.3483872785093	39.824238926666	43.0390857698452
	39.9871741554388	39.0648733099061	42.3440399888092	45.0035308952466
CNGA4	41.483412492426	41.0226232127351	38.941855825269	41.1507980856436
	40.8873907150245	38.9661430990729	39.0458123429605	45.0972184964705
	41.2424637338424	38.7008388965825	37.4642807402741	38.4829727716328
CNGB1	50.5670783769975	42.8542642792862	50.2869791217819	52.5307002738469
	52.2089306840125	44.3648993568405	59.9792284253187	63.7002949745101
	49.8304479381623	58.5087296620514	46.0905079010237	51.0013344286851
CNGB3	49.540449125788	49.4067286971348	47.646800205189	46.8954576545929
	44.9936360641969	39.3841861812262	34.5633623842083	40.5158867964016
	40.9901805728485	41.7405682794166	37.3687900733549	38.9496515344837

CNIH 307.754078087321 340.311633794489 301.139592414061 365.860501246879
296.39460008432 467.351570171652 169.061093538895 200.082941320005
296.664472126617 154.93193897888 165.29964739949 372.02379581293
CNIH2 42.2618615571566 43.7644428186265 50.1579724208315 44.6024422254257
45.7349698678081 42.157627240486 44.7980740332174 44.4455094736026
49.532546810992 42.5664883002833 44.2527566343661 39.8873706855774
CNIH3 51.0225132036905 51.3800036046133 39.1799070031819 49.3654837541353
38.2496050953547 41.554942319225 58.2315917325629 55.6904617926772
39.0775870687758 53.1993028936883 56.5402453093703 44.2527566343661
CNIH4 320.812668695531 295.856741468603 388.580556899321 427.972184856393
277.284338888467 191.955732544426 552.860979481958 448.508714709248
671.959673037066 286.497342437243 289.17237603899 499.550408983803
CNKSR1 49.6241203903354 88.3636380348802 70.8606919488782
46.4555908533915 64.3571664944642 40.9506062495231 54.8074655158941
63.287067794195 62.9922928277769 63.5107036952143 62.6048727240291
52.0359304386476
CNKSR2 41.9407041937973 41.2598458726002 45.3974838013476
42.2496337895968 44.7829632472754 42.9695585740813 45.7452456977857
44.9977782988116 42.9020321355927 45.3530683538086 43.1931731665131
40.4639532172338
CNKSR3 111.691813916343 130.239763076999 90.8831233784641
85.1847490113446 139.847976281228 159.22638189198 109.615289274998
66.4207938873541 94.7110944331075 207.741669973713 218.013913036142
249.220968977184
CNN1 191.394143505573 112.854520785284 95.5022104475922 79.1000897258245
81.2359429678503 4617.45430247751 33.3231576795423 142.309369995384
41.4636047919453 3153.71394627237 3114.05035137552 3234.36469403715
CNN2 421.971171686029 353.751797948675 295.69493803986 404.674869177442
399.66182195331 150.31039735991 150.925721934159 108.213380368446
301.257549182222 170.560304063093 204.273912273411 249.101015988111
CNN3 1315.60546296361 425.776214512634 307.264133974364 651.078746026341
508.474794786176 1127.96214301065 651.969296681551 675.832363405287
551.1195044975 1000.65752290829 831.578043520215 1186.06822787922
CNNM1 44.7044140979108 44.2105195734073 44.6032218589199 47.3574589211166
67.6011992144113 60.2867996701867 47.2927436772465 55.3122077514658
63.1091746640396 66.5422130374814 68.878562323012 57.2350830635912
CNNM2 40.4139947039718 46.6996902348633 46.0727230219512 49.9014504704021
43.0669898157628 43.1826328292558 43.0177448037924 38.7023886970298
40.9901805728485 41.162473449625 42.7815195866158 43.2115532536912
CNNM3 81.2359429678503 99.864496342189 96.6895594961001 101.867388894697
98.7869013773721 72.7213787264217 97.4515987591398 95.2915739972533
96.7422527189063 99.3142021810133 102.87193290903 88.0877175295544
CNNM4 88.8760662992208 60.4646208319121 74.4508307047509 75.9099205260748
76.5375674421131 58.0095518210499 63.2726414544553 96.4971762611189
75.5681913475287 69.1623287497421 70.7045582269595 58.4200819624972
CNO 143.6667863386 148.458171821142 139.883681090758 103.349712762251
153.660685857642 102.322851896214 152.555556133364 99.1438953564062
161.500218853268 129.550925624841 127.051878502711 122.084249177093
CNOT1 222.728316525835 354.158105175308 432.355300448283 263.706801812277
155.519517541864 228.328635242684 268.808342880355 250.308866488702
388.74199767336 345.443895623911 309.102633242573 261.421333472097
CNOT10 114.666088291556 122.44445355345 144.079981029616
128.635811501506 227.059460958349 106.067766766889 192.470747766298

164.312946712409 136.843285454801 128.952325188088 104.486555491331
90.689813277023
CNOT2 276.267583952979 243.609178976074 228.809697062726 188.713487905549
193.703214707909 250.866560791828 244.109166881519 187.277044459942
437.542517888395 247.416016413788 255.483653108786 311.301613620475
CNOT3 107.413918714848 177.878878992191 127.694844372277 154.718907273467
214.732968482214 139.758805241617 234.172977976583 157.082769443568
314.609371608151 156.591349262922 169.223049028418 101.156215957297
CNOT4 110.973625869849 88.8772406115484 90.2790229242453 88.9641595603127
87.8967913380047 98.6706537801886 80.8285585008048 80.5237920647059
116.007007037602 119.634256037186 96.0770202999531 106.454367084321
CNOT6 195.018775118528 153.42135363926 206.649471678786 145.526972410673
146.196470375622 175.114727506747 176.492987902471 209.438270055157
219.103786570566 245.27542001049 234.8262403783 187.026092504992
CNOT6L 52.2236479204029 57.9151768280253 55.4057622584684
65.9703614596867 52.8190882379316 46.0364000143537 47.8947595316832
59.7078429195904 67.9753615619786 69.6511571901941 60.9446532180211
56.6888826857797
CNOT7 97.5104526799755 111.643323610073 137.360931076154 124.350816245276
139.712472369259 129.675613712326 111.97030382494 75.7947206223934
107.087870999702 67.9259304928255 89.2185019250322 138.728648830528
CNOT8 99.8754776648876 84.6655522075709 97.1271615802556 107.242476674629
91.8935361535002 83.7971204489995 81.7434024567356 104.089998273812
106.907589984092 85.3718124658316 69.7644070698124 116.295564219236
CNP 46.5549623118323 41.0678614026819 44.0451487108213 44.265834798964
36.5393857306744 43.9724628401189 44.4639405506967 46.3685439301234
46.7760357462635 44.8997235283254 49.1067959632109 42.357910063876
CNPY3 130.261747462753 133.192618821838 92.0606634977068 161.870972980732
113.747633871556 99.2749350936362 154.166621635012 79.1039287828151
156.433762355183 100.151116279734 95.39134217438 109.889322825648
CNPY4 75.8122107270212 53.5893790024601 51.2776048338 63.1604223919703
66.8129352840907 78.9521593412581 55.012605207172 56.9719061986797
60.8635925649592 63.3872214856708 60.6505672212119 66.6118713143653
CNR1 39.4710743516517 41.554942319225 45.989212296035 40.646702696033
46.8674366115656 49.2050538150106 36.3194282084339 40.7311497914871
44.1519466943185 40.4136535208406 37.3687900733549 46.2625470410195
CNR2 37.8386378737561 42.5269793679335 43.4709415392696 52.1201052574162
42.4864576619427 40.8634043028528 40.1235656101329 42.4580504587581
42.357910063876 37.8217989841918 42.8263096141654 42.4607795638552
CNTD1 46.6348258763289 47.765848710116 43.4709415392696 40.9607305725196
40.2449343247539 42.750861693377 43.0930293831497 44.8997235283254
38.8271346375425 46.1390887492649 43.5710871617107 44.0451487108213
CNTD2 46.7293238583888 41.2598458726002 49.7350276169552 44.6905691366696
44.0451487108213 44.6524308640784 58.2315917325629 45.1714698794331
52.5772854102326 47.4767851750188 41.9908237694988 42.1537476443423
CNTF 41.4508241771315 36.3110701990588 38.4043916290561 34.4382958501338
43.6587492470348 37.3799542961839 39.1539892144371 38.8495095797881
39.9444191873552 39.7835513496873 37.7384958256497 37.6557714476853
CNTFR 41.3848620620968 38.4829727716328 41.9666541498638 39.2459792718908
88.0877175295544 64.1538986629848 41.9820792173615 43.4544762830742
48.4869909888679 43.9910347090126 42.0750889188255 40.0136663626344
CNTLN 54.2648201582657 55.8678615468603 42.640829914102 57.8277957325085
42.7107928951766 68.6424653037264 48.6395795163502 45.3872406432794
39.6609238036815 52.2256177934134 47.299647867533 51.5741598315033

CNTN1 69.3971407893143 42.3711524005238 41.785676764591 40.2455972565968
43.6587492470348 111.626292905866 44.2166118829877 43.4544762830742
46.824299585607 59.1496467460507 53.2083147523758 69.9144520559912
CNTN2 42.7150227340364 36.3110701990588 42.157627240486 45.8844325913247
43.3332476888305 49.5424425850549 42.7431359850339 43.9366081640576
46.1101825640108 46.325232414659 40.6373400186325 41.863927676552
CNTN3 50.1533437545239 47.2770121962655 171.501864800548 44.0451487108213
55.4030910454028 71.3932787152381 81.2136969156029 41.0381860307445
59.4236178078244 68.8167148906225 69.7215372275927 79.8331445290301
CNTN4 41.0871827071116 41.1262212547458 42.7815195866158 37.1259822167035
39.5317722761104 48.5251714843637 39.4859911554583 42.157627240486
40.5465626311506 41.5435611403119 43.995454742337 43.4709415392696
CNTN5 40.2543341073001 38.4757888225478 40.6737160001291 38.2041059863839
35.2729706573192 35.2067831864574 38.8417151851134 38.745552403895
37.3600014814388 43.2988017098387 38.5668610487033 39.2839132447749
CNTN6 53.1309801459151 48.8584917918986 47.9035532886869 43.5627649099623
55.4030910454028 47.7556758818053 50.8388984085636 49.3880946790306
53.9287524861617 51.2318935933116 51.7538628098347 47.9566297250148
CNTNAP1 89.7554896946341 79.271901834289 70.7238101239421
56.7927450229377 67.9032387662129 97.4967204298499 38.5066108855005
53.4109882045447 44.8997235283254 68.0556717366572 63.3544794716167
57.2350830635912
CNTNAP2 40.8681871019812 36.5219488315447 38.6727842796202
43.1847628331565 40.3452320167286 111.669813610213 46.0228732507872
180.53214143537 41.863927676552 41.8613406887824 44.0409178714232
43.9541718254769
CNTNAP3 36.3649677956953 35.4484652445078 40.4070046183108
47.8164193292842 39.2608760193011 49.5424425850549 38.7337086062875
44.6107027295193 34.4716904283753 44.6918600441328 44.0409178714232
44.7401523707775
CNTNAP3B 42.8596678196469 41.159706131351 43.9217273706293
42.7431359850339 40.8351751854504 40.4779565600984 42.6786833450085
39.4710743516517 42.992884918158 45.642304356051 45.8628493605663
44.3977035913759
CNTNAP4 41.0043775553254 42.9887069977696 45.645828394758
47.2009420656854 42.039525923161 41.0012755104374 47.5130990528612
47.2368301203382 51.7289068180135 40.7620472083159 43.3085358214154
42.5365257225985
CNTNAP5 44.1737502492846 42.1372626664516 43.1931731665131
44.0991023527481 40.3452320167286 43.7630587657205 42.629412460409
46.9454141780379 40.9460396298042 42.9358255168086 43.0710577955487
40.2669497398702
CNTROB 55.5276281380084 54.5374188496807 69.4967762263181
56.2508140932179 63.3638417360105 58.1798515131249 63.3924515518667
69.8941497035747 54.4912806027381 73.3479915711031 74.280612516205
58.6481644777552
COASY 236.910422092851 253.605641311356 285.97627172608 175.936150174315
289.472303440614 145.865118592916 270.253329297593 114.719721626067
278.140612054333 300.412043023998 261.081844108932 255.800286504974
COBL 42.6036491382658 47.4076235350059 47.0940613507321 47.6687908290818
42.4864576619427 59.4949177415847 39.6535880152834 141.942507674009
47.3791873993182 52.6879797806876 59.6608518093582 44.4475838947656
COBLL1 292.065366215278 755.106456358949 569.244038644604
413.516548092483 507.498146970824 486.757854744053 334.016152807947

312.7217964763 615.838713743621 1676.22158135012 1060.64261592822
1149.02954135912
COBRA1 247.47601939651 214.03015073442 152.200107222761
164.757702308188 179.756604242873 187.689343320587 356.948763043851
146.196470375622 199.035083736742 204.992039590717 220.743173420176
153.177725521199
COCH 45.5758521267508 68.2184093305248 40.4070046183108 65.2001674269291
70.0133734659853 47.417512242919 71.599955759893 75.9659252679925
69.6511571901941 47.4767851750188 39.0788811357542 43.2776458914333
COG1 90.1830062843449 84.1224653052549 116.184007615285 87.2643859655159
84.4230484528318 95.6783002876222 77.4046818339539 72.692416146584
86.0735583144514 89.07557840469 94.8036856848998 81.6458112867541
COG2 165.775214685061 161.844470327205 230.763782802004 151.344720108793
201.301705082976 166.431392410887 149.156754860718 185.563590013028
212.945601743319 274.994470779348 239.350719871688 191.361705136492
COG3 89.7554896946341 65.4493288628375 75.4317740345967 62.2878942799819
58.1576967525216 72.207833811212 65.5800543140431 55.9303347977781
71.1441318672051 79.9126192278104 92.563449632567 85.3930226794591
COG4 148.32293918881 112.306016029829 189.164097331837 83.4756142147167
82.7394574977114 100.802977734227 156.833474582387 148.906660152784
141.924244643825 131.779333469955 126.920579478593 100.567309348016
COG5 63.61404854326 73.0385284042351 77.5718089378881 71.2572485987604
80.1809075683154 71.5249259772583 78.1623301184313 70.3935694362568
72.9752827496503 60.5548506034677 56.432280646473 76.0709828871754
COG6 189.47090669378 150.262748333939 148.521769674605 129.785929766258
95.1131101630937 129.091447141381 136.293724800658 69.7729022157455
121.737071786167 108.111248374368 106.140215438654 128.873145026756
COG7 83.7215159198897 99.4123924179498 129.2860308084 88.3459116251233
80.6200293361016 88.6287852712744 89.2913927776683 97.4421930425128
80.4554147910354 120.447568925399 96.5451512198207 97.9497090233081
COG8 55.5276281380084 54.4190527512508 70.6592421895338 50.0526763693329
57.1935729219207 54.2503023961401 64.4736987797413 56.6910078150745
92.8206287414481 45.7911932481722 49.319499137678 44.6484005362281
COIL 188.887559923238 173.511692216696 198.623556176842 138.581471125405
263.832968124343 171.302214895226 281.262083731954 220.054040993848
263.096895937991 198.288493088076 202.708232693973 176.670909065664
COL10A1 496.303918649238 40.2493681589104 39.8299340037009
85.3754356809649 39.6535880152834 42.9695585740813 42.7431359850339
41.6466169548846 45.4799270968969 36.4651112441576 39.8184668132023
37.8490162439785
COL11A1 361.776841675393 41.7614975630362 39.6705473876526
59.3618843678775 40.8873907150245 45.3424709970969 39.7264117629268
42.8482181313226 40.5497440764264 40.3418326960047 40.3104838541906
43.2177497174408
COL11A2 42.5193208629123 39.0592366229872 42.6981470312295
41.4606714407332 43.6782245625941 41.0970259989281 40.1662958063877
40.4625704251354 44.1070537445005 40.650742261428 39.6719190054379
43.8291226513066
COL12A1 546.287195443189 106.691530592115 76.6346767983285
120.068630086702 190.548265163011 109.25950270396 94.5261917871909
80.4334958198115 67.1702819938085 75.257352283328 65.4137646609535
86.2488614616838
COL13A1 67.6767845246581 52.683711208052 57.2674335547631
60.0856496605043 54.802736966312 60.1685144469519 32.2796484885898

49.239733414453 39.3750969348308 67.3601172371813 72.9488994273323
43.9541718254769
COL14A1   78.3851687801705 80.8285585008048 64.5232087947596
91.9185612536409 72.5027069303875 189.451890957713 51.0013344286851
53.4109882045447 52.5772854102326 87.5967418612698 75.8976034525022
50.1579724208315
COL15A1   686.154904023493 293.583101558009 302.20349084931
708.839262759372 189.890451486324 230.197573134703 73.3042538589134
186.984157187427 59.5929655877036 127.937436596431 116.938715935808
182.448444305445
COL16A1   346.844545554188 83.4103205764925 56.3471340054074
193.897678586782 179.994715581707 259.456935785521 53.7423898357583
145.335751135441 67.4304134449444 233.07404514708 252.206029058968
230.975122271137
COL17A1   47.1962846565261 211.926421068313 254.920345078558
63.9570596163747 43.5544347011646 46.7386599624801 35.2729706573192
69.1523164481201 135.644934816665 222.336600180634 176.318618244066
249.451753363306
COL18A1   167.920131968611 93.7260389087192 171.386815652277
112.659254685552 96.7351644023249 105.267519627444 45.1694246393676
84.9280683258012 61.8824218975538 97.2865629929716 106.985301705486
69.9767092409915
COL19A1   42.0833588105728 39.5050383901884 37.3687900733549
40.3180310664156 38.9496515344837 55.2515150764173 39.1539892144371
39.3113207075992 41.8197029429821 40.7620472083159 44.8372404608604
41.8200132437731
COL1A1   13656.2581082905 1670.01014610464 1448.17151189672
2771.14077845128 3502.80810319252 746.252816568691 225.875798191473
842.3367709562   252.134800295199 507.375704144286 363.002913525646
195.654315886452
COL1A2   8827.37298329149 1260.45604038487 982.428740433235
2337.7738990994 1855.0119962662 1450.53108586858 124.841811761412
634.226367147025 170.327588764719 824.946988188872 370.027120412227
371.513106715598
COL20A1   46.7293238583888 48.1627097465883 48.1331309338997
55.6481366056639 46.8674366115656 49.1013967533186 49.2546642180676
50.1833483241864 51.8083208406279 52.2256177934134 49.7363778831003
45.6692241948108
COL21A1   37.7384958256497 38.9338521051669 42.4580504587581
37.0732977341185 38.4540849632254 44.5544727111028 43.9450023643394
39.853866204927 41.7627910693483 42.0621425894624 44.9713479185144
41.4508241771315
COL22A1   78.0020909283414 114.796237914478 44.2405728317107
63.0153175111747 51.0107745412152 43.7630587657205 40.2154497375715
78.4815128101967 105.63434679637 147.673181207232 44.6286581923295
55.3563533836982
COL23A1   57.50915811428   46.1569135229922 46.8267540220952
63.3006977162657 52.6922486991742 45.1873487463472 47.4439407315335
49.085741080241 47.5564423692142 46.8954576545929 45.5707650534259
43.6753843488695
COL24A1   68.9745034946434 55.2182004838121 52.3432130135266
63.0153175111747 51.1368583672022 57.2767879969284 45.3597029614942
59.7744650739434 41.9635525160083 53.3059058105836 73.7873098190297
49.7336769474446

COL25A1 38.1169703906207 38.6807508413673 36.0674080694129
38.5855564939014 37.7576900881741 37.0180934800573 38.1154766911051
39.4134201808101 39.0470836345045 39.4710743516517 34.6814739995051
37.3600014814388
COL27A1 50.315768338317 112.944044862693 55.4502804236424
48.1662933382789 48.6224651100733 50.8533737532786 50.6774883370457
50.628152951493 48.6494451061637 42.0621425894624 49.2206713037152
49.2064066614865
COL28 42.6036491382658 38.7344853699576 42.2577689927563 41.3027031037343
42.7982502551634 35.4142595025086 41.706879587091 42.8482181313226
35.9645119596869 48.3693338695392 39.7342718293432 44.4475838947656
COL2A1 39.5676051939342 41.3277288245157 40.7620472083159
39.5050383901884 38.1644569189523 47.417512242919 44.3440839686558
44.7044140979108 40.4220196471124 42.8936990772838 44.553780984244
39.3789825717286
COL3A1 9488.06491634484 1252.94510628089 934.48498309932
2992.62035058532 3043.12489526581 1083.50383916482 156.061711961514
1110.39060103557 192.216758313982 661.880230068693 451.603052501528
495.453743436613
COL4A1 1526.94101719128 1173.47285626656 1649.78627644882
1285.49870339222 1335.4111951034 851.813216452015 245.194730981772
539.188951045594 232.147792636393 488.183286350725 396.167219131922
756.801598119483
COL4A2 163.126491735808 152.36389833079 210.958578914607
170.184914156265 138.032032026715 217.658421476562 64.4736987797413
87.9468208580228 55.3122077514658 91.4684013366068 143.357170805323
142.680436288759
COL4A3 40.5654775358427 41.7614975630362 42.4890076400051
47.9915414951747 41.1507980856436 44.9370339275041 51.2318935933116
47.7508139534968 41.5635499110307 44.0719548209593 44.4688999098904
42.9887069977696
COL4A3BP 79.953657052127 103.569150754726 120.582525405708
192.058030454066 73.9456720099807 105.814959179397 96.0539709962851
171.957278903005 80.0113001016496 174.469283513638 203.109765192983
129.938114164087
COL4A4 38.304249186058 34.0382693188794 34.806104801006
39.3750969348308 38.2496050953547 46.8954576545929 39.1539892144371
41.2551966132486 40.4220196471124 39.4710743516517 46.8954576545929
42.357910063876
COL4A5 118.143444770806 113.102823481296 497.591040004541
66.5010880718463 561.855286945959 467.764770176415 94.9808934532248
202.627009037401 549.117045813593 868.899376368472 631.110081413363
753.491584605447
COL4A6 37.8891397738265 51.5822637582617 95.3300728806601
51.4968903591058 185.165420581989 148.42234459694 50.6774883370457
51.1334099137617 119.512426152446 127.741016067967 133.487946450906
149.515611848932
COL5A1 2113.73880184695 353.213447404132 228.134171304754
466.454993547103 553.094571132092 387.999689038448 55.2088411137193
235.544416908354 70.5699797830702 165.42634596698 176.687970262952
137.438636654385
COL5A2 2559.659041589 318.920760335454 223.801865347687
928.230419226078 529.650395907046 308.568617575909 68.673864919178

286.927016665685 121.449647909665 223.669853276931 156.95090716929
155.928417606775
COL5A3 65.9273663561152 42.3550738181144 34.5718473203083
61.8997157694697 39.7568367479301 48.9826868640753 37.830876898596
42.5435283762933 36.197424053556 44.6918600441328 39.5399188536544
41.863927676552
COL6A1 2337.7738990994 359.733786489623 206.791464145857
763.947361966861 410.257694738546 1242.75259587926 75.8779316255794
392.622487173359 95.65872032796 953.143041145545 894.486674059951
481.145001682177
COL6A2 924.316085565917 201.977265056102 159.76714232068
366.129480083279 242.631544185516 826.490051619826 46.7704450730477
163.742560819575 53.0357938671646 410.623529765253 462.762837284748
225.618853905635
COL6A3 5601.18872540083 526.750242877914 303.670126704469
1557.42399344934 1099.52344340557 1676.22158135012 83.8811735017279
1618.92099084622 147.023857419777 1342.10592868516 895.735191953679
569.244038644604
COL7A1 667.583623414832 1123.99110499679 2733.80542760533
982.428740433235 911.661341702443 121.682085315899 310.120875196999
436.497820504526 1174.92419086759 190.27040993548 247.47601939651
135.824520574227
COL8A1 260.964562714558 114.674541610026 79.4053001255904
204.502683458676 208.410194427484 84.8808332740321 45.0241191707651
214.03015073442 51.7549258776884 82.743286503787 156.311985997533
70.3629652777935
COL8A2 222.126944340681 67.2649356062263 54.7350451926204
117.439175394651 54.2683296643972 116.793627324217 57.8870110456492
86.6586095017998 58.0139597055449 98.6039579639704 80.7986033188909
92.8280881907307
COL9A1 36.321475353804 39.8455060654531 41.0718387273629
40.7481155008596 42.6022652963326 37.7427315853995 38.4456289125816
39.3113207075992 40.7847110517594 39.1832968442811 40.4960693332473
36.3916581215097
COL9A2 87.4335749014848 161.205086002542 167.229755109155
100.001150612051 127.908440924429 113.090530993447 191.09175268141
131.39588569388 205.55797140415 296.176349059192 186.002828997122
207.741669973713
COL9A3 43.4626049853157 41.0678614026819 46.8267540220952
35.9521941716188 44.3557408297309 46.0364000143537 38.941855825269
43.0390857698452 46.9739160257217 42.0621425894624 40.547835538778
43.1805016690297
COLEC10 42.0532901689036 41.554942319225 44.8872784170036
34.7266486944713 42.3685136297603 42.6575535390785 45.3597029614942
39.9576138799997 40.3316153483289 38.4187686307607 42.6366765186182
45.4799270968969
COLEC11 39.0325254636537 45.7317208802318 40.0998167098057
40.0195349109823 66.1650385535549 60.1756087584664 38.8732216254738
38.9207612933107 41.7117861328546 45.731442524732 54.9765783642973
43.6285248402465
COLEC12 210.667709831149 68.4278345554565 64.0759817636265
127.596368103014 58.6481644777552 358.979854534484 48.8319884367456
61.130286531713 41.6616444147804 120.19033659661 121.449647909665
117.260454792593

COLQ 46.6701649454386 47.8947595316832 48.1997158668729 52.6947911480086
46.1513019317765 46.3203995372658 43.2721716572958 57.6336931026499
43.0990926708606 51.2952403053335 52.7817052791259 46.5797464401782
COMMD1 399.410299413602 230.927097824131 354.861943266984
435.325038193179 256.258779434769 314.609371608151 209.860757709908
614.088027946471 392.008276951929 442.853589679801 334.016152807947
367.685570437603
COMMD10 142.840631375936 155.200138285298 177.80178351875
177.27613997459 90.3041737295567 179.378838766172 87.6018870099686
152.608998839745 188.582370265555 110.649342465555 127.634504077212
198.623556176842
COMMD2 159.306689057291 118.67727980117 130.703767898434
94.3229189690855 197.205643078662 126.485649460666 222.40005420611
146.426853713038 182.06733830953 98.8595497262968 106.527144354592
144.348025939706
COMMD3 717.997180920384 999.13039066242 1040.102982885
1046.42391243532 820.147156401172 762.855719373733 874.419252731128
602.441843384617 1139.81870068001 704.378495149104 628.403814060213
949.278493776928
COMMD4 323.780380243759 213.285274542564 153.711194034581
233.294970051779 307.754078087321 145.170590045628 390.202312411165
284.575587310164 243.06561740595 221.325534445978 185.312851727212
150.709301569371
COMMD5 177.779514321673 131.347995391441 98.0473811574767
163.261867589623 84.4821163646146 91.6978282652998 96.8141712830545
76.6389264422388 126.404988409795 100.188736523729 93.0801307192343
78.0133696753253
COMMD6 401.45848063233 445.016226732679 598.970109654466
545.514314651238 556.417356025042 1097.92519921873 382.538365722661
377.679662622526 556.728676214581 679.559864419351 689.738256133922
1075.04105984319
COMMD7 588.972273484465 889.638287212686 855.157599239267
649.908096675489 605.970682363975 537.471287449696 970.379875059112
1055.00793806075 1115.27580736237 1259.2978319548 1107.96086673001
802.650020720921
COMMD8 76.3933911546385 133.558028920419 111.227016020927
139.554519110944 95.1131101630937 115.143810463938 97.2124452343726
81.9218437790485 124.986782737446 64.5093306432163 78.806097512943
117.823678167648
COMMD9 139.712472369259 134.099684731177 184.123415143164
223.826005691932 152.608998839745 124.22494803053 155.099281008688
262.534449975056 154.752920877816 144.427680369554 127.811684080184
127.694844372277
COMP 973.363245512859 63.5932443357808 90.8831233784641 194.666769955149
48.7288537840562 47.8636003588181 41.1507980856436 260.426488908824
36.5393857306744 45.7911932481722 42.8263096141654 49.2064066614865
COMT 619.950241162173 1271.62760896761 1506.33570635551 437.015713629277
428.8440159628 363.324816966828 572.19538884395 646.791777335656
660.295993198711 768.888528247493 708.839262759372 617.271611375695
COMTD1 120.20612443357 132.84476910529 98.3240331951021
64.6945454069792 93.6591201235745 70.5699797830702 168.91210897306
99.8277095030346 363.002913525646 82.7229468743536 105.814959179397
89.5687388187015

COP1 44.4639405506967 42.6939394537076 48.9944486068347 82.1437429222046
39.1087466732192 46.6397391251762 48.6395795163502 42.4580504587581
42.4784375889044 45.0155543555707 42.357910063876 44.0246886382298
COPA 1250.15619347362 1081.97502255142 1194.10154988793 991.630429625437
1317.29255644089 938.151864971251 1537.76265834076 812.386878774445
2201.39729904166 638.848775860866 668.961646543849 770.715044136717
COPB1 329.437593022939 411.254448823371 294.620463776036 474.729653426958
296.532363090593 367.451332073134 278.507764368929 355.04502859751
370.917477324773 282.802560118785 274.081959734368 414.293172794592
COPB2 567.827069916244 570.997661726825 429.725397949276 539.32996290794
686.154904023493 451.13133250897 495.359878931451 423.254211470935
399.21864362319 218.217851814655 267.261896105785 355.351648424266
COPE 467.884559162462 538.153124145211 510.379313524019 599.670771796585
640.407302868904 477.558242266308 500.672147150237 397.745727767552
581.396808977873 302.422475486532 373.626843592567 351.039814818516
COPG 510.525676243746 335.330937634207 292.065366215278 400.760646547012
389.994289973076 252.870708814507 348.913213159222 399.536040890627
352.600793906035 281.262083731954 338.415128695208 277.014526407597
COPG2 71.6849360818041 83.8850034281243 59.1040441894478 52.5307002738469
84.5572894783679 54.0382756991549 58.5087296620514 55.5578334877425
114.535461066963 82.475816811779 63.4323398447015 94.8717345601966
COPS2 44.436995101921 45.8980298854198 44.3648993568405 43.7410507935043
45.9104478407175 51.5522731200852 46.4388841354122 45.1714698794331
49.8304479381623 47.0055981216687 44.4688999098904 46.9867599298218
COPS3 357.254500791202 430.618167106529 410.623529765253 472.522115719366
482.5816387921 497.591040004541 511.527348356071 346.621778258188
440.282299601096 359.185856400148 362.268767190535 401.45848063233
COPS4 175.028390974125 182.219366179899 261.6201598868 190.960237766104
177.958475301951 274.994470779348 156.917623325793 191.394143505573
179.629622763381 155.270252661048 165.942795019496 294.7772562683
COPS5 810.382224368065 891.080174768895 535.449763420086 447.448900931272
715.662312404643 652.480459437977 557.717744882602 409.554706384667
952.16345035163 451.520414426616 444.738154053278 582.017297217368
COPS6 160.702832529922 177.878878992191 217.575115287224 211.926421068313
214.127390534906 296.532363090593 204.490115029568 242.792365320043
258.789217436101 204.273912273411 217.848539722853 223.267781866783
COPS7A 451.437791473506 459.546279672649 837.959914510114
359.465715235071 436.757445186987 409.978564041362 472.522115719366
689.483531595728 718.832367050609 659.666977039793 821.013687795434
590.139974503603
COPS7B 190.570409327756 191.853798887866 227.567182783564
149.260740603715 368.913147706435 175.619398238068 178.306763765612
237.809876921353 195.430996540859 237.039707901886 223.749512763927
190.81617208799
COPS8 103.252085427319 74.8893925448138 87.539553421006 88.1494891612632
72.2665537421443 128.300809076274 67.4421428140671 77.0703146713865
82.4164506744 77.0927030014721 81.0295607064541 99.4546022538476
COPZ1 260.85727391155 324.320137371059 312.984737387254 271.206999109791
303.323289734056 328.46798329618 329.763197973922 473.223156200183
476.653723741096 322.509777895373 254.472291205905 319.954889553226
COPZ2 99.3956523785983 48.8584917918986 47.0940613507321 92.8026641003757
51.0107745412152 127.115038236278 43.9450023643394 59.7744650739434
70.9462008788871 89.3989848564067 83.0072253231162 86.2118355769367

COQ10A 83.1614238528477 87.6763065012524 90.8831233784641
105.234356571498 88.9038123445083 121.236881661832 94.1289383735162
100.370937669662 111.643323610073 111.248876636045 90.807818984128
88.6771566133367
COQ10B 440.282299601096 563.065549077627 507.620619345969
705.283979182062 439.106576928682 609.797179957996 285.187681119318
371.393488010768 455.081394085527 346.285308869994 414.794323433931
550.861615347663
COQ2 315.276749671783 217.065944053174 198.788202490193 355.04502859751
264.88963894526 222.40005420611 228.439524702578 229.47693624489
292.389429869034 101.138003125297 143.84904170479 177.133999361532
COQ3 70.2541961995753 82.0237575809051 106.243765443714 73.0371862899659
98.9988668602468 86.915098846441 131.258204302177 107.576033955049
134.335592890814 125.393232344778 120.289145237975 132.684895918889
COQ5 273.807348011122 307.550936236018 295.127975044241 309.06234888929
178.156332261759 332.490954010208 294.7772562683 233.381378925304
528.242677613584 322.509777895373 296.39460008432 335.167786298149
COQ6 81.6647246398712 73.3270037834227 121.562769658314 66.0397471691747
85.3681894044625 91.8986731227209 80.882176929636 77.7615826856723
107.490296194878 109.758297200083 98.1835264890769 98.3733183587283
COQ7 49.3463416794995 68.2521461582047 66.5285423077808 46.614092935749
54.4586176399381 69.7677752398541 59.1774824918359 52.7787010032051
58.9056300364078 71.9124897002253 80.3500323413132 66.6841624416436
COQ9 368.727384734032 413.31638457585 730.168814097451 258.973633899103
240.731386634957 471.297503316471 455.444228161475 294.849830545881
609.520481504834 679.559864419351 530.996217072759 519.351335801817
CORIN 79.0304119352941 45.7317208802318 48.1997158668729 47.2009420656854
42.269054475455 50.4022543872993 39.0458123429605 71.7336217321663
40.137818555383 44.2580151149956 43.0016969050339 37.6557714476853
CORO1A 95.1513370156467 310.057823337984 172.237164781821
682.479411898549 99.4458090623294 153.279284223069 150.985239447761
158.613823276652 58.2401724760744 77.6056319020116 94.9001252449925
74.5886835786452
CORO1B 207.380865650247 415.05124917015 490.652353306749
272.36028618845 337.363827785838 192.318724749487 429.576301511638
198.129308943666 314.782545392841 243.843941346211 244.736442646499
204.659954879641
CORO1C 199.937572174194 101.882591864061 133.47090252916
303.670126704469 125.62281563325 478.296779356592 124.44575078108
140.698550434486 165.662539866309 317.835330028579 271.813877560354
354.973265582488
CORO2A 78.2644076847509 239.385229462309 209.967057706137
55.3141112947345 101.065203693106 57.6511495792664 172.95999061341
146.789051265805 203.228211195516 126.897002134472 143.773311381452
244.070778149931
CORO2B 52.1788489555942 41.2262949117288 44.9758339877851
42.357910063876 43.5836473788312 55.2515150764173 43.7585538437696
50.8160430472218 40.2615278292875 62.3233491851723 64.0948886658997
50.0159415986047
CORO6 69.0975200641705 40.3384318592985 40.5734458834617 44.7980740332174
47.5647821785529 82.9449579428926 48.6395795163502 47.0940613507321
44.5303781239209 90.6805895192707 86.2670415958076 59.5080588072694

CORO7 98.8719561245756 130.691004726616 123.597489107285 202.983984966874
94.1575209300592 103.139459009593 122.720768249799 103.631449539809
102.706563820232 99.548445157506 107.840833498938 68.4278345554565
CORT 47.0940613507321 40.9731067463382 43.1931731665131 40.3180310664156
48.2748123114598 46.1513019317765 40.4359177791566 43.5544347011646
45.4799270968969 42.1622837416589 35.4386677371439 41.9430737035925
COTL1 35.3227485563945 43.5182502375207 40.931294690669 42.9358255168086
37.0526522140242 37.2686490268265 46.4388841354122 35.7973685350741
38.5252456306656 34.9138625012006 39.153226699662 38.5279745308203
COX10 153.767031974664 294.51382100172 226.785831708938 175.271140689255
308.025419469334 173.745808259419 265.570814613305 144.079981029616
228.254228465645 258.973633899103 212.803471204964 238.246319539302
COX11 48.8333989709933 53.5281825103894 42.2577689927563 44.0451487108213
50.2163892855092 52.4351670989268 44.2166118829877 50.7364620410977
58.9870089759222 52.3304586399695 53.0798381965427 57.0524725494273
COX11P 42.2618615571566 39.0102066700839 41.6550823405646
41.0219290963778 39.7210115145672 48.3686935595826 48.739479557328
40.047314525032 46.2027193115397 38.7008388965825 38.2293008890613
39.3789825717286
COX15 73.4331096065698 70.4233710555202 76.2945357381722 95.8590592250688
84.0996028277101 88.9738853594498 99.7529382840954 79.0373265781543
81.2136969156029 112.17141283151 110.540953771795 97.6552689677199
COX17 1012.91803181784 1231.77360353254 1016.13142851085 1114.15989325456
1182.10714525377 1126.31998762007 1529.52516575545 1180.7204565402
1110.39060103557 502.557430906865 733.358170858378 1050.05355467301
COX18 44.5798743103596 43.896638326678 44.2733573199954 45.334106390212
45.1899271235974 46.263826584899 49.1067959632109 44.0390019242862
42.7150227340364 46.3810615657263 46.5650556481721 46.1592873342534
COX19 137.843308428621 101.759714275976 108.928854330744 117.699959435983
140.076084766156 77.9085946827079 80.0733119200914 163.261867589623
98.2512963929558 310.821299796803 206.234676343851 172.698530496379
COX4I1 5776.17749026501 5733.54417656207 7627.46814033012
4597.60989502097 4550.56817942699 4381.51418239047 8144.52133665388
4597.60989502097 7980.71309230447 6258.22561934986 6673.00322263275
5566.52516420571
COX4I2 58.8224410806741 71.5249259772583 66.1541420734416
44.550322123994 63.686652234709 65.3096524773304 46.1634822993009
59.8760452220989 47.3791873993182 45.1569741609597 56.4873776344986
46.917149434588
COX4NB 85.9970657980042 70.2553917409773 97.1271615802556
84.0899854321388 94.4129246851651 102.020047144427 137.547711779867
56.8094693588238 115.63300104804 76.5375674421131 83.6610321130843
71.1595608255838
COX5A 1383.53136710469 2096.51999792183 1027.05274681749 1191.01873119866
2225.65559724391 1184.75786736273 2581.34849558524 1611.35913846618
1842.15643221012 1019.67015337432 1110.39060103557 1554.42895572029
COX5B 1776.78784112613 2067.66516256598 2465.65854657837 1493.20520979227
2108.03351084959 2771.14077845128 1985.3999418878 2108.03351084959
2992.62035058532 1347.15664445545 1756.86714809925 2096.51999792183
COX6A1 559.9670724278 670.958499409833 723.853348464335
523.25106120389 562.798580332885 603.737787922337 853.117321142008
589.685739519627 1249.41227924272 457.202737754842 495.705224781189
630.150754732971

COX6A2 40.2543341073001 34.6306468196142 41.2365089689635
38.3166554965434 40.9950639693255 38.1169703906207 39.2533668167483
43.4544762830742 34.6720534245631 41.5435611403119 38.4689132737495
40.0557788909923
COX6B1 1789.07186722972 1533.38581753491 1999.21832626916
1408.59798540643 2248.64628069362 1428.47642890543 2854.82112538867
3088.44063817936 3234.36469403715 1466.88103496067 1436.67510926345
1817.14004766564
COX6B2 39.4710743516517 41.0678614026819 43.401782064778
44.550322123994 45.1137938810047 44.4619248757502 42.9803717121865
44.5658219748173 46.8873421271259 43.4885245598939 40.4504608195666
44.0246886382298
COX6BP1 47.5647821785529 45.9266745571724 48.4890615340603
42.2496337895968 42.7982502551634 39.2985791324641 45.4799270968969
47.5950227477807 49.3780606970538 40.6022003765153 43.5710871617107
42.8864415494484
COX6C 3263.05269150231 2854.82112538867 2178.59848996529 1963.91365010531
3800.19142219719 2506.05102956991 4328.723715755 2150.8715161597
3562.96193568469 2417.02418518222 2160.22713775462 2280.42953098693
COX7A1 210.880198736298 96.0838701986116 169.194312808717
230.664926508522 112.100074154279 1134.52018899688 47.1345949868029
101.272774711455 53.6446045619513 736.405815842011 615.838713743621
551.490493022416
COX7A2 1829.30985071028 2201.39729904166 2184.70038900784
2108.03351084959 1776.78784112613 1839.09068171097 2272.91548405176
2160.22713775462 1999.21832626916 1509.04753111016 1182.10714525377
2892.02001412369
COX7B 239.96421556928 250.959527806799 316.419421649196 339.854688605186
249.729778471447 360.668308246913 335.002151198999 646.323419285676
680.279119780451 315.186937216422 307.922754395377 542.069156178791
COX7B2 38.957687788868 40.148780650831 41.528370959196
36.0481934423077 51.2525124735102 45.6129956328415 36.5352572191451
41.8417024632349 543.640122689871 38.8507804383261 44.7926323492519
41.5536168238478
COX7C 2934.35616793443 3776.54943917367 4617.45430247751 3153.71394627237
3592.44409869527 4550.56817942699 2640.06306087033 4820.70258471362
5811.22276358172 3854.42494497831 4200.38495514552 5895.03230740197
COX8A 1144.34555072807 2733.80542760533 2672.94421680196 1850.06063216244
4328.723715755 2374.1766901821 3391.65926812276 2237.08790397818
3124.84336032646 1307.19925493293 1683.24873205783 1835.21901524331
COX8C 39.3709357615493 40.5400384479766 43.1931731665131 41.6564075217517
42.269054475455 35.9305494063137 47.1345949868029 41.3506832276112
37.9273653326866 39.8873706855774 45.8180710486329 41.1554481567603
CP 45.5758521267508 45.0905928998779 66.288032400574 46.3203995372658
42.1650127805175 42.750861693377 43.0930293831497 49.085741080241
85.7842344710572 42.0621425894624 40.723990462047 45.1735152121056
CP110 131.645714321903 157.615972646659 145.14768503072 114.224091812993
176.397140445822 170.475212920685 254.983975710833 184.964039515715
147.023857419777 151.451107406752 161.439879456216 177.845412804098
CPA1 41.3387790895716 32.5482839399637 39.1799070031819 44.7980740332174
37.1565984182084 45.7300501624017 34.0777108441756 36.3405039629059
38.6322728994674 36.3612409063792 40.723990462047 42.2577689927563

CPA2	42.442231667357	43.8495248991589	40.4882741869718	42.4580504587581
	39.859345745831	43.3785777889265	40.7910160811527	41.1575777497576
	39.3750969348308	43.0919221375514	39.6403089864678	42.1537476443423
CPA3	93.3605294458382	76.4439037944547	58.5212649095879	176.816741656659
	47.2074332636566	814.294297312057	45.8980298854198	62.6386437936516
	41.6616444147804	186.965367827758	127.863441809606	145.509901200987
CPA4	39.266565827316	41.6466169548846	81.4931910857436	39.2780515621926
	48.6224651100733	42.5660695551615	35.8227939208521	52.7787010032051
	43.4656637536244	42.8936990772838	43.5710871617107	43.2776458914333
CPA5	43.5710871617107	45.3486182278177	39.4710743516517	37.29566077996
	35.9058393060637	42.2577689927563	38.5066108855005	37.2237132058632
	44.2607789091176	43.7303813515864	37.4642807402741	46.3411353377615
CPA6	29.9775071862804	41.8073910693411	36.2683886571585	38.3166554965434
	37.4470183315826	35.0970060242246	36.3194282084339	37.86982638952
	39.9444191873552	40.1179210534118	48.6184776976167	46.5650556481721
CPAMD8	35.0202257459026	33.5924201549249	42.157627240486	
	42.4580504587581	39.0564222526273	41.2567470068038	40.4359177791566
	47.9942520395434	35.9645119596869	49.7259371799014	45.9104478407175
	46.2625470410195			
CPB1	37.1485601150873	39.0894172982439	41.6079958749402	36.890925044696
	37.956732373465	52.7156971154405	35.7055410599146	38.3684202018524
	33.802253812079	34.6720534245631	37.6484782516569	37.8490162439785
CPB2	40.604930211629	39.9311316520751	42.5406888281114	40.2563544693508
	43.1892285249551	37.8414621798121	40.2154497375715	41.2040352443154
	41.074824727477	40.9607305725196	39.0775870687758	38.9891430655409
CPD	411.73316510051	234.615033303459	315.487514440864	514.261598258395
	287.494740260715	280.526935074437	271.138311888031	262.37779288036
	518.527535205975	398.229956085523	402.794855141389	389.583938820073
CPE	88.2427413480277	60.6080172588224	66.737740728688	66.224559122062
	53.5594683028749	84.2728210732532	44.8643898647322	66.5234828659154
	82.3364971911816	96.577572948724	98.8922486048877	94.4769283676944
CPEB1	45.2875173643252	41.6003109209356	41.6977706798234	40.0195349109823
	38.8775560911841	66.0516224379323	39.9397419279057	38.745552403895
	41.1367478979484	50.1059857872211	53.3325829174829	46.2961166449607
CPEB2	51.1375316857287	52.0268699306396	53.4088637045445	61.0069245485604
	44.4489662507893	80.0042268333735	43.0269218496197	60.7488007939604
	47.7086093893038	64.7269156363245	71.3932787152381	84.8808332740321
CPEB3	55.8709511400674	56.3560498705517	65.4137646609535	107.242476674629
	48.87875862356	98.2843999216669	58.5588286084726	82.8218647837178
	64.7450441264824	132.959812230068	126.204310123186	86.0832675838966
CPEB4	53.3967242431461	53.812632592983	52.9111155236961	93.4853597306163
	49.7235523248653	75.8976034525022	41.8315602975157	46.3685439301234
	52.8502823653368	42.0621425894624	45.310660704531	59.987045425047
CPLX1	44.9896851005259	60.4646208319121	70.8606919488782	69.5544840164738
	96.8490167966217	57.4777075615651	45.8980298854198	47.9942520395434
	46.6944238390932	44.0409178714232	52.902043370862	42.0424641526302
CPLX2	38.8322787989855	38.9338521051669	41.0752399035779	41.353786352147
	37.9058271053655	41.3177250076876	36.3194282084339	40.1985790927248
	41.1742244762318	39.7453420559776	41.231791494452	40.6134262962375
CPLX3	38.5007204246293	36.6968906669565	35.2838283525874	33.9645433044281
	35.3327761997046	36.8059561571675	44.2166118829877	40.6171774724171
	39.4762546236449	35.6208308220005	33.8558232866797	35.5523662009011

CPLX4	41.2424637338424	41.0678614026819	39.6451483836089	43.074472921841
	39.4693871570028	45.7300501624017	37.7136011474903	42.357910063876
	44.7058233759912	44.1379638573455	41.4508241771315	43.7672102682067
CPM	56.7868634876032	51.4240355732132	50.1860191642359	59.0965219753805
	48.2129341876251	47.2505395290129	50.6006409539328	64.4707714725499
	48.8067321016961	46.614092935749	49.6314545328943	48.4869909888679
CPN1	34.8099089553046	41.9894412416874	43.4709415392696	39.853175885492
	41.467005949568	45.6129956328415	38.6243997507646	54.6067620335514
	39.5806465938526	38.2069499294002	44.2142581488029	45.6692241948108
CPN2	41.0970259989281	39.6995529770239	44.1452943804044	46.8954576545929
	42.3685136297603	42.4155142987678	42.4939802311136	40.1474543172332
	43.6670755115848	47.0797760763156	45.5707650534259	43.5738557773903
CPNE1	172.237164781821	170.87055409699	174.559216176175	116.547937420457
	228.254228465645	142.789889238338	197.895987726243	133.11655169884
	109.922909644299	228.571334092678	226.785831708938	87.8505898087072
CPNE2	49.2595762436995	49.4982025424177	50.0748292497578	45.4634652319753
	45.1137938810047	51.8103411703261	59.9792284253187	47.6952179922944
	53.9287524861617	44.7958122466628	43.4709415392696	46.4499258373233
CPNE3	1139.81870068001	1707.92349760322	987.427672417105	530.996217072759
	1963.91365010531	1125.31394383117	917.631567606105	588.247843264181
	990.431472174705	872.305108096296	949.278493776928	947.656220635965
CPNE4	40.8681871019812	46.29198852834	50.0050760710506	35.6027791404152
	49.8304479381623	43.9724628401189	40.4359177791566	50.3082459161777
	49.641159251878	39.4710743516517	38.4689132737495	35.9604262434141
CPNE5	42.6036491382658	50.6229884142872	49.6347864008749	80.4007868743886
	41.467005949568	44.6524308640784	41.253645578517	41.8417024632349
	39.9444191873552	36.5806287103157	35.5179217192467	39.5418280570561
CPNE6	40.9558083259517	39.8695929627783	50.2869791217819	46.0228732507872
	40.148780650831	58.1798515131249	43.3262148375525	41.9315419791594
	38.3418933808844	42.5664883002833	47.7535797917441	43.1805016690297
CPNE7	41.8767957887205	40.6695495527761	40.9856185999216	42.8742421276144
	43.9869272640159	41.4508241771315	42.006843375895	40.6783687486496
	42.2319133247744	36.5377349417874	39.9648352258645	40.0164662097472
CPNE8	73.0970339739187	51.4413099192899	53.7316344826448	115.913030448537
	48.1986674201502	112.721024337362	44.2166118829877	58.5406981871153
	111.501058276821	111.97030382494	100.499763255098	141.572530741416
CPNE9	39.9697332660833	37.4540772310118	34.5718473203083	38.5252456306656
	33.3231576795423	39.132725377815	51.0013344286851	36.5393857306744
	39.2809305246091	38.957687788868	41.2598458726002	34.4570158133896
CPO	41.1523487516364	42.1607322031054	35.9305494063137	39.7411455670172
	42.6022652963326	41.7627910693483	35.9058393060637	37.7612949798895
	44.8997235283254	40.8970401536578	42.911855821602	41.7575097298927
CPS1	49.6241203903354	39.3999056968598	44.2405728317107	44.265834798964
	44.2607789091176	58.8605811695948	39.3585350448719	43.0390857698452
	127.596368103014	57.2813205536176	51.4413099192899	39.6352613956998
CPSF1	271.961673480206	250.176074707634	235.682605856428	138.336991036544
	377.491895827114	157.020685801527	216.004059887037	120.660602388292
	184.048982286364	190.977045076469	139.796374175417	153.237127853072
CPSF2	142.933216800073	101.681822254799	143.706880936186	99.1438953564062
	142.309369995384	125.364451149941	126.826715174564	92.227126770051
	178.858202962948	133.893108529325	159.667153030627	120.571680725698
CPSF3	601.829364668202	252.649651285136	309.994784298282	317.325808319372
	448.446274966018	224.635749257133	648.885062676884	1017.1803754111
	312.045709918301	243.489225650886	245.730139853029	297.950627905767

CPSF3L 89.4747568145717 105.515662220161 97.2615918872914
80.0814445394033 138.728648830528 93.7924763988692 113.430943555215
104.089998273812 110.626479311082 118.854313907627 119.434418653131
96.4913003384143
CPSF4 414.062259640915 280.402165030951 306.895449954881 195.765247956108
528.840700144811 244.736442646499 355.92328557644 384.919835924792
515.409979010663 332.137622104526 293.702642091269 267.85990321012
CPSF6 49.7188990489616 56.6927078228789 57.6371756925687 42.9358255168086
73.0433648756396 46.7800033479223 60.2399886747451 60.419911425109
60.0736861987141 61.9882398986333 52.902043370862 64.531467526747
CPT1A 50.1533437545239 51.7676177022233 45.1846041469628 51.5913529948887
52.9433495155796 51.340484930067 50.9160746031872 47.8277364640334
49.2879849674517 46.9711270147079 50.8485682149772 53.9918882088235
CPT1B 53.5643281930979 79.7061415085215 65.7002710293248 61.1630754250653
67.9531693869426 54.0969604236265 57.4047331041592 74.7127063822938
62.275808835675 69.3468840908665 76.4389426517528 63.8558858499025
CPT1C 69.4501305462809 50.8812920005251 46.8267540220952 66.5921967967793
173.18824792041 63.2343637481705 49.2546642180676 84.8569316561572
52.0583273941813 54.4825201506931 60.2867996701867 55.6351010690528
CPT2 327.631923082696 415.364456363434 368.972794063004 192.264432082651
264.495576211074 122.15017220566 311.301613620475 201.163076338483
227.535855856645 212.152751915521 245.585026913915 271.138311888031
CPVL 249.246628143531 169.168332370229 109.659686701319 925.486088550061
103.530430972911 631.110081413363 169.790843803064 160.171133984141
93.3718038663074 190.214361156876 97.8389686233842 105.63434679637
CPXCR1 42.7613994022988 44.3529919625344 41.6977706798234
38.5252456306656 43.844855336215 40.7616966228404 46.6267962186599
41.3012916486748 42.846832824953 40.1179210534118 40.8681871019812
42.1860813954352
CPXM1 240.841214170409 59.1938299291358 45.8844325913247 148.059385111127
60.4122350478242 121.969082013079 39.0458123429605 60.0835531094681
41.9635525160083 54.2830238965339 49.1868644496737 57.934622029119
CPXM2 144.799432299339 98.0023132593711 79.0962508551502 207.957319374194
73.3533274030352 574.847203528544 53.7423898357583 79.2701322339022
49.9162522057712 238.338646661062 217.263889616995 431.727858902449
CPZ 81.2992152187529 53.8909465631671 56.6888826857797 69.2685201884906
82.4164506744 102.322851896214 48.6395795163502 54.629382710848
44.8681974656149 78.3186115281795 65.088713927995 47.4713070963518
CR1 41.5635499110307 41.0212349917651 40.6210258686042 65.4068498877665
39.4141123790339 44.7022992640631 42.6995880296584 40.9861485852278
38.2981083038519 39.3866822630357 38.3684202018524 39.4830039788612
CR2 38.4344387289957 49.7827734675743 40.5289532121417 112.17141283151
38.745552403895 39.779935291659 40.0052661202207 42.6008809597607
42.0621425894624 43.3438593008468 40.7670908011804 41.6550823405646
CRABP1 36.1232327599945 40.0527893189096 40.4070046183108
40.1804147506786 57.5685357492202 45.3882138838137 50.8388984085636
70.7805103907761 45.3756389170606 41.8613406887824 49.0361180089431
38.2350720635382
CRABP2 1490.1549980903 97.3813557754832 1418.94307844471
123.841740274811 1442.72006787259 326.70962514228 1126.31998762007
1351.49940447914 1858.94179312821 305.786520619504 282.445037659981
288.816567137193

CRADD 77.4999637278723 132.730723037077 134.952392466784 208.7739716139
80.6200293361016 127.115038236278 133.877825732645 89.9227845135785
183.336550845401 109.326279686847 121.195506364658 137.034037711549
CRAMP1L 127.694844372277 180.255771684507 246.820301517765
102.786429761295 136.504157082236 122.405413080836 123.561242462957
80.9092480872499 94.2275161759277 129.272985305607 135.490875023792
115.441414762268
CRAT 66.979105340228 74.0614295574729 58.9135553403521 45.5869891570251
53.4751250621694 69.2001494999588 123.608921653229 102.926945888985
70.7003843117511 69.5853093594169 73.2234405348435 57.4844872128664
CRB1 39.4710743516517 44.4639405506967 39.6833800000587 43.0016969050339
40.5235471987417 37.4782441311626 43.4544762830742 40.0965255448873
40.0870341983328 42.7275315121163 40.1742127891824 39.4830039788612
CRB2 39.5676051939342 43.9421822132391 44.9758339877851 43.4410741327254
41.2740822675592 34.0777108441756 37.9430145836712 42.157627240486
40.4220196471124 41.9666541498638 39.5399188536544 41.5536168238478
CRB3 189.015214517448 297.662970257144 184.235914591152 53.1249776278067
291.086512261106 80.099836448859 280.275836993258 192.264432082651
239.229093340475 131.319040057576 113.322929203297 97.0589984189239
CRBN 132.684895918889 209.931773196136 195.672836308456 210.38953368311
165.411942074375 406.417924060562 162.735117558467 246.869486382264
164.146682514925 238.510952165358 196.267504470029 217.065944053174
CRCT1 40.8681871019812 99.9752275166514 101.2615980834 69.6511571901941
44.8897737954573 49.1013967533186 45.6042625176633 61.9787117490846
42.1594905198929 45.1569741609597 42.4580504587581 44.1452943804044
CREB1 2733.80542760533 1459.69929955338 1796.47241860175 1939.66920192372
1201.00042684883 865.63777294494 1945.33618761041 3234.36469403715
1087.75682788799 2878.82618761804 2721.97551918611 1975.28674300761
CREB3 48.5835484742456 41.9894412416874 46.2625470410195 40.1804147506786
46.1804647841331 43.7630587657205 45.4799270968969 50.5070012045444
45.2897806651929 43.4885245598939 48.2050207071554 52.1186591838715
CREB3L1 40.148780650831 43.3458769998118 43.5710871617107
42.8235313007746 38.0598374201407 40.5281496800812 44.9863726205428
47.5950227477807 40.8970401536578 44.6918600441328 46.0905079010237
36.449101775775
CREB3L2 784.951880532127 683.099079617304 487.261132702908
936.978983469829 351.700618429121 347.196922795214 282.646286144563
738.059797123491 291.42149037871 378.964387825252 380.736562250846
389.364650157102
CREB3L3 41.6354649482853 32.2892762042267 41.2365089689635
45.0798408927912 38.3561682218911 36.6996187567892 36.8369172900902
40.931294690669 34.5633623842083 42.2614524730856 34.3633615460307
34.806104801006
CREB3L4 52.502055890816 53.4109882045447 41.785676764591
38.2041059863839 40.5710637884029 44.5544727111028 62.6885730386156
56.2441895183416 52.4539007817929 48.0211752672327 46.7386599624801
51.3244198342335
CREB5 43.120110422996 42.1607322031054 43.3427991470083 77.6049044026375
41.4307783284114 60.6481332392553 44.3977035913759 45.7995623076688
38.1679953604288 44.298892175909 46.3715909653832 53.6895401581575
CREBBP 155.378768637562 159.046483157634 190.099949303905
190.099949303905 151.255948388415 146.87603018486 118.67727980117
110.487281983469 187.147252401703 187.331614826395 195.864827360933
164.122105098254

CREBL1 41.2424637338424 45.0115870036541 40.4070046183108
39.2780515621926 42.7982502551634 43.6670755115848 43.1931731665131
48.6441988290952 40.0403898620771 40.6022003765153 45.5707650534259
48.6795178347248
CREBL2 41.5421429082231 42.2577689927563 42.5406888281114
40.8681871019812 37.1565984182084 37.9003288112261 32.6530576979948
42.6450179841129 36.9060997041453 49.1928206164702 40.9157352260901
35.1451084093692
CREBZF 69.7442393277127 101.940584589951 89.2285288333749
56.7927450229377 136.04447103201 62.0660509811111 95.6697427870979
81.2850357499192 92.1817062395346 60.2877434874622 81.4702220063796
89.5687388187015
CREG1 307.264133974364 576.140066807763 484.050852319927 1086.12171444149
668.584452504579 958.473187253938 389.686780706341 231.154569340492
323.705254926762 560.413725186282 489.622327794168 812.814329906663
CREG2 44.9896851005259 36.6968906669565 47.4740726032707 45.4634652319753
37.6527901109005 37.1317006162938 37.7136011474903 42.357910063876
40.8970401536578 44.8997235283254 44.4688999098904 39.1553169409075
CRELD1 88.4385631861141 139.022985518859 133.259195601344
74.9236800269025 139.758805241617 94.4618437533343 88.4385631861141
97.9497090233081 76.3168793429802 146.737709403779 105.15507796881
130.775349446374
CRELD2 641.462632112151 624.097858948785 713.243132093773
663.904584382358 868.899376368472 469.553640187203 339.679549262298
414.794323433931 597.191426276771 321.21879937379 487.64491967937
323.002018912848
CREM 42.2455707947223 39.5050383901884 37.5597671487621 39.1706564265495
41.5668884168669 39.6251842352088 42.1607322031054 42.0494116081998
38.0213051227859 38.2069499294002 45.9104478407175 42.357910063876
CRH 51.8781065691038 878.863554510943 59.7483827511172 46.8954576545929
5811.22276358172 977.776395810338 374.723141438448 3602.30252142918
171.770578381558 62.1035858593388 56.4873776344986 46.7182294568603
CRHBP 41.9407041937973 43.683792219729 44.9758339877851 46.614092935749
38.8834538955 56.1671440556142 45.4799270968969 43.3262148375525
44.8997235283254 43.9783383422823 46.0513515682555 41.3948836161924
CRHR1 56.2508140932179 52.153603364404 53.2046867327331 46.7577982595088
64.5531442955004 53.2046867327331 50.6774883370457 58.0892402609917
59.0216019867146 56.1912096308988 55.8173045043348 56.2804354745795
CRHR2 33.3307770183871 35.4484652445078 37.4642807402741 34.3595588146954
29.9775071862804 35.8227939208521 36.634024014558 37.4484310268909
38.0213051227859 40.8049408996274 35.1470994647364 36.2582325396375
CRIM1 56.0477607422828 122.531570028682 96.4740168904128 52.3004056941891
77.4769634299218 110.312681023811 97.4515987591398 62.1934012861178
64.1267046511632 72.3297091475814 84.8084949516218 157.133627517432
CRIM2 47.8249696958403 39.9697332660833 43.7630587657205 43.074472921841
40.7779767565889 41.8613406887824 39.5496902678788 44.797320243931
39.6609238036815 40.2112371512838 43.7585538437696 32.3796054199032
CRIP1 814.991871217268 249.704644191338 289.17237603899 783.440408023061
80.257554904218 908.328776521114 206.676666936979 953.143041145545
139.04252811433 288.237195966495 261.6201598868 501.452602126588
CRIP2 1110.39060103557 588.736435715365 787.671342652389 277.548019244696
153.920604317629 552.162906190653 474.155196153863 418.410951118141
741.908952472992 432.001201940414 478.296779356592 349.90835241442

CRIP3 43.6706764143908 41.2598458726002 48.7039705048129 39.066284740632
41.8806343997257 40.4359177791566 39.7576136507593 40.7311497914871
40.2274522635576 45.5776159432079 43.5710871617107 41.1554481567603
CRIPAK 96.4033477961156 172.39907939484 228.134171304754
120.436827608155 102.085154509866 107.309776354951 105.382126899524
90.1430468048409 96.1710806345503 127.937436596431 106.654782265295
99.6243435693941
CRIPT 179.994715581707 209.750053979826 273.981867154936 199.522267292552
140.719373122712 175.876518898179 167.872060678879 307.329227013816
166.097017297742 159.844078593375 167.920131968611 281.262083731954
CRISP1 41.7397731376692 42.6939394537076 38.6727842796202
41.3027031037343 38.4135023167287 42.7107928951766 38.7337086062875
42.7431359850339 41.9635525160083 39.5637454855233 38.1835938174625
42.1537476443423
CRISP2 36.6480005877129 46.6996902348633 43.9450023643394
44.1483976313959 42.3685136297603 44.3648993568405 52.2476631126873
42.157627240486 42.6366765186182 44.0409178714232 44.3763859390287
41.1554481567603
CRISP3 36.9060997041453 41.2598458726002 41.437237454134
38.8495095797881 40.9950639693255 36.6996187567892 38.7337086062875
37.86982638952 42.9020321355927 40.4136535208406 46.824299585607
39.5418280570561
CRISPLD1 43.5710871617107 45.3756389170606 43.5420658412177
46.0905079010237 43.0723817756832 45.1873487463472 44.7255207589606
46.4672637826582 61.2510119532409 44.9673049606589 42.046575509735
42.0709558258414
CRISPLD2 813.242005949542 314.992390390165 162.151812425949
586.281182112872 421.445268560815 1052.16097214718 196.200780398883
758.462554576949 171.302214895226 641.837188326604 869.879392791086
761.922486104216
CRK 224.60637398191 245.769212889294 237.039707901886 211.451463038315
286.11425202743 271.275703732022 226.090904721424 146.087016079575
280.034472186873 158.590601962928 188.887559923238 219.918644151214
CRKL 685.386956092142 588.972273484465 625.805967352327 577.993850127555
768.888528247493 538.716947884528 612.014486689487 605.970682363975
953.143041145545 893.185203928127 781.223434214089 801.351998609551
CRKRS 79.0304119352941 201.486224052899 183.746651924774 454.293803256904
102.085154509866 60.5803031036931 90.8691042645133 106.292775688671
78.9521593412581 80.0651801266824 76.4389426517528 82.8275497073451
CRLF1 44.7044140979108 51.119812302881 49.3780606970538 44.9370339275041
59.1948983423381 57.0775902639215 46.3057385979341 43.7396619462033
51.9545473262296 52.0999467564713 53.2083147523758 57.8111054584154
CRLF2 41.5787026298957 40.1989248349075 42.7815195866158 42.0102671859606
44.4088785101698 42.0574848021044 42.6786833450085 42.1812299265547
41.9180247412837 38.745552403895 41.0441816173391 43.2312848495114
CRLF3 93.8361140725228 129.414877519657 97.0155195562193 174.368595478868
98.9657264176664 106.418690759491 134.19834305339 75.9659252679925
98.0023132593711 61.5112659162073 85.33395043174 93.0714382925187
CRLS1 380.098235864256 398.641868389511 359.83890067786 374.978401470437
709.730555484551 469.78210773245 499.356036793973 263.706801812277
396.93169345515 179.767874494671 275.928723831899 501.148339791372
CRMP1 53.6741873729867 54.1443658899385 48.2415341573501 52.8041782638549
48.0234896130991 47.428201097025 43.1563544904505 52.6529161327083
45.0017158586743 45.8424887733913 45.4799270968969 44.2925355984056

CRNKL1 51.8781065691038 57.4054069675016 63.6334154351075
60.1837189855762 87.6178717341967 69.5645254789371 57.7805922926828
53.838484549134 52.0583273941813 64.7269156363245 71.1607965028029
59.5080588072694
CRNN 51.1740855632044 56.1355483199198 51.3268475761593 71.7167037224069
58.6481644777552 41.9543948167375 45.3597029614942 127.596368103014
55.7522234735038 50.5252048155459 50.3221380889396 45.5800840647989
CROCC 48.3814421939888 47.5647821785529 52.7005092555102 54.6089451252049
69.3468840908665 72.149478755262 56.4809146784765 66.5234828659154
57.7865070833351 66.4510869175159 78.7260936224802 60.1837189855762
CROP 91.903810379105 115.441414762268 95.9485602132766 80.6317356144732
112.202543937743 85.7208881534518 89.2913927776683 111.910324041953
106.042585997437 133.314819245044 124.611407530723 123.841740274811
CROT 89.1632548302738 239.023368693467 419.054753590648 64.6945454069792
105.825228126465 81.9983163893373 200.002687565519 201.427008153788
226.235985918654 504.281506941044 433.89783033465 457.202737754842
CRP 40.3478825593224 43.683792219729 40.4070046183108 46.3203995372658
39.3667161654772 40.5281496800812 40.4359177791566 40.7311497914871
42.6366765186182 37.8217989841918 42.0750889188255 36.9976231206067
CRSP2 48.2731865958235 46.5650556481721 49.4129140341868 46.3884811643547
46.2437291185739 51.2270031175419 50.7568454312473 47.5414586146928
51.6225091954608 49.7543329469677 49.2965344002582 56.5500409758451
CRSP6 58.9201382292404 52.2501726671381 52.9111155236961 37.5382094267692
47.0954207417646 51.8103411703261 59.8273072817267 41.1575777497576
51.6731332961917 48.8623729374733 51.6414075056938 46.8129509799753
CRSP9 82.8485522398913 57.8005007012205 75.0801071257739 66.130427690095
58.174555206394 73.4820932957859 47.9665737016961 63.9851732498913
67.1278109419819 65.0423160790157 65.6824363141962 73.7986685173579
CRTAC1 44.436995101921 7127.13565491501 4128.92158369026
167.16650846037 331.935072199058 175.550329937952 164.580141651883
62.5528124447176 203.373397813051 3114.05035137552 2892.02001412369
517.216393517181
CRTAM 38.7537364570873 37.5469247328848 44.1452943804044 42.0102671859606
50.7615314686244 39.9397419279057 38.8417151851134 34.8949555140527
37.8319142847141 37.4302011516587 38.5668610487033 41.2437378488025
CRTAP 117.355761277966 121.696160351886 153.992331037033 129.352472514981
238.164418453962 458.47341225284 87.0685571351947 84.1922656126304
112.362505628749 123.817018264773 164.780216383719 181.705809767061
CRTC1 67.4846787600629 60.8608693323868 83.4679576894911 51.5913529948887
79.6516904169771 67.8208976798364 60.9648845690177 72.9074911694002
66.3209349903185 72.2443746456494 85.5839487225537 52.7530873093837
CRTC2 58.8224410806741 54.2924642952939 52.6275131390249 59.4674205670657
50.3713170502557 46.6872654833579 54.8074655158941 71.5062409407193
59.9108736364178 59.9354531144375 52.022479439889 64.531467526747
CRX 40.4639532172338 43.1805016690297 43.6670755115848 50.2163892855092
39.6535880152834 40.8634043028528 49.2546642180676 38.4540849632254
41.3803269429468 35.6208308220005 41.9083581529276 46.3411353377615
CRY1 125.713216265584 94.1050519074545 73.0686482917042 109.144663527001
89.1012752492647 105.414552529943 71.599955759893 101.681822254799
117.565415291934 54.0917531668054 75.1562329744435 137.302487233881
CRY2 66.0303950772736 66.1288889125299 75.7464604972652 63.1604223919703
53.4109882045447 128.776189744323 50.0088611244948 91.5258329186709
68.3157692917711 165.42634596698 171.176393131741 116.17375251898

CRYAA 42.3433931248155 43.7644428186265 41.1367478979484 39.9697332660833
40.8873907150245 39.3841861812262 41.1507980856436 44.8997235283254
38.9834959368496 39.5637454855233 41.9908237694988 37.4554876386402
CRYAB 155.270252661048 91.1741538903522 86.2895084312264 152.679927202674
100.90948905426 1656.16581692717 51.9421032049606 109.481322353402
48.0163377492228 623.574706535278 1337.53997230271 1611.35913846618
CRYBA1 46.5549623118323 33.4967065724734 48.6037684272115
39.7411455670172 41.3755760434934 41.159706131351 32.4466419672036
34.3865306339645 42.992884918158 42.9866214102525 38.2795150354677
39.3575468494941
CRYBA2 44.5282181324797 45.1694246393676 44.014441886238
41.1507980856436 43.1847628331565 42.6575535390785 41.1886975241176
48.9826868640753 42.6366765186182 44.2580151149956 41.8168470823451
39.5418280570561
CRYBA4 39.3709357615493 38.0226905622357 36.5474283500601
42.4580504587581 38.4540849632254 38.9378717994178 40.4359177791566
40.5158867964016 37.2810767789306 40.3235655331796 43.9450023643394
39.5418280570561
CRYBB1 45.1912496459517 40.8880530359822 40.7620472083159
41.5464765641928 38.8456942580061 49.2050538150106 37.1643693306382
40.1474543172332 39.1790517315259 38.6454098044474 40.6373400186325
38.6454098044474
CRYBB2 84.0388582916959 68.3372228877569 51.3268475761593
53.8086134112366 58.2780916793348 55.6242009731278 93.5928272702304
158.081235081686 77.9028721785469 62.5036823572932 57.8111054584154
59.4465684293847
CRYBB3 39.3709357615493 39.3999056968598 40.4070046183108
39.066284740632 40.8873907150245 35.2067831864574 37.4772922422734
39.1652965788406 42.357910063876 42.6793753550854 37.6484782516569
36.449101775775
CRYBG3 44.5282181324797 46.1569135229922 42.9104454767916
42.8235313007746 35.1411239266238 47.5733603903505 38.4005795891396
37.6557714476853 38.7298222155532 35.0668743708816 38.956280281655
40.6614700944853
CRYGA 44.0328992213727 48.1627097465883 49.9256753636167 43.9282059368202
43.6587492470348 44.6524308640784 47.8947595316832 50.9933795450314
44.4395580493118 39.2623423164488 50.3221380889396 41.4508241771315
CRYGB 39.266565827316 38.4330755397458 38.4043916290561 38.8495095797881
41.9842695818285 38.1169703906207 39.4600924554505 38.3684202018524
39.3750969348308 30.8150126829901 41.6336636285875 37.8490162439785
CRYGC 40.3478825593224 41.159706131351 39.8299340037009 47.8164193292842
38.1644569189523 42.357910063876 40.5585676679482 41.2551966132486
41.5635499110307 40.4136535208406 42.5426057453993 44.6484005362281
CRYGD 38.304249186058 37.2810767789306 40.5734458834617 37.29566077996
40.0599807618105 40.9506062495231 46.5650556481721 42.157627240486
38.2215591930186 46.1390887492649 41.9083581529276 40.4639532172338
CRYGN 42.6036491382658 36.6002100641539 38.1421709520616 46.3203995372658
30.7606118575469 39.7394181011603 36.08956004634 38.079222289614
42.8036731479932 40.7063426028871 36.8282383070068 34.4570158133896
CRYGS 70.083473392272 74.621822948059 78.9701459806641 74.2429851177794
61.8471691369569 57.8005007012205 47.2927436772465 54.5125550937898
56.0496524902806 78.59966540743 66.274247400793 66.9239565096247

CRYL1 93.8361140725228 82.7351935657355 98.4930941487534 98.2447263268855
67.7155610106537 153.515845356859 83.2162112994735 113.528908699366
77.2413564369342 113.169111095305 105.953705702398 134.633769604568
CRYM 45.4321966598751 39.1982470626863 38.5470982022687 40.8681871019812
41.1868052124344 54.2924642952939 40.3850714987723 55.6351010690528
40.5465626311506 47.611018474151 47.2134341420411 43.2115532536912
CRYZ 221.195872577098 594.184150517102 225.559405596065 109.726577835621
107.646103699516 313.233036704221 364.795587956819 228.185436206823
97.4062608315831 656.355333007615 461.83420090894 632.577797921655
CRYZL1 111.6348079332 143.068901273486 183.311305255895
93.4180535865914 129.754481922118 176.064476306748 115.861269747317
123.908152883436 104.986694941968 194.207518642389 172.221482103025
196.561401686392
CS 378.358853591082 432.355300448283 433.637087338002 340.370474635434
442.080188140864 325.563540852801 542.069156178791 515.409979010663
410.808988840343 375.095491465679 319.722393409101 383.779263351825
CSAD 88.3335649692467 113.624887028422 147.043419185015 72.4834467838798
88.3954618645976 73.8105012820769 69.1414574152997 243.549194928524
98.0023132593711 92.9043863423147 101.954354107731 120.221914344372
CSAG1 40.9558083259517 42.2900457798748 40.0557788909923 40.5484717678957
46.4732328055735 38.7578265341627 37.2892641289258 41.9820792173615
150.636903991608 39.3698811593364 42.911855821602 40.716553247236
CSAG2 36.4564050256915 41.6466169548846 33.553634633839 36.4564050256915
35.0181575202066 35.314469458379 38.8417151851134 39.5466975353131
46.1101825640108 39.6719190054379 34.8383995317036 38.3291713594599
CSAG3A 46.1673159982664 45.4143854024453 44.696023817801
40.7481155008596 44.2607789091176 44.6524308640784 43.3262148375525
41.9820792173615 112.202543937743 43.781101409552 48.2050207071554
39.3789825717286
CSAG3B 38.5372368196365 40.4882741869718 42.2577689927563
41.6186264038868 39.1177164316495 40.650742261428 43.5627649099623
43.0944129868777 141.784776736918 42.7211272990786 42.2618615571566
43.4390398501329
CSDA 1213.3477481374 2077.74475301595 2337.7738990994 1320.7894611262
1526.94101719128 2513.15722262176 1526.94101719128 946.081492716653
1848.50311456636 1545.76489731307 1835.21901524331 2689.80835574654
CSDC2 64.9367707455078 39.8695929627783 42.5406888281114 43.074472921841
44.7829632472754 92.8131698915896 49.3854238365325 48.0582855095143
43.5669306063196 77.4046818339539 91.8414653633828 69.51607174053
CSDE1 60.9009676344258 59.2251746728404 66.8288956287896 63.8694991450134
66.7426485694967 96.3976093220249 66.1940959364976 59.5830868881343
62.6885730386156 76.9603875266956 78.5325880060762 79.2989156727401
CSE1L 358.779372054826 361.023743912857 409.899024157206 242.080992016235
503.661461720233 344.133384411423 589.395944599882 559.9670724278
540.304257899761 329.282615395786 277.796753761378 447.248719481853
CSF1 42.4607795638552 38.8561375693335 40.0998167098057 55.6481366056639
44.931941140132 50.5761139779524 43.3889851892323 41.6972107715212
42.0621425894624 41.0529409675663 45.6895223823001 44.0451487108213
CSF1R 431.727858902449 431.018580441405 533.820505121067 1377.24483375075
265.860397918913 1086.12171444149 229.428422408702 289.17237603899
83.4465817617201 383.966991071954 227.243009473121 185.312851727212
CSF2 43.9309845324767 40.6380495994768 44.3344900481112 61.0845101400379
35.5220586859301 43.0860383229288 46.0228732507872 54.5125550937898
40.7847110517594 37.0320432433314 37.1059647239814 37.8490162439785

CSF2RA 481.905052451245 232.740032714891 259.220844287491 731.071171171638 184.377148097206 163.946447772618 331.173265739654 719.815240381415 174.263925108623 443.269396125834 441.5479786095 359.628703006773
CSF2RB 37.0320432433314 41.159706131351 41.528370959196 135.138667957956 45.8051846088552 44.5544727111028 47.1345949868029 43.2551508830106 35.1155486926999 39.5637454855233 39.153226699662 39.1553169409075
CSF3 39.1919930809584 38.0226905622357 40.641503836284 44.265834798964 39.2036764702265 62.9126261960734 39.4859911554583 42.4864576619427 38.7537364570873 39.3698811593364 36.0013326338316 36.9976231206067
CSF3R 67.1387898740161 84.3995736970187 106.537423775385 1676.22158135012 99.2341244350412 145.865118592916 283.88971280023 62.105802634526 47.1098984140276 54.691568065544 50.7903824729818 55.916393897815
CSGALNACT1 55.8218917166321 49.2050538150106 47.0055981216687 47.0874209746031 43.004943518025 61.6219790276408 35.2163900400329 38.4540849632254 37.1820543575123 65.3377940117969 77.4625808567081 53.5643281930979
CSGALNACT2 37.6397652536077 38.9338521051669 37.4642807402741 39.066284740632 37.2567512265363 51.294537308199 35.9894133432376 40.7311497914871 34.4716904283753 38.957687788868 36.9060997041453 40.0557788909923
CSGLCA-T 99.6265783263109 103.883220270094 158.321459945365 150.336199715245 105.317399386109 73.688423848106 81.7434024567356 80.9092480872499 100.874437079608 84.5055910320032 86.6798536307388 87.5074297754031
CSH1 39.3303857231606 43.4653857912983 42.7815195866158 41.1886975241176 38.9863369890964 41.554942319225 42.629412460409 38.6454098044474 40.0952076421149 40.650742261428 39.4736654224088 40.3691324547049
CSH2 38.7537364570873 43.3972849968032 50.5089777244781 40.646702696033 38.6454098044474 40.8091178323174 39.1539892144371 41.2098211956301 39.4762546236449 43.401782064778 40.4094901248527 39.5792604499113
CSHL1 44.3754281580557 44.6918600441328 44.9107703930879 41.7614975630362 46.4343327861696 43.4215162178364 44.0256815382334 43.8783254873901 41.7117861328546 44.3648993568405 44.6286581923295 41.4061152929154
CSK 733.358170858378 792.880882528835 343.304521604977 667.710357746751 964.268294825556 289.213144287085 1247.50618540769 673.522667186915 764.571579067138 458.47341225284 388.132295362791 306.294404016622
CSMD1 40.9157352260901 41.6466169548846 43.9450023643394 43.9282059368202 38.7864161493321 40.1428591998404 36.4564050256915 41.8767957887205 36.4476883897928 42.233842834629 42.0750889188255 36.064718696392
CSMD2 48.8941062980393 39.0102066700839 36.2683886571585 40.7481155008596 44.5668141895992 46.8954576545929 32.4466419672036 37.86982638952 37.8319142847141 40.5090849993014 38.6662194645633 31.9386613757097
CSMD3 36.3649677956953 40.591923288644 39.3344387353654 39.0031581123233 40.8351751854504 42.5089442785482 40.2649309204478 37.1317006162938 39.853866204927 36.9795961564828 41.4918397757335 36.7172300749476
CSN1S1 43.0462952354436 42.0709558258414 38.6414316877471 38.2599935139101 37.4470183315826 40.1930209223556 43.8835874380262 40.6723334103791 36.8127605061228 37.3687900733549 38.745552403895 38.9834959368496
CSN1S2A 43.2724556187214 45.4882438975269 45.3974838013476 47.481302504066 44.4489662507893 42.8598805316863 43.3262148375525

38.8495095797881 42.6366765186182 44.7958122466628 46.0095710163378
40.7704587752083
CSN2 35.1307934330047 41.8848959280916 45.3974838013476 34.9221408381038
35.3327761997046 39.8455060654531 44.2166118829877 33.9687743313429
45.0017158586743 39.7835513496873 42.911855821602 38.6454098044474
CSN3 36.2496364442264 42.3550738181144 41.6079958749402 45.5869891570251
40.5710637884029 41.3553369544097 41.3594868055879 41.5464765641928
49.1940901905636 38.3092241935352 39.5399188536544 37.4554876386402
CSNK1A1 85.9555871743678 110.431464700539 128.635811501506
108.589036223189 99.3142021810133 118.341308125578 76.5505585423421
82.7269382676336 116.007007037602 73.4019696977096 78.2839612497413
118.899737794318
CSNK1A1L 39.1590964332473 41.6466169548846 42.5890635915791
40.646702696033 43.3896803974186 41.3553369544097 42.4939802311136
43.2890595186135 42.6762317063737 38.957687788868 44.7752571196612
42.7782216342806
CSNK1D 248.599530435666 348.964065997095 353.751797948675
440.421077161585 329.282615395786 191.141778714144 368.793978305607
325.172633427119 366.326747630465 202.795613942391 194.207518642389
176.839214683647
CSNK1E 323.957165066178 325.275883498219 207.741669973713
184.087380444203 312.40869171788 284.437758089699 608.886783362316
446.902178094675 257.325282051981 437.84407370064 368.318359434313
341.397811775105
CSNK1G1 69.0975200641705 59.0216019867146 50.3334140358007
68.1040271620925 66.6118713143653 56.432280646473 63.4685821260797
77.441946514938 65.8926843080114 76.5699943825825 64.3824529624475
79.3315961732103
CSNK1G2 284.013140015092 370.150839052727 370.240482282297
210.958578914607 394.255628555051 200.019523376661 292.86576686019
288.190015621249 317.483708873867 193.364522980854 286.996922972683
222.696947669812
CSNK1G3 65.9900865113645 69.4820178305563 83.2737524877952
69.9101676350298 82.97559574338 79.5270065657539 70.2288685232877
65.9143782252686 88.9641595603127 64.1675023456571 70.5704019848974
89.8808683919332
CSNK2A1 102.380096190518 113.051477801522 98.0023132593711
70.5992463788381 161.008205823493 83.4033736999037 144.976378707447
75.9136854087044 96.5175130622216 68.8167148906225 76.7806009547111
95.5724734464786
CSNK2A1P 85.7983996395757 107.70358681113 102.00080367858
88.3459116251233 127.701556328274 82.8652108020571 118.816772641597
94.339011229577 82.4975645442705 72.834275688835 71.0369238830779
73.0464199386528
CSNK2A2 285.626835802986 303.670126704469 371.042302128933
218.917221711155 187.430528830941 377.409970666607 314.533750876361
182.685579297058 377.847116683974 423.608694638779 347.750020791938
357.800871454252
CSNK2B 1423.29307392266 802.650020720921 968.405745860353
869.879392791086 968.405745860353 794.806085783439 1017.1803754111
602.047768767953 929.862002014478 950.740991182127 758.462554576949
596.84269596976

CSPG4 56.6951078698291 53.812632592983 74.0913177754682 51.223859150798
66.7426485694967 112.23915619297 34.1889222434768 47.4740726032707
43.9450023643394 104.288073521816 96.8490167966217 80.1461294346211
CSPG4LYP1 36.8072833828297 38.8336427948187 42.157627240486
44.4367387274922 42.9090346219023 42.357910063876 45.223075311113
37.86982638952 39.7537274655115 34.6720534245631 37.4642807402741
36.064718696392
CSPG4LYP2 40.3478825593224 34.8137085420423 39.6451483836089
37.7469621225553 36.3390947880186 38.2293008890613 40.9157352260901
37.2237132058632 40.5912213523345 47.0055981216687 44.7926323492519
39.9519547348106
CSPG5 40.3478825593224 44.3529919625344 35.6139301508335 44.6905691366696
44.7829632472754 37.2686490268265 42.1607322031054 41.2551966132486
40.0403898620771 39.6719190054379 37.0180934800573 38.745552403895
CSPP1 96.8783070574131 92.9453353261232 80.5537984388921 74.1068645821427
110.869341159607 78.0013795192762 77.9028721785469 60.0835531094681
90.1032802171477 78.4289643729837 75.5799609581534 88.8330881965986
CSRP1 1080.43016747082 671.959673037066 675.079525434102 603.224267841548
382.744696166534 7323.77427153966 387.867128019374 842.3367709562
560.954913956997 5776.17749026501 5895.03230740197 4665.45637589484
CSRP2 87.6178717341967 142.863673914134 68.3234331335073 83.4756142147167
67.7155610106537 197.421622315466 140.264270771578 70.2541961995753
211.829374574045 73.2445837559745 69.7215372275927 134.239606718101
CSRP2BP 61.141483572676 74.0995052892511 77.2979451706651
68.534940191185 110.973625869849 87.8081021392626 110.909109947659
73.2870828361337 73.6512983066046 76.2533853403813 87.9871827215149
80.0042268333735
CSRP3 37.1485601150873 38.8336427948187 43.6670755115848 35.314469458379
38.0598374201407 37.7000400506818 39.1539892144371 38.3684202018524
36.197424053556 34.4875013487777 41.3553369544097 35.7451401627836
CST1 41.2424637338424 139.022985518859 147.858425171297 43.5627649099623
44.5668141895992 37.4782441311626 64.5722284870633 207.215265436327
58.6431245059655 169.838827072973 47.4630625313159 454.551748488397
CST11 41.678190413467 36.0366673493555 39.8656165405284 37.4642807402741
36.8059561571675 36.4434672195785 38.1169703906207 35.2184611663232
37.9837233601316 39.2623423164488 36.5359455830388 41.4508241771315
CST2 35.5179217192467 38.3322849790657 33.4240466670776 37.7469621225553
29.4322896559759 36.1190722948669 33.4316681623175 33.0038344711883
36.6312676076142 35.195364621675 35.860430241131 32.3796054199032
CST3 2435.70120719503 6938.87250165681 6819.74871209328 4632.08655821299
5601.18872540083 9181.13240708506 3021.24285327836 2678.43227619157
5112.4354472796 4503.77675488367 5218.25814076207 4986.44888767011
CST4 34.8099089553046 44.0409178714232 36.8730514568925 44.6131679221578
41.467005949568 43.4681646416585 39.6123051184545 37.3875631460917
42.992884918158 40.3458948319669 40.1742127891824 39.6833800000587
CST5 35.2267149016596 36.9994058279362 36.8730514568925 43.074472921841
39.2608760193011 39.3841861812262 42.7431359850339 38.5457716679071
40.3316153483289 44.0409178714232 39.8184668132023 39.3575468494941
CST6 80.8943070653517 81.0766789453664 78.8968326770219 88.4439480366515
67.1702819938085 43.6960408897486 73.3042538589134 91.6253858792439
63.7665341104267 42.5300425828351 51.4941811056642 38.6060558847357
CST7 70.083473392272 83.014860007187 72.2016884438365 334.527003257901
58.9875400392427 73.0247140074919 118.483499958227 71.0593419018503
47.5564423692142 64.1097859982912 60.3431719501866 56.1136936405786

CST8 43.6706764143908 41.6466169548846 38.6727842796202 47.481302504066
36.5393857306744 38.2293008890613 41.0381860307445 39.0620487644635
40.0403898620771 41.9666541498638 44.0409178714232 38.8495095797881
CST9 48.6621527194963 55.2893984191264 49.3000844711116 52.3004056941891
70.5676924620725 53.1052228246467 68.1125016199524 59.9968592852122
62.9922928277769 59.8130420783374 55.4694986901287 52.1918130336397
CST9L 38.957687788868 39.1982470626863 31.9895827886879 38.5252456306656
31.8228237796461 40.244271403831 37.4772922422734 39.853866204927
44.7058233759912 45.0155543555707 37.5583886316038 38.2350720635382
CSTA 38.5648843914189 71.3527183280827 47.0055981216687 73.3725472767369
58.5450868286167 44.9896851005259 35.9058393060637 45.642304356051
61.3733228199803 44.8997235283254 48.6184776976167 55.4635297192076
CSTB 2196.59044510709 4858.39551231606 8050.256095383 4569.48246031279
5354.58328030315 2621.65272077828 6483.09366281532 2091.0611913538
9852.7965902602 1588.70376317303 2712.36664370642 3520.7579823657
CSTF1 40.0079769153431 41.1090481599939 45.3675390992851 41.6045220683604
43.990679726464 42.6575535390785 43.4885245598939 39.4270866851816
37.9837233601316 41.1222709786153 41.8168470823451 42.828737648529
CSTF2 188.61489831342 213.028412890553 160.733526836282 137.955007835039
165.613176704286 113.716666562787 365.792144095604 353.598144765197
232.402174615698 163.048439401341 142.945182805248 151.111664091983
CSTF2T 62.1448806673973 68.921126710047 55.6865353018811
56.9299697426319 60.6400800519777 73.3506594385504 46.7100796279384
47.3791873993182 72.1591028956359 68.534940191185 58.7389869173495
74.4365426051588
CSTF3 119.434418653131 180.562005428669 222.66558323175 110.785049776763
252.280105035425 116.817127851913 174.275409358652 236.759651096748
188.513581321162 194.827750623463 153.940127525369 153.626578526803
CSTL1 44.8914371498528 36.6968906669565 40.931294690669 38.9534635879195
42.4864576619427 39.9397419279057 42.629412460409 48.4670130605028
44.8003345643334 38.8507804383261 44.4688999098904 38.0452862258803
CT45-1 39.0620487644635 39.3681232503406 42.8768096578102
38.745552403895 40.3124262005404 38.3956445058349 39.4069390012996
37.1317006162938 40.2752425854368 37.5672792721124 40.4504608195666
35.903067617318
CT45-2 42.7150227340364 40.3384318592985 43.8495248991589
37.5382094267692 45.6895223823001 40.5281496800812 37.0564573649223
38.5457716679071 42.1594905198929 46.1390887492649 35.860430241131
41.2437378488025
CT45-4 44.3819828231602 42.8142224866049 38.3626655809423
56.6458456890715 38.4540849632254 41.2040352443154 41.7656868106485
43.3729486804084 70.9462008788871 39.9576138799997 37.8766103688718
38.6454098044474
CT45-5 41.0608773166058 38.380619572367 41.0705134231814
38.7035509484261 38.1096479110295 42.0082685530808 42.2577689927563
40.4359177791566 52.2631308081333 39.7835513496873 39.9899430628542
38.1421709520616
CT45-6 39.8662795144616 41.6466169548846 40.0998167098057
46.0228732507872 50.6470461863549 41.554942319225 44.6032218589199
38.745552403895 39.7537274655115 42.0621425894624 38.1835938174625
42.357910063876
CT47.1 40.4179476219497 41.2598458726002 43.6670755115848
42.1273928203021 42.3536549114165 42.8598805316863 40.2601451506332

42.0494116081998 43.5669306063196 44.0719548209593 43.8002456102021
43.0322714179517
CT47.6 44.5282181324797 45.620219291723 47.4740726032707
41.0726336841311 43.1847628331565 43.7630587657205 47.0874209746031
38.8495095797881 39.3506426665347 43.3465699185405 39.8577530602176
43.0930293831497
CT47.7 41.0608773166058 43.9421822132391 43.0016969050339
39.0156305603334 45.0693869004277 42.5660695551615 43.0930293831497
43.1931731665131 42.2248608921879 46.441301934328 40.2317542073211
39.7411455670172
CTAG1A 39.3275194442012 38.7344853699576 40.3452320167286
38.8025302527057 38.2496050953547 38.7340969861754 41.1507980856436
39.8812234363661 129.101365145456 40.5544182411001 36.5584787902344
37.8490162439785
CTAG1B 38.7229679986571 39.3667161654772 40.4070046183108
40.5484717678957 40.0145811166794 37.9003288112261 40.3302268293707
40.8833572570383 44.0519245668737 41.7405682794166 42.7275315121163
39.3575468494941
CTAG2 41.483412492426 40.3384318592985 41.1742244762318 38.9534635879195
39.5595233162192 36.8527889114786 43.3832826368789 38.7864161493321
41.134180463103 41.4397539024976 40.9583127801887 40.6614700944853
CTAGE1 40.0557788909923 38.8336427948187 40.6312687664188
35.5395819665562 39.859345745831 42.4155142987678 38.977631784251
36.4931866273171 38.5252456306656 40.1179210534118 38.1835938174625
38.1421709520616
CTAGE5 42.8638014565441 45.3022591001932 43.3465699185405
46.1074305700679 42.0472847316468 42.6211340086915 42.5660695551615
41.4061152929154 44.5678064264717 45.3954000575181 44.4414964828926
46.0106098064475
CTAGE6 57.6913861717513 54.9144272974449 54.7350451926204
49.085741080241 47.6884372645373 43.0860383229288 49.6011834770324
45.4882438975269 61.6255834422379 59.9876575158981 60.2867996701867
60.9446532180211
CTBP1 143.536318305185 245.060552951511 188.789206373516 183.469819903584
197.598956071422 207.380865650247 218.510816443243 114.623641209561
249.101015988111 172.221482103025 192.922622120507 167.430013807712
CTBP2 41.8409927585705 45.8284004440739 51.267956161763 46.0905079010237
46.2478069975709 51.6558970571712 53.6294062560285 41.4061152929154
47.6582396278888 44.5303781239209 45.9104478407175 51.1897992768608
CTBS 100.489477730896 107.034189847828 121.650910373947 193.703214707909
70.2565873027242 87.4239220649478 81.1284422173774 75.0377156190383
59.1514832343922 62.9205616877344 70.0965466297064 100.289042948128
CTCF 205.24499293304 216.976116788572 246.09270373893 191.771063132796
187.002948435359 222.773939093637 251.65044168003 182.52420078643
251.374750265492 137.658868649032 195.740109330843 184.105396912056
CTCFL 43.4626049853157 42.0709558258414 41.785676764591 42.5664883002833
43.9450023643394 38.3418933808844 39.6535880152834 40.3357777530419
37.0126425326534 39.9948390976317 34.6366195121669 42.6758568421347
CTDP1 52.7817052791259 63.6287257620165 58.0218262176065 66.7750249658122
46.8022627504268 46.7293238583888 55.117521379579 53.4768201292155
78.1448136847078 68.0556717366572 65.7924490713262 53.9918882088235
CTDSP1 158.769954040462 181.2179249806 253.764071470001
170.128899555014 140.719373122712 183.165559564289 123.336184278054

111.501058276821 245.630020408551 230.879083362388 176.138603484209
145.72829095462
CTDSP2 544.355979695242 535.313561995599 450.759762047652
450.139987981805 423.608694638779 728.023307553872 312.015358620043
395.298033031033 503.529187388654 568.905368101326 489.983164420779
532.581631305218
CTDSPL 180.679129876248 285.826380698286 416.323909993454
109.503117647487 689.228901128857 539.188951045594 402.886581458688
168.883366756191 415.759723627205 709.284769121309 493.705301673753
404.674869177442
CTDSPL2 67.9531693869426 63.2475738480323 54.0170817319522
65.524821699432 71.5345397102823 74.6375749739389 74.621822948059
71.4473248804357 86.9274372004388 52.2832912331043 63.9915902128079
68.5174271063894
CTF1 60.1705241170421 58.6249721386149 66.0411740221276 44.9370339275041
70.9638091288679 62.2818512646893 60.3928833351886 45.7476095696859
57.9713749600418 92.9894133685392 70.0965466297064 66.0786923699684
CTF8 43.9577120438279 46.8059596594267 47.4740726032707 43.3744503319109
48.2081129576405 42.157627240486 44.4074592653814 41.496883371243
48.1174861108648 42.6211340086915 45.4953384153622 43.5367669381526
CTGF 2663.19221471348 1313.92053019421 1135.43644148537 1387.28483887704
782.639346040155 1842.15643221012 87.0685571351947 1084.23003918768
138.50445813694 408.353336724969 1712.55038816005 1738.00955642263
CTGLF1 56.921728372453 53.9275079525238 70.18107651423
54.2886483704949 50.3713170502557 57.1752846228778 50.8388984085636
53.4109882045447 52.7512110769042 52.5522545249206 56.7765567227989
53.2046867327331
CTGLF3 509.879213331349 561.855286945959 1019.67015337432
355.568857981155 449.427251674566 316.419421649196 368.660803187338
363.269936098507 455.444228161475 670.271164195182 775.515071756239
566.548446430954
CTH 61.9944005465335 42.9358255168086 40.0998167098057 47.8931773060789
47.3172462673426 43.3356152004262 49.9162522057712 45.5051950394186
41.1262212547458 43.0156099182214 45.9504618460054 44.5432192783161
CTHRC1 1664.00821059181 173.328468320432 105.204861495017
498.284928805673 243.952697457779 260.426488908824 67.9599395822006
146.355277438643 59.0563795877672 130.313381811168 78.0909302258507
79.8331445290301
CTLA4 44.9896851005259 63.1780854659428 53.2874286456249 91.2715671868725
38.307092962865 43.5474493120701 50.7487159427973 47.2340913300786
42.1594905198929 40.769767732912 39.3895791800635 45.0583552831013
CTNNA1 1405.20888023653 1279.90288378214 1413.36279821142
789.511653564341 823.933615182634 1211.8832855325 1147.19483787501
969.805093523651 1679.12630849964 1084.23003918768 1135.43644148537
1448.17151189672
CTNNA2 40.5654775358427 40.4359177791566 42.7303139017786
42.1273928203021 40.8873907150245 44.2607789091176 46.5650556481721
46.1673159982664 35.860430241131 41.2276963324981 44.7926323492519
39.1553169409075
CTNNA3 40.5654775358427 38.4330755397458 41.785676764591
39.5637454855233 39.7162631015511 44.3194383179647 39.6158343667921
36.9355798143571 39.4433784795209 39.9948390976317 41.3553369544097
39.5418280570561

CTNNAL1 85.1950626054282 101.020328028769 63.955655956484
92.3405202477427 89.847560289665 223.501516374076 142.846665249354
91.8207081349104 87.4335749014848 92.6169846049935 88.1614325571117
144.300051706447
CTNNB1 67.0384187945533 79.7882961549974 96.1596075221747
69.1126897800223 89.3680268066234 93.5045354990096 60.7397678774507
77.8039806950172 60.9801094225351 52.4752641497096 64.9559356780521
92.5391033814713
CTNNBIP1 95.8734933265537 73.2842501003097 79.3066495980045
62.1776700757427 83.8679756709676 66.8632294055679 92.0880591999243
71.6202385170491 97.5962784863854 84.8276953169119 72.0073363366137
76.3824005526731
CTNNBL1 243.489225650886 171.205726660714 189.984886903595
250.65667565007 151.848243044839 299.524171071613 258.726193861248
194.862177434028 247.547769833089 132.604567913122 188.582370265555
162.23769718358
CTNND1 47.5647821785529 47.6229101519945 52.6782411631338
44.3590588464211 48.3195541395349 40.1880266484871 50.0954869074666
68.4229528414965 48.1489308918472 58.4262160879174 59.2455550633943
54.3587260982091
CTNND2 39.266565827316 39.1982470626863 37.0642268327439
38.4208429103799 39.859345745831 39.9397419279057 34.6720534245631
40.931294690669 42.7150227340364 37.5343260962758 37.9946685306274
38.8495095797881
CTNS 58.3702889417801 67.4280399954084 74.0913177754682 105.997702773537
62.0925921750472 56.393737074538 63.2726414544553 51.9447450229707
54.2089559589959 66.7585874672879 64.4953675557046 55.8775622338124
CTPS 271.138311888031 186.600931545083 126.578693258328 102.971363152434
323.705254926762 293.755623192857 318.069132873489 295.917955889194
226.580055691897 136.859309317164 134.697808298417 162.540906501584
CTPS2 125.835967514239 136.883475451494 113.388372364837 69.4281256780421
108.868692628326 117.598001907902 194.225364352044 107.818253499703
122.170704886985 155.332764598031 139.731620245421 135.19133529995
CTR9 60.1705241170421 68.307513091369 65.8455652907556 60.8581462216607
55.2214187655851 68.2614370871382 62.2818512646893 48.7814131120945
71.1441318672051 58.037539503209 62.0789181257889 59.987045425047
CTRB1 38.0089018744739 37.5469247328848 39.9101908547397 33.0463640202858
35.0181575202066 35.8227939208521 36.08956004634 33.6816578730693
38.2215591930186 39.3698811593364 36.5359455830388 43.5738557773903
CTRB2 44.6231541342 36.5219488315447 35.9305494063137 43.1847628331565
42.3536549114165 43.3785777889265 42.1157692936428 36.6452480995946
44.4395580493118 39.0266821712099 44.7926323492519 45.223075311113
CTRC 71.3900857589616 59.9682734755814 63.3544794716167 65.524821699432
70.18594926052 61.9432904657734 71.3527183280827 99.5192591352063
60.8635925649592 46.8954576545929 58.2315917325629 51.2318935933116
CTRL 46.5549623118323 59.6359245937831 50.9933795450314 57.0629653735694
41.7929491793697 39.132725377815 64.7023558190673 55.5578334877425
50.3245489035833 45.4614234442527 51.4413099192899 51.9447450229707
CTSA 513.891389190131 337.013669560645 426.276230164353 997.492549573274
282.764445883135 280.526935074437 301.139592414061 357.44124276077
328.46798329618 293.410413883219 239.96421556928 210.958578914607
CTSB 2978.46032143413 721.266942608349 900.517711546397 3761.48462220262
522.377154567257 1059.63540694644 1107.96086673001 1394.28860933629
349.480794966845 1673.60994518271 1580.58484708669 1305.35453202355

CTSC 262.534449975056 250.308866488702 256.843419520398 641.837188326604
437.84407370064 222.202580186782 177.425508756002 170.223657280542
266.323732730581 78.8968326770219 68.2585858486846 130.192692323611
CTSD 989.0602710707 809.577874019028 959.549658414082 2133.78836857697
583.053232396803 491.284423079101 516.992846073349 572.43115808475
1483.40077183826 821.922096305069 1076.05074395483 783.440408023061
CTSE 45.9090659324223 656.355333007615 1440.41726370246 48.3892457696815
77.007438266156 67.5812474586277 58.8993834673475 52.8297102061403
71.4864243594056 62.5610562594275 90.807818984128 62.58750768974
CTSF 110.048921728774 67.8208976798364 91.3890908971311 87.9098670472958
107.427186954956 367.685570437603 60.7992483126292 219.431561967403
100.178375869002 183.311305255895 207.768377497439 119.124583755587
CTSG 59.8248482438562 58.1027665533767 71.5484567134601 133.541341560868
43.9450023643394 2067.66516256598 41.8315602975157 62.1934012861178
36.4476883897928 287.423909789368 234.615033303459 69.1099722732115
CTSH 1349.09482344633 1817.14004766564 2002.0536042686 2126.41018877615
2911.10227993347 700.477686061791 1384.80615431656 935.815928529014
3244.27810937143 2160.22713775462 2206.44541412808 2448.64580552439
CTSK 1516.33074288338 227.206974078547 86.7823828209946 1118.62223628039
180.916465721523 1521.20630233299 64.7023558190673 277.164276391513
70.0801020070064 423.804472820231 249.051882344771 228.984484917083
CTSL1 1521.20630233299 276.86542239106 335.569876624791 3562.96193568469
484.482971152057 1087.75682788799 784.951880532127 1102.03940742413
249.632045894263 414.794323433931 521.029507993615 582.511224532378
CTSL2 77.4999637278723 42.6939394537076 42.5406888281114 38.2041059863839
51.1368583672022 40.650742261428 54.6414321010929 71.0593419018503
62.370559099758 40.6022003765153 40.723990462047 42.2577689927563
CTSL3 44.436995101921 40.716553247236 45.989212296035 45.0798408927912
40.148780650831 45.3882138838137 41.8315602975157 38.956280281655
42.357910063876 38.8507804383261 44.0409178714232 37.2810767789306
CTSO 128.635811501506 98.4175422336425 141.154520472188 131.57403028894
85.7842344710572 180.916465721523 83.2162112994735 134.743068051122
87.7881690937563 143.173898886465 135.824520574227 118.115557136662
CTSS 86.8262463672891 69.640968387662 86.7823828209946 481.145001682177
42.269054475455 90.1080159649841 77.4046818339539 61.130286531713
54.1745577382276 71.9124897002253 59.6608518093582 70.18107651423
CTSW 43.844855336215 57.0082102559228 65.9476039189301 53.2046867327331
43.4544762830742 51.1621379498014 54.4934782594218 63.61404854326
50.5785738734431 61.0935621637178 52.4393177875001 51.6660481331154
CTSZ 226.882600557693 176.07173250529 77.4246856935596 222.16475904496
114.230358276599 81.6949047529395 86.6798536307388 220.896780388946
72.5027069303875 80.191847692094 74.3766944386886 123.978615472736
CTTN 275.061444728555 484.482971152057 327.878405194732 142.880684604126
366.228100574684 252.097980496533 375.697071471585 319.043862813161
363.215063520042 416.246271724181 356.133736281036 326.250872625748
CTTNBP2 39.6609238036815 41.159706131351 38.8775560911841
37.9875302985336 44.0451487108213 53.0290605707162 43.8206425661746
56.6910078150745 46.8873421271259 41.9666541498638 43.844855336215
33.5578650644246
CTTNBP2NL 67.1387898740161 71.0340758556049 61.0370242377391
61.2213001749372 71.9737561190706 66.8632294055679 74.416117986276
71.9771425105735 86.6516906104905 50.4234835272833 57.6831182451733
76.6707262000712

CTXN1 543.975237512604 439.770250139925 97.5104526799755 60.4646208319121
651.078746026341 545.514314651238 471.734457300752 301.525915314179
240.934550408797 334.583159766096 240.431987635418 155.82546212052
CTXN3 38.8336427948187 38.0834507308871 38.0057289062111 41.3027031037343
38.7864161493321 40.5281496800812 41.4742337714895 42.3110002461296
44.8997235283254 39.1177164316495 41.7627910693483 43.3262148375525
CUBN 43.6706764143908 48.7847528954724 56.4779645313958 48.4916117779992
46.0977687311222 42.750861693377 35.4623691645629 44.9977782988116
37.1820543575123 42.6793753550854 37.4642807402741 39.9519547348106
CUEDC1 160.187478921145 105.796515736125 116.426770366736
164.917385239962 112.23915619297 259.033660186329 113.039124313172
143.042664581646 104.626873082138 167.799243454967 226.407063891022
171.705718494906
CUEDC2 207.943044411392 224.1418262489 312.984737387254
226.057278804077 232.847812981531 292.99497881914 382.940842652744
211.451463038315 209.004669589169 302.79352975792 273.032063533642
279.448591201269
CUGBP1 50.0802438185974 67.1209520838357 60.5678281953819
42.5193208629123 72.9700748169457 49.4157684139858 59.3549213146532
58.9081750508316 58.5285779508926 52.2256177934134 56.1822680431676
64.8254097948745
CUGBP2 42.175859643566 53.1278271804774 52.5786055354475
139.989008296272 37.8305309577781 109.943883439481 46.6198823890826
43.1567024035183 40.2274522635576 60.9905272201951 71.1894129030602
52.1186591838715
CUL1 122.136867360309 93.1067450332473 114.039396407323 108.991044950132
146.969565088793 142.731630650904 81.0331497830643 88.9038123445083
159.306689057291 119.301672388541 110.626479311082 141.376779431031
CUL2 221.972553229362 225.654863797316 251.854446030066 267.024267633341
328.855843739232 331.390452289344 214.073500272341 245.630020408551
315.960715273629 194.355765743782 204.344278991396 255.284866561352
CUL3 45.9090659324223 41.8073910693411 40.4070046183108 45.5869891570251
38.6454098044474 49.641159251878 38.8417151851134 43.5544347011646
39.1790517315259 45.4614234442527 47.6125796208753 41.4508241771315
CUL4A 224.914980996667 165.548537317115 202.833844963097 192.700843009002
251.105933757167 213.590067519021 222.453547273598 182.343696914747
215.08694410402 165.613176704286 183.820203505757 186.600931545083
CUL4B 72.3388488936832 74.0039764902057 64.6446465846487 91.0346886951891
78.0077300979911 89.680151549258 72.6959688212342 74.4508307047509
66.8632294055679 96.0236731791095 75.2391243965793 90.3041737295567
CUL5 181.45962058846 252.949225605064 171.623830248512 194.395766807005
222.92930674999 338.475097952023 193.262615637464 175.909221705119
160.283895105835 256.364301298918 233.96534264787 221.123510823097
CUL7 51.2851682978581 46.9919960628953 50.0748292497578 44.9370339275041
51.940083408061 42.357910063876 42.0605906240248 45.642304356051
42.1594905198929 51.3351387089044 52.2229654768933 48.5123041297835
CUTA 786.57695312967 470.676006277762 618.293140877956 510.525676243746
880.992328229414 514.261598258395 796.359355922952 534.629009719855
497.290172759099 610.853753376105 547.729200972776 482.5816387921
CUTC 72.9154861179393 87.6763065012524 114.611557317665 122.863635062993
82.0149542477384 326.055799445169 100.756165194192 71.9000032422349
103.869437847206 225.251124162268 208.7739716139 208.743876555152

CUTL1 172.237164781821 220.217797955745 194.666769955149 192.264432082651
217.995354056264 223.316311787046 129.414877519657 197.13726107504
226.168990555083 189.737472782071 245.127632785841 226.567000755017
CUX1 44.2350046179299 44.6402215707065 46.9578963946094 38.0365987358005
41.948762493127 49.5888934105046 43.4870236357993 40.2906799807722
47.27543600687 41.1678908661272 42.3440399888092 42.7303139017786
CUX2 32.5418171758086 33.8237407699472 43.9450023643394 42.8235313007746
41.1868052124344 39.3841861812262 35.4623691645629 36.1375121409002
35.5220586859301 39.4710743516517 43.7585538437696 37.6557714476853
CUZD1 41.8409927585705 49.6841415268611 47.5673419710251 45.7397504535169
39.7568367479301 46.0364000143537 42.629412460409 41.6466169548846
45.0997987980157 48.3693338695392 45.4799270968969 45.8546719310325
CWC15 257.194689822426 217.995354056264 249.776078532596 295.848968688724
265.520773157991 333.566613571224 341.078728185917 345.615640348066
340.662853333208 253.267901237295 248.012529897462 368.913147706435
CWF19L1 369.717462266442 360.523229691604 361.776841675393
445.421601813221 368.078954546259 464.583498621466 579.795835370595
445.421601813221 264.659866487253 412.29192088057 397.108901822735
291.42149037871
CWF19L2 103.945335792682 87.8681250426295 83.0959169307948
107.111886993156 105.739423435604 169.554408876006 82.9431583622036
163.917208464731 104.409397223301 143.173898886465 155.942366087388
131.496769528338
CX3CL1 301.904682664871 70.5699797830702 162.352052340273
58.5406981871153 69.1862663389904 235.925374041038 333.648836970147
101.516595046503 47.798305839406 154.707548614663 185.109323245018
93.7924763988692
CX3CR1 42.3620028137457 52.0268699306396 51.0539624358342
64.6945454069792 47.3791873993182 51.702461863489 54.6414321010929
43.4544762830742 45.2421183842044 63.1394716239975 51.6869155634183
44.0246886382298
CXADR 205.314410185216 695.255317861845 1055.35144552575 45.0798408927912
926.61614755153 192.240593720489 595.790464730847 429.329962597147
1180.7204565402 831.578043520215 866.712228831149 793.225863118464
CXCL1 43.2724556187214 44.7829632472754 58.637422084759 194.012210675469
72.1154333023225 38.9378717994178 92.3034609409756 52.8622615027833
52.7512110769042 38.528621456378 40.3452320167286 39.1553169409075
CXCL10 567.827069916244 244.529657015078 230.117814622646
376.453560620912 65.9413235523618 84.1922656126304 98.2843999216669
241.455752070163 54.7033747046992 52.6879797806876 66.8188271427672
47.1060577218993
CXCL11 41.9407041937973 42.6022652963326 39.8299340037009
39.853175885492 41.0476065156912 40.244271403831 41.7614975630362
42.9301944191913 41.9083581529276 42.1317134987795 44.057571056322
38.4829727716328
CXCL12 196.17328008185 66.7256057011273 62.9313387640547
164.435300801872 49.8966674037224 960.526372655577 43.0119734905129
62.7950861902067 43.713206937726 205.187554095744 199.57367025082
79.2525964926483
CXCL13 54.3554233228353 96.5102825310977 61.5080959417997
311.810240385503 37.2567512265363 36.2768299545794 44.0687697278114
49.2019743609873 46.3203995372658 36.1550293353654 38.5668610487033
33.3192849177028

CXCL14 72.1066584199167 393.648308810931 80.8400209263587
72.4834467838798 125.786923817078 260.010554284184 46.1634822993009
143.653706909456 84.8235093165193 94.5788538659874 91.1363393944977
68.0751111916102
CXCL16 374.601792595646 214.342804055754 236.307055572306
1050.05355467301 181.298709635609 192.763875975315 257.194689822426
295.841196113053 203.53267380432 223.219262492785 214.051824405993
197.841210387578
CXCL17 117.20232751191 48.4980626712175 190.977045076469
48.665578118898 49.7235523248653 44.5544727111028 41.0381860307445
44.8997235283254 180.475056805817 52.6879797806876 47.0110068985119
55.8775622338124
CXCL2 46.9939712192224 57.1595768494068 79.0962508551502 895.110715324701
59.9326265577462 168.91210897306 187.656257079035 89.5130163419167
40.8970401536578 34.064698871541 38.2795150354677 39.4490233720848
CXCL3 39.7576136507593 39.5050383901884 41.8751578329365 32.3671711013873
41.2740822675592 44.9896851005259 36.4098694708483 37.33030683349
41.4636047919453 41.9666541498638 31.1150868775826 36.8690774823319
CXCL5 48.4459451445651 45.0905928998779 133.163926420863 205.650076927315
43.004943518025 41.2567470068038 95.8734933265537 49.1399035787209
43.0456011922275 38.7008388965825 39.4749602565407 37.5222959113204
CXCL6 44.1737502492846 51.3800036046133 80.7338782543351 130.455608913636
82.1344514651393 45.5758521267508 283.21175871127 60.847641921163
169.75692324104 43.401782064778 41.5668884168669 46.0106098064475
CXCL9 400.327164003217 183.613579501306 89.2285288333749 171.800185639868
45.3675390992851 43.8582747295766 57.51482768194 217.78290286894
43.5669306063196 40.7063426028871 44.1148826235374 40.7704587752083
CXCR3 40.148780650831 43.7644428186265 50.9010149857308 64.898351831341
42.4864576619427 38.6414316877471 54.339958888486 37.4484310268909
41.2870505273664 50.3388505346542 42.170702295259 33.939263369489
CXCR4 145.883224917856 122.702789229752 147.727354881531 1305.35453202355
78.1761025629963 74.9661773375536 100.066535982734 101.39385858723
86.8113410380324 51.6778366958973 70.7045582269595 58.174555206394
CXCR5 44.5798743103596 61.0144918827511 52.9111155236961 66.3188075271379
47.4548471240051 50.2549436338147 42.1372626664516 49.7309743973184
44.5678064264717 53.3577647219133 47.5733603903505 44.7401523707775
CXCR6 55.8218917166321 59.3406363431414 51.9214790114499 117.948047796034
51.2525124735102 53.0290605707162 52.0942589368478 50.7364620410977
41.4636047919453 47.611018474151 48.6184776976167 44.9070250604284
CXCR7 417.4609354305 340.962490240304 1093.04440713629 288.508774004052
1111.13061382818 1478.26733379357 79.8456892021695 393.648308810931
198.821443323024 644.368005872231 484.350946851556 714.990611155096
CXorf1 41.3387790895716 41.7337506166619 43.0016969050339
43.9282059368202 40.8873907150245 45.3882138838137 41.9393838138203
39.3709357615493 40.9901805728485 42.9866214102525 38.1835938174625
43.6753843488695
CXorf12 79.4528447794527 93.8009890556664 81.1249338842464
110.25582862843 87.7337304906377 73.1169003714219 116.122324921267
78.6911549149983 108.815245119101 87.3815040456105 73.3321469094819
68.6047087267554
CXorf15 44.7915315085948 52.7493349111555 59.1040441894478
52.6947911480086 71.0593419018503 48.9826868640753 64.8367546135053
46.7980335276155 52.0583273941813 41.5435611403119 42.7275315121163
48.4535991392809

CXorf20 37.2474303302343 38.7344853699576 38.1421709520616
39.6292788039793 41.5668884168669 43.1180113655693 41.0381860307445
40.6723334103791 38.6322728994674 39.6251842352088 38.4251036088595
40.716553247236
CXorf21 38.7229679986571 38.7344853699576 41.8751578329365
38.8964578286293 42.5449473689701 41.408887993053 37.5424455284588
45.2806199505029 38.6510172759977 39.3113207075992 39.8184668132023
41.2905987859365
CXorf22 50.0466349801009 40.7952353240579 41.6977706798234
36.5393857306744 45.6895223823001 42.9695585740813 39.7576136507593
46.4672637826582 45.2897806651929 37.8217989841918 43.844855336215
37.0953622406687
CXorf23 51.2851682978581 46.9919960628953 41.2365089689635
47.9915414951747 38.745552403895 58.4435847520801 53.1884505015411
58.3046521633424 57.5883027758003 45.9004543328976 57.0154839296649
50.7487159427973
CXorf24 42.6036491382658 45.1390218677367 42.1901757067073
40.1362682639916 40.4321207743917 43.0144844590744 37.7136011474903
44.6560353921013 37.6383530120621 38.8173773425835 43.9802831041664
41.1066323911308
CXorf26 118.186590679218 172.39907939484 177.261319004133
177.584394977912 119.493400362694 146.133951379664 154.612425659555
222.368324930003 197.232697438521 158.091115152384 183.690616959682
190.053043382767
CXorf27 53.3059058105836 41.2598458726002 41.785676764591
40.9607305725196 46.0043807840705 46.7800033479223 46.0228732507872
47.2009420656854 40.9901805728485 44.4652605017595 43.9450023643394
46.917149434588
CXorf30 41.3387790895716 41.554942319225 44.6032218589199
38.2041059863839 35.9058393060637 34.8956628705329 36.7356360093603
40.5158867964016 38.5252456306656 32.1011040606811 37.0180934800573
40.0557788909923
CXorf34 157.658760470424 154.612425659555 139.543015878846
109.726577835621 96.221685383385 113.377818029985 205.991317474325
182.219366179899 121.36340610316 123.46775722643 165.322633260592
122.720768249799
CXorf36 37.2851729259549 37.8685585043426 42.3536549114165
36.264823544143 36.2691013181987 37.9951997351435 36.2561152084183
35.1738399826814 37.4059035942248 39.1621898366355 37.0180934800573
37.2291018661279
CXorf38 99.9752275166514 204.039190380778 205.991317474325
220.79051545138 92.9747592911324 81.6949047529395 205.991317474325
203.228211195516 133.244526666651 77.268323196327 99.1952492360239
121.782045054657
CXorf39 66.6547247910487 50.8812920005251 58.2986825661503
50.2163892855092 48.9627346956003 43.5752393599169 65.8986841086198
77.0262811825953 85.1114448808779 50.22432299342 50.4740079109854
50.2163892855092
CXorf40A 130.985178898633 130.615540145155 130.455608913636
122.863635062993 137.720182545143 102.863599315528 144.515737827731
186.818097299665 140.719373122712 86.5926014007562 93.1238695478498
125.81889994067
CXorf40B 80.3730667493235 88.8039637390786 99.4184778430267
88.051652677564 105.589475270494 76.1991031349451 104.8505815954

101.766641669845 95.2956103624468 65.3377940117969 67.1477341610979
82.1365334012643
CXorf41 41.8409927585705 39.7835513496873 38.4043916290561
43.3078542962968 37.8679241954337 41.4508241771315 41.3594868055879
40.4359177791566 37.10170285405 44.7958122466628 36.43924066428
38.6454098044474
CXorf42 49.4151347850687 48.5905518770847 43.6670755115848
47.3574589211166 40.8873907150245 41.554942319225 46.0228732507872
44.5029690493211 43.0930293831497 40.8970401536578 44.1148826235374
43.6753843488695
CXorf45 61.356483470053 84.0691608193405 110.540953771795
72.89602312859 72.5027069303875 64.3383155834968 49.2546642180676
62.6984594468418 61.9682048429354 80.2774079897733 74.6474346708746
90.2425981161833
CXorf48 42.4763932431258 44.2384941015805 41.5529539017093
42.3887531663733 44.3557408297309 41.7994927517569 41.9918605927928
40.0842340147135 42.357910063876 41.7405682794166 39.9899430628542
41.8291484190524
CXorf50 37.4554876386402 41.8073910693411 38.3166554965434
41.5464765641928 33.5412189543102 37.8102105794655 35.9894133432376
38.5457716679071 42.1594905198929 39.5637454855233 39.6403089864678
37.8490162439785
CXorf55 39.6609238036815 41.8848959280916 45.4865173433646
44.4367387274922 41.7929491793697 39.4953494583071 41.3594868055879
38.6454098044474 41.9635525160083 39.8873706855774 37.9230440150891
39.6352613956998
CXorf56 59.7179445506128 58.6249721386149 52.5353664902258
49.5424425850549 67.1278109419819 61.2387559102636 56.9320717885002
58.676230030835 63.3694113050568 44.4652605017595 49.8393069675192
60.5948704279864
CXorf57 49.540449125788 376.289659542649 559.9670724278
75.2475238554276 316.073008787218 95.5920087912438 490.652353306749
552.162906190653 524.972936438175 190.592556065939 176.064476306748
197.325473090277
CXorf58 41.8409927585705 38.9338521051669 40.301483914446
37.9875302985336 37.3508690072156 39.4953494583071 39.0458123429605
33.6816578730693 37.9273653326866 38.3092241935352 38.745552403895
37.9607839934735
CXorf59 33.0463640202858 36.4002279956519 36.6522278218553
37.0732977341185 32.5318884330468 33.5578650644246 39.4600924554505
36.6452480995946 35.7973685350741 37.5343260962758 30.9159908723475
38.6454098044474
CXorf61 58.3702889417801 40.5400384479766 39.991353369336
38.2041059863839 38.3561682218911 36.1190722948669 37.6036146841933
34.2814697823693 45.0997987980157 38.0078357808393 37.0180934800573
38.3291713594599
CXXC1 354.973265582488 922.047328788961 1021.71286017481 470.453915397461
265.101739636461 317.127679512963 779.028332922339 374.978401470437
836.472724085903 802.036670929858 757.833254346899 559.296669575666
CXXC4 31.9468644542969 41.4508241771315 39.1799070031819 37.0732977341185
40.148780650831 36.8059561571675 39.3585350448719 29.9617615620508
52.2631308081333 39.7835513496873 42.911855821602 37.0953622406687

CXXC5 2077.74475301595 242.396401163598 387.039093527273 480.260282672252
200.019523376661 320.889609846069 229.428422408702 416.149496747947
564.468049419762 239.590143292142 263.44529683749 160.283895105835
CXXC6 46.263826584899 46.4861258030402 46.8267540220952 44.6905691366696
37.4470183315826 41.0582900711341 42.9803717121865 39.0620487644635
54.7033747046992 40.1179210534118 42.5426057453993 41.4508241771315
CXYorf2 49.085741080241 45.7317208802318 50.6004078448405
54.4586176399381 42.4864576619427 44.7804080899431 51.9421032049606
61.8665316900291 41.5635499110307 53.0357938671646 48.1352470945488
45.0035308952466
CYB561 84.5678487053881 122.674230293606 92.9323002425821
57.4595416719581 101.156215957297 66.3558126170575 105.598516887691
102.380096190518 126.528654651607 75.7801512652102 81.0994894489593
71.3068231787348
CYB561D2 133.229859346689 207.087723777954 126.017900743748
266.717224821545 203.092045210928 110.757899996422 240.11295387591
139.062073456923 197.232697438521 142.846665249354 155.225701588645
115.064217549212
CYB5A 247.723546145775 1450.53108586858 1621.89443989657 278.303493506139
545.514314651238 853.464809072841 1932.2647526188 746.252816568691
1561.83892117664 1041.84536202049 1254.34397143795 2167.91218703382
CYB5B 1164.71243740122 1087.75682788799 1756.86714809925 681.474524031866
1106.57048989685 728.679189702033 1382.25775340179 1252.94510628089
1570.45778081591 1046.42391243532 979.468753716758 1749.06320703181
CYB5D1 129.785929766258 163.43197105141 69.3468840908665
321.454824353597 239.924746743429 191.730944258418 121.737071786167
192.922622120507 84.158736606096 138.997932973328 135.308001652191
117.355761277966
CYB5D2 132.838429478396 235.593721159442 245.996655628334
153.153205095999 97.6205705034407 197.205643078662 264.855020003755
136.650986373497 137.302487233881 155.075313035867 163.66696652324
131.942576661072
CYB5R1 177.779514321673 169.168332370229 1039.06999658544
175.009248614892 273.934014944673 158.334581991361 151.621572940457
238.510952165358 309.344766670365 1026.51177853708 851.813216452015
799.803676957263
CYB5R2 76.0532592222027 63.2804984998739 116.122324921267
48.309866878365 69.6330393135101 50.560825686187 129.143252341926
76.8393340617473 111.202737864873 131.537639104397 94.8036856848998
95.0278507757286
CYB5R3 1094.37961997684 1114.15989325456 1457.72260892651
1064.35745220225 936.978983469829 2077.74475301595 933.968759959712
1018.35211991691 1497.73669971925 1907.47560253019 2113.73880184695
1683.24873205783
CYB5R4 65.7587943905649 108.213380368446 69.1938570643524
248.414210905199 49.1928206164702 90.5359675350156 84.7782034884529
100.370937669662 85.3718124658316 56.9463858443402 77.4625808567081
80.8366627364071
CYBA 1588.70376317303 2911.10227993347 2854.82112538867 4200.38495514552
1615.83220398811 1325.2479053325 3776.54943917367 677.668455095171
2733.80542760533 744.080606078086 1317.29255644089 1057.44729389649
CYBASC3 174.685146336531 210.807094064209 200.318682162912
339.262360833465 339.601844139729 136.711122812062 272.545033107746

202.192457390868 191.182530250155 229.971492699272 162.000275136397
192.732356915298
CYBB 92.084526929319 92.3659612653822 90.1284518302601 723.210613328966
53.9074374788981 164.722166245312 78.1623301184313 78.3542840447759
46.8873421271259 45.9004543328976 43.7585538437696 61.7502624458447
CYBRD1 477.558242266308 134.300169542594 85.1661952927159
402.221227674218 119.572497267498 1731.19220270448 84.8808332740321
178.881586334601 126.930102684077 885.224377292125 702.737741114234
755.106456358949
CYC1 681.474524031866 2528.6392272518 1069.3975598703 643.491217668415
1554.42895572029 1009.47177197543 1157.65264077595 561.2769907009
1932.2647526188 1330.19215036764 1214.73798692268 954.116675685628
CYCS 63.1291723738553 108.649424858333 58.5212649095879 65.3191216270241
143.399636653204 172.777241940677 63.1604223919703 51.1694248620208
86.8463723709337 91.9185612536409 105.814959179397 95.6731932757489
CYCSL1 108.649424858333 103.513421757522 119.846620703059
151.111664091983 194.951677338012 138.581471125405 149.551650453179
182.079641528208 172.192366002756 103.001949900533 107.818253499703
189.451890957713
CYFIP1 151.740176274202 177.212612563444 149.515611848932
167.774103692331 137.286091889421 148.608392563633 152.294357984689
104.950400251642 147.9291840243 90.6191520659197 103.695493532556
87.0468045816651
CYFIP2 68.6889514573956 111.97030382494 136.613915120582
201.959257379662 66.5187917407182 115.277469628296 189.984886903595
226.970981973829 80.1505160355175 170.327588764719 167.702870734995
414.539381761279
CYGB 121.744701580607 251.803102904399 183.028254350275 205.154378853199
264.718875269207 424.085665569421 42.4939802311136 261.929727465462
77.4186160113063 564.468049419762 833.136660528149 242.899629257173
CYHR1 108.156702405177 79.3971417347713 81.6486527718661 52.8502823653368
89.8035133597498 64.9498081060676 53.3111230705754 52.8622615027833
77.7907484334847 83.946822476147 79.2701322339022 59.9326265577462
CYLC1 38.4330755397458 39.6493706695391 43.0016969050339 43.3078542962968
42.9655396732962 46.0905079010237 42.6913548400127 44.8727287473605
44.619512785899 45.2023300009435 45.447310733898 44.5432192783161
CYLC2 51.0225132036905 42.7775752471711 46.0727230219512 63.7909104347097
38.9496515344837 48.8744644207821 52.8496345990125 51.7538628098347
46.6944238390932 55.9283039581662 52.5930185400362 55.7067742610401
CYLD 52.109723558511 55.5294887653184 60.4347860127923 73.5834042105313
45.2556760873335 57.013135787334 48.0283573444179 51.6660481331154
47.0804681460898 55.1804535845496 60.3254877099749 59.1514832343922
CYLN2 123.25443049561 77.9992448636936 79.3066495980045 110.112236384679
82.7394574977114 116.900364583878 104.932630862885 139.177720567217
103.264100180823 118.93749473833 142.846665249354 86.8194399159684
CYorf14 44.9370339275041 61.2691385007595 43.917817324545
44.0991023527481 42.3536549114165 56.566634814242 81.7434024567356
72.5835488194631 53.7886950010803 67.3601172371813 65.13722010157
65.9350437838896
CYorf15A 68.3234331335073 225.201522281399 47.2055133646385
151.99110418225 56.0477607422828 119.474339360208 210.768576894328
207.087723777954 151.344720108793 113.26670675749 118.951970882732
171.5709313654

CYorf15B 56.9360840563854 90.500550952567 46.4150464549477
74.1068645821427 48.5000946421718 54.0104259381604 73.0433648756396
102.818879165949 65.9254098858352 84.3672289945664 74.2180011215707
90.4622684763614
CYorf16 42.9104454767916 47.087835967892 43.4709415392696
46.1673159982664 41.0885562160946 43.7630587657205 51.4349390827895
45.642304356051 43.9450023643394 42.9866214102525 41.9083581529276
49.3087627580849
CYP11A1 126.204310123186 47.4740726032707 50.8303921211581
48.1662933382789 96.4112435621587 63.6894484284172 61.2183034191703
56.4779645313958 59.6700171760426 48.0211752672327 47.8988096575648
52.3896785600751
CYP11B1 40.308802735799 38.7578265341627 37.5222959113204
39.4569929845912 37.10170285405 41.0012755104374 42.6913548400127
42.7965563818112 38.9496515344837 39.9519547348106 39.8577530602176
42.4607795638552
CYP11B2 36.9060997041453 38.4330755397458 43.3085358214154
40.9607305725196 39.0564222526273 41.4508241771315 39.8695929627783
41.0381860307445 43.1805016690297 41.9666541498638 38.8507804383261
42.0424641526302
CYP17A1 34.1336988442837 39.6995529770239 42.2577689927563
42.2496337895968 41.0885562160946 39.2248354955045 38.8417151851134
42.5435283762933 41.5635499110307 42.5664883002833 38.8507804383261
39.3575468494941
CYP19A1 39.1094280636983 38.6633191400449 39.7411455670172
40.3180310664156 39.4693871570028 39.3483872785093 43.7570510687482
38.610211769318 41.5635499110307 42.0217776707284 40.9624000010053
40.3691324547049
CYP1A1 35.4287975493553 40.2493681589104 37.3687900733549
44.7980740332174 44.9936360641969 39.3841861812262 2721.97551918611
42.5435283762933 43.2761454096265 805.309720893073 108.945545005203
1425.37639004849
CYP1A2 37.4554876386402 37.10170285405 40.7620472083159
37.7469621225553 43.7517118200398 41.0582900711341 7075.82842238887
42.4580504587581 38.9834959368496 55.4037482627222 40.6373400186325
51.4308501933893
CYP1B1 145.561345203804 60.7495043977707 51.7138050344355
396.167219131922 45.2556760873335 301.728842562175 959.549658414082
63.1604223919703 60.9594847577747 771.554891868456 144.427680369554
973.363245512859
CYP20A1 124.998771394658 155.310176479895 130.313381811168
142.048367760597 99.2662616001616 152.565430903885 86.8596934326046
106.454367084321 122.988056802699 91.0309075163394 106.816835590007
138.292681458047
CYP21A2 60.8357760968871 47.5647821785529 44.1452943804044
42.0102671859606 40.148780650831 57.1752846228778 45.3597029614942
50.0526763693329 40.5912213523345 47.611018474151 43.401782064778
46.3411353377615
CYP24A1 40.5654775358427 44.2105195734073 104.950400251642
38.6414316877471 39.859345745831 42.357910063876 39.3585350448719
52.8622615027833 86.6910280940418 47.4767851750188 66.5539062525344
98.7331384143685
CYP26A1 42.5478935075651 40.716553247236 41.1742244762318
75.5510570035251 43.4805066565809 38.8336427948187 41.706879587091

39.5954353681031 43.9104473952446 40.9470770920425 41.3234441859498
38.3736012215232
CYP26B1 56.6086202928268 74.9059650336395 45.1569741609597
61.8997157694697 44.9936360641969 80.8333046859586 36.7356360093603
62.7950861902067 41.074824727477 50.0564735608363 57.9864701726379
87.6598268114592
CYP26C1 33.0463640202858 41.4508241771315 38.0452862258803
33.1137701681495 39.9697332660833 42.6575535390785 33.2425023465738
36.2539950294089 38.9006788676407 37.6383530120621 35.2339574411711
35.7451401627836
CYP27A1 204.659954879641 81.1416376689959 68.1780382173625
872.305108096296 59.6922302604696 236.727577348109 202.192457390868
94.2678678253657 54.1745577382276 294.372319595103 322.440680131096
166.652850759559
CYP27B1 69.5462764096011 80.6083247572711 66.288032400574
115.299308625328 63.25187762908 44.0804700754786 50.8388984085636
93.3718038663074 60.0736861987141 48.1392888470269 40.3452320167286
50.8442982601142
CYP27C1 52.502055890816 43.683792219729 49.3000844711116
33.9645433044281 37.1565984182084 43.8582747295766 39.7576136507593
41.6466169548846 38.7298222155532 50.6486940895674 40.1742127891824
63.9371474698484
CYP2A13 42.8561766050988 39.5050383901884 40.8420925848403
45.5869891570251 39.528971131769 36.9625898230551 40.0052661202207
41.0381860307445 41.8197029429821 42.0621425894624 43.1724740315182
39.1553169409075
CYP2A6 41.1523487516364 37.1685072112618 40.4882741869718
42.6913548400127 41.6806984441849 43.1826328292558 44.6032218589199
42.8482181313226 43.0930293831497 39.2623423164488 49.7363778831003
42.4607795638552
CYP2A7 39.1059888887285 42.5269793679335 40.244271403831
41.8778873678551 41.467005949568 37.5887129572848 39.7162631015511
42.3110002461296 43.6670755115848 44.4652605017595 40.4094901248527
42.8864415494484
CYP2B6 40.6957787921451 39.5050383901884 38.6414316877471
38.042028333747 39.859345745831 43.9366081640576 39.7162631015511
39.9941474864076 39.1467972692656 39.4710743516517 42.7775752471711
43.5738557773903
CYP2B7P1 41.5421429082231 39.2907645923217 40.6737160001291
40.646702696033 43.1252272136667 41.159706131351 40.2154497375715
37.2237132058632 38.7298222155532 40.5090849993014 37.3687900733549
40.2669497398702
CYP2C18 45.9090659324223 184.750252804038 45.2313723727673
45.334106390212 39.4693871570028 43.7630587657205 46.1634822993009
43.6434576713809 52.1186591838715 45.5776159432079 44.8872784170036
40.0557788909923
CYP2C19 37.1485601150873 42.9358255168086 44.3344900481112
38.0958223740111 43.004943518025 40.7616966228404 43.8206425661746
40.6171774724171 42.7150227340364 40.7063426028871 39.0550139257658
41.7575097298927
CYP2C8 43.0930293831497 45.5842286511645 51.7138050344355
33.4819739460148 40.5710637884029 39.6251842352088 41.8315602975157
40.2382908228517 34.6720534245631 55.1089864632456 45.0589947942306
50.7487159427973

CYP2C9   39.3709357615493 82.2766822827861 39.1799070031819
      40.7481155008596 40.3452320167286 37.2686490268265 43.1931731665131
      41.6466169548846 41.1742244762318 40.6022003765153 37.2892641289258
      41.3481355128089
CYP2D6   43.1001512135266 40.5852276210998 49.501977696496
      45.8102081251786 43.004943518025 42.0016830644258 40.3302268293707
      42.4580504587581 45.7873395324682 45.0155543555707 45.0985088502363
      47.1712755747298
CYP2D7P1   52.3660712483179 43.1464706017434 43.0602678767669
      50.2163892855092 60.5806691666221 43.3269087731399 47.5130990528612
      53.9918882088235 48.3686935595826 49.0841460667818 45.0589947942306
      45.6692241948108
CYP2E1   41.8409927585705 47.6229101519945 42.640829914102
      48.665578118898 43.6587492470348 48.4758590019384 42.0605906240248
      45.5581356216957 39.4762546236449 59.6863210839828 54.113533179847
      50.4847465816184
CYP2F1   44.7044140979108 41.6466169548846 36.1042012504758
      38.2599935139101 40.5235471987417 42.8598805316863 42.4939802311136
      40.3357777530419 41.2870505273664 40.0557788909923 39.5792604499113
      40.9149601380325
CYP2J2   306.294404016622 987.427672417105 377.626746989976
      44.4367387274922 303.139550962377 83.3664149246525 363.269936098507
      113.528908699366 991.630429625437 637.565156571985 571.790034754076
      879.708199333257
CYP2R1   86.2021418088902 89.2913927776683 161.974232441096
      114.124846622876 103.968071466212 79.6895408438974 72.8653205184014
      76.4637616585578 115.328304312071 125.393232344778 90.2624299533482
      99.2432911346944
CYP2S1   71.957942079488 68.7436392042714 77.4906145838899
      107.005820817545 48.7814131120945 38.6704566269103 54.2445399731644
      76.5699943825825 42.8036731479932 194.278792348966 60.9446532180211
      195.765247956108
CYP2U1   42.3620028137457 41.6069493017095 43.2721716572958
      40.6892234267786 40.8873907150245 54.5222652584532 36.7356360093603
      47.9942520395434 50.9612733880858 62.3556396662215 53.3078448066074
      52.153603364404
CYP2W1   34.3473914754963 34.15544299189   44.1452943804044
      32.9046688061235 42.4864576619427 43.9724628401189 39.7576136507593
      39.6474008369935 38.9006788676407 44.6918600441328 34.174642681781
      31.9386613757097
CYP39A1   44.7044140979108 51.8610060995363 48.8623729374733
      43.9282059368202 40.7779767565889 62.4450952595811 43.4885245598939
      38.9271399577966 48.4869909888679 53.9412154719864 45.5707650534259
      49.1200355938198
CYP3A4   39.5676051939342 37.1685072112618 41.437237454134
      34.1051821768421 35.4484652445078 42.357910063876 38.0598374201407
      39.853866204927 36.6312676076142 43.6033221838149 38.1835938174625
      42.0424641526302
CYP3A43   43.1267841876606 42.0500582486532 41.6977706798234
      42.5193208629123 41.0885562160946 39.0620487644635 42.3194600586894
      39.8016018354453 44.1519466943185 43.781101409552 43.9802831041664
      43.1235886043549
CYP3A5   92.1908585323292 89.0203733091224 392.898761909406
      42.9358255168086 59.8102324370568 48.7781962164988 42.1607322031054

152.555556133364 292.99497881914 349.90835241442 265.570814613305
343.382663050351
CYP3A7 43.5710871617107 38.2263227947189 40.5734458834617
36.6501141438378 36.9060997041453 37.7000400506818 37.2892641289258
40.931294690669 45.6582729799009 40.4136535208406 46.4205654140754
40.2669497398702
CYP46A1 43.2724556187214 39.2907645923217 37.9482157381008
41.4273122914951 47.6884372645373 50.8533737532786 41.253645578517
40.2382908228517 38.8271346375425 43.2988017098387 40.547835538778
46.6397391251762
CYP4A11 37.7384958256497 38.4330755397458 43.9450023643394
43.5627649099623 42.7107928951766 40.8634043028528 41.0381860307445
41.2551966132486 44.2607789091176 38.528621456378 44.6286581923295
40.6614700944853
CYP4A22 41.4508241771315 38.1835938174625 35.6765064335425
44.0451487108213 43.4885245598939 42.2577689927563 41.253645578517
40.1474543172332 37.9837233601316 37.3179578037963 39.6403089864678
37.6557714476853
CYP4B1 139.49198349194 839.850453078119 453.652548085387
39.3750969348308 245.630020408551 114.643475335002 313.528012643479
412.421643885499 1012.91803181784 304.877336204875 639.391575249793
209.178131628825
CYP4F11 56.0477607422828 82.5344501505114 231.304658157247
38.8495095797881 143.563236821003 48.3686935595826 101.716697533604
134.83544047809 88.3799086910448 139.814194128548 122.15017220566
61.9882398986333
CYP4F12 70.7238101239421 317.892699374852 383.705244574204
43.3078542962968 333.122044191102 111.532958750074 326.70962514228
139.436084808369 573.832332097619 1237.93130196998 1395.33665255999
353.751797948675
CYP4F2 38.304249186058 47.6914221838216 61.1745030100746
38.3668692419741 52.6226745117396 42.9695585740813 46.6267962186599
50.9933795450314 46.4164127487052 50.294087888475 41.9083581529276
39.2469742642337
CYP4F22 49.085741080241 925.486088550061 811.142611128461
42.8235313007746 397.798003824167 94.7008981904016 667.583623414832
149.260740603715 464.209794165593 617.539783972955 1416.47763911039
200.450523205251
CYP4F3 42.442231667357 54.7855575216687 56.6458456890715
82.8888969977394 52.8190882379316 52.3746819771982 64.8367546135053
41.6466169548846 112.414189401193 67.1926218405833 80.4601303882402
59.7999569757275
CYP4F8 44.3355304829989 46.7591817286632 50.5089777244781
36.6501141438378 99.8699868526053 36.6271061451613 92.4534854832186
142.545268664218 84.0388582916959 51.4454142248566 51.5822637582617
39.2469742642337
CYP4V2 136.155022329495 79.6516904169771 72.4913548133238
147.388500451427 112.311249826645 188.008141649466 86.2118355769367
99.2432911346944 106.799510275842 277.993522959259 191.141778714144
121.259683158193
CYP4X1 39.2076937845359 50.0642388903563 173.677648729894
37.651298468202 85.1661952927159 63.499841172847 48.739479557328
180.349984862124 161.948193932343 96.1825551158198 83.247335725921
87.5759422500374

CYP4Z1 55.4452315601595 39.8463497940802 46.6588655218735
38.1412400762431 47.3716857595991 37.8481704275343 42.7431359850339
60.6168105212811 44.4395580493118 39.9612063794301 41.398124064512
41.903370741929
CYP4Z2P 53.6429962450784 43.2907292669418 48.9741705041438
43.9551059970289 47.608637584681 43.9724628401189 41.7513290046251
58.6249721386149 44.8003345643334 43.6662436706167 45.4538655633538
44.7941491078266
CYP51A1 53.6741873729867 50.8114699212931 48.1331309338997
50.2163892855092 51.940083408061 53.2046867327331 59.5205055437765
73.362936710475 99.6340486428397 55.0016608333828 55.2374601141719
68.8529879402902
CYP7A1 40.4639532172338 41.2598458726002 40.4882741869718
38.5252456306656 40.5710637884029 38.3418933808844 38.4005795891396
40.1474543172332 42.0621425894624 42.5664883002833 45.2265319826548
38.5279745308203
CYP7B1 34.2180669723984 43.1805016690297 37.5597671487621
40.7481155008596 41.7929491793697 45.4882438975269 37.2892641289258
39.853866204927 40.7847110517594 45.0155543555707 42.5426057453993
41.0657892043496
CYP8B1 38.6454098044474 40.7952353240579 41.1367478979484
39.3750969348308 40.2449343247539 37.1317006162938 40.5585676679482
46.5717043290731 42.5406888281114 38.3092241935352 43.4709415392696
45.4799270968969
CYR61 2611.32405374107 1506.33570635551 616.265771322835 1076.05074395483
333.421080658854 1917.90025587049 162.735117558467 580.444451816014
103.349712762251 256.941243394182 1638.16317738035 532.043503787542
CYS1 43.2724556187214 40.931294690669 38.195965820384 42.0621425894624
39.2968590261598 37.9430145836712 43.1602899171656 41.2098211956301
39.2497056720346 39.7411455670172 38.0365987358005 42.4607795638552
CYSLTR1 47.5647821785529 65.5367689605146 57.7415362494584
158.478919789334 47.0954207417646 62.9688525319822 49.3854238365325
51.8347877274018 46.1101825640108 46.441301934328 47.3791873993182
48.3648494350286
CYSLTR2 33.4240466670776 43.9421822132391 36.6522278218553
40.3180310664156 42.269054475455 40.9506062495231 35.3362091871364
34.8949555140527 38.2215591930186 32.7876527533679 38.3684202018524
38.6454098044474
CYTH1 71.2963721541702 68.6074516459969 67.0421310720934 140.175068871414
55.6991707857987 73.0247140074919 54.0170817319522 59.0262390168156
60.5608545741456 57.4665857273093 66.3481553496766 56.1875519519366
CYTH3 75.0237373605584 54.3554233228353 58.2986825661503 47.1332119496722
63.104563489528 72.2544131004774 49.540449125788 48.1591264215184
56.7991853808475 56.057906531592 57.2008272301399 53.1182952086284
CYTL1 44.1294459089314 64.7650773032999 40.7620472083159 90.2790229242453
52.8190882379316 68.4323624797175 38.1793598983615 42.7431359850339
40.137818555383 58.6439719071099 65.4493288628375 51.5741598315033
CYYR1 79.6581393791238 78.6705291023488 135.782043043499 123.281175151414
64.193536444915 132.364260893831 44.2166118829877 85.7732262204681
41.7627910693483 104.672760332135 152.456314990408 110.731862140996
D21S2056E 30.9241129241985 37.5469247328848 32.2796484885898
36.7892997857376 37.8679241954337 35.2067831864574 33.9750800030909
34.3865306339645 34.6720534245631 38.4187686307607 38.956280281655
33.6902293666089

D2HGDH 469.14509810985 382.050584680669 865.63777294494
252.583117397109 250.799060762208 193.981651711507 228.439524702578
137.218635091665 416.40156274382 864.302844775203 886.397061577531
716.754656773542
D4S234E 40.5654775358427 693.056793637199 1533.38581753491
69.3525901554637 206.08791147394 62.9688525319822 78.0020909283414
93.7090094630558 352.768184657577 504.676944739425 524.711981433682
343.94159167652
DAAM1 101.383978726419 142.863673914134 132.52749757795 105.125095381533
69.9021277921574 149.295715963625 84.5055910320032 207.957319374194
151.912182086236 133.549684980003 160.303575545559 184.750252804038
DAAM2 64.1882546112044 47.9665737016961 46.2625470410195 56.9299697426319
50.3713170502557 68.839885767937 64.8367546135053 78.8250642061439
38.3418933808844 79.262977183608 70.0402992940342 75.9136854087044
DAB1 42.7150227340364 38.8336427948187 37.0642268327439 41.6564075217517
37.4470183315826 34.9849896310235 37.7136011474903 40.1474543172332
35.9645119596869 39.8873706855774 44.3763859390287 35.3525469364579
DAB2 290.087040648659 235.682605856428 164.735191308776 521.874349042344
117.76002524375 468.573956652904 147.321968565095 1366.59129560817
57.404059248727 129.613152757362 203.870471031175 174.559216176175
DAB2IP 42.442231667357 46.8236022402545 38.8403878304539
43.6275678329783 46.6972735797732 44.4619248757502 46.7100796279384
44.4489662507893 44.2836121830547 45.9573418095075 42.9699559565161
42.8864415494484
DACH1 41.2836317141451 43.1560065801875 40.641503836284 54.2059913044076
43.0860383229288 45.3882138838137 40.8600431177315 56.6910078150745
41.0522938990031 61.6084234634424 67.2575005672188 48.5123041297835
DACH2 45.3872406432794 57.7757446006652 71.1521292570086 44.1483976313959
53.6907197364586 45.9266745571724 41.5817737564592 44.4031868028216
47.6195009345747 60.0372015630618 54.2999306485758 56.9379721393904
DACT1 107.836867810972 51.6060290544385 42.3536549114165 46.7577982595088
44.2607789091176 75.0801071257739 41.6520912742594 74.4696354388511
42.1594905198929 54.302003946825 51.5822637582617 55.4071190623241
DACT2 46.5549623118323 46.5650556481721 34.4716904283753 44.6905691366696
42.6022652963326 66.6320017520888 56.9320717885002 43.1567024035183
38.9834959368496 105.212030988223 59.2643906275025 62.3997407955361
DACT3 126.10194284238 80.0502531022254 78.080034922779 100.001150612051
76.6914642534934 529.025813945023 63.0119737420787 72.692416146584
55.8510296341925 390.740971459177 290.087040648659 228.703786640132
DAD1 2108.03351084959 1029.12917222294 1234.59589464045 1607.73526966988
959.549658414082 1147.19483787501 793.570993808328 805.590413262364
1221.31634960736 733.358170858378 1057.44729389649 1670.01014610464
DAG1 151.175959542368 171.705718494906 160.853643241489 78.0020909283414
230.344359535334 123.855293655347 215.109108265052 140.199798713912
243.15571937138 198.652987129375 190.921576470336 150.562062400737
DAGLA 52.9240098541574 46.2286164776607 39.0775870687758 54.4586176399381
65.3933030211114 48.5835484742456 44.7255207589606 43.844855336215
57.5883027758003 38.2069499294002 42.0750889188255 36.064718696392
DAGLB 166.644251737703 137.302487233881 181.2179249806 187.802182308625
196.561401686392 104.80882886573 192.171116215791 186.161177110898
179.322661675979 173.150885069619 152.132756414024 141.784776736918
DAK 58.5759178381027 99.2824152850072 113.539485042978 56.2508140932179
110.599131324937 58.0910843932181 117.811967515956 118.075168790221
98.2512963929558 94.233414682323 95.5325488969517 75.7140064167134

DALRD3	46.441301934328	52.8455400026194	49.2595762436995	
	47.5733603903505	50.7116312517035	46.1974343793412	43.1602899171656
	41.2551966132486	51.7289068180135	46.0713306971594	46.0513515682555
	49.0655215909632			
DAND5	41.5421429082231	45.5842286511645	45.989212296035	40.3180310664156
	40.5710637884029	47.7556758818053	41.8315602975157	42.8482181313226
	40.7847110517594	42.2614524730856	41.8168470823451	37.6557714476853
DAO	43.4626049853157	40.7952353240579	38.9790184198145	41.5464765641928
	36.5393857306744	42.5660695551615	46.1634822993009	34.9580369221164
	48.4869909888679	45.0155543555707	46.972521499515	44.3521099857368
DAOA	38.5007204246293	43.0227387991135	40.301483914446	40.7481155008596
	39.1650142708257	38.2293008890613	43.9450023643394	37.2237132058632
	43.2761454096265	37.3179578037963	38.8507804383261	39.7411455670172
DAP	245.811158468638	346.844545554188	293.883582116334	210.255784687052
	170.450752247109	199.294913752704	645.702744916884	264.659866487253
	284.351747562807	297.701126167694	338.72596574658	347.247950836937
DAP3	542.684866424241	544.355979695242	740.423874031773	374.026381773215
	780.966123176259	557.717744882602	451.364230598722	386.98657211459
	471.297503316471	353.868749867979	467.351570171652	566.548446430954
DAPK1	158.042994416158	311.985010273921	95.5022104475922	131.141908315555
	117.893268074013	103.856444152519	146.117756310709	212.813224717262
	121.142787642824	131.779333469955	125.383837024985	92.084526929319
DAPK2	46.0023068797812	46.0713306971594	48.6037684272115	48.4916117779992
	46.0977687311222	44.2607789091176	43.9450023643394	53.4109882045447
	63.1944558106132	90.6805895192707	62.9336909080086	52.1186591838715
DAPK3	133.742988105942	86.6854406823303	83.5149142053137	77.6574950328663
	99.1438953564062	130.506891299903	106.317388820963	123.800965644574
	104.288073521816	101.138003125297	119.956819012894	73.7335870746827
DAPL1	35.2267149016596	40.148780650831	195.430996540859	35.49696651597
	37.2567512265363	40.5281496800812	42.2577689927563	40.047314525032
	79.1576305054224	139.814194128548	76.8713035584472	202.081933301612
DAPP1	911.661341702443	508.688028777582	632.019639546131	874.419252731128
	382.872356030213	337.363827785838	781.923984640049	1423.29307392266
	456.023620287811	1217.21440297138	1327.93833415582	1009.47177197543
DARC	39.5676051939342	139.554519110944	141.293219914385	429.329962597147
	51.2525124735102	939.453799186397	66.4258159334241	139.925369282704
	33.802253812079	122.824055084598	175.84382217093	447.953017125817
DARS	1921.48678333837	1704.0522304387	2045.24530722676	1490.1549980903
	1835.21901524331	1886.05449875658	1333.01876209753	1561.83892117664
	1985.3999418878	2771.14077845128	2300.20602168385	1848.50311456636
DARS2	112.351686948586	68.9833517108866	115.256208610595	60.9379216295576
	138.50445813694	48.7781962164988	97.4515987591398	72.5835488194631
	101.81885073944	75.0665411373616	69.9652162720742	73.3321469094819
DAXX	163.43197105141	112.944044862693	102.500172212095	89.1290937615998
	118.376686726902	72.8472187581193	131.645714321903	88.0827215704624
	97.0348634204639	127.265200736376	102.304422999511	94.3953345963359
DAZ1	43.5710871617107	33.8237407699472	39.4710743516517	40.1804147506786
	40.3452320167286	39.2985791324641	40.7910160811527	40.7311497914871
	39.853866204927	42.4594149893798	40.0766543123257	45.3763275588341
DAZ2	43.2724556187214	41.0226232127351	42.7303139017786	43.1275490164761
	40.4321207743917	45.3233134695905	39.700936357233	43.0930293831497
	40.3316153483289	48.7414853386615	44.9713479185144	39.8873706855774

```
DAZ4  43.2028319630577 40.6380495994768 42.4580504587581 43.26160898693
      42.7107928951766 39.0266821712099 43.1931731665131 43.0895173605825
      44.7804080899431 38.8507804383261 41.7272875295469 42.4162492820012
DAZAP1      82.9875926750034 132.400225103653 100.567309348016
      68.3157692917711 185.378190201718 89.1290937615998 119.747995520476
      91.0354017559834 135.800775637089 77.1767494799711 105.814959179397
      89.847560289665
DAZAP2      1297.78424326775 2978.46032143413 2348.48320928245
      3854.42494497831 1046.42391243532 1835.21901524331 2101.59244895554
      2013.08489754172 2966.53151444114 1855.0119962662  2266.34088015654
      2705.15813218168
DAZL  40.308802735799  37.4772922422734 34.6346303305453 39.1335009052403
      39.3999056968598 36.9625898230551 41.5817737564592 36.489383074099
      38.9496515344837 42.0621425894624 35.1102845029216 41.2437378488025
DBC1  39.1590964332473 40.716553247236  43.0971787292711 47.2009420656854
      37.2567512265363 40.040778349137  38.7337086062875 38.6454098044474
      37.9273653326866 46.7646877499666 50.2203578628217 36.449101775775
DBF4  51.8781065691038 45.1694246393676 45.5883690965192 44.6905691366696
      41.6806984441849 43.6670755115848 41.5817737564592 38.4540849632254
      43.5669306063196 45.1569741609597 44.7926323492519 40.0557788909923
DBF4B 38.2496050953547 40.7952353240579 39.6833800000587 42.7489468897708
      43.0723817756832 39.8785903668981 40.5585676679482 43.7396619462033
      44.2607789091176 42.5193208629123 40.6723334103791 44.8372404608604
DBH   44.0328992213727 35.8227939208521 38.0452862258803 33.3231576795423
      41.5668884168669 42.8598805316863 39.5496902678788 37.2237132058632
      33.4698406117406 39.2623423164488 41.0050831223107 39.9519547348106
DBI   628.84789420227  798.033975337132 786.57695312967  1149.02954135912
      1045.20336057833 497.290172759099 841.850722284484 2045.24530722676
      1642.75555340648 422.237766234202 537.841677481011 760.745188431719
DBN1  257.993462737264 85.0017995689256 79.3066495980045 110.202401893789
      106.8866235778   247.416016413788 158.408644964483 238.025903833797
      73.3506594385504 188.432979046693 158.449570606242 171.485221601137
DBNDD1      119.795228959021 92.2095095678497 76.6346767983285
      35.7674462337723 116.817127851913 59.2643906275025 204.944787289897
      113.767996184128 117.072965367702 57.8585041080995 58.133863696399
      52.0863423572635
DBNDD2      286.927016665685 731.071171171638 306.487935692954
      615.442484911026 237.632703283663 1137.99307039386 756.801598119483
      245.853111206859 100.344513700137 806.34906276292  557.275332269647
      715.662312404643
DBNL  416.052744271365 373.46798029115  442.711822532078 506.75912743556
      347.750020791938 383.578947165143 324.126628444078 460.602626145229
      396.167219131922 460.865101118125 440.728435508514 288.687391623935
DBP   48.0201377454632 50.7487159427973 51.223859150798  56.7927450229377
      60.7495043977707 62.2818512646893 59.3549213146532 46.9986083378817
      116.949813289388 78.0658258913875 64.3032703931013 40.5703581862752
DBR1  53.8856501960117 56.939860285007  51.4308501933893 45.4882438975269
      51.4092491522288 46.3203995372658 47.5130990528612 47.6952179922944
      55.0748878261048 42.5193208629123 52.6547683625018 45.9104478407175
DBT   124.599352191013 168.897476166126 148.3842032507   97.2865629929716
      148.582198572949 133.877825732645 149.231260844854 112.428734515811
      144.300051706447 161.7095047889   179.897685693038 178.592741163095
```

DBX1 40.6559419616644 37.1685072112618 34.6977013306396 40.0698728584484
36.2048942610546 38.2293008890613 35.9894133432376 37.33030683349
41.1742244762318 38.8507804383261 38.3684202018524 30.5239050647746
DBX2 39.512606833112 40.6380495994768 46.4569566520107 38.1421709520616
42.4493032770053 42.4155142987678 40.3302268293707 40.8833572570383
41.0476065156912 42.2614524730856 41.134180463103 40.8098117292322
DC2 406.964801954621 452.797096652556 325.275883498219 446.613670519745
538.427311162325 298.031228833741 292.47671022688 179.322661675979
481.145001682177 129.161393794351 137.360931076154 361.158558609751
DC36 46.9939712192224 49.1283590719461 62.1893884200745 43.8002456102021
48.5000946421718 45.2852528185486 45.8980298854198 49.5185112777773
56.702421372165 46.1390887492649 47.9942520395434 50.8442982601142
DCAKD 407.336051853854 284.437758089699 196.465477936749 214.073500272341
284.795064481553 297.023913988137 491.8684831079 578.456468865003
353.810269076023 979.468753716758 659.666977039793 683.75696650704
DCAMKL3 47.9175508887533 46.4385386401326 42.7303139017786
49.5424425850549 39.9697332660833 42.157627240486 43.4544762830742
46.0633179231318 42.4540072551568 43.7303813515864 39.153226699662
41.1554481567603
DCBLD1 60.8357760968871 80.4822328865 63.7744603935682
71.2572485987604 61.3077641493507 51.8103411703261 56.3376046940285
57.9713749600418 61.6839343510302 62.3233491851723 53.0798381965427
65.524821699432
DCBLD2 220.682149473323 65.1291617069903 75.2237417063897
136.891616331257 86.4195039096886 189.111092737256 176.520449609867
144.813029178278 118.816772641597 199.858233616116 134.697808298417
136.947616119439
DCC 42.1537476443423 42.4493032770053 41.437237454134 45.7397504535169
38.6454098044474 38.5252456306656 40.4359177791566 39.1652965788406
39.1790517315259 43.9450023643394 47.6884372645373 45.3763275588341
DCD 41.9407041937973 45.5842286511645 41.3366779545815 39.853175885492
41.9842695818285 38.8336427948187 45.223075311113 44.7044140979108
37.5469247328848 43.2988017098387 41.9083581529276 44.5432192783161
DCDC1 36.0144420013819 36.9994058279362 40.0998167098057 41.0726336841311
41.2740822675592 38.7340969861754 42.1607322031054 40.5158867964016
41.5635499110307 42.6793753550854 41.2598458726002 39.5418280570561
DCDC2 41.0608773166058 44.2630383225933 38.5045333213784 38.745552403895
41.467005949568 41.3553369544097 85.5027511448958 78.253478446856
42.9020321355927 46.441301934328 40.4504608195666 47.6938628797168
DCDC2B 46.5549623118323 38.0226905622357 41.2365089689635
40.0698728584484 37.4470183315826 39.0266821712099 42.8684958525042
42.4580504587581 35.6139301508335 40.8970401536578 46.1673159982664
44.6484005362281
DCDC5 59.4448586839717 61.5144360539876 71.2681122787939 82.0292880742189
86.033725825968 72.0073363366137 83.5149142053137 67.3976855661302
67.3230935444252 65.9189023794337 67.6699686085575 79.2525964926483
DCHS1 54.5358897781765 51.6060290544385 54.5191471810265 57.2856683017619
57.1935729219207 60.7488007939604 53.0277416531043 47.9942520395434
56.1733549054832 63.6197695952215 60.1466326805616 47.3992733452821
DCHS2 40.4179476219497 40.2493681589104 41.2365089689635 40.1804147506786
40.0145811166794 41.9069736908705 39.700936357233 38.5457716679071
38.0702079388879 39.3113207075992 44.3763859390287 37.3600014814388

DCI 83.1614238528477 114.41703170676 114.734157967466 80.0042268333735
105.589475270494 125.934747056908 135.737771384104 103.199200862158
179.886168478179 116.099186392258 139.071851755156 113.624887028422
DCK 77.0301627655229 101.699631771662 80.3060865378414 111.071209057746
75.7947206223934 86.3185927557402 86.4114854104675 70.4262478188233
86.6910280940418 60.7495043977707 61.0979269700412 72.9580216367379
DCLK1 71.0733129130898 56.3263223563484 53.4088637045445 56.2508140932179
53.8260140375214 142.03031230486 64.3669451425581 71.0197205018137
58.7382738968248 65.429256309049 67.6699686085575 69.9767092409915
DCLK2 63.6287257620165 45.7979032939211 48.8623729374733 48.7646976789783
47.0954207417646 58.9056300364078 40.8681871019812 69.9144520559912
36.5474283500601 55.3477556906605 54.6738135880328 55.7802841481067
DCLRE1A 56.921728372453 76.1682394916873 85.5839487225537
56.6458456890715 86.5513342734384 58.1798515131249 87.7107079534841
74.9541456286637 86.6516906104905 84.5055910320032 76.7063975550116
78.0586153423713
DCLRE1B 44.1294459089314 47.087835967892 44.6032218589199
41.6564075217517 43.3332476888305 39.9397419279057 47.1345949868029
46.9986083378817 49.3780606970538 37.4302011516587 42.0750889188255
43.4709415392696
DCLRE1C 118.923419920947 83.8658743784404 91.1363393944977
158.769954040462 83.6438735616921 93.3718038663074 137.658868649032
180.844663304872 95.1037791424727 156.197625493702 160.605530211736
125.197314240529
DCN 3421.24021192326 595.790464730847 268.144525192544 2009.75680599899
923.470702344816 7785.77078492993 104.480361196422 681.474524031866
95.0350792557735 3905.8371644287 2650.09507972593 2978.46032143413
DCP1A 92.084526929319 96.6963826355216 93.310557793228 132.52749757795
93.5717680539357 125.81889994067 122.514153407264 77.523734787944
101.46584765758 164.176142753482 134.994839576312 143.278668481091
DCP1B 44.6231541342 51.7983110178095 51.6159542017038 60.1837189855762
53.4109882045447 71.7021670182761 60.7992483126292 57.037639245179
50.6750785584772 71.2409890549638 64.3824529624475 55.5712957590038
DCP2 226.970981973829 206.931452638325 177.65469867262 246.274692971423
156.174166381986 143.932614622477 185.165420581989 234.495626550751
212.187855950414 280.075938355178 247.04098109003 242.792365320043
DCPS 92.3659612653822 127.701556328274 150.545989010484 102.605473054584
175.362103430164 104.059057437473 160.605530211736 140.698550434486
160.755701876741 160.056757080676 155.225701588645 167.639975497722
DCST1 45.6517320730042 40.6380495994768 41.8751578329365 41.0726336841311
39.859345745831 39.8455060654531 42.4939802311136 46.3685439301234
41.074824727477 38.1042454705119 40.0766543123257 40.7704587752083
DCST2 43.0001432913058 38.7344853699576 39.3709357615493 36.264823544143
41.6806984441849 42.0574848021044 42.2577689927563 44.7044140979108
45.0017158586743 43.4885245598939 37.2018803984555 40.8681871019812
DCT 40.2543341073001 44.5152708955623 42.9104454767916 41.3027031037343
37.956732373465 41.159706131351 49.1067959632109 40.931294690669
44.8997235283254 40.4136535208406 40.9157352260901 42.6758568421347
DCTD 303.744307989358 545.827286197333 318.069132873489 257.506228440451
504.281506941044 307.754078087321 415.05124917015 228.08291792003
310.900527198512 352.246108891022 426.622204035624 342.146013966482
DCTN1 159.248015095017 123.720102870283 151.795609904857 138.486962379511
132.81898450496 197.103673938152 122.642481894309 219.238235572894
134.968128884298 211.897320355251 221.083802855232 173.288267403695

DCTN2 991.630429625437 808.857027942253 1087.75682788799 851.108027634251
682.35345164708 1260.45604038487 1196.18785007729 1591.51486001004
1228.1735887278 1061.61186520958 1174.92419086759 1103.90449800536
DCTN3 342.300139006451 344.133384411423 265.790391813663 314.782545392841
271.50380898418 406.104588997462 387.631477822638 258.726193861248
200.986069552443 279.782392390288 266.581466060604 258.375785708747
DCTN4 100.057155418659 71.4197504261453 78.5325880060762 102.971363152434
74.0735632658567 94.7008981904016 68.4380822448123 109.326279686847
105.549249728506 151.451107406752 127.708268637068 123.978615472736
DCTN5 213.140641182603 212.945601743319 308.931677425404 223.316311787046
229.697141930047 231.893992191022 181.064239330482 270.947794977829
251.184621899165 247.547769833089 308.931677425404 354.38923772101
DCTN6 127.50316411791 126.775432305478 197.813848976726 168.561260174501
82.5170409503063 211.596652798395 141.69593269802 80.191847692094
114.5696064637 110.164155258574 127.78061946174 192.003788858976
DCUN1D1 59.7961440557352 58.4134572546373 64.3655212368264
69.5544840164738 81.6949047529395 79.4916415027015 60.726977722583
57.3286965007828 64.0903425729408 42.8936990772838 50.0303076717704
59.3082373468217
DCUN1D2 44.7044140979108 47.7962548556026 53.1144624658538
47.0874209746031 46.3607182640185 43.4263583096036 53.1884505015411
45.9604707098953 46.6944238390932 47.0055981216687 48.2910530887214
46.0633179231318
DCUN1D3 143.761359357965 110.666686474986 139.693327116993
573.832332097619 115.229070906222 215.955919625615 137.547711779867
206.072527867605 112.414189401193 155.519517541864 182.188883821722
187.462631817772
DCUN1D4 125.402628368587 97.2803806915572 138.036631806718
114.979124960177 141.180682718758 257.194689822426 135.012695962236
126.930102684077 140.78326016606 311.361885152025 235.682605856428
217.6996233059
DCUN1D5 205.898127436939 252.775631043952 246.274692971423
184.50283792606 452.969595001684 246.151211515664 311.668075784386
189.950108471943 351.439404852971 113.82565808481 106.443777149496
182.551901102169
DCX 43.9309845324767 45.5587966941116 40.9818899755491 41.6045220683604
45.6895223823001 50.0404995046748 44.6809101024392 41.6916508480321
45.0997987980157 41.8613406887824 42.6366765186182 42.0709558258414
DCXR 239.466489599238 284.620447030305 250.866560791828 182.37433006793
443.863346367342 187.238252376588 489.622327794168 343.262403838579
474.267141780289 331.935072199058 359.83890067786 230.520772006119
DDA1 406.104588997462 593.345148568451 509.004054793277 391.187784616808
995.397944432196 392.51606894011 585.771656887036 501.148339791372
398.139730642919 321.818380385895 388.580556899321 302.422475486532
DDAH1 107.466490458133 86.8262463672891 61.1076362129586 55.6481366056639
72.2665537421443 112.23915619297 195.262845535544 226.168990555083
65.0810479794187 223.501516374076 168.326515533208 233.263058459431
DDAH2 59.220967994972 41.9894412416874 41.9666541498638 39.3750969348308
39.3667161654772 120.231435193839 75.164719839017 67.8061298261394
46.6944238390932 59.3618843678775 70.4204944097269 53.4735821194294
DDB1 452.139416552986 474.155196153863 544.355979695242 426.622204035624
928.230419226078 454.02808608679 797.019657306322 883.06773454117
606.481020597082 782.639346040155 697.57030127315 506.585006700107

DDB2	39.1590964332473	50.3586212130973	50.5089777244781	43.3078542962968
	43.6587492470348	44.9896851005259	45.7452456977857	36.0306975524309
	46.4861258030402	47.1001757441025	46.8954576545929	47.2131144508464
DDC	41.2424637338424	45.4143854024453	46.9040704638073	41.4273122914951
	46.3150221024046	45.6129956328415	47.1345949868029	44.797320243931
	43.8482802038826	43.6033221838149	43.401782064778	44.9070250604284
DDEF1	332.137622104526	241.706783147271	172.381808887528	585.771656887036
	202.459251553516	219.702703529829	214.369365247268	311.301613620475
	162.735117558467	187.331614826395	181.381479219186	207.245428311682
DDEF2	132.838429478396	105.34055372228	56.6888826857797	83.1614238528477
	72.828269171954	89.4747568145717	162.486448421519	342.73665541549
	91.2488561751625	101.351907645804	80.6032289311256	113.491158368594
DDHD1	36.5393857306744	36.4564050256915	41.1367478979484	39.2336402665402
	39.3999056968598	39.9777866059804	40.0052661202207	37.7612949798895
	39.0458123429605	38.8173773425835	39.9586075662602	36.8209886489281
DDHD2	92.6545309624315	95.0738959950301	89.8354888041611	130.775349446374
	143.084029849218	131.727983826985	141.784776736918	115.132600341395
	75.4572860692649	106.049736669661	97.7415824971555	101.832945816193
DDI1	36.0144420013819	43.9421822132391	41.0356368374708	41.1886975241176
	46.6397391251762	29.0303154053123	38.4005795891396	39.6474008369935
	37.0126425326534	42.0621425894624	39.6403089864678	37.8490162439785
DDI2	39.0237504776589	44.9863726205428	39.5954353681031	41.7614975630362
	42.3536549114165	43.1180113655693	40.968974769473	39.7509165247419
	43.9204029310411	41.0057887014368	38.7337086062875	40.2237056928605
DDIT3	141.471672990444	78.258486366912	80.4060693489113	334.527003257901
	58.5450868286167	183.243195292141	100.499763255098	125.484233748017
	215.955919625615	214.281036491237	214.281036491237	151.641746442323
DDIT4	2323.81110653289	315.960715273629	4597.60989502097	2036.72286752819
	1896.06380611235	719.815240381415	1423.29307392266	663.372296297652
	13656.2581082905	1801.44003951248	3143.97163345458	4277.93019582362
DDIT4L	104.986694941968	51.524711857679	49.9256753636167	
	45.334106390212	62.8270556619309	104.059057437473	52.4111687156893
	43.3262148375525	52.0583273941813	76.7137782332689	55.5567699051046
	42.4607795638552			
DDN	45.2875173643252	38.9338521051669	38.2280071951674	44.4367387274922
	68.307513091369	36.4986986135769	46.3057385979341	38.3684202018524
	39.9444191873552	39.8873706855774	41.9083581529276	40.7704587752083
DDO	43.2239171482126	48.9800181621986	51.7663108516261	39.5748670524951
	39.6535880152834	43.006072841282	39.6222000738266	42.0778399888414
	41.1742244762318	39.7397636748413	42.0750889188255	42.4322338033273
DDOST	560.413725186282	906.833938613875	849.412637017524	459.92905629125
	1152.55315876935	558.65313710197	911.661341702443	996.520340280883
	1018.35211991691	621.980260052085	678.684833360104	539.32996290794
DDR1	235.682605856428	246.313146942736	245.356135592468	48.4916117779992
	167.095818680513	67.2397076992564	227.170944398338	142.789889238338
	357.574415292533	260.185079572615	246.568253751149	209.31241212773
DDR2	60.0793579902362	50.3586212130973	43.2721716572958	79.7061415085215
	49.0828761839116	173.08677862231	42.8684958525042	55.0698932795613
	43.7078399718247	80.3915591207869	102.304422999511	84.5354986509039
DDT	594.611678976716	959.549658414082	479.318513108472	666.816487335088
	1588.70376317303	884.036280777358	2108.03351084959	700.010354583503
	928.230419226078	796.359355922952	792.022171185649	846.773659307087

DDX1 1470.84169670955 862.031722874406 788.232434009173 696.529869192906
1313.92053019421 1120.83891847181 2004.94135236716 1925.20977162704
923.470702344816 906.833938613875 889.638287212686 906.833938613875
DDX10 225.50098418159 169.603165827302 139.445008123768 128.786137360755
332.322497133752 214.980684661682 184.808179948172 292.156216736355
170.750057881363 170.255635587223 179.214713709599 176.064476306748
DDX11 54.0536858212253 52.6398559444048 67.7235257493648 42.8899872337087
54.1745577382276 51.1621379498014 63.2113624676341 47.6397712007919
63.2409684531772 54.7061116419212 49.4145011642763 54.3820590274321
DDX12 46.7728879680671 43.1524467741439 47.8360317478571 42.7577133601564
43.1914709389238 39.6575367310267 45.402658694824 43.0963492115408
46.4520905520834 47.9885084629869 43.6897331183502 42.434729228822
DDX17 130.336599857526 154.455976468088 181.2179249806 154.93193897888
113.388372364837 118.010166045155 140.128033028434 155.956315815754
116.318299211619 217.743953847269 193.569399061809 172.777241940677
DDX18 427.451886998067 537.213035625492 355.67721196018 453.652548085387
667.837116137964 565.411298018845 362.927960895643 377.491895827114
446.613670519745 372.473625569296 451.290681710534 427.540236966999
DDX19-DDX19L 51.413025495745 48.7847528954724 47.5673419710251
51.0175677516029 57.8039717833131 43.8582747295766 46.5650556481721
48.7814131120945 48.4869909888679 42.8936990772838 55.3147456054175
42.7782216342806
DDX19A 183.2003374677 146.268519891864 187.711309781257
141.043326064913 170.829628173552 181.531005915656 184.536327776767
232.048938339214 165.992057520727 166.129723762515 226.580055691897
170.593589984253
DDX19B 153.237127853072 197.598956071422 286.68367978144
165.72797054597 166.25909484446 159.937192675974 182.31306890697
179.694413858411 172.574996256912 146.840603547323 130.72385089567
129.605603092944
DDX20 40.3478825593224 43.2649781354792 42.3536549114165 41.3027031037343
39.9697332660833 48.0350549043063 42.7431359850339 41.9315419791594
46.0106098064475 49.8393069675192 49.5923712603197 46.4499258373233
DDX21 197.776069056927 310.900527198512 188.008141649466 410.808988840343
398.641868389511 332.490954010208 215.800662077432 166.1490536844
602.726022292973 105.53472751588 152.843785599892 290.710970844525
DDX23 347.486523804413 346.438778257772 300.732955139561 253.140146266702
259.332340491331 305.959643033169 434.223722811041 369.032450063274
502.67939538729 300.762511215441 350.796638646601 265.570814613305
DDX24 477.263677441074 221.450737998571 321.686871145301 326.963277148673
331.474920376907 602.441843384617 275.096637886925 241.017227991238
333.493839176426 499.744856832174 439.558287621096 398.049525642349
DDX25 43.5710871617107 38.6704566269103 42.108130037421 43.9744941494111
40.9416717019827 38.6704566269103 40.0052661202207 41.6466169548846
41.5635499110307 40.9558083259517 43.0971787292711 44.2527566343661
DDX26B 42.3620028137457 48.2220547806679 50.5505693464794
42.2496337895968 49.6507190068324 41.9069736908705 45.6042625176633
50.3972033014227 45.4799270968969 48.0211752672327 43.0710577955487
48.6795178347248
DDX27 80.6434122355417 91.3941563889513 71.0340758556049 55.8709511400674
71.7263663921367 89.2185019250322 109.615289274998 113.917429432477
79.856662992182 68.6788460999238 80.7198077526005 61.8529316708431

DDX28	73.1931742801095	61.5429260998857	68.8098607236981	45.4634652319753
	68.9766943946607	89.680151549258	115.98265270452	78.1448136847078
	79.7882961549974	89.8035133597498	75.9802606133139	71.0340758556049
DDX31	65.6165636276827	71.0593419018503	58.8188963227059	57.8277957325085
	65.8319449883221	61.5635554619635	78.9900928241723	56.3106156221316
	61.5807110546718	61.3529667465225	63.664265793911	58.9836017745712
DDX39	277.367257933324	445.644244692294	352.339572553069	450.139987981805
	1057.44729389649	301.357530757236	495.359878931451	550.990544834571
	551.915864019927	196.427458710993	251.803102904399	172.540737848092
DDX3X	295.5163490537	368.162822151919	337.51861578231	488.277500837251
	287.494740260715	528.242677613584	281.906398109158	368.913147706435
	462.362735276132	387.631477822638	310.900527198512	503.529187388654
DDX3Y	42.3433931248155	40.2493681589104	32.771863438151	42.357910063876
	38.9496515344837	38.3418933808844	34.1889222434768	40.3357777530419
	38.9006788676407	38.2069499294002	36.5359455830388	40.1700117452801
DDX4	43.4626049853157	40.716553247236	38.1421709520616	42.8235313007746
	36.08956004634	38.6414316877471	40.7910160811527	41.4508241771315
	38.9834959368496	45.9004543328976	39.9899430628542	38.5279745308203
DDX41	347.829279462722	121.744701580607	241.599353653369	212.813224717262
	242.236207196941	147.704325041249	185.924923239764	220.337850438943
	230.927097824131	203.651890041185	230.833432994593	169.818067865324
DDX42	298.252355588344	221.76904201992	239.924746743429	193.806701229141
	272.828896268482	368.318359434313	274.731304343034	282.196234758364
	291.5513298383	350.106705208362	355.00914527669	313.046160403661
DDX43	45.4799270968969	44.5884811528117	39.7411455670172	50.2163892855092
	42.9090346219023	44.6524308640784	103.118579484715	47.2864663693787
	40.6957787921451	44.4652605017595	41.3553369544097	42.2577689927563
DDX46	80.3730667493235	121.236881661832	111.077646506425	103.139459009593
	88.3954618645976	202.01900875263	132.172127966602	90.6347108049639
	110.467254789988	109.192770642308	118.951970882732	116.547937420457
DDX47	310.900527198512	312.40869171788	318.369837408344	222.368324930003
	328.63511311303	311.241353755918	321.61848221455	316.604316194398
	513.57613902662	262.534449975056	270.947794977829	481.905052451245
DDX49	41.3387790895716	39.5050383901884	48.9944486068347	51.949834638817
	40.6723334103791	49.3172204533818	62.801431502597	37.4484310268909
	40.5912213523345	38.745552403895	49.7363778831003	46.8129509799753
DDX5	2164.51925341969	2791.75465171135	2712.36664370642	2232.61002288121
	2354.00192115549	2488.32876359853	2018.28984520892	1879.12037123353
	2300.20602168385	1679.12630849964	1950.93175239958	2513.15722262176
DDX50	179.994715581707	168.307676704652	168.640033281185	120.284321694275
	141.410183156424	380.639020028302	224.665128374218	152.272399644814
	559.763116301175	172.416351632442	178.306763765612	191.482692108417
DDX51	1975.28674300761	1064.35745220225	1182.10714525377	1347.15664445545
	922.047328788961	684.238999553691	1440.41726370246	2191.09005731751
	795.381998233245	2220.90215179682	2077.74475301595	1689.77066894108
DDX52	92.9323002425821	87.036020750455	76.093116738739	67.0402749076279
	110.074666148474	69.4214927857472	113.539485042978	146.534713287435
	115.044790975532	88.7928773498158	86.9543262608386	90.385018479478
DDX53	43.0930293831497	43.0227387991135	41.0356368374708	39.5050383901884
	39.3667161654772	39.8455060654531	40.4359177791566	39.7509165247419
	39.9444191873552	40.8049408896274	37.3687900733549	39.1553169409075
DDX54	116.042856605255	112.542326463419	70.0955779023465	87.6763065012524
	120.825113658705	69.1523164481201	100.289042948128	60.6706478075023
	121.737071786167	67.9259304928255	72.1090339831747	68.0751111916102

DDX55	161.48562926756	178.675242612997	123.162013735234	136.155022329495
	151.451107406752	164.097531361519	156.719353642505	99.9896394343368
	192.861174667738	145.050655626219	163.971002049561	172.192366002756
DDX56	539.471011648536	355.67721196018	440.728435508514	234.873589896708
	558.855313555287	289.909646207746	454.939614583134	408.353336724969
	487.540800793233	565.72800641089	488.089090043145	350.106705208362
DDX58	61.5882375235292	65.8082111065882	63.1450855271137	66.0397471691747
	48.2748123114598	69.9819944829311	62.0404348860702	53.1052228246467
	46.0106098064475	63.0119737420787	66.6788758487969	63.3578893149245
DDX59	79.4832790698673	73.4687357112674	76.826527950236	69.5544840164738
	55.6735944873518	71.5616860050001	63.8294437579951	87.4307271341692
	57.4317879234275	68.4137459386155	61.2822647324366	77.6653462778309
DDX6	43.4626049853157	41.0678614026819	40.4882741869718	50.5310598656998
	38.8456942580061	41.9543948167375	45.1017494688414	58.5406981871153
	50.401623258584	53.4109882045447	40.4504608195666	45.9693232070002
DDX60	72.0334593545916	162.287617963536	136.766779297051	94.5501397951415
	42.6022652963326	101.177486898065	72.4651758215622	66.6320017520888
	82.5344501505114	55.6069214673486	70.8670844767965	55.4635297192076
DEADC1	48.1125722136997	49.4982025424177	44.6032218589199	
	43.6726153508978	44.0451487108213	42.9695585740813	49.6872467499977
	44.7044140979108	41.9635525160083	45.0155543555707	47.299647867533
	42.9887069977696			
DEAF1	74.7206530065877	65.3319639385976	70.0059240383657	62.7108230507424
	69.0975200641705	61.8571521441451	66.3775183389603	60.419911425109
	66.6479153633977	73.7133884699889	73.0403099404	68.2708120569221
DEC1	35.9305494063137	40.8880530359822	38.3166554965434	35.9521941716188
	39.7568367479301	36.1190722948669	40.3302268293707	36.43924066428
	39.5806465938526	39.1621898366355	40.1742127891824	45.1735152121056
DECR1	426.276230164353	815.810781070699	553.328261478032	295.014183084118
	353.598144765197	211.451463038315	380.541502795438	401.202348352506
	512.951288394692	360.668308246913	329.978705095448	344.657779779036
DECR2	162.272853632419	130.239763076999	242.751164798133	95.7571966348067
	200.384444538476	133.514641336669	296.842555066902	92.681224908389
	221.22852405053	152.72007888133	151.255948388415	121.851515410237
DEDD	171.877918435017	111.845105854608	142.49850553035	113.528908699366
	161.948193932343	105.997702773537	117.181144429729	95.6731932757489
	370.917477324773	133.314819245044	105.53472751588	105.267519627444
DEDD2	282.764445883135	164.735191308776	177.958475301951	283.393280512514
	165.522304431988	121.236881661832	216.782658096982	211.685526927495
	265.728967648382	190.592556065939	241.736113354506	208.878921620914
DEF6	69.6449298794042	94.1289383735162	116.709906902201	147.52790076468
	71.2787833369055	61.9432904657734	91.5258329186709	68.4659783447092
	99.9896394343368	120.447568925399	112.565968152932	91.531459931855
DEF8	138.82907142482	119.124583755587	149.139710097196	166.847053797281
	151.775928080246	144.046404221564	157.001241754359	101.516595046503
	98.3126451737573	108.251353392321	109.155021443311	122.914175013196
DEFA1	37.55264801907	36.5219488315447	47.0055981216687	100.606802642042
	38.4540849632254	50.2163892855092	46.7100796279384	38.5457716679071
	34.5633623842083	94.0777119695783	41.4508241771315	47.6040671292013
DEFA3	41.3387790895716	38.9338521051669	38.2280071951674	64.0903425729408
	40.9950639693255	41.8613406887824	46.7100796279384	37.7612949798895
	42.5406888281114	76.7137782332689	38.2795150354677	43.0930293831497

DEFA4 42.9104454767916 45.4882438975269 53.0104469636221 46.7577982595088
48.3814421939888 45.6129956328415 50.5252048155459 43.0390857698452
50.401623258584 46.8954576545929 37.9946685306274 43.2776458914333
DEFA5 37.2474303302343 37.8217989841918 41.8751578329365 40.4359177791566
41.1868052124344 40.650742261428 34.9344532055397 43.5544347011646
40.7847110517594 37.2089297656361 45.390192977585 42.57175366709
DEFA6 33.4240466670776 33.5924201549249 43.4709415392696 35.1451084093692
40.0599807618105 38.5252456306656 44.6032218589199 34.806104801006
43.1805016690297 38.745552403895 37.0180934800573 37.4554876386402
DEFB1 141.087571926258 48.4497719907777 307.754078087321 82.7394574977114
103.619396986593 46.7800033479223 48.1747307200218 110.287699369482
129.754481922118 383.578947165143 122.469733727349 533.644397257453
DEFB103A 42.7150227340364 45.6533175604486 42.7303139017786
41.3027031037343 37.4470183315826 41.554942319225 50.6774883370457
42.7431359850339 44.7058233759912 43.7303813515864 40.1742127891824
42.1537476443423
DEFB103B 44.7044140979108 40.2493681589104 42.2577689927563
70.2541961995753 44.2607789091176 37.2686490268265 41.9393838138203
42.7431359850339 42.6762317063737 40.1179210534118 43.3085358214154
43.4709415392696
DEFB104A 39.5676051939342 42.8542642792862 42.157627240486
40.7481155008596 35.2163900400329 41.7627910693483 42.0605906240248
39.9576138799997 47.9035532886869 39.4710743516517 42.911855821602
42.4607795638552
DEFB104B 34.6977013306396 39.6995529770239 35.7105247506212
39.6292788039793 41.3755760434934 39.132725377815 35.7055410599146
36.5393857306744 33.9456837661864 41.3321861371202 40.723990462047
41.0657892043496
DEFB105A 36.1232327599945 34.0382693188794 44.0451487108213
36.9912468186322 44.0451487108213 41.4508241771315 42.629412460409
41.6466169548846 38.3418933808844 44.7958122466628 40.1742127891824
41.3481355128089
DEFB106A 35.0202257459026 39.6995529770239 37.8386378737561
37.1894304543751 39.4693871570028 41.2567470068038 39.6535880152834
36.5393857306744 42.6366765186182 36.9102644736882 39.7342718293432
36.3454320848591
DEFB106B 45.6517320730042 46.29198852834 33.1559412791294
49.3654837541353 45.5809132232681 38.2293008890613 43.6753843488695
40.5158867964016 47.3791873993182 47.9056710878325 46.6442692119997
44.3521099857368
DEFB107A 52.0299937917925 46.4385386401326 47.9035532886869
44.1483976313959 46.6397391251762 44.3648993568405 45.223075311113
46.7980335276155 45.0017158586743 41.5435611403119 45.9104478407175
44.5432192783161
DEFB108B 35.7012997405508 43.896638326678 39.5118967430322
40.5484717678957 37.3785704939904 40.2945586900975 45.8980298854198
37.7612949798895 41.1742244762318 42.3606390470387 42.8263096141654
37.6557714476853
DEFB109 42.8036731479932 40.6380495994768 38.8775560911841
46.0228732507872 42.0709558258414 41.0582900711341 39.1539892144371
42.2577689927563 38.2215591930186 43.6033221838149 41.9083581529276
42.0424641526302
DEFB110 34.8099089553046 41.8848959280916 39.9101908547397
38.6414316877471 40.2449343247539 47.6803619768589 44.8643898647322

34.9580369221164 42.0621425894624 36.8059561571675 37.5583886316038
38.2350720635382
DEFB111 39.3709357615493 42.4493032770053 41.8751578329365
37.1894304543751 37.5556299264699 37.2686490268265 42.9803717121865
40.4359177791566 43.9450023643394 42.0621425894624 40.9157352260901
39.9519547348106
DEFB112 37.0320432433314 39.9697332660833 36.5474283500601
37.9875302985336 38.2496050953547 39.6251842352088 41.0381860307445
41.1575777497576 37.1820543575123 34.7308008643142 32.9879066304034
37.8490162439785
DEFB114 41.9407041937973 36.5219488315447 38.0958223740111
38.042028333747 37.4937394063612 36.8059561571675 41.4742337714895
40.5640952422779 38.4970433581463 37.0740019672788 39.153226699662
40.5703581862752
DEFB118 41.4508241771315 42.6939394537076 45.310660704531
42.1273928203021 40.148780650831 43.1826328292558 45.7452456977857
43.5544347011646 39.9444191873552 43.0919221375514 45.6348103033479
39.6352613956998
DEFB119 45.0283997415478 50.8114699212931 56.8168526942685
43.1275490164761 60.3768221891131 54.0104259381604 50.1185085888209
55.3664856922473 50.4974478884077 49.7728344236992 51.5573930741898
47.1060577218993
DEFB121 32.821392987958 36.3110701990588 32.2133093774284
33.9645433044281 39.1650142708257 35.0970060242246 36.197424053556
40.3357777530419 33.2715481910159 37.4302011516587 31.3142073726046
37.7526485343328
DEFB122 37.1485601150873 41.159706131351 38.8775560911841
37.8442841690502 37.0526522140242 36.6271061451613 44.0687697278114
44.5029690493211 39.5806465938526 37.2089297656361 42.170702295259
41.2437378488025
DEFB123 38.957687788868 42.6022652963326 37.1643693306382
40.0698728584484 41.467005949568 37.9951997351435 42.7431359850339
38.8495095797881 42.6366765186182 37.1275334308172 42.357910063876
39.7411455670172
DEFB124 40.6559419616644 40.148780650831 51.6159542017038
46.3203995372658 41.3755760434934 45.0882356578404 40.9157352260901
42.2577689927563 38.6322728994674 46.2286164776607 40.9157352260901
41.4508241771315
DEFB125 44.7915315085948 49.5443425465542 42.5406888281114
61.2213001749372 51.0107745412152 42.9695585740813 44.3440839686558
42.5435283762933 51.7549258776884 42.6793753550854 47.5439485437351
46.8129509799753
DEFB126 112.211717641877 38.6414316877471 37.7384958256497
31.1401844809132 46.9906120932727 35.9305494063137 41.706879587091
49.2019743609873 40.137818555383 38.2069499294002 35.5179217192467
37.2810767789306
DEFB127 36.0144420013819 40.3384318592985 40.4882741869718
39.5050383901884 39.9697332660833 37.7000400506818 40.6750540874299
41.7419877611072 38.9006788676407 35.6208308220005 29.9617615620508
36.7762265039864
DEFB128 42.2455707947223 42.0709558258414 47.4740726032707
36.9912468186322 42.7982502551634 44.1737502492846 45.8980298854198
43.6434576713809 46.581796165231 40.5090849993014 41.8168470823451
45.0985088502363

DEFB129 44.6231541342 43.0227387991135 34.8956628705329
39.1706564265495 41.5668884168669 42.0574848021044 42.1607322031054
40.2382908228517 38.2215591930186 42.5664883002833 43.3085358214154
43.7672102682067
DEFB131 51.6558970571712 46.7980335276155 42.9641759692926
52.2089306840125 44.3557408297309 50.9724155054821 48.5544398725959
52.5586058896479 48.7358147030082 47.5543691541633 46.29198852834
44.0451487108213
DEFB134 45.9090659324223 39.8695929627783 42.5406888281114
39.066284740632 39.859345745831 38.2293008890613 32.2133093774284
39.6474008369935 40.5912213523345 37.4302011516587 34.8383995317036
34.6720534245631
DEFB136 40.0557788909923 40.2493681589104 40.5734458834617
38.4208429103799 39.0564222526273 38.2293008890613 35.8227939208521
41.5464765641928 38.6322728994674 35.0668743708816 37.6484782516569
39.7411455670172
DEFB137 43.844855336215 36.5219488315447 37.0642268327439
42.8235313007746 41.0885562160946 38.6414316877471 44.3440839686558
44.5029690493211 40.2274522635576 42.0621425894624 41.2598458726002
40.5703581862752
DEFB32 40.2087556903277 37.1685072112618 40.148780650831
41.2551966132486 39.7210115145672 35.4142595025086 42.3194600586894
40.2770232552555 39.0775870687758 37.4642807402741 37.9230440150891
41.7575097298927
DEFB4 30.9241129241985 41.3610369241157 44.5134109184888 176.816741656659
42.9090346219023 40.9506062495231 38.1793598983615 40.7311497914871
36.9060997041453 36.5806287103157 36.2435874904689 36.5474283500601
DEGS1 253.333114749013 157.856158440506 195.968119243879 486.044304392285
221.015209694864 404.175196605897 230.787791652978 241.202687910704
227.598514023545 239.96421556928 225.201522281399 335.818251279914
DEGS2 48.6621527194963 58.3164247463092 153.165464817911 45.334106390212
46.9906120932727 42.2577689927563 52.8496345990125 63.5295088521896
128.05521008708 135.540738841602 148.043320937247 65.1251519077199
DEK 290.956194006554 298.089522641269 309.593721653575 280.239058266018
472.982369155078 425.776214512634 1756.86714809925 554.163106145921
464.99944459917 183.799353414581 195.221917652149 340.607959072657
DENND1A 79.7102196041395 53.4735821194294 47.5216423686268
83.2083460752986 60.7942162859036 55.2336859873958 77.2906963563431
50.5734308316823 51.4749724393807 53.3325829174829 49.319499137678
49.7069910376039
DENND1B 43.9309845324767 39.6995529770239 39.7411455670172
35.49696651597 38.3561682218911 37.2686490268265 35.2163900400329
46.4672637826582 35.9645119596869 43.4885245598939 44.9713479185144
43.8575940017119
DENND1C 35.7012997405508 48.8584917918986 41.785676764591
61.2213001749372 44.2607789091176 45.0882356578404 50.0088611244948
43.6434576713809 39.853866204927 46.7646877499666 43.844855336215
41.6550823405646
DENND2A 223.501516374076 63.4570909617664 68.9522412475296
68.9937449813561 98.0143500562821 227.739219647972 132.541333041312
134.743068051122 47.6195009345747 145.819204020818 136.250317107253
83.2925226467532
DENND2C 55.9249770393176 42.7275315121163 47.8263530599296
41.4273122914951 46.0977687311222 48.4758590019384 41.8751578329365

47.5439485437351 94.6734338650799 115.735359157053 119.015431849218
104.313754953569
DENND2D 81.3544712349691 886.397061577531 486.911782180966
327.283431584541 186.54041506387 101.693848821246 189.204794959564
111.714563124738 293.086409068972 468.153823329361 429.65084326307
586.508419046212
DENND3 62.1448806673973 55.9644250454789 86.1563473462864
231.990562664196 45.8051846088552 66.1082848714279 63.7721358300879
52.1788489555942 48.3119523820103 52.3304586399695 48.9754340235127
49.4786466722535
DENND4A 46.0023068797812 52.6999535920753 47.0055981216687
55.9563971339766 53.0006136299899 57.0775902639215 50.0088611244948
46.0633179231318 52.2216613535546 53.4109882045447 48.0987228351168
60.8662233098324
DENND4C 461.123856163975 332.322497133752 215.645475520124
231.824347524066 227.504982571709 310.763904671301 501.585246203512
497.591040004541 322.888035865453 1291.71781703042 872.305108096296
543.975237512604
DENND5B 63.8626921347231 62.6236991757723 47.646800205189
100.001150612051 57.8039717833131 107.646103699516 51.5967098638347
59.1107691694766 60.9594847577747 51.9547984751408 45.9104478407175
48.6037684272115
DENR 410.402285012885 336.390195295291 325.769076169162 286.927016665685
357.762505796238 285.322305931794 390.202312411165 413.723725202787
421.971171686029 439.558287621096 393.648308810931 380.988918630713
DEPDC1 48.4849194706125 52.8264142735929 45.0985088502363
44.9370339275041 56.2305155589239 36.1190722948669 63.6695826053997
58.9081750508316 43.5669306063196 40.1613888954735 41.0476065156912
46.4499258373233
DEPDC1B 57.6285775590145 62.7743500747415 45.0985088502363
37.651298468202 66.7977843897621 41.554942319225 63.7721358300879
56.1838945110653 71.8576824255215 41.0057887014368 41.3553369544097
37.2291018661279
DEPDC4 37.4554876386402 44.5544727111028 44.2733573199954
41.4727452533295 42.3685136297603 38.1169703906207 45.0283997415478
42.0494116081998 44.1519466943185 41.1222709786153 45.3597029614942
42.3152792870779
DEPDC5 50.4289272827382 51.0744829992368 50.1860191642359
45.5163397926116 47.0954207417646 48.2067028894909 56.566634814242
57.1362071277099 52.2269052457335 56.1186281675573 57.7786197537899
49.5265949137207
DEPDC6 193.981651711507 90.3188163079532 128.596547789044
49.260242314197 45.6895223823001 68.6424653037264 51.9950964009224
70.5322939116406 169.889618506937 75.257352283328 75.8056934060866
73.2234405348435
DEPDC7 47.8832476959752 42.6022652963326 42.2857372794461
40.4359177791566 47.1719672813096 42.0902716047029 41.1507980856436
43.3729486804084 49.2879849674517 41.483412492426 43.0016969050339
43.9379963884747
DERA 820.147156401172 457.860665614779 444.618035487523 574.467486140819
278.773982275525 467.764770176415 730.168814097451 365.401746744015
618.892379887961 336.44013572926 337.084861453686 549.67905283016

DERL1 422.702925141293 515.409979010663 251.374750265492 393.209708455013
264.495576211074 199.035083736742 177.27613997459 169.557757933574
332.830805115112 158.308338986864 190.647389246848 273.934014944673
DERL2 171.021755568144 121.036604456449 143.251956814524 179.07886477603
103.619396986593 102.711934290382 169.633672522325 133.060894083935
150.789911774534 105.05842887748 106.985301705486 124.18058128276
DERL3 48.5123041297835 50.2696827764943 46.7058093820839 54.6089451252049
44.2120774064277 41.159706131351 44.8643898647322 44.8997235283254
38.8495095797881 42.9866214102525 40.4504608195666 40.7704587752083
DERPC 69.6449298794042 73.1640951333742 75.2977438265751 66.5921967967793
70.3677225924146 62.6839349907803 78.6402083787251 50.3082459161777
69.0037087543684 47.3791873993182 51.1483696410762 52.6922486991742
DES 89.0495215957248 62.5528124447176 57.50915811428 90.2790229242453
58.8961770381033 2689.80835574654 39.2533668167483 106.140215438654
39.9444191873552 3176.96480659196 3478.67753301901 2225.65559724391
DET1 79.953657052127 74.1282220615085 66.288032400574 78.0020909283414
77.4769634299218 104.17651993553 87.0685571351947 72.93977835851
73.0403099404 93.9018973330737 102.471939526704 86.7710020879725
DEXI 240.476103976894 300.412043023998 414.539381761279 151.740176274202
274.398580571984 400.8670859408 179.886168478179 244.498602338731
260.062749979574 326.303950252592 305.959643033169 280.275836993258
DFFA 81.0766789453664 99.4123924179498 109.649431670339 66.4510869175159
224.030034866938 112.007118067348 150.562062400737 97.2750076101755
89.9227845135785 85.5439309325541 101.008449241109 107.513442725922
DFFB 73.7800733276454 64.3640973625938 72.6182747525443 57.4595416719581
103.913834330352 56.9320717885002 62.801431502597 60.0372015630618
60.9311907846259 59.3299869707613 62.8753829543444 66.4229480716045
DFNA5 224.202855268674 135.836577310513 180.128769312512 456.023620287811
67.6011992144113 325.172633427119 148.770228614661 165.640362974584
75.5681913475287 296.933220681369 224.450710664296 493.322302312578
DFNB31 79.8844295847317 48.3699741879723 54.5191471810265
57.0629653735694 48.6224651100733 51.294537308199 49.8281348927244
54.3820590274321 49.8304479381623 57.4665857273093 55.0404566626821
52.8455400026194
DFNB59 41.2424637338424 42.5624638829203 45.7452456977857
39.2406797798509 43.9450023643394 40.4753617660107 39.8695929627783
45.223075311113 44.476519804187 46.1390887492649 41.9083581529276
43.7410507935043
DGAT1 134.519244495139 176.07173250529 149.832238573027 120.980558571109
152.792457231813 100.883604376864 95.8149149211251 123.506372870621
134.749803376372 173.694418385132 117.586275152761 109.922909644299
DGAT2 42.1537476443423 57.2231446406982 68.3234331335073 66.5921967967793
47.5647821785529 45.3882138838137 48.8319884367456 72.121833189378
57.678188678075 76.8998454389745 54.1963147679251 60.2550788540864
DGAT2L3 42.0532901689036 40.148780650831 37.9482157381008
42.6913548400127 48.5000946421718 44.4619248757502 41.4742337714895
45.8669209716694 45.0997987980157 40.6022003765153 41.1638563416981
42.2577689927563
DGAT2L4 42.442231667357 39.3999056968598 41.3366779545815
43.074472921841 40.9950639693255 46.1513019317765 42.8684958525042
37.33030683349 42.1594905198929 36.2435874904689 38.079222289614
43.3744503319109
DGAT2L6 46.6348258763289 35.3307157989724 37.0642268327439
43.074472921841 37.8679241954337 43.7630587657205 38.7337086062875

40.4359177791566 32.4931776430014 38.4187686307607 32.775958132716
39.7411455670172
DGCR14 58.4920021605316 58.8281931439093 56.8168526942685
51.1055541075029 63.0383605052305 65.0774755750423 60.6548851977964
59.5080588072694 65.4784797364469 62.8753829543444 66.7826466218147
63.6254894053045
DGCR2 288.816567137193 250.086163187797 184.536327776767 147.285007921741
190.242383482058 172.102568448455 183.702709922933 298.848001894468
309.994784298282 369.258587948416 368.078954546259 198.360449141425
DGCR5 60.4593810615882 41.554942319225 41.0356368374708 43.9282059368202
43.9450023643394 41.0582900711341 45.4799270968969 67.8061298261394
39.9444191873552 41.1222709786153 39.2542963855807 43.3744503319109
DGCR6 298.54401957868 178.249889756228 213.933347509147 251.905799624693
211.654245727798 324.506136847875 312.589169184897 276.399037769005
273.849703944739 243.27954361539 190.487609390723 149.45630414346
DGCR8 197.171449112363 300.121875447445 210.843643231855 121.562769658314
179.994715581707 153.992331037033 303.281551940647 193.782527748199
195.893183546434 230.142262693206 145.09364961191 125.661351053514
DGKA 49.9890328304193 99.2432911346944 185.874151272261 64.2690842803282
47.6206419177008 50.5499559337071 51.540723788287 60.6237094449088
84.7782034884529 118.288549974348 93.7444359334292 112.23915619297
DGKB 37.55264801907 37.3255455874046 36.769073068743 41.5937402943136
37.8357959615237 47.6251784943271 37.9430145836712 38.7981173437121
40.5491080069964 43.3438593008468 44.0627228016455 44.0299389687796
DGKD 41.7397731376692 41.2598458726002 42.4917160307066 43.1131704517186
42.7982502551634 37.0740019672788 41.4397539024976 40.6628537260412
37.9793521263255 44.2580151149956 43.8923243437146 40.5207392413115
DGKE 38.2083710281545 45.4143854024453 43.0971787292711 45.8844325913247
39.7568367479301 38.3418933808844 36.5352572191451 50.3082459161777
48.8067321016961 36.3612409063792 37.8372168909583 35.4484652445078
DGKG 39.5676051939342 48.4980626712175 35.9305494063137 38.745552403895
46.6397391251762 49.4157684139858 45.223075311113 38.3684202018524
50.7619345314571 44.3648993568405 41.8168470823451 42.9887069977696
DGKH 37.55264801907 39.2907645923217 40.6312687664188 38.6414316877471
40.831207953647 40.5805725503899 40.4941260038116 39.6995529770239
40.9901805728485 38.151911051187 39.4749602565407 45.6233434183356
DGKI 46.0023068797812 38.6414316877471 41.3366779545815 42.9358255168086
38.6454098044474 44.0804700754786 38.4005795891396 39.4710743516517
43.0930293831497 37.6383530120621 46.6442692119997 38.745552403895
DGKK 41.1523487516364 40.6380495994768 42.157627240486 37.8442841690502
43.1252272136667 40.3357777530419 39.1539892144371 41.0381860307445
37.8319142847141 42.1622837416589 38.2795150354677 38.5279745308203
DGKQ 112.865496075486 225.056827821165 311.241353755918 142.545268664218
218.958199087236 97.2865629929716 86.4898752526885 122.748811531184
136.843285454801 161.793366042179 125.891602778425 121.696160351886
DGKZ 39.6349162264993 41.1262212547458 41.263535866568 38.6414316877471
39.4693871570028 39.3638967048812 42.0016830644258 42.6808744813778
37.8685585043426 38.9271399577966 40.0394833064005 37.8115992568331
DGUOK 488.908231529696 564.929183025034 492.519023728038 384.522115820048
450.759762047652 327.984902171255 497.591040004541 520.415257194417
495.705224781189 515.216890329893 432.669787408904 424.539111965342
DHCR24 318.069132873489 62.3102413989199 91.5230424957075
64.2130157241811 139.08163074096 63.2343637481705 212.822978676596

141.376779431031 219.918644151214 50.6486940895674 51.838002904753
56.5225014032155
DHCR7 238.077051775147 172.720244584464 240.086349973689 160.883501367581
428.8440159628 100.650129064849 374.297474141009 301.198565023795
2951.88559311416 132.107655569343 127.2070318269 109.294124176508
DHDDS 128.635811501506 97.095992167029 106.443777149496 97.3066473777771
165.684719727205 95.9391851122771 115.441414762268 149.950764820034
107.148000219752 146.90802741139 131.451150955309 101.250422688816
DHDH 57.6285775590145 38.2263227947189 66.737740728688 54.4586176399381
142.731630650904 59.5979861826984 107.748050894857 71.7336217321663
134.968128884298 57.6791750431251 55.2374601141719 61.7502624458447
DHFR 48.1125722136997 50.7487159427973 46.4569566520107 42.4580504587581
43.844855336215 49.9699131196797 41.706879587091 43.2551508830106
51.0062122023297 36.5806287103157 42.6366765186182 34.1509644696334
DHFRL1 67.3269632644926 63.9179552265502 63.3544794716167
64.2926204688673 57.9161791762225 77.8490019489375 69.779139490544
65.7833930916483 74.7934385405346 98.9222919411067 81.8648220673828
71.9771425105735
DHH 38.8561375693335 40.8880530359822 45.3974838013476 38.6414316877471
40.8873907150245 47.0027453663546 39.3585350448719 36.0306975524309
40.6957787921451 44.2580151149956 39.8184668132023 43.5738557773903
DHODH 45.0283997415478 50.8460732110055 51.4308501933893 40.2455972565968
45.1137938810047 50.164201758929 47.2927436772465 43.0930293831497
51.3511088648386 42.1317134987795 44.7114813656401 45.9104478407175
DHPS 214.03015073442 292.065366215278 311.810240385503 240.731386634957
346.438778257772 284.795064481553 290.031202636467 180.53214143537
269.184000351658 355.7118155732 301.025314855652 322.678762360476
DHRS1 330.550018306903 270.947794977829 503.793770799548 184.235914591152
262.579533452771 233.294970051779 119.277678423944 321.069136463599
154.551927302005 383.966991071954 232.320396482547 346.621778258188
DHRS12 51.8781065691038 57.4844872128664 64.4512696384839
59.2778887402357 47.1719672813096 68.5174271063894 64.4127389689053
53.4768201292155 53.0596635022268 66.4958411147116 59.897987870592
51.2623287419357
DHRS13 47.9175508887533 45.2556760873335 47.7737810047679
36.0481934423077 51.1368583672022 36.0306975524309 56.9320717885002
47.5950227477807 76.8864157610323 63.7836577144372 51.2318935933116
65.2771529943605
DHRS2 319.871452890512 1199.29782389306 1118.62223628039 53.7155925246916
744.080606078086 185.139615829489 494.733185937057 707.4124863603
565.72800641089 1554.42895572029 1573.61108703981 1224.80240464583
DHRS3 266.439343650315 253.398345052444 473.223156200183 272.36028618845
301.904682664871 409.724772942357 361.945511835039 220.621142396402
1490.1549980903 1418.94307844471 821.922096305069 1796.47241860175
DHRS4 231.198764780818 265.026710493466 326.500804513133 412.421643885499
173.035987237848 326.357036514613 335.569876624791 345.294930735691
295.312692092025 470.231929311877 358.779372054826 356.318517117856
DHRS4L2 76.1417635449123 85.8945498067934 129.609377870182
117.228320608065 66.1650385535549 98.9560329133205 128.635811501506
105.414552529943 129.252597914828 86.500678864429 89.4747568145717
115.622641633992
DHRS7 423.608694638779 537.841677481011 491.8684831079 570.204236909398
264.263104842911 365.459728613746 376.141477833569 382.538365722661
284.351747562807 343.990395610093 476.421959423835 577.993850127555

DHRS7B 141.79653024085 123.433224929087 130.10148014895
103.961956810572 126.79509061532 109.468741823602 120.96329303892
93.3718038663074 105.71315237465 133.43526498879 125.891602778425
149.515611848932
DHRS8 47.0940613507321 45.7317208802318 47.7737810047679 47.481302504066
42.6022652963326 40.9506062495231 48.9826868640753 45.7476095696859
48.0163377492228 46.325232414659 37.8372168909583 48.1627097465883
DHRS9 142.840631375936 56.2508140932179 57.6801614250432 123.04622719856
39.512606833112 76.0532592222027 41.1742244762318 55.6282425338897
44.298892175909 47.0474493061198 39.0550139257658 43.0322714179517
DHRSX 71.0340758556049 64.0096843640535 47.0940613507321 54.8412943008635
46.8674366115656 67.4846787600629 58.2315917325629 67.0904616289706
57.9713749600418 62.8201270538435 61.6702707724448 48.8670821164567
DHTKD1 54.1745577382276 50.8114699212931 57.0427273538849
45.7397504535169 66.7426485694967 49.1013967533186 73.0433648756396
58.3046521633424 77.9978216366573 49.7259371799014 52.5930185400362
57.0524725494273
DHX15 461.394007153992 854.712488657987 671.959673037066 569.377890112069
792.022171185649 570.824732318332 613.50943414129 459.182372959714
723.531909526537 618.892379887961 478.682734715193 784.268074988084
DHX16 412.694325761831 390.834121304799 385.512974999198 244.435346243998
498.284928805673 301.867441371793 415.251775687794 315.163147652199
320.391231952436 325.275883498219 330.096171705212 305.735573572576
DHX29 216.247225354243 233.72882333861 221.972553229362 191.182530250155
207.943044411392 263.67173636178 124.687212034276 185.874151272261
223.749512763927 208.553134986809 271.50380898418 269.745248423773
DHX30 134.646159826262 186.301404367238 171.920449949522 122.284299477218
260.062749979574 170.390108862804 289.17237603899 176.363810387584
181.064239330482 167.410607476565 233.72882333861 145.791398829369
DHX32 312.40869171788 219.134220280612 215.15351875081 186.64040927334
321.359569119182 223.801865347687 325.450452751375 257.80636663638
221.910075604221 274.02972772427 246.820301517765 327.948814478441
DHX33 152.86270446968 130.121040883877 197.205643078662 107.637618676176
237.039707901886 126.128760255542 186.759757179509 140.288771064502
123.817018264773 186.965367827758 159.827302305842 134.567947469561
DHX34 80.2070411865083 71.7533140056576 77.7907484334847 58.106070377898
92.7836745985234 55.0618818132967 68.2708120569221 68.5328071841649
68.4720753153522 65.813547096222 56.6086202928268 58.7157078555305
DHX35 89.8567957512307 77.9992448636936 69.4967762263181 63.9570596163747
94.1575209300592 82.1974756449763 99.7529382840954 96.0654949568324
80.6434122355417 89.1516394894484 85.5839487225537 85.9286114777649
DHX36 128.436305192918 125.383837024985 87.2316853416374 119.747995520476
172.192366002756 107.576033955049 120.465621774337 119.965950077259
128.923092916744 91.6713614282714 109.144663527001 117.520762118299
DHX37 120.630151942485 129.190555260332 105.052418151358 105.790224260281
130.936053902005 90.2227706363325 142.789889238338 115.482982633699
156.738940207775 113.008760911729 138.070735985503 99.5531381996521
DHX38 91.0346886951891 118.752208441493 154.248946683126 108.025082988807
91.6253858792439 119.015431849218 107.610858427836 107.844799332742
110.517563101892 95.2490714726663 100.234661597218 92.084526929319
DHX40 93.2133061608764 92.7427427543974 103.808640431626 69.1931891474114
91.2107065307656 99.8323169037315 120.825113658705 97.7415824971555
135.226924329183 79.9335381350929 82.4005336009159 91.2107065307656

DHX57 45.5342598239058 45.0905928998779 49.3780606970538 43.4933087941017
53.6334631215581 43.6960408897486 52.9531187309739 47.0540401406168
49.0593794153708 43.5599890586394 49.5551703887595 51.5437850416773
DHX58 64.2690842803282 113.491158368594 117.228320608065 103.349712762251
64.193536444915 80.2112932898502 86.5564881117807 94.2678678253657
93.7090094630558 111.056599389048 102.027922691582 73.1447139654971
DHX8 70.083473392272 69.640968387662 62.4683876804812 56.0794100106654
75.8306078651291 65.4643755537228 81.9504925867759 88.4439480366515
85.5711312226989 79.0249585610565 71.8314470868658 62.6868461044048
DHX9 71.4937262725923 82.3344892042646 82.1974756449763 67.9728793995736
94.2275161759277 85.3086977427271 91.9291200887492 64.9191509252662
85.9596109950132 51.7343702554597 60.7516146716018 68.4278345554565
DIABLO 489.983164420779 337.363827785838 387.867128019374
323.576613035353 365.860501246879 314.856937113103 517.664764759512
477.924466983414 541.631969808467 478.799250340246 412.880824530845
330.096171705212
DIAPH1 269.546586017659 182.80711310964 244.837105178705
322.774093041134 219.073357087203 195.403882457038 252.280105035425
280.680953068339 303.139550962377 259.456935785521 229.47693624489
216.821078573868
DIAPH2 61.0468099710416 62.6984594468418 73.3448790478335
78.4289643729837 71.6537776654915 94.0140258999054 76.0041771381018
101.560929859785 75.7427148831879 102.136265168978 98.0473811574767
95.5325488969517
DIAPH3 45.6001828766679 51.7983110178095 47.9635720235482
43.2504382247503 54.8609068340124 42.0106197763828 58.6431245059655
64.0227655842024 56.7429967489965 41.8613406887824 39.0950295411867
44.7937332169644
DICER1 83.5069968129733 88.4036642243438 123.281175151414
104.103899614521 135.644934816665 121.487591828005 84.4596873416863
67.9778438150245 95.7639712596394 71.5948944013406 81.4061378176246
85.7208881534518
DIDO1 62.0967404944031 71.3015974749703 90.0552783827917 63.4531374125233
97.9305595485282 70.5025097213784 71.5836731059913 59.7744650739434
59.8400088701028 71.1607965028029 73.5161632100822 65.8332717237037
DIMT1L 286.996922972683 327.786619565391 339.980317745427
260.383969219887 373.914455300185 369.032450063274 209.068725760522
236.565670642447 329.557502151159 368.005081137665 320.452418537108
445.307046237791
DIO1 39.3303857231606 38.5808034535515 39.2707964550441 36.5099686711015
37.9058271053655 39.8455060654531 40.2512356243714 41.4123755032424
38.9006788676407 37.7497202946736 38.5668610487033 39.6938066205531
DIO2 148.403272698474 42.0709558258414 39.0775870687758 59.3618843678775
51.7495306734657 72.2544131004774 36.8369172900902 77.1707015554489
48.7646976789783 37.6383530120621 43.8002456102021 44.4067063477243
DIO3 52.2236479204029 40.3384318592985 43.401782064778 34.1051821768421
41.8806343997257 55.9351706773613 39.7576136507593 46.2779564481158
39.1790517315259 45.9004543328976 61.0979269700412 47.3172462673426
DIP2A 47.420942961753 53.7820518773759 82.6036671634626 50.7855742231932
65.1271567764953 51.4931742685391 50.1027491117756 50.2391482329046
55.8046062733709 54.0917531668054 51.5612024992699 51.7382401683029
DIP2B 263.706801812277 197.515728627482 215.831300220615 326.250872625748
155.01005229951 151.225738885272 194.703024752864 253.072560237983
284.942182200489 192.41551362197 170.932645135423 164.780216383719

DIP2C 130.848965290163 75.9802606133139 66.737740728688 66.7750249658122
71.1607965028029 169.554408876006 101.065203693106 94.7718338968252
70.4933405598359 118.027323513177 146.087016079575 110.223625398261
DIRAS1 41.8409927585705 36.6968906669565 38.0452862258803
38.745552403895 36.2048942610546 31.2812535042976 36.197424053556
48.1591264215184 29.9775071862804 37.7315666632629 36.2435874904689
39.8497227447888
DIRAS2 40.2087556903277 42.9020321355927 38.941855825269
38.6414316877471 39.0564222526273 41.0012755104374 64.8367546135053
40.6171774724171 43.8106375505041 39.0266821712099 39.6833800000587
40.3691324547049
DIRAS3 49.2595762436995 39.1982470626863 43.4544762830742
47.8164193292842 43.3332476888305 47.2505395290129 46.8613212048981
44.8727287473605 39.9777866059804 43.6670755115848 44.7752571196612
38.9834959368496
DIRC1 37.6397652536077 38.5252456306656 38.1421709520616 38.5252456306656
35.5220586859301 33.895835691032 45.1017494688414 43.7396619462033
38.1169703906207 38.6454098044474 42.2618615571566 40.4639532172338
DIRC2 234.727123301682 285.254985583609 226.785831708938 250.210134743225
252.487015579489 128.559660070271 327.878405194732 365.860501246879
185.360921731157 204.502683458676 212.576203581294 307.922754395377
DIS3 45.8108990837624 50.0425298550559 55.2141529491512 42.9358255168086
46.7508829977587 55.0009147092272 44.9863726205428 36.7460922671619
49.7336769474446 48.1392888470269 49.2206713037152 54.0374522273907
DIS3L 274.398580571984 266.129007472128 159.506157112627 217.39432336811
364.962649416567 274.398580571984 367.158502379417 300.186402341399
209.099378471892 376.931257615162 328.548716133094 253.841638589385
DIS3L2 87.8746766762445 95.4942268742467 81.382229980722
88.1494891612632 101.017461500409 77.7481370715949 109.339287213971
105.308648028552 97.4062608315831 128.521748930472 149.536891697595
96.6957892186886
DISC1 39.2076937845359 43.9783383422823 43.8495248991589 43.3772464014781
39.3999056968598 42.297291190584 42.4493032770053 42.6786833450085
35.5220586859301 43.3405153487994 42.911855821602 40.9022255200169
DISC2 42.7150227340364 40.5400384479766 39.3709357615493 39.5050383901884
37.2567512265363 42.4580504587581 34.4570158133896 37.1317006162938
37.6383530120621 42.1622837416589 36.2435874904689 39.5418280570561
DISP1 65.0721641331632 56.5319193881205 61.841867306916 63.3006977162657
43.5544347011646 131.319040057576 57.4047331041592 57.8765910878975
56.3441077510744 116.441764463618 98.3322659713024 79.8331445290301
DISP2 54.3554233228353 53.7332797903102 90.4765857081732 58.9168466928565
44.2607789091176 42.2577689927563 36.5352572191451 40.5158867964016
41.9635525160083 82.966263655525 89.2185019250322 41.7575097298927
DIXDC1 57.2529265661628 53.6816179316072 60.9621846036092
62.8201270538435 62.3990310986805 230.014127346507 46.4959793353184
51.3472428383325 57.678188678075 88.8568707880669 100.413031488199
83.5069968129733
dJ222E13.2 72.1408243034509 65.485076402841 74.2122914028677
47.481302504066 61.1216330559316 54.3478800757394 66.3209349903185
67.8061298261394 60.0736861987141 53.4109882045447 65.342842101283
54.720204938924
dJ341D10.1 86.9816932158186 62.6984594468418 63.5107036952143
135.436347750159 60.4122350478242 86.6246917308185 41.8315602975157

71.7336217321663 54.998510746958 51.6778366958973 61.4121672148248 75.4053820110486
DKC1 556.092573451208 734.143790759359 583.053232396803 393.556480397634 1716.82239359769 564.468049419762 780.708896888794 952.16345035163 775.931494648677 477.023373302787 575.268446033282 516.704027135707
DKFZP434B0335 52.109723558511 61.7572675712557 65.2670315837785 72.6824533717231 44.5668141895992 51.3886035574416 56.3376046940285 73.362936710475 53.7442594289333 46.0119912782855 58.8961770381033 54.4313108506244
DKFZP434B061 39.4250105372135 42.1372626664516 40.4882741869718 41.7614975630362 42.3536549114165 42.0016830644258 37.6502409810897 39.8016018354453 39.7162631015511 42.4594149893798 42.7275315121163 43.8336862998829
DKFZp434E1119 33.9561816279577 36.89524015739 42.3536549114165 43.3078542962968 35.4484652445078 43.0860383229288 36.634024014558 44.5029690493211 38.2215591930186 38.528621456378 41.0808257270221 37.6557714476853
DKFZP434I0714 49.085741080241 37.1685072112618 47.0940613507321 45.0798408927912 45.6895223823001 44.8872784170036 42.4939802311136 37.5511560880902 47.798305839406 49.1928206164702 46.6442692119997 41.863927676552
DKFZp434I1020 42.9104454767916 42.4493032770053 41.9287023391451 38.5252456306656 42.269054475455 39.6251842352088 48.1069139823064 41.5464765641928 41.9083581529276 41.4397539024976 39.1878770155069 41.2437378488025
DKFZp434J1015 36.7356360093603 44.1239711172417 49.3780606970538 46.0228732507872 48.3814421939888 46.263826584899 48.3411413510851 42.5435283762933 41.074824727477 34.9138625012006 42.2618615571566 49.904979214379
DKFZp434K191 163.877163712385 111.333692153793 112.727277807495 168.737634511775 134.981483569602 75.8976034525022 144.227861902786 290.956194006554 133.074560812703 209.658186168638 238.205365476671 166.834334710682
DKFZP434L187 35.2267149016596 38.9338521051669 42.157627240486 42.0102671859606 29.9775071862804 38.8336427948187 36.4098694708483 40.6171774724171 41.9635525160083 42.3606390470387 38.3684202018524 38.5279745308203
DKFZp434M131 41.9407041937973 38.9534635879195 43.4544762830742 42.0102671859606 43.683792219729 42.2114134935898 41.8799523546812 41.9315419791594 47.417512242919 41.5435611403119 40.8225507267584 43.1235886043549
DKFZp434N035 52.8427643366617 57.7757446006652 48.2314980343598 45.1151758109609 51.5165373796787 47.9503724044453 51.5229124652769 45.8669209716694 53.8716214229685 48.5374448344297 44.9713479185144 44.1063440402552
DKFZP434P211 40.3478825593224 45.2556760873335 45.989212296035 37.7469621225553 44.4489662507893 43.0860383229288 42.1607322031054 46.2779564481158 41.074824727477 51.4454142248566 42.7275315121163 47.5025821041537
DKFZp451A211 33.4902421268456 36.0366673493555 41.528370959196 43.3078542962968 43.1252272136667 46.4648868771662 40.2154497375715 38.6454098044474 42.0621425894624 44.3648993568405 42.170702295259 38.2350720635382

DKFZp451M2119 39.4710743516517 45.1694246393676 44.7829632472754 40.0698728584484 34.7018263647985 43.5752393599169 40.2154497375715 42.6450179841129 42.1594905198929 45.6868589181768 45.0589947942306 47.2131144508464
DKFZP564C196 48.6621527194963 42.4493032770053 37.9482157381008 39.7411455670172 46.3150221024046 41.554942319225 52.2476631126873 42.2577689927563 41.2870505273664 42.6793753550854 48.3847843498476 41.863927676552
DKFZP564J102 44.1294459089314 51.6869155634183 42.157627240486 44.4367387274922 40.6723334103791 145.335751135441 73.3042538589134 53.9465584609317 58.5561424827901 76.5375674421131 77.2834482217973 61.5300817099814
DKFZp564N2472 40.9558083259517 37.6383530120621 46.2625470410195 38.6414316877471 38.9496515344837 34.0777108441756 38.0598374201407 38.1835938174625 42.8036731479932 42.3606390470387 42.5426057453993 40.0557788909923
DKFZP564O0523 69.0975200641705 75.5775406974686 82.3344892042646 56.2508140932179 67.4534389579576 68.7342341724306 51.0013344286851 46.1673159982664 66.9499465764301 48.7414853386615 60.1466326805616 57.8111054584154
DKFZP564O0823 458.47341225284 316.831358204198 102.053879707074 215.131274710043 121.354667375673 513.891389190131 119.747995520476 315.276749671783 81.4783226003925 738.364399569625 680.279119780451 176.461729584652
DKFZP586H2123 66.4399491500833 97.6326743942878 75.5103237141193 78.6344248102932 148.514056973195 468.153823329361 39.4600924554505 216.45007990575 56.9621724830201 461.83420090894 304.298036741211 135.323750661937
DKFZP586I1420 98.0864897427459 82.0897118579449 95.0738959950301 81.588696805606 70.8749669449099 71.2168693013526 77.9493708990494 92.3388129097482 119.572497267498 174.961427864327 127.1365842097 114.623641209561
DKFZp667M2411 54.1745577382276 55.2893984191264 58.4137028673494 54.1240583139871 50.6470461863549 57.1752846228778 66.1940959364976 50.9933795450314 59.3580517977711 46.325232414659 43.844855336215 51.0013344286851
DKFZP686C2281 45.4799270968969 37.4540772310118 33.1559412791294 41.1886975241176 37.1565984182084 37.7000400506818 40.2154497375715 39.4710743516517 34.9428206949566 37.4302011516587 34.3633615460307 39.2469742642337
DKFZp686D0972 37.9387791625384 40.716553247236 39.9101908547397 30.9241129241985 39.5595233162192 36.8059561571675 37.3799542961839 35.4526946152955 36.197424053556 34.9138625012006 38.1835938174625 37.0953622406687
DKFZP686E2158 46.6348258763289 50.2696827764943 50.3950981988762 47.8164193292842 54.802736966312 42.157627240486 37.7136011474903 41.4508241771315 53.838484549134 46.325232414659 48.1352470945488 50.0571060701893
DKFZp686E2433 42.3433931248155 47.4740726032707 46.4569566520107 56.6458456890715 38.3561682218911 42.157627240486 37.3799542961839 42.6450179841129 44.4395580493118 43.7303813515864 41.3553369544097 44.5432192783161
DKFZp686I15217 70.9419509323007 51.4288869214337 61.7502624458447 58.4476706144086 56.9360840563854 56.3525213736952 47.209353240759

50.5070012045444 71.2963721541702 54.4190527512508 56.0477607422828 54.8630527326985
DKFZp686J0529 40.0945258957481 39.4569929845912 39.991353369336 36.2202703635318 39.4092846492473 37.7441423440745 41.6520912742594 38.0268447621448 41.2437378488025 39.6205203043973 36.9060997041453 41.9820792173615
DKFZp686K1684 47.5647821785529 47.1332119496722 50.1860191642359 53.593974682612 45.5809132232681 45.1873487463472 47.8947595316832 52.9813204449021 47.4699236780425 41.6373771565199 46.824299585607 47.3992733452821
DKFZp686O1327 40.4639532172338 40.5400384479766 43.0016969050339 41.5464765641928 47.2074332636566 40.4359177791566 42.2577689927563 37.9837233601316 40.8970401536578 45.2556760873335 39.8184668132023 38.5279745308203
DKFZp686O24166 54.2648201582657 49.1283590719461 52.2132783757422 200.036360605006 82.0149542477384 59.8102324370568 58.8993834673475 61.5000955267432 58.6431245059655 56.685925255591 65.9273663561152 58.6481644777552
DKFZp761E198 42.442231667357 72.6888636455541 42.0621425894624 72.0694688774804 48.5000946421718 48.260931742163 71.0369238830779 60.7942162859036 51.3511088648386 45.2556760873335 50.6473771879221 55.2449003905956
DKFZp761O2018 43.4626049853157 42.3550738181144 42.7303139017786 45.1944222548351 45.4751076037511 41.4508241771315 43.3262148375525 39.9576138799997 43.2761454096265 46.325232414659 40.4504608195666 46.8129509799753
DKFZp761P0423 218.999184133546 128.256151920468 149.397632479523 176.492987902471 77.7140342228764 70.2553917409773 174.007271403635 142.846665249354 107.309776354951 113.26670675749 123.368987753537 114.488672372067
DKFZp779B1634 47.0940613507321 43.9421822132391 45.310660704531 38.9534635879195 53.8260140375214 46.1513019317765 44.4639405506967 41.1575777497576 41.2870505273664 40.4136535208406 38.1835938174625 40.7704587752083
DKFZP781G0119 34.9138625012006 37.10170285405 41.528370959196 39.3750969348308 40.4166536885831 38.9661430990729 43.2721716572958 42.4580504587581 39.9444191873552 41.6724739851995 38.6662194645633 36.7762265039864
DKFZp781N1041 42.7150227340364 44.2105195734073 47.7737810047679 42.6913548400127 39.4693871570028 47.0940613507321 44.4639405506967 43.7396619462033 50.1603795873336 45.5776159432079 45.0589947942306 43.8575940017119
DKK1 40.308802735799 51.6527806785838 81.6486527718661 58.1798515131249 174.537032518715 74.7669841575348 49.3067361345568 181.983177572371 91.4121563150184 80.2381651206227 143.215491185309 77.6056319020116
DKK2 49.1868644496737 40.4772513262052 43.8495248991589 47.8947595316832 41.0476065156912 36.6271061451613 40.2154497375715 39.853866204927 44.6717213146407 42.6366765186182 37.6921755067309 41.5132248356707
DKK3 274.02972772427 210.73006676203 161.500218853268 152.996027678733 254.519518210024 120.705229050133 86.6023527423111 178.422506138888 83.7019398654324 262.37779288036 146.389547177092 123.387418707738
DKK4 42.442231667357 58.5406981871153 58.0218262176065 40.5484717678957 47.4548471240051 48.260931742163 92.8579489575841 40.5158867964016 281.262083731954 56.5379973844443 49.5923712603197 58.3586374083245

DKKL1 43.1805016690297 44.7829632472754 41.8751578329365 42.2496337895968
49.0828761839116 42.0574848021044 44.3440839686558 49.3880946790306
47.6195009345747 45.9004543328976 40.9157352260901 45.1735152121056
DLAT 104.462230406058 140.128033028434 134.394743648475 111.347228208501
144.139555313429 119.647202975116 129.810477623628 107.232979387172
110.074666148474 88.9738853594498 97.2004257862686 152.200107222761
DLC1 48.1952521496489 45.4143854024453 51.223859150798 64.0395697320109
77.1287755867582 61.4944752562671 37.9946685306274 70.0133734659853
39.3506426665347 346.116601612353 264.071486075874 109.649431670339
DLD 98.4084364053686 136.377736482671 133.576489624155 137.955007835039
134.394743648475 137.286091889421 82.9431583622036 107.971806573919
179.158053533144 88.7461361855771 105.589475270494 174.985336606027
DLEC1 36.6962082197711 41.7656868106485 40.4882741869718 42.0636939252645
46.4775637378703 39.132725377815 38.1154766911051 41.3506832276112
39.9444191873552 39.5317722761104 39.9519547348106 38.2013340576059
DLEU1 45.3872406432794 50.9099078696349 50.0487097583953 44.4367387274922
74.2653431904071 50.8930565655395 49.9032148112001 43.4544762830742
57.1401776589964 52.7972156492746 48.4928863469279 64.9418945936106
DLEU2 44.7520317855773 64.408176016319 54.2670850492947 46.3203995372658
62.4624150544853 44.4619248757502 57.4622430729629 43.1567024035183
47.417512242919 41.3321861371202 42.6776449468246 43.7672102682067
DLEU2L 38.2083710281545 44.7829632472754 38.4043916290561
41.3027031037343 41.467005949568 38.5252456306656 41.1507980856436
46.5717043290731 39.0775870687758 43.2988017098387 35.6970528713709
38.3291713594599
DLEU7 39.6609238036815 39.1982470626863 40.5734458834617 43.3078542962968
41.0885562160946 38.2293008890613 36.7356360093603 39.5466975353131
39.0775870687758 38.957687788868 43.3085358214154 34.1509644696334
DLG1 34.9138625012006 38.5252456306656 33.7737772393677 42.2496337895968
37.4470183315826 39.4953494583071 38.4005795891396 35.6825474397235
33.5578650644246 36.0481934423077 42.8263096141654 36.449101775775
DLG2 43.2135791259317 44.8529259387308 38.4456289125816 44.7401523707775
42.6022652963326 61.3309323244567 37.4386615664066 42.8936990772838
55.1023658447049 85.0653700751969 63.4610447573417 54.4992295592001
DLG3 50.5165823866945 62.3439114913889 51.8122261766744 38.5855564939014
46.4775637378703 51.9885249508283 73.3881007284998 58.6010484516703
61.5080959417997 48.1392888470269 50.6473771879221 48.4535991392809
DLG4 110.88526946931 55.3477556906605 90.1284518302601 79.5838979704243
84.7782034884529 81.9983163893373 57.51482768194 83.2873728545705
87.8724689898823 75.0665411373616 111.186223357396 96.9944911507111
DLG5 32.742439449766 39.0894172982439 46.3411353377615 36.4564050256915
39.0564222526273 41.8613406887824 38.8417151851134 37.33030683349
46.6944238390932 41.8613406887824 42.8263096141654 49.0655215909632
DLG7 76.1417635449123 97.3813557754832 67.895735332714 45.1944222548351
108.607805039005 40.244271403831 83.8811735017279 117.074650076861
123.933814581281 37.6383530120621 32.6463318577756 37.6557714476853
DLGAP1 41.0608773166058 40.931294690669 38.5045333213784
40.1804147506786 41.3277288245157 41.7256940298128 39.8695929627783
35.5179217192467 37.5723609429239 39.2623423164488 36.43924066428
39.1878770155069
DLGAP2 50.5670783769975 59.7098454699348 48.8941062980393
62.6048727240291 47.6884372645373 51.3886035574416 56.4809146784765
51.7538628098347 61.0094935318105 54.1683282066029 57.4078217542368
57.1441484662045

DLGAP3 41.0608773166058 36.4002279956519 36.8730514568925
36.1562979552173 37.4470183315826 48.3686935595826 43.5627649099623
40.3357777530419 39.853866204927 36.5806287103157 42.5426057453993
36.9976231206067
DLGAP4 128.766242896259 91.7629175712824 88.2959958615748
91.6033269431304 82.1344514651393 134.581057139589 140.866324039667
109.398472684587 86.033725825968 133.798096986107 123.586057618734
92.9043863423147
DLK1 41.9407041937973 36.9994058279362 43.8495248991589 41.9620007529243
49.8304479381623 58.3627863274274 39.4916414839923 43.9604529016594
36.3649677956953 39.8455060654531 40.5824352835642 40.3316153483289
DLK2 41.8409927585705 41.5996102310128 45.1798080655905 38.9074189954657
56.2937283136959 41.554942319225 42.7431359850339 39.5215827396813
59.7483827511172 42.3413611855132 40.8746128314437 41.1066323911308
DLL1 154.847446197442 247.511892014861 106.912316023598 123.608921653229
132.555169949054 139.121494956408 65.524821699432 154.707548614663
42.357910063876 121.236881661832 158.334581991361 132.508880040274
DLL3 42.6575535390785 42.2900457798748 38.0958223740111 41.8778873678551
48.5666902992029 36.2768299545794 37.2316700720288 118.736188485344
36.3352882516399 39.3698811593364 37.3687900733549 39.2469742642337
DLL4 44.5282181324797 40.716553247236 56.4779645313958 39.2780515621926
48.9627346956003 39.7394181011603 41.1507980856436 42.357910063876
41.1742244762318 44.1379638573455 43.4709415392696 43.2776458914333
DLST 238.893145911331 141.611097024526 220.054040993848 207.540972220982
169.061093538895 207.726011653345 249.995119246929 170.932645135423
215.800662077432 264.548218296413 297.187417426993 287.423909789368
DLX1 41.2773842727759 49.4493009852254 41.528370959196 42.6222445667107
40.5710637884029 40.2945586900975 40.4967168818294 42.6913548400127
45.0017158586743 40.7620472083159 42.4162492820012 43.2177497174408
DLX2 36.6480005877129 37.7384958256497 35.8265993915844 44.7980740332174
40.6723334103791 41.3553369544097 39.5496902678788 39.3709357615493
40.4220196471124 40.8049408996274 37.1059647239814 42.0424641526302
DLX3 66.2154717217675 43.4396866157171 51.5292723244077 49.3654837541353
56.8307116636372 35.314469458379 34.6720534245631 81.8491450876942
38.5252456306656 58.115198353247 56.8436081694537 45.6692241948108
DLX4 44.1834366106487 43.4681646416585 41.0356368374708 40.4359177791566
39.5111866657136 42.2577689927563 43.3910673889849 40.7752992161136
40.6533975461148 44.7463386786906 42.4098506918871 44.5029690493211
DLX5 51.413025495745 358.30596676183 166.608353896248 43.5627649099623
191.298525555624 53.4466111440611 209.467874193952 195.168348967505
152.054785478928 129.434864701718 77.3999378505514 40.9683275553869
DLX6 39.6609238036815 41.8848959280916 43.9450023643394 44.9370339275041
38.745552403895 43.4681646416585 42.8684958525042 41.0381860307445
45.7452456977857 44.2580151149956 40.4504608195666 35.5523662009011
DMAP1 227.474113475825 207.0155359394 200.615897175992 165.121380058529
209.497482517298 191.361705136492 305.554059609771 250.866560791828
268.144525192544 313.115027781064 293.410413883219 175.985530451862
DMBT1 209.967057706137 45.7576020206129 44.5134109184888 47.3574589211166
45.4751076037511 45.9980734733005 63.2726414544553 63.8008894786105
62.5327652825303 54.3163440690038 58.7389869173495 285.053017606956
DMBX1 51.5033727579864 54.7083331985678 42.7303139017786 44.4367387274922
69.805066579191 37.9003288112261 45.8980298854198 53.838484549134
101.681822254799 47.1001757441025 42.170702295259 49.1200355938198

DMC1 796.359355922952 448.787414220553 523.25106120389 855.602941621804
364.439669236186 337.736535374405 695.684209526222 1459.69929955338
391.397318564183 1211.8832855325 940.781370938596 790.786577596971
DMD 44.2807847072108 44.3529919625344 46.0238646636779 43.4721084347804
47.5057905459278 50.5868854091439 43.8594026120797 48.5989831621201
43.8012040086323 47.5595864930347 48.0987228351168 52.6275131390249
DMGDH 37.2474303302343 41.6069493017095 42.3887531663733 43.7410507935043
43.4885245598939 44.1350486752012 42.8684958525042 41.4812544772644
39.1790517315259 43.401782064778 44.1533327883807 40.9683275553869
DMKN 178.323615832679 260.383969219887 56.8595344107711 49.0816063338953
320.038347979923 86.5616422570166 413.31638457585 255.559793355361
87.6726059613969 233.435884567494 234.495626550751 205.772128732705
DMP1 38.5332184743306 42.8542642792862 41.528370959196 39.2336402665402
43.7783560914188 40.244271403831 39.9519547348106 41.8767957887205
43.2761454096265 38.8173773425835 38.7337086062875 39.0536056496869
DMPK 54.8153573978511 56.8373628733077 67.40251451399 46.3919862398545
62.2354226918761 109.029196594263 44.0137388752186 54.3820590274321
51.9545473262296 79.262977183608 75.854888525664 62.3102413989199
DMRT1 38.6454098044474 33.939263369489 35.9305494063137 40.646702696033
37.1565984182084 43.5752393599169 42.0605906240248 33.9687743313429
44.8003345643334 39.9948390976317 35.860430241131 32.3796054199032
DMRT2 42.9104454767916 81.2182827624857 41.6079958749402 43.3078542962968
41.6806984441849 42.750861693377 52.8496345990125 44.4031868028216
50.0526763693329 40.6533975461148 40.723990462047 39.4916414839923
DMRT3 44.436995101921 34.8137085420423 44.0451487108213 36.264823544143
38.4540849632254 40.4359177791566 41.0381860307445 54.8412943008635
43.8482802038826 39.2623423164488 39.0550139257658 38.745552403895
DMRTA1 42.442231667357 44.0409178714232 44.4311655729487
41.1886975241176 43.6587492470348 42.157627240486 38.0598374201407
38.2782436497441 46.0106098064475 44.2580151149956 52.1788489555942
43.6753843488695
DMRTA2 38.8561375693335 44.3529919625344 37.6557714476853
43.8002456102021 64.9072331703985 40.7616966228404 55.4004611309636
37.7612949798895 61.8445915808329 44.1379638573455 41.2598458726002
42.4607795638552
DMRTB1 39.0620487644635 39.7835513496873 38.1421709520616
38.8495095797881 43.4544762830742 41.554942319225 37.6036146841933
43.5544347011646 37.6383530120621 39.7835513496873 37.6484782516569
37.6557714476853
DMRTC2 38.5007204246293 36.4002279956519 37.8386378737561
33.3231576795423 34.7018263647985 27.4352294118391 37.6036146841933
35.9023742468744 41.863927676552 34.5718473203083 38.8507804383261
37.7526485343328
DMTF1 139.855023065402 124.214669663291 124.992776922316 121.510087314197
123.114162330406 133.930007857002 97.1759226403408 147.704325041249
119.634256037186 172.506486240017 154.612425659555 168.640033281185
DMWD 111.522812019895 108.019575675653 87.2316853416374 40.5484717678957
124.051109959228 71.9329028989527 56.9320717885002 67.5206810636627
76.8864157610323 82.475816811779 86.915098846441 69.1099722732115
DMXL1 38.5007204246293 38.3322849790657 42.5406888281114 35.9521941716188
36.5393857306744 35.8227939208521 35.4623691645629 43.3262148375525
36.197424053556 42.5664883002833 50.7903824729818 42.4607795638552

DMXL2 64.4415447415281 74.6847594297153 66.9239565096247 178.675242612997
79.3889841821749 73.3506594385504 75.4053820110486 58.9081750508316
64.9030399249327 46.0119912782855 51.7538628098347 54.720204938924
DNA2 63.1291723738553 57.5941849631773 60.726977722583 56.2508140932179
64.6374507338031 51.6558970571712 71.4414688202373 70.1211450522292
104.725576313447 57.7805922926828 53.6895401581575 54.7727101923179
DNAH1 44.7915315085948 55.6323803479191 56.4779645313958 63.6334154351075
62.588825192561 46.6872654833579 45.1017494688414 56.2441895183416
40.4997186069494 65.2361525412756 66.4251023257203 57.934622029119
DNAH10 47.691150920844 43.1358833499227 42.8768096578102
44.550322123994 44.6160092538827 46.1859571883455 43.3889851892323
40.8793215723066 42.9020321355927 43.3438593008468 44.0409178714232
45.2189250687483
DNAH11 44.0328992213727 40.8880530359822 29.8304689114955
35.3829784221072 37.956732373465 31.9222834447339 34.5633623842083
37.33030683349 37.9273653326866 32.5178504369282 36.2435874904689
36.5474283500601
DNAH12L 45.4799270968969 41.159706131351 39.1799070031819
43.8002456102021 38.5512429290626 36.6271061451613 36.197424053556
46.695848687694 38.9834959368496 44.3648993568405 39.9070785037411
40.6614700944853
DNAH17 44.5282181324797 36.89524015739 39.6451483836089
61.7502624458447 39.859345745831 43.0860383229288 46.9929836306816
42.357910063876 42.2577689927563 45.6868589181768 45.9104478407175
41.1554481567603
DNAH2 41.8409927585705 40.716553247236 40.1985790927248 45.5869891570251
134.258351436533 49.9699131196797 73.3042538589134 86.7710020879725
53.6446045619513 53.0357938671646 55.4694986901287 57.5372732453319
DNAH3 36.0144420013819 37.5469247328848 33.8558232866797 39.1706564265495
40.7779767565889 40.1428591998404 35.3362091871364 40.7311497914871
39.9444191873552 36.0481934423077 37.8372168909583 40.0557788909923
DNAH5 51.5033727579864 48.1627097465883 45.1569741609597 38.9534635879195
47.2074332636566 37.9951997351435 66.5422130374814 52.7787010032051
47.1744296124496 38.745552403895 39.3667161654772 50.4847465816184
DNAH7 53.1309801459151 50.543526404059 39.6451483836089 50.7046706880325
44.7829632472754 44.3648993568405 52.561573697155 42.157627240486
54.0192060888782 52.0999467564713 43.0971787292711 45.3763275588341
DNAH8 45.3872406432794 41.554942319225 43.7630587657205 39.066284740632
40.3452320167286 42.157627240486 39.4600924554505 38.6454098044474
38.1169703906207 40.1179210534118 40.1742127891824 41.1554481567603
DNAH9 40.6994262313917 38.8561375693335 39.4987030972173 40.646702696033
41.0608773166058 37.8442841690502 39.3585350448719 40.6628537260412
39.3442917208474 41.683412864532 39.2542963855807 42.5294887665766
DNAHL1 38.2083710281545 35.0222940937513 37.5597671487621
38.2041059863839 37.2567512265363 37.2686490268265 33.8558232866797
39.4710743516517 40.0403898620771 35.3849221682845 37.7384958256497
43.4709415392696
DNAI1 46.263826584899 37.2810767789306 40.4070046183108 43.1847628331565
42.269054475455 41.9543948167375 43.0930293831497 39.0620487644635
43.9450023643394 45.6868589181768 53.1505787220552 45.3763275588341
DNAI2 58.3702889417801 50.3586212130973 57.6371756925687 48.309866878365
60.9710783023013 73.8105012820769 54.339958888486 53.7378346199838
63.1944558106132 83.877343750194 60.7516146716018 69.6579723433329

DNAJA1 1541.9572099378 1638.16317738035 661.718946652592
1578.32969650385 924.900902050757 1029.12917222294 1615.83220398811
1362.12587130194 818.608913659251 589.395944599882 669.776067547468
1106.57048989685
DNAJA2 471.59454368268 474.379113836506 665.510487460287
389.583938820073 350.451704069255 533.820505121067 527.686970237622
568.638529128128 461.500892766266 714.075528895169 603.224267841548
668.584452504579
DNAJA3 371.393488010768 626.390544175817 764.571579067138
250.866560791828 617.808073077341 416.40156274382 459.546279672649
250.504350952583 667.837116137964 531.69595054137 602.047768767953
551.915864019927
DNAJA4 319.722393409101 1359.9708539999 880.992328229414
196.065114892186 626.390544175817 246.151211515664 197.040881690021
244.070778149931 633.819901764391 643.9329225331 716.048536335117
726.42773300581
DNAJB1 408.080620074472 333.566613571224 390.202312411165
332.137622104526 451.13133250897 478.799250340246 261.6201598868
205.378123452174 467.351570171652 359.296873000994 431.864508795316
228.21982974423
DNAJB11 708.005870621064 619.337575854369 576.830397705584
766.337559135016 1169.14891282789 513.266895252699 624.954583057431
665.510487460287 754.700295017212 276.321098534247 308.64836180781
515.216890329893
DNAJB12 64.863894209343 69.9431447778061 73.0855167819246
78.59966540743 57.1010233600552 61.2387559102636 76.3583555666815
56.3263223563484 112.131738588405 64.5232087947596 68.4261608261522
57.8111054584154
DNAJB13 43.6706764143908 44.0409178714232 38.0452862258803
44.9370339275041 46.6397391251762 40.4359177791566 39.0458123429605
44.7044140979108 39.5806465938526 46.0119912782855 40.6373400186325
38.6454098044474
DNAJB14 49.1217929709337 62.358854072801 58.3608287133675
69.7610390623756 55.9715651431881 61.3149617644002 50.2626654591654
53.8732900241064 56.2181240696479 50.4855887590421 57.2849292129038
65.1251519077199
DNAJB2 573.394745674984 343.892794667047 294.966924715506
264.855020003755 384.280434953152 266.702252514277 313.115027781064
606.276674926567 398.139730642919 610.502562878788 596.84269596976
396.93169345515
DNAJB4 44.1294459089314 41.8848959280916 43.3085358214154
43.4410741327254 38.8456942580061 48.8744644207821 38.8775560911841
41.9315419791594 38.1169703906207 41.9097689475185 44.9713479185144
59.0457625163577
DNAJB5 50.5291432329798 44.3867827945599 44.5658219748173
54.0332376648108 45.3675390992851 84.1195048659476 43.6753843488695
51.0318873780319 47.3213303943727 78.4590546118913 67.8010199320754
69.0503036666628
DNAJB6 416.717470974721 359.185856400148 299.890705180422
353.751797948675 446.37035118289 508.081726674689 231.198764780818
164.205608279417 439.770250139925 314.856937113103 296.598410253206
445.875994521817
DNAJB7 36.4564050256915 46.8236022402545 40.4882741869718
39.066284740632 40.7779767565889 38.6414316877471 42.2577689927563

43.5544347011646 39.2809305246091 41.9666541498638 39.4410515050885 39.4490233720848
DNAJB8 36.2496364442264 47.087835967892 47.0055981216687 41.6564075217517 38.2496050953547 40.650742261428 44.7255207589606 46.5717043290731 49.9951674107458 38.745552403895 43.401782064778 48.0350549043063
DNAJB9 117.934830767297 149.665486488005 129.2860308084 564.929183025034 132.257197568848 227.206974078547 101.065203693106 149.899176549323 130.313381811168 170.804822846738 149.629940171074 198.522465152099
DNAJC1 50.7390160399184 46.1569135229922 53.2046867327331 52.3004056941891 41.0885562160946 59.0686629877982 44.4639405506967 43.1567024035183 57.9713749600418 46.0119912782855 47.299647867533 47.9566297250148
DNAJC10 105.15507796881 123.455270940015 107.754432832706 137.496822743899 127.701556328274 114.901312275861 102.457537266105 164.462951076718 114.039396407323 94.233414682323 107.044681610056 105.293665242737
DNAJC11 60.7371790125714 83.1979076141836 68.4720753153522 52.3004056941891 82.1344514651393 49.5424425850549 54.9765783642973 78.6911549149983 64.830448408529 47.8207974930031 55.6972661824064 55.9555622193202
DNAJC12 57.2364979699541 40.9291911731327 38.9790184198145 61.8445915808329 65.5618920437306 98.8719561245756 57.9583667603818 46.4329203134841 107.538934056558 53.1124375923907 46.7386599624801 55.8087097725119
DNAJC13 129.336439341458 114.965999573704 145.847014515246 155.287591646469 149.665486488005 161.180847265279 140.51841571801 129.179537795204 118.488996952258 131.973056760893 111.874193363836 136.883475451494
DNAJC14 112.644426507762 126.389908470189 94.6935403697218 94.5501397951415 87.0414124990547 82.8218647837178 100.756165194192 130.673639258681 153.059039745191 96.2385885003071 107.391994714261 110.302475164041
DNAJC15 102.25509551881 103.883220270094 101.494432845825 121.562769658314 62.046151104685 214.245832611737 137.547711779867 72.9752827496503 163.299997237276 136.461760992832 120.046457114882 207.540972220982
DNAJC16 41.0608773166058 61.7572675712557 56.410923639913 46.0228732507872 59.4830424460714 49.9699131196797 47.5963750520409 51.3472428383325 52.0583273941813 44.1379638573455 49.6706535876636 54.1090536870282
DNAJC17 107.251974803232 74.3729155936862 65.8455652907556 72.89602312859 73.6965494979349 66.1082848714279 84.0691608193405 81.5582217750731 98.6825522152097 78.5141640924736 66.1229691315616 83.1874704625702
DNAJC18 53.3059058105836 48.109747794187 57.2107492415265 54.2886483704949 45.8051846088552 71.7021670182761 49.2546642180676 51.8347877274018 43.4656637536244 64.2174369755376 62.5036823572932 60.2550788540864
DNAJC19 80.3730667493235 88.4740208892504 76.450993090133 80.0042268333735 106.009130381007 141.808284719109 84.607833108004 90.2915974511786 85.5711312226989 83.946822476147 96.1307490576512 106.70211644918

DNAJC2 45.0985088502363 48.1627097465883 38.8775560911841
41.3027031037343 39.2608760193011 41.6590564937735 43.0930293831497
50.1833483241864 46.9739160257217 41.7405682794166 44.9713479185144
40.4639532172338
DNAJC21 55.2412344988347 56.8760943789568 54.5620104778414
46.3203995372658 53.8260140375214 78.8741966117641 65.4561038762234
77.8946473414547 60.2509851471485 58.0218262176065 56.6086202928268
67.7965120293445
DNAJC22 60.4593810615882 47.6229101519945 40.0998167098057
56.9299697426319 66.1650385535549 42.750861693377 57.1070449050465
61.8665316900291 48.6494451061637 38.528621456378 42.4580504587581
37.7526485343328
DNAJC24 83.877343750194 115.221134337342 94.5055588176321
107.337113678019 82.0149542477384 160.853643241489 75.8779316255794
72.5835488194631 92.0711167984454 94.1575209300592 98.7446830481164
111.357508398822
DNAJC25 77.4999637278723 76.861722336069 60.4809187893259
78.5141640924736 82.9322141700549 118.57174795047 71.193768715137
92.9043863423147 70.5699797830702 102.771611905219 104.528976428975
117.355761277966
DNAJC27 68.6627996695828 67.6169355376864 68.0649030501515
55.8709511400674 72.1154333023225 134.239606718101 71.5249259772583
115.052948380817 82.3364971911816 121.236881661832 102.993620677265
89.5687388187015
DNAJC28 42.175859643566 39.5050383901884 40.5734458834617
42.1679276329916 41.4479871197761 39.9871741554388 39.3585350448719
38.6005272730664 41.9180247412837 39.6205203043973 39.9671443011409
37.9202704407098
DNAJC3 52.2236479204029 57.9509155144056 52.1242102345408
134.952392466784 51.3511088648386 45.3882138838137 63.5738862549306
64.120219344545 62.370559099758 53.8260140375214 52.6960621424156
46.8129509799753
DNAJC4 44.1294459089314 52.7493349111555 62.5610562594275
48.4916117779992 66.1650385535549 67.5812474586277 55.8019623191927
58.3046521633424 43.5669306063196 78.0658258913875 66.2027948645675
57.3697604614404
DNAJC5 113.113062709739 124.605379715077 82.5344501505114
124.350816245276 102.913039837094 82.260686909201 94.7980740999988
91.5258329186709 106.317388820963 66.274247400793 75.9802606133139
81.3082994649699
DNAJC5B 40.148780650831 36.7976562409506 43.401782064778
54.2886483704949 39.7568367479301 39.4953494583071 40.7910160811527
39.4710743516517 42.9020321355927 33.9967481174035 39.6403089864678
37.1747156131306
DNAJC5G 32.6285977114039 38.1312950900467 38.0452862258803
40.3180310664156 45.2556760873335 43.0860383229288 47.7390230243778
41.7419877611072 38.5252456306656 39.5637454855233 36.2435874904689
41.863927676552
DNAJC6 37.6817357354748 39.5050383901884 38.741573807882
41.1886975241176 38.8834538955 40.244271403831 39.3585350448719
41.1575777497576 44.7752571196612 44.1861590259178 40.4995052849302
36.1190722948669
DNAJC7 225.618853905635 175.28322146764 145.791398829369
200.591114641608 169.693594531146 177.602690452814 214.691909801388

257.050603883498 283.747865829282 178.219279777774 183.311305255895
190.161505611036
DNAJC8 1442.72006787259 1041.84536202049 1224.80240464583
1585.90718459092 1829.30985071028 1896.06380611235 1557.42399344934
1529.52516575545 1591.51486001004 1537.76265834076 1769.18069954113
1426.87261062373
DNAJC9 498.608204176238 483.882804472844 328.855843739232
218.727705098677 489.983164420779 250.545098051392 643.71203221441
355.642611713396 1254.34397143795 245.0324923457 213.768352764473
270.600852806342
DNAL1 53.3343962424887 48.4724651632766 49.4067286971348 48.665578118898
50.2953638261991 60.3254877099749 48.6395795163502 60.4809187893259
55.2485665256307 52.6040246568056 51.1905212004904 57.2767879969284
DNAL4 432.945510073269 597.996273468244 400.973553604192 210.5650813431
364.337811621248 273.275823600027 856.790359133679 662.848386826617
465.981133154896 500.241231327789 574.467486140819 434.841521967958
DNALI1 48.9906383294199 101.804757613639 47.5673419710251
48.309866878365 62.9553576299033 117.823678167648 103.316970096472
58.9081750508316 61.2510119532409 104.672760332135 102.559678338485
86.0230765383602
DNASE1 63.7818442618767 49.83247261979 56.8595344107711
42.5664883002833 49.452985143568 47.2009420656854 40.6750540874299
48.7814131120945 52.0583273941813 43.401782064778 47.299647867533
48.6037684272115
DNASE1L1 76.3933911546385 78.6705291023488 102.786429761295
87.3870406334559 109.14984236229 70.2021753365478 72.3095457386378
66.6320017520888 75.2779848783367 58.037539503209 61.6878176260761
71.5836731059913
DNASE1L2 53.2046867327331 67.1926218405833 47.646800205189
45.7397504535169 61.4366256816323 42.9695585740813 50.0088611244948
49.2019743609873 56.2441895183416 44.0409178714232 42.911855821602
42.8864415494484
DNASE1L3 45.8108990837624 122.292794686468 42.9104454767916
85.3754356809649 52.2089306840125 49.5424425850549 49.6872467499977
50.7364620410977 44.619512785899 44.3648993568405 42.170702295259
53.3059058105836
DNASE2 493.705301673753 536.013150313097 474.155196153863
839.850453078119 773.781266760336 279.175841990291 385.918487165555
401.347734852083 371.513106715598 401.347734852083 347.196922795214
348.206465194205
DNASE2B 38.0698669928229 40.9298924777825 44.6484005362281
43.8479689858951 42.9090346219023 41.0582900711341 44.1350486752012
41.7419877611072 42.6679886757687 39.4012855594431 40.5084378738092
42.6758568421347
DNCL1 3282.4852236415 3421.24021192326 2771.14077845128 2257.78227736778
3681.41904661708 2934.35616793443 3761.48462220262 2357.96534075689
4036.89580181979 1932.2647526188 1928.53890809223 3010.38998829232
DNCL2A 39.0620487644635 38.5252456306656 44.696023817801
52.1201052574162 50.2163892855092 53.4466111440611 45.3597029614942
44.293922970134 42.9020321355927 36.1550293353654 39.2542963855807
45.5800840647989
DND1 45.0985088502363 41.554942319225 43.0971787292711 52.5307002738469
51.940083408061 40.650742261428 48.6395795163502 43.2551508830106
43.3744503319109 38.2069499294002 42.911855821602 40.9683275553869

DNER 44.5282181324797 41.9894412416874 45.310660704531 41.4273122914951
41.0885562160946 63.6894484284172 50.0088611244948 37.4484310268909
35.6970528713709 62.2025996860055 54.7883681830995 49.1200355938198
DNHD1 49.3463416794995 46.6996902348633 53.5698357689852 50.0526763693329
57.6791750431251 52.2089306840125 38.7337086062875 47.9942520395434
52.2631308081333 45.9004543328976 58.2941346100716 53.2046867327331
DNHD2 41.9407041937973 38.2263227947189 37.2838080935695 36.890925044696
38.2496050953547 45.3882138838137 34.9344532055397 41.2551966132486
36.2733735494625 44.6918600441328 41.6336636285875 35.5523662009011
DNHL1 43.9309845324767 41.8073910693411 42.640829914102 40.8084239472012
41.0476065156912 40.4359177791566 41.7614975630362 44.6511073781104
40.2274522635576 48.9826868640753 47.9942520395434 44.4951343330035
DNLZ 484.651969259966 200.082941320005 104.704892803169 208.553134986809
250.545098051392 255.762638489554 378.964387825252 188.408367998553
238.246319539302 188.042978085327 224.14182624489 166.931246502269
DNM1 66.5500089527674 41.2598458726002 42.640829914102 44.550322123994
53.9074374788981 63.1091746640396 40.5585676679482 47.0940613507321
52.4539007817929 57.2813205536176 62.5036823572932 45.4799270968969
DNM1L 129.091447141381 215.290701336046 181.873969432903 133.765486891316
174.469283513638 184.340249262704 215.175765091556 110.214922820817
146.681796324406 85.9910479975356 112.565968152932 167.824386984618
DNM2 172.381808887528 261.51893439249 508.081726674689 265.320447667174
208.897032780128 127.352997770688 224.249210397247 241.24267509333
307.974082654351 343.990395610093 410.257694738546 158.853244260329
DNM3 46.6348258763289 36.5219488315447 41.8751578329365 47.0874209746031
48.3814421939888 44.3648993568405 46.7100796279384 41.7419877611072
34.6720534245631 47.0055981216687 52.022479439889 40.4639532172338
DNMBP 44.9896851005259 45.6533175604486 47.0940613507321 44.4367387274922
46.9906120932727 49.4157684139858 39.4600924554505 43.844855336215
42.5406888281114 40.4136535208406 45.1432412071268 42.7782216342806
DNMT1 368.972794063004 605.338871441599 424.87896140215 386.170232791927
904.487756674518 345.876877384089 554.551745559065 334.016152807947
721.887210480162 189.890451486324 315.487514440864 347.908556198025
DNMT3A 41.5787026298957 44.3874750238889 43.5367669381526
44.265834798964 44.3201481615451 52.2089306840125 40.7340599105623
42.6808744813778 40.3704050658025 42.9285286409526 46.794716003113
46.1980780531506
DNMT3B 50.6109448604627 54.6089451252049 56.0245669026819
42.6082786891193 60.6628365130306 46.0817481577316 70.3454355021212
64.0476795495651 63.955655956484 67.5206810636627 58.4134572546373
50.6937447392501
DNMT3L 38.6213341190836 39.6995529770239 40.6312687664188
42.1273928203021 43.5836473788312 41.9069736908705 40.2154497375715
36.1375121409002 39.5411918095433 46.5650556481721 41.2267153672559
41.2905987859365
DNPEP 68.2614370871382 75.3462586192013 58.5212649095879 46.4555908533915
85.0017995689256 69.4214927857472 61.3620062177524 69.5741225986433
87.6018870099686 64.0162246399972 60.4620008899889 86.4937293696262
DNTT 40.8681871019812 35.9305494063137 42.9641759692926 45.6706505553383
40.2449343247539 39.7697110924474 40.369768755239 40.2382908228517
42.9413736814915 42.6793753550854 39.4410515050885 41.1066323911308
DNTTIP1 281.602712448925 202.103107745135 170.105455298636
282.328238928883 192.377506427627 286.68367978144 311.055487966495

177.688415930665 179.767874494671 177.173301602325 195.58977358589
163.844659359002
DNTTIP2 40.4639532172338 43.5182502375207 33.7737772393677
37.5382094267692 44.6834648302383 36.2015763253919 39.1539892144371
41.3506832276112 37.3600014814388 38.6454098044474 38.3684202018524
44.6484005362281
DOC2A 42.3620028137457 40.4772513262052 40.7910160811527 38.5252456306656
61.8471691369569 43.0177448037924 42.2577689927563 44.4031868028216
39.8048943625718 41.7932258301997 43.6629145624937 42.6278608611758
DOC2B 46.5549623118323 46.0713306971594 51.5292723244077 41.5464765641928
47.6884372645373 48.260931742163 41.3594868055879 68.580705761277
41.863927676552 45.1569741609597 47.5439485437351 44.0451487108213
DOCK1 110.362432903746 101.58345410371 127.49459343016 80.8728961146267
103.619396986593 169.18132209076 98.0023132593711 115.221134337342
93.9756797434207 168.522052983892 159.268245287939 172.192366002756
DOCK10 111.041991642022 127.82585786161 118.720812623965
425.026540236907 54.0126916508417 93.9805435116156 72.4651758215622
85.6673237797982 48.8067321016961 74.3981029634178 78.3542840447759
67.5047089908724
DOCK11 90.6805895192707 66.0479079234982 69.5937318502259
223.168185178205 48.0234896130991 177.515053183327 66.6894494536346
80.8333046859586 51.2776048338 76.1382217104599 85.6301407137205
132.188774889597
DOCK2 120.837993273922 184.859183550225 184.201497822259 589.395944599882
84.4230484528318 200.351560652506 119.015431849218 132.663052961193
48.3119523820103 67.3934788330567 63.9617548533553 83.7829283976292
DOCK3 37.55264801907 37.920964499903 37.7384958256497 39.2780515621926
72.4060089486729 42.750861693377 37.830876898596 40.7311497914871
40.5912213523345 47.0055981216687 44.1148826235374 44.6484005362281
DOCK4 36.8072833828297 40.8880530359822 44.7829632472754 44.4367387274922
39.2608760193011 41.159706131351 36.197424053556 36.8400213077707
40.4997186069494 42.8936990772838 36.7283081422707 37.2810767789306
DOCK5 61.7551416187554 44.9370339275041 49.3000844711116 97.2220312216969
45.9104478407175 41.7627910693483 55.4004611309636 57.9713749600418
43.8482802038826 52.6879797806876 54.2999306485758 54.293880614303
DOCK6 140.853812988562 173.819948756127 273.070365538675 96.5813769696577
131.156876010639 86.7776945455037 82.9431583622036 128.146059846414
172.252848888699 135.007055711266 140.610918488396 126.838679504229
DOCK7 116.35075921851 104.740168626646 83.3826639718546 87.114638423256
113.491158368594 81.6472320169489 99.9896394343368 92.3532155362085
117.239774193934 76.0532592222027 73.6679006272617 92.6888475289584
DOCK8 76.2368668439875 158.806855832703 82.8205269728224 354.861943266984
48.2748123114598 64.0985088513857 101.46584765758 142.714450804595
64.4588422949237 107.840833498938 110.287699369482 107.337113678019
DOCK9 73.0970339739187 80.4334958198115 99.1502352565568 59.2778887402357
67.8233625051884 82.7269382676336 51.9421032049606 60.5571721986885
77.4186160113063 59.8130420783374 78.3542840447759 79.9447969637409
DOHH 54.1745577382276 55.6972661824064 40.7620472083159 44.4367387274922
73.4535007027931 50.2163892855092 58.2315917325629 54.9919060580109
48.8067321016961 58.9069025298754 51.2318935933116 53.655233033193
DOK1 35.8208542506595 38.0226905622357 42.0621425894624 32.9046688061235
32.9756367838345 37.4782441311626 32.1218888714537 37.0390589468421
39.4762546236449 38.6454098044474 40.723990462047 37.7526485343328

DOK2 42.466333667839 45.6948619492056 46.8267540220952 48.309866878365
37.7170995811297 43.7078399718247 40.7376840028647 32.8834169462007
36.3649677956953 39.1177164316495 41.9495387255163 38.745552403895
DOK3 44.9896851005259 48.4497719907777 46.4569566520107 102.786429761295
35.2163900400329 39.2248354955045 37.830876898596 38.5457716679071
41.2870505273664 40.3235655331796 34.174642681781 41.4508241771315
DOK4 106.219480656782 157.040132256772 144.17470770145 68.4261608261522
58.5450868286167 106.186051982612 93.2133061608764 80.9092480872499
100.591821617326 130.812376632261 102.993620677265 100.677422836631
DOK5 42.9104454767916 44.0409178714232 36.178547300352 39.1706564265495
38.0598374201407 44.3648993568405 43.5627649099623 41.1575777497576
41.2870505273664 35.8304004211528 43.9450023643394 41.7575097298927
DOK6 52.6960621424156 44.6918600441328 44.6032218589199 37.9875302985336
47.9128504683618 173.357715186701 44.9863726205428 44.0390019242862
43.3744503319109 56.8201201798712 64.1267046511632 48.1627097465883
DOK7 59.046385957117 58.7111274254142 55.8810837854139 43.5627649099623
58.4383854340181 43.4681646416585 42.2577689927563 54.0507720792049
61.6839343510302 62.3233491851723 61.1801493883012 50.7487159427973
DOLK 201.180226922613 129.299077627671 117.355761277966 138.189579100482
150.221698290502 97.9586375307737 144.227861902786 135.361059757705
134.024691118762 140.199798713912 112.007118067348 94.6167194649276
DOLPP1 103.561100886469 108.083751469991 82.1344514651393
66.0397471691747 129.613152757362 64.5187138759372 163.011116329807
89.6313467455698 140.187433247353 107.501868837432 102.380096190518
99.7529382840954
DOM3Z 97.8141040140486 105.699748702315 107.501868837432 104.335120306753
123.800965644574 85.0275638433103 135.540738841602 91.6777754418002
112.743602566917 104.928805304068 116.080897948094 95.1224420992193
DONSON 75.7342400123505 84.5572894783679 102.607924735378
61.5515639412892 126.578693258328 69.64983895075 131.955454394903
74.0831311663524 119.634256037186 50.9506778292488 51.2623287419357
65.1251519077199
DOPEY1 51.5888395924843 84.6158251687884 89.7299583757595
65.524821699432 51.5888395924843 85.8059456975413 69.6301237585571
65.0656365694468 75.9909523932333 66.0786923699684 81.8648220673828
83.0460206754856
DOPEY2 226.672191323896 97.1634390603131 155.617122020913
160.702832529922 119.167027972122 82.5279746006378 181.45962058846
293.300203370043 129.143252341926 146.046179302584 148.608392563633
120.641774274815
DOT1L 41.1523487516364 43.1805016690297 42.3536549114165 46.7577982595088
49.452985143568 46.0364000143537 49.6872467499977 45.1714698794331
47.27543600687 41.3321861371202 42.8263096141654 44.9070250604284
DPAGT1 176.687970262952 205.627744150936 163.66696652324
136.711122812062 280.680953068339 120.02765867291 195.190044833888
168.225699005719 185.079035617027 130.812376632261 148.178364767218
189.705164944697
DPCR1 39.7576136507593 36.7976562409506 34.0382693188794 44.4367387274922
43.004943518025 40.4359177791566 33.1392460259336 39.7509165247419
39.1790517315259 39.4710743516517 43.0016969050339 43.2776458914333
DPEP1 40.4639532172338 34.6306468196142 44.2405728317107 48.1662933382789
42.1650127805175 36.3845892764084 47.2927436772465 37.9837233601316
43.5669306063196 43.1839109702714 40.1742127891824 44.9070250604284

DPEP2	48.8333989709933	70.0470864733496	192.632227878708	259.43283734195
	44.147557390293	78.1952428431942	80.6634117492762	96.5813769696577
	51.8083208406279	233.96534264787	74.9541456286637	177.535502927617
DPEP3	46.0920218698606	47.6914221838216	35.8265993915844	44.7980740332174
	49.8304479381623	53.7442594289333	47.2927436772465	48.1591264215184
	40.9901805728485	42.1622837416589	40.9157352260901	45.1735152121056
DPF1	51.1055541075029	46.1569135229922	45.4865173433646	43.6726153508978
	50.2163892855092	39.6251842352088	45.8980298854198	44.8997235283254
	45.4799270968969	42.785429206422	41.7272875295469	41.0657892043496
DPF2	206.514263103112	339.679549262298	250.65667565007	197.171449112363
	548.217757366495	311.810240385503	332.745139672471	202.529846569324
	318.609131605258	368.005081137665	388.336994961328	226.719775835218
DPF3	38.0089018744739	37.920964499903	44.0451487108213	45.1944222548351
	35.5220586859301	45.4882438975269	44.8643898647322	42.7431359850339
	43.5669306063196	40.2112371512838	42.170702295259	46.8129509799753
DPH1	58.651329686676	67.6169355376864	61.841867306916	51.223859150798
	62.1836961153924	62.7515429046888	65.8986841086198	68.1311562787361
	67.0290776715331	55.4037482627222	61.940656536312	56.2804354745795
DPH2	128.281765653795	124.691222601355	85.417321091835	70.8088594316677
	194.189674572424	81.8426140611297	100.423344358771	117.723425661764
	115.087963545095	88.1199908664065	78.2839612497413	74.2473868510319
DPH3	104.17651993553	85.7208881534518	105.726287089142	165.496075703734
	207.710354513208	104.860298453006	109.130081850144	121.510087314197
	87.9297641099558	46.7168353198002	52.9405267581903	86.9924799927707
DPH5	144.266359480086	101.634888749356	148.42234459694	107.478392667404
	138.070735985503	212.032445918948	96.2959923858457	122.036614711384
	128.827672077002	157.7623351008	136.408012600787	203.092045210928
DPM1	406.500583302121	359.940393399742	347.420750861056	340.105993325165
	547.729200972776	318.768560912915	438.22469176123	384.180564204564
	443.269396125834	169.18132209076	240.841214170409	410.979796414875
DPM2	112.733531624555	88.3212200388592	74.9820339135242	83.764882413379
	109.23113111292	75.6382797289563	148.333778341005	79.1039287828151
	96.5851811404251	72.3822545261582	75.4466520363487	75.7464604972652
DPM3	550.642253702646	302.20349084931	302.978457293429	261.826480747537
	456.467726693355	455.774407260725	904.487756674518	300.703401968169
	400.327164003217	409.312313055794	351.439404852971	298.282957415786
DPP10	43.781101409552	37.3634897565502	41.3708420203838	44.0451487108213
	41.1868052124344	40.040778349137	42.4493032770053	41.0844534776541
	39.4762546236449	45.0864985243943	41.9495387255163	37.9607839934735
DPP3	92.6545309624315	156.591349262922	153.544554979202	89.2503940049313
	155.252915611651	61.1630754250653	151.912182086236	137.785462971848
	109.889322825648	76.2533853403813	92.9453353261232	89.2385568686014
DPP4	52.9609836781499	47.2770121962655	56.3471340054074	45.4634652319753
	44.6834648302383	45.8325181856483	44.8643898647322	43.6434576713809
	47.27543600687	41.4397539024976	43.844855336215	47.0130468198828
DPP6	38.957687788868	39.9697332660833	38.2280071951674	39.4652349255591
	40.7273974755431	40.5940318808889	38.2933870238743	39.8965727231863
	39.8016018354453	37.5576991642607	40.1362682639916	38.8495095797881
DPP7	153.901083585889	260.426488908824	167.653939596625	212.92335057985
	192.763875975315	160.080366694799	139.758805241617	116.35075921851
	121.064136717339	138.362914843774	157.095926797414	156.864177272437
DPP8	70.5619719048506	74.7370458648253	61.4944752562671	66.7008819035152
	72.3388488936832	84.3040298294164	70.3935694362568	68.8698243753339
	84.7899210293383	83.0700092537618	87.5915970147626	82.7229468743536

DPP9 276.399037769005 202.957014793417 235.047133588022 276.029709051798
251.105933757167 198.15553991394 295.014183084118 299.428590398636
231.555264329444 413.723725202787 330.550018306903 242.396401163598
DPPA2 38.7537364570873 40.3384318592985 43.7630587657205 42.9358255168086
49.0828761839116 36.4986986135769 40.9157352260901 39.853866204927
40.137818555383 43.401782064778 39.6403089864678 40.8681871019812
DPPA3 39.8234920704779 39.8455060654531 38.4456289125816 44.6905691366696
41.7426502114261 44.0399598874356 42.22763273471 44.6107027295193
37.9273653326866 40.0557788909923 39.9070785037411 40.0557788909923
DPPA4 42.442231667357 40.8880530359822 41.0356368374708 42.6913548400127
44.9936360641969 35.5158505219112 40.4359177791566 35.5798430594271
39.1790517315259 36.2435874904689 41.7272875295469 44.1452943804044
DPPA5 37.6397652536077 41.2598458726002 45.3974838013476 42.1273928203021
39.6535880152834 41.7627910693483 38.6243997507646 41.3506832276112
50.401623258584 32.6203270051744 42.2618615571566 41.4508241771315
DPRX 40.7609952437622 40.3384318592985 41.437237454134 34.8137085420423
41.6806984441849 39.4953494583071 38.5066108855005 43.4544762830742
38.0213051227859 37.920964499903 40.9157352260901 36.2582325396375
DPRXP4 40.4639532172338 42.3550738181144 46.2625470410195
43.4812166409346 45.1137938810047 43.1826328292558 43.9450023643394
42.9887069977696 39.1467972692656 45.6868589181768 45.7328341667151
40.1235656101329
DPT 205.627744150936 43.0227387991135 38.9790184198145 66.9218519793499
39.7568367479301 1271.62760896761 40.2154497375715 41.5464765641928
37.0126425326534 596.84269596976 432.355300448283 142.945182805248
DPY19L1 245.060552951511 250.244199415895 373.626843592567
234.113622230498 208.060345754611 206.899594905509 171.853856533964
173.800190134221 277.878824242495 193.830224000034 235.222472959869
338.966238813782
DPY19L2 45.8108990837624 39.6995529770239 45.989212296035
40.4359177791566 42.4864576619427 65.813547096222 42.7431359850339
42.6450179841129 46.9739160257217 46.5650556481721 44.1148826235374
39.5418280570561
DPY19L2P2 39.6609238036815 38.5252456306656 44.5134109184888
46.1074305700679 38.7004513165111 42.0574848021044 41.0381860307445
45.4882438975269 43.8482802038826 40.8970401536578 42.357910063876
41.6031169661721
DPY19L2P4 43.0930293831497 39.9697332660833 43.9450023643394
46.0228732507872 42.7982502551634 37.2686490268265 43.8206425661746
42.7431359850339 46.8873421271259 38.528621456378 41.5421429082231
45.4799270968969
DPY19L3 45.9090659324223 59.3406363431414 51.223859150798
39.066284740632 68.0414563597154 54.2503023961401 57.7805922926828
54.5125550937898 57.1863195337055 41.7405682794166 41.1638563416981
52.8455400026194
DPY19L4 98.4084364053686 95.9750691854349 90.5553202429233
75.9099205260748 152.608998839745 89.2185019250322 101.065203693106
81.7765447802116 141.067111495922 122.576931376426 121.423421895502
130.865421554474
DPY30 424.709002690579 322.371597171026 390.834121304799 289.112248695929
306.487935692954 312.795596385258 560.802447943839 616.912306849151
398.43592055172 204.370291548631 223.749512763927 478.296779356592

DPYD 53.2046867327331 46.29198852834 48.3847843498476 109.726577835621
43.844855336215 96.034706957068 46.7100796279384 44.6107027295193
50.8485682149772 68.4137459386155 67.6271456814495 62.58750768974
DPYS 34.1336988442837 39.5050383901884 41.2365089689635 42.9358255168086
36.7356360093603 38.6414316877471 42.9803717121865 37.5511560880902
39.1790517315259 36.8059561571675 37.8372168909583 41.7575097298927
DPYSL2 698.595302020108 400.973553604192 342.643344197936
1053.67279913417 301.257549182222 1707.92349760322 157.843337785472
366.716775127375 223.562130663956 895.735191953679 935.815928529014
982.428740433235
DPYSL3 1611.35913846618 201.180226922613 153.711194034581
640.944514118411 144.799432299339 3296.66056024983 186.620669365322
440.728435508514 266.323732730581 2640.06306087033 1813.65521720537
1595.69503047506
DPYSL4 129.785929766258 159.393207489822 42.9104454767916
45.1944222548351 83.0497574039035 58.4435847520801 53.0277416531043
49.6203214143464 41.5635499110307 45.0155543555707 45.5707650534259
50.8442982601142
DPYSL5 46.3390850717989 38.2263227947189 37.5597671487621
39.9697332660833 41.1868052124344 38.4330755397458 41.3594868055879
47.8947595316832 45.8325181856483 48.8623729374733 40.1742127891824
37.0953622406687
DQX1 36.7356360093603 51.3800036046133 54.9071823326024 42.357910063876
42.269054475455 47.9503724044453 37.7136011474903 41.9315419791594
43.7630587657205 38.6454098044474 43.0971787292711 49.6629259325705
DR1 91.5417574979683 82.3344892042646 92.3034609409756 82.0292880742189
101.867388894697 94.8884063433735 95.5117928427637 108.57858133896
105.825228126465 78.0658258913875 93.2484615907966 108.479856042609
DRAM 251.803102904399 234.078434290473 188.513581321162 510.82619253142
138.895316905243 213.140641182603 146.117756310709 148.403272698474
164.642060754649 208.457587834567 214.873872837015 339.210875653066
DRAP1 566.318259999405 654.682263833024 790.786577596971 781.223434214089
539.879151334921 795.381998233245 873.451893437179 757.257495810249
781.223434214089 534.881442644054 606.123659335794 513.57613902662
DRD1 39.7576136507593 43.8495248991589 41.437237454134 35.3829784221072
34.4570158133896 36.0306975524309 38.5066108855005 38.956280281655
42.1594905198929 39.9948390976317 42.911855821602 39.0536056496869
DRD1IP 37.7384958256497 41.159706131351 37.3687900733549
41.1886975241176 40.5710637884029 41.0582900711341 37.830876898596
54.6067620335514 37.2810767789306 38.4187686307607 37.7384958256497
38.745552403895
DRD2 37.6786104343959 40.0527893189096 37.9946685306274 40.8681871019812
36.08956004634 40.3357777530419 41.0995198212729 35.1169296995348
38.4344387289956 37.7668944701279 37.1617810121175 38.8146043755011
DRD3 41.9407041937973 41.8848959280916 38.5045333213784 38.4571887275378
38.5115309709704 41.7627910693483 40.7910160811527 38.3684202018524
39.8763736543526 45.642304356051 40.6783687486496 44.0977148249302
DRD4 135.00141569592 82.1344514651393 112.60230393988 68.6379135603075
53.3086645804786 63.3612993417558 59.8273072817267 116.434267173815
112.805898501551 72.1090339831747 96.0126406688619 66.295597720304
DRD5 39.266565827316 42.0709558258414 45.063792968927 42.2496337895968
42.0709558258414 41.554942319225 46.8613212048981 40.831207953647
41.4636047919453 43.6033221838149 41.4508241771315 43.6753843488695

DRG1 357.254500791202 302.50850157207 317.0659599072 276.029709051798
314.281125546552 426.015657750123 431.727858902449 354.158105175308
449.427251674566 288.237195966495 295.194516063502 347.145902252055
DRG2 99.7403508491191 173.977605406146 121.120217132666 112.957927208627
148.544012961815 150.242221910229 151.266092912661 132.931052537195
144.098339910117 179.064697219173 207.671418362257 124.628895737993
DRGX 39.7576136507593 38.3322849790657 42.7303139017786 41.5464765641928
51.7983110178095 43.5752393599169 40.1235656101329 47.2009420656854
44.1519466943185 44.2580151149956 39.2542963855807 40.4639532172338
DRP2 39.266565827316 34.2618170189676 38.2280071951674 32.554751989205
35.0181575202066 34.0777108441756 36.08956004634 35.9023742468744
38.3418933808844 34.064698871541 37.3687900733549 37.0953622406687
DSC1 41.8833988894167 42.7275315121163 40.1985790927248 39.5050383901884
40.9950639693255 40.8091178323174 38.5066108855005 41.5464765641928
40.8420925848403 41.2276963324981 40.4094901248527 37.6557714476853
DSC2 61.6966068000136 46.8954576545929 56.3471340054074 89.815123920749
49.7526478730976 51.9421032049606 52.561573697155 54.720204938924
60.2276476399612 45.3894119568143 46.29198852834 49.4786466722535
DSC3 42.466333667839 41.9894412416874 38.0958223740111 40.3691324547049
44.7829632472754 48.260931742163 41.1886975241176 53.4109882045447
36.5474283500601 49.8393069675192 54.3554233228353 56.1136936405786
DSCAM 48.0201377454632 46.1074305700679 41.4534762662066 45.4977402232805
45.8749253192609 44.4367387274922 45.6042625176633 49.7748385529588
46.8873421271259 46.6099721451275 48.783481283579 45.9132137835627
DSCAML1 41.3387790895716 39.8695929627783 41.6079958749402
36.3604951341921 38.5512429290626 38.1169703906207 39.2533668167483
44.1311654985682 35.4332080966594 41.5435611403119 44.7926323492519
38.9496515344837
DSCC1 46.8954576545929 52.5380506348717 36.769073068743 36.5393857306744
53.3086645804786 42.8598805316863 47.8947595316832 35.1738399826814
36.2733735494625 38.1042454705119 43.0971787292711 38.3291713594599
DSCR10 43.0001432913058 40.8880530359822 46.7508829977587
43.9282059368202 45.1137938810047 43.1826328292558 45.223075311113
46.0633179231318 49.3780606970538 41.0057887014368 50.4740079109854
45.0985088502363
DSCR1L1 37.8386378737561 38.4330755397458 43.1931731665131
40.646702696033 50.3713170502557 39.6251842352088 51.1541843174981
37.5511560880902 44.4395580493118 39.5637454855233 47.7535797917441
43.0930293831497
DSCR3 142.309369995384 308.841311505431 242.503720442197 289.716911324484
309.022069786139 206.559526386642 240.841214170409 120.552647575158
171.191059267941 165.42634596698 136.482957391332 206.41879845205
DSCR4 40.0557788909923 38.1312950900467 38.2280071951674 41.1886975241176
36.3390947880186 44.8872784170036 38.6243997507646 38.8495095797881
38.9006788676407 45.5776159432079 43.844855336215 38.4330755397458
DSCR5 43.5710871617107 40.9731067463382 38.9790184198145 40.5484717678957
36.6354013629791 46.8954576545929 49.1067959632109 34.1806289878747
35.6139301508335 40.1179210534118 46.2560238004757 45.8546719310325
DSCR6 43.6706764143908 130.615540145155 54.6051538001037 36.0481934423077
207.380865650247 61.4944752562671 412.421643885499 97.9497090233081
265.176790020229 65.6721629145797 60.5608545741456 183.526640731647
DSCR8 44.6231541342 43.5599890586394 40.8860076320216 39.066284740632
42.5435283762933 40.800866389165 42.3074504864935 42.6008809597607
43.0496526069072 44.9603714724456 42.4956274276424 40.7063426028871

DSCR9 43.844855336215 44.8529259387308 36.8730514568925 41.6564075217517
44.9936360641969 39.3841861812262 44.9863726205428 34.806104801006
38.3418933808844 38.0078357808393 37.1059647239814 36.8690774823319
DSE 114.935101645207 62.8315857560764 63.8740412329753 418.693470287372
68.8570376191283 138.23147386089 81.8868841896039 71.8613906497084
56.970501040174 92.1725548553384 84.8084949516218 108.616118600231
DSEL 45.9090659324223 42.2577689927563 44.6032218589199 43.26160898693
41.2740822675592 44.8872784170036 40.8600431177315 41.599960574499
41.8994956106406 41.2665909203775 44.2925355984056 41.2870505273664
DSG1 36.8072833828297 35.4484652445078 39.3709357615493 42.2496337895968
39.6535880152834 36.8059561571675 38.0598374201407 38.956280281655
38.6322728994674 37.0320432433314 36.9060997041453 33.5578650644246
DSG2 72.4966502412724 121.542387231625 232.879882989322 38.0958223740111
50.6470461863549 43.5752393599169 99.7529382840954 103.366147685418
135.323750661937 58.115198353247 97.4829901136163 213.309547409053
DSG3 37.1485601150873 47.9665737016961 73.4122412764173 102.483081702338
36.3390947880186 36.3845892764084 42.1607322031054 73.8374823243007
91.5370872909881 52.2256177934134 40.4504608195666 47.6040671292013
DSG4 40.0557788909923 48.9899488302637 56.0477607422828 51.223859150798
42.0709558258414 50.8533737532786 39.6535880152834 40.831207953647
49.0986627051784 58.2818491355907 50.8826620053386 49.0655215909632
DSN1 86.2021418088902 77.8946473414547 79.1649337367773 85.5959901987745
97.3188285021124 69.2685201884906 151.621572940457 118.736188485344
97.9586375307737 57.6791750431251 65.9273663561152 64.650642183463
DSP 403.92758879201 106.521012342668 56.2937283136959 53.0284011078097
48.9627346956003 48.7019915522206 78.4937314423253 79.2185250088688
149.856848496725 74.4365426051588 72.842552380479 71.4197504261453
DSPP 40.4639532172338 40.2493681589104 40.4882741869718 37.651298468202
44.0451487108213 42.8598805316863 39.4600924554505 35.3608605269276
37.0126425326534 43.401782064778 41.2598458726002 39.7411455670172
DST 46.5728197403493 55.3864000014316 48.9775486706613 41.7243656302479
46.8039881499971 46.554154914004 43.7617721656171 50.7855742231932
50.280793313765 67.7831020218081 61.5144360539876 73.5950442915143
DSTN 448.383843915421 543.268021785502 576.367042159347 367.158502379417
476.653723741096 2294.75523373329 596.384327208699 547.729200972776
333.731080636948 1448.17151189672 1275.78715071615 1679.12630849964
DTD1 88.7928773498158 146.046179302584 160.605530211736 132.52749757795
179.994715581707 138.12608134713 227.361044585582 138.170787392253
138.50445813694 101.693848821246 130.775349446374 117.520762118299
DTL 56.0477607422828 54.6089451252049 59.4431489877328 45.1944222548351
80.1294564809102 44.7804080899431 59.3549213146532 64.7318794662958
65.7924490713262 40.8049408996274 38.4689132737495 39.3575468494941
DTNA 48.8333989709933 50.4487395010341 51.4704882732949 59.6804124924694
45.9104478407175 230.486681581762 40.7340599105623 42.6450179841129
50.628152951493 56.2508140932179 64.2690842803282 85.1718404558939
DTNB 118.762101740563 52.2501726671381 69.1938570643524 57.0629653735694
56.6951078698291 51.0506441018166 121.737071786167 111.714563124738
89.6736229308069 101.463033319045 95.4841914842335 73.9986015964321
DTNBP1 81.2850357499192 56.4978377936863 61.7572675712557
81.4621222177298 66.8129352840907 81.1761078123004 66.7826466218147
79.2701322339022 67.9667104519753 71.1607965028029 66.7759319948677
66.1334450017707

DTWD1 73.6362523983338 67.8688083153991 53.0290605707162 72.8120269375546
69.236458110243 113.6408244935 68.8005532048281 63.287067794195
67.4775348162369 68.6095266659765 66.3481553496766 91.4786089554886
DTWD2 353.868749867979 191.141778714144 209.438270055157 342.643344197936
162.424734330982 116.900364583878 256.302432791187 667.583623414832
153.237127853072 450.515644056585 469.893430869091 357.574415292533
DTX1 47.8249696958403 43.3458769998118 40.301483914446 45.7397504535169
40.6723334103791 43.4681646416585 38.2933870238743 46.8954576545929
42.5406888281114 40.5090849993014 36.0481934423077 45.0035308952466
DTX2 216.744244428166 177.133999361532 175.550329937952 224.509915754308
192.171116215791 113.839604953506 162.160380044249 186.325752658912
310.821299796803 156.770274308736 205.24499293304 169.790843803064
DTX3 101.81885073944 53.2841144999258 72.6731815563159 53.593974682612
80.7321212028516 92.6036872789676 78.4937314423253 116.852483297949
77.5480089703916 99.6265783263109 123.368987753537 72.8306652548968
DTX3L 54.931538325692 52.8796369426118 49.9256753636167 53.9517841438975
49.5666074434383 44.9896851005259 46.1634822993009 44.0390019242862
55.6617952786921 41.2276963324981 40.9157352260901 42.357910063876
DTX4 36.6480005877129 42.5269793679335 37.4642807402741 51.3851157261451
49.7235523248653 42.6575535390785 38.5066108855005 42.8482181313226
45.8325181856483 40.4136535208406 40.2649309204478 39.3575468494941
DTYMK 39.0620487644635 37.5469247328848 40.8420925848403 38.8495095797881
43.2484996523549 40.040778349137 38.7337086062875 41.6466169548846
36.6312676076142 33.7883338848419 38.6662194645633 44.7401523707775
DUB3 43.4626049853157 42.3550738181144 42.7303139017786 41.6564075217517
48.0234896130991 35.4142595025086 44.4639405506967 45.8669209716694
38.9006788676407 40.1179210534118 38.5668610487033 41.0657892043496
DULLARD 108.400712514743 97.5849495690144 116.426770366736
122.702789229752 91.5258329186709 98.6108785181637 86.0796981361148
79.9597944271831 89.5239792174887 105.598516887691 111.071209057746
105.74849082793
DUOX1 41.1914475733743 86.1924491308234 92.6036872789676 42.1607322031054
59.4236178078244 45.0241191707651 67.8919712859031 53.6887223745125
78.0133696753253 207.710354513208 120.618530729822 140.309952483162
DUOX2 37.7384958256497 47.765848710116 96.7948977485109 45.334106390212
39.9697332660833 45.6129956328415 46.7100796279384 46.3685439301234
47.0804681460898 46.8954576545929 41.9083581529276 67.4197263416701
DUOXA1 43.0930293831497 111.248876636045 165.029434090892
39.066284740632 54.0126916508417 39.3841861812262 144.863548409034
81.4881766238989 89.5239792174887 410.058119360034 239.117228656704
183.526640731647
DUOXA2 47.3791873993182 51.9421032049606 235.195902632101
60.1837189855762 44.0451487108213 54.2503023961401 74.2690269784394
54.6067620335514 64.0541263553251 48.4890615340603 37.9230440150891
103.071727621257
DUPD1 42.9104454767916 41.2598458726002 40.301483914446 42.9358255168086
39.6535880152834 39.3841861812262 41.706879587091 41.8417024632349
37.7384958256497 37.3179578037963 37.2018803984555 41.5536168238478
DUS1L 110.048921728774 145.694779669933 101.131391476546 66.9218519793499
132.684895918889 71.3932787152381 149.551650453179 89.140025661759
111.939677225005 96.9051967257119 105.589475270494 78.0006681166995
DUS2L 117.20232751191 154.067791946816 237.359267889848 117.228320608065
180.100430567626 102.980622026276 310.900527198512 149.330073823683
173.476817564711 199.970127219451 302.588009553875 150.801729138762

DUS3L 126.682296930289 154.355197155161 190.675425748381 139.101561420631
326.303950252592 195.654315886452 157.843337785472 123.219313750907
275.061444728555 149.173801572239 210.485586903711 151.975559850216
DUS4L 90.4512650366645 91.1741538903522 102.607924735378 67.8978240198575
134.713725350463 86.9709077763908 107.748050894857 112.211717641877
118.155986485411 91.9185612536409 87.4273245333478 82.0869179287671
DUSP1 2992.62035058532 4784.36905964712 2488.32876359853 12125.3293116195
1094.91235708087 6027.02412617194 2697.96877833185 3575.78813099057
265.728967648382 1914.9556343048 3001.25236839028 1917.90025587049
DUSP10 60.394307846869 71.334759229964 57.423873475544
105.34055372228 51.5338331947847 51.3438109941698 52.20169560459
51.9819543312733 95.1423940686828 45.9301227940101 47.8284278211109
44.5432192783161
DUSP11 174.492066109537 141.370796803908 175.508659149068
134.646159826262 176.936216428226 129.909461876076 142.03031230486
152.920586451578 170.150808455621 127.398722392954 144.18700333025
155.6723334711
DUSP12 356.318517117856 284.105466865264 323.295794446334
171.238235624104 331.239629083778 232.740032714891 270.036719921343
221.325534445978 261.326713935356 176.506718222092 205.346264347637
210.255784687052
DUSP13 44.2350046179299 41.4123755032424 43.2721716572958
41.2507419031133 41.3387790895716 40.2851212785573 39.9361851865743
40.7826380785043 40.0753122697556 43.7927887844247 45.0926028738813
43.2177497174408
DUSP14 103.139459009593 101.277664841914 97.4515987591398
84.8948024208923 172.349874473605 111.760213205992 167.229755109155
159.727829541783 173.891042920642 124.362644216178 97.1271615802556
149.899176549323
DUSP15 43.7105673651665 41.159706131351 37.8386378737561
38.4571887275378 39.3866822630357 43.2805590023412 42.3074504864935
38.956280281655 41.0381860307445 37.3179578037963 41.1367478979484
38.2795150354677
DUSP16 65.3531590366085 97.3813557754832 75.0377156190383
79.4528447794527 100.90948905426 62.843734579668 92.6545309624315
157.856158440506 84.673375308972 99.6946103680372 106.771536005741
110.731862140996
DUSP18 89.2913927776683 77.4046818339539 116.080897948094
89.9284109077375 58.5450868286167 73.688423848106 74.7534423194688
146.892027926888 127.345094323724 118.600699776727 97.4062608315831
108.299918384297
DUSP19 3869.93390477952 1855.0119962662 1963.91365010531
3088.44063817936 1390.12380748446 1395.33665255999 2978.46032143413
4406.55576617324 1421.44237064813 4015.88651424568 3966.51586153125
3114.05035137552
DUSP2 58.7404123614695 324.936044300546 135.323750661937 161.48562926756
58.1242391848692 50.6360662655392 109.615289274998 62.1934012861178
64.2174369755376 574.847203528544 155.050936910986 72.0334593545916
DUSP21 41.2424637338424 45.4882438975269 42.7303139017786
43.3078542962968 41.467005949568 42.357910063876 49.8281348927244
38.956280281655 42.7150227340364 41.0057887014368 39.3667161654772
42.4607795638552
DUSP22 322.371597171026 137.785462971848 235.315897087189
263.068504548549 185.72957253374 272.930242946726 115.801367301965

177.494605794577 135.490875023792 201.875082892488 203.334053441063
223.040734249612
DUSP23 545.514314651238 568.371815312895 1030.31910195147
751.953794043451 912.757335169259 534.629009719855 931.401929277001
585.054570630252 1033.99699402259 684.865133028228 754.700295017212
598.970109654466
DUSP26 63.7818442618767 67.5417980425932 56.4779645313958
66.9218519793499 56.8307116636372 57.8944021095265 61.8571521441451
58.676230030835 65.2125527940945 85.8795177524168 82.5225075943931
69.4383198054368
DUSP27 38.0089018744739 39.3999056968598 44.6032218589199
36.4038860800164 44.9384441371181 41.6178506967511 39.9519547348106
40.7311497914871 37.8631570409126 38.957687788868 44.553780984244
38.8964578286293
DUSP28 116.547937420457 174.088328509793 134.725028732282
136.392873701653 78.7320333941149 126.017900743748 131.258204302177
109.922909644299 131.859371655356 124.815448107572 139.204639906937
126.115065200084
DUSP3 623.574706535278 292.47671022688 332.060941890092 647.868022835702
304.577891085373 1040.102982885 218.354538624978 622.435313940734
270.600852806342 589.685739519627 825.558929673308 864.302844775203
DUSP4 46.8141313516997 67.6767845246581 75.1853590860646 64.0903425729408
48.3287893866569 41.0012755104374 50.2016912396771 63.8008894786105
46.9739160257217 68.534940191185 79.7691160370015 53.1491634301682
DUSP5 430.618167106529 1099.52344340557 376.141477833569 624.954583057431
566.318259999405 330.892010103508 444.169882251299 2712.36664370642
231.859167242606 294.908371819124 431.864508795316 698.350485654135
DUSP5P 49.3463416794995 43.7644428186265 40.301483914446
37.8129870676592 34.9428206949566 39.066284740632 45.223075311113
41.5934628038429 44.8003345643334 40.2112371512838 36.2435874904689
40.6614700944853
DUSP6 79.8844295847317 109.581139823852 140.030757545662 102.880267177687
64.3571664944642 81.6472320169489 62.2948511451314 120.142759111608
95.3754438202863 50.6486940895674 77.523734787944 90.0942781313264
DUSP7 40.5654775358427 45.4882438975269 55.5567699051046 44.7980740332174
44.9936360641969 38.4330755397458 39.3585350448719 41.2551966132486
48.2123120250343 37.0320432433314 41.0808257270221 37.4554876386402
DUSP8 51.2851682978581 72.7987593815919 59.7483827511172 49.7350276169552
64.193536444915 58.7404123614695 46.0228732507872 50.1833483241864
47.0804681460898 53.1993028936883 71.2370255410834 66.9239565096247
DUSP9 44.9896851005259 52.1062000766427 32.9174234020987 43.9282059368202
46.7508829977587 40.040778349137 38.941855825269 38.5457716679071
43.1805016690297 45.1569741609597 43.1931731665131 43.7672102682067
DUT 72.5752072120178 82.214837116747 62.7053729334675 54.7231416857164
81.929796716054 75.8976034525022 113.322929203297 75.9659252679925
123.597489107285 78.4428909886495 72.909783842776 74.4365426051588
DUX1 41.5421429082231 44.1239711172417 43.8495248991589 40.3180310664156
40.8873907150245 44.4619248757502 42.4939802311136 41.5464765641928
37.7384958256497 39.6719190054379 46.3370338074996 40.1700117452801
DUX2 42.1537476443423 37.4540772310118 39.8299340037009 39.6292788039793
39.3667161654772 38.2293008890613 41.4742337714895 44.7044140979108
40.5912213523345 35.8304004211528 37.9946685306274 43.2776458914333

DUX3 42.442231667357 33.6902293666089 39.991353369336 42.8235313007746
37.2567512265363 41.0582900711341 37.9430145836712 37.33030683349
43.4656637536244 45.6868589181768 44.7926323492519 43.1805016690297
DUX4 36.064718696392 35.6013646758709 34.9303524605169 36.6501141438378
35.3930760356466 35.2067831864574 33.8558232866797 38.9271399577966
35.161618680127 37.1685072112618 36.7283081422707 35.7451401627836
DUX4C 38.1169703906207 40.7952353240579 43.3085358214154 49.3654837541353
40.0599807618105 39.132725377815 42.8684958525042 43.2551508830106
44.2607789091176 39.9948390976317 40.0766543123257 39.7411455670172
DUX5 38.0089018744739 40.0527893189096 42.8270038153139 43.3078542962968
33.4316681623175 39.4953494583071 36.4098694708483 35.5798430594271
36.6312676076142 34.7308008643142 35.9563099719284 37.0953622406687
DUXA 37.2474303302343 34.6306468196142 41.6079958749402 37.4223289687135
36.6354013629791 39.7394181011603 41.3594868055879 38.745552403895
37.3600014814388 35.6208308220005 38.079222289614 39.0536056496869
DUXAP3   524.451156145427 324.658458599373 331.885872312948
538.153124145211 255.483653108786 233.577690223567 461.83420090894
849.412637017524 221.76904201992 701.168333137671 682.479411898549
542.901822331329
DVL1 51.7098181032862 59.3709786862962 51.6558970571712 42.2655533936307
66.8288956287896 52.5799250974039 48.260931742163 45.4861719648925
52.8190882379316 44.0527419856743 43.6919270353542 47.3716857595991
DVL2 109.251112569215 118.251739134889 108.616118600231 102.971363152434
117.893268074013 134.554839076558 169.168332370229 97.5962784863854
116.287075019475 163.360824752796 144.771861156788 104.192417269189
DVL3 116.426770366736 132.24963384417 178.738800467958 102.365615739399
215.033807819308 132.985150338484 95.2218627805387 122.748811531184
136.736085245679 126.470889051684 131.68956899407 141.255712367708
DYDC1 36.4564050256915 43.0227387991135 40.0998167098057 38.1421709520616
41.5407238526121 35.9305494063137 42.3194600586894 38.3269307390572
37.3600014814388 38.0540928703622 36.8282383070068 34.3409660445447
DYDC2 42.7150227340364 43.3458769998118 44.3344900481112 39.7411455670172
41.2740822675592 40.040778349137 43.6753843488695 49.7309743973184
48.9931786790166 36.6893251782922 48.6184776976167 49.3087627580849
DYM   208.504992019122 287.363928615517 250.359859946258 280.239058266018
136.582966744378 396.63268714181 215.645475520124 198.951182954651
407.161973433666 226.970981973829 205.346264347637 304.258586051126
DYNC1H1   541.105845300009 318.920760335454 314.458148321064
372.982648892131 339.262360833465 377.221676837377 374.601792595646
804.592753722875 478.799250340246 445.307046237791 461.83420090894
468.153823329361
DYNC1I1   72.4094549259929 113.491158368594 39.0775870687758
41.1886975241176 80.8238125943267 315.186937216422 72.8653205184014
59.0262390168156 78.5174639327946 130.812376632261 114.389392280707
110.223625398261
DYNC1I2   488.183286350725 533.820505121067 579.482255191456
404.86512250958 475.466074293565 644.368005872231 350.796638646601
431.353025968649 352.997455210484 331.001195453225 446.613670519745
552.480471244789
DYNC1LI1   43.3638918705225 47.6914221838216 42.5406888281114
51.949834638817 47.4548471240051 53.8461734834857 47.8947595316832
46.5717043290731 40.8970401536578 50.22432299342   45.6348103033479
53.2046867327331

DYNC1LI2 1473.52943897204 927.61341837276 1051.58050264114
740.709094645073 439.664256107097 1771.43541011426 1059.63540694644
2323.81110653289 830.295872113419 1615.83220398811 1296.28036613191
1416.47763911039
DYNC2H1 47.9175508887533 45.0115870036541 44.4311655729487
45.334106390212 63.4753448572245 67.3469281984384 38.941855825269
42.157627240486 57.7865070833351 45.2556760873335 57.115361349315
59.2035499250243
DYNC2LI1 67.7280831060202 58.3912728421768 82.0925058822176
49.4786466722535 64.447308523693 75.7502062965698 72.5075777230597
67.0120793767211 71.5948944013406 61.9080950559794 58.037539503209
77.4307558516809
DYNLL1 1282.9181829891 1601.52194699063 1459.69929955338
1077.94530110976 1813.65521720537 1468.75291542154 1430.73068989193
1184.75786736273 1430.73068989193 926.61614755153 902.691776563423
1333.01876209753
DYNLL2 146.658116519622 173.434174644937 163.38302606725
141.423857667761 207.540972220982 143.607457611143 294.090159716249
218.701583105141 258.446095955014 105.29989739819 133.86254468037
120.483677329061
DYNLRB1 396.167219131922 347.750020791938 320.13475752314
302.012602514613 252.870708814507 445.307046237791 488.089090043145
337.736535374405 313.399524593316 348.862367731887 355.568857981155
445.016226732679
DYNLRB2 66.8544766980562 43.5873188565519 53.6837370963602
51.5913529948887 41.7929491793697 53.4466111440611 50.8388984085636
51.2623287419357 62.1796792097014 54.7827221235949 54.1963147679251
57.8111054584154
DYNLT1 806.827205415602 621.012072500314 623.574706535278
603.737787922337 396.261110334324 478.566247444176 630.53864514662
495.705224781189 492.902027204364 370.088974562672 392.898761909406
669.597878196875
DYNLT3 159.52633805025 161.156612172544 118.556124582201
131.955454394903 81.6099927983382 186.450759425396 194.189674572424
241.557912972813 162.000275136397 97.3870880869036 125.446628066221
156.242688079359
DYRK1A 75.949657568749 93.1668080716761 117.626009607247
110.009834478125 108.718422641679 103.179198287911 81.454335582503
90.8236750745974 117.520762118299 72.5516795240251 97.9806802695685
120.92335773864
DYRK1B 67.8208976798364 66.0147097736612 53.0290605707162
46.3919862398545 61.6452971581503 51.1621379498014 99.4633962228768
101.086264355915 63.5195937348531 95.0026207727863 79.0940380276977
46.6681271693877
DYRK2 88.9038123445083 123.114162330406 105.726287089142 112.707612911654
86.9816932158186 75.6614221167993 147.557451530784 161.817972012879
127.708268637068 103.913834330352 101.494432845825 103.955842514553
DYRK3 47.4167233775693 45.4744187174325 43.0971787292711 46.1673159982664
44.0060504971009 50.543526404059 42.4393920571519 51.4749724393807
41.8225292427334 43.2484996523549 41.3122147844752 41.809704036355
DYRK4 119.167027972122 155.59062961332 156.770274308736 207.087723777954
133.151333210366 194.985223342082 169.859588818306 129.091447141381
355.67721196018 190.77754486426 177.602690452814 207.741669973713

DYSF 179.040571908629 124.490684428024 199.007437365676 349.589955244523
94.0777119695783 257.711912004 111.025105784166 102.717305041352
53.1249776278067 160.53293875611 157.189139558365 80.4404044229341
DYSFIP1 38.2496050953547 44.0409178714232 39.7697110924474
48.9944486068347 45.1137938810047 37.9951997351435 41.0871827071116
42.1860813954352 44.9645354363967 44.7958122466628 42.6366765186182
46.1088006335399
DYX1C1 37.8830265336755 39.1982470626863 33.8015914967864
42.5269793679335 44.1070537445005 46.0867976914262 40.4359177791566
41.5817737564592 41.7627910693483 40.9506062495231 41.7272875295469
43.9541718254769
DZIP1 40.6559419616644 41.9455842688118 40.0998167098057 42.2496337895968
49.1263312877155 56.6172748471831 41.8751578329365 43.4885245598939
43.5669306063196 43.401782064778 43.401782064778 39.4830039788612
DZIP1L 47.3791873993182 44.4411734611229 41.6079958749402
43.074472921841 43.004943518025 45.1873487463472 42.0605906240248
50.7364620410977 40.2274522635576 58.7198210787055 54.7883681830995
53.1182952086284
DZIP3 42.0532901689036 44.6918600441328 43.401782064778 47.3574589211166
47.6884372645373 49.1013967533186 39.0458123429605 37.5511560880902
45.3756389170606 39.3698811593364 46.8954576545929 48.4535991392809
E2F1 42.7150227340364 39.5050383901884 42.640829914102 36.1562979552173
42.269054475455 40.9506062495231 40.9157352260901 45.4757965005055
36.2733735494625 39.7835513496873 38.079222289614 38.745552403895
E2F2 150.031554835542 143.653706909456 98.5746416750459 119.670948133232
144.578232267116 49.2050538150106 172.015856827154 310.860910973626
166.553853424708 39.4710743516517 47.1477056711335 47.5025821041537
E2F3 739.104939969224 318.690767783738 271.334774589829 418.927102569912
975.02086340962 193.15756263121 1886.05449875658 1139.81870068001
326.805803266857 266.323732730581 231.472806936915 276.399037769005
E2F4 271.393858310441 350.278532614563 387.311743342483 253.802852066439
213.204385896543 215.109108265052 277.164276391513 254.920345078558
322.229113548162 247.843927763772 270.89711217076 244.955741307927
E2F5 145.09364961191 290.535354193244 132.307107503819 77.3864518736634
414.062259640915 98.403191200835 241.31366702947 95.0350792557735
110.467254789988 59.2628513357357 78.7260936224802 88.2604063995148
E2F6 162.37682406314 93.1067450332473 118.467231612638 94.1680559848644
205.605413799808 100.017263238916 165.220069268347 189.650905999631
119.095094052197 96.6833301748676 99.0095802833291 109.795084617739
E2F7 56.3471340054074 57.6831182451733 51.1347974668251 53.593974682612
48.5000946421718 39.6251842352088 102.499011190932 46.5717043290731
101.954354107731 48.7414853386615 47.8988096575648 47.0130468198828
E2F8 47.1962846565261 70.2553917409773 63.3544794716167 40.5484717678957
57.0504111569238 44.7804080899431 45.8980298854198 39.6474008369935
65.2125527940945 39.3698811593364 43.0016969050339 41.4508241771315
E4F1 328.341232263338 372.982648892131 494.733185937057 210.73006676203
370.41288705812 250.866560791828 421.791344753995 240.887877769007
351.700618429121 397.798003824167 406.668753694984 282.646286144563
EAF1 56.8168526942685 63.4570909617664 42.2577689927563 80.7168944828052
56.2305155589239 43.5752393599169 63.928488869459 63.7002949745101
60.164635768614 46.7646877499666 56.6884575165011 60.7495043977707
EAF2 49.6241203903354 79.271901834289 56.939860285007 210.5650813431
55.4919197421746 64.7905331739366 116.555666104916 65.4714272652857
69.2001494999588 59.9354531144375 50.2203578628217 60.0940570991178

EARS2	66.0303950772736	75.0873720488265	108.512469038407	58.5406981871153
	112.60230393988	58.0095518210499	57.8870110456492	95.5822406197756
	97.4062608315831	81.0880833950735	85.8277891453447	96.0539709962851
EBAG9	161.553725645675	232.113067048781	192.356440003154	172.451522479253
	274.648263378054	181.772001042799	143.084029849218	88.9038123445083
	143.084029849218	130.058147569795	125.588228083829	159.667153030627
EBF1	55.5276281380084	59.0709442090059	54.2670850492947	75.2475238554276
	49.1928206164702	315.065866567211	35.7055410599146	58.676230030835
	38.9006788676407	168.7696164772	160.962078065068	91.8935361535002
EBF2	38.8336427948187	37.3634897565502	41.0718387273629	41.0726336841311
	38.8834538955	51.3886035574416	35.7681934515502	37.33030683349
	39.7162631015511	39.3113207075992	40.6373400186325	40.716553247236
EBF3	58.4920021605316	51.0175677516029	51.9214790114499	85.1847490113446
	46.6397391251762	130.865421554474	43.8206425661746	59.8760452220989
	42.8036731479932	97.2865629929716	83.0554837248934	60.4872675915085
EBI2	105.197692490363	205.051252996628	97.7620313090597	1263.76838179117
	82.1344514651393	221.195872577098	153.797431763616	252.243064327969
	46.2718838939055	57.9141744971757	51.4413099192899	81.066448172567
EBI3	44.0328992213727	68.6074516459969	100.305262467668	129.190555260332
	60.9710783023013	78.730129036128	65.3694267397114	51.555506218204
	42.8036731479932	44.6918600441328	45.310660704531	47.2131144508464
EBNA1BP2	889.638287212686	793.570993808328	783.440408023061	
	431.353025968649	1430.73068989193	582.017297217368	934.48498309932
	866.712228831149	829.152040996817	471.046851874447	526.750242877914
	644.733901442573			
EBP	157.06144940334	131.413578749264	128.996351661564	84.0899854321388
	146.840603547323	82.260686909201	187.656257079035	141.830305704504
	239.023368693467	78.7543199947688	89.9227845135785	101.156215957297
EBPL	254.983975710833	616.912306849151	496.163796158189	143.13189461696
	603.737787922337	393.944898808828	438.50123471613	160.264217082275
	443.863346367342	197.52820443061	230.117814622646	267.232315058148
ECAT1	41.6354649482853	42.6022652963326	41.9666541498638	40.0698728584484
	42.3685136297603	38.2293008890613	35.9058393060637	35.4526946152955
	38.3418933808844	38.1042454705119	37.0180934800573	37.0953622406687
ECAT8	40.148780650831	47.087835967892	42.8270038153139	45.1944222548351
	35.5220586859301	37.9951997351435	41.3594868055879	35.5798430594271
	39.0775870687758	37.5343260962758	44.0409178714232	46.7182294568603
ECD	91.9459432547337	102.304422999511	100.937651342589	87.799979434508
	95.4964717109139	112.60230393988	87.7844808613265	77.3367966395554
	244.285061669874	69.5853093594169	73.5527788467294	92.0269919454947
ECE1	50.6473771879221	53.3683962805281	59.7483827511172	63.9570596163747
	60.2886873195135	48.4758590019384	53.1884505015411	54.3820590274321
	54.7033747046992	46.8954576545929	48.7502645382856	52.2832912331043
ECE2	65.6914955077809	52.4518619184838	52.4485219671976	51.3064929551674
	121.668011907792	61.3005673792118	49.1067959632109	50.3972033014227
	61.940656536312	43.7707113107752	45.5055402477919	46.1088006335399
ECEL1	43.3638918705225	42.6022652963326	52.9111155236961	43.074472921841
	69.0975200641705	44.7804080899431	49.2546642180676	58.676230030835
	51.5888395924843	40.2112371512838	43.7585538437696	36.1647506311964
ECGF1	657.031980628955	624.954583057431	779.996851709716	2225.65559724391
	493.705301673753	492.902027204364	548.047774435177	843.333981146512
	241.052700961064	173.780433758335	232.147792636393	210.73006676203

ECH1 1163.0370991733 1097.92519921873 1626.71747158994 710.579735673002
1271.62760896761 1436.67510926345 2113.73880184695 1621.89443989657
1611.35913846618 1287.41527826233 1858.94179312821 1362.12587130194
ECHDC1 46.29198852834 52.7948184519106 48.8623729374733
50.1185085888209 50.7116312517035 67.5812474586277 46.8613212048981
43.9366081640576 50.3745056312199 56.6086202928268 50.5188367542048
64.650642183463
ECHDC2 1868.51164332659 1111.13061382818 1239.68399088145
244.560715635796 732.454837219705 736.405815842011 801.351998609551
1047.33749100342 770.715044136717 1963.91365010531 1375.14388134842
1411.12613289991
ECHDC3 49.085741080241 73.5534417023453 46.1653991089885
153.153205095999 52.9433495155796 76.4439037944547 58.5087296620514
49.8344973836834 46.3203995372658 185.139615829489 218.013913036142
63.2113624676341
ECHS1 497.290172759099 897.198663109723 716.434968700383 307.705431227338
897.198663109723 540.304257899761 4093.70002166179 1810.67209360676
794.806085783439 766.647626178442 875.787532301293 653.089523541803
ECM1 82.9875926750034 55.3477556906605 53.0630927250439 63.3006977162657
57.1935729219207 69.0530953932111 41.7614975630362 60.9801094225351
43.9204029310411 67.2811786080606 54.5711704180349 54.7953298048942
ECM2 121.149803296368 55.7405934254857 52.1062000766427 69.734208465775
55.4345579421782 180.349984862124 42.0605906240248 44.7044140979108
45.3756389170606 104.504444994123 65.7587943905649 55.6351010690528
ECOP 146.287473652123 102.150058925164 108.263706890599 147.811784706898
80.9160262677505 76.1991031349451 85.9179359178373 66.3558126170575
131.766252431636 57.1095070128372 67.8010199320754 85.4635831413678
ECSIT 65.0721641331632 60.9311907846259 59.5444359507702 64.6945454069792
97.0055689383756 91.9459432547337 104.341160312305 89.9227845135785
106.165518810891 107.987729161223 131.045615546105 83.1874704625702
ECT2 181.738902391483 134.646159826262 86.500678864429 94.5501397951415
211.829374574045 69.2685201884906 288.687391623935 218.531478946522
190.442443946259 66.0224755625662 61.1801493883012 89.1771576058145
EDA 42.2674195935107 43.0227387991135 45.4684895894515 41.0337778763237
44.9461972583557 41.2067319684816 44.7967171120305 45.7476095696859
45.4799270968969 43.9135824999529 44.4403335343562 42.5267710131643
EDA2R 40.3478825593224 40.7952353240579 53.4088637045445 39.5050383901884
44.7829632472754 44.2607789091176 38.941855825269 42.8482181313226
37.9273653326866 40.7063426028871 36.9060997041453 39.6352613956998
EDAR 44.0328992213727 41.554942319225 51.3268475761593 38.3166554965434
43.3332476888305 30.0771630269067 39.5496902678788 42.4580504587581
45.0997987980157 66.6332042895961 71.0369238830779 57.5372732453319
EDARADD 43.9309845324767 99.5058761858884 124.841811761412
42.2496337895968 78.8320129013551 48.9379556849582 80.5457814044192
72.0443425395404 89.8234189405239 112.760972238512 89.1632548302738
86.4937293696262
EDC3 120.837993273922 87.8681250426295 67.3469281984384 85.5959901987745
122.412295645862 81.5782824513693 174.179617483251 113.277043026153
95.2018660650205 151.175959542368 97.8825824657468 107.799236230743
EDC4 339.418989022736 347.552309199761 465.266372824212 300.935346370468
301.525915314179 248.924819383887 432.355300448283 572.968592371501
322.509777895373 439.241483765416 442.380936516984 266.439343650315

EDEM1 85.6170780640867 136.140128421585 85.9692990407066 138.895316905243
127.60426512782 63.8740412329753 72.2577390063259 64.7318794662958
89.7862835647016 74.4696354388511 72.3822545261582 73.408861359425
EDEM2 118.762101740563 156.65747550889 163.16724817696 215.645475520124
118.10944558223 105.814959179397 126.500059295217 123.410319693175
139.436084808369 153.901083585889 151.758051124404 145.267770598988
EDEM3 56.2971962699581 53.5893790024601 67.2811786080606 54.8412943008635
54.4586176399381 53.3949676275848 55.3141112947345 54.4602501503065
60.3597249466312 55.2998422481855 55.6494029744804 60.4358541520815
EDF1 1559.50766982801 938.151864971251 779.333013700435 1102.03940742413
900.517711546397 1411.12613289991 1485.68317899614 1043.26325848938
989.0602710707 1364.06060391538 1159.77262479002 1147.19483787501
EDG1 55.7246872330059 65.8082111065882 70.2577825186943 82.7394574977114
46.4343327861696 87.7460859694899 44.2166118829877 49.2901038327894
40.5912213523345 61.6670071424359 63.2684764441521 80.4404044229341
EDG4 131.277614500948 261.653821192385 195.168348967505 115.63300104804
247.676607756929 59.0686629877982 150.789911774534 239.070294068861
172.39907939484 157.121355906194 175.271140689255 142.585892496231
EDG7 48.3814421939888 71.0935990523782 73.6323065529916 42.0102671859606
57.9161791762225 46.6872654833579 45.1017494688414 62.8885806455128
52.9482338979681 50.7855742231932 51.2318935933116 55.7067742610401
EDIL3 49.7827734675743 41.6466169548846 38.6727842796202 43.9282059368202
38.3561682218911 39.132725377815 35.4623691645629 43.5544347011646
41.4636047919453 39.6719190054379 38.2795150354677 38.0452862258803
EDN1 63.3717459766798 77.5737637002275 65.8455652907556 225.088911324737
46.0977687311222 62.9688525319822 158.862722884436 240.696431900526
50.8485682149772 47.1001757441025 48.8088340735921 63.4685821260797
EDN2 72.0334593545916 44.1239711172417 79.3066495980045 40.1804147506786
56.4685270445642 39.2248354955045 55.2088411137193 53.3094843669564
124.915754138992 50.22432299342 45.8180710486329 44.3521099857368
EDN3 42.2618615571566 37.3179578037963 40.244271403831 35.249124243346
39.9283177178222 39.9397419279057 39.0788811357542 39.2248354955045
35.6970528713709 41.1222709786153 39.0788811357542 35.0668743708816
EDNRA 171.501864800548 67.6169355376864 62.3245608177257 127.986477405999
90.0942781313264 146.737709403779 46.9929836306816 102.626604917608
44.8003345643334 177.047048502104 200.318682162912 73.9192243586276
EDNRB 47.3289285415289 49.0186200069087 46.1859571883455 62.9247740568696
57.0504111569238 65.2014839770255 48.1069139823064 51.7941211802143
43.8482802038826 50.7237607003817 53.3185097662527 53.2340637435567
EEA1 49.9419897484381 60.6080172588224 48.8941062980393 67.7121534934164
71.8706847571669 62.1857038870283 59.9792284253187 56.6910078150745
85.1920126487447 53.6429962450784 59.2643906275025 61.4366256816323
EED 51.462029007278 52.8509301396005 56.446325212547 49.1786207920603
52.0583273941813 46.5557478175976 56.6537562775715 50.3452972893313
60.8635925649592 56.685925255591 56.8875898013449 58.2879293130296
EEF1A1 13230.9863405074 18219.5845257111 18219.5845257111
13230.9863405074 11410.3307451176 18219.5845257111 11176.402230735
10617.4323014458 12125.3293116195 12770.8282719641 16661.6081002448
18219.5845257111
EEF1A2 855.602941621804 1055.00793806075 80.8400209263587
56.2508140932179 3361.67247904403 472.982369155078 92.8579489575841
456.757277476069 381.426926151034 516.704027135707 950.740991182127
378.626656077546

EEF1B2 825.558929673308 1031.4414268329 726.42773300581
1136.88710104691 1266.41536472956 1320.7894611262 758.462554576949
920.504890206997 841.850722284484 975.02086340962 947.656220635965
1195.24078948059
EEF1D 730.168814097451 684.865133028228 571.790034754076 665.122091380755
630.53864514662 1313.92053019421 610.678132881994 519.351335801817
555.044139654886 688.168272082335 1059.63540694644 775.931494648677
EEF1E1 210.096514889445 186.724171695644 128.146059846414
124.611407530723 366.228100574684 105.317399386109 163.995560004001
111.803238196297 254.425072964926 87.1434190022564 110.973625869849
164.511418506192
EEF1G 1888.71160604243 2448.64580552439 4344.25847766877 1900.96666834569
6356.05807792831 4168.41947709165 3263.05269150231 2581.34849558524
888.56944380788 2036.72286752819 2122.39240932934 2830.73694055758
EEF2 3575.78813099057 3749.78281766196 3399.75820494572 2745.8277126123
5636.93532853955 5438.34345794521 2348.48320928245 4168.41947709165
2689.80835574654 6356.05807792831 5074.07305640514 3653.59181300011
EEF2K 158.156217755244 175.673196925527 289.05213385509 203.759116151774
235.593721159442 561.509463439533 205.991317474325 372.525928228911
260.15500351311 534.056256675345 360.523229691604 308.728126648231
EEFSEC 44.2350046179299 46.1569135229922 46.5650556481721
44.4367387274922 43.3332476888305 44.8872784170036 50.3519150282938
51.7538628098347 56.0496524902806 49.4006068471301 52.5930185400362
48.1627097465883
EEPD1 57.0006212637829 49.4067286971348 44.1452943804044 62.3936376596431
56.359578588344 73.2751502965172 53.7423898357583 54.2877981508636
66.5298067946102 56.5379973844443 58.4816190076338 57.8111054584154
EFCAB1 38.5648843914189 51.119812302881 49.3780606970538
38.4208429103799 42.3685136297603 95.3300728806601 51.0013344286851
40.2382908228517 41.2870505273664 51.7594352155304 49.532546810992
51.8562635270111
EFCAB2 39.3709357615493 37.2810767789306 36.1042012504758
39.1706564265495 37.7576900881741 36.6271061451613 37.2892641289258
39.3709357615493 40.0403898620771 42.4594149893798 39.3667161654772
35.9604262434141
EFCAB3 39.7576136507593 46.7591817286632 42.9104454767916
47.9915414951747 42.0709558258414 39.4953494583071 43.5627649099623
41.7419877611072 44.8997235283254 48.2596564318071 44.9713479185144
47.0130468198828
EFCAB4A 457.681756480301 670.271164195182 785.79878416775
127.789065386943 468.153823329361 139.758805241617 347.908556198025
421.279008280368 498.284928805673 291.867370164212 327.631923082696
179.874652000664
EFCAB4B 49.085741080241 35.1224289925572 42.640829914102
39.853175885492 42.9090346219023 37.9951997351435 51.3064929551674
45.8669209716694 40.0403898620771 45.7911932481722 41.8168470823451
44.9070250604284
EFCAB5 35.195364621675 43.2028319630577 37.9030782766955
38.0365987358005 41.2740822675592 39.1650142708257 38.7803273497182
39.6474008369935 36.7267557061938 37.0698056696061 40.3452320167286
38.2795150354677
EFCAB6 41.3708420203838 43.5873188565519 41.7442113152211
47.481302504066 39.6137246345257 43.2805590023412 38.4428081526233

42.2577689927563 40.2669497398702 46.824299585607 48.1352470945488
43.6753843488695
EFCAB7 46.441301934328 48.8870563092695 42.3536549114165
51.6869155634183 46.0493141231657 50.6915708372926 51.5967098638347
47.2864663693787 56.2441895183416 46.8954576545929 45.2265319826548
46.4499258373233
EFCBP1 40.2543341073001 38.4330755397458 38.7745734037492
36.264823544143 44.8897737954573 106.486195938039 57.4047331041592
39.0620487644635 36.2733735494625 43.6033221838149 47.7535797917441
40.0557788909923
EFEMP1 118.854313907627 82.8652108020571 45.989212296035
93.7260389087192 56.7784166497706 641.462632112151 43.0269218496197
63.2068062699831 43.3118137410238 250.308866488702 226.168990555083
268.144525192544
EFEMP2 506.585006700107 239.229093340475 161.588263691127
256.941243394182 270.303708972854 729.755556260103 241.486113826383
167.25176386043 97.0348634204639 380.015934356432 349.767857026458
267.024267633341
EFHA1 222.336600180634 280.920208331594 268.38649000825 324.658458599373
179.874652000664 241.08817915181 263.330471155421 239.838643553139
216.782658096982 350.278532614563 236.791729190996 324.994911416877
EFHA2 40.5235471987417 43.0227387991135 44.3344900481112 43.8495248991589
43.3896803974186 40.5805725503899 46.0228732507872 40.8833572570383
43.2163597439147 45.0155543555707 41.5668884168669 43.9366081640576
EFHB 43.632365631458 43.1464706017434 39.991353369336 50.7046706880325
38.9919142475596 42.9695585740813 42.1157692936428 41.6916508480321
38.9006788676407 45.0864985243943 46.4753015651202 39.6833800000587
EFHC1 73.8374823243007 62.1642339365777 67.0421310720934 61.7502624458447
57.1935729219207 61.8187341713197 53.4921736109774 61.2343203799193
58.9870089759222 72.6549171886783 55.6151308605445 71.381069076765
EFHC2 35.1307934330047 42.8542642792862 55.7764940944098 49.1786207920603
38.2496050953547 50.3245489035833 39.5496902678788 36.3405039629059
39.7537274655115 46.1390887492649 42.170702295259 36.449101775775
EFHD1 322.169157552725 96.3918711895208 131.586389151079 57.4595416719581
92.5135737873857 273.108672916862 75.0665411373616 63.287067794195
45.3756389170606 130.261747462753 114.979124960177 183.286063142727
EFHD2 1103.90449800536 1442.72006787259 1247.50618540769 1824.79010714035
1319.32406112346 686.154904023493 1896.06380611235 1907.47560253019
1656.16581692717 1480.95754707554 1398.21942794938 1144.34555072807
EFNA1 432.355300448283 152.187430924861 307.445116836976 100.168016285684
227.404530886674 87.8967913380047 189.80672643731 744.080606078086
529.650395907046 594.184150517102 531.36214636517 794.806085783439
EFNA2 36.5393857306744 39.6123051184545 36.2683886571585 41.6564075217517
40.8873907150245 35.8227939208521 38.7337086062875 43.4544762830742
41.2870505273664 38.8507804383261 34.5633623842083 36.449101775775
EFNA3 43.6706764143908 38.0226905622357 37.8386378737561 42.5664883002833
38.745552403895 40.3357777530419 35.9058393060637 36.7460922671619
45.0017158586743 36.4651112441576 34.7308008643142 39.0536056496869
EFNA4 133.067727272864 80.3060865378414 113.454378667943 81.7967230859562
143.913007288353 74.3729155936862 131.738693662183 105.589475270494
116.76301232629 79.5488891958168 77.8220670093392 81.4165540307337
EFNA5 33.5994525227062 42.5269793679335 40.931294690669 44.4367387274922
41.5668884168669 44.3648993568405 44.9863726205428 47.2009420656854
44.8003345643334 45.6868589181768 39.6403089864678 45.8546719310325

EFNB1 67.3816715975056 89.3342384381684 209.31241212773 91.7068354379889
95.7639712596394 79.863426244511 107.466490458133 90.2915974511786
217.875223856197 226.456072107079 196.267504470029 165.797721197832
EFNB2 541.105845300009 336.679355848349 619.950241162173 181.814283147675
243.403622361932 129.190555260332 226.785831708938 349.83809766755
158.998384690038 100.413031488199 126.682296930289 96.3918711895208
EFNB3 55.6268253440479 36.3110701990588 42.4580504587581 53.3967242431461
118.885205874725 44.0804700754786 73.6855877240541 52.7787010032051
100.178375869002 47.1001757441025 87.8967913380047 44.2527566343661
EFR3A 538.427311162325 502.67939538729 378.795484311455 594.184150517102
230.554867472663 573.394745674984 168.561260174501 222.985386635178
506.585006700107 454.02808608679 502.435496018532 477.263677441074
EFS 61.4416707689893 149.213844580441 99.5125674355716 46.4555908533915
92.2001835784782 77.2834482217973 100.499763255098 48.1591264215184
114.230358276599 90.3439862484238 89.2185019250322 70.6903168772326
EFTUD1 78.6911549149983 78.4692960800668 58.3773276500015
100.248038733326 72.0674952635945 73.4037256268744 81.6713911430463
79.9126192278104 86.3185927557402 85.311632806912 79.4832790698673
103.199200862158
EFTUD2 630.53864514662 472.982369155078 428.958127252533
397.850286751468 726.42773300581 263.096895937991 578.601842980701
583.421166842011 653.089523541803 418.927102569912 419.360641528378
280.622184239624
EGF 42.8036731479932 40.9731067463382 37.6557714476853 50.5310598656998
42.7107928951766 43.9724628401189 44.7255207589606 38.079222289614
40.3316153483289 42.4594149893798 43.3085358214154 43.2776458914333
EGFL6 98.7869013773721 83.7019398654324 90.1284518302601 71.7167037224069
74.7557519831859 85.2260422401241 50.0088611244948 63.664265793911
93.6464901357407 56.284748518655 48.9906383294199 48.9944486068347
EGFL7 53.7079631941275 55.6323803479191 52.9111155236961 46.7577982595088
48.5000946421718 58.4984917725442 43.3262148375525 43.6753843488695
53.2735797144627 48.6124127998952 57.115361349315 46.917149434588
EGFL8 48.2910530887214 41.8073910693411 48.0191002461099 35.9521941716188
37.1565984182084 49.2050538150106 36.197424053556 38.8495095797881
45.0017158586743 39.4710743516517 38.2795150354677 40.0557788909923
EGFLAM 69.8712342353417 70.8687598033783 115.530384771495
80.2979179444897 88.1861828863404 65.0774755750423 42.5750559787101
51.1694248620208 81.588696805606 47.1001757441025 50.5499559337071
44.696023817801
EGFR 76.5505585423421 58.2957814921605 79.2525964926483 40.7910160811527
99.2898960399974 60.2528550551807 59.0457625163577 59.1533197797534
68.8353800542132 68.9833517108866 72.5559426064896 98.9657264176664
EGLN1 74.3135387703106 65.8082111065882 62.0008474085033 82.5279746006378
86.033725825968 74.0995052892511 66.1940959364976 53.4109882045447
96.0838701986116 42.9866214102525 53.4088637045445 70.5322939116406
EGLN2 314.281125546552 220.337850438943 189.451890957713 330.096171705212
330.814531248695 278.303493506139 332.322497133752 304.777565078155
270.303708972854 258.589426470951 240.217589414972 231.733564500645
EGLN3 56.2508140932179 46.6996902348633 54.3554233228353 45.0798408927912
46.4343327861696 47.8636003588181 61.3620062177524 51.0724630072723
76.1869593072029 61.1851254827343 59.0537479001842 70.8546350449171
EGR1 1888.71160604243 2816.7754199004 266.634368304904 1260.45604038487
277.993522959259 2045.24530722676 1234.59589464045 2791.75465171135
369.032450063274 700.477686061791 1718.44944271916 438.681666410245

EGR2 349.767857026458 434.523449093691 52.5353664902258 296.753500238226
53.3086645804786 392.51606894011 99.3797043236938 280.117410663595
82.8150137756969 56.1912096308988 55.9269493191353 49.0655215909632
EGR3 54.4140381300469 63.1137861754989 48.7646976789783 76.0975301977715
45.6895223823001 61.1751736985663 45.8980298854198 58.8477194579185
57.678188678075 50.0564735608363 52.6960621424156 48.0080291106034
EGR4 43.3638918705225 40.0527893189096 41.437237454134 37.7469621225553
35.3327761997046 33.7818132446042 43.3262148375525 38.5457716679071
43.0930293831497 44.1379638573455 39.2542963855807 40.0557788909923
EHBP1 160.015674121695 136.377736482671 196.760671234837 109.310200749289
212.99813190918 489.622327794168 112.805898501551 165.640362974584
127.49459343016 208.804071011518 184.048982286364 204.554792089177
EHD1 457.202737754842 331.087219418047 333.421080658854 979.468753716758
725.680256063016 407.818713538416 605.970682363975 686.154904023493
273.460165751162 403.796135347069 388.920739791844 312.795596385258
EHD2 66.8544766980562 45.4882438975269 58.4137028673494 73.3725472767369
52.0583273941813 66.5234828659154 40.3302268293707 45.2806199505029
48.9931786790166 117.033529761587 81.0562186907518 73.2234405348435
EHD3 38.1169703906207 46.4861258030402 41.437237454134 40.0698728584484
33.7494761745342 40.040778349137 40.6750540874299 44.0390019242862
38.3418933808844 47.3791873993182 53.4921736109774 39.7411455670172
EHD4 332.230531176052 556.417356025042 342.829992044257 468.98857415608
515.216890329893 241.599353653369 664.453262499828 919.542051089664
468.98857415608 522.49156295512 494.162646055732 375.757961593064
EHF 76.7820250461545 389.994289973076 381.31252208432 40.1804147506786
306.578554914328 80.099836448859 309.102633242573 288.508774004052
444.937258895152 87.9206275762349 104.486555491331 262.624624672404
EHHADH 54.6235981131919 98.2747494745452 75.8201286658785
58.9168466928565 69.3468840908665 59.2643906275025 88.3335649692467
63.9110569727542 83.105117076029 48.1392888470269 59.4188882103773
74.0913177754682
EHMT1 53.2282811950376 52.4554584270038 52.5353664902258 50.4397478643191
50.0580547772867 51.0988785553434 55.6151308605445 47.2009420656854
49.4275543103572 52.5909491535773 53.9262634476065 48.1002535934643
EHMT2 56.227813871963 54.0052948715814 53.2735797144627 39.1335009052403
62.3862792065884 47.417512242919 53.6294062560285 51.0724630072723
59.3164315859919 58.7198210787055 56.8760943789568 42.3152792870779
EI24 303.93332785291 561.2769907009 499.744856832174 172.540737848092
754.700295017212 449.564281713066 518.936896882424 353.751797948675
455.774407260725 340.311633794489 467.645011859047 379.79657860938
EID1 59.5000730792772 53.8499082625091 45.8844325913247 55.2214187655851
58.2780916793348 93.1067450332473 45.3597029614942 54.2877981508636
49.0986627051784 72.5153335566211 57.0154839296649 58.2879293130296
EID2 93.0714382925187 94.5261917871909 93.310557793228 72.4834467838798
138.895316905243 104.059057437473 117.706922397135 78.0020909283414
127.115038236278 62.9205616877344 67.8570354010492 91.40570426491
EID2B 1266.41536472956 739.541009338849 792.022171185649 1239.68399088145
511.792908607692 522.811631783729 1051.58050264114 1457.72260892651
640.407302868904 1640.74027400791 1466.88103496067 1069.3975598703
EID3 39.0620487644635 43.0227387991135 36.4564050256915 37.29566077996
46.1804647841331 42.9695585740813 37.2892641289258 40.3357777530419
36.3649677956953 39.5637454855233 46.0095710163378 40.2669497398702

EIF1 936.39727542747 1421.44237064813 746.252816568691 1611.35913846618
745.219025960188 1796.47241860175 1163.0370991733 902.691776563423
1114.15989325456 1239.68399088145 1473.52943897204 1776.78784112613
EIF1AD 38.0089018744739 43.4396866157171 44.9758339877851
36.3604951341921 37.0526522140242 38.7340969861754 39.7576136507593
36.5393857306744 37.9273653326866 42.8936990772838 37.8372168909583
41.7575097298927
EIF1AX 61.7551416187554 71.8156159183499 68.6704961638492
60.2709072709358 65.4589642146912 93.1668080716761 90.4160198134865
74.1686961093736 76.9535968101411 53.1993028936883 61.7572675712557
68.6788460999238
EIF1AY 78.7450149191851 168.561260174501 41.528370959196
227.317566600324 46.0043807840705 235.075347885884 238.584514797019
75.719886024949 119.45528139824 164.373677562376 150.262748333939
234.232348771362
EIF1B 121.204795052893 132.946528291039 127.694844372277 205.876395674271
381.426926151034 250.545098051392 144.227861902786 107.232979387172
139.905295316081 106.418690759491 163.280931300437 296.271895016431
EIF2A 292.389429869034 471.874412428677 485.698327133705 465.887689708274
492.710488250721 661.718946652592 400.760646547012 334.349204174886
319.408264906288 342.559364715094 393.337170271481 669.597878196875
EIF2AK1 957.682898193927 920.023344692409 1130.2486883492
566.318259999405 1621.89443989657 611.776512504215 1122.44489338159
1059.63540694644 1598.78859278229 861.729405234231 751.150916058018
725.680256063016
EIF2AK2 230.975122271137 402.794855141389 335.943994595615
338.846080983216 234.873589896708 305.119894105297 517.216393517181
797.019657306322 175.619398238068 248.414210905199 441.976123154763
363.002913525646
EIF2AK3 50.9381343674699 59.4908919662362 55.3812135024656
57.4595416719581 46.7508829977587 51.0506441018166 48.6395795163502
63.8008894786105 60.8635925649592 76.4439037944547 70.7045582269595
65.1251519077199
EIF2AK4 215.68113524702 130.35828139117 146.658116519622
216.744244428166 136.04447103201 109.738537924214 176.839214683647
358.194667053419 143.894266185526 242.966185793816 234.615033303459
229.659465419141
EIF2B1 252.535061916875 271.478148993789 399.80505620721
236.851068206869 396.261110334324 340.311633794489 300.412043023998
192.058030454066 351.039814818516 213.590067519021 264.407051461506
298.089522641269
EIF2B2 284.013140015092 158.349509235411 226.567000755017
256.55408314817 369.258587948416 253.072560237983 196.760671234837
183.66684597416 258.038442651578 193.01447006167 185.54813083393
308.183340806373
EIF2B4 456.757277476069 301.594300620246 354.634819362978
270.922452389109 367.451332073134 355.495119544081 359.296873000994
593.345148568451 316.419421649196 455.444228161475 365.860501246879
318.690767783738
EIF2B5 163.877163712385 174.609448758071 258.757703729905
166.304210248623 279.632025097952 136.711122812062 159.667153030627
220.054040993848 204.200566808626 293.410413883219 280.202284365004
220.010492114456

EIF2C1 117.76002524375 92.5391033814713 97.4515987591398
104.99788782377 87.3957386451235 107.309776354951 120.96329303892
171.205726660714 107.222176166001 125.114613377133 101.540333720181
102.163854544231
EIF2C2 204.039190380778 326.70962514228 147.424598105182
369.032450063274 297.475572902965 204.477547372897 419.46223841313
254.519518210024 147.405190988088 164.917385239962 247.246045048229
183.364949777567
EIF2C3 45.334106390212 48.5905518770847 50.4662238169527
68.8167148906225 50.8875036861067 52.1408852421186 57.5955320223035
74.7794253940974 55.5086026637973 53.8260140375214 53.0419743379342
53.1491634301682
EIF2C4 43.3638918705225 53.565717255469 59.2012779686869
59.4674205670657 62.1836961153924 59.5979861826984 57.2758638871082
58.676230030835 70.9462008788871 68.1674425673171 55.854474649572
73.1008013275549
EIF2S1 81.8104679118753 53.4735821194294 57.7415362494584
76.0975301977715 63.686652234709 86.0630164348952 74.9059650336395
73.5950442915143 84.0899854321388 79.262977183608 72.6162848018047
71.8539743926881
EIF2S2 75.8976034525022 70.8546350449171 62.3936376596431
46.7577982595088 77.2608629053098 79.6895408438974 73.0433648756396
50.9933795450314 85.1920126487447 71.9124897002253 71.6849360818041
79.2525964926483
EIF2S3 475.159378115819 1071.64675274806 766.647626178442
625.805967352327 729.342532316666 1382.25775340179 711.773459577386
565.411298018845 781.923984640049 728.257836227637 929.862002014478
935.815928529014
EIF3A 141.255712367708 162.000275136397 136.236041346288 223.826005691932
166.834334710682 194.355765743782 178.306763765612 208.3497732496
129.252597914828 261.569542242954 199.035083736742 272.545033107746
EIF3B 1330.19215036764 994.404707955031 858.228943026906 663.372296297652
2167.91218703382 1033.99699402259 1236.32289322872 1194.10154988793
1578.32969650385 1132.19026676901 1103.90449800536 704.15545332905
EIF3C 59.6008106247114 45.5842286511645 51.9885249508283 43.4410741327254
68.0414563597154 59.0686629877982 39.5496902678788 50.9933795450314
52.9482338979681 59.4969799685856 53.4921736109774 51.3244198342335
EIF3D 891.080174768895 790.786577596971 1057.44729389649 876.959744461441
845.41973083225 1135.43644148537 1149.90692951795 1223.01485352248
1219.52011418558 1428.47642890543 1301.07436212673 1197.13566108661
EIF3E 3999.32180022337 4632.08655821299 4015.88651424568 2498.54878472152
2331.12401106076 4784.36905964712 2488.32876359853 2755.23573327276
2440.77408185413 4406.55576617324 4368.11345948444 4503.77675488367
EIF3EIP 706.914069768466 804.592753722875 737.604036996153
657.310970474676 844.431085166353 2013.08489754172 583.421166842011
537.841677481011 899.223276238109 888.56944380788 1245.45371155527
1490.1549980903
EIF3F 661.269882993465 1470.84169670955 1287.41527826233 875.463242737778
1516.33074288338 1529.52516575545 734.705859548078 816.746599467159
1178.52115916913 1136.88710104691 1012.0373748933 1421.44237064813
EIF3G 657.936229435025 1058.61208471961 1310.03307063841 865.63777294494
1519.01001038342 1296.28036613191 798.996494242432 755.106456358949
947.656220635965 1111.13061382818 1401.46625360427 953.143041145545

EIF3H 999.862452730252 1062.93431683428 755.852629128252 604.951648398924
1223.01485352248 1369.13346566895 471.297503316471 494.300858189605
817.631505579476 832.181997440212 850.205634919459 981.039763059681
EIF3I 500.794458547464 672.515393219419 875.787532301293 576.140066807763
1219.52011418558 654.682263833024 675.832363405287 1007.03747721563
801.351998609551 472.982369155078 619.950241162173 877.316304736545
EIF3IP1 42.0532901689036 41.0678614026819 38.6727842796202
38.3166554965434 41.3755760434934 43.8582747295766 44.0687697278114
42.4580504587581 41.2870505273664 39.0648733099061 49.1067959632109
42.7782216342806
EIF3J 97.1271615802556 102.605473054584 77.3367966395554 101.867388894697
143.399636653204 126.128760255542 95.9298109273155 118.075168790221
104.220256076979 73.7133884699889 77.8669914727921 112.316483867372
EIF3K 2374.1766901821 834.14766574977 1020.75813028358 910.898566308567
1738.00955642263 1457.72260892651 1303.33517942343 1282.9181829891
1473.52943897204 1232.7632290973 1271.62760896761 1178.52115916913
EIF3M 209.038077542654 398.43592055172 397.94989372796 275.096637886925
356.628599887098 370.495645380436 222.01145815859 260.232090974645
354.38923772101 212.00247895282 224.871193467078 500.794458547464
EIF4A1 5018.90384962952 6258.22561934986 6186.45345364665
6258.22561934986 4784.36905964712 4299.4451139256 5855.6498679729
4550.56817942699 4200.38495514552 3575.78813099057 4216.69417265779
4259.50675863393
EIF4A2 2892.02001412369 2126.41018877615 1824.79010714035
1337.53997230271 3217.83121827744 3361.67247904403 1580.58484708669
1855.0119962662 2337.7738990994 4752.94498024732 3838.19449971537
5112.4354472796
EIF4A3 802.650020720921 1001.99175009124 974.296760034984
1413.36279821142 1459.69929955338 724.881002237334 1578.32969650385
899.223276238109 1343.66876893729 635.050535522135 613.060733526013
738.059797123491
EIF4B 958.473187253938 1379.51119430451 1245.45371155527 943.73554571528
853.464809072841 2101.59244895554 601.611039798626 1285.49870339222
1205.82775512007 1524.01520893763 1839.09068171097 1963.91365010531
EIF4E 42.1537476443423 42.6022652963326 38.6727842796202 41.253645578517
41.6383327669122 40.4779565600984 40.8617699346501 43.3729486804084
40.8503192605024 41.4397539024976 38.5306320917724 44.4004151500264
EIF4E1B 42.0532901689036 40.8880530359822 37.5597671487621
38.745552403895 43.844855336215 43.7630587657205 39.2533668167483
40.931294690669 40.3316153483289 35.4990901121893 45.7328341667151
38.745552403895
EIF4E2 217.065944053174 155.942366087388 136.766779297051
173.013258797349 167.746168796779 149.691911022686 133.487946450906
200.019523376661 288.190015621249 106.752660061588 115.962193866339
163.20801480093
EIF4E3 85.7983996395757 112.714318425036 92.4896666046729
147.811784706898 54.4586176399381 324.658458599373 156.770274308736
101.01459505339 93.310557793228 153.42135363926 152.72007888133
188.272588632909
EIF4EBP1 51.1740855632044 55.2182004838121 54.0536858212253
51.0175677516029 45.4751076037511 58.9676332048764 66.0350709576651
54.8412943008635 67.235655687817 50.4234835272833 50.3876754565799
42.57175366709

EIF4EBP2 203.913062066149 188.042978085327 176.07173250529
185.165420581989 193.094756585577 211.897320355251 200.698372627272
259.958369064691 500.794458547464 236.374695859748 218.917221711155
255.986778613019
EIF4EBP3 43.0001432913058 51.909968572392 46.02045129728
50.6262443356159 48.7847528954724 53.3185097662527 48.5544398725959
49.8344973836834 53.9634127270611 59.9876575158981 54.4140381300469
50.6141827957642
EIF4ENIF1 115.894467332637 143.068901273486 121.120217132666
106.035435807364 112.990752518897 167.319747725488 127.588471566929
89.7184505543918 140.288771064502 143.563236821003 159.022432105333
148.312100828662
EIF4G1 346.621778258188 362.506098778318 359.465715235071
257.662291702982 590.709061039001 360.962191673108 570.204236909398
452.969595001684 440.93348620369 310.358667418222 471.046851874447
288.190015621249
EIF4G2 1152.55315876935 1712.55038816005 1683.24873205783
1090.86617066541 1411.12613289991 1249.41227924272 1114.15989325456
1465.08435775014 2232.61002288121 1266.41536472956 1351.49940447914
1478.26733379357
EIF4G3 156.382146982116 145.607100979838 187.862483922786
205.876395674271 203.334053441063 169.554408876006 180.902423012987
382.872356030213 170.150808455621 143.706880936186 177.460053912279
206.857208722995
EIF4H 653.089523541803 829.152040996817 874.419252731128 719.815240381415
835.171475474151 1030.31910195147 698.105755081829 624.097858948785
1213.3477481374 544.355979695242 677.481235695517 705.283979182062
EIF5 64.120219344545 80.4822328865 134.489077146096 139.768680518677
91.0327980861321 154.166621635012 129.909461876076 87.3634135323569
87.8724689898823 66.9218519793499 92.0711167984454 86.3458707176254
EIF5A 1061.61186520958 443.863346367342 628.403814060213 849.412637017524
1191.93354266135 343.94159167652 457.097617649604 456.467726693355
1067.08316477623 267.657143781939 883.06773454117 492.519023728038
EIF5A2 52.6960621424156 44.3977035913759 48.3847843498476
47.2009420656854 46.6397391251762 58.9676332048764 45.223075311113
56.7569186600983 44.1211971585961 65.3672880026114 61.227809931332
53.0446815750355
EIF5AL1 46.263826584899 50.164201758929 54.9071823326024
49.1786207920603 43.3332476888305 48.1591264215184 49.1067959632109
49.5185112777773 49.4786466722535 45.0155543555707 47.8988096575648
48.3648494350286
EIF5B 147.719172115976 130.239763076999 123.077231445204 120.980558571109
212.688298502868 178.092166249096 143.152895211088 181.093943398293
145.28255999556 128.749209040345 117.439175394651 144.500148287951
EIF6 351.439404852971 507.307494640785 406.500583302121 237.161912299581
446.162602155964 254.82779318297 688.168272082335 476.421959423835
594.611678976716 340.553073657744 353.076989769287 300.703401968169
ELA1 39.5676051939342 40.3384318592985 41.437237454134 42.8235313007746
38.5512429290626 45.8325181856483 39.0458123429605 39.5466975353131
35.7973685350741 44.1379638573455 40.3452320167286 45.6692241948108
ELA2 43.6706764143908 39.0894172982439 41.437237454134 45.5869891570251
42.4864576619427 60.7488007939604 33.9750800030909 40.931294690669
42.4540072551568 51.2318935933116 56.3106156221316 45.8546719310325

ELA2A 35.5179217192467 37.4540772310118 36.178547300352 39.2780515621926
39.4693871570028 39.132725377815 43.0930293831497 39.0620487644635
43.3744503319109 41.9666541498638 42.0750889188255 38.9496515344837
ELA2B 43.6706764143908 48.3077814647455 44.1452943804044 42.4580504587581
38.9496515344837 37.5887129572848 45.7452456977857 43.3262148375525
45.0997987980157 43.401782064778 46.1673159982664 38.9496515344837
ELA3A 39.4250105372135 39.932537801639 40.8420925848403 41.4727452533295
43.6587492470348 42.0847226073592 42.22763273471 42.5435283762933
42.6762317063737 39.6251842352088 41.0476065156912 40.2669497398702
ELA3B 40.0557788909923 42.6939394537076 42.5406888281114 52.3004056941891
39.7568367479301 45.6129956328415 46.8613212048981 43.2551508830106
44.1519466943185 40.8049408996274 40.9157352260901 47.6938628797168
ELAC1 53.0290605707162 53.9275079525238 59.6565330440648 51.3851157261451
52.0583273941813 51.4931742685391 47.8947595316832 45.642304356051
54.998510746958 40.3235655331796 52.5930185400362 47.3172462673426
ELAC2 117.612004923867 174.275409358652 139.08163074096 115.913030448537
157.774096599452 161.65846687852 212.353676518748 141.055218279065
179.953662481428 149.933827818309 167.824386984618 127.694844372277
ELAVL1 86.6655289455628 92.6608453151206 63.1450855271137
44.1483976313959 115.408418935236 53.9517841438975 55.4004611309636
57.8765910878975 67.591570323687 79.4916415027015 61.3529667465225
71.2491135258076
ELAVL2 38.957687788868 44.6918600441328 42.640829914102
44.1483976313959 40.148780650831 43.7630587657205 39.3585350448719
35.6825474397235 39.0775870687758 39.8873706855774 43.844855336215
37.4554876386402
ELAVL3 43.1267841876606 42.2577689927563 42.2577689927563
38.0958223740111 46.1513019317765 43.0177448037924 37.7136011474903
42.0847226073592 42.4540072551568 47.3010040326346 45.310660704531
44.4475838947656
ELAVL4 43.7585538437696 40.148780650831 39.2707964550441
47.0874209746031 41.467005949568 45.1873487463472 42.0605906240248
53.9465584609317 41.1742244762318 41.1222709786153 44.2925355984056
41.6550823405646
ELF1 459.182372959714 556.728676214581 529.977491403949 838.974043046676
322.678762360476 342.475405815033 356.318517117856 255.284866561352
425.895919304186 464.671881584567 513.57613902662 571.170643523692
ELF2 91.7761668960824 114.321083441061 112.977333058979 130.515102593803
120.721482177994 144.757886965525 121.650910373947 80.7664399131413
129.179537795204 155.730834354163 127.232160404726 138.859992681388
ELF3 788.232434009173 1296.28036613191 1183.62228577053 64.0903425729408
806.827205415602 128.414712393554 2672.94421680196 3253.86278287661
1907.47560253019 1001.99175009124 1211.8832855325 1850.06063216244
ELF4 156.242688079359 471.59454368268 362.724027984972 282.035361446993
506.672059588135 148.683947515074 323.906340044066 364.962649416567
355.495119544081 316.373650015333 233.850715388018 269.361536809057
ELF5 47.0940613507321 77.6653462778309 163.280931300437 40.2455972565968
69.805066579191 46.6872654833579 136.979357314656 730.168814097451
245.439289067575 187.923050034941 198.682422442825 107.799236230743
ELFN2 123.521401665865 38.0226905622357 39.6451483836089 40.646702696033
43.4544762830742 45.8325181856483 37.6036146841933 43.7396619462033
40.6957787921451 38.4187686307607 38.3684202018524 39.0536056496869

```
ELK1  227.535855856645 160.973602503707 143.383133346874 207.087723777954
      191.454637685756 160.459991480576 254.756894822368 196.389446842567
      229.659465419141 140.276520383146 179.552011806692 118.376686726902
ELK3  43.0930293831497 48.4497719907777 48.8100349749783 46.3203995372658
      51.940083408061  44.8872784170036 44.6032218589199 45.3872406432794
      43.8482802038826 42.0621425894624 50.1649089793069 44.5432192783161
ELK4  41.1790463122372 45.4737296536645 44.6032218589199 42.4061338151892
      49.904979214379  43.9724628401189 49.1928206164702 49.1263312877155
      47.1079792035761 48.1976189962334 45.9104478407175 44.3927670230328
ELL   109.029196594263 127.82585786161  103.64431747876  209.89649461562
      171.270750760376 149.249181701692 101.46584765758  114.230358276599
      112.014137492029 102.294142438313 126.079949368634 104.02653369229
ELL2  95.4841914842335 88.5706300816555 122.748811531184 364.141443387516
      70.2565873027242 97.4062608315831 69.6301237585571 104.351256270039
      110.219274023648 77.3367966395554 80.9821967801845 106.356074933606
ELL3  67.6767845246581 43.8495248991589 51.1347974668251 41.8778873678551
      63.4753448572245 58.0910843932181 54.0170817319522 55.5578334877425
      54.7966692984192 53.4109882045447 48.2050207071554 39.0536056496869
ELMO1 48.8333989709933 52.4212131710421 55.5567699051046 101.008449241109
      49.7458652239071 73.5161632100822 45.5506835119207 52.5481233846246
      49.532546810992  47.3010040326346 54.761682143135  52.3406235100961
ELMO2 114.493869251904 100.300863150751 114.674541610026 118.762101740563
      87.5074297754031 116.35075921851  97.6552689677199 84.3995736970187
      95.6731932757489 114.488672372067 109.456162739433 113.367264677544
ELMO3 199.54796711653  204.370291548631 369.355222586283 53.3967242431461
      102.170521765252 51.0506441018166 182.219366179899 226.767370335792
      234.308728996238 252.583117397109 212.803471204964 221.450737998571
ELMOD1      42.2455707947223 43.0971787292711 42.5406888281114
      47.9915414951747 45.1137938810047 44.0804700754786 44.7255207589606
      42.5435283762933 39.2809305246091 42.785429206422  41.9908237694988
      40.2669497398702
ELMOD2      84.8139090643412 74.8071157013401 83.5742879296932
      114.876346000655 92.8644908993284 60.9822577063534 89.1012752492647
      105.965906809429 112.414189401193 69.8941497035747 74.0420372528779
      71.501028931626
ELN   57.2008272301399 56.6617674371349 54.1157382341041 46.7577982595088
      48.1473938886239 73.8105012820769 39.7162631015511 56.4779645313958
      42.2248608921879 53.5498255316009 48.8088340735921 59.6757573886627
ELOF1 60.9710783023013 68.2521461582047 56.3471340054074 80.4007868743886
      96.221685383385  78.0586153423713 71.8466937395628 66.7530718914083
      98.4636016442768 63.5107036952143 70.2215218476683 70.25295977615
ELOVL1      96.8783070574131 110.487281983469 109.251112569215
      68.1992221684165 95.010312646142  70.1074782747874 125.512567429299
      126.764572234324 155.075313035867 96.1044453515408 121.487591828005
      92.2530518251152
ELOVL2      34.2180669723984 34.6306468196142 30.3888349004556
      37.4223289687135 41.0885562160946 55.4694986901287 37.0564573649223
      46.695848687694  36.4476883897928 35.6208308220005 42.357910063876
      45.5800840647989
ELOVL3      64.4415447415281 34.3715326040967 40.931294690669
      38.0958223740111 42.6022652963326 44.1737502492846 39.8695929627783
      45.3872406432794 42.357910063876  43.1839109702714 41.6336636285875
      41.7575097298927
```

ELOVL4 59.6008106247114 46.29198852834 48.7039705048129
85.9087280306456 54.6820069269589 70.5699797830702 65.2361525412756
88.4439480366515 57.7865070833351 87.0573871033894 77.1900613562529
60.7495043977707
ELOVL5 183.690616959682 93.584904661285 87.455991111513
240.476103976894 180.648144492464 198.682422442825 123.868848519181
86.4898752526885 214.316246155274 98.9988668602468 80.9821967801845
135.259585457958
ELOVL6 141.577762652792 219.838542502016 180.663636520073
60.9379216295576 137.034037711549 58.0910843932181 262.9072085191
562.798580332885 185.563590013028 193.951097560903 196.844428284247
221.325534445978
ELOVL7 38.1169703906207 40.0527893189096 38.2280071951674
39.066284740632 38.9496515344837 40.040778349137 43.5627649099623
39.853866204927 40.3316153483289 45.2556760873335 40.723990462047
43.2776458914333
ELP2 182.52420078643 259.626847216881 263.765914589758 143.526781144716
151.848243044839 247.134338953977 214.51120218554 261.756974562481
283.21175871127 245.27542001049 253.764071470001 270.110273110996
ELP3 144.863548409034 129.605603092944 173.308366696429 148.964204638494
76.8964050504238 185.360921731157 211.164957509884 108.463445585898
135.706144105357 328.962069934895 311.301613620475 230.014127346507
ELP4 56.8168526942685 91.8568043052819 125.036267601897 106.8866235778
102.282066142307 114.142157259903 96.1825551158198 105.474920850981
117.191735492199 97.4769942713663 97.712117589297 94.9831937714438
ELSPBP1 40.148780650831 43.5182502375207 41.6977706798234
39.1706564265495 38.6454098044474 42.6575535390785 42.629412460409
45.2806199505029 42.0621425894624 44.8997235283254 37.2018803984555
43.1805016690297
EMCN 46.5549623118323 48.7084357480922 56.7776874358774 66.0397471691747
53.3086645804786 71.9329028989527 42.7431359850339 50.628152951493
40.5912213523345 40.5090849993014 61.8445915808329 63.4685821260797
EMD 440.421077161585 530.710127703061 533.468347491982 568.905368101326
1294.42501356365 788.950470077369 848.079114196986 798.996494242432
747.577738135496 760.745188431719 757.257495810249 548.601467852543
EME1 50.4872122867759 51.7495306734657 39.9101908547397 37.1894304543751
44.6834648302383 46.7800033479223 39.6535880152834 47.2864663693787
44.619512785899 35.93950183586 40.547835538778 38.2350720635382
EME2 42.3433931248155 42.6022652963326 53.6837370963602 40.4359177791566
43.3332476888305 37.5887129572848 40.6750540874299 44.8997235283254
32.6039814489621 40.8970401536578 39.153226699662 36.1647506311964
EMG1 306.895449954881 235.33716196909 257.506228440451 240.476103976894
273.540763256424 290.535354193244 446.613670519745 512.821381202441
448.508714709248 311.668075784386 315.880072026714 401.956364010068
EMID1 56.6951078698291 49.83247261979 47.9035532886869 49.1786207920603
81.6486527718661 49.8472708021525 68.4380822448123 75.2101993995344
46.9739160257217 54.8979897528792 49.532546810992 41.3481355128089
EMILIN1 262.679844527518 92.3659612653822 54.4384603094793
151.601402122351 104.599492214295 278.850071205987 37.7136011474903
106.58628367621 41.074824727477 173.308366696429 189.737472782071
71.501028931626
EMILIN2 132.838429478396 235.222472959869 116.709906902201
761.341795853104 201.574654530622 268.54298415337 287.627417889988

118.49449420132 95.0917871457813 159.554916039519 128.70380102034
135.592113666246
EMILIN3 43.5710871617107 42.1607322031054 44.6032218589199
37.9875302985336 39.859345745831 50.0748292497578 40.6750540874299
36.3405039629059 40.4220196471124 38.6454098044474 39.5399188536544
43.7672102682067
EML1 96.143327683221 63.0245691276326 59.9043023855039 66.3075095149783
58.7239345900248 203.586637054746 53.9412154719864 83.3826639718546
65.2670315837785 101.403739410832 98.2180003781859 93.146085697072
EML3 363.324816966828 634.226367147025 546.287195443189 551.915864019927
607.042450917813 377.679662622526 569.966632625157 452.351360652434
429.453114391562 631.632335640448 579.482255191456 406.500583302121
EML4 384.919835924792 241.486113826383 266.905855859261 369.451882513347
157.493875535302 145.741469970609 312.19993077414 294.048088688381
282.445037659981 338.72596574658 335.167786298149 386.640440174303
EML5 41.4508241771315 41.2262949117288 39.1480645318984 39.7840818430159
41.9397336249742 39.2985791324641 39.9559575274857 36.2539950294089
40.8420925848403 44.7520317855773 39.6833800000587 41.2437378488025
EMP1 987.427672417105 311.502922515027 515.856807472321 1223.01485352248
249.308868215205 1907.47560253019 160.117375210168 1182.10714525377
558.855313555287 459.021889198824 303.670126704469 504.847507464269
EMP2 39.3709357615493 38.2263227947189 38.9790184198145 37.0732977341185
31.8228237796461 40.9506062495231 37.4772922422734 39.1652965788406
38.2215591930186 37.6383530120621 51.3103617600342 40.1700117452801
EMP3 910.898566308567 407.564154455615 424.182335137838 2357.96534075689
250.545098051392 2024.4791883541 277.878824242495 317.892699374852
167.410607476565 826.024359447649 769.685677484067 638.064750551015
EMR1 41.9407041937973 47.1332119496722 48.2883694323339 61.7502624458447
38.4540849632254 40.7616966228404 44.4639405506967 39.9576138799997
41.2870505273664 47.0055981216687 40.8225507267584 42.57175366709
EMR2 81.6486527718661 71.4197504261453 66.4079972657486 664.453262499828
109.017269529278 85.3718124658316 71.9474405671289 70.9702772728923
63.6222532132037 50.0564735608363 49.2206713037152 48.27609330316
EMR3 37.6817357354748 40.8476436931481 35.6139301508335 87.114638423256
32.1011040606811 42.357910063876 42.8684958525042 35.5179217192467
35.860430241131 36.1014515012722 35.046946054314 34.2597466305328
EMR4 43.1267841876606 41.6466169548846 39.2356332888903 47.8164193292842
37.7235613375742 40.3981965583868 43.3262148375525 39.5466975353131
38.2981083038519 42.3288275690551 42.409203830483 41.3837720590283
EMX1 44.436995101921 46.1131345131683 41.1742244762318 42.5193208629123
40.1151953732637 43.7078399718247 41.7614975630362 38.1835938174625
46.3203995372658 37.1275334308172 40.3452320167286 40.8681871019812
EMX2 42.3433931248155 44.5544727111028 41.8389336728199 39.6833800000587
49.1263312877155 48.3686935595826 110.379403044901 115.328304312071
50.0526763693329 69.4677195088043 65.7587943905649 43.3262148375525
EN1 38.0089018744739 39.5050383901884 39.8299340037009 35.6928955245309
40.6723334103791 34.2773859804062 39.2533668167483 42.7431359850339
40.0403898620771 32.7131023641972 39.6403089864678 37.6557714476853
EN2 36.2496364442264 33.0553208959996 45.063792968927 42.1273928203021
44.5668141895992 37.4782441311626 41.706879587091 34.3865306339645
40.4220196471124 43.844855336215 38.8507804383261 35.3525469364579
ENAH 69.0226263333556 46.857923273572 48.0191002461099 45.9379019161109
60.4593810615882 172.317945975659 51.5967098638347 59.8760452220989
67.8208976798364 124.76401857899 128.70380102034 151.775928080246

ENAM 36.0144420013819 38.1312950900467 39.5450079747895 41.4273122914951
37.3508690072156 45.6129956328415 44.6032218589199 40.831207953647
39.0775870687758 41.3321861371202 41.9908237694988 39.2469742642337
ENC1 355.423376744934 207.99040155018 243.803668806533 173.694418385132
283.88971280023 89.1290937615998 279.521995263196 389.994289973076
198.585368334563 195.403882457038 282.802560118785 110.13638939902
ENDOD1 129.185046410316 65.6721629145797 72.5835488194631
54.1240583139871 69.8804970538895 101.383978726419 53.8687221233237
93.0474674148534 95.9999165639785 76.011923854897 95.2915739972533
95.3145142003966
ENDOG 133.067727272864 142.066425511614 155.650716735976 92.4781449270918
214.127390534906 116.35075921851 246.20973320244 136.650986373497
200.174103125152 99.9951025176909 131.757828130578 111.357508398822
ENDOGL1 120.381190859636 51.119812302881 77.7907484334847
479.696469083953 145.572606290643 230.879083362388 67.8919712859031
140.175068871414 54.4912806027381 66.5422130374814 58.6464918752765
57.934622029119
ENG 183.799353414581 221.847615564378 271.008929897376 455.223217772744
197.205643078662 176.670909065664 93.5928272702304 154.111066539468
101.954354107731 123.46775722643 217.263889616995 145.847014515246
ENO1 7476.94487791412 5074.07305640514 5776.17749026501 5776.17749026501
5479.94045861143 3099.21690111993 8259.38594893529 5112.4354472796
11410.3307451176 3399.75820494572 3391.65926812276 3800.19142219719
ENO2 460.865101118125 119.647202975116 109.374838860558 118.316810638061
98.0143500562821 137.802147212644 266.717224821545 141.261334415286
282.035361446993 183.311305255895 153.595112580363 173.100153934025
ENO3 73.252713742627 52.3896785600751 54.5191471810265 58.3475983630509
57.9903227926667 50.0404995046748 65.3071954258488 52.3565394429739
43.0456011922275 71.4356132400211 55.5276281380084 54.7953298048942
ENOPH1 301.025314855652 464.209794165593 356.727720725014
271.436000380202 332.137622104526 276.666571115104 458.47341225284
278.850071205987 430.743220222202 169.838827072973 210.096514889445
369.797483212264
ENOSF1 128.436305192918 191.09175268141 164.343309332109
126.911056987607 150.43149196048 120.618530729822 144.227861902786
147.017279035061 304.658728492827 169.633672522325 221.163225922749
138.916915463074
ENOX1 40.7609952437622 34.0382693188794 34.9849896310235 38.2041059863839
34.6227169056411 40.650742261428 41.1507980856436 42.0494116081998
36.6312676076142 37.920964499903 42.6366765186182 49.3984571719129
ENOX2 53.3059058105836 64.7219521869942 56.8595344107711 50.9643363406241
80.0637650599841 68.1969125201241 72.8653205184014 50.7964800730186
72.4796479443344 64.8549209064366 58.6464918752765 61.2685222381296
ENPEP 48.5835484742456 44.7200265825016 45.645828394758 45.4882438975269
44.7829632472754 49.3654837541353 40.6750540874299 45.1151758109609
41.9097689475185 39.3698811593364 42.4580504587581 40.5703581862752
ENPP1 43.9309845324767 47.8400694856726 52.0742951366864 49.9014504704021
43.9450023643394 41.554942319225 49.2546642180676 52.407151732257
47.0804681460898 56.685925255591 52.6960621424156 48.9733758107788
ENPP2 60.394307846869 103.189199090363 284.437758089699 199.858233616116
66.0411740221276 174.263925108623 57.6643188499117 85.8116307540247
52.3187919317561 134.152059562322 275.222714101589 131.380698046552

ENPP3 37.2474303302343 43.0227387991135 42.5406888281114 47.481302504066
42.0709558258414 56.1671440556142 42.8684958525042 42.0494116081998
43.3744503319109 45.0155543555707 40.2649309204478 41.5536168238478
ENPP4 91.6253858792439 101.523741547876 67.6373573669424 100.001150612051
108.063001800611 101.540333720181 82.6381941930783 64.0476795495651
91.8414653633828 63.7836577144372 72.1090339831747 83.2925226467532
ENPP5 60.4593810615882 57.2871072823547 52.3432130135266 46.1673159982664
55.1563703466328 55.4694986901287 62.0404348860702 73.0394191668858
84.0899854321388 92.261357059727 72.1090339831747 72.2211523775459
ENPP6 45.1912496459517 41.2598458726002 44.0451487108213 42.6913548400127
53.9074374788981 46.263826584899 42.357910063876 43.6434576713809
70.178974380507 48.7414853386615 61.0979269700412 45.0035308952466
ENPP7 37.9387791625384 43.6587492470348 40.1985790927248 36.0481934423077
42.4864576619427 39.3565586789274 41.9393838138203 41.0381860307445
45.0666179335087 42.2614524730856 42.357910063876 43.3744503319109
ENSA 144.515737827731 233.435884567494 270.543988412382 127.50316411791
235.764999941441 188.582370265555 220.743173420176 262.088864041385
217.065944053174 95.2915739972533 114.5696064637 195.146655512672
ENTHD1 36.9060997041453 41.2598458726002 38.5045333213784
38.4208429103799 37.0526522140242 39.7394181011603 35.6056062164318
35.1738399826814 34.3842972343227 33.6188027862739 37.2018803984555
32.8921051249019
ENTPD1 184.989940464859 159.747484721905 189.705164944697
294.155188719236 110.599131324937 227.404530886674 86.7968607218397
106.398825125486 60.7480970026669 74.6847594297153 84.1112814779578
83.5069968129733
ENTPD2 39.0237504776589 44.3529919625344 37.634370327756
39.2780515621926 40.7779767565889 40.650742261428 40.4967168818294
37.4484310268909 38.3981095229401 41.9097689475185 34.8383995317036
40.6216891610774
ENTPD3 65.4561038762234 258.726193861248 238.584514797019
50.4487395010341 183.311305255895 55.6735944873518 53.6294062560285
126.218285718433 91.4888405899074 165.613176704286 203.109765192983
214.873872837015
ENTPD4 97.0254711947754 77.9992448636936 140.030757545662
92.2530518251152 64.4999048115671 58.5530199658661 81.9504925867759
68.8058577447202 82.1974756449763 52.5522545249206 76.5505585423421
69.6579723433329
ENTPD5 44.3355304829989 48.4497719907777 48.8100349749783
41.3027031037343 39.6535880152834 40.1428591998404 49.1067959632109
45.1714698794331 44.1519466943185 55.7930787690756 50.4740079109854
57.934622029119
ENTPD6 199.54796711653 134.099684731177 172.317945975659
99.8063480813138 170.593589984253 102.980622026276 124.599352191013
111.367789538268 130.249044369793 128.291287011686 120.837993273922
111.607814098243
ENTPD7 46.9939712192224 54.7083331985678 49.6347864008749
52.5307002738469 56.2305155589239 39.8455060654531 54.0170817319522
61.5000955267432 62.7205631511304 57.9713749600418 66.2027948645675
66.4626619269287
ENTPD8 37.7766325325437 42.7775752471711 37.4642807402741
41.4812544772644 39.9042726867255 37.3356865956886 36.5352572191451
37.9344616270526 37.409945204123 38.745552403895 41.1367478979484
39.983244606774

ENY2 1450.53108586858 743.094853186365 382.744696166534 764.571579067138
1263.76838179117 744.421618408497 750.151825182993 540.304257899761
779.996851709716 338.000998124097 353.531051185171 563.927521628693
EOMES 45.0985088502363 41.8848959280916 46.9040704638073 74.7732045170597
43.6587492470348 41.159706131351 36.197424053556 44.9977782988116
31.7063377004409 45.2556760873335 35.7953070087288 29.0303154053123
EP300 105.45765455249 170.20428461603 163.028918580618 180.648144492464
152.608998839745 125.184689092836 111.714563124738 105.822362552251
137.764748752401 111.399831013724 147.06837658726 114.396745444915
EP400 336.159430045964 246.820301517765 197.373541848064 203.152852313746
163.028918580618 197.325473090277 230.787791652978 150.572774995368
298.54401957868 257.506228440451 280.275836993258 181.420545696696
EP400NL 46.1513019317765 45.0905928998779 49.8344973836834
39.1335009052403 46.7058093820839 43.2805590023412 45.1694246393676
42.4926864381766 40.7460990419215 40.8970401536578 43.5258090162298
43.1805016690297
EPAS1 690.568858101531 1311.88650141004 2009.75680599899 1245.45371155527
720.552354470517 1423.29307392266 146.117756310709 484.968116511768
484.218958528411 878.863554510943 933.968759959712 751.953794043451
EPB41 39.721757876562 53.6895401581575 43.26160898693 42.8768096578102
44.5152708955623 42.0902716047029 48.0283573444179 46.6456668510218
50.401623258584 39.3260254992968 45.390192977585 39.1553169409075
EPB41L1 54.7786516309154 50.4872122867759 47.2962360458401
42.8235313007746 53.4751250621694 46.3203995372658 70.1845639720491
87.0116305167987 75.7502062965698 66.8920765290571 63.3121284680843
61.5300817099814
EPB41L2 96.6833301748676 82.5344501505114 87.5146535320072
80.8078050043929 68.2428802339852 194.278792348966 78.4023698691285
70.6568303458436 48.1174861108648 106.679905803492 112.613079254002
86.0230765383602
EPB41L3 117.869393694564 90.791818798144 108.928854330744
634.514100741768 73.3533274030352 304.658728492827 99.2824152850072
180.916465721523 44.8003345643334 178.961579489906 137.802147212644
189.705164944697
EPB41L4A 54.931538325692 49.5443425465542 56.607199625498
56.6458456890715 37.4470183315826 42.5660695551615 54.1707158323527
61.3077641493507 54.7033747046992 53.4109882045447 57.9864701726379
59.2035499250243
EPB41L4B 50.379587451669 53.4109882045447 41.3366779545815
45.0017158586743 46.8086806989607 43.7630587657205 52.4876329865056
47.4439407315335 54.4912806027381 49.461728580617 50.0303076717704
45.7979032939211
EPB41L5 55.4004611309636 50.1027491117756 54.1952533541951
47.6687908290818 58.3564461855595 78.6948922624369 49.3854238365325
48.3590401011623 65.644767142472 69.4677195088043 61.9976238937207
62.58750768974
EPB42 38.0089018744739 36.5219488315447 39.991353369336 36.6501141438378
40.4639532172338 40.1428591998404 35.8227939208521 37.0390589468421
38.5252456306656 36.2435874904689 37.9946685306274 32.7043743065388
EPB49 55.3153799233744 61.6878176260761 43.7630587657205 48.309866878365
46.0043807840705 56.1671440556142 67.8919712859031 54.3820590274321
55.0748878261048 73.5527788467294 74.466989900636 59.0894921452893

EPC1	54.2648201582657	73.7873098190297	48.3847843498476	54.2886483704949
	61.8471691369569	77.0226403968683	48.9826868640753	49.2019743609873
	66.4251023257203	69.2963913574682	84.2960453806012	73.845912534353
EPC2	42.5193208629123	42.3550738181144	44.5134109184888	40.1804147506786
	48.1473938886239	50.9724155054821	44.7255207589606	45.642304356051
	39.7537274655115	49.3000844711116	56.7765567227989	48.3648494350286
EPDR1	355.04502859751	197.841210387578	51.6159542017038	215.645475520124
	69.805066579191	297.404152949522	103.545764794149	145.630337418553
	114.719721626067	363.947777340854	217.39432336811	229.837344196558
EPGN	52.0299937917925	55.0897019602305	55.5567699051046	69.5544840164738
	58.2780916793348	56.3525213736952	61.6544855697851	49.6203214143464
	69.9767092409915	61.9882398986333	46.824299585607	59.2035499250243
EPHA1	252.535061916875	279.521995263196	373.46798029115	61.0845101400379
	210.200786815048	53.7442594289333	150.374739898477	162.106596527489
	535.585999498708	148.144200968432	126.920579478593	99.1769280484821
EPHA10	52.7948184519106	41.5132248356707	46.6588655218735	
	47.6687908290818	47.5647821785529	46.1513019317765	43.1602899171656
	47.0362584198466	45.3756389170606	48.5374448344297	45.8180710486329
	42.9887069977696			
EPHA2	91.6826894933058	139.999709303614	209.363955566338	50.7046706880325
	132.257197568848	62.1857038870283	65.0031119608637	343.990395610093
	188.750770811584	85.7732262204681	112.565968152932	92.9600461444587
EPHA3	42.3606390470387	42.6939394537076	43.1709743883785	44.1483976313959
	44.7114813656401	70.8674195845729	41.3594868055879	44.293922970134
	44.8003345643334	45.8505603625223	53.5453975444568	43.2312848495114
EPHA4	88.6663961027926	57.5941849631773	54.1637401903523	68.9937449813561
	61.3077641493507	58.2531879680192	61.5000955267432	61.2343203799193
	122.748811531184	61.8529316708431	72.842552380479	51.0647893605839
EPHA5	39.8662795144616	39.9339434067497	32.0500360504817	36.7128997214853
	37.4881665029302	37.0740019672788	38.941855825269	37.0795845518148
	35.3227485563945	37.8490162439785	35.4676843900423	34.9732824977751
EPHA6	41.1523487516364	41.5061991514748	41.8389336728199	41.8125315089117
	40.6387590440575	42.4155142987678	40.1613888954735	41.9680383884948
	39.4524820354904	39.6333888530279	42.9699559565161	41.9701179220033
EPHA7	39.9697332660833	43.8495248991589	38.0452862258803	41.4273122914951
	41.6806984441849	37.2686490268265	46.4388841354122	40.3357777530419
	41.3803269429468	43.4885245598939	39.2542963855807	44.3521099857368
EPHA8	40.8681871019812	44.3187284857534	45.4634652319753	43.4877740913713
	61.0468099710416	47.2505395290129	40.2154497375715	48.8333989709933
	44.9645354363967	44.1861590259178	42.7275315121163	46.8129509799753
EPHB1	48.4849194706125	44.2630383225933	51.3268475761593	69.6511571901941
	44.9936360641969	46.0364000143537	98.0023132593711	61.3864512710242
	52.3579564953125	40.8970401536578	105.953705702398	43.4709415392696
EPHB2	50.8875036861067	37.2103401831788	36.131485826118	42.1273928203021
	48.1986674201502	42.750861693377	48.1146464339794	49.8666125578518
	55.4635297192076	44.3648993568405	36.131485826118	42.8864415494484
EPHB3	101.002303802745	50.7487159427973	44.696023817801	50.8567345921473
	56.5933259268408	53.8461734834857	58.3675164086197	72.3822545261582
	61.5807110546718	76.4439037944547	68.878562323012	52.4992300612807
EPHB4	281.201604778921	98.0967511777819	292.93036571522	174.117816184497
	315.065866567211	111.240804216088	159.359051158123	255.126812649967
	297.187417426993	154.707548614663	146.969565088793	134.024691118762

EPHB6 57.6285775590145 225.460800739619 233.468474830926 81.382229980722
97.7153995525346 90.5359675350156 65.3694267397114 105.474920850981
301.79813401066 259.276586396099 157.020685801527 111.874193363836
EPHX1 123.868848519181 72.7987593815919 84.5998418020787 63.7909104347097
107.770004093887 205.121208974511 102.380096190518 137.403148427792
99.2341244350412 177.912351477808 336.737571879156 200.986069552443
EPHX2 43.6706764143908 49.2050538150106 80.257554904218 52.3004056941891
57.4163094213712 83.7019398654324 43.3262148375525 40.3357777530419
49.9162522057712 76.6346767983285 69.3645322033576 74.9294208313176
EPM2A 41.5668884168669 45.5587966941116 41.924725226806 39.2780515621926
43.5544347011646 55.3202238943712 35.0797534809405 42.157627240486
41.9083581529276 52.2256177934134 48.9906383294199 51.9447450229707
EPM2AIP1 203.913062066149 266.634368304904 186.450759425396
180.873540853324 734.143790759359 366.515210930763 211.164957509884
133.060894083935 194.985223342082 375.697071471585 344.985739524681
215.869541768982
EPN1 1005.45493369789 1466.88103496067 1591.51486001004 1087.75682788799
2435.70120719503 1301.07436212673 1588.70376317303 1296.28036613191
1975.28674300761 1076.05074395483 1259.2978319548 920.504890206997
EPN2 80.3266484520306 87.3870406334559 76.9904391375645 55.8709511400674
70.4262478188233 86.9924799927707 143.973063517706 70.2021753365478
73.4535007027931 84.9863403576284 81.2850357499192 69.9767092409915
EPN3 59.5000730792772 170.082014272945 151.601402122351 38.745552403895
111.256949641793 51.9421032049606 74.5591290353333 69.0819394834919
158.195749606017 80.8593396441328 91.3723635213821 64.7346994616044
EPO 40.4639532172338 43.5182502375207 36.0306975524309 38.5252456306656
44.147557390293 38.7340969861754 39.5496902678788 53.5271601314404
42.8036731479932 42.3606390470387 43.5710871617107 41.9430737035925
EPOR 66.0303950772736 48.8584917918986 41.0356368374708 45.5869891570251
40.2449343247539 43.8582747295766 42.8684958525042 81.3978207813439
46.4164127487052 82.2431666775699 76.8713035584472 78.7496673195407
EPPB9 43.3638918705225 36.89524015739 41.1367478979484 36.0481934423077
46.6397391251762 45.0882356578404 49.6872467499977 42.157627240486
45.2897806651929 46.325232414659 39.9899430628542 48.0350549043063
EPPK1 41.5421429082231 42.7775752471711 36.5474283500601 37.9875302985336
44.2607789091176 41.0582900711341 38.4005795891396 36.43924066428
33.6288663955758 43.1839109702714 34.8383995317036 33.1559412791294
EPR1 45.7356815244513 40.4359177791566 41.437237454134 39.1706564265495
39.859345745831 45.8325181856483 56.7780520416533 47.3791873993182
42.357910063876 42.6793753550854 38.3684202018524 42.0424641526302
EPRS 337.948887427898 514.261598258395 586.026364123617 622.964590694346
644.368005872231 579.260404515899 659.126408705362 591.192020333181
601.375421126664 434.223722811041 488.908231529696 489.622327794168
EPS15 116.426770366736 110.093465196133 88.9928368134295 118.316810638061
71.5345397102823 167.05554959607 105.247736284747 101.39385858723
86.2563919366781 156.591349262922 150.082905944963 145.01315947031
EPS15L1 70.9419509323007 63.2804984998739 64.1080166181228
75.6103053700122 99.0647786948132 79.0940380276977 76.0041771381018
63.9851732498913 57.7865070833351 62.3233491851723 68.3157692917711
63.1443731864055
EPS8 71.2963721541702 99.9752275166514 160.973602503707 53.0284011078097
66.4047895961325 122.036614711384 90.2915974511786 119.86041265821
158.408644964483 190.592556065939 144.098339910117 162.02498480452

EPS8L1 48.3451401499671 56.0183341691645 67.8452645288969
41.1886975241176 50.5707478277575 42.9125608028481 49.319499137678
44.553780984244 70.9214288336034 46.6025757239946 46.2859819541831
46.8752988519617
EPS8L2 38.932557955217 43.683792219729 37.5303767522393
45.2640606138546 38.6134012710484 41.2181878103506 40.3850714987723
40.4737825400374 39.8022382792104 42.2455707947223 40.4504608195666
41.1554481567603
EPS8L3 40.5654775358427 41.8534933577029 39.4710743516517
44.7980740332174 40.9950639693255 42.750861693377 43.6753843488695
39.5954353681031 37.6630259432398 46.3810615657263 38.3684202018524
47.1060577218993
EPSTI1 410.058119360034 391.187784616808 370.240482282297
648.380417557148 117.689172019617 155.888873407786 513.57613902662
176.653849515825 113.148617122619 113.873117749074 168.482495410546
111.227016020927
EPX 35.6111578834671 37.3634897565502 38.4043916290561 38.3166554965434
42.4864576619427 41.4508241771315 35.9058393060637 46.9986083378817
40.6957787921451 38.0078357808393 38.6662194645633 36.2582325396375
EPYC 204.659954879641 39.7835513496873 41.2365089689635 58.7338159410728
36.2048942610546 38.4330755397458 35.3362091871364 41.2551966132486
43.7630587657205 34.064698871541 35.6970528713709 33.5578650644246
ERAF 41.1523487516364 45.7317208802318 39.5450079747895 46.4555908533915
35.7973685350741 44.4619248757502 46.1634822993009 45.642304356051
49.7336769474446 53.8260140375214 45.8180710486329 36.6603338274678
ERAL1 343.892794667047 275.928723831899 244.226756210656 186.64040927334
418.01691355558 225.794026573235 483.20917825594 363.002913525646
419.182443507848 261.569542242954 295.917955889194 227.567182783564
ERAP1 43.1805016690297 51.2405397507224 55.9927099883616 46.9919960628953
43.1252272136667 43.1826328292558 40.7910160811527 45.4882438975269
48.4614401417022 43.1839109702714 45.390192977585 44.4951343330035
ERAP2 322.509777895373 323.295794446334 82.9449579428926 335.818251279914
141.740347756446 179.886168478179 51.7714254834195 299.052630634189
78.4428909886495 72.6549171886783 87.4850071876743 62.8485724004124
ERAS 41.9407041937973 40.4359177791566 42.157627240486 42.9358255168086
43.004943518025 40.244271403831 39.3585350448719 37.1317006162938
37.0126425326534 40.5090849993014 43.7585538437696 41.3481355128089
ERBB2 547.729200972776 348.123960682064 374.723141438448 60.7397678774507
262.579533452771 115.913030448537 690.568858101531 895.735191953679
818.608913659251 474.155196153863 348.450810225807 304.977139992391
ERBB2IP 50.3422285917 53.0063130674306 50.3452972893313
58.3475983630509 56.6557577134933 52.3187919317561 50.4260283682427
48.7039705048129 62.1796792097014 46.2768056702424 51.6869155634183
55.1109784383525
ERBB3 182.601427407211 249.942109342324 146.789051265805 44.484692294383
108.063001800611 53.155979669047 171.33368481035 274.648263378054
267.024267633341 133.893108529325 141.567299023381 133.69701250532
ERBB4 47.1962846565261 48.275452803061 53.4743535852344 57.8277957325085
40.4639532172338 49.1013967533186 56.7780520416533 54.2877981508636
51.0062122023297 49.9493230064613 51.6414075056938 51.9447450229707
ERC1 41.8772051675749 41.768427504666 44.0014471264417 40.4967168818294
41.8524296959457 45.5779686360641 47.209353240759 43.4885245598939
43.3744503319109 46.8954576545929 47.3501115999636 45.8546719310325

ERC2 38.957687788868 43.4396866157171 39.3709357615493 41.4273122914951
36.9060997041453 39.2248354955045 39.5496902678788 36.9355798143571
40.2274522635576 38.4187686307607 36.43924066428 39.9519547348106
ERCC1 163.067962559455 128.436305192918 175.056828374791 210.485586903711
179.694413858411 205.378123452174 193.01447006167 268.54298415337
192.356440003154 179.342338803631 201.657389063895 175.28322146764
ERCC2 106.219480656782 80.257554904218 168.225699005719 67.7121534934164
128.749209040345 92.2772139468085 75.8779316255794 105.743957034577
75.891637602914 113.388372364837 87.7844808613265 69.6021553604792
ERCC3 243.015896514493 247.676607756929 229.697141930047 183.613579501306
232.081000478989 197.868575583033 157.463465828715 198.54718783437
152.942332679485 185.94424664238 190.548265163011 187.16833117416
ERCC4 42.8561766050988 48.2870269886682 50.5505693464794 46.8249967544573
53.4751250621694 53.7236425500919 44.7255207589606 51.4931742685391
44.2142581488029 44.8561781465248 47.2134341420411 47.3172462673426
ERCC5 432.669787408904 454.701322158431 434.682456437647 413.990148966099
305.554059609771 300.412043023998 276.747699321595 274.731304343034
366.515210930763 537.471287449696 469.427640339806 402.412173952792
ERCC6 56.2508140932179 46.3635803515159 50.2869791217819 42.8235313007746
39.0564222526273 42.6575535390785 46.4388841354122 46.1673159982664
48.9931786790166 48.1392888470269 38.5668610487033 51.3244198342335
ERCC6L 44.9896851005259 44.6388394563024 46.7508829977587
41.6564075217517 58.2780916793348 40.1428591998404 55.9821365674554
53.9918882088235 59.4791385941479 40.6559419616644 38.956280281655
40.5703581862752
ERCC8 46.8613212048981 47.6229101519945 52.2695562895186 47.2862299990937
51.7983110178095 51.9421032049606 43.8605301756239 45.8108990837624
46.1572972254663 41.785676764591 43.0785028570404 46.9867599298218
EREG 37.3463875295726 41.2598458726002 50.5089777244781 58.5406981871153
40.3452320167286 45.7300501624017 46.5650556481721 50.5070012045444
47.1744296124496 45.0155543555707 39.2542963855807 43.0930293831497
ERF 92.843017373656 89.4111455826308 109.594402843796 110.431464700539
112.830467116678 119.14126712577 90.0682241853516 101.888001006345
141.501071047479 111.056599389048 117.074650076861 80.0381580139229
ERG 43.1180113655693 44.9877531846933 43.0697338561114 55.4180794172794
46.8674366115656 46.4343327861696 44.7915315085948 44.2580151149956
44.014761416117 46.7646877499666 44.2446125027849 44.7915315085948
ERGIC1 79.5898953726467 68.6627996695828 59.9690744676539
106.067766766889 73.2144205844199 71.2370255410834 63.928488869459
93.7090094630558 72.487245822532 68.4527140982234 69.1743062945144
82.9191817146388
ERGIC2 78.6705291023488 78.9203273221608 76.5699943825825
122.412295645862 76.209239524646 92.47006193987 93.3897477314201
89.5130163419167 88.8772406115484 74.1228645930185 74.1425714439424
102.035798846699
ERGIC3 692.630044242985 689.228901128857 964.268294825556
512.691506909762 864.302844775203 592.399245288911 716.434968700383
651.795139128843 684.865133028228 744.762787024945 878.863554510943
843.078502001733
ERH 375.354746298695 144.954155897343 236.178265404264 235.723799298966
263.832968124343 330.096171705212 180.502282381416 366.998712124711
364.337811621248 198.056931905227 288.687391623935 316.548339196872
ERICH1 119.434418653131 153.515845356859 169.951840840983
205.021644155954 269.113996642102 189.451890957713 208.504992019122

103.530430972911 102.648943280704 91.2016867646161 129.005213737493
128.230543301378
ERLIN1 43.1805016690297 52.5868047927666 67.4280399954084
47.6687908290818 44.0451487108213 45.0882356578404 43.6753843488695
42.8482181313226 58.7382738968248 64.3383155834968 58.0800636554122
64.1607001437806
ERLIN2 61.0278709944136 59.5080588072694 75.8779316255794
60.0765286625112 72.8868488438309 62.3102413989199 66.4258159334241
81.0057400164851 59.7118480874431 101.036327583799 92.4896666046729
87.9638809147835
ERMAP 55.5751438859404 53.9634127270611 66.3188075271379 43.7540438144592
57.0504111569238 50.6360662655392 54.4313108506244 45.5581356216957
57.678188678075 74.0614295574729 65.088713927995 57.934622029119
ERMN 40.8681871019812 40.716553247236 38.4428081526233 41.3027031037343
40.6258566559207 41.7196307387855 39.2809305246091 38.3194261431961
44.3734692860793 40.3519145017156 41.9908237694988 42.8263096141654
ERMP1 111.522812019895 76.1682394916873 70.56368853915 45.5869891570251
116.099186392258 46.8954576545929 70.3935694362568 166.535245140901
135.592113666246 132.107655569343 154.355197155161 223.267781866783
ERN1 76.4983049841207 86.5414905689606 85.8945498067934 233.294970051779
101.46584765758 71.6694991209816 96.5030525416431 115.691017747672
127.034476771265 72.4834467838798 73.1262465119304 64.6194898810562
ERN2 45.4799270968969 47.8400694856726 101.2615980834 38.2041059863839
43.7517118200398 42.2577689927563 34.9344532055397 45.4757965005055
93.0888239577809 55.1089864632456 53.7475011049549 45.0985088502363
ERO1L 369.258587948416 244.529657015078 358.779372054826 238.246319539302
201.456613927613 80.5478617156852 179.522572239758 153.544554979202
424.87896140215 126.470889051684 115.761674855716 220.938982373755
ERO1LB 43.4100411978366 45.5842286511645 60.5678281953819
43.4877740913713 42.3536549114165 51.8103411703261 40.2601451506332
43.2890595186135 43.3744503319109 47.611018474151 50.1649089793069
58.5478485621444
ERP27 276.795797048951 139.758805241617 131.882285871258 72.0694688774804
147.704325041249 58.5530199658661 559.9670724278 891.080174768895
341.535750991033 219.616289279562 392.409679550956 193.676161965973
ERP29 954.116675685628 1269.36837611919 1105.00321488322 893.185203928127
1071.64675274806 796.359355922952 1366.59129560817 844.431085166353
1981.03929621057 1261.91478863258 1242.75259587926 1194.10154988793
ERRFI1 960.526372655577 822.822769402853 555.044139654886
445.644244692294 626.749986744506 919.542051089664 684.238999553691
1440.41726370246 693.727004855319 323.095468495074 360.15483218921
309.994784298282
ERV3 84.7058085717914 126.69706456173 65.9476039189301 52.3004056941891
234.873589896708 80.099836448859 92.4534854832186 322.594259063038
68.4229528414965 57.9713749600418 51.2318935933116 54.5921287593554
ERVK6 42.5193208629123 41.9894412416874 41.528370959196 51.5913529948887
41.3755760434934 43.1826328292558 39.0458123429605 42.357910063876
43.1805016690297 40.2112371512838 40.9157352260901 43.7672102682067
ERVWE1 63.4610447573417 43.3972849968032 45.9227107476843
45.1151758109609 48.1986674201502 45.9266745571724 48.5361215129944
45.4352530863365 53.9287524861617 46.8292269513767 45.390192977585
41.1554481567603

ESAM	166.511915820497	168.225699005719	294.372319595103	181.200738629391
	113.866742186841	160.234018459018	79.909124697198	139.177720567217
	49.641159251878	72.0443425395404	110.334176022265	94.3953345963359
ESCO1	41.4508241771315	40.3384318592985	41.6079958749402	45.8844325913247
	43.3332476888305	45.3882138838137	46.7100796279384	56.9299697426319
	64.4098139218465	45.6868589181768	47.3791873993182	49.3087627580849
ESCO2	36.4564050256915	42.2577689927563	39.6451483836089	35.9521941716188
	40.0599807618105	36.2768299545794	42.7431359850339	37.9837233601316
	38.5252456306656	40.2112371512838	40.6373400186325	37.1747156131306
ESD	218.245471601353	178.858202962948	204.862161704394	245.769212889294
	207.18510695093	494.162646055732	154.132233967972	183.887843429281
	164.642060754649	284.868613843826	259.43283734195	444.858305070415
ESF1	36.8072833828297	46.7591817286632	43.3085358214154	39.2780515621926
	38.4540849632254	42.9695585740813	38.941855825269	38.6454098044474
	39.3750969348308	43.6033221838149	44.4688999098904	35.0222940937513
ESM1	191.771063132796	128.146059846414	148.32293918881	44.265834798964
	88.3954618645976	41.809704036355	44.8643898647322	47.7508139534968
	69.1564181547342	43.3465699185405	39.0788811357542	38.8132170743603
ESPL1	76.3933911546385	75.2824161164996	71.8706847571669	84.0899854321388
	84.0996028277101	66.295597720304	114.526825145076	147.875578127733
	98.3798838151482	57.6791750431251	52.5930185400362	63.9371474698484
ESPN	94.080489738662	45.3486182278177	54.1637401903523	45.1944222548351
	46.0043807840705	43.2805590023412	67.6767845246581	64.3284163241986
	88.6368226476614	46.7646877499666	43.4709415392696	47.8931773060789
ESPNL	48.4849194706125	40.9731067463382	44.6032218589199	46.4555908533915
	40.9950639693255	42.750861693377	33.7574445386307	37.2237132058632
	40.6957787921451	54.8979897528792	48.6184776976167	48.6037684272115
ESPNP	38.304249186058	37.920964499903	33.9435456570176	38.3166554965434
	38.4540849632254	34.9849896310235	38.1793598983615	36.7460922671619
	40.6957787921451	36.5806287103157	38.1835938174625	45.2783024981315
ESR1	64.9367707455078	36.7976562409506	42.3536549114165	63.4531374125233
	41.0885562160946	84.9099930524863	36.9355798143571	61.9787117490846
	65.7321440516177	90.5603308057889	58.8188963227059	54.293880614303
ESR2	40.8091178323174	52.0268699306396	44.6511073781104	43.3078542962968
	43.3332476888305	42.4580504587581	40.7376840028647	48.5681478665796
	45.5779686360641	41.8613406887824	48.3847843498476	39.8497227447888
ESRRA	125.786923817078	165.160683489444	125.794379028627	106.582060920252
	244.837105178705	104.305801811472	136.062864107443	148.770228614661
	166.648551193168	173.391542207362	161.526970033915	136.355036262681
ESRRAP2	78.1313732870053	117.835389983392	97.0516272072238	
	65.524821699432	187.767803297395	73.8105012820769	99.9896394343368
	141.261334415286	93.4980947651211	113.388372364837	101.434947182387
	88.1733775711738			
ESRRB	43.9309845324767	43.1805016690297	39.3709357615493	40.4359177791566
	47.3172462673426	46.7800033479223	46.0228732507872	45.4757965005055
	44.5303781239209	42.2614524730856	40.547835538778	48.1627097465883
ESRRG	39.6349162264993	41.6972107715212	46.0727230219512	39.2320896829616
	42.8414096678708	46.3150221024046	50.2016912396771	41.6825727187466
	56.0121021290387	49.904979214379	46.7386599624801	43.9541718254769
ESSPL	36.0144420013819	36.1232327599945	35.5220586859301	40.9607305725196
	37.3508690072156	32.325260431418	31.098971689775	33.7818132446042
	39.2809305246091	39.8873706855774	37.0180934800573	35.9604262434141

ESX1	41.1523487516364	39.0102066700839	43.0016969050339	43.3078542962968
	41.6806984441849	37.4782441311626	41.0381860307445	42.357910063876
	39.1790517315259	35.3849221682845	39.8184668132023	40.5703581862752
ETAA1	34.5886782974123	47.8400694856726	37.0642268327439	37.651298468202
	43.5544347011646	38.1169703906207	41.253645578517	46.4672637826582
	45.3756389170606	39.1621898366355	41.7272875295469	36.1647506311964
ETF1	271.008929897376	127.82585786161	146.997252473598	309.39599482851
	167.578081645481	281.602712448925	174.007271403635	295.127975044241
	181.45962058846	284.013140015092	241.849566505679	297.662970257144
ETFA	493.322302312578	979.468753716758	586.026364123617	467.645011859047
	1387.28483887704	603.737787922337	637.035929429013	419.054753590648
	1223.01485352248	246.568253751149	386.170232791927	721.266942608349
ETFB	166.129723762515	366.326747630465	512.691506909762	216.45007990575
	394.990145259101	309.593721653575	332.184073392054	270.303708972854
	491.100053064936	335.943994595615	370.917477324773	308.393270347486
ETFDH	89.9580440276828	151.601402122351	221.743315748689	120.436827608155
	120.309345127552	159.071016200059	117.439175394651	92.804679821289
	173.582309251499	113.546568515215	180.398903465014	216.45007990575
ETHE1	44.5282181324797	43.3458769998118	45.6989664108554	41.7614975630362
	58.037539503209	45.6129956328415	50.3519150282938	48.3590401011623
	51.7549258776884	43.6033221838149	47.9942520395434	50.4847465816184
ETNK1	51.3331184755498	59.1570467228834	61.0935621637178	83.5320423407318
	57.4709287098477	47.2927436772465	63.3924515518667	68.580705761277
	66.5141009463313	52.022479439889	51.0013344286851	55.117521379579
ETNK2	70.2541961995753	54.4793574349739	89.1115172961797	47.8164193292842
	50.5110359971301	47.0027453663546	54.9765783642973	67.0904616289706
	74.4958282816601	90.1696131791736	75.3075812535743	71.9369353731849
ETS1	376.931257615162	537.471287449696	506.585006700107	638.305455110267
	200.174103125152	199.181938253995	161.573327074375	337.51861578231
	208.804071011518	448.383843915421	564.19772079287	512.951288394692
ETS2	278.378459771943	1184.75786736273	1137.99307039386	1566.12726152736
	411.431488342465	959.549658414082	665.122091380755	596.384327208699
	1020.75813028358	403.796135347069	538.153124145211	792.022171185649
ETV1	37.8386378737561	31.3542159583213	36.3604951341921	39.9697332660833
	38.9496515344837	40.4359177791566	34.2773859804062	39.9576138799997
	40.3316153483289	39.8873706855774	36.3390947880186	36.6603338274678
ETV2	37.55264801907	41.6466169548846	36.9795961564828	34.5425384901279
	35.9058393060637	41.6590564937735	36.7356360093603	40.7311497914871
	43.2761454096265	33.5412189543102	38.8507804383261	39.4490233720848
ETV3	56.3471340054074	69.5544840164738	84.2385359528743	68.6379135603075
	73.4535007027931	51.6053353177789	71.193768715137	113.277043026153
	53.1249776278067	56.4553747079294	58.8961770381033	39.6352613956998
ETV3L	40.8681871019812	36.0366673493555	41.2365089689635	40.4359177791566
	37.4470183315826	40.8634043028528	38.6243997507646	40.7311497914871
	40.137818555383	40.2112371512838	37.3687900733549	39.3575468494941
ETV4	113.491158368594	76.375769561992	52.8041782638549	43.074472921841
	91.9685593100867	48.8744644207821	72.6959688212342	77.6174893940285
	173.391542207362	46.3810615657263	47.4630625313159	98.1220820761255
ETV5	158.532307373857	64.7650773032999	67.1729762600915	122.702789229752
	106.527144354592	107.682738819549	85.7299543466172	62.1934012861178
	129.909461876076	91.0346886951891	72.6450859740011	90.689813277023
ETV6	174.492066109537	148.879760620316	126.981964766008	207.087723777954
	140.598583034034	114.389392280707	286.291779906932	225.50098418159
	245.060552951511	208.67074469962	156.69976952483	248.691595276298

ETV7	46.6348258763289	58.5406981871153	62.0967404944031	48.856176220627
	69.0975200641705	40.9506062495231	57.51482768194	49.9332280449549
	69.1129431075514	61.4569050766419	54.4825201506931	56.9379721393904
EVC	78.7450149191851	43.5873188565519	50.8601672542371	42.7613994022988
	48.6707521528908	66.4399491500833	49.0497778708674	46.7980335276155
	40.9423087727566	64.428683021978	65.4589642146912	56.6146179789815
EVC2	46.263826584899	49.83247261979	48.4890615340603	51.7739270955254
	49.9493230064613	50.0748292497578	49.8281348927244	41.7419877611072
	51.6731332961917	57.2813205536176	54.6089451252049	49.8253396161069
EVI1	103.945335792682	1342.10592868516	1529.52516575545	174.860894067053
	692.630044242985	322.109212713082	768.354209323884	448.196293409768
	1236.32289322872	1501.67042548971	1817.14004766564	1307.19925493293
EVI2A	62.1448806673973	49.2443202281556	46.2028394323277	188.156490868789
	43.7517118200398	56.9621724830201	53.7423898357583	44.797320243931
	43.0342930070984	45.8424887733913	44.6734266378263	50.0571060701893
EVI2B	76.3933911546385	95.2292899846287	80.815808403022	496.791391622565
	60.2886873195135	92.7427427543974	91.5258329186709	52.8264142735929
	47.1744296124496	50.007830364606	50.5381435455868	56.6146179789815
EVI5	199.858233616116	121.432592327754	147.858425171297	203.979798245524
	110.869341159607	109.352296288717	147.321968565095	294.567137562864
	108.42710653018	214.03015073442	254.639522735592	187.462631817772
EVI5L	119.095094052197	92.1646038105091	234.778900405354	99.5958921252583
	212.353676518748	170.172720177618	73.8313212063649	101.681822254799
	165.311139930529	115.509409633975	126.375315221007	112.113501370522
EVL	744.762787024945	532.981045084589	861.427193617946	669.419736252343
	548.217757366495	688.168272082335	516.213108543512	608.005012673672
	522.49156295512	624.51670158046	714.075528895169	434.523449093691
EVPL	277.936167684143	720.552354470517	560.878675769733	51.223859150798
	516.848416430243	117.907440794926	410.058119360034	454.402224020514
	422.817392818858	510.675912282067	391.187784616808	287.184421338984
EVX1	36.6480005877129	31.0594903826598	36.1042012504758	36.7892997857376
	37.2567512265363	44.5544727111028	39.8695929627783	43.5544347011646
	36.6312676076142	36.5806287103157	35.6041926503637	40.0557788909923
EVX2	34.3473914754963	35.5012138354521	39.1480645318984	35.4355497822498
	38.4540849632254	38.2971553419489	38.7337086062875	38.9074189954657
	38.5252456306656	41.4397539024976	35.8150308232314	34.8510844670321
EWSR1	428.958127252533	603.224267841548	339.52415679299	355.568857981155
	416.717470974721	398.049525642349	615.442484911026	402.221227674218
	576.830397705584	429.725397949276	463.84209540456	369.451882513347
EXDL1	46.6348258763289	39.9697332660833	36.6522278218553	33.5578650644246
	42.269054475455	37.5887129572848	37.830876898596	36.5393857306744
	37.8319142847141	38.4187686307607	38.745552403895	42.0424641526302
EXDL2	193.981651711507	131.537639104397	175.271140689255	133.1387411908
	149.551650453179	179.285390561592	158.321459945365	114.959624673123
	186.042094228091	166.734730009916	162.352052340273	178.060342847557
EXO1	48.3556695727124	55.6972661824064	50.0445602878169	42.7613994022988
	74.3523005665671	42.6187506958386	72.8653205184014	66.8364940429355
	69.6511571901941	41.3735882187951	39.7342718293432	41.9721968713295
EXOC1	75.1328739717464	78.3488426553071	116.547937420457	84.5678487053881
	79.5175547131776	84.056083317311	65.1757400078647	69.2787430208317
	84.5678487053881	72.2443746456494	77.0395487594084	84.4349803529132
EXOC2	100.891869710611	96.1710806345503	99.8231023149756	96.5813769696577
	128.844013736935	99.6821042055758	69.8505620106065	68.0342444360948
	75.6614221167993	105.009081898873	96.8490167966217	102.971363152434

EXOC3 257.325282051981 197.232697438521 178.904972763325 236.242651711871
461.258911881166 335.655540947167 375.818861583151 228.026217504784
220.398778650109 292.800712773283 323.453481668415 167.639975497722
EXOC3L 50.0466349801009 45.0905928998779 53.5698357689852
50.5310598656998 44.0451487108213 43.6670755115848 39.8695929627783
40.831207953647 47.27543600687 47.1001757441025 48.5292718280498
43.0930293831497
EXOC3L2 43.1805016690297 46.6996902348633 50.9010149857308
42.6913548400127 40.2449343247539 38.4330755397458 38.8417151851134
44.0390019242862 40.2274522635576 43.844855336215 44.0409178714232
40.2669497398702
EXOC4 60.7595361363354 66.991625911443 72.3508448479791 64.6945454069792
65.6003544901554 58.2108487642117 66.6894494536346 65.7536901239228
77.6052681514721 72.5153335566211 79.7020636215451 74.2984889814139
EXOC5 55.3153799233744 60.6080172588224 44.8872784170036 57.0629653735694
50.2163892855092 56.6172748471831 44.0687697278114 49.6203214143464
61.3733228199803 65.2361525412756 55.6151308605445 52.3896785600751
EXOC6 51.1347974668251 60.7495043977707 51.1347974668251 66.4510869175159
54.1731035633439 52.902043370862 66.1940959364976 58.2043532960091
59.0850755856409 57.165971310746 59.897987870592 57.9941756686635
EXOC7 360.668308246913 303.139550962377 326.250872625748 163.126491735808
295.848968688724 253.980053738171 321.61848221455 274.259417498569
342.643344197936 329.978705095448 228.615793354146 234.727123301682
EXOC8 40.148780650831 46.3635803515159 44.6032218589199 42.0102671859606
42.7982502551634 42.5660695551615 39.1539892144371 40.3357777530419
42.0621425894624 41.2276963324981 42.2618615571566 33.6902293666089
EXOD1 43.5710871617107 44.8529259387308 59.4431489877328 46.1673159982664
43.5544347011646 45.2852528185486 46.4388841354122 54.0507720792049
42.357910063876 47.9056710878325 44.7926323492519 40.9683275553869
EXOSC1 158.590601962928 214.586915093492 285.689076149891
235.294634126766 253.207750345072 216.699423864285 239.96421556928
249.995119246929 213.380798420955 202.667616796658 198.432987946699
241.849566505679
EXOSC10 207.0155359394 228.499788774214 223.911125639211
176.594203350958 307.656792056993 210.958578914607 292.247095517604
256.165801611585 242.966185793816 177.55994726993 214.369365247268
180.255771684507
EXOSC2 75.8122107270212 105.563773939475 74.4508307047509
73.1447139654971 91.0327980861321 112.23915619297 161.439879456216
80.2680308979464 107.961407367823 88.3335649692467 94.6300022444815
82.3254961129575
EXOSC3 206.571946834892 184.808179948172 92.2095095678497
148.333778341005 275.314994786888 161.948193932343 277.367257933324
117.547659489742 161.768763813052 116.891649452816 148.964204638494
186.372887375058
EXOSC4 207.943044411392 177.067668324062 123.908152883436
117.071280682786 301.728842562175 126.319731178054 155.239308003808
120.850874262069 195.968119243879 85.8795177524168 100.774098314706
79.7238173467616
EXOSC5 199.079207384598 198.652987129375 208.67074469962
140.662969373546 503.661461720233 166.734730009916 353.463970335835
268.936151471133 298.988745247509 173.5606219477 181.912232315225
167.319747725488

EXOSC6 293.355303451014 531.69595054137 648.885062676884
217.575115287224 318.609131605258 279.841798764418 407.818713538416
773.781266760336 399.21864362319 334.583159766096 316.917793468502
306.174148874454
EXOSC7 170.450752247109 260.581111456578 220.398778650109
181.983177572371 413.067407579649 209.467874193952 226.926786963011
203.109765192983 328.295200683554 208.22938545273 257.506228440451
295.194516063502
EXOSC8 106.804269453898 119.088986733238 110.302475164041
105.934378627264 117.689172019617 134.375403847987 170.327588764719
110.599131324937 182.643498505575 123.704998437432 126.920579478593
139.472956453414
EXOSC9 68.3234331335073 80.257554904218 68.6566757444066
86.7823828209946 81.1416376689959 84.2880616879945 95.6697427870979
78.3542840447759 74.4623288220969 73.1407519625966 73.9126724639466
81.7566533443175
EXPH5 45.6517320730042 50.8812920005251 47.0940613507321 38.6414316877471
45.8051846088552 40.5281496800812 56.6537562775715 51.1694248620208
42.4540072551568 47.4767851750188 50.5188367542048 46.7182294568603
EXT1 222.426799131736 213.830284761604 270.156612086731 155.287591646469
337.736535374405 159.747484721905 143.497887869284 156.719353642505
326.250872625748 206.989423961124 197.720854621428 294.220232101389
EXT2 87.5759422500374 98.4930941487534 103.010279797396 86.5414905689606
90.5860767851498 100.087328133967 73.3042538589134 98.5828998914961
86.503774181307 98.2180003781859 93.4494731509092 103.010279797396
EXTL1 50.315768338317 41.2598458726002 43.3085358214154 54.8412943008635
47.4548471240051 43.8582747295766 51.1541843174981 43.9366081640576
45.0017158586743 55.9283039581662 47.0940613507321 39.8497227447888
EXTL2 129.525395445221 111.581224629293 122.052907748249 66.3188075271379
101.2970835312 135.527591897994 84.4045322633219 76.5532859161089
78.2393202422664 95.65872032796 103.14577985944 103.876328830067
EXTL3 160.566474370004 95.6662924228901 134.725028732282 127.986477405999
108.063001800611 158.843766201769 92.6545309624315 86.2118355769367
108.815245119101 176.506718222092 173.328468320432 136.355036262681
EYA1 40.7063426028871 39.4569929845912 38.9790184198145 39.4637566422506
40.1207285185295 40.9506062495231 39.4600924554505 39.8812234363661
38.7537364570873 42.785429206422 40.1422119692495 41.5916331278821
EYA2 57.0504111569238 57.6634521289201 115.761674855716 44.6286581923295
43.0826863247766 45.017247592678 51.4349390827895 69.9984754033658
155.287591646469 60.0856496605043 54.0669061445608 53.8803543493797
EYA3 45.9504618460054 43.0227387991135 52.8427643366617 54.9765783642973
74.8893925448138 43.8582747295766 56.5914743235484 68.4261608261522
53.5677764726461 42.0217776707284 47.396715991429 45.5435778478662
EYA4 39.266565827316 41.2176232897704 39.7683846616421 49.7350276169552
42.2331334096358 57.5398008565764 39.2809305246091 42.8036731479932
39.5377896745682 43.0919221375514 48.7049943037859 46.2072687763147
EZH1 49.7188990489616 43.1805016690297 49.4573631027613 59.0965219753805
47.8075998240396 52.1024135605001 47.4439407315335 45.9604707098953
48.0163377492228 52.8928296360463 49.7363778831003 46.7182294568603
EZH2 63.0266170942566 73.3395284923099 66.9239565096247 67.2714646016644
83.0497574039035 45.1873487463472 99.1769280484821 56.032814067931
115.414481087246 41.1222709786153 40.5750645132377 42.7303139017786

F10	39.8662795144616	40.6380495994768	40.8420925848403	44.0451487108213
	37.4470183315826	291.232601825013	40.6750540874299	39.853866204927
	34.3842972343227	131.380698046552	94.2275161759277	57.445796273665
F11	40.308802735799	42.2114134935898	45.3675390992851	43.367124590178
	43.7517118200398	40.5805725503899	44.2166118829877	39.3113207075992
	44.0256815382334	38.957687788868	43.2551508830106	44.0451487108213
F11R	68.8698243753339	88.2427413480277	85.3718124658316	73.5834042105313
	79.7238173467616	49.0497778708674	75.5830548489892	73.0855167819246
	199.404836798715	103.343354834837	85.2598847559951	102.49785018292
F12	58.3702889417801	73.845912534353	76.5699943825825	55.6481366056639
	218.958199087236	56.1671440556142	87.6018870099686	111.404796541459
	186.67069792963	56.5379973844443	60.6505672212119	90.9361642101537
F13A1	77.6056319020116	150.114520857436	179.040571908629	132.205423909251
	94.3450965454031	521.874349042344	76.7483302168542	75.3462586192013
	47.3791873993182	80.6634117492762	65.6010220593397	196.709620264975
F13B	37.6397652536077	41.8073910693411	38.0452862258803	38.9534635879195
	36.7356360093603	41.159706131351	41.1507980856436	40.931294690669
	34.0382693188794	35.8304004211528	38.1835938174625	38.3291713594599
F2R	11176.402230735	7681.71833395626	9060.10165509099	8259.38594893529
	6749.14636741447	5018.90384962952	9852.7965902602	10072.2414399735
	6873.84878164654	9488.06491634484	9060.10165509099	7627.46814033012
F2RL1	115.321592104747	134.099684731177	111.465167479654	85.1847490113446
	83.1225347811557	46.0905079010237	127.148417780972	124.94083215058
	209.178131628825	229.26498093497	214.127390534906	393.648308810931
F2RL2	39.1059888887285	41.8073910693411	38.5045333213784	41.7614975630362
	40.6308525754377	35.5523662009011	40.0603121770344	38.956280281655
	36.8127605061228	37.6637152940566	36.5359455830388	37.3600014814388
F2RL3	48.2549905619119	48.0296332183129	54.3554233228353	54.6089451252049
	50.2953638261991	43.1180113655693	47.2927436772465	50.8160430472218
	48.8067321016961	47.9707112435212	46.29198852834	45.3763275588341
F3	52.9849979441253	53.0383634135393	47.2505395290129	60.4007755900733
	108.337776546183	72.4651758215622	36.03517591974	99.1438953564062
	61.3216070188812	47.0055981216687	73.155721596638	55.7802841481067
F5	43.9309845324767	39.5050383901884	43.9450023643394	74.0093517743852
	43.4544762830742	38.6414316877471	110.589130460258	45.5581356216957
	45.2897806651929	52.2256177934134	63.0763092522598	53.2046867327331
F7	39.2958230580497	41.6972107715212	42.2857372794461	41.0726336841311
	37.5044776781216	40.5710637884029	42.106632087165	40.781946829774
	37.6383530120621	43.119837808537	38.6322728994674	40.1249533205858
F8	42.442231667357	41.3366779545815	41.1367478979484	42.5321207892864
	44.0920305031178	51.5522731200852	40.0676341303791	41.6045220683604
	43.7078399718247	42.1317134987795	45.4799270968969	47.0775611502299
F8A1	153.384919558071	94.0532777456537	108.700838142885	133.541341560868
	217.293240552946	103.343354834837	158.769954040462	127.418173390084
	186.965367827758	115.509409633975	109.076358658606	92.8006484232438
F8A3	50.9381343674699	53.0063130674306	52.8041782638549	45.8145523469421
	56.8307116636372	45.4607426241257	47.2927436772465	42.3887531663733
	46.7414272831181	40.1613888954735	45.4799270968969	43.6753843488695
F9	46.8141313516997	41.0678614026819	47.9035532886869	42.0102671859606
	46.0977687311222	54.9108046955355	49.6872467499977	49.5185112777773
	44.8003345643334	46.2286164776607	44.553780984244	45.1735152121056
FA2H	44.436995101921	61.8492980174846	59.9690744676539	35.8472302065546
	41.9842695818285	40.650742261428	63.7721358300879	90.716460692818
	78.055431384988	42.6793753550854	50.6473771879221	49.5539622682049

FAAH 165.775214685061 123.768417863139 101.693848821246 43.5627649099623
125.052841200703 45.6129956328415 160.917024416961 229.47693624489
208.023488988931 209.860757709908 219.64979849407 145.14768503072
FAAH2 41.2424637338424 59.4253780950488 52.3432130135266 35.8472302065546
42.1650127805175 41.2567470068038 44.9863726205428 45.8669209716694
61.0094935318105 68.2708120569221 61.7572675712557 54.4992295592001
FABP1 38.6454098044474 39.3999056968598 37.2838080935695 44.4367387274922
40.2449343247539 44.5544727111028 40.6750540874299 44.1311654985682
44.4395580493118 46.2286164776607 39.3667161654772 44.5432192783161
FABP2 47.4678477186801 40.9731067463382 45.989212296035 52.3004056941891
43.7517118200398 45.6129956328415 40.5585676679482 50.3972033014227
42.0621425894624 50.8826620053386 42.7275315121163 43.1805016690297
FABP3 42.6036491382658 37.7384958256497 35.2838283525874 35.9521941716188
35.3327761997046 45.9266745571724 43.3262148375525 39.2672712041774
37.4540772310118 41.8613406887824 43.9450023643394 49.9692385479389
FABP4 34.8099089553046 67.2649356062263 228.134171304754 43.6726153508978
45.3675390992851 175.028390974125 37.2892641289258 84.0899854321388
91.8414653633828 485.698327133705 416.40156274382 1243.32650314893
FABP5 56.5319193881205 1604.80031304031 986.40790007743 207.833867050257
407.818713538416 141.69593269802 202.395470754957 104.351256270039
516.848416430243 239.792011765323 248.390047812338 508.474794786176
FABP5L3 40.4639532172338 55.8046062733709 39.8299340037009
49.7350276169552 49.7235523248653 42.4580504587581 40.3302268293707
44.4031868028216 34.8552366193358 46.6598879466655 43.9450023643394
50.656598108034
FABP5L7 42.7150227340364 39.3999056968598 44.0451487108213
43.3078542962968 46.5557478175976 38.6414316877471 42.0605906240248
42.2577689927563 39.2809305246091 41.2276963324981 37.7384958256497
43.0930293831497
FABP6 66.6118713143653 146.087016079575 351.764130340952 41.8291484190524
186.54041506387 68.7342341724306 62.0404348860702 141.108035324183
229.428422408702 108.04376659067 63.7024494337186 90.99086730704
FABP7 43.3638918705225 38.9338521051669 39.9101908547397 49.1786207920603
40.148780650831 49.9699131196797 41.706879587091 39.5466975353131
42.6366765186182 38.6454098044474 39.5399188536544 42.2577689927563
FABP9 39.4250105372135 38.5252456306656 39.6451483836089 40.1362682639916
39.4693871570028 40.8091178323174 43.7585538437696 42.2577689927563
40.8420925848403 40.5571841955033 37.0180934800573 41.0226232127351
FADD 203.53267380432 231.361506886859 255.598083334598 177.067668324062
253.764071470001 164.62751527755 271.138311888031 166.734730009916
219.34503297115 206.514263103112 185.874151272261 175.378964448101
FADS1 78.1313732870053 55.9644250454789 56.2441895183416 241.08817915181
188.943404504867 87.0685571351947 270.662475158799 126.470889051684
502.32041794318 68.2708120569221 62.5036823572932 84.5998418020787
FADS2 46.263826584899 42.7775752471711 43.5710871617107 67.7121534934164
60.5290966885203 57.8005007012205 97.29969628425 41.4508241771315
141.017696819844 45.0155543555707 46.8954576545929 42.4607795638552
FADS3 44.0328992213727 38.5252456306656 39.8299340037009 37.1894304543751
45.4751076037511 46.8954576545929 41.253645578517 45.9604707098953
42.5406888281114 42.9866214102525 44.7114813656401 46.0633179231318
FADS6 38.385865183581 43.0227387991135 35.9305494063137 33.8111782249716
34.6227169056411 45.4882438975269 32.7676164492555 34.3865306339645
37.1820543575123 40.8970401536578 38.1835938174625 39.2469742642337

FAF1 195.340273658052 136.22176708108 204.344278991396 133.86254468037
236.791729190996 204.862161704394 206.103296228683 189.398959817735
237.632703283663 165.706343725482 166.208731611481 152.86270446968
FAF2 632.019639546131 302.422475486532 463.022816882856 427.281740164881
412.421643885499 301.357530757236 397.154258378193 445.016226732679
604.951648398924 667.583623414832 549.407911286514 561.2769907009
FAH 104.832753086298 95.6662924228901 89.8354888041611 146.573018487871
167.319747725488 123.768417863139 102.852046099268 128.146059846414
158.884110481815 105.29989739819 109.844156801015 121.562769658314
FAHD1 85.5881739836215 123.506372870621 125.27802197261 76.3693529894936
100.114114765033 78.4815128101967 110.048921728774 58.9533374723051
88.1910986077499 85.3057627795203 95.2915739972533 104.486555491331
FAHD2A 52.109723558511 48.7847528954724 48.1997158668729
48.665578118898 51.0107745412152 55.401776072578 52.4111687156893
52.2416165827554 55.1253465069845 50.0564735608363 48.5292718280498
51.3872161199373
FAHD2B 158.702003835578 100.403196213407 169.557757933574
134.567947469561 191.730944258418 173.640820092779 193.60925192949
213.830284761604 226.580055691897 200.483228109704 265.367588517131
197.103673938152
FAIM 50.2203578628217 52.5645416722441 62.1642339365777 50.133362781069
51.8446107597512 53.8461734834857 57.51482768194 49.2458645560476
59.9326265577462 43.844855336215 46.3370338074996 55.0226286701624
FAIM2 43.0930293831497 45.9266745571724 47.2055133646385 48.9944486068347
40.6723334103791 148.144200968432 36.634024014558 40.4359177791566
42.1594905198929 85.8795177524168 88.5511408870489 54.5921287593554
FAIM3 60.6441065119394 159.463273263774 86.1563473462864 231.824347524066
58.6481644777552 52.8421046802739 103.913834330352 108.083751469991
51.2776048338 60.9905272201951 56.1379957867124 95.6731932757489
FALZ 54.0170817319522 53.6429962450784 46.6588655218735 65.524821699432
57.9161791762225 58.8605811695948 50.5252048155459 43.844855336215
51.3511088648386 41.9666541498638 46.8954576545929 52.9609836781499
FAM100A 164.404486294867 172.206923437536 139.620154641549
148.255880793905 147.285007921741 154.633672616367 179.522572239758
116.67447696188 209.31241212773 181.2179249806 211.926421068313
100.049462976601
FAM100B 133.067727272864 149.284007723184 125.794379028627
215.345160952019 158.464244518317 87.0685571351947 228.185436206823
231.893992191022 206.103296228683 109.192770642308 117.8523906126
99.6243435693941
FAM101A 44.6231541342 44.6918600441328 35.2838283525874
34.9221408381038 37.6527901109005 36.1190722948669 37.9430145836712
33.6816578730693 41.7627910693483 38.6454098044474 37.8372168909583
38.1421709520616
FAM101B 49.3000844711116 50.5110359971301 49.4573631027613
53.7155925246916 52.3034212084073 52.5330333302271 42.9803717121865
44.8727287473605 42.4540072551568 45.3894119568143 48.9184531207247
45.9159813905292
FAM102A 122.316617618658 79.3614922672599 68.3234331335073
78.5141640924736 65.5468832395274 85.4429281595877 156.061711961514
127.708268637068 116.17887995398 128.996351661564 94.7008981904016
101.086264355915
FAM102B 57.7877627794725 55.0897019602305 50.2869791217819
133.1387411908 45.8051846088552 102.499011190932 72.6959688212342

108.213380368446 100.874437079608 131.645714321903 100.45351084408
128.230543301378
FAM103A1 104.914715637173 77.4307558516809 79.6133633483649
94.1680559848644 117.389308836013 79.6895408438974 92.1455386560941
105.382126899524 101.954354107731 104.824641162138 89.5887648181505
108.007227281411
FAM104A 229.159869771704 231.472806936915 301.457545514208
237.237762964658 248.963785838427 235.648018909 253.980053738171
291.5513298383 311.301613620475 317.560970617018 268.936151471133
255.087214364926
FAM104B 75.1701698483856 78.5174639327946 65.9476039189301
75.4466520363487 65.6003544901554 105.414552529943 99.1769280484821
85.2849531543957 97.4062608315831 141.386644354989 120.980558571109
112.113501370522
FAM105A 40.9558083259517 42.4493032770053 39.0775870687758
42.4580504587581 57.5685357492202 51.702461863489 35.4623691645629
39.1652965788406 41.863927676552 41.8613406887824 44.3763859390287
53.3059058105836
FAM105B 123.608921653229 97.8764110456132 105.376705821141
136.644759501653 116.22441634196 85.6038071277319 185.079035617027
98.4405692451144 140.187433247353 108.525500270474 109.677588309718
95.9391851122771
FAM106A 42.5534565507139 39.0102066700839 45.1569741609597
40.0698728584484 41.467005949568 41.4508241771315 37.1643693306382
43.5544347011646 38.9496515344837 39.7411455670172 38.079222289614
37.9607839934735
FAM107A 40.9558083259517 80.4822328865 111.735319884512
97.8309399470621 67.8233625051884 1041.84536202049 35.7055410599146
78.8250642061439 38.2215591930186 288.923207993626 658.282562295783
264.161390133378
FAM107B 149.933827818309 116.967446594767 142.012216533823
397.020287751914 131.632115558243 135.985408824299 207.380865650247
643.218735985767 192.861174667738 182.976132230487 242.932905246176
312.469507672374
FAM108A1 38.4330755397458 45.1390218677367 40.148780650831
40.6892234267786 41.8806343997257 41.7256940298128 36.4098694708483
40.831207953647 43.2761454096265 42.233842834629 41.9083581529276
41.863927676552
FAM108A3 84.8569316561572 123.674164545425 143.068901273486
117.699959435983 196.17328008185 145.170590045628 92.5391033814713
64.3284163241986 133.074560812703 69.6511571901941 76.0420039542595
76.2533853403813
FAM108B1 61.0831990510269 84.0305689846475 59.2455550633943
47.2862299990937 63.1646322810687 51.7594352155304 66.603126065489
66.5234828659154 84.3526980051279 59.1496467460507 58.0095518210499
67.9410473129639
FAM108C1 1033.99699402259 1125.31394383117 466.695081390256
331.533249823589 568.371815312895 127.115038236278 805.87120346756
1384.80615431656 1064.35745220225 727.181489302632 558.451033789799
1232.7632290973
FAM109A 114.148080724618 80.173718023183 71.0340758556049
73.1447139654971 75.9136854087044 67.6817814091697 104.341160312305
62.5528124447176 87.2282539094452 73.1407519625966 61.3529667465225
55.3563533836982

FAM109B 75.1701698483856 86.9709077763908 65.6620676514157
67.1961800572759 70.3677225924146 90.1080159649841 68.878562323012
48.6441988290952 58.4891791448514 72.2443746456494 57.0154839296649
62.6868461044048
FAM10A4 101.523741547876 122.0305509548 138.036631806718
116.663501139305 90.7345603183196 166.511915820497 84.5354986509039
123.597489107285 125.801834682035 153.059039745191 159.147713331732
191.896416838806
FAM10A6 46.8141313516997 45.4143854024453 42.640829914102
43.5627649099623 44.0451487108213 44.7804080899431 36.8369172900902
42.157627240486 38.1169703906207 45.1569741609597 40.3452320167286
44.0451487108213
FAM10A7 47.3791873993182 62.0404348860702 61.2679059816982
44.4912617551615 53.6210959113222 81.909815795953 41.253645578517
48.6441988290952 58.9870089759222 46.9598474201212 55.7868713984479
68.5174271063894
FAM110A 176.653849515825 152.972693071526 171.33368481035
81.0486107101125 199.634544522948 70.7782698163955 183.584085676174
157.639656300073 138.170787392253 172.416351632442 197.923403667051
172.869292380866
FAM110B 51.1055541075029 40.7952353240579 44.8872784170036
56.6458456890715 50.7615314686244 62.6839349907803 38.5066108855005
42.8482181313226 44.8003345643334 80.2774079897733 70.8670844767965
53.1182952086284
FAM110C 47.6397712007919 54.6089451252049 56.2441895183416
43.074472921841 47.9128504683618 39.8455060654531 38.4005795891396
46.8954576545929 57.8772482723463 45.9004543328976 39.4410515050885
51.0013344286851
FAM111A 141.649461526745 255.126812649967 266.905855859261
195.968119243879 85.9392883641626 118.806070800059 195.672836308456
325.769076169162 222.40005420611 233.326886009799 185.395460281034
168.492959515167
FAM111B 44.1294459089314 41.4508241771315 44.3344900481112
46.614092935749 41.7929491793697 35.7034203372528 44.3440839686558
44.797320243931 48.2123120250343 43.1839109702714 41.3553369544097
41.6550823405646
FAM113A 115.557690369703 99.571118823509 148.255880793905
75.7666827480067 151.724932189601 60.4065050472082 82.7853677822046
80.3860547572521 89.2056902297352 109.045804556923 113.169111095305
104.936456561176
FAM113B 1149.02954135912 300.732955139561 446.902178094675
303.139550962377 112.721024337362 123.61359914862 150.374739898477
191.394143505573 270.156612086731 328.341232263338 249.995119246929
392.622487173359
FAM114A1 66.7759319948677 79.4710406831985 64.6111899743678
59.4674205670657 110.869341159607 109.832638804839 58.5087296620514
50.5070012045444 70.5699797830702 65.0928075335306 64.5347406608537
84.7947249929138
FAM114A2 52.5792653122861 44.8529259387308 57.7415362494584
58.7338159410728 42.1650127805175 53.565717255469 46.5650556481721
55.3122077514658 48.3119523820103 53.4109882045447 50.0303076717704
60.5948704279864
FAM115A 1524.01520893763 612.441960343995 733.358170858378
927.61341837276 888.56944380788 964.268294825556 1075.04105984319

1806.22468459769 598.686989781235 1824.79010714035 2002.0536042686
1349.09482344633
FAM116A 85.9970657980042 113.198141130994 97.6663462144457
114.611557317665 98.403191200835 120.231435193839 131.496769528338
123.022511916727 128.616776019243 182.448444305445 153.028210322286
146.389547177092
FAM116B 153.028210322286 127.043177339038 113.539485042978
145.526972410673 141.180682718758 81.0880833950735 107.148000219752
150.405742308999 100.874437079608 128.873145026756 119.691133736693
108.479856042609
FAM117A 35.9305494063137 44.0409178714232 45.063792968927
44.265834798964 53.8260140375214 47.2009420656854 46.8613212048981
40.2382908228517 46.581796165231 53.5498255316009 51.3103617600342
43.7672102682067
FAM118A 88.0186381834147 161.326580647514 66.737740728688
47.8164193292842 108.251353392321 66.1082848714279 92.8579489575841
735.60603239712 163.011116329807 65.6721629145797 83.0072253231162
80.4404044229341
FAM118B 59.8248482438562 47.9128504683618 55.6865353018811
49.1786207920603 63.686652234709 58.4435847520801 51.0013344286851
53.1052228246467 61.8824218975538 51.5855515705917 55.6151308605445
62.8485724004124
FAM119A 1130.2486883492 807.223772452842 686.154904023493
1035.67547271468 461.83420090894 500.549865625701 941.514118803572
1390.12380748446 527.439095170064 1712.55038816005 1425.37639004849
1084.23003918768
FAM119B 158.478919789334 90.6269311015532 146.87603018486
106.8866235778 128.532373805894 105.317399386109 117.634238804678
101.738441694376 102.926945888985 152.649165330093 121.449647909665
122.914175013196
FAM120A 781.223434214089 846.773659307087 635.050535522135
688.920755848722 894.486674059951 587.88126282119 807.620534408416
995.397944432196 714.075528895169 1078.86166154061 949.923102632043
999.862452730252
FAM120B 464.671881584567 482.238595345574 460.994460486229
374.601792595646 338.000998124097 383.705244574204 287.39391763763
244.736442646499 233.468474830926 548.217757366495 415.364456363434
371.709014310561
FAM120C 43.5710871617107 39.9697332660833 44.5134109184888
40.7481155008596 40.4639532172338 39.9397419279057 40.0052661202207
43.3262148375525 44.1519466943185 40.8049408996274 48.4717951775049
46.1592873342534
FAM122A 59.5711567415697 62.6984594468418 54.1637401903523
58.8224410806741 56.5094373275922 72.5230902197916 63.2726414544553
50.9099078696349 64.830448408529 49.4006068471301 58.5450868286167
58.4598072919688
FAM122B 133.626390404589 179.241252961119 144.079981029616
97.6774247176874 292.065366215278 106.317388820963 135.323750661937
187.147252401703 164.882513036349 108.525500270474 118.10944558223
145.659132843873
FAM122C 45.7356815244513 43.5182502375207 43.7630587657205
43.5627649099623 41.8806343997257 42.2577689927563 38.6243997507646
45.3872406432794 43.7630587657205 42.0621425894624 39.153226699662
43.6753843488695

FAM123A 41.8409927585705 44.3529919625344 39.8617019260946
45.8051846088552 41.1868052124344 43.5752393599169 50.9246396457383
45.7476095696859 45.6582729799009 43.2988017098387 40.1422119692495
42.8864415494484
FAM124A 37.7384958256497 37.7384958256497 43.401782064778
43.1847628331565 40.8873907150245 35.314469458379 37.7136011474903
37.5511560880902 35.3227485563945 38.6454098044474 36.7283081422707
36.5474283500601
FAM124B 52.2236479204029 51.524711857679 62.7605715573218
84.6435656850129 57.8039717833131 50.3245489035833 49.3854238365325
47.2864663693787 43.1805016690297 52.7972156492746 56.233217375698
53.3904307221902
FAM125A 372.982648892131 277.12373983597 525.687522832693
264.924262411772 606.481020597082 263.799439226561 253.072560237983
351.8239141662 499.356036793973 336.679355848349 475.621740714165
319.954889553226
FAM125B 50.420938814754 124.156774983746 60.7530213660442
57.5666238219027 57.9968895221633 82.034818940429 69.0530953932111
65.1041577962151 70.2725751014865 120.533617429145 105.197692490363
88.4036642243438
FAM126A 43.5710871617107 41.0678614026819 42.640829914102
44.0991023527481 42.1386542162789 51.2318935933116 43.6753843488695
40.6663433492193 41.2870505273664 43.6033221838149 37.8710943171479
45.9693232070002
FAM126B 62.3042205863409 68.009873198833 76.889690218685
106.8866235778 63.4427357028556 70.4482288143151 70.6592421895338
63.5295088521896 77.0244607682203 60.1912192309911 72.0389007415456
68.3604212141462
FAM127A 547.442608452594 437.280601907944 590.709061039001
409.312313055794 675.832363405287 952.16345035163 527.97309138633
497.290172759099 395.298033031033 915.936211119242 728.257836227637
611.538630852232
FAM127B 186.891912776245 143.233722839451 159.440571032045
94.1680559848644 231.085533860683 163.126491735808 255.884444464408
175.41042153356 178.289913291111 204.318269745079 176.43047680293
157.774096599452
FAM127C 56.3471340054074 57.0082102559228 73.1262465119304
49.7350276169552 68.5888253504788 55.1987351219665 66.1940959364976
57.037639245179 70.5699797830702 75.3333024462557 65.342842101283
68.9885481504003
FAM128A 46.9939712192224 39.9697332660833 39.7411455670172
39.5050383901884 39.4693871570028 37.9951997351435 41.706879587091
38.745552403895 36.0741672064298 37.8217989841918 38.745552403895
36.3454320848591
FAM128B 36.7356360093603 38.7344853699576 46.5650556481721
37.9875302985336 43.5544347011646 36.1190722948669 40.3302268293707
35.7973685350741 39.1790517315259 39.0648733099061 40.547835538778
36.5474283500601
FAM129A 47.1962846565261 70.0470864733496 146.658116519622
468.153823329361 57.2960245538881 861.427193617946 88.7029126110977
73.5950442915143 47.798305839406 249.411435337932 357.668448180268
532.981045084589
FAM129B 549.407911286514 487.749060801066 297.187417426993
347.552309199761 302.20349084931 247.676607756929 413.918050849658

366.129480083279 483.416873464931 331.306005726359 333.122044191102
362.506098778318
FAM129C 41.5421429082231 39.7835513496873 41.6079958749402
48.1662933382789 41.6806984441849 43.4681646416585 42.357910063876
42.8482181313226 39.6609238036815 38.8507804383261 38.8507804383261
42.6758568421347
FAM12A 49.085741080241 46.0713306971594 42.8270038153139
45.0798408927912 48.1473938886239 49.5424425850549 43.3262148375525
44.5029690493211 51.3511088648386 49.0841460667818 53.3078448066074
49.5539622682049
FAM12B 42.8036731479932 40.4359177791566 40.4882741869718
34.2080003860523 37.956732373465 41.554942319225 39.2533668167483
43.3262148375525 43.3744503319109 44.2580151149956 39.6403089864678
42.7782216342806
FAM130A1 121.449647909665 123.768417863139 110.734219768388
103.961956810572 112.391188787919 102.24202987437 109.255307556049
141.376779431031 137.395516155158 89.4747568145717 84.9601800557888
99.6243435693941
FAM130A2 43.0930293831497 44.9370339275041 46.4569566520107
52.1201052574162 31.3942305191093 47.8636003588181 42.9803717121865
43.7396619462033 43.9450023643394 55.2214187655851 47.8284278211109
39.0536056496869
FAM131A 101.448989278761 107.585504340548 112.007118067348
97.0813208326187 57.0504111569238 181.2179249806 65.0031119608637
116.547937420457 41.7627910693483 96.5102825310977 92.9043863423147
120.392561825049
FAM131B 43.9309845324767 48.7084357480922 39.0775870687758
43.5627649099623 44.7829632472754 46.7800033479223 44.9863726205428
49.7309743973184 36.197424053556 45.2556760873335 47.299647867533
42.4607795638552
FAM131C 44.1737502492846 44.2630383225933 40.6737160001291
44.265834798964 44.5668141895992 44.9370339275041 41.1886975241176
51.8347877274018 55.1253465069845 43.6670755115848 40.4094901248527
42.2577689927563
FAM132A 53.1309801459151 125.676172266736 46.5650556481721
50.8567345921473 66.1650385535549 47.6803619768589 43.0930293831497
42.5435283762933 55.4030910454028 41.8613406887824 44.7114813656401
45.0985088502363
FAM133A 38.7537364570873 42.4493032770053 41.437237454134
37.651298468202 41.0885562160946 39.8455060654531 36.9355798143571
40.047314525032 39.2809305246091 43.9450023643394 35.6970528713709
36.7762265039864
FAM133B 108.156702405177 97.2004257862686 104.557637353314
103.447730395424 101.789162379972 131.990661474974 113.322929203297
166.488589768213 114.174791258883 134.908982962131 151.425712904293
123.247207157645
FAM134A 168.78407943637 108.39424630351 132.307107503819
148.529482776555 141.410183156424 106.317388820963 115.691017747672
198.705875080904 168.036319777283 355.00914527669 182.576662575358
167.746168796779
FAM134B 77.9820921829024 42.5269793679335 39.4304889666789
65.524821699432 37.2949506366592 63.1780854659428 71.4564394972198
59.7744650739434 67.1278109419819 59.4969799685856 49.319499137678
48.8670821164567

FAM134C 50.0466349801009 51.0175677516029 39.0775870687758
39.7411455670172 39.9697332660833 40.1428591998404 38.7337086062875
38.079222289614 43.9450023643394 44.1379638573455 49.1067959632109
49.3087627580849
FAM135A 51.413025495745 79.508103983962 74.5528557785378
47.2740099216972 61.6452971581503 66.9499465764301 58.7585623394335
55.8087097725119 67.8688083153991 67.0489032883986 80.4239296853518
75.3696105759836
FAM136A 341.535750991033 250.621261212308 233.850715388018
200.401133392259 318.609131605258 183.002191434723 316.745819100367
381.533380474563 244.786768738229 203.353724675525 169.889618506937
189.516455814322
FAM139A 38.5007204246293 40.3104838541906 40.5386195997545
38.1421709520616 38.3561682218911 38.1169703906207 42.1372626664516
37.9273653326866 44.9645354363967 45.6868589181768 40.547835538778
41.8200132437731
FAM13A1 58.3453537594243 85.2364387652201 97.0666517150668
52.9352788093583 64.3571664944642 103.67004664951 56.4199442234707
56.6188723715434 117.626009607247 67.4256666294142 67.1477341610979
72.9580216367379
FAM13C1 36.4179024367746 43.9421822132391 36.5099686711015
41.5464765641928 40.7215101318274 42.8181679217665 38.5512429290626
39.5968185906126 38.5019918517688 41.9666541498638 40.6710390406154
39.1011392200677
FAM148A 43.7585538437696 34.9262464079777 40.8420925848403
47.0874209746031 35.7973685350741 36.0306975524309 37.2892641289258
33.6816578730693 32.7294532998931 37.920964499903 34.8383995317036
31.6419446793642
FAM149A 51.1740855632044 58.9128486419884 37.8386378737561
52.6947911480086 53.4109882045447 300.803955139552 95.9298109273155
89.140025661759 100.076931518374 156.456239358718 135.824520574227
93.4180535865914
FAM14A 92.1908585323292 55.0897019602305 91.8533850418261
142.945182805248 62.588825192561 114.526825145076 56.3376046940285
74.383456365584 67.1278109419819 92.6888475289584 79.0304119352941
71.501028931626
FAM14B 46.9939712192224 42.0709558258414 41.0356368374708
43.4410741327254 38.3561682218911 39.4953494583071 36.9355798143571
42.2577689927563 40.137818555383 41.7405682794166 37.9946685306274
42.1537476443423
FAM150A 46.441301934328 363.324816966828 57.7415362494584
39.1706564265495 80.1294564809102 41.2567470068038 169.970634268117
51.0724630072723 74.6277165793069 74.2379710965903 118.385089099623
315.592187396219
FAM151A 36.1232327599945 35.0668743708816 39.6833800000587
40.0698728584484 41.7929491793697 39.6251842352088 41.8751578329365
37.5511560880902 37.7384958256497 41.9666541498638 40.0766543123257
36.2582325396375
FAM151B 43.6706764143908 44.3529919625344 42.640829914102
44.7980740332174 39.859345745831 41.3553369544097 34.1889222434768
46.2779564481158 40.5912213523345 40.5090849993014 36.6298807161755
45.2783024981315
FAM152A 172.720244584464 199.404836798715 157.281394920992
162.932479392087 195.205980592503 236.488376031891 166.910579215196

191.284436365696 239.569514501529 178.219279777774 171.938863440177
211.625447303889
FAM152B 221.195872577098 320.966569449558 268.808342880355
289.332274319357 297.332750143067 163.360824752796 227.739219647972
234.333935191237 330.391945158969 130.62791061956 152.66454549157
190.161505611036
FAM153A 42.7150227340364 41.690833180291 44.2405728317107
45.8145523469421 46.7508829977587 45.2852528185486 42.8684958525042
44.7044140979108 45.0017158586743 37.3179578037963 42.3413611855132
40.8098117292321
FAM153B 84.0388582916959 59.8102324370568 73.0128142320238
93.1668080716761 60.5290966885203 63.1091746640396 85.5027511448958
143.822560762049 61.9682048429354 123.301491241188 120.20612443357
120.807573842238
FAM153C 43.8582747295766 40.8476436931481 41.8389336728199
42.0621425894624 41.9842695818285 40.9506062495231 42.357910063876
40.188873541274 46.0633179231318 39.8455060654531 40.0766543123257
41.9430737035925
FAM154A 43.0930293831497 35.5310164682026 38.8775560911841
42.8235313007746 39.2608760193011 38.9378717994178 47.2927436772465
38.6454098044474 42.9020321355927 39.2623423164488 41.4508241771315
42.2577689927563
FAM154B 45.1912496459517 33.939263369489 41.785676764591
38.4208429103799 42.3685136297603 42.5660695551615 35.9894133432376
36.1375121409002 39.1790517315259 36.1550293353654 44.2925355984056
38.9496515344837
FAM155A 40.3478825593224 37.5469247328848 36.178547300352
39.066284740632 39.1650142708257 37.1317006162938 39.3585350448719
32.7676164492555 41.1742244762318 35.0668743708816 37.0180934800573
39.1553169409075
FAM155B 42.7150227340364 38.3322849790657 43.8495248991589
41.1886975241176 42.7982502551634 47.0027453663546 42.357910063876
45.7476095696859 49.2879849674517 43.2988017098387 45.4799270968969
40.8681871019812
FAM156A 150.031554835542 235.764999941441 283.484056482174
137.831070407907 170.527024636624 115.441414762268 153.139323479953
148.333778341005 149.330073823683 173.08677862231 164.262531918679
167.430013807712
FAM158A 127.961989181945 72.6888636455541 76.1493261430901
107.637618676176 95.4533981420022 87.0685571351947 111.558107942386
137.403148427792 149.856848496725 118.410983614453 113.791456064084
85.8154958329408
FAM159A 46.1673159982664 47.087835967892 51.6159542017038
62.8201270538435 46.0977687311222 49.1013967533186 51.1541843174981
48.4670130605028 42.992884918158 50.1649089793069 41.4508241771315
37.0953622406687
FAM160A2 139.436084808369 182.219366179899 200.144817111902
135.553887060537 131.973056760893 152.17335005192 115.691017747672
78.6911549149983 126.232262861308 124.683201596193 127.051878502711
98.403191200835
FAM161B 40.2543341073001 42.1607322031054 44.1452943804044
42.8235313007746 38.1644569189523 45.3882138838137 43.8206425661746
33.3307770183871 38.4344387289956 39.1621898366355 36.43924066428
43.0930293831497

FAM162A 129.014076422249 181.966839676473 178.156332261759
104.672760332135 199.442733280533 138.82907142482 136.883475451494
129.925137664962 277.878824242495 205.672652429972 136.686164935569
281.840536529928
FAM162B 75.0704619826557 96.577572948724 136.530734456168
124.350816245276 121.552188287725 154.355197155161 44.8643898647322
220.621142396402 64.4098139218465 148.654594696193 295.69493803986
108.213380368446
FAM163B 44.9370339275041 40.7602938767524 36.5474283500601
37.5764485234171 36.6850522695274 38.861780967827 37.6036146841933
38.1835938174625 39.2497056720346 41.5804111566887 39.5399188536544
45.4799270968969
FAM164A 92.1455386560941 124.064628168507 139.758805241617
100.654701845984 79.6133633483649 164.917385239962 119.277678423944
124.890309370032 100.671408579579 100.638737800637 140.35042464015
128.635811501506
FAM164C 76.6868184563568 42.9358255168086 43.5420658412177
41.2551966132486 56.7602749462766 44.7044140979108 56.0515443021293
66.3558126170575 51.8083208406279 52.3304586399695 48.042366934301
47.5025821041537
FAM166A 38.5007204246293 47.8400694856726 43.1931731665131
34.6468794865286 45.1137938810047 44.4619248757502 39.6535880152834
46.1673159982664 40.9901805728485 42.1622837416589 39.153226699662
43.9541718254769
FAM167B 42.9104454767916 36.9994058279362 37.1643693306382
39.9697332660833 42.3685136297603 41.7627910693483 33.2425023465738
45.642304356051 31.2688290305385 37.7315666632629 38.745552403895
36.9976231206067
FAM168A 41.7397731376692 40.5400384479766 49.5424425850549
43.8002456102021 43.9450023643394 40.244271403831 46.8613212048981
39.9576138799997 43.2761454096265 38.528621456378 38.079222289614
40.8681871019812
FAM168B 68.9745034946434 64.0903425729408 64.5232087947596
72.6824533717231 86.2836002982515 143.152895211088 67.8919712859031
63.8008894786105 63.4531374125233 100.849815617918 92.8089247593564
97.1634390603131
FAM169A 46.3390850717989 43.3458769998118 43.7085219318425
40.1362682639916 40.0599807618105 38.5707420487455 43.3832826368789
41.2884376047238 44.2142581488029 41.9666541498638 43.844855336215
42.6278608611758
FAM169B 49.7827734675743 46.0023068797812 38.7745734037492
38.3166554965434 38.1644569189523 39.132725377815 36.7356360093603
34.1806289878747 39.3750969348308 38.745552403895 39.4410515050885
36.6603338274678
FAM171A1 91.8088250889427 134.099684731177 160.733526836282
315.163147652199 369.451882513347 269.681925095165 448.648042823935
75.3462586192013 50.8485682149772 179.767874494671 272.879564902621
119.700522283289
FAM171B 35.8208542506595 44.2105195734073 43.0016969050339
42.9358255168086 40.0599807618105 37.2686490268265 42.2577689927563
36.6452480995946 36.4476883897928 40.4136535208406 47.2134341420411
39.0536056496869
FAM173A 218.552143403657 240.786294140861 260.426488908824
149.629940171074 170.184914156265 149.856848496725 149.832238573027

117.369735504926 210.768576894328 173.891042920642 228.026217504784
167.229755109155
FAM173B 115.557690369703 88.8039637390786 100.937651342589
66.5921967967793 85.0017995689256 82.3364971911816 99.3797043236938
77.2834482217973 130.184962393833 82.1062834830477 78.963270461629
97.2316181542823
FAM174A 119.243515595981 201.616017553422 189.80672643731
152.250444470971 137.818833473709 118.67727980117 98.2074490157865
151.775928080246 136.686164935569 91.9919654450652 117.439175394651
147.239329753205
FAM174B 159.797219482154 469.30167430316 339.980317745427
73.5834042105313 203.152852313746 99.5531381996521 273.764998628636
188.943404504867 832.181997440212 1059.63540694644 927.921867516809
934.48498309932
FAM175B 158.042994416158 187.836567614419 185.378190201718
213.830284761604 175.362103430164 195.403882457038 216.303036873073
137.496822743899 144.898757916905 219.771890732457 175.345244033253
171.176393131741
FAM176B 116.9812483023 90.0420794458477 83.3826639718546
133.1387411908 74.9113162056974 93.3718038663074 93.748984888348
72.8378863017531 57.1016072897358 84.8999904158793 85.5175308034109
76.7791768896805
FAM177A1 1497.73669971925 1103.90449800536 1305.35453202355
1242.75259587926 1090.86617066541 906.833938613875 1135.43644148537
1756.86714809925 820.147156401172 1776.78784112613 1511.33844030783
1398.21942794938
FAM177B 41.4508241771315 39.5050383901884 41.6977706798234
50.5310598656998 41.2740822675592 38.9378717994178 37.0564573649223
34.8949555140527 34.6720534245631 42.2614524730856 41.8168470823451
40.9683275553869
FAM178B 74.4565795414712 44.3529919625344 37.0642268327439
46.1673159982664 37.8679241954337 53.3185097662527 48.0283573444179
44.4031868028216 46.9739160257217 54.1683282066029 49.3901004211918
50.0571060701893
FAM179B 60.6441065119394 62.1642339365777 56.6888826857797
57.4595416719581 49.9493230064613 52.3187919317561 50.8388984085636
73.362936710475 55.8510296341925 82.1062834830477 70.9319024596628
77.3260618801358
FAM181A 41.2424637338424 45.4882438975269 44.3344900481112
39.9697332660833 48.3814421939888 39.2248354955045 41.4742337714895
36.3405039629059 53.2046867327331 44.1379638573455 43.7585538437696
44.5432192783161
FAM184A 44.6231541342 40.148780650831 45.8844325913247
47.8164193292842 49.32293280121 80.0243450227647 67.3976855661302
57.1362071277099 43.9450023643394 64.0162246399972 54.0374522273907
63.2699016184868
FAM186B 41.4508241771315 39.0894172982439 41.6079958749402
43.4410741327254 39.859345745831 35.2067831864574 48.1747307200218
47.6952179922944 47.1744296124496 44.2580151149956 49.7363778831003
38.745552403895
FAM18B 95.1224420992193 167.824386984618 154.386505379733
114.78317743597 111.742106978992 112.753674408958 106.418690759491
74.9541456286637 127.634504077212 92.6290175610859 109.901105821696
155.174579191838

FAM18B2 53.4249147153548 73.4899394858239 65.5442341130748
60.620259884953 60.7495043977707 58.1576967525216 46.7704450730477
48.5089868936195 48.8584917918986 46.6598879466655 51.0492389286271
70.5322939116406
FAM19A1 36.3649677956953 37.5469247328848 40.4070046183108
35.49696651597 39.1650142708257 52.0299937917925 36.9355798143571
38.5457716679071 43.3744503319109 41.8613406887824 42.2618615571566
35.4484652445078
FAM19A2 45.1648925137409 47.1856919329105 42.8723769353017
47.0874209746031 42.4864576619427 49.5923712603197 46.7100796279384
48.5129953113273 47.3791873993182 45.4882438975269 44.3440839686558
43.6753843488695
FAM19A3 40.6559419616644 37.6813837424257 38.5470982022687
41.1886975241176 40.9950639693255 39.167836466228 39.0735041982358
37.9244295089948 38.7298222155532 40.8970401536578 36.9060997041453
38.5279745308203
FAM19A4 43.7228551318284 43.0227387991135 41.1860481368243
43.3554905202712 43.0860383229288 40.4779565600984 43.0930293831497
41.5817737564592 47.4356803320662 41.483412492426 44.1478805895017
44.9645354363967
FAM19A5 48.4849194706125 43.0227387991135 43.6670755115848
40.8681871019812 40.2449343247539 42.8598805316863 37.7136011474903
37.86982638952 44.0519245668737 44.0409178714232 43.844855336215
40.9683275553869
FAM20A 55.4004611309636 46.8954576545929 43.0971787292711
71.5249259772583 42.7107928951766 51.6053353177789 44.6032218589199
42.8482181313226 39.4762546236449 37.8217989841918 41.9908237694988
47.6040671292013
FAM20B 125.091427687365 202.395470754957 278.067057781084
91.3144101155547 175.136856246782 288.284384035759 128.024853362102
165.905008152959 175.820979889343 146.997252473598 209.497482517298
331.885872312948
FAM20C 164.847648206552 68.5251167137806 50.2203578628217
168.897476166126 71.3752456831662 141.924244643825 45.4164642922183
85.7013212907541 46.2779564481158 67.1926218405833 79.7238173467616
89.0203733091224
FAM21A 45.7356815244513 56.8373628733077 62.4683876804812
69.1931891474114 54.1225249245036 62.7515429046888 59.9792284253187
42.0494116081998 45.2897806651929 37.2089297656361 43.3085358214154
36.3454320848591
FAM21B 46.4669243193596 47.30559806758 50.646715186951
49.4794462948437 47.0417926275315 45.889677919201 44.0137388752186
43.1931731665131 46.2027193115397 43.0322714179517 42.59352468383
44.6067118282704
FAM21C 44.9896851005259 49.8666125578518 51.223859150798
55.9423752286714 48.2129341876251 56.6837988821564 45.9389765191262
46.528592862002 50.8485682149772 44.1976966800856 47.1041363185974
49.5272831893699
FAM22A 40.0557788909923 43.4667754607514 41.437237454134
45.1944222548351 44.4031868028216 41.9543948167375 49.1067959632109
47.0940613507321 43.3744503319109 50.1185085888209 43.9450023643394
44.4067063477243
FAM22B 39.266565827316 39.1982470626863 45.6989664108554
42.357910063876 39.7568367479301 41.9543948167375 50.0088611244948

39.7509165247419 46.2027193115397 45.7911932481722 39.8184668132023
44.7401523707775
FAM22C 44.2350046179299 39.7835513496873 43.6670755115848
41.7614975630362 42.9090346219023 45.2852528185486 43.1931731665131
50.857522488428 43.9450023643394 39.5637454855233 44.4688999098904
42.357910063876
FAM22F 43.7585538437696 45.6533175604486 39.991353369336
48.1662933382789 48.9627346956003 43.7630587657205 46.0228732507872
46.695848687694 45.5779686360641 45.2556760873335 46.4753015651202
43.0930293831497
FAM22G 37.0320432433314 38.9338521051669 39.9101908547397
39.5050383901884 39.4693871570028 40.650742261428 44.2166118829877
41.1575777497576 37.7384958256497 41.8613406887824 40.3452320167286
39.8497227447888
FAM23B 37.1485601150873 42.5269793679335 45.6989664108554
56.4578213622965 44.8897737954573 46.7800033479223 41.4742337714895
44.797320243931 42.5406888281114 46.2286164776607 42.170702295259
39.1553169409075
FAM24A 42.1537476443423 35.3307157989724 36.6522278218553
44.550322123994 35.1411239266238 41.9543948167375 41.1507980856436
41.1575777497576 41.074824727477 42.0621425894624 41.1638563416981
39.7411455670172
FAM24B 54.2648201582657 74.4062547641529 70.18107651423
53.4785152499923 57.4568403979509 49.8472708021525 78.4937314423253
70.0697974278875 61.0094935318105 65.7321440516177 58.4816190076338
63.0266170942566
FAM26A 41.1523487516364 32.742439449766 40.0998167098057
38.9534635879195 37.4470183315826 34.2773859804062 41.0381860307445
42.4580504587581 39.6609238036815 37.0320432433314 33.4071187618571
40.0557788909923
FAM26C 69.1641374421455 62.3983214098965 50.7502704822135
87.114638423256 61.3077641493507 67.5812474586277 84.607833108004
65.5820437889838 72.5993974280876 58.2818491355907 56.6884575165011
59.6757573886627
FAM26D 40.8681871019812 40.148780650831 42.2577689927563
39.7411455670172 38.6454098044474 39.7394181011603 37.7136011474903
39.5466975353131 41.5635499110307 43.401782064778 38.079222289614
36.5474283500601
FAM26F 44.7044140979108 53.3683962805281 46.7508829977587
68.8167148906225 44.9936360641969 52.3187919317561 58.5087296620514
47.3791873993182 52.4539007817929 42.1622837416589 47.6125796208753
47.6938628797168
FAM27L 40.148780650831 50.8812920005251 39.7411455670172
41.0726336841311 45.8051846088552 41.8613406887824 43.3262148375525
35.4526946152955 45.0997987980157 38.8507804383261 42.357910063876
41.3481355128089
FAM29A 40.6062943725118 38.9338521051669 38.0958223740111
47.7362556248858 40.1978874031072 36.6522278218553 41.8751578329365
36.0306975524309 37.3043739460234 41.9666541498638 43.5710871617107
38.745552403895
FAM33A 67.4846787600629 57.8405769800773 59.8273072817267
50.0526763693329 63.955655956484 59.8102324370568 83.5149142053137
95.0350792557735 91.9291200887492 66.4510869175159 58.3586374083245
62.0967404944031

FAM35A 49.540449125788 48.8870563092695 48.2883694323339
58.0218262176065 44.8351976221735 49.5923712603197 48.4859552186772
53.5643281930979 51.0492389286271 54.0917531668054 53.1505787220552
48.3648494350286
FAM36A 55.1306642449627 62.0908175930716 54.4384603094793
60.4646208319121 71.1607965028029 183.96029495885 69.3468840908665
37.7612949798895 63.2753814090696 84.4349803529132 77.1900613562529
96.1710806345503
FAM38A 722.862413305704 769.685677484067 828.403901345618
400.327164003217 613.914396149629 470.004780385708 548.966115135286
609.243908604949 428.515605971056 496.303918649238 546.287195443189
428.515605971056
FAM38B 60.9710783023013 53.4735821194294 55.3812135024656
48.856176220627 50.5110359971301 65.4643755537228 44.3440839686558
67.3976855661302 52.6561576154021 68.0556717366572 65.2295773603116
51.9447450229707
FAM39DP 373.172504097546 633.819901764391 417.201611973051
195.221917652149 672.515393219419 250.41086379231 289.332274319357
1588.70376317303 207.30460556799 435.094968715663 503.793770799548
458.47341225284
FAM39E 1296.28036613191 2545.03032507597 1950.93175239958
617.091932961591 2865.25344854768 963.224016480187 1313.92053019421
4784.36905964712 862.031722874406 1813.65521720537 2101.59244895554
1588.70376317303
FAM3A 454.185408362583 335.655540947167 372.642038290698 266.323732730581
824.946988188872 428.206419080492 434.682456437647 435.209988251425
431.168903637888 482.5816387921 441.247306467645 530.996217072759
FAM3B 45.7700386532539 178.323615832679 319.328571608303 39.3750969348308
45.6409129284202 43.4681646416585 145.883224917856 104.704892803169
135.92805172353 218.056839407813 297.475572902965 549.407911286514
FAM3C 121.682085315899 108.019575675653 124.112216491975 99.9057667432209
158.071355628454 139.294987640071 71.1521292570086 74.9146278608114
205.854666205308 61.9882398986333 79.2824265044758 126.911056987607
FAM3D 251.268123356533 311.361885152025 684.865133028228 50.8567345921473
52.8190882379316 55.6242009731278 78.8250642061439 179.186381381821
79.0276852011359 87.9206275762349 74.3766944386886 60.7495043977707
FAM40A 73.7133884699889 81.3696465421645 74.4508307047509
49.9014504704021 65.6824363141962 66.295597720304 63.2726414544553
62.6984594468418 86.1741504863717 58.9069025298754 70.3381994440425
67.3183506012892
FAM40B 123.608921653229 62.801431502597 73.4611178120841
129.190555260332 78.6807873825307 57.9559082408796 96.8490167966217
196.760671234837 64.447308523693 140.199798713912 137.410781124396
103.343354834837
FAM41C 42.9104454767916 44.5152708955623 43.4709415392696
42.9358255168086 43.5544347011646 42.8598805316863 45.6042625176633
41.7419877611072 42.8036731479932 45.0155543555707 39.9899430628542
41.5536168238478
FAM43A 165.107387190373 115.840734064724 107.501868837432
127.789065386943 173.838110431513 176.816741656659 70.7238101239421
179.974187861013 70.8606919488782 209.068725760522 224.266559865201
133.823107212467
FAM43B 48.1952521496489 42.8542642792862 48.6037684272115
64.0903425729408 43.1252272136667 110.935994444429 36.3194282084339

43.7396619462033 42.8036731479932 46.7646877499666 48.2910530887214 46.4499258373233
FAM44A 42.9104454767916 36.0366673493555 40.4882741869718 36.7892997857376 38.6454098044474 39.7394181011603 39.2533668167483 40.931294690669 48.3119523820103 43.2988017098387 38.6662194645633 42.8864415494484
FAM44B 448.508714709248 162.782568037255 194.469250918013 212.219384864571 465.887689708274 435.632247999054 314.992390390165 500.241231327789 458.861461526841 418.213885929509 343.828445728322 349.371668774717
FAM45A 136.657213529097 231.085533860683 216.976116788572 295.473526251997 157.658760470424 198.432987946699 262.215263489321 149.173801572239 134.749803376372 152.585423208022 127.345094323724 159.12163104195
FAM45B 40.8681871019812 42.9358255168086 41.0356368374708 48.665578118898 48.3814421939888 38.2293008890613 37.4772922422734 43.6434576713809 50.3245489035833 38.8507804383261 40.1742127891824 46.3411353377615
FAM46A 528.512401493184 696.946621824188 371.393488010768 371.167168940365 182.885934774318 1094.91235708087 295.25359816523 779.333013700435 294.220232101389 905.76958446454 873.451893437179 515.007705025879
FAM46B 48.8941062980393 46.2286164776607 64.6111899743678 46.3203995372658 45.2556760873335 357.347859577591 35.9894133432376 116.103491885674 60.164635768614 222.453547273598 271.275703732022 131.496769528338
FAM46C 93.9756797434207 524.972936438175 285.826380698286 1539.35554229258 56.359578588344 134.646159826262 93.9805435116156 140.853812988562 134.968128884298 117.293778127059 91.6826894933058 133.43526498879
FAM46D 41.2424637338424 38.0226905622357 44.2405728317107 42.8235313007746 43.9450023643394 40.1428591998404 43.6753843488695 36.9355798143571 41.6616444147804 40.1179210534118 38.6662194645633 42.9887069977696
FAM47A 37.1485601150873 39.2907645923217 41.0356368374708 40.1804147506786 37.956732373465 36.3845892764084 41.253645578517 36.8400213077707 38.1169703906207 38.2069499294002 36.43924066428 40.3691324547049
FAM47B 40.6559419616644 40.6723334103791 40.6312687664188 40.7481155008596 45.9104478407175 39.4953494583071 38.3346870469391 39.8965727231863 42.38204124321 42.785429206422 41.9083581529276 44.293922970134
FAM47C 46.8141313516997 44.6918600441328 43.6670755115848 46.0905079010237 37.10170285405 40.1428591998404 43.0269218496197 42.4926864381766 39.0775870687758 39.6719190054379 43.0016969050339 38.0958223740111
FAM48A 67.6373573669424 65.6010220593397 63.8740412329753 63.9570596163747 67.1702819938085 68.6889514573956 74.9059650336395 52.6782411631338 75.2977438265751 64.3640973625938 59.9682734755814 74.8893925448138
FAM48B1 41.9407041937973 45.0115870036541 40.4882741869718 42.4580504587581 42.6022652963326 43.2805590023412 44.0687697278114 44.797320243931 40.8970401536578 49.5265949137207 41.7272875295469 40.4639532172338

FAM49A 53.3059058105836 39.6995529770239 47.5673419710251
90.5216273844682 42.0709558258414 47.0940613507321 47.4439407315335
66.1288889125299 44.4395580493118 40.4136535208406 43.0971787292711
47.6040671292013
FAM49B 58.8936382809165 74.6847594297153 54.2670850492947
117.598001907902 54.9249759425252 53.565717255469 69.1414574152997
52.1062000766427 65.6876778328592 55.4037482627222 52.5930185400362
60.2550788540864
FAM50A 1069.3975598703 686.154904023493 676.672146226382
1115.27580736237 2588.47871581066 927.61341837276 1626.71747158994
1111.89793143647 735.460645553438 823.933615182634 827.365231733511
776.670493028756
FAM50B 97.7140868175135 51.6869155634183 107.211374033204
93.3216420803293 182.343696914747 141.594428857706 42.1607322031054
45.9604707098953 43.6670755115848 118.600699776727 122.258194040815
106.020559220488
FAM53A 43.0001432913058 42.6022652963326 37.6557714476853
38.9534635879195 38.0598374201407 39.132725377815 32.6530576979948
39.7509165247419 39.853866204927 39.4710743516517 41.8168470823451
45.9693232070002
FAM53B 85.417321091835 118.143444770806 92.3034609409756
130.585540374294 90.5359675350156 94.5644670313964 75.3394781641786
93.787204465284 63.4889805083459 97.6023731094756 94.2275161759277
68.0751111916102
FAM53C 309.293546994308 241.486113826383 186.655552987116
363.324816966828 304.33749254654 301.941928552396 290.956194006554
256.302432791187 262.024495926094 198.190904631121 188.336316985251
178.060342847557
FAM54A 56.3471340054074 64.8367546135053 56.9958049366454
53.7155925246916 65.2116399196759 43.8582747295766 68.0529308175297
48.1591264215184 55.6617952786921 47.0797760763156 40.8225507267584
47.8931773060789
FAM55A 46.7293238583888 39.0102066700839 39.8299340037009
42.4580504587581 42.9090346219023 45.8325181856483 40.7910160811527
43.5544347011646 42.357910063876 42.4594149893798 40.4504608195666
43.6753843488695
FAM55B 54.6235981131919 37.8217989841918 41.3366779545815
45.8844325913247 41.467005949568 43.8582747295766 41.3594868055879
38.2782436497441 40.137818555383 40.6022003765153 40.6373400186325
43.3744503319109
FAM55C 44.0328992213727 56.7826399116207 51.4308501933893
98.3948200992292 49.452985143568 71.1607965028029 37.4772922422734
47.9942520395434 44.5303781239209 50.6486940895674 61.0094935318105
51.138877923954
FAM55D 76.5124583559262 40.1073950092563 38.9790184198145
43.9282059368202 36.9702228618069 38.8336427948187 41.1507980856436
39.2151045217645 41.4369824439795 38.4187686307607 41.9083581529276
39.1914386799476
FAM57A 144.711001798454 338.966238813782 297.701126167694
166.304210248623 312.045709918301 177.27613997459 582.782165453802
311.901423694475 395.298033031033 315.748168057656 424.87896140215
274.887090359131
FAM58A 205.627744150936 201.236491769447 196.623560874725
228.499788774214 598.404003732481 304.298036741211 291.327030793085

283.747865829282 295.999383698826 146.997252473598 194.536619596804 160.100408020768
FAM58B 44.9896851005259 41.3610369241157 41.3366779545815 38.6414316877471 43.1252272136667 38.3418933808844 42.4939802311136 38.8495095797881 39.2809305246091 46.7646877499666 40.6373400186325 46.917149434588
FAM59A 51.5888395924843 129.190555260332 83.5149142053137 45.5869891570251 58.9875400392427 55.6242009731278 85.0342118119783 123.978615472736 61.4842573582507 97.1201502245778 94.9001252449925 92.3388129097482
FAM59B 56.0477607422828 49.83247261979 37.9482157381008 42.2496337895968 56.5933259268408 49.2050538150106 63.928488869459 50.5070012045444 56.6079099547057 48.6124127998952 47.299647867533 49.7336769474446
FAM5B 44.3355304829989 43.683792219729 34.6977013306396 44.265834798964 41.3755760434934 33.895835691032 46.0228732507872 44.6107027295193 48.3119523820103 42.2614524730856 43.3085358214154 46.3411353377615
FAM5C 43.1267841876606 53.8499082625091 41.9666541498638 45.7397504535169 37.3785704939904 41.2567470068038 38.4005795891396 35.3608605269276 47.417512242919 41.3321861371202 42.6366765186182 40.9683275553869
FAM62A 261.260859494893 416.559486912165 301.257549182222 247.966757890598 225.690879436355 302.79352975792 305.279475274196 544.355979695242 406.104588997462 599.670771796585 431.168903637888 397.108901822735
FAM62B 1914.9556343048 1106.57048989685 1561.83892117664 1129.19103016599 919.542051089664 2448.64580552439 891.080174768895 694.590958116425 855.157599239267 1829.30985071028 1652.85734308754 1436.67510926345
FAM62C 50.1533437545239 38.7344853699576 37.1643693306382 37.4223289687135 45.8051846088552 48.8744644207821 46.1634822993009 42.2577689927563 44.1519466943185 46.1390887492649 35.6970528713709 40.4639532172338
FAM63A 223.826005691932 153.515845356859 153.364107550338 175.028390974125 124.338989399317 123.25443049561 207.513604688444 567.393665550133 148.63149183467 324.658458599373 312.530335465784 230.197573134703
FAM63B 50.5670783769975 46.6314009405034 47.7737810047679 46.8954576545929 43.844855336215 48.260931742163 55.695361644141 69.1973371867031 49.641159251878 48.4890615340603 55.6151308605445 52.8455400026194
FAM64A 116.103491885674 84.1922656126304 78.450972383987 52.5307002738469 186.301404367238 50.5148383400703 240.6614822416 168.522052983892 115.132600341395 41.1222709786153 42.0750889188255 39.2469742642337
FAM65A 309.802432824498 427.199940706169 479.079946537291 358.979854534484 219.555240189863 417.201611973051 302.79352975792 338.355170063433 229.226876896347 286.497342437243 331.677243885539 273.320144423733
FAM65B 100.663054866186 104.072423445726 135.90862211552 1335.4111951034 74.3523005665671 183.393353108744 169.970634268117 69.4677195088043 45.0017158586743 78.6859709780134 72.4834467838798 55.9555622193202
FAM66E 46.0920218698606 41.8073910693411 43.6670755115848 42.8235313007746 37.8679241954337 42.157627240486 42.0605906240248

46.2779564481158 41.9635525160083 35.2880714952489 42.357910063876
42.6758568421347
FAM69A 57.1046180136491 58.5406981871153 78.5325880060762
55.8709511400674 57.2960245538881 61.4944752562671 51.7714254834195
47.5950227477807 48.5792058061372 63.9129423266349 57.4622430729629
60.0940570991178
FAM69B 73.0358442003587 54.5839369292543 41.8751578329365
55.8709511400674 60.9009676344258 70.380703483689 37.4345152884462
70.9377012403023 38.2215591930186 47.7779356299965 48.0283573444179
43.6285248402465
FAM70A 40.5654775358427 37.6786104343959 43.5420658412177
42.0102671859606 39.6123051184545 39.066284740632 39.8299340037009
40.5640952422779 40.4997186069494 42.8414096678708 44.2142581488029
39.8873706855774
FAM70B 69.3418521417182 49.7741754763712 55.3345906164297
75.4466520363487 49.5666074434383 50.8083040235059 44.5480632661672
46.5717043290731 46.824299585607 43.2988017098387 46.7902798502321
48.5632819314123
FAM71A 46.1673159982664 54.6793272901524 45.6989664108554
34.8137085420423 39.859345745831 39.9397419279057 36.08956004634
45.4757965005055 36.3649677956953 37.5343260962758 39.9070785037411
43.2776458914333
FAM71C 43.2724556187214 45.0905928998779 39.991353369336
41.0726336841311 43.5544347011646 38.9378717994178 39.4600924554505
44.4031868028216 45.2897806651929 41.6373771565199 48.1352470945488
41.5536168238478
FAM71D 41.5421429082231 39.9697332660833 41.9666541498638
44.550322123994 41.8806343997257 41.3553369544097 40.1235656101329
39.3709357615493 43.2761454096265 40.5090849993014 37.9230440150891
36.3454320848591
FAM71E1 38.6454098044474 80.257554904218 73.2144205844199
43.8002456102021 220.054040993848 79.4053001255904 85.1227830573138
102.980622026276 72.6675345946258 85.9910479975356 82.8205269728224
85.6038071277319
FAM71F1 36.8072833828297 43.0227387991135 33.0260566875355
36.264823544143 33.4316681623175 33.6859433472087 37.1643693306382
35.9023742468744 41.1742244762318 36.4651112441576 34.4716904283753
36.2582325396375
FAM71F2 35.4287975493553 39.8695929627783 41.6079958749402
45.334106390212 36.6354013629791 41.554942319225 40.5585676679482
41.0381860307445 41.5635499110307 41.8613406887824 39.4410515050885
39.7411455670172
FAM73A 253.841638589385 179.974187861013 171.689014874037
249.37112382902 114.5696064637 105.267519627444 162.902264837659
531.36214636517 130.515102593803 317.004252313348 319.092568066532
245.691073028699
FAM73B 91.5417574979683 133.911556922212 101.804757613639
64.3746985855249 110.411217828763 82.4084917533632 111.367789538268
100.616027485975 79.1576305054224 144.290966198687 122.469733727349
98.3142231283069
FAM75A1 42.8036731479932 40.3384318592985 41.785676764591
50.378948785456 42.269054475455 46.574682061365 39.7576136507593
44.4031868028216 43.7630587657205 40.5090849993014 40.8225507267584
32.3796054199032

FAM75A2 37.6397652536077 33.6902293666089 42.640829914102
39.9697332660833 35.6434124419782 42.0574848021044 37.9430145836712
41.8417024632349 40.4220196471124 39.5637454855233 34.4716904283753
37.3600014814388
FAM75A3 45.8108990837624 38.1312950900467 43.8495248991589
44.0451487108213 44.0451487108213 46.4648868771662 39.5496902678788
40.7311497914871 41.7627910693483 42.9866214102525 44.0409178714232
44.5432192783161
FAM75A7 42.7150227340364 42.6022652963326 36.1042012504758
42.1273928203021 40.148780650831 39.6251842352088 37.830876898596
40.831207953647 42.1594905198929 45.1569741609597 43.0971787292711
41.4508241771315
FAM76A 45.2875173643252 49.6841415268611 50.2869791217819
42.0102671859606 49.9493230064613 45.3882138838137 46.8613212048981
52.3068458655445 43.5669306063196 49.3000844711116 44.2142581488029
46.1592873342534
FAM76B 77.6056319020116 69.8983723094667 66.8632294055679
55.2214187655851 68.307513091369 69.9819944829311 79.1432909353319
48.4670130605028 74.6955454743937 81.537796828766 72.308304085263
70.6903168772326
FAM78A 42.9104454767916 42.9358255168086 35.2101597814352
54.8412943008635 44.0451487108213 41.9543948167375 43.0930293831497
45.7476095696859 39.1790517315259 42.8936990772838 37.4642807402741
39.7411455670172
FAM78B 46.9939712192224 81.3696465421645 47.7737810047679
46.614092935749 148.99515914482 52.6226745117396 123.868848519181
47.2009420656854 45.8325181856483 66.6332042895961 63.3544794716167
44.7401523707775
FAM7A1 34.2180669723984 43.8495248991589 38.0452862258803
40.8681871019812 42.4864576619427 37.7000400506818 41.253645578517
39.7509165247419 35.1155486926999 44.8997235283254 47.4630625313159
41.3481355128089
FAM7A2 40.6130792100165 41.1131693269222 40.1474543172332
42.1679276329916 41.6383327669122 41.0057887014368 41.7656868106485
42.2577689927563 40.3704050658025 42.3433931248155 40.547835538778
43.2312848495114
FAM7A3 40.1925952773317 38.9338521051669 39.6894175734463
39.1350512687938 38.745552403895 41.4186558780356 41.645120086609
40.1812622331588 43.331653186429 39.3016855924751 40.0394833064005
41.3012916486748
FAM80A 73.0970339739187 43.683792219729 49.2041300973417
44.9370339275041 43.2484996523549 50.9724155054821 56.7780520416533
72.2610650652674 78.1448136847078 48.9826868640753 44.553780984244
53.3904307221902
FAM80B 48.7646976789783 38.0226905622357 48.0191002461099
45.4634652319753 40.7779767565889 45.2852528185486 56.3376046940285
53.2046867327331 43.1805016690297 60.8730416435467 57.0690683036183
62.0967404944031
FAM81A 48.3814421939888 43.1805016690297 38.2280071951674
45.4634652319753 37.4470183315826 41.4508241771315 39.4600924554505
67.0904616289706 43.6670755115848 38.1042454705119 43.0016969050339
40.6614700944853
FAM81B 41.8409927585705 45.4882438975269 40.8420925848403
40.7481155008596 41.1868052124344 45.2852528185486 38.2933870238743

44.5029690493211 46.1101825640108 43.1839109702714 37.9230440150891
42.2577689927563
FAM82A1 42.8036731479932 38.3322849790657 36.9795961564828
45.0798408927912 40.3452320167286 38.1169703906207 42.7431359850339
39.853866204927 37.0126425326534 46.1390887492649 34.8383995317036
45.3763275588341
FAM82A2 142.585892496231 182.551901102169 116.709906902201
118.762101740563 128.04037427867 162.721587349434 134.335592890814
158.613823276652 174.752830194939 134.285947677624 138.859992681388
190.977045076469
FAM82B 39.5676051939342 74.1148641558178 61.841867306916
54.8412943008635 69.0975200641705 46.6872654833579 44.0687697278114
42.6450179841129 40.6957787921451 45.4614234442527 57.8991270166934
47.3992733452821
FAM83A 139.356696282462 51.3084273211359 315.163147652199
42.2496337895968 45.8628493605663 43.1826328292558 42.2577689927563
44.571915452417 228.571334092678 44.0784174571411 41.7614975630362
41.8264657291934
FAM83B 51.9545473262296 85.6763929745253 80.1665291227124
44.265834798964 78.6286416672119 46.1513019317765 72.6959688212342
60.2691630545162 99.5901498757707 96.9051967257119 67.8570354010492
88.6771566133367
FAM83C 40.148780650831 150.583488352207 91.3890908971311
43.4410741327254 138.170787392253 49.1013967533186 63.0119737420787
54.3820590274321 125.383837024985 70.7003843117511 62.5036823572932
51.9447450229707
FAM83D 235.888544042065 185.874151272261 94.717335243904
40.4359177791566 265.101739636461 110.272925554245 275.928723831899
102.980622026276 199.937572174194 120.60052672409 122.316617618658
146.509308841361
FAM83E 115.894467332637 43.683792219729 37.5597671487621
37.9875302985336 38.3561682218911 41.159706131351 38.941855825269
51.0724630072723 43.2761454096265 39.1621898366355 49.6706535876636
42.357910063876
FAM83F 53.3059058105836 47.3536182445315 41.2365089689635
43.1847628331565 52.0583273941813 55.9351706773613 43.0930293831497
47.5950227477807 53.3932110698117 48.6124127998952 47.6884372645373
44.5432192783161
FAM83G 34.6977013306396 40.7952353240579 42.9104454767916
38.6414316877471 36.9060997041453 41.2567470068038 38.6243997507646
40.5158867964016 36.6312676076142 37.6383530120621 39.3667161654772
37.9607839934735
FAM83H 559.559234461244 766.027617496754 464.209794165593
78.0020909283414 407.161973433666 92.3477214133335 325.224254365274
338.000998124097 612.014486689487 486.044304392285 448.508714709248
232.440458525975
FAM84A 63.1291723738553 50.8114699212931 50.6004078448405
58.7338159410728 40.9950639693255 34.0045980663271 50.2016912396771
70.7805103907761 62.1796792097014 48.2596564318071 49.2206713037152
54.6659845741133
FAM84B 335.612706052778 784.268074988084 212.395308461139
102.706563820232 532.196768917735 119.866221751572 209.986313475002
252.870708814507 508.081726674689 201.959257379662 196.760671234837
247.905335219826

FAM86A 61.0468099710416 70.4233710555202 65.0656365694468
55.5276281380084 95.9485602132766 62.5542376788358 62.5506324864207
42.7431359850339 67.4775348162369 47.611018474151 47.8676131893619
61.1751736985663
FAM86B1 51.1055541075029 68.8195659637992 62.3245608177257
53.7332797903102 80.6966079421763 61.0311116373365 62.7542357937492
53.4109882045447 62.1796792097014 58.3498430530295 58.2315917325629
59.1391087632509
FAM86C 54.8439819445945 75.3462586192013 65.2670315837785
57.8277957325085 78.7320333941149 70.8674195845729 77.5813459454823
63.287067794195 71.787754116845 82.3488166506716 90.0223113095186
67.6138276415458
FAM87B 44.2350046179299 47.087835967892 45.5883690965192
51.949834638817 50.6470461863549 41.0582900711341 44.8643898647322
46.9986083378817 47.9035532886869 50.8826620053386 48.2050207071554
45.9693232070002
FAM89A 90.1430468048409 138.12608134713 63.4427357028556
97.2220312216969 173.288267403695 95.6783002876222 128.70380102034
95.3631043301461 74.466989900636 73.0186209472046 93.0092452285816
113.336158464071
FAM89B 443.269396125834 260.824949424185 226.262409390222
345.811673759417 319.596352961751 347.829279462722 566.318259999405
428.958127252533 303.93332785291 385.36587480414 290.761706869722
178.249889756228
FAM8A1 57.7877627794725 48.4497719907777 52.1242102345408
67.1961800572759 74.0735632658567 74.6375749739389 74.0039764902057
68.3372228877569 58.174555206394 69.4131195465674 61.1801493883012
59.2035499250243
FAM90A1 44.7044140979108 48.4980626712175 45.989212296035
47.3574589211166 55.8173045043348 42.8598805316863 63.928488869459
41.4508241771315 51.0062122023297 51.3351387089044 47.299647867533
43.0930293831497
FAM90A10 40.5654775358427 44.7829632472754 34.6977013306396
40.7481155008596 35.4484652445078 40.7616966228404 39.1539892144371
41.2551966132486 39.7537274655115 41.4397539024976 36.8282383070068
36.2582325396375
FAM90A11P 42.9104454767916 41.4508241771315 39.991353369336
47.0874209746031 39.5595233162192 41.3553369544097 44.7255207589606
40.831207953647 40.4220196471124 36.2435874904689 41.4508241771315
41.1554481567603
FAM90A12 37.3463875295726 42.1607322031054 40.0998167098057
48.309866878365 42.9090346219023 43.7630587657205 40.1235656101329
36.8400213077707 38.7298222155532 39.8873706855774 40.8225507267584
41.2437378488025
FAM90A20 33.7777950030073 36.4002279956519 41.0356368374708
37.29566077996 40.2449343247539 31.8228237796461 35.4623691645629
38.1835938174625 39.6609238036815 36.8059561571675 35.7953070087288
34.806104801006
FAM90A2P 43.3057780183445 45.1296099011771 42.9703533426259
44.4997132318204 46.2532601271565 42.9079055704748 49.2032063970136
42.2900457798748 45.3490330087217 43.2496075797541 42.9690414573209
45.4339481422072
FAM90A6P 41.2700204291848 40.5852276210998 43.3465699185405
44.6131679221578 42.3685136297603 42.4155142987678 40.5018032884134

42.0494116081998 43.6039443285137 42.1622837416589 42.6920011548175
40.6127321267617
FAM90A8 40.6559419616644 39.6995529770239 37.7384958256497
37.9875302985336 38.5512429290626 45.1873487463472 40.9157352260901
37.6557714476853 38.9834959368496 46.441301934328 43.0971787292711
42.8864415494484
FAM91A1 82.5344501505114 85.5135159896819 67.1299518044849
80.4007868743886 75.9136854087044 62.0660509811111 63.3924515518667
67.6767845246581 78.4428909886495 51.5855515705917 46.7386599624801
57.5372732453319
FAM91A2 47.4767851750188 42.0546888913836 44.7829632472754
44.6905691366696 42.2510526338368 47.2927436772465 44.1350486752012
44.5544727111028 44.20782151793 44.571915452417 48.2492481167112
41.1138630151981
FAM92A1 63.7818442618767 61.3428092991286 61.2153068100932
53.593974682612 98.6553805168141 110.13638939902 52.8496345990125
57.1362071277099 57.8772482723463 44.0409178714232 52.104880481326
59.0894921452893
FAM92B 29.4159269895179 38.0226905622357 32.771863438151
33.9645433044281 34.0045980663271 35.9305494063137 35.6056062164318
36.1375121409002 31.859589438824 31.8890957468737 35.6041926503637
35.3525469364579
FAM96A 379.098697147575 548.815225941887 280.920208331594
615.838713743621 471.046851874447 215.175765091556 406.668753694984
256.941243394182 594.184150517102 179.378838766172 199.776860110557
320.13475752314
FAM96B 275.882262550383 365.401746744015 390.647843814517
446.37035118289 189.425423538906 335.167786298149 477.410937135204
549.117045813593 288.284384035759 277.665554240228 306.115856873599
336.490083577377
FAM98A 192.536546957155 134.099684731177 124.94083215058
155.564141715819 165.974848305379 131.57403028894 152.36389833079
327.238337722366 115.63300104804 192.861174667738 174.148714092395
164.205608279417
FAM98B 40.4261913313642 42.8071549997047 41.3366779545815
40.2427205947092 44.931941140132 43.006072841282 40.6301588195752
37.8245699136327 40.7847110517594 45.8424887733913 42.357910063876
46.4499258373233
FAM98C 306.66244801017 82.4362183543935 101.494432845825
83.014860007187 89.2285288333749 99.5531381996521 123.247207157645
121.050369804136 122.23209417744 112.644426507762 104.986694941968
102.899135663993
FAM99A 37.7384958256497 38.7344853699576 33.9435456570176
37.4223289687135 40.7779767565889 34.3887641786747 36.9355798143571
35.2642250366721 35.6139301508335 34.9138625012006 35.6041926503637
32.3796054199032
FAM9A 37.9387791625384 36.89524015739 42.3536549114165 37.9875302985336
41.7929491793697 43.0860383229288 43.0930293831497 37.86982638952
32.4931776430014 36.6893251782922 36.131485826118 39.1553169409075
FAM9B 38.5648843914189 40.0266778690352 41.9666541498638 39.6292788039793
35.5895375758021 42.0016830644258 40.2154497375715 43.490078970589
41.2424637338424 45.1569741609597 37.9946685306274 43.5420658412177

FAM9C	47.0940613507321	42.0500582486532	43.8153459269057	42.7489468897708
	39.9088079891223	36.1605270099982	44.2166118829877	40.3691324547049
	36.87840425987	39.6251842352088	41.3234441859498	39.5418280570561
FANCA	43.7783560914188	43.0227387991135	47.5673419710251	39.3513480332078
	41.7395080432869	39.8455060654531	41.4742337714895	46.4385386401326
	42.9454180086838	39.3957535885631	41.3553369544097	43.1131704517186
FANCB	49.7827734675743	51.3800036046133	46.0727230219512	46.4555908533915
	48.1986674201502	42.5193208629123	54.339958888486	58.5406981871153
	55.898892031444	45.3530683538086	41.0050831223107	46.2625470410195
FANCC	45.3872406432794	46.8954576545929	46.1653991089885	46.3203995372658
	46.6397391251762	37.4782441311626	41.4742337714895	46.4672637826582
	47.4699236780425	41.5435611403119	42.5426057453993	46.0633179231318
FANCD2	69.3418521417182	77.5737637002275	55.4848084120513	
	48.856176220627	90.8109585999257	44.7058233759912	88.5511408870489
	94.1762630490912	74.9179396623258	52.390086161937	46.6442692119997
	54.4753345912615			
FANCE	79.5531316151328	63.7721358300879	74.9113162056974	64.898351831341
	96.5030525416431	61.1022920882065	80.6634117492762	89.7806631815779
	75.0310858627394	88.8568707880669	86.3374157614394	83.5069968129733
FANCF	43.0001432913058	48.8584917918986	43.401782064778	48.4916117779992
	43.4544762830742	43.2805590023412	48.4859552186772	37.2237132058632
	54.1745577382276	42.4594149893798	41.5421429082231	47.3992733452821
FANCG	152.585423208022	160.134344197725	106.175784900392	99.0647786948132
	156.822939275312	96.2490896573253	237.237762964658	190.442443946259
	135.490875023792	123.704998437432	95.0604882262201	130.865421554474
FANCI	65.4561038762234	64.9959712888709	51.9885249508283	48.309866878365
	71.9737561190706	37.8102105794655	121.441763452598	88.715256326632
	75.0310858627394	44.7958122466628	41.6336636285875	51.4308501933893
FANCL	65.8703820581684	79.7238173467616	93.7782490229256	60.3481762331447
	66.6118713143653	63.3612993417558	89.2120958473987	73.8027902447225
	83.6862086874286	56.284748518655	58.4816190076338	86.3289616331597
FANCM	41.4508241771315	41.3610369241157	38.5045333213784	30.6485078361448
	34.6227169056411	39.2248354955045	41.0381860307445	37.86982638952
	39.9444191873552	40.5090849993014	34.174642681781	42.8864415494484
FANK1	43.844855336215	41.554942319225	43.1931731665131	50.7046706880325
	39.3667161654772	42.5660695551615	45.1017494688414	46.0633179231318
	42.7150227340364	41.9666541498638	45.6348103033479	46.917149434588
FAP	587.385368995676	68.921126710047	65.2670315837785	355.568857981155
	78.0077300979911	98.9560329133205	47.4439407315335	76.3509533017572
	43.6670755115848	51.4454142248566	49.3901004211918	58.2251348002272
FAR1	89.3989848564067	167.678403380953	207.342732103088	140.276520383146
	167.49597550111	165.220069268347	150.242221910229	88.0827215704624
	190.442443946259	315.186937216422	248.482699989877	214.691909801388
FAR2	140.226992517573	195.968119243879	68.6095266659765	151.99110418225
	101.46584765758	75.4227827818591	307.133157118667	172.506486240017
	173.891042920642	46.1390887492649	51.4413099192899	46.8129509799753
FARP1	173.925818790068	126.564008534823	95.7125018118399	81.3364476638024
	140.465247047676	87.8505898087072	181.2179249806	112.281286038716
	100.43365828852	243.403622361932	172.777241940677	222.336600180634
FARP2	57.0006212637829	56.3560498705517	64.3312319756553	81.8727530460686
	58.037539503209	54.9108046955355	66.1940959364976	92.4953883194488
	65.7924490713262	101.759714275976	84.1922656126304	71.1595608255838

FARS2 330.168672676816 156.259762538052 169.99300253813 164.612971085488
393.337170271481 225.918246061369 155.018308238778 204.039190380778
300.186402341399 187.580695643466 198.623556176842 162.30238363798
FARSA 106.521012342668 107.413918714848 118.619819027443 101.708164294697
234.873589896708 91.8295472317386 111.202737864873 137.218635091665
139.590367312743 111.511934617893 131.757828130578 94.6167194649276
FARSB 45.7356815244513 55.6972661824064 45.2313723727673 44.7980740332174
64.193536444915 53.1052228246467 50.0088611244948 48.4670130605028
50.8485682149772 43.2988017098387 54.4140381300469 47.8931773060789
FARSLB 16661.6081002448 10351.5373412096 12125.3293116195
10996.2779403491 8050.256095383 5932.92478996609 12770.8282719641
16661.6081002448 8259.38594893529 13230.9863405074 15358.8899487375
10072.2414399735
FAS 39.3100256336821 46.3370338074996 43.3465699185405 44.7401523707775
40.9950639693255 46.263826584899 39.8695929627783 45.2806199505029
45.4154248354367 42.7275315121163 40.9683275553869 41.8200132437731
FASLG 42.6036491382658 39.2907645923217 49.8344973836834 45.5869891570251
43.7517118200398 41.6590564937735 48.0283573444179 37.2237132058632
42.0621425894624 46.8954576545929 43.3085358214154 38.6454098044474
FASN 771.554891868456 1738.00955642263 1925.20977162704 284.942182200489
2944.3433949897 625.435083064752 2032.18819581205 416.149496747947
3001.25236839028 275.611524861938 390.331295238889 292.389429869034
FASTK 148.529482776555 156.95090716929 185.658246392943 104.462230406058
178.012676342712 116.20672562701 111.256949641793 120.357621336736
220.337850438943 137.916048669047 140.128033028434 122.23209417744
FASTKD1 65.0721641331632 64.643220133046 67.1299518044849
74.2429851177794 100.645556491457 50.2163892855092 62.1970616836737
85.2849531543957 72.5993974280876 83.1874704625702 73.155721596638
74.6734358603424
FASTKD2 82.291097567232 84.6811991332281 61.356483470053
55.2214187655851 105.187381311145 87.0685571351947 85.2088054619229
70.6568303458436 73.5541045639348 58.2818491355907 72.842552380479
76.4580830432622
FASTKD3 79.105698465489 80.7409247380392 71.6573554353691
62.9364422691302 92.4619796591359 63.1604223919703 98.1371527802953
58.364744007152 90.1430468048409 50.9506778292488 52.6960621424156
65.2125527940945
FASTKD5 222.202580186782 215.08694410402 180.663636520073
191.182530250155 293.649670545233 143.265312025262 295.430709655678
134.537040608687 191.298525555624 159.046483157634 160.733526836282
133.43526498879
FAT 346.438778257772 392.237227927376 460.994460486229 118.10944558223
164.312946712409 128.414712393554 230.444295657577 275.096637886925
472.752186426584 382.538365722661 354.015808474189 357.254500791202
FAT2 42.1537476443423 51.7495306734657 96.3741876853801 43.074472921841
39.1650142708257 42.357910063876 46.5650556481721 42.357910063876
140.961217055867 102.607924735378 73.9701365395921 86.2118355769367
FAT3 42.8036731479932 43.2649781354792 40.6737160001291 43.9282059368202
37.0526522140242 48.6621527194963 36.7356360093603 43.7396619462033
40.9901805728485 77.0226403968683 67.8570354010492 52.9609836781499
FAT4 38.5332184743306 42.6939394537076 37.0642268327439 40.0322420615846
42.8510579683751 41.3553369544097 39.2010256871265 43.844855336215
40.0870341983328 42.4594149893798 40.0766543123257 39.2469742642337

FATE1 43.6706764143908 44.0409178714232 38.4043916290561 39.6292788039793
37.5556299264699 43.2805590023412 41.3594868055879 39.7509165247419
41.863927676552 36.9102644736882 35.2339574411711 37.5556299264699
FAU 3202.8848520816 3726.73487426505 3884.2191293028 3999.32180022337
5438.34345794521 6483.09366281532 5218.25814076207 3776.54943917367
4406.55576617324 4299.4451139256 4550.56817942699 4344.25847766877
FBF1 44.8378856799291 41.7614975630362 48.1682125052975 45.8102081251786
43.4544762830742 43.2312848495114 41.7614975630362 44.5029690493211
45.4154248354367 40.1613888954735 45.6706505553383 47.0130468198828
FBL 572.968592371501 516.349618186681 578.601842980701 431.168903637888
1817.14004766564 759.566075437072 1359.9708539999 981.039763059681
970.379875059112 1012.0373748933 822.822769402853 828.403901345618
FBLIM1 52.8427643366617 57.2231446406982 57.1595768494068
44.604338271769 49.452985143568 42.623655455508 40.9784333688086
51.3472428383325 53.0357938671646 47.9853554913436 48.4717951775049
47.8931773060789
FBLN1 909.843512894628 624.51670158046 180.288784077653 472.982369155078
915.102542160713 2237.08790397818 1279.02702402372 5636.93532853955
1130.2486883492 2280.42953098693 1914.9556343048 960.526372655577
FBLN2 181.814283147675 81.2673471767743 75.7140064167134 148.059385111127
76.3334723210842 751.150916058018 36.0366673493555 102.942921317489
60.4837400912239 351.039814818516 245.004434952963 364.628602969366
FBLN5 102.797268694325 67.9259304928255 51.5292723244077 78.2301707073642
70.9638091288679 484.482971152057 46.0817481577316 110.599131324937
47.5183209236505 280.680953068339 254.322611819303 216.654612568853
FBLN7 48.0201377454632 49.0697565324841 46.9040704638073 44.550322123994
54.802736966312 42.157627240486 37.4772922422734 43.5544347011646
39.2809305246091 45.6868589181768 60.4620008899889 55.4635297192076
FBN1 59.987045425047 46.9919960628953 37.8386378737561 57.6511495792664
40.4639532172338 79.8026267036553 45.8980298854198 46.5717043290731
36.8127605061228 58.115198353247 45.310660704531 56.8534370691413
FBN2 228.328635242684 69.5544840164738 51.4308501933893 85.3754356809649
134.468276542607 46.6872654833579 114.755820699611 224.509915754308
97.1271615802556 51.8412522234186 80.0651801266824 49.904979214379
FBN3 44.436995101921 46.0713306971594 37.5597671487621 47.2009420656854
39.2608760193011 41.6590564937735 39.6535880152834 44.293922970134
43.7630587657205 41.2276963324981 43.0016969050339 39.5418280570561
FBP1 385.064309855006 1465.08435775014 699.488969175268 2903.00880209344
610.678132881994 190.81617208799 1950.93175239958 485.310097523194
603.737787922337 3124.84336032646 2978.46032143413 2844.5506310828
FBP2 44.0328992213727 41.4508241771315 43.3085358214154 43.1847628331565
46.4343327861696 45.1873487463472 53.4921736109774 36.9355798143571
44.8003345643334 45.2556760873335 42.2618615571566 41.9430737035925
FBS1 75.0704619826557 78.7282247242035 74.9113162056974 89.6115806694241
72.6675345946258 59.4949177415847 67.1926218405833 137.403148427792
62.7205631511304 85.2634032339854 63.0763092522598 65.0495374451854
FBXL10 198.432987946699 158.349509235411 119.051596183968
146.931674964658 189.542991004953 90.3531863862054 197.171449112363
165.220069268347 207.768377497439 135.169625016001 110.992997780544
118.244019427757
FBXL12 40.8681871019812 57.4538469832241 57.7415362494584
42.1273928203021 75.9909523932333 45.8325181856483 48.3411413510851
44.5029690493211 44.4395580493118 43.844855336215 53.7475011049549
51.138877923954

FBXL13 146.007510897838 41.2598458726002 54.7855575216687
56.9299697426319 45.3675390992851 52.1024135605001 35.4623691645629
36.3405039629059 42.6366765186182 46.6598879466655 41.9083581529276
44.2527566343661
FBXL14 46.9939712192224 41.3610369241157 59.4431489877328
53.0284011078097 45.8051846088552 51.1621379498014 46.0228732507872
43.844855336215 58.0722340887264 54.1683282066029 57.115361349315
47.7962548556026
FBXL15 135.106756607914 165.468411096566 170.184914156265
183.028254350275 130.758455807465 200.986069552443 184.087380444203
98.0352049248004 138.50445813694 197.52820443061 204.7424893289
143.112509176873
FBXL16 56.121460370892 42.6022652963326 44.4311655729487
48.856176220627 45.6895223823001 41.9543948167375 41.1507980856436
47.6952179922944 47.9035532886869 43.4885245598939 38.3684202018524
39.6352613956998
FBXL17 34.8099089553046 40.148780650831 44.6032218589199
37.1894304543751 59.4830424460714 48.1591264215184 39.6535880152834
38.079222289614 38.7298222155532 43.401782064778 40.3452320167286
48.1627097465883
FBXL18 38.957687788868 37.8217989841918 43.8495248991589
41.7614975630362 44.8897737954573 37.9003288112261 40.6750540874299
40.2382908228517 42.2577689927563 41.7405682794166 40.547835538778
44.0451487108213
FBXL19 41.1523487516364 41.9894412416874 39.6451483836089
39.9697332660833 40.2449343247539 39.2985791324641 43.6753843488695
43.9366081640576 38.9834959368496 40.7063426028871 36.9060997041453
41.3481355128089
FBXL2 66.1229691315616 57.1595768494068 52.8041782638549 43.3078542962968
68.4961024593552 70.1074782747874 60.5125774827425 114.342447939197
60.8635925649592 79.8511759086625 73.3321469094819 69.2213838763756
FBXL20 175.876518898179 81.5782824513693 107.501868837432
93.9228336493696 132.946528291039 75.0310858627394 241.706783147271
186.450759425396 131.068763088422 124.683201596193 112.281286038716
133.43526498879
FBXL21 38.304249186058 42.2577689927563 43.0971787292711
38.6414316877471 40.8873907150245 42.357910063876 40.6750540874299
37.86982638952 44.4395580493118 45.1569741609597 44.1148826235374
42.4607795638552
FBXL22 49.2595762436995 43.3458769998118 49.8344973836834
44.9370339275041 34.0045980663271 108.493693862509 49.540449125788
42.357910063876 43.4656637536244 95.5156363110528 96.5451512198207
57.5372732453319
FBXL3 42.6575535390785 50.164201758929 43.6670755115848 46.9919960628953
45.9104478407175 56.7429967489965 41.1886975241176 44.1311654985682
53.4604353973381 43.2988017098387 48.720085661472 53.3059058105836
FBXL4 43.5710871617107 40.0527893189096 42.3536549114165 45.4634652319753
49.0828761839116 41.0582900711341 42.7431359850339 41.8417024632349
42.992884918158 41.4397539024976 43.1931731665131 43.0930293831497
FBXL5 87.8505898087072 125.559679916646 122.904323796484 186.301404367238
89.5597629049747 204.251854342995 100.756165194192 75.3174199658042
146.15014824361 93.6787442149817 112.281286038716 130.177232923004

FBXL6 152.441506175141 160.134344197725 143.383133346874 72.9752827496503
181.45962058846 70.2553917409773 110.684033203048 85.5316122504411
130.039674722115 76.5816788793344 85.1603116461516 67.7662831916293
FBXL7 44.7044140979108 32.0823591288671 34.9849896310235 40.1804147506786
33.7494761745342 47.2009420656854 40.3302268293707 29.4322896559759
38.7298222155532 36.2435874904689 41.9083581529276 39.5418280570561
FBXL8 78.3851687801705 48.4980626712175 39.6451483836089 40.1804147506786
45.6895223823001 44.4619248757502 65.0031119608637 49.085741080241
51.6731332961917 47.0055981216687 47.2134341420411 37.8490162439785
FBXO10 54.2999306485758 43.6587492470348 41.528370959196
55.7616447490886 44.4031868028216 46.4648868771662 49.2546642180676
41.8417024632349 41.8197029429821 49.015238049903 50.0808824383821
45.8546719310325
FBXO11 167.653939596625 124.362644216178 148.477784236365
157.189139558365 116.426770366736 160.566474370004 126.682296930289
202.957014793417 159.827302305842 174.303754116679 155.795095438102
158.364437886751
FBXO15 48.2910530887214 42.1607322031054 43.1931731665131
45.334106390212 48.0234896130991 45.9266745571724 46.9929836306816
49.7309743973184 47.5564423692142 59.4309046716701 54.6089451252049
54.7953298048942
FBXO16 41.2424637338424 48.7847528954724 48.9944486068347
45.7397504535169 40.148780650831 40.650742261428 40.9157352260901
41.2551966132486 39.4762546236449 50.22432299342 50.3876754565799
47.5025821041537
FBXO17 45.1628509319299 42.81562453604 43.0971787292711
43.7517118200398 45.7407938943718 66.1448688879431 44.1430395960362
37.8238773905853 38.5026273921892 59.0216019867146 55.4694986901287
52.9159950879327
FBXO18 159.667153030627 175.936150174315 150.480087842118
192.632227878708 243.27954361539 168.288592321667 213.204385896543
143.042664581646 177.779514321673 181.45962058846 181.298709635609
147.689479458785
FBXO2 41.9918605927928 46.2677540712845 53.9256409473463 47.6687908290818
55.0767527801658 43.9366081640576 40.9157352260901 46.4672637826582
53.9612654850046 44.0719548209593 66.3481553496766 64.7346994616044
FBXO21 249.002758392739 349.90835241442 253.267901237295
134.258351436533 501.585246203512 285.511923726609 348.65230953701
226.288835947947 371.866371754287 363.269936098507 317.406465928295
315.880072026714
FBXO22 64.668912004173 69.9984754033658 58.8188963227059
58.9987341458519 80.0637650599841 67.4421428140671 77.2906963563431
59.4674205670657 70.3117160953476 51.3851157261451 53.6122024523788
67.6983931011126
FBXO22OS 41.0608773166058 46.2286164776607 45.7968648044613
45.8844325913247 43.844855336215 42.0574848021044 43.9450023643394
37.1317006162938 39.3750969348308 40.4136535208406 44.3763859390287
42.0424641526302
FBXO24 38.8322787989855 37.8565969651679 38.2523289394497
40.0195349109823 35.9058393060637 37.6411775481424 40.9157352260901
41.8737744676674 40.4220196471124 40.8049408996274 40.5865217310152
41.4061152929154
FBXO25 45.2875173643252 42.3550738181144 52.2132783757422
53.3039668850879 56.432280646473 52.3746819771982 42.2577689927563

44.3521099857368 48.692302914163 49.5265949137207 45.9104478407175
50.7487159427973
FBXO27 41.8409927585705 46.8954576545929 44.5134109184888
38.6414316877471 50.5110359971301 38.4330755397458 53.1884505015411
41.4508241771315 40.7847110517594 51.1169368198437 46.2560238004757
44.9070250604284
FBXO28 105.621640443267 179.322661675979 187.711309781257
157.979422616649 153.028210322286 120.850874262069 175.74424749223
199.57367025082 142.34144664795 98.9222919411067 120.881290860109
155.6723334711
FBXO3 57.0581769379589 71.8887764487177 77.720889005668 58.6481644777552
65.3019328668633 88.2427413480277 58.3164247463092 68.7342341724306
79.4916415027015 57.4102366425512 64.4837166864084 77.2413564369342
FBXO30 62.9672234302539 58.3627863274274 60.1874689914535
72.3607760798558 60.5806691666221 100.962000280931 65.644767142472
53.1052228246467 50.1603795873336 83.9236799134394 80.5508300225954
85.3431175579775
FBXO31 201.006934353364 190.854807132673 221.847615564378
161.870972980732 147.704325041249 411.047310950296 203.808195515747
184.025325436339 184.377148097206 381.426926151034 466.935292808402
242.282363793567
FBXO32 327.193250073326 98.9988668602468 88.9928368134295
151.111664091983 102.430234282331 667.837116137964 91.1707965298745
95.2292899846287 133.487946450906 175.345244033253 228.185436206823
163.493389267264
FBXO33 208.22938545273 306.487935692954 242.751164798133
410.808988840343 217.848539722853 302.143385957116 288.447603611962
237.100802227545 365.517719684028 211.790532633462 270.303708972854
246.09270373893
FBXO34 278.378459771943 142.731630650904 136.125236142909
191.482692108417 169.970634268117 120.392561825049 187.086662645246
249.776078532596 303.281551940647 235.613437037284 266.07996221256
394.073743195755
FBXO36 35.4287975493553 43.0227387991135 41.437237454134
43.3078542962968 40.3452320167286 39.6251842352088 39.7576136507593
40.2382908228517 38.5252456306656 42.9866214102525 45.7328341667151
35.3525469364579
FBXO38 103.010279797396 115.357402077859 139.385367339095
125.62281563325 92.0606634977068 98.0023132593711 81.7967230859562
87.3085516526362 102.899135663993 110.399579382196 120.046457114882
126.838679504229
FBXO39 41.6354649482853 36.89524015739 40.7620472083159
40.3180310664156 37.0526522140242 41.2567470068038 39.1539892144371
40.931294690669 39.3750969348308 37.1275334308172 37.9230440150891
38.6454098044474
FBXO4 55.7896684777901 58.9128486419884 63.6838561637637 68.087403590986
64.193536444915 63.5295088521896 85.5625396380282 56.9719061986797
63.5489745357606 57.5713378330876 57.6831182451733 61.9036392303558
FBXO40 39.9697332660833 38.5252456306656 37.1643693306382
38.3166554965434 40.7779767565889 39.8455060654531 39.5496902678788
37.9837233601316 41.9635525160083 42.5664883002833 43.5710871617107
43.7672102682067
FBXO41 42.8036731479932 47.6914221838216 41.6977706798234
47.2009420656854 51.0107745412152 48.0350549043063 46.9929836306816

47.6952179922944 40.5912213523345 44.6918600441328 49.8393069675192
47.7962548556026
FBXO42 39.9697332660833 39.0894172982439 34.1806289878747
41.4273122914951 38.1644569189523 37.9951997351435 29.9775071862804
41.3506832276112 32.8704945047196 29.745266251498 36.8282383070068
31.8554734753953
FBXO43 40.7609952437622 40.6723334103791 40.0998167098057
42.0102671859606 42.9090346219023 40.1428591998404 42.4939802311136
40.5640952422779 43.6670755115848 43.7303813515864 44.3440839686558
41.9430737035925
FBXO44 39.3303857231606 46.6075785304453 56.8903641597175
42.5664883002833 51.3033654237232 41.0582900711341 45.2806199505029
42.985929467107 44.4812539947185 44.6217658428521 42.0750889188255
36.2131679586121
FBXO46 110.440072569721 137.016387648703 100.386011517899
84.5411149237799 151.01326330819 80.9821967801845 131.258204302177
98.8478655411492 123.368987753537 85.2893380711323 107.325456368022
75.561262816077
FBXO47 43.0001432913058 33.1817617228171 42.0621425894624
41.8778873678551 37.6527901109005 38.5252456306656 41.9393838138203
41.3506832276112 36.197424053556 38.957687788868 39.153226699662
38.5279745308203
FBXO48 41.9407041937973 42.7775752471711 40.1985790927248
45.0017158586743 45.2556760873335 40.4359177791566 39.9519547348106
46.2779564481158 43.3744503319109 42.8936990772838 45.447310733898
46.9867599298218
FBXO5 78.812106589086 78.258486366912 60.7985356140269 42.357910063876
94.0140258999054 60.6548851977964 83.9873645051122 49.2019743609873
68.6379135603075 42.7275315121163 44.8372404608604 51.1740855632044
FBXO6 124.599352191013 176.963666283432 138.362914843774 183.918927595056
118.376686726902 120.231435193839 178.675242612997 129.309742451628
137.916048669047 87.5967418612698 102.027922691582 98.3142231283069
FBXO7 131.79241580689 120.533617429145 140.992072231913 131.027905075083
113.107942979653 137.034037711549 140.175068871414 114.533239859288
132.684895918889 118.027323513177 106.10668428032 123.205099929475
FBXO8 106.804269453898 133.329721547016 138.568626520133 125.934747056908
94.6432868887401 187.689343320587 95.0190813062982 83.3826639718546
135.138667957956 108.39424630351 130.524308471577 175.876518898179
FBXO9 42.0532901689036 43.3458769998118 41.437237454134 38.0512214519387
39.512606833112 44.1737502492846 40.745874931373 39.4102279045485
43.5669306063196 39.6719190054379 42.59352468383 41.3948836161924
FBXW10 37.2474303302343 38.2263227947189 37.8386378737561
38.2041059863839 36.8059561571675 34.5886782974123 42.629412460409
43.844855336215 42.7150227340364 42.2614524730856 37.2892641289258
33.0217499673686
FBXW11 134.581057139589 108.111248374368 136.843285454801
115.557690369703 122.482560090133 146.42128243217 108.407179111715
113.30970148672 125.290426098227 124.204392146484 139.862070204656
147.471179343348
FBXW12 46.441301934328 36.3110701990588 36.8730514568925
38.9534635879195 38.3561682218911 37.5887129572848 43.1931731665131
40.4359177791566 38.9834959368496 37.6383530120621 36.3390947880186
38.3291713594599

FBXW2 116.685453817071 143.233722839451 89.4747568145717 146.719419166639
114.888828460086 122.904323796484 132.364260893831 116.547937420457
114.935101645207 93.2226507128199 120.142759111608 86.5564881117807
FBXW4 114.666088291556 199.604105066243 225.960701908311 106.8866235778
110.869341159607 216.943972794603 133.576489624155 159.52633805025
158.091115152384 305.684635013927 271.50380898418 238.246319539302
FBXW5 91.5202521578181 82.0102749744973 72.9051985681184 114.959624673123
77.3931945683613 90.1830062843449 104.773064067069 81.8868841896039
80.8078050043929 82.0149542477384 88.8664680253555 74.4365426051588
FBXW7 56.0477607422828 76.034392064609 67.4534389579576 118.714977712617
80.428712610358 66.4841955249254 85.9692990407066 70.355653090662
68.0556717366572 82.1062834830477 67.4421428140671 69.8505620106065
FBXW8 52.9749819616038 50.7046706880325 58.1734393141894 73.0070079786149
59.8698813493379 60.0765286625112 52.0942589368478 58.676230030835
56.6079099547057 56.3441077510744 55.6494029744804 66.3558126170575
FBXW9 77.3752541676627 87.3385669984704 102.607924735378 71.2572485987604
228.747320301945 60.5803031036931 125.052841200703 129.309742451628
108.589036223189 87.7918574811458 98.2581979415778 77.7506984396769
FCAMR 39.0237504776589 42.466333667839 39.1799070031819 38.5707420487455
41.0885562160946 39.4512099697623 40.4359177791566 44.1311654985682
39.7162631015511 40.3458948319669 39.7342718293432 41.9430737035925
FCAR 319.092568066532 141.808284719109 138.972884942374 330.550018306903
146.90802741139 132.144567805599 281.016474354764 616.265771322835
141.108035324183 468.381233506231 538.427311162325 418.01691355558
FCER1A 53.7507429765038 71.5249259772583 65.2670315837785
190.647389246848 50.8875036861067 224.749607192071 60.9648845690177
77.8946473414547 76.1869593072029 213.162949250252 82.1659654020827
93.1067450332473
FCER1G 607.431621448599 198.922575188741 269.801170545042
3941.3269626913 194.703024752864 695.255317861845 340.662853333208
270.394857925086 84.5998418020787 127.213248611204 91.3723635213821
301.79813401066
FCER2 40.7609952437622 39.6123051184545 36.1042012504758 60.9379216295576
38.0598374201407 46.6872654833579 43.1931731665131 42.4580504587581
36.3649677956953 37.7315666632629 41.8168470823451 38.1421709520616
FCF1 106.356074933606 66.6615349144199 85.8945498067934 87.799979434508
82.6983700200815 70.3309641302984 112.930164222877 107.232979387172
84.1254258487497 95.5156363110528 109.005343769033 98.4699679900651
FCGBP 56.3947991135032 72.2527203451447 94.5055588176321 41.7614975630362
80.3768086728545 49.9242614590886 79.7564155881026 44.1702105889066
143.053774297331 133.998375673985 96.1825551158198 108.479856042609
FCGR1A 78.2644076847509 42.8542642792862 40.1985790927248
166.304210248623 39.7568367479301 64.2174369755376 42.629412460409
47.6952179922944 39.4762546236449 43.2988017098387 41.3553369544097
39.9519547348106
FCGR1B 83.8113149043718 55.3951994031724 54.6820069269589
229.971492699272 46.3607182640185 120.149188463323 58.5588286084726
51.9447450229707 42.38204124321 43.7303813515864 37.9553529562144
48.9944486068347
FCGR2A 138.599959012396 89.4504034432321 85.0162042963215
827.365231733511 96.3918711895208 190.099949303905 142.03031230486
67.3976855661302 48.2123120250343 49.3654837541353 52.3187919317561
68.1513215633609

FCGR2B 50.0466349801009 40.4003119241613 40.2455972565968
102.899135663993 38.0598374201407 57.5398008565764 41.5421429082231
38.7023886970298 39.2566275301834 40.8970401536578 37.8372168909583
44.7401523707775
FCGR2C 36.4564050256915 38.2263227947189 35.4484652445078
37.4223289687135 40.9950639693255 40.8634043028528 38.1793598983615
39.9576138799997 45.2897806651929 39.6719190054379 40.3452320167286
38.4330755397458
FCGR3A 42.466333667839 43.0971787292711 44.7255207589606
46.7577982595088 49.1263312877155 41.1090481599939 41.8799523546812
44.2527566343661 48.1578509702472 45.6355219961567 43.7585538437696
47.3992733452821
FCGR3B 72.9154861179393 54.113533179847 62.6885730386156
389.994289973076 50.6470461863549 89.1290937615998 119.846620703059
49.7922355293199 50.2223403889892 51.1169368198437 49.9338905397767
48.8319884367456
FCGRT 454.551748488397 493.705301673753 355.495119544081 1073.84987604662
424.085665569421 1349.09482344633 366.326747630465 425.776214512634
397.798003824167 571.170643523692 584.724083441601 465.981133154896
FCHO1 70.9419509323007 98.2280780221631 59.2012779686869 71.0211264570699
146.551751666276 48.8744644207821 63.928488869459 110.287699369482
60.164635768614 39.7835513496873 44.3763859390287 42.0424641526302
FCHO2 50.9381343674699 54.6793272901524 56.6888826857797 72.89602312859
51.2525124735102 54.1440452292081 55.695361644141 56.3560498705517
62.370559099758 78.5141640924736 57.4622430729629 67.3183506012892
FCHSD1 39.4250105372135 39.8695929627783 48.5460684639932
41.4636047919453 45.310660704531 39.3565586789274 38.3291713594599
38.745552403895 45.9732108120834 45.4882438975269 45.6706505553383
41.6031169661721
FCHSD2 116.547937420457 127.525206038902 124.214669663291
220.597255695837 92.4350913779886 175.114727506747 118.483499958227
97.9497090233081 96.0838701986116 127.213248611204 117.586275152761
126.682296930289
FCN1 88.6663961027926 188.457593309678 280.034472186873 2091.0611913538
104.309778306722 398.43592055172 408.216955625565 124.94083215058
44.8003345643334 70.4895190282848 60.0105984819248 55.9555622193202
FCN2 39.4250105372135 40.4772513262052 39.5954353681031 38.4699622716254
39.2968590261598 38.4330755397458 37.9946685306274 40.831207953647
38.6322728994674 37.3687900733549 37.9230440150891 39.8497227447888
FCN3 49.7827734675743 55.4106986624448 56.8168526942685 63.5195937348531
44.3557408297309 37.5469247328848 37.9430145836712 44.1642484298135
37.5723609429239 40.5090849993014 41.9908237694988 39.5748670524951
FCRL1 39.3709357615493 41.4508241771315 40.6737160001291 42.1273928203021
38.745552403895 38.7340969861754 41.1507980856436 40.931294690669
42.0621425894624 41.3321861371202 43.5710871617107 43.7672102682067
FCRL2 41.7397731376692 44.1519466943185 44.2771355310435 47.0874209746031
41.8407088454059 40.650742261428 42.3074504864935 43.1889516931076
41.6616444147804 42.0217776707284 41.1638563416981 41.6086894860496
FCRL3 41.6354649482853 45.7023929486654 43.826317180678 44.0451487108213
41.9495387255163 42.0574848021044 42.2577689927563 44.1561004152248
41.9180247412837 40.8600431177315 41.1638563416981 41.0254123215328
FCRL4 43.9309845324767 39.9697332660833 36.0306975524309 39.066284740632
38.4540849632254 34.7983399096627 41.0381860307445 38.8495097797881
37.5469247328848 35.93950183586 36.43924066428 33.5578650644246

FCRL5 45.1569741609597 46.0713306971594 54.3554233228353 119.253559091088
42.3536549114165 48.6621527194963 46.8613212048981 48.9826868640753
45.2897806651929 42.1622837416589 40.1742127891824 45.9104478407175
FCRL6 43.844855336215 41.554942319225 48.1331309338997 43.074472921841
47.8075998240396 43.3785777889265 40.7910160811527 44.0390019242862
34.1294694030979 42.4594149893798 42.4580504587581 46.7182294568603
FCRLA 36.6480005877129 53.9275079525238 43.7630587657205 114.520410790135
72.6675345946258 42.750861693377 43.1931731665131 51.2623287419357
64.643220133046 37.0320432433314 37.0180934800573 38.0452862258803
FCRLB 148.558544568518 689.738256133922 757.257495810249 213.768352764473
281.07879968121 89.4747568145717 342.829992044257 68.9522412475296
454.185408362583 102.294142438313 138.349952332963 105.05842887748
FDFT1 414.293172794592 574.847203528544 646.791777335656 384.080719411412
689.738256133922 409.312313055794 670.271164195182 610.853753376105
1211.8832855325 570.204236909398 484.350946851556 830.295872113419
FDPS 116.426770366736 63.1780854659428 76.0240426902567 65.7321440516177
87.3957386451235 61.7091923488929 113.692418102755 95.1037791424727
89.2627798562694 69.4131195465674 69.7855392178808 80.4404044229341
FDPSL2A 36.2496364442264 43.0227387991135 44.4311655729487
41.7614975630362 48.7288537840562 39.7394181011603 42.2577689927563
41.7419877611072 45.4799270968969 39.0648733099061 46.3370338074996
48.7814131120945
FDX1 40.3478825593224 61.3838877877366 55.094385444315 53.2046867327331
51.2525124735102 55.6242009731278 51.0013344286851 48.6441988290952
62.6386437936516 50.1649089793069 54.7883681830995 59.987045425047
FDX1L 144.562563943103 107.176733647834 110.734219768388 108.486774731927
222.05036990665 162.049698241573 184.269633116351 180.128769312512
150.685575301101 186.101626144637 190.703466372947 139.847976281228
FDXR 39.0620487644635 43.3458769998118 37.634370327756 37.9875302985336
31.7270223471062 38.5252456306656 37.9430145836712 38.6454098044474
36.9487027386087 37.5597671487621 41.3145333832821 38.8132170743603
FECH 54.1437245703767 46.0713306971594 58.7783310649709 60.0856496605043
45.1137938810047 84.7816603427127 61.8571521441451 47.2864663693787
74.1031848443392 72.42732668079 67.5417980425932 64.4766262198475
FEM1A 340.962490240304 386.640440174303 403.796135347069 315.163147652199
585.237875919074 266.439343650315 263.170107025525 409.312313055794
348.123960682064 401.45848063233 295.848968688724 241.222680673439
FEM1B 60.8357760968871 74.2653431904071 59.6565330440648 74.4365426051588
72.6675345946258 68.3234331335073 76.3583555666815 110.983311402531
72.4220552950926 66.1851600557127 78.3542840447759 65.2771529943605
FEM1C 162.735117558467 115.221134337342 101.935321521672 197.540681021755
89.6313467455698 210.437554813031 107.895235897156 160.479699603187
143.761359357965 203.759116151774 134.776408077533 174.718984988254
FEN1 364.141443387516 235.33716196909 155.730834354163 188.336316985251
608.579654909865 104.986694941968 731.071171171638 391.658764737446
385.827237353675 83.5895816521251 79.4832790698673 91.2297566574871
FER 48.7646976789783 56.6927078228789 58.637422084759 67.474556589181
48.0234896130991 76.331185787832 45.1017494688414 59.7744650739434
65.7321440516177 63.197604174872 61.3149617644002 68.1513215633609
FER1L3 516.704027135707 350.106705208362 731.071171171638
435.094968715663 402.571146447946 770.715044136717 601.139894733857
385.36587480414 482.071795051342 680.279119780451 899.223276238109
985.328482076985

FER1L4 334.813163079302 905.76958446454 1430.73068989193
173.100153934025 313.233036704221 75.0685015344103 272.828896268482
481.145001682177 420.401914084346 665.122091380755 629.301646370726
484.218958528411
FER1L5 45.1569741609597 42.2577689927563 45.4865173433646
44.7980740332174 42.9090346219023 41.8613406887824 46.3611042575528
43.4885245598939 44.5678064264717 43.8943892311541 45.7328341667151
45.5342598239058
FERD3L 43.3638918705225 40.7952353240579 43.8495248991589
40.0698728584484 38.2496050953547 43.0860383229288 40.9157352260901
42.9301944191913 48.8067321016961 40.8970401536578 46.1673159982664
40.7704587752083
FERMT1 55.6268253440479 309.39599482851 256.215133513379
58.3475983630509 302.978457293429 76.5888466260562 224.976641879383
67.6082563445866 128.05521008708 186.64040927334 207.489116930705
195.893183546434
FERMT2 632.577797921655 147.841274204532 204.251854342995
372.473625569296 224.749607192071 1723.37649434054 126.079949368634
254.322611819303 140.187433247353 1188.3486464341 1043.26325848938
1325.2479053325
FERMT3 108.881703882906 122.952515297686 96.9158008116945
572.667024472672 62.8270556619309 140.629562742418 106.25367135266
78.253478446856 53.0704519012492 62.0500352705887 62.1796792097014
57.6860752168769
FES 133.067727272864 164.941191767024 139.543015878846 318.846373031568
90.5359675350156 205.991317474325 278.773982275525 224.069916407012
100.937651342589 162.272853632419 112.13936276116 108.213380368446
FETUB 46.3390850717989 37.10170285405 42.5406888281114 42.357910063876
44.3557408297309 43.9724628401189 45.6042625176633 38.745552403895
45.8325181856483 41.7405682794166 41.0050831223107 37.6557714476853
FEV 40.2543341073001 34.0382693188794 47.4740726032707 42.2496337895968
41.467005949568 38.1169703906207 49.1067959632109 38.745552403895
41.074824727477 37.7315666632629 41.3553369544097 47.2131144508464
FEZ1 81.5966838909987 84.4111182389028 81.0766789453664 74.1425714439424
98.8821018441815 191.631209717054 203.373397813051 67.4534389579576
63.5896512396512 114.972374827795 109.922909644299 163.585662735512
FEZ2 629.301646370726 330.391945158969 431.727858902449 419.46223841313
238.584514797019 560.802447943839 419.993482471924 479.079946537291
274.134201700259 499.356036793973 472.982369155078 594.611678976716
FEZF1 37.7384958256497 36.7976562409506 40.931294690669 38.4208429103799
41.3755760434934 36.1190722948669 45.1017494688414 38.5457716679071
43.9450023643394 38.4187686307607 37.9230440150891 41.0657892043496
FEZF2 35.9305494063137 43.5873188565519 41.1367478979484 35.9521941716188
36.6354013629791 37.0180934800573 40.1235656101329 39.2672712041774
36.8127605061228 36.3612409063792 37.2892641289258 41.0657892043496
FFAR1 45.7356815244513 42.5269793679335 43.9450023643394 56.4578213622965
44.5668141895992 38.9378717994178 37.3799542961839 45.2806199505029
41.7627910693483 46.1390887492649 52.5154971256566 40.0557788909923
FFAR2 42.2455707947223 60.2547250212462 54.0536858212253 645.114504971255
51.7983110178095 82.4900927793125 192.470747766298 48.272324215504
46.0106098064475 48.2596564318071 46.4753015651202 38.1421709520616
FFAR3 45.7356815244513 43.7644428186265 42.5406888281114 69.1931891474114
48.6224651100733 44.7804080899431 53.1884505015411 42.9301944191913
45.1899271235974 44.6918600441328 44.553780984244 45.9693232070002

FGA	69.9984754033658	47.6534909074889	47.3501115999636	48.9277656327075
	48.9627346956003	50.8114699212931	52.3406235100961	56.5402453093703
	53.5546088535897	54.7105548454293	51.8699412145606	51.0013344286851
FGB	67.0163226819964	43.683792219729	43.8495248991589	43.7410507935043
	37.956732373465	40.8970401536578	42.0605906240248	41.4905114972525
	45.3407340000202	43.8943892311541	38.8507804383261	43.4709415392696
FGD1	68.2614370871382	55.9644250454789	68.4720753153522	62.3936376596431
	64.193536444915	78.9521593412581	128.542999559673	76.6868184563568
	69.2001494999588	83.1874704625702	60.5608545741456	61.4366256816323
FGD2	76.3933911546385	166.25909484446	78.3378279021752	236.374695859748
	60.7495043977707	81.1416376689959	118.376686726902	96.2680061150588
	74.8706881113318	125.786923817078	87.539553421006	119.993761317523
FGD3	110.88526946931	298.313562383092	225.35760689842	429.65084326307
	174.332103484796	104.305801811472	604.951648398924	493.322302312578
	213.309547409053	475.621740714165	445.875994521817	439.770250139925
FGD4	47.3791873993182	41.8073910693411	44.6032218589199	120.068630086702
	40.3452320167286	42.9695585740813	48.3411413510851	39.3709357615493
	56.5094373275922	49.7259371799014	46.7386599624801	48.6037684272115
FGD5	105.45765455249	144.046404221564	300.121875447445	157.095926797414
	89.0203733091224	223.078923630983	54.8074655158941	119.088986733238
	66.7750249658122	101.954354107731	122.688508930699	81.6458112867541
FGD6	55.7246872330059	53.7332797903102	65.2670315837785	47.9915414951747
	51.5888395924843	60.5803031036931	57.1070449050465	56.0496524902806
	60.164635768614	40.4136535208406	45.7328341667151	56.2804354745795
FGF1	47.1962846565261	38.2263227947189	45.5561520014275	45.4634652319753
	43.6782245625941	38.7340969861754	39.3895791800635	42.9301944191913
	44.014761416117	41.0529409675663	43.3085358214154	45.0035308952466
FGF10	39.5676051939342	41.7337506166619	47.0055981216687	42.5664883002833
	41.1868052124344	48.3686935595826	41.253645578517	41.2551966132486
	39.6609238036815	41.2276963324981	40.8225507267584	46.3411353377615
FGF11	82.6983700200815	49.4067286971348	64.2404293863468	49.3654837541353
	42.1650127805175	50.4022543872993	50.0088611244948	53.7378346199838
	97.2425832658887	46.5650556481721	45.8180710486329	50.379587451669
FGF12	41.8890826572422	43.7644428186265	40.3384318592985	38.2516470858206
	46.1804647841331	39.9871741554388	48.5721799977026	50.7964800730186
	46.7353388797135	41.9219580093938	41.7614975630362	41.4061152929154
FGF13	46.144656267199	40.0877366724081	40.1572444265517	38.1464799985979
	40.2903961886288	54.1859679451613	44.6032218589199	41.4508241771315
	40.7847110517594	39.9948390976317	42.0507048990507	40.7063426028871
FGF14	39.4250105372135	37.6383530120621	38.0057289062111	39.6705473876526
	39.0564222526273	42.357910063876	42.629412460409	43.490078970589
	35.1155486926999	41.3641352453779	40.4504608195666	40.2237056928605
FGF16	33.9561816279577	36.4002279956519	35.2101597814352	35.2143190355414
	38.9496515344837	37.5887129572848	35.3362091871364	34.806104801006
	41.863927676552	35.0668743708816	37.5583886316038	34.806104801006
FGF17	39.6609238036815	41.554942319225	39.6451483836089	44.265834798964
	41.1868052124344	40.040778349137	38.941855825269	42.9301944191913
	46.6944238390932	49.3000844711116	55.3942006156899	42.1537476443423
FGF18	49.0462740100128	45.4757965005055	39.3303857231606	48.055683217958
	45.7407938943718	61.1022920882065	44.8643898647322	52.3579564953125
	39.7162631015511	52.3304586399695	45.7792335738285	44.4475838947656
FGF19	43.0930293831497	46.6996902348633	39.8299340037009	38.3166554965434
	41.1868052124344	43.0860383229288	43.8206425661746	42.7431359850339
	45.2897806651929	36.3612409063792	40.3452320167286	44.7401523707775

FGF2 39.3100256336821 42.3550738181144 41.6977706798234 43.5962371290832
37.6119337673806 66.1650385535549 40.6216891610774 45.3389970694851
43.3744503319109 46.8954576545929 51.0492389286271 53.4735821194294
FGF20 38.3459820930759 42.1372626664516 42.9641759692926 40.3180310664156
42.0709558258414 41.554942319225 44.8643898647322 40.931294690669
42.3138951003676 42.0621425894624 41.2598458726002 44.1452943804044
FGF21 44.0328992213727 41.9894412416874 42.9641759692926 43.074472921841
40.7273974755431 42.2577689927563 42.9803717121865 41.3012916486748
39.2497056720346 41.4397539024976 42.0750889188255 39.7844133452634
FGF22 43.3638918705225 42.1607322031054 42.9104454767916 40.4359177791566
40.0599807618105 37.9003288112261 40.5585676679482 41.2551966132486
38.2215591930186 38.4187686307607 38.4689132737495 37.2810767789306
FGF23 41.7835997867848 38.3874422458351 41.0718387273629 39.6705473876526
37.1565984182084 41.0909708790194 43.1602899171656 43.7731874801003
40.5912213523345 42.3606390470387 39.5399188536544 44.1063440402552
FGF3 42.442231667357 40.716553247236 42.5406888281114 39.1706564265495
41.7929491793697 36.2768299545794 37.2892641289258 41.0381860307445
47.1744296124496 36.6893251782922 40.4504608195666 42.1537476443423
FGF4 33.5994525227062 32.742439449766 33.553634633839 40.3180310664156
80.1294564809102 40.650742261428 36.197424053556 40.047314525032
35.860430241131 34.8137085420423 32.406093465115 36.449101775775
FGF5 41.2424637338424 42.8542642792862 43.3465699185405 42.281606728072
46.9906120932727 42.808889470973 44.8643898647322 44.0736170668178
44.4395580493118 43.8943892311541 42.2510526338368 43.5420658412177
FGF6 41.7835997867848 37.2810767789306 41.4613628725541 35.1738399826814
38.9496515344837 39.5581166303303 42.5660695551615 38.6454098044474
37.241386734757 31.6981395162348 39.9070785037411 35.2448979801094
FGF7 37.3463875295726 39.6123051184545 42.3536549114165 37.4223289687135
42.0709558258414 60.2867996701867 40.5585676679482 37.5511560880902
38.0213051227859 45.0155543555707 48.3847843498476 48.7814131120945
FGF8 43.9309845324767 37.1485601150873 42.7881985796277 42.7489468897708
40.4321207743917 38.385865183581 42.0605906240248 40.7799172560245
40.6503965580437 39.3303857231606 40.1742127891824 40.7704587752083
FGF9 39.0620487644635 42.8542642792862 42.640829914102 37.9875302985336
43.1252272136667 77.9085946827079 39.7576136507593 48.0582855095143
38.9834959368496 151.912182086236 217.721787448311 85.9286114777649
FGFBP1 174.860894067053 39.5050383901884 44.4311655729487
54.2886483704949 142.1823033942 45.2852528185486 60.0981687048269
487.749060801066 877.673009984357 37.8217989841918 39.9070785037411
44.9070250604284
FGFBP2 38.6454098044474 39.2907645923217 48.1331309338997
60.7741670229412 39.6535880152834 47.2927436772465 43.5627649099623
41.0381860307445 43.0930293831497 51.4454142248566 52.022479439889
42.8864415494484
FGFBP3 38.5648843914189 38.9338521051669 46.3411353377615
39.6292788039793 49.7235523248653 39.0266821712099 49.540449125788
45.1714698794331 47.27543600687 43.2988017098387 42.7275315121163
41.863927676552
FGFR1 47.2258994932516 44.0578434007778 42.59352468383 39.3750969348308
44.5152708955623 54.1773909134233 41.2884376047238 40.8880530359822
40.1751033251565 41.6657053704762 40.6876726817234 45.4171570229279
FGFR1OP 70.083473392272 71.7624419474395 71.5484567134601
68.6379135603075 72.2665537421443 57.2767879969284 74.9059650336395

75.2101993995344 60.0736861987141 50.3388505346542 56.6884575165011
55.3563533836982
FGFR1OP2 41.4508241771315 46.29198852834 45.1569741609597
61.5515639412892 44.5668141895992 51.3886035574416 60.5125774827425
42.9301944191913 69.1129431075514 42.8936990772838 40.723990462047
57.2350830635912
FGFR2 39.7576136507593 43.0227387991135 43.1931731665131 40.4359177791566
44.8897737954573 46.1513019317765 51.4349390827895 38.4540849632254
46.0106098064475 38.0078357808393 42.0750889188255 39.8497227447888
FGFR3 111.186223357396 2146.1548633579 3941.3269626913 87.2643859655159
1009.47177197543 170.829628173552 347.003621791919 143.278668481091
1704.0522304387 1455.64106630257 634.782261466407 574.847203528544
FGFR4 46.3390850717989 41.159706131351 41.5562673496867 41.4812544772644
63.1443731864055 43.006072841282 49.6078549993388 53.0338164320907
42.6679886757687 46.3838120599769 57.8405769800773 42.8864415494484
FGFRL1 78.1952428431942 72.7213787264217 68.1209771323232
63.0153175111747 108.700838142885 65.8703820581684 111.874193363836
72.7596094170237 82.0237575809051 97.2004257862686 80.2381651206227
93.955760127914
FGG 117.699959435983 41.5061991514748 36.1301597296871 36.6046828105314
39.5111866657136 39.167836466228 37.9905182666856 39.8048943625718
36.9565987097341 39.2010256871265 41.863927676552 40.4220196471124
FGL1 43.4173515108896 42.9838432714922 41.3667878979108 40.8211872708071
38.9919142475596 41.5195355066893 42.0016830644258 42.0847226073592
42.2331334096358 42.3413611855132 40.7792705767601 39.3016855924751
FGL2 96.8783070574131 210.5650813431 105.965906809429 632.019639546131
86.2836002982515 1178.52115916913 133.720493104769 140.853812988562
43.4656637536244 349.096725048776 230.73977644867 147.689479458785
FGR 87.9098670472958 93.0714382925187 113.929486370331 1032.44965920118
71.6354627933716 137.605565493336 145.073599404007 66.7530718914083
106.486195938039 81.4881766238989 76.2738693944687 100.465129764507
FH 331.306005726359 329.120125961774 340.962490240304 308.393270347486
387.311743342483 358.194667053419 481.492404948275 626.570239685127
396.63268714181 343.677907141241 360.668308246913 334.349204174886
FHDC1 53.2046867327331 79.9567256807679 62.4683876804812 55.8709511400674
66.4047895961325 63.6894484284172 84.3526980051279 85.0308877626746
69.5741225986433 67.8233625051884 52.6960621424156 73.0333258448698
FHIT 39.3709357615493 46.3635803515159 53.1491634301682 43.8002456102021
51.0107745412152 43.6670755115848 45.223075311113 34.3865306339645
47.4699236780425 44.6918600441328 45.9104478407175 48.1627097465883
FHL1 72.8120269375546 64.0096843640535 51.4308501933893 59.987045425047
54.9249759425252 1061.61186520958 40.4359177791566 63.7002949745101
86.2563919366781 488.908231529696 635.906934423716 653.880422442079
FHL2 851.108027634251 964.268294825556 708.839262759372 229.379918828848
644.733901442573 292.86576686019 381.480149599459 560.413725186282
693.056793637199 523.25106120389 583.053232396803 717.997180920384
FHL3 240.476103976894 102.490465676619 136.657213529097 137.677598837555
143.084029849218 287.363928615517 99.7529382840954 95.8297699573536
137.197328985851 169.18132209076 190.375732394043 130.121040883877
FHL5 47.6397712007919 46.9919960628953 51.9885249508283 40.7481155008596
40.148780650831 68.4323624797175 42.629412460409 39.4710743516517
43.5669306063196 46.2286164776607 44.2142581488029 46.7182294568603

FHOD1 415.759723627205 103.707931766117 177.067668324062 193.327267186222
90.9521112485099 91.7437837246405 64.4736987797413 157.856158440506
111.845105854608 112.375316615894 108.407179111715 129.538159906075
FHOD3 115.691017747672 123.039116845742 99.8231023149756 63.4531374125233
52.0583273941813 188.912691033518 87.8746766762445 65.3019328668633
87.036020750455 183.057478637596 103.607345835114 149.812291220906
FIBCD1 135.106756607914 44.7829632472754 50.8303921211581
54.8412943008635 41.5668884168669 51.8103411703261 47.7390230243778
47.4740726032707 50.0526763693329 43.1839109702714 44.3763859390287
51.7833333848627
FIBIN 56.6951078698291 42.7775752471711 41.8751578329365 38.9534635879195
38.6454098044474 52.2089306840125 44.2166118829877 42.2577689927563
38.4344387289956 46.8954576545929 47.7535797917441 43.3744503319109
FIBP 197.923403667051 199.208033538936 239.117228656704 208.023488988931
273.231509963261 174.752830194939 411.431488342465 334.527003257901
244.560715635796 200.401133392259 194.951677338012 165.061411701316
FICD 44.436995101921 57.8405769800773 51.1347974668251 81.382229980722
63.955655956484 61.3620062177524 70.2635892414678 46.0633179231318
56.3441077510744 44.0409178714232 56.233217375698 71.5836731059913
FIG4 102.25509551881 179.09303345382 153.364107550338 152.545682001987
80.7321212028516 136.250317107253 144.37233706688 471.734457300752
91.0346886951891 169.18132209076 142.417106615034 135.718484174661
FIGF 41.6354649482853 39.6123051184545 37.5597671487621 42.357910063876
43.004943518025 62.3497263884167 38.2933870238743 37.2237132058632
40.2274522635576 42.785429206422 34.7308008643142 44.3521099857368
FIGLA 39.5676051939342 43.1805016690297 39.3709357615493 53.2046867327331
39.3667161654772 39.2248354955045 42.7431359850339 38.079222289614
43.1805016690297 36.1550293353654 36.6298807161755 34.1509644696334
FIGN 40.4639532172338 51.8610060995363 43.1931731665131 43.3078542962968
46.6397391251762 52.6226745117396 50.3519150282938 55.3122077514658
41.7627910693483 42.2614524730856 46.3370338074996 42.1537476443423
FIGNL1 57.2960245538881 62.3524254253348 55.6865353018811
46.8249967544573 93.7924763988692 65.0774755750423 75.7342400123505
57.2878071731043 67.7344875174123 51.3351387089044 49.9791966667317
48.6447083765118
FILIP1 59.1311136551108 46.4385386401326 56.939860285007
58.0218262176065 60.0736861987141 350.451704069255 46.4388841354122
52.3068458655445 61.1560835966725 105.598516887691 161.439879456216
129.091447141381
FILIP1L 363.67768546508 141.960773054307 124.691222601355
201.875082892488 123.918925337643 501.585246203512 53.6294062560285
109.922909644299 55.898892031444 146.955326324436 158.071355628454
431.168903637888
FIP1L1 81.9258201510475 81.0331497830643 86.6186787663248
96.1596075221747 114.972374827795 86.8691342571214 79.9971782971285
101.156215957297 103.869437847206 71.334759229964 89.4747568145717
104.305801811472
FIS 44.9896851005259 45.8669209716694 44.6032218589199 46.3203995372658
39.4693871570028 47.2009420656854 41.253645578517 46.5717043290731
49.1940901905636 44.6918600441328 48.042366934301 45.0035308952466
FIS1 395.298033031033 328.176884082116 339.262360833465 408.353336724969
281.906398109158 680.279119780451 272.663785805238 294.296266023705
382.425576479893 607.431621448599 640.78586776574 498.608204176238

FIT1 41.0608773166058 49.1283590719461 44.8872784170036 64.3746985855249
43.3332476888305 44.8872784170036 45.4799270968969 40.1474543172332
48.0163377492228 45.7911932481722 45.2265319826548 33.8196020406676
FIZ1 67.6767845246581 72.4325984501758 70.0059240383657 53.593974682612
80.7321212028516 78.6402083787251 72.0568616357755 77.7615826856723
94.4421289055611 97.6023731094756 96.981661155293 71.381069076765
FJX1 62.8635815439291 115.221134337342 186.866378606003 66.224559122062
166.652850759559 55.6242009731278 63.2726414544553 66.1288889125299
42.5406888281114 86.2118355769367 81.6472320169489 98.0307965290686
FKBP10 57.2008272301399 43.8495248991589 41.2365089689635
56.6458456890715 66.1650385535549 51.4931742685391 39.0458123429605
40.3357777530419 32.039934894666 47.611018474151 44.2925355984056
42.57175366709
FKBP11 308.231730523135 479.079946537291 550.642253702646
1370.76188322111 451.13133250897 190.161505611036 194.278792348966
223.669853276931 275.061444728555 187.430528830941 162.721587349434
162.143245260314
FKBP14 699.488969175268 536.290060852815 482.79099112085
597.191426276771 380.736562250846 316.185342210304 480.660242705293
979.468753716758 368.660803187338 904.487756674518 1001.99175009124
728.679189702033
FKBP15 98.0307965290686 86.3374157614394 68.3234331335073
192.41551362197 70.7760293129408 71.7585486695516 91.3607028449916
77.118454956956 90.7037473522607 93.6787442149817 94.5716601751149
77.4769634299218
FKBP1A 266.985013788569 345.876877384089 407.564154455615
370.917477324773 569.511773053233 329.437593022939 380.736562250846
267.608189207303 189.542991004953 223.911125639211 254.756894822368
317.245171206724
FKBP1B 39.9697332660833 41.9894412416874 45.5883690965192
37.9875302985336 45.3675390992851 44.7804080899431 62.3556396662215
44.0390019242862 39.2809305246091 49.9493230064613 48.2050207071554
48.0350549043063
FKBP2 81.2136969156029 125.835967514239 137.294289316915 164.176142753482
154.355197155161 120.881290860109 168.503424269691 92.5903918620825
118.587373377593 88.5907320965949 132.760054781304 122.331946672266
FKBP3 95.042308285667 88.5706300816555 100.708709225818 80.1809075683154
89.7661004674344 83.0554837248934 75.7969486011633 111.573111498905
109.241121384212 77.2979451706651 78.8595005348182 106.878946016737
FKBP4 139.999709303614 168.869258524932 159.268245287939 101.708164294697
189.137593177772 127.701556328274 232.048938339214 167.943170737481
264.924262411772 164.097531361519 137.555275829874 173.694418385132
FKBP5 84.8139090643412 98.2747494745452 237.754990288051 722.514380928291
144.976378707447 485.871284967735 102.626604917608 78.8250642061439
49.2879849674517 140.276520383146 213.768352764473 187.711309781257
FKBP6 44.1294459089314 47.1332119496722 42.157627240486 43.3078542962968
42.7982502551634 42.0574848021044 35.4623691645629 39.853866204927
43.8482802038826 39.3698811593364 44.553780984244 40.4639532172338
FKBP7 46.3390850717989 42.9020321355927 43.2721716572958 40.5484717678957
42.1386542162789 55.8938720883203 43.1602899171656 42.6022652963326
43.0930293831497 46.0119912782855 40.3180310664156 46.0106098064475
FKBP8 52.8427643366617 48.7084357480922 48.6037684272115 52.8502823653368
46.3150221024046 61.4944752562671 60.2399886747451 50.9933795450314
54.0192060888782 63.0119737420787 60.2867996701867 51.138877923954

FKBP9 42.2455707947223 39.6493706695391 43.7630587657205 43.074472921841
46.1804647841331 46.3715909653832 45.7452456977857 39.8965727231863
44.619512785899 41.8613406887824 40.3180310664156 40.716553247236
FKBP9L 303.458085392002 230.286815921497 229.792906003602
173.780433758335 196.465477936749 274.259417498569 133.798096986107
189.245501343165 222.336600180634 246.869486382264 235.222472959869
206.41879845205
FKBPL 96.2248868829355 49.2934904960601 56.939860285007 42.9358255168086
95.1131101630937 48.6621527194963 86.3237770387663 62.8885806455128
74.4365426051588 56.8201201798712 52.3187919317561 49.0655215909632
FKRP 84.4443931765378 65.2702686717535 67.8452645288969 62.5106969730855
95.2833228196616 74.7669841575348 64.7023558190673 70.18107651423
69.1129431075514 68.9115937148858 73.7060605978536 63.7744603935682
FKSG2 44.3355304829989 42.0709558258414 41.0356368374708 41.1886975241176
43.3332476888305 45.0882356578404 43.9450023643394 43.1567024035183
40.3316153483289 44.0409178714232 38.6662194645633 42.57175366709
FKSG24 57.4376528157926 52.5868047927666 59.5444359507702
54.1240583139871 101.156215957297 57.013135787334 54.8074655158941
53.1052228246467 64.7116910405493 60.2877434874622 54.5711704180349
37.1747156131306
FKSG30 579.482255191456 668.961646543849 646.013007560085
837.959914510114 487.749060801066 744.421618408497 331.591589534469
368.727384734032 489.622327794168 273.170084535508 353.751797948675
709.730555484551
FKSG44 43.0930293831497 41.2598458726002 44.9758339877851
42.9358255168086 39.7568367479301 49.1013967533186 42.2577689927563
34.9580369221164 46.7760357462635 51.9547984751408 42.4580504587581
45.2783024981315
FKSG49 41.9407041937973 33.8237407699472 38.4043916290561
38.0958223740111 33.5412189543102 38.7340969861754 37.6036146841933
37.9837233601316 44.4395580493118 41.5435611403119 46.0095710163378
42.357910063876
FKSG83 38.6454098044474 44.0409178714232 50.0748292497578
45.4634652319753 49.7235523248653 41.9543948167375 46.3057385979341
54.8412943008635 48.0163377492228 47.2299678280724 41.2598458726002
47.7962548556026
FKTN 2357.96534075689 1089.08276262641 1280.77934331703 1829.30985071028
1161.71671016964 825.558929673308 1698.18614771363 2911.10227993347
1103.90449800536 2689.80835574654 2551.57660786358 1925.20977162704
FLAD1 229.971492699272 183.336550845401 252.775631043952 121.668011907792
339.854688605186 135.169625016001 236.440703574276 247.193935816149
190.099949303905 142.192315159235 124.112216491975 118.966448789057
FLCN 46.2265798401055 45.7538339834602 43.5710871617107 53.1341332984713
45.8669209716694 44.0299389687796 45.9635923776719 45.7106033460585
48.2123120250343 42.8496759570945 43.8002456102021 45.1296099011771
FLG 43.5710871617107 46.3635803515159 36.1042012504758 36.4564050256915
42.1650127805175 41.8613406887824 37.0564573649223 37.86982638952
42.4540072551568 37.4302011516587 34.9428206949566 38.8495095797881
FLG2 43.7164681443605 40.4772513262052 39.0470836345045 37.0273924363983
43.0723817756832 40.3946758935842 41.253645578517 39.5466975353131
40.4220196471124 40.7620472083159 41.0050831223107 38.6454098044474
FLI1 33.5994525227062 39.0894172982439 42.5406888281114 44.550322123994
39.859345745831 39.9397419279057 36.08956004634 36.2539950294089
43.9450023643394 37.3179578037963 29.0303154053123 49.4786466722535

FLII 165.606015336587 334.016152807947 353.076989769287 297.701126167694
215.131274710043 331.001195453225 277.014526407597 176.057220407246
204.992039590717 302.083293019113 330.241189572237 200.790001763897
FLJ00312 38.0709016691086 42.5564081946232 42.108130037421
40.4831048079412 42.8596678196469 38.2293008890613 40.2601451506332
41.6799375298398 41.0476065156912 40.4136535208406 39.9586075662602
39.1553169409075
FLJ10081 246.274692971423 189.111092737256 156.433762355183
187.802182308625 194.896610327943 247.416016413788 151.795609904857
232.113067048781 234.615033303459 358.879599295105 312.154320305976
280.117410663595
FLJ10120 46.441301934328 46.7591817286632 44.2405728317107
41.6564075217517 41.5668884168669 47.3791873993182 42.4939802311136
54.0507720792049 48.8067321016961 53.5498255316009 49.7363778831003
51.0013344286851
FLJ10213 95.1513370156467 68.0388928513985 112.727277807495
122.024487499513 84.4230484528318 67.9306669919176 105.666698877424
86.5926014007562 99.3797043236938 135.973016473297 119.434418653131
99.3344676796884
FLJ10246 52.8427643366617 60.9755936952178 54.2670850492947
58.3475983630509 73.5541045639348 52.0299937917925 72.5956443098684
79.1039287828151 53.7442594289333 53.8260140375214 62.358854072801
60.0940570991178
FLJ10324 40.5235471987417 41.8848959280916 47.6040671292013
40.2455972565968 45.9104478407175 56.796621176582 43.0269218496197
50.1833483241864 40.4518431978241 46.0119912782855 47.1970991228159
46.0633179231318
FLJ10357 54.2648201582657 45.1694246393676 47.2055133646385
62.1776700757427 46.5557478175976 49.3172204533818 38.7337086062875
42.5435283762933 41.7627910693483 55.6069214673486 50.0808824383821
53.3904307221902
FLJ10374 41.8409927585705 53.7332797903102 46.9040704638073
44.4367387274922 49.7235523248653 40.9506062495231 54.0170817319522
36.6452480995946 57.5883027758003 47.0055981216687 45.9104478407175
49.904979214379
FLJ10404 51.9380636897023 48.3077814647455 50.3452972893313
40.8084239472012 50.2953638261991 48.8744644207821 43.8835874380262
51.4360013601805 48.3119523820103 52.2256177934134 54.5222652584532
48.0350549043063
FLJ10490 46.0920218698606 46.9919960628953 51.223859150798
51.7739270955254 55.4919197421746 41.8613406887824 57.4047331041592
45.7476095696859 41.2870505273664 44.8997235283254 44.2142581488029
42.1537476443423
FLJ10781 36.3649677956953 42.5269793679335 38.3166554965434
37.4223289687135 50.5110359971301 52.6226745117396 41.706879587091
62.4219686957673 33.3643994200057 39.9948390976317 55.6151308605445
38.5279745308203
FLJ10803 47.1962846565261 47.9128504683618 43.401782064778
41.8778873678551 46.5557478175976 48.7781962164988 45.4799270968969
62.105802634526 53.5677764726461 42.1622837416589 49.7363778831003
47.2131144508464
FLJ10916 235.127539195661 74.9956591643367 161.268186656743
54.6089451252049 146.405413944753 210.22828395255 113.692418102755

160.917024416961 67.9176876913772 373.330920144642 366.326747630465 150.242221910229
FLJ10986 137.696331574552 120.465621774337 203.82788889213 117.228320608065 141.410183156424 133.365282773876 258.079468517805 205.154378853199 365.792144095604 297.990925644625 285.390768830888 218.013913036142
FLJ10996 44.8914371498528 43.2649781354792 41.2365089689635 56.6458456890715 44.6834648302383 44.1737502492846 48.4859552186772 49.085741080241 51.5888395924843 40.3235655331796 46.0095710163378 45.0985088502363
FLJ11151 37.0320432433314 46.3635803515159 48.2883694323339 51.949834638817 44.7829632472754 45.3882138838137 47.8947595316832 46.8954576545929 43.6670755115848 41.1222709786153 42.2618615571566 50.4847465816184
FLJ11235 72.8120269375546 59.1938299291358 76.2533853403813 53.2046867327331 51.2525124735102 61.4944752562671 83.6935756500468 68.1311562787361 73.967017874248 77.775644509079 74.280612516205 71.7408778060956
FLJ11292 44.7915315085948 49.0697565324841 45.6989664108554 47.6687908290818 43.6587492470348 45.7300501624017 42.8684958525042 46.1673159982664 49.0986627051784 48.3693338695392 45.7328341667151 47.1060577218993
FLJ11783 43.5710871617107 43.1805016690297 43.0016969050339 38.2041059863839 44.9936360641969 43.1826328292558 47.1345949868029 59.8760452220989 37.10170285405 65.6721629145797 52.5154971256566 46.2625470410195
FLJ11795 41.7397731376692 46.4861258030402 42.640829914102 43.074472921841 47.4548471240051 43.0860383229288 44.0687697278114 48.4670130605028 40.2274522635576 40.3235655331796 44.1148826235374 46.0633179231318
FLJ12078 48.1952521496489 45.4882438975269 53.9612654850046 73.7124988616836 39.1650142708257 45.0882356578404 39.0458123429605 51.3472428383325 51.9545473262296 124.986782737446 82.8205269728224 83.1874704625702
FLJ12331 38.8561375693335 42.3550738181144 42.8270038153139 44.9370339275041 42.6022652963326 39.2985791324641 33.3231576795423 36.43924066428 37.9273653326866 37.1275334308172 39.8184668132023 44.9070250604284
FLJ12334 40.4639532172338 48.3699741879723 40.7620472083159 48.309866878365 51.1368583672022 41.7627910693483 40.3302268293707 42.2577689927563 42.2577689927563 40.5090849993014 37.4642807402741 41.9430737035925
FLJ12355 51.6558970571712 49.6841415268611 52.5353664902258 36.3604951341921 54.4586176399381 48.4758590019384 80.7563265626648 75.5830548489892 81.8983491901699 58.3813211109331 59.501103909345 49.3984571719129
FLJ12529 44.3355304829989 43.7644428186265 36.0306975524309 48.309866878365 48.0234896130991 46.4648868771662 35.6056062164318 44.9977782988116 39.7537274655115 44.8997235283254 44.1148826235374 45.8546719310325
FLJ12684 33.1666036387511 59.9682734755814 84.5998418020787 43.8002456102021 84.6563174894574 43.1826328292558 79.7564155881026 98.4405692451144 74.5694657001572 48.9826868640753 49.0361180089431 54.3587260982091

FLJ12688 42.7150227340364 45.9266745571724 53.4743535852344
55.4180794172794 53.1884505015411 48.5835484742456 46.7100796279384
40.2382908228517 55.4030910454028 38.957687788868 48.8088340735921
44.5432192783161
FLJ12949 85.1950626054282 93.8968899420039 89.5597629049747
86.1703662107384 130.515102593803 77.0226403968683 105.945216149549
131.258204302177 121.84142967836 110.09868737254 116.388069891158
91.1363393944977
FLJ12993 44.7044140979108 48.2220547806679 50.6987358483181
38.6414316877471 47.5647821785529 50.9724155054821 44.7255207589606
43.9366081640576 50.6750785584772 46.8954576545929 46.3370338074996
50.7487159427973
FLJ13224 43.515290338756 44.7829632472754 43.4709415392696
40.3180310664156 42.6022652963326 38.9378717994178 41.7614975630362
44.1642484298135 40.7847110517594 47.7800121097582 39.8577530602176
45.7979032939211
FLJ13305 51.0225132036905 46.29198852834 52.7005092555102
46.614092935749 53.8260140375214 54.1440452292081 63.1604223919703
51.8347877274018 47.6195009345747 50.3388505346542 48.5292718280498
47.6040671292013
FLJ13611 39.4710743516517 40.716553247236 41.0356368374708
48.665578118898 39.9697332660833 43.9724628401189 51.3064929551674
42.357910063876 41.2870505273664 44.0409178714232 40.9157352260901
42.7782216342806
FLJ13614 37.9387791625384 39.9697332660833 35.8265993915844
38.745552403895 38.8456942580061 41.159706131351 38.5066108855005
43.3262148375525 43.0930293831497 38.957687788868 43.5710871617107
42.57175366709
FLJ14082 46.6348258763289 44.6918600441328 49.8344973836834
41.5464765641928 44.7829632472754 39.9397419279057 47.5963750520409
52.7787010032051 54.7033747046992 54.1683282066029 51.524711857679
41.9430737035925
FLJ14107 49.9419897484381 49.2934904960601 53.4743535852344
42.357910063876 40.2449343247539 49.7458652239071 48.8319884367456
43.5544347011646 49.1940901905636 54.7827221235949 60.394307846869
59.0894921452893
FLJ14166 72.3388488936832 44.9370339275041 51.0539624358342
49.3654837541353 57.5040500497076 37.5887129572848 63.5738862549306
45.9604707098953 43.1805016690297 47.8207974930031 45.8180710486329
51.4968903591058
FLJ14186 48.2910530887214 45.0905928998779 43.5710871617107
43.9282059368202 45.2556760873335 52.3187919317561 44.2166118829877
44.9977782988116 44.2607789091176 41.4397539024976 45.9104478407175
45.1735152121056
FLJ14213 70.5096706089299 51.0175677516029 48.7039705048129
107.337113678019 50.0580547772867 52.7156971154405 43.1931731665131
54.0507720792049 42.1594905198929 96.5102825310977 97.3188285021124
93.955760127914
FLJ14712 54.1745577382276 42.6022652963326 44.9758339877851
44.6905691366696 80.3500323413132 42.2577689927563 42.4939802311136
39.7509165247419 56.432280646473 43.0919221375514 40.2649309204478
44.5432192783161
FLJ14816 43.0001432913058 35.7393138893354 41.2365089689635
42.4580504587581 40.8873907150245 37.0180934800573 40.7910160811527

43.5544347011646 40.7847110517594 38.3092241935352 36.43924066428
39.6352613956998
FLJ16124 41.6354649482853 42.0709558258414 43.7630587657205
37.0732977341185 36.9060997041453 39.132725377815 47.5963750520409
43.844855336215 51.7549258776884 43.844855336215 36.2435874904689
40.9683275553869
FLJ16165 34.5886782974123 40.7952353240579 45.5883690965192
39.1706564265495 37.7576900881741 41.0582900711341 54.8074655158941
48.4670130605028 42.5406888281114 36.3612409063792 40.3452320167286
37.1747156131306
FLJ16171 38.957687788868 41.9894412416874 41.8751578329365
38.3166554965434 39.4693871570028 43.3785777889265 40.6750540874299
39.0620487644635 40.8970401536578 43.6033221838149 42.170702295259
43.0930293831497
FLJ16323 48.2910530887214 46.29198852834 42.640829914102
45.5869891570251 44.6834648302383 46.4648868771662 42.7431359850339
42.0494116081998 44.0519245668737 45.5776159432079 42.170702295259
46.3411353377615
FLJ16331 37.4554876386402 48.4980626712175 41.1367478979484
48.4916117779992 50.6470461863549 54.0382756991549 48.0283573444179
41.8417024632349 35.5220586859301 36.8059561571675 42.4580504587581
43.1805016690297
FLJ16369 35.8208542506595 41.2598458726002 40.301483914446
40.7481155008596 34.806104801006 44.6524308640784 42.629412460409
34.806104801006 43.5669306063196 38.2069499294002 39.2542963855807
38.3291713594599
FLJ16686 43.4626049853157 44.5884811528117 41.437237454134
44.265834798964 45.9104478407175 40.5281496800812 42.2577689927563
43.9366081640576 42.2577689927563 47.2299678280724 45.7328341667151
44.4475838947656
FLJ16734 58.7404123614695 46.29198852834 49.3780606970538
59.987045425047 47.6884372645373 47.7556758818053 54.0170817319522
48.3590401011623 46.7760357462635 96.1044453515408 79.7020636215451
58.2879293130296
FLJ16793 42.1537476443423 45.4143854024453 44.696023817801
38.8495095797881 54.4586176399381 51.9421032049606 67.6767845246581
61.8665316900291 56.1172088979505 43.844855336215 49.1067959632109
39.8497227447888
FLJ20021 101.351907645804 67.0402749076279 85.4536891882639
112.743602566917 85.7842344710572 109.221269056557 120.721482177994
69.4677195088043 108.503081044355 128.436305192918 124.350816245276
97.5534379346899
FLJ20125 54.691568065544 54.2924642952939 55.2141529491512
54.1240583139871 42.7107928951766 67.4846787600629 52.8496345990125
46.1673159982664 60.3977304934451 50.6486940895674 55.3147456054175
62.0967404944031
FLJ20160 169.301010687893 287.565588186962 257.194689822426
140.894046694804 133.765486891316 132.059215102854 141.830305704504
314.281125546552 181.340089705052 187.430528830941 237.161912299581
276.267583952979
FLJ20184 35.2267149016596 34.9262464079777 31.0126568324233
46.0228732507872 42.7982502551634 36.2768299545794 36.8369172900902
36.3405039629059 31.2688290305385 42.1622837416589 37.7384958256497
37.4554876386402

FLJ20209 45.4799270968969 43.5873188565519 49.6347864008749
43.9282059368202 47.9128504683618 40.3357777530419 44.4639405506967
44.797320243931 46.8873421271259 55.0016608333828 43.844855336215
39.3575468494941
FLJ20254 145.073599404007 81.7367617812869 75.561262816077
105.934378627264 84.8751572193902 87.8967913380047 89.4747568145717
121.172652675767 87.6726059613969 95.4072431786073 133.067727272864
84.4349803529132
FLJ20273 69.3418521417182 107.836867810972 72.0243286940589
178.379117285008 75.6471706262645 55.7197525213035 99.9896394343368
186.161177110898 103.139459009593 71.0619469948187 85.8277891453447
113.716666562787
FLJ20297 43.5710871617107 37.1685072112618 41.2365089689635
45.1944222548351 38.745552403895 33.7818132446042 33.5578650644246
42.7431359850339 42.992884918158 42.9866214102525 38.745552403895
42.57175366709
FLJ20309 41.3387790895716 44.5884811528117 43.5710871617107
43.1847628331565 44.5668141895992 41.2567470068038 52.8496345990125
41.8417024632349 43.3744503319109 46.6598879466655 45.9104478407175
48.6795178347248
FLJ20323 131.083141370546 99.4123924179498 80.257554904218
125.676172266736 151.724932189601 116.35075921851 115.087963545095
131.67159377585 138.50445813694 134.051011731533 121.831344781285
128.365609268093
FLJ20397 41.6354649482853 44.2460658853547 42.377095964597
40.4826391343033 44.5668141895992 41.2226607554583 42.8027734291654
38.6964057457624 44.0183974528298 40.4455351423846 42.3375909167899
43.2776458914333
FLJ20433 55.3153799233744 48.5905518770847 55.8810837854139
36.9912468186322 43.3332476888305 56.2736599756843 45.8980298854198
58.8022152133979 37.3600014814388 75.0665411373616 88.3725577276628
80.6200293361016
FLJ20444 57.0006212637829 66.0147097736612 56.2181240696479
49.1786207920603 68.8570376191283 52.3579564953125 68.9603421415748
59.526516100518 49.8512010216731 126.115065200084 72.3822545261582
59.0216019867146
FLJ20464 42.1537476443423 43.5182502375207 36.769073068743
37.651298468202 40.0599807618105 35.6125439901743 38.7337086062875
40.6171774724171 41.4636047919453 41.0057887014368 37.7384958256497
40.9683275553869
FLJ20489 74.2151462073098 95.7639712596394 96.2739664178142
80.1809075683154 79.7808217577226 121.120217132666 67.5206810636627
113.064858029749 113.546568515215 154.355197155161 146.892027926888
118.288549974348
FLJ20581 40.308802735799 43.5182502375207 39.1480645318984
40.646702696033 45.1137938810047 43.4681646416585 38.941855825269
41.5934628038429 40.6957787921451 38.9207612933107 42.9634662926872
42.8864415494484
FLJ20628 67.0577715520007 74.9956591643367 63.2475738480323
53.0284011078097 99.8699868526053 66.4015820814547 94.6952414258466
106.78552222475 69.3300058073438 60.7495043977707 61.0979269700412
75.7991766454243
FLJ20674 49.4786466722535 65.7457567201236 57.2107492415265
41.6045220683604 65.8319449883221 45.4882438975269 51.3064929551674

63.0748373122119 54.9426456410692 56.1912096308988 53.0177144132449
65.4435809676217
FLJ20699 118.05982513603 119.434418653131 115.256208610595
121.104663867264 144.799432299339 114.643475335002 99.5844079573552
124.76401857899 132.369828264246 193.703214707909 182.37433006793
116.374125502825
FLJ20712 40.6559419616644 42.1607322031054 41.8751578329365
41.8778873678551 36.2048942610546 41.8613406887824 37.1643693306382
39.0620487644635 42.992884918158 41.1222709786153 47.2134341420411
34.6720534245631
FLJ20718 132.604567913122 128.70380102034 109.696102117537
154.718907273467 100.489477730896 106.186051982612 111.025105784166
105.1770711426 141.180682718758 93.3897477314201 113.169111095305
108.082240435027
FLJ20850 41.8409927585705 48.109747794187 40.8420925848403
43.074472921841 46.6397391251762 42.357910063876 42.2577689927563
39.853866204927 52.7512110769042 41.0057887014368 39.9070785037411
44.4475838947656
FLJ20920 52.0299937917925 84.2880616879945 92.804679821289
55.2214187655851 136.582966744378 54.3478800757394 127.724641303
179.07886477603 131.517202728816 232.516262874696 202.325629133577
165.573693886454
FLJ20972 47.0940613507321 40.9731067463382 43.0971787292711
40.5484717678957 45.1137938810047 41.9543948167375 44.9863726205428
39.7509165247419 50.4974478884077 46.8954576545929 46.4753015651202
45.5800840647989
FLJ21075 42.7150227340364 41.554942319225 42.157627240486
41.4273122914951 46.8674366115656 40.8634043028528 46.3057385979341
42.7431359850339 46.9739160257217 45.2556760873335 39.8184668132023
41.4508241771315
FLJ21438 105.05842887748 193.830224000034 195.018775118528
330.241189572237 74.9661773375536 70.8674195845729 75.0665411373616
114.533239859288 57.8772482723463 79.262977183608 113.612874206329
61.5300817099814
FLJ21511 37.2474303302343 48.0296332183129 47.9035532886869
40.9607305725196 45.6895223823001 44.3648993568405 66.4258159334241
101.156215957297 42.4540072551568 62.2025996860055 67.7280831060202
58.0218262176065
FLJ21687 40.3478825593224 44.7829632472754 44.0451487108213
30.2675713925613 44.7829632472754 43.6670755115848 39.4600924554505
34.8949555140527 51.2066664465185 44.571915452417 42.170702295259
51.3244198342335
FLJ21767 39.8662795144616 43.1805016690297 45.0115870036541
43.8495248991589 47.8697093824642 48.829194318411 39.8695929627783
44.6107027295193 39.3750969348308 46.5650556481721 42.7775752471711
46.3411353377615
FLJ21839 48.3814421939888 44.6918600441328 40.0998167098057
41.5464765641928 43.9450023643394 40.5281496800812 37.3799542961839
43.1567024035183 40.8970401536578 43.9450023643394 45.390192977585
43.0930293831497
FLJ21865 186.965367827758 251.983470875355 330.470972281607
81.8727530460686 261.51893439249 100.883604376864 108.558977253184
260.383969219887 136.451598654243 217.743953847269 262.735075993242
188.767243497644

FLJ21986 72.1408243034509 73.845912534353 51.9885249508283
132.205423909251 61.5236837309467 793.225863118464 41.9393838138203
163.38302606725 52.9482338979681 518.936896882424 378.12777235743
437.542517888395
FLJ22167 54.5358897781765 53.6429962450784 66.737740728688
47.3574589211166 63.686652234709 50.2163892855092 59.3549213146532
54.2877981508636 66.2552876795581 56.8201201798712 49.1067959632109
55.1804535845496
FLJ22184 180.592281750722 92.0880591999243 79.2073371826617
40.4359177791566 148.608392563633 50.2163892855092 111.558107942386
233.850715388018 72.5346469941833 55.4037482627222 62.2025996860055
48.6037684272115
FLJ22222 80.7462144785541 75.7140064167134 78.270329450618
70.1597323687098 75.2923395421694 72.961444366037 106.049736669661
55.7469393723377 101.103458852775 63.5716064500377 74.6662312145629
72.7882384002423
FLJ22269 36.5393857306744 35.9305494063137 40.301483914446
30.2675713925613 42.1650127805175 41.0582900711341 38.0598374201407
37.9837233601316 39.1790517315259 34.8137085420423 38.745552403895
35.8429598823818
FLJ22374 78.5216977073842 62.801431502597 58.5212649095879
55.6481366056639 101.987604729928 95.5920087912438 60.7992483126292
59.7744650739434 73.8800799416166 96.1044453515408 101.065203693106
79.2525964926483
FLJ22447 42.8482181313226 43.1358833499227 43.9217273706293
50.4733460479197 42.9775348174342 41.5146360101556 41.4397539024976
43.6740007496465 42.7606905460304 43.0290260867894 43.3085358214154
39.9519547348106
FLJ22531 41.3387790895716 42.2114134935898 39.7411455670172
43.0016969050339 43.2484996523549 41.3094485574845 44.6032218589199
40.6171774724171 40.1738310955128 39.4710743516517 39.8184668132023
45.6692241948108
FLJ22639 67.9531693869426 55.8046062733709 79.4998723172096
56.4578213622965 97.2316181542823 41.554942319225 55.8019623191927
60.1796637353976 63.5195937348531 68.4137459386155 88.5511408870489
67.6138276415458
FLJ22662 418.410951118141 2013.08489754172 784.951880532127
626.570239685127 294.7772562683 567.393665550133 640.407302868904
151.266092912661 1093.04440713629 947.656220635965 798.033975337132
708.005870621064
FLJ22795 138.401109242778 148.144200968432 58.112348043634
49.3654837541353 113.994256868581 58.2531879680192 132.090336898032
156.123501229126 123.480244775709 245.853111206859 131.126942328593
109.339287213971
FLJ23152 49.085741080241 45.4143854024453 45.2313723727673
48.9944486068347 46.4343327861696 45.3882138838137 195.512986297442
61.9787117490846 46.1101825640108 46.7646877499666 47.3791873993182
52.3896785600751
FLJ23172 33.6902293666089 33.939263369489 36.178547300352
36.0481934423077 35.1411239266238 34.9849896310235 34.4570158133896
38.8495095797881 40.0403898620771 43.6033221838149 35.6970528713709
36.449101775775
FLJ23356 40.7609952437622 38.0226905622357 34.1806289878747
43.5627649099623 40.0599807618105 39.2985791324641 38.2933870238743

35.9023742468744 37.9273653326866 43.7303813515864 40.547835538778
37.9607839934735
FLJ23584 59.6651708873036 52.3187919317561 49.1067959632109
51.3851157261451 64.9072331703985 71.8706847571669 58.7585623394335
55.8087097725119 52.8502823653368 64.8549209064366 58.691436315511
53.9431600945419
FLJ23754 48.8333989709933 48.5905518770847 47.2962360458401
51.5913529948887 44.8897737954573 48.5835484742456 46.1634822993009
44.1311654985682 40.9901805728485 43.6033221838149 47.6125796208753
48.4535991392809
FLJ23834 39.9697332660833 38.9338521051669 40.7620472083159
46.614092935749 46.9906120932727 43.3785777889265 40.9157352260901
45.7476095696859 39.0775870687758 40.7063426028871 40.1742127891824
42.357910063876
FLJ25006 44.380620848621 62.1913948207302 72.6182747525443
45.7397504535169 71.5345397102823 45.1873487463472 65.524821699432
64.4156641487994 49.9162522057712 56.8201201798712 57.0690683036183
63.2475738480323
FLJ25037 32.0823591288671 40.5400384479766 37.6557714476853
42.1273928203021 43.4544762830742 37.3799542961839 37.1643693306382
36.1375121409002 34.4716904283753 53.1993028936883 40.8225507267584
38.745552403895
FLJ25076 43.4100411978366 38.5252456306656 41.9287023391451
37.0732977341185 37.4470183315826 39.3841861812262 37.9430145836712
34.3865306339645 38.6510172759977 38.528621456378 38.2293008890613
38.7012264805355
FLJ25169 42.2455707947223 54.8979897528792 45.310660704531
42.2496337895968 41.6806984441849 51.0506441018166 44.8643898647322
48.3590401011623 40.9901805728485 45.6868589181768 43.401782064778
48.1627097465883
FLJ25328 35.2267149016596 43.4396866157171 37.7384958256497
37.9875302985336 42.269054475455 37.9003288112261 39.5496902678788
44.797320243931 41.2870505273664 33.2987269789328 36.5359455830388
38.5279745308203
FLJ25404 47.4678477186801 42.5269793679335 45.5883690965192
48.4916117779992 57.1935729219207 43.0860383229288 46.0228732507872
77.523734787944 36.197424053556 43.7303813515864 45.4799270968969
47.2131144508464
FLJ25439 46.7293238583888 40.4359177791566 41.785676764591
39.9697332660833 41.467005949568 43.7630587657205 40.1235656101329
44.797320243931 38.6322728994674 48.2596564318071 41.4508241771315
47.2131144508464
FLJ25715 42.2455707947223 42.2577689927563 48.8100349749783
41.6564075217517 46.0977687311222 43.8582747295766 45.1017494688414
44.0390019242862 49.1940901905636 46.7646877499666 43.844855336215
47.2131144508464
FLJ25758 44.1294459089314 40.6380495994768 38.2280071951674
46.4555908533915 45.2556760873335 39.2248354955045 40.9157352260901
40.7311497914871 40.8970401536578 39.8873706855774 43.0016969050339
43.2776458914333
FLJ25770 34.4021554788966 37.3634897565502 35.2838283525874
40.3180310664156 37.0526522140242 37.2686490268265 35.8227939208521
39.0620487644635 37.0126425326534 34.6720534245631 36.3390947880186
34.1509644696334

FLJ25778 44.436995101921 69.1414574152997 66.5285423077808
57.4595416719581 46.8674366115656 56.0477607422828 78.6402083787251
42.9301944191913 53.1249776278067 50.6486940895674 54.8532558909227
54.4992295592001
FLJ25996 47.1962846565261 48.7084357480922 61.2153068100932
40.8681871019812 48.0234896130991 38.4330755397458 42.629412460409
49.9332280449549 62.9922928277769 73.3479915711031 65.6010220593397
58.6481644777552
FLJ26056 44.2350046179299 42.6939394537076 42.2577689927563
39.066284740632 42.9090346219023 43.2805590023412 39.6535880152834
43.5544347011646 41.3803269429468 41.0057887014368 37.2018803984555
39.6352613956998
FLJ26850 44.7044140979108 43.2649781354792 43.9450023643394
41.0726336841311 31.5599106749655 40.9506062495231 41.1507980856436
40.047314525032 38.9006788676407 36.6893251782922 44.2142581488029
40.0557788909923
FLJ27255 44.6231541342 46.1569135229922 36.769073068743
42.357910063876 36.5393857306744 44.3648993568405 40.5585676679482
36.3405039629059 38.2215591930186 40.5090849993014 39.2542963855807
47.3992733452821
FLJ27354 37.0320432433314 40.8880530359822 35.4484652445078
44.1483976313959 45.1137938810047 39.3841861812262 42.0605906240248
39.0620487644635 35.2143190355414 38.0078357808393 40.723990462047
40.6614700944853
FLJ27365 53.2046867327331 65.485076402841 81.8648220673828
42.9358255168086 47.8075998240396 48.4758590019384 39.8695929627783
52.1788489555942 38.7298222155532 42.4594149893798 47.4630625313159
46.5650556481721
FLJ27465 44.0707087774725 45.4757965005055 47.2505395290129
45.8844325913247 44.9936360641969 44.0804700754786 46.6267962186599
48.7814131120945 47.0804681460898 50.007830364606 47.2543830677329
44.3521099857368
FLJ30058 43.2724556187214 45.1694246393676 41.9666541498638
42.5664883002833 53.6907197364586 53.7442594289333 53.4921736109774
40.7311497914871 56.432280646473 42.5664883002833 49.319499137678
50.3118072462443
FLJ30092 45.3451294405285 46.4035310644846 46.7508829977587
44.0860067869564 48.4779133727101 40.650742261428 45.1017494688414
41.7419877611072 41.1367478979484 43.2346122959037 41.6336636285875
43.0456011922275
FLJ30375 40.7609952437622 42.4493032770053 42.640829914102
41.3027031037343 44.7829632472754 39.8455060654531 43.6753843488695
38.8495095797881 43.0930293831497 42.0621425894624 43.6629145624937
38.3291713594599
FLJ30430 41.6354649482853 41.8848959280916 42.2577689927563
41.4273122914951 39.7568367479301 39.2248354955045 37.9430145836712
42.4580504587581 46.8873421271259 41.9666541498638 44.7926323492519
41.863927676552
FLJ30679 41.5421429082231 44.5884811528117 40.5734458834617
46.614092935749 38.0598374201407 45.1873487463472 47.7390230243778
42.5435283762933 48.3119523820103 47.0055981216687 43.3085358214154
43.6753843488695
FLJ30719 39.9697332660833 37.10170285405 41.437237454134
36.6501141438378 36.6354013629791 32.771863438151 32.9962272670005

35.5798430594271 36.5393857306744 34.9138625012006 37.5583886316038
36.9976231206067
FLJ30851 44.5798743103596 43.1464706017434 45.3974838013476
48.4916117779992 43.844855336215 42.6575535390785 42.8036731479932
43.6434576713809 42.0621425894624 43.6033221838149 46.1673159982664
43.1805016690297
FLJ31132 50.4872122867759 61.9753438182501 54.0536858212253
52.6947911480086 68.0414563597154 43.0860383229288 65.8986841086198
59.9968592852122 67.8208976798364 57.5713378330876 54.7883681830995
48.4535991392809
FLJ31222 42.8036731479932 45.7979032939211 38.0452862258803
42.357910063876 40.4639532172338 38.7340969861754 51.5967098638347
43.4544762830742 49.7336769474446 40.3235655331796 40.9157352260901
42.57175366709
FLJ31401 39.2076937845359 42.3711524005238 39.7411455670172
36.7186180663551 41.4397539024976 41.3553369544097 41.4742337714895
39.853866204927 39.1790517315259 43.0919221375514 35.5395819665562
38.5921548841176
FLJ31568 45.3872406432794 46.0023068797812 37.4642807402741
43.6726153508978 44.4489662507893 40.3946758935842 39.2533668167483
50.8160430472218 47.209353240759 46.8292269513767 42.2510526338368
43.5738557773903
FLJ31813 40.8681871019812 43.0227387991135 43.0971787292711
46.7577982595088 41.7929491793697 46.6872654833579 44.7255207589606
45.4757965005055 42.5406888281114 42.2614524730856 37.7384958256497
44.1452943804044
FLJ31945 42.0532901689036 40.3384318592985 40.0998167098057
40.5484717678957 44.9384441371181 42.157627240486 42.629412460409
42.7965563818112 44.1519466943185 42.8414096678708 39.0061493066838
45.1735152121056
FLJ32011 47.2862299990938 43.683792219729 45.5883690965192
40.5484717678957 39.9697332660833 48.0350549043063 47.8947595316832
43.3262148375525 46.4164127487052 44.3648993568405 46.0095710163378
43.7672102682067
FLJ32065 41.9407041937973 44.7829632472754 35.5220586859301
34.5425384901279 39.9697332660833 40.9506062495231 42.0605906240248
42.9301944191913 45.4799270968969 37.7315666632629 37.8372168909583
39.8497227447888
FLJ32154 37.9387791625384 32.2133093774284 37.6557714476853
33.8111782249716 40.5710637884029 38.1169703906207 39.4600924554505
44.5029690493211 35.1155486926999 34.8137085420423 38.956280281655
40.8681871019812
FLJ32214 40.7609952437622 45.7979032939211 42.3536549114165
45.8844325913247 47.5647821785529 41.159706131351 43.4544762830742
39.6474008369935 31.859589438824 43.2988017098387 39.9899430628542
35.1451084093692
FLJ32252 36.1232327599945 50.6229884142872 40.0998167098057
40.9607305725196 45.3675390992851 37.9003288112261 39.2533668167483
42.7431359850339 45.2897806651929 41.2276963324981 38.2795150354677
39.6352613956998
FLJ32255 42.1537476443423 49.9085372049438 64.5232087947596
91.7068354379889 46.0977687311222 50.6360662655392 54.8074655158941
44.0390019242862 47.798305839406 53.1993028936883 47.9942520395434
43.9541718254769

FLJ32310 42.3433931248155 44.3529919625344 39.991353369336
44.1483976313959 42.269054475455 47.9503724044453 46.9929836306816
42.8482181313226 43.8482802038826 44.2580151149956 47.0940613507321
45.0985088502363
FLJ32569 42.466333667839 144.500148287951 489.622327794168
40.4967168818294 284.241284057415 72.6182747525443 83.0647477737971
39.853866204927 121.650910373947 46.2768056702424 64.7598303406649
44.0246886382298
FLJ32658 39.4710743516517 43.2649781354792 39.991353369336
45.7397504535169 40.4639532172338 42.0574848021044 43.4544762830742
44.797320243931 43.1805016690297 39.4710743516517 44.2925355984056
42.9887069977696
FLJ32679 44.436995101921 40.0952076421149 44.1070537445005
44.6246431218094 43.0895173605825 44.3194383179647 42.1046072075724
39.9892510336916 40.4485515631112 41.1688470149988 41.9430737035925
41.1066323911308
FLJ32682 41.4508241771315 37.4540772310118 42.3536549114165
39.853175885492 38.5512429290626 43.4681646416585 40.9157352260901
36.1375121409002 39.3750969348308 38.745552403895 44.0409178714232
43.6753843488695
FLJ32784 41.5421429082231 43.3972849968032 45.5883690965192
41.3027031037343 42.7489468897708 39.5581166303303 41.7614975630362
41.4061152929154 41.9635525160083 41.9666541498638 43.3085358214154
40.716553247236
FLJ32810 42.9104454767916 39.3999056968598 39.7411455670172
44.7980740332174 42.6022652963326 40.7063426028871 46.6267962186599
43.0390857698452 41.134180463103 43.5627649099623 39.8577530602176
41.3481355128089
FLJ32867 44.5282181324797 41.4508241771315 39.9101908547397
42.0102671859606 39.1650142708257 43.4681646416585 42.357910063876
37.7612949798895 41.3803269429468 43.2988017098387 43.9450023643394
34.6720534245631
FLJ32955 47.2862299990938 49.5443425465542 44.8872784170036
50.378948785456 45.5809132232681 46.3715909653832 51.1541843174981
45.642304356051 42.4540072551568 50.3388505346542 47.0940613507321
44.4475838947656
FLJ33360 43.3638918705225 30.6802399384205 38.6005272730664
30.2675713925613 41.467005949568 41.9543948167375 43.5627649099623
35.1738399826814 37.3600014814388 45.9004543328976 41.9083581529276
40.8681871019812
FLJ33387 50.7390160399184 40.7952353240579 46.3411353377615
46.8954576545929 43.9450023643394 44.1737502492846 45.4799270968969
52.5909491535773 40.5912213523345 42.9866214102525 41.3553369544097
41.7575097298927
FLJ33534 38.0089018744739 43.5873188565519 42.3536549114165
45.8844325913247 48.0234896130991 43.5752393599169 46.8613212048981
46.0633179231318 44.619512785899 68.0556717366572 46.2560238004757
41.3481355128089
FLJ33544 46.8954576545929 47.2770121962655 46.2625470410195
43.074472921841 47.9128504683618 43.9724628401189 51.7714254834195
43.2551508830106 45.3756389170606 46.1390887492649 37.9946685306274
45.1735152121056
FLJ33590 46.7293238583888 43.0227387991135 44.7829632472754
60.3481762331447 40.7779767565889 42.4580504587581 41.8315602975157

50.9933795450314 46.581796165231 41.5435611403119 51.5822637582617 48.1627097465883
FLJ33651 44.2350046179299 44.1239711172417 49.5424425850549 42.8235313007746 43.9450023643394 41.554942319225 43.4544762830742 42.157627240486 39.0775870687758 47.9056710878325 45.0589947942306 38.0452862258803
FLJ33706 44.3355304829989 44.5152708955623 45.6989664108554 42.8235313007746 38.745552403895 47.3791873993182 42.1607322031054 40.1474543172332 40.4997186069494 47.0055981216687 44.4688999098904 45.0985088502363
FLJ33708 34.3473914754963 43.5873188565519 39.2707964550441 35.8472302065546 41.6806984441849 35.6125439901743 37.6036146841933 37.1317006162938 41.9635525160083 41.7405682794166 40.2649309204478 36.9976231206067
FLJ33996 57.2008272301399 45.7317208802318 52.7005092555102 49.7350276169552 52.5645416722441 41.0582900711341 55.0897019602305 55.8087097725119 47.1744296124496 51.9547984751408 52.104880481326 45.4799270968969
FLJ34047 46.6348258763289 45.4882438975269 39.5450079747895 45.5869891570251 41.0885562160946 42.8598805316863 37.0564573649223 46.7980335276155 39.9444191873552 39.8873706855774 45.7328341667151 49.3087627580849
FLJ34306 41.6354649482853 38.7344853699576 36.178547300352 40.8681871019812 40.7779767565889 38.3418933808844 35.4623691645629 37.4484310268909 39.6609238036815 38.957687788868 38.6662194645633 42.4607795638552
FLJ34503 35.8208542506595 33.3069622728735 35.3649449469275 40.8681871019812 45.2556760873335 41.4508241771315 44.0687697278114 42.8482181313226 37.3600014814388 40.1179210534118 43.1931731665131 45.5800840647989
FLJ34690 43.6706764143908 38.5252456306656 48.1331309338997 39.6292788039793 41.6806984441849 43.6670755115848 48.8319884367456 47.5950227477807 42.992884918158 43.844855336215 39.2542963855807 33.4316681623175
FLJ34870 43.6706764143908 40.9731067463382 41.8751578329365 38.8495095797881 44.2607789091176 39.6251842352088 43.9450023643394 42.6450179841129 44.8003345643334 40.8049408996274 41.1638563416981 40.4639532172338
FLJ34931 39.7576136507593 36.4002279956519 46.1653991089885 38.2041059863839 49.9493230064613 34.2773859804062 40.6750540874299 38.6454098044474 46.581796165231 43.2988017098387 43.0016969050339 41.4508241771315
FLJ34969 37.9387791625384 38.8336427948187 45.6989664108554 41.4273122914951 44.6834648302383 36.2015763253919 41.0381860307445 40.931294690669 44.3440839686558 44.8997235283254 38.5668610487033 38.745552403895
FLJ35220 47.9175508887533 39.6995529770239 45.310660704531 50.8567345921473 44.0451487108213 59.4949177415847 41.253645578517 53.9465584609317 42.992884918158 67.8233625051884 61.940656536312 57.3697604614404
FLJ35258 192.058030454066 61.1022920882065 52.3432130135266 79.4528447794527 48.3814421939888 220.573371581491 41.1507980856436 98.2074490157865 47.3791873993182 168.366869831376 139.693327116993 72.2211523775459

FLJ35282 35.2267149016596 41.3610369241157 39.8299340037009
41.0726336841311 40.6723334103791 37.7000400506818 31.6981395162348
43.6434576713809 37.9273653326866 39.2623423164488 32.0500360504817
44.6484005362281
FLJ35409 36.7356360093603 44.2630383225933 41.1367478979484
39.9697332660833 42.3685136297603 42.2577689927563 40.4359177791566
43.2551508830106 38.3418933808844 38.0078357808393 39.5399188536544
40.1700117452801
FLJ35424 43.5710871617107 43.2149698150931 47.154615851079
43.006072841282 40.7779767565889 43.5286328921307 45.1017494688414
41.599960574499 43.1087085278313 42.9285286409526 45.9104478407175
43.024125424819
FLJ35429 40.2543341073001 44.0409178714232 46.1653991089885
46.0228732507872 46.7508829977587 42.357910063876 43.0930293831497
40.5158867964016 48.1174861108648 37.7315666632629 51.838002904753
42.0424641526302
FLJ35740 39.0620487644635 39.0102066700839 44.0451487108213
37.29566077996 37.6527901109005 43.1826328292558 36.634024014558
37.7612949798895 38.9834959368496 38.3092241935352 40.2649309204478
39.4490233720848
FLJ35767 45.6517320730042 45.0905928998779 39.6451483836089
43.6726153508978 45.5809132232681 42.2577689927563 42.629412460409
42.8482181313226 41.3803269429468 49.9493230064613 45.2265319826548
40.6614700944853
FLJ35773 43.6706764143908 68.307513091369 45.6989664108554
54.8412943008635 40.8873907150245 41.7627910693483 53.3111230705754
83.5767232665142 57.404059248727 43.6033221838149 47.2134341420411
43.9541718254769
FLJ35776 40.0988483617359 42.0709558258414 40.6312687664188
46.3203995372658 43.683792219729 40.7063426028871 41.8315602975157
38.4540849632254 40.137818555383 41.9097689475185 41.1638563416981
42.3117490140079
FLJ35785 43.0545608384625 41.413768254554 42.640829914102
42.6913548400127 43.5880694884799 40.1428591998404 41.8315602975157
42.1812299265547 42.2577689927563 41.3735882187951 40.7776803630095
44.0977148249302
FLJ35801 64.4415447415281 77.268323196327 66.1541420734416
55.2214187655851 75.5510570035251 77.2834482217973 83.0647477737971
53.3094843669564 99.9057667432209 53.3059058105836 47.9942520395434
55.7067742610401
FLJ35816 40.6559419616644 44.4411734611229 44.5134109184888
41.5464765641928 52.9433495155796 41.8613406887824 48.6395795163502
45.2806199505029 41.7627910693483 40.2112371512838 43.401782064778
40.0557788909923
FLJ35848 42.0833588105728 42.1607322031054 40.0998167098057
44.265834798964 38.7004513165111 42.5193208629123 43.3262148375525
48.2109175258674 41.7627910693483 42.6793753550854 42.2618615571566
42.7782216342806
FLJ35880 39.7576136507593 46.8954576545929 47.0940613507321
37.8442841690502 52.2089306840125 39.8455060654531 35.4623691645629
72.8378863017531 41.7627910693483 42.5664883002833 41.0808257270221
42.8864415494484
FLJ35894 32.4552405279874 36.89524015739 37.7384958256497
45.7397504535169 34.3633615460307 38.7340969861754 36.3194282084339

34.8949555140527 34.4716904283753 40.5090849993014 43.3085358214154 44.9070250604284
FLJ35934 48.1952521496489 39.5050383901884 46.4569566520107 40.7481155008596 45.1137938810047 54.1440452292081 44.2166118829877 59.220967994972 41.7627910693483 51.7594352155304 52.902043370862 48.7814131120945
FLJ35946 45.0985088502363 35.5310164682026 43.9450023643394 34.1051821768421 37.956732373465 41.159706131351 38.6243997507646 38.956280281655 44.4395580493118 37.0320432433314 40.0766543123257 38.2350720635382
FLJ36031 40.2543341073001 40.5400384479766 40.4070046183108 33.1817617228171 38.5512429290626 39.0266821712099 38.4005795891396 35.0510338774593 44.7058233759912 34.7308008643142 37.2892641289258 40.6614700944853
FLJ36032 38.5332184743306 38.1811246394237 39.991353369336 41.5464765641928 39.5595233162192 37.0180934800573 44.6809101024392 40.8833572570383 42.2577689927563 42.3606390470387 39.153226699662 38.1421709520616
FLJ36070 42.8036731479932 45.7317208802318 45.1569741609597 50.7046706880325 71.4183281327686 47.4699236780425 45.3597029614942 46.9986083378817 51.5888395924843 52.5522545249206 57.2979215534929 47.8931773060789
FLJ36116 47.6397712007919 48.9899488302637 47.7737810047679 52.3004056941891 41.6806984441849 44.7804080899431 41.3594868055879 47.0940613507321 50.3245489035833 48.9826868640753 44.8872784170036 43.5738557773903
FLJ36144 38.2403867579549 37.6383530120621 38.4043916290561 40.0698728584484 39.4693871570028 35.2463080299457 40.9647597231175 36.7030475121778 39.5377896745682 42.3433931248155 39.3895791800635 40.5281496800812
FLJ36157 45.8108990837624 40.148780650831 42.5406888281114 35.49696651597 41.3755760434934 42.157627240486 44.8643898647322 39.6474008369935 39.4762546236449 45.4614234442527 43.844855336215 40.2669497398702
FLJ36166 38.1169703906207 34.9262464079777 42.4580504587581 38.0958223740111 37.8679241954337 36.8059561571675 43.4544762830742 37.2237132058632 39.1790517315259 38.4187686307607 38.4689132737495 37.0953622406687
FLJ36208 50.379587451669 47.765848710116 49.7350276169552 58.5406981871153 49.5666074434383 49.9699131196797 55.0897019602305 41.4508241771315 53.838484549134 53.1993028936883 56.8436081694537 54.4313108506244
FLJ36492 42.3433931248155 38.9338521051669 41.1367478979484 43.1847628331565 47.6884372645373 37.5887129572848 39.1539892144371 43.7396619462033 40.0403898620771 45.5776159432079 39.7342718293432 44.7401523707775
FLJ36644 37.55264801907 50.0425298550559 38.6727842796202 42.2496337895968 45.6895223823001 44.3648993568405 53.4921736109774 39.6474008369935 48.1174861108648 46.2286164776607 40.1742127891824 39.9519547348106
FLJ36701 42.2455707947223 42.7775752471711 45.2313723727673 39.2780515621926 41.9842695818285 44.9896851005259 38.7337086062875 47.5950227477807 39.5806465938526 42.0621425894624 40.6373400186325 39.0536056496869

FLJ36848 41.4508241771315 43.5873188565519 37.9482157381008 35.314469458379 33.3231576795423 52.4351670989268 33.4316681623175 38.745552403895 43.2761454096265 39.0648733099061 36.0481934423077 42.2577689927563
FLJ37078 67.4846787600629 51.4413099192899 52.3432130135266 60.620259884953 66.4047895961325 44.5544727111028 63.5738862549306 112.506613718654 101.46584765758 79.7389069715403 63.4323398447015 54.9919060580109
FLJ37201 41.3387790895716 41.8848959280916 41.6079958749402 42.357910063876 43.844855336215 43.0860383229288 38.8417151851134 43.7396619462033 35.5220586859301 40.6022003765153 41.9908237694988 41.2437378488025
FLJ37228 40.8681871019812 39.3999056968598 42.5406888281114 42.357910063876 43.6587492470348 41.0582900711341 39.0458123429605 41.6466169548846 48.9931786790166 40.8970401536578 46.1673159982664 42.0424641526302
FLJ37307 38.385865183581 42.1607322031054 40.6737160001291 42.357910063876 40.8873907150245 37.3799542961839 35.9894133432376 42.4580504587581 41.1742244762318 41.3321861371202 41.1638563416981 40.2669497398702
FLJ37396 62.8635815439291 48.1627097465883 52.8041782638549 75.2475238554276 53.0419743379342 58.9676332048764 67.2811786080606 88.1861828863404 54.5952754712007 76.5375674421131 70.3381994440425 63.3578893149245
FLJ37453 42.2455707947223 53.6429962450784 48.1997158668729 47.9915414951747 56.5933259268408 48.7781962164988 81.2136969156029 48.6441988290952 54.2825877218965 49.7259371799014 50.8826620053386 43.7672102682067
FLJ37464 40.5654775358427 52.8264142735929 64.5232087947596 49.7350276169552 46.1804647841331 39.132725377815 39.4600924554505 79.1039287828151 44.7058233759912 80.3915591207869 64.0541263553251 59.4465684293847
FLJ37543 39.266565827316 35.0222940937513 33.7737772393677 37.7469621225553 42.9090346219023 44.4619248757502 45.6042625176633 37.9837233601316 42.5406888281114 41.3321861371202 40.723990462047 44.7401523707775
FLJ37587 64.1882546112044 37.3634897565502 42.9104454767916 43.3078542962968 42.4864576619427 34.1806289878747 42.629412460409 39.2672712041774 42.357910063876 42.0621425894624 37.8372168909583 44.1452943804044
FLJ37673 41.5421429082231 45.1694246393676 44.1452943804044 37.9875302985336 41.8806343997257 45.4882438975269 39.3585350448719 43.6434576713809 40.7847110517594 41.1222709786153 46.6442692119997 43.1805016690297
FLJ38377 48.0201377454632 41.0678614026819 39.0775870687758 42.357910063876 40.6723334103791 41.8613406887824 40.9157352260901 41.6466169548846 48.3119523820103 44.8997235283254 48.6184776976167 41.0657892043496
FLJ38379 37.5979144071815 35.1527542166156 40.0998167098057 35.4371093426046 36.2048942610546 37.4498437754934 35.7666990316043 38.0240760521689 36.6312676076142 37.3674112783848 36.178547300352 39.8497227447888
FLJ38482 84.2728210732532 149.832238573027 194.469250918013 158.225884127529 237.401487995882 124.22494803053 134.908982962131

59.3164315859919 127.78061946174 90.8188865732032 104.02653369229
109.017269529278
FLJ38717 107.325456368022 55.6323803479191 80.7338782543351
93.1668080716761 52.9433495155796 49.9699131196797 70.7238101239421
84.7642634532073 75.0310858627394 88.6608345370342 77.2834482217973
84.7058085717914
FLJ38723 42.0532901689036 41.6466169548846 43.4709415392696
42.9358255168086 42.4864576619427 29.4322896559759 52.0942589368478
39.1652965788406 46.0106098064475 44.4652605017595 37.9946685306274
42.6758568421347
FLJ38773 41.6354649482853 36.7976562409506 42.9104454767916
41.4273122914951 40.3452320167286 39.0266821712099 43.9450023643394
40.7311497914871 37.2810767789306 38.8507804383261 40.2649309204478
38.1421709520616
FLJ38969 50.2203578628217 46.0713306971594 47.4740726032707
48.4916117779992 56.359578588344 48.6621527194963 58.0139597055449
54.5125550937898 51.1055541075029 46.1390887492649 42.5426057453993
46.917149434588
FLJ38973 42.8036731479932 44.2105195734073 38.4043916290561
38.6414316877471 53.0419743379342 35.7034203372528 42.2577689927563
43.6434576713809 40.0403898620771 34.064698871541 42.7275315121163
44.3521099857368
FLJ39061 39.8234920704779 39.1553169409075 40.5734458834617
44.9370339275041 41.2740822675592 39.9777866059804 43.6753843488695
41.2551966132486 45.6080994842498 44.6235437216725 41.9908237694988
42.2577689927563
FLJ39080 45.7356815244513 46.4385386401326 49.2041300973417
49.5424425850549 52.0583273941813 46.574682061365 45.1017494688414
49.2019743609873 52.1186591838715 47.611018474151 41.3553369544097
41.7575097298927
FLJ39632 66.6547247910487 152.608998839745 127.265200736376
46.8954576545929 219.838542502016 49.2050538150106 41.5421429082231
79.2701322339022 195.303781999387 48.2596564318071 48.1682125052975
40.7704587752083
FLJ39639 45.4799270968969 49.4067286971348 72.6731815563159
84.8948024208923 54.4586176399381 66.8632294055679 49.8281348927244
49.3880946790306 42.7150227340364 74.2379710965903 64.9959712888709
52.6922486991742
FLJ39653 75.0704619826557 59.6359245937831 101.693848821246
56.0794100106654 95.6182216943758 55.540888910486 70.5534237141767
55.1109784383525 111.291087721139 142.444971930298 89.4747568145717
88.8330881965986
FLJ39660 45.0985088502363 42.5269793679335 38.5045333213784
42.1273928203021 56.6951078698291 44.5544727111028 36.3194282084339
50.0526763693329 48.8842909466002 37.2089297656361 36.6298807161755
35.7451401627836
FLJ39739 41.4918397757335 38.7476648567356 41.8291484190524
40.5998758007868 38.3085139808968 38.7679739198116 41.1033381844231
38.6151680924119 39.4433784795209 40.3548989788016 40.0766543123257
39.6682560919759
FLJ39827 132.257197568848 114.876346000655 77.3367966395554
72.4834467838798 135.567012693522 120.618530729822 128.966814822678
83.9236799134394 139.49198349194 119.301672388541 101.540333720181
88.8330881965986

FLJ40113 43.1087085278313 44.9888573005713 44.1933547401128
40.7988621011332 45.4008983015065 40.7013550424464 42.629412460409
43.375692472202 45.6057977304398 45.2089696897205 47.1978679867866
48.303052868477
FLJ40125 53.8856501960117 44.0409178714232 43.1931731665131
49.5424425850549 38.2496050953547 45.0882356578404 37.7136011474903
52.5098795466615 44.0519245668737 56.1912096308988 39.6403089864678
95.1224420992193
FLJ40142 82.1062834830477 83.6351558609087 70.3309641302984
56.6458456890715 103.356071080819 66.7768390362438 83.374777060444
56.3560498705517 78.9203273221608 64.1097859982912 69.2293488487819
71.5484567134601
FLJ40194 37.7384958256497 46.1569135229922 42.3536549114165
36.6501141438378 38.745552403895 37.2686490268265 37.4772922422734
37.0390589468421 34.6720534245631 39.0648733099061 38.5668610487033
35.4484652445078
FLJ40235 44.1294459089314 42.6939394537076 35.4484652445078
44.7980740332174 41.2740822675592 35.0970060242246 39.6535880152834
36.3405039629059 38.5252456306656 42.3606390470387 38.3684202018524
43.1805016690297
FLJ40243 37.6397652536077 38.3322849790657 33.553634633839
33.1817617228171 38.2496050953547 37.1317006162938 36.3194282084339
35.0510338774593 35.2143190355414 35.2880714952489 36.131485826118
35.6427313468066
FLJ40244 41.2424637338424 40.716553247236 35.7105247506212
43.6726153508978 39.0564222526273 41.159706131351 40.0052661202207
37.86982638952 43.9450023643394 41.9666541498638 39.4410515050885
41.863927676552
FLJ40288 39.3709357615493 43.0971787292711 39.3709357615493
38.6414316877471 38.745552403895 38.9378717994178 37.1643693306382
46.1673159982664 36.7267557061938 38.957687788868 44.4688999098904
38.8495095797881
FLJ40292 35.1307934330047 39.6995529770239 42.9104454767916
37.0732977341185 43.5544347011646 40.650742261428 33.8558232866797
39.4710743516517 33.2715481910159 42.6793753550854 40.547835538778
43.2776458914333
FLJ40296 41.7884142319151 40.148780650831 40.0557788909923
40.5061122751948 38.7153183948786 40.5703581862752 40.8714654152472
41.7419877611072 42.8542642792862 40.2579040125416 38.5233040786032
40.6614700944853
FLJ40330 39.7576136507593 42.2900457798748 39.991353369336
41.8291484190524 42.039525923161 39.266565827316 40.0052661202207
44.7044140979108 44.5678064264717 39.1177164316495 39.1878770155069
43.2177497174408
FLJ40411 39.3709357615493 40.5400384479766 42.157627240486
44.9370339275041 39.0564222526273 42.0574848021044 42.1607322031054
39.3709357615493 47.9035532886869 40.4136535208406 42.7275315121163
39.6352613956998
FLJ40448 46.3390850717989 41.0678614026819 36.769073068743
63.0153175111747 40.4639532172338 44.3648993568405 46.4388841354122
46.4672637826582 53.2046867327331 45.4614234442527 51.9302027391447
57.8111054584154
FLJ40453 37.0320432433314 39.4629815741023 39.3709357615493
38.5707420487455 38.2083710281545 39.6251842352088 38.1169703906207

37.7217193704498 42.4540072551568 41.4659679379482 39.0061493066838
37.8490162439785
FLJ40473 38.2083710281545 44.4411734611229 42.8270038153139
42.8235313007746 41.0885562160946 43.1826328292558 43.6753843488695
39.9576138799997 43.1805016690297 42.9866214102525 40.6373400186325
43.8575940017119
FLJ40504 680.837950890159 307.264133974364 276.214079735778
53.8732900241064 194.355765743782 69.5645254789371 338.053116855608
1712.55038816005 312.154320305976 246.274692971423 253.527553472516
160.499410146402
FLJ40712 43.5710871617107 42.1607322031054 43.3085358214154
38.0958223740111 39.0564222526273 47.4699236780425 50.2016912396771
49.8344973836834 41.6616444147804 52.2256177934134 44.6286581923295
42.7782216342806
FLJ40722 117.539037885384 88.4385631861141 112.159199477006
130.908206625954 74.3523005665671 70.6311537856734 108.806649898122
166.36685654692 89.4111455826308 160.360785581153 145.936763882526
121.969082013079
FLJ40852 54.6235981131919 49.3581578587582 50.6004078448405
51.7739270955254 45.2556760873335 40.7616966228404 47.5963750520409
56.8094693588238 53.838484549134 69.0530953932111 52.7817052791259
50.8999542937724
FLJ41046 43.4626049853157 42.5269793679335 37.5597671487621
39.2780515621926 45.1137938810047 40.040778349137 35.6056062164318
48.272324215504 41.6616444147804 42.6793753550854 43.3085358214154
47.3992733452821
FLJ41170 43.844855336215 37.4540772310118 44.4311655729487
41.5464765641928 45.1137938810047 41.554942319225 38.7337086062875
44.5029690493211 42.6366765186182 44.6918600441328 36.8282383070068
38.8495095797881
FLJ41200 39.3709357615493 42.6939394537076 35.2101597814352
49.7350276169552 35.7973685350741 47.0940613507321 41.3594868055879
52.407151732257 48.2123120250343 45.5776159432079 43.0016969050339
41.5536168238478
FLJ41327 45.9090659324223 41.8848959280916 48.6037684272115
40.5484717678957 32.9756367838345 39.0266821712099 39.4600924554505
38.3684202018524 39.0775870687758 41.7405682794166 39.9070785037411
35.3525469364579
FLJ41352 39.6349162264993 42.2192468749755 39.991353369336
37.8072702808148 41.0448919024758 43.9351969768311 41.8887298788489
41.0808257270221 41.1367478979484 39.1621898366355 39.5748670524951
38.2795150354677
FLJ41423 235.33716196909 147.20900462938 4232.402174615698
278.453446231317 188.670053901101 171.689014874037 222.81957100654
259.456935785521 161.008205823493 250.504350952583 217.263889616995
178.661935292895
FLJ41481 45.0985088502363 42.5269793679335 38.6727842796202
40.7481155008596 40.148780650831 43.4681646416585 42.629412460409
45.8669209716694 39.2809305246091 47.8207974930031 30.7315474127249
34.4570158133896
FLJ41603 69.6449298794042 44.0409178714232 43.6670755115848
56.0794100106654 46.8674366115656 69.7677752398541 45.4799270968969
50.1833483241864 51.8083208406279 102.136265168978 70.2215218476683
61.6311118308635

FLJ41649 42.8036731479932 33.0553208959996 39.991353369336
38.745552403895 36.8059561571675 38.3418933808844 38.5066108855005
40.7311497914871 41.4636047919453 39.4710743516517 43.5710871617107
41.6550823405646
FLJ41733 40.4639532172338 36.3110701990588 36.8730514568925
37.9875302985336 45.4751076037511 37.2686490268265 38.0598374201407
43.7396619462033 37.4540772310118 36.3612409063792 41.9908237694988
38.9496515344837
FLJ41766 53.4921736109774 47.2770121962655 59.2012779686869
44.1483976313959 52.2089306840125 58.9676332048764 49.540449125788
45.2806199505029 40.8970401536578 52.7972156492746 60.6505672212119
50.7487159427973
FLJ41821 35.4287975493553 37.5469247328848 38.3166554965434
41.6564075217517 34.7018263647985 34.8956628705329 40.1235656101329
37.9837233601316 34.7722083264266 35.93950183586 39.2542963855807
30.8150126829901
FLJ41841 38.2083710281545 42.2577689927563 39.1799070031819
34.5425384901279 37.6527901109005 47.5733603903505 39.5496902678788
39.1652965788406 41.4636047919453 41.7405682794166 38.2795150354677
39.5418280570561
FLJ41856 41.0608773166058 38.5252456306656 43.0971787292711
38.4208429103799 42.6022652963326 42.750861693377 33.8558232866797
35.2642250366721 43.4656637536244 39.7835513496873 44.553780984244
41.5536168238478
FLJ42102 34.6977013306396 39.1982470626863 38.4043916290561
38.2041059863839 40.5710637884029 38.1169703906207 37.3799542961839
38.079222289614 38.6322728994674 38.4187686307607 33.6902293666089
39.8497227447888
FLJ42133 44.9896851005259 41.4508241771315 40.6737160001291
42.357910063876 45.5809132232681 39.8455060654531 50.0088611244948
36.7460922671619 44.1519466943185 46.7646877499666 45.2265319826548
45.0035308952466
FLJ42177 39.5676051939342 45.8669209716694 35.5220586859301
46.4555908533915 42.4864576619427 37.9003288112261 37.7136011474903
63.1604223919703 38.5252456306656 47.2299678280724 49.2206713037152
43.2776458914333
FLJ42220 45.0985088502363 43.5873188565519 37.1643693306382
39.853175885492 40.5710637884029 38.6414316877471 36.197424053556
36.6452480995946 42.1594905198929 44.2580151149956 43.0016969050339
38.8495095797881
FLJ42258 43.2135791259317 47.9665737016961 40.6737160001291
46.614092935749 44.6834648302383 49.1718303518849 45.6042625176633
43.9366081640576 44.2607789091176 46.0119912782855 45.6706505553383
43.5738557773903
FLJ42280 61.356483470053 43.3458769998118 34.9849896310235
39.9697332660833 39.3667161654772 38.4330755397458 30.561906108551
41.6466169548846 42.1594905198929 43.9450023643394 36.3390947880186
42.4607795638552
FLJ42289 42.5193208629123 39.7835513496873 40.0998167098057
44.1483976313959 54.1225249245036 38.1169703906207 36.5352572191451
36.5393857306744 44.5303781239209 41.1222709786153 39.9070785037411
40.6614700944853
FLJ42291 41.9407041937973 43.2649781354792 39.5450079747895
47.0874209746031 40.5710637884029 35.2067831864574 49.2546642180676

40.3357777530419 45.0017158586743 47.9056710878325 41.0050831223107
44.9070250604284
FLJ42393 59.2903915056319 47.8400694856726 49.4573631027613
48.665578118898 56.1186281675573 56.3525213736952 56.0515443021293
56.3560498705517 53.2046867327331 60.7495043977707 55.9269493191353
55.7067742610401
FLJ42418 43.0001432913058 46.5650556481721 46.6588655218735
50.7046706880325 44.147557390293 43.5752393599169 45.8980298854198
45.8669209716694 42.4540072551568 43.7303813515864 42.170702295259
39.8497227447888
FLJ42709 38.0089018744739 46.0023068797812 41.3366779545815
51.5913529948887 45.2556760873335 43.9724628401189 42.8684958525042
40.7311497914871 43.8482802038826 42.3606390470387 43.3085358214154
43.1805016690297
FLJ42842 38.2083710281545 39.0102066700839 40.8420925848403
39.9697332660833 46.0977687311222 41.2567470068038 38.5066108855005
40.7311497914871 42.0621425894624 40.4136535208406 39.3667161654772
42.57175366709
FLJ42875 47.2862299990938 43.4396866157171 42.9104454767916
52.8502823653368 49.8304479381623 49.8472708021525 67.2811786080606
84.607833108004 42.5406888281114 46.1390887492649 46.824299585607
48.5123041297835
FLJ42953 43.5710871617107 42.6022652963326 42.640829914102
46.7577982595088 50.5110359971301 44.3648993568405 40.6750540874299
39.853866204927 40.5912213523345 42.8936990772838 44.6286581923295
44.0451487108213
FLJ42957 44.5282181324797 42.8542642792862 37.3687900733549
40.7481155008596 37.4470183315826 42.8598805316863 39.5496902678788
40.931294690669 41.3803269429468 37.3179578037963 41.6336636285875
39.9519547348106
FLJ42986 50.4872122867759 42.9887069977696 39.9101908547397
44.8784374362948 42.1650127805175 37.4782441311626 37.1643693306382
40.047314525032 42.4540072551568 41.4397539024976 36.6298807161755
35.2101597814352
FLJ43093 41.4508241771315 50.0425298550559 45.989212296035
56.9299697426319 40.0599807618105 67.8208976798364 43.4544762830742
43.9366081640576 40.7847110517594 42.8936990772838 41.7272875295469
49.4786466722535
FLJ43276 39.8662795144616 37.10170285405 39.8299340037009
37.7469621225553 41.2740822675592 37.5887129572848 37.8877750141557
39.4223544019653 38.3418933808844 40.2493681589104 39.8184668132023
37.4554876386402
FLJ43582 44.7915315085948 41.8848959280916 42.4580504587581
50.2163892855092 47.3172462673426 44.7804080899431 46.1634822993009
45.3872406432794 46.4861258030402 43.6033221838149 51.3103617600342
37.8490162439785
FLJ43663 57.6285775590145 47.765848710116 50.9010149857308
91.9185612536409 53.8260140375214 59.9876575158981 41.3594868055879
59.8760452220989 47.798305839406 45.4614234442527 50.8826620053386
61.7502624458447
FLJ43692 128.374733248762 220.85458646522 293.516547661658
47.9915414951747 236.440703574276 77.2834482217973 90.7037473522607
172.89937021234 334.016152807947 182.80711310964 110.729504563799
234.727123301682

FLJ43752 43.7585538437696 43.4396866157171 45.310660704531
43.4410741327254 54.5683518971261 44.6524308640784 52.7002314230604
46.1673159982664 43.5669306063196 44.1379638573455 40.6373400186325
48.6795178347248
FLJ43763 40.7609952437622 34.2618170189676 48.1331309338997
39.9697332660833 40.8873907150245 40.9506062495231 40.7910160811527
40.5158867964016 39.7537274655115 33.8984830800739 37.3687900733549
41.863927676552
FLJ43806 38.957687788868 42.0709558258414 40.0998167098057
43.6726153508978 44.5668141895992 43.5752393599169 43.0930293831497
43.6434576713809 39.7537274655115 41.5435611403119 40.9157352260901
35.8429598823818
FLJ43826 35.5179217192467 40.0527893189096 48.1997158668729
40.9607305725196 38.6454098044474 40.244271403831 44.2166118829877
36.3405039629059 37.3600014814388 40.2112371512838 35.4386677371439
45.2783024981315
FLJ43860 42.3620028137457 39.1539892144371 40.931294690669
38.1472552468971 42.2455707947223 41.6045220683604 40.9811982338594
40.4359177791566 44.2142581488029 41.2651195687914 43.8855280305245
41.3481355128089
FLJ43870 42.0532901689036 43.9421822132391 43.401782064778
40.7481155008596 44.0451487108213 43.4681646416585 39.5496902678788
44.7044140979108 42.4540072551568 35.4990901121893 40.4504608195666
44.3521099857368
FLJ43879 48.8941062980393 48.0296332183129 42.5406888281114
46.614092935749 46.0043807840705 44.9896851005259 36.7356360093603
48.3590401011623 40.4997186069494 47.611018474151 44.9713479185144
41.863927676552
FLJ43950 45.4507786740825 46.6578431194852 43.3567925731226
46.1074305700679 43.5836473788312 43.1358833499227 43.4844452775022
43.8801268237207 45.6582729799009 45.2949878966345 41.8168470823451
42.297291190584
FLJ43980 37.3463875295726 38.1312950900467 37.4642807402741
41.1886975241176 42.4864576619427 35.7034203372528 42.0605906240248
40.4359177791566 44.1519466943185 42.6793753550854 34.9428206949566
38.8495095797881
FLJ43987 40.4639532172338 42.4493032770053 39.3709357615493
37.7469621225553 39.4693871570028 42.9695585740813 37.2892641289258
38.8495095797881 40.5912213523345 44.2580151149956 38.6662194645633
37.6557714476853
FLJ44005 43.844855336215 48.109747794187 40.6737160001291
46.1673159982664 43.004943518025 43.9724628401189 43.8206425661746
44.6107027295193 49.1940901905636 38.4187686307607 43.401782064778
40.4639532172338
FLJ44006 40.3478825593224 39.6995529770239 40.4070046183108
40.4359177791566 39.1650142708257 39.7394181011603 46.0228732507872
45.0972184964705 38.0213051227859 36.3612409063792 38.6662194645633
34.5633623842083
FLJ44048 43.844855336215 44.5884811528117 52.3432130135266
43.1847628331565 44.9936360641969 38.6414316877471 39.7576136507593
39.5466975353131 36.9060997041453 75.0665411373616 36.43924066428
38.3291713594599
FLJ44060 43.0930293831497 34.9262464079777 29.4322896559759
38.9534635879195 43.1252272136667 41.4508241771315 42.2577689927563

47.8947595316832 43.9450023643394 40.5090849993014 37.7384958256497
41.3481355128089
FLJ44076 44.1294459089314 36.6968906669565 40.8420925848403
38.9534635879195 41.0885562160946 42.8598805316863 40.5585676679482
39.7509165247419 43.5669306063196 46.6598879466655 39.7342718293432
42.57175366709
FLJ44112 36.1232327599945 38.2263227947189 38.6727842796202
42.4580504587581 38.745552403895 47.2009420656854 42.357910063876
42.5435283762933 39.9444191873552 43.0919221375514 41.0050831223107
38.5279745308203
FLJ44124 1020.75813028358 629.532332373083 851.813216452015
836.472724085903 464.671881584567 542.467997217663 749.49934348018
1394.28860933629 440.421077161585 1090.86617066541 1307.19925493293
1004.8679675716
FLJ44186 42.3433931248155 44.3529919625344 37.1643693306382
44.9370339275041 50.8875036861067 41.9543948167375 43.3262148375525
40.7311497914871 44.8003345643334 41.2276963324981 45.4799270968969
43.8575940017119
FLJ44216 35.9305494063137 36.89524015739 46.5650556481721
31.3142073726046 39.6535880152834 42.6575535390785 33.3231576795423
35.2642250366721 37.0126425326534 29.9617615620508 37.7384958256497
34.806104801006
FLJ44290 42.2455707947223 41.9894412416874 42.0621425894624
45.4634652319753 44.147557390293 40.7616966228404 42.7431359850339
43.9366081640576 48.1174861108648 42.5664883002833 42.4580504587581
41.7575097298927
FLJ44313 40.0557788909923 37.5469247328848 39.0775870687758
37.651298468202 31.9468644542969 34.0777108441756 31.197411437683
37.7612949798895 36.197424053556 38.957687788868 38.3684202018524
42.0424641526302
FLJ44379 42.0532901689036 45.0905928998779 33.6816578730693
34.5425384901279 35.4484652445078 38.1169703906207 40.0052661202207
33.8558232866797 44.7058233759912 35.4990901121893 35.7953070087288
34.6720534245631
FLJ44385 50.315768338317 40.8880530359822 39.2707964550441
36.7892997857376 38.4540849632254 35.5158505219112 35.7055410599146
41.8417024632349 33.5578650644246 38.8507804383261 43.844855336215
30.6242215267208
FLJ44450 38.1169703906207 38.6414316877471 42.2577689927563
38.6414316877471 48.8381507187986 35.5158505219112 42.4939802311136
38.2782436497441 44.619512785899 40.5090849993014 37.7384958256497
44.0451487108213
FLJ44477 42.0532901689036 41.8073910693411 40.8420925848403
46.1673159982664 44.8897737954573 50.6360662655392 55.9276266345493
44.2159020605784 40.0403898620771 43.0919221375514 47.8284278211109
43.4709415392696
FLJ44606 38.5648843914189 46.29198852834 54.4384603094793
41.3027031037343 37.7576900881741 45.1873487463472 51.0013344286851
50.5070012045444 49.0986627051784 46.325232414659 43.0016969050339
48.8670821164567
FLJ44635 39.2076937845359 37.3634897565502 41.0356368374708
39.7411455670172 39.2114264935239 37.1317006162938 34.3135545118996
40.7799172560245 39.5806465938526 41.2598458726002 40.3452320167286
36.1647506311964

FLJ44653 38.7537364570873 41.4508241771315 42.2577689927563
42.4580504587581 43.004943518025 45.6129956328415 34.4570158133896
38.3684202018524 45.1899271235974 42.785429206422 46.2560238004757
41.9430737035925
FLJ44674 38.5007204246293 34.15544299189 41.437237454134
29.8304689114955 37.2567512265363 38.2293008890613 36.634024014558
43.1567024035183 38.9006788676407 41.2276963324981 40.0766543123257
39.3575468494941
FLJ44790 41.9407041937973 39.8695929627783 37.5597671487621
36.890925044696 41.8806343997257 42.5660695551615 45.7452456977857
34.1806289878747 44.3440839686558 49.0841460667818 40.723990462047
49.0655215909632
FLJ44815 37.7384958256497 40.6380495994768 43.0971787292711
39.853175885492 33.2021437980841 36.9060997041453 37.830876898596
39.4710743516517 42.357910063876 42.9866214102525 43.9450023643394
38.0452862258803
FLJ44817 33.5994525227062 45.0905928998779 46.7508829977587
42.4580504587581 38.6454098044474 40.244271403831 40.4359177791566
39.9576138799997 37.6383530120621 40.6022003765153 38.745552403895
40.1700117452801
FLJ44861 37.1485601150873 44.5152708955623 38.0452862258803
42.9358255168086 31.5599106749655 38.4330755397458 44.6032218589199
43.3262148375525 36.197424053556 38.2069499294002 35.1470994647364
38.4330755397458
FLJ44881 44.9896851005259 34.2618170189676 40.7620472083159
41.8778873678551 40.0599807618105 39.132725377815 43.8206425661746
38.2782436497441 44.1519466943185 44.1379638573455 44.0409178714232
41.9430737035925
FLJ44955 42.3433931248155 47.1332119496722 35.6139301508335
36.3604951341921 37.2567512265363 50.6360662655392 38.1793598983615
50.9933795450314 40.4997186069494 37.1275334308172 40.4504608195666
38.5279745308203
FLJ45032 39.3709357615493 40.6380495994768 50.8303921211581
40.0698728584484 56.0642694832108 39.2248354955045 39.7576136507593
47.3172462673426 47.5057905459278 39.6719190054379 42.9634662926872
44.0451487108213
FLJ45079 46.263826584899 38.7344853699576 38.6005272730664
41.7614975630362 41.0885562160946 38.2293008890613 39.2533668167483
38.956280281655 35.9645119596869 45.6868589181768 34.6366195121669
36.3454320848591
FLJ45121 42.7150227340364 38.7344853699576 45.063792968927
42.0102671859606 37.8679241954337 40.4359177791566 42.0605906240248
42.357910063876 38.7298222155532 42.8936990772838 41.0808257270221
43.0930293831497
FLJ45139 38.957687788868 39.9697332660833 42.0621425894624
35.49696651597 35.9894133432376 37.9003288112261 43.0930293831497
43.7396619462033 37.5469247328848 36.8059561571675 40.8225507267584
37.8490162439785
FLJ45202 38.8561375693335 43.2649781354792 43.0971787292711
42.6913548400127 40.6723334103791 41.2567470068038 40.2154497375715
40.6171774724171 37.2810767789306 39.4710743516517 45.1432412071268
48.27609330316
FLJ45224 46.7293238583888 48.6447083765118 49.2041300973417
69.1931891474114 44.5668141895992 45.3882138838137 56.3376046940285

46.1673159982664 42.7150227340364 47.611018474151 46.6442692119997
44.2527566343661
FLJ45244 79.0304119352941 53.8499082625091 104.250582652805
59.987045425047 68.1674425673171 62.3497263884167 72.1401612718245
73.2037966614621 78.6048653630165 115.98265270452 90.3114947219972
87.8967913380047
FLJ45248 41.2424637338424 36.9994058279362 41.8751578329365
45.4634652319753 43.5544347011646 36.3845892764084 52.4111687156893
43.7396619462033 44.4395580493118 38.4187686307607 46.972521499515
42.2577689927563
FLJ45256 50.315768338317 45.4143854024453 44.9758339877851
53.0284011078097 50.6470461863549 43.7630587657205 49.3854238365325
55.6904617926772 41.863927676552 51.8412522234186 51.5967098638347
45.6233434183356
FLJ45300 43.0930293831497 45.4143854024453 42.7303139017786
49.3654837541353 43.844855336215 43.7630587657205 41.3594868055879
35.9023742468744 33.6902293666089 50.7855742231932 43.6629145624937
43.3744503319109
FLJ45337 58.5759178381027 62.9904770954043 60.6567691532561
55.6481366056639 52.3242437351389 61.8187341713197 66.8475050031365
57.9713749600418 62.6386437936516 58.6439719071099 59.6608518093582
50.1579724208315
FLJ45422 38.5007204246293 43.3972849968032 40.7910160811527
49.7350276169552 43.3332476888305 46.7386599624801 43.2721716572958
43.1567024035183 48.3119523820103 45.8471859855979 44.8372404608604
43.4709415392696
FLJ45445 40.0557788909923 42.0709558258414 42.281606728072
46.6849259108754 48.7288537840562 41.3135088038412 45.6706505553383
43.1931731665131 40.6957787921451 43.6670755115848 44.4329005595031
43.0930293831497
FLJ45455 40.2543341073001 42.3550738181144 42.4580504587581
42.5664883002833 46.7508829977587 39.6251842352088 51.9421032049606
43.1567024035183 44.8003345643334 44.571915452417 40.8225507267584
45.5800840647989
FLJ45537 37.3463875295726 43.683792219729 39.0470836345045
42.7489468897708 41.8441156763056 43.7630587657205 41.4742337714895
40.7311497914871 40.8970401536578 40.7063426028871 42.5023941254529
40.5235471987417
FLJ45684 42.8036731479932 47.3536182445315 40.4882741869718
40.3180310664156 42.9090346219023 39.132725377815 38.941855825269
44.9977782988116 41.1742244762318 39.9948390976317 37.9230440150891
47.3992733452821
FLJ45717 41.3387790895716 38.6414316877471 40.931294690669
35.9521941716188 52.9433495155796 45.7300501624017 50.5252048155459
47.7889447429963 47.7086093893038 41.5435611403119 54.2999306485758
38.745552403895
FLJ45721 42.1537476443423 36.6002100641539 39.7411455670172
41.8778873678551 43.2484996523549 44.9896851005259 33.2425023465738
39.4710743516517 46.9739160257217 40.8970401536578 37.6484782516569
46.4499258373233
FLJ45803 43.4626049853157 45.0115870036541 59.2778887402357
47.9915414951747 54.4586176399381 54.7083331985678 65.524821699432
55.421880601441 93.8042295944812 89.1516394894484 108.599492114105
71.6502000742477

FLJ45831 44.9896851005259 41.9894412416874 39.8299340037009
38.2041059863839 41.0885562160946 43.1826328292558 38.8417151851134
39.7509165247419 40.137818555383 39.9948390976317 43.0016969050339
37.5556299264699
FLJ45832 44.0328992213727 44.5884811528117 40.4882741869718
42.8235313007746 50.3713170502557 43.2805590023412 39.5496902678788
38.2782436497441 44.4395580493118 41.0057887014368 40.1742127891824
41.7575097298927
FLJ45964 34.4021554788966 40.0527893189096 42.4580504587581
42.1273928203021 36.9060997041453 41.554942319225 43.5627649099623
39.1652965788406 42.9020321355927 38.745552403895 42.7275315121163
35.9604262434141
FLJ45966 44.9370339275041 45.6948619492056 46.02045129728
57.165971310746 47.27543600687 51.0071565607171 52.561573697155
49.239733414453 49.8472708021525 47.3791873993182 46.824299585607
49.6203214143464
FLJ45974 40.9558083259517 40.4359177791566 41.2365089689635
42.0102671859606 43.7517118200398 37.9951997351435 54.339958888486
40.7311497914871 43.4656637536244 47.3791873993182 41.7272875295469
48.7814131120945
FLJ45983 43.5710871617107 44.2105195734073 44.696023817801
35.3829784221072 40.6723334103791 33.7818132446042 39.2533668167483
38.6454098044474 44.1519466943185 40.8970401536578 42.4580504587581
38.745552403895
FLJ45994 43.844855336215 42.5269793679335 42.3536549114165
46.3203995372658 40.3452320167286 42.5660695551615 39.2533668167483
44.7044140979108 41.2870505273664 41.7405682794166 41.9083581529276
42.7782216342806
FLJ46010 39.7576136507593 37.7384958256497 44.0451487108213
39.7411455670172 40.4639532172338 41.9543948167375 44.2166118829877
43.4544762830742 43.5669306063196 41.7405682794166 42.2618615571566
41.6550823405646
FLJ46020 44.436995101921 43.4396866157171 50.0050760710506
39.6292788039793 43.6587492470348 40.650742261428 39.3585350448719
43.2551508830106 46.4164127487052 45.9004543328976 45.2265319826548
46.4499258373233
FLJ46026 41.4508241771315 38.3322849790657 34.8956628705329
42.9358255168086 42.1650127805175 40.4359177791566 43.9450023643394
40.047314525032 36.9060997041453 44.1379638573455 37.7384958256497
36.064718696392
FLJ46082 34.4896350146096 49.6203214143464 42.157627240486
36.890925044696 57.6791750431251 42.9695585740813 44.9863726205428
36.1375121409002 41.3803269429468 36.3612409063792 41.5421429082231
42.7782216342806
FLJ46109 38.7537364570873 38.5921548841176 37.4642807402741
35.3457527182429 39.512606833112 37.9430145836712 38.7337086062875
37.86982638952 34.0382693188794 35.0668743708816 35.7431996538283
39.6352613956998
FLJ46154 41.8409927585705 36.9994058279362 36.9795961564828
43.1847628331565 33.5412189543102 35.5158505219112 38.7337086062875
39.4710743516517 40.4997186069494 34.8137085420423 35.9563099719284
36.8690774823319
FLJ46156 37.3463875295726 39.0894172982439 43.401782064778
45.7397504535169 46.1804647841331 39.4953494583071 40.4359177791566

39.2672712041774 45.8325181856483 38.957687788868 42.357910063876
38.0452862258803
FLJ46210 37.4554876386402 41.3610369241157 38.1421709520616
43.3078542962968 39.859345745831 35.2067831864574 38.8417151851134
37.7612949798895 36.7267557061938 42.785429206422 38.3684202018524
38.4330755397458
FLJ46230 40.8681871019812 44.3529919625344 39.1799070031819
47.9915414951747 42.7107928951766 46.3715909653832 40.6750540874299
45.3872406432794 39.4762546236449 48.0211752672327 45.1432412071268
41.7575097298927
FLJ46257 44.3355304829989 49.83247261979   40.6737160001291
41.5464765641928 42.7107928951766 38.6414316877471 42.2577689927563
43.1567024035183 46.4164127487052 44.0409178714232 43.6629145624937
37.1747156131306
FLJ46284 39.9697332660833 40.8880530359822 42.2577689927563
42.5664883002833 38.2496050953547 42.5660695551615 40.0052661202207
38.956280281655 47.3791873993182 45.0155543555707 39.6403089864678
46.2625470410195
FLJ46300 46.441301934328 43.7644428186265 43.7630587657205
43.9282059368202 49.32293280121   50.0748292497578 44.4639405506967
49.9332280449549 48.3119523820103 50.5252048155459 43.9450023643394
45.4799270968969
FLJ46309 928.230419226078 647.868022835702 655.389317052689
923.470702344816 466.454993547103 419.993482471924 835.171475474151
1595.69503047506 521.874349042344 1453.77801653969 1529.52516575545
987.427672417105
FLJ46321 44.1294459089314 39.5573415356949 45.4634652319753
45.8844325913247 44.9384441371181 41.554942319225 43.2721716572958
39.2248354955045 39.8873706855774 44.1379638573455 43.1724740315182
42.0424641526302
FLJ46347 40.8681871019812 38.8336427948187 39.7411455670172
37.1894304543751 34.9062568194127 36.8059561571675 39.8695929627783
39.5466975353131 37.1820543575123 42.1622837416589 40.2649309204478
39.6352613956998
FLJ46358 59.5000730792772 39.7835513496873 49.1067959632109
36.0481934423077 42.269054475455 42.750861693377 40.3302268293707
40.5158867964016 42.9020321355927 40.2112371512838 45.310660704531
39.9519547348106
FLJ46361 40.1145011765998 42.4649374149262 38.0452862258803
45.390192977585 39.9182914210537 38.8336427948187 43.0930293831497
44.4031868028216 41.7117861328546 39.4710743516517 42.2455707947223
41.2870505273664
FLJ46363 43.1805016690297 45.9266745571724 47.0940613507321
42.2496337895968 46.7508829977587 38.8336427948187 41.9393838138203
42.9301944191913 45.6582729799009 47.0055981216687 51.1483696410762
49.0655215909632
FLJ46380 41.7397731376692 47.2770121962655 56.1434024065079
42.1273928203021 44.8897737954573 45.7300501624017 44.6032218589199
49.7309743973184 45.8325181856483 45.2556760873335 49.8393069675192
42.1537476443423
FLJ46481 43.5710871617107 40.7952353240579 46.0727230219512
46.7577982595088 44.4489662507893 48.8744644207821 44.9863726205428
44.8997235283254 44.1519466943185 43.1839109702714 45.9104478407175
48.27609330316

FLJ46836 32.2133093774284 34.8137085420423 37.1643693306382
36.5393857306744 33.7494761745342 35.8227939208521 35.4623691645629
35.0510338774593 39.3750969348308 34.6720534245631 32.5482839399637
44.0451487108213
FLJ46838 55.6268253440479 43.9421822132391 52.3432130135266
50.378948785456 41.9842695818285 51.4931742685391 41.706879587091
46.5717043290731 40.3316153483289 46.6598879466655 48.8088340735921
44.6484005362281
FLJ46906 100.403196213407 62.9904770954043 65.3531590366085
49.1786207920603 60.9710783023013 48.5835484742456 102.913039837094
68.9522412475296 69.9767092409915 51.4454142248566 62.436851776671
51.0013344286851
FLJ90036 40.2543341073001 38.3322849790657 41.3366779545815
40.0698728584484 38.5512429290626 39.7394181011603 44.0687697278114
36.2539950294089 45.7452456977857 36.1550293353654 47.0940613507321
39.2469742642337
FLJ90086 44.7044140979108 50.7487159427973 50.0748292497578
36.4564050256915 51.2525124735102 40.244271403831 46.3057385979341
43.844855336215 49.4786466722535 50.3388505346542 54.4825201506931
50.8999542937724
FLJ90231 37.6397652536077 41.4223563635976 44.014441886238
40.7481155008596 40.9950639693255 42.2114134935898 42.3194600586894
43.6753843488695 46.581796165231 41.3321861371202 40.4094901248527
44.2527566343661
FLJ90650 43.9309845324767 37.4540772310118 39.5450079747895
40.8681871019812 35.9894133432376 46.6872654833579 40.7910160811527
37.7612949798895 49.1940901905636 51.0055825931636 46.0905079010237
47.6040671292013
FLJ90680 40.7609952437622 42.0709558258414 38.7745734037492
43.6726153508978 51.7983110178095 45.7300501624017 43.4544762830742
42.5435283762933 47.1744296124496 38.4187686307607 46.1673159982664
45.7562276914441
FLJ90757 44.5282181324797 54.113533179847 51.5292723244077
58.7338159410728 55.8173045043348 47.6803619768589 48.0283573444179
54.8412943008635 55.6617952786921 53.4109882045447 47.0940613507321
44.7401523707775
FLNA 58.1027665533767 49.7363778831003 47.0940613507321 50.0526763693329
56.6951078698291 93.6752479496469 40.7910160811527 50.857522488428
41.7627910693483 94.233414682323 126.620595729562 91.531459931855
FLNB 644.368005872231 875.139073253298 1250.15619347362 402.412173952792
477.143510243862 674.270050283701 1012.91803181784 510.946850892298
946.081492716653 1445.73674428909 1405.20888023653 1310.03307063841
FLNC 137.818833473709 50.2163892855092 53.3127618245672 76.0975301977715
62.8270556619309 841.316771679857 41.0381860307445 145.050655626219
45.9227107476843 1533.38581753491 1557.42399344934 1545.76489731307
FLOT1 474.155196153863 262.735075993242 215.15351875081 912.757335169259
268.097966292089 645.114504971255 564.468049419762 225.690879436355
326.250872625748 393.648308810931 379.473739290565 526.146757036152
FLOT2 879.708199333257 1762.93413846062 1640.74027400791 1273.52492984569
1401.46625360427 1081.97502255142 3399.75820494572 2374.1766901821
2755.23573327276 3761.48462220262 3099.21690111993 2337.7738990994
FLRT1 40.0557788909923 44.0409178714232 37.6557714476853 35.7674462337723
39.3667161654772 37.2686490268265 42.0605906240248 41.4508241771315
37.0126425326534 41.6373771565199 42.2618615571566 41.2437378488025

FLRT2 52.310270746917 47.3536182445315 49.8344973836834 99.5958921252583
42.6022652963326 133.514641336669 43.6753843488695 46.7980335276155
39.6609238036815 81.2992152187529 72.1090339831747 65.7853960266587
FLRT3 74.0913177754682 69.2406343120143 135.567012693522 48.055683217958
194.189674572424 96.3976093220249 194.469250918013 101.272774711455
165.141030604713 90.7551885299639 100.120990656033 78.0586153423713
FLT1 39.8662795144616 45.6533175604486 41.785676764591 48.9944486068347
37.5556299264699 41.159706131351 34.9344532055397 42.357910063876
46.1101825640108 39.4710743516517 39.9070785037411 40.1700117452801
FLT3 46.8141313516997 48.109747794187 40.1985790927248 53.593974682612
52.2089306840125 36.1190722948669 41.1507980856436 40.5158867964016
40.0403898620771 47.1001757441025 42.5426057453993 47.1060577218993
FLT3LG 60.4593810615882 67.7189686993694 69.6554376880691
66.7750249658122 67.9032387662129 94.6167194649276 65.644767142472
53.2046867327331 85.6441969757275 80.3915591207869 82.1659654020827
70.5322939116406
FLT4 41.3848620620968 44.7829632472754 42.8663534227844 46.614092935749
41.0608773166058 45.9994850850293 43.1563544904505 41.1575777497576
43.2761454096265 40.2112371512838 41.9083581529276 41.2437378488025
FLVCR1 95.3300728806601 67.8999127712557 79.6675791471526
61.2213001749372 86.8871118411414 49.5424425850549 123.061728345479
126.620595729562 70.2541961995753 47.7180816682562 53.4088637045445
59.8760452220989
FLVCR2 87.5146535320072 68.6074516459969 95.7820879892969
220.36831243882 88.2427413480277 63.8740412329753 89.2913927776683
90.1430468048409 53.0357938671646 62.2025996860055 56.5402453093703
53.655233033193
FLYWCH1 50.7774722329703 48.6795178347248 52.9736397524094
42.2496337895968 46.4343327861696 54.7806868394478 39.0788811357542
40.7799172560245 53.0034632721031 59.0216019867146 51.2318935933116
56.4873776344986
FLYWCH2 86.5564881117807 64.9959712888709 95.9811314739351
84.0899854321388 92.9747592911324 112.60230393988 111.924999671217
75.656434170689 126.500059295217 88.9738853594498 90.0223113095186
81.7566533443175
FMN2 37.55264801907 38.4330755397458 38.6005272730664 36.7892997857376
41.3755760434934 40.650742261428 44.8643898647322 44.2159020605784
41.9635525160083 39.6719190054379 48.042366934301 41.2437378488025
FMNL1 48.9906383294199 43.9421822132391 38.8775560911841 85.5959901987745
41.3755760434934 41.3553369544097 54.6414321010929 45.3872406432794
44.8997235283254 41.0057887014368 42.357910063876 39.7411455670172
FMNL2 58.2401724760744 50.0425298550559 46.614092935749 67.8233625051884
46.7508829977587 56.7429967489965 52.9433495155796 61.3522631937571
48.8067321016961 53.1309801459151 56.5402453093703 66.2305843819029
FMNL3 48.0201377454632 46.2625470410195 51.4308501933893 55.1531662914834
43.8738578522009 45.4882438975269 44.5375569361112 42.0778399888414
41.9635525160083 44.7672565800508 44.4408504417009 42.6758568421347
FMO1 148.758736522703 41.8848959280916 36.9795961564828 87.2643859655159
37.5556299264699 60.4991141099239 46.8613212048981 42.2577689927563
44.8997235283254 35.6208308220005 46.7386599624801 40.1700117452801
FMO2 69.7442393277127 48.9899488302637 44.0451487108213 45.8844325913247
48.2748123114598 142.55778399096 53.4921736109774 49.3880946790306
48.3119523820103 50.7855742231932 49.2206713037152 49.904979214379

FMO3 73.4611178120841 68.0556717366572 63.3544794716167 55.0604256312495
40.7273974755431 95.042308285667 41.9393838138203 41.1575777497576
41.134180463103 46.8954576545929 49.4145011642763 45.7979032939211
FMO4 83.877343750194 61.6219790276408 50.1860191642359 74.8706881113318
57.8039717833131 82.1144251872199 60.7992483126292 68.1311562787361
57.1863195337055 79.0249585610565 76.2226219333049 59.2035499250243
FMO5 51.2851682978581 70.3629652777935 57.7415362494584 41.3027031037343
45.6895223823001 49.9699131196797 44.7255207589606 61.5824518349507
53.1249776278067 50.7855742231932 51.5822637582617 54.720204938924
FMO6P 45.0985088502363 43.826317180678 41.2679739649179 50.3127977239083
48.4724651632766 47.0027453663546 43.3262148375525 46.7508829977587
51.5888395924843 49.2317401696588 51.6414075056938 48.9906383294199
FMO9P 44.7915315085948 632.019639546131 96.6895594961001 42.8235313007746
311.668075784386 85.1544284060549 777.854349637516 277.548019244696
161.326580647514 225.056827821165 432.945510073269 111.166893747402
FMOD 177.985573759136 69.8505620106065 65.1185966859561 213.590067519021
54.0126916508417 155.435700005184 46.1634822993009 81.1284422173774
44.1519466943185 109.326279686847 96.981661155293 90.2425981161833
FMR1 38.304249186058 41.0678614026819 41.785676764591 42.6913548400127
39.6535880152834 38.9378717994178 41.253645578517 38.3684202018524
40.137818555383 38.1042454705119 39.0550139257658 41.4508241771315
FMR1NB 39.9697332660833 42.3550738181144 38.9790184198145
39.3750969348308 37.7576900881741 41.2567470068038 38.6243997507646
39.9576138799997 42.6366765186182 42.9866214102525 40.1742127891824
37.3600014814388
FN1 48.3556695727124 38.1713217245067 42.0960619209699 44.550322123994
38.6880635921304 44.0804700754786 36.8989711187139 45.9159813905292
40.8503192605024 43.1839109702714 42.1516028411593 41.5083179498332
FN3K 45.0985088502363 49.0697565324841 46.0727230219512 43.6726153508978
43.7517118200398 42.9695585740813 43.6753843488695 38.079222289614
44.4395580493118 43.844855336215 47.4630625313159 42.7782216342806
FN3KRP 456.023620287811 146.432425205891 252.347641176529
104.335120306753 376.453560620912 356.318517117856 428.729935028911
184.964039515715 285.511923726609 238.246319539302 198.056931905227
231.859167242606
FNBP1 597.191426276771 389.131545459708 249.195312452368 716.434968700383
179.322661675979 1652.85734308754 239.272650332949 408.739953606815
134.265039211045 1252.94510628089 1139.81870068001 1061.61186520958
FNBP1L 125.503897988367 89.4747568145717 110.540953771795
43.8495248991589 72.2016884438365 68.5689807788431 129.965747812294
103.133138547093 105.376705821141 100.076931518374 74.5654028199926
101.065203693106
FNBP4 453.260719561292 913.704049709264 940.781370938596 542.901822331329
645.114504971255 454.185408362583 474.379113836506 410.402285012885
475.621740714165 807.620534408416 772.368030410394 679.309039810384
FNDC1 401.202348352506 50.7487159427973 44.4652605017595 129.592927998658
42.9655396732962 41.554942319225 38.3346870469391 40.2382908228517
37.1506761793345 47.4534495228738 44.4329005595031 41.1868052124344
FNDC3A 62.8885806455128 86.7076796107636 68.4720753153522
117.149897485203 66.0411740221276 87.4623442823265 70.6225093592927
62.2982003555312 67.0290776715331 69.1623287497421 72.5993974280876
79.5898953726467

FNDC4	73.6362523983338	46.3635803515159	50.9933795450314	53.8086134112366
	45.5809132232681	58.4435847520801	106.878946016737	79.7170181855209
	44.5303781239209	46.5650556481721	49.532546810992	42.4607795638552
FNDC5	46.5549623118323	49.2050538150106	40.8420925848403	37.1894304543751
	57.4163094213712	111.240804216088	48.0283573444179	67.3976855661302
	43.2761454096265	77.0226403968683	59.0537479001842	45.1735152121056
FNDC7	45.6517320730042	45.0905928998779	45.3974838013476	41.4273122914951
	39.859345745831	43.3785777889265	45.3597029614942	40.6171774724171
	42.357910063876	43.401782064778	41.5421429082231	38.4330755397458
FNDC8	46.0920218698606	40.148780650831	37.1643693306382	40.8681871019812
	41.3755760434934	40.3357777530419	39.7576136507593	37.0390589468421
	42.357910063876	42.0621425894624	40.2649309204478	42.6758568421347
FNIP1	91.0346886951891	68.3864191351179	81.7117547901526	139.847976281228
	74.8706881113318	84.9601800557888	63.6695826053997	68.8698243753339
	89.2627798562694	82.5344501505114	79.8701900696361	84.8443836237906
FNTA	306.487935692954	273.849703944739	280.117410663595	177.425508756002
	317.004252313348	356.628599887098	176.687970262952	142.03031230486
	230.594000005581	192.700843009002	190.921576470336	327.283431584541
FNTB	184.162452344446	100.066535982734	162.758841068655	146.405413944753
	125.503897988367	132.604567913122	166.33117014492	103.366147685418
	171.770578381558	203.979798245524	206.559526386642	146.61436309209
FOLH1	51.2851682978581	49.1283590719461	47.646800205189	46.0228732507872
	49.7235523248653	46.0905079010237	47.209353240759	49.085741080241
	44.2142581488029	46.4944619663072	46.8712941728147	45.3267183347084
FOLR1	57.9679239164609	41.9455842688118	43.8289240531545	41.2575219633134
	42.6575535390785	41.3553369544097	38.6537063231682	42.8071549997047
	43.9450023643394	41.6724739851995	39.4410515050885	40.9250062559315
FOLR2	57.934905173625	66.5141009463313	76.1493261430901	95.5022104475922
	67.7155610106537	334.438091900861	49.1067959632109	63.8008894786105
	46.7760357462635	55.3153799233744	46.6442692119997	61.6311118308635
FOLR3	41.5421429082231	44.2105195734073	38.6005272730664	94.5501397951415
	47.2074332636566	44.6524308640784	78.8250642061439	43.4544762830742
	39.9444191873552	35.2880714952489	49.0361180089431	38.2350720635382
FOS	2354.00192115549	5156.59948231705	3618.78958408675	5218.25814076207
	425.60854194674	8050.256095383	2384.00264993279	3343.07293372655
	1425.37639004849	1405.20888023653	1981.03929621057	1055.00793806075
FOSB	824.946988188872	7785.77078492993	753.491584605447	2440.77408185413
	440.282299601096	3854.42494497831	1105.00321488322	9938.5091387622
	909.843512894628	326.902009704746	548.047774435177	156.433762355183
FOSL1	53.8856501960117	80.7198077526005	62.0008474085033	59.0965219753805
	74.5758811681995	67.8208976798364	83.0647477737971	117.612004923867
	109.594402843796	61.4569050766419	41.1638563416981	54.0374522273907
FOSL2	44.3355304829989	47.087835967892	48.0191002461099	63.9570596163747
	40.3452320167286	49.5424425850549	52.561573697155	75.7947206223934
	55.3122077514658	53.1993028936883	51.0492389286271	59.0894921452893
FOXA1	668.773022931608	2551.57660786358	1151.25293766167	65.524821699432
	1114.15989325456	169.18132209076	1077.94530110976	825.558929673308
	1813.65521720537	615.442484911026	651.621028098039	576.140066807763
FOXA2	39.5676051939342	37.2117500129028	64.6748464612845	39.1350512687938
	36.3838763632982	37.5887129572848	35.7772039808118	44.2159020605784
	38.8507804383261	34.9566647540594	40.5084378738092	37.4085590601111
FOXA3	44.436995101921	44.0409178714232	39.6451483836089	43.5627649099623
	72.2665537421443	53.8461734834857	46.3057385979341	45.8669209716694
	45.3756389170606	42.0621425894624	44.7926323492519	44.3521099857368

FOXB1 37.7726313569706 38.957687788868 40.5386195997545 45.0798408927912
40.7273974755431 40.9506062495231 38.0598374201407 40.931294690669
42.1594905198929 40.3235655331796 46.0905079010237 42.1537476443423
FOXB2 40.4639532172338 42.6939394537076 30.8570661199184 39.7411455670172
33.8558232866797 33.7818132446042 32.4466419672036 37.7612949798895
41.5635499110307 38.4187686307607 38.2795150354677 33.939263369489
FOXC1 372.239177888445 558.855313555287 547.729200972776 361.091144969609
542.467997217663 625.805967352327 647.868022835702 876.959744461441
614.603743904512 546.548431253433 626.749986744506 565.411298018845
FOXC2 52.502055890816 43.1805016690297 49.2041300973417 47.3574589211166
46.9906120932727 54.1440452292081 46.7100796279384 59.6720198281342
48.7358147030082 55.7930787690756 46.1673159982664 50.1579724208315
FOXD1 84.6279355865799 43.683792219729 43.4709415392696 43.6726153508978
39.1650142708257 42.6575535390785 45.8980298854198 45.4757965005055
55.9423752286714 35.6208308220005 34.4716904283753 38.5279745308203
FOXD2 84.1254258487497 56.6146179789815 82.4362183543935 43.4410741327254
50.6470461863549 46.7800033479223 58.0139597055449 65.9143782252686
90.3916996175708 53.0357938671646 55.854474649572 43.8575940017119
FOXD3 36.3649677956953 42.2577689927563 35.4484652445078 42.1273928203021
39.7568367479301 37.3799542961839 47.7390230243778 46.695848687694
42.2577689927563 43.401782064778 38.6662194645633 38.1421709520616
FOXD4 62.0104160416544 66.3026572113793 51.7138050344355 59.987045425047
53.1100587888055 49.1718303518849 69.3468840908665 64.2174369755376
84.8694815443111 54.0374522273907 45.5055402477919 50.3118072462443
FOXD4L1 54.4825201506931 58.6249721386149 45.4865173433646
43.6726153508978 45.9104478407175 46.6872654833579 48.6395795163502
48.272324215504 61.7559532093027 43.401782064778 43.5710871617107
41.2437378488025
FOXD4L2 42.0778399888414 42.6939394537076 42.2732555030699
38.2516470858206 37.6013534795056 39.0676956106495 40.0557788909923
42.381482941987 40.137818555383 40.4488698263442 37.2523512295476
41.9820792173615
FOXD4L3 37.55264801907 38.0226905622357 36.6522278218553
36.3604951341921 43.1252272136667 44.9896851005259 33.9750800030909
36.6452480995946 34.0382693188794 38.6454098044474 38.745552403895
39.2469742642337
FOXE1 46.5549623118323 43.3016395799961 43.917817324545 41.6045220683604
42.8510579683751 42.9695585740813 41.9741368971524 48.9826868640753
47.5810366106247 47.3791873993182 44.9081227425497 42.6278608611758
FOXE3 38.7229679986571 39.1982470626863 41.1742244762318 44.0991023527481
42.1386542162789 35.6517244402762 40.1235656101329 37.2237132058632
39.3506426665347 40.8049408996274 38.7337086062875 46.6397391251762
FOXF1 68.8698243753339 64.5347406608537 49.5424425850549 57.2856683017619
71.2787833369055 231.685652845234 37.9430145836712 72.4834467838798
47.0804681460898 98.9657264176664 196.267504470029 113.130838519437
FOXF2 73.3448790478335 70.5699797830702 90.954592973767 47.0874209746031
77.6174893940285 56.9320717885002 48.3411413510851 96.4971762611189
81.552417816742 64.428683021978 88.4594321382477 50.3118072462443
FOXG1 31.8228237796461 43.0227387991135 40.5734458834617 36.0481934423077
43.3332476888305 34.6977013306396 34.9344532055397 38.4540849632254
34.3842972343227 34.1648535095605 41.4508241771315 38.4330755397458
FOXH1 43.9309845324767 47.5647821785529 56.4779645313958 43.6726153508978
46.9906120932727 46.1513019317765 40.1235656101329 50.3972033014227
45.0017158586743 47.1001757441025 47.1477056711335 42.9887069977696

FOXI1	40.2543341073001	40.148780650831	43.9450023643394	42.2496337895968
	43.2484996523549	38.1699123824764	39.6158343667921	42.9301944191913
	46.9062313932642	42.6366765186182	43.4709415392696	41.7575097298927
FOXI2	41.9407041937973	43.1805016690297	39.0775870687758	47.9915414951747
	35.9894133432376	43.2805590023412	45.4799270968969	39.1652965788406
	44.8997235283254	44.3648993568405	47.9942520395434	48.1627097465883
FOXJ1	36.9060997041453	67.6169355376864	34.4716904283753	39.5050383901884
	38.8456942580061	38.9378717994178	67.5206810636627	39.1652965788406
	55.2114969675545	42.785429206422	37.4642807402741	46.1592873342534
FOXJ2	408.353336724969	207.30460556799	301.728842562175	439.770250139925
	298.721050521396	447.448900931272	339.210875653066	310.255767356827
	350.796638646601	583.915973546284	523.679443405512	419.271533050938
FOXJ3	518.074074000609	356.079406408042	362.268767190535	386.640440174303
	343.382663050351	377.573838771285	379.79657860938	627.914461428624
	465.430886956232	525.16535463798	555.044139654886	423.608694638779
FOXK1	121.8863522969	195.221917652149	110.431464700539	101.065203693106
	140.41738844565	158.532307373857	92.5391033814713	85.3681894044625
	104.573463811298	94.0140258999054	95.7263252232278	87.4623442823265
FOXK2	45.7356815244513	47.1332119496722	42.6861857638473	43.9577120438279
	45.3051051665498	46.1513019317765	47.4439407315335	40.2906799807722
	48.4614401417022	42.5664883002833	43.6629145624937	42.3214282169749
FOXL1	42.0532901689036	40.148780650831	41.6079958749402	46.7577982595088
	41.1868052124344	41.4508241771315	48.4859552186772	36.2539950294089
	36.197424053556	41.0057887014368	39.9899430628542	43.9541718254769
FOXL2	66.8188271427672	39.3565586789274	36.769073068743	39.2336402665402
	39.512606833112	38.1699123824764	43.26160898693	51.8347877274018
	42.6615964978834	39.3275194442012	38.4689132737495	41.8036925068356
FOXM1	93.6717518147993	84.8084949516218	60.1444334141838	49.9672547361866
	112.159199477006	43.3785777889265	173.925818790068	113.917429432477
	169.367702213351	40.3458948319669	36.7892997857376	37.4554876386402
FOXN1	41.8409927585705	37.2810767789306	37.1643693306382	42.5664883002833
	40.0599807618105	34.6977013306396	38.6243997507646	44.7044140979108
	36.0741672064298	35.2880714952489	37.3687900733549	36.9976231206067
FOXN2	217.976796656266	147.017279035061	184.964039515715	503.793770799548
	117.260454792593	228.845881709556	137.081911344143	279.782392390288
	197.617554897454	211.625447303889	201.180226922613	159.506157112627
FOXN3	43.0462952354436	37.7384958256497	41.7419877611072	47.1332119496722
	41.467005949568	46.574682061365	40.5585676679482	42.0847226073592
	40.2739700860114	42.02667925031	40.2649309204478	43.0930293831497
FOXN4	42.3433931248155	44.6918600441328	37.6557714476853	38.745552403895
	38.9496515344837	37.9003288112261	38.6243997507646	39.0620487644635
	38.4344387289956	38.1042454705119	40.3452320167286	39.0536056496869
FOXO1	306.66244801017	222.368324930003	213.073369426342	383.642090672435
	172.869292380866	351.883708151967	274.994470779348	166.734730009916
	143.251956814524	303.189681281366	292.47671022688	333.648836970147
FOXO3	277.588692595406	161.25239489071	234.615033303459	447.709872889701
	128.616776019243	457.202737754842	227.598514023545	292.86576686019
	241.51647940043	390.978292094212	454.02808608679	324.877187846959
FOXO4	52.2236479204029	81.1189042503645	82.3344892042646	43.4410741327254
	79.6516904169771	177.27613997459	87.455991111513	60.2691630545162
	102.457537266105	103.264100180823	105.277476070937	82.4362183543935
FOXP1	39.9697332660833	46.6314009405034	43.401782064778	40.646702696033
	39.7568367479301	41.2098211956301	45.4040191352687	44.293922970134
	44.1258877591681	52.1788489555942	52.4111687156893	49.7336769474446

FOXP2 41.3785995857154 44.3529919625344 46.614092935749 43.074472921841
38.4135023167287 61.3973540098375 42.629412460409 38.9074189954657
40.0870341983328 37.920964499903 42.8263096141654 46.9867599298218
FOXP3 34.0467249875782 35.6434124419782 39.991353369336 36.264823544143
38.3561682218911 34.8956628705329 37.4772922422734 37.33030683349
36.8127605061228 39.4710743516517 34.8383995317036 45.3763275588341
FOXP4 58.5759178381027 49.4982025424177 43.7630587657205 49.260242314197
44.2120774064277 44.0399598874356 43.3832826368789 47.9363875418976
48.1174861108648 42.1622837416589 45.5055402477919 46.8129509799753
FOXQ1 393.209708455013 3707.73750609316 2903.00880209344 147.689479458785
4036.89580181979 554.163106145921 3153.71394627237 2009.75680599899
3055.1209535841 4368.11345948444 2621.65272077828 2039.89728774097
FOXR1 38.6454098044474 40.2093230376545 42.0136658108872 45.2606665758581
42.2455707947223 41.6178506967511 40.9811982338594 41.2098211956301
40.7460990419215 38.5782171290591 39.6894175734463 43.0930293831497
FOXR2 38.304249186058 36.5219488315447 35.2101597814352 34.7266486944713
38.6454098044474 36.2015763253919 39.3585350448719 37.86982638952
38.5252456306656 34.4875013487777 37.9946685306274 40.6614700944853
FOXRED1 231.859167242606 159.268245287939 177.757247913754
142.309369995384 222.696947669812 144.593933058291 262.024495926094
293.151956467056 154.287396750021 204.229798794441 175.009248614892
116.874313720275
FOXRED2 86.3793067659925 74.0831311663524 72.7071934110997
59.0965219753805 111.304424389891 75.6382797289563 103.707931766117
123.219313750907 76.3790849853724 83.8113149043718 89.737418965763
64.9114266867808
FOXS1 48.2910530887214 46.8954576545929 44.8872784170036 38.4208429103799
40.9950639693255 40.040778349137 39.4600924554505 33.1477313087177
36.7267557061938 41.9666541498638 41.8168470823451 36.7762265039864
FPGS 40.4639532172338 42.8542642792862 38.7429005059745 41.1886975241176
39.9697332660833 41.9918605927928 44.6032218589199 42.8036731479932
43.8159843786397 40.6573239942138 40.9696219937838 44.7401523707775
FPGT 43.0001432913058 45.0115870036541 36.8730514568925 37.1894304543751
38.0598374201407 39.132725377815 37.6036146841933 39.5466975353131
40.2274522635576 37.8217989841918 43.6629145624937 46.2625470410195
FPR1 57.1046180136491 76.861722336069 95.0026207727863 2581.34849558524
62.9553576299033 256.116479208201 303.93332785291 64.120219344545
42.9020321355927 42.785429206422 42.170702295259 66.9239565096247
FPR2 41.8409927585705 44.4758281923423 56.9133235865341 1524.01520893763
55.6991707857987 61.2387559102636 131.645714321903 42.7431359850339
40.2274522635576 37.2686490268265 38.4689132737495 41.7575097298927
FPR3 205.480081236149 83.7739049195868 55.6865353018811 449.059703234741
57.9161791762225 90.99086730704 70.5534237141767 107.427186954956
48.3119523820103 49.0841460667818 45.1432412071268 70.8546350449171
FRAG1 88.9589979655541 109.943883439481 145.607100979838 56.4578213622965
141.055218279065 68.1969125201241 100.891869710611 93.0474674148534
140.01041112896 157.020685801527 132.851109034738 128.032613585193
FRAP1 294.155188719236 277.878824242495 295.856741468603 253.605641311356
356.079406408042 205.650076927315 265.570814613305 474.379113836506
238.120731107691 201.027801320306 244.070778149931 236.661422875826
FRAS1 65.8703820581684 110.626479311082 48.2492481167112 43.2504382247503
53.2173939357349 45.4882438975269 61.4267636950869 71.136135376296
72.7934987008399 43.9694307523562 47.3935171631611 41.1868052124344

FRAT1 46.68364047986 64.4357814748414 53.7316344826448 68.1992221684165
54.8830461467967 52.6747142992864 59.8273072817267 48.7039705048129
56.7635847444447 57.3562426592112 50.2016912396771 46.0106098064475
FRAT2 240.6614822416 700.010354583503 171.5709313654 366.615979176657
283.161835288074 165.606015336587 486.265784810787 196.247893540766
313.115027781064 198.190904631121 204.477547372897 172.540737848092
FREM1 38.385865183581 57.4054069675016 37.8386378737561 34.8137085420423
34.2638875325204 60.4065050472082 42.4939802311136 36.2539950294089
36.9060997041453 51.1169368198437 47.4630625313159 35.3525469364579
FREM2 40.9558083259517 54.4793574349739 43.0602678767669 42.1622837416589
50.9643363406241 41.7256940298128 62.7488501311848 48.7039705048129
46.7760357462635 45.9426591985894 46.7902798502321 40.8681871019812
FREM3 40.4639532172338 43.5182502375207 41.8751578329365 40.5484717678957
42.9090346219023 47.6803619768589 44.6032218589199 39.3709357615493
44.8997235283254 42.5664883002833 42.911855821602 42.8864415494484
FREQ 124.37984507038 184.340249262704 78.7103847663605 120.068630086702
184.162452344446 710.579735673002 156.061711961514 83.9236799134394
69.4677195088043 386.271174243357 358.30596676183 254.756894822368
FRG1 105.247736284747 111.72494102259 123.597489107285 192.264432082651
98.6553805168141 127.701556328274 151.266092912661 176.492987902471
132.205423909251 168.366869831376 138.032032026715 135.592113666246
FRG2 40.2543341073001 40.3946758935842 35.3227485563945 39.3455642782085
37.1935273352159 39.066284740632 37.7555770011351 38.1835938174625
35.9096438988171 40.1179210534118 37.2892641289258 38.0452862258803
FRG2B 39.2076937845359 39.9697332660833 39.5118967430322 36.890925044696
39.9088079891223 43.3269087731399 42.4939802311136 39.1652965788406
40.2739700860114 41.4659679379482 41.6657053704762 41.8200132437731
FRK 63.6767189846426 43.0227387991135 47.646800205189 37.0732977341185
35.9058393060637 40.9506062495231 41.253645578517 43.6434576713809
47.0804681460898 53.0357938671646 57.8991270166934 51.4308501933893
FRMD1 40.5654775358427 39.9697332660833 39.4710743516517 33.3231576795423
39.3667161654772 42.5660695551615 36.634024014558 35.3608605269276
37.5469247328848 39.5637454855233 37.1059647239814 36.064718696392
FRMD3 57.7877627794725 73.1640951333742 68.1780382173625 52.3004056941891
49.9493230064613 113.716666562787 55.5578334877425 61.6839343510302
49.1940901905636 107.610858427836 162.000275136397 71.2491135258076
FRMD4A 89.2913927776683 116.295564219236 107.413918714848
115.63300104804 78.1275793755619 95.2367177680328 94.8766888888828
65.5820437889838 83.105117076029 121.633811243877 103.199200862158
97.7140868175135
FRMD4B 42.7150227340364 41.7656868106485 37.8990009552441
40.6907735219854 38.8775560911841 43.0860383229288 46.6324636890843
39.8048943625718 39.8984580390722 39.1233194644391 39.0905363288848
41.7575097298927
FRMD5 44.7044140979108 42.466333667839 41.7419877611072 45.9441733841041
40.4166536885831 43.5752393599169 53.4921736109774 48.6441988290952
44.3440839686558 43.128936937037 41.9083581529276 38.8132170743603
FRMD6 217.721787448311 201.145927216439 254.239142284878 111.227016020927
164.547318765586 190.487609390723 79.0741890905715 94.2678678253657
206.481424743576 207.18510695093 184.377148097206 184.50283792606
FRMD7 34.4021554788966 37.10170285405 36.5474283500601 38.8495095797881
38.3561682218911 38.6414316877471 32.1218888714537 37.2237132058632
40.0403898620771 36.1550293353654 42.7275315121163 42.4607795638552

FRMPD1 46.0920218698606 50.6229884142872 47.3861758958576
42.4580504587581 48.5000946421718 40.3357777530419 51.3064929551674
47.5950227477807 42.9020321355927 45.7911932481722 51.6414075056938
43.4709415392696
FRMPD2 41.2693383984535 41.9363480239047 40.0421053671784
42.264777344908 40.3124262005404 42.5089442785482 43.3744503319109
41.7808320192909 39.5377896745682 40.8434758668556 39.7563187199181
42.9887069977696
FRMPD2L1 36.9721666683086 40.8420925848403 40.047314525032
39.7411455670172 36.3867274293191 37.3356865956886 35.1714932603619
38.079222289614 40.5491080069964 37.4656588382066 39.3895791800635
39.901614985832
FRMPD2L2 42.2610433929745 41.4178149669293 39.5930464310561
42.2941793046964 40.7176735294868 43.3367435704657 43.2585069008027
41.3082479425828 44.0137388752186 41.8613406887824 41.1349567921107
40.5243798943351
FRMPD3 55.1306642449627 40.6380495994768 38.6005272730664
41.8778873678551 42.7982502551634 41.3553369544097 39.1539892144371
45.0972184964705 42.357910063876 44.0409178714232 38.4689132737495
44.8372404608604
FRMPD4 45.2875173643252 43.5873188565519 51.4308501933893
46.4555908533915 44.0451487108213 46.3715909653832 53.0277416531043
42.6450179841129 44.0519245668737 45.5776159432079 48.6767028205398
48.6037684272115
FRRS1 44.5282181324797 50.6774883370457 48.8100349749783 53.3967242431461
44.5092684716474 48.7070804960074 45.8980298854198 49.8344973836834
52.6192478923607 44.3303034026662 46.3370338074996 46.7182294568603
FRS2 41.4508241771315 43.4681646416585 43.0971787292711 41.9635525160083
41.3440240757217 42.5089442785482 38.2307941336596 45.8669209716694
46.1579366516298 43.2346122959037 44.9056256433399 40.1700117452801
FRS3 98.1495112518979 99.571118823509 128.680405667403 66.5921967967793
87.2282539094452 89.9789975503195 97.0155195562193 60.5571721986885
82.4467808843629 73.8313212063649 111.760213205992 88.2604063995148
FRY 46.5549623118323 53.1278271804774 48.9944486068347 43.4410741327254
46.7508829977587 52.6226745117396 43.6753843488695 45.1714698794331
49.7336769474446 59.8130420783374 57.2008272301399 57.6860752168769
FRYL 66.3845271542459 69.4820178305563 96.2490896573253 93.4042243540384
67.0738693349694 66.4015820814547 72.3070624532091 81.528980339501
82.1509530759594 101.277664841914 92.8089247593564 92.5011103881887
FRZB 217.976796656266 129.081183929988 116.184007615285 164.612971085488
86.8871118411414 124.032371632524 41.0381860307445 121.729442469889
52.5772854102326 98.501431740607 81.4702220063796 56.361708620372
FSBP 40.8681871019812 47.5647821785529 44.4311655729487 49.3654837541353
45.6895223823001 44.0804700754786 41.253645578517 40.931294690669
47.798305839406 45.0155543555707 42.0750889188255 43.5738557773903
FSCB 41.483412492426 39.9697332660833 39.3709357615493 43.0016969050339
41.9397336249742 37.4782441311626 40.7910160811527 38.132587347852
41.6616444147804 41.7405682794166 39.0550139257658 41.5536168238478
FSCN1 1712.55038816005 2191.09005731751 1839.09068171097 950.740991182127
3114.05035137552 986.40790007743 714.990611155096 505.018127833201
3575.78813099057 672.515393219419 771.554891868456 1313.92053019421
FSCN2 47.7779356299965 44.2630383225933 38.0452862258803 39.5637454855233
42.9090346219023 40.5805725503899 45.4164642922183 47.3172462673426
39.6413313949444 44.2580151149956 42.911855821602 44.6484005362281

FSCN3 41.5421429082231 39.0102066700839 41.0356368374708 43.9282059368202
37.5556299264699 46.574682061365 41.3594868055879 42.357910063876
44.7058233759912 45.5776159432079 43.1931731665131 39.0536056496869
FSD1 45.162170179003 43.6615266096323 44.0215298143291 43.0001432913058
71.8706847571669 43.7630587657205 55.5086026637973 64.6748464612845
45.5265270192861 42.3606390470387 41.0516468406389 43.8336862998829
FSD1CL 41.7397731376692 42.9358255168086 41.528370959196
43.074472921841 40.7779767565889 40.5281496800812 43.0930293831497
36.0306975524309 37.9273653326866 37.4302011516587 36.5359455830388
42.4607795638552
FSD1L 44.2350046179299 46.0713306971594 43.0971787292711 44.265834798964
41.7929491793697 42.357910063876 48.6395795163502 44.2159020605784
41.1742244762318 40.6022003765153 37.9946685306274 39.0536056496869
FSD2 41.4508241771315 41.8073910693411 39.991353369336 36.1562979552173
43.5544347011646 33.3470732872681 42.0605906240248 46.0633179231318
33.4698406117406 35.7202909649682 39.9070785037411 31.7413650507468
FSHB 39.5676051939342 38.7578265341627 39.4710743516517 40.3691324547049
41.2740822675592 39.132725377815 36.7892997857376 39.3709357615493
34.8099089553046 38.1421709520616 38.745552403895 37.8490162439785
FSHR 42.3620028137457 42.7775752471711 42.2577689927563 42.6913548400127
38.6454098044474 41.7256940298128 40.5585676679482 38.3684202018524
40.3662560853635 40.5571841955033 42.4580504587581 38.5279745308203
FSIP1 40.8681871019812 48.109747794187 45.2313723727673 41.8778873678551
44.2607789091176 48.5835484742456 45.3597029614942 37.2237132058632
46.581796165231 59.5785089386245 54.8532558909227 75.5751205142867
FSIP2 41.3387790895716 45.2556760873335 48.9944486068347 44.7980740332174
43.004943518025 44.9896851005259 48.1747307200218 45.5581356216957
45.1899271235974 38.2069499294002 41.6336636285875 43.8575940017119
FST 103.695493532556 583.421166842011 64.1442136854628 80.3204519680998
177.515053183327 184.105396912056 95.5117928427637 289.909646207746
175.165302282522 114.190951215203 100.120990656033 124.280024888206
FSTL1 442.080188140864 139.191263237202 177.261319004133 227.243009473121
225.960701908311 622.964590694346 112.281286038716 162.833785414988
78.055431384988 285.187681119318 286.538414026118 540.913422859469
FSTL3 113.866742186841 64.5347406608537 103.316970096472 81.1189042503645
103.423003544792 146.931674964658 48.1747307200218 56.9299697426319
61.6839343510302 70.0907269565408 73.7873098190297 160.100408020768
FSTL4 40.9558083259517 45.2098033805679 44.8872784170036 41.0219290963778
48.1473938886239 40.8634043028528 39.4916414839923 41.0381860307445
44.619512785899 51.7343702554597 49.4593570700221 47.3172462673426
FSTL5 41.2424637338424 37.5469247328848 38.8775560911841 36.890925044696
34.806104801006 28.6786952763294 32.9962272670005 35.4526946152955
312.795596385258 35.4990901121893 35.6970528713709 32.7800533388945
FTCD 38.5007204246293 41.4223563635976 37.2291018661279 40.2455972565968
49.3984571719129 38.4330755397458 35.0305278582179 40.2819379659218
35.2604260353347 38.2069499294002 43.6294818685075 40.8681871019812
FTH1 599.670771796585 266.381531918483 344.578407722941 1658.86829668934
398.049525642349 704.15545332905 710.831173303701 447.709872889701
305.554059609771 511.164722585602 548.387793019855 465.595459259015
FTHL11 384.522115820048 156.311985997533 199.57367025082
909.843512894628 237.974766880957 320.684862879691 313.046160403661
306.487935692954 237.864776225441 223.562130663956 215.869541768982
275.673080691335

FTHL12 854.712488657987 397.745727767552 522.125691279772
2061.66570137425 674.270050283701 901.793120584383 879.708199333257
684.238999553691 480.660242705293 406.964801954621 528.512401493184
608.579654909865
FTHL17 36.7356360093603 36.5219488315447 37.0642268327439
39.7411455670172 39.0564222526273 41.554942319225 33.5578650644246
40.3357777530419 48.8067321016961 34.6720534245631 39.9070785037411
41.6550823405646
FTHL2 171.770578381558 96.2280884890067 118.155986485411 382.190644764853
126.360723656793 175.441884261362 157.219840854561 137.658868649032
109.714619050525 107.325456368022 124.791747507205 132.364260893831
FTHL3P 308.728126648231 135.29225447532 176.492987902471
793.570993808328 214.127390534906 357.839241226513 250.41086379231
252.870708814507 163.20801480093 175.441884261362 156.822939275312
233.96534264787
FTHL7 5932.92478996609 3399.75820494572 4200.38495514552 10483.5643876539
4381.51418239047 5047.91205452316 5895.03230740197 3428.74022048394
3707.73750609316 2354.00192115549 2424.0414102425 3099.21690111993
FTHL8 483.882804472844 243.952697457779 267.170081805168 1151.25293766167
368.246708866999 492.519023728038 426.933064567744 380.015934356432
307.264133974364 219.388293203286 250.41086379231 338.053116855608
FTL 18219.5845257111 8937.3846214861 10351.5373412096 16661.6081002448
14226.3743910711 14226.3743910711 12456.731302703 8259.38594893529
9582.11974031559 7127.13565491501 7562.42822724655 9060.10165509099
FTMT 41.7397731376692 34.5023764006976 38.8775560911841 36.3604951341921
40.8873907150245 37.8102105794655 38.7337086062875 40.831207953647
34.9428206949566 43.2988017098387 38.6662194645633 37.3600014814388
FTO 118.05982513603 90.7255100542123 165.775214685061 89.0203733091224
95.6182216943758 155.378768637562 118.483499958227 103.199200862158
100.076931518374 135.706144105357 140.853812988562 131.779333469955
FTSJ1 81.638176606529 94.3773551105819 82.8205269728224 74.3292739001607
111.742106978992 80.4404044229341 100.802977734227 71.8314470868658
90.8109585999257 60.9446532180211 65.1496567852393 58.9373124129662
FTSJ2 45.1569741609597 48.0296332183129 52.1636737767351 47.0874209746031
62.5535250577176 47.2009420656854 49.0441221285767 48.4670130605028
50.6346949248834 42.3606390470387 46.4753015651202 50.6141827957642
FTSJ3 41.1523487516364 49.4067286971348 57.7415362494584 40.5484717678957
51.0107745412152 52.9482338979681 45.6042625176633 45.8669209716694
49.2879849674517 53.5498255316009 51.9302027391447 49.0655215909632
FTSJD1 117.612004923867 127.701556328274 104.557637353314
97.6774247176874 97.1271615802556 93.1067450332473 92.3034609409756
120.850874262069 129.538159906075 79.1074681877535 91.8414653633828
104.089998273812
FUBP1 56.921728372453 55.1766423847208 66.8632294055679 54.2886483704949
68.9038222450855 51.6053353177789 61.6544855697851 95.9391851122771
46.1101825640108 61.1851254827343 56.9320717885002 73.408861359425
FUBP3 197.660480001139 198.54718783437 133.720493104769 152.996027678733
187.21383075601 223.562130663956 152.898843315675 129.965747812294
142.1823033942 126.218285718433 192.085063824686 177.379924616924
FUCA1 272.36028618845 776.670493028756 413.067407579649 397.633328080609
351.439404852971 223.669853276931 266.381531918483 467.645011859047
275.975192937951 444.937258895152 450.271658272512 802.036670929858

FUCA2 125.713216265584 141.611097024526 110.684033203048 128.860357469791
128.657014567195 94.7604013126314 124.687212034276 120.980558571109
90.9570747667407 78.0020909283414 88.9038123445083 112.944044862693
FUK 132.117013391364 149.055511050346 264.975481501401 115.468303687641
151.175959542368 83.2083460752986 136.883475451494 210.282066967005
167.799243454967 252.649651285136 165.598854278557 152.72007888133
FUNDC1 89.1632548302738 128.005663945917 135.604088792552
78.1583955490018 80.9160262677505 84.7113556042844 114.230358276599
124.051109959228 176.594203350958 119.747995520476 105.052418151358
147.902378647394
FUNDC2 42.1537476443423 43.4396866157171 42.4580504587581
44.6905691366696 46.5557478175976 60.7488007939604 38.2933870238743
40.831207953647 46.2027193115397 47.0055981216687 51.3103617600342
48.8670821164567
FURIN 128.436305192918 106.951862149262 81.4931910857436 177.067668324062
79.1822591641672 83.2083460752986 102.380096190518 130.058147569795
91.6328359149182 87.3815040456105 76.4389426517528 80.3500323413132
FUS 103.010279797396 66.3026572113793 64.0162246399972 77.2218991550552
65.3933030211114 67.0290776715331 69.4677195088043 76.3509533017572
58.4200819624972 83.6524523974491 84.8084949516218 58.9069025298754
FUSIP1 51.1347974668251 76.9303701148105 73.8949517122417
66.1273501707704 86.3793067659925 63.6894484284172 84.4821163646146
59.6720198281342 59.4714233251027 51.1621379498014 56.6379362051304
64.5845827915699
FUT1 37.55264801907 38.8336427948187 36.6522278218553 41.5464765641928
41.5668884168669 37.4782441311626 43.6753843488695 40.2382908228517
41.1742244762318 42.785429206422 36.5359455830388 43.9541718254769
FUT10 48.1125722136997 45.7979032939211 45.1569741609597 43.1847628331565
43.3332476888305 44.8872784170036 42.4493032770053 47.4740726032707
41.0476065156912 47.2299678280724 45.0589947942306 48.3648494350286
FUT11 37.4554876386402 45.0115870036541 42.2577689927563 30.9241129241985
44.4489662507893 47.3791873993182 38.1793598983615 32.354741557861
47.27543600687 37.6383530120621 40.6373400186325 39.7411455670172
FUT2 112.644426507762 39.2907645923217 39.0775870687758 37.4223289687135
36.8059561571675 38.3418933808844 64.5722284870633 78.0020909283414
140.648209468568 35.7202909649682 51.3861659123096 42.2577689927563
FUT3 243.27954361539 114.056623754207 72.0243286940589 48.856176220627
163.234938974457 48.5835484742456 124.112216491975 205.991317474325
351.700618429121 45.7911932481722 47.4630625313159 47.6938628797168
FUT4 126.682296930289 52.0268699306396 50.6987358483181 130.908206625954
44.9936360641969 63.4610447573417 101.065203693106 110.983311402531
75.0704619826557 75.5861488664745 88.7461361855771 61.1022920882065
FUT5 46.6348258763289 41.3610369241157 42.157627240486 43.8002456102021
42.1650127805175 39.2985791324641 40.9157352260901 40.047314525032
41.5635499110307 45.9004543328976 49.1067959632109 46.0633179231318
FUT6 71.7997882389171 66.9524703393018 60.4809187893259 74.4365426051588
54.802736966312 47.1477056711335 79.9597944271831 89.7448801760766
61.3216070188812 82.966263655525 75.0704619826557 76.6094442444074
FUT7 46.3390850717989 43.0971787292711 38.1421709520616 43.8002456102021
44.9936360641969 44.6524308640784 37.0564573649223 38.6454098044474
42.6366765186182 39.7835513496873 38.6662194645633 43.3744503319109
FUT8 80.8285585008048 49.015238049903 62.7605715573218 89.7554896946341
45.0693869004277 54.4753345912615 47.1345949868029 57.1863195337055
61.2510119532409 54.0374522273907 59.0047891751497 54.6235981131919

FUT9	40.5654775358427	37.5469247328848	35.2101597814352	41.8778873678551
	41.7419877611072	36.9060997041453	38.5066108855005	43.2890595186135
	43.0930293831497	40.4518431978241	43.8002456102021	42.9887069977696
FUZ	51.1740855632044	61.6878176260761	57.2960245538881	53.0284011078097
	83.0497574039035	58.0910843932181	68.5725871332774	57.7805922926828
	69.8899273527731	73.9732553364283	66.8842530423095	50.5511827666954
FVT1	275.811139268285	235.593721159442	192.700843009002	260.964562714558
	114.740276704399	263.937966521917	171.853856533964	144.578232267116
	213.204385896543	288.065258507995	245.127632785841	343.262403838579
FXC1	88.0979122006842	110.837476359817	112.60230393988	146.268519891864
	107.770004093887	126.911056987607	121.441763452598	103.734064904898
	107.538934056558	96.6833301748676	91.6826894933058	109.594402843796
FXN	48.2549905619119	61.8124171056747	70.2141759405882	47.3574589211166
	59.526516100518	57.4777075615651	60.0517078759277	51.9447450229707
	70.5171002055327	85.6038071277319	70.2215218476683	57.2008272301399
FXR1	160.853643241489	145.607100979838	153.901083585889	159.248015095017
	202.01900875263	251.027270265657	160.203825526096	171.468580016846
	164.642060754649	165.797721197832	152.555556133364	224.109805046747
FXR2	102.443884864631	108.083751469991	88.0368652545541	98.9560329133205
	94.8408264309872	86.6246917308185	104.341160312305	112.122619608668
	99.0815700721606	123.608921653229	116.993403211005	86.3888090084564
FXYD1	41.8792703207442	41.9397336249742	43.1931731665131	45.4977402232805
	41.0608773166058	140.961217055867	42.1607322031054	44.6708453138373
	42.357910063876	66.1541420734416	66.7256057011273	50.2163892855092
FXYD2	42.5478935075651	41.2598458726002	42.0233072093141	80.4007868743886
	40.2449343247539	42.1046072075724	42.5750559787101	41.8767957887205
	42.6366765186182	39.7390725304842	40.6857324478814	42.1860813954352
FXYD3	46.6781384361786	179.522572239758	344.737170118121	41.253645578517
	95.0604882262201	53.4921736109774	80.4007868743886	76.5444845463963
	190.592556065939	103.521926015878	85.0342118119783	107.232979387172
FXYD4	44.0328992213727	52.9603588177082	38.8775560911841	37.8442841690502
	55.0031529299037	38.4330755397458	149.978825406418	253.462941029119
	71.6573554353691	38.528621456378	44.2142581488029	47.8931773060789
FXYD5	349.589955244523	199.858233616116	299.665413950641	639.778130093087
	167.626012561905	268.54298415337	539.471011648536	366.515210930763
	336.795797976236	350.917244438732	348.65230953701	378.12777235743
FXYD6	102.770627167954	53.3683962805281	51.8122261766744	119.591054636527
	47.6884372645373	769.685677484067	47.7390230243778	52.5098795466615
	40.4997186069494	293.410413883219	259.553931603133	172.39907939484
FXYD7	42.3433931248155	41.9894412416874	41.8751578329365	46.1673159982664
	46.0043807840705	39.7394181011603	51.4349390827895	39.2672712041774
	36.5393857306744	44.0409178714232	39.2542963855807	40.0557788909923
FYB	71.7624419474395	75.0873720488265	97.4515987591398	312.795596385258
	52.8843091010101	76.4439037944547	85.1227830573138	75.719886024949
	52.2216613535546	47.9707112435212	56.0794100106654	46.9867599298218
FYCO1	131.645714321903	189.80672643731	161.739131587013	115.299308625328
	173.925818790068	482.5816387921	105.549249728506	142.309369995384
	106.418690759491	495.453743436613	367.235521372008	385.512974999198
FYN	69.8231400816467	72.8306652548968	118.05982513603	144.578232267116
	52.6922486991742	207.833867050257	61.9504984796308	114.78317743597
	50.7196000170597	97.0442565553353	100.393361901962	110.860844149372
FYTTD1		64.4895418579465	70.5699797830702	71.6931243081178
	75.3630857139861	81.3364476638024	104.704892803169	76.3583555666815

	91.2647023876304	75.3761360028971	91.036114822363	86.1779349281961
	89.9929484069346			
FZD1	81.8569832022381	85.5797236700758	51.1687217969783	54.4586176399381
	61.7566103867825	56.3293881184854	56.9320717885002	49.7309743973184
	66.7357366223349	57.6791750431251	54.3554233228353	54.1941919612524
FZD10	41.7397731376692	44.8529259387308	48.6037684272115	42.1273928203021
	39.1650142708257	42.750861693377	34.2773859804062	35.4526946152955
	53.6446045619513	37.3179578037963	41.5421429082231	46.6397391251762
FZD2	298.252355588344	67.1926218405833	54.0536858212253	162.151812425949
	280.075938355178	86.6910280940418	244.837105178705	162.181462827314
	205.854666205308	74.3187437598836	114.979124960177	50.5511827666954
FZD3	71.7624419474395	64.8903340853337	69.4047473173218	47.8164193292842
	47.0954207417646	77.2834482217973	99.1769280484821	63.0748373122119
	113.655306804539	96.7422527189063	104.313754953569	108.299918384297
FZD4	127.232160404726	181.565843182713	340.491194032068	201.782740241526
	119.72858253254	289.909646207746	84.7782034884529	112.613079254002
	119.167027972122	146.311363191836	168.326515533208	113.130838519437
FZD5	48.2910530887214	62.6236991757723	50.9010149857308	37.7469621225553
	65.1757400078647	43.5752393599169	88.0664425843955	66.4207938873541
	95.4942268742467	63.7002949745101	61.4121672148248	72.4651758215622
FZD6	41.1523487516364	73.6855877240541	69.24946602508	43.5627649099623
	67.6011992144113	45.7300501624017	52.7002314230604	53.9465584609317
	94.8408264309872	73.7133884699889	78.806097512943	84.2728210732532
FZD7	119.277678423944	70.2553917409773	91.4581948567397	77.1614179978243
	77.7140342228764	134.489077146096	84.5678487053881	91.3607028449916
	81.6508259258669	153.019365845424	167.626012561905	102.971363152434
FZD8	52.2236479204029	45.9266745571724	45.1569741609597	41.7614975630362
	41.0885562160946	55.9351706773613	40.3302268293707	42.8482181313226
	45.1899271235974	46.8954576545929	44.0409178714232	44.3521099857368
FZD9	70.083473392272	44.1239711172417	43.4709415392696	48.1662933382789
	98.4847140916184	55.540888910486	52.561573697155	52.5909491535773
	82.7229468743536	49.7259371799014	52.5930185400362	37.4554876386402
FZR1	51.1055541075029	54.6089451252049	47.646800205189	51.7739270955254
	58.2780916793348	55.3202238943712	47.4439407315335	41.7419877611072
	46.7760357462635	57.5713378330876	56.9320717885002	48.1627097465883
G0S2	49.6241203903354	45.3486182278177	142.614006543733	2191.09005731751
	81.4621222177298	123.768417863139	525.522657246248	56.8094693588238
	45.5779686360641	55.0016608333828	53.4921736109774	50.2163892855092
G3BP1	88.5140510969737	83.6351558609087	88.4211119834434	103.139459009593
	132.205423909251	120.392561825049	94.3537052300317	80.1144023887293
	91.2769094348604	69.4677195088043	83.8637731385607	97.1271615802556
G3BP2	241.24267509333	250.504350952583	224.914980996667	192.171116215791
	204.992039590717	218.013913036142	145.630337418553	159.248015095017
	197.515728627482	145.232878862479	154.045685048215	236.307055572306
G6PC	40.4639532172338	39.5637454855233	43.401782064778	40.646702696033
	44.6231541342	43.1180113655693	39.5496902678788	41.2098211956301
	39.6609238036815	40.4518431978241	36.6603338274678	39.1878770155069
G6PC2	41.7397731376692	40.1989248349075	39.991353369336	39.0031581123233
	42.6022652963326	39.6705473876526	40.6750540874299	41.8792703207442
	41.7627910693483	42.9285286409526	40.723990462047	38.6454098044474
G6PC3	202.128650032341	211.701234819986	169.557757933574	221.163225922749
	168.247603128156	147.424598105182	328.176884082116	277.993522959259
	397.798003824167	270.202959012185	355.319959025243	157.133627517432

G6PD 234.308728996238 116.426770366736 207.768377497439 203.46147333933
459.92905629125 167.449422388453 197.040881690021 175.529493306927
230.594000005581 155.693953208351 143.399636653204 133.43526498879
GAA 168.492959515167 84.5180930520475 219.238235572894 169.818067865324
150.801729138762 101.103458852775 108.213380368446 161.603201688692
247.676607756929 236.727577348109 226.090904721424 170.128899555014
GAB1 44.5732978059923 42.3550738181144 40.1474543172332 42.4580504587581
40.0230205296014 47.2410131431265 39.8390535343912 42.6975474602554
42.5750559787101 44.6918600441328 45.310660704531 47.0797760763156
GAB2 57.9038523094718 50.1860191642359 48.0191002461099 111.626292905866
48.0901896064056 72.6824533717231 73.1654226019863 68.0342444360948
45.9693232070002 56.9463858443402 57.0427273538849 64.9890714157316
GAB3 49.085741080241 47.1332119496722 47.2055133646385 90.2790229242453
42.1650127805175 52.2089306840125 42.4939802311136 39.0620487644635
40.4220196471124 47.9056710878325 47.4630625313159 45.6692241948108
GAB4 40.9558083259517 47.2770121962655 45.7968648044613 44.4367387274922
40.8873907150245 43.4681646416585 44.8643898647322 42.157627240486
40.7847110517594 43.401782064778 44.4688999098904 46.4499258373233
GABARAP 74.0913177754682 60.0601788842942 50.3950981988762
63.0153175111747 50.3713170502557 55.9351706773613 68.1125016199524
74.2517888452553 50.2223403889892 73.9732553364283 60.6505672212119
65.524821699432
GABARAPL1 379.79657860938 211.451463038315 201.616017553422
614.807633538557 140.076084766156 731.071171171638 233.501069644324
693.727004855319 197.617554897454 727.788854407888 666.816487335088
780.708896888794
GABARAPL2 446.902178094675 504.676944739425 663.372296297652
567.534792333871 345.615640348066 433.447319956981 357.254500791202
300.560071753181 334.170764774986 272.930242946726 357.111253129662
495.26603220907
GABBR1 64.668912004173 52.2973903538281 73.8949517122417
56.4578213622965 52.6058831807321 60.3093790894864 57.3032522296189
60.7502080097304 51.631710130416 76.3168793429802 79.1209792219434
70.8546350449171
GABBR2 90.7551885299639 61.3428092991286 42.7303139017786
46.8954576545929 86.5513342734384 42.9695585740813 299.476376921942
805.590413262364 41.4636047919453 45.5776159432079 45.390192977585
40.5703581862752
GABPA 62.5968484440453 84.7222416138393 86.5616422570166 76.5411379733694
82.2348885628237 95.1000239210194 72.8653205184014 53.838484549134
80.2381651206227 54.1683282066029 70.2606858200937 73.5599394565136
GABPAP 41.9969132604035 40.4014349193189 42.9104454767916
44.8793734665306 40.8346075138147 42.7982502551634 42.5487932018953
41.7419877611072 43.2264757900051 41.7405682794166 43.9663150905585
42.7393803617562
GABPB2 1387.28483887704 787.671342652389 920.504890206997
1147.19483787501 757.833254346899 557.094756796515 1132.19026676901
1649.78627644882 743.463635670141 1658.86829668934 1707.92349760322
1123.99110499679
GABRA1 40.6559419616644 35.2143190355414 40.4070046183108
37.9875302985336 37.7576900881741 36.2015763253919 38.2933870238743
37.4484310268909 39.1790517315259 39.1621898366355 33.1645364749371
38.3291713594599

GABRA2 38.304249186058 39.1982470626863 42.9104454767916
35.3829784221072 35.0181575202066 40.650742261428 45.7452456977857
40.2382908228517 41.2870505273664 43.4885245598939 41.7272875295469
39.7411455670172
GABRA3 36.8072833828297 41.3610369241157 41.6977706798234
43.3078542962968 42.269054475455 41.7627910693483 39.7576136507593
46.1673159982664 38.5252456306656 39.7835513496873 37.4642807402741
41.4508241771315
GABRA4 41.0608773166058 37.8217989841918 36.0306975524309
36.0481934423077 40.5710637884029 36.3845892764084 43.1931731665131
33.7818132446042 35.4332080966594 43.7303813515864 42.2618615571566
41.6550823405646
GABRA5 44.0707087774725 42.8542642792862 39.9101908547397
41.8291484190524 41.9842695818285 35.4526946152955 40.8681871019812
39.2248354955045 39.7537274655115 41.3641352453779 41.0050831223107
44.1063440402552
GABRA6 34.4021554788966 37.2810767789306 36.9795961564828
36.4564050256915 35.6434124419782 41.8613406887824 36.9355798143571
34.9580369221164 37.5469247328848 42.4594149893798 38.956280281655
38.8495095797881
GABRB1 41.5421429082231 41.159706131351 38.0452862258803
40.8681871019812 39.859345745831 39.132725377815 41.4742337714895
41.4508241771315 39.7537274655115 36.1550293353654 44.4688999098904
43.3744503319109
GABRB2 45.4507786740825 38.6414316877471 37.7000400506818
37.5764485234171 41.2424637338424 39.7394181011603 41.5817737564592
36.9355798143571 38.9834959368496 38.8295492252264 40.8681871019812
42.357910063876
GABRB3 39.3257528345627 36.9406635096502 39.6938066205531
40.3530367065738 42.2307216849747 39.8929881312378 43.4544762830742
45.7995623076688 42.7635040180321 39.2069469784204 42.5884861805987
42.0772855634209
GABRD 43.5710871617107 45.0905928998779 52.6275131390249 44.0451487108213
44.0451487108213 47.6803619768589 48.4859552186772 44.0390019242862
40.4997186069494 41.3321861371202 50.4740079109854 42.57175366709
GABRE 63.4610447573417 50.4487395010341 51.8122261766744 48.9386616209853
204.039190380778 53.565717255469 46.9929836306816 55.8579198774476
87.6018870099686 162.467159261789 99.126949393699 87.7460859694899
GABRG1 43.2724556187214 35.0222940937513 42.7303139017786
37.1894304543751 45.2556760873335 41.554942319225 41.4742337714895
31.9468644542969 40.4997186069494 38.957687788868 38.2795150354677
37.8490162439785
GABRG2 38.7537364570873 44.9370339275041 37.5222959113204
39.5637454855233 46.0977687311222 48.1310137493545 47.6810611485413
41.1575777497576 39.4762546236449 40.8049408996274 41.4860822396574
43.5420658412177
GABRG3 42.0532901689036 41.8848959280916 41.437237454134
43.3078542962968 36.9702228618069 41.159706131351 41.5817737564592
41.1575777497576 39.4762546236449 46.0119912782855 42.2618615571566
41.9430737035925
GABRP 54.7786516309154 46.29198852834 43.0971787292711 53.3967242431461
45.1137938810047 49.5424425850549 42.0605906240248 78.9361044544626
62.9922928277769 41.2276963324981 44.1148826235374 50.3118072462443

GABRQ 38.304249186058 38.2263227947189 34.806104801006 38.745552403895
35.9058393060637 39.132725377815 33.6696565339534 39.3709357615493
38.6322728994674 36.3612409063792 37.4642807402741 31.5556621809114
GABRR1 39.3709357615493 46.7591817286632 42.0621425894624
35.49696651597 52.3242437351389 47.0027453663546 53.7423898357583
43.2551508830106 38.4344387289956 35.7202909649682 41.5421429082231
37.2810767789306
GABRR2 42.2455707947223 37.10170285405 38.7745734037492
40.4359177791566 46.7508829977587 38.3418933808844 38.941855825269
37.33030683349 39.2809305246091 35.4990901121893 39.7342718293432
46.1592873342534
GABRR3 42.1537476443423 45.0115870036541 35.0970060242246
38.5252456306656 34.5301949364147 37.7000400506818 33.7574445386307
38.3684202018524 38.5252456306656 37.0320432433314 37.4642807402741
37.6557714476853
GAD1 80.7227211275429 76.1382217104599 52.0359304386476 41.4606714407332
61.2070368742244 40.1846227436522 94.952392592003 71.9200120168048
50.0526763693329 79.3775933954493 69.11736279512 61.4884606677516
GAD2 35.8208542506595 37.2810767789306 36.1042012504758 36.7892997857376
37.4470183315826 36.4986986135769 34.6720534245631 45.3872406432794
46.2027193115397 39.4710743516517 37.3687900733549 39.8497227447888
GADD45A 261.878099018309 431.727858902449 506.75912743556
226.35806628102 201.875082892488 253.333114749013 381.480149599459
224.509915754308 196.17328008185 182.246578375636 323.855522995794
496.163796158189
GADD45B 83.7215159198897 75.885672222265 74.5886835786452
210.255784687052 74.8013460023603 841.850722284484 94.1289383735162
249.411435337932 73.4122412764173 72.7502212566295 70.9319024596628
105.220978559157
GADD45G 75.6471706262645 61.6878176260761 45.310660704531
84.2804410361247 50.5110359971301 99.2749350936362 79.5707751325986
95.491982090349 59.5093038629766 79.262977183608 88.7928773498158
61.2685222381296
GADD45GIP1 37.8386378737561 47.5647821785529 41.528370959196
44.1483976313959 39.4693871570028 43.2805590023412 35.6056062164318
41.2551966132486 52.1788489555942 40.5090849993014 44.2925355984056
35.3525469364579
GADL1 41.2424637338424 36.6968906669565 37.2838080935695 39.2780515621926
40.3452320167286 37.7000400506818 42.4939802311136 40.4359177791566
35.6139301508335 42.8936990772838 38.079222289614 35.3525469364579
GAGE1 43.7585538437696 43.2649781354792 43.0971787292711 41.7614975630362
41.8524296959457 41.6178506967511 41.8315602975157 42.9887069977696
41.3468610075772 41.5696109388121 42.3350406618256 41.5132248356707
GAGE12G 36.3649677956953 45.0905928998779 48.4890615340603
46.1673159982664 44.9936360641969 43.8582747295766 40.1235656101329
42.9301944191913 39.2809305246091 45.9004543328976 47.2134341420411
44.3521099857368
GAGE12I 40.308802735799 43.2649781354792 42.9104454767916
43.6726153508978 43.4544762830742 45.0882356578404 44.5390641547628
40.047314525032 42.6762317063737 38.745552403895 41.3553369544097
42.0424641526302
GAGE2C 44.6231541342 41.9894412416874 46.0727230219512
41.8778873678551 44.8897737954573 44.6524308640784 52.561573697155

48.272324215504 45.3756389170606 42.2614524730856 42.2618615571566
35.6427313468066
GAGE4 41.0608773166058 42.0500582486532 39.6451483836089 39.4569929845912
42.6575535390785 41.7627910693483 40.4359177791566 38.5457716679071
42.9020321355927 39.7411455670172 38.3684202018524 45.063792968927
GAGE5 43.6706764143908 44.9370339275041 40.0599807618105 41.353786352147
42.2510526338368 40.4779565600984 43.607701594929 40.5158867964016
40.7493885015617 43.6656890131156 46.0459873800125 40.5207392413115
GAGE6 41.6698172204688 41.9397336249742 40.4882741869718 40.2563544693508
42.5435283762933 40.5281496800812 42.551356054931 39.9084621723809
41.6285300488826 44.0409178714232 36.7950674999881 38.3880780363593
GAGE7 37.55264801907 43.5873188565519 45.6989664108554 46.1673159982664
47.9128504683618 45.1873487463472 51.3064929551674 40.931294690669
49.9162522057712 46.8954576545929 47.8988096575648 45.2783024981315
GAGE8 32.2133093774284 42.5269793679335 34.9849896310235 35.314469458379
40.7779767565889 33.5578650644246 36.197424053556 39.2672712041774
39.1790517315259 35.3849221682845 36.2435874904689 42.6758568421347
GAK 190.854807132673 244.226756210656 286.11425202743 243.27954361539
208.189151365575 164.547318765586 261.374019422095 195.482216656634
277.204818876581 229.188779190649 238.697159169213 171.829798001437
GAL 53.8856501960117 38.1312950900467 48.1997158668729 36.5393857306744
828.403901345618 128.126336084436 62.3556396662215 63.287067794195
63.2753814090696 37.3179578037963 44.4688999098904 44.2527566343661
GAL3ST1 42.9104454767916 49.6841415268611 36.8730514568925
37.651298468202 42.3685136297603 42.6575535390785 39.5496902678788
39.853866204927 33.5578650644246 42.785429206422 37.4642807402741
39.7411455670172
GAL3ST2 50.9381343674699 54.0461131287999 44.7829632472754
40.646702696033 44.3557408297309 53.6858208094027 47.2927436772465
49.9332280449549 58.2401724760744 52.6879797806876 38.745552403895
51.7833333848627
GAL3ST3 45.8108990837624 40.5400384479766 42.5890635915791
45.8844325913247 42.2455707947223 43.2312848495114 42.3194600586894
39.4223544019653 43.0456011922275 42.5664883002833 42.3413611855132
40.1235656101329
GAL3ST4 43.1805016690297 46.0713306971594 37.2838080935695
43.3078542962968 33.0463640202858 44.4619248757502 37.7136011474903
42.2577689927563 37.1820543575123 38.745552403895 39.5399188536544
35.4484652445078
GALC 130.506891299903 115.414481087246 327.878405194732 279.485290822376
82.3181867658154 124.751020763508 62.0404348860702 74.7127063822938
102.797268694325 147.719172115976 130.10148014895 123.978615472736
GALE 81.763263551338 82.0102749744973 123.039116845742 64.7830058555944
94.5055588176321 48.9826868640753 107.610858427836 87.4850071876743
126.071077511531 97.3870880869036 97.1634390603131 88.2604063995148
GALIG 101.935321521672 84.8084949516218 78.080034922779 68.9937449813561
42.269054475455 58.2531879680192 44.2166118829877 63.287067794195
50.4974478884077 50.1649089793069 56.0477607422828 51.7833333848627
GALK1 506.173054520807 262.579533452771 377.626746989976 614.088027946471
377.491895827114 339.418989022736 445.421601813221 499.744856832174
454.701322158431 234.8262403783 260.618790701505 217.065944053174
GALK2 55.4366435285754 77.8039806950172 65.8986841086198 57.3837827230023
94.233414682323 71.0935990523782 70.9702772728923 58.1242391848692
74.2517888452553 53.1182952086284 57.4078217542368 60.3217748365169

GALM 298.252355588344 206.234676343851 142.262349841307 235.075347885884
134.981483569602 111.669813610213 439.770250139925 351.239677010973
243.609178976074 223.356945665761 194.207518642389 164.435300801872
GALNAC4S-6ST 273.460165751162 92.4953883194488 84.3755392205436
549.67905283016 97.1271615802556 171.877918435017 63.2726414544553
72.121833189378 64.7766649630041 75.2053836356882 107.538934056558
110.731862140996
GALNS 49.6241203903354 47.087835967892 48.7039705048129 45.1944222548351
46.8674366115656 49.4157684139858 38.7337086062875 49.9332280449549
47.4699236780425 45.1569741609597 51.1483696410762 43.6753843488695
GALNT1 86.6655289455628 150.051297690759 114.039396407323
76.8521423078922 100.413031488199 61.7566103867825 67.1299518044849
86.3237770387663 141.924244643825 83.9873645051122 72.5835488194631
80.6200293361016
GALNT10 62.1448806673973 46.5372362927461 51.0902940392974
56.5583372864784 48.7738999860475 51.1183745411436 40.1572444265517
54.4602501503065 50.8720311700491 46.0119912782855 46.3563690926644
54.1618170692792
GALNT11 140.369714831352 137.818833473709 169.344094528032
144.239350177363 148.654594696193 207.726011653345 109.062415049404
142.545268664218 110.599131324937 208.943204358024 254.159017331462
261.878099018309
GALNT12 53.4921736109774 293.583101558009 80.354283778818
61.0845101400379 74.8013460023603 122.758873982957 153.901083585889
86.915098846441 91.0952554578659 67.8233625051884 70.0402992940342
119.833821068193
GALNT13 43.9309845324767 38.3322849790657 44.2405728317107
44.7980740332174 36.2048942610546 45.1873487463472 41.706879587091
40.047314525032 41.7627910693483 44.7958122466628 45.0589947942306
43.7672102682067
GALNT14 40.7609952437622 61.3428092991286 57.8111054584154
35.0448343468967 42.4864576619427 42.357910063876 48.3411413510851
52.7787010032051 57.404059248727 46.5650556481721 47.4630625313159
50.1579724208315
GALNT17 44.7915315085948 45.5842286511645 50.6987358483181
42.357910063876 38.4540849632254 40.244271403831 56.9320717885002
47.3791873993182 46.4164127487052 46.7646877499666 54.7061116419212
44.1452943804044
GALNT2 45.4799270968969 46.9919960628953 50.9010149857308
36.1562979552173 39.859345745831 49.641159251878 46.1634822993009
47.4740726032707 38.9834959368496 38.3092241935352 51.524711857679
46.8129509799753
GALNT3 130.002385558757 97.2004257862686 68.0649030501515
113.783910320605 99.571118823509 79.0940380276977 135.997802304719
164.122105098254 89.0684909049451 173.150885069619 168.78407943637
124.791747507205
GALNT4 69.5462764096011 76.9303701148105 72.7987593815919
65.8926843080114 49.1928206164702 63.3612993417558 65.3694267397114
86.5926014007562 115.221134337342 63.0119737420787 59.8070012006708
68.0751111916102
GALNT5 49.4151347850687 41.6466169548846 37.9482157381008
42.0102671859606 39.9697332660833 34.9849896310235 45.3597029614942
37.33030683349 39.3750969348308 33.6902293666089 34.9428206949566
38.2350720635382

GALNT6 49.6241203903354 45.3486182278177 56.7776874358774
60.9379216295576 54.6820069269589 43.1826328292558 56.4809146784765
61.5000955267432 51.2066664465185 38.3092241935352 45.4799270968969
42.2577689927563
GALNT7 38.304249186058 39.2907645923217 33.1559412791294
38.8495095797881 33.6330903323612 36.6271061451613 30.6643697826563
33.0038344711883 37.0126425326534 33.8984830800739 34.7308008643142
35.3525469364579
GALNT8 43.7585538437696 41.9894412416874 44.5134109184888
40.5484717678957 40.6723334103791 39.2248354955045 41.3594868055879
44.797320243931 39.853866204927 37.3179578037963 44.0409178714232
36.9976231206067
GALNT9 39.1590964332473 43.074472921841 41.2365089689635
41.948762493127 40.6723334103791 41.5083179498332 40.0052661202207
38.4187686307607 41.6373771565199 40.0633222311529 41.6336636285875
40.2175332777639
GALNTL1 47.7779356299965 45.1390218677367 45.645828394758
44.3440839686558 51.0107745412152 108.654797121478 48.9088046212958
46.7508829977587 46.9739160257217 60.1466326805616 50.4740079109854
62.3997407955361
GALNTL2 59.3923286524971 36.6002100641539 42.3536549114165
47.2009420656854 39.9697332660833 83.8726595797859 40.2154497375715
44.293922970134 44.0519245668737 84.6563174894574 117.282216847547
156.433762355183
GALNTL4 64.0759817636265 131.917451557976 156.174166381986
82.7394574977114 105.317399386109 92.1151486847283 42.357910063876
64.8336014343435 78.055431384988 61.5635554619635 93.4144098590192
142.492693744683
GALNTL5 36.4564050256915 38.8336427948187 35.7105247506212
44.7980740332174 36.3390947880186 39.2248354955045 38.5066108855005
33.9687743313429 39.2809305246091 38.745552403895 36.9060997041453
41.6550823405646
GALP 39.3709357615493 43.7644428186265 39.7411455670172 42.6913548400127
37.0526522140242 42.6575535390785 45.7452456977857 41.0381860307445
39.9444191873552 39.8873706855774 39.4410515050885 42.357910063876
GALR1 38.2496050953547 43.9783383422823 39.044540595942 37.0732977341185
40.1151953732637 42.6211340086915 44.7255207589606 38.8495095797881
40.3662560853635 39.9948390976317 36.131485826118 38.8964578286293
GALR2 49.8472708021525 52.1967540852276 39.4710743516517 38.745552403895
80.428712610358 51.702461863489 74.7534423194688 80.2680308979464
51.5888395924843 43.9450023643394 47.2134341420411 42.57175366709
GALR3 43.0001432913058 37.1685072112618 38.35720098134 41.8291484190524
39.8198309498737 41.7627910693483 43.7585538437696 40.7311497914871
39.9444191873552 37.9661004970253 44.9081227425497 43.9282059368202
GALT 82.9467575626263 96.8240908274121 91.5230424957075 80.4007868743886
102.840494180617 77.5889289318438 88.655273320123 97.095992167029
60.0736861987141 131.90520406914 105.686346729469 93.6787442149817
GAMT 59.5000730792772 62.8635815439291 142.012216533823 63.5343940247764
72.6675345946258 124.841811761412 45.9426591985894 64.120219344545
118.93749473833 192.318724749487 158.862722884436 143.590160385066
GAN 45.3872406432794 35.2143190355414 43.5710871617107 41.1886975241176
41.2740822675592 42.357910063876 41.253645578517 41.0381860307445
49.1940901905636 40.8970401536578 37.2892641289258 42.8864415494484

GANAB 85.6964911093755 101.351907645804 119.545018066492 90.9106103192162
124.37984507038 80.3805507705979 107.748050894857 104.822963389102
112.488156568824 79.9810256029665 108.12482131423 99.0545942554244
GANC 37.4554876386402 43.4396866157171 41.437237454134 40.8681871019812
41.5668884168669 44.7804080899431 41.0381860307445 48.5681478665796
45.7452456977857 45.1569741609597 43.9450023643394 45.9693232070002
GAP43 47.1962846565261 44.1239711172417 43.0016969050339 36.9912468186322
40.5710637884029 43.0860383229288 39.8695929627783 49.8344973836834
39.9444191873552 48.7414853386615 43.401782064778 37.2810767789306
GAPDH 3313.98659031004 2498.54878472152 3361.67247904403 2921.68921750799
2779.37660132136 1932.2647526188 3602.30252142918 1731.19220270448
6673.00322263275 2196.59044510709 1925.20977162704 2393.42629339901
GAPDHS 38.6454098044474 40.6723334103791 39.2388980107385
46.3919862398545 37.6119337673806 40.7616966228404 42.5660695551615
41.4061152929154 39.9791973792384 41.5435611403119 40.5824352835642
42.1860813954352
GAPVD1 287.565588186962 225.794026573235 165.775214685061
232.516262874696 221.123510823097 231.198764780818 246.313146942736
276.084235934753 160.950554451488 266.687281047486 276.59189773139
248.924819383887
GARNL1 86.9032093551446 102.220824629896 102.228965899392
100.90948905426 81.1214257028304 139.121494956408 73.5993692960562
87.0116305167987 103.264100180823 162.628603963754 132.3192754634
140.648209468568
GARNL3 61.7551416187554 70.4933405598359 70.3613168347492
41.4273122914951 55.6069214673486 133.798096986107 76.0041771381018
50.5070012045444 45.2897806651929 153.059039745191 109.677588309718
74.7557519831859
GARNL4 94.2762661604561 151.175959542368 102.942921317489
68.1992221684165 87.1397220361868 53.9517841438975 124.252483407658
104.351256270039 60.3351200884626 60.6779753362314 62.3990310986805
59.8400088701028
GARS 550.861615347663 567.393665550133 438.50123471613 619.337575854369
666.265306908449 459.182372959714 666.816487335088 464.352441400358
749.49934348018 250.621261212308 311.149068752273 361.023743912857
GART 112.644426507762 177.688415930665 167.872060678879 118.186590679218
439.376432049616 118.186590679218 164.580141651883 150.262748333939
180.562005428669 81.6508259258669 90.3531863862054 132.851109034738
GAS1 574.847203528544 72.3297091475814 51.6660481331154 256.116479208201
64.6835308827804 505.294880430281 55.6268253440479 49.6203214143464
43.9450023643394 224.871193467078 218.819478568299 151.425712904293
GAS2 37.7805366040928 55.7067742610401 34.0927410219282 36.890925044696
37.956732373465 38.385865183581 36.03517591974 36.9948667320377
40.3316153483289 54.5641201866931 40.4995052849302 42.828737648529
GAS2L1 56.3471340054074 49.0697565324841 50.4778868815688
47.0033848638449 54.5683518971261 47.0027453663546 47.3605100090439
46.8954576545929 54.6235981131919 48.2596564318071 48.042366934301
46.3411353377615
GAS2L2 39.3100256336821 35.2143190355414 41.1742244762318
38.5252456306656 39.7210115145672 39.266565827316 38.8775560911841
39.4223544019653 44.5678064264717 40.2615278292875 35.3870574564807
41.4508241771315
GAS2L3 48.2910530887214 64.5347406608537 44.6032218589199
73.0371862899659 49.8304479381623 45.0882356578404 64.0985088513857

86.0944261788554 52.7512110769042 58.8022152133979 57.4078217542368
54.4992295592001
GAS5 36.5393857306744 37.8217989841918 39.3709357615493 43.8002456102021
35.2163900400329 38.1169703906207 40.2154497375715 41.5464765641928
38.7298222155532 38.3092241935352 47.3791873993182 40.0557788909923
GAS6 323.576613035353 225.35760689842 155.252915611651 347.247950836937
115.735359157053 809.577874019028 66.2978052628661 158.884110481815
74.1696173738106 527.037018015766 616.265771322835 316.419421649196
GAS7 63.340012816413 43.1567024035183 41.785676764591 62.5106969730855
43.7858612339862 121.336830564295 41.5464765641928 46.0106098064475
42.7522261624826 65.9574246586409 68.8362790228976 81.3082994649699
GAS8 118.358996104366 183.854020356927 189.164097331837 77.3864518736634
124.664792638889 130.648278937122 169.859588818306 96.5813769696577
117.732966326465 149.59360999995 137.016387648703 105.05842887748
GAST 45.1912496459517 47.8400694856726 51.3268475761593 65.8926843080114
51.2525124735102 50.3245489035833 61.5000955267432 50.5070012045444
54.998510746958 50.8826620053386 47.2134341420411 43.9541718254769
GATA1 41.8409927585705 45.4143854024453 37.6557714476853 42.5664883002833
41.3755760434934 42.6575535390785 38.941855825269 37.6557714476853
40.9901805728485 43.2988017098387 36.131485826118 40.5703581862752
GATA2 77.7665223272661 435.632247999054 374.427492246826 59.6153684374766
155.174579191838 86.915098846441 86.4114854104675 84.0899854321388
71.8576824255215 413.516548092483 413.918050849658 92.9600461444587
GATA3 82.291097567232 646.791777335656 648.380417557148 52.6947911480086
876.959744461441 129.81569370219 1330.19215036764 361.57814355509
866.712228831149 850.205634919459 820.147156401172 592.205084997276
GATA4 39.5676051939342 38.1312950900467 39.7411455670172 41.8778873678551
41.467005949568 39.3841861812262 42.9803717121865 39.1652965788406
45.5779686360641 42.2614524730856 37.2892641289258 40.1700117452801
GATA5 38.5007204246293 45.8669209716694 42.8270038153139 45.1944222548351
45.1137938810047 120.618530729822 45.223075311113 61.5824518349507
45.1899271235974 120.274848052394 97.4829901136163 77.6056319020116
GATA6 42.0532901689036 43.8495248991589 35.8265993915844 41.6564075217517
40.2449343247539 44.5544727111028 36.8369172900902 40.5158867964016
39.853866204927 40.8970401536578 43.4709415392696 41.5536168238478
GATAD1 81.537796828766 61.6219790276408 73.1262465119304
59.8102324370568 60.5290966885203 68.6424653037264 62.3556396662215
78.9361044544626 81.6508259258669 91.2016867646161 83.1184264089873
96.3918711895208
GATAD2A 455.223217772744 575.268446033282 536.73426937255
513.733739926914 1243.32650314893 444.618035487523 563.927521628693
490.876152145712 466.454993547103 394.437597863822 495.26603220907
382.872356030213
GATAD2B 75.3761360028971 94.9831937714438 111.357508398822
93.7782490229256 89.0203733091224 81.3978207813439 91.5258329186709
107.682738819549 85.9087280306456 88.9738853594498 110.214922820817
89.8035133597498
GATC 78.5216977073842 62.3983214098965 71.0340758556049 50.0526763693329
75.7464604972652 58.5530199658661 92.4534854832186 53.2046867327331
77.1317865202676 46.2286164776607 53.9517841438975 70.6903168772326
GATM 63.6767189846426 75.7257660897685 126.578693258328 49.5424425850549
319.596352961751 200.986069552443 68.673864919178 60.5571721986885
204.009492151839 108.63277045284 137.484063876244 153.569831699238

GATS	149.933827818309	81.7367617812869	97.3066473777771	68.8167148906225
	99.1438953564062	167.410607476565	92.3034609409756	106.871269007146
	100.476750028832	218.727705098677	172.83517237888	109.832638804839
GBA	321.949943388959	237.161912299581	477.924466983414	589.685739519627
	229.992809034971	225.056827821165	249.37112382902	324.320137371059
	202.360546931172	199.208033538936	196.389446842567	156.02592319461
GBA2	62.5528124447176	69.3106426188075	56.7272804480684	78.7450149191851
	65.8319449883221	63.3612993417558	71.7546556028831	68.7227011958449
	55.6617952786921	75.4836539148799	70.4204944097269	76.4889974392545
GBA3	39.7320564340365	38.4330755397458	38.8417151851134	45.8844325913247
	39.1087466732192	45.7300501624017	41.0381860307445	39.5954353681031
	149.620814280822	38.3501188844251	43.9802831041664	42.5365257225985
GBAP	45.2875173643252	42.0709558258414	42.0621425894624	46.614092935749
	46.1804647841331	55.1037284831588	56.2181240696479	43.1567024035183
	41.1742244762318	41.6373771565199	42.170702295259	48.4535991392809
GBAS	171.33368481035	128.806903044733	129.179537795204	140.894046694804
	159.416887501941	232.147792636393	206.261432452217	178.249889756228
	189.915336406793	169.633672522325	156.433762355183	208.529062113623
GBE1	120.630151942485	92.1908585323292	124.397048303668	186.195424728755
	95.6182216943758	150.009352890706	93.5928272702304	71.6202385170491
	194.570513601724	87.3815040456105	124.986782737446	156.65747550889
GBF1	83.6080176270653	110.093465196133	118.854313907627	104.158950889938
	95.6182216943758	87.1735705257803	95.5117928427637	89.3868257841517
	74.3394153125605	117.634238804678	142.515628954457	101.2825552085
GBGT1	50.315768338317	53.7332797903102	45.310660704531	119.253559091088
	45.1137938810047	57.3797835448761	57.7805922926828	55.1933004802607
	50.6750785584772	53.0357938671646	59.4188882103773	54.1941919612524
GBL	104.059057437473	134.300169542594	130.10148014895	122.548989126048
	133.38594323309	123.908152883436	147.010700944686	79.7170181855209
	180.916465721523	155.123252685926	134.504159974855	92.084526929319
GBP1	295.69493803986	272.405201392009	164.262531918679	579.038638774301
	71.0204234759627	157.189139558365	172.95999061341	328.548716133094
	62.8635815439291	76.9603875266956	83.6351558609087	83.4679576894911
GBP2	1425.37639004849	1440.41726370246	1679.12630849964	1134.52018899688
	479.318513108472	1180.7204565402	1199.29782389306	2280.42953098693
	192.41551362197	2113.73880184695	1621.89443989657	1354.50256889331
GBP3	49.8472708021525	53.2046867327331	48.7039705048129	50.378948785456
	57.1935729219207	50.9724155054821	52.7002314230604	65.4714272652857
	53.9287524861617	51.3351387089044	50.7159667633757	55.1804535845496
GBP4	197.868575583033	288.023490151593	223.801865347687	292.389429869034
	69.2787430208317	163.299997237276	269.184000351658	558.855313555287
	103.869437847206	153.685754085722	205.876395674271	139.472956453414
GBP5	128.923092916744	91.2647023876304	70.8606919488782	529.977491403949
	45.4751076037511	55.9351706773613	69.3468840908665	80.2680308979464
	45.0997987980157	45.2556760873335	56.1379957867124	40.4639532172338
GBP6	38.1169703906207	49.4982025424177	40.3397581924196	41.4727452533295
	40.9416717019827	42.750861693377	37.8877750141557	39.2672712041774
	39.1790517315259	40.6022003765153	40.9683275553869	40.8681871019812
GBP7	35.1307934330047	41.2598458726002	41.9666541498638	36.6501141438378
	46.6397391251762	39.0266821712099	40.9157352260901	42.0494116081998
	38.6322728994674	44.2580151149956	36.131485826118	41.7575097298927
GBX2	42.0532901689036	50.543526404059	49.7350276169552	45.0798408927912
	52.6922486991742	44.4619248757502	46.9929836306816	46.9986083378817
	53.9287524861617	54.7827221235949	47.9942520395434	50.656598108034

GC	37.2474303302343	42.1607322031054	39.6451483836089	40.5484717678957
	36.4434672195785	37.9951997351435	52.8496345990125	36.7460922671619
	40.7847110517594	37.7315666632629	36.0481934423077	37.2810767789306
GCA	235.514830602053	162.000275136397	177.494605794577	1144.34555072807
	152.942332679485	275.611524861938	402.886581458688	205.806939441015
	104.626873082138	100.756165194192	109.386655134289	162.02498480452
GCAT	107.176733647834	98.5097700382506	107.161099000408	63.6334154351075
	259.332340491331	95.5920087912438	283.643027929014	154.482760264318
	97.4062608315831	106.418690759491	93.2484615907966	85.6964911093755
GCC1	124.22494803053	93.6717518147993	93.1349769600388	112.585860083115
	112.830467116678	95.3300728806601	91.7761668960824	125.197314240529
	152.441506175141	129.434864701718	104.076098789396	96.8141712830545
GCC2	44.2350046179299	47.5176439135793	47.4356803320662	41.1886975241176
	41.7395080432869	46.8436815733702	37.7136011474903	40.3662560853635
	42.4688861147508	39.3016855924751	39.2542963855807	50.3118072462443
GCDH	71.4276813927272	70.7003843117511	84.1895471533806	66.8475050031365
	181.772001042799	85.3718124658316	86.5564881117807	84.1639779987105
	82.5344501505114	71.4356132400211	73.9701365395921	67.8335962843732
GCET2	38.6454098044474	42.7261365779149	41.3749135519808	45.4634652319753
	39.9182914210537	38.1644569189523	39.7576136507593	39.1236645052911
	40.3704050658025	38.9074189954657	42.337073256895	40.9243125941198
GCG	47.1962846565261	43.2649781354792	44.2405728317107	41.8778873678551
	56.5933259268408	43.4681646416585	46.5650556481721	44.4031868028216
	43.0930293831497	39.9948390976317	43.3085358214154	48.3648494350286
GCGR	37.8386378737561	41.4508241771315	44.1452943804044	37.4223289687135
	46.9906120932727	39.0266821712099	37.6036146841933	43.4544762830742
	48.1174861108648	39.2623423164488	42.7275315121163	41.6550823405646
GCH1	54.931538325692	73.6323065529916	58.691436315511	86.6411972262415
	46.4775637378703	46.4648868771662	51.6731332961917	53.9060479865677
	59.1716224344299	48.2596564318071	48.5792058061372	61.0025829061063
GCHFR	429.65084326307	188.734299563005	113.287380238064	459.546279672649
	203.417430809331	112.60230393988	315.592187396219	135.259585457958
	513.266895252699	146.046179302584	111.874193363836	129.185046410316
GCK	35.6420502646498	36.8127605061228	40.7952353240579	38.0365987358005
	34.7542091589235	35.7608926435784	40.9647597231175	37.6126279002007
	38.1548766517774	37.920964499903	39.8184668132023	39.4888702378044
GCKR	44.0328992213727	48.6447083765118	52.9111155236961	42.0102671859606
	46.7508829977587	42.6575535390785	86.7968607218397	60.2691630545162
	43.5669306063196	43.0919221375514	44.2142581488029	44.5432192783161
GCLC	73.3950348855139	249.195312452368	119.124583755587	53.0284011078097
	159.248015095017	103.199200862158	109.726577835621	62.105802634526
	134.713725350463	142.34144664795	175.378964448101	177.019082240886
GCLM	136.786772972738	148.255880793905	120.771048750176	141.423857667761
	145.572606290643	99.6821042055758	179.522572239758	143.875527523263
	126.038716672534	72.0443425395404	79.6581393791238	117.76002524375
GCM1	42.1537476443423	39.8695929627783	43.401782064778	42.0102671859606
	43.3332476888305	40.5281496800812	37.4772922422734	39.9576138799997
	50.1603795873336	43.0919221375514	43.0971787292711	39.1553169409075
GCM2	41.3387790895716	40.716553247236	38.9790184198145	39.1706564265495
	41.8806343997257	46.3715909653832	39.8695929627783	43.5544347011646
	36.3649677956953	38.3092241935352	39.0550139257658	43.1805016690297
GCN1L1	132.838429478396	142.863673914134	114.224091812993	
	119.015431849218	162.569762535436	112.873697337138	181.772001042799

290.087040648659 197.617554897454 183.799353414581 155.942366087388
143.962643695789
GCNT1 85.8895787710259 52.0770287673248 69.4047473173218 57.4595416719581
43.1847628331565 45.6129956328415 46.5650556481721 52.0503062610092
65.564930068976 43.781101409552 45.1778228816327 44.9734311311394
GCNT2 60.8942621888353 41.6466169548846 45.1569741609597 42.6087443864502
42.7463525131552 41.9543948167375 43.4844452775022 41.413768254554
48.7720246202612 43.7707113107752 44.6708453138373 42.1957516560122
GCNT3 1167.25317435052 42.0709558258414 41.8751578329365 49.1786207920603
45.6895223823001 42.357910063876 44.4639405506967 82.5170409503063
45.7452456977857 43.2988017098387 49.1067959632109 73.9192243586276
Gcom1 41.9918605927928 51.6060290544385 42.5864346233886 43.6726153508978
46.572043843509 90.7101037998119 42.1335978926921 43.3696672906922
47.3316207658941 49.5265949137207 57.2777121216586 57.6549040984583
GCOM1 47.3791873993182 65.9574246586409 45.3974838013476 45.3882138838137
60.4122350478242 267.782994219864 46.7800033479223 55.0698932795613
58.9469577494939 64.8549209064366 93.3718038663074 88.2604063995148
GCS1 145.694779669933 135.540738841602 157.592292551459 85.9087280306456
241.599353653369 58.4435847520801 116.555666104916 114.533239859288
127.937436596431 103.001949900533 110.440072569721 112.585860083115
GCSH 54.7980088246886 51.0744829992368 54.4059109100338 43.8479689858951
57.6791750431251 54.3478800757394 59.7863401969736 43.4812166409346
48.6494451061637 47.0627306565247 46.5908542339034 46.5045747284981
GCUD2 41.4508241771315 50.3586212130973 54.2670850492947 38.0958223740111
54.5683518971261 43.4681646416585 51.0013344286851 56.1404433602127
49.3780606970538 43.844855336215 40.0766543123257 36.3454320848591
GDA 42.442231667357 46.5650556481721 45.4634652319753 42.1622837416589
43.004943518025 43.0144844590744 41.706879587091 36.9355798143571
52.3579564953125 42.6793753550854 39.9586075662602 43.0930293831497
GDAP1 42.6575535390785 44.5884811528117 54.3068448962404 43.074472921841
52.6922486991742 53.63657997649 41.9393838138203 41.3012916486748
45.1569741609597 46.441301934328 39.9586075662602 44.8372404608604
GDAP1L1 44.1294459089314 44.8529259387308 45.3974838013476
40.9607305725196 34.9062568194127 39.6251842352088 43.6753843488695
42.2577689927563 44.7058233759912 41.9666541498638 35.6970528713709
38.6454098044474
GDAP2 83.1614238528477 79.2185250088688 70.0059240383657 78.270329450618
78.9088042058577 56.7429967489965 77.4046818339539 83.1702007661831
88.5564369981649 78.839411865787 68.7091708087448 63.6254894053045
GDE1 176.341212868114 244.907057340556 235.315897087189 169.144145421022
173.357715186701 203.497070457832 230.444295657577 275.811139268285
207.726011653345 240.217589414972 234.8262403783 301.025314855652
GDF1 41.7397731376692 38.1713217245067 37.8386378737561 38.1392356671704
42.0709558258414 39.0648733099061 41.253645578517 41.9315419791594
39.8984580390722 40.0599807618105 41.0808257270221 37.5236834932416
GDF10 47.9175508887533 34.9262464079777 33.6816578730693 41.5464765641928
40.0599807618105 52.1024135605001 45.7452456977857 43.1567024035183
40.8970401536578 39.7835513496873 42.2618615571566 41.863927676552
GDF11 33.7777950030073 31.6295801599123 31.3901365951213 36.4564050256915
36.3390947880186 31.7413650507468 34.0777108441756 32.8834169462007
37.5469247328848 31.3901365951213 30.9159908723475 35.9604262434141
GDF15 684.865133028228 3838.19449971537 175.056828374791 157.518879763075
1468.75291542154 237.974766880957 1237.93130196998 3490.63949257581
2018.28984520892 2663.19221471348 5047.91205452316 2650.09507972593

GDF2 41.3387790895716 39.1982470626863 43.5710871617107 43.074472921841
40.0599807618105 40.650742261428 40.4359177791566 44.797320243931
38.9006788676407 42.5664883002833 44.2142581488029 44.6484005362281
GDF3 41.1523487516364 45.3486182278177 41.9666541498638 40.8681871019812
49.5666074434383 41.8613406887824 35.9058393060637 48.5681478665796
44.4395580493118 41.8613406887824 39.9899430628542 40.2669497398702
GDF5 42.7150227340364 39.7835513496873 39.7411455670172 41.0726336841311
41.8806343997257 39.8455060654531 38.941855825269 37.6557714476853
38.0213051227859 40.6022003765153 41.6336636285875 41.1554481567603
GDF6 43.2135791259317 41.0678614026819 42.108130037421 46.8954576545929
41.1868052124344 40.8970401536578 41.5464765641928 42.157627240486
40.7460990419215 36.6396388746561 38.956280281655 38.0452862258803
GDF7 37.8386378737561 38.2263227947189 40.931294690669 41.3027031037343
44.9936360641969 40.040778349137 39.7576136507593 46.5717043290731
38.9006788676407 41.3321861371202 40.4504608195666 43.9541718254769
GDF9 44.0328992213727 40.148780650831 41.437237454134 45.0798408927912
46.1804647841331 43.1826328292558 52.2476631126873 48.87875862356
41.7627910693483 43.2988017098387 43.6629145624937 52.4992300612807
GDI1 228.054565950253 228.571334092678 178.400810392873 208.30969642805
228.328635242684 208.7739716139 318.768560912915 334.90764380842
134.152059562322 365.401746744015 356.948763043851 237.401487995882
GDI2 735.460645553438 522.49156295512 382.940842652744 590.709061039001
562.258108316474 492.13077103258 534.755211286733 666.265306908449
731.071171171638 524.047008889711 473.688869614777 592.989860385203
GDNF 36.3087372569546 40.7568164083581 41.9335763813389 40.3589492801501
37.7115233202949 38.5655198999985 37.8933603299805 38.610211769318
40.7493885015617 36.8059561571675 41.8613406887824 36.5474283500601
GDPD1 86.2021418088902 55.0897019602305 65.4137646609535 72.5956443098684
72.5559426064896 72.4651758215622 78.7182388025668 127.87945182399
64.6748464612845 121.236881661832 119.872031126474 90.6191520659197
GDPD2 42.6036491382658 90.1632837282491 130.533514998688 36.7892997857376
47.5647821785529 41.7627910693483 43.3262148375525 41.3506832276112
65.564930068976 43.844855336215 41.1638563416981 40.7704587752083
GDPD3 65.7259084183743 103.289476128549 63.2936377704897 44.4954892619459
53.4751250621694 43.7078399718247 91.3144101155547 339.313853828248
60.7942162859036 51.0055825931636 61.4800543360853 51.5338331947847
GDPD4 40.7609952437622 42.5269793679335 36.9795961564828 39.1706564265495
40.8873907150245 38.6414316877471 45.6042625176633 43.6434576713809
42.5406888281114 41.3321861371202 42.7275315121163 39.2469742642337
GDPD5 117.612004923867 51.7495306734657 65.9476039189301 89.1290937615998
45.1137938810047 84.3755392205436 52.561573697155 81.1284422173774
51.8083208406279 90.9570747667407 63.5195937348531 67.1675878355908
GEFT 41.5421429082231 43.4681646416585 37.8386378737561 39.1350512687938
41.7503755215516 52.502055890816 44.3440839686558 44.5029690493211
43.3165374444543 52.0231832690887 46.1364790186809 47.2131144508464
GEM 112.721024337362 71.5726787111921 54.2059913044076 94.9785931907153
70.8288090024328 592.989860385203 41.645120086609 114.623641209561
41.0381860307445 65.6165636276827 74.7669841575348 163.742560819575
GEMIN4 305.199674259628 331.390452289344 294.7772562683
184.087380444203 510.379313524019 230.09336914921 419.054753590648
292.389429869034 210.166909564076 330.096171705212 321.686871145301
258.375785708747
GEMIN5 64.7766649630041 48.4980626712175 76.2533853403813
58.9168466928565 85.5362566454799 79.4053001255904 61.2183034191703

66.4207938873541 70.8606919488782 63.3872214856708 61.940656536312
70.5322939116406
GEMIN6 104.704892803169 89.1382266158262 128.327641457403
93.6212502203405 213.590067519021 105.414552529943 182.079641528208
174.584330660466 97.7114610828769 86.8691342571214 83.0072253231162
128.230543301378
GEMIN7 40.5235471987417 38.6414316877471 42.3536549114165
37.9230440150891 40.3021896270845 42.808889470973 42.22763273471
40.188873541274 40.0403898620771 39.5637454855233 40.4504608195666
41.9083581529276
GEMIN8 64.2322994035968 67.4477906495278 78.6402083787251
43.5627649099623 50.5707478277575 47.8029841385514 80.354283778818
59.3923286524971 64.9030399249327 74.6847594297153 72.4325984501758
71.9771425105735
GEN1 40.8091178323174 47.7246924920997 40.7620472083159 39.0156305603334
43.9450023643394 44.812853905056 39.0788811357542 57.3286965007828
48.5136862664526 40.8970401536578 36.5675023356094 43.0930293831497
GFAP 37.6397652536077 42.6022652963326 43.9450023643394 35.3829784221072
39.4693871570028 39.9397419279057 39.1539892144371 42.357910063876
40.0403898620771 37.8217989841918 32.775958132716 42.357910063876
GFER 34.8099089553046 37.2810767789306 38.7745734037492 31.8431345631453
35.3327761997046 33.4492509999281 34.806104801006 39.3709357615493
28.768101860452 34.4875013487777 39.6403089864678 33.0217499673686
GFI1 58.8224410806741 57.4538469832241 51.0539624358342 68.1992221684165
37.5556299264699 41.0582900711341 43.5627649099623 49.085741080241
41.863927676552 38.1042454705119 50.5810338885769 43.9541718254769
GFI1B 47.1962846565261 45.9266745571724 45.1569741609597 36.6501141438378
39.9697332660833 50.0748292497578 52.2476631126873 47.6952179922944
45.9227107476843 44.571915452417 46.824299585607 41.1554481567603
GFM1 194.395766807005 187.086662645246 190.675425748381 185.924923239764
496.163796158189 248.563278344035 189.80672643731 232.147792636393
225.960701908311 228.778506556448 287.494740260715 281.602712448925
GFM2 45.8108990837624 63.8399092891728 64.847834518734 51.1313686723929
56.6557577134933 60.9822577063534 57.9583667603818 48.1591264215184
51.9545473262296 45.2023300009435 43.0710577955487 54.4644832805303
GFOD1 118.05982513603 60.1685144469519 104.557637353314 202.578421978121
87.1109435144663 199.294913752704 40.9157352260901 95.2915739972533
79.1576305054224 111.248876636045 150.009352890706 117.052403700291
GFOD2 168.288592321667 90.9570747667407 106.058751335133 49.3654837541353
85.1661952927159 65.6914955077809 130.211586013657 288.597205372736
78.055431384988 83.5069968129733 97.3188285021124 70.5322939116406
GFPT1 263.21493129287 172.285394357607 126.485649460666 115.132600341395
144.578232267116 103.64431747876 164.312946712409 215.68113524702
139.768680518677 98.1835264890769 94.3872450727268 136.998739859195
GFPT2 167.578081645481 45.1694246393676 43.5710871617107 160.973602503707
55.3100442506299 540.721068846198 50.6774883370457 98.2074490157865
44.619512785899 101.197890058764 93.0801307192343 239.885284409329
GFRA1 40.3180310664156 41.3177250076876 42.381482941987 39.7938220956242
40.6723334103791 51.8762098404619 42.3360569471619 42.3982765397961
43.1087085278313 42.8304701368556 42.7815195866158 44.7915315085948
GFRA2 67.3816715975056 83.7019398654324 74.6901522573522 91.0346886951891
75.6471706262645 181.586195566181 77.7615826856723 89.6313467455698
58.4891791448514 84.4349803529132 83.3729277601518 80.4404044229341

GFRA3	47.8249696958403	40.7952353240579	48.1331309338997	58.0218262176065
	113.367264677544	75.7502062965698	132.24963384417	132.3192754634
	64.4588422949237	42.5664883002833	48.2910530887214	47.5025821041537
GFRA4	50.2203578628217	39.6493706695391	44.7255207589606	42.4061338151892
	49.8865955311391	42.808889470973	44.0687697278114	45.642304356051
	47.945438269111	44.1861590259178	42.4580504587581	54.1090536870282
GFRAL	36.9060997041453	39.932537801639	39.5954353681031	38.042028333747
	40.0599807618105	40.7616966228404	40.6750540874299	37.0932890050788
	38.6510172759977	41.8613406887824	36.6603338274678	44.1063440402552
GGA1	3379.67054115975	2934.35616793443	2911.10227993347	2779.37660132136
	2083.69812106867	2054.64068255575	3234.36469403715	3690.00752647453
	2225.65559724391	3421.24021192326	3618.78958408675	2921.68921750799
GGA2	124.866058211181	154.775578306203	160.973602503707	178.156332261759
	130.002385558757	120.231435193839	79.3861418060845	103.856444152519
	107.808744445896	252.347641176529	161.326580647514	125.512567429299
GGA3	63.3518043114428	74.9294208313176	76.8521423078922	65.2125527940945
	82.4164506744	63.6894484284172	65.524821699432	54.0507720792049
	74.6590272639023	81.0214543140606	82.8205269728224	69.8505620106065
GGCT	388.920739791844	218.273094883881	127.789065386943	90.0942781313264
	233.07404514708	132.473918053428	254.322611819303	430.283061862756
	205.772128732705	91.9919654450652	125.07621933584	129.014076422249
GGCX	80.0113001016496	95.7639712596394	116.295564219236	69.6511571901941
	99.6821042055758	67.9306669919176	97.8309399470621	94.1762630490912
	118.762101740563	71.5948944013406	87.8967913380047	100.120990656033
GGH	100.159565926265	186.891912776245	104.076098789396	102.605473054584
	131.156876010639	52.4351670989268	194.703024752864	160.117375210168
	239.792011765323	54.5641201866931	52.3304586399695	48.3119523820103
GGN	72.9154861179393	44.2630383225933	41.437237454134	40.1804147506786
	46.6397391251762	40.3357777530419	43.9450023643394	42.2577689927563
	40.8970401536578	39.7835513496873	40.4504608195666	41.1554481567603
GGNBP2		447.709872889701	454.402224020514	426.933064567744
	408.991719312986	425.435793468733	480.13416648493	524.972936438175
	388.336994961328	537.471287449696	583.053232396803	585.771656887036
	571.790034754076			
GGPS1	119.502912878938	116.663501139305	179.378838766172	119.39177524986
	131.766252431636	169.554408876006	116.080897948094	108.025082988807
	145.75465017845	118.027323513177	111.501058276821	159.618353114269
GGT1	47.6933206236304	53.1699994703719	45.2689965528388	55.9927099883616
	44.9272279146193	51.294537308199	45.2759158146116	41.9741368971524
	46.3426705495666	45.731999237426	46.3607182640185	42.6350119662243
GGT3P	48.1952521496489	44.8529259387308	42.0621425894624	43.1847628331565
	39.4693871570028	40.8634043028528	41.3594868055879	43.4544762830742
	40.137818555383	38.0078357808393	43.5710871617107	41.4508241771315
GGT5	49.9419897484381	44.0409178714232	48.7039705048129	48.309866878365
	50.3713170502557	48.7781962164988	44.8643898647322	49.7309743973184
	50.1603795873336	51.6778366958973	50.1649089793069	51.0013344286851
GGT6	210.437554813031	388.580556899321	170.619111196551	44.550322123994
	135.41852974791	56.1671440556142	268.613247119583	201.427008153788
	302.083293019113	681.474524031866	414.539381761279	241.599353653369
GGT7	36.0144420013819	49.1283590719461	51.8122261766744	40.7481155008596
	49.0828761839116	42.8598805316863	45.4799270968969	43.6434576713809
	43.5669306063196	46.441301934328	46.2560238004757	47.0130468198828

GGTA1 38.5007204246293 35.1224289925572 39.6451483836089 40.4359177791566
35.7973685350741 44.1737502492846 37.9430145836712 40.831207953647
36.4476883897928 35.7202909649682 35.860430241131 40.8681871019812
GGTL3 40.0557788909923 49.9338905397767 52.6275131390249 42.2496337895968
50.4260283682427 42.808889470973 47.0874209746031 50.628152951493
48.692302914163 56.685925255591 51.7538628098347 52.0359304386476
GGTLC1 43.1180113655693 44.2391871310123 46.6025757239946
44.3425264236664 46.0553319515625 42.357910063876 46.0905079010237
41.8417024632349 40.4544974307172 42.785429206422 47.2134341420411
44.5973052166071
GGTLC2 41.2424637338424 40.6380495994768 41.2365089689635
69.6511571901941 44.4489662507893 47.0940613507321 48.4859552186772
37.9837233601316 37.10170285405 38.6454098044474 42.357910063876
38.2350720635382
GH1 40.7609952437622 37.0390589468421 40.7222199182322 43.4410741327254
41.0885562160946 38.8336427948187 43.4885245598939 41.1575777497576
42.4540072551568 44.7958122466628 44.1148826235374 42.4229201717624
GH2 40.0557788909923 42.4642390990506 40.6312687664188 37.7146582037126
40.8873907150245 41.809704036355 39.6229463459737 41.3945375181463
38.4970433581463 45.2556760873335 39.6403089864678 43.5420658412177
GHDC 102.044838876428 96.5102825310977 84.3755392205436 97.8309399470621
116.362441774156 108.493693862509 113.692418102755 107.094663242259
139.905295316081 187.086662645246 182.866502637283 90.1632837282491
GHITM 836.472724085903 1180.7204565402 1060.64261592822 1296.28036613191
954.116675685628 1673.60994518271 1450.53108586858 1050.05355467301
968.405745860353 1387.28483887704 1273.52492984569 1839.09068171097
GHR 52.0299937917925 42.6022652963326 40.4882741869718 39.3750969348308
45.4751076037511 133.96497573455 38.4005795891396 54.5125550937898
49.5539622682049 120.60052672409 92.2772139468085 62.7669079605627
GHRH 33.3307770183871 42.4493032770053 41.528370959196 45.7397504535169
35.9058393060637 41.3553369544097 35.2163900400329 39.2672712041774
41.9635525160083 43.2988017098387 43.4709415392696 44.2527566343661
GHRHR 37.2865372090014 33.876326795925 36.4098694708483 37.7469621225553
35.7112361794431 37.3207811788937 36.3194282084339 32.6817359372544
40.0403898620771 34.6720534245631 32.2989067914223 38.4741160538511
GHRL 46.3390850717989 52.1062000766427 45.8844325913247 58.5406981871153
39.9697332660833 41.3553369544097 49.540449125788 41.5464765641928
37.10170285405 49.5265949137207 39.7342718293432 40.3691324547049
GHSR 44.0328992213727 38.3874422458351 42.157627240486 43.8002456102021
45.8669209716694 38.6414316877471 38.1793598983615 41.3012916486748
46.0633179231318 43.7303813515864 39.6833800000587 44.1861590259178
GIF 37.2474303302343 41.554942319225 32.7043743065388 39.066284740632
46.8674366115656 42.9695585740813 38.7337086062875 37.1317006162938
41.9635525160083 40.7063426028871 38.079222289614 36.064718696392
GIGYF1 47.2862299990938 47.3536182445315 45.5883690965192
42.1273928203021 46.9906120932727 41.6590564937735 39.6535880152834
45.8669209716694 40.7847110517594 51.9547984751408 44.7114813656401
47.6040671292013
GIMAP1 38.957687788868 40.5400384479766 41.437237454134
42.0102671859606 46.1804647841331 38.8336427948187 39.8695929627783
40.1474543172332 41.863927676552 38.957687788868 45.6348103033479
47.1060577218993
GIMAP2 45.3872406432794 42.2900457798748 46.7800033479223
58.106070377898 37.6527901109005 41.5132248356707 45.223075311113

40.5158867964016 53.8716214229685 46.5650556481721 42.5426057453993 46.7182294568603
GIMAP4 257.993462737264 223.397586938079 307.002236094206 918.083610591772 130.515102593803 365.459728613746 108.558977253184 134.085457101528 57.9713749600418 192.240593720489 214.484877083166 167.999042735556
GIMAP5 98.4084364053686 91.8207081349104 97.0516272072238 260.824949424185 58.2780916793348 111.939677225005 41.8315602975157 60.9009676344258 45.0997987980157 55.1089864632456 88.6460475038549 63.7744603935682
GIMAP6 57.5685357492202 75.633664798698 67.40251451399 123.493308132159 50.7116312517035 77.8490019489375 44.6809101024392 45.063792968927 50.7258407289028 67.1278109419819 69.2787430208317 70.2021753365478
GIMAP7 49.8472708021525 68.1040271620925 56.9958049366454 134.952392466784 48.5000946421718 72.7934987008399 40.2601451506332 47.5439485437351 40.0403898620771 67.9922604709859 79.8701900696361 72.0334593545916
GIMAP8 74.3766944386886 84.2880616879945 94.2275161759277 182.219366179899 58.2780916793348 117.250114037308 47.2927436772465 60.1796637353976 47.9035532886869 75.0665411373616 90.807818984128 71.0340758556049
GINS1 37.3463875295726 39.6995529770239 40.931294690669 34.7266486944713 37.7576900881741 34.9849896310235 42.0605906240248 40.931294690669 40.3316153483289 39.6719190054379 36.5359455830388 40.9683275553869
GINS2 170.223657280542 137.551493752877 165.613176704286 49.5424425850549 212.688298502868 66.7278011183421 468.98857415608 156.276838862671 386.372142079981 55.7930787690756 56.6884575165011 138.972884942374
GINS3 126.253578620746 86.8262463672891 142.262349841307 70.7202372217584 71.8706847571669 74.9006142438341 160.117375210168 95.9391851122771 136.644759501653 83.764882413379 84.9601800557888 98.7331384143685
GINS4 87.1735705257803 104.650071846586 68.0649030501515 65.7321440516177 122.904323796484 66.7278011183421 161.929470244265 413.41645422004 69.4677195088043 53.6429962450784 51.2318935933116 56.1136936405786
GIP 50.1533437545239 41.159706131351 37.4642807402741 43.1847628331565 42.9090346219023 42.4580504587581 41.8315602975157 39.7509165247419 37.5469247328848 41.2276963324981 38.079222289614 40.0557788909923
GIPC1 568.371815312895 712.80113322987 738.66912772732 195.635797217403 667.583623414832 303.239819890428 529.210992541911 759.184121753305 858.975085087497 592.010988342149 644.924175130155 425.435793468733
GIPC2 41.9918605927928 39.3999056968598 40.439898672334 40.5858746708995 36.2048942610546 37.33030683349 38.7745734037492 38.745552403895 40.5912213523345 35.1266109638107 40.4094901248527 38.5921548841176
GIPC3 34.0467249875782 39.3999056968598 38.2280071951674 36.4564050256915 41.2740822675592 43.1826328292558 38.4005795891396 36.7460922671619 37.2810767789306 40.6022003765153 34.3633615460307 36.6603338274678
GIPR 37.8386378737561 36.6968906669565 42.9104454767916 35.6928955245309 41.7929491793697 37.9003288112261 38.4005795891396 41.8417024632349 44.1519466943185 49.1928206164702 38.745552403895 36.9976231206067
GIT1 72.4094549259929 70.5171002055327 77.0301627655229 84.6435656850129 85.6236091398006 53.565717255469 100.756165194192 127.008218054726 101.103458852775 103.001949900533 79.5743379965984 58.6481644777552

GIT2	62.2150049214174	62.3983214098965	69.303129494807	91.3475300950304
	64.6374507338031	64.720130807316	68.4380822448123	48.272324215504
	69.2001494999588	52.0329620305531	55.2152456171699	63.8949010662614
GIYD1	79.2665546280232	85.0308877626746	149.185115531381	110.25582862843
	121.696160351886	71.0369238830779	81.9504925867759	179.874652000664
	98.3158011081832	87.7918574811458	83.0554837248934	68.188634555556
GIYD2	46.8954576545929	44.8044806324595	60.5390917151449	46.546116650292
	49.8304479381623	43.4263583096036	41.9918605927928	54.3820590274321
	49.0986627051784	42.2614524730856	44.6286581923295	47.1060577218993
GJA1	613.50943414129	109.844156801015	214.927272114101	181.488068017883
	241.599353653369	279.521995263196	119.905655993777	167.746168796779
	434.223722811041	380.736562250846	371.709014310561	670.271164195182
GJA10	39.3709357615493	37.920964499903	41.528370959196	39.066284740632
	37.7576900881741	44.3648993568405	39.1539892144371	36.5393857306744
	39.5806465938526	41.8613406887824	40.6373400186325	42.8864415494484
GJA3	41.8409927585705	41.159706131351	41.6977706798234	41.4727452533295
	37.0526522140242	41.0012755104374	43.7585538437696	47.7508139534968
	38.1169703906207	42.9358255168086	44.1533327883807	41.2905987859365
GJA4	81.9258201510475	126.253578620746	201.163076338483	108.330778160202
	84.8751572193902	88.9738853594498	60.9648845690177	87.9468208580228
	48.6494451061637	57.4665857273093	53.2083147523758	63.7744603935682
GJA5	53.3059058105836	46.3370338074996	49.6347864008749	41.8778873678551
	47.2862299990937	44.9896851005259	44.1407835440612	45.5842286511645
	40.9512974748357	42.1317134987795	42.1537476443423	43.3744503319109
GJA8	46.263826584899	39.6995529770239	42.157627240486	43.1847628331565
	43.7517118200398	43.1826328292558	44.8643898647322	38.1835938174625
	48.5792058061372	45.1569741609597	48.9754340235127	45.2783024981315
GJA9	40.3478825593224	40.3384318592985	43.3085358214154	35.49696651597
	39.859345745831	38.5252456306656	41.5817737564592	35.1738399826814
	49.7336769474446	39.5637454855233	45.1432412071268	45.6692241948108
GJB1	42.7150227340364	39.8695929627783	38.4043916290561	44.4367387274922
	42.9090346219023	41.0582900711341	39.2533668167483	39.1652965788406
	41.7627910693483	40.6022003765153	39.8184668132023	41.2437378488025
GJB2	1223.01485352248	550.642253702646	1718.44944271916	172.39907939484
	878.863554510943	127.596368103014	208.97126697486	277.164276391513
	530.996217072759	83.6524523974491	73.4853298204744	231.637751095711
GJB3	59.4346711476105	56.6146179789815	69.11736279512	40.4359177791566
	55.7575080755355	40.5281496800812	56.5914743235484	51.213375233854
	84.8443836237906	78.270329450618	56.9733113918431	57.9941756686635
GJB4	48.8941062980393	63.5932443357808	56.1434024065079	36.9912468186322
	38.6454098044474	41.7627910693483	32.7676164492555	45.4757965005055
	44.3440839686558	39.1621898366355	44.1148826235374	53.840976437122
GJB5	41.5421429082231	40.8880530359822	52.7005092555102	36.3604951341921
	33.143488395779	37.9951997351435	40.9157352260901	36.9355798143571
	40.4997186069494	34.3674468322242	43.3085358214154	41.3481355128089
GJB6	51.8122261766744	264.407051461506	338.586852166449	37.3600014814388
	303.670126704469	70.5025097213784	126.389908470189	89.9227845135785
	246.351606918353	58.2167393576361	59.9199304173784	79.990130381874
GJB7	47.6397712007919	64.9959712888709	41.9666541498638	37.5382094267692
	55.1563703466328	41.7627910693483	51.7714254834195	52.5909491535773
	54.7033747046992	39.4710743516517	49.6706535876636	41.3481355128089
GJC1	2803.11698341125	1634.61795848352	2357.96534075689	2354.00192115549
	1640.74027400791	1208.33498233518	2545.03032507597	3562.96193568469
	1585.90718459092	3379.67054115975	3244.27810937143	2663.19221471348

GJC2 70.8288090024328 61.9459245072385 165.992057520727 91.0346886951891
54.4586176399381 229.745018977185 43.4544762830742 64.4156641487994
44.0519245668737 97.2865629929716 126.375315221007 103.233768723364
GJC3 37.6397652536077 38.6414316877471 37.8386378737561 38.5252456306656
34.9062568194127 45.0882356578404 35.8227939208521 41.3506832276112
46.3203995372658 35.8304004211528 34.174642681781 40.2669497398702
GJD2 35.2267149016596 35.3307157989724 37.2838080935695 28.6786952763294
36.9702228618069 38.3418933808844 37.3799542961839 40.4359177791566
34.9428206949566 34.1648535095605 35.046946054314 31.1733235029813
GJD3 75.2475238554276 381.6928205836 98.8595497262968 72.89602312859
67.0163226819964 59.3618843678775 89.9284109077375 62.4219686957673
82.0081006332668 64.7269156363245 83.7392835102145 52.5752569429057
GJD4 39.7576136507593 35.6434124419782 34.8956628705329 37.7469621225553
39.0564222526273 40.1428591998404 34.5633623842083 42.9301944191913
39.6609238036815 38.0078357808393 40.9157352260901 36.2582325396375
GK 96.2248868829355 118.923419920947 119.979854891563 1366.59129560817
104.754762973117 84.1922656126304 209.658186168638 192.356440003154
129.754481922118 49.5635427311883 50.133362781069 53.840976437122
GK2 102.513192587614 87.0685571351947 93.4180535865914 108.025082988807
81.7765447802116 89.680151549258 94.3537052300317 103.366147685418
96.2385885003071 72.834275688835 87.7844808613265 73.9192243586276
GK5 54.931538325692 67.8208976798364 71.0340758556049 64.898351831341
88.6368226476614 66.295597720304 59.5205055437765 67.8061298261394
59.3580517977711 71.5032996449402 70.3381994440425 78.8968326770219
GKAP1 37.3463875295726 46.0023068797812 42.2577689927563 39.6292788039793
45.8051846088552 45.8325181856483 50.3519150282938 40.6171774724171
39.5806465938526 45.7911932481722 43.0016969050339 44.7401523707775
GKN1 42.1537476443423 62.6236991757723 39.0775870687758 34.9221408381038
46.7508829977587 41.8613406887824 37.6036146841933 40.2382908228517
41.074824727477 139.414044294473 47.4630625313159 224.450710664296
GKN2 36.8072833828297 43.4396866157171 37.1643693306382 42.5664883002833
44.5668141895992 40.7616966228404 37.830876898596 42.9301944191913
41.4636047919453 35.6208308220005 45.8180710486329 44.1452943804044
GLA 274.134201700259 734.705859548078 139.08163074096 1294.42501356365
415.251775687794 208.7739716139 270.89711217076 540.721068846198
228.469654751393 183.311305255895 167.05554959607 198.129308943666
GLB1 229.379918828848 434.223722811041 388.132295362791 478.110586929303
588.064524478384 258.120500906788 352.917938567734 301.941928552396
516.349618186681 233.72882333861 178.249889756228 262.534449975056
GLB1L 64.0759817636265 46.5650556481721 46.4569566520107 77.3864518736634
44.147557390293 57.2767879969284 52.7002314230604 49.5185112777773
47.27543600687 59.1496467460507 54.0374522273907 58.2879293130296
GLB1L2 161.573327074375 66.7256057011273 86.6186787663248
46.614092935749 135.737771384104 71.9329028989527 63.5738862549306
174.416924448198 72.6675345946258 83.6524523974491 61.3529667465225
75.4053820110486
GLCCI1 62.4317959077388 71.5249259772583 63.5107036952143
121.204795052893 77.3931945683613 53.2046867327331 52.0942589368478
56.6910078150745 70.2541961995753 62.1035858593388 56.3106156221316
59.7999569757275
GLCE 106.878946016737 71.6537776654915 63.3544794716167 103.139459009593
124.903031106573 107.148000219752 85.5027511448958 115.577618925646
143.251956814524 127.49459343016 131.319040057576 116.927619635255

GLDC 44.6231541342 49.4982025424177 39.3709357615493 50.8567345921473
50.2163892855092 36.3845892764084 87.0685571351947 63.3768687067745
47.3791873993182 190.375732394043 197.040881690021 137.239944506217
GLDN 50.379587451669 54.9392038576957 54.3554233228353 82.2766822827861
43.1252272136667 50.0748292497578 55.4004611309636 56.5914743235484
42.5406888281114 218.056839407813 119.070289990926 114.488672372067
GLE1 134.62138052303 151.740176274202 90.6516730646251 111.186223357396
96.5615807039306 111.404796541459 142.172292334093 105.822362552251
106.907589984092 119.015431849218 132.679840262941 117.934830767297
GLG1 1115.27580736237 631.632335640448 999.496354673223 797.019657306322
767.921811623919 663.372296297652 461.394007153992 1093.04440713629
884.036280777358 962.079396754147 841.316771679857 663.904584382358
GLI1 61.356483470053 54.2445399731644 66.0411740221276 50.5310598656998
72.5027069303875 55.9351706773613 78.1623301184313 54.0507720792049
60.5608545741456 69.1623287497421 65.088713927995 57.0524725494273
GLI2 45.9090659324223 43.4396866157171 39.3709357615493 40.1804147506786
37.6527901109005 52.7156971154405 38.7337086062875 44.2159020605784
42.2577689927563 47.611018474151 44.553780984244 39.1553169409075
GLI3 64.7766649630041 48.4980626712175 70.2577825186943 55.0604256312495
44.9936360641969 74.0995052892511 39.1539892144371 51.6660481331154
71.1894129030602 80.191847692094 87.3342335690393 66.295597720304
GLI4 52.7570461169969 79.5838979704243 101.131391476546 47.481302504066
92.6736029146947 62.4450952595811 56.4809146784765 59.9968592852122
97.5394336319916 81.1925839235551 77.2834482217973 65.2125527940945
GLIPR1 92.3659612653822 68.7826057564876 60.5678281953819
337.441212908704 94.6432868887401 130.02102880365 57.6643188499117
74.8893925448138 51.9545473262296 57.5713378330876 57.0154839296649
72.0334593545916
GLIPR1L1 43.3638918705225 46.4385386401326 46.9040704638073
40.646702696033 41.2740822675592 43.1826328292558 39.6535880152834
46.2779564481158 42.4540072551568 45.5776159432079 44.8872784170036
45.0985088502363
GLIPR1L2 40.0557788909923 40.6380495994768 50.8303921211581
47.0874209746031 35.9894133432376 38.9378717994178 40.4359177791566
39.7509165247419 40.4997186069494 45.2556760873335 40.1742127891824
43.6753843488695
GLIPR2 574.14982128922 222.01145815859 235.047133588022
1645.71527614542 169.693594531146 1050.05355467301 369.451882513347
415.477167615547 115.954295099681 790.786577596971 610.502562878788
474.379113836506
GLIS1 40.4639532172338 42.9358255168086 36.6522278218553 40.1804147506786
39.3667161654772 42.357910063876 41.3594868055879 42.2577689927563
42.9020321355927 41.1222709786153 40.547835538778 40.8681871019812
GLIS2 38.0089018744739 41.9894412416874 38.1421709520616 39.7411455670172
44.2607789091176 30.8824580894159 42.4939802311136 45.3872406432794
40.8970401536578 44.6918600441328 35.046946054314 41.1554481567603
GLIS3 52.6554629843702 38.4866431306822 43.8249538325648 51.1470623296569
45.6895223823001 45.1873487463472 57.0178321687063 64.1675023456571
44.206798768753 53.4751250621694 55.0024069676601 56.1875519519366
GLMN 97.5464355320239 80.0502531022254 74.8071157013401 61.5515639412892
92.6036872789676 77.2048580540589 93.9472621843566 113.528908699366
78.3082773071531 88.7461361855771 77.8669914727921 78.4641751788842

GLO1 1031.94541988402 1164.71243740122 809.577874019028 575.057786209737
2039.89728774097 1114.7177106695 1369.13346566895 1004.28134410536
1050.05355467301 527.97309138633 600.34379987143 1051.58050264114
GLOD4 252.870708814507 269.963186760811 237.754990288051 203.152852313746
209.281870980564 237.632703283663 301.941928552396 233.07404514708
230.73977644867 284.868613843826 266.439343650315 276.267583952979
GLOD5 38.7537364570873 37.10170285405 40.301483914446 35.6928955245309
37.2567512265363 38.4330755397458 43.1931731665131 31.5766875248365
35.0305278582179 40.1179210534118 45.310660704531 40.6614700944853
GLP1R 39.8662795144616 44.5152708955623 46.9040704638073 43.6726153508978
44.8897737954573 37.4782441311626 41.4742337714895 47.6952179922944
42.0621425894624 51.2318935933116 41.8168470823451 42.57175366709
GLP2R 39.9697332660833 40.9731067463382 39.9101908547397 39.1706564265495
43.7517118200398 56.9320717885002 40.3302268293707 36.2539950294089
43.6670755115848 42.6793753550854 37.9230440150891 39.3575468494941
GLRA1 36.9060997041453 47.765848710116 38.7745734037492 45.1944222548351
41.2740822675592 38.1169703906207 41.1507980856436 39.2672712041774
44.4395580493118 43.7303813515864 45.2265319826548 42.0424641526302
GLRA2 37.3463875295726 42.0709558258414 36.9795961564828 43.3078542962968
38.9496515344837 38.4330755397458 38.941855825269 39.3709357615493
39.7537274655115 36.6893251782922 36.2435874904689 42.7782216342806
GLRA3 41.678190413467 38.7344853699576 41.4613628725541 39.6833800000587
40.7779767565889 41.2098211956301 36.5729485312886 38.079222289614
40.5912213523345 43.9450023643394 43.0971787292711 39.4830039788612
GLRB 63.7818442618767 47.5647821785529 38.9790184198145 45.0798408927912
45.3675390992851 46.574682061365 78.6402083787251 45.0972184964705
62.7205631511304 49.8393069675192 51.0492389286271 53.0446815750355
GLRX 96.2248868829355 78.1623301184313 153.940127525369 407.063375756017
62.6860949544223 141.043326064913 67.6767845246581 114.342447939197
59.198088069573 81.7171463878604 78.1351673826829 135.012695962236
GLRX2 58.8987159047296 63.1394716239975 76.2193772627406 69.9101676350298
72.983338488605 64.4156641487994 77.1521355570024 53.838484549134
76.6027293497456 53.8803543493797 59.5830868881343 64.863894209343
GLRX5 1440.41726370246 767.489657259345 827.365231733511 573.394745674984
1416.47763911039 1204.34388579291 1027.59400018637 1372.51401036753
1291.71781703042 787.671342652389 885.224377292125 905.76958446454
GLS 143.084029849218 93.6717518147993 112.159199477006 146.573018487871
63.686652234709 100.650129064849 60.7992483126292 77.0262811825953
95.4942268742467 143.053774297331 112.881899194726 179.756604242873
GLS2 44.1737502492846 48.6447083765118 45.4634652319753 40.7481155008596
44.6202070855297 40.4753617660107 44.2166118829877 41.7877325563821
55.1804535845496 49.5635427311883 43.1724740315182 41.7575097298927
GLT1D1 38.304249186058 41.8848959280916 37.1643693306382
35.8472302065546 43.2484996523549 37.1317006162938 34.9344532055397
32.6039814489621 37.4540772310118 34.8137085420423 37.2892641289258
40.2669497398702
GLT25D1 913.704049709264 444.858305070415 565.72800641089
868.899376368472 1362.12587130194 588.972273484465 461.607803139448
549.54346533588 429.854222129819 428.206419080492 440.351682914344
343.94159167652
GLT25D2 42.3433931248155 42.2577689927563 40.5734458834617
50.2163892855092 44.147557390293 73.5743059777365 41.706879587091
40.7311497914871 59.5093038629766 81.2992152187529 57.6171094774963
46.1592873342534

GLT6D1 42.6036491382658 43.5873188565519 42.3536549114165
39.2780515621926 47.8075998240396 39.0266821712099 40.5585676679482
36.7460922671619 42.9020321355927 44.3648993568405 39.6403089864678
42.9887069977696
GLT8D1 49.3067361345568 49.3172204533818 53.2046867327331
42.6222445667107 61.3851695159988 50.9724155054821 45.5506835119207
55.2544637845647 54.6183681000147 46.3635803515159 50.133362781069
52.5353664902258
GLT8D2 332.933704863586 84.1224653052549 54.9071823326024
169.99300253813 117.689172019617 362.123479430887 44.2166118829877
108.366007746533 40.8970401536578 135.007055711266 125.07621933584
81.8983491901699
GLT8D3 73.0970339739187 81.2801740824235 94.3953345963359
75.4466520363487 79.5175547131776 65.6914955077809 116.74427460886
72.3822545261582 108.815245119101 136.461760992832 134.994839576312
117.823678167648
GLT8D4 40.5654775358427 40.3384318592985 38.2280071951674
36.4564050256915 47.0954207417646 42.157627240486 39.2533668167483
40.3357777530419 38.7298222155532 38.957687788868 35.046946054314
44.7401523707775
GLTP 1058.61208471961 3391.65926812276 1864.63057767867 807.620534408416
1375.14388134842 811.142611128461 1413.36279821142 1470.84169670955
1652.85734308754 1881.7281441986 1566.12726152736 1164.71243740122
GLTPD1 80.7462144785541 86.1924491308234 76.450993090133
45.4634652319753 105.825228126465 57.1752846228778 116.434267173815
110.599131324937 95.9999165639785 85.1950626054282 80.6550905475507
63.8558858499025
GLTPD2 41.0608773166058 39.7835513496873 38.1421709520616
41.5464765641928 36.4434672195785 43.0860383229288 45.3597029614942
40.5158867964016 44.1519466943185 40.5090849993014 38.8507804383261
34.806104801006
GLTSCR1 114.148080724618 122.203872796773 125.62281563325
73.3725472767369 168.11088055793 90.407200101342 86.6798536307388
75.8976034525022 133.074560812703 70.2565873027242 78.3542840447759
83.7019398654324
GLTSCR2 3001.25236839028 3253.86278287661 4148.07163380544
2002.0536042686 3869.93390477952 5601.18872540083 2220.90215179682
3021.24285327836 3478.67753301901 7235.0789252269 8144.52133665388
4784.36905964712
GLUD1 364.337811621248 510.232992765054 638.305455110267 482.5816387921
478.566247444176 679.810781641519 569.511773053233 728.257836227637
349.480794966845 871.238169066998 676.672146226382 758.462554576949
GLUD2 52.310270746917 54.0052948715814 52.3432130135266 73.7124988616836
53.9074374788981 52.9482338979681 72.8653205184014 50.3082459161777
55.5751438859404 56.1912096308988 54.113533179847 52.7530873093837
GLUL 46.9939712192224 46.1749315148684 51.8122261766744 106.771536005741
44.20782151793 60.6400800519777 56.0515443021293 44.9977782988116
46.0679399375659 46.0119912782855 47.4870742154624 52.4992300612807
GLYAT 37.1485601150873 37.0431286518306 38.8403878304539 37.9116800161496
40.5207392413115 37.7000400506818 37.6527901109005 39.9927637255545
38.1713217245067 36.1000756050843 39.1982470626863 35.4484652445078
GLYATL1 41.9407041937973 39.2907645923217 46.0727230219512
43.8002456102021 41.3755760434934 35.8227939208521 48.1747307200218

41.1575777497576 45.0997987980157 50.5252048155459 48.6184776976167
41.9430737035925
GLYATL2 40.7609952437622 39.5050383901884 37.3687900733549
42.2496337895968 40.148780650831 40.4359177791566 42.1607322031054
37.86982638952 42.357910063876 38.957687788868 44.7926323492519
42.8864415494484
GlyBP 42.9104454767916 45.0115870036541 42.3536549114165 49.1786207920603
42.4864576619427 45.9266745571724 44.3440839686558 43.1567024035183
45.6582729799009 43.844855336215 43.844855336215 45.0035308952466
GLYCAM1 40.9558083259517 36.7976562409506 39.7411455670172
47.4320009861631 40.188873541274 43.4215162178364 43.3744503319109
42.1887890806988 42.8036731479932 42.6211340086915 42.6913548400127
42.4162492820012
GLYCTK 58.5759178381027 68.8195659637992 75.651446553407
72.4834467838798 101.738441694376 52.7156971154405 87.7107079534841
51.9447450229707 72.1591028956359 82.2431666775699 69.2787430208317
61.6311118308635
gm127 38.957687788868 45.8669209716694 38.5045333213784 40.1804147506786
41.9842695818285 43.0860383229288 42.0605906240248 37.7612949798895
42.6366765186182 45.7911932481722 39.153226699662 39.9519547348106
GM2A 167.848222139162 77.8946473414547 166.33117014492 247.843927763772
65.0605897122361 102.711934290382 55.9276266345493 135.997802304719
120.001312008518 158.478919789334 139.385367339095 129.268600210689
GMCL1 71.6849360818041 106.809028844031 96.9569547595983 83.3905516293346
96.9476136220382 73.4122412764173 92.0658899997157 86.7710020879725
65.2881198932621 56.8875898013449 67.5963845975985 85.5570725984366
GMCL1L 39.9697332660833 38.5252456306656 36.9795961564828
38.745552403895 39.1650142708257 44.4619248757502 38.2933870238743
42.157627240486 41.863927676552 32.2133093774284 36.6298807161755
44.2527566343661
GMDS 519.563402444472 583.053232396803 368.246708866999 295.014183084118
1134.52018899688 287.981727851442 533.820505121067 341.97050042695
355.00914527669 372.473625569296 383.966991071954 368.525388695227
GMEB1 65.0721641331632 86.9032093551446 71.2246788429078 110.431464700539
91.8935361535002 79.1257499539205 107.015362116555 93.1067450332473
89.9340376539354 67.0489032883986 76.6232527679845 72.7502212566295
GMEB2 203.759116151774 170.128899555014 146.42128243217 124.611407530723
167.746168796779 149.249181701692 193.395549316561 199.776860110557
168.492959515167 140.484790183918 185.54813083393 137.132535402637
GMFB 502.435496018532 367.158502379417 238.338646661062 580.444451816014
184.50283792606 413.067407579649 278.067057781084 621.012072500314
260.581111456578 436.627613548713 386.372142079981 406.104588997462
GMFG 84.1254258487497 136.081259669601 97.3929166450379 490.876152145712
71.5345397102823 122.904323796484 108.007227281411 116.852483297949
47.3791873993182 160.53293875611 152.555556133364 275.314994786888
GMIP 94.3674661847143 137.424708183613 112.60230393988 168.11088055793
120.465621774337 61.1022920882065 91.6978282652998 153.165464817911
81.1416376689959 93.0626550693989 93.2484615907966 79.3875629814087
GML 34.2180669723984 34.9262464079777 37.2838080935695 43.1847628331565
41.7929491793697 36.6996187567892 40.4359177791566 36.8400213077707
39.4762546236449 37.3179578037963 39.6403089864678 43.5738557773903
GMNN 99.6265783263109 154.232642598588 116.547937420457 68.6379135603075
136.429803886927 61.2387559102636 654.682263833024 210.768576894328
142.492693744683 113.759627906335 86.582851157193 132.985150338484

GMPPA	284.942182200489	163.946447772618	211.468527316402	270.487135967976
	205.806939441015	164.62751527755	176.43047680293	224.373975368936
	180.994353525666	161.948193932343	157.927114480316	132.985150338484
GMPPB	67.8335962843732	105.446338599737	77.8490019489375	108.138395958121
	76.0532592222027	72.5956443098684	140.465247047676	76.1769573071745
	63.340012816413	58.3813211109331	71.1894129030602	75.1953706943085
GMPR	41.6354649482853	37.8217989841918	48.9944486068347	49.1786207920603
	68.5888253504788	53.565717255469	54.4934782594218	52.1788489555942
	61.8445915808329	53.9412154719864	49.2206713037152	56.1136936405786
GMPR2	134.285947677624	146.87603018486	199.404836798715	157.677866955988
	151.047739254277	127.265200736376	106.140215438654	100.45351084408
	124.22494803053	149.494335028498	143.278668481091	182.685579297058
GNA11	340.553073657744	401.45848063233	369.952313383216	171.800185639868
	531.36214636517	538.290200196057	429.983084929605	501.585246203512
	358.498678171092	429.983084929605	647.297964303385	343.677907141241
GNA12	101.759714275976	79.4916415027015	79.4442697175874	163.995560004001
	143.173898886465	141.808284719109	71.3527183280827	70.5322939116406
	77.7506984396769	87.539553421006	83.7829283976292	97.0254711947754
GNA13	175.798140574991	152.031470139119	143.822560762049	442.546348599648
	175.259060743495	187.422036538629	220.621142396402	206.676666936979
	248.797398220982	106.219480656782	124.687212034276	162.486448421519
GNA14	76.2368668439875	57.4538469832241	51.5292723244077	48.665578118898
	50.5110359971301	69.4214927857472	68.878562323012	56.5914743235484
	62.9922928277769	53.0357938671646	50.0303076717704	64.2769859135548
GNA15	305.735573572576	663.372296297652	654.682263833024	1048.98028527123
	612.843402058384	255.762638489554	701.168333137671	337.156068385541
	906.833938613875	411.254448823371	313.660064080622	504.847507464269
GNAI1	43.515290338756	56.9312835667828	54.3068448962404	43.7540438144592
	45.1137938810047	51.702461863489	41.3094485574845	43.1567024035183
	57.1016072897358	64.7598303406649	66.0282565447846	67.6699686085575
GNAI2	592.399245288911	798.996494242432	846.096424249173	1152.55315876935
	480.660242705293	949.923102632043	990.431472174705	437.148137705103
	540.037371261388	713.685405035516	814.294297312057	527.037018015766
GNAI3	97.3929166450379	77.9992448636936	100.824660338235	177.722828589884
	91.7629175712824	79.1922819549042	123.455270940015	103.631449539809
	132.57180783668	102.880267177687	97.0348634204639	120.02765867291
GNAL	41.9823787224629	41.8848959280916	39.5450079747895	44.2222841572272
	41.8407088454059	47.5733603903505	43.2645805835667	40.6171774724171
	39.8763736543526	43.449858008666	44.9056256433399	50.8812920005251
GNAO1	40.6130792100165	39.15937777386	40.6737160001291	41.6186264038868
	40.9450021938518	51.0120959440979	41.4244721780607	43.3262148375525
	39.5806465938526	48.8623729374733	45.6895223823001	45.5265270192861
GNAQ	87.3342335690393	86.1924491308234	75.1477470681238	166.608353896248
	87.2282539094452	134.749803376372	92.3034609409756	79.1039287828151
	76.1019439127881	65.0928075335306	68.7091708087448	105.515662220161
GNAS	994.404707955031	1354.50256889331	1749.06320703181	1019.67015337432
	1642.75555340648	1470.84169670955	1604.80031304031	1347.15664445545
	1588.70376317303	1093.84714207969	1303.33517942343	1430.73068989193
GNAT1	42.3433931248155	45.7317208802318	49.965271003193	48.309866878365
	42.2455707947223	47.1477056711335	46.1634822993009	44.9977782988116
	44.476519804187	41.7405682794166	46.8954576545929	40.2237056928605
GNAT2	48.6621527194963	45.4143854024453	47.9035532886869	46.0228732507872
	40.6723334103791	41.159706131351	40.4359177791566	42.9301944191913
	40.137818555383	35.7202909649682	39.7342718293432	45.6692241948108

GNAT3 37.8386378737561 33.6533600935671 37.3404647796601 39.5050383901884
42.7489468897708 36.2315828396556 40.4359177791566 41.6466169548846
42.2577689927563 37.2686490268265 37.9946685306274 34.0382693188794
GNAZ 32.9174234020987 31.3542159583213 36.178547300352 41.3027031037343
38.1644569189523 43.0860383229288 37.7136011474903 41.8417024632349
34.3842972343227 35.6208308220005 35.6970528713709 43.7672102682067
GNB1 1364.06060391538 1626.71747158994 1493.20520979227 1513.74711975248
1506.33570635551 1921.48678333837 2369.95344896703 1580.58484708669
1744.56688411113 1704.0522304387 1762.93413846062 1549.93897368117
GNB1L 54.6235981131919 61.0144918827511 55.094385444315 68.4261608261522
72.828269171954 51.1621379498014 78.6402083787251 78.5375244257332
58.8201021345225 51.1169368198437 62.436851776671 53.3059058105836
GNB2 103.139459009593 114.719721626067 137.696331574552 134.099684731177
85.4420599474339 101.46584765758 101.898612603107 94.6432868887401
142.066425511614 113.26670675749 96.2248868829355 89.2832797334932
GNB2L1 5696.39636379286 3634.98300564068 6873.84878164654
3905.8371644287 5074.07305640514 4717.02689540013 4858.39551231606
5525.13078364599 4482.5285743878 6483.09366281532 6873.84878164654
6938.87250165681
GNB3 46.8954576545929 48.6447083765118 39.8299340037009 42.1273928203021
48.9627346956003 44.0804700754786 35.6056062164318 41.8417024632349
38.6322728994674 45.1569741609597 45.0589947942306 40.5703581862752
GNB4 56.657759220121 48.3699741879723 50.7774722329703 119.124583755587
46.0043807840705 57.1752846228778 48.0283573444179 54.8412943008635
41.8197029429821 50.4234835272833 55.2152456171699 48.9906383294199
GNB5 43.6706764143908 47.2354741623061 45.5587966941116 40.137818555383
44.4489662507893 68.0881175685974 64.9072331703985 43.781101409552
58.5363098745941 45.087888302889 58.5363098745941 61.6702707724448
GNE 167.430013807712 80.8285585008048 53.0104469636221 77.5698542248061
76.3790849853724 121.613731804111 91.0154871536701 84.5055910320032
71.5327770882433 106.752660061588 107.610858427836 190.61997068475
GNG10 99.3142021810133 102.150058925164 102.734042853587 275.549982777518
80.4895102121889 152.17335005192 101.410859084491 68.4659783447092
83.1874704625702 55.6865353018811 68.878562323012 175.165302282522
GNG11 206.814831223864 171.536394606897 204.944787289897 378.626656077546
181.565843182713 714.990611155096 69.779139490544 156.337202322452
79.7061415085215 346.924074555334 402.08877403329 407.161973433666
GNG12 127.072685504735 83.2873728545705 150.925721934159 71.9062462001972
113.866742186841 250.308866488702 101.549779766678 55.421880601441
67.7662831916293 57.5713378330876 71.3527183280827 152.996027678733
GNG13 40.8681871019812 39.7835513496873 42.8270038153139 44.6905691366696
38.9496515344837 40.8634043028528 58.2315917325629 39.2672712041774
40.0403898620771 42.1622837416589 41.0808257270221 43.1805016690297
GNG2 52.9240098541574 53.7332797903102 51.9214790114499 83.877343750194
54.9249759425252 105.525194437454 56.7780520416533 51.2623287419357
42.8036731479932 45.9004543328976 51.3103617600342 43.0930293831497
GNG3 36.3649677956953 43.7644428186265 38.5045333213784 35.6928955245309
38.745552403895 40.650742261428 36.08956004634 38.3684202018524
40.2274522635576 40.2112371512838 40.3452320167286 34.3409660445447
GNG4 88.9589979655541 42.1607322031054 36.3604951341921 40.3180310664156
45.2556760873335 44.9896851005259 56.0515443021293 40.931294690669
47.5564423692142 45.5776159432079 41.3553369544097 40.7704587752083

GNG5 223.826005691932 222.617854741466 224.976641879383 225.589127792587
142.389246750828 173.640820092779 231.060688044554 194.666769955149
98.9712341137316 93.3897477314201 115.496195377983 185.924923239764
GNG7 43.844855336215 56.463481075522 48.8941062980393 66.224559122062
104.351256270039 141.364814429951 41.8315602975157 51.6660481331154
44.1519466943185 94.4129246851651 84.9601800557888 60.4872675915085
GNG8 41.5421429082231 44.0409178714232 45.8844325913247 41.8778873678551
41.467005949568 42.5660695551615 39.2533668167483 43.0390857698452
43.3744503319109 39.0648733099061 40.547835538778 40.2669497398702
GNGT1 40.148780650831 48.109747794187 35.9305494063137 48.9944486068347
38.2496050953547 44.6524308640784 47.2927436772465 48.7814131120945
39.853866204927 49.3000844711116 47.9942520395434 44.6484005362281
GNGT2 47.1962846565261 43.7644428186265 39.4710743516517 82.5279746006378
45.6895223823001 44.8872784170036 46.4388841354122 43.0390857698452
42.6366765186182 45.3530683538086 49.7363778831003 46.917149434588
GNL1 216.866451320811 158.01463829244 224.827414462257 188.099292617901
203.82788889213 180.447835336714 196.267504470029 157.082769443568
207.380865650247 258.30549459038 233.692490031492 188.767243497644
GNL2 659.666977039793 483.20917825594 395.875593488613 440.282299601096
749.49934348018 477.023373302787 531.869698775629 1147.19483787501
444.169882251299 338.000998124097 376.141477833569 432.945510073269
GNL3 171.073367662623 391.658764737446 296.598410253206 162.447872391923
429.329962597147 205.187554095744 361.632211543977 394.00931573564
210.255784687052 153.42135363926 172.574996256912 284.105466865264
GNL3L 127.352997770688 315.799449362526 343.016541928592 216.1413998895
260.185079572615 146.634440537624 446.162602155964 333.122044191102
120.123385036416 151.451107406752 215.68113524702 197.660480001139
GNLY 61.2130609867477 49.2050538150106 62.6203392459133 223.826005691932
41.948762493127 46.5557478175976 60.9648845690177 55.8775622338124
42.2331334096358 66.8764304705723 51.1055541075029 38.4938120203865
GNMT 38.6454098044474 39.6995529770239 46.5650556481721 42.357910063876
41.6806984441849 38.4330755397458 40.5585676679482 38.745552403895
41.2870505273664 39.6719190054379 37.6484782516569 36.3454320848591
GNPAT 192.058030454066 161.65846687852 158.478919789334 227.986601498805
192.171116215791 272.36028618845 196.034087004464 253.237824005249
166.983092667334 150.649377863375 154.509548705054 291.232601825013
GNPDA1 703.315796616275 433.194113859912 483.313014703768
873.451893437179 643.9329225331 495.359878931451 917.631567606105
505.650842529816 700.477686061791 866.712228831149 853.464809072841
1048.98028527123
GNPDA2 59.8248482438562 67.1926218405833 56.6458456890715
74.2429851177794 71.2787833369055 116.122324921267 58.0969258817391
50.7364620410977 64.9030399249327 67.6733764807973 78.963270461629
86.0230765383602
GNPNAT1 47.4678477186801 49.0697565324841 53.1491634301682
39.9697332660833 45.6895223823001 42.357910063876 39.4600924554505
40.1474543172332 50.4974478884077 40.4136535208406 37.1059647239814
42.57175366709
GNPTAB 92.4896666046729 165.640362974584 167.824386984618
158.478919789334 125.197314240529 102.559678338485 97.1271615802556
81.2228688683149 136.190823415283 91.5230424957075 111.714563124738
93.6717518147993

GNPTG 255.241295723095 317.406465928295 805.590413262364 303.139550962377
312.530335465784 340.553073657744 256.116479208201 203.559653641339
294.966924715506 419.563859911367 444.738154053278 276.550265506988
GNRH1 68.2614370871382 52.9603588177082 51.6159542017038 56.4578213622965
47.8075998240396 48.5835484742456 44.9863726205428 48.6441988290952
52.2631308081333 85.1188125003595 70.3381994440425 77.1059855034406
GNRH2 39.9182914210537 52.2832912331043 44.6032218589199 40.2455972565968
42.2455707947223 41.554942319225 44.1407835440612 45.2806199505029
44.7058233759912 46.3810615657263 45.0985088502363 49.6629259325705
GNRHR 37.1485601150873 38.4330755397458 31.5151915411476 40.7481155008596
42.1650127805175 33.5578650644246 43.5627649099623 33.8558232866797
35.9645119596869 44.6918600441328 36.2435874904689 41.7575097298927
GNRHR2 34.9138625012006 38.0226905622357 35.7105247506212
37.651298468202 45.1137938810047 38.2293008890613 39.0458123429605
35.0510338774593 40.4220196471124 42.1622837416589 35.4386677371439
37.6557714476853
GNS 2101.59244895554 2108.03351084959 2944.3433949897 3361.67247904403
1204.34388579291 1330.19215036764 1656.16581692717 1521.20630233299
2160.22713775462 2588.47871581066 2331.12401106076 1950.93175239958
GOLGA1 90.1056480599534 86.3374157614394 80.8400209263587
86.0630164348952 89.1012752492647 115.659933682533 104.932630862885
93.7090094630558 94.9831937714438 117.746495007818 120.418406532204
80.8366627364071
GOLGA2 194.225364352044 111.071209057746 96.577572948724
118.10944558223 110.729504563799 119.474339360208 146.840603547323
119.738288633084 100.937651342589 134.519244495139 169.144145421022
125.383837024985
GOLGA2L1 40.5654775358427 42.0709558258414 35.8265993915844
41.0726336841311 39.3667161654772 36.4986986135769 42.0605906240248
37.2237132058632 40.6957787921451 38.6454098044474 30.1388203764747
45.2783024981315
GOLGA2LY1 45.9090659324223 40.716553247236 41.6079958749402
41.7614975630362 43.4544762830742 40.244271403831 34.806104801006
43.4544762830742 40.7847110517594 38.2069499294002 41.0050831223107
42.4607795638552
GOLGA3 997.492549573274 572.81778857651 396.355023788839
479.318513108472 319.871452890512 510.82619253142 417.086922265732
362.196116025799 496.791391622565 552.860979481958 514.261598258395
436.627613548713
GOLGA4 62.0553450433433 70.1831787109201 60.7530213660442
75.6103053700122 140.719373122712 98.6108785181637 75.0665411373616
64.7318794662958 88.6368226476614 72.3297091475814 72.1090339831747
82.5956705829173
GOLGA5 403.428042132639 207.489116930705 260.331534765939
280.034472186873 263.492132556601 256.766675299448 202.494545985006
149.515611848932 226.882600557693 186.301404367238 164.642060754649
231.304658157247
GOLGA6 42.2604978533738 40.2973086894872 42.0164252051496
42.8768096578102 46.5557478175976 42.357910063876 42.4939802311136
42.0778399888414 43.5934599686083 42.638061478325 41.8168470823451
42.1915582886043
GOLGA6B 44.9896851005259 48.0570064280814 46.9711270147079
41.3567204694281 51.7495306734657 50.0748292497578 40.5585676679482

43.1567024035183 49.0655215909632 50.4234835272833 46.1673159982664 58.3285450269055
GOLGA7 226.168990555083 192.003788858976 171.021755568144 251.4927207704 170.128899555014 283.111920665176 145.459578481501 334.639325701194 129.925137664962 169.527304857688 152.972693071526 199.687268345276
GOLGA8A 63.200752695983 96.3120603627576 85.5839487225537 88.5511408870489 143.773311381452 51.0506441018166 89.1012752492647 62.2982003555312 72.7071934110997 85.6763929745253 123.032006903803 68.8529879402902
GOLGA8B 247.007210336963 185.924923239764 151.848243044839 134.776408077533 361.023743912857 106.951862149262 168.70463124443 213.204385896543 177.935411895372 237.359267889848 257.761541860837 216.606583011866
GOLGA8E 39.2192721306168 41.159706131351 38.7429005059745 39.066284740632 38.9990801113267 40.3357777530419 39.4600924554505 41.9721968713295 39.5377896745682 39.9479806809296 38.5239513822269 41.3844657483749
GOLGA8F 34.4021554788966 41.554942319225 42.4580504587581 45.0798408927912 39.1650142708257 41.8613406887824 39.5496902678788 43.0390857698452 40.9901805728485 40.4136535208406 41.7272875295469 41.6550823405646
GOLGA8G 40.2973086894872 41.7656868106485 36.9795961564828 37.7469621225553 42.8414096678708 39.8724170885333 37.108950999591 40.4737825400374 41.0381860307445 38.9271399577966 39.6403089864678 40.1249533205858
GOLGB1 143.383133346874 127.525206038902 124.664792638889 159.835690229505 153.660685857642 112.421316052392 129.810477623628 149.792346524401 107.682738819549 130.812376632261 151.652543512032 95.8149149211251
GOLM1 130.985178898633 66.4079972657486 70.4014100046391 59.987045425047 48.2748123114598 65.7587943905649 87.1735705257803 79.1922819549042 57.9713749600418 54.2830238965339 55.7868713984479 67.8919712859031
GOLPH3 472.159979716705 441.247306467645 300.121875447445 375.697071471585 343.828445728322 424.539111965342 624.51670158046 484.651969259966 626.390544175817 191.253860976433 311.301613620475 360.342857415756
GOLPH3L 105.15507796881 117.305340546242 137.424708183613 82.7394574977114 106.951862149262 120.508644792138 116.913991315353 144.898757916905 118.862494537893 168.7696164772 134.40110873422 162.721587349434
GOLPH4 53.8856501960117 109.738537924214 51.5292723244077 104.528976428975 57.0006212637829 101.789162379972 44.2166118829877 49.2901038327894 48.9931786790166 44.8997235283254 53.2083147523758 58.7585623394335
GOLSYN 66.3095900379311 43.9421822132391 38.1421709520616 45.7397504535169 43.1252272136667 101.383978726419 40.7910160811527 45.8669209716694 45.4799270968969 52.2256177934134 71.0369238830779 60.6653636336244
GOLT1A 184.048982286364 104.072423445726 128.219615202232 47.6687908290818 199.208033538936 51.3886035574416 264.451310132096 143.526781144716 199.181938253995 593.801086643125 472.159979716705 755.852629128252

GOLT1B 73.1931742801095 52.8796369426118 50.2869791217819
46.0228732507872 52.5645416722441 49.3172204533818 49.6872467499977
56.4779645313958 56.702421372165 56.5379973844443 46.1673159982664
47.3172462673426
GON4L 74.2151462073098 63.3948709726117 87.455991111513 69.734208465775
92.2001835784782 60.5803031036931 71.5249259772583 72.8073366137855
71.3527183280827 79.3427669741608 88.0186381834147 66.53831442217
GOPC 89.5687388187015 69.8983723094667 74.4508307047509 73.5834042105313
66.6118713143653 69.9819944829311 73.0433648756396 67.2714646016644
69.2001494999588 91.1057573288203 73.2234405348435 74.2748495231478
GORASP1 205.854666205308 286.497342437243 304.33749254654
211.685526927495 730.168814097451 283.951419701286 318.768560912915
270.78959225422 304.33749254654 414.539381761279 371.280311231075
329.677454924062
GORASP2 423.804472820231 426.933064567744 343.591727067749
344.578407722941 375.095491465679 303.93332785291 284.66531382201
276.795797048951 280.159844300266 315.748168057656 286.758099833009
369.032450063274
GOSR1 59.7863401969736 61.8492980174846 55.8243694410835 48.1662933382789
69.9021277921574 53.63657997649 61.0207757595269 46.4672637826582
65.3672880026114 54.9527624170441 56.7272804480684 56.2804354745795
GOSR2 64.0759817636265 56.4917096880521 63.4249771379563 54.6089451252049
71.2787833369055 69.9214859673839 65.6958831220258 66.5539062525344
77.0145316377298 48.9826868640753 54.1440452292081 62.7977686460693
GOT1 166.910579215196 254.874064929741 234.004293901222 195.221917652149
346.844545554188 218.217851814655 374.297474141009 241.706783147271
232.77788697408 249.195312452368 256.892326800908 246.242210944848
GOT1L1 43.0001432913058 38.9338521051669 38.5045333213784
41.3027031037343 39.4693871570028 44.8872784170036 37.1643693306382
39.2672712041774 42.9020321355927 45.4614234442527 41.7272875295469
42.0424641526302
GOT2 714.532923535126 1213.3477481374 2206.44541412808 446.902178094675
556.728676214581 451.13133250897 1307.19925493293 949.278493776928
1580.58484708669 1372.51401036753 1598.78859278229 1337.53997230271
GP1BA 68.3234331335073 67.9697948557876 68.8098607236981 94.5501397951415
70.0133734659853 73.8105012820769 95.096268847843 61.7572675712557
65.0810479794187 64.6446465846487 68.0414563597154 54.4313108506244
GP2 50.8388984085636 43.2649781354792 35.2838283525874 41.1886975241176
50.2163892855092 35.314469458379 38.7337086062875 35.2642250366721
33.0463640202858 41.1222709786153 37.3687900733549 40.4639532172338
GP5 45.0985088502363 40.9731067463382 40.7620472083159 36.5393857306744
35.6434124419782 40.8634043028528 37.9430145836712 37.0390589468421
41.2870505273664 35.2880714952489 40.3452320167286 39.6352613956998
GP6 44.2350046179299 46.4091752823403 51.8122261766744 49.9672547361866
42.3685136297603 46.0364000143537 45.3597029614942 44.9631458622215
46.0106098064475 46.2286164776607 47.1970991228159 43.1805016690297
GP9 35.6111578834671 41.554942319225 36.3604951341921 36.3604951341921
35.0181575202066 38.5252456306656 40.2154497375715 34.1806289878747
36.9060997041453 32.7876527533679 39.7342718293432 35.9604262434141
GPA33 43.7585538437696 45.3486182278177 46.1653991089885 49.1786207920603
40.8873907150245 48.6621527194963 50.6774883370457 48.1591264215184
46.7760357462635 38.3092241935352 41.6336636285875 42.7782216342806

GPAA1 423.254211470935 583.915973546284 383.371300272181 174.559216176175
363.215063520042 233.00902546331 331.390452289344 237.401487995882
449.059703234741 260.766438659762 418.810270137243 228.984484917083
GPAM 60.7371790125714 62.843734579668 62.2200723764909 60.8581462216607
86.2118355769367 104.361353204648 70.9567983915276 93.5769827229864
59.6922302604696 89.9615463738575 84.4821163646146 82.7394574977114
GPATC4 44.6231541342 47.2055133646385 48.2883694323339
46.7577982595088 47.8075998240396 49.1013967533186 41.8315602975157
48.6441988290952 42.1594905198929 46.6598879466655 46.1673159982664
48.8670821164567
GPATCH1 110.214922820817 87.036020750455 100.305262467668
91.5202521578181 101.987604729928 102.020047144427 159.359051158123
113.444217634165 120.771048750176 111.056599389048 130.64028226556
100.677422836631
GPATCH2 70.7238101239421 169.970634268117 129.747577660612
85.1847490113446 111.033548392096 75.4227827818591 104.341160312305
129.309742451628 82.8150137756969 111.511934617893 99.9752275166514
85.7732262204681
GPATCH3 64.546412747238 63.7721358300879 72.2016884438365
70.8835636550472 95.2833228196616 74.3729155936862 77.9028721785469
96.5813769696577 90.4957279507369 89.1516394894484 95.4841914842335
63.1443731864055
GPATCH4 107.176733647834 88.8772406115484 94.3329263062576
78.9479233685889 218.819478568299 148.964204638494 104.541813290553
90.8109585999257 95.6215575902122 82.3488166506716 81.8242150474253
123.608921653229
GPATCH8 48.0201377454632 44.2105195734073 42.157627240486
53.3967242431461 50.2163892855092 40.9506062495231 43.0930293831497
47.4740726032707 46.4164127487052 57.165971310746 44.2925355984056
42.8864415494484
GPBAR1 46.8712941728147 54.5374188496807 52.9736397524094
62.7108230507424 57.0006212637829 118.410983614453 41.5464765641928
48.3871747110233 45.2392954271967 78.0020909283414 74.8473087205709
79.5898953726467
GPBP1 165.349426587915 154.811704749343 174.058845828966 217.127708207497
132.341766267192 202.795613942391 130.379966531543 132.997204273031
200.698372627272 105.414552529943 116.018801676087 165.797721197832
GPBP1L1 118.762101740563 80.882176929636 86.754538970161
77.2906963563431 104.141384807311 87.0045333192221 81.454335582503
98.2074490157865 106.165518810891 86.7076796107636 95.2490714726663
91.1741538903522
GPC1 93.1759373099875 143.822560762049 140.276520383146 58.3475983630509
79.9161139112432 52.4351670989268 66.9239565096247 103.071727621257
156.088284999883 117.634238804678 95.0604882262201 80.8366627364071
GPC2 59.7179445506128 47.8400694856726 41.9666541498638 44.4367387274922
71.1607965028029 33.7818132446042 51.4349390827895 59.0262390168156
50.7619345314571 46.1390887492649 43.4709415392696 44.5432192783161
GPC3 70.3757673501097 254.874064929741 152.200107222761 53.3967242431461
52.3242437351389 92.681224908389 46.3057385979341 43.2551508830106
50.2223403889892 85.3431175579775 74.1425714439424 67.1675878355908
GPC4 127.232160404726 78.5174639327946 49.9256753636167 79.1000897258245
145.572606290643 143.679866958604 74.1353964055485 132.730723037077
126.682296930289 126.670728021682 105.953705702398 197.232697438521

GPC5 43.844855336215 40.2493681589104 43.1931731665131 45.7397504535169
43.2484996523549 57.8005007012205 38.8417151851134 45.1714698794331
43.9450023643394 48.8623729374733 45.6348103033479 41.4508241771315
GPC6 106.521012342668 56.2851430356168 62.0967404944031 99.8063480813138
82.6157566292355 96.034706957068 88.9430141071732 85.8277891453447
66.8632294055679 77.676210875358 75.8056934060866 68.5174271063894
GPD1 36.1232327599945 39.1982470626863 41.1367478979484 43.1847628331565
39.7568367479301 43.7630587657205 44.8643898647322 38.6454098044474
41.3803269429468 46.0119912782855 34.5633623842083 38.8495095797881
GPD1L 212.945601743319 803.548811322583 641.462632112151 176.816741656659
1245.45371155527 804.592753722875 267.70610731202 1327.93833415582
635.288331955846 1506.33570635551 1173.47285626656 540.037371261388
GPD2 44.8914371498528 51.7495306734657 43.7085219318425 49.9672547361866
43.004943518025 43.7078399718247 49.540449125788 49.5544686779125
48.4497719907777 42.5193208629123 41.1638563416981 49.0986627051784
GPER 50.7903824729818 58.4262160879174 79.9391673512018 68.4906691348625
55.1563703466328 80.7067505750848 42.8684958525042 44.571915452417
53.9287524861617 98.2843999216669 72.9648672559089 51.138877923954
GPHA2 32.821392987958 42.9358255168086 34.1806289878747 40.646702696033
36.2048942610546 40.9506062495231 30.4430902995779 31.3302670771182
36.8127605061228 35.0082729554459 35.6970528713709 43.7672102682067
GPHB5 41.6354649482853 44.7829632472754 48.4890615340603 45.334106390212
40.9950639693255 45.3882138838137 43.4544762830742 41.1575777497576
47.9035532886869 53.0357938671646 46.5650556481721 46.1592873342534
GPHN 51.2851682978581 56.3560498705517 73.7133884699889 75.4466520363487
66.2702569771609 64.0354923953233 57.4622430729629 47.8947595316832
73.5834042105313 69.0530953932111 68.6199296680241 62.5036823572932
GPI 766.647626178442 528.840700144811 640.0926391761 588.247843264181
971.856840043688 545.827286197333 1182.10714525377 588.972273484465
2903.00880209344 529.430648638878 531.869698775629 490.301992866742
GPIHBP1 40.8163035350222 44.311722896416 45.6989664108554
42.9358255168086 43.0860383229288 59.5711567415697 41.2040352443154
44.4489662507893 43.0496526069072 49.9493230064613 47.2569851908386
39.9519547348106
GPKOW 62.4317959077388 56.463481075522 62.3245608177257 57.0629653735694
62.8270556619309 57.1752846228778 83.0647477737971 77.2048580540589
85.8154958329408 80.2774079897733 77.5698542248061 58.6481644777552
GPLD1 39.266565827316 38.5921548841176 37.5597671487621 36.264823544143
52.6226745117396 42.2830035667142 42.629412460409 46.0633179231318
43.6039443285137 38.6454098044474 43.9802831041664 38.8964578286293
GPM6A 40.9558083259517 42.4649374149262 41.7364024833895 50.378948785456
44.6253529423632 45.6663865711866 42.0605906240248 43.9755735391884
46.5549623118323 43.9450023643394 44.7765484590865 44.7915315085948
GPM6B 53.5698357689852 43.5873188565519 45.0129098817151 55.7764940944098
42.2510526338368 93.2870243833082 50.2471139112912 45.2806199505029
40.7493885015617 50.2750065554387 54.5374188496807 47.7390230243778
GPN1 560.954913956997 357.800871454252 309.39599482851 348.165210494253
483.416873464931 240.887877769007 554.163106145921 846.773659307087
332.830805115112 266.439343650315 252.134800295199 301.594300620246
GPN2 286.398178392901 244.435346243998 290.316757790733 187.601223003233
337.013669560645 148.770228614661 385.918487165555 416.559486912165
340.429325650126 374.096934824025 279.841798764418 254.373837233228

GPNMB 1037.51323185348 171.992427227088 148.158262886253 2196.59044510709
186.697432898147 1257.19844370057 352.768184657577 362.506098778318
605.970682363975 583.421166842011 322.109212713082 409.724772942357
GPR1 358.30596676183 141.180682718758 184.750252804038 279.782392390288
127.2070318269 147.151416108234 235.826763901522 488.463605278409
160.950554451488 377.491895827114 373.802562320876 298.771150048863
GPR101 41.6354649482853 40.9731067463382 38.5045333213784
41.0726336841311 43.9450023643394 33.895835691032 40.5585676679482
45.7476095696859 38.7298222155532 43.844855336215 41.2598458726002
43.2776458914333
GPR103 45.6517320730042 47.6914221838216 47.9035532886869
44.6905691366696 38.0598374201407 42.2577689927563 43.8206425661746
42.357910063876 42.357910063876 42.4594149893798 41.1638563416981
44.6484005362281
GPR107 44.8914371498528 46.6314009405034 40.8420925848403
44.6905691366696 46.3150221024046 45.1873487463472 57.6643188499117
45.4757965005055 45.1899271235974 40.4136535208406 43.1931731665131
43.5738557773903
GPR108 114.535461066963 89.0898346470643 134.896696950857
174.252441615373 144.799432299339 129.091447141381 96.2959923858457
123.800965644574 128.024853362102 153.595112580363 160.264217082275
162.23769718358
GPR109A 52.6960621424156 60.1685144469519 63.5107036952143
700.010354583503 45.4751076037511 43.2805590023412 126.826715174564
37.2237132058632 65.9254098858352 45.2556760873335 44.9713479185144
58.5087296620514
GPR109B 50.0697497247754 52.2381815246326 56.2000979702695
389.131545459708 50.5868854091439 46.263826584899 102.499011190932
48.8467058855408 56.1733549054832 50.8826620053386 49.8872574072587
52.7972156492746
GPR110 96.5615807039306 77.9992448636936 64.6835308827804
39.5522630869979 104.220256076979 45.030514396298 108.806649898122
48.8524897151151 104.313754953569 63.3872214856708 63.7963501146829
114.073853703538
GPR111 39.7576136507593 44.7829632472754 44.4311655729487
36.3604951341921 40.5710637884029 38.5252456306656 39.2533668167483
39.3709357615493 40.4997186069494 42.8936990772838 39.9070785037411
31.8554734753953
GPR112 50.1533437545239 45.6533175604486 40.5734458834617
41.1886975241176 42.0709558258414 53.3185097662527 37.0564573649223
37.7612949798895 41.1742244762318 48.0211752672327 49.4593570700221
42.4607795638552
GPR113 41.8409927585705 45.3486182278177 47.5673419710251
43.4410741327254 47.0954207417646 42.8598805316863 49.6872467499977
45.642304356051 52.2631308081333 47.4767851750188 48.6767028205398
47.6938628797168
GPR114 43.3638918705225 74.621822948059 64.1080166181228
148.059385111127 52.8190882379316 47.7556758818053 45.7452456977857
51.7538628098347 46.0106098064475 47.7180816682562 51.1483696410762
42.7782216342806
GPR115 61.6544855697851 80.8285585008048 66.9239565096247
40.1804147506786 55.9283039581662 38.7340969861754 71.5249259772583
38.079222289614 79.3861418060845 60.2877434874622 69.1129431075514
60.0940570991178

GPR116 226.785831708938 265.570814613305 428.729935028911
285.187681119318 183.311305255895 218.217851814655 89.1012752492647
196.247893540766 83.0534943004588 128.230543301378 209.61080762285
128.981582396627
GPR119 51.7607563002005 50.6229884142872 53.3127618245672
65.3191216270241 41.5668884168669 49.8472708021525 50.2016912396771
50.5070012045444 58.9056300364078 49.1928206164702 52.104880481326
52.3896785600751
GPR12 40.8681871019812 35.9305494063137 39.9101908547397 46.614092935749
38.0598374201407 42.5660695551615 43.5627649099623 40.3357777530419
39.0775870687758 35.0082729554459 38.956280281655 44.4475838947656
GPR120 55.5276281380084 43.0971787292711 36.4564050256915
36.890925044696 47.0954207417646 38.6414316877471 35.8227939208521
40.4359177791566 35.6139301508335 46.6598879466655 34.7308008643142
38.4330755397458
GPR123 41.0608773166058 36.89524015739 41.6977706798234
38.2041059863839 51.940083408061 32.9046688061235 43.1931731665131
40.5158867964016 37.10170285405 47.2299678280724 39.7342718293432
36.064718696392
GPR124 103.356071080819 82.8205269728224 65.6620676514157
107.111886993156 72.5027069303875 185.703644892356 45.3597029614942
66.211494935811 44.5303781239209 161.180847265279 154.19962858342
109.241121384212
GPR125 64.1882546112044 46.9498651027418 48.3609527732104
44.6131679221578 56.1838945110653 48.260931742163 61.9432904657734
64.0766831552464 52.1379596312084 123.261654256925 71.2708400614309
82.1062834830477
GPR126 68.8698243753339 146.432425205891 89.2285288333749
37.0732977341185 60.5290966885203 53.8461734834857 83.8811735017279
145.526972410673 99.2341244350412 121.45753287862 146.691463448295
142.585892496231
GPR128 45.0985088502363 45.8284004440739 42.4890076400051
40.0698728584484 36.7837318601824 40.7616966228404 52.8496345990125
41.9820792173615 42.1594905198929 45.6355219961567 41.7272875295469
37.9607839934735
GPR132 53.593974682612 58.9128486419884 61.0370242377391
296.532363090593 46.0977687311222 51.9421032049606 66.6894494536346
50.5070012045444 38.9834959368496 42.4594149893798 46.0905079010237
41.7575097298927
GPR133 38.1169703906207 37.8217989841918 37.3687900733549
34.5425384901279 46.6397391251762 40.040778349137 37.3799542961839
40.1474543172332 39.3750969348308 34.9138625012006 37.9946685306274
35.5523662009011
GPR135 39.7576136507593 37.3634897565502 43.1660110696573
40.9607305725196 36.0439235933921 40.5281496800812 40.3302268293707
39.3709357615493 43.4656637536244 39.7835513496873 36.4689948499058
42.357910063876
GPR137 393.464673405634 509.463928291526 768.354209323884
336.679355848349 776.670493028756 297.187417426993 605.338871441599
574.847203528544 549.407911286514 540.037371261388 445.421601813221
332.659496278775
GPR137B 193.981651711507 162.287617963536 259.906934432819
318.069132873489 158.718808397001 143.391384762612 141.924244643825

163.742560819575 140.288771064502 152.054785478928 128.766242896259
225.120999402048
GPR137C 47.2862299990938 46.6996902348633 46.8267540220952
45.334106390212 42.9090346219023 47.2927436772465 49.8281348927244
46.695848687694 49.641159251878 51.5855515705917 45.1432412071268
38.8495095797881
GPR139 38.385865183581 38.8336427948187 35.5220586859301
38.6414316877471 40.6723334103791 48.1591264215184 37.9430145836712
44.9977782988116 33.8558232866797 35.93950183586 39.153226699662
40.8681871019812
GPR141 43.781101409552 44.1533327883807 43.0971787292711
76.7137782332689 44.3557408297309 41.6590564937735 47.3661873039785
43.7731874801003 43.3262148375525 46.441301934328 41.0808257270221
44.1452943804044
GPR142 44.9896851005259 44.0409178714232 37.0642268327439
40.5484717678957 42.7107928951766 44.9896851005259 40.1235656101329
36.1375121409002 37.3600014814388 39.6719190054379 41.1638563416981
44.3521099857368
GPR143 34.2180669723984 44.6918600441328 37.4642807402741
47.6687908290818 52.8190882379316 40.7616966228404 40.5585676679482
44.8997235283254 36.197424053556 35.93950183586 42.4580504587581
45.7562276914441
GPR144 44.2350046179299 40.4359177791566 38.8775560911841
43.4410741327254 38.5512429290626 43.4681646416585 39.1539892144371
45.0972184964705 46.4164127487052 41.8613406887824 43.4709415392696
40.1700117452801
GPR146 50.7390160399184 47.5647821785529 44.3344900481112
45.0798408927912 39.6535880152834 59.5979861826984 44.4639405506967
46.4672637826582 54.1745577382276 47.2299678280724 50.1649089793069
45.1735152121056
GPR148 38.2083710281545 39.1982470626863 39.0775870687758
42.0102671859606 39.4693871570028 39.2985791324641 37.7136011474903
44.6107027295193 41.074824727477 37.6383530120621 37.1059647239814
42.2577689927563
GPR149 40.6559419616644 42.9887069977696 42.640829914102
41.4636047919453 43.9450023643394 38.385865183581 42.1157692936428
41.1575777497576 44.7058233759912 39.2623423164488 42.0750889188255
38.3834435509221
GPR15 45.3872406432794 49.3581578587582 40.4882741869718 42.5664883002833
41.6806984441849 43.6670755115848 45.4799270968969 41.4508241771315
42.1594905198929 44.1379638573455 44.1148826235374 42.4607795638552
GPR150 42.2455707947223 38.3322849790657 40.301483914446
43.5627649099623 40.5710637884029 45.3882138838137 36.9355798143571
41.7419877611072 45.4799270968969 35.6208308220005 43.844855336215
40.1700117452801
GPR151 37.4554876386402 40.5400384479766 38.1421709520616
35.0448343468967 37.5556299264699 34.9849896310235 38.7337086062875
35.6825474397235 35.4332080966594 36.3612409063792 37.7384958256497
34.4570158133896
GPR152 41.3387790895716 39.1982470626863 42.4580504587581
45.0798408927912 39.9697332660833 39.0266821712099 38.7337086062875
39.853866204927 43.8482802038826 43.6033221838149 38.079222289614
37.6557714476853

GPR153 43.5710871617107 47.6229101519945 46.4569566520107
40.1804147506786 52.3242437351389 45.6129956328415 49.6872467499977
44.8997235283254 45.1899271235974 45.7911932481722 49.1868644496737
45.3763275588341
GPR155 39.9182914210537 42.4493032770053 37.2291018661279
41.7614975630362 43.844855336215 37.9951997351435 40.9157352260901
41.3012916486748 39.853866204927 36.9848592422398 40.7725311293918
42.1860813954352
GPR156 39.3709357615493 42.1607322031054 39.8299340037009
39.2780515621926 40.9950639693255 35.7034203372528 38.8417151851134
37.33030683349 40.8970401536578 39.4710743516517 38.4689132737495
48.8670821164567
GPR157 39.4710743516517 42.7775752471711 41.437237454134
37.1894304543751 41.5668884168669 33.2552842693684 38.941855825269
37.4484310268909 33.4698406117406 35.4990901121893 36.43924066428
44.4475838947656
GPR158 40.3478825593224 40.0527893189096 39.991353369336
40.8681871019812 37.2567512265363 37.3799542961839 44.6032218589199
35.2642250366721 36.4476883897928 35.8304004211528 35.4386677371439
47.6040671292013
GPR158L1 45.9090659324223 42.4493032770053 44.8872784170036
39.6292788039793 42.7982502551634 40.244271403831 47.8947595316832
40.7311497914871 38.1169703906207 43.2988017098387 40.547835538778
39.1553169409075
GPR160 71.2963721541702 98.9988668602468 62.3245608177257
112.23915619297 49.5666074434383 48.0350549043063 201.027801320306
172.676819138152 139.04252811433 67.9259304928255 75.9802606133139
114.719721626067
GPR161 40.2543341073001 41.9894412416874 41.924725226806
43.1275490164761 40.5303289242683 46.3203995372658 51.2417443769676
46.7508829977587 40.9460396298042 39.3999056968598 42.357910063876
48.4849194706125
GPR162 59.8838591118356 53.9568457310634 53.9612654850046
88.6287852712744 71.4864243594056 68.9562915755919 50.3519150282938
55.7405934254857 49.1940901905636 65.3672880026114 68.0414563597154
52.6151169127166
GPR17 37.3463875295726 39.3999056968598 41.2365089689635 43.8002456102021
43.5544347011646 39.7394181011603 47.8947595316832 36.9355798143571
43.6670755115848 40.8049408996274 44.1148826235374 41.3481355128089
GPR171 40.2543341073001 44.1239711172417 41.785676764591
35.314469458379 40.2449343247539 38.5252456306656 40.4359177791566
42.0494116081998 44.619512785899 39.5637454855233 42.911855821602
40.4639532172338
GPR172A 746.252816568691 876.959744461441 526.44841348248
380.188301866313 713.685405035516 231.472806936915 511.067537752967
360.342857415756 602.047768767953 232.815747390118 259.812381486915
213.520663841371
GPR172B 45.0985088502363 53.0383634135393 66.5285423077808
48.856176220627 53.8260140375214 50.4022543872993 55.4004611309636
41.4508241771315 75.6382797289563 105.962195935529 102.993620677265
61.5300817099814
GPR173 39.4710743516517 44.3874750238889 45.3974838013476
43.9577120438279 43.844855336215 47.2009420656854 40.4359177791566

38.8964578286293 41.6285300488826 41.3752447964111 39.9899430628542
41.0183489151839
GPR174 39.4710743516517 41.4508241771315 41.1367478979484
41.7144525110048 39.7210115145672 38.861780967827 40.4359177791566
39.6474008369935 38.2874316439311 40.6559419616644 38.7337086062875
39.7844133452634
GPR175 103.945335792682 96.3918711895208 97.1271615802556
151.225738885272 154.111066539468 106.78552222475 167.872060678879
123.597489107285 123.674164545425 128.873145026756 131.68956899407
105.589475270494
GPR176 49.085741080241 41.8848959280916 48.6037684272115
47.481302504066 45.6895223823001 43.6670755115848 41.706879587091
50.5070012045444 50.9010149857308 42.3606390470387 44.3763859390287
44.1452943804044
GPR177 110.146992754585 75.3761360028971 212.813224717262
118.428477744376 191.668846959973 307.264133974364 184.087380444203
179.974187861013 97.3443444704601 242.503720442197 291.232601825013
303.458085392002
GPR179 42.5193208629123 40.2493681589104 39.2707964550441
44.1483976313959 43.5544347011646 36.6271061451613 42.629412460409
46.7980335276155 42.5406888281114 47.2299678280724 47.4630625313159
47.3992733452821
GPR18 36.6480005877129 43.8495248991589 38.2280071951674 65.524821699432
42.6022652963326 38.2293008890613 40.7910160811527 41.1575777497576
39.9444191873552 43.844855336215 43.6629145624937 40.1700117452801
GPR180 94.8260360398356 86.1924491308234 91.2769094348604
97.0813208326187 72.828269171954 70.6691645786819 73.4820932957859
71.0593419018503 75.4572860692649 69.2963913574682 57.9864701726379
90.9361642101537
GPR182 38.0089018744739 40.2493681589104 39.1799070031819
38.9534635879195 36.08956004634 44.8872784170036 36.5352572191451
41.3506832276112 39.1790517315259 38.1042454705119 38.2795150354677
35.2448979801094
GPR19 44.6717213146407 45.7979032939211 48.0654860391912 51.4802440299586
46.7058093820839 42.4580504587581 53.0932869234775 47.8947595316832
44.5678064264717 44.1379638573455 41.3553369544097 44.4475838947656
GPR20 35.4287975493553 41.7337506166619 36.8730514568925 41.3027031037343
46.1804647841331 44.8872784170036 40.3302268293707 39.2672712041774
40.9901805728485 40.1179210534118 38.1835938174625 40.3691324547049
GPR21 49.540449125788 40.4359177791566 44.8872784170036 48.4916117779992
44.6834648302383 47.2927436772465 39.8695929627783 42.357910063876
37.9273653326866 45.1569741609597 45.310660704531 43.1805016690297
GPR22 39.8662795144616 41.4508241771315 46.0727230219512 41.8778873678551
38.8456942580061 43.6670755115848 43.6753843488695 44.797320243931
40.3316153483289 42.6793753550854 44.1148826235374 45.4799270968969
GPR25 32.4552405279874 39.0894172982439 37.5597671487621 35.1451084093692
38.5512429290626 44.7804080899431 37.1643693306382 33.2467975244196
35.9645119596869 42.3606390470387 41.8168470823451 30.9241129241985
GPR26 38.2083710281545 39.2907645923217 38.2280071951674 38.0958223740111
41.0885562160946 39.9397419279057 34.806104801006 41.2551966132486
38.3418933808844 39.8873706855774 37.9230440150891 41.2437378488025
GPR27 77.3752541676627 75.3462586192013 92.4350913779886 84.2804410361247
97.5394336319916 73.8105012820769 85.5027511448958 87.9468208580228
93.0888239577809 95.4072431786073 82.7309298534989 81.1189042503645

GPR3 50.5670783769975 45.4143854024453 41.2365089689635 44.0451487108213
56.8307116636372 57.4777075615651 45.7452456977857 59.220967994972
44.619512785899 38.8507804383261 45.0589947942306 46.3411353377615
GPR31 42.0532901689036 39.6995529770239 44.0451487108213 42.1273928203021
43.7517118200398 45.8325181856483 36.634024014558 38.4540849632254
39.3750969348308 40.8970401536578 36.2435874904689 35.7451401627836
GPR32 38.1169703906207 31.498275130159 37.9482157381008 41.1886975241176
39.7568367479301 43.7630587657205 42.8684958525042 37.86982638952
44.2607789091176 41.7405682794166 40.0766543123257 42.0424641526302
GPR34 45.0985088502363 46.9919960628953 46.4053780043903 50.9339938653926
40.5207392413115 63.805429165532 43.1931731665131 43.2939209072046
38.6558726824244 40.4544974307172 39.0550139257658 46.917149434588
GPR35 52.9240098541574 57.7757446006652 36.5474283500601 50.8567345921473
38.745552403895 43.9724628401189 35.8227939208521 44.293922970134
43.0930293831497 42.5664883002833 44.9713479185144 41.1554481567603
GPR37 58.5759178381027 42.6022652963326 40.5734458834617 54.1240583139871
44.3557408297309 73.5743059777365 47.4439407315335 44.2159020605784
66.3209349903185 49.0841460667818 59.0537479001842 43.6753843488695
GPR37L1 42.0532901689036 37.4540772310118 38.6727842796202
35.9521941716188 36.2048942610546 39.3841861812262 40.9157352260901
42.2577689927563 45.6582729799009 42.3606390470387 37.8372168909583
36.6603338274678
GPR39 37.1485601150873 42.0709558258414 41.3366779545815 44.6905691366696
44.0451487108213 37.5887129572848 42.8684958525042 42.6450179841129
41.5635499110307 41.9666541498638 43.4709415392696 40.2669497398702
GPR4 76.7820250461545 121.84142967836 120.289145237975 78.5141640924736
69.493532577364 78.0586153423713 43.8206425661746 81.1284422173774
50.2223403889892 49.4006068471301 57.8991270166934 51.2318935933116
GPR42 39.3709357615493 44.8529259387308 36.3604951341921 139.925369282704
42.3685136297603 58.0095518210499 46.1634822993009 51.555506218204
40.137818555383 36.9102644736882 43.3085358214154 37.3600014814388
GPR44 38.6454098044474 40.2493681589104 37.6557714476853 43.9282059368202
41.2740822675592 39.4953494583071 39.2533668167483 40.6171774724171
36.9060997041453 45.2556760873335 40.4504608195666 42.7782216342806
GPR45 39.6609238036815 34.0382693188794 41.6079958749402 44.1483976313959
41.1868052124344 46.574682061365 36.8369172900902 38.745552403895
39.1790517315259 40.8970401536578 34.9428206949566 36.449101775775
GPR50 38.8561375693335 36.2103905554984 37.0642268327439 39.6292788039793
42.9090346219023 39.3841861812262 46.3057385979341 38.8495095797881
36.6312676076142 40.4136535208406 40.3452320167286 36.1647506311964
GPR52 39.5676051939342 37.7384958256497 35.8265993915844 40.9607305725196
40.5235471987417 42.5660695551615 38.4456289125816 38.2182800228728
41.8197029429821 35.1266109638107 38.8132170743603 39.3789825717286
GPR55 43.3638918705225 49.83247261979 39.5450079747895 38.9534635879195
43.844855336215 38.8336427948187 41.3594868055879 41.2551966132486
38.1169703906207 44.6918600441328 43.0016969050339 40.8681871019812
GPR56 1075.04105984319 1291.71781703042 2237.08790397818 226.288835947947
350.796638646601 219.64979849407 720.552354470517 628.403814060213
1335.4111951034 1228.1735887278 1111.13061382818 789.511653564341
GPR6 40.4639532172338 45.4882438975269 42.0621425894624 37.7469621225553
39.5595233162192 46.6872654833579 45.1017494688414 42.0494116081998
41.3803269429468 44.8997235283254 35.046946054314 38.9496515344837

GPR61 35.3227485563945 38.9338521051669 37.4642807402741 40.1804147506786
46.7508829977587 33.3470732872681 40.0052661202207 40.4359177791566
42.0621425894624 39.1621898366355 42.6366765186182 34.9138625012006
GPR62 43.4626049853157 36.0366673493555 40.6737160001291 35.314469458379
37.6527901109005 41.8613406887824 39.0458123429605 39.7509165247419
35.6139301508335 40.4136535208406 35.860430241131 39.1553169409075
GPR63 42.442231667357 44.6918600441328 40.931294690669 46.4555908533915
45.5809132232681 48.0350549043063 46.3057385979341 44.0390019242862
43.0930293831497 45.3530683538086 38.3684202018524 45.8546719310325
GPR64 44.5282181324797 43.3458769998118 44.5134109184888 48.3772965749984
36.3390947880186 44.8872784170036 41.253645578517 44.4031868028216
43.0930293831497 48.5374448344297 45.447310733898 41.1554481567603
GPR65 47.5647821785529 49.3103031996204 45.2640606138546 305.959643033169
45.5198200633333 69.8832723783498 54.0170817319522 45.223075311113
44.8997235283254 39.6251842352088 44.7926323492519 44.8872784170036
GPR68 147.719172115976 59.6359245937831 52.5353664902258 136.392873701653
54.1225249245036 56.7429967489965 43.6753843488695 67.6767845246581
43.4656637536244 63.3872214856708 55.5567699051046 46.8129509799753
GPR75 39.9697332660833 43.0227387991135 39.0775870687758 42.6913548400127
40.4639532172338 37.2686490268265 42.9803717121865 44.797320243931
40.8970401536578 39.7835513496873 49.1067959632109 36.1647506311964
GPR77 37.8386378737561 36.89524015739 36.178547300352 37.7469621225553
37.956732373465 35.314469458379 38.6243997507646 34.3865306339645
35.9645119596869 37.4302011516587 33.4071187618571 33.8196020406676
GPR78 46.0920218698606 44.6918600441328 49.5424425850549 42.4580504587581
41.6806984441849 45.4882438975269 47.2927436772465 46.2779564481158
46.2027193115397 46.8954576545929 39.0550139257658 41.6550823405646
GPR81 59.8248482438562 107.005820817545 70.7760293129408 49.5424425850549
100.386011517899 45.8325181856483 122.924027019519 48.87875862356
115.954295099681 124.017319373302 135.308001652191 92.6545309624315
GPR82 43.6706764143908 42.2114134935898 44.4652605017595 45.0017158586743
42.7982502551634 42.0847226073592 41.253645578517 38.9074189954657
42.38204124321 39.528971131769 41.9083581529276 39.9519547348106
GPR83 39.7320564340365 40.3384318592985 42.2577689927563 42.6913548400127
36.8442943492616 40.8970401536578 37.9430145836712 43.3262148375525
39.8873706855774 34.8137085420423 40.4995052849302 40.716553247236
GPR84 74.7955767483841 45.4143854024453 56.410923639913 363.108973029004
45.1137938810047 45.1873487463472 127.724641303 43.1567024035183
41.9635525160083 44.0409178714232 42.5426057453993 38.745552403895
GPR85 36.9060997041453 36.5219488315447 40.6737160001291 36.6501141438378
42.1650127805175 42.2577689927563 38.2933870238743 40.5158867964016
40.5912213523345 43.2988017098387 45.310660704531 38.1421709520616
GPR87 53.3967242431461 44.9370339275041 40.7620472083159 40.7481155008596
43.7517118200398 41.8613406887824 45.8980298854198 41.6466169548846
57.4777075615651 53.0357938671646 49.0361180089431 58.4200819624972
GPR88 40.9558083259517 35.6434124419782 36.5474283500601 45.0798408927912
40.6723334103791 42.5660695551615 38.7337086062875 40.6171774724171
40.6957787921451 41.3321861371202 40.2649309204478 39.7411455670172
GPR89A 62.7669079605627 69.5544840164738 99.6821042055758
58.9168466928565 126.897002134472 66.1082848714279 85.2088054619229
63.3768687067745 86.5616422570166 45.5776159432079 42.2618615571566
65.2125527940945
GPR89B 83.0534943004588 83.0574731969818 140.629562742418
63.1604223919703 144.799432299339 84.3755392205436 87.455991111513

92.4953883194488 105.247736284747 50.7855742231932 57.4622430729629
88.9641595603127
GPR89C 59.3923286524971 70.7702047405718 84.1401040507557
57.2856683017619 105.492189976437 66.9499465764301 69.2322821603564
57.8765910878975 82.4005336009159 41.2598458726002 49.0795382664902
68.6627996695828
GPR97 40.2543341073001 41.5191828467556 41.4528134188983 126.14245679817
41.8441156763056 47.8636003588181 57.0325515903232 42.6022652963326
42.1218529375265 40.8607340038818 41.8645741488184 42.9285286409526
GPR98 58.2401724760744 185.312851727212 451.603052501528 46.3203995372658
77.0301627655229 44.1350486752012 71.0369238830779 38.6088282130727
259.220844287491 110.164155258574 90.9641417408475 151.01326330819
GPRASP1 38.5007204246293 33.3069622728735 42.4580504587581
37.651298468202 33.9142669203111 37.3799542961839 36.9355798143571
40.7311497914871 39.7537274655115 41.3321861371202 35.046946054314
37.6557714476853
GPRASP2 48.8704059222613 58.3475983630509 67.3469281984384
53.3967242431461 55.540888910486 88.0277512472211 55.4004611309636
63.61404854326 58.4891791448514 101.138003125297 78.7260936224802
87.7295604723036
GPRC5A 359.733786489623 52.8796369426118 61.356483470053
44.9370339275041 61.1216330559316 60.4065050472082 92.6545309624315
91.3607028449916 197.070092523652 47.2299678280724 57.2979215534929
78.4023698691285
GPRC5B 51.5888395924843 39.0102066700839 42.9104454767916
46.8954576545929 48.0234896130991 45.3882138838137 53.0277416531043
58.676230030835 45.1899271235974 62.6984594468418 53.1505787220552
45.0985088502363
GPRC5C 174.058845828966 136.613915120582 115.995213598177
49.5424425850549 166.910579215196 65.813547096222 190.187931547806
180.53214143537 375.036941898486 276.550265506988 173.856274004533
268.38649000825
GPRC5D 45.0985088502363 41.6466169548846 43.5710871617107
45.8844325913247 41.6806984441849 44.6524308640784 42.629412460409
37.86982638952 39.6609238036815 43.0919221375514 38.079222289614
40.1700117452801
GPRC6A 43.844855336215 39.8695929627783 44.8872784170036
42.357910063876 48.6224651100733 44.9896851005259 39.0458123429605
41.4508241771315 38.8271346375425 44.4652605017595 44.7926323492519
40.2669497398702
GPRIN1 60.8357760968871 38.9338521051669 37.0642268327439
40.8681871019812 42.7107928951766 39.4953494583071 44.2166118829877
51.2623287419357 48.9931786790166 36.5806287103157 41.8168470823451
42.0424641526302
GPRIN3 43.5710871617107 42.7775752471711 40.7620472083159
89.7554896946341 41.8806343997257 40.7616966228404 42.8684958525042
49.6203214143464 43.9450023643394 41.1222709786153 46.6442692119997
40.9683275553869
GPS1 210.437554813031 176.318618244066 272.315378390681 181.029293055674
295.917955889194 196.228284571015 327.193250073326 267.85990321012
346.844545554188 224.173852018278 247.356027979377 150.636903991608
GPS2 177.65469867262 147.841274204532 188.979306100283 213.204385896543
148.043320937247 214.774035015293 237.582746634246 158.081235081686
154.111066539468 179.158053533144 206.559526386642 156.65747550889

GPSM1 189.984886903595 94.1680559848644 56.0121021290387 75.5431466087264
150.362006499825 96.3976093220249 82.7083356310191 137.878417253635
75.2627855393565 67.2968280876013 80.7563265626648 68.9258122619781
GPSM2 110.729504563799 124.890309370032 140.504336039225 63.6334154351075
128.230543301378 97.0666517150668 215.452117252205 144.898757916905
262.9072085191 82.6036671634626 75.5799609581534 127.486023318527
GPSM3 58.4920021605316 115.840734064724 82.8205269728224 344.38057912034
45.8051846088552 66.8632294055679 86.9185256838836 37.4484310268909
44.3440839686558 67.9259304928255 75.8056934060866 65.7853960266587
GPSN2 672.515393219419 807.223772452842 1205.82775512007 567.978225839768
1178.52115916913 574.467486140819 831.578043520215 647.297964303385
1615.83220398811 810.021622878441 1109.17675353971 860.845934376155
GPT 65.9273663561152 59.2643906275025 42.8270038153139 38.9534635879195
46.0043807840705 44.0804700754786 69.9214859673839 63.287067794195
56.1733549054832 55.0016608333828 53.6895401581575 46.3411353377615
GPT2 307.002236094206 535.585999498708 209.251334289712 96.3741876853801
303.93332785291 103.856444152519 878.863554510943 395.755223372329
416.246271724181 202.278172239082 161.793366042179 113.198141130994
GPX1 901.793120584383 875.787532301293 696.113365766871 1526.94101719128
1000.65752290829 748.913487947791 932.849256536912 550.100918659768
865.63777294494 867.856842601788 714.990611155096 822.822769402853
GPX2 119.853516482249 1287.41527826233 1559.50766982801 85.3754356809649
404.440365771217 127.596368103014 1573.61108703981 483.882804472844
2138.23653387331 3749.78281766196 3428.74022048394 6089.64191751349
GPX3 185.771041814711 509.004054793277 178.559242961756 321.454824353597
141.577762652792 2633.34100695103 74.416117986276 193.395549316561
72.9752827496503 1689.77066894108 1533.38581753491 1801.44003951248
GPX4 1907.47560253019 1928.53890809223 1731.19220270448 2164.51925341969
3343.07293372655 3253.86278287661 2405.8635632802 2220.90215179682
2588.47871581066 2678.43227619157 2598.78910128081 2575.86919902296
GPX5 40.2973086894872 41.0276561769647 40.6737160001291 42.2674195935107
38.8456942580061 40.4681810792098 38.8804689450815 37.9837233601316
39.8089320768344 39.0648733099061 40.4967168818294 41.6972107715212
GPX6 49.1868644496737 44.3529919625344 47.0940613507321 46.614092935749
52.9433495155796 39.4953494583071 44.9863726205428 42.157627240486
38.1169703906207 49.5265949137207 45.4799270968969 44.0451487108213
GPX7 143.194693525776 136.293724800658 109.251112569215 169.818067865324
274.994470779348 131.451150955309 123.247207157645 170.850089909832
110.219274023648 119.493400362694 114.215602320391 95.3145142003966
GRAMD1A 118.49449420132 87.4623442823265 124.032371632524
188.713487905549 80.257554904218 132.985150338484 126.500059295217
130.792245267896 67.6983931011126 114.972374827795 113.879493668284
119.263603432127
GRAMD1C 43.6706764143908 52.8796369426118 52.2132783757422
44.9370339275041 40.4639532172338 42.750861693377 50.8388984085636
47.8947595316832 43.9450023643394 62.2025996860055 50.3876754565799
58.6481644777552
GRAMD2 133.344625514809 43.0227387991135 51.8122261766744
46.1673159982664 47.8075998240396 36.3845892764084 40.7910160811527
42.8482181313226 38.4344387289956 41.4397539024976 39.8184668132023
58.112348043634
GRAMD3 178.643141452509 273.981867154936 332.745139672471
95.6215575902122 158.57392852619 102.711934290382 138.748233664345

195.740109330843 248.599530435666 268.072653271299 225.056827821165
219.103786570566
GRAMD4 159.937192675974 132.588447812638 113.102823481296
181.488068017883 243.15571937138 110.540953771795 96.9051967257119
130.533514998688 129.042495848264 86.500678864429 89.8354888041611
98.1220820761255
GRAP 50.8826620053386 57.493798982912 67.9531693869426 66.3188075271379
46.4831858524246 49.7458652239071 43.8664428040913 47.131828953123
42.9332735344421 46.0727230219512 46.5441096202919 45.7258895124195
GRAP2 40.148780650831 42.4493032770053 37.2838080935695 37.29566077996
39.859345745831 41.3553369544097 41.253645578517 32.1260264272477
37.0126425326534 39.1621898366355 40.1742127891824 42.0424641526302
GRASP 59.1311136551108 67.5417980425932 78.3378279021752 143.13189461696
48.1473938886239 97.7114610828769 54.9765783642973 85.9474373829081
49.3780606970538 57.4665857273093 84.4349803529132 47.9566297250148
GRB10 55.0667638017798 78.0355379907991 73.5527788467294 60.8662233098324
58.7460488758053 105.371285021629 56.4809146784765 49.3217779272599
73.0855167819246 89.6670947876322 99.0095802833291 66.3944891131864
GRB14 42.8447320211178 49.4982025424177 48.97669757548 45.2606665758581
44.4125980509332 44.9432309117725 55.7405934254857 43.282722195001
48.6071208947792 45.6223076037521 52.1519713442163 49.3654837541353
GRB2 106.804269453898 141.924244643825 130.313381811168 311.055487966495
131.67159377585 127.2070318269 140.662969373546 95.6731932757489
123.868848519181 101.277664841914 102.993620677265 97.2535496140648
GRB7 144.928534157065 108.542237471193 108.700838142885 40.5991006167568
82.260686909201 44.5544727111028 167.602045390279 177.82359682336
187.656257079035 93.8630393470857 84.3589195870713 91.8935361535002
GREB1 41.1928222537953 42.1995942415177 43.6670755115848 41.1886975241176
44.0451487108213 40.9950639693255 43.0930293831497 42.2941793046964
41.9097689475185 43.2988017098387 40.547835538778 43.4201407956821
GREM1 433.194113859912 100.708709225818 47.5673419710251 230.486681581762
59.6496385170473 64.4065381524425 43.5627649099623 42.8482181313226
44.476519804187 43.8943892311541 45.310660704531 50.5165823866945
GREM2 39.3709357615493 40.8880530359822 37.1643693306382 39.9697332660833
39.6535880152834 41.8613406887824 39.7576136507593 56.6910078150745
38.1169703906207 43.2988017098387 43.4709415392696 43.6753843488695
GRHL1 50.206880585207 58.2571968311834 54.8265655909623 41.2567470068038
53.9451047872024 42.0574848021044 51.3064929551674 59.499042267068
50.7364620410977 47.1863897448202 46.972521499515 50.8442982601142
GRHL2 88.5140510969737 71.4197504261453 97.9004351588426 44.550322123994
282.445037659981 58.0095518210499 236.565670642447 45.2806199505029
129.613152757362 88.9738853594498 52.104880481326 64.1607001437806
GRHL3 42.4607795638552 194.435776102991 86.6655289455628 39.6609238036815
118.993842599424 53.9517841438975 249.822387177805 115.643361390258
118.556124582201 62.0347191940821 82.9431583622036 56.6537562775715
GRHPR 141.471672990444 94.8766888888828 74.0913177754682 92.084526929319
114.78317743597 151.088035451997 120.582525405708 82.3364971911816
87.1471161251724 122.024487499513 120.142759111608 126.620595729562
GRIA1 37.4554876386402 42.6022652963326 37.7384958256497 42.1273928203021
36.4434672195785 43.4681646416585 38.2933870238743 39.2672712041774
46.9739160257217 36.4651112441576 39.5399188536544 43.8575940017119
GRIA2 37.8386378737561 44.1239711172417 44.4311655729487 41.6564075217517
38.0598374201407 41.2567470068038 45.6042625176633 40.4359177791566
45.7452456977857 44.6918600441328 44.553780984244 40.2669497398702

GRIA3 43.8654109265055 43.8495248991589 43.0971787292711 42.9358255168086
43.9450023643394 45.9266745571724 51.0889740983457 46.7414272831181
44.8003345643334 42.3433931248155 45.6677978788306 40.4639532172338
GRIA4 34.9138625012006 39.1553169409075 35.8894141225349 40.1362682639916
36.4434672195785 39.6609238036815 39.3999056968598 37.6058760248614
40.7847110517594 37.1275334308172 36.368850660757 42.6278608611758
GRID1 40.5654775358427 39.9697332660833 42.4580504587581 38.9534635879195
37.0526522140242 37.3799542961839 39.3585350448719 39.9576138799997
41.863927676552 42.0621425894624 44.8872784170036 34.9138625012006
GRID2 40.8681871019812 42.8542642792862 38.8775560911841 42.1273928203021
37.0526522140242 37.1317006162938 38.7337086062875 42.157627240486
38.2215591930186 40.2112371512838 38.8507804383261 35.1451084093692
GRIK1 39.3303857231606 40.6380495994768 38.1055732602504 36.9912468186322
37.1565984182084 40.3034574135048 37.3523615196416 40.6171774724171
40.4220196471124 40.8546470680011 35.1470994647364 42.1537476443423
GRIK2 43.1413768296576 40.8546470680011 39.7411455670172 40.4359177791566
41.0885562160946 39.2576865880886 41.0808257270221 42.9301944191913
44.563243534964 39.7460325887874 43.1724740315182 43.2776458914333
GRIK3 34.4021554788966 40.6380495994768 40.1985790927248 40.7481155008596
43.6587492470348 40.244271403831 37.7136011474903 41.3506832276112
38.4344387289956 40.3235655331796 42.911855821602 33.6902293666089
GRIK4 37.1485601150873 39.9697332660833 43.6670755115848 40.9607305725196
37.2567512265363 37.3799542961839 39.1539892144371 48.4670130605028
42.9020321355927 35.93950183586 39.6403089864678 35.5523662009011
GRIK5 45.0985088502363 44.2630383225933 40.0998167098057 43.3078542962968
41.9842695818285 48.6621527194963 38.6243997507646 45.2806199505029
43.7630587657205 41.2276963324981 47.8988096575648 34.9138625012006
GRIN1 48.9600850533759 55.6050719338367 57.6371756925687 64.8336014343435
59.2861879506028 53.7155925246916 51.8812208789107 49.0231541118532
61.1560835966725 61.2431917618973 59.3615334058994 58.9836017745712
GRIN2A 39.3709357615493 42.6022652963326 39.3709357615493
34.5425384901279 43.844855336215 40.650742261428 37.6036146841933
39.4710743516517 36.4476883897928 33.8984830800739 35.6041926503637
39.3575468494941
GRIN2B 35.3227485563945 34.8137085420423 36.769073068743
31.8431345631453 33.8558232866797 35.314469458379 33.6696565339534
35.7973685350741 38.6322728994674 32.9981302187884 44.2925355984056
37.6557714476853
GRIN2C 42.0532901689036 41.6466169548846 35.4484652445078
43.5627649099623 46.0977687311222 46.263826584899 37.830876898596
40.4359177791566 44.1519466943185 38.4187686307607 33.0553208959996
38.4330755397458
GRIN2D 44.436995101921 46.4861258030402 39.4710743516517
43.9282059368202 47.2074332636566 41.4508241771315 43.6753843488695
42.9301944191913 42.5406888281114 31.6981395162348 36.131485826118
39.4490233720848
GRIN3A 44.6231541342 35.9305494063137 40.931294690669
38.3166554965434 42.0709558258414 36.6271061451613 41.9393838138203
39.6474008369935 33.1602385985469 38.745552403895 41.2598458726002
43.9541718254769
GRIN3B 53.593974682612 46.4861258030402 39.2707964550441
49.3654837541353 51.4527545197672 43.2805590023412 38.6243997507646
43.1567024035183 44.619512785899 52.3304586399695 41.3553369544097
40.5703581862752

GRINA 365.517719684028 518.527535205975 402.794855141389 829.152040996817
323.048740324895 290.371909722404 381.198152331624 288.335410596699
336.679355848349 384.919835924792 396.493831159481 191.116764060948
GRINL1A 45.8718712965322 49.7293832352456 48.5544398725959
47.2867027408452 47.608637584681 52.0038114601109 47.3773591105902
44.6107027295193 53.0208478294567 42.023861273923 47.9233782645011
47.9136803939857
GRIP1 43.2724556187214 41.8534933577029 42.7881985796277 39.2406797798509
43.844855336215 41.4508241771315 41.8315602975157 40.4359177791566
45.4799270968969 37.7598032934773 41.2598458726002 41.2437378488025
GRIP2 37.1485601150873 40.4904698474072 39.7697110924474 40.1804147506786
39.5357194705749 38.2971553419489 42.4939802311136 39.9576138799997
41.1241827976738 42.233842834629 39.9899430628542 42.7275315121163
GRIPAP1 533.820505121067 335.002151198999 385.827237353675
487.540800793233 288.065258507995 260.279110870904 502.205366225368
788.950470077369 307.754078087321 892.323580265478 860.265067346622
710.579735673002
GRK1 48.6621527194963 47.5647821785529 42.2577689927563 44.0451487108213
44.2607789091176 39.2248354955045 39.8695929627783 40.5158867964016
40.4997186069494 46.1390887492649 40.8225507267584 45.1735152121056
GRK4 44.7520317855773 45.5506835119207 41.3755760434934 44.4978265168756
42.3536549114165 47.4699236780425 42.0605906240248 46.9986083378817
41.2870505273664 44.9536076475946 43.5258090162298 50.3334140358007
GRK5 51.9545473262296 62.843734579668 60.1444334141838 68.6379135603075
76.9788081114727 124.362644216178 45.7452456977857 63.61404854326
50.0802438185974 78.839411865787 67.9922604709859 83.8679756709676
GRK6 67.7189686993694 75.854888525664 63.6894484284172 96.0126406688619
65.6003544901554 61.6219790276408 87.8746766762445 79.0373265781543
91.0154871536701 58.7460488758053 57.1441484662045 55.1804535845496
GRK7 32.3120809246762 34.5023764006976 42.0621425894624 38.5252456306656
34.6227169056411 36.0306975524309 32.4466419672036 38.1835938174625
38.8271346375425 38.957687788868 36.9060997041453 27.0325093564553
GRLF1 37.5058442851529 41.2276963324981 39.3344387353654 43.3744503319109
38.7937049365871 42.8598805316863 40.6130792100165 39.0102066700839
41.3481355128089 40.8462613935508 41.3884410579735 38.9074189954657
GRM1 38.5007204246293 36.9994058279362 38.9790184198145 39.2780515621926
41.5668884168669 40.9506062495231 40.1235656101329 40.2382908228517
42.4540072551568 38.3092241935352 37.4642807402741 37.4554876386402
GRM2 64.3502363726659 54.8439819445945 57.423873475544 48.309866878365
47.6884372645373 45.9266745571724 67.3976855661302 52.1788489555942
54.4912806027381 48.4890615340603 55.0404566626821 50.4847465816184
GRM3 44.8378856799291 43.5627649099623 37.5222959113204 44.7980740332174
41.9397336249742 41.0012755104374 43.9450023643394 44.5658219748173
40.8970401536578 38.6835649080278 43.9063490244748 41.2437378488025
GRM4 68.9745034946434 42.5269793679335 38.0452862258803 46.4555908533915
55.3100442506299 44.9896851005259 49.2546642180676 45.642304356051
46.1101825640108 39.7835513496873 40.723990462047 42.9887069977696
GRM5 31.4517139158876 39.0102066700839 43.5710871617107 37.9875302985336
40.0599807618105 44.0804700754786 34.0777108441756 44.1311654985682
37.8319142847141 39.6719190054379 38.079222289614 37.3600014814388
GRM6 35.3227485563945 35.1224289925572 33.4240466670776 39.7411455670172
33.4316681623175 36.6996187567892 36.9355798143571 35.0510338774593
35.5220586859301 41.1222709786153 39.6403089864678 38.0452862258803

GRM7 41.3387790895716 40.9298924777825 43.0971787292711 44.0451487108213
40.0230205296014 41.9097689475185 46.0905079010237 43.781101409552
41.7627910693483 44.0578434007778 44.1478805895017 42.357910063876
GRM8 47.0940613507321 52.502055890816 56.1434024065079 44.550322123994
50.5110359971301 46.4648868771662 40.5585676679482 45.9604707098953
39.9444191873552 65.9189023794337 47.7535797917441 44.1452943804044
GRN 619.337575854369 623.574706535278 966.803172864162 1157.65264077595
649.487838020327 397.850286751468 454.701322158431 527.97309138633
945.065986430527 440.728435508514 448.383843915421 338.475097952023
GRP 75.9909523932333 39.3667161654772 36.9795961564828 38.9534635879195
36.4809590448114 41.2567470068038 39.3999056968598 41.7419877611072
39.4433784795209 37.8217989841918 41.7651272076914 40.9683275553869
GRPEL1 146.173307474688 352.600793906035 228.328635242684
220.11066803442 429.983084929605 311.901423694475 258.851412005402
162.993316022946 292.065366215278 141.386644354989 168.561260174501
258.757703729905
GRPEL2 149.629940171074 159.667153030627 160.264217082275
116.852483297949 175.362103430164 137.551493752877 155.406719968961
207.600413958886 225.725485439572 176.07173250529 149.076166432567
121.259683158193
GRPR 44.2350046179299 44.2630383225933 42.5406888281114 41.7144525110048
44.7829632472754 40.7063426028871 42.7431359850339 43.5544347011646
42.5864346233886 37.0740019672788 38.6335676551747 41.863927676552
GRRP1 52.4518619184838 75.2519194195288 71.6849360818041 109.310200749289
70.9638091288679 156.088284999883 57.9583667603818 76.1493261430901
47.945438269111 69.4131195465674 145.28255999556 70.5903356985988
GRSF1 435.094968715663 517.664764759512 317.892699374852 252.870708814507
291.596692248591 277.878824242495 284.942182200489 234.495626550751
486.757854744053 404.599730419192 301.867441371793 282.38663225575
GRTP1 82.6246523811721 164.146682514925 87.6960277122561 46.0228732507872
138.349952332963 60.065511140146 138.748233664345 82.9322141700549
121.449647909665 110.219274023648 145.583868248675 96.3918711895208
GRWD1 320.330057050615 220.010492114456 226.262409390222 162.833785414988
354.015808474189 223.801865347687 188.750770811584 255.800286504974
306.746364062777 205.944717184567 321.069136463599 196.17328008185
GRXCR1 42.5193208629123 39.5050383901884 41.6079958749402
37.8442841690502 46.4343327861696 45.6129956328415 41.3594868055879
42.6450179841129 40.6957787921451 38.3092241935352 43.7585538437696
43.5738557773903
GSC 49.4151347850687 39.0102066700839 37.8386378737561 58.3475983630509
42.9090346219023 49.8472708021525 38.7337086062875 49.085741080241
36.8127605061228 35.0668743708816 39.0550139257658 37.0953622406687
GSC2 38.5648843914189 38.9338521051669 39.4710743516517 42.2496337895968
38.0598374201407 37.3799542961839 34.5633623842083 32.4466419672036
49.9162522057712 41.9666541498638 37.5583886316038 40.7704587752083
GSDM1 42.0532901689036 47.4076235350059 50.5089777244781 40.646702696033
55.1563703466328 54.3478800757394 40.7910160811527 38.2782436497441
44.0519245668737 43.844855336215 44.4688999098904 45.9693232070002
GSDMA 41.9407041937973 57.0875538213735 42.4580504587581 48.4916117779992
38.5512429290626 51.702461863489 45.8980298854198 46.1673159982664
42.6366765186182 42.1622837416589 43.5710871617107 42.8864415494484
GSDMB 135.41852974791 243.672418126374 285.322305931794 79.9126192278104
187.147252401703 68.7342341724306 179.767874494671 287.565588186962
251.027270265657 168.869258524932 255.126812649967 180.447835336714

GSDMC	152.86270446968	46.8236022402545	81.5782824513693	54.1240583139871
	46.3150221024046	39.2985791324641	40.6750540874299	48.9826868640753
	52.2631308081333	46.0119912782855	47.1477056711335	93.523394623588
GSDMD	557.094756796515	446.757901018137	336.679355848349	327.283431584541
	245.960009147234	222.773939093637	298.924873508403	191.811190401876
	286.996922972683	328.080879084015	308.183340806373	243.763402918148
GSG1	40.0557788909923	41.7656868106485	39.7411455670172	39.3427411763067
	40.8873907150245	41.5146360101556	43.4544762830742	40.2906799807722
	42.8414096678708	36.5806287103157	41.6616444147804	38.7119440509409
GSG1L	51.7607563002005	61.6878176260761	56.8595344107711	91.7068354379889
	72.828269171954	65.6914955077809	73.6855877240541	54.5125550937898
	64.3425836997629	64.9959712888709	51.6414075056938	62.3997407955361
GSG2	54.8439819445945	49.6203214143464	51.0539624358342	43.5627649099623
	60.9710783023013	39.2985791324641	68.5725871332774	52.0503062610092
	55.7522234735038	42.785429206422	38.2795150354677	41.2437378488025
GSK3A	48.3814421939888	47.6229101519945	47.4740726032707	37.651298468202
	48.6224651100733	43.9724628401189	46.9929836306816	44.2159020605784
	43.4656637536244	41.4397539024976	49.7363778831003	47.3172462673426
GSK3B	181.966839676473	158.449570606242	191.668846959973	284.795064481553
	235.593721159442	114.142157259903	267.523307072523	246.965844426351
	262.024495926094	169.838827072973	132.007307754378	146.389547177092
GSN	169.061093538895	112.733531624555	122.952515297686	220.11066803442
	67.3568864741196	1554.42895572029	130.812376632261	88.6368226476614
	67.7898066939846	776.670493028756	609.243908604949	433.637087338002
GSPT1	1126.31998762007	1086.12171444149	1630.25992078321	858.228943026906
	1287.41527826233	619.950241162173	1224.80240464583	1858.94179312821
	1255.10866316499	856.196444531264	723.853348464335	1024.97546870349
GSPT2	78.7450149191851	131.79241580689	58.4137028673494	135.553887060537
	127.008218054726	136.377736482671	126.63987648161	112.930164222877
	131.929700183999	138.765621737736	106.20479137406	127.093495914275
GSR	74.9491421278749	61.8124171056747	88.9255521472874	64.3746985855249
	57.8039717833131	69.64983895075	82.5050369860039	70.2541961995753
	83.9387217886961	68.2708120569221	65.0359415104302	72.8306652548968
GSS	293.355303451014	256.165801611585	290.316757790733	152.545682001987
	287.932410610182	151.088035451997	316.373650015333	206.559526386642
	278.303493506139	279.782392390288	246.313146942736	227.439319520245
GSTA1	44.7520317855773	43.5182502375207	91.4581948567397	37.651298468202
	40.831207953647	38.1169703906207	54.4934782594218	49.2019743609873
	86.754538970161	40.1613888954735	42.1537476443423	42.4607795638552
GSTA2	42.0532901689036	40.7602938767524	69.4967762263181	37.3600014814388
	38.4135023167287	42.5660695551615	48.739479557328	40.2770232552555
	75.2779848783367	41.4659679379482	39.4749602565407	41.863927676552
GSTA3	44.4639405506967	44.1239711172417	40.7620472083159	48.4916117779992
	46.8086806989607	42.1046072075724	43.5627649099623	43.7731874801003
	43.1805016690297	43.1839109702714	44.9081227425497	45.6233434183356
GSTA4	67.2437599548936	76.5411379733694	89.2285288333749	60.620259884953
	105.825228126465	126.485649460666	59.6780848951767	61.7572675712557
	72.0655217037557	103.521926015878	91.6826894933058	73.0333258448698
GSTA5	35.9305494063137	37.3634897565502	40.301483914446	38.8495095797881
	39.4693871570028	40.1428591998404	43.9450023643394	39.9576138799997
	41.7627910693483	38.8507804383261	35.046946054314	39.7411455670172
GSTCD	72.5516795240251	79.5270065657539	62.8753829543444	85.7013212907541
	80.2027893085758	96.4740168904128	73.5700402711904	71.5345397102823
	94.1680559848644	84.8193235226936	91.7296524744575	85.6582555450821

GSTK1 1188.3486464341 1129.71973548345 2568.97574985073 962.079396754147
1280.77934331703 738.059797123491 629.763102939071 882.105892529265
1199.29782389306 1483.40077183826 1247.50618540769 1046.42391243532
GSTM1 147.033637977077 282.68881112438 257.050603883498 640.407302868904
7562.42822724655 1561.83892117664 228.809697062726 10351.5373412096
123.729687262037 4550.56817942699 451.437791473506 246.965844426351
GSTM1L 38.957687788868 42.0709558258414 42.9104454767916
39.6292788039793 43.3332476888305 41.159706131351 41.5817737564592
49.2901038327894 40.4220196471124 40.6022003765153 42.0750889188255
39.7411455670172
GSTM2 151.175959542368 374.601792595646 357.668448180268 779.333013700435
8144.52133665388 1925.20977162704 101.410859084491 10996.2779403491
89.7184505543918 5636.93532853955 623.574706535278 331.173265739654
GSTM3 76.0532592222027 51.4413099192899 46.3411353377615 60.4646208319121
187.002948435359 511.067537752967 70.7238101239421 102.00080367858
46.4861258030402 1035.67547271468 381.198152331624 179.581456201363
GSTM4 52.3660712483179 61.657921069449 54.9944485073259 49.085741080241
81.6949047529395 69.6021553604792 58.9656737189764 63.8008894786105
50.7619345314571 67.2437599548936 47.1970991228159 52.7847097260565
GSTM5 44.7044140979108 44.6918600441328 39.6451483836089 39.7411455670172
41.467005949568 196.17328008185 43.6753843488695 51.9447450229707
38.2215591930186 152.880772824839 129.236597599908 60.8662233098324
GSTO1 971.856840043688 1335.4111951034 1097.92519921873 2944.3433949897
738.059797123491 1123.99110499679 897.198663109723 831.578043520215
698.595302020108 1282.9181829891 858.228943026906 1372.51401036753
GSTO2 58.1027665533767 267.608189207303 430.868309653765 48.4916117779992
257.325282051981 73.1169003714219 297.105654460146 106.140215438654
304.49707512712 574.467486140819 377.573838771285 401.202348352506
GSTP1 1390.12380748446 3618.78958408675 2424.0414102425 1686.40194722823
4048.33132153903 2280.42953098693 6356.05807792831 3502.80810319252
4344.25847766877 3428.74022048394 3999.32180022337 3786.90274075023
GSTT1 317.127679512963 36.89524015739   358.402309513947 295.014183084118
69.0975200641705 698.105755081829 1339.29022988209 714.990611155096
482.071795051342 37.4302011516587 445.016226732679 727.788854407888
GSTT2 47.0940613507321 48.6447083765118 50.0526763693329 39.5637454855233
86.6854406823303 49.5424425850549 49.7462241109067 49.1330497909043
51.0062122023297 63.1394716239975 63.5716064500377 51.3872161199373
GSTTP1 38.385865183581 37.8217989841918 35.8265993915844
47.481302504066 38.6454098044474 37.3799542961839 41.253645578517
37.86982638952   38.7298222155532 39.2623423164488 41.3553369544097
36.9976231206067
GSTTP2 59.0965219753805 50.8559467080729 54.8317492967714
48.1662933382789 50.6987358483181 45.8325181856483 64.0476795495651
79.7170181855209 47.3102170376006 77.5413980345198 67.4760456862775
59.2720151666582
GSTZ1 50.7903824729818 48.4980626712175 60.5060523570927 54.9961068897605
47.7448435500604 46.2027193115397 54.2625553617642 51.6060290544385
50.2931656908095 72.369223065749 70.8835636550472 56.2387806424733
GSX1 34.4896350146096 43.7644428186265 46.7508829977587 31.6357375926754
35.2163900400329 34.4896350146096 38.2933870238743 34.5886782974123
33.3643994200057 43.0919221375514 41.0050831223107 33.0217499673686
GSX2 33.4902421268456 38.8336427948187 39.991353369336 40.3180310664156
36.8059561571675 38.3418933808844 38.7337086062875 33.4534810696886
39.6609238036815 40.7063426028871 35.6041926503637 45.8546719310325

GTDC1 54.1745577382276 51.6591854913499 54.2978574294868 56.5583372864784 49.2495730217753 57.013135787334 44.9272279146193 54.9169289331775 49.1940901905636 49.904979214379 53.0446815750355 53.9431600945419
GTF2A1 42.0778399888414 41.0269624684418 43.4709415392696 41.0726336841311 40.5207392413115 40.5912213523345 42.7431359850339 43.5738557773903 41.5635499110307 43.5599890586394 45.0071770692626 37.7357214460683
GTF2A1L 41.8409927585705 40.579168499767 40.7256433650856 39.6292788039793 41.5668884168669 47.798305839406 39.824238926666 42.4580504587581 41.0381860307445 44.858845220285 44.5851823170508 46.1005394461164
GTF2A2 413.31638457585 327.786619565391 218.313812956524 303.458085392002 432.945510073269 351.700618429121 470.676006277762 404.524605612471 355.351648424266 176.318618244066 203.870471031175 415.15150032168
GTF2B 275.549982777518 226.090904721424 191.298525555624 239.229093340475 198.754967214875 227.404530886674 213.622816589927 265.467750156221 118.93749473833 150.829277770546 159.667153030627 211.625447303889
GTF2E1 115.221134337342 95.9750691854349 93.6591201235745 118.49449420132 126.711833914667 98.8478655411492 93.9805435116156 81.2850357499192 102.457537266105 81.7171463878604 70.3381994440425 96.8141712830545
GTF2E2 715.662312404643 469.893430869091 802.036670929858 558.855313555287 357.029998842776 531.69595054137 584.889303693522 344.314901683001 534.292112344761 571.790034754076 479.507453857294 734.143790759359
GTF2F1 186.818097299665 278.629995328901 210.485586903711 219.103786570566 421.445268560815 199.57367025082 259.332340491331 261.6201598868 209.986313475002 303.323289734056 284.795064481553 197.660480001139
GTF2F2 637.824136761292 294.620463776036 283.484056482174 290.316757790733 315.799449362526 384.401256392893 368.972794063004 326.605198138576 353.598144765197 318.768560912915 296.271895016431 368.246708866999
GTF2H1 52.0756619340684 52.8455400026194 55.2141529491512 49.9672547361866 55.6617952786921 59.2643906275025 43.4544762830742 53.6210959113222 53.5224822626949 55.2214187655851 57.6171094774963 60.6441065119394
GTF2H2 42.9104454767916 49.0697565324841 45.310660704531 43.6726153508978 42.7107928951766 41.8613406887824 45.223075311113 43.844855336215 42.0621425894624 39.0648733099061 44.0409178714232 41.4508241771315
GTF2H3 53.4921736109774 73.6036945547683 139.883681090758 70.0402992940342 156.822939275312 90.407200101342 76.3583555666815 64.893664076381 70.4262478188233 46.7646877499666 68.7091708087448 61.9882398986333
GTF2H4 322.169157552725 205.695230410884 228.134171304754 138.053682842992 352.997455210484 184.466097348908 363.602775933416 209.178131628825 263.706801812277 186.522518256229 166.608353896248 135.398193235699
GTF2H5 846.773659307087 277.5073518536 401.817698574441 273.231509963261 244.736442646499 426.622204035624 242.58992925232 409.899024157206 298.54401957868 311.1022748407 272.36028618845 593.345148568451

GTF2I 50.8930565655395 74.2984889814139 74.5173894803763 44.1483976313959
70.2565873027242 54.9108046955355 55.9656969281284 46.7980335276155
66.8188271427672 50.4234835272833 47.1477056711335 57.0006212637829
GTF2IP1 160.962078065068 369.258587948416 328.176884082116
108.660169650259 260.383969219887 175.193752938517 198.497745548245
151.088035451997 270.756849919679 96.2799270895937 140.175068871414
212.945601743319
GTF2IRD1 91.1363393944977 87.6763065012524 95.0738959950301
46.614092935749 109.398472684587 79.7564155881026 98.5663841503803
107.325456368022 159.554916039519 74.1228645930185 76.9904391375645
68.7091708087448
GTF2IRD2 54.3554233228353 56.463481075522 63.9148277361329
44.7980740332174 71.0593419018503 86.3888090084564 63.1604223919703
46.5717043290731 57.2716485658185 53.4109882045447 52.5154971256566
61.2108152457954
GTF2IRD2B 136.843285454801 176.816741656659 141.376779431031
111.626292905866 171.829798001437 141.594428857706 205.695230410884
85.8277891453447 287.247399926139 79.8511759086625 87.7253906521723
95.2367177680328
GTF2IRD2P 46.0398513158469 50.9643363406241 47.646800205189
43.2504382247503 54.3668215761835 65.5029051840465 45.3597029614942
43.3262148375525 52.8502823653368 58.8605811695948 60.1756087584664
61.2441059007934
GTF3A 949.278493776928 705.283979182062 818.927157206447 1137.99307039386
439.664256107097 823.933615182634 784.268074988084 1053.67279913417
744.762787024945 756.801598119483 673.522667186915 979.468753716758
GTF3C1 173.838110431513 130.35828139117 168.482495410546
81.0057400164851 179.322661675979 121.613731804111 114.230358276599
210.200786815048 135.706144105357 115.853867920678 160.499410146402
85.3431175579775
GTF3C2 288.386446189379 332.830805115112 239.792011765323
171.501864800548 326.121479451246 172.506486240017 313.399524593316
314.609371608151 254.579513401792 244.498602338731 210.38953368311
179.953662481428
GTF3C3 185.227637763623 228.747320301945 221.598637828404
141.852330109472 210.73006676203 157.109085253328 137.764748752401
171.058650956601 188.750770811584 174.058845828966 179.064697219173
191.482692108417
GTF3C4 37.6397652536077 41.0678614026819 42.2577689927563
45.5869891570251 40.148780650831 43.2805590023412 39.4600924554505
40.7311497914871 38.4344387289956 38.3092241935352 45.9104478407175
43.6753843488695
GTF3C5 260.124930930243 216.404466544919 137.831070407907
154.287396750021 254.639522735592 126.485649460666 384.992066112903
175.798140574991 258.657801126493 244.389359320438 233.07404514708
192.861174667738
GTF3C6 208.67074469962 135.377859777529 130.230482445573
114.734157967466 83.764882413379 108.323780226301 120.327382682483
328.387270297393 105.376705821141 69.8941497035747 80.7198077526005
108.337776546183
GTPBP1 50.6473771879221 48.7847528954724 43.3085358214154
45.1944222548351 37.0526522140242 47.6803619768589 41.706879587091
49.8344973836834 52.8502823653368 49.3000844711116 45.2265319826548
47.2131144508464

GTPBP10 44.1737502492846 50.9895771651972 45.3675390992851 42.8235313007746 46.4343327861696 44.6024422254257 42.2577689927563 49.085741080241 48.4497719907777 39.9519547348106 45.1778228816327 42.9887069977696
GTPBP2 67.0163226819964 59.0709442090059 54.9944485073259 61.5515639412892 59.6922302604696 50.0748292497578 57.51482768194 50.7364620410977 64.7116910405493 58.8022152133979 52.902043370862 58.3586374083245
GTPBP3 106.454367084321 136.04447103201 139.022985518859 72.4834467838798 260.740251007349 83.5274372175811 107.40295615514 137.156113501125 120.19033659661 121.023370640285 113.6408244935 105.699748702315
GTPBP4 561.855286945959 610.502562878788 419.741944629785 372.131471268346 1173.47285626656 427.066481790415 776.670493028756 345.942093303061 542.069156178791 323.095468495074 297.701126167694 440.93348620369
GTPBP5 45.0985088502363 41.8848959280916 46.3411353377615 47.481302504066 42.6022652963326 46.1513019317765 45.223075311113 47.4740726032707 45.4799270968969 47.8207974930031 39.7342718293432 47.6040671292013
GTPBP6 537.342146022804 507.851120677089 402.221227674218 406.500583302121 351.290929267326 550.100918659768 617.271611375695 667.837116137964 683.75696650704 608.579654909865 704.942710834565 437.148137705103
GTPBP8 61.6858759579955 74.0420372528779 85.8059456975413 74.3292739001607 113.518333340953 79.4053001255904 78.3082773071531 69.5225793065367 72.3095457386378 59.4969799685856 63.8558858499025 73.4687357112674
GTSCR1 41.4508241771315 40.5400384479766 44.696023817801 45.7397504535169 42.6022652963326 45.1873487463472 46.0228732507872 45.8669209716694 42.9020321355927 42.0621425894624 40.6373400186325 39.8497227447888
GTSE1 71.6849360818041 71.2370255410834 58.112348043634 55.6481366056639 104.072423445726 48.260931742163 84.3526980051279 69.0819394834919 88.8772406115484 39.7835513496873 46.6442692119997 47.2131144508464
GTSF1 37.7384958256497 62.3983214098965 42.2577689927563 41.3027031037343 36.6354013629791 52.3187919317561 39.3585350448719 38.3684202018524 268.38649000825 42.8936990772838 39.3667161654772 37.6557714476853
GTSF1L 40.2087556903277 36.2561152084183 38.4456289125816 41.0219290963778 40.4639532172338 36.0537855103446 37.5469247328848 38.4128104869434 39.4762546236449 38.4187686307607 38.1253290586431 39.6352613956998
GUCA1A 39.266565827316 43.5182502375207 44.6032218589199 43.6726153508978 41.9842695818285 43.0860383229288 42.0605906240248 45.3872406432794 45.3756389170606 57.2813205536176 51.2318935933116 42.9887069977696
GUCA1B 53.8133030091912 48.4497719907777 45.5883690965192 39.7840818430159 42.4493032770053 44.3648993568405 46.9929836306816 51.4360013601805 54.5374188496807 51.7594352155304 48.5292718280498 60.4872675915085
GUCA1C 49.8472708021525 47.2055133646385 42.640829914102 51.949834638817 50.0580547772867 50.2163892855092 45.3597029614942 54.5125550937898 50.7619345314571 43.6033221838149 48.3847843498476 52.4992300612807

GUCA2A 41.0608773166058 50.164201758929 43.7630587657205
39.3750969348308 42.7982502551634 39.9397419279057 45.7452456977857
46.5717043290731 44.2607789091176 42.8936990772838 41.0050831223107
38.4330755397458
GUCA2B 40.9558083259517 40.2493681589104 36.2683886571585
44.0451487108213 44.5668141895992 37.8102105794655 40.5585676679482
45.5581356216957 43.2761454096265 40.8970401536578 45.2265319826548
41.4508241771315
GUCY1A2 72.4966502412724 46.8954576545929 49.1067959632109
57.4595416719581 44.7829632472754 43.1826328292558 44.9863726205428
43.0390857698452 44.0519245668737 48.4890615340603 43.9450023643394
43.4709415392696
GUCY1A3 115.995213598177 77.8946473414547 71.2681122787939
143.973063517706 60.7495043977707 90.1080159649841 60.4593810615882
57.2350830635912 57.3005868295668 51.2786187688564 52.022479439889
46.4952206446229
GUCY1B2 58.8224410806741 41.2598458726002 73.4962349716902
51.3851157261451 41.2740822675592 44.1737502492846 65.524821699432
73.746472097049 43.8482802038826 62.1035858593388 62.6048727240291
50.8442982601142
GUCY1B3 58.3702889417801 45.2556760873335 49.3000844711116
48.856176220627 55.6069214673486 49.1013967533186 47.1345949868029
43.1567024035183 50.0526763693329 44.4652605017595 46.824299585607
46.917149434588
GUCY2C 40.8681871019812 40.5400384479766 43.7630587657205
43.6726153508978 40.9950639693255 37.9951997351435 39.0458123429605
42.0494116081998 37.3600014814388 39.0648733099061 41.0808257270221
44.2527566343661
GUCY2D 40.148780650831 43.7644428186265 43.4709415392696
41.0726336841311 43.6587492470348 40.040778349137 40.1235656101329
44.797320243931 44.2607789091176 47.0055981216687 41.6336636285875
49.3984571719129
GUCY2E 42.1537476443423 43.4396866157171 44.2405728317107
40.5484717678957 47.9128504683618 41.7627910693483 39.2533668167483
41.4508241771315 37.2810767789306 43.6033221838149 45.0589947942306
39.6352613956998
GUCY2F 33.0463640202858 42.6022652963326 41.437237454134
38.3166554965434 38.6454098044474 42.8598805316863 39.8695929627783
36.9355798143571 39.1790517315259 41.5435611403119 41.6336636285875
41.0657892043496
GUF1 59.7179445506128 89.8234189405239 75.8201286658785 81.382229980722
85.6236091398006 62.2818512646893 72.8653205184014 118.93749473833
77.9978216366573 59.2628513357357 57.0154839296649 67.9850517608624
GUK1 900.517711546397 1050.05355467301 1436.67510926345 1033.99699402259
788.232434009173 1575.71609894038 1041.84536202049 787.671342652389
917.179747195874 1019.67015337432 943.73554571528 740.994425128952
GULP1 49.8472708021525 42.6939394537076 39.5450079747895 45.4634652319753
46.6397391251762 87.539553421006 43.9450023643394 45.7476095696859
40.5912213523345 47.7180816682562 48.2910530887214 43.2776458914333
GUSB 221.847615564378 361.158558609751 457.502917254598 453.260719561292
346.116601612353 292.156216736355 343.764108830519 290.448089141121
615.442484911026 277.837785971587 215.399634344016 317.892699374852
GUSBL1 139.543015878846 239.792011765323 244.343381048643
194.225364352044 161.65846687852 164.780216383719 186.840847924376

155.872353189452 233.96534264787 147.371811804618 199.634544522948
148.71330612986
GUSBL2 57.6285775590145 50.8812920005251 60.6567691532561
71.9062462001972 69.493532577364 59.3618843678775 93.748984888348
96.6957892186886 58.174555206394 57.4665857273093 73.0247140074919
67.5047089908724
GVIN1 56.6086202928268 88.6663961027926 79.3066495980045 297.023913988137
55.6991707857987 48.1591264215184 51.0013344286851 88.4439480366515
47.0804681460898 83.1874704625702 148.440257128143 49.2064066614865
GYG1 237.161912299581 394.00931573564 408.080620074472 524.972936438175
426.622204035624 739.541009338849 362.927960895643 294.966924715506
316.831358204198 449.564281713066 447.709872889701 711.773459577386
GYG2 69.5000200266713 40.7616966228404 48.7039705048129 41.353786352147
64.7050732790408 64.9498081060676 80.2680308979464 87.2437977005922
58.4016768664589 49.0138356310956 48.6441988290952 54.8325386119165
GYLTL1B 36.0144420013819 51.0175677516029 50.6987358483181
36.7892997857376 65.8319449883221 44.0804700754786 60.0981687048269
41.3506832276112 53.0357938671646 54.6089451252049 51.7538628098347
39.6352613956998
GYPA 34.1336988442837 30.9403229851671 35.6139301508335 38.5252456306656
32.8704945047196 37.2686490268265 35.4623691645629 31.0594903826598
35.0305278582179 34.3674468322242 34.064698871541 33.4316681623175
GYPB 37.0320432433314 36.0366673493555 33.0260566875355 38.9534635879195
39.0564222526273 40.1428591998404 41.0381860307445 39.1652965788406
38.8271346375425 34.4875013487777 34.9428206949566 39.9519547348106
GYPC 154.309544187593 120.149188463323 93.8630393470857 414.293172794592
81.588696805606 624.097858948785 70.4325064623268 108.881703882906
50.0822130724389 320.684862879691 298.771150048863 223.562130663956
GYPE 42.2598158199183 42.1607322031054 39.4936330441769 42.4162492820012
47.6884372645373 43.0860383229288 40.7333121467448 38.5066108855005
41.7233690251839 39.8455060654531 41.1418034290776 41.2905987859365
GYS1 102.044838876428 110.431464700539 105.451996424326 106.398825125486
156.433762355183 78.3488426553071 87.8746766762445 69.4677195088043
203.788504042093 123.918925337643 106.771536005741 82.4362183543935
GYS2 41.0608773166058 37.3634897565502 37.6557714476853 50.378948785456
41.2740822675592 39.3841861812262 41.1507980856436 42.6450179841129
41.2870505273664 47.3791873993182 41.1638563416981 36.3454320848591
GZF1 49.6241203903354 55.3477556906605 59.1040441894478 86.7823828209946
48.6224651100733 50.8533737532786 63.2726414544553 45.5581356216957
50.5785738734431 51.0055825931636 51.9302027391447 49.2064066614865
GZMA 69.6449298794042 56.2851430356168 80.6674009289482 187.331614826395
41.5668884168669 52.8421046802739 52.8496345990125 68.3372228877569
47.5564423692142 57.5713378330876 67.8570354010492 46.7182294568603
GZMB 111.760213205992 148.016994658777 143.173898886465 578.601842980701
49.32293280121 71.9329028989527 114.230358276599 228.907776306695
48.2123120250343 61.0935621637178 62.5036823572932 48.3648494350286
GZMH 68.1780382173625 46.6996902348633 106.443777149496 165.820230766206
52.5645416722441 49.2050538150106 71.8466937395628 72.121833189378
41.5635499110307 77.775644509079 145.193498675059 43.4709415392696
GZMK 48.7646976789783 75.2824161164996 102.053879707074 246.044674996861
45.6895223823001 56.0477607422828 39.8695929627783 61.6839343510302
42.992884918158 62.3233491851723 49.5923712603197 43.9541718254769

GZMM 42.8036731479932 45.4143854024453 50.9933795450314 59.987045425047
50.3713170502557 44.3648993568405 48.6395795163502 54.720204938924
44.2607789091176 47.2299678280724 51.6414075056938 51.6660481331154
H19 676.672146226382 10072.2414399735 7393.77508374057 250.993396750789
248.414210905199 5354.58328030315 367.685570437603 193.981651711507
2892.02001412369 2498.54878472152 1323.73429724066 1718.44944271916
H1F0 1835.21901524331 928.230419226078 793.570993808328 444.937258895152
463.022816882856 664.453262499828 818.290793784784 1041.84536202049
1864.63057767867 758.462554576949 1147.19483787501 373.330920144642
H1FNT 40.6559419616644 43.3458769998118 45.310660704531 42.4580504587581
48.2748123114598 44.5544727111028 42.0605906240248 41.3506832276112
41.1742244762318 41.8613406887824 43.1931731665131 41.2437378488025
H1FOO 41.1523487516364 48.2220547806679 41.1367478979484 41.1886975241176
46.1804647841331 39.2985791324641 40.2154497375715 37.6557714476853
36.6312676076142 42.5664883002833 39.9899430628542 39.9519547348106
H1FX 222.05036990665 406.500583302121 271.138311888031 494.300858189605
289.975375372371 264.407051461506 468.153823329361 382.050584680669
382.120608305668 430.743220222202 504.676944739425 181.266908509671
H2AFB1 41.0970259989281 40.8880530359822 45.3675390992851
48.4916117779992 41.9397336249742 44.4311655729487 51.4349390827895
42.357910063876 44.5303781239209 51.6303029075494 47.9942520395434
45.5342598239058
H2AFB2 35.0202257459026 30.6802399384205 36.9795961564828
37.651298468202 40.6723334103791 40.244271403831 37.3799542961839
34.064698871541 46.6944238390932 37.1275334308172 46.0905079010237
40.8681871019812
H2AFB3 35.0202257459026 37.920964499903 38.7745734037492
39.7411455670172 36.2048942610546 37.1317006162938 33.9750800030909
34.2814697823693 36.8127605061228 44.4652605017595 33.8558232866797
40.2669497398702
H2AFJ 428.515605971056 375.354746298695 361.945511835039 210.133037772969
384.522115820048 201.236491769447 558.855313555287 397.520960156896
318.609131605258 193.395549316561 239.590143292142 242.673166257518
H2AFV 52.0299937917925 51.9421032049606 43.4709415392696 46.614092935749
55.0031529299037 57.678188678075 48.1747307200218 46.5717043290731
51.8824384192988 51.1169368198437 46.972521499515 43.7672102682067
H2AFX 77.6056319020116 106.595042669127 60.5678281953819 43.6726153508978
117.893268074013 53.565717255469 116.555666104916 87.8724689898823
112.488156568824 42.8936990772838 53.6334631215581 60.3816817102326
H2AFY 752.837247653926 650.328627263899 615.442484911026 970.379875059112
655.389317052689 439.376432049616 821.922096305069 864.302844775203
1041.84536202049 461.607803139448 492.902027204364 746.847753035399
H2AFY2 688.168272082335 1152.55315876935 887.118866402149
269.745248423773 895.735191953679 409.978564041362 1228.1735887278
872.305108096296 3190.0108307463 654.682263833024 731.071171171638
574.467486140819
H2AFZ 1291.71781703042 2243.24648907474 1698.18614771363 1351.49940447914
1796.47241860175 1693.5377672895 3253.86278287661 1598.78859278229
2934.35616793443 790.094848284547 841.850722284484 1769.18069954113
H2BFM 39.5676051939342 43.3458769998118 40.4070046183108 59.8102324370568
41.1868052124344 38.1169703906207 50.5252048155459 41.5464765641928
40.4220196471124 46.7646877499666 41.8168470823451 48.7814131120945
H2BFWT 47.5647821785529 39.2907645923217 34.1806289878747
43.6726153508978 41.5668884168669 42.357910063876 51.4349390827895

39.2672712041774 46.6944238390932 35.93950183586 38.3684202018524
40.9683275553869
H2BFXP 41.5421429082231 42.9358255168086 40.4882741869718
42.2496337895968 36.4434672195785 41.6590564937735 35.4623691645629
42.2577689927563 40.4997186069494 38.6454098044474 37.1059647239814
37.3600014814388
H3F3A 4820.70258471362 7022.41612496714 7785.77078492993 8050.256095383
4665.45637589484 3681.41904661708 8441.71894028273 6819.74871209328
5733.54417656207 2844.5506310828 3361.67247904403 3707.73750609316
H3F3B 316.185342210304 434.841521967958 332.230531176052 444.937258895152
125.221639689345 528.242677613584 394.00931573564 344.314901683001
412.694325761831 645.702744916884 518.936896882424 451.603052501528
H6PD 56.0836516733215 51.1541843174981 45.6989664108554 52.2089306840125
46.3150221024046 48.5136862664526 42.0605906240248 54.2301964914419
46.2718838939055 43.2438688757519 50.2750065554387 54.1941919612524
HAAO 44.8914371498528 40.2493681589104 47.2962360458401 50.7046706880325
40.4639532172338 52.6226745117396 44.7255207589606 43.1567024035183
43.2761454096265 46.325232414659 54.113533179847 45.3763275588341
HABP2 40.3478825593224 46.29198852834 37.3687900733549 39.3750969348308
45.2556760873335 37.0180934800573 36.634024014558 35.3608605269276
39.3750969348308 42.2614524730856 40.8225507267584 39.0536056496869
HABP4 73.9578111605052 58.2571968311834 58.4137028673494 79.1998092110487
65.6556779936059 161.38599012777 70.0196904419961 83.5767232665142
59.1223319112238 108.976748006885 119.157952832454 117.626009607247
HACE1 44.7915315085948 35.3307157989724 38.6727842796202 44.265834798964
44.3557408297309 46.7800033479223 54.1707158323527 42.2577689927563
42.6366765186182 39.4710743516517 44.7114813656401 44.1452943804044
HACL1 154.730266766226 231.733564500645 200.615897175992 205.605413799808
369.877521477669 183.584085676174 350.365107642944 291.867370164212
238.819980744789 203.759116151774 144.427680369554 170.184914156265
HADH 260.766438659762 687.862452802453 458.230500859847 144.116701129933
354.015808474189 452.351360652434 994.404707955031 495.453743436613
1111.89793143647 636.367246129079 648.380417557148 883.06773454117
HADH2 112.644426507762 113.929486370331 119.979854891563 131.57403028894
141.410183156424 128.923092916744 175.632783805966 146.892027926888
192.536546957155 97.2865629929716 97.9497090233081 101.017461500409
HADHA 158.429106463866 125.290426098227 122.258194040815 165.958820888591
203.417430809331 204.251854342995 202.325629133577 382.872356030213
183.820203505757 251.803102904399 209.178131628825 224.030034866938
HADHB 175.876518898179 158.806855832703 213.232693288132 150.941470539313
139.620154641549 178.44531401852 195.512986297442 313.921236120658
203.109765192983 144.063191647372 164.642060754649 240.975885654236
HAGH 217.189489936251 218.531478946522 234.308728996238 201.616017553422
187.836567614419 251.299662213686 219.838542502016 129.394893423994
197.13726107504 205.830801439839 199.480636964499 268.072653271299
HAGHL 52.1589578279164 51.4931742685391 51.9214790114499 53.593974682612
95.7263252232278 44.9896851005259 43.8973899364093 47.945438269111
51.7356912981073 45.087888302889 48.7720246202612 43.2776458914333
HAL 42.0532901689036 43.683792219729 42.8723769353017 56.7106584977089
44.7210190486327 47.0027453663546 45.6706505553383 40.188873541274
43.8482802038826 43.5652644712197 41.134180463103 40.5703581862752
HAMP 56.121460370892 43.7644428186265 81.6486527718661 361.57814355509
41.467005949568 39.4953494583071 58.3675164086197 57.9713749600418
44.7058233759912 40.1179210534118 40.3452320167286 39.7411455670172

HAND1 49.3463416794995 35.6434124419782 39.2707964550441 38.0958223740111
37.6527901109005 489.983164420779 45.1017494688414 41.9315419791594
87.7337304906377 161.910748720932 195.221917652149 179.994715581707
HAND2 44.380620848621 42.5660695551615 43.3465699185405 41.7144525110048
44.7210190486327 73.8651427228332 42.5660695551615 40.7799172560245
44.2607789091176 56.6086202928268 53.6895401581575 60.0517078759277
HAO1 44.380620848621 43.3972849968032 33.8029161403492 42.1622837416589
39.3667161654772 39.6251842352088 38.7337086062875 37.0390589468421
43.3744503319109 36.6893251782922 39.8184668132023 45.0985088502363
HAO2 40.7022020432945 37.3634897565502 43.4544762830742 37.7146582037126
38.7864161493321 41.0012755104374 40.7910160811527 37.4484310268909
35.668043946649 42.5193208629123 39.2542963855807 41.6550823405646
HAP1 37.8891397738265 44.2105195734073 39.3344387353654 40.3180310664156
45.5311794320092 43.9421822132391 54.4934782594218 40.4359177791566
45.0755084221375 42.6793753550854 39.6719190054379 42.1537476443423
HAPLN1 53.593974682612 45.7317208802318 163.476051754305
38.5252456306656 55.6069214673486 42.2577689927563 39.9519547348106
40.7311497914871 43.423558862426 40.4518431978241 40.723990462047
37.8129870676592
HAPLN2 51.0225132036905 42.1607322031054 44.8872784170036
39.5050383901884 40.9950639693255 48.3686935595826 41.253645578517
47.3791873993182 45.0017158586743 54.6089451252049 48.6767028205398
48.9733758107788
HAPLN3 114.288115730578 66.8693882433359 46.2625470410195
98.2447263268855 47.4548471240051 63.5738862549306 59.6780848951767
64.893664076381 69.5741225986433 47.2299678280724 37.9946685306274
42.2577689927563
HAPLN4 50.5670783769975 47.6914221838216 43.9450023643394
54.4586176399381 41.467005949568 44.6524308640784 46.1634822993009
47.7889447429963 47.0804681460898 53.4109882045447 39.4410515050885
43.5738557773903
HAR1A 45.3872406432794 45.2556760873335 40.931294690669 46.4555908533915
42.7107928951766 45.7300501624017 47.5963750520409 44.0390019242862
43.0930293831497 42.0621425894624 52.2759825608835 46.4499258373233
HARBI1 47.5647821785529 56.5319193881205 55.8810837854139
62.1776700757427 58.4383854340181 53.565717255469 50.0088611244948
43.1567024035183 54.7966692984192 45.0155543555707 48.6767028205398
47.2131144508464
HARS 437.693269824303 260.185079572615 351.290929267326 356.226114718307
347.003621791919 360.962191673108 318.069132873489 508.688028777582
294.090159716249 468.98857415608 424.87896140215 347.552309199761
HARS2 301.025314855652 227.895442198856 278.303493506139 206.28819203182
219.737294408079 165.992057520727 199.57367025082 330.814531248695
253.398345052444 354.634819362978 323.295794446334 318.995164994001
HAS1 40.5654775358427 34.3715326040967 39.2707964550441 39.853175885492
40.8873907150245 89.8567957512307 40.4359177791566 42.357910063876
35.0305278582179 44.8997235283254 35.9563099719284 43.5738557773903
HAS2 45.4614234442527 45.7317208802318 42.0136658108872 46.8249967544573
48.7847528954724 62.5177123761087 41.3094485574845 45.4216391637116
61.6183748238619 49.9085372049438 44.5818127765109 49.1718303518849
HAS3 58.8224410806741 604.357590751951 597.191426276771 48.665578118898
235.222472959869 64.5187138759372 225.618853905635 262.824698774297
873.451893437179 309.802432824498 224.976641879383 515.409979010663

HAT1	80.7067505750848	76.889690218685	74.0913177754682	93.3216420803293
	80.3500323413132	81.8426140611297	80.0733119200914	83.9236799134394
	73.1215732923523	69.6511571901941	66.0282565447846	83.4033736999037
HAVCR2	397.108901822735	179.241252961119	184.964039515715	
	1533.38581753491	102.913039837094	190.81617208799	171.073367662623
	303.323289734056	62.275808835675	86.7076796107636	89.9227845135785
	142.012216533823			
HAX1	95.096268847843	79.909124697198	110.181320436731	135.323750661937
	106.276392548097	114.243522751687	104.650071846586	193.196385709124
	105.598516887691	108.213380368446	100.83436298853	115.761674855716
HBA2	1839.09068171097	2456.86796652313	2598.78910128081	11410.3307451176
	1081.97502255142	9582.11974031559	10072.2414399735	664.178866783226
	503.793770799548	10996.2779403491	10996.2779403491	1640.74027400791
HBB	1585.90718459092	1801.44003951248	1762.93413846062	11176.402230735
	1539.35554229258	7168.83136860899	10483.5643876539	516.704027135707
	504.676944739425	12125.3293116195	8703.02873382565	1806.22468459769
HBBP1	40.2543341073001	43.0971787292711	46.4569566520107	45.7397504535169
	38.1644569189523	42.157627240486	44.4639405506967	44.4031868028216
	44.0519245668737	43.2988017098387	42.170702295259	40.1700117452801
HBD	41.6354649482853	41.8073910693411	41.528370959196	47.3574589211166
	39.2608760193011	43.1826328292558	37.7136011474903	37.4484310268909
	37.9273653326866	60.4837400912239	46.0095710163378	36.449101775775
HBE1	42.2455707947223	35.4484652445078	35.0970060242246	41.8778873678551
	46.4343327861696	38.3418933808844	40.9157352260901	51.4360013601805
	48.4869909888679	41.5435611403119	38.1835938174625	36.8690774823319
HBEGF	163.43197105141	355.351648424266	157.020685801527	1545.76489731307
	138.728648830528	403.92758879201	201.360008430353	469.30167430316
	109.828760284519	66.274247400793	86.7730065232892	94.1128427766432
HBG1	33.9561816279577	43.0971787292711	42.7303139017786	51.3851157261451
	40.4639532172338	63.1091746640396	38.5066108855005	40.3357777530419
	45.2897806651929	131.083141370546	49.3901004211918	41.863927676552
HBG2	46.263826584899	47.6914221838216	48.6037684272115	48.856176220627
	52.0583273941813	86.2563919366781	48.4859552186772	44.797320243931
	44.0519245668737	165.992057520727	48.9754340235127	39.7411455670172
HBM	40.7609952437622	35.0222940937513	40.1985790927248	46.8954576545929
	51.7983110178095	43.4681646416585	47.1345949868029	43.1567024035183
	43.8482802038826	58.8022152133979	50.7903824729818	43.1805016690297
HBP1	180.475056805817	153.279284223069	155.908644253547	291.94914449218
	132.555169949054	249.729778471447	130.585540374294	145.232878862479
	173.11353027932	195.635797217403	191.284436365696	164.262531918679
HBQ1	42.2455707947223	45.3486182278177	36.4564050256915	43.3078542962968
	91.2681347227088	41.159706131351	41.9393838138203	45.4757965005055
	39.6609238036815	44.6918600441328	45.0589947942306	37.2810767789306
HBS1L	40.8681871019812	40.8476436931481	39.5118967430322	45.1151758109609
	39.512606833112	43.6427759607073	43.4544762830742	43.4885245598939
	45.1569741609597	41.0608773166058	43.9450023643394	40.6614700944853
HBXIP	704.601607617837	539.188951045594	647.868022835702	684.865133028228
	477.558242266308	456.467726693355	677.481235695517	675.079525434102
	1188.3486464341	415.477167615547	428.958127252533	810.382224368065
HBZ	39.3709357615493	39.1982470626863	39.9101908547397	47.481302504066
	45.2556760873335	42.4580504587581	37.0564573649223	37.33030683349
	38.6322728994674	42.3606390470387	40.9157352260901	44.0451487108213

HCCA2 119.714551585774 118.143444770806 116.949813289388 139.385367339095
97.5394336319916 228.571334092678 118.736188485344 109.649431670339
145.267770598988 227.404530886674 216.45007990575 128.230543301378
HCCS 73.6855877240541 79.856662992182 67.9850517608624 72.0112676564347
70.506090074243 92.2772139468085 78.6402083787251 71.5484567134601
88.9321928608078 62.9604757922393 62.9886614153696 82.7083356310191
HCFC1 424.182335137838 759.94822128584 510.379313524019 352.997455210484
982.428740433235 471.874412428677 602.726022292973 568.638529128128
661.269882993465 440.421077161585 506.585006700107 473.688869614777
HCFC1R1 615.011590811277 261.023196824723 355.67721196018
394.764369100963 247.298165265093 547.072513229674 297.990925644625
198.010793115588 271.900886756161 811.142611128461 518.527535205975
367.158502379417
HCFC2 44.8914371498528 46.1569135229922 49.7350276169552 52.1201052574162
51.3511088648386 54.8265655909623 44.4639405506967 52.5909491535773
58.5561424827901 50.22432299342 53.7475011049549 50.7487159427973
HCG18 44.9896851005259 53.0383634135393 44.5134109184888 43.4410741327254
65.0605897122361 41.6590564937735 46.7100796279384 46.8954576545929
49.9162522057712 42.3606390470387 46.0095710163378 40.7704587752083
HCG22 42.3433931248155 43.0227387991135 49.3000844711116 42.5664883002833
41.8806343997257 47.9503724044453 46.7100796279384 45.3872406432794
44.0519245668737 48.2596564318071 36.5359455830388 43.3744503319109
HCG27 39.0620487644635 47.1332119496722 49.2041300973417 66.5921967967793
45.6895223823001 50.7516773099639 47.2927436772465 47.5950227477807
54.113533179847 53.0357938671646 44.1148826235374 37.1747156131306
HCG2P7 1457.72260892651 707.4124863603 848.079114196986
1025.7433359938 494.733185937057 572.968592371501 1243.32650314893
1824.79010714035 769.685677484067 1621.89443989657 1731.19220270448
1117.26060212107
HCG3 40.8681871019812 40.716553247236 33.4240466670776 38.9534635879195
39.6535880152834 37.0180934800573 37.6036146841933 37.7612949798895
39.2809305246091 39.3698811593364 41.0808257270221 40.0557788909923
HCG4 468.381233506231 999.862452730252 602.047768767953 884.036280777358
219.737294408079 203.497070457832 545.827286197333 188.567635000971
377.573838771285 101.681822254799 202.529846569324 114.520410790135
HCG4P6 45.3872406432794 41.0678614026819 44.0451487108213
43.8002456102021 44.2607789091176 39.4953494583071 50.0088611244948
38.956280281655 39.4762546236449 45.7911932481722 37.2018803984555
40.9683275553869
HCG9 39.6609238036815 39.3999056968598 41.3366779545815 40.8681871019812
38.8456942580061 48.260931742163 40.4359177791566 36.8400213077707
42.2577689927563 43.844855336215 40.0766543123257 40.4639532172338
HCK 94.9365530062692 91.8568043052819 79.3066495980045 953.629734158221
65.3019328668633 195.740109330843 170.150808455621 78.0020909283414
56.6079099547057 49.7259371799014 50.2203578628217 74.3442318693865
HCLS1 266.129007472128 412.694325761831 794.806085783439 2435.70120719503
270.202959012185 669.19065220034 366.129480083279 252.616382150668
67.8208976798364 315.186937216422 232.019748665806 238.647490689325
HCN1 36.2496364442264 33.8237407699472 45.063792968927 32.9046688061235
42.7982502551634 40.5281496800812 39.6535880152834 39.4710743516517
40.9901805728485 39.6719190054379 43.9450023643394 36.064718696392
HCN2 41.0970259989281 36.6002100641539 36.0306975524309 43.8002456102021
40.0599807618105 41.4508241771315 42.8684958525042 40.6723334103791
41.2870505273664 43.6033221838149 38.1253290586431 41.9741368971524

HCN3 73.0970339739187 68.4278345554565 135.323750661937 46.1673159982664
92.8644908993284 48.4758590019384 84.0691608193405 69.7729022157455
82.6157566292355 86.2118355769367 61.0094935318105 77.1954301283261
HCN4 47.8249696958403 47.6229101519945 45.2313723727673 42.357910063876
47.0954207417646 41.554942319225 48.0283573444179 44.1311654985682
48.2123120250343 51.6778366958973 44.7926323492519 51.6660481331154
HCP5 396.355023788839 1129.71973548345 684.238999553691 648.885062676884
161.890859629246 131.126942328593 600.007191467065 347.145902252055
421.146145642412 222.8744321245 1017.1803754111 202.708232693973
HCRT 44.8914371498528 46.3635803515159 45.1569741609597 54.6089451252049
46.7508829977587 47.0940613507321 48.6395795163502 45.1714698794331
48.5792058061372 56.057906531592 47.9942520395434 49.6629259325705
HCRTR1 35.6111578834671 38.5252456306656 39.991353369336
44.1483976313959 44.147557390293 43.8582747295766 51.7714254834195
41.6466169548846 39.7537274655115 41.5435611403119 39.4410515050885
45.5800840647989
HCRTR2 41.3387790895716 44.5152708955623 45.3974838013476
40.1804147506786 46.0043807840705 40.8634043028528 44.9863726205428
45.642304356051 47.9035532886869 46.7646877499666 40.6373400186325
46.8129509799753
HCST 208.804071011518 212.250918463598 190.214361156876 1359.9708539999
89.4111455826308 548.047774435177 180.502282381416 153.165464817911
65.0423160790157 116.67447696188 187.439021508049 103.133138547093
HDAC1 782.28158356937 1529.52516575545 1333.01876209753 715.662312404643
2133.78836857697 737.604036996153 2196.59044510709 1425.37639004849
1896.06380611235 1529.52516575545 1354.50256889331 1301.07436212673
HDAC10 56.8168526942685 73.6855877240541 62.0008474085033
53.2046867327331 62.6860949544223 45.1873487463472 63.0119737420787
49.5185112777773 58.7382738968248 59.9354531144375 57.8991270166934
55.9555622193202
HDAC11 61.6544855697851 62.801431502597 40.4882741869718
40.0698728584484 46.0977687311222 44.8872784170036 42.7431359850339
64.5257016285499 46.1101825640108 54.1683282066029 47.0940613507321
54.5921287593554
HDAC2 563.065549077627 735.60603239712 415.477167615547 302.79352975792
294.7772562683 319.871452890512 740.423874031773 1848.50311456636
421.146145642412 343.346644539158 345.529757315326 517.869378941762
HDAC3 189.890451486324 177.067668324062 271.334774589829 173.925818790068
215.907790093087 176.670909065664 193.782527748199 187.923050034941
247.356027979377 269.745248423773 190.187931547806 202.957014793417
HDAC4 69.7442393277127 63.1780854659428 49.3000844711116 80.1809075683154
42.3685136297603 164.847648206552 43.3262148375525 57.7805922926828
48.5792058061372 84.1639779987105 88.9538366702651 79.9447969637409
HDAC5 42.3433931248155 43.3016395799961 40.7620472083159 41.9620007529243
38.3561682218911 49.7149784386993 41.3094485574845 50.7364620410977
40.6503965580437 50.5252048155459 48.6494451061637 51.3244198342335
HDAC6 140.226992517573 74.4696354388511 105.515662220161 113.979504140326
84.5572894783679 93.9228336493696 128.766242896259 117.723425661764
89.0203733091224 181.983177572371 127.986477405999 109.492219458129
HDAC7A 48.7646976789783 55.1359824959234 49.8899020044306
44.0451487108213 52.3242437351389 45.6706505553383 39.8695929627783
51.4360013601805 48.0436466478804 56.4917096880521 50.7903824729818
51.973762640055

HDAC8 74.7955767483841 53.2046867327331 64.1080166181228 43.8002456102021
58.6481644777552 49.7458652239071 61.8571521441451 80.4577725550904
68.5599263000733 65.5349012839562 73.845912534353 72.4651758215622
HDAC9 39.4987030972173 38.1725197931105 41.3743834747446 41.1418034290776
38.8043560812482 38.5808034535515 42.2099119257029 39.0048202613914
39.0481003400271 42.11120345157 42.5856588364738 41.2001840592033
HDC 72.9154861179393 74.4696354388511 64.2404293863468 78.7450149191851
33.2552842693684 533.468347491982 40.3302268293707 68.0342444360948
39.7537274655115 80.7338782543351 69.6097529955737 52.6922486991742
HDDC2 778.723771259404 572.81778857651 528.512401493184 516.992846073349
347.486523804413 766.027617496754 558.451033789799 524.451156145427
401.347734852083 695.255317861845 558.855313555287 591.192020333181
HDDC3 130.058147569795 161.326580647514 101.403739410832 125.801834682035
154.111066539468 96.3976093220249 255.126812649967 176.057220407246
165.662539866309 139.645717741863 115.676508349703 147.689479458785
HDGF 701.551782736435 521.874349042344 505.294880430281 324.506136847875
703.315796616275 267.261896105785 625.435083064752 496.163796158189
436.497820504526 341.666968456707 314.006301513986 355.92328557644
HDGF2 126.981964766008 124.112216491975 130.615540145155 116.76301232629
196.034087004464 171.058650956601 193.01447006167 124.605379715077
156.276838862671 141.784776736918 133.594952879573 109.525921548011
HDGFL1 40.3478825593224 38.0226905622357 40.301483914446
43.9744941494111 40.9950639693255 46.1974343793412 43.2721716572958
40.7799172560245 41.8197029429821 40.2112371512838 40.5824352835642
41.6550823405646
HDGFRP3 50.379587451669 84.2484477706194 54.6820069269589
47.6687908290818 46.0977687311222 105.945216149549 93.5928272702304
92.4953883194488 72.3095457386378 79.4158467034017 91.983638239817
117.934830767297
HDHD1A 125.540457221408 96.8240908274121 161.326580647514
86.0630164348952 119.572497267498 80.5478617156852 95.0190813062982
97.4421930425128 156.79660458088 126.670728021682 105.589475270494
134.489077146096
HDHD2 67.6767845246581 64.643220133046 66.737740728688 64.3746985855249
58.7460488758053 73.5743059777365 56.9320717885002 95.491982090349
75.2337946941103 104.288073521816 98.6706537801886 113.277043026153
HDHD3 140.369714831352 80.257554904218 68.4720753153522 53.8086134112366
82.6157566292355 71.2787833369055 99.5844079573552 72.121833189378
99.5192591352063 102.294142438313 117.8523906126 85.1114448808779
HDLBP 48.1125722136997 47.4630625313159 50.6486940895674 50.2950101030503
50.1092226717582 50.7516773099639 40.7451277736087 45.9090659324223
50.4463723277824 44.7958122466628 47.0110068985119 46.0201052072883
HDX 42.9104454767916 49.0697565324841 41.0356368374708 45.4634652319753
36.08956004634 43.9724628401189 40.4359177791566 43.4544762830742
48.5792058061372 42.785429206422 43.1931731665131 47.3992733452821
HEATR1 150.362006499825 193.196385709124 293.410413883219
148.529482776555 521.874349042344 177.27613997459 230.787791652978
268.047342640491 202.459251553516 134.936657884035 158.901175732866
157.518879763075
HEATR2 83.6524523974491 106.691530592115 101.2615980834
72.4834467838798 197.205643078662 97.7415824971555 120.327382682483
90.716460692818 127.547251770359 67.7189686993694 78.9088042058577
86.1433529921684

HEATR3 88.7928773498158 77.775644509079 74.4508307047509
111.522812019895 72.2016884438365 57.3171834644877 80.7563265626648
66.7530718914083 83.6395145977203 64.0624077013652 69.8314582428552
69.6449298794042
HEATR4 49.7188990489616 35.3307157989724 43.0971787292711
37.4223289687135 42.7107928951766 42.0574848021044 46.7100796279384
44.4031868028216 41.3803269429468 37.2089297656361 37.2018803984555
36.449101775775
HEATR5B 113.026772174726 145.027715477051 168.11088055793
131.347995391441 99.7668527666705 127.634504077212 143.251956814524
155.693953208351 119.747995520476 142.515628954457 243.489225650886
156.770274308736
HEATR6 67.2437599548936 75.5775406974686 69.1938570643524
65.8926843080114 93.6591201235745 67.4846787600629 87.6018870099686
79.7170181855209 77.9028721785469 70.7805103907761 82.6602736441839
67.3183506012892
HEBP1 251.268123356533 242.673166257518 239.569514501529 221.743315748689
160.203825526096 311.432395849636 264.116434279258 234.043251639296
272.475108279834 337.156068385541 288.923207993626 345.294930735691
HEBP2 605.145228948011 900.517711546397 569.511773053233 268.936151471133
288.869882644391 447.248719481853 736.846933148564 468.573956652904
435.094968715663 385.827237353675 483.882804472844 470.231929311877
HECA 98.9463403584348 62.8885806455128 67.3469281984384 91.0346886951891
66.0411740221276 62.7515429046888 71.8466937395628 72.3822545261582
78.8968326770219 92.6888475289584 76.7063975550116 100.049462976601
HECTD1 513.57613902662 877.673009984357 1012.0373748933
587.060466667911 685.386956092142 629.301646370726 406.500583302121
515.856807472321 846.773659307087 798.033975337132 787.951838387453
787.671342652389
HECTD2 42.0847226073592 45.1486243270611 42.3536549114165
41.3027031037343 48.4670130605028 53.1457020450516 43.2776458914333
51.402003176055 42.6366765186182 40.7063426028871 52.8190882379316
47.6040671292013
HECTD3 186.301404367238 131.112230954034 246.274692971423
121.737071786167 152.456314990408 111.760213205992 161.573327074375
235.315897087189 166.983092667334 191.926072400139 153.436974478757
123.114162330406
HECW1 34.4896350146096 35.5310164682026 32.821392987958 35.0448343468967
35.0181575202066 32.6368705146258 31.9682164860619 41.5464765641928
34.7722083264266 39.5637454855233 32.2133093774284 40.2669497398702
HECW2 91.8733417805775 58.7111274254142 82.051509943308 106.398825125486
66.4047895961325 51.3886035574416 48.6395795163502 67.5206810636627
42.8036731479932 45.4614234442527 55.1099824417989 43.2776458914333
HEG1 110.729504563799 71.8576824255215 105.376705821141 134.952392466784
67.6011992144113 116.51037006692 52.0942589368478 68.2309359831273
52.5772854102326 76.826527950236 77.9085946827079 83.1874704625702
HEL308 75.1701698483856 66.1863819168127 60.6567691532561
80.1809075683154 64.9072331703985 75.353039684456 58.7585623394335
54.8412943008635 58.3564461855595 75.9037617394486 73.6362523983338
72.7502212566295
HELB 49.2595762436995 53.9275079525238 46.9040704638073 80.7168944828052
43.1252272136667 50.8533737532786 60.5125774827425 46.3685439301234
43.3744503319109 39.3698811593364 43.9450023643394 45.7562276914441

HELLS 47.1216490839161 60.6548851977964 46.1987217359283 49.4742925789913
62.9553576299033 41.6178506967511 86.3237770387663 57.7125861123012
65.0810479794187 46.441301934328 44.9713479185144 44.4475838947656
HELT 42.442231667357 39.3999056968598 35.9305494063137 43.074472921841
41.8806343997257 41.8613406887824 42.357910063876 40.5158867964016
41.074824727477 38.528621456378 39.0550139257658 39.5418280570561
HELZ 147.65688475427 166.208731611481 141.784776736918 186.195424728755
160.973602503707 202.529846569324 250.276530863694 177.111143479957
235.101442092497 353.598144765197 389.686780706341 236.407697413249
HEMGN 42.7150227340364 41.6799375298398 39.050952280947 40.4893720023062
40.5710637884029 39.6595129100865 38.3389087628055 41.0381860307445
40.9901805728485 40.8970401536578 38.4304896304026 40.5281496800812
HEMK1 54.6235981131919 75.9802606133139 69.11736279512 60.3481762331447
71.5345397102823 52.5330333302271 61.8571521441451 55.1933004802607
55.9423752286714 79.6133633483649 65.485076402841 64.863894209343
HEPACAM 38.7537364570873 40.3384318592985 44.825830634601
43.2504382247503 40.0145811166794 49.641159251878 44.4045701012801
40.8833572570383 42.2248608921879 40.2493681589104 36.9768361759916
36.1190722948669
HEPACAM2 40.6994262313917 42.2013327019987 39.1553169409075
39.9182914210537 42.4864576619427 43.9421822132391 43.5898425390297
38.5073031329596 38.8507804383261 39.8873706855774 44.8872784170036
48.6795178347248
HEPH 111.803238196297 39.4629815741023 45.7452456977857 50.3127977239083
37.3508690072156 71.0369238830779 41.4369824439795 46.2779564481158
37.241386734757 64.5845827915699 53.73245713018 50.6895263967098
HERC1 168.91210897306 148.544012961815 132.760054781304 242.282363793567
146.701131209299 260.656475394736 182.079641528208 241.557912972813
139.712472369259 207.957319374194 232.113067048781 197.103673938152
HERC2 156.844010597219 144.633447485038 114.533239859288 138.895316905243
168.883366756191 151.088035451997 203.53267380432 152.555556133364
157.7623351008 150.829277770546 153.364107550338 146.287473652123
HERC2P2 43.4079968811658 43.5627649099623 45.5533570914817
42.1725002904014 45.5001421579961 42.297291190584 43.6084517252627
46.2306893704071 44.6569942625774 42.8297772945791 43.6294818685075
44.0451487108213
HERC2P4 44.3355304829989 37.4540772310118 38.6727842796202
40.1804147506786 38.1644569189523 38.4330755397458 41.8799523546812
45.642304356051 37.6630259432398 43.3465699185405 38.9240290061433
38.745552403895
HERC3 64.3502363726659 75.0873720488265 69.5937318502259 61.0845101400379
64.193536444915 83.0554837248934 43.4544762830742 51.8347877274018
74.7934385405346 61.0935621637178 61.940656536312 77.3260618801358
HERC4 98.3126451737573 87.4273245333478 98.7535408247698 97.0055689383756
79.3539393480885 84.1254258487497 66.3209349903185 90.8109585999257
211.019451518444 94.0777119695783 88.715256326632 109.979856686854
HERC5 124.599352191013 144.412165495719 104.950400251642 421.791344753995
76.6914642534934 193.875712862166 239.838643553139 267.70610731202
103.349712762251 211.421222959783 207.087723777954 145.572606290643
HERC6 111.304424389891 222.453547273598 238.584514797019 138.053682842992
52.8190882379316 134.375403847987 101.716697533604 163.917208464731
160.605530211736 134.869029698594 127.49459343016 63.1443731864055
HERPUD1 301.594300620246 482.5816387921 611.776512504215
1173.47285626656 342.643344197936 346.285308869994 252.649651285136

209.497482517298 456.992521713593 285.322305931794 282.961489041903
290.546420262364
HERPUD2 74.9491421278749 85.8087881787018 101.131391476546
119.502912878938 82.3181867658154 102.899135663993 83.7829283976292
61.3077641493507 92.7427427543974 63.2684764441521 66.0664586519214
71.4518820434918
HERV-FRD 43.0930293831497 46.4861258030402 42.4580504587581
64.3746985855249 44.5668141895992 60.5803031036931 54.339958888486
45.8669209716694 53.0357938671646 52.2256177934134 43.7585538437696
44.5432192783161
HES1 129.938114164087 487.065758294354 305.786520619504 72.89602312859
168.75362473684 85.6038071277319 427.281740164881 289.909646207746
393.001025840896 479.696469083953 475.310458821086 480.13416648493
HES2 91.2647023876304 47.1856919329105 49.5424425850549 46.0228732507872
41.1507980856436 39.8785903668981 42.7065584433947 49.2901038327894
45.7206695113999 41.3641352453779 39.2542963855807 40.1700117452801
HES3 41.7397731376692 44.2630383225933 43.3085358214154 42.2496337895968
43.9450023643394 48.3686935595826 42.1607322031054 44.7044140979108
42.4540072551568 40.1179210534118 40.2649309204478 43.1805016690297
HES4 552.162906190653 1792.42490815074 468.153823329361 617.091932961591
1282.9181829891 375.036941898486 1405.20888023653 908.328776521114
583.690350347288 1174.92419086759 1161.71671016964 922.047328788961
HES5 45.3872406432794 116.967446594767 169.874602999057 37.4223289687135
60.4122350478242 41.0582900711341 38.2933870238743 42.4580504587581
48.0163377492228 62.1035858593388 77.622501706827 47.5025821041537
HES6 143.6667863386 102.760704801607 66.288032400574 56.4578213622965
83.1614238528477 78.3488426553071 143.6667863386 180.679129876248
109.241121384212 57.3562426592112 51.0492389286271 61.9882398986333
HES7 34.4896350146096 34.0382693188794 37.2838080935695 34.1051821768421
40.5710637884029 39.6251842352088 34.9344532055397 34.806104801006
35.4332080966594 33.2987269789328 36.0481934423077 33.8196020406676
HESX1 37.2474303302343 46.1569135229922 38.9790184198145 46.0228732507872
51.5888395924843 46.3715909653832 50.8388984085636 43.2551508830106
43.6670755115848 40.8970401536578 40.3452320167286 41.2437378488025
HEXA 55.5276281380084 45.5842286511645 49.2041300973417 51.5913529948887
51.4527545197672 46.7800033479223 42.4939802311136 48.4670130605028
51.2066664465185 51.9547984751408 47.3791873993182 42.357910063876
HEXB 1604.80031304031 1228.1735887278 1465.08435775014 3681.41904661708
1310.03307063841 1626.71747158994 614.603743904512 1117.26060212107
1014.6400720037 1164.71243740122 1071.64675274806 1171.207937488
HEXDC 43.6706764143908 47.6914221838216 48.7039705048129 37.29566077996
45.2556760873335 43.2805590023412 40.9157352260901 46.4672637826582
51.5229124652769 45.4614234442527 52.6960621424156 47.7962548556026
HEXIM1 42.3433931248155 45.3486182278177 45.1569741609597
42.1273928203021 45.1137938810047 46.7800033479223 39.2533668167483
46.4672637826582 41.4636047919453 47.4767851750188 47.2134341420411
46.5650556481721
HEXIM2 80.8943070653517 56.0477607422828 82.1344514651393
44.1483976313959 47.6884372645373 52.5330333302271 56.4809146784765
52.5909491535773 80.3805507705979 69.9928775398776 61.6084234634424
46.0633179231318
HEY1 159.204751627727 122.758873982957 96.6294773538052 78.4289643729837
86.7823828209946 64.2512089157122 82.0174591986046 52.7247447185469
113.929486370331 362.506098778318 111.558107942386 255.598083334598

HEY2 76.7820250461545 79.4710406831985 95.5022104475922 44.1483976313959
50.8875036861067 58.9676332048764 42.1607322031054 42.2577689927563
45.9227107476843 48.6124127998952 48.5292718280498 48.8670821164567
HEYL 220.192552354287 125.52123746909 154.111066539468 107.478392667404
166.317689650501 76.4439037944547 57.1070449050465 229.621795088195
72.0655217037557 57.6791750431251 66.4251023257203 62.3102413989199
HFE 41.0180685293199 40.841391053665 39.5954353681031 38.6727842796202
44.0920305031178 40.5940318808889 40.6750540874299 38.791462837305
41.1241827976738 41.0608773166058 39.3133871964036 41.9842695818285
HFE2 39.721757876562 42.1257888816374 40.641503836284 37.5996339040239
39.7568367479301 36.7342282170419 38.2933870238743 44.571915452417
42.3060097375421 37.5672792721124 41.0808257270221 41.9820792173615
HFM1 41.678190413467 43.3016395799961 38.195965820384 41.0219290963778
46.3607182640185 45.2457367460834 43.9450023643394 43.1931731665131
38.8271346375425 40.6559419616644 39.9899430628542 44.4951343330035
HGD 40.9558083259517 39.6493706695391 43.0971787292711 45.5869891570251
48.2748123114598 39.2985791324641 42.629412460409 37.5511560880902
35.860430241131 42.3606390470387 41.2267153672559 39.5792604499113
HGF 39.1919930809584 38.4330755397458 41.6564075217517 43.4410741327254
36.0424990731169 43.9724628401189 39.9397419279057 41.7932258301997
36.3649677956953 39.2623423164488 40.3452320167286 41.809704036355
HGFAC 38.2083710281545 43.7644428186265 39.9101908547397 31.6357375926754
40.9950639693255 35.4142595025086 36.4098694708483 41.1575777497576
40.2274522635576 34.3674468322242 34.9428206949566 39.8497227447888
HGS 845.41973083225 915.519281747943 1077.94530110976 621.012072500314
1111.89793143647 693.727004855319 1255.10866316499 827.365231733511
1176.37732422904 917.179747195874 910.898566308567 648.885062676884
HGSNAT 49.4786466722535 50.2303640563218 44.6511073781104
39.853175885492 51.0107745412152 44.7804080899431 39.2010256871265
48.0283573444179 40.4220196471124 40.2615278292875 39.7576136507593
40.6216891610774
HHAT 43.6706764143908 40.3384318592985 46.5650556481721 34.3595588146954
44.0451487108213 37.3799542961839 38.2933870238743 41.7419877611072
43.5669306063196 48.1392888470269 41.3553369544097 48.0350549043063
HHATL 41.3387790895716 35.2143190355414 33.281531405287 37.7469621225553
38.5512429290626 40.040778349137 36.3194282084339 37.1317006162938
42.1594905198929 41.8613406887824 43.3085358214154 43.1805016690297
HHCM 42.0532901689036 36.9994058279362 37.3687900733549 44.550322123994
38.6454098044474 41.9543948167375 41.4742337714895 38.3684202018524
40.137818555383 45.3530683538086 40.0766543123257 42.8864415494484
HHEX 44.7915315085948 73.9376772899278 43.4709415392696 60.620259884953
57.4163094213712 57.2767879969284 80.354283778818 45.0972184964705
44.1519466943185 57.6791750431251 56.6884575165011 45.4799270968969
HHIP 40.7609952437622 40.148780650831 39.1480645318984 38.6835649080278
38.8456942580061 38.2971553419489 41.7614975630362 41.3506832276112
38.6322728994674 41.6724739851995 40.4995052849302 39.6352613956998
HHIPL1 42.8036731479932 48.4497719907777 39.8299340037009
44.0451487108213 46.4343327861696 42.750861693377 47.1345949868029
45.2806199505029 40.4220196471124 49.3000844711116 42.7275315121163
41.3481355128089
HHIPL2 38.7537364570873 45.5842286511645 33.4240466670776
41.0726336841311 34.0927410219282 49.7458652239071 40.7910160811527
33.0038344711883 38.3418933808844 35.2880714952489 43.0971787292711
39.2469742642337

HHLA1 38.304249186058 39.0102066700839 42.640829914102 42.8235313007746
38.3561682218911 43.7630587657205 40.5585676679482 41.2551966132486
36.9060997041453 38.6454098044474 46.6442692119997 39.9519547348106
HHLA2 39.8662795144616 37.5469247328848 37.8386378737561 38.745552403895
42.3685136297603 42.4580504587581 54.6414321010929 40.4359177791566
40.137818555383 39.6719190054379 39.5399188536544 37.3600014814388
HHLA3 45.2875173643252 44.9849915901931 43.6080766584829 40.0337926471478
43.8738578522009 45.996661384888 40.9784333688086 44.7044140979108
41.6285300488826 50.4806959685631 41.2250007054033 47.7596919358651
HIAT1 74.8637649912543 120.001312008518 112.261145599693 109.933396041701
98.6553805168141 106.035435807364 91.8251275769679 64.4801713556664
98.6177995580785 63.0748373122119 78.0909302258507 100.962000280931
HIATL1   124.002268940785 180.100430567626 108.019575675653
124.112216491975 174.021676013865 112.60230393988 177.494605794577
92.9043863423147 151.848243044839 100.927111474879 128.024853362102
120.980558571109
HIBADH   313.080592198795 341.601353423389 342.475405815033
241.222680673439 401.956364010068 596.84269596976 302.50850157207
312.245547906683 442.853589679801 568.371815312895 503.529187388654
586.508419046212
HIBCH 262.024495926094 348.862367731887 505.018127833201 149.426965431854
473.223156200183 248.321641540666 182.80711310964 289.25391828279
338.72596574658 348.206465194205 298.54401957868 694.590958116425
HIC1 65.7587943905649 49.9547657591437 46.6588655218735 61.8997157694697
43.4544762830742 84.1922656126304 46.8613212048981 56.0496524902806
44.2607789091176 55.4037482627222 63.1566669782858 57.6860752168769
HIC2 152.585423208022 521.029507993615 94.2275161759277 127.789065386943
446.757901018137 103.955842514553 261.374019422095 152.031470139119
146.90802741139 80.3915591207869 60.6505672212119 61.9882398986333
HIF1A 94.6167194649276 72.4913548133238 93.0538726751588 239.569514501529
106.186051982612 83.5274372175811 84.8569316561572 87.799979434508
88.9641595603127 63.5489745357606 75.1246064433857 134.132985360393
HIF1AN   95.4841914842335 83.9760574088975 76.7791768896805
91.7068354379889 81.2359429678503 84.5913809068614 122.988056802699
92.4953883194488 86.2563919366781 127.213248611204 103.491711140137
119.512426152446
HIF3A 41.7904796446211 39.9339434067497 40.301483914446 39.6705473876526
39.6023310244105 41.4186558780356 43.0441025203913 40.6723334103791
43.098454794209 41.6724739851995 43.3085358214154 40.807035645835
HIG2 104.232196493024 63.6966761077037 117.556281726503 43.8002456102021
67.222434502867 55.6242009731278 94.4618437533343 45.642304356051
162.895543924742 67.8233625051884 49.6706535876636 366.515210930763
HIGD1A   316.327885002562 879.708199333257 751.953794043451
383.371300272181 765.145138238955 659.126408705362 411.114836576804
451.820169649522 346.285308869994 274.994470779348 330.391945158969
814.991871217268
HIGD1B   61.5882375235292 50.2696827764943 85.5839487225537
48.9944486068347 44.0451487108213 45.2852528185486 40.9157352260901
57.445796273665 44.4395580493118 57.9713749600418 63.5195937348531
50.8999542937724
HIGD2A   478.566247444176 380.736562250846 558.855313555287
479.079946537291 486.044304392285 885.224377292125 370.41288705812
343.892794667047 582.511224532378 539.188951045594 817.631505579476
713.685405035516

HIGD2BP 36.5099686711015 40.4772513262052 40.301483914446
37.5382094267692 36.5849073973352 39.0620487644635 41.4186558780356
38.7864161493321 40.9901805728485 36.7439906400209 36.8730514568925
40.716553247236
HILS1 41.6354649482853 40.716553247236 39.7411455670172 39.7411455670172
39.6535880152834 39.3841861812262 42.357910063876 41.9315419791594
38.9834959368496 47.7180816682562 39.7342718293432 39.1553169409075
HINT1 1723.37649434054 2122.39240932934 2384.00264993279 1868.51164332659
1712.55038816005 2405.8635632802 1561.83892117664 1266.41536472956
4093.70002166179 1769.18069954113 1232.7632290973 2803.11698341125
HINT2 299.188277423715 358.979854534484 222.570136481811 288.12763031226
284.351747562807 342.829992044257 638.305455110267 414.062259640915
393.464673405634 387.039093527273 375.818861583151 280.820745089796
HINT3 62.1875461262634 67.3519071522322 53.2046867327331 70.3646137594581
53.1100587888055 48.4758590019384 63.928488869459 52.9813204449021
51.2423106372758 44.7958122466628 42.5086338413299 57.5372732453319
HIP1 88.2427413480277 69.5544840164738 98.3240331951021 99.8063480813138
83.0497574039035 89.9789975503195 79.1432909353319 69.5741225986433
83.4465817617201 57.2813205536176 62.1796792097014 79.1209792219434
HIP1R 48.7213586071409 76.9904391375645 58.9870089759222 41.0219290963778
58.9201382292404 42.5193208629123 72.2377222744463 48.1591264215184
72.3757385026596 60.562215295948 61.5635554619635 55.1109784383525
HIP2 95.8002081462966 166.317689650501 142.614006543733 122.952515297686
153.139323479953 108.806649898122 111.025105784166 113.528908699366
164.262531918679 84.1030588938732 93.7444359334292 126.360723656793
HIPK1 40.3478825593224 38.957687788868 38.5045333213784 42.3060097375421
39.3999056968598 40.2903961886288 40.6336801346817 43.3696672906922
39.3605785355553 39.2623423164488 38.4251036088595 38.1898990106602
HIPK2 73.1931742801095 76.093116738739 84.9970776435852 126.528654651607
82.9322141700549 85.1544284060549 80.5457814044192 120.286733441947
76.3790849853724 101.877183008944 145.09364961191 110.947379383048
HIPK3 49.3000844711116 42.8542642792862 42.1901757067073 62.1776700757427
41.9842695818285 57.4777075615651 42.357910063876 47.7889447429963
52.6922486991742 48.9088046212958 45.5055402477919 51.2318935933116
HIPK4 39.4710743516517 40.5400384479766 39.1799070031819 37.651298468202
40.0599807618105 40.3357777530419 48.1747307200218 41.2551966132486
40.4997186069494 41.7405682794166 41.5421429082231 47.1060577218993
HIRA 42.7150227340364 45.7979032939211 46.1653991089885 40.0698728584484
42.7107928951766 43.5752393599169 42.9803717121865 36.0306975524309
41.7627910693483 33.4316681623175 43.0016969050339 43.6753843488695
HIRIP3 94.080489738662 85.5135159896819 151.601402122351
81.6780581904253 164.072453709966 202.01900875263 128.391264770998
215.869541768982 99.6340486428397 164.287737381703 157.095926797414
122.292794686468
HISPPD1 42.0532901689036 36.9994058279362 38.6005272730664
41.3027031037343 38.9496515344837 42.0574848021044 40.3302268293707
37.1317006162938 37.10170285405 41.3321861371202 36.9060997041453
38.5279745308203
HISPPD2A 69.6449298794042 76.6936010119768 60.2382194733872
55.3141112947345 91.4121563150184 65.6260887743331 74.2690269784394
89.1217605204013 91.8935361535002 88.4897895318663 82.291097567232
76.1019439127881
HIST1H1A 46.8954576545929 41.7337506166619 36.4564050256915
43.9282059368202 43.9450023643394 46.0364000143537 55.695361644141

42.4580504587581 55.2114969675545 46.441301934328 47.9942520395434
43.1805016690297
HIST1H1B 39.7576136507593 40.2493681589104 43.0971787292711
47.2009420656854 37.6527901109005 44.3648993568405 38.1793598983615
38.2782436497441 37.10170285405 43.0919221375514 39.9899430628542
40.1700117452801
HIST1H1C 1090.86617066541 120.001312008518 167.746168796779
224.450710664296 762.388959943712 197.569816397993 725.680256063016
1135.43644148537 867.856842601788 65.9189023794337 81.9727730469029
52.8455400026194
HIST1H1D 46.0920218698606 40.3384318592985 45.2313723727673
50.0526763693329 44.8897737954573 43.2805590023412 51.9421032049606
45.1714698794331 42.8036731479932 41.5435611403119 43.6629145624937
44.3521099857368
HIST1H1E 33.4902421268456 37.10170285405 34.2890785915814
33.4240466670776 37.2567512265363 35.5158505219112 39.8695929627783
38.4540849632254 40.8970401536578 34.4875013487777 42.357910063876
32.4951076826876
HIST1H1T 39.6609238036815 38.0226905622357 33.0260566875355
39.5050383901884 56.359578588344 44.1737502492846 42.2577689927563
37.33030683349 38.8271346375425 39.4710743516517 34.7308008643142
39.2469742642337
HIST1H2AA 43.7585538437696 45.0115870036541 43.0971787292711
46.4555908533915 41.8806343997257 43.8582747295766 44.4639405506967
41.4508241771315 39.2809305246091 39.9948390976317 42.8263096141654
45.0985088502363
HIST1H2AB 43.7585538437696 36.0366673493555 37.7384958256497
35.9521941716188 37.7576900881741 35.7034203372528 47.8947595316832
35.6825474397235 39.3750969348308 32.0298369224145 35.046946054314
33.5578650644246
HIST1H2AC 959.549658414082 116.882934971483 128.032613585193
240.476103976894 1039.58636143171 212.353676518748 506.585006700107
208.3497732496 241.148224648134 53.8260140375214 69.4877749654481
56.8534370691413
HIST1H2AD 41.1523487516364 40.8880530359822 41.2365089689635
45.0798408927912 50.3713170502557 35.9305494063137 47.8947595316832
37.86982638952 39.4762546236449 35.2880714952489 38.2795150354677
41.3481355128089
HIST1H2AE 39.4710743516517 49.0697565324841 52.7005092555102
53.8086134112366 100.205124008648 45.0882356578404 61.3620062177524
44.293922970134 78.9521593412581 43.844855336215 37.6484782516569
34.4570158133896
HIST1H2AG 53.593974682612 44.1533327883807 41.3708420203838
38.9934686082455 50.2163892855092 40.8970401536578 62.6236991757723
39.5954353681031 50.6346949248834 36.5352572191451 38.079222289614
37.925121819624
HIST1H2AH 42.7150227340364 44.1239711172417 41.2365089689635
39.1706564265495 44.0451487108213 47.2927436772465 39.8695929627783
40.5158867964016 39.0775870687758 39.3698811593364 42.7275315121163
37.3600014814388
HIST1H2AI 43.0001432913058 45.4143854024453 46.7508829977587
45.1944222548351 61.7566103867825 46.3715909653832 40.2154497375715
37.6557714476853 49.4786466722535 43.2988017098387 41.1638563416981
40.1700117452801

HIST1H2AJ 39.266565827316 38.0226905622357 42.8270038153139
44.1483976313959 35.7973685350741 38.9378717994178 38.6243997507646
42.8482181313226 45.7452456977857 44.1379638573455 41.6336636285875
43.3744503319109
HIST1H2AK 41.9407041937973 35.8227939208521 36.3604951341921
38.0958223740111 40.2449343247539 39.6251842352088 33.5578650644246
36.7460922671619 38.9834959368496 34.7308008643142 36.9060997041453
35.5523662009011
HIST1H2AM 83.877343750194 50.2163892855092 54.6051538001037
44.7980740332174 77.1287755867582 39.2248354955045 74.7534423194688
48.9826868640753 60.6706478075023 42.2614524730856 38.745552403895
36.7762265039864
HIST1H2BA 38.8561375693335 38.9338521051669 39.0775870687758
42.4580504587581 39.2608760193011 42.5660695551615 40.5585676679482
38.2782436497441 42.7150227340364 38.4187686307607 35.860430241131
41.7575097298927
HIST1H2BB 35.5179217192467 41.8848959280916 39.5450079747895
37.9875302985336 39.2608760193011 35.9305494063137 39.6535880152834
35.2642250366721 40.0403898620771 35.0082729554459 38.745552403895
38.3291713594599
HIST1H2BC 70.3757673501097 35.4484652445078 42.0621425894624
52.3004056941891 89.1012752492647 40.1428591998404 82.3887053739195
72.3822545261582 58.0722340887264 39.9948390976317 42.8263096141654
40.6614700944853
HIST1H2BD 1084.23003918768 88.9038123445083 258.757703729905
138.728648830528 1051.58050264114 192.922622120507 862.031722874406
455.444228161475 451.290681710534 90.0043438096205 97.6023731094756
60.7103211409901
HIST1H2BE 51.6558970571712 46.6314009405034 45.6989664108554
48.9944486068347 51.7983110178095 38.5252456306656 45.8980298854198
52.8622615027833 55.0748878261048 40.3235655331796 37.8372168909583
40.3691324547049
HIST1H2BF 71.2963721541702 48.7084357480922 47.7737810047679
58.1798515131249 96.8490167966217 44.1737502492846 87.6018870099686
48.6441988290952 89.1217605204013 43.2988017098387 49.4593570700221
45.3763275588341
HIST1H2BG 46.441301934328 83.2873728545705 97.5849495690144
82.0292880742189 100.205124008648 52.8421046802739 42.357910063876
47.9942520395434 132.294940663192 41.5435611403119 50.1649089793069
49.1200355938198
HIST1H2BH 41.6354649482853 70.0470864733496 52.0742951366864
44.7980740332174 155.712392689325 52.7156971154405 99.7529382840954
57.2350830635912 35.2143190355414 40.8049408996274 41.0808257270221
47.9566297250148
HIST1H2BI 34.3473914754963 33.939263369489 35.2838283525874
34.2080003860523 36.6354013629791 40.1428591998404 36.5352572191451
35.4526946152955 39.4762546236449 33.6188027862739 35.046946054314
35.6427313468066
HIST1H2BJ 192.058030454066 105.204861495017 152.612579631539
169.425386468707 262.215263489321 198.010793115588 197.325473090277
136.913434348036 179.378838766172 177.27613997459 117.8523906126
163.917208464731
HIST1H2BK 2966.53151444114 329.282615395786 936.978983469829
900.517711546397 6309.53691418065 1545.76489731307 3343.07293372655

1604.80031304031 1559.50766982801 206.610705620712 284.105466865264
235.764999941441
HIST1H2BL 42.9104454767916 37.6383530120621 36.3604951341921
37.8442841690502 37.8679241954337 35.9305494063137 39.3585350448719
43.0390857698452 43.6670755115848 31.6981395162348 38.6662194645633
35.7451401627836
HIST1H2BM 46.441301934328 45.0905928998779 45.989212296035
42.2496337895968 46.8674366115656 48.260931742163 53.1884505015411
46.7980335276155 50.401623258584 49.6203214143464 46.7386599624801
56.1875519519366
HIST1H2BN 49.7827734675743 46.9919960628953 50.1860191642359
39.9697332660833 54.802736966312 47.0940613507321 61.0979269700412
58.8022152133979 42.2577689927563 40.8970401536578 40.8225507267584
41.4508241771315
HIST1H2BO 40.9558083259517 41.6466169548846 44.3344900481112
39.3750969348308 51.4527545197672 35.9305494063137 41.4742337714895
40.4359177791566 41.3803269429468 42.0621425894624 32.6463318577756
39.7411455670172
HIST1H3A 41.5421429082231 49.9547657591437 50.3950981988762
45.7397504535169 64.193536444915 54.8265655909623 78.0020909283414
46.695848687694 54.0192060888782 49.4006068471301 47.2134341420411
41.3481355128089
HIST1H3B 51.9545473262296 33.0553208959996 37.6557714476853
35.8472302065546 57.5040500497076 36.0306975524309 46.1634822993009
42.4580504587581 38.5252456306656 40.5090849993014 38.2795150354677
39.2469742642337
HIST1H3C 47.7267190613906 50.543526404059 50.6004078448405
52.1201052574162 78.0077300979911 43.7630587657205 64.0985088513857
46.8954576545929 56.5094373275922 43.4885245598939 42.6366765186182
46.4499258373233
HIST1H3D 64.668912004173 48.4980626712175 43.0016969050339
44.9370339275041 95.6182216943758 61.3620062177524 74.0039764902057
67.9103729751746 69.6511571901941 44.6918600441328 54.4825201506931
49.1200355938198
HIST1H3F 48.7646976789783 37.1685072112618 42.5406888281114
42.8235313007746 161.180847265279 51.294537308199 57.8870110456492
41.0381860307445 57.4777075615651 31.3222361955842 39.0550139257658
42.6758568421347
HIST1H3G 42.9104454767916 49.3581578587582 57.423873475544
41.5464765641928 352.997455210484 76.1991031349451 154.08990201795
40.931294690669 56.432280646473 46.325232414659 43.1931731665131
39.9519547348106
HIST1H3H 93.7352369697358 55.1766423847208 58.2986825661503
63.7909104347097 527.563018145855 109.468741823602 73.8313212063649
46.5717043290731 105.036862798568 43.401782064778 44.2142581488029
40.2669497398702
HIST1H3I 45.7356815244513 47.765848710116 54.7855575216687
58.9168466928565 50.5110359971301 44.3648993568405 47.5963750520409
45.9604707098953 51.7549258776884 44.7958122466628 40.8225507267584
45.9693232070002
HIST1H3J 53.7507429765038 34.2618170189676 47.2055133646385
38.0958223740111 41.6806984441849 40.1428591998404 47.1345949868029
41.2551966132486 36.7267557061938 39.3698811593364 39.5399188536544
43.5738557773903

HIST1H4A 41.0608773166058 36.6002100641539 39.7411455670172
44.265834798964 42.4864576619427 42.0574848021044 44.9863726205428
39.1652965788406 32.2133093774284 39.3698811593364 42.0750889188255
39.0536056496869
HIST1H4B 49.7827734675743 46.9919960628953 48.8100349749783
58.3475983630509 52.0583273941813 44.1737502492846 52.8496345990125
45.2806199505029 51.1055541075029 38.0078357808393 44.4688999098904
43.8575940017119
HIST1H4C 2424.0414102425 3001.25236839028 2024.4791883541
2220.90215179682 3478.67753301901 2816.7754199004 7127.13565491501
3786.90274075023 3592.44409869527 2083.69812106867 3176.96480659196
1598.78859278229
HIST1H4D 42.442231667357 34.7135536792489 40.7620472083159
41.0726336841311 43.5544347011646 43.0860383229288 45.1017494688414
38.6454098044474 44.2607789091176 39.3698811593364 36.43924066428
38.1421709520616
HIST1H4E 72.4966502412724 40.148780650831 64.9072331703985
43.8002456102021 181.966839676473 53.565717255469 84.9182861692273
86.5926014007562 76.3790849853724 39.3698811593364 46.4753015651202
39.6352613956998
HIST1H4F 44.0328992213727 40.2493681589104 44.3344900481112
39.6292788039793 43.844855336215 39.0266821712099 43.6753843488695
41.4508241771315 39.9444191873552 40.8049408996274 41.4508241771315
39.0536056496869
HIST1H4G 43.0930293831497 44.1239711172417 39.6451483836089
42.1273928203021 39.9697332660833 41.6590564937735 38.7337086062875
39.9576138799997 37.1820543575123 42.4594149893798 46.4753015651202
36.064718696392
HIST1H4H 1178.52115916913 46.6996902348633 55.5567699051046
76.7806009547111 308.393270347486 89.680151549258 192.700843009002
195.376772135023 176.492987902471 40.8049408996274 42.8263096141654
47.9566297250148
HIST1H4I 61.5882375235292 43.2649781354792 42.3536549114165
39.2780515621926 45.9104478407175 38.4330755397458 61.6544855697851
42.7431359850339 37.7384958256497 35.0668743708816 41.2598458726002
43.7672102682067
HIST1H4J 47.3791873993182 50.2696827764943 54.6820069269589
47.6687908290818 44.3557408297309 47.9503724044453 41.0381860307445
56.3560498705517 45.2897806651929 40.1179210534118 42.5426057453993
42.1537476443423
HIST1H4K 112.787082951803 191.853798887866 139.08163074096
158.071355628454 56.2305155589239 70.8674195845729 149.551650453179
197.205643078662 93.0888239577809 64.2174369755376 57.7786197537899
68.9258122619781
HIST1H4L 36.6480005877129 42.0709558258414 33.7737772393677
39.3750969348308 37.2567512265363 37.7000400506818 41.706879587091
36.2539950294089 39.9444191873552 38.2069499294002 30.1388203764747
39.6352613956998
HIST2H2AA3 839.850453078119 407.818713538416 572.968592371501
830.295872113419 1693.5377672895 358.498678171092 1487.80306986156
456.992521713593 725.680256063016 175.578169606663 385.36587480414
78.8181161234296
HIST2H2AB 43.4626049853157 39.0102066700839 47.2055133646385
43.9282059368202 47.4548471240051 41.8613406887824 43.8206425661746

41.7419877611072 40.7847110517594 35.195364621675 41.5421429082231
40.7704587752083
HIST2H2AC 818.927157206447 415.759723627205 561.855286945959
902.691776563423 1604.80031304031 407.161973433666 1670.01014610464
481.492404948275 613.740813446728 207.957319374194 347.420750861056
88.5638310461364
HIST2H2BE 313.528012643479 143.517244617936 184.048982286364
71.1583251698218 180.648144492464 72.6182747525443 218.868344683409
113.917429432477 143.693373312575 101.954354107731 191.454637685756
59.4465684293847
HIST2H2BF 60.4593810615882 56.3560498705517 61.2153068100932
55.6481366056639 83.5069968129733 49.9699131196797 67.8919712859031
61.8665316900291 74.3394153125605 55.9283039581662 80.0113001016496
45.0985088502363
HIST2H3C 38.5007204246293 44.5152708955623 47.7737810047679
55.6481366056639 82.1344514651393 50.8533737532786 43.0930293831497
42.0494116081998 35.6139301508335 43.401782064778 36.6298807161755
36.449101775775
HIST2H4A 114.148080724618 47.6229101519945 55.7764940944098
55.0604256312495 100.774098314706 53.2046867327331 81.454335582503
58.4200819624972 76.9953921834156 51.1169368198437 43.3085358214154
44.6484005362281
HIST3H2A 252.313870846326 112.944044862693 64.9072331703985
46.4555908533915 255.559793355361 105.036862798568 200.002687565519
76.5444845463963 144.898757916905 54.2830238965339 61.1801493883012
49.3087627580849
HIST3H2BB 49.1868644496737 42.6939394537076 42.7303139017786
38.4208429103799 59.4830424460714 44.6524308640784 59.9792284253187
50.0526763693329 49.641159251878 40.7063426028871 46.6442692119997
39.4490233720848
HIST3H3 39.9697332660833 42.5269793679335 42.640829914102
41.7614975630362 36.7356360093603 40.650742261428 41.253645578517
47.3791873993182 47.1744296124496 39.5637454855233 41.2598458726002
46.3411353377615
HIST4H4 49.4151347850687 45.6533175604486 39.4710743516517
43.6726153508978 47.4548471240051 36.6271061451613 38.5066108855005
36.9355798143571 44.3440839686558 37.5343260962758 35.6970528713709
41.6550823405646
HIT-40 44.6231541342 37.6383530120621 39.0775870687758
37.29566077996 39.2608760193011 40.3357777530419 38.4005795891396
44.0390019242862 36.9060997041453 39.2623423164488 40.3452320167286
48.7814131120945
HIVEP1 130.703767898434 70.9179240997848 72.2797007105795
184.50283792606 104.80882886573 111.939677225005 99.9896394343368
134.83544047809 108.718422641679 73.9732553364283 91.8414653633828
102.655050997714
HIVEP2 70.9419509323007 59.1938299291358 69.9179689232341
75.2475238554276 81.2359429678503 80.2112932898502 76.7483302168542
77.523734787944 81.0169370911764 71.7464249053513 69.1945249877407
75.4935574755043
HIVEP3 40.5654775358427 36.89524015739 36.5474283500601
35.8472302065546 39.2608760193011 41.3553369544097 44.2166118829877
41.1575777497576 46.4164127487052 36.0481934423077 48.6767028205398
38.4330755397458

HJURP 176.816741656659 112.944044862693 80.943990104641 68.0471049167726
172.676819138152 47.0027453663546 188.099292617901 187.277044459942
145.213187433836 40.5090849993014 39.9899430628542 45.8546719310325
HK1 477.924466983414 589.395944599882 892.323580265478 489.622327794168
421.971171686029 566.318259999405 437.84407370064 298.252355588344
1408.59798540643 644.733901442573 643.9329225331 534.292112344761
HK2 176.492987902471 80.0502531022254 63.2475738480323 114.674541610026
76.9788081114727 60.2867996701867 58.8361533901971 104.822963389102
143.173898886465 70.4895190282848 68.3157692917711 148.312100828662
HK3 96.0236731791095 63.6966761077037 60.1874689914535 495.26603220907
53.3086645804786 78.9521593412581 92.1455386560941 62.9922928277769
40.0403898620771 42.0621425894624 43.401782064778 46.3411353377615
HKDC1 82.5344501505114 48.4980626712175 44.3344900481112 39.2780515621926
39.859345745831 36.2768299545794 43.9450023643394 41.9315419791594
37.7384958256497 39.7835513496873 37.4642807402741 42.8864415494484
HKR1 85.0753342967819 58.8281931439093 82.8205269728224 61.8997157694697
58.5450868286167 63.5738862549306 96.7125250803971 81.8491450876942
68.6379135603075 114.796237914478 86.2118355769367 85.1114448808779
HLA-A 5387.08056045279 7627.46814033012 7235.0789252269 7393.77508374057
2697.96877833185 3313.98659031004 4784.36905964712 3592.44409869527
5354.58328030315 2830.73694055758 5156.59948231705 3001.25236839028
HLA-A29.1 871.771475356705 1012.91803181784 47.2055133646385
3176.96480659196 1122.44489338159 648.885062676884 231.333080775773
1132.19026676901 1925.20977162704 343.262403838579 848.079114196986
471.874412428677
HLA-B 2474.22199172028 6186.45345364665 4427.89837060471 6483.09366281532
1069.3975598703 1338.4148149889 2816.7754199004 1125.31394383117
2002.0536042686 1055.00793806075 2911.10227993347 1060.64261592822
HLA-C 52.8427643366617 127.1365842097 80.531334073336 126.420070148631
49.1928206164702 49.8135461150818 74.0995052892511 77.3367966395554
74.220854748627 61.6255834422379 85.9286114777649 71.7408778060956
HLA-DMA 731.670315613547 677.481235695517 574.847203528544
2417.02418518222 436.35723605534 600.34379987143 858.228943026906
672.759115794267 905.76958446454 306.115856873599 646.791777335656
288.552986300755
HLA-DMB 867.856842601788 505.650842529816 444.471597593258
2384.00264993279 194.703024752864 540.037371261388 419.741944629785
766.027617496754 307.445116836976 292.247095517604 374.026381773215
221.383645621978
HLA-DOA 180.80065212092 113.26670675749 95.1666040574435
458.351940464467 78.7320333941149 113.612874206329 175.41042153356
205.510539955583 62.370559099758 100.413031488199 138.349952332963
50.379587451669
HLA-DOB 48.2910530887214 66.4300949831964 57.2960245538881
158.071355628454 58.5450868286167 38.3418933808844 42.0605906240248
54.0507720792049 45.9227107476843 44.4652605017595 42.5426057453993
43.6753843488695
HLA-DPA1 1301.07436212673 1525.47741161586 913.230569760993
5177.04105968809 379.865108456536 1307.19925493293 671.959673037066
974.296760034984 319.871452890512 364.962649416567 484.651969259966
392.237227927376
HLA-DPB1 61.2130609867477 69.1414574152997 54.7855575216687
234.308728996238 44.2607789091176 59.2643906275025 83.6935756500468

70.6568303458436 41.4636047919453 41.6373771565199 55.9269493191353 44.7401523707775
HLA-DPB2 39.8234920704779 40.3104838541906 39.0470836345045 41.6045220683604 38.3085139808968 39.3565586789274 36.8369172900902 45.3389970694851 39.2809305246091 34.614788807503 41.4508241771315 39.5748670524951
HLA-DQA1 965.530928317269 582.017297217368 327.283431584541 2803.11698341125 136.582966744378 186.265538054701 487.261132702908 551.915864019927 99.6340486428397 91.6713614282714 149.312893905408 119.833821068193
HLA-DQA2 42.442231667357 45.7979032939211 46.2625470410195 47.481302504066 42.7982502551634 41.8613406887824 45.3597029614942 42.5435283762933 49.1940901905636 44.1379638573455 36.2435874904689 40.3691324547049
HLA-DQB1 99.9752275166514 206.703865774092 132.84476910529 511.261925898948 65.5029051840465 106.317388820963 114.526825145076 80.5508300225954 53.6446045619513 70.7003843117511 42.170702295259 51.0647893605839
HLA-DQB2 51.7607563002005 65.8082111065882 48.3847843498476 92.8026641003757 40.7779767565889 41.8613406887824 54.9765783642973 54.8412943008635 88.1910986077499 66.6332042895961 48.3847843498476 38.8495095797881
HLA-DRA 3502.80810319252 2588.47871581066 1595.69503047506 11795.0477101407 949.278493776928 2892.02001412369 4015.88651424568 2061.66570137425 2045.24530722676 848.079114196986 1188.3486464341 868.899376368472
HLA-DRB1 491.468862309918 44.9370339275041 36.0306975524309 1969.8871504719 43.844855336215 38.2293008890613 728.679189702033 241.118200030836 1099.52344340557 147.579873410455 544.946796643478 146.61436309209
HLA-DRB3 157.356512456687 154.067791946816 78.450972383987 1000.65752290829 58.6481644777552 138.12608134713 191.631209717054 91.0354017559834 218.675464231277 81.915829566737 138.637058021129 133.244526666651
HLA-DRB4 466.935292808402 396.355023788839 221.450737998571 3296.66056024983 129.014076422249 154.551927302005 1053.67279913417 361.158558609751 568.371815312895 106.752660061588 327.844083226326 90.2790229242453
HLA-DRB5 39.5676051939342 39.0102066700839 46.7508829977587 41.3027031037343 47.3172462673426 38.8336427948187 1517.66978539206 793.570993808328 38.7298222155532 335.167786298149 1738.00955642263 54.5921287593554
HLA-DRB6 137.395516155158 176.687970262952 85.9692990407066 448.196293409768 89.3456573431171 229.379918828848 231.824347524066 112.261145599693 162.352052340273 75.6471706262645 121.135772395548 64.2769859135548
HLA-E 207.710354513208 363.527881831453 453.260719561292 726.804513440787 164.462951076718 290.956194006554 180.902423012987 204.502683458676 206.312848708143 177.722828589884 385.064309855006 181.266908509671
HLA-F 326.250872625748 602.726022292973 494.300858189605 704.601607617837 159.071016200059 221.383645621978 352.339572553069 188.567635000971 316.548339196872 126.319731178054 311.985010273921 182.37433006793

HLA-G 90.9521112485099 104.740168626646 93.4180535865914 146.405413944753
58.5450868286167 67.1729762600915 81.6458112867541 71.9771425105735
84.158736606096 75.5171857162768 90.2790229242453 69.4383198054368
HLA-H 1465.08435775014 2678.43227619157 2232.61002288121 2721.97551918611
676.672146226382 970.379875059112 1377.24483375075 724.881002237334
1201.00042684883 543.975237512604 1478.26733379357 604.951648398924
HLCS 55.2515150764173 63.7226299242322 65.6165636276827 51.1313686723929
106.035435807364 58.8090653936339 65.4784797364469 61.6839343510302
83.3905516293346 74.6847594297153 65.088713927995 65.8959431201418
HLF 39.9697332660833 41.554942319225 43.4709415392696 55.0604256312495
37.956732373465 101.693848821246 40.2154497375715 43.6434576713809
34.9428206949566 66.9218519793499 77.2834482217973 47.6040671292013
HLTF 104.27034824534 133.86254468037 135.00141569592 84.8948024208923
188.120766710975 123.176449895397 131.010196997585 117.948047796034
149.173801572239 115.116519358318 100.802977734227 119.833821068193
HLX 64.1882546112044 60.6779753362314 56.0800798275077 129.190555260332
40.5235471987417 70.7782698163955 46.7800033479223 46.0633179231318
46.4569566520107 51.8412522234186 63.9371474698484 44.0451487108213
HLXB9 50.379587451669 39.2907645923217 45.3974838013476 43.074472921841
45.9104478407175 44.7804080899431 42.4939802311136 43.3262148375525
52.2631308081333 38.745552403895 44.9713479185144 44.9070250604284
HM13 90.8831233784641 87.1183334887694 92.3659612653822 148.654594696193
161.075134901675 72.6182747525443 98.4699679900651 57.50915811428
89.7002002853947 49.4006068471301 55.4294204189628 65.084620579901
HMBOX1 66.4399491500833 56.3560498705517 66.9239565096247
64.898351831341 51.5888395924843 47.8636003588181 66.8475050031365
64.8336014343435 51.3511088648386 95.6182216943758 92.1646038105091
62.8485724004124
HMBS 93.0714382925187 96.7280766051052 111.735319884512 85.0308877626746
149.09682467711 69.5080454202415 125.588228083829 76.9904391375645
116.694275999729 74.9006142438341 75.7502062965698 77.1521355570024
HMCN1 44.7044140979108 34.3715326040967 40.1985790927248 48.9944486068347
42.9090346219023 38.4330755397458 42.0605906240248 43.7396619462033
41.5635499110307 35.4990901121893 39.4410515050885 44.4475838947656
HMG1L1 84.6279355865799 91.5202521578181 111.077646506425
99.5125674355716 150.31039735991 120.508644792138 123.868848519181
96.9476136220382 118.450965500771 80.2774079897733 82.7309298534989
114.363816429963
HMG20A 60.1705241170421 70.9179240997848 51.9214790114499
66.4047895961325 74.6833250738044 69.7677752398541 63.0119737420787
54.1731035633439 79.2824265044758 102.490465676619 89.9227845135785
79.2525964926483
HMG20B 562.531738167208 500.794458547464 753.976423227915
343.591727067749 869.879392791086 403.335980469043 432.807626784742
550.642253702646 490.301992866742 633.819901764391 569.966632625157
362.724027984972
HMG2L1 48.2549905619119 52.4351670989268 45.8445073504868
41.7614975630362 51.2525124735102 50.8533737532786 48.0283573444179
42.7431359850339 45.1569741609597 39.7835513496873 45.6348103033479
49.0986627051784
HMG4L 42.2455707947223 40.716553247236 48.0191002461099 44.7980740332174
40.4639532172338 41.6590564937735 47.4439407315335 46.4672637826582
46.6944238390932 47.4767851750188 51.4413099192899 46.917149434588

HMGA1 219.431561967403 147.602298697211 131.045615546105 98.3142231283069
218.354538624978 66.4015820814547 173.11353027932 94.0749342825094
201.301705082976 80.1210785681103 64.0541263553251 80.1210785681103
HMGA2 40.6559419616644 43.2932256291516 40.1474543172332 45.4634652319753
43.3832826368789 41.3177250076876 40.7340599105623 40.7311497914871
40.1725584225343 39.4710743516517 38.8034431562381 42.1537476443423
HMGB1 49.1868644496737 50.8812920005251 46.0727230219512 47.2009420656854
56.359578588344 66.4015820814547 56.7780520416533 50.9099078696349
69.5741225986433 58.2818491355907 64.5044423866259 52.4351670989268
HMGB2 151.601402122351 220.597255695837 132.52749757795 192.356440003154
193.15756263121 160.853643241489 398.538881167487 214.537530518962
361.57814355509 95.1000239210194 87.3815040456105 113.388372364837
HMGB3 170.850089909832 136.517445122438 86.9924799927707 80.1809075683154
184.750252804038 67.5812474586277 154.386505379733 241.849566505679
173.711189659585 87.6598268114592 75.6883778954949 61.7502624458447
HMGB4 37.1485601150873 40.148780650831 42.0621425894624 40.8681871019812
41.3755760434934 39.2248354955045 39.7162631015511 37.33030683349
41.5635499110307 41.9666541498638 44.5195179655313 43.3744503319109
HMGCL 105.802035886293 127.337191367244 125.383837024985 110.431464700539
96.7351644023249 115.143810463938 215.907790093087 167.746168796779
156.884346540644 182.246578375636 137.802147212644 133.067727272864
HMGCLL1 39.3303857231606 37.7384958256497 43.3085358214154
40.646702696033 39.0916553915607 39.7394181011603 43.1931731665131
40.9808244695721 40.9423087727566 42.0217776707284 41.4508241771315
39.7938220956242
HMGCR 150.925721934159 249.246628143531 181.966839676473 234.45840519702
172.777241940677 110.637910297739 302.422475486532 326.902009704746
468.267514612816 217.425223098994 243.803668806533 208.529062113623
HMGCS1 440.728435508514 214.342804055754 279.632025097952
304.777565078155 691.94603958547 271.50380898418 1850.06063216244
1516.33074288338 1106.57048989685 363.269936098507 409.899024157206
380.015934356432
HMGCS2 40.9558083259517 4048.33132153903 4717.02689540013
59.4674205670657 60.1730663840476 111.760213205992 1090.86617066541
3999.32180022337 1487.80306986156 1343.66876893729 1591.51486001004
1297.78424326775
HMGN1 1658.86829668934 2830.73694055758 2803.11698341125 1029.12917222294
2640.06306087033 1255.10866316499 3800.19142219719 2133.78836857697
2911.10227993347 710.579735673002 892.323580265478 1401.46625360427
HMGN2 196.247893540766 332.659496278775 272.022473793857 220.743173420176
313.528012643479 374.026381773215 515.856807472321 307.974082654351
510.525676243746 163.20801480093 183.393353108744 221.847615564378
HMGN3 40.7609952437622 41.7337506166619 42.1901757067073 43.8495248991589
42.5449473689701 39.066284740632 42.0605906240248 40.4359177791566
43.3262148375525 42.3413611855132 39.3086414047188 40.716553247236
HMGN4 337.363827785838 792.022171185649 343.591727067749 403.664724682094
1154.6386362 576.001941308305 2248.64628069362 1178.52115916913
374.723141438448 404.599730419192 420.401914084346 638.848775860866
HMHA1 55.5276281380084 58.6249721386149 41.528370959196 81.8727530460686
51.5888395924843 50.8533737532786 43.1931731665131 62.1934012861178
42.7150227340364 48.6124127998952 42.7275315121163 39.7411455670172
HMHB1 41.9407041937973 34.6306468196142 36.8730514568925 47.2009420656854
34.6227169056411 35.314469458379 40.5585676679482 35.0510338774593
37.6383530120621 37.8217989841918 51.9302027391447 35.6427313468066

HMMR 74.8637649912543 100.43365828852 112.924613715969 47.5733603903505
157.856158440506 44.6024422254257 132.24963384417 128.937837181433
98.0473811574767 41.6724739851995 37.7963032976457 43.5738557773903
HMOX1 301.728842562175 106.160416826673 121.195506364658 321.454824353597
206.703865774092 155.435700005184 522.49156295512 569.966632625157
93.6124140212565 59.4969799685856 68.6199296680241 72.3297091475814
HMOX2 39.6609238036815 41.7337506166619 41.8751578329365 32.7960913111995
39.4693871570028 44.9896851005259 40.1235656101329 38.5457716679071
41.863927676552 38.528621456378 35.2339574411711 38.3291713594599
HMP19 41.0608773166058 44.4411734611229 39.3709357615493 43.9282059368202
37.7576900881741 36.6996187567892 32.1218888714537 40.1474543172332
41.4636047919453 39.4710743516517 37.6484782516569 36.9976231206067
HMX1 47.4678477186801 44.4411734611229 42.0621425894624 47.3574589211166
49.0828761839116 42.4580504587581 43.5627649099623 44.5029690493211
44.0519245668737 49.9493230064613 45.0589947942306 47.5025821041537
HMX2 46.0920218698606 44.7829632472754 52.5353664902258 52.6947911480086
51.4527545197672 46.3715909653832 42.4939802311136 49.2901038327894
48.3954967265007 43.7303813515864 36.6298807161755 43.6753843488695
HMX3 39.1590964332473 45.4143854024453 40.301483914446 38.2041059863839
38.1644569189523 37.8102105794655 40.1235656101329 44.1311654985682
40.9901805728485 41.7405682794166 41.8168470823451 37.4554876386402
HN1 73.3448790478335 100.774098314706 83.1874704625702 48.856176220627
130.615540145155 59.8102324370568 107.325456368022 70.9702772728923
154.93193897888 41.4397539024976 48.1352470945488 69.2213838763756
HN1L 63.5693267269002 76.093116738739 57.7415362494584 48.9944486068347
59.1948983423381 52.6226745117396 70.5534237141767 54.6067620335514
75.1328739717464 98.4084364053686 89.8354888041611 90.4160198134865
HNF4A 44.0566054055178 40.4359177791566 40.7873632847691 45.2806199505029
41.4479871197761 44.7804080899431 40.5585676679482 40.1985790927248
39.5424643148065 42.6211340086915 43.3085358214154 42.7303139017786
HNF4G 43.0001432913058 48.9259041798949 42.9104454767916 37.8442841690502
41.467005949568 47.7556758818053 44.2166118829877 39.0620487644635
56.3441077510744 46.1390887492649 40.8225507267584 45.7562276914441
HNMT 119.311590524726 79.990130381874 111.522812019895 138.972884942374
77.4769634299218 130.506891299903 62.2613190008072 88.1068080491008
91.7579171517883 141.471672990444 121.782045054657 133.86254468037
HNRNPA0 441.397616937015 370.568550943181 268.683528469759
217.575115287224 348.165210494253 428.729935028911 226.090904721424
263.587821561589 256.55408314817 300.762511215441 421.013324906569
489.232537791775
HNRNPA1 55.3153799233744 65.2295773603116 67.895735332714
45.334106390212 76.301438841111 82.7853677822046 55.0154661412165
55.3664856922473 75.0310858627394 45.4614234442527 58.7722169145704
82.3254961129575
HNRNPA3 54.0990839134272 58.1027665533767 54.4384603094793
40.7481155008596 64.9072331703985 111.760213205992 54.9765783642973
114.533239859288 53.2046867327331 47.2299678280724 66.3481553496766
81.0169370911764
HNRNPA3P1 40.8681871019812 39.5050383901884 43.0016969050339
33.6816578730693 37.1565984182084 53.8461734834857 35.7055410599146
41.2551966132486 36.6312676076142 38.528621456378 33.4071187618571
47.6938628797168
HNRNPAB 1204.34388579291 989.0602710707 1195.24078948059
759.94822128584 1382.25775340179 1076.05074395483 1257.19844370057

1319.32406112346 1370.76188322111 537.213035625492 753.491584605447
997.492549573274
HNRNPC 111.041991642022 310.821299796803 247.193935816149
281.840536529928 249.308868215205 252.097980496533 218.868344683409
138.783011990219 191.896416838806 196.427458710993 205.672652429972
253.398345052444
HNRNPCL1 43.2135791259317 42.2114134935898 42.0621425894624
39.7840818430159 43.004943518025 39.3565586789274 39.7162631015511
38.5457716679071 43.1180113655693 38.8173773425835 39.9586075662602
39.0916553915607
HNRNPD 1287.41527826233 1263.76838179117 1638.16317738035
1025.7433359938 1485.68317899614 1359.9708539999 1829.30985071028
1533.38581753491 2178.59848996529 1301.07436212673 1359.9708539999
1604.80031304031
HNRNPF 122.849949163123 86.5926014007562 78.7103847663605
84.0899854321388 85.7842344710572 75.5561597374812 134.567947469561
151.266092912661 73.6965494979349 70.9702772728923 74.280612516205
79.1209792219434
HNRNPH2 57.6913861717513 73.6855877240541 59.9690744676539
69.1931891474114 63.955655956484 84.4433277732938 55.2998422481855
49.2019743609873 82.8318970266303 45.7371044724173 71.5249259772583
72.2211523775459
HNRNPL 132.37539586883 169.603165827302 148.906660152784
131.347995391441 157.161381086926 84.1922656126304 223.219262492785
161.075134901675 240.841214170409 122.702789229752 112.048972076936
196.844428284247
HNRNPU 298.031228833741 198.922575188741 245.769212889294
333.731080636948 201.236491769447 256.302432791187 282.115786635657
528.840700144811 203.178221073902 566.318259999405 665.122091380755
445.644244692294
HNRNPUL2 277.069127740648 197.421622315466 219.134220280612
198.729330487352 252.616382150668 239.350719871688 356.318517117856
450.972039573066 300.412043023998 360.248832535446 384.727543700024
280.920208331594
HNRPA1L-2 135.106756607914 156.864177272437 126.319731178054
86.3663084195994 162.30238363798 157.568088397305 124.44575078108
187.422036538629 173.013258797349 123.095695502815 149.386774957393
240.841214170409
HNRPA1P4 434.060745987869 409.554706384667 383.705244574204
241.849566505679 642.946369683508 611.776512504215 415.906208143859
592.399245288911 536.512119139536 393.944898808828 529.977491403949
831.578043520215
HNRPA2B1 1698.18614771363 1945.33618761041 1716.82239359769
1280.77934331703 2384.00264993279 1186.06822787922 2167.91218703382
1950.93175239958 2575.86919902296 1057.44729389649 1287.41527826233
1744.56688411113
HNRPC 82.6246523811721 63.5222607845861 75.7464604972652 86.3663084195994
102.282066142307 77.0226403968683 86.9185256838836 98.9657264176664
95.2956103624468 78.6859709780134 72.544822622412 121.851515410237
HNRPDL 219.918644151214 323.705254926762 430.618167106529
147.444007777385 310.900527198512 184.536327776767 192.171116215791
173.18824792041 196.096147690942 207.513604688444 287.363928615517
292.735673136799

HNRPH1 97.6205705034407 132.098298410135 167.430013807712
104.784718169822 120.309345127552 137.10896135739 88.1413320312949
95.8734933265537 100.824660338235 51.6778366958973 77.3640580819535
100.623973215137
HNRPH3 97.3929166450379 79.4442697175874 79.6133633483649
77.7973642829228 75.9397606694785 163.877163712385 107.015362116555
78.8968326770219 146.007510897838 111.347228208501 104.986694941968
157.639656300073
HNRPK 270.756849919679 421.611594456877 251.65044168003 284.66531382201
314.458148321064 381.198152331624 269.857104259782 266.905855859261
299.136348099827 195.790389809892 221.76904201992 356.133736281036
HNRPLL 48.8941062980393 61.3428092991286 42.3536549114165
62.6048727240291 47.4548471240051 65.6914955077809 53.0277416531043
50.857522488428 59.4236178078244 48.3693338695392 52.6960621424156
65.6213260281822
HNRPM 659.126408705362 1303.33517942343 889.638287212686 804.592753722875
1337.53997230271 940.781370938596 1073.84987604662 957.682898193927
1294.42501356365 625.805967352327 903.756866967447 790.094848284547
HNRPR 500.431738940003 1060.64261592822 644.368005872231 520.415257194417
1303.33517942343 745.219025960188 1012.0373748933 593.801086643125
880.992328229414 323.453481668415 580.109585240053 512.691506909762
HNRPUL1 666.265306908449 748.913487947791 721.887210480162
502.67939538729 1052.16097214718 597.191426276771 872.305108096296
727.788854407888 950.740991182127 686.154904023493 593.801086643125
484.968116511768
HNRPUL2 192.41551362197 170.999578495858 217.189489936251
177.27613997459 353.463970335835 249.584588371496 246.433145017965
268.265540230814 255.284866561352 344.314901683001 265.860397918913
247.676607756929
HNT 160.264217082275 65.2966707319433 51.5796347007399 85.7013212907541
68.7342341724306 111.347228208501 40.6130792100165 71.2168693013526
42.6679886757687 61.227809931332 70.9970523709176 41.6086894860496
HOM-TES-103 41.5421429082231 47.1332119496722 42.4890076400051
42.8235313007746 38.3085139808968 50.560825686187 43.8206425661746
41.0381860307445 38.0702079388879 43.6033221838149 39.3086414047188
41.1554481567603
HOMER1 44.6231541342 47.4740726032707 38.6727842796202
35.6027791404152 42.9090346219023 38.7340969861754 40.4359177791566
42.7431359850339 45.1899271235974 39.9948390976317 38.5668610487033
39.9519547348106
HOMER2 95.9750691854349 46.2286164776607 51.371775055873
62.9364422691302 70.8515676385543 51.5522731200852 56.2181240696479
128.070047614488 63.8357465390633 47.611018474151 47.3915967256105
51.0647893605839
HOMER3 138.67794356779 111.025105784166 150.221698290502
136.786772972738 206.791464145857 116.685453817071 143.913007288353
138.349952332963 168.036319777283 114.136234102574 109.076358658606
88.9641595603127
HOMEZ 40.5654775358427 38.6414316877471 48.1331309338997 38.9534635879195
42.0709558258414 43.4681646416585 39.4600924554505 38.6454098044474
43.1805016690297 37.0320432433314 43.1931731665131 40.5703581862752
HOOK1 288.190015621249 147.209004629384 159.268245287939 95.6215575902122
153.405734390061 72.8472187581193 293.755623192857 150.114520857436
133.178271848651 112.02980664709 132.851109034738 102.38706554984

HOOK2 132.117013391364 243.403622361932 303.390680076861 78.7450149191851
311.985010273921 100.423344358771 203.729732499418 138.085451866914
233.263058459431 210.50519205965 195.376772135023 185.378190201718
HOOK3 69.1945249877407 69.4347592040783 60.2528550551807 70.506090074243
61.2130609867477 69.8231400816467 59.6780848951767 56.7569186600983
61.5190597187008 48.665578118898 56.8056093987178 61.7502624458447
HOPX 334.527003257901 498.608204176238 187.439021508049 145.267770598988
67.6699686085575 199.776860110557 63.4889805083459 215.109108265052
62.2175706144204 90.0043438096205 95.1468654370952 109.25950270396
HORMAD1 38.3418933808844 42.5269793679335 41.4613628725541
41.6564075217517 36.8400213077707 38.9661430990729 55.1240750633045
42.0494116081998 41.1742244762318 40.1613888954735 41.2267153672559
40.7704587752083
HORMAD2 46.6348258763289 39.0102066700839 45.1569741609597
40.4359177791566 39.0564222526273 39.4953494583071 41.3594868055879
31.4517139158876 36.5393857306744 41.8613406887824 47.4630625313159
39.7411455670172
HOXA1 39.1590964332473 39.1982470626863 44.825830634601 38.8495095797881
44.4031868028216 44.1737502492846 42.810624012519 41.4905114972525
41.0476065156912 41.1222709786153 41.6657053704762 40.4639532172338
HOXA10 92.3307175266839 43.8336862998829 47.646800205189
40.8211872708071 40.1047531921147 80.4404044229341 51.6869155634183
40.7752992161136 44.9580538951069 71.5345397102823 78.4289643729837
76.5758364080953
HOXA11 53.6741873729867 38.2263227947189 41.6079958749402
44.9370339275041 37.5556299264699 53.8461734834857 59.1774824918359
46.1673159982664 51.1055541075029 68.4137459386155 82.0206083292981
59.0894921452893
HOXA13 135.567012693522 66.1288889125299 70.2577825186943
42.8235313007746 78.9088042058577 118.899737794318 46.7100796279384
54.6067620335514 84.4938528830721 81.1925839235551 127.49459343016
123.022511916727
HOXA2 45.0283997415478 44.3977035913759 44.6032218589199 42.1273928203021
49.9493230064613 47.8029841385514 41.0381860307445 41.4508241771315
51.8396275386273 50.9506778292488 49.8783924642347 49.1718303518849
HOXA3 39.512606833112 41.8566550719218 41.3755760434934 39.4652349255591
42.5449473689701 41.5083179498332 41.9918605927928 39.5466975353131
41.4636047919453 55.5206840401643 45.0985088502363 40.520044947212
HOXA4 50.5670783769975 52.1967540852276 61.0370242377391 43.3078542962968
83.0497574039035 70.5025097213784 54.9765783642973 55.3122077514658
97.2425832658887 93.5511826850067 79.7691160370015 74.0913177754682
HOXA5 190.130724966304 175.820979889343 360.342857415756 82.2766822827861
605.145228948011 326.055799445169 271.393858310441 283.111920665176
636.137048640939 1369.94743248835 645.702744916884 814.294297312057
HOXA6 72.4966502412724 55.8678615468603 81.2136969156029 46.614092935749
114.093655272795 61.7091923488929 67.3976855661302 55.9303347977781
60.5608545741456 103.607345835114 94.8036856848998 77.1954301283261
HOXA7 41.8409927585705 41.3610369241157 39.991353369336 35.2143190355414
39.3667161654772 41.9543948167375 43.1931731665131 42.357910063876
42.2577689927563 43.7303813515864 41.5421429082231 41.1554481567603
HOXA9 115.691017747672 46.1797803908218 49.9547657591437 41.6564075217517
45.1137938810047 75.2337946941103 42.629412460409 51.1293275125104
46.4164127487052 276.399037769005 175.74424749223 203.946427426126

HOXB1 68.4352167527791 62.6984594468418 67.5417980425932 86.6411972262415
62.5106969730855 56.3525213736952 66.0350709576651 56.3560498705517
66.5298067946102 65.813547096222 52.104880481326 57.6860752168769
HOXB13 47.6397712007919 38.4330755397458 37.5597671487621
39.9697332660833 41.8806343997257 39.3841861812262 38.4005795891396
43.7396619462033 43.8482802038826 49.3000844711116 48.8744644207821
41.863927676552
HOXB2 194.716672574835 129.605603092944 49.3780606970538 68.1992221684165
50.0580547772867 62.9688525319822 64.232967574797 50.628152951493
213.140641182603 49.4006068471301 72.0389007415456 80.6200293361016
HOXB3 55.0078788655089 56.6146179789815 47.0055981216687 47.0874209746031
37.8679241954337 40.3357777530419 45.3597029614942 38.5457716679071
59.2566572428546 46.325232414659 52.6960621424156 58.2251348002272
HOXB4 57.7877627794725 52.0268699306396 44.0451487108213 43.3078542962968
58.4383854340181 48.8744644207821 52.2476631126873 56.4779645313958
84.8235093165193 63.197604174872 57.8991270166934 67.9141812398066
HOXB5 526.750242877914 142.03031230486 56.607199625498 45.5869891570251
148.71330612986 63.3612993417558 66.0350709576651 138.349952332963
200.41782363596 340.662853333208 343.591727067749 286.456276735451
HOXB6 78.0586153423713 50.7487159427973 46.6025757239946 55.4180794172794
53.6334631215581 50.2163892855092 52.9405267581903 53.3094843669564
55.4553297468768 61.6670071424359 57.2437208536568 54.5468959212611
HOXB7 339.679549262298 468.573956652904 140.369714831352 118.49449420132
389.248080358801 151.99110418225 347.750020791938 548.217757366495
837.959914510114 371.167168940365 435.325038193179 372.982648892131
HOXB8 366.326747630465 60.8608693323868 51.6159542017038 40.9607305725196
40.148780650831 42.8598805316863 41.5817737564592 124.051109959228
120.123385036416 105.71315237465 99.432142513601 123.455270940015
HOXB9 40.0557788909923 41.7337506166619 42.4580504587581 39.3750969348308
45.3675390992851 41.9543948167375 40.3302268293707 49.7309743973184
40.8970401536578 38.745552403895 38.745552403895 42.8864415494484
HOXC10 43.4626049853157 43.5873188565519 41.9666541498638
50.7046706880325 46.6397391251762 42.0574848021044 45.1017494688414
43.2551508830106 42.5406888281114 40.8970401536578 45.0589947942306
41.0657892043496
HOXC11 43.6706764143908 48.8584917918986 44.3344900481112
50.5310598656998 48.5000946421718 46.263826584899 41.9393838138203
55.3122077514658 48.8842909466002 45.1569741609597 49.9338905397767
49.7336769474446
HOXC12 36.7356360093603 32.2892762042267 43.0016969050339
40.1804147506786 41.9842695818285 41.8613406887824 36.9355798143571
36.9355798143571 34.3842972343227 36.5806287103157 45.0589947942306
28.3262844159644
HOXC13 42.0532901689036 52.3187919317561 77.3367966395554
44.9370339275041 89.1012752492647 39.4953494583071 47.7390230243778
47.5950227477807 88.2639730639822 64.0162246399972 50.5810338885769
38.5279745308203
HOXC4 39.4250105372135 73.6855877240541 48.9720399485413 46.3203995372658
56.2305155589239 39.7394181011603 45.3597029614942 42.5435283762933
44.4395580493118 40.5090849993014 40.723990462047 39.7411455670172
HOXC5 37.4554876386402 40.148780650831 38.5045333213784 45.334106390212
38.3561682218911 40.7616966228404 40.6750540874299 40.047314525032
37.2810767789306 42.6793753550854 44.2142581488029 39.1553169409075

HOXC6 66.603126065489 85.4437963805637 41.9363480239047 50.378948785456
99.1952492360239 59.4309046716701 47.6810611485413 96.0126406688619
125.209476374199 47.4356803320662 44.4414964828926 51.4466290643537
HOXC8 51.7607563002005 62.0908175930716 46.3411353377615 47.2009420656854
155.956315815754 68.3234331335073 48.8319884367456 70.8749669449099
89.7862835647016 46.0119912782855 42.911855821602 48.9733758107788
HOXC9 38.957687788868 54.1773909134233 37.7384958256497 36.264823544143
45.4751076037511 37.1317006162938 37.2892641289258 58.676230030835
35.1155486926999 39.4710743516517 32.6463318577756 39.0536056496869
HOXD1 38.6454098044474 112.250150357875 160.134344197725 42.8235313007746
169.194312808717 61.1022920882065 78.8250642061439 45.2806199505029
145.936763882526 318.369837408344 236.536058101486 307.754078087321
HOXD10 43.3638918705225 40.716553247236 46.9040704638073
40.7481155008596 76.8964050504238 39.4953494583071 36.7356360093603
39.853866204927 42.2577689927563 42.9866214102525 43.1931731665131
42.0424641526302
HOXD11 35.8208542506595 39.3999056968598 35.2101597814352
37.9875302985336 61.6452971581503 32.771863438151 40.2154497375715
38.956280281655 53.9287524861617 42.5664883002833 38.5668610487033
35.9604262434141
HOXD12 63.9851732498913 64.5845827915699 74.6901522573522
73.3725472767369 64.4999048115671 151.522338087219 66.8475050031365
62.6386437936516 77.6496445815897 82.966263655525 70.6138659908885
65.7853960266587
HOXD13 86.0796981361148 60.2547250212462 64.9072331703985
84.4422623834916 73.0433648756396 61.1022920882065 94.5644670313964
77.0262811825953 80.5437011468815 68.0556717366572 64.7598303406649
62.4743608775767
HOXD3 48.1125722136997 54.7855575216687 72.6731815563159 56.4578213622965
72.828269171954 47.2927436772465 66.0350709576651 56.5914743235484
60.9594847577747 62.2025996860055 55.2374601141719 53.3059058105836
HOXD4 44.3355304829989 52.3187919317561 55.5567699051046 43.6726153508978
78.1275793755619 45.9266745571724 76.3583555666815 49.9332280449549
57.2716485658185 38.1042454705119 43.5710871617107 43.3744503319109
HOXD8 38.6454098044474 42.1607322031054 42.2577689927563 41.4273122914951
43.7517118200398 37.4782441311626 44.7255207589606 40.4359177791566
49.0986627051784 44.2580151149956 39.6403089864678 52.7530873093837
HOXD9 40.4639532172338 42.7775752471711 57.8111054584154 41.1886975241176
47.2074332636566 41.4508241771315 42.357910063876 46.5717043290731
46.9739160257217 53.1993028936883 43.4709415392696 46.2625470410195
HP 49.3463416794995 38.2263227947189 42.8270038153139 47.2009420656854
41.7929491793697 41.9543948167375 37.4772922422734 43.4544762830742
40.8970401536578 45.5776159432079 40.4504608195666 49.2064066614865
HP1BP3 65.7587943905649 84.6158251687884 86.500678864429
55.4180794172794 105.187381311145 89.8567957512307 85.3227908896318
108.838406710326 77.0703146713865 51.9547984751408 55.1099824417989
87.5759422500374
HPCA 41.8409927585705 47.4740726032707 38.7745734037492 44.0451487108213
47.4548471240051 39.4953494583071 47.7390230243778 42.5435283762933
44.1519466943185 41.9666541498638 47.8988096575648 40.4639532172338
HPCAL1 445.421601813221 162.993316022946 191.09175268141
326.357036514613 262.865950409375 189.164097331837 267.427827956058
422.104447912999 150.480087842118 111.803238196297 127.676574457297
97.5104526799755

HPCAL4 33.8558232866797 38.0226905622357 37.7384958256497
37.1894304543751 32.9756367838345 52.4351670989268 42.2577689927563
36.2539950294089 39.1790517315259 26.6357008041456 37.6484782516569
39.5418280570561
HPD 45.2875173643252 35.4484652445078 46.8267540220952 47.6687908290818
46.8674366115656 43.3785777889265 41.5817737564592 40.047314525032
41.1742244762318 43.0919221375514 46.7386599624801 44.6484005362281
HPDL 38.957687788868 44.4411734611229 48.4890615340603 45.5869891570251
101.086264355915 44.9896851005259 65.644767142472 39.7509165247419
36.3649677956953 43.1839109702714 41.7272875295469 42.357910063876
HPGD 46.9939712192224 2488.32876359853 901.793120584383 117.071280682786
303.458085392002 406.668753694984 222.336600180634 330.241189572237
1027.59400018637 583.690350347288 672.515393219419 1214.73798692268
HPN 78.59966540743 44.4411734611229 41.1860481368243 43.0001432913058
55.1563703466328 45.3205181880004 44.2688720583707 39.6833800000587
39.0775870687758 42.4301183791899 40.9624000010053 40.2669497398702
HPR 40.148780650831 38.3322849790657 41.9666541498638 42.2496337895968
42.269054475455 51.5522731200852 39.5496902678788 41.8417024632349
41.4397539024976 41.4659679379482 41.9495387255163 43.5420658412177
HPRT1 292.247095517604 287.689260887067 363.215063520042 274.962689159729
418.810270137243 226.057278804077 340.553073657744 211.019451518444
588.247843264181 96.7422527189063 117.689172019617 266.702252514277
HPS1 66.8920765290571 63.9743186148507 79.9161139112432 74.9113162056974
61.2070368742244 53.8461734834857 65.9703614596867 56.4506749914215
51.2066664465185 52.0231832690887 59.3379918468928 57.8863370356541
HPS3 83.5069968129733 123.281175151414 91.2769094348604 86.6411972262415
89.6313467455698 81.5782824513693 82.6381941930783 59.6720198281342
100.294952875307 58.5087296620514 65.2295773603116 81.3082994649699
HPS4 49.8278140263915 49.1054636573278 49.0720076433727 42.8723769353017
51.0724630072723 43.3785777889265 46.4944619663072 44.2159020605784
46.1579366516298 46.8954576545929 45.1432412071268 45.5265270192861
HPS5 72.6450859740011 86.1924491308234 83.0262409967927 221.354588127022
76.4580830432622 92.3477214133335 72.230225336191 68.6199296680241
74.6277165793069 77.8620267163666 81.1350396749303 86.4937293696262
HPS6 316.548339196872 500.313640131475 459.737628144571 272.11060969089
428.206419080492 247.007210336963 496.791391622565 228.660261263355
336.159430045964 338.000998124097 318.846373031568 218.217851814655
HPSE 48.4459451445651 42.9887069977696 44.2405728317107 101.46584765758
44.4489662507893 42.6575535390785 42.2577689927563 45.4882438975269
41.5296458719756 41.9666541498638 42.7775752471711 41.5536168238478
HPSE2 44.8914371498528 40.0527893189096 37.8386378737561 42.6913548400127
40.8873907150245 42.8598805316863 44.0687697278114 43.1567024035183
36.7267557061938 47.4767851750188 43.5710871617107 44.2527566343661
HPX 48.4849194706125 43.0227387991135 41.9666541498638 43.3078542962968
40.9950639693255 42.4580504587581 43.3262148375525 39.5466975353131
42.9020321355927 42.785429206422 39.3667161654772 39.8497227447888
HR 40.9141850646578 38.9338521051669 44.4311655729487 37.5764485234171
39.7210115145672 38.385865183581 37.3508690072156 38.7864161493321
44.476519804187 41.2276963324981 42.5864346233886 38.6454098044474
HRAS 64.2690842803282 147.405190988088 152.72007888133 68.3157692917711
112.100074154279 84.8694815443111 122.23209417744 83.9760574088975
191.454637685756 170.901596796384 134.567947469561 117.934830767297
HRASLS 46.8141313516997 37.3634897565502 39.5450079747895
45.4634652319753 56.9360840563854 40.7616966228404 56.0515443021293

100.813818453306 42.0621425894624 48.2596564318071 42.2618615571566
41.0657892043496
HRASLS2 34.4896350146096 39.6123051184545 41.6977706798234
33.9645433044281 43.5544347011646 42.8598805316863 39.8695929627783
39.853866204927 45.2897806651929 37.920964499903 33.8558232866797
41.863927676552
HRASLS3 463.022816882856 148.458171821142 252.280105035425
275.096637886925 437.411540294212 673.002926695262 820.147156401172
2424.0414102425 213.261004438127 319.043862813161 410.808988840343
404.356143472409
HRASLS5 38.2083710281545 37.6383530120621 32.9174234020987
38.6414316877471 31.098971689775 46.8954576545929 36.7356360093603
39.9576138799997 37.8319142847141 38.6454098044474 37.2018803984555
35.4484652445078
HRB 48.3451401499671 59.7686826549947 84.0785633788363 70.2541961995753
63.955655956484 63.7963501146829 64.4736987797413 44.6560353921013
55.2485665256307 47.0055981216687 50.0808824383821 54.0733528420802
HRC 52.310270746917 47.5647821785529 48.7039705048129 42.9358255168086
41.0885562160946 59.5979861826984 43.0930293831497 54.2877981508636
42.992884918158 43.9450023643394 45.1432412071268 41.6550823405646
HRCT1 38.5648843914189 43.8495248991589 44.2405728317107 39.2780515621926
39.4693871570028 55.7197525213035 38.6243997507646 40.831207953647
42.9020321355927 52.4351670989268 94.5111909776371 48.7814131120945
HRES1 36.3649677956953 41.6466169548846 41.528370959196 36.5393857306744
32.2133093774284 36.6996187567892 36.634024014558 38.4540849632254
36.8127605061228 34.9138625012006 37.5583886316038 39.0536056496869
HRG 41.3785995857154 41.159706131351 41.8751578329365 46.0905079010237
40.3021896270845 45.3882138838137 41.0381860307445 41.8417024632349
38.6510172759977 44.6918600441328 42.7775752471711 41.1868052124344
HRH1 54.5358897781765 43.683792219729 42.7303139017786 43.4410741327254
49.32293280121 47.2927436772465 45.8980298854198 43.1567024035183
50.4974478884077 49.5265949137207 51.6414075056938 54.3587260982091
HRH2 34.9138625012006 38.5252456306656 40.5734458834617 41.5464765641928
40.6723334103791 37.5887129572848 42.357910063876 38.3684202018524
42.357910063876 34.4875013487777 41.6336636285875 39.5418280570561
HRH3 33.8558232866797 37.10170285405 37.9482157381008 43.3078542962968
45.8051846088552 39.3841861812262 53.8687221233237 50.1833483241864
43.9450023643394 35.195364621675 44.8872784170036 42.6758568421347
HRH4 44.436995101921 40.3384318592985 45.3974838013476 44.550322123994
45.5809132232681 40.650742261428 39.7576136507593 45.8669209716694
43.5669306063196 44.6918600441328 45.7328341667151 40.3691324547049
HRIHFB2122 61.9504984796308 71.2370255410834 88.6771566133367
52.5307002738469 66.5187917407182 93.3718038663074 78.0020909283414
58.5406981871153 94.5186910848217 56.4553747079294 62.2200723764909
59.6757573886627
HRK 52.109723558511 42.4493032770053 39.1799070031819 46.8954576545929
38.8456942580061 37.4782441311626 38.8417151851134 39.2672712041774
39.7537274655115 32.7131023641972 38.4689132737495 39.5418280570561
HRNBP3 35.3227485563945 40.5400384479766 33.6816578730693
35.8472302065546 41.0885562160946 46.574682061365 36.4098694708483
41.3506832276112 37.1820543575123 40.5090849993014 37.9946685306274
35.7451401627836

HRNR 48.3814421939888 36.89524015739 40.0998167098057 39.5050383901884
45.1137938810047 36.2015763253919 39.8695929627783 45.5581356216957
44.2607789091176 41.6373771565199 42.170702295259 44.4475838947656
HRSP12 121.204795052893 118.619819027443 107.827560253663
87.6763065012524 195.205980592503 108.168107241759 251.803102904399
67.5206810636627 91.3362264594431 66.5976612069354 72.9488994273323
111.607814098243
HS1BP3 126.302564098322 103.569150754726 87.104092810486
159.248015095017 118.806070800059 157.020685801527 173.819948756127
333.566613571224 121.036604456449 232.628747594691 193.897678586782
121.696160351886
HS2ST1 50.7390160399184 45.8669209716694 44.4311655729487
45.4634652319753 46.6397391251762 44.3648993568405 45.3597029614942
49.3880946790306 49.3780606970538 44.0409178714232 45.8180710486329
46.0633179231318
HS3ST1 76.6868184563568 270.600852806342 219.878589678991
74.2429851177794 104.486555491331 64.5187138759372 114.755820699611
175.909221705119 106.97432416825 152.467040827584 168.897476166126
185.165420581989
HS3ST2 128.13619758592 45.8669209716694 41.0356368374708
371.513106715598 47.2074332636566 62.0660509811111 50.3519150282938
56.2441895183416 46.7760357462635 46.1390887492649 47.299647867533
50.4847465816184
HS3ST3A1 206.261432452217 78.9203273221608 47.4740726032707
109.310200749289 45.4751076037511 46.7800033479223 42.8684958525042
49.7309743973184 46.2027193115397 47.9056710878325 37.1059647239814
43.7672102682067
HS3ST3B1 44.8914371498528 45.2556760873335 41.1367478979484
39.2780515621926 53.1884505015411 47.6803619768589 46.3057385979341
46.1673159982664 48.5792058061372 42.8936990772838 41.9083581529276
43.4709415392696
HS3ST4 34.6977013306396 42.3550738181144 39.3709357615493
41.6564075217517 38.2496050953547 39.4953494583071 34.6720534245631
38.956280281655 37.0126425326534 37.920964499903 41.9083581529276
42.4607795638552
HS3ST5 43.3638918705225 57.2231446406982 41.8751578329365
38.9534635879195 40.5710637884029 36.2768299545794 36.9355798143571
46.695848687694 41.6616444147804 37.4302011516587 42.911855821602
40.4639532172338
HS3ST6 54.3554233228353 43.7644428186265 45.8844325913247
38.6414316877471 40.0599807618105 44.4619248757502 42.1607322031054
43.9366081640576 45.0017158586743 59.6863210839828 63.9029785089421
44.1452943804044
HS6ST1 48.8941062980393 50.2163892855092 46.8267540220952
51.3851157261451 59.8102324370568 55.1987351219665 39.0458123429605
49.3880946790306 60.7480970026669 44.571915452417 43.6629145624937
48.5123041297835
HS6ST2 45.2875173643252 89.0642206299985 40.7952353240579
39.6609238036815 100.066535982734 43.6960408897486 43.5898425390297
51.7866317456986 54.2089559589959 57.6234624694326 50.5566644722643
57.764015576249
HS6ST3 34.8099089553046 42.6939394537076 39.1799070031819
40.8681871019812 40.9950639693255 40.7616966228404 43.5627649099623

41.5464765641928 42.8036731479932 43.6033221838149 42.170702295259 44.8372404608604
HSBP1 1077.94530110976 844.431085166353 760.745188431719 631.110081413363 663.904584382358 749.825513359683 1221.31634960736 612.843402058384 1055.69506483576 789.231011942098 802.650020720921 932.849256536912
HSCB 166.188557509096 99.7361599442168 134.394743648475 166.717256080341 120.92335773864 160.171133984141 182.643498505575 168.926743047741 125.383837024985 191.253860976433 182.757684959879 203.946427426126
HSD11B1 58.4394991753791 55.4452315601595 49.4573631027613 132.997204273031 93.9708162269401 82.2266112813039 44.14228771963 57.51482768194 43.6177016821274 86.7076796107636 63.1566669782858 80.5437011468815
HSD11B1L 57.50915811428 56.0477607422828 70.3613168347492 46.9919960628953 78.7320333941149 62.1448806673973 64.3340477503529 62.9922928277769 59.7044210110693 74.5282276561826 74.6847594297153 65.4435809676217
HSD11B2 72.4966502412724 57.4054069675016 56.0477607422828 37.0732977341185 50.0580547772867 47.2927436772465 76.8788592884109 340.20879802231 62.275808835675 54.4825201506931 50.7903824729818 44.0451487108213
HSD17B1 43.6706764143908 59.4908919662362 52.1242102345408 35.9521941716188 56.0477607422828 46.263826584899 86.7968607218397 76.3509533017572 49.9162522057712 46.5650556481721 44.4688999098904 47.3992733452821
HSD17B10 175.632783805966 198.922575188741 172.070926124844 190.442443946259 202.751918610777 220.192552354287 248.355842321304 178.123995338196 322.109212713082 124.815448107572 117.439175394651 139.472956453414
HSD17B11 90.0288824434037 294.220232101389 154.911548311992 194.079940066252 142.03031230486 213.309547409053 104.059057437473 195.58977358589 92.526038865874 81.2992152187529 94.312912693545 145.630337418553
HSD17B12 317.777971035617 542.069156178791 570.204236909398 532.043503787542 327.912730756303 436.757445186987 277.5073518536 418.410951118141 418.410951118141 312.648013979436 418.810270137243 579.482255191456
HSD17B13 43.1805016690297 42.9358255168086 40.8420925848403 39.4569929845912 39.512606833112 40.8970401536578 40.2601451506332 39.1128725343972 40.137818555383 42.6793753550854 40.8681871019812 39.9519547348106
HSD17B14 84.0388582916959 76.7710944291287 82.2766822827861 191.896416838806 81.4621222177298 160.080366694799 112.805898501551 80.4577725550904 54.8946088631931 159.147713331732 151.472676874224 114.935101645207
HSD17B2 46.441301934328 682.479411898549 629.763102939071 42.9358255168086 263.330471155421 54.5952754712007 307.974082654351 232.402174615698 383.705244574204 359.889643461047 577.276184555955 1127.96214301065
HSD17B3 53.6741873729867 41.3610369241157 43.8495248991589 41.5464765641928 41.3755760434934 44.4619248757502 41.5817737564592 43.7396619462033 43.0930293831497 39.7835513496873 41.1638563416981 44.5432192783161
HSD17B4 627.914461428624 571.992675891226 975.02086340962 683.75696650704 481.145001682177 429.854222129819 635.52621743309

644.368005872231 784.951880532127 1047.33749100342 1149.02954135912 1130.2486883492
HSD17B6 144.063191647372 67.0402749076279 48.8941062980393 45.5869891570251 60.0736861987141 309.198075383525 55.4004611309636 54.3820590274321 45.2897806651929 162.272853632419 145.01315947031 192.732356915298
HSD17B7 2678.43227619157 1208.33498233518 1320.7894611262 1598.78859278229 875.139073253298 812.386878774445 2013.08489754172 3818.71752181269 1102.03940742413 3099.21690111993 2878.82618761804 2077.74475301595
HSD17B7P2 44.5282181324797 48.2220547806679 48.2883694323339 45.334106390212 44.6834648302383 34.3887641786747 46.1634822993009 42.7431359850339 50.9010149857308 39.7835513496873 45.7328341667151 43.8575940017119
HSD17B8 76.4637616585578 97.712117589297 238.819980744789 97.3870880869036 96.143327683221 160.336517310154 103.118579484715 102.49785018292 175.378964448101 117.746495007818 153.673219460519 156.95090716929
HSD3B1 42.8036731479932 45.9266745571724 42.4580504587581 49.3654837541353 42.3685136297603 38.5252456306656 46.1634822993009 53.838484549134 43.2761454096265 40.6022003765153 36.7283081422707 39.8497227447888
HSD3B2 36.8072833828297 40.716553247236 36.3604951341921 35.8472302065546 40.8873907150245 42.4580504587581 40.5585676679482 41.4508241771315 37.4540772310118 37.3179578037963 41.9908237694988 41.7575097298927
HSD3B7 446.491994276469 55.9644250454789 113.367264677544 179.322661675979 62.9553576299033 114.526825145076 45.1017494688414 245.960009147234 54.7966692984192 104.409397223301 74.0420372528779 88.3636380348802
HSDL1 84.9075858560758 84.8084949516218 91.8533850418261 70.2541961995753 61.1216330559316 86.915098846441 96.5030525416431 76.0709828871754 78.3542840447759 85.9910479975356 90.807818984128 74.5886835786452
HSDL2 92.084526929319 163.946447772618 93.9472621843566 113.783910320605 155.050936910986 274.134201700259 437.411540294212 213.162949250252 197.813848976726 306.487935692954 301.941928552396 319.092568066532
HSF1 52.8427643366617 61.1022920882065 46.4569566520107 40.7481155008596 49.9493230064613 44.0804700754786 60.7992483126292 37.4484310268909 62.7977686460693 43.844855336215 52.5154971256566 46.0633179231318
HSF2 85.6170780640867 90.6852012808762 65.2670315837785 64.3746985855249 59.0965219753805 95.8905266177905 84.7782034884529 76.6868184563568 81.0169370911764 88.2056147099779 85.6989061660348 104.704892803169
HSF2BP 60.2547250212462 51.119812302881 52.8041782638549 56.0794100106654 91.9685593100867 59.0686629877982 74.9059650336395 53.6210959113222 76.7820250461545 53.4109882045447 49.532546810992 44.5432192783161
HSF4 45.334106390212 47.6629888215036 77.6385786739426 37.0732977341185 41.8441156763056 48.9393675671956 39.5496902678788 40.9861485852278 47.798305839406 39.4710743516517 45.5707650534259 42.8864415494484
HSF5 40.4639532172338 40.0145811166794 34.9849896310235 41.0219290963778 41.1507980856436 34.8179347896484 41.7614975630362 39.6474008369935 39.6609238036815 43.0177448037924 40.2317542073211 37.6557714476853

HSFX1 37.5100503181769 40.148780650831 41.3366779545815 43.074472921841
42.3685136297603 43.3269087731399 41.1507980856436 47.1345949868029
44.0519245668737 43.6033221838149 42.5864346233886 44.4951343330035
HSFY1 40.018129854 39.932537801639 43.8153459269057 36.6062356000449
40.7273974755431 44.0804700754786 44.9863726205428 41.4905114972525
41.863927676552 37.3179578037963 39.4410515050885 48.7226309877005
HSFY2 40.2945586900975 43.5605449153511 37.9951997351435 43.3701335735446
39.3124560362182 38.8668526610189 42.0605906240248 40.047314525032
40.137818555383 38.0436833784139 38.1318121140453 38.4774616639726
HSGT1 53.6741873729867 68.7826057564876 57.6371756925687 67.474556589181
64.7488184936482 80.2112932898502 75.287377665837 48.272324215504
101.681822254799 56.057906531592 68.4261608261522 62.4743608775767
HSH2D 50.9381343674699 145.193498675059 107.309776354951 149.792346524401
84.2158827857221 51.9421032049606 84.9182861692273 116.184007615285
87.3468807607655 97.7620313090597 93.9018973330737 81.7566533443175
HSN2 40.8681871019812 38.6414316877471 46.2625470410195 42.1273928203021
41.7929491793697 45.3882138838137 49.3854238365325 46.8954576545929
52.9482338979681 43.1839109702714 51.524711857679 50.5511827666954
HSP90AA1 2405.8635632802 1501.67042548971 2126.41018877615
1559.50766982801 1102.03940742413 1598.78859278229 2009.75680599899
1975.28674300761 1810.67209360676 2164.51925341969 1939.66920192372
1868.51164332659
HSP90AB1 4128.92158369026 3296.66056024983 2755.23573327276
1159.77262479002 3296.66056024983 2196.59044510709 2237.08790397818
1448.17151189672 3010.38998829232 2474.22199172028 3296.66056024983
2374.1766901821
HSP90AB2P 38.8336427948187 41.6069493017095 41.528370959196
38.7879667686732 41.7426502114261 38.7578265341627 36.3604951341921
40.1474543172332 42.5864346233886 39.9519547348106 40.6723334103791
38.4829727716328
HSP90AB4P 46.0920218698606 43.6587492470348 37.0642268327439
38.6835649080278 51.7495306734657 39.066284740632 42.629412460409
46.0106098064475 46.6944238390932 40.8970401536578 39.3086414047188
41.9430737035925
HSP90AB6P 44.9896851005259 45.3486182278177 46.6588655218735
42.9358255168086 45.8051846088552 45.4882438975269 45.1017494688414
40.7311497914871 39.9444191873552 43.401782064778 46.3370338074996
43.2776458914333
HSP90B1 1718.44944271916 2294.75523373329 1907.47560253019
2966.53151444114 2733.80542760533 1445.73674428909 2331.12401106076
1221.31634960736 2374.1766901821 1161.71671016964 1012.91803181784
1789.07186722972
HSP90B3P 37.0320432433314 41.8073910693411 40.301483914446
42.0102671859606 34.9062568194127 35.9305494063137 41.4742337714895
43.2551508830106 41.2870505273664 42.6793753550854 36.6298807161755
42.9887069977696
HSPA12A 60.9710783023013 45.2098033805679 42.8270038153139
53.4785152499923 47.3791873993182 91.1707965298745 40.3622285487287
56.2441895183416 44.5678064264717 92.2095095678497 71.8613906497084
70.3935694362568
HSPA12B 97.4769942713663 67.8999127712557 77.0301627655229
93.6212502203405 47.5647821785529 157.250548147161 43.4544762830742
55.5578334877425 41.6616444147804 104.288073521816 86.0100701849272
59.5875857071693

HSPA14 119.095094052197 153.595112580363 159.827302305842
149.426965431854 187.982920458868 144.954155897343 220.36831243882
125.786923817078 218.958199087236 94.1575209300592 96.1307490576512
111.071209057746
HSPA1A 621.012072500314 430.283061862756 976.566026758227
963.224016480187 143.679866958604 1068.10627239579 1064.35745220225
107.971806573919 56.3441077510744 1107.96086673001 1255.10866316499
671.458899624447
HSPA1B 4503.77675488367 2374.1766901821 2146.1548633579
1247.50618540769 6606.35767508139 3546.97585966225 3575.78813099057
857.509349403807 2184.70038900784 2323.81110653289 2791.75465171135
1226.43929953115
HSPA1L 61.8529316708431 56.0477607422828 52.6275131390249
72.4834467838798 56.8307116636372 60.7488007939604 62.9165938167938
45.3872406432794 63.1944558106132 72.2443746456494 60.2165699959135
65.6213260281822
HSPA2 76.6094442444074 105.34055372228 159.12163104195 113.051477801522
154.551927302005 290.174315362911 84.0691608193405 93.299474822664
88.1910986077499 138.840039960639 291.086512261106 179.629622763381
HSPA4 207.710354513208 169.723009455614 182.188883821722 184.898658656843
196.760671234837 127.986477405999 161.205086002542 296.842555066902
236.307055572306 168.640033281185 143.357170805323 192.318724749487
HSPA4L 51.9545473262296 45.3486182278177 40.1985790927248
44.0451487108213 52.6922486991742 61.6219790276408 50.3519150282938
42.4580504587581 48.8842909466002 51.3351387089044 44.7926323492519
57.6860752168769
HSPA5 406.33528162722 195.303781999387 142.769349424534 268.419751472464
138.50445813694 220.36831243882 247.966757890598 162.181462827314
281.688688268815 230.053744836012 159.393207489822 199.970127219451
HSPA6 223.356945665761 122.0305509548 103.856444152519 883.06773454117
313.046160403661 410.623529765253 179.994715581707 78.3542840447759
65.1129635608639 61.9882398986333 66.0664586519214 86.1741504863717
HSPA8 2621.65272077828 2892.02001412369 2435.70120719503 2559.659041589
3099.21690111993 2096.51999792183 1969.8871504719 1578.32969650385
2248.64628069362 1221.31634960736 1342.10592868516 1107.96086673001
HSPA9 425.304961783517 313.711588658312 393.944898808828 291.64206171681
354.861943266984 382.190644764853 271.961673480206 416.559486912165
496.444080712528 409.554706384667 429.65084326307 510.82619253142
HSPB1 1945.33618761041 2878.82618761804 6258.22561934986 987.427672417105
4015.88651424568 3707.73750609316 2588.47871581066 4665.45637589484
6555.04068292972 4632.08655821299 5387.08056045279 3966.51586153125
HSPB2 80.8943070653517 54.4793574349739 57.0427273538849 78.9479233685889
50.2163892855092 211.897320355251 48.3411413510851 53.6210959113222
46.6944238390932 113.26670675749 118.467231612638 92.084526929319
HSPB3 43.9309845324767 50.4022543872993 43.6670755115848 45.7397504535169
48.1473938886239 51.4931742685391 40.0052661202207 45.1714698794331
39.0775870687758 77.775644509079 41.9908237694988 85.9286114777649
HSPB6 73.3950348855139 97.3066473777771 51.4308501933893 71.2572485987604
70.0133734659853 1939.66920192372 39.7576136507593 77.523734787944
45.0997987980157 924.316085565917 753.976423227915 742.726253630865
HSPB7 70.3757673501097 48.3699741879723 42.9104454767916 54.6089451252049
46.9906120932727 422.237766234202 43.3262148375525 60.7942162859036
42.1594905198929 321.359569119182 339.52415679299 302.885979412198

HSPB8 102.863599315528 60.9755936952178 56.3471340054074 60.1837189855762
52.8190882379316 475.310458821086 38.0598374201407 165.440751113862
64.7116910405493 459.021889198824 372.982648892131 279.901217752341
HSPB9 37.4554876386402 41.6466169548846 38.6727842796202 40.8681871019812
39.4693871570028 43.5752393599169 46.3057385979341 38.3684202018524
35.5220586859301 42.6793753550854 36.9060997041453 39.7411455670172
HSPBAP1 95.5724734464786 82.1344514651393 74.0913177754682
172.89937021234 125.503897988367 98.1701560122734 97.0155195562193
131.67159377585 81.1416376689959 123.841740274811 91.4514026506253
81.6458112867541
HSPBP1 48.4614401417022 69.0975200641705 63.3006977162657
43.6275678329783 118.748969134194 75.8331683850176 82.034818940429
59.4188882103773 81.8426140611297 48.8044288137598 55.5313494549746
55.9555622193202
HSPC047 48.2910530887214 54.113533179847 93.1349769600388
65.524821699432 63.8315840603904 70.2553917409773 46.7100796279384
57.1362071277099 44.8997235283254 97.4769942713663 79.1922819549042
60.8662233098324
HSPC072 40.0557788909923 39.2907645923217 42.7303139017786
37.5382094267692 41.0885562160946 37.5887129572848 37.4772922422734
39.6474008369935 35.7973685350741 41.1222709786153 38.1835938174625
43.4709415392696
HSPC105 40.6559419616644 50.7487159427973 94.8260360398356
43.4410741327254 59.3611824459962 41.2567470068038 58.8993834673475
39.1652965788406 47.7086093893038 53.3059058105836 51.524711857679
61.4366256816323
HSPC111 114.666088291556 56.6927078228789 89.3568413748778
66.9218519793499 135.344261822716 101.909225305064 73.6855877240541
95.1037791424727 104.80882886573 67.1278109419819 63.5195937348531
95.9391851122771
HSPC152 535.799532339187 748.372940917543 834.14766574977
633.819901764391 1448.17151189672 954.116675685628 1024.97546870349
587.88126282119 910.898566308567 495.26603220907 641.462632112151
909.843512894628
HSPC159 55.6268253440479 67.8208976798364 61.9293105089291
76.450993090133 72.6675345946258 62.5542376788358 73.4820932957859
111.910324041953 64.4098139218465 35.195364621675 51.4413099192899
47.8931773060789
HSPC171 353.674963012657 224.945809325258 347.908556198025
373.547403496629 116.540209248478 198.922575188741 349.767857026458
330.391945158969 327.844083226326 228.907776306695 216.943972794603
176.064476306748
HSPC268 811.142611128461 488.908231529696 526.44841348248
534.755211286733 282.764445883135 305.399644617845 625.805967352327
1204.34388579291 403.243939813779 1075.04105984319 960.526372655577
744.080606078086
HSPCAL3 36.064718696392 35.0222940937513 40.7910160811527
39.853175885492 33.0851917920412 39.7697110924474 40.9157352260901
44.0736170668178 40.1738310955128 37.2089297656361 42.5023941254529
39.4830039788612
HSPD1 749.49934348018 783.440408023061 598.404003732481 464.99944459917
997.492549573274 712.80113322987 723.210613328966 645.114504971255
617.808073077341 450.972039573066 445.307046237791 851.813216452015

HSPE1 670.958499409833 596.84269596976 518.732175662818 373.802562320876
913.704049709264 454.939614583134 491.468862309918 478.296779356592
879.708199333257 241.278168450381 304.777565078155 821.013687795434
HSPG2 48.5835484742456 48.7084357480922 59.8273072817267 50.0526763693329
46.9906120932727 53.565717255469 43.8206425661746 47.0940613507321
41.7627910693483 42.5664883002833 43.9450023643394 45.2783024981315
HSPH1 453.652548085387 729.342532316666 562.258108316474 493.705301673753
1208.33498233518 480.386431986343 943.73554571528 425.026540236907
687.862452802453 449.564281713066 483.416873464931 1067.08316477623
HSZFP36 106.219480656782 223.1171195512 141.784776736918
82.7394574977114 152.608998839745 113.090530993447 257.506228440451
492.902027204364 212.436948565474 356.948763043851 243.672418126374
204.992039590717
HTATIP 49.7350276169552 56.0642694832108 53.3127618245672
53.9517841438975 49.8304479381623 56.2229687620557 66.7750249658122
49.9951674107458 59.5093038629766 54.8979897528792 65.4493288628375
59.9174456081704
HTATIP2 153.61084474789 450.972039573066 471.874412428677
367.808375988242 176.861690566909 210.22828395255 251.905799624693
326.055799445169 301.457545514208 180.63535336811 209.31241212773
326.121479451246
HTATSF1 41.9407041937973 35.5310164682026 36.0306975524309
40.8681871019812 39.4693871570028 42.357910063876 40.0052661202207
40.2382908228517 37.9273653326866 38.3092241935352 46.4205654140754
44.5432192783161
HTF9C 44.6717213146407 45.620219291723 47.7737810047679 46.7577982595088
46.4343327861696 47.7086093893038 40.1613888954735 43.3838505886856
48.0163377492228 42.8414096678708 47.9269108813022 46.8129509799753
HTN1 39.9697332660833 36.89524015739 39.4710743516517 36.3604951341921
35.5220586859301 33.895835691032 35.7055410599146 38.2782436497441
35.2143190355414 41.0057887014368 37.2018803984555 37.2810767789306
HTN3 42.3620028137457 41.159706131351 42.2577689927563 39.1706564265495
41.7419877611072 42.6575535390785 48.739479557328 42.6913548400127
48.3119523820103 46.441301934328 41.9495387255163 40.716553247236
HTR1A 35.2267149016596 38.7344853699576 38.3166554965434 43.8002456102021
40.4639532172338 37.8102105794655 37.9430145836712 40.831207953647
40.3316153483289 43.0919221375514 39.8184668132023 42.0424641526302
HTR1B 39.0620487644635 41.554942319225 42.5406888281114 38.4208429103799
40.0599807618105 37.5887129572848 41.8315602975157 37.86982638952
38.9834959368496 39.2623423164488 39.153226699662 44.0451487108213
HTR1D 43.3638918705225 43.9421822132391 39.0775870687758 41.7614975630362
40.6723334103791 45.1873487463472 37.3799542961839 46.8954576545929
41.3803269429468 39.3698811593364 54.7061116419212 44.8372404608604
HTR1E 40.3478825593224 47.9128504683618 46.6588655218735 43.1847628331565
51.0107745412152 48.3686935595826 41.1507980856436 46.7980335276155
46.7760357462635 48.9826868640753 50.3221380889396 49.0655215909632
HTR1F 46.5549623118323 43.8495248991589 44.5134109184888 42.8235313007746
39.3667161654772 41.0582900711341 47.2927436772465 46.695848687694
45.5779686360641 42.6793753550854 42.4580504587581 39.2469742642337
HTR2A 241.706783147271 274.134201700259 202.833844963097 217.721787448311
408.353336724969 219.878589678991 156.917623325793 218.531478946522
227.206974078547 204.944787289897 224.871193467078 232.628747594691

HTR2B	58.3702889417801	45.6533175604486	40.931294690669	46.0228732507872
	42.1650127805175	46.0364000143537	38.941855825269	54.720204938924
	39.9444191873552	55.4037482627222	50.9815930710768	42.4607795638552
HTR2C	37.1485601150873	39.5050383901884	43.1931731665131	40.4359177791566
	41.6806984441849	36.5584787902344	44.0137388752186	41.7877325563821
	47.27543600687	40.2493681589104	41.6336636285875	40.8681871019812
HTR3A	42.5506742787391	43.3030298816484	43.8495248991589	40.6857324478814
	43.6782245625941	42.0902716047029	45.8381398279472	42.1887890806988
	42.2331334096358	44.8997235283254	41.7614975630362	41.5083179498332
HTR3B	38.6454098044474	39.1982470626863	42.7303139017786	42.4580504587581
	50.2163892855092	37.0180934800573	45.3597029614942	41.0381860307445
	41.9635525160083	42.1622837416589	41.5421429082231	41.6550823405646
HTR3C	42.1537476443423	38.8336427948187	40.6737160001291	37.651298468202
	38.4540849632254	39.3841861812262	34.4570158133896	41.8417024632349
	36.7267557061938	44.6918600441328	41.6336636285875	40.9683275553869
HTR3D	43.5710871617107	40.2493681589104	42.8723769353017	40.3180310664156
	46.8674366115656	41.809704036355	45.223075311113	42.0847226073592
	42.6366765186182	41.2598458726002	42.5426057453993	44.293922970134
HTR3E	40.6559419616644	42.6939394537076	36.1042012504758	36.9912468186322
	41.0885562160946	33.6859433472087	40.2154497375715	35.7973685350741
	39.3750969348308	40.2112371512838	42.5426057453993	39.1553169409075
HTR4	39.8163644409395	41.848202185821	41.437237454134	39.9084621723809
	40.7376840028647	39.6251842352088	40.2504888274144	37.7612949798895
	37.4040000342773	37.7705653029461	37.3404647796601	39.1807622935084
HTR5A	33.7777950030073	36.6968906669565	40.1985790927248	45.334106390212
	35.6434124419782	38.8336427948187	37.4772922422734	43.1567024035183
	40.7847110517594	37.5343260962758	38.8507804383261	38.4330755397458
HTR6	39.7576136507593	45.7317208802318	44.3344900481112	40.9607305725196
	41.8806343997257	41.2567470068038	39.8695929627783	36.8400213077707
	38.5252456306656	41.7405682794166	39.6403089864678	34.6720534245631
HTR7	40.6559419616644	47.0981389355244	44.8872784170036	41.1507980856436
	49.0828761839116	39.6609238036815	37.6502409810897	40.3357777530419
	44.9645354363967	47.7800121097582	42.6366765186182	45.9104478407175
HTR7P	37.0320432433314	38.8336427948187	39.5450079747895	39.5050383901884
	40.9950639693255	48.8744644207821	39.5496902678788	44.8997235283254
	40.4997186069494	34.2638875325204	33.6080693875435	37.8490162439785
HTRA1	4216.69417265779	1040.102982885	1154.6386362	788.950470077369
	898.114119553014	2039.89728774097	132.903804194161	321.26586939039
	411.184636774653	1129.19103016599	1144.34555072807	853.947671456736
HTRA2	95.65872032796	74.7370458648253	94.5055588176321	97.6774247176874
	108.525500270474	120.392561825049	114.056623754207	99.0202948657926
	79.9391673512018	74.6847594297153	75.0704619826557	74.8706881113318
HTRA3	54.931538325692	41.4508241771315	44.0451487108213	39.5050383901884
	46.4343327861696	73.8105012820769	41.5817737564592	38.079222289614
	40.4220196471124	47.611018474151	46.4205654140754	61.9036392303558
HTRA4	43.515290338756	46.4569566520107	40.4882741869718	79.0039595317374
	48.0234896130991	42.5193208629123	41.3594868055879	44.9977782988116
	37.8319142847141	39.6251842352088	41.4508241771315	46.3411353377615
HUNK	38.8561375693335	41.8073910693411	41.6079958749402	46.8954576545929
	38.1644569189523	38.5252456306656	49.6872467499977	40.6171774724171
	51.2066664465185	42.0621425894624	39.2542963855807	41.7575097298927
HUS1	40.9141850646578	37.4181006952079	35.2838283525874	37.3600014814388
	34.5301949364147	37.3799542961839	34.3842972343227	34.5592213228896
	33.4698406117406	36.5352572191451	35.5179217192467	34.9138625012006

HUS1B	55.5276281380084	48.0296332183129	52.2132783757422	54.8412943008635
	50.2163892855092	54.0382756991549	52.561573697155	45.2806199505029
	63.8740412329753	47.3791873993182	45.9104478407175	53.840976437122
HUWE1	109.5145190042	69.3106426188075	94.0749342825094	75.2475238554276
	79.5175547131776	79.2701322339022	113.692418102755	200.203393423652
	130.455608913636	139.814194128548	143.693373312575	112.316483867372
HVCN1	48.0201377454632	46.1797803908218	44.3648993568405	69.2963913574682
	48.87875862356	47.0027453663546	45.3597029614942	38.132587347852
	48.6494451061637	46.0119912782855	45.6706505553383	44.4067063477243
HYAL1	43.1131704517186	47.2055133646385	52.7570461169969	50.0526763693329
	43.2855454938964	59.217781407734	39.4916414839923	48.0211752672327
	42.2577689927563	49.1928206164702	50.8826620053386	50.5962520938628
HYAL2	100.159565926265	95.2996468986128	188.734299563005	133.365282773876
	134.519244495139	93.2484615907966	98.0473811574767	63.0186614577099
	123.729687262037	68.8167148906225	88.9189119296399	64.0985088513857
HYAL3	51.7607563002005	47.8400694856726	59.7483827511172	74.2429851177794
	112.721024337362	60.2083550387718	82.6381941930783	80.9092480872499
	94.2678678253657	60.2877434874622	52.3761286517985	50.3118072462443
HYAL4	43.3638918705225	38.0226905622357	45.989212296035	33.8111782249716
	47.0954207417646	43.9724628401189	37.9430145836712	44.1311654985682
	39.7537274655115	43.0919221375514	39.9899430628542	53.2046867327331
HYDIN	41.1801554437554	41.5094719913597	41.2656653454411	40.4792096791853
	41.4473192663203	40.7166777560261	41.2092937200453	42.8006000954471
	41.534060591545	40.1655646127146	41.4508241771315	40.2249301774382
HYI	77.3752541676627	53.8499082625091	52.8041782638549	59.8102324370568
	57.5685357492202	77.8490019489375	99.3797043236938	53.6210959113222
	76.3790849853724	98.2843999216669	73.5788549535062	65.8959431201418
HYLS1	85.5362566454799	94.0532777456537	70.56368853915	66.4047895961325
	134.394743648475	88.6287852712744	113.692418102755	75.2101993995344
	143.997817398894	76.3583555666815	66.2027948645675	60.8662233098324
HYMAI	40.6559419616644	34.7135536792489	36.3604951341921	45.0798408927912
	39.2608760193011	46.8954576545929	47.2927436772465	42.157627240486
	42.357910063876	43.4885245598939	39.2542963855807	39.7411455670172
HYOU1	166.644251737703	141.808284719109	135.106756607914	164.312946712409
	172.676819138152	99.5192591352063	144.227861902786	248.438376348617
	188.750770811584	148.144200968432	167.578081645481	127.239392811099
HYPB	43.844855336215	42.0709558258414	38.5045333213784	49.1786207920603
	45.3675390992851	43.7630587657205	38.941855825269	40.1474543172332
	39.3750969348308	40.6022003765153	39.5399188536544	43.8575940017119
HYPK	1401.46625360427	540.037371261388	621.489785652602	796.359355922952
	485.310097523194	435.325038193179	923.470702344816	2039.89728774097
	566.77872642437	1744.56688411113	1670.01014610464	1163.0370991733
IAH1	578.601842980701	361.945511835039	286.497342437243	319.659366973269
	583.690350347288	532.043503787542	665.510487460287	722.514380928291
	360.668308246913	242.751164798133	220.337850438943	444.618035487523
IAPP	43.844855336215	41.159706131351	40.5734458834617	44.4367387274922
	39.9697332660833	44.3648993568405	37.9430145836712	42.357910063876
	45.1899271235974	46.8954576545929	41.1638563416981	42.9887069977696
IARS	116.490154128114	152.555556133364	105.598516887691	113.248260252658
	206.337508331566	159.667153030627	129.538159906075	94.1050519074545
	136.272019225585	94.5055588176321	100.734145862519	156.02592319461
IARS2	224.109805046747	306.578554914328	428.515605971056	225.088911324737
	343.016541928592	252.583117397109	223.669853276931	292.156216736355
	501.148339791372	250.993396750789	275.407306413402	439.376432049616

IBSP 41.7397731376692 44.0409178714232 43.1931731665131 38.3166554965434
45.5809132232681 42.5660695551615 42.4939802311136 50.7364620410977
46.0106098064475 45.6868589181768 53.6334631215581 44.8372404608604
IBTK 168.492959515167 179.322661675979 132.985150338484 174.252441615373
74.0735632658567 151.775928080246 133.576489624155 173.357715186701
157.870937745052 221.450737998571 186.600931545083 230.649032296928
ICA1 198.623556176842 238.205365476671 172.416351632442 141.364814429951
141.567299023381 84.5913809068614 232.147792636393 367.235521372008
195.672836308456 285.97627172608 283.643027929014 243.489225650886
ICA1L 45.2875173643252 43.0829701603126 39.3344387353654 40.4893720023062
49.8304479381623 53.9517841438975 43.1696495767273 50.7364620410977
45.7226406704445 49.5542154724118 48.4916117779992 47.7596919358651
ICAM1 33.8558232866797 40.2493681589104 35.0970060242246 37.7469621225553
36.5393857306744 35.8227939208521 39.8695929627783 38.079222289614
36.197424053556 40.7063426028871 38.2795150354677 42.2577689927563
ICAM2 158.308338986864 270.756849919679 256.116479208201 390.331295238889
263.125290391542 404.599730419192 187.331614826395 99.9896394343368
194.225364352044 225.725485439572 341.142651038591 409.899024157206
ICAM3 221.383645621978 330.037433174255 340.311633794489 1398.21942794938
317.560970617018 228.08291792003 290.524288545599 235.544416908354
203.729732499418 194.469250918013 148.043320937247 123.022511916727
ICAM4 44.3355304829989 38.8336427948187 39.6451483836089 74.8706881113318
42.9090346219023 41.6590564937735 42.57175366709 44.0687697278114
38.1713217245067 42.6211340086915 46.3022643383713 47.4713070963518
ICAM5 41.4508241771315 39.6995529770239 36.2683886571585 44.550322123994
39.7568367479301 39.7394181011603 39.7576136507593 41.0381860307445
34.7722083264266 36.2435874904689 37.9946685306274 43.2776458914333
ICF45 39.9697332660833 46.0023068797812 44.6032218589199 41.8778873678551
39.2608760193011 53.4466111440611 43.9450023643394 50.0526763693329
51.5229124652769 55.5206840401643 51.4413099192899 51.2318935933116
ICHTHYIN 42.7150227340364 204.039190380778 166.608353896248
78.1583955490018 177.334352189317 68.6424653037264 124.204392146484
154.482760264318 115.683762821211 330.737070535671 280.117410663595
209.794541970107
ICK 77.2218991550552 88.2959958615748 79.2250098540901 48.9386616209853
60.3481762331447 81.6949047529395 61.8571521441451 55.1933004802607
68.0010662646828 61.0935621637178 65.963892742019 75.0136718534495
ICMT 133.981674663494 105.446338599737 107.251974803232 88.3459116251233
110.074666148474 124.751020763508 115.557690369703 105.1770711426
125.588228083829 99.1438953564062 96.2799270895937 104.928805304068
ICOS 49.1868644496737 76.5411379733694 79.4998723172096 142.048367760597
43.4544762830742 43.4681646416585 62.1970616836737 66.1288889125299
47.0804681460898 41.7405682794166 46.0095710163378 43.8575940017119
ICOSLG 57.1046180136491 57.6831182451733 48.6037684272115
78.7450149191851 81.6486527718661 57.8005007012205 72.5956443098684
70.0697974278875 68.1674425673171 63.197604174872 69.8899273527731
47.5025821041537
ICT1 145.30915313164 143.590160385066 140.276520383146 109.144663527001
183.918927595056 130.953274536639 188.887559923238 145.335751135441
204.992039590717 90.9570747667407 117.234047261125 98.2074490157865
ID1 168.345356470411 1963.91365010531 1900.96666834569 132.684895918889
813.242005949542 236.851068206869 1395.33665255999 287.981727851442
1645.71527614542 1945.33618761041 2393.42629339901 933.968759959712

ID2 243.952697457779 1127.96214301065 292.247095517604 410.257694738546
169.322551237636 759.566075437072 516.213108543512 886.397061577531
221.383645621978 399.66182195331 435.632247999054 209.61080762285
ID2B 40.6559419616644 42.3550738181144 31.9895827886879 36.890925044696
44.147557390293 40.244271403831 39.7576136507593 45.2806199505029
38.0213051227859 37.920964499903 33.6902293666089 39.6352613956998
ID3 444.471597593258 3055.1209535841 2405.8635632802 380.988918630713
993.626865290891 899.223276238109 464.99944459917 239.838643553139
579.482255191456 3010.38998829232 3465.3013599062 601.611039798626
ID4 41.1523487516364 49.9085372049438 45.5883690965192 32.9879066304034
41.6806984441849 44.5544727111028 41.5817737564592 39.7509165247419
39.5806465938526 46.1390887492649 48.1352470945488 39.1553169409075
IDE 59.220967994972 122.849949163123 102.87193290903 78.7450149191851
97.7153995525346 78.730129036128 83.8811735017279 80.7067505750848
89.4747568145717 59.9354531144375 58.4816190076338 107.440379066528
IDH1 512.691506909762 1105.00321488322 728.679189702033 326.70962514228
679.309039810384 276.029709051798 1305.35453202355 2215.01815680371
1242.75259587926 608.005012673672 1019.67015337432 1097.92519921873
IDH2 1184.18994044558 418.927102569912 229.379918828848 443.269396125834
435.209988251425 392.008276951929 1470.84169670955 942.991198007376
748.913487947791 399.027079798213 344.578407722941 312.469507672374
IDH3A 130.261747462753 123.597489107285 82.6983700200815 144.239350177363
106.125117937458 182.079641528208 116.913991315353 99.1438953564062
132.473918053428 135.323750661937 139.945446129591 161.526970033915
IDH3B 414.794323433931 403.428042132639 478.682734715193 315.696895080135
588.064524478384 494.733185937057 513.266895252699 296.664472126617
324.506136847875 360.523229691604 385.675278463448 373.172504097546
IDH3G 192.41551362197 224.249210397247 238.025903833797 248.414210905199
289.213144287085 212.622278823808 315.799449362526 190.548265163011
226.288835947947 213.162949250252 223.911125639211 140.05110691912
IDI1 74.3135387703106 71.2370255410834 74.4508307047509 137.677598837555
70.6903168772326 83.3664149246525 105.802035886293 100.616027485975
109.654559065946 50.5252048155459 63.1566669782858 76.3824005526731
IDI2 42.9104454767916 43.1805016690297 38.8775560911841 35.314469458379
34.9062568194127 36.4986986135769 36.8369172900902 39.5466975353131
37.0126425326534 39.4710743516517 36.9060997041453 40.1700117452801
IDS 147.782301091454 131.955454394903 118.507569961357 242.673166257518
107.211374033204 168.17913316957 120.231435193839 195.893183546434
81.3858672064773 124.064628168507 115.962193866339 115.001179779307
IDUA 106.027997253013 222.202580186782 397.154258378193 137.555275829874
488.183286350725 148.582198572949 147.758917745955 156.123501229126
237.632703283663 204.710892826638 304.658728492827 182.976132230487
IER2 64.668912004173 88.3636380348802 43.6670755115848 44.0451487108213
50.5110359971301 94.2678678253657 85.2088054619229 82.9322141700549
53.0357938671646 52.0999467564713 55.1099824417989 50.8999542937724
IER3 750.151825182993 450.515644056585 248.321641540666 3313.98659031004
511.261925898948 566.77872642437 867.856842601788 1024.97546870349
155.908644253547 219.878589678991 183.90338485542 662.848386826617
IER3IP1 80.5437011468815 143.728143059207 92.4350913779886
113.783910320605 61.1216330559316 85.9636350040248 81.2136969156029
93.421697456291 117.228320608065 72.2443746456494 76.2226219333049
116.547937420457

IER5 183.799353414581 255.884444464408 231.472806936915 285.322305931794
116.362441774156 137.424708183613 175.41042153356 161.422918681934
145.083624161597 111.248876636045 113.68110396901 110.52925781809
IER5L 281.840536529928 122.0305509548 78.1683130800805 86.3663084195994
109.758297200083 111.760213205992 192.264432082651 220.054040993848
107.148000219752 71.2409890549638 102.471939526704 62.6868461044048
IFFO1 106.219480656782 80.7409247380392 77.4906145838899 139.847976281228
62.1836961153924 133.314819245044 44.3440839686558 57.50915811428
41.3803269429468 93.1238695478498 97.3188285021124 61.3851695159988
IFI16 212.945601743319 223.911125639211 408.080620074472 442.546348599648
87.3957386451235 413.82087661958 76.4889974392545 125.091427687365
341.206585871289 136.629336440719 166.009268520425 246.433145017965
IFI27 945.065986430527 4328.723715755 5047.91205452316 540.721068846198
334.639325701194 802.650020720921 1428.47642890543 669.776067547468
2771.14077845128 397.199620114138 727.181489302632 284.437758089699
IFI30 178.269900400454 102.760704801607 99.9841766494502 1428.47642890543
150.43149196048 110.869341159607 121.945468001236 167.034910927177
81.2249082772437 54.7827221235949 43.3085358214154 79.8331445290301
IFI35 183.165559564289 267.066062068411 253.980053738171 351.883708151967
133.893108529325 227.404530886674 225.725485439572 226.407063891022
260.383969219887 266.9457657143 257.948490663615 201.530434441408
IFI44 176.341212868114 189.015214517448 604.951648398924 209.89649461562
65.8319449883221 198.324467851365 238.893145911331 231.333080775773
97.7114610828769 127.937436596431 151.975559850216 137.378222527303
IFI44L 86.3054012048636 261.326713935356 359.628703006773
79.5838979704243 65.3019328668633 133.610670717456 161.500218853268
131.929700183999 54.7966692984192 117.033529761587 130.890690835631
94.6167194649276
IFI6 494.300858189605 1037.51323185348 801.351998609551 472.159979716705
355.319959025243 312.530335465784 1379.51119430451 1031.4414268329
316.256605575595 526.146757036152 507.375704144286 331.306005726359
IFIH1 57.0006212637829 73.7873098190297 78.1683130800805 199.349867699158
57.6791750431251 78.1952428431942 58.3675164086197 93.0474674148534
65.7924490713262 68.534940191185 74.0420372528779 74.5886835786452
IFIT1 78.6344248102932 128.596547789044 85.4536891882639 104.528976428975
52.3242437351389 151.36347984261 68.4380822448123 79.1922819549042
54.113533179847 81.3895045947915 99.3142021810133 47.6040671292013
IFIT1L 41.6354649482853 39.1982470626863 35.6139301508335
39.3750969348308 37.5556299264699 34.5886782974123 35.7055410599146
38.079222289614 37.10170285405 34.9138625012006 36.2435874904689
36.3454320848591
IFIT2 95.65872032796 96.3918711895208 82.6983700200815 607.042450917813
61.4366256816323 121.86893260878 125.752132815786 84.8569316561572
66.8632294055679 113.336158464071 123.768417863139 77.8620267163666
IFIT3 104.725576313447 124.397048303668 104.832753086298 254.792341536652
51.4230529326653 97.0348634204639 103.394669961417 74.1686961093736
79.5488891958168 80.5981334271235 85.9596109950132 61.356483470053
IFIT5 44.8914371498528 48.3699741879723 43.401782064778 50.8567345921473
44.5668141895992 50.5148383400703 44.7255207589606 41.9315419791594
41.9635525160083 47.2299678280724 51.9302027391447 47.2131144508464
IFITM1 952.16345035163 679.309039810384 593.345148568451
2054.64068255575 563.573152902421 879.708199333257 557.094756796515
617.271611375695 320.038347979923 549.407911286514 774.8306350567
580.444451816014

IFITM2 3176.96480659196 1307.19925493293 1339.29022988209
7168.83136860899 1991.4199819544 3941.3269626913 1506.33570635551
1107.96086673001 995.397944432196 1490.1549980903 2225.65559724391
2294.75523373329
IFITM3 3546.97585966225 1557.42399344934 1634.61795848352
3520.7579823657 2528.6392272518 4752.94498024732 727.788854407888
1301.07436212673 1214.73798692268 1910.5347560855 2513.15722262176
2435.70120719503
IFITM4P 42.8561766050988 43.0971787292711 44.6032218589199
46.8954576545929 48.8381507187986 46.263826584899 45.7452456977857
43.2890595186135 48.4614401417022 40.4136535208406 45.3597029614942
43.9541718254769
IFITM5 42.6036491382658 37.3634897565502 45.5883690965192
40.0698728584484 43.7517118200398 37.1317006162938 46.3057385979341
45.2806199505029 40.4997186069494 45.6868589181768 38.4689132737495
43.3744503319109
IFLTD1 36.3649677956953 43.3458769998118 39.3709357615493
37.651298468202 35.3327761997046 43.6670755115848 39.7576136507593
36.0306975524309 39.2809305246091 32.9003644440608 35.7953070087288
36.1647506311964
IFNA1 34.9138625012006 39.3999056968598 38.7745734037492 47.2009420656854
39.2608760193011 37.7000400506818 40.0052661202207 37.4484310268909
39.3750969348308 41.2276963324981 38.2795150354677 40.8681871019812
IFNA10 43.6706764143908 48.4497719907777 64.0162246399972
40.7481155008596 40.3452320167286 50.4022543872993 40.4359177791566
40.6171774724171 40.4220196471124 56.1912096308988 43.844855336215
49.904979214379
IFNA13 43.4100411978366 39.7835513496873 42.3536549114165
37.1894304543751 42.6022652963326 41.7256940298128 43.1931731665131
41.9315419791594 43.2163597439147 42.6366765186182 43.6919270353542
41.5132248356707
IFNA14 45.0283997415478 55.4635297192076 47.1629799536297
45.0798408927912 45.1137938810047 45.0882356578404 52.561573697155
50.0526763693329 45.3407340000202 46.441301934328 46.1390887492649
45.4218947614732
IFNA16 40.6559419616644 44.3529919625344 44.9758339877851
43.4410741327254 49.1928206164702 41.554942319225 39.3585350448719
39.2672712041774 38.1169703906207 41.8613406887824 47.0110068985119
49.904979214379
IFNA17 42.2455707947223 37.8217989841918 34.2890785915814
39.6292788039793 38.1644569189523 38.9378717994178 36.197424053556
42.9301944191913 38.4344387289956 39.7835513496873 35.2339574411711
39.6352613956998
IFNA2 38.5007204246293 40.8880530359822 35.2838283525874 37.8442841690502
40.7779767565889 36.6996187567892 41.1507980856436 41.0381860307445
40.9901805728485 38.4187686307607 35.6970528713709 40.9683275553869
IFNA21 45.0985088502363 44.4411734611229 51.223859150798
54.6089451252049 41.2740822675592 47.3791873993182 43.3262148375525
42.9301944191913 42.4540072551568 46.6598879466655 46.4753015651202
45.6692241948108
IFNA4 39.5676051939342 45.2098033805679 39.8656165405284 40.0322420615846
42.9090346219023 43.3269087731399 44.3440839686558 39.7509165247419
40.137818555383 44.7520317855773 46.5308571957384 49.8472708021525

IFNA5 39.266565827316 40.148780650831 37.4642807402741 37.7469621225553
43.3332476888305 38.9378717994178 39.1539892144371 35.5798430594271
35.6139301508335 36.1550293353654 38.8507804383261 36.8690774823319
IFNA6 33.5994525227062 43.4396866157171 34.9849896310235 29.4322896559759
31.6519589095843 41.554942319225 42.629412460409 36.43924066428
38.9006788676407 35.3849221682845 38.956280281655 35.9604262434141
IFNA7 36.1232327599945 41.6069493017095 39.991353369336 36.2117792304273
44.0451487108213 41.0012755104374 39.4012855594431 35.4526946152955
40.0870341983328 43.844855336215 40.547835538778 40.1235656101329
IFNA8 108.945545005203 65.1291617069903 58.8936382809165 80.9160262677505
77.3367966395554 63.6172860741922 57.4622430729629 69.8777218396476
70.178974380507 68.0556717366572 66.5310713054733 66.1229691315616
IFNAR1 137.52864596636 110.960501850409 109.832638804839
254.322611819303 124.490684428024 157.463465828715 139.472956453414
144.239350177363 162.895543924742 210.807094064209 191.141778714144
222.426799131736
IFNAR2 57.8944021095265 81.5782824513693 110.112236384679
165.684719727205 97.3188285021124 73.4037256268744 116.993403211005
90.9712092640278 106.042585997437 69.1623287497421 77.720889005668
104.486555491331
IFNB1 42.2455707947223 37.6383530120621 33.6816578730693 39.5050383901884
37.6527901109005 33.2552842693684 33.3231576795423 32.229154316188
30.493888765264 38.528621456378 40.8225507267584 36.3454320848591
IFNE1 45.8108990837624 48.275452803061 59.8838591118356 44.7980740332174
55.6069214673486 48.0080291106034 45.4164642922183 43.7731874801003
57.404059248727 64.5093306432163 55.9401754632145 73.4876346170053
IFNG 46.5549623118323 41.3610369241157 44.6032218589199 68.4261608261522
41.8806343997257 51.0506441018166 40.5585676679482 46.8954576545929
36.8127605061228 46.5650556481721 48.3847843498476 37.8490162439785
IFNGR1 1554.42895572029 777.854349637516 1141.25078954012
2067.66516256598 624.954583057431 637.824136761292 735.60603239712
352.292837622545 425.435793468733 845.41973083225 637.565156571985
927.61341837276
IFNGR2 1327.93833415582 1679.12630849964 2721.97551918611
2892.02001412369 3124.84336032646 1638.16317738035 2096.51999792183
1242.75259587926 3361.67247904403 1149.90692951795 1007.03747721563
1876.51621222292
IFNK 35.0202257459026 39.0894172982439 38.7745734037492 36.6501141438378
36.8059561571675 37.1317006162938 38.6243997507646 38.2782436497441
36.197424053556 34.8137085420423 36.7283081422707 30.8150126829901
IFNW1 45.8574226904273 41.6466169548846 43.917817324545 41.2551966132486
43.8835874380262 42.0016830644258 41.253645578517 44.9977782988116
40.547835538778 45.7328341667151 46.972521499515 42.5365257225985
IFP38 58.8224410806741 82.0237575809051 86.2330249146534 76.2591822114739
86.6854406823303 85.0275638433103 63.7721358300879 73.9624143741222
87.4335749014848 105.212030988223 98.4699679900651 104.192417269189
IFRD1 51.4660044978406 48.3699741879723 47.0940613507321 91.1834104205004
65.1757400078647 77.3640580819535 66.979105340228 73.8875154527631
60.5948704279864 51.5238121536228 61.8683444248482 66.6615349144199
IFRD2 82.1323695817857 93.2133061608764 86.9032093551446 65.524821699432
202.957014793417 97.7114610828769 117.260454792593 60.0835531094681
120.465621774337 67.9259304928255 75.5799609581534 87.4273245333478
IFRG15 45.6517320730042 47.2770121962655 50.0050760710506
50.8567345921473 42.4864576619427 42.9695585740813 44.4639405506967

42.0494116081998 40.137818555383 40.6022003765153 47.1477056711335
45.2783024981315
IFT122 75.8122107270212 50.9895771651972 57.0427273538849
50.9293205467648 59.7406133873058 64.3383155834968 73.1262465119304
65.3477484008479 54.2356999525029 70.9196764450443 59.2837628958562
54.4992295592001
IFT140 62.9952378379206 160.973602503707 205.449627031002
57.8277957325085 85.5362566454799 64.7905331739366 79.5707751325986
49.2901038327894 115.328304312071 245.853111206859 206.146732318529
136.011269830829
IFT172 81.2359429678503 61.6219790276408 63.5107036952143
59.8102324370568 59.8102324370568 69.7677752398541 105.666698877424
166.1490536844 76.1869593072029 81.8426140611297 88.3725577276628
79.4710406831985
IFT52 78.7045070574092 71.6931243081178 66.2167164788023 56.8404854356062
71.8314470868658 103.569150754726 92.3787087536027 85.4790745407113
53.3472907280113 71.9737561190706 65.6010220593397 67.6138276415458
IFT57 49.1868644496737 64.0903425729408 48.4890615340603 52.6947911480086
64.3571664944642 122.284299477218 67.6767845246581 56.8094693588238
63.4531374125233 107.420552630045 87.129027106167 85.5175308034109
IFT74 55.1306642449627 84.8084949516218 62.9967097752591 75.4466520363487
70.0133734659853 73.5743059777365 80.354283778818 98.2074490157865
67.5047089908724 96.3495443565738 100.234661597218 110.302475164041
IFT80 39.7576136507593 42.9020321355927 38.35720098134 40.4359177791566
48.1473938886239 37.7427315853995 36.3194282084339 40.4669141685209
42.7150227340364 40.7063426028871 40.8225507267584 38.9834959368496
IFT81 44.7463386786906 48.4085972463773 45.7968648044613 41.3027031037343
56.8903641597175 52.5330333302271 50.2471139112912 44.7470332010482
49.531907107685 44.7958122466628 48.0258040705037 48.1002535934643
IFT88 49.3914518899324 46.4861258030402 50.4778868815688 55.1531662914834
41.7405682794166 67.222434502867 51.8812208789107 50.007830364606
47.8374975107249 53.7196173866021 63.8399092891728 56.3427558115088
IGBP1 203.228211195516 244.707381127491 230.763782802004 195.482216656634
138.170787392253 256.55408314817 131.645714321903 222.985386635178
255.559793355361 253.267901237295 236.536058101486 411.342959058326
IGF1 45.1912496459517 43.4396866157171 44.7829632472754 43.074472921841
44.147557390293 76.1453055441256 41.7614975630362 46.8482302725294
39.3506426665347 42.9358255168086 47.6884372645373 56.4199442234707
IGF1R 47.9175508887533 51.3800036046133 46.8267540220952 64.2130157241811
48.6224651100733 45.6129956328415 41.0381860307445 45.642304356051
57.0301170427216 52.6879797806876 52.8489752025797 51.0647893605839
IGF2 101.523741547876 360.900649927623 480.386431986343 43.9798942439645
448.787414220553 105.125095381533 65.7321440516177 43.9518547354262
44.4812539947185 44.6217658428521 47.7180816682562 48.5708927206434
IGF2AS 36.2496364442264 42.8542642792862 43.1931731665131
39.1706564265495 42.4864576619427 40.650742261428 37.9430145836712
30.4430902995779 35.3227485563945 39.5637454855233 34.5633623842083
39.9519547348106
IGF2BP1 39.3709357615493 39.0102066700839 37.1643693306382
40.9607305725196 41.6806984441849 32.771863438151 34.806104801006
32.6039814489621 39.3750969348308 35.4990901121893 37.5583886316038
34.9138625012006
IGF2BP2 210.200786815048 65.5367689605146 83.5742879296932
166.304210248623 61.6452971581503 76.0240426902567 115.610095595509

100.616027485975 111.845105854608 169.18132209076 128.300809076274
114.016824404095
IGF2BP3 99.8754776648876 53.0383634135393 76.9373289454707
61.5515639412892 51.6869155634183 44.4619248757502 61.0979269700412
58.4200819624972 64.2174369755376 53.1993028936883 48.3847843498476
48.4535991392809
IGF2R 1120.83891847181 586.281182112872 613.740813446728 1231.77360353254
493.322302312578 506.585006700107 532.981045084589 479.318513108472
356.133736281036 556.417356025042 612.441960343995 456.023620287811
IGFALS 46.0023068797812 44.2105195734073 40.1985790927248
43.6726153508978 41.2740822675592 45.2852528185486 42.8684958525042
40.7311497914871 39.0775870687758 41.6373771565199 41.4508241771315
37.1747156131306
IGFBP1 44.380620848621 44.8955824420372 42.785429206422
38.0958223740111 36.5393857306744 44.0399598874356 41.9393838138203
38.4128104869434 41.1742244762318 45.3530683538086 38.6662194645633
37.9607839934735
IGFBP2 235.593721159442 330.241189572237 278.067057781084
129.190555260332 166.983092667334 81.3978207813439 84.7782034884529
225.016731278437 373.802562320876 1390.12380748446 1090.86617066541
1081.97502255142
IGFBP3 1519.01001038342 2417.02418518222 1377.24483375075
129.320408155239 861.427193617946 379.377005627283 902.691776563423
502.205366225368 2650.09507972593 489.232537791775 267.657143781939
177.379924616924
IGFBP4 1310.03307063841 1021.71286017481 761.922486104216
1224.80240464583 615.442484911026 1319.32406112346 440.282299601096
489.232537791775 874.419252731128 1359.9708539999 1228.1735887278
1810.67209360676
IGFBP5 2272.91548405176 438.681666410245 1445.73674428909
958.473187253938 1357.41745370754 3070.83113612925 61.3149617644002
1345.37091574015 132.57180783668 2934.35616793443 2921.68921750799
2384.00264993279
IGFBP6 242.396401163598 161.48562926756 86.6186787663248
313.399524593316 120.721482177994 4406.55576617324 60.7992483126292
223.168185178205 209.524518082089 2892.02001412369 1956.75875151802
1780.14552702126
IGFBP7 2440.77408185413 2215.01815680371 3690.00752647453
2045.24530722676 1999.21832626916 3749.78281766196 263.068504548549
790.786577596971 275.061444728555 1519.01001038342 1487.80306986156
1896.06380611235
IGFBPL1 37.0320432433314 41.8073910693411 36.769073068743
34.8137085420423 42.269054475455 39.8455060654531 41.8315602975157
40.4359177791566 39.3750969348308 35.0082729554459 42.0750889188255
34.806104801006
IGFL1 43.5710871617107 88.4740208892504 41.6079958749402 67.7121534934164
40.148780650831 44.4619248757502 95.9298109273155 260.964562714558
54.7966692984192 44.6918600441328 162.143245260314 37.9607839934735
IGFL2 87.9251957244202 36.89524015739 42.2577689927563 43.4410741327254
40.4639532172338 41.7627910693483 40.9157352260901 41.6466169548846
40.0403898620771 44.6918600441328 37.4642807402741 36.8690774823319
IGFL3 60.0793579902362 46.6996902348633 49.3780606970538 42.4580504587581
46.4343327861696 33.5578650644246 49.3854238365325 38.2782436497441
46.2027193115397 45.1569741609597 46.824299585607 43.5738557773903

IGFL4 39.3709357615493 39.9697332660833 40.1985790927248 42.9358255168086
40.4639532172338 44.2607789091176 41.4742337714895 39.2672712041774
41.6616444147804 35.7202909649682 38.3684202018524 40.2669497398702
IGFN1 36.9060997041453 37.2810767789306 33.9435456570176 32.554751989205
39.7568367479301 37.3799542961839 35.3362091871364 37.86982638952
38.6322728994674 39.7835513496873 39.6403089864678 34.5633623842083
IGHMBP2 56.1912096308988 57.1595768494068 47.0055981216687
59.6153684374766 59.1948983423381 48.7781962164988 62.801431502597
67.8061298261394 54.2825877218965 70.7003843117511 69.036463612998
55.3563533836982
IGJ 45.9090659324223 468.98857415608 413.067407579649 302.50850157207
37.956732373465 74.3729155936862 65.524821699432 72.9752827496503
35.2143190355414 36.9102644736882 40.4504608195666 41.7575097298927
IGLL1 224.749607192071 2663.19221471348 2417.02418518222 3202.8848520816
53.8658229797154 142.55778399096 658.282562295783 536.013150313097
55.3619977587354 43.9118340045283 43.3085358214154 54.2625553617642
IGLL3 36.6975731268334 94.7498756176388 118.358996104366 293.883582116334
41.2424637338424 39.4953494583071 44.4639405506967 47.6952179922944
40.3316153483289 39.9948390976317 42.046575509735 44.4067063477243
IGSF1 41.0136069709699 40.4003119241613 38.9790184198145 43.3554905202712
47.3172462673426 41.628211174823 42.2577689927563 41.4702213972048
45.7792335738285 45.2994802465737 42.1550412794904 41.4061152929154
IGSF10 43.5710871617107 36.2103905554984 36.5474283500601
35.49696651597 40.5710637884029 60.5803031036931 39.5496902678788
38.8495095797881 35.6970528713709 47.3791873993182 45.7328341667151
41.9430737035925
IGSF11 42.8596678196469 53.9275079525238 44.4612332327353
45.0798408927912 57.1021912253879 48.7019915522206 59.9266636679987
44.6484005362281 54.5518174219283 46.7646877499666 44.1642484298135
43.5367669381526
IGSF2 43.6706764143908 44.2105195734073 49.9256753636167 50.8567345921473
48.6224651100733 40.7616966228404 54.339958888486 44.8997235283254
45.8325181856483 44.4652605017595 44.2925355984056 46.5650556481721
IGSF21 55.0078788655089 47.765848710116 50.6987358483181
52.3004056941891 44.5668141895992 80.9821967801845 50.2016912396771
45.9604707098953 43.6670755115848 50.8826620053386 52.2229654768933
49.2064066614865
IGSF22 42.8036731479932 48.6447083765118 42.7303139017786
40.4359177791566 50.2163892855092 40.7616966228404 44.6032218589199
37.7612949798895 45.1899271235974 42.5664883002833 42.911855821602
46.4499258373233
IGSF3 149.691911022686 231.942272401651 137.580418363812 50.3127977239083
97.4137766910365 43.2312848495114 119.846620703059 144.324036829708
188.077820976107 255.126812649967 184.808179948172 225.35760689842
IGSF5 46.0920218698606 49.0697565324841 50.2869791217819 46.8954576545929
45.9104478407175 41.4508241771315 47.2927436772465 86.3237770387663
40.7847110517594 48.2596564318071 43.7585538437696 50.8442982601142
IGSF6 54.1745577382276 54.1773909134233 45.7968648044613 536.973599140459
40.8873907150245 55.8243694410835 56.7780520416533 53.1052228246467
43.5669306063196 42.8936990772838 36.5359455830388 49.5539622682049
IGSF8 59.046385957117 50.6229884142872 50.9933795450314 46.0228732507872
59.9326265577462 43.2805590023412 46.9929836306816 49.6203214143464
66.603126065489 52.0999467564713 50.5188367542048 49.5539622682049

IGSF9 78.8357122967692 87.9152471471338 55.094385444315 35.9521941716188
144.139555313429 43.8582747295766 157.843337785472 104.089998273812
101.46584765758 88.6608345370342 57.7786197537899 43.8575940017119
IGSF9B 41.8409927585705 40.8880530359822 43.1931731665131
40.3180310664156 46.8674366115656 60.4065050472082 40.7910160811527
45.8669209716694 42.9020321355927 54.4825201506931 56.1379957867124
45.0035308952466
IHH 39.4710743516517 49.9547657591437 38.6727842796202 37.7469621225553
38.1644569189523 36.4986986135769 44.0687697278114 41.2551966132486
37.3600014814388 36.2435874904689 45.5707650534259 42.4607795638552
IHPK1 134.19834305339 167.229755109155 142.933216800073 193.806701229141
187.733278812798 171.501864800548 204.862161704394 123.455270940015
154.752920877816 141.087571926258 147.719172115976 117.574549567
IHPK2 123.739272396278 125.290426098227 106.309161354804 59.7044210110693
166.752205770965 75.5561597374812 125.184689092836 67.0256385495669
138.910687241655 59.6246428440926 76.2226219333049 85.215646983121
IHPK3 47.6397712007919 40.6380495994768 47.646800205189 42.4580504587581
54.6820069269589 40.244271403831 47.8947595316832 47.6952179922944
45.0017158586743 60.4837400912239 43.0016969050339 37.0953622406687
IIP45 83.374777060444 79.1432909353319 80.7338782543351 88.8330881965986
139.989008296272 68.3234331335073 95.096268847843 104.541813290553
80.3500323413132 63.3006977162657 61.1801493883012 59.9576867319572
IK 250.244199415895 229.881790983135 234.556113280762 307.329227013816
188.943404504867 399.410299413602 219.737294408079 246.965844426351
245.004434952963 206.768099707991 248.482699989877 351.239677010973
IKBKAP 43.632365631458 51.0175677516029 44.825830634601
43.8495248991589 48.5666902992029 49.7149784386993 50.0088611244948
45.5581356216957 48.0163377492228 42.7275315121163 51.0013344286851
45.4218947614732
IKBKB 84.511841810844 86.4898752526885 89.2285288333749 78.9479233685889
66.3075095149783 66.2167164788023 87.275548276476 89.1012752492647
77.720889005668 92.3388129097482 88.9836122218372 95.2218627805387
IKBKE 92.6545309624315 121.729442469889 143.383133346874 124.490684428024
85.1661952927159 51.9421032049606 138.23147386089 73.2037966614621
106.361958225818 112.919063481867 96.3495443565738 82.3254961129575
IKBKG 216.488139476342 268.745928429069 409.554706384667 269.582598918998
397.199620114138 245.0324923457 208.7739716139 177.958475301951
212.945601743319 165.311139930529 191.668846959973 167.319747725488
IKIP 51.524711857679 55.2893984191264 51.4704882732949 61.6510502615429
55.094385444315 51.2207153770095 48.3411413510851 43.4812166409346
49.420862176345 41.162473449625 42.6913548400127 45.0583552831013
IKZF1 48.4849194706125 54.0461131287999 72.5835488194631 143.822560762049
42.0709558258414 48.0350549043063 52.0942589368478 49.6203214143464
39.9444191873552 41.6373771565199 48.1352470945488 45.5800840647989
IKZF2 45.3872406432794 55.3178018558518 46.6241521517453 43.4410741327254
47.282591828791 43.7630587657205 52.0942589368478 49.882563505629
52.5215406262267 46.7646877499666 48.7213586071409 50.8999542937724
IKZF3 47.2862299990938 59.6359245937831 59.1533197797534 55.8709511400674
55.0767527801658 43.2805590023412 62.1970616836737 45.7476095696859
49.0986627051784 40.9558083259517 48.0270808941036 46.1088006335399
IKZF4 67.0577715520007 69.3525901554637 64.0162246399972 59.0965219753805
67.7155610106537 105.197692490363 65.644767142472 78.0020909283414
65.0810479794187 76.8998454389745 78.6402083787251 62.2025996860055

IKZF5 55.0078788655089 89.19837608959 81.4931910857436 60.7741670229412
89.4747568145717 80.3805507705979 56.7780520416533 54.0507720792049
66.4251023257203 59.8130420783374 60.7516146716018 72.7015808995427
IL10 475.621740714165 257.948490663615 310.188313944313 506.585006700107
212.00247895282 208.7739716139 356.628599887098 755.852629128252
192.058030454066 668.584452504579 659.126408705362 455.444228161475
IL10RA 64.0759817636265 74.1148641558178 66.737740728688
242.503720442197 52.4370718282084 63.8740412329753 51.0013344286851
73.6855877240541 43.0930293831497 52.7972156492746 49.0361180089431
52.4992300612807
IL10RB 304.133852957499 439.558287621096 390.978292094212
738.66912772732 299.052630634189 254.639522735592 345.942093303061
289.05213385509 370.150839052727 239.229093340475 267.427827956058
385.36587480414
IL11 76.5505585423421 46.8954576545929 38.6005272730664 51.949834638817
58.7460488758053 36.4986986135769 35.0305278582179 290.81245174957
34.5633623842083 32.1260264272477 35.6041926503637 37.2810767789306
IL11RA 85.1950626054282 71.6849360818041 69.0503036666628
82.4084917533632 78.4289643729837 181.123652339134 57.6913861717513
93.8009890556664 60.2001412023436 202.060761076547 186.350104132737
111.166893747402
IL12A 43.1805016690297 46.2286164776607 43.5710871617107 42.357910063876
47.4548471240051 37.8102105794655 38.1793598983615 40.3357777530419
40.4997186069494 43.9450023643394 49.1868644496737 47.6040671292013
IL12B 35.8208542506595 40.0527893189096 41.785676764591 48.4916117779992
40.5710637884029 37.0180934800573 42.0605906240248 40.7311497914871
40.8970401536578 39.6719190054379 41.6336636285875 42.8864415494484
IL12RB1 43.6434576713809 47.3536182445315 43.9063490244748
46.6986977330569 39.2192721306168 40.9043306643373 41.1507980856436
44.95701484183 41.7627910693483 40.8462613935508 39.8184668132023
42.171601283248
IL12RB2 43.5710871617107 49.6203214143464 41.3366779545815
40.3180310664156 43.7517118200398 51.3886035574416 47.2927436772465
48.5681478665796 43.3744503319109 44.4652605017595 43.1931731665131
43.5738557773903
IL13 36.5393857306744 31.1853651446075 35.8265993915844 35.8472302065546
39.0564222526273 40.650742261428 33.3231576795423 36.9355798143571
39.4762546236449 35.2880714952489 33.6902293666089 40.0557788909923
IL13RA1 668.773022931608 330.737070535671 374.601792595646
723.853348464335 298.031228833741 387.311743342483 325.949742917126
393.209708455013 431.353025968649 322.109212713082 291.785618741057
386.372142079981
IL13RA2 71.6849360818041 44.2105195734073 43.0971787292711
68.0471049167726 79.3889841821749 43.2805590023412 41.9393838138203
148.514056973195 211.253227661874 41.2276963324981 36.6298807161755
39.8497227447888
IL15 50.5291432329798 53.6741873729867 49.2427759486929 64.4895418579465
48.4662973897407 54.3720504123075 45.7452456977857 50.3082459161777
57.1595768494068 45.7424985641244 49.9791966667317 47.7962548556026
IL15RA 51.138877923954 48.3464815242327 48.3570240922825
47.8164193292842 49.5210706664792 60.1042270393382 47.6853839342179
47.8947595316832 48.6224651100733 48.5571567616945 50.2203578628217
51.392860749191

IL16 47.0940613507321 52.2269052457335 46.7800033479223 67.8978240198575
42.7489468897708 55.1037284831588 48.5544398725959 48.0283573444179
42.5406888281114 54.8346842758068 54.0374522273907 42.828737648529
IL17A 38.1169703906207 41.9894412416874 37.5597671487621 41.6564075217517
35.2163900400329 40.040778349137 40.5585676679482 42.357910063876
38.2215591930186 40.7063426028871 35.3362091871364 38.5279745308203
IL17B 40.0557788909923 40.716553247236 40.1985790927248 42.9358255168086
42.1650127805175 40.244271403831 42.4939802311136 41.5464765641928
40.3316153483289 40.2112371512838 43.844855336215 42.0424641526302
IL17C 49.6241203903354 45.6533175604486 59.0308764112247 51.3851157261451
69.493532577364 39.9397419279057 49.6872467499977 122.482560090133
48.0163377492228 42.3606390470387 45.7328341667151 39.4490233720848
IL17D 108.815245119101 58.3164247463092 52.4485219671976 51.7739270955254
89.4747568145717 266.581466060604 112.113501370522 181.093943398293
61.8824218975538 128.157032116902 115.40235710164 80.0919469092368
IL17F 37.0320432433314 38.0226905622357 41.785676764591 60.620259884953
38.8456942580061 42.2577689927563 46.9929836306816 37.6557714476853
40.5912213523345 42.785429206422 44.4688999098904 35.9604262434141
IL17RA 55.6268253440479 53.2046867327331 55.7764940944098
101.560929859785 48.0234896130991 48.0350549043063 60.6548851977964
55.5578334877425 53.4604353973381 50.8826620053386 56.3884556031093
57.445796273665
IL17RB 35.9305494063137 57.8759339109109 49.2595762436995
87.6763065012524 74.1282220615085 41.3553369544097 72.8653205184014
39.5466975353131 51.5888395924843 40.8970401536578 40.1742127891824
46.7591817286632
IL17RC 60.9710783023013 55.5789922793469 55.6119508705485
52.4256436890829 53.9391973360983 57.0427273538849 60.3217748365169
67.474556589181 48.3679250034853 62.7743500747415 62.5506324864207
48.1627097465883
IL17RD 240.086349973689 143.997817398894 133.673536028938
216.911833562615 110.869341159607 125.81889994067 171.623830248512
377.221676837377 113.287380238064 275.407306413402 237.443715611786
208.023488988931
IL17RE 42.3433931248155 44.2630383225933 42.2732555030699
43.2706707408262 48.6707521528908 43.5474493120701 43.2776458914333
42.2577689927563 40.5491080069964 40.0599807618105 46.3563690926644
41.1801554437554
IL17REL 44.5282181324797 39.8695929627783 40.4070046183108
45.1944222548351 39.6535880152834 43.4681646416585 42.357910063876
40.931294690669 45.1899271235974 38.0078357808393 38.5668610487033
44.1452943804044
IL18 5733.54417656207 2167.91218703382 3421.24021192326 3707.73750609316
2545.03032507597 1591.51486001004 4036.89580181979 5932.92478996609
2621.65272077828 6555.04068292972 5354.58328030315 4128.92158369026
IL18BP 97.1406120094484 73.0855167819246 67.686778662624
159.76714232068 53.9074374788981 58.6711059433922 46.2395419410743
69.8647472333595 46.1569135229922 47.1635381911975 54.113533179847
52.7924213633885
IL18R1 48.2910530887214 60.6080172588224 61.6878176260761
255.197732321307 71.4183281327686 93.6752479496469 79.1432909353319
66.6320017520888 67.1278109419819 45.0155543555707 51.1483696410762
56.361708620372

IL18RAP 46.441301934328 62.0908175930716 87.6178717341967
642.946369683508 48.8381507187986 144.500148287951 174.007271403635
52.6960621424156 45.9227107476843 36.2435874904689 61.8445915808329
46.4499258373233
IL19 41.8409927585705 47.8400694856726 39.991353369336 46.546116650292
39.6535880152834 43.5752393599169 113.692418102755 40.3357777530419
44.4395580493118 41.9097689475185 41.9083581529276 40.5235471987417
IL1A 48.5835484742456 481.49240494827 110.626479311082 1169.14891282789
332.882251013334 79.1922819549042 482.238595345574 268.419751472464
89.1217605204013 72.42732668079 96.5451512198207 224.976641879383
IL1B 165.775214685061 160.425627898607 473.688869614777 10351.5373412096
207.833867050257 339.210875653066 2184.70038900784 164.435300801872
70.0801020070064 67.6169355376864 67.7280831060202 146.196470375622
IL1F10 39.0620487644635 43.5873188565519 40.8846826807717
41.4273122914951 41.8806343997257 38.6704566269103 40.968974769473
38.8495095797881 36.4476883897928 42.1317134987795 38.8132170743603
40.8098117292322
IL1F5 50.9895771651972 39.5050383901884 42.1901757067073 54.6089451252049
41.6806984441849 40.7616966228404 38.8417151851134 45.5581356216957
39.2809305246091 43.7303813515864 39.0550139257658 40.3316153483289
IL1F6 46.0920218698606 36.0366673493555 37.9482157381008 43.8002456102021
38.0598374201407 43.1826328292558 46.3057385979341 41.4508241771315
41.6616444147804 41.1222709786153 44.4688999098904 39.5418280570561
IL1F7 46.4760131160091 48.4980626712175 50.9933795450314 53.0284011078097
47.965492554043 44.5180992653789 75.0294180790249 68.3372228877569
46.4499258373233 49.7568909515845 37.801577367436 44.0977148249302
IL1F8 43.2028319630577 38.4678643044777 42.9104454767916 45.1094678231052
41.6383327669122 40.8634043028528 39.8695929627783 44.797320243931
37.6610682780061 39.7835513496873 41.5421429082231 41.0183489151839
IL1F9 38.2083710281545 46.8236022402545 63.2475738480323 72.2577390063259
42.3685136297603 41.6590564937735 46.3057385979341 47.4740726032707
38.9834959368496 40.8049408996274 39.153226699662 37.3600014814388
IL1R1 42.3433931248155 34.3715326040967 36.0306975524309 51.949834638817
44.6834648302383 46.0364000143537 36.4098694708483 67.3976855661302
36.8127605061228 40.2112371512838 47.6125796208753 49.8253396161069
IL1R2 42.9613924925696 45.7562276914441 72.8472187581193 903.756866967447
65.6824363141962 54.5952754712007 99.0545942554244 40.9901805728485
45.7226406704445 43.119837808537 39.6719190054379 45.2265319826548
IL1RAP 46.2190348814788 43.2149698150931 51.9447450229707
81.4367600457584 45.4751076037511 41.6086894860496 44.2795065595546
43.0944129868777 45.5265270192861 44.5317661630912 43.2649781354792
48.2220547806679
IL1RAPL1 64.9367707455078 42.4493032770053 41.2365089689635
45.5869891570251 45.1137938810047 152.630871384615 44.7255207589606
49.3880946790306 41.7627910693483 108.39424630351 79.9567256807679
47.3172462673426
IL1RAPL2 39.4710743516517 32.6416008433981 43.6670755115848
38.0958223740111 41.7929491793697 43.1826328292558 37.7136011474903
40.6171774724171 41.6616444147804 40.2112371512838 38.5668610487033
38.4330755397458
IL1RL1 43.632365631458 41.4223563635976 40.6440776327492
59.897987870592 50.4260283682427 52.9953193251303 40.5585676679482
59.9515570764012 40.4518431978241 41.7405682794166 41.6609976002931
45.1193245439293

IL1RL2 42.442231667357 44.7829632472754 41.785676764591
81.0057400164851 52.9433495155796 49.641159251878 51.7714254834195
39.3709357615493 44.8003345643334 43.7303813515864 42.170702295259
43.2776458914333
IL1RN 109.029196594263 356.628599887098 173.391542207362 2266.34088015654
104.541813290553 90.1830062843449 738.66912772732 279.901217752341
196.228284571015 151.088035451997 114.464626286414 97.6023731094756
IL2 43.1805016690297 41.4508241771315 40.5734458834617 36.0481934423077
35.0181575202066 37.5887129572848 41.253645578517 36.8400213077707
37.8319142847141 41.1222709786153 38.2795150354677 40.8681871019812
IL20RA 134.869029698594 77.0244607682203 89.6568705925966
44.6905691366696 74.3523005665671 43.8582747295766 63.5738862549306
48.1591264215184 58.2401724760744 88.6608345370342 69.4877749654481
80.3500323413132
IL20RB 60.0793579902362 57.4054069675016 475.621740714165
50.2163892855092 46.3150221024046 51.0506441018166 50.0088611244948
41.2551966132486 71.4864243594056 53.6429962450784 45.5707650534259
47.9566297250148
IL21 38.8561375693335 41.554942319225 45.8844325913247 41.8778873678551
38.5512429290626 41.6590564937735 43.3262148375525 42.157627240486
42.0621425894624 45.0155543555707 33.5370783156523 40.6614700944853
IL21R 45.0283997415478 46.7591817286632 43.2721716572958 44.2238927021522
44.7359392883974 40.2062851265091 41.4417765092034 44.6107027295193
42.8036731479932 42.1454750198208 39.0769398584742 41.2437378488025
IL22 40.3478825593224 39.2542963855807 43.4544762830742 41.7614975630362
42.9090346219023 45.4607426241257 46.8613212048981 41.5934628038429
39.5806465938526 40.3458948319669 43.1931731665131 39.6833800000587
IL22RA1 39.7576136507593 44.5152708955623 40.6737160001291
40.9607305725196 35.7973685350741 39.6251842352088 39.8695929627783
41.0381860307445 41.6616444147804 39.0648733099061 41.8168470823451
40.4639532172338
IL22RA2 41.9974588626347 38.8336427948187 39.7411455670172
39.6609238036815 38.2069499294002 40.3034574135048 38.4005795891396
39.4710743516517 40.4220196471124 39.0648733099061 39.4762546236449
38.5814307557439
IL23A 49.540449125788 58.4476706144086 56.939860285007 129.190555260332
44.9936360641969 42.5660695551615 165.606015336587 108.700838142885
48.0163377492228 43.1839109702714 38.8507804383261 69.303129494807
IL23R 41.6354649482853 44.4411734611229 41.8389336728199 42.8235313007746
38.1042454705119 43.1826328292558 37.2316700720288 39.1652965788406
42.0621425894624 38.151911051187 39.8184668132023 37.8129870676592
IL24 61.3005673792118 38.956280281655 42.3962308437248 230.664926508522
45.1137938810047 37.0698056696061 45.1741601472623 113.388372364837
40.6533975461148 38.957687788868 39.2907645923217 38.745552403895
IL25 39.7576136507593 41.413768254554 37.4352614195366 41.0273093212371
40.1207285185295 41.9097689475185 39.6222000738266 36.4945692854432
43.2761454096265 38.9271399577966 40.1341976911042 40.0136663626344
IL26 42.8036731479932 41.554942319225 39.4710743516517 41.5464765641928
46.4343327861696 40.650742261428 40.9157352260901 41.2551966132486
40.2274522635576 44.7958122466628 43.401782064778 37.9607839934735
IL27 35.9305494063137 37.4540772310118 35.8265993915844 47.3574589211166
38.0598374201407 41.6590564937735 33.9750800030909 44.5029690493211
37.4540772310118 40.2112371512838 37.9946685306274 39.2469742642337

IL27RA 150.505000526934 113.624887028422 78.5325880060762
277.837785971587 116.882934971483 64.5187138759372 87.3508610599789
94.6432868887401 151.047739254277 58.115198353247 54.4825201506931
57.290967307936
IL28A 50.8388984085636 43.5182502375207 43.4709415392696 40.9607305725196
42.269054475455 36.4986986135769 40.5585676679482 44.293922970134
43.3744503319109 47.0055981216687 42.911855821602 40.4639532172338
IL28B 37.8386378737561 39.8695929627783 37.1643693306382 43.3078542962968
39.0564222526273 224.249210397247 40.7910160811527 38.2782436497441
41.6616444147804 191.482692108417 216.526205739151 151.344720108793
IL28RA 54.6235981131919 131.010196997585 80.0183739952818
76.2591822114739 68.8362790228976 42.9695585740813 144.863548409034
70.1211450522292 73.9416745419036 73.1931742801095 70.4204944097269
77.1059855034406
IL29 40.9157352260901 39.2542963855807 41.3366779545815 40.3180310664156
40.4166536885831 43.7630587657205 42.357910063876 46.695848687694
37.3057614736707 39.2623423164488 43.5258090162298 37.8490162439785
IL2RA 38.2083710281545 49.83247261979 57.165971310746 64.2130157241811
39.9088079891223 45.5758521267508 39.4916414839923 46.8482302725294
36.4816715148518 33.7238325555193 33.6902293666089 36.9162349182365
IL2RB 56.921728372453 107.836867810972 69.3468840908665 117.699959435983
48.2748123114598 33.895835691032 41.3594868055879 60.0835531094681
46.1101825640108 34.5718473203083 49.2206713037152 40.5703581862752
IL2RG 37.7384958256497 40.0527893189096 39.991353369336 68.9937449813561
37.3508690072156 43.9724628401189 42.4939802311136 44.4031868028216
36.0741672064298 39.9948390976317 41.9908237694988 41.5536168238478
IL3 39.5676051939342 39.7835513496873 38.6727842796202 31.1401844809132
41.5668884168669 46.1513019317765 41.706879587091 38.956280281655
39.9444191873552 39.5637454855233 38.8507804383261 38.0452862258803
IL31 40.8681871019812 37.2810767789306 37.3687900733549 32.7960913111995
38.6454098044474 36.4986986135769 34.9344532055397 33.8558232866797
35.860430241131 39.8873706855774 31.1150868775826 41.9430737035925
IL31RA 44.6231541342 39.8695929627783 39.1799070031819
42.4580504587581 43.1252272136667 47.2927436772465 43.1931731665131
42.2577689927563 43.8482802038826 48.0211752672327 44.7926323492519
44.7401523707775
IL32 149.076166432567 88.9836122218372 83.955786676926 111.939677225005
56.9360840563854 62.8946975076799 62.6885730386156 72.121833189378
52.5307002738469 56.057906531592 61.4800543360853 55.5276281380084
IL33 43.6706764143908 69.9872801240574 67.4811066936124 132.838429478396
46.5557478175976 348.65230953701 48.8319884367456 50.5070012045444
46.581796165231 84.4349803529132 94.5111909776371 138.336991036544
IL34 57.2960245538881 55.0009147092272 46.1653991089885 43.8002456102021
42.9090346219023 47.2009420656854 48.8319884367456 43.4544762830742
46.3203995372658 60.8730416435467 44.2142581488029 45.0985088502363
IL3RA 53.0290605707162 46.0023068797812 52.4485219671976 94.9785931907153
44.4489662507893 52.7156971154405 46.0228732507872 45.2806199505029
46.3203995372658 50.8826620053386 50.2203578628217 49.8253396161069
IL4 39.3709357615493 39.8455060654531 42.0233072093141 41.5464765641928
43.6587492470348 44.0399598874356 40.8681871019812 41.3506832276112
41.5296458719756 42.3606390470387 42.5864346233886 35.9016808898213
IL4I1 62.0553450433433 49.5443425465542 43.6039443285137 175.378964448101
45.9104478407175 46.574682061365 56.6537562775715 47.2864663693787
43.8482802038826 43.128936937037 38.9240290061433 41.7072675309997

IL4R	134.407474121421	100.891869710611	235.544416908354	212.99813190918
	99.6243435693941	135.11808699414	89.4747568145717	123.739272396278
	124.646386399592	133.38594323309	140.35042464015	170.829628173552
IL5	41.0043775553254	40.5852276210998	44.4652605017595	42.357910063876
	45.5198200633333	40.3946758935842	41.706879587091	43.2890595186135
	46.2027193115397	43.2438688757519	41.5421429082231	43.6434576713809
IL5RA	43.9309845324767	46.7980335276155	43.7630587657205	56.1780366332082
	40.4166536885831	42.8598805316863	42.6913548400127	43.3838505886856
	43.6039443285137	49.6203214143464	44.1533327883807	41.863927676552
IL6	162.124919858334	142.614006543733	117.074650076861	353.868749867979
	100.114114765033	1689.77066894108	234.873589896708	355.821515437298
	37.2810767789306	48.0211752672327	46.0905079010237	62.2025996860055
IL6R	43.9309845324767	46.0323106800336	46.3150221024046	82.3768788448119
	42.6022652963326	47.3375061488583	44.4639405506967	41.2884376047238
	40.4997186069494	39.8873706855774	43.7585538437696	42.0709558258414
IL6ST	39.3100256336821	37.958111840848	41.1742244762318	46.0905079010237
	39.3667161654772	51.3886035574416	45.5629520869561	44.1311654985682
	35.860430241131	41.5435611403119	43.0710577955487	44.7871262003648
IL7	41.9918605927928	54.3554233228353	50.8303921211581	47.2009420656854
	44.9384441371181	57.8585041080995	43.4544762830742	40.3691324547049
	44.0519245668737	45.4614234442527	43.401782064778	47.6040671292013
IL7R	106.140215438654	134.908982962131	203.619260934048	808.857027942253
	87.9206275762349	62.4914906186314	126.204310123186	88.8039637390786
	43.3262148375525	51.3907321092333	59.6922302604696	71.5249259772583
IL8	1487.80306986156	399.410299413602	2150.8715161597	18219.5845257111
	707.4124863603	400.093769705765	10235.3288951482	1664.00821059181
	823.933615182634	102.194935174057	61.1801493883012	67.3183506012892
IL8RA	33.8558232866797	44.1239711172417	46.7508829977587	154.019005732316
	41.2740822675592	49.1013967533186	40.9157352260901	35.1738399826814
	40.0403898620771	31.5892202809227	34.9428206949566	35.0222940937513
IL8RB	44.7044140979108	50.4022543872993	55.094385444315	489.983164420779
	46.0977687311222	81.2314452281031	100.829511546674	49.6203214143464
	46.7760357462635	47.611018474151	53.1505787220552	58.112348043634
IL8RBP	43.7585538437696	46.7591817286632	48.2883694323339	
	45.8844325913247	45.6895223823001	47.0027453663546	44.2166118829877
	38.079222289614	44.0519245668737	37.920964499903	36.6298807161755
	45.1735152121056			
IL9	40.9558083259517	40.3104838541906	40.6312687664188	42.1273928203021
	42.7982502551634	41.7256940298128	43.6025443656008	37.7612949798895
	37.1820543575123	37.0740019672788	37.7963032976457	40.9149601380325
IL9R	46.0023068797812	39.2907645923217	39.1799070031819	38.9534635879195
	40.4639532172338	41.2567470068038	39.6535880152834	44.293922970134
	41.3803269429468	39.7835513496873	42.357910063876	37.2810767789306
ILDR1	90.5503099572843	139.022985518859	206.989423961124	36.9912468186322
	95.4533981420022	37.2686490268265	134.713725350463	78.8250642061439
	129.37368123079	188.533240079423	165.598854278557	126.620595729562
ILF2	1285.49870339222	954.116675685628	1285.49870339222	741.908952472992
	2122.39240932934	761.922486104216	1204.34388579291	1969.8871504719
	1167.25317435052	481.492404948275	462.362735276132	677.855726232203
ILF3	111.845105854608	220.85458646522	182.905368976291	162.401599565682
	335.002151198999	135.889195284785	216.102691704365	186.655552987116
	174.263925108623	160.360785581153	172.433936159136	141.649461526745

ILK 315.960715273629 264.718875269207 305.048508698231 336.159430045964
194.278792348966 822.822769402853 211.279069257808 190.993853866124
223.168185178205 841.316771679857 666.265306908449 580.109585240053
ILKAP 102.717305041352 85.2598847559951 93.8361140725228 98.4636016442768
67.0163226819964 104.129763935677 104.462230406058 109.922909644299
114.520410790135 130.691004726616 128.219615202232 101.403739410832
ILVBL 363.215063520042 592.989860385203 703.315796616275 236.536058101486
320.330057050615 256.766675299448 262.624624672404 332.933704863586
634.226367147025 494.300858189605 429.329962597147 321.949943388959
IMAA 8259.38594893529 6089.64191751349 7562.42822724655 7235.0789252269
5855.6498679729 4235.59743465057 7168.83136860899 6186.45345364665
5566.52516420571 6873.84878164654 6134.89208517976 5971.45293864208
IMMP2L 82.4263339218052 99.6265783263109 138.568626520133
58.3475983630509 94.5055588176321 145.741469970609 81.6458112867541
106.78552222475 126.826715174564 111.771533072688 98.3322659713024
135.527591897994
IMMT 225.300736968225 243.803668806533 209.89649461562 255.800286504974
189.890451486324 240.696431900526 245.691073028699 257.325282051981
331.591589534469 296.664472126617 316.604316194398 337.948887427898
IMP3 1041.84536202049 1457.72260892651 678.996864855592 658.282562295783
2232.61002288121 765.145138238955 1425.37639004849 1083.50383916482
1693.5377672895 883.06773454117 917.179747195874 872.305108096296
IMP4 648.380417557148 932.849256536912 946.081492716653 738.059797123491
1664.00821059181 680.837950890159 826.024359447649 985.328482076985
759.184121753305 653.089523541803 775.931494648677 843.078502001733
IMP5 36.3649677956953 42.2577689927563 41.9666541498638 44.265834798964
47.4548471240051 45.0882356578404 44.2166118829877 47.0940613507321
43.1805016690297 39.1621898366355 37.4642807402741 40.3691324547049
IMPA1 91.2297566574871 123.176449895397 95.6182216943758 107.637618676176
125.786923817078 98.8478655411492 108.945545005203 80.5508300225954
107.961407367823 119.545018066492 116.882934971483 146.997252473598
IMPA2 219.737294408079 119.176103802958 157.774096599452 166.304210248623
189.425423538906 768.354209323884 536.73426937255 258.446095955014
356.025084823326 286.758099833009 430.868309653765 589.685739519627
IMPACT 41.0608773166058 43.5182502375207 39.4710743516517
42.992884918158 41.1507980856436 47.9503724044453 40.968974769473
44.5029690493211 48.8524897151151 43.4544762830742 46.9919960628953
45.6233434183356
IMPAD1 124.041740442038 122.292794686468 95.3754438202863
90.0942781313264 145.77302334589 86.1633564934019 135.866407965991
110.09868737254 138.362914843774 67.1278109419819 82.3364971911816
90.0223113095186
IMPDH1 134.936657884035 117.811967515956 133.38594323309
183.028254350275 118.720812623965 114.389392280707 142.714450804595
87.7295604723036 152.679927202674 81.6508259258669 68.3799828461475
102.605473054584
IMPDH2 284.66531382201 732.454837219705 997.492549573274
227.817928402227 1521.20630233299 700.010354583503 679.309039810384
347.829279462722 682.227514643175 977.776395810338 686.154904023493
975.02086340962
IMPG1 40.2087556903277 39.0102066700839 40.9818899755491 38.1421709520616
37.4470183315826 39.1664256481434 37.4386615664066 42.4580504587581
37.3043739460234 41.5804111566887 40.0766543123257 41.7072675309997

IMPG2 53.6741873729867 42.7775752471711 49.6347864008749 49.3654837541353
42.1650127805175 41.9543948167375 52.2476631126873 47.9942520395434
47.4699236780425 46.5650556481721 50.5810338885769 46.3411353377615
INA 325.769076169162 628.84789420227 285.459248157609 39.2780515621926
416.052744271365 86.6246917308185 641.103199748891 194.666769955149
495.26603220907 195.482216656634 147.388500451427 84.3464768820344
INADL 92.2857885595617 59.046385957117 79.4998723172096 40.1272810917202
56.8307116636372 47.0940613507321 59.8273072817267 45.6941710526465
101.627551729492 131.195005779516 80.4554147910354 90.0942781313264
INCA 46.5549623118323 40.7952353240579 41.6977706798234 52.8502823653368
41.3755760434934 35.7034203372528 36.5352572191451 42.2577689927563
45.6582729799009 33.9967481174035 43.5710871617107 42.357910063876
INCA1 46.0920218698606 42.7775752471711 33.281531405287 36.7892997857376
39.1650142708257 38.8336427948187 40.0052661202207 40.047314525032
39.0775870687758 38.957687788868 36.7283081422707 33.1559412791294
INCENP 74.2653431904071 71.0340758556049 55.5567699051046
52.3004056941891 127.008218054726 50.560825686187 75.7969486011633
79.6133633483649 67.0695260207402 50.8388984085636 45.4799270968969
50.4168556990205
INDO 90.6805895192707 65.5367689605146 72.4913548133238 232.13042919324
53.3086645804786 45.3882138838137 48.0283573444179 72.5835488194631
68.5599263000733 38.3092241935352 41.2598458726002 40.9683275553869
INDOL1 45.0985088502363 48.109747794187 48.1997158668729
66.224559122062 51.940083408061 46.574682061365 41.5817737564592
54.5125550937898 48.9931786790166 45.7911932481722 43.1931731665131
43.3744503319109
INE1 40.7609952437622 36.6968906669565 39.991353369336 36.4564050256915
40.0599807618105 42.157627240486 44.7255207589606 42.4580504587581
40.4220196471124 35.7202909649682 43.4709415392696 41.863927676552
INF2 185.94424664238 125.676172266736 200.615897175992 126.14245679817
65.8319449883221 95.4393274079354 130.72385089567 193.569399061809
212.062417120966 175.876518898179 185.667983376361 158.25602438933
ING1 53.6396970125549 59.3845821287714 50.9612733880858 52.7924213633885
54.6089451252049 50.3245489035833 57.5685357492202 48.3119523820103
54.4912806027381 48.6705421229977 46.5650556481721 49.6203214143464
ING2 101.877183008944 103.189199090363 140.853812988562 87.9098670472958
106.58628367621 128.126336084436 127.655537534156 75.3761360028971
142.262349841307 74.3482661085192 85.3930226794591 100.151116279734
ING3 66.2154717217675 83.0316824964745 71.0820747228972 98.3142231283069
67.3136079922956 96.4913003384143 65.5800543140431 57.6034762511847
81.7566533443175 73.5657748119622 79.4158467034017 67.8335962843732
ING4 57.2008272301399 48.6184776976167 61.2153068100932 50.3127977239083
54.4586176399381 54.0969604236265 64.9191509252662 69.7729022157455
56.0286903335634 68.75304552302 58.6010484516703 52.8974363028468
ING5 43.4100411978366 43.9783383422823 43.8052993451586 48.9482359914659
43.1826328292558 46.5578790409334 43.8206425661746 40.3357777530419
43.6269210434851 41.4659679379482 39.5399188536544 45.4164642922183
INGX 40.6559419616644 42.2577689927563 41.8751578329365 42.357910063876
36.9060997041453 40.650742261428 46.1634822993009 49.5185112777773
41.3803269429468 42.1622837416589 44.2142581488029 42.2577689927563
INHA 41.9407041937973 40.5400384479766 38.6727842796202 42.8235313007746
40.6723334103791 41.2567470068038 43.9450023643394 42.7431359850339
40.6957787921451 38.4187686307607 38.745552403895 37.9607839934735

INHBA 39.5676051939342 38.0226905622357 33.281531405287 42.2496337895968
37.5556299264699 38.3418933808844 35.6056062164318 40.831207953647
43.3744503319109 36.4651112441576 36.131485826118 39.2469742642337
INHBB 73.9192243586276 58.1027665533767 83.955786676926 57.0629653735694
70.7760293129408 82.3364971911816 52.8496345990125 78.8250642061439
50.5785738734431 54.1683282066029 64.0541263553251 80.1461294346211
INHBC 38.5648843914189 45.0115870036541 45.7968648044613 42.8235313007746
42.269054475455 38.3418933808844 46.5650556481721 42.6450179841129
42.9020321355927 43.9450023643394 41.9908237694988 38.9496515344837
INHBE 36.8072833828297 38.2263227947189 36.3604951341921 37.29566077996
29.6891949090828 43.8582747295766 40.3302268293707 35.9023742468744
44.1519466943185 36.4651112441576 44.4688999098904 37.4554876386402
INMT 66.3095900379311 52.4351670989268 51.223859150798 63.7909104347097
47.5647821785529 165.322633260592 39.3585350448719 62.4219686957673
44.7058233759912 155.406719968961 109.844156801015 93.0092452285816
INO80B 73.8374823243007 76.2478685333968 66.5285423077808
70.2541961995753 76.301438841111 65.3096524773304 86.2118355769367
87.4850071876743 71.0820747228972 76.3583555666815 82.0206083292981
61.9882398986333
INO80C 112.351686948586 301.867441371793 290.761706869722
153.037055310357 146.483908799607 136.517445122438 168.145003400654
145.791398829369 352.019218177371 223.040734249612 243.403622361932
226.090904721424
INO80E 191.503068870249 182.905368976291 271.813877560354
145.391705645319 267.359645500185 205.279698624867 212.664574366477
309.344766670365 127.974232708236 234.333935191237 216.1413998895
183.364949777567
INOC1 68.8005532048281 69.7855392178808 59.8838591118356 76.9373289454707
81.6099927983382 86.3888090084564 78.8250642061439 81.3978207813439
76.4796910268368 87.3815040456105 91.8414653633828 74.5528557785378
INPP1 175.632783805966 268.265540230814 437.84407370064 198.951182954651
468.381233506231 263.799439226561 254.472291205905 245.127632785841
555.910110984847 525.687522832693 511.261925898948 606.378839153932
INPP4A 48.2910530887214 47.2354741623061 44.3635754464295
41.3027031037343 48.8381507187986 44.2607789091176 46.4838970014049
57.6234624694326 47.0027453663546 40.9607305725196 47.6333826891425
43.0930293831497
INPP4B 77.7665223272661 139.311807081199 187.002948435359
80.7168944828052 115.132600341395 62.0660509811111 89.4747568145717
89.0275988550016 182.219366179899 96.8240908274121 93.5982028088194
168.561260174501
INPP5A 72.2797007105795 122.924027019519 108.512469038407
79.3614922672599 112.753674408958 195.968119243879 95.4764015137059
80.1144023887293 84.2880616879945 146.691463448295 162.721587349434
115.970093171058
INPP5B 78.5216977073842 75.7801512652102 73.845912534353
79.5838979704243 70.3677225924146 90.8335894699136 71.6890300780549
110.599131324937 72.1591028956359 88.9738853594498 101.434947182387
78.7496673195407
INPP5D 75.8976034525022 84.7222416138393 121.737071786167
99.4184778430267 49.9493230064613 66.5234828659154 48.9826868640753
80.5508300225954 56.8622704143595 70.7003843117511 66.6118713143653
63.4685821260797

INPP5E 184.162452344446 126.253578620746 62.5610562594275
76.6232527679845 153.920604317629 87.539553421006 180.902423012987
73.0394191668858 104.00520458442 107.792406701643 76.7063975550116
91.6328359149182
INPP5F 49.9791966667317 49.2443202281556 46.1335462158451
56.3441077510744 45.8051846088552 46.9395507483816 48.3411413510851
45.0693869004277 48.5238922337712 50.1059857872211 46.5650556481721
47.7390230243778
INPPL1 843.333981146512 384.919835924792 474.379113836506
289.332274319357 583.421166842011 335.569876624791 648.885062676884
740.994425128952 385.675278463448 433.767439244344 513.266895252699
376.453560620912
INS 39.9697332660833 39.1982470626863 44.2405728317107 45.4634652319753
42.269054475455 41.2567470068038 32.8745892439217 40.3357777530419
40.4220196471124 43.9450023643394 43.401782064778 48.6037684272115
INS-IGF2 41.9407041937973 50.9643363406241 87.3634135323569
40.8084239472012 72.828269171954 45.6706505553383 38.2933870238743
35.1738399826814 32.325260431418 40.8970401536578 40.0766543123257
36.5219488315447
INSC 38.6213341190836 46.0713306971594 42.108130037421 38.745552403895
50.4260283682427 41.6590564937735 45.1017494688414 40.5158867964016
39.0458123429605 42.8936990772838 41.0476065156912 43.3262148375525
INSIG1 108.734392572981 118.288549974348 89.7002002853947
304.33749254654 96.8490167966217 147.093338225478 129.2860308084
223.531821464447 354.158105175308 65.813547096222 132.090336898032
119.124583755587
INSIG2 323.780380243759 199.208033538936 151.067886009549
239.425856083354 171.597378768542 178.904972763325 138.923143963742
127.596368103014 293.410413883219 142.846665249354 155.617122020913
224.976641879383
INSL3 43.6706764143908 40.5400384479766 49.6347864008749 47.0874209746031
39.2608760193011 45.2852528185486 41.5817737564592 43.1567024035183
38.3418933808844 38.6454098044474 35.7953070087288 38.4330755397458
INSL4 42.2455707947223 40.7952353240579 41.437237454134 41.0726336841311
45.2556760873335 37.8102105794655 39.2533668167483 37.0390589468421
41.1742244762318 38.6454098044474 42.7275315121163 45.2783024981315
INSL5 44.436995101921 38.0226905622357 42.640829914102 40.7481155008596
38.8456942580061 37.2686490268265 40.6750540874299 43.1567024035183
45.5779686360641 38.1042454705119 41.9908237694988 47.6040671292013
INSL6 43.7920955808549 121.8863522969 44.5544727111028 45.1944222548351
39.2968590261598 44.812853905056 42.4939802311136 42.357910063876
42.9413736814915 37.3179578037963 45.6706505553383 43.4290452504928
INSM1 57.8765910878975 47.8400694856726 41.6977706798234 44.9370339275041
44.147557390293 44.4619248757502 40.6750540874299 51.0724630072723
48.8842909466002 45.1569741609597 43.0016969050339 53.3904307221902
INSM2 44.0328992213727 37.7384958256497 46.0727230219512 49.3654837541353
40.5710637884029 39.4953494583071 41.1507980856436 38.4540849632254
41.3803269429468 37.0320432433314 47.3791873993182 49.904979214379
INSR 36.8072833828297 36.6002100641539 38.3166554965434 43.8002456102021
38.9496515344837 42.357910063876 37.6036146841933 41.2551966132486
38.9006788676407 35.2880714952489 42.7275315121163 37.9607839934735
INSRR 40.2543341073001 39.7835513496873 37.7384958256497 36.4564050256915
40.5710637884029 35.8227939208521 39.3585350448719 39.853866204927
38.7298222155532 41.7405682794166 37.7384958256497 34.0382693188794

INTS1	148.925503526873	157.615972646659	162.151812425949	121.423421895502
	198.226275659825	127.49459343016	183.431582521592	201.427008153788
	151.775928080246	170.150808455621	152.329124189473	105.796515736125
INTS10	330.168672676816	174.537032518715	235.925374041038	
	250.210134743225	168.75362473684	312.40869171788	250.176074707634
	150.405742308999	280.159844300266	389.364650157102	380.278389209918
	362.268767190535			
INTS12	234.495626550751	224.412339736769	231.778951567627	
	281.262083731954	215.504612948048	244.466972245417	247.193935816149
	171.705718494906	271.478148993789	164.580141651883	165.942795019496
	231.085533860683			
INTS2	56.5094373275922	82.2766822827861	76.0240426902567	76.450993090133
	85.7842344710572	62.843734579668	83.0647477737971	53.9465584609317
	80.8728961146267	63.197604174872	78.7260936224802	71.8539743926881
INTS3	365.401746744015	319.408264906288	413.516548092483	222.985386635178
	576.830397705584	318.995164994001	351.637117984528	633.385474385877
	307.974082654351	512.255695437272	516.992846073349	474.729653426958
INTS4	82.6602736441839	97.9004351588426	111.320157744612	111.703187941409
	105.406282523992	104.409397223301	105.802035886293	93.0474674148534
	89.1217605204013	99.9057667432209	90.3531863862054	91.8251275769679
INTS5	139.436084808369	101.277664841914	130.703767898434	100.344513700137
	229.992809034971	96.034706957068	158.862722884436	153.037055310357
	171.938863440177	114.321083441061	127.345094323724	96.5813769696577
INTS6	85.9392883641626	122.405413080836	113.454378667943	238.616000665585
	110.869341159607	109.943883439481	107.015362116555	144.515737827731
	143.517244617936	139.814194128548	129.697400781396	146.046179302584
INTS7	68.9745034946434	64.0903425729408	62.0967404944031	53.0284011078097
	53.4109882045447	51.4931742685391	66.0350709576651	60.419911425109
	71.9771425105735	51.1169368198437	57.0690683036183	52.3896785600751
INTS8	93.7352369697358	89.5887648181505	79.7733480606334	62.6048727240291
	122.316617618658	67.6817814091697	74.5591290353333	85.2849531543957
	133.842824493862	90.6805895192707	70.7760293129408	86.2974544521879
INTS9	197.515728627482	171.992427227088	249.308868215205	185.924923239764
	167.943170737481	195.168348967505	209.068725760522	117.612004923867
	193.094756585577	239.023368693467	279.448591201269	235.075347885884
INTU	64.3173126779926	77.8220670093392	64.9839099669442	46.614092935749
	67.3568864741196	57.8944021095265	58.6431245059655	49.1399035787209
	55.1253465069845	77.9085946827079	68.3419511055057	75.6428949408034
INVS	61.1630754250653	64.6629780915974	52.1024135605001	55.0604256312495
	55.9283039581662	66.5500089527674	61.2822647324366	62.1934012861178
	51.7098181032862	54.4825201506931	56.9320717885002	64.0541263553251
IPF1	42.3433931248155	45.3486182278177	42.2577689927563	40.1804147506786
	40.3452320167286	32.4301586830653	50.8388984085636	41.7419877611072
	42.7150227340364	40.2112371512838	42.4580504587581	42.2577689927563
IPMK	36.6480005877129	37.8217989841918	36.4564050256915	36.5393857306744
	42.3685136297603	41.8613406887824	38.4005795891396	37.6557714476853
	39.0775870687758	36.9102644736882	32.406093465115	40.0557788909923
IPO11	294.155188719236	293.410413883219	319.328571608303	373.547403496629
	275.846698617064	356.628599887098	288.447603611962	334.170764774986
	328.855843739232	389.481124074714	325.450452751375	293.040690378216
IPO13	138.50445813694	83.4951332849212	91.3890908971311	75.2475238554276
	112.990752518897	95.5022104475922	94.1289383735162	167.034910927177
	131.068763088422	126.546330358591	104.626873082138	89.6670947876322

IPO4	148.016994658777	120.3698209682	115.256208610595	92.4781449270918
	184.50283792606	107.91849682084	86.5564881117807	90.3916996175708
	108.937199348319	72.961444366037	80.6550905475507	70.5322939116406
IPO5	90.9521112485099	73.7873098190297	73.6323065529916	75.0377156190383
	115.827601697699	155.270252661048	83.6935756500468	54.0507720792049
	74.220854748627	77.6056319020116	83.9236799134394	111.166893747402
IPO7	46.9939712192224	58.4891791448514	47.2962360458401	53.9517841438975
	57.4163094213712	107.034189847828	47.2927436772465	55.5578334877425
	71.787754116845	46.2286164776607	57.2008272301399	86.4498396868205
IPO8	119.993761317523	98.9017950719795	175.362103430164	114.876346000655
	58.037539503209	230.554867472663	126.930102684077	66.5234828659154
	85.6441969757275	143.894266185526	89.5887648181505	226.235985918654
IPO9	127.352997770688	114.617965877564	126.578693258328	94.080489738662
	145.083624161597	103.734064904898	160.015674121695	118.600699776727
	123.480244775709	79.7389069715403	92.0711167984454	89.8035133597498
IPP	85.2893380711323	71.4197504261453	63.2475738480323	54.8412943008635
	46.6397391251762	77.2834482217973	58.2315917325629	112.613079254002
	69.6511571901941	102.38706554984	91.2202310968307	83.1874704625702
IPPK	57.7877627794725	68.0388928513985	54.9071823326024	60.1837189855762
	58.9875400392427	60.1042270393382	58.0139597055449	51.7538628098347
	54.998510746958	54.0374522273907	53.5698357689852	52.9609836781499
IQCA1	50.4260283682427	44.2630383225933	121.214084453032	50.2163892855092
	146.311363191836	55.7868713984479	37.6036146841933	43.1567024035183
	41.2424637338424	72.5918913856713	71.3932787152381	79.4710406831985
IQCB1	100.765131355505	102.020047144427	111.025105784166	100.654701845984
	112.60230393988	87.3429006429216	132.24963384417	84.3526980051279
	104.480361196422	90.3439862484238	86.0709878693269	98.2581979415778
IQCC	44.0328992213727	44.9370339275041	49.5424425850549	38.4208429103799
	59.9326265577462	43.2805590023412	55.9276266345493	52.1788489555942
	49.3780606970538	53.5498255316009	45.390192977585	37.7526485343328
IQCD	54.5358897781765	39.1982470626863	41.9666541498638	44.4367387274922
	39.859345745831	44.4619248757502	44.0687697278114	60.0835531094681
	45.7452456977857	46.0119912782855	42.0750889188255	51.138877923954
IQCE	47.6397712007919	44.6918600441328	42.4580504587581	41.0726336841311
	42.7107928951766	45.8325181856483	40.1235656101329	44.2159020605784
	45.4799270968969	49.0841460667818	47.2134341420411	46.5650556481721
IQCF1	39.9697332660833	35.4484652445078	41.1367478979484	40.8681871019812
	42.7982502551634	40.1428591998404	41.706879587091	41.6466169548846
	40.4997186069494	36.4651112441576	41.0808257270221	40.6614700944853
IQCF2	43.3638918705225	38.7344853699576	40.301483914446	49.3654837541353
	38.2496050953547	43.3785777889265	40.4359177791566	45.5581356216957
	44.8003345643334	40.4136535208406	42.0750889188255	43.1805016690297
IQCF3	43.5710871617107	38.2263227947189	40.0998167098057	47.8164193292842
	39.9697332660833	44.1737502492846	41.253645578517	40.5158867964016
	39.0775870687758	39.9948390976317	41.1638563416981	40.0557788909923
IQCG	50.2203578628217	48.7847528954724	56.0477607422828	54.4586176399381
	43.2484996523549	57.4777075615651	48.9826868640753	57.1362071277099
	44.619512785899	47.7180816682562	50.7903824729818	57.0524725494273
IQCH	40.5235471987417	40.6380495994768	40.301483914446	38.042028333747
	41.2740822675592	39.0266821712099	41.0871827071116	39.512606833112
	42.2577689927563	38.8507804383261	42.7275315121163	38.8834538955
IQCK	94.2762661604561	128.766242896259	151.740176274202	95.0873056549336
	104.704892803169	120.618530729822	101.250422688816	139.294987640071
	81.7566533443175	138.401109242778	140.51841571801	137.976238593619

IQGAP1 235.127539195661 179.605537867711 117.869393694564
264.263104842911 105.945216149549 257.851199206959 146.534713287435
183.086707591185 167.799243454967 214.927272114101 184.964039515715
267.70610731202
IQGAP2 66.7759319948677 51.8610060995363 50.0748292497578
150.082905944963 52.6922486991742 78.3488426553071 51.7714254834195
73.362936710475 53.0357938671646 50.22432299342 48.1352470945488
61.0025829061063
IQGAP3 41.3387790895716 49.2050538150106 53.5698357689852
40.8681871019812 49.1928206164702 49.5424425850549 59.0457625163577
44.9977782988116 50.1603795873336 40.7063426028871 46.2560238004757
45.9693232070002
IQSEC1 152.21278457652 115.221134337342 117.228320608065
305.619340338331 166.168385855406 135.985408824299 107.015362116555
174.148714092395 86.6516906104905 136.20629436945 120.046457114882
113.491158368594
IQSEC2 66.7759319948677 56.2305155589239 74.0913177754682
73.1447139654971 58.2780916793348 59.2643906275025 73.3042538589134
68.1311562787361 74.6277165793069 91.8251275769679 84.1922656126304
69.6021553604792
IQSEC3 44.6231541342 45.6533175604486 46.1987217359283
42.8848887336829 42.9090346219023 44.0804700754786 46.9929836306816
45.2806199505029 46.9062313932642 40.1179210534118 40.3452320167286
39.8497227447888
IQUB 37.8386378737561 42.4493032770053 34.8956628705329 34.3595588146954
39.7568367479301 45.8325181856483 37.1643693306382 41.8417024632349
39.7537274655115 41.1222709786153 40.4504608195666 47.3992733452821
IQWD1 128.521748930472 123.039116845742 245.923368125413 115.195645860902
193.806701229141 181.814283147675 103.657181265842 111.643323610073
135.41852974791 112.316483867372 134.853649092952 158.918242816842
IRAK1 303.323289734056 304.41727337972 245.853111206859 438.22469176123
865.63777294494 261.195021649777 348.450810225807 444.471597593258
290.087040648659 193.15756263121 192.171116215791 212.622278823808
IRAK1BP1 44.0707087774725 66.9218519793499 56.7272804480684
51.1313686723929 44.8351976221735 58.8090653936339 61.5635554619635
54.3820590274321 65.429256309049 51.0055825931636 52.6547683625018
57.8111054584154
IRAK2 110.362432903746 114.056623754207 317.245171206724 654.682263833024
602.441843384617 117.019543736429 254.472291205905 557.455966274117
221.910075604221 527.439095170064 460.340300658834 615.838713743621
IRAK3 72.3388488936832 48.3077814647455 72.9051985681184 718.414652618295
53.8260140375214 94.7008981904016 85.5027511448958 55.6904617926772
52.0583273941813 69.4131195465674 62.9336909080086 86.6105153696374
IRAK4 81.2359429678503 79.1822591641672 96.6895594961001 116.295564219236
49.0828761839116 70.5699797830702 76.7483302168542 98.0352049248004
97.2425832658887 67.8233625051884 75.0704619826557 86.6854406823303
IREB2 108.734392572981 157.250548147161 119.634256037186 179.322661675979
196.29493890075 363.947777340854 150.562062400737 129.925137664962
158.308338986864 203.152852313746 250.832808506455 201.711142068161
IRF1 366.915943380617 451.364230598722 296.271895016431 818.927157206447
147.945429978177 314.36962447353 315.592187396219 276.795797048951
336.44013572926 104.928805304068 170.307603160524 159.306689057291

IRF2 46.0023068797812 50.6526511991732 47.2962360458401 48.055683217958
45.7452456977857 51.702461863489 50.8388984085636 43.6434576713809
50.9010149857308 49.9493230064613 52.8297102061403 50.0571060701893
IRF2BP1 100.057155418659 68.2521461582047 77.6971278685226
69.1931891474114 84.2158827857221 66.2167164788023 83.374777060444
103.366147685418 115.88156897199 136.859309317164 109.844156801015
70.9377012403023
IRF2BP2 123.46775722643 132.52749757795 133.47090252916
170.901596796384 165.613176704286 171.302214895226 127.588471566929
118.783682318205 170.287619901365 99.0596863442346 113.322929203297
97.1634390603131
IRF3 144.063191647372 170.20428461603 237.161912299581 150.529917336161
277.5073518536 118.089250727398 195.512986297442 187.189412320761
202.578421978121 227.686495419561 270.036719921343 208.878921620914
IRF4 39.9697332660833 46.0023068797812 43.3085358214154 47.9915414951747
37.2567512265363 44.4619248757502 40.5585676679482 45.4757965005055
41.6616444147804 38.6454098044474 35.2339574411711 35.3525469364579
IRF5 71.0733129130898 86.9329220905001 65.0359415104302 59.7240416362194
67.2779942308321 51.8812208789107 80.4113521705019 41.7877325563821
59.5093038629766 56.1912096308988 45.9104478407175 42.4229201717624
IRF6 89.8234189405239 184.218705402964 267.608189207303 61.141483572676
186.795349445177 80.9160262677505 336.490083577377 166.208731611481
289.25391828279 228.328635242684 229.130963999337 218.446312290024
IRF7 65.2001674269291 135.436347750159 123.729687262037 285.459248157609
87.5074297754031 91.1271533616476 130.946383858668 95.3300728806601
74.6590272639023 75.5561597374812 62.9336909080086 74.1282220615085
IRF8 135.323750661937 241.952522667413 136.456679728935 926.61614755153
72.6675345946258 176.43047680293 85.2088054619229 154.829574442056
44.5303781239209 67.9259304928255 62.7743500747415 76.6707262000712
IRF9 759.184121753305 1069.3975598703 970.379875059112 826.490051619826
320.504100222566 614.807633538557 611.538630852232 522.49156295512
577.993850127555 508.688028777582 778.723771259404 491.8684831079
IRGC 41.3387790895716 42.7775752471711 36.1042012504758 44.1483976313959
42.7982502551634 39.2985791324641 33.6696565339534 42.0494116081998
37.4540772310118 36.3612409063792 38.745552403895 43.5738557773903
IRGM 44.0328992213727 43.4396866157171 47.0940613507321 50.7046706880325
52.2089306840125 45.1873487463472 41.8315602975157 46.1673159982664
44.8003345643334 43.844855336215 44.2142581488029 43.7672102682067
IRGQ 34.4021554788966 40.5400384479766 40.5734458834617 35.8472302065546
46.1804647841331 38.2293008890613 39.0458123429605 42.2577689927563
37.4540772310118 41.4397539024976 40.0766543123257 34.4570158133896
IRS1 91.31756001203 164.312946712409 310.763904671301 48.309866878365
300.762511215441 107.70358681113 111.714563124738 74.383456365584
327.984902171255 160.056757080676 176.816741656659 232.193215437232
IRS2 99.6265783263109 86.7710020879725 60.0856496605043 694.590958116425
66.3075095149783 130.336599857526 79.2701322339022 80.8333046859586
42.7150227340364 126.071077511531 131.156876010639 155.050936910986
IRS4 49.6241203903354 47.3536182445315 43.7630587657205 41.7614975630362
48.7288537840562 43.5752393599169 42.629412460409 51.4360013601805
45.6582729799009 45.6868589181768 49.6706535876636 42.357910063876
IRX1 51.9545473262296 50.6229884142872 43.6670755115848 46.4555908533915
47.5647821785529 35.8227939208521 57.51482768194 53.2046867327331
54.8946088631931 52.7972156492746 56.0477607422828 52.5752569429057

IRX2 44.7044140979108 37.7384958256497 37.2838080935695 45.334106390212
118.376686726902 48.5835484742456 35.3362091871364 43.1567024035183
43.6670755115848 45.5776159432079 58.6464918752765 68.4278345554565
IRX3 455.223217772744 274.962689159729 1261.91478863258 108.779357041421
379.377005627283 99.2749350936362 790.094848284547 100.370937669662
3761.48462220262 58.9836017745712 77.2834482217973 63.7744603935682
IRX4 38.5007204246293 178.525751043592 43.0016969050339 48.856176220627
58.2780916793348 48.4758590019384 39.8695929627783 61.016921238986
52.7512110769042 36.8059561571675 44.2142581488029 44.2527566343661
IRX5 45.7017023767682 70.0965466297064 62.1893884200745 41.8291484190524
47.8075998240396 43.2312848495114 51.3064929551674 37.1317006162938
95.2018660650205 44.3648993568405 42.8742421276144 43.1805016690297
IRX6 43.4626049853157 43.9421822132391 40.4070046183108 43.8002456102021
45.3675390992851 43.7630587657205 42.7431359850339 41.1575777497576
37.0126425326534 45.1569741609597 52.104880481326 42.6758568421347
ISCA1 71.2246788429078 91.4581948567397 59.8273072817267 88.4717765234098
88.1733775711738 113.759627906335 66.4958411147116 54.2301964914419
78.7543199947688 72.1451513995841 72.2377222744463 88.5274823465707
ISCA1L 64.720130807316 71.9539136431119 56.4779645313958
58.2401724760744 100.824660338235 91.640286556353 74.621822948059
73.8875154527631 79.8196996067124 54.0374522273907 67.3976855661302
83.5791586743002
ISCA2 116.426770366736 59.5534127075228 91.9375312869437 84.0899854321388
97.7153995525346 80.287662312208 82.5050369860039 66.9340967279385
94.2275161759277 77.9085946827079 76.375769561992 94.1128427766432
ISCU 429.983084929605 633.385474385877 627.914461428624 635.906934423716
467.645011859047 895.735191953679 372.642038290698 358.979854534484
600.34379987143 611.538630852232 624.51670158046 667.583623414832
ISG15 282.603767561794 838.974043046676 254.425072964926 196.247893540766
150.43149196048 126.981964766008 384.280434953152 983.200149701633
148.467977704907 98.0352049248004 145.09364961191 110.860844149372
ISG20 134.113913870501 356.079406408042 316.660303090646 1561.83892117664
170.450752247109 144.046404221564 329.437593022939 285.626835802986
196.709620264975 88.0664425843955 119.209804934394 80.0381580139229
ISG20L1 130.703767898434 135.997802304719 93.5717680539357
117.228320608065 146.405413944753 120.980558571109 119.124583755587
149.792346524401 90.7037473522607 72.6549171886783 85.2598847559951
107.276913085701
ISG20L2 146.658116519622 243.489225650886 225.559405596065
215.131274710043 235.222472959869 140.264270771578 190.960237766104
135.259585457958 133.244526666651 105.29989739819 162.721587349434
123.978615472736
ISL1 38.7537364570873 39.3999056968598 42.0621425894624 52.3004056941891
41.6806984441849 51.3886035574416 38.941855825269 42.5435283762933
41.6616444147804 116.311176407088 98.2581979415778 82.1365334012643
ISL2 40.8681871019812 46.0713306971594 36.4564050256915 36.1562979552173
41.1868052124344 46.263826584899 43.1931731665131 46.5717043290731
43.4656637536244 40.1179210534118 42.8263096141654 37.8490162439785
ISLR 254.159017331462 44.3088822189408 41.0752399035779 55.1149136190465
42.7982502551634 88.0664425843955 40.5585676679482 62.7515429046888
39.1433524273815 105.15507796881 73.0464199386528 59.6291051806275
ISLR2 50.9381343674699 42.5269793679335 43.0971787292711 43.9282059368202
41.1868052124344 45.1873487463472 42.8684958525042 45.7476095696859
39.9444191873552 43.1839109702714 39.0550139257658 40.7704587752083

ISOC1 78.2644076847509 230.486681581762 170.527024636624 91.3144101155547
136.875335055866 95.0026207727863 118.27400376579 133.69701250532
181.844123841452 99.2432911346944 132.760054781304 213.711954283504
ISOC2 122.136867360309 133.192618821838 146.658116519622 122.412295645862
283.747865829282 172.676819138152 148.016994658777 144.034489386072
270.156612086731 144.835567449189 163.844659359002 115.229070906222
ISX 45.6517320730042 45.4882438975269 39.9101908547397 43.6726153508978
43.7517118200398 46.3715909653832 42.357910063876 48.9826868640753
42.992884918158 38.8507804383261 42.7275315121163 48.3648494350286
ISY1 156.242688079359 208.67074469962 199.181938253995 259.958369064691
299.428590398636 247.298165265093 241.148224648134 141.572530741416
194.703024752864 155.01005229951 178.688550924272 173.212430860273
ISYNA1 107.836867810972 181.420545696696 159.667153030627
55.4180794172794 283.161835288074 75.2735539010032 131.39588569388
64.3284163241986 169.367702213351 161.870972980732 123.868848519181
154.08990201795
ITCH 94.696942512529 94.5261917871909 82.3344892042646 120.068630086702
78.3542840447759 103.139459009593 223.013058725367 140.559370165331
137.395516155158 206.989423961124 121.936597605894 159.12163104195
ITFG1 168.737634511775 251.184621899165 384.727543700024 209.89649461562
131.496769528338 188.912691033518 114.056623754207 112.714318425036
174.350348527098 117.20232751191 135.92805172353 167.095818680513
ITFG2 209.524518082089 246.568253751149 249.776078532596 160.973602503707
191.896416838806 221.015209694864 247.007210336963 229.745018977185
391.658764737446 289.25391828279 356.410753280729 194.570513601724
ITFG3 450.271658272512 439.955651727755 468.573956652904 193.327267186222
241.736113354506 387.039093527273 389.481124074714 190.053043382767
517.664764759512 477.924466983414 396.493831159481 328.962069934895
ITGA1 67.1387898740161 61.8492980174846 88.1797799963114 62.8201270538435
85.7842344710572 91.4446109489387 44.9863726205428 55.1933004802607
45.1899271235974 77.0226403968683 70.2215218476683 101.888001006345
ITGA10 45.3872406432794 47.6914221838216 60.4809187893259
91.5202521578181 47.2074332636566 64.9498081060676 49.3854238365325
48.5681478665796 45.5779686360641 60.4837400912239 66.8842530423095
55.4635297192076
ITGA11 245.060552951511 56.7569186600983 50.0487097583953
94.9785931907153 62.5517224560726 73.0686482917042 43.0269218496197
81.8491450876942 45.5779686360641 66.0224755625662 74.8473087205709
48.7226309877005
ITGA2 97.7140868175135 152.187430924861 216.63059645927 94.4501786945034
137.965622805944 45.3882138838137 68.673864919178 96.7474444319428
149.040814842403 116.76301232629 168.67163443217 217.263889616995
ITGA2B 36.0144420013819 34.7135536792489 38.6727842796202
45.1944222548351 60.8550020364269 31.1150868775826 42.0605906240248
39.9576138799997 44.4395580493118 42.1622837416589 37.9230440150891
42.4607795638552
ITGA3 301.904682664871 255.68068369366 542.467997217663 126.485649460666
132.341766267192 124.628895737993 164.847648206552 192.922622120507
476.421959423835 429.329962597147 391.528019827951 556.417356025042
ITGA4 51.6558970571712 65.3694267397114 51.8122261766744 77.0774476767901
48.3814421939888 52.3187919317561 48.3411413510851 46.4672637826582
45.4799270968969 47.3791873993182 43.1931731665131 44.3521099857368

ITGA5 531.36214636517 125.90746674767 237.4882538723 659.126408705362
185.378190201718 1151.25293766167 78.3082773071531 213.590067519021
135.800775637089 603.224267841548 637.824136761292 560.802447943839
ITGA6 43.9477262001082 53.6895401581575 53.9612654850046 43.26160898693
45.2556760873335 42.157627240486 42.3074504864935 45.8669209716694
45.9693232070002 54.5518174219283 51.592378351965 56.8056093987178
ITGA7 48.8333989709933 46.29198852834 47.3861758958576 52.8502823653368
49.8304479381623 60.4065050472082 47.7390230243778 51.2623287419357
51.0062122023297 54.8979897528792 52.7817052791259 56.7711602236706
ITGA8 41.4508241771315 45.7979032939211 42.8270038153139 41.6564075217517
51.5888395924843 51.702461863489 37.6036146841933 51.3472428383325
40.6957787921451 83.0700092537618 119.691133736693 59.4465684293847
ITGA9 38.8561375693335 48.2220547806679 39.5450079747895 46.4555908533915
53.5594683028749 58.6439719071099 48.3411413510851 45.1714698794331
45.8325181856483 46.0119912782855 47.299647867533 47.6938628797168
ITGAD 35.0202257459026 42.3550738181144 42.4580504587581 47.8164193292842
38.745552403895 36.3845892764084 41.0381860307445 38.079222289614
34.5633623842083 41.9666541498638 40.4504608195666 43.0930293831497
ITGAE 202.325629133577 163.028918580618 156.294411442126 173.536155357688
115.597550918377 134.749803376372 211.964446599144 159.884578124679
145.363725698076 168.522052983892 216.004059887037 217.848539722853
ITGAL 51.5888395924843 79.6516904169771 78.080034922779 184.898658656843
44.6834648302383 48.9826868640753 51.9421032049606 61.5824518349507
39.3750969348308 55.6865353018811 72.308304085263 43.7672102682067
ITGAM 97.2535496140648 81.3696465421645 61.841867306916 554.551745559065
47.8075998240396 198.15553991394 101.898612603107 55.5578334877425
49.9951674107458 55.0016608333828 49.7363778831003 80.0919469092368
ITGAV 450.008356194664 252.206029058968 276.699609951994 299.136348099827
229.188779190649 236.178265404264 95.5117928427637 177.379924616924
192.291576499908 210.50519205965 244.226756210656 349.767857026458
ITGAX 90.407200101342 51.9819543312733 53.5698357689852 391.658764737446
47.4548471240051 50.4022543872993 64.5281945586501 58.2491793807176
45.2897806651929 41.5838486090904 41.8613406887824 39.7763627041882
ITGB1 995.397944432196 626.749986744506 698.105755081829 640.944514118411
557.094756796515 836.472724085903 427.540236966999 472.522115719366
643.491217668415 597.191426276771 515.216890329893 835.171475474151
ITGB1BP1 228.882072078733 124.280024888206 147.727354881531
132.205423909251 195.790389809892 222.985386635178 231.893992191022
305.279475274196 154.752920877816 149.02612008344 147.010700944686
192.922622120507
ITGB1BP2 40.3478825593224 41.8848959280916 39.0775870687758
40.5484717678957 40.2449343247539 48.7781962164988 43.8206425661746
44.0390019242862 44.8003345643334 45.9004543328976 46.7386599624801
50.3118072462443
ITGB1BP3 39.8662795144616 39.7835513496873 39.3709357615493
42.5193208629123 40.0145811166794 41.2567470068038 44.9094465483072
39.512606833112 40.3316153483289 41.1222709786153 43.9450023643394
41.5961238651523
ITGB2 1263.76838179117 714.990611155096 586.735744054398 4036.89580181979
328.962069934895 757.833254346899 697.57030127315 427.363555286404
91.5370872909881 123.247207157645 132.518188482163 216.45007990575
ITGB3 48.6621527194963 44.3529919625344 43.3085358214154 51.3851157261451
43.5544347011646 46.7800033479223 44.0687697278114 43.0390857698452
40.3316153483289 45.3530683538086 41.9908237694988 59.7999569757275

ITGB3BP 60.1705241170421 91.3941563889513 47.646800205189
55.0604256312495 78.1275793755619 52.5330333302271 75.164719839017
60.5571721986885 124.56803502087 64.6446465846487 49.6706535876636
88.0186381834147
ITGB4 86.7710020879725 214.073500272341 517.216393517181 67.9728793995736
158.769954040462 79.4916415027015 141.131275984303 143.215491185309
384.522115820048 247.843927763772 192.98559983091 260.766438659762
ITGB4BP 1122.44489338159 2689.80835574654 2133.78836857697
815.810781070699 1549.93897368117 832.181997440212 2934.35616793443
1468.75291542154 2551.57660786358 1375.14388134842 1453.77801653969
1086.12171444149
ITGB5 670.271164195182 147.371811804618 154.355197155161 180.288784077653
237.237762964658 284.105466865264 183.336550845401 193.703214707909
215.290701336046 288.508774004052 252.583117397109 336.390195295291
ITGB6 44.3355304829989 53.0383634135393 58.112348043634 42.8235313007746
43.3332476888305 40.650742261428 41.5817737564592 37.7612949798895
45.2897806651929 38.957687788868 42.7275315121163 48.3648494350286
ITGB7 113.866742186841 198.103081445734 71.4473248804357 224.373975368936
53.6907197364586 50.8533737532786 64.4736987797413 152.17335005192
108.42710653018 101.954354107731 105.406282523992 66.295597720304
ITGB8 47.8249696958403 42.3550738181144 34.3633615460307 45.7397504535169
41.467005949568 40.650742261428 41.706879587091 46.5717043290731
44.619512785899 45.0155543555707 41.2598458726002 44.2527566343661
ITGBL1 41.9407041937973 41.3610369241157 44.2405728317107
43.5627649099623 39.0564222526273 39.4953494583071 41.4742337714895
48.87875862356 43.7630587657205 47.7180816682562 47.0110068985119
43.7672102682067
ITIH1 43.9309845324767 28.5895665533366 37.5597671487621 40.4359177791566
34.4570158133896 38.5252456306656 35.6056062164318 33.5578650644246
41.2870505273664 40.8049408896274 43.6629145624937 39.3575468494941
ITIH2 37.6397652536077 120.418406532204 40.4882741869718 32.1218888714537
36.6354013629791 38.3418933808844 40.7910160811527 35.5798430594271
38.5252456306656 34.4875013487777 40.0766543123257 36.064718696392
ITIH3 32.9174234020987 35.0222940937513 33.281531405287 33.6816578730693
37.1565984182084 34.7983399096627 35.1307934330047 37.5511560880902
39.9444191873552 32.9981302187884 37.6484782516569 35.1451084093692
ITIH4 40.7609952437622 50.3586212130973 41.0356368374708 41.7614975630362
40.6723334103791 43.9724628401189 39.6535880152834 49.7309743973184
40.6957787921451 71.2963721541702 58.133863696399 53.3059058105836
ITIH5 2878.82618761804 1850.06063216244 2196.59044510709 2160.22713775462
1925.20977162704 1221.31634960736 2071.90741263566 2892.02001412369
1557.42399344934 2854.82112538867 2559.659041589 2243.24648907474
ITIH5L 44.3355304829989 39.0102066700839 44.3344900481112
40.0698728584484 44.7829632472754 39.3841861812262 34.6720534245631
45.7476095696859 42.1594905198929 39.5637454855233 37.5583886316038
39.9519547348106
ITK 71.6849360818041 96.6359641162026 132.985150338484 185.54813083393
55.1563703466328 56.4819802857598 55.695361644141 80.1144023887293
46.581796165231 54.2830238965339 74.7669841575348 52.2832912331043
ITLN1 54.2648201582657 49.3581578587582 41.3366779545815 60.9379216295576
44.147557390293 67.8208976798364 277.796753761378 75.4935574755043
45.7452456977857 81.0880833950735 184.105396912056 59.987045425047

ITLN2 43.6706764143908 46.6314009405034 34.2890785915814 43.3078542962968
35.9058393060637 43.0860383229288 42.7431359850339 41.6466169548846
45.5779686360641 47.611018474151 45.4799270968969 37.4554876386402
ITM2A 73.9986015964321 94.7008981904016 77.5718089378881 260.185079572615
46.572043843509 875.139073253298 70.6592421895338 110.746059249478
52.6561576154021 538.716947884528 347.003621791919 263.636675573982
ITM2B 1052.91661429671 2721.97551918611 3124.84336032646 2122.39240932934
1055.69506483576 1991.4199819544 1855.0119962662 603.737787922337
2004.94135236716 1195.24078948059 1630.25992078321 1855.0119962662
ITM2C 1335.4111951034 1921.48678333837 1009.47177197543 578.311131274653
1470.84169670955 563.927521628693 2323.81110653289 915.102542160713
2488.32876359853 1845.34855798442 1891.37245670852 1907.47560253019
ITPA 166.821616593685 174.469283513638 163.995560004001 112.039388952208
233.326886009799 101.020328028769 193.897678586782 134.908982962131
101.46584765758 118.483499958227 102.750783393253 95.3145142003966
ITPK1 148.925503526873 65.6010220593397 82.2766822827861 154.287396750021
79.1039287828151 128.414712393554 135.012695962236 63.4570909617664
102.559678338485 158.478919789334 185.109323245018 128.559660070271
ITPKA 81.7117547901526 49.4067286971348 53.7792717770978 72.2577390063259
44.8897737954573 57.0775902639215 65.8986841086198 58.676230030835
63.3694113050568 48.3693338695392 53.4921736109774 50.2163892855092
ITPKB 121.104663867264 107.513442725922 140.719373122712 129.592927998658
92.3034609409756 392.237227927376 94.8766888888828 115.328304312071
89.3342384381684 311.985010273921 281.906398109158 136.355036262681
ITPKC 60.2547250212462 57.9509155144056 74.2769881586739 62.3936376596431
68.0414563597154 153.626578526803 69.1414574152997 90.2915974511786
100.476750028832 73.6240672213673 77.7714622826402 67.7662831916293
ITPR1 89.7554896946341 75.8280474316969 72.5835488194631 328.721532812392
57.5685357492202 450.759762047652 121.945468001236 75.2101993995344
116.388069891158 169.18132209076 160.056757080676 241.706783147271
ITPR2 53.3967242431461 51.0175677516029 50.9933795450314 96.7799695976296
50.0580547772867 56.2736599756843 59.5205055437765 48.0582855095143
63.955655956484 58.9836017745712 54.2999306485758 53.1182952086284
ITPR3 1408.59798540643 1384.80615431656 805.590413262364 439.241483765416
752.837247653926 323.576613035353 1249.41227924272 500.241231327789
637.565156571985 484.968116511768 446.37035118289 615.227000137328
ITPRIPL1 40.0270326792985 40.3021896270845 38.6727842796202
42.8768096578102 41.5668884168669 40.3034574135048 40.7910160811527
40.047314525032 38.8271346375425 41.4606714407332 39.9899430628542
42.7303139017786
ITPRIPL2 211.421222959783 607.431621448599 750.151825182993
385.439417884202 245.194730981772 283.111920665176 190.053043382767
261.326713935356 206.571946834892 250.993396750789 361.023743912857
399.66182195331
ITSN1 87.1183334887694 85.1950626054282 111.409762290528 91.2715671868725
90.775821431366 217.065944053174 113.198141130994 93.1668080716761
114.535461066963 136.736085245679 128.04037427867 133.040933717958
ITSN2 40.8681871019812 44.5152708955623 38.6005272730664 40.7481155008596
36.2048942610546 39.6251842352088 40.6216891610774 39.9576138799997
41.7233690251839 40.6559419616644 38.1835938174625 42.934549734195
IVD 139.999709303614 138.314834472957 145.250323683031 125.333208461509
338.000998124097 132.059215102854 130.506891299903 135.706144105357
124.064628168507 150.572774995368 206.41879845205 147.388500451427

IVL 38.957687788868 40.716553247236 41.0356368374708 35.49696651597
40.3452320167286 34.9849896310235 33.4316681623175 38.079222289614
44.8997235283254 42.5664883002833 46.8954576545929 42.4607795638552
IVNS1ABP 143.383133346874 124.738024302128 163.16724817696
699.488969175268 117.165519915808 115.321592104747 197.703414428714
111.522812019895 141.594428857706 103.607345835114 103.826044798469
159.393207489822
IWS1 195.482216656634 173.236617176892 193.758357282411 156.69976952483
229.512022912803 193.395549316561 147.758917745955 201.977265056102
218.675464231277 149.173801572239 166.097017297742 244.109166881519
IYD 39.5676051939342 39.7835513496873 40.931294690669 43.5627649099623
37.4470183315826 43.6670755115848 44.4639405506967 44.2159020605784
40.8970401536578 36.5806287103157 43.5710871617107 41.4508241771315
IZUMO1 38.1169703906207 39.2907645923217 38.9790184198145
46.1673159982664 38.2496050953547 41.554942319225 46.8613212048981
37.0390589468421 40.6957787921451 43.1839109702714 40.4504608195666
38.745552403895
JAG1 131.483899499125 183.002191434723 262.824698774297 183.918927595056
159.697488404476 157.568088397305 204.659954879641 116.967446594767
116.461730749198 378.12777235743 362.615047009897 264.924262411772
JAG2 119.947688643528 116.094881058504 365.401746744015 92.3787087536027
139.905295316081 66.7585874672879 51.5119005564951 71.4564394972198
156.497017347274 147.782301091454 136.552025379187 97.5534379346899
JAK1 163.62024333166 206.450109223116 255.365088438518 320.889609846069
140.853812988562 220.36831243882 134.796819654047 104.089998273812
186.522518256229 76.1382217104599 114.5696064637 219.805214090875
JAK2 51.0225132036905 59.9682734755814 67.5417980425932 96.7799695976296
54.1225249245036 81.8426140611297 51.5967098638347 70.2541961995753
54.113533179847 65.813547096222 73.6362523983338 66.4626619269287
JAK3 42.442231667357 40.4359177791566 46.6588655218735 40.0698728584484
39.4693871570028 40.1428591998404 36.5352572191451 32.4466419672036
38.9834959368496 36.0481934423077 40.2649309204478 40.0557788909923
JAKMIP1 37.7384958256497 40.5400384479766 35.3649449469275
38.5252456306656 36.6354013629791 38.2293008890613 34.806104801006
33.0038344711883 42.357910063876 33.2987269789328 33.8558232866797
39.5418280570561
JAKMIP2 46.0023068797812 42.3550738181144 44.2405728317107
69.5544840164738 40.0599807618105 45.6129956328415 41.4742337714895
43.9366081640576 40.0403898620771 41.7405682794166 48.4717951775049
44.0451487108213
JAM2 77.4999637278723 66.3026572113793 72.5835488194631 119.670948133232
50.2163892855092 245.923368125413 40.4359177791566 62.1934012861178
46.1101825640108 149.719453306304 107.538934056558 147.471179343348
JAM3 521.710190052245 177.29096218424 165.992057520727 253.605641311356
145.77302334589 1466.88103496067 71.3527183280827 239.96421556928
66.3209349903185 898.668526777346 795.381998233245 598.404003732481
JARID1A 141.471672990444 134.258351436533 153.028210322286
236.536058101486 123.768417863139 130.506891299903 216.45007990575
219.134220280612 206.899594905509 175.876518898179 169.75692324104
126.753713093485
JARID1B 713.685405035516 382.251727747377 758.462554576949
232.944023917808 628.84789420227 213.799316520531 399.027079798213
470.004780385708 668.584452504579 552.162906190653 422.931891494195
413.067407579649

JARID1C 54.3554233228353 60.2547250212462 48.8941062980393
43.8002456102021 53.6907197364586 47.3791873993182 53.3111230705754
61.5000955267432 70.178974380507 52.5522545249206 53.0798381965427
56.7711602236706
JARID1D 73.8374823243007 206.103296228683 35.8265993915844
158.862722884436 57.5685357492202 132.84476910529 125.891602778425
143.215491185309 101.2825552085 177.55994726993 168.326515533208
160.566474370004
JARID2 211.109957714241 138.738440901853 139.543015878846
201.959257379662 308.568617575909 119.558756877523 196.844428284247
526.146757036152 217.617227445082 124.017319373302 138.859992681388
117.260454792593
JAZF1 68.3234331335073 52.1062000766427 52.3432130135266 83.2925226467532
56.359578588344 140.921774805792 46.0228732507872 64.120219344545
50.9010149857308 112.551747307723 89.8354888041611 100.188736523729
JDP2 38.957687788868 39.0102066700839 45.7968648044613 56.9299697426319
41.1868052124344 51.1621379498014 39.2533668167483 58.0892402609917
47.0804681460898 43.401782064778 42.170702295259 44.8372404608604
JMJD1A 77.4999637278723 66.8026147385682 57.2960245538881
72.6824533717231 50.6470461863549 50.3245489035833 65.8986841086198
50.857522488428 123.674164545425 93.2226507128199 68.7091708087448
78.8968326770219
JMJD1B 120.837993273922 109.386655134289 124.397048303668
99.2432911346944 73.0433648756396 121.120217132666 99.1769280484821
88.715256326632 140.441315708048 150.829277770546 132.3192754634
113.7763650775
JMJD1C 176.138603484209 101.759714275976 95.5530572805104
195.108184885541 93.8630393470857 158.532307373857 106.582060920252
109.374838860558 155.650716735976 109.795084617739 106.035435807364
138.859992681388
JMJD2A 78.7450149191851 59.8102324370568 57.50915811428
41.7614975630362 55.4919197421746 47.3791873993182 55.4004611309636
55.1933004802607 68.4229528414965 72.5153335566211 73.5788549535062
49.0655215909632
JMJD2B 41.8409927585705 38.6704566269103 41.3708420203838
37.7469621225553 40.1978874031072 38.5252456306656 38.237657348649
44.5029690493211 43.4656637536244 43.4544762830742 40.5824352835642
35.8108713625055
JMJD2C 46.8141313516997 53.565717255469 49.8344973836834
41.7614975630362 42.4864576619427 52.7156971154405 55.2088411137193
53.5271601314404 51.5888395924843 49.8393069675192 54.5711704180349
59.4465684293847
JMJD2D 38.385865183581 44.7829632472754 38.8775560911841
42.2496337895968 41.1868052124344 35.7034203372528 37.1643693306382
43.9366081640576 40.0403898620771 37.6383530120621 39.9899430628542
37.2810767789306
JMJD3 49.085741080241 77.1737254584648 53.8724557170772 110.666686474986
47.8075998240396 52.5330333302271 62.801431502597 77.2834482217973
54.113533179847 54.8979897528792 47.4630625313159 47.8931773060789
JMJD4 54.7786516309154 78.5174639327946 70.4794854241035 55.4180794172794
73.4535007027931 61.4944752562671 121.441763452598 90.1430468048409
84.3526980051279 79.8511759086625 71.9658486648999 57.5372732453319

JMJD5	43.0001432913058	44.1239711172417	48.7039705048129	41.5464765641928
	43.004943518025	43.5752393599169	43.5627649099623	48.6441988290952
	44.5303781239209	47.8207974930031	46.824299585607	45.6692241948108
JMJD6	53.4921736109774	53.9275079525238	51.9490810272761	83.764882413379
	57.9161791762225	65.5226841723958	74.466989900636	54.2301964914419
	53.6210959113222	56.4553747079294	59.9682734755814	61.3851695159988
JMY	48.8333989709933	59.4908919662362	53.9612654850046	71.9062462001972
	52.2089306840125	56.2736599756843	51.7714254834195	65.0656365694468
	71.1894129030602	71.2409890549638	63.7963501146829	56.9379721393904
JOSD1	101.103458852775	112.316483867372	88.6771566133367	243.015896514493
	103.720371491639	107.148000219752	141.630277976624	145.972133104142
	119.167027972122	108.788705797019	89.4747568145717	136.20629436945
JOSD2	85.1950626054282	85.8945498067934	71.4473248804357	101.560929859785
	99.8699868526053	120.392561825049	81.0331497830643	114.719721626067
	106.165518810891	116.67447696188	125.786923817078	85.3431175579775
JOSD3	41.8409927585705	46.4385386401326	42.4580504587581	48.856176220627
	44.6834648302383	46.3715909653832	44.9863726205428	45.1714698794331
	42.5406888281114	42.8936990772838	42.357910063876	51.0013344286851
JPH1	48.6621527194963	47.2055133646385	78.7103847663605	45.5869891570251
	67.0163226819964	61.1022920882065	72.4651758215622	61.5824518349507
	108.098254669378	113.090530993447	87.129027106167	136.883475451494
JPH2	49.4742925789913	43.4681646416585	44.0028192158386	40.3180310664156
	47.973869237177	152.294357984689	44.9272279146193	47.3226997815962
	43.4290452504928	89.7299583757595	88.4897895318663	104.704892803169
JPH3	154.067791946816	45.4882438975269	46.2625470410195	47.3574589211166
	63.3638417360105	59.9174456081704	48.1747307200218	48.0582855095143
	45.0017158586743	44.571915452417	42.0750889188255	44.0451487108213
JPH4	35.2267149016596	43.2649781354792	44.5134109184888	36.0481934423077
	42.7107928951766	41.554942319225	45.1017494688414	36.2539950294089
	35.9645119596869	49.5265949137207	43.0971787292711	45.0985088502363
JRK	45.4507786740825	43.5873188565519	43.0016969050339	40.1804147506786
	43.3896803974186	44.8872784170036	42.5660695551615	44.0390019242862
	43.1180113655693	41.4659679379482	45.2685999173781	44.4067063477243
JRKL	53.1309801459151	53.3094843669564	58.2251348002272	48.1662933382789
	61.4366256816323	60.4065050472082	53.4921736109774	55.3122077514658
	54.4912806027381	53.0357938671646	52.022479439889	58.2251348002272
JSRP1	45.3872406432794	86.6411972262415	67.3469281984384	450.972039573066
	70.6903168772326	47.9503724044453	102.77259665192	121.172652675767
	62.9922928277769	45.3530683538086	44.2925355984056	41.0657892043496
JTB	3010.38998829232	3869.93390477952	3343.07293372655	2513.15722262176
	3653.59181300011	2248.64628069362	3465.3013599062	4093.70002166179
	2611.32405374107	1683.24873205783	1480.95754707554	1676.22158135012
JTV1	321.171736253617	345.529757315326	317.0659599072	300.703401968169
	561.2769907009	310.120875196999	443.269396125834	344.887504955742
	429.453114391562	199.294913752704	277.204818876581	277.12373983597
JUB	36.9721666683086	45.2134010996333	42.7303139017786	40.9022255200169
	44.4954892619459	41.1194494565538	40.7451277736087	37.7315666632629
	42.992884918158	43.6701228397031	46.5650556481721	42.0424641526302
JUN	1501.67042548971	2354.00192115549	1208.33498233518	832.181997440212
	260.964562714558	3800.19142219719	2160.22713775462	1679.12630849964
	752.837247653926	1601.52194699063	3166.48568397766	1357.41745370754
JUNB	510.82619253142	2039.89728774097	1197.13566108661	1418.94307844471
	625.435083064752	2663.19221471348	760.745188431719	799.803676957263
	608.886783362316	1096.1705171588	1311.88650141004	460.04552567416

JUND 4717.02689540013 7075.82842238887 4216.69417265779 4277.93019582362
3562.96193568469 7393.77508374057 5354.58328030315 7562.42822724655
3490.63949257581 4986.44888767011 6819.74871209328 4717.02689540013
JUP 479.696469083953 741.908952472992 853.947671456736 103.808640431626
901.793120584383 206.103296228683 995.397944432196 764.571579067138
1197.13566108661 964.268294825556 694.590958116425 328.176884082116
KAAG1 40.2543341073001 38.0226905622357 38.6727842796202 46.614092935749
46.8674366115656 43.2805590023412 43.3262148375525 40.1474543172332
35.7973685350741 35.2880714952489 42.5426057453993 37.8490162439785
KAL1 66.0303950772736 43.5873188565519 45.8844325913247 57.2856683017619
40.8873907150245 53.2046867327331 39.5496902678788 44.797320243931
39.1790517315259 44.2580151149956 38.3684202018524 42.2577689927563
KALRN 40.7063426028871 42.3138951003676 39.3344387353654 42.1607322031054
38.8775560911841 43.2219358930127 42.442231667357 41.599960574499
39.1790517315259 44.8997235283254 40.9157352260901 38.6948848106802
KANK1 78.1313732870053 246.568253751149 71.6050174762551 77.7973642829228
114.935101645207 279.448591201269 152.47776741936 119.545018066492
88.0877175295544 301.457545514208 241.31366702947 226.023657887826
KANK2 147.498355916596 108.734392572981 134.271727318694 104.8505815954
92.7836745985234 728.023307553872 65.8986841086198 75.2101993995344
85.7111041637416 282.445037659981 315.276749671783 241.706783147271
KARS 232.019748665806 248.194954741095 294.048088688381 228.933483421328
220.79051545138 242.135519563833 254.983975710833 271.529471399941
235.101442092497 153.812414753676 188.042978085327 196.760671234837
KAT2A 333.566613571224 429.576301511638 583.421166842011 160.777879976503
1307.19925493293 235.33716196909 325.769076169162 353.076989769287
331.935072199058 697.837976820516 450.865888317246 457.502917254598
KAT5 42.442231667357 51.3084273211359 52.2132783757422 47.6687908290818
45.3675390992851 53.3185097662527 52.2476631126873 48.9826868640753
51.1055541075029 52.7972156492746 59.1443775199516 53.655233033193
KATNA1 175.125791527244 166.535245140901 176.27162536342
137.176719695561 163.809567262808 208.30969642805 173.343091136741
147.557451530784 126.826715174564 144.17470770145 126.204310123186
149.24022100428
KATNAL1 50.0466349801009 39.2623423164488 46.3057385979341
47.7462490950965 41.8806343997257 77.4578596822431 47.8179475230844
46.8339790776308 40.6533975461148 59.6565330440648 56.3884556031093
60.2989690950183
KATNAL2 38.957687788868 44.3187284857534 44.6511073781104
38.1421709520616 47.3791873993182 46.852437033077 47.5130990528612
37.86982638952 50.5368663784059 47.7800121097582 47.481302504066
47.1060577218993
KATNB1 121.449647909665 162.628603963754 207.513604688444
107.111886993156 124.791747507205 92.8131698915896 203.228211195516
87.6435494489518 137.916048669047 96.8240908274121 92.4619796591359
88.4819048592858
KAZALD1 54.7786516309154 54.0052948715814 37.8386378737561
43.3078542962968 41.6806984441849 140.063595285844 39.0458123429605
41.4508241771315 46.6944238390932 80.3915591207869 66.5539062525344
43.6753843488695
KBTBD10 40.3478825593224 44.9370339275041 42.2837084302963
45.7397504535169 38.2083710281545 156.008696165877 39.3585350448719
40.7311497914871 36.4816715148518 72.5918913856713 64.9401081242429
57.2008272301399

KBTBD11 70.7238101239421 66.4300949831964 60.0856496605043
218.056839407813 76.209239524646 120.123385036416 53.0277416531043
101.681822254799 46.4164127487052 317.406465928295 214.562221385406
169.18132209076
KBTBD2 291.086512261106 247.074756460224 211.019451518444
302.20349084931 175.529493306927 224.412339736769 190.592556065939
222.368324930003 264.071486075874 266.178061771978 262.215263489321
275.882262550383
KBTBD3 46.441301934328 46.1797803908218 50.656598108034
47.3574589211166 47.7508139534968 49.0361180089431 57.1070449050465
43.3838505886856 50.4974478884077 56.8201201798712 52.7466456080101
63.5896512396512
KBTBD4 56.7635847444447 69.2082505043031 79.2250098540901
68.4906691348625 68.2585858486846 69.5080454202415 66.1940959364976
57.7805922926828 77.6174893940285 70.4092514463186 62.1796792097014
66.8703540124724
KBTBD5 31.197411437683 39.7835513496873 47.4740726032707
36.6501141438378 37.956732373465 42.157627240486 40.6750540874299
44.1311654985682 38.2215591930186 40.1179210534118 41.7272875295469
38.4330755397458
KBTBD6 43.3638918705225 41.7614975630362 39.8299340037009
38.8495095797881 41.4397539024976 47.4699236780425 40.968974769473
39.4322247076926 44.1211971585961 41.7932258301997 42.8263096141654
43.9282059368202
KBTBD7 49.540449125788 51.1541843174981 47.4740726032707
52.5307002738469 44.6834648302383 47.0504142893738 51.3702158075274
48.0283573444179 61.7559532093027 60.1466326805616 54.2625553617642
61.340259048151
KBTBD8 53.8856501960117 52.683711208052 48.0191002461099
130.673639258681 51.5888395924843 54.8265655909623 55.2088411137193
66.6320017520888 56.0496524902806 66.3956936248447 84.1112814779578
52.1918130336397
KBTBD9 39.512606833112 68.4278345554565 82.3344892042646
44.7401523707775 51.0107745412152 51.294537308199 42.357910063876
41.7877325563821 57.7865070833351 68.4659783447092 65.2295773603116
73.4535007027931
KCMF1 50.8388984085636 55.5937992407324 52.4146320099098 48.3823941067574
46.146585045771 60.8161720561234 52.6654109976514 45.1714698794331
55.4698797365479 48.1392888470269 50.6109448604627 54.155542621974
KCNA1 40.096207471219 36.9398167613724 40.5734458834617 39.2780515621926
41.3755760434934 41.4508241771315 39.0788811357542 40.931294690669
41.074824727477 42.8936990772838 41.7272875295469 40.8681871019812
KCNA10 39.4710743516517 37.5469247328848 36.9795961564828
41.1886975241176 39.1650142708257 40.5281496800812 38.1793598983615
41.3506832276112 36.2733735494625 35.6208308220005 34.5633623842083
40.4639532172338
KCNA2 40.6559419616644 36.89524015739 35.4484652445078 33.8111782249716
38.1644569189523 44.8872784170036 36.9355798143571 36.8400213077707
41.074824727477 40.2112371512838 37.9946685306274 36.1647506311964
KCNA3 40.2543341073001 43.3458769998118 37.5597671487621 39.6292788039793
39.0564222526273 38.8336427948187 40.3302268293707 34.6977013306396
34.0382693188794 43.9450023643394 37.2892641289258 39.1553169409075

KCNA4 39.3709357615493 39.5050383901884 39.3709357615493 44.265834798964
39.859345745831 41.3553369544097 37.1643693306382 34.806104801006
42.357910063876 45.4614234442527 42.170702295259 43.6753843488695
KCNA5 38.0089018744739 51.119812302881 43.8495248991589 47.6687908290818
36.9060997041453 95.5022104475922 32.8745892439217 40.7311497914871
36.3649677956953 51.0055825931636 64.5722284870633 32.7043743065388
KCNA6 36.8072833828297 40.9731067463382 37.7384958256497 41.1886975241176
38.0598374201407 30.7058829591553 34.4570158133896 44.293922970134
35.6970528713709 38.4187686307607 37.9230440150891 38.8495095797881
KCNA7 34.1336988442837 37.8217989841918 40.4882741869718 37.9875302985336
43.004943518025 43.0860383229288 38.5066108855005 41.0381860307445
40.5912213523345 37.5343260962758 39.6403089864678 43.3744503319109
KCNAB1 39.0237504776589 43.8140132746242 40.6737160001291
39.5522630869979 40.6251465440216 53.565717255469 41.1886975241176
42.2577689927563 42.0621425894624 44.6367584399805 48.3847843498476
42.297291190584
KCNAB2 37.8877750141557 44.9863726205428 43.3085358214154
49.9014504704021 43.683792219729 40.650742261428 39.700936357233
42.5435283762933 41.9097689475185 43.2438688757519 43.9802831041664
37.8490162439785
KCNAB3 41.0608773166058 43.7644428186265 51.0539624358342
48.856176220627 44.0451487108213 39.4953494583071 58.6431245059655
42.7431359850339 47.798305839406 47.611018474151 46.7386599624801
46.4499258373233
KCNB1 38.0089018744739 38.7344853699576 38.3166554965434 52.8502823653368
43.1252272136667 59.7009992986607 41.5817737564592 40.931294690669
36.2733735494625 53.3059058105836 66.737740728688 49.0655215909632
KCNB2 41.4508241771315 41.4508241771315 38.6727842796202 42.1273928203021
43.004943518025 36.8059561571675 46.3057385979341 41.9315419791594
35.9645119596869 37.6383530120621 43.9450023643394 40.5703581862752
KCNC1 38.7537364570873 42.2577689927563 41.0356368374708 36.5393857306744
40.3452320167286 35.4142595025086 44.7255207589606 41.1575777497576
40.7847110517594 38.3092241935352 38.2795150354677 38.6454098044474
KCNC2 42.6561897411324 40.6380495994768 38.2280071951674 41.5937402943136
42.6022652963326 40.7616966228404 44.1350486752012 41.0381860307445
39.1790517315259 44.9603714724456 42.4238335236362 41.0183489151839
KCNC3 37.1485601150873 39.6995529770239 39.6451483836089 38.3166554965434
40.5710637884029 37.3799542961839 41.1507980856436 45.1714698794331
42.2577689927563 36.3612409063792 39.5399188536544 40.2669497398702
KCNC4 39.0237504776589 38.5728560353959 42.3536549114165 39.7840818430159
40.6380495994768 39.1664256481434 42.9214252283214 40.9784333688086
41.3803269429468 42.1317134987795 37.7963032976457 39.9519547348106
KCND1 50.2203578628217 45.0905928998779 47.9035532886869 44.265834798964
35.9058393060637 46.4648868771662 42.9803717121865 43.1567024035183
48.2123120250343 41.0057887014368 45.2265319826548 44.9070250604284
KCND2 62.0553450433433 41.159706131351 45.4865173433646 43.1847628331565
46.9906120932727 45.2852528185486 43.8206425661746 46.1673159982664
48.0163377492228 45.3530683538086 38.3684202018524 41.7575097298927
KCND3 45.039075581593 44.6402215707065 45.7407938943718 42.2674195935107
47.3172462673426 41.9946838598688 42.9176765336717 42.0778399888414
41.863927676552 39.8411395375591 41.9083581529276 42.2577689927563
KCNE1 38.1169703906207 35.6434124419782 34.3633615460307 38.4208429103799
41.9842695818285 34.8956628705329 37.2892641289258 29.9617615620508
35.5220586859301 37.3179578037963 39.153226699662 33.5578650644246

KCNE1L 45.2875173643252 47.087835967892 32.6121532029976
52.6947911480086 39.9697332660833 46.0364000143537 50.6774883370457
39.2672712041774 43.9450023643394 37.5343260962758 49.9338905397767
39.0536056496869
KCNE2 47.7267190613906 40.148780650831 39.7411455670172 47.8164193292842
42.1650127805175 37.4782441311626 42.357910063876 41.8417024632349
39.5806465938526 42.0621425894624 39.6403089864678 38.0452862258803
KCNE3 39.5676051939342 41.9894412416874 39.9101908547397 39.3750969348308
36.2048942610546 41.9543948167375 43.6753843488695 40.7311497914871
43.8482802038826 46.1390887492649 42.6366765186182 36.064718696392
KCNE4 51.0225132036905 41.8848959280916 40.4070046183108 39.6292788039793
48.6224651100733 66.2167164788023 37.0564573649223 40.1474543172332
39.9444191873552 41.4397539024976 41.8168470823451 44.3521099857368
KCNF1 136.355036262681 34.7135536792489 47.0055981216687 34.2080003860523
61.7566103867825 41.7627910693483 97.5962784863854 114.230358276599
41.6616444147804 91.8251275769679 95.0604882262201 40.8681871019812
KCNG1 55.9496022482843 53.7820518773759 50.1860191642359 44.604338271769
154.707548614663 62.4808812951288 315.960715273629 161.870972980732
66.2134832989335 59.2628513357357 53.7848321213704 51.3709954257843
KCNG2 44.1737502492846 45.7317208802318 47.7052132264218 48.9155368657318
47.27543600687 47.6265906358519 57.3199610379543 50.857522488428
44.3440839686558 45.0864985243943 45.7792335738285 46.7591817286632
KCNG3 38.4330755397458 42.466333667839 43.7085219318425 42.1622837416589
42.3685136297603 38.2293008890613 42.2577689927563 43.6434576713809
40.4997186069494 38.151911051187 40.0766543123257 37.7526485343328
KCNG4 39.8662795144616 33.939263369489 32.1218888714537 36.2117792304273
38.745552403895 34.5592213228896 32.8745892439217 34.4590891021041
36.1457789072601 34.3674468322242 33.2774014136617 35.0222940937513
KCNH1 39.4710743516517 38.0226905622357 41.6977706798234 47.481302504066
40.1151953732637 40.4753617660107 41.706879587091 36.2015763253919
38.5655198999985 41.3641352453779 40.1362682639916 39.0916553915607
KCNH2 46.441301934328 41.9390340055841 40.7977328778231 45.505885458784
43.7858612339862 50.6360662655392 40.8546470680011 42.9845402973781
44.4672195495228 54.3554233228353 50.8248871185694 48.1627097465883
KCNH3 46.8141313516997 44.9370339275041 40.4882741869718 47.2009420656854
42.4864576619427 46.3715909653832 48.0283573444179 41.3506832276112
41.1742244762318 44.4652605017595 49.0361180089431 38.6454098044474
KCNH4 42.1537476443423 44.7829632472754 73.6323065529916 44.265834798964
47.0954207417646 42.750861693377 45.1017494688414 42.8482181313226
54.2825877218965 172.416351632442 93.1564463081702 56.8534370691413
KCNH5 42.1679276329916 41.2214779019897 40.931294690669 43.0016969050339
37.8679241954337 40.1428591998404 38.977631784251 42.357910063876
45.0017158586743 36.4351122729314 41.6657053704762 40.8681871019812
KCNH6 1359.9708539999 949.923102632043 860.265067346622 915.936211119242
694.590958116425 577.276184555955 1180.7204565402 1749.06320703181
714.990611155096 1004.28134410536 1698.18614771363 1094.91235708087
KCNH7 36.3207931130288 34.8956628705329 38.6005272730664 39.6292788039793
37.1565984182084 39.132725377815 39.4069390012996 40.7752992161136
39.4512099697623 38.8507804383261 37.8766103688718 39.5806465938526
KCNH8 48.3814421939888 48.9899488302637 44.1452943804044 49.1786207920603
47.4548471240051 42.6575535390785 50.8388984085636 46.0633179231318
42.992884918158 40.7063426028871 44.3763859390287 45.7562276914441
KCNIP1 39.3709357615493 39.7453420559776 39.1799070031819
37.4699641111076 41.1568680200968 45.3882138838137 40.0668875818227

43.2855454938964 41.2870505273664 41.8613406887824 35.195364621675
37.1747156131306
KCNIP2 35.9305494063137 40.2493681589104 38.0057289062111
37.5764485234171 39.859345745831 37.1894304543751 40.6750540874299
41.8417024632349 41.6340461942423 41.6373771565199 37.8372168909583
38.0452862258803
KCNIP3 48.9906383294199 49.7283221493206 54.4384603094793
42.0102671859606 44.7829632472754 62.1334409150387 43.4544762830742
46.9986083378817 46.4623302792651 51.909968572392 61.4467162706417
52.2216613535546
KCNIP4 39.5068753428772 38.7537364570873 38.198620622679
40.3180310664156 38.745552403895 46.0867976914262 40.1613888954735
39.4083414165222 38.5583217392131 39.8455060654531 42.04117016009
41.2870505273664
KCNJ1 41.0136069709699 40.5400384479766 43.1931731665131 40.3589492801501
43.0669898157628 42.2857372794461 41.4742337714895 41.3094485574845
39.5424643148065 41.800198520267 40.0766543123257 36.6176478999851
KCNJ10 46.0023068797812 43.1805016690297 42.0621425894624
50.5310598656998 38.4540849632254 45.7300501624017 41.706879587091
42.5435283762933 41.2870505273664 40.5090849993014 42.7275315121163
45.0985088502363
KCNJ11 36.8072833828297 40.716553247236 44.7829632472754
43.4410741327254 44.2607789091176 43.7630587657205 38.5066108855005
43.9366081640576 45.5779686360641 36.2435874904689 41.2598458726002
35.3525469364579
KCNJ12 40.4639532172338 36.6968906669565 35.4484652445078
43.3078542962968 36.08956004634 41.4508241771315 46.9929836306816
34.2814697823693 36.9060997041453 36.2435874904689 42.170702295259
38.4330755397458
KCNJ13 54.3554233228353 42.6939394537076 40.1985790927248
44.9370339275041 39.859345745831 39.2985791324641 42.4939802311136
40.6171774724171 43.0930293831497 41.1222709786153 44.2142581488029
40.9683275553869
KCNJ14 49.0434455879335 42.8978721487095 43.8067278754098
38.3166554965434 39.6535880152834 45.1296099011771 42.4939802311136
46.852437033077 45.4799270968969 49.8393069675192 46.0095710163378
43.423558862426
KCNJ15 47.1277338930448 59.9968592852122 94.7980740999988
102.268579941461 70.2354761751695 42.4058515827212 65.8986841086198
121.172652675767 61.0429374109454 58.5530199658661 56.3884556031093
80.6844261396045
KCNJ16 53.2398469202731 46.3635803515159 47.9035532886869
53.9517841438975 49.6507190068324 48.4758590019384 57.1070449050465
46.2779564481158 54.155542621974 46.3810615657263 44.9081227425497
45.7206695113999
KCNJ2 77.9057333780847 58.174555206394 75.561262816077 200.401133392259
73.4535007027931 65.3096524773304 44.0687697278114 49.9332280449549
42.2577689927563 43.2988017098387 41.6336636285875 40.2669497398702
KCNJ3 44.436995101921 38.1312950900467 39.5450079747895 39.9697332660833
39.1650142708257 39.2985791324641 39.8695929627783 40.7311497914871
42.357910063876 40.7063426028871 40.9157352260901 44.2527566343661
KCNJ4 42.668311948721 39.7835513496873 39.4710743516517 39.0266821712099
40.1846227436522 40.8125871172472 41.0829574574765 39.0102066700839
39.7210115145672 41.3752447964111 38.7376588373126 40.2669497398702

KCNJ5 47.8249696958403 38.6704566269103 43.6039443285137 56.3471340054074
44.7210190486327 51.2318935933116 47.0874209746031 43.0930293831497
48.7358147030082 42.2614524730856 45.2265319826548 45.6233434183356
KCNJ6 40.8681871019812 38.0226905622357 34.806104801006 45.7397504535169
48.3814421939888 36.8059561571675 37.6036146841933 44.0390019242862
42.992884918158 38.528621456378 45.390192977585 39.4490233720848
KCNJ8 139.712472369259 64.3312319756553 48.7039705048129 91.1834104205004
61.7566103867825 139.121494956408 51.3064929551674 62.9922928277769
75.891637602914 96.2385885003071 82.5225075943931 89.3868257841517
KCNJ9 39.5676051939342 39.7835513496873 37.6557714476853 41.6564075217517
45.1137938810047 39.8455060654531 37.830876898596 44.2159020605784
41.9635525160083 40.8970401536578 37.2018803984555 38.4330755397458
KCNK1 113.866742186841 187.689343320587 129.352472514981 49.5424425850549
77.5698542248061 54.5952754712007 78.7182388025668 82.1344514651393
145.865118592916 63.9129423266349 58.0969258817391 76.2533853403813
KCNK10 40.5235471987417 43.6608324511589 41.8441156763056
39.4433784795209 40.9416717019827 40.9506062495231 42.1157692936428
42.5003198766385 43.423558862426 44.6305275729869 42.357910063876
41.7072675309997
KCNK12 129.653830303132 90.7037473522607 40.1985790927248
79.1000897258245 128.230543301378 47.2009420656854 86.2118355769367
85.9474373829081 44.2607789091176 41.6373771565199 35.2339574411711
44.2527566343661
KCNK13 50.6473771879221 42.6022652963326 39.991353369336
46.614092935749 38.4540849632254 36.9060997041453 33.8558232866797
37.6557714476853 36.7267557061938 35.6208308220005 41.8168470823451
38.2350720635382
KCNK15 33.5994525227062 42.3550738181144 41.6977706798234
36.3604951341921 42.269054475455 48.0350549043063 36.8369172900902
39.3709357615493 39.0775870687758 34.5718473203083 48.8744644207821
39.4490233720848
KCNK16 44.7044140979108 43.8495248991589 41.1367478979484
42.1273928203021 41.5668884168669 42.4580504587581 46.4388841354122
43.3262148375525 44.3440839686558 45.9004543328976 36.131485826118
41.2437378488025
KCNK17 44.2350046179299 56.9312835667828 45.1569741609597
38.9534635879195 61.4366256816323 43.4681646416585 42.357910063876
46.1673159982664 39.6609238036815 49.6203214143464 79.2701322339022
50.4847465816184
KCNK18 44.0328992213727 41.4508241771315 41.0356368374708
41.8778873678551 45.1137938810047 39.6251842352088 43.4544762830742
46.7980335276155 36.8127605061228 47.2299678280724 42.6366765186182
41.4508241771315
KCNK2 41.8833988894167 38.2263227947189 40.3397581924196 43.632365631458
40.2861860225841 45.1411314881341 36.2496364442264 39.5893773029164
41.2457941494573 46.7203198047684 44.825830634601 43.0930293831497
KCNK3 60.3381680822012 49.6203214143464 44.1452943804044 58.0218262176065
53.3086645804786 64.5187138759372 57.1070449050465 79.7170181855209
50.5785738734431 60.2877434874622 59.2106652388061 53.840976437122
KCNK4 40.6559419616644 42.2900457798748 38.8356349277867 40.8084239472012
40.4091351714709 40.7952353240579 42.6758568421347 42.3110002461296
43.2163597439147 44.1758318057918 42.7775752471711 44.3927670230328

KCNK5 59.8248482438562 45.1694246393676 44.2405728317107 41.1886975241176
51.3511088648386 45.2852528185486 47.8947595316832 51.3472428383325
43.9450023643394 39.0648733099061 53.8856501960117 44.7401523707775
KCNK6 212.302291274084 78.7103847663605 91.8533850418261 105.686346729469
61.7009619624746 137.802147212644 88.3954618645976 129.550925624841
72.3515874601288 88.7461361855771 102.304422999511 108.937199348319
KCNK7 45.7700386532539 44.2105195734073 44.8872784170036 57.4595416719581
43.004943518025 44.2607789091176 56.0515443021293 57.610757185978
49.3780606970538 46.8954576545929 44.3440839686558 41.5132248356707
KCNK9 41.1523487516364 40.148780650831 39.8299340037009 41.6564075217517
36.9702228618069 40.650742261428 35.8227939208521 44.7044140979108
37.7384958256497 41.0057887014368 42.357910063876 35.1451084093692
KCNMA1 52.6960621424156 43.4396866157171 44.8872784170036
68.534940191185 44.1070537445005 176.43047680293 44.0263754734997
61.3536703073558 40.4997186069494 135.757061334489 111.501058276821
116.17375251898
KCNMB1 172.506486240017 154.067791946816 153.462055740758
307.705431227338 116.22441634196 1604.80031304031 53.0277416531043
126.360723656793 50.7619345314571 1060.64261592822 970.379875059112
918.083610591772
KCNMB2 42.1679276329916 42.1995942415177 42.640829914102
40.1804147506786 40.5235471987417 45.6706505553383 40.4359177791566
39.8965727231863 41.8245543174822 41.5804111566887 39.9899430628542
41.176305927372
KCNMB3 55.8810837854139 48.275452803061 42.8663534227844
52.0470560622094 57.6234624694326 49.0497778708674 57.2758638871082
65.963892742019 51.631710130416 54.9392038576957 46.824299585607
50.2549436338147
KCNMB4 64.7766649630041 59.7098454699348 43.3085358214154
42.0102671859606 49.5666074434383 65.3096524773304 146.534713287435
44.293922970134 84.7899210293383 53.9412154719864 43.3085358214154
46.8129509799753
KCNN1 39.0620487644635 34.9262464079777 42.8270038153139 43.5627649099623
38.3561682218911 41.159706131351 39.6535880152834 41.6466169548846
43.7630587657205 36.5806287103157 42.5426057453993 47.8931773060789
KCNN2 50.8736372975089 43.7410507935043 48.3847843498476 42.357910063876
43.7783560914188 50.2163892855092 50.0088611244948 40.831207953647
43.8482802038826 52.3935328534772 50.3672856609022 48.3119523820103
KCNN3 46.7704450730477 46.7980335276155 51.4308501933893 45.505885458784
40.831207953647 74.9820339135242 44.8643898647322 44.5658219748173
46.2027193115397 60.6266092582071 59.0850755856409 45.7979032939211
KCNN4 57.50915811428 84.1224653052549 85.3718124658316 79.4528447794527
70.4607501403122 55.1987351219665 139.177720567217 84.607833108004
75.5681913475287 85.0218838801011 113.791456064084 61.6311118308635
KCNQ1 46.4669243193596 50.7774722329703 50.7903824729818 46.1074305700679
55.6069214673486 46.263826584899 52.9352788093583 47.834436078111
53.9612654850046 49.3473947496935 48.2391540335232 52.2381815246326
KCNQ1DN 33.3307770183871 39.1982470626863 36.8730514568925
44.1483976313959 35.9894133432376 38.5252456306656 37.830876898596
38.1835938174625 43.5669306063196 32.2463318211725 31.6063174764616
41.2437378488025
KCNQ2 58.9880711073443 41.7614975630362 43.6670755115848 41.3567204694281
42.960000415838 43.1180113655693 43.4544762830742 42.3207901205146
42.589641010388 44.1976966800856 42.8263096141654 40.6127321267617

KCNQ3 54.931538325692 50.7487159427973 46.1653991089885 44.550322123994
41.6806984441849 47.0027453663546 41.4742337714895 57.2350830635912
53.5677764726461 49.8393069675192 52.2229654768933 49.6629259325705
KCNQ4 39.6392866043607 38.8653023707909 44.014441886238 43.632365631458
39.2608760193011 38.1814775034697 38.1207627353276 37.1859370336728
38.8514156961532 41.1222709786153 40.1742127891824 38.6067491721451
KCNQ5 39.7576136507593 41.5132248356707 43.7630587657205 38.2599935139101
39.6123051184545 38.5707420487455 39.2780515621926 36.1375121409002
39.6413313949444 38.528621456378 42.6366765186182 41.3481355128089
KCNRG 49.540449125788 52.153603364404 47.8263530599296 44.2166118829877
45.0693869004277 37.5887129572848 41.6466169548846 43.2551508830106
46.7414272831181 45.8471859855979 47.299647867533 42.2577689927563
KCNS1 48.1125722136997 63.7721358300879 70.4794854241035 49.5424425850549
72.9154861179393 44.2607789091176 50.0088611244948 110.09868737254
52.4539007817929 121.557478857883 68.1513215633609 55.4635297192076
KCNS2 40.7609952437622 45.0115870036541 42.157627240486 43.8002456102021
46.1804647841331 40.9506062495231 42.9803717121865 42.9301944191913
39.5806465938526 55.2214187655851 37.8372168909583 40.1700117452801
KCNS3 219.582785177134 122.143519601825 116.374125502825 43.4877740913713
71.7263663921367 65.1009848240098 147.187118389181 259.626847216881
67.4775348162369 64.7269156363245 59.2903915056319 77.9028721785469
KCNT1 73.2842501003097 38.0226905622357 36.5474283500601 38.6414316877471
112.990752518897 47.2009420656854 39.5496902678788 141.376779431031
37.10170285405   39.1621898366355 38.3684202018524 38.3291713594599
KCNT2 42.3433931248155 37.8685585043426 39.3303857231606 39.2406797798509
37.1565984182084 41.809704036355 39.0759686770589 39.3113207075992
36.0123765382627 47.1785588364488 41.862634402486 48.2179100336632
KCNV1 41.7397731376692 37.7384958256497 35.8265993915844 43.074472921841
38.1644569189523 40.1428591998404 41.4742337714895 43.4544762830742
39.0775870687758 39.6719190054379 39.6403089864678 43.2776458914333
KCTD1 41.3785995857154 40.0527893189096 48.8623729374733 39.2780515621926
47.1635381911975 41.6178506967511 43.8206425661746 41.6916508480321
46.7414272831181 47.3010040326346 46.201352759435 48.9733758107788
KCTD10 189.47090669378 111.333692153793 116.839274984089
123.077231445204 238.338646661062 241.486113826383 88.8039637390786
79.4916415027015 105.969617813287 306.294404016622 324.994911416877
141.572530741416
KCTD11 61.9504984796308 45.4882438975269 59.7483827511172
44.265834798964 51.3511088648386 49.3172204533818 53.4921736109774
47.8947595316832 62.5542376788358 52.0999467564713 64.0541263553251
59.7999569757275
KCTD12 78.7450149191851 82.8205269728224 87.9871827215149
226.785831708938 58.8188963227059 214.100443708064 61.3620062177524
66.1288889125299 45.3407340000202 70.0470864733496 74.6110659745477
106.486195938039
KCTD13 269.044011137638 129.299077627671 232.147792636393
166.983092667334 312.795596385258 162.30238363798 115.861269747317
437.542517888395 112.131738588405 176.861690566909 175.652989203497
116.547937420457
KCTD14 47.6397712007919 57.9509155144056 86.500678864429
46.4555908533915 44.3557408297309 46.263826584899 117.138945281875
37.86982638952   77.4186160113063 48.9826868640753 59.7269454048338
103.302837652919

KCTD15 84.9075858560758 60.6080172588224 126.911056987607
63.1604223919703 64.9072331703985 82.5956705829173 44.8643898647322
78.0020909283414 80.0113001016496 63.7836577144372 66.0282565447846
71.1595608255838
KCTD16 33.0463640202858 42.2577689927563 45.8844325913247
39.7411455670172 43.6587492470348 41.2567470068038 39.6535880152834
40.5158867964016 46.1101825640108 41.2276963324981 41.6336636285875
34.806104801006
KCTD17 35.8208542506595 38.5252456306656 46.9040704638073
43.6726153508978 46.3150221024046 48.0350549043063 48.1747307200218
46.9986083378817 36.6312676076142 37.1275334308172 41.6336636285875
38.3291713594599
KCTD18 55.0078788655089 69.8983723094667 71.5484567134601
46.3203995372658 57.4163094213712 74.4508307047509 51.3064929551674
60.7942162859036 72.5993974280876 57.0581769379589 61.940656536312
63.6966761077037
KCTD19 44.7044140979108 38.7344853699576 44.1452943804044
38.2041059863839 42.9090346219023 42.6575535390785 52.0942589368478
44.293922970134 39.4762546236449 39.4710743516517 38.6662194645633
41.0657892043496
KCTD2 68.1506072825075 56.3106156221316 59.499042267068 49.8274931621249
54.9796412320083 75.9298650598599 58.3675164086197 53.3472907280113
66.5539062525344 78.9420136903401 80.1809075683154 64.531467526747
KCTD20 262.327538467461 298.420882349382 295.856741468603
486.757854744053 239.070294068861 433.194113859912 388.580556899321
233.232472394727 171.689014874037 223.040734249612 273.807348011122
277.796753761378
KCTD21 131.990661474974 114.41703170676 131.156876010639
189.650905999631 134.981483569602 185.899535522689 105.382126899524
119.86041265821 130.313381811168 148.358988653522 147.388500451427
170.780021121783
KCTD3 201.427008153788 127.937436596431 169.001200403896 105.234356571498
138.895316905243 245.356135592468 176.363810387584 227.843903680287
193.758357282411 190.888188882162 215.131274710043 205.650076927315
KCTD4 42.7150227340364 37.920964499903 40.7620472083159 40.5484717678957
32.9756367838345 36.4986986135769 40.6750540874299 38.6454098044474
47.0804681460898 43.4885245598939 36.7283081422707 39.1553169409075
KCTD5 263.937966521917 214.980684661682 147.689479458785 128.485596739193
275.314994786888 153.685754085722 312.015358620043 264.855020003755
276.795797048951 148.781721594419 197.040881690021 151.975559850216
KCTD6 84.2104160850167 82.966263655525 89.0642206299985 80.6550905475507
107.693162310852 131.045615546105 107.148000219752 92.6230008876347
88.0055507173538 107.54932544455 99.4633962228768 105.63434679637
KCTD7 44.5282181324797 45.0905928998779 40.301483914446 50.7046706880325
41.1868052124344 44.6524308640784 44.2166118829877 57.9713749600418
46.3203995372658 52.5522545249206 51.0492389286271 47.9566297250148
KCTD8 36.5393857306744 41.7337506166619 38.8775560911841 40.9607305725196
38.4540849632254 43.8582747295766 37.6036146841933 40.831207953647
46.1101825640108 44.7958122466628 44.6286581923295 43.1805016690297
KCTD9 73.9986015964321 54.9392038576957 70.0955779023465 86.3663084195994
55.1563703466328 124.628895737993 77.4046818339539 68.9522412475296
62.1796792097014 171.877918435017 125.221639689345 95.614885914917
KDELC1 56.0477607422828 47.2770121962655 43.1931731665131
48.665578118898 58.037539503209 59.0686629877982 45.4799270968969

41.4508241771315 48.9931786790166 37.920964499903 49.7363778831003
49.904979214379
KDELC2 249.889110678139 148.198469375575 162.628603963754
156.150710793563 132.81898450496 316.745819100367 119.124583755587
183.182947690657 154.287396750021 202.983984966874 184.218705402964
150.396608064863
KDELR1 1018.35211991691 460.162024551004 683.099079617304
392.622487173359 810.021622878441 385.675278463448 403.92758879201
564.929183025034 756.801598119483 532.35007819883 614.603743904512
486.622591654868
KDELR2 588.247843264181 608.005012673672 485.139076884109
539.32996290794 965.530928317269 326.902009704746 506.75912743556
373.077564417927 519.563402444472 185.395460281034 205.672652429972
312.923326422701
KDELR3 219.737294408079 69.6975740412402 53.7792717770978
129.070921534555 121.354667375673 92.2001835784782 117.369735504926
139.554519110944 71.1583251698218 57.5713378330876 52.6677145042939
65.9273663561152
KDR 43.6706764143908 49.4982025424177 43.401782064778 39.9697332660833
45.3675390992851 42.357910063876 39.1539892144371 45.0972184964705
46.0106098064475 48.0211752672327 44.553780984244 41.4508241771315
KEAP1 154.509548705054 170.287619901365 198.682422442825 128.885933852704
327.844083226326 182.37433006793 168.91210897306 130.002385558757
208.743876555152 162.782568037255 189.035121001539 149.5664106654
KEL 44.7044140979108 37.920964499903 39.2707964550441 46.8954576545929
37.5556299264699 39.8455060654531 40.3302268293707 39.3709357615493
52.5772854102326 45.7911932481722 40.6373400186325 44.6484005362281
KENAE 54.2648201582657 66.0479079234982 69.24946602508 56.7927450229377
56.5933259268408 44.3648993568405 69.3468840908665 50.628152951493
59.0563795877672 47.2299678280724 53.4921736109774 50.4847465816184
KERA 43.844855336215 39.4629815741023 44.1915202657512 41.6045220683604
40.9416717019827 44.1737502492846 39.7576136507593 39.1128725343972
38.6322728994674 37.1275334308172 40.1742127891824 46.8129509799753
KGFLP1 41.4508241771315 41.2598458726002 45.8844325913247
42.1622837416589 39.6123051184545 41.4508241771315 41.5464765641928
40.188873541274 36.6680209229535 38.6454098044474 40.8225507267584
48.5632819314123
KHDC1 42.1537476443423 36.89524015739 40.4882741869718 46.8954576545929
39.0564222526273 54.1440452292081 49.540449125788 40.047314525032
43.5669306063196 44.8997235283254 50.0808824383821 45.6692241948108
KHDRBS1 616.265771322835 1109.17675353971 866.712228831149
706.914069768466 1032.44965920118 1152.55315876935 1183.62228577053
970.379875059112 1144.34555072807 517.216393517181 711.08269990525
788.950470077369
KHDRBS2 36.4564050256915 40.2493681589104 39.5450079747895
42.5664883002833 36.9702228618069 35.2067831864574 37.9430145836712
38.8495095797881 37.7384958256497 36.4651112441576 44.4688999098904
40.5703581862752
KHDRBS3 444.618035487523 560.413725186282 62.4683876804812
109.726577835621 101.225705082674 189.769786121649 72.4651758215622
319.596352961751 48.1578509702472 126.670728021682 147.023857419777
158.718808397001

KHK 43.0545608384625 43.5873188565519 43.26160898693 46.4555908533915
52.3660712483179 49.5888934105046 48.2748123114598 47.428201097025
48.042366934301 46.824299585607 44.6892782664938 42.2577689927563
KHSRP 207.710354513208 226.553946570328 249.308868215205 176.816741656659
526.750242877914 130.953274536639 284.105466865264 267.107863045114
343.677907141241 183.526640731647 166.608353896248 165.942795019496
KIAA0020 426.933064567744 464.495132469257 238.819980744789
278.140612054333 534.881442644054 444.169882251299 538.153124145211
472.159979716705 337.627557996504 291.596692248591 345.529757315326
388.132295362791
KIAA0040 53.8856501960117 52.9603588177082 50.5089777244781
59.6153684374766 44.147557390293 58.2531879680192 48.8319884367456
53.2046867327331 46.2027193115397 53.7196173866021 62.8408273986904
47.3172462673426
KIAA0082 41.2424637338424 46.8954576545929 49.8344973836834
42.6913548400127 41.8806343997257 50.7516773099639 41.3594868055879
40.047314525032 47.7086093893038 48.2596564318071 51.4413099192899
43.9541718254769
KIAA0090 235.33716196909 187.238252376588 187.002948435359
152.792457231813 242.932905246176 137.286091889421 159.571799428131
295.430709655678 155.148913816397 193.15756263121 198.288493088076
138.783011990219
KIAA0100 50.6473771879221 51.0175677516029 46.3411353377615
37.7469621225553 45.8051846088552 39.0266821712099 46.8613212048981
37.33030683349 41.863927676552 36.0481934423077 43.6629145624937
38.8495095797881
KIAA0101 4406.55576617324 2712.36664370642 3313.98659031004
3399.75820494572 2892.02001412369 2071.90741263566 3999.32180022337
3838.19449971537 2921.68921750799 3690.00752647453 3490.63949257581
3143.97163345458
KIAA0125 40.6559419616644 53.4735821194294 49.9256753636167
112.743602566917 45.2556760873335 42.9695585740813 39.3585350448719
39.6474008369935 40.8970401536578 42.3606390470387 41.1638563416981
43.3744503319109
KIAA0133 57.934905173625 57.2871072823547 67.0421310720934
71.9062462001972 96.6128615107975 57.8005007012205 74.2690269784394
73.362936710475 71.5327770882433 62.3997407955361 60.3431719501866
58.2251348002272
KIAA0141 153.436974478757 113.929486370331 158.478919789334
161.48562926756 116.099186392258 128.300809076274 93.5928272702304
104.351256270039 126.826715174564 222.05036990665 227.535855856645
155.378768637562
KIAA0146 89.3989848564067 171.920449949522 127.265200736376
73.3725472767369 104.80882886573 69.7677752398541 119.369994922092
95.1037791424727 99.3797043236938 100.076931518374 112.753674408958
127.345094323724
KIAA0152 707.4124863603 383.578947165143 412.162238678684
386.98657211459 371.393488010768 369.584648534533 418.01691355558
427.818758455214 384.280434953152 601.611039798626 542.467997217663
534.629009719855
KIAA0174 788.950470077369 764.571579067138 911.661341702443
657.310970474676 571.790034754076 524.972936438175 687.862452802453
1228.1735887278 762.855719373733 1102.03940742413 938.151864971251
808.857027942253

KIAA0182 347.074754731148 108.991044950132 233.07404514708
141.577762652792 108.815245119101 138.581471125405 53.0277416531043
161.422918681934 119.82102280033 220.454785391748 180.15711251649
149.352994264999
KIAA0194 282.840679491909 113.491158368594 165.440751113862
207.741669973713 204.944787289897 142.846665249354 136.883475451494
243.549194928524 178.961579489906 190.592556065939 200.591114641608
209.038077542654
KIAA0195 244.498602338731 268.265540230814 181.516519907011
120.284321694275 106.276392548097 100.650129064849 183.702709922933
159.884578124679 171.135041358339 174.469283513638 143.973063517706
114.142157259903
KIAA0196 343.016541928592 382.808520776835 284.575587310164
308.728126648231 548.815225941887 313.594031411352 231.685652845234
134.307645598718 270.44099301072 173.308366696429 184.964039515715
242.135519563833
KIAA0226 44.7915315085948 45.4143854024453 47.5673419710251
44.9370339275041 43.3332476888305 46.4648868771662 47.4439407315335
48.4670130605028 48.8067321016961 43.401782064778 44.7926323492519
41.3481355128089
KIAA0232 48.5835484742456 67.0402749076279 55.3122077514658
54.6089451252049 52.8190882379316 58.6439719071099 60.0981687048269
46.0633179231318 61.0094935318105 66.0786923699684 69.7855392178808
53.2046867327331
KIAA0240 318.527516338791 182.219366179899 202.627009037401
222.16475904496 112.830467116678 213.140641182603 164.580141651883
131.808634405319 183.526640731647 385.594118191769 303.281551940647
228.384073242481
KIAA0241 100.663054866186 59.4908919662362 68.7091708087448
75.6103053700122 94.1575209300592 46.3715909653832 66.8475050031365
90.9010016862442 104.900627862215 69.7729022157455 66.6788758487969
58.112348043634
KIAA0247 192.632227878708 118.439721088864 129.938114164087
511.067537752967 105.589475270494 168.78407943637 78.8250642061439
120.142759111608 79.5488891958168 143.053774297331 137.955007835039
141.572530741416
KIAA0251 56.0477607422828 70.4607501403122 77.7615826856723
49.4786466722535 67.4534389579576 56.0477607422828 54.8074655158941
51.3872161199373 58.865217170468 41.9219580093938 44.9713479185144
51.2318935933116
KIAA0256 213.799316520531 157.040132256772 131.045615546105
190.854807132673 107.294098632695 241.08817915181 164.234067632827
191.6998930948 138.81810375553 240.975885654236 233.263058459431
244.707381127491
KIAA0258 38.2083710281545 43.4396866157171 40.931294690669
39.7411455670172 38.745552403895 43.4681646416585 42.1607322031054
40.6171774724171 37.2810767789306 38.3092241935352 37.1059647239814
38.1421709520616
KIAA0261 87.1735705257803 113.102823481296 99.5531381996521
89.9284109077375 92.9747592911324 116.900364583878 82.2650787041549
75.0377156190383 90.2227706363325 66.274247400793 89.0684909049451
95.7383730980717
KIAA0284 53.1309801459151 47.1332119496722 48.3847843498476
42.1273928203021 51.5888395924843 44.8872784170036 54.6414321010929

55.6904617926772 49.0986627051784 46.7646877499666 49.319499137678
49.5539622682049
KIAA0317 41.2424637338424 43.2649781354792 40.4882741869718
49.9014504704021 36.6354013629791 41.7627910693483 44.6032218589199
42.4580504587581 35.2143190355414 47.0055981216687 43.5710871617107
44.5432192783161
KIAA0319 38.1169703906207 40.2493681589104 40.301483914446
41.8778873678551 40.6723334103791 36.2768299545794 47.1345949868029
37.5511560880902 38.2215591930186 35.195364621675 43.5710871617107
39.5418280570561
KIAA0319L 65.5495324730507 88.2568398791731 97.9806802695685
70.6138659908885 83.6000059297772 59.7629007954215 76.0709828871754
78.59966540743 76.8864157610323 73.4899394858239 74.5065528806984
71.9369353731849
KIAA0323 168.288592321667 165.336029381509 115.256208610595
99.2432911346944 96.6128615107975 111.532958750074 83.2162112994735
88.5511408870489 77.5480089703916 118.854313907627 101.28981910937
140.226992517573
KIAA0329 39.9697332660833 44.5884811528117 42.4580504587581
55.4180794172794 43.844855336215 45.6129956328415 37.0564573649223
40.1474543172332 47.4699236780425 47.4767851750188 44.1148826235374
48.27609330316
KIAA0355 363.002913525646 305.684635013927 502.205366225368
366.615979176657 260.185079572615 292.433066791716 359.940393399742
320.504100222566 379.473739290565 357.668448180268 326.250872625748
328.721532812392
KIAA0363 63.7818442618767 45.7576020206129 45.7968648044613
49.3654837541353 53.2090401010575 112.721024337362 46.4944619663072
47.7508139534968 62.7977686460693 91.5230424957075 74.3766944386886
76.3334723210842
KIAA0367 76.4637616585578 50.0425298550559 44.7829632472754
94.3229189690855 42.7982502551634 846.773659307087 44.2166118829877
68.9522412475296 39.3750969348308 567.534792333871 525.687522832693
556.092573451208
KIAA0368 133.314819245044 122.143519601825 93.5717680539357
132.205423909251 87.7844808613265 179.767874494671 100.289042948128
120.001312008518 135.843624468413 139.218017862207 161.133104660281
140.853812988562
KIAA0391 96.6833301748676 234.078434290473 202.060761076547
150.801729138762 135.737771384104 121.744701580607 139.177720567217
178.496935603128 157.334378544568 165.311139930529 161.890859629246
174.332103484796
KIAA0406 79.8844295847317 73.3950348855139 73.3448790478335
60.3481762331447 91.7629175712824 64.3383155834968 118.483499958227
80.3860547572521 73.7873098190297 70.9702772728923 68.7091708087448
70.3309641302984
KIAA0408 65.4561038762234 51.3084273211359 60.9621846036092
60.4646208319121 61.7566103867825 57.8944021095265 65.524821699432
101.272774711455 59.6700171760426 91.2715671868725 89.2185019250322
83.0460206754856
KIAA0409 67.6011992144113 81.7367617812869 72.2797007105795
63.6334154351075 96.332996264667 118.302679462326 62.801431502597
78.8250642061439 80.0733119200914 95.5156363110528 108.512469038407
99.7529382840954

KIAA0415 78.0020909283414 61.3838877877366 61.5824518349507 87.2643859655159 62.9553576299033 55.3202238943712 62.3556396662215 63.9110569727542 54.7966692984192 61.8529316708431 58.2941346100716 46.917149434588
KIAA0427 142.492693744683 107.585504340548 107.034189847828 102.177189421378 76.5375674421131 223.219262492785 91.5258329186709 98.2074490157865 79.9391673512018 220.597255695837 199.776860110557 213.073369426342
KIAA0430 485.310097523194 492.902027204364 651.969296681551 468.477585169219 324.994911416877 689.228901128857 316.745819100367 474.729653426958 414.293172794592 726.42773300581 745.219025960188 777.854349637516
KIAA0460 76.2368668439875 65.2001674269291 69.4967762263181 65.8926843080114 85.2849531543957 84.5913809068614 89.9284109077375 133.930007857002 86.7730065232892 141.567299023381 129.299077627671 88.5638310461364
KIAA0467 39.0620487644635 43.5873188565519 43.0016969050339 39.9697332660833 45.4751076037511 37.2686490268265 42.629412460409 40.2382908228517 43.9450023643394 41.4397539024976 39.7342718293432 36.6603338274678
KIAA0492 95.3300728806601 57.8405769800773 57.2960245538881 59.8102324370568 42.1650127805175 47.7556758818053 57.4047331041592 87.2437977005922 62.9922928277769 74.1228645930185 65.7587943905649 57.6860752168769
KIAA0494 376.141477833569 442.080188140864 537.997378276053 301.525915314179 276.360065404067 372.421330252979 302.667538432677 431.727858902449 562.798580332885 657.59007878546 622.964590694346 502.205366225368
KIAA0495 56.1912096308988 47.3536182445315 34.6977013306396 60.1837189855762 53.5594683028749 58.5530199658661 44.6032218589199 57.9713749600418 32.9756367838345 57.3562426592112 56.4873776344986 59.6757573886627
KIAA0513 90.8236750745974 56.6146179789815 77.5718089378881 160.360785581153 57.0504111569238 130.336599857526 46.1634822993009 58.0892402609917 49.9162522057712 120.837993273922 104.873452487222 91.3112603277315
KIAA0514 52.7367271988171 42.2577689927563 61.7502624458447 41.8778873678551 47.8697093824642 45.3328270847467 88.8772406115484 88.1331756561668 69.779139490544 48.3693338695392 51.4995997550889 48.1125722136997
KIAA0527 39.3100256336821 39.6493706695391 40.0998167098057 44.265834798964 45.3675390992851 43.0144844590744 39.7162631015511 42.3110002461296 39.853866204927 43.8710182120571 39.8184668132023 41.8200132437731
KIAA0528 187.580695643466 195.765247956108 183.746651924774 256.258779434769 128.749209040345 203.353724675525 210.485586903711 236.340873296213 324.658458599373 317.835330028579 300.186402341399 218.917221711155
KIAA0556 133.453860783605 155.942366087388 192.264432082651 95.3070802596041 142.863673914134 106.486195938039 105.247736284747 181.586195566181 92.4350913779886 97.9675668521083 103.316970096472 91.531459931855
KIAA0562 201.800572658224 128.402988047056 124.397048303668 120.068630086702 156.976072444343 118.302679462326 164.580141651883

235.315897087189 137.10896135739 187.21383075601 154.93193897888
123.978615472736
KIAA0564 57.4376528157926 86.1303605978972 80.7894026811963
55.3141112947345 84.7058085717914 56.8875898013449 76.1162400934059
57.2856683017619 77.034044544055 77.8620267163666 78.1132840562953
93.7444359334292
KIAA0565 43.0462952354436 41.4123755032424 45.7486514056858
41.948762493127 43.0723817756832 42.4580504587581 43.3262148375525
43.0390857698452 45.1899271235974 42.4247468951743 44.825830634601
42.8256152216734
KIAA0586 67.9531693869426 75.41408189458 82.6983700200815
53.3967242431461 67.0163226819964 69.7677752398541 49.540449125788
67.5206810636627 60.3977304934451 76.011923854897 72.6162848018047
73.6512983066046
KIAA0644 141.852330109472 49.2050538150106 52.7005092555102
54.6089451252049 57.4163094213712 64.3383155834968 46.3057385979341
48.6441988290952 48.6494451061637 73.8313212063649 49.0361180089431
42.6758568421347
KIAA0649 91.8733417805775 106.309161354804 61.841867306916
59.2778887402357 77.6174893940285 69.0530953932111 123.247207157645
75.3462586192013 68.0796261751272 76.826527950236 81.7500436717038
67.5047089908724
KIAA0652 47.8249696958403 48.3699741879723 47.3861758958576
43.8002456102021 43.9450023643394 42.8598805316863 42.9803717121865
45.0972184964705 56.2441895183416 48.6124127998952 48.2910530887214
49.8253396161069
KIAA0664 241.175454742029 328.387270297393 323.705254926762
100.774098314706 348.862367731887 144.290966198687 615.011590811277
379.473739290565 292.389429869034 347.196922795214 399.733432664718
207.928770428477
KIAA0672 135.567012693522 73.9376772899278 67.8010199320754
66.7750249658122 56.5933259268408 104.914715637173 112.805898501551
148.403272698474 113.873117749074 132.604567913122 125.891602778425
95.491982090349
KIAA0692 43.309444338099 40.7952353240579 41.2793038361528
40.6897401973581 40.3124262005404 39.2623423164488 38.8742176213726
38.1281002984366 41.433180957815 38.4970433581463 39.9070785037411
39.085216569009
KIAA0738 41.0608773166058 44.7829632472754 42.4580504587581
41.5464765641928 37.7576900881741 45.1873487463472 41.4742337714895
36.43924066428 38.9006788676407 43.6033221838149 42.911855821602
43.1805016690297
KIAA0746 101.211796615204 455.223217772744 501.234159111283
390.906200053021 91.9685593100867 81.0880833950735 192.171116215791
131.483899499125 343.677907141241 616.912306849151 551.915864019927
615.227000137328
KIAA0748 41.7397731376692 48.0296332183129 46.1653991089885
41.5464765641928 46.1804647841331 50.6360662655392 39.7576136507593
39.2672712041774 40.6957787921451 38.1042454705119 39.8184668132023
40.2669497398702
KIAA0753 81.537796828766 63.4570909617664 62.4683876804812
51.7739270955254 57.4163094213712 61.6219790276408 69.9214859673839
65.1637149462661 57.8772482723463 78.7543199947688 73.0247140074919
70.9377012403023

KIAA0754 41.1523487516364 40.7952353240579 43.3085358214154
38.5252456306656 38.8456942580061 44.4619248757502 40.0052661202207
40.931294690669 42.1594905198929 40.3235655331796 40.6373400186325
37.6557714476853
KIAA0773 45.4507786740825 43.8845577235486 43.6670755115848
40.0322420615846 47.5002668436865 42.7107928951766 42.8036731479932
41.2551966132486 44.5678064264717 46.1390887492649 42.5426057453993
43.4709415392696
KIAA0774 39.1590964332473 44.4612332327353 45.0958954736034
38.7864161493321 44.4954892619459 41.3012916486748 43.2776458914333
41.5817737564592 44.1070537445005 39.9559575274857 45.8180710486329
44.5973052166071
KIAA0776 42.0532901689036 55.4635297192076 69.5937318502259
55.6481366056639 51.1368583672022 54.3478800757394 46.7100796279384
41.7419877611072 44.1519466943185 43.7303813515864 45.310660704531
46.1592873342534
KIAA0802 49.3463416794995 65.2464683900404 66.603126065489
55.8709511400674 58.5450868286167 49.7458652239071 57.4047331041592
49.5185112777773 65.9254098858352 60.7495043977707 56.233217375698
61.0025829061063
KIAA0831 117.76002524375 98.5746416750459 90.4765857081732
74.2429851177794 63.955655956484 111.009050621499 61.2183034191703
115.577618925646 95.3507664364654 154.149426842591 106.443777149496
87.2175634803271
KIAA0853 36.6480005877129 35.3307157989724 36.0306975524309
38.3166554965434 43.1252272136667 40.650742261428 36.08956004634
45.3872406432794 39.5806465938526 38.2069499294002 40.2649309204478
39.8497227447888
KIAA0859 407.564154455615 182.685579297058 268.097966292089
199.181938253995 573.832332097619 176.520449609867 362.196116025799
235.682605856428 187.862483922786 215.399634344016 201.875082892488
165.797721197832
KIAA0888 43.3638918705225 42.3550738181144 40.1985790927248
40.4359177791566 39.6123051184545 42.357910063876 45.6706505553383
42.0847226073592 42.0621425894624 44.8997235283254 39.1878770155069
40.7704587752083
KIAA0889 51.413025495745 56.8373628733077 50.9010149857308
48.665578118898 57.5040500497076 53.8461734834857 57.1070449050465
54.720204938924 53.9287524861617 49.7259371799014 52.104880481326
49.0655215909632
KIAA0892 116.426770366736 181.420545696696 155.099281008688
102.706563820232 199.54796711653 119.015431849218 103.75895351937
71.6202385170491 99.6946103680372 131.645714321903 100.937651342589
107.693162310852
KIAA0895 39.4710743516517 40.6380495994768 32.5052327840481
36.0481934423077 45.2556760873335 55.3202238943712 59.5205055437765
52.3068458655445 78.7182388025668 43.0919221375514 40.4504608195666
47.6040671292013
KIAA0907 239.229093340475 210.38953368311 265.520773157991
151.740176274202 373.077564417927 159.554916039519 159.571799428131
183.77850568836 193.380035526465 336.795797976236 279.841798764418
290.371909722404
KIAA0913 577.276184555955 423.804472820231 425.60854194674
508.581400606496 227.817928402227 359.889643461047 309.022069786139

285.626835802986 727.181489302632 545.827286197333 518.074074000609 326.357036514613
KIAA0922 72.0334593545916 63.9179552265502 75.9174504780598 122.548989126048 76.5375674421131 91.2681347227088 67.5206810636627 91.9919654450652 67.3230935444252 66.0786923699684 60.9446532180211 58.4200819624972
KIAA0947 48.1460386356622 48.4497719907777 48.8670821164567 53.9517841438975 47.2740099216972 51.4931742685391 57.2008272301399 53.2695955993896 60.7605365979891 54.2830238965339 54.1963147679251 52.2832912331043
KIAA0953 39.8662795144616 42.0709558258414 43.5710871617107 43.8002456102021 47.8075998240396 39.132725377815 52.0942589368478 46.8954576545929 46.581796165231 46.0119912782855 41.3553369544097 45.4799270968969
KIAA0999 67.0577715520007 102.655050997714 91.0443726041992 167.626012561905 77.9085946827079 177.515053183327 118.27400376579 108.213380368446 105.328975917946 144.17470770145 161.326580647514 134.633769604568
KIAA1009 59.046385957117 62.5528124447176 73.4962349716902 57.8277957325085 48.1473938886239 77.9085946827079 64.0985088513857 54.9919060580109 58.174555206394 68.1674425673171 65.5386366902998 67.0782129304647
KIAA1012 125.465429498178 211.625447303889 188.386331007465 262.215263489321 110.214922820817 184.50283792606 173.694418385132 206.481424743576 236.10027586633 162.467159261789 197.13726107504 187.331614826395
KIAA1024 55.9249770393176 47.4740726032707 42.9104454767916 47.481302504066 41.3755760434934 36.6271061451613 40.1235656101329 52.3068458655445 39.6609238036815 47.4767851750188 47.7535797917441 40.0557788909923
KIAA1026 56.3047629466121 53.0798381965427 56.9733113918431 47.0874209746031 62.5106969730855 54.2886483704949 59.4346711476105 78.6100656626189 51.9950964009224 49.1263312877155 58.4816190076338 50.8056327587768
KIAA1033 39.6609238036815 36.4002279956519 33.553634633839 31.7207795618545 32.5318884330468 36.1190722948669 33.4316681623175 33.0038344711883 37.5469247328848 45.1569741609597 32.2133093774284 36.449101775775
KIAA1086 46.6348258763289 41.6466169548846 40.931294690669 42.357910063876 41.8806343997257 40.3357777530419 45.1017494688414 47.3791873993182 39.2809305246091 40.6022003765153 41.6336636285875 39.9519547348106
KIAA1107 52.9240098541574 50.2696827764943 49.2041300973417 45.1944222548351 42.6022652963326 46.7800033479223 60.6548851977964 52.9813204449021 54.8946088631931 70.8749669449099 57.2979215534929 59.7999569757275
KIAA1109 42.6036491382658 44.7829632472754 43.1931731665131 44.0451487108213 46.0043807840705 45.6129956328415 43.1931731665131 49.2019743609873 40.2274522635576 44.4652605017595 46.3370338074996 44.4475838947656
KIAA1128 103.302837652919 90.0420794458477 105.914573205761 119.72858253254 71.9737561190706 233.90802199627 71.7546556028831 83.2240772671045 89.140025661759 123.536432289877 141.370796803908 178.400810392873

KIAA1143 48.9906383294199 50.8114699212931 53.5139000636716
54.4586176399381 63.6254894053045 47.3375061488583 63.2726414544553
50.6666199323963 58.4891791448514 45.6355219961567 45.8180710486329
49.2731519807085
KIAA1147 106.70211644918 128.356485935893 209.794541970107
171.623830248512 138.50445813694 91.5618002664085 117.033529761587
115.761674855716 104.103899614521 171.5709313654 110.146992754585
154.945533851823
KIAA1160 100.63191957178 111.939677225005 99.7529382840954
131.238796973323 140.504336039225 131.451150955309 142.515628954457
104.587564056763 122.0305509548 103.385447205588 114.5696064637
116.475941571644
KIAA1161 42.2455707947223 40.148780650831 40.5734458834617
32.3671711013873 32.771863438151 47.2009420656854 35.9894133432376
36.5393857306744 42.9020321355927 39.8873706855774 50.9815930710768
40.5703581862752
KIAA1166 40.7609952437622 48.8584917918986 37.8386378737561
44.4367387274922 45.4751076037511 40.5281496800812 45.223075311113
41.2551966132486 46.3203995372658 47.611018474151 43.401782064778
49.904979214379
KIAA1191 483.000434270291 281.07879968121 393.464673405634
241.599353653369 291.596692248591 452.139416552986 271.674332647296
331.762920362224 313.233036704221 451.290681710534 423.804472820231
497.591040004541
KIAA1199 251.65044168003 116.374125502825 54.5191471810265
337.156068385541 107.094663242259 45.0882356578404 57.7805922926828
91.3607028449916 94.8408264309872 50.6486940895674 44.7926323492519
129.336439341458
KIAA1202 41.7397731376692 64.9959712888709 73.0128142320238
55.4180794172794 38.9496515344837 50.7516773099639 40.2154497375715
41.4508241771315 40.7847110517594 54.0374522273907 51.7538628098347
38.745552403895
KIAA1210 43.1805016690297 36.5219488315447 38.8775560911841
41.0726336841311 49.0828761839116 47.0027453663546 36.3194282084339
37.9837233601316 38.4344387289956 39.8873706855774 43.844855336215
39.8497227447888
KIAA1211 44.0760877044901 46.1569135229922 43.4558010540988
47.2797818740024 41.9495387255163 42.2006337556379 49.7495619589928
47.5529788356555 46.3576476245409 41.6657053704762 42.1516028411593
44.1915202657512
KIAA1217 240.314054155006 90.3691010312535 92.3034609409756
57.6511495792664 86.8871118411414 63.6894484284172 110.223625398261
103.366147685418 141.017696819844 174.584330660466 132.973097496421
127.694844372277
KIAA1219 110.609133094021 87.6763065012524 150.801729138762
100.774098314706 153.028210322286 75.353039684456 120.02765867291
205.314410185216 158.884110481815 123.095695502815 125.494065483032
128.365609268093
KIAA1239 36.1831439734485 35.6434124419782 33.9435456570176
41.6045220683604 38.9496515344837 39.9777866059804 37.4772922422734
40.831207953647 41.4636047919453 43.1839109702714 43.0016969050339
36.1647506311964
KIAA1244 61.2130609867477 55.7405934254857 51.9214790114499
41.7614975630362 41.9842695818285 42.5660695551615 54.339958888486

55.5578334877425 47.5564423692142 45.1569741609597 51.3861659123096
67.4197263416701
KIAA1245 40.9133663634149 41.1250236884325 41.1654477997049
43.8550481835503 40.5349168203034 41.2226607554583 44.6861785933669
41.9741368971524 39.2594097769139 41.1697539052486 40.970657220687
41.7022936121242
KIAA1257 43.2724556187214 40.5852276210998 38.6374490609417
39.6292788039793 37.6527901109005 38.1699123824764 42.22763273471
40.2770232552555 35.5631236789214 37.920964499903 39.7576136507593
41.2437378488025
KIAA1267 187.580695643466 199.480636964499 162.151812425949
205.695230410884 187.347386400244 150.405742308999 192.318724749487
231.472806936915 250.866560791828 439.955651727755 348.123960682064
292.86576686019
KIAA1271 55.7246872330059 66.0147097736612 61.6878176260761
57.4595416719581 83.0497574039035 61.3620062177524 72.6959688212342
55.9303347977781 45.9227107476843 65.2361525412756 96.6969760559963
76.7791768896805
KIAA1274 42.5193208629123 41.0678614026819 38.8775560911841
40.9607305725196 38.6454098044474 36.1190722948669 59.5205055437765
39.2672712041774 38.3418933808844 32.9003644440608 36.0481934423077
39.8497227447888
KIAA1276 57.2960245538881 53.2046867327331 51.8122261766744
56.7927450229377 54.5683518971261 50.7516773099639 54.0170817319522
59.5830868881343 54.7966692984192 53.8260140375214 57.5430365699386
50.2163892855092
KIAA1279 114.605149116084 142.49850553035 129.938114164087
109.933396041701 144.139555313429 130.10148014895 114.711404298369
84.7642634532073 248.058310353348 84.8999904158793 81.382229980722
117.678385591938
KIAA1285 42.9104454767916 47.765848710116 40.7620472083159
33.1137701681495 47.4548471240051 43.6670755115848 46.5650556481721
41.8417024632349 57.0301170427216 45.7911932481722 51.4413099192899
48.4535991392809
KIAA1303 43.0462952354436 44.8529259387308 46.4150464549477
45.0017158586743 44.7210190486327 51.8103411703261 50.8388984085636
51.7538628098347 56.8622704143595 44.4652605017595 49.1495645678651
44.0451487108213
KIAA1324 44.7915315085948 45.2556760873335 46.7508829977587
110.666686474986 37.0526522140242 44.0804700754786 72.3515874601288
45.8669209716694 62.8635815439291 45.6868589181768 39.153226699662
43.4709415392696
KIAA1324L 53.1309801459151 180.902423012987 190.403032965372
59.6153684374766 68.7911855725594 84.096146903568 91.7761668960824
143.399636653204 125.383837024985 144.609635554528 132.007307754378
140.226992517573
KIAA1328 46.7293238583888 45.5842286511645 46.2625470410195
47.8164193292842 50.6470461863549 51.1621379498014 40.1235656101329
51.0724630072723 54.4912806027381 70.7003843117511 58.5588286084726
54.9919060580109
KIAA1333 42.8036731479932 53.2046867327331 45.4865173433646
42.0102671859606 52.9433495155796 50.9724155054821 44.7255207589606
32.4466419672036 50.9010149857308 53.9412154719864 49.9338905397767
49.2064066614865

KIAA1345 48.1952521496489 43.683792219729 47.5673419710251
41.4273122914951 50.0580547772867 51.702461863489 44.0687697278114
44.4031868028216 49.9162522057712 43.0919221375514 44.2142581488029
46.1592873342534
KIAA1370 154.232642598588 145.526972410673 129.501808300251
153.595112580363 130.417782238512 122.036614711384 133.453860783605
152.272399644814 169.001200403896 127.61938370689 134.335592890814
118.376686726902
KIAA1377 39.5676051939342 41.6466169548846 35.2838283525874
38.745552403895 39.0564222526273 43.4681646416585 42.357910063876
41.3506832276112 37.2810767789306 43.844855336215 43.0971787292711
45.6692241948108
KIAA1383 45.6517320730042 61.6878176260761 53.4088637045445
58.5406981871153 55.6069214673486 44.6524308640784 55.4004611309636
46.5717043290731 61.2510119532409 51.1169368198437 44.1148826235374
50.2163892855092
KIAA1394 44.8914371498528 45.6533175604486 49.9256753636167
49.9014504704021 45.3675390992851 43.8582747295766 41.253645578517
43.1567024035183 38.6322728994674 42.1622837416589 43.401782064778
34.9138625012006
KIAA1407 54.2999306485758 55.9007879696845 77.1111458359361
54.7350451926204 59.9876575158981 59.9876575158981 67.8919712859031
60.2691630545162 53.6837370963602 77.9820921829024 78.4962825977573
62.2685634967698
KIAA1409 39.3100256336821 43.3458769998118 46.1653991089885
42.0621425894624 44.0451487108213 43.8308097066396 41.4369824439795
42.2577689927563 43.4656637536244 50.294087888475 38.5252456306656
45.4799270968969
KIAA1429 140.961217055867 119.700522283289 91.6033269431304
95.8590592250688 208.553134986809 148.3842032507 96.0539709962851
83.2240772671045 118.089250727398 108.788705797019 104.873452487222
100.289042948128
KIAA1432 42.5193208629123 43.4396866157171 43.7630587657205
44.7980740332174 43.5544347011646 42.750861693377 46.5650556481721
37.7612949798895 40.6957787921451 38.2069499294002 49.8393069675192
41.3481355128089
KIAA1462 45.2875173643252 36.0366673493555 49.8344973836834
47.2009420656854 46.0977687311222 41.0582900711341 42.8684958525042
41.3506832276112 39.2809305246091 51.5855515705917 47.7535797917441
41.6550823405646
KIAA1467 62.928986708012 91.3941563889513 80.4060693489113
56.9299697426319 88.3954618645976 62.843734579668 85.2088054619229
64.7318794662958 97.8764110456132 137.831070407907 104.626873082138
132.59650761791
KIAA1468 83.2737524877952 101.192523227755 130.184962393833
86.9274372004388 70.0133734659853 60.4991141099239 102.00080367858
110.983311402531 168.655833116537 143.563236821003 154.19962858342
125.512567429299
KIAA1486 44.5282181324797 36.89524015739 43.4709415392696
42.2496337895968 35.6434124419782 44.8872784170036 38.1793598983615
40.3357777530419 44.8997235283254 49.1928206164702 42.6366765186182
45.4799270968969
KIAA1522 197.325473090277 326.121479451246 298.607482741423
69.3525901554637 356.503013319738 78.5276518966943 219.555240189863

210.605371352247 269.044011137638 217.263889616995 171.829798001437
217.533011278692
KIAA1524 42.466333667839 43.3016395799961 37.7000400506818
43.074472921841 49.6507190068324 39.4953494583071 38.7337086062875
49.6629259325705 46.4861258030402 41.6373771565199 42.6776449468246
38.4330755397458
KIAA1529 40.2543341073001 44.3529919625344 40.1985790927248
37.8442841690502 39.2608760193011 41.2567470068038 44.7255207589606
43.7396619462033 37.6383530120621 41.6373771565199 41.2598458726002
37.8490162439785
KIAA1530 51.2851682978581 60.2165699959135 64.2174369755376
69.4281256780421 55.9669688396835 49.3581578587582 54.4384603094793
59.3923286524971 52.5120528339416 71.9805290614073 71.1163541406788
60.5594938829162
KIAA1539 150.685575301101 94.5261917871909 106.537423775385
180.128769312512 62.6860949544223 86.915098846441 67.0455170947389
80.1144023887293 76.2591822114739 118.726647822102 91.2202310968307
88.9641595603127
KIAA1542 43.0001432913058 52.4351670989268 43.6670755115848
44.4367387274922 47.3172462673426 41.8613406887824 57.7805922926828
45.642304356051 54.0192060888782 49.7259371799014 53.8856501960117
45.0035308952466
KIAA1543 63.1291723738553 70.9179240997848 86.3793067659925
53.7155925246916 76.7556711670518 43.3269087731399 83.8811735017279
70.9266654533052 83.1702007661831 72.1687283197901 67.9599395822006
55.5276281380084
KIAA1545 190.993853866124 100.774098314706 87.9871827215149
80.1809075683154 71.5345397102823 130.716532316693 114.148080724618
62.6984594468418 93.4980947651211 129.754481922118 123.032006903803
88.6771566133367
KIAA1549 40.2543341073001 44.4411734611229 42.157627240486
37.7469621225553 45.5809132232681 41.2567470068038 47.7390230243778
42.9301944191913 45.8325181856483 37.8217989841918 40.2649309204478
38.9496515344837
KIAA1553 75.9909523932333 51.2079772365211 45.3623491537646
40.5484717678957 53.0965951260419 41.0057887014368 55.1359824959234
79.9126192278104 49.7868528436838 44.8522313252892 41.6616444147804
44.1915202657512
KIAA1571 47.2862299990938 34.6306468196142 38.6727842796202
43.6726153508978 42.7982502551634 41.6590564937735 39.8695929627783
42.8482181313226 45.9227107476843 50.22432299342 38.079222289614
39.2469742642337
KIAA1586 39.3709357615493 44.0409178714232 42.157627240486
44.4367387274922 47.6884372645373 46.7800033479223 42.4939802311136
42.0494116081998 48.1174861108648 50.5252048155459 46.6442692119997
45.9693232070002
KIAA1598 204.251854342995 71.0935990523782 87.9152471471338
269.857104259782 93.7924763988692 112.873697337138 116.555666104916
208.67074469962 93.8042295944812 145.28255999556 107.044681610056
120.73773749436
KIAA1600 157.906415100288 220.454785391748 176.871009816977
187.601223003233 103.199200862158 210.958578914607 146.701131209299
133.314819245044 119.333788465158 276.214079735778 245.730139853029
225.300736968225

KIAA1602 173.391542207362 104.650071846586 116.295564219236
112.368910939752 111.399831013724 108.718422641679 121.737071786167
196.623560874725 123.597489107285 126.814751973459 133.1387411908
78.5108643908344
KIAA1604 51.413025495745 40.9731067463382 42.640829914102
43.8002456102021 44.5668141895992 43.9724628401189 44.7255207589606
46.695848687694 43.6670755115848 45.4614234442527 45.310660704531
44.9070250604284
KIAA1609 77.2643026985097 78.3658211474135 78.080034922779
39.3750969348308 51.5888395924843 45.4882438975269 47.8947595316832
44.6107027295193 56.7991853808475 42.0621425894624 67.6271456814495
73.6512983066046
KIAA1614 43.9309845324767 45.3486182278177 49.1067959632109
37.4223289687135 34.6227169056411 48.3686935595826 47.2927436772465
45.1714698794331 44.8997235283254 42.9866214102525 41.4508241771315
49.1200355938198
KIAA1618 64.6316818494785 73.1262465119304 62.2685634967698
100.344513700137 58.037539503209 68.2428802339852 74.6572522248684
105.563773939475 68.8353800542132 69.3497375926383 77.118454956956
80.7664399131413
KIAA1622 37.4554876386402 42.2577689927563 40.5734458834617
38.5252456306656 36.3390947880186 35.9305494063137 45.1017494688414
34.8949555140527 36.2733735494625 39.0648733099061 37.7384958256497
36.5474283500601
KIAA1627 51.2851682978581 45.2556760873335 50.7502704822135
39.9697332660833 52.3242437351389 54.2503023961401 50.6774883370457
39.7509165247419 54.4912806027381 37.3179578037963 49.8393069675192
52.1918130336397
KIAA1632 49.965271003193 54.7350451926204 51.7663108516261
64.898351831341 40.8270141118472 46.8436815733702 43.5898425390297
52.7107145265067 54.0192060888782 59.4019188643872 57.2979215534929
52.7972156492746
KIAA1641 50.5252048155459 44.6918600441328 41.0356368374708
45.6602029784334 42.2455707947223 44.1386689648017 42.7958064399619
61.8061006855512 40.9416717019827 47.4356803320662 46.5421017969405
47.8472928419101
KIAA1644 49.5666074434383 43.3016395799961 42.1901757067073
43.367124590178 41.2424637338424 105.317399386109 36.5352572191451
40.5158867964016 39.8048943625718 75.2053836356882 84.8808332740321
71.9369353731849
KIAA1660 34.9138625012006 41.6466169548846 45.7968648044613
46.3203995372658 44.3557408297309 44.7804080899431 46.9929836306816
41.3506832276112 42.2577689927563 46.441301934328 42.170702295259
36.6603338274678
KIAA1666 49.6751542251638 46.29198852834 50.5089777244781
55.6481366056639 48.7814131120945 43.8308097066396 47.5130990528612
42.9301944191913 48.1174861108648 54.4190527512508 47.1098984140276
43.2776458914333
KIAA1671 233.789761419387 132.946528291039 123.597489107285
93.3216420803293 175.922685424474 219.134220280612 168.345356470411
199.522267292552 228.469654751393 252.206029058968 222.696947669812
202.230726475934
KIAA1683 46.6348258763289 54.2445399731644 54.2670850492947
43.3078542962968 39.1650142708257 55.3202238943712 60.3928833351886

55.8087097725119 66.3209349903185 61.6670071424359 47.5439485437351
55.1804535845496
KIAA1688 384.727543700024 224.60637398191 130.848965290163
61.5515639412892 205.772128732705 72.149478755262 271.813877560354
216.744244428166 195.740109330843 80.5388767883646 94.428252478069
67.8335962843732
KIAA1704 40.2543341073001 40.5400384479766 42.3887531663733
42.0621425894624 42.4493032770053 42.8598805316863 41.706879587091
43.6753843488695 41.6340461942423 44.3303034026662 41.7272875295469
40.6216891610774
KIAA1712 104.335120306753 123.350559552455 116.490154128114
103.607345835114 125.383837024985 134.749803376372 117.634238804678
110.715042245563 120.825113658705 185.079035617027 165.640362974584
158.532307373857
KIAA1715 109.326279686847 112.02980664709 110.061090703983
103.734064904898 95.9075629352341 109.795084617739 130.058147569795
123.114162330406 137.438636654385 144.348025939706 143.497887869284
99.3344676796884
KIAA1737 207.380865650247 118.507569961357 133.893108529325
127.225812893692 143.639231185825 244.907057340556 158.702003835578
165.322633260592 180.916465721523 271.104193102849 306.895449954881
220.192552354287
KIAA1751 191.141778714144 127.352997770688 150.636903991608
200.986069552443 120.392561825049 119.253559091088 169.144145421022
299.665413950641 107.348865210074 335.167786298149 283.30253361072
227.059460958349
KIAA1754 214.342804055754 140.288771064502 169.344094528032
605.970682363975 157.312247745825 211.701234819986 121.737071786167
281.452208033175 108.27606179864 105.212030988223 115.496195377983
138.599959012396
KIAA1755 38.0089018744739 35.2143190355414 39.1799070031819
36.264823544143 42.1650127805175 31.1150868775826 42.7431359850339
36.8400213077707 36.3649677956953 39.0648733099061 35.6970528713709
34.1509644696334
KIAA1772 40.9558083259517 36.7976562409506 36.3604951341921
48.9944486068347 42.7982502551634 39.9397419279057 44.9863726205428
41.5464765641928 44.4395580493118 43.7303813515864 46.2560238004757
50.4847465816184
KIAA1787 116.685453817071 84.4596873416863 99.6101168315098
79.1000897258245 95.6182216943758 90.99086730704 98.5663841503803
100.887736959095 79.1074681877535 107.610858427836 99.6288131333575
73.9192243586276
KIAA1797 116.311176407088 116.295564219236 71.6573554353691
81.6780581904253 95.6182216943758 110.399579382196 78.4937314423253
75.4935574755043 82.4467808843629 100.076931518374 99.6288131333575
96.5813769696577
KIAA1804 43.2724556187214 51.2405397507224 47.0940613507321
43.7540438144592 53.9074374788981 43.5416683728269 42.6913548400127
44.7044140979108 48.5136862664526 43.401782064778 43.401782064778
43.5738557773903
KIAA1826 137.696331574552 139.999709303614 102.330999847292
165.606015336587 195.512986297442 181.950503247343 143.761359357965
102.626604917608 113.218291314173 170.420427857495 172.83517237888
153.992331037033

KIAA1841 35.4287975493553 35.1224289925572 32.821392987958
34.4382958501338 29.6891949090828 34.9849896310235 33.2425023465738
34.5886782974123 30.9241129241985 30.3383368296776 31.6063174764616
31.372171135652
KIAA1843 44.7044140979108 40.2493681589104 36.3604951341921
39.5050383901884 39.1650142708257 39.0266821712099 43.6753843488695
41.1575777497576 42.9020321355927 41.3321861371202 43.4709415392696
52.3896785600751
KIAA1853 42.3433931248155 37.5469247328848 40.6737160001291
57.2856683017619 42.4864576619427 37.7000400506818 42.1607322031054
42.2577689927563 42.1594905198929 36.2435874904689 38.745552403895
36.8690774823319
KIAA1862 60.8357760968871 67.2649356062263 54.2670850492947
53.9517841438975 76.6914642534934 52.9482338979681 77.118454956956
58.0892402609917 58.174555206394 51.9547984751408 61.7572675712557
58.4200819624972
KIAA1875 55.5276281380084 113.929486370331 56.8595344107711
48.309866878365 77.6174893940285 44.8872784170036 58.2315917325629
57.037639245179 61.7865043876727 60.7495043977707 58.8961770381033
55.0767527801658
KIAA1881 55.2182004838121 115.840734064724 220.743173420176
85.1847490113446 279.448591201269 599.670771796585 102.626604917608
131.068763088422 195.430996540859 327.283431584541 437.542517888395
309.06234888929
KIAA1906 40.9558083259517 41.8848959280916 42.9104454767916
38.3166554965434 43.3332476888305 41.0582900711341 42.4939802311136
45.7476095696859 50.0526763693329 49.6203214143464 45.310660704531
48.4535991392809
KIAA1919 42.1537476443423 40.716553247236 41.8751578329365
41.0726336841311 40.148780650831 42.750861693377 35.9058393060637
42.0494116081998 40.6957787921451 46.8954576545929 42.2618615571566
44.8372404608604
KIAA1920 46.1673159982664 41.554942319225 40.931294690669
41.8778873678551 41.5668884168669 42.9695585740813 42.7431359850339
40.3357777530419 44.5303781239209 45.4614234442527 45.9104478407175
41.5536168238478
KIAA1922 39.0620487644635 40.5400384479766 44.0451487108213
39.5050383901884 40.7779767565889 43.8582747295766 46.7100796279384
35.4526946152955 39.2809305246091 40.3235655331796 40.2649309204478
41.2437378488025
KIAA1949 262.679844527518 257.584248252408 207.833867050257
668.961646543849 162.902264837659 350.796638646601 211.019451518444
150.262748333939 108.27606179864 130.812376632261 128.559660070271
148.570371099987
KIAA1957 43.6706764143908 42.1607322031054 43.1931731665131
40.3180310664156 37.4470183315826 48.6621527194963 43.6753843488695
41.6466169548846 43.2761454096265 40.6022003765153 44.9713479185144
43.3744503319109
KIAA1958 42.9104454767916 43.9421822132391 43.3085358214154
39.5050383901884 51.7983110178095 45.8325181856483 58.0969258817391
44.6107027295193 39.9444191873552 41.3321861371202 49.8393069675192
49.0655215909632
KIAA1967 84.3338099925204 59.0457625163577 66.8188271427672
53.8086134112366 57.6791750431251 60.3381680822012 60.8942621888353

58.0353195691931 73.5950442915143 73.2956678446902 79.7992359774565
76.7063975550116
KIAA1975 43.0001432913058 46.1569135229922 37.7384958256497
45.334106390212 41.9842695818285 42.750861693377 40.9157352260901
42.4580504587581 48.8842909466002 47.3791873993182 47.0940613507321
45.0985088502363
KIAA1984 47.6397712007919 99.864496342189 69.3468840908665
54.8412943008635 42.269054475455 50.9724155054821 43.3262148375525
40.931294690669 44.619512785899 54.7827221235949 46.4753015651202
45.7562276914441
KIAA2010 170.307603160524 99.2341244350412 194.827750623463
183.028254350275 141.055218279065 145.791398829369 128.208688034402
109.573199664599 195.543760781509 150.829277770546 141.672695206961
157.133627517432
KIAA2013 425.776214512634 247.511892014861 284.868613843826
232.740032714891 357.029998842776 268.808342880355 296.463473585355
356.948763043851 298.313562383092 298.607482741423 263.125290391542
200.450523205251
KIAA2018 41.9407041937973 33.4967065724734 33.1559412791294
37.29566077996 34.4570158133896 37.2686490268265 35.3362091871364
47.4740726032707 38.3418933808844 34.2638875325204 34.5633623842083
42.0424641526302
KIAA2022 36.5393857306744 39.0894172982439 38.6005272730664
38.0958223740111 32.0568298766078 36.1190722948669 39.7576136507593
36.43924066428 39.9444191873552 39.9948390976317 34.064698871541
29.9775071862804
KIAA2026 81.8569832022381 104.796373568883 74.6514558411477
108.838406710326 75.3580625318157 97.3310111513156 79.6581393791238
84.3040298294164 82.7471156870608 95.9485602132766 113.964753321316
101.2825552085
KIDINS220 550.100918659768 344.38057912034 256.55408314817
299.890705180422 295.430709655678 448.196293409768 450.865888317246
869.879392791086 413.990148966099 343.418685340038 404.86512250958
315.139359883556
KIF11 75.2923395421694 147.471179343348 96.9569547595983 73.5834042105313
137.509582795609 55.4345579421782 195.262845535544 109.922909644299
149.950764820034 42.233842834629 49.6706535876636 42.57175366709
KIF12 47.5647821785529 47.4740726032707 41.8751578329365 44.4367387274922
47.3172462673426 46.3715909653832 51.4349390827895 49.085741080241
45.4799270968969 45.7911932481722 48.042366934301 37.4554876386402
KIF13A 36.1232327599945 41.554942319225 41.2365089689635
47.8164193292842 49.32293280121 43.3785777889265 43.3262148375525
55.1933004802607 40.6957787921451 43.9450023643394 47.6125796208753
43.1805016690297
KIF13B 341.666968456707 144.633447485038 217.575115287224
157.518879763075 69.0975200641705 166.648551193168 107.466490458133
134.407474121421 79.0276852011359 312.154320305976 228.882072078733
246.965844426351
KIF14 92.843017373656 70.1831787109201 71.5484567134601 49.9014504704021
119.291755076832 55.7197525213035 87.6018870099686 98.8478655411492
78.055431384988 41.6373771565199 45.5707650534259 48.0350549043063
KIF15 55.1306642449627 68.5251167137806 61.6878176260761 46.1673159982664
134.981483569602 57.013135787334 125.997088252817 83.3826639718546
68.2372031645261 44.0409178714232 45.4799270968969 49.3984571719129

KIF16B 56.5094373275922 67.8208976798364 84.7851173379268
67.1961800572759 56.1733549054832 73.2144205844199 72.0112676564347
53.9465584609317 57.0690683036183 67.5047089908724 69.3645322033576
82.8888969977394
KIF17 43.0001432913058 45.4143854024453 38.3166554965434 43.8002456102021
35.6434124419782 43.0860383229288 44.7255207589606 42.5435283762933
37.10170285405 44.0409178714232 47.5439485437351 51.8562635270111
KIF18A 41.0970259989281 45.5587966941116 41.0356368374708
42.281606728072 44.5092684716474 47.3791873993182 45.6706505553383
47.3172462673426 55.2485665256307 41.0608773166058 40.4094901248527
41.8200132437731
KIF18B 39.7576136507593 44.3187284857534 44.1915202657512
43.074472921841 43.8835874380262 39.3841861812262 41.8315602975157
39.6995529770239 44.8003345643334 38.3092241935352 36.6603338274678
39.9941474864076
KIF19 42.6036491382658 44.5152708955623 43.7630587657205 40.1804147506786
40.2449343247539 42.9695585740813 41.3594868055879 38.3684202018524
39.5806465938526 36.0481934423077 41.2598458726002 42.1537476443423
KIF1A 59.5000730792772 45.2556760873335 39.9101908547397 53.3967242431461
68.4961024593552 45.7300501624017 49.1067959632109 199.23413224268
37.10170285405 40.6022003765153 40.1742127891824 40.7704587752083
KIF1B 179.09303345382 82.8205269728224 86.3458707176254 139.625593914573
102.597012252626 144.116701129933 110.88526946931 183.526640731647
98.4763347474813 201.145927216439 149.8814624626 148.514056973195
KIF1C 53.7507429765038 65.3694267397114 56.2441895183416 42.8235313007746
50.0580547772867 58.0095518210499 50.5252048155459 49.8344973836834
42.0621425894624 52.8928296360463 57.5430365699386 49.2064066614865
KIF20A 157.658760470424 235.101442092497 173.08677862231
84.4422623834916 326.963277148673 60.1042270393382 219.036267470696
177.958475301951 331.239629083778 41.2276963324981 37.2018803984555
48.4535991392809
KIF20B 141.364814429951 115.321592104747 115.468303687641
105.790224260281 153.279284223069 91.9459432547337 152.17335005192
143.653706909456 176.02137177982 86.2118355769367 82.0206083292981
64.7346994616044
KIF21A 58.4920021605316 61.1452879517526 57.9279989359711
48.309866878365 63.1443731864055 62.3497263884167 64.0985088513857
108.945545005203 93.8042295944812 57.5713378330876 64.898351831341
71.381069076765
KIF21B 45.1479346191482 45.6243792564364 45.8409758448533
44.4367387274922 42.3685136297603 41.3012916486748 45.1679144865704
50.7964800730186 40.9901805728485 40.5090849993014 40.9696219937838
41.5916331278821
KIF23 64.4415447415281 69.4583814833055 53.2046867327331 44.3440839686558
87.6178717341967 40.650742261428 78.1204313889396 66.5694839794116
79.1305209735569 42.0621425894624 41.5421429082231 45.3763275588341
KIF24 46.9939712192224 48.0570064280814 43.0016969050339 38.4699622716254
45.6895223823001 40.8091178323174 52.1918130336397 51.7121586468362
50.2223403889892 39.8873706855774 42.4162492820012 43.3262148375525
KIF25 48.5123041297835 46.29198852834 42.5864346233886 42.6913548400127
44.4031868028216 50.6915708372926 41.3094485574845 42.8936990772838
52.8190882379316 50.4234835272833 43.6629145624937 52.8974363028468
KIF26B 71.4937262725923 46.7980335276155 42.3536549114165
50.6262443356159 44.4031868028216 42.4155142987678 46.5650556481721

53.4109882045447 48.4869909888679 41.6724739851995 44.6834648302383
44.7401523707775
KIF27 42.5615835912214 46.3319769551314 41.4727452533295 44.8854076849881
41.7530073084919 42.4580504587581 43.1593895157816 43.0877002403108
40.2630560420191 43.149950256043 42.2518289437063 45.1390218677367
KIF2A 157.658760470424 154.984589017187 131.156876010639 117.699959435983
131.365512154718 89.4747568145717 166.009268520425 99.5192591352063
158.493140172253 93.8630393470857 102.027922691582 136.20629436945
KIF2B 39.7162631015511 42.0709558258414 41.9666541498638 40.0322420615846
42.3685136297603 41.2567470068038 42.7431359850339 39.1652965788406
44.293922970134 43.8710182120571 41.0808257270221 41.3481355128089
KIF2C 135.829530518199 101.426462865382 73.6323065529916 58.0218262176065
157.161381086926 52.6226745117396 217.848539722853 146.426853713038
158.408644964483 46.0119912782855 43.401782064778 51.2318935933116
KIF3A 35.8208542506595 42.2577689927563 38.6727842796202 34.3595588146954
34.5301949364147 39.0266821712099 34.806104801006 37.4484310268909
34.0382693188794 33.8984830800739 39.5399188536544 35.4484652445078
KIF3B 112.506613718654 111.109515944561 110.112236384679 97.5849495690144
133.541341560868 135.11808699414 106.712703356565 97.0589984189239
126.711833914667 131.645714321903 130.524308471577 118.966448789057
KIF3C 55.9249770393176 61.1022920882065 53.0630927250439 50.378948785456
52.8190882379316 54.5952754712007 90.0682241853516 169.790843803064
64.7116910405493 53.5498255316009 48.6767028205398 42.9887069977696
KIF4A 63.5693267269002 61.1022920882065 50.8303921211581 42.1273928203021
86.033725825968 40.5281496800812 81.9504925867759 69.1973371867031
125.946162432491 40.4136535208406 40.3452320167286 35.4484652445078
KIF5A 42.0532901689036 41.690833180291 45.4865173433646 42.281606728072
42.7489468897708 42.157627240486 42.4939802311136 46.5717043290731
42.0126083977597 41.3321861371202 42.6776449468246 48.8319884367456
KIF5B 151.936097714553 237.532800487032 194.317275235015 157.095926797414
210.73006676203 388.458756841292 147.010700944686 147.017279035061
266.905855859261 76.2533853403813 120.679433440203 229.547114945412
KIF5C 103.808640431626 126.054896053572 52.5353664902258 51.2973486610884
54.3116862772198 56.9635892586579 120.582525405708 98.2074490157865
51.1055541075029 231.824347524066 142.647217549267 112.220892096054
KIF6 58.2902084992082 49.9085372049438 51.7138050344355 42.5664883002833
52.9433495155796 45.0882356578404 59.8273072817267 53.1052228246467
55.2114969675545 54.4825201506931 54.0374522273907 50.656598108034
KIF7 71.3900857589616 57.4054069675016 58.8188963227059 59.0965219753805
98.6553805168141 80.8333046859586 72.8653205184014 74.0831311663524
61.5807110546718 82.6036671634626 96.1307490576512 69.2213838763756
KIF9 39.2076937845359 47.5203201376024 39.1799070031819 37.8442841690502
40.4321207743917 42.5660695551615 43.3262148375525 41.4061152929154
42.992884918158 45.4882438975269 39.1878770155069 40.5703581862752
KIFAP3 279.632025097952 118.143444770806 147.945429978177
154.287396750021 101.156215957297 178.269900400454 136.629336440719
172.89937021234 97.8009303343756 184.65212689433 153.595112580363
178.98901256658
KIFC1 532.981045084589 349.096725048776 168.388385941598 98.5663841503803
370.64147085217 91.8295472317386 647.297964303385 343.50519530761
375.036941898486 62.3997407955361 49.2206713037152 59.4465684293847
KIFC2 132.604567913122 306.94883838074 256.55408314817 75.9099205260748
132.117013391364 56.6172748471831 120.231435193839 78.9900928241723
130.533514998688 190.888188882162 211.654245727798 123.114162330406

KIFC3 47.5647821785529 44.0409178714232 39.5450079747895 43.9282059368202
38.1644569189523 38.8336427948187 51.5967098638347 35.3608605269276
46.6944238390932 36.1550293353654 39.5399188536544 39.3575468494941
KIN 45.5758521267508 43.0971787292711 44.1452943804044 41.5464765641928
47.8075998240396 46.263826584899 48.9826868640753 43.3262148375525
41.5635499110307 45.1569741609597 46.7386599624801 44.1452943804044
KIR2DL1 41.7835997867848 39.3667161654772 40.439898672334
41.6045220683604 38.2083710281545 40.5281496800812 41.5817737564592
42.7965563818112 39.8048943625718 45.2556760873335 37.1604861088456
40.9149601380325
KIR2DL3 43.6706764143908 44.2105195734073 43.0971787292711
46.3203995372658 40.3452320167286 39.2248354955045 45.6706505553383
50.3344433595166 41.074824727477 46.8292269513767 39.9070785037411
39.2469742642337
KIR2DL4 44.2760426402717 52.502055890816 41.7364024833895
47.2009420656854 44.5152708955623 44.2335949843431 45.656343062947
50.0991178780329 44.9580538951069 44.9603714724456 42.5153507543837
46.4499258373233
KIR2DL5A 37.3925854497082 39.4693871570028 40.5443356330931
41.8203235468665 38.9953561482209 43.3350508941896 41.74510997753
40.6783687486496 39.2406797798509 39.5196946310402 40.0400013787864
38.9126372022985
KIR2DL5B 38.8336427948187 41.0678614026819 40.8846826807717
44.550322123994 39.3999056968598 41.159706131351 38.5474297403025
38.7215839727591 42.2577689927563 43.0919221375514 39.3667161654772
43.1235886043549
KIR2DS1 40.9558083259517 41.9894412416874 37.3404647796601
38.6414316877471 44.2607789091176 44.4311655729487 41.0871827071116
39.8965727231863 41.2424637338424 40.3235655331796 36.5359455830388
39.1553169409075
KIR2DS2 38.2083710281545 40.6380495994768 43.9450023643394
33.9645433044281 46.7508829977587 40.244271403831 40.1235656101329
40.1474543172332 40.4220196471124 35.6208308220005 41.1638563416981
42.6758568421347
KIR2DS3 42.9104454767916 35.3307157989724 36.3604951341921
37.3600014814388 39.5595233162192 35.5523662009011 42.0605906240248
37.7217193704498 41.1742244762318 36.2435874904689 37.5343260962758
41.9741368971524
KIR2DS4 37.4554876386402 42.1607322031054 37.2838080935695
34.3595588146954 40.5710637884029 41.3553369544097 35.6056062164318
35.0510338774593 42.0621425894624 36.2435874904689 39.9899430628542
43.5738557773903
KIR2DS5 42.6036491382658 39.5050383901884 42.4580504587581
43.3078542962968 40.5710637884029 40.7616966228404 47.4439407315335
42.8482181313226 38.6322728994674 38.528621456378 47.6125796208753
42.8864415494484
KIR3DL1 43.632365631458 44.6388394563024 40.8420925848403
41.8778873678551 37.6119337673806 42.2577689927563 41.4186558780356
44.1642484298135 40.8420925848403 40.3235655331796 41.2267153672559
39.3789825717286
KIR3DL2 50.5670783769975 45.1694246393676 40.4882741869718
45.0798408927912 46.1804647841331 45.6129956328415 43.8206425661746
43.3262148375525 46.7760357462635 45.4614234442527 43.7585538437696
43.4709415392696

KIR3DL3 40.4179476219497 36.7976562409506 42.6920011548175
44.7980740332174 43.5836473788312 38.385865183581 38.8417151851134
40.8833572570383 43.3262148375525 43.8943892311541 38.8173773425835
42.7782216342806
KIR3DP1 39.9697332660833 44.6918600441328 40.4070046183108
40.3180310664156 43.3332476888305 42.2577689927563 38.6243997507646
45.2806199505029 50.401623258584 42.8936990772838 43.0971787292711
38.3291713594599
KIR3DX1 40.0557788909923 42.7775752471711 42.0621425894624
40.1804147506786 41.7929491793697 42.5660695551615 43.5627649099623
46.2779564481158 43.7630587657205 48.3693338695392 45.310660704531
43.2776458914333
KIRREL 38.2083710281545 43.5873188565519 38.7745734037492
38.8495095797881 41.8806343997257 38.5252456306656 38.5066108855005
43.3262148375525 39.2809305246091 44.2580151149956 37.6484782516569
42.357910063876
KIRREL2 40.148780650831 41.413768254554 40.1572444265517
44.4367387274922 40.5710637884029 39.1650142708257 43.9450023643394
38.7981173437121 43.0930293831497 37.6383530120621 40.7776803630095
41.1801554437554
KIRREL3 38.8561375693335 36.4002279956519 33.6816578730693
40.4359177791566 36.5393857306744 37.2686490268265 37.1643693306382
36.9355798143571 40.9901805728485 39.6719190054379 36.2435874904689
42.4607795638552
KISS1 51.7607563002005 48.7847528954724 57.7415362494584 40.7481155008596
65.3019328668633 40.1428591998404 116.913991315353 71.9771425105735
58.6431245059655 47.7180816682562 65.088713927995 83.105117076029
KISS1R 135.106756607914 49.83247261979 50.2869791217819
43.9282059368202 138.895316905243 48.5835484742456 47.1345949868029
42.2577689927563 188.36429659391 41.0057887014368 41.0050831223107
40.0557788909923
KIT 76.3435517544167 64.3640973625938 180.222765336208 101.250422688816
63.955655956484 451.437791473506 174.559216176175 116.694275999729
360.342857415756 214.127390534906 165.349426587915 344.985739524681
KITLG 51.6558970571712 44.7255207589606 45.1912496459517 41.0726336841311
38.1644569189523 107.513442725922 39.5496902678788 42.8899872337087
46.7483258592567 59.0336440347545 52.4752641497096 49.5143331736386
KL 43.1805016690297 43.8495248991589 46.9040704638073 45.1944222548351
39.4693871570028 55.7197525213035 47.5963750520409 44.797320243931
44.5303781239209 54.1683282066029 55.854474649572 45.7562276914441
KLB 47.2862299990938 44.5884811528117 48.8941062980393 40.5484717678957
58.7460488758053 52.8421046802739 42.357910063876 44.5029690493211
44.8003345643334 40.2112371512838 45.6348103033479 44.6484005362281
KLC1 195.127419251015 109.386655134289 164.462951076718 152.146012870339
135.106756607914 234.615033303459 185.264053754114 191.482692108417
188.912691033518 209.401109514058 240.314054155006 182.80711310964
KLC2 45.0241191707651 48.7084357480922 45.8844325913247 40.1321264723693
52.0042367882776 47.6280023169526 65.2966707319433 44.9977782988116
43.1235886043549 43.4885245598939 54.2999306485758 46.7577982595088
KLC3 43.2028319630577 39.9697332660833 42.6861857638473 41.8190986513051
50.8460732110055 41.7309289572934 51.0572809855463 56.2441895183416
48.0163377492228 38.3092241935352 38.9953561482209 42.0778399888414

KLC4	42.267010764315	47.0130468198828	40.3458948319669	40.1385934541471
	42.4514311150237	38.0712544534799	43.26160898693	41.3012916486748
	41.2870505273664	41.6724739851995	38.2293008890613	44.0970208808899
KLF1	42.442231667357	39.6995529770239	39.4710743516517	37.0732977341185
	40.9950639693255	47.2009420656854	44.4639405506967	52.3068458655445
	46.6944238390932	50.7855742231932	45.9104478407175	42.2577689927563
KLF10	60.8357760968871	58.6249721386149	69.8832723783498	72.7934987008399
	53.4921736109774	102.212684008752	48.4859552186772	47.7889447429963
	68.7952490738858	42.0217776707284	48.8088340735921	53.3904307221902
KLF11	234.556113280762	135.836577310513	109.901105821696	191.59357986481
	106.383332356527	258.913621521866	222.16475904496	335.655540947167
	137.764748752401	195.146655512672	207.768377497439	194.435776102991
KLF12	47.3388629326073	47.8020258652388	49.5923712603197	64.4512696384839
	41.5335058664597	59.9546218258435	44.4639405506967	41.5464765641928
	52.0042367882776	52.390086161937	49.3533917376456	49.0048900199083
KLF13	225.827961496781	148.333778341005	149.639066617945	324.320137371059
	74.2283328056604	233.468474830926	67.7965120293445	120.552647575158
	81.3858672064773	99.3142021810133	119.846620703059	137.239944506217
KLF14	35.0202257459026	39.9697332660833	43.0971787292711	42.2496337895968
	41.2740822675592	43.7630587657205	40.3302268293707	41.5464765641928
	42.1594905198929	40.2112371512838	36.0481934423077	44.7401523707775
KLF15	53.3967242431461	46.8954576545929	52.7005092555102	57.2856683017619
	80.428712610358	71.7585486695516	79.7564155881026	49.3880946790306
	51.5888395924843	75.3333024462557	73.845912534353	80.0381580139229
KLF16	43.1805016690297	39.6123051184545	39.5450079747895	36.3604951341921
	41.8806343997257	40.3357777530419	42.8684958525042	40.5158867964016
	45.7452456977857	41.6373771565199	44.7926323492519	38.3291713594599
KLF17	43.0001432913058	38.5252456306656	38.9790184198145	39.7411455670172
	38.8456942580061	43.2805590023412	35.2163900400329	37.4484310268909
	42.5406888281114	41.1222709786153	36.131485826118	40.3691324547049
KLF2	797.019657306322	1433.1813604069	1956.75875151802	1442.72006787259
	164.462951076718	3484.65337999238	341.302185430613	471.734457300752
	95.8075612515186	959.549658414082	1408.59798540643	718.832367050609
KLF3	47.2862299990938	49.4493009852254	44.825830634601	39.5637454855233
	52.4370718282084	44.5544727111028	42.7431359850339	49.9951674107458
	54.9426456410692	42.6793753550854	43.4709415392696	43.6434576713809
KLF4	61.2691385007595	392.008276951929	84.7851173379268	192.98559983091
	58.2780916793348	336.904716156626	82.8485522398913	144.116701129933
	73.9986015964321	106.160416826673	91.9375312869437	99.9951025176909
KLF5	51.413025495745	76.093116738739	63.5107036952143	35.9521941716188
	63.25187762908	49.4157684139858	71.193768715137	63.3768687067745
	133.453860783605	87.9206275762349	70.8670844767965	67.7662831916293
KLF6	583.690350347288	756.801598119483	594.611678976716	1183.62228577053
	410.402285012885	936.978983469829	1401.46625360427	1716.82239359769
	383.578947165143	379.280296622892	790.094848284547	644.368005872231
KLF7	47.3791873993182	36.9994058279362	33.6816578730693	40.7481155008596
	38.2496050953547	48.260931742163	34.806104801006	36.43924066428
	39.4762546236449	39.8873706855774	41.5421429082231	38.8495095797881
KLF8	65.5495324730507	49.6841415268611	48.4890615340603	76.7806009547111
	52.8190882379316	66.295597720304	66.3209349903185	97.1271615802556
	62.275808835675	62.6984594468418	66.8842530423095	67.1675878355908
KLF9	323.295794446334	228.328635242684	372.642038290698	993.626865290891
	398.229956085523	1769.18069954113	168.145003400654	432.669787408904
	165.942795019496	465.266372824212	578.311131274653	833.136660528149

KLHDC1 40.0988483617359 41.9894412416874 41.1742244762318
46.3203995372658 43.004943518025 44.7044140979108 39.3999056968598
45.1151758109609 42.0126083977597 41.6724739851995 41.5668884168669
39.9941474864076
KLHDC10 49.0462740100128 50.543526404059 52.4485219671976
42.7489468897708 52.3242437351389 51.9421032049606 48.3411413510851
45.223075311113 46.2027193115397 39.8873706855774 46.824299585607
46.6397391251762
KLHDC2 226.262409390222 302.667538432677 280.202284365004
198.010793115588 142.731630650904 372.239177888445 58.5087296620514
286.243476131448 246.869486382264 432.669787408904 288.023490151593
395.546110689003
KLHDC3 552.860979481958 323.855522995794 350.796638646601
258.589426470951 454.02808608679 396.355023788839 566.77872642437
266.687281047486 369.717462266442 344.985739524681 366.915943380617
410.157894910571
KLHDC4 118.614027673431 121.236881661832 156.08027400493
96.9476136220382 176.670909065664 108.847044476692 134.024691118762
126.882948837851 99.9057667432209 122.924027019519 131.045615546105
68.2309359831273
KLHDC5 141.649461526745 119.088986733238 97.3066473777771
115.048869605875 112.391188787919 510.525676243746 138.436822676252
117.723425661764 119.700522283289 267.475563253966 283.747865829282
316.185342210304
KLHDC6 34.5886782974123 59.0709442090059 57.423873475544
37.9875302985336 37.956732373465 33.6859433472087 41.4742337714895
36.7460922671619 44.8997235283254 98.501431740607 54.8532558909227
45.8546719310325
KLHDC7A 34.8099089553046 40.4359177791566 44.4311655729487
44.6905691366696 41.1868052124344 43.0860383229288 44.0687697278114
49.5185112777773 39.853866204927 50.7855742231932 43.4709415392696
41.0657892043496
KLHDC7B 38.6454098044474 45.0905928998779 39.1799070031819
39.7411455670172 42.4864576619427 37.9951997351435 50.3519150282938
39.6474008369935 47.27543600687 38.4187686307607 39.9899430628542
42.0424641526302
KLHDC8A 39.1590964332473 38.7344853699576 28.3262844159644
31.7207795618545 31.098971689775 37.3799542961839 35.1307934330047
36.8400213077707 38.9006788676407 33.2987269789328 36.3390947880186
36.6603338274678
KLHDC8B 258.30549459038 101.759714275976 246.500690127803
307.329227013816 613.914396149629 448.383843915421 521.874349042344
135.11808699414 136.843285454801 492.13077103258 404.524605612471
335.569876624791
KLHDC9 42.0532901689036 81.528980339501 136.236041346288
42.357910063876 82.3181867658154 83.2083460752986 111.924999671217
54.7911541269672 340.105993325165 111.669813610213 105.503925445543
100.774098314706
KLHL1 42.9104454767916 39.3999056968598 43.8495248991589 43.9282059368202
41.5668884168669 40.040778349137 39.1539892144371 41.0381860307445
47.7086093893038 43.7303813515864 41.4508241771315 43.3744503319109
KLHL10 36.6480005877129 32.7800533388945 44.0451487108213
39.6292788039793 46.0977687311222 44.8872784170036 38.7337086062875

37.6557714476853 40.5912213523345 39.3698811593364 37.7384958256497 37.9607839934735
KLHL11 45.7356815244513 45.3036821110221 41.6550823405646 41.3027031037343 48.1473938886239 41.9543948167375 43.0269218496197 47.3791873993182 43.2163597439147 41.1222709786153 45.0589947942306 39.8497227447888
KLHL12 173.476817564711 135.138667957956 203.729732499418 136.04447103201 149.792346524401 150.405742308999 150.737482160358 139.554519110944 143.822560762049 156.096296406009 159.095553026723 173.035987237848
KLHL13 46.0023068797812 42.2900457798748 52.3432130135266 39.3455642782085 41.4397539024976 68.7780894982816 36.5729485312886 42.9301944191913 47.0804681460898 69.1623287497421 60.2165699959135 55.3563533836982
KLHL14 41.0970259989281 41.554942319225 38.35720098134 46.0228732507872 37.10170285405 39.7697110924474 41.9393838138203 42.4580504587581 40.4220196471124 39.3999056968598 40.8681871019812 40.5703581862752
KLHL15 43.2724556187214 40.8880530359822 41.528370959196 58.5406981871153 36.7356360093603 43.2805590023412 51.0013344286851 47.2009420656854 41.7627910693483 43.4885245598939 35.6041926503637 40.8681871019812
KLHL17 59.046385957117 61.0144918827511 45.5574745588721 53.4785152499923 64.1080166181228 46.5387044784895 56.393737074538 63.5295088521896 48.0163377492228 53.3059058105836 53.9918882088235 52.9609836781499
KLHL18 61.2130609867477 65.6721629145797 54.4384603094793 56.6458456890715 81.4621222177298 61.4944752562671 58.8993834673475 62.4219686957673 71.0820747228972 77.1767494799711 66.5063354430003 67.6138276415458
KLHL2 57.0006212637829 49.83247261979 58.5212649095879 116.295564219236 54.1225249245036 49.1013967533186 48.4859552186772 48.1591264215184 58.4200819624972 65.429256309049 68.0414563597154 82.0124493733776
KLHL20 81.0295607064541 96.1710806345503 120.401643722713 79.7937658787441 103.080153415433 153.626578526803 90.1500004950245 58.8022152133979 68.3157692917711 78.5141640924736 99.8323169037315 93.6260859565297
KLHL21 67.3816715975056 76.3583555666815 64.9072331703985 106.8866235778 122.531570028682 143.478533731355 60.7992483126292 77.2048580540589 60.7942162859036 122.23209417744 116.304054038726 103.955842514553
KLHL22 310.821299796803 136.517445122438 124.032371632524 175.936150174315 183.165559564289 239.117228656704 313.763121699885 203.946427426126 161.7095047889 215.15351875081 238.510952165358 136.355036262681
KLHL23 45.3872406432794 56.3560498705517 50.6004078448405 43.1847628331565 69.6330393135101 69.4214927857472 51.1541843174981 39.853866204927 40.3316153483289 48.9826868640753 46.2560238004757 53.4735821194294
KLHL24 211.596652798395 227.059460958349 153.028210322286 187.802182308625 203.82788889213 174.933650466403 328.46798329618 230.787791652978 242.899629257173 357.668448180268 253.398345052444 319.248898194029

KLHL25 43.7585538437696 46.3635803515159 37.1643693306382
39.2780515621926 42.0709558258414 40.3357777530419 56.4809146784765
55.6904617926772 48.3954967265007 51.4454142248566 58.9676332048764
47.9566297250148
KLHL26 59.1311136551108 48.3699741879723 56.2441895183416
56.4578213622965 86.4195039096886 64.3383155834968 46.0228732507872
41.7419877611072 60.0736861987141 60.6266092582071 65.342842101283
59.5080588072694
KLHL28 96.3232257371077 82.0897118579449 74.2769881586739
111.071209057746 59.3611824459962 89.8567957512307 60.6548851977964
170.172720177618 81.066448172567 131.380698046552 148.514056973195
129.675613712326
KLHL3 96.4971762611189 158.806855832703 188.900125060451 134.725028732282
120.046457114882 109.607667469015 70.3935694362568 126.882948837851
157.7623351008 222.453547273598 136.377736482671 168.407377937447
KLHL30 45.3872406432794 42.1607322031054 43.1931731665131
42.0102671859606 38.3561682218911 40.7616966228404 37.1643693306382
42.2577689927563 41.9635525160083 46.1390887492649 42.8263096141654
43.5738557773903
KLHL31 39.7576136507593 37.2810767789306 43.401782064778
38.8495095797881 38.745552403895 37.3799542961839 33.5578650644246
34.1806289878747 37.7384958256497 34.3674468322242 31.6063174764616
38.9496515344837
KLHL32 39.6609238036815 42.3550738181144 41.785676764591
42.4580504587581 39.3667161654772 43.0860383229288 43.5627649099623
37.6557714476853 39.7537274655115 39.8873706855774 34.3633615460307
41.3481355128089
KLHL34 45.0985088502363 41.4508241771315 44.9758339877851
36.3604951341921 39.7568367479301 35.4142595025086 35.6056062164318
36.0306975524309 42.2577689927563 40.6022003765153 43.844855336215
35.9604262434141
KLHL35 74.3135387703106 55.0009147092272 91.0443726041992
66.224559122062 60.1730663840476 67.8208976798364 68.0157730844432
78.6911549149983 64.830448408529 59.1496467460507 60.6505672212119
58.9069025298754
KLHL36 162.569762535436 204.370291548631 235.925374041038
155.287591646469 217.189489936251 113.090530993447 202.795613942391
113.064858029749 215.15351875081 99.8699868526053 104.398842509776
125.92333271598
KLHL4 40.8091178323174 37.2103401831788 37.5597671487621 37.4223289687135
37.0526522140242 47.0940613507321 39.1539892144371 38.4128104869434
37.4937394063612 39.6251842352088 40.9683275553869 43.1235886043549
KLHL5 188.272588632909 113.388372364837 124.777882272835 334.527003257901
209.363955566338 321.686871145301 112.60230393988 140.465247047676
125.735441231594 152.545682001987 170.999578495858 254.639522735592
KLHL6 51.5888395924843 61.2108152457954 53.3127618245672 166.752205770965
42.6022652963326 45.3882138838137 40.4359177791566 46.9986083378817
42.9020321355927 51.0055825931636 48.2050207071554 47.7962548556026
KLHL7 63.7066330986416 58.174555206394 62.2685634967698 62.8201270538435
64.2848885182563 73.4208306290635 64.7023558190673 64.1777305915695
65.6003544901554 61.3005673792118 57.3697604614404 66.4229480716045
KLHL8 173.603999265239 191.6998930948 192.43245357593 250.993396750789
257.993462737264 212.219384864571 174.252441615373 288.816567137193
212.353676518748 338.586852166449 297.950627905767 213.204385896543

KLHL9 225.088911324737 213.140641182603 122.924027019519 165.820230766206
135.737771384104 281.906398109158 208.189151365575 126.360723656793
165.662539866309 226.262409390222 236.139267415604 255.598083334598
KLK1 38.304249186058 41.2598458726002 39.3709357615493 41.4273122914951
43.5544347011646 56.9320717885002 46.3057385979341 49.085741080241
45.9227107476843 56.1912096308988 46.4753015651202 52.0359304386476
KLK10 48.6621527194963 41.3277288245157 42.8270038153139 40.3180310664156
47.8075998240396 43.0177448037924 44.2733573199954 42.157627240486
46.6491671626143 43.7303813515864 43.6919270353542 39.8497227447888
KLK11 78.328947112997 46.9578963946094 44.2733573199954 46.1074305700679
41.2424637338424 46.3203995372658 47.2927436772465 88.8039637390786
48.2768592317085 46.1390887492649 57.8991270166934 43.6434576713809
KLK12 45.7356815244513 42.5269793679335 40.6312687664188 36.4098694708483
40.5710637884029 38.8336427948187 37.5469247328848 39.5083491499018
43.6670755115848 41.6724739851995 37.2018803984555 36.7762265039864
KLK13 71.5484567134601 34.8137085420423 29.4322896559759 38.5252456306656
37.5556299264699 41.9543948167375 37.4772922422734 38.745552403895
40.7847110517594 32.9981302187884 33.2774014136617 35.2448979801094
KLK14 42.5193208629123 40.3384318592985 44.0451487108213 40.5484717678957
45.3675390992851 42.0574848021044 40.7910160811527 43.1567024035183
36.0741672064298 36.5806287103157 40.723990462047 37.5556299264699
KLK15 37.3952038370286 39.6123051184545 36.4098694708483 39.6609238036815
39.4092846492473 39.9871741554388 38.4428081526233 36.075550657169
40.2274522635576 38.528621456378 42.6366765186182 42.6208563330129
KLK2 41.3387790895716 42.2114134935898 43.8153459269057 42.281606728072
37.5556299264699 44.3648993568405 39.3585350448719 41.7419877611072
44.7771939752086 39.1832968442811 46.5952288260503 42.7782216342806
KLK3 42.5193208629123 38.2263227947189 40.5289532121417 39.853175885492
40.4098450813524 38.3956445058349 37.9430145836712 38.7981173437121
36.7657614042911 38.6454098044474 37.1617810121175 39.4888702378044
KLK4 81.2359429678503 36.6968906669565 39.7411455670172 40.3180310664156
30.1388203764747 37.0180934800573 43.9450023643394 55.9303347977781
37.9273653326866 38.4187686307607 36.131485826118 35.5523662009011
KLK5 55.7246872330059 40.3384318592985 41.2365089689635 46.614092935749
42.9090346219023 41.7196307387855 41.294200401933 39.0102066700839
41.4636047919453 42.7211272990786 40.3248602167143 34.3409660445447
KLK6 156.382146982116 40.148780650831 37.0187570354067 72.9752827496503
41.9842695818285 40.7616966228404 39.5496902678788 44.293922970134
38.8495095797881 40.6533975461148 41.0050831223107 39.9941474864076
KLK7 45.9090659324223 48.9259041798949 44.6032218589199 59.8102324370568
45.1899271235974 44.7044140979108 36.4564050256915 46.0633179231318
44.5678064264717 45.3530683538086 46.5650556481721 48.0350549043063
KLK8 46.0398513158469 44.5544727111028 43.3465699185405 42.1273928203021
39.6535880152834 38.7340969861754 41.5817737564592 43.7731874801003
40.5912213523345 40.5090849993014 39.153226699662 40.7704587752083
KLK9 33.9561816279577 37.10170285405 44.0451487108213 38.0958223740111
38.5512429290626 41.4508241771315 40.2154497375715 43.844855336215
38.0213051227859 46.8954576545929 38.3684202018524 41.9430737035925
KLKB1 36.2496364442264 36.6002100641539 36.8730514568925 35.0448343468967
34.4570158133896 44.3648993568405 38.1793598983615 37.0390589468421
35.860430241131 45.9004543328976 41.9908237694988 33.6902293666089
KLKBL4 37.3463875295726 38.9338521051669 40.1985790927248
38.745552403895 42.1650127805175 37.5887129572848 42.0605906240248

	34.6977013306396	37.5469247328848	41.0057887014368	32.2133093774284
	41.1554481567603			
KLKP1	37.8386378737561	39.0894172982439	39.7411455670172	43.1847628331565
	36.08956004634	37.1317006162938	42.357910063876	41.8417024632349
	40.6957787921451	39.3698811593364	41.9908237694988	38.6454098044474
KLRA1	40.0557788909923	47.5647821785529	48.8941062980393	46.8954576545929
	42.4864576619427	42.4580504587581	38.2933870238743	42.357910063876
	55.5086026637973	45.6868589181768	38.8507804383261	47.1060577218993
KLRB1	89.4747568145717	91.640286556353	146.543232229227	567.534792333871
	44.9936360641969	75.4227827818591	73.0433648756396	78.0020909283414
	46.581796165231	101.197890058764	96.4278951689874	94.8717345601966
KLRC1	40.9558083259517	42.8142224866049	41.7442113152211	41.7217466249678
	41.2740822675592	40.800866389165	42.2577689927563	42.4864576619427
	41.3481355128089	42.1414432370095	41.9083581529276	45.1735152121056
KLRC2	35.290191727027	38.2263227947189	39.0775870687758	37.7146582037126
	37.0105110201759	38.4330755397458	45.0283997415478	35.9489900178061
	37.2810767789306	39.9519547348106	43.9450023643394	39.7844133452634
KLRC3	41.0136069709699	43.4031723677362	41.1678908661272	42.8878262561526
	41.7929491793697	40.9967936671247	40.6130792100165	38.4085832916721
	41.4417765092034	39.6333888530279	42.911855821602	40.5703581862752
KLRC4	32.6285977114039	41.159706131351	43.9450023643394	48.9944486068347
	40.4639532172338	38.8336427948187	41.3594868055879	46.1673159982664
	42.5406888281114	45.5776159432079	42.6366765186182	43.3744503319109
KLRD1	45.6989664108554	43.2028319630577	49.7350276169552	60.0981687048269
	37.292108575069	44.6524308640784	40.3850714987723	44.0687697278114
	41.809704036355	46.1756277225778	47.6125796208753	45.0035308952466
KLRF1	37.6817357354748	43.0227387991135	62.2200723764909	43.074472921841
	43.9994592139885	42.6211340086915	39.7576136507593	40.931294690669
	127.232160404726	43.5652644712197	48.2910530887214	41.1554481567603
KLRG1	42.8539910132415	45.8284004440739	49.33855351691	84.2013403597582
	42.7982502551634	61.8187341713197	54.6414321010929	42.8482181313226
	43.3262148375525	45.4882438975269	46.4753015651202	47.2862299990938
KLRG2	49.4151347850687	51.119812302881	48.4890615340603	49.5424425850549
	62.588825192561	55.401776072578	62.6885730386156	51.1694248620208
	65.9254098858352	54.6089451252049	56.6884575165011	55.2449003905956
KLRK1	43.3638918705225	40.8880530359822	42.2577689927563	39.9697332660833
	40.7779767565889	49.3172204533818	44.7255207589606	44.7044140979108
	44.8003345643334	46.5650556481721	42.5426057453993	48.3648494350286
KMO	42.2455707947223	41.159706131351	37.1643693306382	62.3936376596431
	46.8674366115656	40.040778349137	37.9430145836712	42.4580504587581
	33.2715481910159	42.3606390470387	34.174642681781	38.745552403895
KNDC1	45.8669209716694	40.1073950092563	43.0971787292711	41.1886975241176
	39.4693871570028	45.5772632530809	39.1878770155069	39.853866204927
	39.9871741554388	41.3321861371202	42.589641010388	41.1138630151981
KNG1	39.7576136507593	46.4861258030402	40.931294690669	42.0102671859606
	47.2074332636566	38.9378717994178	39.0458123429605	42.357910063876
	44.0519245668737	44.8997235283254	37.5583886316038	48.3648494350286
KNTC1	198.754967214875	163.16724817696	168.522052983892	172.720244584464
	175.136856246782	78.0586153423713	292.389429869034	140.559370165331
	200.566335168667	91.6713614282714	74.8473087205709	116.007007037602
KPNA1	149.832238573027	114.617965877564	150.801729138762	162.932479392087
	189.137593177772	111.240804216088	110.837476359817	162.513675180449
	120.26537515666	116.311176407088	109.503117647487	134.869029698594

KPNA2 71.2246788429078 81.0057400164851 60.7530213660442 49.260242314197
121.945468001236 57.6034762511847 110.223625398261 73.1045688753569
128.391264770998 44.1379638573455 46.2560238004757 52.1186591838715
KPNA3 91.31756001203 97.8764110456132 84.5998418020787 115.468303687641
92.3034609409756 121.744701580607 89.7242042805798 77.6174893940285
114.404099081799 99.3142021810133 104.715234047628 131.717764850211
KPNA4 449.059703234741 295.856741468603 280.563427715895 464.025908364057
563.927521628693 694.590958116425 382.744696166534 525.35784336478
312.795596385258 612.843402058384 524.047008889711 621.489785652602
KPNA5 47.7267190613906 42.6939394537076 42.8270038153139 44.550322123994
40.0599807618105 45.9266745571724 44.3440839686558 41.2551966132486
43.7630587657205 46.441301934328 46.1673159982664 47.7962548556026
KPNA6 386.934057829091 360.668308246913 444.858305070415 290.81245174957
525.687522832693 421.013324906569 372.642038290698 774.8306350567
444.937258895152 717.997180920384 739.541009338849 469.30167430316
KPNB1 1411.12613289991 1236.32289322872 1163.0370991733 1163.0370991733
1413.36279821142 1327.93833415582 1704.0522304387 1261.91478863258
1783.87583818834 775.515071756239 1040.97380790412 878.863554510943
KPRP 37.4554876386402 46.0023068797812 49.6347864008749 40.7481155008596
40.4639532172338 40.650742261428 38.4005795891396 40.2382908228517
40.4997186069494 41.2276963324981 32.406093465115 41.3481355128089
KPTN 45.2875173643252 43.896638326678 45.0985088502363 39.1706564265495
42.8510579683751 45.9980734733005 40.369768755239 44.4031868028216
50.0802438185974 43.4885245598939 48.042366934301 44.0451487108213
KRAS 86.4937293696262 77.6174893940285 80.7338782543351 95.3070802596041
62.3862792065884 88.6287852712744 98.1371527802953 73.1045688753569
122.203872796773 66.8693882433359 69.5544840164738 74.7794253940974
KRBA2 47.9175508887533 66.3904351698776 60.8357760968871 56.0794100106654
51.4527545197672 51.294537308199 54.0170817319522 47.5950227477807
40.137818555383 95.5156363110528 93.7977486287985 76.1453055441256
KRCC1 92.3034609409756 72.2016884438365 86.0761288363404 95.4393274079354
57.8039717833131 119.947688643528 70.1184116246724 108.025082988807
76.5699943825825 83.7215159198897 88.1614325571117 108.649424858333
KREMEN1 47.4964755141472 61.5236837309467 65.3531590366085
55.7418331108915 49.6733735285073 53.4921736109774 59.7629007954215
46.3685439301234 58.2941346100716 50.1314528043038 51.4413099192899
54.8360406467395
KREMEN2 54.2931724501801 47.664340982223 43.4558010540988
45.0798408927912 50.977432027367 48.303052868477 49.6872467499977
44.081163104701 45.3756389170606 40.6573239942138 39.3667161654772
40.8254641406268
KRI1 55.9249770393176 57.0875538213735 55.9459886217809 46.7577982595088
73.2607982527105 48.260931742163 56.0515443021293 55.5578334877425
60.164635768614 54.7827221235949 52.7817052791259 54.1090536870282
KRIT1 66.5500089527674 84.1224653052549 106.679905803492 74.4851052263387
84.0305689846475 102.430234282331 87.539553421006 68.580705761277
95.5530572805104 68.1209771323232 69.7215372275927 82.6602736441839
KRR1 58.2902084992082 88.9321928608078 66.4079972657486 61.7502624458447
70.7760293129408 92.9820860411485 69.1414574152997 56.3560498705517
75.2337946941103 50.0564735608363 53.4088637045445 68.0751111916102
KRT1 43.6706764143908 52.3187919317561 39.0775870687758 44.6905691366696
40.4639532172338 65.3096524773304 42.8684958525042 45.2806199505029
39.2809305246091 46.7646877499666 40.4504608195666 37.1747156131306

KRT10 443.863346367342 647.297964303385 593.801086643125 287.247399926139
811.142611128461 486.487366153374 965.530928317269 378.626656077546
932.849256536912 352.47015903013 581.093069802913 568.638529128128
KRT12 41.5421429082231 44.1239711172417 78.1683130800805 41.6564075217517
44.2607789091176 37.2686490268265 46.5650556481721 65.3019328668633
45.1899271235974 39.6719190054379 40.6373400186325 42.6758568421347
KRT126P 44.9370339275041 44.9863726205428 45.310660704531
42.281606728072 47.4548471240051 45.2852528185486 43.4544762830742
46.6384636367629 44.6717213146407 44.6918600441328 45.9938357766653
42.6278608611758
KRT13 54.3554233228353 1018.35211991691 5733.54417656207 356.628599887098
7393.77508374057 1716.82239359769 506.173054520807 3634.98300564068
18219.5845257111 7681.71833395626 3190.0108307463 4058.32430616734
KRT14 42.8036731479932 164.511418506192 93.9472621843566 75.0377156190383
53.4109882045447 44.1737502492846 45.8980298854198 56.8094693588238
53.2046867327331 42.4594149893798 43.3085358214154 41.4508241771315
KRT15 53.8856501960117 237.974766880957 331.533249823589 38.0958223740111
55.4919197421746 45.2852528185486 52.0942589368478 90.0223113095186
452.797096652556 70.0907269565408 52.104880481326 70.8546350449171
KRT16 145.419126322238 87.8681250426295 147.424598105182 84.5411149237799
85.352285669007 47.4699236780425 65.2361525412756 121.37214545992
138.581471125405 55.1804535845496 47.1477056711335 62.8050945727712
KRT17 339.088535171641 1194.10154988793 2506.05102956991 411.114836576804
1634.61795848352 259.626847216881 987.427672417105 1436.67510926345
1792.42490815074 913.704049709264 447.348799009312 1149.90692951795
KRT18 51.413025495745 70.3213394542625 63.6334154351075 43.607701594929
62.7488501311848 42.5193208629123 76.0041771381018 75.0377156190383
96.2058974277481 43.9450023643394 43.5258090162298 48.9733758107788
KRT19 7627.46814033012 8630.67463560037 16661.6081002448 361.776841675393
7476.94487791412 1559.50766982801 7681.71833395626 3399.75820494572
14226.3743910711 4036.89580181979 4093.70002166179 3379.67054115975
KRT2 43.3638918705225 39.0894172982439 40.4882741869718 41.5464765641928
37.0526522140242 32.2133093774284 39.3585350448719 35.2642250366721
44.4395580493118 36.3612409063792 38.2795150354677 48.6795178347248
KRT20 82.5344501505114 327.631923082696 186.229678647062 42.0102671859606
115.048869605875 57.0775902639215 228.08291792003 423.989018031638
105.434580236651 44.7958122466628 58.9676332048764 39.9941474864076
KRT222P 41.6354649482853 40.716553247236 39.4710743516517
47.3574589211166 36.6354013629791 72.0674952635945 43.4544762830742
40.7311497914871 42.9020321355927 70.7805103907761 53.0177144132449
55.3563533836982
KRT23 69.6449298794042 51.340484930067 49.2041300973417 52.3004056941891
53.6907197364586 52.2089306840125 118.27400376579 54.6476581536041
75.2337946941103 59.6246428440926 52.5154971256566 52.3304586399695
KRT24 41.3387790895716 47.6229101519945 37.7384958256497 49.9014504704021
43.7517118200398 131.126942328593 36.7356360093603 44.1311654985682
47.798305839406 60.9905272201951 55.4694986901287 94.1128427766432
KRT25 41.0608773166058 37.10170285405 37.2838080935695 37.7469621225553
40.5710637884029 39.3841861812262 39.4600924554505 35.2642250366721
38.6322728994674 35.93950183586 34.174642681781 36.3454320848591
KRT26 41.2773842727759 40.0984333134909 38.5442780220289 54.1240583139871
39.3667161654772 38.3418933808844 41.5464765641928 45.063792968927
42.2577689927563 37.7598032934773 39.3086414047188 41.0226232127351

KRT27 39.6609238036815 37.6383530120621 34.3633615460307 38.8495095797881
40.8873907150245 64.6835308827804 40.7910160811527 35.6825474397235
40.4220196471124 36.5806287103157 44.2142581488029 36.1647506311964
KRT28 36.1232327599945 41.7337506166619 43.9450023643394 38.745552403895
43.4544762830742 38.5252456306656 34.0777108441756 41.8417024632349
35.860430241131 39.2623423164488 38.2795150354677 40.6614700944853
KRT3 44.5282181324797 44.2630383225933 43.7630587657205 48.9944486068347
40.0599807618105 43.7630587657205 44.8643898647322 46.8954576545929
41.4636047919453 43.401782064778 38.8507804383261 47.9566297250148
KRT32 31.4517139158876 35.6434124419782 40.4070046183108 38.5252456306656
34.806104801006 36.8059561571675 27.4352294118391 35.1738399826814
36.7267557061938 38.1042454705119 34.4716904283753 37.5556299264699
KRT33A 41.483412492426 42.9887069977696 44.696023817801
39.853175885492 41.1507980856436 44.7044140979108 45.1694246393676
43.6753843488695 47.6582396278888 42.785429206422 42.4580504587581
42.1537476443423
KRT33B 44.8914371498528 41.159706131351 50.3950981988762
36.0481934423077 40.2449343247539 38.4330755397458 42.0605906240248
42.6450179841129 39.5806465938526 41.5435611403119 33.5370783156523
45.3763275588341
KRT34 39.266565827316 39.2907645923217 33.9435456570176 37.0732977341185
49.5666074434383 40.9506062495231 31.098971689775 38.745552403895
39.7537274655115 44.2580151149956 39.5399188536544 40.8681871019812
KRT36 43.3638918705225 40.0527893189096 43.401782064778 46.0228732507872
40.8873907150245 39.4953494583071 41.0381860307445 46.5717043290731
46.581796165231 45.1569741609597 42.8263096141654 40.8681871019812
KRT37 35.6111578834671 35.4484652445078 40.4070046183108 40.9607305725196
41.2740822675592 37.2686490268265 41.253645578517 34.6977013306396
37.0126425326534 36.0481934423077 39.5399188536544 37.3600014814388
KRT38 44.6231541342 38.4330755397458 44.2405728317107 39.7411455670172
38.1644569189523 38.9378717994178 42.4939802311136 40.6171774724171
36.197424053556 41.7405682794166 36.131485826118 37.1747156131306
KRT39 41.678190413467 37.3179578037963 40.0998167098057 42.6222445667107
40.9416717019827 40.2945586900975 41.253645578517 44.4031868028216
43.4656637536244 43.6033221838149 43.1931731665131 38.6454098044474
KRT4 49.2595762436995 48.275452803061 71.3527183280827 44.0451487108213
51.7983110178095 33.2552842693684 39.8695929627783 202.360546931172
65.3672880026114 40.4136535208406 39.6403089864678 34.806104801006
KRT40 30.493888765264 34.0382693188794 36.3604951341921 42.0102671859606
37.5556299264699 30.493888765264 31.6981395162348 42.5435283762933
38.7298222155532 35.3849221682845 35.6041926503637 42.357910063876
KRT5 40.3478825593224 127.043177339038 561.2769907009 93.4853597306163
54.3116862772198 42.0574848021044 42.2577689927563 164.96500173066
757.545320379227 164.917385239962 53.8856501960117 57.1441484662045
KRT6A 368.972794063004 379.79657860938 947.656220635965 729.342532316666
56.9360840563854 48.5835484742456 47.7390230243778 661.269882993465
310.188313944313 48.7414853386615 43.5710871617107 42.6758568421347
KRT6B 50.1833483241864 44.8969632710144 46.0241950824438 57.8277957325085
42.352235481637 40.4359177791566 43.4870236357993 49.6203214143464
45.0755084221375 42.5153507543837 42.4238335236362 37.0390589468421
KRT6C 71.6849360818041 65.3694267397114 149.515611848932 39.1706564265495
44.7829632472754 37.9951997351435 45.3597029614942 144.324036829708
56.9320717885002 39.4710743516517 43.5710871617107 38.9496515344837

KRT7	428.105433953405	2184.70038900784	5525.13078364599	194.395766807005
	2712.36664370642	401.679080975109	3884.2191293028	3166.48568397766
	5696.39636379286	2071.90741263566	1539.35554229258	1263.76838179117
KRT71	41.4508241771315	40.5400384479766	35.7105247506212	37.651298468202
	43.2484996523549	44.1737502492846	43.0930293831497	40.931294690669
	43.0930293831497	44.2580151149956	37.6484782516569	36.7762265039864
KRT72	39.1590964332473	44.6918600441328	46.2625470410195	39.7411455670172
	45.4751076037511	42.157627240486	40.6750540874299	42.4580504587581
	47.27543600687	41.7405682794166	45.0589947942306	46.5650556481721
KRT73	41.9407041937973	48.7084357480922	40.0998167098057	44.7980740332174
	46.1804647841331	42.357910063876	44.4639405506967	37.33030683349
	37.1820543575123	44.571915452417	37.1059647239814	43.6753843488695
KRT74	39.3709357615493	37.10170285405	31.6419446793642	38.8495095797881
	32.2133093774284	36.9060997041453	38.941855825269	38.3684202018524
	39.9444191873552	40.7063426028871	38.8507804383261	31.1733235029813
KRT75	45.5758521267508	38.6414316877471	40.7620472083159	44.4367387274922
	45.1137938810047	37.3799542961839	37.2892641289258	38.1835938174625
	41.074824727477	37.3179578037963	38.8507804383261	43.7672102682067
KRT76	39.6609238036815	44.1239711172417	41.2365089689635	41.5464765641928
	39.859345745831	39.3841861812262	39.2533668167483	37.5511560880902
	41.1742244762318	41.9666541498638	41.0050831223107	38.5279745308203
KRT77	41.7397731376692	45.1694246393676	72.7987593815919	44.6905691366696
	37.8679241954337	38.1169703906207	39.2533668167483	42.6450179841129
	44.5303781239209	34.9138625012006	40.9157352260901	38.3291713594599
KRT78	37.7384958256497	37.4540772310118	41.2365089689635	41.8778873678551
	37.5556299264699	37.2686490268265	44.9863726205428	39.4710743516517
	43.9450023643394	39.8873706855774	40.8225507267584	47.3992733452821
KRT79	42.442231667357	41.159706131351	38.2280071951674	46.1673159982664
	43.3332476888305	46.6872654833579	43.8206425661746	43.3262148375525
	40.6957787921451	39.9948390976317	43.3085358214154	37.4554876386402
KRT8	2225.65559724391	1413.36279821142	1416.47763911039	96.2385885003071
	564.929183025034	197.569816397993	1202.11455437028	2474.22199172028
	1465.08435775014	648.380417557148	954.116675685628	367.931222555457
KRT80	98.2280780221631	105.739423435604	46.5650556481721	43.9744941494111
	74.6833250738044	43.3785777889265	105.166073980781	145.335751135441
	125.835967514239	57.2231446406982	85.1603116461516	87.4623442823265
KRT81	38.5648843914189	51.119812302881	36.178547300352	42.9358255168086
	33.7494761745342	40.4359177791566	38.2933870238743	41.9315419791594
	40.9901805728485	42.9866214102525	42.911855821602	38.4330755397458
KRT82	44.6231541342	36.2103905554984	40.5734458834617	39.2780515621926
	41.2740822675592	38.4330755397458	49.540449125788	37.1317006162938
	39.5806465938526	41.3321861371202	39.8184668132023	38.745552403895
KRT83	37.2474303302343	41.8848959280916	47.9035532886869	40.646702696033
	44.5668141895992	47.7556758818053	56.2181240696479	43.9366081640576
	40.2274522635576	49.4006068471301	46.972521499515	41.863927676552
KRT84	42.9104454767916	36.1232327599945	40.4882741869718	47.8164193292842
	43.1252272136667	37.8102105794655	35.3362091871364	39.2672712041774
	39.853866204927	36.2435874904689	38.4689132737495	39.9519547348106
KRT85	36.3649677956953	40.148780650831	41.437237454134	41.8778873678551
	40.148780650831	42.4580504587581	46.8613212048981	38.1835938174625
	34.8552366193358	41.9666541498638	42.911855821602	39.9519547348106
KRT86	68.4352167527791	64.408176016319	60.6567691532561	51.0175677516029
	50.5110359971301	41.4508241771315	68.878562323012	103.631449539809
	53.3932110698117	65.429256309049	87.4273245333478	65.3585701203568

KRT9 59.987045425047 51.2405397507224 46.9040704638073 63.1604223919703
50.8875036861067 49.5424425850549 59.0457625163577 68.2309359831273
51.1055541075029 62.1035858593388 59.9682734755814 55.1804535845496
KRTAP1-3 41.7397731376692 40.9731067463382 44.8872784170036
39.1706564265495 41.5668884168669 39.8455060654531 36.4098694708483
49.2901038327894 38.5252456306656 38.957687788868 43.0971787292711
38.745552403895
KRTAP1-5 45.3872406432794 43.0971787292711 45.6989664108554
45.334106390212 41.3755760434934 41.159706131351 39.6535880152834
47.7889447429963 47.7086093893038 44.6918600441328 39.6403089864678
38.2350720635382
KRTAP10-1 42.442231667357 37.1685072112618 42.5406888281114
39.1706564265495 40.3452320167286 39.0266821712099 42.357910063876
39.5466975353131 40.8970401536578 36.3612409063792 39.153226699662
41.3481355128089
KRTAP10-10 42.8482181313226 42.985929467107 44.6032218589199
39.5558610526094 45.6355219961567 44.7058233759912 43.8664428040913
42.6450179841129 43.5669306063196 47.0055981216687 44.1642484298135
43.4390398501329
KRTAP10-11 43.2724556187214 47.4740726032707 48.4890615340603
42.5664883002833 39.6535880152834 49.7458652239071 42.4939802311136
43.5544347011646 45.9227107476843 45.1569741609597 49.4593570700221
44.6484005362281
KRTAP10-12 39.0620487644635 35.3307157989724 35.7105247506212
40.9607305725196 40.7779767565889 38.2293008890613 34.1889222434768
38.2782436497441 35.5220586859301 36.1550293353654 36.3390947880186
35.2448979801094
KRTAP10-2 45.4507786740825 43.2649781354792 41.8751578329365
39.7411455670172 40.6308525754377 38.385865183581 45.017247592678
41.3012916486748 40.2739700860114 39.7411455670172 42.8263096141654
39.1553169409075
KRTAP10-3 41.9407041937973 38.6414316877471 36.0306975524309
39.5050383901884 42.3685136297603 48.260931742163 46.3057385979341
37.9837233601316 46.4164127487052 44.0409178714232 39.4410515050885
38.9496515344837
KRTAP10-4 34.2180669723984 40.6380495994768 43.1931731665131
45.5869891570251 39.1650142708257 40.1428591998404 42.357910063876
39.0620487644635 38.7298222155532 48.4890615340603 42.7275315121163
37.2810767789306
KRTAP10-5 32.2133093774284 36.3110701990588 34.0382693188794
37.4223289687135 37.4470183315826 39.8455060654531 32.2133093774284
35.7973685350741 26.6357008041456 40.8970401536578 39.7342718293432
40.1700117452801
KRTAP10-7 34.4896350146096 35.2143190355414 34.5718473203083
37.5382094267692 35.5220586859301 39.9397419279057 37.0564573649223
37.4484310268909 36.8127605061228 33.1559412791294 33.6080693875435
38.4330755397458
KRTAP10-8 40.5654775358427 44.4411734611229 37.1643693306382
41.8778873678551 47.0954207417646 46.574682061365 46.9929836306816
39.0620487644635 38.4344387289956 43.2988017098387 49.532546810992
40.0557788909923
KRTAP10-9 43.2135791259317 43.6587492470348 44.9758339877851
43.7540438144592 43.6587492470348 44.5099734963815 45.4799270968969

45.223075311113 39.2497056720346 46.2768056702424 41.3553369544097 44.2527566343661
KRTAP11-1 38.8561375693335 44.4411734611229 42.9104454767916 42.9358255168086 43.7517118200398 43.5752393599169 36.197424053556 41.7419877611072 40.5912213523345 45.1569741609597 38.4689132737495 43.5738557773903
KRTAP12-1 43.0930293831497 46.4385386401326 43.4709415392696 49.5424425850549 42.3685136297603 42.4580504587581 50.2016912396771 47.5950227477807 44.0519245668737 43.401782064778 48.042366934301 47.3992733452821
KRTAP12-2 35.9305494063137 44.0409178714232 43.4709415392696 44.0451487108213 51.2525124735102 36.2768299545794 39.0458123429605 43.6434576713809 43.3744503319109 42.4594149893798 46.7386599624801 41.6550823405646
KRTAP12-3 38.1169703906207 42.2577689927563 39.5450079747895 35.7674462337723 42.1650127805175 38.9378717994178 38.7337086062875 39.1652965788406 41.074824727477 41.3321861371202 41.2598458726002 41.9430737035925
KRTAP12-4 37.2474303302343 38.3322849790657 37.9482157381008 47.481302504066 41.467005949568 35.8227939208521 39.7576136507593 39.2672712041774 45.3756389170606 40.4136535208406 40.1742127891824 41.4508241771315
KRTAP13-1 41.187562301961 46.5421017969405 42.7881985796277 40.8084239472012 42.8510579683751 45.4607426241257 50.4260283682427 44.8997235283254 46.7414272831181 42.233842834629 43.8002456102021 42.1860813954352
KRTAP13-2 38.5007204246293 42.1607322031054 44.2405728317107 44.550322123994 44.9936360641969 37.5887129572848 37.3799542961839 39.7509165247419 39.1790517315259 42.0621425894624 40.6373400186325 38.3291713594599
KRTAP13-3 40.3478825593224 40.0527893189096 42.157627240486 41.0726336841311 44.4489662507893 45.2852528185486 41.706879587091 43.1567024035183 46.0106098064475 39.3698811593364 36.9060997041453 45.0035308952466
KRTAP13-4 40.7609952437622 42.7775752471711 38.4043916290561 35.8472302065546 41.1868052124344 48.1591264215184 40.9157352260901 41.6466169548846 42.9020321355927 47.611018474151 43.7585538437696 39.2469742642337
KRTAP15-1 34.5886782974123 36.9994058279362 38.6005272730664 40.646702696033 40.9950639693255 39.2248354955045 41.9393838138203 30.2675713925613 36.8127605061228 36.0481934423077 36.3390947880186 42.7782216342806
KRTAP17-1 44.7915315085948 37.7384958256497 38.3166554965434 42.357910063876 40.8873907150245 39.132725377815 35.6056062164318 36.1375121409002 40.4220196471124 39.9948390976317 36.9060997041453 39.7411455670172
KRTAP19-1 35.2267149016596 40.7952353240579 35.5220586859301 36.0481934423077 40.148780650831 39.9397419279057 37.6036146841933 38.2782436497441 38.6322728994674 44.1379638573455 36.7283081422707 38.4330755397458
KRTAP19-2 43.1805016690297 41.0678614026819 37.9482157381008 43.074472921841 37.4470183315826 32.2133093774284 44.4639405506967 47.3791873993182 40.3316153483289 36.8059561571675 38.079222289614 44.2527566343661

KRTAP19-3 35.8208542506595 39.6123051184545 47.0055981216687
33.1817617228171 41.8806343997257 38.1169703906207 40.3302268293707
36.5393857306744 42.992884918158 41.1222709786153 39.6403089864678
35.2448979801094
KRTAP19-4 38.385865183581 40.7952353240579 38.4043916290561
41.7614975630362 37.6527901109005 44.7804080899431 38.7337086062875
39.9576138799997 40.8970401536578 42.5664883002833 41.8168470823451
39.1553169409075
KRTAP19-5 36.5393857306744 38.5252456306656 35.2838283525874
32.2212308728305 34.0927410219282 41.3553369544097 31.4474799869345
33.5578650644246 37.9273653326866 38.1042454705119 32.1218888714537
35.0222940937513
KRTAP19-6 51.1740855632044 53.0383634135393 53.9612654850046
176.27162536342 48.0234896130991 56.6172748471831 51.3064929551674
55.8087097725119 46.1101825640108 50.0564735608363 51.2318935933116
55.9555622193202
KRTAP19-7 45.3872406432794 37.5469247328848 37.3687900733549
39.853175885492 39.4693871570028 39.4953494583071 40.7910160811527
42.9301944191913 38.7298222155532 37.5343260962758 42.2618615571566
42.57175366709
KRTAP2-1 42.8539910132415 41.0226232127351 38.941855825269
39.3455642782085 39.3999056968598 38.9378717994178 42.4939802311136
37.86982638952 36.7604001262514 41.3641352453779 39.9070785037411
45.6233434183356
KRTAP2-2 39.512606833112 40.3384318592985 39.3709357615493
42.0621425894624 38.6213341190836 34.8956628705329 44.9863726205428
37.33030683349 39.2809305246091 39.5637454855233 36.3810228376302
37.3057614736707
KRTAP20-1 39.1590964332473 42.5269793679335 38.9790184198145
40.9607305725196 39.5595233162192 37.7000400506818 36.08956004634
44.5029690493211 49.3780606970538 41.7405682794166 39.0550139257658
40.7704587752083
KRTAP20-2 38.8561375693335 44.0409178714232 49.2041300973417
44.265834798964 43.2484996523549 37.2686490268265 39.2533668167483
42.9301944191913 42.8036731479932 41.1222709786153 45.9104478407175
45.2783024981315
KRTAP21-1 42.442231667357 44.1239711172417 44.3344900481112
47.3574589211166 45.1137938810047 48.9826868640753 45.223075311113
47.6952179922944 46.0106098064475 47.0055981216687 43.844855336215
43.6753843488695
KRTAP21-2 46.7293238583888 36.3110701990588 41.9666541498638
40.7481155008596 44.7829632472754 39.7394181011603 42.9803717121865
46.695848687694 47.0804681460898 39.5637454855233 41.0808257270221
53.2046867327331
KRTAP22-1 39.3709357615493 39.6995529770239 35.2101597814352
36.3604951341921 43.1252272136667 40.4359177791566 39.7576136507593
34.1806289878747 40.6957787921451 38.0078357808393 38.4689132737495
36.9976231206067
KRTAP23-1 39.6609238036815 40.148780650831 41.437237454134
48.1662933382789 46.4343327861696 38.3418933808844 42.9803717121865
40.1474543172332 40.9901805728485 43.4885245598939 40.3452320167286
37.4554876386402
KRTAP24-1 42.8036731479932 40.148780650831 43.4709415392696
38.8495095797881 38.1644569189523 40.9506062495231 49.540449125788

40.1474543172332 46.2027193115397 42.6793753550854 43.0016969050339
39.0536056496869
KRTAP26-1 44.2350046179299 40.7952353240579 41.437237454134
41.1886975241176 35.4484652445078 46.1513019317765 39.3585350448719
46.3685439301234 44.4395580493118 39.8873706855774 44.6286581923295
44.5432192783161
KRTAP27-1 42.466333667839 42.6450179841129 43.7630587657205
45.8102081251786 40.5235471987417 42.9104454767916 39.9559575274857
43.0930293831497 42.357910063876 45.3530683538086 40.5824352835642
39.5792604499113
KRTAP3-1 36.6062356000449 42.0709558258414 40.6312687664188
38.0540928703622 41.4397539024976 41.7627910693483 37.830876898596
39.853866204927 40.1738310955128 39.528971131769 40.723990462047
44.3521099857368
KRTAP3-2 40.3180310664156 40.5400384479766 40.7952353240579
40.5991006167568 42.9775348174342 41.8890826572422 41.1818632392526
41.6825727187466 41.7117861328546 41.3321861371202 41.1367478979484
41.809704036355
KRTAP4-1 59.9232969481163 43.4396866157171 46.4569566520107
40.9607305725196 57.5685357492202 38.5252456306656 55.2088411137193
37.86982638952 45.3756389170606 41.9666541498638 41.7272875295469
39.1553169409075
KRTAP4-11 38.2083710281545 46.8236022402545 36.0306975524309
35.6027791404152 39.3667161654772 41.6590564937735 44.4639405506967
40.4359177791566 37.1820543575123 37.8217989841918 41.4508241771315
38.9496515344837
KRTAP4-12 43.2724556187214 41.2598458726002 45.5883690965192
40.9607305725196 46.0043807840705 38.3418933808844 37.1643693306382
36.3405039629059 41.9635525160083 41.1222709786153 40.8225507267584
42.7782216342806
KRTAP4-2 37.1485601150873 36.5219488315447 40.931294690669
45.7397504535169 39.4693871570028 34.0045980663271 39.0458123429605
38.8495095797881 37.0126425326534 40.8049408996274 39.2542963855807
41.9430737035925
KRTAP4-3 38.385865183581 40.6380495994768 36.8730514568925
44.6905691366696 42.0709558258414 36.6271061451613 38.2933870238743
45.8669209716694 38.2215591930186 40.6022003765153 45.310660704531
38.6454098044474
KRTAP4-4 41.7397731376692 41.4508241771315 40.6737160001291
40.5484717678957 37.2567512265363 40.040778349137 41.8315602975157
36.2539950294089 35.9645119596869 41.1222709786153 41.9083581529276
44.0451487108213
KRTAP4-5 40.9558083259517 33.1817617228171 37.7384958256497
38.9534635879195 38.8456942580061 39.7394181011603 40.4359177791566
31.3302670771182 43.9450023643394 40.4136535208406 34.7308008643142
41.3481355128089
KRTAP4-7 41.9407041937973 41.8073910693411 42.0621425894624
44.265834798964 46.2478069975709 42.357910063876 42.22763273471
38.3684202018524 41.863927676552 38.3501188844251 41.3553369544097
42.828737648529
KRTAP4-8 40.3478825593224 46.7591817286632 40.1985790927248
37.4223289687135 42.7982502551634 46.6872654833579 43.6753843488695
41.6466169548846 43.0930293831497 43.6033221838149 38.1835938174625
43.6753843488695

KRTAP5-1 39.5676051939342 34.6306468196142 40.5734458834617
37.7469621225553 43.004943518025 40.5281496800812 37.3799542961839
34.9580369221164 42.992884918158 39.6719190054379 42.170702295259
36.2582325396375
KRTAP5-10 42.442231667357 44.3529919625344 43.6670755115848
41.7614975630362 39.2608760193011 37.7000400506818 37.1643693306382
36.1375121409002 32.1218888714537 40.2112371512838 37.3687900733549
47.2131144508464
KRTAP5-11 44.7915315085948 37.920964499903 41.437237454134
46.7577982595088 39.4693871570028 40.3357777530419 40.9157352260901
41.1575777497576 40.2274522635576 40.8049408996274 40.2649309204478
41.1554481567603
KRTAP5-2 32.2133093774284 51.9421032049606 49.2041300973417
41.4273122914951 36.5393857306744 38.1169703906207 43.3262148375525
38.6454098044474 46.3203995372658 56.3441077510744 49.0361180089431
50.5511827666954
KRTAP5-3 38.7537364570873 44.5152708955623 37.0642268327439
46.3203995372658 34.0927410219282 32.2133093774284 36.197424053556
42.157627240486 31.372171135652 37.1275334308172 38.4689132737495
33.3192849177028
KRTAP5-4 38.304249186058 40.4359177791566 34.4716904283753
34.8137085420423 37.5556299264699 39.8455060654531 40.5585676679482
46.3685439301234 42.4540072551568 39.8873706855774 39.6403089864678
36.7762265039864
KRTAP5-5 42.8036731479932 40.2493681589104 40.931294690669
38.6414316877471 37.956732373465 41.8613406887824 42.357910063876
37.6557714476853 38.3418933808844 45.1569741609597 39.2542963855807
39.7411455670172
KRTAP5-6 35.7012997405508 42.4493032770053 43.5710871617107
33.6816578730693 36.7356360093603 41.159706131351 38.941855825269
39.2672712041774 44.2607789091176 40.3235655331796 38.8507804383261
36.7762265039864
KRTAP5-7 40.3478825593224 43.5182502375207 40.0998167098057
40.7481155008596 39.1650142708257 40.8634043028528 43.4544762830742
42.7431359850339 42.1594905198929 43.6033221838149 42.5426057453993
41.2437378488025
KRTAP5-8 33.8558232866797 37.7384958256497 41.3366779545815
38.8495095797881 38.5512429290626 39.7394181011603 38.4005795891396
38.079222289614 38.3418933808844 42.2614524730856 40.3452320167286
43.1805016690297
KRTAP5-9 39.1590964332473 46.1569135229922 37.2838080935695
35.314469458379 37.7576900881741 40.3357777530419 38.0598374201407
36.7460922671619 33.4698406117406 43.7303813515864 42.5426057453993
42.57175366709
KRTAP6-1 46.0023068797812 40.5400384479766 36.3604951341921
36.9912468186322 38.8456942580061 40.244271403831 39.2533668167483
40.047314525032 38.8271346375425 37.1275334308172 39.4410515050885
42.1537476443423
KRTAP6-2 41.1523487516364 39.6995529770239 46.2625470410195
46.4555908533915 39.0564222526273 49.641159251878 42.7431359850339
38.6454098044474 42.1594905198929 45.7911932481722 45.5707650534259
40.8681871019812
KRTAP6-3 39.3709357615493 46.3635803515159 36.769073068743
38.2041059863839 34.1686574241133 37.9003288112261 48.9826868640753

40.047314525032 39.7537274655115 35.0668743708816 43.1931731665131
37.4554876386402
KRTAP7-1   43.1805016690297 41.7337506166619 50.2869791217819
53.2046867327331 44.9936360641969 42.5660695551615 43.8206425661746
38.5457716679071 47.4699236780425 40.7063426028871 47.6884372645373
[truncated: 427,695 more chars]
