# Supplementary material for: Novel robust biomarkers for human bladder cancer based on activation of intracellular signaling pathways
Source: Oncotarget. 2014 Sep 16;5(19):9022–32. doi: 10.18632/oncotarget.2493 (PMC4253415; doi:10.18632/oncotarget.2493)
Supplement: Supplementary file 11 [file oncotarget-05-9022-s011.pdf]

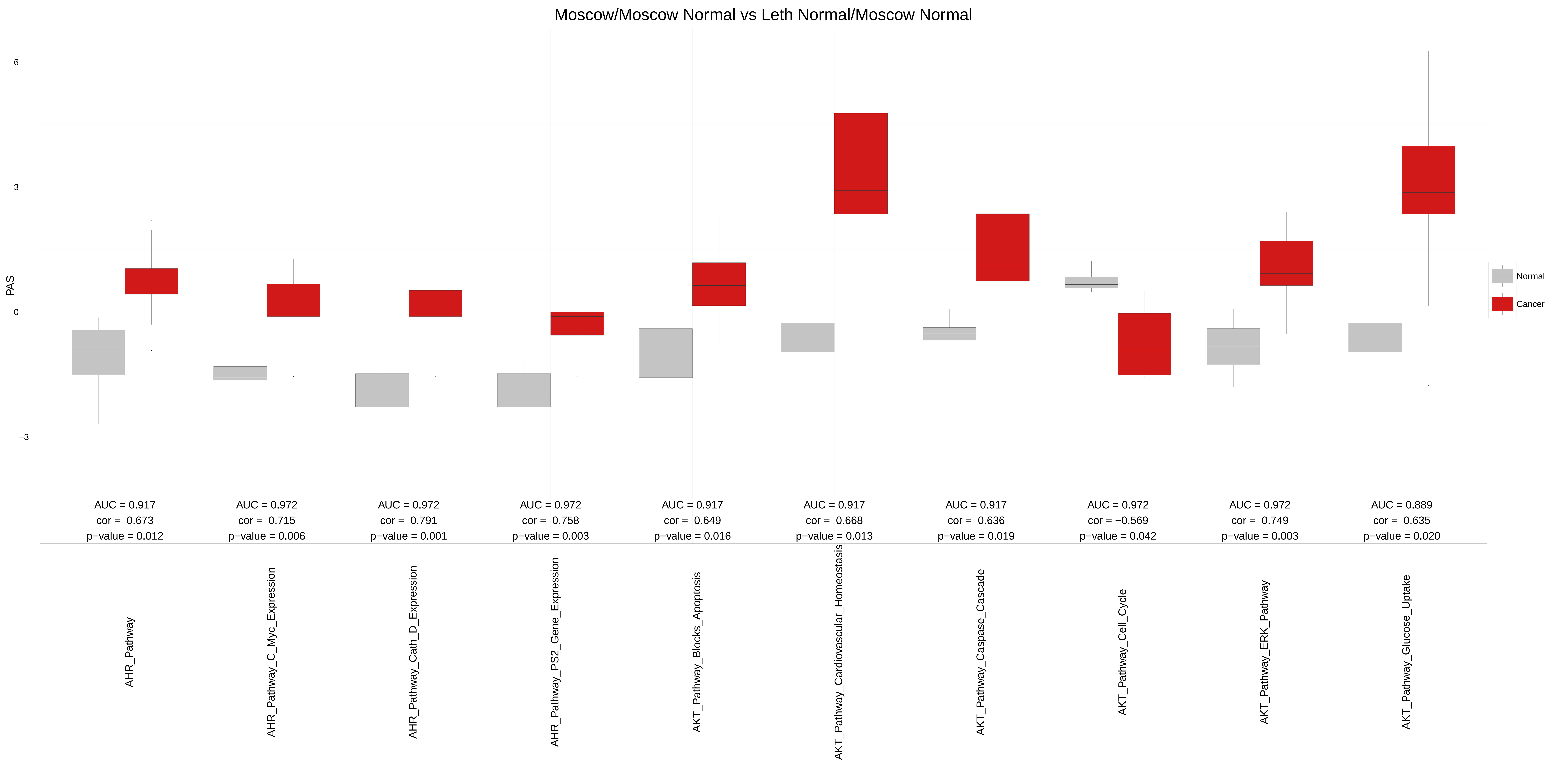
Moscow/Moscow Normal vs Leth Normal/Moscow Normal
PAS
6
3
0
−3
Normal
Cancer
AUC = 0.917
cor = 0.673
p−value = 0.012
AUC = 0.972
cor = 0.715
p−value = 0.006
AUC = 0.972
cor = 0.791
p−value = 0.001
AUC = 0.972
cor = 0.758
p−value = 0.003
AUC = 0.917
cor = 0.649
p−value = 0.016
AUC = 0.917
cor = 0.668
p−value = 0.013
AUC = 0.917
cor = 0.636
p−value = 0.019
AUC = 0.972
cor = −0.569
p−value = 0.042
AUC = 0.972
cor = 0.749
p−value = 0.003
AUC = 0.889
cor = 0.635
p−value = 0.020
AHR_Pathway
AHR_Pathway_C_Myc_Expression
AHR_Pathway_Cath_D_Expression
AHR_Pathway_PS2_Gene_Expression
AKT_Pathway_Blocks_Apoptosis
AKT_Pathway_Cardiovascular_Homeostasis
AKT_Pathway_Caspase_Cascade
AKT_Pathway_Cell_Cycle
AKT_Pathway_ERK_Pathway
AKT_Pathway_Glucose_Uptake

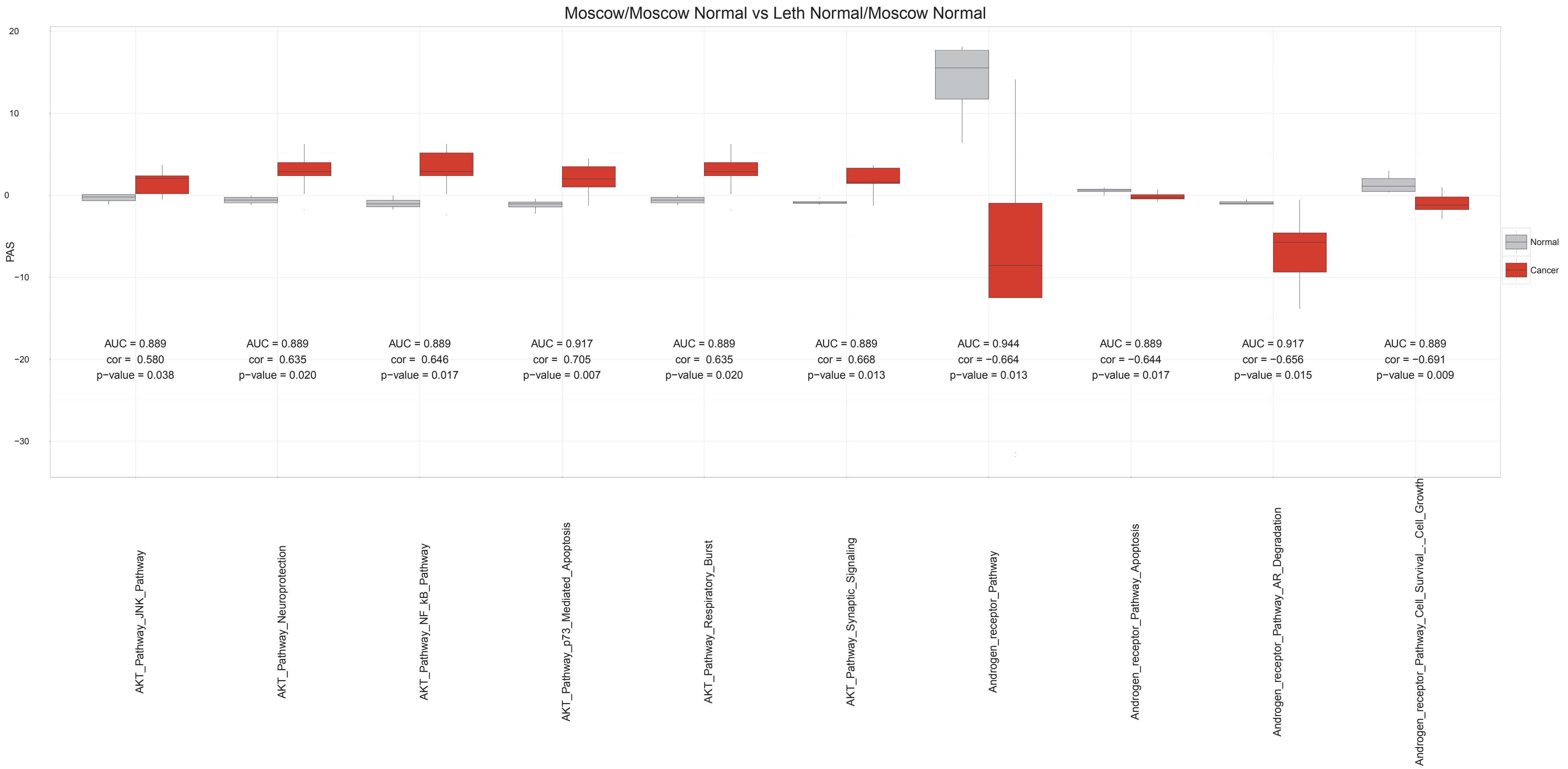
Moscow/Moscow Normal vs Leth Normal/Moscow Normal
20
10
0
PAS
−10
−20
−30
Normal
Cancer
AUC = 0.889
cor = 0.580
p−value = 0.038
AUC = 0.889
cor = 0.635
p−value = 0.020
AUC = 0.889
cor = 0.646
p−value = 0.017
AUC = 0.917
cor = 0.705
p−value = 0.007
AUC = 0.889
cor = 0.635
p−value = 0.020
AUC = 0.889
cor = 0.668
p−value = 0.013
AUC = 0.944
cor = −0.664
p−value = 0.013
AUC = 0.889
cor = −0.644
p−value = 0.017
AUC = 0.917
cor = −0.656
p−value = 0.015
AUC = 0.889
cor = −0.691
p−value = 0.009
AKT_Pathway_JNK_Pathway
AKT_Pathway_Neuroprotection
AKT_Pathway_NF_kB_Pathway
AKT_Pathway_p73_Mediated_Apoptosis
AKT_Pathway_Respiratory_Burst
AKT_Pathway_Synaptic_Signaling
Androgen_receptor_Pathway
Androgen_receptor_Pathway_Apoptosis
Androgen_receptor_Pathway_AR_Degradation
Androgen_receptor_Pathway_Cell_Survival_._Cell_Growth

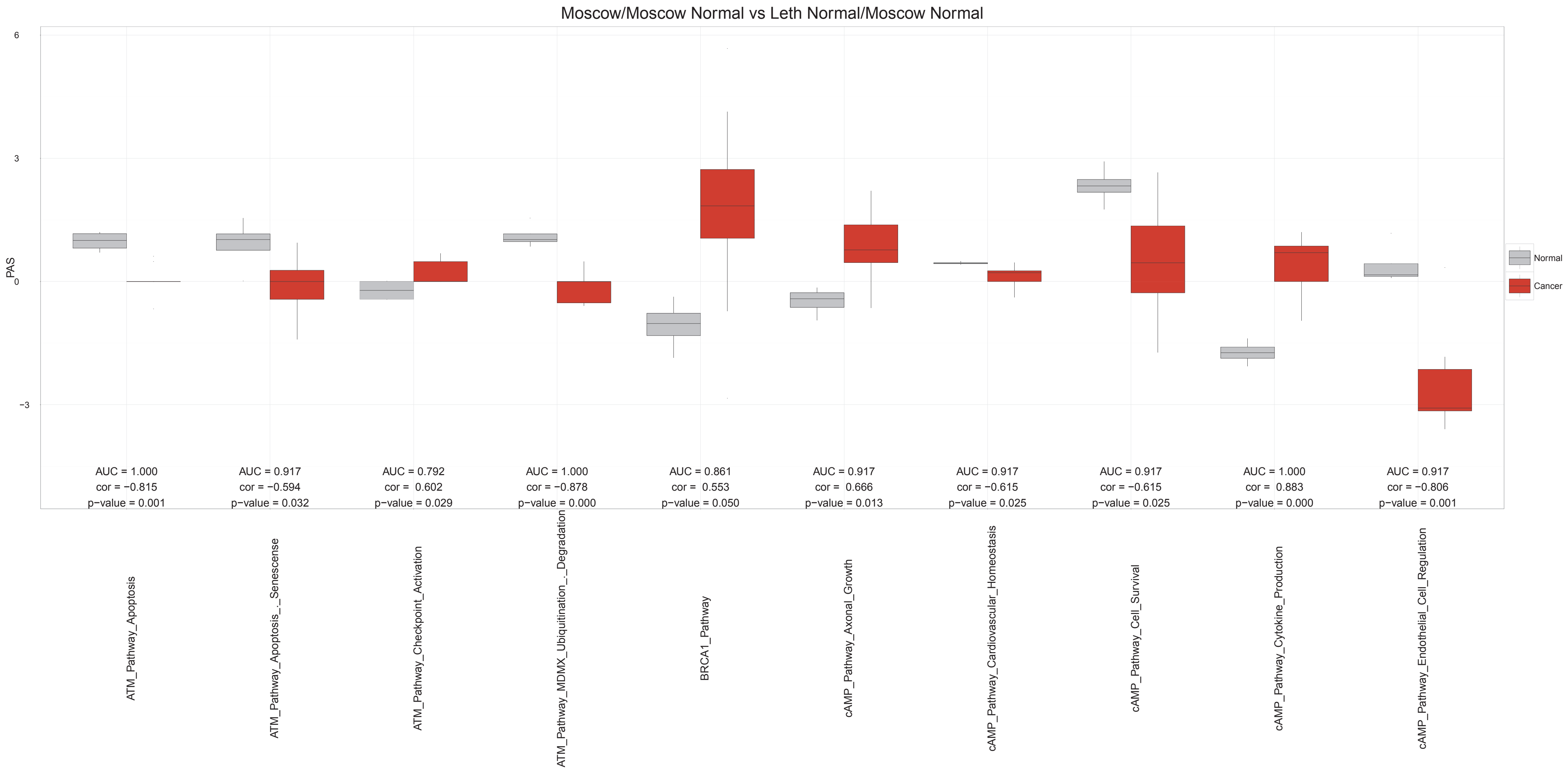

Moscow/Moscow Normal vs Leth Normal/Moscow Normal
PAS
6
3
0
−3
Normal
Cancer
AUC = 1.000
cor = −0.815
p−value = 0.001
AUC = 0.917
cor = −0.594
p−value = 0.032
AUC = 0.792
cor = 0.602
p−value = 0.029
AUC = 1.000
cor = −0.878
p−value = 0.000
AUC = 0.861
cor = 0.553
p−value = 0.050
AUC = 0.917
cor = 0.666
p−value = 0.013
AUC = 0.917
cor = −0.615
p−value = 0.025
AUC = 0.917
cor = −0.615
p−value = 0.025
AUC = 1.000
cor = 0.883
p−value = 0.000
AUC = 0.917
cor = −0.806
p−value = 0.001
ATM_Pathway_Apoptosis
ATM_Pathway_Apoptosis_._Senescense
ATM_Pathway_Checkpoint_Activation
ATM_Pathway_MDMX_Ubiquitination_._Degradation
BRCA1_Pathway
cAMP_Pathway_Axonal_Growth
cAMP_Pathway_Cardiovascular_Homeostasis
cAMP_Pathway_Cell_Survival
cAMP_Pathway_Cytokine_Production
cAMP_Pathway_Endothelial_Cell_Regulation

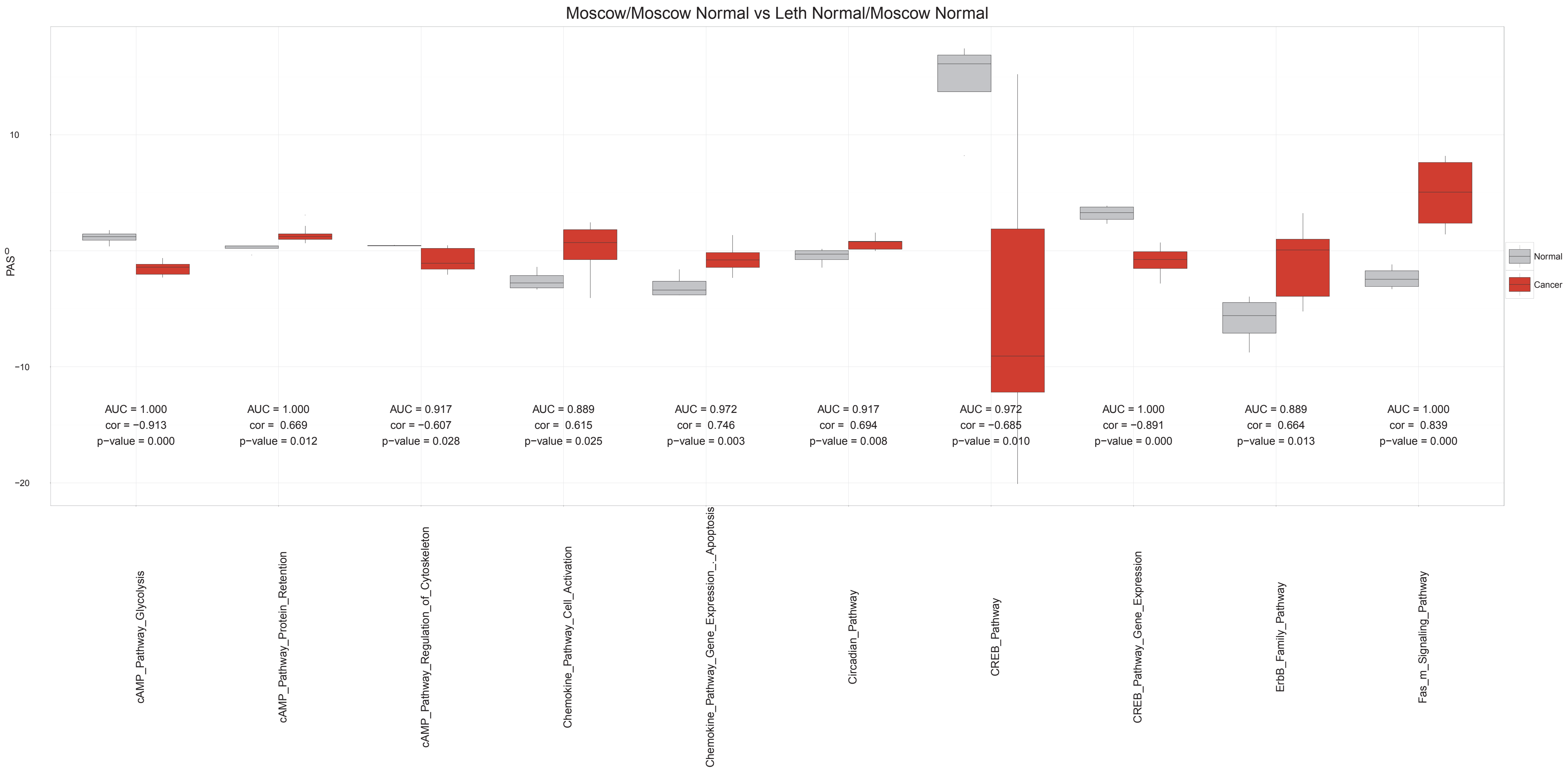
Moscow/Moscow Normal vs Leth Normal/Moscow Normal
PAS
10
0
−10
−20
Normal
Cancer
AUC = 1.000
cor = −0.913
p−value = 0.000
AUC = 1.000
cor = 0.669
p−value = 0.012
AUC = 0.917
cor = −0.607
p−value = 0.028
AUC = 0.889
cor = 0.615
p−value = 0.025
AUC = 0.972
cor = 0.746
p−value = 0.003
AUC = 0.917
cor = 0.694
p−value = 0.008
AUC = 0.972
cor = −0.685
p−value = 0.010
AUC = 1.000
cor = −0.891
p−value = 0.000
AUC = 0.889
cor = 0.664
p−value = 0.013
AUC = 1.000
cor = 0.839
p−value = 0.000
cAMP_Pathway_Glycolysis
cAMP_Pathway_Protein_Retention
cAMP_Pathway_Regulation_of_Cytoskeleton
Chemokine_Pathway_Cell_Activation
Chemokine_Pathway_Gene_Expression_._Apoptosis
Circadian_Pathway
CREB_Pathway
CREB_Pathway_Gene_Expression
ErbB_Family_Pathway
Fas_m_Signaling_Pathway

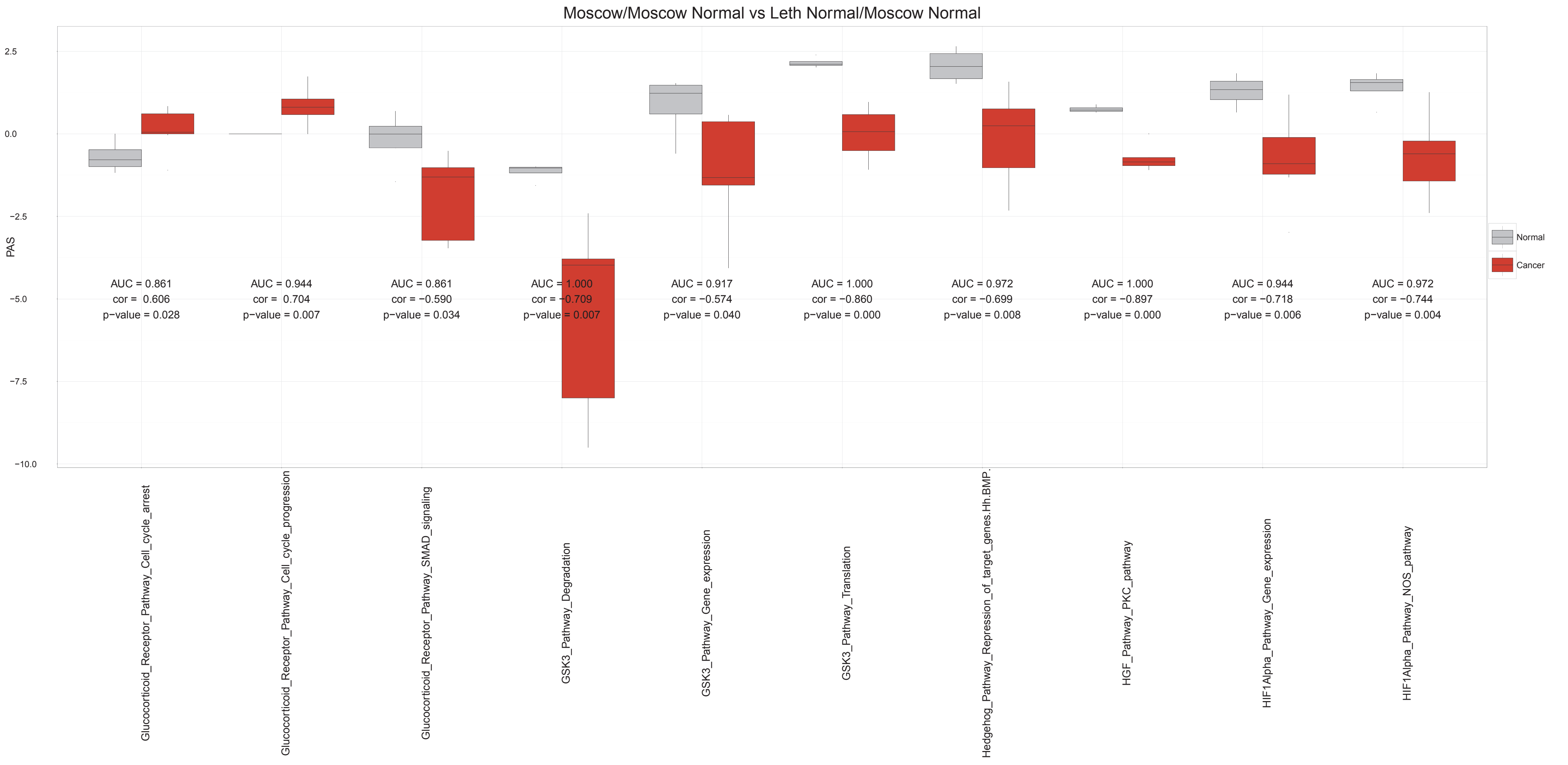

Moscow/Moscow Normal vs Leth Normal/Moscow Normal
PAS
2.5
0.0
−2.5
−5.0
−7.5
−10.0
Normal
Cancer
AUC = 0.861
cor = 0.606
p−value = 0.028
AUC = 0.944
cor = 0.704
p−value = 0.007
AUC = 0.861
cor = −0.590
p−value = 0.034
AUC = 1.000
cor = −0.709
p−value = 0.007
AUC = 0.917
cor = −0.574
p−value = 0.040
AUC = 1.000
cor = −0.860
p−value = 0.000
AUC = 0.972
cor = −0.699
p−value = 0.008
AUC = 1.000
cor = −0.897
p−value = 0.000
AUC = 0.944
cor = −0.718
p−value = 0.006
AUC = 0.972
cor = −0.744
p−value = 0.004
Glucocorticoid_Receptor_Pathway_Cell_cycle_arrest
Glucocorticoid_Receptor_Pathway_Cell_cycle_progression
Glucocorticoid_Receptor_Pathway_SMAD_signaling
GSK3_Pathway_Degradation
GSK3_Pathway_Gene_expression
GSK3_Pathway_Translation
Hedgehog_Pathway_Repression_of_target_genes.Hh.BMP.
HGF_Pathway_PKC_pathway
HIF1Alpha_Pathway_Gene_expression
HIF1Alpha_Pathway_NOS_pathway

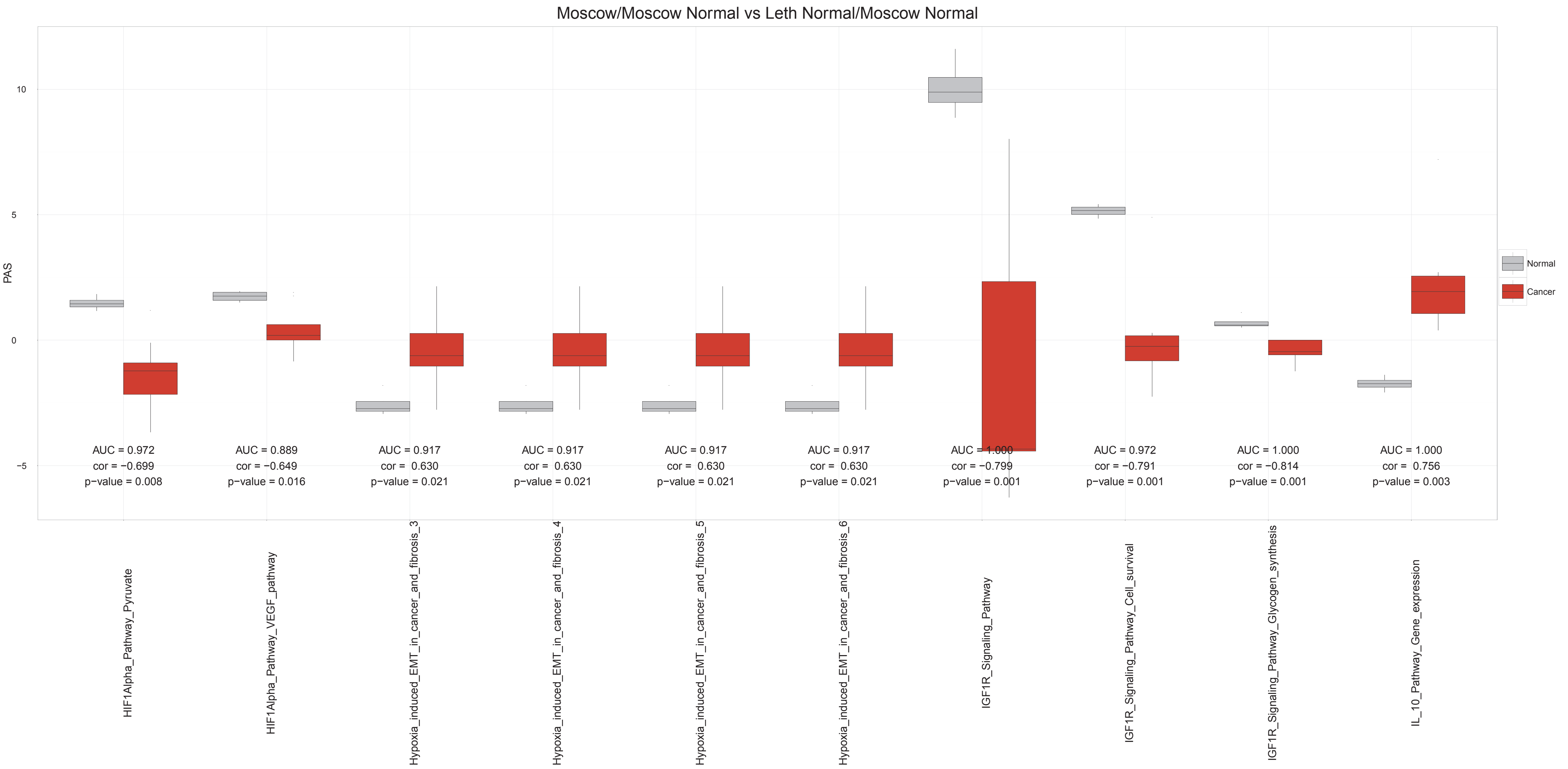

Moscow/Moscow Normal vs Leth Normal/Moscow Normal
PAS
10
5
0
−5
Normal
Cancer
AUC = 0.972
cor = −0.699
p−value = 0.008
AUC = 0.889
cor = −0.649
p−value = 0.016
AUC = 0.917
cor = 0.630
p−value = 0.021
AUC = 0.917
cor = 0.630
p−value = 0.021
AUC = 0.917
cor = 0.630
p−value = 0.021
AUC = 0.917
cor = 0.630
p−value = 0.021
AUC = 1.000
cor = −0.799
p−value = 0.001
AUC = 0.972
cor = −0.791
p−value = 0.001
AUC = 1.000
cor = −0.814
p−value = 0.001
AUC = 1.000
cor = 0.756
p−value = 0.003
HIF1Alpha_Pathway_Pyruvate
HIF1Alpha_Pathway_VEGF_pathway
Hypoxia_induced_EMT_in_cancer_and_fibrosis_3
Hypoxia_induced_EMT_in_cancer_and_fibrosis_4
Hypoxia_induced_EMT_in_cancer_and_fibrosis_5
Hypoxia_induced_EMT_in_cancer_and_fibrosis_6
IGF1R_Signaling_Pathway
IGF1R_Signaling_Pathway_Cell_survival
IGF1R_Signaling_Pathway_Glycogen_synthesis
IL_10_Pathway_Gene_expression

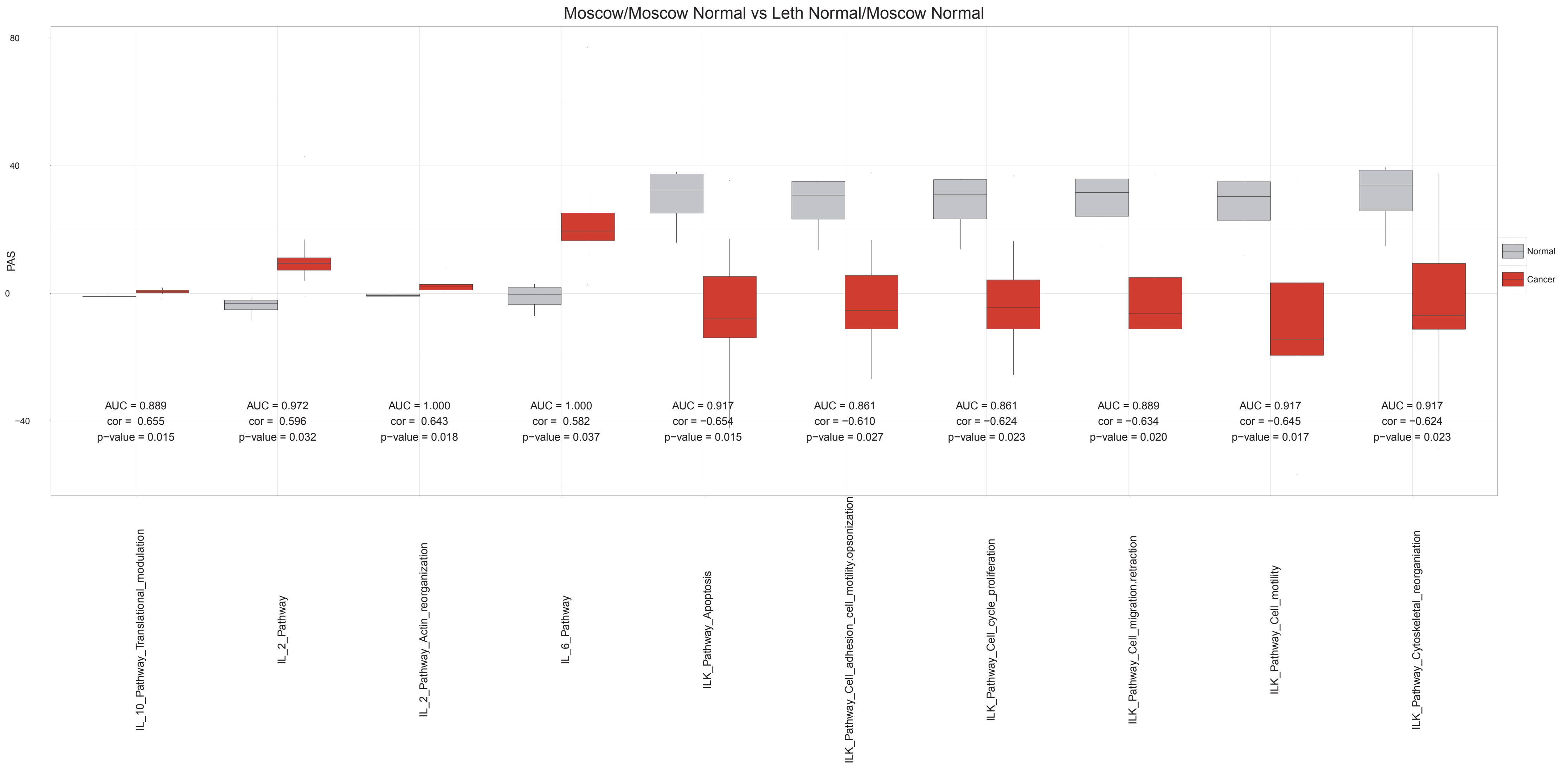
Moscow/Moscow Normal vs Leth Normal/Moscow Normal
PAS
80
40
0
−40
Normal
Cancer
AUC = 0.889
cor = 0.655
p−value = 0.015
AUC = 0.972
cor = 0.596
p−value = 0.032
AUC = 1.000
cor = 0.643
p−value = 0.018
AUC = 1.000
cor = 0.582
p−value = 0.037
AUC = 0.917
cor = −0.654
p−value = 0.015
AUC = 0.861
cor = −0.610
p−value = 0.027
AUC = 0.861
cor = −0.624
p−value = 0.023
AUC = 0.889
cor = −0.634
p−value = 0.020
AUC = 0.917
cor = −0.645
p−value = 0.017
AUC = 0.917
cor = −0.624
p−value = 0.023
IL_10_Pathway_Translational_modulation
IL_2_Pathway
IL_2_Pathway_Actin_reorganization
IL_6_Pathway
ILK_Pathway_Apoptosis
ILK_Pathway_Cell_adhesion_cell_motility.opsonization
ILK_Pathway_Cell_cycle_proliferation
ILK_Pathway_Cell_migration.retraction
ILK_Pathway_Cell_motility
ILK_Pathway_Cytoskeletal_reorganiation

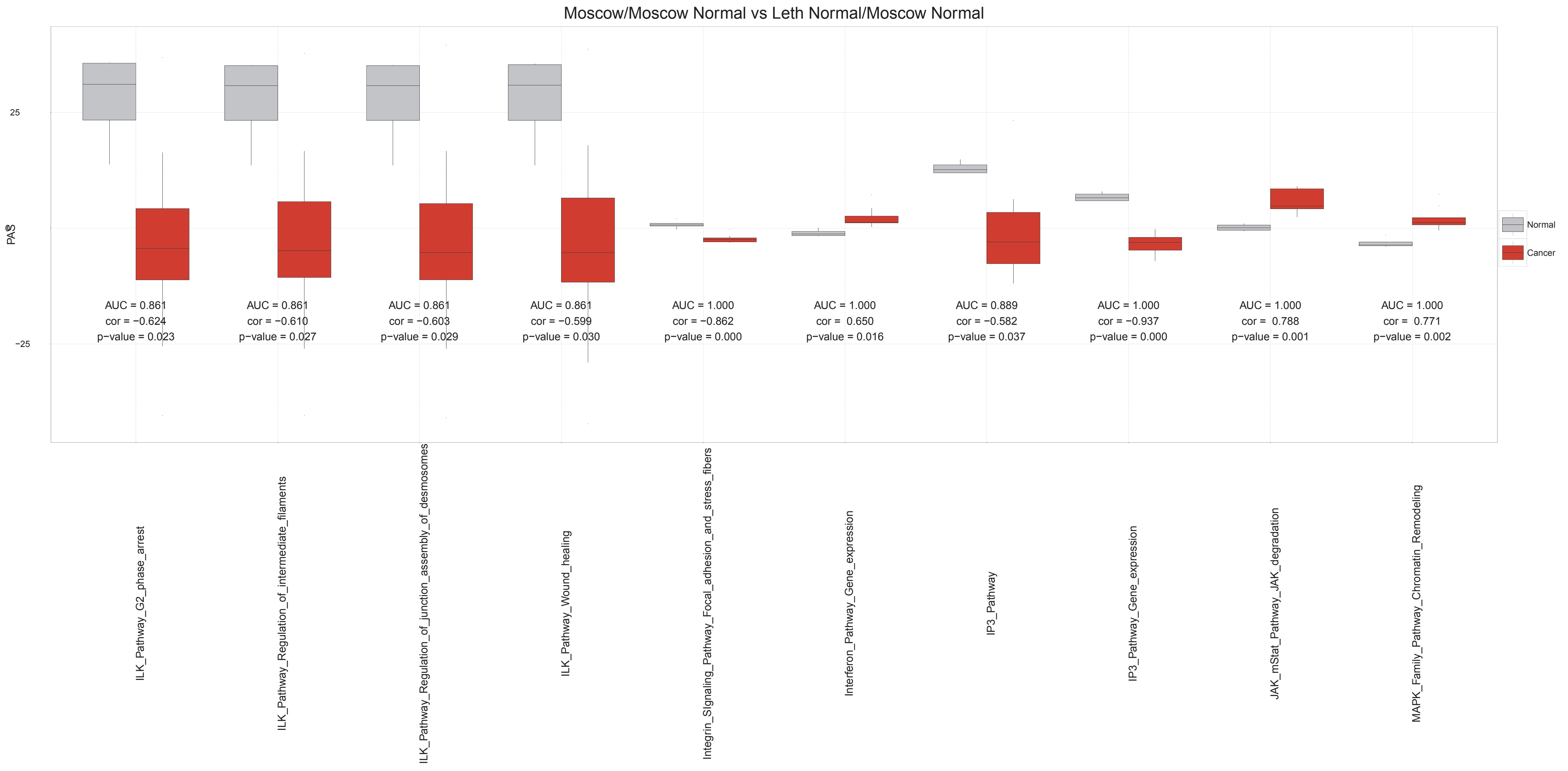

Moscow/Moscow Normal vs Leth Normal/Moscow Normal
25
-25
PAS
Normal
Cancer
AUC = 0.861
cor = −0.624
p−value = 0.023
AUC = 0.861
cor = −0.610
p−value = 0.027
AUC = 0.861
cor = −0.603
p−value = 0.029
AUC = 0.861
cor = −0.599
p−value = 0.030
AUC = 1.000
cor = −0.862
p−value = 0.000
AUC = 1.000
cor = 0.650
p−value = 0.016
AUC = 0.889
cor = −0.582
p−value = 0.037
AUC = 1.000
cor = −0.937
p−value = 0.000
AUC = 1.000
cor = 0.788
p−value = 0.001
AUC = 1.000
cor = 0.771
p−value = 0.002
ILK_Pathway_G2_phase_arrest
ILK_Pathway_Regulation_of_intermediate_filaments
ILK_Pathway_Regulation_of_junction_assembly_of_desmosomes
ILK_Pathway_Wound_healing
Integrin_SIgnaling_Pathway_Focal_adhesion_and_stress_fibers
Interferon_Pathway_Gene_expression
IP3_Pathway
IP3_Pathway_Gene_expression
JAK_mStat_Pathway_JAK_degradation
MAPK_Family_Pathway_Chromatin_Remodeling

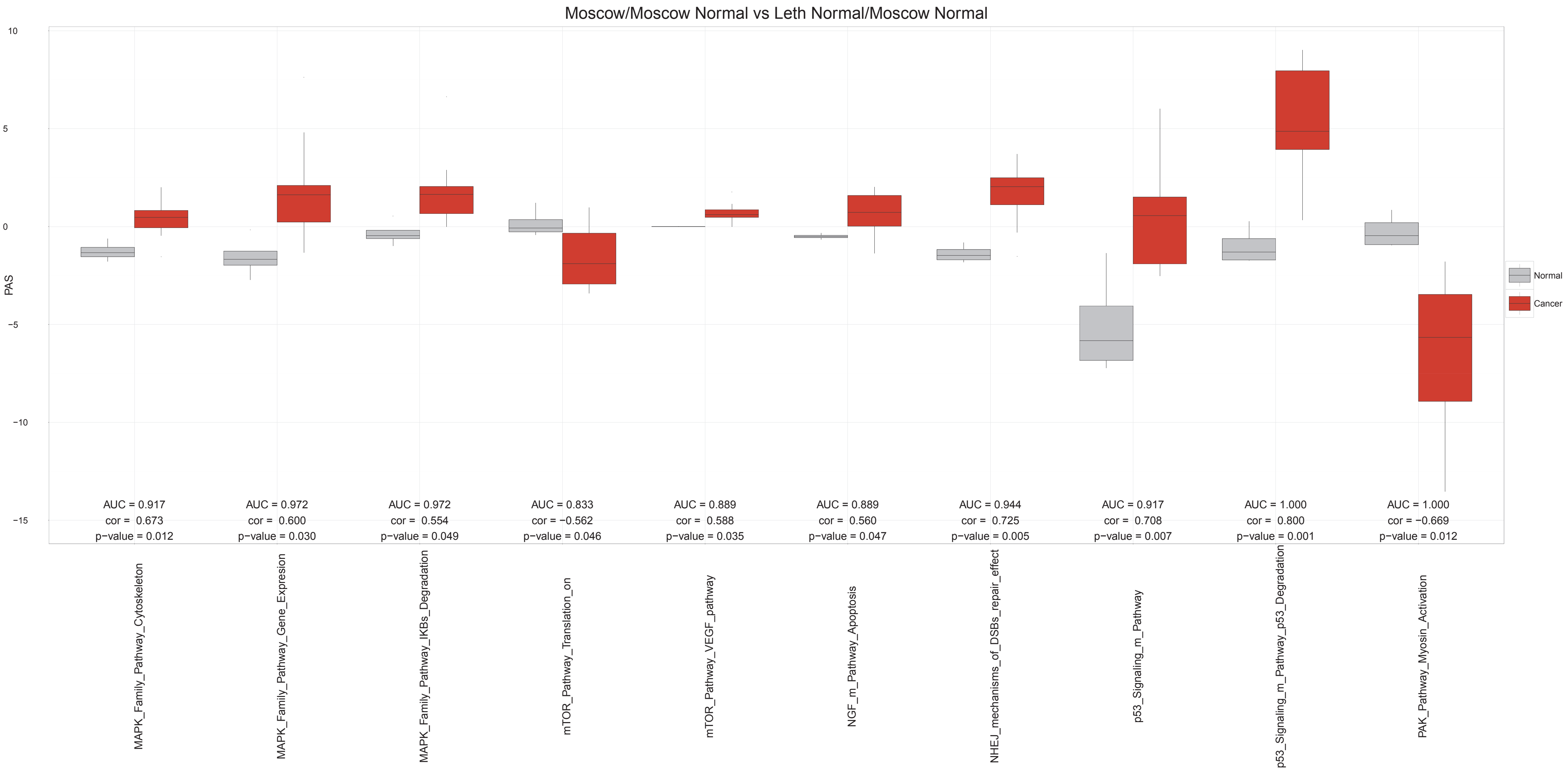
Moscow/Moscow Normal vs Leth Normal/Moscow Normal
PAS
10
5
0
−5
−10
−15
Normal
Cancer
AUC = 0.917
cor = 0.673
p−value = 0.012
AUC = 0.972
cor = 0.600
p−value = 0.030
AUC = 0.972
cor = 0.554
p−value = 0.049
AUC = 0.833
cor = −0.562
p−value = 0.046
AUC = 0.889
cor = 0.588
p−value = 0.035
AUC = 0.889
cor = 0.560
p−value = 0.047
AUC = 0.944
cor = 0.725
p−value = 0.005
AUC = 0.917
cor = 0.708
p−value = 0.007
AUC = 1.000
cor = 0.800
p−value = 0.001
AUC = 1.000
cor = −0.669
p−value = 0.012
MAPK_Family_Pathway_Cytoskeleton
MAPK_Family_Pathway_Gene_Expresion
MAPK_Family_Pathway_IKBs_Degradation
mTOR_Pathway_Translation_on
mTOR_Pathway_VEGF_pathway
NGF_m_Pathway_Apoptosis
NHEJ_mechanisms_of_DSBs_repair_effect
p53_Signaling_m_Pathway
p53_Signaling_m_Pathway_p53_Degradation
PAK_Pathway_Myosin_Activation

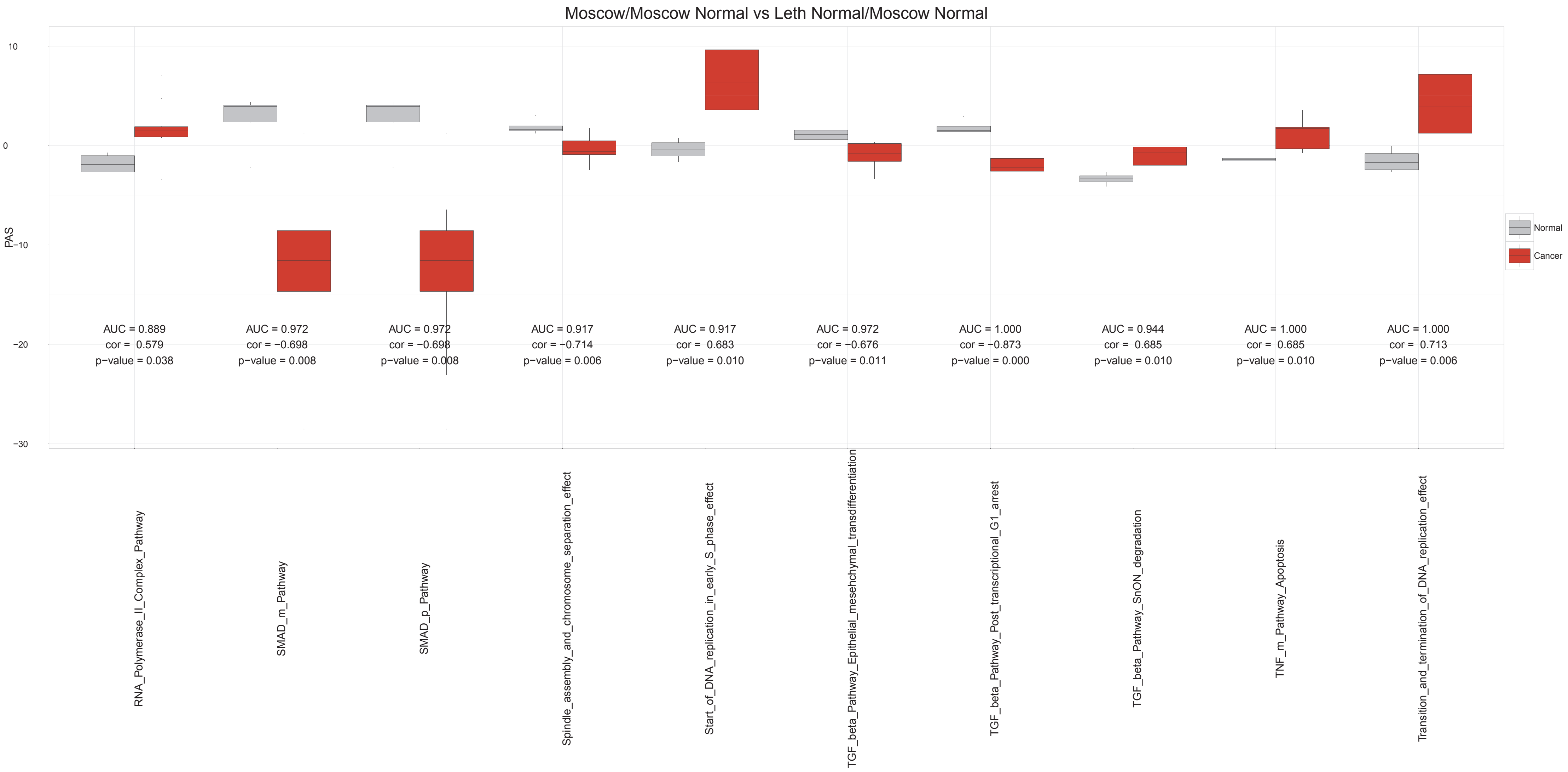
Moscow/Moscow Normal vs Leth Normal/Moscow Normal
PAS
10
0
−10
−20
−30
Normal
Cancer
AUC = 0.889
cor = 0.579
p−value = 0.038
AUC = 0.972
cor = −0.698
p−value = 0.008
AUC = 0.972
cor = −0.698
p−value = 0.008
AUC = 0.917
cor = −0.714
p−value = 0.006
AUC = 0.917
cor = 0.683
p−value = 0.010
AUC = 0.972
cor = −0.676
p−value = 0.011
AUC = 1.000
cor = −0.873
p−value = 0.000
AUC = 0.944
cor = 0.685
p−value = 0.010
AUC = 1.000
cor = 0.685
p−value = 0.010
AUC = 1.000
cor = 0.713
p−value = 0.006
RNA_Polymerase_II_Complex_Pathway
SMAD_m_Pathway
SMAD_p_Pathway
Spindle_assembly_and_chromosome_separation_effect
Start_of_DNA_replication_in_early_S_phase_effect
TGF_beta_Pathway_Epithelial_mesehchymal_transdifferentiation
TGF_beta_Pathway_Post_transcripional_G1_arrest
TGF_beta_Pathway_SnON_degradation
TNF_m_Pathway_Apoptosis
Transition_and_termination_of_DNA_replication_effect

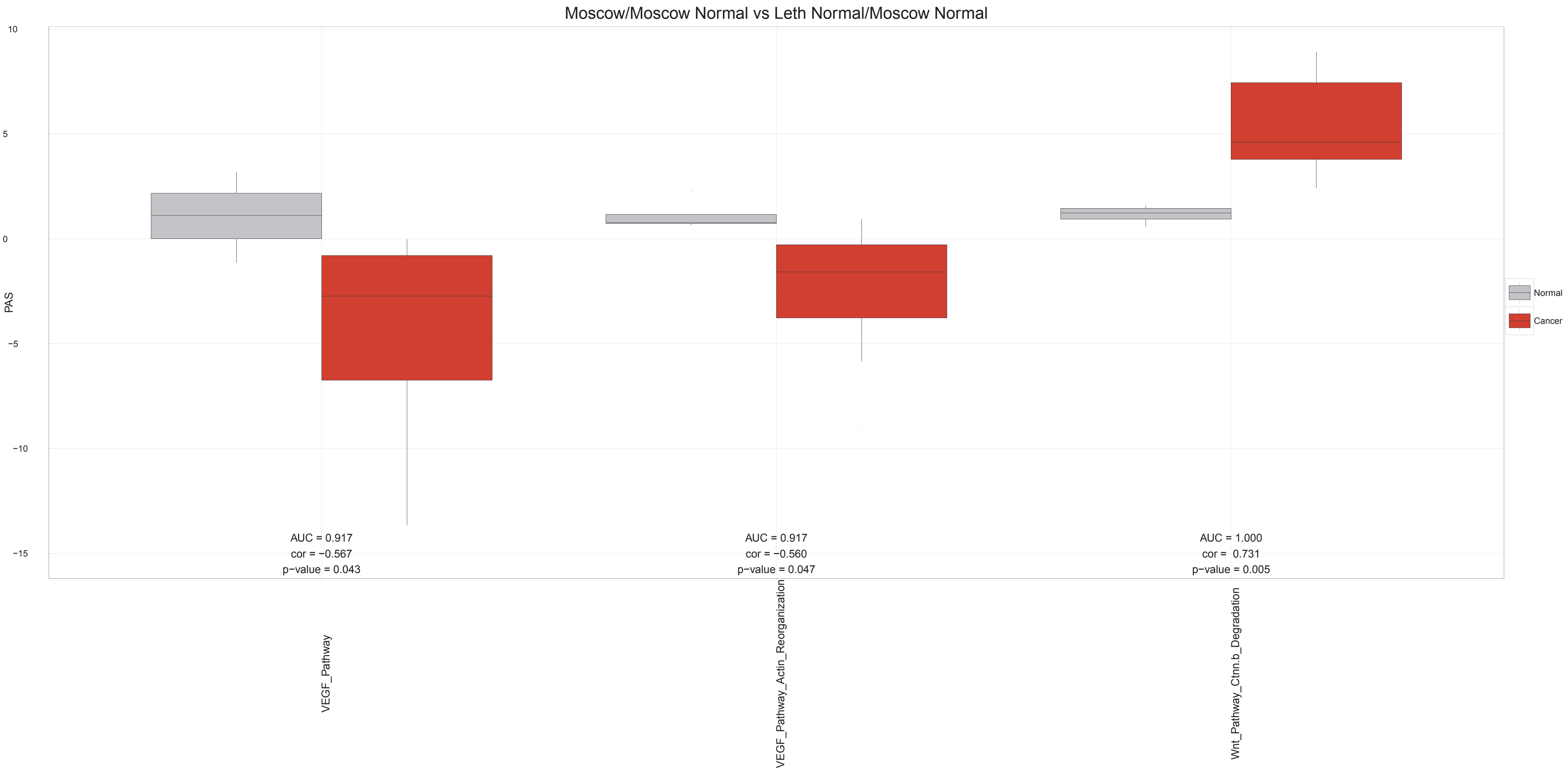
Moscow/Moscow Normal vs Leth Normal/Moscow Normal
10
5
0
−5
−10
−15
PAS
AUC = 0.917
cor = −0.567
p−value = 0.043
AUC = 0.917
cor = −0.560
p−value = 0.047
AUC = 1.000
cor = 0.731
p−value = 0.005
VEGF_Pathway
VEGF_Pathway_Actin_Reorganization
Wnt_Pathway_Ctnn.b_Degradation
Normal
Cancer
