# Supplementary material for: Novel robust biomarkers for human bladder cancer based on activation of intracellular signaling pathways
Source: Oncotarget. 2014 Sep 16;5(19):9022–32. doi: 10.18632/oncotarget.2493 (PMC4253415; doi:10.18632/oncotarget.2493)
Supplement: Supplementary file 12 [file oncotarget-05-9022-s012.pdf]

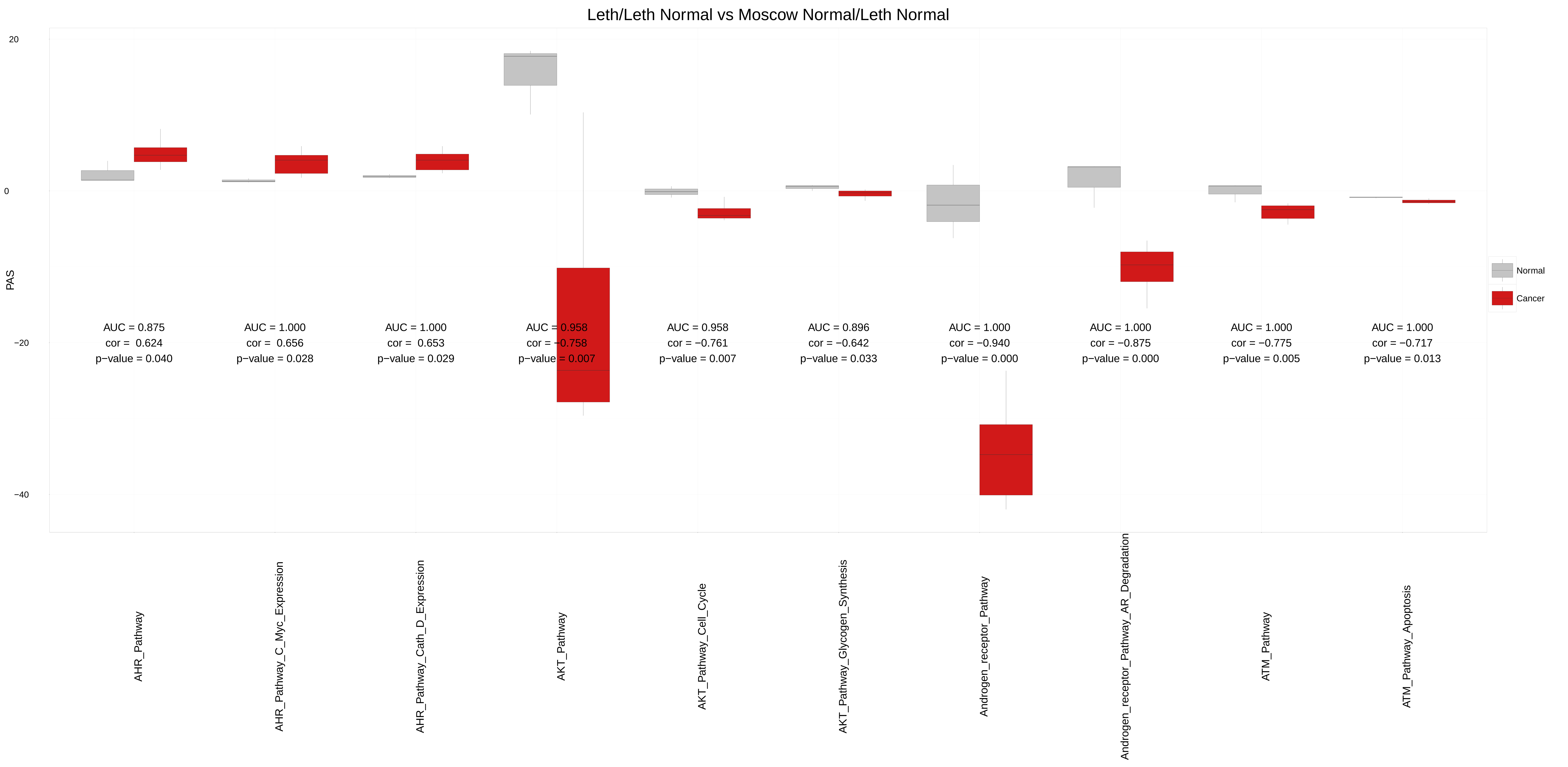

Leth/Leth Normal vs Moscow Normal/Leth Normal
20
0
−20
−40
PAS
AUC = 0.875
cor = 0.624
p−value = 0.040
AUC = 1.000
cor = 0.656
p−value = 0.028
AUC = 1.000
cor = 0.653
p−value = 0.029
AUC = 0.958
cor = −0.758
p−value = 0.007
AUC = 0.958
cor = −0.761
p−value = 0.007
AUC = 0.896
cor = −0.642
p−value = 0.033
AUC = 1.000
cor = −0.940
p−value = 0.000
AUC = 1.000
cor = −0.875
p−value = 0.000
AUC = 1.000
cor = −0.775
p−value = 0.005
AUC = 1.000
cor = −0.717
p−value = 0.013
Normal
Cancer
AHR_Pathway
AHR_Pathway_C_Myc_Expression
AHR_Pathway_Cath_D_Expression
AKT_Pathway
AKT_Pathway_Cell_Cycle
AKT_Pathway_Glycogen_Synthesis
Androgen_receptor_Pathway
Androgen_receptor_Pathway_AR_Degradation
ATM_Pathway
ATM_Pathway_Apoptosis

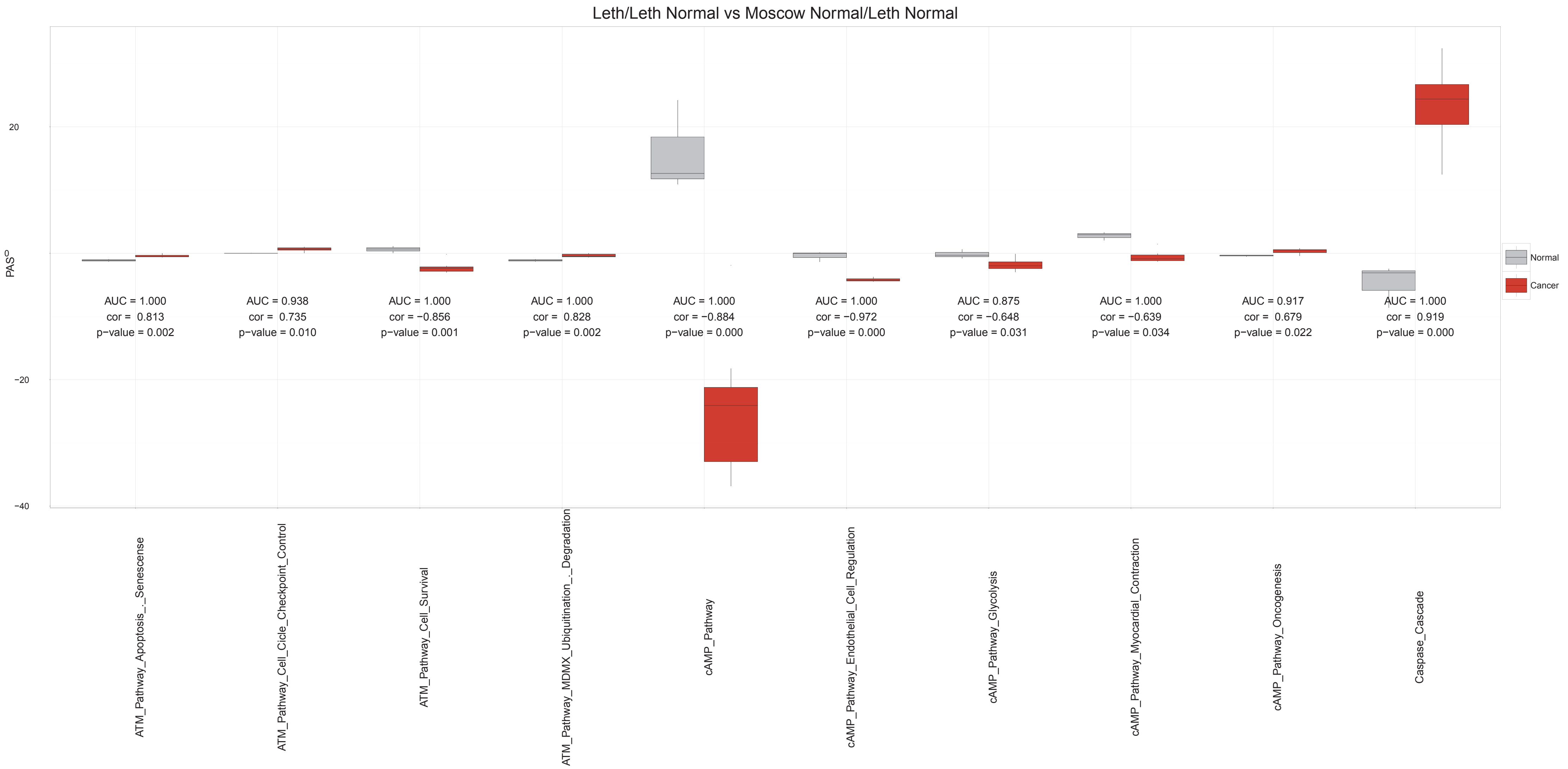

Leth/Leth Normal vs Moscow Normal/Leth Normal
PAS°
20
−20
−40
Normal
Cancer
AUC = 1.000
cor = 0.813
p−value = 0.002
AUC = 0.938
cor = 0.735
p−value = 0.010
AUC = 1.000
cor = −0.856
p−value = 0.001
AUC = 1.000
cor = 0.828
p−value = 0.002
AUC = 1.000
cor = −0.884
p−value = 0.000
AUC = 1.000
cor = −0.972
p−value = 0.000
AUC = 0.875
cor = −0.648
p−value = 0.031
AUC = 1.000
cor = −0.639
p−value = 0.034
AUC = 0.917
cor = 0.679
p−value = 0.022
AUC = 1.000
cor = 0.919
p−value = 0.000
ATM_Pathway_Apoptosis_._Senescense
ATM_Pathway_Cell_Cicle_Checkpoint_Control
ATM_Pathway_Cell_Survival
ATM_Pathway_MDMX_Ubiquitination_._Degradation
cAMP_Pathway
cAMP_Pathway_Endothelial_Cell_Regulation
cAMP_Pathway_Glycolysis
cAMP_Pathway_Myocardial_Contraction
cAMP_Pathway_Oncogenesis
Caspase_Cascade

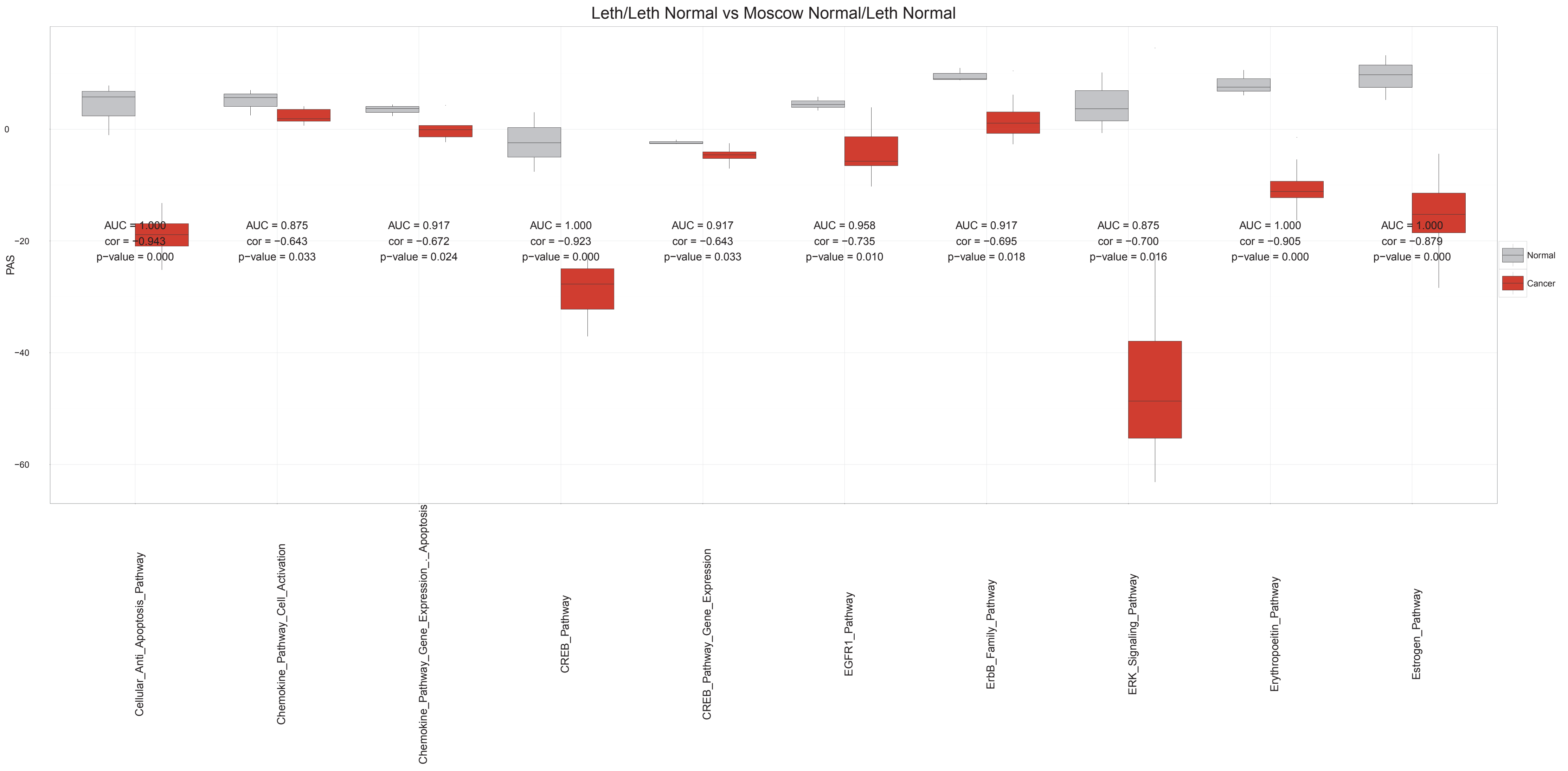

Leth/Leth Normal vs Moscow Normal/Leth Normal
PAS
0
−20
−40
−60
AUC = 1.000
cor = −0.943
p−value = 0.000
AUC = 0.875
cor = −0.643
p−value = 0.033
AUC = 0.917
cor = −0.672
p−value = 0.024
AUC = 1.000
cor = −0.923
p−value = 0.000
AUC = 0.917
cor = −0.643
p−value = 0.033
AUC = 0.958
cor = −0.735
p−value = 0.010
AUC = 0.917
cor = −0.695
p−value = 0.018
AUC = 0.875
cor = −0.700
p−value = 0.016
AUC = 1.000
cor = −0.905
p−value = 0.000
AUC = 1.000
cor = −0.879
p−value = 0.000
Normal
Cancer
Cellular_Anti_Apoptosis_Pathway
Chemokine_Pathway_Cell_Activation
Chemokine_Pathway_Gene_Expression_._Apoptosis
CREB_Pathway
CREB_Pathway_Gene_Expression
EGFR1_Pathway
ErbB_Family_Pathway
ERK_Signaling_Pathway
Erythropoeitin_Pathway
Estrogen_Pathway

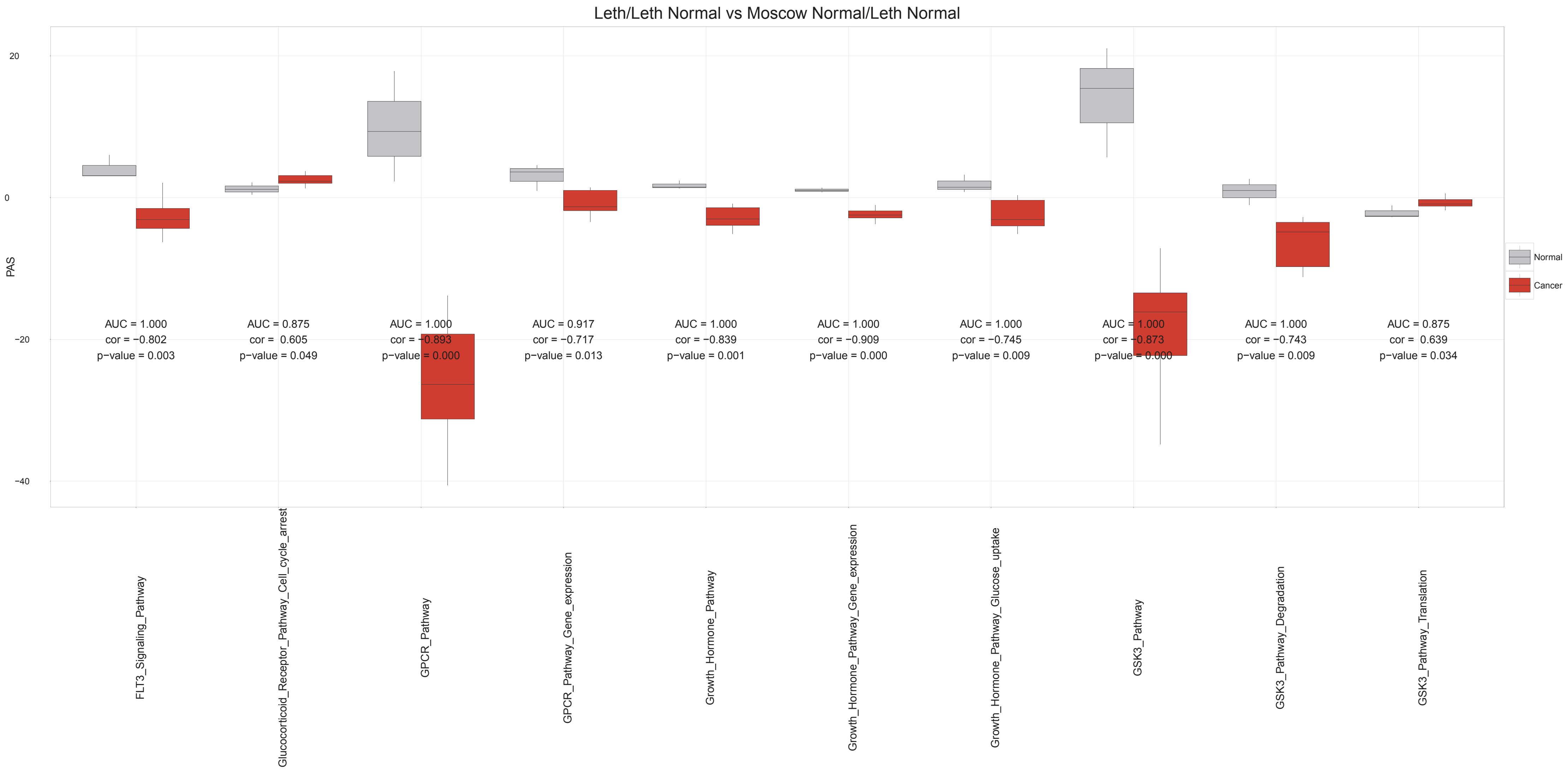

Leth/Leth Normal vs Moscow Normal/Leth Normal
PAS
20
0
-20
-40
Normal
Cancer
AUC = 1.000
cor = −0.802
p−value = 0.003
AUC = 0.875
cor = 0.605
p−value = 0.049
AUC = 1.000
cor = −0.893
p−value = 0.000
AUC = 0.917
cor = −0.717
p−value = 0.013
AUC = 1.000
cor = −0.839
p−value = 0.001
AUC = 1.000
cor = −0.909
p−value = 0.000
AUC = 1.000
cor = −0.745
p−value = 0.009
AUC = 1.000
cor = −0.873
p−value = 0.000
AUC = 1.000
cor = −0.743
p−value = 0.009
AUC = 0.875
cor = 0.639
p−value = 0.034
FLT3_Signaling_Pathway
Glucocorticoid_Receptor_Pathway_Cell_cycle_arrest
GPCR_Pathway
GPCR_Pathway_Gene_expression
Growth_Hormone_Pathway
Growth_Hormone_Pathway_Gene_expression
Growth_Hormone_Pathway_Glucose_uptake
GSK3_Pathway
GSK3_Pathway_Degradation
GSK3_Pathway_Translation

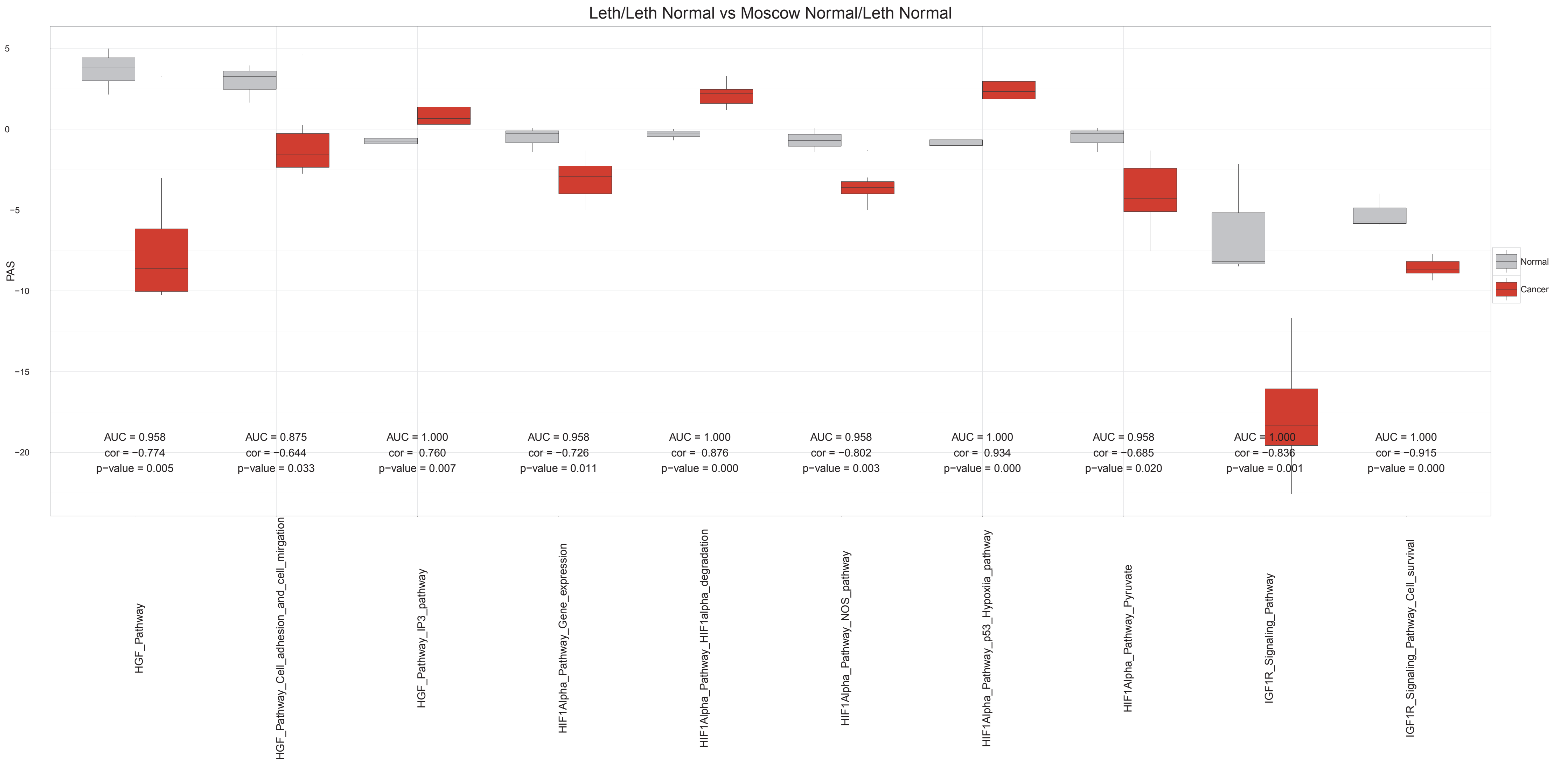

Leth/Leth Normal vs Moscow Normal/Leth Normal
PAS
5
0
−5
−10
−15
−20
Normal
Cancer
AUC = 0.958
cor = −0.774
p−value = 0.005
AUC = 0.875
cor = −0.644
p−value = 0.033
AUC = 1.000
cor = 0.760
p−value = 0.007
AUC = 0.958
cor = −0.726
p−value = 0.011
AUC = 1.000
cor = 0.876
p−value = 0.000
AUC = 0.958
cor = −0.802
p−value = 0.003
AUC = 1.000
cor = 0.934
p−value = 0.000
AUC = 0.958
cor = −0.685
p−value = 0.020
AUC = 1.000
cor = −0.836
p−value = 0.001
AUC = 1.000
cor = −0.915
p−value = 0.000
HGF_Pathway
HGF_Pathway_Cell_adhesion_and_cell_mirgation
HGF_Pathway_IP3_pathway
HIF1Alpha_Pathway_Gene_expression
HIF1Alpha_Pathway_HIF1alpha_degradation
HIF1Alpha_Pathway_NOS_pathway
HIF1Alpha_Pathway_p53_Hypoxiia_pathway
HIF1Alpha_Pathway_Pyruvate
IGF1R_Signaling_Pathway
IGF1R_Signaling_Pathway_Cell_survival

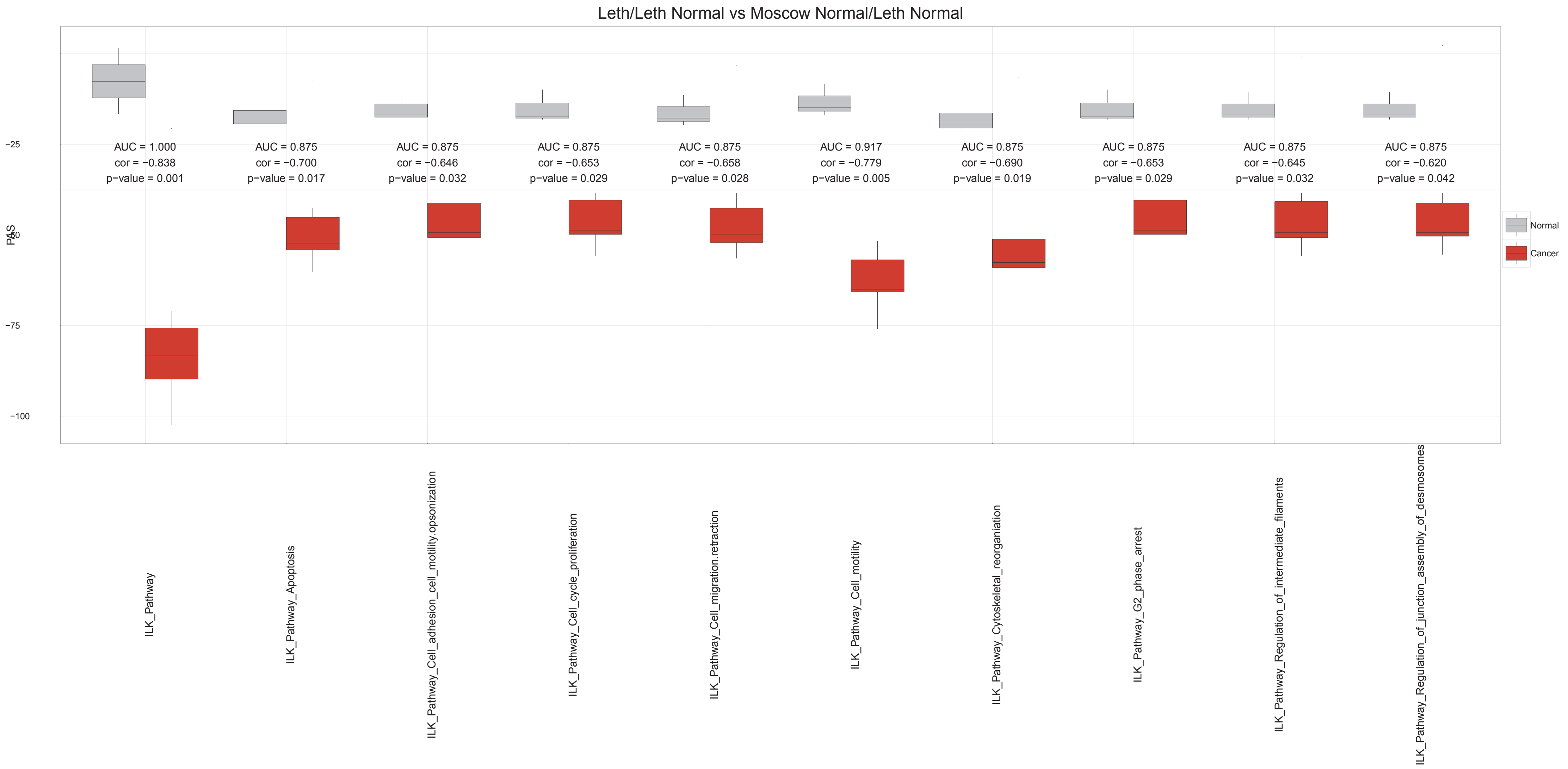

Leth/Leth Normal vs Moscow Normal/Leth Normal
PAS
−25
−50
−75
−100
AUC = 1.000
cor = −0.838
p−value = 0.001
AUC = 0.875
cor = −0.700
p−value = 0.017
AUC = 0.875
cor = −0.646
p−value = 0.032
AUC = 0.875
cor = −0.653
p−value = 0.029
AUC = 0.875
cor = −0.658
p−value = 0.028
AUC = 0.917
cor = −0.779
p−value = 0.005
AUC = 0.875
cor = −0.690
p−value = 0.019
AUC = 0.875
cor = −0.653
p−value = 0.029
AUC = 0.875
cor = −0.645
p−value = 0.032
AUC = 0.875
cor = −0.620
p−value = 0.042
Normal
Cancer
ILK_Pathway
ILK_Pathway_Apoptosis
ILK_Pathway_Cell_adhesion_cell_motility.opsonization
ILK_Pathway_Cell_cycle_proliferation
ILK_Pathway_Cell_migration.retraction
ILK_Pathway_Cell_motility
ILK_Pathway_Cytoskeletal_reorganiation
ILK_Pathway_G2_phase_arrest
ILK_Pathway_Regulation_of_intermediate_filaments
ILK_Pathway_Regulation_of_junction_assembly_of_desmosomes

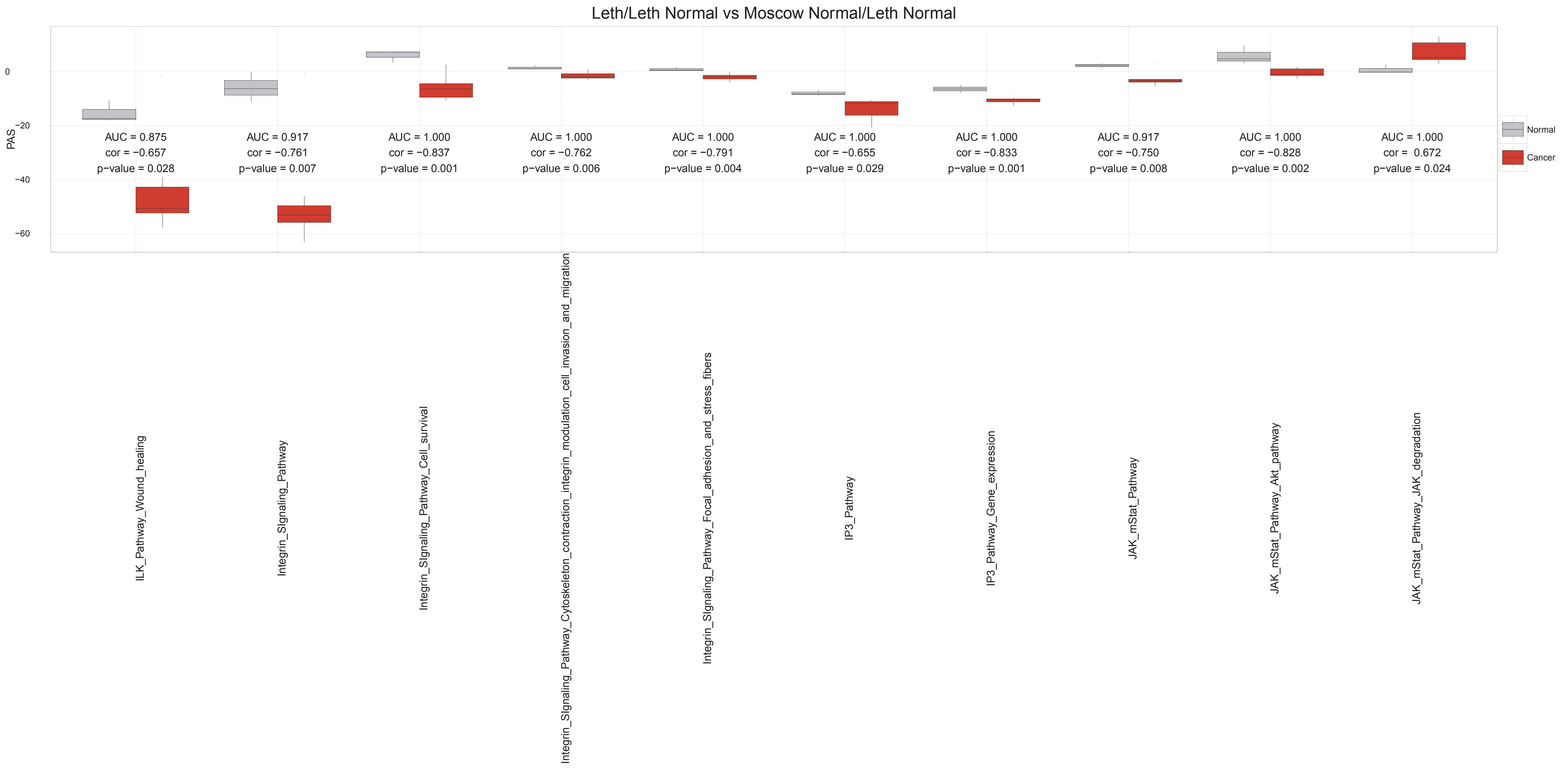

Leth/Leth Normal vs Moscow Normal/Leth Normal
PAS
0
-20
-40
-60
AUC = 0.875
cor = −0.657
p−value = 0.028
AUC = 0.917
cor = −0.761
p−value = 0.007
AUC = 1.000
cor = −0.837
p−value = 0.001
AUC = 1.000
cor = −0.762
p−value = 0.006
AUC = 1.000
cor = −0.791
p−value = 0.004
AUC = 1.000
cor = −0.655
p−value = 0.029
AUC = 1.000
cor = −0.833
p−value = 0.001
AUC = 0.917
cor = −0.750
p−value = 0.008
AUC = 1.000
cor = −0.828
p−value = 0.002
AUC = 1.000
cor = 0.672
p−value = 0.024
Normal
Cancer
ILK_Pathway_Wound_healing
Integrin_SIgnaling_Pathway
Integrin_SIgnaling_Pathway_Cell_survival
Integrin_SIgnaling_Pathway_Cytoskeleton_contraction_integrin_modulation_cell_invasion_and_migration
Integrin_SIgnaling_Pathway_Focal_adhesion_and_stress_fibers
IP3_Pathway
IP3_Pathway_Gene_expression
JAK_mStat_Pathway
JAK_mStat_Pathway_Akt_pathway
JAK_mStat_Pathway_JAK_degradation

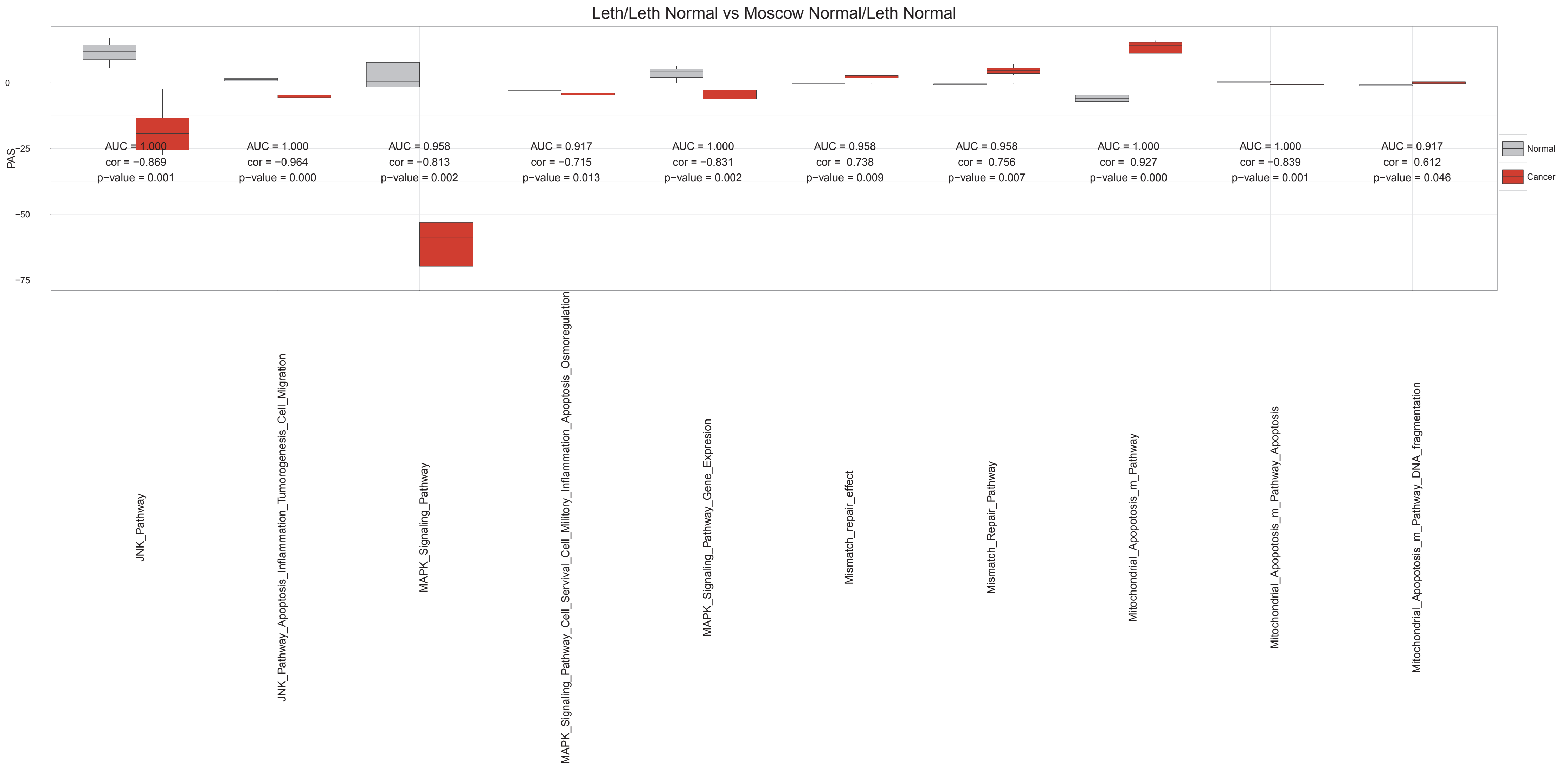

Leth/Leth Normal vs Moscow Normal/Leth Normal
PAS
0
−25
−50
−75
AUC = 1.000
cor = −0.869
p−value = 0.001
AUC = 1.000
cor = −0.964
p−value = 0.000
AUC = 0.958
cor = −0.813
p−value = 0.002
AUC = 0.917
cor = −0.715
p−value = 0.013
AUC = 1.000
cor = −0.831
p−value = 0.002
AUC = 0.958
cor = 0.738
p−value = 0.009
AUC = 0.958
cor = 0.756
p−value = 0.007
AUC = 1.000
cor = 0.927
p−value = 0.000
AUC = 1.000
cor = −0.839
p−value = 0.001
AUC = 0.917
cor = 0.612
p−value = 0.046
Normal
Cancer
JNK_Pathway
JNK_Pathway_Apoptosis_Inflammation_Tumorogenesis_Cell_Migration
MAPK_Signaling_Pathway
MAPK_Signaling_Pathway_Cell_Servival_Cell_Militory_Inflammation_Apoptosis_Osmoregulation
MAPK_Signaling_Pathway_Gene_Expresion
Mismatch_repair_effect
Mismatch_Repair_Pathway
Mitochondrial_Apoptosis_m_Pathway
Mitochondrial_Apoptosis_m_Pathway_Apoptosis
Mitochondrial_Apoptosis_m_Pathway_DNA_fragmentation

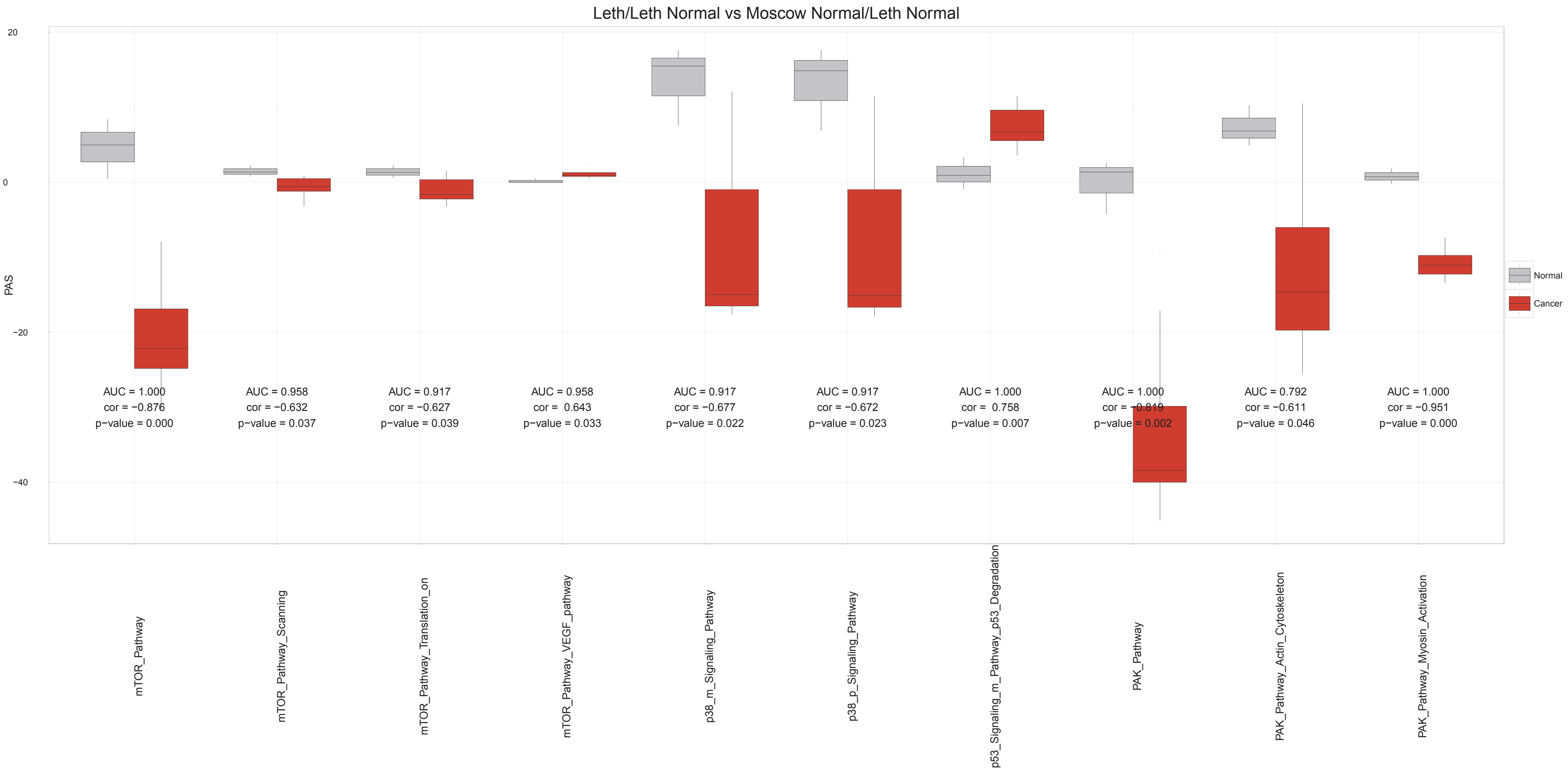

Leth/Leth Normal vs Moscow Normal/Leth Normal
20
0
-20
-40
PAS
Normal
Cancer
AUC = 1.000
cor = −0.876
p−value = 0.000
AUC = 0.958
cor = −0.632
p−value = 0.037
AUC = 0.917
cor = −0.627
p−value = 0.039
AUC = 0.958
cor = 0.643
p−value = 0.033
AUC = 0.917
cor = −0.677
p−value = 0.022
AUC = 0.917
cor = −0.672
p−value = 0.023
AUC = 1.000
cor = 0.758
p−value = 0.007
AUC = 1.000
cor = −0.819
p−value = 0.002
AUC = 0.792
cor = −0.611
p−value = 0.046
AUC = 1.000
cor = −0.951
p−value = 0.000
mTOR_Pathway
mTOR_Pathway_Scanning
mTOR_Pathway_Translation_on
mTOR_Pathway_VEGF_pathway
p38_m_Signaling_Pathway
p38_p_Signaling_Pathway
p53_Signaling_m_Pathway_p53_Degradation
PAK_Pathway
PAK_Pathway_Actin_Cytoskeleton
PAK_Pathway_Myosin_Activation

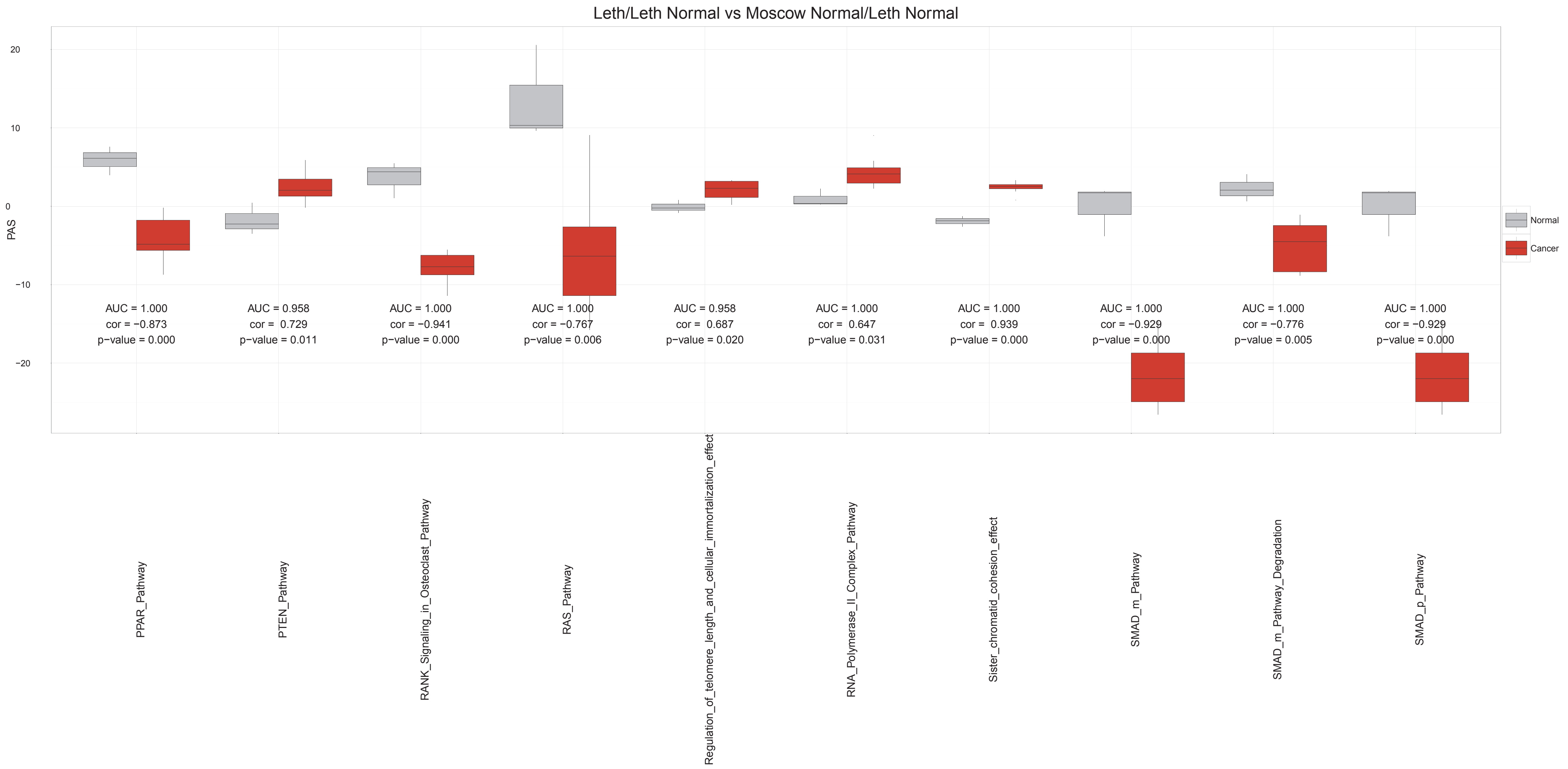

Leth/Leth Normal vs Moscow Normal/Leth Normal
PAS
20
10
0
−10
−20
Normal
Cancer
AUC = 1.000
cor = −0.873
p−value = 0.000
AUC = 0.958
cor = 0.729
p−value = 0.011
AUC = 1.000
cor = −0.941
p−value = 0.000
AUC = 1.000
cor = −0.767
p−value = 0.006
AUC = 0.958
cor = 0.687
p−value = 0.020
AUC = 1.000
cor = 0.647
p−value = 0.031
AUC = 1.000
cor = 0.939
p−value = 0.000
AUC = 1.000
cor = −0.929
p−value = 0.000
AUC = 1.000
cor = −0.776
p−value = 0.005
AUC = 1.000
cor = −0.929
p−value = 0.000
PPAR_Pathway
PTEN_Pathway
RANK_Signaling_in_Osteoclast_Pathway
RAS_Pathway
Regulation_of_telomere_length_and_cellular_immortalization_effect
RNA_Polymerase_II_Complex_Pathway
Sister_chromatid_cohesion_effect
SMAD_m_Pathway
SMAD_m_Pathway_Degradation
SMAD_p_Pathway

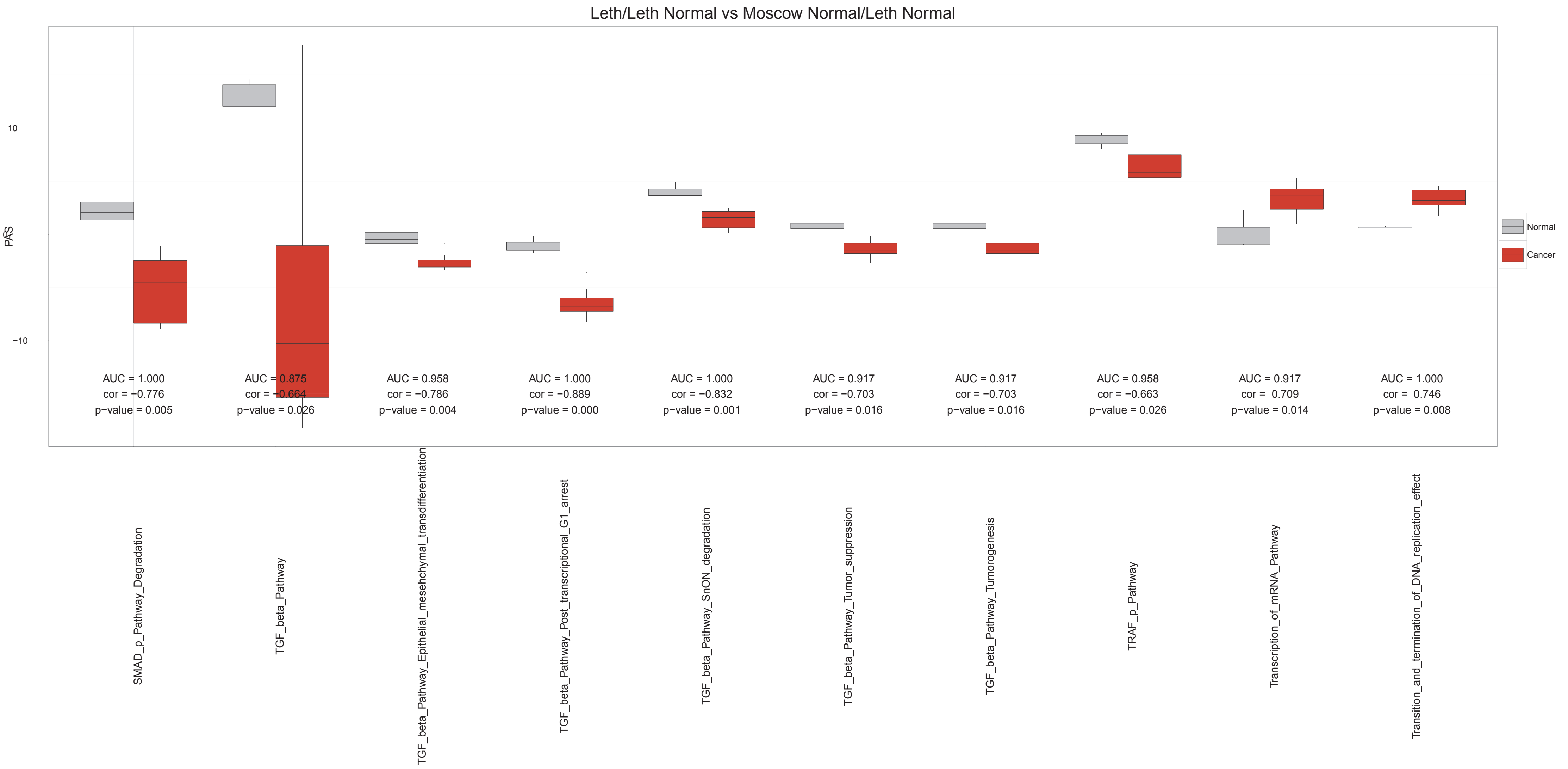

Leth/Leth Normal vs Moscow Normal/Leth Normal
PAS
10
−10
AUC = 1.000
cor = −0.776
p−value = 0.005
AUC = 0.875
cor = −0.664
p−value = 0.026
AUC = 0.958
cor = −0.786
p−value = 0.004
AUC = 1.000
cor = −0.889
p−value = 0.000
AUC = 1.000
cor = −0.832
p−value = 0.001
AUC = 0.917
cor = −0.703
p−value = 0.016
AUC = 0.917
cor = −0.703
p−value = 0.016
AUC = 0.958
cor = −0.663
p−value = 0.026
AUC = 0.917
cor = 0.709
p−value = 0.014
AUC = 1.000
cor = 0.746
p−value = 0.008
Normal
Cancer
SMAD_p_Pathway_Degradation
TGF_beta_Pathway
TGF_beta_Pathway_Epithelial_mesehchymal_transdifferentiation
TGF_beta_Pathway_Post_transcriptional_G1_arrest
TGF_beta_Pathway_SnON_degradation
TGF_beta_Pathway_Tumor_suppression
TGF_beta_Pathway_Tumorogenesis
TRAF_p_Pathway
Transcription_of_mRNA_Pathway
Transition_and_termination_of_DNA_replication_effect

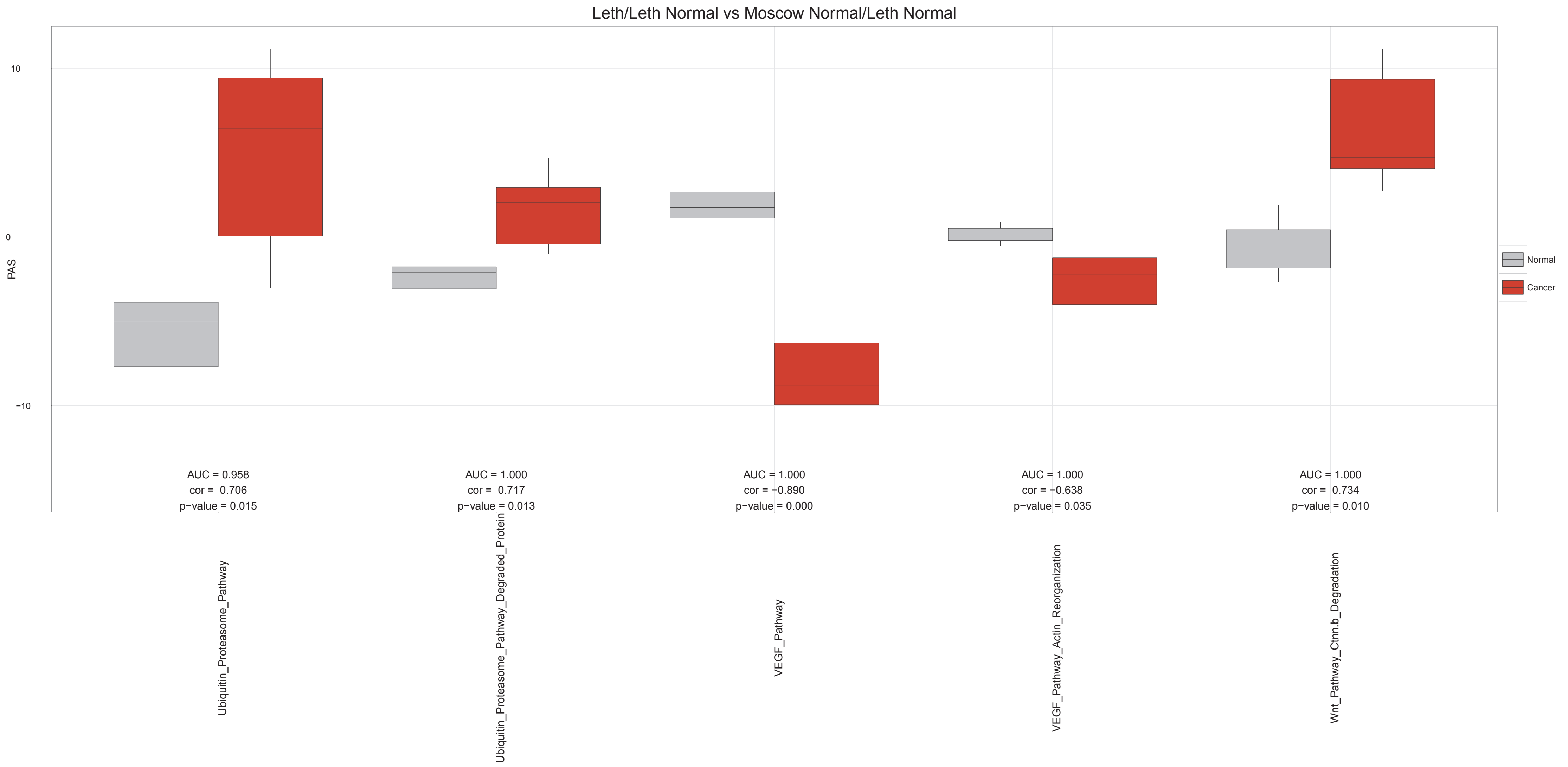

Leth/Leth Normal vs Moscow Normal/Leth Normal
10
0
−10
PAS
Normal
Cancer
AUC = 0.958
cor = 0.706
p−value = 0.015
AUC = 1.000
cor = 0.717
p−value = 0.013
AUC = 1.000
cor = −0.890
p−value = 0.000
AUC = 1.000
cor = −0.638
p−value = 0.035
AUC = 1.000
cor = 0.734
p−value = 0.010
Ubiquitin_Proteasome_Pathway
Ubiquitin_Proteasome_Pathway_Degraded_Protein
VEGF_Pathway
VEGF_Pathway_Actin_Reorganization
Wnt_Pathway_Ctnn.b_Degradation
